# Plastid phylogenomic insights into relationships of all flowering plant families

**DOI:** 10.1186/s12915-021-01166-2

**Published:** 2021-10-29

**Authors:** Hong-Tao Li, Yang Luo, Lu Gan, Peng-Fei Ma, Lian-Ming Gao, Jun-Bo Yang, Jie Cai, Matthew A. Gitzendanner, Peter W. Fritsch, Ting Zhang, Jian-Jun Jin, Chun-Xia Zeng, Hong Wang, Wen-Bin Yu, Rong Zhang, Michelle van der Bank, Richard G. Olmstead, Peter M. Hollingsworth, Mark W. Chase, Douglas E. Soltis, Pamela S. Soltis, Ting-Shuang Yi, De-Zhu Li

**Affiliations:** 1 Germplasm Bank of Wild Species, Kunming Institute of Botany, Chinese Academy of Sciences, Kunming, 650201 Yunnan China; 2 Kunming College of Life Science, University of Chinese Academy of Sciences, Kunming, 650201 Yunnan China; 3 CAS Key Laboratory for Plant Diversity and Biogeography of East Asia, Kunming Institute of Botany, Chinese Academy of Sciences, Kunming, 650201 Yunnan China; 4 Lijiang Forest Ecosystem National Observation and Research Station, Kunming Institute of Botany, Chinese Academy of Sciences, Lijiang, 674100 Yunnan China; 5 Florida Museum of Natural History, University of Florida, Gainesville, FL 32611 USA; 6 Biodiversity Institute, University of Florida, Gainesville, FL 32611 USA; 7 Botanical Research Institute of Texas, 1700 University Drive, Fort Worth, TX 76017 USA; 8 Department of Ecology, Evolution and Environmental Biology, Columbia University, New York, NY 10025 USA; 9 Center for Integrative Conservation, Xishuangbanna Tropical Botanical Garden, Chinese Academy of Sciences, Mengla, 666303 Yunnan China; 10 Department of Botany & Plant Biotechnology, University of Johannesburg, PO Box 524, Auckland Park, Johannesburg, Gauteng 2006 South Africa; 11 Department of Biology and Burke Museum, University of Washington, Seattle, WA 98195-5325 USA; 12 Royal Botanic Garden Edinburgh, Edinburgh, EH3 5LR Scotland, UK; 13 Royal Botanic Gardens, Kew, Richmond, Surrey TW9 3DS England, UK; 14 Department of Environment and Agriculture, Curtin University, Bentley, Western Australia 6102 Australia; 15 Department of Biology, University of Florida, Gainesville, FL 32611 USA

**Keywords:** *Mesangiospermae*, Tree of life, Interfamilial relationships, Plastome, PPA II

## Abstract

**Background:**

Flowering plants (angiosperms) are dominant components of global terrestrial ecosystems, but phylogenetic relationships at the familial level and above remain only partially resolved, greatly impeding our full understanding of their evolution and early diversification. The plastome, typically mapped as a circular genome, has been the most important molecular data source for plant phylogeny reconstruction for decades.

**Results:**

Here, we assembled by far the largest plastid dataset of angiosperms, composed of 80 genes from 4792 plastomes of 4660 species in 2024 genera representing all currently recognized families. Our phylogenetic tree (PPA II) is essentially congruent with those of previous plastid phylogenomic analyses but generally provides greater clade support. In the PPA II tree, 75% of nodes at or above the ordinal level and 78% at or above the familial level were resolved with high bootstrap support (BP ≥ 90). We obtained strong support for many interordinal and interfamilial relationships that were poorly resolved previously within the core eudicots, such as Dilleniales, Saxifragales, and Vitales being resolved as successive sisters to the remaining rosids, and Santalales, Berberidopsidales, and Caryophyllales as successive sisters to the asterids. However, the placement of magnoliids, although resolved as sister to all other *Mesangiospermae*, is not well supported and disagrees with topologies inferred from nuclear data. Relationships among the five major clades of *Mesangiospermae* remain intractable despite increased sampling, probably due to an ancient rapid radiation.

**Conclusions:**

We provide the most comprehensive dataset of plastomes to date and a well-resolved phylogenetic tree, which together provide a strong foundation for future evolutionary studies of flowering plants.

**Supplementary Information:**

The online version contains supplementary material available at 10.1186/s12915-021-01166-2.

## Background

Angiosperms, or flowering plants, are by far the largest, most diverse, and most species-rich clade of green plants, with estimates of the number of species ranging from ~295,000 [1] to ~370,000 [2]. Traditionally, angiosperms were divided into two fundamental groups on the basis of cotyledon numbers, i.e., monocotyledons (monocots or Monocotyledoneae) and dicotyledons (dicots or Dicotyledoneae). Toward the end of the twentieth century, in several morphologically based cladistic analyses (e.g., [3, 4]), the monocots remained as a well-defined group with uniaperturate or uniaperturate-derived pollen, but the traditionally defined dicots were recovered as non-monophyletic. The majority of “dicots” formed a well-supported clade termed the tricolpates or eudicots [5] based on their triaperturate or triaperturate-derived pollen. These findings were corroborated in subsequent DNA-based phylogenetic studies, and the composition and placement of many of the remaining highly heterogeneous non-eudicots were clarified [6–11]. Among extant angiosperms, three small clades, Amborellales (1 species), Nymphaeales (88 species), and Austrobaileyales (94 species), collectively referred to as the ANA grade, represent the first-branching clades [8, 9]. The remainder belongs to a highly supported clade referred to as core angiosperms or *Mesangiospermae* ([12], comprising over 99.9% of extant angiosperm species), which was resolved into five clades (e.g., [13–17]): eudicots (~210,600 species), monocots (~74,300 species), magnoliids (*Magnoliidae* of [12]; ~10,800 species), Chloranthales (77 species), and Ceratophyllales (four species) [1, 12, 13]. These findings provide a firm understanding of the major clades of angiosperms, reflected in the widely accepted classification of the Angiosperm Phylogeny Group (APG; most recently, APG IV [18]).

During the past three decades, many molecular phylogenetic studies have achieved great progress in clarifying the backbone relationships of angiosperms [7, 9–11, 13, 14, 19–27]. However, the phylogenetic relationships among the eight major clades have remained controversial, hindering our understanding of the origin and early diversification of angiosperms. The debate on whether Amborellales alone or Amborellales + Nymphaeales are sisters to all other extant angiosperms is resolved, with all recent studies supporting Amborellales alone as a sister (e.g., [13, 28–30]). In contrast, the relationships among the five clades of *Mesangiospermae* are far more uncertain, and many contrasting topologies have been recovered from different datasets (nuclear, plastid, or mitochondrial), analytical methods (e.g., concatenation vs. coalescent), and taxon sampling [15, 21, 27, 31–33]. Furthermore, recent phylogenetic analyses with broad taxon sampling but genes from different genomes (2351 angiosperm plastomes in [13]; 682 angiosperm transcriptomes in [28] and 3099 angiosperm samples with target sequence capture data in [34]) yield conflicting topologies. Analyses based on recently sequenced genomes from key major clades of Ceratophyllales, magnoliids, and Nymphaeales [29, 35–39], with their limited taxon sampling, highlight this phylogenetic complexity with major highly conflicting signal.

Through a series of phylogenetic studies that applied broad taxon sampling with a small number of genes [9, 19, 24, 40], more limited taxon sampling with a large number of genes [15, 20–22], or both extensive taxon sampling and many genes [13, 28, 34, 41], great progress has been realized in resolving relationships among the eudicot clades, long recognized taxonomically as angiosperm orders and families (APG IV, which we will use here for discussion). Hereafter, these clades are also referred to as orders and families following APG IV for clarity and simplicity. However, these analyses either could not resolve or did not produce congruent results for certain parts of the angiosperm tree: (1) the placements of Dilleniales, Saxifragales, Vitales, Santalales, Berberidopsidales, and Caryophyllales in the core eudicots; (2) the interordinal relationships within asterids; and (3) the phylogenetic position and inter ordinal relationships of the Celastrales-Oxalidales-Malpighiales (COM) clade. Moreover, some interfamilial relationships within orders such as Malpighiales, Saxifragales, Commelinales, and Rosales were also not fully resolved.

Phylogenetic analyses based on plastid genes, and more recently complete or nearly complete plastomes, have led the way in reconstructing the phylogenetic backbone for angiosperms over the past three decades [6, 19, 23–25, 27, 42]. Plastomes, usually mapped as circular genomes, have numerous advantages for phylogenetic reconstruction, including mostly uniparental inheritance and a relatively conserved rate of evolution [41]. Recent advances in sequencing technology have made the acquisition of complete plastomes both practical and cost-effective, and an explosion of plastid phylogenomic studies has provided critical insights into historically difficult relationships of the major angiosperm subclades [22, 26, 43–45]. Our previous work [13], the then-largest plastid phylogenomic angiosperm (PPA) tree comprising 2351 angiosperm species representing 353 families and all 64 then-recognized orders, provided a significant advance towards a robust familial-level tree for angiosperms. However, 63 angiosperm families recognized by APG IV [18] and other 10 of 17 newly recognized families recorded by the Angiosperm Phylogeny Website (hereafter abbreviated as APW, last accessed May 23, 2019, [46]) but not recognized by APG IV were missing from the PPA tree, the remaining seven newly recognized families by APW were previously sampled in the PPA tree as genera of other families. These 73 omissions have precluded a full assessment of phylogenetic relationships among all angiosperm families.

In this study, we aim to better resolve evolutionary relationships of angiosperms at the familial level and above by analyzing the largest plastome dataset ever assembled for this purpose. Compared to our previous PPA project [13], the number of angiosperm plastomes has greatly increased from 2694 (1390 genera) to 4627 (2024 genera), a 66.3% increase in samples and a 45.6% increase in generic coverage, and all 433 recognized angiosperm families in APW [46], which provides narrower family circumscriptions than those of the APG system based on recent publications, were sampled accordingly. Our goals are to consolidate plastome-based phylogenetic relationships of the major clades recognized as families, orders, or more inclusive clades, provide additional perspectives on the early evolutionary history of angiosperms, and provide a robust plastome-based topology for comparison with studies based on the nuclear genome.

## Results

### Characteristics of the dataset

**Fig. 1:**
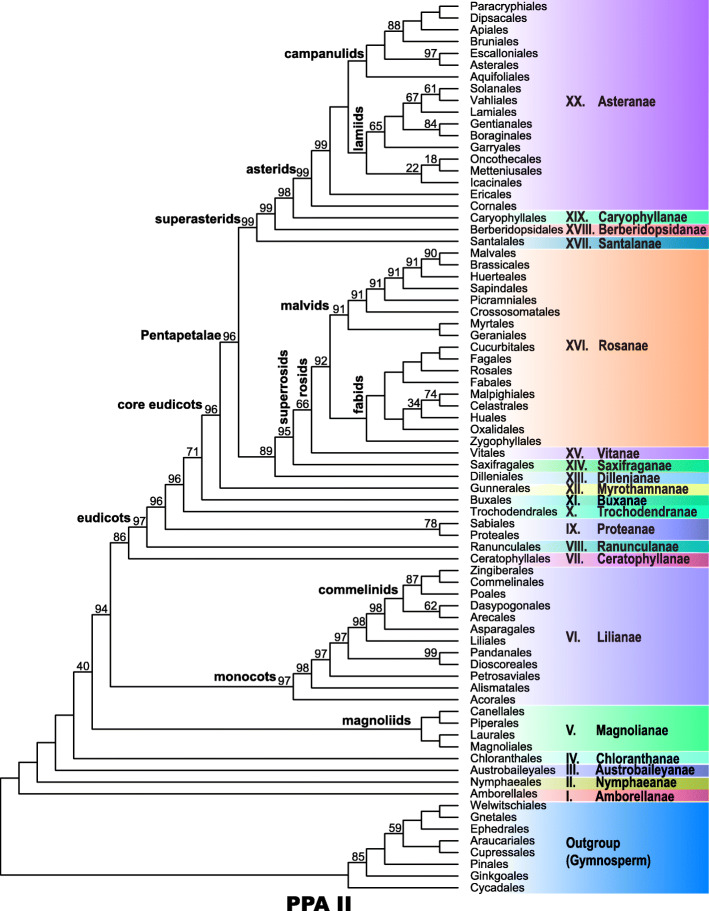
Relationships of 68 angiosperm orders in PPA II, based on maximum likelihood analysis of 80 plastid genes and 4782 samples. Bootstrap percentages less than 100 are shown. Twenty clades (labeled with roman numerals) are listed in Additional file 15: Table S2. Eight gymnosperm orders are included

**Fig. 2:**
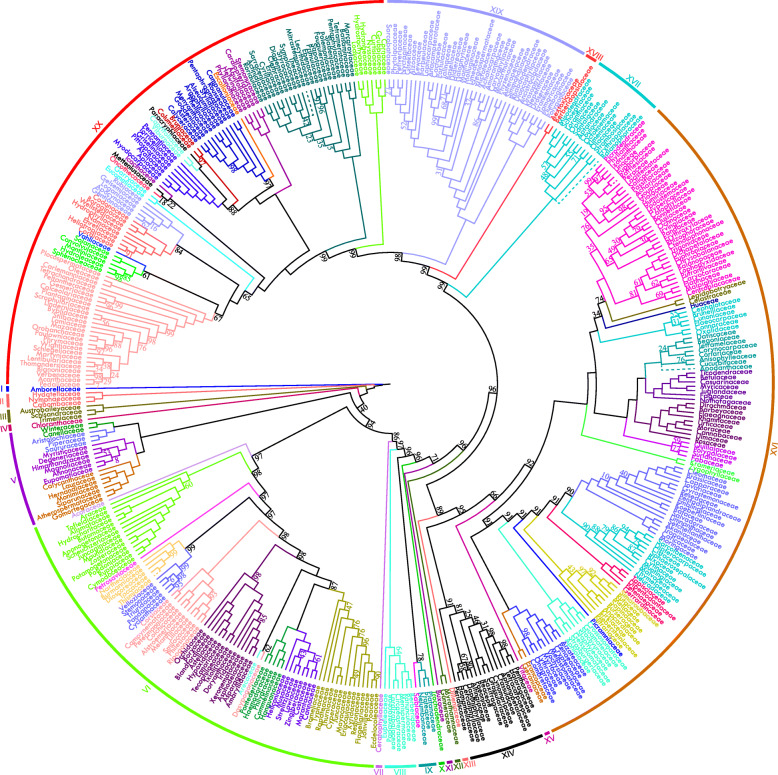
Relationships of 428 angiosperm families in PPA II, based on ML analysis of 80 plastid genes and 4782 samples. Bootstrap percentages less than 100 are shown. Five problematic families (Rafflesiaceae, Apodanthaceae, Balanophoraceae, Mitrastemonaceae, and Thismiaceae) are shown in dashed lines (see the “Results” section for details). Twenty clades (labeled with roman numerals) are listed in Additional file 15: Table S2

Our dataset comprised 4792 samples for initial analysis, including 4627 samples representing 4498 angiosperm species from all currently recognized families and orders of angiosperms and 165 samples representing 162 gymnosperm species as the outgroup (Additional file 1: Table S1). The taxonomic circumscription within seed plants followed APW [46]. Using our 86 newly sequenced plastomes representing 57 angiosperm families along with the recently issued plastomes from GenBank, we completed the representatives of 73 families absent from previous work [13] in the current analysis. The alignment of 80 genes from 4792 taxa had < 10% gaps/missing data. To our knowledge, this is the first phylogenomic study to include all currently recognized angiosperm families in APW [46] with plastome data. Overall, plastid phylogenomic analyses resulted in a tree referred to herein as the “PPA II tree” (Figs. 1 and 2; Additional files 2, 3, 4, 5, 6: Figs. S1–S5) with 75% of angiosperm nodes at or above the ordinal level and 78% at or above the familial level receiving bootstrap percentages (BP) ≥ 90.

### The impact of heterotrophic taxa on phylogenetic inferences

Five heterotrophic families lacked clear phylogenetic positions in our analyses (Additional files 6, 7, 8: Figs. S5–S7). One of these, Rafflesiaceae, was nested within its host family Vitaceae of Vitales with moderate support (BP = 83); similar relationships were also recovered by Molina et al. [47], which suggests that these plastid gene sequences are from the host plant. Thus, Rafflesiaceae were excluded from subsequent analyses. Four other heterotrophic families, Apodanthaceae, Balanophoraceae, Mitrastemonaceae, and Thismiaceae, with long branches, formed a strongly supported “clade” (BP = 100) within Saxifragales, as sister to another holoparasitic family, Cynomoriaceae, with moderate support (BP = 73) (Additional file 8: Fig. S7). Upon removal of Cynomoriaceae, this “clade” was sister to fully mycoheterotrophic *Epipogium* (Orchidaceae), again with a long branch (Additional file 9: Fig. S8a). However, when both Cynomoriaceae and *Epipogium* were removed (Additional file 9: Fig. S8b), these four families formed a “clade” with long-branched *Sarracenia* (Sarraceniaceae) and the long branch persisted upon the successive deletion of its sister in one earlier analysis (Additional file 9: Figs. S8c to S8i). The extremely long branch lengths involving these taxa suggest a typical case of long-branch attraction, which has been used to explain unusual phylogenetic positions of some heterotrophic plants [48]. Phylogenetic analysis excluding the other four families (Apodanthaceae, Balanophoraceae, Mitrastemonaceae, and Thismiaceae) produced trees that were largely congruent with previous analyses. Moreover, removing these four families plus Cynomoriaceae significantly increased support for many nodes, especially deeper nodes in both monocots and asterids (Figs. 1 and 2 and Additional files 2, 3, 4, 5: Figs. S1–S4).

Other fully heterotrophic families seem to have consistent phylogenetic positions as resolved in previous studies. For example, Triuridaceae were supported as a member of Pandanales, Corsiaceae, and Campynemataceae formed a strongly supported (BP = 100) clade sister to all other Liliales, and Cytinaceae and Muntingiaceae formed a clade in Malvales. Phylogenetic positions of partially heterotrophic families such as Burmanniaceae (with both partially and fully mycoheterotrophic plants) and Krameriaceae (hemiparasites) that have retained a larger number of putatively functional plastid genes were resolved with high support.

### Phylogenetic relationships at the ordinal level and above

In PPA II, the angiosperm clade received 100 bootstrap support (Figs. 1 and 2, Additional files 2, 3, 4, 5: Figs. S1–S4). Amborellales, Nymphaeales, and Austrobaileyales were supported as successive sisters to *Mesangiospermae* (BP = 100 for all). Although *Mesangiospermae* were strongly supported (BP =100), relationships among its five major clades (Chloranthales, magnoliids, monocots, Ceratophyllales, and eudicots) were not fully resolved. Chloranthales, magnoliids, monocots, and Ceratophyllales were successive sisters of eudicots with BP of 100, 40, 94, and 86, respectively.

Our results provided strong support (BP = 100) for the monophyly of magnoliids and their four orders, which were further resolved into strongly supported Canellales + Piperales and Laurales + Magnoliales (both pairs BP = 100). However, two interfamilial relationships within Magnoliales were only weakly supported.

Acorales, followed by Alismatales, Petrosaviales, Dioscoreales + Pandanales, Liliales, and Asparagales were strongly supported as successive sisters to the commelinid clade (support at each node; BP = 97, 98, 97, 97, 98, 98, respectively). Within the commelinid clade (BP = 100), the weakly supported (BP = 62) clade of Dasypogonaceae + Arecales was sister (BP = 98) to a strongly supported (BP = 100) clade, within which a clade (BP = 87) comprising Poales was sister to a strongly supported (BP = 100) clade comprising Commelinales and Zingiberales. However, some interfamilial relationships within Zingiberales and Poales received low support.

The monophyly of eudicots received strong support (BP = 97), with Ranunculales sister to all other eudicots, followed by Proteales + Sabiaceae, Trochodendrales, and Buxales with strong to moderate support as successive sisters to the core eudicots (support at each node; BP = 96, 96, 71, respectively). Core eudicots were strongly supported (BP = 96), among which Gunnerales were sister to a highly supported (BP = 96) Pentapetalae, which comprised a moderately supported (BP = 89) Dilleniales + superrosids clade and strongly supported (BP = 99) superasterids.

Within Dilleniales + superrosids, Dilleniales, Saxifragales, and Vitales were weakly to strongly supported as successive sisters to the remaining rosids (support at each node; BP = 89, 95, 66, respectively). The strongly supported (BP = 92) rosids, excluding Vitales, were further divided into malvids (BP = 91) and fabids (BP = 100). Within malvids, Geraniales + Myrtales were supported as sister to the rest (BP = 91), and then Crossosomatales, Picramniales, Sapindales, and Huerteales were strongly supported (support at each node; BP = 91, 91, 91, 91, respectively) as successive sisters to Malvales + Brassicales (BS = 90). Zygophyllales were sister to the remaining fabid clade, which was further divided into a strongly supported (BP = 100) nitrogen-fixing clade and a strongly supported (BP = 100) COM clade. Within the nitrogen-fixing clade, Fabales, Rosales, and Cucurbitales were successive sisters to Fagales (all BP = 100). Interordinal relationships of the COM clade were poorly resolved, with Huales falling in an isolated position away from Oxalidales.

Within the superasterids, Santalales were sister to the rest, and Berberidopsidales and Caryophyllales were strongly supported (support at each node; BP = 98, 99, respectively) as successive sisters of asterids, within which Cornales were sister (BP = 99) to Ericales + remaining asterids (BP = 99). The remaining asterids (BP = 100) were resolved into two strongly supported clades, campanulids and lamiids (each BP = 100). Within campanulids, Aquifoliales, Escalloniales + Asterales, Bruniales, Apiales, and Dipsacales were successive sisters to Paracryphiales, and all interordinal campanulid relationships were well supported (BP > 85), whereas most interordinal lamiid relationships were weakly supported.

### Major phylogenetic relationships at the familial level

**Fig. 3:**
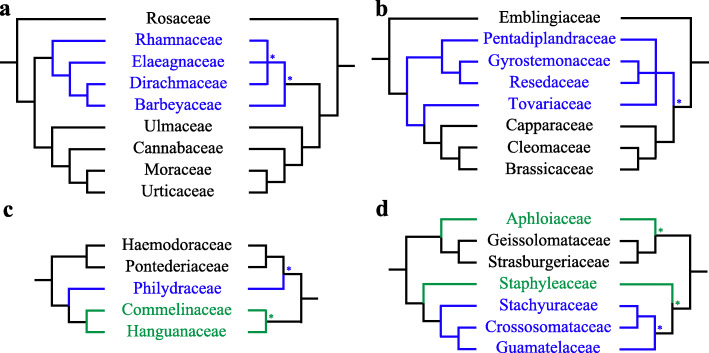
Familial phylogenetic relationships in PPA II (left) versus APW (right) of Rosales (**a**), partial Brassicales (**b**), Commelinales (**c**), and Crossosomatales (**d**). All nodes in PPA II have 100 bootstrap percentages. Asterisks (*) represent the nodes with low BP in APW. The blue lines show different phylogenetic positions between PPA II and APW, and the green lines show increased support in PPA II

All families with more than one sample included except Aristolochiaceae were resolved as monophyletic, and all families except Hamamelidaceae (BP = 67) were strongly supported (BP ≥ 98). To compare relationships at the interfamilial level from the current PPA II to those of previous studies, we refer to APW [46], which represents the most comprehensive current overview of interfamilial relationships based on previous studies. Our tree was largely consistent with the tree summarized in APW [46], but some incongruence was present (see Additional file 9: Fig. S8 and discussion in Additional file 14: Additional Text). Our analyses clarified some previously unresolved polytomies noted in APW (Additional file 4: Fig. S3), such as relationships among Rhamnaceae, Elaeagnaceae, Barbeyaceae, and Dirachmaceae in Rosales (Fig. 3a) [49–51], relationships among Pentadiplandraceae, Resedaceae + Gyrostemonaceae, Tovariaceae, and [Capparaceae [Cleomaceae + Brassicaceae]] in Brassicales (Fig. 3b) [50, 52–54], relationships among Meliaceae, Simaroubaceae, and Rutaceae in Sapindales [27, 50, 55, 56], relationships among Campynemataceae, Corsiaceae, and Melanthiaceae in Liliales [57–60], as well as some relationships within Malvales [50, 61, 62] and others in Cornales [63–65] (see Additional file 9: Fig. S8 and discussion in Additional file 14: Additional Text).

Our study also greatly improved support for the positions of many families, with all interfamilial relationships of 27 orders (over half of the 49 non-monofamilial orders of extant angiosperms), such as Asparagales, Asterales, Commelinales, Crossosomatales, Fagales, Myrtales, and Rosales, being strongly supported (BP ≥ 85; Fig. 3c, d for examples, also see Fig. 2, Additional files 3, 4: Figs. S2, S3). Additionally, phylogenetic relationships of the 73 families unsampled in [13] were generally clarified (see Additional file 4: Fig. S3 for details), usually with strong support, such as Corsiaceae sister to Campynemataceae (Liliales, BP = 100), Ixioliriaceae sister to Tecophilaeaceae (Asparagales, BP = 100), Circaeasteraceae sister to Lardizabalaceae (Ranunculales, BP = 100), Anisophylleaceae sister to Cucurbitaceae (Cucurbitales, BP = 76), Stachyuraceae sister to Guamatelaceae + Crossosomataceae (Crossosomatales, BP = 100), Petenaeaceae sister to Tapisciaceae + Dipentodontaceae (Huerteales, BP = 100), Pentadiplandraceae sister to Gyrostemonaceae + Resedaceae (Brassicales, BP = 100), Tovariaceae sister to Capparaceae plus Cleomaceae + Brassicaceae (Brassicales, BP = 100), Macarthuriaceae sister to Caryophyllaceae + Achatocarpaceae + Amaranthaceae (Caryophyllales, BP = 86), Loasaceae sister to Hydrostachyaceae (Cornales, BP = 100), and Namaceae sister to [Ehretiaceae [Cordiaceae + Heliotropiaceae]] (Boraginales, BP = 100). However, intractable interfamilial relationships remained in Poales [45, 66], Saxifragales [67, 68], Cucurbitales [49, 50], Oxalidales [27, 50], Malpighiales [69, 70], Santalales [71, 72], Ericales [73, 74], and Lamiales [75, 76] (see Fig. 2, Additional file 3: Fig. S2 and discussion for details in Additional file 14: Additional Text).

### Phylogenetic evaluation and comparison of angiosperm family trees

Maximum likelihood (ML) and ASTRAL trees of 428 families (i.e., with five heterotrophic families removed) included in the subdataset generally showed consistent relationships with strong support, only slightly different at some nodes with weak or moderate support (Additional file 10: Fig. S9). Under Quartet Sampling (QS) evaluation, analyses of a pruned plastome dataset indicated strong support for monophyly of the majority of orders (Additional file 11: Fig. S10), but with some alternative relationships among some orders or families. Our results showed that bootstrap values and concordance factors could provide some different information about each branch in the tree, but they tended to display a similar pattern (Additional file 12: Fig. S11). Meanwhile, estimates of gene and site concordance factors (gCF and sCF) were generally correlated across the ML tree of angiosperms, but we note that both measures fell well below standard measures of bootstrap support (Additional file 13: Fig. S12).

## Discussion

A plastid phylogenomic analysis including all recognized families provides an unparalleled opportunity to address interfamilial relationships of angiosperms and their associated patterns of phenotypic evolution. Our results are largely congruent with previous analyses [27] but provide higher support for many relationships among major clades, including those recognized as orders and families, and a complete phylogenetic framework of angiosperms at the familial level. Overall, our study represents the first phylogenetic analysis using complete plastomes and a large sampling of all recognized angiosperm families (except Rafflesiaceae and four other heterotrophic families due to the complete or large number of gene losses in their plastomes), from which the phylogenetic relationships among angiosperm families, orders, and high-level clades could be addressed in a single phylogenetic tree. The higher support for many nodes may be attributed to the much better sampling of representative clades. The monophyly of the angiosperms and their division into eight major clades was supported. Amborellales, Nymphaeales, and Austrobaileyales were resolved as successive sisters to the remaining angiosperms, consistent with current understanding [13, 28–30]. The monophyly of *Mesangiospermae* received 100 BP, and a topology of [Chloranthales [magnoliids [monocots [Ceratophyllales + eudicots]]]] was well supported except for the weakly supported position of magnoliids.

**Fig. 4:**
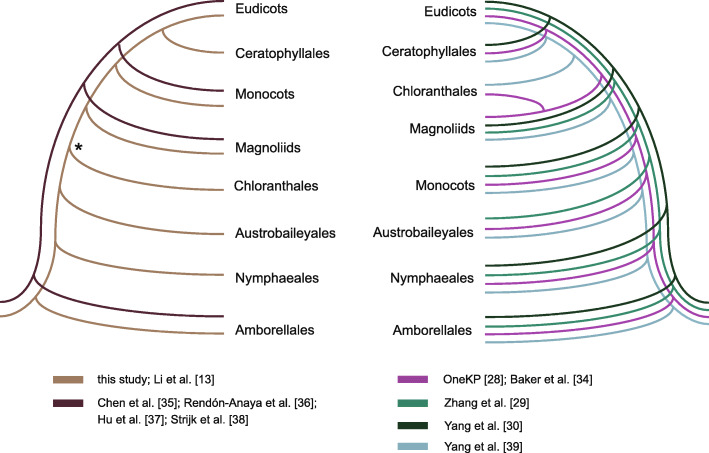
Two contrasting topologies for the eight major lineages of angiosperms (Amborellales, Nymphaeales, Austrobaileyales, Ceratophyllales, Chloranthales, magnoliids, monocots, and eudicots) based on the plastid (left, light brown) [13] and nuclear (right) [28–30, 34, 39] genome-scale datasets. Four recent studies with new nuclear genomes sequenced from different species of magnoliids (left, dark brown) [35–38] also resolved the same topology as that of the plastid phylogeny. The asterisk indicates that this node was weakly supported

This backbone plastid topology reviewed above has been consistently recovered in previous plastid phylogenomic studies [13, 21, 22]. Recent nuclear phylogenetic analyses have produced multiple topologies [13, 28–30, 34–39]. Notably, for the three clades with the highest species diversity, monocots are more closely related to eudicots than to magnoliids in the plastid tree, whereas magnoliids and eudicots are more closely related in recent nuclear trees (Fig. 4). A recent study [32] using 38 mitochondrial genes of 91 angiosperm taxa representing seven of eight major angiosperm clades (except Ceratophyllales) found that relationships among these major clades were congruent with those of the plastid tree. Nuclear-organellar discordance regarding relationships among the five major *Mesangiospermae* clades, particularly those among monocots, magnoliids, and eudicots, may imply both rapid radiation as well as reticulate evolution in the early history of angiosperms [13, 28, 39]. More genomic data, particularly those of Chloranthales and Austrobaileyales, should be explored to address this question.

Most angiosperm interordinal relationships have been clarified on the basis of plastome analyses. For the long-controversial phylogenetic positions of a few early-diverging orders in Pentapetalae, our study and most recent plastid phylogenomic studies [13, 28] have supported Dilleniales, Saxifragales, or Vitales as successive sisters of the remaining rosids, and Santalales, Berberidopsidales, and Caryophyllales as successive sisters to the asterids. However, phylogenetic analyses of nuclear data showed substantial discordance regarding the phylogenetic positions of these orders [28, 30, 34, 77]. Dilleniales have been supported as sister to superrosids, superasterids, the remaining Pentapetalae, Gunnerales, or Caryophyllales in recent studies using nuclear gene sequence data [26, 28, 30, 78]. The uncertain position of Dilleniales hampers an accurate understanding of the origin of key trait innovations, such as pentamerous flowers and the distinction between sepals and petals in eudicots. The rapid diversification of core eudicots following two rounds of whole-genome duplication (WGD) currently hinders the confident resolution of relationships [28, 30, 79, 80].

Our study and most recent plastid phylogenomic analyses [13, 45] support the placement of the COM clade (Celastrales, Huales, Oxalidales, Malpighiales) within the fabids, but other analyses based on mitochondrial and nuclear data [15, 28, 31, 33, 81, 82] supported the COM clade within the malvids. Incomplete lineage sorting and/or ancient introgressive hybridization may be the cause of the conflicting positions for this clade [83]. All three topologies among the three large orders (Celastrales, Oxalidales, Malpighiales) within the COM clade were reported in previous studies [83], and our study also failed to resolve relationships among these three orders relative to unplaced Huales (consisting only of Huaceae). Our study provided good support for the phylogenetic positions of Escalloniales, Asterales, Boraginales, Gentianales, Vahliales, Solanales, and Lamiales within asterids. Nevertheless, our analysis did not confidently resolve some interordinal relationships, especially those within lamiids.

Our study did clarify some long-controversial interfamilial relationships within Poales, Saxifragales, Brassicales, Caryophyllales, etc. (please refer to Additional file 14: additional text for more detailed discussion). However, some previously unresolved interfamilial relationships within Saxifragales, Malpighiales, Ericales, and Lamiales [50, 68, 70, 84, 85] remain unresolved in the current study. Families of these orders may have experienced rapid radiations, which may not be resolved by plastome data. Whereas plastome data have generally been considered to represent uniparental phylogenetic history [86, 87], more complex plastome evolution has been found in Fabaceae [86]. Previous empirical and simulated analyses have suggested that reliable inference of species trees requires the use of large numbers of nuclear loci [87–89]. Increased sampling with hundreds of single-copy nuclear genes may be needed to fully resolve these recalcitrant familial relationships.

Huaceae were placed as sister to the remaining members of Oxalidales in several previous studies, sometimes with relatively high support (BP > 80) [69, 81, 88], so that APG III [89] tentatively included Huaceae in Oxalidales. However, both our previous work [13] and current study strongly supported (BP = 100) the monophyly of Oxalidales (excluding Huaceae), and Huaceae were placed as sister to Celastrales + Malpighiales with weak support in this study (BP = 34) here. In APG IV [18], Dasypogonaceae, Sabiaceae, and Oncothecaceae were placed in Arecales, Proteales, and Icacinales, respectively, according to the plastid phylogenomic studies of Barrett et al. [90], Sun et al. [44], and Stull et al. [43]. Nevertheless, in recent studies [45] and our study with denser taxon sampling, support for the monophyly of Arecales and Proteales was relatively low (BP < 80). In addition, a poor resolution was also apparent in the weakly supported assemblage of Icacinales, Oncothecaceae, and Metteniusales (BP < 25). These residual issues in angiosperm phylogeny need to be settled. Thus, we suggest separating Dasypogonales from Arecales, Sabiales from Proteales, Huales from Oxalidales, and Oncothecales from Icacinales, as the monophyly of all other orders in our tree received strong support (BP≥90).

All recognized families in our study received > 95 BP support, with the exception of Aristolochiaceae and Hamamelidaceae. Aristolochiaceae were found to be paraphyletic in the current study with *Aristolochia* sister to [Saururaceae + Piperaceae] and [*Saruma* + *Asarum*] sister to that clade (Additional file 5:Fig. S4). However, we did not sample Hydnoraceae and Lactoridaceae, both recognized previously by APG III [89] but not APG IV [18]. The monophyly of Hamamelidaceae was weakly supported (BP = 67). These two cases should be the focus of further studies.

## Conclusions

Our plastid phylogenomic analysis, which included representatives of all recognized angiosperm families [46], greatly clarified many deep phylogenetic relationships, particularly those at and above the familial level. The robust phylogenetic backbone presented here will provide a firm basis for future evolutionary studies of flowering plants. Our analyses further indicate that recalcitrant relationships among the five major clades of *Mesangiospermae* and interfamilial relationships such as those of Malpighiales and a few other orders could not be resolved exclusively through increased taxonomic sampling and greater amounts of plastid data but must include the analyses of large numbers of single-copy nuclear genes.

## Methods

### Taxon sampling

To reconstruct the phylogenetic relationships of angiosperms at the family level, 4627 samples representing 4498 species, 2024 genera, 416 families, and 64 orders recognized by APG IV [18], and 17 additional families recognized by APW [46], were included in the analyses. In addition, 165 samples from 162 species, 77 genera, 12 families, and eight orders of gymnosperms comprised the outgroup. The dataset consisted of 86 newly sequenced plastomes with Illumina HiSeq2500, 2425 samples from our previous work [13, 91], and an additional 2281 plastomes from GenBank (released from January 1, 2017, to April 30, 2019) (Additional file 1: Table S1). The final sampling of 4792 taxa includes representatives of all 72 orders and 445 families of seed plants (Additional file 1: Tables S1 and Additional file 15: Table S2). Order and family circumscriptions of seed plants are as in APW [46].

### Molecular techniques

Total genomic DNA was extracted using a modified CTAB protocol [92] from leaf tissue of herbarium specimens and silica-dried materials. The DNA samples were sheared into fragments and used to construct short-insert (500 bp) libraries in accordance with the manufacturer’s manual (Illumina, San Diego, CA, USA). Paired-end sequencing of 150 bp was conducted on an Illumina HiSeq 2500. High-quality Illumina sequencing reads were assembled using the GetOrganelle toolkit [93]. The assembled plastomes were annotated using PGA [94] and manually adjusted in Geneious v9.1.8 [95]. Data from complete plastid genomes in GenBank as of April 30, 2019, were downloaded and re-annotated using PGA. For some incomplete plastomes, we used scripts to obtain assembled sequences by mapping contigs to a reference and then extracting the annotated gene fragments.

### Phylogenetic inference

All alignments of protein-coding exons and rRNA genes were performed using PASTA [96] before being further locally re-aligned in Geneious v9.1.8 using MAFFT v7.394 [97] and MUSCLE v3.8.425 [98]. Three genes, *infA*, *ycf1*, and *ycf2*, were difficult to align and were thus excluded from the phylogenetic analysis. We conducted analyses with and without the inclusion of five heterotrophic families, i.e., Apodanthaceae, Balanophoraceae, Mitrastemonaceae, Rafflesiaceae, and Thismiaceae, given that their plastome sequences are highly reduced and that the retained sequences have unusually high substitution rates that strongly hamper proper alignment and may cause long-branch attraction artifacts in many focal clades. However, for the completeness of the PPA tree, these families were included in the figures following their placement in APW. All aligned genes were concatenated into a supermatrix with a length of 89,357 bp. Maximum likelihood (ML) analyses were performed with RAxML v8.2.12 [99] under the GTRGAMMA model for a partitioned supermatrix. Searches for the best trees were conducted by starting from random trees, and bootstrap percentages were obtained with 1000 non-parametric bootstrap replicates.

To further evaluate the phylogenetic relationships of the backbone tree of angiosperm families, we generated a subdataset of 431 species representing 428 angiosperm families and two outgroup taxa using the Python package ete3 v3.1.2 [100] and pxrms from the phyx package [101]. Maximum likelihood analyses were conducted with RAxML v.8.1.2 [99] including 500 rapid bootstraps and a search for the best-scoring tree, employing the GTRGAMMA model. We evaluated clade/branch support under various metrics of branch support including Quartet Sampling [102] with 1000 replicates, gene concordance factors (gCF) [103] and site concordance factors (sCF) [104] from IQtree v2.0 [105], and internode certainty all (ICA) [106] from RAxML v.8.1.2 [99]. We compared the angiosperm phylogeny estimated with the concatenated approach and that resulted from the multispecies coalescent-based approach [107, 108] based on 80 single-gene trees from RAxML with local posterior probabilities (LPP) [109] to assess clade/branch support. Two multispecies coalescent-based analyses were executed in which all bipartitions were included and bipartitions with <10 bootstrap support were collapsed prior to the analyses.

## Supplementary Information

**Additional file 1: Table S1.** Species sampled in this study. The 4792 individuals sampled including 86 newly sequenced plastomes, involving 4498 angiosperm species and 162 gymnosperm species.

**Additional file 2: Figure S1.** Phylogenetic tree of 4782 plastomes of 68 orders of angiosperms.

**Additional file 3: Figure S2.** Phylogenetic tree of 4782 plastomes of 445 families (including 12 gymnosperm families) of seed plants. Five problematic families (Rafflesiaceae, Apodanthaceae, Balanophoraceae, Mitrastemonaceae, and Thismiaceae) were added manually (see Results for details).

**Additional file 4: Figure S3.** Angiosperm family-level phylogenetic relationships in PPA II versus APW. Red: different phylogenetic positions between PPA II and APW; green: resolved nodes in PPA II relative to APW. Different phylogenetic positions between PPA II and APW with bootstrap values < 50 in PPA are not shown.

**Additional file 5: Figure S4.** Phylogenetic tree of 4782 plastomes (with ten plastomes of five problematic families excluded) of 4650 species of seed plants. Bootstrap values are shown.

**Additional file 6: Figure S5.** Phylogenetic tree of 4792 plastomes of 76 orders (including eight gymnosperm orders) of seed plants.

**Additional file 7: Figure S6.** Phylogenetic tree of 4792 plastomes of 445 families (including 12 gymnosperm families) of seed plants.

**Additional file 8: Figure S7.** Phylogenetic tree of 4792 plastomes of 4660 species of seed plants. All bootstrap values are shown.

**Additional file 9: Figure S8.** Phylogenetic tree of 4792 plastomes with successive removal of the long branch forming a sister relationship with a ‘clade’ of Mitrastemonaceae, Thismiaceae, Apodanthaceae, and Balanophoraceae.

**Additional file 10: Figure S9.** Topologies of a pruned Maximum Likelihood phylogeny of 431 representative species (“ML431_pruned”) and ASTRAL analysis (“astral431_BS10”) of a 43-species subdataset of angiosperms using 80 plastid genes. The ML bootstrap percentages and ASTRAL local posterior probabilities are shown, respectively.

**Additional file 11: Figure S10.** Family relationships within the pruned angiosperm phylogeny: nodes by Quartet Concordance (QC) scores for internal branches: green (QC > 0.2), blue (0.2 ≥ QC > 0), orange (0 ≥ QC ≥ −0.05, or red (QC < −0.05). QC/Quartet Differential (QD)/Quartet Informativeness (QI) scores are shown for all internal branches.

**Additional file 12: Figure S11.** Plot showing the relationship between gene and site concordance factors (gCF and sCF) relative to bootstrap support from the pruned angiosperm phylogeny.

**Additional file 13: Figure S12.** Family relationships within the pruned angiosperm subdataset. In this tree, bootstrap/gCF/sCF scores are shown for each branch.

**Additional file 14: Additional Text**. Overview of angiosperm phylogeny at the familial level.

**Additional file 15: Table S2**. Summary of all recognized 433 families, 68 orders, and more inclusive clades for flowering plants, with numbers of known genera and species.

## Data Availability

Sequence alignments underlying analyses and phylogenetic trees are available from figshare (10.6084/m9.figshare.16573115) [110]. Raw reads of 86 new genome skims used in this study are available at the NCBI SRA database as Bioproject PRJNA767934 (https://www.ncbi.nlm.nih.gov/sra/PRJNA767934) [111].
