# Supplementary figures and images for: Plastid phylogenomic insights into relationships of all flowering plant families
Source: BMC Biol. 2021 Oct 29;19:232. doi: 10.1186/s12915-021-01166-2 (PMC8555322; doi:10.1186/s12915-021-01166-2)

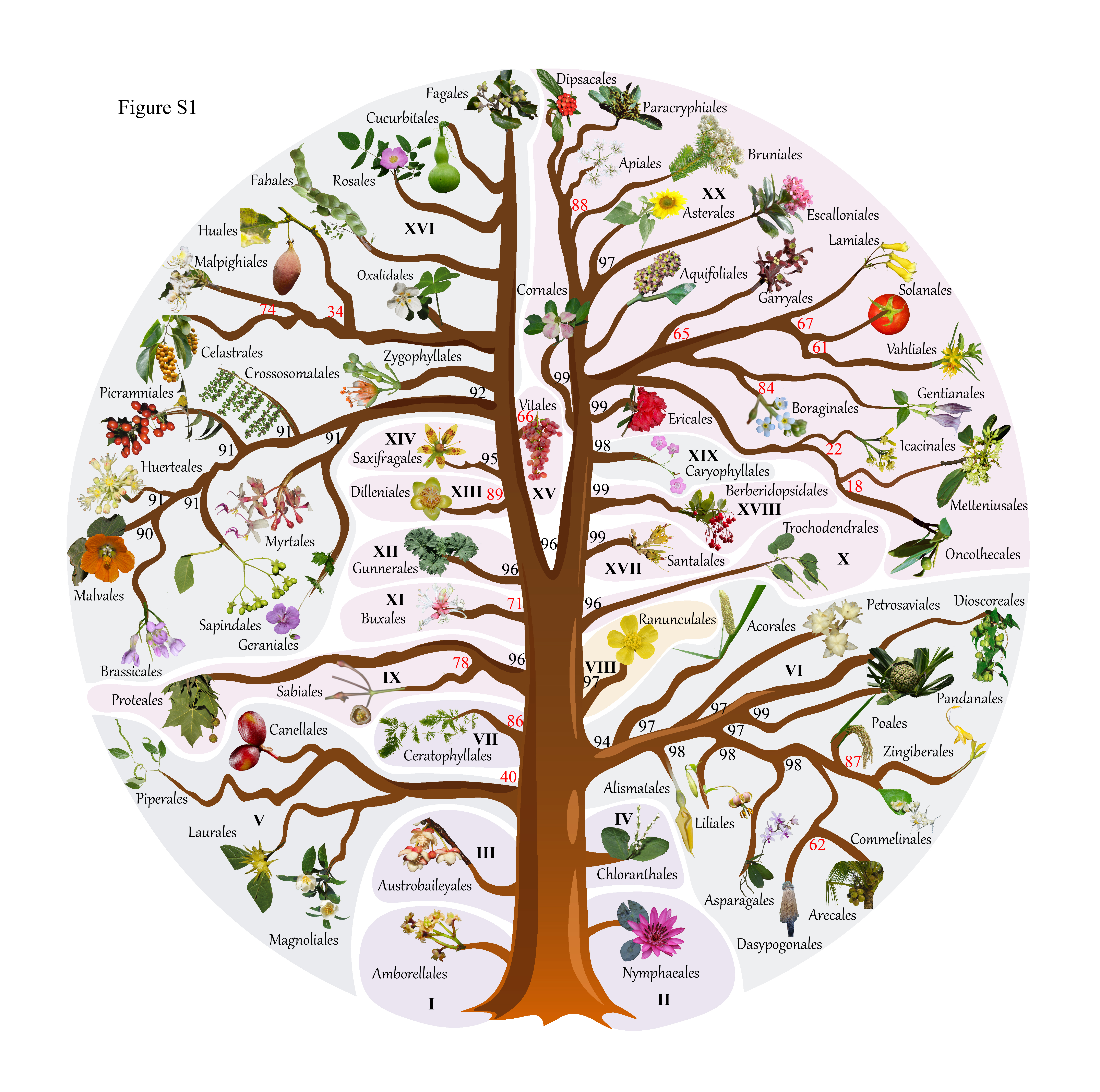

Supplement: Supplementary file 2 — Additional file 2: Figure S1. Phylogenetic tree of 4782 plastomes of 68 orders of angiosperms. [file 12915_2021_1166_MOESM2_ESM.jpg]

[illegible]

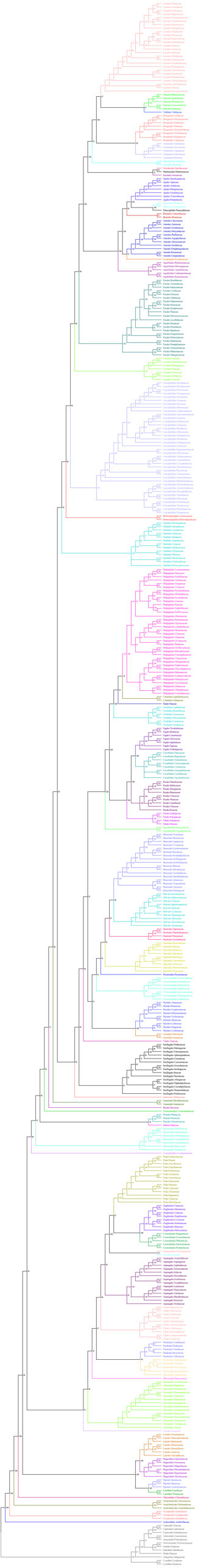

Supplement: Supplementary file 3 — Additional file 3: Figure S2. Phylogenetic tree of 4782 plastomes of 445 families (including 12 gymnosperm families) of seed plants. Five problematic families (Rafflesiaceae, Apodanthaceae, Balanophoraceae, Mitrastemonaceae, and Thismiaceae) were added manually (see Results for details). [file 12915_2021_1166_MOESM3_ESM.pdf]

[Figure S4]

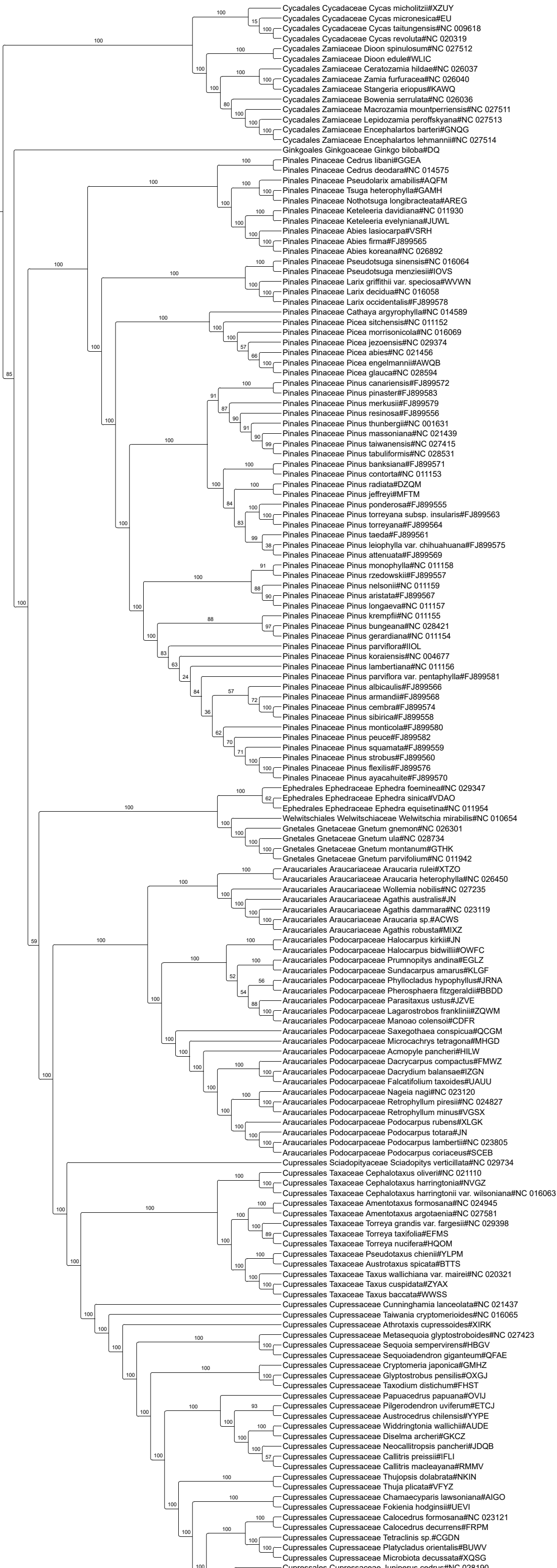

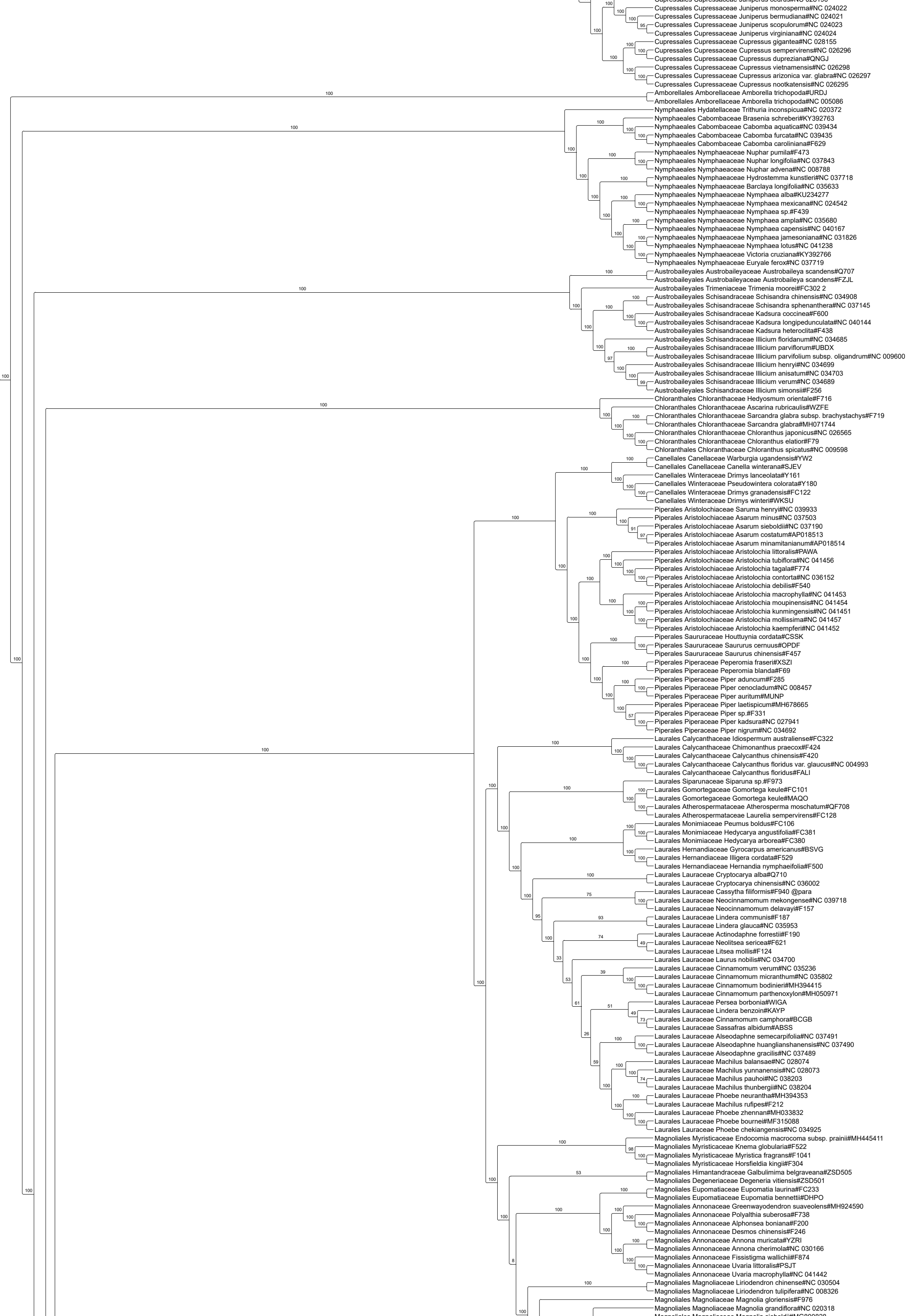

Supplement: Supplementary file 5 — Additional file 5: Figure S4. Phylogenetic tree of 4782 plastomes (with ten plastomes of five problematic families excluded) of 4650 species of seed plants. Bootstrap values are shown. [file 12915_2021_1166_MOESM5_ESM.pdf]

[Figure S5]

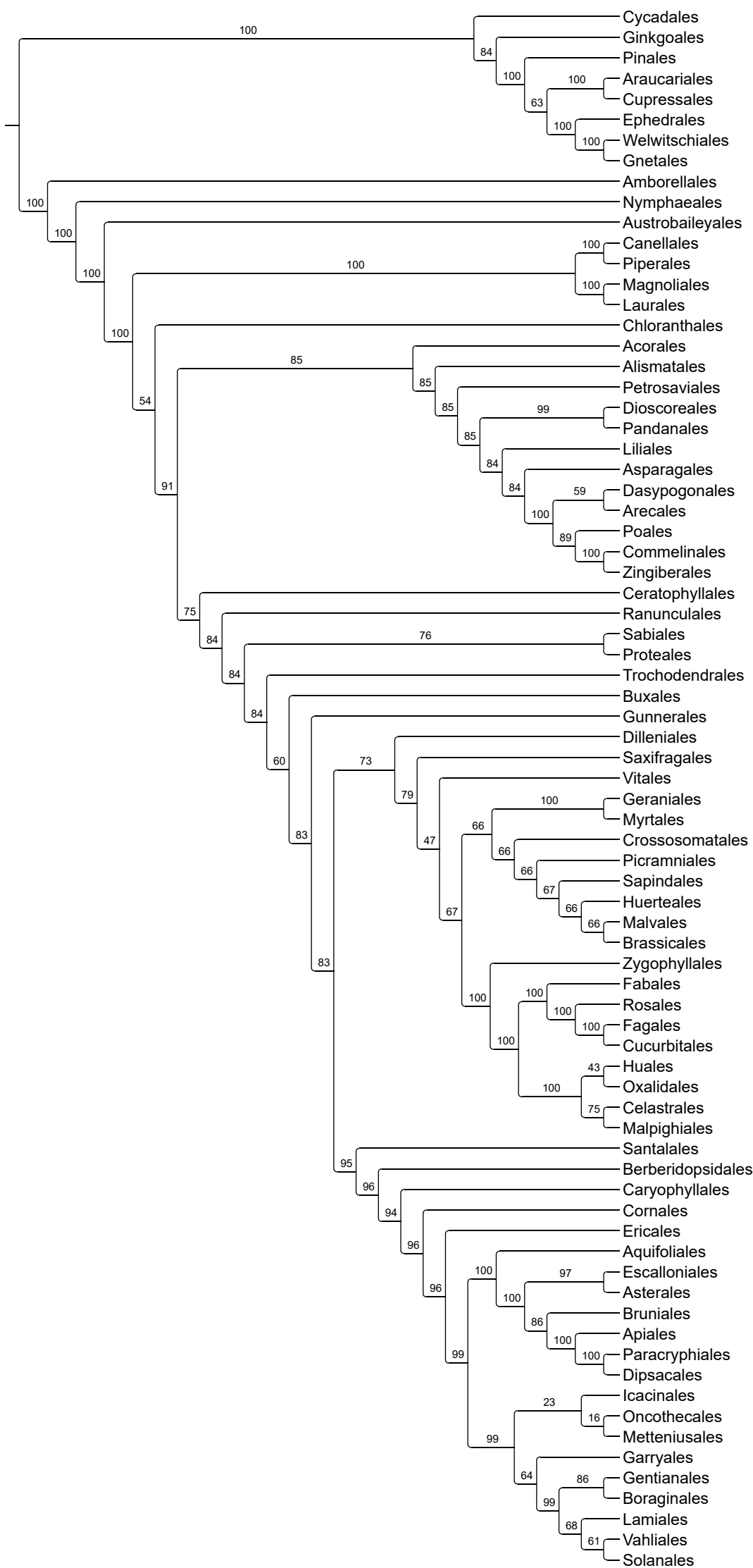

Supplement: Supplementary file 6 — Additional file 6: Figure S5. Phylogenetic tree of 4792 plastomes of 76 orders (including eight gymnosperm orders) of seed plants. [file 12915_2021_1166_MOESM6_ESM.pdf]

[Figure S6]

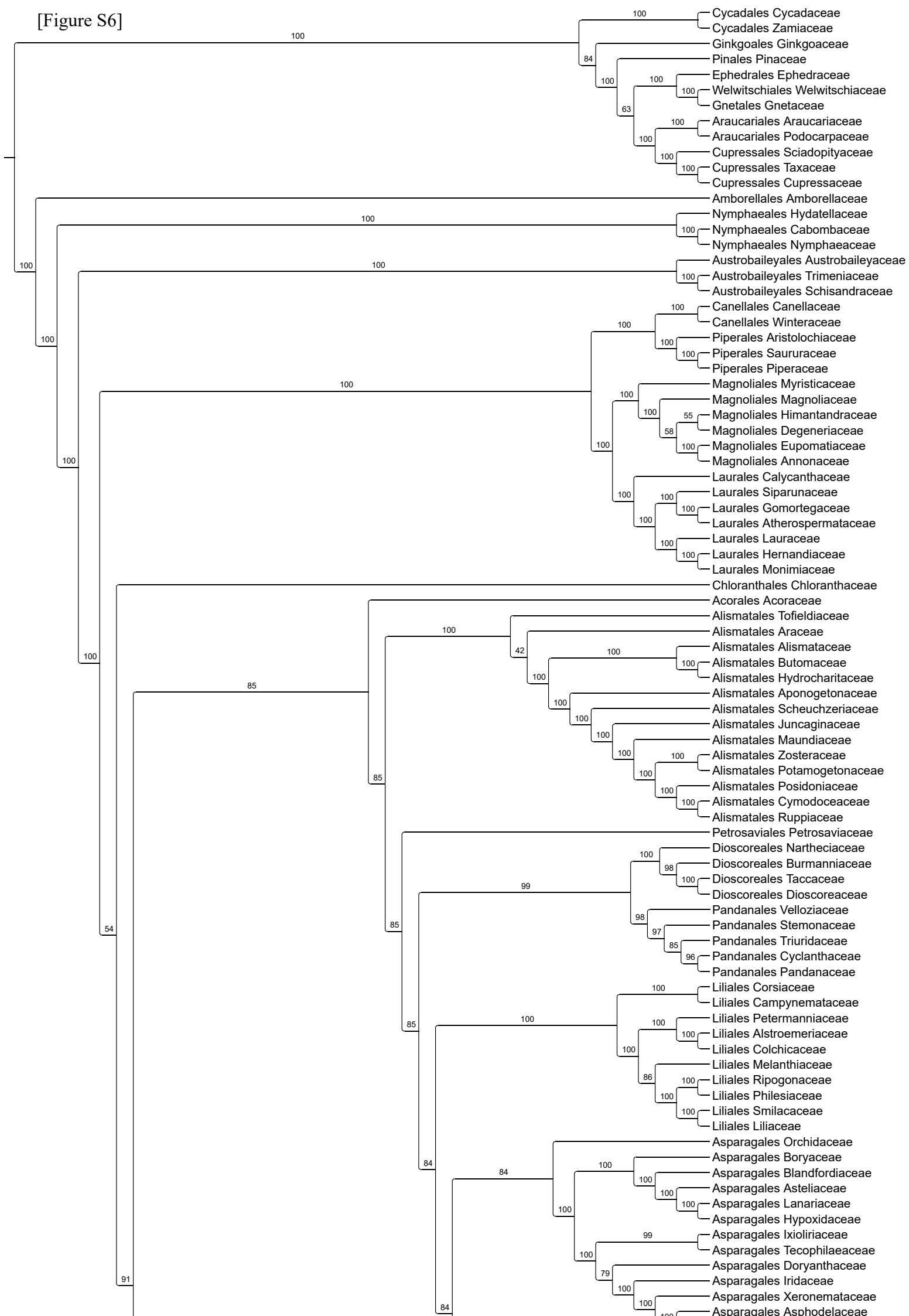

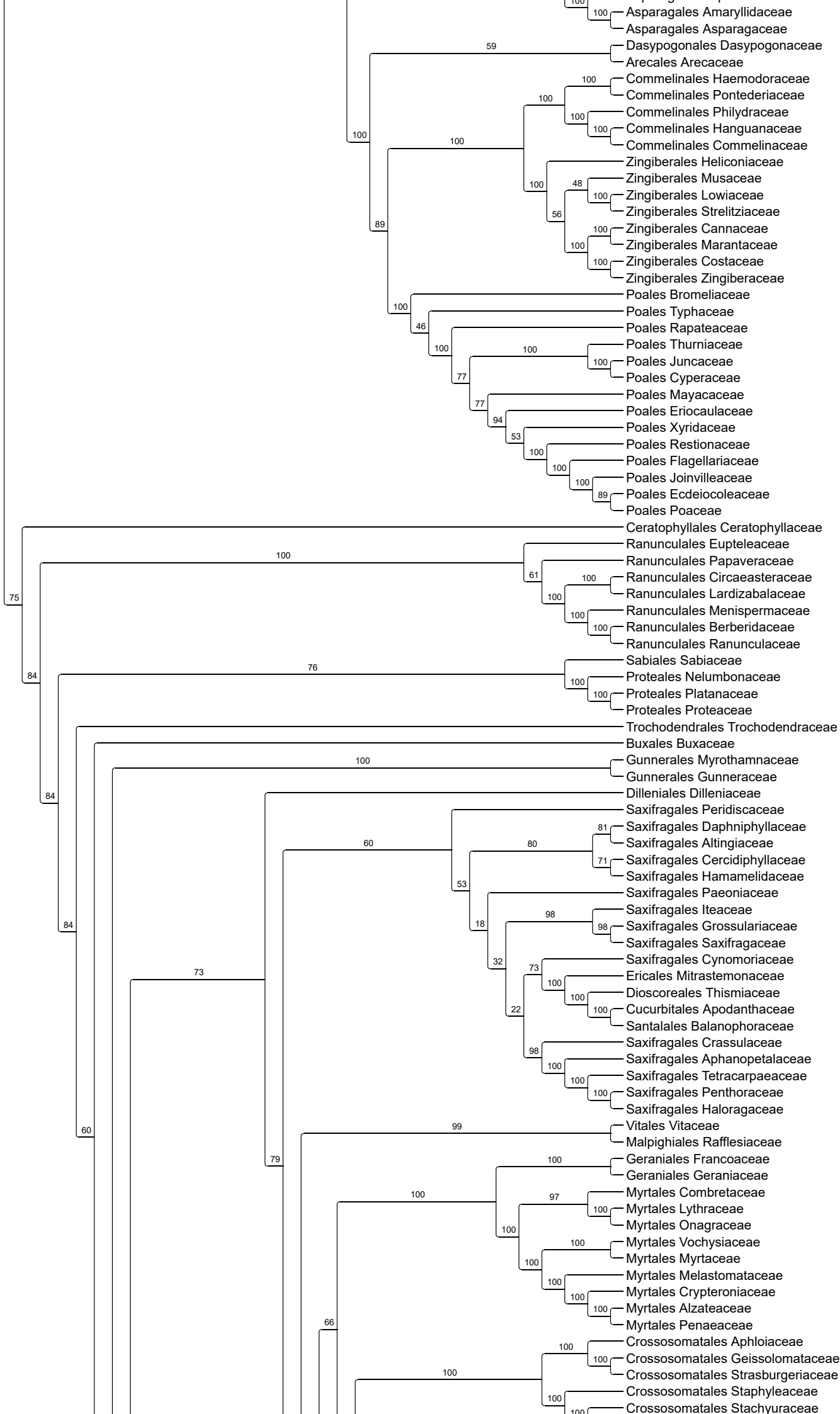

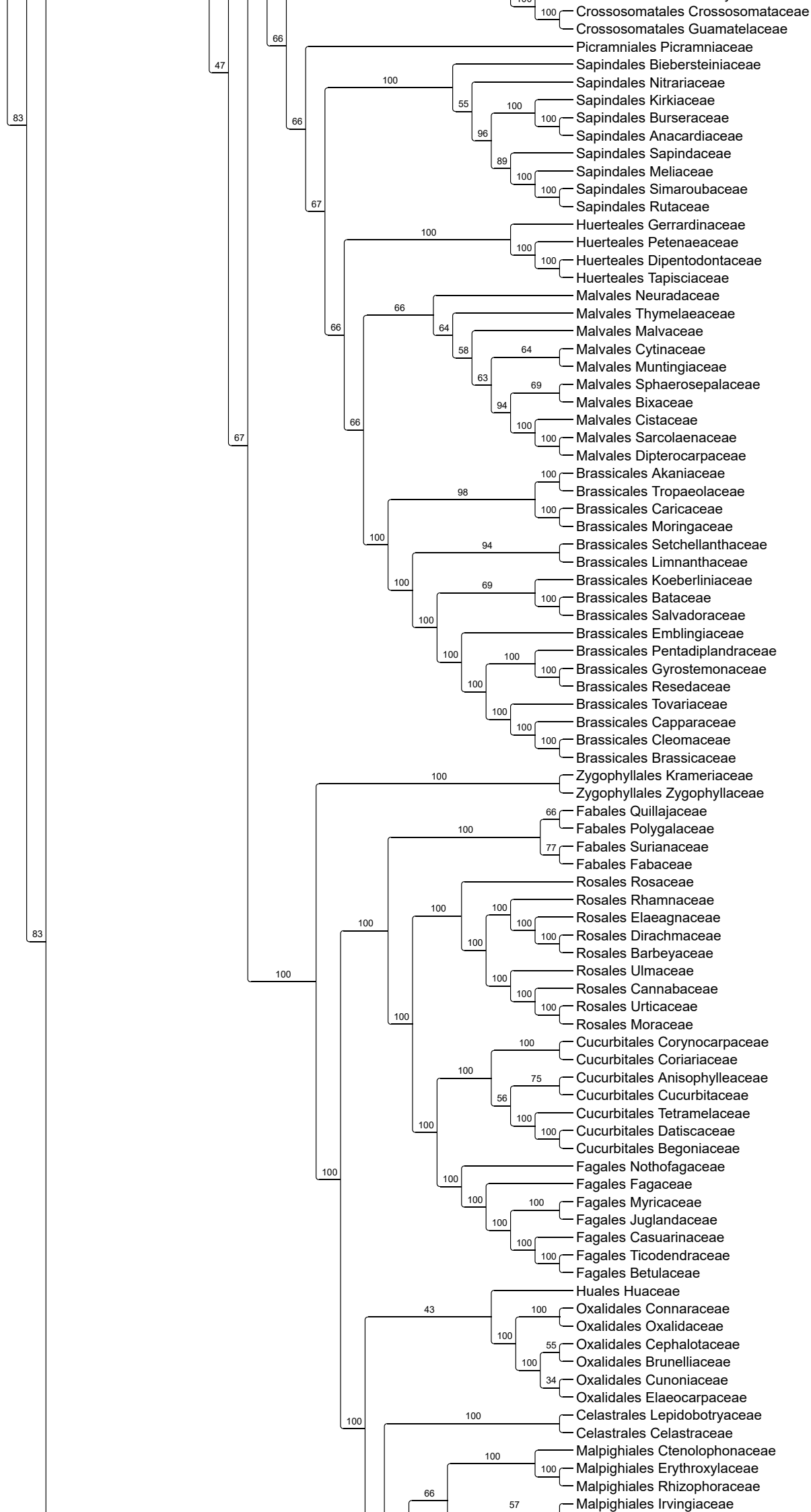

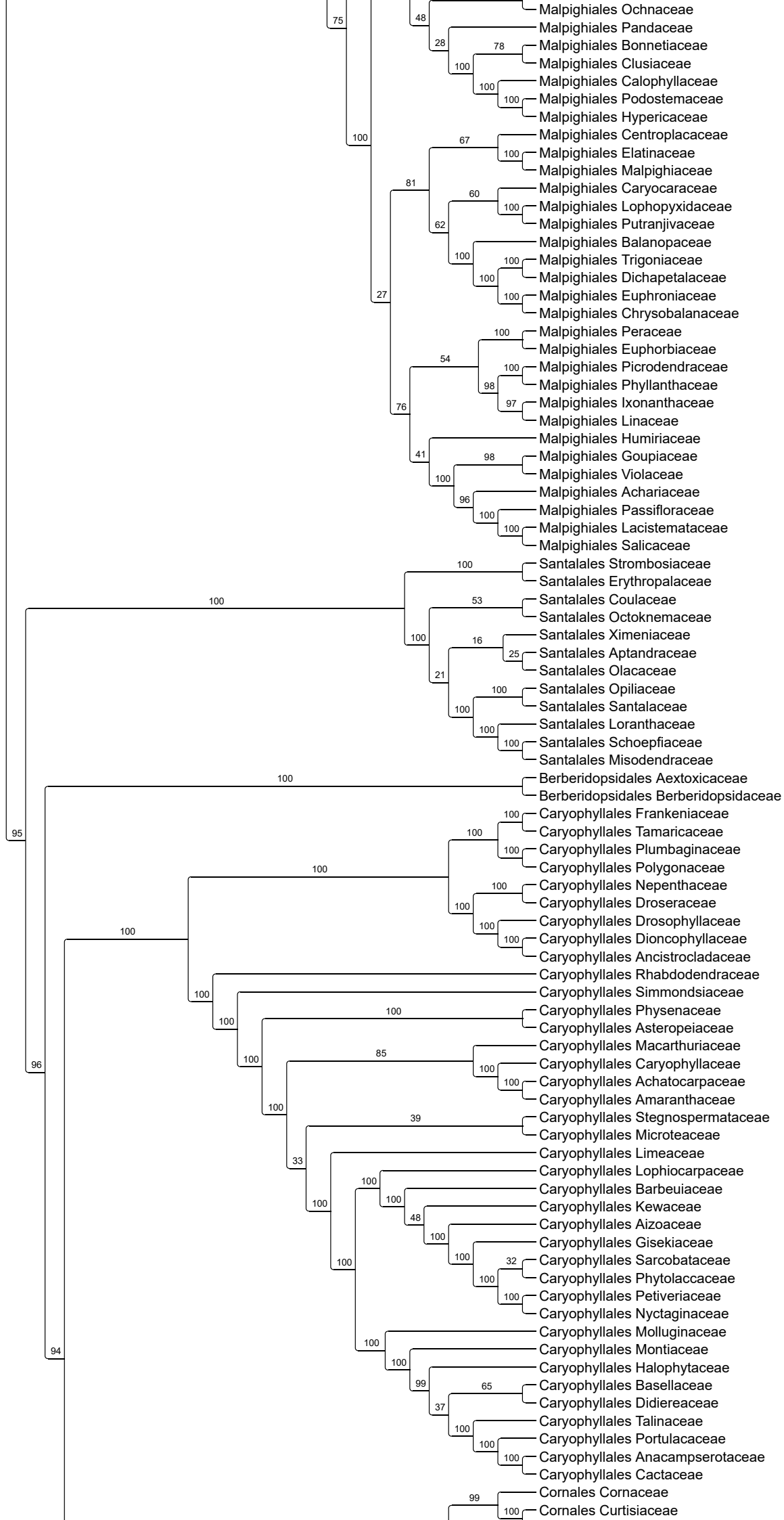

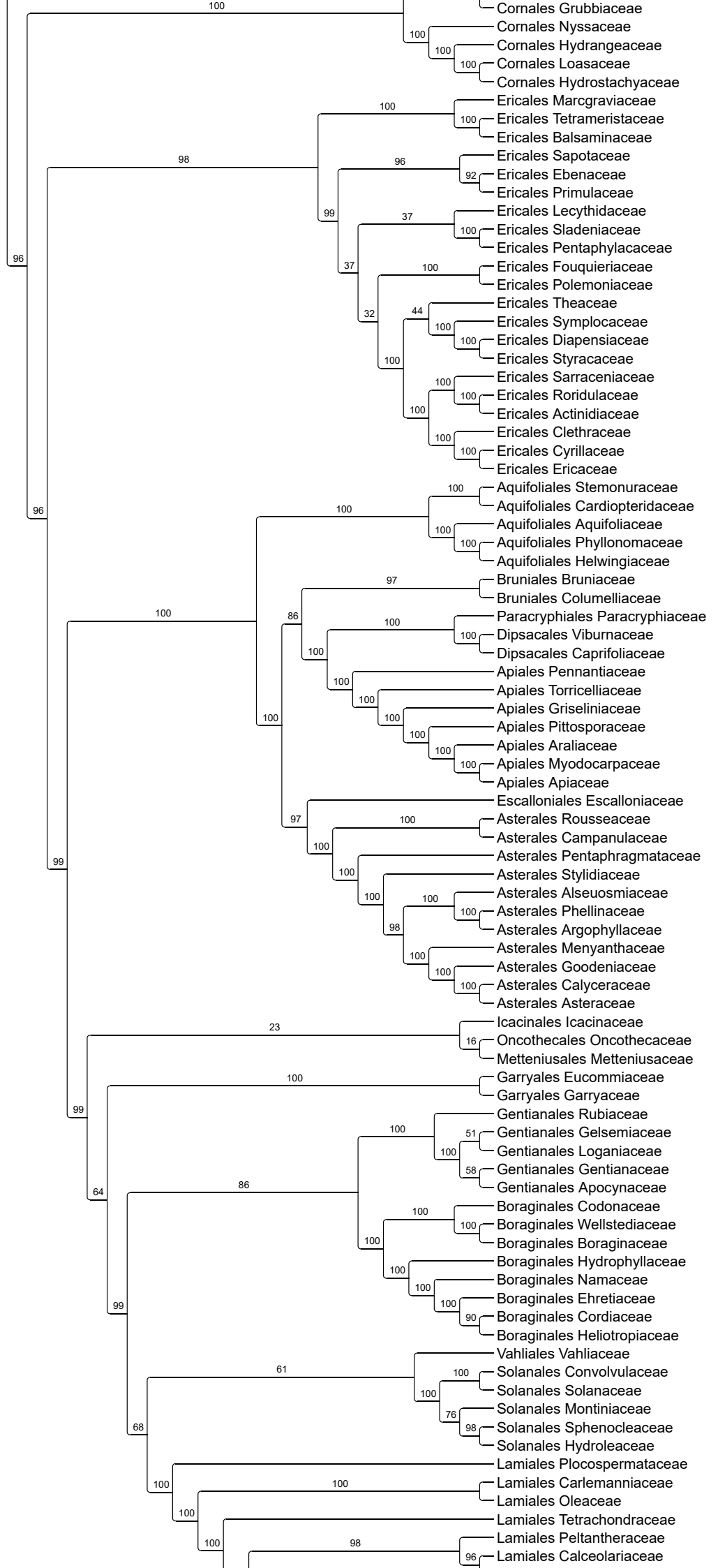

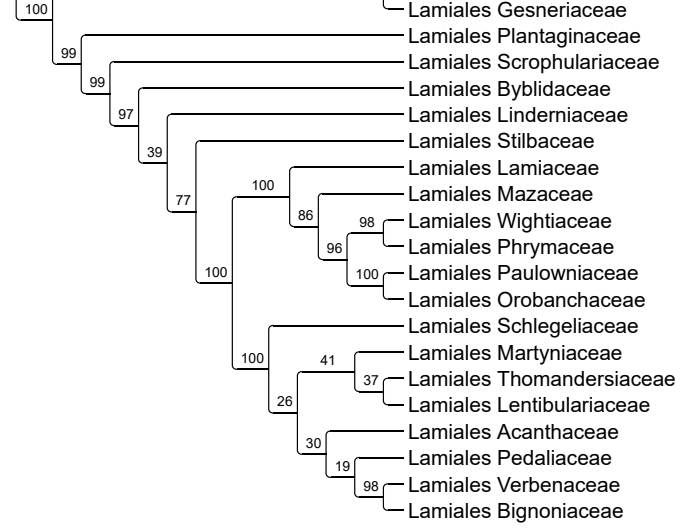

Supplement: Supplementary file 7 — Additional file 7: Figure S6. Phylogenetic tree of 4792 plastomes of 445 families (including 12 gymnosperm families) of seed plants. [file 12915_2021_1166_MOESM7_ESM.pdf]

[Figure S7]

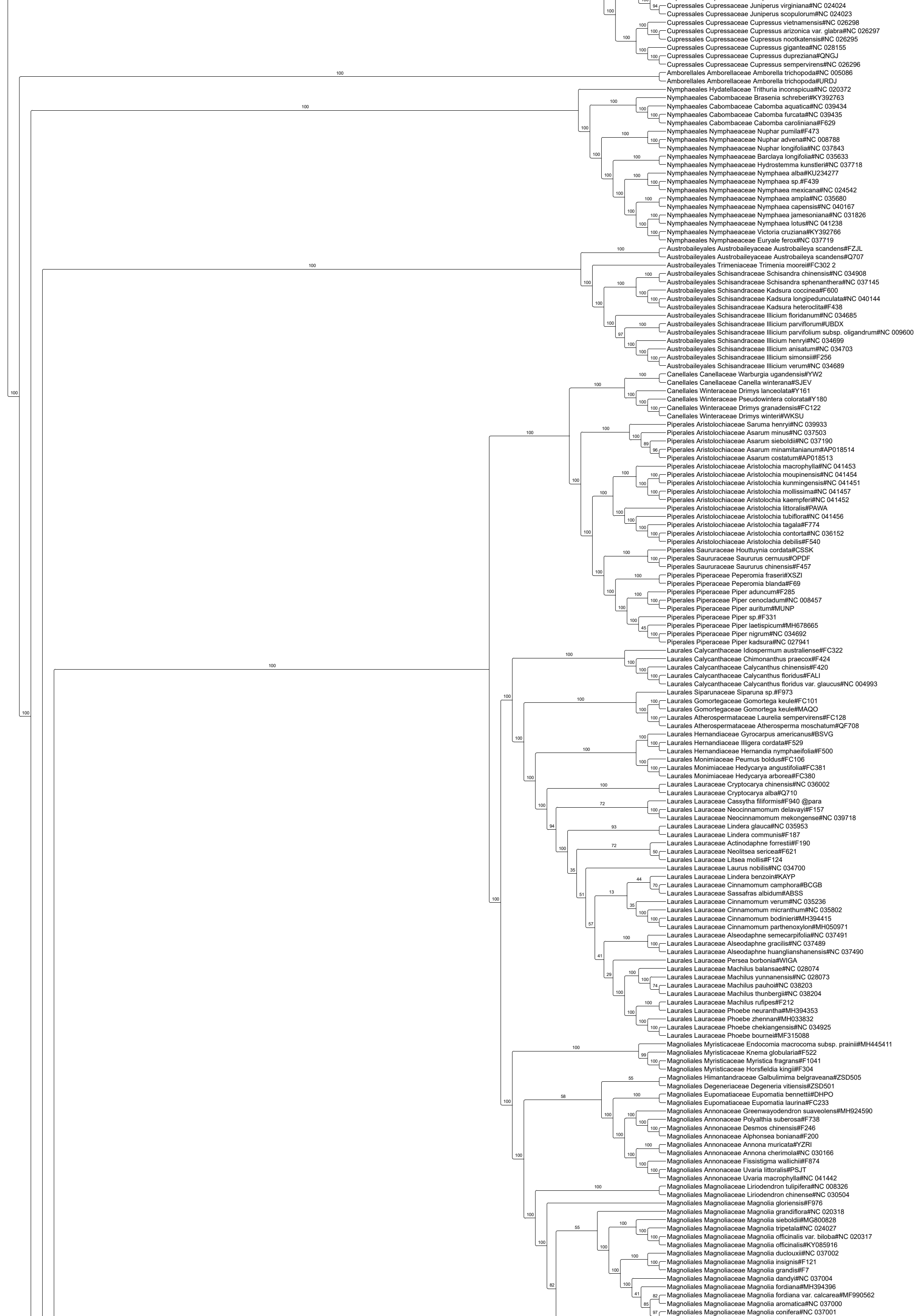

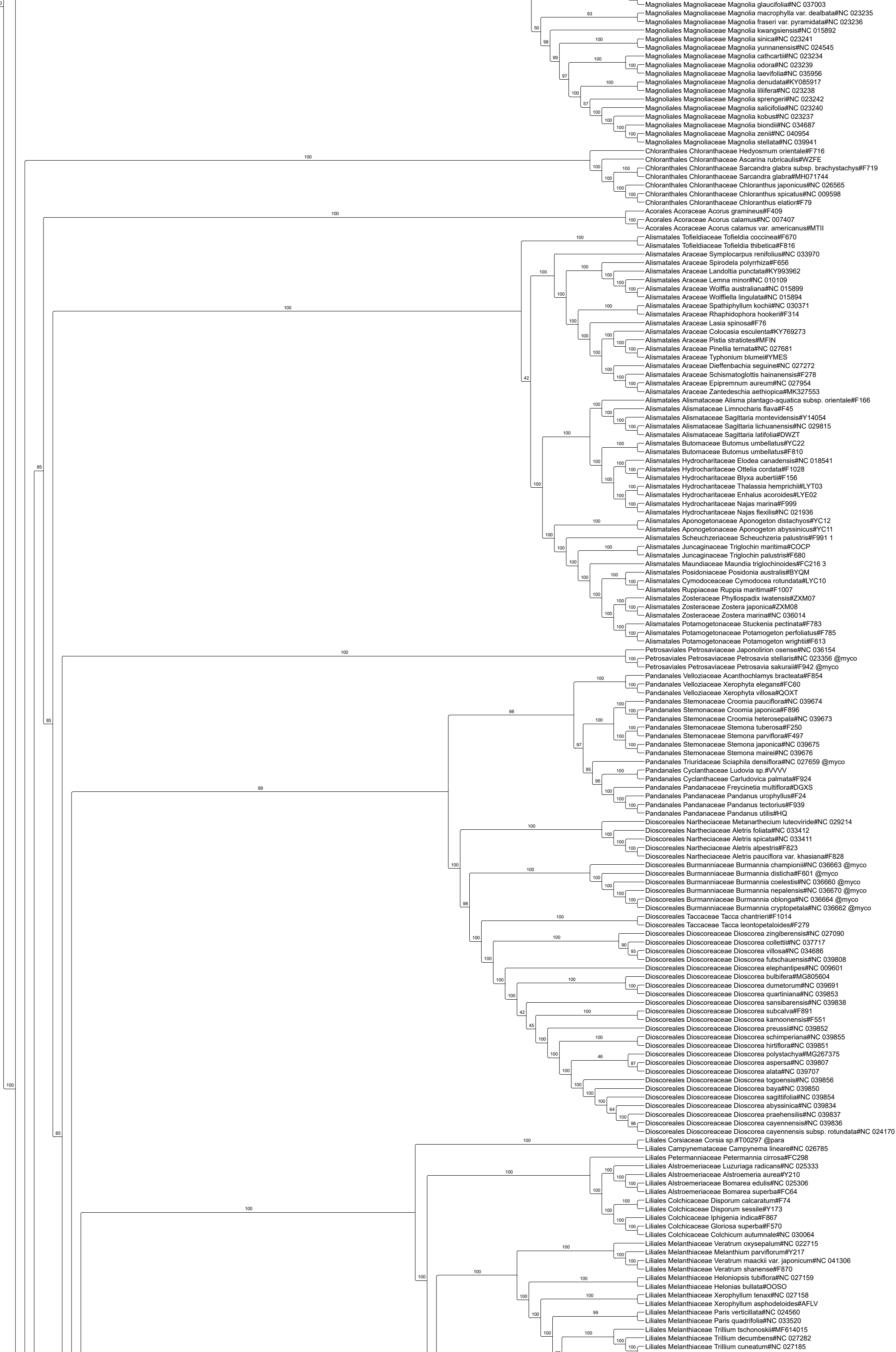

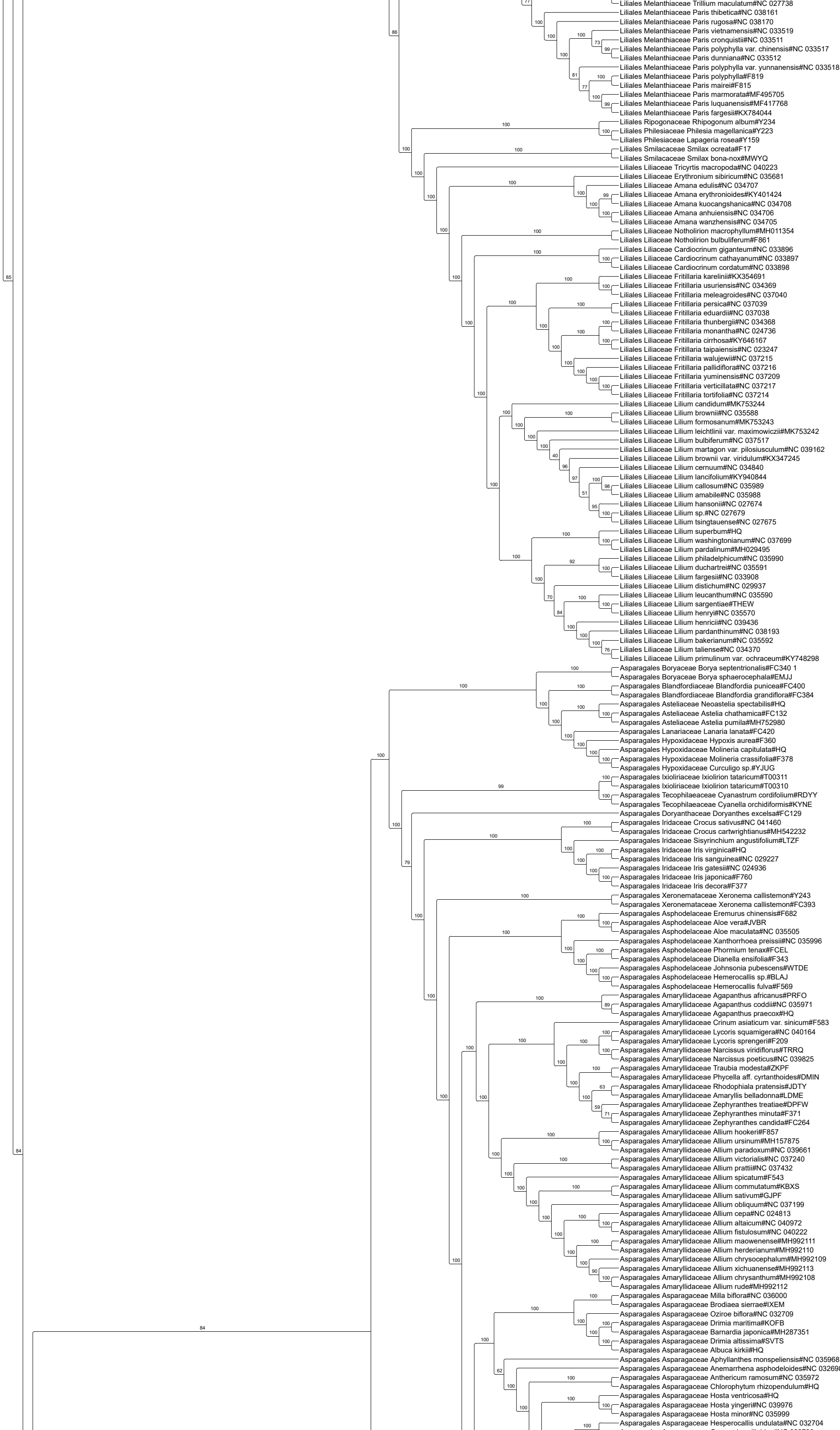

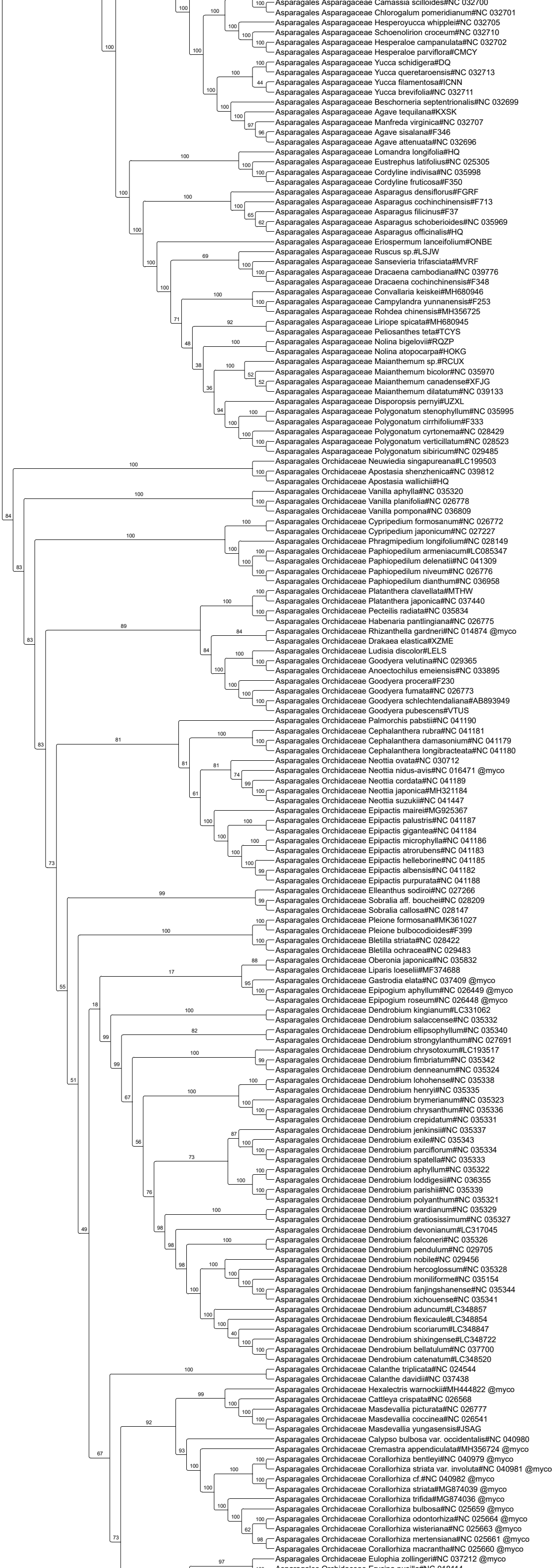

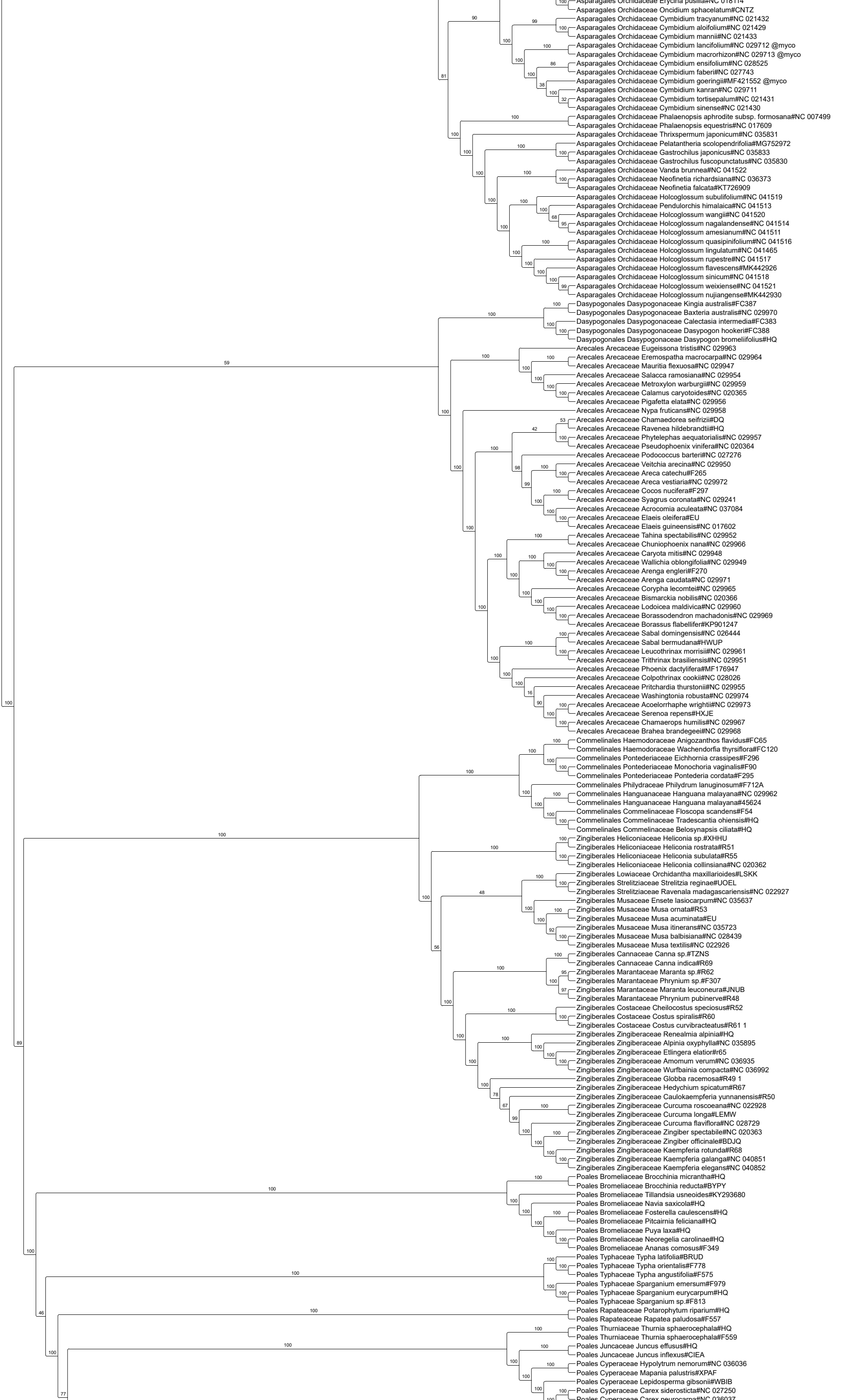

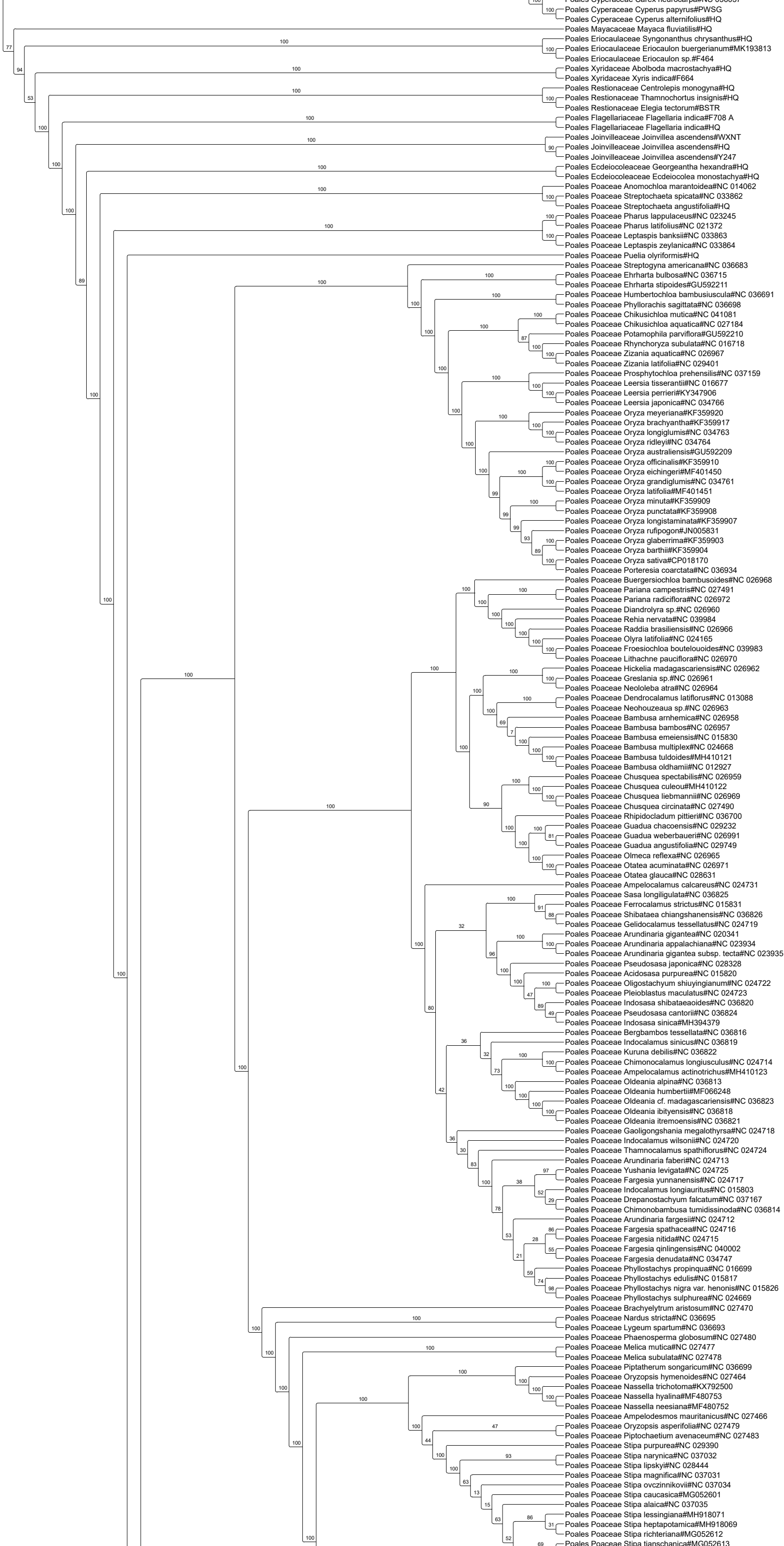

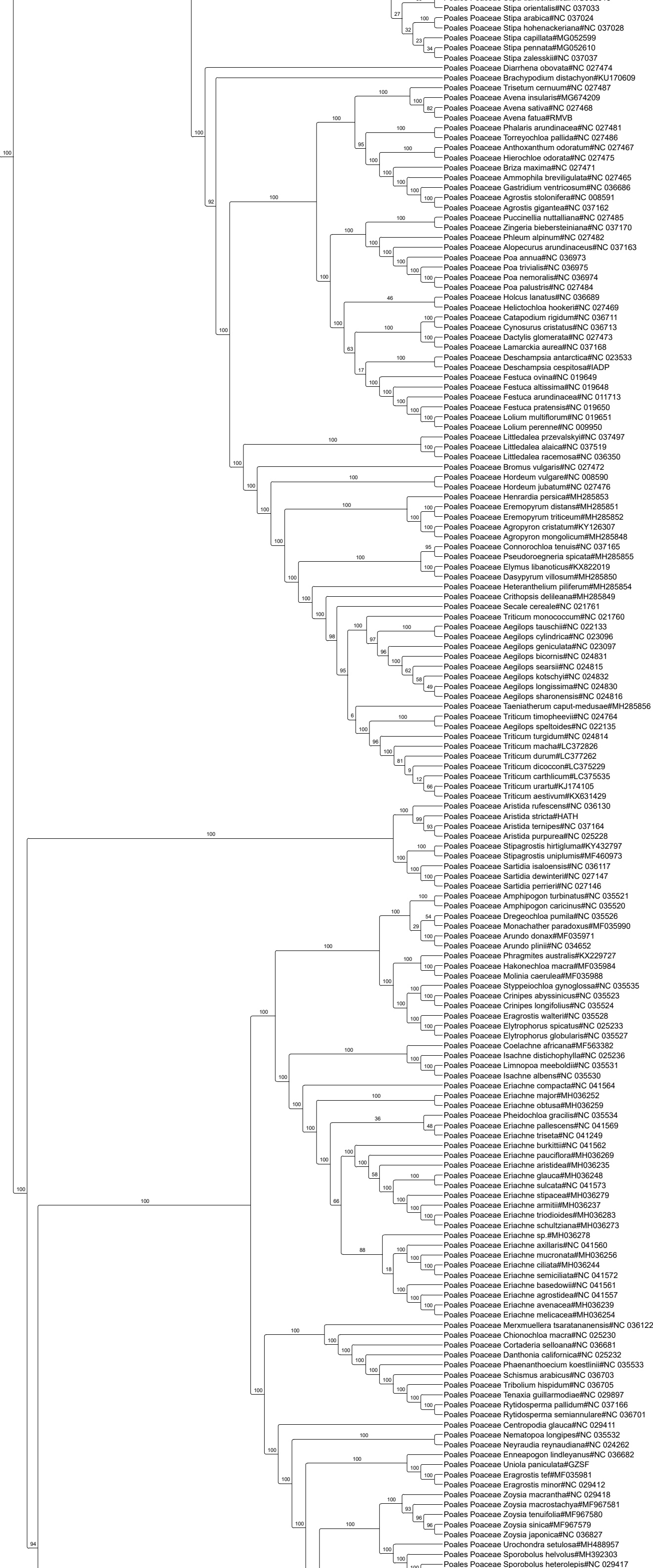

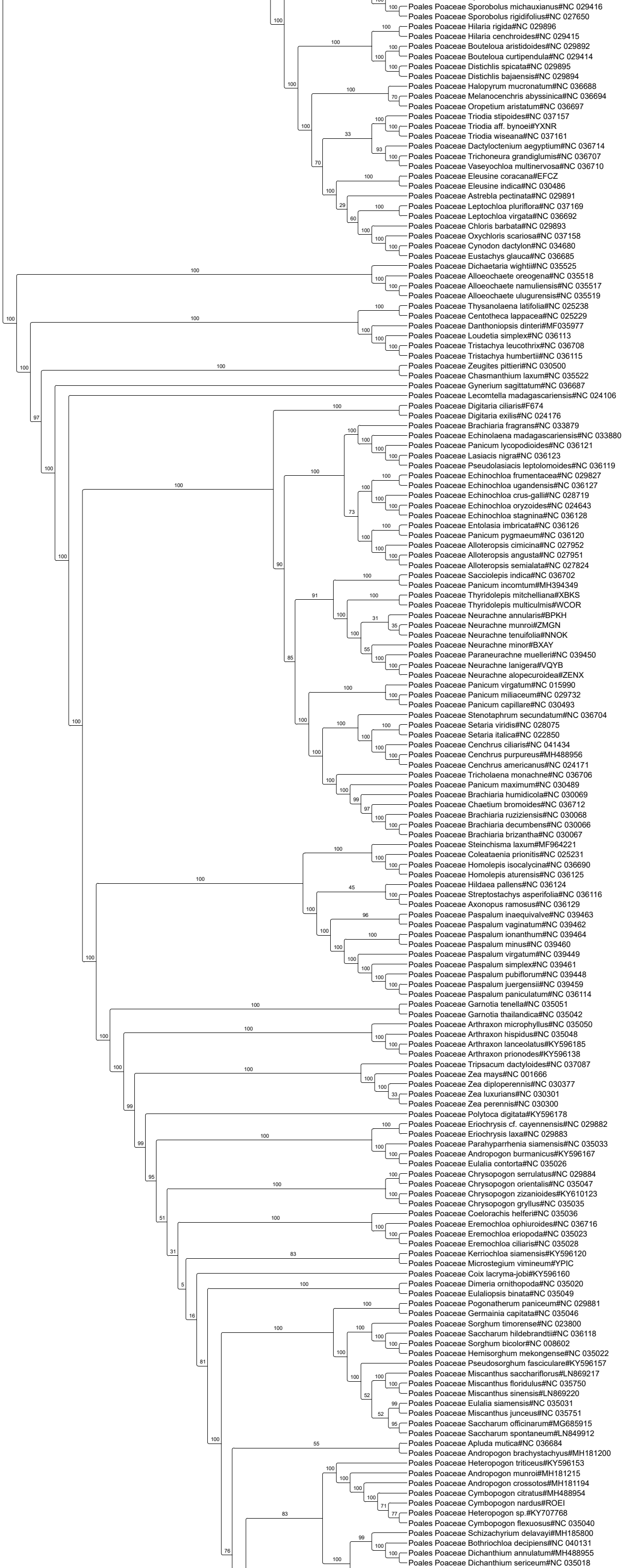

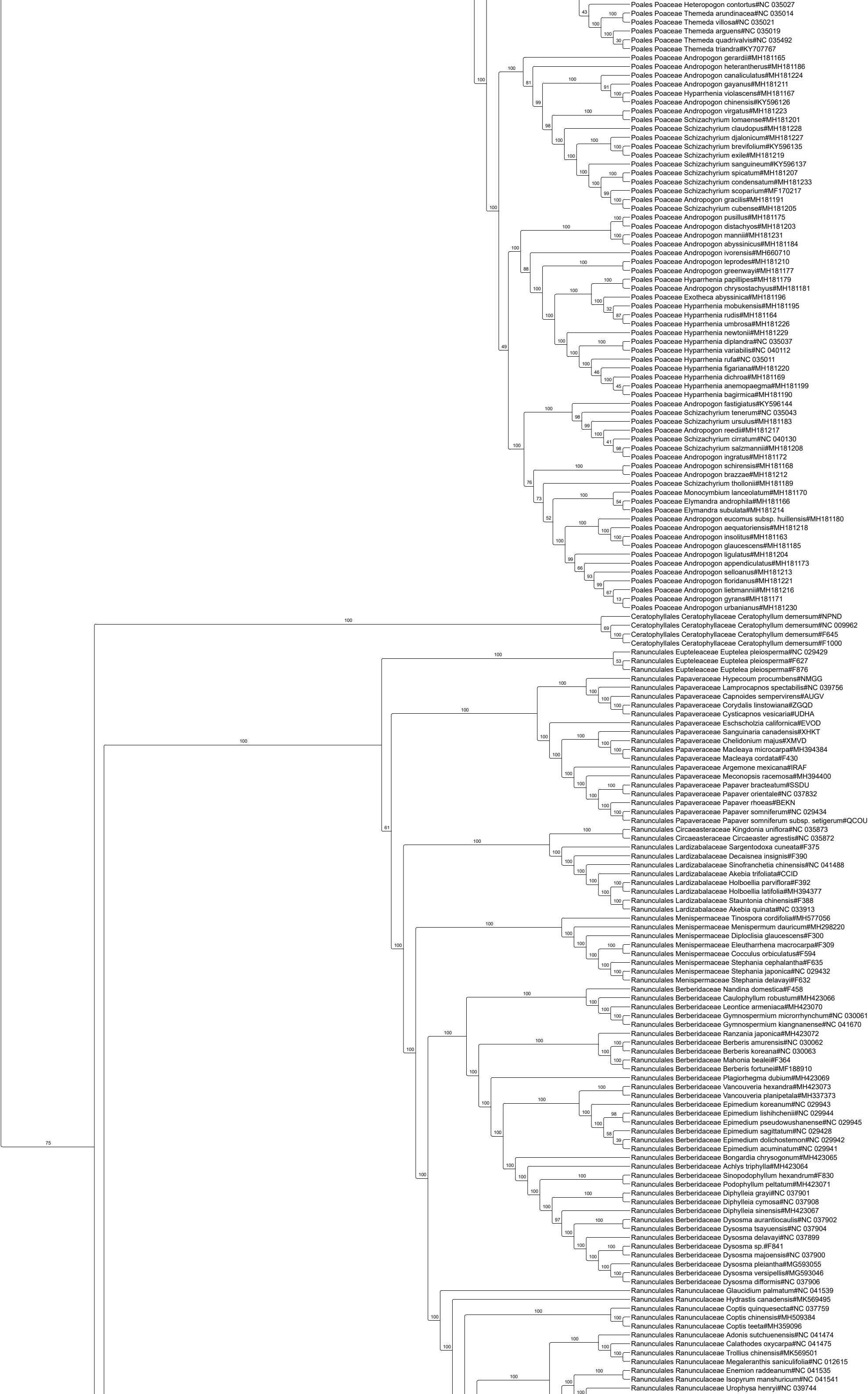

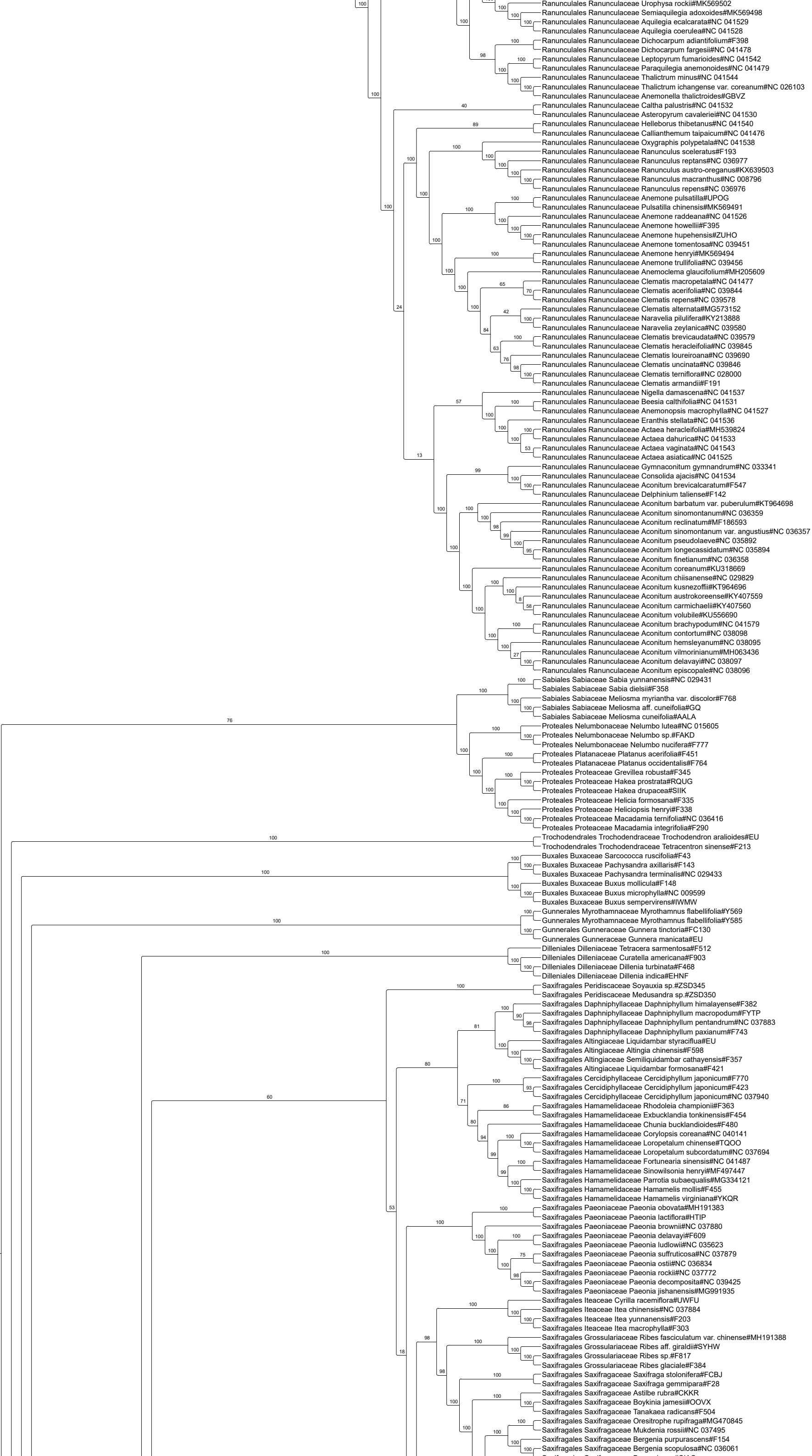

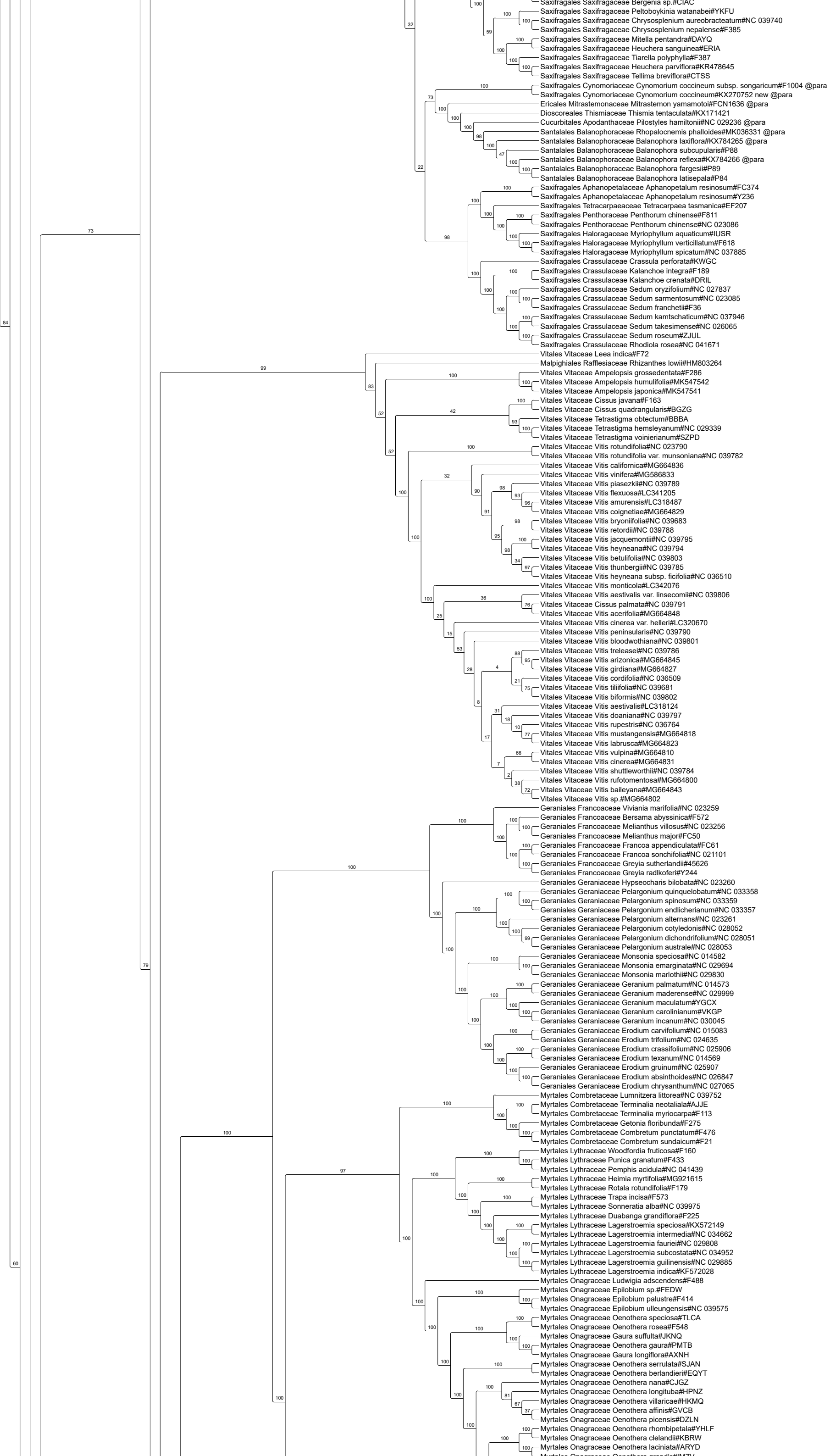

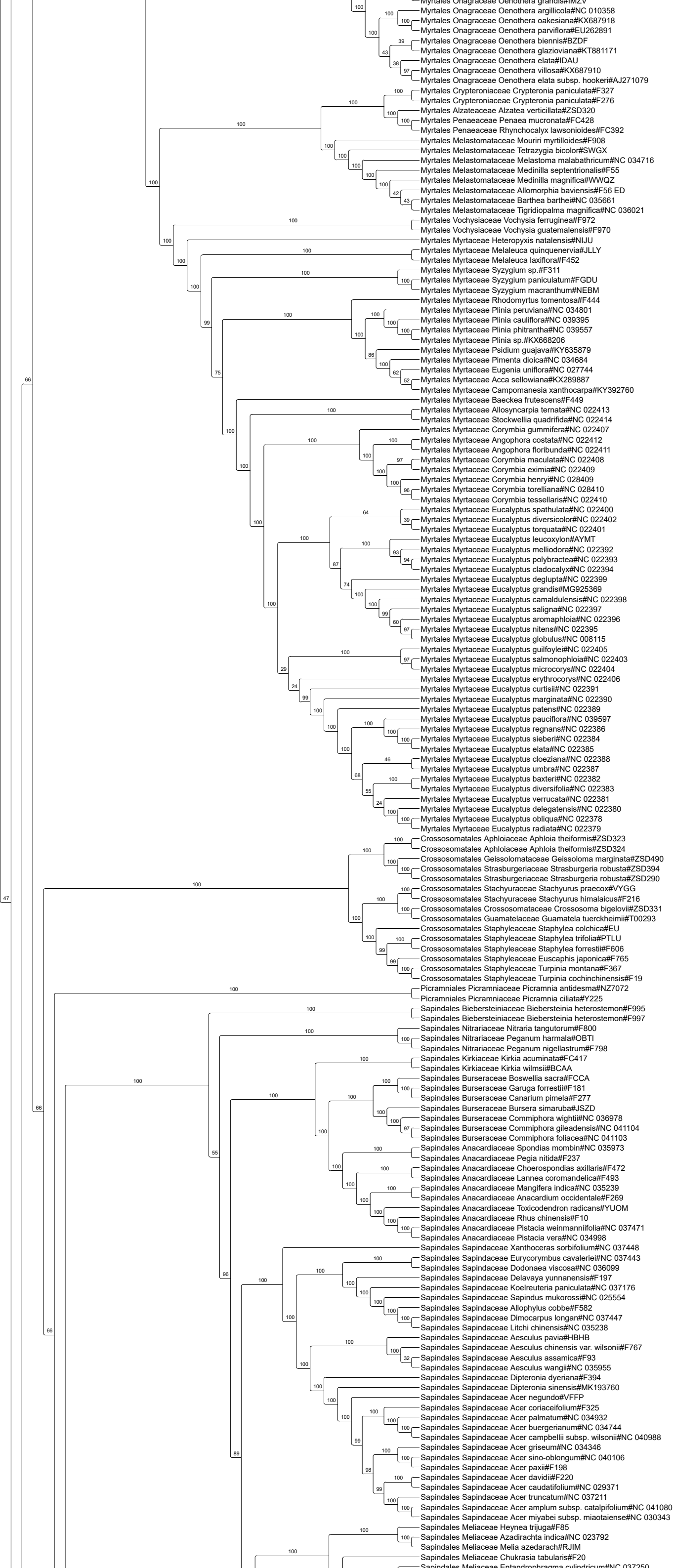

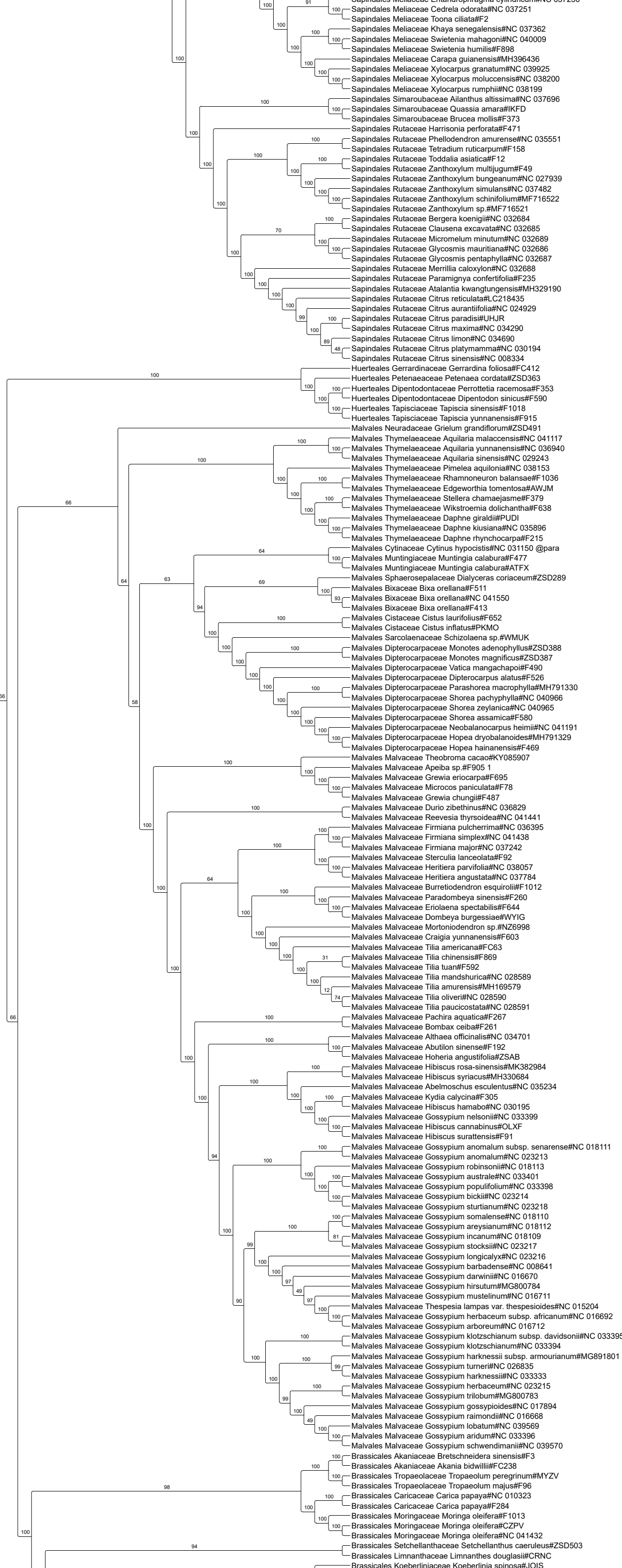

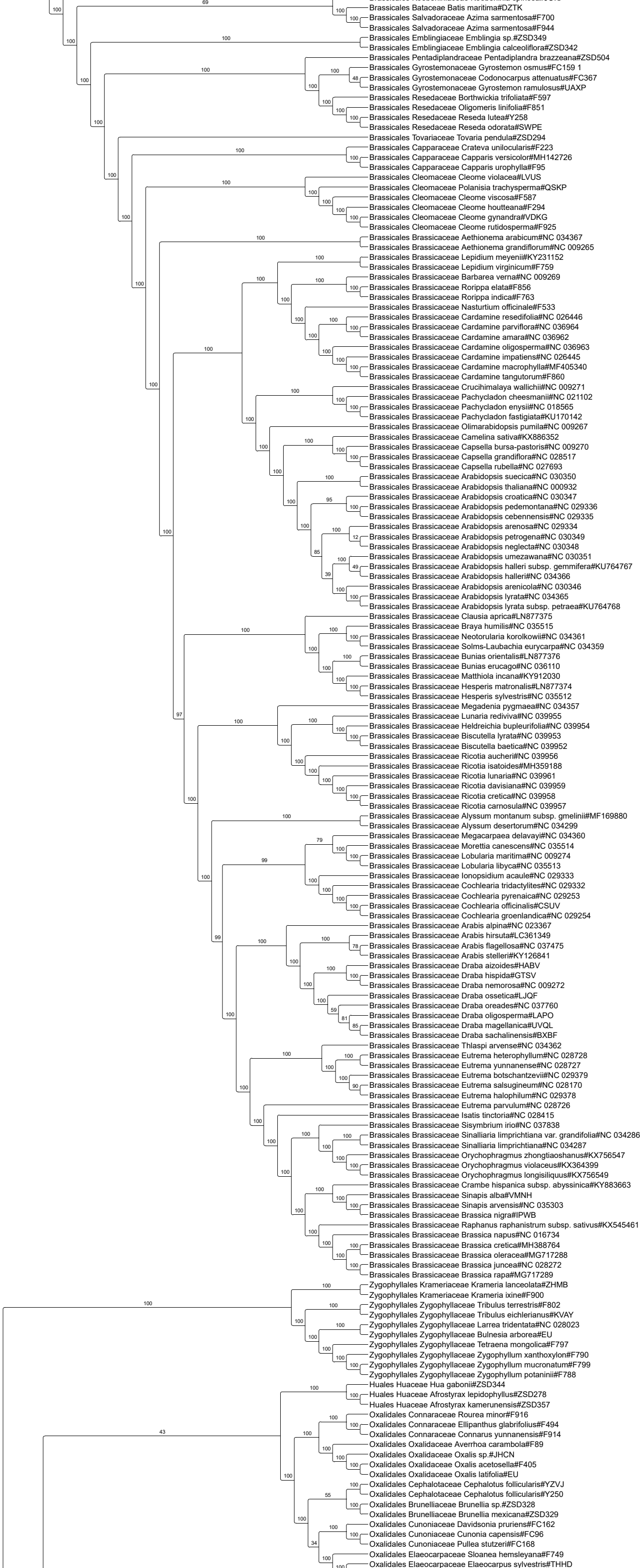

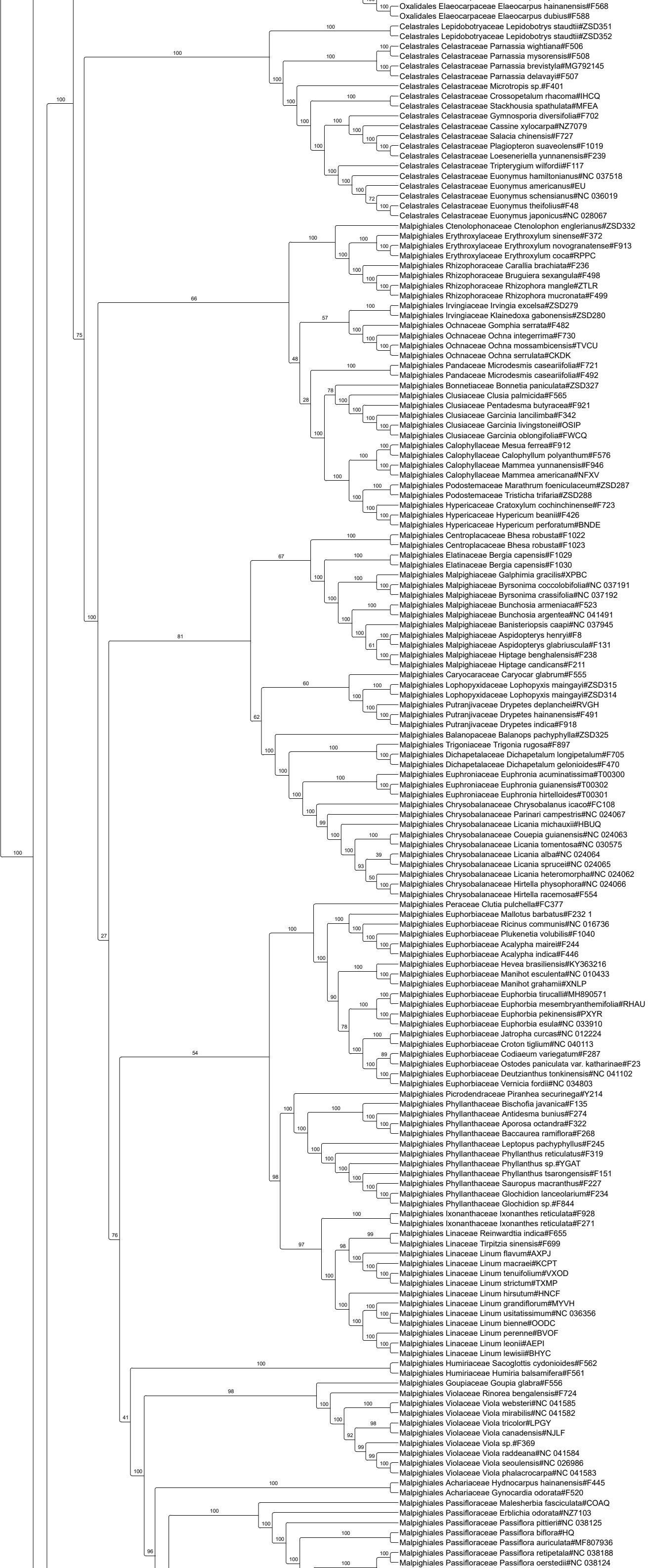

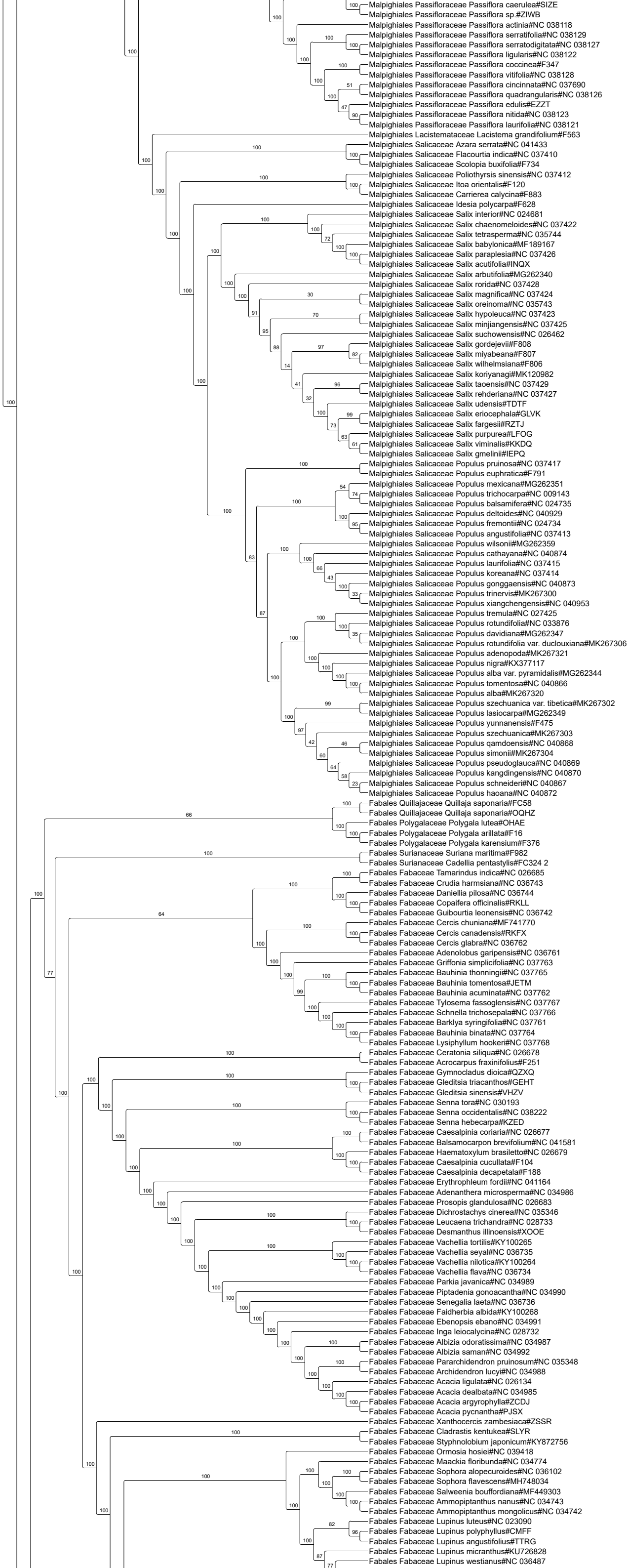

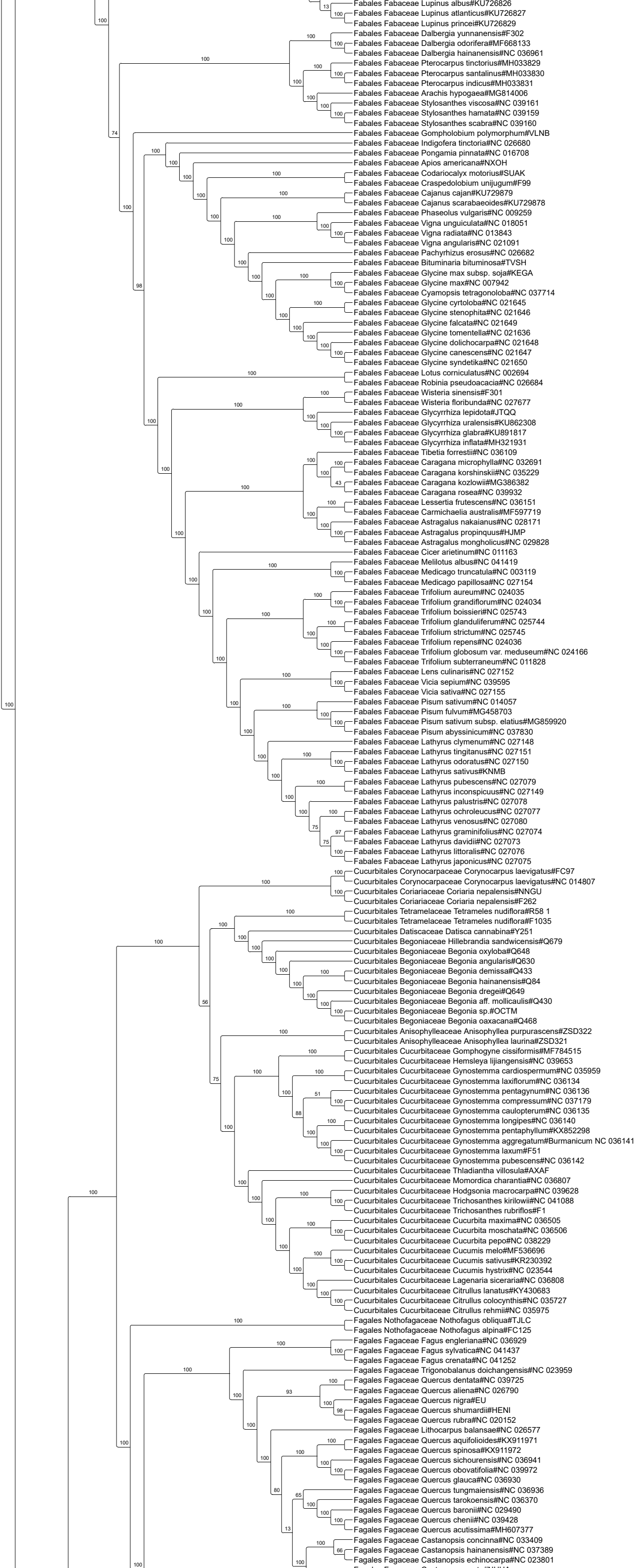

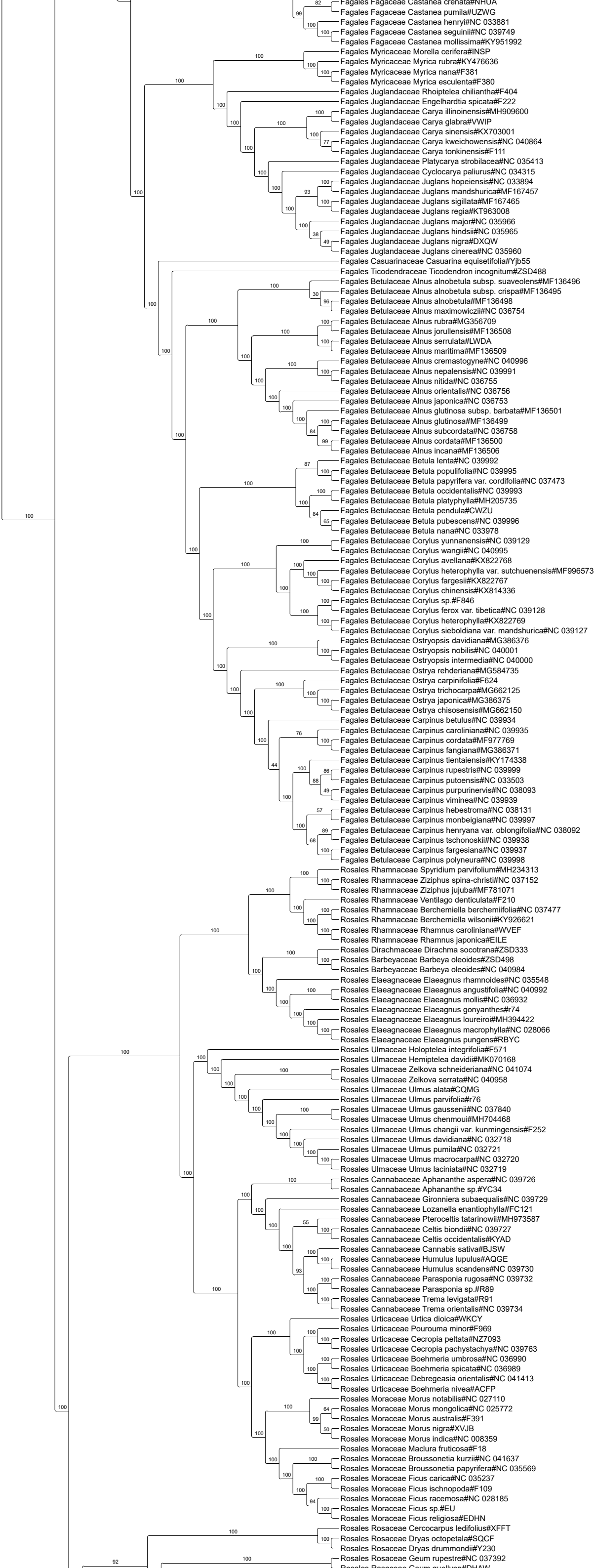

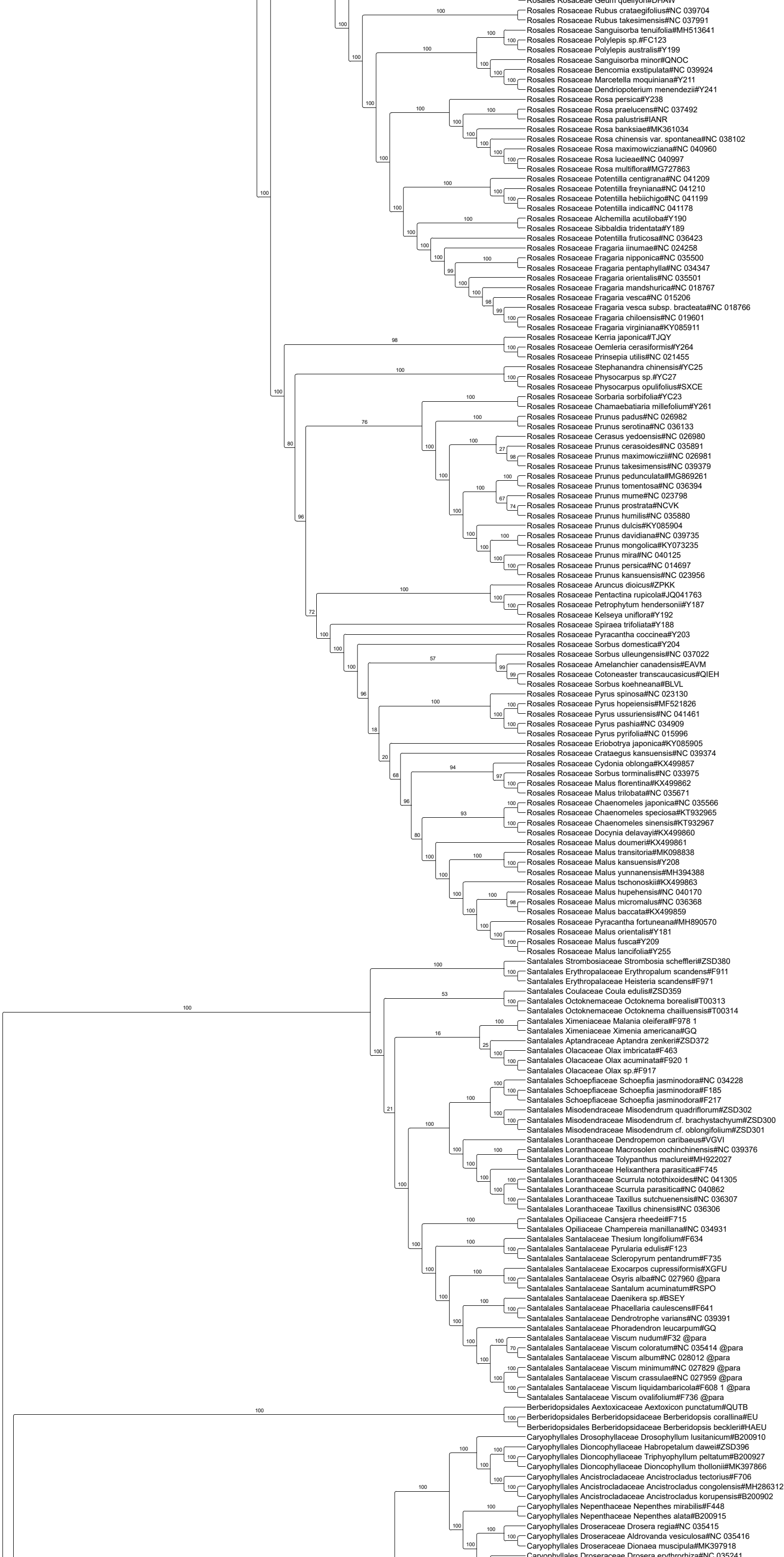

95

96

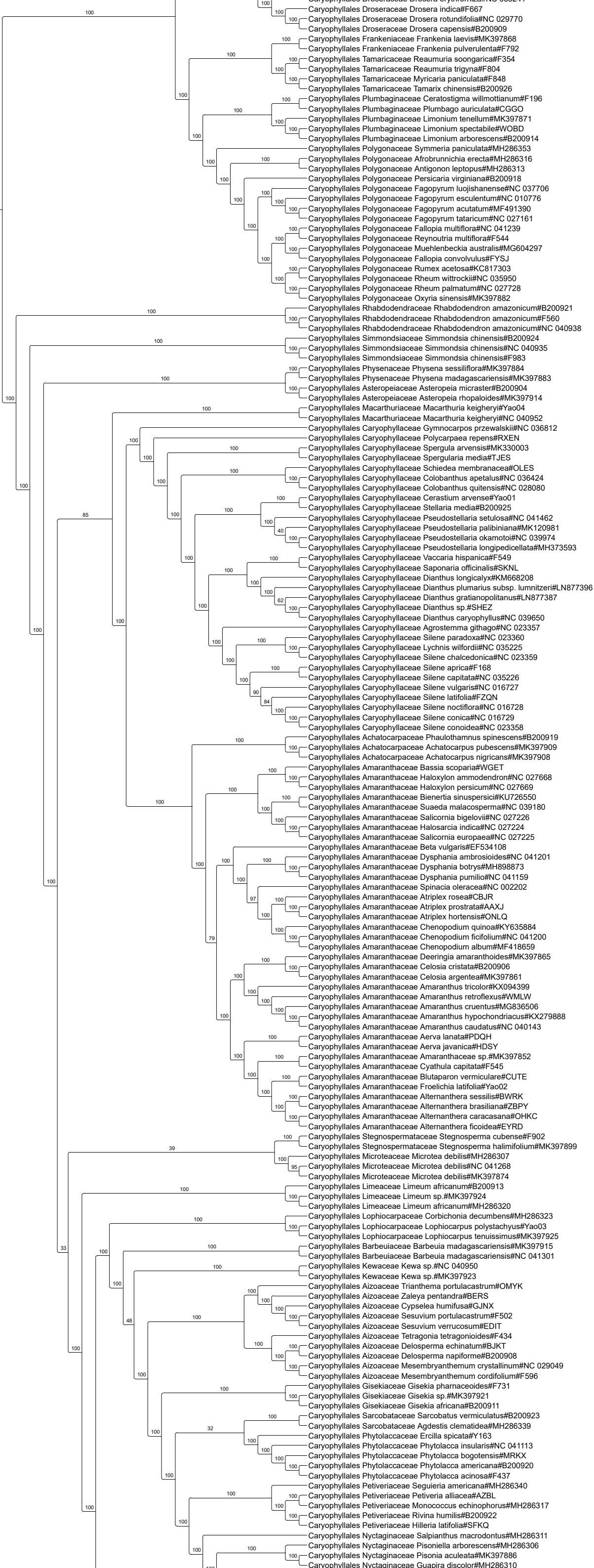

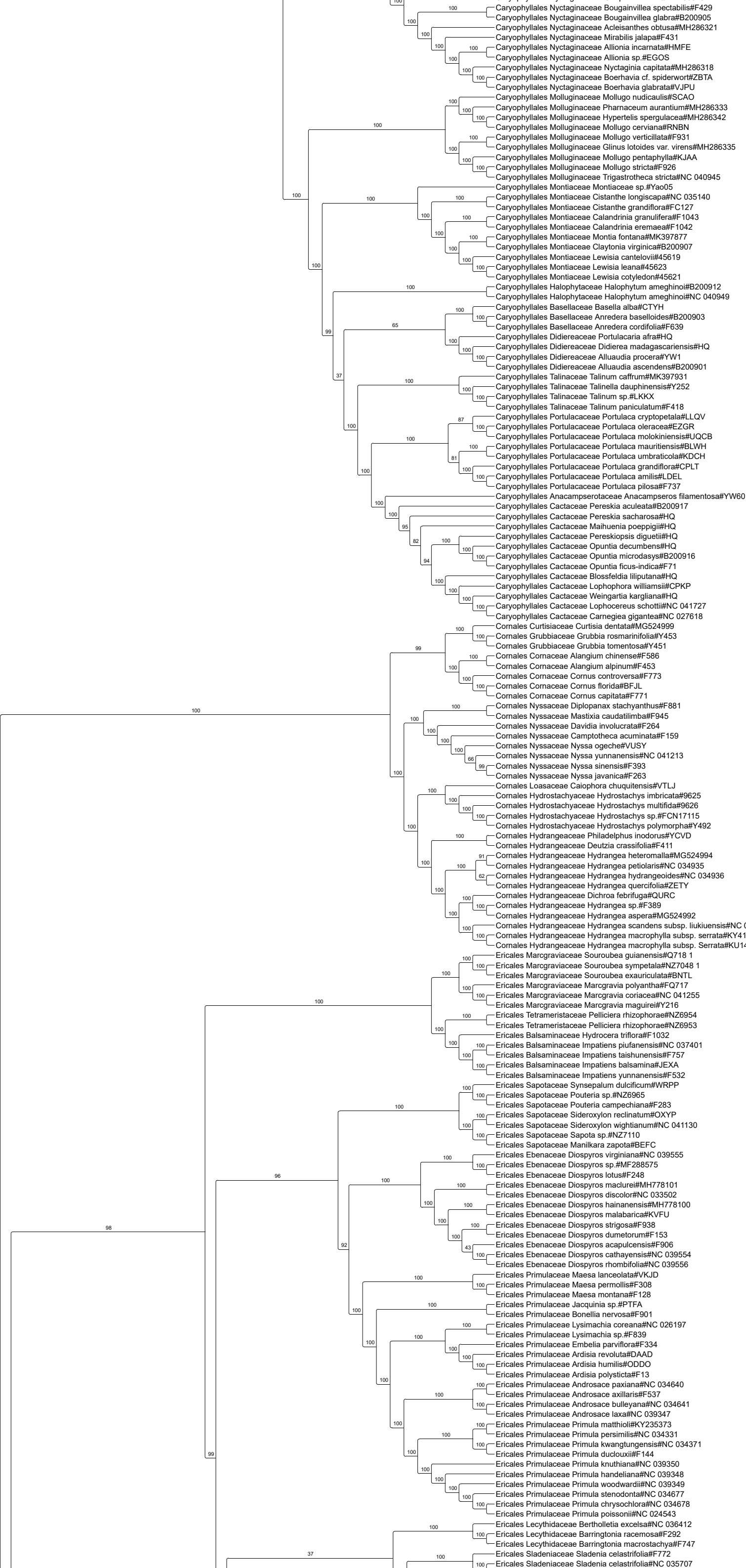

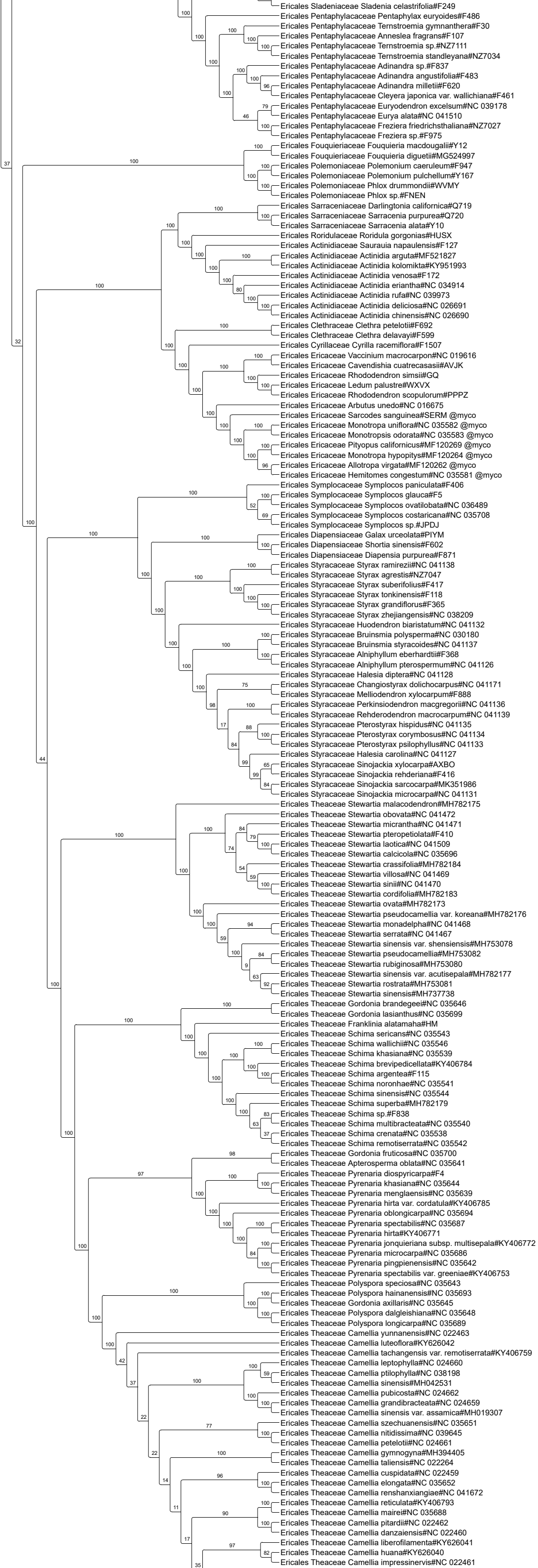

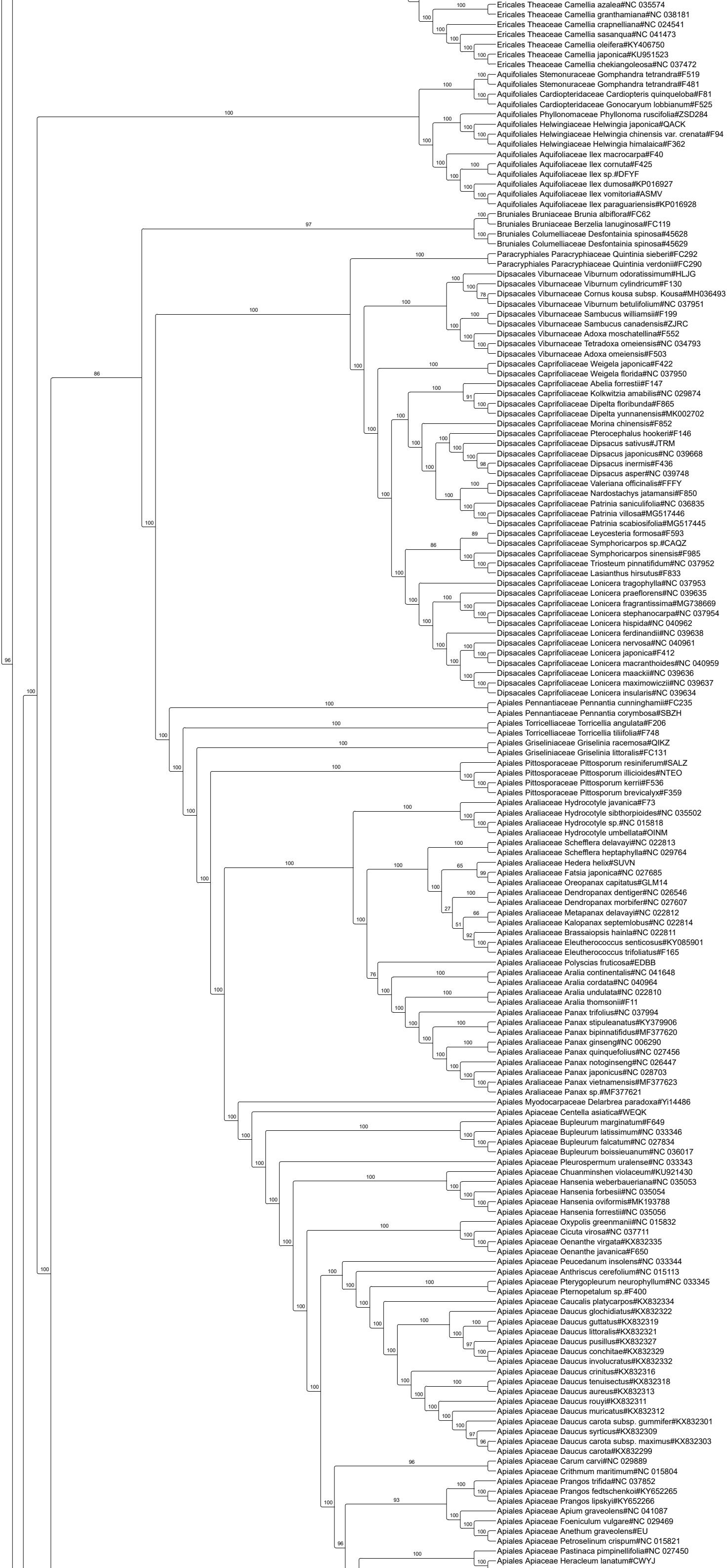

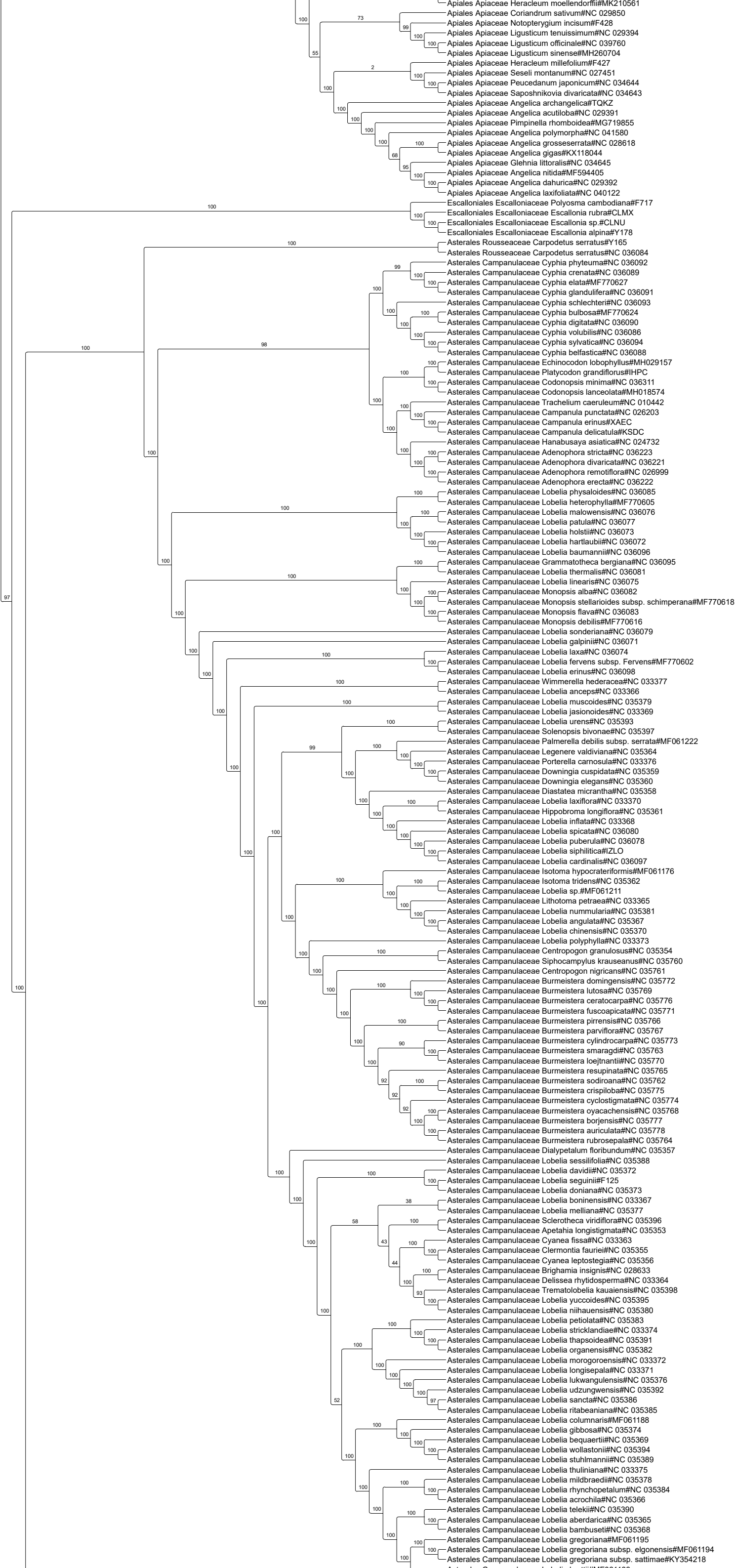

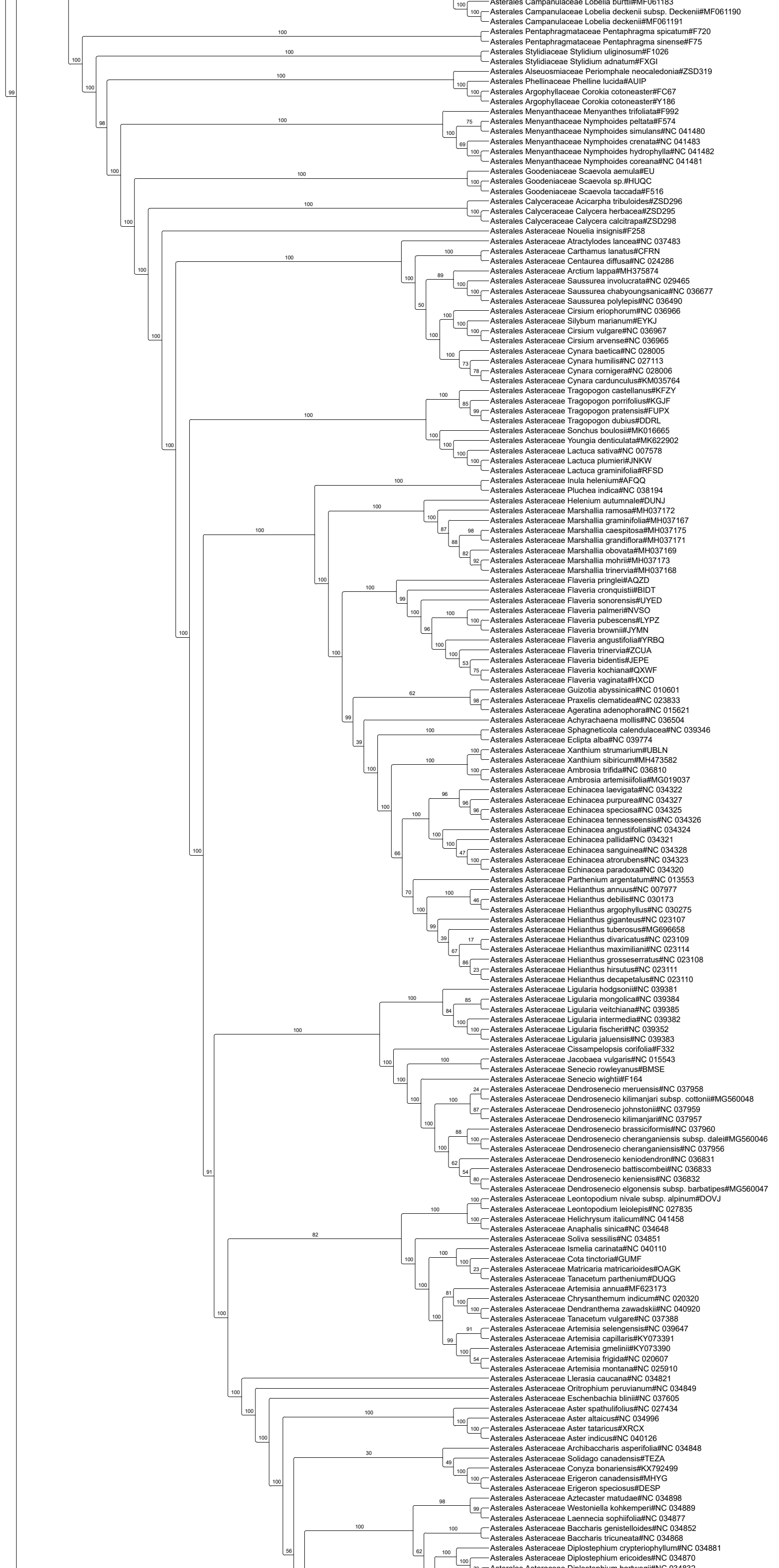

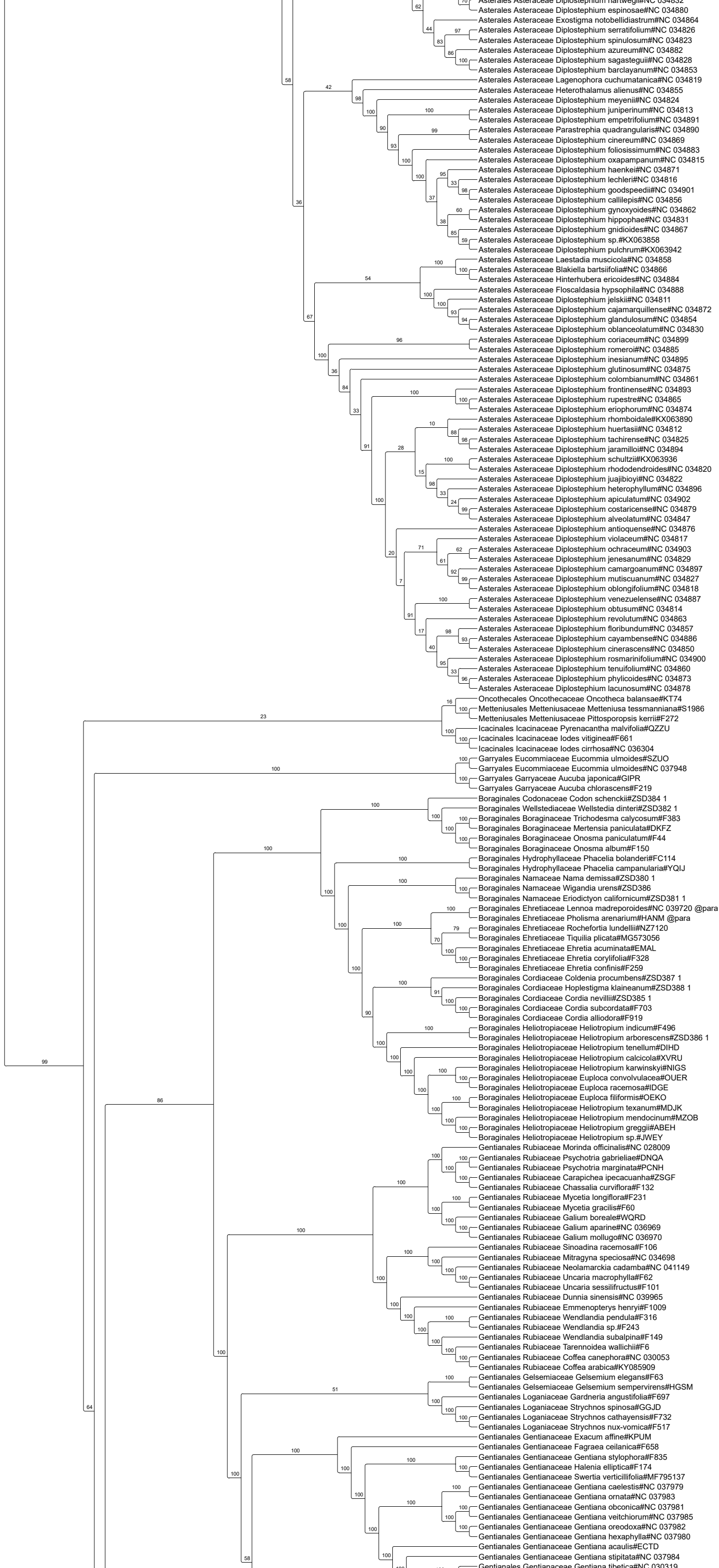

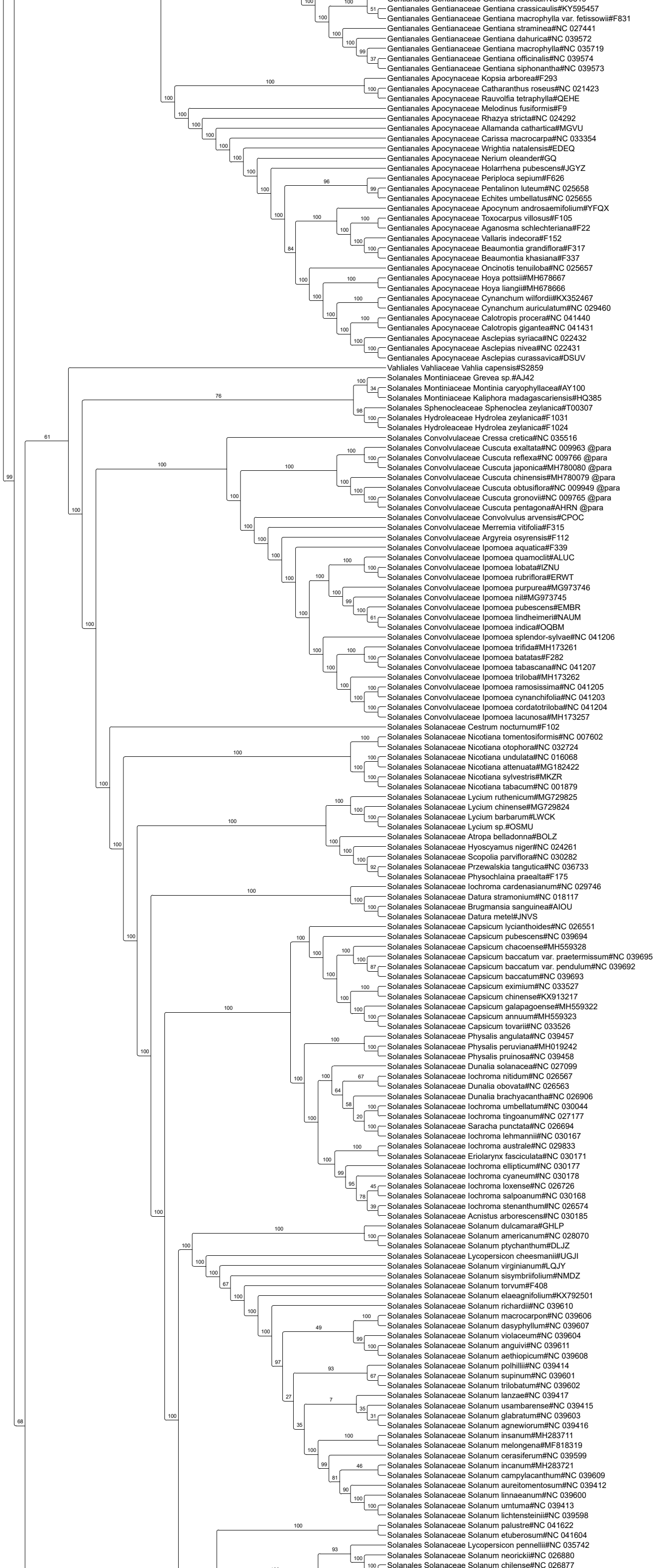

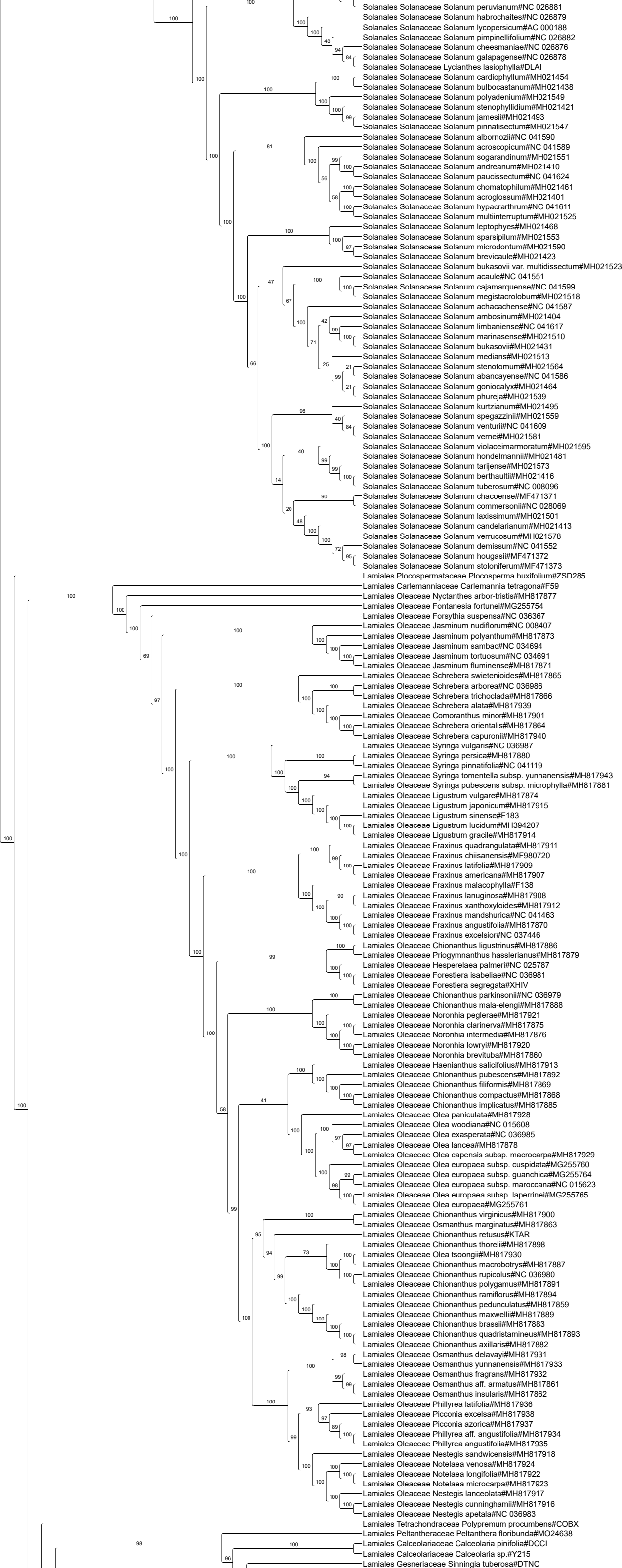

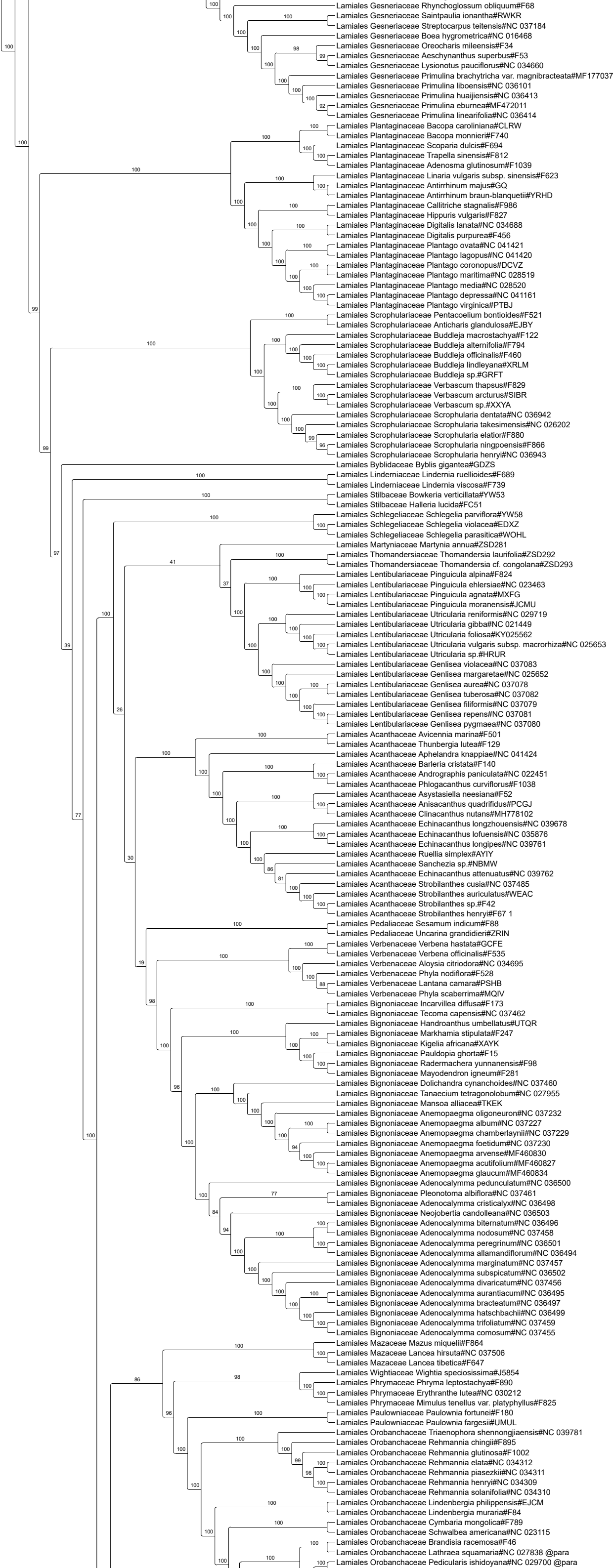

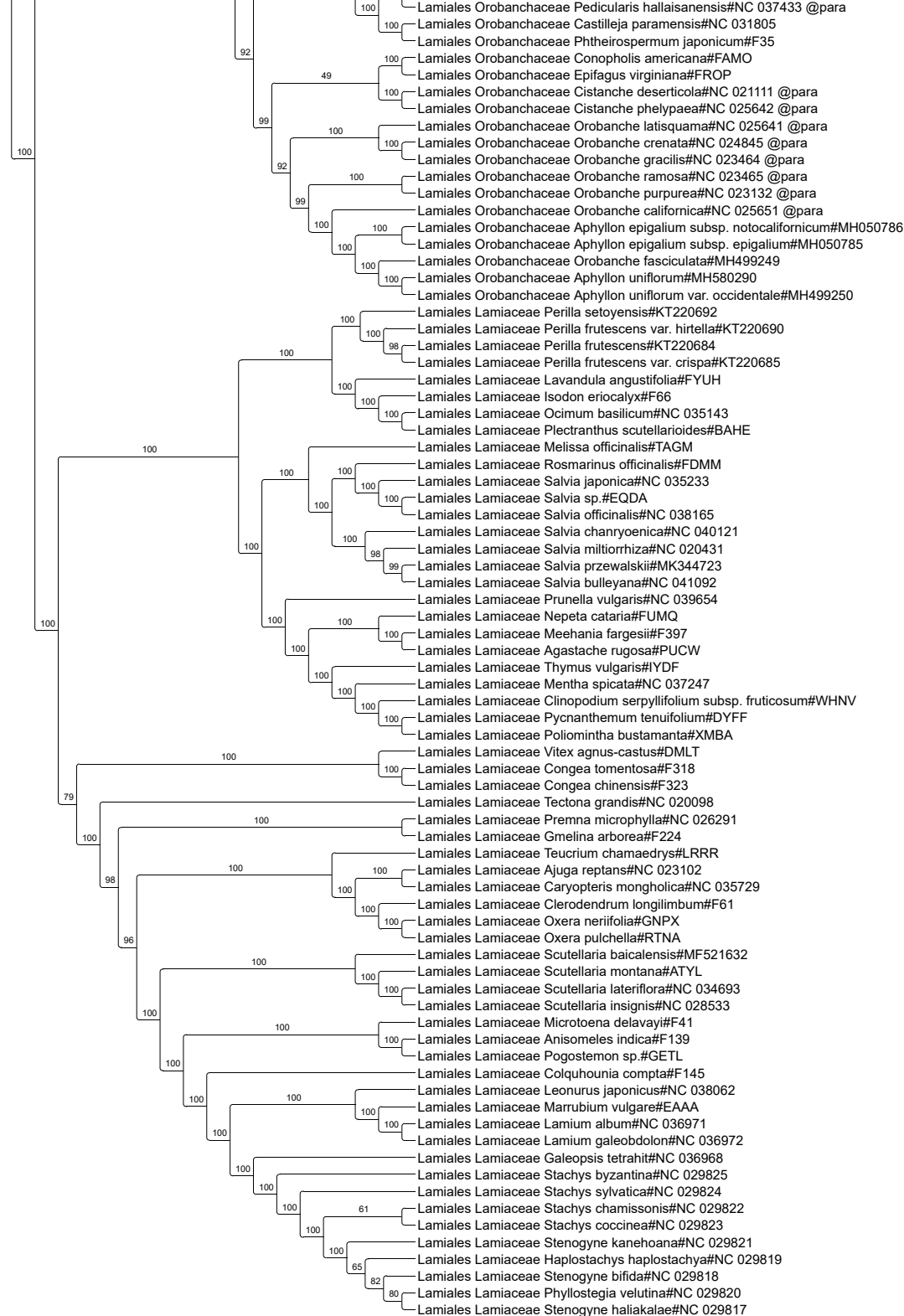

Supplement: Supplementary file 8 — Additional file 8: Figure S7. Phylogenetic tree of 4792 plastomes of 4660 species of seed plants. All bootstrap values are shown. [file 12915_2021_1166_MOESM8_ESM.pdf]

QS score(QC)

- QC > 0.2
- 0 < QC ≤ 0.2
- -0.05 < QC ≤ 0
- QC ≤ -0.05

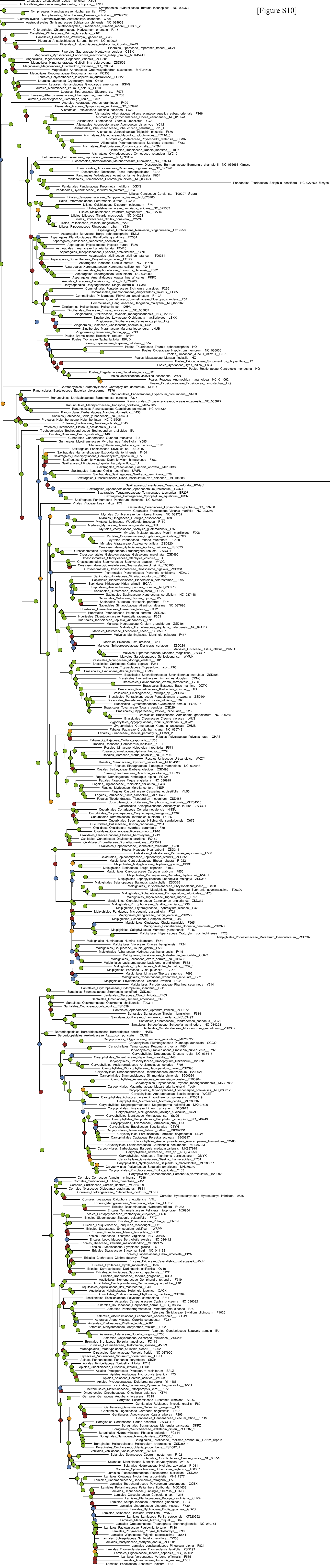

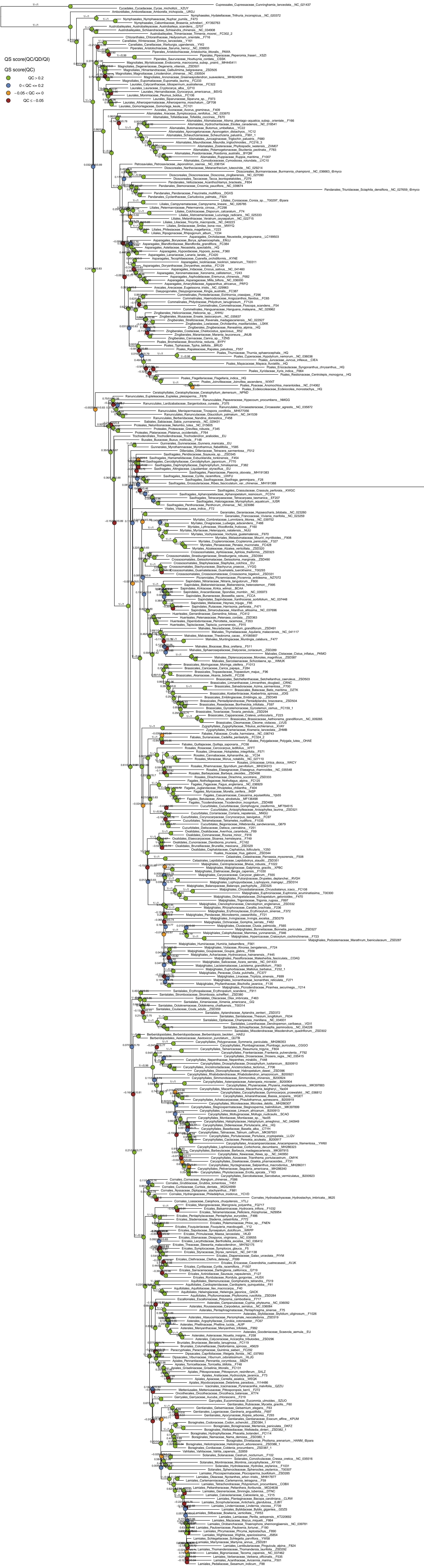

Supplement: Supplementary file 11 — Additional file 11: Figure S10. Family relationships within the pruned angiosperm phylogeny: nodes by Quartet Concordance (QC) scores for internal branches: green (QC > 0.2), blue (0.2 ≥ QC > 0), orange (0 ≥ QC ≥ −0.05, or red (QC < −0.05). QC/Quartet Differential (QD)/Quartet Informativeness (QI) scores are shown for all internal branches. [file 12915_2021_1166_MOESM11_ESM.pdf]

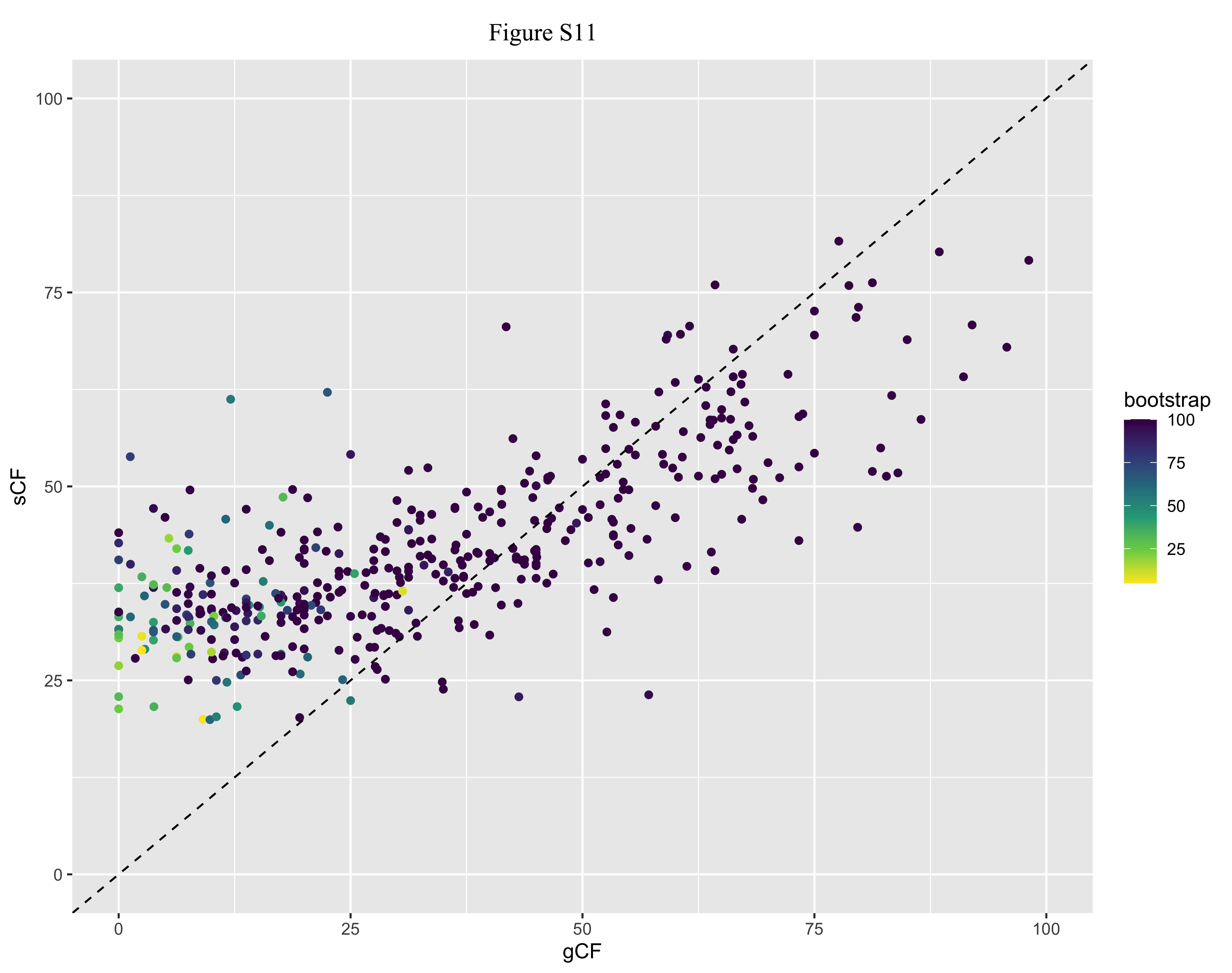

Supplement: Supplementary file 12 — Additional file 12: Figure S11. Plot showing the relationship between gene and site concordance factors (gCF and sCF) relative to bootstrap support from the pruned angiosperm phylogeny. [file 12915_2021_1166_MOESM12_ESM.jpg]

[Figure S12]

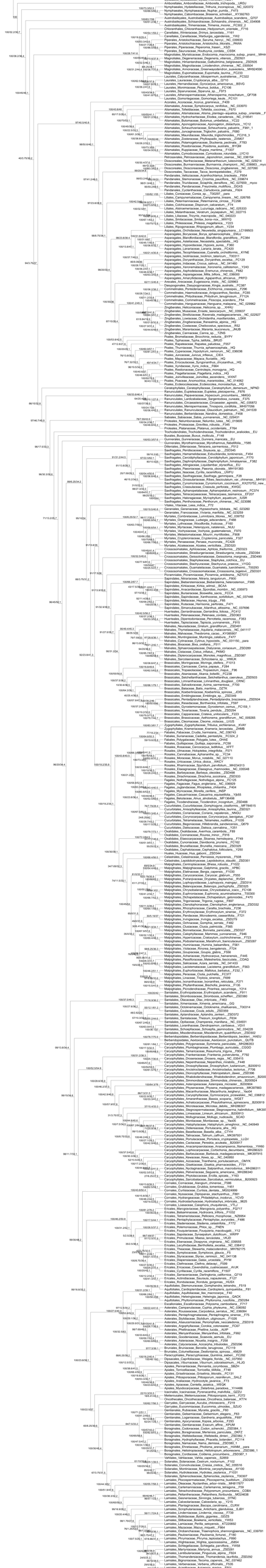

Supplement: Supplementary file 13 — Additional file 13: Figure S12. Family relationships within the pruned angiosperm subdataset. In this tree, bootstrap/gCF/sCF scores are shown for each branch. [file 12915_2021_1166_MOESM13_ESM.pdf]
