# Supplementary material for: Plastid phylogenomic insights into relationships of all flowering plant families
Source: BMC Biol. 2021 Oct 29;19:232. doi: 10.1186/s12915-021-01166-2 (PMC8555322; doi:10.1186/s12915-021-01166-2)

[Figure S3]

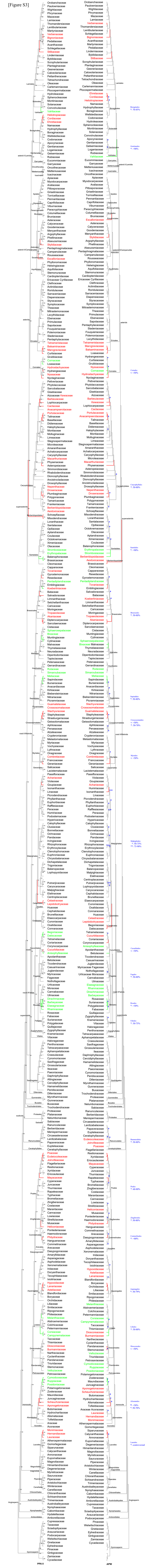

Supplement: Supplementary file 4 — Additional file 4: Figure S3. Angiosperm family-level phylogenetic relationships in PPA II versus APW. Red: different phylogenetic positions between PPA II and APW; green: resolved nodes in PPA II relative to APW. Different phylogenetic positions between PPA II and APW with bootstrap values < 50 in PPA are not shown. [file 12915_2021_1166_MOESM4_ESM.pdf]
