# Supplementary material for: Plastid phylogenomic insights into relationships of all flowering plant families
Source: BMC Biol. 2021 Oct 29;19:232. doi: 10.1186/s12915-021-01166-2 (PMC8555322; doi:10.1186/s12915-021-01166-2)
Supplement: Supplementary file 14 — Additional file 14: Additional Text. Overview of angiosperm phylogeny at the familial level. [file 12915_2021_1166_MOESM14_ESM.docx]

**[Additional Text]**

**Plastid phylogenomic insights into relationships of all flowering plant families**

Hong-Tao Li^1,2†^, Yang Luo^3†^, Lu Gan^1,2†^, Peng-Fei Ma^1,2†^, Lian-Ming Gao^2,3,4†^, Jun-Bo Yang^1,2†^, Jie Cai^1†^, Matthew A. Gitzendanner^5,6^, Peter W. Fritsch^7^, Ting Zhang^1^, Jian-Jun Jin^1,8^, Chun-Xia Zeng^1^, Hong Wang^2,3^, Wen-Bin Yu^9^, Rong Zhang^1^, Michelle van der Bank^10^, Richard G. Olmstead^11^, Peter M. Hollingsworth^12^, Mark W. Chase^13,14^, Douglas E. Soltis^5,6^, Pamela S. Soltis^5,6,15^, Ting-Shuang Yi^1,2*^ and De-Zhu Li^1,2,3*^

^1^ Germplasm Bank of Wild Species, Kunming Institute of Botany, Chinese Academy of Sciences, Kunming, Yunnan 650201, China

^2^ Kunming College of Life Science, University of Chinese Academy of Sciences, Kunming, Yunnan 650201, China

^3^ CAS Key Laboratory for Plant Diversity and Biogeography of East Asia, Kunming Institute of Botany, Chinese Academy of Sciences, Kunming, Yunnan 650201, China

^4^ Lijiang Forest Ecosystem National Observation and Research Station, Kunming Institute of Botany, Chinese Academy of Sciences, Lijiang 674100, Yunnan, China

^5^ Florida Museum of Natural History, University of Florida, Gainesville, FL 32611, USA

^6^ Biodiversity Institute, University of Florida, Gainesville, FL 32611, USA

^7^ Botanical Research Institute of Texas, 1700 University Drive, Fort Worth, TX 76017, USA

^8^ Department of Ecology, Evolution and Environmental Biology, Columbia University, New York, NY 10025, USA

^9^ Center for Integrative Conservation, Xishuangbanna Tropical Botanical Garden, Chinese Academy of Sciences, Mengla, Yunnan 666303, China

^10^ Department of Botany & Plant Biotechnology, University of Johannesburg, PO Box 524 Auckland Park, Johannesburg, Gauteng 2006, South Africa

^11^ Department of Biology and Burke Museum, University of Washington, Seattle, WA 98195‐5325, USA

^12^ Royal Botanic Garden Edinburgh, Edinburgh EH3 5LR, Scotland, UK

^13^ Royal Botanic Gardens, Kew, Richmond, Surrey TW9 3DS, England, UK

^14^ Department of Environment and Agriculture, Curtin University, Bentley, Western Australia 6102, Australia

^15^ Department of Biology, University of Florida, Gainesville, FL 32611, USA

* Corresponding authors: [dzl@mail.kib.ac.cn](mailto:dzl@mail.kib.ac.cn), [tingshuangyi@mail.kib.ac.cn](mailto:tingshuangyi@mail.kib.ac.cn)

^†^Hong-Tao Li, Yang Luo, Lu Gan, Peng-Fei Ma, Lian-Ming Gao, Jun-Bo Yang and Jie Cai contributed equally to this work.

**Overview of angiosperm phylogeny at the familial level**

**Table of Contents**

[Overview 2](#_Toc81991451)

[Amborellales, Nymphaeales, and Austrobaileyales 2](#_Toc81991452)

[Chloranthales 3](#_Toc81991453)

[Magnoliids 3](#_Toc81991454)

[Monocots 4](#_Toc81991455)

[Ceratophyllales 7](#_Toc81991456)

[Eudicots 8](#_Toc81991457)

[Rosids (excluding Vitales) 10](#_Toc81991458)

[Asterids 15](#_Toc81991459)

[References 2](#_Toc81991460)0

# Overview

Our results, when integrated with previously published studies of angiosperm phylogeny, shed new light on angiosperm phylogeny. Through sampling all recognized families and the use of plastome sequences, our results clarify most interfamilial relationships with high support at most nodes within angiosperms (see Additional files 3–4: Figs. S2 and S3). Here we discuss our major findings at the level of family and above comparing with previous major studies, especially the recent nuclear phylogenetic study by Baker et al. [1] using 353 nuclear genes and 3,099 samples representing 64 orders, 404 families and 2,333 genera.

# Amborellales, Nymphaeales, and Austrobaileyales

Our results supported *Amborella trichopoda*, the sole species of Amborellales, as sister to all other living angiosperms, followed in succession by Nymphaeales and then Austrobaileyales (all nodes with 100 bootstrap percentages support, BP; see Additional file 2: Fig. S1). In some early studies, Amborellales and Nymphaeales appeared as sister taxa, and together they were sister to the remaining angiosperms (e.g., [2]). However, almost all recent plastid and nuclear phylogenomic analyses have supported *Amborella* alone as sister to all other extant angiosperms (e.g., [1,3–5]).

Within Nymphaeales, Hydatellaceae were sister (BP = 100) to [Cabombaceae + Nymphaeaceae]. Within Austrobaileyales, Austrobaileyaceae were sister (BP = 100) to [Trimeniaceae + Schisandraceae]. The same interfamilial relationships within Austrobaileyales and Nymphaeales were strongly supported in most previous studies (e.g., [6–8]). The same interfamilial relationships were recovered in nuclear phylogenomic analysis for Nymphaeales, but different interfamilial relationships were recovered in Austrobaileyales, with Trimeniaceae being sister to [Austrobaileyaceae + Schisandraceae] (local posterior probability, LPP = 1) [1]. Gruenstaeudl [9] pointed to a possible close affinity of *Nuphar* and Cabombaceae, but the monophyly of Nymphaeaceae was strongly supported here (BP = 100, see Additional file 5: Fig. S4).

# Chloranthales

Chloranthales were placed as sister to magnoliids in previous plastid phylogenomic studies with sparse taxon sampling [10–12], but recent studies using plastome data with broader taxon sampling have suggested that Chloranthales are sister to other mesangiosperms, with weak support ([6]; current study BP = 40). The former relationship was strongly supported in a recent nuclear phylogenomic analysis [1].

# Magnoliids

Magnoliids comprise four orders: Magnoliales, Laurales, Piperales, and Canellales. Our results provided strong support (BP = 100) for the monophyly of each order, for the sister relationship between Canellales and Piperales, and for the sister relationship between Laurales and Magnoliales. These relationships have been supported in most phylogenetic studies (e.g., [1, 3, 8]).

Myristicaceae were strongly supported (BP = 100) as sister to a strongly supported (BP = 100) clade of the remaining Magnoliales. Within the latter clade, only [Annonaceae + Eupomatiaceae] was strongly supported (BP = 100); the other two nodes (i.e., Magnoliaceae as sister to [Annonaceae + Eupomatiaceae] and the sister relationship between Degeneriaceae and Himantandraceae) received low support. Although the clade of [Degeneriaceae + Myristicaceae] was highly supported in Soltis *et al.* [8], the phylogenetic positions of Magnoliaceae, Himantandraceae, and Degeneriaceae were poorly resolved in many previous studies (e.g., [7, 8, 13, 14]). However, Baker *et al*. [1] obtained a well-resolved topology of [Myristicaceae [[Eupomatiaceae + Annonaceae] [Magnoliaceae [Himantandraceae + Degeneriaceae]]]].

All interfamilial relationships within Laurales received 100 BP support here (see Additional file 3: Fig. S2). Baker *et al.* [1] recovered well-resolved interfamilial relationships within Laurales, but their results supported a non-monophyletic Hernandiaceae with *Hernandia nymphaeifolia* being sister to other Laurales, and *Gyrocarpus americanus* sister to [Monimiaceae + Lauraceae]. Our results exhibited a monophyletic Hernandiaceae (three species sampled, including the above two), and [Lauraceae [Hernandiaceae + Monimiaceae]] with strong support (BP = 100) (see Additional file 5: Fig. S4), although most previous studies did not confidently resolve the latter relationship (e.g., [3, 8, 15]), and the analysis of Baker *et al*. [1] recovered a moderately supported clade of Lauraceae and Monimiaceae. Within Piperales, the circumscription of Aristolochiaceae is still controversial because of the placements of Hydnoraceae and Lactoridaceae ([14, 16, 17]). Aristolochiaceae were found to be paraphyletic in the current study with *Aristolochia* sister to [Saururaceae + Piperaceae] and [*Saruma* + *Asarum*] sister to that clade (Additional file 5: Fig. S4). The sister relationship between Saururaceae and Piperaceae was strongly (BP = 100) supported in our results and also in previous studies [1, 3, 8, 14]. We did not sample *Lactoris* or Hydnoroideae (formerly Lactoridaceae and Hydnoraceae [18]), and *Lactoris fernandeziana* was remarkably resolved as sister to [Ceratophyllales [Chloranthales + Magnoliids]] in Baker *et al.* [1] (LPP = 1).

# Monocots

Monocots are one of the best-supported major clades of angiosperms, comprising about 25% of angiosperm diversity [19, 20]. Most interordinal relationships have been clarified in previous studies, but a few remain ambiguous [21]. For example, the basalmost node of monocots, the positions of Liliales and Asparagales, and the relative positions of Dasypogonaceae and Arecales within commelinids are inconsistent or strongly conflicting among some nuclear, plastid, and mitochondrial phylogenomic analyses (e.g., [22–27]). Most recently, Givnish *et al.* [20] provided a greatly improved understanding of monocot phylogenetics based on plastome-scale data. Givnish *et al.* [20] and our results show the same topology at the ordinal level but with slight differences in the position of a few families (i.e., Thismiaceae in Dioscoreales, Triuridaceae in Pandanales, Smilacaceae in Liliales, Philydraceae in Commelinales, Musaceae and Heliconiaceae in Zingiberales).

**Acorales and Alismatales**—Consistent with Ross *et al.* [28] and Givnish *et al.* [20], our results provided 100 BP support for all interfamilial relationships within Alismatales with the exception of the positions of Araceae and Tofieldiaceae (Additional file 3: Fig. S2). The current study and nearly all previous studies (e.g., [1, 8, 20, 21, 29]) supported *Acorus* as sister to the remaining monocots, although the small family Tofieldiaceae were placed as sister to all other monocots in a few analyses using nuclear, plastid, and mitochondrial loci (e.g., [30, 31]). The overwhelming evidence, from both the plastid (this study; [8, 13, 29, 31]) and nuclear genomes (e.g., [1, 3]), supports *Acorus* at this position.

**Dioscoreales and Pandanales**—The phylogenetic position of Thismiaceae remains controversial; some studies suggested the paraphyly of this family, and *Afrothismia* (formerly a member of Thismiaceae) was found to be sister to the clade of *Tacca* plus other Thismiaceae [32–34]. APG IV [16] recognized this family as a member of Burmanniaceae *s.l.* because of discrepancies in the placements of Thismiaceae and Burmanniaceae in published studies. The placement of Thismiaceae was not resolved in our tree; however, the sister relationship of Thismiaceae and Taccaceae has received some support in previous studies [20, 35], unfortunately, *Afrothismia* was not included in these analyses.

Previous organellar phylogenomic analyses found strong support for Triuridaceae as sister to [Pandanaceae + Cyclanthaceae] [20, 35, 36, 37], and Li *et al.* [6] also obtained the same relationship with strong support (BP = 88) [6], but the phylogenetic position of Triuridaceae was not resolved with confidence in the current study. The nuclear phylogenomic analysis of Baker *et al.* [1] supported (LPP = 0.9) a sister relationship between Triuridaceae and [Stemonaceae [Pandanaceae + Cyclanthaceae]].

**Petrosaviales, Liliales, and Asparagales**—Petrosaviales nested within Asparagales as sister to Orchidaceae (BP = 90) in Li *et al.* [6] but were sister to all monocots except Acorales and Alismatales (BP = 97) in the current study (Additional file 2: Fig. S1), in agreement with the majority of previous studies [1, 8, 20, 21, 29]. The previously unusual placement of *Petrosavia* in Li *et al.* [6] has been resolved by the additional sampling of the non-mycoheterotrophic genus *Japonolirion*, which is the closest photosynthetic relative of *Petrosavia*.

Corsiaceae were not included in our previous analysis [6]; the current study placed Corsiaceae as sister to Campynemataceae, which accords with some recent plastid phylogenomic studies [20, 35, 38]. The position of Smilacaceae was not resolved in Givnish *et al.* [20], and this family was recovered as sister to [Philesiaceae + Ripogonaceae] in Baker *et al.* [1] (LPP = 0.94), but both the current analysis and our previous work [6] placed this family as sister to Liliaceae with strongly support (BP = 100).

The overall interfamilial relationships of Asparagales were well resolved (100 BP for each node; see Additional file 3: Fig. S2), except for the position of Doryanthaceae, which was resolved as sister to the clade of Iridaceae-Asparagaceae (BP = 85). Support for the position of Doryanthaceae, and the relationships of [Asteliaceae [Lanariaceae + Hypoxidaceae]] was weak to moderate (BP < 80) in Givnish *et al.* [20], and our results have substantially increased support for these relationships (85 BP for the former, and 100 BP for the latter). Baker *et al.* [1] obtained the same family-level topology of Asparagales with strong support (LPP = 1) except for the clade of Boryaceae-Hypoxidaceae (0.91), the placement of Blandfordiaceae sister to the clade of [Asteliaceae [Lanariaceae + Hypoxidaceae]] (0.14), and that of Doryanthaceae as sister to the clade of Iridaceae-Asparagaceae (0.72).

**Commelinids**—Consistent with the results of Baker *et al.* [1], Givnish *et al.* [20, 29], and Barrett *et al.* [22, 39], our results support the Dasypogonaceae + Arecaceae clade as sister to the remaining commelinids (BP = 87), which were further resolved with Poales as sister to a clade (BP = 100) of Commelinales and Zingiberales.

Relationships within Commelinales were well resolved and supported (100 BP for all nodes of interfamilial relationships), including the previously problematic placement of Philydraceae, which were here strongly supported (BP = 100) as sister to [Hanguanaceae + Commelinaceae]. Philydraceae were supported as sister to [Haemodoraceae + Pontederiaceae] with weak support (BP = 50–64) in Givnish *et al.* [20]. However, Baker *et al.* [1] found the relationships [*Pontederia cordata* [[Commelinaceae + Hanguanaceae] [Philydraceae [*P. diversifolia* + Haemodoraceae]]]] in Commelinales, and another species of *Pontederia* was sister to the remaining commelinids. The monophyly of *Pontederia* needs careful examination. Within Zingiberales, the Lowiaceae + Strelitziaceae clade and the clade comprising four families (the Cannaceae + Marantaceae clade as sister to the Costaceae + Zingiberaceae clade) were well supported (BP = 100). The close relationship of Heliconiaceae and the Lowiaceae + Strelitziaceae clade has been suggested by most studies, including recent nuclear phylogenomic analyses [1, 3, 20, 22, 39, 40], but our results weakly supported (BP = 61) Heliconiaceae as sister to the remaining Zingiberales.

In Poales, the position of Typhaceae and the relationships among the grade of xyrid families (Eriocaulaceae, Mayacaceae, Xyridaceae) have long been controversial (e.g., [21, 29]). Our results and those from some previous phylogenomic analyses [20, 41] supported the sister relationship of Eriocaulaceae and Xyridaceae, although with weak support (BP = 49) in our analyses. In agreement with the plastome-based studies of Givnish *et al.* [20] and Li *et al.* [6], the Ecdeiocoleaceae + Poaceae clade was strongly supported here (BP = 90), whereas the nuclear phylogenomic studies of McKain *et al.* [41] and Baker *et al.* [1] supported the Joinvilleaceae + Ecdeiocoleaceae clade as sister to Poaceae.

# Ceratophyllales

Ceratophyllales were early suggested to be sister to all other angiosperms in the benchmark study of Chase *et al.* [42], but now they are typically resolved as sister to the eudicots with both nuclear (e.g., [1,3–5]) and plastid data (e.g., [6]; and the current study: BP = 86). However, Baker *et al.* [1] found Ceratophyllales to be sister to the Chloranthales + magnoliids clade with strong support (LPP = 1).

# Eudicots

**Basal eudicots**—Ranunculales are sister to all other eudicots, followed successively by Proteales, Trochodendrales, and Buxales. Relationships in this sequence are generally well resolved with strong support (BP > 95), except for the relative position of Buxales and Trochodendrales. Our study found Buxales rather than Trochodendrales as sister to core eudicots with moderate support (BP = 71, see Additional file 3: Fig. S2). The phylogenetic positions of these two orders remained unresolved or weakly supported in Sun *et al.* [43], but they form a well supported clade and sister to the core eudicots in Baker *et al.* [1].

Within Ranunculales, Eupteleaceae and Papaveraceae were successive sisters to a well-supported clade of [[Circaeasteraceae + Lardizabalaceae] [Menispermaceae [Berberidaceae + Ranunculaceae]]], although the position of Papaveraceae received only moderate support (BP = 64). Eupteleaceae and Papaveraceae formed a weakly supported clade (BP = 34) in Li *et al.* [6]. However, Baker *et al.* [1] obtained the relationships of [Circaeasteraceae [Eupteleaceae [Papaveraceae [Lardizabalaceae [Menispermaceae [Berberidaceae + Ranunculaceae]]]]]] with strong support, except for the placement of Menispermaceae sister to the clade of Berberidaceae + Ranunculaceae (LPP = 0.84).

**Gunnerales, Dilleniales, Saxifragales, and** **Vitales**—Gunnerales were placed as sister to all other core eudicots in our results (BP = 96), in accordance with many other molecular studies [4,6-8], although the [Gunnerales + Dilleniales] clade was weakly supported in Zhu *et al.* [44] (BP = 59; the combined four-gene analysis), Baker *et al.* [1] (LPP = 0.21), and One Thousand Plant Transcriptomes Initiative [3] (LPP = 0.51; the ASTRAL analyses). In our tree, Dilleniales were sister to the [Saxifragales [Vitales + remaining rosids]] clade (BP = 96).

Interfamilial relationships within Saxifragales are still inconsistent across multiple studies (e.g. [8, 45–47]). Our results found strong support for Peridiscaceae as sister to the remaining Saxifragales (BP = 91), consistent with the result of Soltis *et al.* [8]. However, the results of Baker *et al.* [1] suggested that Cynomoriaceae are sister to all other Saxifragales with weak support (LPP = 0.35). Multiple nodes in our tree received relatively low support (e.g., the positions of Cercidiphyllaceae, Paeoniaceae, and Cynomoriaceae), except for the sister relationship between Daphniphyllaceae and Altingiaceae (BP = 80) and the interfamilial relationships in the clades of [Iteaceae [Grossulariaceae + Saxifragaceae]] (BP = 100) and [Crassulaceae [Aphanopetalaceae [Tetracarpaeaceae [Penthoraceae + Haloragaceae]]]] (BP > 95). The latter two clades with the same topology were also found in Baker *et al.* [1]. In the recent comprehensive study of Folk *et al.* [48] that used 301 low-copy nuclear loci and 627 species representing all families of Saxifragales, the positions of Peridiscaceae, Cercidiphyllaceae, and Cynomoriaceae were still unresolved, but other interfamilial relationships received strong support. Compared to the results of Folk *et al.* [48], our results received higher support for the position of Peridiscaceae, and the positions of Aphanopetalaceae and Tetracarpaeaceae were switched (although these positions exhibit strong support in both studies). Folk *et al.* [48] found strong support for a position of Paeoniaceae as sister to [Daphniphyllaceae [Cercidiphyllaceae [Altingiaceae + Hamamelidaceae]]], Baker *et al.* [1] also found a similar topology with different relative positions of Cercidiphyllaceae and Altingiaceae, whereas we found low to moderate support (BP = 58–80) for the different topology of [[Daphniphyllaceae + Altingiaceae] [Cercidiphyllaceae + Hamamelidaceae]] and low support for the position of Paeoniaceae.

Vitales, considered part of the rosid clade [19], have been placed in various positions in different molecular studies, either as sister to Saxifragales [10, 49], Santalales [3], Saxifragales plus rosids [1, 4, 50], or remaining rosids [8, 51]. The latter relationship was well supported by the results of Li *et al.* [6] and those of the current study.

## Rosids (excluding Vitales)

Two major clades, the fabids (BP = 100) and malvids (BP = 91), were well supported (Additional file 2: Fig. S1). Within fabids, Zygophyllales were sister to the remaining fabids (BP = 100), which were further resolved into the nitrogen-fixing clade (Cucurbitales, Fabales, Fagales, and Rosales) and the COM clade (Celastrales, Oxalidales, Huales, and Malpighiales). For the nitrogen-fixing clade, the topology of [Fabales [Rosales [Fagales + Cucurbitales]]] was found, with high support (BP = 100) at all nodes, in agreement with various multigene studies (e.g., [8, 51, 52]). For the COM clade, the relationships of [Oxalidales [Huales [Celastrales + Malpighiales]]] were found; however, the position of Huales received only 31 BP support. Within malvids, the Geraniales + Myrtales clade was highly supported as sister to the remainder of this clade (BP = 91), and then Crossosomatales, Picramniales, Sapindales, and Huerteales were strongly supported (BP = 91, 91, 91, 91, respectively) as successive sisters to the Malvales + Brassicales clade. These results were consistent with those of Wang *et al.* [51] and Soltis *et al.* [8].

Based on the recent analyses of nuclear genes used in the One Thousand Plant Transcriptomes Initiative [3], the Geraniales + Crossosomatales clade was sister to the rest of the rosids; then [[Fabales + Fagales] [Rosales + Cucurbitales]], the Zygophyllales + Myrtales clade, and the Celastrales + Malpighiales clade were successive sisters to the clade of five orders (Oxalidales, Huerteales, Sapindales, Brassicales, and Malvales), which have slightly different positions of Huerteales in results based on different analytical methods. In the results of Baker *et al.* [1], Geraniales and Malpighiales were not monophyletic, and most ordinal relationships with rosids were generally weakly supported, especially around the basal groups and the COM clade. The conflicting overall phylogenetic pattern of rosids between nuclear and plastid trees (e.g., the COM clade) is evident in the rosid clade. Of these, the unstable placement of COM may have been caused by ancient lineage sorting or hybridization [53].

**The COM clade**—Oxalidales were divided into two well-supported clades, i.e., the Oxalidaceae + Connaraceae clade (BP = 100) and the Brunelliaceae + Cephalotaceae + Cunoniaceae + Elaeocarpaceae clade (BP = 100). Li *et al.* [6] found the topology of [Cephalotaceae [Cunoniaceae + Elaeocarpaceae]] with high support (BP = 100), but our results resolved the clade into two weakly supported subclades of [Cephalotaceae + Brunelliaceae] (BP = 55) and [Cunoniaceae + Elaeocarpaceae] (BP = 33). However, Baker *et al.* [1] found the set of relationships of [[Oxalidaceae + Connaraceae] [Cephalotaceae [Cunoniaceae [Cephalotaceae [Brunelliaceae + Elaeocarpaceae]]]]] with strong support (LPP ≥ 0.98).

Interfamilial relationships within Malpighiales have been particularly problematic. Three major clades were recovered by Li *et al.* [6] and the current study (see Additional file 3: Fig. S2), i.e., the Humiriaceae to Lacistemataceae clade (BP = 76), the Ctenolophonaceae to Podostemaceae clade (BP = 65), and the Centroplacaceae to Trigoniaceae clade (BP = 81), all of which are consistent with the benchmark study of Xi *et al.* [54]. However, both our results and the PPA tree [6] could not resolve the polytomies and poorly supported nodes in Xi *et al.* [54] and yielded weak support for most nodes. Baker *et al.* [1] and Cai *et al.* [55] have greatly improved the resolution of interfamilial relationships of this order using large nuclear gene data sets.

**The nitrogen-fixing clade**—Relationships among four families of Fabales (Polygalaceae, Quillajaceae, Surianaceae, and Fabaceae) were poorly resolved and weakly supported in previous studies (e.g., [3, 6, 8, 45, 56, 57]). Baker *et al.* [1] found the relationships [Polygalaceae [Quillajaceae + Fabaceae] with good support (LPP ≥ 0.95), but the Surianaceae were not sampled in their study. Our results found two weakly or moderately supported family pairs [Polygalaceae + Quillajaceae] (BP = 59) and [Surianaceae + Fabaceae] (BP = 77).

Interfamilial relationships within Rosales were well resolved here with 100 BP at all nodes (see Additional files 3–4: Figs. S2 and S3). Rosaceae were sister to the remaining Rosales, which were further resolved into two well-supported clades, i.e., [Rhamnaceae [Elaeagnaceae [Dirachmaceae + Barbeyaceae]]] and [Ulmaceae [Cannabaceae [Urticaceae + Moraceae]]]. Zhang *et al.* [58] obtained the same topology, but with lower support for relationships within [Elaeagnaceae [Barbeyaceae + Dirachmaceae]], and Baker *et al.* [1] also found a similar topology but the position of Rhamnaceae was unresolved.

In Fagales, our results supported Nothofagaceae and Fagaceae as successive sisters to the rest of this order (BP = 100), which consists of two well-supported (BP = 100) clades, i.e., [Myricaceae + Juglandaceae] and [Casuarinaceae [Ticodendraceae + Betulaceae]]. These results agreed with Li *et al.* [56] and with nuclear analyses from the One Thousand Plant Transcriptomes Initiative [3] and Baker *et al.* [1] (Ticodendraceae were not sampled in the former two studies), but differed from the topology revealed by Sun *et al.* [52] for the position of Myricaceae, which was supported as sister to [Casuarinaceae [Betulaceae + Ticodendraceae]].

The interfamilial relationships within Cucurbitales have been poorly resolved in previous studies (e.g., [8, 52]). Two well-supported (BP = 100) clades of [Coriariaceae + Corynocarpaceae] and [Tetramelaceae [Datiscaceae + Begoniaceae]] and one moderately supported (BP = 76) clade of [Anisophyllaceae + Cucurbitaceae] were found in the current study, but relationships among the three clades remain unclear. Baker *et al*. [1] recovered a rather different topology [Corynocarpaceae [Anisophyllaceae [Coriariaceae [Cucurbitaceae [Datiscaceae [Tetramelaceae + Begoniaceae]]]]]] with strong support (LPP ≥ 0.98), except for the relationship between Cucurbitaceae and the clade of [Datiscaceae [Tetramelaceae + Begoniaceae] (0.54).

**Malvids**— The position of Combretaceae within Myrtales was unclear in previous studies (e.g., [52, 59]), but our results supported the sister relationship of Combretaceae and the Onagraceae + Lythraceae clade (BP = 98) (see Additional files 3–4: Figs. S2 and S3). The remaining Myrtales were resolved into two strongly supported (BP = 100) clades of [Vochysiaceae + Myrtaceae] and [Melastomataceae [Crypteroniaceae [Alzateaceae + Penaeaceae]]]; all interfamilial relationships of the latter clade received 100 BP (see Additional file 3: Fig. S2). However, Baker *et al.* [1] found strong support for the sister relationship between Combretaceae and other Myrtales (LPP = 1).

In Crossosomatales, two well-supported (BP = 100) clades were recovered: [Aphloiaceae [Strasburgeriaceae + Geissolomataceae]] and [Staphyleaceae [Stachyuraceae [Crossosomataceae + Guamatelaceae]]]. However, Baker *et al.* [3] found the relationships of [Guamatelaceae [Staphyleaceae [Crossosomataceae + Stachyuraceae]]] with good support (LPP ≥ 0.95). As compared with previous studies (e.g., [52, 60]), the current study improved support for the positions of Aphloiaceae and Geissolomataceae and recovered Guamatelaceae as sister to Crossosomataceae with strong support (BP = 100, see Additional files 3–4: Figs. S2 and S3).

In Huerteales, Sun *et al.* [52] recovered the relationships [[Gerrardinaceae + Petenaeaceae] [Tapisciaceae + Dipentodontaceae]], and the sister relationship between Gerrardinaceae and Petenaeaceae was not strongly supported (BP = 73). However, our study recovered the topology of [[Gerrardinaceae [Petenaeaceae [Tapisciaceae + Dipentodontaceae]]] with 100 BP support at all nodes. Baker *et al.* [1] obtained the same topology but the position of Petenaeaceae was not fully supported (LPP = 0.92).

Within Sapindales, our results weakly supported Nitrariaceae and Biebersteiniaceae as successive sisters to a clade comprising the remaining Sapindales. Nitrariaceae and Biebersteiniaceae have been supported at these positions, but their relative positions were controversial and weakly supported ([1, 45, 52, 61]). Some relationships among other families of this order were also weakly supported in previous studies, especially the position of Sapindaceae and the relationships among Meliaceae, Simaroubaceae, and Rutaceae [1, 8, 52, 61]. Aside from the uncertain positions of Nitrariaceae and Biebersteiniaceae, relationships within the remaining Sapindales were well resolved (BP > 90) in the current study. Sapindaceae were sister (BP = 92) to [Meliaceae [Simaroubaceae + Rutaceae]], and together they were well supported (BP = 97) as sister to the strongly supported (BP = 100) [Kirkiaceae [Burseraceae + Anacardiaceae]] clade (see Additional files 3–4: Figs. S2 and S3).

Previous studies placed Neuradaceae as sister to the rest of Malvales, and the remaining nine families were placed in six unresolved clades, i.e., Thymelaeaceae, Sphaerosepalaceae, Bixaceae, Malvaceae, the Cytinaceae + Muntingiaceae clade, and the CSD clade of [Cistaceae + [Sarcolaenaceae + Dipterocarpaceae]] (see Le Péchon and Gigord [62] for a summary). Baker *et al.* [1] recovered the rather different topology of [Thymelaeaceae [[Malvaceae + Muntingiaceae] [Sphaerosepalaceae [Bixaceae [Datiscaceae [Tetramelaceae + Begoniaceae]]]]]] with strong support (LPP ≥ 0.98), except for the sister relationship between Cucurbitaceae and the clade of [Datiscaceae [Tetramelaceae + Begoniaceae] (LPP = 0.54). Our analyses resolved the relationships of these six clades with moderate to strong support, i.e., Thymelaeaceae, Malvaceae, the Cytinaceae + Muntingiaceae clade, and the Sphaerosepalaceae + Bixaceae clade were moderately to strongly supported (BP = 88, 79, 86, 94, respectively) as successive sisters to a well-supported (BP = 100) clade of CSD (see Additional files 3–4: Figs. S2 and S3). Baker *et al.* [1] found Sphaerosepalaceae and Bixaceae as successive sisters to the CSD clade with strong support (LPP = 1) and also obtained a sister relationship of Malvaceae and Muntingiaceae (0.96) but the Cytinaceae were not included in their analysis.

Our analysis recovered a well-supported (BP = 100) Brassicales clade. Most clades within Brassicales are quite well supported in the current study, but the positions of Limnanthaceae and Koeberliniaceae remain unstable. Within this clade, [[Akaniaceae + Tropaeolaceae] [Caricaceae + Moringaceae]], Setchellanthaceae, Limnanthaceae, the Bataceae + Salvadoraceae clade, and Koeberliniaceae were successive sisters (BP = 100, 10, 100, 40, respectively) to the core Brassicales, among which all interfamilial relationships were well resolved with 100 BP support (see Additional file 3: Fig. S2). Our results are congruent with those from the phylotranscriptomic study of Edger *et al.* [63] and the plastid phylogenomic study of Edger *et al.* [61]. Baker *et al.* [1] also suggested similar relationships, but recovered a different placement of Tovariaceae.

**Santalales, Berberidopsidales, and Caryophyllales**—Our results placed Santalales, Berberidopsidales, and Caryophyllales as the successive sisters (BP = 99, 99, 98, respectively) to the strongly supported (BP = 99) clade of asterids. The same relationships were also recovered in the plastid phylogenomic study of Gitzendanner *et al.* [40]. However, Berberidopsidales were resolved as sister to core eudicots (excluding Gunneraceae), or asterids, and Santalales were sister to Vitales or rosids (including Vitales and Saxifragales) in different analyses of nuclear genes conducted by the One Thousand Plant Transcriptomes Initiative [3]; Caryophyllales and Santalales were successive sisters to rosids, and Berberidopsidales were placed as sister to asterids in Baker *et al.* [1].

Interfamilial relationships of Santalales have not been well resolved in previous studies (e.g., [65, 66]). Our study also could not fully resolve the relationships with strong support; however, the sister relationship between Strombosiaceae and Erythropalaceae, and all nodes of [[Opiliaceae + Santalaceae] [Loranthaceae [Misodendraceae + Schoepfiaceae]]] received 100 BP support (see Additional files 3–4: Figs. S2 and S3). The circumscription and placement of Balanophoraceae were particularly problematic (e.g., [65, 67, 68]); this family was also unresolved within the Santalales clade in our results (see Additional file 9: Figs. S8a–h).

Our estimate of the phylogeny of the Caryophyllales is largely congruent with the extensive plastid phylogenomic analysis of Yao *et al.* [69] except for some weakly supported nodes in both studies. Similar to the results of Yao *et al.* [69], the positions of Kewaceae, Microteaceae, Sarcobataceae, Stegnospermataceae, and the Halophytaceae + Basellaceae + Didiereaceae clade were not strongly supported (BP < 75). These families have been placed in a quite different position within Caryophyllales in the nuclear phylogenomic analysis of Baker *et al.* [1]. The recent phylotranscriptomic study of Yang *et al.* [70] with 26 Caryophyllales families also yielded a similar topology of our plastome analysis and recovered a strongly supported clade of [Sarcobataceae [Phytolaccaceae [Petiveriaceae + Nyctaginaceae]]]. Yang *et al.* [70] found a strongly supported topology of [Cactaceae [Portulacaceae + Anacampserotaceae]], whereas Yao *et al.* [69], Baker *et al.* [1], and our study yielded a strongly supported topology of [Portulacaceae [Cactaceae +Anacampserotaceae]].

## Asterids

Cornales and Ericales were recovered as successive sisters to all other asterids, and the latter were divided into a lamiid clade and a campanulid clade; all of these clades received strong support (BP > 98) in our study (see Additional file 2: Fig. S1 and Additional file 4: Fig. S3). These results are consistent with those from many previous molecular analyses (e.g., [8, 10, 45, 71]), but nuclear phylogenetic analyses have instead yielded a clade of [Cornales + Ericales] as sister to the remaining asterids (e.g., [3, 50, 72]). The recent analyses of Stull *et al.* [73] found conflict between genes from the plastid and nuclear genome and suggested that this conflict might result from ancient hybridization, allopolyploidy, or lineage sorting.

The relationships among the orders of campanulids were well resolved in our results (see Additional file 2: Figs. S1 and Additional file 4: Figs. S3). Aquifoliales, the Escalloniales + Asterales clade, Bruniales, and Apiales were successive sisters to the Paracryphiales + Dipsacales clade (BP = 100, 100, 88, 100, respectively), consistent with the study of Soltis *et al.* [8]. However, Aquifoliales have been placed as sister to either Garryales or core lamiids (Gentianales, Solanales, Vahliaceae, Boraginales, and Lamiales) in analyses of nuclear genes (e.g., [3, 50, 72]). Escalloniales have been recovered as sister to Asterales [8], the campanulids with Asterales and Aquifoliales being excluded [74], Dipsacales [3], or the clade of Dipsacales, Apiales, and Paracryphiales [1]. Our results found strong support (BP = 97) for the sister relationship between Escalloniales and Asterales.

The deep-level relationships of lamiids are among the most recalcitrant relationships within angiosperms; almost all nodes for the interordinal relationships received lower than 85 BP support in our results (see Additional file 2: Fig. S1). Stull *et al.* [71] resolved basal lamiid relationships with strong support using plastome data, but the current study yielded a topology of [[Icacinales [Oncothecaceae + Metteniusales]] [Garryales + core lamiids]] with negligible to weak support (BP = 18–65). Within core lamiids, a topology of [Gentianales [[Solanales + Vahliaceae] [Boraginales + Lamiales]]] was recovered in the multiple gene analysis of Refulio-Rodriguez and Olmstead [75], whereas the plastome-scale analysis of Stull *et al.* [71] recovered a topology of [[Boraginales + Gentianales] [Solanales [Lamiales + Vahliales]]]. The recent phylotranscriptomic study of the One Thousand Plant Transcriptomes Initiative [3] provided a topology of [Lamiales [Solanales [Boraginales + Gentianales]]] (Vahliales were not sampled). However, our data recovered a topology of [[Boraginales + Gentianales] [Lamiales [Vahliales + Solanales]]] with weak to moderate support (BP = 61–84).

**Cornales and Ericales**—Cornales were resolved into two well-supported (BS = 100) clades of [Cornaceae [Curtisiaceae + Grubbiaceae]] and [Nyssaceae [Hydrangeaceae [Hydrostachyaceae + Loasaceae]]], in agreement with the plastid phylogenomic study of Fu *et al.* [76] (Additional files 3–4: Figs. S2 and S3). However, Hydrostachyaceae were placed outside Cornales but sister to other asterids with strong support (LPP = 1) in Baker *et al.* [1].

Within Ericales, the well-supported balsaminoid clade of [Marcgraviaceae [Tetrameristaceae + Balsaminaceae]] was sister to the remaining Ericales (BP = 100). The latter was further divided into five unresolved clades: the Sladeniaceae + Pentaphylacaceae clade (BP = 100), the polemonioid clade (BP = 100) of [Fouquieriaceae + Polemoniaceae], the primuloid clade (BP = 96) of [Sapotaceae [Ebenaceae + Primulaceae]], Lecythidaceae, and the theoid clade (BP = 100), which comprises Theaceae, the styracoid clade (BP = 100) of [Symplocaceae [Diapensiaceae + Styracaceae]], the ericoid clade (BP = 100) of [Clethraceae [Cyrillaceae + Ericaceae]], and the sarracenioid clade (BP = 100) of [Sarraceniaceae [Actinidiaceae + Roridulaceae]]. Overall, our results are largely similar to those from the earlier studies of Schönenberger *et al.* [77] and Rose *et al.* [78], but provided higher support for most relationships. Relationships suggested by Baker *et al.* [1] are rather different, and interfamilial relationships with the earliest-diverging clade of [Balsaminaceae [Tetrameristaceae + Marcgraviaceae]] and the clade of [[[Symplocaceae [Diapensiaceae + Styracaceae]] [Sarraceniaceae [Actinidiaceae + Roridulaceae] [[Clethraceae [Cyrillaceae + Ericaceae]]]] were generally strongly supported (all nodes ≥ 0.98 LPP, except the sister relationship of Cyrillaceae + Ericaceae, which was 0.93).

**Campanulids**—Within Aquifoliales, the relationships of [[Cardiopteridaceae + Stemonuraceae] [Aquifoliaceae [Phyllonomaceae + Helwingiaceae]]] were quite well-supported in our analysis (BP = 100), congruent with studies of Tank and Donoghue [74] and Soltis *et al.* [8].

The phylogenetic analysis of Tank and Donoghue [74], based on ten plastid genes, resolved the interfamilial relationships of Asterales, and we obtained a largely congruent topology except for the position of Stylidiaceae (see Additional files 3–4: Figs. S2 and S3). Stylidiaceae were sister (BP = 98) to the clade of Alseuosmiaceae to Asteraceae; however, Tank and Donoghue [74] placed Stylidiaceae as the well-supported sister to the clade of [Menyanthaceae [Goodeniaceae [Calyceraceae + Asteraceae]]]. Based on the addition of mitochondrial genes, the study of Soltis *et al.* [8] recovered Pentaphragmataceae and the Roussaceae + Campanulaceae clade as successive sisters to the clade comprising the remaining Asterales, albeit with weak support. The nuclear phylogenomic analysis of Baker *et al.* [1] found Roussaceae and Campanulaceae as successive sisters to other Asterales with strong support (LPP ≥ 0.98).

Within Apiales, Pennantiaceae, Torricelliaceae, Griseliniaceae, and Pittosporaceae were successive sisters to the clade of [Araliaceae [Myodocarpaceae + Apiaceae]], and all nodes for interfamilial relationships received 100 BP support (see Additional file 4: Fig. S3). These results are consistent with other analyses that provided strong support for these relationships (e.g., [1, 3, 8, 74]).

**Lamiids**—Within Gentianales, Rubiaceae were strongly supported (BP = 100) as sister to the strongly supported (BP = 100) clade of [Gelsemiaceae [Loganiaceae [Gentianaceae + Apocynaceae]]], among which the interfamilial relationships were poorly resolved (BP = 16, 62) (see Additional file 3: Fig. S2). The same topology was revealed by Chen *et al.* [45]. Various relationships among these four families, including [[Apocynaceae + Gentianaceae] [Loganiaceae + Gelsemiaceae]] [75], [Gelsemiaceae [Loganiaceae [Gentianaceae + Apocynaceae]]] [8], [Gentianaceae [Gelsemiaceae [Loganiaceae + Apocynaceae]]] [79], [Gentianaceae [Apocynaceae [Gelsemiaceae + Loganiaceae]]] [80], and [[Gentianaceae + Gelsemiaceae] [Apocynaceae + Loganiaceae]] [1, 3], have been recovered in previous studies, but all clades received weak to moderate support except those from the nuclear analysis of Baker *et al.* [1].

Within Boraginales (= Boraginaceae sensu APG IV [16]), two well-supported clades (BP = 100) were resolved, i.e., [Codonaceae, [Wellstediaceae + Boraginaceae]] and [Hydrophyllaceae [Namaceae [Ehretiaceae [Cordiaceae + Heliotropiaceae]]]], with all interfamilial relationships receiving strong support (100 BP for all, except 91 BP for the clade of Cordiaceae and Ehretiaceae) (see Additional file 3: Fig. S2). These results are consistent with those of Refulio-Rodriguez and Olmstead [75] and Weigend *et al.* [81]. All four genera from the three small families Coldeniaceae, Hoplestigmataceae, and Lennoaceae recognized by the Boraginales Working Group [82] were also sampled in the current study. *Coldenia* and *Hoplestigma* were successive sisters to *Cordia* (BP = 100, 92, respectively). *Lennoa* and *Pholisma* (members of Lennoaceae of Boraginales Working Group [82]) formed a well-supported clade, and they were sister to the weakly support clade (BP = 61) of the remaining Ehretiaceae.

Within Solanales, the strongly supported (BP = 100) clade of [Convolvulaceae + Solanaceae] was sister to the clade (BP = 85) of [Montiniaceae [Sphenocleaceae + Hydroleaceae]]. Soltis *et al.* [8] and Refulio-Rodriguez and Olmstead [75] also recovered the same relationships with strong support. However, Baker *et al.* [1] found the relationships [Hydroleaceae [Garryales [Icacinales [Montiniaceae [Vahliales [Lamiales [Gentianales [Solanales + Boraginales]]]]]]]]; Montiniaceae and Hydroleaceae were separated from other Solanales (Sphenocleaceae were not included).

Within Lamiales, Plocospermataceae, [Carlemanniaceae + Oleaceae], and Tetrachondraceae were successive sisters to core lamiales (as defined by Schaferhoff *et al.* [83]), with [Peltantheraceae [Calceolariaceae + Gesneriaceae]], Plantaginaceae, Scrophulariaceae, [Byblidaceae + Linderniaceae], Stilbaceae as successive sisters to the strongly supported (BP = 100) “higher core Lamiales” (HCL, defined by Schaferhoff *et al.* [83]) (Additional files 3–4: Figs. S2 and S3). The sister relationship between Carlemanniaceae and Oleaceae and the interfamilial relationships of [Peltantheraceae [Calceolariaceae + Gesneriaceae]] also received high support. Byblidaceae and Linderniaceae formed a weakly supported clade (BP = 56) that was sister to the moderately supported clade (BP = 76) of [Stilbaceae + HCL] (BP = 100). Lamiaceae and Mazaceae were successive sisters to the clade of [[Wightiaceae + Phrymaceae] [Paulowniaceae + Orobanchaceae]], and these relationships received strong support (BP > 85). However, the interfamilial relationships of the strongly supported (BP = 100) clade of [Schlegeliaceae + Martyniaceae + Lentibulariaceae + Thomandersiaceae + Pedaliaceae + Acanthaceae + Verbenaceae + Bignoniaceae] were poorly resolved and weakly supported except for the strongly supported sister relationship between Verbenaceae and Bignoniaceae (BP = 98). Previous studies also failed to resolve these relationships [1, 75, 80, 83].

# References

1. Baker WJ, Bailey P, Barber V, Barker A, Bellot S, Bishop D, Botigue LR, Brewer G, Carruthers T, Clarkson JJ et al. A comprehensive phylogenomic platform for exploring the angiosperm tree of life. Syst Biol. 2021; doi:10.1093/sysbio/syab035.

2. Xi Z, Liu L, Rest JS, Davis CC. Coalescent versus concatenation methods and the placement of *Amborella* as sister to water lilies. Syst Biol. 2014;63:919–932. doi:10.1093/sysbio/syu055.

3. One Thousand Plant Transcriptomes Initiative. One thousand plant transcriptomes and the phylogenomics of green plants. Nature. 2019;574:679–685. doi:10.1038/s41586-019-1693-2.

4. Yang LX, Su DY, Chang X, Foster CSP, Sun L, Huang C-H, et al. Phylogenomic insights into deep phylogeny of angiosperms based on broad nuclear gene sampling. Plant Commun. 2020;1:100027. doi:10.1016/j.xplc.2020.100027.

5. Yang YZ, Sun PC, Lv LK, Wang D, Ru D, Li Y, et al. Prickly waterlily and rigid hornwort genomes shed light on early angiosperm evolution. Nat Plants. 2020;6:215–222. doi:10.1038/s41477-020-0594-6.

6. Li HT, Yi TS, Gao LM, Ma PF, Zhang T, Yang JB, et al. Origin of angiosperms and the puzzle of the Jurassic gap. Nat Plants. 2019;5:461–470. doi:10.1038/s41477-019-0421-0.

7. Magallón S, Gómez‐Acevedo S, Sánchez‐Reyes LL, Hernández‐Hernández T. A metacalibrated time‐tree documents the early rise of flowering plant phylogenetic diversity. New Phytol. 2015;207:437–453. doi:10.1111/nph.13264.

8. Soltis DE, Smith SA, Cellinese N, Wurdack KJ, Tank DC, Brockington SF, et al. Angiosperm phylogeny: 17 genes, 640 taxa. Am J Bot. 2011;98:704–730. doi:10.3732/ajb.1000404.

9. Gruenstaeudl M. Why the monophyly of Nymphaeaceae currently remains indeterminate: an assessment based on gene-wise plastid phylogenomics. Plant Syst Evol. 2019;305:827–836. doi:10.1007/s00606-019-01610-5.

10. Gitzendanner MA, Soltis PS, Wong GK, Ruhfel BR, Soltis DE. Plastid phylogenomic analysis of green plants: a billion years of evolutionary history. Am J Bot. 2018;105:291–301. doi:10.1002/ajb2.1048.

11. Jansen RK, Cai Z, Raubeson LA, Daniell H, Leebens-Mack J, Müller KF, et al. Analysis of 81 genes from 64 plastid genomes resolves relationships in angiosperms and identifies genome-scale evolutionary patterns. Proc Natl Acad Sci USA. 2007;104:19369–19374. doi:10.1073/pnas.0709121104.

12. Moore MJ, Bell CD, Soltis PS, Soltis DE. Using plastid genome-scale data to resolve enigmatic relationships among basal angiosperms. Proc Natl Acad Sci USA. 2007;104:19363–19368. doi:10.1073/pnas.0708072104.

13. Qiu YL, Li LB, Wang B, Xue J-Y, Hendry TA, Li R-Q, et al. Angiosperm phylogeny inferred from sequences of four mitochondrial genes. J Syst Evol. 2010;48:391–425. doi:10.1111/j.1759-6831.2010.00097.x.

14. Massoni J, Forest F, Sauquet H. Increased sampling of both genes and taxa improves resolution of phylogenetic relationships within Magnoliidae, a large and early-diverging clade of angiosperms. Mol Phylogenet Evol. 2014;70:84–93. doi:10.1016/j.ympev.2013.09.010.

15. Qiu YL, Li LB, Hendry TA, Li R, Taylor DW, Issa MJ, et al. Reconstructing the basal angiosperm phylogeny: evaluating information content of mitochondrial genes. Taxon. 2006, 55:837–856. doi:10.2307/25065680.

16. APG IV. An update of the Angiosperm Phylogeny Group classification for the orders and families of flowering plants APG IV. Bot J Linn Soc. 2016;181:1–20. doi:10.1111/boj.12385.

17. Naumann J, Salomo K, Der JP, Wafula EK, Bolin JF, Maass E, et al. Single-copy nuclear genes place haustorial Hydnoraceae within Piperales and reveal a Cretaceous origin of multiple parasitic angiosperm lineages. PLoS One 2013, 8:e79204. doi:10.1371/journal.pone.0079204.

18. APG III. An update of the Angiosperm Phylogeny Group classification for the orders and families of flowering plants APG III. Bot J Linn Soc. 2009;161:105–121. doi:10.1111/j.1095-8339.2009.00996.x.

19. Chase MW. Monocot relationships: an overview. Am J Bot. 2004;91:1645–1655. doi:10.3732/ajb.91.10.1645.

20. Givnish TJ, Zuluaga A, Spalink D, Soto Gomez M, Lam VKY, Saarela JM, et al. Monocot plastid phylogenomics, timeline, net rates of species diversification, the power of multi-gene analyses, and a functional model for the origin of monocots. Am J Bot. 2018;105:1–23. doi:10.1002/ajb2.1178.

21. Chase MW, Fay MF, Devey DS, Maurin O, RØnsted N, Davies TJ, et al. Multigene analyses of monotoc relationships: a summary. Aliso. 2006;22:63–75.

22. Barrett CF, Specht CD, Leebens-Mack J, Stevenson DW, Zomlefer WB, Davis JI. Resolving ancient radiations: can complete plastid gene sets elucidate deep relationships among the tropical gingers (Zingiberales)? Ann Bot. 2014;113:119–133. doi:10.1093/aob/mct264.

23. Givnish TJ, Pires JC, Graham SW, McPherson MA, Prince LM, Patterson TB, et al. Phylogenetic relationships of monocots based on the highly informative plastid gene *ndh*F: evidence for widespread concerted convergence. Aliso. 2006;22:28–51. doi: 10.5642/aliso.20062201.04.

24. Graham SW, Zgurski JM, McPherson MA, Cherniawsky DM, Saarela JM, Horne EF, et al. Robust inference of monocot deep phylogeny using an expanded multigene plastid data set. Aliso. 2006;22:3–21. doi: 10.5642/aliso.20062201.02.

25. Petersen G, Seberg O, Davis JI, Goldman DH, Stevenson DW, Campbell LM, et al. Mitochondrial data in monocot phylogenetics. Aliso. 2006;22:52–62. doi: 10.5642/aliso.20062201.05.

26. Tamura MN, Yamashita J, Fuse S, Haraguchi M. Molecular phylogeny of monocotyledons inferred from combined analysis of plastid *mat*K and *rbc*L gene sequences. J Plant Res. 2004;117:109–120. doi:10.1007/s10265-003-0133-3.

27. Davis JI, Stevenson DW, Petersen G, Seberg O, Campbell LM, Freudenstein JV, et al. A phylogeny of the monocots, as inferred from *rbc*L and *atp*A sequence variation, and a comparison of methods for calculating jackknife and bootstrap values. Syst Bot. 2004;29:467–510. doi:Doi 10.1600/0363644041744365.

28. Ross TG, Barrett CF, Soto Gomez M, Lam VK, Henriquez CL, Les DH, et al. Plastid phylogenomics and molecular evolution of Alismatales. Cladistics. 2016;32:160–178. doi:10.1111/cla.12133.

29. Givnish TJ, Ames M, McNeal JR, McKain MR, Steele PR, Depamphilis CW, et al. Assembling the tree of the monocotyledons: plastome sequence phylogeny and evolution of Poales. Ann Mo Bot Gard. 2010;97:584–617. doi:10.3417/2010023.

30. Petersen G, Seberg O, Cuenca A, Stevenson DW, Thadeo M, Davis JI, et al. Phylogeny of the Alismatales (Monocotyledons) and the relationship of *Acorus* (Acorales?). Cladistics. 2016;32:141–159. doi:10.1111/cla.12120.

31. Thadeo M, Hampilos KE, Stevenson DW. Anatomy of fleshy fruits in the monocots. Am J Bot. 2015;102:1757–1779. doi:10.3732/ajb.1500204.

32. Merckx V, Bidartondo MI. Breakdown and delayed cospeciation in the arbuscular mycorrhizal mutualism. P Roy Soc B-Biol Sci. 2008;275:1029–1035. doi:10.1098/rspb.2007.1622.

33. Merckx V, Bakker FT, Huysmans S, Smets E. Bias and conﬂict in phylogenetic inference of myco-heterotrophic plants: a case study in Thismiaceae. Cladistics 2009;22:64–77. doi:10.1111/j.1096-0031.2008.00241.x.

34. Merckx V, Smets EF. *Thismia americana*, the 101st anniversary of a botanical mystery. Int J Plant Sci. 2014;175:165–175. doi:10.1086/674315.

35. Lam VKY, Darby H, Merckx V, Lim G, Yukawa T, Neubig KM, et al. Phylogenomic inference in extremis: a case study with mycoheterotroph plastomes. Am J Bot. 2018;105:480–494. doi:10.1002/ajb2.1070.

36. Lam VKY, Soto Gomez M, Graham SW. The highly reduced plastome of mycoheterotrophic *Sciaphila* (Triuridaceae) is colinear with its green relatives and is under strong purifying selection. Genome Biol Evol. 2015;7:2220–2236. doi:10.1093/gbe/evv134.

37. Soto Gomez M, Lin Q, da Silva Leal E, Gallaher TJ, Scherberich D, Mennes CB, et al. A bi‐organellar phylogenomic study of Pandanales: inference of higher‐order relationships and unusual rate‐variation patterns. 2020; 36:481–504. doi:10.1111/cla.12417.

38. Mennes CB, Lam VKY, Rudall PJ, Lyon SP, Graham SW, Smets EF, et al. Ancient Gondwana break-up explains the distribution of the mycoheterotrophic family Corsiaceae (Liliales). J Biogeogr. 2015;42:1123–1136. doi:10.1111/jbi.12486.

39. Barrett CF, Baker WJ, Comer JR, Conran JG, Lahmeyer SC, Leebens-Mack JH, et al. Plastid genomes reveal support for deep phylogenetic relationships and extensive rate variation among palms and other commelinid monocots. New Phytol. 2016;209:855–870. doi:10.1111/nph.13617.

40. Sass C, Iles WJ, Barrett CF, Smith SY, Specht CD. Revisiting the Zingiberales: using multiplexed exon capture to resolve ancient and recent phylogenetic splits in a charismatic plant lineage. PeerJ. 2016;4:e1584. doi:10.7717/peerj.1584.

41. McKain MR, Tang H, McNeal JR, Ayyampalayam S, Davis JI, dePamphilis CW, et al. A phylogenomic assessment of ancient polyploidy and genome evolution across the Poales. Genome Biol Evol. 2016;8:1150–1164. doi:10.1093/gbe/evw060.

42. Chase MW, Soltis DE, Olmstead RG, Morgan D, Les DH, Mishler BD, et al. Phylogenetics of seed plants: an analysis of nucleotide sequences from the plastid gene *rbc*L. Ann Mo Bot Gard. 1993;80:528–580. doi:10.2307/2399846.

43. Sun YX, Moore MJ, Zhang SJ, Soltis PS, Soltis DE, Zhao T, et al. Phylogenomic and structural analyses of 18 complete plastomes across all families of early-diverging eudicots, including an angiosperm-wide analysis of IR gene content evolution. Mol Phylogenet Evol. 2016;96:93–101. doi:10.1016/j.ympev.2015.12.006.

44. Zhu XY, Chase MW, Qiu YL, Kong HZ, Dilcher DL, Li JH, et al. Mitochondrial *mat*R sequences help to resolve deep phylogenetic relationships in rosids. BMC Evol Biol. 2007;7:217. doi:10.1186/1471-2148-7-217.

45. Chen ZD, Yang T, Lin L, Lu L-M, Li H-L, Sun M, et al. Tree of life for the genera of Chinese vascular plants. J Syst Evol. 2016;54:273–276. doi:10.1111/jse.12219.

46. Dong WP, Xu C, Wu P, Cheng T, Yu J, Zhou S, et al. Resolving the systematic positions of enigmatic taxa: manipulating the chloroplast genome data of Saxifragales. Mol Phylogenet Evol. 2018;126:321–330. doi:10.1016/j.ympev.2018.04.033.

47. Soltis DE, Mort ME, Latvis M, Mavrodiev EV, O'Meara BC, Soltis PS, et al. Phylogenetic relationships and character evolution analysis of Saxifragales using a supermatrix approach. Am J Bot. 2013;100:916–929. doi:10.3732/ajb.1300044.

48. Folk RA, Stubbs RL, Mort ME, Cellinese N, Allen JM, Soltis PS, et al. Rates of niche and phenotype evolution lag behind diversification in a temperate radiation. Proc Natl Acad Sci USA. 2019;116:10874–10882. doi:10.1073/pnas.1817999116.

49. Moore MJ, Soltis PS, Bell CD, Burleigh JG, Soltis DE. Phylogenetic analysis of 83 plastid genes further resolves the early diversification of eudicots. Proc Natl Acad Sci USA. 2010;107:4623–4628. doi:10.1073/pnas.0907801107.

50. Zeng L, Zhang N, Zhang Q, Endress PK, Huang J, Ma H. Resolution of deep eudicot phylogeny and their temporal diversification using nuclear genes from transcriptomic and genomic datasets. New Phytol. 2017;214:1338–1354. doi:10.1111/nph.14503.

51. Wang H, Moore MJ, Soltis PS, Bell CD, Brockington SF, Alexandre R, et al. Rosid radiation and the rapid rise of angiosperm-dominated forests. Proc Natl Acad Sci USA. 2009;106:3853–3858. doi:10.1073/pnas.0813376106.

52. Sun M, Naeem RH, Su JX, Cao ZY, Burleigh JG, Soltis PS, et al. Phylogeny of the Rosidae: a dense taxon sampling analysis. J Syst Evol. 2016;54:363–391. doi:10.1111/jse.12211.

53. Sun M, Soltis DE, Soltis PS, Zhu X, Burleigh JG, Chen Z. Deep phylogenetic incongruence in the angiosperm clade Rosidae. Mol Phylogenet Evol. 2015;83:156–166. doi:10.1016/j.ympev.2014.11.003.

54. Xi Z, Ruhfel BR, Schaefer H, Amorim AM, Sugumaran M, Wurdack KJ, et al. Phylogenomics and a posteriori data partitioning resolve the Cretaceous angiosperm radiation Malpighiales. Proc Natl Acad Sci USA. 2012;109:17519–17524. doi:10.1073/pnas.1205818109.

55. Cai L, Xi Z, Lemmon EM, Lemmon AR, Mast A, Buddenhagen CE, et al. The perfect storm: Gene tree estimation error, incomplete lineage sorting, and ancient gene flow explain the most recalcitrant ancient angiosperm clade, Malpighiales. 2021, 70:491–507. doi:10.1093/sysbio/syaa083.

56. Li HL, Wang W, Mortimer PE, Li RQ, Li DZ, Hyde KD, et al. Large-scale phylogenetic analyses reveal multiple gains of actinorhizal nitrogen-fixing symbioses in angiosperms associated with climate change. Sci Rep. 2015;5:14023. doi:10.1038/srep14023.

57. Li HL, Wang W, Li RQ, Zhang JB, Sun M, Naeem R, et al. Global versus Chinese perspectives on the phylogeny of the N-fixing clade. J Syst Evol. 2016;54:392–399. doi:10.1111/jse.12201.

58. Zhang SD, Soltis DE, Yang Y, Li DZ, Yi TS. Multi-gene analysis provides a well-supported phylogeny of Rosales. Mol Phylogenet Evol. 2011;60:21–28. doi:10.1016/j.ympev.2011.04.008.

59. Kriebel R, Khabbazian M, Sytsma KJ. A continuous morphological approach to study the evolution of pollen in a phylogenetic context: an example with the order Myrtales. PLoS One. 2017;12:e0187228. doi:10.1371/journal.pone.0187228.

60. Oh SH. Phylogeny and systematics of Crossosomatales as inferred from chloroplast *atp*B, *mat*K, and *rbc*L sequences. Korean J Pl Taxon. 2010;40:208–217.

61. Muellner-Riehl AN, Weeks A, Clayton JW, Buerki S, Nauheimer L, Chiang Y-C, et al. Molecular phylogenetics and molecular clock dating of Sapindales based on plastid *rbc*L, *atp*B and *trn*L-*trn*F DNA sequences. Taxon. 2016;65:1019–1036. doi:10.12705/655.5.

62. Le Péchon T, Gigord LD. On the relevance of molecular tools for taxonomic revision in Malvales, Malvaceae *s.l.*, and Dombeyoideae. In: Walker JM, editor. Methods in Molecular Biology*.* vol. 1115, 2014/01/15 edn; 2014. p. 337–363. doi:10.1007/978-1-62703-767-9_17.

63. Edger PP, Heidel-Fischer HM, Bekaert M, Jadranka Rota GG, Platts AE, Heckel DG, et al. The butterfly plant arms-race escalated by genome duplications. Proc Natl Acad Sci USA. 2015;112:8362–8366. doi:10.1073/pnas.1503926112.

64. Edger PP, Hall JC, Harkess A, Tang M, Coombs J, Mohammadin S, et al. Brassicales phylogeny inferred from 72 plastid genes: a reanalysis of the phylogenetic localization of two paleopolyploid events and origin of novel chemical defenses. Am J Bot. 2018;105:463–469. doi:10.1002/ajb2.1040.

65. Su HJ, Hu JM, Anderson FE, Der JP, Nickrent DL. Phylogenetic relationships of Santalales with insights into the origins of holoparasitic Balanophoraceae. Taxon. 2015;64:491–506. doi:10.12705/643.2.

66. Nickrent DL, Anderson F, Kuijt J. Inflorescence evolution in Santalales: integrating morphological characters and molecular phylogenetics. Am J Bot. 2019;106:402–414. doi:10.1002/ajb2.1250.

67. Su HJ, Hu JM. Rate heterogeneity in six protein-coding genes from the holoparasite *Balanophora* (Balanophoraceae) and other taxa of Santalales. Ann Bot. 2012;110:1137–1147. doi:10.1093/aob/mcs197.

68. Chen X, Fang D, Wu C, Liu B, Liu Y, Sahu SK, et al. Comparative plastome analysis of root- and stem-feeding parasites of Santalales untangle the footprints of feeding mode and lifestyle transitions. Genome Biol Evol. 2020;12:3663–3676. doi:10.1093/gbe/evz271.

69. Yao G, Jin JJ, Li HT, Yang JB, Mandala VS, Croley M, et al. Plastid phylogenomic insights into the evolution of Caryophyllales. Mol Phylogenet Evol. 2019;134:74–86. doi:10.1016/j.ympev.2018.12.023.

70. Yang Y, Moore MJ, Brockington SF, Mikenas J, Olivieri J, Walker JF, et al. Improved transcriptome sampling pinpoints 26 ancient and more recent polyploidy events in Caryophyllales, including two allopolyploidy events. New Phytol. 2018;217:855–870. doi:10.1111/nph.14812.

71. Stull GW, Duno de Stefano R, Soltis DE, Soltis PS. Resolving basal lamiid phylogeny and the circumscription of Icacinaceae with a plastome-scale data set. Am J Bot. 2015;102:1794–1813. doi:10.3732/ajb.1500298.

72. Zhang N, Zeng LP, Shan HY, Ma H. Highly conserved low‐copy nuclear genes as effective markers for phylogenetic analyses in angiosperms. New Phytol. 2012;195:923–937. doi:10.1111/j.1469-8137.2012.04212.x.

73. Stull GW, Soltis PS, Soltis DE, Gitzendanner MA, Smith SA. Nuclear phylogenomic analyses of asterids conflict with plastome trees and support novel relationships among major lineages. Am J Bot. 2020;107:790–805. doi:10.1002/ajb2.1468.

74. Tank DC, Donoghue MJ. Phylogeny and phylogenetic nomenclature of the Campanulidae based on an expanded sample of genes and taxa. Syst Bot. 2010;35:425–441. doi:10.1600/036364410791638306.

75. Refulio-Rodriguez NF, Olmstead RG. Phylogeny of Lamiidae. Am J Bot. 2014;101:287–299. doi:10.3732/ajb.1300394.

76. Fu CN, Mo ZQ, Yang JB, Ge XJ, Li DZ, Xiang QJ, et al. Plastid phylogenomics and biogeographic analysis support a trans-Tethyan origin and rapid early radiation of Cornales in the Mid-Cretaceous. Mol Phylogenet Evol. 2019;140:106601. doi:10.1016/j.ympev.2019.106601.

77. Schönenberger J, Anderberg AA, Sytsma KJ. Molecular phylogenetics and patterns of floral evolution in the Ericales. Int J Plant Sci. 2005;166:265–288. doi: 10.1086/427198.

78. Rose JP, Kleist TJ, Lofstrand SD, Drew BT, Schoenenberger J, Sytsma KJ. Phylogeny, historical biogeography, and diversification of angiosperm order Ericales suggest ancient Neotropical and East Asian connections. Mol Phylogenet Evol. 2018;122:59–79. doi:10.1016/j.ympev.2018.01.014.

79. Yang LL, Li HL, Wei L, Yang T, Kuang DY, Li MH, et al. A supermatrix approach provides a comprehensive genus-level phylogeny for Gentianales. J Syst Evol. 2016;54:400–415. doi:10.1111/jse.12192.

80. Luna JA, Richardson JE, Nishii K, Clark JL, Möller M. The family placement of *Cyrtandromoea*. Syst Bot. 2019;44:616–630. doi:10.1600/036364419x15620113920653.

81. Weigend M, Luebert F, Gottschling M, Couvreur TL, Hilger HH, Miller JS. From capsules to nutlets: phylogenetic relationships in the Boraginales. Cladistics. 2014;30:508–518. doi:10.1111/cla.12061.

82. Boraginales Working Group. Familial classification of the Boraginales. Taxan. 2016;65:502–522. doi:10.12705/653.5.

83. Schäferhoff B, Fleischmann A, Fischer E, Albach DC, Borsch T, Heubl G, et al. Towards resolving Lamiales relationships: insights from rapidly evolving chloroplast sequences. BMC Evol Biol. 2010;10:352. doi:10.1186/1471-2148-10-352.
