# Supplementary material for: Plastid phylogenomic insights into relationships of all flowering plant families
Source: BMC Biol. 2021 Oct 29;19:232. doi: 10.1186/s12915-021-01166-2 (PMC8555322; doi:10.1186/s12915-021-01166-2)
Supplement: Supplementary file 9 — Additional file 9: Figure S8. Phylogenetic tree of 4792 plastomes with successive removal of the long branch forming a sister relationship with a ‘clade’ of Mitrastemonaceae, Thismiaceae, Apodanthaceae, and Balanophoraceae. [file 12915_2021_1166_MOESM9_ESM.pdf]

[Figure S8a]

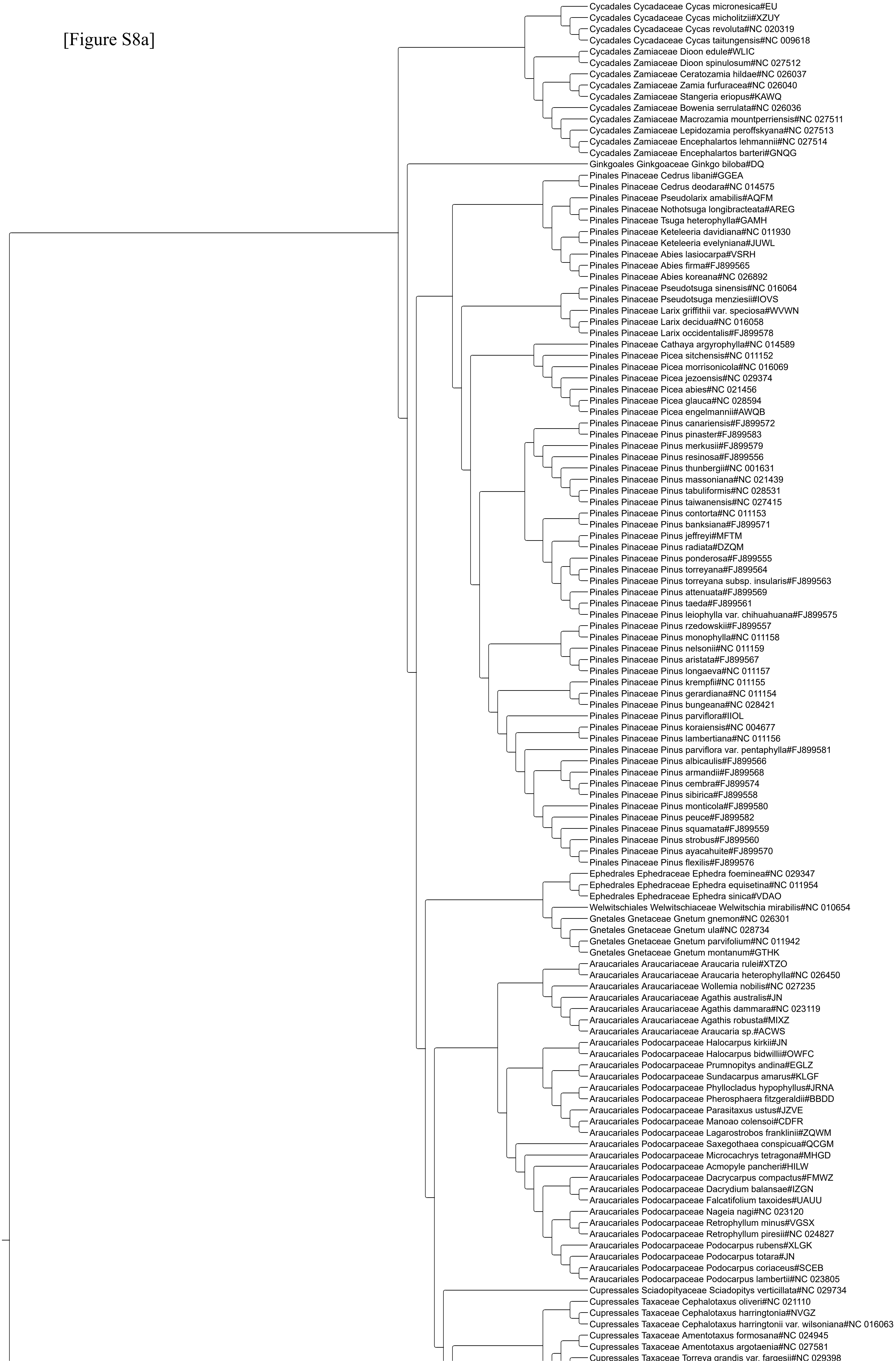

Cycadales Cycadaceae Cycas micronesica#EU
Cycadales Cycadaceae Cycas micholitzii#XZUY
Cycadales Cycadaceae Cycas revoluta#NC 020319
Cycadales Cycadaceae Cycas taitungensis#NC 009618
Cycadales Zamiaceae Dioon edule#WLIC
Cycadales Zamiaceae Dioon spinulosum#NC 027512
Cycadales Zamiaceae Ceratozamia hildae#NC 026037
Cycadales Zamiaceae Zamia furfuracea#NC 026040
Cycadales Zamiaceae Stangeria eriopus#KAWQ
Cycadales Zamiaceae Bowenia serrulata#NC 026036
Cycadales Zamiaceae Macrozamia mountperriensis#NC 027511
Cycadales Zamiaceae Lepidozamia peroffskyana#NC 027513
Cycadales Zamiaceae Encephalartos lehmannii#NC 027514
Cycadales Zamiaceae Encephalartos barteri#GNQG
Ginkgoales Ginkgoaceae Ginkgo biloba#DQ
Pinales Pinaceae Cedrus libani#GGEA
Pinales Pinaceae Cedrus deodara#NC 014575
Pinales Pinaceae Pseudolarix amabilis#AQFM
Pinales Pinaceae Nothotsuga longibracteata#AREG
Pinales Pinaceae Tsuga heterophylla#GAMH
Pinales Pinaceae Keteleeria davidiana#NC 011930
Pinales Pinaceae Keteleeria evelyniana#JUWL
Pinales Pinaceae Abies lasiocarpa#VSRH
Pinales Pinaceae Abies firma#FJ899565
Pinales Pinaceae Abies koreana#NC 026892
Pinales Pinaceae Pseudotsuga sinensis#NC 016064
Pinales Pinaceae Pseudotsuga menziesii#IOVS
Pinales Pinaceae Larix griffithii var. speciosa#WVWN
Pinales Pinaceae Larix decidua#NC 016058
Pinales Pinaceae Larix occidentalis#FJ899578
Pinales Pinaceae Cathaya argyrophylla#NC 014589
Pinales Pinaceae Picea sitchensis#NC 011152
Pinales Pinaceae Picea morrisonicola#NC 016069
Pinales Pinaceae Picea jezoensis#NC 029374
Pinales Pinaceae Picea abies#NC 021456
Pinales Pinaceae Picea glauca#NC 028594
Pinales Pinaceae Picea engelmannii#AWQB
Pinales Pinaceae Pinus canariensis#FJ899572
Pinales Pinaceae Pinus pinaster#FJ899583
Pinales Pinaceae Pinus merkusii#FJ899579
Pinales Pinaceae Pinus resinosa#FJ899556
Pinales Pinaceae Pinus thunbergii#NC 001631
Pinales Pinaceae Pinus massoniana#NC 021439
Pinales Pinaceae Pinus tabuliformis#NC 028531
Pinales Pinaceae Pinus taiwanensis#NC 027415
Pinales Pinaceae Pinus contorta#NC 011153
Pinales Pinaceae Pinus banksiana#FJ899571
Pinales Pinaceae Pinus jeffreyi#MFTM
Pinales Pinaceae Pinus radiata#DZQM
Pinales Pinaceae Pinus ponderosa#FJ899555
Pinales Pinaceae Pinus torreyana#FJ899564
Pinales Pinaceae Pinus torreyana subsp. insularis#FJ899563
Pinales Pinaceae Pinus attenuata#FJ899569
Pinales Pinaceae Pinus taeda#FJ899561
Pinales Pinaceae Pinus leiophylla var. chihuahuana#FJ899575
Pinales Pinaceae Pinus rzedowskii#FJ899557
Pinales Pinaceae Pinus monophylla#NC 011158
Pinales Pinaceae Pinus nelsonii#NC 011159
Pinales Pinaceae Pinus aristata#FJ899567
Pinales Pinaceae Pinus longaeva#NC 011157
Pinales Pinaceae Pinus krempfii#NC 011155
Pinales Pinaceae Pinus gerardiana#NC 011154
Pinales Pinaceae Pinus bungeana#NC 028421
Pinales Pinaceae Pinus parviflora#IIOL
Pinales Pinaceae Pinus koraiensis#NC 004677
Pinales Pinaceae Pinus lambertiana#NC 011156
Pinales Pinaceae Pinus parviflora var. pentaphylla#FJ899581
Pinales Pinaceae Pinus albicaulis#FJ899566
Pinales Pinaceae Pinus armandii#FJ899568
Pinales Pinaceae Pinus cembra#FJ899574
Pinales Pinaceae Pinus sibirica#FJ899558
Pinales Pinaceae Pinus monticola#FJ899580
Pinales Pinaceae Pinus peuce#FJ899582
Pinales Pinaceae Pinus squamata#FJ899559
Pinales Pinaceae Pinus strobus#FJ899560
Pinales Pinaceae Pinus ayacahuite#FJ899570
Pinales Pinaceae Pinus flexilis#FJ899576
Ephedrales Ephedraceae Ephedra foeminea#NC 029347
Ephedrales Ephedraceae Ephedra equisetina#NC 011954
Ephedrales Ephedraceae Ephedra sinica#VDAO
Welwitschiales Welwitschiaceae Welwitschia mirabilis#NC 010654
Gnetales Gnetaceae Gnetum gnemon#NC 026301
Gnetales Gnetaceae Gnetum ula#NC 028734
Gnetales Gnetaceae Gnetum parvifolium#NC 011942
Gnetales Gnetaceae Gnetum montanum#GTHK
Araucariales Araucariaceae Araucaria rulei#XTZO
Araucariales Araucariaceae Araucaria heterophylla#NC 026450
Araucariales Araucariaceae Wollemia nobilis#NC 027235
Araucariales Araucariaceae Agathis australis#JN
Araucariales Araucariaceae Agathis dammara#NC 023119
Araucariales Araucariaceae Agathis robusta#MIXZ
Araucariales Araucariaceae Araucaria sp.#ACWS
Araucariales Podocarpaceae Halocarpus kirkii#JN
Araucariales Podocarpaceae Halocarpus bidwillii#OWFC
Araucariales Podocarpaceae Prumnopitys andina#EGLZ
Araucariales Podocarpaceae Sundacarpus amarus#KLGF
Araucariales Podocarpaceae Phyllocladus hypophyllus#JRNA
Araucariales Podocarpaceae Pherosphaera fitzgeraldii#BBDD
Araucariales Podocarpaceae Parasitaxus ustus#JZVE
Araucariales Podocarpaceae Manoao colensoi#CDFR
Araucariales Podocarpaceae Lagarostrobos franklinii#ZQWM
Araucariales Podocarpaceae Saxegothaea conspicua#QCGM
Araucariales Podocarpaceae Microcachrys tetragona#MHGD
Araucariales Podocarpaceae Acmopyle pancheri#HILW
Araucariales Podocarpaceae Dacrycarpus compactus#FMWZ
Araucariales Podocarpaceae Dacrydium balansae#IZGN
Araucariales Podocarpaceae Falcatifolium taxoides#UAUU
Araucariales Podocarpaceae Nageia nagi#NC 023120
Araucariales Podocarpaceae Retrophyllum minus#VGSX
Araucariales Podocarpaceae Retrophyllum piresii#NC 024827
Araucariales Podocarpaceae Podocarpus rubens#XLGK
Araucariales Podocarpaceae Podocarpus totara#JN
Araucariales Podocarpaceae Podocarpus coriaceus#SCEB
Araucariales Podocarpaceae Podocarpus lambertii#NC 023805
Cupressales Sciadopityaceae Sciadopitys verticillata#NC 029734
Cupressales Taxaceae Cephalotaxus oliveri#NC 021110
Cupressales Taxaceae Cephalotaxus harringtonia#NVGZ
Cupressales Taxaceae Cephalotaxus harringtonii var. wilsoniana#NC 016063
Cupressales Taxaceae Amentotaxus formosana#NC 024945
Cupressales Taxaceae Amentotaxus argotaenia#NC 027581
Cupressales Taxaceae Torreya grandis var. fargesii#NC 029398

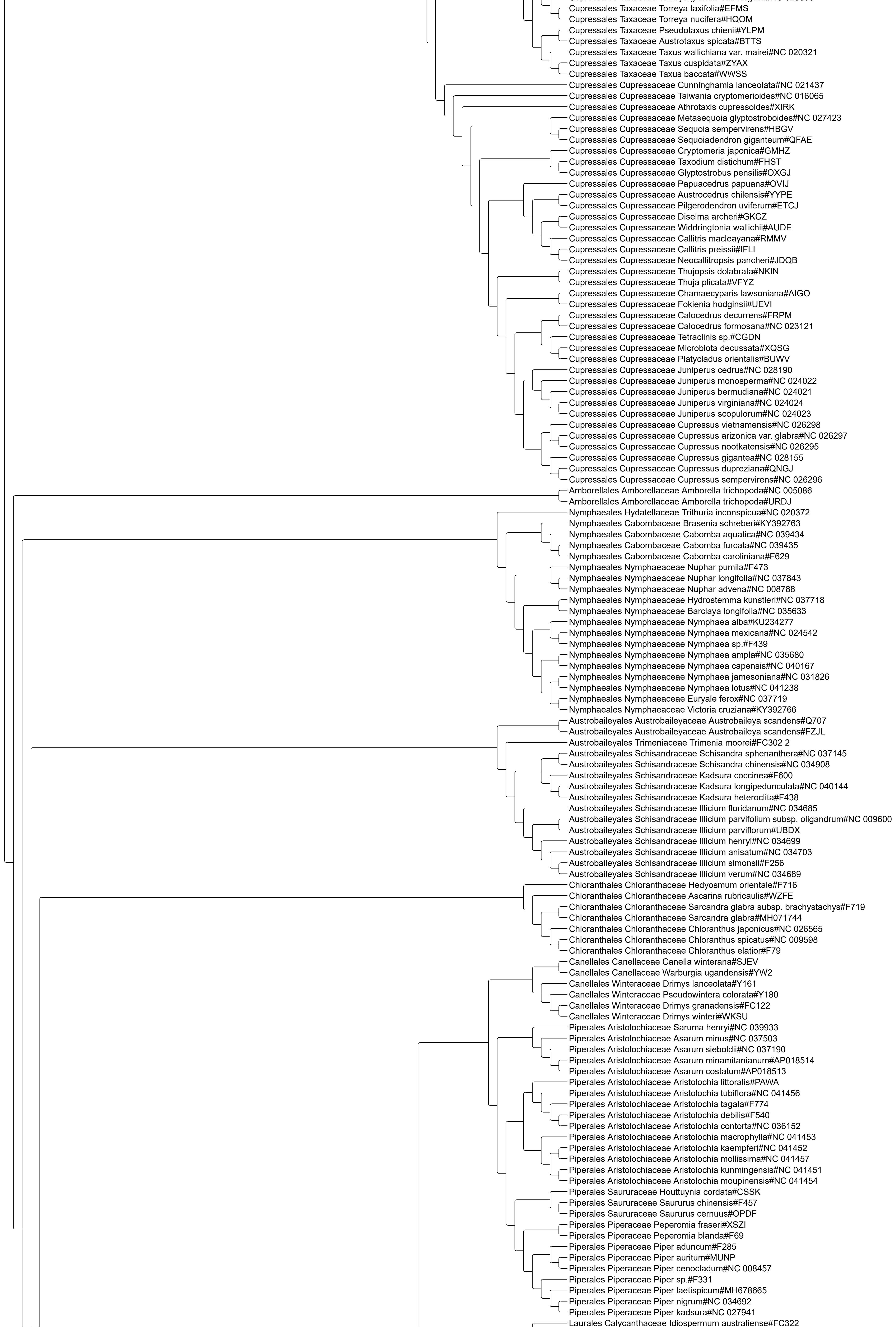

Cupressales Taxaceae Torreya taxifolia#EFMS
Cupressales Taxaceae Torreya nucifera#HQOM
Cupressales Taxaceae Pseudotaxus chienii#YLPM
Cupressales Taxaceae Austrotaxus spicata#BTTS
Cupressales Taxaceae Taxus wallichiana var. mairei#NC 020321
Cupressales Taxaceae Taxus cuspidata#ZYAX
Cupressales Taxaceae Taxus baccata#WWSS
Cupressales Cupressaceae Cunninghamia lanceolata#NC 021437
Cupressales Cupressaceae Taiwania cryptomerioides#NC 016065
Cupressales Cupressaceae Athrotaxis cupressoides#XIRK
Cupressales Cupressaceae Metasequoia glyptostroboides#NC 027423
Cupressales Cupressaceae Sequoia sempervirens#HBGV
Cupressales Cupressaceae Sequoiadendron giganteum#QFAE
Cupressales Cupressaceae Cryptomeria japonica#GMHZ
Cupressales Cupressaceae Taxodium distichum#FHST
Cupressales Cupressaceae Glyptostrobus pensilis#OXGJ
Cupressales Cupressaceae Papuacedrus papuana#OVIJ
Cupressales Cupressaceae Austrocedrus chilensis#YYPE
Cupressales Cupressaceae Pilgerodendron uviferum#ETCJ
Cupressales Cupressaceae Diselma archeri#GKCZ
Cupressales Cupressaceae Widdringtonia wallichii#AUDE
Cupressales Cupressaceae Callitris macleayana#RMMV
Cupressales Cupressaceae Callitris preissii#IFLI
Cupressales Cupressaceae Neocallitropsis pancheri#JDQB
Cupressales Cupressaceae Thujopsis dolabrata#NKIN
Cupressales Cupressaceae Thuja plicata#VFYZ
Cupressales Cupressaceae Chamaecyparis lawsoniana#AIGO
Cupressales Cupressaceae Fokienia hodginsii#UEVI
Cupressales Cupressaceae Calocedrus decurrens#FRPM
Cupressales Cupressaceae Calocedrus formosana#NC 023121
Cupressales Cupressaceae Tetraclinis sp.#CGDN
Cupressales Cupressaceae Microbiota decussata#XQSG
Cupressales Cupressaceae Platycladus orientalis#BUWV
Cupressales Cupressaceae Juniperus cedrus#NC 028190
Cupressales Cupressaceae Juniperus monosperma#NC 024022
Cupressales Cupressaceae Juniperus bermudiana#NC 024021
Cupressales Cupressaceae Juniperus virginiana#NC 024024
Cupressales Cupressaceae Juniperus scopulorum#NC 024023
Cupressales Cupressaceae Cupressus vietnamensis#NC 026298
Cupressales Cupressaceae Cupressus arizonica var. glabra#NC 026297
Cupressales Cupressaceae Cupressus nootkatensis#NC 026295
Cupressales Cupressaceae Cupressus gigantea#NC 028155
Cupressales Cupressaceae Cupressus dupreziana#QNGJ
Cupressales Cupressaceae Cupressus sempervirens#NC 026296
Amborellales Amborellaceae Amborella trichopoda#NC 005086
Amborellales Amborellaceae Amborella trichopoda#URDJ
Nymphaeales Hydatellaceae Trithuria inconspicua#NC 020372
Nymphaeales Cabombaceae Brasenia schreberi#KY392763
Nymphaeales Cabombaceae Cabomba aquatica#NC 039434
Nymphaeales Cabombaceae Cabomba furcata#NC 039435
Nymphaeales Cabombaceae Cabomba caroliniana#F629
Nymphaeales Nymphaeaceae Nuphar pumila#F473
Nymphaeales Nymphaeaceae Nuphar longifolia#NC 037843
Nymphaeales Nymphaeaceae Nuphar advena#NC 008788
Nymphaeales Nymphaeaceae Hydrostemma kunstleri#NC 037718
Nymphaeales Nymphaeaceae Barclaya longifolia#NC 035633
Nymphaeales Nymphaeaceae Nymphaea alba#KU234277
Nymphaeales Nymphaeaceae Nymphaea mexicana#NC 024542
Nymphaeales Nymphaeaceae Nymphaea sp.#F439
Nymphaeales Nymphaeaceae Nymphaea ampla#NC 035680
Nymphaeales Nymphaeaceae Nymphaea capensis#NC 040167
Nymphaeales Nymphaeaceae Nymphaea jamesoniana#NC 031826
Nymphaeales Nymphaeaceae Nymphaea lotus#NC 041238
Nymphaeales Nymphaeaceae Euryale ferox#NC 037719
Nymphaeales Nymphaeaceae Victoria cruziana#KY392766
Austrobaileyales Austrobaileyaceae Austrobaileya scandens#Q707
Austrobaileyales Austrobaileyaceae Austrobaileya scandens#FZJL
Austrobaileyales Trimeniaceae Trimenia moorei#FC302 2
Austrobaileyales Schisandraceae Schisandra sphenanthera#NC 037145
Austrobaileyales Schisandraceae Schisandra chinensis#NC 034908
Austrobaileyales Schisandraceae Kadsura coccinea#F600
Austrobaileyales Schisandraceae Kadsura longipedunculata#NC 040144
Austrobaileyales Schisandraceae Kadsura heteroclita#F438
Austrobaileyales Schisandraceae Illicium floridanum#NC 034685
Austrobaileyales Schisandraceae Illicium parvifolium subsp. oligandrum#NC 009600
Austrobaileyales Schisandraceae Illicium parviflorum#UBDX
Austrobaileyales Schisandraceae Illicium henryi#NC 034699
Austrobaileyales Schisandraceae Illicium anisatum#NC 034703
Austrobaileyales Schisandraceae Illicium simonsii#F256
Austrobaileyales Schisandraceae Illicium verum#NC 034689
Chloranthales Chloranthaceae Hedyosmum orientale#F716
Chloranthales Chloranthaceae Ascarina rubricaulis#WZFE
Chloranthales Chloranthaceae Sarcandra glabra subsp. brachystachys#F719
Chloranthales Chloranthaceae Sarcandra glabra#MH071744
Chloranthales Chloranthaceae Chloranthus japonicus#NC 026565
Chloranthales Chloranthaceae Chloranthus spicatus#NC 009598
Chloranthales Chloranthaceae Chloranthus elatior#F79
Canellales Canellaceae Canella winterana#SJEV
Canellales Canellaceae Warburgia ugandensis#YW2
Canellales Winteraceae Drimys lanceolata#Y161
Canellales Winteraceae Pseudowintera colorata#Y180
Canellales Winteraceae Drimys granadensis#FC122
Canellales Winteraceae Drimys winteri#WKSU
Piperales Aristolochiaceae Saruma henryi#NC 039933
Piperales Aristolochiaceae Asarum minus#NC 037503
Piperales Aristolochiaceae Asarum sieboldii#NC 037190
Piperales Aristolochiaceae Asarum minamitanianum#AP018514
Piperales Aristolochiaceae Asarum costatum#AP018513
Piperales Aristolochiaceae Aristolochia littoralis#PAWA
Piperales Aristolochiaceae Aristolochia tubiflora#NC 041456
Piperales Aristolochiaceae Aristolochia tagala#F774
Piperales Aristolochiaceae Aristolochia debilis#F540
Piperales Aristolochiaceae Aristolochia contorta#NC 036152
Piperales Aristolochiaceae Aristolochia macrophylla#NC 041453
Piperales Aristolochiaceae Aristolochia kaempferi#NC 041452
Piperales Aristolochiaceae Aristolochia mollissima#NC 041457
Piperales Aristolochiaceae Aristolochia kunmingensis#NC 041451
Piperales Aristolochiaceae Aristolochia moupinensis#NC 041454
Piperales Saururaceae Houttuynia cordata#CSSK
Piperales Saururaceae Saururus chinensis#F457
Piperales Saururaceae Saururus cernuus#OPDF
Piperales Piperaceae Peperomia fraseri#XSZI
Piperales Piperaceae Peperomia blanda#F69
Piperales Piperaceae Piper aduncum#F285
Piperales Piperaceae Piper auritum#MUNP
Piperales Piperaceae Piper cenocladum#NC 008457
Piperales Piperaceae Piper sp.#F331
Piperales Piperaceae Piper laetispicum#MH678665
Piperales Piperaceae Piper nigrum#NC 034692
Piperales Piperaceae Piper kadsura#NC 027941
Laurales Calycanthaceae Idiospermum australiense#FC322

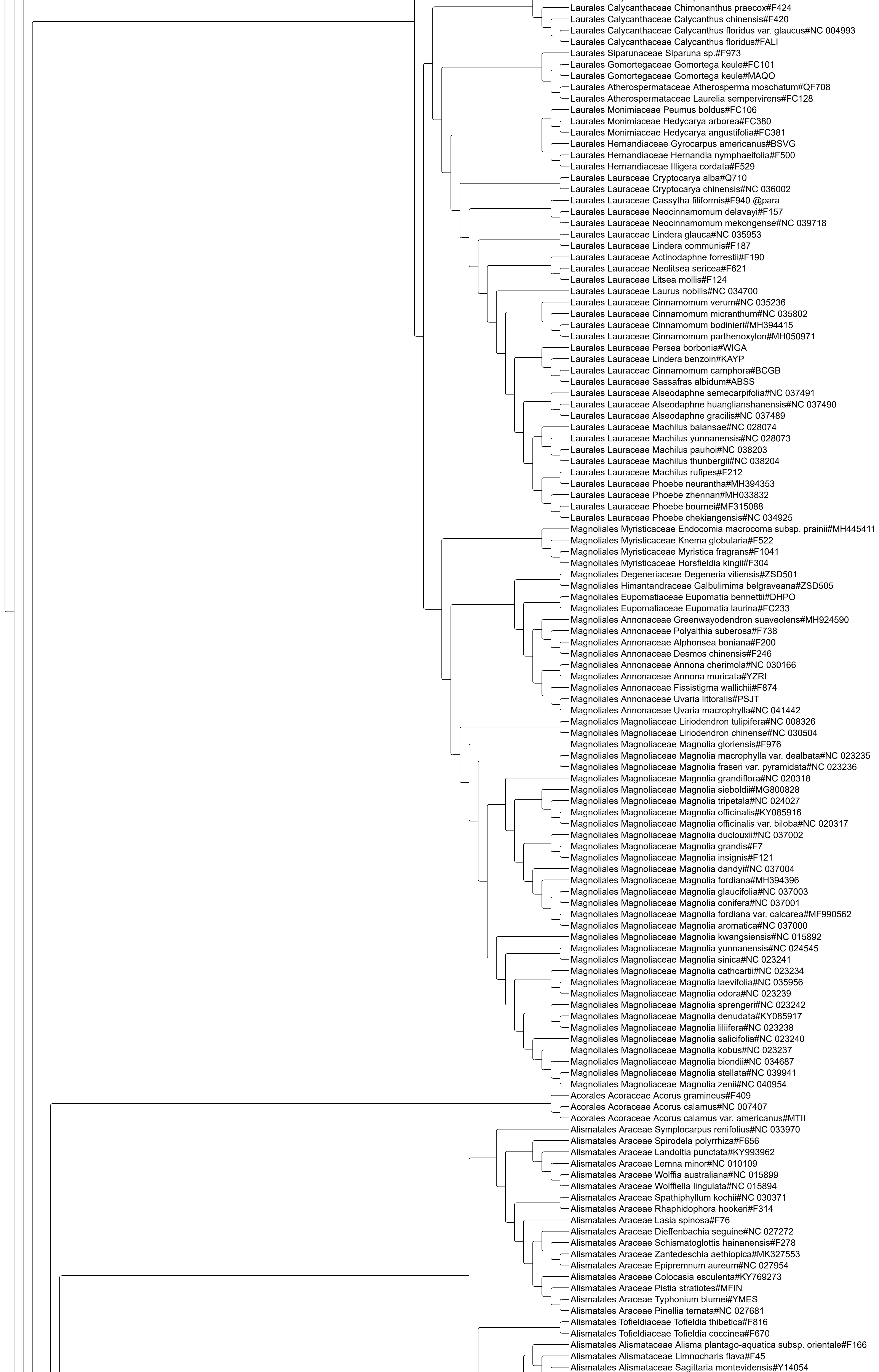
Laurales Calycanthaceae Chimonanthus praecox#F424
Laurales Calycanthaceae Calycanthus chinensis#F420
Laurales Calycanthaceae Calycanthus floridus var. glaucus#NC 004993
Laurales Calycanthaceae Calycanthus floridus#FALI
Laurales Siparunaceae Siparuna sp.#F973
Laurales Gomortegaceae Gomortega keule#FC101
Laurales Gomortegaceae Gomortega keule#MAQO
Laurales Atherospermataceae Atherosperma moschatum#QF708
Laurales Atherospermataceae Laurelia sempervirens#FC128
Laurales Monimiaceae Peumus boldus#FC106
Laurales Monimiaceae Hedycarya arborea#FC380
Laurales Monimiaceae Hedycarya angustifolia#FC381
Laurales Hernandiaceae Gyrocarpus americanus#BSVG
Laurales Hernandiaceae Hernandia nymphaeifolia#F500
Laurales Hernandiaceae Illigera cordata#F529
Laurales Lauraceae Cryptocarya alba#Q710
Laurales Lauraceae Cryptocarya chinensis#NC 036002
Laurales Lauraceae Cassytha filiformis#F940 @para
Laurales Lauraceae Neocinnamomum delavayi#F157
Laurales Lauraceae Neocinnamomum mekongense#NC 039718
Laurales Lauraceae Lindera glauca#NC 035953
Laurales Lauraceae Lindera communis#F187
Laurales Lauraceae Actinodaphne forrestii#F190
Laurales Lauraceae Neolitsea sericea#F621
Laurales Lauraceae Litsea mollis#F124
Laurales Lauraceae Laurus nobilis#NC 034700
Laurales Lauraceae Cinnamomum verum#NC 035236
Laurales Lauraceae Cinnamomum micranthum#NC 035802
Laurales Lauraceae Cinnamomum bodinieri#MH394415
Laurales Lauraceae Cinnamomum parthenoxylon#MH050971
Laurales Lauraceae Persea borbonia#WIGA
Laurales Lauraceae Lindera benzoin#KAYP
Laurales Lauraceae Cinnamomum camphora#BCGB
Laurales Lauraceae Sassafras albidum#ABSS
Laurales Lauraceae Alseodaphne semecarpifolia#NC 037491
Laurales Lauraceae Alseodaphne huanglianshanensis#NC 037490
Laurales Lauraceae Alseodaphne gracilis#NC 037489
Laurales Lauraceae Machilus balansae#NC 028074
Laurales Lauraceae Machilus yunnanensis#NC 028073
Laurales Lauraceae Machilus pauhoi#NC 038203
Laurales Lauraceae Machilus thunbergii#NC 038204
Laurales Lauraceae Machilus rufipes#F212
Laurales Lauraceae Phoebe neurantha#MH394353
Laurales Lauraceae Phoebe zhennan#MH033832
Laurales Lauraceae Phoebe bournei#MF315088
Laurales Lauraceae Phoebe chekiangensis#NC 034925
Magnoliales Myristicaceae Endocomia macrocoma subsp. prainii#MH445411
Magnoliales Myristicaceae Knema globularia#F522
Magnoliales Myristicaceae Myristica fragrans#F1041
Magnoliales Myristicaceae Horsfieldia kingii#F304
Magnoliales Degeneriaceae Degeneria vitiensis#ZSD501
Magnoliales Himantandraceae Galbulimima belgraveana#ZSD505
Magnoliales Eupomatiaceae Eupomatia bennettii#DHPO
Magnoliales Eupomatiaceae Eupomatia laurina#FC233
Magnoliales Annonaceae Greenwayodendron suaveolens#MH924590
Magnoliales Annonaceae Polyalthia suberosa#F738
Magnoliales Annonaceae Alphonsea boniana#F200
Magnoliales Annonaceae Desmos chinensis#F246
Magnoliales Annonaceae Annona cherimola#NC 030166
Magnoliales Annonaceae Annona muricata#YZRI
Magnoliales Annonaceae Fissistigma wallichii#F874
Magnoliales Annonaceae Uvaria littoralis#PSJT
Magnoliales Annonaceae Uvaria macrophylla#NC 041442
Magnoliales Magnoliaceae Liriodendron tulipifera#NC 008326
Magnoliales Magnoliaceae Liriodendron chinense#NC 030504
Magnoliales Magnoliaceae Magnolia gloriensis#F976
Magnoliales Magnoliaceae Magnolia macrophylla var. dealbata#NC 023235
Magnoliales Magnoliaceae Magnolia fraseri var. pyramidata#NC 023236
Magnoliales Magnoliaceae Magnolia grandiflora#NC 020318
Magnoliales Magnoliaceae Magnolia sieboldii#MG800828
Magnoliales Magnoliaceae Magnolia tripetala#NC 024027
Magnoliales Magnoliaceae Magnolia officinalis#KY085916
Magnoliales Magnoliaceae Magnolia officinalis var. biloba#NC 020317
Magnoliales Magnoliaceae Magnolia duclouxii#NC 037002
Magnoliales Magnoliaceae Magnolia grandis#F7
Magnoliales Magnoliaceae Magnolia insignis#F121
Magnoliales Magnoliaceae Magnolia dandyi#NC 037004
Magnoliales Magnoliaceae Magnolia fordiana#MH394396
Magnoliales Magnoliaceae Magnolia glaucifolia#NC 037003
Magnoliales Magnoliaceae Magnolia conifera#NC 037001
Magnoliales Magnoliaceae Magnolia fordiana var. calcarea#MF990562
Magnoliales Magnoliaceae Magnolia aromatica#NC 037000
Magnoliales Magnoliaceae Magnolia kwangsiensis#NC 015892
Magnoliales Magnoliaceae Magnolia yunnanensis#NC 024545
Magnoliales Magnoliaceae Magnolia sinica#NC 023241
Magnoliales Magnoliaceae Magnolia cathcartii#NC 023234
Magnoliales Magnoliaceae Magnolia laevifolia#NC 035956
Magnoliales Magnoliaceae Magnolia odora#NC 023239
Magnoliales Magnoliaceae Magnolia sprengeri#NC 023242
Magnoliales Magnoliaceae Magnolia denudata#KY085917
Magnoliales Magnoliaceae Magnolia liliifera#NC 023238
Magnoliales Magnoliaceae Magnolia salicifolia#NC 023240
Magnoliales Magnoliaceae Magnolia kobus#NC 023237
Magnoliales Magnoliaceae Magnolia biondii#NC 034687
Magnoliales Magnoliaceae Magnolia stellata#NC 039941
Magnoliales Magnoliaceae Magnolia zenii#NC 040954
Acorales Acoraceae Acorus gramineus#F409
Acorales Acoraceae Acorus calamus#NC 007407
Acorales Acoraceae Acorus calamus var. americanus#MTII
Alismatales Araceae Symplocarpus renifolius#NC 033970
Alismatales Araceae Spirodela polyrrhiza#F656
Alismatales Araceae Landoltia punctata#KY993962
Alismatales Araceae Lemna minor#NC 010109
Alismatales Araceae Wolffia australiana#NC 015899
Alismatales Araceae Wolffiella lingulata#NC 015894
Alismatales Araceae Spathiphyllum kochii#NC 030371
Alismatales Araceae Rhaphidophora hookeri#F314
Alismatales Araceae Lasia spinosa#F76
Alismatales Araceae Dieffenbachia seguine#NC 027272
Alismatales Araceae Schismatoglottis hainanensis#F278
Alismatales Araceae Zantedeschia aethiopica#MK327553
Alismatales Araceae Epipremnum aureum#NC 027954
Alismatales Araceae Colocasia esculenta#KY769273
Alismatales Araceae Pistia stratiotes#MFIN
Alismatales Araceae Typhonium blumei#YMES
Alismatales Araceae Pinellia ternata#NC 027681
Alismatales Tofieldiaceae Tofieldia thibetica#F816
Alismatales Tofieldiaceae Tofieldia coccinea#F670
Alismatales Alismataceae Alisma plantago-aquatica subsp. orientale#F166
Alismatales Alismataceae Limnocharis flava#F45
Alismatales Alismataceae Sagittaria montevidensis#Y14054

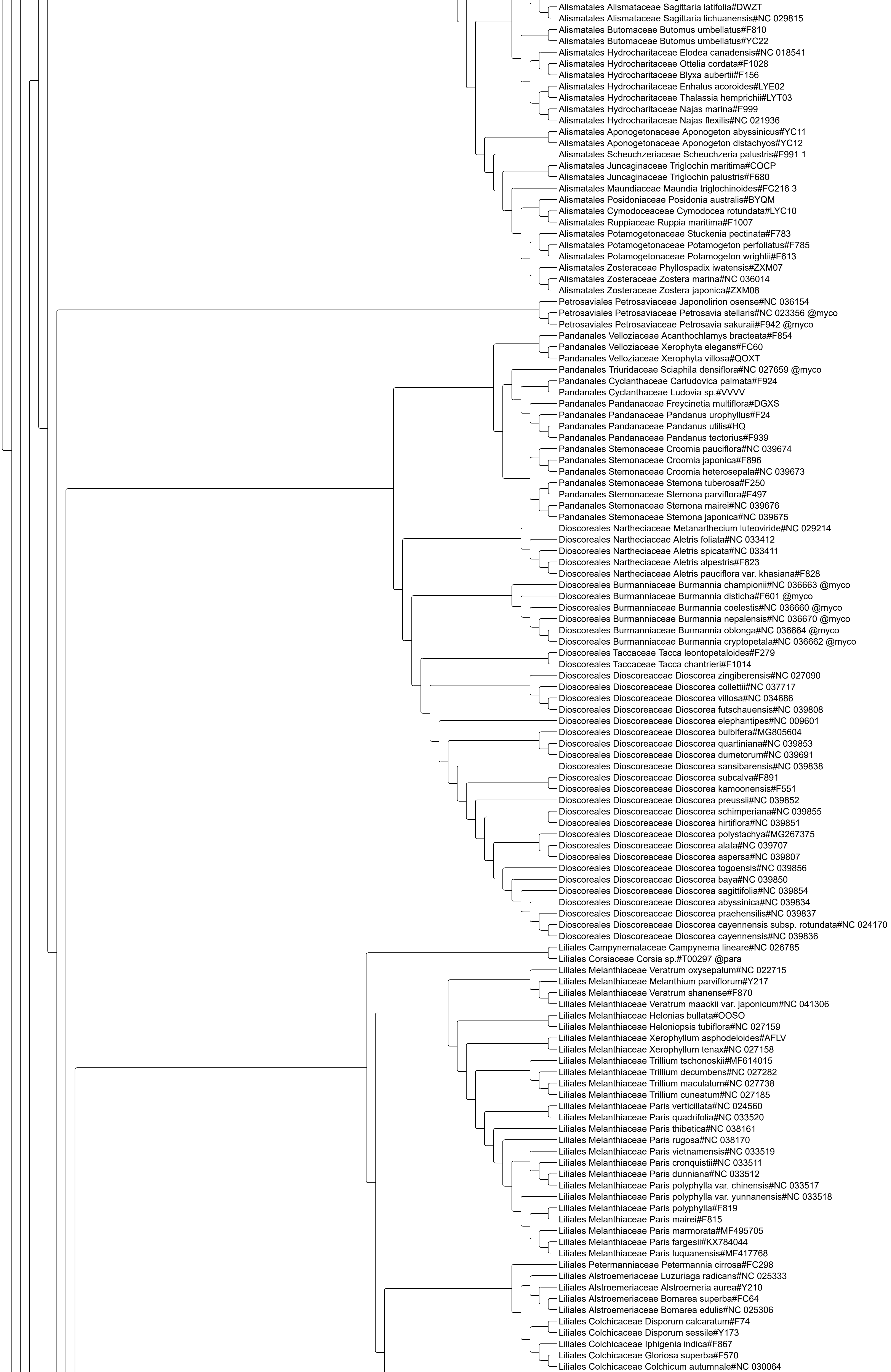
Alismatales Alismataceae Sagittaria latifolia#DWZT
Alismatales Alismataceae Sagittaria lichuanensis#NC 029815
Alismatales Butomaceae Butomus umbellatus#F810
Alismatales Butomaceae Butomus umbellatus#YC22
Alismatales Hydrocharitaceae Elodea canadensis#NC 018541
Alismatales Hydrocharitaceae Ottelia cordata#F1028
Alismatales Hydrocharitaceae Blyxa aubertii#F156
Alismatales Hydrocharitaceae Enhalus acoroides#LYE02
Alismatales Hydrocharitaceae Thalassia hemprichii#LYT03
Alismatales Hydrocharitaceae Najas marina#F999
Alismatales Hydrocharitaceae Najas flexilis#NC 021936
Alismatales Aponogetonaceae Aponogeton abyssinicus#YC11
Alismatales Aponogetonaceae Aponogeton distachyos#YC12
Alismatales Scheuchzeriaceae Scheuchzeria palustris#F991 1
Alismatales Juncaginaceae Triglochin maritima#COCP
Alismatales Juncaginaceae Triglochin palustris#F680
Alismatales Maundiaceae Maundia triglochinoides#FC216 3
Alismatales Posidoniaceae Posidonia australis#BYQM
Alismatales Cymodoceaceae Cymodocea rotundata#LYC10
Alismatales Ruppiaceae Ruppia maritima#F1007
Alismatales Potamogetonaceae Stuckenia pectinata#F783
Alismatales Potamogetonaceae Potamogeton perfoliatus#F785
Alismatales Potamogetonaceae Potamogeton wrightii#F613
Alismatales Zosteraceae Phyllospadix iwatensis#ZXM07
Alismatales Zosteraceae Zostera marina#NC 036014
Alismatales Zosteraceae Zostera japonica#ZXM08
Petrosaviales Petrosaviaceae Japonolirion osense#NC 036154
Petrosaviales Petrosaviaceae Petrosavia stellaris#NC 023356 @myco
Petrosaviales Petrosaviaceae Petrosavia sakuraii#F942 @myco
Pandanales Velloziaceae Acanthochlamys bracteata#F854
Pandanales Velloziaceae Xerophyta elegans#FC60
Pandanales Velloziaceae Xerophyta villosa#QOXT
Pandanales Triuridaceae Sciaphila densiflora#NC 027659 @myco
Pandanales Cyclanthaceae Carludovica palmata#F924
Pandanales Cyclanthaceae Ludovia sp.#VVVV
Pandanales Pandanaceae Freycinetia multiflora#DGXS
Pandanales Pandanaceae Pandanus urophyllus#F24
Pandanales Pandanaceae Pandanus utilis#HQ
Pandanales Pandanaceae Pandanus tectorius#F939
Pandanales Stemonaceae Croomia pauciflora#NC 039674
Pandanales Stemonaceae Croomia japonica#F896
Pandanales Stemonaceae Croomia heterosepala#NC 039673
Pandanales Stemonaceae Stemona tuberosa#F250
Pandanales Stemonaceae Stemona parviflora#F497
Pandanales Stemonaceae Stemona mairei#NC 039676
Pandanales Stemonaceae Stemona japonica#NC 039675
Dioscoreales Nartheciaceae Metanarthecium luteoviride#NC 029214
Dioscoreales Nartheciaceae Aletris foliata#NC 033412
Dioscoreales Nartheciaceae Aletris spicata#NC 033411
Dioscoreales Nartheciaceae Aletris alpestris#F823
Dioscoreales Nartheciaceae Aletris pauciflora var. khasiana#F828
Dioscoreales Burmanniaceae Burmannia championii#NC 036663 @myco
Dioscoreales Burmanniaceae Burmannia disticha#F601 @myco
Dioscoreales Burmanniaceae Burmannia coelestis#NC 036660 @myco
Dioscoreales Burmanniaceae Burmannia nepalensis#NC 036670 @myco
Dioscoreales Burmanniaceae Burmannia oblonga#NC 036664 @myco
Dioscoreales Burmanniaceae Burmannia cryptopetala#NC 036662 @myco
Dioscoreales Taccaceae Tacca leontopetaloides#F279
Dioscoreales Taccaceae Tacca chantrieri#F1014
Dioscoreales Dioscoreaceae Dioscorea zingiberensis#NC 027090
Dioscoreales Dioscoreaceae Dioscorea collettii#NC 037717
Dioscoreales Dioscoreaceae Dioscorea villosa#NC 034686
Dioscoreales Dioscoreaceae Dioscorea futschauensis#NC 039808
Dioscoreales Dioscoreaceae Dioscorea elephantipes#NC 009601
Dioscoreales Dioscoreaceae Dioscorea bulbifera#MG805604
Dioscoreales Dioscoreaceae Dioscorea quartiniana#NC 039853
Dioscoreales Dioscoreaceae Dioscorea dumetorum#NC 039691
Dioscoreales Dioscoreaceae Dioscorea sansibarensis#NC 039838
Dioscoreales Dioscoreaceae Dioscorea subcalva#F891
Dioscoreales Dioscoreaceae Dioscorea kamoonensis#F551
Dioscoreales Dioscoreaceae Dioscorea preussii#NC 039852
Dioscoreales Dioscoreaceae Dioscorea schimperiana#NC 039855
Dioscoreales Dioscoreaceae Dioscorea hirtiflora#NC 039851
Dioscoreales Dioscoreaceae Dioscorea polystachya#MG267375
Dioscoreales Dioscoreaceae Dioscorea alata#NC 039707
Dioscoreales Dioscoreaceae Dioscorea aspersa#NC 039807
Dioscoreales Dioscoreaceae Dioscorea togoensis#NC 039856
Dioscoreales Dioscoreaceae Dioscorea baya#NC 039850
Dioscoreales Dioscoreaceae Dioscorea sagittifolia#NC 039854
Dioscoreales Dioscoreaceae Dioscorea abyssinica#NC 039834
Dioscoreales Dioscoreaceae Dioscorea praehensilis#NC 039837
Dioscoreales Dioscoreaceae Dioscorea cayennensis subsp. rotundata#NC 024170
Dioscoreales Dioscoreaceae Dioscorea cayennensis#NC 039836
Liliales Campynemataceae Campynema lineare#NC 026785
Liliales Corsiaceae Corsia sp.#T00297 @para
Liliales Melanthiaceae Veratrum oxysepalum#NC 022715
Liliales Melanthiaceae Melanthium parviflorum#Y217
Liliales Melanthiaceae Veratrum shanense#F870
Liliales Melanthiaceae Veratrum maackii var. japonicum#NC 041306
Liliales Melanthiaceae Helonias bullata#OOSO
Liliales Melanthiaceae Heloniopsis tubiflora#NC 027159
Liliales Melanthiaceae Xerophyllum asphodeloides#AFLV
Liliales Melanthiaceae Xerophyllum tenax#NC 027158
Liliales Melanthiaceae Trillium tschonoskii#MF614015
Liliales Melanthiaceae Trillium decumbens#NC 027282
Liliales Melanthiaceae Trillium maculatum#NC 027738
Liliales Melanthiaceae Trillium cuneatum#NC 027185
Liliales Melanthiaceae Paris verticillata#NC 024560
Liliales Melanthiaceae Paris quadrifolia#NC 033520
Liliales Melanthiaceae Paris thibetica#NC 038161
Liliales Melanthiaceae Paris rugosa#NC 038170
Liliales Melanthiaceae Paris vietnamensis#NC 033519
Liliales Melanthiaceae Paris cronquistii#NC 033511
Liliales Melanthiaceae Paris dunniana#NC 033512
Liliales Melanthiaceae Paris polyphylla var. chinensis#NC 033517
Liliales Melanthiaceae Paris polyphylla var. yunnanensis#NC 033518
Liliales Melanthiaceae Paris polyphylla#F819
Liliales Melanthiaceae Paris mairei#F815
Liliales Melanthiaceae Paris marmorata#MF495705
Liliales Melanthiaceae Paris fargesii#KX784044
Liliales Melanthiaceae Paris luquanensis#MF417768
Liliales Petermanniaceae Petermannia cirrosa#FC298
Liliales Alstroemeriaceae Luzuriaga radicans#NC 025333
Liliales Alstroemeriaceae Alstroemeria aurea#Y210
Liliales Alstroemeriaceae Bomarea superba#FC64
Liliales Alstroemeriaceae Bomarea edulis#NC 025306
Liliales Colchicaceae Disporum calcaratum#F74
Liliales Colchicaceae Disporum sessile#Y173
Liliales Colchicaceae Iphigenia indica#F867
Liliales Colchicaceae Gloriosa superba#F570
Liliales Colchicaceae Colchicum autumnale#NC 030064

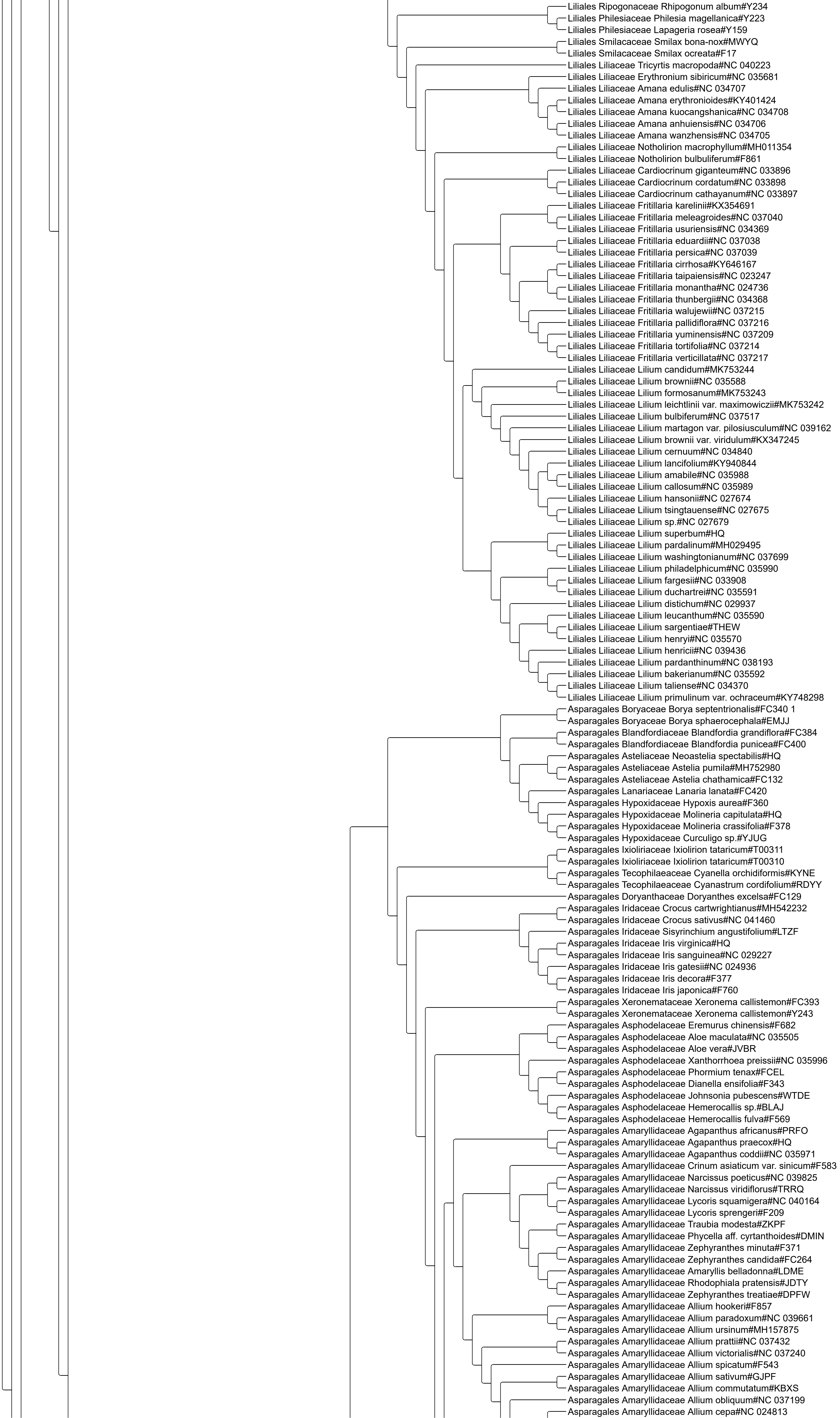
Liliales Ripogonaceae Rhipogonum album#Y234
Liliales Philesiaceae Philesia magellanica#Y223
Liliales Philesiaceae Lapageria rosea#Y159
Liliales Smilacaceae Smilax bona-nox#MWYQ
Liliales Smilacaceae Smilax ocreata#F17
Liliales Liliaceae Tricyrtis macropoda#NC 040223
Liliales Liliaceae Erythronium sibiricum#NC 035681
Liliales Liliaceae Amana edulis#NC 034707
Liliales Liliaceae Amana erythronioides#KY401424
Liliales Liliaceae Amana kuocangshanica#NC 034708
Liliales Liliaceae Amana anhuiensis#NC 034706
Liliales Liliaceae Amana wanzhensis#NC 034705
Liliales Liliaceae Notholirion macrophyllum#MH011354
Liliales Liliaceae Notholirion bulbuliferum#F861
Liliales Liliaceae Cardiocrinum giganteum#NC 033896
Liliales Liliaceae Cardiocrinum cordatum#NC 033898
Liliales Liliaceae Cardiocrinum cathayanum#NC 033897
Liliales Liliaceae Fritillaria karelinii#KX354691
Liliales Liliaceae Fritillaria meleagroides#NC 037040
Liliales Liliaceae Fritillaria usuriensis#NC 034369
Liliales Liliaceae Fritillaria eduardii#NC 037038
Liliales Liliaceae Fritillaria persica#NC 037039
Liliales Liliaceae Fritillaria cirrhosa#KY646167
Liliales Liliaceae Fritillaria taipaiensis#NC 023247
Liliales Liliaceae Fritillaria monantha#NC 024736
Liliales Liliaceae Fritillaria thunbergii#NC 034368
Liliales Liliaceae Fritillaria walujewii#NC 037215
Liliales Liliaceae Fritillaria pallidiflora#NC 037216
Liliales Liliaceae Fritillaria yuminensis#NC 037209
Liliales Liliaceae Fritillaria tortifolia#NC 037214
Liliales Liliaceae Fritillaria verticillata#NC 037217
Liliales Liliaceae Lilium candidum#MK753244
Liliales Liliaceae Lilium brownii#NC 035588
Liliales Liliaceae Lilium formosanum#MK753243
Liliales Liliaceae Lilium leichtlinii var. maximowiczii#MK753242
Liliales Liliaceae Lilium bulbiferum#NC 037517
Liliales Liliaceae Lilium martagon var. pilosiusculum#NC 039162
Liliales Liliaceae Lilium brownii var. viridulum#KX347245
Liliales Liliaceae Lilium cernuum#NC 034840
Liliales Liliaceae Lilium lancifolium#KY940844
Liliales Liliaceae Lilium amabile#NC 035988
Liliales Liliaceae Lilium callosum#NC 035989
Liliales Liliaceae Lilium hansonii#NC 027674
Liliales Liliaceae Lilium tsingtauense#NC 027675
Liliales Liliaceae Lilium sp.#NC 027679
Liliales Liliaceae Lilium superbum#HQ
Liliales Liliaceae Lilium pardalinum#MH029495
Liliales Liliaceae Lilium washingtonianum#NC 037699
Liliales Liliaceae Lilium philadelphicum#NC 035990
Liliales Liliaceae Lilium fargesii#NC 033908
Liliales Liliaceae Lilium duchartrei#NC 035591
Liliales Liliaceae Lilium distichum#NC 029937
Liliales Liliaceae Lilium leucanthum#NC 035590
Liliales Liliaceae Lilium sargentiae#THEW
Liliales Liliaceae Lilium henryi#NC 035570
Liliales Liliaceae Lilium henricii#NC 039436
Liliales Liliaceae Lilium pardanthinum#NC 038193
Liliales Liliaceae Lilium bakerianum#NC 035592
Liliales Liliaceae Lilium taliense#NC 034370
Liliales Liliaceae Lilium primulinum var. ochraceum#KY748298
Asparagales Boryaceae Borya septentrionalis#FC340 1
Asparagales Boryaceae Borya sphaerocephala#EMJJ
Asparagales Blandfordiaceae Blandfordia grandiflora#FC384
Asparagales Blandfordiaceae Blandfordia punicea#FC400
Asparagales Asteliaceae Neoastelia spectabilis#HQ
Asparagales Asteliaceae Astelia pumila#MH752980
Asparagales Asteliaceae Astelia chathamica#FC132
Asparagales Lanariaceae Lanaria lanata#FC420
Asparagales Hypoxidaceae Hypoxis aurea#F360
Asparagales Hypoxidaceae Molineria capitulata#HQ
Asparagales Hypoxidaceae Molineria crassifolia#F378
Asparagales Hypoxidaceae Curculigo sp.#YJUG
Asparagales Ixioliriaceae Ixiolirion tataricum#T00311
Asparagales Ixioliriaceae Ixiolirion tataricum#T00310
Asparagales Tecophilaeaceae Cyanella orchidiformis#KYNE
Asparagales Tecophilaeaceae Cyanastrum cordifolium#RDYY
Asparagales Doryanthaceae Doryanthes excelsa#FC129
Asparagales Iridaceae Crocus cartwrightianus#MH542232
Asparagales Iridaceae Crocus sativus#NC 041460
Asparagales Iridaceae Sisyrinchium angustifolium#LTZF
Asparagales Iridaceae Iris virginica#HQ
Asparagales Iridaceae Iris sanguinea#NC 029227
Asparagales Iridaceae Iris gatesii#NC 024936
Asparagales Iridaceae Iris decora#F377
Asparagales Iridaceae Iris japonica#F760
Asparagales Xeronemataceae Xeronema callistemon#FC393
Asparagales Xeronemataceae Xeronema callistemon#Y243
Asparagales Asphodelaceae Eremurus chinensis#F682
Asparagales Asphodelaceae Aloe maculata#NC 035505
Asparagales Asphodelaceae Aloe vera#JVBR
Asparagales Asphodelaceae Xanthorrhoea preissii#NC 035996
Asparagales Asphodelaceae Phormium tenax#FCEL
Asparagales Asphodelaceae Dianella ensifolia#F343
Asparagales Asphodelaceae Johnsonia pubescens#WTDE
Asparagales Asphodelaceae Hemerocallis sp.#BLAJ
Asparagales Asphodelaceae Hemerocallis fulva#F569
Asparagales Amaryllidaceae Agapanthus africanus#PRFO
Asparagales Amaryllidaceae Agapanthus praecox#HQ
Asparagales Amaryllidaceae Agapanthus coddii#NC 035971
Asparagales Amaryllidaceae Crinum asiaticum var. sinicum#F583
Asparagales Amaryllidaceae Narcissus poeticus#NC 039825
Asparagales Amaryllidaceae Narcissus viridiflorus#TRRQ
Asparagales Amaryllidaceae Lycoris squamigera#NC 040164
Asparagales Amaryllidaceae Lycoris sprengeri#F209
Asparagales Amaryllidaceae Traubia modesta#ZKPF
Asparagales Amaryllidaceae Phycella aff. cyrtanthoides#DMIN
Asparagales Amaryllidaceae Zephyranthes minuta#F371
Asparagales Amaryllidaceae Zephyranthes candida#FC264
Asparagales Amaryllidaceae Amaryllis belladonna#LDME
Asparagales Amaryllidaceae Rhodophiala pratensis#JDTY
Asparagales Amaryllidaceae Zephyranthes treatiae#DPFW
Asparagales Amaryllidaceae Allium hookeri#F857
Asparagales Amaryllidaceae Allium paradoxum#NC 039661
Asparagales Amaryllidaceae Allium ursinum#MH157875
Asparagales Amaryllidaceae Allium prattii#NC 037432
Asparagales Amaryllidaceae Allium victorialis#NC 037240
Asparagales Amaryllidaceae Allium spicatum#F543
Asparagales Amaryllidaceae Allium sativum#GJPF
Asparagales Amaryllidaceae Allium commutatum#KBXS
Asparagales Amaryllidaceae Allium obliquum#NC 037199
Asparagales Amaryllidaceae Allium cepa#NC 024813

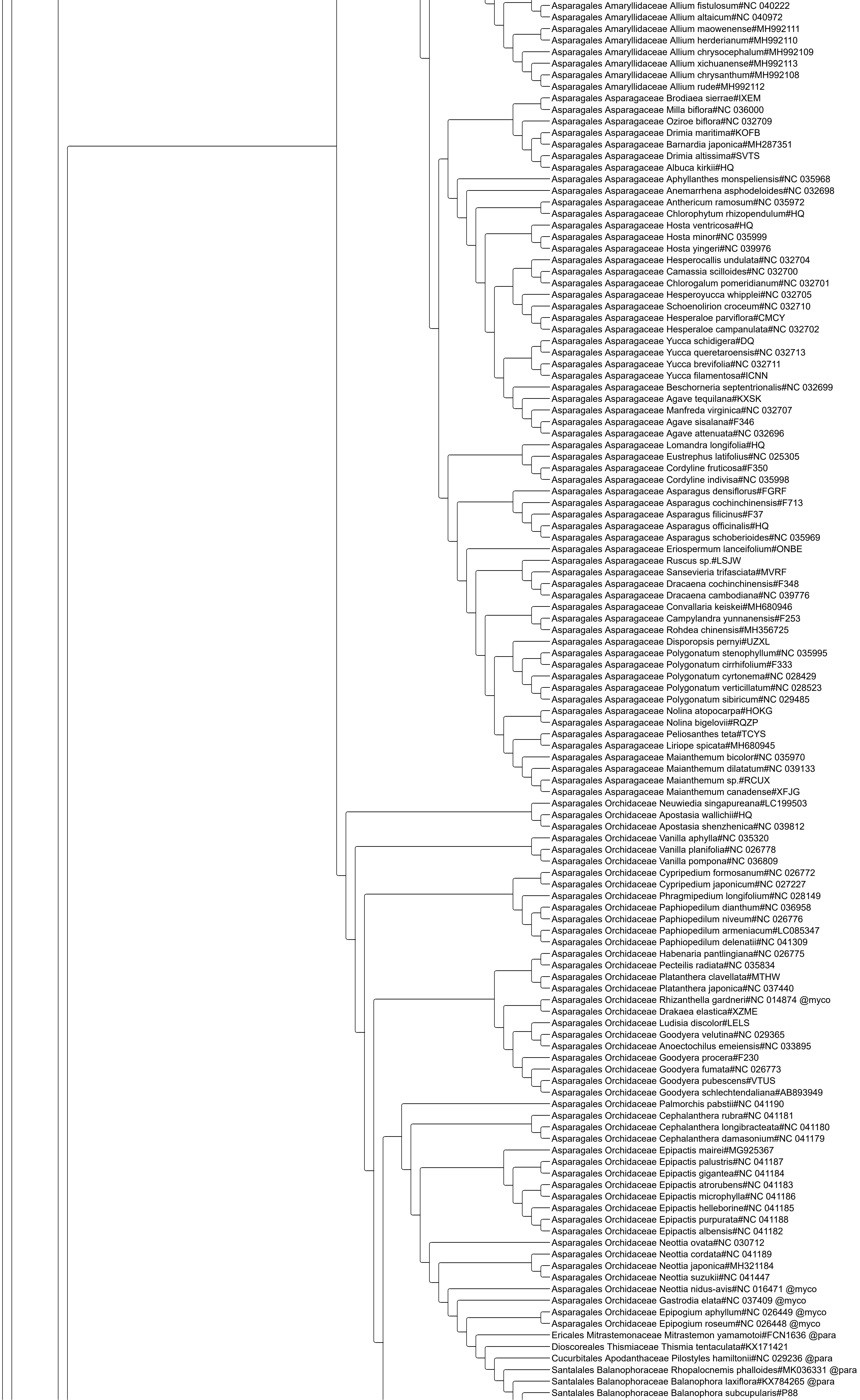
Asparagales Amaryllidaceae Allium fistulosum#NC 040222
Asparagales Amaryllidaceae Allium altaicum#NC 040972
Asparagales Amaryllidaceae Allium maowenense#MH992111
Asparagales Amaryllidaceae Allium herderianum#MH992110
Asparagales Amaryllidaceae Allium chrysocephalum#MH992109
Asparagales Amaryllidaceae Allium xichuanense#MH992113
Asparagales Amaryllidaceae Allium chrysanthum#MH992108
Asparagales Amaryllidaceae Allium rude#MH992112
Asparagales Asparagaceae Brodiaea sierrae#IXEM
Asparagales Asparagaceae Milla biflora#NC 036000
Asparagales Asparagaceae Oziroe biflora#NC 032709
Asparagales Asparagaceae Drimia maritima#KOFB
Asparagales Asparagaceae Barnardia japonica#MH287351
Asparagales Asparagaceae Drimia altissima#SVTS
Asparagales Asparagaceae Albuca kirkii#HQ
Asparagales Asparagaceae Aphyllanthes monspeliensis#NC 035968
Asparagales Asparagaceae Anemarrhena asphodeloides#NC 032698
Asparagales Asparagaceae Anthericum ramosum#NC 035972
Asparagales Asparagaceae Chlorophytum rhizopendulum#HQ
Asparagales Asparagaceae Hosta ventricosa#HQ
Asparagales Asparagaceae Hosta minor#NC 035999
Asparagales Asparagaceae Hosta yingeri#NC 039976
Asparagales Asparagaceae Hesperocallis undulata#NC 032704
Asparagales Asparagaceae Camassia scilloides#NC 032700
Asparagales Asparagaceae Chlorogalum pomeridianum#NC 032701
Asparagales Asparagaceae Hesperoyucca whipplei#NC 032705
Asparagales Asparagaceae Schoenolirion croceum#NC 032710
Asparagales Asparagaceae Hesperaloe parviflora#CMCY
Asparagales Asparagaceae Hesperaloe campanulata#NC 032702
Asparagales Asparagaceae Yucca schidigera#DQ
Asparagales Asparagaceae Yucca queretaroensis#NC 032713
Asparagales Asparagaceae Yucca brevifolia#NC 032711
Asparagales Asparagaceae Yucca filamentosa#ICNN
Asparagales Asparagaceae Beschorneria septentrionalis#NC 032699
Asparagales Asparagaceae Agave tequilana#KXSK
Asparagales Asparagaceae Manfreda virginica#NC 032707
Asparagales Asparagaceae Agave sisalana#F346
Asparagales Asparagaceae Agave attenuata#NC 032696
Asparagales Asparagaceae Lomandra longifolia#HQ
Asparagales Asparagaceae Eustrephus latifolius#NC 025305
Asparagales Asparagaceae Cordyline fruticosa#F350
Asparagales Asparagaceae Cordyline indivisa#NC 035998
Asparagales Asparagaceae Asparagus densiflorus#FGRF
Asparagales Asparagaceae Asparagus cochinchinensis#F713
Asparagales Asparagaceae Asparagus filicinus#F37
Asparagales Asparagaceae Asparagus officinalis#HQ
Asparagales Asparagaceae Asparagus schoberioides#NC 035969
Asparagales Asparagaceae Eriospermum lanceifolium#ONBE
Asparagales Asparagaceae Ruscus sp.#LSJW
Asparagales Asparagaceae Sansevieria trifasciata#MVRF
Asparagales Asparagaceae Dracaena cochinchinensis#F348
Asparagales Asparagaceae Dracaena cambodiana#NC 039776
Asparagales Asparagaceae Convallaria keiskei#MH680946
Asparagales Asparagaceae Campylandra yunnanensis#F253
Asparagales Asparagaceae Rohdea chinensis#MH356725
Asparagales Asparagaceae Disporopsis pernyi#UZXL
Asparagales Asparagaceae Polygonatum stenophyllum#NC 035995
Asparagales Asparagaceae Polygonatum cirrhifolium#F333
Asparagales Asparagaceae Polygonatum cyrtonema#NC 028429
Asparagales Asparagaceae Polygonatum verticillatum#NC 028523
Asparagales Asparagaceae Polygonatum sibiricum#NC 029485
Asparagales Asparagaceae Nolina atopocarpa#HOKG
Asparagales Asparagaceae Nolina bigelovii#RQZP
Asparagales Asparagaceae Peliosanthes teta#TCYS
Asparagales Asparagaceae Liriope spicata#MH680945
Asparagales Asparagaceae Maianthemum bicolor#NC 035970
Asparagales Asparagaceae Maianthemum dilatatum#NC 039133
Asparagales Asparagaceae Maianthemum sp.#RCUX
Asparagales Asparagaceae Maianthemum canadense#XFJG
Asparagales Orchidaceae Neuwiedia singapureana#LC199503
Asparagales Orchidaceae Apostasia wallichii#HQ
Asparagales Orchidaceae Apostasia shenzhenica#NC 039812
Asparagales Orchidaceae Vanilla aphylla#NC 035320
Asparagales Orchidaceae Vanilla planifolia#NC 026778
Asparagales Orchidaceae Vanilla pompona#NC 036809
Asparagales Orchidaceae Cypripedium formosanum#NC 026772
Asparagales Orchidaceae Cypripedium japonicum#NC 027227
Asparagales Orchidaceae Phragmipedium longifolium#NC 028149
Asparagales Orchidaceae Paphiopedilum dianthum#NC 036958
Asparagales Orchidaceae Paphiopedilum niveum#NC 026776
Asparagales Orchidaceae Paphiopedilum armeniacum#LC085347
Asparagales Orchidaceae Paphiopedilum delenatii#NC 041309
Asparagales Orchidaceae Habenaria pantlingiana#NC 026775
Asparagales Orchidaceae Pecteilis radiata#NC 035834
Asparagales Orchidaceae Platanthera clavellata#MTHW
Asparagales Orchidaceae Platanthera japonica#NC 037440
Asparagales Orchidaceae Rhizanthella gardneri#NC 014874 @myco
Asparagales Orchidaceae Drakaea elastica#XZME
Asparagales Orchidaceae Ludisia discolor#LELS
Asparagales Orchidaceae Goodyera velutina#NC 029365
Asparagales Orchidaceae Anoectochilus emeiensis#NC 033895
Asparagales Orchidaceae Goodyera procera#F230
Asparagales Orchidaceae Goodyera fumata#NC 026773
Asparagales Orchidaceae Goodyera pubescens#VTUS
Asparagales Orchidaceae Goodyera schlechtendaliana#AB893949
Asparagales Orchidaceae Palmorchis pabstii#NC 041190
Asparagales Orchidaceae Cephalanthera rubra#NC 041181
Asparagales Orchidaceae Cephalanthera longibracteata#NC 041180
Asparagales Orchidaceae Cephalanthera damasonium#NC 041179
Asparagales Orchidaceae Epipactis mairei#MG925367
Asparagales Orchidaceae Epipactis palustris#NC 041187
Asparagales Orchidaceae Epipactis gigantea#NC 041184
Asparagales Orchidaceae Epipactis atrorubens#NC 041183
Asparagales Orchidaceae Epipactis microphylla#NC 041186
Asparagales Orchidaceae Epipactis helleborine#NC 041185
Asparagales Orchidaceae Epipactis purpurata#NC 041188
Asparagales Orchidaceae Epipactis albensis#NC 041182
Asparagales Orchidaceae Neottia ovata#NC 030712
Asparagales Orchidaceae Neottia cordata#NC 041189
Asparagales Orchidaceae Neottia japonica#MH321184
Asparagales Orchidaceae Neottia suzukii#NC 041447
Asparagales Orchidaceae Neottia nidus-avis#NC 016471 @myco
Asparagales Orchidaceae Gastrodia elata#NC 037409 @myco
Asparagales Orchidaceae Epipogium aphyllum#NC 026449 @myco
Asparagales Orchidaceae Epipogium roseum#NC 026448 @myco
Ericales Mitrastemonaceae Mitrastemon yamamotoi#FCN1636 @para
Dioscoreales Thismiaceae Thismia tentaculata#KX171421
Cucurbitales Apodanthaceae Pilostyles hamiltonii#NC 029236 @para
Santalales Balanophoraceae Rhopalocnemis phalloides#MK036331 @para
Santalales Balanophoraceae Balanophora laxiflora#KX784265 @para
Santalales Balanophoraceae Balanophora subcupularis#P88

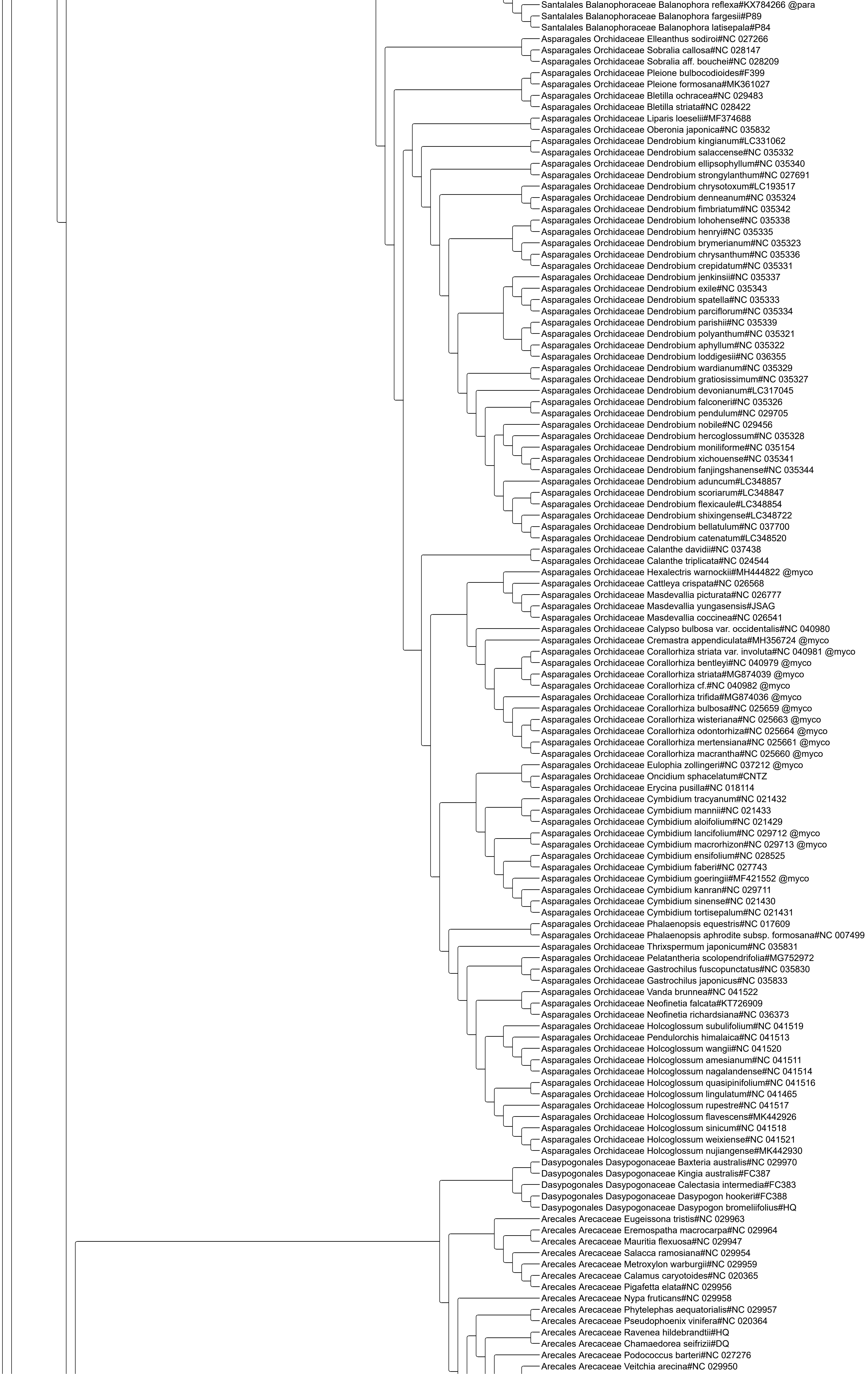

Santalales Balanophoraceae Balanophora reflexa#KX784266 @para
Santalales Balanophoraceae Balanophora fargesii#P89
Santalales Balanophoraceae Balanophora latisepala#P84
Asparagales Orchidaceae Elleanthus sodiroi#NC 027266
Asparagales Orchidaceae Sobralia callosa#NC 028147
Asparagales Orchidaceae Sobralia aff. bouchei#NC 028209
Asparagales Orchidaceae Pleione bulbocodioides#F399
Asparagales Orchidaceae Pleione formosana#MK361027
Asparagales Orchidaceae Bletilla ochracea#NC 029483
Asparagales Orchidaceae Bletilla striata#NC 028422
Asparagales Orchidaceae Liparis loeselii#MF374688
Asparagales Orchidaceae Oberonia japonica#NC 035832
Asparagales Orchidaceae Dendrobium kingianum#LC331062
Asparagales Orchidaceae Dendrobium salaccense#NC 035332
Asparagales Orchidaceae Dendrobium ellipsophyllum#NC 035340
Asparagales Orchidaceae Dendrobium strongylanthum#NC 027691
Asparagales Orchidaceae Dendrobium chrysotoxum#LC193517
Asparagales Orchidaceae Dendrobium denneanum#NC 035324
Asparagales Orchidaceae Dendrobium fimbriatum#NC 035342
Asparagales Orchidaceae Dendrobium lohohense#NC 035338
Asparagales Orchidaceae Dendrobium henryi#NC 035335
Asparagales Orchidaceae Dendrobium brymerianum#NC 035323
Asparagales Orchidaceae Dendrobium chrysanthum#NC 035336
Asparagales Orchidaceae Dendrobium crepidatum#NC 035331
Asparagales Orchidaceae Dendrobium jenkinsii#NC 035337
Asparagales Orchidaceae Dendrobium exile#NC 035343
Asparagales Orchidaceae Dendrobium spatella#NC 035333
Asparagales Orchidaceae Dendrobium parciflorum#NC 035334
Asparagales Orchidaceae Dendrobium parishii#NC 035339
Asparagales Orchidaceae Dendrobium polyanthum#NC 035321
Asparagales Orchidaceae Dendrobium aphyllum#NC 035322
Asparagales Orchidaceae Dendrobium loddigesii#NC 036355
Asparagales Orchidaceae Dendrobium wardianum#NC 035329
Asparagales Orchidaceae Dendrobium gratiosissimum#NC 035327
Asparagales Orchidaceae Dendrobium devonianum#LC317045
Asparagales Orchidaceae Dendrobium falconeri#NC 035326
Asparagales Orchidaceae Dendrobium pendulum#NC 029705
Asparagales Orchidaceae Dendrobium nobile#NC 029456
Asparagales Orchidaceae Dendrobium hercoglossum#NC 035328
Asparagales Orchidaceae Dendrobium moniliforme#NC 035154
Asparagales Orchidaceae Dendrobium xichouense#NC 035341
Asparagales Orchidaceae Dendrobium fanjingshanense#NC 035344
Asparagales Orchidaceae Dendrobium aduncum#LC348857
Asparagales Orchidaceae Dendrobium scoriarum#LC348847
Asparagales Orchidaceae Dendrobium flexicaule#LC348854
Asparagales Orchidaceae Dendrobium shixingense#LC348722
Asparagales Orchidaceae Dendrobium bellatulum#NC 037700
Asparagales Orchidaceae Dendrobium catenatum#LC348520
Asparagales Orchidaceae Calanthe davidii#NC 037438
Asparagales Orchidaceae Calanthe triplicata#NC 024544
Asparagales Orchidaceae Hexalectris warnockii#MH444822 @myco
Asparagales Orchidaceae Cattleya crispata#NC 026568
Asparagales Orchidaceae Masdevallia picturata#NC 026777
Asparagales Orchidaceae Masdevallia yungasensis#JSAG
Asparagales Orchidaceae Masdevallia coccinea#NC 026541
Asparagales Orchidaceae Calypso bulbosa var. occidentalis#NC 040980
Asparagales Orchidaceae Cremastra appendiculata#MH356724 @myco
Asparagales Orchidaceae Corallorhiza striata var. involuta#NC 040981 @myco
Asparagales Orchidaceae Corallorhiza bentleyi#NC 040979 @myco
Asparagales Orchidaceae Corallorhiza striata#MG874039 @myco
Asparagales Orchidaceae Corallorhiza cf.#NC 040982 @myco
Asparagales Orchidaceae Corallorhiza trifida#MG874036 @myco
Asparagales Orchidaceae Corallorhiza bulbosa#NC 025659 @myco
Asparagales Orchidaceae Corallorhiza wisteriana#NC 025663 @myco
Asparagales Orchidaceae Corallorhiza odontorhiza#NC 025664 @myco
Asparagales Orchidaceae Corallorhiza mertensiana#NC 025661 @myco
Asparagales Orchidaceae Corallorhiza macrantha#NC 025660 @myco
Asparagales Orchidaceae Eulophia zollingeri#NC 037212 @myco
Asparagales Orchidaceae Oncidium sphacelatum#CNTZ
Asparagales Orchidaceae Erycina pusilla#NC 018114
Asparagales Orchidaceae Cymbidium tracyanum#NC 021432
Asparagales Orchidaceae Cymbidium mannii#NC 021433
Asparagales Orchidaceae Cymbidium aloifolium#NC 021429
Asparagales Orchidaceae Cymbidium lancifolium#NC 029712 @myco
Asparagales Orchidaceae Cymbidium macrorhizon#NC 029713 @myco
Asparagales Orchidaceae Cymbidium ensifolium#NC 028525
Asparagales Orchidaceae Cymbidium faberi#NC 027743
Asparagales Orchidaceae Cymbidium goeringii#MF421552 @myco
Asparagales Orchidaceae Cymbidium kanran#NC 029711
Asparagales Orchidaceae Cymbidium sinense#NC 021430
Asparagales Orchidaceae Cymbidium tortisepalum#NC 021431
Asparagales Orchidaceae Phalaenopsis equestris#NC 017609
Asparagales Orchidaceae Phalaenopsis aphrodite subsp. formosana#NC 007499
Asparagales Orchidaceae Thrixspermum japonicum#NC 035831
Asparagales Orchidaceae Pelatantheria scolopendrifolia#MG752972
Asparagales Orchidaceae Gastrochilus fuscopunctatus#NC 035830
Asparagales Orchidaceae Gastrochilus japonicus#NC 035833
Asparagales Orchidaceae Vanda brunnea#NC 041522
Asparagales Orchidaceae Neofinetia falcata#KT726909
Asparagales Orchidaceae Neofinetia richardsiana#NC 036373
Asparagales Orchidaceae Holcoglossum subulifolium#NC 041519
Asparagales Orchidaceae Pendulorchis himalaica#NC 041513
Asparagales Orchidaceae Holcoglossum wangii#NC 041520
Asparagales Orchidaceae Holcoglossum amesianum#NC 041511
Asparagales Orchidaceae Holcoglossum nagalandense#NC 041514
Asparagales Orchidaceae Holcoglossum quasipinifolium#NC 041516
Asparagales Orchidaceae Holcoglossum lingulatum#NC 041465
Asparagales Orchidaceae Holcoglossum rupestre#NC 041517
Asparagales Orchidaceae Holcoglossum flavescens#MK442926
Asparagales Orchidaceae Holcoglossum sinicum#NC 041518
Asparagales Orchidaceae Holcoglossum weixiense#NC 041521
Asparagales Orchidaceae Holcoglossum nujiangense#MK442930
Dasypogonales Dasypogonaceae Baxteria australis#NC 029970
Dasypogonales Dasypogonaceae Kingia australis#FC387
Dasypogonales Dasypogonaceae Calectasia intermedia#FC383
Dasypogonales Dasypogonaceae Dasypogon hookeri#FC388
Dasypogonales Dasypogonaceae Dasypogon bromeliifolius#HQ
Arecales Arecaceae Eugeissona tristis#NC 029963
Arecales Arecaceae Eremospatha macrocarpa#NC 029964
Arecales Arecaceae Mauritia flexuosa#NC 029947
Arecales Arecaceae Salacca ramosiana#NC 029954
Arecales Arecaceae Metroxylon warburgii#NC 029959
Arecales Arecaceae Calamus caryotoides#NC 020365
Arecales Arecaceae Pigafetta elata#NC 029956
Arecales Arecaceae Nypa fruticans#NC 029958
Arecales Arecaceae Phytelephas aequatorialis#NC 029957
Arecales Arecaceae Pseudophoenix vinifera#NC 020364
Arecales Arecaceae Ravenea hildebrandtii#HQ
Arecales Arecaceae Chamaedorea seifrizii#DQ
Arecales Arecaceae Podococcus barteri#NC 027276
Arecales Arecaceae Veitchia arecina#NC 029950

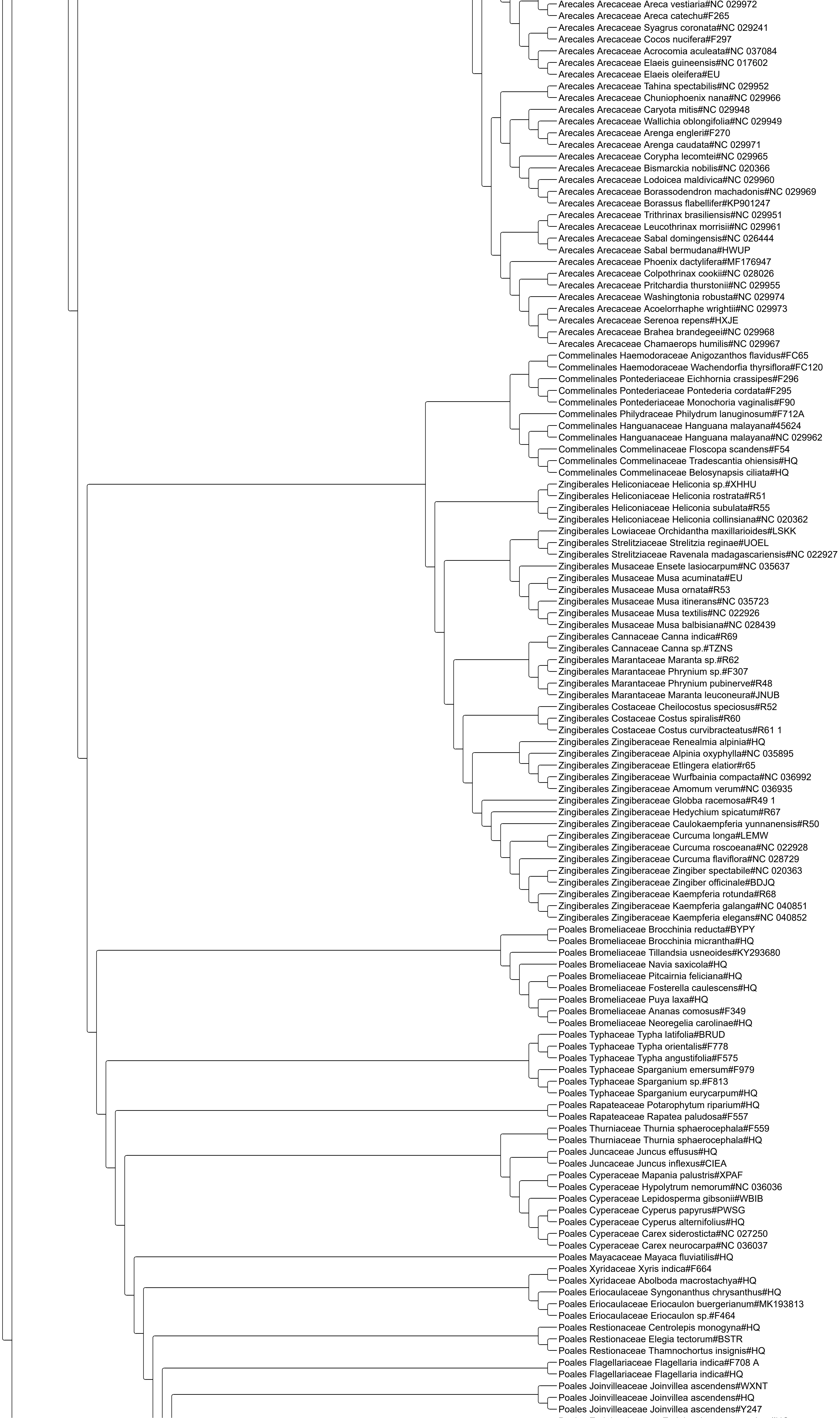
Arecales Arecaceae Areca vestiaria#NC 029972
Arecales Arecaceae Areca catechu#F265
Arecales Arecaceae Syagrus coronata#NC 029241
Arecales Arecaceae Cocos nucifera#F297
Arecales Arecaceae Acrocomia aculeata#NC 037084
Arecales Arecaceae Elaeis guineensis#NC 017602
Arecales Arecaceae Elaeis oleifera#EU
Arecales Arecaceae Tahina spectabilis#NC 029952
Arecales Arecaceae Chuniophoenix nana#NC 029966
Arecales Arecaceae Caryota mitis#NC 029948
Arecales Arecaceae Wallichia oblongifolia#NC 029949
Arecales Arecaceae Arenga engleri#F270
Arecales Arecaceae Arenga caudata#NC 029971
Arecales Arecaceae Corypha lecomtei#NC 029965
Arecales Arecaceae Bismarckia nobilis#NC 020366
Arecales Arecaceae Lodoicea maldivica#NC 029960
Arecales Arecaceae Borassodendron machadonis#NC 029969
Arecales Arecaceae Borassus flabellifer#KP901247
Arecales Arecaceae Trithrinax brasiliensis#NC 029951
Arecales Arecaceae Leucothrinax morrisii#NC 029961
Arecales Arecaceae Sabal domingensis#NC 026444
Arecales Arecaceae Sabal bermudana#HWUP
Arecales Arecaceae Phoenix dactylifera#MF176947
Arecales Arecaceae Colpothrinax cookii#NC 028026
Arecales Arecaceae Pritchardia thurstonii#NC 029955
Arecales Arecaceae Washingtonia robusta#NC 029974
Arecales Arecaceae Acoelorrhaphe wrightii#NC 029973
Arecales Arecaceae Serenoa repens#HXJE
Arecales Arecaceae Brahea brandegeei#NC 029968
Arecales Arecaceae Chamaerops humilis#NC 029967
Commelinales Haemodoraceae Anigozanthos flavidus#FC65
Commelinales Haemodoraceae Wachendorfia thyrsiflora#FC120
Commelinales Pontederiaceae Eichhornia crassipes#F296
Commelinales Pontederiaceae Pontederia cordata#F295
Commelinales Pontederiaceae Monochoria vaginalis#F90
Commelinales Philydraceae Philydrum lanuginosum#F712A
Commelinales Hanguanaceae Hanguana malayana#45624
Commelinales Hanguanaceae Hanguana malayana#NC 029962
Commelinales Commelinaceae Floscopa scandens#F54
Commelinales Commelinaceae Tradescantia ohiensis#HQ
Commelinales Commelinaceae Belosynapsis ciliata#HQ
Zingiberales Heliconiaceae Heliconia sp.#XHHU
Zingiberales Heliconiaceae Heliconia rostrata#R51
Zingiberales Heliconiaceae Heliconia subulata#R55
Zingiberales Heliconiaceae Heliconia collinsiana#NC 020362
Zingiberales Lowiaceae Orchidantha maxillarioides#LSKK
Zingiberales Strelitziaceae Strelitzia reginae#UOEL
Zingiberales Strelitziaceae Ravenala madagascariensis#NC 022927
Zingiberales Musaceae Ensete lasiocarpum#NC 035637
Zingiberales Musaceae Musa acuminata#EU
Zingiberales Musaceae Musa ornata#R53
Zingiberales Musaceae Musa itinerans#NC 035723
Zingiberales Musaceae Musa textilis#NC 022926
Zingiberales Musaceae Musa balbisiana#NC 028439
Zingiberales Cannaceae Canna indica#R69
Zingiberales Cannaceae Canna sp.#TZNS
Zingiberales Marantaceae Maranta sp.#R62
Zingiberales Marantaceae Phrynium sp.#F307
Zingiberales Marantaceae Phrynium pubinerve#R48
Zingiberales Marantaceae Maranta leuconeura#JNUB
Zingiberales Costaceae Cheilocostus speciosus#R52
Zingiberales Costaceae Costus spiralis#R60
Zingiberales Costaceae Costus curvibracteatus#R61 1
Zingiberales Zingiberaceae Renealmia alpinia#HQ
Zingiberales Zingiberaceae Alpinia oxyphylla#NC 035895
Zingiberales Zingiberaceae Etlingera elatior#r65
Zingiberales Zingiberaceae Wurfbainia compacta#NC 036992
Zingiberales Zingiberaceae Amomum verum#NC 036935
Zingiberales Zingiberaceae Globba racemosa#R49 1
Zingiberales Zingiberaceae Hedychium spicatum#R67
Zingiberales Zingiberaceae Caulokaempferia yunnanensis#R50
Zingiberales Zingiberaceae Curcuma longa#LEMW
Zingiberales Zingiberaceae Curcuma roscoeana#NC 022928
Zingiberales Zingiberaceae Curcuma flaviflora#NC 028729
Zingiberales Zingiberaceae Zingiber spectabile#NC 020363
Zingiberales Zingiberaceae Zingiber officinale#BDJQ
Zingiberales Zingiberaceae Kaempferia rotunda#R68
Zingiberales Zingiberaceae Kaempferia galanga#NC 040851
Zingiberales Zingiberaceae Kaempferia elegans#NC 040852
Poales Bromeliaceae Brocchinia reducta#BYPY
Poales Bromeliaceae Brocchinia micrantha#HQ
Poales Bromeliaceae Tillandsia usneoides#KY293680
Poales Bromeliaceae Navia saxicola#HQ
Poales Bromeliaceae Pitcairnia feliciana#HQ
Poales Bromeliaceae Fosterella caulescens#HQ
Poales Bromeliaceae Puya laxa#HQ
Poales Bromeliaceae Ananas comosus#F349
Poales Bromeliaceae Neoregelia carolinae#HQ
Poales Typhaceae Typha latifolia#BRUD
Poales Typhaceae Typha orientalis#F778
Poales Typhaceae Typha angustifolia#F575
Poales Typhaceae Sparganium emersum#F979
Poales Typhaceae Sparganium sp.#F813
Poales Typhaceae Sparganium eurycarpum#HQ
Poales Rapateaceae Potarophytum riparium#HQ
Poales Rapateaceae Rapatea paludosa#F557
Poales Thurniaceae Thurnia sphaerocephala#F559
Poales Thurniaceae Thurnia sphaerocephala#HQ
Poales Juncaceae Juncus effusus#HQ
Poales Juncaceae Juncus inflexus#CIEA
Poales Cyperaceae Mapania palustris#XPAF
Poales Cyperaceae Hypolytrum nemorum#NC 036036
Poales Cyperaceae Lepidosperma gibsonii#WBIB
Poales Cyperaceae Cyperus papyrus#PWSG
Poales Cyperaceae Cyperus alternifolius#HQ
Poales Cyperaceae Carex siderosticta#NC 027250
Poales Cyperaceae Carex neurocarpa#NC 036037
Poales Mayacaceae Mayaca fluviatilis#HQ
Poales Xyridaceae Xyris indica#F664
Poales Xyridaceae Abolboda macrostachya#HQ
Poales Eriocaulaceae Syngonanthus chrysanthus#HQ
Poales Eriocaulaceae Eriocaulon buergerianum#MK193813
Poales Eriocaulaceae Eriocaulon sp.#F464
Poales Restionaceae Centrolepis monogyna#HQ
Poales Restionaceae Elegia tectorum#BSTR
Poales Restionaceae Thamnochortus insignis#HQ
Poales Flagellariaceae Flagellaria indica#F708 A
Poales Flagellariaceae Flagellaria indica#HQ
Poales Joinvilleaceae Joinvillea ascendens#WXNT
Poales Joinvilleaceae Joinvillea ascendens#HQ
Poales Joinvilleaceae Joinvillea ascendens#Y247

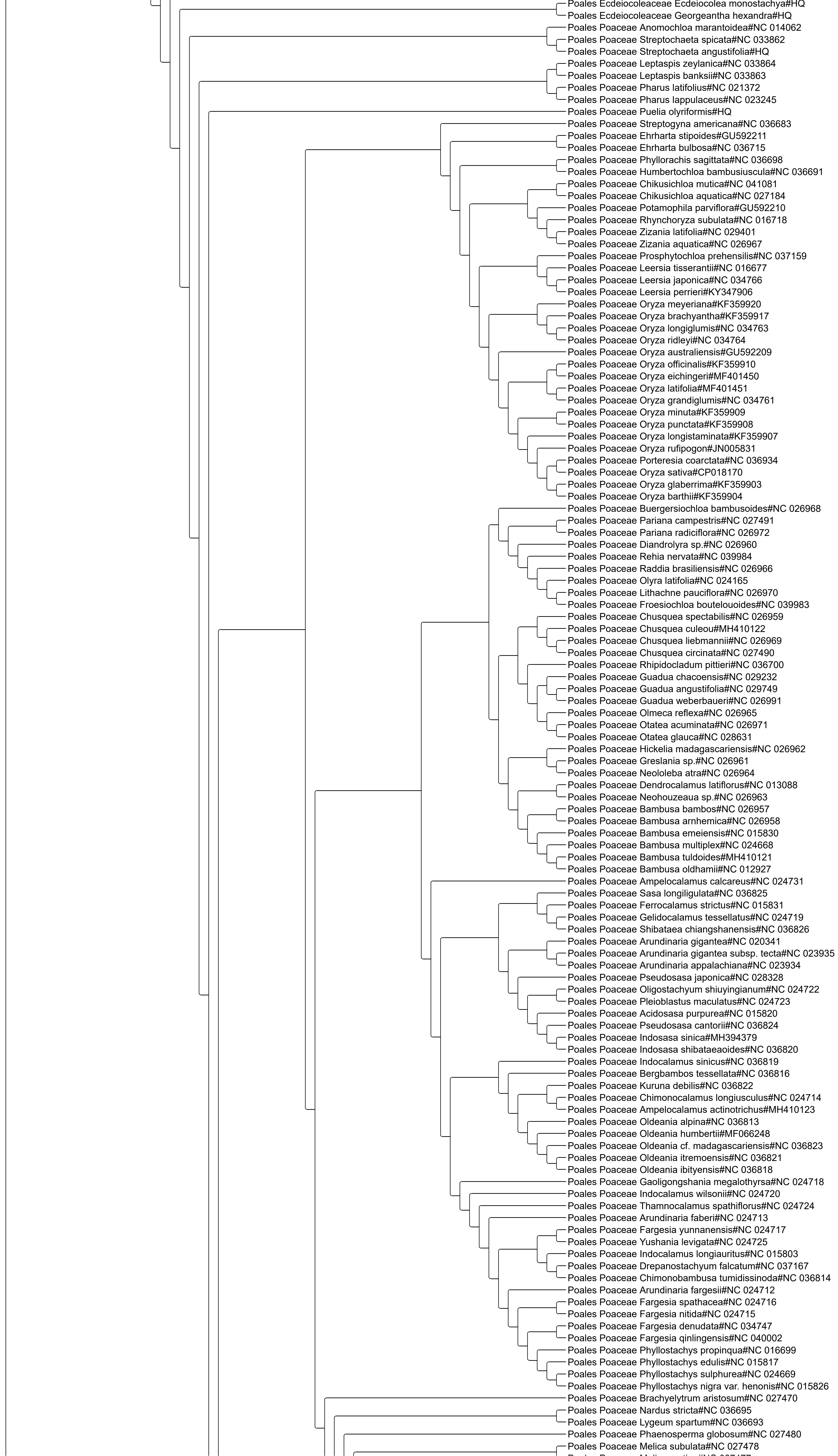
Poales Ecdeiocoleaceae Ecdeiocolea monostachya#HQ
Poales Ecdeiocoleaceae Georgeantha hexandra#HQ
Poales Poaceae Anomochloa marantoidea#NC 014062
Poales Poaceae Streptochaeta spicata#NC 033862
Poales Poaceae Streptochaeta angustifolia#HQ
Poales Poaceae Leptaspis zeylanica#NC 033864
Poales Poaceae Leptaspis banksii#NC 033863
Poales Poaceae Pharus latifolius#NC 021372
Poales Poaceae Pharus lappulaceus#NC 023245
Poales Poaceae Puelia olyriformis#HQ
Poales Poaceae Streptogyna americana#NC 036683
Poales Poaceae Ehrharta stipoides#GU592211
Poales Poaceae Ehrharta bulbosa#NC 036715
Poales Poaceae Phyllorachis sagittata#NC 036698
Poales Poaceae Humbertochloa bambusiuscula#NC 036691
Poales Poaceae Chikusichloa mutica#NC 041081
Poales Poaceae Chikusichloa aquatica#NC 027184
Poales Poaceae Potamophila parviflora#GU592210
Poales Poaceae Rhynchoryza subulata#NC 016718
Poales Poaceae Zizania latifolia#NC 029401
Poales Poaceae Zizania aquatica#NC 026967
Poales Poaceae Prosphytochloa prehensilis#NC 037159
Poales Poaceae Leersia tisserantii#NC 016677
Poales Poaceae Leersia japonica#NC 034766
Poales Poaceae Leersia perrieri#KY347906
Poales Poaceae Oryza meyeriana#KF359920
Poales Poaceae Oryza brachyantha#KF359917
Poales Poaceae Oryza longiglumis#NC 034763
Poales Poaceae Oryza ridleyi#NC 034764
Poales Poaceae Oryza australiensis#GU592209
Poales Poaceae Oryza officinalis#KF359910
Poales Poaceae Oryza eichingeri#MF401450
Poales Poaceae Oryza latifolia#MF401451
Poales Poaceae Oryza grandiglumis#NC 034761
Poales Poaceae Oryza minuta#KF359909
Poales Poaceae Oryza punctata#KF359908
Poales Poaceae Oryza longistaminata#KF359907
Poales Poaceae Oryza rufipogon#JN005831
Poales Poaceae Porteresia coarctata#NC 036934
Poales Poaceae Oryza sativa#CP018170
Poales Poaceae Oryza glaberrima#KF359903
Poales Poaceae Oryza barthii#KF359904
Poales Poaceae Buergersiochloa bambusoides#NC 026968
Poales Poaceae Pariana campestris#NC 027491
Poales Poaceae Pariana radiciflora#NC 026972
Poales Poaceae Diandrolyra sp.#NC 026960
Poales Poaceae Rehia nervata#NC 039984
Poales Poaceae Raddia brasiliensis#NC 026966
Poales Poaceae Olyra latifolia#NC 024165
Poales Poaceae Lithachne pauciflora#NC 026970
Poales Poaceae Froesiochloa boutelouoides#NC 039983
Poales Poaceae Chusquea spectabilis#NC 026959
Poales Poaceae Chusquea culeou#MH410122
Poales Poaceae Chusquea liebmannii#NC 026969
Poales Poaceae Chusquea circinata#NC 027490
Poales Poaceae Rhipidocladum pittieri#NC 036700
Poales Poaceae Guadua chacoensis#NC 029232
Poales Poaceae Guadua angustifolia#NC 029749
Poales Poaceae Guadua weberbaueri#NC 026991
Poales Poaceae Olmeca reflexa#NC 026965
Poales Poaceae Otatea acuminata#NC 026971
Poales Poaceae Otatea glauca#NC 028631
Poales Poaceae Hickelia madagascariensis#NC 026962
Poales Poaceae Greslania sp.#NC 026961
Poales Poaceae Neololeba atra#NC 026964
Poales Poaceae Dendrocalamus latiflorus#NC 013088
Poales Poaceae Neohouzeaua sp.#NC 026963
Poales Poaceae Bambusa bambos#NC 026957
Poales Poaceae Bambusa arnhemica#NC 026958
Poales Poaceae Bambusa emeiensis#NC 015830
Poales Poaceae Bambusa multiplex#NC 024668
Poales Poaceae Bambusa tuldoides#MH410121
Poales Poaceae Bambusa oldhamii#NC 012927
Poales Poaceae Ampelocalamus calcareus#NC 024731
Poales Poaceae Sasa longiligulata#NC 036825
Poales Poaceae Ferrocalamus strictus#NC 015831
Poales Poaceae Gelidocalamus tessellatus#NC 024719
Poales Poaceae Shibataea chiangshanensis#NC 036826
Poales Poaceae Arundinaria gigantea#NC 020341
Poales Poaceae Arundinaria gigantea subsp. tecta#NC 023935
Poales Poaceae Arundinaria appalachiana#NC 023934
Poales Poaceae Pseudosasa japonica#NC 028328
Poales Poaceae Oligostachyum shiuyingianum#NC 024722
Poales Poaceae Pleioblastus maculatus#NC 024723
Poales Poaceae Acidosasa purpurea#NC 015820
Poales Poaceae Pseudosasa cantorii#NC 036824
Poales Poaceae Indosasa sinica#MH394379
Poales Poaceae Indosasa shibataeaoides#NC 036820
Poales Poaceae Indocalamus sinicus#NC 036819
Poales Poaceae Bergbambos tessellata#NC 036816
Poales Poaceae Kuruna debilis#NC 036822
Poales Poaceae Chimonocalamus longiusculus#NC 024714
Poales Poaceae Ampelocalamus actinotrichus#MH410123
Poales Poaceae Oldeania alpina#NC 036813
Poales Poaceae Oldeania humbertii#MF066248
Poales Poaceae Oldeania cf. madagascariensis#NC 036823
Poales Poaceae Oldeania itremoensis#NC 036821
Poales Poaceae Oldeania ibityensis#NC 036818
Poales Poaceae Gaoligongshania megalothyrsa#NC 024718
Poales Poaceae Indocalamus wilsonii#NC 024720
Poales Poaceae Thamnocalamus spathiflorus#NC 024724
Poales Poaceae Arundinaria faberi#NC 024713
Poales Poaceae Fargesia yunnanensis#NC 024717
Poales Poaceae Yushania levigata#NC 024725
Poales Poaceae Indocalamus longiauritus#NC 015803
Poales Poaceae Drepanostachyum falcatum#NC 037167
Poales Poaceae Chimonobambusa tumidissinoda#NC 036814
Poales Poaceae Arundinaria fargesii#NC 024712
Poales Poaceae Fargesia spathacea#NC 024716
Poales Poaceae Fargesia nitida#NC 024715
Poales Poaceae Fargesia denudata#NC 034747
Poales Poaceae Fargesia qinlingensis#NC 040002
Poales Poaceae Phyllostachys propinqua#NC 016699
Poales Poaceae Phyllostachys edulis#NC 015817
Poales Poaceae Phyllostachys sulphurea#NC 024669
Poales Poaceae Phyllostachys nigra var. henonis#NC 015826
Poales Poaceae Brachyelytrum aristosum#NC 027470
Poales Poaceae Nardus stricta#NC 036695
Poales Poaceae Lygeum spartum#NC 036693
Poales Poaceae Phaenosperma globosum#NC 027480
Poales Poaceae Melica subulata#NC 027478

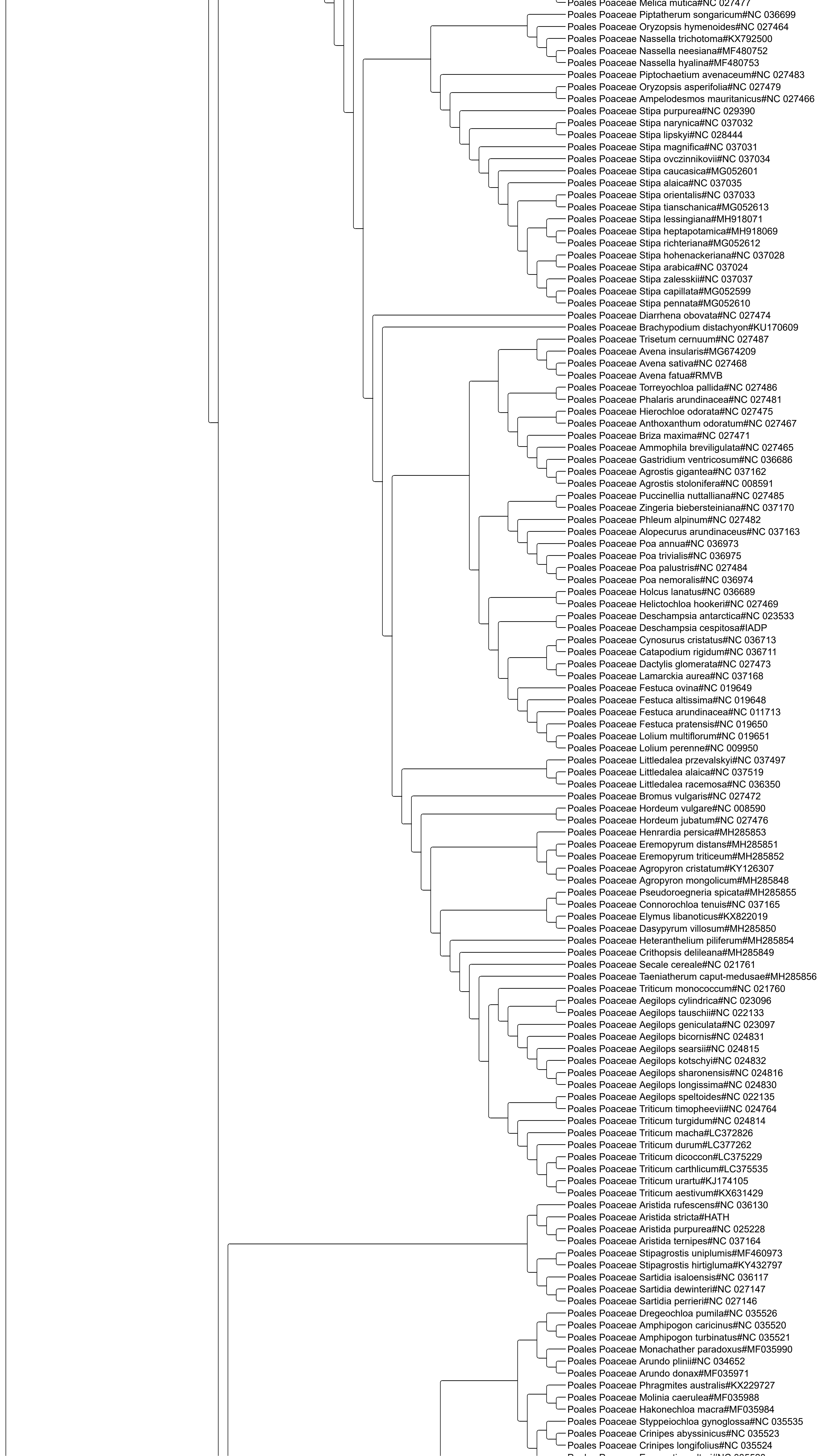
Poales Poaceae Melica mutica#NC 027477
Poales Poaceae Piptatherum songaricum#NC 036699
Poales Poaceae Oryzopsis hymenoides#NC 027464
Poales Poaceae Nassella trichotoma#KX792500
Poales Poaceae Nassella neesiana#MF480752
Poales Poaceae Nassella hyalina#MF480753
Poales Poaceae Piptochaetium avenaceum#NC 027483
Poales Poaceae Oryzopsis asperifolia#NC 027479
Poales Poaceae Ampelodesmos mauritanicus#NC 027466
Poales Poaceae Stipa purpurea#NC 029390
Poales Poaceae Stipa narynica#NC 037032
Poales Poaceae Stipa lipskyi#NC 028444
Poales Poaceae Stipa magnifica#NC 037031
Poales Poaceae Stipa ovczinnikovii#NC 037034
Poales Poaceae Stipa caucasica#MG052601
Poales Poaceae Stipa alaica#NC 037035
Poales Poaceae Stipa orientalis#NC 037033
Poales Poaceae Stipa tianschanica#MG052613
Poales Poaceae Stipa lessingiana#MH918071
Poales Poaceae Stipa heptapotamica#MH918069
Poales Poaceae Stipa richteriana#MG052612
Poales Poaceae Stipa hohenackeriana#NC 037028
Poales Poaceae Stipa arabica#NC 037024
Poales Poaceae Stipa zalesskii#NC 037037
Poales Poaceae Stipa capillata#MG052599
Poales Poaceae Stipa pennata#MG052610
Poales Poaceae Diarrhena obovata#NC 027474
Poales Poaceae Brachypodium distachyon#KU170609
Poales Poaceae Trisetum cernuum#NC 027487
Poales Poaceae Avena insularis#MG674209
Poales Poaceae Avena sativa#NC 027468
Poales Poaceae Avena fatua#RMVB
Poales Poaceae Torreyochloa pallida#NC 027486
Poales Poaceae Phalaris arundinacea#NC 027481
Poales Poaceae Hierochloe odorata#NC 027475
Poales Poaceae Anthoxanthum odoratum#NC 027467
Poales Poaceae Briza maxima#NC 027471
Poales Poaceae Ammophila breviligulata#NC 027465
Poales Poaceae Gastridium ventricosum#NC 036686
Poales Poaceae Agrostis gigantea#NC 037162
Poales Poaceae Agrostis stolonifera#NC 008591
Poales Poaceae Puccinellia nuttalliana#NC 027485
Poales Poaceae Zingeria biebersteiniana#NC 037170
Poales Poaceae Phleum alpinum#NC 027482
Poales Poaceae Alopecurus arundinaceus#NC 037163
Poales Poaceae Poa annua#NC 036973
Poales Poaceae Poa trivialis#NC 036975
Poales Poaceae Poa palustris#NC 027484
Poales Poaceae Poa nemoralis#NC 036974
Poales Poaceae Holcus lanatus#NC 036689
Poales Poaceae Helictochloa hookeri#NC 027469
Poales Poaceae Deschampsia antarctica#NC 023533
Poales Poaceae Deschampsia cespitosa#IADP
Poales Poaceae Cynosurus cristatus#NC 036713
Poales Poaceae Catapodium rigidum#NC 036711
Poales Poaceae Dactylis glomerata#NC 027473
Poales Poaceae Lamarckia aurea#NC 037168
Poales Poaceae Festuca ovina#NC 019649
Poales Poaceae Festuca altissima#NC 019648
Poales Poaceae Festuca arundinacea#NC 011713
Poales Poaceae Festuca pratensis#NC 019650
Poales Poaceae Lolium multiflorum#NC 019651
Poales Poaceae Lolium perenne#NC 009950
Poales Poaceae Littledalea przevalskyi#NC 037497
Poales Poaceae Littledalea alaica#NC 037519
Poales Poaceae Littledalea racemosa#NC 036350
Poales Poaceae Bromus vulgaris#NC 027472
Poales Poaceae Hordeum vulgare#NC 008590
Poales Poaceae Hordeum jubatum#NC 027476
Poales Poaceae Henrardia persica#MH285853
Poales Poaceae Eremopyrum distans#MH285851
Poales Poaceae Eremopyrum triticeum#MH285852
Poales Poaceae Agropyron cristatum#KY126307
Poales Poaceae Agropyron mongolicum#MH285848
Poales Poaceae Pseudoroegneria spicata#MH285855
Poales Poaceae Connorochloa tenuis#NC 037165
Poales Poaceae Elymus libanoticus#KX822019
Poales Poaceae Dasypyrum villosum#MH285850
Poales Poaceae Heteranthelium piliferum#MH285854
Poales Poaceae Crithopsis delileana#MH285849
Poales Poaceae Secale cereale#NC 021761
Poales Poaceae Taeniatherum caput-medusae#MH285856
Poales Poaceae Triticum monococcum#NC 021760
Poales Poaceae Aegilops cylindrica#NC 023096
Poales Poaceae Aegilops tauschii#NC 022133
Poales Poaceae Aegilops geniculata#NC 023097
Poales Poaceae Aegilops bicornis#NC 024831
Poales Poaceae Aegilops searsii#NC 024815
Poales Poaceae Aegilops kotschyi#NC 024832
Poales Poaceae Aegilops sharonensis#NC 024816
Poales Poaceae Aegilops longissima#NC 024830
Poales Poaceae Aegilops speltoides#NC 022135
Poales Poaceae Triticum timopheevii#NC 024764
Poales Poaceae Triticum turgidum#NC 024814
Poales Poaceae Triticum macha#LC372826
Poales Poaceae Triticum durum#LC377262
Poales Poaceae Triticum dicoccon#LC375229
Poales Poaceae Triticum carthlicum#LC375535
Poales Poaceae Triticum urartu#KJ174105
Poales Poaceae Triticum aestivum#KX631429
Poales Poaceae Aristida rufescens#NC 036130
Poales Poaceae Aristida stricta#HATH
Poales Poaceae Aristida purpurea#NC 025228
Poales Poaceae Aristida ternipes#NC 037164
Poales Poaceae Stipagrostis uniplumis#MF460973
Poales Poaceae Stipagrostis hirtigluma#KY432797
Poales Poaceae Sartidia isaloensis#NC 036117
Poales Poaceae Sartidia dewinteri#NC 027147
Poales Poaceae Sartidia perrieri#NC 027146
Poales Poaceae Dregeochloa pumila#NC 035526
Poales Poaceae Amphipogon caricinus#NC 035520
Poales Poaceae Amphipogon turbinatus#NC 035521
Poales Poaceae Monachather paradoxus#MF035990
Poales Poaceae Arundo plinii#NC 034652
Poales Poaceae Arundo donax#MF035971
Poales Poaceae Phragmites australis#KX229727
Poales Poaceae Molinia caerulea#MF035988
Poales Poaceae Hakonechloa macra#MF035984
Poales Poaceae Styppeiochloa gynoglossa#NC 035535
Poales Poaceae Crinipes abyssinicus#NC 035523
Poales Poaceae Crinipes longifolius#NC 035524

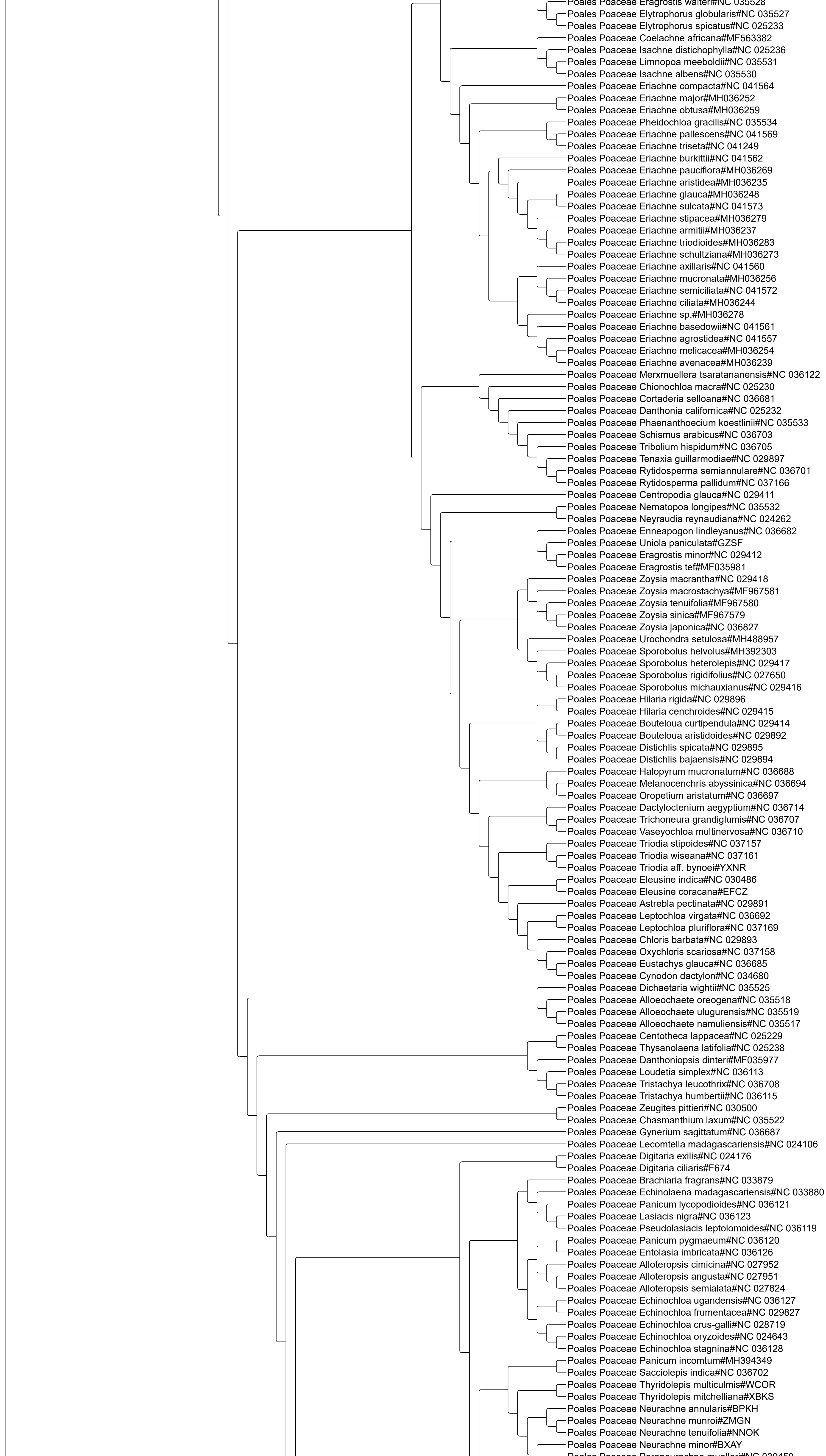

Poales Poaceae Eragrostis walteri#NC 035528
Poales Poaceae Elytrophorus globularis#NC 035527
Poales Poaceae Elytrophorus spicatus#NC 025233
Poales Poaceae Coelachne africana#MF563382
Poales Poaceae Isachne distichophylla#NC 025236
Poales Poaceae Limnopoa meeboldii#NC 035531
Poales Poaceae Isachne albens#NC 035530
Poales Poaceae Eriachne compacta#NC 041564
Poales Poaceae Eriachne major#MH036252
Poales Poaceae Eriachne obtusa#MH036259
Poales Poaceae Pheidochloa gracilis#NC 035534
Poales Poaceae Eriachne pallescens#NC 041569
Poales Poaceae Eriachne triseta#NC 041249
Poales Poaceae Eriachne burkittii#NC 041562
Poales Poaceae Eriachne pauciflora#MH036269
Poales Poaceae Eriachne aristidea#MH036235
Poales Poaceae Eriachne glauca#MH036248
Poales Poaceae Eriachne sulcata#NC 041573
Poales Poaceae Eriachne stipacea#MH036279
Poales Poaceae Eriachne armitii#MH036237
Poales Poaceae Eriachne triodioides#MH036283
Poales Poaceae Eriachne schultziana#MH036273
Poales Poaceae Eriachne axillaris#NC 041560
Poales Poaceae Eriachne mucronata#MH036256
Poales Poaceae Eriachne semiciliata#NC 041572
Poales Poaceae Eriachne ciliata#MH036244
Poales Poaceae Eriachne sp.#MH036278
Poales Poaceae Eriachne basedowii#NC 041561
Poales Poaceae Eriachne agrostidea#NC 041557
Poales Poaceae Eriachne melicacea#MH036254
Poales Poaceae Eriachne avenacea#MH036239
Poales Poaceae Merxmuellera tsaratananensis#NC 036122
Poales Poaceae Chionochloa macra#NC 025230
Poales Poaceae Cortaderia selloana#NC 036681
Poales Poaceae Danthonia californica#NC 025232
Poales Poaceae Phaenanthoecium koestlinii#NC 035533
Poales Poaceae Schismus arabicus#NC 036703
Poales Poaceae Tribolium hispidum#NC 036705
Poales Poaceae Tenaxia guillarmodiae#NC 029897
Poales Poaceae Rytidosperma semiannulare#NC 036701
Poales Poaceae Rytidosperma pallidum#NC 037166
Poales Poaceae Centropodia glauca#NC 029411
Poales Poaceae Nematopoa longipes#NC 035532
Poales Poaceae Neyraudia reynaudiana#NC 024262
Poales Poaceae Enneapogon lindleyanus#NC 036682
Poales Poaceae Uniola paniculata#GZSF
Poales Poaceae Eragrostis minor#NC 029412
Poales Poaceae Eragrostis tef#MF035981
Poales Poaceae Zoysia macrantha#NC 029418
Poales Poaceae Zoysia macrostachya#MF967581
Poales Poaceae Zoysia tenuifolia#MF967580
Poales Poaceae Zoysia sinica#MF967579
Poales Poaceae Zoysia japonica#NC 036827
Poales Poaceae Urochondra setulosa#MH488957
Poales Poaceae Sporobolus helvolus#MH392303
Poales Poaceae Sporobolus heterolepis#NC 029417
Poales Poaceae Sporobolus rigidifolius#NC 027650
Poales Poaceae Sporobolus michauxianus#NC 029416
Poales Poaceae Hilaria rigida#NC 029896
Poales Poaceae Hilaria cenchroides#NC 029415
Poales Poaceae Bouteloua curtipendula#NC 029414
Poales Poaceae Bouteloua aristidoides#NC 029892
Poales Poaceae Distichlis spicata#NC 029895
Poales Poaceae Distichlis bajaensis#NC 029894
Poales Poaceae Halopyrum mucronatum#NC 036688
Poales Poaceae Melanocenchris abyssinica#NC 036694
Poales Poaceae Oropetium aristatum#NC 036697
Poales Poaceae Dactyloctenium aegyptium#NC 036714
Poales Poaceae Trichoneura grandiglumis#NC 036707
Poales Poaceae Vaseyochloa multinervosa#NC 036710
Poales Poaceae Triodia stipoides#NC 037157
Poales Poaceae Triodia wiseana#NC 037161
Poales Poaceae Triodia aff. bynoei#YXNR
Poales Poaceae Eleusine indica#NC 030486
Poales Poaceae Eleusine coracana#EFCZ
Poales Poaceae Astrebla pectinata#NC 029891
Poales Poaceae Leptochloa virgata#NC 036692
Poales Poaceae Leptochloa pluriflora#NC 037169
Poales Poaceae Chloris barbata#NC 029893
Poales Poaceae Oxychloris scariosa#NC 037158
Poales Poaceae Eustachys glauca#NC 036685
Poales Poaceae Cynodon dactylon#NC 034680
Poales Poaceae Dichaetaria wightii#NC 035525
Poales Poaceae Alloeochaete oreogena#NC 035518
Poales Poaceae Alloeochaete ulugurensis#NC 035519
Poales Poaceae Alloeochaete namuliensis#NC 035517
Poales Poaceae Centotheca lappacea#NC 025229
Poales Poaceae Thysanolaena latifolia#NC 025238
Poales Poaceae Danthoniopsis dinteri#MF035977
Poales Poaceae Loudetia simplex#NC 036113
Poales Poaceae Tristachya leucothrix#NC 036708
Poales Poaceae Tristachya humbertii#NC 036115
Poales Poaceae Zeugites pittieri#NC 030500
Poales Poaceae Chasmanthium laxum#NC 035522
Poales Poaceae Gynerium sagittatum#NC 036687
Poales Poaceae Lecomtella madagascariensis#NC 024106
Poales Poaceae Digitaria exilis#NC 024176
Poales Poaceae Digitaria ciliaris#F674
Poales Poaceae Brachiaria fragrans#NC 033879
Poales Poaceae Echinolaena madagascariensis#NC 033880
Poales Poaceae Panicum lycopodioides#NC 036121
Poales Poaceae Lasiacis nigra#NC 036123
Poales Poaceae Pseudolasiacis leptolomoides#NC 036119
Poales Poaceae Panicum pygmaeum#NC 036120
Poales Poaceae Entolasia imbricata#NC 036126
Poales Poaceae Alloteropsis cimicina#NC 027952
Poales Poaceae Alloteropsis angusta#NC 027951
Poales Poaceae Alloteropsis semialata#NC 027824
Poales Poaceae Echinochloa ugandensis#NC 036127
Poales Poaceae Echinochloa frumentacea#NC 029827
Poales Poaceae Echinochloa crus-galli#NC 028719
Poales Poaceae Echinochloa oryzoides#NC 024643
Poales Poaceae Echinochloa stagnina#NC 036128
Poales Poaceae Panicum incomtum#MH394349
Poales Poaceae Sacciolepis indica#NC 036702
Poales Poaceae Thyridolepis multiculmis#WCOR
Poales Poaceae Thyridolepis mitchelliana#XBKS
Poales Poaceae Neurachne annularis#BPKH
Poales Poaceae Neurachne munroi#ZMGN
Poales Poaceae Neurachne tenuifolia#NNOK
Poales Poaceae Neurachne minor#BXAY

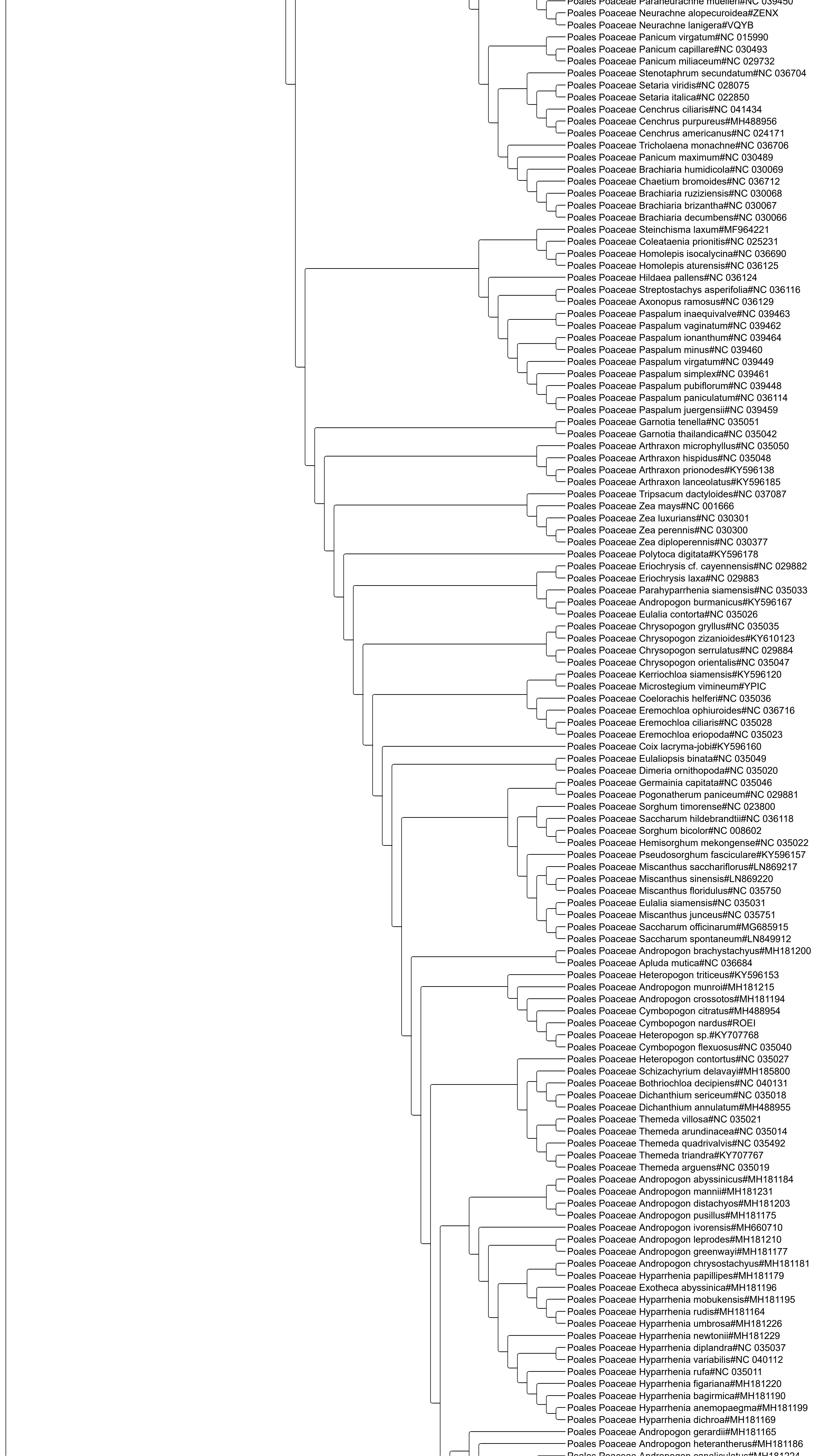
Poales Poaceae Neurachne alopecuroidea#ZENX
Poales Poaceae Neurachne lanigera#VQYB
Poales Poaceae Panicum virgatum#NC 015990
Poales Poaceae Panicum capillare#NC 030493
Poales Poaceae Panicum miliaceum#NC 029732
Poales Poaceae Stenotaphrum secundatum#NC 036704
Poales Poaceae Setaria viridis#NC 028075
Poales Poaceae Setaria italica#NC 022850
Poales Poaceae Cenchrus ciliaris#NC 041434
Poales Poaceae Cenchrus purpureus#MH488956
Poales Poaceae Cenchrus americanus#NC 024171
Poales Poaceae Tricholaena monachne#NC 036706
Poales Poaceae Panicum maximum#NC 030489
Poales Poaceae Brachiaria humidicola#NC 030069
Poales Poaceae Chaetium bromoides#NC 036712
Poales Poaceae Brachiaria ruziziensis#NC 030068
Poales Poaceae Brachiaria brizantha#NC 030067
Poales Poaceae Brachiaria decumbens#NC 030066
Poales Poaceae Steinchisma laxum#MF964221
Poales Poaceae Coleataenia prionitis#NC 025231
Poales Poaceae Homolepis isocalycina#NC 036690
Poales Poaceae Homolepis aturensis#NC 036125
Poales Poaceae Hildaea pallens#NC 036124
Poales Poaceae Streptostachys asperifolia#NC 036116
Poales Poaceae Axonopus ramosus#NC 036129
Poales Poaceae Paspalum inaequivalve#NC 039463
Poales Poaceae Paspalum vaginatum#NC 039462
Poales Poaceae Paspalum ionanthum#NC 039464
Poales Poaceae Paspalum minus#NC 039460
Poales Poaceae Paspalum virgatum#NC 039449
Poales Poaceae Paspalum simplex#NC 039461
Poales Poaceae Paspalum pubiflorum#NC 039448
Poales Poaceae Paspalum paniculatum#NC 036114
Poales Poaceae Paspalum juergensii#NC 039459
Poales Poaceae Garnotia tenella#NC 035051
Poales Poaceae Garnotia thailandica#NC 035042
Poales Poaceae Arthraxon microphyllus#NC 035050
Poales Poaceae Arthraxon hispidus#NC 035048
Poales Poaceae Arthraxon prionodes#KY596138
Poales Poaceae Arthraxon lanceolatus#KY596185
Poales Poaceae Tripsacum dactyloides#NC 037087
Poales Poaceae Zea mays#NC 001666
Poales Poaceae Zea luxurians#NC 030301
Poales Poaceae Zea perennis#NC 030300
Poales Poaceae Zea diploperennis#NC 030377
Poales Poaceae Polytoca digitata#KY596178
Poales Poaceae Eriochrysis cf. cayennensis#NC 029882
Poales Poaceae Eriochrysis laxa#NC 029883
Poales Poaceae Parahyparrhenia siamensis#NC 035033
Poales Poaceae Andropogon burmanicus#KY596167
Poales Poaceae Eulalia contorta#NC 035026
Poales Poaceae Chrysopogon gryllus#NC 035035
Poales Poaceae Chrysopogon zizanioides#KY610123
Poales Poaceae Chrysopogon serrulatus#NC 029884
Poales Poaceae Chrysopogon orientalis#NC 035047
Poales Poaceae Kerriochloa siamensis#KY596120
Poales Poaceae Microstegium vimineum#YPIC
Poales Poaceae Coelorachis helferi#NC 035036
Poales Poaceae Eremochloa ophiuroides#NC 036716
Poales Poaceae Eremochloa ciliaris#NC 035028
Poales Poaceae Eremochloa eriopoda#NC 035023
Poales Poaceae Coix lacryma-jobi#KY596160
Poales Poaceae Eulaliopsis binata#NC 035049
Poales Poaceae Dimeria ornithopoda#NC 035020
Poales Poaceae Germainia capitata#NC 035046
Poales Poaceae Pogonatherum paniceum#NC 029881
Poales Poaceae Sorghum timorense#NC 023800
Poales Poaceae Saccharum hildebrandtii#NC 036118
Poales Poaceae Sorghum bicolor#NC 008602
Poales Poaceae Hemisorghum mekongense#NC 035022
Poales Poaceae Pseudosorghum fasciculare#KY596157
Poales Poaceae Miscanthus sacchariflorus#LN869217
Poales Poaceae Miscanthus sinensis#LN869220
Poales Poaceae Miscanthus floridulus#NC 035750
Poales Poaceae Eulalia siamensis#NC 035031
Poales Poaceae Miscanthus junceus#NC 035751
Poales Poaceae Saccharum officinarum#MG685915
Poales Poaceae Saccharum spontaneum#LN849912
Poales Poaceae Andropogon brachystachyus#MH181200
Poales Poaceae Apluda mutica#NC 036684
Poales Poaceae Heteropogon triticeus#KY596153
Poales Poaceae Andropogon munroi#MH181215
Poales Poaceae Andropogon crossotos#MH181194
Poales Poaceae Cymbopogon citratus#MH488954
Poales Poaceae Cymbopogon nardus#ROEI
Poales Poaceae Heteropogon sp.#KY707768
Poales Poaceae Cymbopogon flexuosus#NC 035040
Poales Poaceae Heteropogon contortus#NC 035027
Poales Poaceae Schizachyrium delavayi#MH185800
Poales Poaceae Bothriochloa decipiens#NC 040131
Poales Poaceae Dichanthium sericeum#NC 035018
Poales Poaceae Dichanthium annulatum#MH488955
Poales Poaceae Themeda villosa#NC 035021
Poales Poaceae Themeda arundinacea#NC 035014
Poales Poaceae Themeda quadrivalvis#NC 035492
Poales Poaceae Themeda triandra#KY707767
Poales Poaceae Themeda arguens#NC 035019
Poales Poaceae Andropogon abyssinicus#MH181184
Poales Poaceae Andropogon mannii#MH181231
Poales Poaceae Andropogon distachyos#MH181203
Poales Poaceae Andropogon pusillus#MH181175
Poales Poaceae Andropogon ivorensis#MH660710
Poales Poaceae Andropogon leprodes#MH181210
Poales Poaceae Andropogon greenwayi#MH181177
Poales Poaceae Andropogon chrysostachyus#MH181181
Poales Poaceae Hyparrhenia papillipes#MH181179
Poales Poaceae Exotheca abyssinica#MH181196
Poales Poaceae Hyparrhenia mobukensis#MH181195
Poales Poaceae Hyparrhenia rudis#MH181164
Poales Poaceae Hyparrhenia umbrosa#MH181226
Poales Poaceae Hyparrhenia newtonii#MH181229
Poales Poaceae Hyparrhenia diplandra#NC 035037
Poales Poaceae Hyparrhenia variabilis#NC 040112
Poales Poaceae Hyparrhenia rufa#NC 035011
Poales Poaceae Hyparrhenia figariana#MH181220
Poales Poaceae Hyparrhenia bagirmica#MH181190
Poales Poaceae Hyparrhenia anemopaegma#MH181199
Poales Poaceae Hyparrhenia dichroa#MH181169
Poales Poaceae Andropogon gerardii#MH181165
Poales Poaceae Andropogon heteranthus#MH181186

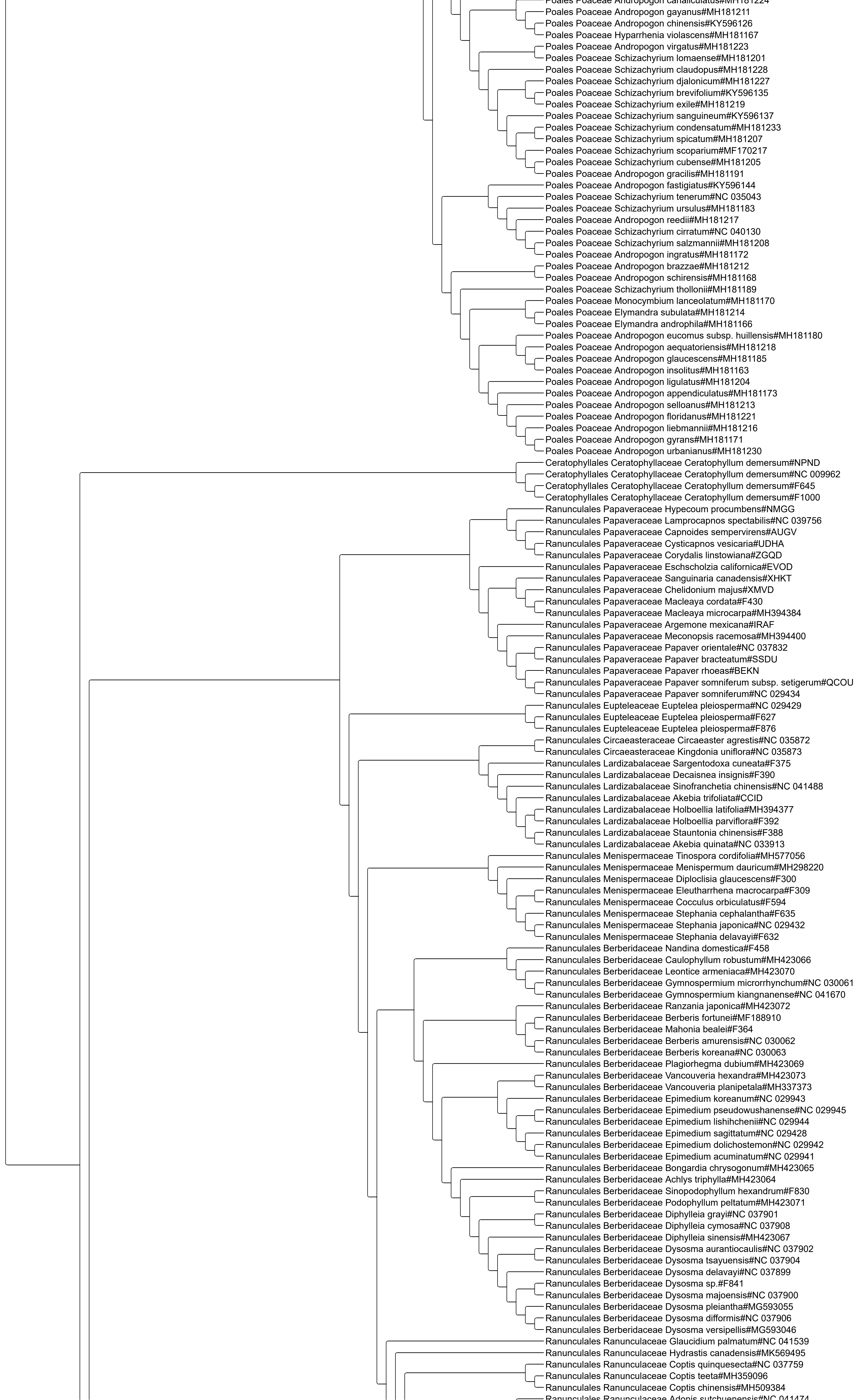
Poales Poaceae Andropogon gayanus#MH181211
Poales Poaceae Andropogon chinensis#KY596126
Poales Poaceae Hyparrhenia violascens#MH181167
Poales Poaceae Andropogon virgatus#MH181223
Poales Poaceae Schizachyrium lomaense#MH181201
Poales Poaceae Schizachyrium claudopus#MH181228
Poales Poaceae Schizachyrium djalonicum#MH181227
Poales Poaceae Schizachyrium brevifolium#KY596135
Poales Poaceae Schizachyrium exile#MH181219
Poales Poaceae Schizachyrium sanguineum#KY596137
Poales Poaceae Schizachyrium condensatum#MH181233
Poales Poaceae Schizachyrium spicatum#MH181207
Poales Poaceae Schizachyrium scoparium#MF170217
Poales Poaceae Schizachyrium cubense#MH181205
Poales Poaceae Andropogon gracilis#MH181191
Poales Poaceae Andropogon fastigiatus#KY596144
Poales Poaceae Schizachyrium tenerum#NC 035043
Poales Poaceae Schizachyrium ursulus#MH181183
Poales Poaceae Andropogon reedii#MH181217
Poales Poaceae Schizachyrium cirratum#NC 040130
Poales Poaceae Schizachyrium salzmannii#MH181208
Poales Poaceae Andropogon ingratus#MH181172
Poales Poaceae Andropogon brazzae#MH181212
Poales Poaceae Andropogon schirensis#MH181168
Poales Poaceae Schizachyrium thollonii#MH181189
Poales Poaceae Monocymbium lanceolatum#MH181170
Poales Poaceae Elymandra subulata#MH181214
Poales Poaceae Elymandra androphila#MH181166
Poales Poaceae Andropogon eucomus subsp. huillensis#MH181180
Poales Poaceae Andropogon aequatoriensis#MH181218
Poales Poaceae Andropogon glaucescens#MH181185
Poales Poaceae Andropogon insolitus#MH181163
Poales Poaceae Andropogon ligulatus#MH181204
Poales Poaceae Andropogon appendiculatus#MH181173
Poales Poaceae Andropogon selloanus#MH181213
Poales Poaceae Andropogon floridanus#MH181221
Poales Poaceae Andropogon liebmannii#MH181216
Poales Poaceae Andropogon gyrans#MH181171
Poales Poaceae Andropogon urbanianus#MH181230
Ceratophyllales Ceratophyllaceae Ceratophyllum demersum#NPND
Ceratophyllales Ceratophyllaceae Ceratophyllum demersum#NC 009962
Ceratophyllales Ceratophyllaceae Ceratophyllum demersum#F645
Ceratophyllales Ceratophyllaceae Ceratophyllum demersum#F1000
Ranunculales Papaveraceae Hypecoum procumbens#NMGG
Ranunculales Papaveraceae Lamprocapnos spectabilis#NC 039756
Ranunculales Papaveraceae Capnoides sempervirens#AUGV
Ranunculales Papaveraceae Cysticapnos vesicaria#UDHA
Ranunculales Papaveraceae Corydalis linstowiana#ZGQD
Ranunculales Papaveraceae Eschscholzia californica#EVOD
Ranunculales Papaveraceae Sanguinaria canadensis#XHKT
Ranunculales Papaveraceae Chelidonium majus#XMVD
Ranunculales Papaveraceae Macleaya cordata#F430
Ranunculales Papaveraceae Macleaya microcarpa#MH394384
Ranunculales Papaveraceae Argemone mexicana#IRAF
Ranunculales Papaveraceae Meconopsis racemosa#MH394400
Ranunculales Papaveraceae Papaver orientale#NC 037832
Ranunculales Papaveraceae Papaver bracteatum#SSDU
Ranunculales Papaveraceae Papaver rhoeas#BEKN
Ranunculales Papaveraceae Papaver somniferum subsp. setigerum#QCOU
Ranunculales Papaveraceae Papaver somniferum#NC 029434
Ranunculales Eupteleaceae Euptelea pleiosperma#NC 029429
Ranunculales Eupteleaceae Euptelea pleiosperma#F627
Ranunculales Eupteleaceae Euptelea pleiosperma#F876
Ranunculales Circaeasteraceae Circaeaster agrestis#NC 035872
Ranunculales Circaeasteraceae Kingdonia uniflora#NC 035873
Ranunculales Lardizabalaceae Sargentodoxa cuneata#F375
Ranunculales Lardizabalaceae Decaisnea insignis#F390
Ranunculales Lardizabalaceae Sinofranchetia chinensis#NC 041488
Ranunculales Lardizabalaceae Akebia trifoliata#CCID
Ranunculales Lardizabalaceae Holboellia latifolia#MH394377
Ranunculales Lardizabalaceae Holboellia parviflora#F392
Ranunculales Lardizabalaceae Stauntonia chinensis#F388
Ranunculales Lardizabalaceae Akebia quinata#NC 033913
Ranunculales Menispermaceae Tinospora cordifolia#MH577056
Ranunculales Menispermaceae Menispermum dauricum#MH298220
Ranunculales Menispermaceae Diploclisia glaucescens#F300
Ranunculales Menispermaceae Eleutharrhena macrocarpa#F309
Ranunculales Menispermaceae Cocculus orbiculatus#F594
Ranunculales Menispermaceae Stephania cephalantha#F635
Ranunculales Menispermaceae Stephania japonica#NC 029432
Ranunculales Menispermaceae Stephania delavayi#F632
Ranunculales Berberidaceae Nandina domestica#F458
Ranunculales Berberidaceae Caulophyllum robustum#MH423066
Ranunculales Berberidaceae Leontice armeniaca#MH423070
Ranunculales Berberidaceae Gymnospermium microrrhynchum#NC 030061
Ranunculales Berberidaceae Gymnospermium kiangnanense#NC 041670
Ranunculales Berberidaceae Ranzania japonica#MH423072
Ranunculales Berberidaceae Berberis fortunei#MF188910
Ranunculales Berberidaceae Mahonia bealei#F364
Ranunculales Berberidaceae Berberis amurensis#NC 030062
Ranunculales Berberidaceae Berberis koreana#NC 030063
Ranunculales Berberidaceae Plagiorhegma dubium#MH423069
Ranunculales Berberidaceae Vancouveria hexandra#MH423073
Ranunculales Berberidaceae Vancouveria planipetala#MH337373
Ranunculales Berberidaceae Epimedium koreanum#NC 029943
Ranunculales Berberidaceae Epimedium pseudowushanense#NC 029945
Ranunculales Berberidaceae Epimedium lishihchenii#NC 029944
Ranunculales Berberidaceae Epimedium sagittatum#NC 029428
Ranunculales Berberidaceae Epimedium dolichostemon#NC 029942
Ranunculales Berberidaceae Epimedium acuminatum#NC 029941
Ranunculales Berberidaceae Bongardia chrysogonum#MH423065
Ranunculales Berberidaceae Achlys triphylla#MH423064
Ranunculales Berberidaceae Sinopodophyllum hexandrum#F830
Ranunculales Berberidaceae Podophyllum peltatum#MH423071
Ranunculales Berberidaceae Diphylleia grayi#NC 037901
Ranunculales Berberidaceae Diphylleia cymosa#NC 037908
Ranunculales Berberidaceae Diphylleia sinensis#MH423067
Ranunculales Berberidaceae Dysosma aurantiocaulis#NC 037902
Ranunculales Berberidaceae Dysosma tsayuensis#NC 037904
Ranunculales Berberidaceae Dysosma delavayi#NC 037899
Ranunculales Berberidaceae Dysosma sp.#F841
Ranunculales Berberidaceae Dysosma majoensis#NC 037900
Ranunculales Berberidaceae Dysosma pleiantha#MG593055
Ranunculales Berberidaceae Dysosma difformis#NC 037906
Ranunculales Berberidaceae Dysosma versipellis#MG593046
Ranunculales Ranunculaceae Glaucidium palmatum#NC 041539
Ranunculales Ranunculaceae Hydrastis canadensis#MK569495
Ranunculales Ranunculaceae Coptis quinquesecta#NC 037759
Ranunculales Ranunculaceae Coptis teeta#MH359096
Ranunculales Ranunculaceae Coptis chinensis#MH509384

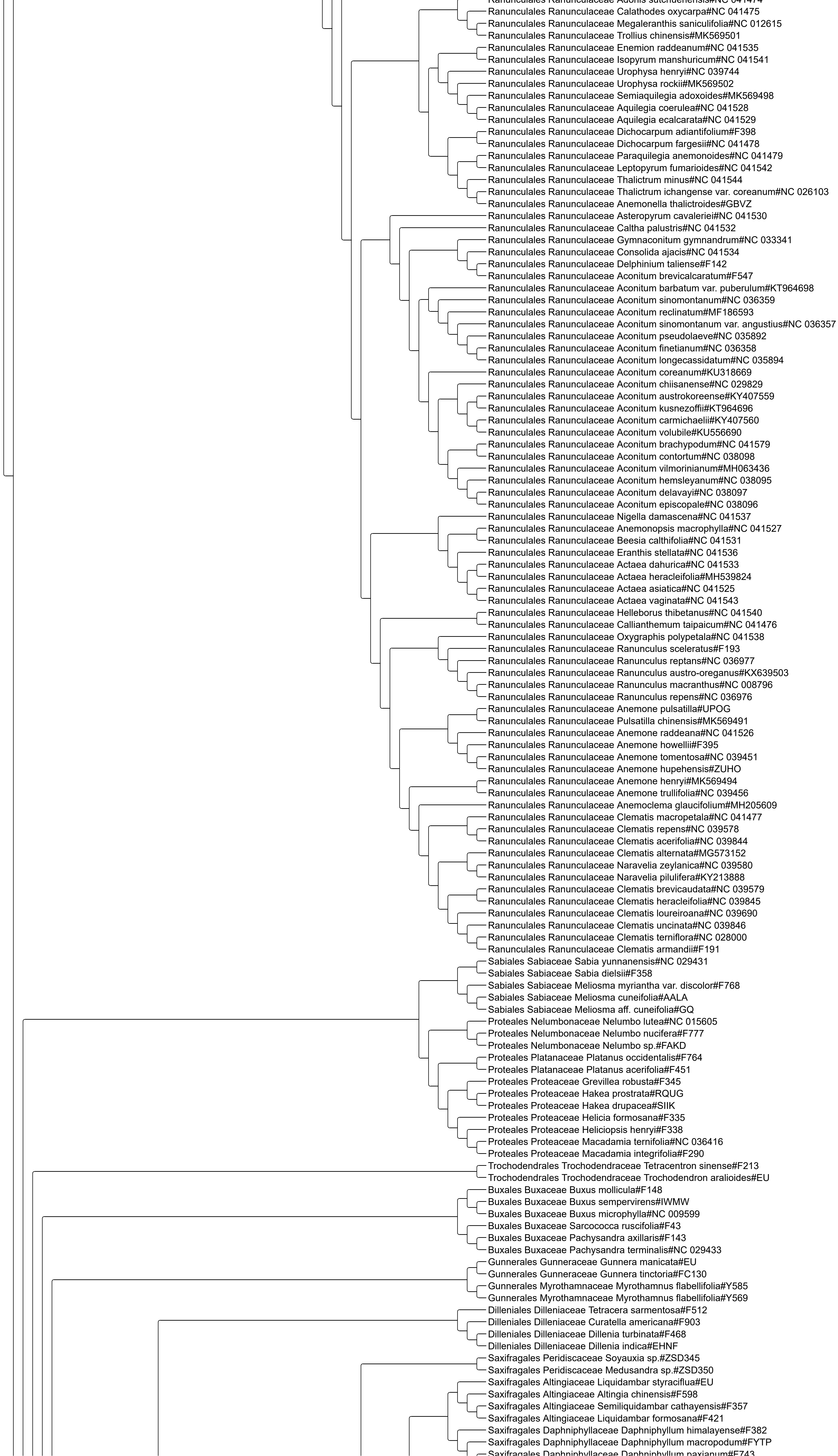
Ranunculales Ranunculaceae Adonis sutchuenensis#NC 041474
Ranunculales Ranunculaceae Calathodes oxycarpa#NC 041475
Ranunculales Ranunculaceae Megaleranthis saniculifolia#NC 012615
Ranunculales Ranunculaceae Trollius chinensis#MK569501
Ranunculales Ranunculaceae Enemion raddeanum#NC 041535
Ranunculales Ranunculaceae Isopyrum manshuricum#NC 041541
Ranunculales Ranunculaceae Urophysa henryi#NC 039744
Ranunculales Ranunculaceae Urophysa rockii#MK569502
Ranunculales Ranunculaceae Semiaquilegia adoxoides#MK569498
Ranunculales Ranunculaceae Aquilegia coerulea#NC 041528
Ranunculales Ranunculaceae Aquilegia ecalcarata#NC 041529
Ranunculales Ranunculaceae Dichocarpum adiantifolium#F398
Ranunculales Ranunculaceae Dichocarpum fargesii#NC 041478
Ranunculales Ranunculaceae Paraquilegia anemonoides#NC 041479
Ranunculales Ranunculaceae Leptopyrum fumarioides#NC 041542
Ranunculales Ranunculaceae Thalictrum minus#NC 041544
Ranunculales Ranunculaceae Thalictrum ichangense var. coreanum#NC 026103
Ranunculales Ranunculaceae Anemonella thalictroides#GBVZ
Ranunculales Ranunculaceae Asteropyrum cavaleriei#NC 041530
Ranunculales Ranunculaceae Caltha palustris#NC 041532
Ranunculales Ranunculaceae Gymnaconitum gymnandrum#NC 033341
Ranunculales Ranunculaceae Consolida ajacis#NC 041534
Ranunculales Ranunculaceae Delphinium taliense#F142
Ranunculales Ranunculaceae Aconitum brevicalcaratum#F547
Ranunculales Ranunculaceae Aconitum barbatum var. puberulum#KT964698
Ranunculales Ranunculaceae Aconitum sinomontanum#NC 036359
Ranunculales Ranunculaceae Aconitum reclinatum#MF186593
Ranunculales Ranunculaceae Aconitum sinomontanum var. angustius#NC 036357
Ranunculales Ranunculaceae Aconitum pseudolaeve#NC 035892
Ranunculales Ranunculaceae Aconitum finetianum#NC 036358
Ranunculales Ranunculaceae Aconitum longecassidatum#NC 035894
Ranunculales Ranunculaceae Aconitum coreanum#KU318669
Ranunculales Ranunculaceae Aconitum chiisanense#NC 029829
Ranunculales Ranunculaceae Aconitum austrokoreense#KY407559
Ranunculales Ranunculaceae Aconitum kusnezoffii#KT964696
Ranunculales Ranunculaceae Aconitum carmichaelii#KY407560
Ranunculales Ranunculaceae Aconitum volubile#KU556690
Ranunculales Ranunculaceae Aconitum brachypodum#NC 041579
Ranunculales Ranunculaceae Aconitum contortum#NC 038098
Ranunculales Ranunculaceae Aconitum vilmorinianum#MH063436
Ranunculales Ranunculaceae Aconitum hemsleyanum#NC 038095
Ranunculales Ranunculaceae Aconitum delavayi#NC 038097
Ranunculales Ranunculaceae Aconitum episcopale#NC 038096
Ranunculales Ranunculaceae Nigella damascena#NC 041537
Ranunculales Ranunculaceae Anemonopsis macrophylla#NC 041527
Ranunculales Ranunculaceae Beesia calthifolia#NC 041531
Ranunculales Ranunculaceae Eranthis stellata#NC 041536
Ranunculales Ranunculaceae Actaea dahurica#NC 041533
Ranunculales Ranunculaceae Actaea heracleifolia#MH539824
Ranunculales Ranunculaceae Actaea asiatica#NC 041525
Ranunculales Ranunculaceae Actaea vaginata#NC 041543
Ranunculales Ranunculaceae Helleborus thibetanus#NC 041540
Ranunculales Ranunculaceae Callianthemum taipaicum#NC 041476
Ranunculales Ranunculaceae Oxygraphis polypetala#NC 041538
Ranunculales Ranunculaceae Ranunculus sceleratus#F193
Ranunculales Ranunculaceae Ranunculus reptans#NC 036977
Ranunculales Ranunculaceae Ranunculus austro-oreganus#KX639503
Ranunculales Ranunculaceae Ranunculus macranthus#NC 008796
Ranunculales Ranunculaceae Ranunculus repens#NC 036976
Ranunculales Ranunculaceae Anemone pulsatilla#UPOG
Ranunculales Ranunculaceae Pulsatilla chinensis#MK569491
Ranunculales Ranunculaceae Anemone raddeana#NC 041526
Ranunculales Ranunculaceae Anemone howellii#F395
Ranunculales Ranunculaceae Anemone tomentosa#NC 039451
Ranunculales Ranunculaceae Anemone hupehensis#ZUHO
Ranunculales Ranunculaceae Anemone henryi#MK569494
Ranunculales Ranunculaceae Anemone trullifolia#NC 039456
Ranunculales Ranunculaceae Anemoclema glaucifolium#MH205609
Ranunculales Ranunculaceae Clematis macropetala#NC 041477
Ranunculales Ranunculaceae Clematis repens#NC 039578
Ranunculales Ranunculaceae Clematis acerifolia#NC 039844
Ranunculales Ranunculaceae Clematis alternata#MG573152
Ranunculales Ranunculaceae Naravelia zeylanica#NC 039580
Ranunculales Ranunculaceae Naravelia pilulifera#KY213888
Ranunculales Ranunculaceae Clematis brevicaudata#NC 039579
Ranunculales Ranunculaceae Clematis heracleifolia#NC 039845
Ranunculales Ranunculaceae Clematis loureiroana#NC 039690
Ranunculales Ranunculaceae Clematis uncinata#NC 039846
Ranunculales Ranunculaceae Clematis terniflora#NC 028000
Ranunculales Ranunculaceae Clematis armandii#F191
Sabiales Sabiaceae Sabia yunnanensis#NC 029431
Sabiales Sabiaceae Sabia dielsii#F358
Sabiales Sabiaceae Meliosma myriantha var. discolor#F768
Sabiales Sabiaceae Meliosma cuneifolia#AALA
Sabiales Sabiaceae Meliosma aff. cuneifolia#GQ
Proteales Nelumbonaceae Nelumbo lutea#NC 015605
Proteales Nelumbonaceae Nelumbo nucifera#F777
Proteales Nelumbonaceae Nelumbo sp.#FAKD
Proteales Platanaceae Platanus occidentalis#F764
Proteales Platanaceae Platanus acerifolia#F451
Proteales Proteaceae Grevillea robusta#F345
Proteales Proteaceae Hakea prostrata#RQUG
Proteales Proteaceae Hakea drupacea#SIIK
Proteales Proteaceae Helicia formosana#F335
Proteales Proteaceae Heliciopsis henryi#F338
Proteales Proteaceae Macadamia ternifolia#NC 036416
Proteales Proteaceae Macadamia integrifolia#F290
Trochodendrales Trochodendraceae Tetracentron sinense#F213
Trochodendrales Trochodendraceae Trochodendron aralioides#EU
Buxales Buxaceae Buxus mollicula#F148
Buxales Buxaceae Buxus sempervirens#IWMW
Buxales Buxaceae Buxus microphylla#NC 009599
Buxales Buxaceae Sarcococca ruscifolia#F43
Buxales Buxaceae Pachysandra axillaris#F143
Buxales Buxaceae Pachysandra terminalis#NC 029433
Gunnerales Gunneraceae Gunnera manicata#EU
Gunnerales Gunneraceae Gunnera tinctoria#FC130
Gunnerales Myrothamnaceae Myrothamnus flabellifolia#Y585
Gunnerales Myrothamnaceae Myrothamnus flabellifolia#Y569
Dilleniales Dilleniaceae Tetracera sarmentosa#F512
Dilleniales Dilleniaceae Curatella americana#F903
Dilleniales Dilleniaceae Dillenia turbinata#F468
Dilleniales Dilleniaceae Dillenia indica#EHNF
Saxifragales Peridiscaceae Soyauxia sp.#ZSD345
Saxifragales Peridiscaceae Medusandra sp.#ZSD350
Saxifragales Altingiaceae Liquidambar styraciflua#EU
Saxifragales Altingiaceae Altingia chinensis#F598
Saxifragales Altingiaceae Semiliquidambar cathayensis#F357
Saxifragales Altingiaceae Liquidambar formosana#F421
Saxifragales Daphniphyllaceae Daphniphyllum himalayense#F382
Saxifragales Daphniphyllaceae Daphniphyllum macropodum#FYTP
Saxifragales Daphniphyllaceae Daphniphyllum paxianum#F743

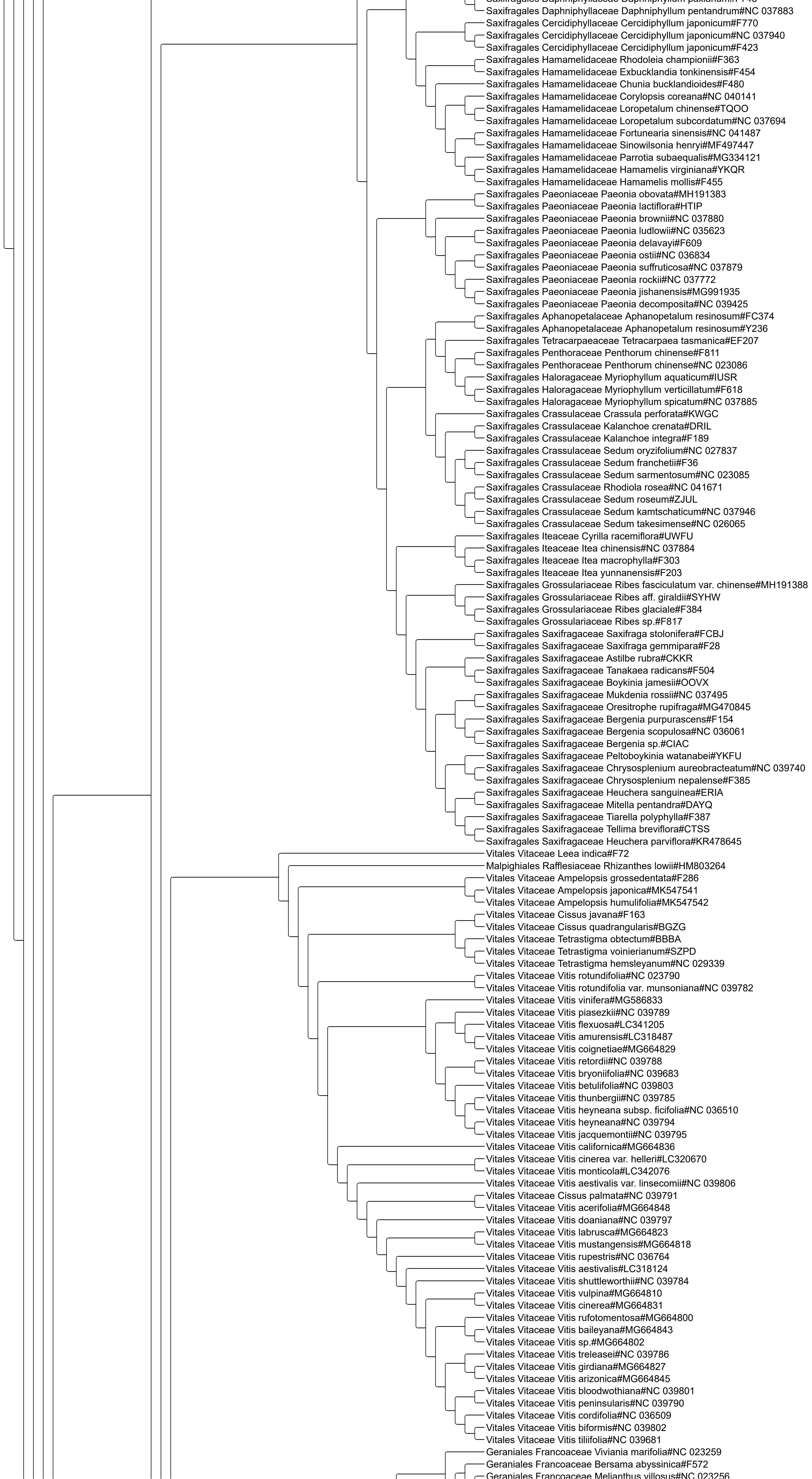

Saxifragales Daphniphyllaceae Daphniphyllum pentandrum#NC 037883
Saxifragales Cercidiphyllaceae Cercidiphyllum japonicum#F770
Saxifragales Cercidiphyllaceae Cercidiphyllum japonicum#NC 037940
Saxifragales Cercidiphyllaceae Cercidiphyllum japonicum#F423
Saxifragales Hamamelidaceae Rhodoleia championii#F363
Saxifragales Hamamelidaceae Exbucklandia tonkinensis#F454
Saxifragales Hamamelidaceae Chunia bucklandioides#F480
Saxifragales Hamamelidaceae Corylopsis coreana#NC 040141
Saxifragales Hamamelidaceae Loropetalum chinense#TQOO
Saxifragales Hamamelidaceae Loropetalum subcordatum#NC 037694
Saxifragales Hamamelidaceae Fortunearia sinensis#NC 041487
Saxifragales Hamamelidaceae Sinowilsonia henryi#MF497447
Saxifragales Hamamelidaceae Parrotia subaequalis#MG334121
Saxifragales Hamamelidaceae Hamamelis virginiana#YKQR
Saxifragales Hamamelidaceae Hamamelis mollis#F455
Saxifragales Paeoniaceae Paeonia obovata#MH191383
Saxifragales Paeoniaceae Paeonia lactiflora#HTIP
Saxifragales Paeoniaceae Paeonia brownii#NC 037880
Saxifragales Paeoniaceae Paeonia ludlowii#NC 035623
Saxifragales Paeoniaceae Paeonia delavayi#F609
Saxifragales Paeoniaceae Paeonia ostii#NC 036834
Saxifragales Paeoniaceae Paeonia suffruticosa#NC 037879
Saxifragales Paeoniaceae Paeonia rockii#NC 037772
Saxifragales Paeoniaceae Paeonia jishanensis#MG991935
Saxifragales Paeoniaceae Paeonia decomposita#NC 039425
Saxifragales Aphanopetalaceae Aphanopetalum resinosum#FC374
Saxifragales Aphanopetalaceae Aphanopetalum resinosum#Y236
Saxifragales Tetracarpaeaceae Tetracarpaea tasmanica#EF207
Saxifragales Penthoraceae Penthorum chinense#F811
Saxifragales Penthoraceae Penthorum chinense#NC 023086
Saxifragales Haloragaceae Myriophyllum aquaticum#IUSR
Saxifragales Haloragaceae Myriophyllum verticillatum#F618
Saxifragales Haloragaceae Myriophyllum spicatum#NC 037885
Saxifragales Crassulaceae Crassula perforata#KWGC
Saxifragales Crassulaceae Kalanchoe crenata#DRIL
Saxifragales Crassulaceae Kalanchoe integra#F189
Saxifragales Crassulaceae Sedum oryzifolium#NC 027837
Saxifragales Crassulaceae Sedum franchetii#F36
Saxifragales Crassulaceae Sedum sarmentosum#NC 023085
Saxifragales Crassulaceae Rhodiola rosea#NC 041671
Saxifragales Crassulaceae Sedum roseum#ZJUL
Saxifragales Crassulaceae Sedum kamtschaticum#NC 037946
Saxifragales Crassulaceae Sedum takesimense#NC 026065
Saxifragales Iteaceae Cyrilla racemiflora#UWFU
Saxifragales Iteaceae Itea chinensis#NC 037884
Saxifragales Iteaceae Itea macrophylla#F303
Saxifragales Iteaceae Itea yunnanensis#F203
Saxifragales Grossulariaceae Ribes fasciculatum var. chinense#MH191388
Saxifragales Grossulariaceae Ribes aff. giraldii#SYHW
Saxifragales Grossulariaceae Ribes glaciale#F384
Saxifragales Grossulariaceae Ribes sp.#F817
Saxifragales Saxifragaceae Saxifraga stolonifera#FCBJ
Saxifragales Saxifragaceae Saxifraga gemmipara#F28
Saxifragales Saxifragaceae Astilbe rubra#CKKR
Saxifragales Saxifragaceae Tanakaea radicans#F504
Saxifragales Saxifragaceae Boykinia jamesii#OOVX
Saxifragales Saxifragaceae Mukdenia rossii#NC 037495
Saxifragales Saxifragaceae Oresitrophe rupifraga#MG470845
Saxifragales Saxifragaceae Bergenia purpurascens#F154
Saxifragales Saxifragaceae Bergenia scopulosa#NC 036061
Saxifragales Saxifragaceae Bergenia sp.#CIAC
Saxifragales Saxifragaceae Peltoboykinia watanabei#YKFU
Saxifragales Saxifragaceae Chrysosplenium aureobracteatum#NC 039740
Saxifragales Saxifragaceae Chrysosplenium nepalense#F385
Saxifragales Saxifragaceae Heuchera sanguinea#ERIA
Saxifragales Saxifragaceae Mitella pentandra#DAYQ
Saxifragales Saxifragaceae Tiarella polyphylla#F387
Saxifragales Saxifragaceae Tellima breviflora#CTSS
Saxifragales Saxifragaceae Heuchera parviflora#KR478645
Vitales Vitaceae Leea indica#F72
Malpighiales Rafflesiaceae Rhizanthes lowii#HM803264
Vitales Vitaceae Ampelopsis grossedentata#F286
Vitales Vitaceae Ampelopsis japonica#MK547541
Vitales Vitaceae Ampelopsis humulifolia#MK547542
Vitales Vitaceae Cissus javana#F163
Vitales Vitaceae Cissus quadrangularis#BGZG
Vitales Vitaceae Tetrastigma obtectum#BBBA
Vitales Vitaceae Tetrastigma voinierianum#SZPD
Vitales Vitaceae Tetrastigma hemsleyanum#NC 029339
Vitales Vitaceae Vitis rotundifolia#NC 023790
Vitales Vitaceae Vitis rotundifolia var. munsoniana#NC 039782
Vitales Vitaceae Vitis vinifera#MG586833
Vitales Vitaceae Vitis piasezkii#NC 039789
Vitales Vitaceae Vitis flexuosa#LC341205
Vitales Vitaceae Vitis amurensis#LC318487
Vitales Vitaceae Vitis coignetiae#MG664829
Vitales Vitaceae Vitis retordii#NC 039788
Vitales Vitaceae Vitis bryoniifolia#NC 039683
Vitales Vitaceae Vitis betulifolia#NC 039803
Vitales Vitaceae Vitis thunbergii#NC 039785
Vitales Vitaceae Vitis heyneana subsp. ficifolia#NC 036510
Vitales Vitaceae Vitis heyneana#NC 039794
Vitales Vitaceae Vitis jacquemontii#NC 039795
Vitales Vitaceae Vitis californica#MG664836
Vitales Vitaceae Vitis cinerea var. helleri#LC320670
Vitales Vitaceae Vitis monticola#LC342076
Vitales Vitaceae Vitis aestivalis var. linsecomii#NC 039806
Vitales Vitaceae Cissus palmata#NC 039791
Vitales Vitaceae Vitis acerifolia#MG664848
Vitales Vitaceae Vitis doaniana#NC 039797
Vitales Vitaceae Vitis labrusca#MG664823
Vitales Vitaceae Vitis mustangensis#MG664818
Vitales Vitaceae Vitis rupestris#NC 036764
Vitales Vitaceae Vitis aestivalis#LC318124
Vitales Vitaceae Vitis shuttleworthii#NC 039784
Vitales Vitaceae Vitis vulpina#MG664810
Vitales Vitaceae Vitis cinerea#MG664831
Vitales Vitaceae Vitis rufotomentosa#MG664800
Vitales Vitaceae Vitis baileyana#MG664843
Vitales Vitaceae Vitis sp.#MG664802
Vitales Vitaceae Vitis treleasei#NC 039786
Vitales Vitaceae Vitis girdiana#MG664827
Vitales Vitaceae Vitis arizonica#MG664845
Vitales Vitaceae Vitis bloodwothiana#NC 039801
Vitales Vitaceae Vitis peninsularis#NC 039790
Vitales Vitaceae Vitis cordifolia#NC 036509
Vitales Vitaceae Vitis biformis#NC 039802
Vitales Vitaceae Vitis tiliifolia#NC 039681
Geraniales Francoaceae Viviania marifolia#NC 023259
Geraniales Francoaceae Bersama abyssinica#F572
Geraniales Francoaceae Melianthus villosus#NC 023256

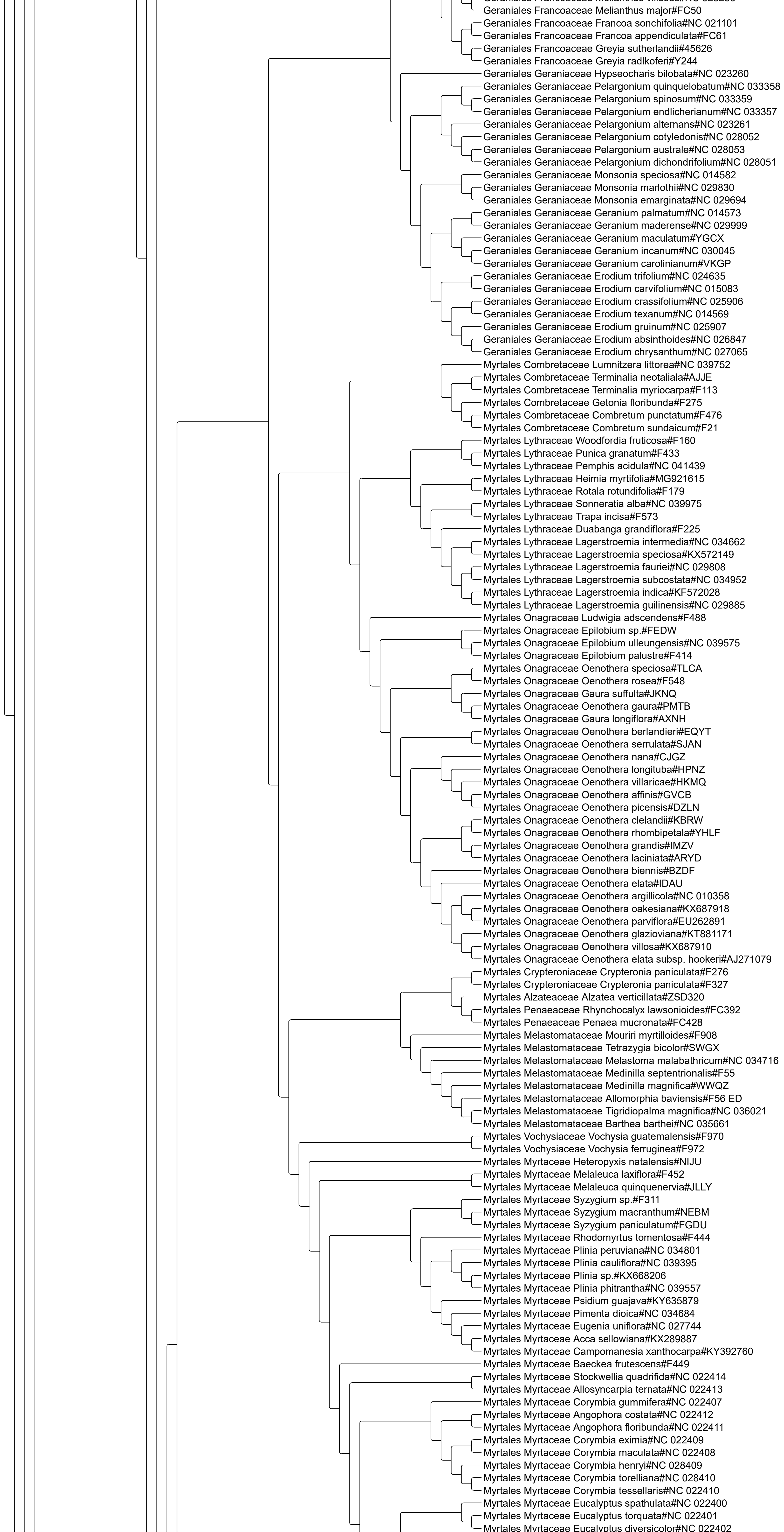
Geraniales Francoaceae Melianthus major#FC50
Geraniales Francoaceae Francoa sonchifolia#NC 021101
Geraniales Francoaceae Francoa appendiculata#FC61
Geraniales Francoaceae Greyia sutherlandii#45626
Geraniales Francoaceae Greyia radlkoferi#Y244
Geraniales Geraniaceae Hypseocharis bilobata#NC 023260
Geraniales Geraniaceae Pelargonium quinquelobatum#NC 033358
Geraniales Geraniaceae Pelargonium spinosum#NC 033359
Geraniales Geraniaceae Pelargonium endlicherianum#NC 033357
Geraniales Geraniaceae Pelargonium alternans#NC 023261
Geraniales Geraniaceae Pelargonium cotyledonis#NC 028052
Geraniales Geraniaceae Pelargonium australe#NC 028053
Geraniales Geraniaceae Pelargonium dichondrifolium#NC 028051
Geraniales Geraniaceae Monsonia speciosa#NC 014582
Geraniales Geraniaceae Monsonia marlothii#NC 029830
Geraniales Geraniaceae Monsonia emarginata#NC 029694
Geraniales Geraniaceae Geranium palmatum#NC 014573
Geraniales Geraniaceae Geranium maderense#NC 029999
Geraniales Geraniaceae Geranium maculatum#YGCX
Geraniales Geraniaceae Geranium incanum#NC 030045
Geraniales Geraniaceae Geranium carolinianum#VKGP
Geraniales Geraniaceae Erodium trifolium#NC 024635
Geraniales Geraniaceae Erodium carvifolium#NC 015083
Geraniales Geraniaceae Erodium crassifolium#NC 025906
Geraniales Geraniaceae Erodium texanum#NC 014569
Geraniales Geraniaceae Erodium gruinum#NC 025907
Geraniales Geraniaceae Erodium absinthoides#NC 026847
Geraniales Geraniaceae Erodium chrysanthum#NC 027065
Myrtales Combretaceae Lumnitzera littorea#NC 039752
Myrtales Combretaceae Terminalia neotaliala#AJJE
Myrtales Combretaceae Terminalia myriocarpa#F113
Myrtales Combretaceae Getonia floribunda#F275
Myrtales Combretaceae Combretum punctatum#F476
Myrtales Combretaceae Combretum sundaicum#F21
Myrtales Lythraceae Woodfordia fruticosa#F160
Myrtales Lythraceae Punica granatum#F433
Myrtales Lythraceae Pemphis acidula#NC 041439
Myrtales Lythraceae Heimia myrtifolia#MG921615
Myrtales Lythraceae Rotala rotundifolia#F179
Myrtales Lythraceae Sonneratia alba#NC 039975
Myrtales Lythraceae Trapa incisa#F573
Myrtales Lythraceae Duabanga grandiflora#F225
Myrtales Lythraceae Lagerstroemia intermedia#NC 034662
Myrtales Lythraceae Lagerstroemia speciosa#KX572149
Myrtales Lythraceae Lagerstroemia fauriei#NC 029808
Myrtales Lythraceae Lagerstroemia subcostata#NC 034952
Myrtales Lythraceae Lagerstroemia indica#KF572028
Myrtales Lythraceae Lagerstroemia guilinensis#NC 029885
Myrtales Onagraceae Ludwigia adscendens#F488
Myrtales Onagraceae Epilobium sp.#FEDW
Myrtales Onagraceae Epilobium ulleungensis#NC 039575
Myrtales Onagraceae Epilobium palustre#F414
Myrtales Onagraceae Oenothera speciosa#TLCA
Myrtales Onagraceae Oenothera rosea#F548
Myrtales Onagraceae Gaura suffulta#JKNQ
Myrtales Onagraceae Oenothera gaura#PMTB
Myrtales Onagraceae Gaura longiflora#AXNH
Myrtales Onagraceae Oenothera berlandieri#EQYT
Myrtales Onagraceae Oenothera serrulata#SJAN
Myrtales Onagraceae Oenothera nana#CJGZ
Myrtales Onagraceae Oenothera longituba#HPNZ
Myrtales Onagraceae Oenothera villaricae#HKMQ
Myrtales Onagraceae Oenothera affinis#GVCB
Myrtales Onagraceae Oenothera picensis#DZLN
Myrtales Onagraceae Oenothera clelandii#KBRW
Myrtales Onagraceae Oenothera rhombipetala#YHLF
Myrtales Onagraceae Oenothera grandis#IMZV
Myrtales Onagraceae Oenothera laciniata#ARYD
Myrtales Onagraceae Oenothera biennis#BZDF
Myrtales Onagraceae Oenothera elata#IDAU
Myrtales Onagraceae Oenothera argillicola#NC 010358
Myrtales Onagraceae Oenothera oakesiana#KX687918
Myrtales Onagraceae Oenothera parviflora#EU262891
Myrtales Onagraceae Oenothera glazioviana#KT881171
Myrtales Onagraceae Oenothera villosa#KX687910
Myrtales Onagraceae Oenothera elata subsp. hookeri#AJ271079
Myrtales Crypteroniaceae Crypteronia paniculata#F276
Myrtales Crypteroniaceae Crypteronia paniculata#F327
Myrtales Alzateaceae Alzatea verticillata#ZSD320
Myrtales Penaeaceae Rhynchocalyx lawsonioides#FC392
Myrtales Penaeaceae Penaea mucronata#FC428
Myrtales Melastomataceae Mouriri myrtilloides#F908
Myrtales Melastomataceae Tetrazygia bicolor#SWGX
Myrtales Melastomataceae Melastoma malabathricum#NC 034716
Myrtales Melastomataceae Medinilla septentrionalis#F55
Myrtales Melastomataceae Medinilla magnifica#WWQZ
Myrtales Melastomataceae Allomorphia baviensis#F56 ED
Myrtales Melastomataceae Tigridiopalma magnifica#NC 036021
Myrtales Melastomataceae Barthea barthei#NC 035661
Myrtales Vochysiaceae Vochysia guatemalensis#F970
Myrtales Vochysiaceae Vochysia ferruginea#F972
Myrtales Myrtaceae Heteropyxis natalensis#NIJU
Myrtales Myrtaceae Melaleuca laxiflora#F452
Myrtales Myrtaceae Melaleuca quinquenervia#JLLY
Myrtales Myrtaceae Syzygium sp.#F311
Myrtales Myrtaceae Syzygium macranthum#NEBM
Myrtales Myrtaceae Syzygium paniculatum#FGDU
Myrtales Myrtaceae Rhodomyrtus tomentosa#F444
Myrtales Myrtaceae Plinia peruviana#NC 034801
Myrtales Myrtaceae Plinia cauliflora#NC 039395
Myrtales Myrtaceae Plinia sp.#KX668206
Myrtales Myrtaceae Plinia phitrantha#NC 039557
Myrtales Myrtaceae Psidium guajava#KY635879
Myrtales Myrtaceae Pimenta dioica#NC 034684
Myrtales Myrtaceae Eugenia uniflora#NC 027744
Myrtales Myrtaceae Acca sellowiana#KX289887
Myrtales Myrtaceae Campomanesia xanthocarpa#KY392760
Myrtales Myrtaceae Baeckea frutescens#F449
Myrtales Myrtaceae Stockwellia quadrifida#NC 022414
Myrtales Myrtaceae Allosyncarpia ternata#NC 022413
Myrtales Myrtaceae Corymbia gummifera#NC 022407
Myrtales Myrtaceae Angophora costata#NC 022412
Myrtales Myrtaceae Angophora floribunda#NC 022411
Myrtales Myrtaceae Corymbia eximia#NC 022409
Myrtales Myrtaceae Corymbia maculata#NC 022408
Myrtales Myrtaceae Corymbia henryi#NC 028409
Myrtales Myrtaceae Corymbia torelliana#NC 028410
Myrtales Myrtaceae Corymbia tessellaris#NC 022410
Myrtales Myrtaceae Eucalyptus spathulata#NC 022400
Myrtales Myrtaceae Eucalyptus torquata#NC 022401

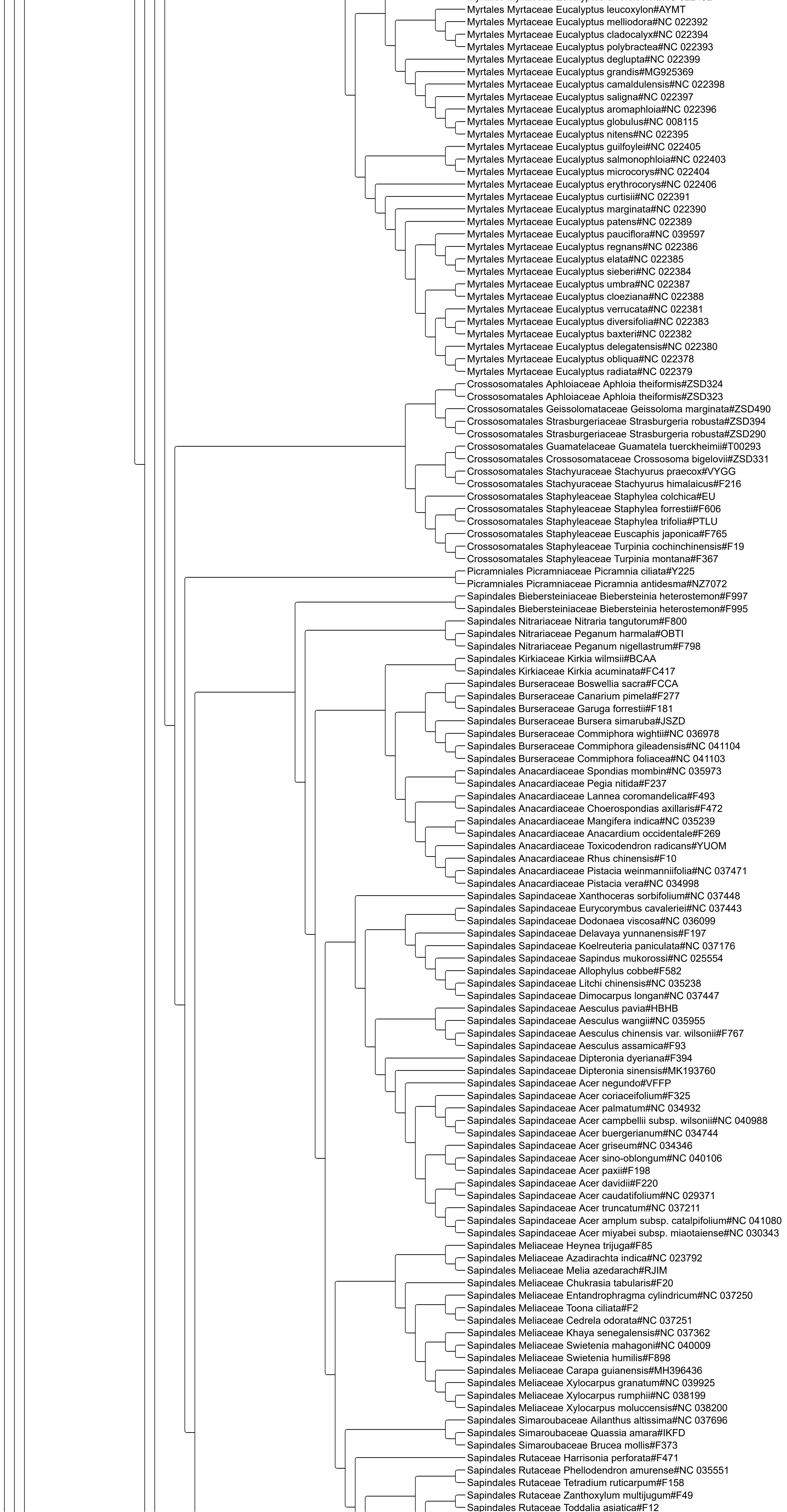
Myrtales Myrtaceae Eucalyptus leucoxylon#AYMT
Myrtales Myrtaceae Eucalyptus melliodora#NC 022392
Myrtales Myrtaceae Eucalyptus cladocalyx#NC 022394
Myrtales Myrtaceae Eucalyptus polybractea#NC 022393
Myrtales Myrtaceae Eucalyptus deglupta#NC 022399
Myrtales Myrtaceae Eucalyptus grandis#MG925369
Myrtales Myrtaceae Eucalyptus camaldulensis#NC 022398
Myrtales Myrtaceae Eucalyptus saligna#NC 022397
Myrtales Myrtaceae Eucalyptus aromaphloia#NC 022396
Myrtales Myrtaceae Eucalyptus globulus#NC 008115
Myrtales Myrtaceae Eucalyptus nitens#NC 022395
Myrtales Myrtaceae Eucalyptus guilfoylei#NC 022405
Myrtales Myrtaceae Eucalyptus salmonophloia#NC 022403
Myrtales Myrtaceae Eucalyptus microcorys#NC 022404
Myrtales Myrtaceae Eucalyptus erythrocorys#NC 022406
Myrtales Myrtaceae Eucalyptus curtisii#NC 022391
Myrtales Myrtaceae Eucalyptus marginata#NC 022390
Myrtales Myrtaceae Eucalyptus patens#NC 022389
Myrtales Myrtaceae Eucalyptus pauciflora#NC 039597
Myrtales Myrtaceae Eucalyptus regnans#NC 022386
Myrtales Myrtaceae Eucalyptus elata#NC 022385
Myrtales Myrtaceae Eucalyptus sieberi#NC 022384
Myrtales Myrtaceae Eucalyptus umbra#NC 022387
Myrtales Myrtaceae Eucalyptus cloeziana#NC 022388
Myrtales Myrtaceae Eucalyptus verrucata#NC 022381
Myrtales Myrtaceae Eucalyptus diversifolia#NC 022383
Myrtales Myrtaceae Eucalyptus baxteri#NC 022382
Myrtales Myrtaceae Eucalyptus delegatensis#NC 022380
Myrtales Myrtaceae Eucalyptus obliqua#NC 022378
Myrtales Myrtaceae Eucalyptus radiata#NC 022379
Crossosomatales Aphloiaceae Aphloia theiformis#ZSD324
Crossosomatales Aphloiaceae Aphloia theiformis#ZSD323
Crossosomatales Geissolomataceae Geissoloma marginata#ZSD490
Crossosomatales Strasburgeriaceae Strasburgeria robusta#ZSD394
Crossosomatales Strasburgeriaceae Strasburgeria robusta#ZSD290
Crossosomatales Guamatelaceae Guamatela tuerckheimii#T00293
Crossosomatales Crossosomataceae Crossosoma bigelovii#ZSD331
Crossosomatales Stachyuraceae Stachyurus praecox#VYGG
Crossosomatales Stachyuraceae Stachyurus himalaicus#F216
Crossosomatales Staphyleaceae Staphylea colchica#EU
Crossosomatales Staphyleaceae Staphylea forrestii#F606
Crossosomatales Staphyleaceae Staphylea trifolia#PTLU
Crossosomatales Staphyleaceae Euscaphis japonica#F765
Crossosomatales Staphyleaceae Turpinia cochinchinensis#F19
Crossosomatales Staphyleaceae Turpinia montana#F367
Picramniales Picramniaceae Picramnia ciliata#Y225
Picramniales Picramniaceae Picramnia antidesma#NZ7072
Sapindales Biebersteiniaceae Biebersteinia heterostemon#F997
Sapindales Biebersteiniaceae Biebersteinia heterostemon#F995
Sapindales Nitrariaceae Nitraria tangutorum#F800
Sapindales Nitrariaceae Peganum harmala#OBTI
Sapindales Nitrariaceae Peganum nigellastrum#F798
Sapindales Kirkiaceae Kirkia wilmsii#BCAA
Sapindales Kirkiaceae Kirkia acuminata#FC417
Sapindales Burseraceae Boswellia sacra#FCCA
Sapindales Burseraceae Canarium pimela#F277
Sapindales Burseraceae Garuga forrestii#F181
Sapindales Burseraceae Bursera simaruba#JSZD
Sapindales Burseraceae Commiphora wightii#NC 036978
Sapindales Burseraceae Commiphora gileadensis#NC 041104
Sapindales Burseraceae Commiphora foliacea#NC 041103
Sapindales Anacardiaceae Spondias mombin#NC 035973
Sapindales Anacardiaceae Pegia nitida#F237
Sapindales Anacardiaceae Lannea coromandelica#F493
Sapindales Anacardiaceae Choerospondias axillaris#F472
Sapindales Anacardiaceae Mangifera indica#NC 035239
Sapindales Anacardiaceae Anacardium occidentale#F269
Sapindales Anacardiaceae Toxicodendron radicans#YUOM
Sapindales Anacardiaceae Rhus chinensis#F10
Sapindales Anacardiaceae Pistacia weinmanniifolia#NC 037471
Sapindales Anacardiaceae Pistacia vera#NC 034998
Sapindales Sapindaceae Xanthoceras sorbifolium#NC 037448
Sapindales Sapindaceae Eurycorymbus cavaleriei#NC 037443
Sapindales Sapindaceae Dodonaea viscosa#NC 036099
Sapindales Sapindaceae Delavaya yunnanensis#F197
Sapindales Sapindaceae Koelreuteria paniculata#NC 037176
Sapindales Sapindaceae Sapindus mukorossi#NC 025554
Sapindales Sapindaceae Allophylus cobbe#F582
Sapindales Sapindaceae Litchi chinensis#NC 035238
Sapindales Sapindaceae Dimocarpus longan#NC 037447
Sapindales Sapindaceae Aesculus pavia#HBHB
Sapindales Sapindaceae Aesculus wangii#NC 035955
Sapindales Sapindaceae Aesculus chinensis var. wilsonii#F767
Sapindales Sapindaceae Aesculus assamica#F93
Sapindales Sapindaceae Dipteronia dyeriana#F394
Sapindales Sapindaceae Dipteronia sinensis#MK193760
Sapindales Sapindaceae Acer negundo#VFFP
Sapindales Sapindaceae Acer coriaceifolium#F325
Sapindales Sapindaceae Acer palmatum#NC 034932
Sapindales Sapindaceae Acer campbellii subsp. wilsonii#NC 040988
Sapindales Sapindaceae Acer buergerianum#NC 034744
Sapindales Sapindaceae Acer griseum#NC 034346
Sapindales Sapindaceae Acer sino-oblongum#NC 040106
Sapindales Sapindaceae Acer paxii#F198
Sapindales Sapindaceae Acer davidii#F220
Sapindales Sapindaceae Acer caudatifolium#NC 029371
Sapindales Sapindaceae Acer truncatum#NC 037211
Sapindales Sapindaceae Acer amplum subsp. catalpifolium#NC 041080
Sapindales Sapindaceae Acer miyabei subsp. miaotaiense#NC 030343
Sapindales Meliaceae Heynea trijuga#F85
Sapindales Meliaceae Azadirachta indica#NC 023792
Sapindales Meliaceae Melia azedarach#RJIM
Sapindales Meliaceae Chukrasia tabularis#F20
Sapindales Meliaceae Entandrophragma cylindricum#NC 037250
Sapindales Meliaceae Toona ciliata#F2
Sapindales Meliaceae Cedrela odorata#NC 037251
Sapindales Meliaceae Khaya senegalensis#NC 037362
Sapindales Meliaceae Swietenia mahagoni#NC 040009
Sapindales Meliaceae Swietenia humilis#F898
Sapindales Meliaceae Carapa guianensis#MH396436
Sapindales Meliaceae Xylocarpus granatum#NC 039925
Sapindales Meliaceae Xylocarpus rumphii#NC 038199
Sapindales Meliaceae Xylocarpus moluccensis#NC 038200
Sapindales Simaroubaceae Ailanthus altissima#NC 037696
Sapindales Simaroubaceae Quassia amara#IKFD
Sapindales Simaroubaceae Brucea mollis#F373
Sapindales Rutaceae Harrisonia perforata#F471
Sapindales Rutaceae Phellodendron amurense#NC 035551
Sapindales Rutaceae Tetradium ruticarpum#F158
Sapindales Rutaceae Zanthoxylum multijugum#F49
Sapindales Rutaceae Toddalia asiatica#F12

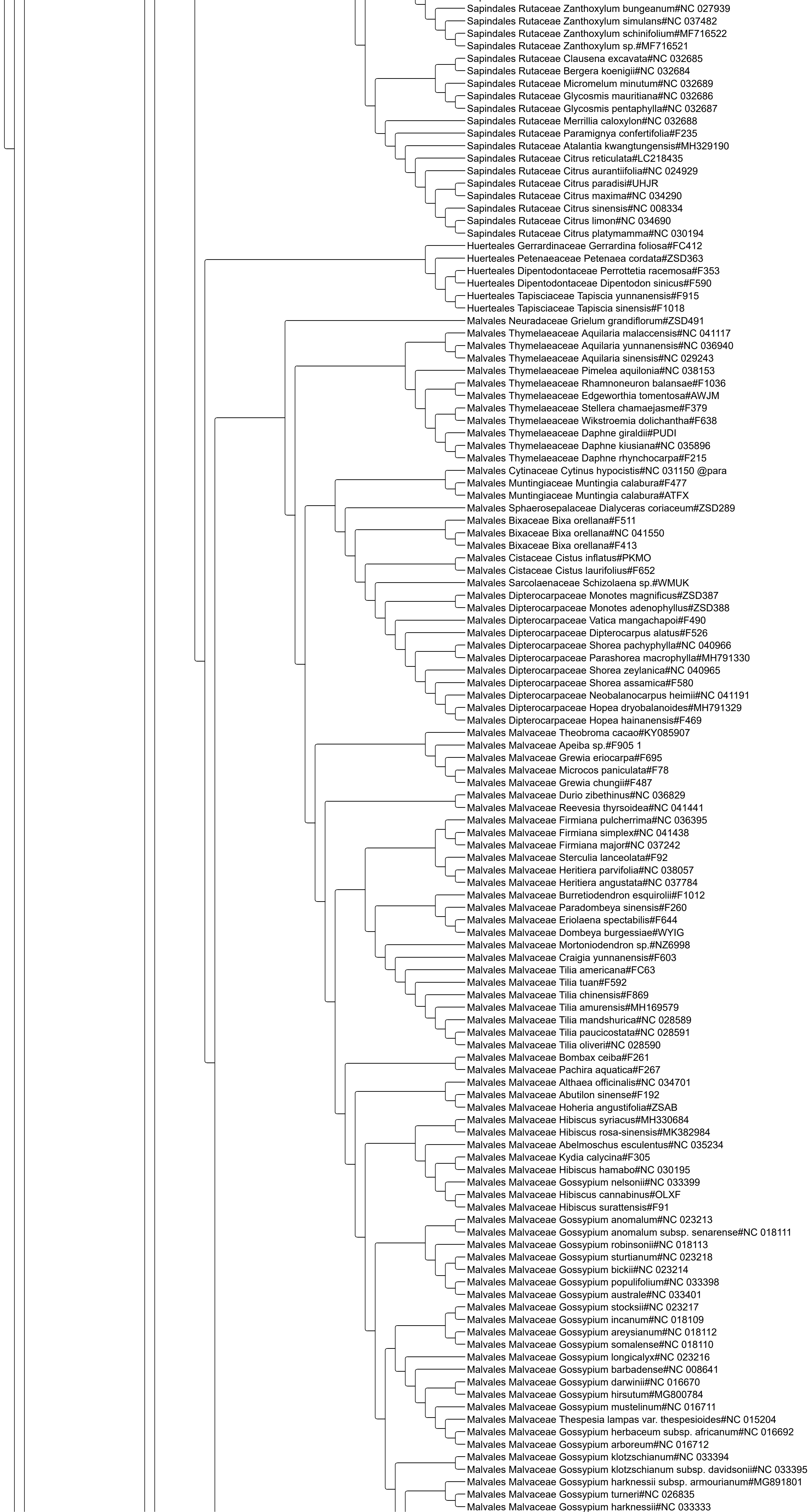
Sapindales Rutaceae Zanthoxylum bungeanum#NC 027939
Sapindales Rutaceae Zanthoxylum simulans#NC 037482
Sapindales Rutaceae Zanthoxylum schinifolium#MF716522
Sapindales Rutaceae Zanthoxylum sp.#MF716521
Sapindales Rutaceae Clausena excavata#NC 032685
Sapindales Rutaceae Bergera koenigii#NC 032684
Sapindales Rutaceae Micromelum minutum#NC 032689
Sapindales Rutaceae Glycosmis mauritiana#NC 032686
Sapindales Rutaceae Glycosmis pentaphylla#NC 032687
Sapindales Rutaceae Merrillia caloxylon#NC 032688
Sapindales Rutaceae Paramignya confertifolia#F235
Sapindales Rutaceae Atalantia kwangtungensis#MH329190
Sapindales Rutaceae Citrus reticulata#LC218435
Sapindales Rutaceae Citrus aurantiifolia#NC 024929
Sapindales Rutaceae Citrus paradisi#UHJR
Sapindales Rutaceae Citrus maxima#NC 034290
Sapindales Rutaceae Citrus sinensis#NC 008334
Sapindales Rutaceae Citrus limon#NC 034690
Sapindales Rutaceae Citrus platymamma#NC 030194
Huerteales Gerrardinaceae Gerrardina foliosa#FC412
Huerteales Petenaeaceae Petenaea cordata#ZSD363
Huerteales Dipentodontaceae Perrottetia racemosa#F353
Huerteales Dipentodontaceae Dipentodon sinicus#F590
Huerteales Tapisciaceae Tapiscia yunnanensis#F915
Huerteales Tapisciaceae Tapiscia sinensis#F1018
Malvales Neuradaceae Grielum grandiflorum#ZSD491
Malvales Thymelaeaceae Aquilaria malaccensis#NC 041117
Malvales Thymelaeaceae Aquilaria yunnanensis#NC 036940
Malvales Thymelaeaceae Aquilaria sinensis#NC 029243
Malvales Thymelaeaceae Pimelea aquilonia#NC 038153
Malvales Thymelaeaceae Rhamnoneuron balansae#F1036
Malvales Thymelaeaceae Edgeworthia tomentosa#AWJM
Malvales Thymelaeaceae Stellera chamaejasme#F379
Malvales Thymelaeaceae Wikstroemia dolichantha#F638
Malvales Thymelaeaceae Daphne giraldii#PUDI
Malvales Thymelaeaceae Daphne kiusiana#NC 035896
Malvales Thymelaeaceae Daphne rhynchocarpa#F215
Malvales Cytinaceae Cytinus hypocistis#NC 031150 @para
Malvales Muntingiaceae Muntingia calabura#F477
Malvales Muntingiaceae Muntingia calabura#ATFX
Malvales Sphaerosepalaceae Dialyceras coriaceum#ZSD289
Malvales Bixaceae Bixa orellana#F511
Malvales Bixaceae Bixa orellana#NC 041550
Malvales Bixaceae Bixa orellana#F413
Malvales Cistaceae Cistus inflatus#PKMO
Malvales Cistaceae Cistus laurifolius#F652
Malvales Sarcolaenaceae Schizolaena sp.#WMUK
Malvales Dipterocarpaceae Monotes magnificus#ZSD387
Malvales Dipterocarpaceae Monotes adenophyllus#ZSD388
Malvales Dipterocarpaceae Vatica mangachapoi#F490
Malvales Dipterocarpaceae Dipterocarpus alatus#F526
Malvales Dipterocarpaceae Shorea pachyphylla#NC 040966
Malvales Dipterocarpaceae Parashorea macrophylla#MH791330
Malvales Dipterocarpaceae Shorea zeylanica#NC 040965
Malvales Dipterocarpaceae Shorea assamica#F580
Malvales Dipterocarpaceae Neobalanocarpus heimii#NC 041191
Malvales Dipterocarpaceae Hopea dryobalanoides#MH791329
Malvales Dipterocarpaceae Hopea hainanensis#F469
Malvales Malvaceae Theobroma cacao#KY085907
Malvales Malvaceae Apeiba sp.#F905 1
Malvales Malvaceae Grewia eriocarpa#F695
Malvales Malvaceae Microcos paniculata#F78
Malvales Malvaceae Grewia chungii#F487
Malvales Malvaceae Durio zibethinus#NC 036829
Malvales Malvaceae Reevesia thyrsoidea#NC 041441
Malvales Malvaceae Firmiana pulcherrima#NC 036395
Malvales Malvaceae Firmiana simplex#NC 041438
Malvales Malvaceae Firmiana major#NC 037242
Malvales Malvaceae Sterculia lanceolata#F92
Malvales Malvaceae Heritiera parvifolia#NC 038057
Malvales Malvaceae Heritiera angustata#NC 037784
Malvales Malvaceae Burretiodendron esquirolii#F1012
Malvales Malvaceae Paradombeya sinensis#F260
Malvales Malvaceae Eriolaena spectabilis#F644
Malvales Malvaceae Dombeya burgessiae#WYIG
Malvales Malvaceae Mortoniodendron sp.#NZ6998
Malvales Malvaceae Craigia yunnanensis#F603
Malvales Malvaceae Tilia americana#FC63
Malvales Malvaceae Tilia tuan#F592
Malvales Malvaceae Tilia chinensis#F869
Malvales Malvaceae Tilia amurensis#MH169579
Malvales Malvaceae Tilia mandshurica#NC 028589
Malvales Malvaceae Tilia paucicostata#NC 028591
Malvales Malvaceae Tilia oliveri#NC 028590
Malvales Malvaceae Bombax ceiba#F261
Malvales Malvaceae Pachira aquatica#F267
Malvales Malvaceae Althaea officinalis#NC 034701
Malvales Malvaceae Abutilon sinense#F192
Malvales Malvaceae Hoheria angustifolia#ZSAB
Malvales Malvaceae Hibiscus syriacus#MH330684
Malvales Malvaceae Hibiscus rosa-sinensis#MK382984
Malvales Malvaceae Abelmoschus esculentus#NC 035234
Malvales Malvaceae Kydia calycina#F305
Malvales Malvaceae Hibiscus hamabo#NC 030195
Malvales Malvaceae Gossypium nelsonii#NC 033399
Malvales Malvaceae Hibiscus cannabinus#OLXF
Malvales Malvaceae Hibiscus surattensis#F91
Malvales Malvaceae Gossypium anomalum#NC 023213
Malvales Malvaceae Gossypium anomalum subsp. senarense#NC 018111
Malvales Malvaceae Gossypium robinsonii#NC 018113
Malvales Malvaceae Gossypium sturtianum#NC 023218
Malvales Malvaceae Gossypium bickii#NC 023214
Malvales Malvaceae Gossypium populifolium#NC 033398
Malvales Malvaceae Gossypium australe#NC 033401
Malvales Malvaceae Gossypium stocksii#NC 023217
Malvales Malvaceae Gossypium incanum#NC 018109
Malvales Malvaceae Gossypium areysianum#NC 018112
Malvales Malvaceae Gossypium somalense#NC 018110
Malvales Malvaceae Gossypium longicalyx#NC 023216
Malvales Malvaceae Gossypium barbadense#NC 008641
Malvales Malvaceae Gossypium darwinii#NC 016670
Malvales Malvaceae Gossypium hirsutum#MG800784
Malvales Malvaceae Gossypium mustelinum#NC 016711
Malvales Malvaceae Thespesia lampas var. thespesioides#NC 015204
Malvales Malvaceae Gossypium herbaceum subsp. africanum#NC 016692
Malvales Malvaceae Gossypium arboreum#NC 016712
Malvales Malvaceae Gossypium klotzschianum#NC 033394
Malvales Malvaceae Gossypium klotzschianum subsp. davidsonii#NC 033395
Malvales Malvaceae Gossypium harknessii subsp. armourianum#MG891801
Malvales Malvaceae Gossypium turneri#NC 026835
Malvales Malvaceae Gossypium harknessii#NC 033333

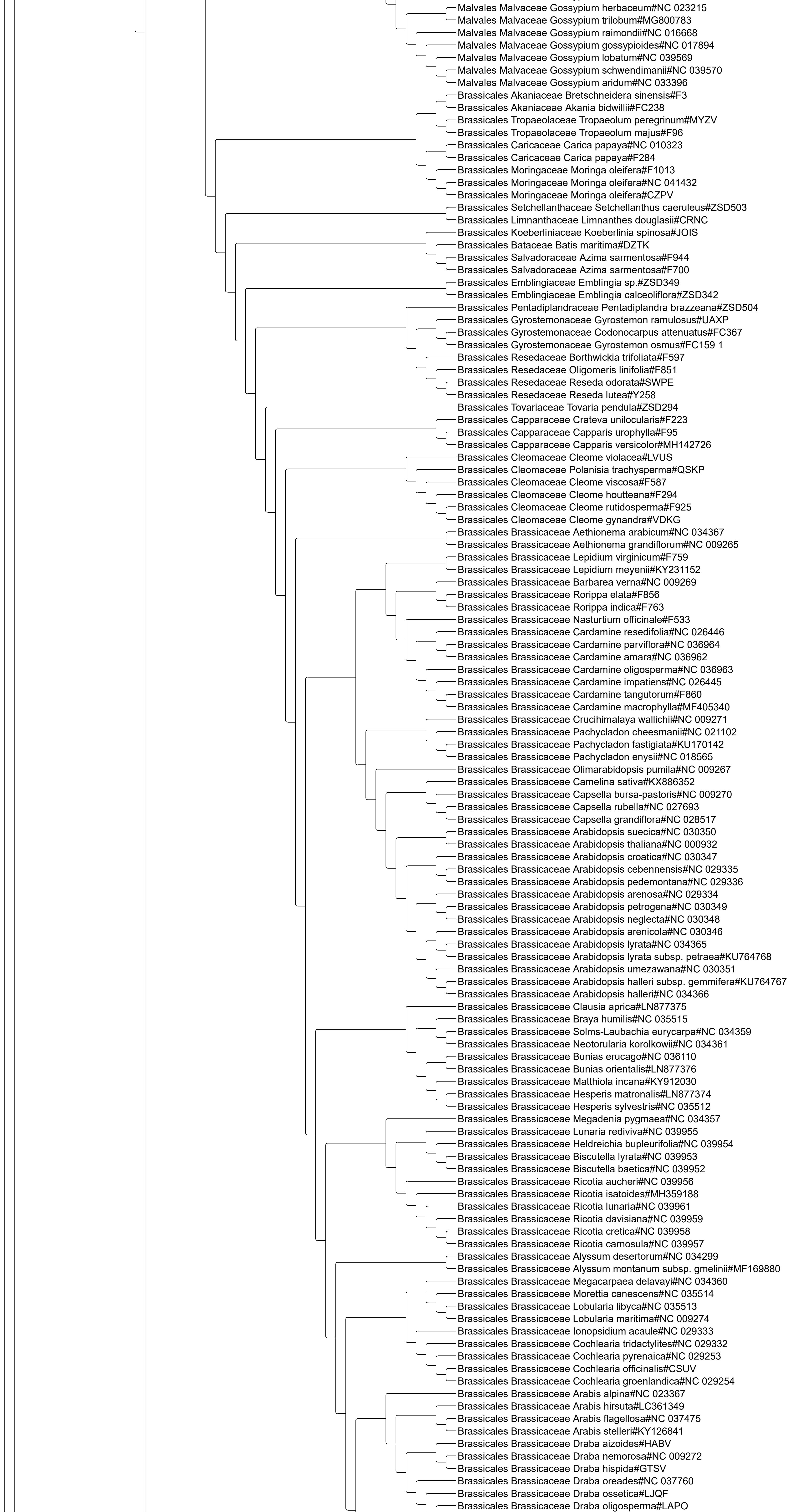
Malvales Malvaceae Gossypium herbaceum#NC 023215
Malvales Malvaceae Gossypium trilobum#MG800783
Malvales Malvaceae Gossypium raimondii#NC 016668
Malvales Malvaceae Gossypium gossypioides#NC 017894
Malvales Malvaceae Gossypium lobatum#NC 039569
Malvales Malvaceae Gossypium schwendimanii#NC 039570
Malvales Malvaceae Gossypium aridum#NC 033396
Brassicales Akaniaceae Bretschneidera sinensis#F3
Brassicales Akaniaceae Akania bidwillii#FC238
Brassicales Tropaeolaceae Tropaeolum peregrinum#MYZV
Brassicales Tropaeolaceae Tropaeolum majus#F96
Brassicales Caricaceae Carica papaya#NC 010323
Brassicales Caricaceae Carica papaya#F284
Brassicales Moringaceae Moringa oleifera#F1013
Brassicales Moringaceae Moringa oleifera#NC 041432
Brassicales Moringaceae Moringa oleifera#CZPV
Brassicales Setchellanthaceae Setchellanthus caeruleus#ZSD503
Brassicales Limnanthaceae Limnanthes douglasii#CRNC
Brassicales Koeberliniaceae Koeberlinia spinosa#JOIS
Brassicales Bataceae Batis maritima#DZTK
Brassicales Salvadoraceae Azima sarmentosa#F944
Brassicales Salvadoraceae Azima sarmentosa#F700
Brassicales Emblingiaceae Emblingia sp.#ZSD349
Brassicales Emblingiaceae Emblingia calceoliflora#ZSD342
Brassicales Pentadiplandraceae Pentadiplandra brazzeana#ZSD504
Brassicales Gyrostemonaceae Gyrostemon ramulosus#UAXP
Brassicales Gyrostemonaceae Codonocarpus attenuatus#FC367
Brassicales Gyrostemonaceae Gyrostemon osmus#FC159 1
Brassicales Resedaceae Borthwickia trifoliata#F597
Brassicales Resedaceae Oligomeris linifolia#F851
Brassicales Resedaceae Reseda odorata#SWPE
Brassicales Resedaceae Reseda lutea#Y258
Brassicales Tovariaceae Tovaria pendula#ZSD294
Brassicales Capparaceae Crateva unilocularis#F223
Brassicales Capparaceae Capparis urophylla#F95
Brassicales Capparaceae Capparis versicolor#MH142726
Brassicales Cleomaceae Cleome violacea#LVUS
Brassicales Cleomaceae Polanisia trachysperma#QSKP
Brassicales Cleomaceae Cleome viscosa#F587
Brassicales Cleomaceae Cleome houtteana#F294
Brassicales Cleomaceae Cleome rutidosperma#F925
Brassicales Cleomaceae Cleome gynandra#VDKG
Brassicales Brassicaceae Aethionema arabicum#NC 034367
Brassicales Brassicaceae Aethionema grandiflorum#NC 009265
Brassicales Brassicaceae Lepidium virginicum#F759
Brassicales Brassicaceae Lepidium meyenii#KY231152
Brassicales Brassicaceae Barbarea verna#NC 009269
Brassicales Brassicaceae Rorippa elata#F856
Brassicales Brassicaceae Rorippa indica#F763
Brassicales Brassicaceae Nasturtium officinale#F533
Brassicales Brassicaceae Cardamine resedifolia#NC 026446
Brassicales Brassicaceae Cardamine parviflora#NC 036964
Brassicales Brassicaceae Cardamine amara#NC 036962
Brassicales Brassicaceae Cardamine oligosperma#NC 036963
Brassicales Brassicaceae Cardamine impatiens#NC 026445
Brassicales Brassicaceae Cardamine tangutorum#F860
Brassicales Brassicaceae Cardamine macrophylla#MF405340
Brassicales Brassicaceae Crucihimalaya wallichii#NC 009271
Brassicales Brassicaceae Pachycladon cheesmanii#NC 021102
Brassicales Brassicaceae Pachycladon fastigiata#KU170142
Brassicales Brassicaceae Pachycladon enysii#NC 018565
Brassicales Brassicaceae Olimarabidopsis pumila#NC 009267
Brassicales Brassicaceae Camelina sativa#KX886352
Brassicales Brassicaceae Capsella bursa-pastoris#NC 009270
Brassicales Brassicaceae Capsella rubella#NC 027693
Brassicales Brassicaceae Capsella grandiflora#NC 028517
Brassicales Brassicaceae Arabidopsis suecica#NC 030350
Brassicales Brassicaceae Arabidopsis thaliana#NC 000932
Brassicales Brassicaceae Arabidopsis croatica#NC 030347
Brassicales Brassicaceae Arabidopsis cebennensis#NC 029335
Brassicales Brassicaceae Arabidopsis pedemontana#NC 029336
Brassicales Brassicaceae Arabidopsis arenosa#NC 029334
Brassicales Brassicaceae Arabidopsis petrogena#NC 030349
Brassicales Brassicaceae Arabidopsis neglecta#NC 030348
Brassicales Brassicaceae Arabidopsis arenicola#NC 030346
Brassicales Brassicaceae Arabidopsis lyrata#NC 034365
Brassicales Brassicaceae Arabidopsis lyrata subsp. petraea#KU764768
Brassicales Brassicaceae Arabidopsis umezawana#NC 030351
Brassicales Brassicaceae Arabidopsis halleri subsp. gemmifera#KU764767
Brassicales Brassicaceae Arabidopsis halleri#NC 034366
Brassicales Brassicaceae Clausia aprica#LN877375
Brassicales Brassicaceae Braya humilis#NC 035515
Brassicales Brassicaceae Solms-Laubachia eurycarpa#NC 034359
Brassicales Brassicaceae Neotorularia korolkowii#NC 034361
Brassicales Brassicaceae Bunias erucago#NC 036110
Brassicales Brassicaceae Bunias orientalis#LN877376
Brassicales Brassicaceae Matthiola incana#KY912030
Brassicales Brassicaceae Hesperis matronalis#LN877374
Brassicales Brassicaceae Hesperis sylvestris#NC 035512
Brassicales Brassicaceae Megadenia pygmaea#NC 034357
Brassicales Brassicaceae Lunaria rediviva#NC 039955
Brassicales Brassicaceae Heldreichia bupleurifolia#NC 039954
Brassicales Brassicaceae Biscutella lyrata#NC 039953
Brassicales Brassicaceae Biscutella baetica#NC 039952
Brassicales Brassicaceae Ricotia aucheri#NC 039956
Brassicales Brassicaceae Ricotia isatoides#MH359188
Brassicales Brassicaceae Ricotia lunaria#NC 039961
Brassicales Brassicaceae Ricotia davisiana#NC 039959
Brassicales Brassicaceae Ricotia cretica#NC 039958
Brassicales Brassicaceae Ricotia carnosula#NC 039957
Brassicales Brassicaceae Alyssum desertorum#NC 034299
Brassicales Brassicaceae Alyssum montanum subsp. gmelinii#MF169880
Brassicales Brassicaceae Megacarpaea delavayi#NC 034360
Brassicales Brassicaceae Morettia canescens#NC 035514
Brassicales Brassicaceae Lobularia libyca#NC 035513
Brassicales Brassicaceae Lobularia maritima#NC 009274
Brassicales Brassicaceae Ionopsidium acaule#NC 029333
Brassicales Brassicaceae Cochlearia tridactylites#NC 029332
Brassicales Brassicaceae Cochlearia pyrenaica#NC 029253
Brassicales Brassicaceae Cochlearia officinalis#CSUV
Brassicales Brassicaceae Cochlearia groenlandica#NC 029254
Brassicales Brassicaceae Arabis alpina#NC 023367
Brassicales Brassicaceae Arabis hirsuta#LC361349
Brassicales Brassicaceae Arabis flagellosa#NC 037475
Brassicales Brassicaceae Arabis stelleri#KY126841
Brassicales Brassicaceae Draba aizoides#HABV
Brassicales Brassicaceae Draba nemorosa#NC 009272
Brassicales Brassicaceae Draba hispida#GTSV
Brassicales Brassicaceae Draba oreades#NC 037760
Brassicales Brassicaceae Draba ossetica#LJQF
Brassicales Brassicaceae Draba oligosperma#LAPO

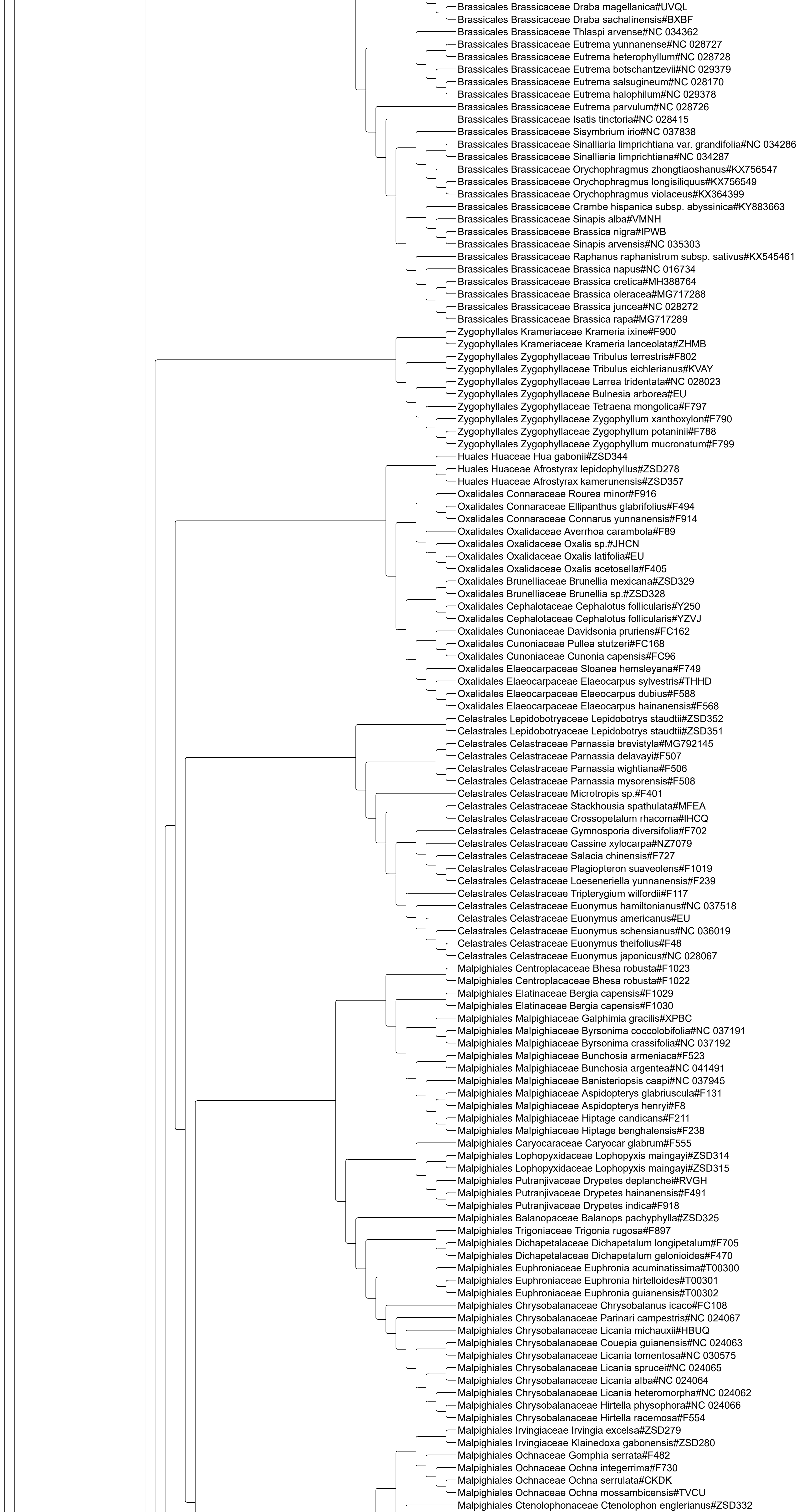
Brassicales Brassicaceae Draba magellanica#UVQL
Brassicales Brassicaceae Draba sachalinensis#BXBF
Brassicales Brassicaceae Thlaspi arvense#NC 034362
Brassicales Brassicaceae Eutrema yunnanense#NC 028727
Brassicales Brassicaceae Eutrema heterophyllum#NC 028728
Brassicales Brassicaceae Eutrema botschantzevii#NC 029379
Brassicales Brassicaceae Eutrema salsugineum#NC 028170
Brassicales Brassicaceae Eutrema halophilum#NC 029378
Brassicales Brassicaceae Eutrema parvulum#NC 028726
Brassicales Brassicaceae Isatis tinctoria#NC 028415
Brassicales Brassicaceae Sisymbrium irio#NC 037838
Brassicales Brassicaceae Sinalliaria limprichtiana var. grandifolia#NC 034286
Brassicales Brassicaceae Sinalliaria limprichtiana#NC 034287
Brassicales Brassicaceae Orychophragmus zhongtiaoshanus#KX756547
Brassicales Brassicaceae Orychophragmus longisiliquus#KX756549
Brassicales Brassicaceae Orychophragmus violaceus#KX364399
Brassicales Brassicaceae Crambe hispanica subsp. abyssinica#KY883663
Brassicales Brassicaceae Sinapis alba#VMNH
Brassicales Brassicaceae Brassica nigra#IPWB
Brassicales Brassicaceae Sinapis arvensis#NC 035303
Brassicales Brassicaceae Raphanus raphanistrum subsp. sativus#KX545461
Brassicales Brassicaceae Brassica napus#NC 016734
Brassicales Brassicaceae Brassica cretica#MH388764
Brassicales Brassicaceae Brassica oleracea#MG717288
Brassicales Brassicaceae Brassica juncea#NC 028272
Brassicales Brassicaceae Brassica rapa#MG717289
Zygophyllales Krameriaceae Krameria ixine#F900
Zygophyllales Krameriaceae Krameria lanceolata#ZHMB
Zygophyllales Zygophyllaceae Tribulus terrestris#F802
Zygophyllales Zygophyllaceae Tribulus eichlerianus#KVAY
Zygophyllales Zygophyllaceae Larrea tridentata#NC 028023
Zygophyllales Zygophyllaceae Bulnesia arborea#EU
Zygophyllales Zygophyllaceae Tetraena mongolica#F797
Zygophyllales Zygophyllaceae Zygophyllum xanthoxylon#F790
Zygophyllales Zygophyllaceae Zygophyllum potaninii#F788
Zygophyllales Zygophyllaceae Zygophyllum mucronatum#F799
Huales Huaceae Hua gabonii#ZSD344
Huales Huaceae Afrostyrax lepidophyllus#ZSD278
Huales Huaceae Afrostyrax kamerunensis#ZSD357
Oxalidales Connaraceae Rourea minor#F916
Oxalidales Connaraceae Ellipanthus glabrifolius#F494
Oxalidales Connaraceae Connarus yunnanensis#F914
Oxalidales Oxalidaceae Averrhoa carambola#F89
Oxalidales Oxalidaceae Oxalis sp.#JHCN
Oxalidales Oxalidaceae Oxalis latifolia#EU
Oxalidales Oxalidaceae Oxalis acetosella#F405
Oxalidales Brunelliaceae Brunellia mexicana#ZSD329
Oxalidales Brunelliaceae Brunellia sp.#ZSD328
Oxalidales Cephalotaceae Cephalotus follicularis#Y250
Oxalidales Cephalotaceae Cephalotus follicularis#YZVJ
Oxalidales Cunoniaceae Davidsonia pruriens#FC162
Oxalidales Cunoniaceae Pullea stutzeri#FC168
Oxalidales Cunoniaceae Cunonia capensis#FC96
Oxalidales Elaeocarpaceae Sloanea hemsleyana#F749
Oxalidales Elaeocarpaceae Elaeocarpus sylvestris#THHD
Oxalidales Elaeocarpaceae Elaeocarpus dubius#F588
Oxalidales Elaeocarpaceae Elaeocarpus hainanensis#F568
Celastrales Lepidobotryaceae Lepidobotrys staudtii#ZSD352
Celastrales Lepidobotryaceae Lepidobotrys staudtii#ZSD351
Celastrales Celastraceae Parnassia brevistyla#MG792145
Celastrales Celastraceae Parnassia delavayi#F507
Celastrales Celastraceae Parnassia wightiana#F506
Celastrales Celastraceae Parnassia mysorensis#F508
Celastrales Celastraceae Microtropis sp.#F401
Celastrales Celastraceae Stackhousia spathulata#MFEA
Celastrales Celastraceae Crossopetalum rhacoma#IHCQ
Celastrales Celastraceae Gymnosporia diversifolia#F702
Celastrales Celastraceae Cassine xylocarpa#NZ7079
Celastrales Celastraceae Salacia chinensis#F727
Celastrales Celastraceae Plagiopteron suaveolens#F1019
Celastrales Celastraceae Loeseneriella yunnanensis#F239
Celastrales Celastraceae Tripterygium wilfordii#F117
Celastrales Celastraceae Euonymus hamiltonianus#NC 037518
Celastrales Celastraceae Euonymus americanus#EU
Celastrales Celastraceae Euonymus schensianus#NC 036019
Celastrales Celastraceae Euonymus theifolius#F48
Celastrales Celastraceae Euonymus japonicus#NC 028067
Malpighiales Centroplacaceae Bhesa robusta#F1023
Malpighiales Centroplacaceae Bhesa robusta#F1022
Malpighiales Elatinaceae Bergia capensis#F1029
Malpighiales Elatinaceae Bergia capensis#F1030
Malpighiales Malpighiaceae Galphimia gracilis#XPBC
Malpighiales Malpighiaceae Byrsonima coccolobifolia#NC 037191
Malpighiales Malpighiaceae Byrsonima crassifolia#NC 037192
Malpighiales Malpighiaceae Bunchosia armeniaca#F523
Malpighiales Malpighiaceae Bunchosia argentea#NC 041491
Malpighiales Malpighiaceae Banisteriopsis caapi#NC 037945
Malpighiales Malpighiaceae Aspidopterys glabriuscula#F131
Malpighiales Malpighiaceae Aspidopterys henryi#F8
Malpighiales Malpighiaceae Hiptage candicans#F211
Malpighiales Malpighiaceae Hiptage benghalensis#F238
Malpighiales Caryocaraceae Caryocar glabrum#F555
Malpighiales Lophopyxidaceae Lophopyxis maingayi#ZSD314
Malpighiales Lophopyxidaceae Lophopyxis maingayi#ZSD315
Malpighiales Putranjivaceae Drypetes deplanchei#RVGH
Malpighiales Putranjivaceae Drypetes hainanensis#F491
Malpighiales Putranjivaceae Drypetes indica#F918
Malpighiales Balanopaceae Balanops pachyphylla#ZSD325
Malpighiales Trigoniaceae Trigonia rugosa#F897
Malpighiales Dichapetalaceae Dichapetalum longipetalum#F705
Malpighiales Dichapetalaceae Dichapetalum gelonioides#F470
Malpighiales Euphroniaceae Euphronia acuminatissima#T00300
Malpighiales Euphroniaceae Euphronia hirtelloides#T00301
Malpighiales Euphroniaceae Euphronia guianensis#T00302
Malpighiales Chrysobalanaceae Chrysobalanus icaco#FC108
Malpighiales Chrysobalanaceae Parinari campestris#NC 024067
Malpighiales Chrysobalanaceae Licania michauxii#HBUQ
Malpighiales Chrysobalanaceae Couepia guianensis#NC 024063
Malpighiales Chrysobalanaceae Licania tomentosa#NC 030575
Malpighiales Chrysobalanaceae Licania sprucei#NC 024065
Malpighiales Chrysobalanaceae Licania alba#NC 024064
Malpighiales Chrysobalanaceae Licania heteromorpha#NC 024062
Malpighiales Chrysobalanaceae Hirtella physophora#NC 024066
Malpighiales Chrysobalanaceae Hirtella racemosa#F554
Malpighiales Irvingiaceae Irvingia excelsa#ZSD279
Malpighiales Irvingiaceae Klainedoxa gabonensis#ZSD280
Malpighiales Ochnaceae Gomphia serrata#F482
Malpighiales Ochnaceae Ochna integerrima#F730
Malpighiales Ochnaceae Ochna serrulata#CKDK
Malpighiales Ochnaceae Ochna mossambicensis#TVCU
Malpighiales Ctenolophonaceae Ctenolophon englerianus#ZSD332

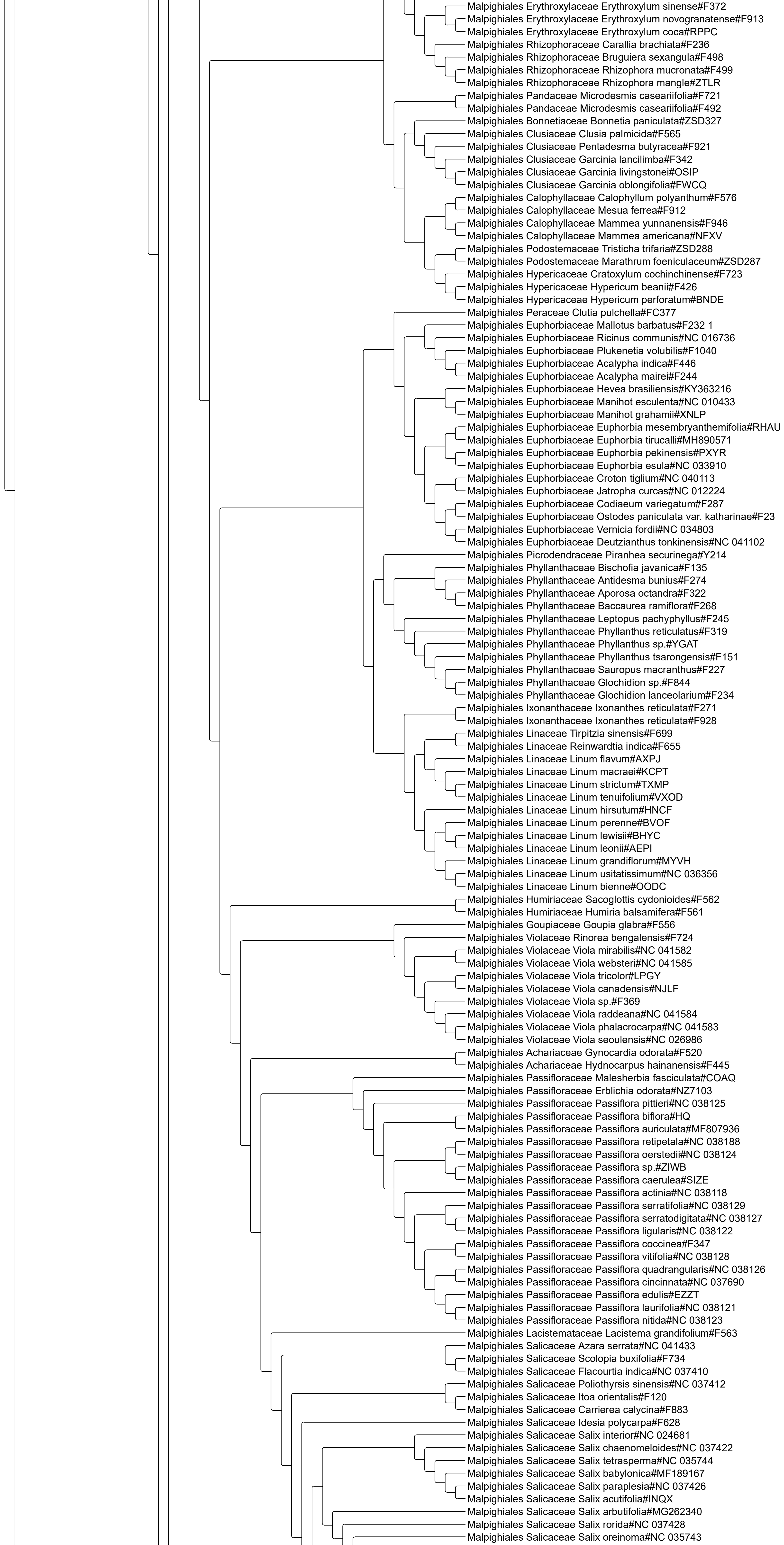
Malpighiales Erythroxylaceae Erythroxylum sinense#F372
Malpighiales Erythroxylaceae Erythroxylum novogranatense#F913
Malpighiales Erythroxylaceae Erythroxylum coca#RPPC
Malpighiales Rhizophoraceae Carallia brachiata#F236
Malpighiales Rhizophoraceae Bruguiera sexangula#F498
Malpighiales Rhizophoraceae Rhizophora mucronata#F499
Malpighiales Rhizophoraceae Rhizophora mangle#ZTLR
Malpighiales Pandaceae Microdesmis caseariifolia#F721
Malpighiales Pandaceae Microdesmis caseariifolia#F492
Malpighiales Bonnetiaceae Bonnetia paniculata#ZSD327
Malpighiales Clusiaceae Clusia palmicida#F565
Malpighiales Clusiaceae Pentadesma butyracea#F921
Malpighiales Clusiaceae Garcinia lancilimba#F342
Malpighiales Clusiaceae Garcinia livingstonei#OSIP
Malpighiales Clusiaceae Garcinia oblongifolia#FWCQ
Malpighiales Calophyllaceae Calophyllum polyanthum#F576
Malpighiales Calophyllaceae Mesua ferrea#F912
Malpighiales Calophyllaceae Mammea yunnanensis#F946
Malpighiales Calophyllaceae Mammea americana#NFXV
Malpighiales Podostemaceae Tristicha trifaria#ZSD288
Malpighiales Podostemaceae Marathrum foeniculaceum#ZSD287
Malpighiales Hypericaceae Cratoxylum cochinchinense#F723
Malpighiales Hypericaceae Hypericum beanii#F426
Malpighiales Hypericaceae Hypericum perforatum#BNDE
Malpighiales Peraceae Clutia pulchella#FC377
Malpighiales Euphorbiaceae Mallotus barbatus#F232 1
Malpighiales Euphorbiaceae Ricinus communis#NC 016736
Malpighiales Euphorbiaceae Plukenetia volubilis#F1040
Malpighiales Euphorbiaceae Acalypha indica#F446
Malpighiales Euphorbiaceae Acalypha mairei#F244
Malpighiales Euphorbiaceae Hevea brasiliensis#KY363216
Malpighiales Euphorbiaceae Manihot esculenta#NC 010433
Malpighiales Euphorbiaceae Manihot grahamii#XNLP
Malpighiales Euphorbiaceae Euphorbia mesembryanthemifolia#RHAU
Malpighiales Euphorbiaceae Euphorbia tirucalli#MH890571
Malpighiales Euphorbiaceae Euphorbia pekinensis#PXYR
Malpighiales Euphorbiaceae Euphorbia esula#NC 033910
Malpighiales Euphorbiaceae Croton tiglium#NC 040113
Malpighiales Euphorbiaceae Jatropha curcas#NC 012224
Malpighiales Euphorbiaceae Codiaeum variegatum#F287
Malpighiales Euphorbiaceae Ostodes paniculata var. katharinae#F23
Malpighiales Euphorbiaceae Vernicia fordii#NC 034803
Malpighiales Euphorbiaceae Deutzianthus tonkinensis#NC 041102
Malpighiales Picrodendraceae Piranhea securinega#Y214
Malpighiales Phyllanthaceae Bischofia javanica#F135
Malpighiales Phyllanthaceae Antidesma bunius#F274
Malpighiales Phyllanthaceae Aporosa octandra#F322
Malpighiales Phyllanthaceae Baccaurea ramiflora#F268
Malpighiales Phyllanthaceae Leptopus pachyphyllus#F245
Malpighiales Phyllanthaceae Phyllanthus reticulatus#F319
Malpighiales Phyllanthaceae Phyllanthus sp.#YGAT
Malpighiales Phyllanthaceae Phyllanthus tsarongensis#F151
Malpighiales Phyllanthaceae Sauropus macranthus#F227
Malpighiales Phyllanthaceae Glochidion sp.#F844
Malpighiales Phyllanthaceae Glochidion lanceolarium#F234
Malpighiales Ixonanthaceae Ixonanthes reticulata#F271
Malpighiales Ixonanthaceae Ixonanthes reticulata#F928
Malpighiales Linaceae Tirpitzia sinensis#F699
Malpighiales Linaceae Reinwardtia indica#F655
Malpighiales Linaceae Linum flavum#AXPJ
Malpighiales Linaceae Linum macraei#KCPT
Malpighiales Linaceae Linum strictum#TXMP
Malpighiales Linaceae Linum tenuifolium#VXOD
Malpighiales Linaceae Linum hirsutum#HNCF
Malpighiales Linaceae Linum perenne#BVOF
Malpighiales Linaceae Linum lewisii#BHYC
Malpighiales Linaceae Linum leonii#AEPI
Malpighiales Linaceae Linum grandiflorum#MYVH
Malpighiales Linaceae Linum usitatissimum#NC 036356
Malpighiales Linaceae Linum bienne#OODC
Malpighiales Humiriaceae Sacoglottis cydonioides#F562
Malpighiales Humiriaceae Humiria balsamifera#F561
Malpighiales Goupiaceae Goupia glabra#F556
Malpighiales Violaceae Rinorea bengalensis#F724
Malpighiales Violaceae Viola mirabilis#NC 041582
Malpighiales Violaceae Viola websteri#NC 041585
Malpighiales Violaceae Viola tricolor#LPGY
Malpighiales Violaceae Viola canadensis#NJLF
Malpighiales Violaceae Viola sp.#F369
Malpighiales Violaceae Viola raddeana#NC 041584
Malpighiales Violaceae Viola phalacrocarpa#NC 041583
Malpighiales Violaceae Viola seoulensis#NC 026986
Malpighiales Achariaceae Gynocardia odorata#F520
Malpighiales Achariaceae Hydnocarpus hainanensis#F445
Malpighiales Passifloraceae Malesherbia fasciculata#COAQ
Malpighiales Passifloraceae Erblichia odorata#NZ7103
Malpighiales Passifloraceae Passiflora pittieri#NC 038125
Malpighiales Passifloraceae Passiflora biflora#HQ
Malpighiales Passifloraceae Passiflora auriculata#MF807936
Malpighiales Passifloraceae Passiflora retipetala#NC 038188
Malpighiales Passifloraceae Passiflora oerstedii#NC 038124
Malpighiales Passifloraceae Passiflora sp.#ZIWB
Malpighiales Passifloraceae Passiflora caerulea#SIZE
Malpighiales Passifloraceae Passiflora actinia#NC 038118
Malpighiales Passifloraceae Passiflora serratifolia#NC 038129
Malpighiales Passifloraceae Passiflora serratodigitata#NC 038127
Malpighiales Passifloraceae Passiflora ligularis#NC 038122
Malpighiales Passifloraceae Passiflora coccinea#F347
Malpighiales Passifloraceae Passiflora vitifolia#NC 038128
Malpighiales Passifloraceae Passiflora quadrangularis#NC 038126
Malpighiales Passifloraceae Passiflora cincinnata#NC 037690
Malpighiales Passifloraceae Passiflora edulis#EZZT
Malpighiales Passifloraceae Passiflora laurifolia#NC 038121
Malpighiales Passifloraceae Passiflora nitida#NC 038123
Malpighiales Lacistemataceae Lacistema grandifolium#F563
Malpighiales Salicaceae Azara serrata#NC 041433
Malpighiales Salicaceae Scolopia buxifolia#F734
Malpighiales Salicaceae Flacourtia indica#NC 037410
Malpighiales Salicaceae Poliothyrsis sinensis#NC 037412
Malpighiales Salicaceae Itoa orientalis#F120
Malpighiales Salicaceae Carrierea calycina#F883
Malpighiales Salicaceae Idesia polycarpa#F628
Malpighiales Salicaceae Salix interior#NC 024681
Malpighiales Salicaceae Salix chaenomeloides#NC 037422
Malpighiales Salicaceae Salix tetrasperma#NC 035744
Malpighiales Salicaceae Salix babylonica#MF189167
Malpighiales Salicaceae Salix paraplesia#NC 037426
Malpighiales Salicaceae Salix acutifolia#INQX
Malpighiales Salicaceae Salix arbutifolia#MG262340
Malpighiales Salicaceae Salix rorida#NC 037428
Malpighiales Salicaceae Salix oreinoma#NC 035743

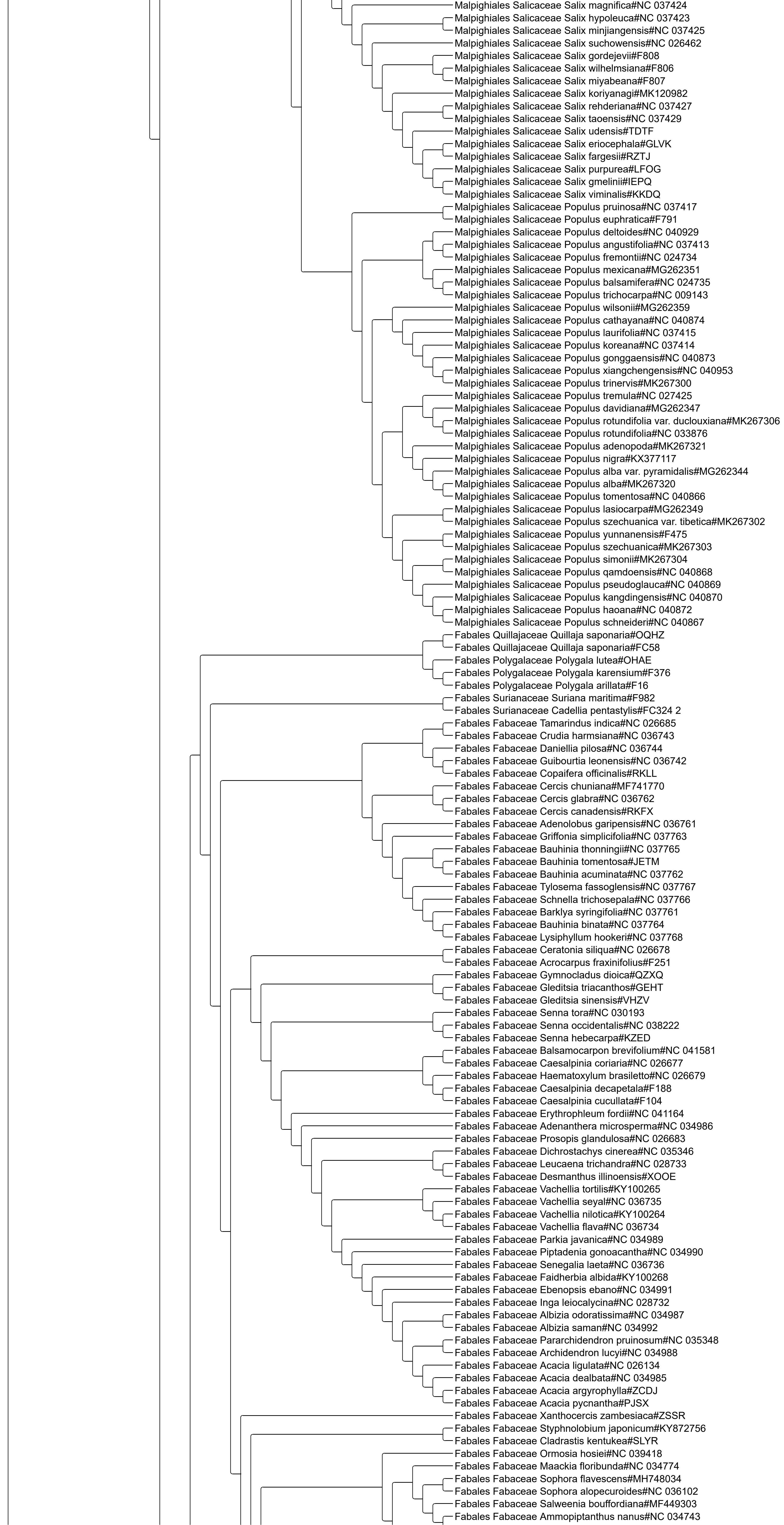

Malpighiales Salicaceae Salix magnifica#NC 037424
Malpighiales Salicaceae Salix hypoleuca#NC 037423
Malpighiales Salicaceae Salix minjiangensis#NC 037425
Malpighiales Salicaceae Salix suchowensis#NC 026462
Malpighiales Salicaceae Salix gordejevii#F808
Malpighiales Salicaceae Salix wilhelmsiana#F806
Malpighiales Salicaceae Salix miyabeana#F807
Malpighiales Salicaceae Salix koriyanagi#MK120982
Malpighiales Salicaceae Salix rehderiana#NC 037427
Malpighiales Salicaceae Salix taoensis#NC 037429
Malpighiales Salicaceae Salix udensis#TDTF
Malpighiales Salicaceae Salix eriocephala#GLVK
Malpighiales Salicaceae Salix fargesii#RZTJ
Malpighiales Salicaceae Salix purpurea#LFOG
Malpighiales Salicaceae Salix gmelinii#IEPQ
Malpighiales Salicaceae Salix viminalis#KKDQ
Malpighiales Salicaceae Populus pruinosa#NC 037417
Malpighiales Salicaceae Populus euphratica#F791
Malpighiales Salicaceae Populus deltoides#NC 040929
Malpighiales Salicaceae Populus angustifolia#NC 037413
Malpighiales Salicaceae Populus fremontii#NC 024734
Malpighiales Salicaceae Populus mexicana#MG262351
Malpighiales Salicaceae Populus balsamifera#NC 024735
Malpighiales Salicaceae Populus trichocarpa#NC 009143
Malpighiales Salicaceae Populus wilsonii#MG262359
Malpighiales Salicaceae Populus cathayana#NC 040874
Malpighiales Salicaceae Populus laurifolia#NC 037415
Malpighiales Salicaceae Populus koreana#NC 037414
Malpighiales Salicaceae Populus gonggaensis#NC 040873
Malpighiales Salicaceae Populus xiangchengensis#NC 040953
Malpighiales Salicaceae Populus trinervis#MK267300
Malpighiales Salicaceae Populus tremula#NC 027425
Malpighiales Salicaceae Populus davidiana#MG262347
Malpighiales Salicaceae Populus rotundifolia var. duclouxiana#MK267306
Malpighiales Salicaceae Populus rotundifolia#NC 033876
Malpighiales Salicaceae Populus adenopoda#MK267321
Malpighiales Salicaceae Populus nigra#KX377117
Malpighiales Salicaceae Populus alba var. pyramidalis#MG262344
Malpighiales Salicaceae Populus alba#MK267320
Malpighiales Salicaceae Populus tomentosa#NC 040866
Malpighiales Salicaceae Populus lasiocarpa#MG262349
Malpighiales Salicaceae Populus szechuanica var. tibetica#MK267302
Malpighiales Salicaceae Populus yunnanensis#F475
Malpighiales Salicaceae Populus szechuanica#MK267303
Malpighiales Salicaceae Populus simonii#MK267304
Malpighiales Salicaceae Populus qamdoensis#NC 040868
Malpighiales Salicaceae Populus pseudoglauca#NC 040869
Malpighiales Salicaceae Populus kangdingensis#NC 040870
Malpighiales Salicaceae Populus haoana#NC 040872
Malpighiales Salicaceae Populus schneideri#NC 040867
Fabales Quillajaceae Quillaja saponaria#OQHZ
Fabales Quillajaceae Quillaja saponaria#FC58
Fabales Polygalaceae Polygala lutea#OHAE
Fabales Polygalaceae Polygala karensium#F376
Fabales Polygalaceae Polygala arillata#F16
Fabales Surianaceae Suriana maritima#F982
Fabales Surianaceae Cadellia pentastylis#FC324 2
Fabales Fabaceae Tamarindus indica#NC 026685
Fabales Fabaceae Crudia harmsiana#NC 036743
Fabales Fabaceae Daniellia pilosa#NC 036744
Fabales Fabaceae Guibourtia leonensis#NC 036742
Fabales Fabaceae Copaifera officinalis#RKLL
Fabales Fabaceae Cercis chuniana#MF741770
Fabales Fabaceae Cercis glabra#NC 036762
Fabales Fabaceae Cercis canadensis#RKFX
Fabales Fabaceae Adenolobus garipensis#NC 036761
Fabales Fabaceae Griffonia simplicifolia#NC 037763
Fabales Fabaceae Bauhinia thonningii#NC 037765
Fabales Fabaceae Bauhinia tomentosa#JETM
Fabales Fabaceae Bauhinia acuminata#NC 037762
Fabales Fabaceae Tylosema fassoglensis#NC 037767
Fabales Fabaceae Schnella trichosepala#NC 037766
Fabales Fabaceae Barklya syringifolia#NC 037761
Fabales Fabaceae Bauhinia binata#NC 037764
Fabales Fabaceae Lysiphyllum hookeri#NC 037768
Fabales Fabaceae Ceratonia siliqua#NC 026678
Fabales Fabaceae Acrocarpus fraxinifolius#F251
Fabales Fabaceae Gymnocladus dioica#QZXQ
Fabales Fabaceae Gleditsia triacanthos#GEHT
Fabales Fabaceae Gleditsia sinensis#VHZV
Fabales Fabaceae Senna tora#NC 030193
Fabales Fabaceae Senna occidentalis#NC 038222
Fabales Fabaceae Senna hebecarpa#KZED
Fabales Fabaceae Balsamocarpon brevifolium#NC 041581
Fabales Fabaceae Caesalpinia coriaria#NC 026677
Fabales Fabaceae Haematoxylum brasiletto#NC 026679
Fabales Fabaceae Caesalpinia decapetala#F188
Fabales Fabaceae Caesalpinia cucullata#F104
Fabales Fabaceae Erythrophleum fordii#NC 041164
Fabales Fabaceae Adenanthera microsperma#NC 034986
Fabales Fabaceae Prosopis glandulosa#NC 026683
Fabales Fabaceae Dichrostachys cinerea#NC 035346
Fabales Fabaceae Leucaena trichandra#NC 028733
Fabales Fabaceae Desmanthus illinoensis#XOOE
Fabales Fabaceae Vachellia tortilis#KY100265
Fabales Fabaceae Vachellia seyal#NC 036735
Fabales Fabaceae Vachellia nilotica#KY100264
Fabales Fabaceae Vachellia flava#NC 036734
Fabales Fabaceae Parkia javanica#NC 034989
Fabales Fabaceae Piptadenia gonoacantha#NC 034990
Fabales Fabaceae Senegalia laeta#NC 036736
Fabales Fabaceae Faidherbia albida#KY100268
Fabales Fabaceae Ebenopsis ebano#NC 034991
Fabales Fabaceae Inga leiocalycina#NC 028732
Fabales Fabaceae Albizia odoratissima#NC 034987
Fabales Fabaceae Albizia saman#NC 034992
Fabales Fabaceae Pararchidendron pruinosum#NC 035348
Fabales Fabaceae Archidendron lucyi#NC 034988
Fabales Fabaceae Acacia ligulata#NC 026134
Fabales Fabaceae Acacia dealbata#NC 034985
Fabales Fabaceae Acacia argyrophylla#ZCDJ
Fabales Fabaceae Acacia pycnantha#PJSX
Fabales Fabaceae Xanthocercis zambesiaca#ZSSR
Fabales Fabaceae Styphnolobium japonicum#KY872756
Fabales Fabaceae Cladrastis kentukea#SLYR
Fabales Fabaceae Ormosia hosiei#NC 039418
Fabales Fabaceae Maackia floribunda#NC 034774
Fabales Fabaceae Sophora flavescens#MH748034
Fabales Fabaceae Sophora alopecuroides#NC 036102
Fabales Fabaceae Salweenia bouffordiana#MF449303
Fabales Fabaceae Ammopiptanthus nanus#NC 034743

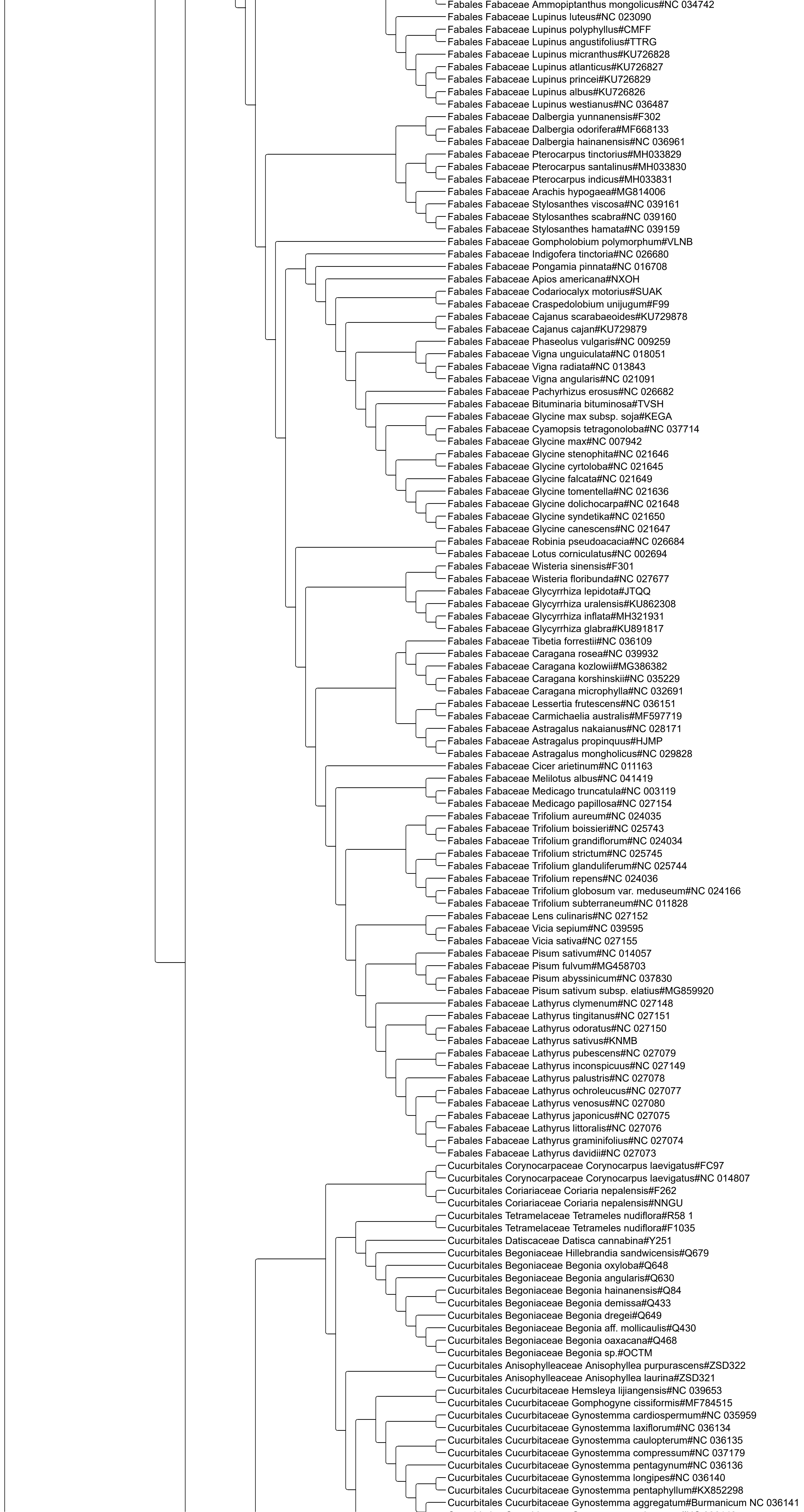

Fabales Fabaceae Ammopiptanthus mongolicus#NC 034742
Fabales Fabaceae Lupinus luteus#NC 023090
Fabales Fabaceae Lupinus polyphyllus#CMFF
Fabales Fabaceae Lupinus angustifolius#TTRG
Fabales Fabaceae Lupinus micranthus#KU726828
Fabales Fabaceae Lupinus atlanticus#KU726827
Fabales Fabaceae Lupinus princei#KU726829
Fabales Fabaceae Lupinus albus#KU726826
Fabales Fabaceae Lupinus westianus#NC 036487
Fabales Fabaceae Dalbergia yunnanensis#F302
Fabales Fabaceae Dalbergia odorifera#MF668133
Fabales Fabaceae Dalbergia hainanensis#NC 036961
Fabales Fabaceae Pterocarpus tinctorius#MH033829
Fabales Fabaceae Pterocarpus santalinus#MH033830
Fabales Fabaceae Pterocarpus indicus#MH033831
Fabales Fabaceae Arachis hypogaea#MG814006
Fabales Fabaceae Stylosanthes viscosa#NC 039161
Fabales Fabaceae Stylosanthes scabra#NC 039160
Fabales Fabaceae Stylosanthes hamata#NC 039159
Fabales Fabaceae Gompholobium polymorphum#VLNB
Fabales Fabaceae Indigofera tinctoria#NC 026680
Fabales Fabaceae Pongamia pinnata#NC 016708
Fabales Fabaceae Apios americana#NXOH
Fabales Fabaceae Codariocalyx motorius#SUAK
Fabales Fabaceae Craspedolobium unijugum#F99
Fabales Fabaceae Cajanus scarabaeoides#KU729878
Fabales Fabaceae Cajanus cajan#KU729879
Fabales Fabaceae Phaseolus vulgaris#NC 009259
Fabales Fabaceae Vigna unguiculata#NC 018051
Fabales Fabaceae Vigna radiata#NC 013843
Fabales Fabaceae Vigna angularis#NC 021091
Fabales Fabaceae Pachyrhizus erosus#NC 026682
Fabales Fabaceae Bituminaria bituminosa#TVSH
Fabales Fabaceae Glycine max subsp. soja#KEGA
Fabales Fabaceae Cyamopsis tetragonoloba#NC 037714
Fabales Fabaceae Glycine max#NC 007942
Fabales Fabaceae Glycine stenophita#NC 021646
Fabales Fabaceae Glycine cyrtoloba#NC 021645
Fabales Fabaceae Glycine falcata#NC 021649
Fabales Fabaceae Glycine tomentella#NC 021636
Fabales Fabaceae Glycine dolichocarpa#NC 021648
Fabales Fabaceae Glycine syndetika#NC 021650
Fabales Fabaceae Glycine canescens#NC 021647
Fabales Fabaceae Robinia pseudoacacia#NC 026684
Fabales Fabaceae Lotus corniculatus#NC 002694
Fabales Fabaceae Wisteria sinensis#F301
Fabales Fabaceae Wisteria floribunda#NC 027677
Fabales Fabaceae Glycyrrhiza lepidota#JTQQ
Fabales Fabaceae Glycyrrhiza uralensis#KU862308
Fabales Fabaceae Glycyrrhiza inflata#MH321931
Fabales Fabaceae Glycyrrhiza glabra#KU891817
Fabales Fabaceae Tibetia forrestii#NC 036109
Fabales Fabaceae Caragana rosea#NC 039932
Fabales Fabaceae Caragana kozlowii#MG386382
Fabales Fabaceae Caragana korshinskii#NC 035229
Fabales Fabaceae Caragana microphylla#NC 032691
Fabales Fabaceae Lessertia frutescens#NC 036151
Fabales Fabaceae Carmichaelia australis#MF597719
Fabales Fabaceae Astragalus nakaianus#NC 028171
Fabales Fabaceae Astragalus propinquus#HJMP
Fabales Fabaceae Astragalus mongholicus#NC 029828
Fabales Fabaceae Cicer arietinum#NC 011163
Fabales Fabaceae Melilotus albus#NC 041419
Fabales Fabaceae Medicago truncatula#NC 003119
Fabales Fabaceae Medicago papillosa#NC 027154
Fabales Fabaceae Trifolium aureum#NC 024035
Fabales Fabaceae Trifolium boissieri#NC 025743
Fabales Fabaceae Trifolium grandiflorum#NC 024034
Fabales Fabaceae Trifolium strictum#NC 025745
Fabales Fabaceae Trifolium glanduliferum#NC 025744
Fabales Fabaceae Trifolium repens#NC 024036
Fabales Fabaceae Trifolium globosum var. meduseum#NC 024166
Fabales Fabaceae Trifolium subterraneum#NC 011828
Fabales Fabaceae Lens culinaris#NC 027152
Fabales Fabaceae Vicia sepium#NC 039595
Fabales Fabaceae Vicia sativa#NC 027155
Fabales Fabaceae Pisum sativum#NC 014057
Fabales Fabaceae Pisum fulvum#MG458703
Fabales Fabaceae Pisum abyssinicum#NC 037830
Fabales Fabaceae Pisum sativum subsp. elatius#MG859920
Fabales Fabaceae Lathyrus clymenum#NC 027148
Fabales Fabaceae Lathyrus tingitanus#NC 027151
Fabales Fabaceae Lathyrus odoratus#NC 027150
Fabales Fabaceae Lathyrus sativus#KNMB
Fabales Fabaceae Lathyrus pubescens#NC 027079
Fabales Fabaceae Lathyrus inconspicuus#NC 027149
Fabales Fabaceae Lathyrus palustris#NC 027078
Fabales Fabaceae Lathyrus ochroleucus#NC 027077
Fabales Fabaceae Lathyrus venosus#NC 027080
Fabales Fabaceae Lathyrus japonicus#NC 027075
Fabales Fabaceae Lathyrus littoralis#NC 027076
Fabales Fabaceae Lathyrus graminifolius#NC 027074
Fabales Fabaceae Lathyrus davidii#NC 027073
Cucurbitales Corynocarpaceae Corynocarpus laevigatus#FC97
Cucurbitales Corynocarpaceae Corynocarpus laevigatus#NC 014807
Cucurbitales Coriariaceae Coriaria nepalensis#F262
Cucurbitales Coriariaceae Coriaria nepalensis#NNGU
Cucurbitales Tetramelaceae Tetrameles nudiflora#R58 1
Cucurbitales Tetramelaceae Tetrameles nudiflora#F1035
Cucurbitales Datiscaceae Datisca cannabina#Y251
Cucurbitales Begoniaceae Hillebrandia sandwicensis#Q679
Cucurbitales Begoniaceae Begonia oxyloba#Q648
Cucurbitales Begoniaceae Begonia angularis#Q630
Cucurbitales Begoniaceae Begonia hainanensis#Q84
Cucurbitales Begoniaceae Begonia demissa#Q433
Cucurbitales Begoniaceae Begonia dregei#Q649
Cucurbitales Begoniaceae Begonia aff. mollicaulis#Q430
Cucurbitales Begoniaceae Begonia oaxacana#Q468
Cucurbitales Begoniaceae Begonia sp.#OCTM
Cucurbitales Anisophylleaceae Anisophyllea purpurascens#ZSD322
Cucurbitales Anisophylleaceae Anisophyllea laurina#ZSD321
Cucurbitales Cucurbitaceae Hemsleya lijiangensis#NC 039653
Cucurbitales Cucurbitaceae Gomphogyne cissiformis#MF784515
Cucurbitales Cucurbitaceae Gynostemma cardiospermum#NC 035959
Cucurbitales Cucurbitaceae Gynostemma laxiflorum#NC 036134
Cucurbitales Cucurbitaceae Gynostemma caulopterum#NC 036135
Cucurbitales Cucurbitaceae Gynostemma compressum#NC 037179
Cucurbitales Cucurbitaceae Gynostemma pentagynum#NC 036136
Cucurbitales Cucurbitaceae Gynostemma longipes#NC 036140
Cucurbitales Cucurbitaceae Gynostemma pentaphyllum#KX852298
Cucurbitales Cucurbitaceae Gynostemma aggregatum#Burmanicum NC 036141

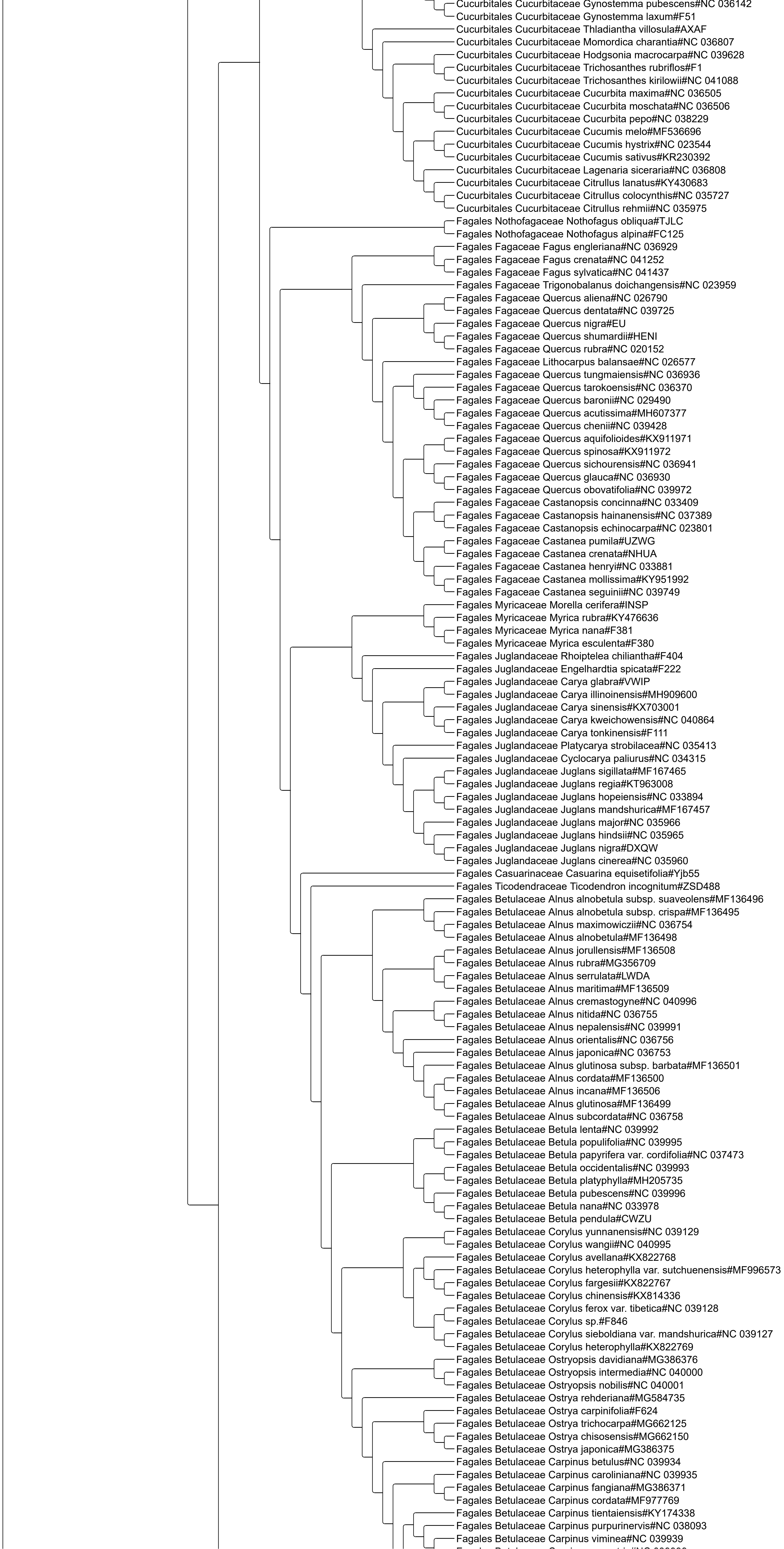

Cucurbitales Cucurbitaceae Gynostemma pubescens#NC 036142
Cucurbitales Cucurbitaceae Gynostemma laxum#F51
Cucurbitales Cucurbitaceae Thladiantha villosula#AXAF
Cucurbitales Cucurbitaceae Momordica charantia#NC 036807
Cucurbitales Cucurbitaceae Hodgsonia macrocarpa#NC 039628
Cucurbitales Cucurbitaceae Trichosanthes rubriflos#F1
Cucurbitales Cucurbitaceae Trichosanthes kirilowii#NC 041088
Cucurbitales Cucurbitaceae Cucurbita maxima#NC 036505
Cucurbitales Cucurbitaceae Cucurbita moschata#NC 036506
Cucurbitales Cucurbitaceae Cucurbita pepo#NC 038229
Cucurbitales Cucurbitaceae Cucumis melo#MF536696
Cucurbitales Cucurbitaceae Cucumis hystrix#NC 023544
Cucurbitales Cucurbitaceae Cucumis sativus#KR230392
Cucurbitales Cucurbitaceae Lagenaria siceraria#NC 036808
Cucurbitales Cucurbitaceae Citrullus lanatus#KY430683
Cucurbitales Cucurbitaceae Citrullus colocynthis#NC 035727
Cucurbitales Cucurbitaceae Citrullus rehmii#NC 035975
Fagales Nothofagaceae Nothofagus obliqua#TJLC
Fagales Nothofagaceae Nothofagus alpina#FC125
Fagales Fagaceae Fagus engleriana#NC 036929
Fagales Fagaceae Fagus crenata#NC 041252
Fagales Fagaceae Fagus sylvatica#NC 041437
Fagales Fagaceae Trigonobalanus doichangensis#NC 023959
Fagales Fagaceae Quercus aliena#NC 026790
Fagales Fagaceae Quercus dentata#NC 039725
Fagales Fagaceae Quercus nigra#EU
Fagales Fagaceae Quercus shumardii#HENI
Fagales Fagaceae Quercus rubra#NC 020152
Fagales Fagaceae Lithocarpus balansae#NC 026577
Fagales Fagaceae Quercus tungmaiensis#NC 036936
Fagales Fagaceae Quercus tarokoensis#NC 036370
Fagales Fagaceae Quercus baronii#NC 029490
Fagales Fagaceae Quercus acutissima#MH607377
Fagales Fagaceae Quercus chenii#NC 039428
Fagales Fagaceae Quercus aquifolioides#KX911971
Fagales Fagaceae Quercus spinosa#KX911972
Fagales Fagaceae Quercus sichourensis#NC 036941
Fagales Fagaceae Quercus glauca#NC 036930
Fagales Fagaceae Quercus obovatifolia#NC 039972
Fagales Fagaceae Castanopsis concinna#NC 033409
Fagales Fagaceae Castanopsis hainanensis#NC 037389
Fagales Fagaceae Castanopsis echinocarpa#NC 023801
Fagales Fagaceae Castanea pumila#UZWG
Fagales Fagaceae Castanea crenata#NHUA
Fagales Fagaceae Castanea henryi#NC 033881
Fagales Fagaceae Castanea mollissima#KY951992
Fagales Fagaceae Castanea seguinii#NC 039749
Fagales Myricaceae Morella cerifera#INSP
Fagales Myricaceae Myrica rubra#KY476636
Fagales Myricaceae Myrica nana#F381
Fagales Myricaceae Myrica esculenta#F380
Fagales Juglandaceae Rhoiptelea chiliantha#F404
Fagales Juglandaceae Engelhardtia spicata#F222
Fagales Juglandaceae Carya glabra#VWIP
Fagales Juglandaceae Carya illinoinensis#MH909600
Fagales Juglandaceae Carya sinensis#KX703001
Fagales Juglandaceae Carya kweichowensis#NC 040864
Fagales Juglandaceae Carya tonkinensis#F111
Fagales Juglandaceae Platycarya strobilacea#NC 035413
Fagales Juglandaceae Cyclocarya paliurus#NC 034315
Fagales Juglandaceae Juglans sigillata#MF167465
Fagales Juglandaceae Juglans regia#KT963008
Fagales Juglandaceae Juglans hopeiensis#NC 033894
Fagales Juglandaceae Juglans mandshurica#MF167457
Fagales Juglandaceae Juglans major#NC 035966
Fagales Juglandaceae Juglans hindsii#NC 035965
Fagales Juglandaceae Juglans nigra#DXQW
Fagales Juglandaceae Juglans cinerea#NC 035960
Fagales Casuarinaceae Casuarina equisetifolia#Yjb55
Fagales Ticodendraceae Ticodendron incognitum#ZSD488
Fagales Betulaceae Alnus alnobetula subsp. suaveolens#MF136496
Fagales Betulaceae Alnus alnobetula subsp. crispa#MF136495
Fagales Betulaceae Alnus maximowiczii#NC 036754
Fagales Betulaceae Alnus alnobetula#MF136498
Fagales Betulaceae Alnus jorullensis#MF136508
Fagales Betulaceae Alnus rubra#MG356709
Fagales Betulaceae Alnus serrulata#LWDA
Fagales Betulaceae Alnus maritima#MF136509
Fagales Betulaceae Alnus cremastogyne#NC 040996
Fagales Betulaceae Alnus nitida#NC 036755
Fagales Betulaceae Alnus nepalensis#NC 039991
Fagales Betulaceae Alnus orientalis#NC 036756
Fagales Betulaceae Alnus japonica#NC 036753
Fagales Betulaceae Alnus glutinosa subsp. barbata#MF136501
Fagales Betulaceae Alnus cordata#MF136500
Fagales Betulaceae Alnus incana#MF136506
Fagales Betulaceae Alnus glutinosa#MF136499
Fagales Betulaceae Alnus subcordata#NC 036758
Fagales Betulaceae Betula lenta#NC 039992
Fagales Betulaceae Betula populifolia#NC 039995
Fagales Betulaceae Betula papyrifera var. cordifolia#NC 037473
Fagales Betulaceae Betula occidentalis#NC 039993
Fagales Betulaceae Betula platyphylla#MH205735
Fagales Betulaceae Betula pubescens#NC 039996
Fagales Betulaceae Betula nana#NC 033978
Fagales Betulaceae Betula pendula#CWZU
Fagales Betulaceae Corylus yunnanensis#NC 039129
Fagales Betulaceae Corylus wangii#NC 040995
Fagales Betulaceae Corylus avellana#KX822768
Fagales Betulaceae Corylus heterophylla var. sutchuenensis#MF996573
Fagales Betulaceae Corylus fargesii#KX822767
Fagales Betulaceae Corylus chinensis#KX814336
Fagales Betulaceae Corylus ferox var. tibetica#NC 039128
Fagales Betulaceae Corylus sp.#F846
Fagales Betulaceae Corylus sieboldiana var. mandshurica#NC 039127
Fagales Betulaceae Corylus heterophylla#KX822769
Fagales Betulaceae Ostryopsis davidiana#MG386376
Fagales Betulaceae Ostryopsis intermedia#NC 040000
Fagales Betulaceae Ostryopsis nobilis#NC 040001
Fagales Betulaceae Ostrya rehderiana#MG584735
Fagales Betulaceae Ostrya carpinifolia#F624
Fagales Betulaceae Ostrya trichocarpa#MG662125
Fagales Betulaceae Ostrya chisosensis#MG662150
Fagales Betulaceae Ostrya japonica#MG386375
Fagales Betulaceae Carpinus betulus#NC 039934
Fagales Betulaceae Carpinus caroliniana#NC 039935
Fagales Betulaceae Carpinus fangiana#MG386371
Fagales Betulaceae Carpinus cordata#MF977769
Fagales Betulaceae Carpinus tientaiensis#KY174338
Fagales Betulaceae Carpinus purpurinervis#NC 038093
Fagales Betulaceae Carpinus viminea#NC 039939

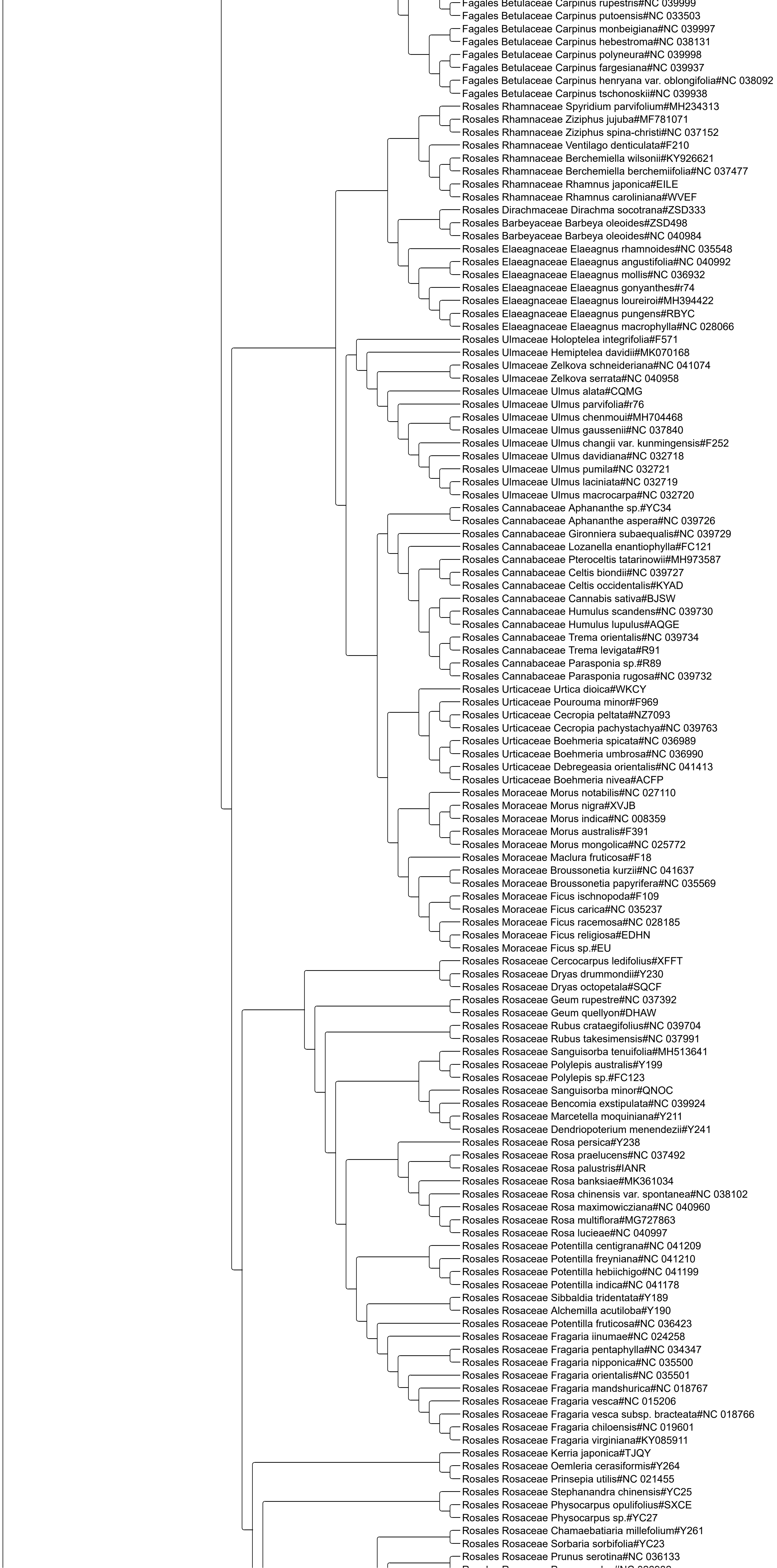
Fagales Betulaceae Carpinus rupestris#NC 039999
Fagales Betulaceae Carpinus putoensis#NC 033503
Fagales Betulaceae Carpinus monbeigiana#NC 039997
Fagales Betulaceae Carpinus hebestroma#NC 038131
Fagales Betulaceae Carpinus polyneura#NC 039998
Fagales Betulaceae Carpinus fargesiana#NC 039937
Fagales Betulaceae Carpinus henryana var. oblongifolia#NC 038092
Fagales Betulaceae Carpinus tschonoskii#NC 039938
Rosales Rhamnaceae Spyridium parvifolium#MH234313
Rosales Rhamnaceae Ziziphus jujuba#MF781071
Rosales Rhamnaceae Ziziphus spina-christi#NC 037152
Rosales Rhamnaceae Ventilago denticulata#F210
Rosales Rhamnaceae Berchemiella wilsonii#KY926621
Rosales Rhamnaceae Berchemiella berchemiifolia#NC 037477
Rosales Rhamnaceae Rhamnus japonica#EILE
Rosales Rhamnaceae Rhamnus caroliniana#WVEF
Rosales Dirachmaceae Dirachma socotrana#ZSD333
Rosales Barbeyaceae Barbeya oleoides#ZSD498
Rosales Barbeyaceae Barbeya oleoides#NC 040984
Rosales Elaeagnaceae Elaeagnus rhamnoides#NC 035548
Rosales Elaeagnaceae Elaeagnus angustifolia#NC 040992
Rosales Elaeagnaceae Elaeagnus mollis#NC 036932
Rosales Elaeagnaceae Elaeagnus gonyanthes#r74
Rosales Elaeagnaceae Elaeagnus loureiroi#MH394422
Rosales Elaeagnaceae Elaeagnus pungens#RBYC
Rosales Elaeagnaceae Elaeagnus macrophylla#NC 028066
Rosales Ulmaceae Holoptelea integrifolia#F571
Rosales Ulmaceae Hemiptelea davidii#MK070168
Rosales Ulmaceae Zelkova schneideriana#NC 041074
Rosales Ulmaceae Zelkova serrata#NC 040958
Rosales Ulmaceae Ulmus alata#CQMG
Rosales Ulmaceae Ulmus parvifolia#r76
Rosales Ulmaceae Ulmus chenmoui#MH704468
Rosales Ulmaceae Ulmus gaussenii#NC 037840
Rosales Ulmaceae Ulmus changii var. kunmingensis#F252
Rosales Ulmaceae Ulmus davidiana#NC 032718
Rosales Ulmaceae Ulmus pumila#NC 032721
Rosales Ulmaceae Ulmus laciniata#NC 032719
Rosales Ulmaceae Ulmus macrocarpa#NC 032720
Rosales Cannabaceae Aphananthe sp.#YC34
Rosales Cannabaceae Aphananthe aspera#NC 039726
Rosales Cannabaceae Gironniera subaequalis#NC 039729
Rosales Cannabaceae Lozanella enantiophylla#FC121
Rosales Cannabaceae Pteroceltis tatarinowii#MH973587
Rosales Cannabaceae Celtis biondii#NC 039727
Rosales Cannabaceae Celtis occidentalis#KYAD
Rosales Cannabaceae Cannabis sativa#BJSW
Rosales Cannabaceae Humulus scandens#NC 039730
Rosales Cannabaceae Humulus lupulus#AQGE
Rosales Cannabaceae Trema orientalis#NC 039734
Rosales Cannabaceae Trema levigata#R91
Rosales Cannabaceae Parasponia sp.#R89
Rosales Cannabaceae Parasponia rugosa#NC 039732
Rosales Urticaceae Urtica dioica#WKCY
Rosales Urticaceae Pourouma minor#F969
Rosales Urticaceae Cecropia peltata#NZ7093
Rosales Urticaceae Cecropia pachystachya#NC 039763
Rosales Urticaceae Boehmeria spicata#NC 036989
Rosales Urticaceae Boehmeria umbrosa#NC 036990
Rosales Urticaceae Debregeasia orientalis#NC 041413
Rosales Urticaceae Boehmeria nivea#ACFP
Rosales Moraceae Morus notabilis#NC 027110
Rosales Moraceae Morus nigra#XVJB
Rosales Moraceae Morus indica#NC 008359
Rosales Moraceae Morus australis#F391
Rosales Moraceae Morus mongolica#NC 025772
Rosales Moraceae Maclura fruticosa#F18
Rosales Moraceae Broussonetia kurzii#NC 041637
Rosales Moraceae Broussonetia papyrifera#NC 035569
Rosales Moraceae Ficus ischnopoda#F109
Rosales Moraceae Ficus carica#NC 035237
Rosales Moraceae Ficus racemosa#NC 028185
Rosales Moraceae Ficus religiosa#EDHN
Rosales Moraceae Ficus sp.#EU
Rosales Rosaceae Cercocarpus ledifolius#XFFT
Rosales Rosaceae Dryas drummondii#Y230
Rosales Rosaceae Dryas octopetala#SQCF
Rosales Rosaceae Geum rupestre#NC 037392
Rosales Rosaceae Geum quellyon#DHAW
Rosales Rosaceae Rubus crataegifolius#NC 039704
Rosales Rosaceae Rubus takesimensis#NC 037991
Rosales Rosaceae Sanguisorba tenuifolia#MH513641
Rosales Rosaceae Polylepis australis#Y199
Rosales Rosaceae Polylepis sp.#FC123
Rosales Rosaceae Sanguisorba minor#QNOC
Rosales Rosaceae Bencomia exstipulata#NC 039924
Rosales Rosaceae Marcetella moquiniana#Y211
Rosales Rosaceae Dendriopoterium menendezii#Y241
Rosales Rosaceae Rosa persica#Y238
Rosales Rosaceae Rosa praelucens#NC 037492
Rosales Rosaceae Rosa palustris#IANR
Rosales Rosaceae Rosa banksiae#MK361034
Rosales Rosaceae Rosa chinensis var. spontanea#NC 038102
Rosales Rosaceae Rosa maximowicziana#NC 040960
Rosales Rosaceae Rosa multiflora#MG727863
Rosales Rosaceae Rosa lucieae#NC 040997
Rosales Rosaceae Potentilla centigrana#NC 041209
Rosales Rosaceae Potentilla freyniana#NC 041210
Rosales Rosaceae Potentilla hebiichigo#NC 041199
Rosales Rosaceae Potentilla indica#NC 041178
Rosales Rosaceae Sibbaldia tridentata#Y189
Rosales Rosaceae Alchemilla acutiloba#Y190
Rosales Rosaceae Potentilla fruticosa#NC 036423
Rosales Rosaceae Fragaria iinumae#NC 024258
Rosales Rosaceae Fragaria pentaphylla#NC 034347
Rosales Rosaceae Fragaria nipponica#NC 035500
Rosales Rosaceae Fragaria orientalis#NC 035501
Rosales Rosaceae Fragaria mandshurica#NC 018767
Rosales Rosaceae Fragaria vesca#NC 015206
Rosales Rosaceae Fragaria vesca subsp. bracteata#NC 018766
Rosales Rosaceae Fragaria chiloensis#NC 019601
Rosales Rosaceae Fragaria virginiana#KY085911
Rosales Rosaceae Kerria japonica#TJQY
Rosales Rosaceae Oemleria cerasiformis#Y264
Rosales Rosaceae Prinsepia utilis#NC 021455
Rosales Rosaceae Stephanandra chinensis#YC25
Rosales Rosaceae Physocarpus opulifolius#SXCE
Rosales Rosaceae Physocarpus sp.#YC27
Rosales Rosaceae Chamaebatiaria millefolium#Y261
Rosales Rosaceae Sorbaria sorbifolia#YC23
Rosales Rosaceae Prunus serotina#NC 036133

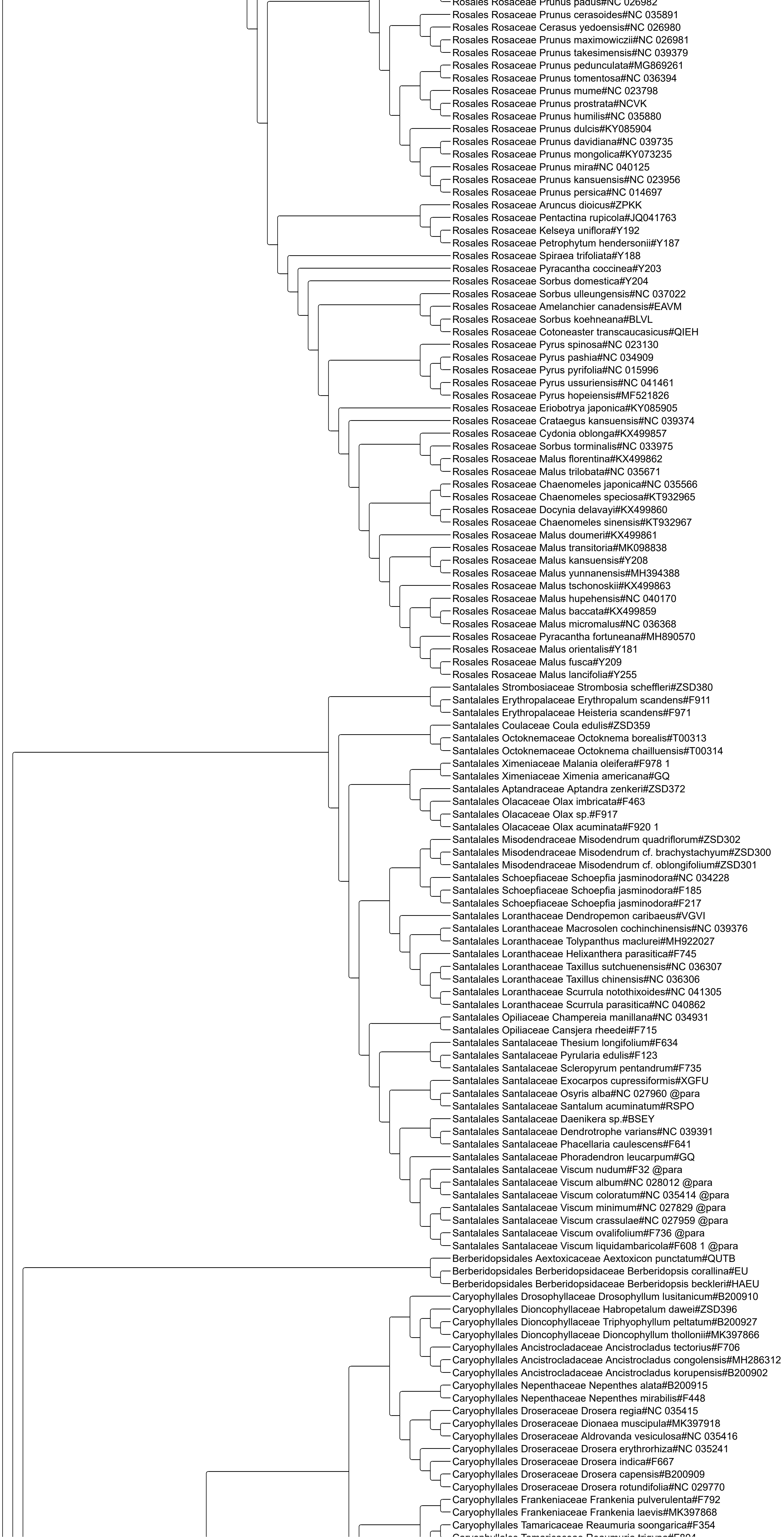
Rosales Rosaceae Prunus padus#NC 026982
Rosales Rosaceae Prunus cerasoides#NC 035891
Rosales Rosaceae Cerasus yedoensis#NC 026980
Rosales Rosaceae Prunus maximowiczii#NC 026981
Rosales Rosaceae Prunus takesimensis#NC 039379
Rosales Rosaceae Prunus pedunculata#MG869261
Rosales Rosaceae Prunus tomentosa#NC 036394
Rosales Rosaceae Prunus mume#NC 023798
Rosales Rosaceae Prunus prostrata#NCVK
Rosales Rosaceae Prunus humilis#NC 035880
Rosales Rosaceae Prunus dulcis#KY085904
Rosales Rosaceae Prunus davidiana#NC 039735
Rosales Rosaceae Prunus mongolica#KY073235
Rosales Rosaceae Prunus mira#NC 040125
Rosales Rosaceae Prunus kansuensis#NC 023956
Rosales Rosaceae Prunus persica#NC 014697
Rosales Rosaceae Aruncus dioicus#ZPKK
Rosales Rosaceae Pentactina rupicola#JQ041763
Rosales Rosaceae Kelseya uniflora#Y192
Rosales Rosaceae Petrophytum hendersonii#Y187
Rosales Rosaceae Spiraea trifoliata#Y188
Rosales Rosaceae Pyracantha coccinea#Y203
Rosales Rosaceae Sorbus domestica#Y204
Rosales Rosaceae Sorbus ulleungensis#NC 037022
Rosales Rosaceae Amelanchier canadensis#EAVM
Rosales Rosaceae Sorbus koehneana#BLVL
Rosales Rosaceae Cotoneaster transcaucasicus#QIEH
Rosales Rosaceae Pyrus spinosa#NC 023130
Rosales Rosaceae Pyrus pashia#NC 034909
Rosales Rosaceae Pyrus pyrifolia#NC 015996
Rosales Rosaceae Pyrus ussuriensis#NC 041461
Rosales Rosaceae Pyrus hopeiensis#MF521826
Rosales Rosaceae Eriobotrya japonica#KY085905
Rosales Rosaceae Crataegus kansuensis#NC 039374
Rosales Rosaceae Cydonia oblonga#KX499857
Rosales Rosaceae Sorbus torminalis#NC 033975
Rosales Rosaceae Malus florentina#KX499862
Rosales Rosaceae Malus trilobata#NC 035671
Rosales Rosaceae Chaenomeles japonica#NC 035566
Rosales Rosaceae Chaenomeles speciosa#KT932965
Rosales Rosaceae Docynia delavayi#KX499860
Rosales Rosaceae Chaenomeles sinensis#KT932967
Rosales Rosaceae Malus doumeri#KX499861
Rosales Rosaceae Malus transitoria#MK098838
Rosales Rosaceae Malus kansuensis#Y208
Rosales Rosaceae Malus yunnanensis#MH394388
Rosales Rosaceae Malus tschonoskii#KX499863
Rosales Rosaceae Malus hupehensis#NC 040170
Rosales Rosaceae Malus baccata#KX499859
Rosales Rosaceae Malus micromalus#NC 036368
Rosales Rosaceae Pyracantha fortuneana#MH890570
Rosales Rosaceae Malus orientalis#Y181
Rosales Rosaceae Malus fusca#Y209
Rosales Rosaceae Malus lancifolia#Y255
Santalales Strombosiaceae Strombosia scheffleri#ZSD380
Santalales Erythropalaceae Erythropalum scandens#F911
Santalales Erythropalaceae Heisteria scandens#F971
Santalales Coulaceae Coula edulis#ZSD359
Santalales Octoknemaceae Octoknema borealis#T00313
Santalales Octoknemaceae Octoknema chailluensis#T00314
Santalales Ximeniaceae Malania oleifera#F978 1
Santalales Ximeniaceae Ximenia americana#GQ
Santalales Aptandraceae Aptandra zenkeri#ZSD372
Santalales Olacaceae Olax imbricata#F463
Santalales Olacaceae Olax sp.#F917
Santalales Olacaceae Olax acuminata#F920 1
Santalales Misodendraceae Misodendrum quadriflorum#ZSD302
Santalales Misodendraceae Misodendrum cf. brachystachyum#ZSD300
Santalales Misodendraceae Misodendrum cf. oblongifolium#ZSD301
Santalales Schoepfiaceae Schoepfia jasminodora#NC 034228
Santalales Schoepfiaceae Schoepfia jasminodora#F185
Santalales Schoepfiaceae Schoepfia jasminodora#F217
Santalales Loranthaceae Dendropemon caribaeus#VGVI
Santalales Loranthaceae Macrosolen cochinchinensis#NC 039376
Santalales Loranthaceae Tolypanthus maclurei#MH922027
Santalales Loranthaceae Helixanthera parasitica#F745
Santalales Loranthaceae Taxillus sutchuenensis#NC 036307
Santalales Loranthaceae Taxillus chinensis#NC 036306
Santalales Loranthaceae Scurrula notothixoides#NC 041305
Santalales Loranthaceae Scurrula parasitica#NC 040862
Santalales Opiliaceae Champereia manillana#NC 034931
Santalales Opiliaceae Cansjera rheedei#F715
Santalales Santalaceae Thesium longifolium#F634
Santalales Santalaceae Pyrularia edulis#F123
Santalales Santalaceae Scleropyrum pentandrum#F735
Santalales Santalaceae Exocarpos cupressiformis#XGFU
Santalales Santalaceae Osyris alba#NC 027960 @para
Santalales Santalaceae Santalum acuminatum#RSPO
Santalales Santalaceae Daenikera sp.#BSEY
Santalales Santalaceae Dendrotrophe varians#NC 039391
Santalales Santalaceae Phacellaria caulescens#F641
Santalales Santalaceae Phoradendron leucarpum#GQ
Santalales Santalaceae Viscum nudum#F32 @para
Santalales Santalaceae Viscum album#NC 028012 @para
Santalales Santalaceae Viscum coloratum#NC 035414 @para
Santalales Santalaceae Viscum minimum#NC 027829 @para
Santalales Santalaceae Viscum crassulae#NC 027959 @para
Santalales Santalaceae Viscum ovalifolium#F736 @para
Santalales Santalaceae Viscum liquidambaricola#F608 1 @para
Berberidopsidales Aextoxicaceae Aextoxicon punctatum#QUTB
Berberidopsidales Berberidopsidaceae Berberidopsis corallina#EU
Berberidopsidales Berberidopsidaceae Berberidopsis beckleri#HAEU
Caryophyllales Drosophyllaceae Drosophyllum lusitanicum#B200910
Caryophyllales Dioncophyllaceae Habropetalum dawei#ZSD396
Caryophyllales Dioncophyllaceae Triphyophyllum peltatum#B200927
Caryophyllales Dioncophyllaceae Dioncophyllum thollonii#MK397866
Caryophyllales Ancistrocladaceae Ancistrocladus tectorius#F706
Caryophyllales Ancistrocladaceae Ancistrocladus congolensis#MH286312
Caryophyllales Ancistrocladaceae Ancistrocladus korupensis#B200902
Caryophyllales Nepenthaceae Nepenthes alata#B200915
Caryophyllales Nepenthaceae Nepenthes mirabilis#F448
Caryophyllales Droseraceae Drosera regia#NC 035415
Caryophyllales Droseraceae Dionaea muscipula#MK397918
Caryophyllales Droseraceae Aldrovanda vesiculosa#NC 035416
Caryophyllales Droseraceae Drosera erythrorhiza#NC 035241
Caryophyllales Droseraceae Drosera indica#F667
Caryophyllales Droseraceae Drosera capensis#B200909
Caryophyllales Droseraceae Drosera rotundifolia#NC 029770
Caryophyllales Frankeniaceae Frankenia pulverulenta#F792
Caryophyllales Frankeniaceae Frankenia laevis#MK397868
Caryophyllales Tamaricaceae Reaumuria soongarica#F354

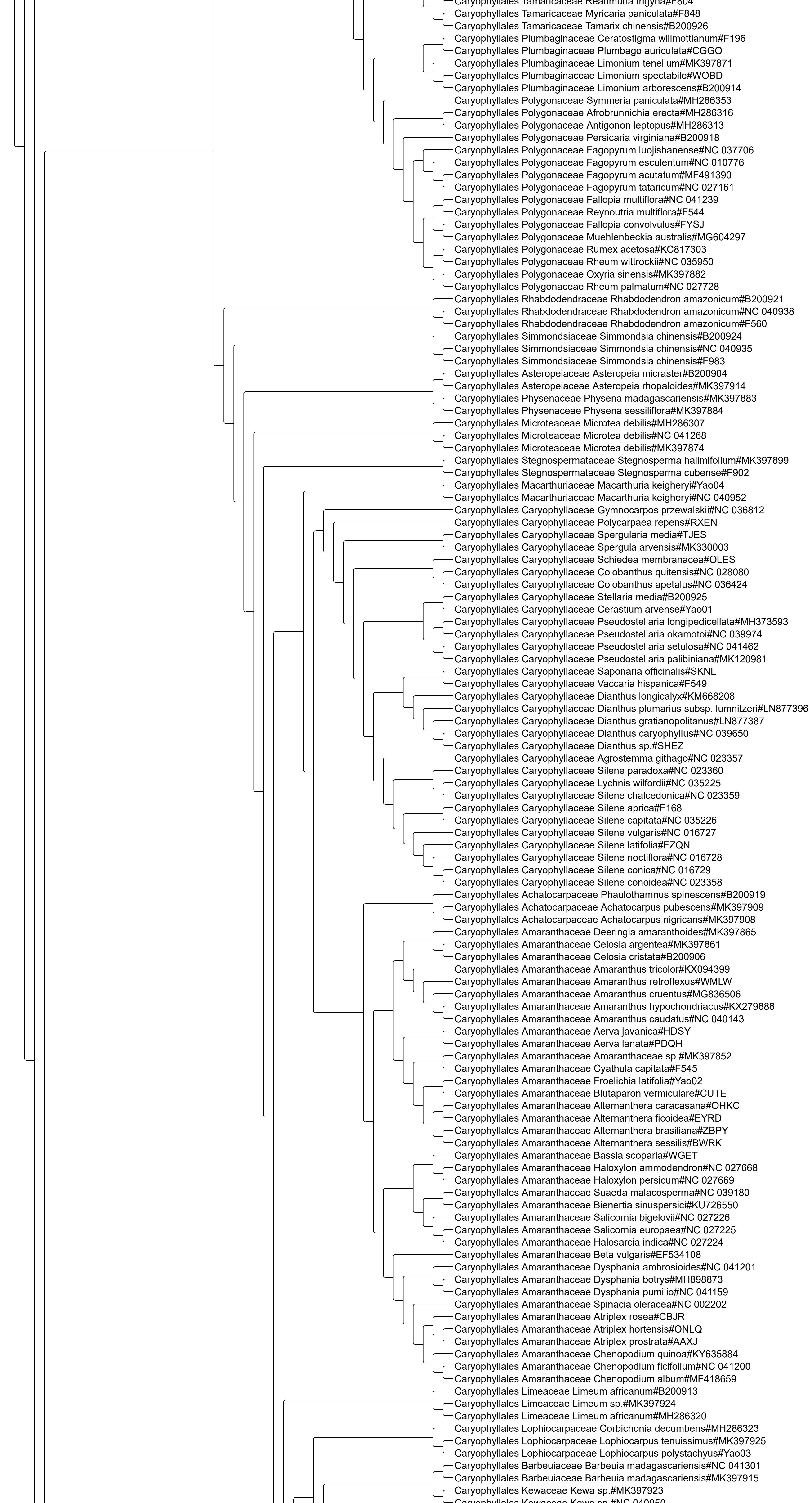
Caryophyllales Tamaricaceae Reaumuria trigyna#F804
Caryophyllales Tamaricaceae Myricaria paniculata#F848
Caryophyllales Tamaricaceae Tamarix chinensis#B200926
Caryophyllales Plumbaginaceae Ceratostigma willmottianum#F196
Caryophyllales Plumbaginaceae Plumbago auriculata#CGGO
Caryophyllales Plumbaginaceae Limonium tenellum#MK397871
Caryophyllales Plumbaginaceae Limonium spectabile#WOBD
Caryophyllales Plumbaginaceae Limonium arborescens#B200914
Caryophyllales Polygonaceae Symmeria paniculata#MH286353
Caryophyllales Polygonaceae Afrobrunnichia erecta#MH286316
Caryophyllales Polygonaceae Antigonon leptopus#MH286313
Caryophyllales Polygonaceae Persicaria virginiana#B200918
Caryophyllales Polygonaceae Fagopyrum luojishanense#NC 037706
Caryophyllales Polygonaceae Fagopyrum esculentum#NC 010776
Caryophyllales Polygonaceae Fagopyrum acutatum#MF491390
Caryophyllales Polygonaceae Fagopyrum tataricum#NC 027161
Caryophyllales Polygonaceae Fallopia multiflora#NC 041239
Caryophyllales Polygonaceae Reynoutria multiflora#F544
Caryophyllales Polygonaceae Fallopia convolvulus#FYSJ
Caryophyllales Polygonaceae Muehlenbeckia australis#MG604297
Caryophyllales Polygonaceae Rumex acetosa#KC817303
Caryophyllales Polygonaceae Rheum wittrockii#NC 035950
Caryophyllales Polygonaceae Oxyria sinensis#MK397882
Caryophyllales Polygonaceae Rheum palmatum#NC 027728
Caryophyllales Rhabdodendraceae Rhabdodendron amazonicum#B200921
Caryophyllales Rhabdodendraceae Rhabdodendron amazonicum#NC 040938
Caryophyllales Rhabdodendraceae Rhabdodendron amazonicum#F560
Caryophyllales Simmondsiaceae Simmondsia chinensis#B200924
Caryophyllales Simmondsiaceae Simmondsia chinensis#NC 040935
Caryophyllales Simmondsiaceae Simmondsia chinensis#F983
Caryophyllales Asteropeiaceae Asteropeia micraster#B200904
Caryophyllales Asteropeiaceae Asteropeia rhopaloides#MK397914
Caryophyllales Physenaceae Physena madagascariensis#MK397883
Caryophyllales Physenaceae Physena sessiliflora#MK397884
Caryophyllales Microteaceae Microtea debilis#MH286307
Caryophyllales Microteaceae Microtea debilis#NC 041268
Caryophyllales Microteaceae Microtea debilis#MK397874
Caryophyllales Stegnospermataceae Stegnosperma halimifolium#MK397899
Caryophyllales Stegnospermataceae Stegnosperma cubense#F902
Caryophyllales Macarthuriaceae Macarthuria keigheryi#Yao04
Caryophyllales Macarthuriaceae Macarthuria keigheryi#NC 040952
Caryophyllales Caryophyllaceae Gymnocarpos przewalskii#NC 036812
Caryophyllales Caryophyllaceae Polycarpaea repens#RXEN
Caryophyllales Caryophyllaceae Spergularia media#TJES
Caryophyllales Caryophyllaceae Spergula arvensis#MK330003
Caryophyllales Caryophyllaceae Schiedea membranacea#OLES
Caryophyllales Caryophyllaceae Colobanthus quitensis#NC 028080
Caryophyllales Caryophyllaceae Colobanthus apetalus#NC 036424
Caryophyllales Caryophyllaceae Stellaria media#B200925
Caryophyllales Caryophyllaceae Cerastium arvense#Yao01
Caryophyllales Caryophyllaceae Pseudostellaria longipedicellata#MH373593
Caryophyllales Caryophyllaceae Pseudostellaria okamotoi#NC 039974
Caryophyllales Caryophyllaceae Pseudostellaria setulosa#NC 041462
Caryophyllales Caryophyllaceae Pseudostellaria palibiniana#MK120981
Caryophyllales Caryophyllaceae Saponaria officinalis#SKNL
Caryophyllales Caryophyllaceae Vaccaria hispanica#F549
Caryophyllales Caryophyllaceae Dianthus longicalyx#KM668208
Caryophyllales Caryophyllaceae Dianthus plumarius subsp. lumnitzeri#LN877396
Caryophyllales Caryophyllaceae Dianthus gratianopolitanus#LN877387
Caryophyllales Caryophyllaceae Dianthus caryophyllus#NC 039650
Caryophyllales Caryophyllaceae Dianthus sp.#SHEZ
Caryophyllales Caryophyllaceae Agrostemma githago#NC 023357
Caryophyllales Caryophyllaceae Silene paradoxa#NC 023360
Caryophyllales Caryophyllaceae Lychnis wilfordii#NC 035225
Caryophyllales Caryophyllaceae Silene chalcedonica#NC 023359
Caryophyllales Caryophyllaceae Silene aprica#F168
Caryophyllales Caryophyllaceae Silene capitata#NC 035226
Caryophyllales Caryophyllaceae Silene vulgaris#NC 016727
Caryophyllales Caryophyllaceae Silene latifolia#FZQN
Caryophyllales Caryophyllaceae Silene noctiflora#NC 016728
Caryophyllales Caryophyllaceae Silene conica#NC 016729
Caryophyllales Caryophyllaceae Silene conoidea#NC 023358
Caryophyllales Achatocarpaceae Phaulothamnus spinescens#B200919
Caryophyllales Achatocarpaceae Achatocarpus pubescens#MK397909
Caryophyllales Achatocarpaceae Achatocarpus nigricans#MK397908
Caryophyllales Amaranthaceae Deeringia amaranthoides#MK397865
Caryophyllales Amaranthaceae Celosia argentea#MK397861
Caryophyllales Amaranthaceae Celosia cristata#B200906
Caryophyllales Amaranthaceae Amaranthus tricolor#KX094399
Caryophyllales Amaranthaceae Amaranthus retroflexus#WMLW
Caryophyllales Amaranthaceae Amaranthus cruentus#MG836506
Caryophyllales Amaranthaceae Amaranthus hypochondriacus#KX279888
Caryophyllales Amaranthaceae Amaranthus caudatus#NC 040143
Caryophyllales Amaranthaceae Aerva javanica#HDSY
Caryophyllales Amaranthaceae Aerva lanata#PDQH
Caryophyllales Amaranthaceae Amaranthaceae sp.#MK397852
Caryophyllales Amaranthaceae Cyathula capitata#F545
Caryophyllales Amaranthaceae Froelichia latifolia#Yao02
Caryophyllales Amaranthaceae Blutaparon vermiculare#CUTE
Caryophyllales Amaranthaceae Alternanthera caracasana#OHKC
Caryophyllales Amaranthaceae Alternanthera ficoidea#EYRD
Caryophyllales Amaranthaceae Alternanthera brasiliana#ZBPY
Caryophyllales Amaranthaceae Alternanthera sessilis#BWRK
Caryophyllales Amaranthaceae Bassia scoparia#WGET
Caryophyllales Amaranthaceae Haloxylon ammodendron#NC 027668
Caryophyllales Amaranthaceae Haloxylon persicum#NC 027669
Caryophyllales Amaranthaceae Suaeda malacosperma#NC 039180
Caryophyllales Amaranthaceae Bienertia sinuspersici#KU726550
Caryophyllales Amaranthaceae Salicornia bigelovii#NC 027226
Caryophyllales Amaranthaceae Salicornia europaea#NC 027225
Caryophyllales Amaranthaceae Halosarcia indica#NC 027224
Caryophyllales Amaranthaceae Beta vulgaris#EF534108
Caryophyllales Amaranthaceae Dysphania ambrosioides#NC 041201
Caryophyllales Amaranthaceae Dysphania botrys#MH898873
Caryophyllales Amaranthaceae Dysphania pumilio#NC 041159
Caryophyllales Amaranthaceae Spinacia oleracea#NC 002202
Caryophyllales Amaranthaceae Atriplex rosea#CBJR
Caryophyllales Amaranthaceae Atriplex hortensis#ONLQ
Caryophyllales Amaranthaceae Atriplex prostrata#AAXJ
Caryophyllales Amaranthaceae Chenopodium quinoa#KY635884
Caryophyllales Amaranthaceae Chenopodium ficifolium#NC 041200
Caryophyllales Amaranthaceae Chenopodium album#MF418659
Caryophyllales Limeaceae Limeum africanum#B200913
Caryophyllales Limeaceae Limeum sp.#MK397924
Caryophyllales Limeaceae Limeum africanum#MH286320
Caryophyllales Lophiocarpaceae Corbichonia decumbens#MH286323
Caryophyllales Lophiocarpaceae Lophiocarpus tenuissimus#MK397925
Caryophyllales Lophiocarpaceae Lophiocarpus polystachyus#Yao03
Caryophyllales Barbeuiaceae Barbeuia madagascariensis#NC 041301
Caryophyllales Barbeuiaceae Barbeuia madagascariensis#MK397915
Caryophyllales Kewaceae Kewa sp.#MK397923

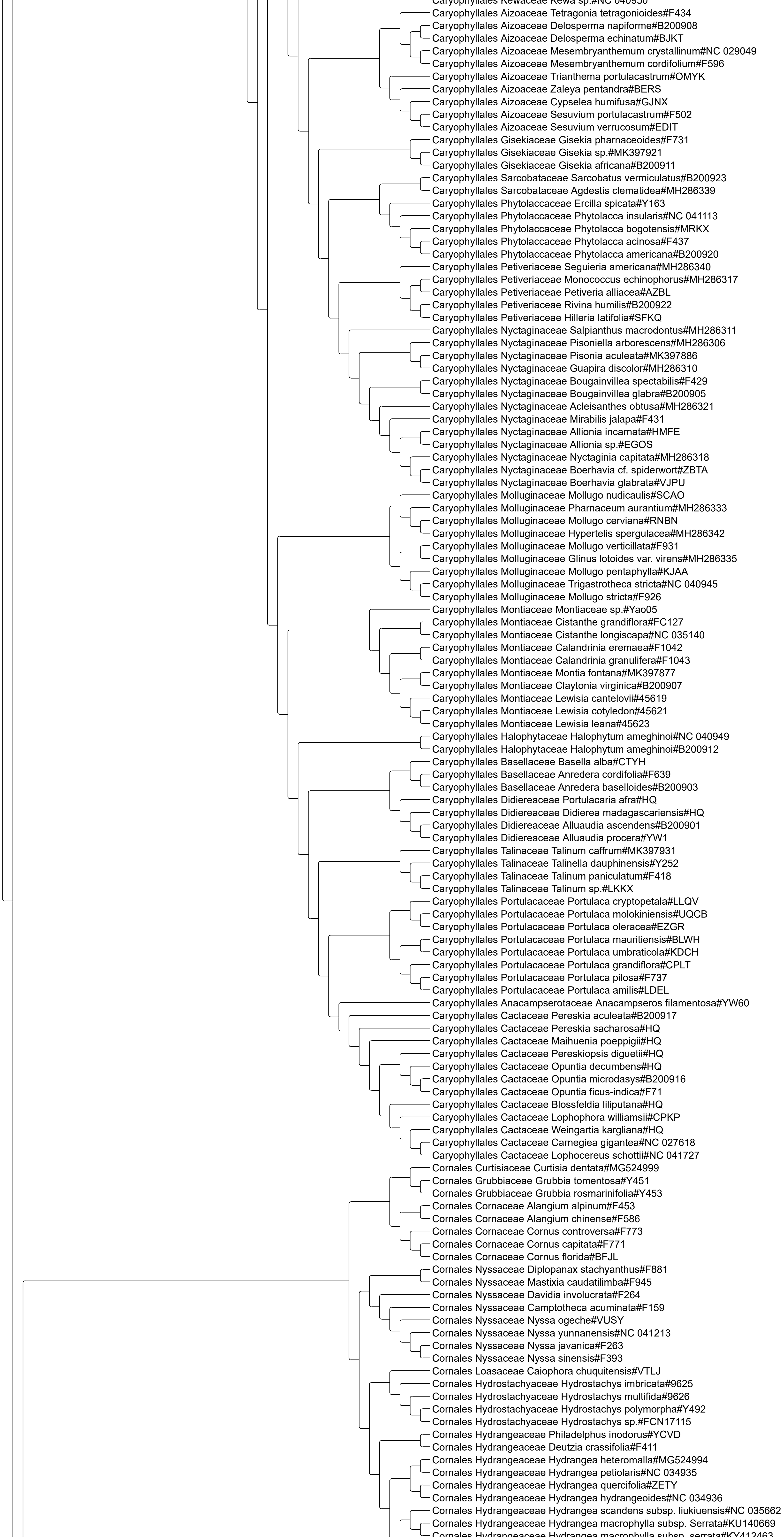
Caryophyllales Aizoaceae Tetragonia tetragonioides#F434
Caryophyllales Aizoaceae Delosperma napiforme#B200908
Caryophyllales Aizoaceae Delosperma echinatum#BJKT
Caryophyllales Aizoaceae Mesembryanthemum crystallinum#NC 029049
Caryophyllales Aizoaceae Mesembryanthemum cordifolium#F596
Caryophyllales Aizoaceae Trianthema portulacastrum#OMYK
Caryophyllales Aizoaceae Zaleya pentandra#BERS
Caryophyllales Aizoaceae Cypselea humifusa#GJNX
Caryophyllales Aizoaceae Sesuvium portulacastrum#F502
Caryophyllales Aizoaceae Sesuvium verrucosum#EDIT
Caryophyllales Gisekiaceae Gisekia pharnaceoides#F731
Caryophyllales Gisekiaceae Gisekia sp.#MK397921
Caryophyllales Gisekiaceae Gisekia africana#B200911
Caryophyllales Sarcobataceae Sarcobatus vermiculatus#B200923
Caryophyllales Sarcobataceae Agdestis clematidea#MH286339
Caryophyllales Phytolaccaceae Ercilla spicata#Y163
Caryophyllales Phytolaccaceae Phytolacca insularis#NC 041113
Caryophyllales Phytolaccaceae Phytolacca bogotensis#MRKX
Caryophyllales Phytolaccaceae Phytolacca acinosa#F437
Caryophyllales Phytolaccaceae Phytolacca americana#B200920
Caryophyllales Petiveriaceae Seguieria americana#MH286340
Caryophyllales Petiveriaceae Monococcus echinophorus#MH286317
Caryophyllales Petiveriaceae Petiveria alliacea#AZBL
Caryophyllales Petiveriaceae Rivina humilis#B200922
Caryophyllales Petiveriaceae Hilleria latifolia#SFKQ
Caryophyllales Nyctaginaceae Salpianthus macrodontus#MH286311
Caryophyllales Nyctaginaceae Pisoniella arborescens#MH286306
Caryophyllales Nyctaginaceae Pisonia aculeata#MK397886
Caryophyllales Nyctaginaceae Guapira discolor#MH286310
Caryophyllales Nyctaginaceae Bougainvillea spectabilis#F429
Caryophyllales Nyctaginaceae Bougainvillea glabra#B200905
Caryophyllales Nyctaginaceae Acleisanthes obtusa#MH286321
Caryophyllales Nyctaginaceae Mirabilis jalapa#F431
Caryophyllales Nyctaginaceae Allionia incarnata#HMFE
Caryophyllales Nyctaginaceae Allionia sp.#EGOS
Caryophyllales Nyctaginaceae Nyctaginia capitata#MH286318
Caryophyllales Nyctaginaceae Boerhavia cf. spiderwort#ZBTA
Caryophyllales Nyctaginaceae Boerhavia glabrata#VJPU
Caryophyllales Molluginaceae Mollugo nudicaulis#SCAO
Caryophyllales Molluginaceae Pharnaceum aurantium#MH286333
Caryophyllales Molluginaceae Mollugo cerviana#RNBN
Caryophyllales Molluginaceae Hypertelis spergulacea#MH286342
Caryophyllales Molluginaceae Mollugo verticillata#F931
Caryophyllales Molluginaceae Glinus lotoides var. virens#MH286335
Caryophyllales Molluginaceae Mollugo pentaphylla#KJAA
Caryophyllales Molluginaceae Trigastrotheca stricta#NC 040945
Caryophyllales Molluginaceae Mollugo stricta#F926
Caryophyllales Montiaceae Montiaceae sp.#Yao05
Caryophyllales Montiaceae Cistanthe grandiflora#FC127
Caryophyllales Montiaceae Cistanthe longiscapa#NC 035140
Caryophyllales Montiaceae Calandrinia eremaea#F1042
Caryophyllales Montiaceae Calandrinia granulifera#F1043
Caryophyllales Montiaceae Montia fontana#MK397877
Caryophyllales Montiaceae Claytonia virginica#B200907
Caryophyllales Montiaceae Lewisia cantelovii#45619
Caryophyllales Montiaceae Lewisia cotyledon#45621
Caryophyllales Montiaceae Lewisia leana#45623
Caryophyllales Halophytaceae Halophytum ameghinoi#NC 040949
Caryophyllales Halophytaceae Halophytum ameghinoi#B200912
Caryophyllales Basellaceae Basella alba#CTYH
Caryophyllales Basellaceae Anredera cordifolia#F639
Caryophyllales Basellaceae Anredera baselloides#B200903
Caryophyllales Didiereaceae Portulacaria afra#HQ
Caryophyllales Didiereaceae Didierea madagascariensis#HQ
Caryophyllales Didiereaceae Alluaudia ascendens#B200901
Caryophyllales Didiereaceae Alluaudia procera#YW1
Caryophyllales Talinaceae Talinum caffrum#MK397931
Caryophyllales Talinaceae Talinella dauphinensis#Y252
Caryophyllales Talinaceae Talinum paniculatum#F418
Caryophyllales Talinaceae Talinum sp.#LKKX
Caryophyllales Portulacaceae Portulaca cryptopetala#LLQV
Caryophyllales Portulacaceae Portulaca molokiniensis#UQCB
Caryophyllales Portulacaceae Portulaca oleracea#EZGR
Caryophyllales Portulacaceae Portulaca mauritiensis#BLWH
Caryophyllales Portulacaceae Portulaca umbraticola#KDCH
Caryophyllales Portulacaceae Portulaca grandiflora#CPLT
Caryophyllales Portulacaceae Portulaca pilosa#F737
Caryophyllales Portulacaceae Portulaca amilis#LDEL
Caryophyllales Anacampserotaceae Anacampseros filamentosa#YW60
Caryophyllales Cactaceae Pereskia aculeata#B200917
Caryophyllales Cactaceae Pereskia sacharosa#HQ
Caryophyllales Cactaceae Maihuenia poeppigii#HQ
Caryophyllales Cactaceae Pereskiopsis diguetii#HQ
Caryophyllales Cactaceae Opuntia decumbens#HQ
Caryophyllales Cactaceae Opuntia microdasys#B200916
Caryophyllales Cactaceae Opuntia ficus-indica#F71
Caryophyllales Cactaceae Blossfeldia liliputana#HQ
Caryophyllales Cactaceae Lophophora williamsii#CPKP
Caryophyllales Cactaceae Weingartia kargliana#HQ
Caryophyllales Cactaceae Carnegiea gigantea#NC 027618
Caryophyllales Cactaceae Lophocereus schottii#NC 041727
Cornales Curtisiaceae Curtisia dentata#MG524999
Cornales Grubbiaceae Grubbia tomentosa#Y451
Cornales Grubbiaceae Grubbia rosmarinifolia#Y453
Cornales Cornaceae Alangium alpinum#F453
Cornales Cornaceae Alangium chinense#F586
Cornales Cornaceae Cornus controversa#F773
Cornales Cornaceae Cornus capitata#F771
Cornales Cornaceae Cornus florida#BFJL
Cornales Nyssaceae Diplopanax stachyanthus#F881
Cornales Nyssaceae Mastixia caudatilimba#F945
Cornales Nyssaceae Davidia involucrata#F264
Cornales Nyssaceae Camptotheca acuminata#F159
Cornales Nyssaceae Nyssa ogeche#VUSY
Cornales Nyssaceae Nyssa yunnanensis#NC 041213
Cornales Nyssaceae Nyssa javanica#F263
Cornales Nyssaceae Nyssa sinensis#F393
Cornales Loasaceae Caiophora chuquitensis#VTLJ
Cornales Hydrostachyaceae Hydrostachys imbricata#9625
Cornales Hydrostachyaceae Hydrostachys multifida#9626
Cornales Hydrostachyaceae Hydrostachys polymorpha#Y492
Cornales Hydrostachyaceae Hydrostachys sp.#FCN17115
Cornales Hydrangeaceae Philadelphus inodorus#YCVD
Cornales Hydrangeaceae Deutzia crassifolia#F411
Cornales Hydrangeaceae Hydrangea heteromalla#MG524994
Cornales Hydrangeaceae Hydrangea petiolaris#NC 034935
Cornales Hydrangeaceae Hydrangea quercifolia#ZETY
Cornales Hydrangeaceae Hydrangea hydrangeoides#NC 034936
Cornales Hydrangeaceae Hydrangea scandens subsp. liukiuensis#NC 035662
Cornales Hydrangeaceae Hydrangea macrophylla subsp. Serrata#KU140669

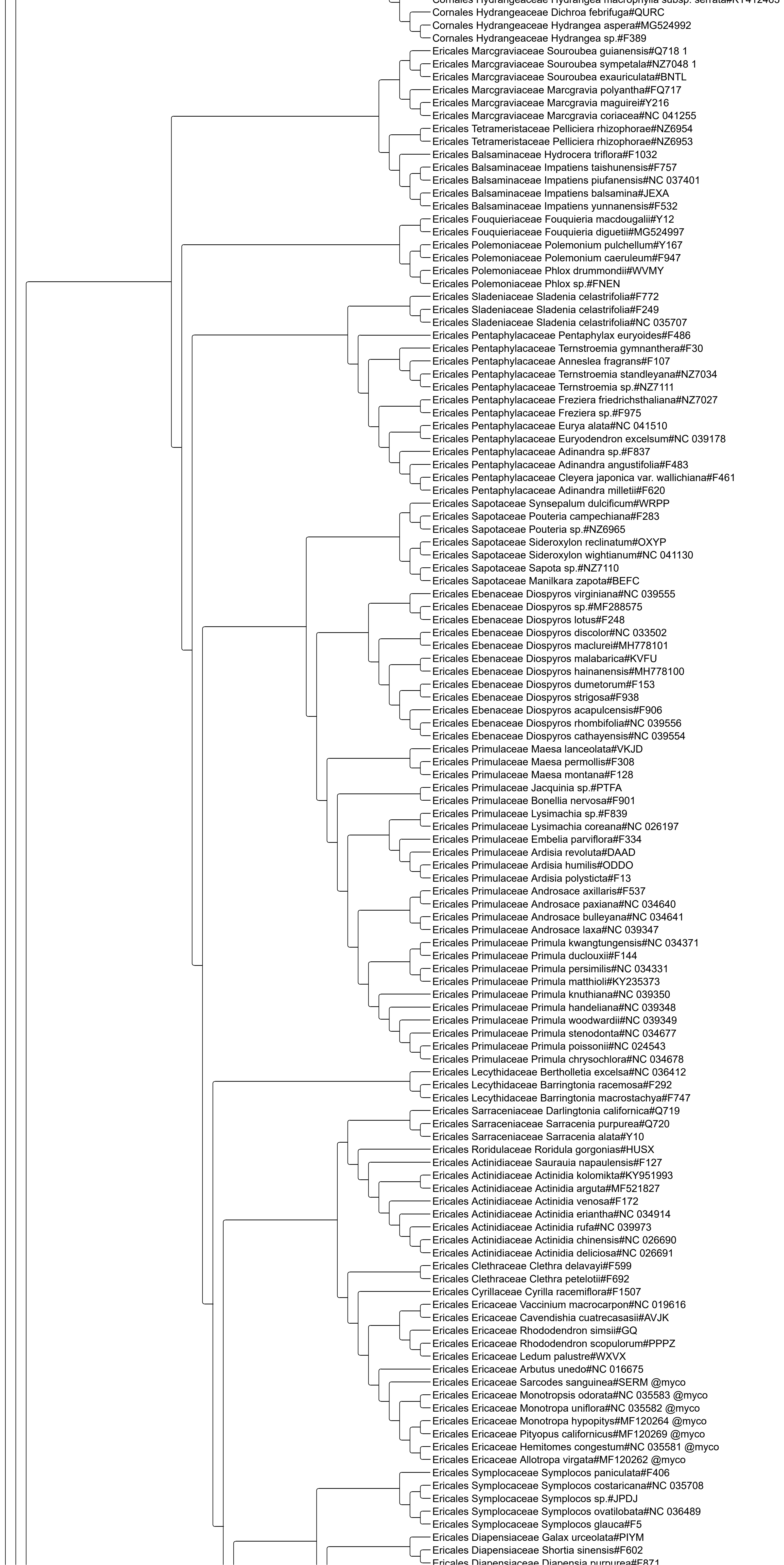
Cornales Hydrangeaceae Hydrangea macrophylla subsp. serrata#KY412465
Cornales Hydrangeaceae Dichroa febrifuga#QURC
Cornales Hydrangeaceae Hydrangea aspera#MG524992
Cornales Hydrangeaceae Hydrangea sp.#F389
Ericales Marcgraviaceae Souroubea guianensis#Q718 1
Ericales Marcgraviaceae Souroubea sympetala#NZ7048 1
Ericales Marcgraviaceae Souroubea exauriculata#BNTL
Ericales Marcgraviaceae Marcgravia polyantha#FQ717
Ericales Marcgraviaceae Marcgravia maguirei#Y216
Ericales Marcgraviaceae Marcgravia coriacea#NC 041255
Ericales Tetrameristaceae Pelliciera rhizophorae#NZ6954
Ericales Tetrameristaceae Pelliciera rhizophorae#NZ6953
Ericales Balsaminaceae Hydrocera triflora#F1032
Ericales Balsaminaceae Impatiens taishunensis#F757
Ericales Balsaminaceae Impatiens piufanensis#NC 037401
Ericales Balsaminaceae Impatiens balsamina#JEXA
Ericales Balsaminaceae Impatiens yunnanensis#F532
Ericales Fouquieriaceae Fouquieria macdougalii#Y12
Ericales Fouquieriaceae Fouquieria diguetii#MG524997
Ericales Polemoniaceae Polemonium pulchellum#Y167
Ericales Polemoniaceae Polemonium caeruleum#F947
Ericales Polemoniaceae Phlox drummondii#WVMY
Ericales Polemoniaceae Phlox sp.#FNEN
Ericales Sladeniaceae Sladenia celastrifolia#F772
Ericales Sladeniaceae Sladenia celastrifolia#F249
Ericales Sladeniaceae Sladenia celastrifolia#NC 035707
Ericales Pentaphylacaceae Pentaphylax euryoides#F486
Ericales Pentaphylacaceae Ternstroemia gymnanthera#F30
Ericales Pentaphylacaceae Anneslea fragrans#F107
Ericales Pentaphylacaceae Ternstroemia standleyana#NZ7034
Ericales Pentaphylacaceae Ternstroemia sp.#NZ7111
Ericales Pentaphylacaceae Freziera friedrichsthaliana#NZ7027
Ericales Pentaphylacaceae Freziera sp.#F975
Ericales Pentaphylacaceae Eurya alata#NC 041510
Ericales Pentaphylacaceae Euryodendron excelsum#NC 039178
Ericales Pentaphylacaceae Adinandra sp.#F837
Ericales Pentaphylacaceae Adinandra angustifolia#F483
Ericales Pentaphylacaceae Cleyera japonica var. wallichiana#F461
Ericales Pentaphylacaceae Adinandra millettii#F620
Ericales Sapotaceae Synsepalum dulcificum#WRPP
Ericales Sapotaceae Pouteria campechiana#F283
Ericales Sapotaceae Pouteria sp.#NZ6965
Ericales Sapotaceae Sideroxylon reclinatum#OXYP
Ericales Sapotaceae Sideroxylon wightianum#NC 041130
Ericales Sapotaceae Sapota sp.#NZ7110
Ericales Sapotaceae Manilkara zapota#BEFC
Ericales Ebenaceae Diospyros virginiana#NC 039555
Ericales Ebenaceae Diospyros sp.#MF288575
Ericales Ebenaceae Diospyros lotus#F248
Ericales Ebenaceae Diospyros discolor#NC 033502
Ericales Ebenaceae Diospyros maclurei#MH778101
Ericales Ebenaceae Diospyros malabarica#KVFU
Ericales Ebenaceae Diospyros hainanensis#MH778100
Ericales Ebenaceae Diospyros dumetorum#F153
Ericales Ebenaceae Diospyros strigosa#F938
Ericales Ebenaceae Diospyros acapulcensis#F906
Ericales Ebenaceae Diospyros rhombifolia#NC 039556
Ericales Ebenaceae Diospyros cathayensis#NC 039554
Ericales Primulaceae Maesa lanceolata#VKJD
Ericales Primulaceae Maesa permollis#F308
Ericales Primulaceae Maesa montana#F128
Ericales Primulaceae Jacquinia sp.#PTFA
Ericales Primulaceae Bonellia nervosa#F901
Ericales Primulaceae Lysimachia sp.#F839
Ericales Primulaceae Lysimachia coreana#NC 026197
Ericales Primulaceae Embelia parviflora#F334
Ericales Primulaceae Ardisia revoluta#DAAD
Ericales Primulaceae Ardisia humilis#ODDO
Ericales Primulaceae Ardisia polysticta#F13
Ericales Primulaceae Androsace axillaris#F537
Ericales Primulaceae Androsace paxiana#NC 034640
Ericales Primulaceae Androsace bulleyana#NC 034641
Ericales Primulaceae Androsace laxa#NC 039347
Ericales Primulaceae Primula kwangtungensis#NC 034371
Ericales Primulaceae Primula duclouxii#F144
Ericales Primulaceae Primula persimilis#NC 034331
Ericales Primulaceae Primula matthioli#KY235373
Ericales Primulaceae Primula knuthiana#NC 039350
Ericales Primulaceae Primula handeliana#NC 039348
Ericales Primulaceae Primula woodwardii#NC 039349
Ericales Primulaceae Primula stenodonta#NC 034677
Ericales Primulaceae Primula poissonii#NC 024543
Ericales Primulaceae Primula chrysochlora#NC 034678
Ericales Lecythidaceae Bertholletia excelsa#NC 036412
Ericales Lecythidaceae Barringtonia racemosa#F292
Ericales Lecythidaceae Barringtonia macrostachya#F747
Ericales Sarraceniaceae Darlingtonia californica#Q719
Ericales Sarraceniaceae Sarracenia purpurea#Q720
Ericales Sarraceniaceae Sarracenia alata#Y10
Ericales Roridulaceae Roridula gorgonias#HUSX
Ericales Actinidiaceae Saurauia napaulensis#F127
Ericales Actinidiaceae Actinidia kolomikta#KY951993
Ericales Actinidiaceae Actinidia arguta#MF521827
Ericales Actinidiaceae Actinidia venosa#F172
Ericales Actinidiaceae Actinidia eriantha#NC 034914
Ericales Actinidiaceae Actinidia rufa#NC 039973
Ericales Actinidiaceae Actinidia chinensis#NC 026690
Ericales Actinidiaceae Actinidia deliciosa#NC 026691
Ericales Clethraceae Clethra delavayi#F599
Ericales Clethraceae Clethra petelotii#F692
Ericales Cyrillaceae Cyrilla racemiflora#F1507
Ericales Ericaceae Vaccinium macrocarpon#NC 019616
Ericales Ericaceae Cavendishia cuatrecasasii#AVJK
Ericales Ericaceae Rhododendron simsii#GQ
Ericales Ericaceae Rhododendron scopulorum#PPPZ
Ericales Ericaceae Ledum palustre#WXVX
Ericales Ericaceae Arbutus unedo#NC 016675
Ericales Ericaceae Sarcodes sanguinea#SERM @myco
Ericales Ericaceae Monotropsis odorata#NC 035583 @myco
Ericales Ericaceae Monotropa uniflora#NC 035582 @myco
Ericales Ericaceae Monotropa hypopitys#MF120264 @myco
Ericales Ericaceae Pityopus californicus#MF120269 @myco
Ericales Ericaceae Hemitomes congestum#NC 035581 @myco
Ericales Ericaceae Allotropa virgata#MF120262 @myco
Ericales Symplocaceae Symplocos paniculata#F406
Ericales Symplocaceae Symplocos costaricana#NC 035708
Ericales Symplocaceae Symplocos sp.#JPDJ
Ericales Symplocaceae Symplocos ovatilobata#NC 036489
Ericales Symplocaceae Symplocos glauca#F5
Ericales Diapensiaceae Galax urceolata#PIYM
Ericales Diapensiaceae Shortia sinensis#F602
Ericales Diapensiaceae Diapensia purpurea#F871

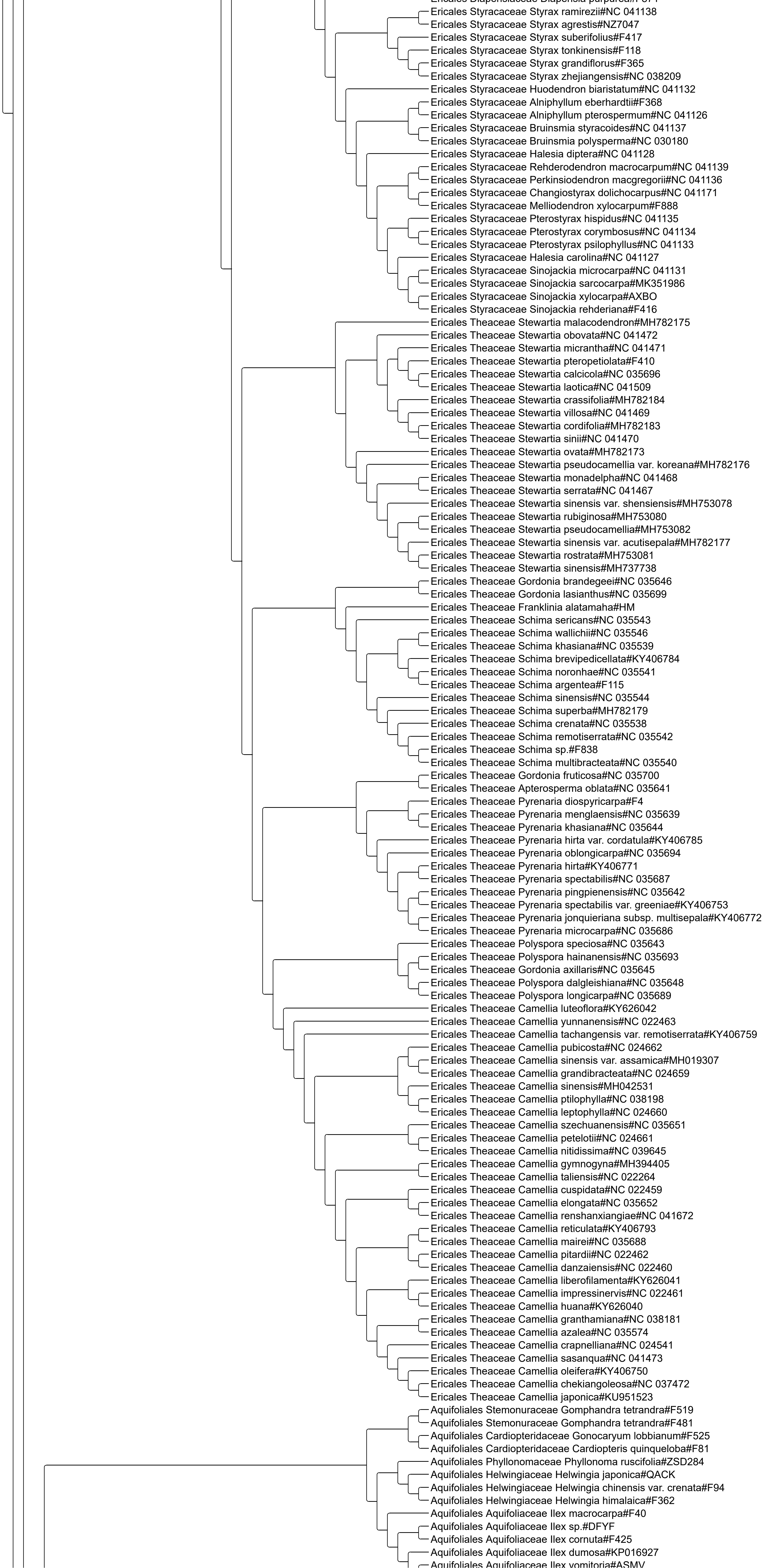
Ericales Styracaceae Styrax ramirezii#NC 041138
Ericales Styracaceae Styrax agrestis#NZ7047
Ericales Styracaceae Styrax suberifolius#F417
Ericales Styracaceae Styrax tonkinensis#F118
Ericales Styracaceae Styrax grandiflorus#F365
Ericales Styracaceae Styrax zhejiangensis#NC 038209
Ericales Styracaceae Huodendron biaristatum#NC 041132
Ericales Styracaceae Alniphyllum eberhardtii#F368
Ericales Styracaceae Alniphyllum pterospermum#NC 041126
Ericales Styracaceae Bruinsmia styracoides#NC 041137
Ericales Styracaceae Bruinsmia polysperma#NC 030180
Ericales Styracaceae Halesia diptera#NC 041128
Ericales Styracaceae Rehderodendron macrocarpum#NC 041139
Ericales Styracaceae Perkinsiodendron macgregorii#NC 041136
Ericales Styracaceae Changiostyrax dolichocarpus#NC 041171
Ericales Styracaceae Melliodendron xylocarpum#F888
Ericales Styracaceae Pterostyrax hispidus#NC 041135
Ericales Styracaceae Pterostyrax corymbosus#NC 041134
Ericales Styracaceae Pterostyrax psilophyllus#NC 041133
Ericales Styracaceae Halesia carolina#NC 041127
Ericales Styracaceae Sinojackia microcarpa#NC 041131
Ericales Styracaceae Sinojackia sarcocarpa#MK351986
Ericales Styracaceae Sinojackia xylocarpa#AXBO
Ericales Styracaceae Sinojackia rehderiana#F416
Ericales Theaceae Stewartia malacodendron#MH782175
Ericales Theaceae Stewartia obovata#NC 041472
Ericales Theaceae Stewartia micrantha#NC 041471
Ericales Theaceae Stewartia pteropetiolata#F410
Ericales Theaceae Stewartia calcicola#NC 035696
Ericales Theaceae Stewartia laotica#NC 041509
Ericales Theaceae Stewartia crassifolia#MH782184
Ericales Theaceae Stewartia villosa#NC 041469
Ericales Theaceae Stewartia cordifolia#MH782183
Ericales Theaceae Stewartia sinii#NC 041470
Ericales Theaceae Stewartia ovata#MH782173
Ericales Theaceae Stewartia pseudocamellia var. koreana#MH782176
Ericales Theaceae Stewartia monadelpha#NC 041468
Ericales Theaceae Stewartia serrata#NC 041467
Ericales Theaceae Stewartia sinensis var. shensiensis#MH753078
Ericales Theaceae Stewartia rubiginosa#MH753080
Ericales Theaceae Stewartia pseudocamellia#MH753082
Ericales Theaceae Stewartia sinensis var. acutisepala#MH782177
Ericales Theaceae Stewartia rostrata#MH753081
Ericales Theaceae Stewartia sinensis#MH737738
Ericales Theaceae Gordonia brandegeei#NC 035646
Ericales Theaceae Gordonia lasianthus#NC 035699
Ericales Theaceae Franklinia alatamaha#HM
Ericales Theaceae Schima sericans#NC 035543
Ericales Theaceae Schima wallichii#NC 035546
Ericales Theaceae Schima khasiana#NC 035539
Ericales Theaceae Schima brevipedicellata#KY406784
Ericales Theaceae Schima noronhae#NC 035541
Ericales Theaceae Schima argentea#F115
Ericales Theaceae Schima sinensis#NC 035544
Ericales Theaceae Schima superba#MH782179
Ericales Theaceae Schima crenata#NC 035538
Ericales Theaceae Schima remotiserrata#NC 035542
Ericales Theaceae Schima sp.#F838
Ericales Theaceae Schima multibracteata#NC 035540
Ericales Theaceae Gordonia fruticosa#NC 035700
Ericales Theaceae Apterosperma oblata#NC 035641
Ericales Theaceae Pyrenaria diospyricarpa#F4
Ericales Theaceae Pyrenaria menglaensis#NC 035639
Ericales Theaceae Pyrenaria khasiana#NC 035644
Ericales Theaceae Pyrenaria hirta var. cordatula#KY406785
Ericales Theaceae Pyrenaria oblongicarpa#NC 035694
Ericales Theaceae Pyrenaria hirta#KY406771
Ericales Theaceae Pyrenaria spectabilis#NC 035687
Ericales Theaceae Pyrenaria pingpienensis#NC 035642
Ericales Theaceae Pyrenaria spectabilis var. greeniae#KY406753
Ericales Theaceae Pyrenaria jonquieriana subsp. multisepala#KY406772
Ericales Theaceae Pyrenaria microcarpa#NC 035686
Ericales Theaceae Polyspora speciosa#NC 035643
Ericales Theaceae Polyspora hainanensis#NC 035693
Ericales Theaceae Gordonia axillaris#NC 035645
Ericales Theaceae Polyspora dalgleishiana#NC 035648
Ericales Theaceae Polyspora longicarpa#NC 035689
Ericales Theaceae Camellia luteoflora#KY626042
Ericales Theaceae Camellia yunnanensis#NC 022463
Ericales Theaceae Camellia tachangensis var. remotiserrata#KY406759
Ericales Theaceae Camellia pubicosta#NC 024662
Ericales Theaceae Camellia sinensis var. assamica#MH019307
Ericales Theaceae Camellia grandibracteata#NC 024659
Ericales Theaceae Camellia sinensis#MH042531
Ericales Theaceae Camellia ptilophylla#NC 038198
Ericales Theaceae Camellia leptophylla#NC 024660
Ericales Theaceae Camellia szechuanensis#NC 035651
Ericales Theaceae Camellia petelotii#NC 024661
Ericales Theaceae Camellia nitidissima#NC 039645
Ericales Theaceae Camellia gymnogyna#MH394405
Ericales Theaceae Camellia taliensis#NC 022264
Ericales Theaceae Camellia cuspidata#NC 022459
Ericales Theaceae Camellia elongata#NC 035652
Ericales Theaceae Camellia renshanxiangiae#NC 041672
Ericales Theaceae Camellia reticulata#KY406793
Ericales Theaceae Camellia mairei#NC 035688
Ericales Theaceae Camellia pitardii#NC 022462
Ericales Theaceae Camellia danzaiensis#NC 022460
Ericales Theaceae Camellia liberofilamenta#KY626041
Ericales Theaceae Camellia impressinervis#NC 022461
Ericales Theaceae Camellia huana#KY626040
Ericales Theaceae Camellia granthamiana#NC 038181
Ericales Theaceae Camellia azalea#NC 035574
Ericales Theaceae Camellia crapnelliana#NC 024541
Ericales Theaceae Camellia sasanqua#NC 041473
Ericales Theaceae Camellia oleifera#KY406750
Ericales Theaceae Camellia chekiangoleosa#NC 037472
Ericales Theaceae Camellia japonica#KU951523
Aquifoliales Stemonuraceae Gomphandra tetrandra#F519
Aquifoliales Stemonuraceae Gomphandra tetrandra#F481
Aquifoliales Cardiopteridaceae Gonocaryum lobbianum#F525
Aquifoliales Cardiopteridaceae Cardiopteris quinqueloba#F81
Aquifoliales Phyllonomaceae Phyllonoma ruscifolia#ZSD284
Aquifoliales Helwingiaceae Helwingia japonica#QACK
Aquifoliales Helwingiaceae Helwingia chinensis var. crenata#F94
Aquifoliales Helwingiaceae Helwingia himalaica#F362
Aquifoliales Aquifoliaceae Ilex macrocarpa#F40
Aquifoliales Aquifoliaceae Ilex sp.#DFYF
Aquifoliales Aquifoliaceae Ilex cornuta#F425
Aquifoliales Aquifoliaceae Ilex dumosa#KP016927

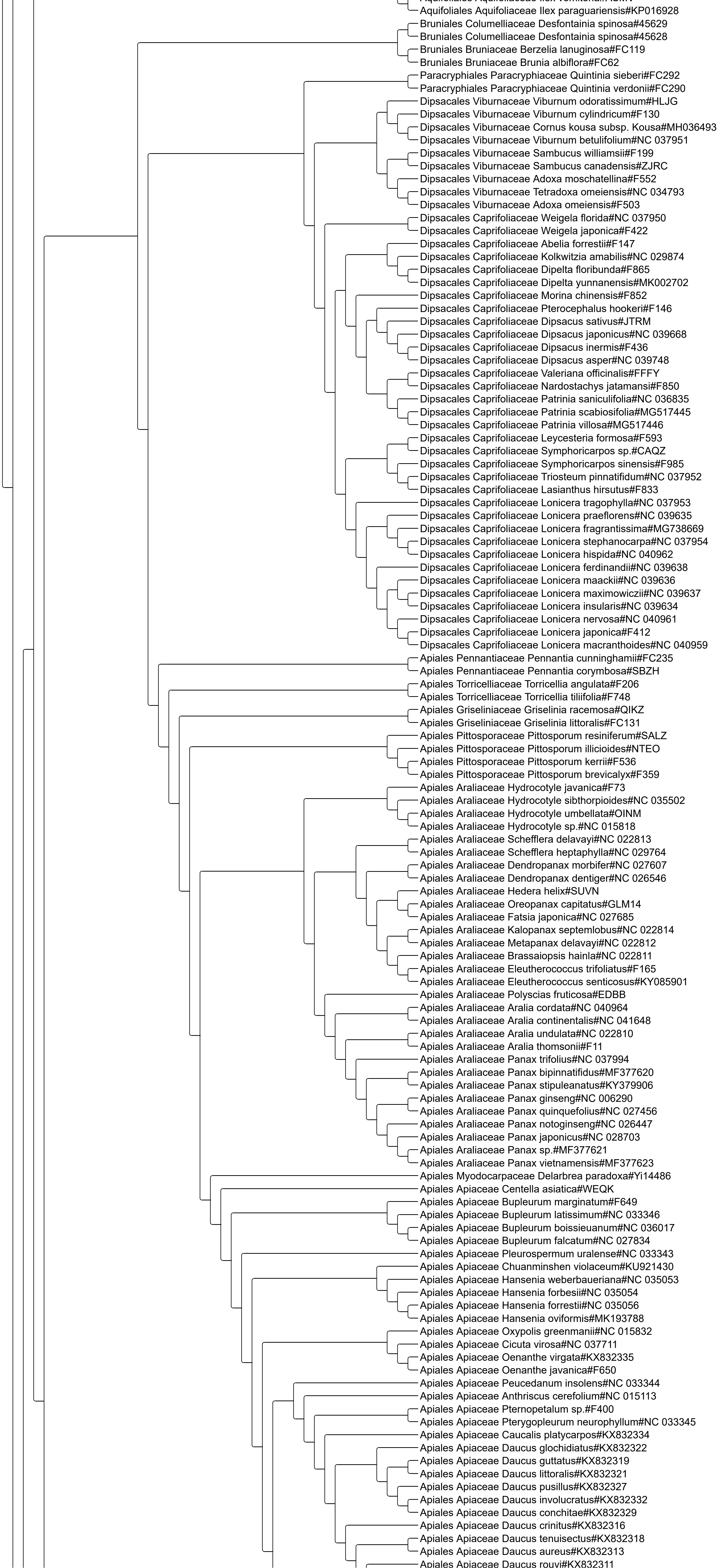

Aquifoliales Aquifoliaceae Ilex paraguariensis#KP016928
Bruniales Columelliaceae Desfontainia spinosa#45629
Bruniales Columelliaceae Desfontainia spinosa#45628
Bruniales Bruniaceae Berzelia lanuginosa#FC119
Bruniales Bruniaceae Brunia albiflora#FC62
Paracryphiales Paracryphiaceae Quintinia sieberi#FC292
Paracryphiales Paracryphiaceae Quintinia verdonii#FC290
Dipsacales Viburnaceae Viburnum odoratissimum#HLJG
Dipsacales Viburnaceae Viburnum cylindricum#F130
Dipsacales Viburnaceae Cornus kousa subsp. Kousa#MH036493
Dipsacales Viburnaceae Viburnum betulifolium#NC 037951
Dipsacales Viburnaceae Sambucus williamsii#F199
Dipsacales Viburnaceae Sambucus canadensis#ZJRC
Dipsacales Viburnaceae Adoxa moschatellina#F552
Dipsacales Viburnaceae Tetradoxa omeiensis#NC 034793
Dipsacales Viburnaceae Adoxa omeiensis#F503
Dipsacales Caprifoliaceae Weigela florida#NC 037950
Dipsacales Caprifoliaceae Weigela japonica#F422
Dipsacales Caprifoliaceae Abelia forrestii#F147
Dipsacales Caprifoliaceae Kolkwitzia amabilis#NC 029874
Dipsacales Caprifoliaceae Dipelta floribunda#F865
Dipsacales Caprifoliaceae Dipelta yunnanensis#MK002702
Dipsacales Caprifoliaceae Morina chinensis#F852
Dipsacales Caprifoliaceae Pterocephalus hookeri#F146
Dipsacales Caprifoliaceae Dipsacus sativus#JTRM
Dipsacales Caprifoliaceae Dipsacus japonicus#NC 039668
Dipsacales Caprifoliaceae Dipsacus inermis#F436
Dipsacales Caprifoliaceae Dipsacus asper#NC 039748
Dipsacales Caprifoliaceae Valeriana officinalis#FFFY
Dipsacales Caprifoliaceae Nardostachys jatamansi#F850
Dipsacales Caprifoliaceae Patrinia saniculifolia#NC 036835
Dipsacales Caprifoliaceae Patrinia scabiosifolia#MG517445
Dipsacales Caprifoliaceae Patrinia villosa#MG517446
Dipsacales Caprifoliaceae Leycesteria formosa#F593
Dipsacales Caprifoliaceae Symphoricarpos sp.#CAQZ
Dipsacales Caprifoliaceae Symphoricarpos sinensis#F985
Dipsacales Caprifoliaceae Triosteum pinnatifidum#NC 037952
Dipsacales Caprifoliaceae Lasianthus hirsutus#F833
Dipsacales Caprifoliaceae Lonicera tragophylla#NC 037953
Dipsacales Caprifoliaceae Lonicera praeflorens#NC 039635
Dipsacales Caprifoliaceae Lonicera fragrantissima#MG738669
Dipsacales Caprifoliaceae Lonicera stephanocarpa#NC 037954
Dipsacales Caprifoliaceae Lonicera hispida#NC 040962
Dipsacales Caprifoliaceae Lonicera ferdinandii#NC 039638
Dipsacales Caprifoliaceae Lonicera maackii#NC 039636
Dipsacales Caprifoliaceae Lonicera maximowiczii#NC 039637
Dipsacales Caprifoliaceae Lonicera insularis#NC 039634
Dipsacales Caprifoliaceae Lonicera nervosa#NC 040961
Dipsacales Caprifoliaceae Lonicera japonica#F412
Dipsacales Caprifoliaceae Lonicera macranthoides#NC 040959
Apiales Pennantiaceae Pennantia cunninghamii#FC235
Apiales Pennantiaceae Pennantia corymbosa#SBZH
Apiales Torricelliaceae Torricellia angulata#F206
Apiales Torricelliaceae Torricellia tiliifolia#F748
Apiales Griseliniaceae Griselinia racemosa#QIKZ
Apiales Griseliniaceae Griselinia littoralis#FC131
Apiales Pittosporaceae Pittosporum resiniferum#SALZ
Apiales Pittosporaceae Pittosporum illicioides#NTEO
Apiales Pittosporaceae Pittosporum kerrii#F536
Apiales Pittosporaceae Pittosporum brevicalyx#F359
Apiales Araliaceae Hydrocotyle javanica#F73
Apiales Araliaceae Hydrocotyle sibthorpioides#NC 035502
Apiales Araliaceae Hydrocotyle umbellata#OINM
Apiales Araliaceae Hydrocotyle sp.#NC 015818
Apiales Araliaceae Schefflera delavayi#NC 022813
Apiales Araliaceae Schefflera heptaphylla#NC 029764
Apiales Araliaceae Dendropanax morbifer#NC 027607
Apiales Araliaceae Dendropanax dentiger#NC 026546
Apiales Araliaceae Hedera helix#SUVN
Apiales Araliaceae Oreopanax capitatus#GLM14
Apiales Araliaceae Fatsia japonica#NC 027685
Apiales Araliaceae Kalopanax septemlobus#NC 022814
Apiales Araliaceae Metapanax delavayi#NC 022812
Apiales Araliaceae Brassaiopsis hainla#NC 022811
Apiales Araliaceae Eleutherococcus trifoliatus#F165
Apiales Araliaceae Eleutherococcus senticosus#KY085901
Apiales Araliaceae Polyscias fruticosa#EDBB
Apiales Araliaceae Aralia cordata#NC 040964
Apiales Araliaceae Aralia continentalis#NC 041648
Apiales Araliaceae Aralia undulata#NC 022810
Apiales Araliaceae Aralia thomsonii#F11
Apiales Araliaceae Panax trifolius#NC 037994
Apiales Araliaceae Panax bipinnatifidus#MF377620
Apiales Araliaceae Panax stipuleanatus#KY379906
Apiales Araliaceae Panax ginseng#NC 006290
Apiales Araliaceae Panax quinquefolius#NC 027456
Apiales Araliaceae Panax notoginseng#NC 026447
Apiales Araliaceae Panax japonicus#NC 028703
Apiales Araliaceae Panax sp.#MF377621
Apiales Araliaceae Panax vietnamensis#MF377623
Apiales Myodocarpaceae Delarbrea paradoxa#Yi14486
Apiales Apiaceae Centella asiatica#WEQK
Apiales Apiaceae Bupleurum marginatum#F649
Apiales Apiaceae Bupleurum latissimum#NC 033346
Apiales Apiaceae Bupleurum boissieuanum#NC 036017
Apiales Apiaceae Bupleurum falcatum#NC 027834
Apiales Apiaceae Pleurospermum uralense#NC 033343
Apiales Apiaceae Chuanminshen violaceum#KU921430
Apiales Apiaceae Hansenia weberbaueriana#NC 035053
Apiales Apiaceae Hansenia forbesii#NC 035054
Apiales Apiaceae Hansenia forrestii#NC 035056
Apiales Apiaceae Hansenia oviformis#MK193788
Apiales Apiaceae Oxypolis greenmanii#NC 015832
Apiales Apiaceae Cicuta virosa#NC 037711
Apiales Apiaceae Oenanthe virgata#KX832335
Apiales Apiaceae Oenanthe javanica#F650
Apiales Apiaceae Peucedanum insolens#NC 033344
Apiales Apiaceae Anthriscus cerefolium#NC 015113
Apiales Apiaceae Pternopetalum sp.#F400
Apiales Apiaceae Pterygopleurum neurophyllum#NC 033345
Apiales Apiaceae Caucalis platycarpos#KX832334
Apiales Apiaceae Daucus glochidiatus#KX832322
Apiales Apiaceae Daucus guttatus#KX832319
Apiales Apiaceae Daucus littoralis#KX832321
Apiales Apiaceae Daucus pusillus#KX832327
Apiales Apiaceae Daucus involucratus#KX832332
Apiales Apiaceae Daucus conchitae#KX832329
Apiales Apiaceae Daucus crinitus#KX832316
Apiales Apiaceae Daucus tenuisectus#KX832318
Apiales Apiaceae Daucus aureus#KX832313
Apiales Apiaceae Daucus rouyi#KX832311

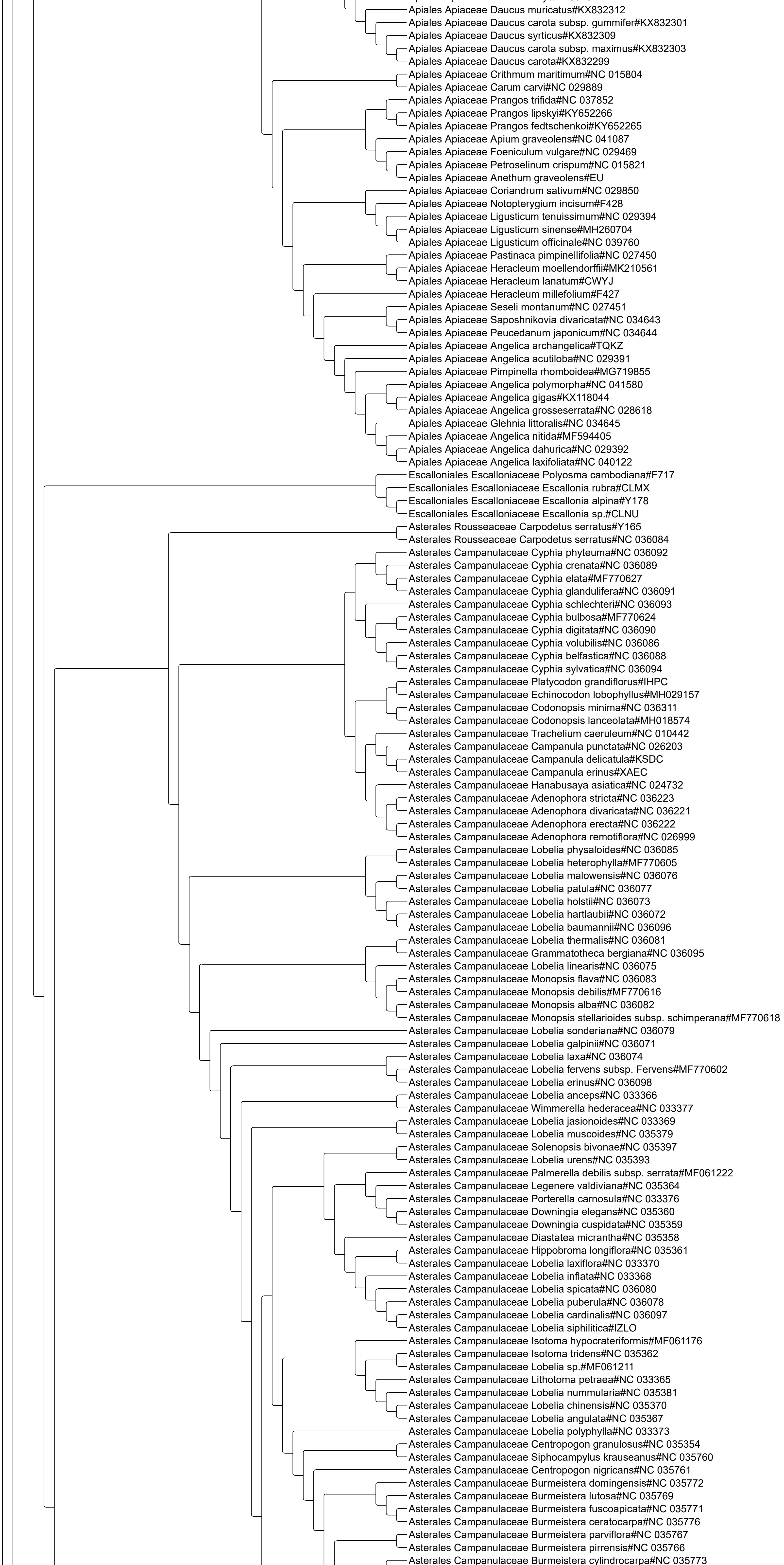

Apiales Apiaceae Daucus muricatus#KX832312
Apiales Apiaceae Daucus carota subsp. gummifer#KX832301
Apiales Apiaceae Daucus syrticus#KX832309
Apiales Apiaceae Daucus carota subsp. maximus#KX832303
Apiales Apiaceae Daucus carota#KX832299
Apiales Apiaceae Crithmum maritimum#NC 015804
Apiales Apiaceae Carum carvi#NC 029889
Apiales Apiaceae Prangos trifida#NC 037852
Apiales Apiaceae Prangos lipskyi#KY652266
Apiales Apiaceae Prangos fedtschenkoi#KY652265
Apiales Apiaceae Apium graveolens#NC 041087
Apiales Apiaceae Foeniculum vulgare#NC 029469
Apiales Apiaceae Petroselinum crispum#NC 015821
Apiales Apiaceae Anethum graveolens#EU
Apiales Apiaceae Coriandrum sativum#NC 029850
Apiales Apiaceae Notopterygium incisum#F428
Apiales Apiaceae Ligusticum tenuissimum#NC 029394
Apiales Apiaceae Ligusticum sinense#MH260704
Apiales Apiaceae Ligusticum officinale#NC 039760
Apiales Apiaceae Pastinaca pimpinellifolia#NC 027450
Apiales Apiaceae Heracleum moellendorffii#MK210561
Apiales Apiaceae Heracleum lanatum#CWYJ
Apiales Apiaceae Heracleum millefolium#F427
Apiales Apiaceae Seseli montanum#NC 027451
Apiales Apiaceae Saposhnikovia divaricata#NC 034643
Apiales Apiaceae Peucedanum japonicum#NC 034644
Apiales Apiaceae Angelica archangelica#TQKZ
Apiales Apiaceae Angelica acutiloba#NC 029391
Apiales Apiaceae Pimpinella rhomboidea#MG719855
Apiales Apiaceae Angelica polymorpha#NC 041580
Apiales Apiaceae Angelica gigas#KX118044
Apiales Apiaceae Angelica grosseserrata#NC 028618
Apiales Apiaceae Glehnia littoralis#NC 034645
Apiales Apiaceae Angelica nitida#MF594405
Apiales Apiaceae Angelica dahurica#NC 029392
Apiales Apiaceae Angelica laxifoliata#NC 040122
Escalloniales Escalloniaceae Polyosma cambodiana#F717
Escalloniales Escalloniaceae Escallonia rubra#CLMX
Escalloniales Escalloniaceae Escallonia alpina#Y178
Escalloniales Escalloniaceae Escallonia sp.#CLNU
Asterales Rousseaceae Carpodetus serratus#Y165
Asterales Rousseaceae Carpodetus serratus#NC 036084
Asterales Campanulaceae Cyphia phyteuma#NC 036092
Asterales Campanulaceae Cyphia crenata#NC 036089
Asterales Campanulaceae Cyphia elata#MF770627
Asterales Campanulaceae Cyphia glandulifera#NC 036091
Asterales Campanulaceae Cyphia schlechteri#NC 036093
Asterales Campanulaceae Cyphia bulbosa#MF770624
Asterales Campanulaceae Cyphia digitata#NC 036090
Asterales Campanulaceae Cyphia volubilis#NC 036086
Asterales Campanulaceae Cyphia belfastica#NC 036088
Asterales Campanulaceae Cyphia sylvatica#NC 036094
Asterales Campanulaceae Platycodon grandiflorus#IHPC
Asterales Campanulaceae Echinocodon lobophyllus#MH029157
Asterales Campanulaceae Codonopsis minima#NC 036311
Asterales Campanulaceae Codonopsis lanceolata#MH018574
Asterales Campanulaceae Trachelium caeruleum#NC 010442
Asterales Campanulaceae Campanula punctata#NC 026203
Asterales Campanulaceae Campanula delicatula#KSDC
Asterales Campanulaceae Campanula erinus#XAEC
Asterales Campanulaceae Hanabusaya asiatica#NC 024732
Asterales Campanulaceae Adenophora stricta#NC 036223
Asterales Campanulaceae Adenophora divaricata#NC 036221
Asterales Campanulaceae Adenophora erecta#NC 036222
Asterales Campanulaceae Adenophora remotiflora#NC 026999
Asterales Campanulaceae Lobelia physaloides#NC 036085
Asterales Campanulaceae Lobelia heterophylla#MF770605
Asterales Campanulaceae Lobelia malowensis#NC 036076
Asterales Campanulaceae Lobelia patula#NC 036077
Asterales Campanulaceae Lobelia holstii#NC 036073
Asterales Campanulaceae Lobelia hartlaubii#NC 036072
Asterales Campanulaceae Lobelia baumannii#NC 036096
Asterales Campanulaceae Lobelia thermalis#NC 036081
Asterales Campanulaceae Grammatotheca bergiana#NC 036095
Asterales Campanulaceae Lobelia linearis#NC 036075
Asterales Campanulaceae Monopsis flava#NC 036083
Asterales Campanulaceae Monopsis debilis#MF770616
Asterales Campanulaceae Monopsis alba#NC 036082
Asterales Campanulaceae Monopsis stellarioides subsp. schimperana#MF770618
Asterales Campanulaceae Lobelia sonderiana#NC 036079
Asterales Campanulaceae Lobelia galpinii#NC 036071
Asterales Campanulaceae Lobelia laxa#NC 036074
Asterales Campanulaceae Lobelia fervens subsp. Fervens#MF770602
Asterales Campanulaceae Lobelia erinus#NC 036098
Asterales Campanulaceae Lobelia anceps#NC 033366
Asterales Campanulaceae Wimmerella hederacea#NC 033377
Asterales Campanulaceae Lobelia jasionoides#NC 033369
Asterales Campanulaceae Lobelia muscoides#NC 035379
Asterales Campanulaceae Solenopsis bivonae#NC 035397
Asterales Campanulaceae Lobelia urens#NC 035393
Asterales Campanulaceae Palmerella debilis subsp. serrata#MF061222
Asterales Campanulaceae Legenere valdiviana#NC 035364
Asterales Campanulaceae Porterella carnosula#NC 033376
Asterales Campanulaceae Downingia elegans#NC 035360
Asterales Campanulaceae Downingia cuspidata#NC 035359
Asterales Campanulaceae Diastatea micrantha#NC 035358
Asterales Campanulaceae Hippobroma longiflora#NC 035361
Asterales Campanulaceae Lobelia laxiflora#NC 033370
Asterales Campanulaceae Lobelia inflata#NC 033368
Asterales Campanulaceae Lobelia spicata#NC 036080
Asterales Campanulaceae Lobelia puberula#NC 036078
Asterales Campanulaceae Lobelia cardinalis#NC 036097
Asterales Campanulaceae Lobelia siphilitica#IZLO
Asterales Campanulaceae Isotoma hypocrateriformis#MF061176
Asterales Campanulaceae Isotoma tridens#NC 035362
Asterales Campanulaceae Lobelia sp.#MF061211
Asterales Campanulaceae Lithotoma petraea#NC 033365
Asterales Campanulaceae Lobelia nummularia#NC 035381
Asterales Campanulaceae Lobelia chinensis#NC 035370
Asterales Campanulaceae Lobelia angulata#NC 035367
Asterales Campanulaceae Lobelia polyphylla#NC 033373
Asterales Campanulaceae Centropogon granulosus#NC 035354
Asterales Campanulaceae Siphocampylus krauseanus#NC 035760
Asterales Campanulaceae Centropogon nigricans#NC 035761
Asterales Campanulaceae Burmeistera domingensis#NC 035772
Asterales Campanulaceae Burmeistera lutosa#NC 035769
Asterales Campanulaceae Burmeistera fuscoapicata#NC 035771
Asterales Campanulaceae Burmeistera ceratocarpa#NC 035776
Asterales Campanulaceae Burmeistera parviflora#NC 035767
Asterales Campanulaceae Burmeistera pirrensis#NC 035766
Asterales Campanulaceae Burmeistera cylindrocarpa#NC 035773

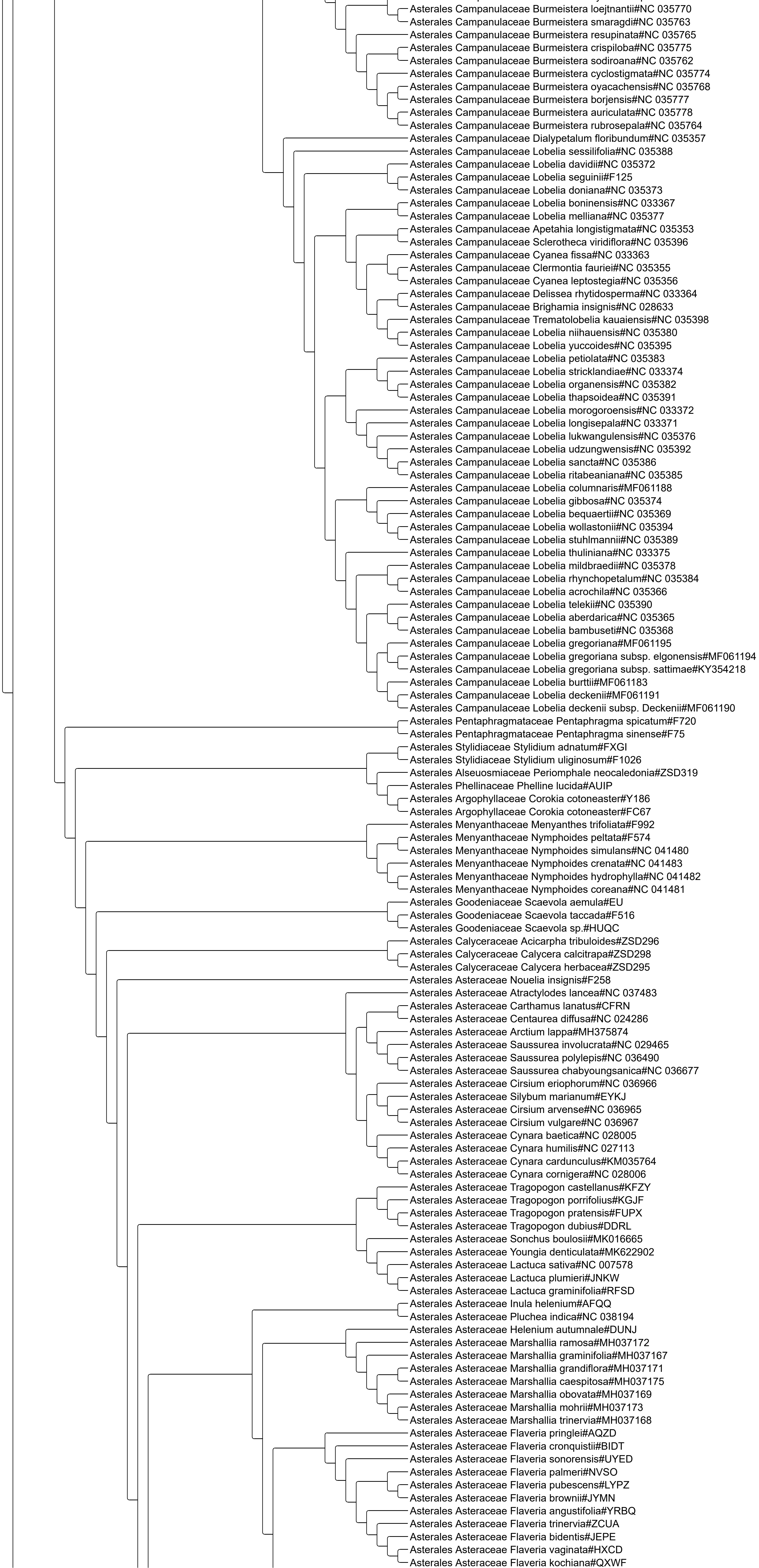
Asterales Campanulaceae Burmeistera loejtnantii#NC 035770
Asterales Campanulaceae Burmeistera smaragdi#NC 035763
Asterales Campanulaceae Burmeistera resupinata#NC 035765
Asterales Campanulaceae Burmeistera crispiloba#NC 035775
Asterales Campanulaceae Burmeistera sodiroana#NC 035762
Asterales Campanulaceae Burmeistera cyclostigmata#NC 035774
Asterales Campanulaceae Burmeistera oyacachensis#NC 035768
Asterales Campanulaceae Burmeistera borjensis#NC 035777
Asterales Campanulaceae Burmeistera auriculata#NC 035778
Asterales Campanulaceae Burmeistera rubrosepala#NC 035764
Asterales Campanulaceae Dialypetalum floribundum#NC 035357
Asterales Campanulaceae Lobelia sessilifolia#NC 035388
Asterales Campanulaceae Lobelia davidii#NC 035372
Asterales Campanulaceae Lobelia seguinii#F125
Asterales Campanulaceae Lobelia doniana#NC 035373
Asterales Campanulaceae Lobelia boninensis#NC 033367
Asterales Campanulaceae Lobelia melliana#NC 035377
Asterales Campanulaceae Apetahia longistigmata#NC 035353
Asterales Campanulaceae Sclerotheca viridiflora#NC 035396
Asterales Campanulaceae Cyanea fissa#NC 033363
Asterales Campanulaceae Clermontia fauriei#NC 035355
Asterales Campanulaceae Cyanea leptostegia#NC 035356
Asterales Campanulaceae Delissea rhytidosperma#NC 033364
Asterales Campanulaceae Brighamia insignis#NC 028633
Asterales Campanulaceae Trematolobelia kauaiensis#NC 035398
Asterales Campanulaceae Lobelia niihauensis#NC 035380
Asterales Campanulaceae Lobelia yuccoides#NC 035395
Asterales Campanulaceae Lobelia petiolata#NC 035383
Asterales Campanulaceae Lobelia stricklandiae#NC 033374
Asterales Campanulaceae Lobelia organensis#NC 035382
Asterales Campanulaceae Lobelia thapsoidea#NC 035391
Asterales Campanulaceae Lobelia morogoroensis#NC 033372
Asterales Campanulaceae Lobelia longisepala#NC 033371
Asterales Campanulaceae Lobelia lukwangulensis#NC 035376
Asterales Campanulaceae Lobelia udzungwensis#NC 035392
Asterales Campanulaceae Lobelia sancta#NC 035386
Asterales Campanulaceae Lobelia ritabeaniana#NC 035385
Asterales Campanulaceae Lobelia columnaris#MF061188
Asterales Campanulaceae Lobelia gibbosa#NC 035374
Asterales Campanulaceae Lobelia bequaertii#NC 035369
Asterales Campanulaceae Lobelia wollastonii#NC 035394
Asterales Campanulaceae Lobelia stuhlmannii#NC 035389
Asterales Campanulaceae Lobelia thuliniana#NC 033375
Asterales Campanulaceae Lobelia mildbraedii#NC 035378
Asterales Campanulaceae Lobelia rhynchopetalum#NC 035384
Asterales Campanulaceae Lobelia acrochila#NC 035366
Asterales Campanulaceae Lobelia telekii#NC 035390
Asterales Campanulaceae Lobelia aberdarica#NC 035365
Asterales Campanulaceae Lobelia bambuseti#NC 035368
Asterales Campanulaceae Lobelia gregoriana#MF061195
Asterales Campanulaceae Lobelia gregoriana subsp. elgonensis#MF061194
Asterales Campanulaceae Lobelia gregoriana subsp. sattimae#KY354218
Asterales Campanulaceae Lobelia burttii#MF061183
Asterales Campanulaceae Lobelia deckenii#MF061191
Asterales Campanulaceae Lobelia deckenii subsp. Deckenii#MF061190
Asterales Pentaphragmataceae Pentaphragma spicatum#F720
Asterales Pentaphragmataceae Pentaphragma sinense#F75
Asterales Stylidiaceae Stylidium adnatum#FXGI
Asterales Stylidiaceae Stylidium uliginosum#F1026
Asterales Alseuosmiaceae Periomphale neocaledonia#ZSD319
Asterales Phellinaceae Phelline lucida#AUIP
Asterales Argophyllaceae Corokia cotoneaster#Y186
Asterales Argophyllaceae Corokia cotoneaster#FC67
Asterales Menyanthaceae Menyanthes trifoliata#F992
Asterales Menyanthaceae Nymphoides peltata#F574
Asterales Menyanthaceae Nymphoides simulans#NC 041480
Asterales Menyanthaceae Nymphoides crenata#NC 041483
Asterales Menyanthaceae Nymphoides hydrophylla#NC 041482
Asterales Menyanthaceae Nymphoides coreana#NC 041481
Asterales Goodeniaceae Scaevola aemula#EU
Asterales Goodeniaceae Scaevola taccada#F516
Asterales Goodeniaceae Scaevola sp.#HUQC
Asterales Calyceraceae Acicarpha tribuloides#ZSD296
Asterales Calyceraceae Calycera calcitrapa#ZSD298
Asterales Calyceraceae Calycera herbacea#ZSD295
Asterales Asteraceae Nouelia insignis#F258
Asterales Asteraceae Atractylodes lancea#NC 037483
Asterales Asteraceae Carthamus lanatus#CFRN
Asterales Asteraceae Centaurea diffusa#NC 024286
Asterales Asteraceae Arctium lappa#MH375874
Asterales Asteraceae Saussurea involucrata#NC 029465
Asterales Asteraceae Saussurea polylepis#NC 036490
Asterales Asteraceae Saussurea chabyoungsanica#NC 036677
Asterales Asteraceae Cirsium eriophorum#NC 036966
Asterales Asteraceae Silybum marianum#EYKJ
Asterales Asteraceae Cirsium arvense#NC 036965
Asterales Asteraceae Cirsium vulgare#NC 036967
Asterales Asteraceae Cynara baetica#NC 028005
Asterales Asteraceae Cynara humilis#NC 027113
Asterales Asteraceae Cynara cardunculus#KM035764
Asterales Asteraceae Cynara cornigera#NC 028006
Asterales Asteraceae Tragopogon castellanus#KFZY
Asterales Asteraceae Tragopogon porrifolius#KGJF
Asterales Asteraceae Tragopogon pratensis#FUPX
Asterales Asteraceae Tragopogon dubius#DDRL
Asterales Asteraceae Sonchus boulosii#MK016665
Asterales Asteraceae Youngia denticulata#MK622902
Asterales Asteraceae Lactuca sativa#NC 007578
Asterales Asteraceae Lactuca plumieri#JNKW
Asterales Asteraceae Lactuca graminifolia#RFSD
Asterales Asteraceae Inula helenium#AFQQ
Asterales Asteraceae Pluchea indica#NC 038194
Asterales Asteraceae Helenium autumnale#DUNJ
Asterales Asteraceae Marshallia ramosa#MH037172
Asterales Asteraceae Marshallia graminifolia#MH037167
Asterales Asteraceae Marshallia grandiflora#MH037171
Asterales Asteraceae Marshallia caespitosa#MH037175
Asterales Asteraceae Marshallia obovata#MH037169
Asterales Asteraceae Marshallia mohrii#MH037173
Asterales Asteraceae Marshallia trinervia#MH037168
Asterales Asteraceae Flaveria pringlei#AQZD
Asterales Asteraceae Flaveria cronquistii#BIDT
Asterales Asteraceae Flaveria sonorensis#UYED
Asterales Asteraceae Flaveria palmeri#NVSO
Asterales Asteraceae Flaveria pubescens#LYPZ
Asterales Asteraceae Flaveria brownii#JYMN
Asterales Asteraceae Flaveria angustifolia#YRBQ
Asterales Asteraceae Flaveria trinervia#ZCUA
Asterales Asteraceae Flaveria bidentis#JEPE
Asterales Asteraceae Flaveria vaginata#HXCD
Asterales Asteraceae Flaveria kochiana#QXWF

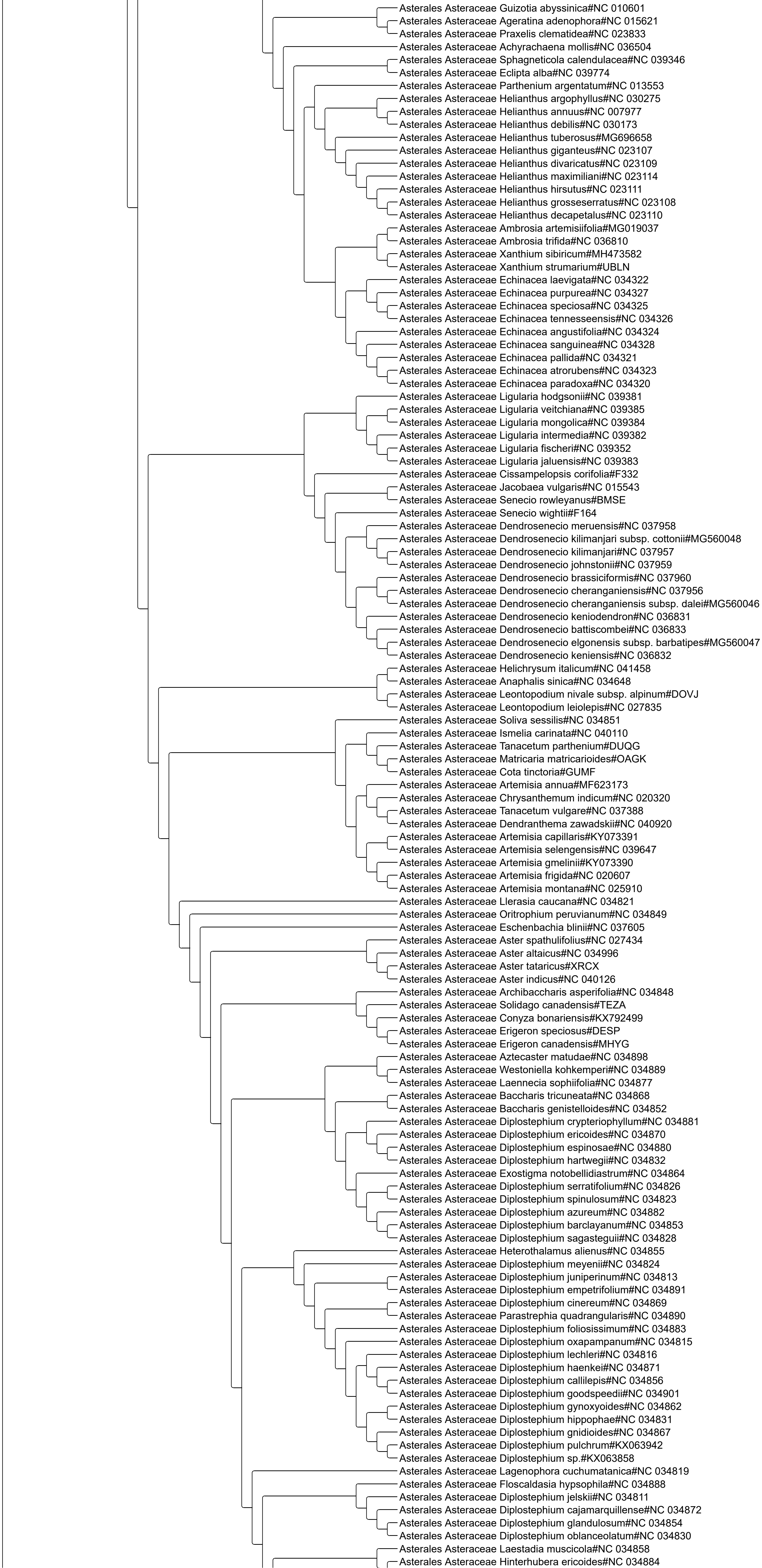

Asterales Asteraceae Guizotia abyssinica#NC 010601
Asterales Asteraceae Ageratina adenophora#NC 015621
Asterales Asteraceae Praxelis clematidea#NC 023833
Asterales Asteraceae Achyrachaena mollis#NC 036504
Asterales Asteraceae Sphagneticola calendulacea#NC 039346
Asterales Asteraceae Eclipta alba#NC 039774
Asterales Asteraceae Parthenium argentatum#NC 013553
Asterales Asteraceae Helianthus argophyllus#NC 030275
Asterales Asteraceae Helianthus annuus#NC 007977
Asterales Asteraceae Helianthus debilis#NC 030173
Asterales Asteraceae Helianthus tuberosus#MG696658
Asterales Asteraceae Helianthus giganteus#NC 023107
Asterales Asteraceae Helianthus divaricatus#NC 023109
Asterales Asteraceae Helianthus maximiliani#NC 023114
Asterales Asteraceae Helianthus hirsutus#NC 023111
Asterales Asteraceae Helianthus grosseserratus#NC 023108
Asterales Asteraceae Helianthus decapetalus#NC 023110
Asterales Asteraceae Ambrosia artemisiifolia#MG019037
Asterales Asteraceae Ambrosia trifida#NC 036810
Asterales Asteraceae Xanthium sibiricum#MH473582
Asterales Asteraceae Xanthium strumarium#UBLN
Asterales Asteraceae Echinacea laevigata#NC 034322
Asterales Asteraceae Echinacea purpurea#NC 034327
Asterales Asteraceae Echinacea speciosa#NC 034325
Asterales Asteraceae Echinacea tennesseensis#NC 034326
Asterales Asteraceae Echinacea angustifolia#NC 034324
Asterales Asteraceae Echinacea sanguinea#NC 034328
Asterales Asteraceae Echinacea pallida#NC 034321
Asterales Asteraceae Echinacea atrorubens#NC 034323
Asterales Asteraceae Echinacea paradoxa#NC 034320
Asterales Asteraceae Ligularia hodgsonii#NC 039381
Asterales Asteraceae Ligularia veitchiana#NC 039385
Asterales Asteraceae Ligularia mongolica#NC 039384
Asterales Asteraceae Ligularia intermedia#NC 039382
Asterales Asteraceae Ligularia fischeri#NC 039352
Asterales Asteraceae Ligularia jaluensis#NC 039383
Asterales Asteraceae Cissampelopsis corifolia#F332
Asterales Asteraceae Jacobaea vulgaris#NC 015543
Asterales Asteraceae Senecio rowleyanus#BMSE
Asterales Asteraceae Senecio wightii#F164
Asterales Asteraceae Dendrosenecio meruensis#NC 037958
Asterales Asteraceae Dendrosenecio kilimanjari subsp. cottonii#MG560048
Asterales Asteraceae Dendrosenecio kilimanjari#NC 037957
Asterales Asteraceae Dendrosenecio johnstonii#NC 037959
Asterales Asteraceae Dendrosenecio brassiciformis#NC 037960
Asterales Asteraceae Dendrosenecio cheranganiensis#NC 037956
Asterales Asteraceae Dendrosenecio cheranganiensis subsp. dalei#MG560046
Asterales Asteraceae Dendrosenecio keniodendron#NC 036831
Asterales Asteraceae Dendrosenecio battiscombei#NC 036833
Asterales Asteraceae Dendrosenecio elgonensis subsp. barbatipes#MG560047
Asterales Asteraceae Dendrosenecio keniensis#NC 036832
Asterales Asteraceae Helichrysum italicum#NC 041458
Asterales Asteraceae Anaphalis sinica#NC 034648
Asterales Asteraceae Leontopodium nivale subsp. alpinum#DOVJ
Asterales Asteraceae Leontopodium leiolepis#NC 027835
Asterales Asteraceae Soliva sessilis#NC 034851
Asterales Asteraceae Ismelia carinata#NC 040110
Asterales Asteraceae Tanacetum parthenium#DUQG
Asterales Asteraceae Matricaria matricarioides#OAGK
Asterales Asteraceae Cota tinctoria#GUMF
Asterales Asteraceae Artemisia annua#MF623173
Asterales Asteraceae Chrysanthemum indicum#NC 020320
Asterales Asteraceae Tanacetum vulgare#NC 037388
Asterales Asteraceae Dendranthema zawadskii#NC 040920
Asterales Asteraceae Artemisia capillaris#KY073391
Asterales Asteraceae Artemisia selengensis#NC 039647
Asterales Asteraceae Artemisia gmelinii#KY073390
Asterales Asteraceae Artemisia frigida#NC 020607
Asterales Asteraceae Artemisia montana#NC 025910
Asterales Asteraceae Llerasia caucana#NC 034821
Asterales Asteraceae Oritrophium peruvianum#NC 034849
Asterales Asteraceae Eschenbachia blinii#NC 037605
Asterales Asteraceae Aster spathulifolius#NC 027434
Asterales Asteraceae Aster altaicus#NC 034996
Asterales Asteraceae Aster tataricus#XRCX
Asterales Asteraceae Aster indicus#NC 040126
Asterales Asteraceae Archibaccharis asperifolia#NC 034848
Asterales Asteraceae Solidago canadensis#TEZA
Asterales Asteraceae Conyza bonariensis#KX792499
Asterales Asteraceae Erigeron speciosus#DESP
Asterales Asteraceae Erigeron canadensis#MHYG
Asterales Asteraceae Aztecaster matudae#NC 034898
Asterales Asteraceae Westoniella kohkemperi#NC 034889
Asterales Asteraceae Laennecia sophiifolia#NC 034877
Asterales Asteraceae Baccharis tricuneata#NC 034868
Asterales Asteraceae Baccharis genistelloides#NC 034852
Asterales Asteraceae Diplostephium crypteriophyllum#NC 034881
Asterales Asteraceae Diplostephium ericoides#NC 034870
Asterales Asteraceae Diplostephium espinosae#NC 034880
Asterales Asteraceae Diplostephium hartwegii#NC 034832
Asterales Asteraceae Exostigma notobellidiastrum#NC 034864
Asterales Asteraceae Diplostephium serratifolium#NC 034826
Asterales Asteraceae Diplostephium spinulosum#NC 034823
Asterales Asteraceae Diplostephium azureum#NC 034882
Asterales Asteraceae Diplostephium barclayanum#NC 034853
Asterales Asteraceae Diplostephium sagasteguii#NC 034828
Asterales Asteraceae Heterothalamus alienus#NC 034855
Asterales Asteraceae Diplostephium meyenii#NC 034824
Asterales Asteraceae Diplostephium juniperinum#NC 034813
Asterales Asteraceae Diplostephium empetrifolium#NC 034891
Asterales Asteraceae Diplostephium cinereum#NC 034869
Asterales Asteraceae Parastrephia quadrangularis#NC 034890
Asterales Asteraceae Diplostephium foliosissimum#NC 034883
Asterales Asteraceae Diplostephium oxapampanum#NC 034815
Asterales Asteraceae Diplostephium lechleri#NC 034816
Asterales Asteraceae Diplostephium haenkei#NC 034871
Asterales Asteraceae Diplostephium callilepis#NC 034856
Asterales Asteraceae Diplostephium goodspeedii#NC 034901
Asterales Asteraceae Diplostephium gynoxyoides#NC 034862
Asterales Asteraceae Diplostephium hippophae#NC 034831
Asterales Asteraceae Diplostephium gnidioides#NC 034867
Asterales Asteraceae Diplostephium pulchrum#KX063942
Asterales Asteraceae Diplostephium sp.#KX063858
Asterales Asteraceae Lagenophora cuchumatanica#NC 034819
Asterales Asteraceae Floscaldasia hypsophila#NC 034888
Asterales Asteraceae Diplostephium jelskii#NC 034811
Asterales Asteraceae Diplostephium cajamarquillense#NC 034872
Asterales Asteraceae Diplostephium glandulosum#NC 034854
Asterales Asteraceae Diplostephium oblanceolatum#NC 034830
Asterales Asteraceae Laestadia muscicola#NC 034858
Asterales Asteraceae Hinterhubera ericoides#NC 034884

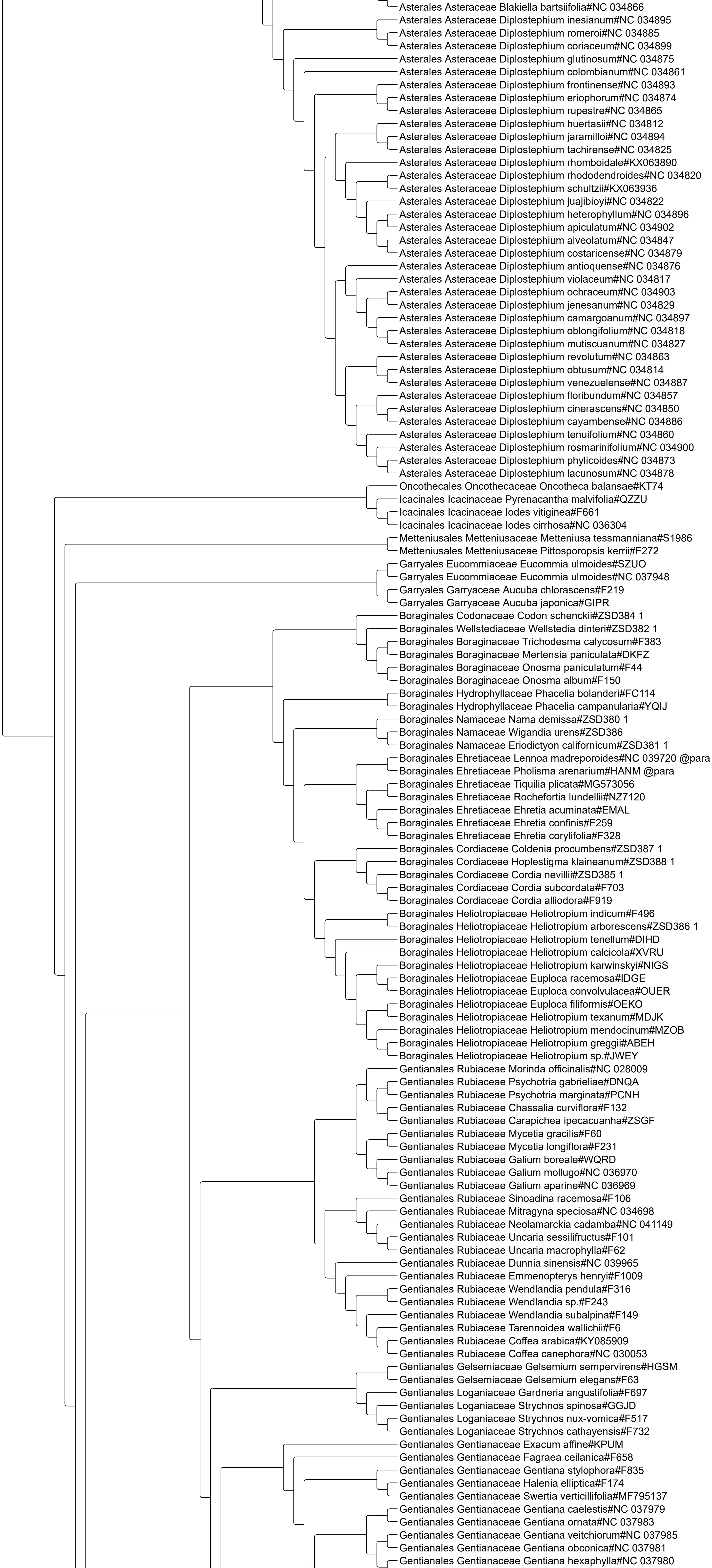
Asterales Asteraceae Blakiella bartsiifolia#NC 034866
Asterales Asteraceae Diplostephium inesianum#NC 034895
Asterales Asteraceae Diplostephium romeroi#NC 034885
Asterales Asteraceae Diplostephium coriaceum#NC 034899
Asterales Asteraceae Diplostephium glutinosum#NC 034875
Asterales Asteraceae Diplostephium colombianum#NC 034861
Asterales Asteraceae Diplostephium frontinense#NC 034893
Asterales Asteraceae Diplostephium eriophorum#NC 034874
Asterales Asteraceae Diplostephium rupestre#NC 034865
Asterales Asteraceae Diplostephium huertasii#NC 034812
Asterales Asteraceae Diplostephium jaramilloi#NC 034894
Asterales Asteraceae Diplostephium tachirense#NC 034825
Asterales Asteraceae Diplostephium rhomboidale#KX063890
Asterales Asteraceae Diplostephium rhododendroides#NC 034820
Asterales Asteraceae Diplostephium schultzii#KX063936
Asterales Asteraceae Diplostephium juajibioyi#NC 034822
Asterales Asteraceae Diplostephium heterophyllum#NC 034896
Asterales Asteraceae Diplostephium apiculatum#NC 034902
Asterales Asteraceae Diplostephium alveolatum#NC 034847
Asterales Asteraceae Diplostephium costaricense#NC 034879
Asterales Asteraceae Diplostephium antioquense#NC 034876
Asterales Asteraceae Diplostephium violaceum#NC 034817
Asterales Asteraceae Diplostephium ochraceum#NC 034903
Asterales Asteraceae Diplostephium jenesanum#NC 034829
Asterales Asteraceae Diplostephium camargoanum#NC 034897
Asterales Asteraceae Diplostephium oblongifolium#NC 034818
Asterales Asteraceae Diplostephium mutiscuanum#NC 034827
Asterales Asteraceae Diplostephium revolutum#NC 034863
Asterales Asteraceae Diplostephium obtusum#NC 034814
Asterales Asteraceae Diplostephium venezuelense#NC 034887
Asterales Asteraceae Diplostephium floribundum#NC 034857
Asterales Asteraceae Diplostephium cinerascens#NC 034850
Asterales Asteraceae Diplostephium cayambense#NC 034886
Asterales Asteraceae Diplostephium tenuifolium#NC 034860
Asterales Asteraceae Diplostephium rosmarinifolium#NC 034900
Asterales Asteraceae Diplostephium phylicoides#NC 034873
Asterales Asteraceae Diplostephium lacunosum#NC 034878
Oncothecales Oncothecaceae Oncotheca balansae#KT74
Icacinales Icacinaceae Pyrenacantha malvifolia#QZZU
Icacinales Icacinaceae Iodes vitiginea#F661
Icacinales Icacinaceae Iodes cirrhosa#NC 036304
Metteniusales Metteniusaceae Metteniusa tessmanniana#S1986
Metteniusales Metteniusaceae Pittosporopsis kerrii#F272
Garryales Eucommiaceae Eucommia ulmoides#SZUO
Garryales Eucommiaceae Eucommia ulmoides#NC 037948
Garryales Garryaceae Aucuba chlorascens#F219
Garryales Garryaceae Aucuba japonica#GIPR
Boraginales Codonaceae Codon schenckii#ZSD384 1
Boraginales Wellstediaceae Wellstedia dinteri#ZSD382 1
Boraginales Boraginaceae Trichodesma calycosum#F383
Boraginales Boraginaceae Mertensia paniculata#DKFZ
Boraginales Boraginaceae Onosma paniculatum#F44
Boraginales Boraginaceae Onosma album#F150
Boraginales Hydrophyllaceae Phacelia bolanderi#FC114
Boraginales Hydrophyllaceae Phacelia campanularia#YQIJ
Boraginales Namaceae Nama demissa#ZSD380 1
Boraginales Namaceae Wigandia urens#ZSD386
Boraginales Namaceae Eriodictyon californicum#ZSD381 1
Boraginales Ehretiaceae Lennoa madreporoides#NC 039720 @para
Boraginales Ehretiaceae Pholisma arenarium#HANM @para
Boraginales Ehretiaceae Tiquilia plicata#MG573056
Boraginales Ehretiaceae Rochefortia lundellii#NZ7120
Boraginales Ehretiaceae Ehretia acuminata#EMAL
Boraginales Ehretiaceae Ehretia confinis#F259
Boraginales Ehretiaceae Ehretia corylifolia#F328
Boraginales Cordiaceae Coldenia procumbens#ZSD387 1
Boraginales Cordiaceae Hoplestigma klaineanum#ZSD388 1
Boraginales Cordiaceae Cordia nevillii#ZSD385 1
Boraginales Cordiaceae Cordia subcordata#F703
Boraginales Cordiaceae Cordia alliodora#F919
Boraginales Heliotropiaceae Heliotropium indicum#F496
Boraginales Heliotropiaceae Heliotropium arborescens#ZSD386 1
Boraginales Heliotropiaceae Heliotropium tenellum#DIHD
Boraginales Heliotropiaceae Heliotropium calcicola#XVRU
Boraginales Heliotropiaceae Heliotropium karwinskyi#NIGS
Boraginales Heliotropiaceae Euploca racemosa#IDGE
Boraginales Heliotropiaceae Euploca convolvulacea#OUER
Boraginales Heliotropiaceae Euploca filiformis#OEKO
Boraginales Heliotropiaceae Heliotropium texanum#MDJK
Boraginales Heliotropiaceae Heliotropium mendocinum#MZOB
Boraginales Heliotropiaceae Heliotropium greggii#ABEH
Boraginales Heliotropiaceae Heliotropium sp.#JWEY
Gentianales Rubiaceae Morinda officinalis#NC 028009
Gentianales Rubiaceae Psychotria gabrieliae#DNQA
Gentianales Rubiaceae Psychotria marginata#PCNH
Gentianales Rubiaceae Chassalia curviflora#F132
Gentianales Rubiaceae Carapichea ipecacuanha#ZSGF
Gentianales Rubiaceae Mycetia gracilis#F60
Gentianales Rubiaceae Mycetia longiflora#F231
Gentianales Rubiaceae Galium boreale#WQRD
Gentianales Rubiaceae Galium mollugo#NC 036970
Gentianales Rubiaceae Galium aparine#NC 036969
Gentianales Rubiaceae Sinoadina racemosa#F106
Gentianales Rubiaceae Mitragyna speciosa#NC 034698
Gentianales Rubiaceae Neolamarckia cadamba#NC 041149
Gentianales Rubiaceae Uncaria sessilifructus#F101
Gentianales Rubiaceae Uncaria macrophylla#F62
Gentianales Rubiaceae Dunnia sinensis#NC 039965
Gentianales Rubiaceae Emmenopterys henryi#F1009
Gentianales Rubiaceae Wendlandia pendula#F316
Gentianales Rubiaceae Wendlandia sp.#F243
Gentianales Rubiaceae Wendlandia subalpina#F149
Gentianales Rubiaceae Tarennoidea wallichii#F6
Gentianales Rubiaceae Coffea arabica#KY085909
Gentianales Rubiaceae Coffea canephora#NC 030053
Gentianales Gelsemiaceae Gelsemium sempervirens#HGSM
Gentianales Gelsemiaceae Gelsemium elegans#F63
Gentianales Loganiaceae Gardneria angustifolia#F697
Gentianales Loganiaceae Strychnos spinosa#GGJD
Gentianales Loganiaceae Strychnos nux-vomica#F517
Gentianales Loganiaceae Strychnos cathayensis#F732
Gentianales Gentianaceae Exacum affine#KPUM
Gentianales Gentianaceae Fagraea ceilanica#F658
Gentianales Gentianaceae Gentiana stylophora#F835
Gentianales Gentianaceae Halenia elliptica#F174
Gentianales Gentianaceae Swertia verticillifolia#MF795137
Gentianales Gentianaceae Gentiana caelestis#NC 037979
Gentianales Gentianaceae Gentiana ornata#NC 037983
Gentianales Gentianaceae Gentiana veitchiorum#NC 037985
Gentianales Gentianaceae Gentiana obconica#NC 037981
Gentianales Gentianaceae Gentiana hexaphylla#NC 037980

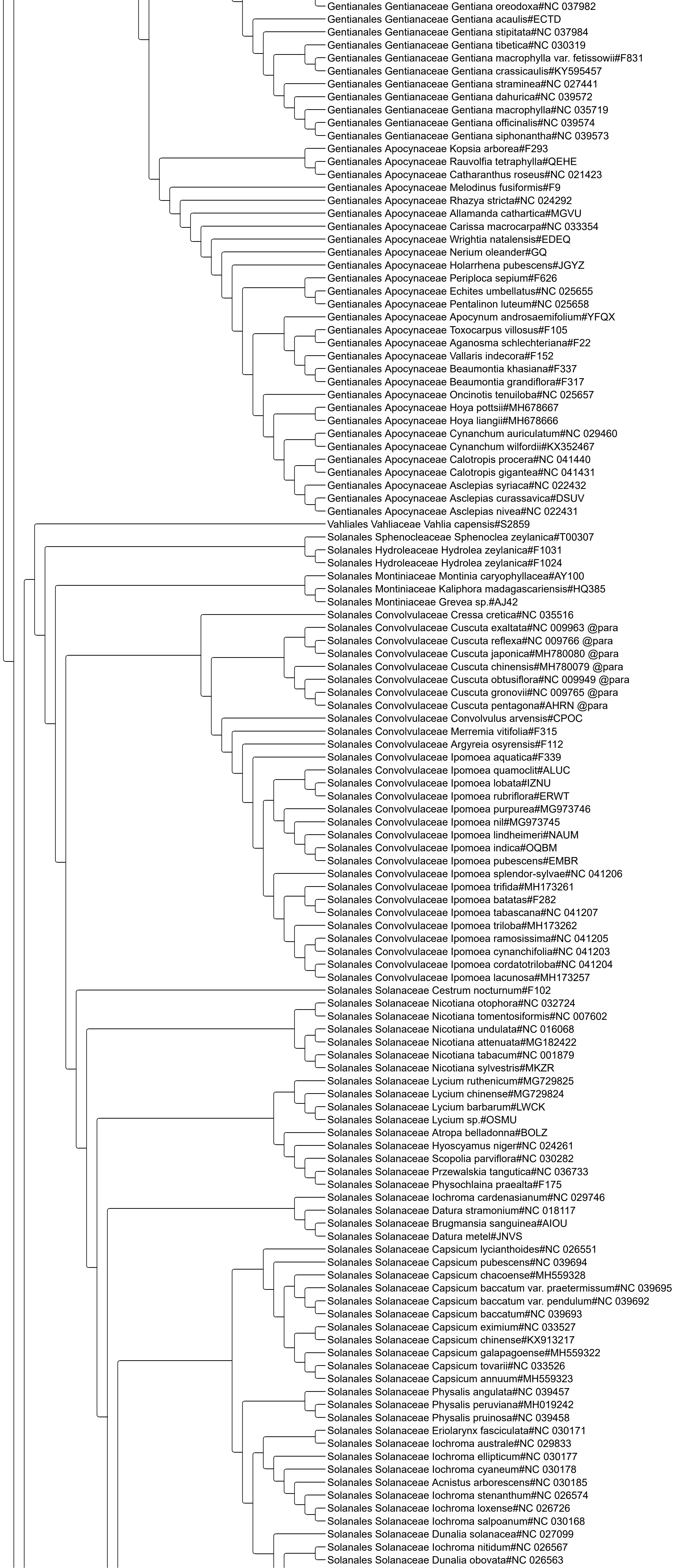
Gentianales Gentianaceae Gentiana oreodoxa#NC 037982
Gentianales Gentianaceae Gentiana acaulis#ECTD
Gentianales Gentianaceae Gentiana stipitata#NC 037984
Gentianales Gentianaceae Gentiana tibetica#NC 030319
Gentianales Gentianaceae Gentiana macrophylla var. fetissowii#F831
Gentianales Gentianaceae Gentiana crassicaulis#KY595457
Gentianales Gentianaceae Gentiana straminea#NC 027441
Gentianales Gentianaceae Gentiana dahurica#NC 039572
Gentianales Gentianaceae Gentiana macrophylla#NC 035719
Gentianales Gentianaceae Gentiana officinalis#NC 039574
Gentianales Gentianaceae Gentiana siphonantha#NC 039573
Gentianales Apocynaceae Kopsia arborea#F293
Gentianales Apocynaceae Rauvolfia tetraphylla#QEHE
Gentianales Apocynaceae Catharanthus roseus#NC 021423
Gentianales Apocynaceae Melodinus fusiformis#F9
Gentianales Apocynaceae Rhazya stricta#NC 024292
Gentianales Apocynaceae Allamanda cathartica#MGVU
Gentianales Apocynaceae Carissa macrocarpa#NC 033354
Gentianales Apocynaceae Wrightia natalensis#EDEQ
Gentianales Apocynaceae Nerium oleander#GQ
Gentianales Apocynaceae Holarrhena pubescens#JGYZ
Gentianales Apocynaceae Periploca sepium#F626
Gentianales Apocynaceae Echites umbellatus#NC 025655
Gentianales Apocynaceae Pentalinon luteum#NC 025658
Gentianales Apocynaceae Apocynum androsaemifolium#YFQX
Gentianales Apocynaceae Toxocarpus villosus#F105
Gentianales Apocynaceae Aganosma schlechteriana#F22
Gentianales Apocynaceae Vallaris indecora#F152
Gentianales Apocynaceae Beaumontia khasiana#F337
Gentianales Apocynaceae Beaumontia grandiflora#F317
Gentianales Apocynaceae Oncinotis tenuiloba#NC 025657
Gentianales Apocynaceae Hoya pottsii#MH678667
Gentianales Apocynaceae Hoya liangii#MH678666
Gentianales Apocynaceae Cynanchum auriculatum#NC 029460
Gentianales Apocynaceae Cynanchum wilfordii#KX352467
Gentianales Apocynaceae Calotropis procera#NC 041440
Gentianales Apocynaceae Calotropis gigantea#NC 041431
Gentianales Apocynaceae Asclepias syriaca#NC 022432
Gentianales Apocynaceae Asclepias curassavica#DSUV
Gentianales Apocynaceae Asclepias nivea#NC 022431
Vahliales Vahliaceae Vahlia capensis#S2859
Solanales Sphenocleaceae Sphenoclea zeylanica#T00307
Solanales Hydroleaceae Hydrolea zeylanica#F1031
Solanales Hydroleaceae Hydrolea zeylanica#F1024
Solanales Montiniaceae Montinia caryophyllacea#AY100
Solanales Montiniaceae Kaliphora madagascariensis#HQ385
Solanales Montiniaceae Grevea sp.#AJ42
Solanales Convolvulaceae Cressa cretica#NC 035516
Solanales Convolvulaceae Cuscuta exaltata#NC 009963 @para
Solanales Convolvulaceae Cuscuta reflexa#NC 009766 @para
Solanales Convolvulaceae Cuscuta japonica#MH780080 @para
Solanales Convolvulaceae Cuscuta chinensis#MH780079 @para
Solanales Convolvulaceae Cuscuta obtusiflora#NC 009949 @para
Solanales Convolvulaceae Cuscuta gronovii#NC 009765 @para
Solanales Convolvulaceae Cuscuta pentagona#AHRN @para
Solanales Convolvulaceae Convolvulus arvensis#CPOC
Solanales Convolvulaceae Merremia vitifolia#F315
Solanales Convolvulaceae Argyreia osyrensis#F112
Solanales Convolvulaceae Ipomoea aquatica#F339
Solanales Convolvulaceae Ipomoea quamoclit#ALUC
Solanales Convolvulaceae Ipomoea lobata#IZNU
Solanales Convolvulaceae Ipomoea rubriflora#ERWT
Solanales Convolvulaceae Ipomoea purpurea#MG973746
Solanales Convolvulaceae Ipomoea nil#MG973745
Solanales Convolvulaceae Ipomoea lindheimeri#NAUM
Solanales Convolvulaceae Ipomoea indica#OQBM
Solanales Convolvulaceae Ipomoea pubescens#EMBR
Solanales Convolvulaceae Ipomoea splendor-sylvae#NC 041206
Solanales Convolvulaceae Ipomoea trifida#MH173261
Solanales Convolvulaceae Ipomoea batatas#F282
Solanales Convolvulaceae Ipomoea tabascana#NC 041207
Solanales Convolvulaceae Ipomoea triloba#MH173262
Solanales Convolvulaceae Ipomoea ramosissima#NC 041205
Solanales Convolvulaceae Ipomoea cynanchifolia#NC 041203
Solanales Convolvulaceae Ipomoea cordatotriloba#NC 041204
Solanales Convolvulaceae Ipomoea lacunosa#MH173257
Solanales Solanaceae Cestrum nocturnum#F102
Solanales Solanaceae Nicotiana otophora#NC 032724
Solanales Solanaceae Nicotiana tomentosiformis#NC 007602
Solanales Solanaceae Nicotiana undulata#NC 016068
Solanales Solanaceae Nicotiana attenuata#MG182422
Solanales Solanaceae Nicotiana tabacum#NC 001879
Solanales Solanaceae Nicotiana sylvestris#MKZR
Solanales Solanaceae Lycium ruthenicum#MG729825
Solanales Solanaceae Lycium chinense#MG729824
Solanales Solanaceae Lycium barbarum#LWCK
Solanales Solanaceae Lycium sp.#OSMU
Solanales Solanaceae Atropa belladonna#BOLZ
Solanales Solanaceae Hyoscyamus niger#NC 024261
Solanales Solanaceae Scopolia parviflora#NC 030282
Solanales Solanaceae Przewalskia tangutica#NC 036733
Solanales Solanaceae Physochlaina praealta#F175
Solanales Solanaceae Iochroma cardenasianum#NC 029746
Solanales Solanaceae Datura stramonium#NC 018117
Solanales Solanaceae Brugmansia sanguinea#AIOU
Solanales Solanaceae Datura metel#JNVS
Solanales Solanaceae Capsicum lycianthoides#NC 026551
Solanales Solanaceae Capsicum pubescens#NC 039694
Solanales Solanaceae Capsicum chacoense#MH559328
Solanales Solanaceae Capsicum baccatum var. praetermissum#NC 039695
Solanales Solanaceae Capsicum baccatum var. pendulum#NC 039692
Solanales Solanaceae Capsicum baccatum#NC 039693
Solanales Solanaceae Capsicum eximium#NC 033527
Solanales Solanaceae Capsicum chinense#KX913217
Solanales Solanaceae Capsicum galapagoense#MH559322
Solanales Solanaceae Capsicum tovarii#NC 033526
Solanales Solanaceae Capsicum annuum#MH559323
Solanales Solanaceae Physalis angulata#NC 039457
Solanales Solanaceae Physalis peruviana#MH019242
Solanales Solanaceae Physalis pruinosa#NC 039458
Solanales Solanaceae Eriolarynx fasciculata#NC 030171
Solanales Solanaceae Iochroma australe#NC 029833
Solanales Solanaceae Iochroma ellipticum#NC 030177
Solanales Solanaceae Iochroma cyaneum#NC 030178
Solanales Solanaceae Acnistus arborescens#NC 030185
Solanales Solanaceae Iochroma stenanthum#NC 026574
Solanales Solanaceae Iochroma loxense#NC 026726
Solanales Solanaceae Iochroma salpoanum#NC 030168
Solanales Solanaceae Dunalia solanacea#NC 027099
Solanales Solanaceae Iochroma nitidum#NC 026567
Solanales Solanaceae Dunalia obovata#NC 026563

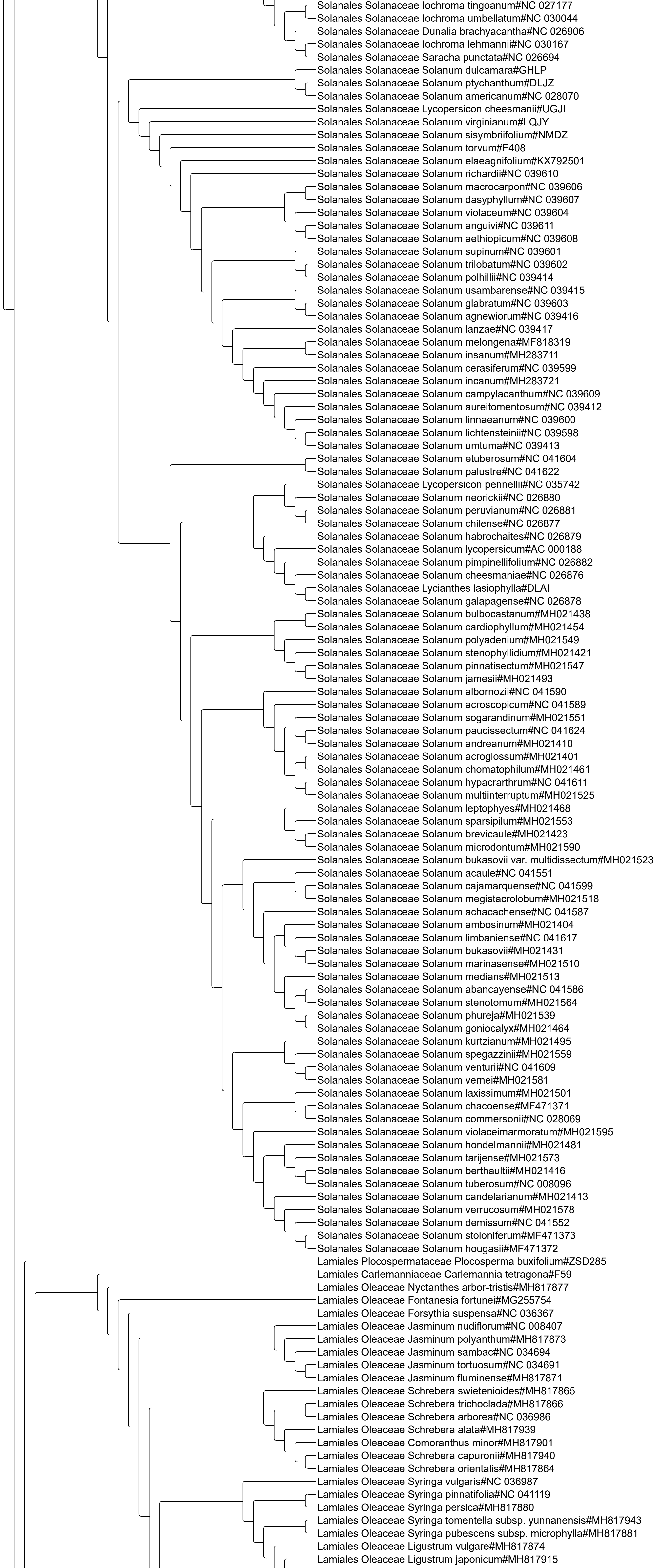

Solanales Solanaceae Iochroma tingoanum#NC 027177
Solanales Solanaceae Iochroma umbellatum#NC 030044
Solanales Solanaceae Dunalia brachyacantha#NC 026906
Solanales Solanaceae Iochroma lehmannii#NC 030167
Solanales Solanaceae Saracha punctata#NC 026694
Solanales Solanaceae Solanum dulcamara#GHLP
Solanales Solanaceae Solanum ptychanthum#DLJZ
Solanales Solanaceae Solanum americanum#NC 028070
Solanales Solanaceae Lycopersicon cheesmanii#UGJI
Solanales Solanaceae Solanum virginianum#LQJY
Solanales Solanaceae Solanum sisymbriifolium#NMDZ
Solanales Solanaceae Solanum torvum#F408
Solanales Solanaceae Solanum elaeagnifolium#KX792501
Solanales Solanaceae Solanum richardii#NC 039610
Solanales Solanaceae Solanum macrocarpon#NC 039606
Solanales Solanaceae Solanum dasyphyllum#NC 039607
Solanales Solanaceae Solanum violaceum#NC 039604
Solanales Solanaceae Solanum anguivi#NC 039611
Solanales Solanaceae Solanum aethiopicum#NC 039608
Solanales Solanaceae Solanum supinum#NC 039601
Solanales Solanaceae Solanum trilobatum#NC 039602
Solanales Solanaceae Solanum polhillii#NC 039414
Solanales Solanaceae Solanum usambarense#NC 039415
Solanales Solanaceae Solanum glabratum#NC 039603
Solanales Solanaceae Solanum agnewiorum#NC 039416
Solanales Solanaceae Solanum lanzae#NC 039417
Solanales Solanaceae Solanum melongena#MF818319
Solanales Solanaceae Solanum insanum#MH283711
Solanales Solanaceae Solanum cerasiferum#NC 039599
Solanales Solanaceae Solanum incanum#MH283721
Solanales Solanaceae Solanum campylacanthum#NC 039609
Solanales Solanaceae Solanum aureitomentosum#NC 039412
Solanales Solanaceae Solanum linnaeanum#NC 039600
Solanales Solanaceae Solanum lichtensteinii#NC 039598
Solanales Solanaceae Solanum umtuma#NC 039413
Solanales Solanaceae Solanum etuberosum#NC 041604
Solanales Solanaceae Solanum palustre#NC 041622
Solanales Solanaceae Lycopersicon pennellii#NC 035742
Solanales Solanaceae Solanum neorickii#NC 026880
Solanales Solanaceae Solanum peruvianum#NC 026881
Solanales Solanaceae Solanum chilense#NC 026877
Solanales Solanaceae Solanum habrochaites#NC 026879
Solanales Solanaceae Solanum lycopersicum#AC 000188
Solanales Solanaceae Solanum pimpinellifolium#NC 026882
Solanales Solanaceae Solanum cheesmaniae#NC 026876
Solanales Solanaceae Lycianthes lasiophylla#DLAI
Solanales Solanaceae Solanum galapagense#NC 026878
Solanales Solanaceae Solanum bulbocastanum#MH021438
Solanales Solanaceae Solanum cardiophyllum#MH021454
Solanales Solanaceae Solanum polyadenium#MH021549
Solanales Solanaceae Solanum stenophyllidium#MH021421
Solanales Solanaceae Solanum pinnatisectum#MH021547
Solanales Solanaceae Solanum jamesii#MH021493
Solanales Solanaceae Solanum albornozii#NC 041590
Solanales Solanaceae Solanum acroscopicum#NC 041589
Solanales Solanaceae Solanum sogarandinum#MH021551
Solanales Solanaceae Solanum paucissectum#NC 041624
Solanales Solanaceae Solanum andreanum#MH021410
Solanales Solanaceae Solanum acroglossum#MH021401
Solanales Solanaceae Solanum chomatophilum#MH021461
Solanales Solanaceae Solanum hypacrarthrum#NC 041611
Solanales Solanaceae Solanum multiinterruptum#MH021525
Solanales Solanaceae Solanum leptophyes#MH021468
Solanales Solanaceae Solanum sparsipilum#MH021553
Solanales Solanaceae Solanum brevicaule#MH021423
Solanales Solanaceae Solanum microdontum#MH021590
Solanales Solanaceae Solanum bukasovii var. multidissectum#MH021523
Solanales Solanaceae Solanum acaule#NC 041551
Solanales Solanaceae Solanum cajamarquense#NC 041599
Solanales Solanaceae Solanum megistacrolobum#MH021518
Solanales Solanaceae Solanum achacachense#NC 041587
Solanales Solanaceae Solanum ambosinum#MH021404
Solanales Solanaceae Solanum limbaniense#NC 041617
Solanales Solanaceae Solanum bukasovii#MH021431
Solanales Solanaceae Solanum marinasense#MH021510
Solanales Solanaceae Solanum medians#MH021513
Solanales Solanaceae Solanum abancayense#NC 041586
Solanales Solanaceae Solanum stenotomum#MH021564
Solanales Solanaceae Solanum phureja#MH021539
Solanales Solanaceae Solanum goniocalyx#MH021464
Solanales Solanaceae Solanum kurtzianum#MH021495
Solanales Solanaceae Solanum spegazzinii#MH021559
Solanales Solanaceae Solanum venturii#NC 041609
Solanales Solanaceae Solanum vernei#MH021581
Solanales Solanaceae Solanum laxissimum#MH021501
Solanales Solanaceae Solanum chacoense#MF471371
Solanales Solanaceae Solanum commersonii#NC 028069
Solanales Solanaceae Solanum violaceimarmoratum#MH021595
Solanales Solanaceae Solanum hondelmannii#MH021481
Solanales Solanaceae Solanum tarijense#MH021573
Solanales Solanaceae Solanum berthaultii#MH021416
Solanales Solanaceae Solanum tuberosum#NC 008096
Solanales Solanaceae Solanum candelarianum#MH021413
Solanales Solanaceae Solanum verrucosum#MH021578
Solanales Solanaceae Solanum demissum#NC 041552
Solanales Solanaceae Solanum stoloniferum#MF471373
Solanales Solanaceae Solanum hougasii#MF471372
Lamiales Plocospermataceae Plocosperma buxifolium#ZSD285
Lamiales Carlemanniaceae Carlemannia tetragona#F59
Lamiales Oleaceae Nyctanthes arbor-tristis#MH817877
Lamiales Oleaceae Fontanesia fortunei#MG255754
Lamiales Oleaceae Forsythia suspensa#NC 036367
Lamiales Oleaceae Jasminum nudiflorum#NC 008407
Lamiales Oleaceae Jasminum polyanthum#MH817873
Lamiales Oleaceae Jasminum sambac#NC 034694
Lamiales Oleaceae Jasminum tortuosum#NC 034691
Lamiales Oleaceae Jasminum fluminense#MH817871
Lamiales Oleaceae Schrebera swietenioides#MH817865
Lamiales Oleaceae Schrebera trichoclada#MH817866
Lamiales Oleaceae Schrebera arborea#NC 036986
Lamiales Oleaceae Schrebera alata#MH817939
Lamiales Oleaceae Comoranthus minor#MH817901
Lamiales Oleaceae Schrebera capuronii#MH817940
Lamiales Oleaceae Schrebera orientalis#MH817864
Lamiales Oleaceae Syringa vulgaris#NC 036987
Lamiales Oleaceae Syringa pinnatifolia#NC 041119
Lamiales Oleaceae Syringa persica#MH817880
Lamiales Oleaceae Syringa tomentella subsp. yunnanensis#MH817943
Lamiales Oleaceae Syringa pubescens subsp. microphylla#MH817881
Lamiales Oleaceae Ligustrum vulgare#MH817874
Lamiales Oleaceae Ligustrum japonicum#MH817915

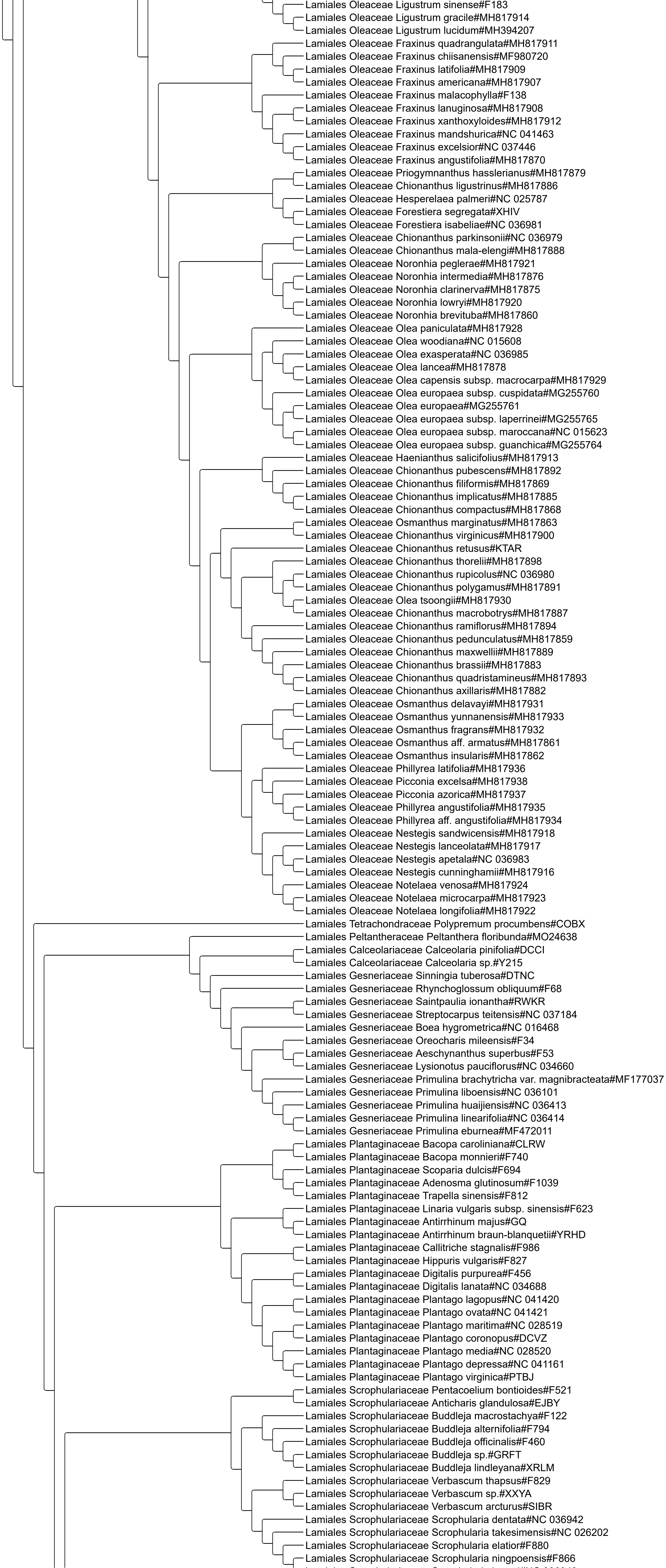

Lamiales Oleaceae Ligustrum sinense#F183
Lamiales Oleaceae Ligustrum gracile#MH817914
Lamiales Oleaceae Ligustrum lucidum#MH394207
Lamiales Oleaceae Fraxinus quadrangulata#MH817911
Lamiales Oleaceae Fraxinus chiisanensis#MF980720
Lamiales Oleaceae Fraxinus latifolia#MH817909
Lamiales Oleaceae Fraxinus americana#MH817907
Lamiales Oleaceae Fraxinus malacophylla#F138
Lamiales Oleaceae Fraxinus lanuginosa#MH817908
Lamiales Oleaceae Fraxinus xanthoxyloides#MH817912
Lamiales Oleaceae Fraxinus mandshurica#NC 041463
Lamiales Oleaceae Fraxinus excelsior#NC 037446
Lamiales Oleaceae Fraxinus angustifolia#MH817870
Lamiales Oleaceae Priogymnanthus hasslerianus#MH817879
Lamiales Oleaceae Chionanthus ligustrinus#MH817886
Lamiales Oleaceae Hesperelaea palmeri#NC 025787
Lamiales Oleaceae Forestiera segregata#XHIV
Lamiales Oleaceae Forestiera isabeliae#NC 036981
Lamiales Oleaceae Chionanthus parkinsonii#NC 036979
Lamiales Oleaceae Chionanthus mala-elengi#MH817888
Lamiales Oleaceae Noronhia peglerae#MH817921
Lamiales Oleaceae Noronhia intermedia#MH817876
Lamiales Oleaceae Noronhia clarinerva#MH817875
Lamiales Oleaceae Noronhia lowryi#MH817920
Lamiales Oleaceae Noronhia brevituba#MH817860
Lamiales Oleaceae Olea paniculata#MH817928
Lamiales Oleaceae Olea woodiana#NC 015608
Lamiales Oleaceae Olea exasperata#NC 036985
Lamiales Oleaceae Olea lancea#MH817878
Lamiales Oleaceae Olea capensis subsp. macrocarpa#MH817929
Lamiales Oleaceae Olea europaea subsp. cuspidata#MG255760
Lamiales Oleaceae Olea europaea#MG255761
Lamiales Oleaceae Olea europaea subsp. laperrinei#MG255765
Lamiales Oleaceae Olea europaea subsp. maroccana#NC 015623
Lamiales Oleaceae Olea europaea subsp. guanchica#MG255764
Lamiales Oleaceae Haenianthus salicifolius#MH817913
Lamiales Oleaceae Chionanthus pubescens#MH817892
Lamiales Oleaceae Chionanthus filiformis#MH817869
Lamiales Oleaceae Chionanthus implicatus#MH817885
Lamiales Oleaceae Chionanthus compactus#MH817868
Lamiales Oleaceae Osmanthus marginatus#MH817863
Lamiales Oleaceae Chionanthus virginicus#MH817900
Lamiales Oleaceae Chionanthus retusus#KTAR
Lamiales Oleaceae Chionanthus thorelii#MH817898
Lamiales Oleaceae Chionanthus rupicolus#NC 036980
Lamiales Oleaceae Chionanthus polygamus#MH817891
Lamiales Oleaceae Olea tsoongii#MH817930
Lamiales Oleaceae Chionanthus macrobotrys#MH817887
Lamiales Oleaceae Chionanthus ramiflorus#MH817894
Lamiales Oleaceae Chionanthus pedunculatus#MH817859
Lamiales Oleaceae Chionanthus maxwellii#MH817889
Lamiales Oleaceae Chionanthus brassii#MH817883
Lamiales Oleaceae Chionanthus quadristamineus#MH817893
Lamiales Oleaceae Chionanthus axillaris#MH817882
Lamiales Oleaceae Osmanthus delavayi#MH817931
Lamiales Oleaceae Osmanthus yunnanensis#MH817933
Lamiales Oleaceae Osmanthus fragrans#MH817932
Lamiales Oleaceae Osmanthus aff. armatus#MH817861
Lamiales Oleaceae Osmanthus insularis#MH817862
Lamiales Oleaceae Phillyrea latifolia#MH817936
Lamiales Oleaceae Picconia excelsa#MH817938
Lamiales Oleaceae Picconia azorica#MH817937
Lamiales Oleaceae Phillyrea angustifolia#MH817935
Lamiales Oleaceae Phillyrea aff. angustifolia#MH817934
Lamiales Oleaceae Nestegis sandwicensis#MH817918
Lamiales Oleaceae Nestegis lanceolata#MH817917
Lamiales Oleaceae Nestegis apetala#NC 036983
Lamiales Oleaceae Nestegis cunninghamii#MH817916
Lamiales Oleaceae Notelaea venosa#MH817924
Lamiales Oleaceae Notelaea microcarpa#MH817923
Lamiales Oleaceae Notelaea longifolia#MH817922
Lamiales Tetrachondraceae Polypremum procumbens#COBX
Lamiales Peltantheraceae Peltanthera floribunda#MO24638
Lamiales Calceolariaceae Calceolaria pinifolia#DCCI
Lamiales Calceolariaceae Calceolaria sp.#Y215
Lamiales Gesneriaceae Sinningia tuberosa#DTNC
Lamiales Gesneriaceae Rhynchoglossum obliquum#F68
Lamiales Gesneriaceae Saintpaulia ionantha#RWKR
Lamiales Gesneriaceae Streptocarpus teitensis#NC 037184
Lamiales Gesneriaceae Boea hygrometrica#NC 016468
Lamiales Gesneriaceae Oreocharis mileensis#F34
Lamiales Gesneriaceae Aeschynanthus superbus#F53
Lamiales Gesneriaceae Lysionotus pauciflorus#NC 034660
Lamiales Gesneriaceae Primulina brachytricha var. magnibracteata#MF177037
Lamiales Gesneriaceae Primulina liboensis#NC 036101
Lamiales Gesneriaceae Primulina huaijiensis#NC 036413
Lamiales Gesneriaceae Primulina linearifolia#NC 036414
Lamiales Gesneriaceae Primulina eburnea#MF472011
Lamiales Plantaginaceae Bacopa caroliniana#CLRW
Lamiales Plantaginaceae Bacopa monnieri#F740
Lamiales Plantaginaceae Scoparia dulcis#F694
Lamiales Plantaginaceae Adenosma glutinosum#F1039
Lamiales Plantaginaceae Trapella sinensis#F812
Lamiales Plantaginaceae Linaria vulgaris subsp. sinensis#F623
Lamiales Plantaginaceae Antirrhinum majus#GQ
Lamiales Plantaginaceae Antirrhinum braun-blanquetii#YRHD
Lamiales Plantaginaceae Callitriche stagnalis#F986
Lamiales Plantaginaceae Hippuris vulgaris#F827
Lamiales Plantaginaceae Digitalis purpurea#F456
Lamiales Plantaginaceae Digitalis lanata#NC 034688
Lamiales Plantaginaceae Plantago lagopus#NC 041420
Lamiales Plantaginaceae Plantago ovata#NC 041421
Lamiales Plantaginaceae Plantago maritima#NC 028519
Lamiales Plantaginaceae Plantago coronopus#DCVZ
Lamiales Plantaginaceae Plantago media#NC 028520
Lamiales Plantaginaceae Plantago depressa#NC 041161
Lamiales Plantaginaceae Plantago virginica#PTBJ
Lamiales Scrophulariaceae Pentacoelium bontioides#F521
Lamiales Scrophulariaceae Anticharis glandulosa#EJBY
Lamiales Scrophulariaceae Buddleja macrostachya#F122
Lamiales Scrophulariaceae Buddleja alternifolia#F794
Lamiales Scrophulariaceae Buddleja officinalis#F460
Lamiales Scrophulariaceae Buddleja sp.#GRFT
Lamiales Scrophulariaceae Buddleja lindleyana#XRLM
Lamiales Scrophulariaceae Verbascum thapsus#F829
Lamiales Scrophulariaceae Verbascum sp.#XXYA
Lamiales Scrophulariaceae Verbascum arcturus#SIBR
Lamiales Scrophulariaceae Scrophularia dentata#NC 036942
Lamiales Scrophulariaceae Scrophularia takesimensis#NC 026202
Lamiales Scrophulariaceae Scrophularia elatior#F880
Lamiales Scrophulariaceae Scrophularia ningpoensis#F866

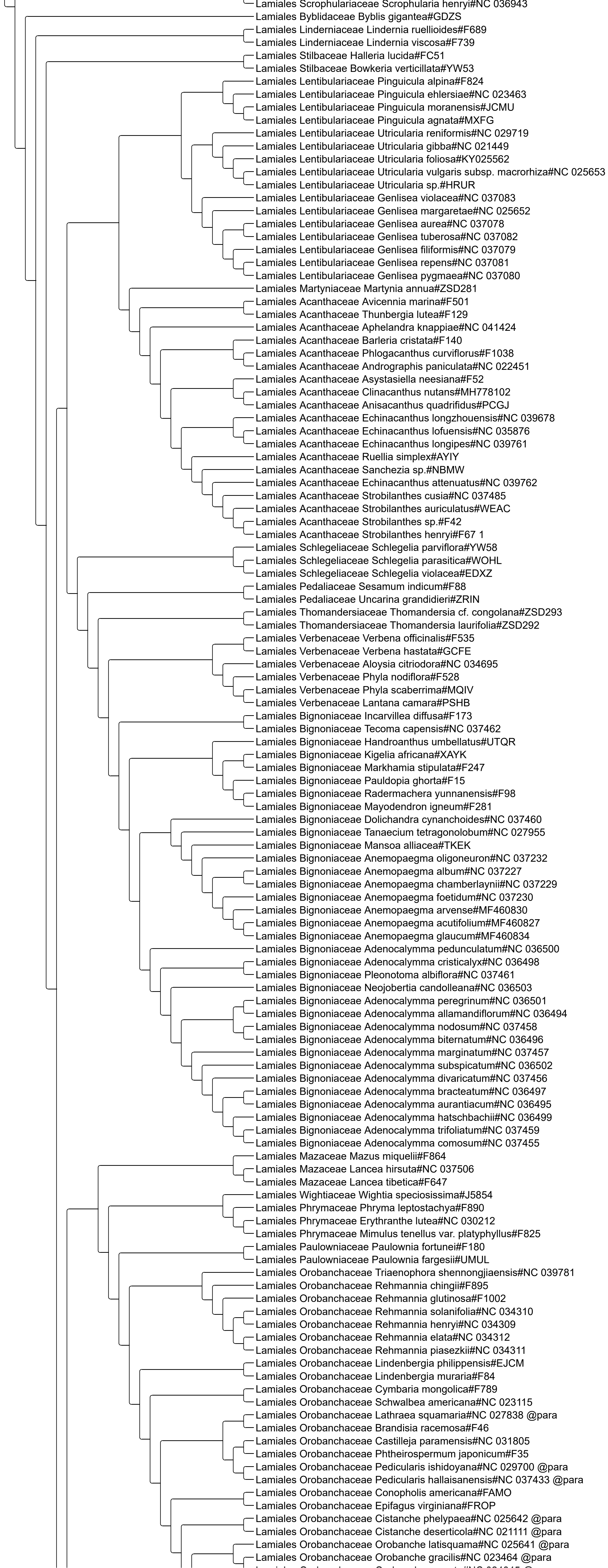

Lamiales Scrophulariaceae Scrophularia henryi#NC 036943
Lamiales Byblidaceae Byblis gigantea#GDZS
Lamiales Linderniaceae Lindernia ruellioides#F689
Lamiales Linderniaceae Lindernia viscosa#F739
Lamiales Stilbaceae Halleria lucida#FC51
Lamiales Stilbaceae Bowkeria verticillata#YW53
Lamiales Lentibulariaceae Pinguicula alpina#F824
Lamiales Lentibulariaceae Pinguicula ehlersiae#NC 023463
Lamiales Lentibulariaceae Pinguicula moranensis#JCMU
Lamiales Lentibulariaceae Pinguicula agnata#MXFG
Lamiales Lentibulariaceae Utricularia reniformis#NC 029719
Lamiales Lentibulariaceae Utricularia gibba#NC 021449
Lamiales Lentibulariaceae Utricularia foliosa#KY025562
Lamiales Lentibulariaceae Utricularia vulgaris subsp. macrorhiza#NC 025653
Lamiales Lentibulariaceae Utricularia sp.#HRUR
Lamiales Lentibulariaceae Genlisea violacea#NC 037083
Lamiales Lentibulariaceae Genlisea margaretae#NC 025652
Lamiales Lentibulariaceae Genlisea aurea#NC 037078
Lamiales Lentibulariaceae Genlisea tuberosa#NC 037082
Lamiales Lentibulariaceae Genlisea filiformis#NC 037079
Lamiales Lentibulariaceae Genlisea repens#NC 037081
Lamiales Lentibulariaceae Genlisea pygmaea#NC 037080
Lamiales Martyniaceae Martynia annua#ZSD281
Lamiales Acanthaceae Avicennia marina#F501
Lamiales Acanthaceae Thunbergia lutea#F129
Lamiales Acanthaceae Aphelandra knappiae#NC 041424
Lamiales Acanthaceae Barleria cristata#F140
Lamiales Acanthaceae Phlogacanthus curviflorus#F1038
Lamiales Acanthaceae Andrographis paniculata#NC 022451
Lamiales Acanthaceae Asystasiella neesiana#F52
Lamiales Acanthaceae Clinacanthus nutans#MH778102
Lamiales Acanthaceae Anisacanthus quadrifidus#PCGJ
Lamiales Acanthaceae Echinacanthus longzhouensis#NC 039678
Lamiales Acanthaceae Echinacanthus lofuensis#NC 035876
Lamiales Acanthaceae Echinacanthus longipes#NC 039761
Lamiales Acanthaceae Ruellia simplex#AYIY
Lamiales Acanthaceae Sanchezia sp.#NBMW
Lamiales Acanthaceae Echinacanthus attenuatus#NC 039762
Lamiales Acanthaceae Strobilanthes cusia#NC 037485
Lamiales Acanthaceae Strobilanthes auriculatus#WEAC
Lamiales Acanthaceae Strobilanthes sp.#F42
Lamiales Acanthaceae Strobilanthes henryi#F67 1
Lamiales Schlegeliaceae Schlegelia parviflora#YW58
Lamiales Schlegeliaceae Schlegelia parasitica#WOHL
Lamiales Schlegeliaceae Schlegelia violacea#EDXZ
Lamiales Pedaliaceae Sesamum indicum#F88
Lamiales Pedaliaceae Uncarina grandidieri#ZRIN
Lamiales Thomandersiaceae Thomandersia cf. congolana#ZSD293
Lamiales Thomandersiaceae Thomandersia laurifolia#ZSD292
Lamiales Verbenaceae Verbena officinalis#F535
Lamiales Verbenaceae Verbena hastata#GCFE
Lamiales Verbenaceae Aloysia citriodora#NC 034695
Lamiales Verbenaceae Phyla nodiflora#F528
Lamiales Verbenaceae Phyla scaberrima#MQIV
Lamiales Verbenaceae Lantana camara#PSHB
Lamiales Bignoniaceae Incarvillea diffusa#F173
Lamiales Bignoniaceae Tecoma capensis#NC 037462
Lamiales Bignoniaceae Handroanthus umbellatus#UTQR
Lamiales Bignoniaceae Kigelia africana#XAYK
Lamiales Bignoniaceae Markhamia stipulata#F247
Lamiales Bignoniaceae Pauldopia ghorta#F15
Lamiales Bignoniaceae Radermachera yunnanensis#F98
Lamiales Bignoniaceae Mayodendron igneum#F281
Lamiales Bignoniaceae Dolichandra cynanchoides#NC 037460
Lamiales Bignoniaceae Tanaecium tetragonolobum#NC 027955
Lamiales Bignoniaceae Mansoa alliacea#TKEK
Lamiales Bignoniaceae Anemopaegma oligoneuron#NC 037232
Lamiales Bignoniaceae Anemopaegma album#NC 037227
Lamiales Bignoniaceae Anemopaegma chamberlaynii#NC 037229
Lamiales Bignoniaceae Anemopaegma foetidum#NC 037230
Lamiales Bignoniaceae Anemopaegma arvense#MF460830
Lamiales Bignoniaceae Anemopaegma acutifolium#MF460827
Lamiales Bignoniaceae Anemopaegma glaucum#MF460834
Lamiales Bignoniaceae Adenocalymma pedunculatum#NC 036500
Lamiales Bignoniaceae Adenocalymma cristicalyx#NC 036498
Lamiales Bignoniaceae Pleonotoma albiflora#NC 037461
Lamiales Bignoniaceae Neojobertia candolleana#NC 036503
Lamiales Bignoniaceae Adenocalymma peregrinum#NC 036501
Lamiales Bignoniaceae Adenocalymma allamandiflorum#NC 036494
Lamiales Bignoniaceae Adenocalymma nodosum#NC 037458
Lamiales Bignoniaceae Adenocalymma biternatum#NC 036496
Lamiales Bignoniaceae Adenocalymma marginatum#NC 037457
Lamiales Bignoniaceae Adenocalymma subspicatum#NC 036502
Lamiales Bignoniaceae Adenocalymma divaricatum#NC 037456
Lamiales Bignoniaceae Adenocalymma bracteatum#NC 036497
Lamiales Bignoniaceae Adenocalymma aurantiacum#NC 036495
Lamiales Bignoniaceae Adenocalymma hatschbachii#NC 036499
Lamiales Bignoniaceae Adenocalymma trifoliatum#NC 037459
Lamiales Bignoniaceae Adenocalymma comosum#NC 037455
Lamiales Mazaceae Mazus miquelii#F864
Lamiales Mazaceae Lancea hirsuta#NC 037506
Lamiales Mazaceae Lancea tibetica#F647
Lamiales Wightiaceae Wightia speciosissima#J5854
Lamiales Phrymaceae Phryma leptostachya#F890
Lamiales Phrymaceae Erythranthe lutea#NC 030212
Lamiales Phrymaceae Mimulus tenellus var. platyphyllus#F825
Lamiales Paulowniaceae Paulownia fortunei#F180
Lamiales Paulowniaceae Paulownia fargesii#UMUL
Lamiales Orobanchaceae Triaenophora shennongjiaensis#NC 039781
Lamiales Orobanchaceae Rehmannia chingii#F895
Lamiales Orobanchaceae Rehmannia glutinosa#F1002
Lamiales Orobanchaceae Rehmannia solanifolia#NC 034310
Lamiales Orobanchaceae Rehmannia henryi#NC 034309
Lamiales Orobanchaceae Rehmannia elata#NC 034312
Lamiales Orobanchaceae Rehmannia piasezkii#NC 034311
Lamiales Orobanchaceae Lindenbergia philippensis#EJCM
Lamiales Orobanchaceae Lindenbergia muraria#F84
Lamiales Orobanchaceae Cymbaria mongolica#F789
Lamiales Orobanchaceae Schwalbea americana#NC 023115
Lamiales Orobanchaceae Lathraea squamaria#NC 027838 @para
Lamiales Orobanchaceae Brandisia racemosa#F46
Lamiales Orobanchaceae Castilleja paramensis#NC 031805
Lamiales Orobanchaceae Phtheirospermum japonicum#F35
Lamiales Orobanchaceae Pedicularis ishidoyana#NC 029700 @para
Lamiales Orobanchaceae Pedicularis hallaisanensis#NC 037433 @para
Lamiales Orobanchaceae Conopholis americana#FAMO
Lamiales Orobanchaceae Epifagus virginiana#FROP
Lamiales Orobanchaceae Cistanche phelypaea#NC 025642 @para
Lamiales Orobanchaceae Cistanche deserticola#NC 021111 @para
Lamiales Orobanchaceae Orobanche latisquama#NC 025641 @para
Lamiales Orobanchaceae Orobanche gracilis#NC 023464 @para

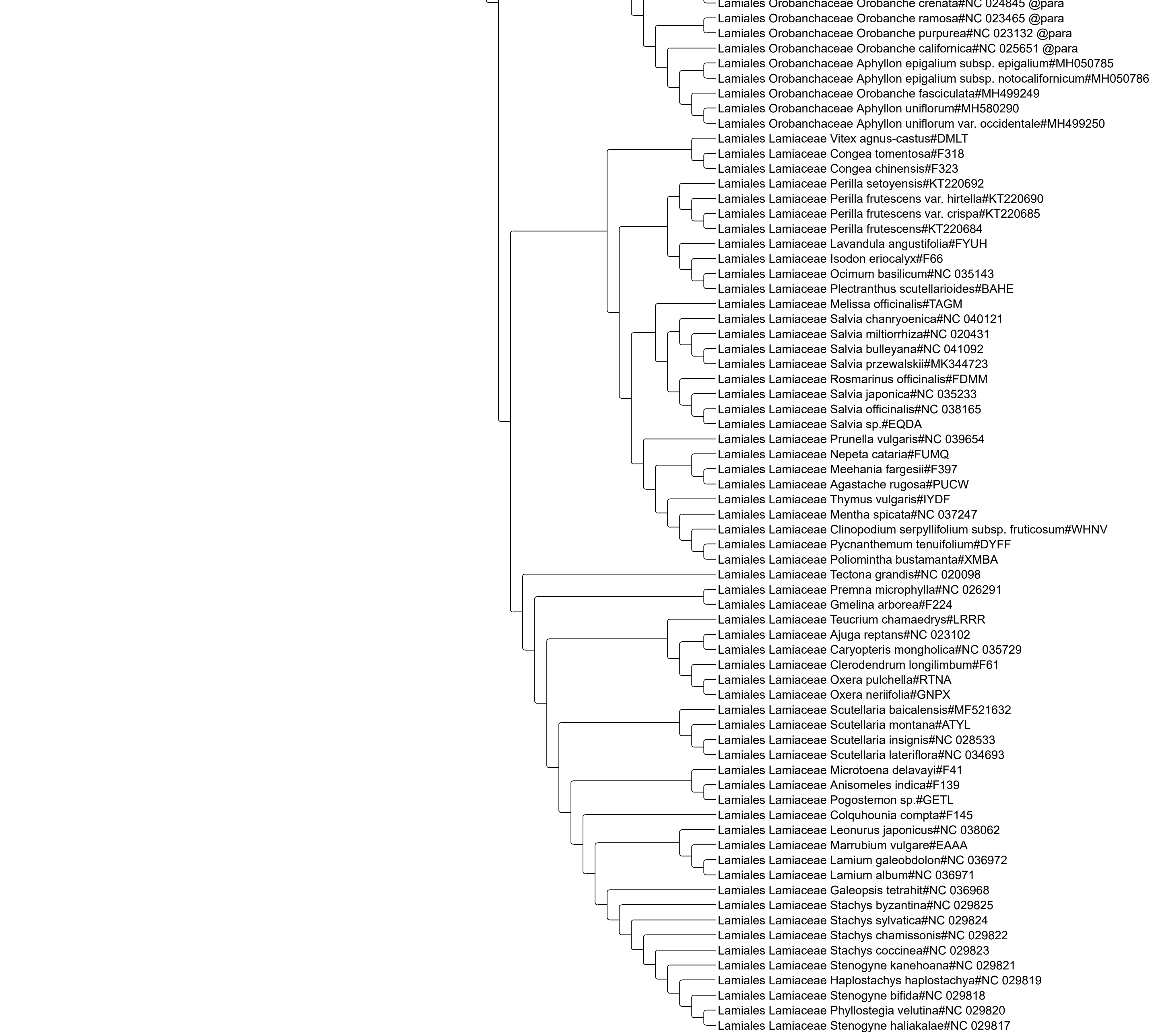
Lamiales Orobanchaceae Orobanche crenata#NC 024845 @para
Lamiales Orobanchaceae Orobanche ramosa#NC 023465 @para
Lamiales Orobanchaceae Orobanche purpurea#NC 023132 @para
Lamiales Orobanchaceae Orobanche californica#NC 025651 @para
Lamiales Orobanchaceae Aphyllon epigalium subsp. epigalium#MH050785
Lamiales Orobanchaceae Aphyllon epigalium subsp. notocalifornicum#MH050786
Lamiales Orobanchaceae Orobanche fasciculata#MH499249
Lamiales Orobanchaceae Aphyllon uniflorum#MH580290
Lamiales Orobanchaceae Aphyllon uniflorum var. occidentale#MH499250
Lamiales Lamiaceae Vitex agnus-castus#DMLT
Lamiales Lamiaceae Congea tomentosa#F318
Lamiales Lamiaceae Congea chinensis#F323
Lamiales Lamiaceae Perilla setoyensis#KT220692
Lamiales Lamiaceae Perilla frutescens var. hirtella#KT220690
Lamiales Lamiaceae Perilla frutescens var. crispa#KT220685
Lamiales Lamiaceae Perilla frutescens#KT220684
Lamiales Lamiaceae Lavandula angustifolia#FYUH
Lamiales Lamiaceae Isodon eriocalyx#F66
Lamiales Lamiaceae Ocimum basilicum#NC 035143
Lamiales Lamiaceae Plectranthus scutellarioides#BAHE
Lamiales Lamiaceae Melissa officinalis#TAGM
Lamiales Lamiaceae Salvia chanryoenica#NC 040121
Lamiales Lamiaceae Salvia miltiorrhiza#NC 020431
Lamiales Lamiaceae Salvia bulleyana#NC 041092
Lamiales Lamiaceae Salvia przewalskii#MK344723
Lamiales Lamiaceae Rosmarinus officinalis#FDMM
Lamiales Lamiaceae Salvia japonica#NC 035233
Lamiales Lamiaceae Salvia officinalis#NC 038165
Lamiales Lamiaceae Salvia sp.#EQDA
Lamiales Lamiaceae Prunella vulgaris#NC 039654
Lamiales Lamiaceae Nepeta cataria#FUMQ
Lamiales Lamiaceae Meehania fargesii#F397
Lamiales Lamiaceae Agastache rugosa#PUCW
Lamiales Lamiaceae Thymus vulgaris#IYDF
Lamiales Lamiaceae Mentha spicata#NC 037247
Lamiales Lamiaceae Clinopodium serpyllifolium subsp. fruticosum#WHNV
Lamiales Lamiaceae Pycnanthemum tenuifolium#DYFF
Lamiales Lamiaceae Poliomintha bustamanta#XMBA
Lamiales Lamiaceae Tectona grandis#NC 020098
Lamiales Lamiaceae Premna microphylla#NC 026291
Lamiales Lamiaceae Gmelina arborea#F224
Lamiales Lamiaceae Teucrium chamaedrys#LRRR
Lamiales Lamiaceae Ajuga reptans#NC 023102
Lamiales Lamiaceae Caryopteris mongholica#NC 035729
Lamiales Lamiaceae Clerodendrum longilimbum#F61
Lamiales Lamiaceae Oxera pulchella#RTNA
Lamiales Lamiaceae Oxera neriifolia#GNPX
Lamiales Lamiaceae Scutellaria baicalensis#MF521632
Lamiales Lamiaceae Scutellaria montana#ATYL
Lamiales Lamiaceae Scutellaria insignis#NC 028533
Lamiales Lamiaceae Scutellaria lateriflora#NC 034693
Lamiales Lamiaceae Microtoena delavayi#F41
Lamiales Lamiaceae Anisomeles indica#F139
Lamiales Lamiaceae Pogostemon sp.#GETL
Lamiales Lamiaceae Colquhounia compta#F145
Lamiales Lamiaceae Leonurus japonicus#NC 038062
Lamiales Lamiaceae Marrubium vulgare#EAAA
Lamiales Lamiaceae Lamium galeobdolon#NC 036972
Lamiales Lamiaceae Lamium album#NC 036971
Lamiales Lamiaceae Galeopsis tetrahit#NC 036968
Lamiales Lamiaceae Stachys byzantina#NC 029825
Lamiales Lamiaceae Stachys sylvatica#NC 029824
Lamiales Lamiaceae Stachys chamissonis#NC 029822
Lamiales Lamiaceae Stachys coccinea#NC 029823
Lamiales Lamiaceae Stenogyne kanehoana#NC 029821
Lamiales Lamiaceae Haplostachys haplostachya#NC 029819
Lamiales Lamiaceae Stenogyne bifida#NC 029818
Lamiales Lamiaceae Phyllostegia velutina#NC 029820
Lamiales Lamiaceae Stenogyne haliakalae#NC 029817

[Figure S8b]

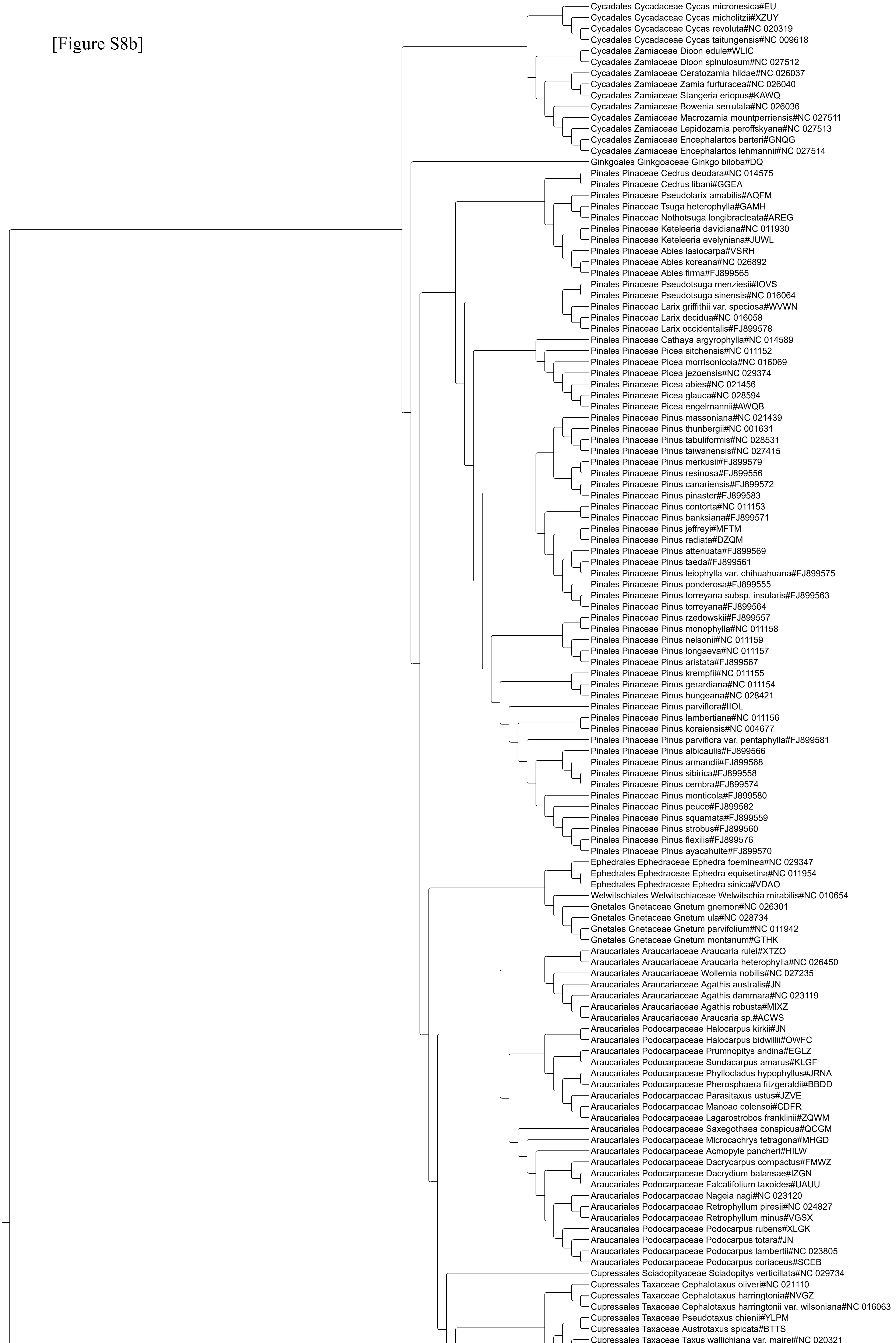

Cycadales Cycadaceae Cycas micronesica#EU
Cycadales Cycadaceae Cycas micholitzii#XZUY
Cycadales Cycadaceae Cycas revoluta#NC 020319
Cycadales Cycadaceae Cycas taitungensis#NC 009618
Cycadales Zamiaceae Dioon edule#WLIC
Cycadales Zamiaceae Dioon spinulosum#NC 027512
Cycadales Zamiaceae Ceratozamia hildae#NC 026037
Cycadales Zamiaceae Zamia furfuracea#NC 026040
Cycadales Zamiaceae Stangeria eriopus#KAWQ
Cycadales Zamiaceae Bowenia serrulata#NC 026036
Cycadales Zamiaceae Macrozamia mountperriensis#NC 027511
Cycadales Zamiaceae Lepidozamia peroffskyana#NC 027513
Cycadales Zamiaceae Encephalartos barteri#GNQG
Cycadales Zamiaceae Encephalartos lehmannii#NC 027514
Ginkgoales Ginkgoaceae Ginkgo biloba#DQ
Pinales Pinaceae Cedrus deodara#NC 014575
Pinales Pinaceae Cedrus libani#GGEA
Pinales Pinaceae Pseudolarix amabilis#AQFM
Pinales Pinaceae Tsuga heterophylla#GAMH
Pinales Pinaceae Nothotsuga longibracteata#AREG
Pinales Pinaceae Keteleeria davidiana#NC 011930
Pinales Pinaceae Keteleeria evelyniana#JUWL
Pinales Pinaceae Abies lasiocarpa#VSRH
Pinales Pinaceae Abies koreana#NC 026892
Pinales Pinaceae Abies firma#FJ899565
Pinales Pinaceae Pseudotsuga menziesii#IOVS
Pinales Pinaceae Pseudotsuga sinensis#NC 016064
Pinales Pinaceae Larix griffithii var. speciosa#WVWN
Pinales Pinaceae Larix decidua#NC 016058
Pinales Pinaceae Larix occidentalis#FJ899578
Pinales Pinaceae Cathaya argyrophylla#NC 014589
Pinales Pinaceae Picea sitchensis#NC 011152
Pinales Pinaceae Picea morrisonicola#NC 016069
Pinales Pinaceae Picea jezoensis#NC 029374
Pinales Pinaceae Picea abies#NC 021456
Pinales Pinaceae Picea glauca#NC 028594
Pinales Pinaceae Picea engelmannii#AWQB
Pinales Pinaceae Pinus massoniana#NC 021439
Pinales Pinaceae Pinus thunbergii#NC 001631
Pinales Pinaceae Pinus tabuliformis#NC 028531
Pinales Pinaceae Pinus taiwanensis#NC 027415
Pinales Pinaceae Pinus merkusii#FJ899579
Pinales Pinaceae Pinus resinosa#FJ899556
Pinales Pinaceae Pinus canariensis#FJ899572
Pinales Pinaceae Pinus pinaster#FJ899583
Pinales Pinaceae Pinus contorta#NC 011153
Pinales Pinaceae Pinus banksiana#FJ899571
Pinales Pinaceae Pinus jeffreyi#MFTM
Pinales Pinaceae Pinus radiata#DZQM
Pinales Pinaceae Pinus attenuata#FJ899569
Pinales Pinaceae Pinus taeda#FJ899561
Pinales Pinaceae Pinus leiophylla var. chihuahuana#FJ899575
Pinales Pinaceae Pinus ponderosa#FJ899555
Pinales Pinaceae Pinus torreyana subsp. insularis#FJ899563
Pinales Pinaceae Pinus torreyana#FJ899564
Pinales Pinaceae Pinus rzedowskii#FJ899557
Pinales Pinaceae Pinus monophylla#NC 011158
Pinales Pinaceae Pinus nelsonii#NC 011159
Pinales Pinaceae Pinus longaeva#NC 011157
Pinales Pinaceae Pinus aristata#FJ899567
Pinales Pinaceae Pinus krempfii#NC 011155
Pinales Pinaceae Pinus gerardiana#NC 011154
Pinales Pinaceae Pinus bungeana#NC 028421
Pinales Pinaceae Pinus parviflora#IIOL
Pinales Pinaceae Pinus lambertiana#NC 011156
Pinales Pinaceae Pinus koraiensis#NC 004677
Pinales Pinaceae Pinus parviflora var. pentaphylla#FJ899581
Pinales Pinaceae Pinus albicaulis#FJ899566
Pinales Pinaceae Pinus armandii#FJ899568
Pinales Pinaceae Pinus sibirica#FJ899558
Pinales Pinaceae Pinus cembra#FJ899574
Pinales Pinaceae Pinus monticola#FJ899580
Pinales Pinaceae Pinus peuce#FJ899582
Pinales Pinaceae Pinus squamata#FJ899559
Pinales Pinaceae Pinus strobus#FJ899560
Pinales Pinaceae Pinus flexilis#FJ899576
Pinales Pinaceae Pinus ayacahuite#FJ899570
Ephedrales Ephedraceae Ephedra foeminea#NC 029347
Ephedrales Ephedraceae Ephedra equisetina#NC 011954
Ephedrales Ephedraceae Ephedra sinica#VDAO
Welwitschiales Welwitschiaceae Welwitschia mirabilis#NC 010654
Gnetales Gnetaceae Gnetum gnemon#NC 026301
Gnetales Gnetaceae Gnetum ula#NC 028734
Gnetales Gnetaceae Gnetum parvifolium#NC 011942
Gnetales Gnetaceae Gnetum montanum#GTHK
Araucariales Araucariaceae Araucaria rulei#XTZO
Araucariales Araucariaceae Araucaria heterophylla#NC 026450
Araucariales Araucariaceae Wollemia nobilis#NC 027235
Araucariales Araucariaceae Agathis australis#JN
Araucariales Araucariaceae Agathis dammara#NC 023119
Araucariales Araucariaceae Agathis robusta#MIXZ
Araucariales Araucariaceae Araucaria sp.#ACWS
Araucariales Podocarpaceae Halocarpus kirkii#JN
Araucariales Podocarpaceae Halocarpus bidwillii#OWFC
Araucariales Podocarpaceae Prumnopitys andina#EGLZ
Araucariales Podocarpaceae Sundacarpus amarus#KLGF
Araucariales Podocarpaceae Phyllocladus hypophyllus#JRNA
Araucariales Podocarpaceae Pherosphaera fitzgeraldii#BBDD
Araucariales Podocarpaceae Parasitaxus ustus#JZVE
Araucariales Podocarpaceae Manoao colensoi#CDFR
Araucariales Podocarpaceae Lagarostrobos franklinii#ZQWM
Araucariales Podocarpaceae Saxegothaea conspicua#QCGM
Araucariales Podocarpaceae Microcachrys tetragona#MHGD
Araucariales Podocarpaceae Acmopyle pancheri#HILW
Araucariales Podocarpaceae Dacrycarpus compactus#FMWZ
Araucariales Podocarpaceae Dacrydium balansae#IZGN
Araucariales Podocarpaceae Falcatifolium taxoides#UAUU
Araucariales Podocarpaceae Nageia nagi#NC 023120
Araucariales Podocarpaceae Retrophyllum piresii#NC 024827
Araucariales Podocarpaceae Retrophyllum minus#VGSX
Araucariales Podocarpaceae Podocarpus rubens#XLGK
Araucariales Podocarpaceae Podocarpus totara#JN
Araucariales Podocarpaceae Podocarpus lambertii#NC 023805
Araucariales Podocarpaceae Podocarpus coriaceus#SCEB
Cupressales Sciadopityaceae Sciadopitys verticillata#NC 029734
Cupressales Taxaceae Cephalotaxus oliveri#NC 021110
Cupressales Taxaceae Cephalotaxus harringtonia#NVGZ
Cupressales Taxaceae Cephalotaxus harringtonii var. wilsoniana#NC 016063
Cupressales Taxaceae Pseudotaxus chienii#YLPM
Cupressales Taxaceae Austrotaxus spicata#BTTS
Cupressales Taxaceae Taxus wallichiana var. mairei#NC 020321

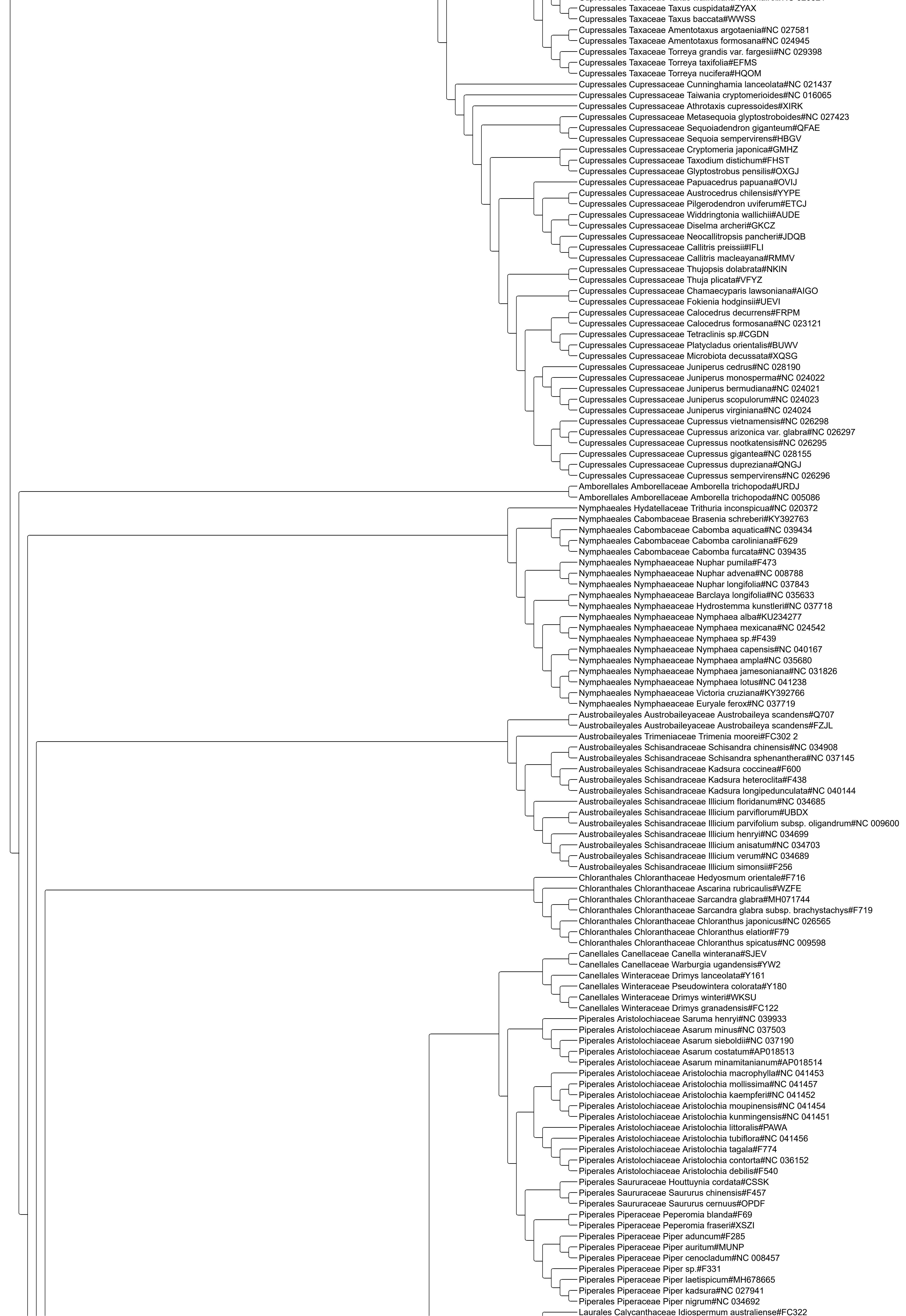

Cupressales Taxaceae Taxus cuspidata#ZYAX
Cupressales Taxaceae Taxus baccata#WWSS
Cupressales Taxaceae Amentotaxus argotaenia#NC 027581
Cupressales Taxaceae Amentotaxus formosana#NC 024945
Cupressales Taxaceae Torreya grandis var. fargesii#NC 029398
Cupressales Taxaceae Torreya taxifolia#EFMS
Cupressales Taxaceae Torreya nucifera#HQOM
Cupressales Cupressaceae Cunninghamia lanceolata#NC 021437
Cupressales Cupressaceae Taiwania cryptomerioides#NC 016065
Cupressales Cupressaceae Athrotaxis cupressoides#XIRK
Cupressales Cupressaceae Metasequoia glyptostroboides#NC 027423
Cupressales Cupressaceae Sequoiadendron giganteum#QFAE
Cupressales Cupressaceae Sequoia sempervirens#HBGV
Cupressales Cupressaceae Cryptomeria japonica#GMHZ
Cupressales Cupressaceae Taxodium distichum#FHST
Cupressales Cupressaceae Glyptostrobus pensilis#OXGJ
Cupressales Cupressaceae Papuacedrus papuana#OVIJ
Cupressales Cupressaceae Austrocedrus chilensis#YYPE
Cupressales Cupressaceae Pilgerodendron uviferum#ETCJ
Cupressales Cupressaceae Widdringtonia wallichii#AUDE
Cupressales Cupressaceae Diselma archeri#GKCZ
Cupressales Cupressaceae Neocallitropsis pancheri#JDQB
Cupressales Cupressaceae Callitris preissii#IFLI
Cupressales Cupressaceae Callitris macleayana#RMMV
Cupressales Cupressaceae Thujopsis dolabrata#NKIN
Cupressales Cupressaceae Thuja plicata#VFYZ
Cupressales Cupressaceae Chamaecyparis lawsoniana#AIGO
Cupressales Cupressaceae Fokienia hodginsii#UEVI
Cupressales Cupressaceae Calocedrus decurrens#FRPM
Cupressales Cupressaceae Calocedrus formosana#NC 023121
Cupressales Cupressaceae Tetraclinis sp.#CGDN
Cupressales Cupressaceae Platycladus orientalis#BUWV
Cupressales Cupressaceae Microbiota decussata#XQSG
Cupressales Cupressaceae Juniperus cedrus#NC 028190
Cupressales Cupressaceae Juniperus monosperma#NC 024022
Cupressales Cupressaceae Juniperus bermudiana#NC 024021
Cupressales Cupressaceae Juniperus scopulorum#NC 024023
Cupressales Cupressaceae Juniperus virginiana#NC 024024
Cupressales Cupressaceae Cupressus vietnamensis#NC 026298
Cupressales Cupressaceae Cupressus arizonica var. glabra#NC 026297
Cupressales Cupressaceae Cupressus nootkatensis#NC 026295
Cupressales Cupressaceae Cupressus gigantea#NC 028155
Cupressales Cupressaceae Cupressus dupreziana#QNGJ
Cupressales Cupressaceae Cupressus sempervirens#NC 026296
Amborellales Amborellaceae Amborella trichopoda#URDJ
Amborellales Amborellaceae Amborella trichopoda#NC 005086
Nymphaeales Hydatellaceae Trithuria inconspicua#NC 020372
Nymphaeales Cabombaceae Brasenia schreberi#KY392763
Nymphaeales Cabombaceae Cabomba aquatica#NC 039434
Nymphaeales Cabombaceae Cabomba caroliniana#F629
Nymphaeales Cabombaceae Cabomba furcata#NC 039435
Nymphaeales Nymphaeaceae Nuphar pumila#F473
Nymphaeales Nymphaeaceae Nuphar advena#NC 008788
Nymphaeales Nymphaeaceae Nuphar longifolia#NC 037843
Nymphaeales Nymphaeaceae Barclaya longifolia#NC 035633
Nymphaeales Nymphaeaceae Hydrostemma kunstleri#NC 037718
Nymphaeales Nymphaeaceae Nymphaea alba#KU234277
Nymphaeales Nymphaeaceae Nymphaea mexicana#NC 024542
Nymphaeales Nymphaeaceae Nymphaea sp.#F439
Nymphaeales Nymphaeaceae Nymphaea capensis#NC 040167
Nymphaeales Nymphaeaceae Nymphaea ampla#NC 035680
Nymphaeales Nymphaeaceae Nymphaea jamesoniana#NC 031826
Nymphaeales Nymphaeaceae Nymphaea lotus#NC 041238
Nymphaeales Nymphaeaceae Victoria cruziana#KY392766
Nymphaeales Nymphaeaceae Euryale ferox#NC 037719
Austrobaileyales Austrobaileyaceae Austrobaileya scandens#Q707
Austrobaileyales Austrobaileyaceae Austrobaileya scandens#FZJL
Austrobaileyales Trimeniaceae Trimenia moorei#FC302 2
Austrobaileyales Schisandraceae Schisandra chinensis#NC 034908
Austrobaileyales Schisandraceae Schisandra sphenanthera#NC 037145
Austrobaileyales Schisandraceae Kadsura coccinea#F600
Austrobaileyales Schisandraceae Kadsura heteroclita#F438
Austrobaileyales Schisandraceae Kadsura longipedunculata#NC 040144
Austrobaileyales Schisandraceae Illicium floridanum#NC 034685
Austrobaileyales Schisandraceae Illicium parviflorum#UBDX
Austrobaileyales Schisandraceae Illicium parvifolium subsp. oligandrum#NC 009600
Austrobaileyales Schisandraceae Illicium henryi#NC 034699
Austrobaileyales Schisandraceae Illicium anisatum#NC 034703
Austrobaileyales Schisandraceae Illicium verum#NC 034689
Austrobaileyales Schisandraceae Illicium simonsii#F256
Chloranthales Chloranthaceae Hedyosmum orientale#F716
Chloranthales Chloranthaceae Ascarina rubricaulis#WZFE
Chloranthales Chloranthaceae Sarcandra glabra#MH071744
Chloranthales Chloranthaceae Sarcandra glabra subsp. brachystachys#F719
Chloranthales Chloranthaceae Chloranthus japonicus#NC 026565
Chloranthales Chloranthaceae Chloranthus elatior#F79
Chloranthales Chloranthaceae Chloranthus spicatus#NC 009598
Canellales Canellaceae Canella winterana#SJEV
Canellales Canellaceae Warburgia ugandensis#YW2
Canellales Winteraceae Drimys lanceolata#Y161
Canellales Winteraceae Pseudowintera colorata#Y180
Canellales Winteraceae Drimys winteri#WKSU
Canellales Winteraceae Drimys granadensis#FC122
Piperales Aristolochiaceae Saruma henryi#NC 039933
Piperales Aristolochiaceae Asarum minus#NC 037503
Piperales Aristolochiaceae Asarum sieboldii#NC 037190
Piperales Aristolochiaceae Asarum costatum#AP018513
Piperales Aristolochiaceae Asarum minamitanianum#AP018514
Piperales Aristolochiaceae Aristolochia macrophylla#NC 041453
Piperales Aristolochiaceae Aristolochia mollissima#NC 041457
Piperales Aristolochiaceae Aristolochia kaempferi#NC 041452
Piperales Aristolochiaceae Aristolochia moupinensis#NC 041454
Piperales Aristolochiaceae Aristolochia kunmingensis#NC 041451
Piperales Aristolochiaceae Aristolochia littoralis#PAWA
Piperales Aristolochiaceae Aristolochia tubiflora#NC 041456
Piperales Aristolochiaceae Aristolochia tagala#F774
Piperales Aristolochiaceae Aristolochia contorta#NC 036152
Piperales Aristolochiaceae Aristolochia debilis#F540
Piperales Saururaceae Houttuynia cordata#CSSK
Piperales Saururaceae Saururus chinensis#F457
Piperales Saururaceae Saururus cernuus#OPDF
Piperales Piperaceae Peperomia blanda#F69
Piperales Piperaceae Peperomia fraseri#XSZI
Piperales Piperaceae Piper aduncum#F285
Piperales Piperaceae Piper auritum#MUNP
Piperales Piperaceae Piper cenocladum#NC 008457
Piperales Piperaceae Piper sp.#F331
Piperales Piperaceae Piper laetispicum#MH678665
Piperales Piperaceae Piper kadsura#NC 027941
Piperales Piperaceae Piper nigrum#NC 034692
Laurales Calycanthaceae Idiospermum australiense#FC322

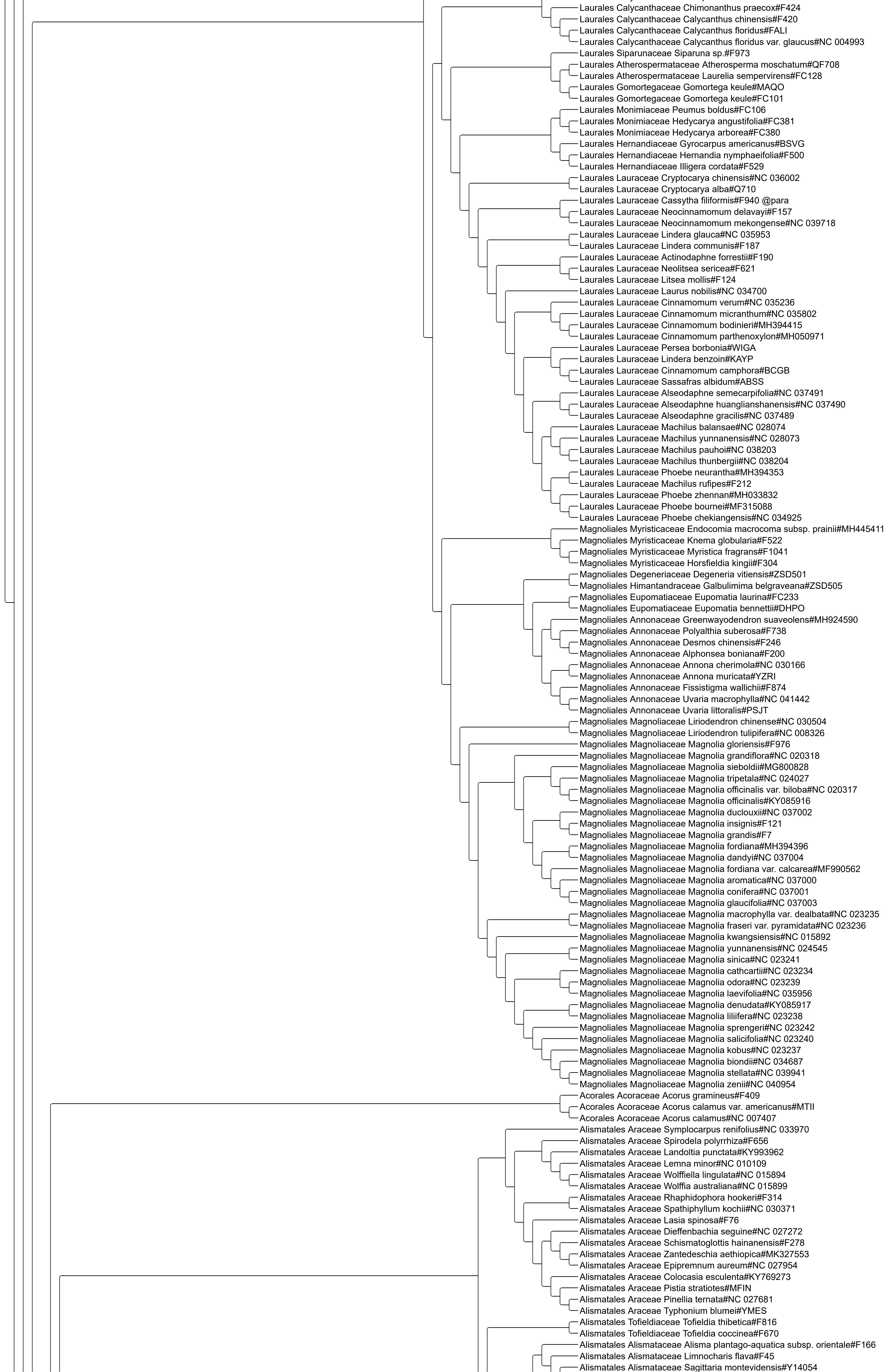
Laurales Calycanthaceae Chimonanthus praecox#F424
Laurales Calycanthaceae Calycanthus chinensis#F420
Laurales Calycanthaceae Calycanthus floridus#FALI
Laurales Calycanthaceae Calycanthus floridus var. glaucus#NC 004993
Laurales Siparunaceae Siparuna sp.#F973
Laurales Atherospermataceae Atherosperma moschatum#QF708
Laurales Atherospermataceae Laurelia sempervirens#FC128
Laurales Gomortegaceae Gomortega keule#MAQO
Laurales Gomortegaceae Gomortega keule#FC101
Laurales Monimiaceae Peumus boldus#FC106
Laurales Monimiaceae Hedycarya angustifolia#FC381
Laurales Monimiaceae Hedycarya arborea#FC380
Laurales Hernandiaceae Gyrocarpus americanus#BSVG
Laurales Hernandiaceae Hernandia nymphaeifolia#F500
Laurales Hernandiaceae Illigera cordata#F529
Laurales Lauraceae Cryptocarya chinensis#NC 036002
Laurales Lauraceae Cryptocarya alba#Q710
Laurales Lauraceae Cassytha filiformis#F940 @para
Laurales Lauraceae Neocinnamomum delavayi#F157
Laurales Lauraceae Neocinnamomum mekongense#NC 039718
Laurales Lauraceae Lindera glauca#NC 035953
Laurales Lauraceae Lindera communis#F187
Laurales Lauraceae Actinodaphne forrestii#F190
Laurales Lauraceae Neolitsea sericea#F621
Laurales Lauraceae Litsea mollis#F124
Laurales Lauraceae Laurus nobilis#NC 034700
Laurales Lauraceae Cinnamomum verum#NC 035236
Laurales Lauraceae Cinnamomum micranthum#NC 035802
Laurales Lauraceae Cinnamomum bodinieri#MH394415
Laurales Lauraceae Cinnamomum parthenoxylon#MH050971
Laurales Lauraceae Persea borbonia#WIGA
Laurales Lauraceae Lindera benzoin#KAYP
Laurales Lauraceae Cinnamomum camphora#BCGB
Laurales Lauraceae Sassafras albidum#ABSS
Laurales Lauraceae Alseodaphne semecarpifolia#NC 037491
Laurales Lauraceae Alseodaphne huanglianshanensis#NC 037490
Laurales Lauraceae Alseodaphne gracilis#NC 037489
Laurales Lauraceae Machilus balansae#NC 028074
Laurales Lauraceae Machilus yunnanensis#NC 028073
Laurales Lauraceae Machilus pauhoi#NC 038203
Laurales Lauraceae Machilus thunbergii#NC 038204
Laurales Lauraceae Phoebe neurantha#MH394353
Laurales Lauraceae Machilus rufipes#F212
Laurales Lauraceae Phoebe zhennan#MH033832
Laurales Lauraceae Phoebe bournei#MF315088
Laurales Lauraceae Phoebe chekiangensis#NC 034925
Magnoliales Myristicaceae Endocomia macrocoma subsp. prainii#MH445411
Magnoliales Myristicaceae Knema globularia#F522
Magnoliales Myristicaceae Myristica fragrans#F1041
Magnoliales Myristicaceae Horsfieldia kingii#F304
Magnoliales Degeneriaceae Degeneria vitiensis#ZSD501
Magnoliales Himantandraceae Galbulimima belgraveana#ZSD505
Magnoliales Eupomatiaceae Eupomatia laurina#FC233
Magnoliales Eupomatiaceae Eupomatia bennettii#DHPO
Magnoliales Annonaceae Greenwayodendron suaveolens#MH924590
Magnoliales Annonaceae Polyalthia suberosa#F738
Magnoliales Annonaceae Desmos chinensis#F246
Magnoliales Annonaceae Alphonsea boniana#F200
Magnoliales Annonaceae Annona cherimola#NC 030166
Magnoliales Annonaceae Annona muricata#YZRI
Magnoliales Annonaceae Fissistigma wallichii#F874
Magnoliales Annonaceae Uvaria macrophylla#NC 041442
Magnoliales Annonaceae Uvaria littoralis#PSJT
Magnoliales Magnoliaceae Liriodendron chinense#NC 030504
Magnoliales Magnoliaceae Liriodendron tulipifera#NC 008326
Magnoliales Magnoliaceae Magnolia gloriensis#F976
Magnoliales Magnoliaceae Magnolia grandiflora#NC 020318
Magnoliales Magnoliaceae Magnolia sieboldii#MG800828
Magnoliales Magnoliaceae Magnolia tripetala#NC 024027
Magnoliales Magnoliaceae Magnolia officinalis var. biloba#NC 020317
Magnoliales Magnoliaceae Magnolia officinalis#KY085916
Magnoliales Magnoliaceae Magnolia duclouxii#NC 037002
Magnoliales Magnoliaceae Magnolia insignis#F121
Magnoliales Magnoliaceae Magnolia grandis#F7
Magnoliales Magnoliaceae Magnolia fordiana#MH394396
Magnoliales Magnoliaceae Magnolia dandyi#NC 037004
Magnoliales Magnoliaceae Magnolia fordiana var. calcarea#MF990562
Magnoliales Magnoliaceae Magnolia aromatica#NC 037000
Magnoliales Magnoliaceae Magnolia conifera#NC 037001
Magnoliales Magnoliaceae Magnolia glaucifolia#NC 037003
Magnoliales Magnoliaceae Magnolia macrophylla var. dealbata#NC 023235
Magnoliales Magnoliaceae Magnolia fraseri var. pyramidata#NC 023236
Magnoliales Magnoliaceae Magnolia kwangsiensis#NC 015892
Magnoliales Magnoliaceae Magnolia yunnanensis#NC 024545
Magnoliales Magnoliaceae Magnolia sinica#NC 023241
Magnoliales Magnoliaceae Magnolia cathcartii#NC 023234
Magnoliales Magnoliaceae Magnolia odora#NC 023239
Magnoliales Magnoliaceae Magnolia laevifolia#NC 035956
Magnoliales Magnoliaceae Magnolia denudata#KY085917
Magnoliales Magnoliaceae Magnolia liliifera#NC 023238
Magnoliales Magnoliaceae Magnolia sprengeri#NC 023242
Magnoliales Magnoliaceae Magnolia salicifolia#NC 023240
Magnoliales Magnoliaceae Magnolia kobus#NC 023237
Magnoliales Magnoliaceae Magnolia biondii#NC 034687
Magnoliales Magnoliaceae Magnolia stellata#NC 039941
Magnoliales Magnoliaceae Magnolia zenii#NC 040954
Acorales Acoraceae Acorus gramineus#F409
Acorales Acoraceae Acorus calamus var. americanus#MTII
Acorales Acoraceae Acorus calamus#NC 007407
Alismatales Araceae Symplocarpus renifolius#NC 033970
Alismatales Araceae Spirodela polyrrhiza#F656
Alismatales Araceae Landoltia punctata#KY993962
Alismatales Araceae Lemna minor#NC 010109
Alismatales Araceae Wolffiella lingulata#NC 015894
Alismatales Araceae Wolffia australiana#NC 015899
Alismatales Araceae Rhaphidophora hookeri#F314
Alismatales Araceae Spathiphyllum kochii#NC 030371
Alismatales Araceae Lasia spinosa#F76
Alismatales Araceae Dieffenbachia seguine#NC 027272
Alismatales Araceae Schismatoglottis hainanensis#F278
Alismatales Araceae Zantedeschia aethiopica#MK327553
Alismatales Araceae Epipremnum aureum#NC 027954
Alismatales Araceae Colocasia esculenta#KY769273
Alismatales Araceae Pistia stratiotes#MFIN
Alismatales Araceae Pinellia ternata#NC 027681
Alismatales Araceae Typhonium blumei#YMES
Alismatales Tofieldiaceae Tofieldia thibetica#F816
Alismatales Tofieldiaceae Tofieldia coccinea#F670
Alismatales Alismataceae Alisma plantago-aquatica subsp. orientale#F166
Alismatales Alismataceae Limnocharis flava#F45
Alismatales Alismataceae Sagittaria montevidensis#Y14054

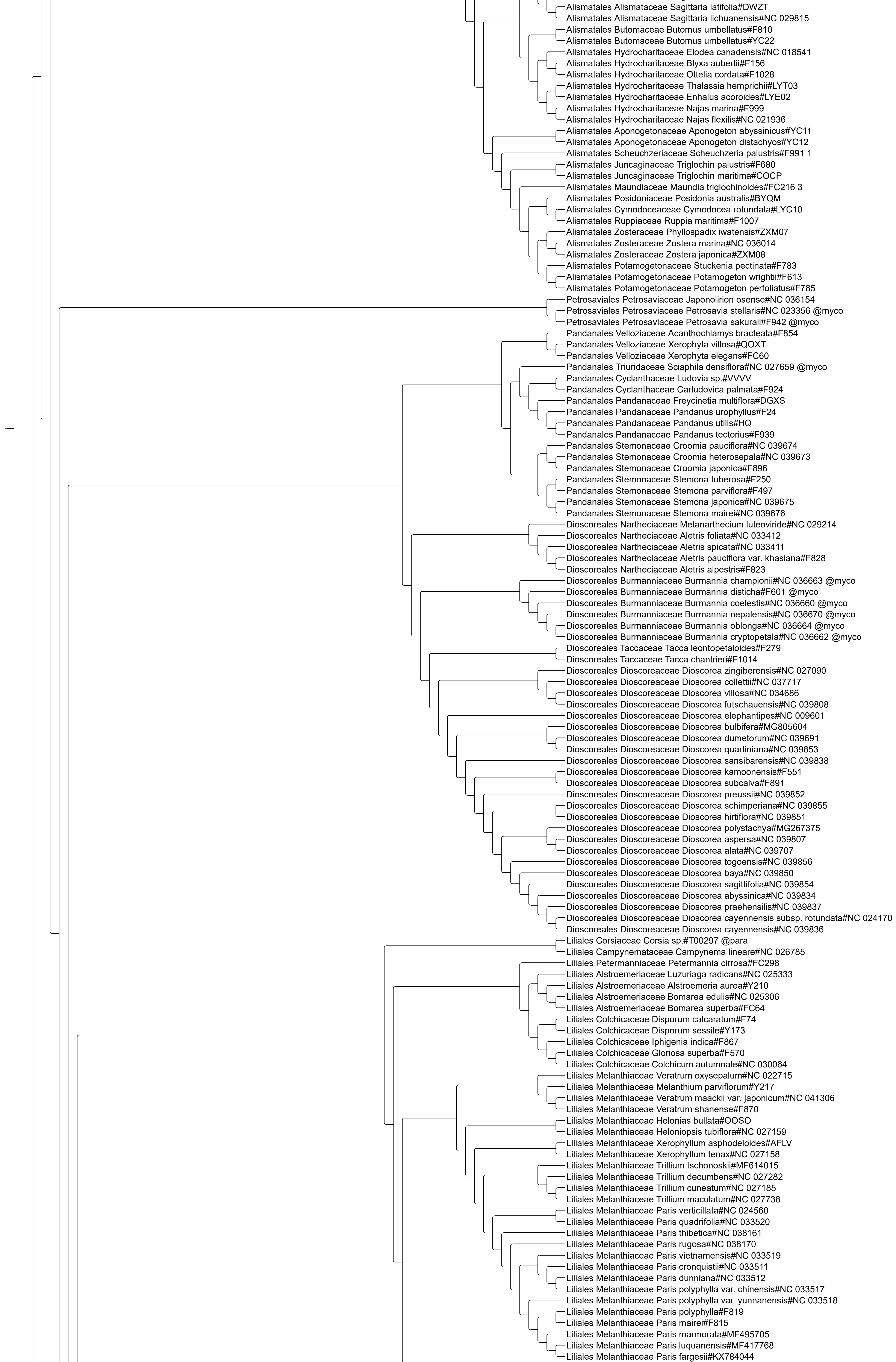

Alismatales Alismataceae Sagittaria latifolia#DWZT
Alismatales Alismataceae Sagittaria lichuanensis#NC 029815
Alismatales Butomaceae Butomus umbellatus#F810
Alismatales Butomaceae Butomus umbellatus#YC22
Alismatales Hydrocharitaceae Elodea canadensis#NC 018541
Alismatales Hydrocharitaceae Blyxa aubertii#F156
Alismatales Hydrocharitaceae Ottelia cordata#F1028
Alismatales Hydrocharitaceae Thalassia hemprichii#LYT03
Alismatales Hydrocharitaceae Enhalus acoroides#LYE02
Alismatales Hydrocharitaceae Najas marina#F999
Alismatales Hydrocharitaceae Najas flexilis#NC 021936
Alismatales Aponogetonaceae Aponogeton abyssinicus#YC11
Alismatales Aponogetonaceae Aponogeton distachyos#YC12
Alismatales Scheuchzeriaceae Scheuchzeria palustris#F991 1
Alismatales Juncaginaceae Triglochin palustris#F680
Alismatales Juncaginaceae Triglochin maritima#COCP
Alismatales Maundiaceae Maundia triglochinoides#FC216 3
Alismatales Posidoniaceae Posidonia australis#BYQM
Alismatales Cymodoceaceae Cymodocea rotundata#LYC10
Alismatales Ruppiaceae Ruppia maritima#F1007
Alismatales Zosteraceae Phyllospadix iwatensis#ZXM07
Alismatales Zosteraceae Zostera marina#NC 036014
Alismatales Zosteraceae Zostera japonica#ZXM08
Alismatales Potamogetonaceae Stuckenia pectinata#F783
Alismatales Potamogetonaceae Potamogeton wrightii#F613
Alismatales Potamogetonaceae Potamogeton perfoliatus#F785
Petrosaviales Petrosaviaceae Japonolirion osense#NC 036154
Petrosaviales Petrosaviaceae Petrosavia stellaris#NC 023356 @myco
Petrosaviales Petrosaviaceae Petrosavia sakuraii#F942 @myco
Pandanales Velloziaceae Acanthochlamys bracteata#F854
Pandanales Velloziaceae Xerophyta villosa#QOXT
Pandanales Velloziaceae Xerophyta elegans#FC60
Pandanales Triuridaceae Sciaphila densiflora#NC 027659 @myco
Pandanales Cyclanthaceae Ludovia sp.#VVVV
Pandanales Cyclanthaceae Carludovica palmata#F924
Pandanales Pandanaceae Freycinetia multiflora#DGXS
Pandanales Pandanaceae Pandanus urophyllus#F24
Pandanales Pandanaceae Pandanus utilis#HQ
Pandanales Pandanaceae Pandanus tectorius#F939
Pandanales Stemonaceae Croomia pauciflora#NC 039674
Pandanales Stemonaceae Croomia heterosepala#NC 039673
Pandanales Stemonaceae Croomia japonica#F896
Pandanales Stemonaceae Stemona tuberosa#F250
Pandanales Stemonaceae Stemona parviflora#F497
Pandanales Stemonaceae Stemona japonica#NC 039675
Pandanales Stemonaceae Stemona mairei#NC 039676
Dioscoreales Nartheciaceae Metanarthecium luteoviride#NC 029214
Dioscoreales Nartheciaceae Aletris foliata#NC 033412
Dioscoreales Nartheciaceae Aletris spicata#NC 033411
Dioscoreales Nartheciaceae Aletris pauciflora var. khasiana#F828
Dioscoreales Nartheciaceae Aletris alpestris#F823
Dioscoreales Burmanniaceae Burmannia championii#NC 036663 @myco
Dioscoreales Burmanniaceae Burmannia disticha#F601 @myco
Dioscoreales Burmanniaceae Burmannia coelestis#NC 036660 @myco
Dioscoreales Burmanniaceae Burmannia nepalensis#NC 036670 @myco
Dioscoreales Burmanniaceae Burmannia oblonga#NC 036664 @myco
Dioscoreales Burmanniaceae Burmannia cryptopetala#NC 036662 @myco
Dioscoreales Taccaceae Tacca leontopetaloides#F279
Dioscoreales Taccaceae Tacca chantrieri#F1014
Dioscoreales Dioscoreaceae Dioscorea zingiberensis#NC 027090
Dioscoreales Dioscoreaceae Dioscorea collettii#NC 037717
Dioscoreales Dioscoreaceae Dioscorea villosa#NC 034686
Dioscoreales Dioscoreaceae Dioscorea futschauensis#NC 039808
Dioscoreales Dioscoreaceae Dioscorea elephantipes#NC 009601
Dioscoreales Dioscoreaceae Dioscorea bulbifera#MG805604
Dioscoreales Dioscoreaceae Dioscorea dumetorum#NC 039691
Dioscoreales Dioscoreaceae Dioscorea quartiniana#NC 039853
Dioscoreales Dioscoreaceae Dioscorea sansibarensis#NC 039838
Dioscoreales Dioscoreaceae Dioscorea kamoonensis#F551
Dioscoreales Dioscoreaceae Dioscorea subcalva#F891
Dioscoreales Dioscoreaceae Dioscorea preussii#NC 039852
Dioscoreales Dioscoreaceae Dioscorea schimperiana#NC 039855
Dioscoreales Dioscoreaceae Dioscorea hirtiflora#NC 039851
Dioscoreales Dioscoreaceae Dioscorea polystachya#MG267375
Dioscoreales Dioscoreaceae Dioscorea aspersa#NC 039807
Dioscoreales Dioscoreaceae Dioscorea alata#NC 039707
Dioscoreales Dioscoreaceae Dioscorea togoensis#NC 039856
Dioscoreales Dioscoreaceae Dioscorea baya#NC 039850
Dioscoreales Dioscoreaceae Dioscorea sagittifolia#NC 039854
Dioscoreales Dioscoreaceae Dioscorea abyssinica#NC 039834
Dioscoreales Dioscoreaceae Dioscorea praehensilis#NC 039837
Dioscoreales Dioscoreaceae Dioscorea cayennensis subsp. rotundata#NC 024170
Dioscoreales Dioscoreaceae Dioscorea cayennensis#NC 039836
Liliales Corsiaceae Corsia sp.#T00297 @para
Liliales Campynemataceae Campynema lineare#NC 026785
Liliales Petermanniaceae Petermannia cirrosa#FC298
Liliales Alstroemeriaceae Luzuriaga radicans#NC 025333
Liliales Alstroemeriaceae Alstroemeria aurea#Y210
Liliales Alstroemeriaceae Bomarea edulis#NC 025306
Liliales Alstroemeriaceae Bomarea superba#FC64
Liliales Colchicaceae Disporum calcaratum#F74
Liliales Colchicaceae Disporum sessile#Y173
Liliales Colchicaceae Iphigenia indica#F867
Liliales Colchicaceae Gloriosa superba#F570
Liliales Colchicaceae Colchicum autumnale#NC 030064
Liliales Melanthiaceae Veratrum oxysepalum#NC 022715
Liliales Melanthiaceae Melanthium parviflorum#Y217
Liliales Melanthiaceae Veratrum maackii var. japonicum#NC 041306
Liliales Melanthiaceae Veratrum shanense#F870
Liliales Melanthiaceae Helonias bullata#OOSO
Liliales Melanthiaceae Heloniopsis tubiflora#NC 027159
Liliales Melanthiaceae Xerophyllum asphodeloides#AFLV
Liliales Melanthiaceae Xerophyllum tenax#NC 027158
Liliales Melanthiaceae Trillium tschonoskii#MF614015
Liliales Melanthiaceae Trillium decumbens#NC 027282
Liliales Melanthiaceae Trillium cuneatum#NC 027185
Liliales Melanthiaceae Trillium maculatum#NC 027738
Liliales Melanthiaceae Paris verticillata#NC 024560
Liliales Melanthiaceae Paris quadrifolia#NC 033520
Liliales Melanthiaceae Paris thibetica#NC 038161
Liliales Melanthiaceae Paris rugosa#NC 038170
Liliales Melanthiaceae Paris vietnamensis#NC 033519
Liliales Melanthiaceae Paris cronquistii#NC 033511
Liliales Melanthiaceae Paris dunniana#NC 033512
Liliales Melanthiaceae Paris polyphylla var. chinensis#NC 033517
Liliales Melanthiaceae Paris polyphylla var. yunnanensis#NC 033518
Liliales Melanthiaceae Paris polyphylla#F819
Liliales Melanthiaceae Paris mairei#F815
Liliales Melanthiaceae Paris marmorata#MF495705
Liliales Melanthiaceae Paris luquanensis#MF417768
Liliales Melanthiaceae Paris fargesii#KX784044

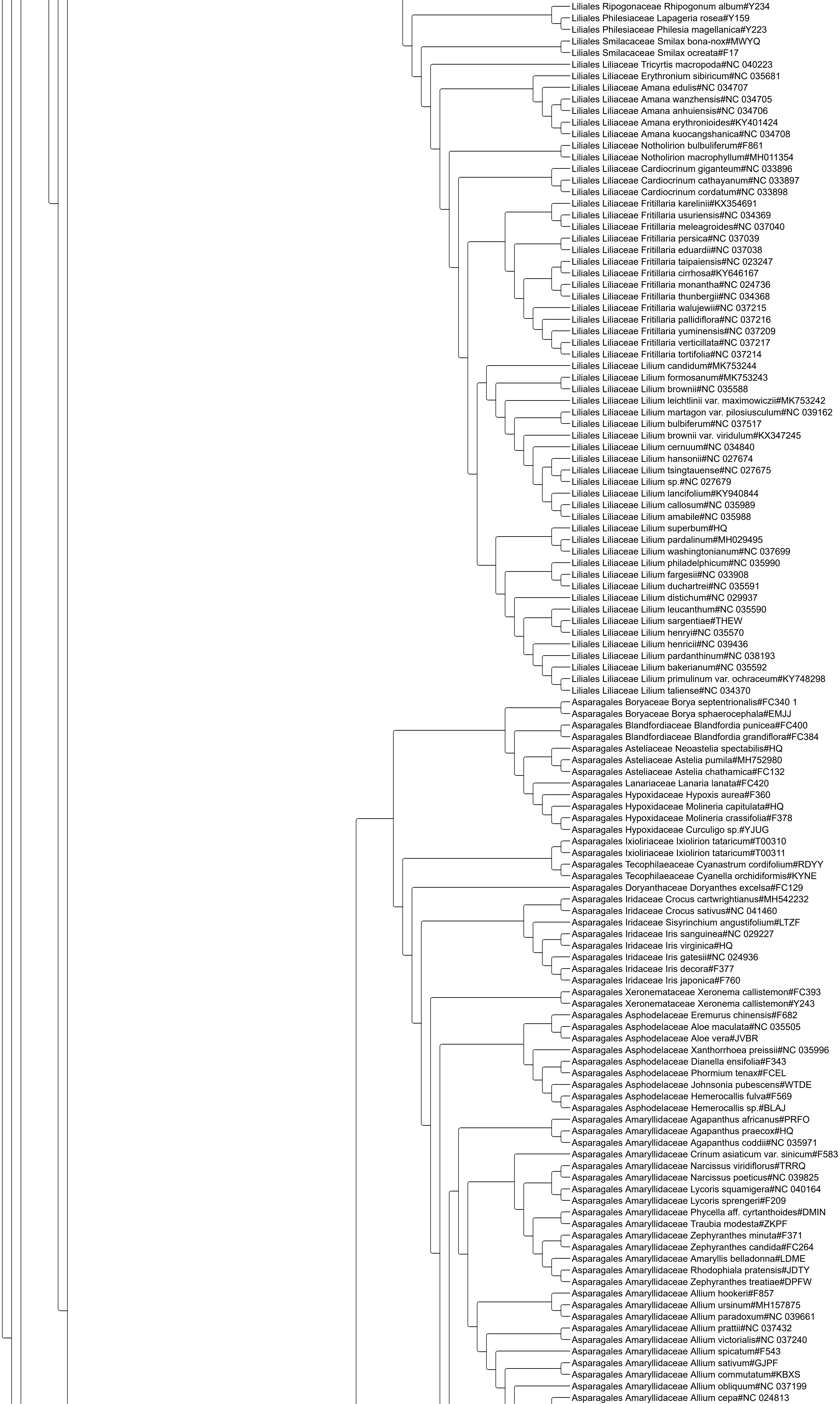

Liliales Ripogonaceae Rhipogonum album#Y234
Liliales Philesiaceae Lapageria rosea#Y159
Liliales Philesiaceae Philesia magellanica#Y223
Liliales Smilacaceae Smilax bona-nox#MWYQ
Liliales Smilacaceae Smilax ocreata#F17
Liliales Liliaceae Tricyrtis macropoda#NC 040223
Liliales Liliaceae Erythronium sibiricum#NC 035681
Liliales Liliaceae Amana edulis#NC 034707
Liliales Liliaceae Amana wanzhensis#NC 034705
Liliales Liliaceae Amana anhuiensis#NC 034706
Liliales Liliaceae Amana erythronioides#KY401424
Liliales Liliaceae Amana kuocangshanica#NC 034708
Liliales Liliaceae Notholirion bulbuliferum#F861
Liliales Liliaceae Notholirion macrophyllum#MH011354
Liliales Liliaceae Cardiocrinum giganteum#NC 033896
Liliales Liliaceae Cardiocrinum cathayanum#NC 033897
Liliales Liliaceae Cardiocrinum cordatum#NC 033898
Liliales Liliaceae Fritillaria karelinii#KX354691
Liliales Liliaceae Fritillaria usuriensis#NC 034369
Liliales Liliaceae Fritillaria meleagroides#NC 037040
Liliales Liliaceae Fritillaria persica#NC 037039
Liliales Liliaceae Fritillaria eduardii#NC 037038
Liliales Liliaceae Fritillaria taipaiensis#NC 023247
Liliales Liliaceae Fritillaria cirrhosa#KY646167
Liliales Liliaceae Fritillaria monantha#NC 024736
Liliales Liliaceae Fritillaria thunbergii#NC 034368
Liliales Liliaceae Fritillaria walujewii#NC 037215
Liliales Liliaceae Fritillaria pallidiflora#NC 037216
Liliales Liliaceae Fritillaria yuminensis#NC 037209
Liliales Liliaceae Fritillaria verticillata#NC 037217
Liliales Liliaceae Fritillaria tortifolia#NC 037214
Liliales Liliaceae Lilium candidum#MK753244
Liliales Liliaceae Lilium formosanum#MK753243
Liliales Liliaceae Lilium brownii#NC 035588
Liliales Liliaceae Lilium leichtlinii var. maximowiczii#MK753242
Liliales Liliaceae Lilium martagon var. pilosiusculum#NC 039162
Liliales Liliaceae Lilium bulbiferum#NC 037517
Liliales Liliaceae Lilium brownii var. viridulum#KX347245
Liliales Liliaceae Lilium cernuum#NC 034840
Liliales Liliaceae Lilium hansonii#NC 027674
Liliales Liliaceae Lilium tsingtauense#NC 027675
Liliales Liliaceae Lilium sp.#NC 027679
Liliales Liliaceae Lilium lancifolium#KY940844
Liliales Liliaceae Lilium callosum#NC 035989
Liliales Liliaceae Lilium amabile#NC 035988
Liliales Liliaceae Lilium superbum#HQ
Liliales Liliaceae Lilium pardalinum#MH029495
Liliales Liliaceae Lilium washingtonianum#NC 037699
Liliales Liliaceae Lilium philadelphicum#NC 035990
Liliales Liliaceae Lilium fargesii#NC 033908
Liliales Liliaceae Lilium duchartrei#NC 035591
Liliales Liliaceae Lilium distichum#NC 029937
Liliales Liliaceae Lilium leucanthum#NC 035590
Liliales Liliaceae Lilium sargentiae#THEW
Liliales Liliaceae Lilium henryi#NC 035570
Liliales Liliaceae Lilium henricii#NC 039436
Liliales Liliaceae Lilium pardanthinum#NC 038193
Liliales Liliaceae Lilium bakerianum#NC 035592
Liliales Liliaceae Lilium primulinum var. ochraceum#KY748298
Liliales Liliaceae Lilium taliense#NC 034370
Asparagales Boryaceae Borya septentrionalis#FC340 1
Asparagales Boryaceae Borya sphaerocephala#EMJJ
Asparagales Blandfordiaceae Blandfordia punicea#FC400
Asparagales Blandfordiaceae Blandfordia grandiflora#FC384
Asparagales Asteliaceae Neoastelia spectabilis#HQ
Asparagales Asteliaceae Astelia pumila#MH752980
Asparagales Asteliaceae Astelia chathamica#FC132
Asparagales Lanariaceae Lanaria lanata#FC420
Asparagales Hypoxidaceae Hypoxis aurea#F360
Asparagales Hypoxidaceae Molineria capitulata#HQ
Asparagales Hypoxidaceae Molineria crassifolia#F378
Asparagales Hypoxidaceae Curculigo sp.#YJUG
Asparagales Ixioliriaceae Ixiolirion tataricum#T00310
Asparagales Ixioliriaceae Ixiolirion tataricum#T00311
Asparagales Tecophilaeaceae Cyanastrum cordifolium#RDYY
Asparagales Tecophilaeaceae Cyanella orchidiformis#KYNE
Asparagales Doryanthaceae Doryanthes excelsa#FC129
Asparagales Iridaceae Crocus cartwrightianus#MH542232
Asparagales Iridaceae Crocus sativus#NC 041460
Asparagales Iridaceae Sisyrinchium angustifolium#LTZF
Asparagales Iridaceae Iris sanguinea#NC 029227
Asparagales Iridaceae Iris virginica#HQ
Asparagales Iridaceae Iris gatesii#NC 024936
Asparagales Iridaceae Iris decora#F377
Asparagales Iridaceae Iris japonica#F760
Asparagales Xeronemataceae Xeronema callistemon#FC393
Asparagales Xeronemataceae Xeronema callistemon#Y243
Asparagales Asphodelaceae Eremurus chinensis#F682
Asparagales Asphodelaceae Aloe maculata#NC 035505
Asparagales Asphodelaceae Aloe vera#JVBR
Asparagales Asphodelaceae Xanthorrhoea preissii#NC 035996
Asparagales Asphodelaceae Dianella ensifolia#F343
Asparagales Asphodelaceae Phormium tenax#FCEL
Asparagales Asphodelaceae Johnsonia pubescens#WTDE
Asparagales Asphodelaceae Hemerocallis fulva#F569
Asparagales Asphodelaceae Hemerocallis sp.#BLAJ
Asparagales Amaryllidaceae Agapanthus africanus#PRFO
Asparagales Amaryllidaceae Agapanthus praecox#HQ
Asparagales Amaryllidaceae Agapanthus coddii#NC 035971
Asparagales Amaryllidaceae Crinum asiaticum var. sinicum#F583
Asparagales Amaryllidaceae Narcissus viridiflorus#TRRQ
Asparagales Amaryllidaceae Narcissus poeticus#NC 039825
Asparagales Amaryllidaceae Lycoris squamigera#NC 040164
Asparagales Amaryllidaceae Lycoris sprengeri#F209
Asparagales Amaryllidaceae Phycella aff. cyrtanthoides#DMIN
Asparagales Amaryllidaceae Traubia modesta#ZKPF
Asparagales Amaryllidaceae Zephyranthes minuta#F371
Asparagales Amaryllidaceae Zephyranthes candida#FC264
Asparagales Amaryllidaceae Amaryllis belladonna#LDME
Asparagales Amaryllidaceae Rhodophiala pratensis#JDTY
Asparagales Amaryllidaceae Zephyranthes treatiae#DPFW
Asparagales Amaryllidaceae Allium hookeri#F857
Asparagales Amaryllidaceae Allium ursinum#MH157875
Asparagales Amaryllidaceae Allium paradoxum#NC 039661
Asparagales Amaryllidaceae Allium prattii#NC 037432
Asparagales Amaryllidaceae Allium victorialis#NC 037240
Asparagales Amaryllidaceae Allium spicatum#F543
Asparagales Amaryllidaceae Allium sativum#GJPF
Asparagales Amaryllidaceae Allium commutatum#KBXS
Asparagales Amaryllidaceae Allium obliquum#NC 037199
Asparagales Amaryllidaceae Allium cepa#NC 024813

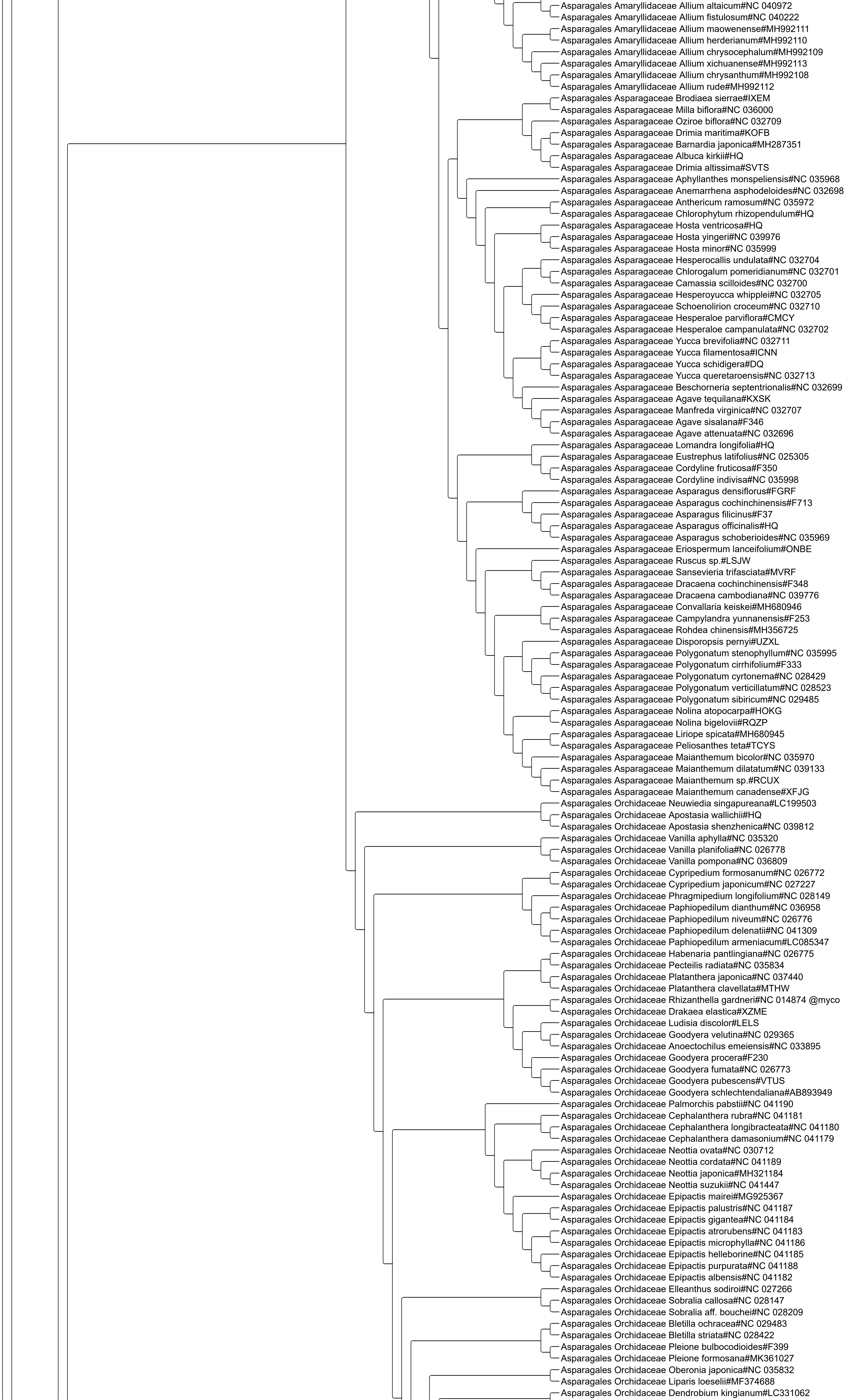
Asparagales Amaryllidaceae Allium altaicum#NC 040972
Asparagales Amaryllidaceae Allium fistulosum#NC 040222
Asparagales Amaryllidaceae Allium maowenense#MH992111
Asparagales Amaryllidaceae Allium herderianum#MH992110
Asparagales Amaryllidaceae Allium chrysocephalum#MH992109
Asparagales Amaryllidaceae Allium xichuanense#MH992113
Asparagales Amaryllidaceae Allium chrysanthum#MH992108
Asparagales Amaryllidaceae Allium rude#MH992112
Asparagales Asparagaceae Brodiaea sierrae#IXEM
Asparagales Asparagaceae Milla biflora#NC 036000
Asparagales Asparagaceae Oziroe biflora#NC 032709
Asparagales Asparagaceae Drimia maritima#KOFB
Asparagales Asparagaceae Barnardia japonica#MH287351
Asparagales Asparagaceae Albuca kirkii#HQ
Asparagales Asparagaceae Drimia altissima#SVTS
Asparagales Asparagaceae Aphyllanthes monspeliensis#NC 035968
Asparagales Asparagaceae Anemarrhena asphodeloides#NC 032698
Asparagales Asparagaceae Anthericum ramosum#NC 035972
Asparagales Asparagaceae Chlorophytum rhizopendulum#HQ
Asparagales Asparagaceae Hosta ventricosa#HQ
Asparagales Asparagaceae Hosta yingeri#NC 039976
Asparagales Asparagaceae Hosta minor#NC 035999
Asparagales Asparagaceae Hesperocallis undulata#NC 032704
Asparagales Asparagaceae Chlorogalum pomeridianum#NC 032701
Asparagales Asparagaceae Camassia scilloides#NC 032700
Asparagales Asparagaceae Hesperoyucca whipplei#NC 032705
Asparagales Asparagaceae Schoenolirion croceum#NC 032710
Asparagales Asparagaceae Hesperaloe parviflora#CMCY
Asparagales Asparagaceae Hesperaloe campanulata#NC 032702
Asparagales Asparagaceae Yucca brevifolia#NC 032711
Asparagales Asparagaceae Yucca filamentosa#ICNN
Asparagales Asparagaceae Yucca schidigera#DQ
Asparagales Asparagaceae Yucca queretaroensis#NC 032713
Asparagales Asparagaceae Beschorneria septentrionalis#NC 032699
Asparagales Asparagaceae Agave tequilana#KXSK
Asparagales Asparagaceae Manfreda virginica#NC 032707
Asparagales Asparagaceae Agave sisalana#F346
Asparagales Asparagaceae Agave attenuata#NC 032696
Asparagales Asparagaceae Lomandra longifolia#HQ
Asparagales Asparagaceae Eustrephus latifolius#NC 025305
Asparagales Asparagaceae Cordyline fruticosa#F350
Asparagales Asparagaceae Cordyline indivisa#NC 035998
Asparagales Asparagaceae Asparagus densiflorus#FGRF
Asparagales Asparagaceae Asparagus cochinchinensis#F713
Asparagales Asparagaceae Asparagus filicinus#F37
Asparagales Asparagaceae Asparagus officinalis#HQ
Asparagales Asparagaceae Asparagus schoberioides#NC 035969
Asparagales Asparagaceae Eriospermum lanceifolium#ONBE
Asparagales Asparagaceae Ruscus sp.#LSJW
Asparagales Asparagaceae Sansevieria trifasciata#MVRF
Asparagales Asparagaceae Dracaena cochinchinensis#F348
Asparagales Asparagaceae Dracaena cambodiana#NC 039776
Asparagales Asparagaceae Convallaria keiskei#MH680946
Asparagales Asparagaceae Campylandra yunnanensis#F253
Asparagales Asparagaceae Rohdea chinensis#MH356725
Asparagales Asparagaceae Disporopsis pernyi#UZXL
Asparagales Asparagaceae Polygonatum stenophyllum#NC 035995
Asparagales Asparagaceae Polygonatum cirrhifolium#F333
Asparagales Asparagaceae Polygonatum cyrtonema#NC 028429
Asparagales Asparagaceae Polygonatum verticillatum#NC 028523
Asparagales Asparagaceae Polygonatum sibiricum#NC 029485
Asparagales Asparagaceae Nolina atopocarpa#HOKG
Asparagales Asparagaceae Nolina bigelovii#RQZP
Asparagales Asparagaceae Liriope spicata#MH680945
Asparagales Asparagaceae Peliosanthes teta#TCYS
Asparagales Asparagaceae Maianthemum bicolor#NC 035970
Asparagales Asparagaceae Maianthemum dilatatum#NC 039133
Asparagales Asparagaceae Maianthemum sp.#RCUX
Asparagales Asparagaceae Maianthemum canadense#XFJG
Asparagales Orchidaceae Neuwiedia singapureana#LC199503
Asparagales Orchidaceae Apostasia wallichii#HQ
Asparagales Orchidaceae Apostasia shenzhenica#NC 039812
Asparagales Orchidaceae Vanilla aphylla#NC 035320
Asparagales Orchidaceae Vanilla planifolia#NC 026778
Asparagales Orchidaceae Vanilla pompona#NC 036809
Asparagales Orchidaceae Cypripedium formosanum#NC 026772
Asparagales Orchidaceae Cypripedium japonicum#NC 027227
Asparagales Orchidaceae Phragmipedium longifolium#NC 028149
Asparagales Orchidaceae Paphiopedilum dianthum#NC 036958
Asparagales Orchidaceae Paphiopedilum niveum#NC 026776
Asparagales Orchidaceae Paphiopedilum delenatii#NC 041309
Asparagales Orchidaceae Paphiopedilum armeniacum#LC085347
Asparagales Orchidaceae Habenaria pantlingiana#NC 026775
Asparagales Orchidaceae Pecteilis radiata#NC 035834
Asparagales Orchidaceae Platanthera japonica#NC 037440
Asparagales Orchidaceae Platanthera clavellata#MTHW
Asparagales Orchidaceae Rhizanthella gardneri#NC 014874 @myco
Asparagales Orchidaceae Drakaea elastica#XZME
Asparagales Orchidaceae Ludisia discolor#LELS
Asparagales Orchidaceae Goodyera velutina#NC 029365
Asparagales Orchidaceae Anoectochilus emeiensis#NC 033895
Asparagales Orchidaceae Goodyera procera#F230
Asparagales Orchidaceae Goodyera fumata#NC 026773
Asparagales Orchidaceae Goodyera pubescens#VTUS
Asparagales Orchidaceae Goodyera schlechtendaliana#AB893949
Asparagales Orchidaceae Palmorchis pabstii#NC 041190
Asparagales Orchidaceae Cephalanthera rubra#NC 041181
Asparagales Orchidaceae Cephalanthera longibracteata#NC 041180
Asparagales Orchidaceae Cephalanthera damasonium#NC 041179
Asparagales Orchidaceae Neottia ovata#NC 030712
Asparagales Orchidaceae Neottia cordata#NC 041189
Asparagales Orchidaceae Neottia japonica#MH321184
Asparagales Orchidaceae Neottia suzukii#NC 041447
Asparagales Orchidaceae Epipactis mairei#MG925367
Asparagales Orchidaceae Epipactis palustris#NC 041187
Asparagales Orchidaceae Epipactis gigantea#NC 041184
Asparagales Orchidaceae Epipactis atrorubens#NC 041183
Asparagales Orchidaceae Epipactis microphylla#NC 041186
Asparagales Orchidaceae Epipactis helleborine#NC 041185
Asparagales Orchidaceae Epipactis purpurata#NC 041188
Asparagales Orchidaceae Epipactis albensis#NC 041182
Asparagales Orchidaceae Elleanthus sodiroi#NC 027266
Asparagales Orchidaceae Sobralia callosa#NC 028147
Asparagales Orchidaceae Sobralia aff. bouchei#NC 028209
Asparagales Orchidaceae Bletilla ochracea#NC 029483
Asparagales Orchidaceae Bletilla striata#NC 028422
Asparagales Orchidaceae Pleione bulbocodioides#F399
Asparagales Orchidaceae Pleione formosana#MK361027
Asparagales Orchidaceae Oberonia japonica#NC 035832
Asparagales Orchidaceae Liparis loeselii#MF374688
Asparagales Orchidaceae Dendrobium kingianum#LC331062

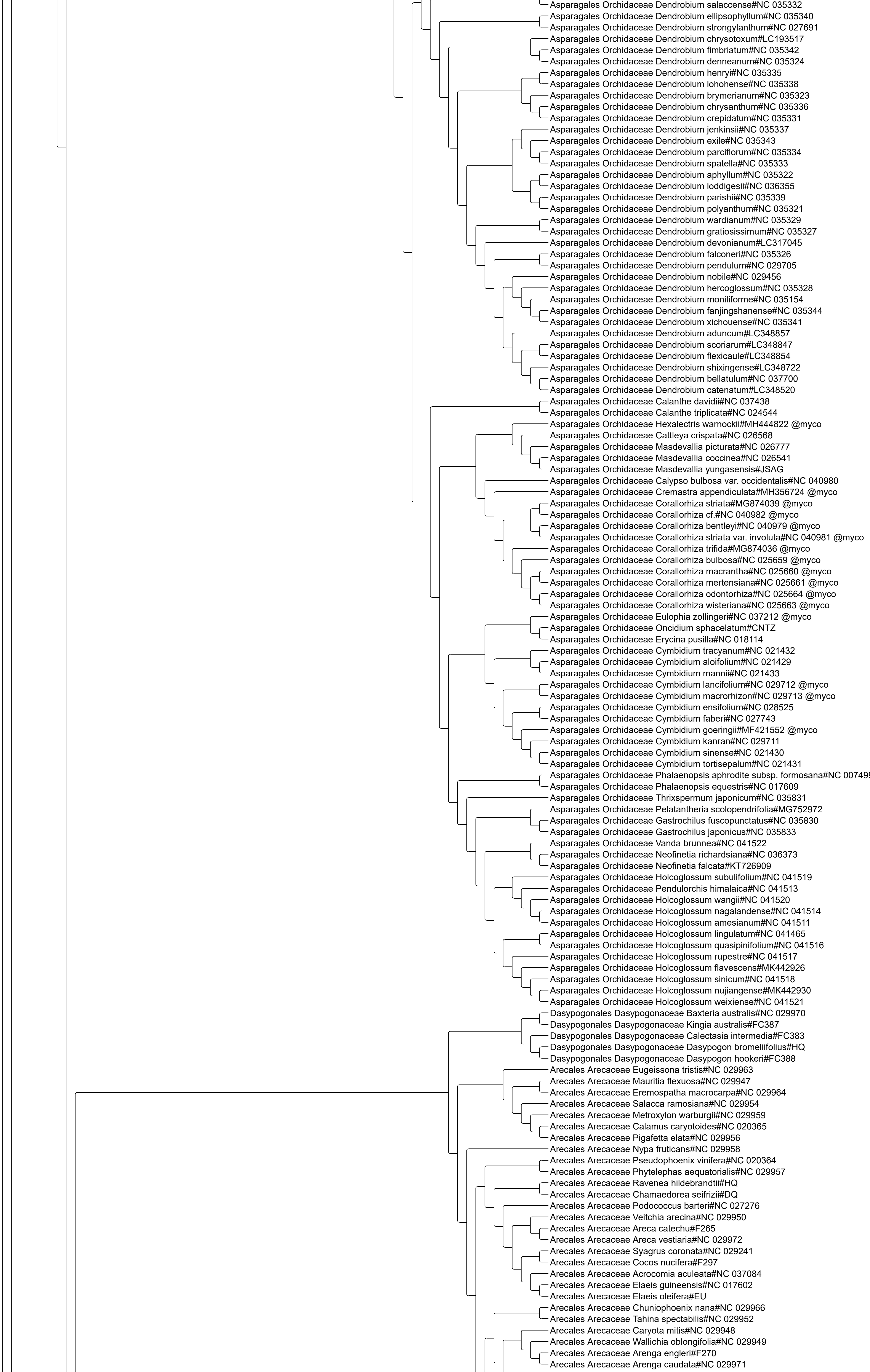
Asparagales Orchidaceae Dendrobium salaccense#NC 035332
Asparagales Orchidaceae Dendrobium ellipsophyllum#NC 035340
Asparagales Orchidaceae Dendrobium strongylanthum#NC 027691
Asparagales Orchidaceae Dendrobium chrysotoxum#LC193517
Asparagales Orchidaceae Dendrobium fimbriatum#NC 035342
Asparagales Orchidaceae Dendrobium denneanum#NC 035324
Asparagales Orchidaceae Dendrobium henryi#NC 035335
Asparagales Orchidaceae Dendrobium lohohense#NC 035338
Asparagales Orchidaceae Dendrobium brymerianum#NC 035323
Asparagales Orchidaceae Dendrobium chrysanthum#NC 035336
Asparagales Orchidaceae Dendrobium crepidatum#NC 035331
Asparagales Orchidaceae Dendrobium jenkinsii#NC 035337
Asparagales Orchidaceae Dendrobium exile#NC 035343
Asparagales Orchidaceae Dendrobium parciflorum#NC 035334
Asparagales Orchidaceae Dendrobium spatella#NC 035333
Asparagales Orchidaceae Dendrobium aphyllum#NC 035322
Asparagales Orchidaceae Dendrobium loddigesii#NC 036355
Asparagales Orchidaceae Dendrobium parishii#NC 035339
Asparagales Orchidaceae Dendrobium polyanthum#NC 035321
Asparagales Orchidaceae Dendrobium wardianum#NC 035329
Asparagales Orchidaceae Dendrobium gratiosissimum#NC 035327
Asparagales Orchidaceae Dendrobium devonianum#LC317045
Asparagales Orchidaceae Dendrobium falconeri#NC 035326
Asparagales Orchidaceae Dendrobium pendulum#NC 029705
Asparagales Orchidaceae Dendrobium nobile#NC 029456
Asparagales Orchidaceae Dendrobium hercoglossum#NC 035328
Asparagales Orchidaceae Dendrobium moniliforme#NC 035154
Asparagales Orchidaceae Dendrobium fanjingshanense#NC 035344
Asparagales Orchidaceae Dendrobium xichouense#NC 035341
Asparagales Orchidaceae Dendrobium aduncum#LC348857
Asparagales Orchidaceae Dendrobium scoriarum#LC348847
Asparagales Orchidaceae Dendrobium flexicaule#LC348854
Asparagales Orchidaceae Dendrobium shixingense#LC348722
Asparagales Orchidaceae Dendrobium bellatulum#NC 037700
Asparagales Orchidaceae Dendrobium catenatum#LC348520
Asparagales Orchidaceae Calanthe davidii#NC 037438
Asparagales Orchidaceae Calanthe triplicata#NC 024544
Asparagales Orchidaceae Hexalectris warnockii#MH444822 @myco
Asparagales Orchidaceae Cattleya crispata#NC 026568
Asparagales Orchidaceae Masdevallia picturata#NC 026777
Asparagales Orchidaceae Masdevallia coccinea#NC 026541
Asparagales Orchidaceae Masdevallia yungasensis#JSAG
Asparagales Orchidaceae Calypso bulbosa var. occidentalis#NC 040980
Asparagales Orchidaceae Cremastra appendiculata#MH356724 @myco
Asparagales Orchidaceae Corallorhiza striata#MG874039 @myco
Asparagales Orchidaceae Corallorhiza cf.#NC 040982 @myco
Asparagales Orchidaceae Corallorhiza bentleyi#NC 040979 @myco
Asparagales Orchidaceae Corallorhiza striata var. involuta#NC 040981 @myco
Asparagales Orchidaceae Corallorhiza trifida#MG874036 @myco
Asparagales Orchidaceae Corallorhiza bulbosa#NC 025659 @myco
Asparagales Orchidaceae Corallorhiza macrantha#NC 025660 @myco
Asparagales Orchidaceae Corallorhiza mertensiana#NC 025661 @myco
Asparagales Orchidaceae Corallorhiza odontorhiza#NC 025664 @myco
Asparagales Orchidaceae Corallorhiza wisteriana#NC 025663 @myco
Asparagales Orchidaceae Eulophia zollingeri#NC 037212 @myco
Asparagales Orchidaceae Oncidium sphacelatum#CNTZ
Asparagales Orchidaceae Erycina pusilla#NC 018114
Asparagales Orchidaceae Cymbidium tracyanum#NC 021432
Asparagales Orchidaceae Cymbidium aloifolium#NC 021429
Asparagales Orchidaceae Cymbidium mannii#NC 021433
Asparagales Orchidaceae Cymbidium lancifolium#NC 029712 @myco
Asparagales Orchidaceae Cymbidium macrorhizon#NC 029713 @myco
Asparagales Orchidaceae Cymbidium ensifolium#NC 028525
Asparagales Orchidaceae Cymbidium faberi#NC 027743
Asparagales Orchidaceae Cymbidium goeringii#MF421552 @myco
Asparagales Orchidaceae Cymbidium kanran#NC 029711
Asparagales Orchidaceae Cymbidium sinense#NC 021430
Asparagales Orchidaceae Cymbidium tortisepalum#NC 021431
Asparagales Orchidaceae Phalaenopsis aphrodite subsp. formosana#NC 007499
Asparagales Orchidaceae Phalaenopsis equestris#NC 017609
Asparagales Orchidaceae Thrixspermum japonicum#NC 035831
Asparagales Orchidaceae Pelatantheria scolopendrifolia#MG752972
Asparagales Orchidaceae Gastrochilus fuscopunctatus#NC 035830
Asparagales Orchidaceae Gastrochilus japonicus#NC 035833
Asparagales Orchidaceae Vanda brunnea#NC 041522
Asparagales Orchidaceae Neofinetia richardsiana#NC 036373
Asparagales Orchidaceae Neofinetia falcata#KT726909
Asparagales Orchidaceae Holcoglossum subulifolium#NC 041519
Asparagales Orchidaceae Pendulorchis himalaica#NC 041513
Asparagales Orchidaceae Holcoglossum wangii#NC 041520
Asparagales Orchidaceae Holcoglossum nagalandense#NC 041514
Asparagales Orchidaceae Holcoglossum amesianum#NC 041511
Asparagales Orchidaceae Holcoglossum lingulatum#NC 041465
Asparagales Orchidaceae Holcoglossum quasipinifolium#NC 041516
Asparagales Orchidaceae Holcoglossum rupestre#NC 041517
Asparagales Orchidaceae Holcoglossum flavescens#MK442926
Asparagales Orchidaceae Holcoglossum sinicum#NC 041518
Asparagales Orchidaceae Holcoglossum nujiangense#MK442930
Asparagales Orchidaceae Holcoglossum weixiense#NC 041521
Dasypogonales Dasypogonaceae Baxteria australis#NC 029970
Dasypogonales Dasypogonaceae Kingia australis#FC387
Dasypogonales Dasypogonaceae Calectasia intermedia#FC383
Dasypogonales Dasypogonaceae Dasypogon bromeliifolius#HQ
Dasypogonales Dasypogonaceae Dasypogon hookeri#FC388
Arecales Arecaceae Eugeissona tristis#NC 029963
Arecales Arecaceae Mauritia flexuosa#NC 029947
Arecales Arecaceae Eremospatha macrocarpa#NC 029964
Arecales Arecaceae Salacca ramosiana#NC 029954
Arecales Arecaceae Metroxylon warburgii#NC 029959
Arecales Arecaceae Calamus caryotoides#NC 020365
Arecales Arecaceae Pigafetta elata#NC 029956
Arecales Arecaceae Nypa fruticans#NC 029958
Arecales Arecaceae Pseudophoenix vinifera#NC 020364
Arecales Arecaceae Phytelephas aequatorialis#NC 029957
Arecales Arecaceae Ravenea hildebrandtii#HQ
Arecales Arecaceae Chamaedorea seifrizii#DQ
Arecales Arecaceae Podococcus barteri#NC 027276
Arecales Arecaceae Veitchia arecina#NC 029950
Arecales Arecaceae Areca catechu#F265
Arecales Arecaceae Areca vestiaria#NC 029972
Arecales Arecaceae Syagrus coronata#NC 029241
Arecales Arecaceae Cocos nucifera#F297
Arecales Arecaceae Acrocomia aculeata#NC 037084
Arecales Arecaceae Elaeis guineensis#NC 017602
Arecales Arecaceae Elaeis oleifera#EU
Arecales Arecaceae Chuniophoenix nana#NC 029966
Arecales Arecaceae Tahina spectabilis#NC 029952
Arecales Arecaceae Caryota mitis#NC 029948
Arecales Arecaceae Wallichia oblongifolia#NC 029949
Arecales Arecaceae Arenga engleri#F270
Arecales Arecaceae Arenga caudata#NC 029971

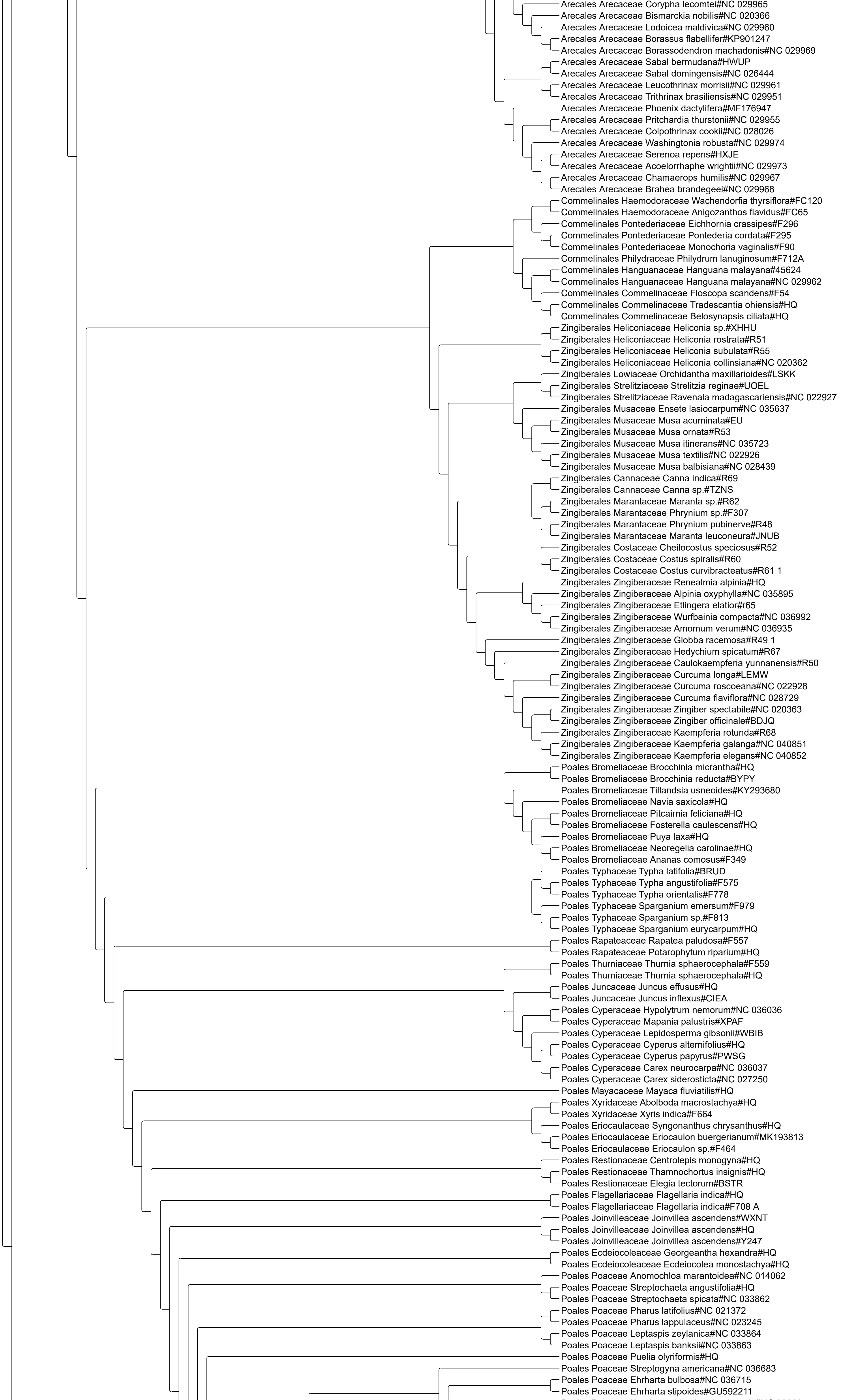

Arecales Arecaceae Corypha lecomtei#NC 029965
Arecales Arecaceae Bismarckia nobilis#NC 020366
Arecales Arecaceae Lodoicea maldivica#NC 029960
Arecales Arecaceae Borassus flabellifer#KP901247
Arecales Arecaceae Borassodendron machadonis#NC 029969
Arecales Arecaceae Sabal bermudana#HWUP
Arecales Arecaceae Sabal domingensis#NC 026444
Arecales Arecaceae Leucothrinax morrisii#NC 029961
Arecales Arecaceae Trithrinax brasiliensis#NC 029951
Arecales Arecaceae Phoenix dactylifera#MF176947
Arecales Arecaceae Pritchardia thurstonii#NC 029955
Arecales Arecaceae Colpothrinax cookii#NC 028026
Arecales Arecaceae Washingtonia robusta#NC 029974
Arecales Arecaceae Serenoa repens#HXJE
Arecales Arecaceae Acoelorrhaphe wrightii#NC 029973
Arecales Arecaceae Chamaerops humilis#NC 029967
Arecales Arecaceae Brahea brandegeei#NC 029968
Commelinales Haemodoraceae Wachendorfia thyrsiflora#FC120
Commelinales Haemodoraceae Anigozanthos flavidus#FC65
Commelinales Pontederiaceae Eichhornia crassipes#F296
Commelinales Pontederiaceae Pontederia cordata#F295
Commelinales Pontederiaceae Monochoria vaginalis#F90
Commelinales Philydraceae Philydrum lanuginosum#F712A
Commelinales Hanguanaceae Hanguana malayana#45624
Commelinales Hanguanaceae Hanguana malayana#NC 029962
Commelinales Commelinaceae Floscopa scandens#F54
Commelinales Commelinaceae Tradescantia ohiensis#HQ
Commelinales Commelinaceae Belosynapsis ciliata#HQ
Zingiberales Heliconiaceae Heliconia sp.#XHHU
Zingiberales Heliconiaceae Heliconia rostrata#R51
Zingiberales Heliconiaceae Heliconia subulata#R55
Zingiberales Heliconiaceae Heliconia collinsiana#NC 020362
Zingiberales Lowiaceae Orchidantha maxillarioides#LSKK
Zingiberales Strelitziaceae Strelitzia reginae#UOEL
Zingiberales Strelitziaceae Ravenala madagascariensis#NC 022927
Zingiberales Musaceae Ensete lasiocarpum#NC 035637
Zingiberales Musaceae Musa acuminata#EU
Zingiberales Musaceae Musa ornata#R53
Zingiberales Musaceae Musa itinerans#NC 035723
Zingiberales Musaceae Musa textilis#NC 022926
Zingiberales Musaceae Musa balbisiana#NC 028439
Zingiberales Cannaceae Canna indica#R69
Zingiberales Cannaceae Canna sp.#TZNS
Zingiberales Marantaceae Maranta sp.#R62
Zingiberales Marantaceae Phrynium sp.#F307
Zingiberales Marantaceae Phrynium pubinerve#R48
Zingiberales Marantaceae Maranta leuconeura#JNUB
Zingiberales Costaceae Cheilocostus speciosus#R52
Zingiberales Costaceae Costus spiralis#R60
Zingiberales Costaceae Costus curvibracteatus#R61 1
Zingiberales Zingiberaceae Renealmia alpinia#HQ
Zingiberales Zingiberaceae Alpinia oxyphylla#NC 035895
Zingiberales Zingiberaceae Etlingera elatior#r65
Zingiberales Zingiberaceae Wurfbainia compacta#NC 036992
Zingiberales Zingiberaceae Amomum verum#NC 036935
Zingiberales Zingiberaceae Globba racemosa#R49 1
Zingiberales Zingiberaceae Hedychium spicatum#R67
Zingiberales Zingiberaceae Caulokaempferia yunnanensis#R50
Zingiberales Zingiberaceae Curcuma longa#LEMW
Zingiberales Zingiberaceae Curcuma roscoeana#NC 022928
Zingiberales Zingiberaceae Curcuma flaviflora#NC 028729
Zingiberales Zingiberaceae Zingiber spectabile#NC 020363
Zingiberales Zingiberaceae Zingiber officinale#BDJQ
Zingiberales Zingiberaceae Kaempferia rotunda#R68
Zingiberales Zingiberaceae Kaempferia galanga#NC 040851
Zingiberales Zingiberaceae Kaempferia elegans#NC 040852
Poales Bromeliaceae Brocchinia micrantha#HQ
Poales Bromeliaceae Brocchinia reducta#BYPY
Poales Bromeliaceae Tillandsia usneoides#KY293680
Poales Bromeliaceae Navia saxicola#HQ
Poales Bromeliaceae Pitcairnia feliciana#HQ
Poales Bromeliaceae Fosterella caulescens#HQ
Poales Bromeliaceae Puya laxa#HQ
Poales Bromeliaceae Neoregelia carolinae#HQ
Poales Bromeliaceae Ananas comosus#F349
Poales Typhaceae Typha latifolia#BRUD
Poales Typhaceae Typha angustifolia#F575
Poales Typhaceae Typha orientalis#F778
Poales Typhaceae Sparganium emersum#F979
Poales Typhaceae Sparganium sp.#F813
Poales Typhaceae Sparganium eurycarpum#HQ
Poales Rapateaceae Rapatea paludosa#F557
Poales Rapateaceae Potarophytum riparium#HQ
Poales Thurniaceae Thurnia sphaerocephala#F559
Poales Thurniaceae Thurnia sphaerocephala#HQ
Poales Juncaceae Juncus effusus#HQ
Poales Juncaceae Juncus inflexus#CIEA
Poales Cyperaceae Hypolytrum nemorum#NC 036036
Poales Cyperaceae Mapania palustris#XPAF
Poales Cyperaceae Lepidosperma gibsonii#WBIB
Poales Cyperaceae Cyperus alternifolius#HQ
Poales Cyperaceae Cyperus papyrus#PWSG
Poales Cyperaceae Carex neurocarpa#NC 036037
Poales Cyperaceae Carex siderosticta#NC 027250
Poales Mayacaceae Mayaca fluviatilis#HQ
Poales Xyridaceae Abolboda macrostachya#HQ
Poales Xyridaceae Xyris indica#F664
Poales Eriocaulaceae Syngonanthus chrysanthus#HQ
Poales Eriocaulaceae Eriocaulon buergerianum#MK193813
Poales Eriocaulaceae Eriocaulon sp.#F464
Poales Restionaceae Centrolepis monogyna#HQ
Poales Restionaceae Thamnochortus insignis#HQ
Poales Restionaceae Elegia tectorum#BSTR
Poales Flagellariaceae Flagellaria indica#HQ
Poales Flagellariaceae Flagellaria indica#F708 A
Poales Joinvilleaceae Joinvillea ascendens#WXNT
Poales Joinvilleaceae Joinvillea ascendens#HQ
Poales Joinvilleaceae Joinvillea ascendens#Y247
Poales Ecdeiocoleaceae Georgeantha hexandra#HQ
Poales Ecdeiocoleaceae Ecdeiocolea monostachya#HQ
Poales Poaceae Anomochloa marantoidea#NC 014062
Poales Poaceae Streptochaeta angustifolia#HQ
Poales Poaceae Streptochaeta spicata#NC 033862
Poales Poaceae Pharus latifolius#NC 021372
Poales Poaceae Pharus lappulaceus#NC 023245
Poales Poaceae Leptaspis zeylanica#NC 033864
Poales Poaceae Leptaspis banksii#NC 033863
Poales Poaceae Puelia olyriformis#HQ
Poales Poaceae Streptogyna americana#NC 036683
Poales Poaceae Ehrharta bulbosa#NC 036715
Poales Poaceae Ehrharta stipoides#GU592211

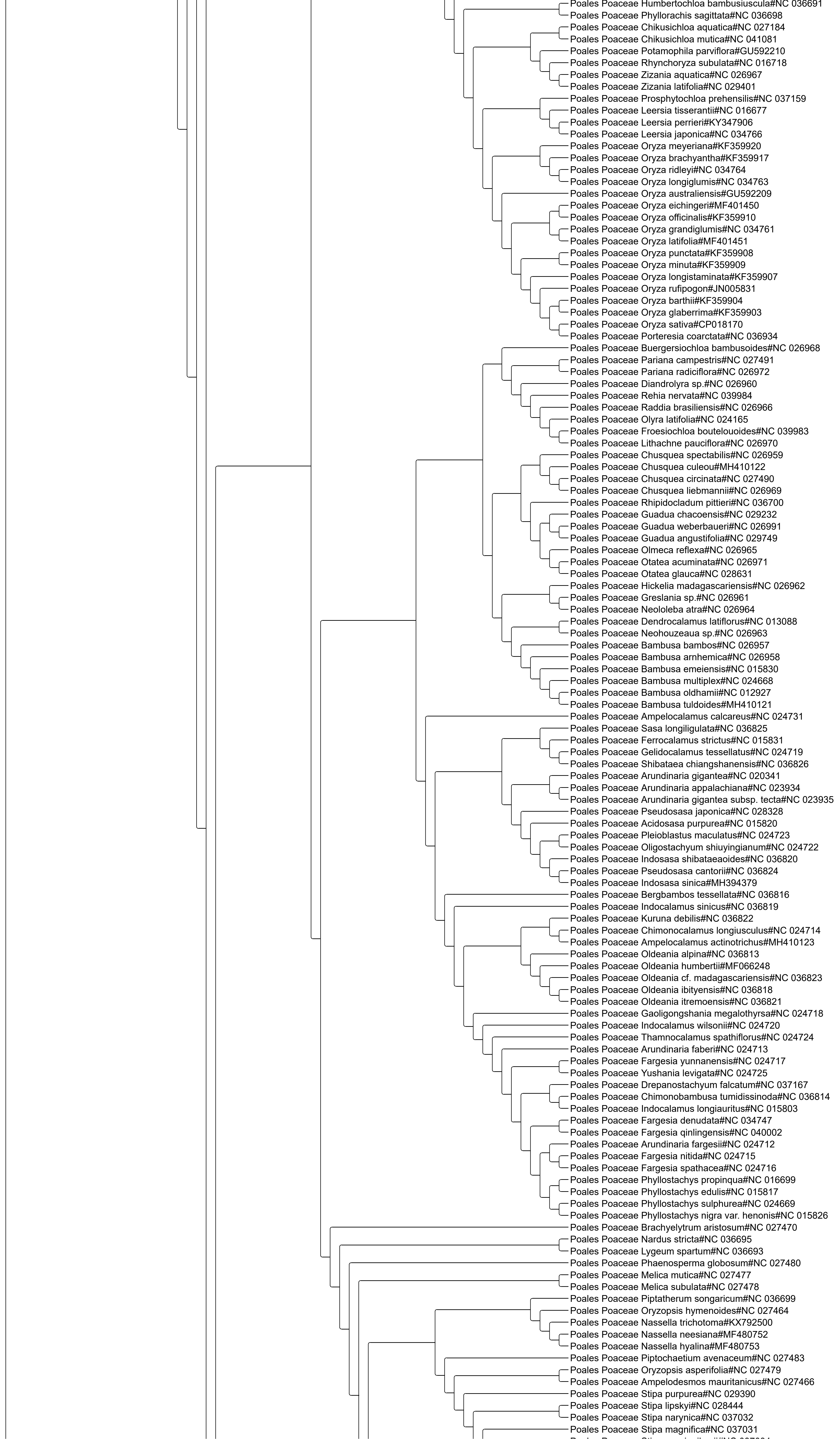
Poales Poaceae Humbertochloa bambusiuscula#NC 036691
Poales Poaceae Phyllorachis sagittata#NC 036698
Poales Poaceae Chikusichloa aquatica#NC 027184
Poales Poaceae Chikusichloa mutica#NC 041081
Poales Poaceae Potamophila parviflora#GU592210
Poales Poaceae Rhynchoryza subulata#NC 016718
Poales Poaceae Zizania aquatica#NC 026967
Poales Poaceae Zizania latifolia#NC 029401
Poales Poaceae Prosphytochloa prehensilis#NC 037159
Poales Poaceae Leersia tisserantii#NC 016677
Poales Poaceae Leersia perrieri#KY347906
Poales Poaceae Leersia japonica#NC 034766
Poales Poaceae Oryza meyeriana#KF359920
Poales Poaceae Oryza brachyantha#KF359917
Poales Poaceae Oryza ridleyi#NC 034764
Poales Poaceae Oryza longiglumis#NC 034763
Poales Poaceae Oryza australiensis#GU592209
Poales Poaceae Oryza eichingeri#MF401450
Poales Poaceae Oryza officinalis#KF359910
Poales Poaceae Oryza grandiglumis#NC 034761
Poales Poaceae Oryza latifolia#MF401451
Poales Poaceae Oryza punctata#KF359908
Poales Poaceae Oryza minuta#KF359909
Poales Poaceae Oryza longistaminata#KF359907
Poales Poaceae Oryza rufipogon#JN005831
Poales Poaceae Oryza barthii#KF359904
Poales Poaceae Oryza glaberrima#KF359903
Poales Poaceae Oryza sativa#CP018170
Poales Poaceae Porteresia coarctata#NC 036934
Poales Poaceae Buergersiochloa bambusoides#NC 026968
Poales Poaceae Pariana campestris#NC 027491
Poales Poaceae Pariana radiciflora#NC 026972
Poales Poaceae Diandrolyra sp.#NC 026960
Poales Poaceae Rehia nervata#NC 039984
Poales Poaceae Raddia brasiliensis#NC 026966
Poales Poaceae Olyra latifolia#NC 024165
Poales Poaceae Froesiochloa boutelouoides#NC 039983
Poales Poaceae Lithachne pauciflora#NC 026970
Poales Poaceae Chusquea spectabilis#NC 026959
Poales Poaceae Chusquea culeou#MH410122
Poales Poaceae Chusquea circinata#NC 027490
Poales Poaceae Chusquea liebmannii#NC 026969
Poales Poaceae Rhipidocladum pittieri#NC 036700
Poales Poaceae Guadua chacoensis#NC 029232
Poales Poaceae Guadua weberbaueri#NC 026991
Poales Poaceae Guadua angustifolia#NC 029749
Poales Poaceae Olmeca reflexa#NC 026965
Poales Poaceae Otatea acuminata#NC 026971
Poales Poaceae Otatea glauca#NC 028631
Poales Poaceae Hickelia madagascariensis#NC 026962
Poales Poaceae Greslania sp.#NC 026961
Poales Poaceae Neololeba atra#NC 026964
Poales Poaceae Dendrocalamus latiflorus#NC 013088
Poales Poaceae Neohouzeaua sp.#NC 026963
Poales Poaceae Bambusa bambos#NC 026957
Poales Poaceae Bambusa arnhemica#NC 026958
Poales Poaceae Bambusa emeiensis#NC 015830
Poales Poaceae Bambusa multiplex#NC 024668
Poales Poaceae Bambusa oldhamii#NC 012927
Poales Poaceae Bambusa tuldoides#MH410121
Poales Poaceae Ampelocalamus calcareus#NC 024731
Poales Poaceae Sasa longiligulata#NC 036825
Poales Poaceae Ferrocalamus strictus#NC 015831
Poales Poaceae Gelidocalamus tessellatus#NC 024719
Poales Poaceae Shibataea chiangshanensis#NC 036826
Poales Poaceae Arundinaria gigantea#NC 020341
Poales Poaceae Arundinaria appalachiana#NC 023934
Poales Poaceae Arundinaria gigantea subsp. tecta#NC 023935
Poales Poaceae Pseudosasa japonica#NC 028328
Poales Poaceae Acidosasa purpurea#NC 015820
Poales Poaceae Pleioblastus maculatus#NC 024723
Poales Poaceae Oligostachyum shiuyingianum#NC 024722
Poales Poaceae Indosasa shibataeaoides#NC 036820
Poales Poaceae Pseudosasa cantorii#NC 036824
Poales Poaceae Indosasa sinica#MH394379
Poales Poaceae Bergbambos tessellata#NC 036816
Poales Poaceae Indocalamus sinicus#NC 036819
Poales Poaceae Kuruna debilis#NC 036822
Poales Poaceae Chimonocalamus longiusculus#NC 024714
Poales Poaceae Ampelocalamus actinotrichus#MH410123
Poales Poaceae Oldeania alpina#NC 036813
Poales Poaceae Oldeania humbertii#MF066248
Poales Poaceae Oldeania cf. madagascariensis#NC 036823
Poales Poaceae Oldeania ibityensis#NC 036818
Poales Poaceae Oldeania itremoensis#NC 036821
Poales Poaceae Gaoligongshania megalothyrsa#NC 024718
Poales Poaceae Indocalamus wilsonii#NC 024720
Poales Poaceae Thamnocalamus spathiflorus#NC 024724
Poales Poaceae Arundinaria faberi#NC 024713
Poales Poaceae Fargesia yunnanensis#NC 024717
Poales Poaceae Yushania levigata#NC 024725
Poales Poaceae Drepanostachyum falcatum#NC 037167
Poales Poaceae Chimonobambusa tumidissinoda#NC 036814
Poales Poaceae Indocalamus longiauritus#NC 015803
Poales Poaceae Fargesia denudata#NC 034747
Poales Poaceae Fargesia qinlingensis#NC 040002
Poales Poaceae Arundinaria fargesii#NC 024712
Poales Poaceae Fargesia nitida#NC 024715
Poales Poaceae Fargesia spathacea#NC 024716
Poales Poaceae Phyllostachys propinqua#NC 016699
Poales Poaceae Phyllostachys edulis#NC 015817
Poales Poaceae Phyllostachys sulphurea#NC 024669
Poales Poaceae Phyllostachys nigra var. henonis#NC 015826
Poales Poaceae Brachyelytrum aristosum#NC 027470
Poales Poaceae Nardus stricta#NC 036695
Poales Poaceae Lygeum spartum#NC 036693
Poales Poaceae Phaenosperma globosum#NC 027480
Poales Poaceae Melica mutica#NC 027477
Poales Poaceae Melica subulata#NC 027478
Poales Poaceae Piptatherum songaricum#NC 036699
Poales Poaceae Oryzopsis hymenoides#NC 027464
Poales Poaceae Nassella trichotoma#KX792500
Poales Poaceae Nassella neesiana#MF480752
Poales Poaceae Nassella hyalina#MF480753
Poales Poaceae Piptochaetium avenaceum#NC 027483
Poales Poaceae Oryzopsis asperifolia#NC 027479
Poales Poaceae Ampelodesmos mauritanicus#NC 027466
Poales Poaceae Stipa purpurea#NC 029390
Poales Poaceae Stipa lipskyi#NC 028444
Poales Poaceae Stipa narynica#NC 037032
Poales Poaceae Stipa magnifica#NC 037031

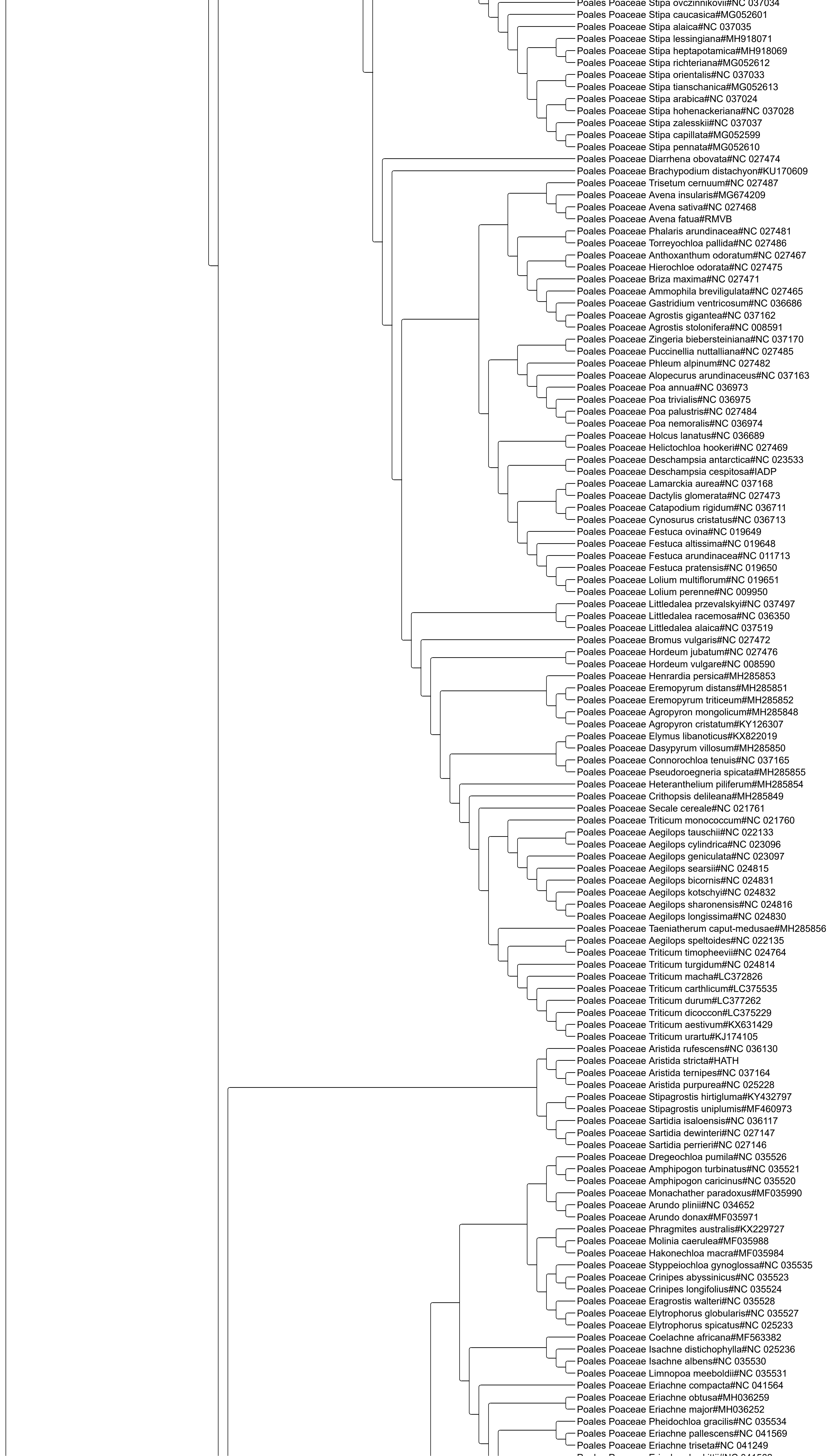

Poales Poaceae Stipa ovczinnikovii#NC 037034
Poales Poaceae Stipa caucasica#MG052601
Poales Poaceae Stipa alaica#NC 037035
Poales Poaceae Stipa lessingiana#MH918071
Poales Poaceae Stipa heptapotamica#MH918069
Poales Poaceae Stipa richteriana#MG052612
Poales Poaceae Stipa orientalis#NC 037033
Poales Poaceae Stipa tianschanica#MG052613
Poales Poaceae Stipa arabica#NC 037024
Poales Poaceae Stipa hohenackeriana#NC 037028
Poales Poaceae Stipa zalesskii#NC 037037
Poales Poaceae Stipa capillata#MG052599
Poales Poaceae Stipa pennata#MG052610
Poales Poaceae Diarrhena obovata#NC 027474
Poales Poaceae Brachypodium distachyon#KU170609
Poales Poaceae Trisetum cernuum#NC 027487
Poales Poaceae Avena insularis#MG674209
Poales Poaceae Avena sativa#NC 027468
Poales Poaceae Avena fatua#RMVB
Poales Poaceae Phalaris arundinacea#NC 027481
Poales Poaceae Torreyochloa pallida#NC 027486
Poales Poaceae Anthoxanthum odoratum#NC 027467
Poales Poaceae Hierochloe odorata#NC 027475
Poales Poaceae Briza maxima#NC 027471
Poales Poaceae Ammophila breviligulata#NC 027465
Poales Poaceae Gastridium ventricosum#NC 036686
Poales Poaceae Agrostis gigantea#NC 037162
Poales Poaceae Agrostis stolonifera#NC 008591
Poales Poaceae Zingeria biebersteiniana#NC 037170
Poales Poaceae Puccinellia nuttalliana#NC 027485
Poales Poaceae Phleum alpinum#NC 027482
Poales Poaceae Alopecurus arundinaceus#NC 037163
Poales Poaceae Poa annua#NC 036973
Poales Poaceae Poa trivialis#NC 036975
Poales Poaceae Poa palustris#NC 027484
Poales Poaceae Poa nemoralis#NC 036974
Poales Poaceae Holcus lanatus#NC 036689
Poales Poaceae Helictochloa hookeri#NC 027469
Poales Poaceae Deschampsia antarctica#NC 023533
Poales Poaceae Deschampsia cespitosa#IADP
Poales Poaceae Lamarckia aurea#NC 037168
Poales Poaceae Dactylis glomerata#NC 027473
Poales Poaceae Catapodium rigidum#NC 036711
Poales Poaceae Cynosurus cristatus#NC 036713
Poales Poaceae Festuca ovina#NC 019649
Poales Poaceae Festuca altissima#NC 019648
Poales Poaceae Festuca arundinacea#NC 011713
Poales Poaceae Festuca pratensis#NC 019650
Poales Poaceae Lolium multiflorum#NC 019651
Poales Poaceae Lolium perenne#NC 009950
Poales Poaceae Littledalea przevalskyi#NC 037497
Poales Poaceae Littledalea racemosa#NC 036350
Poales Poaceae Littledalea alaica#NC 037519
Poales Poaceae Bromus vulgaris#NC 027472
Poales Poaceae Hordeum jubatum#NC 027476
Poales Poaceae Hordeum vulgare#NC 008590
Poales Poaceae Henrardia persica#MH285853
Poales Poaceae Eremopyrum distans#MH285851
Poales Poaceae Eremopyrum triticeum#MH285852
Poales Poaceae Agropyron mongolicum#MH285848
Poales Poaceae Agropyron cristatum#KY126307
Poales Poaceae Elymus libanoticus#KX822019
Poales Poaceae Dasypyrum villosum#MH285850
Poales Poaceae Connorochloa tenuis#NC 037165
Poales Poaceae Pseudoroegneria spicata#MH285855
Poales Poaceae Heteranthelium piliferum#MH285854
Poales Poaceae Crithopsis delileana#MH285849
Poales Poaceae Secale cereale#NC 021761
Poales Poaceae Triticum monococcum#NC 021760
Poales Poaceae Aegilops tauschii#NC 022133
Poales Poaceae Aegilops cylindrica#NC 023096
Poales Poaceae Aegilops geniculata#NC 023097
Poales Poaceae Aegilops searsii#NC 024815
Poales Poaceae Aegilops bicornis#NC 024831
Poales Poaceae Aegilops kotschyi#NC 024832
Poales Poaceae Aegilops sharonensis#NC 024816
Poales Poaceae Aegilops longissima#NC 024830
Poales Poaceae Taeniatherum caput-medusae#MH285856
Poales Poaceae Aegilops speltoides#NC 022135
Poales Poaceae Triticum timopheevii#NC 024764
Poales Poaceae Triticum turgidum#NC 024814
Poales Poaceae Triticum macha#LC372826
Poales Poaceae Triticum carthlicum#LC375535
Poales Poaceae Triticum durum#LC377262
Poales Poaceae Triticum dicoccon#LC375229
Poales Poaceae Triticum aestivum#KX631429
Poales Poaceae Triticum urartu#KJ174105
Poales Poaceae Aristida rufescens#NC 036130
Poales Poaceae Aristida stricta#HATH
Poales Poaceae Aristida ternipes#NC 037164
Poales Poaceae Aristida purpurea#NC 025228
Poales Poaceae Stipagrostis hirtigluma#KY432797
Poales Poaceae Stipagrostis uniplumis#MF460973
Poales Poaceae Sartidia isaloensis#NC 036117
Poales Poaceae Sartidia dewinteri#NC 027147
Poales Poaceae Sartidia perrieri#NC 027146
Poales Poaceae Dregeochloa pumila#NC 035526
Poales Poaceae Amphipogon turbinatus#NC 035521
Poales Poaceae Amphipogon caricinus#NC 035520
Poales Poaceae Monachather paradoxus#MF035990
Poales Poaceae Arundo plinii#NC 034652
Poales Poaceae Arundo donax#MF035971
Poales Poaceae Phragmites australis#KX229727
Poales Poaceae Molinia caerulea#MF035988
Poales Poaceae Hakonechloa macra#MF035984
Poales Poaceae Styppeiochloa gynoglossa#NC 035535
Poales Poaceae Crinipes abyssinicus#NC 035523
Poales Poaceae Crinipes longifolius#NC 035524
Poales Poaceae Eragrostis walteri#NC 035528
Poales Poaceae Elytrophorus globularis#NC 035527
Poales Poaceae Elytrophorus spicatus#NC 025233
Poales Poaceae Coelachne africana#MF563382
Poales Poaceae Isachne distichophylla#NC 025236
Poales Poaceae Isachne albens#NC 035530
Poales Poaceae Limnopoa meeboldii#NC 035531
Poales Poaceae Eriachne compacta#NC 041564
Poales Poaceae Eriachne obtusa#MH036259
Poales Poaceae Eriachne major#MH036252
Poales Poaceae Pheidochloa gracilis#NC 035534
Poales Poaceae Eriachne pallescens#NC 041569
Poales Poaceae Eriachne triseta#NC 041249

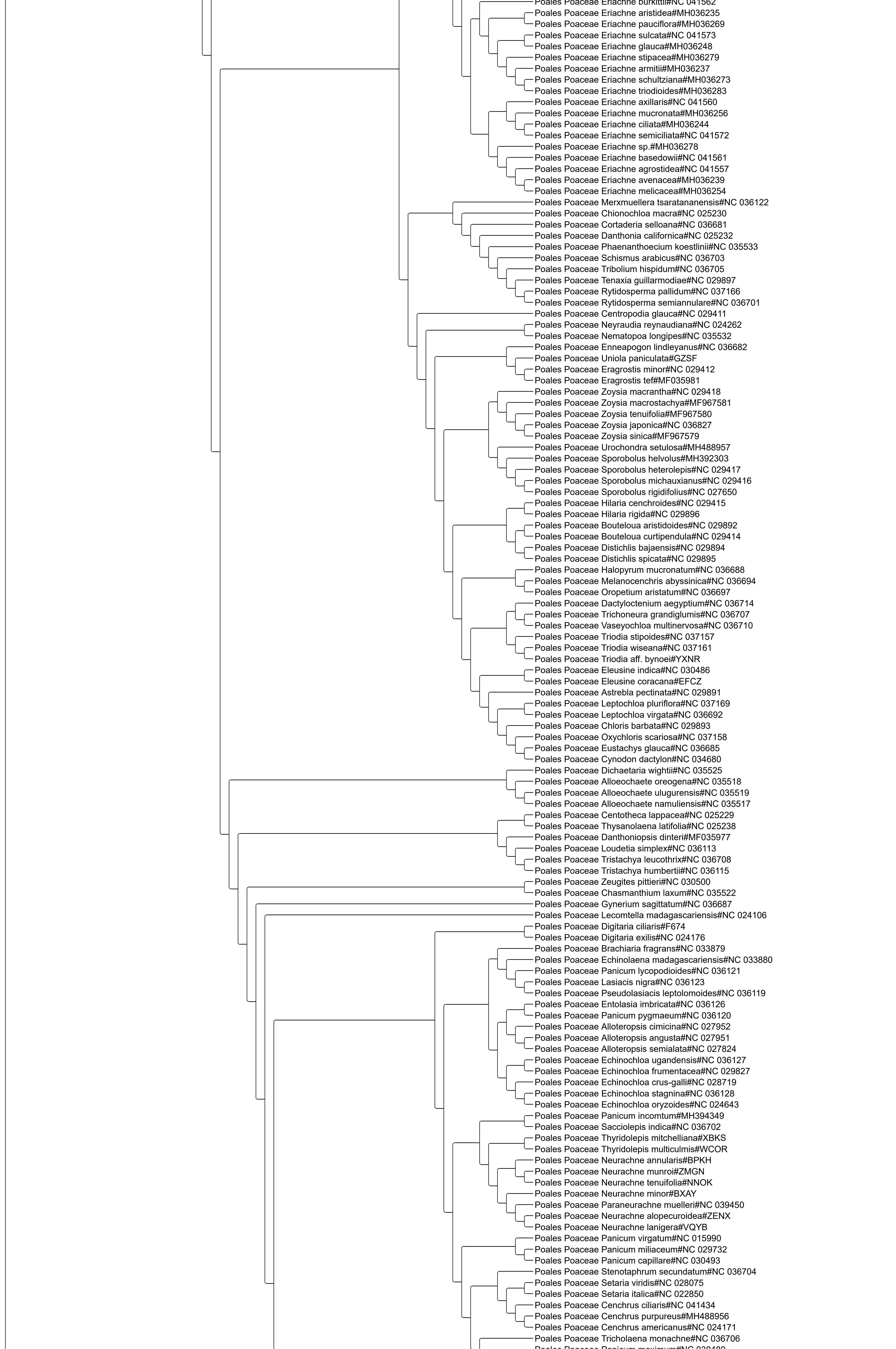
Poales Poaceae Eriachne burkittii#NC 041562
Poales Poaceae Eriachne aristidea#MH036235
Poales Poaceae Eriachne pauciflora#MH036269
Poales Poaceae Eriachne sulcata#NC 041573
Poales Poaceae Eriachne glauca#MH036248
Poales Poaceae Eriachne stipacea#MH036279
Poales Poaceae Eriachne armitii#MH036237
Poales Poaceae Eriachne schultziana#MH036273
Poales Poaceae Eriachne triodioides#MH036283
Poales Poaceae Eriachne axillaris#NC 041560
Poales Poaceae Eriachne mucronata#MH036256
Poales Poaceae Eriachne ciliata#MH036244
Poales Poaceae Eriachne semiciliata#NC 041572
Poales Poaceae Eriachne sp.#MH036278
Poales Poaceae Eriachne basedowii#NC 041561
Poales Poaceae Eriachne agrostidea#NC 041557
Poales Poaceae Eriachne avenacea#MH036239
Poales Poaceae Eriachne melicacea#MH036254
Poales Poaceae Merxmuellera tsaratananensis#NC 036122
Poales Poaceae Chionochloa macra#NC 025230
Poales Poaceae Cortaderia selloana#NC 036681
Poales Poaceae Danthonia californica#NC 025232
Poales Poaceae Phaenanthoecium koestlinii#NC 035533
Poales Poaceae Schismus arabicus#NC 036703
Poales Poaceae Tribolium hispidum#NC 036705
Poales Poaceae Tenaxia guillarmodiae#NC 029897
Poales Poaceae Rytidosperma pallidum#NC 037166
Poales Poaceae Rytidosperma semiannulare#NC 036701
Poales Poaceae Centropodia glauca#NC 029411
Poales Poaceae Neyraudia reynaudiana#NC 024262
Poales Poaceae Nematopoa longipes#NC 035532
Poales Poaceae Enneapogon lindleyanus#NC 036682
Poales Poaceae Uniola paniculata#GZSF
Poales Poaceae Eragrostis minor#NC 029412
Poales Poaceae Eragrostis tef#MF035981
Poales Poaceae Zoysia macrantha#NC 029418
Poales Poaceae Zoysia macrostachya#MF967581
Poales Poaceae Zoysia tenuifolia#MF967580
Poales Poaceae Zoysia japonica#NC 036827
Poales Poaceae Zoysia sinica#MF967579
Poales Poaceae Urochondra setulosa#MH488957
Poales Poaceae Sporobolus helvolus#MH392303
Poales Poaceae Sporobolus heterolepis#NC 029417
Poales Poaceae Sporobolus michauxianus#NC 029416
Poales Poaceae Sporobolus rigidifolius#NC 027650
Poales Poaceae Hilaria cenchroides#NC 029415
Poales Poaceae Hilaria rigida#NC 029896
Poales Poaceae Bouteloua aristidoides#NC 029892
Poales Poaceae Bouteloua curtipendula#NC 029414
Poales Poaceae Distichlis bajaensis#NC 029894
Poales Poaceae Distichlis spicata#NC 029895
Poales Poaceae Halopyrum mucronatum#NC 036688
Poales Poaceae Melanocenchris abyssinica#NC 036694
Poales Poaceae Oropetium aristatum#NC 036697
Poales Poaceae Dactyloctenium aegyptium#NC 036714
Poales Poaceae Trichoneura grandiglumis#NC 036707
Poales Poaceae Vaseyochloa multinervosa#NC 036710
Poales Poaceae Triodia stipoides#NC 037157
Poales Poaceae Triodia wiseana#NC 037161
Poales Poaceae Triodia aff. bynoei#YXNR
Poales Poaceae Eleusine indica#NC 030486
Poales Poaceae Eleusine coracana#EFCZ
Poales Poaceae Astrebla pectinata#NC 029891
Poales Poaceae Leptochloa pluriflora#NC 037169
Poales Poaceae Leptochloa virgata#NC 036692
Poales Poaceae Chloris barbata#NC 029893
Poales Poaceae Oxychloris scariosa#NC 037158
Poales Poaceae Eustachys glauca#NC 036685
Poales Poaceae Cynodon dactylon#NC 034680
Poales Poaceae Dichaetaria wightii#NC 035525
Poales Poaceae Alloeochaete oreogena#NC 035518
Poales Poaceae Alloeochaete ulugurensis#NC 035519
Poales Poaceae Alloeochaete namuliensis#NC 035517
Poales Poaceae Centotheca lappacea#NC 025229
Poales Poaceae Thysanolaena latifolia#NC 025238
Poales Poaceae Danthoniopsis dinteri#MF035977
Poales Poaceae Loudetia simplex#NC 036113
Poales Poaceae Tristachya leucothrix#NC 036708
Poales Poaceae Tristachya humbertii#NC 036115
Poales Poaceae Zeugites pittieri#NC 030500
Poales Poaceae Chasmanthium laxum#NC 035522
Poales Poaceae Gynerium sagittatum#NC 036687
Poales Poaceae Lecomtella madagascariensis#NC 024106
Poales Poaceae Digitaria ciliaris#F674
Poales Poaceae Digitaria exilis#NC 024176
Poales Poaceae Brachiaria fragrans#NC 033879
Poales Poaceae Echinolaena madagascariensis#NC 033880
Poales Poaceae Panicum lycopodioides#NC 036121
Poales Poaceae Lasiacis nigra#NC 036123
Poales Poaceae Pseudolasiacis leptolomoides#NC 036119
Poales Poaceae Entolasia imbricata#NC 036126
Poales Poaceae Panicum pygmaeum#NC 036120
Poales Poaceae Alloteropsis cimicina#NC 027952
Poales Poaceae Alloteropsis angusta#NC 027951
Poales Poaceae Alloteropsis semialata#NC 027824
Poales Poaceae Echinochloa ugandensis#NC 036127
Poales Poaceae Echinochloa frumentacea#NC 029827
Poales Poaceae Echinochloa crus-galli#NC 028719
Poales Poaceae Echinochloa stagnina#NC 036128
Poales Poaceae Echinochloa oryzoides#NC 024643
Poales Poaceae Panicum incomtum#MH394349
Poales Poaceae Sacciolepis indica#NC 036702
Poales Poaceae Thyridolepis mitchelliana#XBKS
Poales Poaceae Thyridolepis multiculmis#WCOR
Poales Poaceae Neurachne annularis#BPKH
Poales Poaceae Neurachne munroi#ZMGN
Poales Poaceae Neurachne tenuifolia#NNOK
Poales Poaceae Neurachne minor#BXAY
Poales Poaceae Paraneurachne muelleri#NC 039450
Poales Poaceae Neurachne alopecuroidea#ZENX
Poales Poaceae Neurachne lanigera#VQYB
Poales Poaceae Panicum virgatum#NC 015990
Poales Poaceae Panicum miliaceum#NC 029732
Poales Poaceae Panicum capillare#NC 030493
Poales Poaceae Stenotaphrum secundatum#NC 036704
Poales Poaceae Setaria viridis#NC 028075
Poales Poaceae Setaria italica#NC 022850
Poales Poaceae Cenchrus ciliaris#NC 041434
Poales Poaceae Cenchrus purpureus#MH488956
Poales Poaceae Cenchrus americanus#NC 024171
Poales Poaceae Tricholaena monachne#NC 036706

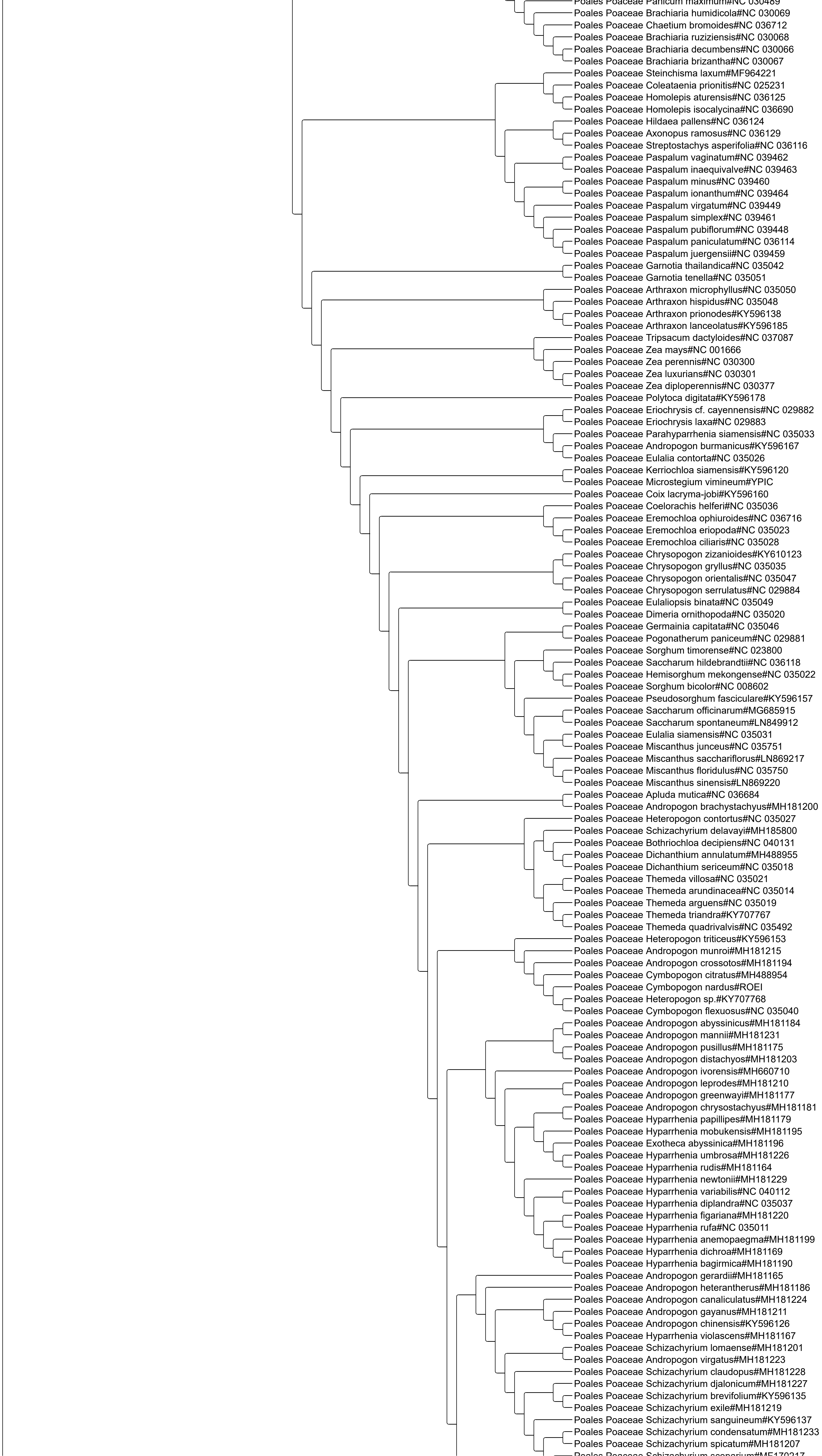
Poales Poaceae Panicum maximum#NC 030489
Poales Poaceae Brachiaria humidicola#NC 030069
Poales Poaceae Chaetium bromoides#NC 036712
Poales Poaceae Brachiaria ruziziensis#NC 030068
Poales Poaceae Brachiaria decumbens#NC 030066
Poales Poaceae Brachiaria brizantha#NC 030067
Poales Poaceae Steinchisma laxum#MF964221
Poales Poaceae Coleataenia prionitis#NC 025231
Poales Poaceae Homolepis aturensis#NC 036125
Poales Poaceae Homolepis isocalycina#NC 036690
Poales Poaceae Hildaea pallens#NC 036124
Poales Poaceae Axonopus ramosus#NC 036129
Poales Poaceae Streptostachys asperifolia#NC 036116
Poales Poaceae Paspalum vaginatum#NC 039462
Poales Poaceae Paspalum inaequivalve#NC 039463
Poales Poaceae Paspalum minus#NC 039460
Poales Poaceae Paspalum ionanthum#NC 039464
Poales Poaceae Paspalum virgatum#NC 039449
Poales Poaceae Paspalum simplex#NC 039461
Poales Poaceae Paspalum pubiflorum#NC 039448
Poales Poaceae Paspalum paniculatum#NC 036114
Poales Poaceae Paspalum juergensii#NC 039459
Poales Poaceae Garnotia thailandica#NC 035042
Poales Poaceae Garnotia tenella#NC 035051
Poales Poaceae Arthraxon microphyllus#NC 035050
Poales Poaceae Arthraxon hispidus#NC 035048
Poales Poaceae Arthraxon prionodes#KY596138
Poales Poaceae Arthraxon lanceolatus#KY596185
Poales Poaceae Tripsacum dactyloides#NC 037087
Poales Poaceae Zea mays#NC 001666
Poales Poaceae Zea perennis#NC 030300
Poales Poaceae Zea luxurians#NC 030301
Poales Poaceae Zea diploperennis#NC 030377
Poales Poaceae Polytoca digitata#KY596178
Poales Poaceae Eriochrysis cf. cayennensis#NC 029882
Poales Poaceae Eriochrysis laxa#NC 029883
Poales Poaceae Parahyparrhenia siamensis#NC 035033
Poales Poaceae Andropogon burmanicus#KY596167
Poales Poaceae Eulalia contorta#NC 035026
Poales Poaceae Kerriochloa siamensis#KY596120
Poales Poaceae Microstegium vimineum#YPIC
Poales Poaceae Coix lacryma-jobi#KY596160
Poales Poaceae Coelorachis helferi#NC 035036
Poales Poaceae Eremochloa ophiuroides#NC 036716
Poales Poaceae Eremochloa eriopoda#NC 035023
Poales Poaceae Eremochloa ciliaris#NC 035028
Poales Poaceae Chrysopogon zizanioides#KY610123
Poales Poaceae Chrysopogon gryllus#NC 035035
Poales Poaceae Chrysopogon orientalis#NC 035047
Poales Poaceae Chrysopogon serrulatus#NC 029884
Poales Poaceae Eulaliopsis binata#NC 035049
Poales Poaceae Dimeria ornithopoda#NC 035020
Poales Poaceae Germainia capitata#NC 035046
Poales Poaceae Pogonatherum paniceum#NC 029881
Poales Poaceae Sorghum timorense#NC 023800
Poales Poaceae Saccharum hildebrandtii#NC 036118
Poales Poaceae Hemisorghum mekongense#NC 035022
Poales Poaceae Sorghum bicolor#NC 008602
Poales Poaceae Pseudosorghum fasciculare#KY596157
Poales Poaceae Saccharum officinarum#MG685915
Poales Poaceae Saccharum spontaneum#LN849912
Poales Poaceae Eulalia siamensis#NC 035031
Poales Poaceae Miscanthus junceus#NC 035751
Poales Poaceae Miscanthus sacchariflorus#LN869217
Poales Poaceae Miscanthus floridulus#NC 035750
Poales Poaceae Miscanthus sinensis#LN869220
Poales Poaceae Apluda mutica#NC 036684
Poales Poaceae Andropogon brachystachyus#MH181200
Poales Poaceae Heteropogon contortus#NC 035027
Poales Poaceae Schizachyrium delavayi#MH185800
Poales Poaceae Bothriochloa decipiens#NC 040131
Poales Poaceae Dichanthium annulatum#MH488955
Poales Poaceae Dichanthium sericeum#NC 035018
Poales Poaceae Themeda villosa#NC 035021
Poales Poaceae Themeda arundinacea#NC 035014
Poales Poaceae Themeda arguens#NC 035019
Poales Poaceae Themeda triandra#KY707767
Poales Poaceae Themeda quadrivalvis#NC 035492
Poales Poaceae Heteropogon triticeus#KY596153
Poales Poaceae Andropogon munroi#MH181215
Poales Poaceae Andropogon crossotos#MH181194
Poales Poaceae Cymbopogon citratus#MH488954
Poales Poaceae Cymbopogon nardus#ROEI
Poales Poaceae Heteropogon sp.#KY707768
Poales Poaceae Cymbopogon flexuosus#NC 035040
Poales Poaceae Andropogon abyssinicus#MH181184
Poales Poaceae Andropogon mannii#MH181231
Poales Poaceae Andropogon pusillus#MH181175
Poales Poaceae Andropogon distachyos#MH181203
Poales Poaceae Andropogon ivorensis#MH660710
Poales Poaceae Andropogon leprodes#MH181210
Poales Poaceae Andropogon greenwayi#MH181177
Poales Poaceae Andropogon chrysostachyus#MH181181
Poales Poaceae Hyparrhenia papillipes#MH181179
Poales Poaceae Hyparrhenia mobukensis#MH181195
Poales Poaceae Exotheca abyssinica#MH181196
Poales Poaceae Hyparrhenia umbrosa#MH181226
Poales Poaceae Hyparrhenia rudis#MH181164
Poales Poaceae Hyparrhenia newtonii#MH181229
Poales Poaceae Hyparrhenia variabilis#NC 040112
Poales Poaceae Hyparrhenia diplandra#NC 035037
Poales Poaceae Hyparrhenia figariana#MH181220
Poales Poaceae Hyparrhenia rufa#NC 035011
Poales Poaceae Hyparrhenia anemopaegma#MH181199
Poales Poaceae Hyparrhenia dichroa#MH181169
Poales Poaceae Hyparrhenia bagirmica#MH181190
Poales Poaceae Andropogon gerardii#MH181165
Poales Poaceae Andropogon heterantherus#MH181186
Poales Poaceae Andropogon canaliculatus#MH181224
Poales Poaceae Andropogon gayanus#MH181211
Poales Poaceae Andropogon chinensis#KY596126
Poales Poaceae Hyparrhenia violascens#MH181167
Poales Poaceae Schizachyrium lomaense#MH181201
Poales Poaceae Andropogon virgatus#MH181223
Poales Poaceae Schizachyrium claudopus#MH181228
Poales Poaceae Schizachyrium djalonicum#MH181227
Poales Poaceae Schizachyrium brevifolium#KY596135
Poales Poaceae Schizachyrium exile#MH181219
Poales Poaceae Schizachyrium sanguineum#KY596137
Poales Poaceae Schizachyrium condensatum#MH181233
Poales Poaceae Schizachyrium spicatum#MH181207

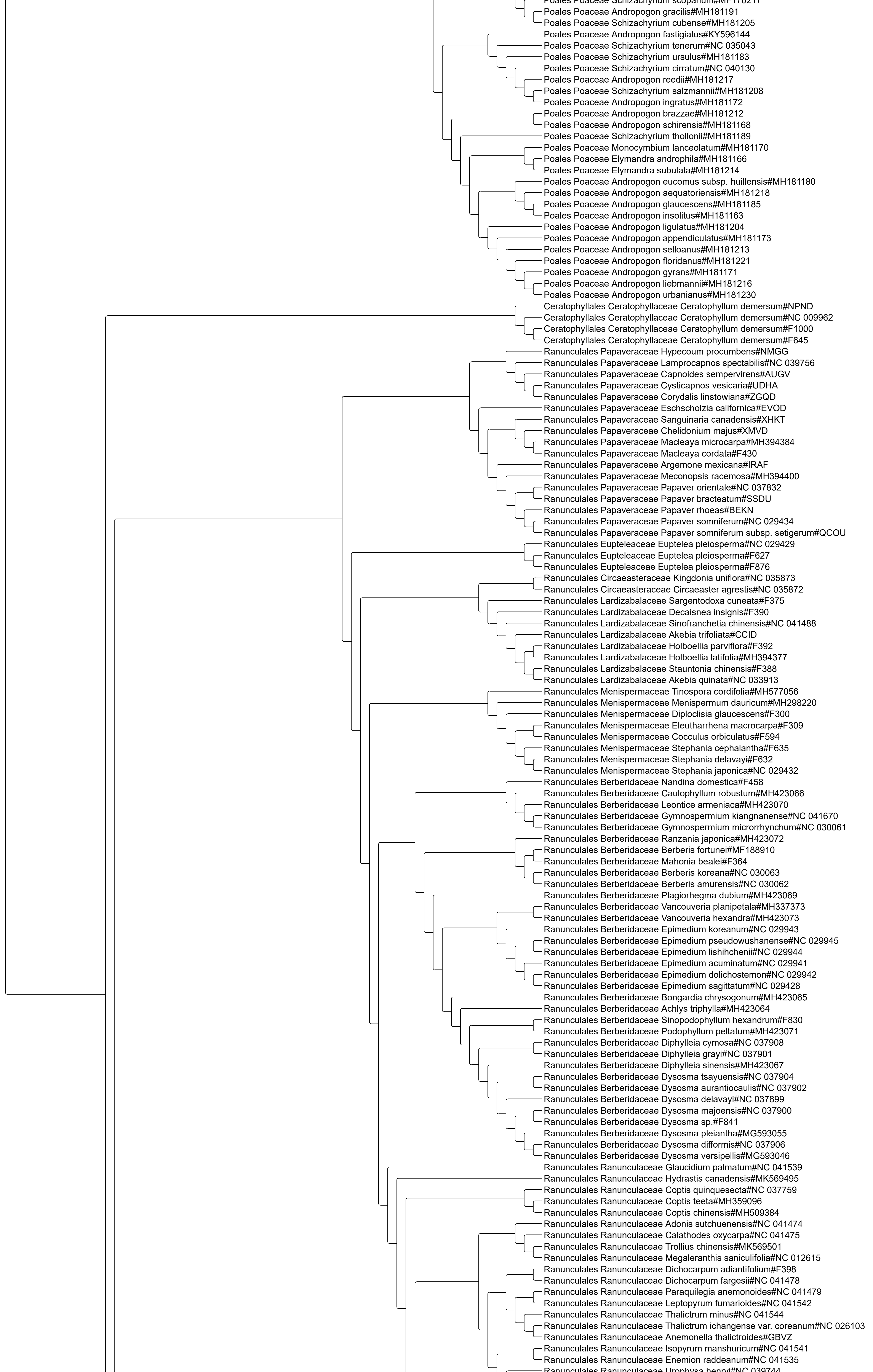
Poales Poaceae Andropogon gracilis#MH181191
Poales Poaceae Schizachyrium cubense#MH181205
Poales Poaceae Andropogon fastigiatus#KY596144
Poales Poaceae Schizachyrium tenerum#NC 035043
Poales Poaceae Schizachyrium ursulus#MH181183
Poales Poaceae Schizachyrium cirratum#NC 040130
Poales Poaceae Andropogon reedii#MH181217
Poales Poaceae Schizachyrium salzmannii#MH181208
Poales Poaceae Andropogon ingratus#MH181172
Poales Poaceae Andropogon brazzae#MH181212
Poales Poaceae Andropogon schirensis#MH181168
Poales Poaceae Schizachyrium thollonii#MH181189
Poales Poaceae Monocymbium lanceolatum#MH181170
Poales Poaceae Elymandra androphila#MH181166
Poales Poaceae Elymandra subulata#MH181214
Poales Poaceae Andropogon eucomus subsp. huillensis#MH181180
Poales Poaceae Andropogon aequatoriensis#MH181218
Poales Poaceae Andropogon glaucescens#MH181185
Poales Poaceae Andropogon insolitus#MH181163
Poales Poaceae Andropogon ligulatus#MH181204
Poales Poaceae Andropogon appendiculatus#MH181173
Poales Poaceae Andropogon selloanus#MH181213
Poales Poaceae Andropogon floridanus#MH181221
Poales Poaceae Andropogon gyrans#MH181171
Poales Poaceae Andropogon liebmannii#MH181216
Poales Poaceae Andropogon urbanianus#MH181230
Ceratophyllales Ceratophyllaceae Ceratophyllum demersum#NPND
Ceratophyllales Ceratophyllaceae Ceratophyllum demersum#NC 009962
Ceratophyllales Ceratophyllaceae Ceratophyllum demersum#F1000
Ceratophyllales Ceratophyllaceae Ceratophyllum demersum#F645
Ranunculales Papaveraceae Hypecoum procumbens#NMGG
Ranunculales Papaveraceae Lamprocapnos spectabilis#NC 039756
Ranunculales Papaveraceae Capnoides sempervirens#AUGV
Ranunculales Papaveraceae Cysticapnos vesicaria#UDHA
Ranunculales Papaveraceae Corydalis linstowiana#ZGQD
Ranunculales Papaveraceae Eschscholzia californica#EVOD
Ranunculales Papaveraceae Sanguinaria canadensis#XHKT
Ranunculales Papaveraceae Chelidonium majus#XMVD
Ranunculales Papaveraceae Macleaya microcarpa#MH394384
Ranunculales Papaveraceae Macleaya cordata#F430
Ranunculales Papaveraceae Argemone mexicana#IRAF
Ranunculales Papaveraceae Meconopsis racemosa#MH394400
Ranunculales Papaveraceae Papaver orientale#NC 037832
Ranunculales Papaveraceae Papaver bracteatum#SSDU
Ranunculales Papaveraceae Papaver rhoeas#BEKN
Ranunculales Papaveraceae Papaver somniferum#NC 029434
Ranunculales Papaveraceae Papaver somniferum subsp. setigerum#QCOU
Ranunculales Eupteleaceae Euptelea pleiosperma#NC 029429
Ranunculales Eupteleaceae Euptelea pleiosperma#F627
Ranunculales Eupteleaceae Euptelea pleiosperma#F876
Ranunculales Circaeasteraceae Kingdonia uniflora#NC 035873
Ranunculales Circaeasteraceae Circaeaster agrestis#NC 035872
Ranunculales Lardizabalaceae Sargentodoxa cuneata#F375
Ranunculales Lardizabalaceae Decaisnea insignis#F390
Ranunculales Lardizabalaceae Sinofranchetia chinensis#NC 041488
Ranunculales Lardizabalaceae Akebia trifoliata#CCID
Ranunculales Lardizabalaceae Holboellia parviflora#F392
Ranunculales Lardizabalaceae Holboellia latifolia#MH394377
Ranunculales Lardizabalaceae Stauntonia chinensis#F388
Ranunculales Lardizabalaceae Akebia quinata#NC 033913
Ranunculales Menispermaceae Tinospora cordifolia#MH577056
Ranunculales Menispermaceae Menispermum dauricum#MH298220
Ranunculales Menispermaceae Diploclisia glaucescens#F300
Ranunculales Menispermaceae Eleutharrhena macrocarpa#F309
Ranunculales Menispermaceae Cocculus orbiculatus#F594
Ranunculales Menispermaceae Stephania cephalantha#F635
Ranunculales Menispermaceae Stephania delavayi#F632
Ranunculales Menispermaceae Stephania japonica#NC 029432
Ranunculales Berberidaceae Nandina domestica#F458
Ranunculales Berberidaceae Caulophyllum robustum#MH423066
Ranunculales Berberidaceae Leontice armeniaca#MH423070
Ranunculales Berberidaceae Gymnospermium kiangnanense#NC 041670
Ranunculales Berberidaceae Gymnospermium microrrhynchum#NC 030061
Ranunculales Berberidaceae Ranzania japonica#MH423072
Ranunculales Berberidaceae Berberis fortunei#MF188910
Ranunculales Berberidaceae Mahonia bealei#F364
Ranunculales Berberidaceae Berberis koreana#NC 030063
Ranunculales Berberidaceae Berberis amurensis#NC 030062
Ranunculales Berberidaceae Plagiorhegma dubium#MH423069
Ranunculales Berberidaceae Vancouveria planipetala#MH337373
Ranunculales Berberidaceae Vancouveria hexandra#MH423073
Ranunculales Berberidaceae Epimedium koreanum#NC 029943
Ranunculales Berberidaceae Epimedium pseudowushanense#NC 029945
Ranunculales Berberidaceae Epimedium lishihchenii#NC 029944
Ranunculales Berberidaceae Epimedium acuminatum#NC 029941
Ranunculales Berberidaceae Epimedium dolichostemon#NC 029942
Ranunculales Berberidaceae Epimedium sagittatum#NC 029428
Ranunculales Berberidaceae Bongardia chrysogonum#MH423065
Ranunculales Berberidaceae Achlys triphylla#MH423064
Ranunculales Berberidaceae Sinopodophyllum hexandrum#F830
Ranunculales Berberidaceae Podophyllum peltatum#MH423071
Ranunculales Berberidaceae Diphylleia cymosa#NC 037908
Ranunculales Berberidaceae Diphylleia grayi#NC 037901
Ranunculales Berberidaceae Diphylleia sinensis#MH423067
Ranunculales Berberidaceae Dysosma tsayuensis#NC 037904
Ranunculales Berberidaceae Dysosma aurantiocaulis#NC 037902
Ranunculales Berberidaceae Dysosma delavayi#NC 037899
Ranunculales Berberidaceae Dysosma majoensis#NC 037900
Ranunculales Berberidaceae Dysosma sp.#F841
Ranunculales Berberidaceae Dysosma pleiantha#MG593055
Ranunculales Berberidaceae Dysosma difformis#NC 037906
Ranunculales Berberidaceae Dysosma versipellis#MG593046
Ranunculales Ranunculaceae Glaucidium palmatum#NC 041539
Ranunculales Ranunculaceae Hydrastis canadensis#MK569495
Ranunculales Ranunculaceae Coptis quinquesecta#NC 037759
Ranunculales Ranunculaceae Coptis teeta#MH359096
Ranunculales Ranunculaceae Coptis chinensis#MH509384
Ranunculales Ranunculaceae Adonis sutchuenensis#NC 041474
Ranunculales Ranunculaceae Calathodes oxycarpa#NC 041475
Ranunculales Ranunculaceae Trollius chinensis#MK569501
Ranunculales Ranunculaceae Megaleranthis saniculifolia#NC 012615
Ranunculales Ranunculaceae Dichocarpum adiantifolium#F398
Ranunculales Ranunculaceae Dichocarpum fargesii#NC 041478
Ranunculales Ranunculaceae Paraquilegia anemonoides#NC 041479
Ranunculales Ranunculaceae Leptopyrum fumarioides#NC 041542
Ranunculales Ranunculaceae Thalictrum minus#NC 041544
Ranunculales Ranunculaceae Thalictrum ichangense var. coreanum#NC 026103
Ranunculales Ranunculaceae Anemonella thalictroides#GBVZ
Ranunculales Ranunculaceae Isopyrum manshuricum#NC 041541
Ranunculales Ranunculaceae Enemion raddeanum#NC 041535

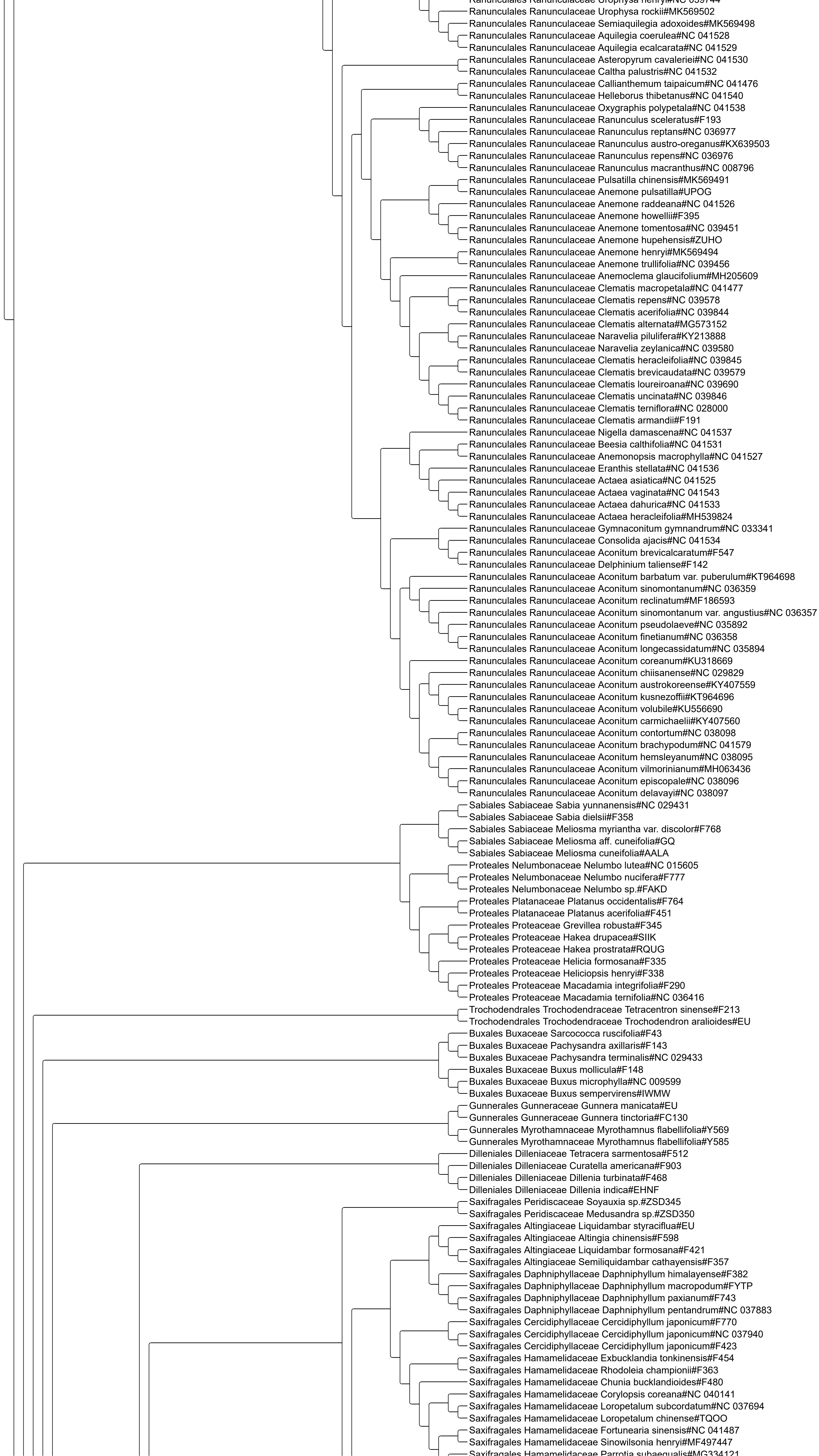

Ranunculales Ranunculaceae Urophysa henryi#NC 039744
Ranunculales Ranunculaceae Urophysa rockii#MK569502
Ranunculales Ranunculaceae Semiaquilegia adoxoides#MK569498
Ranunculales Ranunculaceae Aquilegia coerulea#NC 041528
Ranunculales Ranunculaceae Aquilegia ecalcarata#NC 041529
Ranunculales Ranunculaceae Asteropyrum cavaleriei#NC 041530
Ranunculales Ranunculaceae Caltha palustris#NC 041532
Ranunculales Ranunculaceae Callianthemum taipaicum#NC 041476
Ranunculales Ranunculaceae Helleborus thibetanus#NC 041540
Ranunculales Ranunculaceae Oxygraphis polypetala#NC 041538
Ranunculales Ranunculaceae Ranunculus sceleratus#F193
Ranunculales Ranunculaceae Ranunculus reptans#NC 036977
Ranunculales Ranunculaceae Ranunculus austro-oreganus#KX639503
Ranunculales Ranunculaceae Ranunculus repens#NC 036976
Ranunculales Ranunculaceae Ranunculus macranthus#NC 008796
Ranunculales Ranunculaceae Pulsatilla chinensis#MK569491
Ranunculales Ranunculaceae Anemone pulsatilla#UPOG
Ranunculales Ranunculaceae Anemone raddeana#NC 041526
Ranunculales Ranunculaceae Anemone howellii#F395
Ranunculales Ranunculaceae Anemone tomentosa#NC 039451
Ranunculales Ranunculaceae Anemone hupehensis#ZUHO
Ranunculales Ranunculaceae Anemone henryi#MK569494
Ranunculales Ranunculaceae Anemone trullifolia#NC 039456
Ranunculales Ranunculaceae Anemoclema glaucifolium#MH205609
Ranunculales Ranunculaceae Clematis macropetala#NC 041477
Ranunculales Ranunculaceae Clematis repens#NC 039578
Ranunculales Ranunculaceae Clematis acerifolia#NC 039844
Ranunculales Ranunculaceae Clematis alternata#MG573152
Ranunculales Ranunculaceae Naravelia pilulifera#KY213888
Ranunculales Ranunculaceae Naravelia zeylanica#NC 039580
Ranunculales Ranunculaceae Clematis heracleifolia#NC 039845
Ranunculales Ranunculaceae Clematis brevicaudata#NC 039579
Ranunculales Ranunculaceae Clematis loureiroana#NC 039690
Ranunculales Ranunculaceae Clematis uncinata#NC 039846
Ranunculales Ranunculaceae Clematis terniflora#NC 028000
Ranunculales Ranunculaceae Clematis armandii#F191
Ranunculales Ranunculaceae Nigella damascena#NC 041537
Ranunculales Ranunculaceae Beesia calthifolia#NC 041531
Ranunculales Ranunculaceae Anemonopsis macrophylla#NC 041527
Ranunculales Ranunculaceae Eranthis stellata#NC 041536
Ranunculales Ranunculaceae Actaea asiatica#NC 041525
Ranunculales Ranunculaceae Actaea vaginata#NC 041543
Ranunculales Ranunculaceae Actaea dahurica#NC 041533
Ranunculales Ranunculaceae Actaea heracleifolia#MH539824
Ranunculales Ranunculaceae Gymnaconitum gymnandrum#NC 033341
Ranunculales Ranunculaceae Consolida ajacis#NC 041534
Ranunculales Ranunculaceae Aconitum brevicalcaratum#F547
Ranunculales Ranunculaceae Delphinium taliense#F142
Ranunculales Ranunculaceae Aconitum barbatum var. puberulum#KT964698
Ranunculales Ranunculaceae Aconitum sinomontanum#NC 036359
Ranunculales Ranunculaceae Aconitum reclinatum#MF186593
Ranunculales Ranunculaceae Aconitum sinomontanum var. angustius#NC 036357
Ranunculales Ranunculaceae Aconitum pseudolaeve#NC 035892
Ranunculales Ranunculaceae Aconitum finetianum#NC 036358
Ranunculales Ranunculaceae Aconitum longecassidatum#NC 035894
Ranunculales Ranunculaceae Aconitum coreanum#KU318669
Ranunculales Ranunculaceae Aconitum chiisanense#NC 029829
Ranunculales Ranunculaceae Aconitum austrokoreense#KY407559
Ranunculales Ranunculaceae Aconitum kusnezoffii#KT964696
Ranunculales Ranunculaceae Aconitum volubile#KU556690
Ranunculales Ranunculaceae Aconitum carmichaelii#KY407560
Ranunculales Ranunculaceae Aconitum contortum#NC 038098
Ranunculales Ranunculaceae Aconitum brachypodum#NC 041579
Ranunculales Ranunculaceae Aconitum hemsleyanum#NC 038095
Ranunculales Ranunculaceae Aconitum vilmorinianum#MH063436
Ranunculales Ranunculaceae Aconitum episcopale#NC 038096
Ranunculales Ranunculaceae Aconitum delavayi#NC 038097
Sabiales Sabiaceae Sabia yunnanensis#NC 029431
Sabiales Sabiaceae Sabia dielsii#F358
Sabiales Sabiaceae Meliosma myriantha var. discolor#F768
Sabiales Sabiaceae Meliosma aff. cuneifolia#GQ
Sabiales Sabiaceae Meliosma cuneifolia#AALA
Proteales Nelumbonaceae Nelumbo lutea#NC 015605
Proteales Nelumbonaceae Nelumbo nucifera#F777
Proteales Nelumbonaceae Nelumbo sp.#FAKD
Proteales Platanaceae Platanus occidentalis#F764
Proteales Platanaceae Platanus acerifolia#F451
Proteales Proteaceae Grevillea robusta#F345
Proteales Proteaceae Hakea drupacea#SIIK
Proteales Proteaceae Hakea prostrata#RQUG
Proteales Proteaceae Helicia formosana#F335
Proteales Proteaceae Heliciopsis henryi#F338
Proteales Proteaceae Macadamia integrifolia#F290
Proteales Proteaceae Macadamia ternifolia#NC 036416
Trochodendrales Trochodendraceae Tetracentron sinense#F213
Trochodendrales Trochodendraceae Trochodendron aralioides#EU
Buxales Buxaceae Sarcococca ruscifolia#F43
Buxales Buxaceae Pachysandra axillaris#F143
Buxales Buxaceae Pachysandra terminalis#NC 029433
Buxales Buxaceae Buxus mollicula#F148
Buxales Buxaceae Buxus microphylla#NC 009599
Buxales Buxaceae Buxus sempervirens#IWMW
Gunnerales Gunneraceae Gunnera manicata#EU
Gunnerales Gunneraceae Gunnera tinctoria#FC130
Gunnerales Myrothamnaceae Myrothamnus flabellifolia#Y569
Gunnerales Myrothamnaceae Myrothamnus flabellifolia#Y585
Dilleniales Dilleniaceae Tetracera sarmentosa#F512
Dilleniales Dilleniaceae Curatella americana#F903
Dilleniales Dilleniaceae Dillenia turbinata#F468
Dilleniales Dilleniaceae Dillenia indica#EHNF
Saxifragales Peridiscaceae Soyauxia sp.#ZSD345
Saxifragales Peridiscaceae Medusandra sp.#ZSD350
Saxifragales Altingiaceae Liquidambar styraciflua#EU
Saxifragales Altingiaceae Altingia chinensis#F598
Saxifragales Altingiaceae Liquidambar formosana#F421
Saxifragales Altingiaceae Semiliquidambar cathayensis#F357
Saxifragales Daphniphyllaceae Daphniphyllum himalayense#F382
Saxifragales Daphniphyllaceae Daphniphyllum macropodum#FYTP
Saxifragales Daphniphyllaceae Daphniphyllum paxianum#F743
Saxifragales Daphniphyllaceae Daphniphyllum pentandrum#NC 037883
Saxifragales Cercidiphyllaceae Cercidiphyllum japonicum#F770
Saxifragales Cercidiphyllaceae Cercidiphyllum japonicum#NC 037940
Saxifragales Cercidiphyllaceae Cercidiphyllum japonicum#F423
Saxifragales Hamamelidaceae Exbucklandia tonkinensis#F454
Saxifragales Hamamelidaceae Rhodoleia championii#F363
Saxifragales Hamamelidaceae Chunia bucklandioides#F480
Saxifragales Hamamelidaceae Corylopsis coreana#NC 040141
Saxifragales Hamamelidaceae Loropetalum subcordatum#NC 037694
Saxifragales Hamamelidaceae Loropetalum chinense#TQOO
Saxifragales Hamamelidaceae Fortunearia sinensis#NC 041487
Saxifragales Hamamelidaceae Sinowilsonia henryi#MF497447
Saxifragales Hamamelidaceae Parrotia subaequalis#MG334121

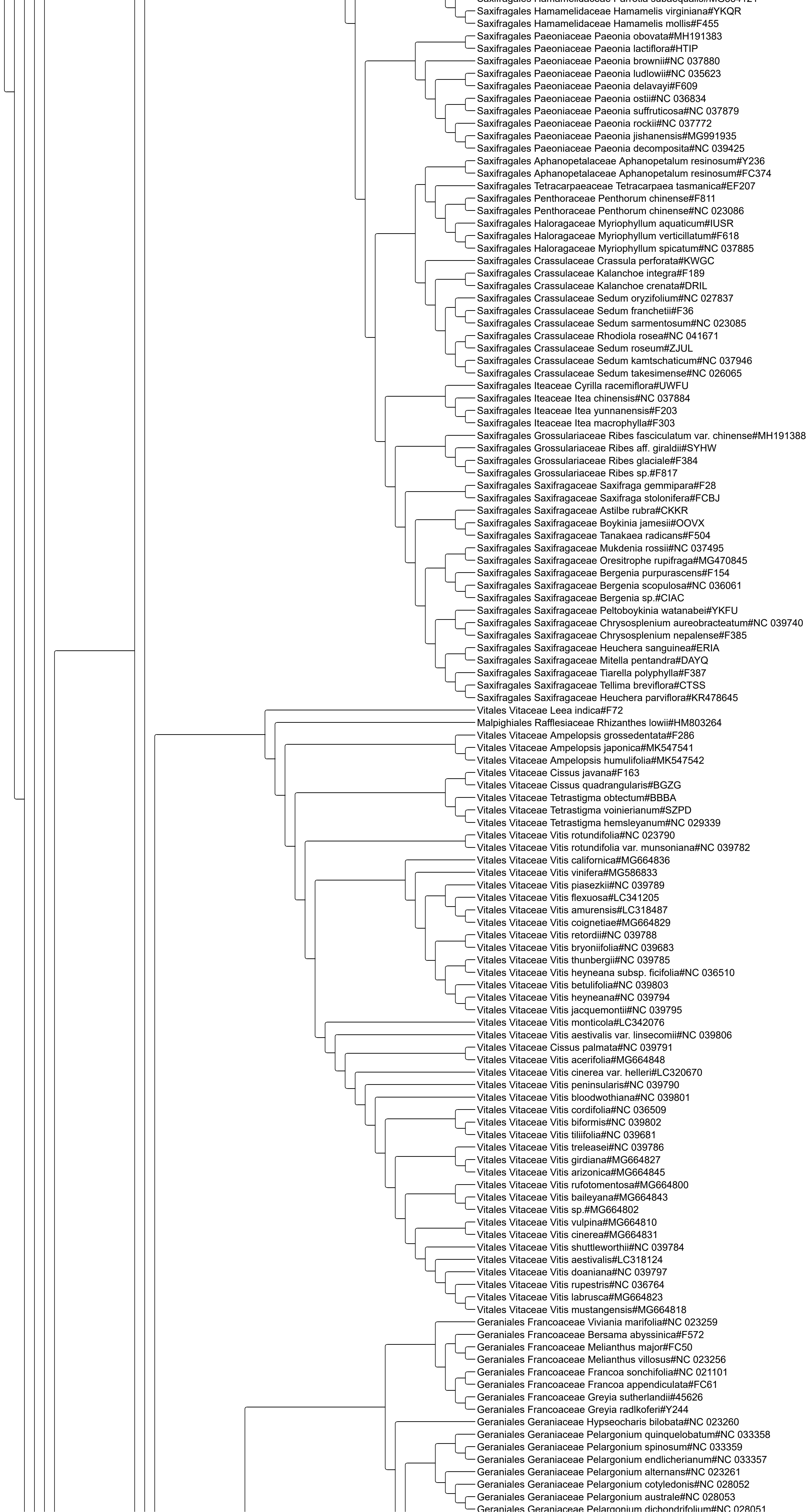

Saxifragales Hamamelidaceae Hamamelis virginiana#YKQR
Saxifragales Hamamelidaceae Hamamelis mollis#F455
Saxifragales Paeoniaceae Paeonia obovata#MH191383
Saxifragales Paeoniaceae Paeonia lactiflora#HTIP
Saxifragales Paeoniaceae Paeonia brownii#NC 037880
Saxifragales Paeoniaceae Paeonia ludlowii#NC 035623
Saxifragales Paeoniaceae Paeonia delavayi#F609
Saxifragales Paeoniaceae Paeonia ostii#NC 036834
Saxifragales Paeoniaceae Paeonia suffruticosa#NC 037879
Saxifragales Paeoniaceae Paeonia rockii#NC 037772
Saxifragales Paeoniaceae Paeonia jishanensis#MG991935
Saxifragales Paeoniaceae Paeonia decomposita#NC 039425
Saxifragales Aphanopetalaceae Aphanopetalum resinosum#Y236
Saxifragales Aphanopetalaceae Aphanopetalum resinosum#FC374
Saxifragales Tetracarpaeaceae Tetracarpaea tasmanica#EF207
Saxifragales Penthoraceae Penthorum chinense#F811
Saxifragales Penthoraceae Penthorum chinense#NC 023086
Saxifragales Haloragaceae Myriophyllum aquaticum#IUSR
Saxifragales Haloragaceae Myriophyllum verticillatum#F618
Saxifragales Haloragaceae Myriophyllum spicatum#NC 037885
Saxifragales Crassulaceae Crassula perforata#KWGC
Saxifragales Crassulaceae Kalanchoe integra#F189
Saxifragales Crassulaceae Kalanchoe crenata#DRIL
Saxifragales Crassulaceae Sedum oryzifolium#NC 027837
Saxifragales Crassulaceae Sedum franchetii#F36
Saxifragales Crassulaceae Sedum sarmentosum#NC 023085
Saxifragales Crassulaceae Rhodiola rosea#NC 041671
Saxifragales Crassulaceae Sedum roseum#ZJUL
Saxifragales Crassulaceae Sedum kamtschaticum#NC 037946
Saxifragales Crassulaceae Sedum takesimense#NC 026065
Saxifragales Iteaceae Cyrilla racemiflora#UWFU
Saxifragales Iteaceae Itea chinensis#NC 037884
Saxifragales Iteaceae Itea yunnanensis#F203
Saxifragales Iteaceae Itea macrophylla#F303
Saxifragales Grossulariaceae Ribes fasciculatum var. chinense#MH191388
Saxifragales Grossulariaceae Ribes aff. giraldii#SYHW
Saxifragales Grossulariaceae Ribes glaciale#F384
Saxifragales Grossulariaceae Ribes sp.#F817
Saxifragales Saxifragaceae Saxifraga gemmipara#F28
Saxifragales Saxifragaceae Saxifraga stolonifera#FCBJ
Saxifragales Saxifragaceae Astilbe rubra#CKKR
Saxifragales Saxifragaceae Boykinia jamesii#OOVX
Saxifragales Saxifragaceae Tanakaea radicans#F504
Saxifragales Saxifragaceae Mukdenia rossii#NC 037495
Saxifragales Saxifragaceae Oresitrophe rupifraga#MG470845
Saxifragales Saxifragaceae Bergenia purpurascens#F154
Saxifragales Saxifragaceae Bergenia scopulosa#NC 036061
Saxifragales Saxifragaceae Bergenia sp.#CIAC
Saxifragales Saxifragaceae Peltoboykinia watanabei#YKFU
Saxifragales Saxifragaceae Chrysosplenium aureobracteatum#NC 039740
Saxifragales Saxifragaceae Chrysosplenium nepalense#F385
Saxifragales Saxifragaceae Heuchera sanguinea#ERIA
Saxifragales Saxifragaceae Mitella pentandra#DAYQ
Saxifragales Saxifragaceae Tiarella polyphylla#F387
Saxifragales Saxifragaceae Tellima breviflora#CTSS
Saxifragales Saxifragaceae Heuchera parviflora#KR478645
Vitales Vitaceae Leea indica#F72
Malpighiales Rafflesiaceae Rhizanthes lowii#HM803264
Vitales Vitaceae Ampelopsis grossedentata#F286
Vitales Vitaceae Ampelopsis japonica#MK547541
Vitales Vitaceae Ampelopsis humulifolia#MK547542
Vitales Vitaceae Cissus javana#F163
Vitales Vitaceae Cissus quadrangularis#BGZG
Vitales Vitaceae Tetrastigma obtectum#BBBA
Vitales Vitaceae Tetrastigma voinierianum#SZPD
Vitales Vitaceae Tetrastigma hemsleyanum#NC 029339
Vitales Vitaceae Vitis rotundifolia#NC 023790
Vitales Vitaceae Vitis rotundifolia var. munsoniana#NC 039782
Vitales Vitaceae Vitis californica#MG664836
Vitales Vitaceae Vitis vinifera#MG586833
Vitales Vitaceae Vitis piasezkii#NC 039789
Vitales Vitaceae Vitis flexuosa#LC341205
Vitales Vitaceae Vitis amurensis#LC318487
Vitales Vitaceae Vitis coignetiae#MG664829
Vitales Vitaceae Vitis retordii#NC 039788
Vitales Vitaceae Vitis bryoniifolia#NC 039683
Vitales Vitaceae Vitis thunbergii#NC 039785
Vitales Vitaceae Vitis heyneana subsp. ficifolia#NC 036510
Vitales Vitaceae Vitis betulifolia#NC 039803
Vitales Vitaceae Vitis heyneana#NC 039794
Vitales Vitaceae Vitis jacquemontii#NC 039795
Vitales Vitaceae Vitis monticola#LC342076
Vitales Vitaceae Vitis aestivalis var. linsecomii#NC 039806
Vitales Vitaceae Cissus palmata#NC 039791
Vitales Vitaceae Vitis acerifolia#MG664848
Vitales Vitaceae Vitis cinerea var. helleri#LC320670
Vitales Vitaceae Vitis peninsularis#NC 039790
Vitales Vitaceae Vitis bloodwothiana#NC 039801
Vitales Vitaceae Vitis cordifolia#NC 036509
Vitales Vitaceae Vitis biformis#NC 039802
Vitales Vitaceae Vitis tiliifolia#NC 039681
Vitales Vitaceae Vitis treleasei#NC 039786
Vitales Vitaceae Vitis girdiana#MG664827
Vitales Vitaceae Vitis arizonica#MG664845
Vitales Vitaceae Vitis rufotomentosa#MG664800
Vitales Vitaceae Vitis baileyana#MG664843
Vitales Vitaceae Vitis sp.#MG664802
Vitales Vitaceae Vitis vulpina#MG664810
Vitales Vitaceae Vitis cinerea#MG664831
Vitales Vitaceae Vitis shuttleworthii#NC 039784
Vitales Vitaceae Vitis aestivalis#LC318124
Vitales Vitaceae Vitis doaniana#NC 039797
Vitales Vitaceae Vitis rupestris#NC 036764
Vitales Vitaceae Vitis labrusca#MG664823
Vitales Vitaceae Vitis mustangensis#MG664818
Geraniales Francoaceae Viviania marifolia#NC 023259
Geraniales Francoaceae Bersama abyssinica#F572
Geraniales Francoaceae Melianthus major#FC50
Geraniales Francoaceae Melianthus villosus#NC 023256
Geraniales Francoaceae Francoa sonchifolia#NC 021101
Geraniales Francoaceae Francoa appendiculata#FC61
Geraniales Francoaceae Greyia sutherlandii#45626
Geraniales Francoaceae Greyia radlkoferi#Y244
Geraniales Geraniaceae Hypseocharis bilobata#NC 023260
Geraniales Geraniaceae Pelargonium quinquelobatum#NC 033358
Geraniales Geraniaceae Pelargonium spinosum#NC 033359
Geraniales Geraniaceae Pelargonium endlicherianum#NC 033357
Geraniales Geraniaceae Pelargonium alternans#NC 023261
Geraniales Geraniaceae Pelargonium cotyledonis#NC 028052
Geraniales Geraniaceae Pelargonium australe#NC 028053
Geraniales Geraniaceae Pelargonium dichondrifolium#NC 028051

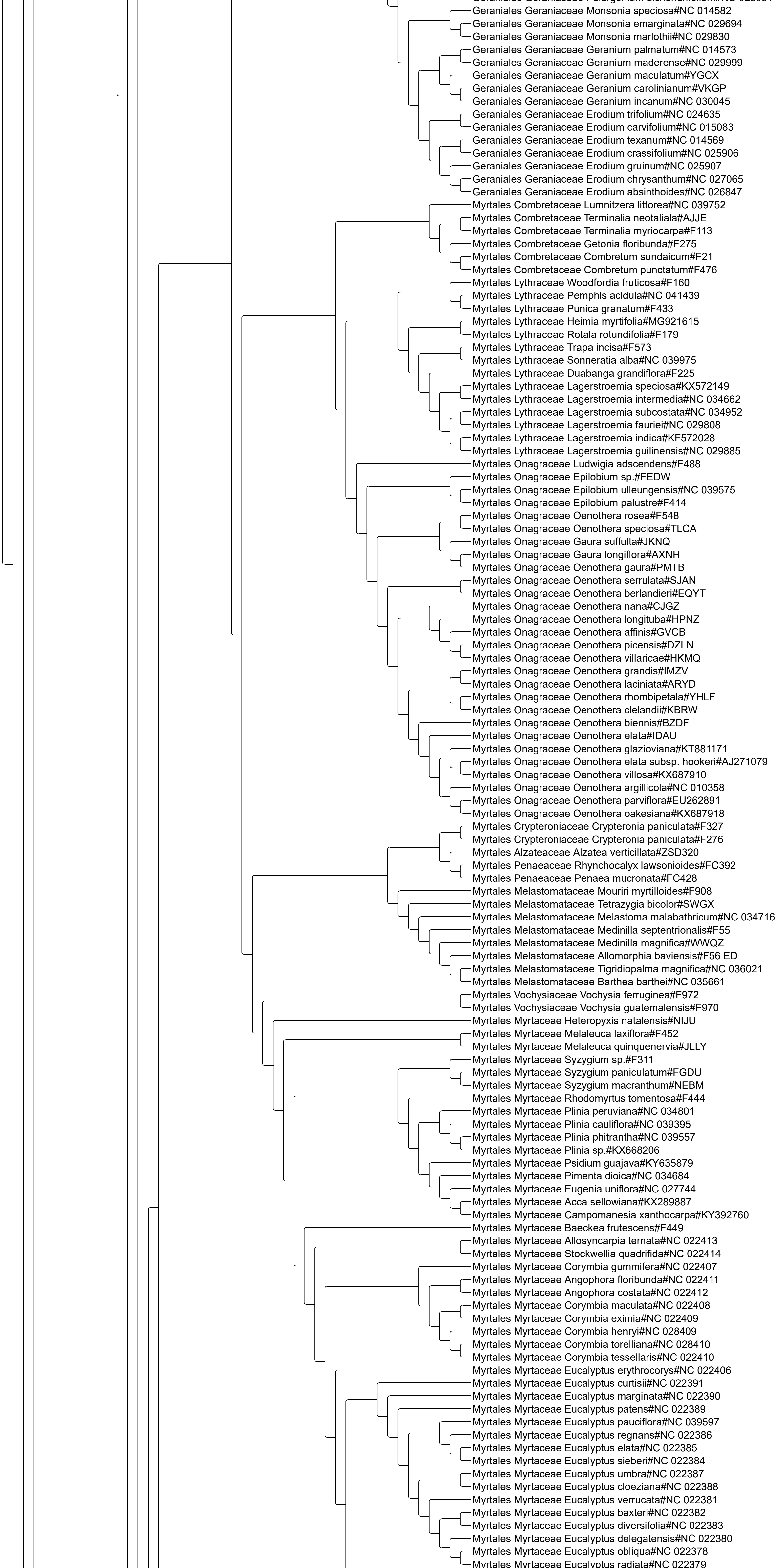
Geraniales Geraniaceae Monsonia speciosa#NC 014582
Geraniales Geraniaceae Monsonia emarginata#NC 029694
Geraniales Geraniaceae Monsonia marlothii#NC 029830
Geraniales Geraniaceae Geranium palmatum#NC 014573
Geraniales Geraniaceae Geranium maderense#NC 029999
Geraniales Geraniaceae Geranium maculatum#YGCX
Geraniales Geraniaceae Geranium carolinianum#VKGP
Geraniales Geraniaceae Geranium incanum#NC 030045
Geraniales Geraniaceae Erodium trifolium#NC 024635
Geraniales Geraniaceae Erodium carvifolium#NC 015083
Geraniales Geraniaceae Erodium texanum#NC 014569
Geraniales Geraniaceae Erodium crassifolium#NC 025906
Geraniales Geraniaceae Erodium gruinum#NC 025907
Geraniales Geraniaceae Erodium chrysanthum#NC 027065
Geraniales Geraniaceae Erodium absinthoides#NC 026847
Myrtales Combretaceae Lumnitzera littorea#NC 039752
Myrtales Combretaceae Terminalia neotaliala#AJJE
Myrtales Combretaceae Terminalia myriocarpa#F113
Myrtales Combretaceae Getonia floribunda#F275
Myrtales Combretaceae Combretum sundaicum#F21
Myrtales Combretaceae Combretum punctatum#F476
Myrtales Lythraceae Woodfordia fruticosa#F160
Myrtales Lythraceae Pemphis acidula#NC 041439
Myrtales Lythraceae Punica granatum#F433
Myrtales Lythraceae Heimia myrtifolia#MG921615
Myrtales Lythraceae Rotala rotundifolia#F179
Myrtales Lythraceae Trapa incisa#F573
Myrtales Lythraceae Sonneratia alba#NC 039975
Myrtales Lythraceae Duabanga grandiflora#F225
Myrtales Lythraceae Lagerstroemia speciosa#KX572149
Myrtales Lythraceae Lagerstroemia intermedia#NC 034662
Myrtales Lythraceae Lagerstroemia subcostata#NC 034952
Myrtales Lythraceae Lagerstroemia fauriei#NC 029808
Myrtales Lythraceae Lagerstroemia indica#KF572028
Myrtales Lythraceae Lagerstroemia guilinensis#NC 029885
Myrtales Onagraceae Ludwigia adscendens#F488
Myrtales Onagraceae Epilobium sp.#FEDW
Myrtales Onagraceae Epilobium ulleungensis#NC 039575
Myrtales Onagraceae Epilobium palustre#F414
Myrtales Onagraceae Oenothera rosea#F548
Myrtales Onagraceae Oenothera speciosa#TLCA
Myrtales Onagraceae Gaura suffulta#JKNQ
Myrtales Onagraceae Gaura longiflora#AXNH
Myrtales Onagraceae Oenothera gaura#PMTB
Myrtales Onagraceae Oenothera serrulata#SJAN
Myrtales Onagraceae Oenothera berlandieri#EQYT
Myrtales Onagraceae Oenothera nana#CJGZ
Myrtales Onagraceae Oenothera longituba#HPNZ
Myrtales Onagraceae Oenothera affinis#GVCB
Myrtales Onagraceae Oenothera picensis#DZLN
Myrtales Onagraceae Oenothera villaricae#HKMQ
Myrtales Onagraceae Oenothera grandis#IMZV
Myrtales Onagraceae Oenothera laciniata#ARYD
Myrtales Onagraceae Oenothera rhombipetala#YHLF
Myrtales Onagraceae Oenothera clelandii#KBRW
Myrtales Onagraceae Oenothera biennis#BZDF
Myrtales Onagraceae Oenothera elata#IDAU
Myrtales Onagraceae Oenothera glazioviana#KT881171
Myrtales Onagraceae Oenothera elata subsp. hookeri#AJ271079
Myrtales Onagraceae Oenothera villosa#KX687910
Myrtales Onagraceae Oenothera argillicola#NC 010358
Myrtales Onagraceae Oenothera parviflora#EU262891
Myrtales Onagraceae Oenothera oakesiana#KX687918
Myrtales Crypteroniaceae Crypteronia paniculata#F327
Myrtales Crypteroniaceae Crypteronia paniculata#F276
Myrtales Alzateaceae Alzatea verticillata#ZSD320
Myrtales Penaeaceae Rhynchocalyx lawsonioides#FC392
Myrtales Penaeaceae Penaea mucronata#FC428
Myrtales Melastomataceae Mouriri myrtilloides#F908
Myrtales Melastomataceae Tetrazygia bicolor#SWGX
Myrtales Melastomataceae Melastoma malabathricum#NC 034716
Myrtales Melastomataceae Medinilla septentrionalis#F55
Myrtales Melastomataceae Medinilla magnifica#WWQZ
Myrtales Melastomataceae Allomorphia baviensis#F56 ED
Myrtales Melastomataceae Tigridiopalma magnifica#NC 036021
Myrtales Melastomataceae Barthea barthei#NC 035661
Myrtales Vochysiaceae Vochysia ferruginea#F972
Myrtales Vochysiaceae Vochysia guatemalensis#F970
Myrtales Myrtaceae Heteropyxis natalensis#NIJU
Myrtales Myrtaceae Melaleuca laxiflora#F452
Myrtales Myrtaceae Melaleuca quinquenervia#JLLY
Myrtales Myrtaceae Syzygium sp.#F311
Myrtales Myrtaceae Syzygium paniculatum#FGDU
Myrtales Myrtaceae Syzygium macranthum#NEBM
Myrtales Myrtaceae Rhodomyrtus tomentosa#F444
Myrtales Myrtaceae Plinia peruviana#NC 034801
Myrtales Myrtaceae Plinia cauliflora#NC 039395
Myrtales Myrtaceae Plinia phitrantha#NC 039557
Myrtales Myrtaceae Plinia sp.#KX668206
Myrtales Myrtaceae Psidium guajava#KY635879
Myrtales Myrtaceae Pimenta dioica#NC 034684
Myrtales Myrtaceae Eugenia uniflora#NC 027744
Myrtales Myrtaceae Acca sellowiana#KX289887
Myrtales Myrtaceae Campomanesia xanthocarpa#KY392760
Myrtales Myrtaceae Baeckea frutescens#F449
Myrtales Myrtaceae Allosyncarpia ternata#NC 022413
Myrtales Myrtaceae Stockwellia quadrifida#NC 022414
Myrtales Myrtaceae Corymbia gummifera#NC 022407
Myrtales Myrtaceae Angophora floribunda#NC 022411
Myrtales Myrtaceae Angophora costata#NC 022412
Myrtales Myrtaceae Corymbia maculata#NC 022408
Myrtales Myrtaceae Corymbia eximia#NC 022409
Myrtales Myrtaceae Corymbia henryi#NC 028409
Myrtales Myrtaceae Corymbia torelliana#NC 028410
Myrtales Myrtaceae Corymbia tessellaris#NC 022410
Myrtales Myrtaceae Eucalyptus erythrocorys#NC 022406
Myrtales Myrtaceae Eucalyptus curtisii#NC 022391
Myrtales Myrtaceae Eucalyptus marginata#NC 022390
Myrtales Myrtaceae Eucalyptus patens#NC 022389
Myrtales Myrtaceae Eucalyptus pauciflora#NC 039597
Myrtales Myrtaceae Eucalyptus regnans#NC 022386
Myrtales Myrtaceae Eucalyptus elata#NC 022385
Myrtales Myrtaceae Eucalyptus sieberi#NC 022384
Myrtales Myrtaceae Eucalyptus umbra#NC 022387
Myrtales Myrtaceae Eucalyptus cloeziana#NC 022388
Myrtales Myrtaceae Eucalyptus verrucata#NC 022381
Myrtales Myrtaceae Eucalyptus baxteri#NC 022382
Myrtales Myrtaceae Eucalyptus diversifolia#NC 022383
Myrtales Myrtaceae Eucalyptus delegatensis#NC 022380
Myrtales Myrtaceae Eucalyptus obliqua#NC 022378
Myrtales Myrtaceae Eucalyptus radiata#NC 022379

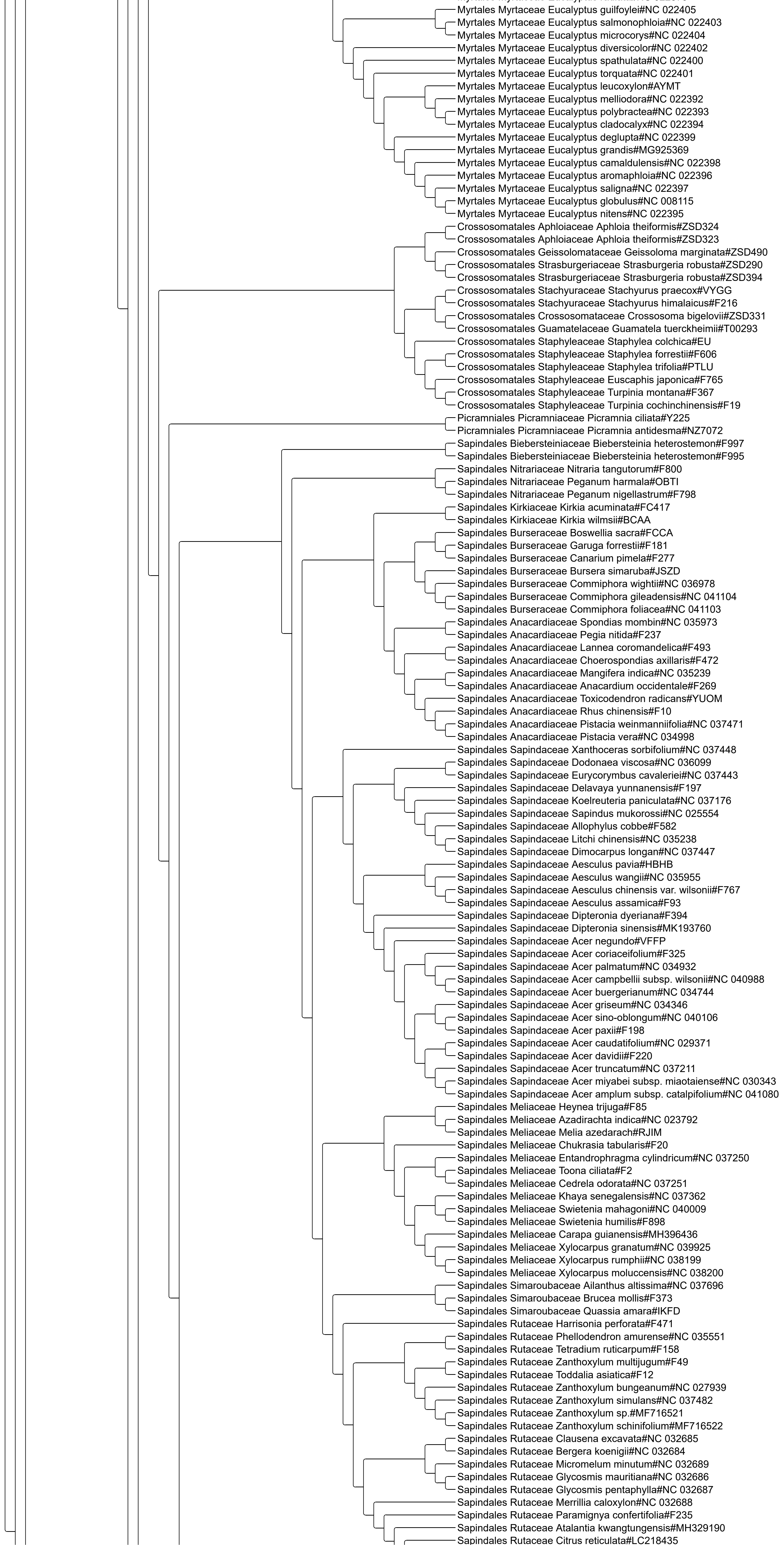
Myrtales Myrtaceae Eucalyptus guilfoylei#NC 022405
Myrtales Myrtaceae Eucalyptus salmonophloia#NC 022403
Myrtales Myrtaceae Eucalyptus microcorys#NC 022404
Myrtales Myrtaceae Eucalyptus diversicolor#NC 022402
Myrtales Myrtaceae Eucalyptus spathulata#NC 022400
Myrtales Myrtaceae Eucalyptus torquata#NC 022401
Myrtales Myrtaceae Eucalyptus leucoxylon#AYMT
Myrtales Myrtaceae Eucalyptus melliodora#NC 022392
Myrtales Myrtaceae Eucalyptus polybractea#NC 022393
Myrtales Myrtaceae Eucalyptus cladocalyx#NC 022394
Myrtales Myrtaceae Eucalyptus deglupta#NC 022399
Myrtales Myrtaceae Eucalyptus grandis#MG925369
Myrtales Myrtaceae Eucalyptus camaldulensis#NC 022398
Myrtales Myrtaceae Eucalyptus aromaphloia#NC 022396
Myrtales Myrtaceae Eucalyptus saligna#NC 022397
Myrtales Myrtaceae Eucalyptus globulus#NC 008115
Myrtales Myrtaceae Eucalyptus nitens#NC 022395
Crossosomatales Aphloiaceae Aphloia theiformis#ZSD324
Crossosomatales Aphloiaceae Aphloia theiformis#ZSD323
Crossosomatales Geissolomataceae Geissoloma marginata#ZSD490
Crossosomatales Strasburgeriaceae Strasburgeria robusta#ZSD290
Crossosomatales Strasburgeriaceae Strasburgeria robusta#ZSD394
Crossosomatales Stachyuraceae Stachyurus praecox#VYGG
Crossosomatales Stachyuraceae Stachyurus himalaicus#F216
Crossosomatales Crossosomataceae Crossosoma bigelovii#ZSD331
Crossosomatales Guamatelaceae Guamatela tuerckheimii#T00293
Crossosomatales Staphyleaceae Staphylea colchica#EU
Crossosomatales Staphyleaceae Staphylea forrestii#F606
Crossosomatales Staphyleaceae Staphylea trifolia#PTLU
Crossosomatales Staphyleaceae Euscaphis japonica#F765
Crossosomatales Staphyleaceae Turpinia montana#F367
Crossosomatales Staphyleaceae Turpinia cochinchinensis#F19
Picramniales Picramniaceae Picramnia ciliata#Y225
Picramniales Picramniaceae Picramnia antidesma#NZ7072
Sapindales Biebersteiniaceae Biebersteinia heterostemon#F997
Sapindales Biebersteiniaceae Biebersteinia heterostemon#F995
Sapindales Nitrariaceae Nitraria tangutorum#F800
Sapindales Nitrariaceae Peganum harmala#OBTI
Sapindales Nitrariaceae Peganum nigellastrum#F798
Sapindales Kirkiaceae Kirkia acuminata#FC417
Sapindales Kirkiaceae Kirkia wilmsii#BCAA
Sapindales Burseraceae Boswellia sacra#FCCA
Sapindales Burseraceae Garuga forrestii#F181
Sapindales Burseraceae Canarium pimela#F277
Sapindales Burseraceae Bursera simaruba#JSZD
Sapindales Burseraceae Commiphora wightii#NC 036978
Sapindales Burseraceae Commiphora gileadensis#NC 041104
Sapindales Burseraceae Commiphora foliacea#NC 041103
Sapindales Anacardiaceae Spondias mombin#NC 035973
Sapindales Anacardiaceae Pegia nitida#F237
Sapindales Anacardiaceae Lannea coromandelica#F493
Sapindales Anacardiaceae Choerospondias axillaris#F472
Sapindales Anacardiaceae Mangifera indica#NC 035239
Sapindales Anacardiaceae Anacardium occidentale#F269
Sapindales Anacardiaceae Toxicodendron radicans#YUOM
Sapindales Anacardiaceae Rhus chinensis#F10
Sapindales Anacardiaceae Pistacia weinmanniifolia#NC 037471
Sapindales Anacardiaceae Pistacia vera#NC 034998
Sapindales Sapindaceae Xanthoceras sorbifolium#NC 037448
Sapindales Sapindaceae Dodonaea viscosa#NC 036099
Sapindales Sapindaceae Eurycorymbus cavaleriei#NC 037443
Sapindales Sapindaceae Delavaya yunnanensis#F197
Sapindales Sapindaceae Koelreuteria paniculata#NC 037176
Sapindales Sapindaceae Sapindus mukorossi#NC 025554
Sapindales Sapindaceae Allophylus cobbe#F582
Sapindales Sapindaceae Litchi chinensis#NC 035238
Sapindales Sapindaceae Dimocarpus longan#NC 037447
Sapindales Sapindaceae Aesculus pavia#HBHB
Sapindales Sapindaceae Aesculus wangii#NC 035955
Sapindales Sapindaceae Aesculus chinensis var. wilsonii#F767
Sapindales Sapindaceae Aesculus assamica#F93
Sapindales Sapindaceae Dipteronia dyeriana#F394
Sapindales Sapindaceae Dipteronia sinensis#MK193760
Sapindales Sapindaceae Acer negundo#VFFP
Sapindales Sapindaceae Acer coriaceifolium#F325
Sapindales Sapindaceae Acer palmatum#NC 034932
Sapindales Sapindaceae Acer campbellii subsp. wilsonii#NC 040988
Sapindales Sapindaceae Acer buergerianum#NC 034744
Sapindales Sapindaceae Acer griseum#NC 034346
Sapindales Sapindaceae Acer sino-oblongum#NC 040106
Sapindales Sapindaceae Acer paxii#F198
Sapindales Sapindaceae Acer caudatifolium#NC 029371
Sapindales Sapindaceae Acer davidii#F220
Sapindales Sapindaceae Acer truncatum#NC 037211
Sapindales Sapindaceae Acer miyabei subsp. miaotaiense#NC 030343
Sapindales Sapindaceae Acer amplum subsp. catalpifolium#NC 041080
Sapindales Meliaceae Heynea trijuga#F85
Sapindales Meliaceae Azadirachta indica#NC 023792
Sapindales Meliaceae Melia azedarach#RJIM
Sapindales Meliaceae Chukrasia tabularis#F20
Sapindales Meliaceae Entandrophragma cylindricum#NC 037250
Sapindales Meliaceae Toona ciliata#F2
Sapindales Meliaceae Cedrela odorata#NC 037251
Sapindales Meliaceae Khaya senegalensis#NC 037362
Sapindales Meliaceae Swietenia mahagoni#NC 040009
Sapindales Meliaceae Swietenia humilis#F898
Sapindales Meliaceae Carapa guianensis#MH396436
Sapindales Meliaceae Xylocarpus granatum#NC 039925
Sapindales Meliaceae Xylocarpus rumphii#NC 038199
Sapindales Meliaceae Xylocarpus moluccensis#NC 038200
Sapindales Simaroubaceae Ailanthus altissima#NC 037696
Sapindales Simaroubaceae Brucea mollis#F373
Sapindales Simaroubaceae Quassia amara#IKFD
Sapindales Rutaceae Harrisonia perforata#F471
Sapindales Rutaceae Phellodendron amurense#NC 035551
Sapindales Rutaceae Tetradium ruticarpum#F158
Sapindales Rutaceae Zanthoxylum multijugum#F49
Sapindales Rutaceae Toddalia asiatica#F12
Sapindales Rutaceae Zanthoxylum bungeanum#NC 027939
Sapindales Rutaceae Zanthoxylum simulans#NC 037482
Sapindales Rutaceae Zanthoxylum sp.#MF716521
Sapindales Rutaceae Zanthoxylum schinifolium#MF716522
Sapindales Rutaceae Clausena excavata#NC 032685
Sapindales Rutaceae Bergera koenigii#NC 032684
Sapindales Rutaceae Micromelum minutum#NC 032689
Sapindales Rutaceae Glycosmis mauritiana#NC 032686
Sapindales Rutaceae Glycosmis pentaphylla#NC 032687
Sapindales Rutaceae Merrillia caloxylon#NC 032688
Sapindales Rutaceae Paramignya confertifolia#F235
Sapindales Rutaceae Atalantia kwangtungensis#MH329190
Sapindales Rutaceae Citrus reticulata#LC218435

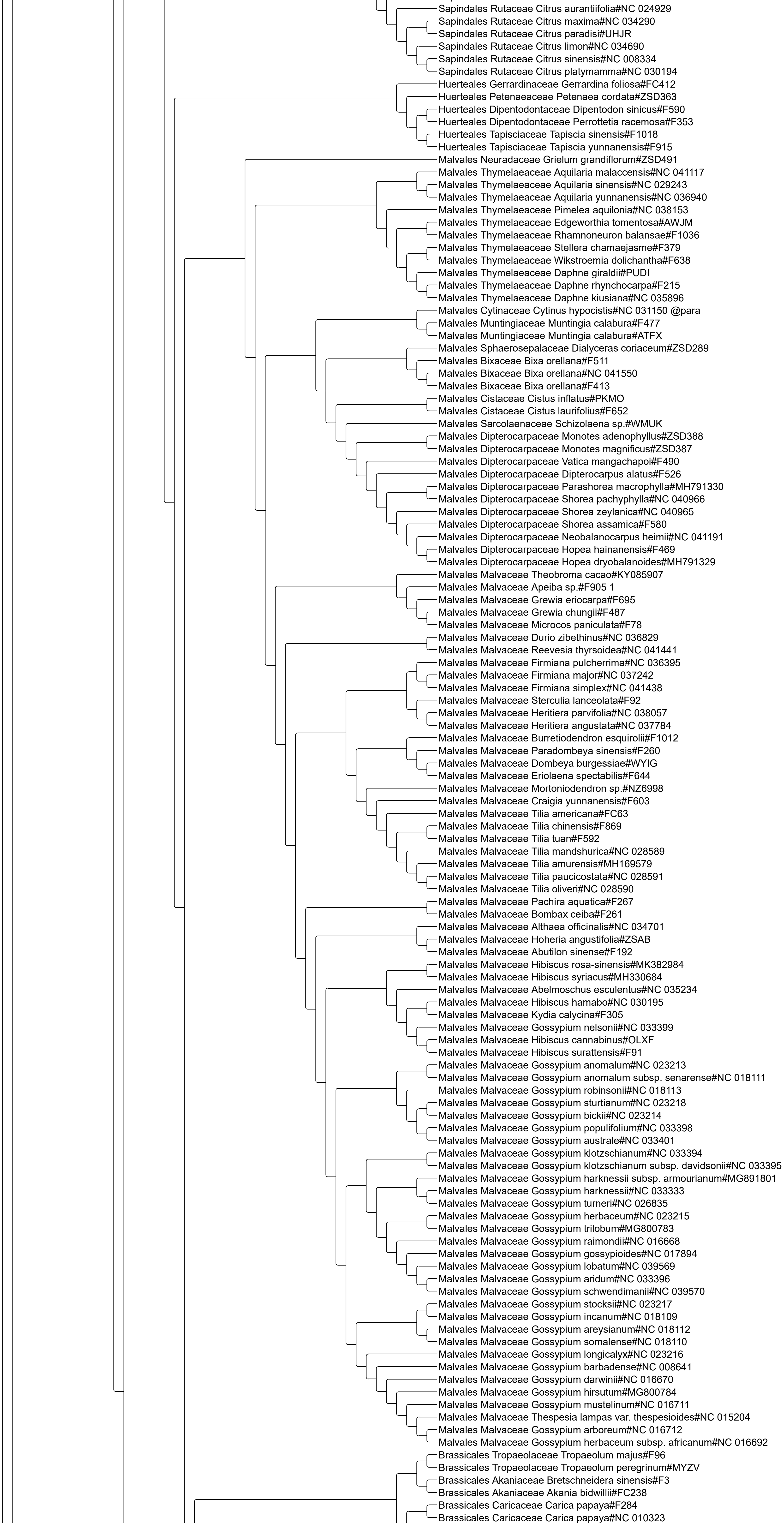
Sapindales Rutaceae Citrus aurantiifolia#NC 024929
Sapindales Rutaceae Citrus maxima#NC 034290
Sapindales Rutaceae Citrus paradisi#UHJR
Sapindales Rutaceae Citrus limon#NC 034690
Sapindales Rutaceae Citrus sinensis#NC 008334
Sapindales Rutaceae Citrus platymamma#NC 030194
Huerteales Gerrardinaceae Gerrardina foliosa#FC412
Huerteales Petenaeaceae Petenaea cordata#ZSD363
Huerteales Dipentodontaceae Dipentodon sinicus#F590
Huerteales Dipentodontaceae Perrottetia racemosa#F353
Huerteales Tapisciaceae Tapiscia sinensis#F1018
Huerteales Tapisciaceae Tapiscia yunnanensis#F915
Malvales Neuradaceae Grielum grandiflorum#ZSD491
Malvales Thymelaeaceae Aquilaria malaccensis#NC 041117
Malvales Thymelaeaceae Aquilaria sinensis#NC 029243
Malvales Thymelaeaceae Aquilaria yunnanensis#NC 036940
Malvales Thymelaeaceae Pimelea aquilonia#NC 038153
Malvales Thymelaeaceae Edgeworthia tomentosa#AWJM
Malvales Thymelaeaceae Rhamnoneuron balansae#F1036
Malvales Thymelaeaceae Stellera chamaejasme#F379
Malvales Thymelaeaceae Wikstroemia dolichantha#F638
Malvales Thymelaeaceae Daphne giraldii#PUDI
Malvales Thymelaeaceae Daphne rhynchocarpa#F215
Malvales Thymelaeaceae Daphne kiusiana#NC 035896
Malvales Cytinaceae Cytinus hypocistis#NC 031150 @para
Malvales Muntingiaceae Muntingia calabura#F477
Malvales Muntingiaceae Muntingia calabura#ATFX
Malvales Sphaerosepalaceae Dialyceras coriaceum#ZSD289
Malvales Bixaceae Bixa orellana#F511
Malvales Bixaceae Bixa orellana#NC 041550
Malvales Bixaceae Bixa orellana#F413
Malvales Cistaceae Cistus inflatus#PKMO
Malvales Cistaceae Cistus laurifolius#F652
Malvales Sarcolaenaceae Schizolaena sp.#WMUK
Malvales Dipterocarpaceae Monotes adenophyllus#ZSD388
Malvales Dipterocarpaceae Monotes magnificus#ZSD387
Malvales Dipterocarpaceae Vatica mangachapoi#F490
Malvales Dipterocarpaceae Dipterocarpus alatus#F526
Malvales Dipterocarpaceae Parashorea macrophylla#MH791330
Malvales Dipterocarpaceae Shorea pachyphylla#NC 040966
Malvales Dipterocarpaceae Shorea zeylanica#NC 040965
Malvales Dipterocarpaceae Shorea assamica#F580
Malvales Dipterocarpaceae Neobalanocarpus heimii#NC 041191
Malvales Dipterocarpaceae Hopea hainanensis#F469
Malvales Dipterocarpaceae Hopea dryobalanoides#MH791329
Malvales Malvaceae Theobroma cacao#KY085907
Malvales Malvaceae Apeiba sp.#F905 1
Malvales Malvaceae Grewia eriocarpa#F695
Malvales Malvaceae Grewia chungii#F487
Malvales Malvaceae Microcos paniculata#F78
Malvales Malvaceae Durio zibethinus#NC 036829
Malvales Malvaceae Reevesia thyrsoidea#NC 041441
Malvales Malvaceae Firmiana pulcherrima#NC 036395
Malvales Malvaceae Firmiana major#NC 037242
Malvales Malvaceae Firmiana simplex#NC 041438
Malvales Malvaceae Sterculia lanceolata#F92
Malvales Malvaceae Heritiera parvifolia#NC 038057
Malvales Malvaceae Heritiera angustata#NC 037784
Malvales Malvaceae Burretiodendron esquirolii#F1012
Malvales Malvaceae Paradombeya sinensis#F260
Malvales Malvaceae Dombeya burgessiae#WYIG
Malvales Malvaceae Eriolaena spectabilis#F644
Malvales Malvaceae Mortoniodendron sp.#NZ6998
Malvales Malvaceae Craigia yunnanensis#F603
Malvales Malvaceae Tilia americana#FC63
Malvales Malvaceae Tilia chinensis#F869
Malvales Malvaceae Tilia tuan#F592
Malvales Malvaceae Tilia mandshurica#NC 028589
Malvales Malvaceae Tilia amurensis#MH169579
Malvales Malvaceae Tilia paucicostata#NC 028591
Malvales Malvaceae Tilia oliveri#NC 028590
Malvales Malvaceae Pachira aquatica#F267
Malvales Malvaceae Bombax ceiba#F261
Malvales Malvaceae Althaea officinalis#NC 034701
Malvales Malvaceae Hoheria angustifolia#ZSAB
Malvales Malvaceae Abutilon sinense#F192
Malvales Malvaceae Hibiscus rosa-sinensis#MK382984
Malvales Malvaceae Hibiscus syriacus#MH330684
Malvales Malvaceae Abelmoschus esculentus#NC 035234
Malvales Malvaceae Hibiscus hamabo#NC 030195
Malvales Malvaceae Kydia calycina#F305
Malvales Malvaceae Gossypium nelsonii#NC 033399
Malvales Malvaceae Hibiscus cannabinus#OLXF
Malvales Malvaceae Hibiscus surattensis#F91
Malvales Malvaceae Gossypium anomalum#NC 023213
Malvales Malvaceae Gossypium anomalum subsp. senarense#NC 018111
Malvales Malvaceae Gossypium robinsonii#NC 018113
Malvales Malvaceae Gossypium sturtianum#NC 023218
Malvales Malvaceae Gossypium bickii#NC 023214
Malvales Malvaceae Gossypium populifolium#NC 033398
Malvales Malvaceae Gossypium australe#NC 033401
Malvales Malvaceae Gossypium klotzschianum#NC 033394
Malvales Malvaceae Gossypium klotzschianum subsp. davidsonii#NC 033395
Malvales Malvaceae Gossypium harknessii subsp. armourianum#MG891801
Malvales Malvaceae Gossypium harknessii#NC 033333
Malvales Malvaceae Gossypium turneri#NC 026835
Malvales Malvaceae Gossypium herbaceum#NC 023215
Malvales Malvaceae Gossypium trilobum#MG800783
Malvales Malvaceae Gossypium raimondii#NC 016668
Malvales Malvaceae Gossypium gossypioides#NC 017894
Malvales Malvaceae Gossypium lobatum#NC 039569
Malvales Malvaceae Gossypium aridum#NC 033396
Malvales Malvaceae Gossypium schwendimanii#NC 039570
Malvales Malvaceae Gossypium stocksii#NC 023217
Malvales Malvaceae Gossypium incanum#NC 018109
Malvales Malvaceae Gossypium areysianum#NC 018112
Malvales Malvaceae Gossypium somalense#NC 018110
Malvales Malvaceae Gossypium longicalyx#NC 023216
Malvales Malvaceae Gossypium barbadense#NC 008641
Malvales Malvaceae Gossypium darwinii#NC 016670
Malvales Malvaceae Gossypium hirsutum#MG800784
Malvales Malvaceae Gossypium mustelinum#NC 016711
Malvales Malvaceae Thespesia lampas var. thespesioides#NC 015204
Malvales Malvaceae Gossypium arboreum#NC 016712
Malvales Malvaceae Gossypium herbaceum subsp. africanum#NC 016692
Brassicales Tropaeolaceae Tropaeolum majus#F96
Brassicales Tropaeolaceae Tropaeolum peregrinum#MYZV
Brassicales Akaniaceae Bretschneidera sinensis#F3
Brassicales Akaniaceae Akania bidwillii#FC238
Brassicales Caricaceae Carica papaya#F284
Brassicales Caricaceae Carica papaya#NC 010323

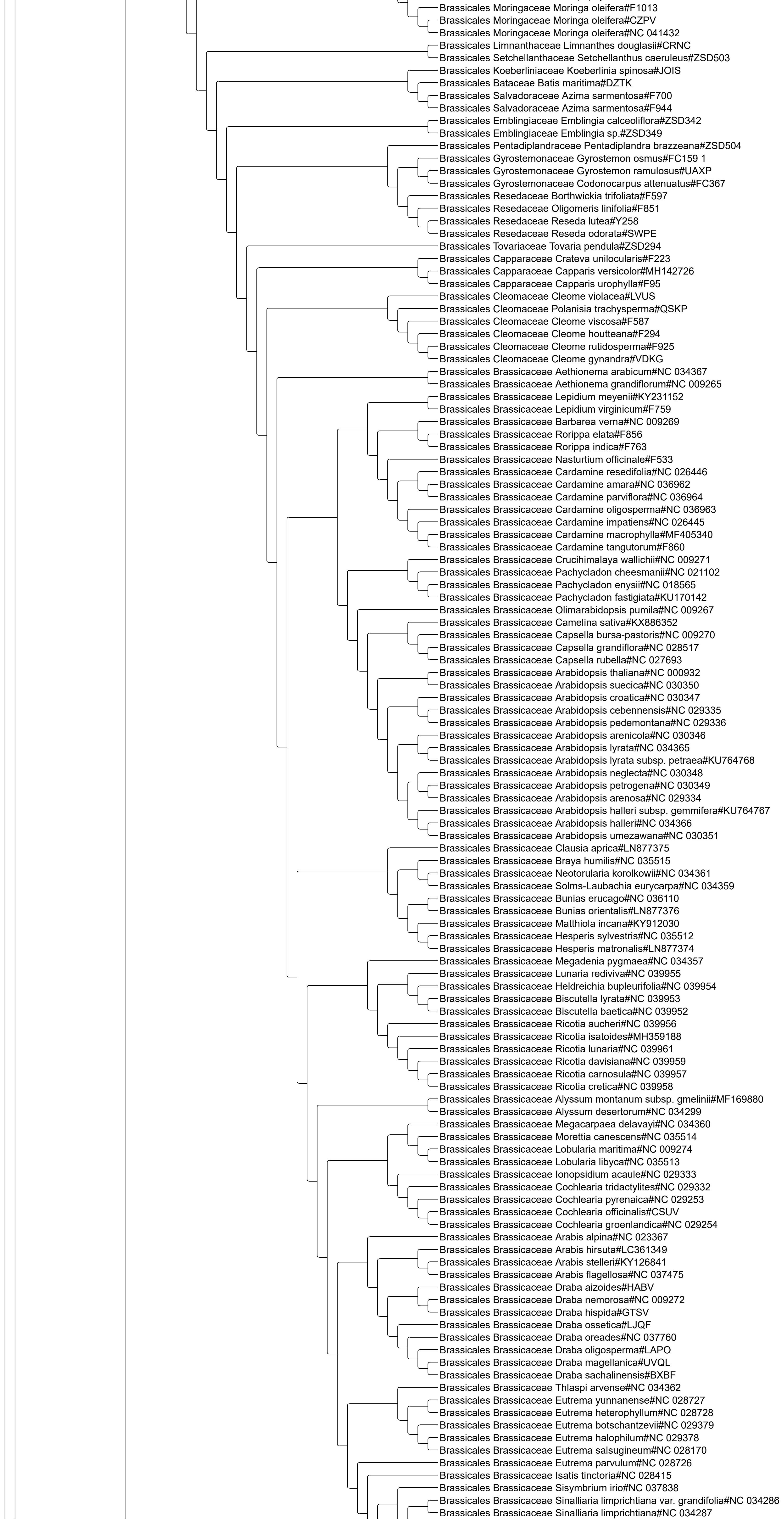
Brassicales Moringaceae Moringa oleifera#F1013
Brassicales Moringaceae Moringa oleifera#CZPV
Brassicales Moringaceae Moringa oleifera#NC 041432
Brassicales Limnanthaceae Limnanthes douglasii#CRNC
Brassicales Setchellanthaceae Setchellanthus caeruleus#ZSD503
Brassicales Koeberliniaceae Koeberlinia spinosa#JOIS
Brassicales Bataceae Batis maritima#DZTK
Brassicales Salvadoraceae Azima sarmentosa#F700
Brassicales Salvadoraceae Azima sarmentosa#F944
Brassicales Emblingiaceae Emblingia calceoliflora#ZSD342
Brassicales Emblingiaceae Emblingia sp.#ZSD349
Brassicales Pentadiplandraceae Pentadiplandra brazzeana#ZSD504
Brassicales Gyrostemonaceae Gyrostemon osmus#FC159 1
Brassicales Gyrostemonaceae Gyrostemon ramulosus#UAXP
Brassicales Gyrostemonaceae Codonocarpus attenuatus#FC367
Brassicales Resedaceae Borthwickia trifoliata#F597
Brassicales Resedaceae Oligomeris linifolia#F851
Brassicales Resedaceae Reseda lutea#Y258
Brassicales Resedaceae Reseda odorata#SWPE
Brassicales Tovariaceae Tovaria pendula#ZSD294
Brassicales Capparaceae Crateva unilocularis#F223
Brassicales Capparaceae Capparis versicolor#MH142726
Brassicales Capparaceae Capparis urophylla#F95
Brassicales Cleomaceae Cleome violacea#LVUS
Brassicales Cleomaceae Polanisia trachysperma#QSKP
Brassicales Cleomaceae Cleome viscosa#F587
Brassicales Cleomaceae Cleome houtteana#F294
Brassicales Cleomaceae Cleome rutidosperma#F925
Brassicales Cleomaceae Cleome gynandra#VDKG
Brassicales Brassicaceae Aethionema arabicum#NC 034367
Brassicales Brassicaceae Aethionema grandiflorum#NC 009265
Brassicales Brassicaceae Lepidium meyenii#KY231152
Brassicales Brassicaceae Lepidium virginicum#F759
Brassicales Brassicaceae Barbarea verna#NC 009269
Brassicales Brassicaceae Rorippa elata#F856
Brassicales Brassicaceae Rorippa indica#F763
Brassicales Brassicaceae Nasturtium officinale#F533
Brassicales Brassicaceae Cardamine resedifolia#NC 026446
Brassicales Brassicaceae Cardamine amara#NC 036962
Brassicales Brassicaceae Cardamine parviflora#NC 036964
Brassicales Brassicaceae Cardamine oligosperma#NC 036963
Brassicales Brassicaceae Cardamine impatiens#NC 026445
Brassicales Brassicaceae Cardamine macrophylla#MF405340
Brassicales Brassicaceae Cardamine tangutorum#F860
Brassicales Brassicaceae Crucihimalaya wallichii#NC 009271
Brassicales Brassicaceae Pachycladon cheesmanii#NC 021102
Brassicales Brassicaceae Pachycladon enysii#NC 018565
Brassicales Brassicaceae Pachycladon fastigiata#KU170142
Brassicales Brassicaceae Olimarabidopsis pumila#NC 009267
Brassicales Brassicaceae Camelina sativa#KX886352
Brassicales Brassicaceae Capsella bursa-pastoris#NC 009270
Brassicales Brassicaceae Capsella grandiflora#NC 028517
Brassicales Brassicaceae Capsella rubella#NC 027693
Brassicales Brassicaceae Arabidopsis thaliana#NC 000932
Brassicales Brassicaceae Arabidopsis suecica#NC 030350
Brassicales Brassicaceae Arabidopsis croatica#NC 030347
Brassicales Brassicaceae Arabidopsis cebennensis#NC 029335
Brassicales Brassicaceae Arabidopsis pedemontana#NC 029336
Brassicales Brassicaceae Arabidopsis arenicola#NC 030346
Brassicales Brassicaceae Arabidopsis lyrata#NC 034365
Brassicales Brassicaceae Arabidopsis lyrata subsp. petraea#KU764768
Brassicales Brassicaceae Arabidopsis neglecta#NC 030348
Brassicales Brassicaceae Arabidopsis petrogena#NC 030349
Brassicales Brassicaceae Arabidopsis arenosa#NC 029334
Brassicales Brassicaceae Arabidopsis halleri subsp. gemmifera#KU764767
Brassicales Brassicaceae Arabidopsis halleri#NC 034366
Brassicales Brassicaceae Arabidopsis umezawana#NC 030351
Brassicales Brassicaceae Clausia aprica#LN877375
Brassicales Brassicaceae Braya humilis#NC 035515
Brassicales Brassicaceae Neotorularia korolkowii#NC 034361
Brassicales Brassicaceae Solms-Laubachia eurycarpa#NC 034359
Brassicales Brassicaceae Bunias erucago#NC 036110
Brassicales Brassicaceae Bunias orientalis#LN877376
Brassicales Brassicaceae Matthiola incana#KY912030
Brassicales Brassicaceae Hesperis sylvestris#NC 035512
Brassicales Brassicaceae Hesperis matronalis#LN877374
Brassicales Brassicaceae Megadenia pygmaea#NC 034357
Brassicales Brassicaceae Lunaria rediviva#NC 039955
Brassicales Brassicaceae Heldreichia bupleurifolia#NC 039954
Brassicales Brassicaceae Biscutella lyrata#NC 039953
Brassicales Brassicaceae Biscutella baetica#NC 039952
Brassicales Brassicaceae Ricotia aucheri#NC 039956
Brassicales Brassicaceae Ricotia isatoides#MH359188
Brassicales Brassicaceae Ricotia lunaria#NC 039961
Brassicales Brassicaceae Ricotia davisiana#NC 039959
Brassicales Brassicaceae Ricotia carnosula#NC 039957
Brassicales Brassicaceae Ricotia cretica#NC 039958
Brassicales Brassicaceae Alyssum montanum subsp. gmelinii#MF169880
Brassicales Brassicaceae Alyssum desertorum#NC 034299
Brassicales Brassicaceae Megacarpaea delavayi#NC 034360
Brassicales Brassicaceae Morettia canescens#NC 035514
Brassicales Brassicaceae Lobularia maritima#NC 009274
Brassicales Brassicaceae Lobularia libyca#NC 035513
Brassicales Brassicaceae Ionopsidium acaule#NC 029333
Brassicales Brassicaceae Cochlearia tridactylites#NC 029332
Brassicales Brassicaceae Cochlearia pyrenaica#NC 029253
Brassicales Brassicaceae Cochlearia officinalis#CSUV
Brassicales Brassicaceae Cochlearia groenlandica#NC 029254
Brassicales Brassicaceae Arabis alpina#NC 023367
Brassicales Brassicaceae Arabis hirsuta#LC361349
Brassicales Brassicaceae Arabis stelleri#KY126841
Brassicales Brassicaceae Arabis flagellosa#NC 037475
Brassicales Brassicaceae Draba aizoides#HABV
Brassicales Brassicaceae Draba nemorosa#NC 009272
Brassicales Brassicaceae Draba hispida#GTSV
Brassicales Brassicaceae Draba ossetica#LJQF
Brassicales Brassicaceae Draba oreades#NC 037760
Brassicales Brassicaceae Draba oligosperma#LAPO
Brassicales Brassicaceae Draba magellanica#UVQL
Brassicales Brassicaceae Draba sachalinensis#BXBF
Brassicales Brassicaceae Thlaspi arvense#NC 034362
Brassicales Brassicaceae Eutrema yunnanense#NC 028727
Brassicales Brassicaceae Eutrema heterophyllum#NC 028728
Brassicales Brassicaceae Eutrema botschantzevii#NC 029379
Brassicales Brassicaceae Eutrema halophilum#NC 029378
Brassicales Brassicaceae Eutrema salsugineum#NC 028170
Brassicales Brassicaceae Eutrema parvulum#NC 028726
Brassicales Brassicaceae Isatis tinctoria#NC 028415
Brassicales Brassicaceae Sisymbrium irio#NC 037838
Brassicales Brassicaceae Sinalliaria limprichtiana var. grandifolia#NC 034286
Brassicales Brassicaceae Sinalliaria limprichtiana#NC 034287

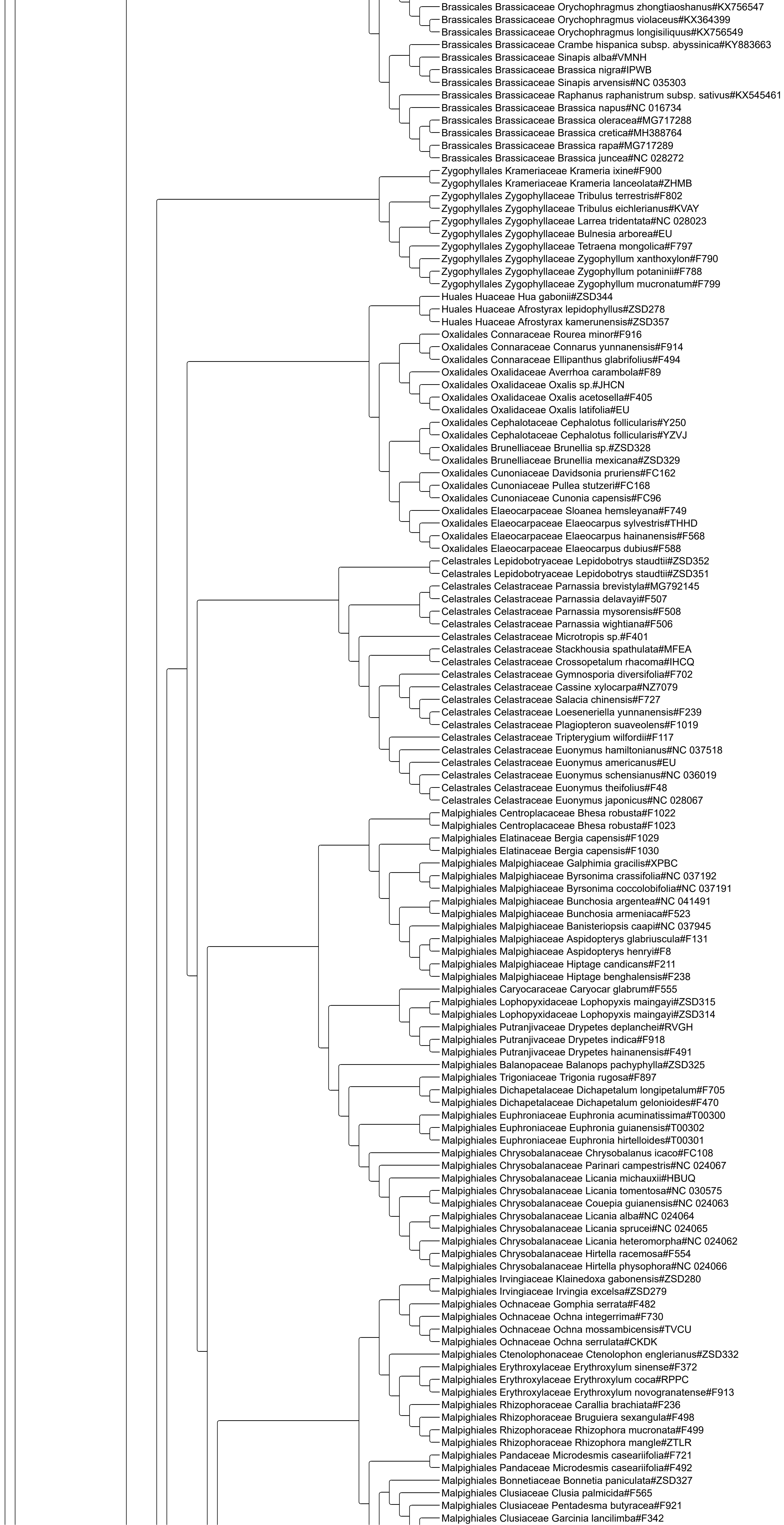

Brassicales Brassicaceae Orychophragmus zhongtiaoshanus#KX756547
Brassicales Brassicaceae Orychophragmus violaceus#KX364399
Brassicales Brassicaceae Orychophragmus longisiliquus#KX756549
Brassicales Brassicaceae Crambe hispanica subsp. abyssinica#KY883663
Brassicales Brassicaceae Sinapis alba#VMNH
Brassicales Brassicaceae Brassica nigra#IPWB
Brassicales Brassicaceae Sinapis arvensis#NC 035303
Brassicales Brassicaceae Raphanus raphanistrum subsp. sativus#KX545461
Brassicales Brassicaceae Brassica napus#NC 016734
Brassicales Brassicaceae Brassica oleracea#MG717288
Brassicales Brassicaceae Brassica cretica#MH388764
Brassicales Brassicaceae Brassica rapa#MG717289
Brassicales Brassicaceae Brassica juncea#NC 028272
Zygophyllales Krameriaceae Krameria ixine#F900
Zygophyllales Krameriaceae Krameria lanceolata#ZHMB
Zygophyllales Zygophyllaceae Tribulus terrestris#F802
Zygophyllales Zygophyllaceae Tribulus eichlerianus#KVAY
Zygophyllales Zygophyllaceae Larrea tridentata#NC 028023
Zygophyllales Zygophyllaceae Bulnesia arborea#EU
Zygophyllales Zygophyllaceae Tetraena mongolica#F797
Zygophyllales Zygophyllaceae Zygophyllum xanthoxylon#F790
Zygophyllales Zygophyllaceae Zygophyllum potaninii#F788
Zygophyllales Zygophyllaceae Zygophyllum mucronatum#F799
Huales Huaceae Hua gabonii#ZSD344
Huales Huaceae Afrostyrax lepidophyllus#ZSD278
Huales Huaceae Afrostyrax kamerunensis#ZSD357
Oxalidales Connaraceae Rourea minor#F916
Oxalidales Connaraceae Connarus yunnanensis#F914
Oxalidales Connaraceae Ellipanthus glabrifolius#F494
Oxalidales Oxalidaceae Averrhoa carambola#F89
Oxalidales Oxalidaceae Oxalis sp.#JHCN
Oxalidales Oxalidaceae Oxalis acetosella#F405
Oxalidales Oxalidaceae Oxalis latifolia#EU
Oxalidales Cephalotaceae Cephalotus follicularis#Y250
Oxalidales Cephalotaceae Cephalotus follicularis#YZVJ
Oxalidales Brunelliaceae Brunellia sp.#ZSD328
Oxalidales Brunelliaceae Brunellia mexicana#ZSD329
Oxalidales Cunoniaceae Davidsonia pruriens#FC162
Oxalidales Cunoniaceae Pullea stutzeri#FC168
Oxalidales Cunoniaceae Cunonia capensis#FC96
Oxalidales Elaeocarpaceae Sloanea hemsleyana#F749
Oxalidales Elaeocarpaceae Elaeocarpus sylvestris#THHD
Oxalidales Elaeocarpaceae Elaeocarpus hainanensis#F568
Oxalidales Elaeocarpaceae Elaeocarpus dubius#F588
Celastrales Lepidobotryaceae Lepidobotrys staudtii#ZSD352
Celastrales Lepidobotryaceae Lepidobotrys staudtii#ZSD351
Celastrales Celastraceae Parnassia brevistyla#MG792145
Celastrales Celastraceae Parnassia delavayi#F507
Celastrales Celastraceae Parnassia mysorensis#F508
Celastrales Celastraceae Parnassia wightiana#F506
Celastrales Celastraceae Microtropis sp.#F401
Celastrales Celastraceae Stackhousia spathulata#MFEA
Celastrales Celastraceae Crossopetalum rhacoma#IHCQ
Celastrales Celastraceae Gymnosporia diversifolia#F702
Celastrales Celastraceae Cassine xylocarpa#NZ7079
Celastrales Celastraceae Salacia chinensis#F727
Celastrales Celastraceae Loeseneriella yunnanensis#F239
Celastrales Celastraceae Plagiopteron suaveolens#F1019
Celastrales Celastraceae Tripterygium wilfordii#F117
Celastrales Celastraceae Euonymus hamiltonianus#NC 037518
Celastrales Celastraceae Euonymus americanus#EU
Celastrales Celastraceae Euonymus schensianus#NC 036019
Celastrales Celastraceae Euonymus theifolius#F48
Celastrales Celastraceae Euonymus japonicus#NC 028067
Malpighiales Centroplacaceae Bhesa robusta#F1022
Malpighiales Centroplacaceae Bhesa robusta#F1023
Malpighiales Elatinaceae Bergia capensis#F1029
Malpighiales Elatinaceae Bergia capensis#F1030
Malpighiales Malpighiaceae Galphimia gracilis#XPBC
Malpighiales Malpighiaceae Byrsonima crassifolia#NC 037192
Malpighiales Malpighiaceae Byrsonima coccolobifolia#NC 037191
Malpighiales Malpighiaceae Bunchosia argentea#NC 041491
Malpighiales Malpighiaceae Bunchosia armeniaca#F523
Malpighiales Malpighiaceae Banisteriopsis caapi#NC 037945
Malpighiales Malpighiaceae Aspidopterys glabriuscula#F131
Malpighiales Malpighiaceae Aspidopterys henryi#F8
Malpighiales Malpighiaceae Hiptage candicans#F211
Malpighiales Malpighiaceae Hiptage benghalensis#F238
Malpighiales Caryocaraceae Caryocar glabrum#F555
Malpighiales Lophopyxidaceae Lophopyxis maingayi#ZSD315
Malpighiales Lophopyxidaceae Lophopyxis maingayi#ZSD314
Malpighiales Putranjivaceae Drypetes deplanchei#RVGH
Malpighiales Putranjivaceae Drypetes indica#F918
Malpighiales Putranjivaceae Drypetes hainanensis#F491
Malpighiales Balanopaceae Balanops pachyphylla#ZSD325
Malpighiales Trigoniaceae Trigonia rugosa#F897
Malpighiales Dichapetalaceae Dichapetalum longipetalum#F705
Malpighiales Dichapetalaceae Dichapetalum gelonioides#F470
Malpighiales Euphroniaceae Euphronia acuminatissima#T00300
Malpighiales Euphroniaceae Euphronia guianensis#T00302
Malpighiales Euphroniaceae Euphronia hirtelloides#T00301
Malpighiales Chrysobalanaceae Chrysobalanus icaco#FC108
Malpighiales Chrysobalanaceae Parinari campestris#NC 024067
Malpighiales Chrysobalanaceae Licania michauxii#HBUQ
Malpighiales Chrysobalanaceae Licania tomentosa#NC 030575
Malpighiales Chrysobalanaceae Couepia guianensis#NC 024063
Malpighiales Chrysobalanaceae Licania alba#NC 024064
Malpighiales Chrysobalanaceae Licania sprucei#NC 024065
Malpighiales Chrysobalanaceae Licania heteromorpha#NC 024062
Malpighiales Chrysobalanaceae Hirtella racemosa#F554
Malpighiales Chrysobalanaceae Hirtella physophora#NC 024066
Malpighiales Irvingiaceae Klainedoxa gabonensis#ZSD280
Malpighiales Irvingiaceae Irvingia excelsa#ZSD279
Malpighiales Ochnaceae Gomphia serrata#F482
Malpighiales Ochnaceae Ochna integerrima#F730
Malpighiales Ochnaceae Ochna mossambicensis#TVCU
Malpighiales Ochnaceae Ochna serrulata#CKDK
Malpighiales Ctenolophonaceae Ctenolophon englerianus#ZSD332
Malpighiales Erythroxylaceae Erythroxylum sinense#F372
Malpighiales Erythroxylaceae Erythroxylum coca#RPPC
Malpighiales Erythroxylaceae Erythroxylum novogranatense#F913
Malpighiales Rhizophoraceae Carallia brachiata#F236
Malpighiales Rhizophoraceae Bruguiera sexangula#F498
Malpighiales Rhizophoraceae Rhizophora mucronata#F499
Malpighiales Rhizophoraceae Rhizophora mangle#ZTLR
Malpighiales Pandaceae Microdesmis caseariifolia#F721
Malpighiales Pandaceae Microdesmis caseariifolia#F492
Malpighiales Bonnetiaceae Bonnetia paniculata#ZSD327
Malpighiales Clusiaceae Clusia palmicida#F565
Malpighiales Clusiaceae Pentadesma butyracea#F921
Malpighiales Clusiaceae Garcinia lancilimba#F342

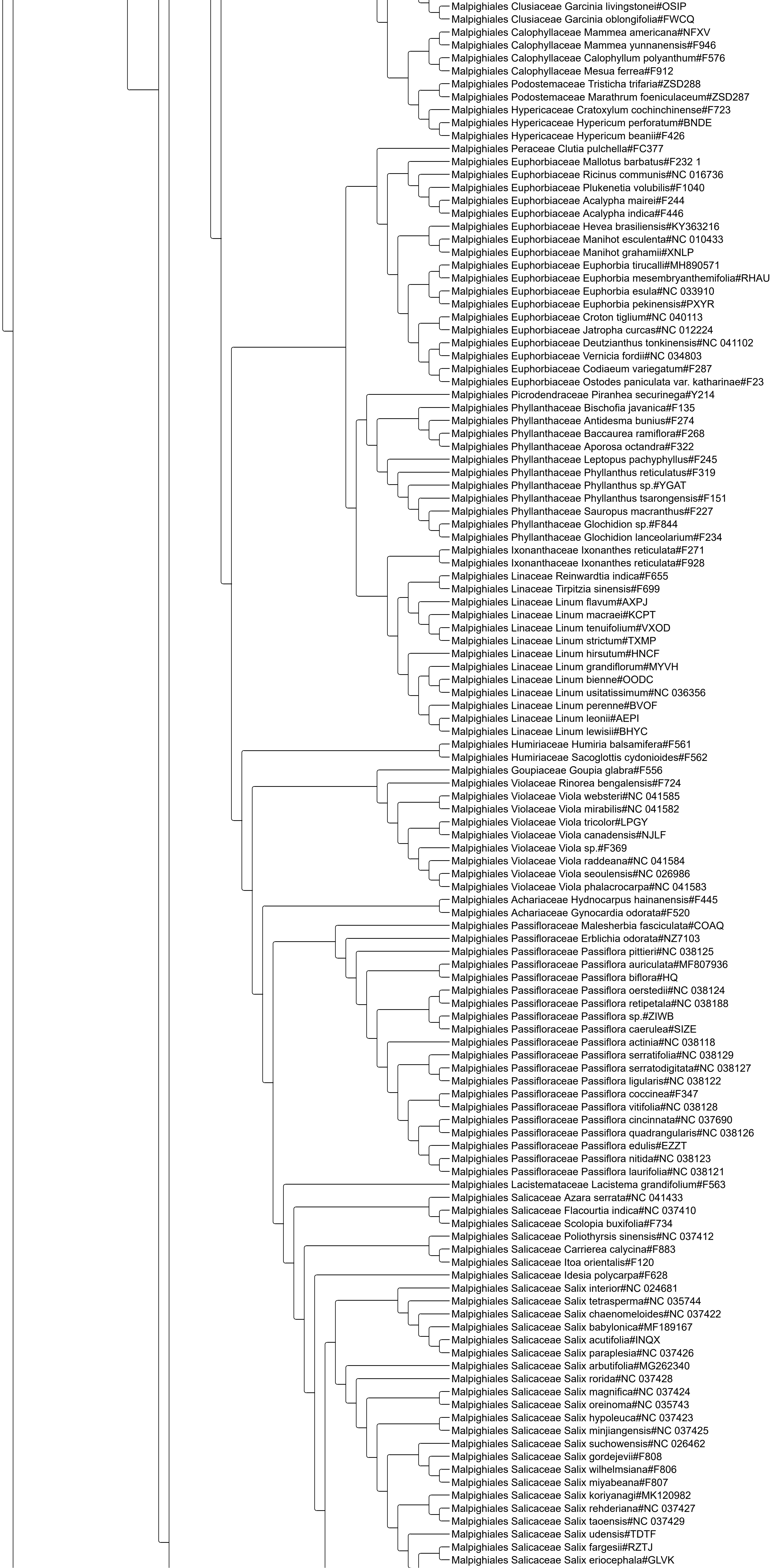
Malpighiales Clusiaceae Garcinia livingstonei#OSIP
Malpighiales Clusiaceae Garcinia oblongifolia#FWCQ
Malpighiales Calophyllaceae Mammea americana#NFXV
Malpighiales Calophyllaceae Mammea yunnanensis#F946
Malpighiales Calophyllaceae Calophyllum polyanthum#F576
Malpighiales Calophyllaceae Mesua ferrea#F912
Malpighiales Podostemaceae Tristicha trifaria#ZSD288
Malpighiales Podostemaceae Marathrum foeniculaceum#ZSD287
Malpighiales Hypericaceae Cratoxylum cochinchinense#F723
Malpighiales Hypericaceae Hypericum perforatum#BNDE
Malpighiales Hypericaceae Hypericum beanii#F426
Malpighiales Peraceae Clutia pulchella#FC377
Malpighiales Euphorbiaceae Mallotus barbatus#F232 1
Malpighiales Euphorbiaceae Ricinus communis#NC 016736
Malpighiales Euphorbiaceae Plukenetia volubilis#F1040
Malpighiales Euphorbiaceae Acalypha mairei#F244
Malpighiales Euphorbiaceae Acalypha indica#F446
Malpighiales Euphorbiaceae Hevea brasiliensis#KY363216
Malpighiales Euphorbiaceae Manihot esculenta#NC 010433
Malpighiales Euphorbiaceae Manihot grahamii#XNLP
Malpighiales Euphorbiaceae Euphorbia tirucalli#MH890571
Malpighiales Euphorbiaceae Euphorbia mesembryanthemifolia#RHAU
Malpighiales Euphorbiaceae Euphorbia esula#NC 033910
Malpighiales Euphorbiaceae Euphorbia pekinensis#PXYR
Malpighiales Euphorbiaceae Croton tiglium#NC 040113
Malpighiales Euphorbiaceae Jatropha curcas#NC 012224
Malpighiales Euphorbiaceae Deutzianthus tonkinensis#NC 041102
Malpighiales Euphorbiaceae Vernicia fordii#NC 034803
Malpighiales Euphorbiaceae Codiaeum variegatum#F287
Malpighiales Euphorbiaceae Ostodes paniculata var. katharinae#F23
Malpighiales Picrodendraceae Piranhea securinega#Y214
Malpighiales Phyllanthaceae Bischofia javanica#F135
Malpighiales Phyllanthaceae Antidesma bunius#F274
Malpighiales Phyllanthaceae Baccaurea ramiflora#F268
Malpighiales Phyllanthaceae Aporosa octandra#F322
Malpighiales Phyllanthaceae Leptopus pachyphyllus#F245
Malpighiales Phyllanthaceae Phyllanthus reticulatus#F319
Malpighiales Phyllanthaceae Phyllanthus sp.#YGAT
Malpighiales Phyllanthaceae Phyllanthus tsarongensis#F151
Malpighiales Phyllanthaceae Sauropus macranthus#F227
Malpighiales Phyllanthaceae Glochidion sp.#F844
Malpighiales Phyllanthaceae Glochidion lanceolarium#F234
Malpighiales Ixonanthaceae Ixonanthes reticulata#F271
Malpighiales Ixonanthaceae Ixonanthes reticulata#F928
Malpighiales Linaceae Reinwardtia indica#F655
Malpighiales Linaceae Tirpitzia sinensis#F699
Malpighiales Linaceae Linum flavum#AXPJ
Malpighiales Linaceae Linum macraei#KCPT
Malpighiales Linaceae Linum tenuifolium#VXOD
Malpighiales Linaceae Linum strictum#TXMP
Malpighiales Linaceae Linum hirsutum#HNCF
Malpighiales Linaceae Linum grandiflorum#MYVH
Malpighiales Linaceae Linum bienne#OODC
Malpighiales Linaceae Linum usitatissimum#NC 036356
Malpighiales Linaceae Linum perenne#BVOF
Malpighiales Linaceae Linum leonii#AEPI
Malpighiales Linaceae Linum lewisii#BHYC
Malpighiales Humiriaceae Humiria balsamifera#F561
Malpighiales Humiriaceae Sacoglottis cydonioides#F562
Malpighiales Goupiaceae Goupia glabra#F556
Malpighiales Violaceae Rinorea bengalensis#F724
Malpighiales Violaceae Viola websteri#NC 041585
Malpighiales Violaceae Viola mirabilis#NC 041582
Malpighiales Violaceae Viola tricolor#LPGY
Malpighiales Violaceae Viola canadensis#NJLF
Malpighiales Violaceae Viola sp.#F369
Malpighiales Violaceae Viola raddeana#NC 041584
Malpighiales Violaceae Viola seoulensis#NC 026986
Malpighiales Violaceae Viola phalacrocarpa#NC 041583
Malpighiales Achariaceae Hydnocarpus hainanensis#F445
Malpighiales Achariaceae Gynocardia odorata#F520
Malpighiales Passifloraceae Malesherbia fasciculata#COAQ
Malpighiales Passifloraceae Erblichia odorata#NZ7103
Malpighiales Passifloraceae Passiflora pittieri#NC 038125
Malpighiales Passifloraceae Passiflora auriculata#MF807936
Malpighiales Passifloraceae Passiflora biflora#HQ
Malpighiales Passifloraceae Passiflora oerstedii#NC 038124
Malpighiales Passifloraceae Passiflora retipetala#NC 038188
Malpighiales Passifloraceae Passiflora sp.#ZIWB
Malpighiales Passifloraceae Passiflora caerulea#SIZE
Malpighiales Passifloraceae Passiflora actinia#NC 038118
Malpighiales Passifloraceae Passiflora serratifolia#NC 038129
Malpighiales Passifloraceae Passiflora serratodigitata#NC 038127
Malpighiales Passifloraceae Passiflora ligularis#NC 038122
Malpighiales Passifloraceae Passiflora coccinea#F347
Malpighiales Passifloraceae Passiflora vitifolia#NC 038128
Malpighiales Passifloraceae Passiflora cincinnata#NC 037690
Malpighiales Passifloraceae Passiflora quadrangularis#NC 038126
Malpighiales Passifloraceae Passiflora edulis#EZZT
Malpighiales Passifloraceae Passiflora nitida#NC 038123
Malpighiales Passifloraceae Passiflora laurifolia#NC 038121
Malpighiales Lacistemataceae Lacistema grandifolium#F563
Malpighiales Salicaceae Azara serrata#NC 041433
Malpighiales Salicaceae Flacourtia indica#NC 037410
Malpighiales Salicaceae Scolopia buxifolia#F734
Malpighiales Salicaceae Poliothyrsis sinensis#NC 037412
Malpighiales Salicaceae Carrierea calycina#F883
Malpighiales Salicaceae Itoa orientalis#F120
Malpighiales Salicaceae Idesia polycarpa#F628
Malpighiales Salicaceae Salix interior#NC 024681
Malpighiales Salicaceae Salix tetrasperma#NC 035744
Malpighiales Salicaceae Salix chaenomeloides#NC 037422
Malpighiales Salicaceae Salix babylonica#MF189167
Malpighiales Salicaceae Salix acutifolia#INQX
Malpighiales Salicaceae Salix paraplesia#NC 037426
Malpighiales Salicaceae Salix arbutifolia#MG262340
Malpighiales Salicaceae Salix rorida#NC 037428
Malpighiales Salicaceae Salix magnifica#NC 037424
Malpighiales Salicaceae Salix oreinoma#NC 035743
Malpighiales Salicaceae Salix hypoleuca#NC 037423
Malpighiales Salicaceae Salix minjiangensis#NC 037425
Malpighiales Salicaceae Salix suchowensis#NC 026462
Malpighiales Salicaceae Salix gordejevii#F808
Malpighiales Salicaceae Salix wilhelmsiana#F806
Malpighiales Salicaceae Salix miyabeana#F807
Malpighiales Salicaceae Salix koriyanagi#MK120982
Malpighiales Salicaceae Salix rehderiana#NC 037427
Malpighiales Salicaceae Salix taoensis#NC 037429
Malpighiales Salicaceae Salix udensis#TDTF
Malpighiales Salicaceae Salix fargesii#RZTJ
Malpighiales Salicaceae Salix eriocephala#GLVK

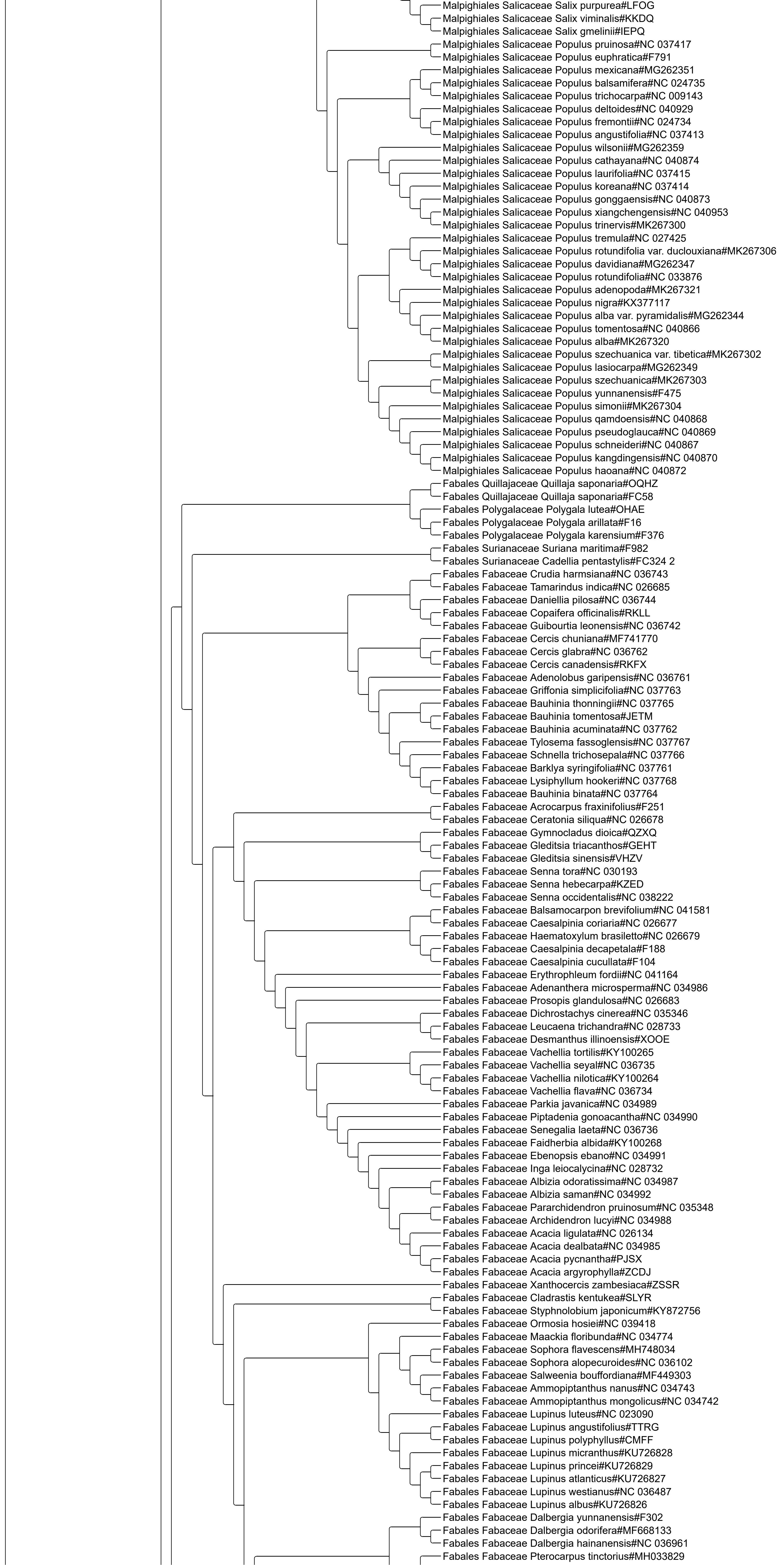

Malpighiales Salicaceae Salix purpurea#LFOG
Malpighiales Salicaceae Salix viminalis#KKDQ
Malpighiales Salicaceae Salix gmelinii#IEPQ
Malpighiales Salicaceae Populus pruinosa#NC 037417
Malpighiales Salicaceae Populus euphratica#F791
Malpighiales Salicaceae Populus mexicana#MG262351
Malpighiales Salicaceae Populus balsamifera#NC 024735
Malpighiales Salicaceae Populus trichocarpa#NC 009143
Malpighiales Salicaceae Populus deltoides#NC 040929
Malpighiales Salicaceae Populus fremontii#NC 024734
Malpighiales Salicaceae Populus angustifolia#NC 037413
Malpighiales Salicaceae Populus wilsonii#MG262359
Malpighiales Salicaceae Populus cathayana#NC 040874
Malpighiales Salicaceae Populus laurifolia#NC 037415
Malpighiales Salicaceae Populus koreana#NC 037414
Malpighiales Salicaceae Populus gonggaensis#NC 040873
Malpighiales Salicaceae Populus xiangchengensis#NC 040953
Malpighiales Salicaceae Populus trinervis#MK267300
Malpighiales Salicaceae Populus tremula#NC 027425
Malpighiales Salicaceae Populus rotundifolia var. duclouxiana#MK267306
Malpighiales Salicaceae Populus davidiana#MG262347
Malpighiales Salicaceae Populus rotundifolia#NC 033876
Malpighiales Salicaceae Populus adenopoda#MK267321
Malpighiales Salicaceae Populus nigra#KX377117
Malpighiales Salicaceae Populus alba var. pyramidalis#MG262344
Malpighiales Salicaceae Populus tomentosa#NC 040866
Malpighiales Salicaceae Populus alba#MK267320
Malpighiales Salicaceae Populus szechuanica var. tibetica#MK267302
Malpighiales Salicaceae Populus lasiocarpa#MG262349
Malpighiales Salicaceae Populus szechuanica#MK267303
Malpighiales Salicaceae Populus yunnanensis#F475
Malpighiales Salicaceae Populus simonii#MK267304
Malpighiales Salicaceae Populus qamdoensis#NC 040868
Malpighiales Salicaceae Populus pseudoglauca#NC 040869
Malpighiales Salicaceae Populus schneideri#NC 040867
Malpighiales Salicaceae Populus kangdingensis#NC 040870
Malpighiales Salicaceae Populus haoana#NC 040872
Fabales Quillajaceae Quillaja saponaria#OQHZ
Fabales Quillajaceae Quillaja saponaria#FC58
Fabales Polygalaceae Polygala lutea#OHAE
Fabales Polygalaceae Polygala arillata#F16
Fabales Polygalaceae Polygala karensium#F376
Fabales Surianaceae Suriana maritima#F982
Fabales Surianaceae Cadellia pentastylis#FC324 2
Fabales Fabaceae Crudia harmsiana#NC 036743
Fabales Fabaceae Tamarindus indica#NC 026685
Fabales Fabaceae Daniellia pilosa#NC 036744
Fabales Fabaceae Copaifera officinalis#RKLL
Fabales Fabaceae Guibourtia leonensis#NC 036742
Fabales Fabaceae Cercis chuniana#MF741770
Fabales Fabaceae Cercis glabra#NC 036762
Fabales Fabaceae Cercis canadensis#RKFX
Fabales Fabaceae Adenolobus garipensis#NC 036761
Fabales Fabaceae Griffonia simplicifolia#NC 037763
Fabales Fabaceae Bauhinia thonningii#NC 037765
Fabales Fabaceae Bauhinia tomentosa#JETM
Fabales Fabaceae Bauhinia acuminata#NC 037762
Fabales Fabaceae Tylosema fassoglensis#NC 037767
Fabales Fabaceae Schnella trichosepala#NC 037766
Fabales Fabaceae Barklya syringifolia#NC 037761
Fabales Fabaceae Lysiphyllum hookeri#NC 037768
Fabales Fabaceae Bauhinia binata#NC 037764
Fabales Fabaceae Acrocarpus fraxinifolius#F251
Fabales Fabaceae Ceratonia siliqua#NC 026678
Fabales Fabaceae Gymnocladus dioica#QZXQ
Fabales Fabaceae Gleditsia triacanthos#GEHT
Fabales Fabaceae Gleditsia sinensis#VHZV
Fabales Fabaceae Senna tora#NC 030193
Fabales Fabaceae Senna hebecarpa#KZED
Fabales Fabaceae Senna occidentalis#NC 038222
Fabales Fabaceae Balsamocarpon brevifolium#NC 041581
Fabales Fabaceae Caesalpinia coriaria#NC 026677
Fabales Fabaceae Haematoxylum brasiletto#NC 026679
Fabales Fabaceae Caesalpinia decapetala#F188
Fabales Fabaceae Caesalpinia cucullata#F104
Fabales Fabaceae Erythrophleum fordii#NC 041164
Fabales Fabaceae Adenanthera microsperma#NC 034986
Fabales Fabaceae Prosopis glandulosa#NC 026683
Fabales Fabaceae Dichrostachys cinerea#NC 035346
Fabales Fabaceae Leucaena trichandra#NC 028733
Fabales Fabaceae Desmanthus illinoensis#XOOE
Fabales Fabaceae Vachellia tortilis#KY100265
Fabales Fabaceae Vachellia seyal#NC 036735
Fabales Fabaceae Vachellia nilotica#KY100264
Fabales Fabaceae Vachellia flava#NC 036734
Fabales Fabaceae Parkia javanica#NC 034989
Fabales Fabaceae Piptadenia gonoacantha#NC 034990
Fabales Fabaceae Senegalia laeta#NC 036736
Fabales Fabaceae Faidherbia albida#KY100268
Fabales Fabaceae Ebenopsis ebano#NC 034991
Fabales Fabaceae Inga leiocalycina#NC 028732
Fabales Fabaceae Albizia odoratissima#NC 034987
Fabales Fabaceae Albizia saman#NC 034992
Fabales Fabaceae Pararchidendron pruinosum#NC 035348
Fabales Fabaceae Archidendron lucyi#NC 034988
Fabales Fabaceae Acacia ligulata#NC 026134
Fabales Fabaceae Acacia dealbata#NC 034985
Fabales Fabaceae Acacia pycnantha#PJSX
Fabales Fabaceae Acacia argyrophylla#ZCDJ
Fabales Fabaceae Xanthocercis zambesiaca#ZSSR
Fabales Fabaceae Cladrastis kentukea#SLYR
Fabales Fabaceae Styphnolobium japonicum#KY872756
Fabales Fabaceae Ormosia hosiei#NC 039418
Fabales Fabaceae Maackia floribunda#NC 034774
Fabales Fabaceae Sophora flavescens#MH748034
Fabales Fabaceae Sophora alopecuroides#NC 036102
Fabales Fabaceae Salweenia bouffordiana#MF449303
Fabales Fabaceae Ammopiptanthus nanus#NC 034743
Fabales Fabaceae Ammopiptanthus mongolicus#NC 034742
Fabales Fabaceae Lupinus luteus#NC 023090
Fabales Fabaceae Lupinus angustifolius#TTRG
Fabales Fabaceae Lupinus polyphyllus#CMFF
Fabales Fabaceae Lupinus micranthus#KU726828
Fabales Fabaceae Lupinus princei#KU726829
Fabales Fabaceae Lupinus atlanticus#KU726827
Fabales Fabaceae Lupinus westianus#NC 036487
Fabales Fabaceae Lupinus albus#KU726826
Fabales Fabaceae Dalbergia yunnanensis#F302
Fabales Fabaceae Dalbergia odorifera#MF668133
Fabales Fabaceae Dalbergia hainanensis#NC 036961
Fabales Fabaceae Pterocarpus tinctorius#MH033829

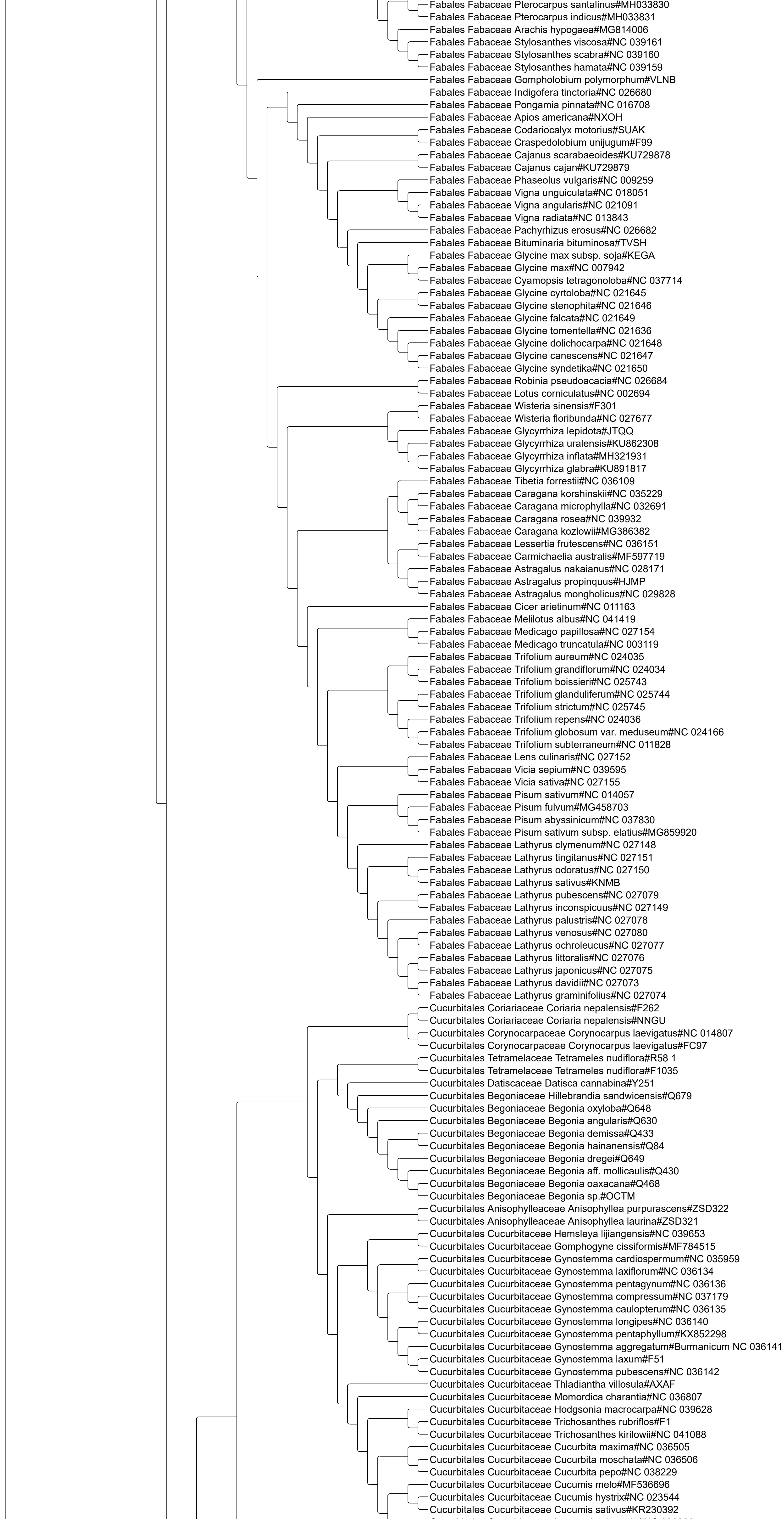
Fabales Fabaceae Pterocarpus santalinus#MH033830
Fabales Fabaceae Pterocarpus indicus#MH033831
Fabales Fabaceae Arachis hypogaea#MG814006
Fabales Fabaceae Stylosanthes viscosa#NC 039161
Fabales Fabaceae Stylosanthes scabra#NC 039160
Fabales Fabaceae Stylosanthes hamata#NC 039159
Fabales Fabaceae Gompholobium polymorphum#VLNB
Fabales Fabaceae Indigofera tinctoria#NC 026680
Fabales Fabaceae Pongamia pinnata#NC 016708
Fabales Fabaceae Apios americana#NXOH
Fabales Fabaceae Codariocalyx motorius#SUAK
Fabales Fabaceae Craspedolobium unijugum#F99
Fabales Fabaceae Cajanus scarabaeoides#KU729878
Fabales Fabaceae Cajanus cajan#KU729879
Fabales Fabaceae Phaseolus vulgaris#NC 009259
Fabales Fabaceae Vigna unguiculata#NC 018051
Fabales Fabaceae Vigna angularis#NC 021091
Fabales Fabaceae Vigna radiata#NC 013843
Fabales Fabaceae Pachyrhizus erosus#NC 026682
Fabales Fabaceae Bituminaria bituminosa#TVSH
Fabales Fabaceae Glycine max subsp. soja#KEGA
Fabales Fabaceae Glycine max#NC 007942
Fabales Fabaceae Cyamopsis tetragonoloba#NC 037714
Fabales Fabaceae Glycine cyrtoloba#NC 021645
Fabales Fabaceae Glycine stenophita#NC 021646
Fabales Fabaceae Glycine falcata#NC 021649
Fabales Fabaceae Glycine tomentella#NC 021636
Fabales Fabaceae Glycine dolichocarpa#NC 021648
Fabales Fabaceae Glycine canescens#NC 021647
Fabales Fabaceae Glycine syndetika#NC 021650
Fabales Fabaceae Robinia pseudoacacia#NC 026684
Fabales Fabaceae Lotus corniculatus#NC 002694
Fabales Fabaceae Wisteria sinensis#F301
Fabales Fabaceae Wisteria floribunda#NC 027677
Fabales Fabaceae Glycyrrhiza lepidota#JTQQ
Fabales Fabaceae Glycyrrhiza uralensis#KU862308
Fabales Fabaceae Glycyrrhiza inflata#MH321931
Fabales Fabaceae Glycyrrhiza glabra#KU891817
Fabales Fabaceae Tibetia forrestii#NC 036109
Fabales Fabaceae Caragana korshinskii#NC 035229
Fabales Fabaceae Caragana microphylla#NC 032691
Fabales Fabaceae Caragana rosea#NC 039932
Fabales Fabaceae Caragana kozlowii#MG386382
Fabales Fabaceae Lessertia frutescens#NC 036151
Fabales Fabaceae Carmichaelia australis#MF597719
Fabales Fabaceae Astragalus nakaianus#NC 028171
Fabales Fabaceae Astragalus propinquus#HJMP
Fabales Fabaceae Astragalus mongholicus#NC 029828
Fabales Fabaceae Cicer arietinum#NC 011163
Fabales Fabaceae Melilotus albus#NC 041419
Fabales Fabaceae Medicago papillosa#NC 027154
Fabales Fabaceae Medicago truncatula#NC 003119
Fabales Fabaceae Trifolium aureum#NC 024035
Fabales Fabaceae Trifolium grandiflorum#NC 024034
Fabales Fabaceae Trifolium boissieri#NC 025743
Fabales Fabaceae Trifolium glanduliferum#NC 025744
Fabales Fabaceae Trifolium strictum#NC 025745
Fabales Fabaceae Trifolium repens#NC 024036
Fabales Fabaceae Trifolium globosum var. meduseum#NC 024166
Fabales Fabaceae Trifolium subterraneum#NC 011828
Fabales Fabaceae Lens culinaris#NC 027152
Fabales Fabaceae Vicia sepium#NC 039595
Fabales Fabaceae Vicia sativa#NC 027155
Fabales Fabaceae Pisum sativum#NC 014057
Fabales Fabaceae Pisum fulvum#MG458703
Fabales Fabaceae Pisum abyssinicum#NC 037830
Fabales Fabaceae Pisum sativum subsp. elatius#MG859920
Fabales Fabaceae Lathyrus clymenum#NC 027148
Fabales Fabaceae Lathyrus tingitanus#NC 027151
Fabales Fabaceae Lathyrus odoratus#NC 027150
Fabales Fabaceae Lathyrus sativus#KNMB
Fabales Fabaceae Lathyrus pubescens#NC 027079
Fabales Fabaceae Lathyrus inconspicuus#NC 027149
Fabales Fabaceae Lathyrus palustris#NC 027078
Fabales Fabaceae Lathyrus venosus#NC 027080
Fabales Fabaceae Lathyrus ochroleucus#NC 027077
Fabales Fabaceae Lathyrus littoralis#NC 027076
Fabales Fabaceae Lathyrus japonicus#NC 027075
Fabales Fabaceae Lathyrus davidii#NC 027073
Fabales Fabaceae Lathyrus graminifolius#NC 027074
Cucurbitales Coriariaceae Coriaria nepalensis#F262
Cucurbitales Coriariaceae Coriaria nepalensis#NNGU
Cucurbitales Corynocarpaceae Corynocarpus laevigatus#NC 014807
Cucurbitales Corynocarpaceae Corynocarpus laevigatus#FC97
Cucurbitales Tetramelaceae Tetrameles nudiflora#R58 1
Cucurbitales Tetramelaceae Tetrameles nudiflora#F1035
Cucurbitales Datiscaceae Datisca cannabina#Y251
Cucurbitales Begoniaceae Hillebrandia sandwicensis#Q679
Cucurbitales Begoniaceae Begonia oxyloba#Q648
Cucurbitales Begoniaceae Begonia angularis#Q630
Cucurbitales Begoniaceae Begonia demissa#Q433
Cucurbitales Begoniaceae Begonia hainanensis#Q84
Cucurbitales Begoniaceae Begonia dregei#Q649
Cucurbitales Begoniaceae Begonia aff. mollicaulis#Q430
Cucurbitales Begoniaceae Begonia oaxacana#Q468
Cucurbitales Begoniaceae Begonia sp.#OCTM
Cucurbitales Anisophylleaceae Anisophyllea purpurascens#ZSD322
Cucurbitales Anisophylleaceae Anisophyllea laurina#ZSD321
Cucurbitales Cucurbitaceae Hemsleya lijiangensis#NC 039653
Cucurbitales Cucurbitaceae Gomphogyne cissiformis#MF784515
Cucurbitales Cucurbitaceae Gynostemma cardiospermum#NC 035959
Cucurbitales Cucurbitaceae Gynostemma laxiflorum#NC 036134
Cucurbitales Cucurbitaceae Gynostemma pentagynum#NC 036136
Cucurbitales Cucurbitaceae Gynostemma compressum#NC 037179
Cucurbitales Cucurbitaceae Gynostemma caulopterum#NC 036135
Cucurbitales Cucurbitaceae Gynostemma longipes#NC 036140
Cucurbitales Cucurbitaceae Gynostemma pentaphyllum#KX852298
Cucurbitales Cucurbitaceae Gynostemma aggregatum#Burmanicum NC 036141
Cucurbitales Cucurbitaceae Gynostemma laxum#F51
Cucurbitales Cucurbitaceae Gynostemma pubescens#NC 036142
Cucurbitales Cucurbitaceae Thladiantha villosula#AXAF
Cucurbitales Cucurbitaceae Momordica charantia#NC 036807
Cucurbitales Cucurbitaceae Hodgsonia macrocarpa#NC 039628
Cucurbitales Cucurbitaceae Trichosanthes rubriflos#F1
Cucurbitales Cucurbitaceae Trichosanthes kirilowii#NC 041088
Cucurbitales Cucurbitaceae Cucurbita maxima#NC 036505
Cucurbitales Cucurbitaceae Cucurbita moschata#NC 036506
Cucurbitales Cucurbitaceae Cucurbita pepo#NC 038229
Cucurbitales Cucurbitaceae Cucumis melo#MF536696
Cucurbitales Cucurbitaceae Cucumis hystrix#NC 023544
Cucurbitales Cucurbitaceae Cucumis sativus#KR230392

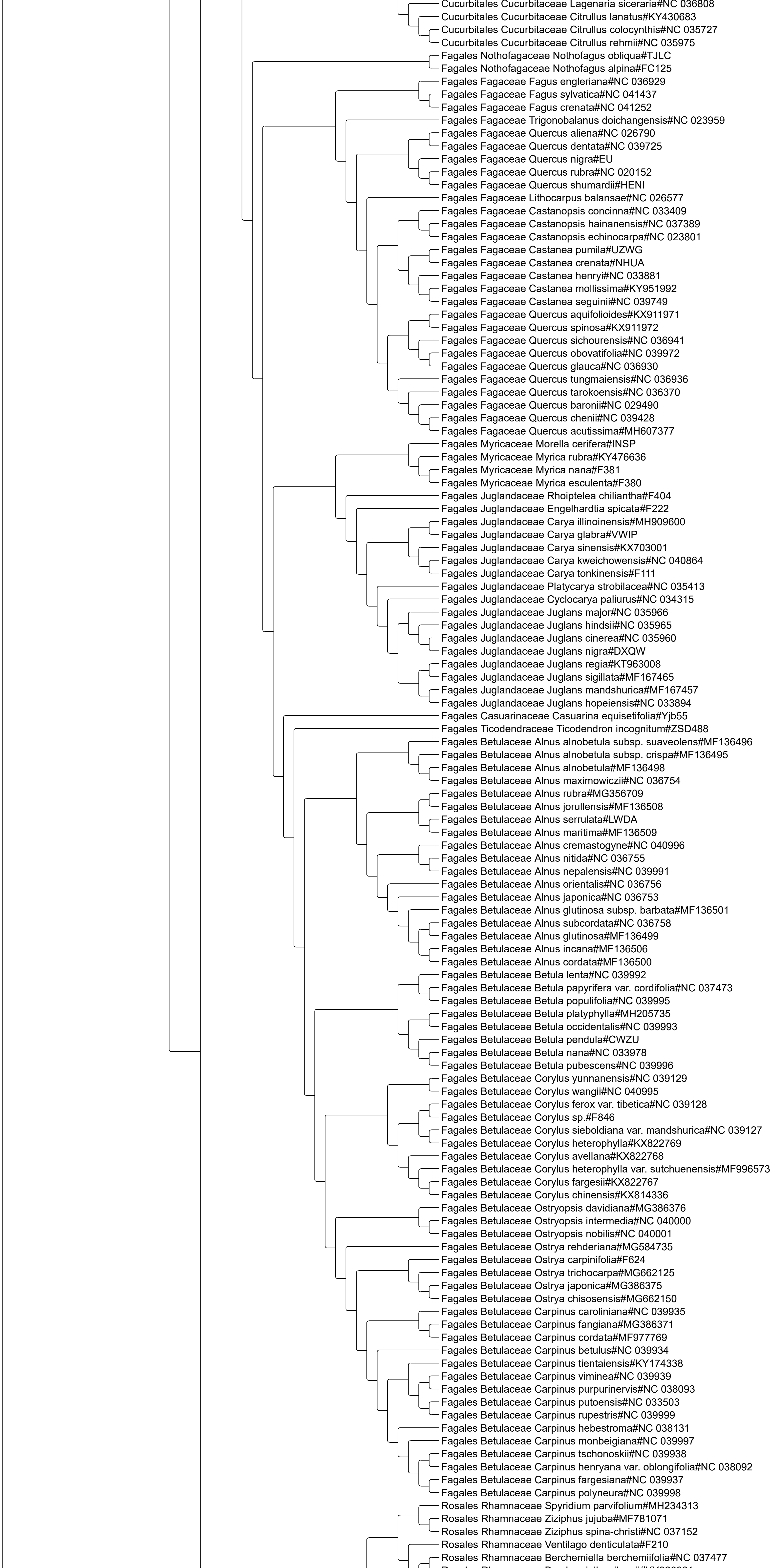

Cucurbitales Cucurbitaceae Lagenaria siceraria#NC 036808
Cucurbitales Cucurbitaceae Citrullus lanatus#KY430683
Cucurbitales Cucurbitaceae Citrullus colocynthis#NC 035727
Cucurbitales Cucurbitaceae Citrullus rehmii#NC 035975
Fagales Nothofagaceae Nothofagus obliqua#TJLC
Fagales Nothofagaceae Nothofagus alpina#FC125
Fagales Fagaceae Fagus engleriana#NC 036929
Fagales Fagaceae Fagus sylvatica#NC 041437
Fagales Fagaceae Fagus crenata#NC 041252
Fagales Fagaceae Trigonobalanus doichangensis#NC 023959
Fagales Fagaceae Quercus aliena#NC 026790
Fagales Fagaceae Quercus dentata#NC 039725
Fagales Fagaceae Quercus nigra#EU
Fagales Fagaceae Quercus rubra#NC 020152
Fagales Fagaceae Quercus shumardii#HENI
Fagales Fagaceae Lithocarpus balansae#NC 026577
Fagales Fagaceae Castanopsis concinna#NC 033409
Fagales Fagaceae Castanopsis hainanensis#NC 037389
Fagales Fagaceae Castanopsis echinocarpa#NC 023801
Fagales Fagaceae Castanea pumila#UZWG
Fagales Fagaceae Castanea crenata#NHUA
Fagales Fagaceae Castanea henryi#NC 033881
Fagales Fagaceae Castanea mollissima#KY951992
Fagales Fagaceae Castanea seguinii#NC 039749
Fagales Fagaceae Quercus aquifolioides#KX911971
Fagales Fagaceae Quercus spinosa#KX911972
Fagales Fagaceae Quercus sichourensis#NC 036941
Fagales Fagaceae Quercus obovatifolia#NC 039972
Fagales Fagaceae Quercus glauca#NC 036930
Fagales Fagaceae Quercus tungmaiensis#NC 036936
Fagales Fagaceae Quercus tarokoensis#NC 036370
Fagales Fagaceae Quercus baronii#NC 029490
Fagales Fagaceae Quercus chenii#NC 039428
Fagales Fagaceae Quercus acutissima#MH607377
Fagales Myricaceae Morella cerifera#INSP
Fagales Myricaceae Myrica rubra#KY476636
Fagales Myricaceae Myrica nana#F381
Fagales Myricaceae Myrica esculenta#F380
Fagales Juglandaceae Rhoiptelea chiliantha#F404
Fagales Juglandaceae Engelhardtia spicata#F222
Fagales Juglandaceae Carya illinoinensis#MH909600
Fagales Juglandaceae Carya glabra#VWIP
Fagales Juglandaceae Carya sinensis#KX703001
Fagales Juglandaceae Carya kweichowensis#NC 040864
Fagales Juglandaceae Carya tonkinensis#F111
Fagales Juglandaceae Platycarya strobilacea#NC 035413
Fagales Juglandaceae Cyclocarya paliurus#NC 034315
Fagales Juglandaceae Juglans major#NC 035966
Fagales Juglandaceae Juglans hindsii#NC 035965
Fagales Juglandaceae Juglans cinerea#NC 035960
Fagales Juglandaceae Juglans nigra#DXQW
Fagales Juglandaceae Juglans regia#KT963008
Fagales Juglandaceae Juglans sigillata#MF167465
Fagales Juglandaceae Juglans mandshurica#MF167457
Fagales Juglandaceae Juglans hopeiensis#NC 033894
Fagales Casuarinaceae Casuarina equisetifolia#Yjb55
Fagales Ticodendraceae Ticodendron incognitum#ZSD488
Fagales Betulaceae Alnus alnobetula subsp. suaveolens#MF136496
Fagales Betulaceae Alnus alnobetula subsp. crispa#MF136495
Fagales Betulaceae Alnus alnobetula#MF136498
Fagales Betulaceae Alnus maximowiczii#NC 036754
Fagales Betulaceae Alnus rubra#MG356709
Fagales Betulaceae Alnus jorullensis#MF136508
Fagales Betulaceae Alnus serrulata#LWDA
Fagales Betulaceae Alnus maritima#MF136509
Fagales Betulaceae Alnus cremastogyne#NC 040996
Fagales Betulaceae Alnus nitida#NC 036755
Fagales Betulaceae Alnus nepalensis#NC 039991
Fagales Betulaceae Alnus orientalis#NC 036756
Fagales Betulaceae Alnus japonica#NC 036753
Fagales Betulaceae Alnus glutinosa subsp. barbata#MF136501
Fagales Betulaceae Alnus subcordata#NC 036758
Fagales Betulaceae Alnus glutinosa#MF136499
Fagales Betulaceae Alnus incana#MF136506
Fagales Betulaceae Alnus cordata#MF136500
Fagales Betulaceae Betula lenta#NC 039992
Fagales Betulaceae Betula papyrifera var. cordifolia#NC 037473
Fagales Betulaceae Betula populifolia#NC 039995
Fagales Betulaceae Betula platyphylla#MH205735
Fagales Betulaceae Betula occidentalis#NC 039993
Fagales Betulaceae Betula pendula#CWZU
Fagales Betulaceae Betula nana#NC 033978
Fagales Betulaceae Betula pubescens#NC 039996
Fagales Betulaceae Corylus yunnanensis#NC 039129
Fagales Betulaceae Corylus wangii#NC 040995
Fagales Betulaceae Corylus ferox var. tibetica#NC 039128
Fagales Betulaceae Corylus sp.#F846
Fagales Betulaceae Corylus sieboldiana var. mandshurica#NC 039127
Fagales Betulaceae Corylus heterophylla#KX822769
Fagales Betulaceae Corylus avellana#KX822768
Fagales Betulaceae Corylus heterophylla var. sutchuenensis#MF996573
Fagales Betulaceae Corylus fargesii#KX822767
Fagales Betulaceae Corylus chinensis#KX814336
Fagales Betulaceae Ostryopsis davidiana#MG386376
Fagales Betulaceae Ostryopsis intermedia#NC 040000
Fagales Betulaceae Ostryopsis nobilis#NC 040001
Fagales Betulaceae Ostrya rehderiana#MG584735
Fagales Betulaceae Ostrya carpinifolia#F624
Fagales Betulaceae Ostrya trichocarpa#MG662125
Fagales Betulaceae Ostrya japonica#MG386375
Fagales Betulaceae Ostrya chisosensis#MG662150
Fagales Betulaceae Carpinus caroliniana#NC 039935
Fagales Betulaceae Carpinus fangiana#MG386371
Fagales Betulaceae Carpinus cordata#MF977769
Fagales Betulaceae Carpinus betulus#NC 039934
Fagales Betulaceae Carpinus tientaiensis#KY174338
Fagales Betulaceae Carpinus viminea#NC 039939
Fagales Betulaceae Carpinus purpurinervis#NC 038093
Fagales Betulaceae Carpinus putoensis#NC 033503
Fagales Betulaceae Carpinus rupestris#NC 039999
Fagales Betulaceae Carpinus hebestroma#NC 038131
Fagales Betulaceae Carpinus monbeigiana#NC 039997
Fagales Betulaceae Carpinus tschonoskii#NC 039938
Fagales Betulaceae Carpinus henryana var. oblongifolia#NC 038092
Fagales Betulaceae Carpinus fargesiana#NC 039937
Fagales Betulaceae Carpinus polyneura#NC 039998
Rosales Rhamnaceae Spyridium parvifolium#MH234313
Rosales Rhamnaceae Ziziphus jujuba#MF781071
Rosales Rhamnaceae Ziziphus spina-christi#NC 037152
Rosales Rhamnaceae Ventilago denticulata#F210
Rosales Rhamnaceae Berchemiella berchemiifolia#NC 037477

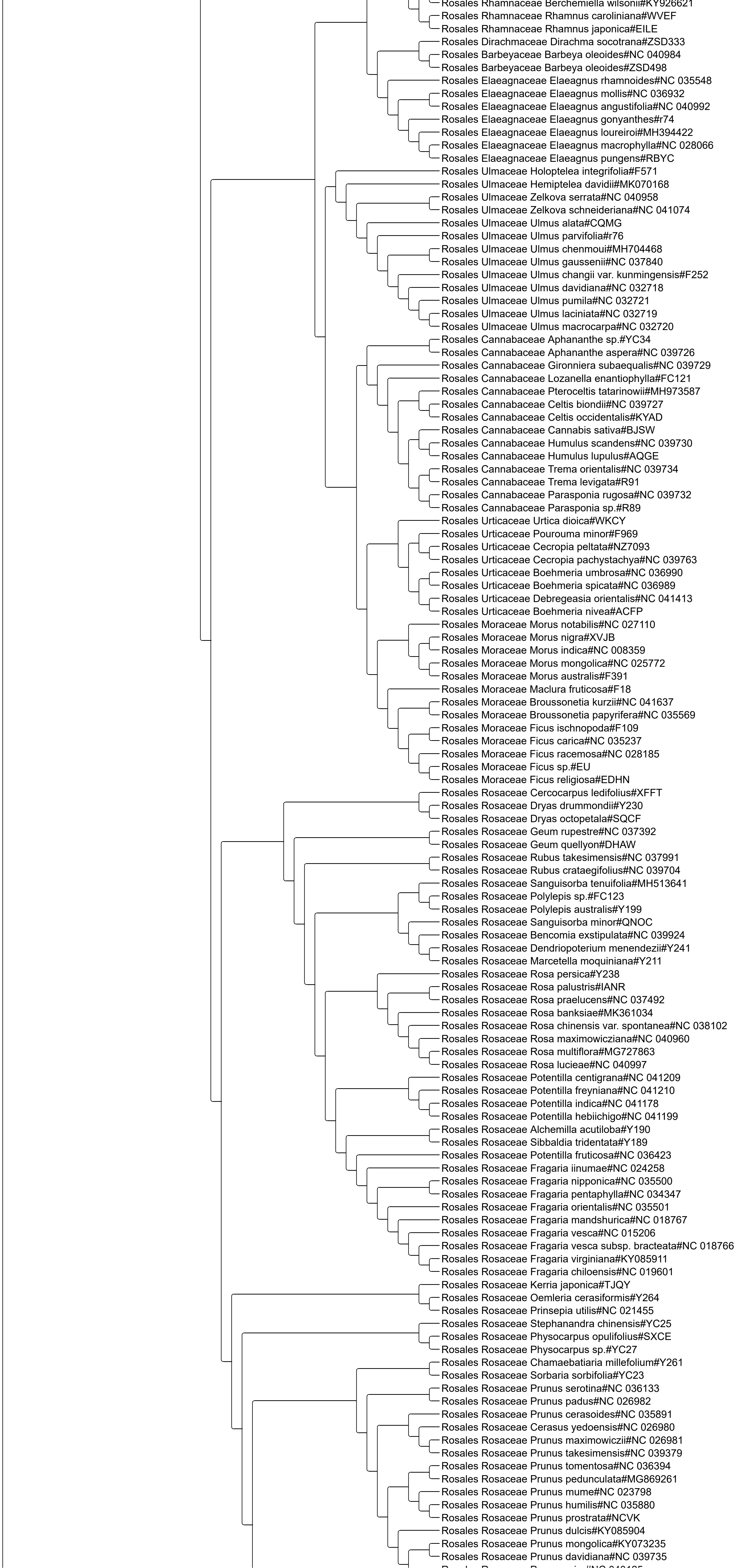
Rosales Rhamnaceae Berchemiella wilsonii#KY926621
Rosales Rhamnaceae Rhamnus caroliniana#WVEF
Rosales Rhamnaceae Rhamnus japonica#EILE
Rosales Dirachmaceae Dirachma socotrana#ZSD333
Rosales Barbeyaceae Barbeya oleoides#NC 040984
Rosales Barbeyaceae Barbeya oleoides#ZSD498
Rosales Elaeagnaceae Elaeagnus rhamnoides#NC 035548
Rosales Elaeagnaceae Elaeagnus mollis#NC 036932
Rosales Elaeagnaceae Elaeagnus angustifolia#NC 040992
Rosales Elaeagnaceae Elaeagnus gonyanthes#r74
Rosales Elaeagnaceae Elaeagnus loureiroi#MH394422
Rosales Elaeagnaceae Elaeagnus macrophylla#NC 028066
Rosales Elaeagnaceae Elaeagnus pungens#RBYC
Rosales Ulmaceae Holoptelea integrifolia#F571
Rosales Ulmaceae Hemiptelea davidii#MK070168
Rosales Ulmaceae Zelkova serrata#NC 040958
Rosales Ulmaceae Zelkova schneideriana#NC 041074
Rosales Ulmaceae Ulmus alata#CQMG
Rosales Ulmaceae Ulmus parvifolia#r76
Rosales Ulmaceae Ulmus chenmoui#MH704468
Rosales Ulmaceae Ulmus gaussenii#NC 037840
Rosales Ulmaceae Ulmus changii var. kunmingensis#F252
Rosales Ulmaceae Ulmus davidiana#NC 032718
Rosales Ulmaceae Ulmus pumila#NC 032721
Rosales Ulmaceae Ulmus laciniata#NC 032719
Rosales Ulmaceae Ulmus macrocarpa#NC 032720
Rosales Cannabaceae Aphananthe sp.#YC34
Rosales Cannabaceae Aphananthe aspera#NC 039726
Rosales Cannabaceae Gironniera subaequalis#NC 039729
Rosales Cannabaceae Lozanella enantiophylla#FC121
Rosales Cannabaceae Pteroceltis tatarinowii#MH973587
Rosales Cannabaceae Celtis biondii#NC 039727
Rosales Cannabaceae Celtis occidentalis#KYAD
Rosales Cannabaceae Cannabis sativa#BJSW
Rosales Cannabaceae Humulus scandens#NC 039730
Rosales Cannabaceae Humulus lupulus#AQGE
Rosales Cannabaceae Trema orientalis#NC 039734
Rosales Cannabaceae Trema levigata#R91
Rosales Cannabaceae Parasponia rugosa#NC 039732
Rosales Cannabaceae Parasponia sp.#R89
Rosales Urticaceae Urtica dioica#WKCY
Rosales Urticaceae Pourouma minor#F969
Rosales Urticaceae Cecropia peltata#NZ7093
Rosales Urticaceae Cecropia pachystachya#NC 039763
Rosales Urticaceae Boehmeria umbrosa#NC 036990
Rosales Urticaceae Boehmeria spicata#NC 036989
Rosales Urticaceae Debregeasia orientalis#NC 041413
Rosales Urticaceae Boehmeria nivea#ACFP
Rosales Moraceae Morus notabilis#NC 027110
Rosales Moraceae Morus nigra#XVJB
Rosales Moraceae Morus indica#NC 008359
Rosales Moraceae Morus mongolica#NC 025772
Rosales Moraceae Morus australis#F391
Rosales Moraceae Maclura fruticosa#F18
Rosales Moraceae Broussonetia kurzii#NC 041637
Rosales Moraceae Broussonetia papyrifera#NC 035569
Rosales Moraceae Ficus ischnopoda#F109
Rosales Moraceae Ficus carica#NC 035237
Rosales Moraceae Ficus racemosa#NC 028185
Rosales Moraceae Ficus sp.#EU
Rosales Moraceae Ficus religiosa#EDHN
Rosales Rosaceae Cercocarpus ledifolius#XFFT
Rosales Rosaceae Dryas drummondii#Y230
Rosales Rosaceae Dryas octopetala#SQCF
Rosales Rosaceae Geum rupestre#NC 037392
Rosales Rosaceae Geum quellyon#DHAW
Rosales Rosaceae Rubus takesimensis#NC 037991
Rosales Rosaceae Rubus crataegifolius#NC 039704
Rosales Rosaceae Sanguisorba tenuifolia#MH513641
Rosales Rosaceae Polylepis sp.#FC123
Rosales Rosaceae Polylepis australis#Y199
Rosales Rosaceae Sanguisorba minor#QNOC
Rosales Rosaceae Bencomia exstipulata#NC 039924
Rosales Rosaceae Dendriopoterium menendezii#Y241
Rosales Rosaceae Marcetella moquiniana#Y211
Rosales Rosaceae Rosa persica#Y238
Rosales Rosaceae Rosa palustris#IANR
Rosales Rosaceae Rosa praelucens#NC 037492
Rosales Rosaceae Rosa banksiae#MK361034
Rosales Rosaceae Rosa chinensis var. spontanea#NC 038102
Rosales Rosaceae Rosa maximowicziana#NC 040960
Rosales Rosaceae Rosa multiflora#MG727863
Rosales Rosaceae Rosa lucieae#NC 040997
Rosales Rosaceae Potentilla centigrana#NC 041209
Rosales Rosaceae Potentilla freyniana#NC 041210
Rosales Rosaceae Potentilla indica#NC 041178
Rosales Rosaceae Potentilla hebiichigo#NC 041199
Rosales Rosaceae Alchemilla acutiloba#Y190
Rosales Rosaceae Sibbaldia tridentata#Y189
Rosales Rosaceae Potentilla fruticosa#NC 036423
Rosales Rosaceae Fragaria iinumae#NC 024258
Rosales Rosaceae Fragaria nipponica#NC 035500
Rosales Rosaceae Fragaria pentaphylla#NC 034347
Rosales Rosaceae Fragaria orientalis#NC 035501
Rosales Rosaceae Fragaria mandshurica#NC 018767
Rosales Rosaceae Fragaria vesca#NC 015206
Rosales Rosaceae Fragaria vesca subsp. bracteata#NC 018766
Rosales Rosaceae Fragaria virginiana#KY085911
Rosales Rosaceae Fragaria chiloensis#NC 019601
Rosales Rosaceae Kerria japonica#TJQY
Rosales Rosaceae Oemleria cerasiformis#Y264
Rosales Rosaceae Prinsepia utilis#NC 021455
Rosales Rosaceae Stephanandra chinensis#YC25
Rosales Rosaceae Physocarpus opulifolius#SXCE
Rosales Rosaceae Physocarpus sp.#YC27
Rosales Rosaceae Chamaebatiaria millefolium#Y261
Rosales Rosaceae Sorbaria sorbifolia#YC23
Rosales Rosaceae Prunus serotina#NC 036133
Rosales Rosaceae Prunus padus#NC 026982
Rosales Rosaceae Prunus cerasoides#NC 035891
Rosales Rosaceae Cerasus yedoensis#NC 026980
Rosales Rosaceae Prunus maximowiczii#NC 026981
Rosales Rosaceae Prunus takesimensis#NC 039379
Rosales Rosaceae Prunus tomentosa#NC 036394
Rosales Rosaceae Prunus pedunculata#MG869261
Rosales Rosaceae Prunus mume#NC 023798
Rosales Rosaceae Prunus humilis#NC 035880
Rosales Rosaceae Prunus prostrata#NCVK
Rosales Rosaceae Prunus dulcis#KY085904
Rosales Rosaceae Prunus mongolica#KY073235
Rosales Rosaceae Prunus davidiana#NC 039735

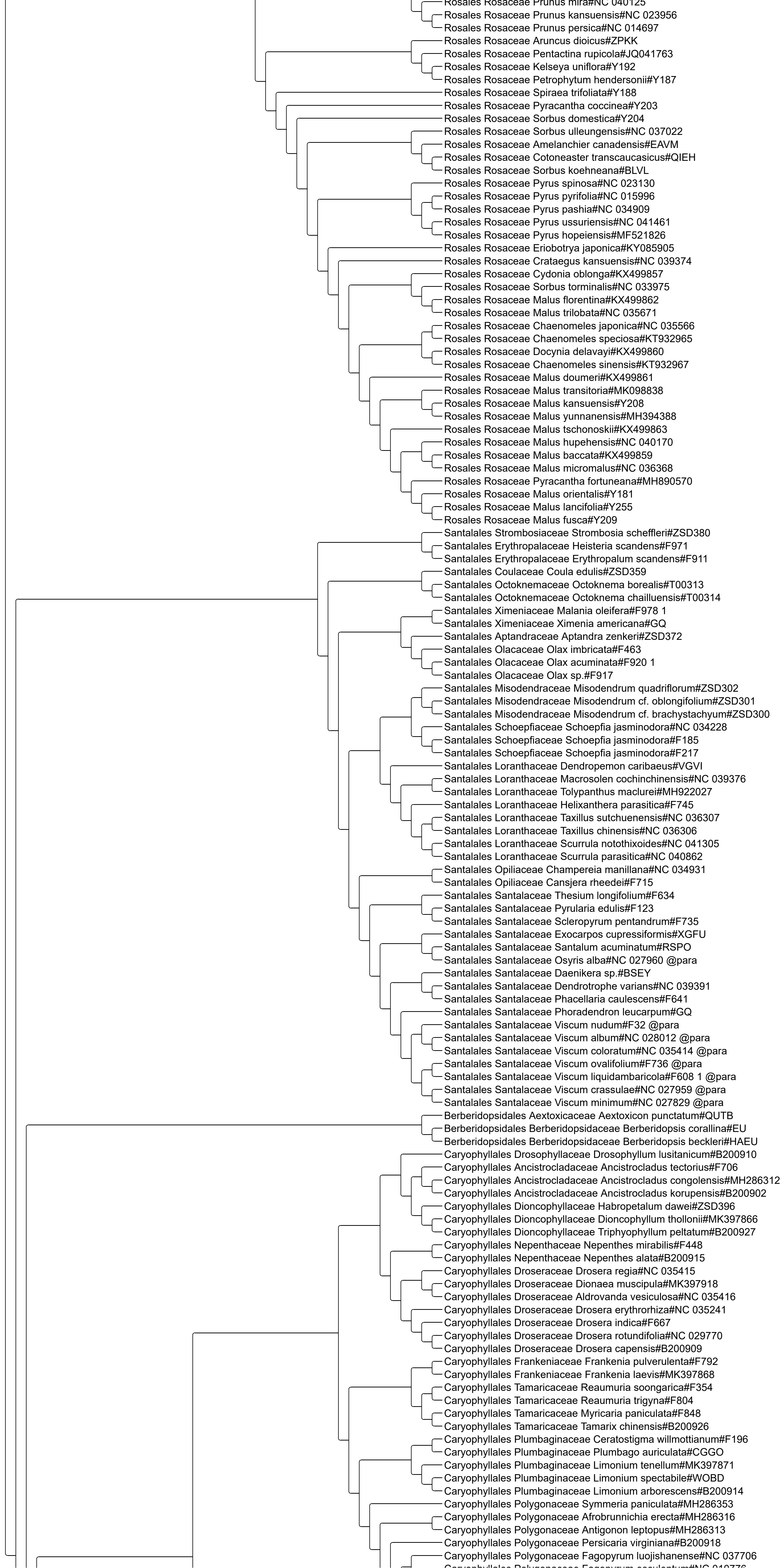

Rosales Rosaceae Prunus mira#NC 040125
Rosales Rosaceae Prunus kansuensis#NC 023956
Rosales Rosaceae Prunus persica#NC 014697
Rosales Rosaceae Aruncus dioicus#ZPKK
Rosales Rosaceae Pentactina rupicola#JQ041763
Rosales Rosaceae Kelseya uniflora#Y192
Rosales Rosaceae Petrophytum hendersonii#Y187
Rosales Rosaceae Spiraea trifoliata#Y188
Rosales Rosaceae Pyracantha coccinea#Y203
Rosales Rosaceae Sorbus domestica#Y204
Rosales Rosaceae Sorbus ulleungensis#NC 037022
Rosales Rosaceae Amelanchier canadensis#EAVM
Rosales Rosaceae Cotoneaster transcaucasicus#QIEH
Rosales Rosaceae Sorbus koehneana#BLVL
Rosales Rosaceae Pyrus spinosa#NC 023130
Rosales Rosaceae Pyrus pyrifolia#NC 015996
Rosales Rosaceae Pyrus pashia#NC 034909
Rosales Rosaceae Pyrus ussuriensis#NC 041461
Rosales Rosaceae Pyrus hopeiensis#MF521826
Rosales Rosaceae Eriobotrya japonica#KY085905
Rosales Rosaceae Crataegus kansuensis#NC 039374
Rosales Rosaceae Cydonia oblonga#KX499857
Rosales Rosaceae Sorbus torminalis#NC 033975
Rosales Rosaceae Malus florentina#KX499862
Rosales Rosaceae Malus trilobata#NC 035671
Rosales Rosaceae Chaenomeles japonica#NC 035566
Rosales Rosaceae Chaenomeles speciosa#KT932965
Rosales Rosaceae Docynia delavayi#KX499860
Rosales Rosaceae Chaenomeles sinensis#KT932967
Rosales Rosaceae Malus doumeri#KX499861
Rosales Rosaceae Malus transitoria#MK098838
Rosales Rosaceae Malus kansuensis#Y208
Rosales Rosaceae Malus yunnanensis#MH394388
Rosales Rosaceae Malus tschonoskii#KX499863
Rosales Rosaceae Malus hupehensis#NC 040170
Rosales Rosaceae Malus baccata#KX499859
Rosales Rosaceae Malus micromalus#NC 036368
Rosales Rosaceae Pyracantha fortuneana#MH890570
Rosales Rosaceae Malus orientalis#Y181
Rosales Rosaceae Malus lancifolia#Y255
Rosales Rosaceae Malus fusca#Y209
Santalales Strombosiaceae Strombosia scheffleri#ZSD380
Santalales Erythropalaceae Heisteria scandens#F971
Santalales Erythropalaceae Erythropalum scandens#F911
Santalales Coulaceae Coula edulis#ZSD359
Santalales Octoknemaceae Octoknema borealis#T00313
Santalales Octoknemaceae Octoknema chailluensis#T00314
Santalales Ximeniaceae Malania oleifera#F978 1
Santalales Ximeniaceae Ximenia americana#GQ
Santalales Aptandraceae Aptandra zenkeri#ZSD372
Santalales Olacaceae Olax imbricata#F463
Santalales Olacaceae Olax acuminata#F920 1
Santalales Olacaceae Olax sp.#F917
Santalales Misodendraceae Misodendrum quadriflorum#ZSD302
Santalales Misodendraceae Misodendrum cf. oblongifolium#ZSD301
Santalales Misodendraceae Misodendrum cf. brachystachyum#ZSD300
Santalales Schoepfiaceae Schoepfia jasminodora#NC 034228
Santalales Schoepfiaceae Schoepfia jasminodora#F185
Santalales Schoepfiaceae Schoepfia jasminodora#F217
Santalales Loranthaceae Dendropemon caribaeus#VGVI
Santalales Loranthaceae Macrosolen cochinchinensis#NC 039376
Santalales Loranthaceae Tolypanthus maclurei#MH922027
Santalales Loranthaceae Helixanthera parasitica#F745
Santalales Loranthaceae Taxillus sutchuenensis#NC 036307
Santalales Loranthaceae Taxillus chinensis#NC 036306
Santalales Loranthaceae Scurrula notothixoides#NC 041305
Santalales Loranthaceae Scurrula parasitica#NC 040862
Santalales Opiliaceae Champereia manillana#NC 034931
Santalales Opiliaceae Cansjera rheedei#F715
Santalales Santalaceae Thesium longifolium#F634
Santalales Santalaceae Pyrularia edulis#F123
Santalales Santalaceae Scleropyrum pentandrum#F735
Santalales Santalaceae Exocarpos cupressiformis#XGFU
Santalales Santalaceae Santalum acuminatum#RSPO
Santalales Santalaceae Osyris alba#NC 027960 @para
Santalales Santalaceae Daenikera sp.#BSEY
Santalales Santalaceae Dendrotrophe varians#NC 039391
Santalales Santalaceae Phacellaria caulescens#F641
Santalales Santalaceae Phoradendron leucarpum#GQ
Santalales Santalaceae Viscum nudum#F32 @para
Santalales Santalaceae Viscum album#NC 028012 @para
Santalales Santalaceae Viscum coloratum#NC 035414 @para
Santalales Santalaceae Viscum ovalifolium#F736 @para
Santalales Santalaceae Viscum liquidambaricola#F608 1 @para
Santalales Santalaceae Viscum crassulae#NC 027959 @para
Santalales Santalaceae Viscum minimum#NC 027829 @para
Berberidopsidales Aextoxicaceae Aextoxicon punctatum#QUTB
Berberidopsidales Berberidopsidaceae Berberidopsis corallina#EU
Berberidopsidales Berberidopsidaceae Berberidopsis beckleri#HAEU
Caryophyllales Drosophyllaceae Drosophyllum lusitanicum#B200910
Caryophyllales Ancistrocladaceae Ancistrocladus tectorius#F706
Caryophyllales Ancistrocladaceae Ancistrocladus congolensis#MH286312
Caryophyllales Ancistrocladaceae Ancistrocladus korupensis#B200902
Caryophyllales Dioncophyllaceae Habropetalum dawei#ZSD396
Caryophyllales Dioncophyllaceae Dioncophyllum thollonii#MK397866
Caryophyllales Dioncophyllaceae Triphyophyllum peltatum#B200927
Caryophyllales Nepenthaceae Nepenthes mirabilis#F448
Caryophyllales Nepenthaceae Nepenthes alata#B200915
Caryophyllales Droseraceae Drosera regia#NC 035415
Caryophyllales Droseraceae Dionaea muscipula#MK397918
Caryophyllales Droseraceae Aldrovanda vesiculosa#NC 035416
Caryophyllales Droseraceae Drosera erythrorhiza#NC 035241
Caryophyllales Droseraceae Drosera indica#F667
Caryophyllales Droseraceae Drosera rotundifolia#NC 029770
Caryophyllales Droseraceae Drosera capensis#B200909
Caryophyllales Frankeniaceae Frankenia pulverulenta#F792
Caryophyllales Frankeniaceae Frankenia laevis#MK397868
Caryophyllales Tamaricaceae Reaumuria soongarica#F354
Caryophyllales Tamaricaceae Reaumuria trigyna#F804
Caryophyllales Tamaricaceae Myricaria paniculata#F848
Caryophyllales Tamaricaceae Tamarix chinensis#B200926
Caryophyllales Plumbaginaceae Ceratostigma willmottianum#F196
Caryophyllales Plumbaginaceae Plumbago auriculata#CGGO
Caryophyllales Plumbaginaceae Limonium tenellum#MK397871
Caryophyllales Plumbaginaceae Limonium spectabile#WOBD
Caryophyllales Plumbaginaceae Limonium arborescens#B200914
Caryophyllales Polygonaceae Symmeria paniculata#MH286353
Caryophyllales Polygonaceae Afrobrunnichia erecta#MH286316
Caryophyllales Polygonaceae Antigonon leptopus#MH286313
Caryophyllales Polygonaceae Persicaria virginiana#B200918
Caryophyllales Polygonaceae Fagopyrum luojishanense#NC 037706

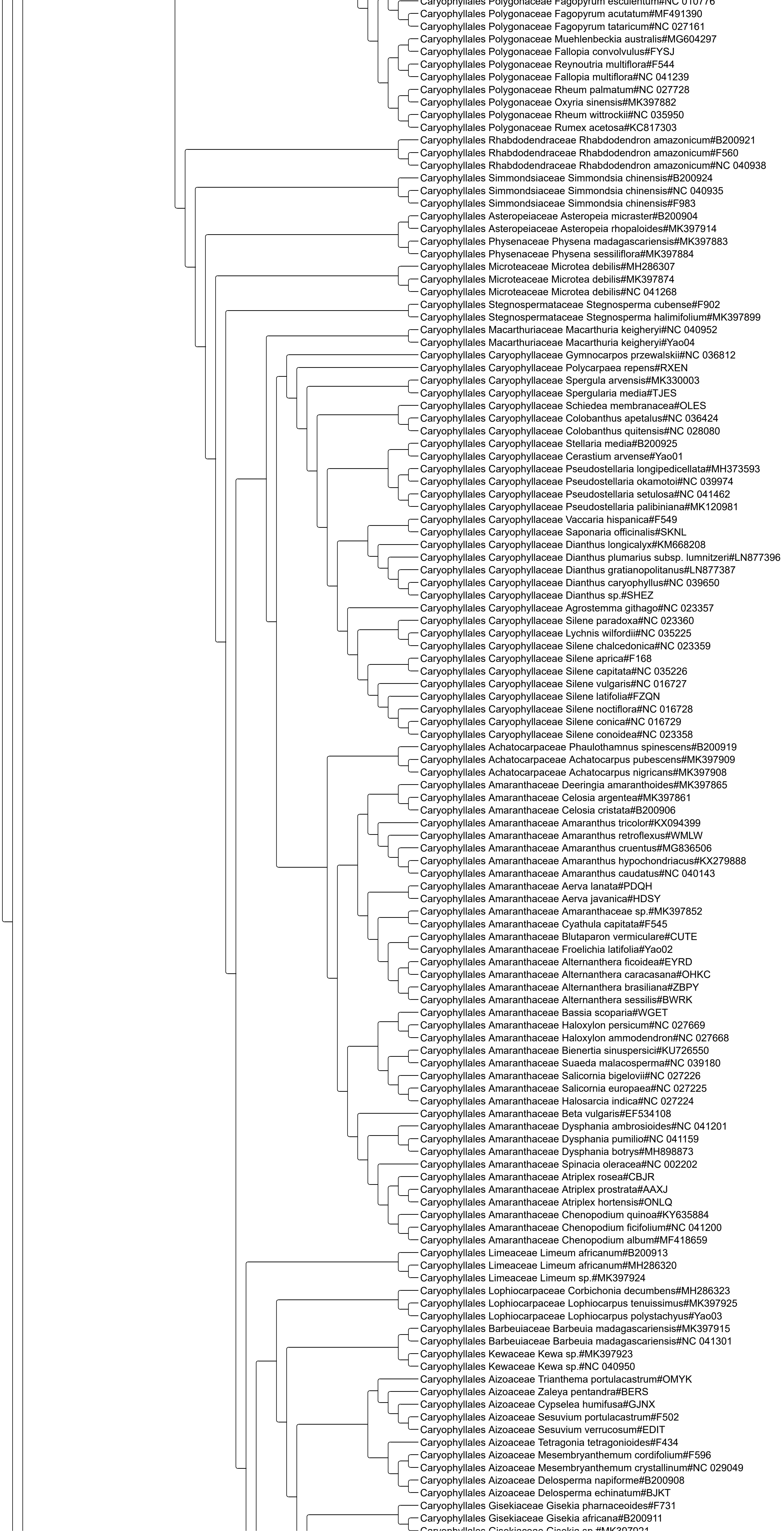
Caryophyllales Polygonaceae Fagopyrum esculentum#NC 010776
Caryophyllales Polygonaceae Fagopyrum acutatum#MF491390
Caryophyllales Polygonaceae Fagopyrum tataricum#NC 027161
Caryophyllales Polygonaceae Muehlenbeckia australis#MG604297
Caryophyllales Polygonaceae Fallopia convolvulus#FYSJ
Caryophyllales Polygonaceae Reynoutria multiflora#F544
Caryophyllales Polygonaceae Fallopia multiflora#NC 041239
Caryophyllales Polygonaceae Rheum palmatum#NC 027728
Caryophyllales Polygonaceae Oxyria sinensis#MK397882
Caryophyllales Polygonaceae Rheum wittrockii#NC 035950
Caryophyllales Polygonaceae Rumex acetosa#KC817303
Caryophyllales Rhabdodendraceae Rhabdodendron amazonicum#B200921
Caryophyllales Rhabdodendraceae Rhabdodendron amazonicum#F560
Caryophyllales Rhabdodendraceae Rhabdodendron amazonicum#NC 040938
Caryophyllales Simmondsiaceae Simmondsia chinensis#B200924
Caryophyllales Simmondsiaceae Simmondsia chinensis#NC 040935
Caryophyllales Simmondsiaceae Simmondsia chinensis#F983
Caryophyllales Asteropeiaceae Asteropeia micraster#B200904
Caryophyllales Asteropeiaceae Asteropeia rhopaloides#MK397914
Caryophyllales Physenaceae Physena madagascariensis#MK397883
Caryophyllales Physenaceae Physena sessiliflora#MK397884
Caryophyllales Microteaceae Microtea debilis#MH286307
Caryophyllales Microteaceae Microtea debilis#MK397874
Caryophyllales Microteaceae Microtea debilis#NC 041268
Caryophyllales Stegnospermataceae Stegnosperma cubense#F902
Caryophyllales Stegnospermataceae Stegnosperma halimifolium#MK397899
Caryophyllales Macarthuriaceae Macarthuria keigheryi#NC 040952
Caryophyllales Macarthuriaceae Macarthuria keigheryi#Yao04
Caryophyllales Caryophyllaceae Gymnocarpos przewalskii#NC 036812
Caryophyllales Caryophyllaceae Polycarpaea repens#RXEN
Caryophyllales Caryophyllaceae Spergula arvensis#MK330003
Caryophyllales Caryophyllaceae Spergularia media#TJES
Caryophyllales Caryophyllaceae Schiedea membranacea#OLES
Caryophyllales Caryophyllaceae Colobanthus apetalus#NC 036424
Caryophyllales Caryophyllaceae Colobanthus quitensis#NC 028080
Caryophyllales Caryophyllaceae Stellaria media#B200925
Caryophyllales Caryophyllaceae Cerastium arvense#Yao01
Caryophyllales Caryophyllaceae Pseudostellaria longipedicellata#MH373593
Caryophyllales Caryophyllaceae Pseudostellaria okamotoi#NC 039974
Caryophyllales Caryophyllaceae Pseudostellaria setulosa#NC 041462
Caryophyllales Caryophyllaceae Pseudostellaria palibiniana#MK120981
Caryophyllales Caryophyllaceae Vaccaria hispanica#F549
Caryophyllales Caryophyllaceae Saponaria officinalis#SKNL
Caryophyllales Caryophyllaceae Dianthus longicalyx#KM668208
Caryophyllales Caryophyllaceae Dianthus plumarius subsp. lumnitzeri#LN877396
Caryophyllales Caryophyllaceae Dianthus gratianopolitanus#LN877387
Caryophyllales Caryophyllaceae Dianthus caryophyllus#NC 039650
Caryophyllales Caryophyllaceae Dianthus sp.#SHEZ
Caryophyllales Caryophyllaceae Agrostemma githago#NC 023357
Caryophyllales Caryophyllaceae Silene paradoxa#NC 023360
Caryophyllales Caryophyllaceae Lychnis wilfordii#NC 035225
Caryophyllales Caryophyllaceae Silene chalcedonica#NC 023359
Caryophyllales Caryophyllaceae Silene aprica#F168
Caryophyllales Caryophyllaceae Silene capitata#NC 035226
Caryophyllales Caryophyllaceae Silene vulgaris#NC 016727
Caryophyllales Caryophyllaceae Silene latifolia#FZQN
Caryophyllales Caryophyllaceae Silene noctiflora#NC 016728
Caryophyllales Caryophyllaceae Silene conica#NC 016729
Caryophyllales Caryophyllaceae Silene conoidea#NC 023358
Caryophyllales Achatocarpaceae Phaulothamnus spinescens#B200919
Caryophyllales Achatocarpaceae Achatocarpus pubescens#MK397909
Caryophyllales Achatocarpaceae Achatocarpus nigricans#MK397908
Caryophyllales Amaranthaceae Deeringia amaranthoides#MK397865
Caryophyllales Amaranthaceae Celosia argentea#MK397861
Caryophyllales Amaranthaceae Celosia cristata#B200906
Caryophyllales Amaranthaceae Amaranthus tricolor#KX094399
Caryophyllales Amaranthaceae Amaranthus retroflexus#WMLW
Caryophyllales Amaranthaceae Amaranthus cruentus#MG836506
Caryophyllales Amaranthaceae Amaranthus hypochondriacus#KX279888
Caryophyllales Amaranthaceae Amaranthus caudatus#NC 040143
Caryophyllales Amaranthaceae Aerva lanata#PDQH
Caryophyllales Amaranthaceae Aerva javanica#HDSY
Caryophyllales Amaranthaceae Amaranthaceae sp.#MK397852
Caryophyllales Amaranthaceae Cyathula capitata#F545
Caryophyllales Amaranthaceae Blutaparon vermiculare#CUTE
Caryophyllales Amaranthaceae Froelichia latifolia#Yao02
Caryophyllales Amaranthaceae Alternanthera ficoidea#EYRD
Caryophyllales Amaranthaceae Alternanthera caracasana#OHKC
Caryophyllales Amaranthaceae Alternanthera brasiliana#ZBPY
Caryophyllales Amaranthaceae Alternanthera sessilis#BWRK
Caryophyllales Amaranthaceae Bassia scoparia#WGET
Caryophyllales Amaranthaceae Haloxylon persicum#NC 027669
Caryophyllales Amaranthaceae Haloxylon ammodendron#NC 027668
Caryophyllales Amaranthaceae Bienertia sinuspersici#KU726550
Caryophyllales Amaranthaceae Suaeda malacosperma#NC 039180
Caryophyllales Amaranthaceae Salicornia bigelovii#NC 027226
Caryophyllales Amaranthaceae Salicornia europaea#NC 027225
Caryophyllales Amaranthaceae Halosarcia indica#NC 027224
Caryophyllales Amaranthaceae Beta vulgaris#EF534108
Caryophyllales Amaranthaceae Dysphania ambrosioides#NC 041201
Caryophyllales Amaranthaceae Dysphania pumilio#NC 041159
Caryophyllales Amaranthaceae Dysphania botrys#MH898873
Caryophyllales Amaranthaceae Spinacia oleracea#NC 002202
Caryophyllales Amaranthaceae Atriplex rosea#CBJR
Caryophyllales Amaranthaceae Atriplex prostrata#AAXJ
Caryophyllales Amaranthaceae Atriplex hortensis#ONLQ
Caryophyllales Amaranthaceae Chenopodium quinoa#KY635884
Caryophyllales Amaranthaceae Chenopodium ficifolium#NC 041200
Caryophyllales Amaranthaceae Chenopodium album#MF418659
Caryophyllales Limeaceae Limeum africanum#B200913
Caryophyllales Limeaceae Limeum africanum#MH286320
Caryophyllales Limeaceae Limeum sp.#MK397924
Caryophyllales Lophiocarpaceae Corbichonia decumbens#MH286323
Caryophyllales Lophiocarpaceae Lophiocarpus tenuissimus#MK397925
Caryophyllales Lophiocarpaceae Lophiocarpus polystachyus#Yao03
Caryophyllales Barbeuiaceae Barbeuia madagascariensis#MK397915
Caryophyllales Barbeuiaceae Barbeuia madagascariensis#NC 041301
Caryophyllales Kewaceae Kewa sp.#MK397923
Caryophyllales Kewaceae Kewa sp.#NC 040950
Caryophyllales Aizoaceae Trianthema portulacastrum#OMYK
Caryophyllales Aizoaceae Zaleya pentandra#BERS
Caryophyllales Aizoaceae Cypselea humifusa#GJNX
Caryophyllales Aizoaceae Sesuvium portulacastrum#F502
Caryophyllales Aizoaceae Sesuvium verrucosum#EDIT
Caryophyllales Aizoaceae Tetragonia tetragonioides#F434
Caryophyllales Aizoaceae Mesembryanthemum cordifolium#F596
Caryophyllales Aizoaceae Mesembryanthemum crystallinum#NC 029049
Caryophyllales Aizoaceae Delosperma napiforme#B200908
Caryophyllales Aizoaceae Delosperma echinatum#BJKT
Caryophyllales Gisekiaceae Gisekia pharnaceoides#F731
Caryophyllales Gisekiaceae Gisekia africana#B200911
Caryophyllales Gisekiaceae Gisekia sp.#MK397921

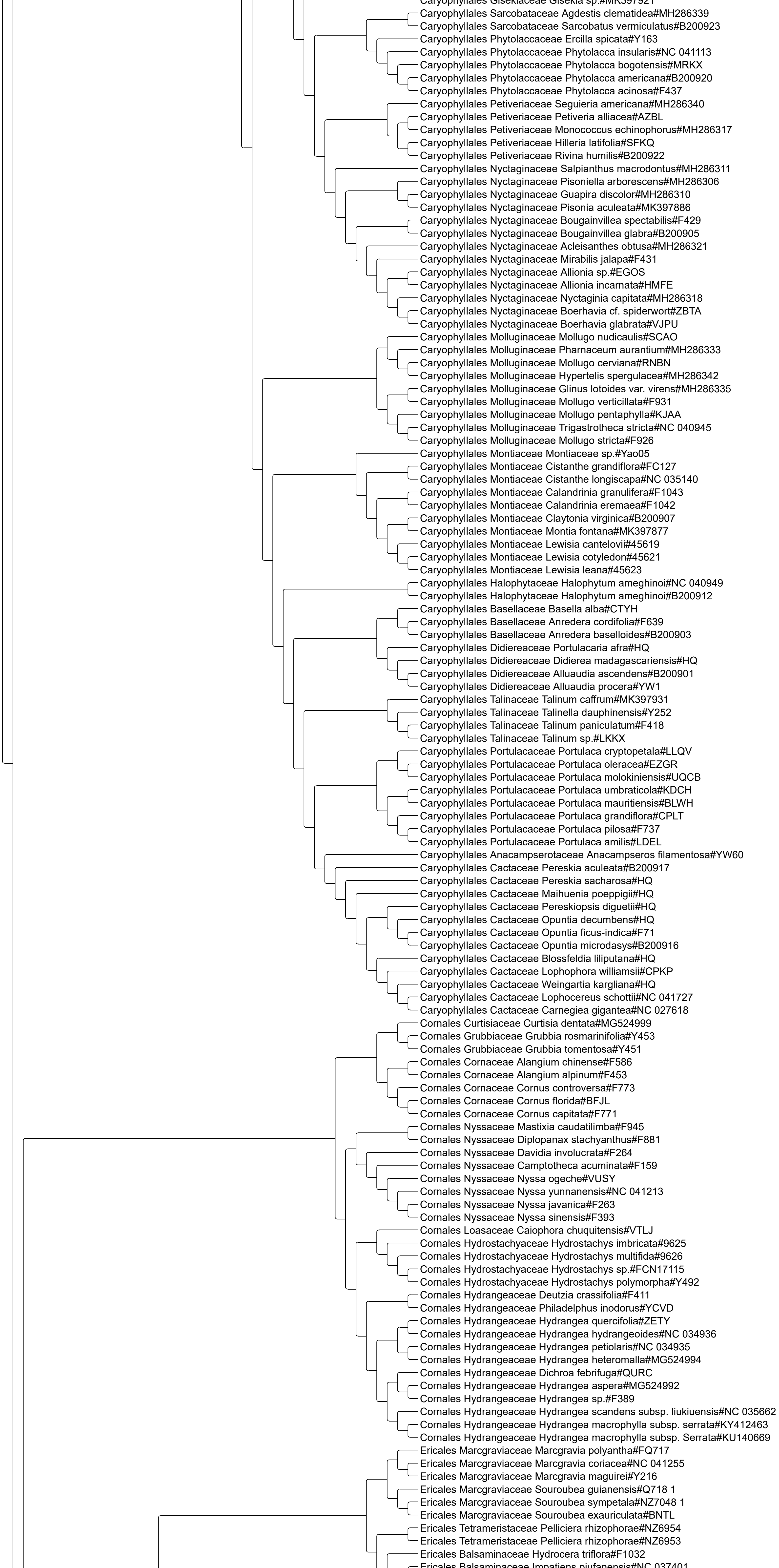
Caryophyllales Gisekiaceae Gisekia sp.#MK397921
Caryophyllales Sarcobataceae Agdestis clematidea#MH286339
Caryophyllales Sarcobataceae Sarcobatus vermiculatus#B200923
Caryophyllales Phytolaccaceae Ercilla spicata#Y163
Caryophyllales Phytolaccaceae Phytolacca insularis#NC 041113
Caryophyllales Phytolaccaceae Phytolacca bogotensis#MRKX
Caryophyllales Phytolaccaceae Phytolacca americana#B200920
Caryophyllales Phytolaccaceae Phytolacca acinosa#F437
Caryophyllales Petiveriaceae Seguieria americana#MH286340
Caryophyllales Petiveriaceae Petiveria alliacea#AZBL
Caryophyllales Petiveriaceae Monococcus echinophorus#MH286317
Caryophyllales Petiveriaceae Hilleria latifolia#SFKQ
Caryophyllales Petiveriaceae Rivina humilis#B200922
Caryophyllales Nyctaginaceae Salpianthus macrodontus#MH286311
Caryophyllales Nyctaginaceae Pisoniella arborescens#MH286306
Caryophyllales Nyctaginaceae Guapira discolor#MH286310
Caryophyllales Nyctaginaceae Pisonia aculeata#MK397886
Caryophyllales Nyctaginaceae Bougainvillea spectabilis#F429
Caryophyllales Nyctaginaceae Bougainvillea glabra#B200905
Caryophyllales Nyctaginaceae Acleisanthes obtusa#MH286321
Caryophyllales Nyctaginaceae Mirabilis jalapa#F431
Caryophyllales Nyctaginaceae Allionia sp.#EGOS
Caryophyllales Nyctaginaceae Allionia incarnata#HMFE
Caryophyllales Nyctaginaceae Nyctaginia capitata#MH286318
Caryophyllales Nyctaginaceae Boerhavia cf. spiderwort#ZBTA
Caryophyllales Nyctaginaceae Boerhavia glabrata#VJPU
Caryophyllales Molluginaceae Mollugo nudicaulis#SCAO
Caryophyllales Molluginaceae Pharnaceum aurantium#MH286333
Caryophyllales Molluginaceae Mollugo cerviana#RNBN
Caryophyllales Molluginaceae Hypertelis spergulacea#MH286342
Caryophyllales Molluginaceae Glinus lotoides var. virens#MH286335
Caryophyllales Molluginaceae Mollugo verticillata#F931
Caryophyllales Molluginaceae Mollugo pentaphylla#KJAA
Caryophyllales Molluginaceae Trigastrotheca stricta#NC 040945
Caryophyllales Molluginaceae Mollugo stricta#F926
Caryophyllales Montiaceae Montiaceae sp.#Yao05
Caryophyllales Montiaceae Cistanthe grandiflora#FC127
Caryophyllales Montiaceae Cistanthe longiscapa#NC 035140
Caryophyllales Montiaceae Calandrinia granulifera#F1043
Caryophyllales Montiaceae Calandrinia eremaea#F1042
Caryophyllales Montiaceae Claytonia virginica#B200907
Caryophyllales Montiaceae Montia fontana#MK397877
Caryophyllales Montiaceae Lewisia cantelovii#45619
Caryophyllales Montiaceae Lewisia cotyledon#45621
Caryophyllales Montiaceae Lewisia leana#45623
Caryophyllales Halophytaceae Halophytum ameghinoi#NC 040949
Caryophyllales Halophytaceae Halophytum ameghinoi#B200912
Caryophyllales Basellaceae Basella alba#CTYH
Caryophyllales Basellaceae Anredera cordifolia#F639
Caryophyllales Basellaceae Anredera baselloides#B200903
Caryophyllales Didiereaceae Portulacaria afra#HQ
Caryophyllales Didiereaceae Didierea madagascariensis#HQ
Caryophyllales Didiereaceae Alluaudia ascendens#B200901
Caryophyllales Didiereaceae Alluaudia procera#YW1
Caryophyllales Talinaceae Talinum caffrum#MK397931
Caryophyllales Talinaceae Talinella dauphinensis#Y252
Caryophyllales Talinaceae Talinum paniculatum#F418
Caryophyllales Talinaceae Talinum sp.#LKKX
Caryophyllales Portulacaceae Portulaca cryptopetala#LLQV
Caryophyllales Portulacaceae Portulaca oleracea#EZGR
Caryophyllales Portulacaceae Portulaca molokiniensis#UQCB
Caryophyllales Portulacaceae Portulaca umbraticola#KDCH
Caryophyllales Portulacaceae Portulaca mauritiensis#BLWH
Caryophyllales Portulacaceae Portulaca grandiflora#CPLT
Caryophyllales Portulacaceae Portulaca pilosa#F737
Caryophyllales Portulacaceae Portulaca amilis#LDEL
Caryophyllales Anacampserotaceae Anacampseros filamentosa#YW60
Caryophyllales Cactaceae Pereskia aculeata#B200917
Caryophyllales Cactaceae Pereskia sacharosa#HQ
Caryophyllales Cactaceae Maihuenia poeppigii#HQ
Caryophyllales Cactaceae Pereskiopsis diguetii#HQ
Caryophyllales Cactaceae Opuntia decumbens#HQ
Caryophyllales Cactaceae Opuntia ficus-indica#F71
Caryophyllales Cactaceae Opuntia microdasys#B200916
Caryophyllales Cactaceae Blossfeldia liliputana#HQ
Caryophyllales Cactaceae Lophophora williamsii#CPKP
Caryophyllales Cactaceae Weingartia kargliana#HQ
Caryophyllales Cactaceae Lophocereus schottii#NC 041727
Caryophyllales Cactaceae Carnegiea gigantea#NC 027618
Cornales Curtisiaceae Curtisia dentata#MG524999
Cornales Grubbiaceae Grubbia rosmarinifolia#Y453
Cornales Grubbiaceae Grubbia tomentosa#Y451
Cornales Cornaceae Alangium chinense#F586
Cornales Cornaceae Alangium alpinum#F453
Cornales Cornaceae Cornus controversa#F773
Cornales Cornaceae Cornus florida#BFJL
Cornales Cornaceae Cornus capitata#F771
Cornales Nyssaceae Mastixia caudatilimba#F945
Cornales Nyssaceae Diplopanax stachyanthus#F881
Cornales Nyssaceae Davidia involucrata#F264
Cornales Nyssaceae Camptotheca acuminata#F159
Cornales Nyssaceae Nyssa ogeche#VUSY
Cornales Nyssaceae Nyssa yunnanensis#NC 041213
Cornales Nyssaceae Nyssa javanica#F263
Cornales Nyssaceae Nyssa sinensis#F393
Cornales Loasaceae Caiophora chuquitensis#VTLJ
Cornales Hydrostachyaceae Hydrostachys imbricata#9625
Cornales Hydrostachyaceae Hydrostachys multifida#9626
Cornales Hydrostachyaceae Hydrostachys sp.#FCN17115
Cornales Hydrostachyaceae Hydrostachys polymorpha#Y492
Cornales Hydrangeaceae Deutzia crassifolia#F411
Cornales Hydrangeaceae Philadelphus inodorus#YCVD
Cornales Hydrangeaceae Hydrangea quercifolia#ZETY
Cornales Hydrangeaceae Hydrangea hydrangeoides#NC 034936
Cornales Hydrangeaceae Hydrangea petiolaris#NC 034935
Cornales Hydrangeaceae Hydrangea heteromalla#MG524994
Cornales Hydrangeaceae Dichroa febrifuga#QURC
Cornales Hydrangeaceae Hydrangea aspera#MG524992
Cornales Hydrangeaceae Hydrangea sp.#F389
Cornales Hydrangeaceae Hydrangea scandens subsp. liukiuensis#NC 035662
Cornales Hydrangeaceae Hydrangea macrophylla subsp. serrata#KY412463
Cornales Hydrangeaceae Hydrangea macrophylla subsp. Serrata#KU140669
Ericales Marcgraviaceae Marcgravia polyantha#FQ717
Ericales Marcgraviaceae Marcgravia coriacea#NC 041255
Ericales Marcgraviaceae Marcgravia maguirei#Y216
Ericales Marcgraviaceae Souroubea guianensis#Q718 1
Ericales Marcgraviaceae Souroubea sympetala#NZ7048 1
Ericales Marcgraviaceae Souroubea exauriculata#BNTL
Ericales Tetrameristaceae Pelliciera rhizophorae#NZ6954
Ericales Tetrameristaceae Pelliciera rhizophorae#NZ6953
Ericales Balsaminaceae Hydrocera triflora#F1032

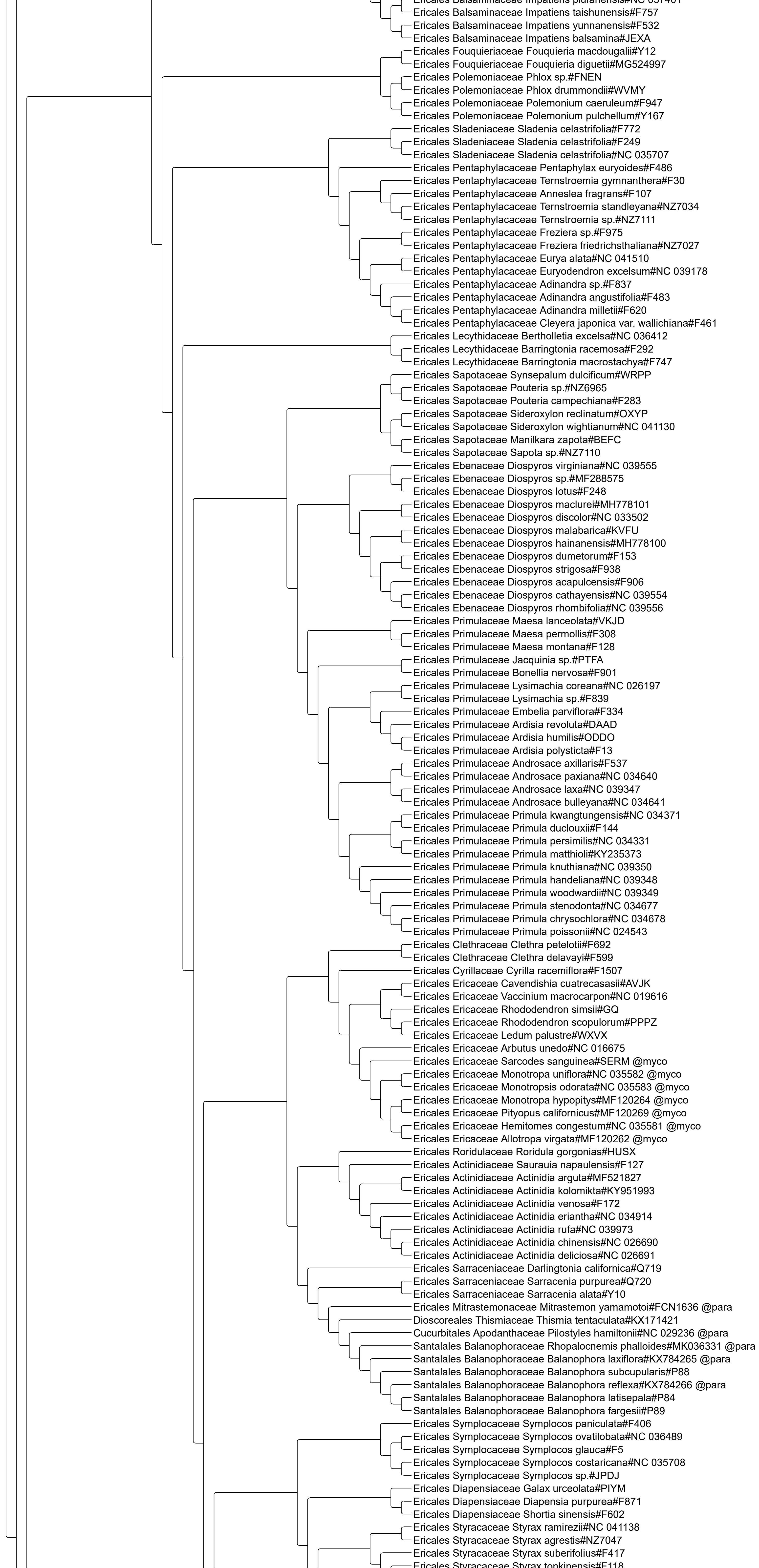

Ericales Balsaminaceae Impatiens taishunensis#F757
Ericales Balsaminaceae Impatiens yunnanensis#F532
Ericales Balsaminaceae Impatiens balsamina#JEXA
Ericales Fouquieriaceae Fouquieria macdougalii#Y12
Ericales Fouquieriaceae Fouquieria diguetii#MG524997
Ericales Polemoniaceae Phlox sp.#FNEN
Ericales Polemoniaceae Phlox drummondii#WVMY
Ericales Polemoniaceae Polemonium caeruleum#F947
Ericales Polemoniaceae Polemonium pulchellum#Y167
Ericales Sladeniaceae Sladenia celastrifolia#F772
Ericales Sladeniaceae Sladenia celastrifolia#F249
Ericales Sladeniaceae Sladenia celastrifolia#NC 035707
Ericales Pentaphylacaceae Pentaphylax euryoides#F486
Ericales Pentaphylacaceae Ternstroemia gymnanthera#F30
Ericales Pentaphylacaceae Anneslea fragrans#F107
Ericales Pentaphylacaceae Ternstroemia standleyana#NZ7034
Ericales Pentaphylacaceae Ternstroemia sp.#NZ7111
Ericales Pentaphylacaceae Freziera sp.#F975
Ericales Pentaphylacaceae Freziera friedrichsthaliana#NZ7027
Ericales Pentaphylacaceae Eurya alata#NC 041510
Ericales Pentaphylacaceae Euryodendron excelsum#NC 039178
Ericales Pentaphylacaceae Adinandra sp.#F837
Ericales Pentaphylacaceae Adinandra angustifolia#F483
Ericales Pentaphylacaceae Adinandra millettii#F620
Ericales Pentaphylacaceae Cleyera japonica var. wallichiana#F461
Ericales Lecythidaceae Bertholletia excelsa#NC 036412
Ericales Lecythidaceae Barringtonia racemosa#F292
Ericales Lecythidaceae Barringtonia macrostachya#F747
Ericales Sapotaceae Synsepalum dulcificum#WRPP
Ericales Sapotaceae Pouteria sp.#NZ6965
Ericales Sapotaceae Pouteria campechiana#F283
Ericales Sapotaceae Sideroxylon reclinatum#OXYP
Ericales Sapotaceae Sideroxylon wightianum#NC 041130
Ericales Sapotaceae Manilkara zapota#BEFC
Ericales Sapotaceae Sapota sp.#NZ7110
Ericales Ebenaceae Diospyros virginiana#NC 039555
Ericales Ebenaceae Diospyros sp.#MF288575
Ericales Ebenaceae Diospyros lotus#F248
Ericales Ebenaceae Diospyros maclurei#MH778101
Ericales Ebenaceae Diospyros discolor#NC 033502
Ericales Ebenaceae Diospyros malabarica#KVFU
Ericales Ebenaceae Diospyros hainanensis#MH778100
Ericales Ebenaceae Diospyros dumetorum#F153
Ericales Ebenaceae Diospyros strigosa#F938
Ericales Ebenaceae Diospyros acapulcensis#F906
Ericales Ebenaceae Diospyros cathayensis#NC 039554
Ericales Ebenaceae Diospyros rhombifolia#NC 039556
Ericales Primulaceae Maesa lanceolata#VKJD
Ericales Primulaceae Maesa permollis#F308
Ericales Primulaceae Maesa montana#F128
Ericales Primulaceae Jacquinia sp.#PTFA
Ericales Primulaceae Bonellia nervosa#F901
Ericales Primulaceae Lysimachia coreana#NC 026197
Ericales Primulaceae Lysimachia sp.#F839
Ericales Primulaceae Embelia parviflora#F334
Ericales Primulaceae Ardisia revoluta#DAAD
Ericales Primulaceae Ardisia humilis#ODDO
Ericales Primulaceae Ardisia polysticta#F13
Ericales Primulaceae Androsace axillaris#F537
Ericales Primulaceae Androsace paxiana#NC 034640
Ericales Primulaceae Androsace laxa#NC 039347
Ericales Primulaceae Androsace bulleyana#NC 034641
Ericales Primulaceae Primula kwangtungensis#NC 034371
Ericales Primulaceae Primula duclouxii#F144
Ericales Primulaceae Primula persimilis#NC 034331
Ericales Primulaceae Primula matthioli#KY235373
Ericales Primulaceae Primula knuthiana#NC 039350
Ericales Primulaceae Primula handeliana#NC 039348
Ericales Primulaceae Primula woodwardii#NC 039349
Ericales Primulaceae Primula stenodonta#NC 034677
Ericales Primulaceae Primula chrysochlora#NC 034678
Ericales Primulaceae Primula poissonii#NC 024543
Ericales Clethraceae Clethra petelotii#F692
Ericales Clethraceae Clethra delavayi#F599
Ericales Cyrillaceae Cyrilla racemiflora#F1507
Ericales Ericaceae Cavendishia cuatrecasasii#AVJK
Ericales Ericaceae Vaccinium macrocarpon#NC 019616
Ericales Ericaceae Rhododendron simsii#GQ
Ericales Ericaceae Rhododendron scopulorum#PPPZ
Ericales Ericaceae Ledum palustre#WXVX
Ericales Ericaceae Arbutus unedo#NC 016675
Ericales Ericaceae Sarcodes sanguinea#SERM @myco
Ericales Ericaceae Monotropa uniflora#NC 035582 @myco
Ericales Ericaceae Monotropsis odorata#NC 035583 @myco
Ericales Ericaceae Monotropa hypopitys#MF120264 @myco
Ericales Ericaceae Pityopus californicus#MF120269 @myco
Ericales Ericaceae Hemitomes congestum#NC 035581 @myco
Ericales Ericaceae Allotropa virgata#MF120262 @myco
Ericales Roridulaceae Roridula gorgonias#HUSX
Ericales Actinidiaceae Saurauia napaulensis#F127
Ericales Actinidiaceae Actinidia arguta#MF521827
Ericales Actinidiaceae Actinidia kolomikta#KY951993
Ericales Actinidiaceae Actinidia venosa#F172
Ericales Actinidiaceae Actinidia eriantha#NC 034914
Ericales Actinidiaceae Actinidia rufa#NC 039973
Ericales Actinidiaceae Actinidia chinensis#NC 026690
Ericales Actinidiaceae Actinidia deliciosa#NC 026691
Ericales Sarraceniaceae Darlingtonia californica#Q719
Ericales Sarraceniaceae Sarracenia purpurea#Q720
Ericales Sarraceniaceae Sarracenia alata#Y10
Ericales Mitrastemonaceae Mitrastemon yamamotoi#FCN1636 @para
Dioscoreales Thismiaceae Thismia tentaculata#KX171421
Cucurbitales Apodanthaceae Pilostyles hamiltonii#NC 029236 @para
Santalales Balanophoraceae Rhopalocnemis phalloides#MK036331 @para
Santalales Balanophoraceae Balanophora laxiflora#KX784265 @para
Santalales Balanophoraceae Balanophora subcupularis#P88
Santalales Balanophoraceae Balanophora reflexa#KX784266 @para
Santalales Balanophoraceae Balanophora latisepala#P84
Santalales Balanophoraceae Balanophora fargesii#P89
Ericales Symplocaceae Symplocos paniculata#F406
Ericales Symplocaceae Symplocos ovatilobata#NC 036489
Ericales Symplocaceae Symplocos glauca#F5
Ericales Symplocaceae Symplocos costaricana#NC 035708
Ericales Symplocaceae Symplocos sp.#JPDJ
Ericales Diapensiaceae Galax urceolata#PIYM
Ericales Diapensiaceae Diapensia purpurea#F871
Ericales Diapensiaceae Shortia sinensis#F602
Ericales Styracaceae Styrax ramirezii#NC 041138
Ericales Styracaceae Styrax agrestis#NZ7047
Ericales Styracaceae Styrax suberifolius#F417
Ericales Styracaceae Styrax tonkinensis#F118

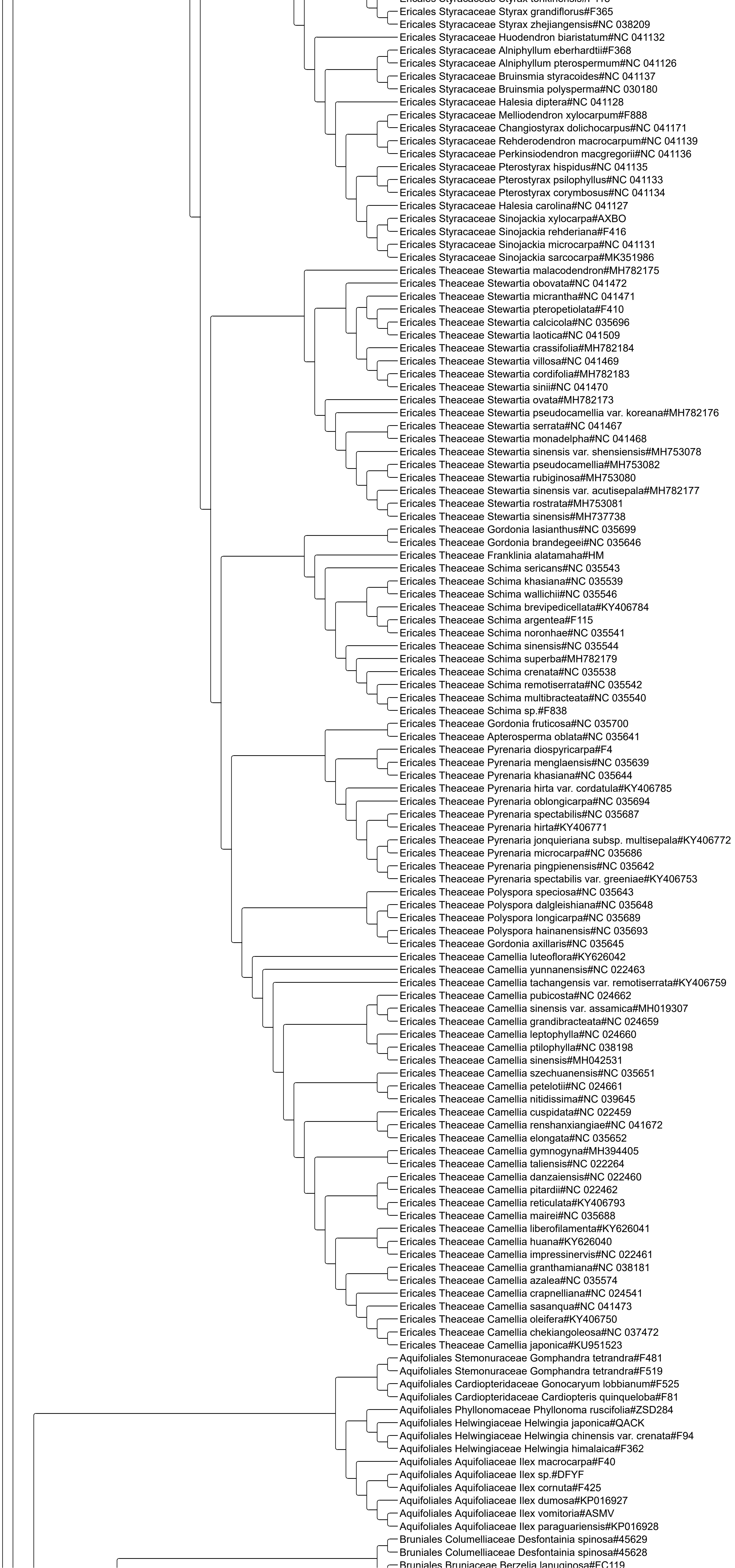

Ericales Styracaceae Styrax grandiflorus#F365
Ericales Styracaceae Styrax zhejiangensis#NC 038209
Ericales Styracaceae Huodendron biaristatum#NC 041132
Ericales Styracaceae Alniphyllum eberhardtii#F368
Ericales Styracaceae Alniphyllum pterospermum#NC 041126
Ericales Styracaceae Bruinsmia styracoides#NC 041137
Ericales Styracaceae Bruinsmia polysperma#NC 030180
Ericales Styracaceae Halesia diptera#NC 041128
Ericales Styracaceae Melliodendron xylocarpum#F888
Ericales Styracaceae Changiostyrax dolichocarpus#NC 041171
Ericales Styracaceae Rehderodendron macrocarpum#NC 041139
Ericales Styracaceae Perkinsiodendron macgregorii#NC 041136
Ericales Styracaceae Pterostyrax hispidus#NC 041135
Ericales Styracaceae Pterostyrax psilophyllus#NC 041133
Ericales Styracaceae Pterostyrax corymbosus#NC 041134
Ericales Styracaceae Halesia carolina#NC 041127
Ericales Styracaceae Sinojackia xylocarpa#AXBO
Ericales Styracaceae Sinojackia rehderiana#F416
Ericales Styracaceae Sinojackia microcarpa#NC 041131
Ericales Styracaceae Sinojackia sarcocarpa#MK351986
Ericales Theaceae Stewartia malacodendron#MH782175
Ericales Theaceae Stewartia obovata#NC 041472
Ericales Theaceae Stewartia micrantha#NC 041471
Ericales Theaceae Stewartia pteropetiolata#F410
Ericales Theaceae Stewartia calcicola#NC 035696
Ericales Theaceae Stewartia laotica#NC 041509
Ericales Theaceae Stewartia crassifolia#MH782184
Ericales Theaceae Stewartia villosa#NC 041469
Ericales Theaceae Stewartia cordifolia#MH782183
Ericales Theaceae Stewartia sinii#NC 041470
Ericales Theaceae Stewartia ovata#MH782173
Ericales Theaceae Stewartia pseudocamellia var. koreana#MH782176
Ericales Theaceae Stewartia serrata#NC 041467
Ericales Theaceae Stewartia monadelpha#NC 041468
Ericales Theaceae Stewartia sinensis var. shensiensis#MH753078
Ericales Theaceae Stewartia pseudocamellia#MH753082
Ericales Theaceae Stewartia rubiginosa#MH753080
Ericales Theaceae Stewartia sinensis var. acutisepala#MH782177
Ericales Theaceae Stewartia rostrata#MH753081
Ericales Theaceae Stewartia sinensis#MH737738
Ericales Theaceae Gordonia lasianthus#NC 035699
Ericales Theaceae Gordonia brandegeei#NC 035646
Ericales Theaceae Franklinia alatamaha#HM
Ericales Theaceae Schima sericans#NC 035543
Ericales Theaceae Schima khasiana#NC 035539
Ericales Theaceae Schima wallichii#NC 035546
Ericales Theaceae Schima brevipedicellata#KY406784
Ericales Theaceae Schima argentea#F115
Ericales Theaceae Schima noronhae#NC 035541
Ericales Theaceae Schima sinensis#NC 035544
Ericales Theaceae Schima superba#MH782179
Ericales Theaceae Schima crenata#NC 035538
Ericales Theaceae Schima remotiserrata#NC 035542
Ericales Theaceae Schima multibracteata#NC 035540
Ericales Theaceae Schima sp.#F838
Ericales Theaceae Gordonia fruticosa#NC 035700
Ericales Theaceae Apterosperma oblata#NC 035641
Ericales Theaceae Pyrenaria diospyricarpa#F4
Ericales Theaceae Pyrenaria menglaensis#NC 035639
Ericales Theaceae Pyrenaria khasiana#NC 035644
Ericales Theaceae Pyrenaria hirta var. cordatula#KY406785
Ericales Theaceae Pyrenaria oblongicarpa#NC 035694
Ericales Theaceae Pyrenaria spectabilis#NC 035687
Ericales Theaceae Pyrenaria hirta#KY406771
Ericales Theaceae Pyrenaria jonquieriana subsp. multisepala#KY406772
Ericales Theaceae Pyrenaria microcarpa#NC 035686
Ericales Theaceae Pyrenaria pingpienensis#NC 035642
Ericales Theaceae Pyrenaria spectabilis var. greeniae#KY406753
Ericales Theaceae Polyspora speciosa#NC 035643
Ericales Theaceae Polyspora dalgleishiana#NC 035648
Ericales Theaceae Polyspora longicarpa#NC 035689
Ericales Theaceae Polyspora hainanensis#NC 035693
Ericales Theaceae Gordonia axillaris#NC 035645
Ericales Theaceae Camellia luteoflora#KY626042
Ericales Theaceae Camellia yunnanensis#NC 022463
Ericales Theaceae Camellia tachangensis var. remotiserrata#KY406759
Ericales Theaceae Camellia pubicosta#NC 024662
Ericales Theaceae Camellia sinensis var. assamica#MH019307
Ericales Theaceae Camellia grandibracteata#NC 024659
Ericales Theaceae Camellia leptophylla#NC 024660
Ericales Theaceae Camellia ptilophylla#NC 038198
Ericales Theaceae Camellia sinensis#MH042531
Ericales Theaceae Camellia szechuanensis#NC 035651
Ericales Theaceae Camellia petelotii#NC 024661
Ericales Theaceae Camellia nitidissima#NC 039645
Ericales Theaceae Camellia cuspidata#NC 022459
Ericales Theaceae Camellia renshanxiangiae#NC 041672
Ericales Theaceae Camellia elongata#NC 035652
Ericales Theaceae Camellia gymnogyna#MH394405
Ericales Theaceae Camellia taliensis#NC 022264
Ericales Theaceae Camellia danzaiensis#NC 022460
Ericales Theaceae Camellia pitardii#NC 022462
Ericales Theaceae Camellia reticulata#KY406793
Ericales Theaceae Camellia mairei#NC 035688
Ericales Theaceae Camellia liberofilamenta#KY626041
Ericales Theaceae Camellia huana#KY626040
Ericales Theaceae Camellia impressinervis#NC 022461
Ericales Theaceae Camellia granthamiana#NC 038181
Ericales Theaceae Camellia azalea#NC 035574
Ericales Theaceae Camellia crapnelliana#NC 024541
Ericales Theaceae Camellia sasanqua#NC 041473
Ericales Theaceae Camellia oleifera#KY406750
Ericales Theaceae Camellia chekiangoleosa#NC 037472
Ericales Theaceae Camellia japonica#KU951523
Aquifoliales Stemonuraceae Gomphandra tetrandra#F481
Aquifoliales Stemonuraceae Gomphandra tetrandra#F519
Aquifoliales Cardiopteridaceae Gonocaryum lobbianum#F525
Aquifoliales Cardiopteridaceae Cardiopteris quinqueloba#F81
Aquifoliales Phyllonomaceae Phyllonoma ruscifolia#ZSD284
Aquifoliales Helwingiaceae Helwingia japonica#QACK
Aquifoliales Helwingiaceae Helwingia chinensis var. crenata#F94
Aquifoliales Helwingiaceae Helwingia himalaica#F362
Aquifoliales Aquifoliaceae Ilex macrocarpa#F40
Aquifoliales Aquifoliaceae Ilex sp.#DFYF
Aquifoliales Aquifoliaceae Ilex cornuta#F425
Aquifoliales Aquifoliaceae Ilex dumosa#KP016927
Aquifoliales Aquifoliaceae Ilex vomitoria#ASMV
Aquifoliales Aquifoliaceae Ilex paraguariensis#KP016928
Bruniales Columelliaceae Desfontainia spinosa#45629
Bruniales Columelliaceae Desfontainia spinosa#45628
Bruniales Bruniaceae Berzelia lanuginosa#FC119

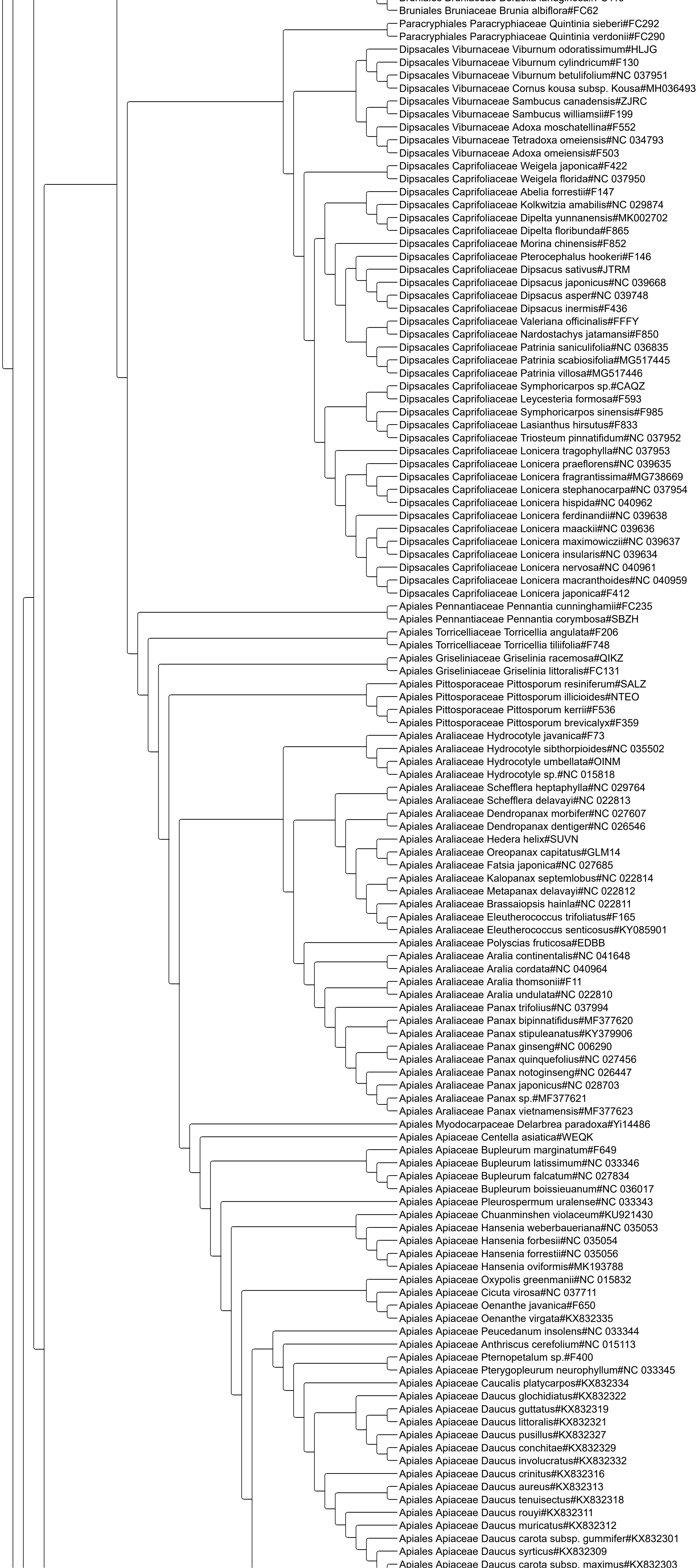

Bruniales Bruniaceae Brunia albiflora#FC62
Paracryphiales Paracryphiaceae Quintinia sieberi#FC292
Paracryphiales Paracryphiaceae Quintinia verdonii#FC290
Dipsacales Viburnaceae Viburnum odoratissimum#HLJG
Dipsacales Viburnaceae Viburnum cylindricum#F130
Dipsacales Viburnaceae Viburnum betulifolium#NC 037951
Dipsacales Viburnaceae Cornus kousa subsp. Kousa#MH036493
Dipsacales Viburnaceae Sambucus canadensis#ZJRC
Dipsacales Viburnaceae Sambucus williamsii#F199
Dipsacales Viburnaceae Adoxa moschatellina#F552
Dipsacales Viburnaceae Tetradoxa omeiensis#NC 034793
Dipsacales Viburnaceae Adoxa omeiensis#F503
Dipsacales Caprifoliaceae Weigela japonica#F422
Dipsacales Caprifoliaceae Weigela florida#NC 037950
Dipsacales Caprifoliaceae Abelia forrestii#F147
Dipsacales Caprifoliaceae Kolkwitzia amabilis#NC 029874
Dipsacales Caprifoliaceae Dipelta yunnanensis#MK002702
Dipsacales Caprifoliaceae Dipelta floribunda#F865
Dipsacales Caprifoliaceae Morina chinensis#F852
Dipsacales Caprifoliaceae Pterocephalus hookeri#F146
Dipsacales Caprifoliaceae Dipsacus sativus#JTRM
Dipsacales Caprifoliaceae Dipsacus japonicus#NC 039668
Dipsacales Caprifoliaceae Dipsacus asper#NC 039748
Dipsacales Caprifoliaceae Dipsacus inermis#F436
Dipsacales Caprifoliaceae Valeriana officinalis#FFFY
Dipsacales Caprifoliaceae Nardostachys jatamansi#F850
Dipsacales Caprifoliaceae Patrinia saniculifolia#NC 036835
Dipsacales Caprifoliaceae Patrinia scabiosifolia#MG517445
Dipsacales Caprifoliaceae Patrinia villosa#MG517446
Dipsacales Caprifoliaceae Symphoricarpos sp.#CAQZ
Dipsacales Caprifoliaceae Leycesteria formosa#F593
Dipsacales Caprifoliaceae Symphoricarpos sinensis#F985
Dipsacales Caprifoliaceae Lasianthus hirsutus#F833
Dipsacales Caprifoliaceae Triosteum pinnatifidum#NC 037952
Dipsacales Caprifoliaceae Lonicera tragophylla#NC 037953
Dipsacales Caprifoliaceae Lonicera praeflorens#NC 039635
Dipsacales Caprifoliaceae Lonicera fragrantissima#MG738669
Dipsacales Caprifoliaceae Lonicera stephanocarpa#NC 037954
Dipsacales Caprifoliaceae Lonicera hispida#NC 040962
Dipsacales Caprifoliaceae Lonicera ferdinandii#NC 039638
Dipsacales Caprifoliaceae Lonicera maackii#NC 039636
Dipsacales Caprifoliaceae Lonicera maximowiczii#NC 039637
Dipsacales Caprifoliaceae Lonicera insularis#NC 039634
Dipsacales Caprifoliaceae Lonicera nervosa#NC 040961
Dipsacales Caprifoliaceae Lonicera macranthoides#NC 040959
Dipsacales Caprifoliaceae Lonicera japonica#F412
Apiales Pennantiaceae Pennantia cunninghamii#FC235
Apiales Pennantiaceae Pennantia corymbosa#SBZH
Apiales Torricelliaceae Torricellia angulata#F206
Apiales Torricelliaceae Torricellia tiliifolia#F748
Apiales Griseliniaceae Griselinia racemosa#QIKZ
Apiales Griseliniaceae Griselinia littoralis#FC131
Apiales Pittosporaceae Pittosporum resiniferum#SALZ
Apiales Pittosporaceae Pittosporum illicioides#NTEO
Apiales Pittosporaceae Pittosporum kerrii#F536
Apiales Pittosporaceae Pittosporum brevicalyx#F359
Apiales Araliaceae Hydrocotyle javanica#F73
Apiales Araliaceae Hydrocotyle sibthorpioides#NC 035502
Apiales Araliaceae Hydrocotyle umbellata#OINM
Apiales Araliaceae Hydrocotyle sp.#NC 015818
Apiales Araliaceae Schefflera heptaphylla#NC 029764
Apiales Araliaceae Schefflera delavayi#NC 022813
Apiales Araliaceae Dendropanax morbifer#NC 027607
Apiales Araliaceae Dendropanax dentiger#NC 026546
Apiales Araliaceae Hedera helix#SUVN
Apiales Araliaceae Oreopanax capitatus#GLM14
Apiales Araliaceae Fatsia japonica#NC 027685
Apiales Araliaceae Kalopanax septemlobus#NC 022814
Apiales Araliaceae Metapanax delavayi#NC 022812
Apiales Araliaceae Brassaiopsis hainla#NC 022811
Apiales Araliaceae Eleutherococcus trifoliatus#F165
Apiales Araliaceae Eleutherococcus senticosus#KY085901
Apiales Araliaceae Polyscias fruticosa#EDBB
Apiales Araliaceae Aralia continentalis#NC 041648
Apiales Araliaceae Aralia cordata#NC 040964
Apiales Araliaceae Aralia thomsonii#F11
Apiales Araliaceae Aralia undulata#NC 022810
Apiales Araliaceae Panax trifolius#NC 037994
Apiales Araliaceae Panax bipinnatifidus#MF377620
Apiales Araliaceae Panax stipuleanatus#KY379906
Apiales Araliaceae Panax ginseng#NC 006290
Apiales Araliaceae Panax quinquefolius#NC 027456
Apiales Araliaceae Panax notoginseng#NC 026447
Apiales Araliaceae Panax japonicus#NC 028703
Apiales Araliaceae Panax sp.#MF377621
Apiales Araliaceae Panax vietnamensis#MF377623
Apiales Myodocarpaceae Delarbrea paradoxa#Yi14486
Apiales Apiaceae Centella asiatica#WEQK
Apiales Apiaceae Bupleurum marginatum#F649
Apiales Apiaceae Bupleurum latissimum#NC 033346
Apiales Apiaceae Bupleurum falcatum#NC 027834
Apiales Apiaceae Bupleurum boissieuanum#NC 036017
Apiales Apiaceae Pleurospermum uralense#NC 033343
Apiales Apiaceae Chuanminshen violaceum#KU921430
Apiales Apiaceae Hansenia weberbaueriana#NC 035053
Apiales Apiaceae Hansenia forbesii#NC 035054
Apiales Apiaceae Hansenia forrestii#NC 035056
Apiales Apiaceae Hansenia oviformis#MK193788
Apiales Apiaceae Oxypolis greenmanii#NC 015832
Apiales Apiaceae Cicuta virosa#NC 037711
Apiales Apiaceae Oenanthe javanica#F650
Apiales Apiaceae Oenanthe virgata#KX832335
Apiales Apiaceae Peucedanum insolens#NC 033344
Apiales Apiaceae Anthriscus cerefolium#NC 015113
Apiales Apiaceae Pternopetalum sp.#F400
Apiales Apiaceae Pterygopleurum neurophyllum#NC 033345
Apiales Apiaceae Caucalis platycarpos#KX832334
Apiales Apiaceae Daucus glochidiatus#KX832322
Apiales Apiaceae Daucus guttatus#KX832319
Apiales Apiaceae Daucus littoralis#KX832321
Apiales Apiaceae Daucus pusillus#KX832327
Apiales Apiaceae Daucus conchitae#KX832329
Apiales Apiaceae Daucus involucratus#KX832332
Apiales Apiaceae Daucus crinitus#KX832316
Apiales Apiaceae Daucus aureus#KX832313
Apiales Apiaceae Daucus tenuisectus#KX832318
Apiales Apiaceae Daucus rouyi#KX832311
Apiales Apiaceae Daucus muricatus#KX832312
Apiales Apiaceae Daucus carota subsp. gummifer#KX832301
Apiales Apiaceae Daucus syrticus#KX832309
Apiales Apiaceae Daucus carota subsp. maximus#KX832303

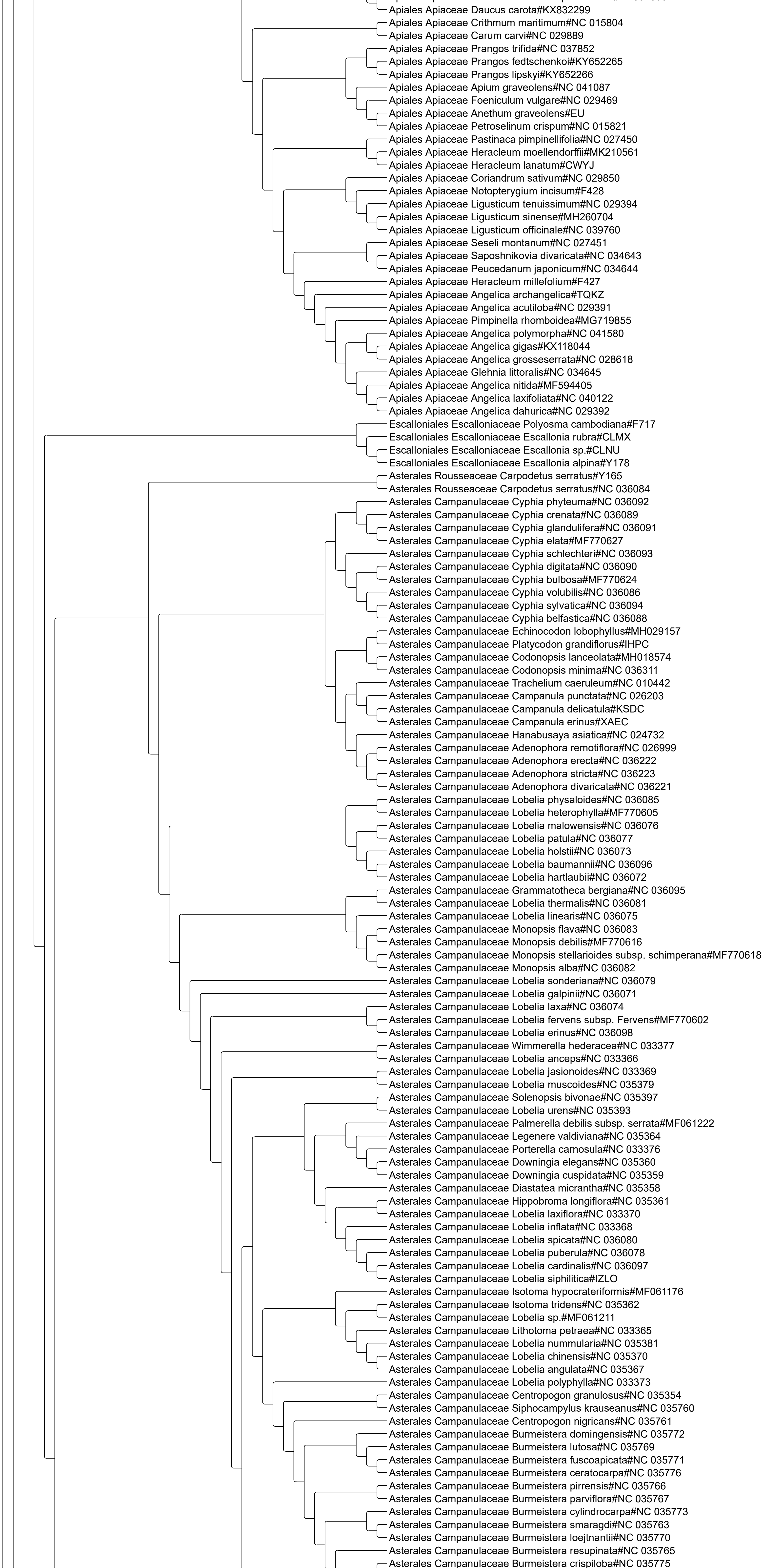
Apiales Apiaceae Daucus carota#KX832299
Apiales Apiaceae Crithmum maritimum#NC 015804
Apiales Apiaceae Carum carvi#NC 029889
Apiales Apiaceae Prangos trifida#NC 037852
Apiales Apiaceae Prangos fedtschenkoi#KY652265
Apiales Apiaceae Prangos lipskyi#KY652266
Apiales Apiaceae Apium graveolens#NC 041087
Apiales Apiaceae Foeniculum vulgare#NC 029469
Apiales Apiaceae Anethum graveolens#EU
Apiales Apiaceae Petroselinum crispum#NC 015821
Apiales Apiaceae Pastinaca pimpinellifolia#NC 027450
Apiales Apiaceae Heracleum moellendorffii#MK210561
Apiales Apiaceae Heracleum lanatum#CWYJ
Apiales Apiaceae Coriandrum sativum#NC 029850
Apiales Apiaceae Notopterygium incisum#F428
Apiales Apiaceae Ligusticum tenuissimum#NC 029394
Apiales Apiaceae Ligusticum sinense#MH260704
Apiales Apiaceae Ligusticum officinale#NC 039760
Apiales Apiaceae Seseli montanum#NC 027451
Apiales Apiaceae Saposhnikovia divaricata#NC 034643
Apiales Apiaceae Peucedanum japonicum#NC 034644
Apiales Apiaceae Heracleum millefolium#F427
Apiales Apiaceae Angelica archangelica#TQKZ
Apiales Apiaceae Angelica acutiloba#NC 029391
Apiales Apiaceae Pimpinella rhomboidea#MG719855
Apiales Apiaceae Angelica polymorpha#NC 041580
Apiales Apiaceae Angelica gigas#KX118044
Apiales Apiaceae Angelica grosseserrata#NC 028618
Apiales Apiaceae Glehnia littoralis#NC 034645
Apiales Apiaceae Angelica nitida#MF594405
Apiales Apiaceae Angelica laxifoliata#NC 040122
Apiales Apiaceae Angelica dahurica#NC 029392
Escalloniales Escalloniaceae Polyosma cambodiana#F717
Escalloniales Escalloniaceae Escallonia rubra#CLMX
Escalloniales Escalloniaceae Escallonia sp.#CLNU
Escalloniales Escalloniaceae Escallonia alpina#Y178
Asterales Rousseaceae Carpodetus serratus#Y165
Asterales Rousseaceae Carpodetus serratus#NC 036084
Asterales Campanulaceae Cyphia phyteuma#NC 036092
Asterales Campanulaceae Cyphia crenata#NC 036089
Asterales Campanulaceae Cyphia glandulifera#NC 036091
Asterales Campanulaceae Cyphia elata#MF770627
Asterales Campanulaceae Cyphia schlechteri#NC 036093
Asterales Campanulaceae Cyphia digitata#NC 036090
Asterales Campanulaceae Cyphia bulbosa#MF770624
Asterales Campanulaceae Cyphia volubilis#NC 036086
Asterales Campanulaceae Cyphia sylvatica#NC 036094
Asterales Campanulaceae Cyphia belfastica#NC 036088
Asterales Campanulaceae Echinocodon lobophyllus#MH029157
Asterales Campanulaceae Platycodon grandiflorus#IHPC
Asterales Campanulaceae Codonopsis lanceolata#MH018574
Asterales Campanulaceae Codonopsis minima#NC 036311
Asterales Campanulaceae Trachelium caeruleum#NC 010442
Asterales Campanulaceae Campanula punctata#NC 026203
Asterales Campanulaceae Campanula delicatula#KSDC
Asterales Campanulaceae Campanula erinus#XAEC
Asterales Campanulaceae Hanabusaya asiatica#NC 024732
Asterales Campanulaceae Adenophora remotiflora#NC 026999
Asterales Campanulaceae Adenophora erecta#NC 036222
Asterales Campanulaceae Adenophora stricta#NC 036223
Asterales Campanulaceae Adenophora divaricata#NC 036221
Asterales Campanulaceae Lobelia physaloides#NC 036085
Asterales Campanulaceae Lobelia heterophylla#MF770605
Asterales Campanulaceae Lobelia malowensis#NC 036076
Asterales Campanulaceae Lobelia patula#NC 036077
Asterales Campanulaceae Lobelia holstii#NC 036073
Asterales Campanulaceae Lobelia baumannii#NC 036096
Asterales Campanulaceae Lobelia hartlaubii#NC 036072
Asterales Campanulaceae Grammatotheca bergiana#NC 036095
Asterales Campanulaceae Lobelia thermalis#NC 036081
Asterales Campanulaceae Lobelia linearis#NC 036075
Asterales Campanulaceae Monopsis flava#NC 036083
Asterales Campanulaceae Monopsis debilis#MF770616
Asterales Campanulaceae Monopsis stellarioides subsp. schimperana#MF770618
Asterales Campanulaceae Monopsis alba#NC 036082
Asterales Campanulaceae Lobelia sonderiana#NC 036079
Asterales Campanulaceae Lobelia galpinii#NC 036071
Asterales Campanulaceae Lobelia laxa#NC 036074
Asterales Campanulaceae Lobelia fervens subsp. Fervens#MF770602
Asterales Campanulaceae Lobelia erinus#NC 036098
Asterales Campanulaceae Wimmerella hederacea#NC 033377
Asterales Campanulaceae Lobelia anceps#NC 033366
Asterales Campanulaceae Lobelia jasionoides#NC 033369
Asterales Campanulaceae Lobelia muscoides#NC 035379
Asterales Campanulaceae Solenopsis bivonae#NC 035397
Asterales Campanulaceae Lobelia urens#NC 035393
Asterales Campanulaceae Palmerella debilis subsp. serrata#MF061222
Asterales Campanulaceae Legenere valdiviana#NC 035364
Asterales Campanulaceae Porterella carnosula#NC 033376
Asterales Campanulaceae Downingia elegans#NC 035360
Asterales Campanulaceae Downingia cuspidata#NC 035359
Asterales Campanulaceae Diastatea micrantha#NC 035358
Asterales Campanulaceae Hippobroma longiflora#NC 035361
Asterales Campanulaceae Lobelia laxiflora#NC 033370
Asterales Campanulaceae Lobelia inflata#NC 033368
Asterales Campanulaceae Lobelia spicata#NC 036080
Asterales Campanulaceae Lobelia puberula#NC 036078
Asterales Campanulaceae Lobelia cardinalis#NC 036097
Asterales Campanulaceae Lobelia siphilitica#IZLO
Asterales Campanulaceae Isotoma hypocrateriformis#MF061176
Asterales Campanulaceae Isotoma tridens#NC 035362
Asterales Campanulaceae Lobelia sp.#MF061211
Asterales Campanulaceae Lithotoma petraea#NC 033365
Asterales Campanulaceae Lobelia nummularia#NC 035381
Asterales Campanulaceae Lobelia chinensis#NC 035370
Asterales Campanulaceae Lobelia angulata#NC 035367
Asterales Campanulaceae Lobelia polyphylla#NC 033373
Asterales Campanulaceae Centropogon granulosus#NC 035354
Asterales Campanulaceae Siphocampylus krauseanus#NC 035760
Asterales Campanulaceae Centropogon nigricans#NC 035761
Asterales Campanulaceae Burmeistera domingensis#NC 035772
Asterales Campanulaceae Burmeistera lutosa#NC 035769
Asterales Campanulaceae Burmeistera fuscoapicata#NC 035771
Asterales Campanulaceae Burmeistera ceratocarpa#NC 035776
Asterales Campanulaceae Burmeistera pirrensis#NC 035766
Asterales Campanulaceae Burmeistera parviflora#NC 035767
Asterales Campanulaceae Burmeistera cylindrocarpa#NC 035773
Asterales Campanulaceae Burmeistera smaragdi#NC 035763
Asterales Campanulaceae Burmeistera loejtnantii#NC 035770
Asterales Campanulaceae Burmeistera resupinata#NC 035765
Asterales Campanulaceae Burmeistera crispiloba#NC 035775

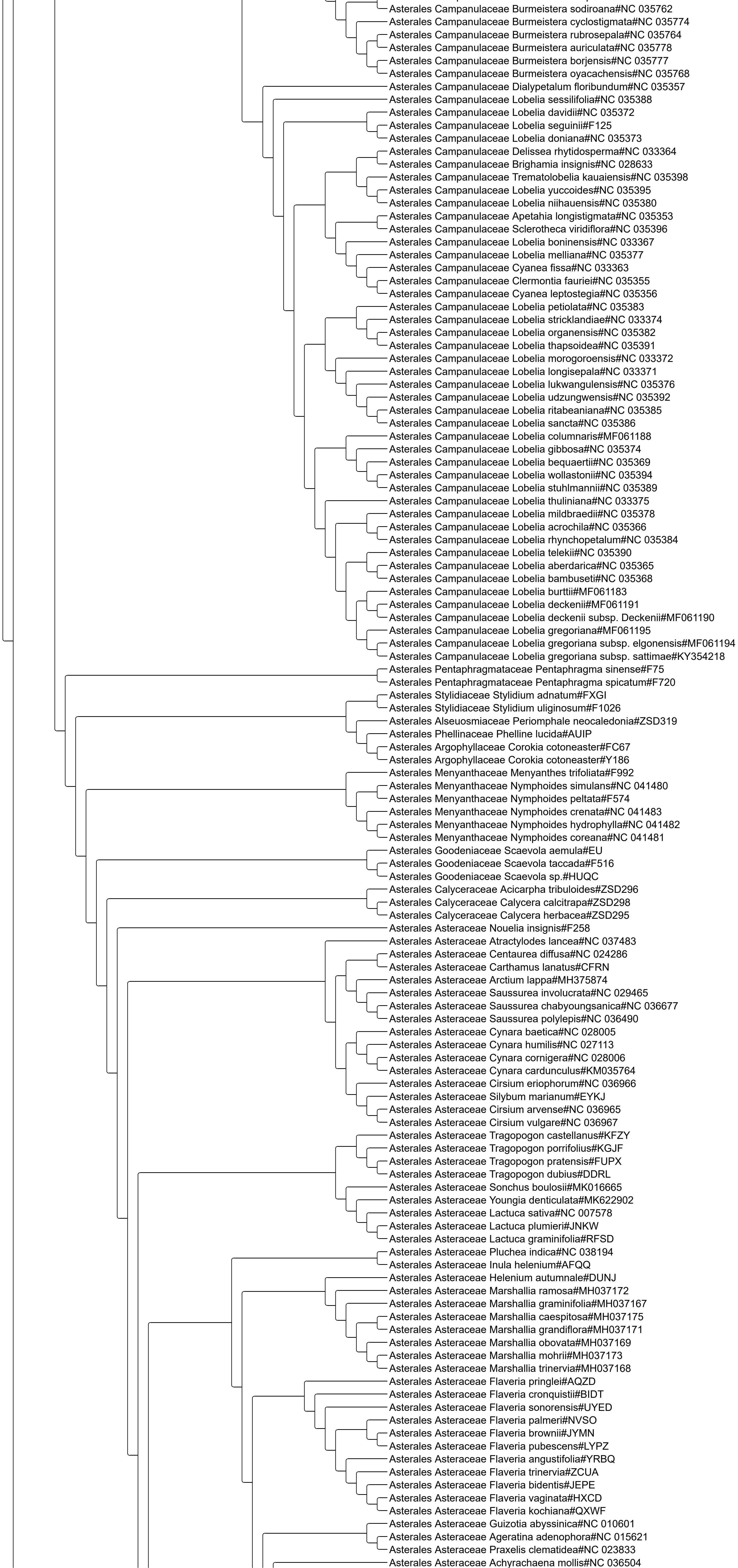
Asterales Campanulaceae Burmeistera sodiroana#NC 035762
Asterales Campanulaceae Burmeistera cyclostigmata#NC 035774
Asterales Campanulaceae Burmeistera rubrosepala#NC 035764
Asterales Campanulaceae Burmeistera auriculata#NC 035778
Asterales Campanulaceae Burmeistera borjensis#NC 035777
Asterales Campanulaceae Burmeistera oyacachensis#NC 035768
Asterales Campanulaceae Dialypetalum floribundum#NC 035357
Asterales Campanulaceae Lobelia sessilifolia#NC 035388
Asterales Campanulaceae Lobelia davidii#NC 035372
Asterales Campanulaceae Lobelia seguinii#F125
Asterales Campanulaceae Lobelia doniana#NC 035373
Asterales Campanulaceae Delissea rhytidosperma#NC 033364
Asterales Campanulaceae Brighamia insignis#NC 028633
Asterales Campanulaceae Trematolobelia kauaiensis#NC 035398
Asterales Campanulaceae Lobelia yuccoides#NC 035395
Asterales Campanulaceae Lobelia niihauensis#NC 035380
Asterales Campanulaceae Apetahia longistigmata#NC 035353
Asterales Campanulaceae Sclerotheca viridiflora#NC 035396
Asterales Campanulaceae Lobelia boninensis#NC 033367
Asterales Campanulaceae Lobelia melliana#NC 035377
Asterales Campanulaceae Cyanea fissa#NC 033363
Asterales Campanulaceae Clermontia fauriei#NC 035355
Asterales Campanulaceae Cyanea leptostegia#NC 035356
Asterales Campanulaceae Lobelia petiolata#NC 035383
Asterales Campanulaceae Lobelia stricklandiae#NC 033374
Asterales Campanulaceae Lobelia organensis#NC 035382
Asterales Campanulaceae Lobelia thapsoidea#NC 035391
Asterales Campanulaceae Lobelia morogoroensis#NC 033372
Asterales Campanulaceae Lobelia longisepala#NC 033371
Asterales Campanulaceae Lobelia lukwangulensis#NC 035376
Asterales Campanulaceae Lobelia udzungwensis#NC 035392
Asterales Campanulaceae Lobelia ritabeaniana#NC 035385
Asterales Campanulaceae Lobelia sancta#NC 035386
Asterales Campanulaceae Lobelia columnaris#MF061188
Asterales Campanulaceae Lobelia gibbosa#NC 035374
Asterales Campanulaceae Lobelia bequaertii#NC 035369
Asterales Campanulaceae Lobelia wollastonii#NC 035394
Asterales Campanulaceae Lobelia stuhlmannii#NC 035389
Asterales Campanulaceae Lobelia thuliniana#NC 033375
Asterales Campanulaceae Lobelia mildbraedii#NC 035378
Asterales Campanulaceae Lobelia acrochila#NC 035366
Asterales Campanulaceae Lobelia rhynchopetalum#NC 035384
Asterales Campanulaceae Lobelia telekii#NC 035390
Asterales Campanulaceae Lobelia aberdarica#NC 035365
Asterales Campanulaceae Lobelia bambuseti#NC 035368
Asterales Campanulaceae Lobelia burttii#MF061183
Asterales Campanulaceae Lobelia deckenii#MF061191
Asterales Campanulaceae Lobelia deckenii subsp. Deckenii#MF061190
Asterales Campanulaceae Lobelia gregoriana#MF061195
Asterales Campanulaceae Lobelia gregoriana subsp. elgonensis#MF061194
Asterales Campanulaceae Lobelia gregoriana subsp. sattimae#KY354218
Asterales Pentaphragmataceae Pentaphragma sinense#F75
Asterales Pentaphragmataceae Pentaphragma spicatum#F720
Asterales Stylidiaceae Stylidium adnatum#FXGI
Asterales Stylidiaceae Stylidium uliginosum#F1026
Asterales Alseuosmiaceae Periomphale neocaledonia#ZSD319
Asterales Phellinaceae Phelline lucida#AUIP
Asterales Argophyllaceae Corokia cotoneaster#FC67
Asterales Argophyllaceae Corokia cotoneaster#Y186
Asterales Menyanthaceae Menyanthes trifoliata#F992
Asterales Menyanthaceae Nymphoides simulans#NC 041480
Asterales Menyanthaceae Nymphoides peltata#F574
Asterales Menyanthaceae Nymphoides crenata#NC 041483
Asterales Menyanthaceae Nymphoides hydrophylla#NC 041482
Asterales Menyanthaceae Nymphoides coreana#NC 041481
Asterales Goodeniaceae Scaevola aemula#EU
Asterales Goodeniaceae Scaevola taccada#F516
Asterales Goodeniaceae Scaevola sp.#HUQC
Asterales Calyceraceae Acicarpha tribuloides#ZSD296
Asterales Calyceraceae Calycera calcitrapa#ZSD298
Asterales Calyceraceae Calycera herbacea#ZSD295
Asterales Asteraceae Nouelia insignis#F258
Asterales Asteraceae Atractylodes lancea#NC 037483
Asterales Asteraceae Centaurea diffusa#NC 024286
Asterales Asteraceae Carthamus lanatus#CFRN
Asterales Asteraceae Arctium lappa#MH375874
Asterales Asteraceae Saussurea involucrata#NC 029465
Asterales Asteraceae Saussurea chabyoungsanica#NC 036677
Asterales Asteraceae Saussurea polylepis#NC 036490
Asterales Asteraceae Cynara baetica#NC 028005
Asterales Asteraceae Cynara humilis#NC 027113
Asterales Asteraceae Cynara cornigera#NC 028006
Asterales Asteraceae Cynara cardunculus#KM035764
Asterales Asteraceae Cirsium eriophorum#NC 036966
Asterales Asteraceae Silybum marianum#EYKJ
Asterales Asteraceae Cirsium arvense#NC 036965
Asterales Asteraceae Cirsium vulgare#NC 036967
Asterales Asteraceae Tragopogon castellanus#KFZY
Asterales Asteraceae Tragopogon porrifolius#KGJF
Asterales Asteraceae Tragopogon pratensis#FUPX
Asterales Asteraceae Tragopogon dubius#DDRL
Asterales Asteraceae Sonchus boulosii#MK016665
Asterales Asteraceae Youngia denticulata#MK622902
Asterales Asteraceae Lactuca sativa#NC 007578
Asterales Asteraceae Lactuca plumieri#JNKW
Asterales Asteraceae Lactuca graminifolia#RFSD
Asterales Asteraceae Pluchea indica#NC 038194
Asterales Asteraceae Inula helenium#AFQQ
Asterales Asteraceae Helenium autumnale#DUNJ
Asterales Asteraceae Marshallia ramosa#MH037172
Asterales Asteraceae Marshallia graminifolia#MH037167
Asterales Asteraceae Marshallia caespitosa#MH037175
Asterales Asteraceae Marshallia grandiflora#MH037171
Asterales Asteraceae Marshallia obovata#MH037169
Asterales Asteraceae Marshallia mohrii#MH037173
Asterales Asteraceae Marshallia trinervia#MH037168
Asterales Asteraceae Flaveria pringlei#AQZD
Asterales Asteraceae Flaveria cronquistii#BIDT
Asterales Asteraceae Flaveria sonorensis#UYED
Asterales Asteraceae Flaveria palmeri#NVSO
Asterales Asteraceae Flaveria brownii#JYMN
Asterales Asteraceae Flaveria pubescens#LYPZ
Asterales Asteraceae Flaveria angustifolia#YRBQ
Asterales Asteraceae Flaveria trinervia#ZCUA
Asterales Asteraceae Flaveria bidentis#JEPE
Asterales Asteraceae Flaveria vaginata#HXCD
Asterales Asteraceae Flaveria kochiana#QXWF
Asterales Asteraceae Guizotia abyssinica#NC 010601
Asterales Asteraceae Ageratina adenophora#NC 015621
Asterales Asteraceae Praxelis clematidea#NC 023833
Asterales Asteraceae Achyrachaena mollis#NC 036504

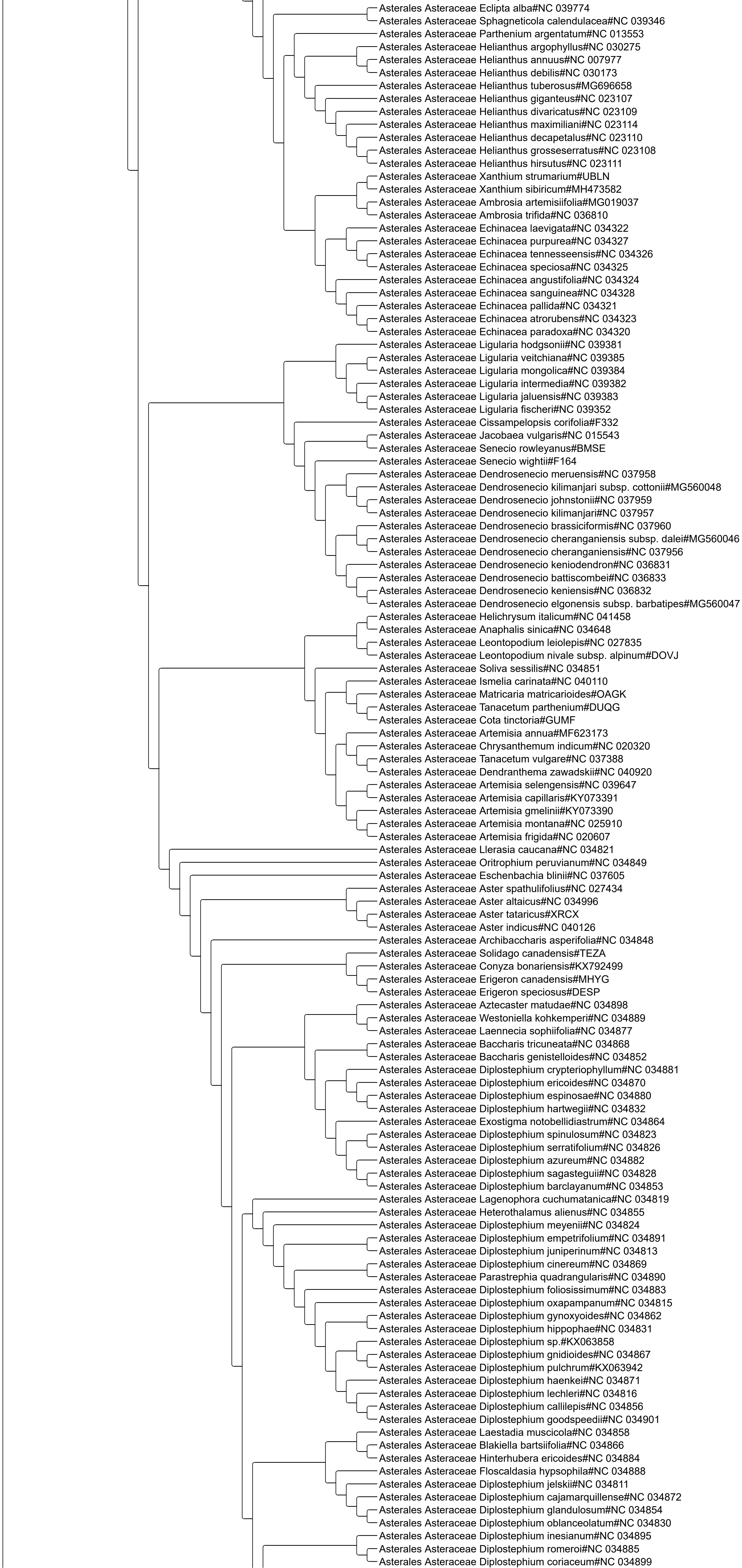
Asterales Asteraceae Eclipta alba#NC 039774
Asterales Asteraceae Sphagneticola calendulacea#NC 039346
Asterales Asteraceae Parthenium argentatum#NC 013553
Asterales Asteraceae Helianthus argophyllus#NC 030275
Asterales Asteraceae Helianthus annuus#NC 007977
Asterales Asteraceae Helianthus debilis#NC 030173
Asterales Asteraceae Helianthus tuberosus#MG696658
Asterales Asteraceae Helianthus giganteus#NC 023107
Asterales Asteraceae Helianthus divaricatus#NC 023109
Asterales Asteraceae Helianthus maximiliani#NC 023114
Asterales Asteraceae Helianthus decapetalus#NC 023110
Asterales Asteraceae Helianthus grosseserratus#NC 023108
Asterales Asteraceae Helianthus hirsutus#NC 023111
Asterales Asteraceae Xanthium strumarium#UBLN
Asterales Asteraceae Xanthium sibiricum#MH473582
Asterales Asteraceae Ambrosia artemisiifolia#MG019037
Asterales Asteraceae Ambrosia trifida#NC 036810
Asterales Asteraceae Echinacea laevigata#NC 034322
Asterales Asteraceae Echinacea purpurea#NC 034327
Asterales Asteraceae Echinacea tennesseensis#NC 034326
Asterales Asteraceae Echinacea speciosa#NC 034325
Asterales Asteraceae Echinacea angustifolia#NC 034324
Asterales Asteraceae Echinacea sanguinea#NC 034328
Asterales Asteraceae Echinacea pallida#NC 034321
Asterales Asteraceae Echinacea atrorubens#NC 034323
Asterales Asteraceae Echinacea paradoxa#NC 034320
Asterales Asteraceae Ligularia hodgsonii#NC 039381
Asterales Asteraceae Ligularia veitchiana#NC 039385
Asterales Asteraceae Ligularia mongolica#NC 039384
Asterales Asteraceae Ligularia intermedia#NC 039382
Asterales Asteraceae Ligularia jaluensis#NC 039383
Asterales Asteraceae Ligularia fischeri#NC 039352
Asterales Asteraceae Cissampelopsis corifolia#F332
Asterales Asteraceae Jacobaea vulgaris#NC 015543
Asterales Asteraceae Senecio rowleyanus#BMSE
Asterales Asteraceae Senecio wightii#F164
Asterales Asteraceae Dendrosenecio meruensis#NC 037958
Asterales Asteraceae Dendrosenecio kilimanjari subsp. cottonii#MG560048
Asterales Asteraceae Dendrosenecio johnstonii#NC 037959
Asterales Asteraceae Dendrosenecio kilimanjari#NC 037957
Asterales Asteraceae Dendrosenecio brassiciformis#NC 037960
Asterales Asteraceae Dendrosenecio cheranganiensis subsp. dalei#MG560046
Asterales Asteraceae Dendrosenecio cheranganiensis#NC 037956
Asterales Asteraceae Dendrosenecio keniodendron#NC 036831
Asterales Asteraceae Dendrosenecio battiscombei#NC 036833
Asterales Asteraceae Dendrosenecio keniensis#NC 036832
Asterales Asteraceae Dendrosenecio elgonensis subsp. barbatipes#MG560047
Asterales Asteraceae Helichrysum italicum#NC 041458
Asterales Asteraceae Anaphalis sinica#NC 034648
Asterales Asteraceae Leontopodium leiolepis#NC 027835
Asterales Asteraceae Leontopodium nivale subsp. alpinum#DOVJ
Asterales Asteraceae Soliva sessilis#NC 034851
Asterales Asteraceae Ismelia carinata#NC 040110
Asterales Asteraceae Matricaria matricarioides#OAGK
Asterales Asteraceae Tanacetum parthenium#DUQG
Asterales Asteraceae Cota tinctoria#GUMF
Asterales Asteraceae Artemisia annua#MF623173
Asterales Asteraceae Chrysanthemum indicum#NC 020320
Asterales Asteraceae Tanacetum vulgare#NC 037388
Asterales Asteraceae Dendranthema zawadskii#NC 040920
Asterales Asteraceae Artemisia selengensis#NC 039647
Asterales Asteraceae Artemisia capillaris#KY073391
Asterales Asteraceae Artemisia gmelinii#KY073390
Asterales Asteraceae Artemisia montana#NC 025910
Asterales Asteraceae Artemisia frigida#NC 020607
Asterales Asteraceae Llerasia caucana#NC 034821
Asterales Asteraceae Oritrophium peruvianum#NC 034849
Asterales Asteraceae Eschenbachia blinii#NC 037605
Asterales Asteraceae Aster spathulifolius#NC 027434
Asterales Asteraceae Aster altaicus#NC 034996
Asterales Asteraceae Aster tataricus#XRCX
Asterales Asteraceae Aster indicus#NC 040126
Asterales Asteraceae Archibaccharis asperifolia#NC 034848
Asterales Asteraceae Solidago canadensis#TEZA
Asterales Asteraceae Conyza bonariensis#KX792499
Asterales Asteraceae Erigeron canadensis#MHYG
Asterales Asteraceae Erigeron speciosus#DESP
Asterales Asteraceae Aztecaster matudae#NC 034898
Asterales Asteraceae Westoniella kohkemperi#NC 034889
Asterales Asteraceae Laennecia sophiifolia#NC 034877
Asterales Asteraceae Baccharis tricuneata#NC 034868
Asterales Asteraceae Baccharis genistelloides#NC 034852
Asterales Asteraceae Diplostephium crypteriophyllum#NC 034881
Asterales Asteraceae Diplostephium ericoides#NC 034870
Asterales Asteraceae Diplostephium espinosae#NC 034880
Asterales Asteraceae Diplostephium hartwegii#NC 034832
Asterales Asteraceae Exostigma notobellidiastrum#NC 034864
Asterales Asteraceae Diplostephium spinulosum#NC 034823
Asterales Asteraceae Diplostephium serratifolium#NC 034826
Asterales Asteraceae Diplostephium azureum#NC 034882
Asterales Asteraceae Diplostephium sagasteguii#NC 034828
Asterales Asteraceae Diplostephium barclayanum#NC 034853
Asterales Asteraceae Lagenophora cuchumatanica#NC 034819
Asterales Asteraceae Heterothalamus alienus#NC 034855
Asterales Asteraceae Diplostephium meyenii#NC 034824
Asterales Asteraceae Diplostephium empetrifolium#NC 034891
Asterales Asteraceae Diplostephium juniperinum#NC 034813
Asterales Asteraceae Diplostephium cinereum#NC 034869
Asterales Asteraceae Parastrephia quadrangularis#NC 034890
Asterales Asteraceae Diplostephium foliosissimum#NC 034883
Asterales Asteraceae Diplostephium oxapampanum#NC 034815
Asterales Asteraceae Diplostephium gynoxyoides#NC 034862
Asterales Asteraceae Diplostephium hippophae#NC 034831
Asterales Asteraceae Diplostephium sp.#KX063858
Asterales Asteraceae Diplostephium gnidioides#NC 034867
Asterales Asteraceae Diplostephium pulchrum#KX063942
Asterales Asteraceae Diplostephium haenkei#NC 034871
Asterales Asteraceae Diplostephium lechleri#NC 034816
Asterales Asteraceae Diplostephium callilepis#NC 034856
Asterales Asteraceae Diplostephium goodspeedii#NC 034901
Asterales Asteraceae Laestadia muscicola#NC 034858
Asterales Asteraceae Blakiella bartsiifolia#NC 034866
Asterales Asteraceae Hinterhubera ericoides#NC 034884
Asterales Asteraceae Floscaldasia hypsophila#NC 034888
Asterales Asteraceae Diplostephium jelskii#NC 034811
Asterales Asteraceae Diplostephium cajamarquillense#NC 034872
Asterales Asteraceae Diplostephium glandulosum#NC 034854
Asterales Asteraceae Diplostephium oblanceolatum#NC 034830
Asterales Asteraceae Diplostephium inesianum#NC 034895
Asterales Asteraceae Diplostephium romeroi#NC 034885
Asterales Asteraceae Diplostephium coriaceum#NC 034899

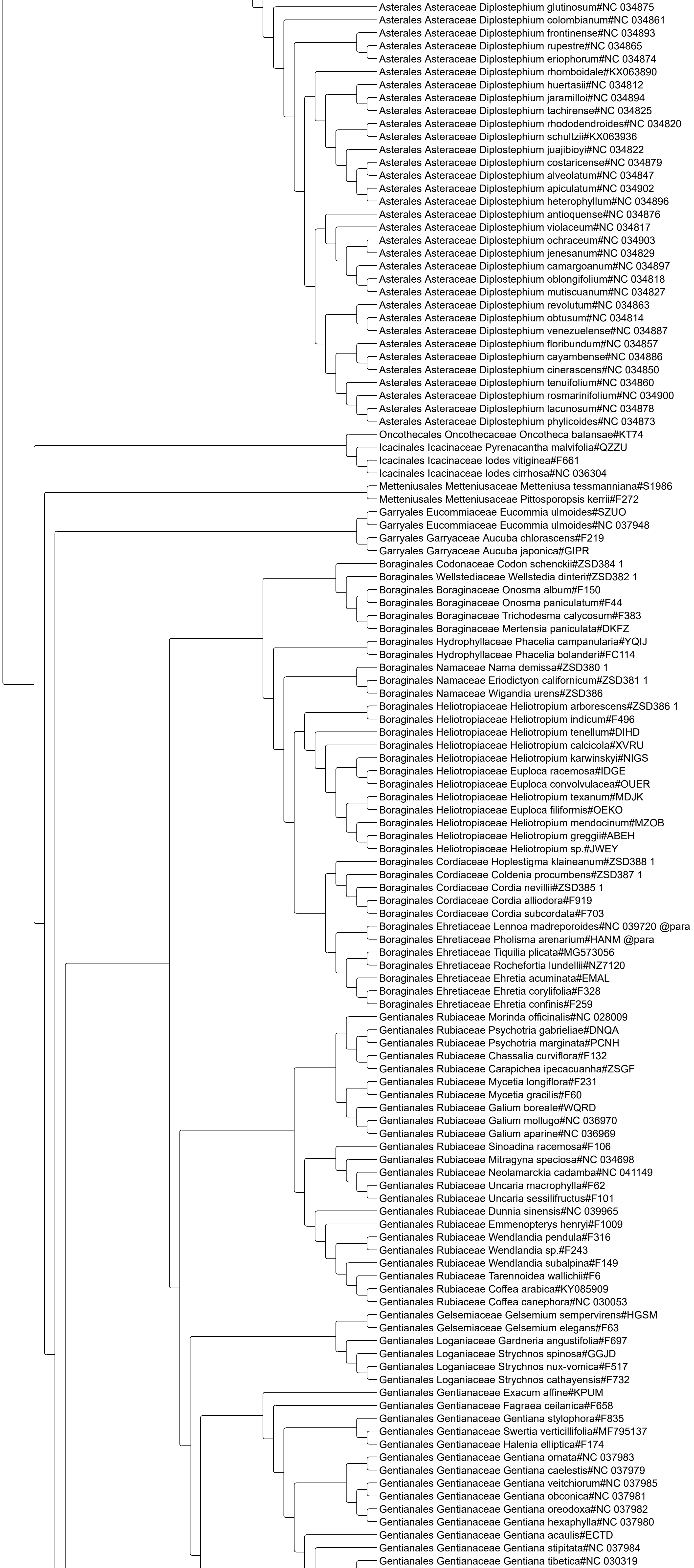
Asterales Asteraceae Diplostephium glutinosum#NC 034875
Asterales Asteraceae Diplostephium colombianum#NC 034861
Asterales Asteraceae Diplostephium frontinense#NC 034893
Asterales Asteraceae Diplostephium rupestre#NC 034865
Asterales Asteraceae Diplostephium eriophorum#NC 034874
Asterales Asteraceae Diplostephium rhomboidale#KX063890
Asterales Asteraceae Diplostephium huertasii#NC 034812
Asterales Asteraceae Diplostephium jaramilloi#NC 034894
Asterales Asteraceae Diplostephium tachirense#NC 034825
Asterales Asteraceae Diplostephium rhododendroides#NC 034820
Asterales Asteraceae Diplostephium schultzii#KX063936
Asterales Asteraceae Diplostephium juajibioyi#NC 034822
Asterales Asteraceae Diplostephium costaricense#NC 034879
Asterales Asteraceae Diplostephium alveolatum#NC 034847
Asterales Asteraceae Diplostephium apiculatum#NC 034902
Asterales Asteraceae Diplostephium heterophyllum#NC 034896
Asterales Asteraceae Diplostephium antioquense#NC 034876
Asterales Asteraceae Diplostephium violaceum#NC 034817
Asterales Asteraceae Diplostephium ochraceum#NC 034903
Asterales Asteraceae Diplostephium jenesanum#NC 034829
Asterales Asteraceae Diplostephium camargoanum#NC 034897
Asterales Asteraceae Diplostephium oblongifolium#NC 034818
Asterales Asteraceae Diplostephium mutiscuanum#NC 034827
Asterales Asteraceae Diplostephium revolutum#NC 034863
Asterales Asteraceae Diplostephium obtusum#NC 034814
Asterales Asteraceae Diplostephium venezuelense#NC 034887
Asterales Asteraceae Diplostephium floribundum#NC 034857
Asterales Asteraceae Diplostephium cayambense#NC 034886
Asterales Asteraceae Diplostephium cinerascens#NC 034850
Asterales Asteraceae Diplostephium tenuifolium#NC 034860
Asterales Asteraceae Diplostephium rosmarinifolium#NC 034900
Asterales Asteraceae Diplostephium lacunosum#NC 034878
Asterales Asteraceae Diplostephium phylicoides#NC 034873
Oncothecales Oncothecaceae Oncotheca balansae#KT74
Icacinales Icacinaceae Pyrenacantha malvifolia#QZZU
Icacinales Icacinaceae Iodes vitiginea#F661
Icacinales Icacinaceae Iodes cirrhosa#NC 036304
Metteniusales Metteniusaceae Metteniusa tessmanniana#S1986
Metteniusales Metteniusaceae Pittosporopsis kerrii#F272
Garryales Eucommiaceae Eucommia ulmoides#SZUO
Garryales Eucommiaceae Eucommia ulmoides#NC 037948
Garryales Garryaceae Aucuba chlorascens#F219
Garryales Garryaceae Aucuba japonica#GIPR
Boraginales Codonaceae Codon schenckii#ZSD384 1
Boraginales Wellstediaceae Wellstedia dinteri#ZSD382 1
Boraginales Boraginaceae Onosma album#F150
Boraginales Boraginaceae Onosma paniculatum#F44
Boraginales Boraginaceae Trichodesma calycosum#F383
Boraginales Boraginaceae Mertensia paniculata#DKFZ
Boraginales Hydrophyllaceae Phacelia campanularia#YQIJ
Boraginales Hydrophyllaceae Phacelia bolanderi#FC114
Boraginales Namaceae Nama demissa#ZSD380 1
Boraginales Namaceae Eriodictyon californicum#ZSD381 1
Boraginales Namaceae Wigandia urens#ZSD386
Boraginales Heliotropiaceae Heliotropium arborescens#ZSD386 1
Boraginales Heliotropiaceae Heliotropium indicum#F496
Boraginales Heliotropiaceae Heliotropium tenellum#DIHD
Boraginales Heliotropiaceae Heliotropium calcicola#XVRU
Boraginales Heliotropiaceae Heliotropium karwinskyi#NIGS
Boraginales Heliotropiaceae Euploca racemosa#IDGE
Boraginales Heliotropiaceae Euploca convolvulacea#OUER
Boraginales Heliotropiaceae Heliotropium texanum#MDJK
Boraginales Heliotropiaceae Euploca filiformis#OEKO
Boraginales Heliotropiaceae Heliotropium mendocinum#MZOB
Boraginales Heliotropiaceae Heliotropium greggii#ABEH
Boraginales Heliotropiaceae Heliotropium sp.#JWEY
Boraginales Cordiaceae Hoplestigma klaineanum#ZSD388 1
Boraginales Cordiaceae Coldenia procumbens#ZSD387 1
Boraginales Cordiaceae Cordia nevillii#ZSD385 1
Boraginales Cordiaceae Cordia alliodora#F919
Boraginales Cordiaceae Cordia subcordata#F703
Boraginales Ehretiaceae Lennoa madreporoides#NC 039720 @para
Boraginales Ehretiaceae Pholisma arenarium#HANM @para
Boraginales Ehretiaceae Tiquilia plicata#MG573056
Boraginales Ehretiaceae Rochefortia lundellii#NZ7120
Boraginales Ehretiaceae Ehretia acuminata#EMAL
Boraginales Ehretiaceae Ehretia corylifolia#F328
Boraginales Ehretiaceae Ehretia confinis#F259
Gentianales Rubiaceae Morinda officinalis#NC 028009
Gentianales Rubiaceae Psychotria gabrieliae#DNQA
Gentianales Rubiaceae Psychotria marginata#PCNH
Gentianales Rubiaceae Chassalia curviflora#F132
Gentianales Rubiaceae Carapichea ipecacuanha#ZSGF
Gentianales Rubiaceae Mycetia longiflora#F231
Gentianales Rubiaceae Mycetia gracilis#F60
Gentianales Rubiaceae Galium boreale#WQRD
Gentianales Rubiaceae Galium mollugo#NC 036970
Gentianales Rubiaceae Galium aparine#NC 036969
Gentianales Rubiaceae Sinoadina racemosa#F106
Gentianales Rubiaceae Mitragyna speciosa#NC 034698
Gentianales Rubiaceae Neolamarckia cadamba#NC 041149
Gentianales Rubiaceae Uncaria macrophylla#F62
Gentianales Rubiaceae Uncaria sessilifructus#F101
Gentianales Rubiaceae Dunnia sinensis#NC 039965
Gentianales Rubiaceae Emmenopterys henryi#F1009
Gentianales Rubiaceae Wendlandia pendula#F316
Gentianales Rubiaceae Wendlandia sp.#F243
Gentianales Rubiaceae Wendlandia subalpina#F149
Gentianales Rubiaceae Tarennoidea wallichii#F6
Gentianales Rubiaceae Coffea arabica#KY085909
Gentianales Rubiaceae Coffea canephora#NC 030053
Gentianales Gelsemiaceae Gelsemium sempervirens#HGSM
Gentianales Gelsemiaceae Gelsemium elegans#F63
Gentianales Loganiaceae Gardneria angustifolia#F697
Gentianales Loganiaceae Strychnos spinosa#GGJD
Gentianales Loganiaceae Strychnos nux-vomica#F517
Gentianales Loganiaceae Strychnos cathayensis#F732
Gentianales Gentianaceae Exacum affine#KPUM
Gentianales Gentianaceae Fagraea ceilanica#F658
Gentianales Gentianaceae Gentiana stylophora#F835
Gentianales Gentianaceae Swertia verticillifolia#MF795137
Gentianales Gentianaceae Halenia elliptica#F174
Gentianales Gentianaceae Gentiana ornata#NC 037983
Gentianales Gentianaceae Gentiana caelestis#NC 037979
Gentianales Gentianaceae Gentiana veitchiorum#NC 037985
Gentianales Gentianaceae Gentiana obconica#NC 037981
Gentianales Gentianaceae Gentiana oreodoxa#NC 037982
Gentianales Gentianaceae Gentiana hexaphylla#NC 037980
Gentianales Gentianaceae Gentiana acaulis#ECTD
Gentianales Gentianaceae Gentiana stipitata#NC 037984
Gentianales Gentianaceae Gentiana tibetica#NC 030319

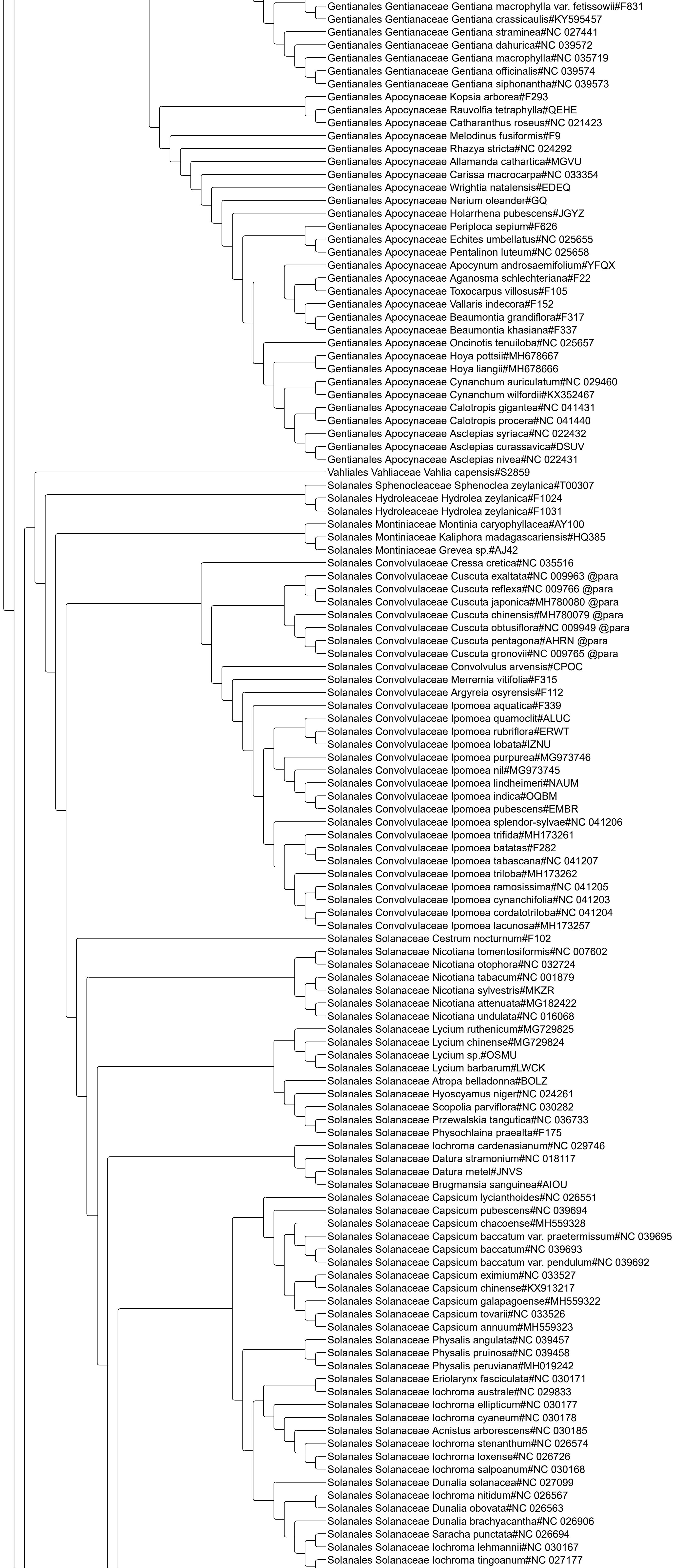
Gentianales Gentianaceae Gentiana macrophylla var. fetissowii#F831
Gentianales Gentianaceae Gentiana crassicaulis#KY595457
Gentianales Gentianaceae Gentiana straminea#NC 027441
Gentianales Gentianaceae Gentiana dahurica#NC 039572
Gentianales Gentianaceae Gentiana macrophylla#NC 035719
Gentianales Gentianaceae Gentiana officinalis#NC 039574
Gentianales Gentianaceae Gentiana siphonantha#NC 039573
Gentianales Apocynaceae Kopsia arborea#F293
Gentianales Apocynaceae Rauvolfia tetraphylla#QEHE
Gentianales Apocynaceae Catharanthus roseus#NC 021423
Gentianales Apocynaceae Melodinus fusiformis#F9
Gentianales Apocynaceae Rhazya stricta#NC 024292
Gentianales Apocynaceae Allamanda cathartica#MGVU
Gentianales Apocynaceae Carissa macrocarpa#NC 033354
Gentianales Apocynaceae Wrightia natalensis#EDEQ
Gentianales Apocynaceae Nerium oleander#GQ
Gentianales Apocynaceae Holarrhena pubescens#JGYZ
Gentianales Apocynaceae Periploca sepium#F626
Gentianales Apocynaceae Echites umbellatus#NC 025655
Gentianales Apocynaceae Pentalinon luteum#NC 025658
Gentianales Apocynaceae Apocynum androsaemifolium#YFQX
Gentianales Apocynaceae Aganosma schlechteriana#F22
Gentianales Apocynaceae Toxocarpus villosus#F105
Gentianales Apocynaceae Vallaris indecora#F152
Gentianales Apocynaceae Beaumontia grandiflora#F317
Gentianales Apocynaceae Beaumontia khasiana#F337
Gentianales Apocynaceae Oncinotis tenuiloba#NC 025657
Gentianales Apocynaceae Hoya pottsii#MH678667
Gentianales Apocynaceae Hoya liangii#MH678666
Gentianales Apocynaceae Cynanchum auriculatum#NC 029460
Gentianales Apocynaceae Cynanchum wilfordii#KX352467
Gentianales Apocynaceae Calotropis gigantea#NC 041431
Gentianales Apocynaceae Calotropis procera#NC 041440
Gentianales Apocynaceae Asclepias syriaca#NC 022432
Gentianales Apocynaceae Asclepias curassavica#DSUV
Gentianales Apocynaceae Asclepias nivea#NC 022431
Vahliales Vahliaceae Vahlia capensis#S2859
Solanales Sphenocleaceae Sphenoclea zeylanica#T00307
Solanales Hydroleaceae Hydrolea zeylanica#F1024
Solanales Hydroleaceae Hydrolea zeylanica#F1031
Solanales Montiniaceae Montinia caryophyllacea#AY100
Solanales Montiniaceae Kaliphora madagascariensis#HQ385
Solanales Montiniaceae Grevea sp.#AJ42
Solanales Convolvulaceae Cressa cretica#NC 035516
Solanales Convolvulaceae Cuscuta exaltata#NC 009963 @para
Solanales Convolvulaceae Cuscuta reflexa#NC 009766 @para
Solanales Convolvulaceae Cuscuta japonica#MH780080 @para
Solanales Convolvulaceae Cuscuta chinensis#MH780079 @para
Solanales Convolvulaceae Cuscuta obtusiflora#NC 009949 @para
Solanales Convolvulaceae Cuscuta pentagona#AHRN @para
Solanales Convolvulaceae Cuscuta gronovii#NC 009765 @para
Solanales Convolvulaceae Convolvulus arvensis#CPOC
Solanales Convolvulaceae Merremia vitifolia#F315
Solanales Convolvulaceae Argyreia osyrensis#F112
Solanales Convolvulaceae Ipomoea aquatica#F339
Solanales Convolvulaceae Ipomoea quamoclit#ALUC
Solanales Convolvulaceae Ipomoea rubriflora#ERWT
Solanales Convolvulaceae Ipomoea lobata#IZNU
Solanales Convolvulaceae Ipomoea purpurea#MG973746
Solanales Convolvulaceae Ipomoea nil#MG973745
Solanales Convolvulaceae Ipomoea lindheimeri#NAUM
Solanales Convolvulaceae Ipomoea indica#OQBM
Solanales Convolvulaceae Ipomoea pubescens#EMBR
Solanales Convolvulaceae Ipomoea splendor-sylvae#NC 041206
Solanales Convolvulaceae Ipomoea trifida#MH173261
Solanales Convolvulaceae Ipomoea batatas#F282
Solanales Convolvulaceae Ipomoea tabascana#NC 041207
Solanales Convolvulaceae Ipomoea triloba#MH173262
Solanales Convolvulaceae Ipomoea ramosissima#NC 041205
Solanales Convolvulaceae Ipomoea cynanchifolia#NC 041203
Solanales Convolvulaceae Ipomoea cordatotriloba#NC 041204
Solanales Convolvulaceae Ipomoea lacunosa#MH173257
Solanales Solanaceae Cestrum nocturnum#F102
Solanales Solanaceae Nicotiana tomentosiformis#NC 007602
Solanales Solanaceae Nicotiana otophora#NC 032724
Solanales Solanaceae Nicotiana tabacum#NC 001879
Solanales Solanaceae Nicotiana sylvestris#MKZR
Solanales Solanaceae Nicotiana attenuata#MG182422
Solanales Solanaceae Nicotiana undulata#NC 016068
Solanales Solanaceae Lycium ruthenicum#MG729825
Solanales Solanaceae Lycium chinense#MG729824
Solanales Solanaceae Lycium sp.#OSMU
Solanales Solanaceae Lycium barbarum#LWCK
Solanales Solanaceae Atropa belladonna#BOLZ
Solanales Solanaceae Hyoscyamus niger#NC 024261
Solanales Solanaceae Scopolia parviflora#NC 030282
Solanales Solanaceae Przewalskia tangutica#NC 036733
Solanales Solanaceae Physochlaina praealta#F175
Solanales Solanaceae Iochroma cardenasianum#NC 029746
Solanales Solanaceae Datura stramonium#NC 018117
Solanales Solanaceae Datura metel#JNVS
Solanales Solanaceae Brugmansia sanguinea#AIOU
Solanales Solanaceae Capsicum lycianthoides#NC 026551
Solanales Solanaceae Capsicum pubescens#NC 039694
Solanales Solanaceae Capsicum chacoense#MH559328
Solanales Solanaceae Capsicum baccatum var. praetermissum#NC 039695
Solanales Solanaceae Capsicum baccatum#NC 039693
Solanales Solanaceae Capsicum baccatum var. pendulum#NC 039692
Solanales Solanaceae Capsicum eximium#NC 033527
Solanales Solanaceae Capsicum chinense#KX913217
Solanales Solanaceae Capsicum galapagoense#MH559322
Solanales Solanaceae Capsicum tovarii#NC 033526
Solanales Solanaceae Capsicum annuum#MH559323
Solanales Solanaceae Physalis angulata#NC 039457
Solanales Solanaceae Physalis pruinosa#NC 039458
Solanales Solanaceae Physalis peruviana#MH019242
Solanales Solanaceae Eriolarynx fasciculata#NC 030171
Solanales Solanaceae Iochroma australe#NC 029833
Solanales Solanaceae Iochroma ellipticum#NC 030177
Solanales Solanaceae Iochroma cyaneum#NC 030178
Solanales Solanaceae Acnistus arborescens#NC 030185
Solanales Solanaceae Iochroma stenanthum#NC 026574
Solanales Solanaceae Iochroma loxense#NC 026726
Solanales Solanaceae Iochroma salpoanum#NC 030168
Solanales Solanaceae Dunalia solanacea#NC 027099
Solanales Solanaceae Iochroma nitidum#NC 026567
Solanales Solanaceae Dunalia obovata#NC 026563
Solanales Solanaceae Dunalia brachyacantha#NC 026906
Solanales Solanaceae Saracha punctata#NC 026694
Solanales Solanaceae Iochroma lehmannii#NC 030167
Solanales Solanaceae Iochroma tingoanum#NC 027177

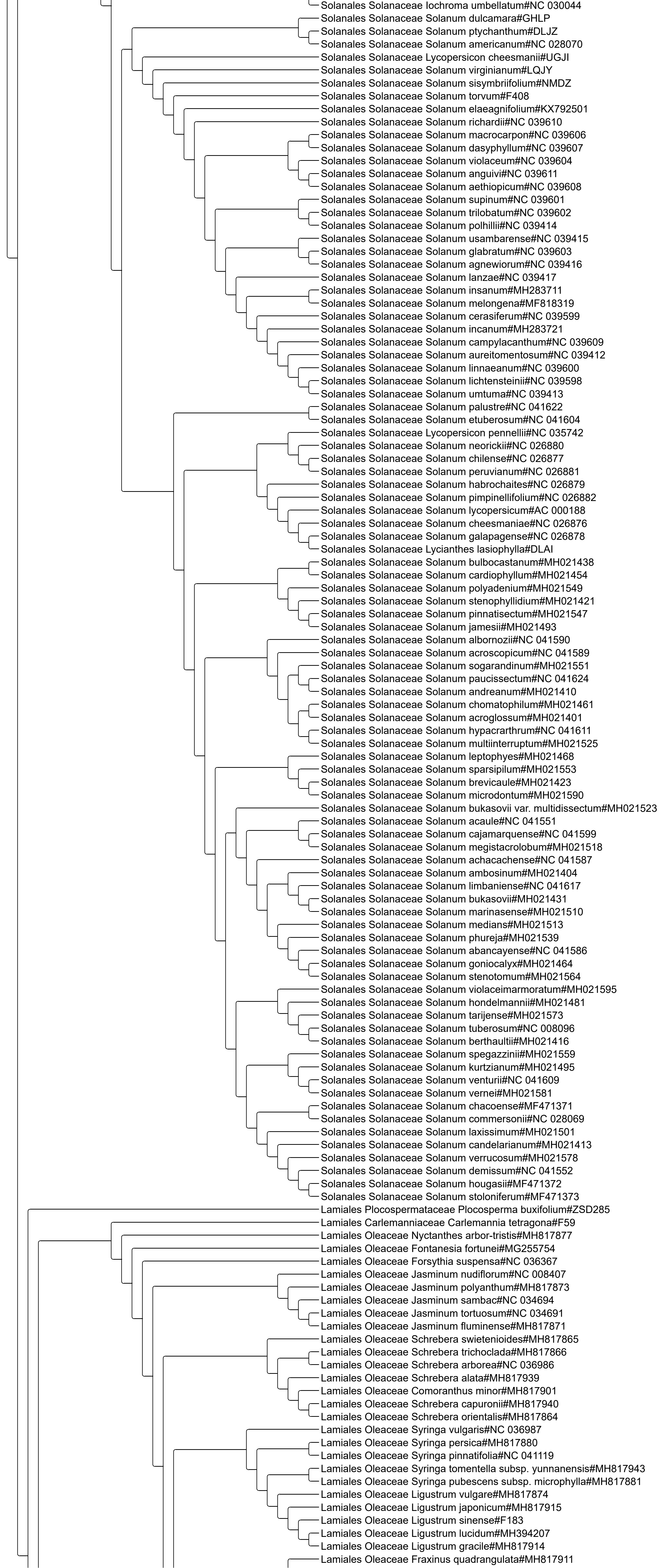
Solanales Solanaceae Iochroma umbellatum#NC 030044
Solanales Solanaceae Solanum dulcamara#GHLP
Solanales Solanaceae Solanum ptychanthum#DLJZ
Solanales Solanaceae Solanum americanum#NC 028070
Solanales Solanaceae Lycopersicon cheesmanii#UGJI
Solanales Solanaceae Solanum virginianum#LQJY
Solanales Solanaceae Solanum sisymbriifolium#NMDZ
Solanales Solanaceae Solanum torvum#F408
Solanales Solanaceae Solanum elaeagnifolium#KX792501
Solanales Solanaceae Solanum richardii#NC 039610
Solanales Solanaceae Solanum macrocarpon#NC 039606
Solanales Solanaceae Solanum dasyphyllum#NC 039607
Solanales Solanaceae Solanum violaceum#NC 039604
Solanales Solanaceae Solanum anguivi#NC 039611
Solanales Solanaceae Solanum aethiopicum#NC 039608
Solanales Solanaceae Solanum supinum#NC 039601
Solanales Solanaceae Solanum trilobatum#NC 039602
Solanales Solanaceae Solanum polhillii#NC 039414
Solanales Solanaceae Solanum usambarense#NC 039415
Solanales Solanaceae Solanum glabratum#NC 039603
Solanales Solanaceae Solanum agnewiorum#NC 039416
Solanales Solanaceae Solanum lanzae#NC 039417
Solanales Solanaceae Solanum insanum#MH283711
Solanales Solanaceae Solanum melongena#MF818319
Solanales Solanaceae Solanum cerasiferum#NC 039599
Solanales Solanaceae Solanum incanum#MH283721
Solanales Solanaceae Solanum campylacanthum#NC 039609
Solanales Solanaceae Solanum aureitomentosum#NC 039412
Solanales Solanaceae Solanum linnaeanum#NC 039600
Solanales Solanaceae Solanum lichtensteinii#NC 039598
Solanales Solanaceae Solanum umtuma#NC 039413
Solanales Solanaceae Solanum palustre#NC 041622
Solanales Solanaceae Solanum etuberosum#NC 041604
Solanales Solanaceae Lycopersicon pennellii#NC 035742
Solanales Solanaceae Solanum neorickii#NC 026880
Solanales Solanaceae Solanum chilense#NC 026877
Solanales Solanaceae Solanum peruvianum#NC 026881
Solanales Solanaceae Solanum habrochaites#NC 026879
Solanales Solanaceae Solanum pimpinellifolium#NC 026882
Solanales Solanaceae Solanum lycopersicum#AC 000188
Solanales Solanaceae Solanum cheesmaniae#NC 026876
Solanales Solanaceae Solanum galapagense#NC 026878
Solanales Solanaceae Lycianthes lasiophylla#DLAI
Solanales Solanaceae Solanum bulbocastanum#MH021438
Solanales Solanaceae Solanum cardiophyllum#MH021454
Solanales Solanaceae Solanum polyadenium#MH021549
Solanales Solanaceae Solanum stenophyllidium#MH021421
Solanales Solanaceae Solanum pinnatisectum#MH021547
Solanales Solanaceae Solanum jamesii#MH021493
Solanales Solanaceae Solanum albornozii#NC 041590
Solanales Solanaceae Solanum acroscopicum#NC 041589
Solanales Solanaceae Solanum sogarandinum#MH021551
Solanales Solanaceae Solanum paucissectum#NC 041624
Solanales Solanaceae Solanum andreanum#MH021410
Solanales Solanaceae Solanum chomatophilum#MH021461
Solanales Solanaceae Solanum acroglossum#MH021401
Solanales Solanaceae Solanum hypacrarthrum#NC 041611
Solanales Solanaceae Solanum multiinterruptum#MH021525
Solanales Solanaceae Solanum leptophyes#MH021468
Solanales Solanaceae Solanum sparsipilum#MH021553
Solanales Solanaceae Solanum brevicaule#MH021423
Solanales Solanaceae Solanum microdontum#MH021590
Solanales Solanaceae Solanum bukasovii var. multidissectum#MH021523
Solanales Solanaceae Solanum acaule#NC 041551
Solanales Solanaceae Solanum cajamarquense#NC 041599
Solanales Solanaceae Solanum megistacrolobum#MH021518
Solanales Solanaceae Solanum achacachense#NC 041587
Solanales Solanaceae Solanum ambosinum#MH021404
Solanales Solanaceae Solanum limbaniense#NC 041617
Solanales Solanaceae Solanum bukasovii#MH021431
Solanales Solanaceae Solanum marinasense#MH021510
Solanales Solanaceae Solanum medians#MH021513
Solanales Solanaceae Solanum phureja#MH021539
Solanales Solanaceae Solanum abancayense#NC 041586
Solanales Solanaceae Solanum goniocalyx#MH021464
Solanales Solanaceae Solanum stenotomum#MH021564
Solanales Solanaceae Solanum violaceimarmoratum#MH021595
Solanales Solanaceae Solanum hondelmannii#MH021481
Solanales Solanaceae Solanum tarijense#MH021573
Solanales Solanaceae Solanum tuberosum#NC 008096
Solanales Solanaceae Solanum berthaultii#MH021416
Solanales Solanaceae Solanum spegazzinii#MH021559
Solanales Solanaceae Solanum kurtzianum#MH021495
Solanales Solanaceae Solanum venturii#NC 041609
Solanales Solanaceae Solanum vernei#MH021581
Solanales Solanaceae Solanum chacoense#MF471371
Solanales Solanaceae Solanum commersonii#NC 028069
Solanales Solanaceae Solanum laxissimum#MH021501
Solanales Solanaceae Solanum candelarianum#MH021413
Solanales Solanaceae Solanum verrucosum#MH021578
Solanales Solanaceae Solanum demissum#NC 041552
Solanales Solanaceae Solanum hougasii#MF471372
Solanales Solanaceae Solanum stoloniferum#MF471373
Lamiales Plocospermataceae Plocosperma buxifolium#ZSD285
Lamiales Carlemanniaceae Carlemannia tetragona#F59
Lamiales Oleaceae Nyctanthes arbor-tristis#MH817877
Lamiales Oleaceae Fontanesia fortunei#MG255754
Lamiales Oleaceae Forsythia suspensa#NC 036367
Lamiales Oleaceae Jasminum nudiflorum#NC 008407
Lamiales Oleaceae Jasminum polyanthum#MH817873
Lamiales Oleaceae Jasminum sambac#NC 034694
Lamiales Oleaceae Jasminum tortuosum#NC 034691
Lamiales Oleaceae Jasminum fluminense#MH817871
Lamiales Oleaceae Schrebera swietenioides#MH817865
Lamiales Oleaceae Schrebera trichoclada#MH817866
Lamiales Oleaceae Schrebera arborea#NC 036986
Lamiales Oleaceae Schrebera alata#MH817939
Lamiales Oleaceae Comoranthus minor#MH817901
Lamiales Oleaceae Schrebera capuronii#MH817940
Lamiales Oleaceae Schrebera orientalis#MH817864
Lamiales Oleaceae Syringa vulgaris#NC 036987
Lamiales Oleaceae Syringa persica#MH817880
Lamiales Oleaceae Syringa pinnatifolia#NC 041119
Lamiales Oleaceae Syringa tomentella subsp. yunnanensis#MH817943
Lamiales Oleaceae Syringa pubescens subsp. microphylla#MH817881
Lamiales Oleaceae Ligustrum vulgare#MH817874
Lamiales Oleaceae Ligustrum japonicum#MH817915
Lamiales Oleaceae Ligustrum sinense#F183
Lamiales Oleaceae Ligustrum lucidum#MH394207
Lamiales Oleaceae Ligustrum gracile#MH817914
Lamiales Oleaceae Fraxinus quadrangulata#MH817911

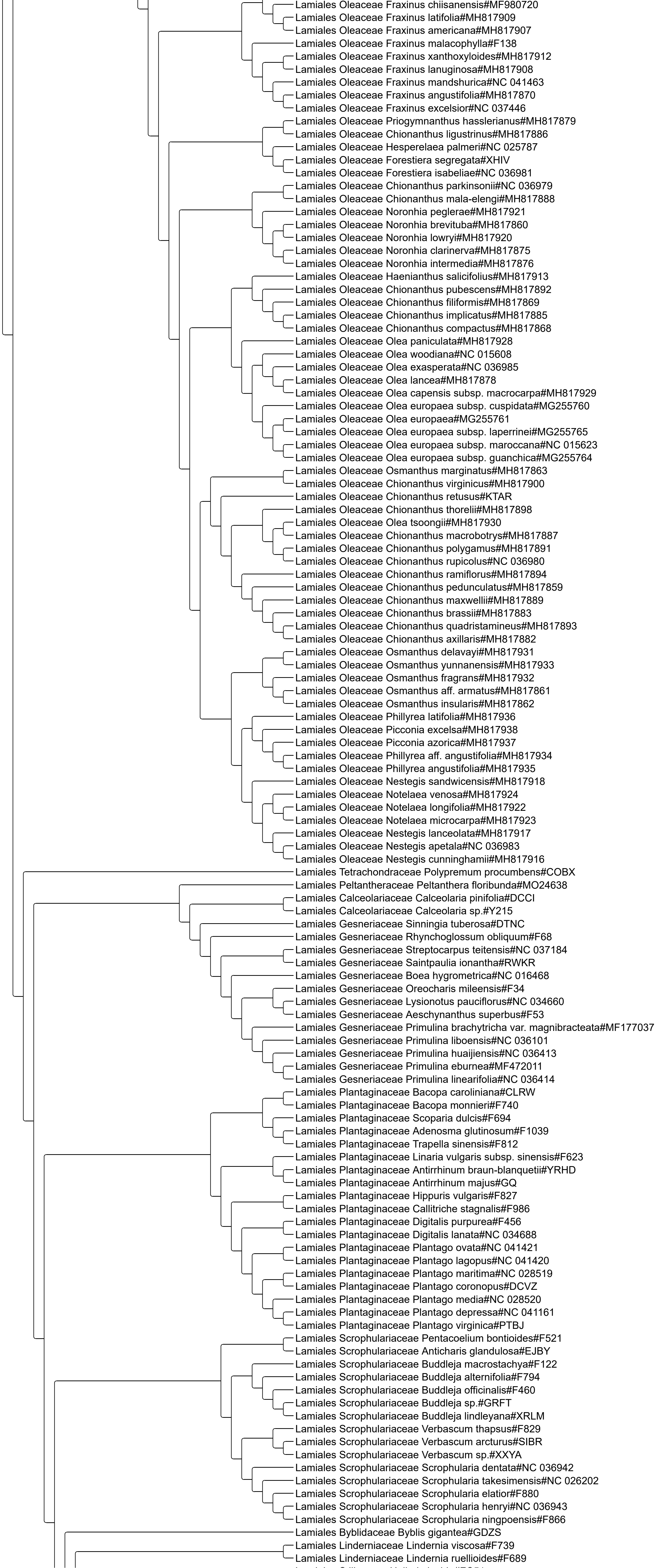

Lamiales Oleaceae Fraxinus chiisanensis#MF980720
Lamiales Oleaceae Fraxinus latifolia#MH817909
Lamiales Oleaceae Fraxinus americana#MH817907
Lamiales Oleaceae Fraxinus malacophylla#F138
Lamiales Oleaceae Fraxinus xanthoxyloides#MH817912
Lamiales Oleaceae Fraxinus lanuginosa#MH817908
Lamiales Oleaceae Fraxinus mandshurica#NC 041463
Lamiales Oleaceae Fraxinus angustifolia#MH817870
Lamiales Oleaceae Fraxinus excelsior#NC 037446
Lamiales Oleaceae Priogymnanthus hasslerianus#MH817879
Lamiales Oleaceae Chionanthus ligustrinus#MH817886
Lamiales Oleaceae Hesperelaea palmeri#NC 025787
Lamiales Oleaceae Forestiera segregata#XHIV
Lamiales Oleaceae Forestiera isabeliae#NC 036981
Lamiales Oleaceae Chionanthus parkinsonii#NC 036979
Lamiales Oleaceae Chionanthus mala-elengi#MH817888
Lamiales Oleaceae Noronhia peglerae#MH817921
Lamiales Oleaceae Noronhia brevituba#MH817860
Lamiales Oleaceae Noronhia lowryi#MH817920
Lamiales Oleaceae Noronhia clarinerva#MH817875
Lamiales Oleaceae Noronhia intermedia#MH817876
Lamiales Oleaceae Haenianthus salicifolius#MH817913
Lamiales Oleaceae Chionanthus pubescens#MH817892
Lamiales Oleaceae Chionanthus filiformis#MH817869
Lamiales Oleaceae Chionanthus implicatus#MH817885
Lamiales Oleaceae Chionanthus compactus#MH817868
Lamiales Oleaceae Olea paniculata#MH817928
Lamiales Oleaceae Olea woodiana#NC 015608
Lamiales Oleaceae Olea exasperata#NC 036985
Lamiales Oleaceae Olea lancea#MH817878
Lamiales Oleaceae Olea capensis subsp. macrocarpa#MH817929
Lamiales Oleaceae Olea europaea subsp. cuspidata#MG255760
Lamiales Oleaceae Olea europaea#MG255761
Lamiales Oleaceae Olea europaea subsp. laperrinei#MG255765
Lamiales Oleaceae Olea europaea subsp. maroccana#NC 015623
Lamiales Oleaceae Olea europaea subsp. guanchica#MG255764
Lamiales Oleaceae Osmanthus marginatus#MH817863
Lamiales Oleaceae Chionanthus virginicus#MH817900
Lamiales Oleaceae Chionanthus retusus#KTAR
Lamiales Oleaceae Chionanthus thorelii#MH817898
Lamiales Oleaceae Olea tsoongii#MH817930
Lamiales Oleaceae Chionanthus macrobotrys#MH817887
Lamiales Oleaceae Chionanthus polygamus#MH817891
Lamiales Oleaceae Chionanthus rupicolus#NC 036980
Lamiales Oleaceae Chionanthus ramiflorus#MH817894
Lamiales Oleaceae Chionanthus pedunculatus#MH817859
Lamiales Oleaceae Chionanthus maxwellii#MH817889
Lamiales Oleaceae Chionanthus brassii#MH817883
Lamiales Oleaceae Chionanthus quadristamineus#MH817893
Lamiales Oleaceae Chionanthus axillaris#MH817882
Lamiales Oleaceae Osmanthus delavayi#MH817931
Lamiales Oleaceae Osmanthus yunnanensis#MH817933
Lamiales Oleaceae Osmanthus fragrans#MH817932
Lamiales Oleaceae Osmanthus aff. armatus#MH817861
Lamiales Oleaceae Osmanthus insularis#MH817862
Lamiales Oleaceae Phillyrea latifolia#MH817936
Lamiales Oleaceae Picconia excelsa#MH817938
Lamiales Oleaceae Picconia azorica#MH817937
Lamiales Oleaceae Phillyrea aff. angustifolia#MH817934
Lamiales Oleaceae Phillyrea angustifolia#MH817935
Lamiales Oleaceae Nestegis sandwicensis#MH817918
Lamiales Oleaceae Notelaea venosa#MH817924
Lamiales Oleaceae Notelaea longifolia#MH817922
Lamiales Oleaceae Notelaea microcarpa#MH817923
Lamiales Oleaceae Nestegis lanceolata#MH817917
Lamiales Oleaceae Nestegis apetala#NC 036983
Lamiales Oleaceae Nestegis cunninghamii#MH817916
Lamiales Tetrachondraceae Polypremum procumbens#COBX
Lamiales Peltantheraceae Peltanthera floribunda#MO24638
Lamiales Calceolariaceae Calceolaria pinifolia#DCCI
Lamiales Calceolariaceae Calceolaria sp.#Y215
Lamiales Gesneriaceae Sinningia tuberosa#DTNC
Lamiales Gesneriaceae Rhynchoglossum obliquum#F68
Lamiales Gesneriaceae Streptocarpus teitensis#NC 037184
Lamiales Gesneriaceae Saintpaulia ionantha#RWKR
Lamiales Gesneriaceae Boea hygrometrica#NC 016468
Lamiales Gesneriaceae Oreocharis mileensis#F34
Lamiales Gesneriaceae Lysionotus pauciflorus#NC 034660
Lamiales Gesneriaceae Aeschynanthus superbus#F53
Lamiales Gesneriaceae Primulina brachytricha var. magnibracteata#MF177037
Lamiales Gesneriaceae Primulina liboensis#NC 036101
Lamiales Gesneriaceae Primulina huaijiensis#NC 036413
Lamiales Gesneriaceae Primulina eburnea#MF472011
Lamiales Gesneriaceae Primulina linearifolia#NC 036414
Lamiales Plantaginaceae Bacopa caroliniana#CLRW
Lamiales Plantaginaceae Bacopa monnieri#F740
Lamiales Plantaginaceae Scoparia dulcis#F694
Lamiales Plantaginaceae Adenosma glutinosum#F1039
Lamiales Plantaginaceae Trapella sinensis#F812
Lamiales Plantaginaceae Linaria vulgaris subsp. sinensis#F623
Lamiales Plantaginaceae Antirrhinum braun-blanquetii#YRHD
Lamiales Plantaginaceae Antirrhinum majus#GQ
Lamiales Plantaginaceae Hippuris vulgaris#F827
Lamiales Plantaginaceae Callitriche stagnalis#F986
Lamiales Plantaginaceae Digitalis purpurea#F456
Lamiales Plantaginaceae Digitalis lanata#NC 034688
Lamiales Plantaginaceae Plantago ovata#NC 041421
Lamiales Plantaginaceae Plantago lagopus#NC 041420
Lamiales Plantaginaceae Plantago maritima#NC 028519
Lamiales Plantaginaceae Plantago coronopus#DCVZ
Lamiales Plantaginaceae Plantago media#NC 028520
Lamiales Plantaginaceae Plantago depressa#NC 041161
Lamiales Plantaginaceae Plantago virginica#PTBJ
Lamiales Scrophulariaceae Pentacoelium bontioides#F521
Lamiales Scrophulariaceae Anticharis glandulosa#EJBY
Lamiales Scrophulariaceae Buddleja macrostachya#F122
Lamiales Scrophulariaceae Buddleja alternifolia#F794
Lamiales Scrophulariaceae Buddleja officinalis#F460
Lamiales Scrophulariaceae Buddleja sp.#GRFT
Lamiales Scrophulariaceae Buddleja lindleyana#XRLM
Lamiales Scrophulariaceae Verbascum thapsus#F829
Lamiales Scrophulariaceae Verbascum arcturus#SIBR
Lamiales Scrophulariaceae Verbascum sp.#XXYA
Lamiales Scrophulariaceae Scrophularia dentata#NC 036942
Lamiales Scrophulariaceae Scrophularia takesimensis#NC 026202
Lamiales Scrophulariaceae Scrophularia elatior#F880
Lamiales Scrophulariaceae Scrophularia henryi#NC 036943
Lamiales Scrophulariaceae Scrophularia ningpoensis#F866
Lamiales Byblidaceae Byblis gigantea#GDZS
Lamiales Linderniaceae Lindernia viscosa#F739
Lamiales Linderniaceae Lindernia ruellioides#F689

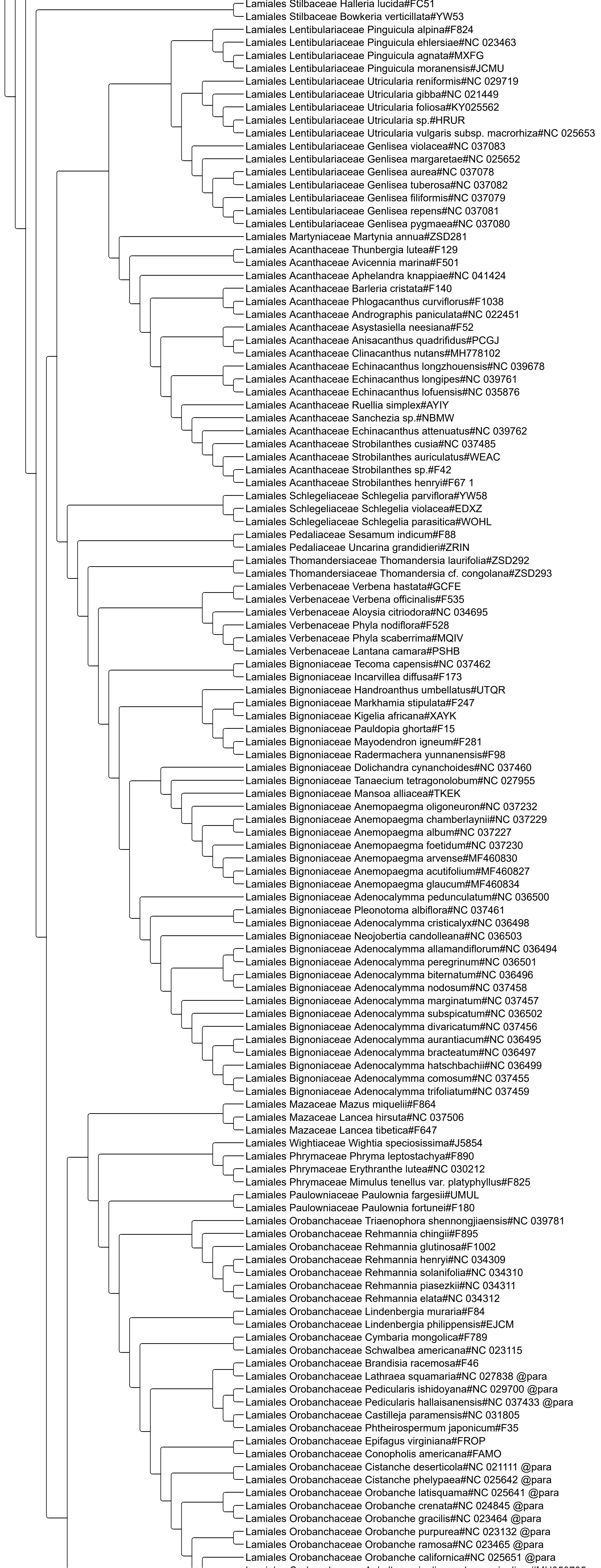
Lamiales Stilbaceae Halleria lucida#FC51
Lamiales Stilbaceae Bowkeria verticillata#YW53
Lamiales Lentibulariaceae Pinguicula alpina#F824
Lamiales Lentibulariaceae Pinguicula ehlersiae#NC 023463
Lamiales Lentibulariaceae Pinguicula agnata#MXFG
Lamiales Lentibulariaceae Pinguicula moranensis#JCMU
Lamiales Lentibulariaceae Utricularia reniformis#NC 029719
Lamiales Lentibulariaceae Utricularia gibba#NC 021449
Lamiales Lentibulariaceae Utricularia foliosa#KY025562
Lamiales Lentibulariaceae Utricularia sp.#HRUR
Lamiales Lentibulariaceae Utricularia vulgaris subsp. macrorhiza#NC 025653
Lamiales Lentibulariaceae Genlisea violacea#NC 037083
Lamiales Lentibulariaceae Genlisea margaretae#NC 025652
Lamiales Lentibulariaceae Genlisea aurea#NC 037078
Lamiales Lentibulariaceae Genlisea tuberosa#NC 037082
Lamiales Lentibulariaceae Genlisea filiformis#NC 037079
Lamiales Lentibulariaceae Genlisea repens#NC 037081
Lamiales Lentibulariaceae Genlisea pygmaea#NC 037080
Lamiales Martyniaceae Martynia annua#ZSD281
Lamiales Acanthaceae Thunbergia lutea#F129
Lamiales Acanthaceae Avicennia marina#F501
Lamiales Acanthaceae Aphelandra knappiae#NC 041424
Lamiales Acanthaceae Barleria cristata#F140
Lamiales Acanthaceae Phlogacanthus curviflorus#F1038
Lamiales Acanthaceae Andrographis paniculata#NC 022451
Lamiales Acanthaceae Asystasiella neesiana#F52
Lamiales Acanthaceae Anisacanthus quadrifidus#PCGJ
Lamiales Acanthaceae Clinacanthus nutans#MH778102
Lamiales Acanthaceae Echinacanthus longzhouensis#NC 039678
Lamiales Acanthaceae Echinacanthus longipes#NC 039761
Lamiales Acanthaceae Echinacanthus lofuensis#NC 035876
Lamiales Acanthaceae Ruellia simplex#AYIY
Lamiales Acanthaceae Sanchezia sp.#NBMW
Lamiales Acanthaceae Echinacanthus attenuatus#NC 039762
Lamiales Acanthaceae Strobilanthes cusia#NC 037485
Lamiales Acanthaceae Strobilanthes auriculatus#WEAC
Lamiales Acanthaceae Strobilanthes sp.#F42
Lamiales Acanthaceae Strobilanthes henryi#F67 1
Lamiales Schlegeliaceae Schlegelia parviflora#YW58
Lamiales Schlegeliaceae Schlegelia violacea#EDXZ
Lamiales Schlegeliaceae Schlegelia parasitica#WOHL
Lamiales Pedaliaceae Sesamum indicum#F88
Lamiales Pedaliaceae Uncarina grandidieri#ZRIN
Lamiales Thomandersiaceae Thomandersia laurifolia#ZSD292
Lamiales Thomandersiaceae Thomandersia cf. congolana#ZSD293
Lamiales Verbenaceae Verbena hastata#GCFE
Lamiales Verbenaceae Verbena officinalis#F535
Lamiales Verbenaceae Aloysia citriodora#NC 034695
Lamiales Verbenaceae Phyla nodiflora#F528
Lamiales Verbenaceae Phyla scaberrima#MQIV
Lamiales Verbenaceae Lantana camara#PSHB
Lamiales Bignoniaceae Tecoma capensis#NC 037462
Lamiales Bignoniaceae Incarvillea diffusa#F173
Lamiales Bignoniaceae Handroanthus umbellatus#UTQR
Lamiales Bignoniaceae Markhamia stipulata#F247
Lamiales Bignoniaceae Kigelia africana#XAYK
Lamiales Bignoniaceae Pauldopia ghorta#F15
Lamiales Bignoniaceae Mayodendron igneum#F281
Lamiales Bignoniaceae Radermachera yunnanensis#F98
Lamiales Bignoniaceae Dolichandra cynanchoides#NC 037460
Lamiales Bignoniaceae Tanaecium tetragonolobum#NC 027955
Lamiales Bignoniaceae Mansoa alliacea#TKEK
Lamiales Bignoniaceae Anemopaegma oligoneuron#NC 037232
Lamiales Bignoniaceae Anemopaegma chamberlaynii#NC 037229
Lamiales Bignoniaceae Anemopaegma album#NC 037227
Lamiales Bignoniaceae Anemopaegma foetidum#NC 037230
Lamiales Bignoniaceae Anemopaegma arvense#MF460830
Lamiales Bignoniaceae Anemopaegma acutifolium#MF460827
Lamiales Bignoniaceae Anemopaegma glaucum#MF460834
Lamiales Bignoniaceae Adenocalymma pedunculatum#NC 036500
Lamiales Bignoniaceae Pleonotoma albiflora#NC 037461
Lamiales Bignoniaceae Adenocalymma cristicalyx#NC 036498
Lamiales Bignoniaceae Neojobertia candolleana#NC 036503
Lamiales Bignoniaceae Adenocalymma allamandiflorum#NC 036494
Lamiales Bignoniaceae Adenocalymma peregrinum#NC 036501
Lamiales Bignoniaceae Adenocalymma biternatum#NC 036496
Lamiales Bignoniaceae Adenocalymma nodosum#NC 037458
Lamiales Bignoniaceae Adenocalymma marginatum#NC 037457
Lamiales Bignoniaceae Adenocalymma subspicatum#NC 036502
Lamiales Bignoniaceae Adenocalymma divaricatum#NC 037456
Lamiales Bignoniaceae Adenocalymma aurantiacum#NC 036495
Lamiales Bignoniaceae Adenocalymma bracteatum#NC 036497
Lamiales Bignoniaceae Adenocalymma hatschbachii#NC 036499
Lamiales Bignoniaceae Adenocalymma comosum#NC 037455
Lamiales Bignoniaceae Adenocalymma trifoliatum#NC 037459
Lamiales Mazaceae Mazus miquelii#F864
Lamiales Mazaceae Lancea hirsuta#NC 037506
Lamiales Mazaceae Lancea tibetica#F647
Lamiales Wightiaceae Wightia speciosissima#J5854
Lamiales Phrymaceae Phryma leptostachya#F890
Lamiales Phrymaceae Erythranthe lutea#NC 030212
Lamiales Phrymaceae Mimulus tenellus var. platyphyllus#F825
Lamiales Paulowniaceae Paulownia fargesii#UMUL
Lamiales Paulowniaceae Paulownia fortunei#F180
Lamiales Orobanchaceae Triaenophora shennongjiaensis#NC 039781
Lamiales Orobanchaceae Rehmannia chingii#F895
Lamiales Orobanchaceae Rehmannia glutinosa#F1002
Lamiales Orobanchaceae Rehmannia henryi#NC 034309
Lamiales Orobanchaceae Rehmannia solanifolia#NC 034310
Lamiales Orobanchaceae Rehmannia piasezkii#NC 034311
Lamiales Orobanchaceae Rehmannia elata#NC 034312
Lamiales Orobanchaceae Lindenbergia muraria#F84
Lamiales Orobanchaceae Lindenbergia philippensis#EJCM
Lamiales Orobanchaceae Cymbaria mongolica#F789
Lamiales Orobanchaceae Schwalbea americana#NC 023115
Lamiales Orobanchaceae Brandisia racemosa#F46
Lamiales Orobanchaceae Lathraea squamaria#NC 027838 @para
Lamiales Orobanchaceae Pedicularis ishidoyana#NC 029700 @para
Lamiales Orobanchaceae Pedicularis hallaisanensis#NC 037433 @para
Lamiales Orobanchaceae Castilleja paramensis#NC 031805
Lamiales Orobanchaceae Phtheirospermum japonicum#F35
Lamiales Orobanchaceae Epifagus virginiana#FROP
Lamiales Orobanchaceae Conopholis americana#FAMO
Lamiales Orobanchaceae Cistanche deserticola#NC 021111 @para
Lamiales Orobanchaceae Cistanche phelypaea#NC 025642 @para
Lamiales Orobanchaceae Orobanche latisquama#NC 025641 @para
Lamiales Orobanchaceae Orobanche crenata#NC 024845 @para
Lamiales Orobanchaceae Orobanche gracilis#NC 023464 @para
Lamiales Orobanchaceae Orobanche purpurea#NC 023132 @para
Lamiales Orobanchaceae Orobanche ramosa#NC 023465 @para
Lamiales Orobanchaceae Orobanche californica#NC 025651 @para

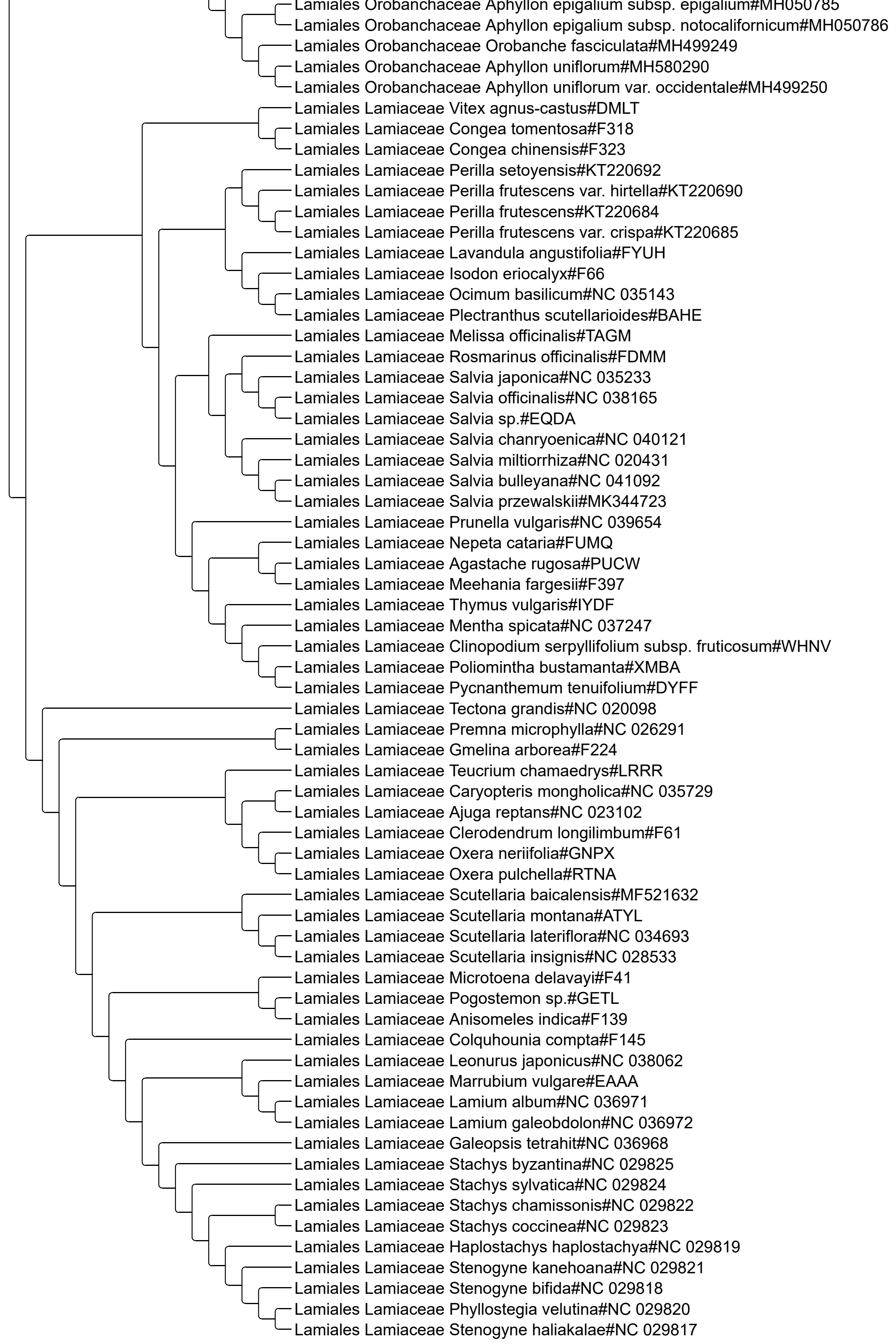
Lamiales Orobanchaceae Aphyllon epigalium subsp. epigalium#MH050785
Lamiales Orobanchaceae Aphyllon epigalium subsp. notocalifornicum#MH050786
Lamiales Orobanchaceae Orobanche fasciculata#MH499249
Lamiales Orobanchaceae Aphyllon uniflorum#MH580290
Lamiales Orobanchaceae Aphyllon uniflorum var. occidentale#MH499250
Lamiales Lamiaceae Vitex agnus-castus#DMLT
Lamiales Lamiaceae Congea tomentosa#F318
Lamiales Lamiaceae Congea chinensis#F323
Lamiales Lamiaceae Perilla setoyensis#KT220692
Lamiales Lamiaceae Perilla frutescens var. hirtella#KT220690
Lamiales Lamiaceae Perilla frutescens#KT220684
Lamiales Lamiaceae Perilla frutescens var. crispa#KT220685
Lamiales Lamiaceae Lavandula angustifolia#FYUH
Lamiales Lamiaceae Isodon eriocalyx#F66
Lamiales Lamiaceae Ocimum basilicum#NC 035143
Lamiales Lamiaceae Plectranthus scutellarioides#BAHE
Lamiales Lamiaceae Melissa officinalis#TAGM
Lamiales Lamiaceae Rosmarinus officinalis#FDMM
Lamiales Lamiaceae Salvia japonica#NC 035233
Lamiales Lamiaceae Salvia officinalis#NC 038165
Lamiales Lamiaceae Salvia sp.#EQDA
Lamiales Lamiaceae Salvia chanryoenica#NC 040121
Lamiales Lamiaceae Salvia miltiorrhiza#NC 020431
Lamiales Lamiaceae Salvia bulleyana#NC 041092
Lamiales Lamiaceae Salvia przewalskii#MK344723
Lamiales Lamiaceae Prunella vulgaris#NC 039654
Lamiales Lamiaceae Nepeta cataria#FUMQ
Lamiales Lamiaceae Agastache rugosa#PUCW
Lamiales Lamiaceae Meehania fargesii#F397
Lamiales Lamiaceae Thymus vulgaris#IYDF
Lamiales Lamiaceae Mentha spicata#NC 037247
Lamiales Lamiaceae Clinopodium serpyllifolium subsp. fruticosum#WHNV
Lamiales Lamiaceae Poliomintha bustamanta#XMBA
Lamiales Lamiaceae Pycnanthemum tenuifolium#DYFF
Lamiales Lamiaceae Tectona grandis#NC 020098
Lamiales Lamiaceae Premna microphylla#NC 026291
Lamiales Lamiaceae Gmelina arborea#F224
Lamiales Lamiaceae Teucrium chamaedrys#LRRR
Lamiales Lamiaceae Caryopteris mongholica#NC 035729
Lamiales Lamiaceae Ajuga reptans#NC 023102
Lamiales Lamiaceae Clerodendrum longilimbum#F61
Lamiales Lamiaceae Oxera neriifolia#GNPX
Lamiales Lamiaceae Oxera pulchella#RTNA
Lamiales Lamiaceae Scutellaria baicalensis#MF521632
Lamiales Lamiaceae Scutellaria montana#ATYL
Lamiales Lamiaceae Scutellaria lateriflora#NC 034693
Lamiales Lamiaceae Scutellaria insignis#NC 028533
Lamiales Lamiaceae Microtoena delavayi#F41
Lamiales Lamiaceae Pogostemon sp.#GETL
Lamiales Lamiaceae Anisomeles indica#F139
Lamiales Lamiaceae Colquhounia compta#F145
Lamiales Lamiaceae Leonurus japonicus#NC 038062
Lamiales Lamiaceae Marrubium vulgare#EAAA
Lamiales Lamiaceae Lamium album#NC 036971
Lamiales Lamiaceae Lamium galeobdolon#NC 036972
Lamiales Lamiaceae Galeopsis tetrahit#NC 036968
Lamiales Lamiaceae Stachys byzantina#NC 029825
Lamiales Lamiaceae Stachys sylvatica#NC 029824
Lamiales Lamiaceae Stachys chamissonis#NC 029822
Lamiales Lamiaceae Stachys coccinea#NC 029823
Lamiales Lamiaceae Haplostachys haplostachya#NC 029819
Lamiales Lamiaceae Stenogyne kanehoana#NC 029821
Lamiales Lamiaceae Stenogyne bifida#NC 029818
Lamiales Lamiaceae Phyllostegia velutina#NC 029820
Lamiales Lamiaceae Stenogyne haliakalae#NC 029817

[Figure S8c]

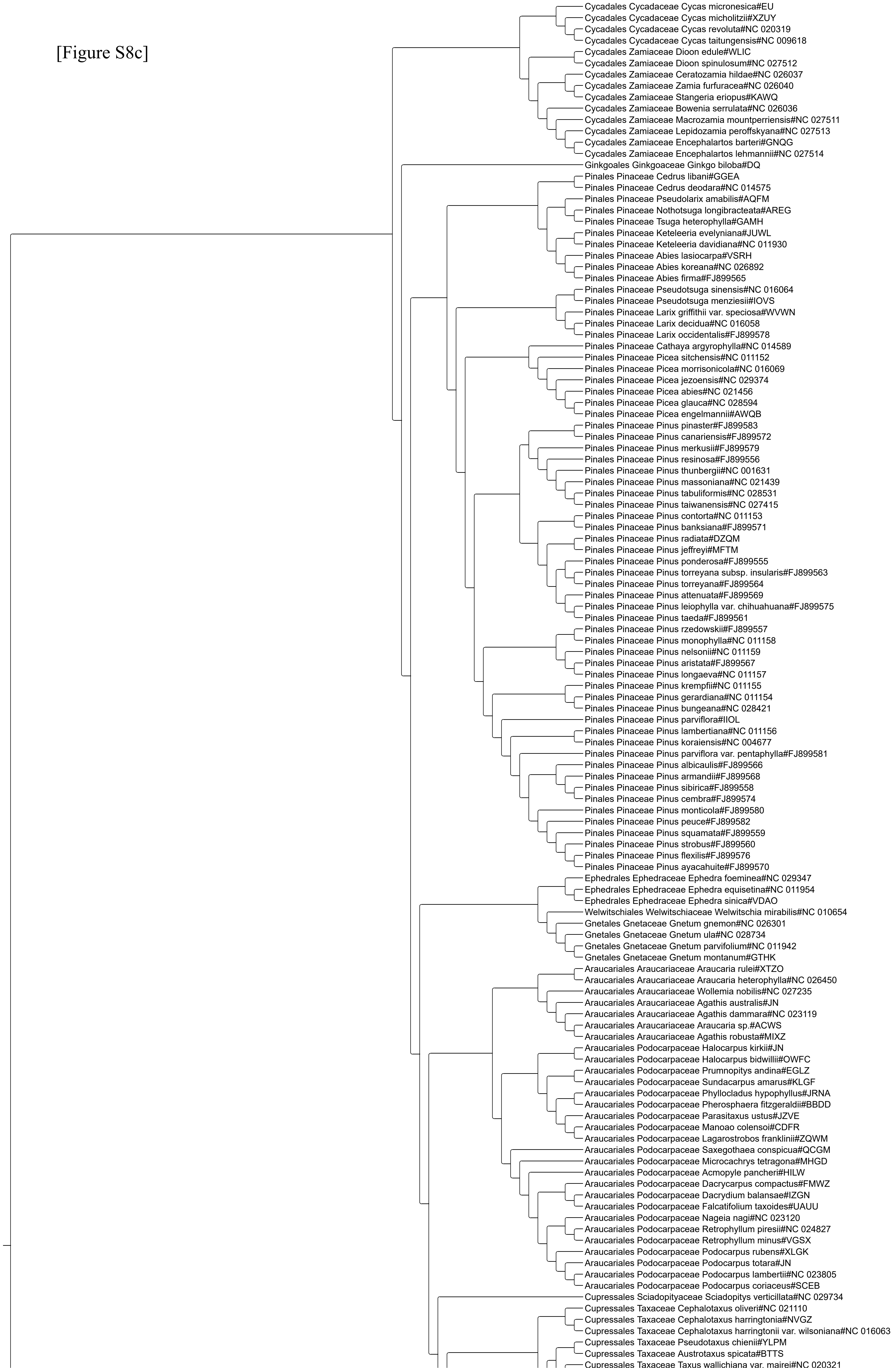

Cycadales Cycadaceae Cycas micronesica#EU
Cycadales Cycadaceae Cycas micholitzii#XZUY
Cycadales Cycadaceae Cycas revoluta#NC 020319
Cycadales Cycadaceae Cycas taitungensis#NC 009618
Cycadales Zamiaceae Dioon edule#WLIC
Cycadales Zamiaceae Dioon spinulosum#NC 027512
Cycadales Zamiaceae Ceratozamia hildae#NC 026037
Cycadales Zamiaceae Zamia furfuracea#NC 026040
Cycadales Zamiaceae Stangeria eriopus#KAWQ
Cycadales Zamiaceae Bowenia serrulata#NC 026036
Cycadales Zamiaceae Macrozamia mountperriensis#NC 027511
Cycadales Zamiaceae Lepidozamia peroffskyana#NC 027513
Cycadales Zamiaceae Encephalartos barteri#GNQG
Cycadales Zamiaceae Encephalartos lehmannii#NC 027514
Ginkgoales Ginkgoaceae Ginkgo biloba#DQ
Pinales Pinaceae Cedrus libani#GGEA
Pinales Pinaceae Cedrus deodara#NC 014575
Pinales Pinaceae Pseudolarix amabilis#AQFM
Pinales Pinaceae Nothotsuga longibracteata#AREG
Pinales Pinaceae Tsuga heterophylla#GAMH
Pinales Pinaceae Keteleeria evelyniana#JUWL
Pinales Pinaceae Keteleeria davidiana#NC 011930
Pinales Pinaceae Abies lasiocarpa#VSRH
Pinales Pinaceae Abies koreana#NC 026892
Pinales Pinaceae Abies firma#FJ899565
Pinales Pinaceae Pseudotsuga sinensis#NC 016064
Pinales Pinaceae Pseudotsuga menziesii#IOVS
Pinales Pinaceae Larix griffithii var. speciosa#WVWN
Pinales Pinaceae Larix decidua#NC 016058
Pinales Pinaceae Larix occidentalis#FJ899578
Pinales Pinaceae Cathaya argyrophylla#NC 014589
Pinales Pinaceae Picea sitchensis#NC 011152
Pinales Pinaceae Picea morrisonicola#NC 016069
Pinales Pinaceae Picea jezoensis#NC 029374
Pinales Pinaceae Picea abies#NC 021456
Pinales Pinaceae Picea glauca#NC 028594
Pinales Pinaceae Picea engelmannii#AWQB
Pinales Pinaceae Pinus pinaster#FJ899583
Pinales Pinaceae Pinus canariensis#FJ899572
Pinales Pinaceae Pinus merkusii#FJ899579
Pinales Pinaceae Pinus resinosa#FJ899556
Pinales Pinaceae Pinus thunbergii#NC 001631
Pinales Pinaceae Pinus massoniana#NC 021439
Pinales Pinaceae Pinus tabuliformis#NC 028531
Pinales Pinaceae Pinus taiwanensis#NC 027415
Pinales Pinaceae Pinus contorta#NC 011153
Pinales Pinaceae Pinus banksiana#FJ899571
Pinales Pinaceae Pinus radiata#DZQM
Pinales Pinaceae Pinus jeffreyi#MFTM
Pinales Pinaceae Pinus ponderosa#FJ899555
Pinales Pinaceae Pinus torreyana subsp. insularis#FJ899563
Pinales Pinaceae Pinus torreyana#FJ899564
Pinales Pinaceae Pinus attenuata#FJ899569
Pinales Pinaceae Pinus leiophylla var. chihuahuana#FJ899575
Pinales Pinaceae Pinus taeda#FJ899561
Pinales Pinaceae Pinus rzedowskii#FJ899557
Pinales Pinaceae Pinus monophylla#NC 011158
Pinales Pinaceae Pinus nelsonii#NC 011159
Pinales Pinaceae Pinus aristata#FJ899567
Pinales Pinaceae Pinus longaeva#NC 011157
Pinales Pinaceae Pinus krempfii#NC 011155
Pinales Pinaceae Pinus gerardiana#NC 011154
Pinales Pinaceae Pinus bungeana#NC 028421
Pinales Pinaceae Pinus parviflora#IIOL
Pinales Pinaceae Pinus lambertiana#NC 011156
Pinales Pinaceae Pinus koraiensis#NC 004677
Pinales Pinaceae Pinus parviflora var. pentaphylla#FJ899581
Pinales Pinaceae Pinus albicaulis#FJ899566
Pinales Pinaceae Pinus armandii#FJ899568
Pinales Pinaceae Pinus sibirica#FJ899558
Pinales Pinaceae Pinus cembra#FJ899574
Pinales Pinaceae Pinus monticola#FJ899580
Pinales Pinaceae Pinus peuce#FJ899582
Pinales Pinaceae Pinus squamata#FJ899559
Pinales Pinaceae Pinus strobus#FJ899560
Pinales Pinaceae Pinus flexilis#FJ899576
Pinales Pinaceae Pinus ayacahuite#FJ899570
Ephedrales Ephedraceae Ephedra foeminea#NC 029347
Ephedrales Ephedraceae Ephedra equisetina#NC 011954
Ephedrales Ephedraceae Ephedra sinica#VDAO
Welwitschiales Welwitschiaceae Welwitschia mirabilis#NC 010654
Gnetales Gnetaceae Gnetum gnemon#NC 026301
Gnetales Gnetaceae Gnetum ula#NC 028734
Gnetales Gnetaceae Gnetum parvifolium#NC 011942
Gnetales Gnetaceae Gnetum montanum#GTHK
Araucariales Araucariaceae Araucaria rulei#XTZO
Araucariales Araucariaceae Araucaria heterophylla#NC 026450
Araucariales Araucariaceae Wollemia nobilis#NC 027235
Araucariales Araucariaceae Agathis australis#JN
Araucariales Araucariaceae Agathis dammara#NC 023119
Araucariales Araucariaceae Araucaria sp.#ACWS
Araucariales Araucariaceae Agathis robusta#MIXZ
Araucariales Podocarpaceae Halocarpus kirkii#JN
Araucariales Podocarpaceae Halocarpus bidwillii#OWFC
Araucariales Podocarpaceae Prumnopitys andina#EGLZ
Araucariales Podocarpaceae Sundacarpus amarus#KLGF
Araucariales Podocarpaceae Phyllocladus hypophyllus#JRNA
Araucariales Podocarpaceae Pherosphaera fitzgeraldii#BBDD
Araucariales Podocarpaceae Parasitaxus ustus#JZVE
Araucariales Podocarpaceae Manoao colensoi#CDFR
Araucariales Podocarpaceae Lagarostrobos franklinii#ZQWM
Araucariales Podocarpaceae Saxegothaea conspicua#QCGM
Araucariales Podocarpaceae Microcachrys tetragona#MHGD
Araucariales Podocarpaceae Acmopyle pancheri#HILW
Araucariales Podocarpaceae Dacrycarpus compactus#FMWZ
Araucariales Podocarpaceae Dacrydium balansae#IZGN
Araucariales Podocarpaceae Falcatifolium taxoides#UAUU
Araucariales Podocarpaceae Nageia nagi#NC 023120
Araucariales Podocarpaceae Retrophyllum piresii#NC 024827
Araucariales Podocarpaceae Retrophyllum minus#VGSX
Araucariales Podocarpaceae Podocarpus rubens#XLGK
Araucariales Podocarpaceae Podocarpus totara#JN
Araucariales Podocarpaceae Podocarpus lambertii#NC 023805
Araucariales Podocarpaceae Podocarpus coriaceus#SCEB
Cupressales Sciadopityaceae Sciadopitys verticillata#NC 029734
Cupressales Taxaceae Cephalotaxus oliveri#NC 021110
Cupressales Taxaceae Cephalotaxus harringtonia#NVGZ
Cupressales Taxaceae Cephalotaxus harringtonii var. wilsoniana#NC 016063
Cupressales Taxaceae Pseudotaxus chienii#YLPM
Cupressales Taxaceae Austrotaxus spicata#BTTS
Cupressales Taxaceae Taxus wallichiana var. mairei#NC 020321

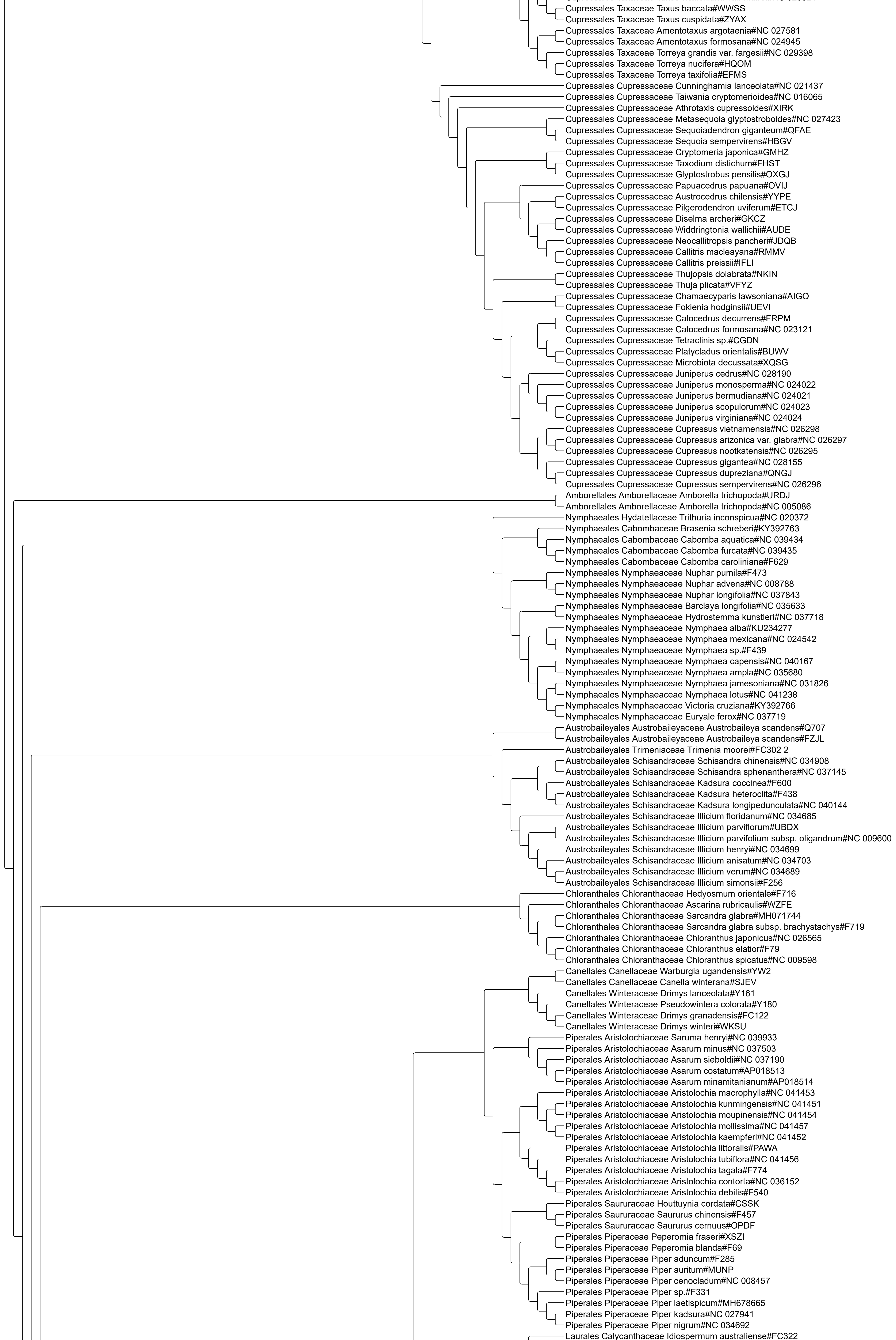

Cupressales Taxaceae Taxus baccata#WWSS
Cupressales Taxaceae Taxus cuspidata#ZYAX
Cupressales Taxaceae Amentotaxus argotaenia#NC 027581
Cupressales Taxaceae Amentotaxus formosana#NC 024945
Cupressales Taxaceae Torreya grandis var. fargesii#NC 029398
Cupressales Taxaceae Torreya nucifera#HQOM
Cupressales Taxaceae Torreya taxifolia#EFMS
Cupressales Cupressaceae Cunninghamia lanceolata#NC 021437
Cupressales Cupressaceae Taiwania cryptomerioides#NC 016065
Cupressales Cupressaceae Athrotaxis cupressoides#XIRK
Cupressales Cupressaceae Metasequoia glyptostroboides#NC 027423
Cupressales Cupressaceae Sequoiadendron giganteum#QFAE
Cupressales Cupressaceae Sequoia sempervirens#HBGV
Cupressales Cupressaceae Cryptomeria japonica#GMHZ
Cupressales Cupressaceae Taxodium distichum#FHST
Cupressales Cupressaceae Glyptostrobus pensilis#OXGJ
Cupressales Cupressaceae Papuacedrus papuana#OVIJ
Cupressales Cupressaceae Austrocedrus chilensis#YYPE
Cupressales Cupressaceae Pilgerodendron uviferum#ETCJ
Cupressales Cupressaceae Diselma archeri#GKCZ
Cupressales Cupressaceae Widdringtonia wallichii#AUDE
Cupressales Cupressaceae Neocallitropsis pancheri#JDQB
Cupressales Cupressaceae Callitris macleayana#RMMV
Cupressales Cupressaceae Callitris preissii#IFLI
Cupressales Cupressaceae Thujopsis dolabrata#NKIN
Cupressales Cupressaceae Thuja plicata#VFYZ
Cupressales Cupressaceae Chamaecyparis lawsoniana#AIGO
Cupressales Cupressaceae Fokienia hodginsii#UEVI
Cupressales Cupressaceae Calocedrus decurrens#FRPM
Cupressales Cupressaceae Calocedrus formosana#NC 023121
Cupressales Cupressaceae Tetraclinis sp.#CGDN
Cupressales Cupressaceae Platycladus orientalis#BUWV
Cupressales Cupressaceae Microbiota decussata#XQSG
Cupressales Cupressaceae Juniperus cedrus#NC 028190
Cupressales Cupressaceae Juniperus monosperma#NC 024022
Cupressales Cupressaceae Juniperus bermudiana#NC 024021
Cupressales Cupressaceae Juniperus scopulorum#NC 024023
Cupressales Cupressaceae Juniperus virginiana#NC 024024
Cupressales Cupressaceae Cupressus vietnamensis#NC 026298
Cupressales Cupressaceae Cupressus arizonica var. glabra#NC 026297
Cupressales Cupressaceae Cupressus nootkatensis#NC 026295
Cupressales Cupressaceae Cupressus gigantea#NC 028155
Cupressales Cupressaceae Cupressus dupreziana#QNGJ
Cupressales Cupressaceae Cupressus sempervirens#NC 026296
Amborellales Amborellaceae Amborella trichopoda#URDJ
Amborellales Amborellaceae Amborella trichopoda#NC 005086
Nymphaeales Hydatellaceae Trithuria inconspicua#NC 020372
Nymphaeales Cabombaceae Brasenia schreberi#KY392763
Nymphaeales Cabombaceae Cabomba aquatica#NC 039434
Nymphaeales Cabombaceae Cabomba furcata#NC 039435
Nymphaeales Cabombaceae Cabomba caroliniana#F629
Nymphaeales Nymphaeaceae Nuphar pumila#F473
Nymphaeales Nymphaeaceae Nuphar advena#NC 008788
Nymphaeales Nymphaeaceae Nuphar longifolia#NC 037843
Nymphaeales Nymphaeaceae Barclaya longifolia#NC 035633
Nymphaeales Nymphaeaceae Hydrostemma kunstleri#NC 037718
Nymphaeales Nymphaeaceae Nymphaea alba#KU234277
Nymphaeales Nymphaeaceae Nymphaea mexicana#NC 024542
Nymphaeales Nymphaeaceae Nymphaea sp.#F439
Nymphaeales Nymphaeaceae Nymphaea capensis#NC 040167
Nymphaeales Nymphaeaceae Nymphaea ampla#NC 035680
Nymphaeales Nymphaeaceae Nymphaea jamesoniana#NC 031826
Nymphaeales Nymphaeaceae Nymphaea lotus#NC 041238
Nymphaeales Nymphaeaceae Victoria cruziana#KY392766
Nymphaeales Nymphaeaceae Euryale ferox#NC 037719
Austrobaileyales Austrobaileyaceae Austrobaileya scandens#Q707
Austrobaileyales Austrobaileyaceae Austrobaileya scandens#FZJL
Austrobaileyales Trimeniaceae Trimenia moorei#FC302 2
Austrobaileyales Schisandraceae Schisandra chinensis#NC 034908
Austrobaileyales Schisandraceae Schisandra sphenanthera#NC 037145
Austrobaileyales Schisandraceae Kadsura coccinea#F600
Austrobaileyales Schisandraceae Kadsura heteroclita#F438
Austrobaileyales Schisandraceae Kadsura longipedunculata#NC 040144
Austrobaileyales Schisandraceae Illicium floridanum#NC 034685
Austrobaileyales Schisandraceae Illicium parviflorum#UBDX
Austrobaileyales Schisandraceae Illicium parvifolium subsp. oligandrum#NC 009600
Austrobaileyales Schisandraceae Illicium henryi#NC 034699
Austrobaileyales Schisandraceae Illicium anisatum#NC 034703
Austrobaileyales Schisandraceae Illicium verum#NC 034689
Austrobaileyales Schisandraceae Illicium simonsii#F256
Chloranthales Chloranthaceae Hedyosmum orientale#F716
Chloranthales Chloranthaceae Ascarina rubricaulis#WZFE
Chloranthales Chloranthaceae Sarcandra glabra#MH071744
Chloranthales Chloranthaceae Sarcandra glabra subsp. brachystachys#F719
Chloranthales Chloranthaceae Chloranthus japonicus#NC 026565
Chloranthales Chloranthaceae Chloranthus elatior#F79
Chloranthales Chloranthaceae Chloranthus spicatus#NC 009598
Canellales Canellaceae Warburgia ugandensis#YW2
Canellales Canellaceae Canella winterana#SJEV
Canellales Winteraceae Drimys lanceolata#Y161
Canellales Winteraceae Pseudowintera colorata#Y180
Canellales Winteraceae Drimys granadensis#FC122
Canellales Winteraceae Drimys winteri#WKSU
Piperales Aristolochiaceae Saruma henryi#NC 039933
Piperales Aristolochiaceae Asarum minus#NC 037503
Piperales Aristolochiaceae Asarum sieboldii#NC 037190
Piperales Aristolochiaceae Asarum costatum#AP018513
Piperales Aristolochiaceae Asarum minamitanianum#AP018514
Piperales Aristolochiaceae Aristolochia macrophylla#NC 041453
Piperales Aristolochiaceae Aristolochia kunmingensis#NC 041451
Piperales Aristolochiaceae Aristolochia moupinensis#NC 041454
Piperales Aristolochiaceae Aristolochia mollissima#NC 041457
Piperales Aristolochiaceae Aristolochia kaempferi#NC 041452
Piperales Aristolochiaceae Aristolochia littoralis#PAWA
Piperales Aristolochiaceae Aristolochia tubiflora#NC 041456
Piperales Aristolochiaceae Aristolochia tagala#F774
Piperales Aristolochiaceae Aristolochia contorta#NC 036152
Piperales Aristolochiaceae Aristolochia debilis#F540
Piperales Saururaceae Houttuynia cordata#CSSK
Piperales Saururaceae Saururus chinensis#F457
Piperales Saururaceae Saururus cernuus#OPDF
Piperales Piperaceae Peperomia fraseri#XSZI
Piperales Piperaceae Peperomia blanda#F69
Piperales Piperaceae Piper aduncum#F285
Piperales Piperaceae Piper auritum#MUNP
Piperales Piperaceae Piper cenocladum#NC 008457
Piperales Piperaceae Piper sp.#F331
Piperales Piperaceae Piper laetispicum#MH678665
Piperales Piperaceae Piper kadsura#NC 027941
Piperales Piperaceae Piper nigrum#NC 034692
Laurales Calycanthaceae Idiospermum australiense#FC322

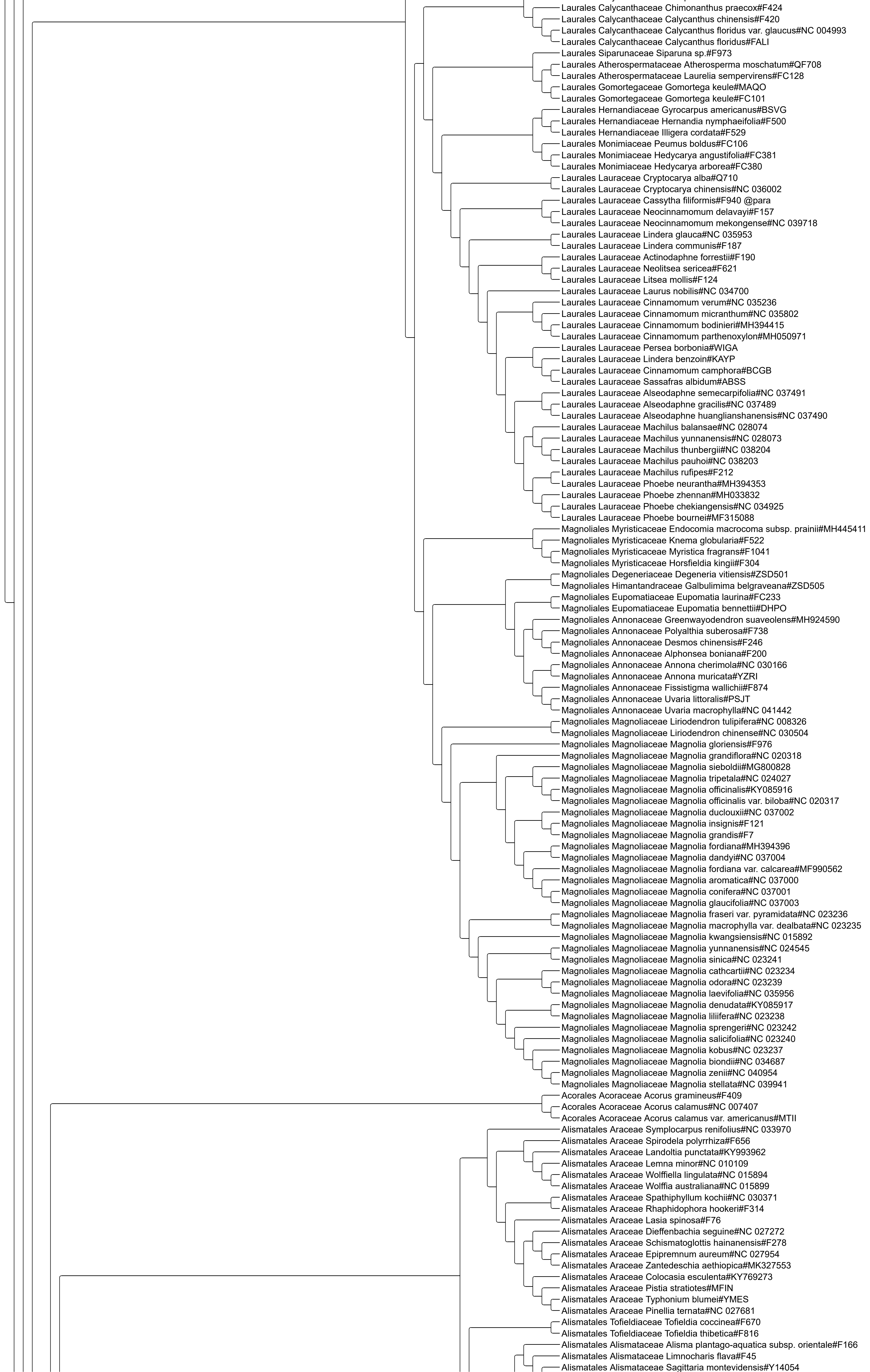

Laurales Calycanthaceae Chimonanthus praecox#F424
Laurales Calycanthaceae Calycanthus chinensis#F420
Laurales Calycanthaceae Calycanthus floridus var. glaucus#NC 004993
Laurales Calycanthaceae Calycanthus floridus#FALI
Laurales Siparunaceae Siparuna sp.#F973
Laurales Atherospermataceae Atherosperma moschatum#QF708
Laurales Atherospermataceae Laurelia sempervirens#FC128
Laurales Gomortegaceae Gomortega keule#MAQO
Laurales Gomortegaceae Gomortega keule#FC101
Laurales Hernandiaceae Gyrocarpus americanus#BSVG
Laurales Hernandiaceae Hernandia nymphaeifolia#F500
Laurales Hernandiaceae Illigera cordata#F529
Laurales Monimiaceae Peumus boldus#FC106
Laurales Monimiaceae Hedycarya angustifolia#FC381
Laurales Monimiaceae Hedycarya arborea#FC380
Laurales Lauraceae Cryptocarya alba#Q710
Laurales Lauraceae Cryptocarya chinensis#NC 036002
Laurales Lauraceae Cassytha filiformis#F940 @para
Laurales Lauraceae Neocinnamomum delavayi#F157
Laurales Lauraceae Neocinnamomum mekongense#NC 039718
Laurales Lauraceae Lindera glauca#NC 035953
Laurales Lauraceae Lindera communis#F187
Laurales Lauraceae Actinodaphne forrestii#F190
Laurales Lauraceae Neolitsea sericea#F621
Laurales Lauraceae Litsea mollis#F124
Laurales Lauraceae Laurus nobilis#NC 034700
Laurales Lauraceae Cinnamomum verum#NC 035236
Laurales Lauraceae Cinnamomum micranthum#NC 035802
Laurales Lauraceae Cinnamomum bodinieri#MH394415
Laurales Lauraceae Cinnamomum parthenoxylon#MH050971
Laurales Lauraceae Persea borbonia#WIGA
Laurales Lauraceae Lindera benzoin#KAYP
Laurales Lauraceae Cinnamomum camphora#BCGB
Laurales Lauraceae Sassafras albidum#ABSS
Laurales Lauraceae Alseodaphne semecarpifolia#NC 037491
Laurales Lauraceae Alseodaphne gracilis#NC 037489
Laurales Lauraceae Alseodaphne huanglianshanensis#NC 037490
Laurales Lauraceae Machilus balansae#NC 028074
Laurales Lauraceae Machilus yunnanensis#NC 028073
Laurales Lauraceae Machilus thunbergii#NC 038204
Laurales Lauraceae Machilus pauhoi#NC 038203
Laurales Lauraceae Machilus rufipes#F212
Laurales Lauraceae Phoebe neurantha#MH394353
Laurales Lauraceae Phoebe zhennan#MH033832
Laurales Lauraceae Phoebe chekiangensis#NC 034925
Laurales Lauraceae Phoebe bournei#MF315088
Magnoliales Myristicaceae Endocomia macrocoma subsp. prainii#MH445411
Magnoliales Myristicaceae Knema globularia#F522
Magnoliales Myristicaceae Myristica fragrans#F1041
Magnoliales Myristicaceae Horsfieldia kingii#F304
Magnoliales Degeneriaceae Degeneria vitiensis#ZSD501
Magnoliales Himantandraceae Galbulimima belgraveana#ZSD505
Magnoliales Eupomatiaceae Eupomatia laurina#FC233
Magnoliales Eupomatiaceae Eupomatia bennettii#DHPO
Magnoliales Annonaceae Greenwayodendron suaveolens#MH924590
Magnoliales Annonaceae Polyalthia suberosa#F738
Magnoliales Annonaceae Desmos chinensis#F246
Magnoliales Annonaceae Alphonsea boniana#F200
Magnoliales Annonaceae Annona cherimola#NC 030166
Magnoliales Annonaceae Annona muricata#YZRI
Magnoliales Annonaceae Fissistigma wallichii#F874
Magnoliales Annonaceae Uvaria littoralis#PSJT
Magnoliales Annonaceae Uvaria macrophylla#NC 041442
Magnoliales Magnoliaceae Liriodendron tulipifera#NC 008326
Magnoliales Magnoliaceae Liriodendron chinense#NC 030504
Magnoliales Magnoliaceae Magnolia gloriensis#F976
Magnoliales Magnoliaceae Magnolia grandiflora#NC 020318
Magnoliales Magnoliaceae Magnolia sieboldii#MG800828
Magnoliales Magnoliaceae Magnolia tripetala#NC 024027
Magnoliales Magnoliaceae Magnolia officinalis#KY085916
Magnoliales Magnoliaceae Magnolia officinalis var. biloba#NC 020317
Magnoliales Magnoliaceae Magnolia duclouxii#NC 037002
Magnoliales Magnoliaceae Magnolia insignis#F121
Magnoliales Magnoliaceae Magnolia grandis#F7
Magnoliales Magnoliaceae Magnolia fordiana#MH394396
Magnoliales Magnoliaceae Magnolia dandyi#NC 037004
Magnoliales Magnoliaceae Magnolia fordiana var. calcarea#MF990562
Magnoliales Magnoliaceae Magnolia aromatica#NC 037000
Magnoliales Magnoliaceae Magnolia conifera#NC 037001
Magnoliales Magnoliaceae Magnolia glaucifolia#NC 037003
Magnoliales Magnoliaceae Magnolia fraseri var. pyramidata#NC 023236
Magnoliales Magnoliaceae Magnolia macrophylla var. dealbata#NC 023235
Magnoliales Magnoliaceae Magnolia kwangsiensis#NC 015892
Magnoliales Magnoliaceae Magnolia yunnanensis#NC 024545
Magnoliales Magnoliaceae Magnolia sinica#NC 023241
Magnoliales Magnoliaceae Magnolia cathcartii#NC 023234
Magnoliales Magnoliaceae Magnolia odora#NC 023239
Magnoliales Magnoliaceae Magnolia laevifolia#NC 035956
Magnoliales Magnoliaceae Magnolia denudata#KY085917
Magnoliales Magnoliaceae Magnolia liliifera#NC 023238
Magnoliales Magnoliaceae Magnolia sprengeri#NC 023242
Magnoliales Magnoliaceae Magnolia salicifolia#NC 023240
Magnoliales Magnoliaceae Magnolia kobus#NC 023237
Magnoliales Magnoliaceae Magnolia biondii#NC 034687
Magnoliales Magnoliaceae Magnolia zenii#NC 040954
Magnoliales Magnoliaceae Magnolia stellata#NC 039941
Acorales Acoraceae Acorus gramineus#F409
Acorales Acoraceae Acorus calamus#NC 007407
Acorales Acoraceae Acorus calamus var. americanus#MTII
Alismatales Araceae Symplocarpus renifolius#NC 033970
Alismatales Araceae Spirodela polyrrhiza#F656
Alismatales Araceae Landoltia punctata#KY993962
Alismatales Araceae Lemna minor#NC 010109
Alismatales Araceae Wolffiella lingulata#NC 015894
Alismatales Araceae Wolffia australiana#NC 015899
Alismatales Araceae Spathiphyllum kochii#NC 030371
Alismatales Araceae Rhaphidophora hookeri#F314
Alismatales Araceae Lasia spinosa#F76
Alismatales Araceae Dieffenbachia seguine#NC 027272
Alismatales Araceae Schismatoglottis hainanensis#F278
Alismatales Araceae Epipremnum aureum#NC 027954
Alismatales Araceae Zantedeschia aethiopica#MK327553
Alismatales Araceae Colocasia esculenta#KY769273
Alismatales Araceae Pistia stratiotes#MFIN
Alismatales Araceae Typhonium blumei#YMES
Alismatales Araceae Pinellia ternata#NC 027681
Alismatales Tofieldiaceae Tofieldia coccinea#F670
Alismatales Tofieldiaceae Tofieldia thibetica#F816
Alismatales Alismataceae Alisma plantago-aquatica subsp. orientale#F166
Alismatales Alismataceae Limnocharis flava#F45
Alismatales Alismataceae Sagittaria montevidensis#Y14054

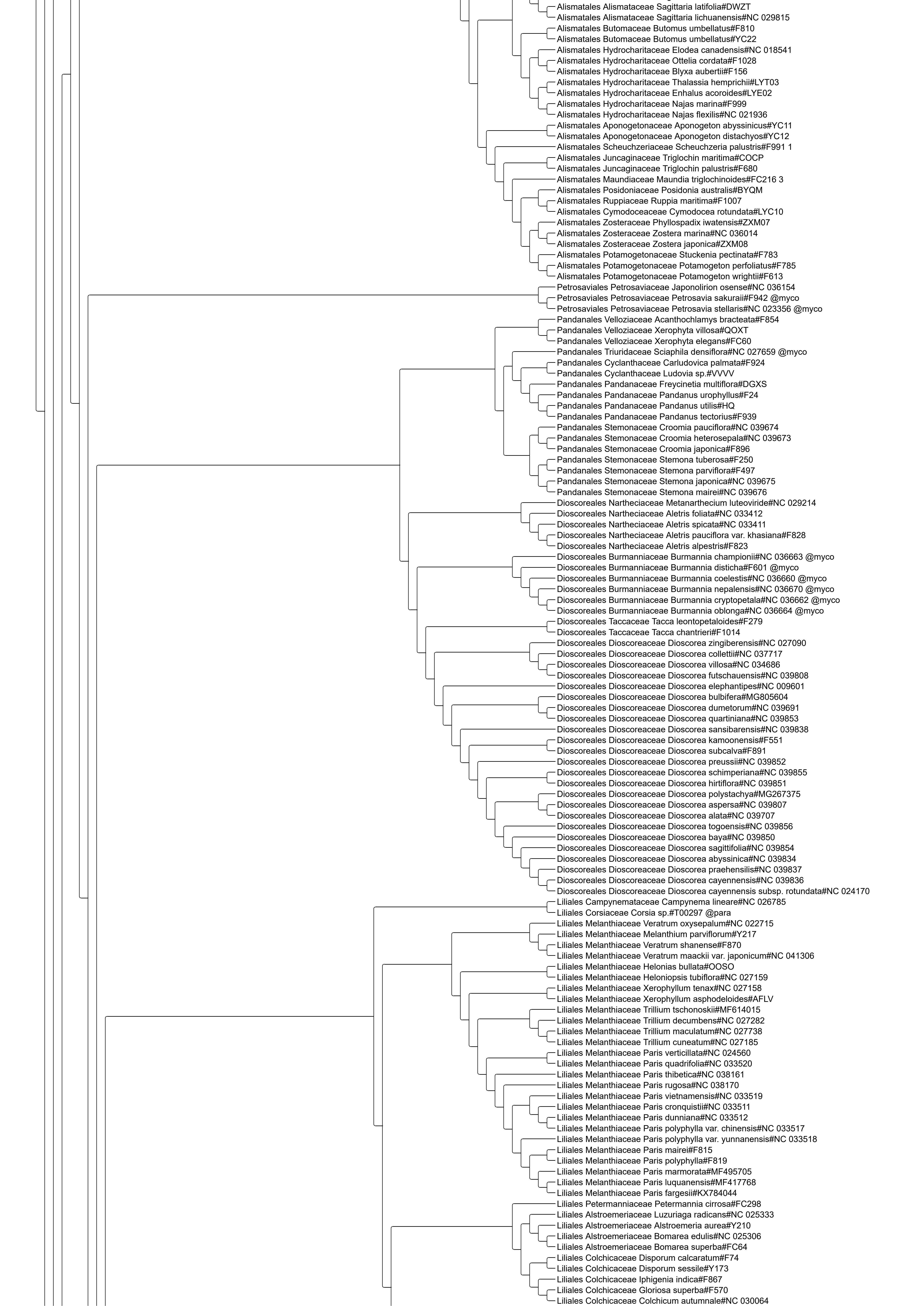
Alismatales Alismataceae Sagittaria latifolia#DWZT
Alismatales Alismataceae Sagittaria lichuanensis#NC 029815
Alismatales Butomaceae Butomus umbellatus#F810
Alismatales Butomaceae Butomus umbellatus#YC22
Alismatales Hydrocharitaceae Elodea canadensis#NC 018541
Alismatales Hydrocharitaceae Ottelia cordata#F1028
Alismatales Hydrocharitaceae Blyxa aubertii#F156
Alismatales Hydrocharitaceae Thalassia hemprichii#LYT03
Alismatales Hydrocharitaceae Enhalus acoroides#LYE02
Alismatales Hydrocharitaceae Najas marina#F999
Alismatales Hydrocharitaceae Najas flexilis#NC 021936
Alismatales Aponogetonaceae Aponogeton abyssinicus#YC11
Alismatales Aponogetonaceae Aponogeton distachyos#YC12
Alismatales Scheuchzeriaceae Scheuchzeria palustris#F991 1
Alismatales Juncaginaceae Triglochin maritima#COCP
Alismatales Juncaginaceae Triglochin palustris#F680
Alismatales Maundiaceae Maundia triglochinoides#FC216 3
Alismatales Posidoniaceae Posidonia australis#BYQM
Alismatales Ruppiaceae Ruppia maritima#F1007
Alismatales Cymodoceaceae Cymodocea rotundata#LYC10
Alismatales Zosteraceae Phyllospadix iwatensis#ZXM07
Alismatales Zosteraceae Zostera marina#NC 036014
Alismatales Zosteraceae Zostera japonica#ZXM08
Alismatales Potamogetonaceae Stuckenia pectinata#F783
Alismatales Potamogetonaceae Potamogeton perfoliatus#F785
Alismatales Potamogetonaceae Potamogeton wrightii#F613
Petrosaviales Petrosaviaceae Japonolirion osense#NC 036154
Petrosaviales Petrosaviaceae Petrosavia sakuraii#F942 @myco
Petrosaviales Petrosaviaceae Petrosavia stellaris#NC 023356 @myco
Pandanales Velloziaceae Acanthochlamys bracteata#F854
Pandanales Velloziaceae Xerophyta villosa#QOXT
Pandanales Velloziaceae Xerophyta elegans#FC60
Pandanales Triuridaceae Sciaphila densiflora#NC 027659 @myco
Pandanales Cyclanthaceae Carludovica palmata#F924
Pandanales Cyclanthaceae Ludovia sp.#VVVV
Pandanales Pandanaceae Freycinetia multiflora#DGXS
Pandanales Pandanaceae Pandanus urophyllus#F24
Pandanales Pandanaceae Pandanus utilis#HQ
Pandanales Pandanaceae Pandanus tectorius#F939
Pandanales Stemonaceae Croomia pauciflora#NC 039674
Pandanales Stemonaceae Croomia heterosepala#NC 039673
Pandanales Stemonaceae Croomia japonica#F896
Pandanales Stemonaceae Stemona tuberosa#F250
Pandanales Stemonaceae Stemona parviflora#F497
Pandanales Stemonaceae Stemona japonica#NC 039675
Pandanales Stemonaceae Stemona mairei#NC 039676
Dioscoreales Nartheciaceae Metanarthecium luteoviride#NC 029214
Dioscoreales Nartheciaceae Aletris foliata#NC 033412
Dioscoreales Nartheciaceae Aletris spicata#NC 033411
Dioscoreales Nartheciaceae Aletris pauciflora var. khasiana#F828
Dioscoreales Nartheciaceae Aletris alpestris#F823
Dioscoreales Burmanniaceae Burmannia championii#NC 036663 @myco
Dioscoreales Burmanniaceae Burmannia disticha#F601 @myco
Dioscoreales Burmanniaceae Burmannia coelestis#NC 036660 @myco
Dioscoreales Burmanniaceae Burmannia nepalensis#NC 036670 @myco
Dioscoreales Burmanniaceae Burmannia cryptopetala#NC 036662 @myco
Dioscoreales Burmanniaceae Burmannia oblonga#NC 036664 @myco
Dioscoreales Taccaceae Tacca leontopetaloides#F279
Dioscoreales Taccaceae Tacca chantrieri#F1014
Dioscoreales Dioscoreaceae Dioscorea zingiberensis#NC 027090
Dioscoreales Dioscoreaceae Dioscorea collettii#NC 037717
Dioscoreales Dioscoreaceae Dioscorea villosa#NC 034686
Dioscoreales Dioscoreaceae Dioscorea futschauensis#NC 039808
Dioscoreales Dioscoreaceae Dioscorea elephantipes#NC 009601
Dioscoreales Dioscoreaceae Dioscorea bulbifera#MG805604
Dioscoreales Dioscoreaceae Dioscorea dumetorum#NC 039691
Dioscoreales Dioscoreaceae Dioscorea quartiniana#NC 039853
Dioscoreales Dioscoreaceae Dioscorea sansibarensis#NC 039838
Dioscoreales Dioscoreaceae Dioscorea kamoonensis#F551
Dioscoreales Dioscoreaceae Dioscorea subcalva#F891
Dioscoreales Dioscoreaceae Dioscorea preussii#NC 039852
Dioscoreales Dioscoreaceae Dioscorea schimperiana#NC 039855
Dioscoreales Dioscoreaceae Dioscorea hirtiflora#NC 039851
Dioscoreales Dioscoreaceae Dioscorea polystachya#MG267375
Dioscoreales Dioscoreaceae Dioscorea aspersa#NC 039807
Dioscoreales Dioscoreaceae Dioscorea alata#NC 039707
Dioscoreales Dioscoreaceae Dioscorea togoensis#NC 039856
Dioscoreales Dioscoreaceae Dioscorea baya#NC 039850
Dioscoreales Dioscoreaceae Dioscorea sagittifolia#NC 039854
Dioscoreales Dioscoreaceae Dioscorea abyssinica#NC 039834
Dioscoreales Dioscoreaceae Dioscorea praehensilis#NC 039837
Dioscoreales Dioscoreaceae Dioscorea cayennensis#NC 039836
Dioscoreales Dioscoreaceae Dioscorea cayennensis subsp. rotundata#NC 024170
Liliales Campynemataceae Campynema lineare#NC 026785
Liliales Corsiaceae Corsia sp.#T00297 @para
Liliales Melanthiaceae Veratrum oxysepalum#NC 022715
Liliales Melanthiaceae Melanthium parviflorum#Y217
Liliales Melanthiaceae Veratrum shanense#F870
Liliales Melanthiaceae Veratrum maackii var. japonicum#NC 041306
Liliales Melanthiaceae Helonias bullata#OOSO
Liliales Melanthiaceae Heloniopsis tubiflora#NC 027159
Liliales Melanthiaceae Xerophyllum tenax#NC 027158
Liliales Melanthiaceae Xerophyllum asphodeloides#AFLV
Liliales Melanthiaceae Trillium tschonoskii#MF614015
Liliales Melanthiaceae Trillium decumbens#NC 027282
Liliales Melanthiaceae Trillium maculatum#NC 027738
Liliales Melanthiaceae Trillium cuneatum#NC 027185
Liliales Melanthiaceae Paris verticillata#NC 024560
Liliales Melanthiaceae Paris quadrifolia#NC 033520
Liliales Melanthiaceae Paris thibetica#NC 038161
Liliales Melanthiaceae Paris rugosa#NC 038170
Liliales Melanthiaceae Paris vietnamensis#NC 033519
Liliales Melanthiaceae Paris cronquistii#NC 033511
Liliales Melanthiaceae Paris dunniana#NC 033512
Liliales Melanthiaceae Paris polyphylla var. chinensis#NC 033517
Liliales Melanthiaceae Paris polyphylla var. yunnanensis#NC 033518
Liliales Melanthiaceae Paris mairei#F815
Liliales Melanthiaceae Paris polyphylla#F819
Liliales Melanthiaceae Paris marmorata#MF495705
Liliales Melanthiaceae Paris luquanensis#MF417768
Liliales Melanthiaceae Paris fargesii#KX784044
Liliales Petermanniaceae Petermannia cirrosa#FC298
Liliales Alstroemeriaceae Luzuriaga radicans#NC 025333
Liliales Alstroemeriaceae Alstroemeria aurea#Y210
Liliales Alstroemeriaceae Bomarea edulis#NC 025306
Liliales Alstroemeriaceae Bomarea superba#FC64
Liliales Colchicaceae Disporum calcaratum#F74
Liliales Colchicaceae Disporum sessile#Y173
Liliales Colchicaceae Iphigenia indica#F867
Liliales Colchicaceae Gloriosa superba#F570
Liliales Colchicaceae Colchicum autumnale#NC 030064

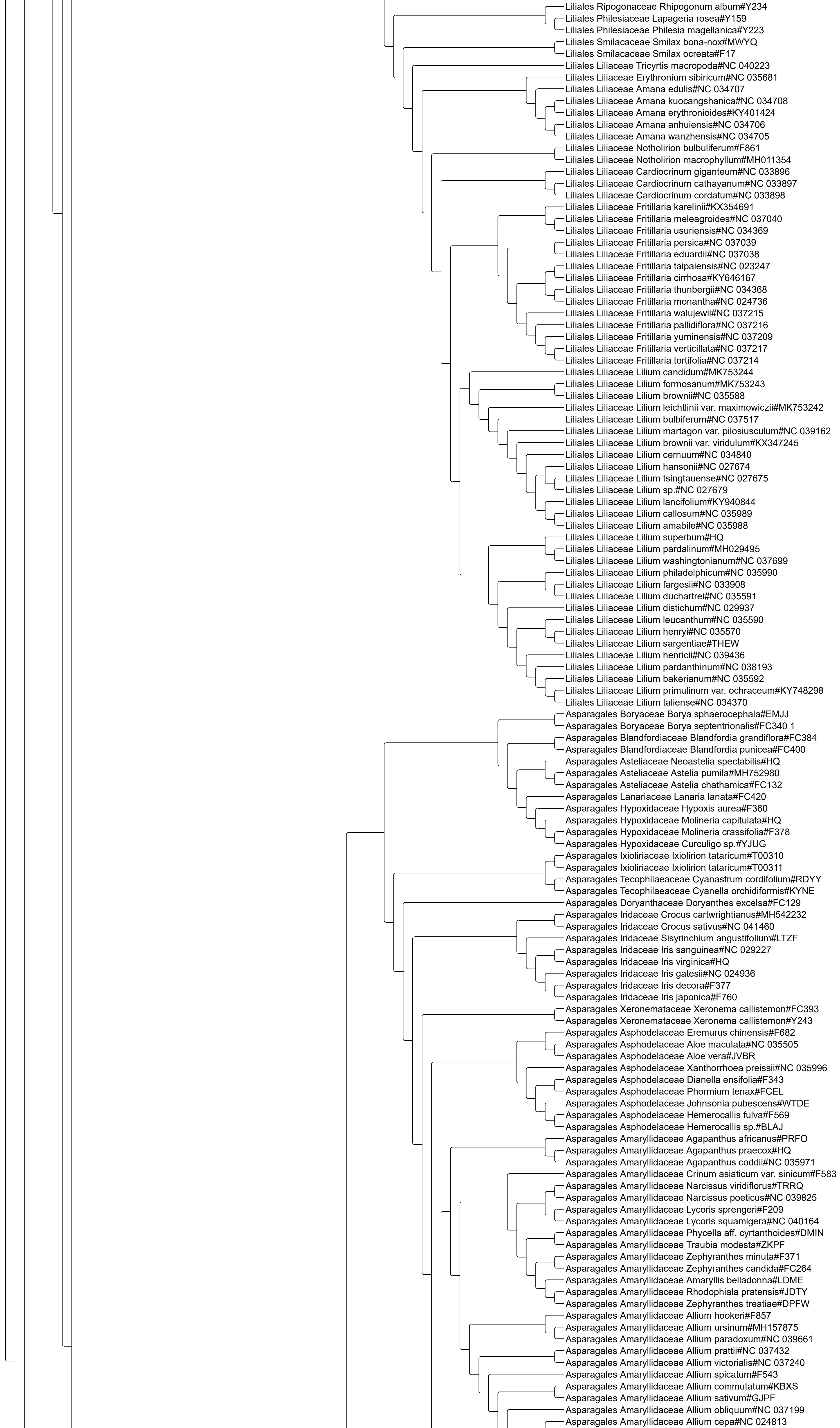

Liliales Ripogonaceae Rhipogonum album#Y234
Liliales Philesiaceae Lapageria rosea#Y159
Liliales Philesiaceae Philesia magellanica#Y223
Liliales Smilacaceae Smilax bona-nox#MWYQ
Liliales Smilacaceae Smilax ocreata#F17
Liliales Liliaceae Tricyrtis macropoda#NC 040223
Liliales Liliaceae Erythronium sibiricum#NC 035681
Liliales Liliaceae Amana edulis#NC 034707
Liliales Liliaceae Amana kuocangshanica#NC 034708
Liliales Liliaceae Amana erythronioides#KY401424
Liliales Liliaceae Amana anhuiensis#NC 034706
Liliales Liliaceae Amana wanzhensis#NC 034705
Liliales Liliaceae Notholirion bulbuliferum#F861
Liliales Liliaceae Notholirion macrophyllum#MH011354
Liliales Liliaceae Cardiocrinum giganteum#NC 033896
Liliales Liliaceae Cardiocrinum cathayanum#NC 033897
Liliales Liliaceae Cardiocrinum cordatum#NC 033898
Liliales Liliaceae Fritillaria karelinii#KX354691
Liliales Liliaceae Fritillaria meleagroides#NC 037040
Liliales Liliaceae Fritillaria usuriensis#NC 034369
Liliales Liliaceae Fritillaria persica#NC 037039
Liliales Liliaceae Fritillaria eduardii#NC 037038
Liliales Liliaceae Fritillaria taipaiensis#NC 023247
Liliales Liliaceae Fritillaria cirrhosa#KY646167
Liliales Liliaceae Fritillaria thunbergii#NC 034368
Liliales Liliaceae Fritillaria monantha#NC 024736
Liliales Liliaceae Fritillaria walujewii#NC 037215
Liliales Liliaceae Fritillaria pallidiflora#NC 037216
Liliales Liliaceae Fritillaria yuminensis#NC 037209
Liliales Liliaceae Fritillaria verticillata#NC 037217
Liliales Liliaceae Fritillaria tortifolia#NC 037214
Liliales Liliaceae Lilium candidum#MK753244
Liliales Liliaceae Lilium formosanum#MK753243
Liliales Liliaceae Lilium brownii#NC 035588
Liliales Liliaceae Lilium leichtlinii var. maximowiczii#MK753242
Liliales Liliaceae Lilium bulbiferum#NC 037517
Liliales Liliaceae Lilium martagon var. pilosiusculum#NC 039162
Liliales Liliaceae Lilium brownii var. viridulum#KX347245
Liliales Liliaceae Lilium cernuum#NC 034840
Liliales Liliaceae Lilium hansonii#NC 027674
Liliales Liliaceae Lilium tsingtauense#NC 027675
Liliales Liliaceae Lilium sp.#NC 027679
Liliales Liliaceae Lilium lancifolium#KY940844
Liliales Liliaceae Lilium callosum#NC 035989
Liliales Liliaceae Lilium amabile#NC 035988
Liliales Liliaceae Lilium superbum#HQ
Liliales Liliaceae Lilium pardalinum#MH029495
Liliales Liliaceae Lilium washingtonianum#NC 037699
Liliales Liliaceae Lilium philadelphicum#NC 035990
Liliales Liliaceae Lilium fargesii#NC 033908
Liliales Liliaceae Lilium duchartrei#NC 035591
Liliales Liliaceae Lilium distichum#NC 029937
Liliales Liliaceae Lilium leucanthum#NC 035590
Liliales Liliaceae Lilium henryi#NC 035570
Liliales Liliaceae Lilium sargentiae#THEW
Liliales Liliaceae Lilium henricii#NC 039436
Liliales Liliaceae Lilium pardanthinum#NC 038193
Liliales Liliaceae Lilium bakerianum#NC 035592
Liliales Liliaceae Lilium primulinum var. ochraceum#KY748298
Liliales Liliaceae Lilium taliense#NC 034370
Asparagales Boryaceae Borya sphaerocephala#EMJJ
Asparagales Boryaceae Borya septentrionalis#FC340 1
Asparagales Blandfordiaceae Blandfordia grandiflora#FC384
Asparagales Blandfordiaceae Blandfordia punicea#FC400
Asparagales Asteliaceae Neoastelia spectabilis#HQ
Asparagales Asteliaceae Astelia pumila#MH752980
Asparagales Asteliaceae Astelia chathamica#FC132
Asparagales Lanariaceae Lanaria lanata#FC420
Asparagales Hypoxidaceae Hypoxis aurea#F360
Asparagales Hypoxidaceae Molineria capitulata#HQ
Asparagales Hypoxidaceae Molineria crassifolia#F378
Asparagales Hypoxidaceae Curculigo sp.#YJUG
Asparagales Ixioliriaceae Ixiolirion tataricum#T00310
Asparagales Ixioliriaceae Ixiolirion tataricum#T00311
Asparagales Tecophilaeaceae Cyanastrum cordifolium#RDYY
Asparagales Tecophilaeaceae Cyanella orchidiformis#KYNE
Asparagales Doryanthaceae Doryanthes excelsa#FC129
Asparagales Iridaceae Crocus cartwrightianus#MH542232
Asparagales Iridaceae Crocus sativus#NC 041460
Asparagales Iridaceae Sisyrinchium angustifolium#LTZF
Asparagales Iridaceae Iris sanguinea#NC 029227
Asparagales Iridaceae Iris virginica#HQ
Asparagales Iridaceae Iris gatesii#NC 024936
Asparagales Iridaceae Iris decora#F377
Asparagales Iridaceae Iris japonica#F760
Asparagales Xeronemataceae Xeronema callistemon#FC393
Asparagales Xeronemataceae Xeronema callistemon#Y243
Asparagales Asphodelaceae Eremurus chinensis#F682
Asparagales Asphodelaceae Aloe maculata#NC 035505
Asparagales Asphodelaceae Aloe vera#JVBR
Asparagales Asphodelaceae Xanthorrhoea preissii#NC 035996
Asparagales Asphodelaceae Dianella ensifolia#F343
Asparagales Asphodelaceae Phormium tenax#FCEL
Asparagales Asphodelaceae Johnsonia pubescens#WTDE
Asparagales Asphodelaceae Hemerocallis fulva#F569
Asparagales Asphodelaceae Hemerocallis sp.#BLAJ
Asparagales Amaryllidaceae Agapanthus africanus#PRFO
Asparagales Amaryllidaceae Agapanthus praecox#HQ
Asparagales Amaryllidaceae Agapanthus coddii#NC 035971
Asparagales Amaryllidaceae Crinum asiaticum var. sinicum#F583
Asparagales Amaryllidaceae Narcissus viridiflorus#TRRQ
Asparagales Amaryllidaceae Narcissus poeticus#NC 039825
Asparagales Amaryllidaceae Lycoris sprengeri#F209
Asparagales Amaryllidaceae Lycoris squamigera#NC 040164
Asparagales Amaryllidaceae Phycella aff. cyrtanthoides#DMIN
Asparagales Amaryllidaceae Traubia modesta#ZKPF
Asparagales Amaryllidaceae Zephyranthes minuta#F371
Asparagales Amaryllidaceae Zephyranthes candida#FC264
Asparagales Amaryllidaceae Amaryllis belladonna#LDME
Asparagales Amaryllidaceae Rhodophiala pratensis#JDTY
Asparagales Amaryllidaceae Zephyranthes treatiae#DPFW
Asparagales Amaryllidaceae Allium hookeri#F857
Asparagales Amaryllidaceae Allium ursinum#MH157875
Asparagales Amaryllidaceae Allium paradoxum#NC 039661
Asparagales Amaryllidaceae Allium prattii#NC 037432
Asparagales Amaryllidaceae Allium victorialis#NC 037240
Asparagales Amaryllidaceae Allium spicatum#F543
Asparagales Amaryllidaceae Allium commutatum#KBXS
Asparagales Amaryllidaceae Allium sativum#GJPF
Asparagales Amaryllidaceae Allium obliquum#NC 037199
Asparagales Amaryllidaceae Allium cepa#NC 024813

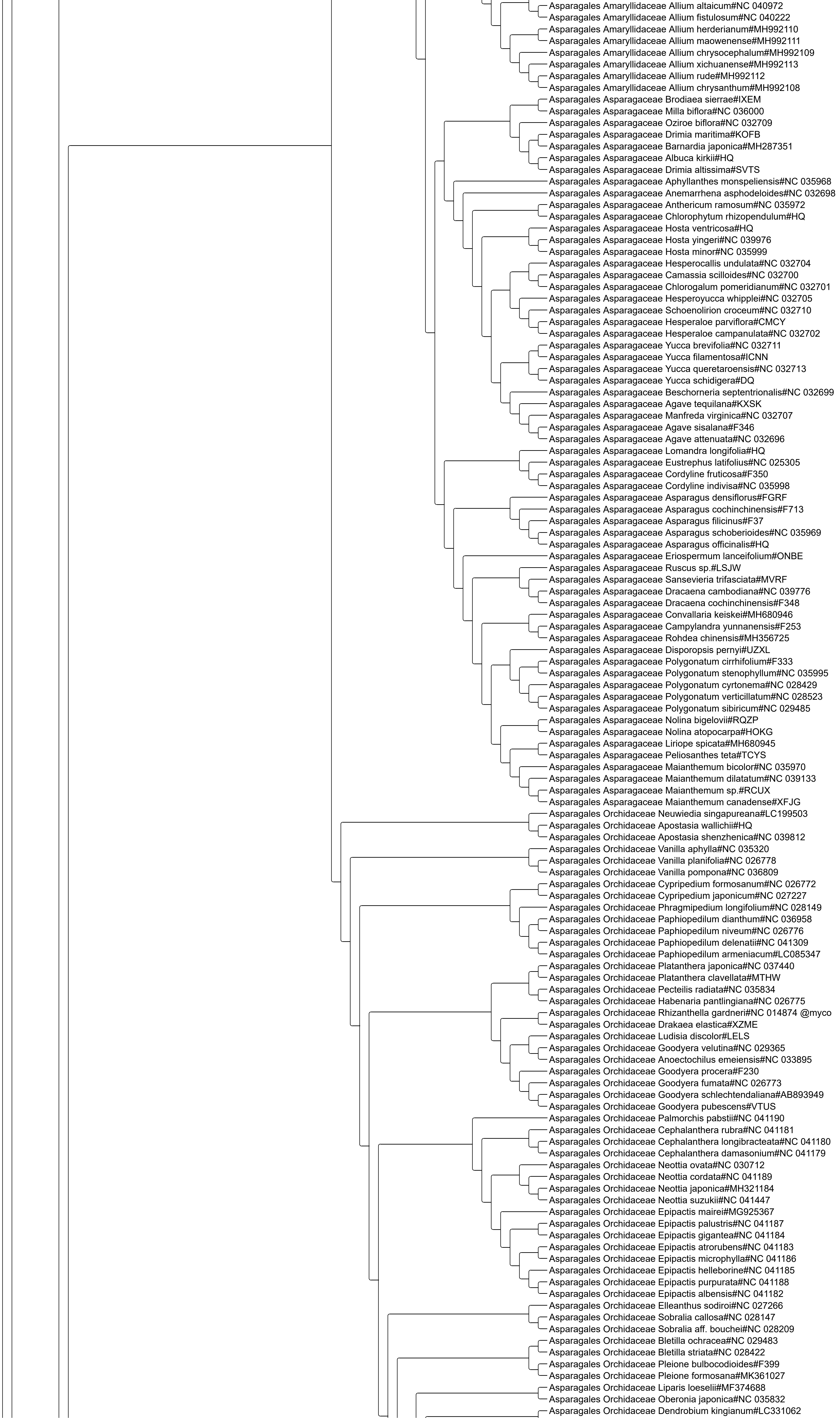

Asparagales Amaryllidaceae Allium altaicum#NC 040972
Asparagales Amaryllidaceae Allium fistulosum#NC 040222
Asparagales Amaryllidaceae Allium herderianum#MH992110
Asparagales Amaryllidaceae Allium maowenense#MH992111
Asparagales Amaryllidaceae Allium chrysocephalum#MH992109
Asparagales Amaryllidaceae Allium xichuanense#MH992113
Asparagales Amaryllidaceae Allium rude#MH992112
Asparagales Amaryllidaceae Allium chrysanthum#MH992108
Asparagales Asparagaceae Brodiaea sierrae#IXEM
Asparagales Asparagaceae Milla biflora#NC 036000
Asparagales Asparagaceae Oziroe biflora#NC 032709
Asparagales Asparagaceae Drimia maritima#KOFB
Asparagales Asparagaceae Barnardia japonica#MH287351
Asparagales Asparagaceae Albuca kirkii#HQ
Asparagales Asparagaceae Drimia altissima#SVTS
Asparagales Asparagaceae Aphyllanthes monspeliensis#NC 035968
Asparagales Asparagaceae Anemarrhena asphodeloides#NC 032698
Asparagales Asparagaceae Anthericum ramosum#NC 035972
Asparagales Asparagaceae Chlorophytum rhizopendulum#HQ
Asparagales Asparagaceae Hosta ventricosa#HQ
Asparagales Asparagaceae Hosta yingeri#NC 039976
Asparagales Asparagaceae Hosta minor#NC 035999
Asparagales Asparagaceae Hesperocallis undulata#NC 032704
Asparagales Asparagaceae Camassia scilloides#NC 032700
Asparagales Asparagaceae Chlorogalum pomeridianum#NC 032701
Asparagales Asparagaceae Hesperoyucca whipplei#NC 032705
Asparagales Asparagaceae Schoenolirion croceum#NC 032710
Asparagales Asparagaceae Hesperaloe parviflora#CMCY
Asparagales Asparagaceae Hesperaloe campanulata#NC 032702
Asparagales Asparagaceae Yucca brevifolia#NC 032711
Asparagales Asparagaceae Yucca filamentosa#ICNN
Asparagales Asparagaceae Yucca queretaroensis#NC 032713
Asparagales Asparagaceae Yucca schidigera#DQ
Asparagales Asparagaceae Beschorneria septentrionalis#NC 032699
Asparagales Asparagaceae Agave tequilana#KXSK
Asparagales Asparagaceae Manfreda virginica#NC 032707
Asparagales Asparagaceae Agave sisalana#F346
Asparagales Asparagaceae Agave attenuata#NC 032696
Asparagales Asparagaceae Lomandra longifolia#HQ
Asparagales Asparagaceae Eustrephus latifolius#NC 025305
Asparagales Asparagaceae Cordyline fruticosa#F350
Asparagales Asparagaceae Cordyline indivisa#NC 035998
Asparagales Asparagaceae Asparagus densiflorus#FGRF
Asparagales Asparagaceae Asparagus cochinchinensis#F713
Asparagales Asparagaceae Asparagus filicinus#F37
Asparagales Asparagaceae Asparagus schoberioides#NC 035969
Asparagales Asparagaceae Asparagus officinalis#HQ
Asparagales Asparagaceae Eriospermum lanceifolium#ONBE
Asparagales Asparagaceae Ruscus sp.#LSJW
Asparagales Asparagaceae Sansevieria trifasciata#MVRF
Asparagales Asparagaceae Dracaena cambodiana#NC 039776
Asparagales Asparagaceae Dracaena cochinchinensis#F348
Asparagales Asparagaceae Convallaria keiskei#MH680946
Asparagales Asparagaceae Campylandra yunnanensis#F253
Asparagales Asparagaceae Rohdea chinensis#MH356725
Asparagales Asparagaceae Disporopsis pernyi#UZXL
Asparagales Asparagaceae Polygonatum cirrhifolium#F333
Asparagales Asparagaceae Polygonatum stenophyllum#NC 035995
Asparagales Asparagaceae Polygonatum cyrtonema#NC 028429
Asparagales Asparagaceae Polygonatum verticillatum#NC 028523
Asparagales Asparagaceae Polygonatum sibiricum#NC 029485
Asparagales Asparagaceae Nolina bigelovii#RQZP
Asparagales Asparagaceae Nolina atopocarpa#HOKG
Asparagales Asparagaceae Liriope spicata#MH680945
Asparagales Asparagaceae Peliosanthes teta#TCYS
Asparagales Asparagaceae Maianthemum bicolor#NC 035970
Asparagales Asparagaceae Maianthemum dilatatum#NC 039133
Asparagales Asparagaceae Maianthemum sp.#RCUX
Asparagales Asparagaceae Maianthemum canadense#XFJG
Asparagales Orchidaceae Neuwiedia singapureana#LC199503
Asparagales Orchidaceae Apostasia wallichii#HQ
Asparagales Orchidaceae Apostasia shenzhenica#NC 039812
Asparagales Orchidaceae Vanilla aphylla#NC 035320
Asparagales Orchidaceae Vanilla planifolia#NC 026778
Asparagales Orchidaceae Vanilla pompona#NC 036809
Asparagales Orchidaceae Cypripedium formosanum#NC 026772
Asparagales Orchidaceae Cypripedium japonicum#NC 027227
Asparagales Orchidaceae Phragmipedium longifolium#NC 028149
Asparagales Orchidaceae Paphiopedilum dianthum#NC 036958
Asparagales Orchidaceae Paphiopedilum niveum#NC 026776
Asparagales Orchidaceae Paphiopedilum delenatii#NC 041309
Asparagales Orchidaceae Paphiopedilum armeniacum#LC085347
Asparagales Orchidaceae Platanthera japonica#NC 037440
Asparagales Orchidaceae Platanthera clavellata#MTHW
Asparagales Orchidaceae Pecteilis radiata#NC 035834
Asparagales Orchidaceae Habenaria pantlingiana#NC 026775
Asparagales Orchidaceae Rhizanthella gardneri#NC 014874 @myco
Asparagales Orchidaceae Drakaea elastica#XZME
Asparagales Orchidaceae Ludisia discolor#LELS
Asparagales Orchidaceae Goodyera velutina#NC 029365
Asparagales Orchidaceae Anoectochilus emeiensis#NC 033895
Asparagales Orchidaceae Goodyera procera#F230
Asparagales Orchidaceae Goodyera fumata#NC 026773
Asparagales Orchidaceae Goodyera schlechtendaliana#AB893949
Asparagales Orchidaceae Goodyera pubescens#VTUS
Asparagales Orchidaceae Palmorchis pabstii#NC 041190
Asparagales Orchidaceae Cephalanthera rubra#NC 041181
Asparagales Orchidaceae Cephalanthera longibracteata#NC 041180
Asparagales Orchidaceae Cephalanthera damasonium#NC 041179
Asparagales Orchidaceae Neottia ovata#NC 030712
Asparagales Orchidaceae Neottia cordata#NC 041189
Asparagales Orchidaceae Neottia japonica#MH321184
Asparagales Orchidaceae Neottia suzukii#NC 041447
Asparagales Orchidaceae Epipactis mairei#MG925367
Asparagales Orchidaceae Epipactis palustris#NC 041187
Asparagales Orchidaceae Epipactis gigantea#NC 041184
Asparagales Orchidaceae Epipactis atrorubens#NC 041183
Asparagales Orchidaceae Epipactis microphylla#NC 041186
Asparagales Orchidaceae Epipactis helleborine#NC 041185
Asparagales Orchidaceae Epipactis purpurata#NC 041188
Asparagales Orchidaceae Epipactis albensis#NC 041182
Asparagales Orchidaceae Elleanthus sodiroi#NC 027266
Asparagales Orchidaceae Sobralia callosa#NC 028147
Asparagales Orchidaceae Sobralia aff. bouchei#NC 028209
Asparagales Orchidaceae Bletilla ochracea#NC 029483
Asparagales Orchidaceae Bletilla striata#NC 028422
Asparagales Orchidaceae Pleione bulbocodioides#F399
Asparagales Orchidaceae Pleione formosana#MK361027
Asparagales Orchidaceae Liparis loeselii#MF374688
Asparagales Orchidaceae Oberonia japonica#NC 035832
Asparagales Orchidaceae Dendrobium kingianum#LC331062

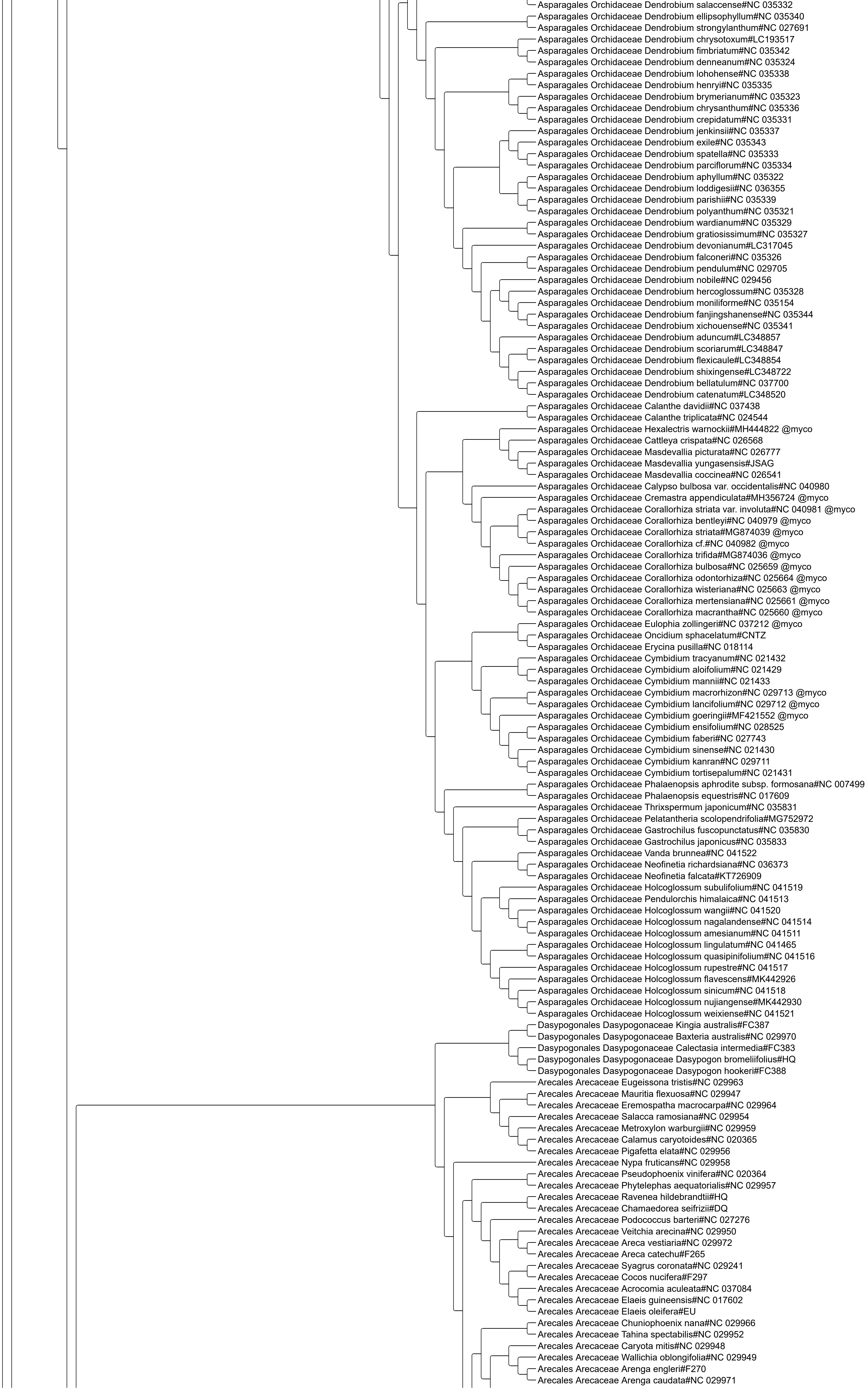

Asparagales Orchidaceae Dendrobium salaccense#NC 035332
Asparagales Orchidaceae Dendrobium ellipsophyllum#NC 035340
Asparagales Orchidaceae Dendrobium strongylanthum#NC 027691
Asparagales Orchidaceae Dendrobium chrysotoxum#LC193517
Asparagales Orchidaceae Dendrobium fimbriatum#NC 035342
Asparagales Orchidaceae Dendrobium denneanum#NC 035324
Asparagales Orchidaceae Dendrobium lohohense#NC 035338
Asparagales Orchidaceae Dendrobium henryi#NC 035335
Asparagales Orchidaceae Dendrobium brymerianum#NC 035323
Asparagales Orchidaceae Dendrobium chrysanthum#NC 035336
Asparagales Orchidaceae Dendrobium crepidatum#NC 035331
Asparagales Orchidaceae Dendrobium jenkinsii#NC 035337
Asparagales Orchidaceae Dendrobium exile#NC 035343
Asparagales Orchidaceae Dendrobium spatella#NC 035333
Asparagales Orchidaceae Dendrobium parciflorum#NC 035334
Asparagales Orchidaceae Dendrobium aphyllum#NC 035322
Asparagales Orchidaceae Dendrobium loddigesii#NC 036355
Asparagales Orchidaceae Dendrobium parishii#NC 035339
Asparagales Orchidaceae Dendrobium polyanthum#NC 035321
Asparagales Orchidaceae Dendrobium wardianum#NC 035329
Asparagales Orchidaceae Dendrobium gratiosissimum#NC 035327
Asparagales Orchidaceae Dendrobium devonianum#LC317045
Asparagales Orchidaceae Dendrobium falconeri#NC 035326
Asparagales Orchidaceae Dendrobium pendulum#NC 029705
Asparagales Orchidaceae Dendrobium nobile#NC 029456
Asparagales Orchidaceae Dendrobium hercoglossum#NC 035328
Asparagales Orchidaceae Dendrobium moniliforme#NC 035154
Asparagales Orchidaceae Dendrobium fanjingshanense#NC 035344
Asparagales Orchidaceae Dendrobium xichouense#NC 035341
Asparagales Orchidaceae Dendrobium aduncum#LC348857
Asparagales Orchidaceae Dendrobium scoriarum#LC348847
Asparagales Orchidaceae Dendrobium flexicaule#LC348854
Asparagales Orchidaceae Dendrobium shixingense#LC348722
Asparagales Orchidaceae Dendrobium bellatulum#NC 037700
Asparagales Orchidaceae Dendrobium catenatum#LC348520
Asparagales Orchidaceae Calanthe davidii#NC 037438
Asparagales Orchidaceae Calanthe triplicata#NC 024544
Asparagales Orchidaceae Hexalectris warnockii#MH444822 @myco
Asparagales Orchidaceae Cattleya crispata#NC 026568
Asparagales Orchidaceae Masdevallia picturata#NC 026777
Asparagales Orchidaceae Masdevallia yungasensis#JSAG
Asparagales Orchidaceae Masdevallia coccinea#NC 026541
Asparagales Orchidaceae Calypso bulbosa var. occidentalis#NC 040980
Asparagales Orchidaceae Cremastra appendiculata#MH356724 @myco
Asparagales Orchidaceae Corallorhiza striata var. involuta#NC 040981 @myco
Asparagales Orchidaceae Corallorhiza bentleyi#NC 040979 @myco
Asparagales Orchidaceae Corallorhiza striata#MG874039 @myco
Asparagales Orchidaceae Corallorhiza cf.#NC 040982 @myco
Asparagales Orchidaceae Corallorhiza trifida#MG874036 @myco
Asparagales Orchidaceae Corallorhiza bulbosa#NC 025659 @myco
Asparagales Orchidaceae Corallorhiza odontorhiza#NC 025664 @myco
Asparagales Orchidaceae Corallorhiza wisteriana#NC 025663 @myco
Asparagales Orchidaceae Corallorhiza mertensiana#NC 025661 @myco
Asparagales Orchidaceae Corallorhiza macrantha#NC 025660 @myco
Asparagales Orchidaceae Eulophia zollingeri#NC 037212 @myco
Asparagales Orchidaceae Oncidium sphacelatum#CNTZ
Asparagales Orchidaceae Erycina pusilla#NC 018114
Asparagales Orchidaceae Cymbidium tracyanum#NC 021432
Asparagales Orchidaceae Cymbidium aloifolium#NC 021429
Asparagales Orchidaceae Cymbidium mannii#NC 021433
Asparagales Orchidaceae Cymbidium macrorhizon#NC 029713 @myco
Asparagales Orchidaceae Cymbidium lancifolium#NC 029712 @myco
Asparagales Orchidaceae Cymbidium goeringii#MF421552 @myco
Asparagales Orchidaceae Cymbidium ensifolium#NC 028525
Asparagales Orchidaceae Cymbidium faberi#NC 027743
Asparagales Orchidaceae Cymbidium sinense#NC 021430
Asparagales Orchidaceae Cymbidium kanran#NC 029711
Asparagales Orchidaceae Cymbidium tortisepalum#NC 021431
Asparagales Orchidaceae Phalaenopsis aphrodite subsp. formosana#NC 007499
Asparagales Orchidaceae Phalaenopsis equestris#NC 017609
Asparagales Orchidaceae Thrixspermum japonicum#NC 035831
Asparagales Orchidaceae Pelatantheria scolopendrifolia#MG752972
Asparagales Orchidaceae Gastrochilus fuscopunctatus#NC 035830
Asparagales Orchidaceae Gastrochilus japonicus#NC 035833
Asparagales Orchidaceae Vanda brunnea#NC 041522
Asparagales Orchidaceae Neofinetia richardsiana#NC 036373
Asparagales Orchidaceae Neofinetia falcata#KT726909
Asparagales Orchidaceae Holcoglossum subulifolium#NC 041519
Asparagales Orchidaceae Pendulorchis himalaica#NC 041513
Asparagales Orchidaceae Holcoglossum wangii#NC 041520
Asparagales Orchidaceae Holcoglossum nagalandense#NC 041514
Asparagales Orchidaceae Holcoglossum amesianum#NC 041511
Asparagales Orchidaceae Holcoglossum lingulatum#NC 041465
Asparagales Orchidaceae Holcoglossum quasipinifolium#NC 041516
Asparagales Orchidaceae Holcoglossum rupestre#NC 041517
Asparagales Orchidaceae Holcoglossum flavescens#MK442926
Asparagales Orchidaceae Holcoglossum sinicum#NC 041518
Asparagales Orchidaceae Holcoglossum nujiangense#MK442930
Asparagales Orchidaceae Holcoglossum weixiense#NC 041521
Dasypogonales Dasypogonaceae Kingia australis#FC387
Dasypogonales Dasypogonaceae Baxteria australis#NC 029970
Dasypogonales Dasypogonaceae Calectasia intermedia#FC383
Dasypogonales Dasypogonaceae Dasypogon bromeliifolius#HQ
Dasypogonales Dasypogonaceae Dasypogon hookeri#FC388
Arecales Arecaceae Eugeissona tristis#NC 029963
Arecales Arecaceae Mauritia flexuosa#NC 029947
Arecales Arecaceae Eremospatha macrocarpa#NC 029964
Arecales Arecaceae Salacca ramosiana#NC 029954
Arecales Arecaceae Metroxylon warburgii#NC 029959
Arecales Arecaceae Calamus caryotoides#NC 020365
Arecales Arecaceae Pigafetta elata#NC 029956
Arecales Arecaceae Nypa fruticans#NC 029958
Arecales Arecaceae Pseudophoenix vinifera#NC 020364
Arecales Arecaceae Phytelephas aequatorialis#NC 029957
Arecales Arecaceae Ravenea hildebrandtii#HQ
Arecales Arecaceae Chamaedorea seifrizii#DQ
Arecales Arecaceae Podococcus barteri#NC 027276
Arecales Arecaceae Veitchia arecina#NC 029950
Arecales Arecaceae Areca vestiaria#NC 029972
Arecales Arecaceae Areca catechu#F265
Arecales Arecaceae Syagrus coronata#NC 029241
Arecales Arecaceae Cocos nucifera#F297
Arecales Arecaceae Acrocomia aculeata#NC 037084
Arecales Arecaceae Elaeis guineensis#NC 017602
Arecales Arecaceae Elaeis oleifera#EU
Arecales Arecaceae Chuniophoenix nana#NC 029966
Arecales Arecaceae Tahina spectabilis#NC 029952
Arecales Arecaceae Caryota mitis#NC 029948
Arecales Arecaceae Wallichia oblongifolia#NC 029949
Arecales Arecaceae Arenga engleri#F270
Arecales Arecaceae Arenga caudata#NC 029971

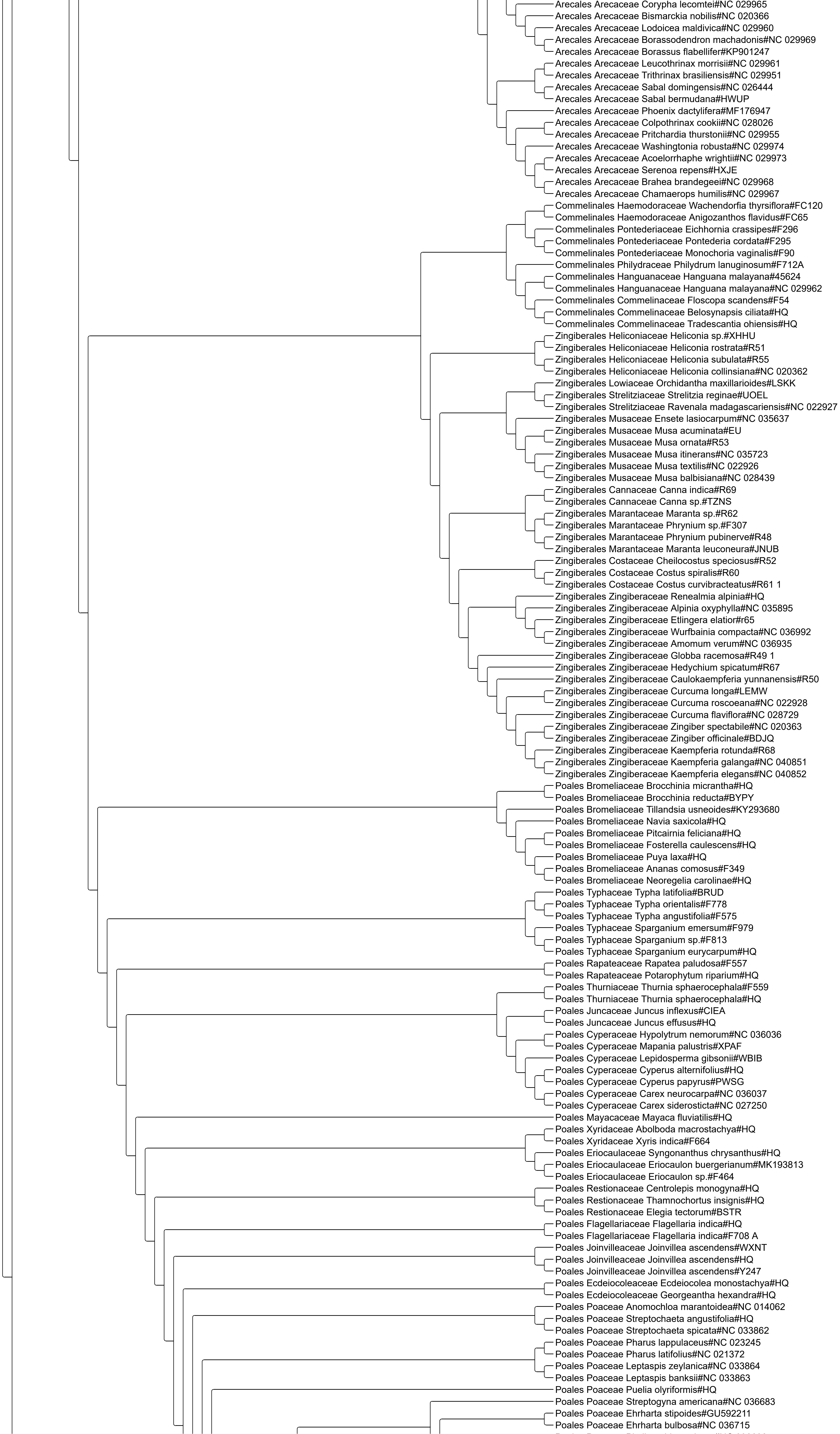
Arecales Arecaceae Corypha lecomtei#NC 029965
Arecales Arecaceae Bismarckia nobilis#NC 020366
Arecales Arecaceae Lodoicea maldivica#NC 029960
Arecales Arecaceae Borassodendron machadonis#NC 029969
Arecales Arecaceae Borassus flabellifer#KP901247
Arecales Arecaceae Leucothrinax morrisii#NC 029961
Arecales Arecaceae Trithrinax brasiliensis#NC 029951
Arecales Arecaceae Sabal domingensis#NC 026444
Arecales Arecaceae Sabal bermudana#HWUP
Arecales Arecaceae Phoenix dactylifera#MF176947
Arecales Arecaceae Colpothrinax cookii#NC 028026
Arecales Arecaceae Pritchardia thurstonii#NC 029955
Arecales Arecaceae Washingtonia robusta#NC 029974
Arecales Arecaceae Acoelorrhaphe wrightii#NC 029973
Arecales Arecaceae Serenoa repens#HXJE
Arecales Arecaceae Brahea brandegeei#NC 029968
Arecales Arecaceae Chamaerops humilis#NC 029967
Commelinales Haemodoraceae Wachendorfia thyrsiflora#FC120
Commelinales Haemodoraceae Anigozanthos flavidus#FC65
Commelinales Pontederiaceae Eichhornia crassipes#F296
Commelinales Pontederiaceae Pontederia cordata#F295
Commelinales Pontederiaceae Monochoria vaginalis#F90
Commelinales Philydraceae Philydrum lanuginosum#F712A
Commelinales Hanguanaceae Hanguana malayana#45624
Commelinales Hanguanaceae Hanguana malayana#NC 029962
Commelinales Commelinaceae Floscopa scandens#F54
Commelinales Commelinaceae Belosynapsis ciliata#HQ
Commelinales Commelinaceae Tradescantia ohiensis#HQ
Zingiberales Heliconiaceae Heliconia sp.#XHHU
Zingiberales Heliconiaceae Heliconia rostrata#R51
Zingiberales Heliconiaceae Heliconia subulata#R55
Zingiberales Heliconiaceae Heliconia collinsiana#NC 020362
Zingiberales Lowiaceae Orchidantha maxillarioides#LSKK
Zingiberales Strelitziaceae Strelitzia reginae#UOEL
Zingiberales Strelitziaceae Ravenala madagascariensis#NC 022927
Zingiberales Musaceae Ensete lasiocarpum#NC 035637
Zingiberales Musaceae Musa acuminata#EU
Zingiberales Musaceae Musa ornata#R53
Zingiberales Musaceae Musa itinerans#NC 035723
Zingiberales Musaceae Musa textilis#NC 022926
Zingiberales Musaceae Musa balbisiana#NC 028439
Zingiberales Cannaceae Canna indica#R69
Zingiberales Cannaceae Canna sp.#TZNS
Zingiberales Marantaceae Maranta sp.#R62
Zingiberales Marantaceae Phrynium sp.#F307
Zingiberales Marantaceae Phrynium pubinerve#R48
Zingiberales Marantaceae Maranta leuconeura#JNUB
Zingiberales Costaceae Cheilocostus speciosus#R52
Zingiberales Costaceae Costus spiralis#R60
Zingiberales Costaceae Costus curvibracteatus#R61 1
Zingiberales Zingiberaceae Renealmia alpinia#HQ
Zingiberales Zingiberaceae Alpinia oxyphylla#NC 035895
Zingiberales Zingiberaceae Etlingera elatior#r65
Zingiberales Zingiberaceae Wurfbainia compacta#NC 036992
Zingiberales Zingiberaceae Amomum verum#NC 036935
Zingiberales Zingiberaceae Globba racemosa#R49 1
Zingiberales Zingiberaceae Hedychium spicatum#R67
Zingiberales Zingiberaceae Caulokaempferia yunnanensis#R50
Zingiberales Zingiberaceae Curcuma longa#LEMW
Zingiberales Zingiberaceae Curcuma roscoeana#NC 022928
Zingiberales Zingiberaceae Curcuma flaviflora#NC 028729
Zingiberales Zingiberaceae Zingiber spectabile#NC 020363
Zingiberales Zingiberaceae Zingiber officinale#BDJQ
Zingiberales Zingiberaceae Kaempferia rotunda#R68
Zingiberales Zingiberaceae Kaempferia galanga#NC 040851
Zingiberales Zingiberaceae Kaempferia elegans#NC 040852
Poales Bromeliaceae Brocchinia micrantha#HQ
Poales Bromeliaceae Brocchinia reducta#BYPY
Poales Bromeliaceae Tillandsia usneoides#KY293680
Poales Bromeliaceae Navia saxicola#HQ
Poales Bromeliaceae Pitcairnia feliciana#HQ
Poales Bromeliaceae Fosterella caulescens#HQ
Poales Bromeliaceae Puya laxa#HQ
Poales Bromeliaceae Ananas comosus#F349
Poales Bromeliaceae Neoregelia carolinae#HQ
Poales Typhaceae Typha latifolia#BRUD
Poales Typhaceae Typha orientalis#F778
Poales Typhaceae Typha angustifolia#F575
Poales Typhaceae Sparganium emersum#F979
Poales Typhaceae Sparganium sp.#F813
Poales Typhaceae Sparganium eurycarpum#HQ
Poales Rapateaceae Rapatea paludosa#F557
Poales Rapateaceae Potarophytum riparium#HQ
Poales Thurniaceae Thurnia sphaerocephala#F559
Poales Thurniaceae Thurnia sphaerocephala#HQ
Poales Juncaceae Juncus inflexus#CIEA
Poales Juncaceae Juncus effusus#HQ
Poales Cyperaceae Hypolytrum nemorum#NC 036036
Poales Cyperaceae Mapania palustris#XPAF
Poales Cyperaceae Lepidosperma gibsonii#WBIB
Poales Cyperaceae Cyperus alternifolius#HQ
Poales Cyperaceae Cyperus papyrus#PWSG
Poales Cyperaceae Carex neurocarpa#NC 036037
Poales Cyperaceae Carex siderosticta#NC 027250
Poales Mayacaceae Mayaca fluviatilis#HQ
Poales Xyridaceae Abolboda macrostachya#HQ
Poales Xyridaceae Xyris indica#F664
Poales Eriocaulaceae Syngonanthus chrysanthus#HQ
Poales Eriocaulaceae Eriocaulon buergerianum#MK193813
Poales Eriocaulaceae Eriocaulon sp.#F464
Poales Restionaceae Centrolepis monogyna#HQ
Poales Restionaceae Thamnochortus insignis#HQ
Poales Restionaceae Elegia tectorum#BSTR
Poales Flagellariaceae Flagellaria indica#HQ
Poales Flagellariaceae Flagellaria indica#F708 A
Poales Joinvilleaceae Joinvillea ascendens#WXNT
Poales Joinvilleaceae Joinvillea ascendens#HQ
Poales Joinvilleaceae Joinvillea ascendens#Y247
Poales Ecdeiocoleaceae Ecdeiocolea monostachya#HQ
Poales Ecdeiocoleaceae Georgeantha hexandra#HQ
Poales Poaceae Anomochloa marantoidea#NC 014062
Poales Poaceae Streptochaeta angustifolia#HQ
Poales Poaceae Streptochaeta spicata#NC 033862
Poales Poaceae Pharus lappulaceus#NC 023245
Poales Poaceae Pharus latifolius#NC 021372
Poales Poaceae Leptaspis zeylanica#NC 033864
Poales Poaceae Leptaspis banksii#NC 033863
Poales Poaceae Puelia olyriformis#HQ
Poales Poaceae Streptogyna americana#NC 036683
Poales Poaceae Ehrharta stipoides#GU592211
Poales Poaceae Ehrharta bulbosa#NC 036715

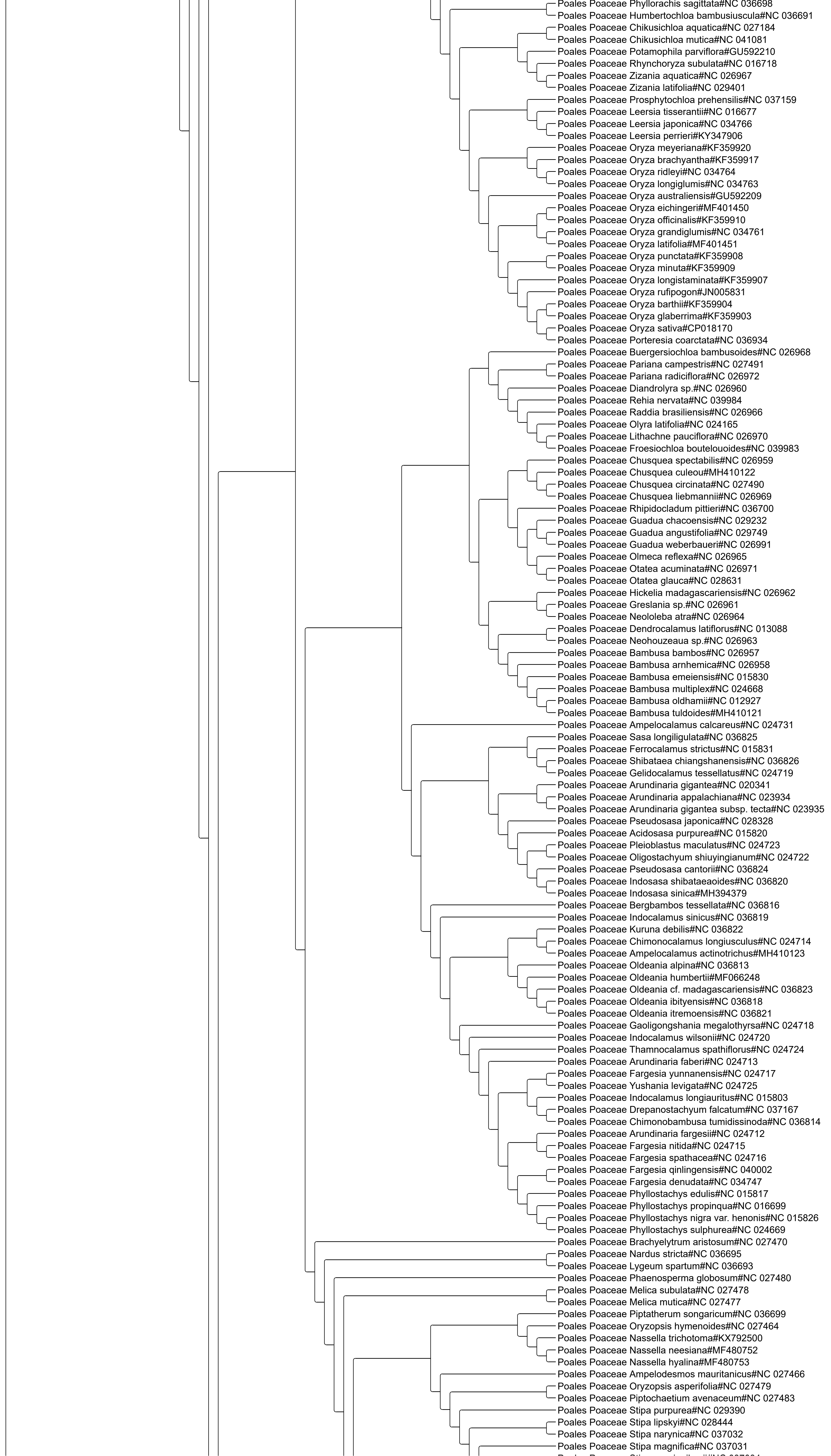
Poales Poaceae Phyllorachis sagittata#NC 036698
Poales Poaceae Humbertochloa bambusiuscula#NC 036691
Poales Poaceae Chikusichloa aquatica#NC 027184
Poales Poaceae Chikusichloa mutica#NC 041081
Poales Poaceae Potamophila parviflora#GU592210
Poales Poaceae Rhynchoryza subulata#NC 016718
Poales Poaceae Zizania aquatica#NC 026967
Poales Poaceae Zizania latifolia#NC 029401
Poales Poaceae Prosphytochloa prehensilis#NC 037159
Poales Poaceae Leersia tisserantii#NC 016677
Poales Poaceae Leersia japonica#NC 034766
Poales Poaceae Leersia perrieri#KY347906
Poales Poaceae Oryza meyeriana#KF359920
Poales Poaceae Oryza brachyantha#KF359917
Poales Poaceae Oryza ridleyi#NC 034764
Poales Poaceae Oryza longiglumis#NC 034763
Poales Poaceae Oryza australiensis#GU592209
Poales Poaceae Oryza eichingeri#MF401450
Poales Poaceae Oryza officinalis#KF359910
Poales Poaceae Oryza grandiglumis#NC 034761
Poales Poaceae Oryza latifolia#MF401451
Poales Poaceae Oryza punctata#KF359908
Poales Poaceae Oryza minuta#KF359909
Poales Poaceae Oryza longistaminata#KF359907
Poales Poaceae Oryza rufipogon#JN005831
Poales Poaceae Oryza barthii#KF359904
Poales Poaceae Oryza glaberrima#KF359903
Poales Poaceae Oryza sativa#CP018170
Poales Poaceae Porteresia coarctata#NC 036934
Poales Poaceae Buergersiochloa bambusoides#NC 026968
Poales Poaceae Pariana campestris#NC 027491
Poales Poaceae Pariana radiciflora#NC 026972
Poales Poaceae Diandrolyra sp.#NC 026960
Poales Poaceae Rehia nervata#NC 039984
Poales Poaceae Raddia brasiliensis#NC 026966
Poales Poaceae Olyra latifolia#NC 024165
Poales Poaceae Lithachne pauciflora#NC 026970
Poales Poaceae Froesiochloa boutelouoides#NC 039983
Poales Poaceae Chusquea spectabilis#NC 026959
Poales Poaceae Chusquea culeou#MH410122
Poales Poaceae Chusquea circinata#NC 027490
Poales Poaceae Chusquea liebmannii#NC 026969
Poales Poaceae Rhipidocladum pittieri#NC 036700
Poales Poaceae Guadua chacoensis#NC 029232
Poales Poaceae Guadua angustifolia#NC 029749
Poales Poaceae Guadua weberbaueri#NC 026991
Poales Poaceae Olmeca reflexa#NC 026965
Poales Poaceae Otatea acuminata#NC 026971
Poales Poaceae Otatea glauca#NC 028631
Poales Poaceae Hickelia madagascariensis#NC 026962
Poales Poaceae Greslania sp.#NC 026961
Poales Poaceae Neololeba atra#NC 026964
Poales Poaceae Dendrocalamus latiflorus#NC 013088
Poales Poaceae Neohouzeaua sp.#NC 026963
Poales Poaceae Bambusa bambos#NC 026957
Poales Poaceae Bambusa arnhemica#NC 026958
Poales Poaceae Bambusa emeiensis#NC 015830
Poales Poaceae Bambusa multiplex#NC 024668
Poales Poaceae Bambusa oldhamii#NC 012927
Poales Poaceae Bambusa tuldoides#MH410121
Poales Poaceae Ampelocalamus calcareus#NC 024731
Poales Poaceae Sasa longiligulata#NC 036825
Poales Poaceae Ferrocalamus strictus#NC 015831
Poales Poaceae Shibataea chiangshanensis#NC 036826
Poales Poaceae Gelidocalamus tessellatus#NC 024719
Poales Poaceae Arundinaria gigantea#NC 020341
Poales Poaceae Arundinaria appalachiana#NC 023934
Poales Poaceae Arundinaria gigantea subsp. tecta#NC 023935
Poales Poaceae Pseudosasa japonica#NC 028328
Poales Poaceae Acidosasa purpurea#NC 015820
Poales Poaceae Pleioblastus maculatus#NC 024723
Poales Poaceae Oligostachyum shiuyingianum#NC 024722
Poales Poaceae Pseudosasa cantorii#NC 036824
Poales Poaceae Indosasa shibataeaoides#NC 036820
Poales Poaceae Indosasa sinica#MH394379
Poales Poaceae Bergbambos tessellata#NC 036816
Poales Poaceae Indocalamus sinicus#NC 036819
Poales Poaceae Kuruna debilis#NC 036822
Poales Poaceae Chimonocalamus longiusculus#NC 024714
Poales Poaceae Ampelocalamus actinotrichus#MH410123
Poales Poaceae Oldeania alpina#NC 036813
Poales Poaceae Oldeania humbertii#MF066248
Poales Poaceae Oldeania cf. madagascariensis#NC 036823
Poales Poaceae Oldeania ibityensis#NC 036818
Poales Poaceae Oldeania itremoensis#NC 036821
Poales Poaceae Gaoligongshania megalothyrsa#NC 024718
Poales Poaceae Indocalamus wilsonii#NC 024720
Poales Poaceae Thamnocalamus spathiflorus#NC 024724
Poales Poaceae Arundinaria faberi#NC 024713
Poales Poaceae Fargesia yunnanensis#NC 024717
Poales Poaceae Yushania levigata#NC 024725
Poales Poaceae Indocalamus longiauritus#NC 015803
Poales Poaceae Drepanostachyum falcatum#NC 037167
Poales Poaceae Chimonobambusa tumidissinoda#NC 036814
Poales Poaceae Arundinaria fargesii#NC 024712
Poales Poaceae Fargesia nitida#NC 024715
Poales Poaceae Fargesia spathacea#NC 024716
Poales Poaceae Fargesia qinlingensis#NC 040002
Poales Poaceae Fargesia denudata#NC 034747
Poales Poaceae Phyllostachys edulis#NC 015817
Poales Poaceae Phyllostachys propinqua#NC 016699
Poales Poaceae Phyllostachys nigra var. henonis#NC 015826
Poales Poaceae Phyllostachys sulphurea#NC 024669
Poales Poaceae Brachyelytrum aristosum#NC 027470
Poales Poaceae Nardus stricta#NC 036695
Poales Poaceae Lygeum spartum#NC 036693
Poales Poaceae Phaenosperma globosum#NC 027480
Poales Poaceae Melica subulata#NC 027478
Poales Poaceae Melica mutica#NC 027477
Poales Poaceae Piptatherum songaricum#NC 036699
Poales Poaceae Oryzopsis hymenoides#NC 027464
Poales Poaceae Nassella trichotoma#KX792500
Poales Poaceae Nassella neesiana#MF480752
Poales Poaceae Nassella hyalina#MF480753
Poales Poaceae Ampelodesmos mauritanicus#NC 027466
Poales Poaceae Oryzopsis asperifolia#NC 027479
Poales Poaceae Piptochaetium avenaceum#NC 027483
Poales Poaceae Stipa purpurea#NC 029390
Poales Poaceae Stipa lipskyi#NC 028444
Poales Poaceae Stipa narynica#NC 037032
Poales Poaceae Stipa magnifica#NC 037031

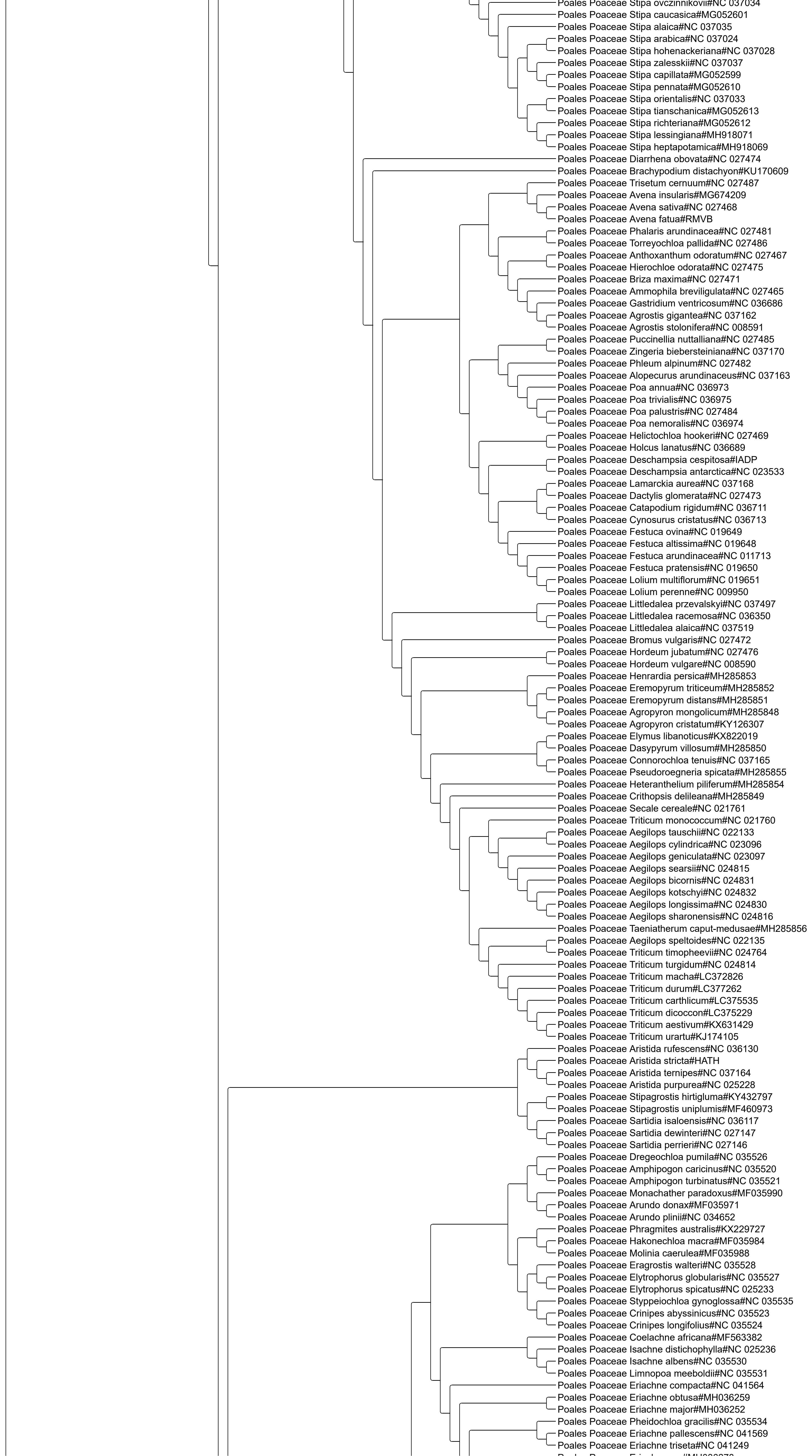
Poales Poaceae Stipa ovczinnikovii#NC 037034
Poales Poaceae Stipa caucasica#MG052601
Poales Poaceae Stipa alaica#NC 037035
Poales Poaceae Stipa arabica#NC 037024
Poales Poaceae Stipa hohenackeriana#NC 037028
Poales Poaceae Stipa zalesskii#NC 037037
Poales Poaceae Stipa capillata#MG052599
Poales Poaceae Stipa pennata#MG052610
Poales Poaceae Stipa orientalis#NC 037033
Poales Poaceae Stipa tianschanica#MG052613
Poales Poaceae Stipa richteriana#MG052612
Poales Poaceae Stipa lessingiana#MH918071
Poales Poaceae Stipa heptapotamica#MH918069
Poales Poaceae Diarrhena obovata#NC 027474
Poales Poaceae Brachypodium distachyon#KU170609
Poales Poaceae Trisetum cernuum#NC 027487
Poales Poaceae Avena insularis#MG674209
Poales Poaceae Avena sativa#NC 027468
Poales Poaceae Avena fatua#RMVB
Poales Poaceae Phalaris arundinacea#NC 027481
Poales Poaceae Torreyochloa pallida#NC 027486
Poales Poaceae Anthoxanthum odoratum#NC 027467
Poales Poaceae Hierochloe odorata#NC 027475
Poales Poaceae Briza maxima#NC 027471
Poales Poaceae Ammophila breviligulata#NC 027465
Poales Poaceae Gastridium ventricosum#NC 036686
Poales Poaceae Agrostis gigantea#NC 037162
Poales Poaceae Agrostis stolonifera#NC 008591
Poales Poaceae Puccinellia nuttalliana#NC 027485
Poales Poaceae Zingeria biebersteiniana#NC 037170
Poales Poaceae Phleum alpinum#NC 027482
Poales Poaceae Alopecurus arundinaceus#NC 037163
Poales Poaceae Poa annua#NC 036973
Poales Poaceae Poa trivialis#NC 036975
Poales Poaceae Poa palustris#NC 027484
Poales Poaceae Poa nemoralis#NC 036974
Poales Poaceae Helictochloa hookeri#NC 027469
Poales Poaceae Holcus lanatus#NC 036689
Poales Poaceae Deschampsia cespitosa#IADP
Poales Poaceae Deschampsia antarctica#NC 023533
Poales Poaceae Lamarckia aurea#NC 037168
Poales Poaceae Dactylis glomerata#NC 027473
Poales Poaceae Catapodium rigidum#NC 036711
Poales Poaceae Cynosurus cristatus#NC 036713
Poales Poaceae Festuca ovina#NC 019649
Poales Poaceae Festuca altissima#NC 019648
Poales Poaceae Festuca arundinacea#NC 011713
Poales Poaceae Festuca pratensis#NC 019650
Poales Poaceae Lolium multiflorum#NC 019651
Poales Poaceae Lolium perenne#NC 009950
Poales Poaceae Littledalea przevalskyi#NC 037497
Poales Poaceae Littledalea racemosa#NC 036350
Poales Poaceae Littledalea alaica#NC 037519
Poales Poaceae Bromus vulgaris#NC 027472
Poales Poaceae Hordeum jubatum#NC 027476
Poales Poaceae Hordeum vulgare#NC 008590
Poales Poaceae Henrardia persica#MH285853
Poales Poaceae Eremopyrum triticeum#MH285852
Poales Poaceae Eremopyrum distans#MH285851
Poales Poaceae Agropyron mongolicum#MH285848
Poales Poaceae Agropyron cristatum#KY126307
Poales Poaceae Elymus libanoticus#KX822019
Poales Poaceae Dasypyrum villosum#MH285850
Poales Poaceae Connorochloa tenuis#NC 037165
Poales Poaceae Pseudoroegneria spicata#MH285855
Poales Poaceae Heteranthelium piliferum#MH285854
Poales Poaceae Crithopsis delileana#MH285849
Poales Poaceae Secale cereale#NC 021761
Poales Poaceae Triticum monococcum#NC 021760
Poales Poaceae Aegilops tauschii#NC 022133
Poales Poaceae Aegilops cylindrica#NC 023096
Poales Poaceae Aegilops geniculata#NC 023097
Poales Poaceae Aegilops searsii#NC 024815
Poales Poaceae Aegilops bicornis#NC 024831
Poales Poaceae Aegilops kotschyi#NC 024832
Poales Poaceae Aegilops longissima#NC 024830
Poales Poaceae Aegilops sharonensis#NC 024816
Poales Poaceae Taeniatherum caput-medusae#MH285856
Poales Poaceae Aegilops speltoides#NC 022135
Poales Poaceae Triticum timopheevii#NC 024764
Poales Poaceae Triticum turgidum#NC 024814
Poales Poaceae Triticum macha#LC372826
Poales Poaceae Triticum durum#LC377262
Poales Poaceae Triticum carthlicum#LC375535
Poales Poaceae Triticum dicoccon#LC375229
Poales Poaceae Triticum aestivum#KX631429
Poales Poaceae Triticum urartu#KJ174105
Poales Poaceae Aristida rufescens#NC 036130
Poales Poaceae Aristida stricta#HATH
Poales Poaceae Aristida ternipes#NC 037164
Poales Poaceae Aristida purpurea#NC 025228
Poales Poaceae Stipagrostis hirtigluma#KY432797
Poales Poaceae Stipagrostis uniplumis#MF460973
Poales Poaceae Sartidia isaloensis#NC 036117
Poales Poaceae Sartidia dewinteri#NC 027147
Poales Poaceae Sartidia perrieri#NC 027146
Poales Poaceae Dregeochloa pumila#NC 035526
Poales Poaceae Amphipogon caricinus#NC 035520
Poales Poaceae Amphipogon turbinatus#NC 035521
Poales Poaceae Monachather paradoxus#MF035990
Poales Poaceae Arundo donax#MF035971
Poales Poaceae Arundo plinii#NC 034652
Poales Poaceae Phragmites australis#KX229727
Poales Poaceae Hakonechloa macra#MF035984
Poales Poaceae Molinia caerulea#MF035988
Poales Poaceae Eragrostis walteri#NC 035528
Poales Poaceae Elytrophorus globularis#NC 035527
Poales Poaceae Elytrophorus spicatus#NC 025233
Poales Poaceae Styppeiochloa gynoglossa#NC 035535
Poales Poaceae Crinipes abyssinicus#NC 035523
Poales Poaceae Crinipes longifolius#NC 035524
Poales Poaceae Coelachne africana#MF563382
Poales Poaceae Isachne distichophylla#NC 025236
Poales Poaceae Isachne albens#NC 035530
Poales Poaceae Limnopoa meeboldii#NC 035531
Poales Poaceae Eriachne compacta#NC 041564
Poales Poaceae Eriachne obtusa#MH036259
Poales Poaceae Eriachne major#MH036252
Poales Poaceae Pheidochloa gracilis#NC 035534
Poales Poaceae Eriachne pallescens#NC 041569
Poales Poaceae Eriachne triseta#NC 041249

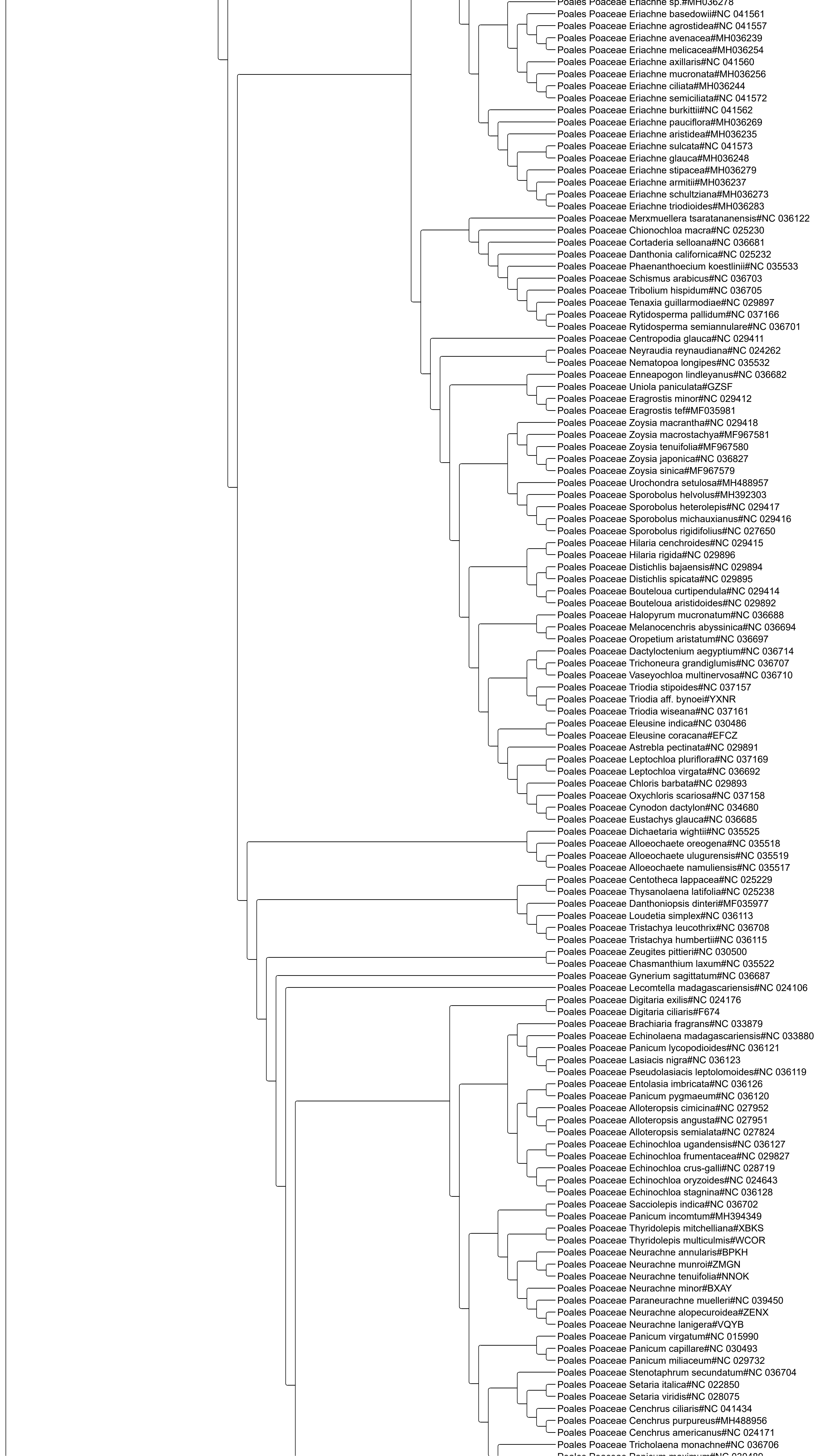

Poales Poaceae Eriachne sp.#MH036278
Poales Poaceae Eriachne basedowii#NC 041561
Poales Poaceae Eriachne agrostidea#NC 041557
Poales Poaceae Eriachne avenacea#MH036239
Poales Poaceae Eriachne melicacea#MH036254
Poales Poaceae Eriachne axillaris#NC 041560
Poales Poaceae Eriachne mucronata#MH036256
Poales Poaceae Eriachne ciliata#MH036244
Poales Poaceae Eriachne semiciliata#NC 041572
Poales Poaceae Eriachne burkittii#NC 041562
Poales Poaceae Eriachne pauciflora#MH036269
Poales Poaceae Eriachne aristidea#MH036235
Poales Poaceae Eriachne sulcata#NC 041573
Poales Poaceae Eriachne glauca#MH036248
Poales Poaceae Eriachne stipacea#MH036279
Poales Poaceae Eriachne armitii#MH036237
Poales Poaceae Eriachne schultziana#MH036273
Poales Poaceae Eriachne triodioides#MH036283
Poales Poaceae Merxmuellera tsaratananensis#NC 036122
Poales Poaceae Chionochloa macra#NC 025230
Poales Poaceae Cortaderia selloana#NC 036681
Poales Poaceae Danthonia californica#NC 025232
Poales Poaceae Phaenanthoecium koestlinii#NC 035533
Poales Poaceae Schismus arabicus#NC 036703
Poales Poaceae Tribolium hispidum#NC 036705
Poales Poaceae Tenaxia guillarmodiae#NC 029897
Poales Poaceae Rytidosperma pallidum#NC 037166
Poales Poaceae Rytidosperma semiannulare#NC 036701
Poales Poaceae Centropodia glauca#NC 029411
Poales Poaceae Neyraudia reynaudiana#NC 024262
Poales Poaceae Nematopoa longipes#NC 035532
Poales Poaceae Enneapogon lindleyanus#NC 036682
Poales Poaceae Uniola paniculata#GZSF
Poales Poaceae Eragrostis minor#NC 029412
Poales Poaceae Eragrostis tef#MF035981
Poales Poaceae Zoysia macrantha#NC 029418
Poales Poaceae Zoysia macrostachya#MF967581
Poales Poaceae Zoysia tenuifolia#MF967580
Poales Poaceae Zoysia japonica#NC 036827
Poales Poaceae Zoysia sinica#MF967579
Poales Poaceae Urochondra setulosa#MH488957
Poales Poaceae Sporobolus helvolus#MH392303
Poales Poaceae Sporobolus heterolepis#NC 029417
Poales Poaceae Sporobolus michauxianus#NC 029416
Poales Poaceae Sporobolus rigidifolius#NC 027650
Poales Poaceae Hilaria cenchroides#NC 029415
Poales Poaceae Hilaria rigida#NC 029896
Poales Poaceae Distichlis bajaensis#NC 029894
Poales Poaceae Distichlis spicata#NC 029895
Poales Poaceae Bouteloua curtipendula#NC 029414
Poales Poaceae Bouteloua aristidoides#NC 029892
Poales Poaceae Halopyrum mucronatum#NC 036688
Poales Poaceae Melanocenchris abyssinica#NC 036694
Poales Poaceae Oropetium aristatum#NC 036697
Poales Poaceae Dactyloctenium aegyptium#NC 036714
Poales Poaceae Trichoneura grandiglumis#NC 036707
Poales Poaceae Vaseyochloa multinervosa#NC 036710
Poales Poaceae Triodia stipoides#NC 037157
Poales Poaceae Triodia aff. bynoei#YXNR
Poales Poaceae Triodia wiseana#NC 037161
Poales Poaceae Eleusine indica#NC 030486
Poales Poaceae Eleusine coracana#EFCZ
Poales Poaceae Astrebla pectinata#NC 029891
Poales Poaceae Leptochloa pluriflora#NC 037169
Poales Poaceae Leptochloa virgata#NC 036692
Poales Poaceae Chloris barbata#NC 029893
Poales Poaceae Oxychloris scariosa#NC 037158
Poales Poaceae Cynodon dactylon#NC 034680
Poales Poaceae Eustachys glauca#NC 036685
Poales Poaceae Dichaetaria wightii#NC 035525
Poales Poaceae Alloeochaete oreogena#NC 035518
Poales Poaceae Alloeochaete ulugurensis#NC 035519
Poales Poaceae Alloeochaete namuliensis#NC 035517
Poales Poaceae Centotheca lappacea#NC 025229
Poales Poaceae Thysanolaena latifolia#NC 025238
Poales Poaceae Danthoniopsis dinteri#MF035977
Poales Poaceae Loudetia simplex#NC 036113
Poales Poaceae Tristachya leucothrix#NC 036708
Poales Poaceae Tristachya humbertii#NC 036115
Poales Poaceae Zeugites pittieri#NC 030500
Poales Poaceae Chasmanthium laxum#NC 035522
Poales Poaceae Gynerium sagittatum#NC 036687
Poales Poaceae Lecomtella madagascariensis#NC 024106
Poales Poaceae Digitaria exilis#NC 024176
Poales Poaceae Digitaria ciliaris#F674
Poales Poaceae Brachiaria fragrans#NC 033879
Poales Poaceae Echinolaena madagascariensis#NC 033880
Poales Poaceae Panicum lycopodioides#NC 036121
Poales Poaceae Lasiacis nigra#NC 036123
Poales Poaceae Pseudolasiacis leptolomoides#NC 036119
Poales Poaceae Entolasia imbricata#NC 036126
Poales Poaceae Panicum pygmaeum#NC 036120
Poales Poaceae Alloteropsis cimicina#NC 027952
Poales Poaceae Alloteropsis angusta#NC 027951
Poales Poaceae Alloteropsis semialata#NC 027824
Poales Poaceae Echinochloa ugandensis#NC 036127
Poales Poaceae Echinochloa frumentacea#NC 029827
Poales Poaceae Echinochloa crus-galli#NC 028719
Poales Poaceae Echinochloa oryzoides#NC 024643
Poales Poaceae Echinochloa stagnina#NC 036128
Poales Poaceae Sacciolepis indica#NC 036702
Poales Poaceae Panicum incomtum#MH394349
Poales Poaceae Thyridolepis mitchelliana#XBKS
Poales Poaceae Thyridolepis multiculmis#WCOR
Poales Poaceae Neurachne annularis#BPKH
Poales Poaceae Neurachne munroi#ZMGN
Poales Poaceae Neurachne tenuifolia#NNOK
Poales Poaceae Neurachne minor#BXAY
Poales Poaceae Paraneurachne muelleri#NC 039450
Poales Poaceae Neurachne alopecuroidea#ZENX
Poales Poaceae Neurachne lanigera#VQYB
Poales Poaceae Panicum virgatum#NC 015990
Poales Poaceae Panicum capillare#NC 030493
Poales Poaceae Panicum miliaceum#NC 029732
Poales Poaceae Stenotaphrum secundatum#NC 036704
Poales Poaceae Setaria italica#NC 022850
Poales Poaceae Setaria viridis#NC 028075
Poales Poaceae Cenchrus ciliaris#NC 041434
Poales Poaceae Cenchrus purpureus#MH488956
Poales Poaceae Cenchrus americanus#NC 024171
Poales Poaceae Tricholaena monachne#NC 036706

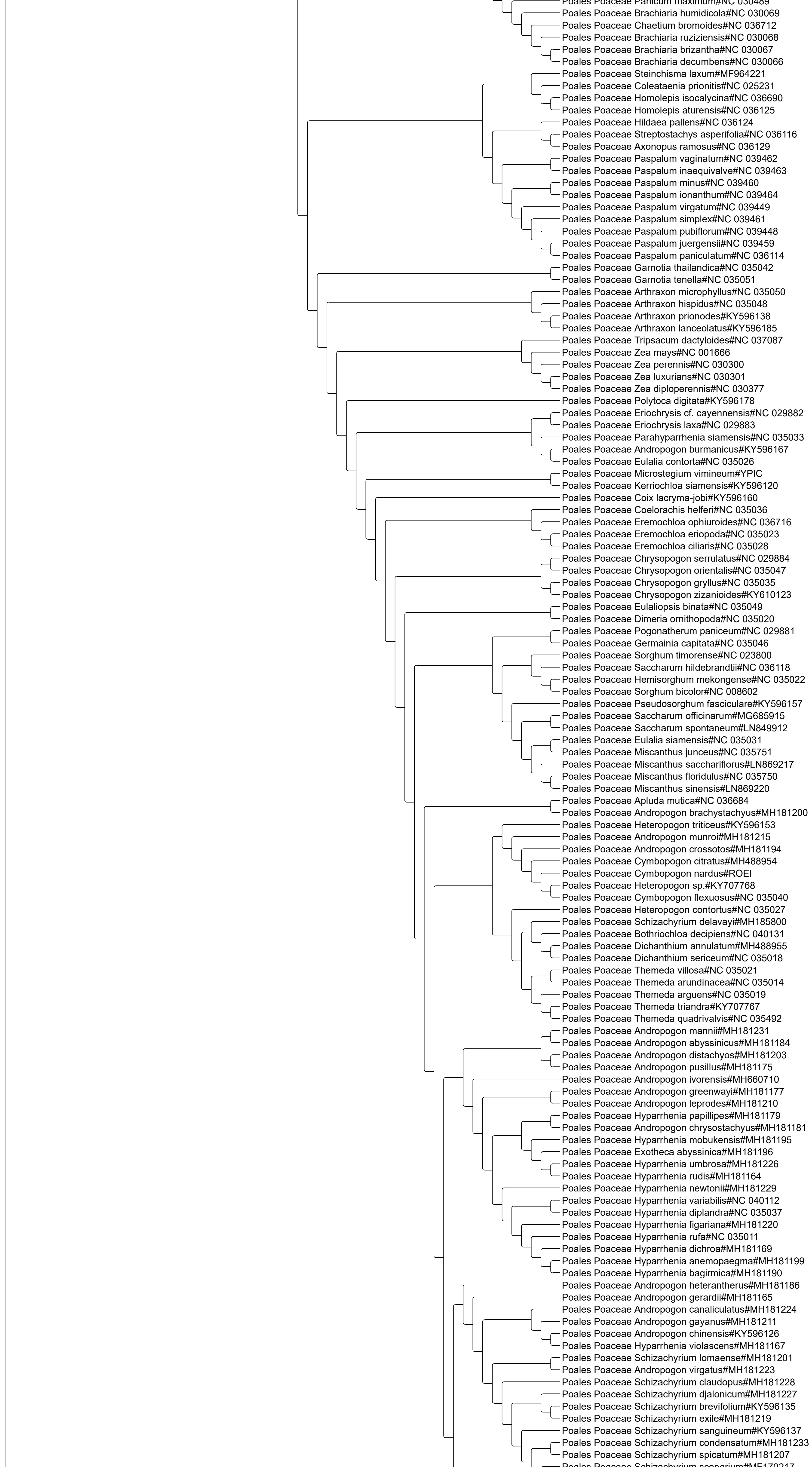

Poales Poaceae Panicum maximum#NC 030489
Poales Poaceae Brachiaria humidicola#NC 030069
Poales Poaceae Chaetium bromoides#NC 036712
Poales Poaceae Brachiaria ruziziensis#NC 030068
Poales Poaceae Brachiaria brizantha#NC 030067
Poales Poaceae Brachiaria decumbens#NC 030066
Poales Poaceae Steinchisma laxum#MF964221
Poales Poaceae Coleataenia prionitis#NC 025231
Poales Poaceae Homolepis isocalycina#NC 036690
Poales Poaceae Homolepis aturensis#NC 036125
Poales Poaceae Hildaea pallens#NC 036124
Poales Poaceae Streptostachys asperifolia#NC 036116
Poales Poaceae Axonopus ramosus#NC 036129
Poales Poaceae Paspalum vaginatum#NC 039462
Poales Poaceae Paspalum inaequivalve#NC 039463
Poales Poaceae Paspalum minus#NC 039460
Poales Poaceae Paspalum ionanthum#NC 039464
Poales Poaceae Paspalum virgatum#NC 039449
Poales Poaceae Paspalum simplex#NC 039461
Poales Poaceae Paspalum pubiflorum#NC 039448
Poales Poaceae Paspalum juergensii#NC 039459
Poales Poaceae Paspalum paniculatum#NC 036114
Poales Poaceae Garnotia thailandica#NC 035042
Poales Poaceae Garnotia tenella#NC 035051
Poales Poaceae Arthraxon microphyllus#NC 035050
Poales Poaceae Arthraxon hispidus#NC 035048
Poales Poaceae Arthraxon prionodes#KY596138
Poales Poaceae Arthraxon lanceolatus#KY596185
Poales Poaceae Tripsacum dactyloides#NC 037087
Poales Poaceae Zea mays#NC 001666
Poales Poaceae Zea perennis#NC 030300
Poales Poaceae Zea luxurians#NC 030301
Poales Poaceae Zea diploperennis#NC 030377
Poales Poaceae Polytoca digitata#KY596178
Poales Poaceae Eriochrysis cf. cayennensis#NC 029882
Poales Poaceae Eriochrysis laxa#NC 029883
Poales Poaceae Parahyparrhenia siamensis#NC 035033
Poales Poaceae Andropogon burmanicus#KY596167
Poales Poaceae Eulalia contorta#NC 035026
Poales Poaceae Microstegium vimineum#YPIC
Poales Poaceae Kerriochloa siamensis#KY596120
Poales Poaceae Coix lacryma-jobi#KY596160
Poales Poaceae Coelorachis helferi#NC 035036
Poales Poaceae Eremochloa ophiuroides#NC 036716
Poales Poaceae Eremochloa eriopoda#NC 035023
Poales Poaceae Eremochloa ciliaris#NC 035028
Poales Poaceae Chrysopogon serrulatus#NC 029884
Poales Poaceae Chrysopogon orientalis#NC 035047
Poales Poaceae Chrysopogon gryllus#NC 035035
Poales Poaceae Chrysopogon zizanioides#KY610123
Poales Poaceae Eulaliopsis binata#NC 035049
Poales Poaceae Dimeria ornithopoda#NC 035020
Poales Poaceae Pogonatherum paniceum#NC 029881
Poales Poaceae Germainia capitata#NC 035046
Poales Poaceae Sorghum timorense#NC 023800
Poales Poaceae Saccharum hildebrandtii#NC 036118
Poales Poaceae Hemisorghum mekongense#NC 035022
Poales Poaceae Sorghum bicolor#NC 008602
Poales Poaceae Pseudosorghum fasciculare#KY596157
Poales Poaceae Saccharum officinarum#MG685915
Poales Poaceae Saccharum spontaneum#LN849912
Poales Poaceae Eulalia siamensis#NC 035031
Poales Poaceae Miscanthus junceus#NC 035751
Poales Poaceae Miscanthus sacchariflorus#LN869217
Poales Poaceae Miscanthus floridulus#NC 035750
Poales Poaceae Miscanthus sinensis#LN869220
Poales Poaceae Apluda mutica#NC 036684
Poales Poaceae Andropogon brachystachyus#MH181200
Poales Poaceae Heteropogon triticeus#KY596153
Poales Poaceae Andropogon munroi#MH181215
Poales Poaceae Andropogon crossotos#MH181194
Poales Poaceae Cymbopogon citratus#MH488954
Poales Poaceae Cymbopogon nardus#ROEI
Poales Poaceae Heteropogon sp.#KY707768
Poales Poaceae Cymbopogon flexuosus#NC 035040
Poales Poaceae Heteropogon contortus#NC 035027
Poales Poaceae Schizachyrium delavayi#MH185800
Poales Poaceae Bothriochloa decipiens#NC 040131
Poales Poaceae Dichanthium annulatum#MH488955
Poales Poaceae Dichanthium sericeum#NC 035018
Poales Poaceae Themeda villosa#NC 035021
Poales Poaceae Themeda arundinacea#NC 035014
Poales Poaceae Themeda arguens#NC 035019
Poales Poaceae Themeda triandra#KY707767
Poales Poaceae Themeda quadrivalvis#NC 035492
Poales Poaceae Andropogon mannii#MH181231
Poales Poaceae Andropogon abyssinicus#MH181184
Poales Poaceae Andropogon distachyos#MH181203
Poales Poaceae Andropogon pusillus#MH181175
Poales Poaceae Andropogon ivorensis#MH660710
Poales Poaceae Andropogon greenwayi#MH181177
Poales Poaceae Andropogon leprodes#MH181210
Poales Poaceae Hyparrhenia papillipes#MH181179
Poales Poaceae Andropogon chrysostachyus#MH181181
Poales Poaceae Hyparrhenia mobukensis#MH181195
Poales Poaceae Exotheca abyssinica#MH181196
Poales Poaceae Hyparrhenia umbrosa#MH181226
Poales Poaceae Hyparrhenia rudis#MH181164
Poales Poaceae Hyparrhenia newtonii#MH181229
Poales Poaceae Hyparrhenia variabilis#NC 040112
Poales Poaceae Hyparrhenia diplandra#NC 035037
Poales Poaceae Hyparrhenia figariana#MH181220
Poales Poaceae Hyparrhenia rufa#NC 035011
Poales Poaceae Hyparrhenia dichroa#MH181169
Poales Poaceae Hyparrhenia anemopaegma#MH181199
Poales Poaceae Hyparrhenia bagirmica#MH181190
Poales Poaceae Andropogon heterantherus#MH181186
Poales Poaceae Andropogon gerardii#MH181165
Poales Poaceae Andropogon canaliculatus#MH181224
Poales Poaceae Andropogon gayanus#MH181211
Poales Poaceae Andropogon chinensis#KY596126
Poales Poaceae Hyparrhenia violascens#MH181167
Poales Poaceae Schizachyrium lomaense#MH181201
Poales Poaceae Andropogon virgatus#MH181223
Poales Poaceae Schizachyrium claudopus#MH181228
Poales Poaceae Schizachyrium djalonicum#MH181227
Poales Poaceae Schizachyrium brevifolium#KY596135
Poales Poaceae Schizachyrium exile#MH181219
Poales Poaceae Schizachyrium sanguineum#KY596137
Poales Poaceae Schizachyrium condensatum#MH181233
Poales Poaceae Schizachyrium spicatum#MH181207

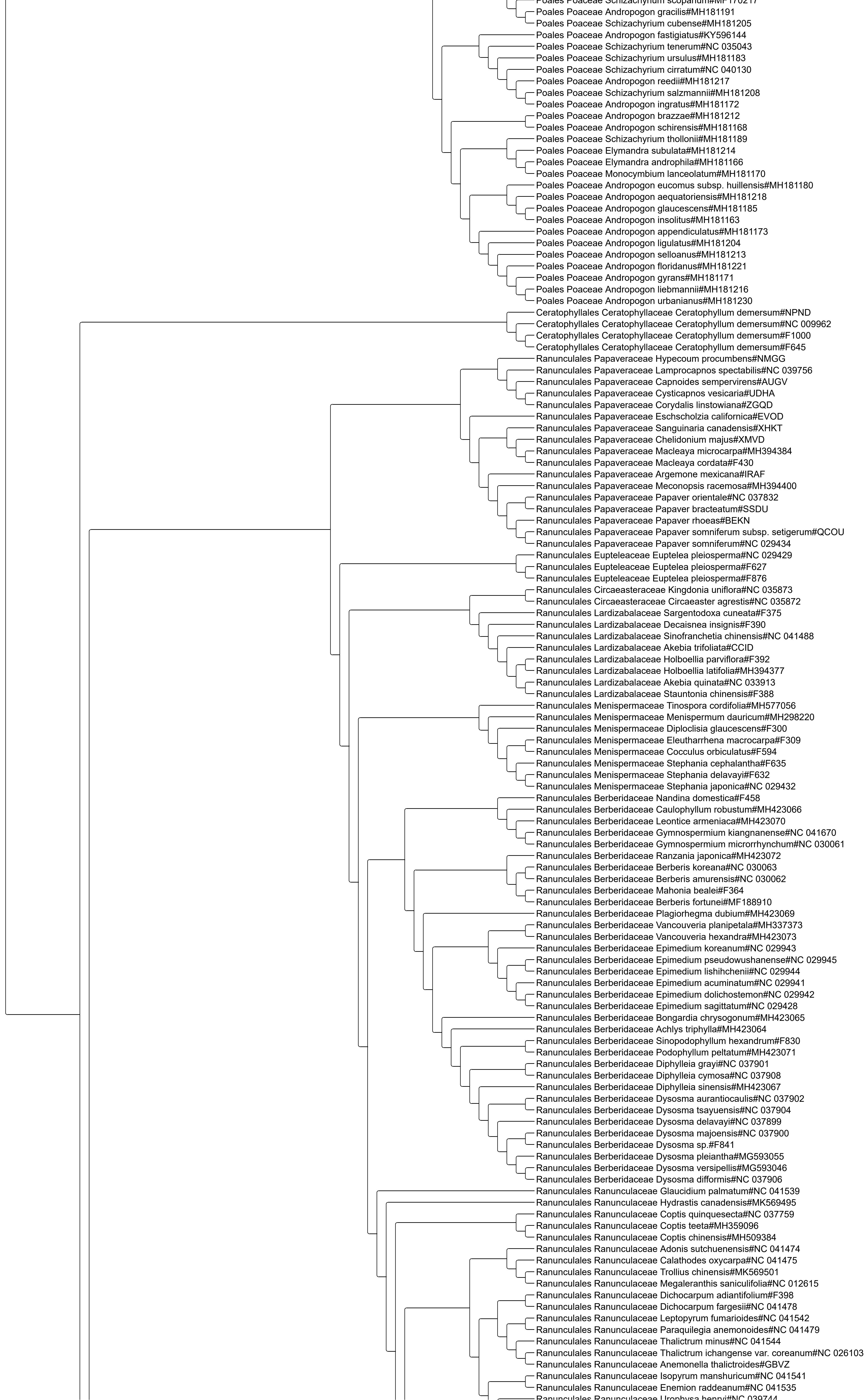
Poales Poaceae Andropogon gracilis#MH181191
Poales Poaceae Schizachyrium cubense#MH181205
Poales Poaceae Andropogon fastigiatus#KY596144
Poales Poaceae Schizachyrium tenerum#NC 035043
Poales Poaceae Schizachyrium ursulus#MH181183
Poales Poaceae Schizachyrium cirratum#NC 040130
Poales Poaceae Andropogon reedii#MH181217
Poales Poaceae Schizachyrium salzmannii#MH181208
Poales Poaceae Andropogon ingratus#MH181172
Poales Poaceae Andropogon brazzae#MH181212
Poales Poaceae Andropogon schirensis#MH181168
Poales Poaceae Schizachyrium thollonii#MH181189
Poales Poaceae Elymandra subulata#MH181214
Poales Poaceae Elymandra androphila#MH181166
Poales Poaceae Monocymbium lanceolatum#MH181170
Poales Poaceae Andropogon eucomus subsp. huillensis#MH181180
Poales Poaceae Andropogon aequatoriensis#MH181218
Poales Poaceae Andropogon glaucescens#MH181185
Poales Poaceae Andropogon insolitus#MH181163
Poales Poaceae Andropogon appendiculatus#MH181173
Poales Poaceae Andropogon ligulatus#MH181204
Poales Poaceae Andropogon selloanus#MH181213
Poales Poaceae Andropogon floridanus#MH181221
Poales Poaceae Andropogon gyrans#MH181171
Poales Poaceae Andropogon liebmannii#MH181216
Poales Poaceae Andropogon urbanianus#MH181230
Ceratophyllales Ceratophyllaceae Ceratophyllum demersum#NPND
Ceratophyllales Ceratophyllaceae Ceratophyllum demersum#NC 009962
Ceratophyllales Ceratophyllaceae Ceratophyllum demersum#F1000
Ceratophyllales Ceratophyllaceae Ceratophyllum demersum#F645
Ranunculales Papaveraceae Hypecoum procumbens#NMGG
Ranunculales Papaveraceae Lamprocapnos spectabilis#NC 039756
Ranunculales Papaveraceae Capnoides sempervirens#AUGV
Ranunculales Papaveraceae Cysticapnos vesicaria#UDHA
Ranunculales Papaveraceae Corydalis linstowiana#ZGQD
Ranunculales Papaveraceae Eschscholzia californica#EVOD
Ranunculales Papaveraceae Sanguinaria canadensis#XHKT
Ranunculales Papaveraceae Chelidonium majus#XMVD
Ranunculales Papaveraceae Macleaya microcarpa#MH394384
Ranunculales Papaveraceae Macleaya cordata#F430
Ranunculales Papaveraceae Argemone mexicana#IRAF
Ranunculales Papaveraceae Meconopsis racemosa#MH394400
Ranunculales Papaveraceae Papaver orientale#NC 037832
Ranunculales Papaveraceae Papaver bracteatum#SSDU
Ranunculales Papaveraceae Papaver rhoeas#BEKN
Ranunculales Papaveraceae Papaver somniferum subsp. setigerum#QCOU
Ranunculales Papaveraceae Papaver somniferum#NC 029434
Ranunculales Eupteleaceae Euptelea pleiosperma#NC 029429
Ranunculales Eupteleaceae Euptelea pleiosperma#F627
Ranunculales Eupteleaceae Euptelea pleiosperma#F876
Ranunculales Circaeasteraceae Kingdonia uniflora#NC 035873
Ranunculales Circaeasteraceae Circaeaster agrestis#NC 035872
Ranunculales Lardizabalaceae Sargentodoxa cuneata#F375
Ranunculales Lardizabalaceae Decaisnea insignis#F390
Ranunculales Lardizabalaceae Sinofranchetia chinensis#NC 041488
Ranunculales Lardizabalaceae Akebia trifoliata#CCID
Ranunculales Lardizabalaceae Holboellia parviflora#F392
Ranunculales Lardizabalaceae Holboellia latifolia#MH394377
Ranunculales Lardizabalaceae Akebia quinata#NC 033913
Ranunculales Lardizabalaceae Stauntonia chinensis#F388
Ranunculales Menispermaceae Tinospora cordifolia#MH577056
Ranunculales Menispermaceae Menispermum dauricum#MH298220
Ranunculales Menispermaceae Diploclisia glaucescens#F300
Ranunculales Menispermaceae Eleutharrhena macrocarpa#F309
Ranunculales Menispermaceae Cocculus orbiculatus#F594
Ranunculales Menispermaceae Stephania cephalantha#F635
Ranunculales Menispermaceae Stephania delavayi#F632
Ranunculales Menispermaceae Stephania japonica#NC 029432
Ranunculales Berberidaceae Nandina domestica#F458
Ranunculales Berberidaceae Caulophyllum robustum#MH423066
Ranunculales Berberidaceae Leontice armeniaca#MH423070
Ranunculales Berberidaceae Gymnospermium kiangnanense#NC 041670
Ranunculales Berberidaceae Gymnospermium microrrhynchum#NC 030061
Ranunculales Berberidaceae Ranzania japonica#MH423072
Ranunculales Berberidaceae Berberis koreana#NC 030063
Ranunculales Berberidaceae Berberis amurensis#NC 030062
Ranunculales Berberidaceae Mahonia bealei#F364
Ranunculales Berberidaceae Berberis fortunei#MF188910
Ranunculales Berberidaceae Plagiorhegma dubium#MH423069
Ranunculales Berberidaceae Vancouveria planipetala#MH337373
Ranunculales Berberidaceae Vancouveria hexandra#MH423073
Ranunculales Berberidaceae Epimedium koreanum#NC 029943
Ranunculales Berberidaceae Epimedium pseudowushanense#NC 029945
Ranunculales Berberidaceae Epimedium lishihchenii#NC 029944
Ranunculales Berberidaceae Epimedium acuminatum#NC 029941
Ranunculales Berberidaceae Epimedium dolichostemon#NC 029942
Ranunculales Berberidaceae Epimedium sagittatum#NC 029428
Ranunculales Berberidaceae Bongardia chrysogonum#MH423065
Ranunculales Berberidaceae Achlys triphylla#MH423064
Ranunculales Berberidaceae Sinopodophyllum hexandrum#F830
Ranunculales Berberidaceae Podophyllum peltatum#MH423071
Ranunculales Berberidaceae Diphylleia grayi#NC 037901
Ranunculales Berberidaceae Diphylleia cymosa#NC 037908
Ranunculales Berberidaceae Diphylleia sinensis#MH423067
Ranunculales Berberidaceae Dysosma aurantiocaulis#NC 037902
Ranunculales Berberidaceae Dysosma tsayuensis#NC 037904
Ranunculales Berberidaceae Dysosma delavayi#NC 037899
Ranunculales Berberidaceae Dysosma majoensis#NC 037900
Ranunculales Berberidaceae Dysosma sp.#F841
Ranunculales Berberidaceae Dysosma pleiantha#MG593055
Ranunculales Berberidaceae Dysosma versipellis#MG593046
Ranunculales Berberidaceae Dysosma difformis#NC 037906
Ranunculales Ranunculaceae Glaucidium palmatum#NC 041539
Ranunculales Ranunculaceae Hydrastis canadensis#MK569495
Ranunculales Ranunculaceae Coptis quinquesecta#NC 037759
Ranunculales Ranunculaceae Coptis teeta#MH359096
Ranunculales Ranunculaceae Coptis chinensis#MH509384
Ranunculales Ranunculaceae Adonis sutchuenensis#NC 041474
Ranunculales Ranunculaceae Calathodes oxycarpa#NC 041475
Ranunculales Ranunculaceae Trollius chinensis#MK569501
Ranunculales Ranunculaceae Megaleranthis saniculifolia#NC 012615
Ranunculales Ranunculaceae Dichocarpum adiantifolium#F398
Ranunculales Ranunculaceae Dichocarpum fargesii#NC 041478
Ranunculales Ranunculaceae Leptopyrum fumarioides#NC 041542
Ranunculales Ranunculaceae Paraquilegia anemonoides#NC 041479
Ranunculales Ranunculaceae Thalictrum minus#NC 041544
Ranunculales Ranunculaceae Thalictrum ichangense var. coreanum#NC 026103
Ranunculales Ranunculaceae Anemonella thalictroides#GBVZ
Ranunculales Ranunculaceae Isopyrum manshuricum#NC 041541
Ranunculales Ranunculaceae Enemion raddeanum#NC 041535

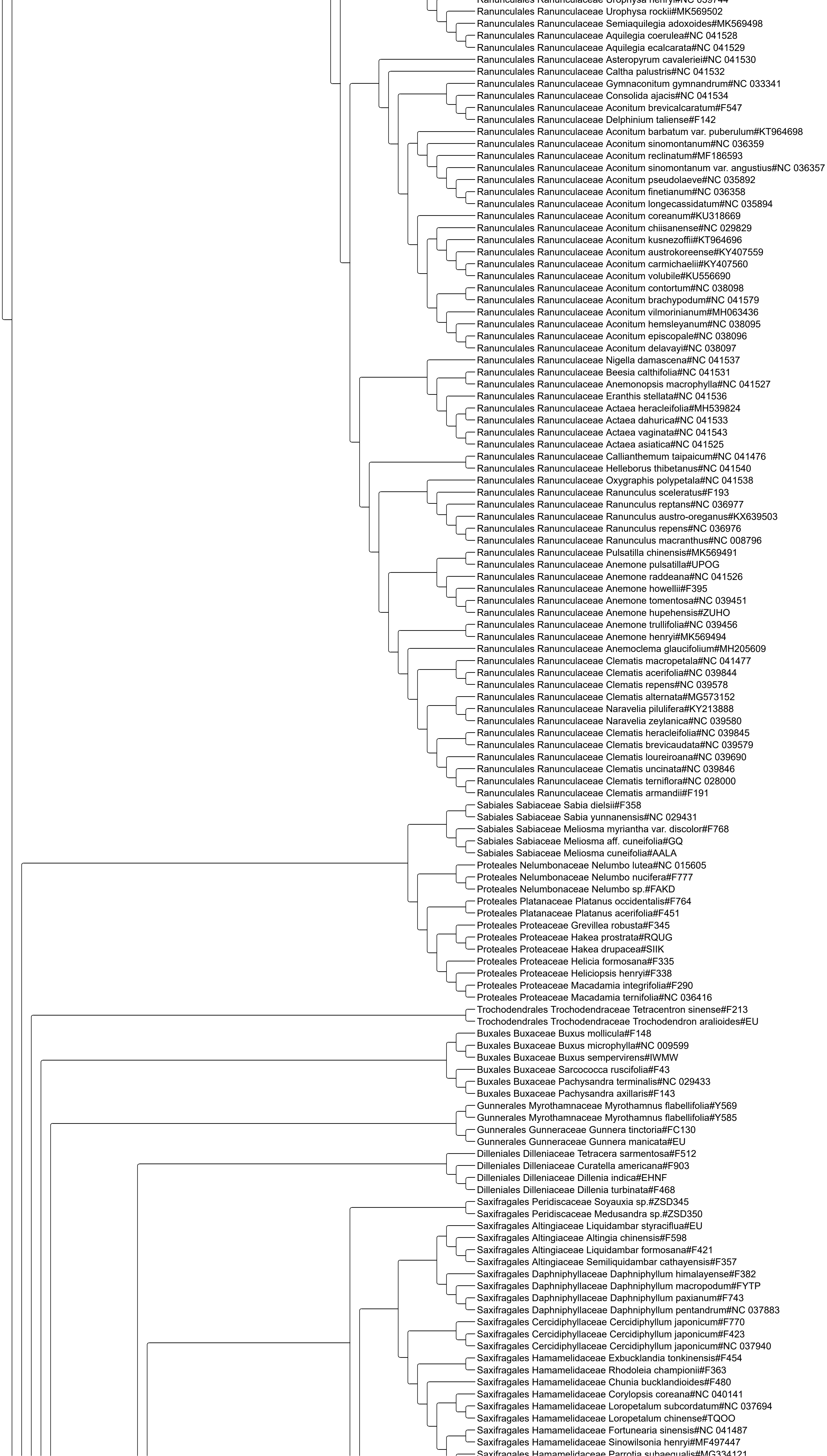
Ranunculales Ranunculaceae Urophysa rockii#MK569502
Ranunculales Ranunculaceae Semiaquilegia adoxoides#MK569498
Ranunculales Ranunculaceae Aquilegia coerulea#NC 041528
Ranunculales Ranunculaceae Aquilegia ecalcarata#NC 041529
Ranunculales Ranunculaceae Asteropyrum cavaleriei#NC 041530
Ranunculales Ranunculaceae Caltha palustris#NC 041532
Ranunculales Ranunculaceae Gymnaconitum gymnandrum#NC 033341
Ranunculales Ranunculaceae Consolida ajacis#NC 041534
Ranunculales Ranunculaceae Aconitum brevicalcaratum#F547
Ranunculales Ranunculaceae Delphinium taliense#F142
Ranunculales Ranunculaceae Aconitum barbatum var. puberulum#KT964698
Ranunculales Ranunculaceae Aconitum sinomontanum#NC 036359
Ranunculales Ranunculaceae Aconitum reclinatum#MF186593
Ranunculales Ranunculaceae Aconitum sinomontanum var. angustius#NC 036357
Ranunculales Ranunculaceae Aconitum pseudolaeve#NC 035892
Ranunculales Ranunculaceae Aconitum finetianum#NC 036358
Ranunculales Ranunculaceae Aconitum longecassidatum#NC 035894
Ranunculales Ranunculaceae Aconitum coreanum#KU318669
Ranunculales Ranunculaceae Aconitum chiisanense#NC 029829
Ranunculales Ranunculaceae Aconitum kusnezoffii#KT964696
Ranunculales Ranunculaceae Aconitum austrokoreense#KY407559
Ranunculales Ranunculaceae Aconitum carmichaelii#KY407560
Ranunculales Ranunculaceae Aconitum volubile#KU556690
Ranunculales Ranunculaceae Aconitum contortum#NC 038098
Ranunculales Ranunculaceae Aconitum brachypodum#NC 041579
Ranunculales Ranunculaceae Aconitum vilmorinianum#MH063436
Ranunculales Ranunculaceae Aconitum hemsleyanum#NC 038095
Ranunculales Ranunculaceae Aconitum episcopale#NC 038096
Ranunculales Ranunculaceae Aconitum delavayi#NC 038097
Ranunculales Ranunculaceae Nigella damascena#NC 041537
Ranunculales Ranunculaceae Beesia calthifolia#NC 041531
Ranunculales Ranunculaceae Anemonopsis macrophylla#NC 041527
Ranunculales Ranunculaceae Eranthis stellata#NC 041536
Ranunculales Ranunculaceae Actaea heracleifolia#MH539824
Ranunculales Ranunculaceae Actaea dahurica#NC 041533
Ranunculales Ranunculaceae Actaea vaginata#NC 041543
Ranunculales Ranunculaceae Actaea asiatica#NC 041525
Ranunculales Ranunculaceae Callianthemum taipaicum#NC 041476
Ranunculales Ranunculaceae Helleborus thibetanus#NC 041540
Ranunculales Ranunculaceae Oxygraphis polypetala#NC 041538
Ranunculales Ranunculaceae Ranunculus sceleratus#F193
Ranunculales Ranunculaceae Ranunculus reptans#NC 036977
Ranunculales Ranunculaceae Ranunculus austro-oreganus#KX639503
Ranunculales Ranunculaceae Ranunculus repens#NC 036976
Ranunculales Ranunculaceae Ranunculus macranthus#NC 008796
Ranunculales Ranunculaceae Pulsatilla chinensis#MK569491
Ranunculales Ranunculaceae Anemone pulsatilla#UPOG
Ranunculales Ranunculaceae Anemone raddeana#NC 041526
Ranunculales Ranunculaceae Anemone howellii#F395
Ranunculales Ranunculaceae Anemone tomentosa#NC 039451
Ranunculales Ranunculaceae Anemone hupehensis#ZUHO
Ranunculales Ranunculaceae Anemone trullifolia#NC 039456
Ranunculales Ranunculaceae Anemone henryi#MK569494
Ranunculales Ranunculaceae Anemoclema glaucifolium#MH205609
Ranunculales Ranunculaceae Clematis macropetala#NC 041477
Ranunculales Ranunculaceae Clematis acerifolia#NC 039844
Ranunculales Ranunculaceae Clematis repens#NC 039578
Ranunculales Ranunculaceae Clematis alternata#MG573152
Ranunculales Ranunculaceae Naravelia pilulifera#KY213888
Ranunculales Ranunculaceae Naravelia zeylanica#NC 039580
Ranunculales Ranunculaceae Clematis heracleifolia#NC 039845
Ranunculales Ranunculaceae Clematis brevicaudata#NC 039579
Ranunculales Ranunculaceae Clematis loureiroana#NC 039690
Ranunculales Ranunculaceae Clematis uncinata#NC 039846
Ranunculales Ranunculaceae Clematis terniflora#NC 028000
Ranunculales Ranunculaceae Clematis armandii#F191
Sabiales Sabiaceae Sabia dielsii#F358
Sabiales Sabiaceae Sabia yunnanensis#NC 029431
Sabiales Sabiaceae Meliosma myriantha var. discolor#F768
Sabiales Sabiaceae Meliosma aff. cuneifolia#GQ
Sabiales Sabiaceae Meliosma cuneifolia#AALA
Proteales Nelumbonaceae Nelumbo lutea#NC 015605
Proteales Nelumbonaceae Nelumbo nucifera#F777
Proteales Nelumbonaceae Nelumbo sp.#FAKD
Proteales Platanaceae Platanus occidentalis#F764
Proteales Platanaceae Platanus acerifolia#F451
Proteales Proteaceae Grevillea robusta#F345
Proteales Proteaceae Hakea prostrata#RQUG
Proteales Proteaceae Hakea drupacea#SIIK
Proteales Proteaceae Helicia formosana#F335
Proteales Proteaceae Heliciopsis henryi#F338
Proteales Proteaceae Macadamia integrifolia#F290
Proteales Proteaceae Macadamia ternifolia#NC 036416
Trochodendrales Trochodendraceae Tetracentron sinense#F213
Trochodendrales Trochodendraceae Trochodendron aralioides#EU
Buxales Buxaceae Buxus mollicula#F148
Buxales Buxaceae Buxus microphylla#NC 009599
Buxales Buxaceae Buxus sempervirens#IWMW
Buxales Buxaceae Sarcococca ruscifolia#F43
Buxales Buxaceae Pachysandra terminalis#NC 029433
Buxales Buxaceae Pachysandra axillaris#F143
Gunnerales Myrothamnaceae Myrothamnus flabellifolia#Y569
Gunnerales Myrothamnaceae Myrothamnus flabellifolia#Y585
Gunnerales Gunneraceae Gunnera tinctoria#FC130
Gunnerales Gunneraceae Gunnera manicata#EU
Dilleniales Dilleniaceae Tetracera sarmentosa#F512
Dilleniales Dilleniaceae Curatella americana#F903
Dilleniales Dilleniaceae Dillenia indica#EHNF
Dilleniales Dilleniaceae Dillenia turbinata#F468
Saxifragales Peridiscaceae Soyauxia sp.#ZSD345
Saxifragales Peridiscaceae Medusandra sp.#ZSD350
Saxifragales Altingiaceae Liquidambar styraciflua#EU
Saxifragales Altingiaceae Altingia chinensis#F598
Saxifragales Altingiaceae Liquidambar formosana#F421
Saxifragales Altingiaceae Semiliquidambar cathayensis#F357
Saxifragales Daphniphyllaceae Daphniphyllum himalayense#F382
Saxifragales Daphniphyllaceae Daphniphyllum macropodum#FYTP
Saxifragales Daphniphyllaceae Daphniphyllum paxianum#F743
Saxifragales Daphniphyllaceae Daphniphyllum pentandrum#NC 037883
Saxifragales Cercidiphyllaceae Cercidiphyllum japonicum#F770
Saxifragales Cercidiphyllaceae Cercidiphyllum japonicum#F423
Saxifragales Cercidiphyllaceae Cercidiphyllum japonicum#NC 037940
Saxifragales Hamamelidaceae Exbucklandia tonkinensis#F454
Saxifragales Hamamelidaceae Rhodoleia championii#F363
Saxifragales Hamamelidaceae Chunia bucklandioides#F480
Saxifragales Hamamelidaceae Corylopsis coreana#NC 040141
Saxifragales Hamamelidaceae Loropetalum subcordatum#NC 037694
Saxifragales Hamamelidaceae Loropetalum chinense#TQOO
Saxifragales Hamamelidaceae Fortunearia sinensis#NC 041487
Saxifragales Hamamelidaceae Sinowilsonia henryi#MF497447

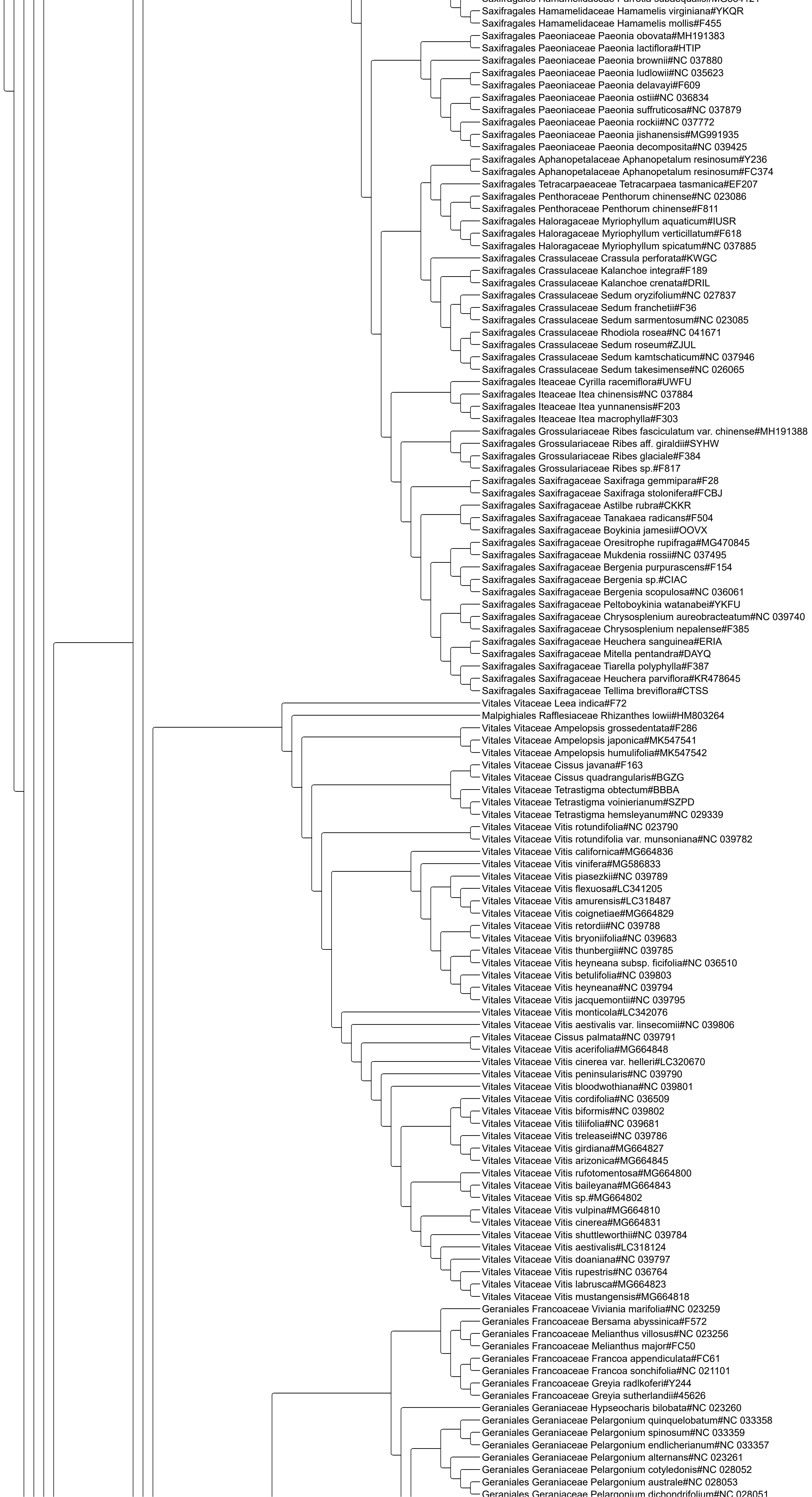

Saxifragales Hamamelidaceae Hamamelis virginiana#YKQR
Saxifragales Hamamelidaceae Hamamelis mollis#F455
Saxifragales Paeoniaceae Paeonia obovata#MH191383
Saxifragales Paeoniaceae Paeonia lactiflora#HTIP
Saxifragales Paeoniaceae Paeonia brownii#NC 037880
Saxifragales Paeoniaceae Paeonia ludlowii#NC 035623
Saxifragales Paeoniaceae Paeonia delavayi#F609
Saxifragales Paeoniaceae Paeonia ostii#NC 036834
Saxifragales Paeoniaceae Paeonia suffruticosa#NC 037879
Saxifragales Paeoniaceae Paeonia rockii#NC 037772
Saxifragales Paeoniaceae Paeonia jishanensis#MG991935
Saxifragales Paeoniaceae Paeonia decomposita#NC 039425
Saxifragales Aphanopetalaceae Aphanopetalum resinosum#Y236
Saxifragales Aphanopetalaceae Aphanopetalum resinosum#FC374
Saxifragales Tetracarpaeaceae Tetracarpaea tasmanica#EF207
Saxifragales Penthoraceae Penthorum chinense#NC 023086
Saxifragales Penthoraceae Penthorum chinense#F811
Saxifragales Haloragaceae Myriophyllum aquaticum#IUSR
Saxifragales Haloragaceae Myriophyllum verticillatum#F618
Saxifragales Haloragaceae Myriophyllum spicatum#NC 037885
Saxifragales Crassulaceae Crassula perforata#KWGC
Saxifragales Crassulaceae Kalanchoe integra#F189
Saxifragales Crassulaceae Kalanchoe crenata#DRIL
Saxifragales Crassulaceae Sedum oryzifolium#NC 027837
Saxifragales Crassulaceae Sedum franchetii#F36
Saxifragales Crassulaceae Sedum sarmentosum#NC 023085
Saxifragales Crassulaceae Rhodiola rosea#NC 041671
Saxifragales Crassulaceae Sedum roseum#ZJUL
Saxifragales Crassulaceae Sedum kamtschaticum#NC 037946
Saxifragales Crassulaceae Sedum takesimense#NC 026065
Saxifragales Iteaceae Cyrilla racemiflora#UWFU
Saxifragales Iteaceae Itea chinensis#NC 037884
Saxifragales Iteaceae Itea yunnanensis#F203
Saxifragales Iteaceae Itea macrophylla#F303
Saxifragales Grossulariaceae Ribes fasciculatum var. chinense#MH191388
Saxifragales Grossulariaceae Ribes aff. giraldii#SYHW
Saxifragales Grossulariaceae Ribes glaciale#F384
Saxifragales Grossulariaceae Ribes sp.#F817
Saxifragales Saxifragaceae Saxifraga gemmipara#F28
Saxifragales Saxifragaceae Saxifraga stolonifera#FCBJ
Saxifragales Saxifragaceae Astilbe rubra#CKKR
Saxifragales Saxifragaceae Tanakaea radicans#F504
Saxifragales Saxifragaceae Boykinia jamesii#OOVX
Saxifragales Saxifragaceae Oresitrophe rupifraga#MG470845
Saxifragales Saxifragaceae Mukdenia rossii#NC 037495
Saxifragales Saxifragaceae Bergenia purpurascens#F154
Saxifragales Saxifragaceae Bergenia sp.#CIAC
Saxifragales Saxifragaceae Bergenia scopulosa#NC 036061
Saxifragales Saxifragaceae Peltoboykinia watanabei#YKFU
Saxifragales Saxifragaceae Chrysosplenium aureobracteatum#NC 039740
Saxifragales Saxifragaceae Chrysosplenium nepalense#F385
Saxifragales Saxifragaceae Heuchera sanguinea#ERIA
Saxifragales Saxifragaceae Mitella pentandra#DAYQ
Saxifragales Saxifragaceae Tiarella polyphylla#F387
Saxifragales Saxifragaceae Heuchera parviflora#KR478645
Saxifragales Saxifragaceae Tellima breviflora#CTSS
Vitales Vitaceae Leea indica#F72
Malpighiales Rafflesiaceae Rhizanthes lowii#HM803264
Vitales Vitaceae Ampelopsis grossedentata#F286
Vitales Vitaceae Ampelopsis japonica#MK547541
Vitales Vitaceae Ampelopsis humulifolia#MK547542
Vitales Vitaceae Cissus javana#F163
Vitales Vitaceae Cissus quadrangularis#BGZG
Vitales Vitaceae Tetrastigma obtectum#BBBA
Vitales Vitaceae Tetrastigma voinierianum#SZPD
Vitales Vitaceae Tetrastigma hemsleyanum#NC 029339
Vitales Vitaceae Vitis rotundifolia#NC 023790
Vitales Vitaceae Vitis rotundifolia var. munsoniana#NC 039782
Vitales Vitaceae Vitis californica#MG664836
Vitales Vitaceae Vitis vinifera#MG586833
Vitales Vitaceae Vitis piasezkii#NC 039789
Vitales Vitaceae Vitis flexuosa#LC341205
Vitales Vitaceae Vitis amurensis#LC318487
Vitales Vitaceae Vitis coignetiae#MG664829
Vitales Vitaceae Vitis retordii#NC 039788
Vitales Vitaceae Vitis bryoniifolia#NC 039683
Vitales Vitaceae Vitis thunbergii#NC 039785
Vitales Vitaceae Vitis heyneana subsp. ficifolia#NC 036510
Vitales Vitaceae Vitis betulifolia#NC 039803
Vitales Vitaceae Vitis heyneana#NC 039794
Vitales Vitaceae Vitis jacquemontii#NC 039795
Vitales Vitaceae Vitis monticola#LC342076
Vitales Vitaceae Vitis aestivalis var. linsecomii#NC 039806
Vitales Vitaceae Cissus palmata#NC 039791
Vitales Vitaceae Vitis acerifolia#MG664848
Vitales Vitaceae Vitis cinerea var. helleri#LC320670
Vitales Vitaceae Vitis peninsularis#NC 039790
Vitales Vitaceae Vitis bloodwothiana#NC 039801
Vitales Vitaceae Vitis cordifolia#NC 036509
Vitales Vitaceae Vitis biformis#NC 039802
Vitales Vitaceae Vitis tiliifolia#NC 039681
Vitales Vitaceae Vitis treleasei#NC 039786
Vitales Vitaceae Vitis girdiana#MG664827
Vitales Vitaceae Vitis arizonica#MG664845
Vitales Vitaceae Vitis rufotomentosa#MG664800
Vitales Vitaceae Vitis baileyana#MG664843
Vitales Vitaceae Vitis sp.#MG664802
Vitales Vitaceae Vitis vulpina#MG664810
Vitales Vitaceae Vitis cinerea#MG664831
Vitales Vitaceae Vitis shuttleworthii#NC 039784
Vitales Vitaceae Vitis aestivalis#LC318124
Vitales Vitaceae Vitis doaniana#NC 039797
Vitales Vitaceae Vitis rupestris#NC 036764
Vitales Vitaceae Vitis labrusca#MG664823
Vitales Vitaceae Vitis mustangensis#MG664818
Geraniales Francoaceae Viviania marifolia#NC 023259
Geraniales Francoaceae Bersama abyssinica#F572
Geraniales Francoaceae Melianthus villosus#NC 023256
Geraniales Francoaceae Melianthus major#FC50
Geraniales Francoaceae Francoa appendiculata#FC61
Geraniales Francoaceae Francoa sonchifolia#NC 021101
Geraniales Francoaceae Greyia radlkoferi#Y244
Geraniales Francoaceae Greyia sutherlandii#45626
Geraniales Geraniaceae Hypseocharis bilobata#NC 023260
Geraniales Geraniaceae Pelargonium quinquelobatum#NC 033358
Geraniales Geraniaceae Pelargonium spinosum#NC 033359
Geraniales Geraniaceae Pelargonium endlicherianum#NC 033357
Geraniales Geraniaceae Pelargonium alternans#NC 023261
Geraniales Geraniaceae Pelargonium cotyledonis#NC 028052
Geraniales Geraniaceae Pelargonium australe#NC 028053

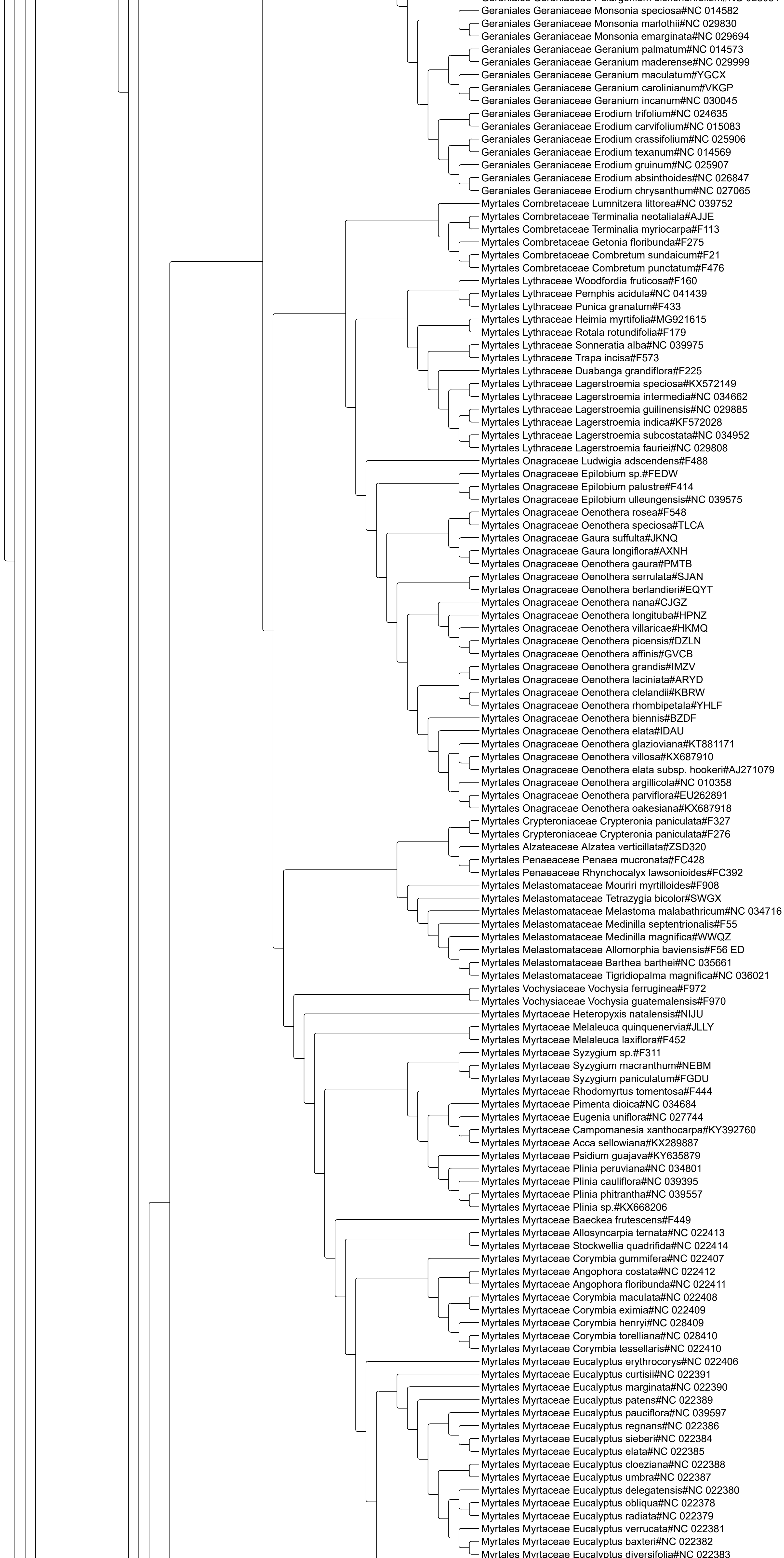

Geraniales Geraniaceae Monsonia speciosa#NC 014582
Geraniales Geraniaceae Monsonia marlothii#NC 029830
Geraniales Geraniaceae Monsonia emarginata#NC 029694
Geraniales Geraniaceae Geranium palmatum#NC 014573
Geraniales Geraniaceae Geranium maderense#NC 029999
Geraniales Geraniaceae Geranium maculatum#YGCX
Geraniales Geraniaceae Geranium carolinianum#VKGP
Geraniales Geraniaceae Geranium incanum#NC 030045
Geraniales Geraniaceae Erodium trifolium#NC 024635
Geraniales Geraniaceae Erodium carvifolium#NC 015083
Geraniales Geraniaceae Erodium crassifolium#NC 025906
Geraniales Geraniaceae Erodium texanum#NC 014569
Geraniales Geraniaceae Erodium gruinum#NC 025907
Geraniales Geraniaceae Erodium absinthoides#NC 026847
Geraniales Geraniaceae Erodium chrysanthum#NC 027065
Myrtales Combretaceae Lumnitzera littorea#NC 039752
Myrtales Combretaceae Terminalia neotaliala#AJJE
Myrtales Combretaceae Terminalia myriocarpa#F113
Myrtales Combretaceae Getonia floribunda#F275
Myrtales Combretaceae Combretum sundaicum#F21
Myrtales Combretaceae Combretum punctatum#F476
Myrtales Lythraceae Woodfordia fruticosa#F160
Myrtales Lythraceae Pemphis acidula#NC 041439
Myrtales Lythraceae Punica granatum#F433
Myrtales Lythraceae Heimia myrtifolia#MG921615
Myrtales Lythraceae Rotala rotundifolia#F179
Myrtales Lythraceae Sonneratia alba#NC 039975
Myrtales Lythraceae Trapa incisa#F573
Myrtales Lythraceae Duabanga grandiflora#F225
Myrtales Lythraceae Lagerstroemia speciosa#KX572149
Myrtales Lythraceae Lagerstroemia intermedia#NC 034662
Myrtales Lythraceae Lagerstroemia guilinensis#NC 029885
Myrtales Lythraceae Lagerstroemia indica#KF572028
Myrtales Lythraceae Lagerstroemia subcostata#NC 034952
Myrtales Lythraceae Lagerstroemia fauriei#NC 029808
Myrtales Onagraceae Ludwigia adscendens#F488
Myrtales Onagraceae Epilobium sp.#FEDW
Myrtales Onagraceae Epilobium palustre#F414
Myrtales Onagraceae Epilobium ulleungensis#NC 039575
Myrtales Onagraceae Oenothera rosea#F548
Myrtales Onagraceae Oenothera speciosa#TLCA
Myrtales Onagraceae Gaura suffulta#JKNQ
Myrtales Onagraceae Gaura longiflora#AXNH
Myrtales Onagraceae Oenothera gaura#PMTB
Myrtales Onagraceae Oenothera serrulata#SJAN
Myrtales Onagraceae Oenothera berlandieri#EQYT
Myrtales Onagraceae Oenothera nana#CJGZ
Myrtales Onagraceae Oenothera longituba#HPNZ
Myrtales Onagraceae Oenothera villaricae#HKMQ
Myrtales Onagraceae Oenothera picensis#DZLN
Myrtales Onagraceae Oenothera affinis#GVCB
Myrtales Onagraceae Oenothera grandis#IMZV
Myrtales Onagraceae Oenothera laciniata#ARYD
Myrtales Onagraceae Oenothera clelandii#KBRW
Myrtales Onagraceae Oenothera rhombipetala#YHLF
Myrtales Onagraceae Oenothera biennis#BZDF
Myrtales Onagraceae Oenothera elata#IDAU
Myrtales Onagraceae Oenothera glazioviana#KT881171
Myrtales Onagraceae Oenothera villosa#KX687910
Myrtales Onagraceae Oenothera elata subsp. hookeri#AJ271079
Myrtales Onagraceae Oenothera argillicola#NC 010358
Myrtales Onagraceae Oenothera parviflora#EU262891
Myrtales Onagraceae Oenothera oakesiana#KX687918
Myrtales Crypteroniaceae Crypteronia paniculata#F327
Myrtales Crypteroniaceae Crypteronia paniculata#F276
Myrtales Alzateaceae Alzatea verticillata#ZSD320
Myrtales Penaeaceae Penaea mucronata#FC428
Myrtales Penaeaceae Rhynchocalyx lawsonioides#FC392
Myrtales Melastomataceae Mouriri myrtilloides#F908
Myrtales Melastomataceae Tetrazygia bicolor#SWGX
Myrtales Melastomataceae Melastoma malabathricum#NC 034716
Myrtales Melastomataceae Medinilla septentrionalis#F55
Myrtales Melastomataceae Medinilla magnifica#WWQZ
Myrtales Melastomataceae Allomorphia baviensis#F56 ED
Myrtales Melastomataceae Barthea barthei#NC 035661
Myrtales Melastomataceae Tigridiopalma magnifica#NC 036021
Myrtales Vochysiaceae Vochysia ferruginea#F972
Myrtales Vochysiaceae Vochysia guatemalensis#F970
Myrtales Myrtaceae Heteropyxis natalensis#NIJU
Myrtales Myrtaceae Melaleuca quinquenervia#JLLY
Myrtales Myrtaceae Melaleuca laxiflora#F452
Myrtales Myrtaceae Syzygium sp.#F311
Myrtales Myrtaceae Syzygium macranthum#NEBM
Myrtales Myrtaceae Syzygium paniculatum#FGDU
Myrtales Myrtaceae Rhodomyrtus tomentosa#F444
Myrtales Myrtaceae Pimenta dioica#NC 034684
Myrtales Myrtaceae Eugenia uniflora#NC 027744
Myrtales Myrtaceae Campomanesia xanthocarpa#KY392760
Myrtales Myrtaceae Acca sellowiana#KX289887
Myrtales Myrtaceae Psidium guajava#KY635879
Myrtales Myrtaceae Plinia peruviana#NC 034801
Myrtales Myrtaceae Plinia cauliflora#NC 039395
Myrtales Myrtaceae Plinia phitrantha#NC 039557
Myrtales Myrtaceae Plinia sp.#KX668206
Myrtales Myrtaceae Baeckea frutescens#F449
Myrtales Myrtaceae Allosyncarpia ternata#NC 022413
Myrtales Myrtaceae Stockwellia quadrifida#NC 022414
Myrtales Myrtaceae Corymbia gummifera#NC 022407
Myrtales Myrtaceae Angophora costata#NC 022412
Myrtales Myrtaceae Angophora floribunda#NC 022411
Myrtales Myrtaceae Corymbia maculata#NC 022408
Myrtales Myrtaceae Corymbia eximia#NC 022409
Myrtales Myrtaceae Corymbia henryi#NC 028409
Myrtales Myrtaceae Corymbia torelliana#NC 028410
Myrtales Myrtaceae Corymbia tessellaris#NC 022410
Myrtales Myrtaceae Eucalyptus erythrocorys#NC 022406
Myrtales Myrtaceae Eucalyptus curtisii#NC 022391
Myrtales Myrtaceae Eucalyptus marginata#NC 022390
Myrtales Myrtaceae Eucalyptus patens#NC 022389
Myrtales Myrtaceae Eucalyptus pauciflora#NC 039597
Myrtales Myrtaceae Eucalyptus regnans#NC 022386
Myrtales Myrtaceae Eucalyptus sieberi#NC 022384
Myrtales Myrtaceae Eucalyptus elata#NC 022385
Myrtales Myrtaceae Eucalyptus cloeziana#NC 022388
Myrtales Myrtaceae Eucalyptus umbra#NC 022387
Myrtales Myrtaceae Eucalyptus delegatensis#NC 022380
Myrtales Myrtaceae Eucalyptus obliqua#NC 022378
Myrtales Myrtaceae Eucalyptus radiata#NC 022379
Myrtales Myrtaceae Eucalyptus verrucata#NC 022381
Myrtales Myrtaceae Eucalyptus baxteri#NC 022382
Myrtales Myrtaceae Eucalyptus diversifolia#NC 022383

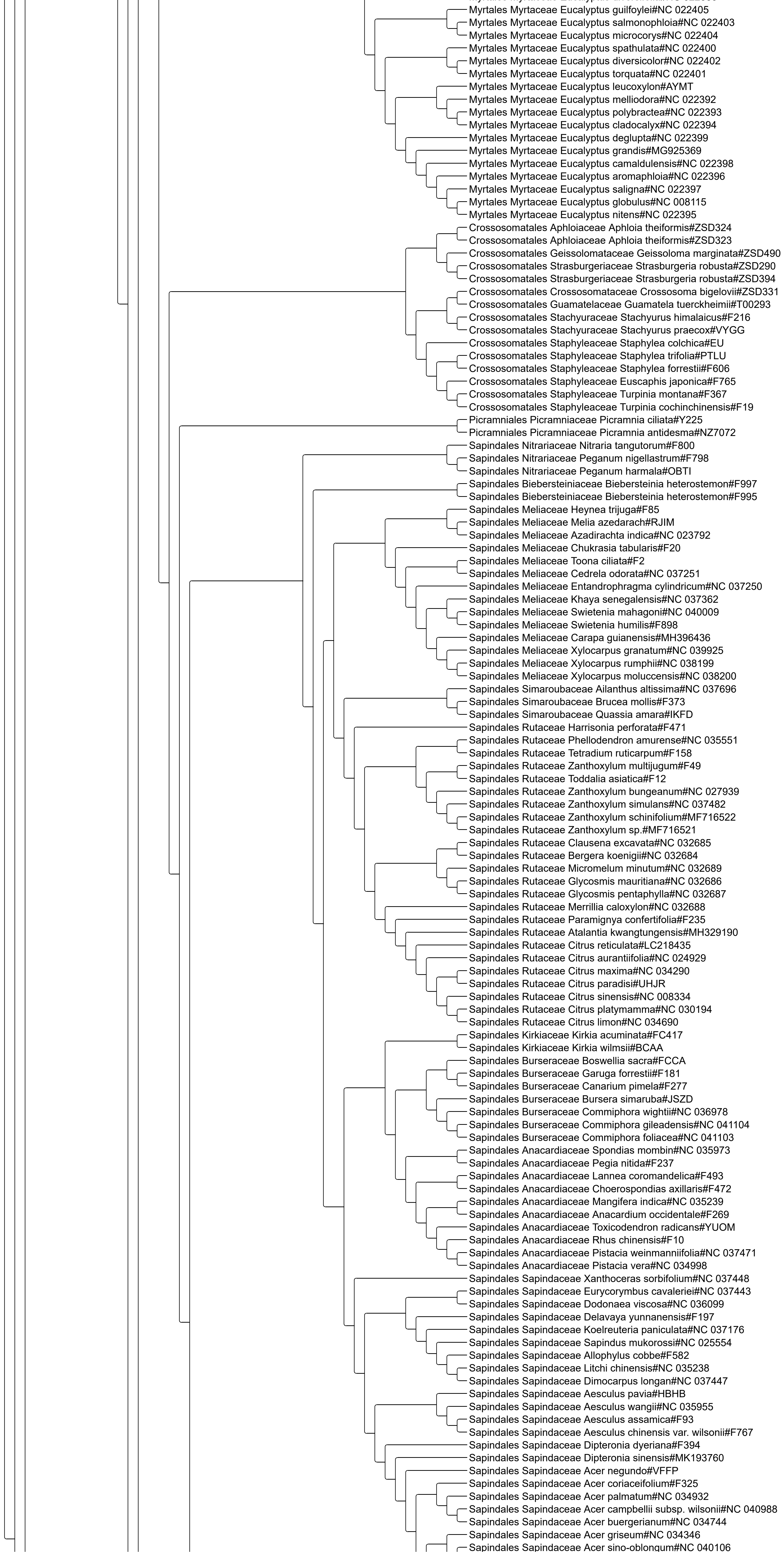
Myrtales Myrtaceae Eucalyptus guilfoylei#NC 022405
Myrtales Myrtaceae Eucalyptus salmonophloia#NC 022403
Myrtales Myrtaceae Eucalyptus microcorys#NC 022404
Myrtales Myrtaceae Eucalyptus spathulata#NC 022400
Myrtales Myrtaceae Eucalyptus diversicolor#NC 022402
Myrtales Myrtaceae Eucalyptus torquata#NC 022401
Myrtales Myrtaceae Eucalyptus leucoxylon#AYMT
Myrtales Myrtaceae Eucalyptus melliodora#NC 022392
Myrtales Myrtaceae Eucalyptus polybractea#NC 022393
Myrtales Myrtaceae Eucalyptus cladocalyx#NC 022394
Myrtales Myrtaceae Eucalyptus deglupta#NC 022399
Myrtales Myrtaceae Eucalyptus grandis#MG925369
Myrtales Myrtaceae Eucalyptus camaldulensis#NC 022398
Myrtales Myrtaceae Eucalyptus aromaphloia#NC 022396
Myrtales Myrtaceae Eucalyptus saligna#NC 022397
Myrtales Myrtaceae Eucalyptus globulus#NC 008115
Myrtales Myrtaceae Eucalyptus nitens#NC 022395
Crossosomatales Aphloiaceae Aphloia theiformis#ZSD324
Crossosomatales Aphloiaceae Aphloia theiformis#ZSD323
Crossosomatales Geissolomataceae Geissoloma marginata#ZSD490
Crossosomatales Strasburgeriaceae Strasburgeria robusta#ZSD290
Crossosomatales Strasburgeriaceae Strasburgeria robusta#ZSD394
Crossosomatales Crossosomataceae Crossosoma bigelovii#ZSD331
Crossosomatales Guamatelaceae Guamatela tuerckheimii#T00293
Crossosomatales Stachyuraceae Stachyurus himalaicus#F216
Crossosomatales Stachyuraceae Stachyurus praecox#VYGG
Crossosomatales Staphyleaceae Staphylea colchica#EU
Crossosomatales Staphyleaceae Staphylea trifolia#PTLU
Crossosomatales Staphyleaceae Staphylea forrestii#F606
Crossosomatales Staphyleaceae Euscaphis japonica#F765
Crossosomatales Staphyleaceae Turpinia montana#F367
Crossosomatales Staphyleaceae Turpinia cochinchinensis#F19
Picramniales Picramniaceae Picramnia ciliata#Y225
Picramniales Picramniaceae Picramnia antidesma#NZ7072
Sapindales Nitrariaceae Nitraria tangutorum#F800
Sapindales Nitrariaceae Peganum nigellastrum#F798
Sapindales Nitrariaceae Peganum harmala#OBTI
Sapindales Biebersteiniaceae Biebersteinia heterostemon#F997
Sapindales Biebersteiniaceae Biebersteinia heterostemon#F995
Sapindales Meliaceae Heynea trijuga#F85
Sapindales Meliaceae Melia azedarach#RJIM
Sapindales Meliaceae Azadirachta indica#NC 023792
Sapindales Meliaceae Chukrasia tabularis#F20
Sapindales Meliaceae Toona ciliata#F2
Sapindales Meliaceae Cedrela odorata#NC 037251
Sapindales Meliaceae Entandrophragma cylindricum#NC 037250
Sapindales Meliaceae Khaya senegalensis#NC 037362
Sapindales Meliaceae Swietenia mahagoni#NC 040009
Sapindales Meliaceae Swietenia humilis#F898
Sapindales Meliaceae Carapa guianensis#MH396436
Sapindales Meliaceae Xylocarpus granatum#NC 039925
Sapindales Meliaceae Xylocarpus rumphii#NC 038199
Sapindales Meliaceae Xylocarpus moluccensis#NC 038200
Sapindales Simaroubaceae Ailanthus altissima#NC 037696
Sapindales Simaroubaceae Brucea mollis#F373
Sapindales Simaroubaceae Quassia amara#IKFD
Sapindales Rutaceae Harrisonia perforata#F471
Sapindales Rutaceae Phellodendron amurense#NC 035551
Sapindales Rutaceae Tetradium ruticarpum#F158
Sapindales Rutaceae Zanthoxylum multijugum#F49
Sapindales Rutaceae Toddalia asiatica#F12
Sapindales Rutaceae Zanthoxylum bungeanum#NC 027939
Sapindales Rutaceae Zanthoxylum simulans#NC 037482
Sapindales Rutaceae Zanthoxylum schinifolium#MF716522
Sapindales Rutaceae Zanthoxylum sp.#MF716521
Sapindales Rutaceae Clausena excavata#NC 032685
Sapindales Rutaceae Bergera koenigii#NC 032684
Sapindales Rutaceae Micromelum minutum#NC 032689
Sapindales Rutaceae Glycosmis mauritiana#NC 032686
Sapindales Rutaceae Glycosmis pentaphylla#NC 032687
Sapindales Rutaceae Merrillia caloxylon#NC 032688
Sapindales Rutaceae Paramignya confertifolia#F235
Sapindales Rutaceae Atalantia kwangtungensis#MH329190
Sapindales Rutaceae Citrus reticulata#LC218435
Sapindales Rutaceae Citrus aurantiifolia#NC 024929
Sapindales Rutaceae Citrus maxima#NC 034290
Sapindales Rutaceae Citrus paradisi#UHJR
Sapindales Rutaceae Citrus sinensis#NC 008334
Sapindales Rutaceae Citrus platymamma#NC 030194
Sapindales Rutaceae Citrus limon#NC 034690
Sapindales Kirkiaceae Kirkia acuminata#FC417
Sapindales Kirkiaceae Kirkia wilmsii#BCAA
Sapindales Burseraceae Boswellia sacra#FCCA
Sapindales Burseraceae Garuga forrestii#F181
Sapindales Burseraceae Canarium pimela#F277
Sapindales Burseraceae Bursera simaruba#JSZD
Sapindales Burseraceae Commiphora wightii#NC 036978
Sapindales Burseraceae Commiphora gileadensis#NC 041104
Sapindales Burseraceae Commiphora foliacea#NC 041103
Sapindales Anacardiaceae Spondias mombin#NC 035973
Sapindales Anacardiaceae Pegia nitida#F237
Sapindales Anacardiaceae Lannea coromandelica#F493
Sapindales Anacardiaceae Choerospondias axillaris#F472
Sapindales Anacardiaceae Mangifera indica#NC 035239
Sapindales Anacardiaceae Anacardium occidentale#F269
Sapindales Anacardiaceae Toxicodendron radicans#YUOM
Sapindales Anacardiaceae Rhus chinensis#F10
Sapindales Anacardiaceae Pistacia weinmanniifolia#NC 037471
Sapindales Anacardiaceae Pistacia vera#NC 034998
Sapindales Sapindaceae Xanthoceras sorbifolium#NC 037448
Sapindales Sapindaceae Eurycorymbus cavaleriei#NC 037443
Sapindales Sapindaceae Dodonaea viscosa#NC 036099
Sapindales Sapindaceae Delavaya yunnanensis#F197
Sapindales Sapindaceae Koelreuteria paniculata#NC 037176
Sapindales Sapindaceae Sapindus mukorossi#NC 025554
Sapindales Sapindaceae Allophylus cobbe#F582
Sapindales Sapindaceae Litchi chinensis#NC 035238
Sapindales Sapindaceae Dimocarpus longan#NC 037447
Sapindales Sapindaceae Aesculus pavia#HBHB
Sapindales Sapindaceae Aesculus wangii#NC 035955
Sapindales Sapindaceae Aesculus assamica#F93
Sapindales Sapindaceae Aesculus chinensis var. wilsonii#F767
Sapindales Sapindaceae Dipteronia dyeriana#F394
Sapindales Sapindaceae Dipteronia sinensis#MK193760
Sapindales Sapindaceae Acer negundo#VFFP
Sapindales Sapindaceae Acer coriaceifolium#F325
Sapindales Sapindaceae Acer palmatum#NC 034932
Sapindales Sapindaceae Acer campbellii subsp. wilsonii#NC 040988
Sapindales Sapindaceae Acer buergerianum#NC 034744
Sapindales Sapindaceae Acer griseum#NC 034346
Sapindales Sapindaceae Acer sino-oblongum#NC 040106

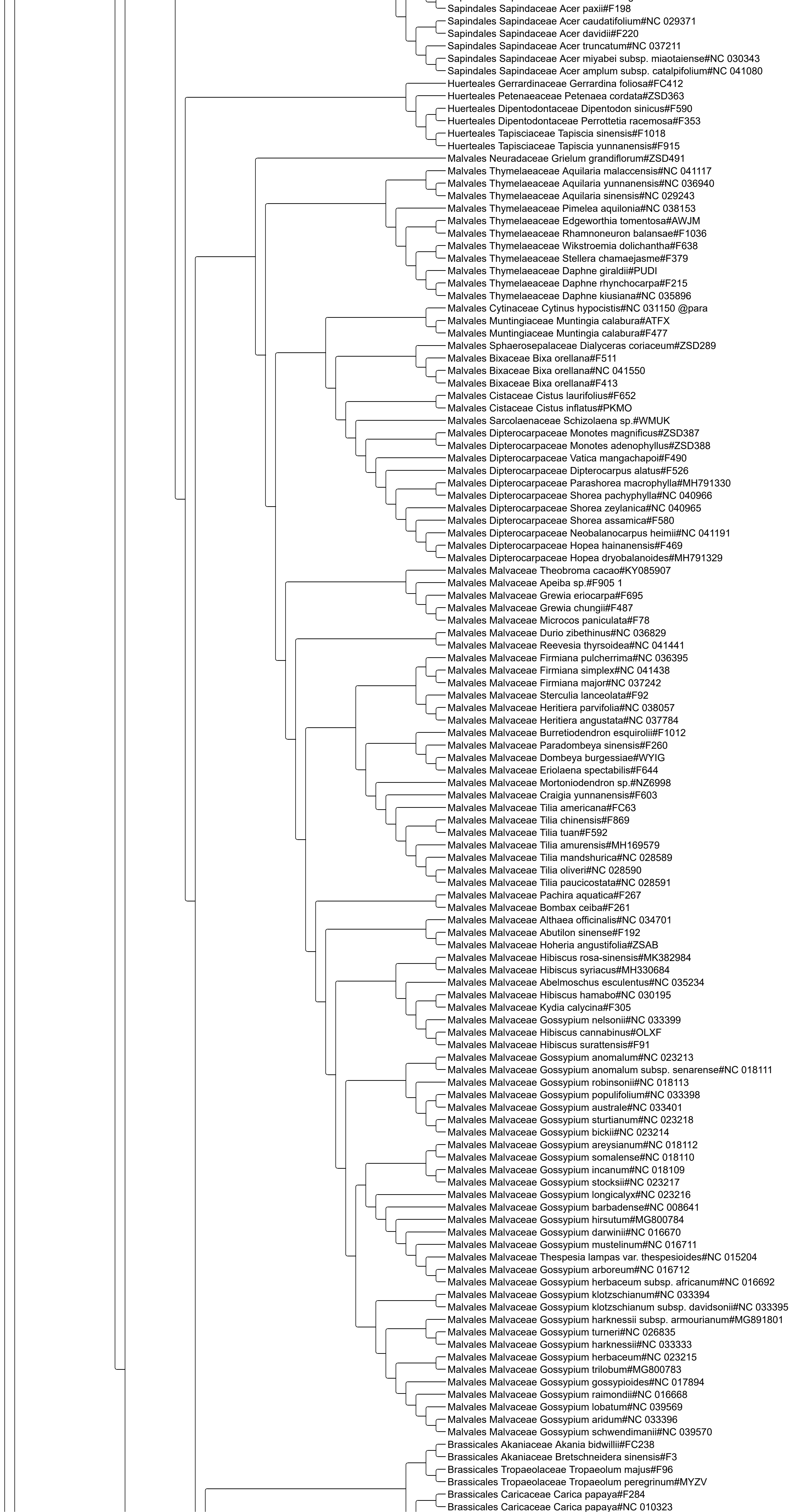
Sapindales Sapindaceae Acer paxii#F198
Sapindales Sapindaceae Acer caudatifolium#NC 029371
Sapindales Sapindaceae Acer davidii#F220
Sapindales Sapindaceae Acer truncatum#NC 037211
Sapindales Sapindaceae Acer miyabei subsp. miaotaiense#NC 030343
Sapindales Sapindaceae Acer amplum subsp. catalpifolium#NC 041080
Huerteales Gerrardinaceae Gerrardina foliosa#FC412
Huerteales Petenaeaceae Petenaea cordata#ZSD363
Huerteales Dipentodontaceae Dipentodon sinicus#F590
Huerteales Dipentodontaceae Perrottetia racemosa#F353
Huerteales Tapisciaceae Tapiscia sinensis#F1018
Huerteales Tapisciaceae Tapiscia yunnanensis#F915
Malvales Neuradaceae Grielum grandiflorum#ZSD491
Malvales Thymelaeaceae Aquilaria malaccensis#NC 041117
Malvales Thymelaeaceae Aquilaria yunnanensis#NC 036940
Malvales Thymelaeaceae Aquilaria sinensis#NC 029243
Malvales Thymelaeaceae Pimelea aquilonia#NC 038153
Malvales Thymelaeaceae Edgeworthia tomentosa#AWJM
Malvales Thymelaeaceae Rhamnoneuron balansae#F1036
Malvales Thymelaeaceae Wikstroemia dolichantha#F638
Malvales Thymelaeaceae Stellera chamaejasme#F379
Malvales Thymelaeaceae Daphne giraldii#PUDI
Malvales Thymelaeaceae Daphne rhynchocarpa#F215
Malvales Thymelaeaceae Daphne kiusiana#NC 035896
Malvales Cytinaceae Cytinus hypocistis#NC 031150 @para
Malvales Muntingiaceae Muntingia calabura#ATFX
Malvales Muntingiaceae Muntingia calabura#F477
Malvales Sphaerosepalaceae Dialyceras coriaceum#ZSD289
Malvales Bixaceae Bixa orellana#F511
Malvales Bixaceae Bixa orellana#NC 041550
Malvales Bixaceae Bixa orellana#F413
Malvales Cistaceae Cistus laurifolius#F652
Malvales Cistaceae Cistus inflatus#PKMO
Malvales Sarcolaenaceae Schizolaena sp.#WMUK
Malvales Dipterocarpaceae Monotes magnificus#ZSD387
Malvales Dipterocarpaceae Monotes adenophyllus#ZSD388
Malvales Dipterocarpaceae Vatica mangachapoi#F490
Malvales Dipterocarpaceae Dipterocarpus alatus#F526
Malvales Dipterocarpaceae Parashorea macrophylla#MH791330
Malvales Dipterocarpaceae Shorea pachyphylla#NC 040966
Malvales Dipterocarpaceae Shorea zeylanica#NC 040965
Malvales Dipterocarpaceae Shorea assamica#F580
Malvales Dipterocarpaceae Neobalanocarpus heimii#NC 041191
Malvales Dipterocarpaceae Hopea hainanensis#F469
Malvales Dipterocarpaceae Hopea dryobalanoides#MH791329
Malvales Malvaceae Theobroma cacao#KY085907
Malvales Malvaceae Apeiba sp.#F905 1
Malvales Malvaceae Grewia eriocarpa#F695
Malvales Malvaceae Grewia chungii#F487
Malvales Malvaceae Microcos paniculata#F78
Malvales Malvaceae Durio zibethinus#NC 036829
Malvales Malvaceae Reevesia thyrsoidea#NC 041441
Malvales Malvaceae Firmiana pulcherrima#NC 036395
Malvales Malvaceae Firmiana simplex#NC 041438
Malvales Malvaceae Firmiana major#NC 037242
Malvales Malvaceae Sterculia lanceolata#F92
Malvales Malvaceae Heritiera parvifolia#NC 038057
Malvales Malvaceae Heritiera angustata#NC 037784
Malvales Malvaceae Burretiodendron esquirolii#F1012
Malvales Malvaceae Paradombeya sinensis#F260
Malvales Malvaceae Dombeya burgessiae#WYIG
Malvales Malvaceae Eriolaena spectabilis#F644
Malvales Malvaceae Mortoniodendron sp.#NZ6998
Malvales Malvaceae Craigia yunnanensis#F603
Malvales Malvaceae Tilia americana#FC63
Malvales Malvaceae Tilia chinensis#F869
Malvales Malvaceae Tilia tuan#F592
Malvales Malvaceae Tilia amurensis#MH169579
Malvales Malvaceae Tilia mandshurica#NC 028589
Malvales Malvaceae Tilia oliveri#NC 028590
Malvales Malvaceae Tilia paucicostata#NC 028591
Malvales Malvaceae Pachira aquatica#F267
Malvales Malvaceae Bombax ceiba#F261
Malvales Malvaceae Althaea officinalis#NC 034701
Malvales Malvaceae Abutilon sinense#F192
Malvales Malvaceae Hoheria angustifolia#ZSAB
Malvales Malvaceae Hibiscus rosa-sinensis#MK382984
Malvales Malvaceae Hibiscus syriacus#MH330684
Malvales Malvaceae Abelmoschus esculentus#NC 035234
Malvales Malvaceae Hibiscus hamabo#NC 030195
Malvales Malvaceae Kydia calycina#F305
Malvales Malvaceae Gossypium nelsonii#NC 033399
Malvales Malvaceae Hibiscus cannabinus#OLXF
Malvales Malvaceae Hibiscus surattensis#F91
Malvales Malvaceae Gossypium anomalum#NC 023213
Malvales Malvaceae Gossypium anomalum subsp. senarense#NC 018111
Malvales Malvaceae Gossypium robinsonii#NC 018113
Malvales Malvaceae Gossypium populifolium#NC 033398
Malvales Malvaceae Gossypium australe#NC 033401
Malvales Malvaceae Gossypium sturtianum#NC 023218
Malvales Malvaceae Gossypium bickii#NC 023214
Malvales Malvaceae Gossypium areysianum#NC 018112
Malvales Malvaceae Gossypium somalense#NC 018110
Malvales Malvaceae Gossypium incanum#NC 018109
Malvales Malvaceae Gossypium stocksii#NC 023217
Malvales Malvaceae Gossypium longicalyx#NC 023216
Malvales Malvaceae Gossypium barbadense#NC 008641
Malvales Malvaceae Gossypium hirsutum#MG800784
Malvales Malvaceae Gossypium darwinii#NC 016670
Malvales Malvaceae Gossypium mustelinum#NC 016711
Malvales Malvaceae Thespesia lampas var. thespesioides#NC 015204
Malvales Malvaceae Gossypium arboreum#NC 016712
Malvales Malvaceae Gossypium herbaceum subsp. africanum#NC 016692
Malvales Malvaceae Gossypium klotzschianum#NC 033394
Malvales Malvaceae Gossypium klotzschianum subsp. davidsonii#NC 033395
Malvales Malvaceae Gossypium harknessii subsp. armourianum#MG891801
Malvales Malvaceae Gossypium turneri#NC 026835
Malvales Malvaceae Gossypium harknessii#NC 033333
Malvales Malvaceae Gossypium herbaceum#NC 023215
Malvales Malvaceae Gossypium trilobum#MG800783
Malvales Malvaceae Gossypium gossypioides#NC 017894
Malvales Malvaceae Gossypium raimondii#NC 016668
Malvales Malvaceae Gossypium lobatum#NC 039569
Malvales Malvaceae Gossypium aridum#NC 033396
Malvales Malvaceae Gossypium schwendimanii#NC 039570
Brassicales Akaniaceae Akania bidwillii#FC238
Brassicales Akaniaceae Bretschneidera sinensis#F3
Brassicales Tropaeolaceae Tropaeolum majus#F96
Brassicales Tropaeolaceae Tropaeolum peregrinum#MYZV
Brassicales Caricaceae Carica papaya#F284
Brassicales Caricaceae Carica papaya#NC 010323

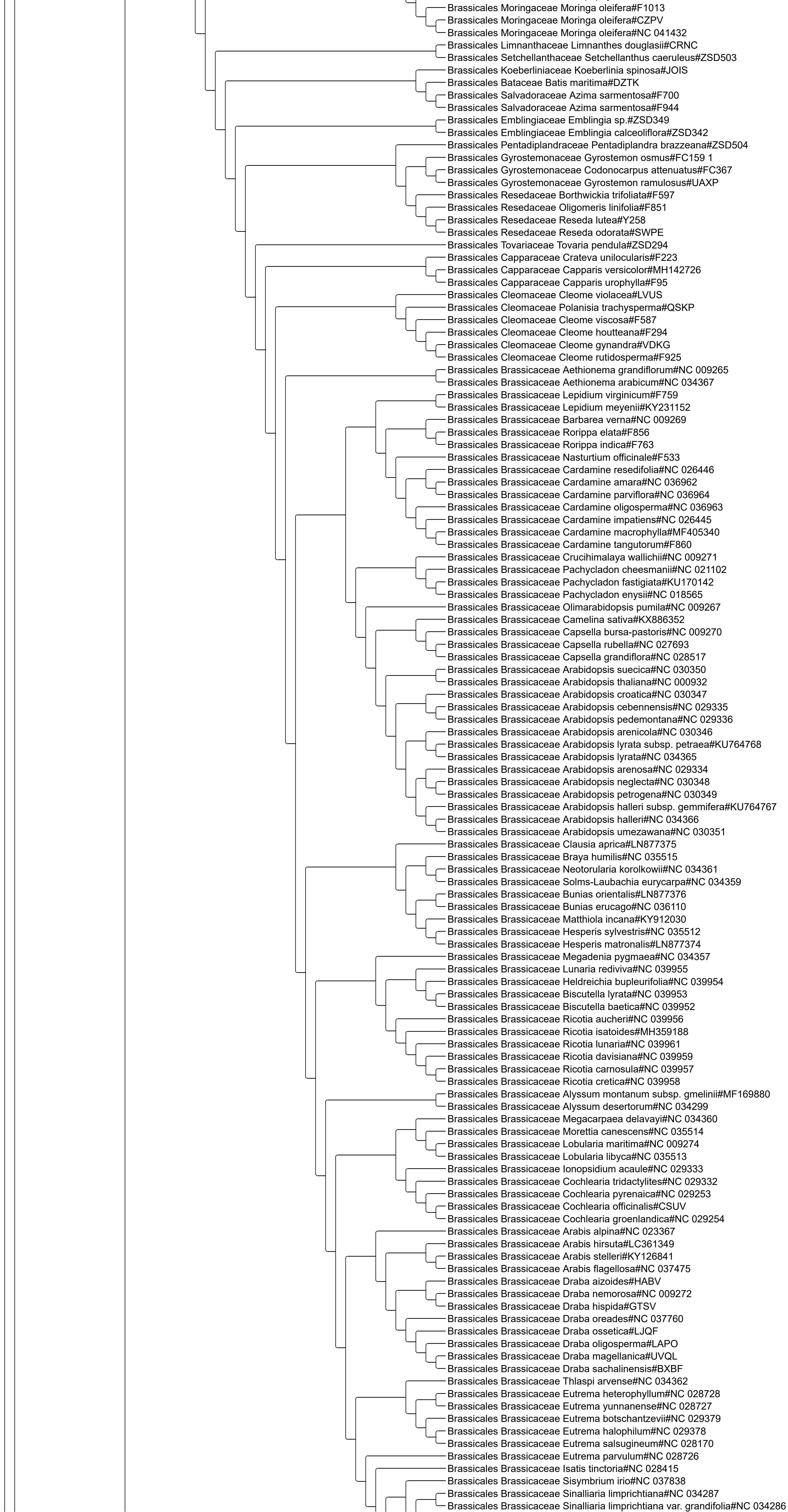
Brassicales Moringaceae Moringa oleifera#F1013
Brassicales Moringaceae Moringa oleifera#CZPV
Brassicales Moringaceae Moringa oleifera#NC 041432
Brassicales Limnanthaceae Limnanthes douglasii#CRNC
Brassicales Setchellanthaceae Setchellanthus caeruleus#ZSD503
Brassicales Koeberliniaceae Koeberlinia spinosa#JOIS
Brassicales Bataceae Batis maritima#DZTK
Brassicales Salvadoraceae Azima sarmentosa#F700
Brassicales Salvadoraceae Azima sarmentosa#F944
Brassicales Emblingiaceae Emblingia sp.#ZSD349
Brassicales Emblingiaceae Emblingia calceoliflora#ZSD342
Brassicales Pentadiplandraceae Pentadiplandra brazzeana#ZSD504
Brassicales Gyrostemonaceae Gyrostemon osmus#FC159 1
Brassicales Gyrostemonaceae Codonocarpus attenuatus#FC367
Brassicales Gyrostemonaceae Gyrostemon ramulosus#UAXP
Brassicales Resedaceae Borthwickia trifoliata#F597
Brassicales Resedaceae Oligomeris linifolia#F851
Brassicales Resedaceae Reseda lutea#Y258
Brassicales Resedaceae Reseda odorata#SWPE
Brassicales Tovariaceae Tovaria pendula#ZSD294
Brassicales Capparaceae Crateva unilocularis#F223
Brassicales Capparaceae Capparis versicolor#MH142726
Brassicales Capparaceae Capparis urophylla#F95
Brassicales Cleomaceae Cleome violacea#LVUS
Brassicales Cleomaceae Polanisia trachysperma#QSKP
Brassicales Cleomaceae Cleome viscosa#F587
Brassicales Cleomaceae Cleome houtteana#F294
Brassicales Cleomaceae Cleome gynandra#VDKG
Brassicales Cleomaceae Cleome rutidosperma#F925
Brassicales Brassicaceae Aethionema grandiflorum#NC 009265
Brassicales Brassicaceae Aethionema arabicum#NC 034367
Brassicales Brassicaceae Lepidium virginicum#F759
Brassicales Brassicaceae Lepidium meyenii#KY231152
Brassicales Brassicaceae Barbarea verna#NC 009269
Brassicales Brassicaceae Rorippa elata#F856
Brassicales Brassicaceae Rorippa indica#F763
Brassicales Brassicaceae Nasturtium officinale#F533
Brassicales Brassicaceae Cardamine resedifolia#NC 026446
Brassicales Brassicaceae Cardamine amara#NC 036962
Brassicales Brassicaceae Cardamine parviflora#NC 036964
Brassicales Brassicaceae Cardamine oligosperma#NC 036963
Brassicales Brassicaceae Cardamine impatiens#NC 026445
Brassicales Brassicaceae Cardamine macrophylla#MF405340
Brassicales Brassicaceae Cardamine tangutorum#F860
Brassicales Brassicaceae Crucihimalaya wallichii#NC 009271
Brassicales Brassicaceae Pachycladon cheesmanii#NC 021102
Brassicales Brassicaceae Pachycladon fastigiata#KU170142
Brassicales Brassicaceae Pachycladon enysii#NC 018565
Brassicales Brassicaceae Olimarabidopsis pumila#NC 009267
Brassicales Brassicaceae Camelina sativa#KX886352
Brassicales Brassicaceae Capsella bursa-pastoris#NC 009270
Brassicales Brassicaceae Capsella rubella#NC 027693
Brassicales Brassicaceae Capsella grandiflora#NC 028517
Brassicales Brassicaceae Arabidopsis suecica#NC 030350
Brassicales Brassicaceae Arabidopsis thaliana#NC 000932
Brassicales Brassicaceae Arabidopsis croatica#NC 030347
Brassicales Brassicaceae Arabidopsis cebennensis#NC 029335
Brassicales Brassicaceae Arabidopsis pedemontana#NC 029336
Brassicales Brassicaceae Arabidopsis arenicola#NC 030346
Brassicales Brassicaceae Arabidopsis lyrata subsp. petraea#KU764768
Brassicales Brassicaceae Arabidopsis lyrata#NC 034365
Brassicales Brassicaceae Arabidopsis arenosa#NC 029334
Brassicales Brassicaceae Arabidopsis neglecta#NC 030348
Brassicales Brassicaceae Arabidopsis petrogena#NC 030349
Brassicales Brassicaceae Arabidopsis halleri subsp. gemmifera#KU764767
Brassicales Brassicaceae Arabidopsis halleri#NC 034366
Brassicales Brassicaceae Arabidopsis umezawana#NC 030351
Brassicales Brassicaceae Clausia aprica#LN877375
Brassicales Brassicaceae Braya humilis#NC 035515
Brassicales Brassicaceae Neotorularia korolkowii#NC 034361
Brassicales Brassicaceae Solms-Laubachia eurycarpa#NC 034359
Brassicales Brassicaceae Bunias orientalis#LN877376
Brassicales Brassicaceae Bunias erucago#NC 036110
Brassicales Brassicaceae Matthiola incana#KY912030
Brassicales Brassicaceae Hesperis sylvestris#NC 035512
Brassicales Brassicaceae Hesperis matronalis#LN877374
Brassicales Brassicaceae Megadenia pygmaea#NC 034357
Brassicales Brassicaceae Lunaria rediviva#NC 039955
Brassicales Brassicaceae Heldreichia bupleurifolia#NC 039954
Brassicales Brassicaceae Biscutella lyrata#NC 039953
Brassicales Brassicaceae Biscutella baetica#NC 039952
Brassicales Brassicaceae Ricotia aucheri#NC 039956
Brassicales Brassicaceae Ricotia isatoides#MH359188
Brassicales Brassicaceae Ricotia lunaria#NC 039961
Brassicales Brassicaceae Ricotia davisiana#NC 039959
Brassicales Brassicaceae Ricotia carnosula#NC 039957
Brassicales Brassicaceae Ricotia cretica#NC 039958
Brassicales Brassicaceae Alyssum montanum subsp. gmelinii#MF169880
Brassicales Brassicaceae Alyssum desertorum#NC 034299
Brassicales Brassicaceae Megacarpaea delavayi#NC 034360
Brassicales Brassicaceae Morettia canescens#NC 035514
Brassicales Brassicaceae Lobularia maritima#NC 009274
Brassicales Brassicaceae Lobularia libyca#NC 035513
Brassicales Brassicaceae Ionopsidium acaule#NC 029333
Brassicales Brassicaceae Cochlearia tridactylites#NC 029332
Brassicales Brassicaceae Cochlearia pyrenaica#NC 029253
Brassicales Brassicaceae Cochlearia officinalis#CSUV
Brassicales Brassicaceae Cochlearia groenlandica#NC 029254
Brassicales Brassicaceae Arabis alpina#NC 023367
Brassicales Brassicaceae Arabis hirsuta#LC361349
Brassicales Brassicaceae Arabis stelleri#KY126841
Brassicales Brassicaceae Arabis flagellosa#NC 037475
Brassicales Brassicaceae Draba aizoides#HABV
Brassicales Brassicaceae Draba nemorosa#NC 009272
Brassicales Brassicaceae Draba hispida#GTSV
Brassicales Brassicaceae Draba oreades#NC 037760
Brassicales Brassicaceae Draba ossetica#LJQF
Brassicales Brassicaceae Draba oligosperma#LAPO
Brassicales Brassicaceae Draba magellanica#UVQL
Brassicales Brassicaceae Draba sachalinensis#BXBF
Brassicales Brassicaceae Thlaspi arvense#NC 034362
Brassicales Brassicaceae Eutrema heterophyllum#NC 028728
Brassicales Brassicaceae Eutrema yunnanense#NC 028727
Brassicales Brassicaceae Eutrema botschantzevii#NC 029379
Brassicales Brassicaceae Eutrema halophilum#NC 029378
Brassicales Brassicaceae Eutrema salsugineum#NC 028170
Brassicales Brassicaceae Eutrema parvulum#NC 028726
Brassicales Brassicaceae Isatis tinctoria#NC 028415
Brassicales Brassicaceae Sisymbrium irio#NC 037838
Brassicales Brassicaceae Sinalliaria limprichtiana#NC 034287
Brassicales Brassicaceae Sinalliaria limprichtiana var. grandifolia#NC 034286

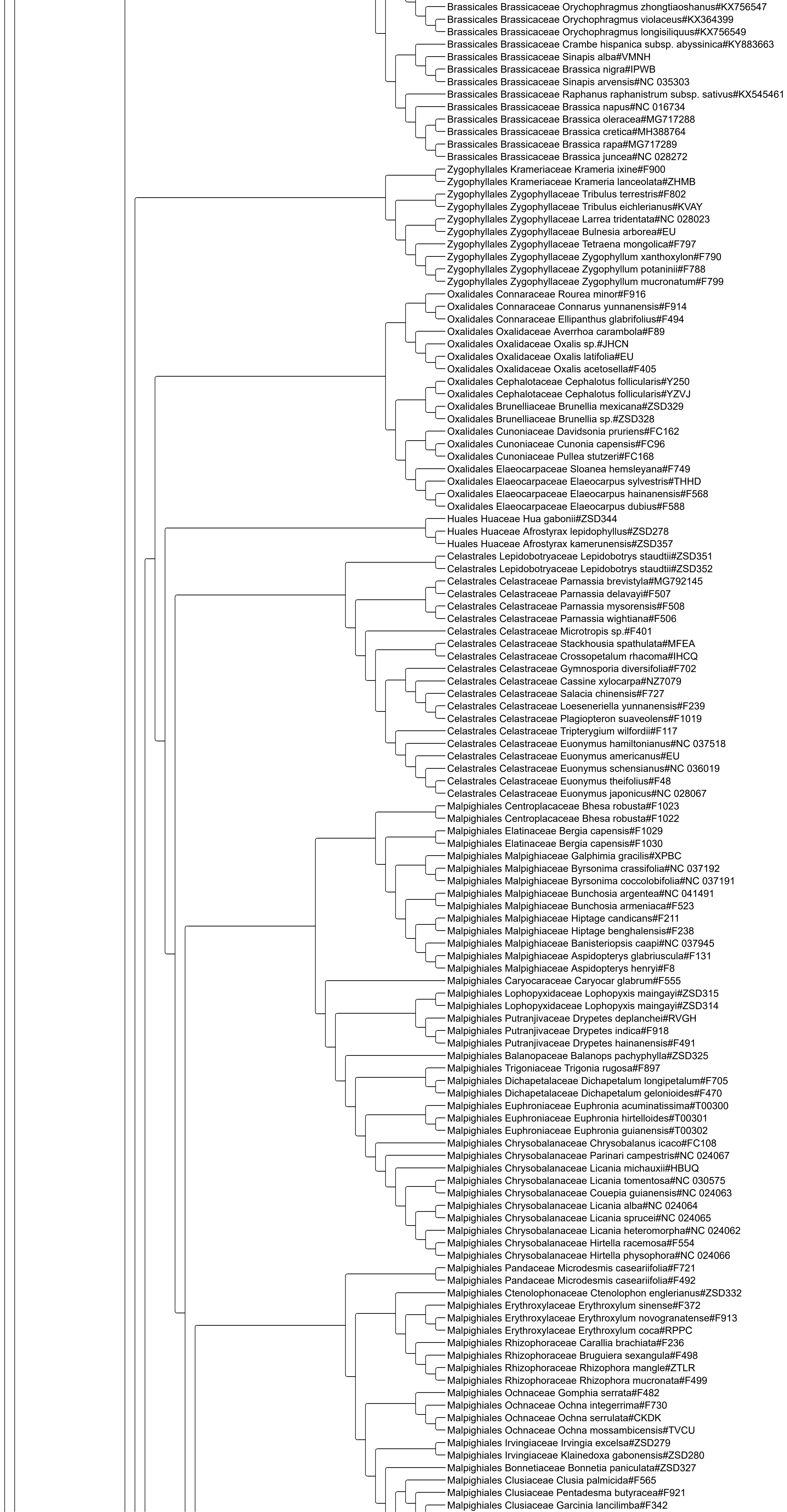
Brassicales Brassicaceae Orychophragmus zhongtiaoshanus#KX756547
Brassicales Brassicaceae Orychophragmus violaceus#KX364399
Brassicales Brassicaceae Orychophragmus longisiliquus#KX756549
Brassicales Brassicaceae Crambe hispanica subsp. abyssinica#KY883663
Brassicales Brassicaceae Sinapis alba#VMNH
Brassicales Brassicaceae Brassica nigra#IPWB
Brassicales Brassicaceae Sinapis arvensis#NC 035303
Brassicales Brassicaceae Raphanus raphanistrum subsp. sativus#KX545461
Brassicales Brassicaceae Brassica napus#NC 016734
Brassicales Brassicaceae Brassica oleracea#MG717288
Brassicales Brassicaceae Brassica cretica#MH388764
Brassicales Brassicaceae Brassica rapa#MG717289
Brassicales Brassicaceae Brassica juncea#NC 028272
Zygophyllales Krameriaceae Krameria ixine#F900
Zygophyllales Krameriaceae Krameria lanceolata#ZHMB
Zygophyllales Zygophyllaceae Tribulus terrestris#F802
Zygophyllales Zygophyllaceae Tribulus eichlerianus#KVAY
Zygophyllales Zygophyllaceae Larrea tridentata#NC 028023
Zygophyllales Zygophyllaceae Bulnesia arborea#EU
Zygophyllales Zygophyllaceae Tetraena mongolica#F797
Zygophyllales Zygophyllaceae Zygophyllum xanthoxylon#F790
Zygophyllales Zygophyllaceae Zygophyllum potaninii#F788
Zygophyllales Zygophyllaceae Zygophyllum mucronatum#F799
Oxalidales Connaraceae Rourea minor#F916
Oxalidales Connaraceae Connarus yunnanensis#F914
Oxalidales Connaraceae Ellipanthus glabrifolius#F494
Oxalidales Oxalidaceae Averrhoa carambola#F89
Oxalidales Oxalidaceae Oxalis sp.#JHCN
Oxalidales Oxalidaceae Oxalis latifolia#EU
Oxalidales Oxalidaceae Oxalis acetosella#F405
Oxalidales Cephalotaceae Cephalotus follicularis#Y250
Oxalidales Cephalotaceae Cephalotus follicularis#YZVJ
Oxalidales Brunelliaceae Brunellia mexicana#ZSD329
Oxalidales Brunelliaceae Brunellia sp.#ZSD328
Oxalidales Cunoniaceae Davidsonia pruriens#FC162
Oxalidales Cunoniaceae Cunonia capensis#FC96
Oxalidales Cunoniaceae Pullea stutzeri#FC168
Oxalidales Elaeocarpaceae Sloanea hemsleyana#F749
Oxalidales Elaeocarpaceae Elaeocarpus sylvestris#THHD
Oxalidales Elaeocarpaceae Elaeocarpus hainanensis#F568
Oxalidales Elaeocarpaceae Elaeocarpus dubius#F588
Huales Huaceae Hua gabonii#ZSD344
Huales Huaceae Afrostyrax lepidophyllus#ZSD278
Huales Huaceae Afrostyrax kamerunensis#ZSD357
Celastrales Lepidobotryaceae Lepidobotrys staudtii#ZSD351
Celastrales Lepidobotryaceae Lepidobotrys staudtii#ZSD352
Celastrales Celastraceae Parnassia brevistyla#MG792145
Celastrales Celastraceae Parnassia delavayi#F507
Celastrales Celastraceae Parnassia mysorensis#F508
Celastrales Celastraceae Parnassia wightiana#F506
Celastrales Celastraceae Microtropis sp.#F401
Celastrales Celastraceae Stackhousia spathulata#MFEA
Celastrales Celastraceae Crossopetalum rhacoma#IHCQ
Celastrales Celastraceae Gymnosporia diversifolia#F702
Celastrales Celastraceae Cassine xylocarpa#NZ7079
Celastrales Celastraceae Salacia chinensis#F727
Celastrales Celastraceae Loeseneriella yunnanensis#F239
Celastrales Celastraceae Plagiopteron suaveolens#F1019
Celastrales Celastraceae Tripterygium wilfordii#F117
Celastrales Celastraceae Euonymus hamiltonianus#NC 037518
Celastrales Celastraceae Euonymus americanus#EU
Celastrales Celastraceae Euonymus schensianus#NC 036019
Celastrales Celastraceae Euonymus theifolius#F48
Celastrales Celastraceae Euonymus japonicus#NC 028067
Malpighiales Centroplacaceae Bhesa robusta#F1023
Malpighiales Centroplacaceae Bhesa robusta#F1022
Malpighiales Elatinaceae Bergia capensis#F1029
Malpighiales Elatinaceae Bergia capensis#F1030
Malpighiales Malpighiaceae Galphimia gracilis#XPBC
Malpighiales Malpighiaceae Byrsonima crassifolia#NC 037192
Malpighiales Malpighiaceae Byrsonima coccolobifolia#NC 037191
Malpighiales Malpighiaceae Bunchosia argentea#NC 041491
Malpighiales Malpighiaceae Bunchosia armeniaca#F523
Malpighiales Malpighiaceae Hiptage candicans#F211
Malpighiales Malpighiaceae Hiptage benghalensis#F238
Malpighiales Malpighiaceae Banisteriopsis caapi#NC 037945
Malpighiales Malpighiaceae Aspidopterys glabriuscula#F131
Malpighiales Malpighiaceae Aspidopterys henryi#F8
Malpighiales Caryocaraceae Caryocar glabrum#F555
Malpighiales Lophopyxidaceae Lophopyxis maingayi#ZSD315
Malpighiales Lophopyxidaceae Lophopyxis maingayi#ZSD314
Malpighiales Putranjivaceae Drypetes deplanchei#RVGH
Malpighiales Putranjivaceae Drypetes indica#F918
Malpighiales Putranjivaceae Drypetes hainanensis#F491
Malpighiales Balanopaceae Balanops pachyphylla#ZSD325
Malpighiales Trigoniaceae Trigonia rugosa#F897
Malpighiales Dichapetalaceae Dichapetalum longipetalum#F705
Malpighiales Dichapetalaceae Dichapetalum gelonioides#F470
Malpighiales Euphroniaceae Euphronia acuminatissima#T00300
Malpighiales Euphroniaceae Euphronia hirtelloides#T00301
Malpighiales Euphroniaceae Euphronia guianensis#T00302
Malpighiales Chrysobalanaceae Chrysobalanus icaco#FC108
Malpighiales Chrysobalanaceae Parinari campestris#NC 024067
Malpighiales Chrysobalanaceae Licania michauxii#HBUQ
Malpighiales Chrysobalanaceae Licania tomentosa#NC 030575
Malpighiales Chrysobalanaceae Couepia guianensis#NC 024063
Malpighiales Chrysobalanaceae Licania alba#NC 024064
Malpighiales Chrysobalanaceae Licania sprucei#NC 024065
Malpighiales Chrysobalanaceae Licania heteromorpha#NC 024062
Malpighiales Chrysobalanaceae Hirtella racemosa#F554
Malpighiales Chrysobalanaceae Hirtella physophora#NC 024066
Malpighiales Pandaceae Microdesmis caseariifolia#F721
Malpighiales Pandaceae Microdesmis caseariifolia#F492
Malpighiales Ctenolophonaceae Ctenolophon englerianus#ZSD332
Malpighiales Erythroxylaceae Erythroxylum sinense#F372
Malpighiales Erythroxylaceae Erythroxylum novogranatense#F913
Malpighiales Erythroxylaceae Erythroxylum coca#RPPC
Malpighiales Rhizophoraceae Carallia brachiata#F236
Malpighiales Rhizophoraceae Bruguiera sexangula#F498
Malpighiales Rhizophoraceae Rhizophora mangle#ZTLR
Malpighiales Rhizophoraceae Rhizophora mucronata#F499
Malpighiales Ochnaceae Gomphia serrata#F482
Malpighiales Ochnaceae Ochna integerrima#F730
Malpighiales Ochnaceae Ochna serrulata#CKDK
Malpighiales Ochnaceae Ochna mossambicensis#TVCU
Malpighiales Irvingiaceae Irvingia excelsa#ZSD279
Malpighiales Irvingiaceae Klainedoxa gabonensis#ZSD280
Malpighiales Bonnetiaceae Bonnetia paniculata#ZSD327
Malpighiales Clusiaceae Clusia palmicida#F565
Malpighiales Clusiaceae Pentadesma butyracea#F921
Malpighiales Clusiaceae Garcinia lancilimba#F342

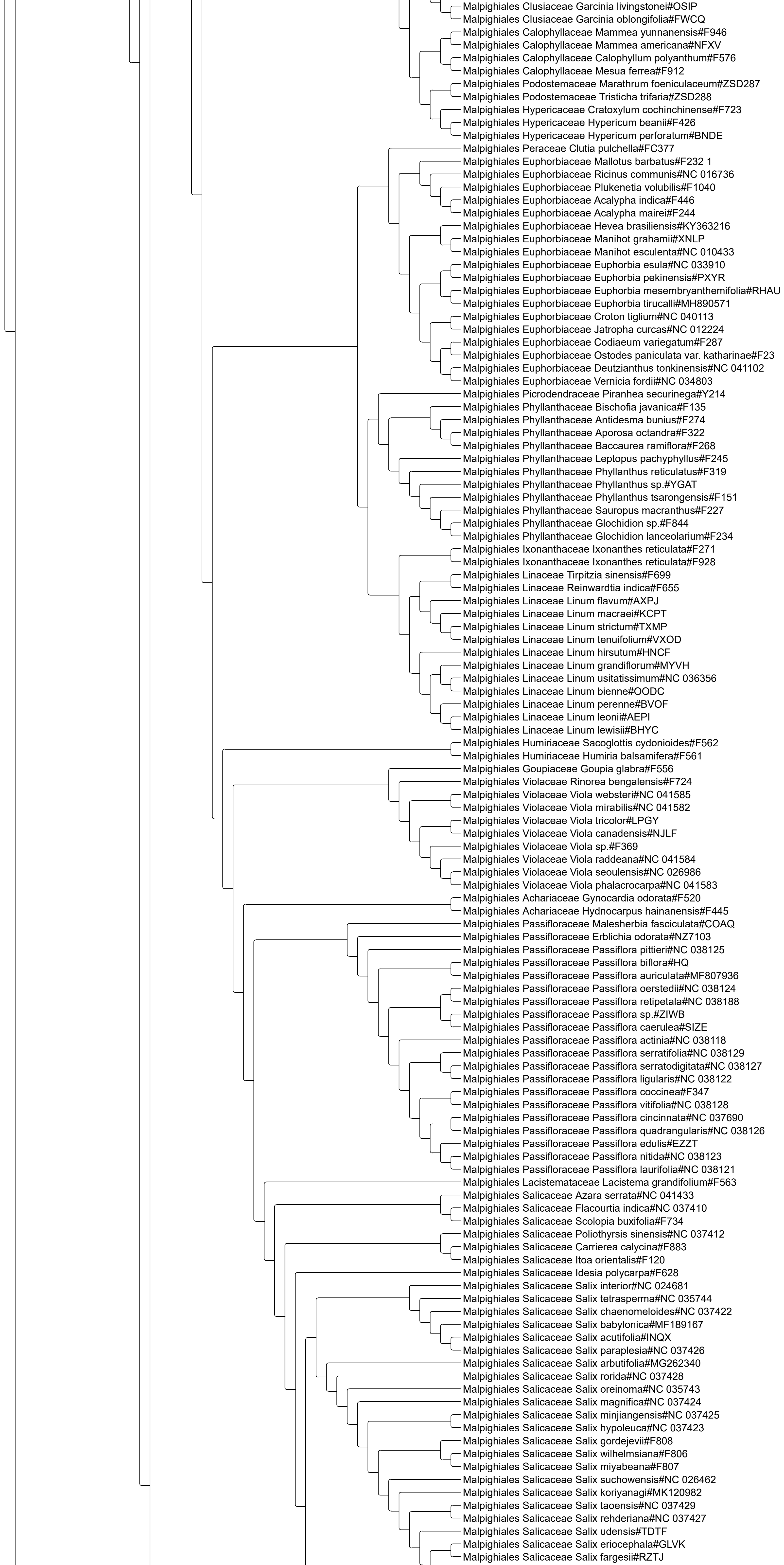
Malpighiales Clusiaceae Garcinia livingstonei#OSIP
Malpighiales Clusiaceae Garcinia oblongifolia#FWCQ
Malpighiales Calophyllaceae Mammea yunnanensis#F946
Malpighiales Calophyllaceae Mammea americana#NFXV
Malpighiales Calophyllaceae Calophyllum polyanthum#F576
Malpighiales Calophyllaceae Mesua ferrea#F912
Malpighiales Podostemaceae Marathrum foeniculaceum#ZSD287
Malpighiales Podostemaceae Tristicha trifaria#ZSD288
Malpighiales Hypericaceae Cratoxylum cochinchinense#F723
Malpighiales Hypericaceae Hypericum beanii#F426
Malpighiales Hypericaceae Hypericum perforatum#BNDE
Malpighiales Peraceae Clutia pulchella#FC377
Malpighiales Euphorbiaceae Mallotus barbatus#F232 1
Malpighiales Euphorbiaceae Ricinus communis#NC 016736
Malpighiales Euphorbiaceae Plukenetia volubilis#F1040
Malpighiales Euphorbiaceae Acalypha indica#F446
Malpighiales Euphorbiaceae Acalypha mairei#F244
Malpighiales Euphorbiaceae Hevea brasiliensis#KY363216
Malpighiales Euphorbiaceae Manihot grahamii#XNLP
Malpighiales Euphorbiaceae Manihot esculenta#NC 010433
Malpighiales Euphorbiaceae Euphorbia esula#NC 033910
Malpighiales Euphorbiaceae Euphorbia pekinensis#PXYR
Malpighiales Euphorbiaceae Euphorbia mesembryanthemifolia#RHAU
Malpighiales Euphorbiaceae Euphorbia tirucalli#MH890571
Malpighiales Euphorbiaceae Croton tiglium#NC 040113
Malpighiales Euphorbiaceae Jatropha curcas#NC 012224
Malpighiales Euphorbiaceae Codiaeum variegatum#F287
Malpighiales Euphorbiaceae Ostodes paniculata var. katharinae#F23
Malpighiales Euphorbiaceae Deutzianthus tonkinensis#NC 041102
Malpighiales Euphorbiaceae Vernicia fordii#NC 034803
Malpighiales Picrodendraceae Piranhea securinega#Y214
Malpighiales Phyllanthaceae Bischofia javanica#F135
Malpighiales Phyllanthaceae Antidesma bunius#F274
Malpighiales Phyllanthaceae Aporosa octandra#F322
Malpighiales Phyllanthaceae Baccaurea ramiflora#F268
Malpighiales Phyllanthaceae Leptopus pachyphyllus#F245
Malpighiales Phyllanthaceae Phyllanthus reticulatus#F319
Malpighiales Phyllanthaceae Phyllanthus sp.#YGAT
Malpighiales Phyllanthaceae Phyllanthus tsarongensis#F151
Malpighiales Phyllanthaceae Sauropus macranthus#F227
Malpighiales Phyllanthaceae Glochidion sp.#F844
Malpighiales Phyllanthaceae Glochidion lanceolarium#F234
Malpighiales Ixonanthaceae Ixonanthes reticulata#F271
Malpighiales Ixonanthaceae Ixonanthes reticulata#F928
Malpighiales Linaceae Tirpitzia sinensis#F699
Malpighiales Linaceae Reinwardtia indica#F655
Malpighiales Linaceae Linum flavum#AXPJ
Malpighiales Linaceae Linum macraei#KCPT
Malpighiales Linaceae Linum strictum#TXMP
Malpighiales Linaceae Linum tenuifolium#VXOD
Malpighiales Linaceae Linum hirsutum#HNCF
Malpighiales Linaceae Linum grandiflorum#MYVH
Malpighiales Linaceae Linum usitatissimum#NC 036356
Malpighiales Linaceae Linum bienne#OODC
Malpighiales Linaceae Linum perenne#BVOF
Malpighiales Linaceae Linum leonii#AEPI
Malpighiales Linaceae Linum lewisii#BHYC
Malpighiales Humiriaceae Sacoglottis cydonioides#F562
Malpighiales Humiriaceae Humiria balsamifera#F561
Malpighiales Goupiaceae Goupia glabra#F556
Malpighiales Violaceae Rinorea bengalensis#F724
Malpighiales Violaceae Viola websteri#NC 041585
Malpighiales Violaceae Viola mirabilis#NC 041582
Malpighiales Violaceae Viola tricolor#LPGY
Malpighiales Violaceae Viola canadensis#NJLF
Malpighiales Violaceae Viola sp.#F369
Malpighiales Violaceae Viola raddeana#NC 041584
Malpighiales Violaceae Viola seoulensis#NC 026986
Malpighiales Violaceae Viola phalacrocarpa#NC 041583
Malpighiales Achariaceae Gynocardia odorata#F520
Malpighiales Achariaceae Hydnocarpus hainanensis#F445
Malpighiales Passifloraceae Malesherbia fasciculata#COAQ
Malpighiales Passifloraceae Erblichia odorata#NZ7103
Malpighiales Passifloraceae Passiflora pittieri#NC 038125
Malpighiales Passifloraceae Passiflora biflora#HQ
Malpighiales Passifloraceae Passiflora auriculata#MF807936
Malpighiales Passifloraceae Passiflora oerstedii#NC 038124
Malpighiales Passifloraceae Passiflora retipetala#NC 038188
Malpighiales Passifloraceae Passiflora sp.#ZIWB
Malpighiales Passifloraceae Passiflora caerulea#SIZE
Malpighiales Passifloraceae Passiflora actinia#NC 038118
Malpighiales Passifloraceae Passiflora serratifolia#NC 038129
Malpighiales Passifloraceae Passiflora serratodigitata#NC 038127
Malpighiales Passifloraceae Passiflora ligularis#NC 038122
Malpighiales Passifloraceae Passiflora coccinea#F347
Malpighiales Passifloraceae Passiflora vitifolia#NC 038128
Malpighiales Passifloraceae Passiflora cincinnata#NC 037690
Malpighiales Passifloraceae Passiflora quadrangularis#NC 038126
Malpighiales Passifloraceae Passiflora edulis#EZZT
Malpighiales Passifloraceae Passiflora nitida#NC 038123
Malpighiales Passifloraceae Passiflora laurifolia#NC 038121
Malpighiales Lacistemataceae Lacistema grandifolium#F563
Malpighiales Salicaceae Azara serrata#NC 041433
Malpighiales Salicaceae Flacourtia indica#NC 037410
Malpighiales Salicaceae Scolopia buxifolia#F734
Malpighiales Salicaceae Poliothyrsis sinensis#NC 037412
Malpighiales Salicaceae Carrierea calycina#F883
Malpighiales Salicaceae Itoa orientalis#F120
Malpighiales Salicaceae Idesia polycarpa#F628
Malpighiales Salicaceae Salix interior#NC 024681
Malpighiales Salicaceae Salix tetrasperma#NC 035744
Malpighiales Salicaceae Salix chaenomeloides#NC 037422
Malpighiales Salicaceae Salix babylonica#MF189167
Malpighiales Salicaceae Salix acutifolia#INQX
Malpighiales Salicaceae Salix paraplesia#NC 037426
Malpighiales Salicaceae Salix arbutifolia#MG262340
Malpighiales Salicaceae Salix rorida#NC 037428
Malpighiales Salicaceae Salix oreinoma#NC 035743
Malpighiales Salicaceae Salix magnifica#NC 037424
Malpighiales Salicaceae Salix minjiangensis#NC 037425
Malpighiales Salicaceae Salix hypoleuca#NC 037423
Malpighiales Salicaceae Salix gordejevii#F808
Malpighiales Salicaceae Salix wilhelmsiana#F806
Malpighiales Salicaceae Salix miyabeana#F807
Malpighiales Salicaceae Salix suchowensis#NC 026462
Malpighiales Salicaceae Salix koriyanagi#MK120982
Malpighiales Salicaceae Salix taoensis#NC 037429
Malpighiales Salicaceae Salix rehderiana#NC 037427
Malpighiales Salicaceae Salix udensis#TDTF
Malpighiales Salicaceae Salix eriocephala#GLVK
Malpighiales Salicaceae Salix fargesii#RZTJ

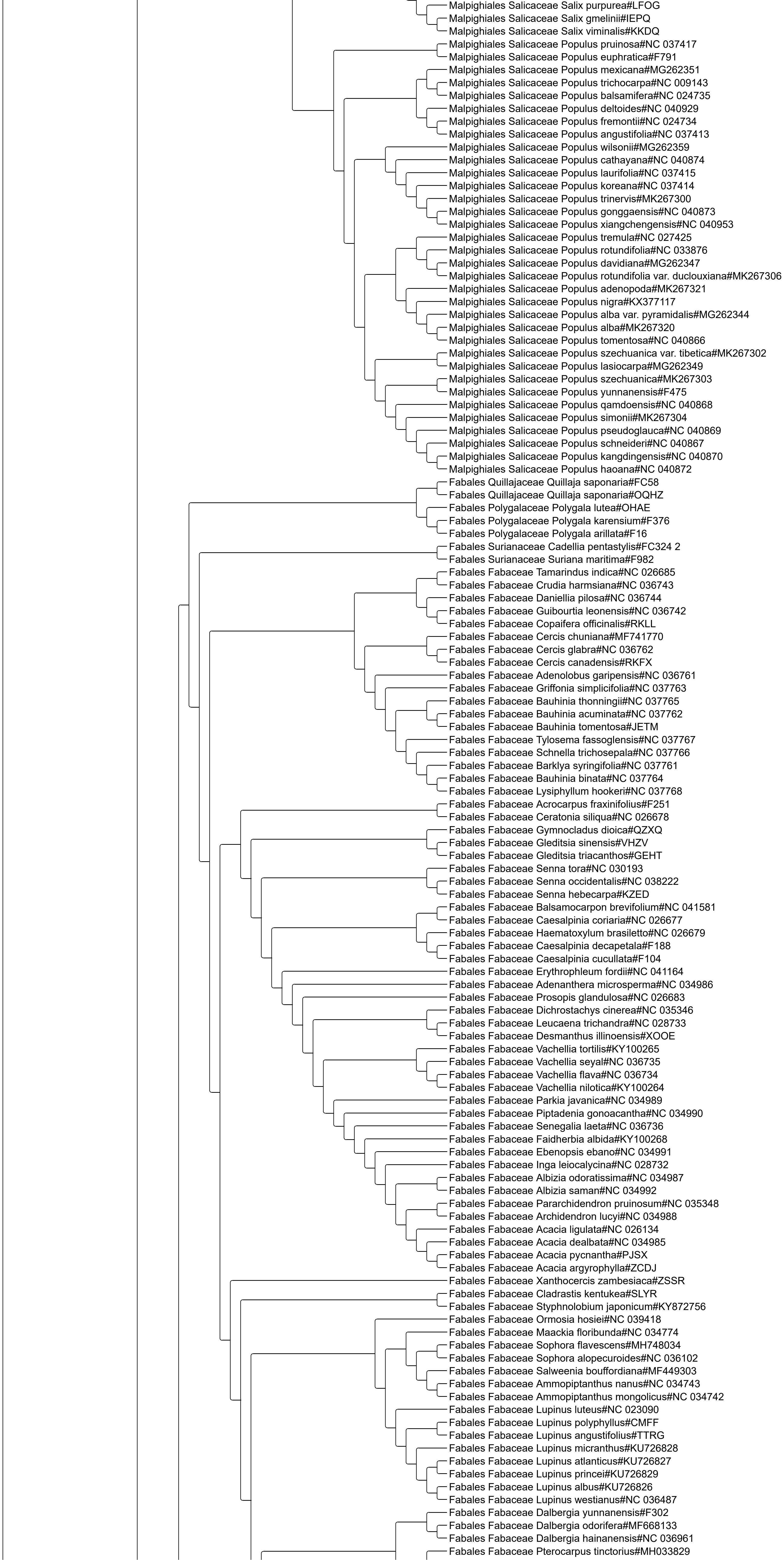
Malpighiales Salicaceae Salix purpurea#LFOG
Malpighiales Salicaceae Salix gmelinii#IEPQ
Malpighiales Salicaceae Salix viminalis#KKDQ
Malpighiales Salicaceae Populus pruinosa#NC 037417
Malpighiales Salicaceae Populus euphratica#F791
Malpighiales Salicaceae Populus mexicana#MG262351
Malpighiales Salicaceae Populus trichocarpa#NC 009143
Malpighiales Salicaceae Populus balsamifera#NC 024735
Malpighiales Salicaceae Populus deltoides#NC 040929
Malpighiales Salicaceae Populus fremontii#NC 024734
Malpighiales Salicaceae Populus angustifolia#NC 037413
Malpighiales Salicaceae Populus wilsonii#MG262359
Malpighiales Salicaceae Populus cathayana#NC 040874
Malpighiales Salicaceae Populus laurifolia#NC 037415
Malpighiales Salicaceae Populus koreana#NC 037414
Malpighiales Salicaceae Populus trinervis#MK267300
Malpighiales Salicaceae Populus gonggaensis#NC 040873
Malpighiales Salicaceae Populus xiangchengensis#NC 040953
Malpighiales Salicaceae Populus tremula#NC 027425
Malpighiales Salicaceae Populus rotundifolia#NC 033876
Malpighiales Salicaceae Populus davidiana#MG262347
Malpighiales Salicaceae Populus rotundifolia var. duclouxiana#MK267306
Malpighiales Salicaceae Populus adenopoda#MK267321
Malpighiales Salicaceae Populus nigra#KX377117
Malpighiales Salicaceae Populus alba var. pyramidalis#MG262344
Malpighiales Salicaceae Populus alba#MK267320
Malpighiales Salicaceae Populus tomentosa#NC 040866
Malpighiales Salicaceae Populus szechuanica var. tibetica#MK267302
Malpighiales Salicaceae Populus lasiocarpa#MG262349
Malpighiales Salicaceae Populus szechuanica#MK267303
Malpighiales Salicaceae Populus yunnanensis#F475
Malpighiales Salicaceae Populus qamdoensis#NC 040868
Malpighiales Salicaceae Populus simonii#MK267304
Malpighiales Salicaceae Populus pseudoglauca#NC 040869
Malpighiales Salicaceae Populus schneideri#NC 040867
Malpighiales Salicaceae Populus kangdingensis#NC 040870
Malpighiales Salicaceae Populus haoana#NC 040872
Fabales Quillajaceae Quillaja saponaria#FC58
Fabales Quillajaceae Quillaja saponaria#OQHZ
Fabales Polygalaceae Polygala lutea#OHAE
Fabales Polygalaceae Polygala karensium#F376
Fabales Polygalaceae Polygala arillata#F16
Fabales Surianaceae Cadellia pentastylis#FC324 2
Fabales Surianaceae Suriana maritima#F982
Fabales Fabaceae Tamarindus indica#NC 026685
Fabales Fabaceae Crudia harmsiana#NC 036743
Fabales Fabaceae Daniellia pilosa#NC 036744
Fabales Fabaceae Guibourtia leonensis#NC 036742
Fabales Fabaceae Copaifera officinalis#RKLL
Fabales Fabaceae Cercis chuniana#MF741770
Fabales Fabaceae Cercis glabra#NC 036762
Fabales Fabaceae Cercis canadensis#RKFX
Fabales Fabaceae Adenolobus garipensis#NC 036761
Fabales Fabaceae Griffonia simplicifolia#NC 037763
Fabales Fabaceae Bauhinia thonningii#NC 037765
Fabales Fabaceae Bauhinia acuminata#NC 037762
Fabales Fabaceae Bauhinia tomentosa#JETM
Fabales Fabaceae Tylosema fassoglensis#NC 037767
Fabales Fabaceae Schnella trichosepala#NC 037766
Fabales Fabaceae Barklya syringifolia#NC 037761
Fabales Fabaceae Bauhinia binata#NC 037764
Fabales Fabaceae Lysiphyllum hookeri#NC 037768
Fabales Fabaceae Acrocarpus fraxinifolius#F251
Fabales Fabaceae Ceratonia siliqua#NC 026678
Fabales Fabaceae Gymnocladus dioica#QZXQ
Fabales Fabaceae Gleditsia sinensis#VHZV
Fabales Fabaceae Gleditsia triacanthos#GEHT
Fabales Fabaceae Senna tora#NC 030193
Fabales Fabaceae Senna occidentalis#NC 038222
Fabales Fabaceae Senna hebecarpa#KZED
Fabales Fabaceae Balsamocarpon brevifolium#NC 041581
Fabales Fabaceae Caesalpinia coriaria#NC 026677
Fabales Fabaceae Haematoxylum brasiletto#NC 026679
Fabales Fabaceae Caesalpinia decapetala#F188
Fabales Fabaceae Caesalpinia cucullata#F104
Fabales Fabaceae Erythrophleum fordii#NC 041164
Fabales Fabaceae Adenanthera microsperma#NC 034986
Fabales Fabaceae Prosopis glandulosa#NC 026683
Fabales Fabaceae Dichrostachys cinerea#NC 035346
Fabales Fabaceae Leucaena trichandra#NC 028733
Fabales Fabaceae Desmanthus illinoensis#XOOE
Fabales Fabaceae Vachellia tortilis#KY100265
Fabales Fabaceae Vachellia seyal#NC 036735
Fabales Fabaceae Vachellia flava#NC 036734
Fabales Fabaceae Vachellia nilotica#KY100264
Fabales Fabaceae Parkia javanica#NC 034989
Fabales Fabaceae Piptadenia gonoacantha#NC 034990
Fabales Fabaceae Senegalia laeta#NC 036736
Fabales Fabaceae Faidherbia albida#KY100268
Fabales Fabaceae Ebenopsis ebano#NC 034991
Fabales Fabaceae Inga leiocalycina#NC 028732
Fabales Fabaceae Albizia odoratissima#NC 034987
Fabales Fabaceae Albizia saman#NC 034992
Fabales Fabaceae Pararchidendron pruinosum#NC 035348
Fabales Fabaceae Archidendron lucyi#NC 034988
Fabales Fabaceae Acacia ligulata#NC 026134
Fabales Fabaceae Acacia dealbata#NC 034985
Fabales Fabaceae Acacia pycnantha#PJSX
Fabales Fabaceae Acacia argyrophylla#ZCDJ
Fabales Fabaceae Xanthocercis zambesiaca#ZSSR
Fabales Fabaceae Cladrastis kentukea#SLYR
Fabales Fabaceae Styphnolobium japonicum#KY872756
Fabales Fabaceae Ormosia hosiei#NC 039418
Fabales Fabaceae Maackia floribunda#NC 034774
Fabales Fabaceae Sophora flavescens#MH748034
Fabales Fabaceae Sophora alopecuroides#NC 036102
Fabales Fabaceae Salweenia bouffordiana#MF449303
Fabales Fabaceae Ammopiptanthus nanus#NC 034743
Fabales Fabaceae Ammopiptanthus mongolicus#NC 034742
Fabales Fabaceae Lupinus luteus#NC 023090
Fabales Fabaceae Lupinus polyphyllus#CMFF
Fabales Fabaceae Lupinus angustifolius#TTRG
Fabales Fabaceae Lupinus micranthus#KU726828
Fabales Fabaceae Lupinus atlanticus#KU726827
Fabales Fabaceae Lupinus princei#KU726829
Fabales Fabaceae Lupinus albus#KU726826
Fabales Fabaceae Lupinus westianus#NC 036487
Fabales Fabaceae Dalbergia yunnanensis#F302
Fabales Fabaceae Dalbergia odorifera#MF668133
Fabales Fabaceae Dalbergia hainanensis#NC 036961
Fabales Fabaceae Pterocarpus tinctorius#MH033829

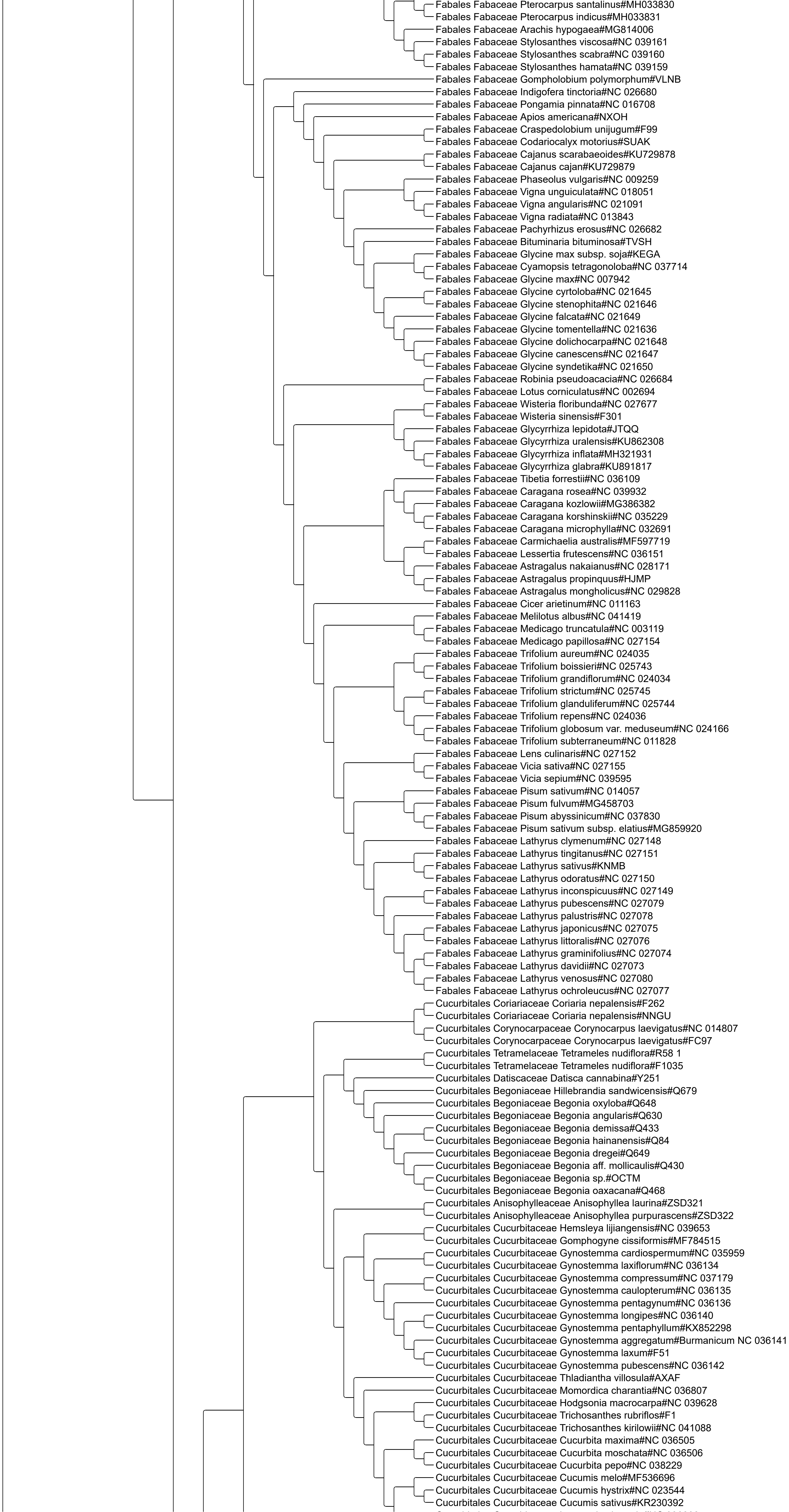

Fabales Fabaceae Pterocarpus santalinus#MH033830
Fabales Fabaceae Pterocarpus indicus#MH033831
Fabales Fabaceae Arachis hypogaea#MG814006
Fabales Fabaceae Stylosanthes viscosa#NC 039161
Fabales Fabaceae Stylosanthes scabra#NC 039160
Fabales Fabaceae Stylosanthes hamata#NC 039159
Fabales Fabaceae Gompholobium polymorphum#VLNB
Fabales Fabaceae Indigofera tinctoria#NC 026680
Fabales Fabaceae Pongamia pinnata#NC 016708
Fabales Fabaceae Apios americana#NXOH
Fabales Fabaceae Craspedolobium unijugum#F99
Fabales Fabaceae Codariocalyx motorius#SUAK
Fabales Fabaceae Cajanus scarabaeoides#KU729878
Fabales Fabaceae Cajanus cajan#KU729879
Fabales Fabaceae Phaseolus vulgaris#NC 009259
Fabales Fabaceae Vigna unguiculata#NC 018051
Fabales Fabaceae Vigna angularis#NC 021091
Fabales Fabaceae Vigna radiata#NC 013843
Fabales Fabaceae Pachyrhizus erosus#NC 026682
Fabales Fabaceae Bituminaria bituminosa#TVSH
Fabales Fabaceae Glycine max subsp. soja#KEGA
Fabales Fabaceae Cyamopsis tetragonoloba#NC 037714
Fabales Fabaceae Glycine max#NC 007942
Fabales Fabaceae Glycine cyrtoloba#NC 021645
Fabales Fabaceae Glycine stenophita#NC 021646
Fabales Fabaceae Glycine falcata#NC 021649
Fabales Fabaceae Glycine tomentella#NC 021636
Fabales Fabaceae Glycine dolichocarpa#NC 021648
Fabales Fabaceae Glycine canescens#NC 021647
Fabales Fabaceae Glycine syndetika#NC 021650
Fabales Fabaceae Robinia pseudoacacia#NC 026684
Fabales Fabaceae Lotus corniculatus#NC 002694
Fabales Fabaceae Wisteria floribunda#NC 027677
Fabales Fabaceae Wisteria sinensis#F301
Fabales Fabaceae Glycyrrhiza lepidota#JTQQ
Fabales Fabaceae Glycyrrhiza uralensis#KU862308
Fabales Fabaceae Glycyrrhiza inflata#MH321931
Fabales Fabaceae Glycyrrhiza glabra#KU891817
Fabales Fabaceae Tibetia forrestii#NC 036109
Fabales Fabaceae Caragana rosea#NC 039932
Fabales Fabaceae Caragana kozlowii#MG386382
Fabales Fabaceae Caragana korshinskii#NC 035229
Fabales Fabaceae Caragana microphylla#NC 032691
Fabales Fabaceae Carmichaelia australis#MF597719
Fabales Fabaceae Lessertia frutescens#NC 036151
Fabales Fabaceae Astragalus nakaianus#NC 028171
Fabales Fabaceae Astragalus propinquus#HJMP
Fabales Fabaceae Astragalus mongholicus#NC 029828
Fabales Fabaceae Cicer arietinum#NC 011163
Fabales Fabaceae Melilotus albus#NC 041419
Fabales Fabaceae Medicago truncatula#NC 003119
Fabales Fabaceae Medicago papillosa#NC 027154
Fabales Fabaceae Trifolium aureum#NC 024035
Fabales Fabaceae Trifolium boissieri#NC 025743
Fabales Fabaceae Trifolium grandiflorum#NC 024034
Fabales Fabaceae Trifolium strictum#NC 025745
Fabales Fabaceae Trifolium glanduliferum#NC 025744
Fabales Fabaceae Trifolium repens#NC 024036
Fabales Fabaceae Trifolium globosum var. meduseum#NC 024166
Fabales Fabaceae Trifolium subterraneum#NC 011828
Fabales Fabaceae Lens culinaris#NC 027152
Fabales Fabaceae Vicia sativa#NC 027155
Fabales Fabaceae Vicia sepium#NC 039595
Fabales Fabaceae Pisum sativum#NC 014057
Fabales Fabaceae Pisum fulvum#MG458703
Fabales Fabaceae Pisum abyssinicum#NC 037830
Fabales Fabaceae Pisum sativum subsp. elatius#MG859920
Fabales Fabaceae Lathyrus clymenum#NC 027148
Fabales Fabaceae Lathyrus tingitanus#NC 027151
Fabales Fabaceae Lathyrus sativus#KNMB
Fabales Fabaceae Lathyrus odoratus#NC 027150
Fabales Fabaceae Lathyrus inconspicuus#NC 027149
Fabales Fabaceae Lathyrus pubescens#NC 027079
Fabales Fabaceae Lathyrus palustris#NC 027078
Fabales Fabaceae Lathyrus japonicus#NC 027075
Fabales Fabaceae Lathyrus littoralis#NC 027076
Fabales Fabaceae Lathyrus graminifolius#NC 027074
Fabales Fabaceae Lathyrus davidii#NC 027073
Fabales Fabaceae Lathyrus venosus#NC 027080
Fabales Fabaceae Lathyrus ochroleucus#NC 027077
Cucurbitales Coriariaceae Coriaria nepalensis#F262
Cucurbitales Coriariaceae Coriaria nepalensis#NNGU
Cucurbitales Corynocarpaceae Corynocarpus laevigatus#NC 014807
Cucurbitales Corynocarpaceae Corynocarpus laevigatus#FC97
Cucurbitales Tetramelaceae Tetrameles nudiflora#R58 1
Cucurbitales Tetramelaceae Tetrameles nudiflora#F1035
Cucurbitales Datiscaceae Datisca cannabina#Y251
Cucurbitales Begoniaceae Hillebrandia sandwicensis#Q679
Cucurbitales Begoniaceae Begonia oxyloba#Q648
Cucurbitales Begoniaceae Begonia angularis#Q630
Cucurbitales Begoniaceae Begonia demissa#Q433
Cucurbitales Begoniaceae Begonia hainanensis#Q84
Cucurbitales Begoniaceae Begonia dregei#Q649
Cucurbitales Begoniaceae Begonia aff. mollicaulis#Q430
Cucurbitales Begoniaceae Begonia sp.#OCTM
Cucurbitales Begoniaceae Begonia oaxacana#Q468
Cucurbitales Anisophylleaceae Anisophyllea laurina#ZSD321
Cucurbitales Anisophylleaceae Anisophyllea purpurascens#ZSD322
Cucurbitales Cucurbitaceae Hemsleya lijiangensis#NC 039653
Cucurbitales Cucurbitaceae Gomphogyne cissiformis#MF784515
Cucurbitales Cucurbitaceae Gynostemma cardiospermum#NC 035959
Cucurbitales Cucurbitaceae Gynostemma laxiflorum#NC 036134
Cucurbitales Cucurbitaceae Gynostemma compressum#NC 037179
Cucurbitales Cucurbitaceae Gynostemma caulopterum#NC 036135
Cucurbitales Cucurbitaceae Gynostemma pentagynum#NC 036136
Cucurbitales Cucurbitaceae Gynostemma longipes#NC 036140
Cucurbitales Cucurbitaceae Gynostemma pentaphyllum#KX852298
Cucurbitales Cucurbitaceae Gynostemma aggregatum#Burmanicum NC 036141
Cucurbitales Cucurbitaceae Gynostemma laxum#F51
Cucurbitales Cucurbitaceae Gynostemma pubescens#NC 036142
Cucurbitales Cucurbitaceae Thladiantha villosula#AXAF
Cucurbitales Cucurbitaceae Momordica charantia#NC 036807
Cucurbitales Cucurbitaceae Hodgsonia macrocarpa#NC 039628
Cucurbitales Cucurbitaceae Trichosanthes rubriflos#F1
Cucurbitales Cucurbitaceae Trichosanthes kirilowii#NC 041088
Cucurbitales Cucurbitaceae Cucurbita maxima#NC 036505
Cucurbitales Cucurbitaceae Cucurbita moschata#NC 036506
Cucurbitales Cucurbitaceae Cucurbita pepo#NC 038229
Cucurbitales Cucurbitaceae Cucumis melo#MF536696
Cucurbitales Cucurbitaceae Cucumis hystrix#NC 023544
Cucurbitales Cucurbitaceae Cucumis sativus#KR230392

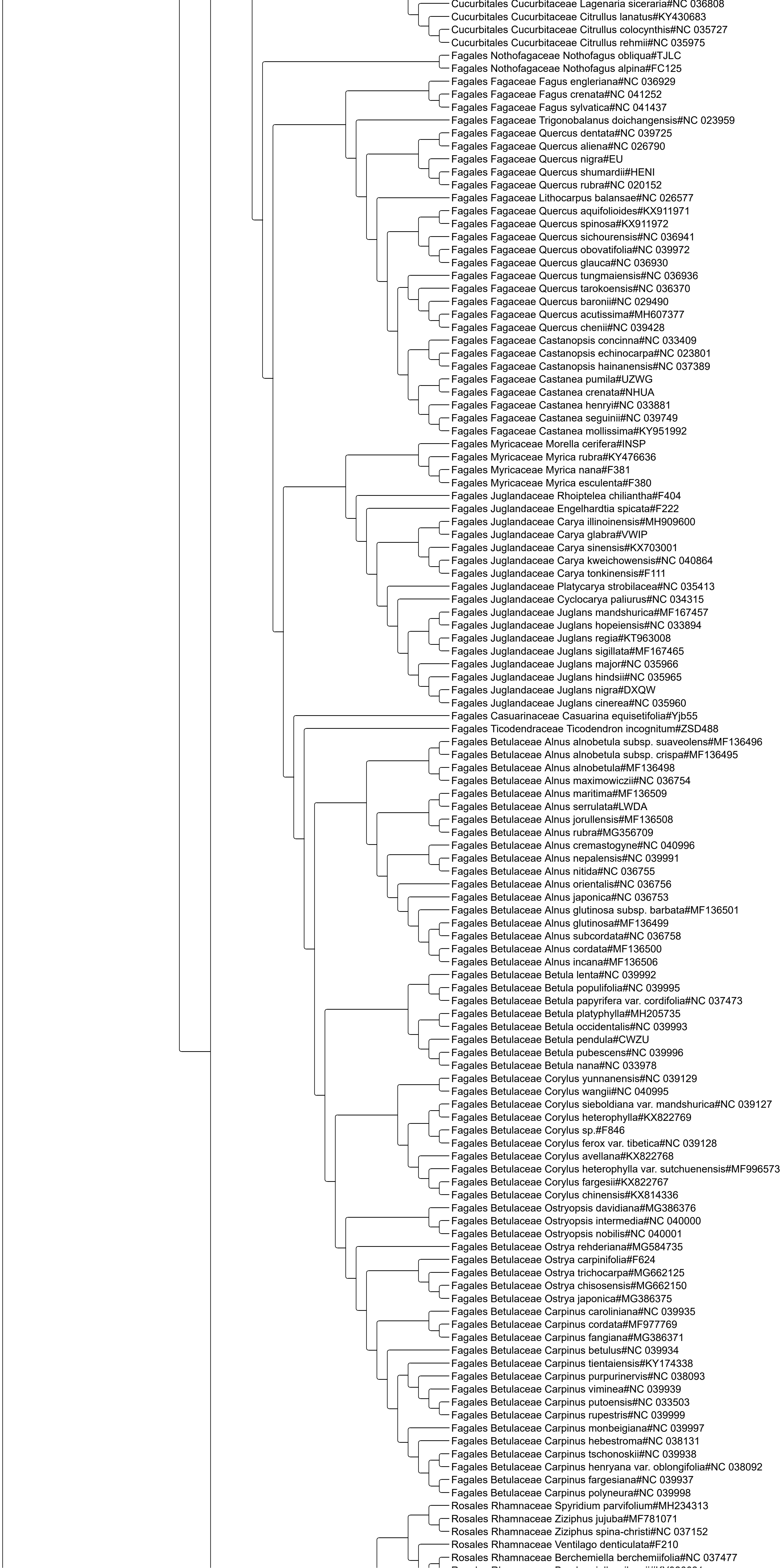
Cucurbitales Cucurbitaceae Lagenaria siceraria#NC 036808
Cucurbitales Cucurbitaceae Citrullus lanatus#KY430683
Cucurbitales Cucurbitaceae Citrullus colocynthis#NC 035727
Cucurbitales Cucurbitaceae Citrullus rehmii#NC 035975
Fagales Nothofagaceae Nothofagus obliqua#TJLC
Fagales Nothofagaceae Nothofagus alpina#FC125
Fagales Fagaceae Fagus engleriana#NC 036929
Fagales Fagaceae Fagus crenata#NC 041252
Fagales Fagaceae Fagus sylvatica#NC 041437
Fagales Fagaceae Trigonobalanus doichangensis#NC 023959
Fagales Fagaceae Quercus dentata#NC 039725
Fagales Fagaceae Quercus aliena#NC 026790
Fagales Fagaceae Quercus nigra#EU
Fagales Fagaceae Quercus shumardii#HENI
Fagales Fagaceae Quercus rubra#NC 020152
Fagales Fagaceae Lithocarpus balansae#NC 026577
Fagales Fagaceae Quercus aquifolioides#KX911971
Fagales Fagaceae Quercus spinosa#KX911972
Fagales Fagaceae Quercus sichourensis#NC 036941
Fagales Fagaceae Quercus obovatifolia#NC 039972
Fagales Fagaceae Quercus glauca#NC 036930
Fagales Fagaceae Quercus tungmaiensis#NC 036936
Fagales Fagaceae Quercus tarokoensis#NC 036370
Fagales Fagaceae Quercus baronii#NC 029490
Fagales Fagaceae Quercus acutissima#MH607377
Fagales Fagaceae Quercus chenii#NC 039428
Fagales Fagaceae Castanopsis concinna#NC 033409
Fagales Fagaceae Castanopsis echinocarpa#NC 023801
Fagales Fagaceae Castanopsis hainanensis#NC 037389
Fagales Fagaceae Castanea pumila#UZWG
Fagales Fagaceae Castanea crenata#NHUA
Fagales Fagaceae Castanea henryi#NC 033881
Fagales Fagaceae Castanea seguinii#NC 039749
Fagales Fagaceae Castanea mollissima#KY951992
Fagales Myricaceae Morella cerifera#INSP
Fagales Myricaceae Myrica rubra#KY476636
Fagales Myricaceae Myrica nana#F381
Fagales Myricaceae Myrica esculenta#F380
Fagales Juglandaceae Rhoiptelea chiliantha#F404
Fagales Juglandaceae Engelhardtia spicata#F222
Fagales Juglandaceae Carya illinoinensis#MH909600
Fagales Juglandaceae Carya glabra#VWIP
Fagales Juglandaceae Carya sinensis#KX703001
Fagales Juglandaceae Carya kweichowensis#NC 040864
Fagales Juglandaceae Carya tonkinensis#F111
Fagales Juglandaceae Platycarya strobilacea#NC 035413
Fagales Juglandaceae Cyclocarya paliurus#NC 034315
Fagales Juglandaceae Juglans mandshurica#MF167457
Fagales Juglandaceae Juglans hopeiensis#NC 033894
Fagales Juglandaceae Juglans regia#KT963008
Fagales Juglandaceae Juglans sigillata#MF167465
Fagales Juglandaceae Juglans major#NC 035966
Fagales Juglandaceae Juglans hindsii#NC 035965
Fagales Juglandaceae Juglans nigra#DXQW
Fagales Juglandaceae Juglans cinerea#NC 035960
Fagales Casuarinaceae Casuarina equisetifolia#Yjb55
Fagales Ticodendraceae Ticodendron incognitum#ZSD488
Fagales Betulaceae Alnus alnobetula subsp. suaveolens#MF136496
Fagales Betulaceae Alnus alnobetula subsp. crispa#MF136495
Fagales Betulaceae Alnus alnobetula#MF136498
Fagales Betulaceae Alnus maximowiczii#NC 036754
Fagales Betulaceae Alnus maritima#MF136509
Fagales Betulaceae Alnus serrulata#LWDA
Fagales Betulaceae Alnus jorullensis#MF136508
Fagales Betulaceae Alnus rubra#MG356709
Fagales Betulaceae Alnus cremastogyne#NC 040996
Fagales Betulaceae Alnus nepalensis#NC 039991
Fagales Betulaceae Alnus nitida#NC 036755
Fagales Betulaceae Alnus orientalis#NC 036756
Fagales Betulaceae Alnus japonica#NC 036753
Fagales Betulaceae Alnus glutinosa subsp. barbata#MF136501
Fagales Betulaceae Alnus glutinosa#MF136499
Fagales Betulaceae Alnus subcordata#NC 036758
Fagales Betulaceae Alnus cordata#MF136500
Fagales Betulaceae Alnus incana#MF136506
Fagales Betulaceae Betula lenta#NC 039992
Fagales Betulaceae Betula populifolia#NC 039995
Fagales Betulaceae Betula papyrifera var. cordifolia#NC 037473
Fagales Betulaceae Betula platyphylla#MH205735
Fagales Betulaceae Betula occidentalis#NC 039993
Fagales Betulaceae Betula pendula#CWZU
Fagales Betulaceae Betula pubescens#NC 039996
Fagales Betulaceae Betula nana#NC 033978
Fagales Betulaceae Corylus yunnanensis#NC 039129
Fagales Betulaceae Corylus wangii#NC 040995
Fagales Betulaceae Corylus sieboldiana var. mandshurica#NC 039127
Fagales Betulaceae Corylus heterophylla#KX822769
Fagales Betulaceae Corylus sp.#F846
Fagales Betulaceae Corylus ferox var. tibetica#NC 039128
Fagales Betulaceae Corylus avellana#KX822768
Fagales Betulaceae Corylus heterophylla var. sutchuenensis#MF996573
Fagales Betulaceae Corylus fargesii#KX822767
Fagales Betulaceae Corylus chinensis#KX814336
Fagales Betulaceae Ostryopsis davidiana#MG386376
Fagales Betulaceae Ostryopsis intermedia#NC 040000
Fagales Betulaceae Ostryopsis nobilis#NC 040001
Fagales Betulaceae Ostrya rehderiana#MG584735
Fagales Betulaceae Ostrya carpinifolia#F624
Fagales Betulaceae Ostrya trichocarpa#MG662125
Fagales Betulaceae Ostrya chisosensis#MG662150
Fagales Betulaceae Ostrya japonica#MG386375
Fagales Betulaceae Carpinus caroliniana#NC 039935
Fagales Betulaceae Carpinus cordata#MF977769
Fagales Betulaceae Carpinus fangiana#MG386371
Fagales Betulaceae Carpinus betulus#NC 039934
Fagales Betulaceae Carpinus tientaiensis#KY174338
Fagales Betulaceae Carpinus purpurinervis#NC 038093
Fagales Betulaceae Carpinus viminea#NC 039939
Fagales Betulaceae Carpinus putoensis#NC 033503
Fagales Betulaceae Carpinus rupestris#NC 039999
Fagales Betulaceae Carpinus monbeigiana#NC 039997
Fagales Betulaceae Carpinus hebestroma#NC 038131
Fagales Betulaceae Carpinus tschonoskii#NC 039938
Fagales Betulaceae Carpinus henryana var. oblongifolia#NC 038092
Fagales Betulaceae Carpinus fargesiana#NC 039937
Fagales Betulaceae Carpinus polyneura#NC 039998
Rosales Rhamnaceae Spyridium parvifolium#MH234313
Rosales Rhamnaceae Ziziphus jujuba#MF781071
Rosales Rhamnaceae Ziziphus spina-christi#NC 037152
Rosales Rhamnaceae Ventilago denticulata#F210
Rosales Rhamnaceae Berchemiella berchemiifolia#NC 037477

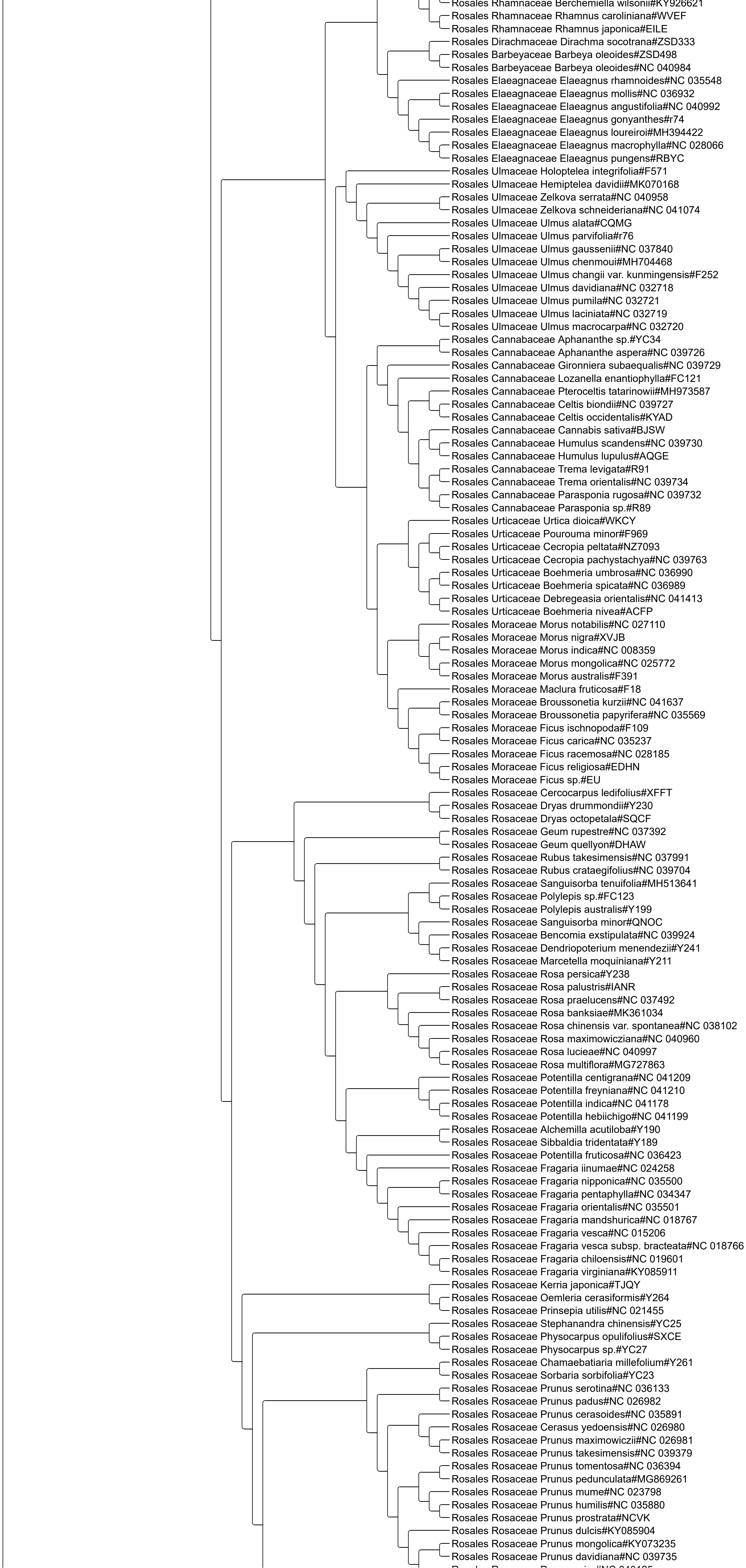
Rosales Rhamnaceae Berchemiella wilsonii#KY926621
Rosales Rhamnaceae Rhamnus caroliniana#WVEF
Rosales Rhamnaceae Rhamnus japonica#EILE
Rosales Dirachmaceae Dirachma socotrana#ZSD333
Rosales Barbeyaceae Barbeya oleoides#ZSD498
Rosales Barbeyaceae Barbeya oleoides#NC 040984
Rosales Elaeagnaceae Elaeagnus rhamnoides#NC 035548
Rosales Elaeagnaceae Elaeagnus mollis#NC 036932
Rosales Elaeagnaceae Elaeagnus angustifolia#NC 040992
Rosales Elaeagnaceae Elaeagnus gonyanthes#r74
Rosales Elaeagnaceae Elaeagnus loureiroi#MH394422
Rosales Elaeagnaceae Elaeagnus macrophylla#NC 028066
Rosales Elaeagnaceae Elaeagnus pungens#RBYC
Rosales Ulmaceae Holoptelea integrifolia#F571
Rosales Ulmaceae Hemiptelea davidii#MK070168
Rosales Ulmaceae Zelkova serrata#NC 040958
Rosales Ulmaceae Zelkova schneideriana#NC 041074
Rosales Ulmaceae Ulmus alata#CQMG
Rosales Ulmaceae Ulmus parvifolia#r76
Rosales Ulmaceae Ulmus gaussenii#NC 037840
Rosales Ulmaceae Ulmus chenmoui#MH704468
Rosales Ulmaceae Ulmus changii var. kunmingensis#F252
Rosales Ulmaceae Ulmus davidiana#NC 032718
Rosales Ulmaceae Ulmus pumila#NC 032721
Rosales Ulmaceae Ulmus laciniata#NC 032719
Rosales Ulmaceae Ulmus macrocarpa#NC 032720
Rosales Cannabaceae Aphananthe sp.#YC34
Rosales Cannabaceae Aphananthe aspera#NC 039726
Rosales Cannabaceae Gironniera subaequalis#NC 039729
Rosales Cannabaceae Lozanella enantiophylla#FC121
Rosales Cannabaceae Pteroceltis tatarinowii#MH973587
Rosales Cannabaceae Celtis biondii#NC 039727
Rosales Cannabaceae Celtis occidentalis#KYAD
Rosales Cannabaceae Cannabis sativa#BJSW
Rosales Cannabaceae Humulus scandens#NC 039730
Rosales Cannabaceae Humulus lupulus#AQGE
Rosales Cannabaceae Trema levigata#R91
Rosales Cannabaceae Trema orientalis#NC 039734
Rosales Cannabaceae Parasponia rugosa#NC 039732
Rosales Cannabaceae Parasponia sp.#R89
Rosales Urticaceae Urtica dioica#WKCY
Rosales Urticaceae Pourouma minor#F969
Rosales Urticaceae Cecropia peltata#NZ7093
Rosales Urticaceae Cecropia pachystachya#NC 039763
Rosales Urticaceae Boehmeria umbrosa#NC 036990
Rosales Urticaceae Boehmeria spicata#NC 036989
Rosales Urticaceae Debregeasia orientalis#NC 041413
Rosales Urticaceae Boehmeria nivea#ACFP
Rosales Moraceae Morus notabilis#NC 027110
Rosales Moraceae Morus nigra#XVJB
Rosales Moraceae Morus indica#NC 008359
Rosales Moraceae Morus mongolica#NC 025772
Rosales Moraceae Morus australis#F391
Rosales Moraceae Maclura fruticosa#F18
Rosales Moraceae Broussonetia kurzii#NC 041637
Rosales Moraceae Broussonetia papyrifera#NC 035569
Rosales Moraceae Ficus ischnopoda#F109
Rosales Moraceae Ficus carica#NC 035237
Rosales Moraceae Ficus racemosa#NC 028185
Rosales Moraceae Ficus religiosa#EDHN
Rosales Moraceae Ficus sp.#EU
Rosales Rosaceae Cercocarpus ledifolius#XFFT
Rosales Rosaceae Dryas drummondii#Y230
Rosales Rosaceae Dryas octopetala#SQCF
Rosales Rosaceae Geum rupestre#NC 037392
Rosales Rosaceae Geum quellyon#DHAW
Rosales Rosaceae Rubus takesimensis#NC 037991
Rosales Rosaceae Rubus crataegifolius#NC 039704
Rosales Rosaceae Sanguisorba tenuifolia#MH513641
Rosales Rosaceae Polylepis sp.#FC123
Rosales Rosaceae Polylepis australis#Y199
Rosales Rosaceae Sanguisorba minor#QNOC
Rosales Rosaceae Bencomia exstipulata#NC 039924
Rosales Rosaceae Dendriopoterium menendezii#Y241
Rosales Rosaceae Marcetella moquiniana#Y211
Rosales Rosaceae Rosa persica#Y238
Rosales Rosaceae Rosa palustris#IANR
Rosales Rosaceae Rosa praelucens#NC 037492
Rosales Rosaceae Rosa banksiae#MK361034
Rosales Rosaceae Rosa chinensis var. spontanea#NC 038102
Rosales Rosaceae Rosa maximowicziana#NC 040960
Rosales Rosaceae Rosa lucieae#NC 040997
Rosales Rosaceae Rosa multiflora#MG727863
Rosales Rosaceae Potentilla centigrana#NC 041209
Rosales Rosaceae Potentilla freyniana#NC 041210
Rosales Rosaceae Potentilla indica#NC 041178
Rosales Rosaceae Potentilla hebiichigo#NC 041199
Rosales Rosaceae Alchemilla acutiloba#Y190
Rosales Rosaceae Sibbaldia tridentata#Y189
Rosales Rosaceae Potentilla fruticosa#NC 036423
Rosales Rosaceae Fragaria iinumae#NC 024258
Rosales Rosaceae Fragaria nipponica#NC 035500
Rosales Rosaceae Fragaria pentaphylla#NC 034347
Rosales Rosaceae Fragaria orientalis#NC 035501
Rosales Rosaceae Fragaria mandshurica#NC 018767
Rosales Rosaceae Fragaria vesca#NC 015206
Rosales Rosaceae Fragaria vesca subsp. bracteata#NC 018766
Rosales Rosaceae Fragaria chiloensis#NC 019601
Rosales Rosaceae Fragaria virginiana#KY085911
Rosales Rosaceae Kerria japonica#TJQY
Rosales Rosaceae Oemleria cerasiformis#Y264
Rosales Rosaceae Prinsepia utilis#NC 021455
Rosales Rosaceae Stephanandra chinensis#YC25
Rosales Rosaceae Physocarpus opulifolius#SXCE
Rosales Rosaceae Physocarpus sp.#YC27
Rosales Rosaceae Chamaebatiaria millefolium#Y261
Rosales Rosaceae Sorbaria sorbifolia#YC23
Rosales Rosaceae Prunus serotina#NC 036133
Rosales Rosaceae Prunus padus#NC 026982
Rosales Rosaceae Prunus cerasoides#NC 035891
Rosales Rosaceae Cerasus yedoensis#NC 026980
Rosales Rosaceae Prunus maximowiczii#NC 026981
Rosales Rosaceae Prunus takesimensis#NC 039379
Rosales Rosaceae Prunus tomentosa#NC 036394
Rosales Rosaceae Prunus pedunculata#MG869261
Rosales Rosaceae Prunus mume#NC 023798
Rosales Rosaceae Prunus humilis#NC 035880
Rosales Rosaceae Prunus prostrata#NCVK
Rosales Rosaceae Prunus dulcis#KY085904
Rosales Rosaceae Prunus mongolica#KY073235
Rosales Rosaceae Prunus davidiana#NC 039735

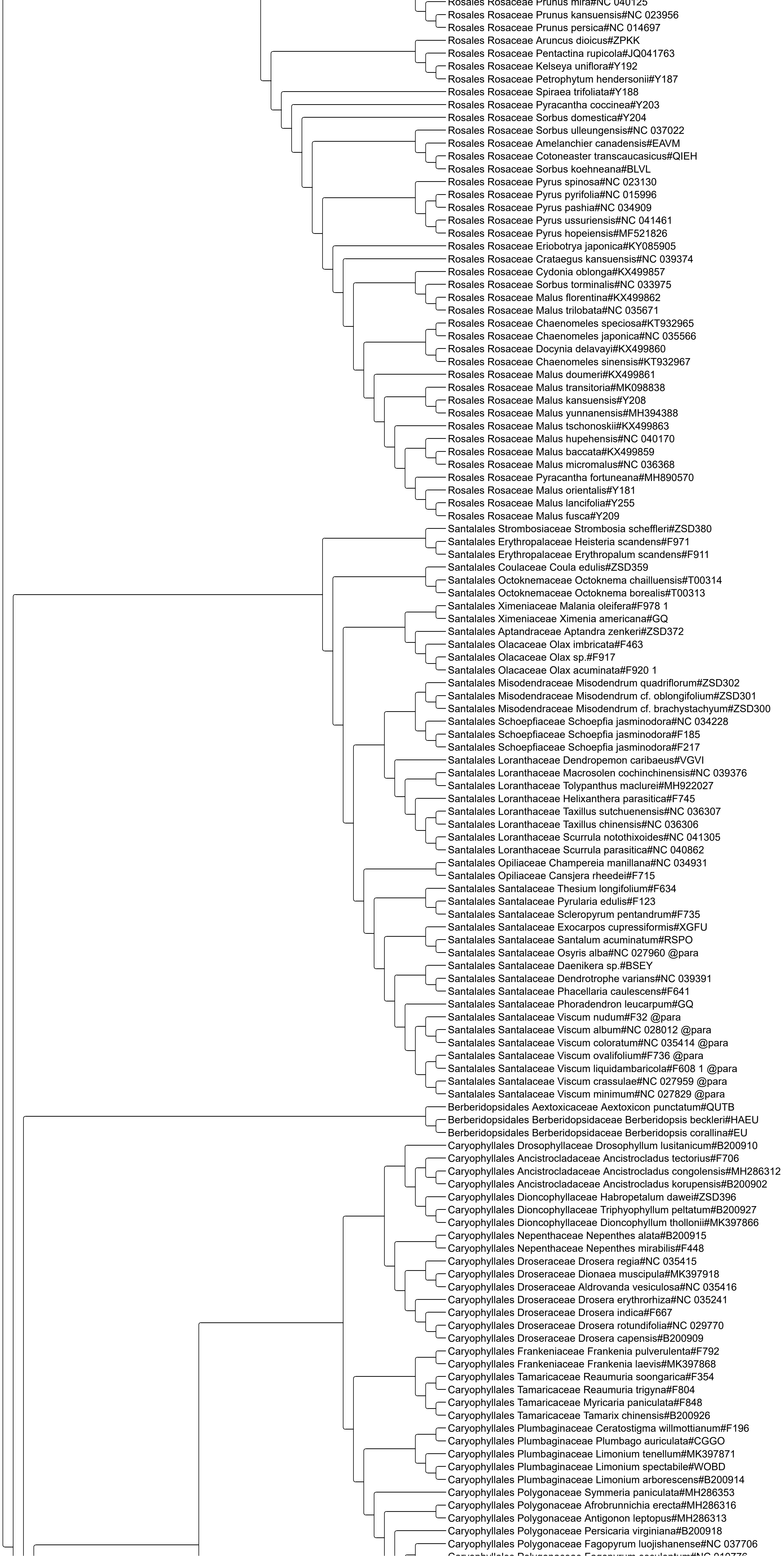

Rosales Rosaceae Prunus mira#NC 040125
Rosales Rosaceae Prunus kansuensis#NC 023956
Rosales Rosaceae Prunus persica#NC 014697
Rosales Rosaceae Aruncus dioicus#ZPKK
Rosales Rosaceae Pentactina rupicola#JQ041763
Rosales Rosaceae Kelseya uniflora#Y192
Rosales Rosaceae Petrophytum hendersonii#Y187
Rosales Rosaceae Spiraea trifoliata#Y188
Rosales Rosaceae Pyracantha coccinea#Y203
Rosales Rosaceae Sorbus domestica#Y204
Rosales Rosaceae Sorbus ulleungensis#NC 037022
Rosales Rosaceae Amelanchier canadensis#EAVM
Rosales Rosaceae Cotoneaster transcaucasicus#QIEH
Rosales Rosaceae Sorbus koehneana#BLVL
Rosales Rosaceae Pyrus spinosa#NC 023130
Rosales Rosaceae Pyrus pyrifolia#NC 015996
Rosales Rosaceae Pyrus pashia#NC 034909
Rosales Rosaceae Pyrus ussuriensis#NC 041461
Rosales Rosaceae Pyrus hopeiensis#MF521826
Rosales Rosaceae Eriobotrya japonica#KY085905
Rosales Rosaceae Crataegus kansuensis#NC 039374
Rosales Rosaceae Cydonia oblonga#KX499857
Rosales Rosaceae Sorbus torminalis#NC 033975
Rosales Rosaceae Malus florentina#KX499862
Rosales Rosaceae Malus trilobata#NC 035671
Rosales Rosaceae Chaenomeles speciosa#KT932965
Rosales Rosaceae Chaenomeles japonica#NC 035566
Rosales Rosaceae Docynia delavayi#KX499860
Rosales Rosaceae Chaenomeles sinensis#KT932967
Rosales Rosaceae Malus doumeri#KX499861
Rosales Rosaceae Malus transitoria#MK098838
Rosales Rosaceae Malus kansuensis#Y208
Rosales Rosaceae Malus yunnanensis#MH394388
Rosales Rosaceae Malus tschonoskii#KX499863
Rosales Rosaceae Malus hupehensis#NC 040170
Rosales Rosaceae Malus baccata#KX499859
Rosales Rosaceae Malus micromalus#NC 036368
Rosales Rosaceae Pyracantha fortuneana#MH890570
Rosales Rosaceae Malus orientalis#Y181
Rosales Rosaceae Malus lancifolia#Y255
Rosales Rosaceae Malus fusca#Y209
Santalales Strombosiaceae Strombosia scheffleri#ZSD380
Santalales Erythropalaceae Heisteria scandens#F971
Santalales Erythropalaceae Erythropalum scandens#F911
Santalales Coulaceae Coula edulis#ZSD359
Santalales Octoknemaceae Octoknema chailluensis#T00314
Santalales Octoknemaceae Octoknema borealis#T00313
Santalales Ximeniaceae Malania oleifera#F978 1
Santalales Ximeniaceae Ximenia americana#GQ
Santalales Aptandraceae Aptandra zenkeri#ZSD372
Santalales Olacaceae Olax imbricata#F463
Santalales Olacaceae Olax sp.#F917
Santalales Olacaceae Olax acuminata#F920 1
Santalales Misodendraceae Misodendrum quadriflorum#ZSD302
Santalales Misodendraceae Misodendrum cf. oblongifolium#ZSD301
Santalales Misodendraceae Misodendrum cf. brachystachyum#ZSD300
Santalales Schoepfiaceae Schoepfia jasminodora#NC 034228
Santalales Schoepfiaceae Schoepfia jasminodora#F185
Santalales Schoepfiaceae Schoepfia jasminodora#F217
Santalales Loranthaceae Dendropemon caribaeus#VGVI
Santalales Loranthaceae Macrosolen cochinchinensis#NC 039376
Santalales Loranthaceae Tolypanthus maclurei#MH922027
Santalales Loranthaceae Helixanthera parasitica#F745
Santalales Loranthaceae Taxillus sutchuenensis#NC 036307
Santalales Loranthaceae Taxillus chinensis#NC 036306
Santalales Loranthaceae Scurrula notothixoides#NC 041305
Santalales Loranthaceae Scurrula parasitica#NC 040862
Santalales Opiliaceae Champereia manillana#NC 034931
Santalales Opiliaceae Cansjera rheedei#F715
Santalales Santalaceae Thesium longifolium#F634
Santalales Santalaceae Pyrularia edulis#F123
Santalales Santalaceae Scleropyrum pentandrum#F735
Santalales Santalaceae Exocarpos cupressiformis#XGFU
Santalales Santalaceae Santalum acuminatum#RSPO
Santalales Santalaceae Osyris alba#NC 027960 @para
Santalales Santalaceae Daenikera sp.#BSEY
Santalales Santalaceae Dendrotrophe varians#NC 039391
Santalales Santalaceae Phacellaria caulescens#F641
Santalales Santalaceae Phoradendron leucarpum#GQ
Santalales Santalaceae Viscum nudum#F32 @para
Santalales Santalaceae Viscum album#NC 028012 @para
Santalales Santalaceae Viscum coloratum#NC 035414 @para
Santalales Santalaceae Viscum ovalifolium#F736 @para
Santalales Santalaceae Viscum liquidambaricola#F608 1 @para
Santalales Santalaceae Viscum crassulae#NC 027959 @para
Santalales Santalaceae Viscum minimum#NC 027829 @para
Berberidopsidales Aextoxicaceae Aextoxicon punctatum#QUTB
Berberidopsidales Berberidopsidaceae Berberidopsis beckleri#HAEU
Berberidopsidales Berberidopsidaceae Berberidopsis corallina#EU
Caryophyllales Drosophyllaceae Drosophyllum lusitanicum#B200910
Caryophyllales Ancistrocladaceae Ancistrocladus tectorius#F706
Caryophyllales Ancistrocladaceae Ancistrocladus congolensis#MH286312
Caryophyllales Ancistrocladaceae Ancistrocladus korupensis#B200902
Caryophyllales Dioncophyllaceae Habropetalum dawei#ZSD396
Caryophyllales Dioncophyllaceae Triphyophyllum peltatum#B200927
Caryophyllales Dioncophyllaceae Dioncophyllum thollonii#MK397866
Caryophyllales Nepenthaceae Nepenthes alata#B200915
Caryophyllales Nepenthaceae Nepenthes mirabilis#F448
Caryophyllales Droseraceae Drosera regia#NC 035415
Caryophyllales Droseraceae Dionaea muscipula#MK397918
Caryophyllales Droseraceae Aldrovanda vesiculosa#NC 035416
Caryophyllales Droseraceae Drosera erythrorhiza#NC 035241
Caryophyllales Droseraceae Drosera indica#F667
Caryophyllales Droseraceae Drosera rotundifolia#NC 029770
Caryophyllales Droseraceae Drosera capensis#B200909
Caryophyllales Frankeniaceae Frankenia pulverulenta#F792
Caryophyllales Frankeniaceae Frankenia laevis#MK397868
Caryophyllales Tamaricaceae Reaumuria soongarica#F354
Caryophyllales Tamaricaceae Reaumuria trigyna#F804
Caryophyllales Tamaricaceae Myricaria paniculata#F848
Caryophyllales Tamaricaceae Tamarix chinensis#B200926
Caryophyllales Plumbaginaceae Ceratostigma willmottianum#F196
Caryophyllales Plumbaginaceae Plumbago auriculata#CGGO
Caryophyllales Plumbaginaceae Limonium tenellum#MK397871
Caryophyllales Plumbaginaceae Limonium spectabile#WOBD
Caryophyllales Plumbaginaceae Limonium arborescens#B200914
Caryophyllales Polygonaceae Symmeria paniculata#MH286353
Caryophyllales Polygonaceae Afrobrunnichia erecta#MH286316
Caryophyllales Polygonaceae Antigonon leptopus#MH286313
Caryophyllales Polygonaceae Persicaria virginiana#B200918
Caryophyllales Polygonaceae Fagopyrum luojishanense#NC 037706

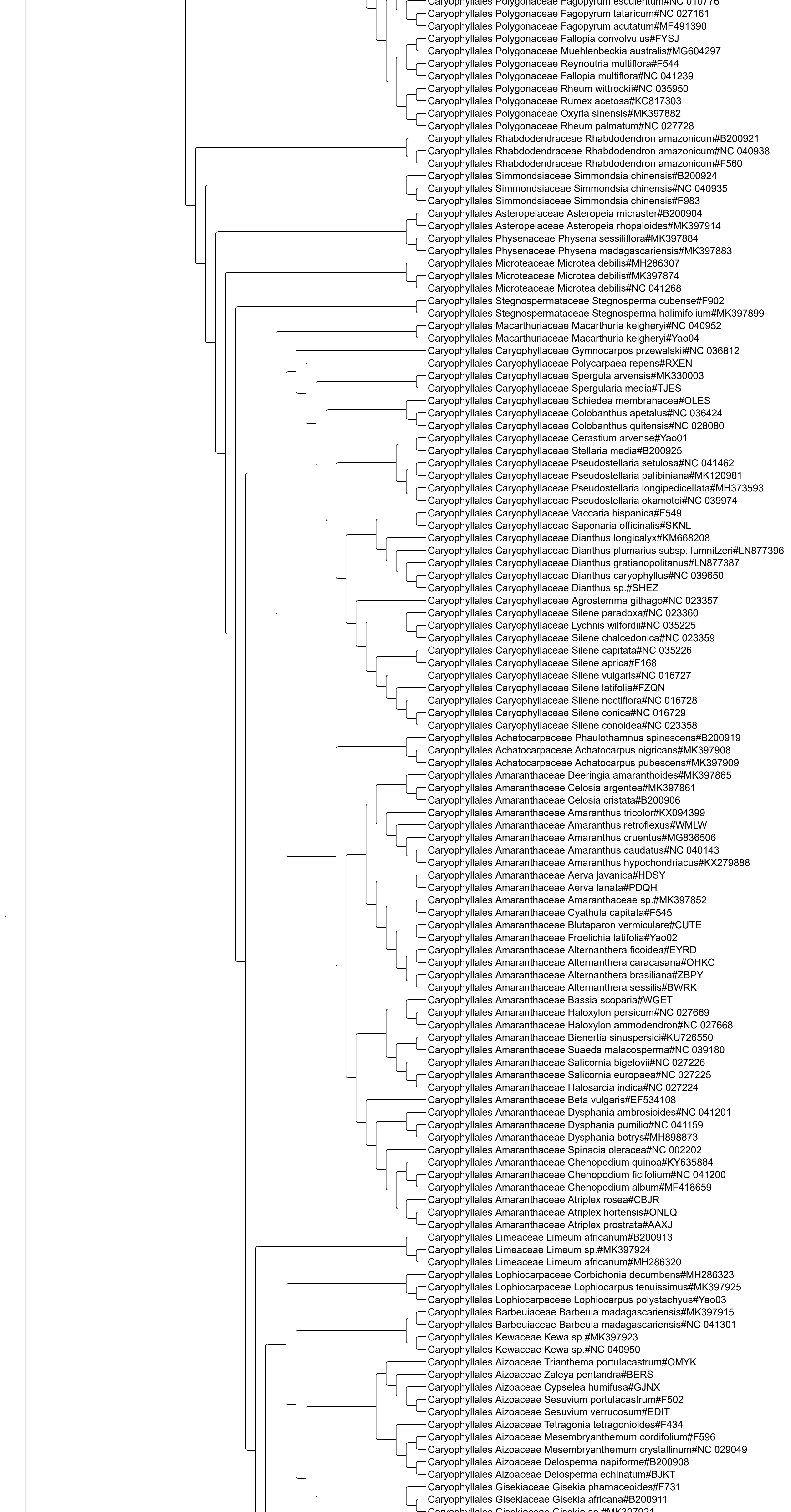
Caryophyllales Polygonaceae Fagopyrum tataricum#NC 027161
Caryophyllales Polygonaceae Fagopyrum acutatum#MF491390
Caryophyllales Polygonaceae Fallopia convolvulus#FYSJ
Caryophyllales Polygonaceae Muehlenbeckia australis#MG604297
Caryophyllales Polygonaceae Reynoutria multiflora#F544
Caryophyllales Polygonaceae Fallopia multiflora#NC 041239
Caryophyllales Polygonaceae Rheum wittrockii#NC 035950
Caryophyllales Polygonaceae Rumex acetosa#KC817303
Caryophyllales Polygonaceae Oxyria sinensis#MK397882
Caryophyllales Polygonaceae Rheum palmatum#NC 027728
Caryophyllales Rhabdodendraceae Rhabdodendron amazonicum#B200921
Caryophyllales Rhabdodendraceae Rhabdodendron amazonicum#NC 040938
Caryophyllales Rhabdodendraceae Rhabdodendron amazonicum#F560
Caryophyllales Simmondsiaceae Simmondsia chinensis#B200924
Caryophyllales Simmondsiaceae Simmondsia chinensis#NC 040935
Caryophyllales Simmondsiaceae Simmondsia chinensis#F983
Caryophyllales Asteropeiaceae Asteropeia micraster#B200904
Caryophyllales Asteropeiaceae Asteropeia rhopaloides#MK397914
Caryophyllales Physenaceae Physena sessiliflora#MK397884
Caryophyllales Physenaceae Physena madagascariensis#MK397883
Caryophyllales Microteaceae Microtea debilis#MH286307
Caryophyllales Microteaceae Microtea debilis#MK397874
Caryophyllales Microteaceae Microtea debilis#NC 041268
Caryophyllales Stegnospermataceae Stegnosperma cubense#F902
Caryophyllales Stegnospermataceae Stegnosperma halimifolium#MK397899
Caryophyllales Macarthuriaceae Macarthuria keigheryi#NC 040952
Caryophyllales Macarthuriaceae Macarthuria keigheryi#Yao04
Caryophyllales Caryophyllaceae Gymnocarpos przewalskii#NC 036812
Caryophyllales Caryophyllaceae Polycarpaea repens#RXEN
Caryophyllales Caryophyllaceae Spergula arvensis#MK330003
Caryophyllales Caryophyllaceae Spergularia media#TJES
Caryophyllales Caryophyllaceae Schiedea membranacea#OLES
Caryophyllales Caryophyllaceae Colobanthus apetalus#NC 036424
Caryophyllales Caryophyllaceae Colobanthus quitensis#NC 028080
Caryophyllales Caryophyllaceae Cerastium arvense#Yao01
Caryophyllales Caryophyllaceae Stellaria media#B200925
Caryophyllales Caryophyllaceae Pseudostellaria setulosa#NC 041462
Caryophyllales Caryophyllaceae Pseudostellaria palibiniana#MK120981
Caryophyllales Caryophyllaceae Pseudostellaria longipedicellata#MH373593
Caryophyllales Caryophyllaceae Pseudostellaria okamotoi#NC 039974
Caryophyllales Caryophyllaceae Vaccaria hispanica#F549
Caryophyllales Caryophyllaceae Saponaria officinalis#SKNL
Caryophyllales Caryophyllaceae Dianthus longicalyx#KM668208
Caryophyllales Caryophyllaceae Dianthus plumarius subsp. lumnitzeri#LN877396
Caryophyllales Caryophyllaceae Dianthus gratianopolitanus#LN877387
Caryophyllales Caryophyllaceae Dianthus caryophyllus#NC 039650
Caryophyllales Caryophyllaceae Dianthus sp.#SHEZ
Caryophyllales Caryophyllaceae Agrostemma githago#NC 023357
Caryophyllales Caryophyllaceae Silene paradoxa#NC 023360
Caryophyllales Caryophyllaceae Lychnis wilfordii#NC 035225
Caryophyllales Caryophyllaceae Silene chalcedonica#NC 023359
Caryophyllales Caryophyllaceae Silene capitata#NC 035226
Caryophyllales Caryophyllaceae Silene aprica#F168
Caryophyllales Caryophyllaceae Silene vulgaris#NC 016727
Caryophyllales Caryophyllaceae Silene latifolia#FZQN
Caryophyllales Caryophyllaceae Silene noctiflora#NC 016728
Caryophyllales Caryophyllaceae Silene conica#NC 016729
Caryophyllales Caryophyllaceae Silene conoidea#NC 023358
Caryophyllales Achatocarpaceae Phaulothamnus spinescens#B200919
Caryophyllales Achatocarpaceae Achatocarpus nigricans#MK397908
Caryophyllales Achatocarpaceae Achatocarpus pubescens#MK397909
Caryophyllales Amaranthaceae Deeringia amaranthoides#MK397865
Caryophyllales Amaranthaceae Celosia argentea#MK397861
Caryophyllales Amaranthaceae Celosia cristata#B200906
Caryophyllales Amaranthaceae Amaranthus tricolor#KX094399
Caryophyllales Amaranthaceae Amaranthus retroflexus#WMLW
Caryophyllales Amaranthaceae Amaranthus cruentus#MG836506
Caryophyllales Amaranthaceae Amaranthus caudatus#NC 040143
Caryophyllales Amaranthaceae Amaranthus hypochondriacus#KX279888
Caryophyllales Amaranthaceae Aerva javanica#HDSY
Caryophyllales Amaranthaceae Aerva lanata#PDQH
Caryophyllales Amaranthaceae Amaranthaceae sp.#MK397852
Caryophyllales Amaranthaceae Cyathula capitata#F545
Caryophyllales Amaranthaceae Blutaparon vermiculare#CUTE
Caryophyllales Amaranthaceae Froelichia latifolia#Yao02
Caryophyllales Amaranthaceae Alternanthera ficoidea#EYRD
Caryophyllales Amaranthaceae Alternanthera caracasana#OHKC
Caryophyllales Amaranthaceae Alternanthera brasiliana#ZBPY
Caryophyllales Amaranthaceae Alternanthera sessilis#BWRK
Caryophyllales Amaranthaceae Bassia scoparia#WGET
Caryophyllales Amaranthaceae Haloxylon persicum#NC 027669
Caryophyllales Amaranthaceae Haloxylon ammodendron#NC 027668
Caryophyllales Amaranthaceae Bienertia sinuspersici#KU726550
Caryophyllales Amaranthaceae Suaeda malacosperma#NC 039180
Caryophyllales Amaranthaceae Salicornia bigelovii#NC 027226
Caryophyllales Amaranthaceae Salicornia europaea#NC 027225
Caryophyllales Amaranthaceae Halosarcia indica#NC 027224
Caryophyllales Amaranthaceae Beta vulgaris#EF534108
Caryophyllales Amaranthaceae Dysphania ambrosioides#NC 041201
Caryophyllales Amaranthaceae Dysphania pumilio#NC 041159
Caryophyllales Amaranthaceae Dysphania botrys#MH898873
Caryophyllales Amaranthaceae Spinacia oleracea#NC 002202
Caryophyllales Amaranthaceae Chenopodium quinoa#KY635884
Caryophyllales Amaranthaceae Chenopodium ficifolium#NC 041200
Caryophyllales Amaranthaceae Chenopodium album#MF418659
Caryophyllales Amaranthaceae Atriplex rosea#CBJR
Caryophyllales Amaranthaceae Atriplex hortensis#ONLQ
Caryophyllales Amaranthaceae Atriplex prostrata#AAXJ
Caryophyllales Limeaceae Limeum africanum#B200913
Caryophyllales Limeaceae Limeum sp.#MK397924
Caryophyllales Limeaceae Limeum africanum#MH286320
Caryophyllales Lophiocarpaceae Corbichonia decumbens#MH286323
Caryophyllales Lophiocarpaceae Lophiocarpus tenuissimus#MK397925
Caryophyllales Lophiocarpaceae Lophiocarpus polystachyus#Yao03
Caryophyllales Barbeuiaceae Barbeuia madagascariensis#MK397915
Caryophyllales Barbeuiaceae Barbeuia madagascariensis#NC 041301
Caryophyllales Kewaceae Kewa sp.#MK397923
Caryophyllales Kewaceae Kewa sp.#NC 040950
Caryophyllales Aizoaceae Trianthema portulacastrum#OMYK
Caryophyllales Aizoaceae Zaleya pentandra#BERS
Caryophyllales Aizoaceae Cypselea humifusa#GJNX
Caryophyllales Aizoaceae Sesuvium portulacastrum#F502
Caryophyllales Aizoaceae Sesuvium verrucosum#EDIT
Caryophyllales Aizoaceae Tetragonia tetragonioides#F434
Caryophyllales Aizoaceae Mesembryanthemum cordifolium#F596
Caryophyllales Aizoaceae Mesembryanthemum crystallinum#NC 029049
Caryophyllales Aizoaceae Delosperma napiforme#B200908
Caryophyllales Aizoaceae Delosperma echinatum#BJKT
Caryophyllales Gisekiaceae Gisekia pharnaceoides#F731
Caryophyllales Gisekiaceae Gisekia africana#B200911

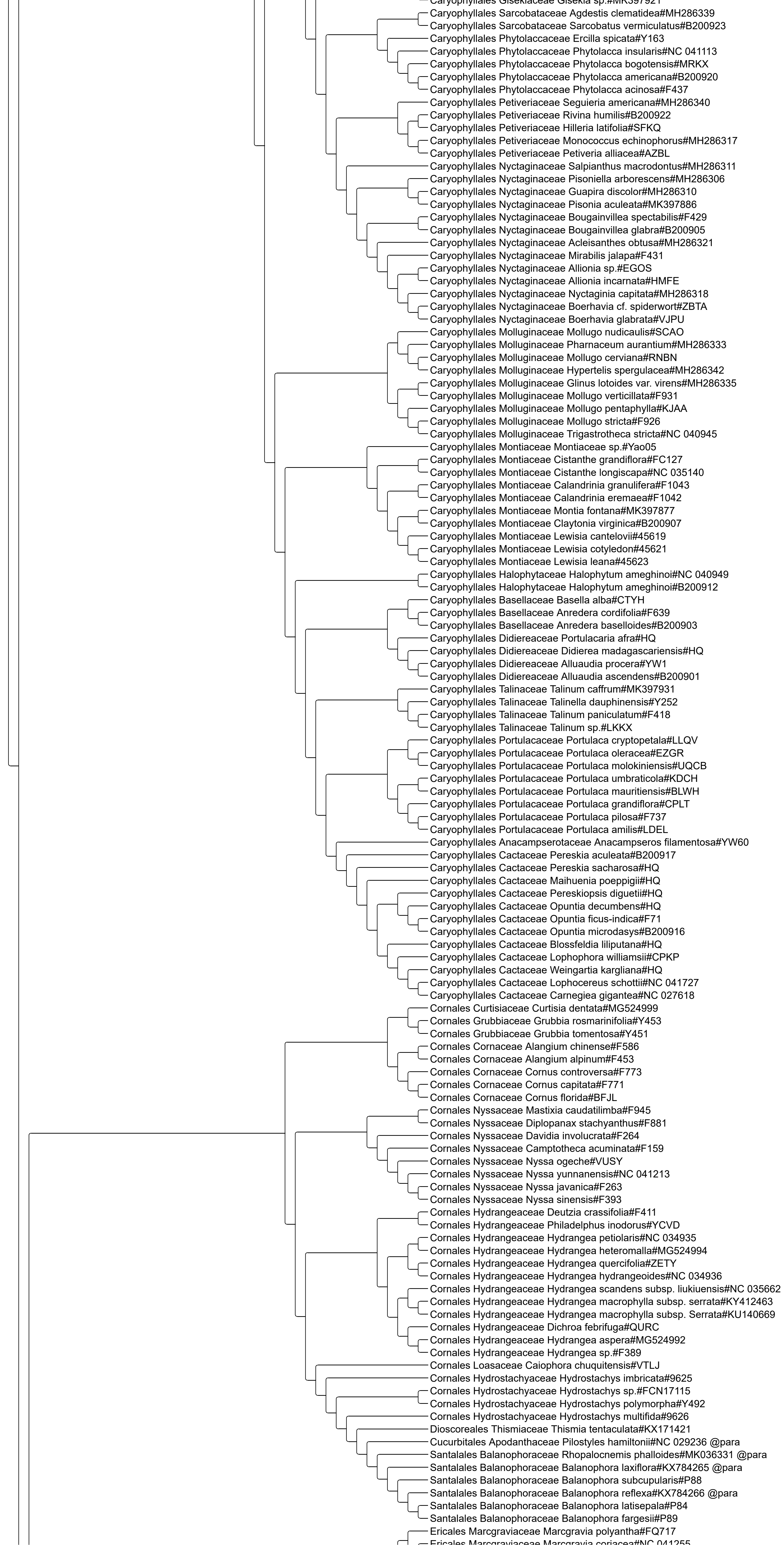

Caryophyllales Gisekiaceae Gisekia sp.#MK397921
Caryophyllales Sarcobataceae Agdestis clematidea#MH286339
Caryophyllales Sarcobataceae Sarcobatus vermiculatus#B200923
Caryophyllales Phytolaccaceae Ercilla spicata#Y163
Caryophyllales Phytolaccaceae Phytolacca insularis#NC 041113
Caryophyllales Phytolaccaceae Phytolacca bogotensis#MRKX
Caryophyllales Phytolaccaceae Phytolacca americana#B200920
Caryophyllales Phytolaccaceae Phytolacca acinosa#F437
Caryophyllales Petiveriaceae Seguieria americana#MH286340
Caryophyllales Petiveriaceae Rivina humilis#B200922
Caryophyllales Petiveriaceae Hilleria latifolia#SFKQ
Caryophyllales Petiveriaceae Monococcus echinophorus#MH286317
Caryophyllales Petiveriaceae Petiveria alliacea#AZBL
Caryophyllales Nyctaginaceae Salpianthus macrodontus#MH286311
Caryophyllales Nyctaginaceae Pisoniella arborescens#MH286306
Caryophyllales Nyctaginaceae Guapira discolor#MH286310
Caryophyllales Nyctaginaceae Pisonia aculeata#MK397886
Caryophyllales Nyctaginaceae Bougainvillea spectabilis#F429
Caryophyllales Nyctaginaceae Bougainvillea glabra#B200905
Caryophyllales Nyctaginaceae Acleisanthes obtusa#MH286321
Caryophyllales Nyctaginaceae Mirabilis jalapa#F431
Caryophyllales Nyctaginaceae Allionia sp.#EGOS
Caryophyllales Nyctaginaceae Allionia incarnata#HMFE
Caryophyllales Nyctaginaceae Nyctaginia capitata#MH286318
Caryophyllales Nyctaginaceae Boerhavia cf. spiderwort#ZBTA
Caryophyllales Nyctaginaceae Boerhavia glabrata#VJPU
Caryophyllales Molluginaceae Mollugo nudicaulis#SCAO
Caryophyllales Molluginaceae Pharnaceum aurantium#MH286333
Caryophyllales Molluginaceae Mollugo cerviana#RNBN
Caryophyllales Molluginaceae Hypertelis spergulacea#MH286342
Caryophyllales Molluginaceae Glinus lotoides var. virens#MH286335
Caryophyllales Molluginaceae Mollugo verticillata#F931
Caryophyllales Molluginaceae Mollugo pentaphylla#KJAA
Caryophyllales Molluginaceae Mollugo stricta#F926
Caryophyllales Molluginaceae Trigastrotheca stricta#NC 040945
Caryophyllales Montiaceae Montiaceae sp.#Yao05
Caryophyllales Montiaceae Cistanthe grandiflora#FC127
Caryophyllales Montiaceae Cistanthe longiscapa#NC 035140
Caryophyllales Montiaceae Calandrinia granulifera#F1043
Caryophyllales Montiaceae Calandrinia eremaea#F1042
Caryophyllales Montiaceae Montia fontana#MK397877
Caryophyllales Montiaceae Claytonia virginica#B200907
Caryophyllales Montiaceae Lewisia cantelovii#45619
Caryophyllales Montiaceae Lewisia cotyledon#45621
Caryophyllales Montiaceae Lewisia leana#45623
Caryophyllales Halophytaceae Halophytum ameghinoi#NC 040949
Caryophyllales Halophytaceae Halophytum ameghinoi#B200912
Caryophyllales Basellaceae Basella alba#CTYH
Caryophyllales Basellaceae Anredera cordifolia#F639
Caryophyllales Basellaceae Anredera baselloides#B200903
Caryophyllales Didiereaceae Portulacaria afra#HQ
Caryophyllales Didiereaceae Didierea madagascariensis#HQ
Caryophyllales Didiereaceae Alluaudia procera#YW1
Caryophyllales Didiereaceae Alluaudia ascendens#B200901
Caryophyllales Talinaceae Talinum caffrum#MK397931
Caryophyllales Talinaceae Talinella dauphinensis#Y252
Caryophyllales Talinaceae Talinum paniculatum#F418
Caryophyllales Talinaceae Talinum sp.#LKKX
Caryophyllales Portulacaceae Portulaca cryptopetala#LLQV
Caryophyllales Portulacaceae Portulaca oleracea#EZGR
Caryophyllales Portulacaceae Portulaca molokiniensis#UQCB
Caryophyllales Portulacaceae Portulaca umbraticola#KDCH
Caryophyllales Portulacaceae Portulaca mauritiensis#BLWH
Caryophyllales Portulacaceae Portulaca grandiflora#CPLT
Caryophyllales Portulacaceae Portulaca pilosa#F737
Caryophyllales Portulacaceae Portulaca amilis#LDEL
Caryophyllales Anacampserotaceae Anacampseros filamentosa#YW60
Caryophyllales Cactaceae Pereskia aculeata#B200917
Caryophyllales Cactaceae Pereskia sacharosa#HQ
Caryophyllales Cactaceae Maihuenia poeppigii#HQ
Caryophyllales Cactaceae Pereskiopsis diguetii#HQ
Caryophyllales Cactaceae Opuntia decumbens#HQ
Caryophyllales Cactaceae Opuntia ficus-indica#F71
Caryophyllales Cactaceae Opuntia microdasys#B200916
Caryophyllales Cactaceae Blossfeldia liliputana#HQ
Caryophyllales Cactaceae Lophophora williamsii#CPKP
Caryophyllales Cactaceae Weingartia kargliana#HQ
Caryophyllales Cactaceae Lophocereus schottii#NC 041727
Caryophyllales Cactaceae Carnegiea gigantea#NC 027618
Cornales Curtisiaceae Curtisia dentata#MG524999
Cornales Grubbiaceae Grubbia rosmarinifolia#Y453
Cornales Grubbiaceae Grubbia tomentosa#Y451
Cornales Cornaceae Alangium chinense#F586
Cornales Cornaceae Alangium alpinum#F453
Cornales Cornaceae Cornus controversa#F773
Cornales Cornaceae Cornus capitata#F771
Cornales Cornaceae Cornus florida#BFJL
Cornales Nyssaceae Mastixia caudatilimba#F945
Cornales Nyssaceae Diplopanax stachyanthus#F881
Cornales Nyssaceae Davidia involucrata#F264
Cornales Nyssaceae Camptotheca acuminata#F159
Cornales Nyssaceae Nyssa ogeche#VUSY
Cornales Nyssaceae Nyssa yunnanensis#NC 041213
Cornales Nyssaceae Nyssa javanica#F263
Cornales Nyssaceae Nyssa sinensis#F393
Cornales Hydrangeaceae Deutzia crassifolia#F411
Cornales Hydrangeaceae Philadelphus inodorus#YCVD
Cornales Hydrangeaceae Hydrangea petiolaris#NC 034935
Cornales Hydrangeaceae Hydrangea heteromalla#MG524994
Cornales Hydrangeaceae Hydrangea quercifolia#ZETY
Cornales Hydrangeaceae Hydrangea hydrangeoides#NC 034936
Cornales Hydrangeaceae Hydrangea scandens subsp. liukiuensis#NC 035662
Cornales Hydrangeaceae Hydrangea macrophylla subsp. serrata#KY412463
Cornales Hydrangeaceae Hydrangea macrophylla subsp. Serrata#KU140669
Cornales Hydrangeaceae Dichroa febrifuga#QURC
Cornales Hydrangeaceae Hydrangea aspera#MG524992
Cornales Hydrangeaceae Hydrangea sp.#F389
Cornales Loasaceae Caiophora chuquitensis#VTLJ
Cornales Hydrostachyaceae Hydrostachys imbricata#9625
Cornales Hydrostachyaceae Hydrostachys sp.#FCN17115
Cornales Hydrostachyaceae Hydrostachys polymorpha#Y492
Cornales Hydrostachyaceae Hydrostachys multifida#9626
Dioscoreales Thismiaceae Thismia tentaculata#KX171421
Cucurbitales Apodanthaceae Pilostyles hamiltonii#NC 029236 @para
Santalales Balanophoraceae Rhopalocnemis phalloides#MK036331 @para
Santalales Balanophoraceae Balanophora laxiflora#KX784265 @para
Santalales Balanophoraceae Balanophora subcupularis#P88
Santalales Balanophoraceae Balanophora reflexa#KX784266 @para
Santalales Balanophoraceae Balanophora latisepala#P84
Santalales Balanophoraceae Balanophora fargesii#P89
Ericales Marcgraviaceae Marcgravia polyantha#FQ717
Ericales Marcgraviaceae Marcgravia coriacea#NC 041255

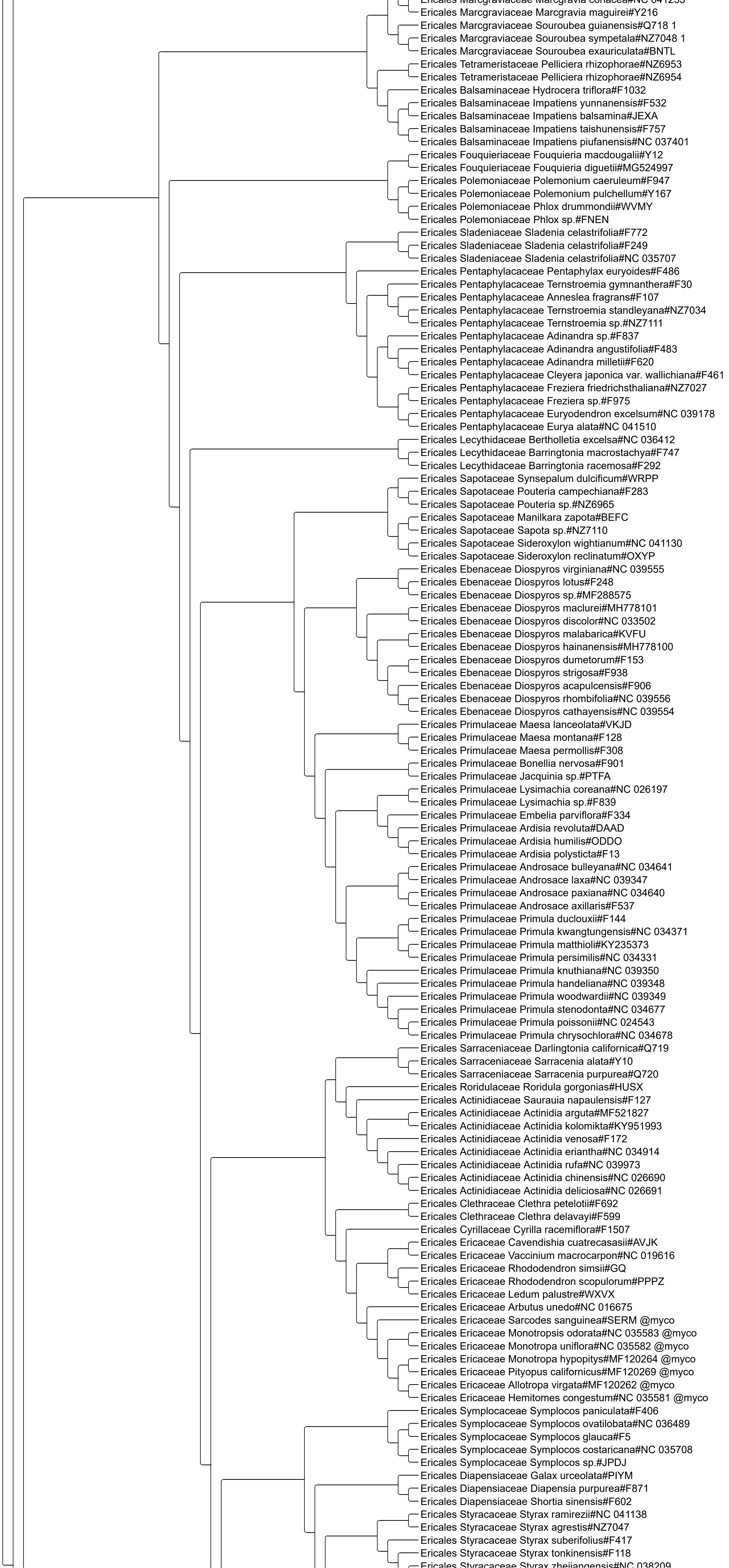
Ericales Marcgraviaceae Marcgravia maguirei#Y216
Ericales Marcgraviaceae Souroubea guianensis#Q718 1
Ericales Marcgraviaceae Souroubea sympetala#NZ7048 1
Ericales Marcgraviaceae Souroubea exauriculata#BNTL
Ericales Tetrameristaceae Pelliciera rhizophorae#NZ6953
Ericales Tetrameristaceae Pelliciera rhizophorae#NZ6954
Ericales Balsaminaceae Hydrocera triflora#F1032
Ericales Balsaminaceae Impatiens yunnanensis#F532
Ericales Balsaminaceae Impatiens balsamina#JEXA
Ericales Balsaminaceae Impatiens taishunensis#F757
Ericales Balsaminaceae Impatiens piufanensis#NC 037401
Ericales Fouquieriaceae Fouquieria macdougalii#Y12
Ericales Fouquieriaceae Fouquieria diguetii#MG524997
Ericales Polemoniaceae Polemonium caeruleum#F947
Ericales Polemoniaceae Polemonium pulchellum#Y167
Ericales Polemoniaceae Phlox drummondii#WVMY
Ericales Polemoniaceae Phlox sp.#FNEN
Ericales Sladeniaceae Sladenia celastrifolia#F772
Ericales Sladeniaceae Sladenia celastrifolia#F249
Ericales Sladeniaceae Sladenia celastrifolia#NC 035707
Ericales Pentaphylacaceae Pentaphylax euryoides#F486
Ericales Pentaphylacaceae Ternstroemia gymnanthera#F30
Ericales Pentaphylacaceae Anneslea fragrans#F107
Ericales Pentaphylacaceae Ternstroemia standleyana#NZ7034
Ericales Pentaphylacaceae Ternstroemia sp.#NZ7111
Ericales Pentaphylacaceae Adinandra sp.#F837
Ericales Pentaphylacaceae Adinandra angustifolia#F483
Ericales Pentaphylacaceae Adinandra milletii#F620
Ericales Pentaphylacaceae Cleyera japonica var. wallichiana#F461
Ericales Pentaphylacaceae Freziera friedrichsthaliana#NZ7027
Ericales Pentaphylacaceae Freziera sp.#F975
Ericales Pentaphylacaceae Euryodendron excelsum#NC 039178
Ericales Pentaphylacaceae Eurya alata#NC 041510
Ericales Lecythidaceae Bertholletia excelsa#NC 036412
Ericales Lecythidaceae Barringtonia macrostachya#F747
Ericales Lecythidaceae Barringtonia racemosa#F292
Ericales Sapotaceae Synsepalum dulcificum#WRPP
Ericales Sapotaceae Pouteria campechiana#F283
Ericales Sapotaceae Pouteria sp.#NZ6965
Ericales Sapotaceae Manilkara zapota#BEFC
Ericales Sapotaceae Sapota sp.#NZ7110
Ericales Sapotaceae Sideroxylon wightianum#NC 041130
Ericales Sapotaceae Sideroxylon reclinatum#OXYP
Ericales Ebenaceae Diospyros virginiana#NC 039555
Ericales Ebenaceae Diospyros lotus#F248
Ericales Ebenaceae Diospyros sp.#MF288575
Ericales Ebenaceae Diospyros maclurei#MH778101
Ericales Ebenaceae Diospyros discolor#NC 033502
Ericales Ebenaceae Diospyros malabarica#KVFU
Ericales Ebenaceae Diospyros hainanensis#MH778100
Ericales Ebenaceae Diospyros dumetorum#F153
Ericales Ebenaceae Diospyros strigosa#F938
Ericales Ebenaceae Diospyros acapulcensis#F906
Ericales Ebenaceae Diospyros rhombifolia#NC 039556
Ericales Ebenaceae Diospyros cathayensis#NC 039554
Ericales Primulaceae Maesa lanceolata#VKJD
Ericales Primulaceae Maesa montana#F128
Ericales Primulaceae Maesa permollis#F308
Ericales Primulaceae Bonellia nervosa#F901
Ericales Primulaceae Jacquinia sp.#PTFA
Ericales Primulaceae Lysimachia coreana#NC 026197
Ericales Primulaceae Lysimachia sp.#F839
Ericales Primulaceae Embelia parviflora#F334
Ericales Primulaceae Ardisia revoluta#DAAD
Ericales Primulaceae Ardisia humilis#ODDO
Ericales Primulaceae Ardisia polysticta#F13
Ericales Primulaceae Androsace bulleyana#NC 034641
Ericales Primulaceae Androsace laxa#NC 039347
Ericales Primulaceae Androsace paxiana#NC 034640
Ericales Primulaceae Androsace axillaris#F537
Ericales Primulaceae Primula duclouxii#F144
Ericales Primulaceae Primula kwangtungensis#NC 034371
Ericales Primulaceae Primula matthioli#KY235373
Ericales Primulaceae Primula persimilis#NC 034331
Ericales Primulaceae Primula knuthiana#NC 039350
Ericales Primulaceae Primula handeliana#NC 039348
Ericales Primulaceae Primula woodwardii#NC 039349
Ericales Primulaceae Primula stenodonta#NC 034677
Ericales Primulaceae Primula poissonii#NC 024543
Ericales Primulaceae Primula chrysochlora#NC 034678
Ericales Sarraceniaceae Darlingtonia californica#Q719
Ericales Sarraceniaceae Sarracenia alata#Y10
Ericales Sarraceniaceae Sarracenia purpurea#Q720
Ericales Roridulaceae Roridula gorgonias#HUSX
Ericales Actinidiaceae Saurauia napaulensis#F127
Ericales Actinidiaceae Actinidia arguta#MF521827
Ericales Actinidiaceae Actinidia kolomikta#KY951993
Ericales Actinidiaceae Actinidia venosa#F172
Ericales Actinidiaceae Actinidia eriantha#NC 034914
Ericales Actinidiaceae Actinidia rufa#NC 039973
Ericales Actinidiaceae Actinidia chinensis#NC 026690
Ericales Actinidiaceae Actinidia deliciosa#NC 026691
Ericales Clethraceae Clethra petelotii#F692
Ericales Clethraceae Clethra delavayi#F599
Ericales Cyrillaceae Cyrilla racemiflora#F1507
Ericales Ericaceae Cavendishia cuatrecasasii#AVJK
Ericales Ericaceae Vaccinium macrocarpon#NC 019616
Ericales Ericaceae Rhododendron simsii#GQ
Ericales Ericaceae Rhododendron scopulorum#PPPZ
Ericales Ericaceae Ledum palustre#WXVX
Ericales Ericaceae Arbutus unedo#NC 016675
Ericales Ericaceae Sarcodes sanguinea#SERM @myco
Ericales Ericaceae Monotropsis odorata#NC 035583 @myco
Ericales Ericaceae Monotropa uniflora#NC 035582 @myco
Ericales Ericaceae Monotropa hypopitys#MF120264 @myco
Ericales Ericaceae Pityopus californicus#MF120269 @myco
Ericales Ericaceae Allotropa virgata#MF120262 @myco
Ericales Ericaceae Hemitomes congestum#NC 035581 @myco
Ericales Symplocaceae Symplocos paniculata#F406
Ericales Symplocaceae Symplocos ovatilobata#NC 036489
Ericales Symplocaceae Symplocos glauca#F5
Ericales Symplocaceae Symplocos costaricana#NC 035708
Ericales Symplocaceae Symplocos sp.#JPDJ
Ericales Diapensiaceae Galax urceolata#PIYM
Ericales Diapensiaceae Diapensia purpurea#F871
Ericales Diapensiaceae Shortia sinensis#F602
Ericales Styracaceae Styrax ramirezii#NC 041138
Ericales Styracaceae Styrax agrestis#NZ7047
Ericales Styracaceae Styrax suberifolius#F417
Ericales Styracaceae Styrax tonkinensis#F118

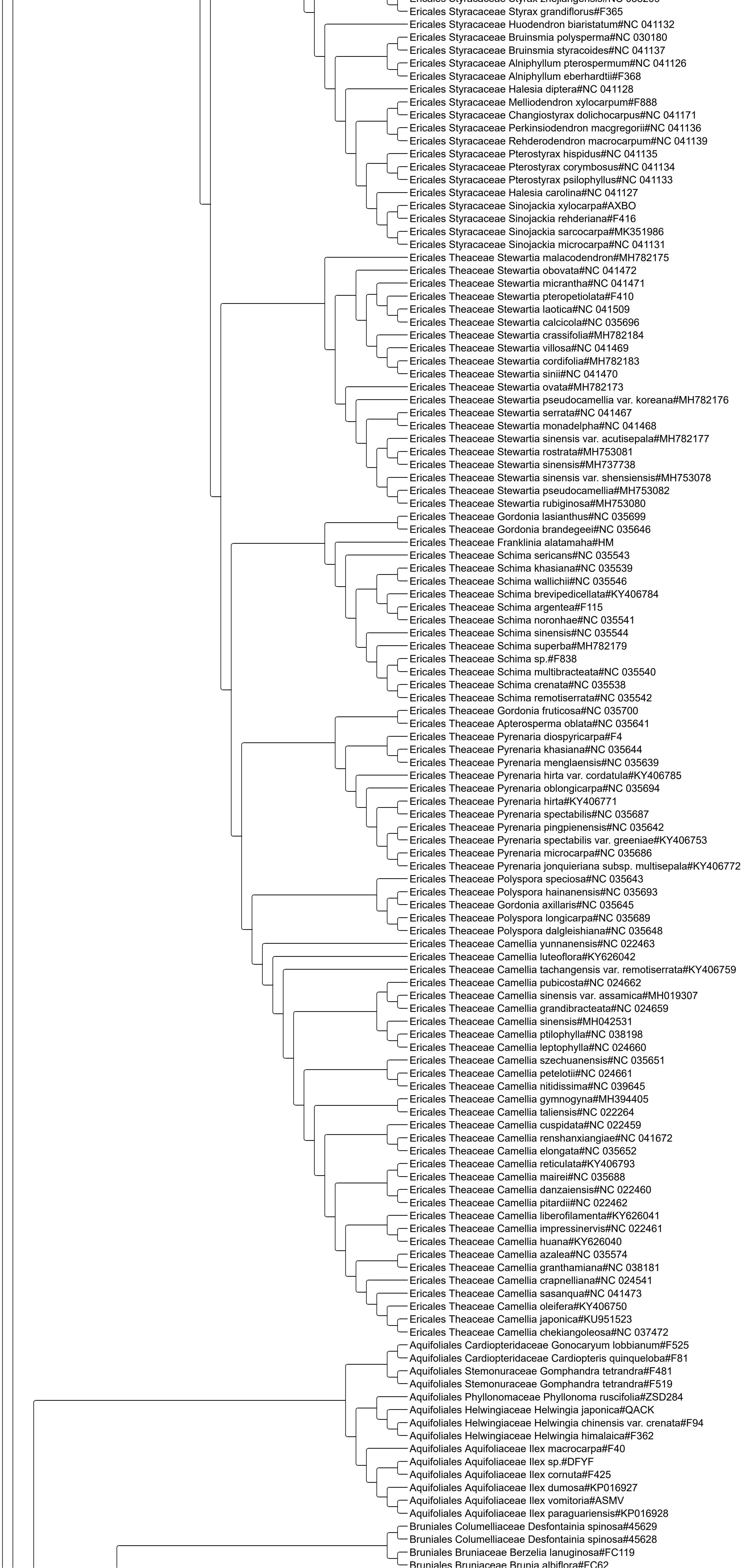

Ericales Styracaceae Styrax grandiflorus#F365
Ericales Styracaceae Huodendron biaristatum#NC 041132
Ericales Styracaceae Bruinsmia polysperma#NC 030180
Ericales Styracaceae Bruinsmia styracoides#NC 041137
Ericales Styracaceae Alniphyllum pterospermum#NC 041126
Ericales Styracaceae Alniphyllum eberhardtii#F368
Ericales Styracaceae Halesia diptera#NC 041128
Ericales Styracaceae Melliodendron xylocarpum#F888
Ericales Styracaceae Changiostyrax dolichocarpus#NC 041171
Ericales Styracaceae Perkinsiodendron macgregorii#NC 041136
Ericales Styracaceae Rehderodendron macrocarpum#NC 041139
Ericales Styracaceae Pterostyrax hispidus#NC 041135
Ericales Styracaceae Pterostyrax corymbosus#NC 041134
Ericales Styracaceae Pterostyrax psilophyllus#NC 041133
Ericales Styracaceae Halesia carolina#NC 041127
Ericales Styracaceae Sinojackia xylocarpa#AXBO
Ericales Styracaceae Sinojackia rehderiana#F416
Ericales Styracaceae Sinojackia sarcocarpa#MK351986
Ericales Styracaceae Sinojackia microcarpa#NC 041131
Ericales Theaceae Stewartia malacodendron#MH782175
Ericales Theaceae Stewartia obovata#NC 041472
Ericales Theaceae Stewartia micrantha#NC 041471
Ericales Theaceae Stewartia pteropetiolata#F410
Ericales Theaceae Stewartia laotica#NC 041509
Ericales Theaceae Stewartia calcicola#NC 035696
Ericales Theaceae Stewartia crassifolia#MH782184
Ericales Theaceae Stewartia villosa#NC 041469
Ericales Theaceae Stewartia cordifolia#MH782183
Ericales Theaceae Stewartia sinii#NC 041470
Ericales Theaceae Stewartia ovata#MH782173
Ericales Theaceae Stewartia pseudocamellia var. koreana#MH782176
Ericales Theaceae Stewartia serrata#NC 041467
Ericales Theaceae Stewartia monadelpha#NC 041468
Ericales Theaceae Stewartia sinensis var. acutisepala#MH782177
Ericales Theaceae Stewartia rostrata#MH753081
Ericales Theaceae Stewartia sinensis#MH737738
Ericales Theaceae Stewartia sinensis var. shensiensis#MH753078
Ericales Theaceae Stewartia pseudocamellia#MH753082
Ericales Theaceae Stewartia rubiginosa#MH753080
Ericales Theaceae Gordonia lasianthus#NC 035699
Ericales Theaceae Gordonia brandegeei#NC 035646
Ericales Theaceae Franklinia alatamaha#HM
Ericales Theaceae Schima sericans#NC 035543
Ericales Theaceae Schima khasiana#NC 035539
Ericales Theaceae Schima wallichii#NC 035546
Ericales Theaceae Schima brevipedicellata#KY406784
Ericales Theaceae Schima argentea#F115
Ericales Theaceae Schima noronhae#NC 035541
Ericales Theaceae Schima sinensis#NC 035544
Ericales Theaceae Schima superba#MH782179
Ericales Theaceae Schima sp.#F838
Ericales Theaceae Schima multibracteata#NC 035540
Ericales Theaceae Schima crenata#NC 035538
Ericales Theaceae Schima remotiserrata#NC 035542
Ericales Theaceae Gordonia fruticosa#NC 035700
Ericales Theaceae Apterosperma oblata#NC 035641
Ericales Theaceae Pyrenaria diospyricarpa#F4
Ericales Theaceae Pyrenaria khasiana#NC 035644
Ericales Theaceae Pyrenaria menglaensis#NC 035639
Ericales Theaceae Pyrenaria hirta var. cordatula#KY406785
Ericales Theaceae Pyrenaria oblongicarpa#NC 035694
Ericales Theaceae Pyrenaria hirta#KY406771
Ericales Theaceae Pyrenaria spectabilis#NC 035687
Ericales Theaceae Pyrenaria pingpienensis#NC 035642
Ericales Theaceae Pyrenaria spectabilis var. greeniae#KY406753
Ericales Theaceae Pyrenaria microcarpa#NC 035686
Ericales Theaceae Pyrenaria jonquieriana subsp. multisepala#KY406772
Ericales Theaceae Polyspora speciosa#NC 035643
Ericales Theaceae Polyspora hainanensis#NC 035693
Ericales Theaceae Gordonia axillaris#NC 035645
Ericales Theaceae Polyspora longicarpa#NC 035689
Ericales Theaceae Polyspora dalgleishiana#NC 035648
Ericales Theaceae Camellia yunnanensis#NC 022463
Ericales Theaceae Camellia luteoflora#KY626042
Ericales Theaceae Camellia tachangensis var. remotiserrata#KY406759
Ericales Theaceae Camellia pubicosta#NC 024662
Ericales Theaceae Camellia sinensis var. assamica#MH019307
Ericales Theaceae Camellia grandibracteata#NC 024659
Ericales Theaceae Camellia sinensis#MH042531
Ericales Theaceae Camellia ptilophylla#NC 038198
Ericales Theaceae Camellia leptophylla#NC 024660
Ericales Theaceae Camellia szechuanensis#NC 035651
Ericales Theaceae Camellia petelotii#NC 024661
Ericales Theaceae Camellia nitidissima#NC 039645
Ericales Theaceae Camellia gymnogyna#MH394405
Ericales Theaceae Camellia taliensis#NC 022264
Ericales Theaceae Camellia cuspidata#NC 022459
Ericales Theaceae Camellia renshanxiangiae#NC 041672
Ericales Theaceae Camellia elongata#NC 035652
Ericales Theaceae Camellia reticulata#KY406793
Ericales Theaceae Camellia mairei#NC 035688
Ericales Theaceae Camellia danzaiensis#NC 022460
Ericales Theaceae Camellia pitardii#NC 022462
Ericales Theaceae Camellia liberofilamenta#KY626041
Ericales Theaceae Camellia impressinervis#NC 022461
Ericales Theaceae Camellia huana#KY626040
Ericales Theaceae Camellia azalea#NC 035574
Ericales Theaceae Camellia granthamiana#NC 038181
Ericales Theaceae Camellia crapnelliana#NC 024541
Ericales Theaceae Camellia sasanqua#NC 041473
Ericales Theaceae Camellia oleifera#KY406750
Ericales Theaceae Camellia japonica#KU951523
Ericales Theaceae Camellia chekiangoleosa#NC 037472
Aquifoliales Cardiopteridaceae Gonocaryum lobbianum#F525
Aquifoliales Cardiopteridaceae Cardiopteris quinqueloba#F81
Aquifoliales Stemonuraceae Gomphandra tetrandra#F481
Aquifoliales Stemonuraceae Gomphandra tetrandra#F519
Aquifoliales Phyllonomaceae Phyllonoma ruscifolia#ZSD284
Aquifoliales Helwingiaceae Helwingia japonica#QACK
Aquifoliales Helwingiaceae Helwingia chinensis var. crenata#F94
Aquifoliales Helwingiaceae Helwingia himalaica#F362
Aquifoliales Aquifoliaceae Ilex macrocarpa#F40
Aquifoliales Aquifoliaceae Ilex sp.#DFYF
Aquifoliales Aquifoliaceae Ilex cornuta#F425
Aquifoliales Aquifoliaceae Ilex dumosa#KP016927
Aquifoliales Aquifoliaceae Ilex vomitoria#ASMV
Aquifoliales Aquifoliaceae Ilex paraguariensis#KP016928
Bruniales Columelliaceae Desfontainia spinosa#45629
Bruniales Columelliaceae Desfontainia spinosa#45628
Bruniales Bruniaceae Berzelia lanuginosa#FC119
Bruniales Bruniaceae Brunia albiflora#FC62

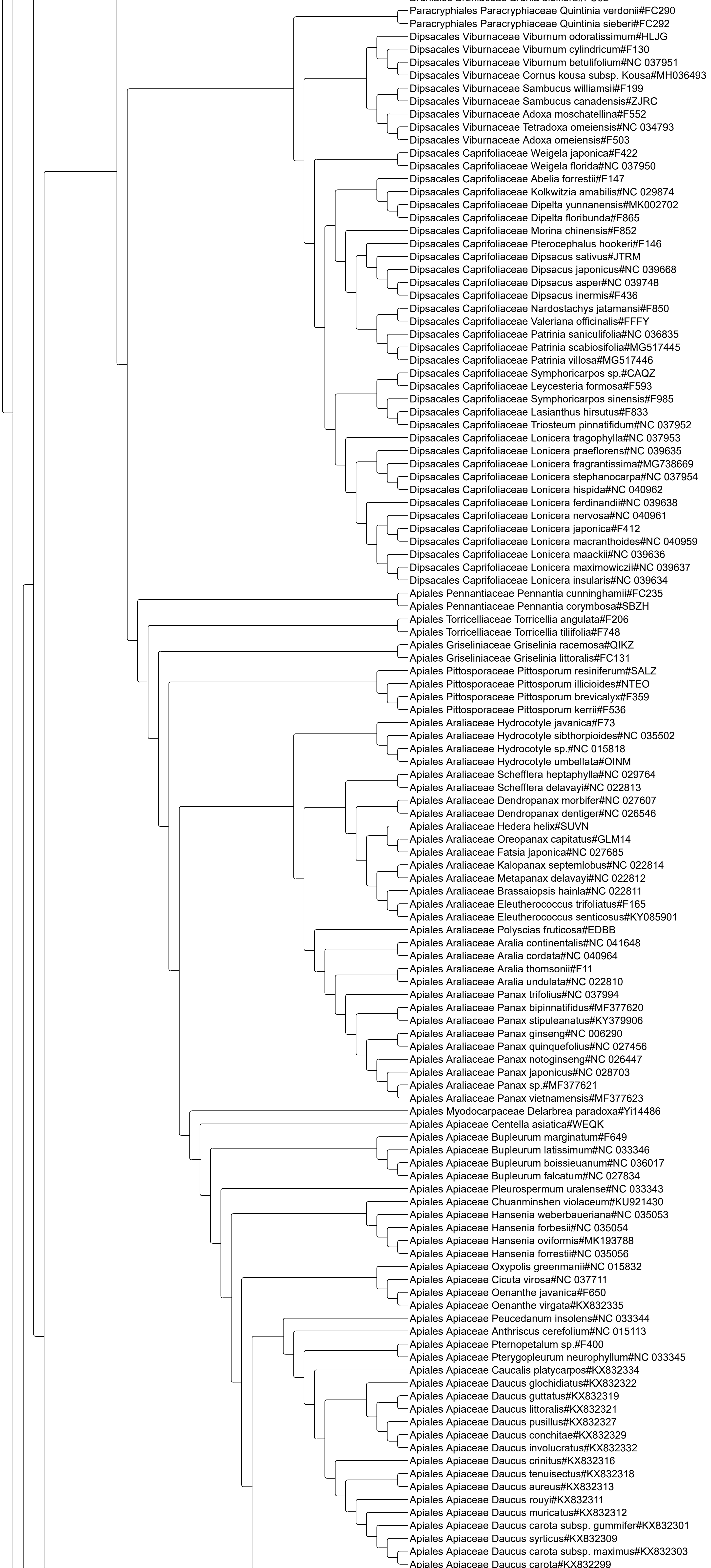

Paracryphiales Paracryphiaceae Quintinia verdonii#FC290
Paracryphiales Paracryphiaceae Quintinia sieberi#FC292
Dipsacales Viburnaceae Viburnum odoratissimum#HLJG
Dipsacales Viburnaceae Viburnum cylindricum#F130
Dipsacales Viburnaceae Viburnum betulifolium#NC 037951
Dipsacales Viburnaceae Cornus kousa subsp. Kousa#MH036493
Dipsacales Viburnaceae Sambucus williamsii#F199
Dipsacales Viburnaceae Sambucus canadensis#ZJRC
Dipsacales Viburnaceae Adoxa moschatellina#F552
Dipsacales Viburnaceae Tetradoxa omeiensis#NC 034793
Dipsacales Viburnaceae Adoxa omeiensis#F503
Dipsacales Caprifoliaceae Weigela japonica#F422
Dipsacales Caprifoliaceae Weigela florida#NC 037950
Dipsacales Caprifoliaceae Abelia forrestii#F147
Dipsacales Caprifoliaceae Kolkwitzia amabilis#NC 029874
Dipsacales Caprifoliaceae Dipelta yunnanensis#MK002702
Dipsacales Caprifoliaceae Dipelta floribunda#F865
Dipsacales Caprifoliaceae Morina chinensis#F852
Dipsacales Caprifoliaceae Pterocephalus hookeri#F146
Dipsacales Caprifoliaceae Dipsacus sativus#JTRM
Dipsacales Caprifoliaceae Dipsacus japonicus#NC 039668
Dipsacales Caprifoliaceae Dipsacus asper#NC 039748
Dipsacales Caprifoliaceae Dipsacus inermis#F436
Dipsacales Caprifoliaceae Nardostachys jatamansi#F850
Dipsacales Caprifoliaceae Valeriana officinalis#FFFY
Dipsacales Caprifoliaceae Patrinia saniculifolia#NC 036835
Dipsacales Caprifoliaceae Patrinia scabiosifolia#MG517445
Dipsacales Caprifoliaceae Patrinia villosa#MG517446
Dipsacales Caprifoliaceae Symphoricarpos sp.#CAQZ
Dipsacales Caprifoliaceae Leycesteria formosa#F593
Dipsacales Caprifoliaceae Symphoricarpos sinensis#F985
Dipsacales Caprifoliaceae Lasianthus hirsutus#F833
Dipsacales Caprifoliaceae Triosteum pinnatifidum#NC 037952
Dipsacales Caprifoliaceae Lonicera tragophylla#NC 037953
Dipsacales Caprifoliaceae Lonicera praeflorens#NC 039635
Dipsacales Caprifoliaceae Lonicera fragrantissima#MG738669
Dipsacales Caprifoliaceae Lonicera stephanocarpa#NC 037954
Dipsacales Caprifoliaceae Lonicera hispida#NC 040962
Dipsacales Caprifoliaceae Lonicera ferdinandii#NC 039638
Dipsacales Caprifoliaceae Lonicera nervosa#NC 040961
Dipsacales Caprifoliaceae Lonicera japonica#F412
Dipsacales Caprifoliaceae Lonicera macranthoides#NC 040959
Dipsacales Caprifoliaceae Lonicera maackii#NC 039636
Dipsacales Caprifoliaceae Lonicera maximowiczii#NC 039637
Dipsacales Caprifoliaceae Lonicera insularis#NC 039634
Apiales Pennantiaceae Pennantia cunninghamii#FC235
Apiales Pennantiaceae Pennantia corymbosa#SBZH
Apiales Torricelliaceae Torricellia angulata#F206
Apiales Torricelliaceae Torricellia tiliifolia#F748
Apiales Griseliniaceae Griselinia racemosa#QIKZ
Apiales Griseliniaceae Griselinia littoralis#FC131
Apiales Pittosporaceae Pittosporum resiniferum#SALZ
Apiales Pittosporaceae Pittosporum illicioides#NTEO
Apiales Pittosporaceae Pittosporum brevicalyx#F359
Apiales Pittosporaceae Pittosporum kerrii#F536
Apiales Araliaceae Hydrocotyle javanica#F73
Apiales Araliaceae Hydrocotyle sibthorpioides#NC 035502
Apiales Araliaceae Hydrocotyle sp.#NC 015818
Apiales Araliaceae Hydrocotyle umbellata#OINM
Apiales Araliaceae Schefflera heptaphylla#NC 029764
Apiales Araliaceae Schefflera delavayi#NC 022813
Apiales Araliaceae Dendropanax morbifer#NC 027607
Apiales Araliaceae Dendropanax dentiger#NC 026546
Apiales Araliaceae Hedera helix#SUVN
Apiales Araliaceae Oreopanax capitatus#GLM14
Apiales Araliaceae Fatsia japonica#NC 027685
Apiales Araliaceae Kalopanax septemlobus#NC 022814
Apiales Araliaceae Metapanax delavayi#NC 022812
Apiales Araliaceae Brassaiopsis hainla#NC 022811
Apiales Araliaceae Eleutherococcus trifoliatus#F165
Apiales Araliaceae Eleutherococcus senticosus#KY085901
Apiales Araliaceae Polyscias fruticosa#EDBB
Apiales Araliaceae Aralia continentalis#NC 041648
Apiales Araliaceae Aralia cordata#NC 040964
Apiales Araliaceae Aralia thomsonii#F11
Apiales Araliaceae Aralia undulata#NC 022810
Apiales Araliaceae Panax trifolius#NC 037994
Apiales Araliaceae Panax bipinnatifidus#MF377620
Apiales Araliaceae Panax stipuleanatus#KY379906
Apiales Araliaceae Panax ginseng#NC 006290
Apiales Araliaceae Panax quinquefolius#NC 027456
Apiales Araliaceae Panax notoginseng#NC 026447
Apiales Araliaceae Panax japonicus#NC 028703
Apiales Araliaceae Panax sp.#MF377621
Apiales Araliaceae Panax vietnamensis#MF377623
Apiales Myodocarpaceae Delarbrea paradoxa#Yi14486
Apiales Apiaceae Centella asiatica#WEQK
Apiales Apiaceae Bupleurum marginatum#F649
Apiales Apiaceae Bupleurum latissimum#NC 033346
Apiales Apiaceae Bupleurum boissieuanum#NC 036017
Apiales Apiaceae Bupleurum falcatum#NC 027834
Apiales Apiaceae Pleurospermum uralense#NC 033343
Apiales Apiaceae Chuanminshen violaceum#KU921430
Apiales Apiaceae Hansenia weberbaueriana#NC 035053
Apiales Apiaceae Hansenia forbesii#NC 035054
Apiales Apiaceae Hansenia oviformis#MK193788
Apiales Apiaceae Hansenia forrestii#NC 035056
Apiales Apiaceae Oxypolis greenmanii#NC 015832
Apiales Apiaceae Cicuta virosa#NC 037711
Apiales Apiaceae Oenanthe javanica#F650
Apiales Apiaceae Oenanthe virgata#KX832335
Apiales Apiaceae Peucedanum insolens#NC 033344
Apiales Apiaceae Anthriscus cerefolium#NC 015113
Apiales Apiaceae Pternopetalum sp.#F400
Apiales Apiaceae Pterygopleurum neurophyllum#NC 033345
Apiales Apiaceae Caucalis platycarpos#KX832334
Apiales Apiaceae Daucus glochidiatus#KX832322
Apiales Apiaceae Daucus guttatus#KX832319
Apiales Apiaceae Daucus littoralis#KX832321
Apiales Apiaceae Daucus pusillus#KX832327
Apiales Apiaceae Daucus conchitae#KX832329
Apiales Apiaceae Daucus involucratus#KX832332
Apiales Apiaceae Daucus crinitus#KX832316
Apiales Apiaceae Daucus tenuisectus#KX832318
Apiales Apiaceae Daucus aureus#KX832313
Apiales Apiaceae Daucus rouyi#KX832311
Apiales Apiaceae Daucus muricatus#KX832312
Apiales Apiaceae Daucus carota subsp. gummifer#KX832301
Apiales Apiaceae Daucus syrticus#KX832309
Apiales Apiaceae Daucus carota subsp. maximus#KX832303
Apiales Apiaceae Daucus carota#KX832299

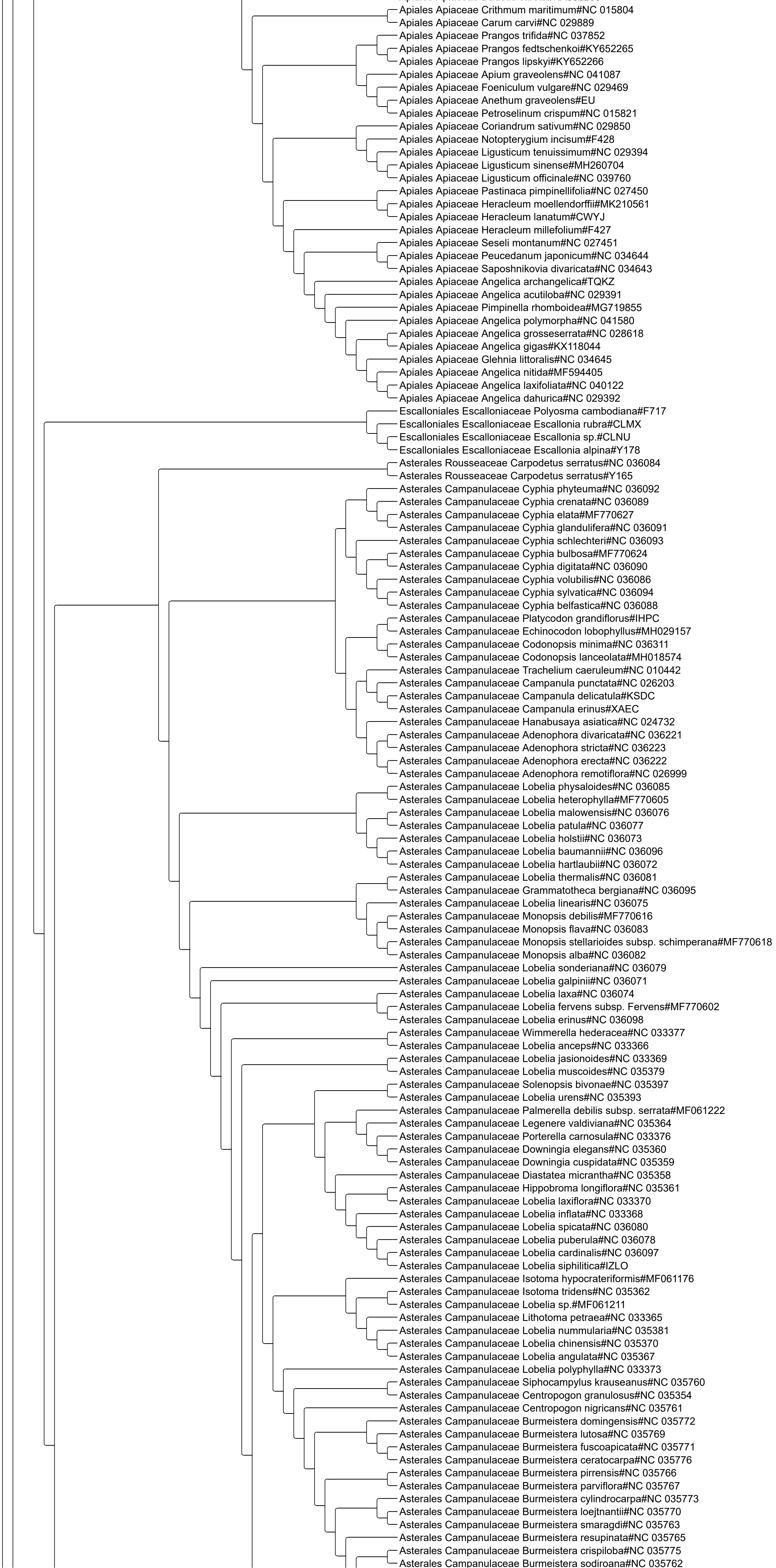
Apiales Apiaceae Crithmum maritimum#NC 015804
Apiales Apiaceae Carum carvi#NC 029889
Apiales Apiaceae Prangos trifida#NC 037852
Apiales Apiaceae Prangos fedtschenkoi#KY652265
Apiales Apiaceae Prangos lipskyi#KY652266
Apiales Apiaceae Apium graveolens#NC 041087
Apiales Apiaceae Foeniculum vulgare#NC 029469
Apiales Apiaceae Anethum graveolens#EU
Apiales Apiaceae Petroselinum crispum#NC 015821
Apiales Apiaceae Coriandrum sativum#NC 029850
Apiales Apiaceae Notopterygium incisum#F428
Apiales Apiaceae Ligusticum tenuissimum#NC 029394
Apiales Apiaceae Ligusticum sinense#MH260704
Apiales Apiaceae Ligusticum officinale#NC 039760
Apiales Apiaceae Pastinaca pimpinellifolia#NC 027450
Apiales Apiaceae Heracleum moellendorffii#MK210561
Apiales Apiaceae Heracleum lanatum#CWYJ
Apiales Apiaceae Heracleum millefolium#F427
Apiales Apiaceae Seseli montanum#NC 027451
Apiales Apiaceae Peucedanum japonicum#NC 034644
Apiales Apiaceae Saposhnikovia divaricata#NC 034643
Apiales Apiaceae Angelica archangelica#TQKZ
Apiales Apiaceae Angelica acutiloba#NC 029391
Apiales Apiaceae Pimpinella rhomboidea#MG719855
Apiales Apiaceae Angelica polymorpha#NC 041580
Apiales Apiaceae Angelica grosseserrata#NC 028618
Apiales Apiaceae Angelica gigas#KX118044
Apiales Apiaceae Glehnia littoralis#NC 034645
Apiales Apiaceae Angelica nitida#MF594405
Apiales Apiaceae Angelica laxifoliata#NC 040122
Apiales Apiaceae Angelica dahurica#NC 029392
Escalloniales Escalloniaceae Polyosma cambodiana#F717
Escalloniales Escalloniaceae Escallonia rubra#CLMX
Escalloniales Escalloniaceae Escallonia sp.#CLNU
Escalloniales Escalloniaceae Escallonia alpina#Y178
Asterales Rousseaceae Carpodetus serratus#NC 036084
Asterales Rousseaceae Carpodetus serratus#Y165
Asterales Campanulaceae Cyphia phyteuma#NC 036092
Asterales Campanulaceae Cyphia crenata#NC 036089
Asterales Campanulaceae Cyphia elata#MF770627
Asterales Campanulaceae Cyphia glandulifera#NC 036091
Asterales Campanulaceae Cyphia schlechteri#NC 036093
Asterales Campanulaceae Cyphia bulbosa#MF770624
Asterales Campanulaceae Cyphia digitata#NC 036090
Asterales Campanulaceae Cyphia volubilis#NC 036086
Asterales Campanulaceae Cyphia sylvatica#NC 036094
Asterales Campanulaceae Cyphia belfastica#NC 036088
Asterales Campanulaceae Platycodon grandiflorus#IHPC
Asterales Campanulaceae Echinocodon lobophyllus#MH029157
Asterales Campanulaceae Codonopsis minima#NC 036311
Asterales Campanulaceae Codonopsis lanceolata#MH018574
Asterales Campanulaceae Trachelium caeruleum#NC 010442
Asterales Campanulaceae Campanula punctata#NC 026203
Asterales Campanulaceae Campanula delicatula#KSDC
Asterales Campanulaceae Campanula erinus#XAEC
Asterales Campanulaceae Hanabusaya asiatica#NC 024732
Asterales Campanulaceae Adenophora divaricata#NC 036221
Asterales Campanulaceae Adenophora stricta#NC 036223
Asterales Campanulaceae Adenophora erecta#NC 036222
Asterales Campanulaceae Adenophora remotiflora#NC 026999
Asterales Campanulaceae Lobelia physaloides#NC 036085
Asterales Campanulaceae Lobelia heterophylla#MF770605
Asterales Campanulaceae Lobelia malowensis#NC 036076
Asterales Campanulaceae Lobelia patula#NC 036077
Asterales Campanulaceae Lobelia holstii#NC 036073
Asterales Campanulaceae Lobelia baumannii#NC 036096
Asterales Campanulaceae Lobelia hartlaubii#NC 036072
Asterales Campanulaceae Lobelia thermalis#NC 036081
Asterales Campanulaceae Grammatotheca bergiana#NC 036095
Asterales Campanulaceae Lobelia linearis#NC 036075
Asterales Campanulaceae Monopsis debilis#MF770616
Asterales Campanulaceae Monopsis flava#NC 036083
Asterales Campanulaceae Monopsis stellarioides subsp. schimperana#MF770618
Asterales Campanulaceae Monopsis alba#NC 036082
Asterales Campanulaceae Lobelia sonderiana#NC 036079
Asterales Campanulaceae Lobelia galpinii#NC 036071
Asterales Campanulaceae Lobelia laxa#NC 036074
Asterales Campanulaceae Lobelia fervens subsp. Fervens#MF770602
Asterales Campanulaceae Lobelia erinus#NC 036098
Asterales Campanulaceae Wimmerella hederacea#NC 033377
Asterales Campanulaceae Lobelia anceps#NC 033366
Asterales Campanulaceae Lobelia jasionoides#NC 033369
Asterales Campanulaceae Lobelia muscoides#NC 035379
Asterales Campanulaceae Solenopsis bivonae#NC 035397
Asterales Campanulaceae Lobelia urens#NC 035393
Asterales Campanulaceae Palmerella debilis subsp. serrata#MF061222
Asterales Campanulaceae Legenere valdiviana#NC 035364
Asterales Campanulaceae Porterella carnosula#NC 033376
Asterales Campanulaceae Downingia elegans#NC 035360
Asterales Campanulaceae Downingia cuspidata#NC 035359
Asterales Campanulaceae Diastatea micrantha#NC 035358
Asterales Campanulaceae Hippobroma longiflora#NC 035361
Asterales Campanulaceae Lobelia laxiflora#NC 033370
Asterales Campanulaceae Lobelia inflata#NC 033368
Asterales Campanulaceae Lobelia spicata#NC 036080
Asterales Campanulaceae Lobelia puberula#NC 036078
Asterales Campanulaceae Lobelia cardinalis#NC 036097
Asterales Campanulaceae Lobelia siphilitica#IZLO
Asterales Campanulaceae Isotoma hypocrateriformis#MF061176
Asterales Campanulaceae Isotoma tridens#NC 035362
Asterales Campanulaceae Lobelia sp.#MF061211
Asterales Campanulaceae Lithotoma petraea#NC 033365
Asterales Campanulaceae Lobelia nummularia#NC 035381
Asterales Campanulaceae Lobelia chinensis#NC 035370
Asterales Campanulaceae Lobelia angulata#NC 035367
Asterales Campanulaceae Lobelia polyphylla#NC 033373
Asterales Campanulaceae Siphocampylus krauseanus#NC 035760
Asterales Campanulaceae Centropogon granulosus#NC 035354
Asterales Campanulaceae Centropogon nigricans#NC 035761
Asterales Campanulaceae Burmeistera domingensis#NC 035772
Asterales Campanulaceae Burmeistera lutosa#NC 035769
Asterales Campanulaceae Burmeistera fuscoapicata#NC 035771
Asterales Campanulaceae Burmeistera ceratocarpa#NC 035776
Asterales Campanulaceae Burmeistera pirrensis#NC 035766
Asterales Campanulaceae Burmeistera parviflora#NC 035767
Asterales Campanulaceae Burmeistera cylindrocarpa#NC 035773
Asterales Campanulaceae Burmeistera loejtnantii#NC 035770
Asterales Campanulaceae Burmeistera smaragdi#NC 035763
Asterales Campanulaceae Burmeistera resupinata#NC 035765
Asterales Campanulaceae Burmeistera crispiloba#NC 035775
Asterales Campanulaceae Burmeistera sodiroana#NC 035762

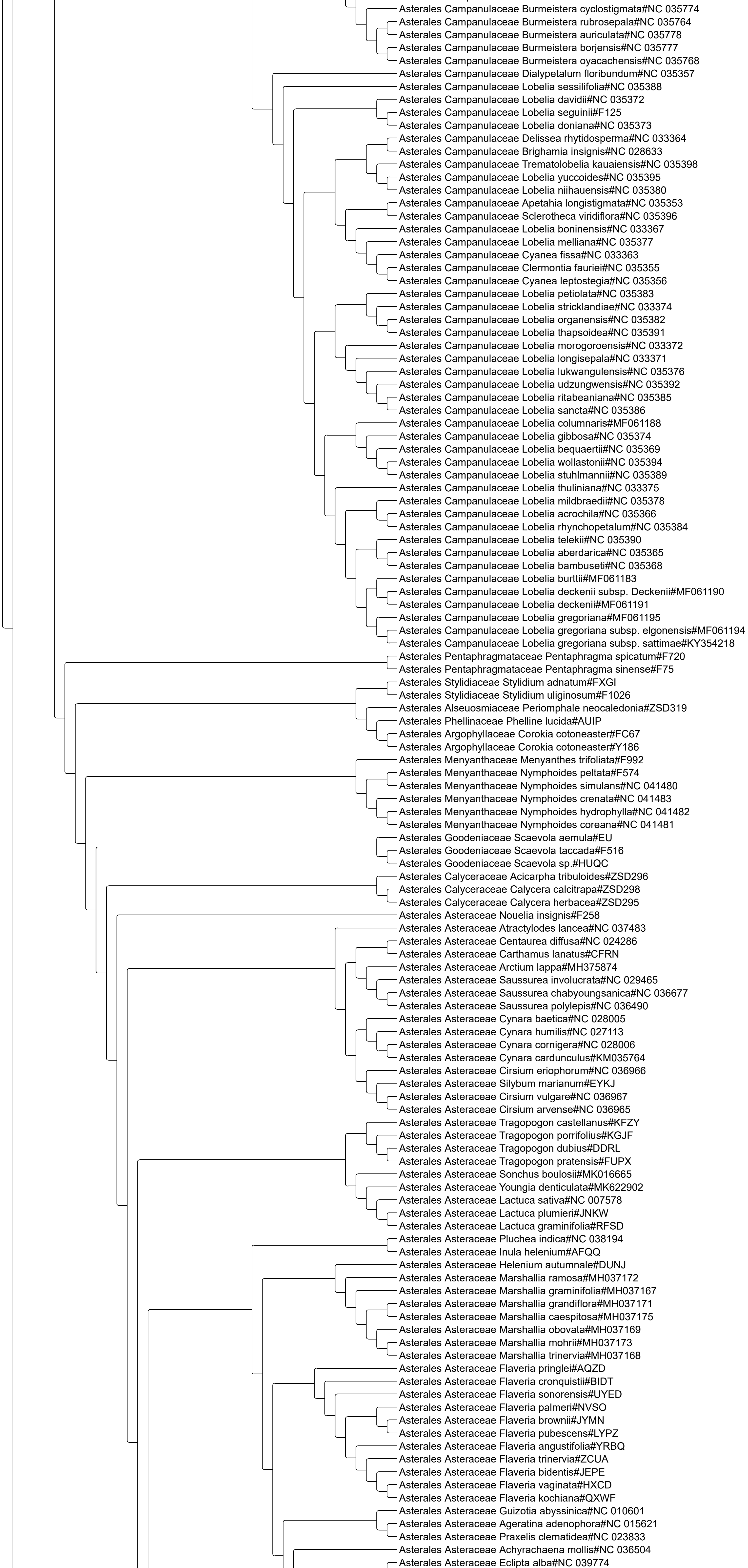

Asterales Campanulaceae Burmeistera cyclostigmata#NC 035774
Asterales Campanulaceae Burmeistera rubrosepala#NC 035764
Asterales Campanulaceae Burmeistera auriculata#NC 035778
Asterales Campanulaceae Burmeistera borjensis#NC 035777
Asterales Campanulaceae Burmeistera oyacachensis#NC 035768
Asterales Campanulaceae Dialypetalum floribundum#NC 035357
Asterales Campanulaceae Lobelia sessilifolia#NC 035388
Asterales Campanulaceae Lobelia davidii#NC 035372
Asterales Campanulaceae Lobelia seguinii#F125
Asterales Campanulaceae Lobelia doniana#NC 035373
Asterales Campanulaceae Delissea rhytidosperma#NC 033364
Asterales Campanulaceae Brighamia insignis#NC 028633
Asterales Campanulaceae Trematolobelia kauaiensis#NC 035398
Asterales Campanulaceae Lobelia yuccoides#NC 035395
Asterales Campanulaceae Lobelia niihauensis#NC 035380
Asterales Campanulaceae Apetahia longistigmata#NC 035353
Asterales Campanulaceae Sclerotheca viridiflora#NC 035396
Asterales Campanulaceae Lobelia boninensis#NC 033367
Asterales Campanulaceae Lobelia melliana#NC 035377
Asterales Campanulaceae Cyanea fissa#NC 033363
Asterales Campanulaceae Clermontia fauriei#NC 035355
Asterales Campanulaceae Cyanea leptostegia#NC 035356
Asterales Campanulaceae Lobelia petiolata#NC 035383
Asterales Campanulaceae Lobelia stricklandiae#NC 033374
Asterales Campanulaceae Lobelia organensis#NC 035382
Asterales Campanulaceae Lobelia thapsoidea#NC 035391
Asterales Campanulaceae Lobelia morogoroensis#NC 033372
Asterales Campanulaceae Lobelia longisepala#NC 033371
Asterales Campanulaceae Lobelia lukwangulensis#NC 035376
Asterales Campanulaceae Lobelia udzungwensis#NC 035392
Asterales Campanulaceae Lobelia ritabeaniana#NC 035385
Asterales Campanulaceae Lobelia sancta#NC 035386
Asterales Campanulaceae Lobelia columnaris#MF061188
Asterales Campanulaceae Lobelia gibbosa#NC 035374
Asterales Campanulaceae Lobelia bequaertii#NC 035369
Asterales Campanulaceae Lobelia wollastonii#NC 035394
Asterales Campanulaceae Lobelia stuhlmannii#NC 035389
Asterales Campanulaceae Lobelia thuliniana#NC 033375
Asterales Campanulaceae Lobelia mildbraedii#NC 035378
Asterales Campanulaceae Lobelia acrochila#NC 035366
Asterales Campanulaceae Lobelia rhynchopetalum#NC 035384
Asterales Campanulaceae Lobelia telekii#NC 035390
Asterales Campanulaceae Lobelia aberdarica#NC 035365
Asterales Campanulaceae Lobelia bambuseti#NC 035368
Asterales Campanulaceae Lobelia burttii#MF061183
Asterales Campanulaceae Lobelia deckenii subsp. Deckenii#MF061190
Asterales Campanulaceae Lobelia deckenii#MF061191
Asterales Campanulaceae Lobelia gregoriana#MF061195
Asterales Campanulaceae Lobelia gregoriana subsp. elgonensis#MF061194
Asterales Campanulaceae Lobelia gregoriana subsp. sattimae#KY354218
Asterales Pentaphragmataceae Pentaphragma spicatum#F720
Asterales Pentaphragmataceae Pentaphragma sinense#F75
Asterales Stylidiaceae Stylidium adnatum#FXGI
Asterales Stylidiaceae Stylidium uliginosum#F1026
Asterales Alseuosmiaceae Periomphale neocaledonia#ZSD319
Asterales Phellinaceae Phelline lucida#AUIP
Asterales Argophyllaceae Corokia cotoneaster#FC67
Asterales Argophyllaceae Corokia cotoneaster#Y186
Asterales Menyanthaceae Menyanthes trifoliata#F992
Asterales Menyanthaceae Nymphoides peltata#F574
Asterales Menyanthaceae Nymphoides simulans#NC 041480
Asterales Menyanthaceae Nymphoides crenata#NC 041483
Asterales Menyanthaceae Nymphoides hydrophylla#NC 041482
Asterales Menyanthaceae Nymphoides coreana#NC 041481
Asterales Goodeniaceae Scaevola aemula#EU
Asterales Goodeniaceae Scaevola taccada#F516
Asterales Goodeniaceae Scaevola sp.#HUQC
Asterales Calyceraceae Acicarpha tribuloides#ZSD296
Asterales Calyceraceae Calycera calcitrapa#ZSD298
Asterales Calyceraceae Calycera herbacea#ZSD295
Asterales Asteraceae Nouelia insignis#F258
Asterales Asteraceae Atractylodes lancea#NC 037483
Asterales Asteraceae Centaurea diffusa#NC 024286
Asterales Asteraceae Carthamus lanatus#CFRN
Asterales Asteraceae Arctium lappa#MH375874
Asterales Asteraceae Saussurea involucrata#NC 029465
Asterales Asteraceae Saussurea chabyoungsanica#NC 036677
Asterales Asteraceae Saussurea polylepis#NC 036490
Asterales Asteraceae Cynara baetica#NC 028005
Asterales Asteraceae Cynara humilis#NC 027113
Asterales Asteraceae Cynara cornigera#NC 028006
Asterales Asteraceae Cynara cardunculus#KM035764
Asterales Asteraceae Cirsium eriophorum#NC 036966
Asterales Asteraceae Silybum marianum#EYKJ
Asterales Asteraceae Cirsium vulgare#NC 036967
Asterales Asteraceae Cirsium arvense#NC 036965
Asterales Asteraceae Tragopogon castellanus#KFZY
Asterales Asteraceae Tragopogon porrifolius#KGJF
Asterales Asteraceae Tragopogon dubius#DDRL
Asterales Asteraceae Tragopogon pratensis#FUPX
Asterales Asteraceae Sonchus boulosii#MK016665
Asterales Asteraceae Youngia denticulata#MK622902
Asterales Asteraceae Lactuca sativa#NC 007578
Asterales Asteraceae Lactuca plumieri#JNKW
Asterales Asteraceae Lactuca graminifolia#RFSD
Asterales Asteraceae Pluchea indica#NC 038194
Asterales Asteraceae Inula helenium#AFQQ
Asterales Asteraceae Helenium autumnale#DUNJ
Asterales Asteraceae Marshallia ramosa#MH037172
Asterales Asteraceae Marshallia graminifolia#MH037167
Asterales Asteraceae Marshallia grandiflora#MH037171
Asterales Asteraceae Marshallia caespitosa#MH037175
Asterales Asteraceae Marshallia obovata#MH037169
Asterales Asteraceae Marshallia mohrii#MH037173
Asterales Asteraceae Marshallia trinervia#MH037168
Asterales Asteraceae Flaveria pringlei#AQZD
Asterales Asteraceae Flaveria cronquistii#BIDT
Asterales Asteraceae Flaveria sonorensis#UYED
Asterales Asteraceae Flaveria palmeri#NVSO
Asterales Asteraceae Flaveria brownii#JYMN
Asterales Asteraceae Flaveria pubescens#LYPZ
Asterales Asteraceae Flaveria angustifolia#YRBQ
Asterales Asteraceae Flaveria trinervia#ZCUA
Asterales Asteraceae Flaveria bidentis#JEPE
Asterales Asteraceae Flaveria vaginata#HXCD
Asterales Asteraceae Flaveria kochiana#QXWF
Asterales Asteraceae Guizotia abyssinica#NC 010601
Asterales Asteraceae Ageratina adenophora#NC 015621
Asterales Asteraceae Praxelis clematidea#NC 023833
Asterales Asteraceae Achyrachaena mollis#NC 036504
Asterales Asteraceae Eclipta alba#NC 039774

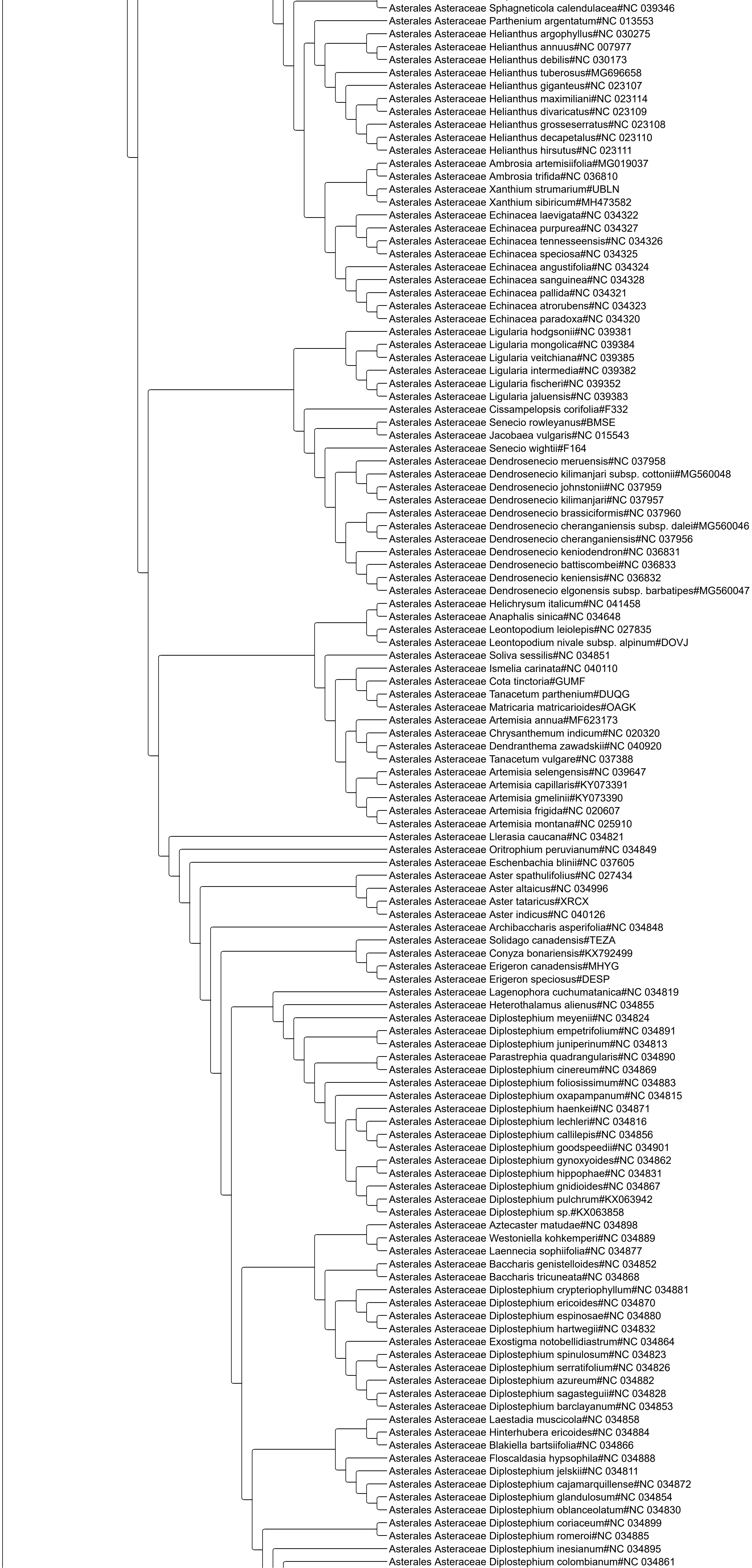
Asterales Asteraceae Sphagneticola calendulacea#NC 039346
Asterales Asteraceae Parthenium argentatum#NC 013553
Asterales Asteraceae Helianthus argophyllus#NC 030275
Asterales Asteraceae Helianthus annuus#NC 007977
Asterales Asteraceae Helianthus debilis#NC 030173
Asterales Asteraceae Helianthus tuberosus#MG696658
Asterales Asteraceae Helianthus giganteus#NC 023107
Asterales Asteraceae Helianthus maximiliani#NC 023114
Asterales Asteraceae Helianthus divaricatus#NC 023109
Asterales Asteraceae Helianthus grosseserratus#NC 023108
Asterales Asteraceae Helianthus decapetalus#NC 023110
Asterales Asteraceae Helianthus hirsutus#NC 023111
Asterales Asteraceae Ambrosia artemisiifolia#MG019037
Asterales Asteraceae Ambrosia trifida#NC 036810
Asterales Asteraceae Xanthium strumarium#UBLN
Asterales Asteraceae Xanthium sibiricum#MH473582
Asterales Asteraceae Echinacea laevigata#NC 034322
Asterales Asteraceae Echinacea purpurea#NC 034327
Asterales Asteraceae Echinacea tennesseensis#NC 034326
Asterales Asteraceae Echinacea speciosa#NC 034325
Asterales Asteraceae Echinacea angustifolia#NC 034324
Asterales Asteraceae Echinacea sanguinea#NC 034328
Asterales Asteraceae Echinacea pallida#NC 034321
Asterales Asteraceae Echinacea atrorubens#NC 034323
Asterales Asteraceae Echinacea paradoxa#NC 034320
Asterales Asteraceae Ligularia hodgsonii#NC 039381
Asterales Asteraceae Ligularia mongolica#NC 039384
Asterales Asteraceae Ligularia veitchiana#NC 039385
Asterales Asteraceae Ligularia intermedia#NC 039382
Asterales Asteraceae Ligularia fischeri#NC 039352
Asterales Asteraceae Ligularia jaluensis#NC 039383
Asterales Asteraceae Cissampelopsis corifolia#F332
Asterales Asteraceae Senecio rowleyanus#BMSE
Asterales Asteraceae Jacobaea vulgaris#NC 015543
Asterales Asteraceae Senecio wightii#F164
Asterales Asteraceae Dendrosenecio meruensis#NC 037958
Asterales Asteraceae Dendrosenecio kilimanjari subsp. cottonii#MG560048
Asterales Asteraceae Dendrosenecio johnstonii#NC 037959
Asterales Asteraceae Dendrosenecio kilimanjari#NC 037957
Asterales Asteraceae Dendrosenecio brassiciformis#NC 037960
Asterales Asteraceae Dendrosenecio cheranganiensis subsp. dalei#MG560046
Asterales Asteraceae Dendrosenecio cheranganiensis#NC 037956
Asterales Asteraceae Dendrosenecio keniodendron#NC 036831
Asterales Asteraceae Dendrosenecio battiscombei#NC 036833
Asterales Asteraceae Dendrosenecio keniensis#NC 036832
Asterales Asteraceae Dendrosenecio elgonensis subsp. barbatipes#MG560047
Asterales Asteraceae Helichrysum italicum#NC 041458
Asterales Asteraceae Anaphalis sinica#NC 034648
Asterales Asteraceae Leontopodium leiolepis#NC 027835
Asterales Asteraceae Leontopodium nivale subsp. alpinum#DOVJ
Asterales Asteraceae Soliva sessilis#NC 034851
Asterales Asteraceae Ismelia carinata#NC 040110
Asterales Asteraceae Cota tinctoria#GUMF
Asterales Asteraceae Tanacetum parthenium#DUQG
Asterales Asteraceae Matricaria matricarioides#OAGK
Asterales Asteraceae Artemisia annua#MF623173
Asterales Asteraceae Chrysanthemum indicum#NC 020320
Asterales Asteraceae Dendranthema zawadskii#NC 040920
Asterales Asteraceae Tanacetum vulgare#NC 037388
Asterales Asteraceae Artemisia selengensis#NC 039647
Asterales Asteraceae Artemisia capillaris#KY073391
Asterales Asteraceae Artemisia gmelinii#KY073390
Asterales Asteraceae Artemisia frigida#NC 020607
Asterales Asteraceae Artemisia montana#NC 025910
Asterales Asteraceae Llerasia caucana#NC 034821
Asterales Asteraceae Oritrophium peruvianum#NC 034849
Asterales Asteraceae Eschenbachia blinii#NC 037605
Asterales Asteraceae Aster spathulifolius#NC 027434
Asterales Asteraceae Aster altaicus#NC 034996
Asterales Asteraceae Aster tataricus#XRCX
Asterales Asteraceae Aster indicus#NC 040126
Asterales Asteraceae Archibaccharis asperifolia#NC 034848
Asterales Asteraceae Solidago canadensis#TEZA
Asterales Asteraceae Conyza bonariensis#KX792499
Asterales Asteraceae Erigeron canadensis#MHYG
Asterales Asteraceae Erigeron speciosus#DESP
Asterales Asteraceae Lagenophora cuchumatanica#NC 034819
Asterales Asteraceae Heterothalamus alienus#NC 034855
Asterales Asteraceae Diplostephium meyenii#NC 034824
Asterales Asteraceae Diplostephium empetrifolium#NC 034891
Asterales Asteraceae Diplostephium juniperinum#NC 034813
Asterales Asteraceae Parastrephia quadrangularis#NC 034890
Asterales Asteraceae Diplostephium cinereum#NC 034869
Asterales Asteraceae Diplostephium foliosissimum#NC 034883
Asterales Asteraceae Diplostephium oxapampanum#NC 034815
Asterales Asteraceae Diplostephium haenkei#NC 034871
Asterales Asteraceae Diplostephium lechleri#NC 034816
Asterales Asteraceae Diplostephium callilepis#NC 034856
Asterales Asteraceae Diplostephium goodspeedii#NC 034901
Asterales Asteraceae Diplostephium gynoxyoides#NC 034862
Asterales Asteraceae Diplostephium hippophae#NC 034831
Asterales Asteraceae Diplostephium gnidioides#NC 034867
Asterales Asteraceae Diplostephium pulchrum#KX063942
Asterales Asteraceae Diplostephium sp.#KX063858
Asterales Asteraceae Aztecaster matudae#NC 034898
Asterales Asteraceae Westoniella kohkemperi#NC 034889
Asterales Asteraceae Laennecia sophiifolia#NC 034877
Asterales Asteraceae Baccharis genistelloides#NC 034852
Asterales Asteraceae Baccharis tricuneata#NC 034868
Asterales Asteraceae Diplostephium crypteriophyllum#NC 034881
Asterales Asteraceae Diplostephium ericoides#NC 034870
Asterales Asteraceae Diplostephium espinosae#NC 034880
Asterales Asteraceae Diplostephium hartwegii#NC 034832
Asterales Asteraceae Exostigma notobellidiastrum#NC 034864
Asterales Asteraceae Diplostephium spinulosum#NC 034823
Asterales Asteraceae Diplostephium serratifolium#NC 034826
Asterales Asteraceae Diplostephium azureum#NC 034882
Asterales Asteraceae Diplostephium sagasteguii#NC 034828
Asterales Asteraceae Diplostephium barclayanum#NC 034853
Asterales Asteraceae Laestadia muscicola#NC 034858
Asterales Asteraceae Hinterhubera ericoides#NC 034884
Asterales Asteraceae Blakiella bartsiifolia#NC 034866
Asterales Asteraceae Floscaldasia hypsophila#NC 034888
Asterales Asteraceae Diplostephium jelskii#NC 034811
Asterales Asteraceae Diplostephium cajamarquillense#NC 034872
Asterales Asteraceae Diplostephium glandulosum#NC 034854
Asterales Asteraceae Diplostephium oblanceolatum#NC 034830
Asterales Asteraceae Diplostephium coriaceum#NC 034899
Asterales Asteraceae Diplostephium romeroi#NC 034885
Asterales Asteraceae Diplostephium inesianum#NC 034895
Asterales Asteraceae Diplostephium colombianum#NC 034861

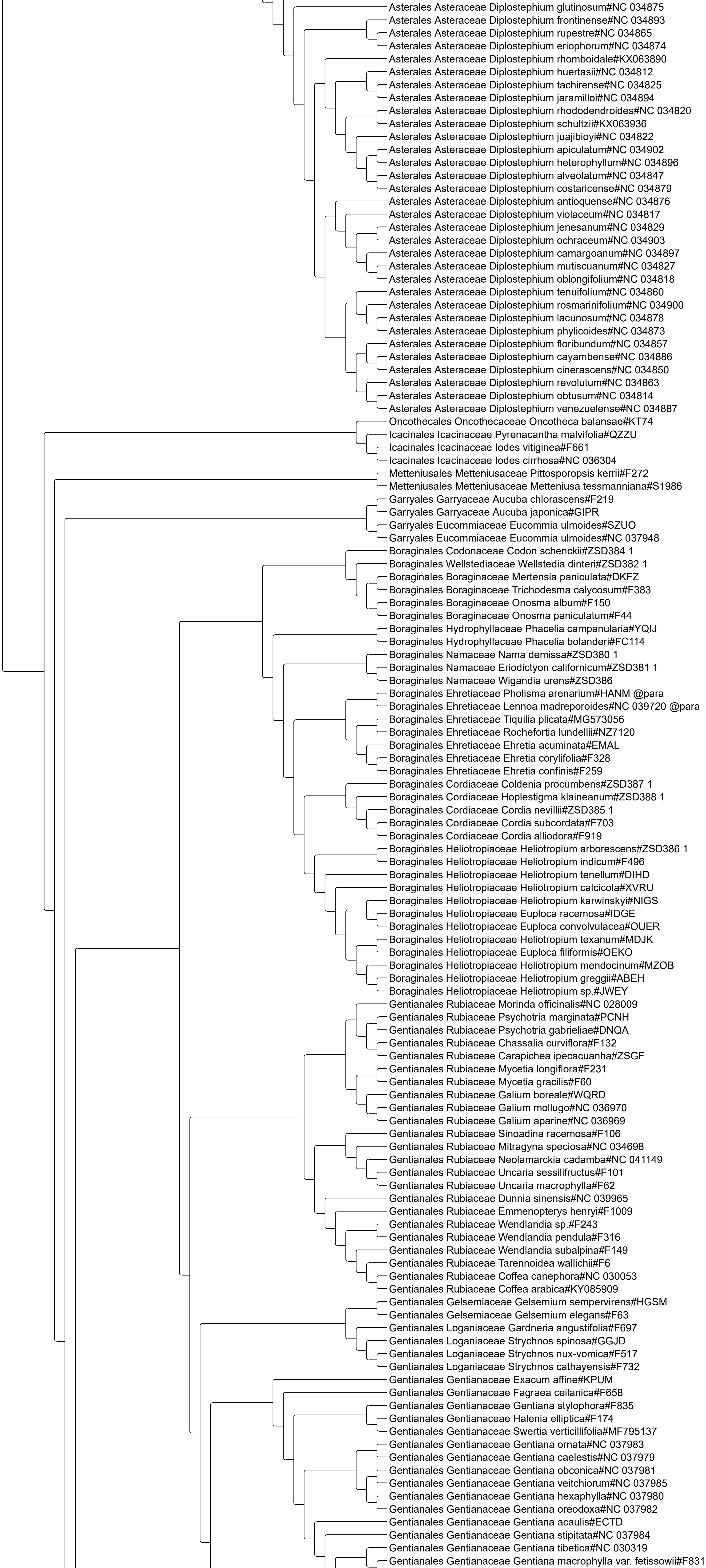
Asterales Asteraceae Diplostephium glutinosum#NC 034875
Asterales Asteraceae Diplostephium frontinense#NC 034893
Asterales Asteraceae Diplostephium rupestre#NC 034865
Asterales Asteraceae Diplostephium eriophorum#NC 034874
Asterales Asteraceae Diplostephium rhomboidale#KX063890
Asterales Asteraceae Diplostephium huertasii#NC 034812
Asterales Asteraceae Diplostephium tachirense#NC 034825
Asterales Asteraceae Diplostephium jaramilloi#NC 034894
Asterales Asteraceae Diplostephium rhododendroides#NC 034820
Asterales Asteraceae Diplostephium schultzii#KX063936
Asterales Asteraceae Diplostephium juajibioyi#NC 034822
Asterales Asteraceae Diplostephium apiculatum#NC 034902
Asterales Asteraceae Diplostephium heterophyllum#NC 034896
Asterales Asteraceae Diplostephium alveolatum#NC 034847
Asterales Asteraceae Diplostephium costaricense#NC 034879
Asterales Asteraceae Diplostephium antioquense#NC 034876
Asterales Asteraceae Diplostephium violaceum#NC 034817
Asterales Asteraceae Diplostephium jenesanum#NC 034829
Asterales Asteraceae Diplostephium ochraceum#NC 034903
Asterales Asteraceae Diplostephium camargoanum#NC 034897
Asterales Asteraceae Diplostephium mutiscuanum#NC 034827
Asterales Asteraceae Diplostephium oblongifolium#NC 034818
Asterales Asteraceae Diplostephium tenuifolium#NC 034860
Asterales Asteraceae Diplostephium rosmarinifolium#NC 034900
Asterales Asteraceae Diplostephium lacunosum#NC 034878
Asterales Asteraceae Diplostephium phylicoides#NC 034873
Asterales Asteraceae Diplostephium floribundum#NC 034857
Asterales Asteraceae Diplostephium cayambense#NC 034886
Asterales Asteraceae Diplostephium cinerascens#NC 034850
Asterales Asteraceae Diplostephium revolutum#NC 034863
Asterales Asteraceae Diplostephium obtusum#NC 034814
Asterales Asteraceae Diplostephium venezuelense#NC 034887
Oncothecales Oncothecaceae Oncotheca balansae#KT74
Icacinales Icacinaceae Pyrenacantha malvifolia#QZZU
Icacinales Icacinaceae Iodes vitiginea#F661
Icacinales Icacinaceae Iodes cirrhosa#NC 036304
Metteniusales Metteniusaceae Pittosporopsis kerrii#F272
Metteniusales Metteniusaceae Metteniusa tessmanniana#S1986
Garryales Garryaceae Aucuba chlorascens#F219
Garryales Garryaceae Aucuba japonica#GIPR
Garryales Eucommiaceae Eucommia ulmoides#SZUO
Garryales Eucommiaceae Eucommia ulmoides#NC 037948
Boraginales Codonaceae Codon schenckii#ZSD384 1
Boraginales Wellstediaceae Wellstedia dinteri#ZSD382 1
Boraginales Boraginaceae Mertensia paniculata#DKFZ
Boraginales Boraginaceae Trichodesma calycosum#F383
Boraginales Boraginaceae Onosma album#F150
Boraginales Boraginaceae Onosma paniculatum#F44
Boraginales Hydrophyllaceae Phacelia campanularia#YQIJ
Boraginales Hydrophyllaceae Phacelia bolanderi#FC114
Boraginales Namaceae Nama demissa#ZSD380 1
Boraginales Namaceae Eriodictyon californicum#ZSD381 1
Boraginales Namaceae Wigandia urens#ZSD386
Boraginales Ehretiaceae Pholisma arenarium#HANM @para
Boraginales Ehretiaceae Lennoa madreporoides#NC 039720 @para
Boraginales Ehretiaceae Tiquilia plicata#MG573056
Boraginales Ehretiaceae Rochefortia lundellii#NZ7120
Boraginales Ehretiaceae Ehretia acuminata#EMAL
Boraginales Ehretiaceae Ehretia corylifolia#F328
Boraginales Ehretiaceae Ehretia confinis#F259
Boraginales Cordiaceae Coldenia procumbens#ZSD387 1
Boraginales Cordiaceae Hoplestigma klaineanum#ZSD388 1
Boraginales Cordiaceae Cordia nevillii#ZSD385 1
Boraginales Cordiaceae Cordia subcordata#F703
Boraginales Cordiaceae Cordia alliodora#F919
Boraginales Heliotropiaceae Heliotropium arborescens#ZSD386 1
Boraginales Heliotropiaceae Heliotropium indicum#F496
Boraginales Heliotropiaceae Heliotropium tenellum#DIHD
Boraginales Heliotropiaceae Heliotropium calcicola#XVRU
Boraginales Heliotropiaceae Heliotropium karwinskyi#NIGS
Boraginales Heliotropiaceae Euploca racemosa#IDGE
Boraginales Heliotropiaceae Euploca convolvulacea#OUER
Boraginales Heliotropiaceae Heliotropium texanum#MDJK
Boraginales Heliotropiaceae Euploca filiformis#OEKO
Boraginales Heliotropiaceae Heliotropium mendocinum#MZOB
Boraginales Heliotropiaceae Heliotropium greggii#ABEH
Boraginales Heliotropiaceae Heliotropium sp.#JWEY
Gentianales Rubiaceae Morinda officinalis#NC 028009
Gentianales Rubiaceae Psychotria marginata#PCNH
Gentianales Rubiaceae Psychotria gabrieliae#DNQA
Gentianales Rubiaceae Chassalia curviflora#F132
Gentianales Rubiaceae Carapichea ipecacuanha#ZSGF
Gentianales Rubiaceae Mycetia longiflora#F231
Gentianales Rubiaceae Mycetia gracilis#F60
Gentianales Rubiaceae Galium boreale#WQRD
Gentianales Rubiaceae Galium mollugo#NC 036970
Gentianales Rubiaceae Galium aparine#NC 036969
Gentianales Rubiaceae Sinoadina racemosa#F106
Gentianales Rubiaceae Mitragyna speciosa#NC 034698
Gentianales Rubiaceae Neolamarckia cadamba#NC 041149
Gentianales Rubiaceae Uncaria sessilifructus#F101
Gentianales Rubiaceae Uncaria macrophylla#F62
Gentianales Rubiaceae Dunnia sinensis#NC 039965
Gentianales Rubiaceae Emmenopterys henryi#F1009
Gentianales Rubiaceae Wendlandia sp.#F243
Gentianales Rubiaceae Wendlandia pendula#F316
Gentianales Rubiaceae Wendlandia subalpina#F149
Gentianales Rubiaceae Tarennoidea wallichii#F6
Gentianales Rubiaceae Coffea canephora#NC 030053
Gentianales Rubiaceae Coffea arabica#KY085909
Gentianales Gelsemiaceae Gelsemium sempervirens#HGSM
Gentianales Gelsemiaceae Gelsemium elegans#F63
Gentianales Loganiaceae Gardneria angustifolia#F697
Gentianales Loganiaceae Strychnos spinosa#GGJD
Gentianales Loganiaceae Strychnos nux-vomica#F517
Gentianales Loganiaceae Strychnos cathayensis#F732
Gentianales Gentianaceae Exacum affine#KPUM
Gentianales Gentianaceae Fagraea ceilanica#F658
Gentianales Gentianaceae Gentiana stylophora#F835
Gentianales Gentianaceae Halenia elliptica#F174
Gentianales Gentianaceae Swertia verticillifolia#MF795137
Gentianales Gentianaceae Gentiana ornata#NC 037983
Gentianales Gentianaceae Gentiana caelestis#NC 037979
Gentianales Gentianaceae Gentiana obconica#NC 037981
Gentianales Gentianaceae Gentiana veitchiorum#NC 037985
Gentianales Gentianaceae Gentiana hexaphylla#NC 037980
Gentianales Gentianaceae Gentiana oreodoxa#NC 037982
Gentianales Gentianaceae Gentiana acaulis#ECTD
Gentianales Gentianaceae Gentiana stipitata#NC 037984
Gentianales Gentianaceae Gentiana tibetica#NC 030319
Gentianales Gentianaceae Gentiana macrophylla var. fetissowii#F831

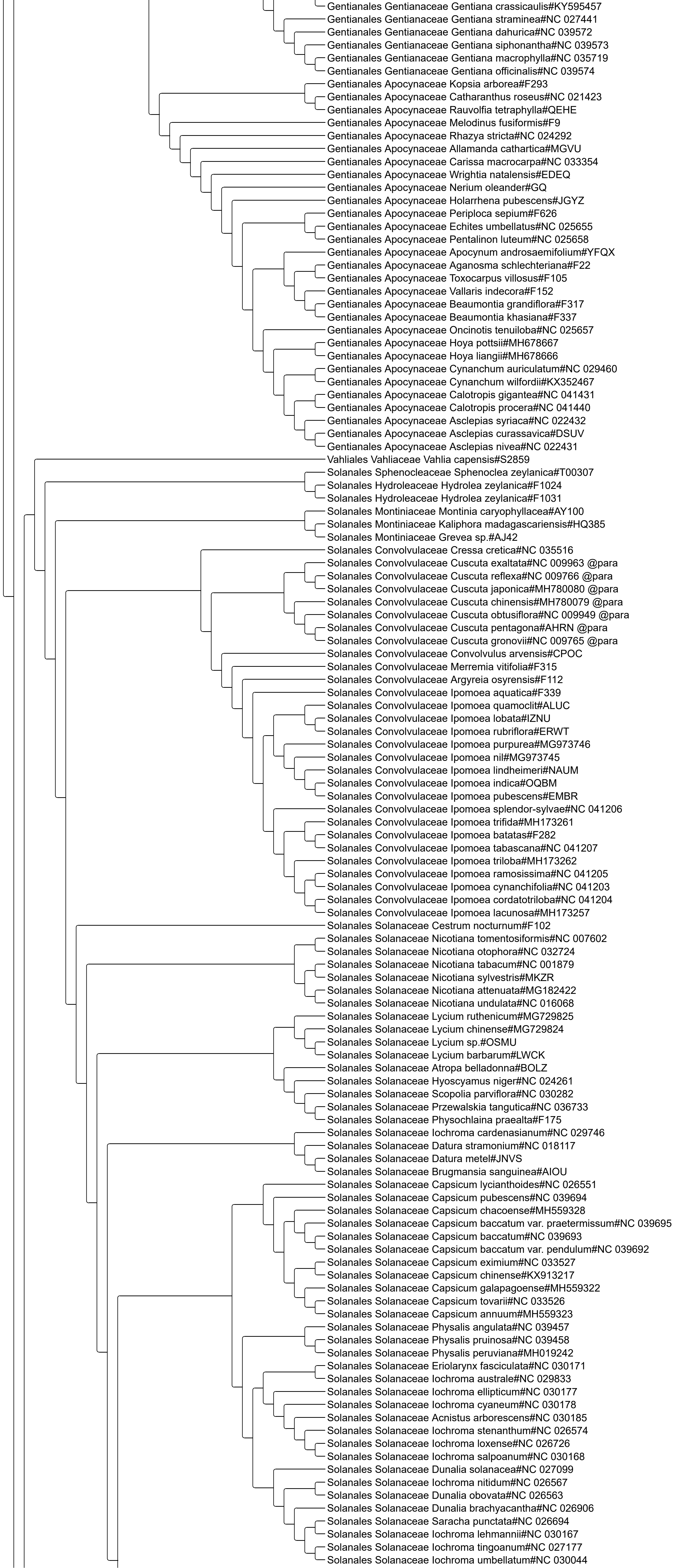

Gentianales Gentianaceae Gentiana crassicaulis#KY595457
Gentianales Gentianaceae Gentiana straminea#NC 027441
Gentianales Gentianaceae Gentiana dahurica#NC 039572
Gentianales Gentianaceae Gentiana siphonantha#NC 039573
Gentianales Gentianaceae Gentiana macrophylla#NC 035719
Gentianales Gentianaceae Gentiana officinalis#NC 039574
Gentianales Apocynaceae Kopsia arborea#F293
Gentianales Apocynaceae Catharanthus roseus#NC 021423
Gentianales Apocynaceae Rauvolfia tetraphylla#QEHE
Gentianales Apocynaceae Melodinus fusiformis#F9
Gentianales Apocynaceae Rhazya stricta#NC 024292
Gentianales Apocynaceae Allamanda cathartica#MGVU
Gentianales Apocynaceae Carissa macrocarpa#NC 033354
Gentianales Apocynaceae Wrightia natalensis#EDEQ
Gentianales Apocynaceae Nerium oleander#GQ
Gentianales Apocynaceae Holarrhena pubescens#JGYZ
Gentianales Apocynaceae Periploca sepium#F626
Gentianales Apocynaceae Echites umbellatus#NC 025655
Gentianales Apocynaceae Pentalinon luteum#NC 025658
Gentianales Apocynaceae Apocynum androsaemifolium#YFQX
Gentianales Apocynaceae Aganosma schlechteriana#F22
Gentianales Apocynaceae Toxocarpus villosus#F105
Gentianales Apocynaceae Vallaris indecora#F152
Gentianales Apocynaceae Beaumontia grandiflora#F317
Gentianales Apocynaceae Beaumontia khasiana#F337
Gentianales Apocynaceae Oncinotis tenuiloba#NC 025657
Gentianales Apocynaceae Hoya pottsii#MH678667
Gentianales Apocynaceae Hoya liangii#MH678666
Gentianales Apocynaceae Cynanchum auriculatum#NC 029460
Gentianales Apocynaceae Cynanchum wilfordii#KX352467
Gentianales Apocynaceae Calotropis gigantea#NC 041431
Gentianales Apocynaceae Calotropis procera#NC 041440
Gentianales Apocynaceae Asclepias syriaca#NC 022432
Gentianales Apocynaceae Asclepias curassavica#DSUV
Gentianales Apocynaceae Asclepias nivea#NC 022431
Vahliales Vahliaceae Vahlia capensis#S2859
Solanales Sphenocleaceae Sphenoclea zeylanica#T00307
Solanales Hydroleaceae Hydrolea zeylanica#F1024
Solanales Hydroleaceae Hydrolea zeylanica#F1031
Solanales Montiniaceae Montinia caryophyllacea#AY100
Solanales Montiniaceae Kaliphora madagascariensis#HQ385
Solanales Montiniaceae Grevea sp.#AJ42
Solanales Convolvulaceae Cressa cretica#NC 035516
Solanales Convolvulaceae Cuscuta exaltata#NC 009963 @para
Solanales Convolvulaceae Cuscuta reflexa#NC 009766 @para
Solanales Convolvulaceae Cuscuta japonica#MH780080 @para
Solanales Convolvulaceae Cuscuta chinensis#MH780079 @para
Solanales Convolvulaceae Cuscuta obtusiflora#NC 009949 @para
Solanales Convolvulaceae Cuscuta pentagona#AHRN @para
Solanales Convolvulaceae Cuscuta gronovii#NC 009765 @para
Solanales Convolvulaceae Convolvulus arvensis#CPOC
Solanales Convolvulaceae Merremia vitifolia#F315
Solanales Convolvulaceae Argyreia osyrensis#F112
Solanales Convolvulaceae Ipomoea aquatica#F339
Solanales Convolvulaceae Ipomoea quamoclit#ALUC
Solanales Convolvulaceae Ipomoea lobata#IZNU
Solanales Convolvulaceae Ipomoea rubriflora#ERWT
Solanales Convolvulaceae Ipomoea purpurea#MG973746
Solanales Convolvulaceae Ipomoea nil#MG973745
Solanales Convolvulaceae Ipomoea lindheimeri#NAUM
Solanales Convolvulaceae Ipomoea indica#OQBM
Solanales Convolvulaceae Ipomoea pubescens#EMBR
Solanales Convolvulaceae Ipomoea splendor-sylvae#NC 041206
Solanales Convolvulaceae Ipomoea trifida#MH173261
Solanales Convolvulaceae Ipomoea batatas#F282
Solanales Convolvulaceae Ipomoea tabascana#NC 041207
Solanales Convolvulaceae Ipomoea triloba#MH173262
Solanales Convolvulaceae Ipomoea ramosissima#NC 041205
Solanales Convolvulaceae Ipomoea cynanchifolia#NC 041203
Solanales Convolvulaceae Ipomoea cordatotriloba#NC 041204
Solanales Convolvulaceae Ipomoea lacunosa#MH173257
Solanales Solanaceae Cestrum nocturnum#F102
Solanales Solanaceae Nicotiana tomentosiformis#NC 007602
Solanales Solanaceae Nicotiana otophora#NC 032724
Solanales Solanaceae Nicotiana tabacum#NC 001879
Solanales Solanaceae Nicotiana sylvestris#MKZR
Solanales Solanaceae Nicotiana attenuata#MG182422
Solanales Solanaceae Nicotiana undulata#NC 016068
Solanales Solanaceae Lycium ruthenicum#MG729825
Solanales Solanaceae Lycium chinense#MG729824
Solanales Solanaceae Lycium sp.#OSMU
Solanales Solanaceae Lycium barbarum#LWCK
Solanales Solanaceae Atropa belladonna#BOLZ
Solanales Solanaceae Hyoscyamus niger#NC 024261
Solanales Solanaceae Scopolia parviflora#NC 030282
Solanales Solanaceae Przewalskia tangutica#NC 036733
Solanales Solanaceae Physochlaina praealta#F175
Solanales Solanaceae Iochroma cardenasianum#NC 029746
Solanales Solanaceae Datura stramonium#NC 018117
Solanales Solanaceae Datura metel#JNVS
Solanales Solanaceae Brugmansia sanguinea#AIOU
Solanales Solanaceae Capsicum lycianthoides#NC 026551
Solanales Solanaceae Capsicum pubescens#NC 039694
Solanales Solanaceae Capsicum chacoense#MH559328
Solanales Solanaceae Capsicum baccatum var. praetermissum#NC 039695
Solanales Solanaceae Capsicum baccatum#NC 039693
Solanales Solanaceae Capsicum baccatum var. pendulum#NC 039692
Solanales Solanaceae Capsicum eximium#NC 033527
Solanales Solanaceae Capsicum chinense#KX913217
Solanales Solanaceae Capsicum galapagoense#MH559322
Solanales Solanaceae Capsicum tovarii#NC 033526
Solanales Solanaceae Capsicum annuum#MH559323
Solanales Solanaceae Physalis angulata#NC 039457
Solanales Solanaceae Physalis pruinosa#NC 039458
Solanales Solanaceae Physalis peruviana#MH019242
Solanales Solanaceae Eriolarynx fasciculata#NC 030171
Solanales Solanaceae Iochroma australe#NC 029833
Solanales Solanaceae Iochroma ellipticum#NC 030177
Solanales Solanaceae Iochroma cyaneum#NC 030178
Solanales Solanaceae Acnistus arborescens#NC 030185
Solanales Solanaceae Iochroma stenanthum#NC 026574
Solanales Solanaceae Iochroma loxense#NC 026726
Solanales Solanaceae Iochroma salpoanum#NC 030168
Solanales Solanaceae Dunalia solanacea#NC 027099
Solanales Solanaceae Iochroma nitidum#NC 026567
Solanales Solanaceae Dunalia obovata#NC 026563
Solanales Solanaceae Dunalia brachyacantha#NC 026906
Solanales Solanaceae Saracha punctata#NC 026694
Solanales Solanaceae Iochroma lehmannii#NC 030167
Solanales Solanaceae Iochroma tingoanum#NC 027177
Solanales Solanaceae Iochroma umbellatum#NC 030044

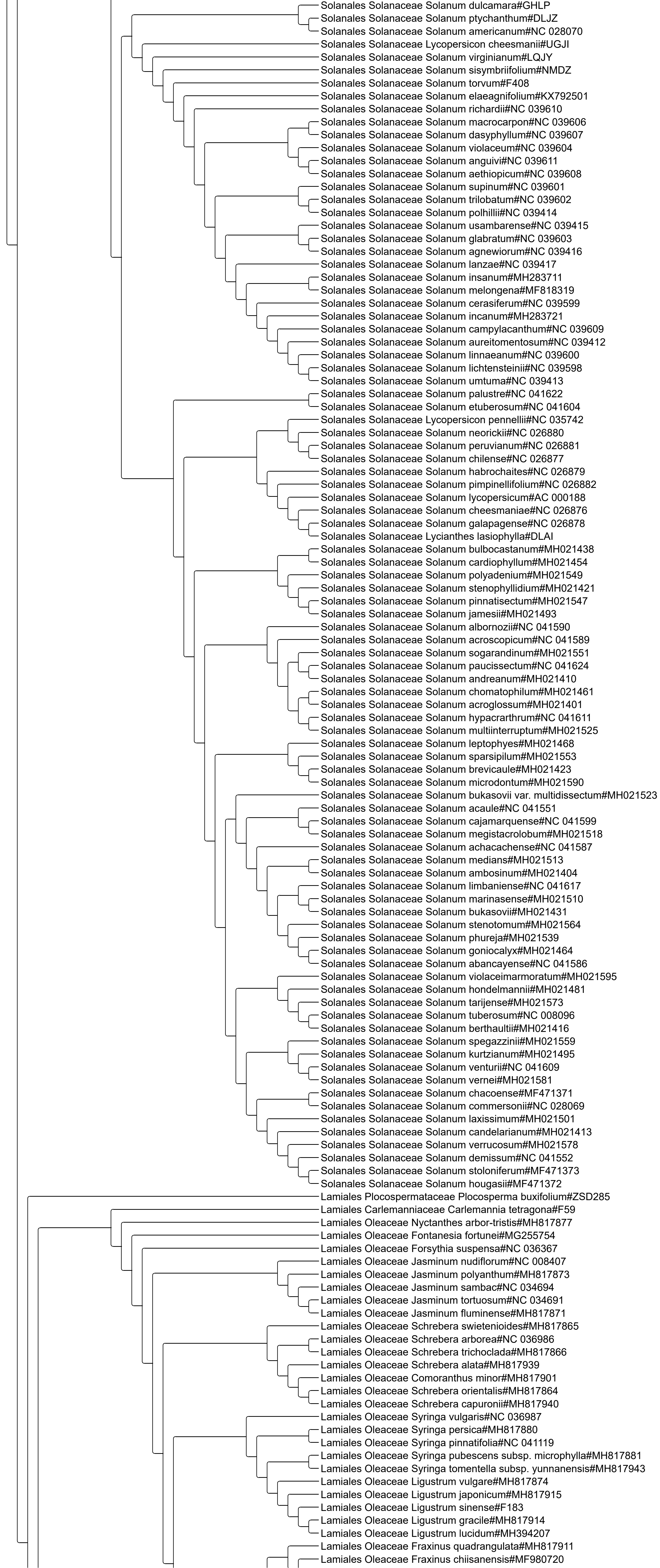
Solanales Solanaceae Solanum dulcamara#GHLP
Solanales Solanaceae Solanum ptychanthum#DLJZ
Solanales Solanaceae Solanum americanum#NC 028070
Solanales Solanaceae Lycopersicon cheesmanii#UGJI
Solanales Solanaceae Solanum virginianum#LQJY
Solanales Solanaceae Solanum sisymbriifolium#NMDZ
Solanales Solanaceae Solanum torvum#F408
Solanales Solanaceae Solanum elaeagnifolium#KX792501
Solanales Solanaceae Solanum richardii#NC 039610
Solanales Solanaceae Solanum macrocarpon#NC 039606
Solanales Solanaceae Solanum dasyphyllum#NC 039607
Solanales Solanaceae Solanum violaceum#NC 039604
Solanales Solanaceae Solanum anguivi#NC 039611
Solanales Solanaceae Solanum aethiopicum#NC 039608
Solanales Solanaceae Solanum supinum#NC 039601
Solanales Solanaceae Solanum trilobatum#NC 039602
Solanales Solanaceae Solanum polhillii#NC 039414
Solanales Solanaceae Solanum usambarense#NC 039415
Solanales Solanaceae Solanum glabratum#NC 039603
Solanales Solanaceae Solanum agnewiorum#NC 039416
Solanales Solanaceae Solanum lanzae#NC 039417
Solanales Solanaceae Solanum insanum#MH283711
Solanales Solanaceae Solanum melongena#MF818319
Solanales Solanaceae Solanum cerasiferum#NC 039599
Solanales Solanaceae Solanum incanum#MH283721
Solanales Solanaceae Solanum campylacanthum#NC 039609
Solanales Solanaceae Solanum aureitomentosum#NC 039412
Solanales Solanaceae Solanum linnaeanum#NC 039600
Solanales Solanaceae Solanum lichtensteinii#NC 039598
Solanales Solanaceae Solanum umtuma#NC 039413
Solanales Solanaceae Solanum palustre#NC 041622
Solanales Solanaceae Solanum etuberosum#NC 041604
Solanales Solanaceae Lycopersicon pennellii#NC 035742
Solanales Solanaceae Solanum neorickii#NC 026880
Solanales Solanaceae Solanum peruvianum#NC 026881
Solanales Solanaceae Solanum chilense#NC 026877
Solanales Solanaceae Solanum habrochaites#NC 026879
Solanales Solanaceae Solanum pimpinellifolium#NC 026882
Solanales Solanaceae Solanum lycopersicum#AC 000188
Solanales Solanaceae Solanum cheesmaniae#NC 026876
Solanales Solanaceae Solanum galapagense#NC 026878
Solanales Solanaceae Lycianthes lasiophylla#DLAI
Solanales Solanaceae Solanum bulbocastanum#MH021438
Solanales Solanaceae Solanum cardiophyllum#MH021454
Solanales Solanaceae Solanum polyadenium#MH021549
Solanales Solanaceae Solanum stenophyllidium#MH021421
Solanales Solanaceae Solanum pinnatisectum#MH021547
Solanales Solanaceae Solanum jamesii#MH021493
Solanales Solanaceae Solanum albornozii#NC 041590
Solanales Solanaceae Solanum acroscopicum#NC 041589
Solanales Solanaceae Solanum sogarandinum#MH021551
Solanales Solanaceae Solanum paucissectum#NC 041624
Solanales Solanaceae Solanum andreanum#MH021410
Solanales Solanaceae Solanum chomatophilum#MH021461
Solanales Solanaceae Solanum acroglossum#MH021401
Solanales Solanaceae Solanum hypacrarthrum#NC 041611
Solanales Solanaceae Solanum multiinterruptum#MH021525
Solanales Solanaceae Solanum leptophyes#MH021468
Solanales Solanaceae Solanum sparsipilum#MH021553
Solanales Solanaceae Solanum brevicaule#MH021423
Solanales Solanaceae Solanum microdontum#MH021590
Solanales Solanaceae Solanum bukasovii var. multidissectum#MH021523
Solanales Solanaceae Solanum acaule#NC 041551
Solanales Solanaceae Solanum cajamarquense#NC 041599
Solanales Solanaceae Solanum megistacrolobum#MH021518
Solanales Solanaceae Solanum achacachense#NC 041587
Solanales Solanaceae Solanum medians#MH021513
Solanales Solanaceae Solanum ambosinum#MH021404
Solanales Solanaceae Solanum limbaniense#NC 041617
Solanales Solanaceae Solanum marinasense#MH021510
Solanales Solanaceae Solanum bukasovii#MH021431
Solanales Solanaceae Solanum stenotomum#MH021564
Solanales Solanaceae Solanum phureja#MH021539
Solanales Solanaceae Solanum goniocalyx#MH021464
Solanales Solanaceae Solanum abancayense#NC 041586
Solanales Solanaceae Solanum violaceimarmoratum#MH021595
Solanales Solanaceae Solanum hondelmannii#MH021481
Solanales Solanaceae Solanum tarijense#MH021573
Solanales Solanaceae Solanum tuberosum#NC 008096
Solanales Solanaceae Solanum berthaultii#MH021416
Solanales Solanaceae Solanum spegazzinii#MH021559
Solanales Solanaceae Solanum kurtzianum#MH021495
Solanales Solanaceae Solanum venturii#NC 041609
Solanales Solanaceae Solanum vernei#MH021581
Solanales Solanaceae Solanum chacoense#MF471371
Solanales Solanaceae Solanum commersonii#NC 028069
Solanales Solanaceae Solanum laxissimum#MH021501
Solanales Solanaceae Solanum candelarianum#MH021413
Solanales Solanaceae Solanum verrucosum#MH021578
Solanales Solanaceae Solanum demissum#NC 041552
Solanales Solanaceae Solanum stoloniferum#MF471373
Solanales Solanaceae Solanum hougasii#MF471372
Lamiales Plocospermataceae Plocosperma buxifolium#ZSD285
Lamiales Carlemanniaceae Carlemannia tetragona#F59
Lamiales Oleaceae Nyctanthes arbor-tristis#MH817877
Lamiales Oleaceae Fontanesia fortunei#MG255754
Lamiales Oleaceae Forsythia suspensa#NC 036367
Lamiales Oleaceae Jasminum nudiflorum#NC 008407
Lamiales Oleaceae Jasminum polyanthum#MH817873
Lamiales Oleaceae Jasminum sambac#NC 034694
Lamiales Oleaceae Jasminum tortuosum#NC 034691
Lamiales Oleaceae Jasminum fluminense#MH817871
Lamiales Oleaceae Schrebera swietenioides#MH817865
Lamiales Oleaceae Schrebera arborea#NC 036986
Lamiales Oleaceae Schrebera trichoclada#MH817866
Lamiales Oleaceae Schrebera alata#MH817939
Lamiales Oleaceae Comoranthus minor#MH817901
Lamiales Oleaceae Schrebera orientalis#MH817864
Lamiales Oleaceae Schrebera capuronii#MH817940
Lamiales Oleaceae Syringa vulgaris#NC 036987
Lamiales Oleaceae Syringa persica#MH817880
Lamiales Oleaceae Syringa pinnatifolia#NC 041119
Lamiales Oleaceae Syringa pubescens subsp. microphylla#MH817881
Lamiales Oleaceae Syringa tomentella subsp. yunnanensis#MH817943
Lamiales Oleaceae Ligustrum vulgare#MH817874
Lamiales Oleaceae Ligustrum japonicum#MH817915
Lamiales Oleaceae Ligustrum sinense#F183
Lamiales Oleaceae Ligustrum gracile#MH817914
Lamiales Oleaceae Ligustrum lucidum#MH394207
Lamiales Oleaceae Fraxinus quadrangulata#MH817911
Lamiales Oleaceae Fraxinus chiisanensis#MF980720

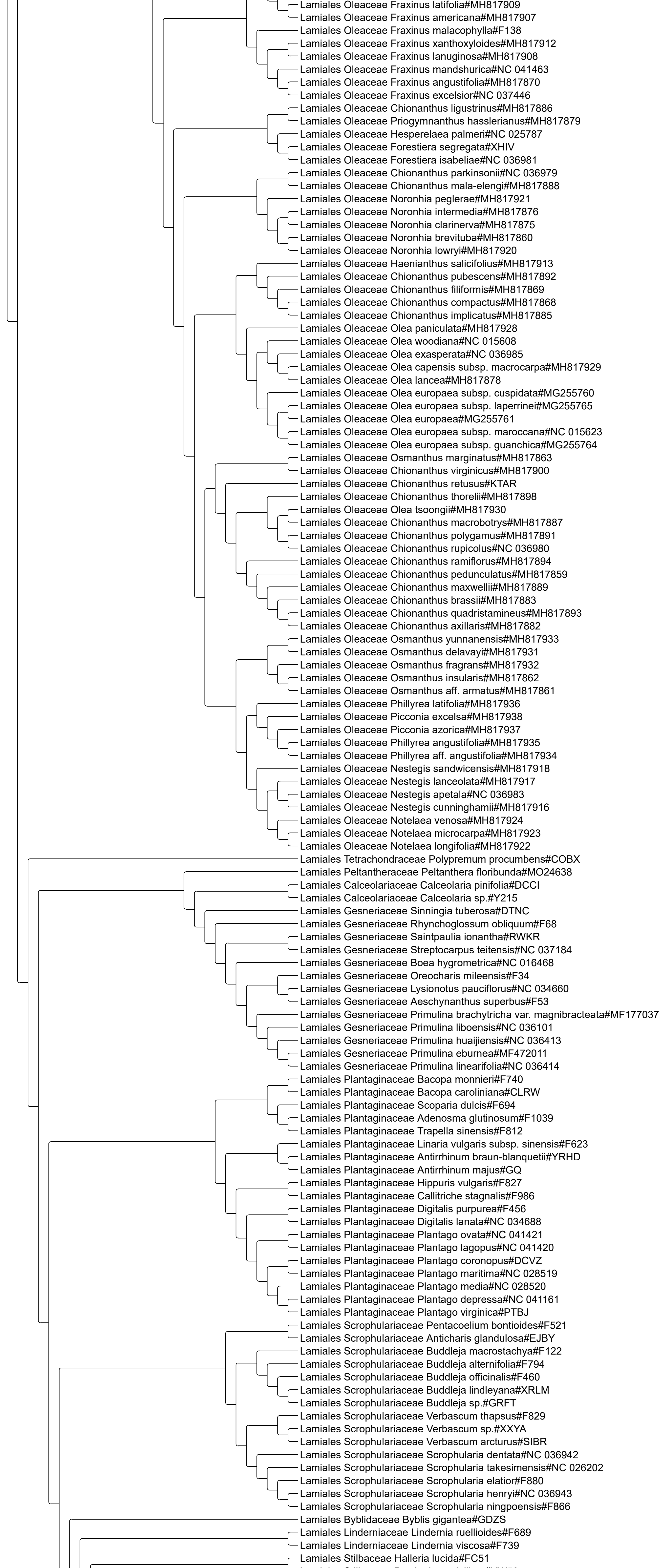
Lamiales Oleaceae Fraxinus latifolia#MH817909
Lamiales Oleaceae Fraxinus americana#MH817907
Lamiales Oleaceae Fraxinus malacophylla#F138
Lamiales Oleaceae Fraxinus xanthoxyloides#MH817912
Lamiales Oleaceae Fraxinus lanuginosa#MH817908
Lamiales Oleaceae Fraxinus mandshurica#NC 041463
Lamiales Oleaceae Fraxinus angustifolia#MH817870
Lamiales Oleaceae Fraxinus excelsior#NC 037446
Lamiales Oleaceae Chionanthus ligustrinus#MH817886
Lamiales Oleaceae Priogymnanthus hasslerianus#MH817879
Lamiales Oleaceae Hesperelaea palmeri#NC 025787
Lamiales Oleaceae Forestiera segregata#XHIV
Lamiales Oleaceae Forestiera isabeliae#NC 036981
Lamiales Oleaceae Chionanthus parkinsonii#NC 036979
Lamiales Oleaceae Chionanthus mala-elengi#MH817888
Lamiales Oleaceae Noronhia peglerae#MH817921
Lamiales Oleaceae Noronhia intermedia#MH817876
Lamiales Oleaceae Noronhia clarinerva#MH817875
Lamiales Oleaceae Noronhia brevituba#MH817860
Lamiales Oleaceae Noronhia lowryi#MH817920
Lamiales Oleaceae Haenianthus salicifolius#MH817913
Lamiales Oleaceae Chionanthus pubescens#MH817892
Lamiales Oleaceae Chionanthus filiformis#MH817869
Lamiales Oleaceae Chionanthus compactus#MH817868
Lamiales Oleaceae Chionanthus implicatus#MH817885
Lamiales Oleaceae Olea paniculata#MH817928
Lamiales Oleaceae Olea woodiana#NC 015608
Lamiales Oleaceae Olea exasperata#NC 036985
Lamiales Oleaceae Olea capensis subsp. macrocarpa#MH817929
Lamiales Oleaceae Olea lancea#MH817878
Lamiales Oleaceae Olea europaea subsp. cuspidata#MG255760
Lamiales Oleaceae Olea europaea subsp. laperrinei#MG255765
Lamiales Oleaceae Olea europaea#MG255761
Lamiales Oleaceae Olea europaea subsp. maroccana#NC 015623
Lamiales Oleaceae Olea europaea subsp. guanchica#MG255764
Lamiales Oleaceae Osmanthus marginatus#MH817863
Lamiales Oleaceae Chionanthus virginicus#MH817900
Lamiales Oleaceae Chionanthus retusus#KTAR
Lamiales Oleaceae Chionanthus thorelii#MH817898
Lamiales Oleaceae Olea tsoongii#MH817930
Lamiales Oleaceae Chionanthus macrobotrys#MH817887
Lamiales Oleaceae Chionanthus polygamus#MH817891
Lamiales Oleaceae Chionanthus rupicolus#NC 036980
Lamiales Oleaceae Chionanthus ramiflorus#MH817894
Lamiales Oleaceae Chionanthus pedunculatus#MH817859
Lamiales Oleaceae Chionanthus maxwellii#MH817889
Lamiales Oleaceae Chionanthus brassii#MH817883
Lamiales Oleaceae Chionanthus quadristamineus#MH817893
Lamiales Oleaceae Chionanthus axillaris#MH817882
Lamiales Oleaceae Osmanthus yunnanensis#MH817933
Lamiales Oleaceae Osmanthus delavayi#MH817931
Lamiales Oleaceae Osmanthus fragrans#MH817932
Lamiales Oleaceae Osmanthus insularis#MH817862
Lamiales Oleaceae Osmanthus aff. armatus#MH817861
Lamiales Oleaceae Phillyrea latifolia#MH817936
Lamiales Oleaceae Picconia excelsa#MH817938
Lamiales Oleaceae Picconia azorica#MH817937
Lamiales Oleaceae Phillyrea angustifolia#MH817935
Lamiales Oleaceae Phillyrea aff. angustifolia#MH817934
Lamiales Oleaceae Nestegis sandwicensis#MH817918
Lamiales Oleaceae Nestegis lanceolata#MH817917
Lamiales Oleaceae Nestegis apetala#NC 036983
Lamiales Oleaceae Nestegis cunninghamii#MH817916
Lamiales Oleaceae Notelaea venosa#MH817924
Lamiales Oleaceae Notelaea microcarpa#MH817923
Lamiales Oleaceae Notelaea longifolia#MH817922
Lamiales Tetrachondraceae Polypremum procumbens#COBX
Lamiales Peltantheraceae Peltanthera floribunda#MO24638
Lamiales Calceolariaceae Calceolaria pinifolia#DCCI
Lamiales Calceolariaceae Calceolaria sp.#Y215
Lamiales Gesneriaceae Sinningia tuberosa#DTNC
Lamiales Gesneriaceae Rhynchoglossum obliquum#F68
Lamiales Gesneriaceae Saintpaulia ionantha#RWKR
Lamiales Gesneriaceae Streptocarpus teitensis#NC 037184
Lamiales Gesneriaceae Boea hygrometrica#NC 016468
Lamiales Gesneriaceae Oreocharis mileensis#F34
Lamiales Gesneriaceae Lysionotus pauciflorus#NC 034660
Lamiales Gesneriaceae Aeschynanthus superbus#F53
Lamiales Gesneriaceae Primulina brachytricha var. magnibracteata#MF177037
Lamiales Gesneriaceae Primulina liboensis#NC 036101
Lamiales Gesneriaceae Primulina huaijiensis#NC 036413
Lamiales Gesneriaceae Primulina eburnea#MF472011
Lamiales Gesneriaceae Primulina linearifolia#NC 036414
Lamiales Plantaginaceae Bacopa monnieri#F740
Lamiales Plantaginaceae Bacopa caroliniana#CLRW
Lamiales Plantaginaceae Scoparia dulcis#F694
Lamiales Plantaginaceae Adenosma glutinosum#F1039
Lamiales Plantaginaceae Trapella sinensis#F812
Lamiales Plantaginaceae Linaria vulgaris subsp. sinensis#F623
Lamiales Plantaginaceae Antirrhinum braun-blanquetii#YRHD
Lamiales Plantaginaceae Antirrhinum majus#GQ
Lamiales Plantaginaceae Hippuris vulgaris#F827
Lamiales Plantaginaceae Callitriche stagnalis#F986
Lamiales Plantaginaceae Digitalis purpurea#F456
Lamiales Plantaginaceae Digitalis lanata#NC 034688
Lamiales Plantaginaceae Plantago ovata#NC 041421
Lamiales Plantaginaceae Plantago lagopus#NC 041420
Lamiales Plantaginaceae Plantago coronopus#DCVZ
Lamiales Plantaginaceae Plantago maritima#NC 028519
Lamiales Plantaginaceae Plantago media#NC 028520
Lamiales Plantaginaceae Plantago depressa#NC 041161
Lamiales Plantaginaceae Plantago virginica#PTBJ
Lamiales Scrophulariaceae Pentacoelium bontioides#F521
Lamiales Scrophulariaceae Anticharis glandulosa#EJBY
Lamiales Scrophulariaceae Buddleja macrostachya#F122
Lamiales Scrophulariaceae Buddleja alternifolia#F794
Lamiales Scrophulariaceae Buddleja officinalis#F460
Lamiales Scrophulariaceae Buddleja lindleyana#XRLM
Lamiales Scrophulariaceae Buddleja sp.#GRFT
Lamiales Scrophulariaceae Verbascum thapsus#F829
Lamiales Scrophulariaceae Verbascum sp.#XXYA
Lamiales Scrophulariaceae Verbascum arcturus#SIBR
Lamiales Scrophulariaceae Scrophularia dentata#NC 036942
Lamiales Scrophulariaceae Scrophularia takesimensis#NC 026202
Lamiales Scrophulariaceae Scrophularia elatior#F880
Lamiales Scrophulariaceae Scrophularia henryi#NC 036943
Lamiales Scrophulariaceae Scrophularia ningpoensis#F866
Lamiales Byblidaceae Byblis gigantea#GDZS
Lamiales Linderniaceae Lindernia ruellioides#F689
Lamiales Linderniaceae Lindernia viscosa#F739
Lamiales Stilbaceae Halleria lucida#FC51

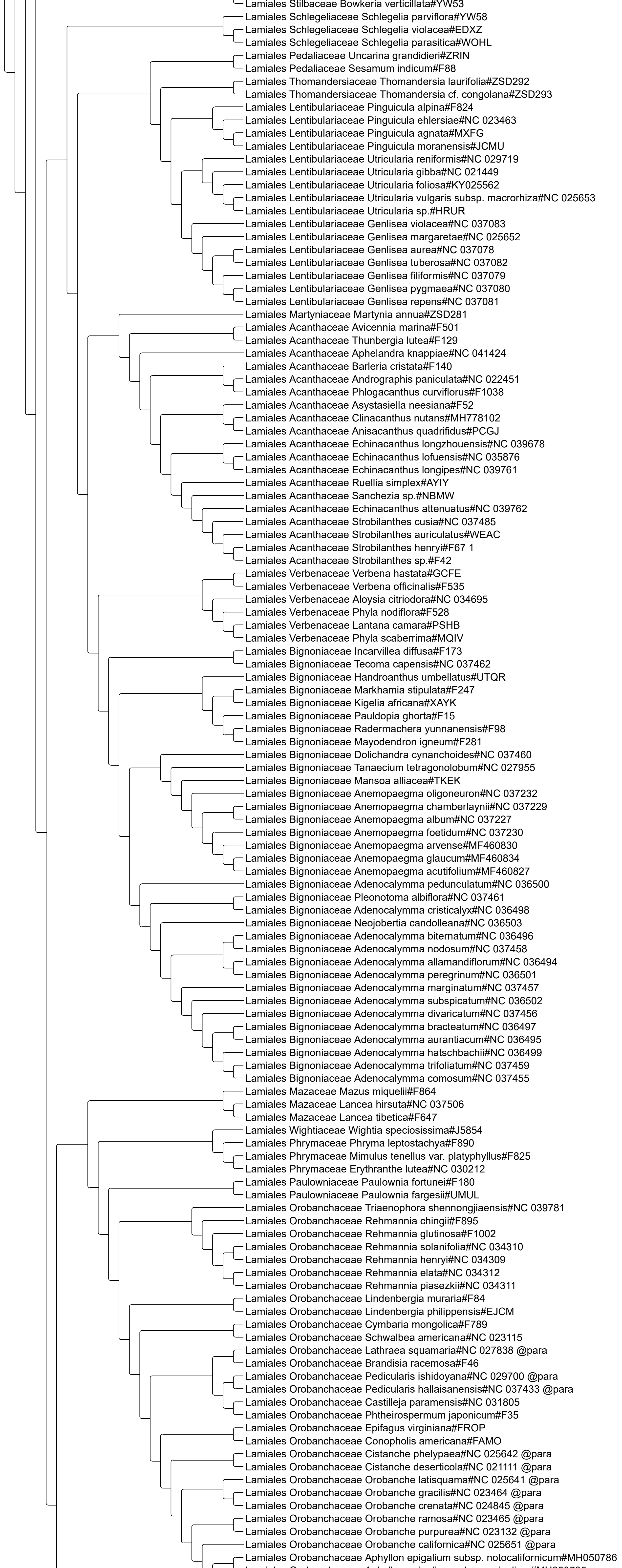

Lamiales Stilbaceae Bowkeria verticillata#YW53
Lamiales Schlegeliaceae Schlegelia parviflora#YW58
Lamiales Schlegeliaceae Schlegelia violacea#EDXZ
Lamiales Schlegeliaceae Schlegelia parasitica#WOHL
Lamiales Pedaliaceae Uncarina grandidieri#ZRIN
Lamiales Pedaliaceae Sesamum indicum#F88
Lamiales Thomandersiaceae Thomandersia laurifolia#ZSD292
Lamiales Thomandersiaceae Thomandersia cf. congolana#ZSD293
Lamiales Lentibulariaceae Pinguicula alpina#F824
Lamiales Lentibulariaceae Pinguicula ehlersiae#NC 023463
Lamiales Lentibulariaceae Pinguicula agnata#MXFG
Lamiales Lentibulariaceae Pinguicula moranensis#JCMU
Lamiales Lentibulariaceae Utricularia reniformis#NC 029719
Lamiales Lentibulariaceae Utricularia gibba#NC 021449
Lamiales Lentibulariaceae Utricularia foliosa#KY025562
Lamiales Lentibulariaceae Utricularia vulgaris subsp. macrorhiza#NC 025653
Lamiales Lentibulariaceae Utricularia sp.#HRUR
Lamiales Lentibulariaceae Genlisea violacea#NC 037083
Lamiales Lentibulariaceae Genlisea margaretae#NC 025652
Lamiales Lentibulariaceae Genlisea aurea#NC 037078
Lamiales Lentibulariaceae Genlisea tuberosa#NC 037082
Lamiales Lentibulariaceae Genlisea filiformis#NC 037079
Lamiales Lentibulariaceae Genlisea pygmaea#NC 037080
Lamiales Lentibulariaceae Genlisea repens#NC 037081
Lamiales Martyniaceae Martynia annua#ZSD281
Lamiales Acanthaceae Avicennia marina#F501
Lamiales Acanthaceae Thunbergia lutea#F129
Lamiales Acanthaceae Aphelandra knappiae#NC 041424
Lamiales Acanthaceae Barleria cristata#F140
Lamiales Acanthaceae Andrographis paniculata#NC 022451
Lamiales Acanthaceae Phlogacanthus curviflorus#F1038
Lamiales Acanthaceae Asystasiella neesiana#F52
Lamiales Acanthaceae Clinacanthus nutans#MH778102
Lamiales Acanthaceae Anisacanthus quadrifidus#PCGJ
Lamiales Acanthaceae Echinacanthus longzhouensis#NC 039678
Lamiales Acanthaceae Echinacanthus lofuensis#NC 035876
Lamiales Acanthaceae Echinacanthus longipes#NC 039761
Lamiales Acanthaceae Ruellia simplex#AYIY
Lamiales Acanthaceae Sanchezia sp.#NBMW
Lamiales Acanthaceae Echinacanthus attenuatus#NC 039762
Lamiales Acanthaceae Strobilanthes cusia#NC 037485
Lamiales Acanthaceae Strobilanthes auriculatus#WEAC
Lamiales Acanthaceae Strobilanthes henryi#F67 1
Lamiales Acanthaceae Strobilanthes sp.#F42
Lamiales Verbenaceae Verbena hastata#GCFE
Lamiales Verbenaceae Verbena officinalis#F535
Lamiales Verbenaceae Aloysia citriodora#NC 034695
Lamiales Verbenaceae Phyla nodiflora#F528
Lamiales Verbenaceae Lantana camara#PSHB
Lamiales Verbenaceae Phyla scaberrima#MQIV
Lamiales Bignoniaceae Incarvillea diffusa#F173
Lamiales Bignoniaceae Tecoma capensis#NC 037462
Lamiales Bignoniaceae Handroanthus umbellatus#UTQR
Lamiales Bignoniaceae Markhamia stipulata#F247
Lamiales Bignoniaceae Kigelia africana#XAYK
Lamiales Bignoniaceae Pauldopia ghorta#F15
Lamiales Bignoniaceae Radermachera yunnanensis#F98
Lamiales Bignoniaceae Mayodendron igneum#F281
Lamiales Bignoniaceae Dolichandra cynanchoides#NC 037460
Lamiales Bignoniaceae Tanaecium tetragonolobum#NC 027955
Lamiales Bignoniaceae Mansoa alliacea#TKEK
Lamiales Bignoniaceae Anemopaegma oligoneuron#NC 037232
Lamiales Bignoniaceae Anemopaegma chamberlaynii#NC 037229
Lamiales Bignoniaceae Anemopaegma album#NC 037227
Lamiales Bignoniaceae Anemopaegma foetidum#NC 037230
Lamiales Bignoniaceae Anemopaegma arvense#MF460830
Lamiales Bignoniaceae Anemopaegma glaucum#MF460834
Lamiales Bignoniaceae Anemopaegma acutifolium#MF460827
Lamiales Bignoniaceae Adenocalymma pedunculatum#NC 036500
Lamiales Bignoniaceae Pleonotoma albiflora#NC 037461
Lamiales Bignoniaceae Adenocalymma cristicalyx#NC 036498
Lamiales Bignoniaceae Neojobertia candolleana#NC 036503
Lamiales Bignoniaceae Adenocalymma biternatum#NC 036496
Lamiales Bignoniaceae Adenocalymma nodosum#NC 037458
Lamiales Bignoniaceae Adenocalymma allamandiflorum#NC 036494
Lamiales Bignoniaceae Adenocalymma peregrinum#NC 036501
Lamiales Bignoniaceae Adenocalymma marginatum#NC 037457
Lamiales Bignoniaceae Adenocalymma subspicatum#NC 036502
Lamiales Bignoniaceae Adenocalymma divaricatum#NC 037456
Lamiales Bignoniaceae Adenocalymma bracteatum#NC 036497
Lamiales Bignoniaceae Adenocalymma aurantiacum#NC 036495
Lamiales Bignoniaceae Adenocalymma hatschbachii#NC 036499
Lamiales Bignoniaceae Adenocalymma trifoliatum#NC 037459
Lamiales Bignoniaceae Adenocalymma comosum#NC 037455
Lamiales Mazaceae Mazus miquelii#F864
Lamiales Mazaceae Lancea hirsuta#NC 037506
Lamiales Mazaceae Lancea tibetica#F647
Lamiales Wightiaceae Wightia speciosissima#J5854
Lamiales Phrymaceae Phryma leptostachya#F890
Lamiales Phrymaceae Mimulus tenellus var. platyphyllus#F825
Lamiales Phrymaceae Erythranthe lutea#NC 030212
Lamiales Paulowniaceae Paulownia fortunei#F180
Lamiales Paulowniaceae Paulownia fargesii#UMUL
Lamiales Orobanchaceae Triaenophora shennongjiaensis#NC 039781
Lamiales Orobanchaceae Rehmannia chingii#F895
Lamiales Orobanchaceae Rehmannia glutinosa#F1002
Lamiales Orobanchaceae Rehmannia solanifolia#NC 034310
Lamiales Orobanchaceae Rehmannia henryi#NC 034309
Lamiales Orobanchaceae Rehmannia elata#NC 034312
Lamiales Orobanchaceae Rehmannia piasezkii#NC 034311
Lamiales Orobanchaceae Lindenbergia muraria#F84
Lamiales Orobanchaceae Lindenbergia philippensis#EJCM
Lamiales Orobanchaceae Cymbaria mongolica#F789
Lamiales Orobanchaceae Schwalbea americana#NC 023115
Lamiales Orobanchaceae Lathraea squamaria#NC 027838 @para
Lamiales Orobanchaceae Brandisia racemosa#F46
Lamiales Orobanchaceae Pedicularis ishidoyana#NC 029700 @para
Lamiales Orobanchaceae Pedicularis hallaisanensis#NC 037433 @para
Lamiales Orobanchaceae Castilleja paramensis#NC 031805
Lamiales Orobanchaceae Phtheirospermum japonicum#F35
Lamiales Orobanchaceae Epifagus virginiana#FROP
Lamiales Orobanchaceae Conopholis americana#FAMO
Lamiales Orobanchaceae Cistanche phelypaea#NC 025642 @para
Lamiales Orobanchaceae Cistanche deserticola#NC 021111 @para
Lamiales Orobanchaceae Orobanche latisquama#NC 025641 @para
Lamiales Orobanchaceae Orobanche gracilis#NC 023464 @para
Lamiales Orobanchaceae Orobanche crenata#NC 024845 @para
Lamiales Orobanchaceae Orobanche ramosa#NC 023465 @para
Lamiales Orobanchaceae Orobanche purpurea#NC 023132 @para
Lamiales Orobanchaceae Orobanche californica#NC 025651 @para
Lamiales Orobanchaceae Aphyllon epigalium subsp. notocalifornicum#MH050786

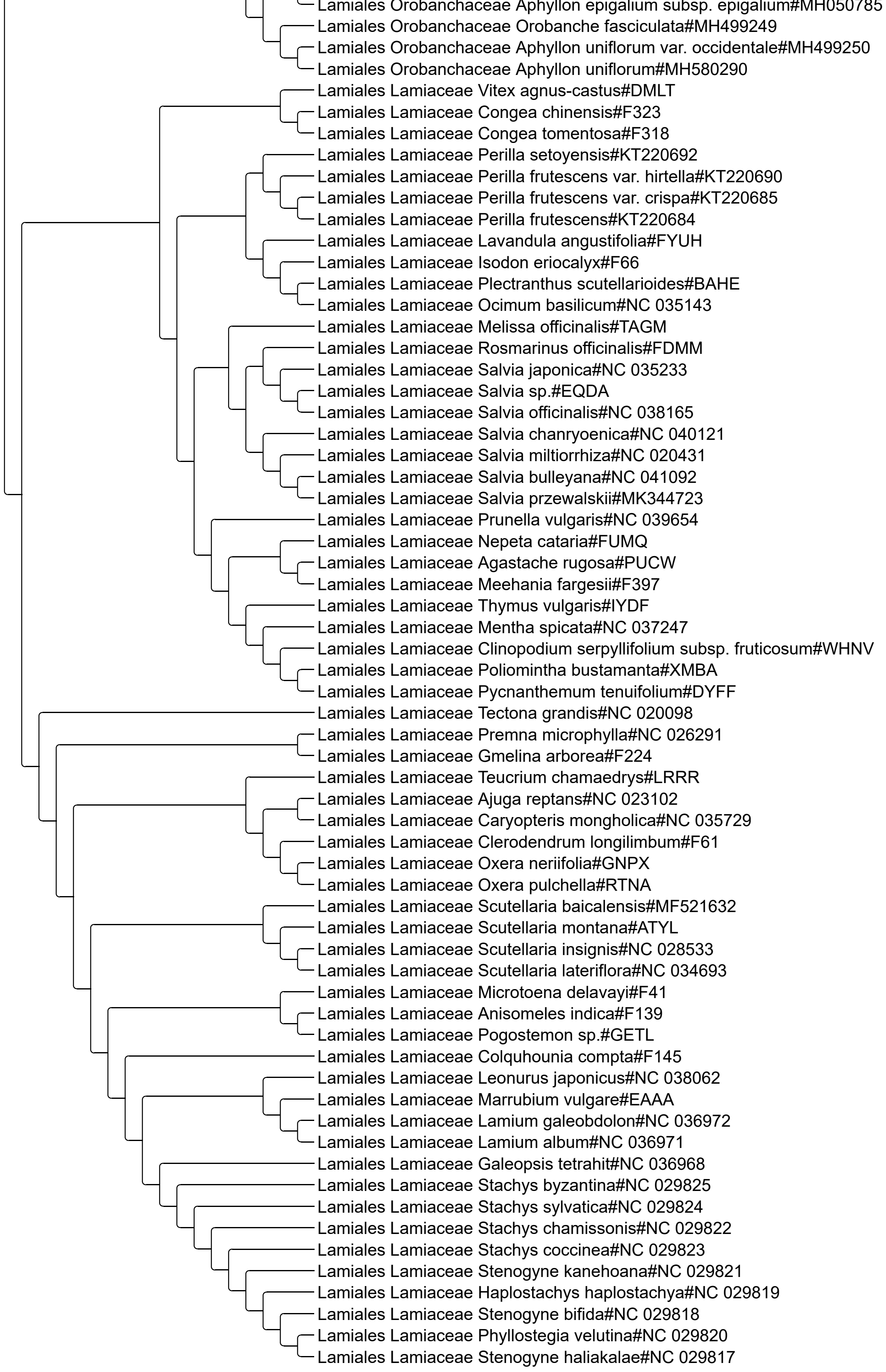
Lamiales Orobanchaceae Aphyllon epigalium subsp. epigalium#MH050785
Lamiales Orobanchaceae Orobanche fasciculata#MH499249
Lamiales Orobanchaceae Aphyllon uniflorum var. occidentale#MH499250
Lamiales Orobanchaceae Aphyllon uniflorum#MH580290
Lamiales Lamiaceae Vitex agnus-castus#DMLT
Lamiales Lamiaceae Congea chinensis#F323
Lamiales Lamiaceae Congea tomentosa#F318
Lamiales Lamiaceae Perilla setoyensis#KT220692
Lamiales Lamiaceae Perilla frutescens var. hirtella#KT220690
Lamiales Lamiaceae Perilla frutescens var. crispa#KT220685
Lamiales Lamiaceae Perilla frutescens#KT220684
Lamiales Lamiaceae Lavandula angustifolia#FYUH
Lamiales Lamiaceae Isodon eriocalyx#F66
Lamiales Lamiaceae Plectranthus scutellarioides#BAHE
Lamiales Lamiaceae Ocimum basilicum#NC 035143
Lamiales Lamiaceae Melissa officinalis#TAGM
Lamiales Lamiaceae Rosmarinus officinalis#FDMM
Lamiales Lamiaceae Salvia japonica#NC 035233
Lamiales Lamiaceae Salvia sp.#EQDA
Lamiales Lamiaceae Salvia officinalis#NC 038165
Lamiales Lamiaceae Salvia chanryoenica#NC 040121
Lamiales Lamiaceae Salvia miltiorrhiza#NC 020431
Lamiales Lamiaceae Salvia bulleyana#NC 041092
Lamiales Lamiaceae Salvia przewalskii#MK344723
Lamiales Lamiaceae Prunella vulgaris#NC 039654
Lamiales Lamiaceae Nepeta cataria#FUMQ
Lamiales Lamiaceae Agastache rugosa#PUCW
Lamiales Lamiaceae Meehania fargesii#F397
Lamiales Lamiaceae Thymus vulgaris#IYDF
Lamiales Lamiaceae Mentha spicata#NC 037247
Lamiales Lamiaceae Clinopodium serpyllifolium subsp. fruticosum#WHNV
Lamiales Lamiaceae Poliomintha bustamanta#XMBA
Lamiales Lamiaceae Pycnanthemum tenuifolium#DYFF
Lamiales Lamiaceae Tectona grandis#NC 020098
Lamiales Lamiaceae Premna microphylla#NC 026291
Lamiales Lamiaceae Gmelina arborea#F224
Lamiales Lamiaceae Teucrium chamaedrys#LRRR
Lamiales Lamiaceae Ajuga reptans#NC 023102
Lamiales Lamiaceae Caryopteris mongholica#NC 035729
Lamiales Lamiaceae Clerodendrum longilimbum#F61
Lamiales Lamiaceae Oxera neriifolia#GNPX
Lamiales Lamiaceae Oxera pulchella#RTNA
Lamiales Lamiaceae Scutellaria baicalensis#MF521632
Lamiales Lamiaceae Scutellaria montana#ATYL
Lamiales Lamiaceae Scutellaria insignis#NC 028533
Lamiales Lamiaceae Scutellaria lateriflora#NC 034693
Lamiales Lamiaceae Microtoena delavayi#F41
Lamiales Lamiaceae Anisomeles indica#F139
Lamiales Lamiaceae Pogostemon sp.#GETL
Lamiales Lamiaceae Colquhounia compta#F145
Lamiales Lamiaceae Leonurus japonicus#NC 038062
Lamiales Lamiaceae Marrubium vulgare#EAAA
Lamiales Lamiaceae Lamium galeobdolon#NC 036972
Lamiales Lamiaceae Lamium album#NC 036971
Lamiales Lamiaceae Galeopsis tetrahit#NC 036968
Lamiales Lamiaceae Stachys byzantina#NC 029825
Lamiales Lamiaceae Stachys sylvatica#NC 029824
Lamiales Lamiaceae Stachys chamissonis#NC 029822
Lamiales Lamiaceae Stachys coccinea#NC 029823
Lamiales Lamiaceae Stenogyne kanehoana#NC 029821
Lamiales Lamiaceae Haplostachys haplostachya#NC 029819
Lamiales Lamiaceae Stenogyne bifida#NC 029818
Lamiales Lamiaceae Phyllostegia velutina#NC 029820
Lamiales Lamiaceae Stenogyne haliakalae#NC 029817

[Figure S8d]

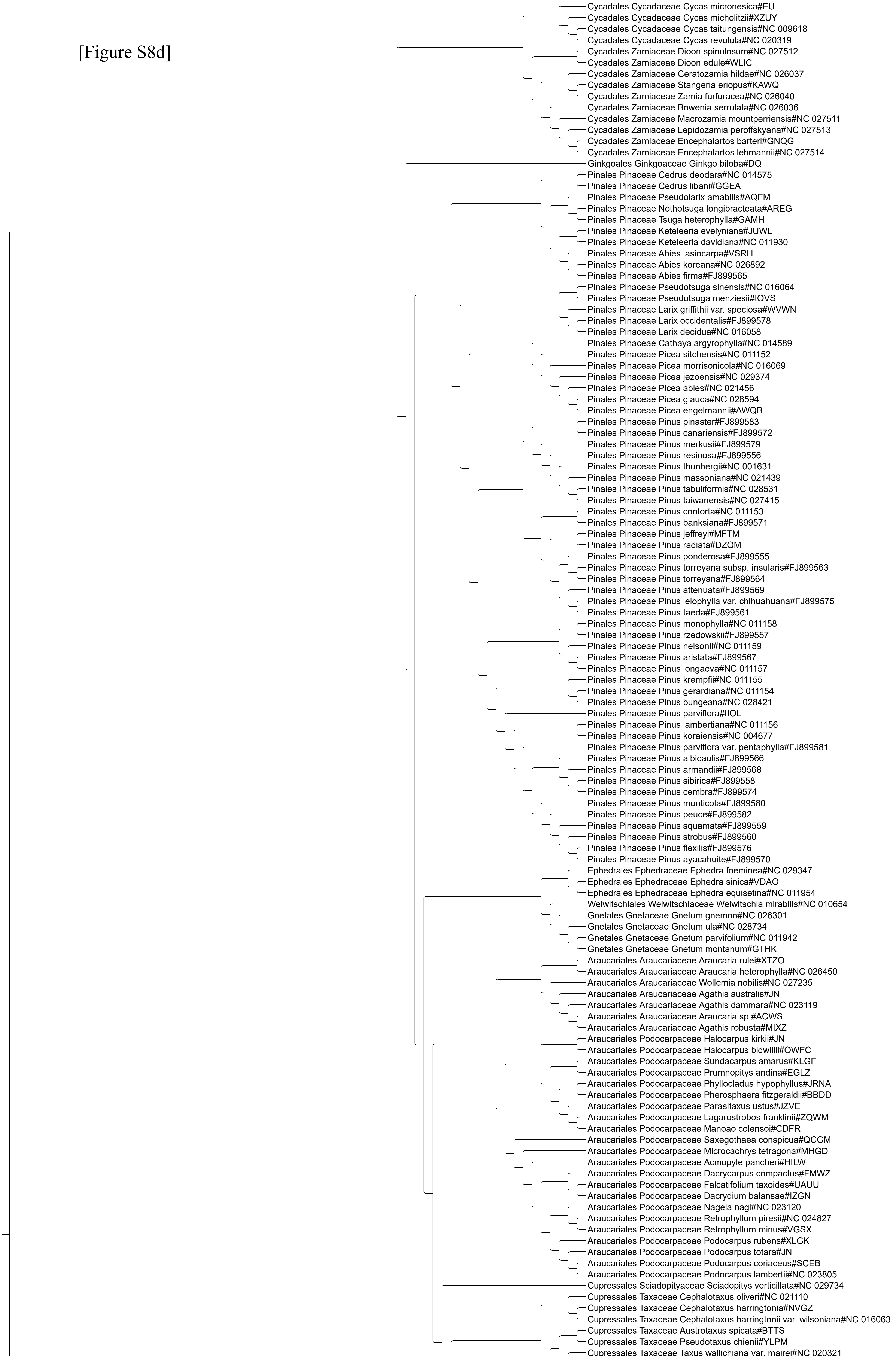
Cycadales Cycadaceae Cycas micronesica#EU
Cycadales Cycadaceae Cycas micholitzii#XZUY
Cycadales Cycadaceae Cycas taitungensis#NC 009618
Cycadales Cycadaceae Cycas revoluta#NC 020319
Cycadales Zamiaceae Dioon spinulosum#NC 027512
Cycadales Zamiaceae Dioon edule#WLIC
Cycadales Zamiaceae Ceratozamia hildae#NC 026037
Cycadales Zamiaceae Stangeria eriopus#KAWQ
Cycadales Zamiaceae Zamia furfuracea#NC 026040
Cycadales Zamiaceae Bowenia serrulata#NC 026036
Cycadales Zamiaceae Macrozamia mountperriensis#NC 027511
Cycadales Zamiaceae Lepidozamia peroffskyana#NC 027513
Cycadales Zamiaceae Encephalartos barteri#GNQG
Cycadales Zamiaceae Encephalartos lehmannii#NC 027514
Ginkgoales Ginkgoaceae Ginkgo biloba#DQ
Pinales Pinaceae Cedrus deodara#NC 014575
Pinales Pinaceae Cedrus libani#GGEA
Pinales Pinaceae Pseudolarix amabilis#AQFM
Pinales Pinaceae Nothotsuga longibracteata#AREG
Pinales Pinaceae Tsuga heterophylla#GAMH
Pinales Pinaceae Keteleeria evelyniana#JUWL
Pinales Pinaceae Keteleeria davidiana#NC 011930
Pinales Pinaceae Abies lasiocarpa#VSRH
Pinales Pinaceae Abies koreana#NC 026892
Pinales Pinaceae Abies firma#FJ899565
Pinales Pinaceae Pseudotsuga sinensis#NC 016064
Pinales Pinaceae Pseudotsuga menziesii#IOVS
Pinales Pinaceae Larix griffithii var. speciosa#WVWN
Pinales Pinaceae Larix occidentalis#FJ899578
Pinales Pinaceae Larix decidua#NC 016058
Pinales Pinaceae Cathaya argyrophylla#NC 014589
Pinales Pinaceae Picea sitchensis#NC 011152
Pinales Pinaceae Picea morrisonicola#NC 016069
Pinales Pinaceae Picea jezoensis#NC 029374
Pinales Pinaceae Picea abies#NC 021456
Pinales Pinaceae Picea glauca#NC 028594
Pinales Pinaceae Picea engelmannii#AWQB
Pinales Pinaceae Pinus pinaster#FJ899583
Pinales Pinaceae Pinus canariensis#FJ899572
Pinales Pinaceae Pinus merkusii#FJ899579
Pinales Pinaceae Pinus resinosa#FJ899556
Pinales Pinaceae Pinus thunbergii#NC 001631
Pinales Pinaceae Pinus massoniana#NC 021439
Pinales Pinaceae Pinus tabuliformis#NC 028531
Pinales Pinaceae Pinus taiwanensis#NC 027415
Pinales Pinaceae Pinus contorta#NC 011153
Pinales Pinaceae Pinus banksiana#FJ899571
Pinales Pinaceae Pinus jeffreyi#MFTM
Pinales Pinaceae Pinus radiata#DZQM
Pinales Pinaceae Pinus ponderosa#FJ899555
Pinales Pinaceae Pinus torreyana subsp. insularis#FJ899563
Pinales Pinaceae Pinus torreyana#FJ899564
Pinales Pinaceae Pinus attenuata#FJ899569
Pinales Pinaceae Pinus leiophylla var. chihuahuana#FJ899575
Pinales Pinaceae Pinus taeda#FJ899561
Pinales Pinaceae Pinus monophylla#NC 011158
Pinales Pinaceae Pinus rzedowskii#FJ899557
Pinales Pinaceae Pinus nelsonii#NC 011159
Pinales Pinaceae Pinus aristata#FJ899567
Pinales Pinaceae Pinus longaeva#NC 011157
Pinales Pinaceae Pinus krempfii#NC 011155
Pinales Pinaceae Pinus gerardiana#NC 011154
Pinales Pinaceae Pinus bungeana#NC 028421
Pinales Pinaceae Pinus parviflora#IIOL
Pinales Pinaceae Pinus lambertiana#NC 011156
Pinales Pinaceae Pinus koraiensis#NC 004677
Pinales Pinaceae Pinus parviflora var. pentaphylla#FJ899581
Pinales Pinaceae Pinus albicaulis#FJ899566
Pinales Pinaceae Pinus armandii#FJ899568
Pinales Pinaceae Pinus sibirica#FJ899558
Pinales Pinaceae Pinus cembra#FJ899574
Pinales Pinaceae Pinus monticola#FJ899580
Pinales Pinaceae Pinus peuce#FJ899582
Pinales Pinaceae Pinus squamata#FJ899559
Pinales Pinaceae Pinus strobus#FJ899560
Pinales Pinaceae Pinus flexilis#FJ899576
Pinales Pinaceae Pinus ayacahuite#FJ899570
Ephedrales Ephedraceae Ephedra foeminea#NC 029347
Ephedrales Ephedraceae Ephedra sinica#VDAO
Ephedrales Ephedraceae Ephedra equisetina#NC 011954
Welwitschiales Welwitschiaceae Welwitschia mirabilis#NC 010654
Gnetales Gnetaceae Gnetum gnemon#NC 026301
Gnetales Gnetaceae Gnetum ula#NC 028734
Gnetales Gnetaceae Gnetum parvifolium#NC 011942
Gnetales Gnetaceae Gnetum montanum#GTHK
Araucariales Araucariaceae Araucaria rulei#XTZO
Araucariales Araucariaceae Araucaria heterophylla#NC 026450
Araucariales Araucariaceae Wollemia nobilis#NC 027235
Araucariales Araucariaceae Agathis australis#JN
Araucariales Araucariaceae Agathis dammara#NC 023119
Araucariales Araucariaceae Araucaria sp.#ACWS
Araucariales Araucariaceae Agathis robusta#MIXZ
Araucariales Podocarpaceae Halocarpus kirkii#JN
Araucariales Podocarpaceae Halocarpus bidwillii#OWFC
Araucariales Podocarpaceae Sundacarpus amarus#KLGF
Araucariales Podocarpaceae Prumnopitys andina#EGLZ
Araucariales Podocarpaceae Phyllocladus hypophyllus#JRNA
Araucariales Podocarpaceae Pherosphaera fitzgeraldii#BBDD
Araucariales Podocarpaceae Parasitaxus ustus#JZVE
Araucariales Podocarpaceae Lagarostrobos franklinii#ZQWM
Araucariales Podocarpaceae Manoao colensoi#CDFR
Araucariales Podocarpaceae Saxegothaea conspicua#QCGM
Araucariales Podocarpaceae Microcachrys tetragona#MHGD
Araucariales Podocarpaceae Acmopyle pancheri#HILW
Araucariales Podocarpaceae Dacrycarpus compactus#FMWZ
Araucariales Podocarpaceae Falcatifolium taxoides#UAUU
Araucariales Podocarpaceae Dacrydium balansae#IZGN
Araucariales Podocarpaceae Nageia nagi#NC 023120
Araucariales Podocarpaceae Retrophyllum piresii#NC 024827
Araucariales Podocarpaceae Retrophyllum minus#VGSX
Araucariales Podocarpaceae Podocarpus rubens#XLGK
Araucariales Podocarpaceae Podocarpus totara#JN
Araucariales Podocarpaceae Podocarpus coriaceus#SCEB
Araucariales Podocarpaceae Podocarpus lambertii#NC 023805
Cupressales Sciadopityaceae Sciadopitys verticillata#NC 029734
Cupressales Taxaceae Cephalotaxus oliveri#NC 021110
Cupressales Taxaceae Cephalotaxus harringtonia#NVGZ
Cupressales Taxaceae Cephalotaxus harringtonii var. wilsoniana#NC 016063
Cupressales Taxaceae Austrotaxus spicata#BTTS
Cupressales Taxaceae Pseudotaxus chienii#YLPM
Cupressales Taxaceae Taxus wallichiana var. mairei#NC 020321

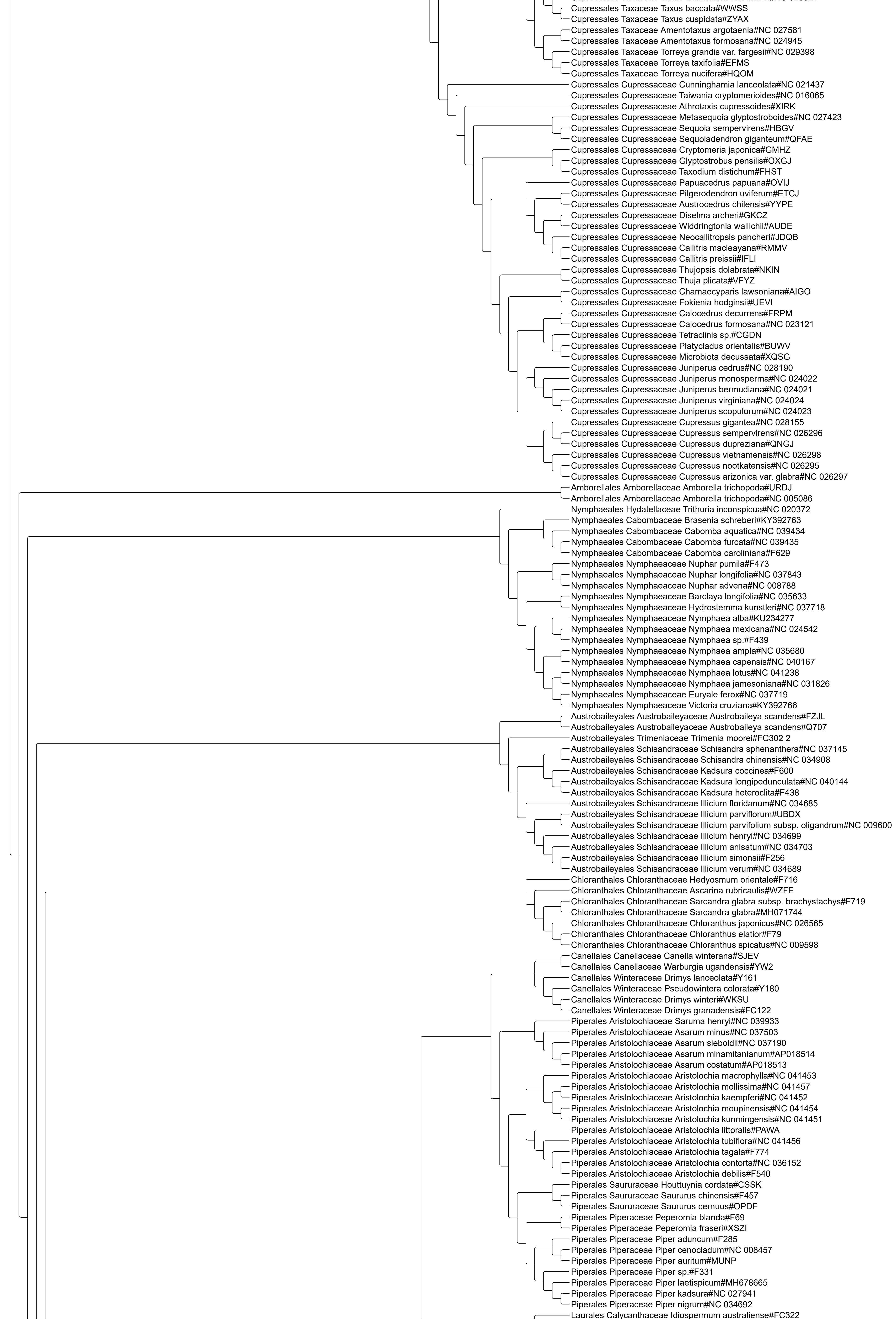
Cupressales Taxaceae Taxus baccata#WWSS
Cupressales Taxaceae Taxus cuspidata#ZYAX
Cupressales Taxaceae Amentotaxus argotaenia#NC 027581
Cupressales Taxaceae Amentotaxus formosana#NC 024945
Cupressales Taxaceae Torreya grandis var. fargesii#NC 029398
Cupressales Taxaceae Torreya taxifolia#EFMS
Cupressales Taxaceae Torreya nucifera#HQOM
Cupressales Cupressaceae Cunninghamia lanceolata#NC 021437
Cupressales Cupressaceae Taiwania cryptomerioides#NC 016065
Cupressales Cupressaceae Athrotaxis cupressoides#XIRK
Cupressales Cupressaceae Metasequoia glyptostroboides#NC 027423
Cupressales Cupressaceae Sequoia sempervirens#HBGV
Cupressales Cupressaceae Sequoiadendron giganteum#QFAE
Cupressales Cupressaceae Cryptomeria japonica#GMHZ
Cupressales Cupressaceae Glyptostrobus pensilis#OXGJ
Cupressales Cupressaceae Taxodium distichum#FHST
Cupressales Cupressaceae Papuacedrus papuana#OVIJ
Cupressales Cupressaceae Pilgerodendron uviferum#ETCJ
Cupressales Cupressaceae Austrocedrus chilensis#YYPE
Cupressales Cupressaceae Diselma archeri#GKCZ
Cupressales Cupressaceae Widdringtonia wallichii#AUDE
Cupressales Cupressaceae Neocallitropsis pancheri#JDQB
Cupressales Cupressaceae Callitris macleayana#RMMV
Cupressales Cupressaceae Callitris preissii#IFLI
Cupressales Cupressaceae Thujopsis dolabrata#NKIN
Cupressales Cupressaceae Thuja plicata#VFYZ
Cupressales Cupressaceae Chamaecyparis lawsoniana#AIGO
Cupressales Cupressaceae Fokienia hodginsii#UEVI
Cupressales Cupressaceae Calocedrus decurrens#FRPM
Cupressales Cupressaceae Calocedrus formosana#NC 023121
Cupressales Cupressaceae Tetraclinis sp.#CGDN
Cupressales Cupressaceae Platycladus orientalis#BUWV
Cupressales Cupressaceae Microbiota decussata#XQSG
Cupressales Cupressaceae Juniperus cedrus#NC 028190
Cupressales Cupressaceae Juniperus monosperma#NC 024022
Cupressales Cupressaceae Juniperus bermudiana#NC 024021
Cupressales Cupressaceae Juniperus virginiana#NC 024024
Cupressales Cupressaceae Juniperus scopulorum#NC 024023
Cupressales Cupressaceae Cupressus gigantea#NC 028155
Cupressales Cupressaceae Cupressus sempervirens#NC 026296
Cupressales Cupressaceae Cupressus dupreziana#QNGJ
Cupressales Cupressaceae Cupressus vietnamensis#NC 026298
Cupressales Cupressaceae Cupressus nootkatensis#NC 026295
Cupressales Cupressaceae Cupressus arizonica var. glabra#NC 026297
Amborellales Amborellaceae Amborella trichopoda#URDJ
Amborellales Amborellaceae Amborella trichopoda#NC 005086
Nymphaeales Hydatellaceae Trithuria inconspicua#NC 020372
Nymphaeales Cabombaceae Brasenia schreberi#KY392763
Nymphaeales Cabombaceae Cabomba aquatica#NC 039434
Nymphaeales Cabombaceae Cabomba furcata#NC 039435
Nymphaeales Cabombaceae Cabomba caroliniana#F629
Nymphaeales Nymphaeaceae Nuphar pumila#F473
Nymphaeales Nymphaeaceae Nuphar longifolia#NC 037843
Nymphaeales Nymphaeaceae Nuphar advena#NC 008788
Nymphaeales Nymphaeaceae Barclaya longifolia#NC 035633
Nymphaeales Nymphaeaceae Hydrostemma kunstleri#NC 037718
Nymphaeales Nymphaeaceae Nymphaea alba#KU234277
Nymphaeales Nymphaeaceae Nymphaea mexicana#NC 024542
Nymphaeales Nymphaeaceae Nymphaea sp.#F439
Nymphaeales Nymphaeaceae Nymphaea ampla#NC 035680
Nymphaeales Nymphaeaceae Nymphaea capensis#NC 040167
Nymphaeales Nymphaeaceae Nymphaea lotus#NC 041238
Nymphaeales Nymphaeaceae Nymphaea jamesoniana#NC 031826
Nymphaeales Nymphaeaceae Euryale ferox#NC 037719
Nymphaeales Nymphaeaceae Victoria cruziana#KY392766
Austrobaileyales Austrobaileyaceae Austrobaileya scandens#FZJL
Austrobaileyales Austrobaileyaceae Austrobaileya scandens#Q707
Austrobaileyales Trimeniaceae Trimenia moorei#FC302 2
Austrobaileyales Schisandraceae Schisandra sphenanthera#NC 037145
Austrobaileyales Schisandraceae Schisandra chinensis#NC 034908
Austrobaileyales Schisandraceae Kadsura coccinea#F600
Austrobaileyales Schisandraceae Kadsura longipedunculata#NC 040144
Austrobaileyales Schisandraceae Kadsura heteroclita#F438
Austrobaileyales Schisandraceae Illicium floridanum#NC 034685
Austrobaileyales Schisandraceae Illicium parviflorum#UBDX
Austrobaileyales Schisandraceae Illicium parvifolium subsp. oligandrum#NC 009600
Austrobaileyales Schisandraceae Illicium henryi#NC 034699
Austrobaileyales Schisandraceae Illicium anisatum#NC 034703
Austrobaileyales Schisandraceae Illicium simonsii#F256
Austrobaileyales Schisandraceae Illicium verum#NC 034689
Chloranthales Chloranthaceae Hedyosmum orientale#F716
Chloranthales Chloranthaceae Ascarina rubricaulis#WZFE
Chloranthales Chloranthaceae Sarcandra glabra subsp. brachystachys#F719
Chloranthales Chloranthaceae Sarcandra glabra#MH071744
Chloranthales Chloranthaceae Chloranthus japonicus#NC 026565
Chloranthales Chloranthaceae Chloranthus elatior#F79
Chloranthales Chloranthaceae Chloranthus spicatus#NC 009598
Canellales Canellaceae Canella winterana#SJEV
Canellales Canellaceae Warburgia ugandensis#YW2
Canellales Winteraceae Drimys lanceolata#Y161
Canellales Winteraceae Pseudowintera colorata#Y180
Canellales Winteraceae Drimys winteri#WKSU
Canellales Winteraceae Drimys granadensis#FC122
Piperales Aristolochiaceae Saruma henryi#NC 039933
Piperales Aristolochiaceae Asarum minus#NC 037503
Piperales Aristolochiaceae Asarum sieboldii#NC 037190
Piperales Aristolochiaceae Asarum minamitanianum#AP018514
Piperales Aristolochiaceae Asarum costatum#AP018513
Piperales Aristolochiaceae Aristolochia macrophylla#NC 041453
Piperales Aristolochiaceae Aristolochia mollissima#NC 041457
Piperales Aristolochiaceae Aristolochia kaempferi#NC 041452
Piperales Aristolochiaceae Aristolochia moupinensis#NC 041454
Piperales Aristolochiaceae Aristolochia kunmingensis#NC 041451
Piperales Aristolochiaceae Aristolochia littoralis#PAWA
Piperales Aristolochiaceae Aristolochia tubiflora#NC 041456
Piperales Aristolochiaceae Aristolochia tagala#F774
Piperales Aristolochiaceae Aristolochia contorta#NC 036152
Piperales Aristolochiaceae Aristolochia debilis#F540
Piperales Saururaceae Houttuynia cordata#CSSK
Piperales Saururaceae Saururus chinensis#F457
Piperales Saururaceae Saururus cernuus#OPDF
Piperales Piperaceae Peperomia blanda#F69
Piperales Piperaceae Peperomia fraseri#XSZI
Piperales Piperaceae Piper aduncum#F285
Piperales Piperaceae Piper cenocladum#NC 008457
Piperales Piperaceae Piper auritum#MUNP
Piperales Piperaceae Piper sp.#F331
Piperales Piperaceae Piper laetispicum#MH678665
Piperales Piperaceae Piper kadsura#NC 027941
Piperales Piperaceae Piper nigrum#NC 034692
Laurales Calycanthaceae Idiospermum australiense#FC322

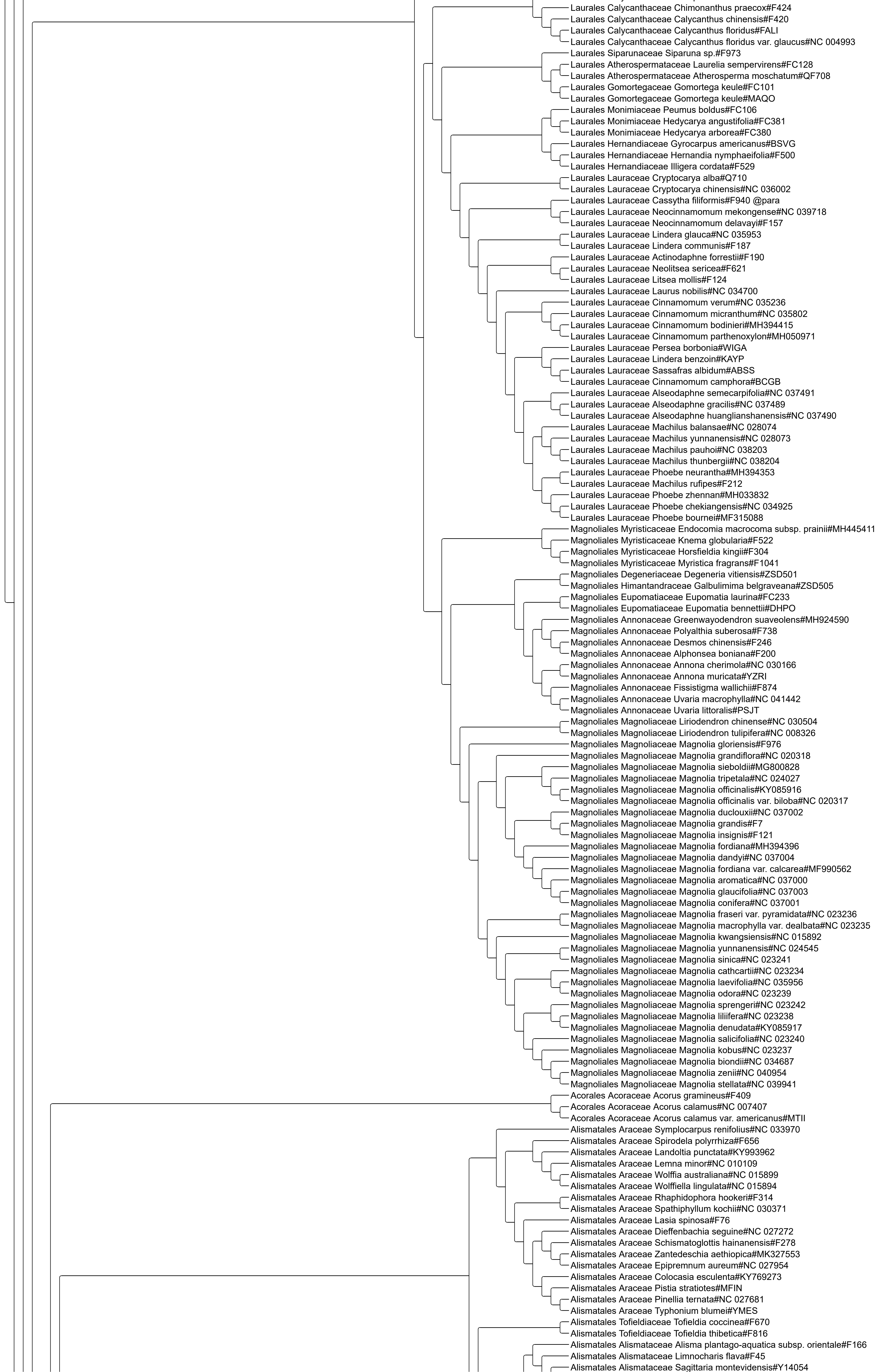
Laurales Calycanthaceae Chimonanthus praecox#F424
Laurales Calycanthaceae Calycanthus chinensis#F420
Laurales Calycanthaceae Calycanthus floridus#FALI
Laurales Calycanthaceae Calycanthus floridus var. glaucus#NC 004993
Laurales Siparunaceae Siparuna sp.#F973
Laurales Atherospermataceae Laurelia sempervirens#FC128
Laurales Atherospermataceae Atherosperma moschatum#QF708
Laurales Gomortegaceae Gomortega keule#FC101
Laurales Gomortegaceae Gomortega keule#MAQO
Laurales Monimiaceae Peumus boldus#FC106
Laurales Monimiaceae Hedycarya angustifolia#FC381
Laurales Monimiaceae Hedycarya arborea#FC380
Laurales Hernandiaceae Gyrocarpus americanus#BSVG
Laurales Hernandiaceae Hernandia nymphaeifolia#F500
Laurales Hernandiaceae Illigera cordata#F529
Laurales Lauraceae Cryptocarya alba#Q710
Laurales Lauraceae Cryptocarya chinensis#NC 036002
Laurales Lauraceae Cassytha filiformis#F940 @para
Laurales Lauraceae Neocinnamomum mekongense#NC 039718
Laurales Lauraceae Neocinnamomum delavayi#F157
Laurales Lauraceae Lindera glauca#NC 035953
Laurales Lauraceae Lindera communis#F187
Laurales Lauraceae Actinodaphne forrestii#F190
Laurales Lauraceae Neolitsea sericea#F621
Laurales Lauraceae Litsea mollis#F124
Laurales Lauraceae Laurus nobilis#NC 034700
Laurales Lauraceae Cinnamomum verum#NC 035236
Laurales Lauraceae Cinnamomum micranthum#NC 035802
Laurales Lauraceae Cinnamomum bodinieri#MH394415
Laurales Lauraceae Cinnamomum parthenoxylon#MH050971
Laurales Lauraceae Persea borbonia#WIGA
Laurales Lauraceae Lindera benzoin#KAYP
Laurales Lauraceae Sassafras albidum#ABSS
Laurales Lauraceae Cinnamomum camphora#BCGB
Laurales Lauraceae Alseodaphne semecarpifolia#NC 037491
Laurales Lauraceae Alseodaphne gracilis#NC 037489
Laurales Lauraceae Alseodaphne huanglianshanensis#NC 037490
Laurales Lauraceae Machilus balansae#NC 028074
Laurales Lauraceae Machilus yunnanensis#NC 028073
Laurales Lauraceae Machilus pauhoi#NC 038203
Laurales Lauraceae Machilus thunbergii#NC 038204
Laurales Lauraceae Phoebe neurantha#MH394353
Laurales Lauraceae Machilus rufipes#F212
Laurales Lauraceae Phoebe zhennan#MH033832
Laurales Lauraceae Phoebe chekiangensis#NC 034925
Laurales Lauraceae Phoebe bournei#MF315088
Magnoliales Myristicaceae Endocomia macrocoma subsp. prainii#MH445411
Magnoliales Myristicaceae Knema globularia#F522
Magnoliales Myristicaceae Horsfieldia kingii#F304
Magnoliales Myristicaceae Myristica fragrans#F1041
Magnoliales Degeneriaceae Degeneria vitiensis#ZSD501
Magnoliales Himantandraceae Galbulimima belgraveana#ZSD505
Magnoliales Eupomatiaceae Eupomatia laurina#FC233
Magnoliales Eupomatiaceae Eupomatia bennettii#DHPO
Magnoliales Annonaceae Greenwayodendron suaveolens#MH924590
Magnoliales Annonaceae Polyalthia suberosa#F738
Magnoliales Annonaceae Desmos chinensis#F246
Magnoliales Annonaceae Alphonsea boniana#F200
Magnoliales Annonaceae Annona cherimola#NC 030166
Magnoliales Annonaceae Annona muricata#YZRI
Magnoliales Annonaceae Fissistigma wallichii#F874
Magnoliales Annonaceae Uvaria macrophylla#NC 041442
Magnoliales Annonaceae Uvaria littoralis#PSJT
Magnoliales Magnoliaceae Liriodendron chinense#NC 030504
Magnoliales Magnoliaceae Liriodendron tulipifera#NC 008326
Magnoliales Magnoliaceae Magnolia gloriensis#F976
Magnoliales Magnoliaceae Magnolia grandiflora#NC 020318
Magnoliales Magnoliaceae Magnolia sieboldii#MG800828
Magnoliales Magnoliaceae Magnolia tripetala#NC 024027
Magnoliales Magnoliaceae Magnolia officinalis#KY085916
Magnoliales Magnoliaceae Magnolia officinalis var. biloba#NC 020317
Magnoliales Magnoliaceae Magnolia duclouxii#NC 037002
Magnoliales Magnoliaceae Magnolia grandis#F7
Magnoliales Magnoliaceae Magnolia insignis#F121
Magnoliales Magnoliaceae Magnolia fordiana#MH394396
Magnoliales Magnoliaceae Magnolia dandyi#NC 037004
Magnoliales Magnoliaceae Magnolia fordiana var. calcarea#MF990562
Magnoliales Magnoliaceae Magnolia aromatica#NC 037000
Magnoliales Magnoliaceae Magnolia glaucifolia#NC 037003
Magnoliales Magnoliaceae Magnolia conifera#NC 037001
Magnoliales Magnoliaceae Magnolia fraseri var. pyramidata#NC 023236
Magnoliales Magnoliaceae Magnolia macrophylla var. dealbata#NC 023235
Magnoliales Magnoliaceae Magnolia kwangsiensis#NC 015892
Magnoliales Magnoliaceae Magnolia yunnanensis#NC 024545
Magnoliales Magnoliaceae Magnolia sinica#NC 023241
Magnoliales Magnoliaceae Magnolia cathcartii#NC 023234
Magnoliales Magnoliaceae Magnolia laevifolia#NC 035956
Magnoliales Magnoliaceae Magnolia odora#NC 023239
Magnoliales Magnoliaceae Magnolia sprengeri#NC 023242
Magnoliales Magnoliaceae Magnolia liliifera#NC 023238
Magnoliales Magnoliaceae Magnolia denudata#KY085917
Magnoliales Magnoliaceae Magnolia salicifolia#NC 023240
Magnoliales Magnoliaceae Magnolia kobus#NC 023237
Magnoliales Magnoliaceae Magnolia biondii#NC 034687
Magnoliales Magnoliaceae Magnolia zenii#NC 040954
Magnoliales Magnoliaceae Magnolia stellata#NC 039941
Acorales Acoraceae Acorus gramineus#F409
Acorales Acoraceae Acorus calamus#NC 007407
Acorales Acoraceae Acorus calamus var. americanus#MTII
Alismatales Araceae Symplocarpus renifolius#NC 033970
Alismatales Araceae Spirodela polyrrhiza#F656
Alismatales Araceae Landoltia punctata#KY993962
Alismatales Araceae Lemna minor#NC 010109
Alismatales Araceae Wolffia australiana#NC 015899
Alismatales Araceae Wolffiella lingulata#NC 015894
Alismatales Araceae Rhaphidophora hookeri#F314
Alismatales Araceae Spathiphyllum kochii#NC 030371
Alismatales Araceae Lasia spinosa#F76
Alismatales Araceae Dieffenbachia seguine#NC 027272
Alismatales Araceae Schismatoglottis hainanensis#F278
Alismatales Araceae Zantedeschia aethiopica#MK327553
Alismatales Araceae Epipremnum aureum#NC 027954
Alismatales Araceae Colocasia esculenta#KY769273
Alismatales Araceae Pistia stratiotes#MFIN
Alismatales Araceae Pinellia ternata#NC 027681
Alismatales Araceae Typhonium blumei#YMES
Alismatales Tofieldiaceae Tofieldia coccinea#F670
Alismatales Tofieldiaceae Tofieldia thibetica#F816
Alismatales Alismataceae Alisma plantago-aquatica subsp. orientale#F166
Alismatales Alismataceae Limnocharis flava#F45
Alismatales Alismataceae Sagittaria montevidensis#Y14054

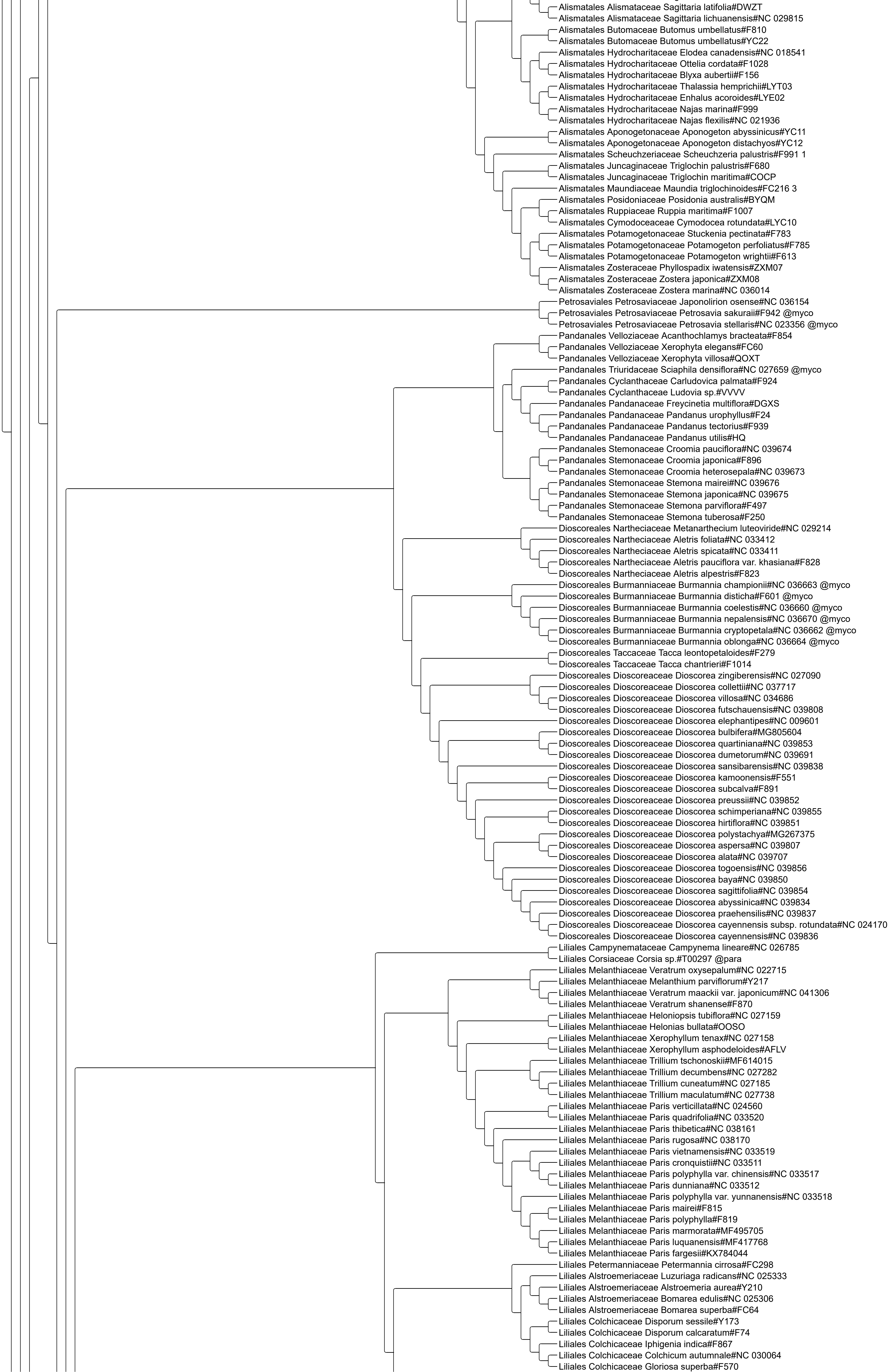
Alismatales Alismataceae Sagittaria latifolia#DWZT
Alismatales Alismataceae Sagittaria lichuanensis#NC 029815
Alismatales Butomaceae Butomus umbellatus#F810
Alismatales Butomaceae Butomus umbellatus#YC22
Alismatales Hydrocharitaceae Elodea canadensis#NC 018541
Alismatales Hydrocharitaceae Ottelia cordata#F1028
Alismatales Hydrocharitaceae Blyxa aubertii#F156
Alismatales Hydrocharitaceae Thalassia hemprichii#LYT03
Alismatales Hydrocharitaceae Enhalus acoroides#LYE02
Alismatales Hydrocharitaceae Najas marina#F999
Alismatales Hydrocharitaceae Najas flexilis#NC 021936
Alismatales Aponogetonaceae Aponogeton abyssinicus#YC11
Alismatales Aponogetonaceae Aponogeton distachyos#YC12
Alismatales Scheuchzeriaceae Scheuchzeria palustris#F991 1
Alismatales Juncaginaceae Triglochin palustris#F680
Alismatales Juncaginaceae Triglochin maritima#COCP
Alismatales Maundiaceae Maundia triglochinoides#FC216 3
Alismatales Posidoniaceae Posidonia australis#BYQM
Alismatales Ruppiaceae Ruppia maritima#F1007
Alismatales Cymodoceaceae Cymodocea rotundata#LYC10
Alismatales Potamogetonaceae Stuckenia pectinata#F783
Alismatales Potamogetonaceae Potamogeton perfoliatus#F785
Alismatales Potamogetonaceae Potamogeton wrightii#F613
Alismatales Zosteraceae Phyllospadix iwatensis#ZXM07
Alismatales Zosteraceae Zostera japonica#ZXM08
Alismatales Zosteraceae Zostera marina#NC 036014
Petrosaviales Petrosaviaceae Japonolirion osense#NC 036154
Petrosaviales Petrosaviaceae Petrosavia sakuraii#F942 @myco
Petrosaviales Petrosaviaceae Petrosavia stellaris#NC 023356 @myco
Pandanales Velloziaceae Acanthochlamys bracteata#F854
Pandanales Velloziaceae Xerophyta elegans#FC60
Pandanales Velloziaceae Xerophyta villosa#QOXT
Pandanales Triuridaceae Sciaphila densiflora#NC 027659 @myco
Pandanales Cyclanthaceae Carludovica palmata#F924
Pandanales Cyclanthaceae Ludovia sp.#VVVV
Pandanales Pandanaceae Freycinetia multiflora#DGXS
Pandanales Pandanaceae Pandanus urophyllus#F24
Pandanales Pandanaceae Pandanus tectorius#F939
Pandanales Pandanaceae Pandanus utilis#HQ
Pandanales Stemonaceae Croomia pauciflora#NC 039674
Pandanales Stemonaceae Croomia japonica#F896
Pandanales Stemonaceae Croomia heterosepala#NC 039673
Pandanales Stemonaceae Stemona mairei#NC 039676
Pandanales Stemonaceae Stemona japonica#NC 039675
Pandanales Stemonaceae Stemona parviflora#F497
Pandanales Stemonaceae Stemona tuberosa#F250
Dioscoreales Nartheciaceae Metanarthecium luteoviride#NC 029214
Dioscoreales Nartheciaceae Aletris foliata#NC 033412
Dioscoreales Nartheciaceae Aletris spicata#NC 033411
Dioscoreales Nartheciaceae Aletris pauciflora var. khasiana#F828
Dioscoreales Nartheciaceae Aletris alpestris#F823
Dioscoreales Burmanniaceae Burmannia championii#NC 036663 @myco
Dioscoreales Burmanniaceae Burmannia disticha#F601 @myco
Dioscoreales Burmanniaceae Burmannia coelestis#NC 036660 @myco
Dioscoreales Burmanniaceae Burmannia nepalensis#NC 036670 @myco
Dioscoreales Burmanniaceae Burmannia cryptopetala#NC 036662 @myco
Dioscoreales Burmanniaceae Burmannia oblonga#NC 036664 @myco
Dioscoreales Taccaceae Tacca leontopetaloides#F279
Dioscoreales Taccaceae Tacca chantrieri#F1014
Dioscoreales Dioscoreaceae Dioscorea zingiberensis#NC 027090
Dioscoreales Dioscoreaceae Dioscorea collettii#NC 037717
Dioscoreales Dioscoreaceae Dioscorea villosa#NC 034686
Dioscoreales Dioscoreaceae Dioscorea futschauensis#NC 039808
Dioscoreales Dioscoreaceae Dioscorea elephantipes#NC 009601
Dioscoreales Dioscoreaceae Dioscorea bulbifera#MG805604
Dioscoreales Dioscoreaceae Dioscorea quartiniana#NC 039853
Dioscoreales Dioscoreaceae Dioscorea dumetorum#NC 039691
Dioscoreales Dioscoreaceae Dioscorea sansibarensis#NC 039838
Dioscoreales Dioscoreaceae Dioscorea kamoonensis#F551
Dioscoreales Dioscoreaceae Dioscorea subcalva#F891
Dioscoreales Dioscoreaceae Dioscorea preussii#NC 039852
Dioscoreales Dioscoreaceae Dioscorea schimperiana#NC 039855
Dioscoreales Dioscoreaceae Dioscorea hirtiflora#NC 039851
Dioscoreales Dioscoreaceae Dioscorea polystachya#MG267375
Dioscoreales Dioscoreaceae Dioscorea aspersa#NC 039807
Dioscoreales Dioscoreaceae Dioscorea alata#NC 039707
Dioscoreales Dioscoreaceae Dioscorea togoensis#NC 039856
Dioscoreales Dioscoreaceae Dioscorea baya#NC 039850
Dioscoreales Dioscoreaceae Dioscorea sagittifolia#NC 039854
Dioscoreales Dioscoreaceae Dioscorea abyssinica#NC 039834
Dioscoreales Dioscoreaceae Dioscorea praehensilis#NC 039837
Dioscoreales Dioscoreaceae Dioscorea cayennensis subsp. rotundata#NC 024170
Dioscoreales Dioscoreaceae Dioscorea cayennensis#NC 039836
Liliales Campynemataceae Campynema lineare#NC 026785
Liliales Corsiaceae Corsia sp.#T00297 @para
Liliales Melanthiaceae Veratrum oxysepalum#NC 022715
Liliales Melanthiaceae Melanthium parviflorum#Y217
Liliales Melanthiaceae Veratrum maackii var. japonicum#NC 041306
Liliales Melanthiaceae Veratrum shanense#F870
Liliales Melanthiaceae Heloniopsis tubiflora#NC 027159
Liliales Melanthiaceae Helonias bullata#OOSO
Liliales Melanthiaceae Xerophyllum tenax#NC 027158
Liliales Melanthiaceae Xerophyllum asphodeloides#AFLV
Liliales Melanthiaceae Trillium tschonoskii#MF614015
Liliales Melanthiaceae Trillium decumbens#NC 027282
Liliales Melanthiaceae Trillium cuneatum#NC 027185
Liliales Melanthiaceae Trillium maculatum#NC 027738
Liliales Melanthiaceae Paris verticillata#NC 024560
Liliales Melanthiaceae Paris quadrifolia#NC 033520
Liliales Melanthiaceae Paris thibetica#NC 038161
Liliales Melanthiaceae Paris rugosa#NC 038170
Liliales Melanthiaceae Paris vietnamensis#NC 033519
Liliales Melanthiaceae Paris cronquistii#NC 033511
Liliales Melanthiaceae Paris polyphylla var. chinensis#NC 033517
Liliales Melanthiaceae Paris dunniana#NC 033512
Liliales Melanthiaceae Paris polyphylla var. yunnanensis#NC 033518
Liliales Melanthiaceae Paris mairei#F815
Liliales Melanthiaceae Paris polyphylla#F819
Liliales Melanthiaceae Paris marmorata#MF495705
Liliales Melanthiaceae Paris luquanensis#MF417768
Liliales Melanthiaceae Paris fargesii#KX784044
Liliales Petermanniaceae Petermannia cirrosa#FC298
Liliales Alstroemeriaceae Luzuriaga radicans#NC 025333
Liliales Alstroemeriaceae Alstroemeria aurea#Y210
Liliales Alstroemeriaceae Bomarea edulis#NC 025306
Liliales Alstroemeriaceae Bomarea superba#FC64
Liliales Colchicaceae Disporum sessile#Y173
Liliales Colchicaceae Disporum calcaratum#F74
Liliales Colchicaceae Iphigenia indica#F867
Liliales Colchicaceae Colchicum autumnale#NC 030064
Liliales Colchicaceae Gloriosa superba#F570

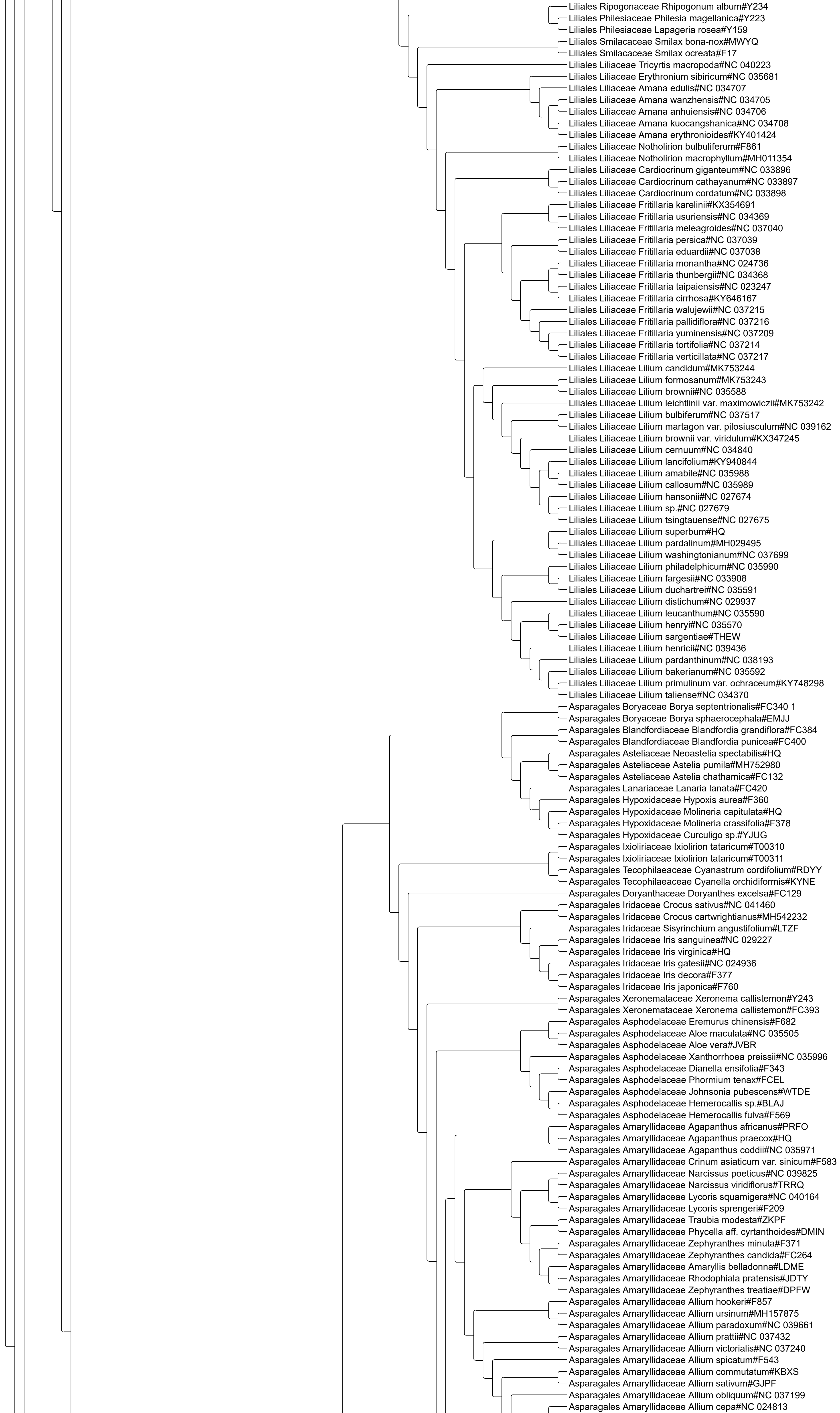
Liliales Ripogonaceae Rhipogonum album#Y234
Liliales Philesiaceae Philesia magellanica#Y223
Liliales Philesiaceae Lapageria rosea#Y159
Liliales Smilacaceae Smilax bona-nox#MWYQ
Liliales Smilacaceae Smilax ocreata#F17
Liliales Liliaceae Tricyrtis macropoda#NC 040223
Liliales Liliaceae Erythronium sibiricum#NC 035681
Liliales Liliaceae Amana edulis#NC 034707
Liliales Liliaceae Amana wanzhensis#NC 034705
Liliales Liliaceae Amana anhuiensis#NC 034706
Liliales Liliaceae Amana kuocangshanica#NC 034708
Liliales Liliaceae Amana erythronioides#KY401424
Liliales Liliaceae Notholirion bulbuliferum#F861
Liliales Liliaceae Notholirion macrophyllum#MH011354
Liliales Liliaceae Cardiocrinum giganteum#NC 033896
Liliales Liliaceae Cardiocrinum cathayanum#NC 033897
Liliales Liliaceae Cardiocrinum cordatum#NC 033898
Liliales Liliaceae Fritillaria karelinii#KX354691
Liliales Liliaceae Fritillaria usuriensis#NC 034369
Liliales Liliaceae Fritillaria meleagroides#NC 037040
Liliales Liliaceae Fritillaria persica#NC 037039
Liliales Liliaceae Fritillaria eduardii#NC 037038
Liliales Liliaceae Fritillaria monantha#NC 024736
Liliales Liliaceae Fritillaria thunbergii#NC 034368
Liliales Liliaceae Fritillaria taipaiensis#NC 023247
Liliales Liliaceae Fritillaria cirrhosa#KY646167
Liliales Liliaceae Fritillaria walujewii#NC 037215
Liliales Liliaceae Fritillaria pallidiflora#NC 037216
Liliales Liliaceae Fritillaria yuminensis#NC 037209
Liliales Liliaceae Fritillaria tortifolia#NC 037214
Liliales Liliaceae Fritillaria verticillata#NC 037217
Liliales Liliaceae Lilium candidum#MK753244
Liliales Liliaceae Lilium formosanum#MK753243
Liliales Liliaceae Lilium brownii#NC 035588
Liliales Liliaceae Lilium leichtlinii var. maximowiczii#MK753242
Liliales Liliaceae Lilium bulbiferum#NC 037517
Liliales Liliaceae Lilium martagon var. pilosiusculum#NC 039162
Liliales Liliaceae Lilium brownii var. viridulum#KX347245
Liliales Liliaceae Lilium cernuum#NC 034840
Liliales Liliaceae Lilium lancifolium#KY940844
Liliales Liliaceae Lilium amabile#NC 035988
Liliales Liliaceae Lilium callosum#NC 035989
Liliales Liliaceae Lilium hansonii#NC 027674
Liliales Liliaceae Lilium sp.#NC 027679
Liliales Liliaceae Lilium tsingtauense#NC 027675
Liliales Liliaceae Lilium superbum#HQ
Liliales Liliaceae Lilium pardalinum#MH029495
Liliales Liliaceae Lilium washingtonianum#NC 037699
Liliales Liliaceae Lilium philadelphicum#NC 035990
Liliales Liliaceae Lilium fargesii#NC 033908
Liliales Liliaceae Lilium duchartrei#NC 035591
Liliales Liliaceae Lilium distichum#NC 029937
Liliales Liliaceae Lilium leucanthum#NC 035590
Liliales Liliaceae Lilium henryi#NC 035570
Liliales Liliaceae Lilium sargentiae#THEW
Liliales Liliaceae Lilium henricii#NC 039436
Liliales Liliaceae Lilium pardanthinum#NC 038193
Liliales Liliaceae Lilium bakerianum#NC 035592
Liliales Liliaceae Lilium primulinum var. ochraceum#KY748298
Liliales Liliaceae Lilium taliense#NC 034370
Asparagales Boryaceae Borya septentrionalis#FC340 1
Asparagales Boryaceae Borya sphaerocephala#EMJJ
Asparagales Blandfordiaceae Blandfordia grandiflora#FC384
Asparagales Blandfordiaceae Blandfordia punicea#FC400
Asparagales Asteliaceae Neoastelia spectabilis#HQ
Asparagales Asteliaceae Astelia pumila#MH752980
Asparagales Asteliaceae Astelia chathamica#FC132
Asparagales Lanariaceae Lanaria lanata#FC420
Asparagales Hypoxidaceae Hypoxis aurea#F360
Asparagales Hypoxidaceae Molineria capitulata#HQ
Asparagales Hypoxidaceae Molineria crassifolia#F378
Asparagales Hypoxidaceae Curculigo sp.#YJUG
Asparagales Ixioliriaceae Ixiolirion tataricum#T00310
Asparagales Ixioliriaceae Ixiolirion tataricum#T00311
Asparagales Tecophilaeaceae Cyanastrum cordifolium#RDYY
Asparagales Tecophilaeaceae Cyanella orchidiformis#KYNE
Asparagales Doryanthaceae Doryanthes excelsa#FC129
Asparagales Iridaceae Crocus sativus#NC 041460
Asparagales Iridaceae Crocus cartwrightianus#MH542232
Asparagales Iridaceae Sisyrinchium angustifolium#LTZF
Asparagales Iridaceae Iris sanguinea#NC 029227
Asparagales Iridaceae Iris virginica#HQ
Asparagales Iridaceae Iris gatesii#NC 024936
Asparagales Iridaceae Iris decora#F377
Asparagales Iridaceae Iris japonica#F760
Asparagales Xeronemataceae Xeronema callistemon#Y243
Asparagales Xeronemataceae Xeronema callistemon#FC393
Asparagales Asphodelaceae Eremurus chinensis#F682
Asparagales Asphodelaceae Aloe maculata#NC 035505
Asparagales Asphodelaceae Aloe vera#JVBR
Asparagales Asphodelaceae Xanthorrhoea preissii#NC 035996
Asparagales Asphodelaceae Dianella ensifolia#F343
Asparagales Asphodelaceae Phormium tenax#FCEL
Asparagales Asphodelaceae Johnsonia pubescens#WTDE
Asparagales Asphodelaceae Hemerocallis sp.#BLAJ
Asparagales Asphodelaceae Hemerocallis fulva#F569
Asparagales Amaryllidaceae Agapanthus africanus#PRFO
Asparagales Amaryllidaceae Agapanthus praecox#HQ
Asparagales Amaryllidaceae Agapanthus coddii#NC 035971
Asparagales Amaryllidaceae Crinum asiaticum var. sinicum#F583
Asparagales Amaryllidaceae Narcissus poeticus#NC 039825
Asparagales Amaryllidaceae Narcissus viridiflorus#TRRQ
Asparagales Amaryllidaceae Lycoris squamigera#NC 040164
Asparagales Amaryllidaceae Lycoris sprengeri#F209
Asparagales Amaryllidaceae Traubia modesta#ZKPF
Asparagales Amaryllidaceae Phycella aff. cyrtanthoides#DMIN
Asparagales Amaryllidaceae Zephyranthes minuta#F371
Asparagales Amaryllidaceae Zephyranthes candida#FC264
Asparagales Amaryllidaceae Amaryllis belladonna#LDME
Asparagales Amaryllidaceae Rhodophiala pratensis#JDTY
Asparagales Amaryllidaceae Zephyranthes treatiae#DPFW
Asparagales Amaryllidaceae Allium hookeri#F857
Asparagales Amaryllidaceae Allium ursinum#MH157875
Asparagales Amaryllidaceae Allium paradoxum#NC 039661
Asparagales Amaryllidaceae Allium prattii#NC 037432
Asparagales Amaryllidaceae Allium victorialis#NC 037240
Asparagales Amaryllidaceae Allium spicatum#F543
Asparagales Amaryllidaceae Allium commutatum#KBXS
Asparagales Amaryllidaceae Allium sativum#GJPF
Asparagales Amaryllidaceae Allium obliquum#NC 037199
Asparagales Amaryllidaceae Allium cepa#NC 024813

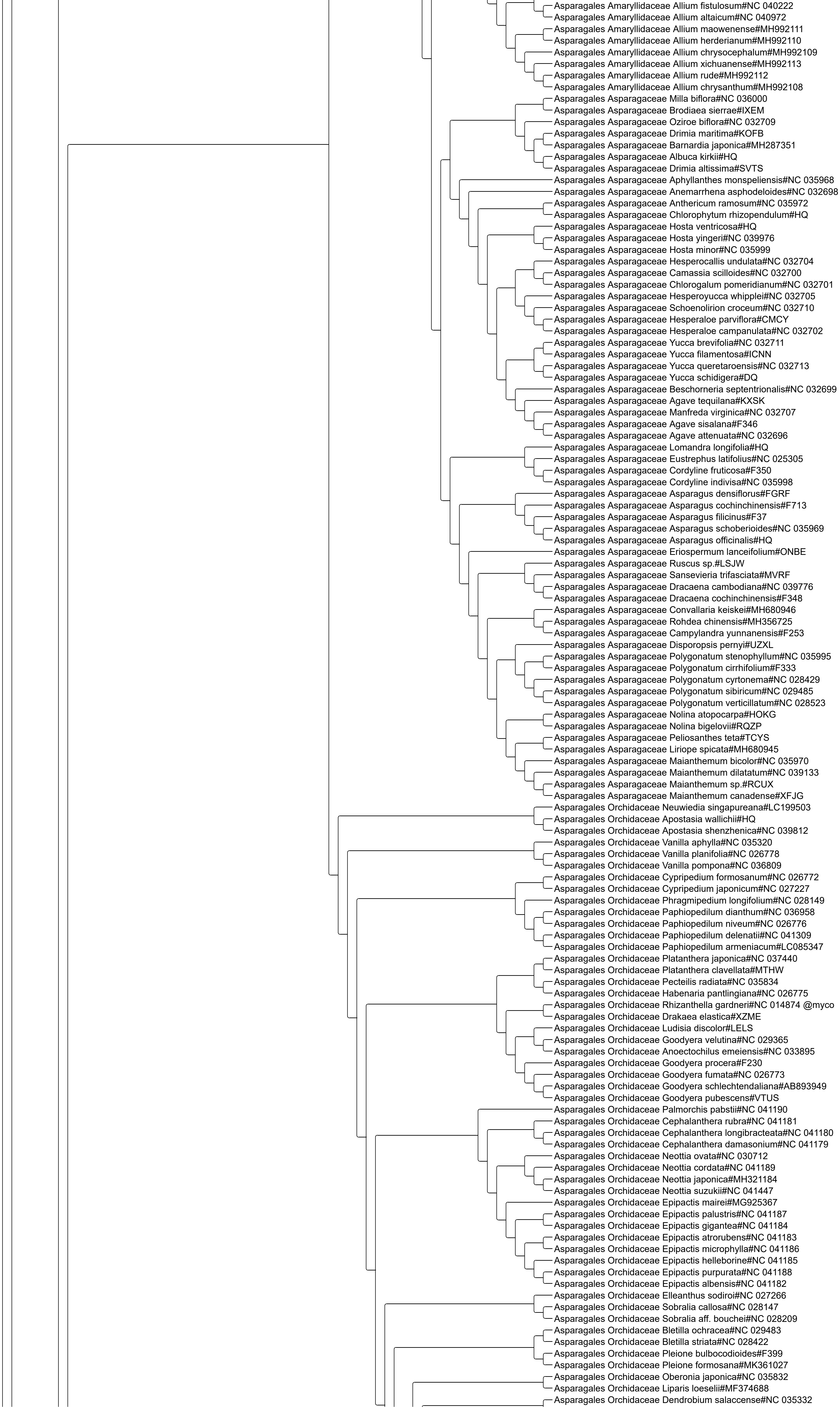
Asparagales Amaryllidaceae Allium fistulosum#NC 040222
Asparagales Amaryllidaceae Allium altaicum#NC 040972
Asparagales Amaryllidaceae Allium maowenense#MH992111
Asparagales Amaryllidaceae Allium herderianum#MH992110
Asparagales Amaryllidaceae Allium chrysocephalum#MH992109
Asparagales Amaryllidaceae Allium xichuanense#MH992113
Asparagales Amaryllidaceae Allium rude#MH992112
Asparagales Amaryllidaceae Allium chrysanthum#MH992108
Asparagales Asparagaceae Milla biflora#NC 036000
Asparagales Asparagaceae Brodiaea sierrae#IXEM
Asparagales Asparagaceae Oziroe biflora#NC 032709
Asparagales Asparagaceae Drimia maritima#KOFB
Asparagales Asparagaceae Barnardia japonica#MH287351
Asparagales Asparagaceae Albuca kirkii#HQ
Asparagales Asparagaceae Drimia altissima#SVTS
Asparagales Asparagaceae Aphyllanthes monspeliensis#NC 035968
Asparagales Asparagaceae Anemarrhena asphodeloides#NC 032698
Asparagales Asparagaceae Anthericum ramosum#NC 035972
Asparagales Asparagaceae Chlorophytum rhizopendulum#HQ
Asparagales Asparagaceae Hosta ventricosa#HQ
Asparagales Asparagaceae Hosta yingeri#NC 039976
Asparagales Asparagaceae Hosta minor#NC 035999
Asparagales Asparagaceae Hesperocallis undulata#NC 032704
Asparagales Asparagaceae Camassia scilloides#NC 032700
Asparagales Asparagaceae Chlorogalum pomeridianum#NC 032701
Asparagales Asparagaceae Hesperoyucca whipplei#NC 032705
Asparagales Asparagaceae Schoenolirion croceum#NC 032710
Asparagales Asparagaceae Hesperaloe parviflora#CMCY
Asparagales Asparagaceae Hesperaloe campanulata#NC 032702
Asparagales Asparagaceae Yucca brevifolia#NC 032711
Asparagales Asparagaceae Yucca filamentosa#ICNN
Asparagales Asparagaceae Yucca queretaroensis#NC 032713
Asparagales Asparagaceae Yucca schidigera#DQ
Asparagales Asparagaceae Beschorneria septentrionalis#NC 032699
Asparagales Asparagaceae Agave tequilana#KXSK
Asparagales Asparagaceae Manfreda virginica#NC 032707
Asparagales Asparagaceae Agave sisalana#F346
Asparagales Asparagaceae Agave attenuata#NC 032696
Asparagales Asparagaceae Lomandra longifolia#HQ
Asparagales Asparagaceae Eustrephus latifolius#NC 025305
Asparagales Asparagaceae Cordyline fruticosa#F350
Asparagales Asparagaceae Cordyline indivisa#NC 035998
Asparagales Asparagaceae Asparagus densiflorus#FGRF
Asparagales Asparagaceae Asparagus cochinchinensis#F713
Asparagales Asparagaceae Asparagus filicinus#F37
Asparagales Asparagaceae Asparagus schoberioides#NC 035969
Asparagales Asparagaceae Asparagus officinalis#HQ
Asparagales Asparagaceae Eriospermum lanceifolium#ONBE
Asparagales Asparagaceae Ruscus sp.#LSJW
Asparagales Asparagaceae Sansevieria trifasciata#MVRF
Asparagales Asparagaceae Dracaena cambodiana#NC 039776
Asparagales Asparagaceae Dracaena cochinchinensis#F348
Asparagales Asparagaceae Convallaria keiskei#MH680946
Asparagales Asparagaceae Rohdea chinensis#MH356725
Asparagales Asparagaceae Campylandra yunnanensis#F253
Asparagales Asparagaceae Disporopsis pernyi#UZXL
Asparagales Asparagaceae Polygonatum stenophyllum#NC 035995
Asparagales Asparagaceae Polygonatum cirrhifolium#F333
Asparagales Asparagaceae Polygonatum cyrtonema#NC 028429
Asparagales Asparagaceae Polygonatum sibiricum#NC 029485
Asparagales Asparagaceae Polygonatum verticillatum#NC 028523
Asparagales Asparagaceae Nolina atopocarpa#HOKG
Asparagales Asparagaceae Nolina bigelovii#RQZP
Asparagales Asparagaceae Peliosanthes teta#TCYS
Asparagales Asparagaceae Liriope spicata#MH680945
Asparagales Asparagaceae Maianthemum bicolor#NC 035970
Asparagales Asparagaceae Maianthemum dilatatum#NC 039133
Asparagales Asparagaceae Maianthemum sp.#RCUX
Asparagales Asparagaceae Maianthemum canadense#XFJG
Asparagales Orchidaceae Neuwiedia singapureana#LC199503
Asparagales Orchidaceae Apostasia wallichii#HQ
Asparagales Orchidaceae Apostasia shenzhenica#NC 039812
Asparagales Orchidaceae Vanilla aphylla#NC 035320
Asparagales Orchidaceae Vanilla planifolia#NC 026778
Asparagales Orchidaceae Vanilla pompona#NC 036809
Asparagales Orchidaceae Cypripedium formosanum#NC 026772
Asparagales Orchidaceae Cypripedium japonicum#NC 027227
Asparagales Orchidaceae Phragmipedium longifolium#NC 028149
Asparagales Orchidaceae Paphiopedilum dianthum#NC 036958
Asparagales Orchidaceae Paphiopedilum niveum#NC 026776
Asparagales Orchidaceae Paphiopedilum delenatii#NC 041309
Asparagales Orchidaceae Paphiopedilum armeniacum#LC085347
Asparagales Orchidaceae Platanthera japonica#NC 037440
Asparagales Orchidaceae Platanthera clavellata#MTHW
Asparagales Orchidaceae Pecteilis radiata#NC 035834
Asparagales Orchidaceae Habenaria pantlingiana#NC 026775
Asparagales Orchidaceae Rhizanthella gardneri#NC 014874 @myco
Asparagales Orchidaceae Drakaea elastica#XZME
Asparagales Orchidaceae Ludisia discolor#LELS
Asparagales Orchidaceae Goodyera velutina#NC 029365
Asparagales Orchidaceae Anoectochilus emeiensis#NC 033895
Asparagales Orchidaceae Goodyera procera#F230
Asparagales Orchidaceae Goodyera fumata#NC 026773
Asparagales Orchidaceae Goodyera schlechtendaliana#AB893949
Asparagales Orchidaceae Goodyera pubescens#VTUS
Asparagales Orchidaceae Palmorchis pabstii#NC 041190
Asparagales Orchidaceae Cephalanthera rubra#NC 041181
Asparagales Orchidaceae Cephalanthera longibracteata#NC 041180
Asparagales Orchidaceae Cephalanthera damasonium#NC 041179
Asparagales Orchidaceae Neottia ovata#NC 030712
Asparagales Orchidaceae Neottia cordata#NC 041189
Asparagales Orchidaceae Neottia japonica#MH321184
Asparagales Orchidaceae Neottia suzukii#NC 041447
Asparagales Orchidaceae Epipactis mairei#MG925367
Asparagales Orchidaceae Epipactis palustris#NC 041187
Asparagales Orchidaceae Epipactis gigantea#NC 041184
Asparagales Orchidaceae Epipactis atrorubens#NC 041183
Asparagales Orchidaceae Epipactis microphylla#NC 041186
Asparagales Orchidaceae Epipactis helleborine#NC 041185
Asparagales Orchidaceae Epipactis purpurata#NC 041188
Asparagales Orchidaceae Epipactis albensis#NC 041182
Asparagales Orchidaceae Elleanthus sodiroi#NC 027266
Asparagales Orchidaceae Sobralia callosa#NC 028147
Asparagales Orchidaceae Sobralia aff. bouchei#NC 028209
Asparagales Orchidaceae Bletilla ochracea#NC 029483
Asparagales Orchidaceae Bletilla striata#NC 028422
Asparagales Orchidaceae Pleione bulbocodioides#F399
Asparagales Orchidaceae Pleione formosana#MK361027
Asparagales Orchidaceae Oberonia japonica#NC 035832
Asparagales Orchidaceae Liparis loeselii#MF374688
Asparagales Orchidaceae Dendrobium salaccense#NC 035332

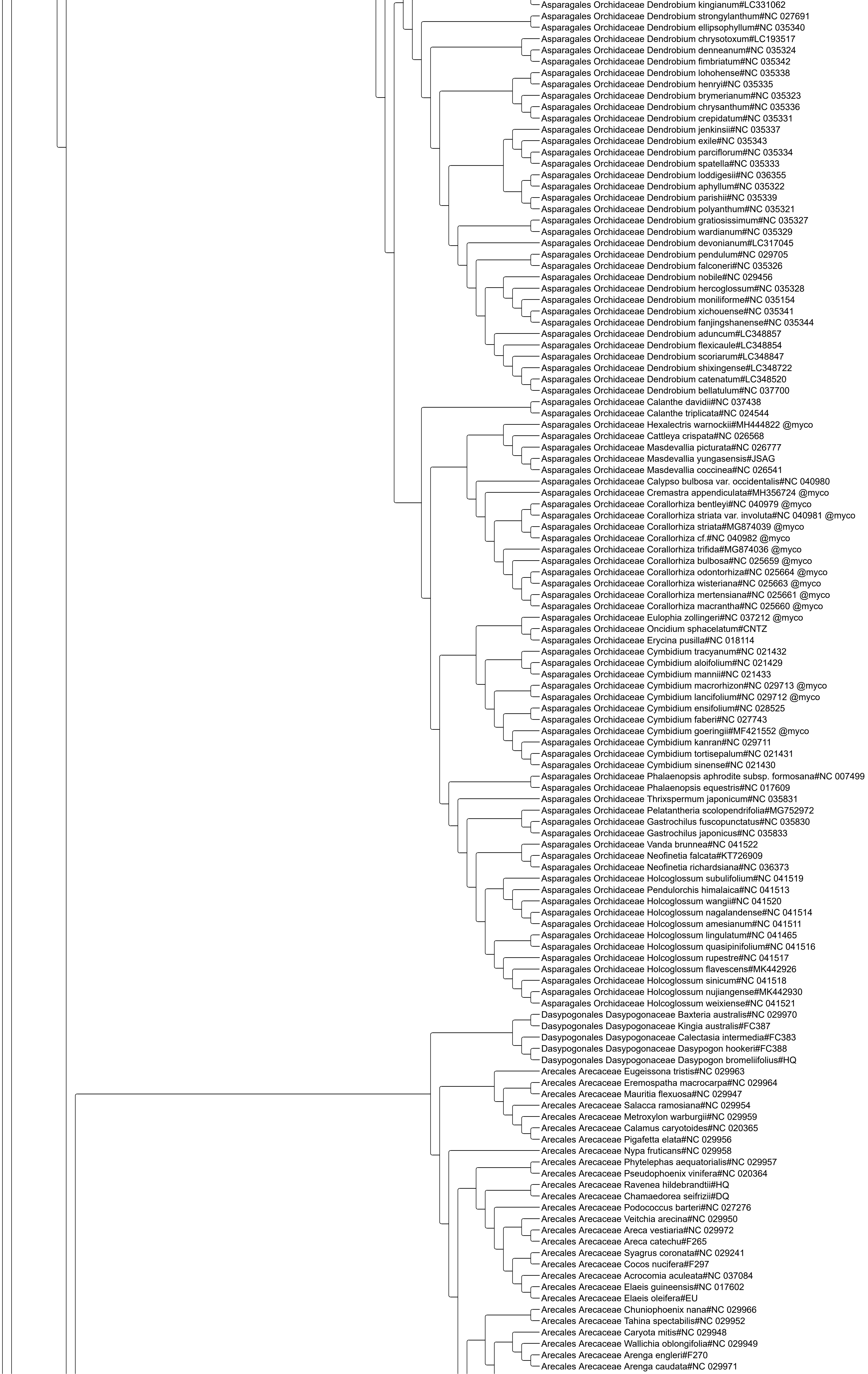

Asparagales Orchidaceae Dendrobium kingianum#LC331062
Asparagales Orchidaceae Dendrobium strongylanthum#NC 027691
Asparagales Orchidaceae Dendrobium ellipsophyllum#NC 035340
Asparagales Orchidaceae Dendrobium chrysotoxum#LC193517
Asparagales Orchidaceae Dendrobium denneanum#NC 035324
Asparagales Orchidaceae Dendrobium fimbriatum#NC 035342
Asparagales Orchidaceae Dendrobium lohohense#NC 035338
Asparagales Orchidaceae Dendrobium henryi#NC 035335
Asparagales Orchidaceae Dendrobium brymerianum#NC 035323
Asparagales Orchidaceae Dendrobium chrysanthum#NC 035336
Asparagales Orchidaceae Dendrobium crepidatum#NC 035331
Asparagales Orchidaceae Dendrobium jenkinsii#NC 035337
Asparagales Orchidaceae Dendrobium exile#NC 035343
Asparagales Orchidaceae Dendrobium parciflorum#NC 035334
Asparagales Orchidaceae Dendrobium spatella#NC 035333
Asparagales Orchidaceae Dendrobium loddigesii#NC 036355
Asparagales Orchidaceae Dendrobium aphyllum#NC 035322
Asparagales Orchidaceae Dendrobium parishii#NC 035339
Asparagales Orchidaceae Dendrobium polyanthum#NC 035321
Asparagales Orchidaceae Dendrobium gratiosissimum#NC 035327
Asparagales Orchidaceae Dendrobium wardianum#NC 035329
Asparagales Orchidaceae Dendrobium devonianum#LC317045
Asparagales Orchidaceae Dendrobium pendulum#NC 029705
Asparagales Orchidaceae Dendrobium falconeri#NC 035326
Asparagales Orchidaceae Dendrobium nobile#NC 029456
Asparagales Orchidaceae Dendrobium hercoglossum#NC 035328
Asparagales Orchidaceae Dendrobium moniliforme#NC 035154
Asparagales Orchidaceae Dendrobium xichouense#NC 035341
Asparagales Orchidaceae Dendrobium fanjingshanense#NC 035344
Asparagales Orchidaceae Dendrobium aduncum#LC348857
Asparagales Orchidaceae Dendrobium flexicaule#LC348854
Asparagales Orchidaceae Dendrobium scoriarum#LC348847
Asparagales Orchidaceae Dendrobium shixingense#LC348722
Asparagales Orchidaceae Dendrobium catenatum#LC348520
Asparagales Orchidaceae Dendrobium bellatulum#NC 037700
Asparagales Orchidaceae Calanthe davidii#NC 037438
Asparagales Orchidaceae Calanthe triplicata#NC 024544
Asparagales Orchidaceae Hexalectris warnockii#MH444822 @myco
Asparagales Orchidaceae Cattleya crispata#NC 026568
Asparagales Orchidaceae Masdevallia picturata#NC 026777
Asparagales Orchidaceae Masdevallia yungasensis#JSAG
Asparagales Orchidaceae Masdevallia coccinea#NC 026541
Asparagales Orchidaceae Calypso bulbosa var. occidentalis#NC 040980
Asparagales Orchidaceae Cremastra appendiculata#MH356724 @myco
Asparagales Orchidaceae Corallorhiza bentleyi#NC 040979 @myco
Asparagales Orchidaceae Corallorhiza striata var. involuta#NC 040981 @myco
Asparagales Orchidaceae Corallorhiza striata#MG874039 @myco
Asparagales Orchidaceae Corallorhiza cf.#NC 040982 @myco
Asparagales Orchidaceae Corallorhiza trifida#MG874036 @myco
Asparagales Orchidaceae Corallorhiza bulbosa#NC 025659 @myco
Asparagales Orchidaceae Corallorhiza odontorhiza#NC 025664 @myco
Asparagales Orchidaceae Corallorhiza wisteriana#NC 025663 @myco
Asparagales Orchidaceae Corallorhiza mertensiana#NC 025661 @myco
Asparagales Orchidaceae Corallorhiza macrantha#NC 025660 @myco
Asparagales Orchidaceae Eulophia zollingeri#NC 037212 @myco
Asparagales Orchidaceae Oncidium sphacelatum#CNTZ
Asparagales Orchidaceae Erycina pusilla#NC 018114
Asparagales Orchidaceae Cymbidium tracyanum#NC 021432
Asparagales Orchidaceae Cymbidium aloifolium#NC 021429
Asparagales Orchidaceae Cymbidium mannii#NC 021433
Asparagales Orchidaceae Cymbidium macrorhizon#NC 029713 @myco
Asparagales Orchidaceae Cymbidium lancifolium#NC 029712 @myco
Asparagales Orchidaceae Cymbidium ensifolium#NC 028525
Asparagales Orchidaceae Cymbidium faberi#NC 027743
Asparagales Orchidaceae Cymbidium goeringii#MF421552 @myco
Asparagales Orchidaceae Cymbidium kanran#NC 029711
Asparagales Orchidaceae Cymbidium tortisepalum#NC 021431
Asparagales Orchidaceae Cymbidium sinense#NC 021430
Asparagales Orchidaceae Phalaenopsis aphrodite subsp. formosana#NC 007499
Asparagales Orchidaceae Phalaenopsis equestris#NC 017609
Asparagales Orchidaceae Thrixspermum japonicum#NC 035831
Asparagales Orchidaceae Pelatantheria scolopendrifolia#MG752972
Asparagales Orchidaceae Gastrochilus fuscopunctatus#NC 035830
Asparagales Orchidaceae Gastrochilus japonicus#NC 035833
Asparagales Orchidaceae Vanda brunnea#NC 041522
Asparagales Orchidaceae Neofinetia falcata#KT726909
Asparagales Orchidaceae Neofinetia richardsiana#NC 036373
Asparagales Orchidaceae Holcoglossum subulifolium#NC 041519
Asparagales Orchidaceae Pendulorchis himalaica#NC 041513
Asparagales Orchidaceae Holcoglossum wangii#NC 041520
Asparagales Orchidaceae Holcoglossum nagalandense#NC 041514
Asparagales Orchidaceae Holcoglossum amesianum#NC 041511
Asparagales Orchidaceae Holcoglossum lingulatum#NC 041465
Asparagales Orchidaceae Holcoglossum quasipinifolium#NC 041516
Asparagales Orchidaceae Holcoglossum rupestre#NC 041517
Asparagales Orchidaceae Holcoglossum flavescens#MK442926
Asparagales Orchidaceae Holcoglossum sinicum#NC 041518
Asparagales Orchidaceae Holcoglossum nujiangense#MK442930
Asparagales Orchidaceae Holcoglossum weixiense#NC 041521
Dasypogonales Dasypogonaceae Baxteria australis#NC 029970
Dasypogonales Dasypogonaceae Kingia australis#FC387
Dasypogonales Dasypogonaceae Calectasia intermedia#FC383
Dasypogonales Dasypogonaceae Dasypogon hookeri#FC388
Dasypogonales Dasypogonaceae Dasypogon bromeliifolius#HQ
Arecales Arecaceae Eugeissona tristis#NC 029963
Arecales Arecaceae Eremospatha macrocarpa#NC 029964
Arecales Arecaceae Mauritia flexuosa#NC 029947
Arecales Arecaceae Salacca ramosiana#NC 029954
Arecales Arecaceae Metroxylon warburgii#NC 029959
Arecales Arecaceae Calamus caryotoides#NC 020365
Arecales Arecaceae Pigafetta elata#NC 029956
Arecales Arecaceae Nypa fruticans#NC 029958
Arecales Arecaceae Phytelephas aequatorialis#NC 029957
Arecales Arecaceae Pseudophoenix vinifera#NC 020364
Arecales Arecaceae Ravenea hildebrandtii#HQ
Arecales Arecaceae Chamaedorea seifrizii#DQ
Arecales Arecaceae Podococcus barteri#NC 027276
Arecales Arecaceae Veitchia arecina#NC 029950
Arecales Arecaceae Areca vestiaria#NC 029972
Arecales Arecaceae Areca catechu#F265
Arecales Arecaceae Syagrus coronata#NC 029241
Arecales Arecaceae Cocos nucifera#F297
Arecales Arecaceae Acrocomia aculeata#NC 037084
Arecales Arecaceae Elaeis guineensis#NC 017602
Arecales Arecaceae Elaeis oleifera#EU
Arecales Arecaceae Chuniophoenix nana#NC 029966
Arecales Arecaceae Tahina spectabilis#NC 029952
Arecales Arecaceae Caryota mitis#NC 029948
Arecales Arecaceae Wallichia oblongifolia#NC 029949
Arecales Arecaceae Arenga engleri#F270
Arecales Arecaceae Arenga caudata#NC 029971

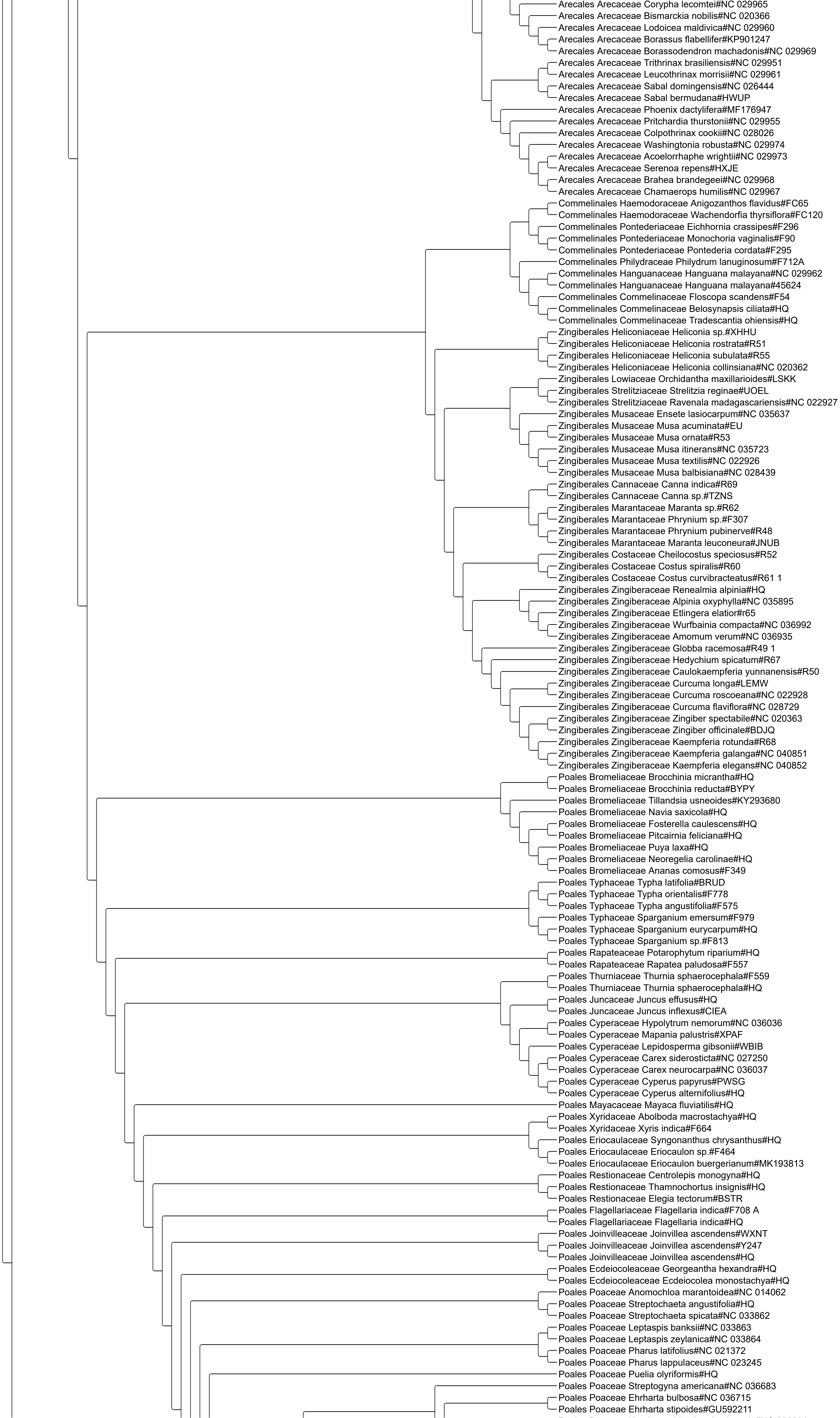

Arecales Arecaceae Corypha lecomtei#NC 029965
Arecales Arecaceae Bismarckia nobilis#NC 020366
Arecales Arecaceae Lodoicea maldivica#NC 029960
Arecales Arecaceae Borassus flabellifer#KP901247
Arecales Arecaceae Borassodendron machadonis#NC 029969
Arecales Arecaceae Trithrinax brasiliensis#NC 029951
Arecales Arecaceae Leucothrinax morrisii#NC 029961
Arecales Arecaceae Sabal domingensis#NC 026444
Arecales Arecaceae Sabal bermudana#HWUP
Arecales Arecaceae Phoenix dactylifera#MF176947
Arecales Arecaceae Pritchardia thurstonii#NC 029955
Arecales Arecaceae Colpothrinax cookii#NC 028026
Arecales Arecaceae Washingtonia robusta#NC 029974
Arecales Arecaceae Acoelorrhaphe wrightii#NC 029973
Arecales Arecaceae Serenoa repens#HXJE
Arecales Arecaceae Brahea brandegeei#NC 029968
Arecales Arecaceae Chamaerops humilis#NC 029967
Commelinales Haemodoraceae Anigozanthos flavidus#FC65
Commelinales Haemodoraceae Wachendorfia thyrsiflora#FC120
Commelinales Pontederiaceae Eichhornia crassipes#F296
Commelinales Pontederiaceae Monochoria vaginalis#F90
Commelinales Pontederiaceae Pontederia cordata#F295
Commelinales Philydraceae Philydrum lanuginosum#F712A
Commelinales Hanguanaceae Hanguana malayana#NC 029962
Commelinales Hanguanaceae Hanguana malayana#45624
Commelinales Commelinaceae Floscopa scandens#F54
Commelinales Commelinaceae Belosynapsis ciliata#HQ
Commelinales Commelinaceae Tradescantia ohiensis#HQ
Zingiberales Heliconiaceae Heliconia sp.#XHHU
Zingiberales Heliconiaceae Heliconia rostrata#R51
Zingiberales Heliconiaceae Heliconia subulata#R55
Zingiberales Heliconiaceae Heliconia collinsiana#NC 020362
Zingiberales Lowiaceae Orchidantha maxillarioides#LSKK
Zingiberales Strelitziaceae Strelitzia reginae#UOEL
Zingiberales Strelitziaceae Ravenala madagascariensis#NC 022927
Zingiberales Musaceae Ensete lasiocarpum#NC 035637
Zingiberales Musaceae Musa acuminata#EU
Zingiberales Musaceae Musa ornata#R53
Zingiberales Musaceae Musa itinerans#NC 035723
Zingiberales Musaceae Musa textilis#NC 022926
Zingiberales Musaceae Musa balbisiana#NC 028439
Zingiberales Cannaceae Canna indica#R69
Zingiberales Cannaceae Canna sp.#TZNS
Zingiberales Marantaceae Maranta sp.#R62
Zingiberales Marantaceae Phrynium sp.#F307
Zingiberales Marantaceae Phrynium pubinerve#R48
Zingiberales Marantaceae Maranta leuconeura#JNUB
Zingiberales Costaceae Cheilocostus speciosus#R52
Zingiberales Costaceae Costus spiralis#R60
Zingiberales Costaceae Costus curvibracteatus#R61 1
Zingiberales Zingiberaceae Renealmia alpinia#HQ
Zingiberales Zingiberaceae Alpinia oxyphylla#NC 035895
Zingiberales Zingiberaceae Etlingera elatior#r65
Zingiberales Zingiberaceae Wurfbainia compacta#NC 036992
Zingiberales Zingiberaceae Amomum verum#NC 036935
Zingiberales Zingiberaceae Globba racemosa#R49 1
Zingiberales Zingiberaceae Hedychium spicatum#R67
Zingiberales Zingiberaceae Caulokaempferia yunnanensis#R50
Zingiberales Zingiberaceae Curcuma longa#LEMW
Zingiberales Zingiberaceae Curcuma roscoeana#NC 022928
Zingiberales Zingiberaceae Curcuma flaviflora#NC 028729
Zingiberales Zingiberaceae Zingiber spectabile#NC 020363
Zingiberales Zingiberaceae Zingiber officinale#BDJQ
Zingiberales Zingiberaceae Kaempferia rotunda#R68
Zingiberales Zingiberaceae Kaempferia galanga#NC 040851
Zingiberales Zingiberaceae Kaempferia elegans#NC 040852
Poales Bromeliaceae Brocchinia micrantha#HQ
Poales Bromeliaceae Brocchinia reducta#BYPY
Poales Bromeliaceae Tillandsia usneoides#KY293680
Poales Bromeliaceae Navia saxicola#HQ
Poales Bromeliaceae Fosterella caulescens#HQ
Poales Bromeliaceae Pitcairnia feliciana#HQ
Poales Bromeliaceae Puya laxa#HQ
Poales Bromeliaceae Neoregelia carolinae#HQ
Poales Bromeliaceae Ananas comosus#F349
Poales Typhaceae Typha latifolia#BRUD
Poales Typhaceae Typha orientalis#F778
Poales Typhaceae Typha angustifolia#F575
Poales Typhaceae Sparganium emersum#F979
Poales Typhaceae Sparganium eurycarpum#HQ
Poales Typhaceae Sparganium sp.#F813
Poales Rapateaceae Potarophytum riparium#HQ
Poales Rapateaceae Rapatea paludosa#F557
Poales Thurniaceae Thurnia sphaerocephala#F559
Poales Thurniaceae Thurnia sphaerocephala#HQ
Poales Juncaceae Juncus effusus#HQ
Poales Juncaceae Juncus inflexus#CIEA
Poales Cyperaceae Hypolytrum nemorum#NC 036036
Poales Cyperaceae Mapania palustris#XPAF
Poales Cyperaceae Lepidosperma gibsonii#WBIB
Poales Cyperaceae Carex siderosticta#NC 027250
Poales Cyperaceae Carex neurocarpa#NC 036037
Poales Cyperaceae Cyperus papyrus#PWSG
Poales Cyperaceae Cyperus alternifolius#HQ
Poales Mayacaceae Mayaca fluviatilis#HQ
Poales Xyridaceae Abolboda macrostachya#HQ
Poales Xyridaceae Xyris indica#F664
Poales Eriocaulaceae Syngonanthus chrysanthus#HQ
Poales Eriocaulaceae Eriocaulon sp.#F464
Poales Eriocaulaceae Eriocaulon buergerianum#MK193813
Poales Restionaceae Centrolepis monogyna#HQ
Poales Restionaceae Thamnochortus insignis#HQ
Poales Restionaceae Elegia tectorum#BSTR
Poales Flagellariaceae Flagellaria indica#F708 A
Poales Flagellariaceae Flagellaria indica#HQ
Poales Joinvilleaceae Joinvillea ascendens#WXNT
Poales Joinvilleaceae Joinvillea ascendens#Y247
Poales Joinvilleaceae Joinvillea ascendens#HQ
Poales Ecdeiocoleaceae Georgeantha hexandra#HQ
Poales Ecdeiocoleaceae Ecdeiocolea monostachya#HQ
Poales Poaceae Anomochloa marantoidea#NC 014062
Poales Poaceae Streptochaeta angustifolia#HQ
Poales Poaceae Streptochaeta spicata#NC 033862
Poales Poaceae Leptaspis banksii#NC 033863
Poales Poaceae Leptaspis zeylanica#NC 033864
Poales Poaceae Pharus latifolius#NC 021372
Poales Poaceae Pharus lappulaceus#NC 023245
Poales Poaceae Puelia olyriformis#HQ
Poales Poaceae Streptogyna americana#NC 036683
Poales Poaceae Ehrharta bulbosa#NC 036715
Poales Poaceae Ehrharta stipoides#GU592211

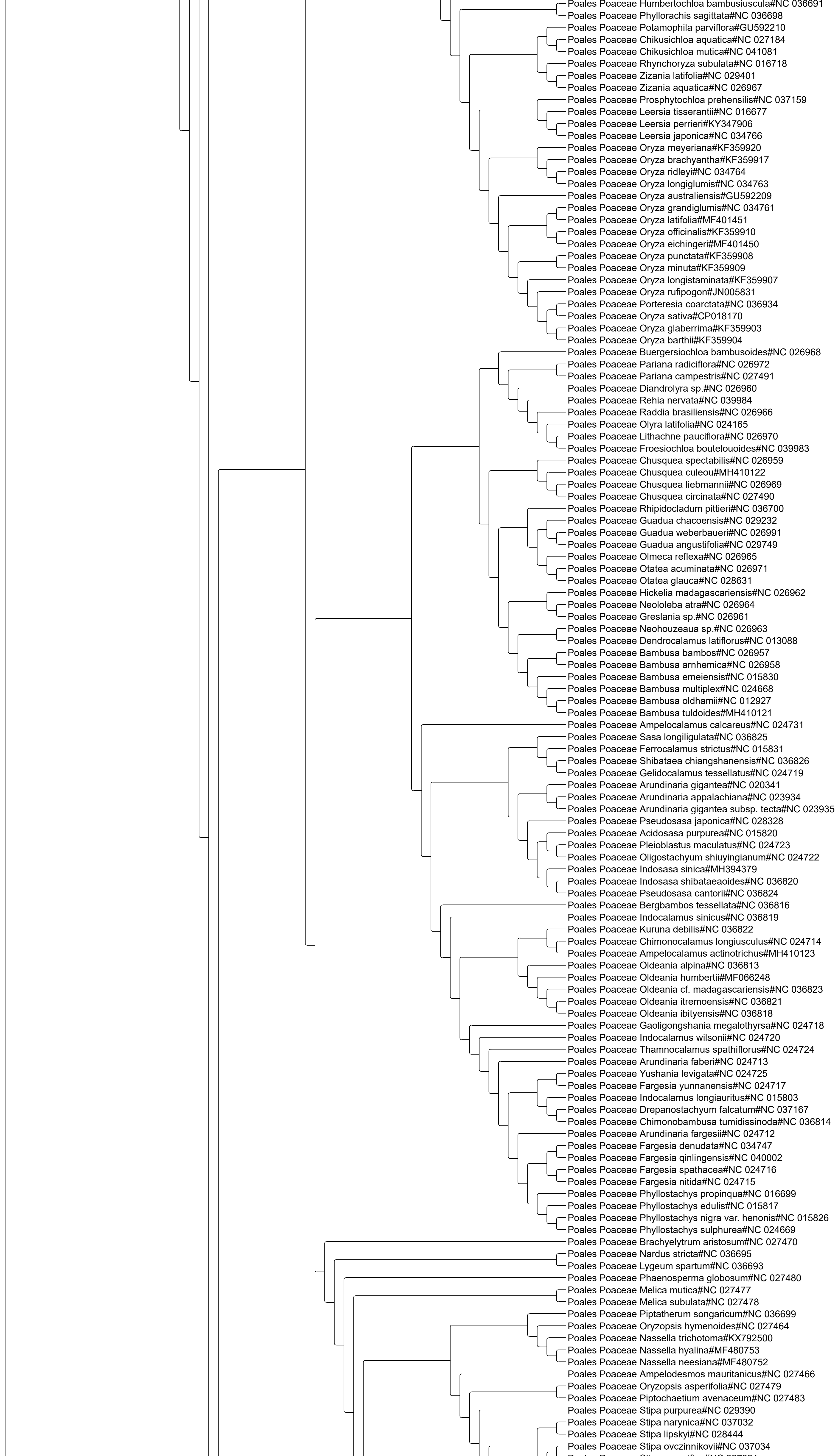
Poales Poaceae Humbertochloa bambusiuscula#NC 036691
Poales Poaceae Phyllorachis sagittata#NC 036698
Poales Poaceae Potamophila parviflora#GU592210
Poales Poaceae Chikusichloa aquatica#NC 027184
Poales Poaceae Chikusichloa mutica#NC 041081
Poales Poaceae Rhynchoryza subulata#NC 016718
Poales Poaceae Zizania latifolia#NC 029401
Poales Poaceae Zizania aquatica#NC 026967
Poales Poaceae Prosphytochloa prehensilis#NC 037159
Poales Poaceae Leersia tisserantii#NC 016677
Poales Poaceae Leersia perrieri#KY347906
Poales Poaceae Leersia japonica#NC 034766
Poales Poaceae Oryza meyeriana#KF359920
Poales Poaceae Oryza brachyantha#KF359917
Poales Poaceae Oryza ridleyi#NC 034764
Poales Poaceae Oryza longiglumis#NC 034763
Poales Poaceae Oryza australiensis#GU592209
Poales Poaceae Oryza grandiglumis#NC 034761
Poales Poaceae Oryza latifolia#MF401451
Poales Poaceae Oryza officinalis#KF359910
Poales Poaceae Oryza eichingeri#MF401450
Poales Poaceae Oryza punctata#KF359908
Poales Poaceae Oryza minuta#KF359909
Poales Poaceae Oryza longistaminata#KF359907
Poales Poaceae Oryza rufipogon#JN005831
Poales Poaceae Porteresia coarctata#NC 036934
Poales Poaceae Oryza sativa#CP018170
Poales Poaceae Oryza glaberrima#KF359903
Poales Poaceae Oryza barthii#KF359904
Poales Poaceae Buergersiochloa bambusoides#NC 026968
Poales Poaceae Pariana radiciflora#NC 026972
Poales Poaceae Pariana campestris#NC 027491
Poales Poaceae Diandrolyra sp.#NC 026960
Poales Poaceae Rehia nervata#NC 039984
Poales Poaceae Raddia brasiliensis#NC 026966
Poales Poaceae Olyra latifolia#NC 024165
Poales Poaceae Lithachne pauciflora#NC 026970
Poales Poaceae Froesiochloa boutelouoides#NC 039983
Poales Poaceae Chusquea spectabilis#NC 026959
Poales Poaceae Chusquea culeou#MH410122
Poales Poaceae Chusquea liebmannii#NC 026969
Poales Poaceae Chusquea circinata#NC 027490
Poales Poaceae Rhipidocladum pittieri#NC 036700
Poales Poaceae Guadua chacoensis#NC 029232
Poales Poaceae Guadua weberbaueri#NC 026991
Poales Poaceae Guadua angustifolia#NC 029749
Poales Poaceae Olmeca reflexa#NC 026965
Poales Poaceae Otatea acuminata#NC 026971
Poales Poaceae Otatea glauca#NC 028631
Poales Poaceae Hickelia madagascariensis#NC 026962
Poales Poaceae Neololeba atra#NC 026964
Poales Poaceae Greslania sp.#NC 026961
Poales Poaceae Neohouzeaua sp.#NC 026963
Poales Poaceae Dendrocalamus latiflorus#NC 013088
Poales Poaceae Bambusa bambos#NC 026957
Poales Poaceae Bambusa arnhemica#NC 026958
Poales Poaceae Bambusa emeiensis#NC 015830
Poales Poaceae Bambusa multiplex#NC 024668
Poales Poaceae Bambusa oldhamii#NC 012927
Poales Poaceae Bambusa tuldoides#MH410121
Poales Poaceae Ampelocalamus calcareus#NC 024731
Poales Poaceae Sasa longiligulata#NC 036825
Poales Poaceae Ferrocalamus strictus#NC 015831
Poales Poaceae Shibataea chiangshanensis#NC 036826
Poales Poaceae Gelidocalamus tessellatus#NC 024719
Poales Poaceae Arundinaria gigantea#NC 020341
Poales Poaceae Arundinaria appalachiana#NC 023934
Poales Poaceae Arundinaria gigantea subsp. tecta#NC 023935
Poales Poaceae Pseudosasa japonica#NC 028328
Poales Poaceae Acidosasa purpurea#NC 015820
Poales Poaceae Pleioblastus maculatus#NC 024723
Poales Poaceae Oligostachyum shiuyingianum#NC 024722
Poales Poaceae Indosasa sinica#MH394379
Poales Poaceae Indosasa shibataeaoides#NC 036820
Poales Poaceae Pseudosasa cantorii#NC 036824
Poales Poaceae Bergbambos tessellata#NC 036816
Poales Poaceae Indocalamus sinicus#NC 036819
Poales Poaceae Kuruna debilis#NC 036822
Poales Poaceae Chimonocalamus longiusculus#NC 024714
Poales Poaceae Ampelocalamus actinotrichus#MH410123
Poales Poaceae Oldeania alpina#NC 036813
Poales Poaceae Oldeania humbertii#MF066248
Poales Poaceae Oldeania cf. madagascariensis#NC 036823
Poales Poaceae Oldeania itremoensis#NC 036821
Poales Poaceae Oldeania ibityensis#NC 036818
Poales Poaceae Gaoligongshania megalothyrsa#NC 024718
Poales Poaceae Indocalamus wilsonii#NC 024720
Poales Poaceae Thamnocalamus spathiflorus#NC 024724
Poales Poaceae Arundinaria faberi#NC 024713
Poales Poaceae Yushania levigata#NC 024725
Poales Poaceae Fargesia yunnanensis#NC 024717
Poales Poaceae Indocalamus longiauritus#NC 015803
Poales Poaceae Drepanostachyum falcatum#NC 037167
Poales Poaceae Chimonobambusa tumidissinoda#NC 036814
Poales Poaceae Arundinaria fargesii#NC 024712
Poales Poaceae Fargesia denudata#NC 034747
Poales Poaceae Fargesia qinlingensis#NC 040002
Poales Poaceae Fargesia spathacea#NC 024716
Poales Poaceae Fargesia nitida#NC 024715
Poales Poaceae Phyllostachys propinqua#NC 016699
Poales Poaceae Phyllostachys edulis#NC 015817
Poales Poaceae Phyllostachys nigra var. henonis#NC 015826
Poales Poaceae Phyllostachys sulphurea#NC 024669
Poales Poaceae Brachyelytrum aristosum#NC 027470
Poales Poaceae Nardus stricta#NC 036695
Poales Poaceae Lygeum spartum#NC 036693
Poales Poaceae Phaenosperma globosum#NC 027480
Poales Poaceae Melica mutica#NC 027477
Poales Poaceae Melica subulata#NC 027478
Poales Poaceae Piptatherum songaricum#NC 036699
Poales Poaceae Oryzopsis hymenoides#NC 027464
Poales Poaceae Nassella trichotoma#KX792500
Poales Poaceae Nassella hyalina#MF480753
Poales Poaceae Nassella neesiana#MF480752
Poales Poaceae Ampelodesmos mauritanicus#NC 027466
Poales Poaceae Oryzopsis asperifolia#NC 027479
Poales Poaceae Piptochaetium avenaceum#NC 027483
Poales Poaceae Stipa purpurea#NC 029390
Poales Poaceae Stipa narynica#NC 037032
Poales Poaceae Stipa lipskyi#NC 028444
Poales Poaceae Stipa ovczinnikovii#NC 037034

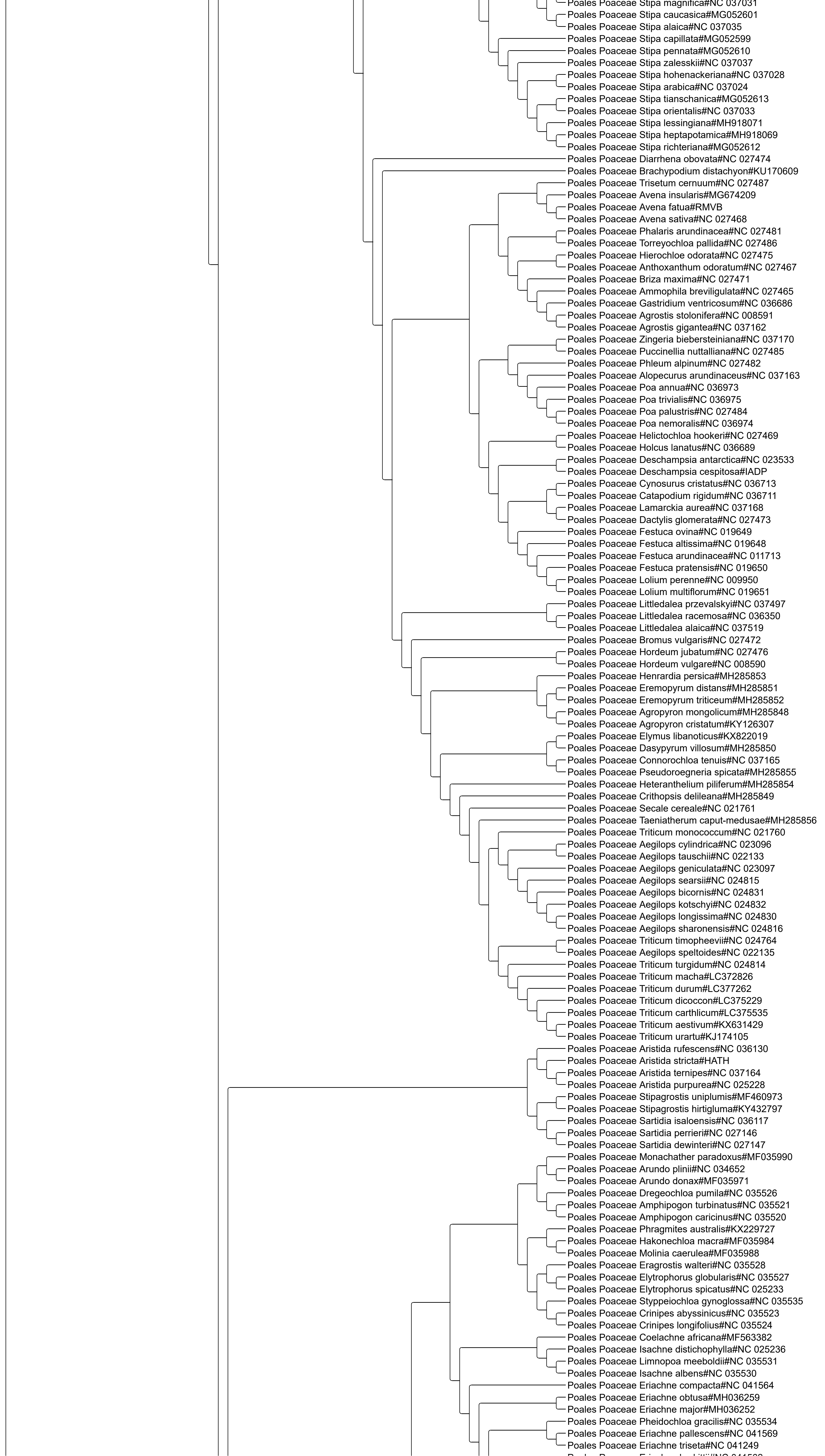

Poales Poaceae Stipa magnifica#NC 037031
Poales Poaceae Stipa caucasica#MG052601
Poales Poaceae Stipa alaica#NC 037035
Poales Poaceae Stipa capillata#MG052599
Poales Poaceae Stipa pennata#MG052610
Poales Poaceae Stipa zalesskii#NC 037037
Poales Poaceae Stipa hohenackeriana#NC 037028
Poales Poaceae Stipa arabica#NC 037024
Poales Poaceae Stipa tianschanica#MG052613
Poales Poaceae Stipa orientalis#NC 037033
Poales Poaceae Stipa lessingiana#MH918071
Poales Poaceae Stipa heptapotamica#MH918069
Poales Poaceae Stipa richteriana#MG052612
Poales Poaceae Diarrhena obovata#NC 027474
Poales Poaceae Brachypodium distachyon#KU170609
Poales Poaceae Trisetum cernuum#NC 027487
Poales Poaceae Avena insularis#MG674209
Poales Poaceae Avena fatua#RMVB
Poales Poaceae Avena sativa#NC 027468
Poales Poaceae Phalaris arundinacea#NC 027481
Poales Poaceae Torreyochloa pallida#NC 027486
Poales Poaceae Hierochloe odorata#NC 027475
Poales Poaceae Anthoxanthum odoratum#NC 027467
Poales Poaceae Briza maxima#NC 027471
Poales Poaceae Ammophila breviligulata#NC 027465
Poales Poaceae Gastridium ventricosum#NC 036686
Poales Poaceae Agrostis stolonifera#NC 008591
Poales Poaceae Agrostis gigantea#NC 037162
Poales Poaceae Zingeria biebersteiniana#NC 037170
Poales Poaceae Puccinellia nuttalliana#NC 027485
Poales Poaceae Phleum alpinum#NC 027482
Poales Poaceae Alopecurus arundinaceus#NC 037163
Poales Poaceae Poa annua#NC 036973
Poales Poaceae Poa trivialis#NC 036975
Poales Poaceae Poa palustris#NC 027484
Poales Poaceae Poa nemoralis#NC 036974
Poales Poaceae Helictochloa hookeri#NC 027469
Poales Poaceae Holcus lanatus#NC 036689
Poales Poaceae Deschampsia antarctica#NC 023533
Poales Poaceae Deschampsia cespitosa#IADP
Poales Poaceae Cynosurus cristatus#NC 036713
Poales Poaceae Catapodium rigidum#NC 036711
Poales Poaceae Lamarckia aurea#NC 037168
Poales Poaceae Dactylis glomerata#NC 027473
Poales Poaceae Festuca ovina#NC 019649
Poales Poaceae Festuca altissima#NC 019648
Poales Poaceae Festuca arundinacea#NC 011713
Poales Poaceae Festuca pratensis#NC 019650
Poales Poaceae Lolium perenne#NC 009950
Poales Poaceae Lolium multiflorum#NC 019651
Poales Poaceae Littledalea przevalskyi#NC 037497
Poales Poaceae Littledalea racemosa#NC 036350
Poales Poaceae Littledalea alaica#NC 037519
Poales Poaceae Bromus vulgaris#NC 027472
Poales Poaceae Hordeum jubatum#NC 027476
Poales Poaceae Hordeum vulgare#NC 008590
Poales Poaceae Henrardia persica#MH285853
Poales Poaceae Eremopyrum distans#MH285851
Poales Poaceae Eremopyrum triticeum#MH285852
Poales Poaceae Agropyron mongolicum#MH285848
Poales Poaceae Agropyron cristatum#KY126307
Poales Poaceae Elymus libanoticus#KX822019
Poales Poaceae Dasypyrum villosum#MH285850
Poales Poaceae Connorochloa tenuis#NC 037165
Poales Poaceae Pseudoroegneria spicata#MH285855
Poales Poaceae Heteranthelium piliferum#MH285854
Poales Poaceae Crithopsis delileana#MH285849
Poales Poaceae Secale cereale#NC 021761
Poales Poaceae Taeniatherum caput-medusae#MH285856
Poales Poaceae Triticum monococcum#NC 021760
Poales Poaceae Aegilops cylindrica#NC 023096
Poales Poaceae Aegilops tauschii#NC 022133
Poales Poaceae Aegilops geniculata#NC 023097
Poales Poaceae Aegilops searsii#NC 024815
Poales Poaceae Aegilops bicornis#NC 024831
Poales Poaceae Aegilops kotschyi#NC 024832
Poales Poaceae Aegilops longissima#NC 024830
Poales Poaceae Aegilops sharonensis#NC 024816
Poales Poaceae Triticum timopheevii#NC 024764
Poales Poaceae Aegilops speltoides#NC 022135
Poales Poaceae Triticum turgidum#NC 024814
Poales Poaceae Triticum macha#LC372826
Poales Poaceae Triticum durum#LC377262
Poales Poaceae Triticum dicoccon#LC375229
Poales Poaceae Triticum carthlicum#LC375535
Poales Poaceae Triticum aestivum#KX631429
Poales Poaceae Triticum urartu#KJ174105
Poales Poaceae Aristida rufescens#NC 036130
Poales Poaceae Aristida stricta#HATH
Poales Poaceae Aristida ternipes#NC 037164
Poales Poaceae Aristida purpurea#NC 025228
Poales Poaceae Stipagrostis uniplumis#MF460973
Poales Poaceae Stipagrostis hirtigluma#KY432797
Poales Poaceae Sartidia isaloensis#NC 036117
Poales Poaceae Sartidia perrieri#NC 027146
Poales Poaceae Sartidia dewinteri#NC 027147
Poales Poaceae Monachather paradoxus#MF035990
Poales Poaceae Arundo plinii#NC 034652
Poales Poaceae Arundo donax#MF035971
Poales Poaceae Dregeochloa pumila#NC 035526
Poales Poaceae Amphipogon turbinatus#NC 035521
Poales Poaceae Amphipogon caricinus#NC 035520
Poales Poaceae Phragmites australis#KX229727
Poales Poaceae Hakonechloa macra#MF035984
Poales Poaceae Molinia caerulea#MF035988
Poales Poaceae Eragrostis walteri#NC 035528
Poales Poaceae Elytrophorus globularis#NC 035527
Poales Poaceae Elytrophorus spicatus#NC 025233
Poales Poaceae Styppeiochloa gynoglossa#NC 035535
Poales Poaceae Crinipes abyssinicus#NC 035523
Poales Poaceae Crinipes longifolius#NC 035524
Poales Poaceae Coelachne africana#MF563382
Poales Poaceae Isachne distichophylla#NC 025236
Poales Poaceae Limnopoa meeboldii#NC 035531
Poales Poaceae Isachne albens#NC 035530
Poales Poaceae Eriachne compacta#NC 041564
Poales Poaceae Eriachne obtusa#MH036259
Poales Poaceae Eriachne major#MH036252
Poales Poaceae Pheidochloa gracilis#NC 035534
Poales Poaceae Eriachne pallescens#NC 041569
Poales Poaceae Eriachne triseta#NC 041249

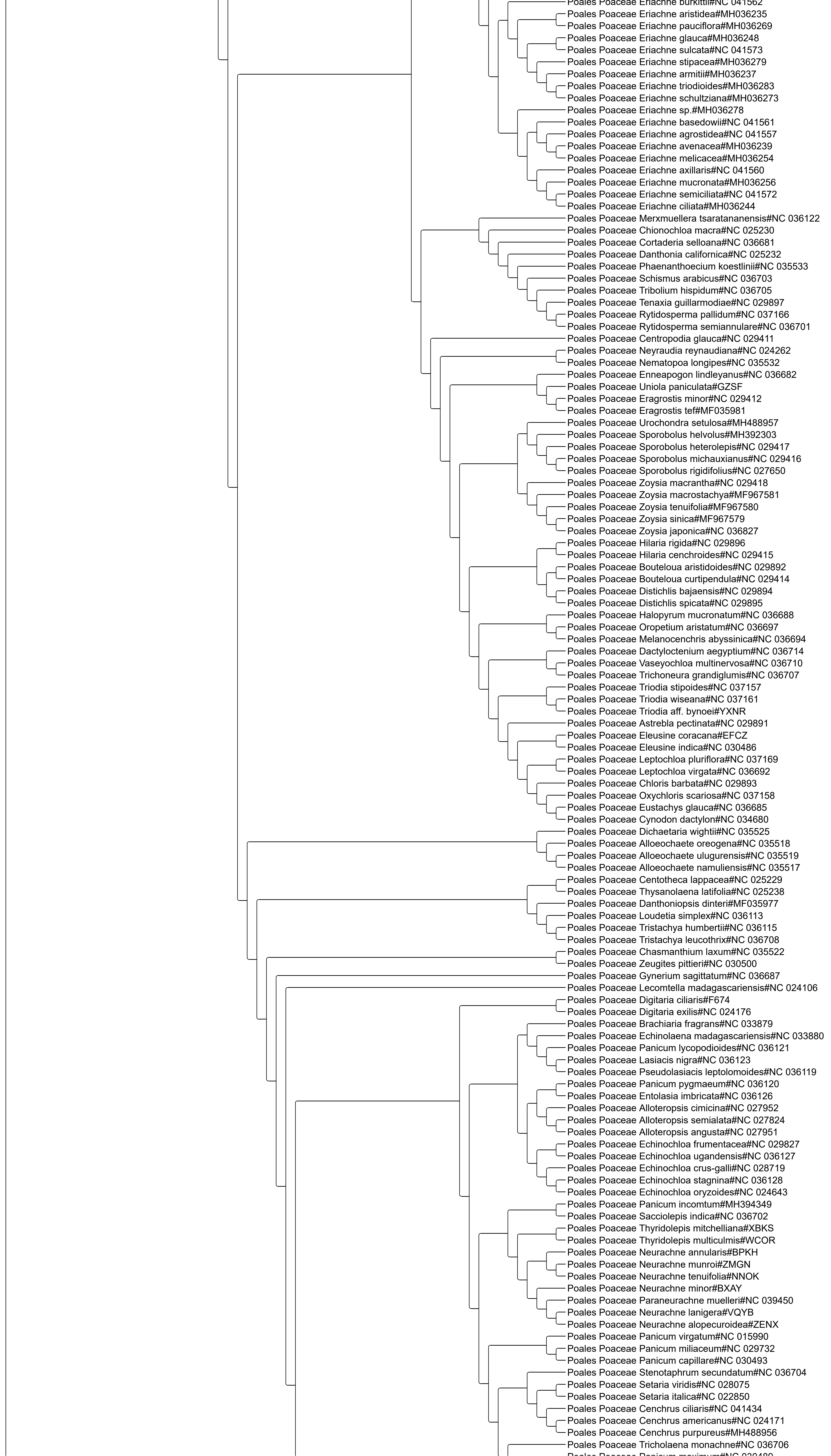

Poales Poaceae Eriachne burkittii#NC 041562
Poales Poaceae Eriachne aristidea#MH036235
Poales Poaceae Eriachne pauciflora#MH036269
Poales Poaceae Eriachne glauca#MH036248
Poales Poaceae Eriachne sulcata#NC 041573
Poales Poaceae Eriachne stipacea#MH036279
Poales Poaceae Eriachne armitii#MH036237
Poales Poaceae Eriachne triodioides#MH036283
Poales Poaceae Eriachne schultziana#MH036273
Poales Poaceae Eriachne sp.#MH036278
Poales Poaceae Eriachne basedowii#NC 041561
Poales Poaceae Eriachne agrostidea#NC 041557
Poales Poaceae Eriachne avenacea#MH036239
Poales Poaceae Eriachne melicacea#MH036254
Poales Poaceae Eriachne axillaris#NC 041560
Poales Poaceae Eriachne mucronata#MH036256
Poales Poaceae Eriachne semiciliata#NC 041572
Poales Poaceae Eriachne ciliata#MH036244
Poales Poaceae Merxmuellera tsaratananensis#NC 036122
Poales Poaceae Chionochloa macra#NC 025230
Poales Poaceae Cortaderia selloana#NC 036681
Poales Poaceae Danthonia californica#NC 025232
Poales Poaceae Phaenanthoecium koestlinii#NC 035533
Poales Poaceae Schismus arabicus#NC 036703
Poales Poaceae Tribolium hispidum#NC 036705
Poales Poaceae Tenaxia guillarmodiae#NC 029897
Poales Poaceae Rytidosperma pallidum#NC 037166
Poales Poaceae Rytidosperma semiannulare#NC 036701
Poales Poaceae Centropodia glauca#NC 029411
Poales Poaceae Neyraudia reynaudiana#NC 024262
Poales Poaceae Nematopoa longipes#NC 035532
Poales Poaceae Enneapogon lindleyanus#NC 036682
Poales Poaceae Uniola paniculata#GZSF
Poales Poaceae Eragrostis minor#NC 029412
Poales Poaceae Eragrostis tef#MF035981
Poales Poaceae Urochondra setulosa#MH488957
Poales Poaceae Sporobolus helvolus#MH392303
Poales Poaceae Sporobolus heterolepis#NC 029417
Poales Poaceae Sporobolus michauxianus#NC 029416
Poales Poaceae Sporobolus rigidifolius#NC 027650
Poales Poaceae Zoysia macrantha#NC 029418
Poales Poaceae Zoysia macrostachya#MF967581
Poales Poaceae Zoysia tenuifolia#MF967580
Poales Poaceae Zoysia sinica#MF967579
Poales Poaceae Zoysia japonica#NC 036827
Poales Poaceae Hilaria rigida#NC 029896
Poales Poaceae Hilaria cenchroides#NC 029415
Poales Poaceae Bouteloua aristidoides#NC 029892
Poales Poaceae Bouteloua curtipendula#NC 029414
Poales Poaceae Distichlis bajaensis#NC 029894
Poales Poaceae Distichlis spicata#NC 029895
Poales Poaceae Halopyrum mucronatum#NC 036688
Poales Poaceae Oropetium aristatum#NC 036697
Poales Poaceae Melanocenchris abyssinica#NC 036694
Poales Poaceae Dactyloctenium aegyptium#NC 036714
Poales Poaceae Vaseyochloa multinervosa#NC 036710
Poales Poaceae Trichoneura grandiglumis#NC 036707
Poales Poaceae Triodia stipoides#NC 037157
Poales Poaceae Triodia wiseana#NC 037161
Poales Poaceae Triodia aff. bynoei#YXNR
Poales Poaceae Astrebla pectinata#NC 029891
Poales Poaceae Eleusine coracana#EFCZ
Poales Poaceae Eleusine indica#NC 030486
Poales Poaceae Leptochloa pluriflora#NC 037169
Poales Poaceae Leptochloa virgata#NC 036692
Poales Poaceae Chloris barbata#NC 029893
Poales Poaceae Oxychloris scariosa#NC 037158
Poales Poaceae Eustachys glauca#NC 036685
Poales Poaceae Cynodon dactylon#NC 034680
Poales Poaceae Dichaetaria wightii#NC 035525
Poales Poaceae Alloeochaete oreogena#NC 035518
Poales Poaceae Alloeochaete ulugurensis#NC 035519
Poales Poaceae Alloeochaete namuliensis#NC 035517
Poales Poaceae Centotheca lappacea#NC 025229
Poales Poaceae Thysanolaena latifolia#NC 025238
Poales Poaceae Danthoniopsis dinteri#MF035977
Poales Poaceae Loudetia simplex#NC 036113
Poales Poaceae Tristachya humbertii#NC 036115
Poales Poaceae Tristachya leucothrix#NC 036708
Poales Poaceae Chasmanthium laxum#NC 035522
Poales Poaceae Zeugites pittieri#NC 030500
Poales Poaceae Gynerium sagittatum#NC 036687
Poales Poaceae Lecomtella madagascariensis#NC 024106
Poales Poaceae Digitaria ciliaris#F674
Poales Poaceae Digitaria exilis#NC 024176
Poales Poaceae Brachiaria fragrans#NC 033879
Poales Poaceae Echinolaena madagascariensis#NC 033880
Poales Poaceae Panicum lycopodioides#NC 036121
Poales Poaceae Lasiacis nigra#NC 036123
Poales Poaceae Pseudolasiacis leptolomoides#NC 036119
Poales Poaceae Panicum pygmaeum#NC 036120
Poales Poaceae Entolasia imbricata#NC 036126
Poales Poaceae Alloteropsis cimicina#NC 027952
Poales Poaceae Alloteropsis semialata#NC 027824
Poales Poaceae Alloteropsis angusta#NC 027951
Poales Poaceae Echinochloa frumentacea#NC 029827
Poales Poaceae Echinochloa ugandensis#NC 036127
Poales Poaceae Echinochloa crus-galli#NC 028719
Poales Poaceae Echinochloa stagnina#NC 036128
Poales Poaceae Echinochloa oryzoides#NC 024643
Poales Poaceae Panicum incomtum#MH394349
Poales Poaceae Sacciolepis indica#NC 036702
Poales Poaceae Thyridolepis mitchelliana#XBKS
Poales Poaceae Thyridolepis multiculmis#WCOR
Poales Poaceae Neurachne annularis#BPKH
Poales Poaceae Neurachne munroi#ZMGN
Poales Poaceae Neurachne tenuifolia#NNOK
Poales Poaceae Neurachne minor#BXAY
Poales Poaceae Paraneurachne muelleri#NC 039450
Poales Poaceae Neurachne lanigera#VQYB
Poales Poaceae Neurachne alopecuroidea#ZENX
Poales Poaceae Panicum virgatum#NC 015990
Poales Poaceae Panicum miliaceum#NC 029732
Poales Poaceae Panicum capillare#NC 030493
Poales Poaceae Stenotaphrum secundatum#NC 036704
Poales Poaceae Setaria viridis#NC 028075
Poales Poaceae Setaria italica#NC 022850
Poales Poaceae Cenchrus ciliaris#NC 041434
Poales Poaceae Cenchrus americanus#NC 024171
Poales Poaceae Cenchrus purpureus#MH488956
Poales Poaceae Tricholaena monachne#NC 036706

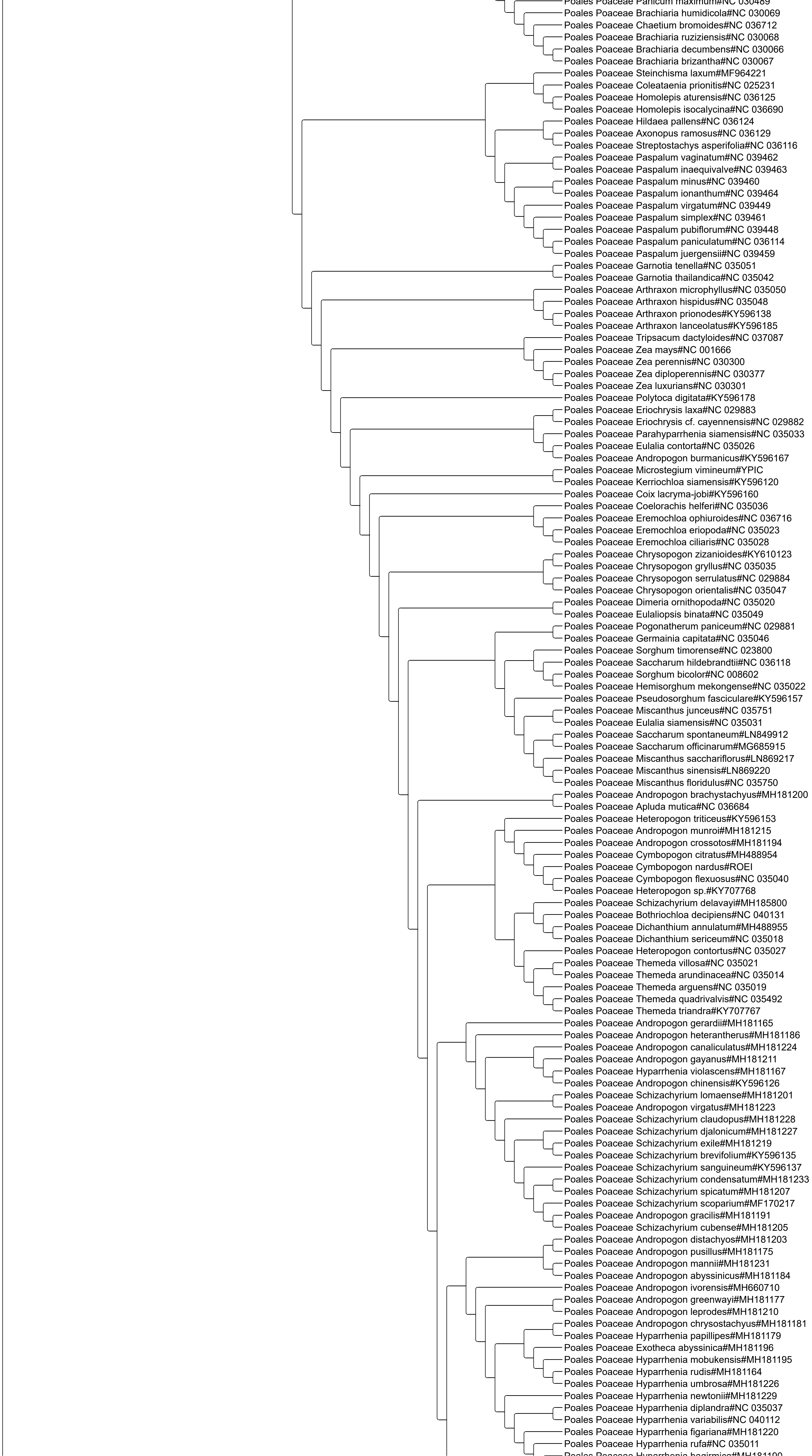
Poales Poaceae Panicum maximum#NC 030489
Poales Poaceae Brachiaria humidicola#NC 030069
Poales Poaceae Chaetium bromoides#NC 036712
Poales Poaceae Brachiaria ruziziensis#NC 030068
Poales Poaceae Brachiaria decumbens#NC 030066
Poales Poaceae Brachiaria brizantha#NC 030067
Poales Poaceae Steinchisma laxum#MF964221
Poales Poaceae Coleataenia prionitis#NC 025231
Poales Poaceae Homolepis aturensis#NC 036125
Poales Poaceae Homolepis isocalycina#NC 036690
Poales Poaceae Hildaea pallens#NC 036124
Poales Poaceae Axonopus ramosus#NC 036129
Poales Poaceae Streptostachys asperifolia#NC 036116
Poales Poaceae Paspalum vaginatum#NC 039462
Poales Poaceae Paspalum inaequivalve#NC 039463
Poales Poaceae Paspalum minus#NC 039460
Poales Poaceae Paspalum ionanthum#NC 039464
Poales Poaceae Paspalum virgatum#NC 039449
Poales Poaceae Paspalum simplex#NC 039461
Poales Poaceae Paspalum pubiflorum#NC 039448
Poales Poaceae Paspalum paniculatum#NC 036114
Poales Poaceae Paspalum juergensii#NC 039459
Poales Poaceae Garnotia tenella#NC 035051
Poales Poaceae Garnotia thailandica#NC 035042
Poales Poaceae Arthraxon microphyllus#NC 035050
Poales Poaceae Arthraxon hispidus#NC 035048
Poales Poaceae Arthraxon prionodes#KY596138
Poales Poaceae Arthraxon lanceolatus#KY596185
Poales Poaceae Tripsacum dactyloides#NC 037087
Poales Poaceae Zea mays#NC 001666
Poales Poaceae Zea perennis#NC 030300
Poales Poaceae Zea diploperennis#NC 030377
Poales Poaceae Zea luxurians#NC 030301
Poales Poaceae Polytoca digitata#KY596178
Poales Poaceae Eriochrysis laxa#NC 029883
Poales Poaceae Eriochrysis cf. cayennensis#NC 029882
Poales Poaceae Parahyparrhenia siamensis#NC 035033
Poales Poaceae Eulalia contorta#NC 035026
Poales Poaceae Andropogon burmanicus#KY596167
Poales Poaceae Microstegium vimineum#YPIC
Poales Poaceae Kerriochloa siamensis#KY596120
Poales Poaceae Coix lacryma-jobi#KY596160
Poales Poaceae Coelorachis helferi#NC 035036
Poales Poaceae Eremochloa ophiuroides#NC 036716
Poales Poaceae Eremochloa eriopoda#NC 035023
Poales Poaceae Eremochloa ciliaris#NC 035028
Poales Poaceae Chrysopogon zizanioides#KY610123
Poales Poaceae Chrysopogon gryllus#NC 035035
Poales Poaceae Chrysopogon serrulatus#NC 029884
Poales Poaceae Chrysopogon orientalis#NC 035047
Poales Poaceae Dimeria ornithopoda#NC 035020
Poales Poaceae Eulaliopsis binata#NC 035049
Poales Poaceae Pogonatherum paniceum#NC 029881
Poales Poaceae Germainia capitata#NC 035046
Poales Poaceae Sorghum timorense#NC 023800
Poales Poaceae Saccharum hildebrandtii#NC 036118
Poales Poaceae Sorghum bicolor#NC 008602
Poales Poaceae Hemisorghum mekongense#NC 035022
Poales Poaceae Pseudosorghum fasciculare#KY596157
Poales Poaceae Miscanthus junceus#NC 035751
Poales Poaceae Eulalia siamensis#NC 035031
Poales Poaceae Saccharum spontaneum#LN849912
Poales Poaceae Saccharum officinarum#MG685915
Poales Poaceae Miscanthus sacchariflorus#LN869217
Poales Poaceae Miscanthus sinensis#LN869220
Poales Poaceae Miscanthus floridulus#NC 035750
Poales Poaceae Andropogon brachystachyus#MH181200
Poales Poaceae Apluda mutica#NC 036684
Poales Poaceae Heteropogon triticeus#KY596153
Poales Poaceae Andropogon munroi#MH181215
Poales Poaceae Andropogon crossotos#MH181194
Poales Poaceae Cymbopogon citratus#MH488954
Poales Poaceae Cymbopogon nardus#ROEI
Poales Poaceae Cymbopogon flexuosus#NC 035040
Poales Poaceae Heteropogon sp.#KY707768
Poales Poaceae Schizachyrium delavayi#MH185800
Poales Poaceae Bothriochloa decipiens#NC 040131
Poales Poaceae Dichanthium annulatum#MH488955
Poales Poaceae Dichanthium sericeum#NC 035018
Poales Poaceae Heteropogon contortus#NC 035027
Poales Poaceae Themeda villosa#NC 035021
Poales Poaceae Themeda arundinacea#NC 035014
Poales Poaceae Themeda arguens#NC 035019
Poales Poaceae Themeda quadrivalvis#NC 035492
Poales Poaceae Themeda triandra#KY707767
Poales Poaceae Andropogon gerardii#MH181165
Poales Poaceae Andropogon heterantherus#MH181186
Poales Poaceae Andropogon canaliculatus#MH181224
Poales Poaceae Andropogon gayanus#MH181211
Poales Poaceae Hyparrhenia violascens#MH181167
Poales Poaceae Andropogon chinensis#KY596126
Poales Poaceae Schizachyrium lomaense#MH181201
Poales Poaceae Andropogon virgatus#MH181223
Poales Poaceae Schizachyrium claudopus#MH181228
Poales Poaceae Schizachyrium djalonicum#MH181227
Poales Poaceae Schizachyrium exile#MH181219
Poales Poaceae Schizachyrium brevifolium#KY596135
Poales Poaceae Schizachyrium sanguineum#KY596137
Poales Poaceae Schizachyrium condensatum#MH181233
Poales Poaceae Schizachyrium spicatum#MH181207
Poales Poaceae Schizachyrium scoparium#MF170217
Poales Poaceae Andropogon gracilis#MH181191
Poales Poaceae Schizachyrium cubense#MH181205
Poales Poaceae Andropogon distachyos#MH181203
Poales Poaceae Andropogon pusillus#MH181175
Poales Poaceae Andropogon mannii#MH181231
Poales Poaceae Andropogon abyssinicus#MH181184
Poales Poaceae Andropogon ivorensis#MH660710
Poales Poaceae Andropogon greenwayi#MH181177
Poales Poaceae Andropogon leprodes#MH181210
Poales Poaceae Andropogon chrysostachyus#MH181181
Poales Poaceae Hyparrhenia papillipes#MH181179
Poales Poaceae Exotheca abyssinica#MH181196
Poales Poaceae Hyparrhenia mobukensis#MH181195
Poales Poaceae Hyparrhenia rudis#MH181164
Poales Poaceae Hyparrhenia umbrosa#MH181226
Poales Poaceae Hyparrhenia newtonii#MH181229
Poales Poaceae Hyparrhenia diplandra#NC 035037
Poales Poaceae Hyparrhenia variabilis#NC 040112
Poales Poaceae Hyparrhenia figariana#MH181220
Poales Poaceae Hyparrhenia rufa#NC 035011

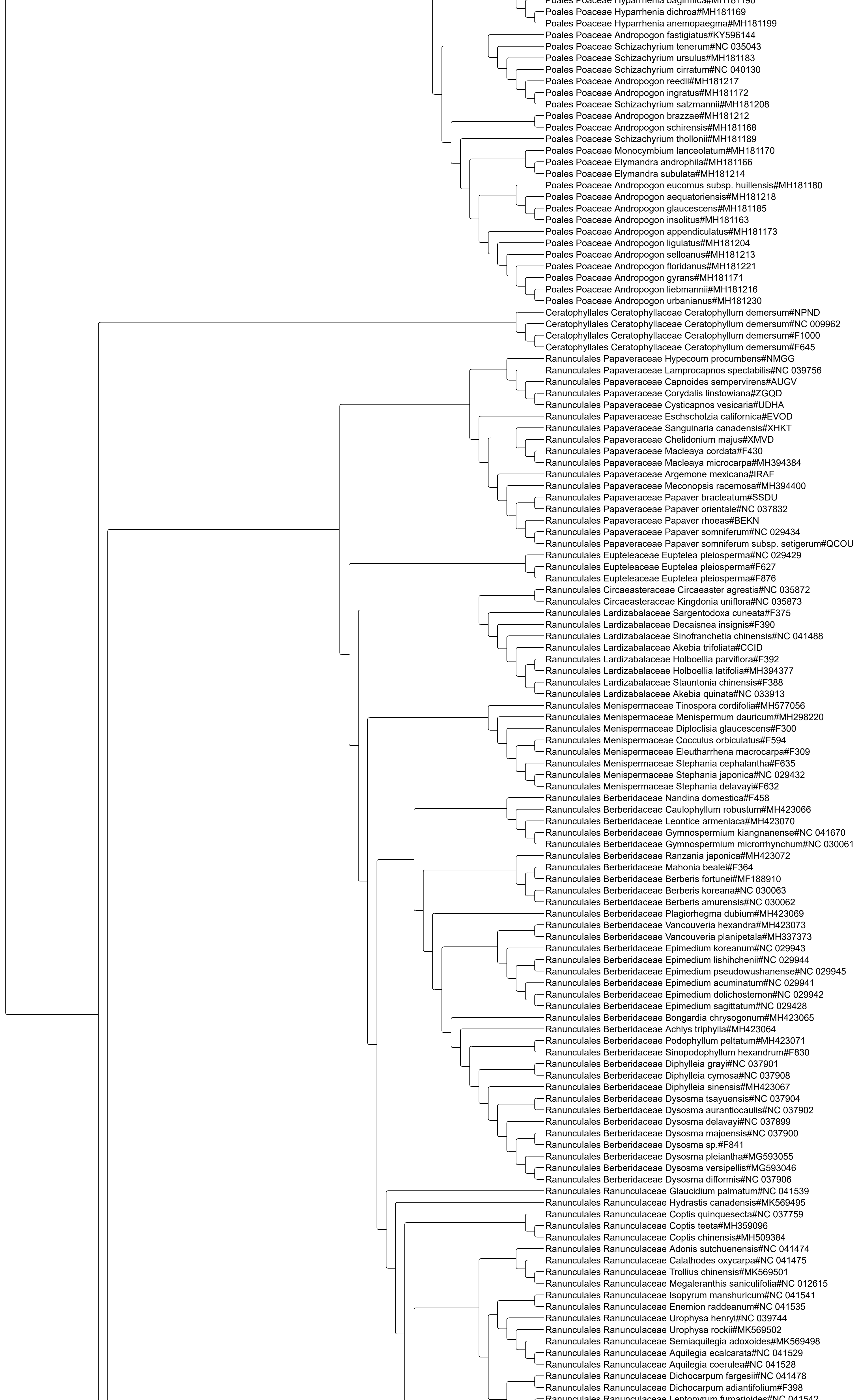
Poales Poaceae Hyparrhenia bagirmica#MH181190
Poales Poaceae Hyparrhenia dichroa#MH181169
Poales Poaceae Hyparrhenia anemopaegma#MH181199
Poales Poaceae Andropogon fastigiatus#KY596144
Poales Poaceae Schizachyrium tenerum#NC 035043
Poales Poaceae Schizachyrium ursulus#MH181183
Poales Poaceae Schizachyrium cirratum#NC 040130
Poales Poaceae Andropogon reedii#MH181217
Poales Poaceae Andropogon ingratus#MH181172
Poales Poaceae Schizachyrium salzmannii#MH181208
Poales Poaceae Andropogon brazzae#MH181212
Poales Poaceae Andropogon schirensis#MH181168
Poales Poaceae Schizachyrium thollonii#MH181189
Poales Poaceae Monocymbium lanceolatum#MH181170
Poales Poaceae Elymandra androphila#MH181166
Poales Poaceae Elymandra subulata#MH181214
Poales Poaceae Andropogon eucomus subsp. huillensis#MH181180
Poales Poaceae Andropogon aequatoriensis#MH181218
Poales Poaceae Andropogon glaucescens#MH181185
Poales Poaceae Andropogon insolitus#MH181163
Poales Poaceae Andropogon appendiculatus#MH181173
Poales Poaceae Andropogon ligulatus#MH181204
Poales Poaceae Andropogon selloanus#MH181213
Poales Poaceae Andropogon floridanus#MH181221
Poales Poaceae Andropogon gyrans#MH181171
Poales Poaceae Andropogon liebmannii#MH181216
Poales Poaceae Andropogon urbanianus#MH181230
Ceratophyllales Ceratophyllaceae Ceratophyllum demersum#NPND
Ceratophyllales Ceratophyllaceae Ceratophyllum demersum#NC 009962
Ceratophyllales Ceratophyllaceae Ceratophyllum demersum#F1000
Ceratophyllales Ceratophyllaceae Ceratophyllum demersum#F645
Ranunculales Papaveraceae Hypecoum procumbens#NMGG
Ranunculales Papaveraceae Lamprocapnos spectabilis#NC 039756
Ranunculales Papaveraceae Capnoides sempervirens#AUGV
Ranunculales Papaveraceae Corydalis linstowiana#ZGQD
Ranunculales Papaveraceae Cysticapnos vesicaria#UDHA
Ranunculales Papaveraceae Eschscholzia californica#EVOD
Ranunculales Papaveraceae Sanguinaria canadensis#XHKT
Ranunculales Papaveraceae Chelidonium majus#XMVD
Ranunculales Papaveraceae Macleaya cordata#F430
Ranunculales Papaveraceae Macleaya microcarpa#MH394384
Ranunculales Papaveraceae Argemone mexicana#IRAF
Ranunculales Papaveraceae Meconopsis racemosa#MH394400
Ranunculales Papaveraceae Papaver bracteatum#SSDU
Ranunculales Papaveraceae Papaver orientale#NC 037832
Ranunculales Papaveraceae Papaver rhoeas#BEKN
Ranunculales Papaveraceae Papaver somniferum#NC 029434
Ranunculales Papaveraceae Papaver somniferum subsp. setigerum#QCOU
Ranunculales Eupteleaceae Euptelea pleiosperma#NC 029429
Ranunculales Eupteleaceae Euptelea pleiosperma#F627
Ranunculales Eupteleaceae Euptelea pleiosperma#F876
Ranunculales Circaeasteraceae Circaeaster agrestis#NC 035872
Ranunculales Circaeasteraceae Kingdonia uniflora#NC 035873
Ranunculales Lardizabalaceae Sargentodoxa cuneata#F375
Ranunculales Lardizabalaceae Decaisnea insignis#F390
Ranunculales Lardizabalaceae Sinofranchetia chinensis#NC 041488
Ranunculales Lardizabalaceae Akebia trifoliata#CCID
Ranunculales Lardizabalaceae Holboellia parviflora#F392
Ranunculales Lardizabalaceae Holboellia latifolia#MH394377
Ranunculales Lardizabalaceae Stauntonia chinensis#F388
Ranunculales Lardizabalaceae Akebia quinata#NC 033913
Ranunculales Menispermaceae Tinospora cordifolia#MH577056
Ranunculales Menispermaceae Menispermum dauricum#MH298220
Ranunculales Menispermaceae Diploclisia glaucescens#F300
Ranunculales Menispermaceae Cocculus orbiculatus#F594
Ranunculales Menispermaceae Eleutharrhena macrocarpa#F309
Ranunculales Menispermaceae Stephania cephalantha#F635
Ranunculales Menispermaceae Stephania japonica#NC 029432
Ranunculales Menispermaceae Stephania delavayi#F632
Ranunculales Berberidaceae Nandina domestica#F458
Ranunculales Berberidaceae Caulophyllum robustum#MH423066
Ranunculales Berberidaceae Leontice armeniaca#MH423070
Ranunculales Berberidaceae Gymnospermium kiangnanense#NC 041670
Ranunculales Berberidaceae Gymnospermium microrrhynchum#NC 030061
Ranunculales Berberidaceae Ranzania japonica#MH423072
Ranunculales Berberidaceae Mahonia bealei#F364
Ranunculales Berberidaceae Berberis fortunei#MF188910
Ranunculales Berberidaceae Berberis koreana#NC 030063
Ranunculales Berberidaceae Berberis amurensis#NC 030062
Ranunculales Berberidaceae Plagiorhegma dubium#MH423069
Ranunculales Berberidaceae Vancouveria hexandra#MH423073
Ranunculales Berberidaceae Vancouveria planipetala#MH337373
Ranunculales Berberidaceae Epimedium koreanum#NC 029943
Ranunculales Berberidaceae Epimedium lishihchenii#NC 029944
Ranunculales Berberidaceae Epimedium pseudowushanense#NC 029945
Ranunculales Berberidaceae Epimedium acuminatum#NC 029941
Ranunculales Berberidaceae Epimedium dolichostemon#NC 029942
Ranunculales Berberidaceae Epimedium sagittatum#NC 029428
Ranunculales Berberidaceae Bongardia chrysogonum#MH423065
Ranunculales Berberidaceae Achlys triphylla#MH423064
Ranunculales Berberidaceae Podophyllum peltatum#MH423071
Ranunculales Berberidaceae Sinopodophyllum hexandrum#F830
Ranunculales Berberidaceae Diphylleia grayi#NC 037901
Ranunculales Berberidaceae Diphylleia cymosa#NC 037908
Ranunculales Berberidaceae Diphylleia sinensis#MH423067
Ranunculales Berberidaceae Dysosma tsayuensis#NC 037904
Ranunculales Berberidaceae Dysosma aurantiocaulis#NC 037902
Ranunculales Berberidaceae Dysosma delavayi#NC 037899
Ranunculales Berberidaceae Dysosma majoensis#NC 037900
Ranunculales Berberidaceae Dysosma sp.#F841
Ranunculales Berberidaceae Dysosma pleiantha#MG593055
Ranunculales Berberidaceae Dysosma versipellis#MG593046
Ranunculales Berberidaceae Dysosma difformis#NC 037906
Ranunculales Ranunculaceae Glaucidium palmatum#NC 041539
Ranunculales Ranunculaceae Hydrastis canadensis#MK569495
Ranunculales Ranunculaceae Coptis quinquesecta#NC 037759
Ranunculales Ranunculaceae Coptis teeta#MH359096
Ranunculales Ranunculaceae Coptis chinensis#MH509384
Ranunculales Ranunculaceae Adonis sutchuenensis#NC 041474
Ranunculales Ranunculaceae Calathodes oxycarpa#NC 041475
Ranunculales Ranunculaceae Trollius chinensis#MK569501
Ranunculales Ranunculaceae Megaleranthis saniculifolia#NC 012615
Ranunculales Ranunculaceae Isopyrum manshuricum#NC 041541
Ranunculales Ranunculaceae Enemion raddeanum#NC 041535
Ranunculales Ranunculaceae Urophysa henryi#NC 039744
Ranunculales Ranunculaceae Urophysa rockii#MK569502
Ranunculales Ranunculaceae Semiaquilegia adoxoides#MK569498
Ranunculales Ranunculaceae Aquilegia ecalcarata#NC 041529
Ranunculales Ranunculaceae Aquilegia coerulea#NC 041528
Ranunculales Ranunculaceae Dichocarpum fargesii#NC 041478
Ranunculales Ranunculaceae Dichocarpum adiantifolium#F398
Ranunculales Ranunculaceae Leptopyrum fumarioides#NC 041542

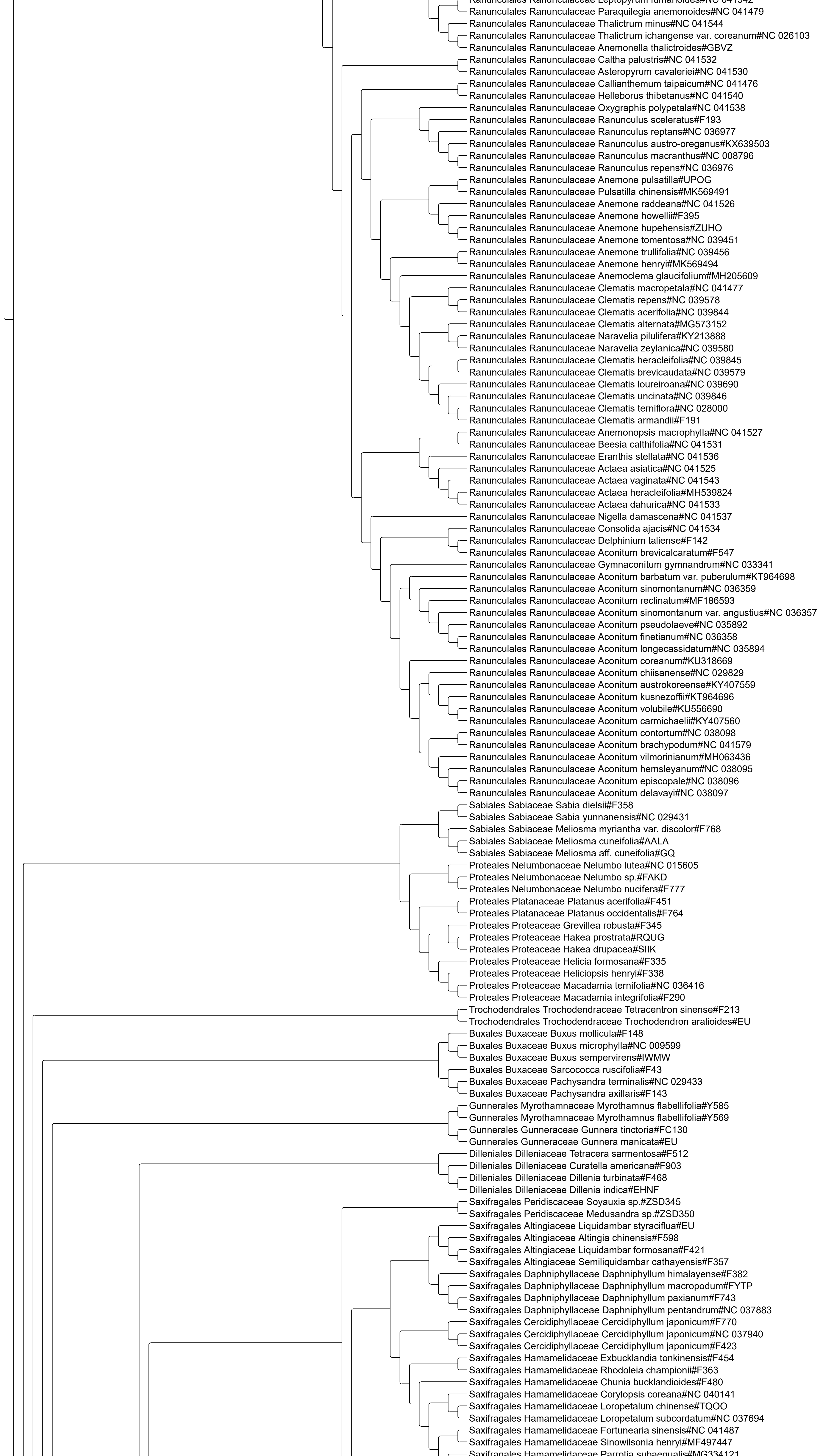
Ranunculales Ranunculaceae Leptopyrum fumarioides#NC 041542
Ranunculales Ranunculaceae Paraquilegia anemonoides#NC 041479
Ranunculales Ranunculaceae Thalictrum minus#NC 041544
Ranunculales Ranunculaceae Thalictrum ichangense var. coreanum#NC 026103
Ranunculales Ranunculaceae Anemonella thalictroides#GBVZ
Ranunculales Ranunculaceae Caltha palustris#NC 041532
Ranunculales Ranunculaceae Asteropyrum cavaleriei#NC 041530
Ranunculales Ranunculaceae Callianthemum taipaicum#NC 041476
Ranunculales Ranunculaceae Helleborus thibetanus#NC 041540
Ranunculales Ranunculaceae Oxygraphis polypetala#NC 041538
Ranunculales Ranunculaceae Ranunculus sceleratus#F193
Ranunculales Ranunculaceae Ranunculus reptans#NC 036977
Ranunculales Ranunculaceae Ranunculus austro-oreganus#KX639503
Ranunculales Ranunculaceae Ranunculus macranthus#NC 008796
Ranunculales Ranunculaceae Ranunculus repens#NC 036976
Ranunculales Ranunculaceae Anemone pulsatilla#UPOG
Ranunculales Ranunculaceae Pulsatilla chinensis#MK569491
Ranunculales Ranunculaceae Anemone raddeana#NC 041526
Ranunculales Ranunculaceae Anemone howellii#F395
Ranunculales Ranunculaceae Anemone hupehensis#ZUHO
Ranunculales Ranunculaceae Anemone tomentosa#NC 039451
Ranunculales Ranunculaceae Anemone trullifolia#NC 039456
Ranunculales Ranunculaceae Anemone henryi#MK569494
Ranunculales Ranunculaceae Anemoclema glaucifolium#MH205609
Ranunculales Ranunculaceae Clematis macropetala#NC 041477
Ranunculales Ranunculaceae Clematis repens#NC 039578
Ranunculales Ranunculaceae Clematis acerifolia#NC 039844
Ranunculales Ranunculaceae Clematis alternata#MG573152
Ranunculales Ranunculaceae Naravelia pilulifera#KY213888
Ranunculales Ranunculaceae Naravelia zeylanica#NC 039580
Ranunculales Ranunculaceae Clematis heracleifolia#NC 039845
Ranunculales Ranunculaceae Clematis brevicaudata#NC 039579
Ranunculales Ranunculaceae Clematis loureiroana#NC 039690
Ranunculales Ranunculaceae Clematis uncinata#NC 039846
Ranunculales Ranunculaceae Clematis terniflora#NC 028000
Ranunculales Ranunculaceae Clematis armandii#F191
Ranunculales Ranunculaceae Anemonopsis macrophylla#NC 041527
Ranunculales Ranunculaceae Beesia calthifolia#NC 041531
Ranunculales Ranunculaceae Eranthis stellata#NC 041536
Ranunculales Ranunculaceae Actaea asiatica#NC 041525
Ranunculales Ranunculaceae Actaea vaginata#NC 041543
Ranunculales Ranunculaceae Actaea heracleifolia#MH539824
Ranunculales Ranunculaceae Actaea dahurica#NC 041533
Ranunculales Ranunculaceae Nigella damascena#NC 041537
Ranunculales Ranunculaceae Consolida ajacis#NC 041534
Ranunculales Ranunculaceae Delphinium taliense#F142
Ranunculales Ranunculaceae Aconitum brevicalcaratum#F547
Ranunculales Ranunculaceae Gymnaconitum gymnandrum#NC 033341
Ranunculales Ranunculaceae Aconitum barbatum var. puberulum#KT964698
Ranunculales Ranunculaceae Aconitum sinomontanum#NC 036359
Ranunculales Ranunculaceae Aconitum reclinatum#MF186593
Ranunculales Ranunculaceae Aconitum sinomontanum var. angustius#NC 036357
Ranunculales Ranunculaceae Aconitum pseudolaeve#NC 035892
Ranunculales Ranunculaceae Aconitum finetianum#NC 036358
Ranunculales Ranunculaceae Aconitum longecassidatum#NC 035894
Ranunculales Ranunculaceae Aconitum coreanum#KU318669
Ranunculales Ranunculaceae Aconitum chiisanense#NC 029829
Ranunculales Ranunculaceae Aconitum austrokoreense#KY407559
Ranunculales Ranunculaceae Aconitum kusnezoffii#KT964696
Ranunculales Ranunculaceae Aconitum volubile#KU556690
Ranunculales Ranunculaceae Aconitum carmichaelii#KY407560
Ranunculales Ranunculaceae Aconitum contortum#NC 038098
Ranunculales Ranunculaceae Aconitum brachypodum#NC 041579
Ranunculales Ranunculaceae Aconitum vilmorinianum#MH063436
Ranunculales Ranunculaceae Aconitum hemsleyanum#NC 038095
Ranunculales Ranunculaceae Aconitum episcopale#NC 038096
Ranunculales Ranunculaceae Aconitum delavayi#NC 038097
Sabiales Sabiaceae Sabia dielsii#F358
Sabiales Sabiaceae Sabia yunnanensis#NC 029431
Sabiales Sabiaceae Meliosma myriantha var. discolor#F768
Sabiales Sabiaceae Meliosma cuneifolia#AALA
Sabiales Sabiaceae Meliosma aff. cuneifolia#GQ
Proteales Nelumbonaceae Nelumbo lutea#NC 015605
Proteales Nelumbonaceae Nelumbo sp.#FAKD
Proteales Nelumbonaceae Nelumbo nucifera#F777
Proteales Platanaceae Platanus acerifolia#F451
Proteales Platanaceae Platanus occidentalis#F764
Proteales Proteaceae Grevillea robusta#F345
Proteales Proteaceae Hakea prostrata#RQUG
Proteales Proteaceae Hakea drupacea#SIIK
Proteales Proteaceae Helicia formosana#F335
Proteales Proteaceae Heliciopsis henryi#F338
Proteales Proteaceae Macadamia ternifolia#NC 036416
Proteales Proteaceae Macadamia integrifolia#F290
Trochodendrales Trochodendraceae Tetracentron sinense#F213
Trochodendrales Trochodendraceae Trochodendron aralioides#EU
Buxales Buxaceae Buxus mollicula#F148
Buxales Buxaceae Buxus microphylla#NC 009599
Buxales Buxaceae Buxus sempervirens#IWMW
Buxales Buxaceae Sarcococca ruscifolia#F43
Buxales Buxaceae Pachysandra terminalis#NC 029433
Buxales Buxaceae Pachysandra axillaris#F143
Gunnerales Myrothamnaceae Myrothamnus flabellifolia#Y585
Gunnerales Myrothamnaceae Myrothamnus flabellifolia#Y569
Gunnerales Gunneraceae Gunnera tinctoria#FC130
Gunnerales Gunneraceae Gunnera manicata#EU
Dilleniales Dilleniaceae Tetracera sarmentosa#F512
Dilleniales Dilleniaceae Curatella americana#F903
Dilleniales Dilleniaceae Dillenia turbinata#F468
Dilleniales Dilleniaceae Dillenia indica#EHNF
Saxifragales Peridiscaceae Soyauxia sp.#ZSD345
Saxifragales Peridiscaceae Medusandra sp.#ZSD350
Saxifragales Altingiaceae Liquidambar styraciflua#EU
Saxifragales Altingiaceae Altingia chinensis#F598
Saxifragales Altingiaceae Liquidambar formosana#F421
Saxifragales Altingiaceae Semiliquidambar cathayensis#F357
Saxifragales Daphniphyllaceae Daphniphyllum himalayense#F382
Saxifragales Daphniphyllaceae Daphniphyllum macropodum#FYTP
Saxifragales Daphniphyllaceae Daphniphyllum paxianum#F743
Saxifragales Daphniphyllaceae Daphniphyllum pentandrum#NC 037883
Saxifragales Cercidiphyllaceae Cercidiphyllum japonicum#F770
Saxifragales Cercidiphyllaceae Cercidiphyllum japonicum#NC 037940
Saxifragales Cercidiphyllaceae Cercidiphyllum japonicum#F423
Saxifragales Hamamelidaceae Exbucklandia tonkinensis#F454
Saxifragales Hamamelidaceae Rhodoleia championii#F363
Saxifragales Hamamelidaceae Chunia bucklandioides#F480
Saxifragales Hamamelidaceae Corylopsis coreana#NC 040141
Saxifragales Hamamelidaceae Loropetalum chinense#TQOO
Saxifragales Hamamelidaceae Loropetalum subcordatum#NC 037694
Saxifragales Hamamelidaceae Fortunearia sinensis#NC 041487
Saxifragales Hamamelidaceae Sinowilsonia henryi#MF497447
Saxifragales Hamamelidaceae Parrotia subaequalis#MG334121

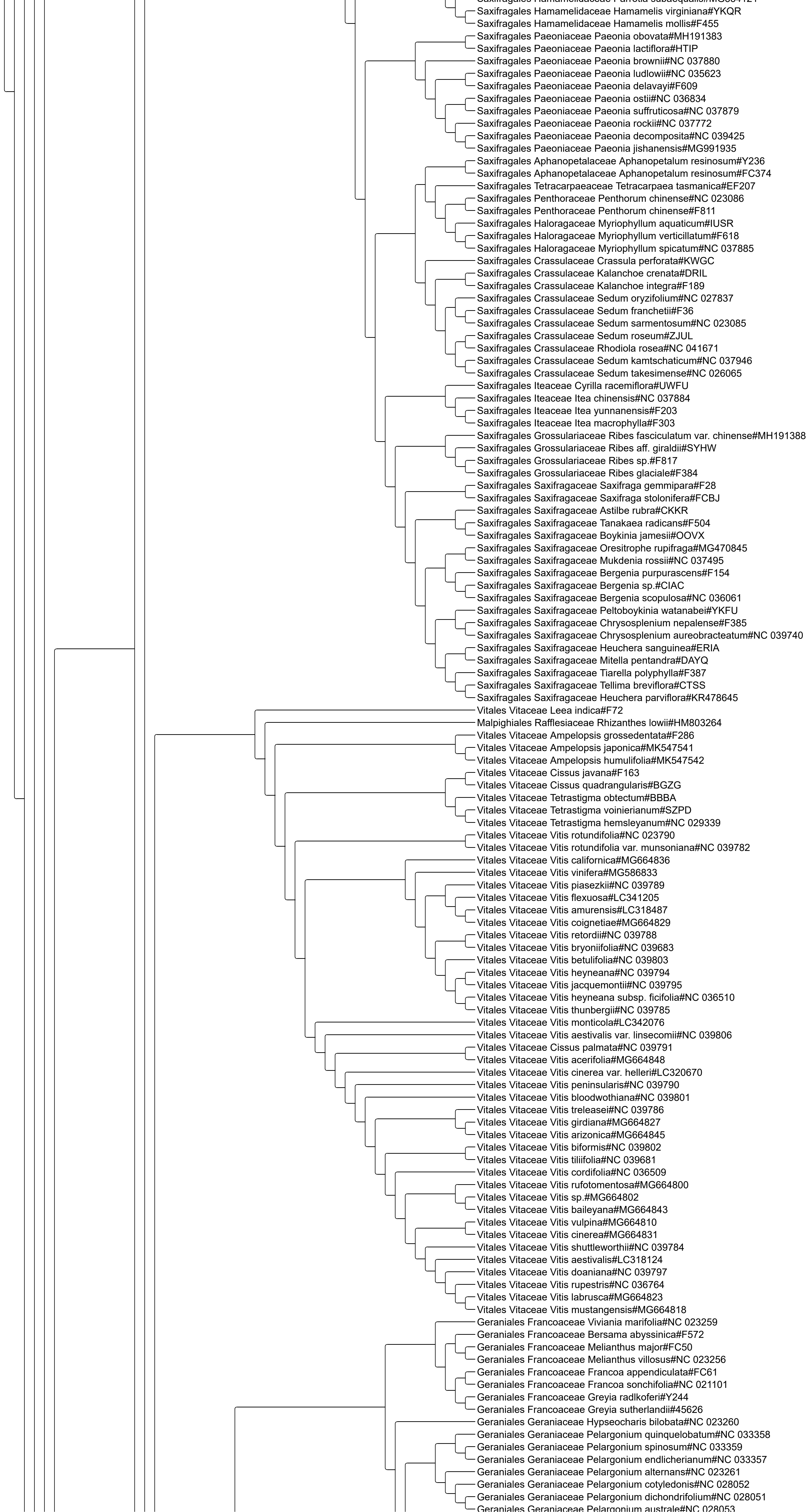

Saxifragales Hamamelidaceae Hamamelis virginiana#YKQR
Saxifragales Hamamelidaceae Hamamelis mollis#F455
Saxifragales Paeoniaceae Paeonia obovata#MH191383
Saxifragales Paeoniaceae Paeonia lactiflora#HTIP
Saxifragales Paeoniaceae Paeonia brownii#NC 037880
Saxifragales Paeoniaceae Paeonia ludlowii#NC 035623
Saxifragales Paeoniaceae Paeonia delavayi#F609
Saxifragales Paeoniaceae Paeonia ostii#NC 036834
Saxifragales Paeoniaceae Paeonia suffruticosa#NC 037879
Saxifragales Paeoniaceae Paeonia rockii#NC 037772
Saxifragales Paeoniaceae Paeonia decomposita#NC 039425
Saxifragales Paeoniaceae Paeonia jishanensis#MG991935
Saxifragales Aphanopetalaceae Aphanopetalum resinosum#Y236
Saxifragales Aphanopetalaceae Aphanopetalum resinosum#FC374
Saxifragales Tetracarpaeaceae Tetracarpaea tasmanica#EF207
Saxifragales Penthoraceae Penthorum chinense#NC 023086
Saxifragales Penthoraceae Penthorum chinense#F811
Saxifragales Haloragaceae Myriophyllum aquaticum#IUSR
Saxifragales Haloragaceae Myriophyllum verticillatum#F618
Saxifragales Haloragaceae Myriophyllum spicatum#NC 037885
Saxifragales Crassulaceae Crassula perforata#KWGC
Saxifragales Crassulaceae Kalanchoe crenata#DRIL
Saxifragales Crassulaceae Kalanchoe integra#F189
Saxifragales Crassulaceae Sedum oryzifolium#NC 027837
Saxifragales Crassulaceae Sedum franchetii#F36
Saxifragales Crassulaceae Sedum sarmentosum#NC 023085
Saxifragales Crassulaceae Sedum roseum#ZJUL
Saxifragales Crassulaceae Rhodiola rosea#NC 041671
Saxifragales Crassulaceae Sedum kamtschaticum#NC 037946
Saxifragales Crassulaceae Sedum takesimense#NC 026065
Saxifragales Iteaceae Cyrilla racemiflora#UWFU
Saxifragales Iteaceae Itea chinensis#NC 037884
Saxifragales Iteaceae Itea yunnanensis#F203
Saxifragales Iteaceae Itea macrophylla#F303
Saxifragales Grossulariaceae Ribes fasciculatum var. chinense#MH191388
Saxifragales Grossulariaceae Ribes aff. giraldii#SYHW
Saxifragales Grossulariaceae Ribes sp.#F817
Saxifragales Grossulariaceae Ribes glaciale#F384
Saxifragales Saxifragaceae Saxifraga gemmipara#F28
Saxifragales Saxifragaceae Saxifraga stolonifera#FCBJ
Saxifragales Saxifragaceae Astilbe rubra#CKKR
Saxifragales Saxifragaceae Tanakaea radicans#F504
Saxifragales Saxifragaceae Boykinia jamesii#OOVX
Saxifragales Saxifragaceae Oresitrophe rupifraga#MG470845
Saxifragales Saxifragaceae Mukdenia rossii#NC 037495
Saxifragales Saxifragaceae Bergenia purpurascens#F154
Saxifragales Saxifragaceae Bergenia sp.#CIAC
Saxifragales Saxifragaceae Bergenia scopulosa#NC 036061
Saxifragales Saxifragaceae Peltoboykinia watanabei#YKFU
Saxifragales Saxifragaceae Chrysosplenium nepalense#F385
Saxifragales Saxifragaceae Chrysosplenium aureobracteatum#NC 039740
Saxifragales Saxifragaceae Heuchera sanguinea#ERIA
Saxifragales Saxifragaceae Mitella pentandra#DAYQ
Saxifragales Saxifragaceae Tiarella polyphylla#F387
Saxifragales Saxifragaceae Tellima breviflora#CTSS
Saxifragales Saxifragaceae Heuchera parviflora#KR478645
Vitales Vitaceae Leea indica#F72
Malpighiales Rafflesiaceae Rhizanthes lowii#HM803264
Vitales Vitaceae Ampelopsis grossedentata#F286
Vitales Vitaceae Ampelopsis japonica#MK547541
Vitales Vitaceae Ampelopsis humulifolia#MK547542
Vitales Vitaceae Cissus javana#F163
Vitales Vitaceae Cissus quadrangularis#BGZG
Vitales Vitaceae Tetrastigma obtectum#BBBA
Vitales Vitaceae Tetrastigma voinierianum#SZPD
Vitales Vitaceae Tetrastigma hemsleyanum#NC 029339
Vitales Vitaceae Vitis rotundifolia#NC 023790
Vitales Vitaceae Vitis rotundifolia var. munsoniana#NC 039782
Vitales Vitaceae Vitis californica#MG664836
Vitales Vitaceae Vitis vinifera#MG586833
Vitales Vitaceae Vitis piasezkii#NC 039789
Vitales Vitaceae Vitis flexuosa#LC341205
Vitales Vitaceae Vitis amurensis#LC318487
Vitales Vitaceae Vitis coignetiae#MG664829
Vitales Vitaceae Vitis retordii#NC 039788
Vitales Vitaceae Vitis bryoniifolia#NC 039683
Vitales Vitaceae Vitis betulifolia#NC 039803
Vitales Vitaceae Vitis heyneana#NC 039794
Vitales Vitaceae Vitis jacquemontii#NC 039795
Vitales Vitaceae Vitis heyneana subsp. ficifolia#NC 036510
Vitales Vitaceae Vitis thunbergii#NC 039785
Vitales Vitaceae Vitis monticola#LC342076
Vitales Vitaceae Vitis aestivalis var. linsecomii#NC 039806
Vitales Vitaceae Cissus palmata#NC 039791
Vitales Vitaceae Vitis acerifolia#MG664848
Vitales Vitaceae Vitis cinerea var. helleri#LC320670
Vitales Vitaceae Vitis peninsularis#NC 039790
Vitales Vitaceae Vitis bloodwothiana#NC 039801
Vitales Vitaceae Vitis treleasei#NC 039786
Vitales Vitaceae Vitis girdiana#MG664827
Vitales Vitaceae Vitis arizonica#MG664845
Vitales Vitaceae Vitis biformis#NC 039802
Vitales Vitaceae Vitis tiliifolia#NC 039681
Vitales Vitaceae Vitis cordifolia#NC 036509
Vitales Vitaceae Vitis rufotomentosa#MG664800
Vitales Vitaceae Vitis sp.#MG664802
Vitales Vitaceae Vitis baileyana#MG664843
Vitales Vitaceae Vitis vulpina#MG664810
Vitales Vitaceae Vitis cinerea#MG664831
Vitales Vitaceae Vitis shuttleworthii#NC 039784
Vitales Vitaceae Vitis aestivalis#LC318124
Vitales Vitaceae Vitis doaniana#NC 039797
Vitales Vitaceae Vitis rupestris#NC 036764
Vitales Vitaceae Vitis labrusca#MG664823
Vitales Vitaceae Vitis mustangensis#MG664818
Geraniales Francoaceae Viviania marifolia#NC 023259
Geraniales Francoaceae Bersama abyssinica#F572
Geraniales Francoaceae Melianthus major#FC50
Geraniales Francoaceae Melianthus villosus#NC 023256
Geraniales Francoaceae Francoa appendiculata#FC61
Geraniales Francoaceae Francoa sonchifolia#NC 021101
Geraniales Francoaceae Greyia radlkoferi#Y244
Geraniales Francoaceae Greyia sutherlandii#45626
Geraniales Geraniaceae Hypseocharis bilobata#NC 023260
Geraniales Geraniaceae Pelargonium quinquelobatum#NC 033358
Geraniales Geraniaceae Pelargonium spinosum#NC 033359
Geraniales Geraniaceae Pelargonium endlicherianum#NC 033357
Geraniales Geraniaceae Pelargonium alternans#NC 023261
Geraniales Geraniaceae Pelargonium cotyledonis#NC 028052
Geraniales Geraniaceae Pelargonium dichondrifolium#NC 028051
Geraniales Geraniaceae Pelargonium australe#NC 028053

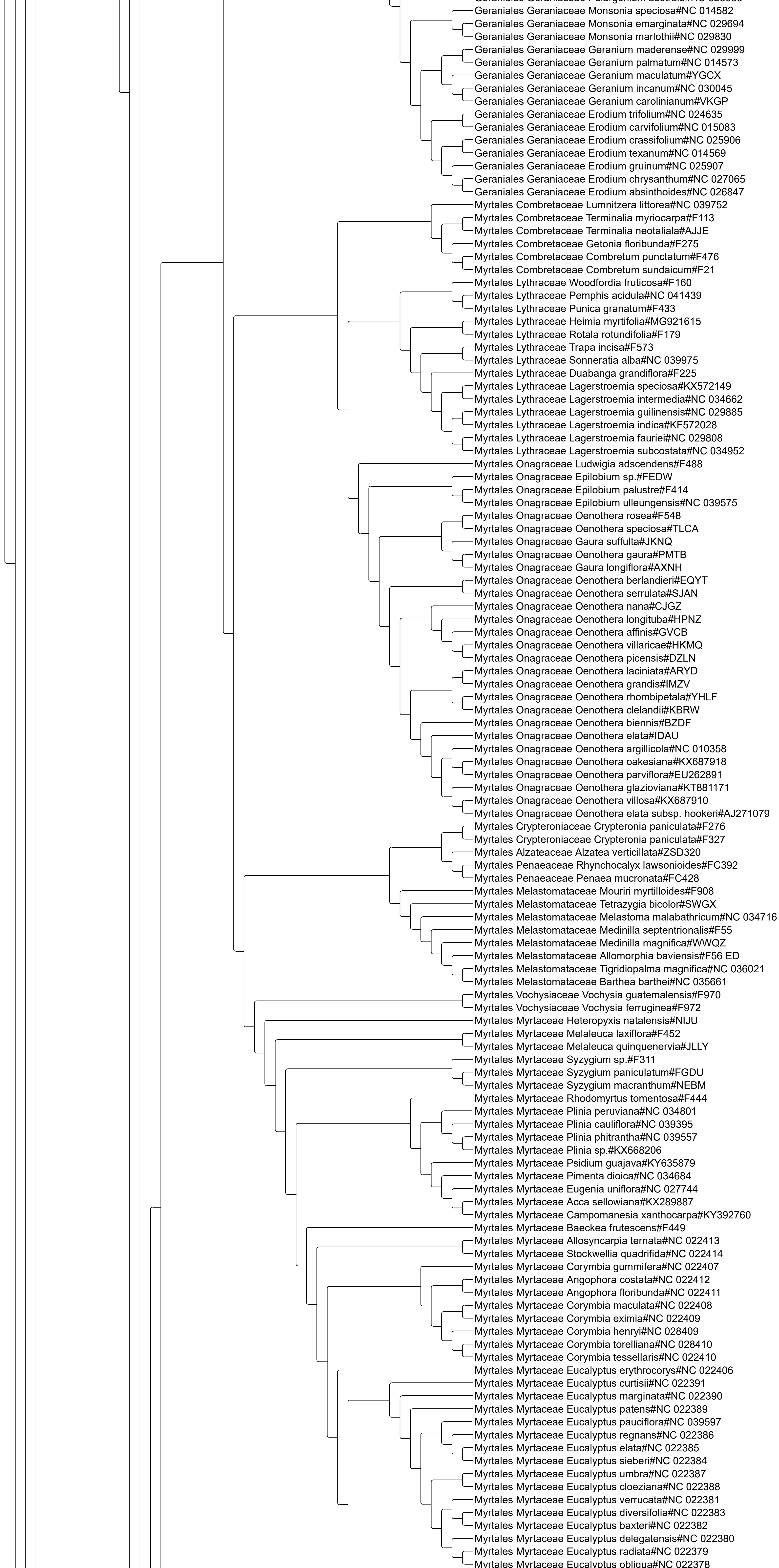
Geraniales Geraniaceae Monsonia speciosa#NC 014582
Geraniales Geraniaceae Monsonia emarginata#NC 029694
Geraniales Geraniaceae Monsonia marlothii#NC 029830
Geraniales Geraniaceae Geranium maderense#NC 029999
Geraniales Geraniaceae Geranium palmatum#NC 014573
Geraniales Geraniaceae Geranium maculatum#YGCX
Geraniales Geraniaceae Geranium incanum#NC 030045
Geraniales Geraniaceae Geranium carolinianum#VKGP
Geraniales Geraniaceae Erodium trifolium#NC 024635
Geraniales Geraniaceae Erodium carvifolium#NC 015083
Geraniales Geraniaceae Erodium crassifolium#NC 025906
Geraniales Geraniaceae Erodium texanum#NC 014569
Geraniales Geraniaceae Erodium gruinum#NC 025907
Geraniales Geraniaceae Erodium chrysanthum#NC 027065
Geraniales Geraniaceae Erodium absinthoides#NC 026847
Myrtales Combretaceae Lumnitzera littorea#NC 039752
Myrtales Combretaceae Terminalia myriocarpa#F113
Myrtales Combretaceae Terminalia neotaliala#AJJE
Myrtales Combretaceae Getonia floribunda#F275
Myrtales Combretaceae Combretum punctatum#F476
Myrtales Combretaceae Combretum sundaicum#F21
Myrtales Lythraceae Woodfordia fruticosa#F160
Myrtales Lythraceae Pemphis acidula#NC 041439
Myrtales Lythraceae Punica granatum#F433
Myrtales Lythraceae Heimia myrtifolia#MG921615
Myrtales Lythraceae Rotala rotundifolia#F179
Myrtales Lythraceae Trapa incisa#F573
Myrtales Lythraceae Sonneratia alba#NC 039975
Myrtales Lythraceae Duabanga grandiflora#F225
Myrtales Lythraceae Lagerstroemia speciosa#KX572149
Myrtales Lythraceae Lagerstroemia intermedia#NC 034662
Myrtales Lythraceae Lagerstroemia guilinensis#NC 029885
Myrtales Lythraceae Lagerstroemia indica#KF572028
Myrtales Lythraceae Lagerstroemia fauriei#NC 029808
Myrtales Lythraceae Lagerstroemia subcostata#NC 034952
Myrtales Onagraceae Ludwigia adscendens#F488
Myrtales Onagraceae Epilobium sp.#FEDW
Myrtales Onagraceae Epilobium palustre#F414
Myrtales Onagraceae Epilobium ulleungensis#NC 039575
Myrtales Onagraceae Oenothera rosea#F548
Myrtales Onagraceae Oenothera speciosa#TLCA
Myrtales Onagraceae Gaura suffulta#JKNQ
Myrtales Onagraceae Oenothera gaura#PMTB
Myrtales Onagraceae Gaura longiflora#AXNH
Myrtales Onagraceae Oenothera berlandieri#EQYT
Myrtales Onagraceae Oenothera serrulata#SJAN
Myrtales Onagraceae Oenothera nana#CJGZ
Myrtales Onagraceae Oenothera longituba#HPNZ
Myrtales Onagraceae Oenothera affinis#GVCB
Myrtales Onagraceae Oenothera villaricae#HKMQ
Myrtales Onagraceae Oenothera picensis#DZLN
Myrtales Onagraceae Oenothera laciniata#ARYD
Myrtales Onagraceae Oenothera grandis#IMZV
Myrtales Onagraceae Oenothera rhombipetala#YHLF
Myrtales Onagraceae Oenothera clelandii#KBRW
Myrtales Onagraceae Oenothera biennis#BZDF
Myrtales Onagraceae Oenothera elata#IDAU
Myrtales Onagraceae Oenothera argillicola#NC 010358
Myrtales Onagraceae Oenothera oakesiana#KX687918
Myrtales Onagraceae Oenothera parviflora#EU262891
Myrtales Onagraceae Oenothera glazioviana#KT881171
Myrtales Onagraceae Oenothera villosa#KX687910
Myrtales Onagraceae Oenothera elata subsp. hookeri#AJ271079
Myrtales Crypteroniaceae Crypteronia paniculata#F276
Myrtales Crypteroniaceae Crypteronia paniculata#F327
Myrtales Alzateaceae Alzatea verticillata#ZSD320
Myrtales Penaeaceae Rhynchocalyx lawsonioides#FC392
Myrtales Penaeaceae Penaea mucronata#FC428
Myrtales Melastomataceae Mouriri myrtilloides#F908
Myrtales Melastomataceae Tetrazygia bicolor#SWGX
Myrtales Melastomataceae Melastoma malabathricum#NC 034716
Myrtales Melastomataceae Medinilla septentrionalis#F55
Myrtales Melastomataceae Medinilla magnifica#WWQZ
Myrtales Melastomataceae Allomorphia baviensis#F56 ED
Myrtales Melastomataceae Tigridiopalma magnifica#NC 036021
Myrtales Melastomataceae Barthea barthei#NC 035661
Myrtales Vochysiaceae Vochysia guatemalensis#F970
Myrtales Vochysiaceae Vochysia ferruginea#F972
Myrtales Myrtaceae Heteropyxis natalensis#NIJU
Myrtales Myrtaceae Melaleuca laxiflora#F452
Myrtales Myrtaceae Melaleuca quinquenervia#JLLY
Myrtales Myrtaceae Syzygium sp.#F311
Myrtales Myrtaceae Syzygium paniculatum#FGDU
Myrtales Myrtaceae Syzygium macranthum#NEBM
Myrtales Myrtaceae Rhodomyrtus tomentosa#F444
Myrtales Myrtaceae Plinia peruviana#NC 034801
Myrtales Myrtaceae Plinia cauliflora#NC 039395
Myrtales Myrtaceae Plinia phitrantha#NC 039557
Myrtales Myrtaceae Plinia sp.#KX668206
Myrtales Myrtaceae Psidium guajava#KY635879
Myrtales Myrtaceae Pimenta dioica#NC 034684
Myrtales Myrtaceae Eugenia uniflora#NC 027744
Myrtales Myrtaceae Acca sellowiana#KX289887
Myrtales Myrtaceae Campomanesia xanthocarpa#KY392760
Myrtales Myrtaceae Baeckea frutescens#F449
Myrtales Myrtaceae Allosyncarpia ternata#NC 022413
Myrtales Myrtaceae Stockwellia quadrifida#NC 022414
Myrtales Myrtaceae Corymbia gummifera#NC 022407
Myrtales Myrtaceae Angophora costata#NC 022412
Myrtales Myrtaceae Angophora floribunda#NC 022411
Myrtales Myrtaceae Corymbia maculata#NC 022408
Myrtales Myrtaceae Corymbia eximia#NC 022409
Myrtales Myrtaceae Corymbia henryi#NC 028409
Myrtales Myrtaceae Corymbia torelliana#NC 028410
Myrtales Myrtaceae Corymbia tessellaris#NC 022410
Myrtales Myrtaceae Eucalyptus erythrocorys#NC 022406
Myrtales Myrtaceae Eucalyptus curtisii#NC 022391
Myrtales Myrtaceae Eucalyptus marginata#NC 022390
Myrtales Myrtaceae Eucalyptus patens#NC 022389
Myrtales Myrtaceae Eucalyptus pauciflora#NC 039597
Myrtales Myrtaceae Eucalyptus regnans#NC 022386
Myrtales Myrtaceae Eucalyptus elata#NC 022385
Myrtales Myrtaceae Eucalyptus sieberi#NC 022384
Myrtales Myrtaceae Eucalyptus umbra#NC 022387
Myrtales Myrtaceae Eucalyptus cloeziana#NC 022388
Myrtales Myrtaceae Eucalyptus verrucata#NC 022381
Myrtales Myrtaceae Eucalyptus diversifolia#NC 022383
Myrtales Myrtaceae Eucalyptus baxteri#NC 022382
Myrtales Myrtaceae Eucalyptus delegatensis#NC 022380
Myrtales Myrtaceae Eucalyptus radiata#NC 022379
Myrtales Myrtaceae Eucalyptus obliqua#NC 022378

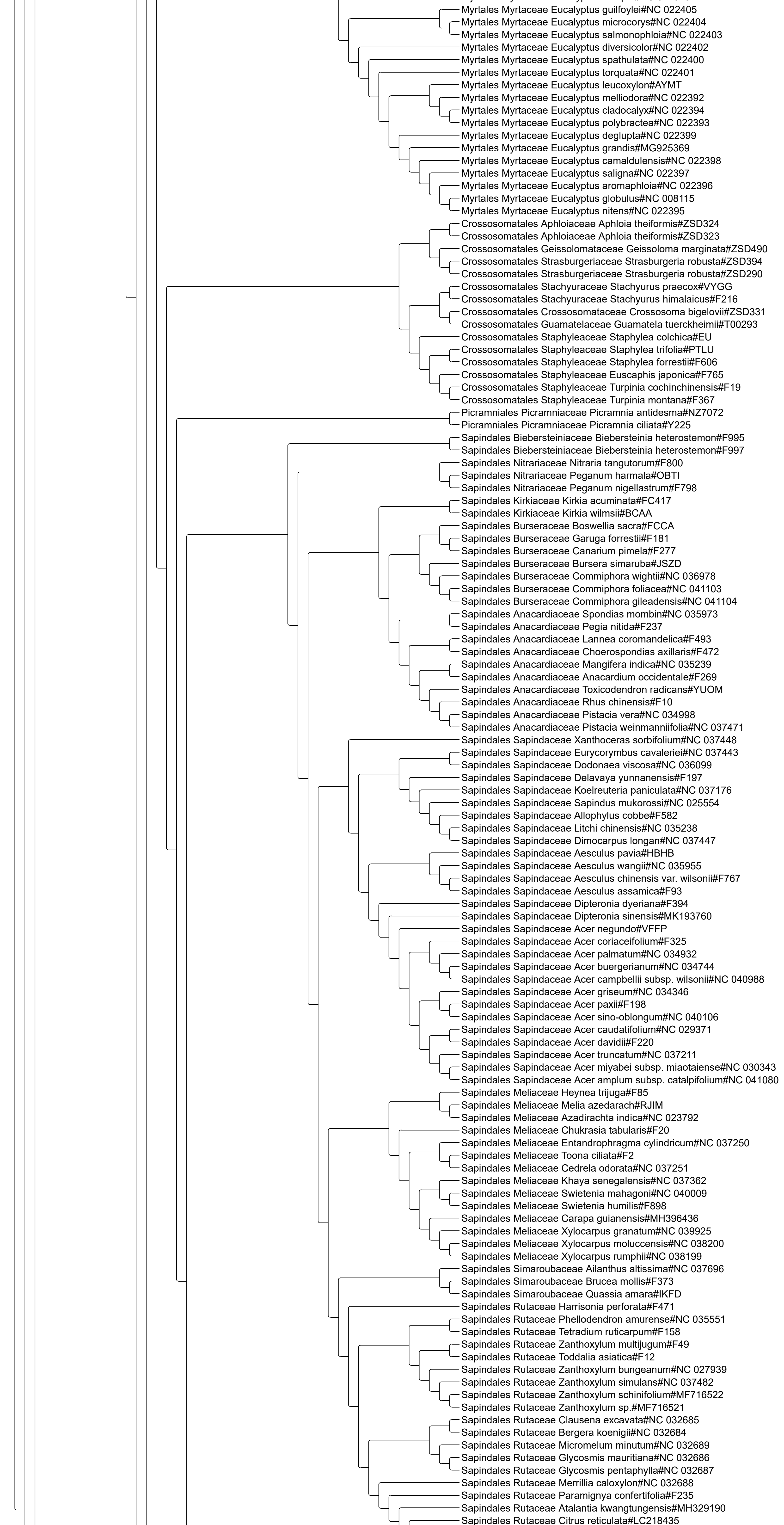

Myrtales Myrtaceae Eucalyptus guilfoylei#NC 022405
Myrtales Myrtaceae Eucalyptus microcorys#NC 022404
Myrtales Myrtaceae Eucalyptus salmonophloia#NC 022403
Myrtales Myrtaceae Eucalyptus diversicolor#NC 022402
Myrtales Myrtaceae Eucalyptus spathulata#NC 022400
Myrtales Myrtaceae Eucalyptus torquata#NC 022401
Myrtales Myrtaceae Eucalyptus leucoxylon#AYMT
Myrtales Myrtaceae Eucalyptus melliodora#NC 022392
Myrtales Myrtaceae Eucalyptus cladocalyx#NC 022394
Myrtales Myrtaceae Eucalyptus polybractea#NC 022393
Myrtales Myrtaceae Eucalyptus deglupta#NC 022399
Myrtales Myrtaceae Eucalyptus grandis#MG925369
Myrtales Myrtaceae Eucalyptus camaldulensis#NC 022398
Myrtales Myrtaceae Eucalyptus saligna#NC 022397
Myrtales Myrtaceae Eucalyptus aromaphloia#NC 022396
Myrtales Myrtaceae Eucalyptus globulus#NC 008115
Myrtales Myrtaceae Eucalyptus nitens#NC 022395
Crossosomatales Aphloiaceae Aphloia theiformis#ZSD324
Crossosomatales Aphloiaceae Aphloia theiformis#ZSD323
Crossosomatales Geissolomataceae Geissoloma marginata#ZSD490
Crossosomatales Strasburgeriaceae Strasburgeria robusta#ZSD394
Crossosomatales Strasburgeriaceae Strasburgeria robusta#ZSD290
Crossosomatales Stachyuraceae Stachyurus praecox#VYGG
Crossosomatales Stachyuraceae Stachyurus himalaicus#F216
Crossosomatales Crossosomataceae Crossosoma bigelovii#ZSD331
Crossosomatales Guamatelaceae Guamatela tuerckheimii#T00293
Crossosomatales Staphyleaceae Staphylea colchica#EU
Crossosomatales Staphyleaceae Staphylea trifolia#PTLU
Crossosomatales Staphyleaceae Staphylea forrestii#F606
Crossosomatales Staphyleaceae Euscaphis japonica#F765
Crossosomatales Staphyleaceae Turpinia cochinchinensis#F19
Crossosomatales Staphyleaceae Turpinia montana#F367
Picramniales Picramniaceae Picramnia antidesma#NZ7072
Picramniales Picramniaceae Picramnia ciliata#Y225
Sapindales Biebersteiniaceae Biebersteinia heterostemon#F995
Sapindales Biebersteiniaceae Biebersteinia heterostemon#F997
Sapindales Nitrariaceae Nitraria tangutorum#F800
Sapindales Nitrariaceae Peganum harmala#OBTI
Sapindales Nitrariaceae Peganum nigellastrum#F798
Sapindales Kirkiaceae Kirkia acuminata#FC417
Sapindales Kirkiaceae Kirkia wilmsii#BCAA
Sapindales Burseraceae Boswellia sacra#FCCA
Sapindales Burseraceae Garuga forrestii#F181
Sapindales Burseraceae Canarium pimela#F277
Sapindales Burseraceae Bursera simaruba#JSZD
Sapindales Burseraceae Commiphora wightii#NC 036978
Sapindales Burseraceae Commiphora foliacea#NC 041103
Sapindales Burseraceae Commiphora gileadensis#NC 041104
Sapindales Anacardiaceae Spondias mombin#NC 035973
Sapindales Anacardiaceae Pegia nitida#F237
Sapindales Anacardiaceae Lannea coromandelica#F493
Sapindales Anacardiaceae Choerospondias axillaris#F472
Sapindales Anacardiaceae Mangifera indica#NC 035239
Sapindales Anacardiaceae Anacardium occidentale#F269
Sapindales Anacardiaceae Toxicodendron radicans#YUOM
Sapindales Anacardiaceae Rhus chinensis#F10
Sapindales Anacardiaceae Pistacia vera#NC 034998
Sapindales Anacardiaceae Pistacia weinmanniifolia#NC 037471
Sapindales Sapindaceae Xanthoceras sorbifolium#NC 037448
Sapindales Sapindaceae Eurycorymbus cavaleriei#NC 037443
Sapindales Sapindaceae Dodonaea viscosa#NC 036099
Sapindales Sapindaceae Delavaya yunnanensis#F197
Sapindales Sapindaceae Koelreuteria paniculata#NC 037176
Sapindales Sapindaceae Sapindus mukorossi#NC 025554
Sapindales Sapindaceae Allophylus cobbe#F582
Sapindales Sapindaceae Litchi chinensis#NC 035238
Sapindales Sapindaceae Dimocarpus longan#NC 037447
Sapindales Sapindaceae Aesculus pavia#HBHB
Sapindales Sapindaceae Aesculus wangii#NC 035955
Sapindales Sapindaceae Aesculus chinensis var. wilsonii#F767
Sapindales Sapindaceae Aesculus assamica#F93
Sapindales Sapindaceae Dipteronia dyeriana#F394
Sapindales Sapindaceae Dipteronia sinensis#MK193760
Sapindales Sapindaceae Acer negundo#VFFP
Sapindales Sapindaceae Acer coriaceifolium#F325
Sapindales Sapindaceae Acer palmatum#NC 034932
Sapindales Sapindaceae Acer buergerianum#NC 034744
Sapindales Sapindaceae Acer campbellii subsp. wilsonii#NC 040988
Sapindales Sapindaceae Acer griseum#NC 034346
Sapindales Sapindaceae Acer paxii#F198
Sapindales Sapindaceae Acer sino-oblongum#NC 040106
Sapindales Sapindaceae Acer caudatifolium#NC 029371
Sapindales Sapindaceae Acer davidii#F220
Sapindales Sapindaceae Acer truncatum#NC 037211
Sapindales Sapindaceae Acer miyabei subsp. miaotaiense#NC 030343
Sapindales Sapindaceae Acer amplum subsp. catalpifolium#NC 041080
Sapindales Meliaceae Heynea trijuga#F85
Sapindales Meliaceae Melia azedarach#RJIM
Sapindales Meliaceae Azadirachta indica#NC 023792
Sapindales Meliaceae Chukrasia tabularis#F20
Sapindales Meliaceae Entandrophragma cylindricum#NC 037250
Sapindales Meliaceae Toona ciliata#F2
Sapindales Meliaceae Cedrela odorata#NC 037251
Sapindales Meliaceae Khaya senegalensis#NC 037362
Sapindales Meliaceae Swietenia mahagoni#NC 040009
Sapindales Meliaceae Swietenia humilis#F898
Sapindales Meliaceae Carapa guianensis#MH396436
Sapindales Meliaceae Xylocarpus granatum#NC 039925
Sapindales Meliaceae Xylocarpus moluccensis#NC 038200
Sapindales Meliaceae Xylocarpus rumphii#NC 038199
Sapindales Simaroubaceae Ailanthus altissima#NC 037696
Sapindales Simaroubaceae Brucea mollis#F373
Sapindales Simaroubaceae Quassia amara#IKFD
Sapindales Rutaceae Harrisonia perforata#F471
Sapindales Rutaceae Phellodendron amurense#NC 035551
Sapindales Rutaceae Tetradium ruticarpum#F158
Sapindales Rutaceae Zanthoxylum multijugum#F49
Sapindales Rutaceae Toddalia asiatica#F12
Sapindales Rutaceae Zanthoxylum bungeanum#NC 027939
Sapindales Rutaceae Zanthoxylum simulans#NC 037482
Sapindales Rutaceae Zanthoxylum schinifolium#MF716522
Sapindales Rutaceae Zanthoxylum sp.#MF716521
Sapindales Rutaceae Clausena excavata#NC 032685
Sapindales Rutaceae Bergera koenigii#NC 032684
Sapindales Rutaceae Micromelum minutum#NC 032689
Sapindales Rutaceae Glycosmis mauritiana#NC 032686
Sapindales Rutaceae Glycosmis pentaphylla#NC 032687
Sapindales Rutaceae Merrillia caloxylon#NC 032688
Sapindales Rutaceae Paramignya confertifolia#F235
Sapindales Rutaceae Atalantia kwangtungensis#MH329190
Sapindales Rutaceae Citrus reticulata#LC218435

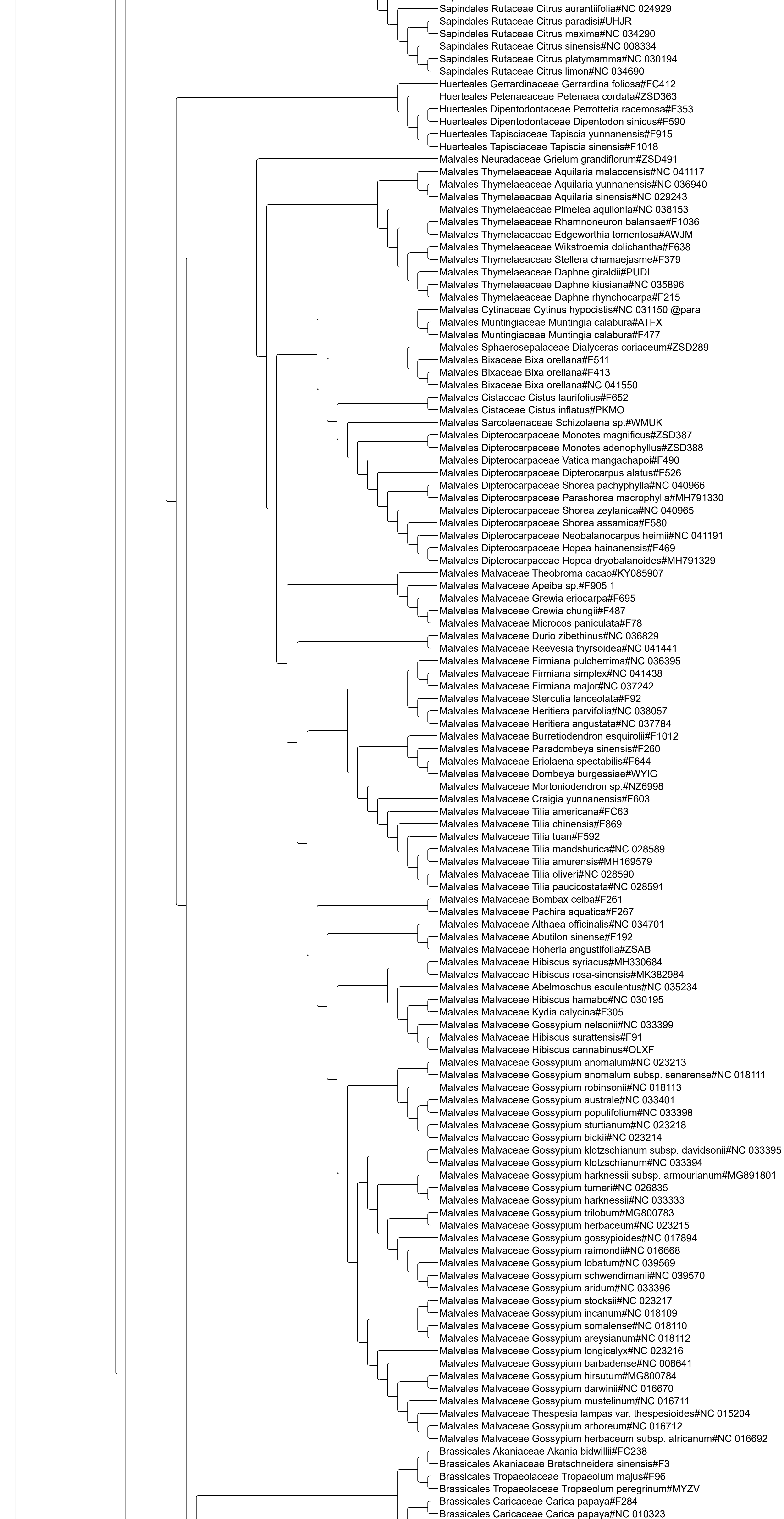
Sapindales Rutaceae Citrus aurantiifolia#NC 024929
Sapindales Rutaceae Citrus paradisi#UHJR
Sapindales Rutaceae Citrus maxima#NC 034290
Sapindales Rutaceae Citrus sinensis#NC 008334
Sapindales Rutaceae Citrus platymamma#NC 030194
Sapindales Rutaceae Citrus limon#NC 034690
Huerteales Gerrardinaceae Gerrardina foliosa#FC412
Huerteales Petenaeaceae Petenaea cordata#ZSD363
Huerteales Dipentodontaceae Perrottetia racemosa#F353
Huerteales Dipentodontaceae Dipentodon sinicus#F590
Huerteales Tapisciaceae Tapiscia yunnanensis#F915
Huerteales Tapisciaceae Tapiscia sinensis#F1018
Malvales Neuradaceae Grielum grandiflorum#ZSD491
Malvales Thymelaeaceae Aquilaria malaccensis#NC 041117
Malvales Thymelaeaceae Aquilaria yunnanensis#NC 036940
Malvales Thymelaeaceae Aquilaria sinensis#NC 029243
Malvales Thymelaeaceae Pimelea aquilonia#NC 038153
Malvales Thymelaeaceae Rhamnoneuron balansae#F1036
Malvales Thymelaeaceae Edgeworthia tomentosa#AWJM
Malvales Thymelaeaceae Wikstroemia dolichantha#F638
Malvales Thymelaeaceae Stellera chamaejasme#F379
Malvales Thymelaeaceae Daphne giraldii#PUDI
Malvales Thymelaeaceae Daphne kiusiana#NC 035896
Malvales Thymelaeaceae Daphne rhynchocarpa#F215
Malvales Cytinaceae Cytinus hypocistis#NC 031150 @para
Malvales Muntingiaceae Muntingia calabura#ATFX
Malvales Muntingiaceae Muntingia calabura#F477
Malvales Sphaerosepalaceae Dialyceras coriaceum#ZSD289
Malvales Bixaceae Bixa orellana#F511
Malvales Bixaceae Bixa orellana#F413
Malvales Bixaceae Bixa orellana#NC 041550
Malvales Cistaceae Cistus laurifolius#F652
Malvales Cistaceae Cistus inflatus#PKMO
Malvales Sarcolaenaceae Schizolaena sp.#WMUK
Malvales Dipterocarpaceae Monotes magnificus#ZSD387
Malvales Dipterocarpaceae Monotes adenophyllus#ZSD388
Malvales Dipterocarpaceae Vatica mangachapoi#F490
Malvales Dipterocarpaceae Dipterocarpus alatus#F526
Malvales Dipterocarpaceae Shorea pachyphylla#NC 040966
Malvales Dipterocarpaceae Parashorea macrophylla#MH791330
Malvales Dipterocarpaceae Shorea zeylanica#NC 040965
Malvales Dipterocarpaceae Shorea assamica#F580
Malvales Dipterocarpaceae Neobalanocarpus heimii#NC 041191
Malvales Dipterocarpaceae Hopea hainanensis#F469
Malvales Dipterocarpaceae Hopea dryobalanoides#MH791329
Malvales Malvaceae Theobroma cacao#KY085907
Malvales Malvaceae Apeiba sp.#F905 1
Malvales Malvaceae Grewia eriocarpa#F695
Malvales Malvaceae Grewia chungii#F487
Malvales Malvaceae Microcos paniculata#F78
Malvales Malvaceae Durio zibethinus#NC 036829
Malvales Malvaceae Reevesia thyrsoidea#NC 041441
Malvales Malvaceae Firmiana pulcherrima#NC 036395
Malvales Malvaceae Firmiana simplex#NC 041438
Malvales Malvaceae Firmiana major#NC 037242
Malvales Malvaceae Sterculia lanceolata#F92
Malvales Malvaceae Heritiera parvifolia#NC 038057
Malvales Malvaceae Heritiera angustata#NC 037784
Malvales Malvaceae Burretiodendron esquirolii#F1012
Malvales Malvaceae Paradombeya sinensis#F260
Malvales Malvaceae Eriolaena spectabilis#F644
Malvales Malvaceae Dombeya burgessiae#WYIG
Malvales Malvaceae Mortoniodendron sp.#NZ6998
Malvales Malvaceae Craigia yunnanensis#F603
Malvales Malvaceae Tilia americana#FC63
Malvales Malvaceae Tilia chinensis#F869
Malvales Malvaceae Tilia tuan#F592
Malvales Malvaceae Tilia mandshurica#NC 028589
Malvales Malvaceae Tilia amurensis#MH169579
Malvales Malvaceae Tilia oliveri#NC 028590
Malvales Malvaceae Tilia paucicostata#NC 028591
Malvales Malvaceae Bombax ceiba#F261
Malvales Malvaceae Pachira aquatica#F267
Malvales Malvaceae Althaea officinalis#NC 034701
Malvales Malvaceae Abutilon sinense#F192
Malvales Malvaceae Hoheria angustifolia#ZSAB
Malvales Malvaceae Hibiscus syriacus#MH330684
Malvales Malvaceae Hibiscus rosa-sinensis#MK382984
Malvales Malvaceae Abelmoschus esculentus#NC 035234
Malvales Malvaceae Hibiscus hamabo#NC 030195
Malvales Malvaceae Kydia calycina#F305
Malvales Malvaceae Gossypium nelsonii#NC 033399
Malvales Malvaceae Hibiscus surattensis#F91
Malvales Malvaceae Hibiscus cannabinus#OLXF
Malvales Malvaceae Gossypium anomalum#NC 023213
Malvales Malvaceae Gossypium anomalum subsp. senarense#NC 018111
Malvales Malvaceae Gossypium robinsonii#NC 018113
Malvales Malvaceae Gossypium australe#NC 033401
Malvales Malvaceae Gossypium populifolium#NC 033398
Malvales Malvaceae Gossypium sturtianum#NC 023218
Malvales Malvaceae Gossypium bickii#NC 023214
Malvales Malvaceae Gossypium klotzschianum subsp. davidsonii#NC 033395
Malvales Malvaceae Gossypium klotzschianum#NC 033394
Malvales Malvaceae Gossypium harknessii subsp. armourianum#MG891801
Malvales Malvaceae Gossypium turneri#NC 026835
Malvales Malvaceae Gossypium harknessii#NC 033333
Malvales Malvaceae Gossypium trilobum#MG800783
Malvales Malvaceae Gossypium herbaceum#NC 023215
Malvales Malvaceae Gossypium gossypioides#NC 017894
Malvales Malvaceae Gossypium raimondii#NC 016668
Malvales Malvaceae Gossypium lobatum#NC 039569
Malvales Malvaceae Gossypium schwendimanii#NC 039570
Malvales Malvaceae Gossypium aridum#NC 033396
Malvales Malvaceae Gossypium stocksii#NC 023217
Malvales Malvaceae Gossypium incanum#NC 018109
Malvales Malvaceae Gossypium somalense#NC 018110
Malvales Malvaceae Gossypium areysianum#NC 018112
Malvales Malvaceae Gossypium longicalyx#NC 023216
Malvales Malvaceae Gossypium barbadense#NC 008641
Malvales Malvaceae Gossypium hirsutum#MG800784
Malvales Malvaceae Gossypium darwinii#NC 016670
Malvales Malvaceae Gossypium mustelinum#NC 016711
Malvales Malvaceae Thespesia lampas var. thespesioides#NC 015204
Malvales Malvaceae Gossypium arboreum#NC 016712
Malvales Malvaceae Gossypium herbaceum subsp. africanum#NC 016692
Brassicales Akaniaceae Akania bidwillii#FC238
Brassicales Akaniaceae Bretschneidera sinensis#F3
Brassicales Tropaeolaceae Tropaeolum majus#F96
Brassicales Tropaeolaceae Tropaeolum peregrinum#MYZV
Brassicales Caricaceae Carica papaya#F284
Brassicales Caricaceae Carica papaya#NC 010323

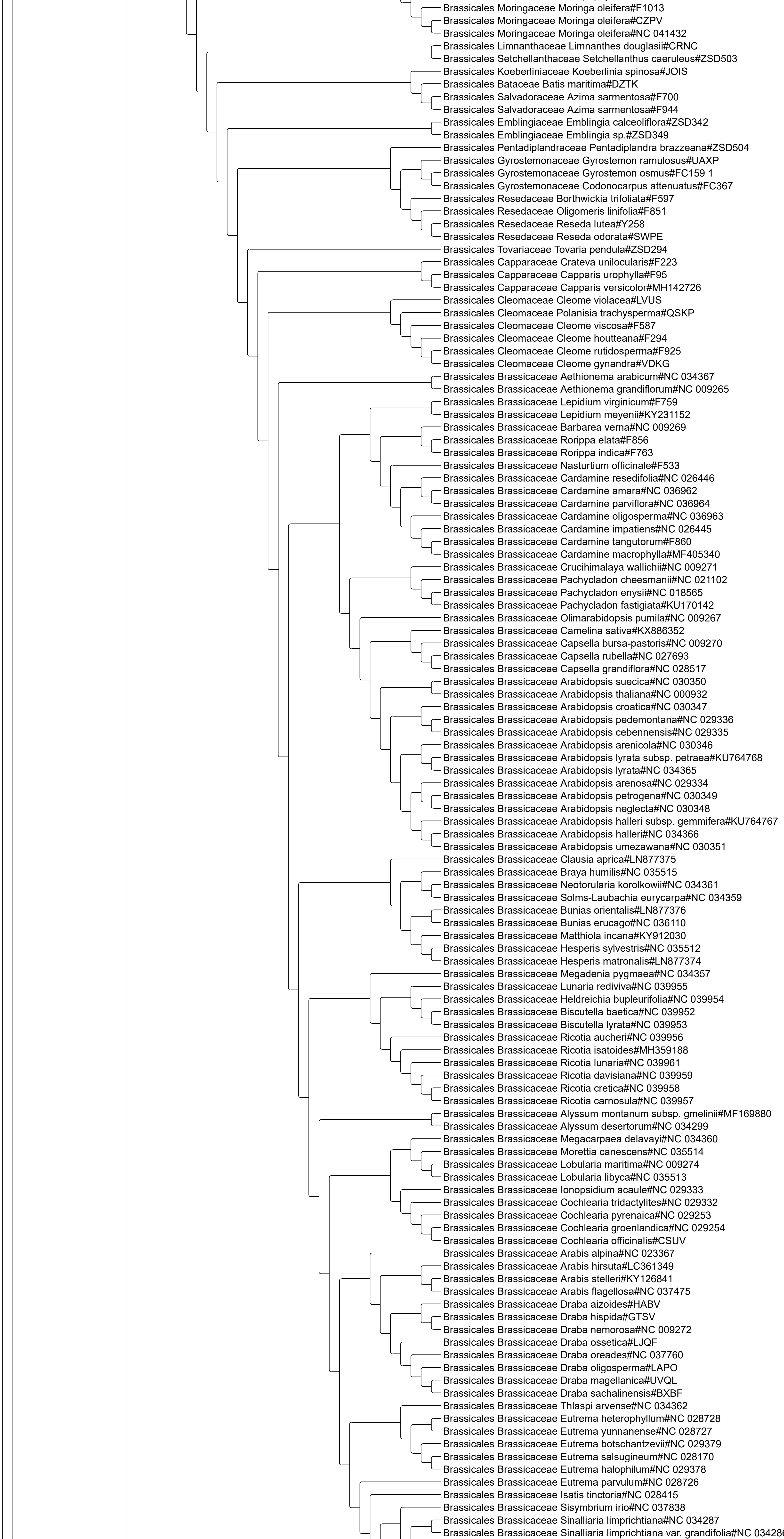
Brassicales Moringaceae Moringa oleifera#F1013
Brassicales Moringaceae Moringa oleifera#CZPV
Brassicales Moringaceae Moringa oleifera#NC 041432
Brassicales Limnanthaceae Limnanthes douglasii#CRNC
Brassicales Setchellanthaceae Setchellanthus caeruleus#ZSD503
Brassicales Koeberliniaceae Koeberlinia spinosa#JOIS
Brassicales Bataceae Batis maritima#DZTK
Brassicales Salvadoraceae Azima sarmentosa#F700
Brassicales Salvadoraceae Azima sarmentosa#F944
Brassicales Emblingiaceae Emblingia calceoliflora#ZSD342
Brassicales Emblingiaceae Emblingia sp.#ZSD349
Brassicales Pentadiplandraceae Pentadiplandra brazzeana#ZSD504
Brassicales Gyrostemonaceae Gyrostemon ramulosus#UAXP
Brassicales Gyrostemonaceae Gyrostemon osmus#FC159 1
Brassicales Gyrostemonaceae Codonocarpus attenuatus#FC367
Brassicales Resedaceae Borthwickia trifoliata#F597
Brassicales Resedaceae Oligomeris linifolia#F851
Brassicales Resedaceae Reseda lutea#Y258
Brassicales Resedaceae Reseda odorata#SWPE
Brassicales Tovariaceae Tovaria pendula#ZSD294
Brassicales Capparaceae Crateva unilocularis#F223
Brassicales Capparaceae Capparis urophylla#F95
Brassicales Capparaceae Capparis versicolor#MH142726
Brassicales Cleomaceae Cleome violacea#LVUS
Brassicales Cleomaceae Polanisia trachysperma#QSKP
Brassicales Cleomaceae Cleome viscosa#F587
Brassicales Cleomaceae Cleome houtteana#F294
Brassicales Cleomaceae Cleome rutidosperma#F925
Brassicales Cleomaceae Cleome gynandra#VDKG
Brassicales Brassicaceae Aethionema arabicum#NC 034367
Brassicales Brassicaceae Aethionema grandiflorum#NC 009265
Brassicales Brassicaceae Lepidium virginicum#F759
Brassicales Brassicaceae Lepidium meyenii#KY231152
Brassicales Brassicaceae Barbarea verna#NC 009269
Brassicales Brassicaceae Rorippa elata#F856
Brassicales Brassicaceae Rorippa indica#F763
Brassicales Brassicaceae Nasturtium officinale#F533
Brassicales Brassicaceae Cardamine resedifolia#NC 026446
Brassicales Brassicaceae Cardamine amara#NC 036962
Brassicales Brassicaceae Cardamine parviflora#NC 036964
Brassicales Brassicaceae Cardamine oligosperma#NC 036963
Brassicales Brassicaceae Cardamine impatiens#NC 026445
Brassicales Brassicaceae Cardamine tangutorum#F860
Brassicales Brassicaceae Cardamine macrophylla#MF405340
Brassicales Brassicaceae Crucihimalaya wallichii#NC 009271
Brassicales Brassicaceae Pachycladon cheesmanii#NC 021102
Brassicales Brassicaceae Pachycladon enysii#NC 018565
Brassicales Brassicaceae Pachycladon fastigiata#KU170142
Brassicales Brassicaceae Olimarabidopsis pumila#NC 009267
Brassicales Brassicaceae Camelina sativa#KX886352
Brassicales Brassicaceae Capsella bursa-pastoris#NC 009270
Brassicales Brassicaceae Capsella rubella#NC 027693
Brassicales Brassicaceae Capsella grandiflora#NC 028517
Brassicales Brassicaceae Arabidopsis suecica#NC 030350
Brassicales Brassicaceae Arabidopsis thaliana#NC 000932
Brassicales Brassicaceae Arabidopsis croatica#NC 030347
Brassicales Brassicaceae Arabidopsis pedemontana#NC 029336
Brassicales Brassicaceae Arabidopsis cebennensis#NC 029335
Brassicales Brassicaceae Arabidopsis arenicola#NC 030346
Brassicales Brassicaceae Arabidopsis lyrata subsp. petraea#KU764768
Brassicales Brassicaceae Arabidopsis lyrata#NC 034365
Brassicales Brassicaceae Arabidopsis arenosa#NC 029334
Brassicales Brassicaceae Arabidopsis petrogena#NC 030349
Brassicales Brassicaceae Arabidopsis neglecta#NC 030348
Brassicales Brassicaceae Arabidopsis halleri subsp. gemmifera#KU764767
Brassicales Brassicaceae Arabidopsis halleri#NC 034366
Brassicales Brassicaceae Arabidopsis umezawana#NC 030351
Brassicales Brassicaceae Clausia aprica#LN877375
Brassicales Brassicaceae Braya humilis#NC 035515
Brassicales Brassicaceae Neotorularia korolkowii#NC 034361
Brassicales Brassicaceae Solms-Laubachia eurycarpa#NC 034359
Brassicales Brassicaceae Bunias orientalis#LN877376
Brassicales Brassicaceae Bunias erucago#NC 036110
Brassicales Brassicaceae Matthiola incana#KY912030
Brassicales Brassicaceae Hesperis sylvestris#NC 035512
Brassicales Brassicaceae Hesperis matronalis#LN877374
Brassicales Brassicaceae Megadenia pygmaea#NC 034357
Brassicales Brassicaceae Lunaria rediviva#NC 039955
Brassicales Brassicaceae Heldreichia bupleurifolia#NC 039954
Brassicales Brassicaceae Biscutella baetica#NC 039952
Brassicales Brassicaceae Biscutella lyrata#NC 039953
Brassicales Brassicaceae Ricotia aucheri#NC 039956
Brassicales Brassicaceae Ricotia isatoides#MH359188
Brassicales Brassicaceae Ricotia lunaria#NC 039961
Brassicales Brassicaceae Ricotia davisiana#NC 039959
Brassicales Brassicaceae Ricotia cretica#NC 039958
Brassicales Brassicaceae Ricotia carnosula#NC 039957
Brassicales Brassicaceae Alyssum montanum subsp. gmelinii#MF169880
Brassicales Brassicaceae Alyssum desertorum#NC 034299
Brassicales Brassicaceae Megacarpaea delavayi#NC 034360
Brassicales Brassicaceae Morettia canescens#NC 035514
Brassicales Brassicaceae Lobularia maritima#NC 009274
Brassicales Brassicaceae Lobularia libyca#NC 035513
Brassicales Brassicaceae Ionopsidium acaule#NC 029333
Brassicales Brassicaceae Cochlearia tridactylites#NC 029332
Brassicales Brassicaceae Cochlearia pyrenaica#NC 029253
Brassicales Brassicaceae Cochlearia groenlandica#NC 029254
Brassicales Brassicaceae Cochlearia officinalis#CSUV
Brassicales Brassicaceae Arabis alpina#NC 023367
Brassicales Brassicaceae Arabis hirsuta#LC361349
Brassicales Brassicaceae Arabis stelleri#KY126841
Brassicales Brassicaceae Arabis flagellosa#NC 037475
Brassicales Brassicaceae Draba aizoides#HABV
Brassicales Brassicaceae Draba hispida#GTSV
Brassicales Brassicaceae Draba nemorosa#NC 009272
Brassicales Brassicaceae Draba ossetica#LJQF
Brassicales Brassicaceae Draba oreades#NC 037760
Brassicales Brassicaceae Draba oligosperma#LAPO
Brassicales Brassicaceae Draba magellanica#UVQL
Brassicales Brassicaceae Draba sachalinensis#BXBF
Brassicales Brassicaceae Thlaspi arvense#NC 034362
Brassicales Brassicaceae Eutrema heterophyllum#NC 028728
Brassicales Brassicaceae Eutrema yunnanense#NC 028727
Brassicales Brassicaceae Eutrema botschantzevii#NC 029379
Brassicales Brassicaceae Eutrema salsugineum#NC 028170
Brassicales Brassicaceae Eutrema halophilum#NC 029378
Brassicales Brassicaceae Eutrema parvulum#NC 028726
Brassicales Brassicaceae Isatis tinctoria#NC 028415
Brassicales Brassicaceae Sisymbrium irio#NC 037838
Brassicales Brassicaceae Sinalliaria limprichtiana#NC 034287
Brassicales Brassicaceae Sinalliaria limprichtiana var. grandifolia#NC 034286

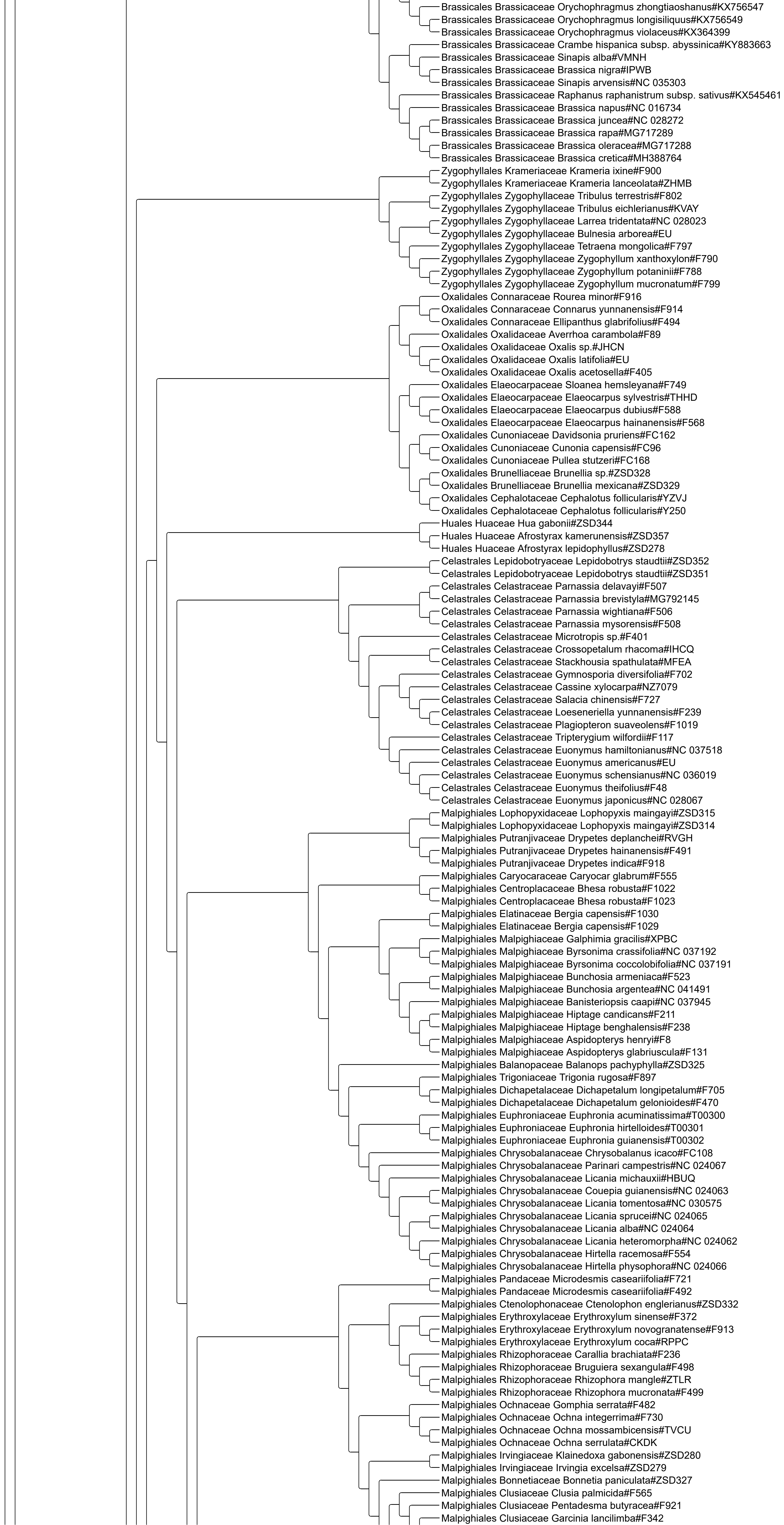
Brassicales Brassicaceae Orychophragmus zhongtiaoshanus#KX756547
Brassicales Brassicaceae Orychophragmus longisiliquus#KX756549
Brassicales Brassicaceae Orychophragmus violaceus#KX364399
Brassicales Brassicaceae Crambe hispanica subsp. abyssinica#KY883663
Brassicales Brassicaceae Sinapis alba#VMNH
Brassicales Brassicaceae Brassica nigra#IPWB
Brassicales Brassicaceae Sinapis arvensis#NC 035303
Brassicales Brassicaceae Raphanus raphanistrum subsp. sativus#KX545461
Brassicales Brassicaceae Brassica napus#NC 016734
Brassicales Brassicaceae Brassica juncea#NC 028272
Brassicales Brassicaceae Brassica rapa#MG717289
Brassicales Brassicaceae Brassica oleracea#MG717288
Brassicales Brassicaceae Brassica cretica#MH388764
Zygophyllales Krameriaceae Krameria ixine#F900
Zygophyllales Krameriaceae Krameria lanceolata#ZHMB
Zygophyllales Zygophyllaceae Tribulus terrestris#F802
Zygophyllales Zygophyllaceae Tribulus eichlerianus#KVAY
Zygophyllales Zygophyllaceae Larrea tridentata#NC 028023
Zygophyllales Zygophyllaceae Bulnesia arborea#EU
Zygophyllales Zygophyllaceae Tetraena mongolica#F797
Zygophyllales Zygophyllaceae Zygophyllum xanthoxylon#F790
Zygophyllales Zygophyllaceae Zygophyllum potaninii#F788
Zygophyllales Zygophyllaceae Zygophyllum mucronatum#F799
Oxalidales Connaraceae Rourea minor#F916
Oxalidales Connaraceae Connarus yunnanensis#F914
Oxalidales Connaraceae Ellipanthus glabrifolius#F494
Oxalidales Oxalidaceae Averrhoa carambola#F89
Oxalidales Oxalidaceae Oxalis sp.#JHCN
Oxalidales Oxalidaceae Oxalis latifolia#EU
Oxalidales Oxalidaceae Oxalis acetosella#F405
Oxalidales Elaeocarpaceae Sloanea hemsleyana#F749
Oxalidales Elaeocarpaceae Elaeocarpus sylvestris#THHD
Oxalidales Elaeocarpaceae Elaeocarpus dubius#F588
Oxalidales Elaeocarpaceae Elaeocarpus hainanensis#F568
Oxalidales Cunoniaceae Davidsonia pruriens#FC162
Oxalidales Cunoniaceae Cunonia capensis#FC96
Oxalidales Cunoniaceae Pullea stutzeri#FC168
Oxalidales Brunelliaceae Brunellia sp.#ZSD328
Oxalidales Brunelliaceae Brunellia mexicana#ZSD329
Oxalidales Cephalotaceae Cephalotus follicularis#YZVJ
Oxalidales Cephalotaceae Cephalotus follicularis#Y250
Huales Huaceae Hua gabonii#ZSD344
Huales Huaceae Afrostyrax kamerunensis#ZSD357
Huales Huaceae Afrostyrax lepidophyllus#ZSD278
Celastrales Lepidobotryaceae Lepidobotrys staudtii#ZSD352
Celastrales Lepidobotryaceae Lepidobotrys staudtii#ZSD351
Celastrales Celastraceae Parnassia delavayi#F507
Celastrales Celastraceae Parnassia brevistyla#MG792145
Celastrales Celastraceae Parnassia wightiana#F506
Celastrales Celastraceae Parnassia mysorensis#F508
Celastrales Celastraceae Microtropis sp.#F401
Celastrales Celastraceae Crossopetalum rhacoma#IHCQ
Celastrales Celastraceae Stackhousia spathulata#MFEA
Celastrales Celastraceae Gymnosporia diversifolia#F702
Celastrales Celastraceae Cassine xylocarpa#NZ7079
Celastrales Celastraceae Salacia chinensis#F727
Celastrales Celastraceae Loeseneriella yunnanensis#F239
Celastrales Celastraceae Plagiopteron suaveolens#F1019
Celastrales Celastraceae Tripterygium wilfordii#F117
Celastrales Celastraceae Euonymus hamiltonianus#NC 037518
Celastrales Celastraceae Euonymus americanus#EU
Celastrales Celastraceae Euonymus schensianus#NC 036019
Celastrales Celastraceae Euonymus theifolius#F48
Celastrales Celastraceae Euonymus japonicus#NC 028067
Malpighiales Lophopyxidaceae Lophopyxis maingayi#ZSD315
Malpighiales Lophopyxidaceae Lophopyxis maingayi#ZSD314
Malpighiales Putranjivaceae Drypetes deplanchei#RVGH
Malpighiales Putranjivaceae Drypetes hainanensis#F491
Malpighiales Putranjivaceae Drypetes indica#F918
Malpighiales Caryocaraceae Caryocar glabrum#F555
Malpighiales Centroplacaceae Bhesa robusta#F1022
Malpighiales Centroplacaceae Bhesa robusta#F1023
Malpighiales Elatinaceae Bergia capensis#F1030
Malpighiales Elatinaceae Bergia capensis#F1029
Malpighiales Malpighiaceae Galphimia gracilis#XPBC
Malpighiales Malpighiaceae Byrsonima crassifolia#NC 037192
Malpighiales Malpighiaceae Byrsonima coccolobifolia#NC 037191
Malpighiales Malpighiaceae Bunchosia armeniaca#F523
Malpighiales Malpighiaceae Bunchosia argentea#NC 041491
Malpighiales Malpighiaceae Banisteriopsis caapi#NC 037945
Malpighiales Malpighiaceae Hiptage candicans#F211
Malpighiales Malpighiaceae Hiptage benghalensis#F238
Malpighiales Malpighiaceae Aspidopterys henryi#F8
Malpighiales Malpighiaceae Aspidopterys glabriuscula#F131
Malpighiales Balanopaceae Balanops pachyphylla#ZSD325
Malpighiales Trigoniaceae Trigonia rugosa#F897
Malpighiales Dichapetalaceae Dichapetalum longipetalum#F705
Malpighiales Dichapetalaceae Dichapetalum gelonioides#F470
Malpighiales Euphroniaceae Euphronia acuminatissima#T00300
Malpighiales Euphroniaceae Euphronia hirtelloides#T00301
Malpighiales Euphroniaceae Euphronia guianensis#T00302
Malpighiales Chrysobalanaceae Chrysobalanus icaco#FC108
Malpighiales Chrysobalanaceae Parinari campestris#NC 024067
Malpighiales Chrysobalanaceae Licania michauxii#HBUQ
Malpighiales Chrysobalanaceae Couepia guianensis#NC 024063
Malpighiales Chrysobalanaceae Licania tomentosa#NC 030575
Malpighiales Chrysobalanaceae Licania sprucei#NC 024065
Malpighiales Chrysobalanaceae Licania alba#NC 024064
Malpighiales Chrysobalanaceae Licania heteromorpha#NC 024062
Malpighiales Chrysobalanaceae Hirtella racemosa#F554
Malpighiales Chrysobalanaceae Hirtella physophora#NC 024066
Malpighiales Pandaceae Microdesmis caseariifolia#F721
Malpighiales Pandaceae Microdesmis caseariifolia#F492
Malpighiales Ctenolophonaceae Ctenolophon englerianus#ZSD332
Malpighiales Erythroxylaceae Erythroxylum sinense#F372
Malpighiales Erythroxylaceae Erythroxylum novogranatense#F913
Malpighiales Erythroxylaceae Erythroxylum coca#RPPC
Malpighiales Rhizophoraceae Carallia brachiata#F236
Malpighiales Rhizophoraceae Bruguiera sexangula#F498
Malpighiales Rhizophoraceae Rhizophora mangle#ZTLR
Malpighiales Rhizophoraceae Rhizophora mucronata#F499
Malpighiales Ochnaceae Gomphia serrata#F482
Malpighiales Ochnaceae Ochna integerrima#F730
Malpighiales Ochnaceae Ochna mossambicensis#TVCU
Malpighiales Ochnaceae Ochna serrulata#CKDK
Malpighiales Irvingiaceae Klainedoxa gabonensis#ZSD280
Malpighiales Irvingiaceae Irvingia excelsa#ZSD279
Malpighiales Bonnetiaceae Bonnetia paniculata#ZSD327
Malpighiales Clusiaceae Clusia palmicida#F565
Malpighiales Clusiaceae Pentadesma butyracea#F921
Malpighiales Clusiaceae Garcinia lancilimba#F342

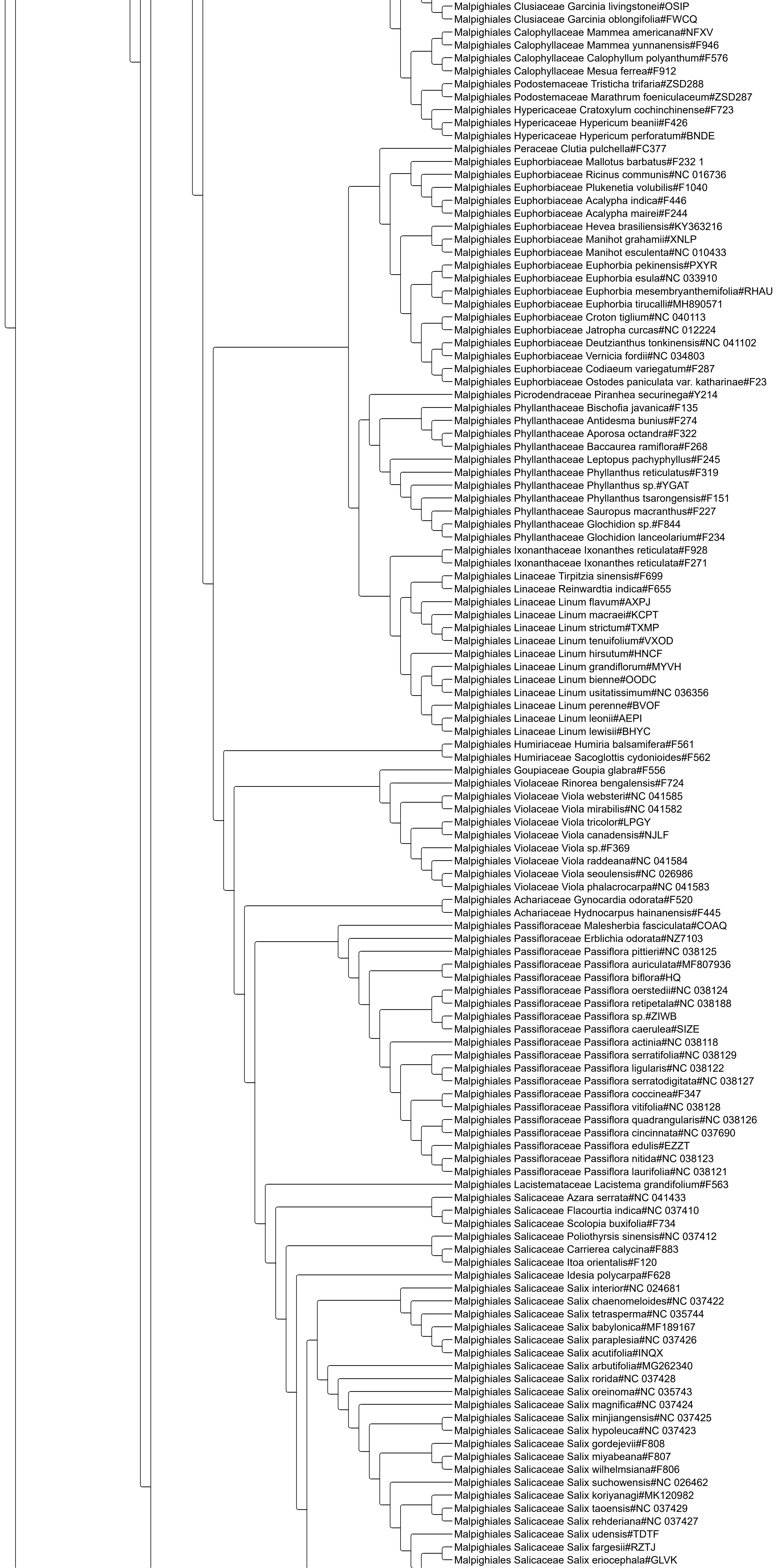

Malpighiales Clusiaceae Garcinia livingstonei#OSIP
Malpighiales Clusiaceae Garcinia oblongifolia#FWCQ
Malpighiales Calophyllaceae Mammea americana#NFXV
Malpighiales Calophyllaceae Mammea yunnanensis#F946
Malpighiales Calophyllaceae Calophyllum polyanthum#F576
Malpighiales Calophyllaceae Mesua ferrea#F912
Malpighiales Podostemaceae Tristicha trifaria#ZSD288
Malpighiales Podostemaceae Marathrum foeniculaceum#ZSD287
Malpighiales Hypericaceae Cratoxylum cochinchinense#F723
Malpighiales Hypericaceae Hypericum beanii#F426
Malpighiales Hypericaceae Hypericum perforatum#BNDE
Malpighiales Peraceae Clutia pulchella#FC377
Malpighiales Euphorbiaceae Mallotus barbatus#F232 1
Malpighiales Euphorbiaceae Ricinus communis#NC 016736
Malpighiales Euphorbiaceae Plukenetia volubilis#F1040
Malpighiales Euphorbiaceae Acalypha indica#F446
Malpighiales Euphorbiaceae Acalypha mairei#F244
Malpighiales Euphorbiaceae Hevea brasiliensis#KY363216
Malpighiales Euphorbiaceae Manihot grahamii#XNLP
Malpighiales Euphorbiaceae Manihot esculenta#NC 010433
Malpighiales Euphorbiaceae Euphorbia pekinensis#PXYR
Malpighiales Euphorbiaceae Euphorbia esula#NC 033910
Malpighiales Euphorbiaceae Euphorbia mesembryanthemifolia#RHAU
Malpighiales Euphorbiaceae Euphorbia tirucalli#MH890571
Malpighiales Euphorbiaceae Croton tiglium#NC 040113
Malpighiales Euphorbiaceae Jatropha curcas#NC 012224
Malpighiales Euphorbiaceae Deutzianthus tonkinensis#NC 041102
Malpighiales Euphorbiaceae Vernicia fordii#NC 034803
Malpighiales Euphorbiaceae Codiaeum variegatum#F287
Malpighiales Euphorbiaceae Ostodes paniculata var. katharinae#F23
Malpighiales Picrodendraceae Piranhea securinega#Y214
Malpighiales Phyllanthaceae Bischofia javanica#F135
Malpighiales Phyllanthaceae Antidesma bunius#F274
Malpighiales Phyllanthaceae Aporosa octandra#F322
Malpighiales Phyllanthaceae Baccaurea ramiflora#F268
Malpighiales Phyllanthaceae Leptopus pachyphyllus#F245
Malpighiales Phyllanthaceae Phyllanthus reticulatus#F319
Malpighiales Phyllanthaceae Phyllanthus sp.#YGAT
Malpighiales Phyllanthaceae Phyllanthus tsarongensis#F151
Malpighiales Phyllanthaceae Sauropus macranthus#F227
Malpighiales Phyllanthaceae Glochidion sp.#F844
Malpighiales Phyllanthaceae Glochidion lanceolarium#F234
Malpighiales Ixonanthaceae Ixonanthes reticulata#F928
Malpighiales Ixonanthaceae Ixonanthes reticulata#F271
Malpighiales Linaceae Tirpitzia sinensis#F699
Malpighiales Linaceae Reinwardtia indica#F655
Malpighiales Linaceae Linum flavum#AXPJ
Malpighiales Linaceae Linum macraei#KCPT
Malpighiales Linaceae Linum strictum#TXMP
Malpighiales Linaceae Linum tenuifolium#VXOD
Malpighiales Linaceae Linum hirsutum#HNCF
Malpighiales Linaceae Linum grandiflorum#MYVH
Malpighiales Linaceae Linum bienne#OODC
Malpighiales Linaceae Linum usitatissimum#NC 036356
Malpighiales Linaceae Linum perenne#BVOF
Malpighiales Linaceae Linum leonii#AEPI
Malpighiales Linaceae Linum lewisii#BHYC
Malpighiales Humiriaceae Humiria balsamifera#F561
Malpighiales Humiriaceae Sacoglottis cydonioides#F562
Malpighiales Goupiaceae Goupia glabra#F556
Malpighiales Violaceae Rinorea bengalensis#F724
Malpighiales Violaceae Viola websteri#NC 041585
Malpighiales Violaceae Viola mirabilis#NC 041582
Malpighiales Violaceae Viola tricolor#LPGY
Malpighiales Violaceae Viola canadensis#NJLF
Malpighiales Violaceae Viola sp.#F369
Malpighiales Violaceae Viola raddeana#NC 041584
Malpighiales Violaceae Viola seoulensis#NC 026986
Malpighiales Violaceae Viola phalacrocarpa#NC 041583
Malpighiales Achariaceae Gynocardia odorata#F520
Malpighiales Achariaceae Hydnocarpus hainanensis#F445
Malpighiales Passifloraceae Malesherbia fasciculata#COAQ
Malpighiales Passifloraceae Erblichia odorata#NZ7103
Malpighiales Passifloraceae Passiflora pittieri#NC 038125
Malpighiales Passifloraceae Passiflora auriculata#MF807936
Malpighiales Passifloraceae Passiflora biflora#HQ
Malpighiales Passifloraceae Passiflora oerstedii#NC 038124
Malpighiales Passifloraceae Passiflora retipetala#NC 038188
Malpighiales Passifloraceae Passiflora sp.#ZIWB
Malpighiales Passifloraceae Passiflora caerulea#SIZE
Malpighiales Passifloraceae Passiflora actinia#NC 038118
Malpighiales Passifloraceae Passiflora serratifolia#NC 038129
Malpighiales Passifloraceae Passiflora ligularis#NC 038122
Malpighiales Passifloraceae Passiflora serratodigitata#NC 038127
Malpighiales Passifloraceae Passiflora coccinea#F347
Malpighiales Passifloraceae Passiflora vitifolia#NC 038128
Malpighiales Passifloraceae Passiflora quadrangularis#NC 038126
Malpighiales Passifloraceae Passiflora cincinnata#NC 037690
Malpighiales Passifloraceae Passiflora edulis#EZZT
Malpighiales Passifloraceae Passiflora nitida#NC 038123
Malpighiales Passifloraceae Passiflora laurifolia#NC 038121
Malpighiales Lacistemataceae Lacistema grandifolium#F563
Malpighiales Salicaceae Azara serrata#NC 041433
Malpighiales Salicaceae Flacourtia indica#NC 037410
Malpighiales Salicaceae Scolopia buxifolia#F734
Malpighiales Salicaceae Poliothyrsis sinensis#NC 037412
Malpighiales Salicaceae Carrierea calycina#F883
Malpighiales Salicaceae Itoa orientalis#F120
Malpighiales Salicaceae Idesia polycarpa#F628
Malpighiales Salicaceae Salix interior#NC 024681
Malpighiales Salicaceae Salix chaenomeloides#NC 037422
Malpighiales Salicaceae Salix tetrasperma#NC 035744
Malpighiales Salicaceae Salix babylonica#MF189167
Malpighiales Salicaceae Salix paraplesia#NC 037426
Malpighiales Salicaceae Salix acutifolia#INQX
Malpighiales Salicaceae Salix arbutifolia#MG262340
Malpighiales Salicaceae Salix rorida#NC 037428
Malpighiales Salicaceae Salix oreinoma#NC 035743
Malpighiales Salicaceae Salix magnifica#NC 037424
Malpighiales Salicaceae Salix minjiangensis#NC 037425
Malpighiales Salicaceae Salix hypoleuca#NC 037423
Malpighiales Salicaceae Salix gordejevii#F808
Malpighiales Salicaceae Salix miyabeana#F807
Malpighiales Salicaceae Salix wilhelmsiana#F806
Malpighiales Salicaceae Salix suchowensis#NC 026462
Malpighiales Salicaceae Salix koriyanagi#MK120982
Malpighiales Salicaceae Salix taoensis#NC 037429
Malpighiales Salicaceae Salix rehderiana#NC 037427
Malpighiales Salicaceae Salix udensis#TDTF
Malpighiales Salicaceae Salix fargesii#RZTJ
Malpighiales Salicaceae Salix eriocephala#GLVK

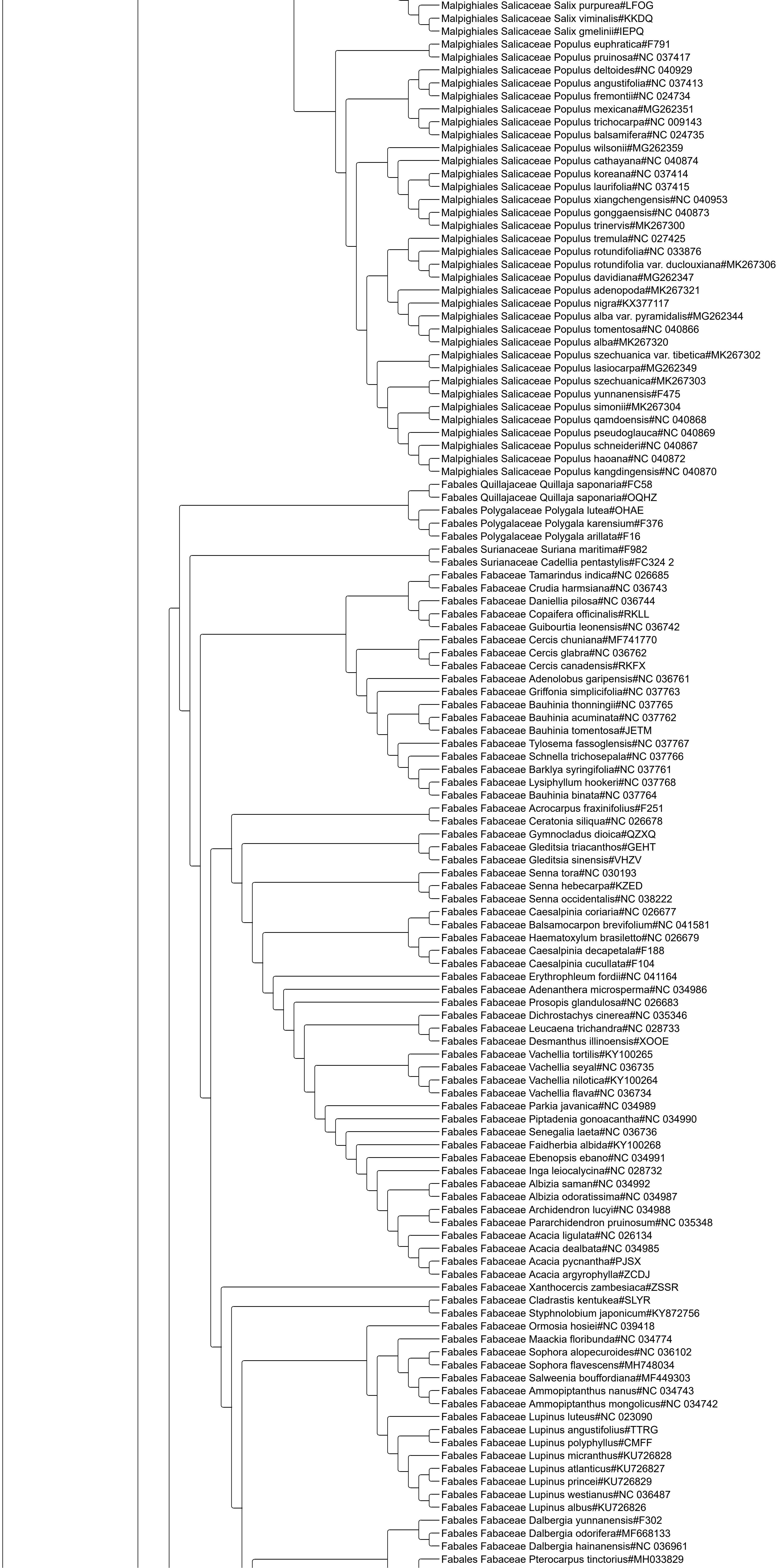
Malpighiales Salicaceae Salix purpurea#LFOG
Malpighiales Salicaceae Salix viminalis#KKDQ
Malpighiales Salicaceae Salix gmelinii#IEPQ
Malpighiales Salicaceae Populus euphratica#F791
Malpighiales Salicaceae Populus pruinosa#NC 037417
Malpighiales Salicaceae Populus deltoides#NC 040929
Malpighiales Salicaceae Populus angustifolia#NC 037413
Malpighiales Salicaceae Populus fremontii#NC 024734
Malpighiales Salicaceae Populus mexicana#MG262351
Malpighiales Salicaceae Populus trichocarpa#NC 009143
Malpighiales Salicaceae Populus balsamifera#NC 024735
Malpighiales Salicaceae Populus wilsonii#MG262359
Malpighiales Salicaceae Populus cathayana#NC 040874
Malpighiales Salicaceae Populus koreana#NC 037414
Malpighiales Salicaceae Populus laurifolia#NC 037415
Malpighiales Salicaceae Populus xiangchengensis#NC 040953
Malpighiales Salicaceae Populus gonggaensis#NC 040873
Malpighiales Salicaceae Populus trinervis#MK267300
Malpighiales Salicaceae Populus tremula#NC 027425
Malpighiales Salicaceae Populus rotundifolia#NC 033876
Malpighiales Salicaceae Populus rotundifolia var. duclouxiana#MK267306
Malpighiales Salicaceae Populus davidiana#MG262347
Malpighiales Salicaceae Populus adenopoda#MK267321
Malpighiales Salicaceae Populus nigra#KX377117
Malpighiales Salicaceae Populus alba var. pyramidalis#MG262344
Malpighiales Salicaceae Populus tomentosa#NC 040866
Malpighiales Salicaceae Populus alba#MK267320
Malpighiales Salicaceae Populus szechuanica var. tibetica#MK267302
Malpighiales Salicaceae Populus lasiocarpa#MG262349
Malpighiales Salicaceae Populus szechuanica#MK267303
Malpighiales Salicaceae Populus yunnanensis#F475
Malpighiales Salicaceae Populus simonii#MK267304
Malpighiales Salicaceae Populus qamdoensis#NC 040868
Malpighiales Salicaceae Populus pseudoglauca#NC 040869
Malpighiales Salicaceae Populus schneideri#NC 040867
Malpighiales Salicaceae Populus haoana#NC 040872
Malpighiales Salicaceae Populus kangdingensis#NC 040870
Fabales Quillajaceae Quillaja saponaria#FC58
Fabales Quillajaceae Quillaja saponaria#OQHZ
Fabales Polygalaceae Polygala lutea#OHAE
Fabales Polygalaceae Polygala karensium#F376
Fabales Polygalaceae Polygala arillata#F16
Fabales Surianaceae Suriana maritima#F982
Fabales Surianaceae Cadellia pentastylis#FC324 2
Fabales Fabaceae Tamarindus indica#NC 026685
Fabales Fabaceae Crudia harmsiana#NC 036743
Fabales Fabaceae Daniellia pilosa#NC 036744
Fabales Fabaceae Copaifera officinalis#RKLL
Fabales Fabaceae Guibourtia leonensis#NC 036742
Fabales Fabaceae Cercis chuniana#MF741770
Fabales Fabaceae Cercis glabra#NC 036762
Fabales Fabaceae Cercis canadensis#RKFX
Fabales Fabaceae Adenolobus garipensis#NC 036761
Fabales Fabaceae Griffonia simplicifolia#NC 037763
Fabales Fabaceae Bauhinia thonningii#NC 037765
Fabales Fabaceae Bauhinia acuminata#NC 037762
Fabales Fabaceae Bauhinia tomentosa#JETM
Fabales Fabaceae Tylosema fassoglensis#NC 037767
Fabales Fabaceae Schnella trichosepala#NC 037766
Fabales Fabaceae Barklya syringifolia#NC 037761
Fabales Fabaceae Lysiphyllum hookeri#NC 037768
Fabales Fabaceae Bauhinia binata#NC 037764
Fabales Fabaceae Acrocarpus fraxinifolius#F251
Fabales Fabaceae Ceratonia siliqua#NC 026678
Fabales Fabaceae Gymnocladus dioica#QZXQ
Fabales Fabaceae Gleditsia triacanthos#GEHT
Fabales Fabaceae Gleditsia sinensis#VHZV
Fabales Fabaceae Senna tora#NC 030193
Fabales Fabaceae Senna hebecarpa#KZED
Fabales Fabaceae Senna occidentalis#NC 038222
Fabales Fabaceae Caesalpinia coriaria#NC 026677
Fabales Fabaceae Balsamocarpon brevifolium#NC 041581
Fabales Fabaceae Haematoxylum brasiletto#NC 026679
Fabales Fabaceae Caesalpinia decapetala#F188
Fabales Fabaceae Caesalpinia cucullata#F104
Fabales Fabaceae Erythrophleum fordii#NC 041164
Fabales Fabaceae Adenanthera microsperma#NC 034986
Fabales Fabaceae Prosopis glandulosa#NC 026683
Fabales Fabaceae Dichrostachys cinerea#NC 035346
Fabales Fabaceae Leucaena trichandra#NC 028733
Fabales Fabaceae Desmanthus illinoensis#XOOE
Fabales Fabaceae Vachellia tortilis#KY100265
Fabales Fabaceae Vachellia seyal#NC 036735
Fabales Fabaceae Vachellia nilotica#KY100264
Fabales Fabaceae Vachellia flava#NC 036734
Fabales Fabaceae Parkia javanica#NC 034989
Fabales Fabaceae Piptadenia gonoacantha#NC 034990
Fabales Fabaceae Senegalia laeta#NC 036736
Fabales Fabaceae Faidherbia albida#KY100268
Fabales Fabaceae Ebenopsis ebano#NC 034991
Fabales Fabaceae Inga leiocalycina#NC 028732
Fabales Fabaceae Albizia saman#NC 034992
Fabales Fabaceae Albizia odoratissima#NC 034987
Fabales Fabaceae Archidendron lucyi#NC 034988
Fabales Fabaceae Pararchidendron pruinosum#NC 035348
Fabales Fabaceae Acacia ligulata#NC 026134
Fabales Fabaceae Acacia dealbata#NC 034985
Fabales Fabaceae Acacia pycnantha#PJSX
Fabales Fabaceae Acacia argyrophylla#ZCDJ
Fabales Fabaceae Xanthocercis zambesiaca#ZSSR
Fabales Fabaceae Cladrastis kentukea#SLYR
Fabales Fabaceae Styphnolobium japonicum#KY872756
Fabales Fabaceae Ormosia hosiei#NC 039418
Fabales Fabaceae Maackia floribunda#NC 034774
Fabales Fabaceae Sophora alopecuroides#NC 036102
Fabales Fabaceae Sophora flavescens#MH748034
Fabales Fabaceae Salweenia bouffordiana#MF449303
Fabales Fabaceae Ammopiptanthus nanus#NC 034743
Fabales Fabaceae Ammopiptanthus mongolicus#NC 034742
Fabales Fabaceae Lupinus luteus#NC 023090
Fabales Fabaceae Lupinus angustifolius#TTRG
Fabales Fabaceae Lupinus polyphyllus#CMFF
Fabales Fabaceae Lupinus micranthus#KU726828
Fabales Fabaceae Lupinus atlanticus#KU726827
Fabales Fabaceae Lupinus princei#KU726829
Fabales Fabaceae Lupinus westianus#NC 036487
Fabales Fabaceae Lupinus albus#KU726826
Fabales Fabaceae Dalbergia yunnanensis#F302
Fabales Fabaceae Dalbergia odorifera#MF668133
Fabales Fabaceae Dalbergia hainanensis#NC 036961
Fabales Fabaceae Pterocarpus tinctorius#MH033829

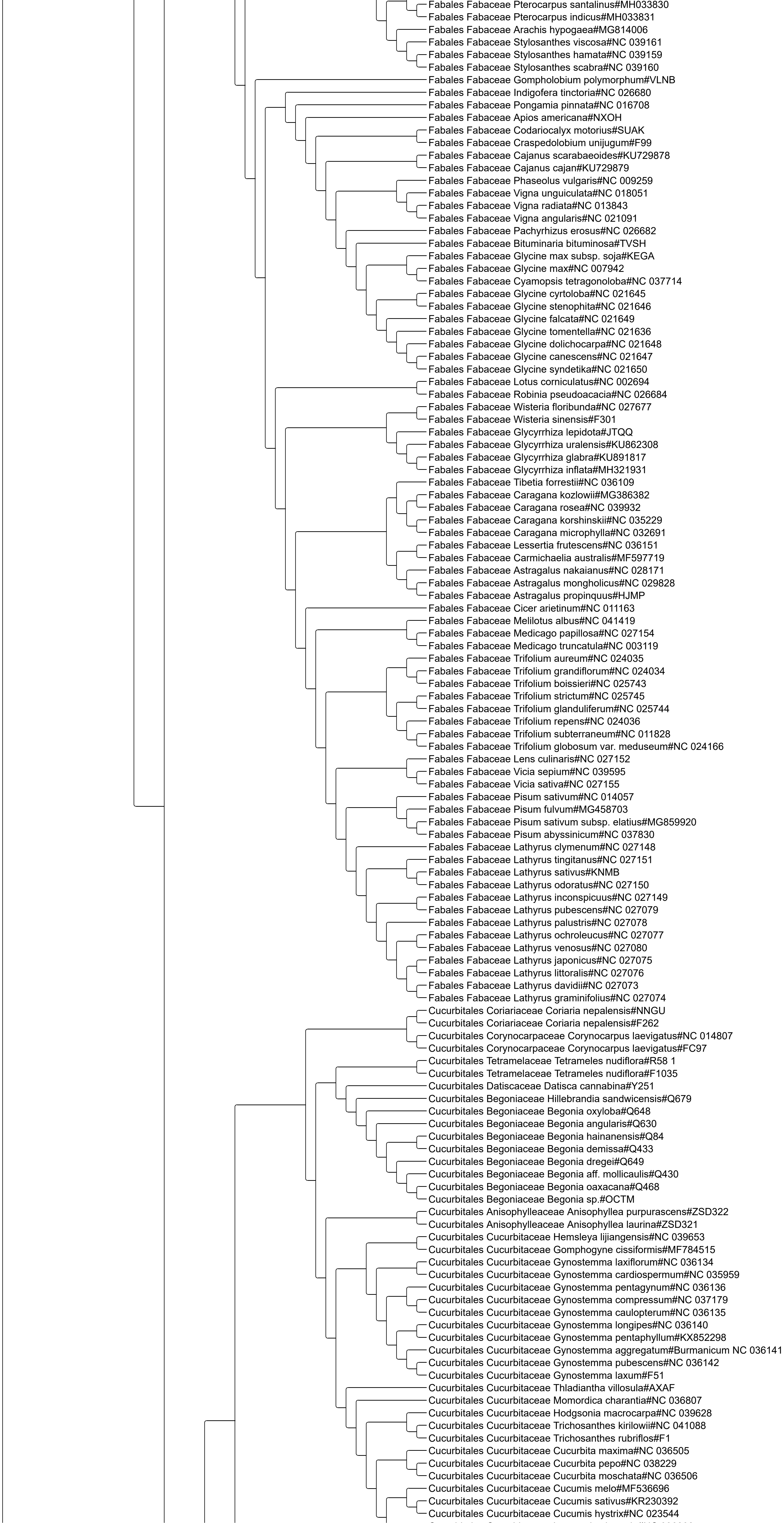

Fabales Fabaceae Pterocarpus santalinus#MH033830
Fabales Fabaceae Pterocarpus indicus#MH033831
Fabales Fabaceae Arachis hypogaea#MG814006
Fabales Fabaceae Stylosanthes viscosa#NC 039161
Fabales Fabaceae Stylosanthes hamata#NC 039159
Fabales Fabaceae Stylosanthes scabra#NC 039160
Fabales Fabaceae Gompholobium polymorphum#VLNB
Fabales Fabaceae Indigofera tinctoria#NC 026680
Fabales Fabaceae Pongamia pinnata#NC 016708
Fabales Fabaceae Apios americana#NXOH
Fabales Fabaceae Codariocalyx motorius#SUAK
Fabales Fabaceae Craspedolobium unijugum#F99
Fabales Fabaceae Cajanus scarabaeoides#KU729878
Fabales Fabaceae Cajanus cajan#KU729879
Fabales Fabaceae Phaseolus vulgaris#NC 009259
Fabales Fabaceae Vigna unguiculata#NC 018051
Fabales Fabaceae Vigna radiata#NC 013843
Fabales Fabaceae Vigna angularis#NC 021091
Fabales Fabaceae Pachyrhizus erosus#NC 026682
Fabales Fabaceae Bituminaria bituminosa#TVSH
Fabales Fabaceae Glycine max subsp. soja#KEGA
Fabales Fabaceae Glycine max#NC 007942
Fabales Fabaceae Cyamopsis tetragonoloba#NC 037714
Fabales Fabaceae Glycine cyrtoloba#NC 021645
Fabales Fabaceae Glycine stenophita#NC 021646
Fabales Fabaceae Glycine falcata#NC 021649
Fabales Fabaceae Glycine tomentella#NC 021636
Fabales Fabaceae Glycine dolichocarpa#NC 021648
Fabales Fabaceae Glycine canescens#NC 021647
Fabales Fabaceae Glycine syndetika#NC 021650
Fabales Fabaceae Lotus corniculatus#NC 002694
Fabales Fabaceae Robinia pseudoacacia#NC 026684
Fabales Fabaceae Wisteria floribunda#NC 027677
Fabales Fabaceae Wisteria sinensis#F301
Fabales Fabaceae Glycyrrhiza lepidota#JTQQ
Fabales Fabaceae Glycyrrhiza uralensis#KU862308
Fabales Fabaceae Glycyrrhiza glabra#KU891817
Fabales Fabaceae Glycyrrhiza inflata#MH321931
Fabales Fabaceae Tibetia forrestii#NC 036109
Fabales Fabaceae Caragana kozlowii#MG386382
Fabales Fabaceae Caragana rosea#NC 039932
Fabales Fabaceae Caragana korshinskii#NC 035229
Fabales Fabaceae Caragana microphylla#NC 032691
Fabales Fabaceae Lessertia frutescens#NC 036151
Fabales Fabaceae Carmichaelia australis#MF597719
Fabales Fabaceae Astragalus nakaianus#NC 028171
Fabales Fabaceae Astragalus mongholicus#NC 029828
Fabales Fabaceae Astragalus propinquus#HJMP
Fabales Fabaceae Cicer arietinum#NC 011163
Fabales Fabaceae Melilotus albus#NC 041419
Fabales Fabaceae Medicago papillosa#NC 027154
Fabales Fabaceae Medicago truncatula#NC 003119
Fabales Fabaceae Trifolium aureum#NC 024035
Fabales Fabaceae Trifolium grandiflorum#NC 024034
Fabales Fabaceae Trifolium boissieri#NC 025743
Fabales Fabaceae Trifolium strictum#NC 025745
Fabales Fabaceae Trifolium glanduliferum#NC 025744
Fabales Fabaceae Trifolium repens#NC 024036
Fabales Fabaceae Trifolium subterraneum#NC 011828
Fabales Fabaceae Trifolium globosum var. meduseum#NC 024166
Fabales Fabaceae Lens culinaris#NC 027152
Fabales Fabaceae Vicia sepium#NC 039595
Fabales Fabaceae Vicia sativa#NC 027155
Fabales Fabaceae Pisum sativum#NC 014057
Fabales Fabaceae Pisum fulvum#MG458703
Fabales Fabaceae Pisum sativum subsp. elatius#MG859920
Fabales Fabaceae Pisum abyssinicum#NC 037830
Fabales Fabaceae Lathyrus clymenum#NC 027148
Fabales Fabaceae Lathyrus tingitanus#NC 027151
Fabales Fabaceae Lathyrus sativus#KNMB
Fabales Fabaceae Lathyrus odoratus#NC 027150
Fabales Fabaceae Lathyrus inconspicuus#NC 027149
Fabales Fabaceae Lathyrus pubescens#NC 027079
Fabales Fabaceae Lathyrus palustris#NC 027078
Fabales Fabaceae Lathyrus ochroleucus#NC 027077
Fabales Fabaceae Lathyrus venosus#NC 027080
Fabales Fabaceae Lathyrus japonicus#NC 027075
Fabales Fabaceae Lathyrus littoralis#NC 027076
Fabales Fabaceae Lathyrus davidii#NC 027073
Fabales Fabaceae Lathyrus graminifolius#NC 027074
Cucurbitales Coriariaceae Coriaria nepalensis#NNGU
Cucurbitales Coriariaceae Coriaria nepalensis#F262
Cucurbitales Corynocarpaceae Corynocarpus laevigatus#NC 014807
Cucurbitales Corynocarpaceae Corynocarpus laevigatus#FC97
Cucurbitales Tetramelaceae Tetrameles nudiflora#R58 1
Cucurbitales Tetramelaceae Tetrameles nudiflora#F1035
Cucurbitales Datiscaceae Datisca cannabina#Y251
Cucurbitales Begoniaceae Hillebrandia sandwicensis#Q679
Cucurbitales Begoniaceae Begonia oxyloba#Q648
Cucurbitales Begoniaceae Begonia angularis#Q630
Cucurbitales Begoniaceae Begonia hainanensis#Q84
Cucurbitales Begoniaceae Begonia demissa#Q433
Cucurbitales Begoniaceae Begonia dregei#Q649
Cucurbitales Begoniaceae Begonia aff. mollicaulis#Q430
Cucurbitales Begoniaceae Begonia oaxacana#Q468
Cucurbitales Begoniaceae Begonia sp.#OCTM
Cucurbitales Anisophylleaceae Anisophyllea purpurascens#ZSD322
Cucurbitales Anisophylleaceae Anisophyllea laurina#ZSD321
Cucurbitales Cucurbitaceae Hemsleya lijiangensis#NC 039653
Cucurbitales Cucurbitaceae Gomphogyne cissiformis#MF784515
Cucurbitales Cucurbitaceae Gynostemma laxiflorum#NC 036134
Cucurbitales Cucurbitaceae Gynostemma cardiospermum#NC 035959
Cucurbitales Cucurbitaceae Gynostemma pentagynum#NC 036136
Cucurbitales Cucurbitaceae Gynostemma compressum#NC 037179
Cucurbitales Cucurbitaceae Gynostemma caulopterum#NC 036135
Cucurbitales Cucurbitaceae Gynostemma longipes#NC 036140
Cucurbitales Cucurbitaceae Gynostemma pentaphyllum#KX852298
Cucurbitales Cucurbitaceae Gynostemma aggregatum#Burmanicum NC 036141
Cucurbitales Cucurbitaceae Gynostemma pubescens#NC 036142
Cucurbitales Cucurbitaceae Gynostemma laxum#F51
Cucurbitales Cucurbitaceae Thladiantha villosula#AXAF
Cucurbitales Cucurbitaceae Momordica charantia#NC 036807
Cucurbitales Cucurbitaceae Hodgsonia macrocarpa#NC 039628
Cucurbitales Cucurbitaceae Trichosanthes kirilowii#NC 041088
Cucurbitales Cucurbitaceae Trichosanthes rubriflos#F1
Cucurbitales Cucurbitaceae Cucurbita maxima#NC 036505
Cucurbitales Cucurbitaceae Cucurbita pepo#NC 038229
Cucurbitales Cucurbitaceae Cucurbita moschata#NC 036506
Cucurbitales Cucurbitaceae Cucumis melo#MF536696
Cucurbitales Cucurbitaceae Cucumis sativus#KR230392
Cucurbitales Cucurbitaceae Cucumis hystrix#NC 023544

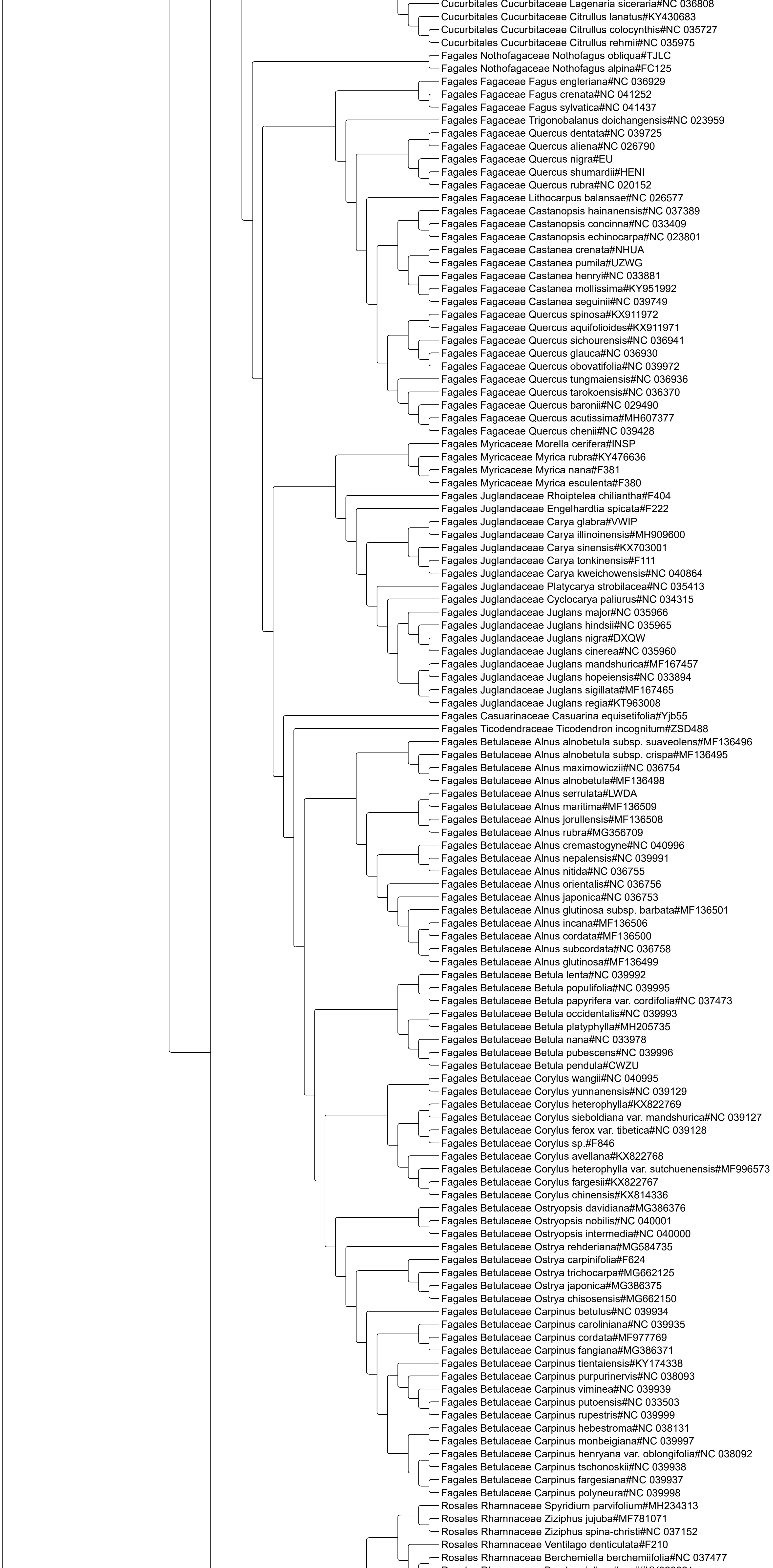

Cucurbitales Cucurbitaceae Lagenaria siceraria#NC 036808
Cucurbitales Cucurbitaceae Citrullus lanatus#KY430683
Cucurbitales Cucurbitaceae Citrullus colocynthis#NC 035727
Cucurbitales Cucurbitaceae Citrullus rehmii#NC 035975
Fagales Nothofagaceae Nothofagus obliqua#TJLC
Fagales Nothofagaceae Nothofagus alpina#FC125
Fagales Fagaceae Fagus engleriana#NC 036929
Fagales Fagaceae Fagus crenata#NC 041252
Fagales Fagaceae Fagus sylvatica#NC 041437
Fagales Fagaceae Trigonobalanus doichangensis#NC 023959
Fagales Fagaceae Quercus dentata#NC 039725
Fagales Fagaceae Quercus aliena#NC 026790
Fagales Fagaceae Quercus nigra#EU
Fagales Fagaceae Quercus shumardii#HENI
Fagales Fagaceae Quercus rubra#NC 020152
Fagales Fagaceae Lithocarpus balansae#NC 026577
Fagales Fagaceae Castanopsis hainanensis#NC 037389
Fagales Fagaceae Castanopsis concinna#NC 033409
Fagales Fagaceae Castanopsis echinocarpa#NC 023801
Fagales Fagaceae Castanea crenata#NHUA
Fagales Fagaceae Castanea pumila#UZWG
Fagales Fagaceae Castanea henryi#NC 033881
Fagales Fagaceae Castanea mollissima#KY951992
Fagales Fagaceae Castanea seguinii#NC 039749
Fagales Fagaceae Quercus spinosa#KX911972
Fagales Fagaceae Quercus aquifolioides#KX911971
Fagales Fagaceae Quercus sichourensis#NC 036941
Fagales Fagaceae Quercus glauca#NC 036930
Fagales Fagaceae Quercus obovatifolia#NC 039972
Fagales Fagaceae Quercus tungmaiensis#NC 036936
Fagales Fagaceae Quercus tarokoensis#NC 036370
Fagales Fagaceae Quercus baronii#NC 029490
Fagales Fagaceae Quercus acutissima#MH607377
Fagales Fagaceae Quercus chenii#NC 039428
Fagales Myricaceae Morella cerifera#INSP
Fagales Myricaceae Myrica rubra#KY476636
Fagales Myricaceae Myrica nana#F381
Fagales Myricaceae Myrica esculenta#F380
Fagales Juglandaceae Rhoiptelea chiliantha#F404
Fagales Juglandaceae Engelhardtia spicata#F222
Fagales Juglandaceae Carya glabra#VWIP
Fagales Juglandaceae Carya illinoinensis#MH909600
Fagales Juglandaceae Carya sinensis#KX703001
Fagales Juglandaceae Carya tonkinensis#F111
Fagales Juglandaceae Carya kweichowensis#NC 040864
Fagales Juglandaceae Platycarya strobilacea#NC 035413
Fagales Juglandaceae Cyclocarya paliurus#NC 034315
Fagales Juglandaceae Juglans major#NC 035966
Fagales Juglandaceae Juglans hindsii#NC 035965
Fagales Juglandaceae Juglans nigra#DXQW
Fagales Juglandaceae Juglans cinerea#NC 035960
Fagales Juglandaceae Juglans mandshurica#MF167457
Fagales Juglandaceae Juglans hopeiensis#NC 033894
Fagales Juglandaceae Juglans sigillata#MF167465
Fagales Juglandaceae Juglans regia#KT963008
Fagales Casuarinaceae Casuarina equisetifolia#Yjb55
Fagales Ticodendraceae Ticodendron incognitum#ZSD488
Fagales Betulaceae Alnus alnobetula subsp. suaveolens#MF136496
Fagales Betulaceae Alnus alnobetula subsp. crispa#MF136495
Fagales Betulaceae Alnus maximowiczii#NC 036754
Fagales Betulaceae Alnus alnobetula#MF136498
Fagales Betulaceae Alnus serrulata#LWDA
Fagales Betulaceae Alnus maritima#MF136509
Fagales Betulaceae Alnus jorullensis#MF136508
Fagales Betulaceae Alnus rubra#MG356709
Fagales Betulaceae Alnus cremastogyne#NC 040996
Fagales Betulaceae Alnus nepalensis#NC 039991
Fagales Betulaceae Alnus nitida#NC 036755
Fagales Betulaceae Alnus orientalis#NC 036756
Fagales Betulaceae Alnus japonica#NC 036753
Fagales Betulaceae Alnus glutinosa subsp. barbata#MF136501
Fagales Betulaceae Alnus incana#MF136506
Fagales Betulaceae Alnus cordata#MF136500
Fagales Betulaceae Alnus subcordata#NC 036758
Fagales Betulaceae Alnus glutinosa#MF136499
Fagales Betulaceae Betula lenta#NC 039992
Fagales Betulaceae Betula populifolia#NC 039995
Fagales Betulaceae Betula papyrifera var. cordifolia#NC 037473
Fagales Betulaceae Betula occidentalis#NC 039993
Fagales Betulaceae Betula platyphylla#MH205735
Fagales Betulaceae Betula nana#NC 033978
Fagales Betulaceae Betula pubescens#NC 039996
Fagales Betulaceae Betula pendula#CWZU
Fagales Betulaceae Corylus wangii#NC 040995
Fagales Betulaceae Corylus yunnanensis#NC 039129
Fagales Betulaceae Corylus heterophylla#KX822769
Fagales Betulaceae Corylus sieboldiana var. mandshurica#NC 039127
Fagales Betulaceae Corylus ferox var. tibetica#NC 039128
Fagales Betulaceae Corylus sp.#F846
Fagales Betulaceae Corylus avellana#KX822768
Fagales Betulaceae Corylus heterophylla var. sutchuenensis#MF996573
Fagales Betulaceae Corylus fargesii#KX822767
Fagales Betulaceae Corylus chinensis#KX814336
Fagales Betulaceae Ostryopsis davidiana#MG386376
Fagales Betulaceae Ostryopsis nobilis#NC 040001
Fagales Betulaceae Ostryopsis intermedia#NC 040000
Fagales Betulaceae Ostrya rehderiana#MG584735
Fagales Betulaceae Ostrya carpinifolia#F624
Fagales Betulaceae Ostrya trichocarpa#MG662125
Fagales Betulaceae Ostrya japonica#MG386375
Fagales Betulaceae Ostrya chisosensis#MG662150
Fagales Betulaceae Carpinus betulus#NC 039934
Fagales Betulaceae Carpinus caroliniana#NC 039935
Fagales Betulaceae Carpinus cordata#MF977769
Fagales Betulaceae Carpinus fangiana#MG386371
Fagales Betulaceae Carpinus tientaiensis#KY174338
Fagales Betulaceae Carpinus purpurinervis#NC 038093
Fagales Betulaceae Carpinus viminea#NC 039939
Fagales Betulaceae Carpinus putoensis#NC 033503
Fagales Betulaceae Carpinus rupestris#NC 039999
Fagales Betulaceae Carpinus hebestroma#NC 038131
Fagales Betulaceae Carpinus monbeigiana#NC 039997
Fagales Betulaceae Carpinus henryana var. oblongifolia#NC 038092
Fagales Betulaceae Carpinus tschonoskii#NC 039938
Fagales Betulaceae Carpinus fargesiana#NC 039937
Fagales Betulaceae Carpinus polyneura#NC 039998
Rosales Rhamnaceae Spyridium parvifolium#MH234313
Rosales Rhamnaceae Ziziphus jujuba#MF781071
Rosales Rhamnaceae Ziziphus spina-christi#NC 037152
Rosales Rhamnaceae Ventilago denticulata#F210
Rosales Rhamnaceae Berchemiella berchemiifolia#NC 037477

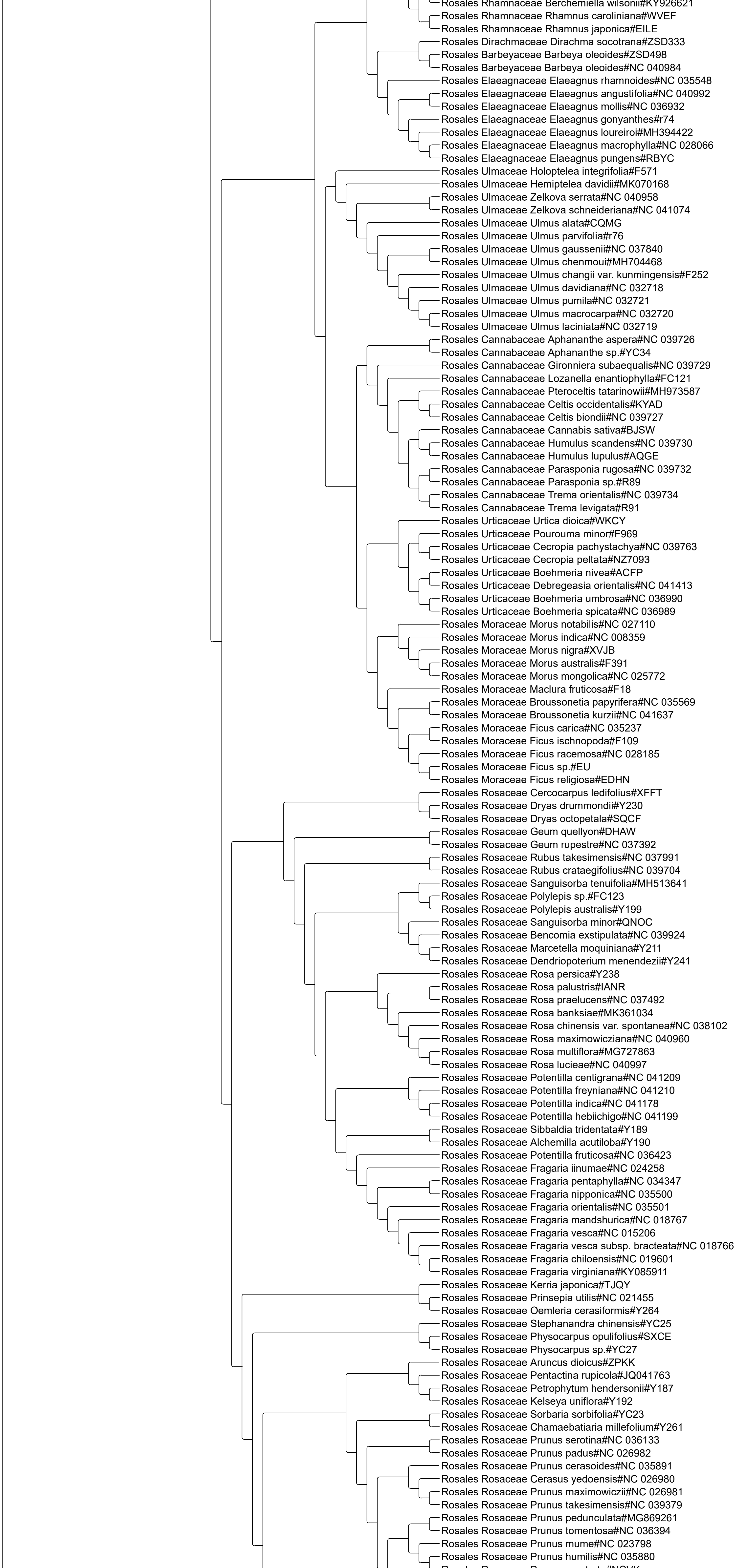

Rosales Rhamnaceae Berchemiella wilsonii#KY926621
Rosales Rhamnaceae Rhamnus caroliniana#WVEF
Rosales Rhamnaceae Rhamnus japonica#EILE
Rosales Dirachmaceae Dirachma socotrana#ZSD333
Rosales Barbeyaceae Barbeya oleoides#ZSD498
Rosales Barbeyaceae Barbeya oleoides#NC 040984
Rosales Elaeagnaceae Elaeagnus rhamnoides#NC 035548
Rosales Elaeagnaceae Elaeagnus angustifolia#NC 040992
Rosales Elaeagnaceae Elaeagnus mollis#NC 036932
Rosales Elaeagnaceae Elaeagnus gonyanthes#r74
Rosales Elaeagnaceae Elaeagnus loureiroi#MH394422
Rosales Elaeagnaceae Elaeagnus macrophylla#NC 028066
Rosales Elaeagnaceae Elaeagnus pungens#RBYC
Rosales Ulmaceae Holoptelea integrifolia#F571
Rosales Ulmaceae Hemiptelea davidii#MK070168
Rosales Ulmaceae Zelkova serrata#NC 040958
Rosales Ulmaceae Zelkova schneideriana#NC 041074
Rosales Ulmaceae Ulmus alata#CQMG
Rosales Ulmaceae Ulmus parvifolia#r76
Rosales Ulmaceae Ulmus gaussenii#NC 037840
Rosales Ulmaceae Ulmus chenmoui#MH704468
Rosales Ulmaceae Ulmus changii var. kunmingensis#F252
Rosales Ulmaceae Ulmus davidiana#NC 032718
Rosales Ulmaceae Ulmus pumila#NC 032721
Rosales Ulmaceae Ulmus macrocarpa#NC 032720
Rosales Ulmaceae Ulmus laciniata#NC 032719
Rosales Cannabaceae Aphananthe aspera#NC 039726
Rosales Cannabaceae Aphananthe sp.#YC34
Rosales Cannabaceae Gironniera subaequalis#NC 039729
Rosales Cannabaceae Lozanella enantiophylla#FC121
Rosales Cannabaceae Pteroceltis tatarinowii#MH973587
Rosales Cannabaceae Celtis occidentalis#KYAD
Rosales Cannabaceae Celtis biondii#NC 039727
Rosales Cannabaceae Cannabis sativa#BJSW
Rosales Cannabaceae Humulus scandens#NC 039730
Rosales Cannabaceae Humulus lupulus#AQGE
Rosales Cannabaceae Parasponia rugosa#NC 039732
Rosales Cannabaceae Parasponia sp.#R89
Rosales Cannabaceae Trema orientalis#NC 039734
Rosales Cannabaceae Trema levigata#R91
Rosales Urticaceae Urtica dioica#WKCY
Rosales Urticaceae Pourouma minor#F969
Rosales Urticaceae Cecropia pachystachya#NC 039763
Rosales Urticaceae Cecropia peltata#NZ7093
Rosales Urticaceae Boehmeria nivea#ACFP
Rosales Urticaceae Debregeasia orientalis#NC 041413
Rosales Urticaceae Boehmeria umbrosa#NC 036990
Rosales Urticaceae Boehmeria spicata#NC 036989
Rosales Moraceae Morus notabilis#NC 027110
Rosales Moraceae Morus indica#NC 008359
Rosales Moraceae Morus nigra#XVJB
Rosales Moraceae Morus australis#F391
Rosales Moraceae Morus mongolica#NC 025772
Rosales Moraceae Maclura fruticosa#F18
Rosales Moraceae Broussonetia papyrifera#NC 035569
Rosales Moraceae Broussonetia kurzii#NC 041637
Rosales Moraceae Ficus carica#NC 035237
Rosales Moraceae Ficus ischnopoda#F109
Rosales Moraceae Ficus racemosa#NC 028185
Rosales Moraceae Ficus sp.#EU
Rosales Moraceae Ficus religiosa#EDHN
Rosales Rosaceae Cercocarpus ledifolius#XFFT
Rosales Rosaceae Dryas drummondii#Y230
Rosales Rosaceae Dryas octopetala#SQCF
Rosales Rosaceae Geum quellyon#DHAW
Rosales Rosaceae Geum rupestre#NC 037392
Rosales Rosaceae Rubus takesimensis#NC 037991
Rosales Rosaceae Rubus crataegifolius#NC 039704
Rosales Rosaceae Sanguisorba tenuifolia#MH513641
Rosales Rosaceae Polylepis sp.#FC123
Rosales Rosaceae Polylepis australis#Y199
Rosales Rosaceae Sanguisorba minor#QNOC
Rosales Rosaceae Bencomia exstipulata#NC 039924
Rosales Rosaceae Marcetella moquiniana#Y211
Rosales Rosaceae Dendriopoterium menendezii#Y241
Rosales Rosaceae Rosa persica#Y238
Rosales Rosaceae Rosa palustris#IANR
Rosales Rosaceae Rosa praelucens#NC 037492
Rosales Rosaceae Rosa banksiae#MK361034
Rosales Rosaceae Rosa chinensis var. spontanea#NC 038102
Rosales Rosaceae Rosa maximowicziana#NC 040960
Rosales Rosaceae Rosa multiflora#MG727863
Rosales Rosaceae Rosa lucieae#NC 040997
Rosales Rosaceae Potentilla centigrana#NC 041209
Rosales Rosaceae Potentilla freyniana#NC 041210
Rosales Rosaceae Potentilla indica#NC 041178
Rosales Rosaceae Potentilla hebiichigo#NC 041199
Rosales Rosaceae Sibbaldia tridentata#Y189
Rosales Rosaceae Alchemilla acutiloba#Y190
Rosales Rosaceae Potentilla fruticosa#NC 036423
Rosales Rosaceae Fragaria iinumae#NC 024258
Rosales Rosaceae Fragaria pentaphylla#NC 034347
Rosales Rosaceae Fragaria nipponica#NC 035500
Rosales Rosaceae Fragaria orientalis#NC 035501
Rosales Rosaceae Fragaria mandshurica#NC 018767
Rosales Rosaceae Fragaria vesca#NC 015206
Rosales Rosaceae Fragaria vesca subsp. bracteata#NC 018766
Rosales Rosaceae Fragaria chiloensis#NC 019601
Rosales Rosaceae Fragaria virginiana#KY085911
Rosales Rosaceae Kerria japonica#TJQY
Rosales Rosaceae Prinsepia utilis#NC 021455
Rosales Rosaceae Oemleria cerasiformis#Y264
Rosales Rosaceae Stephanandra chinensis#YC25
Rosales Rosaceae Physocarpus opulifolius#SXCE
Rosales Rosaceae Physocarpus sp.#YC27
Rosales Rosaceae Aruncus dioicus#ZPKK
Rosales Rosaceae Pentactina rupicola#JQ041763
Rosales Rosaceae Petrophytum hendersonii#Y187
Rosales Rosaceae Kelseya uniflora#Y192
Rosales Rosaceae Sorbaria sorbifolia#YC23
Rosales Rosaceae Chamaebatiaria millefolium#Y261
Rosales Rosaceae Prunus serotina#NC 036133
Rosales Rosaceae Prunus padus#NC 026982
Rosales Rosaceae Prunus cerasoides#NC 035891
Rosales Rosaceae Cerasus yedoensis#NC 026980
Rosales Rosaceae Prunus maximowiczii#NC 026981
Rosales Rosaceae Prunus takesimensis#NC 039379
Rosales Rosaceae Prunus pedunculata#MG869261
Rosales Rosaceae Prunus tomentosa#NC 036394
Rosales Rosaceae Prunus mume#NC 023798
Rosales Rosaceae Prunus humilis#NC 035880

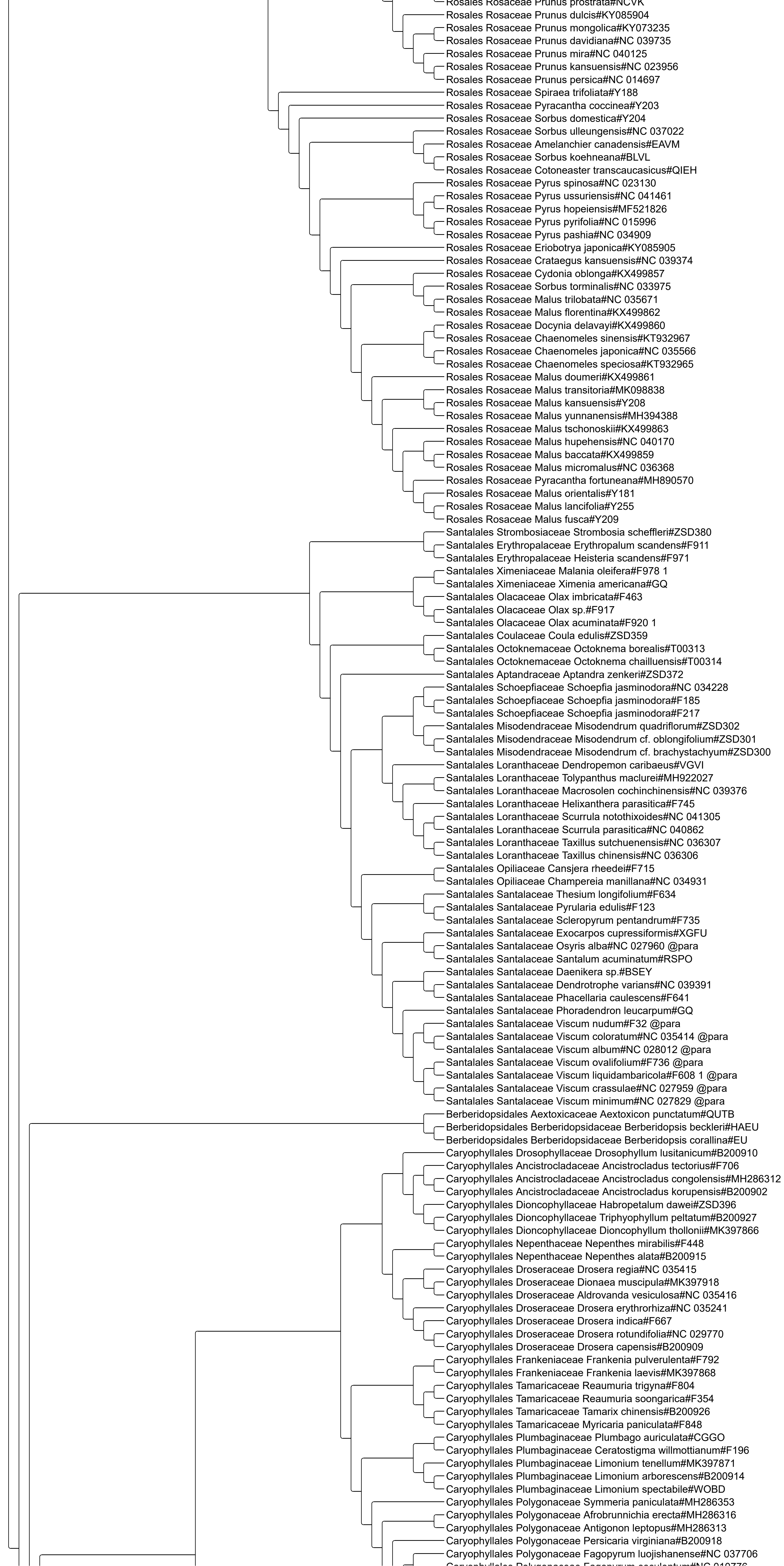
Rosales Rosaceae Prunus prostrata#NCVK
Rosales Rosaceae Prunus dulcis#KY085904
Rosales Rosaceae Prunus mongolica#KY073235
Rosales Rosaceae Prunus davidiana#NC 039735
Rosales Rosaceae Prunus mira#NC 040125
Rosales Rosaceae Prunus kansuensis#NC 023956
Rosales Rosaceae Prunus persica#NC 014697
Rosales Rosaceae Spiraea trifoliata#Y188
Rosales Rosaceae Pyracantha coccinea#Y203
Rosales Rosaceae Sorbus domestica#Y204
Rosales Rosaceae Sorbus ulleungensis#NC 037022
Rosales Rosaceae Amelanchier canadensis#EAVM
Rosales Rosaceae Sorbus koehneana#BLVL
Rosales Rosaceae Cotoneaster transcaucasicus#QIEH
Rosales Rosaceae Pyrus spinosa#NC 023130
Rosales Rosaceae Pyrus ussuriensis#NC 041461
Rosales Rosaceae Pyrus hopeiensis#MF521826
Rosales Rosaceae Pyrus pyrifolia#NC 015996
Rosales Rosaceae Pyrus pashia#NC 034909
Rosales Rosaceae Eriobotrya japonica#KY085905
Rosales Rosaceae Crataegus kansuensis#NC 039374
Rosales Rosaceae Cydonia oblonga#KX499857
Rosales Rosaceae Sorbus torminalis#NC 033975
Rosales Rosaceae Malus trilobata#NC 035671
Rosales Rosaceae Malus florentina#KX499862
Rosales Rosaceae Docynia delavayi#KX499860
Rosales Rosaceae Chaenomeles sinensis#KT932967
Rosales Rosaceae Chaenomeles japonica#NC 035566
Rosales Rosaceae Chaenomeles speciosa#KT932965
Rosales Rosaceae Malus doumeri#KX499861
Rosales Rosaceae Malus transitoria#MK098838
Rosales Rosaceae Malus kansuensis#Y208
Rosales Rosaceae Malus yunnanensis#MH394388
Rosales Rosaceae Malus tschonoskii#KX499863
Rosales Rosaceae Malus hupehensis#NC 040170
Rosales Rosaceae Malus baccata#KX499859
Rosales Rosaceae Malus micromalus#NC 036368
Rosales Rosaceae Pyracantha fortuneana#MH890570
Rosales Rosaceae Malus orientalis#Y181
Rosales Rosaceae Malus lancifolia#Y255
Rosales Rosaceae Malus fusca#Y209
Santalales Strombosiaceae Strombosia scheffleri#ZSD380
Santalales Erythropalaceae Erythropalum scandens#F911
Santalales Erythropalaceae Heisteria scandens#F971
Santalales Ximeniaceae Malania oleifera#F978 1
Santalales Ximeniaceae Ximenia americana#GQ
Santalales Olacaceae Olax imbricata#F463
Santalales Olacaceae Olax sp.#F917
Santalales Olacaceae Olax acuminata#F920 1
Santalales Coulaceae Coula edulis#ZSD359
Santalales Octoknemaceae Octoknema borealis#T00313
Santalales Octoknemaceae Octoknema chailluensis#T00314
Santalales Aptandraceae Aptandra zenkeri#ZSD372
Santalales Schoepfiaceae Schoepfia jasminodora#NC 034228
Santalales Schoepfiaceae Schoepfia jasminodora#F185
Santalales Schoepfiaceae Schoepfia jasminodora#F217
Santalales Misodendraceae Misodendrum quadriflorum#ZSD302
Santalales Misodendraceae Misodendrum cf. oblongifolium#ZSD301
Santalales Misodendraceae Misodendrum cf. brachystachyum#ZSD300
Santalales Loranthaceae Dendropemon caribaeus#VGVI
Santalales Loranthaceae Tolypanthus maclurei#MH922027
Santalales Loranthaceae Macrosolen cochinchinensis#NC 039376
Santalales Loranthaceae Helixanthera parasitica#F745
Santalales Loranthaceae Scurrula notothixoides#NC 041305
Santalales Loranthaceae Scurrula parasitica#NC 040862
Santalales Loranthaceae Taxillus sutchuenensis#NC 036307
Santalales Loranthaceae Taxillus chinensis#NC 036306
Santalales Opiliaceae Cansjera rheedei#F715
Santalales Opiliaceae Champereia manillana#NC 034931
Santalales Santalaceae Thesium longifolium#F634
Santalales Santalaceae Pyrularia edulis#F123
Santalales Santalaceae Scleropyrum pentandrum#F735
Santalales Santalaceae Exocarpos cupressiformis#XGFU
Santalales Santalaceae Osyris alba#NC 027960 @para
Santalales Santalaceae Santalum acuminatum#RSPO
Santalales Santalaceae Daenikera sp.#BSEY
Santalales Santalaceae Dendrotrophe varians#NC 039391
Santalales Santalaceae Phacellaria caulescens#F641
Santalales Santalaceae Phoradendron leucarpum#GQ
Santalales Santalaceae Viscum nudum#F32 @para
Santalales Santalaceae Viscum coloratum#NC 035414 @para
Santalales Santalaceae Viscum album#NC 028012 @para
Santalales Santalaceae Viscum ovalifolium#F736 @para
Santalales Santalaceae Viscum liquidambaricola#F608 1 @para
Santalales Santalaceae Viscum crassulae#NC 027959 @para
Santalales Santalaceae Viscum minimum#NC 027829 @para
Berberidopsidales Aextoxicaceae Aextoxicon punctatum#QUTB
Berberidopsidales Berberidopsidaceae Berberidopsis beckleri#HAEU
Berberidopsidales Berberidopsidaceae Berberidopsis corallina#EU
Caryophyllales Drosophyllaceae Drosophyllum lusitanicum#B200910
Caryophyllales Ancistrocladaceae Ancistrocladus tectorius#F706
Caryophyllales Ancistrocladaceae Ancistrocladus congolensis#MH286312
Caryophyllales Ancistrocladaceae Ancistrocladus korupensis#B200902
Caryophyllales Dioncophyllaceae Habropetalum dawei#ZSD396
Caryophyllales Dioncophyllaceae Triphyophyllum peltatum#B200927
Caryophyllales Dioncophyllaceae Dioncophyllum thollonii#MK397866
Caryophyllales Nepenthaceae Nepenthes mirabilis#F448
Caryophyllales Nepenthaceae Nepenthes alata#B200915
Caryophyllales Droseraceae Drosera regia#NC 035415
Caryophyllales Droseraceae Dionaea muscipula#MK397918
Caryophyllales Droseraceae Aldrovanda vesiculosa#NC 035416
Caryophyllales Droseraceae Drosera erythrorhiza#NC 035241
Caryophyllales Droseraceae Drosera indica#F667
Caryophyllales Droseraceae Drosera rotundifolia#NC 029770
Caryophyllales Droseraceae Drosera capensis#B200909
Caryophyllales Frankeniaceae Frankenia pulverulenta#F792
Caryophyllales Frankeniaceae Frankenia laevis#MK397868
Caryophyllales Tamaricaceae Reaumuria trigyna#F804
Caryophyllales Tamaricaceae Reaumuria soongarica#F354
Caryophyllales Tamaricaceae Tamarix chinensis#B200926
Caryophyllales Tamaricaceae Myricaria paniculata#F848
Caryophyllales Plumbaginaceae Plumbago auriculata#CGGO
Caryophyllales Plumbaginaceae Ceratostigma willmottianum#F196
Caryophyllales Plumbaginaceae Limonium tenellum#MK397871
Caryophyllales Plumbaginaceae Limonium arborescens#B200914
Caryophyllales Plumbaginaceae Limonium spectabile#WOBD
Caryophyllales Polygonaceae Symmeria paniculata#MH286353
Caryophyllales Polygonaceae Afrobrunnichia erecta#MH286316
Caryophyllales Polygonaceae Antigonon leptopus#MH286313
Caryophyllales Polygonaceae Persicaria virginiana#B200918
Caryophyllales Polygonaceae Fagopyrum luojishanense#NC 037706

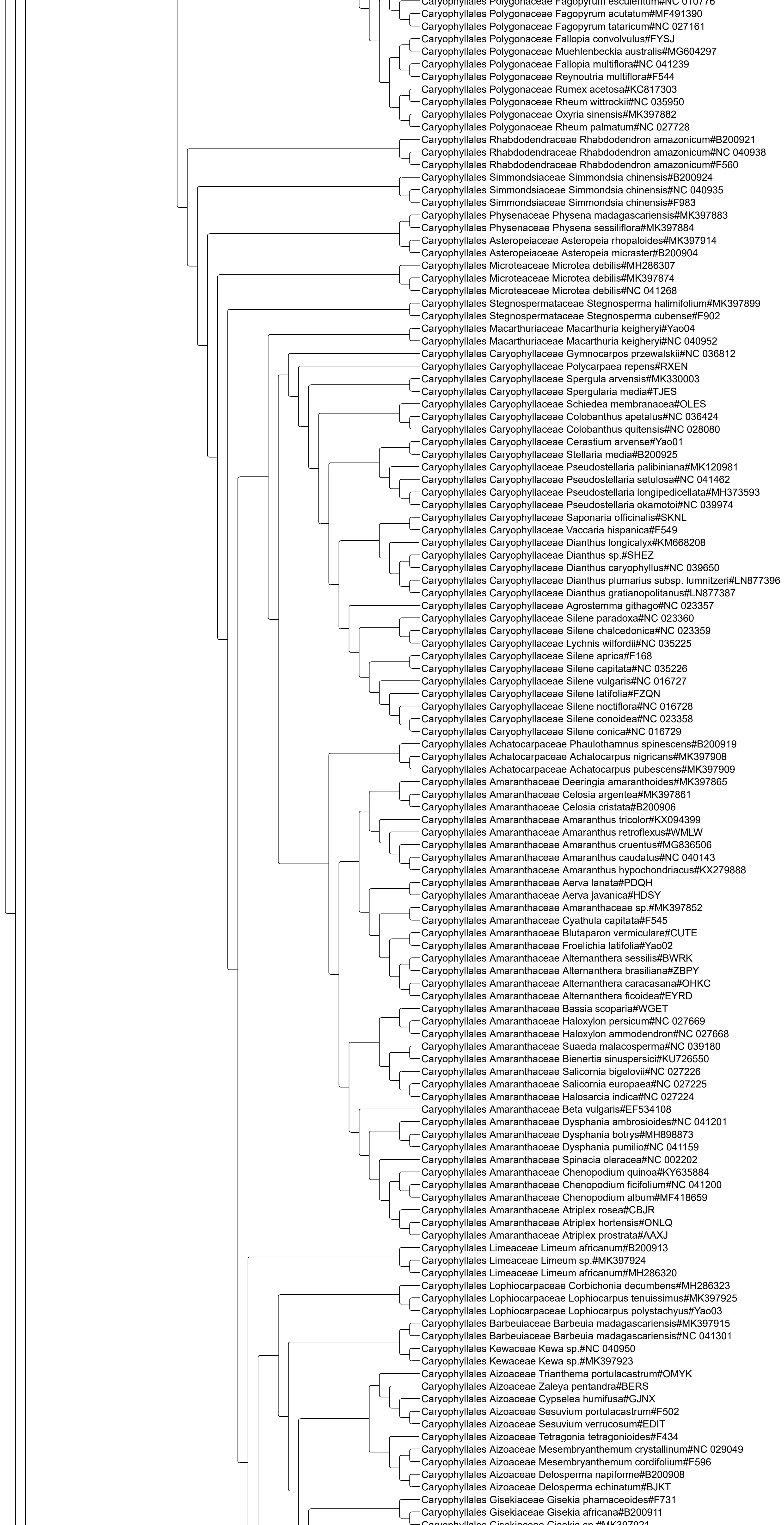

Caryophyllales Polygonaceae Fagopyrum esculentum#NC 010776
Caryophyllales Polygonaceae Fagopyrum acutatum#MF491390
Caryophyllales Polygonaceae Fagopyrum tataricum#NC 027161
Caryophyllales Polygonaceae Fallopia convolvulus#FYSJ
Caryophyllales Polygonaceae Muehlenbeckia australis#MG604297
Caryophyllales Polygonaceae Fallopia multiflora#NC 041239
Caryophyllales Polygonaceae Reynoutria multiflora#F544
Caryophyllales Polygonaceae Rumex acetosa#KC817303
Caryophyllales Polygonaceae Rheum wittrockii#NC 035950
Caryophyllales Polygonaceae Oxyria sinensis#MK397882
Caryophyllales Polygonaceae Rheum palmatum#NC 027728
Caryophyllales Rhabdodendraceae Rhabdodendron amazonicum#B200921
Caryophyllales Rhabdodendraceae Rhabdodendron amazonicum#NC 040938
Caryophyllales Rhabdodendraceae Rhabdodendron amazonicum#F560
Caryophyllales Simmondsiaceae Simmondsia chinensis#B200924
Caryophyllales Simmondsiaceae Simmondsia chinensis#NC 040935
Caryophyllales Simmondsiaceae Simmondsia chinensis#F983
Caryophyllales Physenaceae Physena madagascariensis#MK397883
Caryophyllales Physenaceae Physena sessiliflora#MK397884
Caryophyllales Asteropeiaceae Asteropeia rhopaloides#MK397914
Caryophyllales Asteropeiaceae Asteropeia micraster#B200904
Caryophyllales Microteaceae Microtea debilis#MH286307
Caryophyllales Microteaceae Microtea debilis#MK397874
Caryophyllales Microteaceae Microtea debilis#NC 041268
Caryophyllales Stegnospermataceae Stegnosperma halimifolium#MK397899
Caryophyllales Stegnospermataceae Stegnosperma cubense#F902
Caryophyllales Macarthuriaceae Macarthuria keigheryi#Yao04
Caryophyllales Macarthuriaceae Macarthuria keigheryi#NC 040952
Caryophyllales Caryophyllaceae Gymnocarpos przewalskii#NC 036812
Caryophyllales Caryophyllaceae Polycarpaea repens#RXEN
Caryophyllales Caryophyllaceae Spergula arvensis#MK330003
Caryophyllales Caryophyllaceae Spergularia media#TJES
Caryophyllales Caryophyllaceae Schiedea membranacea#OLES
Caryophyllales Caryophyllaceae Colobanthus apetalus#NC 036424
Caryophyllales Caryophyllaceae Colobanthus quitensis#NC 028080
Caryophyllales Caryophyllaceae Cerastium arvense#Yao01
Caryophyllales Caryophyllaceae Stellaria media#B200925
Caryophyllales Caryophyllaceae Pseudostellaria palibiniana#MK120981
Caryophyllales Caryophyllaceae Pseudostellaria setulosa#NC 041462
Caryophyllales Caryophyllaceae Pseudostellaria longipedicellata#MH373593
Caryophyllales Caryophyllaceae Pseudostellaria okamotoi#NC 039974
Caryophyllales Caryophyllaceae Saponaria officinalis#SKNL
Caryophyllales Caryophyllaceae Vaccaria hispanica#F549
Caryophyllales Caryophyllaceae Dianthus longicalyx#KM668208
Caryophyllales Caryophyllaceae Dianthus sp.#SHEZ
Caryophyllales Caryophyllaceae Dianthus caryophyllus#NC 039650
Caryophyllales Caryophyllaceae Dianthus plumarius subsp. lumnitzeri#LN877396
Caryophyllales Caryophyllaceae Dianthus gratianopolitanus#LN877387
Caryophyllales Caryophyllaceae Agrostemma githago#NC 023357
Caryophyllales Caryophyllaceae Silene paradoxa#NC 023360
Caryophyllales Caryophyllaceae Silene chalcedonica#NC 023359
Caryophyllales Caryophyllaceae Lychnis wilfordii#NC 035225
Caryophyllales Caryophyllaceae Silene aprica#F168
Caryophyllales Caryophyllaceae Silene capitata#NC 035226
Caryophyllales Caryophyllaceae Silene vulgaris#NC 016727
Caryophyllales Caryophyllaceae Silene latifolia#FZQN
Caryophyllales Caryophyllaceae Silene noctiflora#NC 016728
Caryophyllales Caryophyllaceae Silene conoidea#NC 023358
Caryophyllales Caryophyllaceae Silene conica#NC 016729
Caryophyllales Achatocarpaceae Phaulothamnus spinescens#B200919
Caryophyllales Achatocarpaceae Achatocarpus nigricans#MK397908
Caryophyllales Achatocarpaceae Achatocarpus pubescens#MK397909
Caryophyllales Amaranthaceae Deeringia amaranthoides#MK397865
Caryophyllales Amaranthaceae Celosia argentea#MK397861
Caryophyllales Amaranthaceae Celosia cristata#B200906
Caryophyllales Amaranthaceae Amaranthus tricolor#KX094399
Caryophyllales Amaranthaceae Amaranthus retroflexus#WMLW
Caryophyllales Amaranthaceae Amaranthus cruentus#MG836506
Caryophyllales Amaranthaceae Amaranthus caudatus#NC 040143
Caryophyllales Amaranthaceae Amaranthus hypochondriacus#KX279888
Caryophyllales Amaranthaceae Aerva lanata#PDQH
Caryophyllales Amaranthaceae Aerva javanica#HDSY
Caryophyllales Amaranthaceae Amaranthaceae sp.#MK397852
Caryophyllales Amaranthaceae Cyathula capitata#F545
Caryophyllales Amaranthaceae Blutaparon vermiculare#CUTE
Caryophyllales Amaranthaceae Froelichia latifolia#Yao02
Caryophyllales Amaranthaceae Alternanthera sessilis#BWRK
Caryophyllales Amaranthaceae Alternanthera brasiliana#ZBPY
Caryophyllales Amaranthaceae Alternanthera caracasana#OHKC
Caryophyllales Amaranthaceae Alternanthera ficoidea#EYRD
Caryophyllales Amaranthaceae Bassia scoparia#WGET
Caryophyllales Amaranthaceae Haloxylon persicum#NC 027669
Caryophyllales Amaranthaceae Haloxylon ammodendron#NC 027668
Caryophyllales Amaranthaceae Suaeda malacosperma#NC 039180
Caryophyllales Amaranthaceae Bienertia sinuspersici#KU726550
Caryophyllales Amaranthaceae Salicornia bigelovii#NC 027226
Caryophyllales Amaranthaceae Salicornia europaea#NC 027225
Caryophyllales Amaranthaceae Halosarcia indica#NC 027224
Caryophyllales Amaranthaceae Beta vulgaris#EF534108
Caryophyllales Amaranthaceae Dysphania ambrosioides#NC 041201
Caryophyllales Amaranthaceae Dysphania botrys#MH898873
Caryophyllales Amaranthaceae Dysphania pumilio#NC 041159
Caryophyllales Amaranthaceae Spinacia oleracea#NC 002202
Caryophyllales Amaranthaceae Chenopodium quinoa#KY635884
Caryophyllales Amaranthaceae Chenopodium ficifolium#NC 041200
Caryophyllales Amaranthaceae Chenopodium album#MF418659
Caryophyllales Amaranthaceae Atriplex rosea#CBJR
Caryophyllales Amaranthaceae Atriplex hortensis#ONLQ
Caryophyllales Amaranthaceae Atriplex prostrata#AAXJ
Caryophyllales Limeaceae Limeum africanum#B200913
Caryophyllales Limeaceae Limeum sp.#MK397924
Caryophyllales Limeaceae Limeum africanum#MH286320
Caryophyllales Lophiocarpaceae Corbichonia decumbens#MH286323
Caryophyllales Lophiocarpaceae Lophiocarpus tenuissimus#MK397925
Caryophyllales Lophiocarpaceae Lophiocarpus polystachyus#Yao03
Caryophyllales Barbeuiaceae Barbeuia madagascariensis#MK397915
Caryophyllales Barbeuiaceae Barbeuia madagascariensis#NC 041301
Caryophyllales Kewaceae Kewa sp.#NC 040950
Caryophyllales Kewaceae Kewa sp.#MK397923
Caryophyllales Aizoaceae Trianthema portulacastrum#OMYK
Caryophyllales Aizoaceae Zaleya pentandra#BERS
Caryophyllales Aizoaceae Cypselea humifusa#GJNX
Caryophyllales Aizoaceae Sesuvium portulacastrum#F502
Caryophyllales Aizoaceae Sesuvium verrucosum#EDIT
Caryophyllales Aizoaceae Tetragonia tetragonioides#F434
Caryophyllales Aizoaceae Mesembryanthemum crystallinum#NC 029049
Caryophyllales Aizoaceae Mesembryanthemum cordifolium#F596
Caryophyllales Aizoaceae Delosperma napiforme#B200908
Caryophyllales Aizoaceae Delosperma echinatum#BJKT
Caryophyllales Gisekiaceae Gisekia pharnaceoides#F731
Caryophyllales Gisekiaceae Gisekia africana#B200911

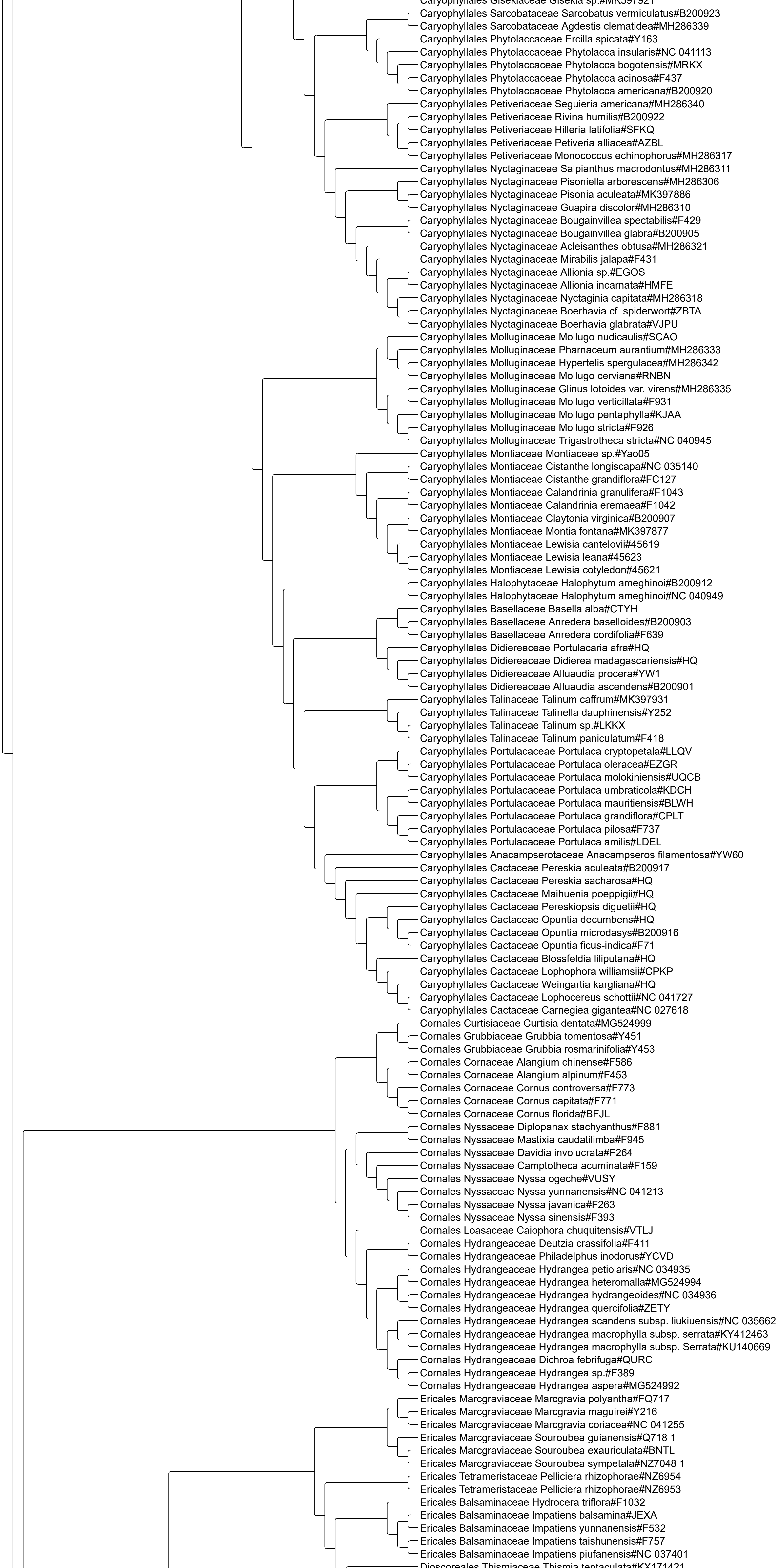
Caryophyllales Sarcobataceae Sarcobatus vermiculatus#B200923
Caryophyllales Sarcobataceae Agdestis clematidea#MH286339
Caryophyllales Phytolaccaceae Ercilla spicata#Y163
Caryophyllales Phytolaccaceae Phytolacca insularis#NC 041113
Caryophyllales Phytolaccaceae Phytolacca bogotensis#MRKX
Caryophyllales Phytolaccaceae Phytolacca acinosa#F437
Caryophyllales Phytolaccaceae Phytolacca americana#B200920
Caryophyllales Petiveriaceae Seguieria americana#MH286340
Caryophyllales Petiveriaceae Rivina humilis#B200922
Caryophyllales Petiveriaceae Hilleria latifolia#SFKQ
Caryophyllales Petiveriaceae Petiveria alliacea#AZBL
Caryophyllales Petiveriaceae Monococcus echinophorus#MH286317
Caryophyllales Nyctaginaceae Salpianthus macrodontus#MH286311
Caryophyllales Nyctaginaceae Pisoniella arborescens#MH286306
Caryophyllales Nyctaginaceae Pisonia aculeata#MK397886
Caryophyllales Nyctaginaceae Guapira discolor#MH286310
Caryophyllales Nyctaginaceae Bougainvillea spectabilis#F429
Caryophyllales Nyctaginaceae Bougainvillea glabra#B200905
Caryophyllales Nyctaginaceae Acleisanthes obtusa#MH286321
Caryophyllales Nyctaginaceae Mirabilis jalapa#F431
Caryophyllales Nyctaginaceae Allionia sp.#EGOS
Caryophyllales Nyctaginaceae Allionia incarnata#HMFE
Caryophyllales Nyctaginaceae Nyctaginia capitata#MH286318
Caryophyllales Nyctaginaceae Boerhavia cf. spiderwort#ZBTA
Caryophyllales Nyctaginaceae Boerhavia glabrata#VJPU
Caryophyllales Molluginaceae Mollugo nudicaulis#SCAO
Caryophyllales Molluginaceae Pharnaceum aurantium#MH286333
Caryophyllales Molluginaceae Hypertelis spergulacea#MH286342
Caryophyllales Molluginaceae Mollugo cerviana#RNBN
Caryophyllales Molluginaceae Glinus lotoides var. virens#MH286335
Caryophyllales Molluginaceae Mollugo verticillata#F931
Caryophyllales Molluginaceae Mollugo pentaphylla#KJAA
Caryophyllales Molluginaceae Mollugo stricta#F926
Caryophyllales Molluginaceae Trigastrotheca stricta#NC 040945
Caryophyllales Montiaceae Montiaceae sp.#Yao05
Caryophyllales Montiaceae Cistanthe longiscapa#NC 035140
Caryophyllales Montiaceae Cistanthe grandiflora#FC127
Caryophyllales Montiaceae Calandrinia granulifera#F1043
Caryophyllales Montiaceae Calandrinia eremaea#F1042
Caryophyllales Montiaceae Claytonia virginica#B200907
Caryophyllales Montiaceae Montia fontana#MK397877
Caryophyllales Montiaceae Lewisia cantelovii#45619
Caryophyllales Montiaceae Lewisia leana#45623
Caryophyllales Montiaceae Lewisia cotyledon#45621
Caryophyllales Halophytaceae Halophytum ameghinoi#B200912
Caryophyllales Halophytaceae Halophytum ameghinoi#NC 040949
Caryophyllales Basellaceae Basella alba#CTYH
Caryophyllales Basellaceae Anredera baselloides#B200903
Caryophyllales Basellaceae Anredera cordifolia#F639
Caryophyllales Didiereaceae Portulacaria afra#HQ
Caryophyllales Didiereaceae Didierea madagascariensis#HQ
Caryophyllales Didiereaceae Alluaudia procera#YW1
Caryophyllales Didiereaceae Alluaudia ascendens#B200901
Caryophyllales Talinaceae Talinum caffrum#MK397931
Caryophyllales Talinaceae Talinella dauphinensis#Y252
Caryophyllales Talinaceae Talinum sp.#LKKX
Caryophyllales Talinaceae Talinum paniculatum#F418
Caryophyllales Portulacaceae Portulaca cryptopetala#LLQV
Caryophyllales Portulacaceae Portulaca oleracea#EZGR
Caryophyllales Portulacaceae Portulaca molokiniensis#UQCB
Caryophyllales Portulacaceae Portulaca umbraticola#KDCH
Caryophyllales Portulacaceae Portulaca mauritiensis#BLWH
Caryophyllales Portulacaceae Portulaca grandiflora#CPLT
Caryophyllales Portulacaceae Portulaca pilosa#F737
Caryophyllales Portulacaceae Portulaca amilis#LDEL
Caryophyllales Anacampserotaceae Anacampseros filamentosa#YW60
Caryophyllales Cactaceae Pereskia aculeata#B200917
Caryophyllales Cactaceae Pereskia sacharosa#HQ
Caryophyllales Cactaceae Maihuenia poeppigii#HQ
Caryophyllales Cactaceae Pereskiopsis diguetii#HQ
Caryophyllales Cactaceae Opuntia decumbens#HQ
Caryophyllales Cactaceae Opuntia microdasys#B200916
Caryophyllales Cactaceae Opuntia ficus-indica#F71
Caryophyllales Cactaceae Blossfeldia liliputana#HQ
Caryophyllales Cactaceae Lophophora williamsii#CPKP
Caryophyllales Cactaceae Weingartia kargliana#HQ
Caryophyllales Cactaceae Lophocereus schottii#NC 041727
Caryophyllales Cactaceae Carnegiea gigantea#NC 027618
Cornales Curtisiaceae Curtisia dentata#MG524999
Cornales Grubbiaceae Grubbia tomentosa#Y451
Cornales Grubbiaceae Grubbia rosmarinifolia#Y453
Cornales Cornaceae Alangium chinense#F586
Cornales Cornaceae Alangium alpinum#F453
Cornales Cornaceae Cornus controversa#F773
Cornales Cornaceae Cornus capitata#F771
Cornales Cornaceae Cornus florida#BFJL
Cornales Nyssaceae Diplopanax stachyanthus#F881
Cornales Nyssaceae Mastixia caudatilimba#F945
Cornales Nyssaceae Davidia involucrata#F264
Cornales Nyssaceae Camptotheca acuminata#F159
Cornales Nyssaceae Nyssa ogeche#VUSY
Cornales Nyssaceae Nyssa yunnanensis#NC 041213
Cornales Nyssaceae Nyssa javanica#F263
Cornales Nyssaceae Nyssa sinensis#F393
Cornales Loasaceae Caiophora chuquitensis#VTLJ
Cornales Hydrangeaceae Deutzia crassifolia#F411
Cornales Hydrangeaceae Philadelphus inodorus#YCVD
Cornales Hydrangeaceae Hydrangea petiolaris#NC 034935
Cornales Hydrangeaceae Hydrangea heteromalla#MG524994
Cornales Hydrangeaceae Hydrangea hydrangeoides#NC 034936
Cornales Hydrangeaceae Hydrangea quercifolia#ZETY
Cornales Hydrangeaceae Hydrangea scandens subsp. liukiuensis#NC 035662
Cornales Hydrangeaceae Hydrangea macrophylla subsp. serrata#KY412463
Cornales Hydrangeaceae Hydrangea macrophylla subsp. Serrata#KU140669
Cornales Hydrangeaceae Dichroa febrifuga#QURC
Cornales Hydrangeaceae Hydrangea sp.#F389
Cornales Hydrangeaceae Hydrangea aspera#MG524992
Ericales Marcgraviaceae Marcgravia polyantha#FQ717
Ericales Marcgraviaceae Marcgravia maguirei#Y216
Ericales Marcgraviaceae Marcgravia coriacea#NC 041255
Ericales Marcgraviaceae Souroubea guianensis#Q718 1
Ericales Marcgraviaceae Souroubea exauriculata#BNTL
Ericales Marcgraviaceae Souroubea sympetala#NZ7048 1
Ericales Tetrameristaceae Pelliciera rhizophorae#NZ6954
Ericales Tetrameristaceae Pelliciera rhizophorae#NZ6953
Ericales Balsaminaceae Hydrocera triflora#F1032
Ericales Balsaminaceae Impatiens balsamina#JEXA
Ericales Balsaminaceae Impatiens yunnanensis#F532
Ericales Balsaminaceae Impatiens taishunensis#F757
Ericales Balsaminaceae Impatiens piufanensis#NC 037401

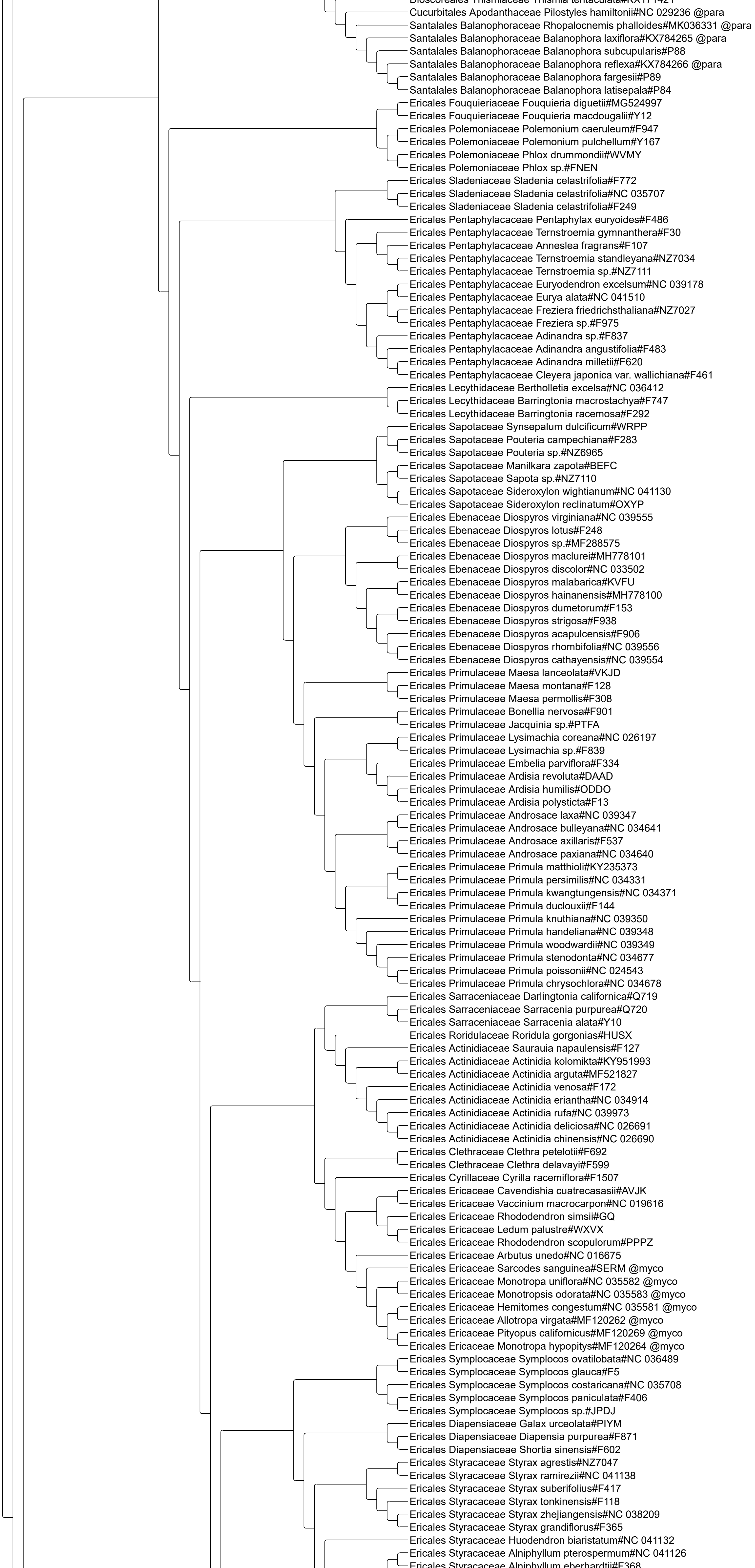

Dioscoreales Thismiaceae Thismia tentaculata#KX171421
Cucurbitales Apodanthaceae Pilostyles hamiltonii#NC 029236 @para
Santalales Balanophoraceae Rhopalocnemis phalloides#MK036331 @para
Santalales Balanophoraceae Balanophora laxiflora#KX784265 @para
Santalales Balanophoraceae Balanophora subcupularis#P88
Santalales Balanophoraceae Balanophora reflexa#KX784266 @para
Santalales Balanophoraceae Balanophora fargesii#P89
Santalales Balanophoraceae Balanophora latisepala#P84
Ericales Fouquieriaceae Fouquieria diguetii#MG524997
Ericales Fouquieriaceae Fouquieria macdougalii#Y12
Ericales Polemoniaceae Polemonium caeruleum#F947
Ericales Polemoniaceae Polemonium pulchellum#Y167
Ericales Polemoniaceae Phlox drummondii#WVMY
Ericales Polemoniaceae Phlox sp.#FNEN
Ericales Sladeniaceae Sladenia celastrifolia#F772
Ericales Sladeniaceae Sladenia celastrifolia#NC 035707
Ericales Sladeniaceae Sladenia celastrifolia#F249
Ericales Pentaphylacaceae Pentaphylax euryoides#F486
Ericales Pentaphylacaceae Ternstroemia gymnanthera#F30
Ericales Pentaphylacaceae Anneslea fragrans#F107
Ericales Pentaphylacaceae Ternstroemia standleyana#NZ7034
Ericales Pentaphylacaceae Ternstroemia sp.#NZ7111
Ericales Pentaphylacaceae Euryodendron excelsum#NC 039178
Ericales Pentaphylacaceae Eurya alata#NC 041510
Ericales Pentaphylacaceae Freziera friedrichsthaliana#NZ7027
Ericales Pentaphylacaceae Freziera sp.#F975
Ericales Pentaphylacaceae Adinandra sp.#F837
Ericales Pentaphylacaceae Adinandra angustifolia#F483
Ericales Pentaphylacaceae Adinandra millettii#F620
Ericales Pentaphylacaceae Cleyera japonica var. wallichiana#F461
Ericales Lecythidaceae Bertholletia excelsa#NC 036412
Ericales Lecythidaceae Barringtonia macrostachya#F747
Ericales Lecythidaceae Barringtonia racemosa#F292
Ericales Sapotaceae Synsepalum dulcificum#WRPP
Ericales Sapotaceae Pouteria campechiana#F283
Ericales Sapotaceae Pouteria sp.#NZ6965
Ericales Sapotaceae Manilkara zapota#BEFC
Ericales Sapotaceae Sapota sp.#NZ7110
Ericales Sapotaceae Sideroxylon wightianum#NC 041130
Ericales Sapotaceae Sideroxylon reclinatum#OXYP
Ericales Ebenaceae Diospyros virginiana#NC 039555
Ericales Ebenaceae Diospyros lotus#F248
Ericales Ebenaceae Diospyros sp.#MF288575
Ericales Ebenaceae Diospyros maclurei#MH778101
Ericales Ebenaceae Diospyros discolor#NC 033502
Ericales Ebenaceae Diospyros malabarica#KVFU
Ericales Ebenaceae Diospyros hainanensis#MH778100
Ericales Ebenaceae Diospyros dumetorum#F153
Ericales Ebenaceae Diospyros strigosa#F938
Ericales Ebenaceae Diospyros acapulcensis#F906
Ericales Ebenaceae Diospyros rhombifolia#NC 039556
Ericales Ebenaceae Diospyros cathayensis#NC 039554
Ericales Primulaceae Maesa lanceolata#VKJD
Ericales Primulaceae Maesa montana#F128
Ericales Primulaceae Maesa permollis#F308
Ericales Primulaceae Bonellia nervosa#F901
Ericales Primulaceae Jacquinia sp.#PTFA
Ericales Primulaceae Lysimachia coreana#NC 026197
Ericales Primulaceae Lysimachia sp.#F839
Ericales Primulaceae Embelia parviflora#F334
Ericales Primulaceae Ardisia revoluta#DAAD
Ericales Primulaceae Ardisia humilis#ODDO
Ericales Primulaceae Ardisia polysticta#F13
Ericales Primulaceae Androsace laxa#NC 039347
Ericales Primulaceae Androsace bulleyana#NC 034641
Ericales Primulaceae Androsace axillaris#F537
Ericales Primulaceae Androsace paxiana#NC 034640
Ericales Primulaceae Primula matthioli#KY235373
Ericales Primulaceae Primula persimilis#NC 034331
Ericales Primulaceae Primula kwangtungensis#NC 034371
Ericales Primulaceae Primula duclouxii#F144
Ericales Primulaceae Primula knuthiana#NC 039350
Ericales Primulaceae Primula handeliana#NC 039348
Ericales Primulaceae Primula woodwardii#NC 039349
Ericales Primulaceae Primula stenodonta#NC 034677
Ericales Primulaceae Primula poissonii#NC 024543
Ericales Primulaceae Primula chrysochlora#NC 034678
Ericales Sarraceniaceae Darlingtonia californica#Q719
Ericales Sarraceniaceae Sarracenia purpurea#Q720
Ericales Sarraceniaceae Sarracenia alata#Y10
Ericales Roridulaceae Roridula gorgonias#HUSX
Ericales Actinidiaceae Saurauia napaulensis#F127
Ericales Actinidiaceae Actinidia kolomikta#KY951993
Ericales Actinidiaceae Actinidia arguta#MF521827
Ericales Actinidiaceae Actinidia venosa#F172
Ericales Actinidiaceae Actinidia eriantha#NC 034914
Ericales Actinidiaceae Actinidia rufa#NC 039973
Ericales Actinidiaceae Actinidia deliciosa#NC 026691
Ericales Actinidiaceae Actinidia chinensis#NC 026690
Ericales Clethraceae Clethra petelotii#F692
Ericales Clethraceae Clethra delavayi#F599
Ericales Cyrillaceae Cyrilla racemiflora#F1507
Ericales Ericaceae Cavendishia cuatrecasasii#AVJK
Ericales Ericaceae Vaccinium macrocarpon#NC 019616
Ericales Ericaceae Rhododendron simsii#GQ
Ericales Ericaceae Ledum palustre#WXVX
Ericales Ericaceae Rhododendron scopulorum#PPPZ
Ericales Ericaceae Arbutus unedo#NC 016675
Ericales Ericaceae Sarcodes sanguinea#SERM @myco
Ericales Ericaceae Monotropa uniflora#NC 035582 @myco
Ericales Ericaceae Monotropsis odorata#NC 035583 @myco
Ericales Ericaceae Hemitomes congestum#NC 035581 @myco
Ericales Ericaceae Allotropa virgata#MF120262 @myco
Ericales Ericaceae Pityopus californicus#MF120269 @myco
Ericales Ericaceae Monotropa hypopitys#MF120264 @myco
Ericales Symplocaceae Symplocos ovatilobata#NC 036489
Ericales Symplocaceae Symplocos glauca#F5
Ericales Symplocaceae Symplocos costaricana#NC 035708
Ericales Symplocaceae Symplocos paniculata#F406
Ericales Symplocaceae Symplocos sp.#JPDJ
Ericales Diapensiaceae Galax urceolata#PIYM
Ericales Diapensiaceae Diapensia purpurea#F871
Ericales Diapensiaceae Shortia sinensis#F602
Ericales Styracaceae Styrax agrestis#NZ7047
Ericales Styracaceae Styrax ramirezii#NC 041138
Ericales Styracaceae Styrax suberifolius#F417
Ericales Styracaceae Styrax tonkinensis#F118
Ericales Styracaceae Styrax zhejiangensis#NC 038209
Ericales Styracaceae Styrax grandiflorus#F365
Ericales Styracaceae Huodendron biaristatum#NC 041132
Ericales Styracaceae Alniphyllum pterospermum#NC 041126
Ericales Styracaceae Alniphyllum eberhardtii#F368

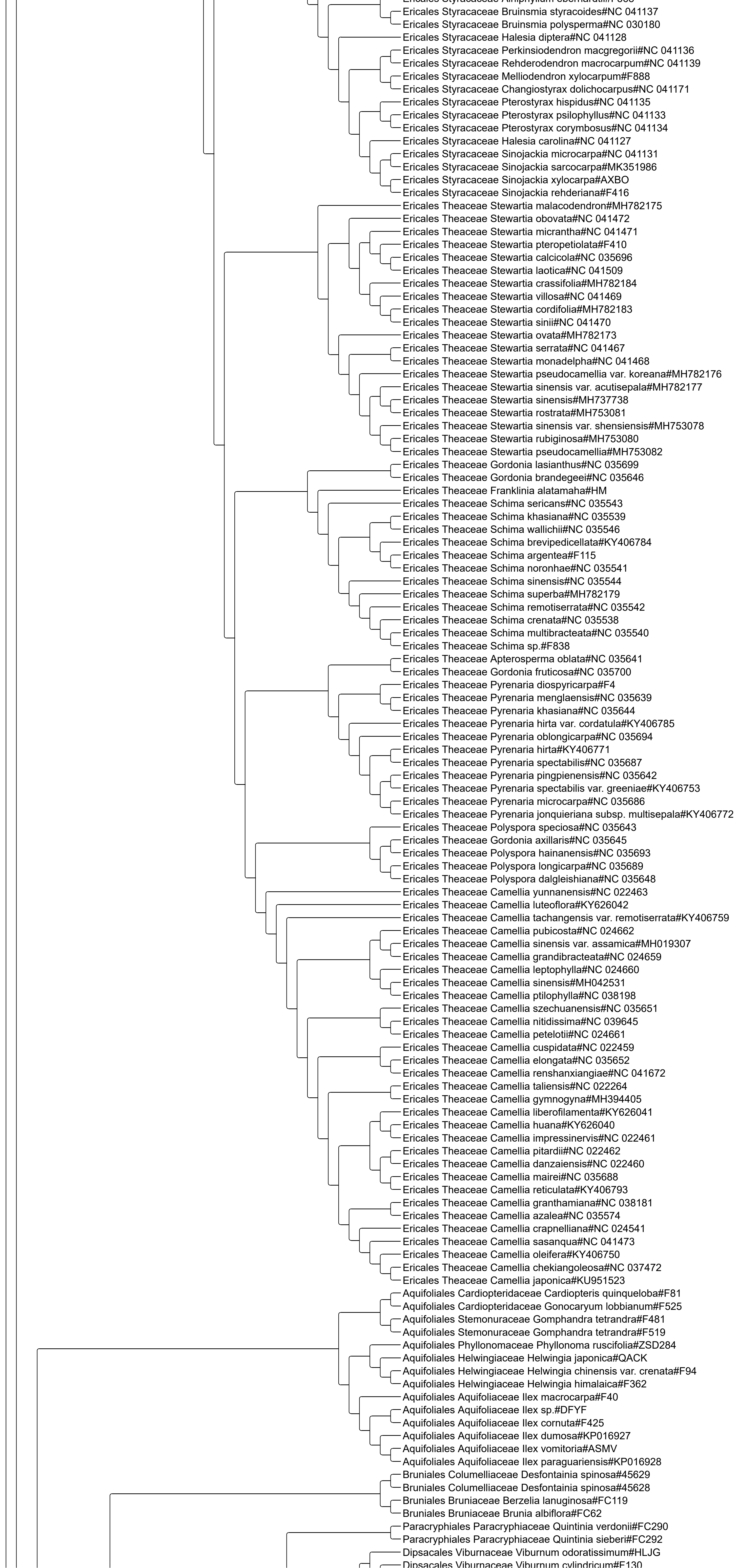
Ericales Styracaceae Bruinsmia styracoides#NC 041137
Ericales Styracaceae Bruinsmia polysperma#NC 030180
Ericales Styracaceae Halesia diptera#NC 041128
Ericales Styracaceae Perkinsiodendron macgregorii#NC 041136
Ericales Styracaceae Rehderodendron macrocarpum#NC 041139
Ericales Styracaceae Melliodendron xylocarpum#F888
Ericales Styracaceae Changiostyrax dolichocarpus#NC 041171
Ericales Styracaceae Pterostyrax hispidus#NC 041135
Ericales Styracaceae Pterostyrax psilophyllus#NC 041133
Ericales Styracaceae Pterostyrax corymbosus#NC 041134
Ericales Styracaceae Halesia carolina#NC 041127
Ericales Styracaceae Sinojackia microcarpa#NC 041131
Ericales Styracaceae Sinojackia sarcocarpa#MK351986
Ericales Styracaceae Sinojackia xylocarpa#AXBO
Ericales Styracaceae Sinojackia rehderiana#F416
Ericales Theaceae Stewartia malacodendron#MH782175
Ericales Theaceae Stewartia obovata#NC 041472
Ericales Theaceae Stewartia micrantha#NC 041471
Ericales Theaceae Stewartia pteropetiolata#F410
Ericales Theaceae Stewartia calcicola#NC 035696
Ericales Theaceae Stewartia laotica#NC 041509
Ericales Theaceae Stewartia crassifolia#MH782184
Ericales Theaceae Stewartia villosa#NC 041469
Ericales Theaceae Stewartia cordifolia#MH782183
Ericales Theaceae Stewartia sinii#NC 041470
Ericales Theaceae Stewartia ovata#MH782173
Ericales Theaceae Stewartia serrata#NC 041467
Ericales Theaceae Stewartia monadelpha#NC 041468
Ericales Theaceae Stewartia pseudocamellia var. koreana#MH782176
Ericales Theaceae Stewartia sinensis var. acutisepala#MH782177
Ericales Theaceae Stewartia sinensis#MH737738
Ericales Theaceae Stewartia rostrata#MH753081
Ericales Theaceae Stewartia sinensis var. shensiensis#MH753078
Ericales Theaceae Stewartia rubiginosa#MH753080
Ericales Theaceae Stewartia pseudocamellia#MH753082
Ericales Theaceae Gordonia lasianthus#NC 035699
Ericales Theaceae Gordonia brandegeei#NC 035646
Ericales Theaceae Franklinia alatamaha#HM
Ericales Theaceae Schima sericans#NC 035543
Ericales Theaceae Schima khasiana#NC 035539
Ericales Theaceae Schima wallichii#NC 035546
Ericales Theaceae Schima brevipedicellata#KY406784
Ericales Theaceae Schima argentea#F115
Ericales Theaceae Schima noronhae#NC 035541
Ericales Theaceae Schima sinensis#NC 035544
Ericales Theaceae Schima superba#MH782179
Ericales Theaceae Schima remotiserrata#NC 035542
Ericales Theaceae Schima crenata#NC 035538
Ericales Theaceae Schima multibracteata#NC 035540
Ericales Theaceae Schima sp.#F838
Ericales Theaceae Apterosperma oblata#NC 035641
Ericales Theaceae Gordonia fruticosa#NC 035700
Ericales Theaceae Pyrenaria diospyricarpa#F4
Ericales Theaceae Pyrenaria menglaensis#NC 035639
Ericales Theaceae Pyrenaria khasiana#NC 035644
Ericales Theaceae Pyrenaria hirta var. cordatula#KY406785
Ericales Theaceae Pyrenaria oblongicarpa#NC 035694
Ericales Theaceae Pyrenaria hirta#KY406771
Ericales Theaceae Pyrenaria spectabilis#NC 035687
Ericales Theaceae Pyrenaria pingpienensis#NC 035642
Ericales Theaceae Pyrenaria spectabilis var. greeniae#KY406753
Ericales Theaceae Pyrenaria microcarpa#NC 035686
Ericales Theaceae Pyrenaria jonquieriana subsp. multisepala#KY406772
Ericales Theaceae Polyspora speciosa#NC 035643
Ericales Theaceae Gordonia axillaris#NC 035645
Ericales Theaceae Polyspora hainanensis#NC 035693
Ericales Theaceae Polyspora longicarpa#NC 035689
Ericales Theaceae Polyspora dalgleishiana#NC 035648
Ericales Theaceae Camellia yunnanensis#NC 022463
Ericales Theaceae Camellia luteoflora#KY626042
Ericales Theaceae Camellia tachangensis var. remotiserrata#KY406759
Ericales Theaceae Camellia pubicosta#NC 024662
Ericales Theaceae Camellia sinensis var. assamica#MH019307
Ericales Theaceae Camellia grandibracteata#NC 024659
Ericales Theaceae Camellia leptophylla#NC 024660
Ericales Theaceae Camellia sinensis#MH042531
Ericales Theaceae Camellia ptilophylla#NC 038198
Ericales Theaceae Camellia szechuanensis#NC 035651
Ericales Theaceae Camellia nitidissima#NC 039645
Ericales Theaceae Camellia petelotii#NC 024661
Ericales Theaceae Camellia cuspidata#NC 022459
Ericales Theaceae Camellia elongata#NC 035652
Ericales Theaceae Camellia renshanxiangiae#NC 041672
Ericales Theaceae Camellia taliensis#NC 022264
Ericales Theaceae Camellia gymnogyna#MH394405
Ericales Theaceae Camellia liberofilamenta#KY626041
Ericales Theaceae Camellia huana#KY626040
Ericales Theaceae Camellia impressinervis#NC 022461
Ericales Theaceae Camellia pitardii#NC 022462
Ericales Theaceae Camellia danzaiensis#NC 022460
Ericales Theaceae Camellia mairei#NC 035688
Ericales Theaceae Camellia reticulata#KY406793
Ericales Theaceae Camellia granthamiana#NC 038181
Ericales Theaceae Camellia azalea#NC 035574
Ericales Theaceae Camellia crapnelliana#NC 024541
Ericales Theaceae Camellia sasanqua#NC 041473
Ericales Theaceae Camellia oleifera#KY406750
Ericales Theaceae Camellia chekiangoleosa#NC 037472
Ericales Theaceae Camellia japonica#KU951523
Aquifoliales Cardiopteridaceae Cardiopteris quinqueloba#F81
Aquifoliales Cardiopteridaceae Gonocaryum lobbianum#F525
Aquifoliales Stemonuraceae Gomphandra tetrandra#F481
Aquifoliales Stemonuraceae Gomphandra tetrandra#F519
Aquifoliales Phyllonomaceae Phyllonoma ruscifolia#ZSD284
Aquifoliales Helwingiaceae Helwingia japonica#QACK
Aquifoliales Helwingiaceae Helwingia chinensis var. crenata#F94
Aquifoliales Helwingiaceae Helwingia himalaica#F362
Aquifoliales Aquifoliaceae Ilex macrocarpa#F40
Aquifoliales Aquifoliaceae Ilex sp.#DFYF
Aquifoliales Aquifoliaceae Ilex cornuta#F425
Aquifoliales Aquifoliaceae Ilex dumosa#KP016927
Aquifoliales Aquifoliaceae Ilex vomitoria#ASMV
Aquifoliales Aquifoliaceae Ilex paraguariensis#KP016928
Bruniales Columelliaceae Desfontainia spinosa#45629
Bruniales Columelliaceae Desfontainia spinosa#45628
Bruniales Bruniaceae Berzelia lanuginosa#FC119
Bruniales Bruniaceae Brunia albiflora#FC62
Paracryphiales Paracryphiaceae Quintinia verdonii#FC290
Paracryphiales Paracryphiaceae Quintinia sieberi#FC292
Dipsacales Viburnaceae Viburnum odoratissimum#HLJG
Dipsacales Viburnaceae Viburnum cylindricum#F130

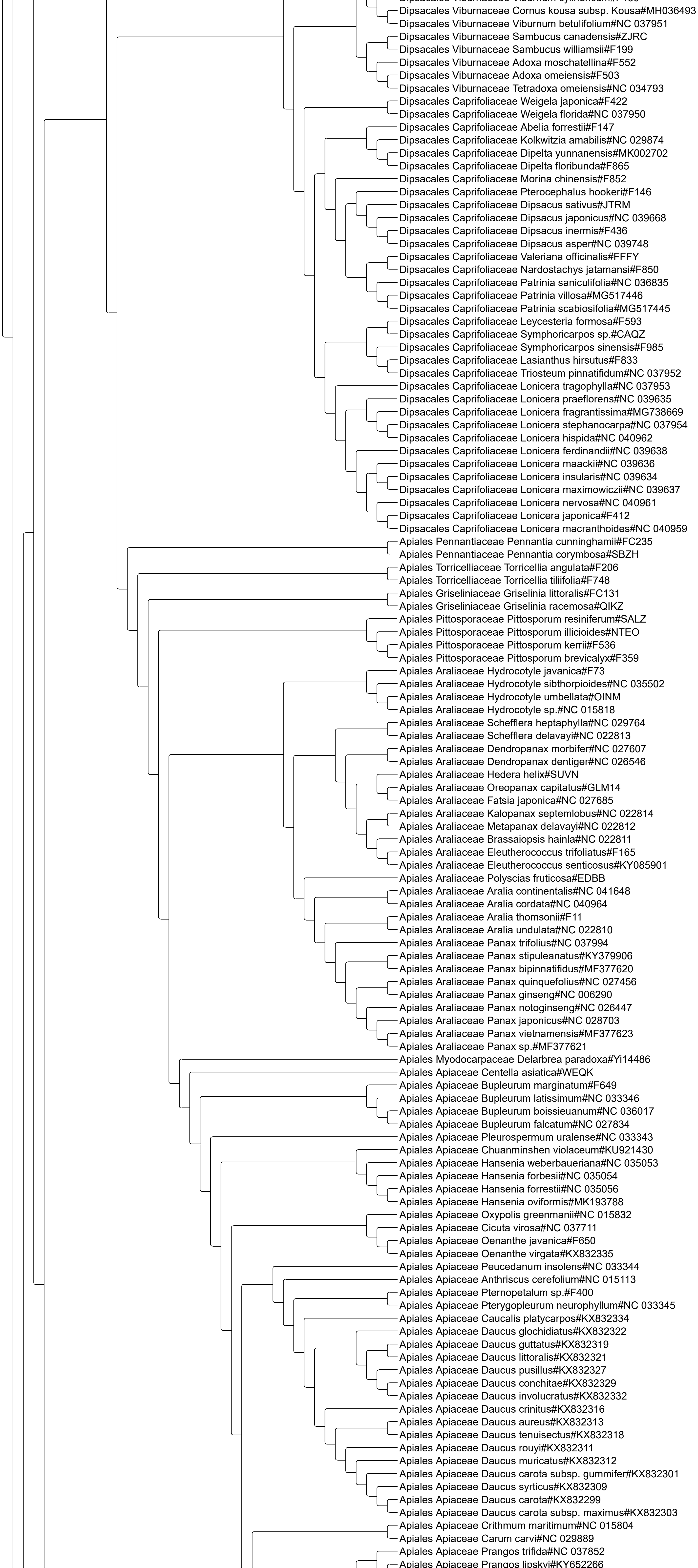

Dipsacales Viburnaceae Cornus kousa subsp. Kousa#MH036493
Dipsacales Viburnaceae Viburnum betulifolium#NC 037951
Dipsacales Viburnaceae Sambucus canadensis#ZJRC
Dipsacales Viburnaceae Sambucus williamsii#F199
Dipsacales Viburnaceae Adoxa moschatellina#F552
Dipsacales Viburnaceae Adoxa omeiensis#F503
Dipsacales Viburnaceae Tetradoxa omeiensis#NC 034793
Dipsacales Caprifoliaceae Weigela japonica#F422
Dipsacales Caprifoliaceae Weigela florida#NC 037950
Dipsacales Caprifoliaceae Abelia forrestii#F147
Dipsacales Caprifoliaceae Kolkwitzia amabilis#NC 029874
Dipsacales Caprifoliaceae Dipelta yunnanensis#MK002702
Dipsacales Caprifoliaceae Dipelta floribunda#F865
Dipsacales Caprifoliaceae Morina chinensis#F852
Dipsacales Caprifoliaceae Pterocephalus hookeri#F146
Dipsacales Caprifoliaceae Dipsacus sativus#JTRM
Dipsacales Caprifoliaceae Dipsacus japonicus#NC 039668
Dipsacales Caprifoliaceae Dipsacus inermis#F436
Dipsacales Caprifoliaceae Dipsacus asper#NC 039748
Dipsacales Caprifoliaceae Valeriana officinalis#FFFY
Dipsacales Caprifoliaceae Nardostachys jatamansi#F850
Dipsacales Caprifoliaceae Patrinia saniculifolia#NC 036835
Dipsacales Caprifoliaceae Patrinia villosa#MG517446
Dipsacales Caprifoliaceae Patrinia scabiosifolia#MG517445
Dipsacales Caprifoliaceae Leycesteria formosa#F593
Dipsacales Caprifoliaceae Symphoricarpos sp.#CAQZ
Dipsacales Caprifoliaceae Symphoricarpos sinensis#F985
Dipsacales Caprifoliaceae Lasianthus hirsutus#F833
Dipsacales Caprifoliaceae Triosteum pinnatifidum#NC 037952
Dipsacales Caprifoliaceae Lonicera tragophylla#NC 037953
Dipsacales Caprifoliaceae Lonicera praeflorens#NC 039635
Dipsacales Caprifoliaceae Lonicera fragrantissima#MG738669
Dipsacales Caprifoliaceae Lonicera stephanocarpa#NC 037954
Dipsacales Caprifoliaceae Lonicera hispida#NC 040962
Dipsacales Caprifoliaceae Lonicera ferdinandii#NC 039638
Dipsacales Caprifoliaceae Lonicera maackii#NC 039636
Dipsacales Caprifoliaceae Lonicera insularis#NC 039634
Dipsacales Caprifoliaceae Lonicera maximowiczii#NC 039637
Dipsacales Caprifoliaceae Lonicera nervosa#NC 040961
Dipsacales Caprifoliaceae Lonicera japonica#F412
Dipsacales Caprifoliaceae Lonicera macranthoides#NC 040959
Apiales Pennantiaceae Pennantia cunninghamii#FC235
Apiales Pennantiaceae Pennantia corymbosa#SBZH
Apiales Torricelliaceae Torricellia angulata#F206
Apiales Torricelliaceae Torricellia tiliifolia#F748
Apiales Griseliniaceae Griselinia littoralis#FC131
Apiales Griseliniaceae Griselinia racemosa#QIKZ
Apiales Pittosporaceae Pittosporum resiniferum#SALZ
Apiales Pittosporaceae Pittosporum illicioides#NTEO
Apiales Pittosporaceae Pittosporum kerrii#F536
Apiales Pittosporaceae Pittosporum brevicalyx#F359
Apiales Araliaceae Hydrocotyle javanica#F73
Apiales Araliaceae Hydrocotyle sibthorpioides#NC 035502
Apiales Araliaceae Hydrocotyle umbellata#OINM
Apiales Araliaceae Hydrocotyle sp.#NC 015818
Apiales Araliaceae Schefflera heptaphylla#NC 029764
Apiales Araliaceae Schefflera delavayi#NC 022813
Apiales Araliaceae Dendropanax morbifer#NC 027607
Apiales Araliaceae Dendropanax dentiger#NC 026546
Apiales Araliaceae Hedera helix#SUVN
Apiales Araliaceae Oreopanax capitatus#GLM14
Apiales Araliaceae Fatsia japonica#NC 027685
Apiales Araliaceae Kalopanax septemlobus#NC 022814
Apiales Araliaceae Metapanax delavayi#NC 022812
Apiales Araliaceae Brassaiopsis hainla#NC 022811
Apiales Araliaceae Eleutherococcus trifoliatus#F165
Apiales Araliaceae Eleutherococcus senticosus#KY085901
Apiales Araliaceae Polyscias fruticosa#EDBB
Apiales Araliaceae Aralia continentalis#NC 041648
Apiales Araliaceae Aralia cordata#NC 040964
Apiales Araliaceae Aralia thomsonii#F11
Apiales Araliaceae Aralia undulata#NC 022810
Apiales Araliaceae Panax trifolius#NC 037994
Apiales Araliaceae Panax stipuleanatus#KY379906
Apiales Araliaceae Panax bipinnatifidus#MF377620
Apiales Araliaceae Panax quinquefolius#NC 027456
Apiales Araliaceae Panax ginseng#NC 006290
Apiales Araliaceae Panax notoginseng#NC 026447
Apiales Araliaceae Panax japonicus#NC 028703
Apiales Araliaceae Panax vietnamensis#MF377623
Apiales Araliaceae Panax sp.#MF377621
Apiales Myodocarpaceae Delarbrea paradoxa#Yi14486
Apiales Apiaceae Centella asiatica#WEQK
Apiales Apiaceae Bupleurum marginatum#F649
Apiales Apiaceae Bupleurum latissimum#NC 033346
Apiales Apiaceae Bupleurum boissieuanum#NC 036017
Apiales Apiaceae Bupleurum falcatum#NC 027834
Apiales Apiaceae Pleurospermum uralense#NC 033343
Apiales Apiaceae Chuanminshen violaceum#KU921430
Apiales Apiaceae Hansenia weberbaueriana#NC 035053
Apiales Apiaceae Hansenia forbesii#NC 035054
Apiales Apiaceae Hansenia forrestii#NC 035056
Apiales Apiaceae Hansenia oviformis#MK193788
Apiales Apiaceae Oxypolis greenmanii#NC 015832
Apiales Apiaceae Cicuta virosa#NC 037711
Apiales Apiaceae Oenanthe javanica#F650
Apiales Apiaceae Oenanthe virgata#KX832335
Apiales Apiaceae Peucedanum insolens#NC 033344
Apiales Apiaceae Anthriscus cerefolium#NC 015113
Apiales Apiaceae Pternopetalum sp.#F400
Apiales Apiaceae Pterygopleurum neurophyllum#NC 033345
Apiales Apiaceae Caucalis platycarpos#KX832334
Apiales Apiaceae Daucus glochidiatus#KX832322
Apiales Apiaceae Daucus guttatus#KX832319
Apiales Apiaceae Daucus littoralis#KX832321
Apiales Apiaceae Daucus pusillus#KX832327
Apiales Apiaceae Daucus conchitae#KX832329
Apiales Apiaceae Daucus involucratus#KX832332
Apiales Apiaceae Daucus crinitus#KX832316
Apiales Apiaceae Daucus aureus#KX832313
Apiales Apiaceae Daucus tenuisectus#KX832318
Apiales Apiaceae Daucus rouyi#KX832311
Apiales Apiaceae Daucus muricatus#KX832312
Apiales Apiaceae Daucus carota subsp. gummifer#KX832301
Apiales Apiaceae Daucus syrticus#KX832309
Apiales Apiaceae Daucus carota#KX832299
Apiales Apiaceae Daucus carota subsp. maximus#KX832303
Apiales Apiaceae Crithmum maritimum#NC 015804
Apiales Apiaceae Carum carvi#NC 029889
Apiales Apiaceae Prangos trifida#NC 037852
Apiales Apiaceae Prangos lipskyi#KY652266

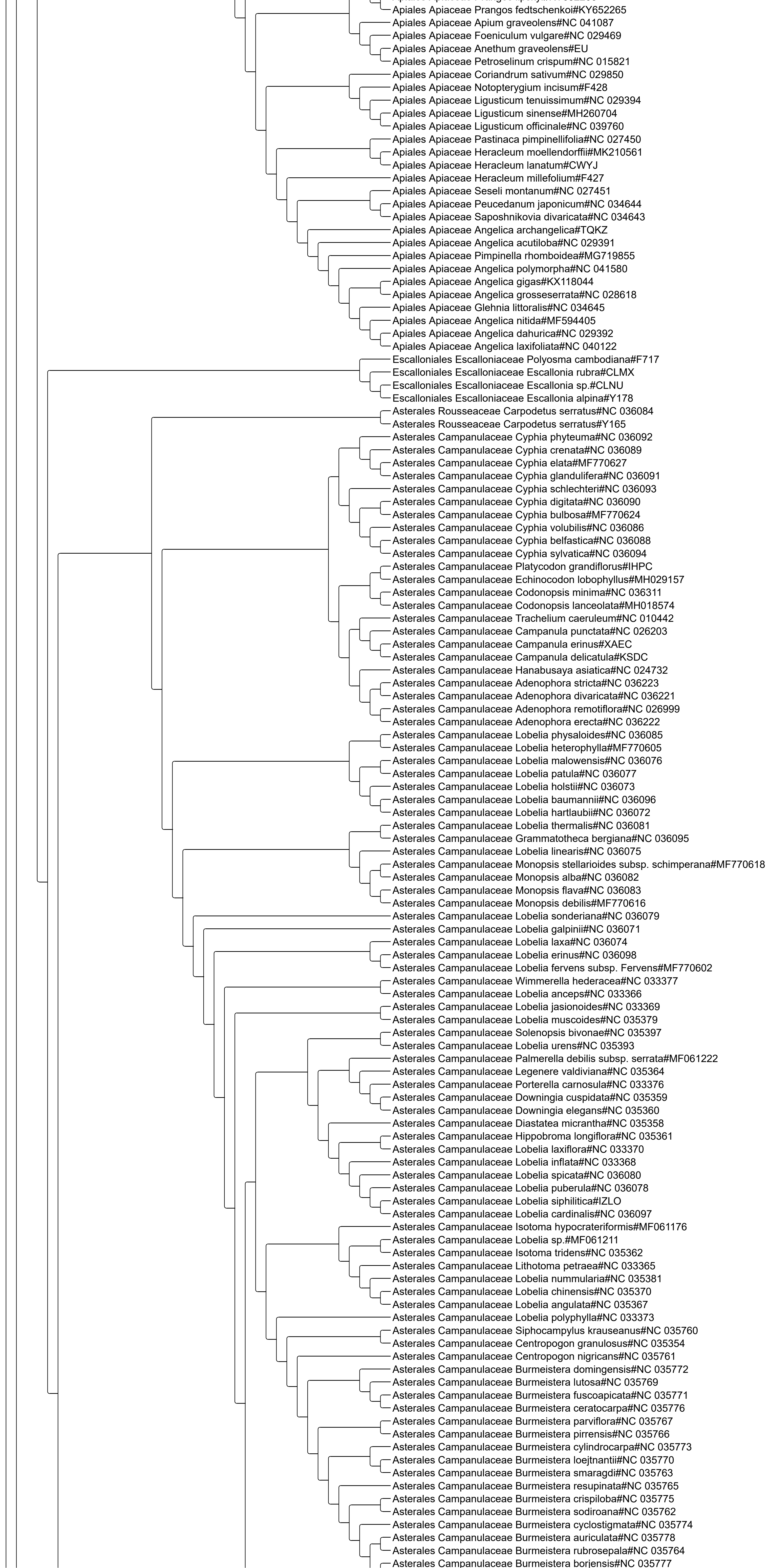

Apiales Apiaceae Prangos fedtschenkoi#KY652265
Apiales Apiaceae Apium graveolens#NC 041087
Apiales Apiaceae Foeniculum vulgare#NC 029469
Apiales Apiaceae Anethum graveolens#EU
Apiales Apiaceae Petroselinum crispum#NC 015821
Apiales Apiaceae Coriandrum sativum#NC 029850
Apiales Apiaceae Notopterygium incisum#F428
Apiales Apiaceae Ligusticum tenuissimum#NC 029394
Apiales Apiaceae Ligusticum sinense#MH260704
Apiales Apiaceae Ligusticum officinale#NC 039760
Apiales Apiaceae Pastinaca pimpinellifolia#NC 027450
Apiales Apiaceae Heracleum moellendorffii#MK210561
Apiales Apiaceae Heracleum lanatum#CWYJ
Apiales Apiaceae Heracleum millefolium#F427
Apiales Apiaceae Seseli montanum#NC 027451
Apiales Apiaceae Peucedanum japonicum#NC 034644
Apiales Apiaceae Saposhnikovia divaricata#NC 034643
Apiales Apiaceae Angelica archangelica#TQKZ
Apiales Apiaceae Angelica acutiloba#NC 029391
Apiales Apiaceae Pimpinella rhomboidea#MG719855
Apiales Apiaceae Angelica polymorpha#NC 041580
Apiales Apiaceae Angelica gigas#KX118044
Apiales Apiaceae Angelica grosseserrata#NC 028618
Apiales Apiaceae Glehnia littoralis#NC 034645
Apiales Apiaceae Angelica nitida#MF594405
Apiales Apiaceae Angelica dahurica#NC 029392
Apiales Apiaceae Angelica laxifoliata#NC 040122
Escalloniales Escalloniaceae Polyosma cambodiana#F717
Escalloniales Escalloniaceae Escallonia rubra#CLMX
Escalloniales Escalloniaceae Escallonia sp.#CLNU
Escalloniales Escalloniaceae Escallonia alpina#Y178
Asterales Rousseaceae Carpodetus serratus#NC 036084
Asterales Rousseaceae Carpodetus serratus#Y165
Asterales Campanulaceae Cyphia phyteuma#NC 036092
Asterales Campanulaceae Cyphia crenata#NC 036089
Asterales Campanulaceae Cyphia elata#MF770627
Asterales Campanulaceae Cyphia glandulifera#NC 036091
Asterales Campanulaceae Cyphia schlechteri#NC 036093
Asterales Campanulaceae Cyphia digitata#NC 036090
Asterales Campanulaceae Cyphia bulbosa#MF770624
Asterales Campanulaceae Cyphia volubilis#NC 036086
Asterales Campanulaceae Cyphia belfastica#NC 036088
Asterales Campanulaceae Cyphia sylvatica#NC 036094
Asterales Campanulaceae Platycodon grandiflorus#IHPC
Asterales Campanulaceae Echinocodon lobophyllus#MH029157
Asterales Campanulaceae Codonopsis minima#NC 036311
Asterales Campanulaceae Codonopsis lanceolata#MH018574
Asterales Campanulaceae Trachelium caeruleum#NC 010442
Asterales Campanulaceae Campanula punctata#NC 026203
Asterales Campanulaceae Campanula erinus#XAEC
Asterales Campanulaceae Campanula delicatula#KSDC
Asterales Campanulaceae Hanabusaya asiatica#NC 024732
Asterales Campanulaceae Adenophora stricta#NC 036223
Asterales Campanulaceae Adenophora divaricata#NC 036221
Asterales Campanulaceae Adenophora remotiflora#NC 026999
Asterales Campanulaceae Adenophora erecta#NC 036222
Asterales Campanulaceae Lobelia physaloides#NC 036085
Asterales Campanulaceae Lobelia heterophylla#MF770605
Asterales Campanulaceae Lobelia malowensis#NC 036076
Asterales Campanulaceae Lobelia patula#NC 036077
Asterales Campanulaceae Lobelia holstii#NC 036073
Asterales Campanulaceae Lobelia baumannii#NC 036096
Asterales Campanulaceae Lobelia hartlaubii#NC 036072
Asterales Campanulaceae Lobelia thermalis#NC 036081
Asterales Campanulaceae Grammatotheca bergiana#NC 036095
Asterales Campanulaceae Lobelia linearis#NC 036075
Asterales Campanulaceae Monopsis stellarioides subsp. schimperana#MF770618
Asterales Campanulaceae Monopsis alba#NC 036082
Asterales Campanulaceae Monopsis flava#NC 036083
Asterales Campanulaceae Monopsis debilis#MF770616
Asterales Campanulaceae Lobelia sonderiana#NC 036079
Asterales Campanulaceae Lobelia galpinii#NC 036071
Asterales Campanulaceae Lobelia laxa#NC 036074
Asterales Campanulaceae Lobelia erinus#NC 036098
Asterales Campanulaceae Lobelia fervens subsp. Fervens#MF770602
Asterales Campanulaceae Wimmerella hederacea#NC 033377
Asterales Campanulaceae Lobelia anceps#NC 033366
Asterales Campanulaceae Lobelia jasionoides#NC 033369
Asterales Campanulaceae Lobelia muscoides#NC 035379
Asterales Campanulaceae Solenopsis bivonae#NC 035397
Asterales Campanulaceae Lobelia urens#NC 035393
Asterales Campanulaceae Palmerella debilis subsp. serrata#MF061222
Asterales Campanulaceae Legenere valdiviana#NC 035364
Asterales Campanulaceae Porterella carnosula#NC 033376
Asterales Campanulaceae Downingia cuspidata#NC 035359
Asterales Campanulaceae Downingia elegans#NC 035360
Asterales Campanulaceae Diastatea micrantha#NC 035358
Asterales Campanulaceae Hippobroma longiflora#NC 035361
Asterales Campanulaceae Lobelia laxiflora#NC 033370
Asterales Campanulaceae Lobelia inflata#NC 033368
Asterales Campanulaceae Lobelia spicata#NC 036080
Asterales Campanulaceae Lobelia puberula#NC 036078
Asterales Campanulaceae Lobelia siphilitica#IZLO
Asterales Campanulaceae Lobelia cardinalis#NC 036097
Asterales Campanulaceae Isotoma hypocrateriformis#MF061176
Asterales Campanulaceae Lobelia sp.#MF061211
Asterales Campanulaceae Isotoma tridens#NC 035362
Asterales Campanulaceae Lithotoma petraea#NC 033365
Asterales Campanulaceae Lobelia nummularia#NC 035381
Asterales Campanulaceae Lobelia chinensis#NC 035370
Asterales Campanulaceae Lobelia angulata#NC 035367
Asterales Campanulaceae Lobelia polyphylla#NC 033373
Asterales Campanulaceae Siphocampylus krauseanus#NC 035760
Asterales Campanulaceae Centropogon granulosus#NC 035354
Asterales Campanulaceae Centropogon nigricans#NC 035761
Asterales Campanulaceae Burmeistera domingensis#NC 035772
Asterales Campanulaceae Burmeistera lutosa#NC 035769
Asterales Campanulaceae Burmeistera fuscoapicata#NC 035771
Asterales Campanulaceae Burmeistera ceratocarpa#NC 035776
Asterales Campanulaceae Burmeistera parviflora#NC 035767
Asterales Campanulaceae Burmeistera pirrensis#NC 035766
Asterales Campanulaceae Burmeistera cylindrocarpa#NC 035773
Asterales Campanulaceae Burmeistera loejtnantii#NC 035770
Asterales Campanulaceae Burmeistera smaragdi#NC 035763
Asterales Campanulaceae Burmeistera resupinata#NC 035765
Asterales Campanulaceae Burmeistera crispiloba#NC 035775
Asterales Campanulaceae Burmeistera sodiroana#NC 035762
Asterales Campanulaceae Burmeistera cyclostigmata#NC 035774
Asterales Campanulaceae Burmeistera auriculata#NC 035778
Asterales Campanulaceae Burmeistera rubrosepala#NC 035764
Asterales Campanulaceae Burmeistera borjensis#NC 035777

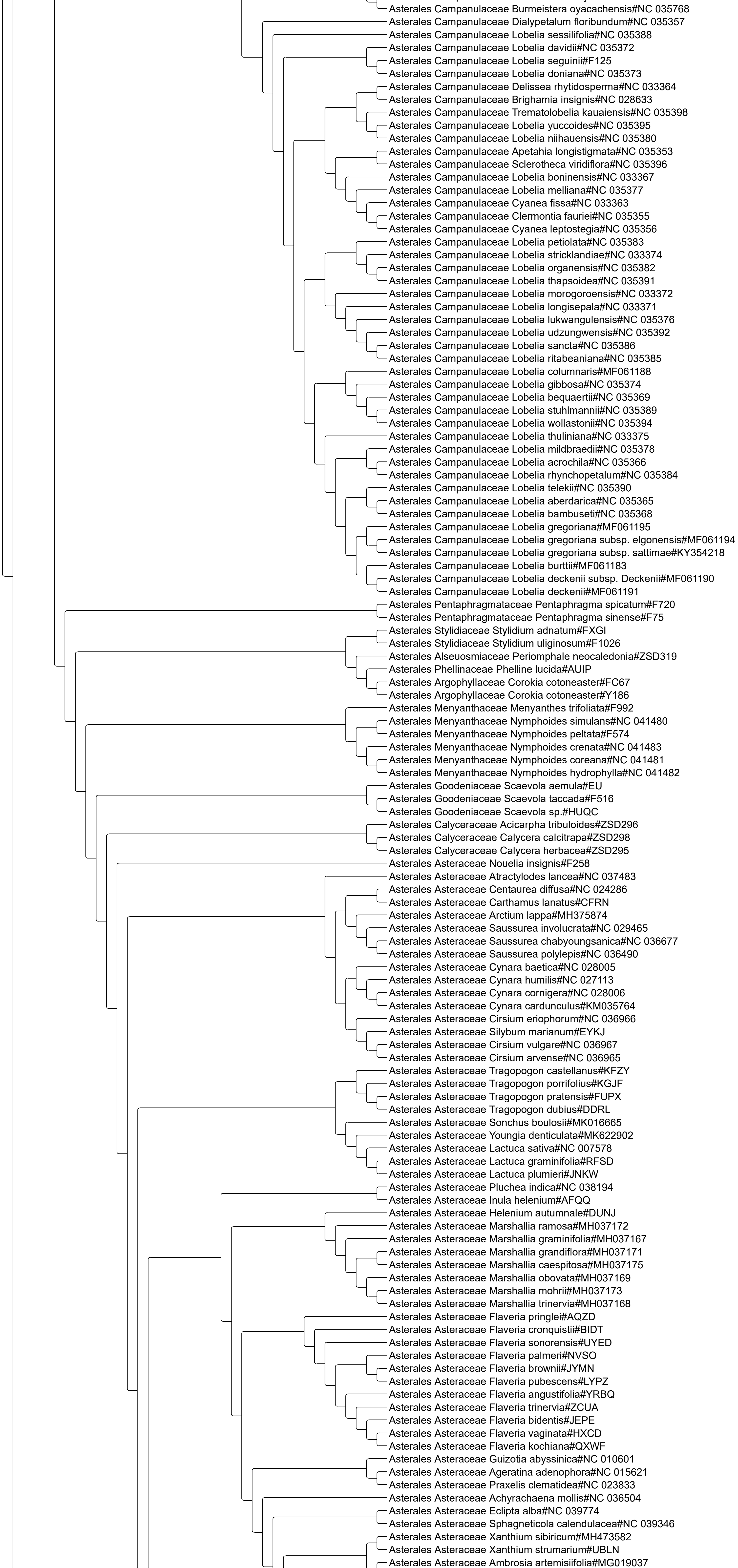

Asterales Campanulaceae Burmeistera oyacachensis#NC 035768
Asterales Campanulaceae Dialypetalum floribundum#NC 035357
Asterales Campanulaceae Lobelia sessilifolia#NC 035388
Asterales Campanulaceae Lobelia davidii#NC 035372
Asterales Campanulaceae Lobelia seguinii#F125
Asterales Campanulaceae Lobelia doniana#NC 035373
Asterales Campanulaceae Delissea rhytidosperma#NC 033364
Asterales Campanulaceae Brighamia insignis#NC 028633
Asterales Campanulaceae Trematolobelia kauaiensis#NC 035398
Asterales Campanulaceae Lobelia yuccoides#NC 035395
Asterales Campanulaceae Lobelia niihauensis#NC 035380
Asterales Campanulaceae Apetahia longistigmata#NC 035353
Asterales Campanulaceae Sclerotheca viridiflora#NC 035396
Asterales Campanulaceae Lobelia boninensis#NC 033367
Asterales Campanulaceae Lobelia melliana#NC 035377
Asterales Campanulaceae Cyanea fissa#NC 033363
Asterales Campanulaceae Clermontia fauriei#NC 035355
Asterales Campanulaceae Cyanea leptostegia#NC 035356
Asterales Campanulaceae Lobelia petiolata#NC 035383
Asterales Campanulaceae Lobelia stricklandiae#NC 033374
Asterales Campanulaceae Lobelia organensis#NC 035382
Asterales Campanulaceae Lobelia thapsoidea#NC 035391
Asterales Campanulaceae Lobelia morogoroensis#NC 033372
Asterales Campanulaceae Lobelia longisepala#NC 033371
Asterales Campanulaceae Lobelia lukwangulensis#NC 035376
Asterales Campanulaceae Lobelia udzungwensis#NC 035392
Asterales Campanulaceae Lobelia sancta#NC 035386
Asterales Campanulaceae Lobelia ritabeaniana#NC 035385
Asterales Campanulaceae Lobelia columnaris#MF061188
Asterales Campanulaceae Lobelia gibbosa#NC 035374
Asterales Campanulaceae Lobelia bequaertii#NC 035369
Asterales Campanulaceae Lobelia stuhlmannii#NC 035389
Asterales Campanulaceae Lobelia wollastonii#NC 035394
Asterales Campanulaceae Lobelia thuliniana#NC 033375
Asterales Campanulaceae Lobelia mildbraedii#NC 035378
Asterales Campanulaceae Lobelia acrochila#NC 035366
Asterales Campanulaceae Lobelia rhynchopetalum#NC 035384
Asterales Campanulaceae Lobelia telekii#NC 035390
Asterales Campanulaceae Lobelia aberdarica#NC 035365
Asterales Campanulaceae Lobelia bambuseti#NC 035368
Asterales Campanulaceae Lobelia gregoriana#MF061195
Asterales Campanulaceae Lobelia gregoriana subsp. elgonensis#MF061194
Asterales Campanulaceae Lobelia gregoriana subsp. sattimae#KY354218
Asterales Campanulaceae Lobelia burttii#MF061183
Asterales Campanulaceae Lobelia deckenii subsp. Deckenii#MF061190
Asterales Campanulaceae Lobelia deckenii#MF061191
Asterales Pentaphragmataceae Pentaphragma spicatum#F720
Asterales Pentaphragmataceae Pentaphragma sinense#F75
Asterales Stylidiaceae Stylidium adnatum#FXGI
Asterales Stylidiaceae Stylidium uliginosum#F1026
Asterales Alseuosmiaceae Periomphale neocaledonia#ZSD319
Asterales Phellinaceae Phelline lucida#AUIP
Asterales Argophyllaceae Corokia cotoneaster#FC67
Asterales Argophyllaceae Corokia cotoneaster#Y186
Asterales Menyanthaceae Menyanthes trifoliata#F992
Asterales Menyanthaceae Nymphoides simulans#NC 041480
Asterales Menyanthaceae Nymphoides peltata#F574
Asterales Menyanthaceae Nymphoides crenata#NC 041483
Asterales Menyanthaceae Nymphoides coreana#NC 041481
Asterales Menyanthaceae Nymphoides hydrophylla#NC 041482
Asterales Goodeniaceae Scaevola aemula#EU
Asterales Goodeniaceae Scaevola taccada#F516
Asterales Goodeniaceae Scaevola sp.#HUQC
Asterales Calyceraceae Acicarpha tribuloides#ZSD296
Asterales Calyceraceae Calycera calcitrapa#ZSD298
Asterales Calyceraceae Calycera herbacea#ZSD295
Asterales Asteraceae Nouelia insignis#F258
Asterales Asteraceae Atractylodes lancea#NC 037483
Asterales Asteraceae Centaurea diffusa#NC 024286
Asterales Asteraceae Carthamus lanatus#CFRN
Asterales Asteraceae Arctium lappa#MH375874
Asterales Asteraceae Saussurea involucrata#NC 029465
Asterales Asteraceae Saussurea chabyoungsanica#NC 036677
Asterales Asteraceae Saussurea polylepis#NC 036490
Asterales Asteraceae Cynara baetica#NC 028005
Asterales Asteraceae Cynara humilis#NC 027113
Asterales Asteraceae Cynara cornigera#NC 028006
Asterales Asteraceae Cynara cardunculus#KM035764
Asterales Asteraceae Cirsium eriophorum#NC 036966
Asterales Asteraceae Silybum marianum#EYKJ
Asterales Asteraceae Cirsium vulgare#NC 036967
Asterales Asteraceae Cirsium arvense#NC 036965
Asterales Asteraceae Tragopogon castellanus#KFZY
Asterales Asteraceae Tragopogon porrifolius#KGJF
Asterales Asteraceae Tragopogon pratensis#FUPX
Asterales Asteraceae Tragopogon dubius#DDRL
Asterales Asteraceae Sonchus boulosii#MK016665
Asterales Asteraceae Youngia denticulata#MK622902
Asterales Asteraceae Lactuca sativa#NC 007578
Asterales Asteraceae Lactuca graminifolia#RFSD
Asterales Asteraceae Lactuca plumieri#JNKW
Asterales Asteraceae Pluchea indica#NC 038194
Asterales Asteraceae Inula helenium#AFQQ
Asterales Asteraceae Helenium autumnale#DUNJ
Asterales Asteraceae Marshallia ramosa#MH037172
Asterales Asteraceae Marshallia graminifolia#MH037167
Asterales Asteraceae Marshallia grandiflora#MH037171
Asterales Asteraceae Marshallia caespitosa#MH037175
Asterales Asteraceae Marshallia obovata#MH037169
Asterales Asteraceae Marshallia mohrii#MH037173
Asterales Asteraceae Marshallia trinervia#MH037168
Asterales Asteraceae Flaveria pringlei#AQZD
Asterales Asteraceae Flaveria cronquistii#BIDT
Asterales Asteraceae Flaveria sonorensis#UYED
Asterales Asteraceae Flaveria palmeri#NVSO
Asterales Asteraceae Flaveria brownii#JYMN
Asterales Asteraceae Flaveria pubescens#LYPZ
Asterales Asteraceae Flaveria angustifolia#YRBQ
Asterales Asteraceae Flaveria trinervia#ZCUA
Asterales Asteraceae Flaveria bidentis#JEPE
Asterales Asteraceae Flaveria vaginata#HXCD
Asterales Asteraceae Flaveria kochiana#QXWF
Asterales Asteraceae Guizotia abyssinica#NC 010601
Asterales Asteraceae Ageratina adenophora#NC 015621
Asterales Asteraceae Praxelis clematidea#NC 023833
Asterales Asteraceae Achyrachaena mollis#NC 036504
Asterales Asteraceae Eclipta alba#NC 039774
Asterales Asteraceae Sphagneticola calendulacea#NC 039346
Asterales Asteraceae Xanthium sibiricum#MH473582
Asterales Asteraceae Xanthium strumarium#UBLN
Asterales Asteraceae Ambrosia artemisiifolia#MG019037

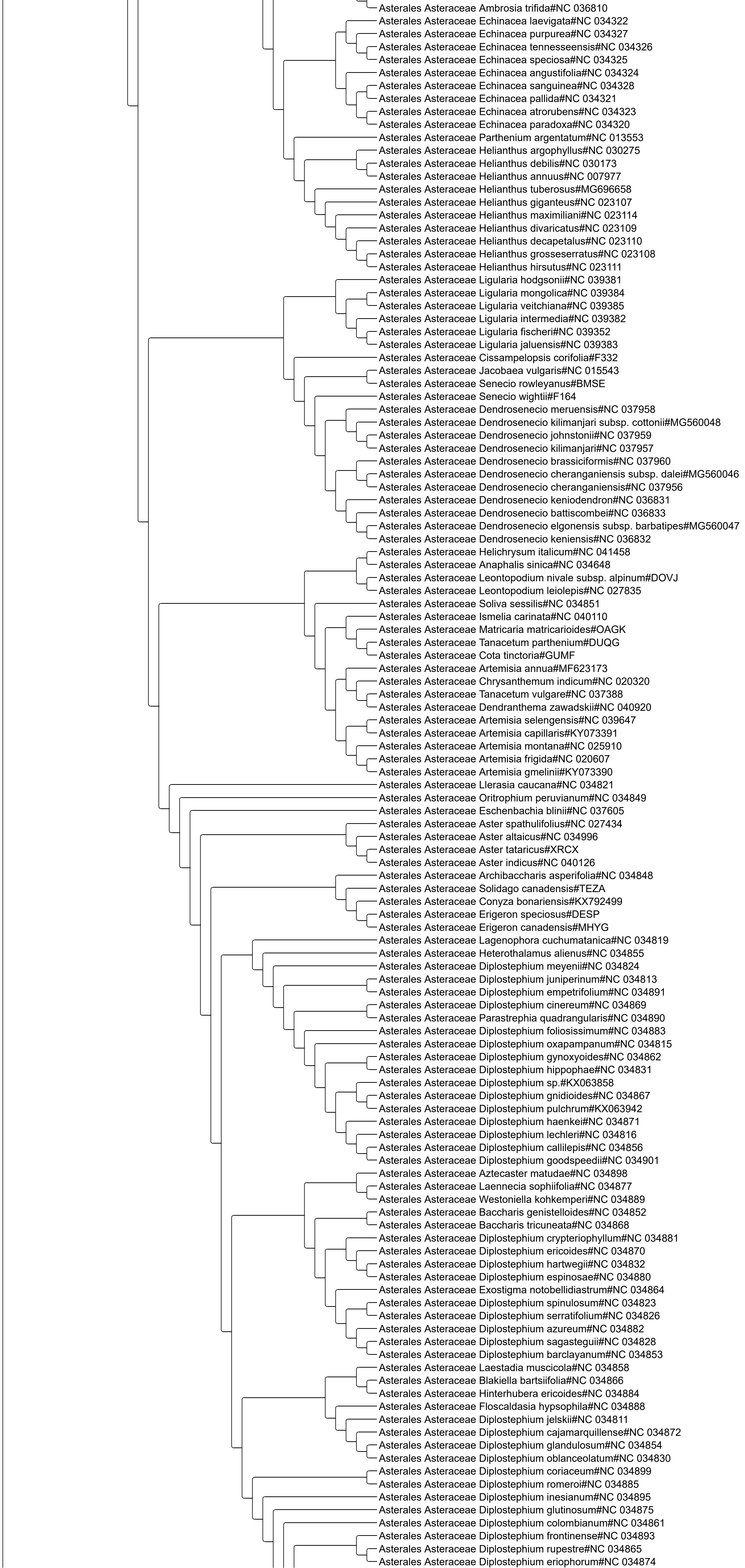
Asterales Asteraceae Ambrosia trifida#NC 036810
Asterales Asteraceae Echinacea laevigata#NC 034322
Asterales Asteraceae Echinacea purpurea#NC 034327
Asterales Asteraceae Echinacea tennesseensis#NC 034326
Asterales Asteraceae Echinacea speciosa#NC 034325
Asterales Asteraceae Echinacea angustifolia#NC 034324
Asterales Asteraceae Echinacea sanguinea#NC 034328
Asterales Asteraceae Echinacea pallida#NC 034321
Asterales Asteraceae Echinacea atrorubens#NC 034323
Asterales Asteraceae Echinacea paradoxa#NC 034320
Asterales Asteraceae Parthenium argentatum#NC 013553
Asterales Asteraceae Helianthus argophyllus#NC 030275
Asterales Asteraceae Helianthus debilis#NC 030173
Asterales Asteraceae Helianthus annuus#NC 007977
Asterales Asteraceae Helianthus tuberosus#MG696658
Asterales Asteraceae Helianthus giganteus#NC 023107
Asterales Asteraceae Helianthus maximiliani#NC 023114
Asterales Asteraceae Helianthus divaricatus#NC 023109
Asterales Asteraceae Helianthus decapetalus#NC 023110
Asterales Asteraceae Helianthus grosseserratus#NC 023108
Asterales Asteraceae Helianthus hirsutus#NC 023111
Asterales Asteraceae Ligularia hodgsonii#NC 039381
Asterales Asteraceae Ligularia mongolica#NC 039384
Asterales Asteraceae Ligularia veitchiana#NC 039385
Asterales Asteraceae Ligularia intermedia#NC 039382
Asterales Asteraceae Ligularia fischeri#NC 039352
Asterales Asteraceae Ligularia jaluensis#NC 039383
Asterales Asteraceae Cissampelopsis corifolia#F332
Asterales Asteraceae Jacobaea vulgaris#NC 015543
Asterales Asteraceae Senecio rowleyanus#BMSE
Asterales Asteraceae Senecio wightii#F164
Asterales Asteraceae Dendrosenecio meruensis#NC 037958
Asterales Asteraceae Dendrosenecio kilimanjari subsp. cottonii#MG560048
Asterales Asteraceae Dendrosenecio johnstonii#NC 037959
Asterales Asteraceae Dendrosenecio kilimanjari#NC 037957
Asterales Asteraceae Dendrosenecio brassiciformis#NC 037960
Asterales Asteraceae Dendrosenecio cheranganiensis subsp. dalei#MG560046
Asterales Asteraceae Dendrosenecio cheranganiensis#NC 037956
Asterales Asteraceae Dendrosenecio keniodendron#NC 036831
Asterales Asteraceae Dendrosenecio battiscombei#NC 036833
Asterales Asteraceae Dendrosenecio elgonensis subsp. barbatipes#MG560047
Asterales Asteraceae Dendrosenecio keniensis#NC 036832
Asterales Asteraceae Helichrysum italicum#NC 041458
Asterales Asteraceae Anaphalis sinica#NC 034648
Asterales Asteraceae Leontopodium nivale subsp. alpinum#DOVJ
Asterales Asteraceae Leontopodium leiolepis#NC 027835
Asterales Asteraceae Soliva sessilis#NC 034851
Asterales Asteraceae Ismelia carinata#NC 040110
Asterales Asteraceae Matricaria matricarioides#OAGK
Asterales Asteraceae Tanacetum parthenium#DUQG
Asterales Asteraceae Cota tinctoria#GUMF
Asterales Asteraceae Artemisia annua#MF623173
Asterales Asteraceae Chrysanthemum indicum#NC 020320
Asterales Asteraceae Tanacetum vulgare#NC 037388
Asterales Asteraceae Dendranthema zawadskii#NC 040920
Asterales Asteraceae Artemisia selengensis#NC 039647
Asterales Asteraceae Artemisia capillaris#KY073391
Asterales Asteraceae Artemisia montana#NC 025910
Asterales Asteraceae Artemisia frigida#NC 020607
Asterales Asteraceae Artemisia gmelinii#KY073390
Asterales Asteraceae Llerasia caucana#NC 034821
Asterales Asteraceae Oritrophium peruvianum#NC 034849
Asterales Asteraceae Eschenbachia blinii#NC 037605
Asterales Asteraceae Aster spathulifolius#NC 027434
Asterales Asteraceae Aster altaicus#NC 034996
Asterales Asteraceae Aster tataricus#XRCX
Asterales Asteraceae Aster indicus#NC 040126
Asterales Asteraceae Archibaccharis asperifolia#NC 034848
Asterales Asteraceae Solidago canadensis#TEZA
Asterales Asteraceae Conyza bonariensis#KX792499
Asterales Asteraceae Erigeron speciosus#DESP
Asterales Asteraceae Erigeron canadensis#MHYG
Asterales Asteraceae Lagenophora cuchumatanica#NC 034819
Asterales Asteraceae Heterothalamus alienus#NC 034855
Asterales Asteraceae Diplostephium meyenii#NC 034824
Asterales Asteraceae Diplostephium juniperinum#NC 034813
Asterales Asteraceae Diplostephium empetrifolium#NC 034891
Asterales Asteraceae Diplostephium cinereum#NC 034869
Asterales Asteraceae Parastrephia quadrangularis#NC 034890
Asterales Asteraceae Diplostephium foliosissimum#NC 034883
Asterales Asteraceae Diplostephium oxapampanum#NC 034815
Asterales Asteraceae Diplostephium gynoxyoides#NC 034862
Asterales Asteraceae Diplostephium hippophae#NC 034831
Asterales Asteraceae Diplostephium sp.#KX063858
Asterales Asteraceae Diplostephium gnidioides#NC 034867
Asterales Asteraceae Diplostephium pulchrum#KX063942
Asterales Asteraceae Diplostephium haenkei#NC 034871
Asterales Asteraceae Diplostephium lechleri#NC 034816
Asterales Asteraceae Diplostephium callilepis#NC 034856
Asterales Asteraceae Diplostephium goodspeedii#NC 034901
Asterales Asteraceae Aztecaster matudae#NC 034898
Asterales Asteraceae Laennecia sophiifolia#NC 034877
Asterales Asteraceae Westoniella kohkemperi#NC 034889
Asterales Asteraceae Baccharis genistelloides#NC 034852
Asterales Asteraceae Baccharis tricuneata#NC 034868
Asterales Asteraceae Diplostephium crypteriophyllum#NC 034881
Asterales Asteraceae Diplostephium ericoides#NC 034870
Asterales Asteraceae Diplostephium hartwegii#NC 034832
Asterales Asteraceae Diplostephium espinosae#NC 034880
Asterales Asteraceae Exostigma notobellidiastrum#NC 034864
Asterales Asteraceae Diplostephium spinulosum#NC 034823
Asterales Asteraceae Diplostephium serratifolium#NC 034826
Asterales Asteraceae Diplostephium azureum#NC 034882
Asterales Asteraceae Diplostephium sagasteguii#NC 034828
Asterales Asteraceae Diplostephium barclayanum#NC 034853
Asterales Asteraceae Laestadia muscicola#NC 034858
Asterales Asteraceae Blakiella bartsiifolia#NC 034866
Asterales Asteraceae Hinterhubera ericoides#NC 034884
Asterales Asteraceae Floscaldasia hypsophila#NC 034888
Asterales Asteraceae Diplostephium jelskii#NC 034811
Asterales Asteraceae Diplostephium cajamarquillense#NC 034872
Asterales Asteraceae Diplostephium glandulosum#NC 034854
Asterales Asteraceae Diplostephium oblanceolatum#NC 034830
Asterales Asteraceae Diplostephium coriaceum#NC 034899
Asterales Asteraceae Diplostephium romeroi#NC 034885
Asterales Asteraceae Diplostephium inesianum#NC 034895
Asterales Asteraceae Diplostephium glutinosum#NC 034875
Asterales Asteraceae Diplostephium colombianum#NC 034861
Asterales Asteraceae Diplostephium frontinense#NC 034893
Asterales Asteraceae Diplostephium rupestre#NC 034865
Asterales Asteraceae Diplostephium eriophorum#NC 034874

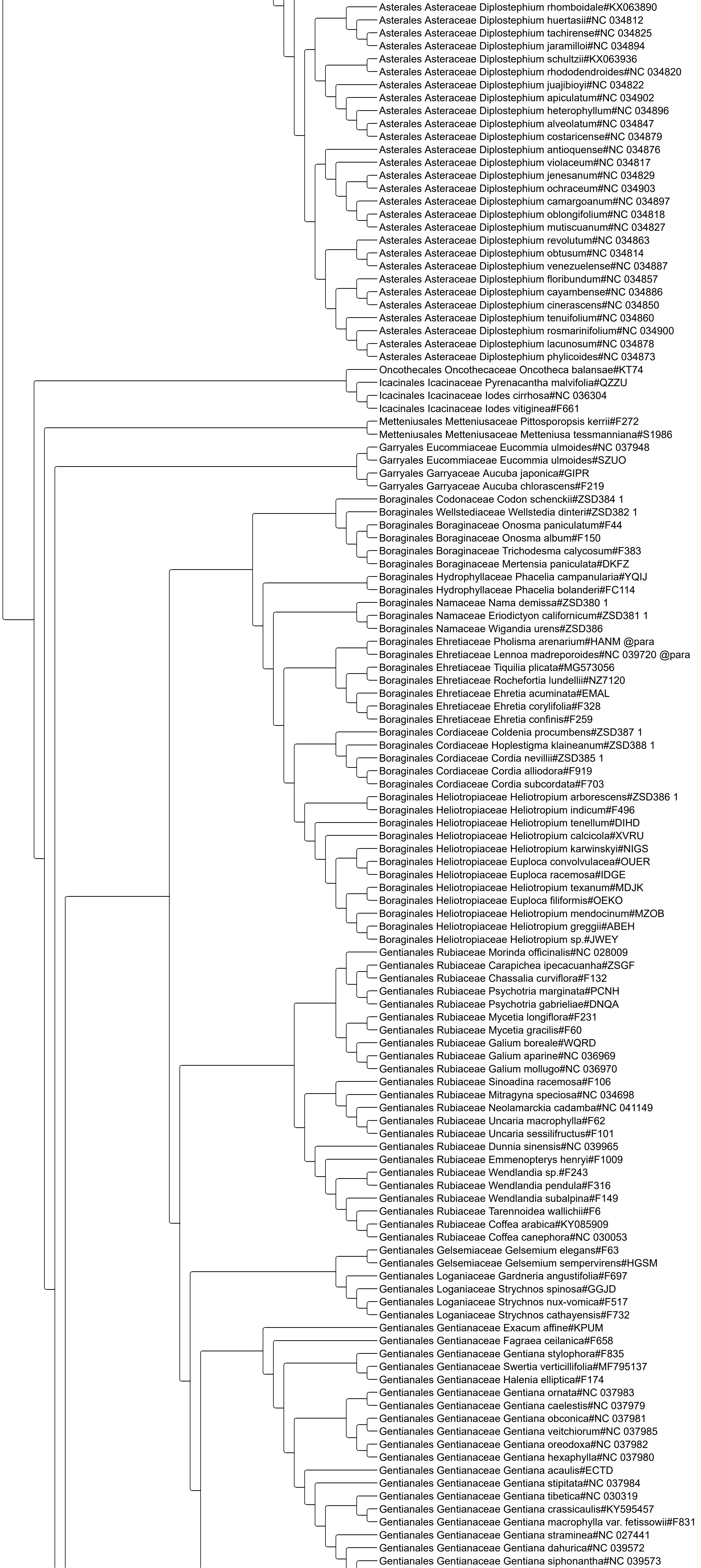
Asterales Asteraceae Diplostephium rhomboidale#KX063890
Asterales Asteraceae Diplostephium huertasii#NC 034812
Asterales Asteraceae Diplostephium tachirense#NC 034825
Asterales Asteraceae Diplostephium jaramilloi#NC 034894
Asterales Asteraceae Diplostephium schultzii#KX063936
Asterales Asteraceae Diplostephium rhododendroides#NC 034820
Asterales Asteraceae Diplostephium juajibioyi#NC 034822
Asterales Asteraceae Diplostephium apiculatum#NC 034902
Asterales Asteraceae Diplostephium heterophyllum#NC 034896
Asterales Asteraceae Diplostephium alveolatum#NC 034847
Asterales Asteraceae Diplostephium costaricense#NC 034879
Asterales Asteraceae Diplostephium antioquense#NC 034876
Asterales Asteraceae Diplostephium violaceum#NC 034817
Asterales Asteraceae Diplostephium jenesanum#NC 034829
Asterales Asteraceae Diplostephium ochraceum#NC 034903
Asterales Asteraceae Diplostephium camargoanum#NC 034897
Asterales Asteraceae Diplostephium oblongifolium#NC 034818
Asterales Asteraceae Diplostephium mutiscuanum#NC 034827
Asterales Asteraceae Diplostephium revolutum#NC 034863
Asterales Asteraceae Diplostephium obtusum#NC 034814
Asterales Asteraceae Diplostephium venezuelense#NC 034887
Asterales Asteraceae Diplostephium floribundum#NC 034857
Asterales Asteraceae Diplostephium cayambense#NC 034886
Asterales Asteraceae Diplostephium cinerascens#NC 034850
Asterales Asteraceae Diplostephium tenuifolium#NC 034860
Asterales Asteraceae Diplostephium rosmarinifolium#NC 034900
Asterales Asteraceae Diplostephium lacunosum#NC 034878
Asterales Asteraceae Diplostephium phylicoides#NC 034873
Oncothecales Oncothecaceae Oncotheca balansae#KT74
Icacinales Icacinaceae Pyrenacantha malvifolia#QZZU
Icacinales Icacinaceae Iodes cirrhosa#NC 036304
Icacinales Icacinaceae Iodes vitiginea#F661
Metteniusales Metteniusaceae Pittosporopsis kerrii#F272
Metteniusales Metteniusaceae Metteniusa tessmanniana#S1986
Garryales Eucommiaceae Eucommia ulmoides#NC 037948
Garryales Eucommiaceae Eucommia ulmoides#SZUO
Garryales Garryaceae Aucuba japonica#GIPR
Garryales Garryaceae Aucuba chlorascens#F219
Boraginales Codonaceae Codon schenckii#ZSD384 1
Boraginales Wellstediaceae Wellstedia dinteri#ZSD382 1
Boraginales Boraginaceae Onosma paniculatum#F44
Boraginales Boraginaceae Onosma album#F150
Boraginales Boraginaceae Trichodesma calycosum#F383
Boraginales Boraginaceae Mertensia paniculata#DKFZ
Boraginales Hydrophyllaceae Phacelia campanularia#YQIJ
Boraginales Hydrophyllaceae Phacelia bolanderi#FC114
Boraginales Namaceae Nama demissa#ZSD380 1
Boraginales Namaceae Eriodictyon californicum#ZSD381 1
Boraginales Namaceae Wigandia urens#ZSD386
Boraginales Ehretiaceae Pholisma arenarium#HANM @para
Boraginales Ehretiaceae Lennoa madreporoides#NC 039720 @para
Boraginales Ehretiaceae Tiquilia plicata#MG573056
Boraginales Ehretiaceae Rochefortia lundellii#NZ7120
Boraginales Ehretiaceae Ehretia acuminata#EMAL
Boraginales Ehretiaceae Ehretia corylifolia#F328
Boraginales Ehretiaceae Ehretia confinis#F259
Boraginales Cordiaceae Coldenia procumbens#ZSD387 1
Boraginales Cordiaceae Hoplestigma klaineanum#ZSD388 1
Boraginales Cordiaceae Cordia nevillii#ZSD385 1
Boraginales Cordiaceae Cordia alliodora#F919
Boraginales Cordiaceae Cordia subcordata#F703
Boraginales Heliotropiaceae Heliotropium arborescens#ZSD386 1
Boraginales Heliotropiaceae Heliotropium indicum#F496
Boraginales Heliotropiaceae Heliotropium tenellum#DIHD
Boraginales Heliotropiaceae Heliotropium calcicola#XVRU
Boraginales Heliotropiaceae Heliotropium karwinskyi#NIGS
Boraginales Heliotropiaceae Euploca convolvulacea#OUER
Boraginales Heliotropiaceae Euploca racemosa#IDGE
Boraginales Heliotropiaceae Heliotropium texanum#MDJK
Boraginales Heliotropiaceae Euploca filiformis#OEKO
Boraginales Heliotropiaceae Heliotropium mendocinum#MZOB
Boraginales Heliotropiaceae Heliotropium greggii#ABEH
Boraginales Heliotropiaceae Heliotropium sp.#JWEY
Gentianales Rubiaceae Morinda officinalis#NC 028009
Gentianales Rubiaceae Carapichea ipecacuanha#ZSGF
Gentianales Rubiaceae Chassalia curviflora#F132
Gentianales Rubiaceae Psychotria marginata#PCNH
Gentianales Rubiaceae Psychotria gabrieliae#DNQA
Gentianales Rubiaceae Mycetia longiflora#F231
Gentianales Rubiaceae Mycetia gracilis#F60
Gentianales Rubiaceae Galium boreale#WQRD
Gentianales Rubiaceae Galium aparine#NC 036969
Gentianales Rubiaceae Galium mollugo#NC 036970
Gentianales Rubiaceae Sinoadina racemosa#F106
Gentianales Rubiaceae Mitragyna speciosa#NC 034698
Gentianales Rubiaceae Neolamarckia cadamba#NC 041149
Gentianales Rubiaceae Uncaria macrophylla#F62
Gentianales Rubiaceae Uncaria sessilifructus#F101
Gentianales Rubiaceae Dunnia sinensis#NC 039965
Gentianales Rubiaceae Emmenopterys henryi#F1009
Gentianales Rubiaceae Wendlandia sp.#F243
Gentianales Rubiaceae Wendlandia pendula#F316
Gentianales Rubiaceae Wendlandia subalpina#F149
Gentianales Rubiaceae Tarennoidea wallichii#F6
Gentianales Rubiaceae Coffea arabica#KY085909
Gentianales Rubiaceae Coffea canephora#NC 030053
Gentianales Gelsemiaceae Gelsemium elegans#F63
Gentianales Gelsemiaceae Gelsemium sempervirens#HGSM
Gentianales Loganiaceae Gardneria angustifolia#F697
Gentianales Loganiaceae Strychnos spinosa#GGJD
Gentianales Loganiaceae Strychnos nux-vomica#F517
Gentianales Loganiaceae Strychnos cathayensis#F732
Gentianales Gentianaceae Exacum affine#KPUM
Gentianales Gentianaceae Fagraea ceilanica#F658
Gentianales Gentianaceae Gentiana stylophora#F835
Gentianales Gentianaceae Swertia verticillifolia#MF795137
Gentianales Gentianaceae Halenia elliptica#F174
Gentianales Gentianaceae Gentiana ornata#NC 037983
Gentianales Gentianaceae Gentiana caelestis#NC 037979
Gentianales Gentianaceae Gentiana obconica#NC 037981
Gentianales Gentianaceae Gentiana veitchiorum#NC 037985
Gentianales Gentianaceae Gentiana oreodoxa#NC 037982
Gentianales Gentianaceae Gentiana hexaphylla#NC 037980
Gentianales Gentianaceae Gentiana acaulis#ECTD
Gentianales Gentianaceae Gentiana stipitata#NC 037984
Gentianales Gentianaceae Gentiana tibetica#NC 030319
Gentianales Gentianaceae Gentiana crassicaulis#KY595457
Gentianales Gentianaceae Gentiana macrophylla var. fetissowii#F831
Gentianales Gentianaceae Gentiana straminea#NC 027441
Gentianales Gentianaceae Gentiana dahurica#NC 039572
Gentianales Gentianaceae Gentiana siphonantha#NC 039573

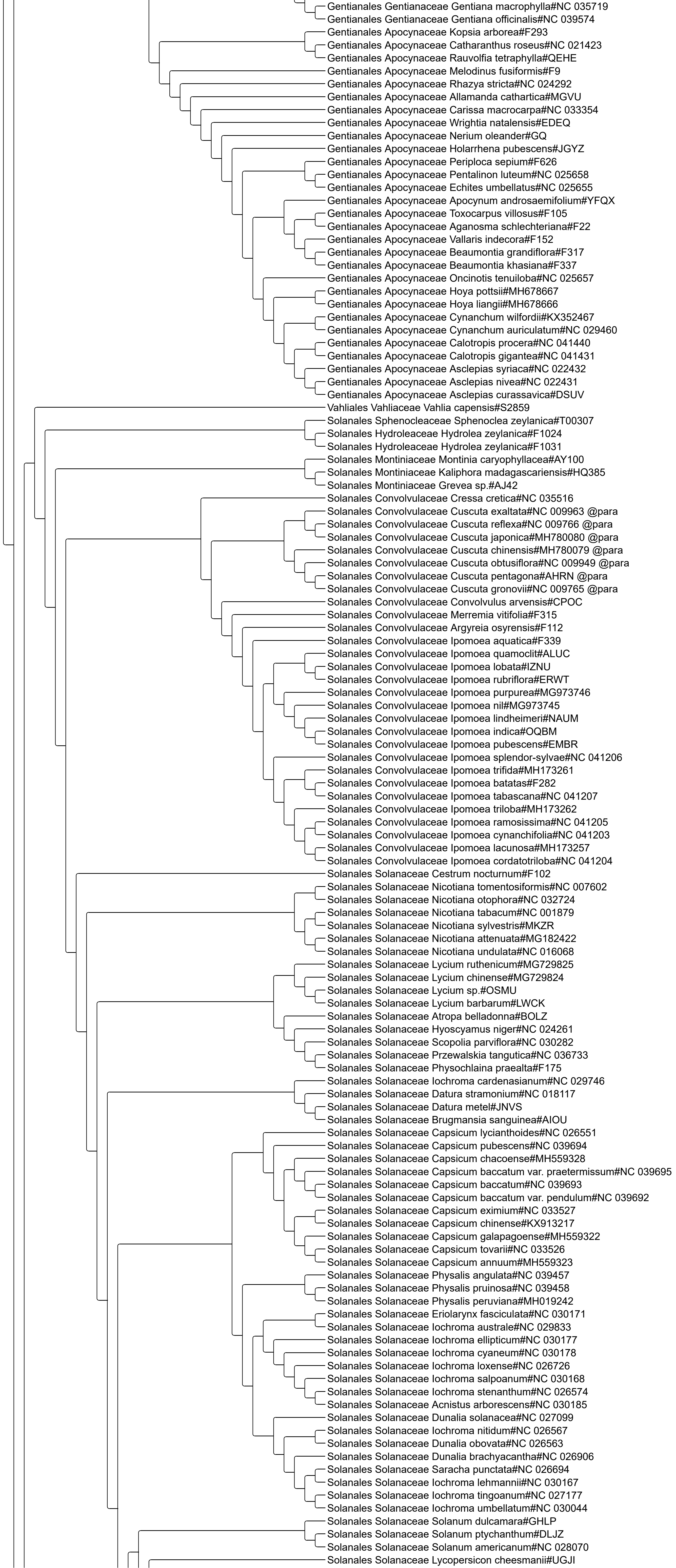
Gentianales Gentianaceae Gentiana macrophylla#NC 035719
Gentianales Gentianaceae Gentiana officinalis#NC 039574
Gentianales Apocynaceae Kopsia arborea#F293
Gentianales Apocynaceae Catharanthus roseus#NC 021423
Gentianales Apocynaceae Rauvolfia tetraphylla#QEHE
Gentianales Apocynaceae Melodinus fusiformis#F9
Gentianales Apocynaceae Rhazya stricta#NC 024292
Gentianales Apocynaceae Allamanda cathartica#MGVU
Gentianales Apocynaceae Carissa macrocarpa#NC 033354
Gentianales Apocynaceae Wrightia natalensis#EDEQ
Gentianales Apocynaceae Nerium oleander#GQ
Gentianales Apocynaceae Holarrhena pubescens#JGYZ
Gentianales Apocynaceae Periploca sepium#F626
Gentianales Apocynaceae Pentalinon luteum#NC 025658
Gentianales Apocynaceae Echites umbellatus#NC 025655
Gentianales Apocynaceae Apocynum androsaemifolium#YFQX
Gentianales Apocynaceae Toxocarpus villosus#F105
Gentianales Apocynaceae Aganosma schlechteriana#F22
Gentianales Apocynaceae Vallaris indecora#F152
Gentianales Apocynaceae Beaumontia grandiflora#F317
Gentianales Apocynaceae Beaumontia khasiana#F337
Gentianales Apocynaceae Oncinotis tenuiloba#NC 025657
Gentianales Apocynaceae Hoya pottsii#MH678667
Gentianales Apocynaceae Hoya liangii#MH678666
Gentianales Apocynaceae Cynanchum wilfordii#KX352467
Gentianales Apocynaceae Cynanchum auriculatum#NC 029460
Gentianales Apocynaceae Calotropis procera#NC 041440
Gentianales Apocynaceae Calotropis gigantea#NC 041431
Gentianales Apocynaceae Asclepias syriaca#NC 022432
Gentianales Apocynaceae Asclepias nivea#NC 022431
Gentianales Apocynaceae Asclepias curassavica#DSUV
Vahliales Vahliaceae Vahlia capensis#S2859
Solanales Sphenocleaceae Sphenoclea zeylanica#T00307
Solanales Hydroleaceae Hydrolea zeylanica#F1024
Solanales Hydroleaceae Hydrolea zeylanica#F1031
Solanales Montiniaceae Montinia caryophyllacea#AY100
Solanales Montiniaceae Kaliphora madagascariensis#HQ385
Solanales Montiniaceae Grevea sp.#AJ42
Solanales Convolvulaceae Cressa cretica#NC 035516
Solanales Convolvulaceae Cuscuta exaltata#NC 009963 @para
Solanales Convolvulaceae Cuscuta reflexa#NC 009766 @para
Solanales Convolvulaceae Cuscuta japonica#MH780080 @para
Solanales Convolvulaceae Cuscuta chinensis#MH780079 @para
Solanales Convolvulaceae Cuscuta obtusiflora#NC 009949 @para
Solanales Convolvulaceae Cuscuta pentagona#AHRN @para
Solanales Convolvulaceae Cuscuta gronovii#NC 009765 @para
Solanales Convolvulaceae Convolvulus arvensis#CPOC
Solanales Convolvulaceae Merremia vitifolia#F315
Solanales Convolvulaceae Argyreia osyrensis#F112
Solanales Convolvulaceae Ipomoea aquatica#F339
Solanales Convolvulaceae Ipomoea quamoclit#ALUC
Solanales Convolvulaceae Ipomoea lobata#IZNU
Solanales Convolvulaceae Ipomoea rubriflora#ERWT
Solanales Convolvulaceae Ipomoea purpurea#MG973746
Solanales Convolvulaceae Ipomoea nil#MG973745
Solanales Convolvulaceae Ipomoea lindheimeri#NAUM
Solanales Convolvulaceae Ipomoea indica#OQBM
Solanales Convolvulaceae Ipomoea pubescens#EMBR
Solanales Convolvulaceae Ipomoea splendor-sylvae#NC 041206
Solanales Convolvulaceae Ipomoea trifida#MH173261
Solanales Convolvulaceae Ipomoea batatas#F282
Solanales Convolvulaceae Ipomoea tabascana#NC 041207
Solanales Convolvulaceae Ipomoea triloba#MH173262
Solanales Convolvulaceae Ipomoea ramosissima#NC 041205
Solanales Convolvulaceae Ipomoea cynanchifolia#NC 041203
Solanales Convolvulaceae Ipomoea lacunosa#MH173257
Solanales Convolvulaceae Ipomoea cordatotriloba#NC 041204
Solanales Solanaceae Cestrum nocturnum#F102
Solanales Solanaceae Nicotiana tomentosiformis#NC 007602
Solanales Solanaceae Nicotiana otophora#NC 032724
Solanales Solanaceae Nicotiana tabacum#NC 001879
Solanales Solanaceae Nicotiana sylvestris#MKZR
Solanales Solanaceae Nicotiana attenuata#MG182422
Solanales Solanaceae Nicotiana undulata#NC 016068
Solanales Solanaceae Lycium ruthenicum#MG729825
Solanales Solanaceae Lycium chinense#MG729824
Solanales Solanaceae Lycium sp.#OSMU
Solanales Solanaceae Lycium barbarum#LWCK
Solanales Solanaceae Atropa belladonna#BOLZ
Solanales Solanaceae Hyoscyamus niger#NC 024261
Solanales Solanaceae Scopolia parviflora#NC 030282
Solanales Solanaceae Przewalskia tangutica#NC 036733
Solanales Solanaceae Physochlaina praealta#F175
Solanales Solanaceae Iochroma cardenasianum#NC 029746
Solanales Solanaceae Datura stramonium#NC 018117
Solanales Solanaceae Datura metel#JNVS
Solanales Solanaceae Brugmansia sanguinea#AIOU
Solanales Solanaceae Capsicum lycianthoides#NC 026551
Solanales Solanaceae Capsicum pubescens#NC 039694
Solanales Solanaceae Capsicum chacoense#MH559328
Solanales Solanaceae Capsicum baccatum var. praetermissum#NC 039695
Solanales Solanaceae Capsicum baccatum#NC 039693
Solanales Solanaceae Capsicum baccatum var. pendulum#NC 039692
Solanales Solanaceae Capsicum eximium#NC 033527
Solanales Solanaceae Capsicum chinense#KX913217
Solanales Solanaceae Capsicum galapagoense#MH559322
Solanales Solanaceae Capsicum tovarii#NC 033526
Solanales Solanaceae Capsicum annuum#MH559323
Solanales Solanaceae Physalis angulata#NC 039457
Solanales Solanaceae Physalis pruinosa#NC 039458
Solanales Solanaceae Physalis peruviana#MH019242
Solanales Solanaceae Eriolarynx fasciculata#NC 030171
Solanales Solanaceae Iochroma australe#NC 029833
Solanales Solanaceae Iochroma ellipticum#NC 030177
Solanales Solanaceae Iochroma cyaneum#NC 030178
Solanales Solanaceae Iochroma loxense#NC 026726
Solanales Solanaceae Iochroma salpoanum#NC 030168
Solanales Solanaceae Iochroma stenanthum#NC 026574
Solanales Solanaceae Acnistus arborescens#NC 030185
Solanales Solanaceae Dunalia solanacea#NC 027099
Solanales Solanaceae Iochroma nitidum#NC 026567
Solanales Solanaceae Dunalia obovata#NC 026563
Solanales Solanaceae Dunalia brachyacantha#NC 026906
Solanales Solanaceae Saracha punctata#NC 026694
Solanales Solanaceae Iochroma lehmannii#NC 030167
Solanales Solanaceae Iochroma tingoanum#NC 027177
Solanales Solanaceae Iochroma umbellatum#NC 030044
Solanales Solanaceae Solanum dulcamara#GHLP
Solanales Solanaceae Solanum ptychanthum#DLJZ
Solanales Solanaceae Solanum americanum#NC 028070
Solanales Solanaceae Lycopersicon cheesmanii#UGJI

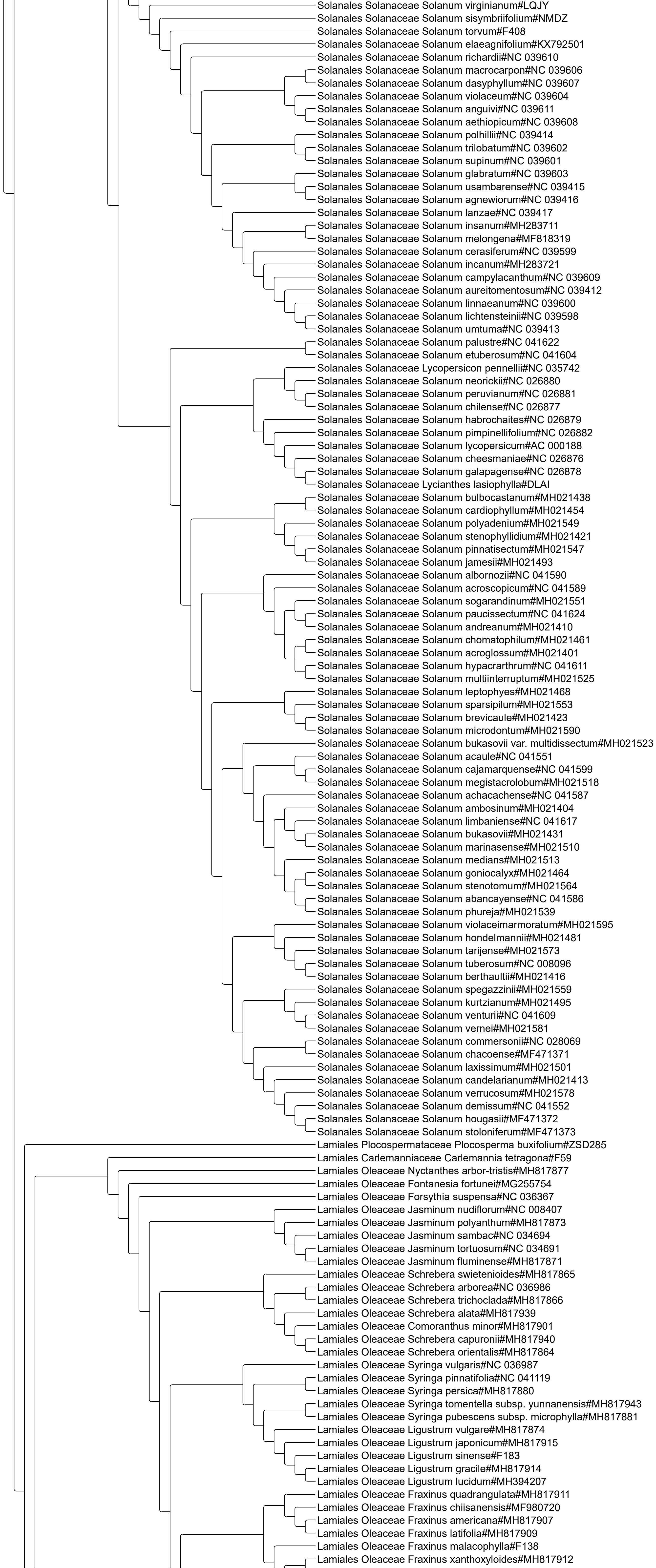

Solanales Solanaceae Solanum virginianum#LQJY
Solanales Solanaceae Solanum sisymbriifolium#NMDZ
Solanales Solanaceae Solanum torvum#F408
Solanales Solanaceae Solanum elaeagnifolium#KX792501
Solanales Solanaceae Solanum richardii#NC 039610
Solanales Solanaceae Solanum macrocarpon#NC 039606
Solanales Solanaceae Solanum dasyphyllum#NC 039607
Solanales Solanaceae Solanum violaceum#NC 039604
Solanales Solanaceae Solanum anguivi#NC 039611
Solanales Solanaceae Solanum aethiopicum#NC 039608
Solanales Solanaceae Solanum polhillii#NC 039414
Solanales Solanaceae Solanum trilobatum#NC 039602
Solanales Solanaceae Solanum supinum#NC 039601
Solanales Solanaceae Solanum glabratum#NC 039603
Solanales Solanaceae Solanum usambarense#NC 039415
Solanales Solanaceae Solanum agnewiorum#NC 039416
Solanales Solanaceae Solanum lanzae#NC 039417
Solanales Solanaceae Solanum insanum#MH283711
Solanales Solanaceae Solanum melongena#MF818319
Solanales Solanaceae Solanum cerasiferum#NC 039599
Solanales Solanaceae Solanum incanum#MH283721
Solanales Solanaceae Solanum campylacanthum#NC 039609
Solanales Solanaceae Solanum aureitomentosum#NC 039412
Solanales Solanaceae Solanum linnaeanum#NC 039600
Solanales Solanaceae Solanum lichtensteinii#NC 039598
Solanales Solanaceae Solanum umtuma#NC 039413
Solanales Solanaceae Solanum palustre#NC 041622
Solanales Solanaceae Solanum etuberosum#NC 041604
Solanales Solanaceae Lycopersicon pennellii#NC 035742
Solanales Solanaceae Solanum neorickii#NC 026880
Solanales Solanaceae Solanum peruvianum#NC 026881
Solanales Solanaceae Solanum chilense#NC 026877
Solanales Solanaceae Solanum habrochaites#NC 026879
Solanales Solanaceae Solanum pimpinellifolium#NC 026882
Solanales Solanaceae Solanum lycopersicum#AC 000188
Solanales Solanaceae Solanum cheesmaniae#NC 026876
Solanales Solanaceae Solanum galapagense#NC 026878
Solanales Solanaceae Lycianthes lasiophylla#DLAI
Solanales Solanaceae Solanum bulbocastanum#MH021438
Solanales Solanaceae Solanum cardiophyllum#MH021454
Solanales Solanaceae Solanum polyadenium#MH021549
Solanales Solanaceae Solanum stenophyllidium#MH021421
Solanales Solanaceae Solanum pinnatisectum#MH021547
Solanales Solanaceae Solanum jamesii#MH021493
Solanales Solanaceae Solanum albornozii#NC 041590
Solanales Solanaceae Solanum acroscopicum#NC 041589
Solanales Solanaceae Solanum sogarandinum#MH021551
Solanales Solanaceae Solanum paucissectum#NC 041624
Solanales Solanaceae Solanum andreanum#MH021410
Solanales Solanaceae Solanum chomatophilum#MH021461
Solanales Solanaceae Solanum acroglossum#MH021401
Solanales Solanaceae Solanum hypacrarthrum#NC 041611
Solanales Solanaceae Solanum multiinterruptum#MH021525
Solanales Solanaceae Solanum leptophyes#MH021468
Solanales Solanaceae Solanum sparsipilum#MH021553
Solanales Solanaceae Solanum brevicaule#MH021423
Solanales Solanaceae Solanum microdontum#MH021590
Solanales Solanaceae Solanum bukasovii var. multidissectum#MH021523
Solanales Solanaceae Solanum acaule#NC 041551
Solanales Solanaceae Solanum cajamarquense#NC 041599
Solanales Solanaceae Solanum megistacrolobum#MH021518
Solanales Solanaceae Solanum achacachense#NC 041587
Solanales Solanaceae Solanum ambosinum#MH021404
Solanales Solanaceae Solanum limbaniense#NC 041617
Solanales Solanaceae Solanum bukasovii#MH021431
Solanales Solanaceae Solanum marinasense#MH021510
Solanales Solanaceae Solanum medians#MH021513
Solanales Solanaceae Solanum goniocalyx#MH021464
Solanales Solanaceae Solanum stenotomum#MH021564
Solanales Solanaceae Solanum abancayense#NC 041586
Solanales Solanaceae Solanum phureja#MH021539
Solanales Solanaceae Solanum violaceimarmoratum#MH021595
Solanales Solanaceae Solanum hondelmannii#MH021481
Solanales Solanaceae Solanum tarijense#MH021573
Solanales Solanaceae Solanum tuberosum#NC 008096
Solanales Solanaceae Solanum berthaultii#MH021416
Solanales Solanaceae Solanum spegazzinii#MH021559
Solanales Solanaceae Solanum kurtzianum#MH021495
Solanales Solanaceae Solanum venturii#NC 041609
Solanales Solanaceae Solanum vernei#MH021581
Solanales Solanaceae Solanum commersonii#NC 028069
Solanales Solanaceae Solanum chacoense#MF471371
Solanales Solanaceae Solanum laxissimum#MH021501
Solanales Solanaceae Solanum candelarianum#MH021413
Solanales Solanaceae Solanum verrucosum#MH021578
Solanales Solanaceae Solanum demissum#NC 041552
Solanales Solanaceae Solanum hougasii#MF471372
Solanales Solanaceae Solanum stoloniferum#MF471373
Lamiales Plocospermataceae Plocosperma buxifolium#ZSD285
Lamiales Carlemanniaceae Carlemannia tetragona#F59
Lamiales Oleaceae Nyctanthes arbor-tristis#MH817877
Lamiales Oleaceae Fontanesia fortunei#MG255754
Lamiales Oleaceae Forsythia suspensa#NC 036367
Lamiales Oleaceae Jasminum nudiflorum#NC 008407
Lamiales Oleaceae Jasminum polyanthum#MH817873
Lamiales Oleaceae Jasminum sambac#NC 034694
Lamiales Oleaceae Jasminum tortuosum#NC 034691
Lamiales Oleaceae Jasminum fluminense#MH817871
Lamiales Oleaceae Schrebera swietenioides#MH817865
Lamiales Oleaceae Schrebera arborea#NC 036986
Lamiales Oleaceae Schrebera trichoclada#MH817866
Lamiales Oleaceae Schrebera alata#MH817939
Lamiales Oleaceae Comoranthus minor#MH817901
Lamiales Oleaceae Schrebera capuronii#MH817940
Lamiales Oleaceae Schrebera orientalis#MH817864
Lamiales Oleaceae Syringa vulgaris#NC 036987
Lamiales Oleaceae Syringa pinnatifolia#NC 041119
Lamiales Oleaceae Syringa persica#MH817880
Lamiales Oleaceae Syringa tomentella subsp. yunnanensis#MH817943
Lamiales Oleaceae Syringa pubescens subsp. microphylla#MH817881
Lamiales Oleaceae Ligustrum vulgare#MH817874
Lamiales Oleaceae Ligustrum japonicum#MH817915
Lamiales Oleaceae Ligustrum sinense#F183
Lamiales Oleaceae Ligustrum gracile#MH817914
Lamiales Oleaceae Ligustrum lucidum#MH394207
Lamiales Oleaceae Fraxinus quadrangulata#MH817911
Lamiales Oleaceae Fraxinus chiisanensis#MF980720
Lamiales Oleaceae Fraxinus americana#MH817907
Lamiales Oleaceae Fraxinus latifolia#MH817909
Lamiales Oleaceae Fraxinus malacophylla#F138
Lamiales Oleaceae Fraxinus xanthoxyloides#MH817912

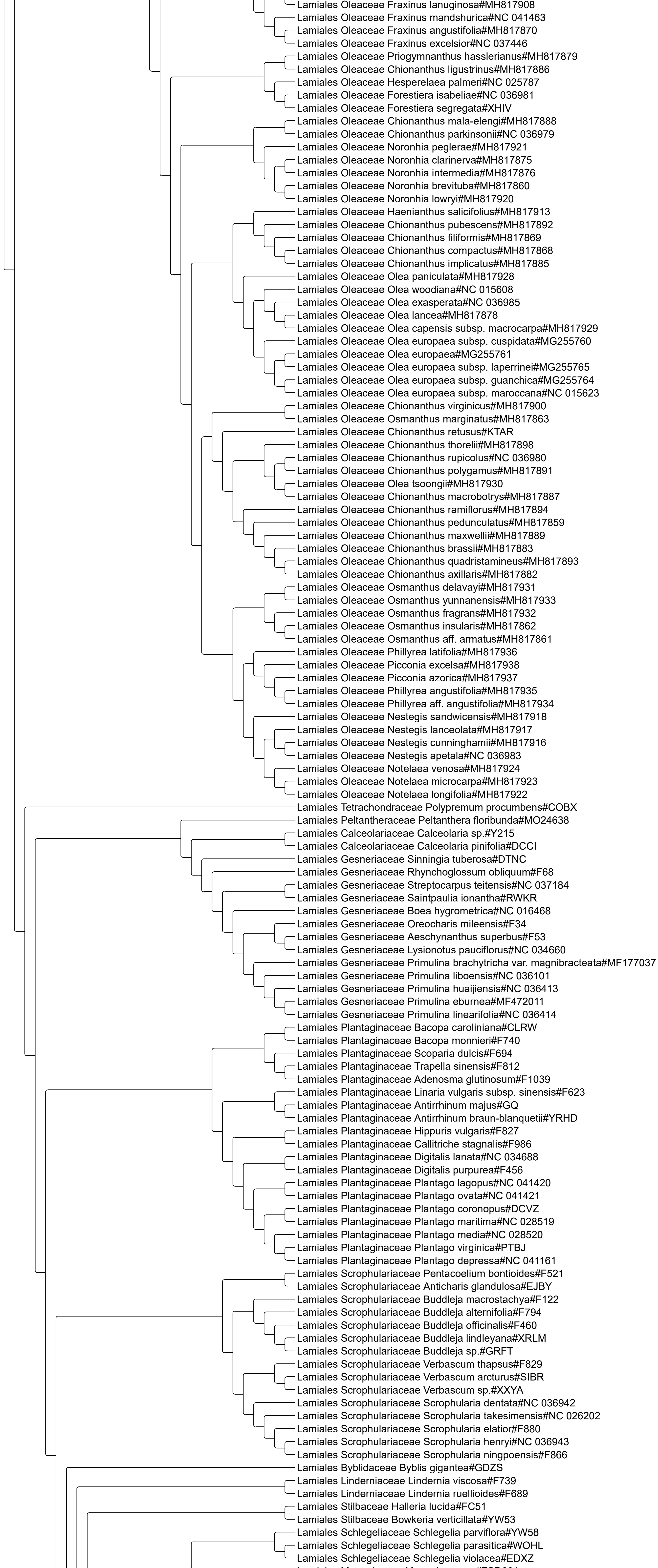
Lamiales Oleaceae Fraxinus lanuginosa#MH817908
Lamiales Oleaceae Fraxinus mandshurica#NC 041463
Lamiales Oleaceae Fraxinus angustifolia#MH817870
Lamiales Oleaceae Fraxinus excelsior#NC 037446
Lamiales Oleaceae Priogymnanthus hasslerianus#MH817879
Lamiales Oleaceae Chionanthus ligustrinus#MH817886
Lamiales Oleaceae Hesperelaea palmeri#NC 025787
Lamiales Oleaceae Forestiera isabeliae#NC 036981
Lamiales Oleaceae Forestiera segregata#XHIV
Lamiales Oleaceae Chionanthus mala-elengi#MH817888
Lamiales Oleaceae Chionanthus parkinsonii#NC 036979
Lamiales Oleaceae Noronhia peglerae#MH817921
Lamiales Oleaceae Noronhia clarinerva#MH817875
Lamiales Oleaceae Noronhia intermedia#MH817876
Lamiales Oleaceae Noronhia brevituba#MH817860
Lamiales Oleaceae Noronhia lowryi#MH817920
Lamiales Oleaceae Haenianthus salicifolius#MH817913
Lamiales Oleaceae Chionanthus pubescens#MH817892
Lamiales Oleaceae Chionanthus filiformis#MH817869
Lamiales Oleaceae Chionanthus compactus#MH817868
Lamiales Oleaceae Chionanthus implicatus#MH817885
Lamiales Oleaceae Olea paniculata#MH817928
Lamiales Oleaceae Olea woodiana#NC 015608
Lamiales Oleaceae Olea exasperata#NC 036985
Lamiales Oleaceae Olea lancea#MH817878
Lamiales Oleaceae Olea capensis subsp. macrocarpa#MH817929
Lamiales Oleaceae Olea europaea subsp. cuspidata#MG255760
Lamiales Oleaceae Olea europaea#MG255761
Lamiales Oleaceae Olea europaea subsp. laperrinei#MG255765
Lamiales Oleaceae Olea europaea subsp. guanchica#MG255764
Lamiales Oleaceae Olea europaea subsp. maroccana#NC 015623
Lamiales Oleaceae Chionanthus virginicus#MH817900
Lamiales Oleaceae Osmanthus marginatus#MH817863
Lamiales Oleaceae Chionanthus retusus#KTAR
Lamiales Oleaceae Chionanthus thorelii#MH817898
Lamiales Oleaceae Chionanthus rupicolus#NC 036980
Lamiales Oleaceae Chionanthus polygamus#MH817891
Lamiales Oleaceae Olea tsoongii#MH817930
Lamiales Oleaceae Chionanthus macrobotrys#MH817887
Lamiales Oleaceae Chionanthus ramiflorus#MH817894
Lamiales Oleaceae Chionanthus pedunculatus#MH817859
Lamiales Oleaceae Chionanthus maxwellii#MH817889
Lamiales Oleaceae Chionanthus brassii#MH817883
Lamiales Oleaceae Chionanthus quadristamineus#MH817893
Lamiales Oleaceae Chionanthus axillaris#MH817882
Lamiales Oleaceae Osmanthus delavayi#MH817931
Lamiales Oleaceae Osmanthus yunnanensis#MH817933
Lamiales Oleaceae Osmanthus fragrans#MH817932
Lamiales Oleaceae Osmanthus insularis#MH817862
Lamiales Oleaceae Osmanthus aff. armatus#MH817861
Lamiales Oleaceae Phillyrea latifolia#MH817936
Lamiales Oleaceae Picconia excelsa#MH817938
Lamiales Oleaceae Picconia azorica#MH817937
Lamiales Oleaceae Phillyrea angustifolia#MH817935
Lamiales Oleaceae Phillyrea aff. angustifolia#MH817934
Lamiales Oleaceae Nestegis sandwicensis#MH817918
Lamiales Oleaceae Nestegis lanceolata#MH817917
Lamiales Oleaceae Nestegis cunninghamii#MH817916
Lamiales Oleaceae Nestegis apetala#NC 036983
Lamiales Oleaceae Notelaea venosa#MH817924
Lamiales Oleaceae Notelaea microcarpa#MH817923
Lamiales Oleaceae Notelaea longifolia#MH817922
Lamiales Tetrachondraceae Polypremum procumbens#COBX
Lamiales Peltantheraceae Peltanthera floribunda#MO24638
Lamiales Calceolariaceae Calceolaria sp.#Y215
Lamiales Calceolariaceae Calceolaria pinifolia#DCCI
Lamiales Gesneriaceae Sinningia tuberosa#DTNC
Lamiales Gesneriaceae Rhynchoglossum obliquum#F68
Lamiales Gesneriaceae Streptocarpus teitensis#NC 037184
Lamiales Gesneriaceae Saintpaulia ionantha#RWKR
Lamiales Gesneriaceae Boea hygrometrica#NC 016468
Lamiales Gesneriaceae Oreocharis mileensis#F34
Lamiales Gesneriaceae Aeschynanthus superbus#F53
Lamiales Gesneriaceae Lysionotus pauciflorus#NC 034660
Lamiales Gesneriaceae Primulina brachytricha var. magnibracteata#MF177037
Lamiales Gesneriaceae Primulina liboensis#NC 036101
Lamiales Gesneriaceae Primulina huaijiensis#NC 036413
Lamiales Gesneriaceae Primulina eburnea#MF472011
Lamiales Gesneriaceae Primulina linearifolia#NC 036414
Lamiales Plantaginaceae Bacopa caroliniana#CLRW
Lamiales Plantaginaceae Bacopa monnieri#F740
Lamiales Plantaginaceae Scoparia dulcis#F694
Lamiales Plantaginaceae Trapella sinensis#F812
Lamiales Plantaginaceae Adenosma glutinosum#F1039
Lamiales Plantaginaceae Linaria vulgaris subsp. sinensis#F623
Lamiales Plantaginaceae Antirrhinum majus#GQ
Lamiales Plantaginaceae Antirrhinum braun-blanquetii#YRHD
Lamiales Plantaginaceae Hippuris vulgaris#F827
Lamiales Plantaginaceae Callitriche stagnalis#F986
Lamiales Plantaginaceae Digitalis lanata#NC 034688
Lamiales Plantaginaceae Digitalis purpurea#F456
Lamiales Plantaginaceae Plantago lagopus#NC 041420
Lamiales Plantaginaceae Plantago ovata#NC 041421
Lamiales Plantaginaceae Plantago coronopus#DCVZ
Lamiales Plantaginaceae Plantago maritima#NC 028519
Lamiales Plantaginaceae Plantago media#NC 028520
Lamiales Plantaginaceae Plantago virginica#PTBJ
Lamiales Plantaginaceae Plantago depressa#NC 041161
Lamiales Scrophulariaceae Pentacoelium bontioides#F521
Lamiales Scrophulariaceae Anticharis glandulosa#EJBY
Lamiales Scrophulariaceae Buddleja macrostachya#F122
Lamiales Scrophulariaceae Buddleja alternifolia#F794
Lamiales Scrophulariaceae Buddleja officinalis#F460
Lamiales Scrophulariaceae Buddleja lindleyana#XRLM
Lamiales Scrophulariaceae Buddleja sp.#GRFT
Lamiales Scrophulariaceae Verbascum thapsus#F829
Lamiales Scrophulariaceae Verbascum arcturus#SIBR
Lamiales Scrophulariaceae Verbascum sp.#XXYA
Lamiales Scrophulariaceae Scrophularia dentata#NC 036942
Lamiales Scrophulariaceae Scrophularia takesimensis#NC 026202
Lamiales Scrophulariaceae Scrophularia elatior#F880
Lamiales Scrophulariaceae Scrophularia henryi#NC 036943
Lamiales Scrophulariaceae Scrophularia ningpoensis#F866
Lamiales Byblidaceae Byblis gigantea#GDZS
Lamiales Linderniaceae Lindernia viscosa#F739
Lamiales Linderniaceae Lindernia ruellioides#F689
Lamiales Stilbaceae Halleria lucida#FC51
Lamiales Stilbaceae Bowkeria verticillata#YW53
Lamiales Schlegeliaceae Schlegelia parviflora#YW58
Lamiales Schlegeliaceae Schlegelia parasitica#WOHL
Lamiales Schlegeliaceae Schlegelia violacea#EDXZ

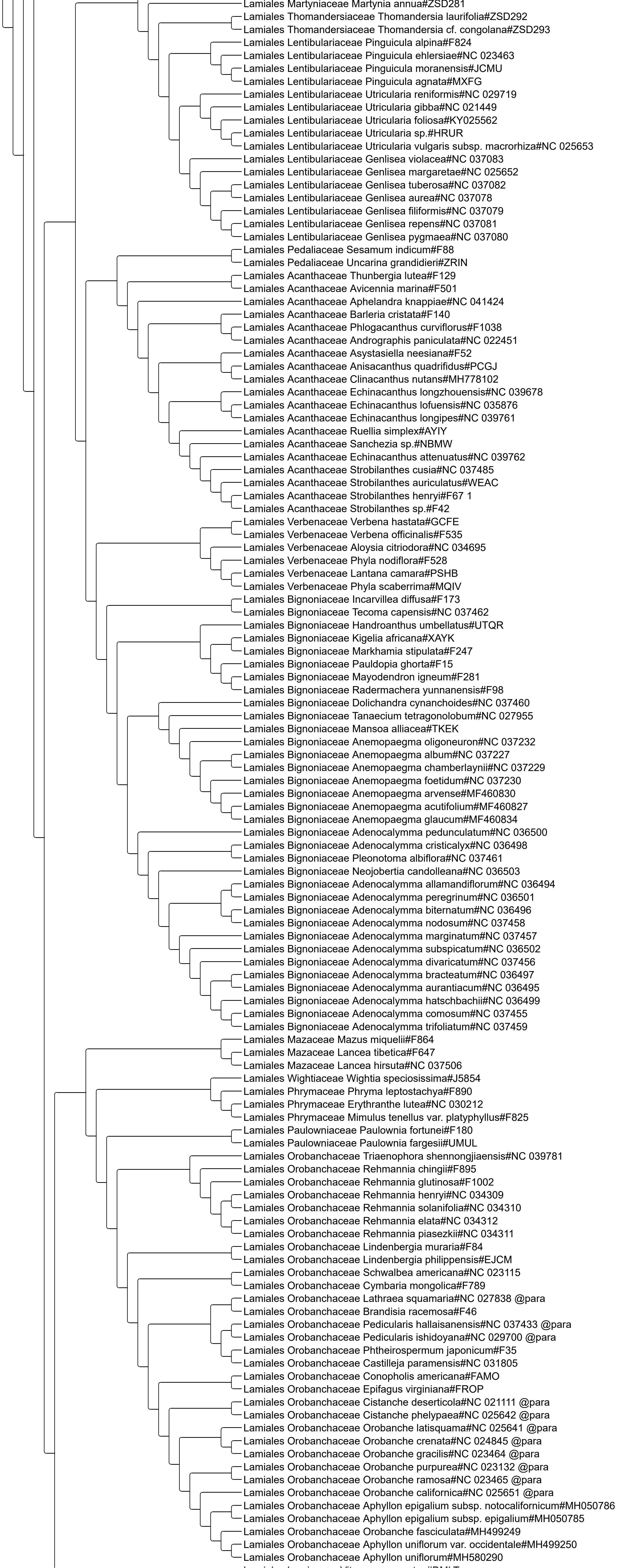
Lamiales Martyniaceae Martynia annua#ZSD281
Lamiales Thomandersiaceae Thomandersia laurifolia#ZSD292
Lamiales Thomandersiaceae Thomandersia cf. congolana#ZSD293
Lamiales Lentibulariaceae Pinguicula alpina#F824
Lamiales Lentibulariaceae Pinguicula ehlersiae#NC 023463
Lamiales Lentibulariaceae Pinguicula moranensis#JCMU
Lamiales Lentibulariaceae Pinguicula agnata#MXFG
Lamiales Lentibulariaceae Utricularia reniformis#NC 029719
Lamiales Lentibulariaceae Utricularia gibba#NC 021449
Lamiales Lentibulariaceae Utricularia foliosa#KY025562
Lamiales Lentibulariaceae Utricularia sp.#HRUR
Lamiales Lentibulariaceae Utricularia vulgaris subsp. macrorhiza#NC 025653
Lamiales Lentibulariaceae Genlisea violacea#NC 037083
Lamiales Lentibulariaceae Genlisea margaretae#NC 025652
Lamiales Lentibulariaceae Genlisea tuberosa#NC 037082
Lamiales Lentibulariaceae Genlisea aurea#NC 037078
Lamiales Lentibulariaceae Genlisea filiformis#NC 037079
Lamiales Lentibulariaceae Genlisea repens#NC 037081
Lamiales Lentibulariaceae Genlisea pygmaea#NC 037080
Lamiales Pedaliaceae Sesamum indicum#F88
Lamiales Pedaliaceae Uncarina grandidieri#ZRIN
Lamiales Acanthaceae Thunbergia lutea#F129
Lamiales Acanthaceae Avicennia marina#F501
Lamiales Acanthaceae Aphelandra knappiae#NC 041424
Lamiales Acanthaceae Barleria cristata#F140
Lamiales Acanthaceae Phlogacanthus curviflorus#F1038
Lamiales Acanthaceae Andrographis paniculata#NC 022451
Lamiales Acanthaceae Asystasiella neesiana#F52
Lamiales Acanthaceae Anisacanthus quadrifidus#PCGJ
Lamiales Acanthaceae Clinacanthus nutans#MH778102
Lamiales Acanthaceae Echinacanthus longzhouensis#NC 039678
Lamiales Acanthaceae Echinacanthus lofuensis#NC 035876
Lamiales Acanthaceae Echinacanthus longipes#NC 039761
Lamiales Acanthaceae Ruellia simplex#AYIY
Lamiales Acanthaceae Sanchezia sp.#NBMW
Lamiales Acanthaceae Echinacanthus attenuatus#NC 039762
Lamiales Acanthaceae Strobilanthes cusia#NC 037485
Lamiales Acanthaceae Strobilanthes auriculatus#WEAC
Lamiales Acanthaceae Strobilanthes henryi#F67 1
Lamiales Acanthaceae Strobilanthes sp.#F42
Lamiales Verbenaceae Verbena hastata#GCFE
Lamiales Verbenaceae Verbena officinalis#F535
Lamiales Verbenaceae Aloysia citriodora#NC 034695
Lamiales Verbenaceae Phyla nodiflora#F528
Lamiales Verbenaceae Lantana camara#PSHB
Lamiales Verbenaceae Phyla scaberrima#MQIV
Lamiales Bignoniaceae Incarvillea diffusa#F173
Lamiales Bignoniaceae Tecoma capensis#NC 037462
Lamiales Bignoniaceae Handroanthus umbellatus#UTQR
Lamiales Bignoniaceae Kigelia africana#XAYK
Lamiales Bignoniaceae Markhamia stipulata#F247
Lamiales Bignoniaceae Pauldopia ghorta#F15
Lamiales Bignoniaceae Mayodendron igneum#F281
Lamiales Bignoniaceae Radermachera yunnanensis#F98
Lamiales Bignoniaceae Dolichandra cynanchoides#NC 037460
Lamiales Bignoniaceae Tanaecium tetragonolobum#NC 027955
Lamiales Bignoniaceae Mansoa alliacea#TKEK
Lamiales Bignoniaceae Anemopaegma oligoneuron#NC 037232
Lamiales Bignoniaceae Anemopaegma album#NC 037227
Lamiales Bignoniaceae Anemopaegma chamberlaynii#NC 037229
Lamiales Bignoniaceae Anemopaegma foetidum#NC 037230
Lamiales Bignoniaceae Anemopaegma arvense#MF460830
Lamiales Bignoniaceae Anemopaegma acutifolium#MF460827
Lamiales Bignoniaceae Anemopaegma glaucum#MF460834
Lamiales Bignoniaceae Adenocalymma pedunculatum#NC 036500
Lamiales Bignoniaceae Adenocalymma cristicalyx#NC 036498
Lamiales Bignoniaceae Pleonotoma albiflora#NC 037461
Lamiales Bignoniaceae Neojobertia candolleana#NC 036503
Lamiales Bignoniaceae Adenocalymma allamandiflorum#NC 036494
Lamiales Bignoniaceae Adenocalymma peregrinum#NC 036501
Lamiales Bignoniaceae Adenocalymma biternatum#NC 036496
Lamiales Bignoniaceae Adenocalymma nodosum#NC 037458
Lamiales Bignoniaceae Adenocalymma marginatum#NC 037457
Lamiales Bignoniaceae Adenocalymma subspicatum#NC 036502
Lamiales Bignoniaceae Adenocalymma divaricatum#NC 037456
Lamiales Bignoniaceae Adenocalymma bracteatum#NC 036497
Lamiales Bignoniaceae Adenocalymma aurantiacum#NC 036495
Lamiales Bignoniaceae Adenocalymma hatschbachii#NC 036499
Lamiales Bignoniaceae Adenocalymma comosum#NC 037455
Lamiales Bignoniaceae Adenocalymma trifoliatum#NC 037459
Lamiales Mazaceae Mazus miquelii#F864
Lamiales Mazaceae Lancea tibetica#F647
Lamiales Mazaceae Lancea hirsuta#NC 037506
Lamiales Wightiaceae Wightia speciosissima#J5854
Lamiales Phrymaceae Phryma leptostachya#F890
Lamiales Phrymaceae Erythranthe lutea#NC 030212
Lamiales Phrymaceae Mimulus tenellus var. platyphyllus#F825
Lamiales Paulowniaceae Paulownia fortunei#F180
Lamiales Paulowniaceae Paulownia fargesii#UMUL
Lamiales Orobanchaceae Triaenophora shennongjiaensis#NC 039781
Lamiales Orobanchaceae Rehmannia chingii#F895
Lamiales Orobanchaceae Rehmannia glutinosa#F1002
Lamiales Orobanchaceae Rehmannia henryi#NC 034309
Lamiales Orobanchaceae Rehmannia solanifolia#NC 034310
Lamiales Orobanchaceae Rehmannia elata#NC 034312
Lamiales Orobanchaceae Rehmannia piasezkii#NC 034311
Lamiales Orobanchaceae Lindenbergia muraria#F84
Lamiales Orobanchaceae Lindenbergia philippensis#EJCM
Lamiales Orobanchaceae Schwalbea americana#NC 023115
Lamiales Orobanchaceae Cymbaria mongolica#F789
Lamiales Orobanchaceae Lathraea squamaria#NC 027838 @para
Lamiales Orobanchaceae Brandisia racemosa#F46
Lamiales Orobanchaceae Pedicularis hallaisanensis#NC 037433 @para
Lamiales Orobanchaceae Pedicularis ishidoyana#NC 029700 @para
Lamiales Orobanchaceae Phtheirospermum japonicum#F35
Lamiales Orobanchaceae Castilleja paramensis#NC 031805
Lamiales Orobanchaceae Conopholis americana#FAMO
Lamiales Orobanchaceae Epifagus virginiana#FROP
Lamiales Orobanchaceae Cistanche deserticola#NC 021111 @para
Lamiales Orobanchaceae Cistanche phelypaea#NC 025642 @para
Lamiales Orobanchaceae Orobanche latisquama#NC 025641 @para
Lamiales Orobanchaceae Orobanche crenata#NC 024845 @para
Lamiales Orobanchaceae Orobanche gracilis#NC 023464 @para
Lamiales Orobanchaceae Orobanche purpurea#NC 023132 @para
Lamiales Orobanchaceae Orobanche ramosa#NC 023465 @para
Lamiales Orobanchaceae Orobanche californica#NC 025651 @para
Lamiales Orobanchaceae Aphyllon epigalium subsp. notocalifornicum#MH050786
Lamiales Orobanchaceae Aphyllon epigalium subsp. epigalium#MH050785
Lamiales Orobanchaceae Orobanche fasciculata#MH499249
Lamiales Orobanchaceae Aphyllon uniflorum var. occidentale#MH499250
Lamiales Orobanchaceae Aphyllon uniflorum#MH580290

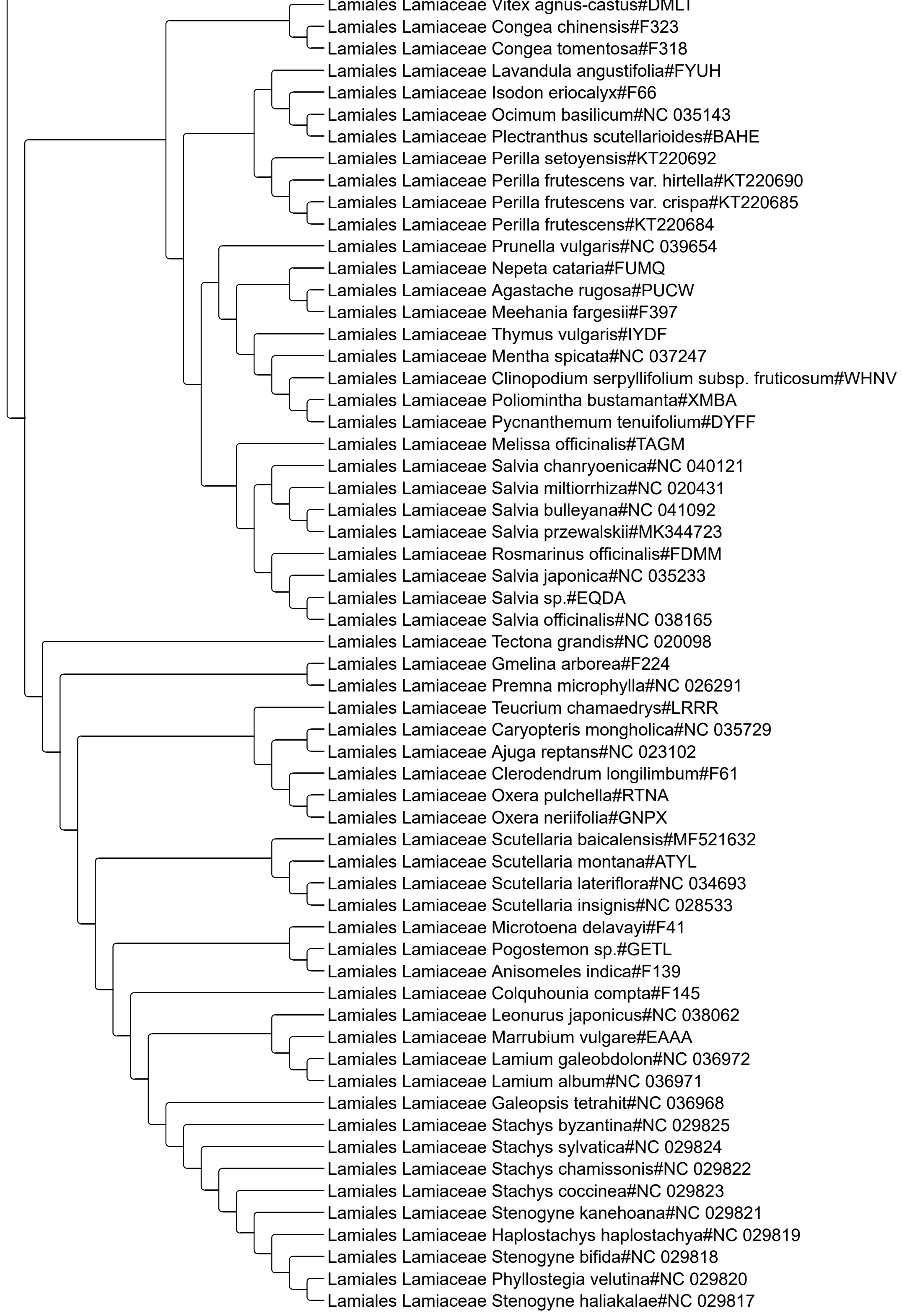

Lamiales Lamiaceae Vitex agnus-castus#DMLT
Lamiales Lamiaceae Congea chinensis#F323
Lamiales Lamiaceae Congea tomentosa#F318
Lamiales Lamiaceae Lavandula angustifolia#FYUH
Lamiales Lamiaceae Isodon eriocalyx#F66
Lamiales Lamiaceae Ocimum basilicum#NC 035143
Lamiales Lamiaceae Plectranthus scutellarioides#BAHE
Lamiales Lamiaceae Perilla setoyensis#KT220692
Lamiales Lamiaceae Perilla frutescens var. hirtella#KT220690
Lamiales Lamiaceae Perilla frutescens var. crispa#KT220685
Lamiales Lamiaceae Perilla frutescens#KT220684
Lamiales Lamiaceae Prunella vulgaris#NC 039654
Lamiales Lamiaceae Nepeta cataria#FUMQ
Lamiales Lamiaceae Agastache rugosa#PUCW
Lamiales Lamiaceae Meehania fargesii#F397
Lamiales Lamiaceae Thymus vulgaris#IYDF
Lamiales Lamiaceae Mentha spicata#NC 037247
Lamiales Lamiaceae Clinopodium serpyllifolium subsp. fruticosum#WHNV
Lamiales Lamiaceae Poliomintha bustamanta#XMBA
Lamiales Lamiaceae Pycnanthemum tenuifolium#DYFF
Lamiales Lamiaceae Melissa officinalis#TAGM
Lamiales Lamiaceae Salvia chanryoenica#NC 040121
Lamiales Lamiaceae Salvia miltiorrhiza#NC 020431
Lamiales Lamiaceae Salvia bulleyana#NC 041092
Lamiales Lamiaceae Salvia przewalskii#MK344723
Lamiales Lamiaceae Rosmarinus officinalis#FDMM
Lamiales Lamiaceae Salvia japonica#NC 035233
Lamiales Lamiaceae Salvia sp.#EQDA
Lamiales Lamiaceae Salvia officinalis#NC 038165
Lamiales Lamiaceae Tectona grandis#NC 020098
Lamiales Lamiaceae Gmelina arborea#F224
Lamiales Lamiaceae Premna microphylla#NC 026291
Lamiales Lamiaceae Teucrium chamaedrys#LRRR
Lamiales Lamiaceae Caryopteris mongholica#NC 035729
Lamiales Lamiaceae Ajuga reptans#NC 023102
Lamiales Lamiaceae Clerodendrum longilimbum#F61
Lamiales Lamiaceae Oxera pulchella#RTNA
Lamiales Lamiaceae Oxera neriifolia#GNPX
Lamiales Lamiaceae Scutellaria baicalensis#MF521632
Lamiales Lamiaceae Scutellaria montana#ATYL
Lamiales Lamiaceae Scutellaria lateriflora#NC 034693
Lamiales Lamiaceae Scutellaria insignis#NC 028533
Lamiales Lamiaceae Microtoena delavayi#F41
Lamiales Lamiaceae Pogostemon sp.#GETL
Lamiales Lamiaceae Anisomeles indica#F139
Lamiales Lamiaceae Colquhounia compta#F145
Lamiales Lamiaceae Leonurus japonicus#NC 038062
Lamiales Lamiaceae Marrubium vulgare#EAAA
Lamiales Lamiaceae Lamium galeobdolon#NC 036972
Lamiales Lamiaceae Lamium album#NC 036971
Lamiales Lamiaceae Galeopsis tetrahit#NC 036968
Lamiales Lamiaceae Stachys byzantina#NC 029825
Lamiales Lamiaceae Stachys sylvatica#NC 029824
Lamiales Lamiaceae Stachys chamissonis#NC 029822
Lamiales Lamiaceae Stachys coccinea#NC 029823
Lamiales Lamiaceae Stenogyne kanehoana#NC 029821
Lamiales Lamiaceae Haplostachys haplostachya#NC 029819
Lamiales Lamiaceae Stenogyne bifida#NC 029818
Lamiales Lamiaceae Phyllostegia velutina#NC 029820
Lamiales Lamiaceae Stenogyne haliakalae#NC 029817

[Figure S8e]

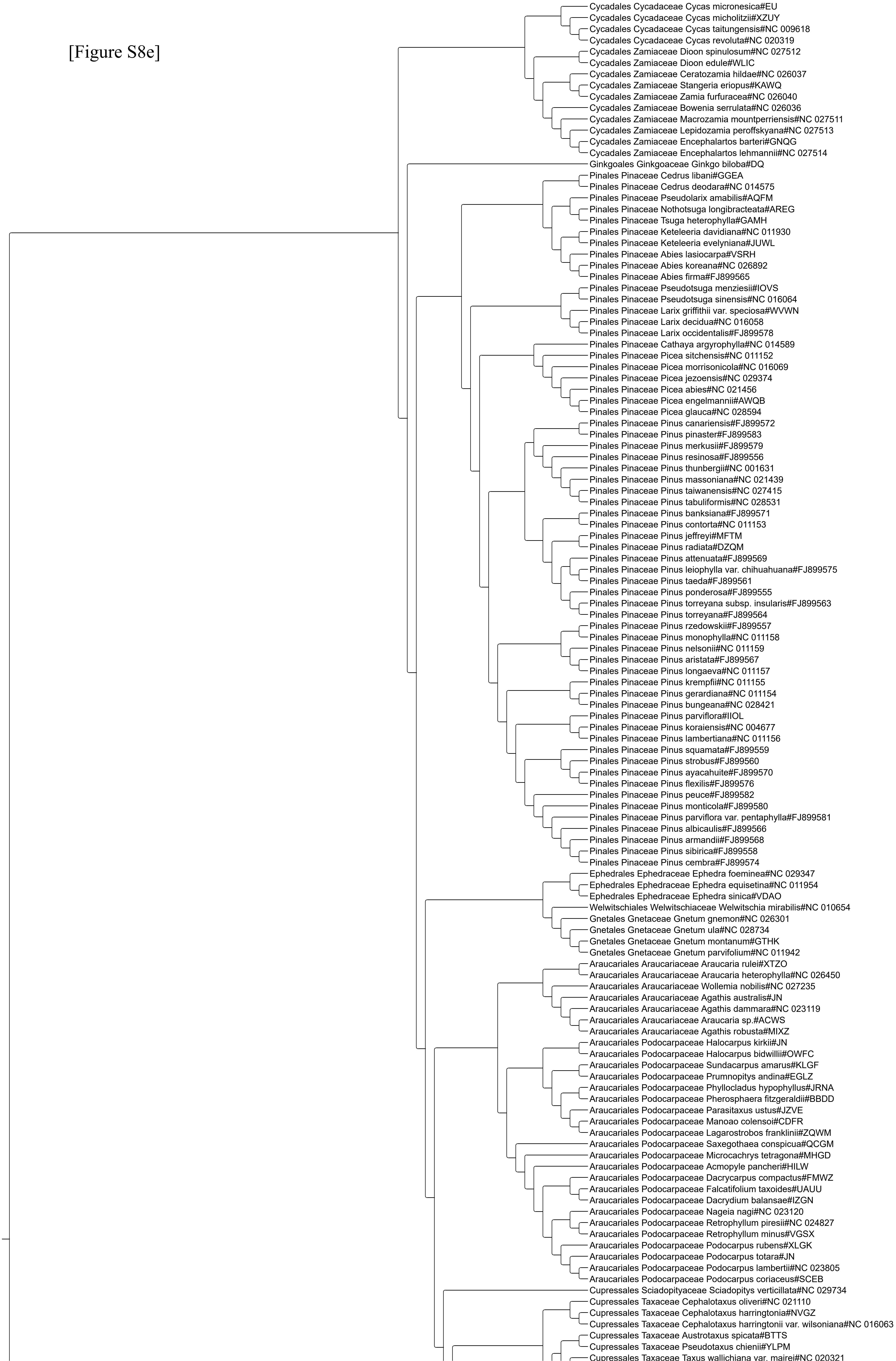

Cycadales Cycadaceae Cycas micronesica#EU
Cycadales Cycadaceae Cycas micholitzii#XZUY
Cycadales Cycadaceae Cycas taitungensis#NC 009618
Cycadales Cycadaceae Cycas revoluta#NC 020319
Cycadales Zamiaceae Dioon spinulosum#NC 027512
Cycadales Zamiaceae Dioon edule#WLIC
Cycadales Zamiaceae Ceratozamia hildae#NC 026037
Cycadales Zamiaceae Stangeria eriopus#KAWQ
Cycadales Zamiaceae Zamia furfuracea#NC 026040
Cycadales Zamiaceae Bowenia serrulata#NC 026036
Cycadales Zamiaceae Macrozamia mountperriensis#NC 027511
Cycadales Zamiaceae Lepidozamia peroffskyana#NC 027513
Cycadales Zamiaceae Encephalartos barteri#GNQG
Cycadales Zamiaceae Encephalartos lehmannii#NC 027514
Ginkgoales Ginkgoaceae Ginkgo biloba#DQ
Pinales Pinaceae Cedrus libani#GGEA
Pinales Pinaceae Cedrus deodara#NC 014575
Pinales Pinaceae Pseudolarix amabilis#AQFM
Pinales Pinaceae Nothotsuga longibracteata#AREG
Pinales Pinaceae Tsuga heterophylla#GAMH
Pinales Pinaceae Keteleeria davidiana#NC 011930
Pinales Pinaceae Keteleeria evelyniana#JUWL
Pinales Pinaceae Abies lasiocarpa#VSRH
Pinales Pinaceae Abies koreana#NC 026892
Pinales Pinaceae Abies firma#FJ899565
Pinales Pinaceae Pseudotsuga menziesii#IOVS
Pinales Pinaceae Pseudotsuga sinensis#NC 016064
Pinales Pinaceae Larix griffithii var. speciosa#WVWN
Pinales Pinaceae Larix decidua#NC 016058
Pinales Pinaceae Larix occidentalis#FJ899578
Pinales Pinaceae Cathaya argyrophylla#NC 014589
Pinales Pinaceae Picea sitchensis#NC 011152
Pinales Pinaceae Picea morrisonicola#NC 016069
Pinales Pinaceae Picea jezoensis#NC 029374
Pinales Pinaceae Picea abies#NC 021456
Pinales Pinaceae Picea engelmannii#AWQB
Pinales Pinaceae Picea glauca#NC 028594
Pinales Pinaceae Pinus canariensis#FJ899572
Pinales Pinaceae Pinus pinaster#FJ899583
Pinales Pinaceae Pinus merkusii#FJ899579
Pinales Pinaceae Pinus resinosa#FJ899556
Pinales Pinaceae Pinus thunbergii#NC 001631
Pinales Pinaceae Pinus massoniana#NC 021439
Pinales Pinaceae Pinus taiwanensis#NC 027415
Pinales Pinaceae Pinus tabuliformis#NC 028531
Pinales Pinaceae Pinus banksiana#FJ899571
Pinales Pinaceae Pinus contorta#NC 011153
Pinales Pinaceae Pinus jeffreyi#MFTM
Pinales Pinaceae Pinus radiata#DZQM
Pinales Pinaceae Pinus attenuata#FJ899569
Pinales Pinaceae Pinus leiophylla var. chihuahuana#FJ899575
Pinales Pinaceae Pinus taeda#FJ899561
Pinales Pinaceae Pinus ponderosa#FJ899555
Pinales Pinaceae Pinus torreyana subsp. insularis#FJ899563
Pinales Pinaceae Pinus torreyana#FJ899564
Pinales Pinaceae Pinus rzedowskii#FJ899557
Pinales Pinaceae Pinus monophylla#NC 011158
Pinales Pinaceae Pinus nelsonii#NC 011159
Pinales Pinaceae Pinus aristata#FJ899567
Pinales Pinaceae Pinus longaeva#NC 011157
Pinales Pinaceae Pinus krempfii#NC 011155
Pinales Pinaceae Pinus gerardiana#NC 011154
Pinales Pinaceae Pinus bungeana#NC 028421
Pinales Pinaceae Pinus parviflora#IIOL
Pinales Pinaceae Pinus koraiensis#NC 004677
Pinales Pinaceae Pinus lambertiana#NC 011156
Pinales Pinaceae Pinus squamata#FJ899559
Pinales Pinaceae Pinus strobus#FJ899560
Pinales Pinaceae Pinus ayacahuite#FJ899570
Pinales Pinaceae Pinus flexilis#FJ899576
Pinales Pinaceae Pinus peuce#FJ899582
Pinales Pinaceae Pinus monticola#FJ899580
Pinales Pinaceae Pinus parviflora var. pentaphylla#FJ899581
Pinales Pinaceae Pinus albicaulis#FJ899566
Pinales Pinaceae Pinus armandii#FJ899568
Pinales Pinaceae Pinus sibirica#FJ899558
Pinales Pinaceae Pinus cembra#FJ899574
Ephedrales Ephedraceae Ephedra foeminea#NC 029347
Ephedrales Ephedraceae Ephedra equisetina#NC 011954
Ephedrales Ephedraceae Ephedra sinica#VDAO
Welwitschiales Welwitschiaceae Welwitschia mirabilis#NC 010654
Gnetales Gnetaceae Gnetum gnemon#NC 026301
Gnetales Gnetaceae Gnetum ula#NC 028734
Gnetales Gnetaceae Gnetum montanum#GTHK
Gnetales Gnetaceae Gnetum parvifolium#NC 011942
Araucariales Araucariaceae Araucaria rulei#XTZO
Araucariales Araucariaceae Araucaria heterophylla#NC 026450
Araucariales Araucariaceae Wollemia nobilis#NC 027235
Araucariales Araucariaceae Agathis australis#JN
Araucariales Araucariaceae Agathis dammara#NC 023119
Araucariales Araucariaceae Araucaria sp.#ACWS
Araucariales Araucariaceae Agathis robusta#MIXZ
Araucariales Podocarpaceae Halocarpus kirkii#JN
Araucariales Podocarpaceae Halocarpus bidwillii#OWFC
Araucariales Podocarpaceae Sundacarpus amarus#KLGF
Araucariales Podocarpaceae Prumnopitys andina#EGLZ
Araucariales Podocarpaceae Phyllocladus hypophyllus#JRNA
Araucariales Podocarpaceae Pherosphaera fitzgeraldii#BBDD
Araucariales Podocarpaceae Parasitaxus ustus#JZVE
Araucariales Podocarpaceae Manoao colensoi#CDFR
Araucariales Podocarpaceae Lagarostrobos franklinii#ZQWM
Araucariales Podocarpaceae Saxegothaea conspicua#QCGM
Araucariales Podocarpaceae Microcachrys tetragona#MHGD
Araucariales Podocarpaceae Acmopyle pancheri#HILW
Araucariales Podocarpaceae Dacrycarpus compactus#FMWZ
Araucariales Podocarpaceae Falcatifolium taxoides#UAUU
Araucariales Podocarpaceae Dacrydium balansae#IZGN
Araucariales Podocarpaceae Nageia nagi#NC 023120
Araucariales Podocarpaceae Retrophyllum piresii#NC 024827
Araucariales Podocarpaceae Retrophyllum minus#VGSX
Araucariales Podocarpaceae Podocarpus rubens#XLGK
Araucariales Podocarpaceae Podocarpus totara#JN
Araucariales Podocarpaceae Podocarpus lambertii#NC 023805
Araucariales Podocarpaceae Podocarpus coriaceus#SCEB
Cupressales Sciadopityaceae Sciadopitys verticillata#NC 029734
Cupressales Taxaceae Cephalotaxus oliveri#NC 021110
Cupressales Taxaceae Cephalotaxus harringtonia#NVGZ
Cupressales Taxaceae Cephalotaxus harringtonii var. wilsoniana#NC 016063
Cupressales Taxaceae Austrotaxus spicata#BTTS
Cupressales Taxaceae Pseudotaxus chienii#YLPM
Cupressales Taxaceae Taxus wallichiana var. mairei#NC 020321

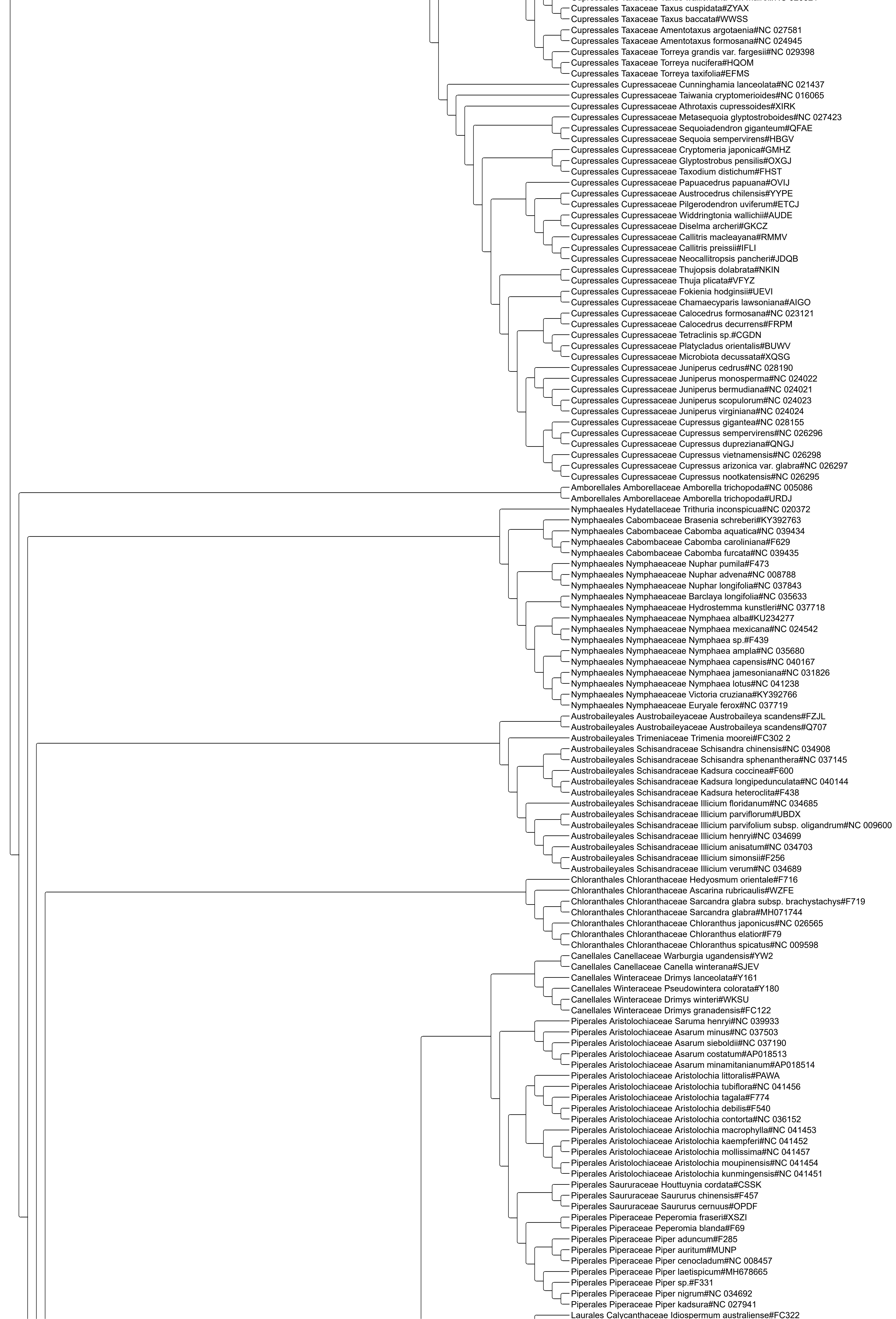
Cupressales Taxaceae Taxus cuspidata#ZYAX
Cupressales Taxaceae Taxus baccata#WWSS
Cupressales Taxaceae Amentotaxus argotaenia#NC 027581
Cupressales Taxaceae Amentotaxus formosana#NC 024945
Cupressales Taxaceae Torreya grandis var. fargesii#NC 029398
Cupressales Taxaceae Torreya nucifera#HQOM
Cupressales Taxaceae Torreya taxifolia#EFMS
Cupressales Cupressaceae Cunninghamia lanceolata#NC 021437
Cupressales Cupressaceae Taiwania cryptomerioides#NC 016065
Cupressales Cupressaceae Athrotaxis cupressoides#XIRK
Cupressales Cupressaceae Metasequoia glyptostroboides#NC 027423
Cupressales Cupressaceae Sequoiadendron giganteum#QFAE
Cupressales Cupressaceae Sequoia sempervirens#HBGV
Cupressales Cupressaceae Cryptomeria japonica#GMHZ
Cupressales Cupressaceae Glyptostrobus pensilis#OXGJ
Cupressales Cupressaceae Taxodium distichum#FHST
Cupressales Cupressaceae Papuacedrus papuana#OVIJ
Cupressales Cupressaceae Austrocedrus chilensis#YYPE
Cupressales Cupressaceae Pilgerodendron uviferum#ETCJ
Cupressales Cupressaceae Widdringtonia wallichii#AUDE
Cupressales Cupressaceae Diselma archeri#GKCZ
Cupressales Cupressaceae Callitris macleayana#RMMV
Cupressales Cupressaceae Callitris preissii#IFLI
Cupressales Cupressaceae Neocallitropsis pancheri#JDQB
Cupressales Cupressaceae Thujopsis dolabrata#NKIN
Cupressales Cupressaceae Thuja plicata#VFYZ
Cupressales Cupressaceae Fokienia hodginsii#UEVI
Cupressales Cupressaceae Chamaecyparis lawsoniana#AIGO
Cupressales Cupressaceae Calocedrus formosana#NC 023121
Cupressales Cupressaceae Calocedrus decurrens#FRPM
Cupressales Cupressaceae Tetraclinis sp.#CGDN
Cupressales Cupressaceae Platycladus orientalis#BUWV
Cupressales Cupressaceae Microbiota decussata#XQSG
Cupressales Cupressaceae Juniperus cedrus#NC 028190
Cupressales Cupressaceae Juniperus monosperma#NC 024022
Cupressales Cupressaceae Juniperus bermudiana#NC 024021
Cupressales Cupressaceae Juniperus scopulorum#NC 024023
Cupressales Cupressaceae Juniperus virginiana#NC 024024
Cupressales Cupressaceae Cupressus gigantea#NC 028155
Cupressales Cupressaceae Cupressus sempervirens#NC 026296
Cupressales Cupressaceae Cupressus dupreziana#QNGJ
Cupressales Cupressaceae Cupressus vietnamensis#NC 026298
Cupressales Cupressaceae Cupressus arizonica var. glabra#NC 026297
Cupressales Cupressaceae Cupressus nootkatensis#NC 026295
Amborellales Amborellaceae Amborella trichopoda#NC 005086
Amborellales Amborellaceae Amborella trichopoda#URDJ
Nymphaeales Hydatellaceae Trithuria inconspicua#NC 020372
Nymphaeales Cabombaceae Brasenia schreberi#KY392763
Nymphaeales Cabombaceae Cabomba aquatica#NC 039434
Nymphaeales Cabombaceae Cabomba caroliniana#F629
Nymphaeales Cabombaceae Cabomba furcata#NC 039435
Nymphaeales Nymphaeaceae Nuphar pumila#F473
Nymphaeales Nymphaeaceae Nuphar advena#NC 008788
Nymphaeales Nymphaeaceae Nuphar longifolia#NC 037843
Nymphaeales Nymphaeaceae Barclaya longifolia#NC 035633
Nymphaeales Nymphaeaceae Hydrostemma kunstleri#NC 037718
Nymphaeales Nymphaeaceae Nymphaea alba#KU234277
Nymphaeales Nymphaeaceae Nymphaea mexicana#NC 024542
Nymphaeales Nymphaeaceae Nymphaea sp.#F439
Nymphaeales Nymphaeaceae Nymphaea ampla#NC 035680
Nymphaeales Nymphaeaceae Nymphaea capensis#NC 040167
Nymphaeales Nymphaeaceae Nymphaea jamesoniana#NC 031826
Nymphaeales Nymphaeaceae Nymphaea lotus#NC 041238
Nymphaeales Nymphaeaceae Victoria cruziana#KY392766
Nymphaeales Nymphaeaceae Euryale ferox#NC 037719
Austrobaileyales Austrobaileyaceae Austrobaileya scandens#FZJL
Austrobaileyales Austrobaileyaceae Austrobaileya scandens#Q707
Austrobaileyales Trimeniaceae Trimenia moorei#FC302 2
Austrobaileyales Schisandraceae Schisandra chinensis#NC 034908
Austrobaileyales Schisandraceae Schisandra sphenanthera#NC 037145
Austrobaileyales Schisandraceae Kadsura coccinea#F600
Austrobaileyales Schisandraceae Kadsura longipedunculata#NC 040144
Austrobaileyales Schisandraceae Kadsura heteroclita#F438
Austrobaileyales Schisandraceae Illicium floridanum#NC 034685
Austrobaileyales Schisandraceae Illicium parviflorum#UBDX
Austrobaileyales Schisandraceae Illicium parvifolium subsp. oligandrum#NC 009600
Austrobaileyales Schisandraceae Illicium henryi#NC 034699
Austrobaileyales Schisandraceae Illicium anisatum#NC 034703
Austrobaileyales Schisandraceae Illicium simonsii#F256
Austrobaileyales Schisandraceae Illicium verum#NC 034689
Chloranthales Chloranthaceae Hedyosmum orientale#F716
Chloranthales Chloranthaceae Ascarina rubricaulis#WZFE
Chloranthales Chloranthaceae Sarcandra glabra subsp. brachystachys#F719
Chloranthales Chloranthaceae Sarcandra glabra#MH071744
Chloranthales Chloranthaceae Chloranthus japonicus#NC 026565
Chloranthales Chloranthaceae Chloranthus elatior#F79
Chloranthales Chloranthaceae Chloranthus spicatus#NC 009598
Canellales Canellaceae Warburgia ugandensis#YW2
Canellales Canellaceae Canella winterana#SJEV
Canellales Winteraceae Drimys lanceolata#Y161
Canellales Winteraceae Pseudowintera colorata#Y180
Canellales Winteraceae Drimys winteri#WKSU
Canellales Winteraceae Drimys granadensis#FC122
Piperales Aristolochiaceae Saruma henryi#NC 039933
Piperales Aristolochiaceae Asarum minus#NC 037503
Piperales Aristolochiaceae Asarum sieboldii#NC 037190
Piperales Aristolochiaceae Asarum costatum#AP018513
Piperales Aristolochiaceae Asarum minamitanianum#AP018514
Piperales Aristolochiaceae Aristolochia littoralis#PAWA
Piperales Aristolochiaceae Aristolochia tubiflora#NC 041456
Piperales Aristolochiaceae Aristolochia tagala#F774
Piperales Aristolochiaceae Aristolochia debilis#F540
Piperales Aristolochiaceae Aristolochia contorta#NC 036152
Piperales Aristolochiaceae Aristolochia macrophylla#NC 041453
Piperales Aristolochiaceae Aristolochia kaempferi#NC 041452
Piperales Aristolochiaceae Aristolochia mollissima#NC 041457
Piperales Aristolochiaceae Aristolochia moupinensis#NC 041454
Piperales Aristolochiaceae Aristolochia kunmingensis#NC 041451
Piperales Saururaceae Houttuynia cordata#CSSK
Piperales Saururaceae Saururus chinensis#F457
Piperales Saururaceae Saururus cernuus#OPDF
Piperales Piperaceae Peperomia fraseri#XSZI
Piperales Piperaceae Peperomia blanda#F69
Piperales Piperaceae Piper aduncum#F285
Piperales Piperaceae Piper auritum#MUNP
Piperales Piperaceae Piper cenocladum#NC 008457
Piperales Piperaceae Piper laetispicum#MH678665
Piperales Piperaceae Piper sp.#F331
Piperales Piperaceae Piper nigrum#NC 034692
Piperales Piperaceae Piper kadsura#NC 027941
Laurales Calycanthaceae Idiospermum australiense#FC322

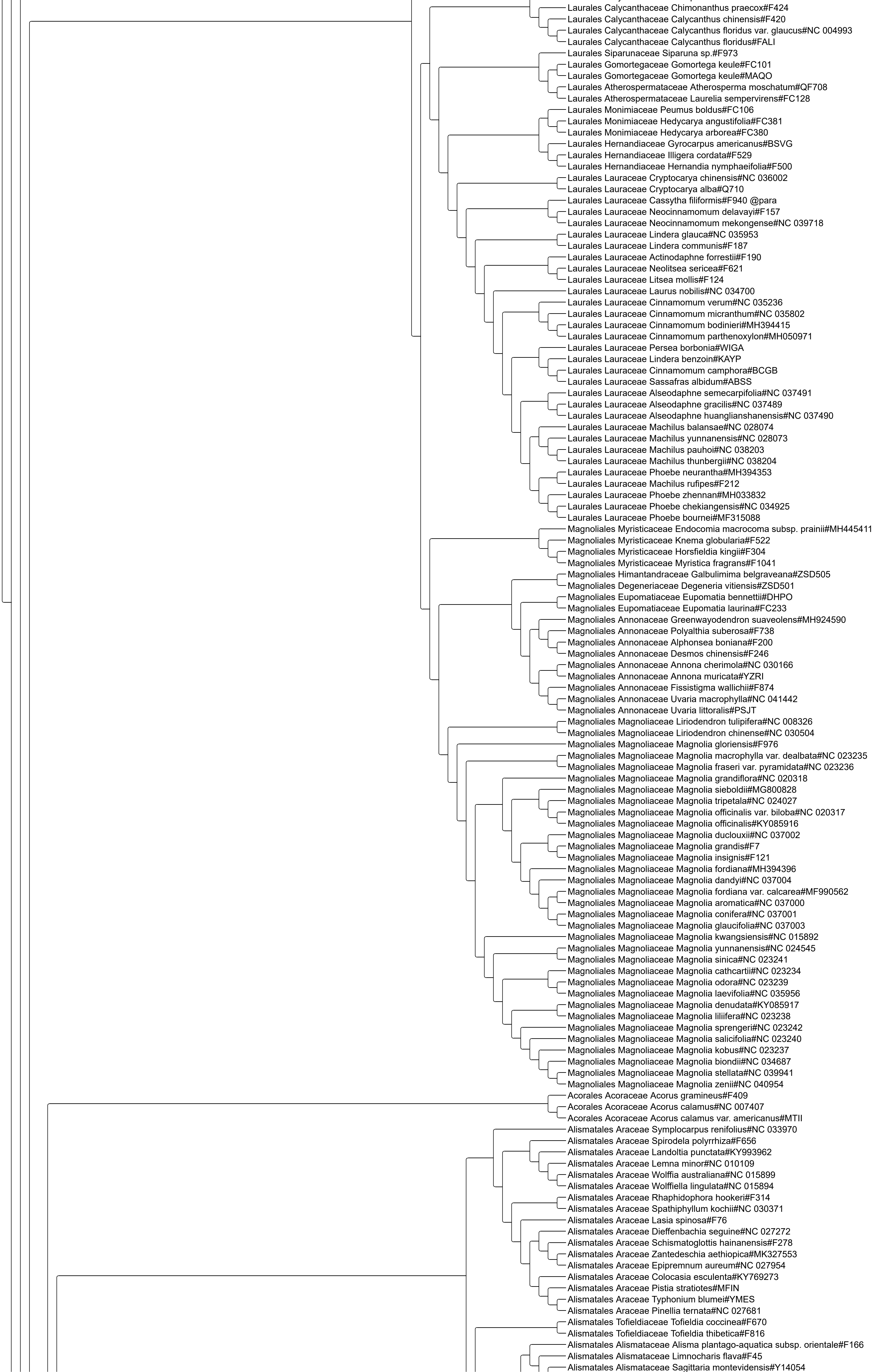
Laurales Calycanthaceae Chimonanthus praecox#F424
Laurales Calycanthaceae Calycanthus chinensis#F420
Laurales Calycanthaceae Calycanthus floridus var. glaucus#NC 004993
Laurales Calycanthaceae Calycanthus floridus#FALI
Laurales Siparunaceae Siparuna sp.#F973
Laurales Gomortegaceae Gomortega keule#FC101
Laurales Gomortegaceae Gomortega keule#MAQO
Laurales Atherospermataceae Atherosperma moschatum#QF708
Laurales Atherospermataceae Laurelia sempervirens#FC128
Laurales Monimiaceae Peumus boldus#FC106
Laurales Monimiaceae Hedycarya angustifolia#FC381
Laurales Monimiaceae Hedycarya arborea#FC380
Laurales Hernandiaceae Gyrocarpus americanus#BSVG
Laurales Hernandiaceae Illigera cordata#F529
Laurales Hernandiaceae Hernandia nymphaeifolia#F500
Laurales Lauraceae Cryptocarya chinensis#NC 036002
Laurales Lauraceae Cryptocarya alba#Q710
Laurales Lauraceae Cassytha filiformis#F940 @para
Laurales Lauraceae Neocinnamomum delavayi#F157
Laurales Lauraceae Neocinnamomum mekongense#NC 039718
Laurales Lauraceae Lindera glauca#NC 035953
Laurales Lauraceae Lindera communis#F187
Laurales Lauraceae Actinodaphne forrestii#F190
Laurales Lauraceae Neolitsea sericea#F621
Laurales Lauraceae Litsea mollis#F124
Laurales Lauraceae Laurus nobilis#NC 034700
Laurales Lauraceae Cinnamomum verum#NC 035236
Laurales Lauraceae Cinnamomum micranthum#NC 035802
Laurales Lauraceae Cinnamomum bodinieri#MH394415
Laurales Lauraceae Cinnamomum parthenoxylon#MH050971
Laurales Lauraceae Persea borbonia#WIGA
Laurales Lauraceae Lindera benzoin#KAYP
Laurales Lauraceae Cinnamomum camphora#BCGB
Laurales Lauraceae Sassafras albidum#ABSS
Laurales Lauraceae Alseodaphne semecarpifolia#NC 037491
Laurales Lauraceae Alseodaphne gracilis#NC 037489
Laurales Lauraceae Alseodaphne huanglianshanensis#NC 037490
Laurales Lauraceae Machilus balansae#NC 028074
Laurales Lauraceae Machilus yunnanensis#NC 028073
Laurales Lauraceae Machilus pauhoi#NC 038203
Laurales Lauraceae Machilus thunbergii#NC 038204
Laurales Lauraceae Phoebe neurantha#MH394353
Laurales Lauraceae Machilus rufipes#F212
Laurales Lauraceae Phoebe zhennan#MH033832
Laurales Lauraceae Phoebe chekiangensis#NC 034925
Laurales Lauraceae Phoebe bournei#MF315088
Magnoliales Myristicaceae Endocomia macrocoma subsp. prainii#MH445411
Magnoliales Myristicaceae Knema globularia#F522
Magnoliales Myristicaceae Horsfieldia kingii#F304
Magnoliales Myristicaceae Myristica fragrans#F1041
Magnoliales Himantandraceae Galbulimima belgraveana#ZSD505
Magnoliales Degeneriaceae Degeneria vitiensis#ZSD501
Magnoliales Eupomatiaceae Eupomatia bennettii#DHPO
Magnoliales Eupomatiaceae Eupomatia laurina#FC233
Magnoliales Annonaceae Greenwayodendron suaveolens#MH924590
Magnoliales Annonaceae Polyalthia suberosa#F738
Magnoliales Annonaceae Alphonsea boniana#F200
Magnoliales Annonaceae Desmos chinensis#F246
Magnoliales Annonaceae Annona cherimola#NC 030166
Magnoliales Annonaceae Annona muricata#YZRI
Magnoliales Annonaceae Fissistigma wallichii#F874
Magnoliales Annonaceae Uvaria macrophylla#NC 041442
Magnoliales Annonaceae Uvaria littoralis#PSJT
Magnoliales Magnoliaceae Liriodendron tulipifera#NC 008326
Magnoliales Magnoliaceae Liriodendron chinense#NC 030504
Magnoliales Magnoliaceae Magnolia gloriensis#F976
Magnoliales Magnoliaceae Magnolia macrophylla var. dealbata#NC 023235
Magnoliales Magnoliaceae Magnolia fraseri var. pyramidata#NC 023236
Magnoliales Magnoliaceae Magnolia grandiflora#NC 020318
Magnoliales Magnoliaceae Magnolia sieboldii#MG800828
Magnoliales Magnoliaceae Magnolia tripetala#NC 024027
Magnoliales Magnoliaceae Magnolia officinalis var. biloba#NC 020317
Magnoliales Magnoliaceae Magnolia officinalis#KY085916
Magnoliales Magnoliaceae Magnolia duclouxii#NC 037002
Magnoliales Magnoliaceae Magnolia grandis#F7
Magnoliales Magnoliaceae Magnolia insignis#F121
Magnoliales Magnoliaceae Magnolia fordiana#MH394396
Magnoliales Magnoliaceae Magnolia dandyi#NC 037004
Magnoliales Magnoliaceae Magnolia fordiana var. calcarea#MF990562
Magnoliales Magnoliaceae Magnolia aromatica#NC 037000
Magnoliales Magnoliaceae Magnolia conifera#NC 037001
Magnoliales Magnoliaceae Magnolia glaucifolia#NC 037003
Magnoliales Magnoliaceae Magnolia kwangsiensis#NC 015892
Magnoliales Magnoliaceae Magnolia yunnanensis#NC 024545
Magnoliales Magnoliaceae Magnolia sinica#NC 023241
Magnoliales Magnoliaceae Magnolia cathcartii#NC 023234
Magnoliales Magnoliaceae Magnolia odora#NC 023239
Magnoliales Magnoliaceae Magnolia laevifolia#NC 035956
Magnoliales Magnoliaceae Magnolia denudata#KY085917
Magnoliales Magnoliaceae Magnolia liliifera#NC 023238
Magnoliales Magnoliaceae Magnolia sprengeri#NC 023242
Magnoliales Magnoliaceae Magnolia salicifolia#NC 023240
Magnoliales Magnoliaceae Magnolia kobus#NC 023237
Magnoliales Magnoliaceae Magnolia biondii#NC 034687
Magnoliales Magnoliaceae Magnolia stellata#NC 039941
Magnoliales Magnoliaceae Magnolia zenii#NC 040954
Acorales Acoraceae Acorus gramineus#F409
Acorales Acoraceae Acorus calamus#NC 007407
Acorales Acoraceae Acorus calamus var. americanus#MTII
Alismatales Araceae Symplocarpus renifolius#NC 033970
Alismatales Araceae Spirodela polyrrhiza#F656
Alismatales Araceae Landoltia punctata#KY993962
Alismatales Araceae Lemna minor#NC 010109
Alismatales Araceae Wolffia australiana#NC 015899
Alismatales Araceae Wolffiella lingulata#NC 015894
Alismatales Araceae Rhaphidophora hookeri#F314
Alismatales Araceae Spathiphyllum kochii#NC 030371
Alismatales Araceae Lasia spinosa#F76
Alismatales Araceae Dieffenbachia seguine#NC 027272
Alismatales Araceae Schismatoglottis hainanensis#F278
Alismatales Araceae Zantedeschia aethiopica#MK327553
Alismatales Araceae Epipremnum aureum#NC 027954
Alismatales Araceae Colocasia esculenta#KY769273
Alismatales Araceae Pistia stratiotes#MFIN
Alismatales Araceae Typhonium blumei#YMES
Alismatales Araceae Pinellia ternata#NC 027681
Alismatales Tofieldiaceae Tofieldia coccinea#F670
Alismatales Tofieldiaceae Tofieldia thibetica#F816
Alismatales Alismataceae Alisma plantago-aquatica subsp. orientale#F166
Alismatales Alismataceae Limnocharis flava#F45
Alismatales Alismataceae Sagittaria montevidensis#Y14054

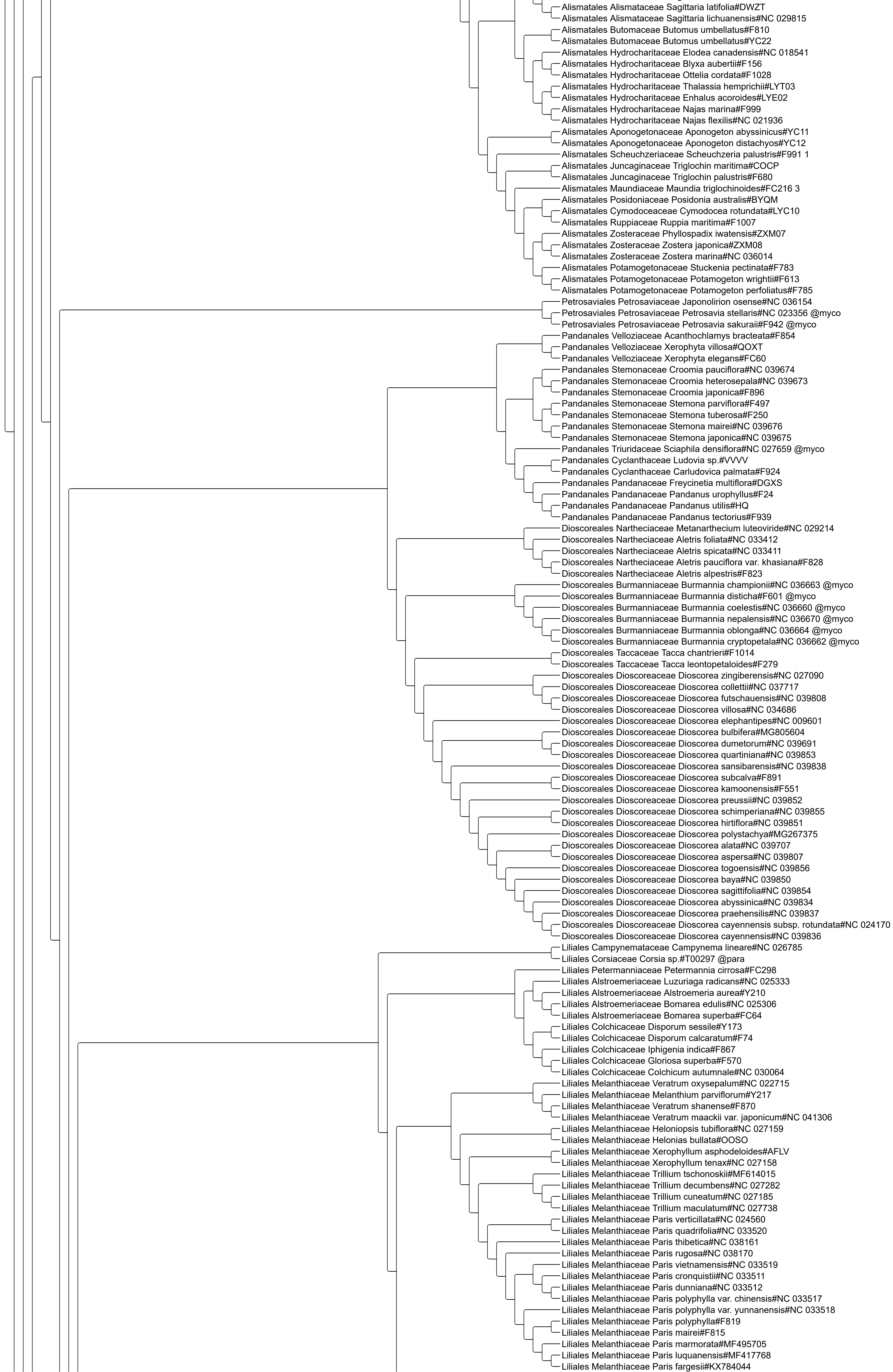
Alismatales Alismataceae Sagittaria latifolia#DWZT
Alismatales Alismataceae Sagittaria lichuanensis#NC 029815
Alismatales Butomaceae Butomus umbellatus#F810
Alismatales Butomaceae Butomus umbellatus#YC22
Alismatales Hydrocharitaceae Elodea canadensis#NC 018541
Alismatales Hydrocharitaceae Blyxa aubertii#F156
Alismatales Hydrocharitaceae Ottelia cordata#F1028
Alismatales Hydrocharitaceae Thalassia hemprichii#LYT03
Alismatales Hydrocharitaceae Enhalus acoroides#LYE02
Alismatales Hydrocharitaceae Najas marina#F999
Alismatales Hydrocharitaceae Najas flexilis#NC 021936
Alismatales Aponogetonaceae Aponogeton abyssinicus#YC11
Alismatales Aponogetonaceae Aponogeton distachyos#YC12
Alismatales Scheuchzeriaceae Scheuchzeria palustris#F991 1
Alismatales Juncaginaceae Triglochin maritima#COCP
Alismatales Juncaginaceae Triglochin palustris#F680
Alismatales Maundiaceae Maundia triglochinoides#FC216 3
Alismatales Posidoniaceae Posidonia australis#BYQM
Alismatales Cymodoceaceae Cymodocea rotundata#LYC10
Alismatales Ruppiaceae Ruppia maritima#F1007
Alismatales Zosteraceae Phyllospadix iwatensis#ZXM07
Alismatales Zosteraceae Zostera japonica#ZXM08
Alismatales Zosteraceae Zostera marina#NC 036014
Alismatales Potamogetonaceae Stuckenia pectinata#F783
Alismatales Potamogetonaceae Potamogeton wrightii#F613
Alismatales Potamogetonaceae Potamogeton perfoliatus#F785
Petrosaviales Petrosaviaceae Japonolirion osense#NC 036154
Petrosaviales Petrosaviaceae Petrosavia stellaris#NC 023356 @myco
Petrosaviales Petrosaviaceae Petrosavia sakuraii#F942 @myco
Pandanales Velloziaceae Acanthochlamys bracteata#F854
Pandanales Velloziaceae Xerophyta villosa#QOXT
Pandanales Velloziaceae Xerophyta elegans#FC60
Pandanales Stemonaceae Croomia pauciflora#NC 039674
Pandanales Stemonaceae Croomia heterosepala#NC 039673
Pandanales Stemonaceae Croomia japonica#F896
Pandanales Stemonaceae Stemona parviflora#F497
Pandanales Stemonaceae Stemona tuberosa#F250
Pandanales Stemonaceae Stemona mairei#NC 039676
Pandanales Stemonaceae Stemona japonica#NC 039675
Pandanales Triuridaceae Sciaphila densiflora#NC 027659 @myco
Pandanales Cyclanthaceae Ludovia sp.#VVVV
Pandanales Cyclanthaceae Carludovica palmata#F924
Pandanales Pandanaceae Freycinetia multiflora#DGXS
Pandanales Pandanaceae Pandanus urophyllus#F24
Pandanales Pandanaceae Pandanus utilis#HQ
Pandanales Pandanaceae Pandanus tectorius#F939
Dioscoreales Nartheciaceae Metanarthecium luteoviride#NC 029214
Dioscoreales Nartheciaceae Aletris foliata#NC 033412
Dioscoreales Nartheciaceae Aletris spicata#NC 033411
Dioscoreales Nartheciaceae Aletris pauciflora var. khasiana#F828
Dioscoreales Nartheciaceae Aletris alpestris#F823
Dioscoreales Burmanniaceae Burmannia championii#NC 036663 @myco
Dioscoreales Burmanniaceae Burmannia disticha#F601 @myco
Dioscoreales Burmanniaceae Burmannia coelestis#NC 036660 @myco
Dioscoreales Burmanniaceae Burmannia nepalensis#NC 036670 @myco
Dioscoreales Burmanniaceae Burmannia oblonga#NC 036664 @myco
Dioscoreales Burmanniaceae Burmannia cryptopetala#NC 036662 @myco
Dioscoreales Taccaceae Tacca chantrieri#F1014
Dioscoreales Taccaceae Tacca leontopetaloides#F279
Dioscoreales Dioscoreaceae Dioscorea zingiberensis#NC 027090
Dioscoreales Dioscoreaceae Dioscorea collettii#NC 037717
Dioscoreales Dioscoreaceae Dioscorea futschauensis#NC 039808
Dioscoreales Dioscoreaceae Dioscorea villosa#NC 034686
Dioscoreales Dioscoreaceae Dioscorea elephantipes#NC 009601
Dioscoreales Dioscoreaceae Dioscorea bulbifera#MG805604
Dioscoreales Dioscoreaceae Dioscorea dumetorum#NC 039691
Dioscoreales Dioscoreaceae Dioscorea quartiniana#NC 039853
Dioscoreales Dioscoreaceae Dioscorea sansibarensis#NC 039838
Dioscoreales Dioscoreaceae Dioscorea subcalva#F891
Dioscoreales Dioscoreaceae Dioscorea kamoonensis#F551
Dioscoreales Dioscoreaceae Dioscorea preussii#NC 039852
Dioscoreales Dioscoreaceae Dioscorea schimperiana#NC 039855
Dioscoreales Dioscoreaceae Dioscorea hirtiflora#NC 039851
Dioscoreales Dioscoreaceae Dioscorea polystachya#MG267375
Dioscoreales Dioscoreaceae Dioscorea alata#NC 039707
Dioscoreales Dioscoreaceae Dioscorea aspersa#NC 039807
Dioscoreales Dioscoreaceae Dioscorea togoensis#NC 039856
Dioscoreales Dioscoreaceae Dioscorea baya#NC 039850
Dioscoreales Dioscoreaceae Dioscorea sagittifolia#NC 039854
Dioscoreales Dioscoreaceae Dioscorea abyssinica#NC 039834
Dioscoreales Dioscoreaceae Dioscorea praehensilis#NC 039837
Dioscoreales Dioscoreaceae Dioscorea cayennensis subsp. rotundata#NC 024170
Dioscoreales Dioscoreaceae Dioscorea cayennensis#NC 039836
Liliales Campynemataceae Campynema lineare#NC 026785
Liliales Corsiaceae Corsia sp.#T00297 @para
Liliales Petermanniaceae Petermannia cirrosa#FC298
Liliales Alstroemeriaceae Luzuriaga radicans#NC 025333
Liliales Alstroemeriaceae Alstroemeria aurea#Y210
Liliales Alstroemeriaceae Bomarea edulis#NC 025306
Liliales Alstroemeriaceae Bomarea superba#FC64
Liliales Colchicaceae Disporum sessile#Y173
Liliales Colchicaceae Disporum calcaratum#F74
Liliales Colchicaceae Iphigenia indica#F867
Liliales Colchicaceae Gloriosa superba#F570
Liliales Colchicaceae Colchicum autumnale#NC 030064
Liliales Melanthiaceae Veratrum oxysepalum#NC 022715
Liliales Melanthiaceae Melanthium parviflorum#Y217
Liliales Melanthiaceae Veratrum shanense#F870
Liliales Melanthiaceae Veratrum maackii var. japonicum#NC 041306
Liliales Melanthiaceae Heloniopsis tubiflora#NC 027159
Liliales Melanthiaceae Helonias bullata#OOSO
Liliales Melanthiaceae Xerophyllum asphodeloides#AFLV
Liliales Melanthiaceae Xerophyllum tenax#NC 027158
Liliales Melanthiaceae Trillium tschonoskii#MF614015
Liliales Melanthiaceae Trillium decumbens#NC 027282
Liliales Melanthiaceae Trillium cuneatum#NC 027185
Liliales Melanthiaceae Trillium maculatum#NC 027738
Liliales Melanthiaceae Paris verticillata#NC 024560
Liliales Melanthiaceae Paris quadrifolia#NC 033520
Liliales Melanthiaceae Paris thibetica#NC 038161
Liliales Melanthiaceae Paris rugosa#NC 038170
Liliales Melanthiaceae Paris vietnamensis#NC 033519
Liliales Melanthiaceae Paris cronquistii#NC 033511
Liliales Melanthiaceae Paris dunniana#NC 033512
Liliales Melanthiaceae Paris polyphylla var. chinensis#NC 033517
Liliales Melanthiaceae Paris polyphylla var. yunnanensis#NC 033518
Liliales Melanthiaceae Paris polyphylla#F819
Liliales Melanthiaceae Paris mairei#F815
Liliales Melanthiaceae Paris marmorata#MF495705
Liliales Melanthiaceae Paris luquanensis#MF417768
Liliales Melanthiaceae Paris fargesii#KX784044

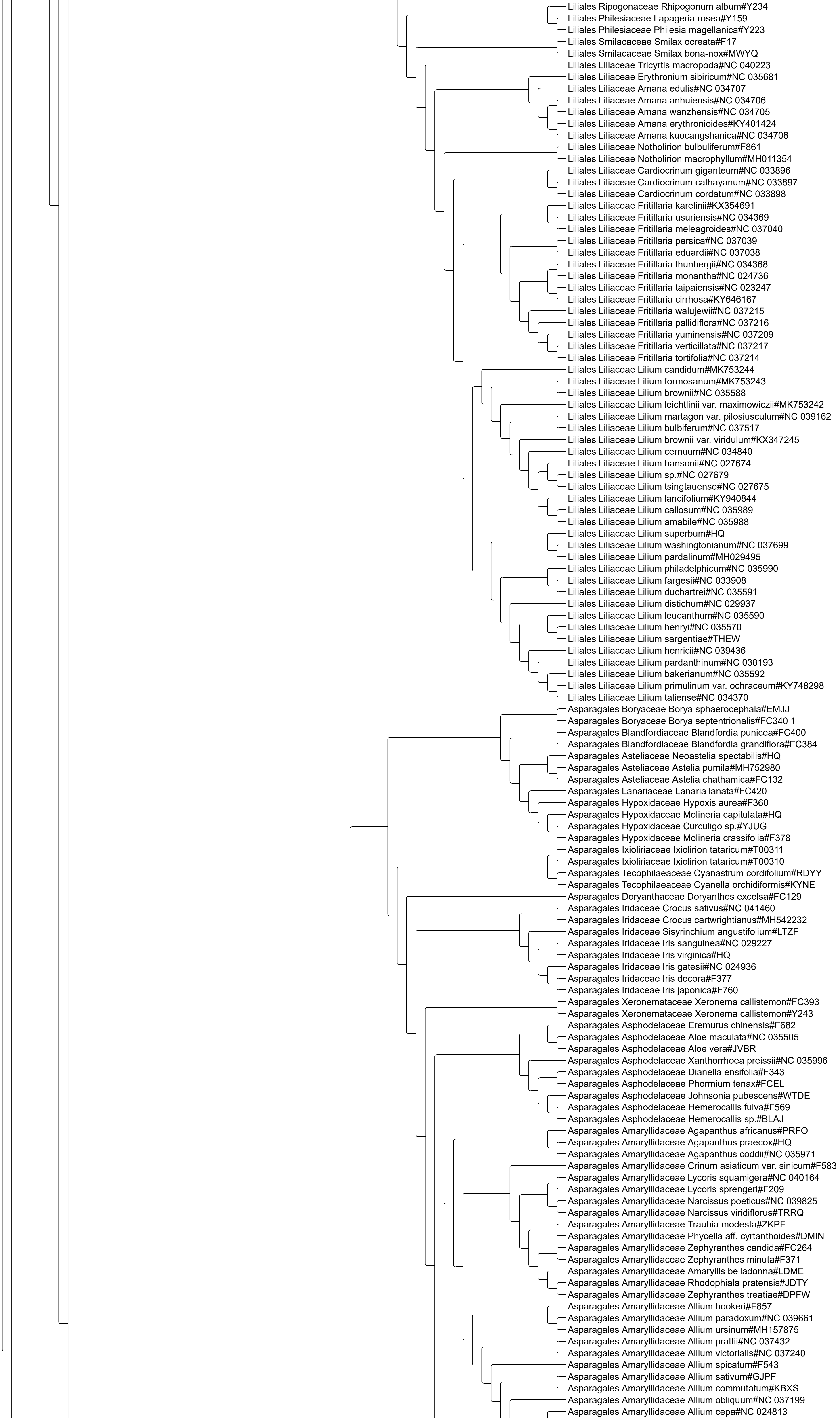

Liliales Ripogonaceae Rhipogonum album#Y234
Liliales Philesiaceae Lapageria rosea#Y159
Liliales Philesiaceae Philesia magellanica#Y223
Liliales Smilacaceae Smilax ocreata#F17
Liliales Smilacaceae Smilax bona-nox#MWYQ
Liliales Liliaceae Tricyrtis macropoda#NC 040223
Liliales Liliaceae Erythronium sibiricum#NC 035681
Liliales Liliaceae Amana edulis#NC 034707
Liliales Liliaceae Amana anhuiensis#NC 034706
Liliales Liliaceae Amana wanzhensis#NC 034705
Liliales Liliaceae Amana erythronioides#KY401424
Liliales Liliaceae Amana kuocangshanica#NC 034708
Liliales Liliaceae Notholirion bulbuliferum#F861
Liliales Liliaceae Notholirion macrophyllum#MH011354
Liliales Liliaceae Cardiocrinum giganteum#NC 033896
Liliales Liliaceae Cardiocrinum cathayanum#NC 033897
Liliales Liliaceae Cardiocrinum cordatum#NC 033898
Liliales Liliaceae Fritillaria karelinii#KX354691
Liliales Liliaceae Fritillaria usuriensis#NC 034369
Liliales Liliaceae Fritillaria meleagroides#NC 037040
Liliales Liliaceae Fritillaria persica#NC 037039
Liliales Liliaceae Fritillaria eduardii#NC 037038
Liliales Liliaceae Fritillaria thunbergii#NC 034368
Liliales Liliaceae Fritillaria monantha#NC 024736
Liliales Liliaceae Fritillaria taipaiensis#NC 023247
Liliales Liliaceae Fritillaria cirrhosa#KY646167
Liliales Liliaceae Fritillaria walujewii#NC 037215
Liliales Liliaceae Fritillaria pallidiflora#NC 037216
Liliales Liliaceae Fritillaria yuminensis#NC 037209
Liliales Liliaceae Fritillaria verticillata#NC 037217
Liliales Liliaceae Fritillaria tortifolia#NC 037214
Liliales Liliaceae Lilium candidum#MK753244
Liliales Liliaceae Lilium formosanum#MK753243
Liliales Liliaceae Lilium brownii#NC 035588
Liliales Liliaceae Lilium leichtlinii var. maximowiczii#MK753242
Liliales Liliaceae Lilium martagon var. pilosiusculum#NC 039162
Liliales Liliaceae Lilium bulbiferum#NC 037517
Liliales Liliaceae Lilium brownii var. viridulum#KX347245
Liliales Liliaceae Lilium cernuum#NC 034840
Liliales Liliaceae Lilium hansonii#NC 027674
Liliales Liliaceae Lilium sp.#NC 027679
Liliales Liliaceae Lilium tsingtauense#NC 027675
Liliales Liliaceae Lilium lancifolium#KY940844
Liliales Liliaceae Lilium callosum#NC 035989
Liliales Liliaceae Lilium amabile#NC 035988
Liliales Liliaceae Lilium superbum#HQ
Liliales Liliaceae Lilium washingtonianum#NC 037699
Liliales Liliaceae Lilium pardalinum#MH029495
Liliales Liliaceae Lilium philadelphicum#NC 035990
Liliales Liliaceae Lilium fargesii#NC 033908
Liliales Liliaceae Lilium duchartrei#NC 035591
Liliales Liliaceae Lilium distichum#NC 029937
Liliales Liliaceae Lilium leucanthum#NC 035590
Liliales Liliaceae Lilium henryi#NC 035570
Liliales Liliaceae Lilium sargentiae#THEW
Liliales Liliaceae Lilium henricii#NC 039436
Liliales Liliaceae Lilium pardanthinum#NC 038193
Liliales Liliaceae Lilium bakerianum#NC 035592
Liliales Liliaceae Lilium primulinum var. ochraceum#KY748298
Liliales Liliaceae Lilium taliense#NC 034370
Asparagales Boryaceae Borya sphaerocephala#EMJJ
Asparagales Boryaceae Borya septentrionalis#FC340 1
Asparagales Blandfordiaceae Blandfordia punicea#FC400
Asparagales Blandfordiaceae Blandfordia grandiflora#FC384
Asparagales Asteliaceae Neoastelia spectabilis#HQ
Asparagales Asteliaceae Astelia pumila#MH752980
Asparagales Asteliaceae Astelia chathamica#FC132
Asparagales Lanariaceae Lanaria lanata#FC420
Asparagales Hypoxidaceae Hypoxis aurea#F360
Asparagales Hypoxidaceae Molineria capitulata#HQ
Asparagales Hypoxidaceae Curculigo sp.#YJUG
Asparagales Hypoxidaceae Molineria crassifolia#F378
Asparagales Ixioliriaceae Ixiolirion tataricum#T00311
Asparagales Ixioliriaceae Ixiolirion tataricum#T00310
Asparagales Tecophilaeaceae Cyanastrum cordifolium#RDYY
Asparagales Tecophilaeaceae Cyanella orchidiformis#KYNE
Asparagales Doryanthaceae Doryanthes excelsa#FC129
Asparagales Iridaceae Crocus sativus#NC 041460
Asparagales Iridaceae Crocus cartwrightianus#MH542232
Asparagales Iridaceae Sisyrinchium angustifolium#LTZF
Asparagales Iridaceae Iris sanguinea#NC 029227
Asparagales Iridaceae Iris virginica#HQ
Asparagales Iridaceae Iris gatesii#NC 024936
Asparagales Iridaceae Iris decora#F377
Asparagales Iridaceae Iris japonica#F760
Asparagales Xeronemataceae Xeronema callistemon#FC393
Asparagales Xeronemataceae Xeronema callistemon#Y243
Asparagales Asphodelaceae Eremurus chinensis#F682
Asparagales Asphodelaceae Aloe maculata#NC 035505
Asparagales Asphodelaceae Aloe vera#JVBR
Asparagales Asphodelaceae Xanthorrhoea preissii#NC 035996
Asparagales Asphodelaceae Dianella ensifolia#F343
Asparagales Asphodelaceae Phormium tenax#FCEL
Asparagales Asphodelaceae Johnsonia pubescens#WTDE
Asparagales Asphodelaceae Hemerocallis fulva#F569
Asparagales Asphodelaceae Hemerocallis sp.#BLAJ
Asparagales Amaryllidaceae Agapanthus africanus#PRFO
Asparagales Amaryllidaceae Agapanthus praecox#HQ
Asparagales Amaryllidaceae Agapanthus coddii#NC 035971
Asparagales Amaryllidaceae Crinum asiaticum var. sinicum#F583
Asparagales Amaryllidaceae Lycoris squamigera#NC 040164
Asparagales Amaryllidaceae Lycoris sprengeri#F209
Asparagales Amaryllidaceae Narcissus poeticus#NC 039825
Asparagales Amaryllidaceae Narcissus viridiflorus#TRRQ
Asparagales Amaryllidaceae Traubia modesta#ZKPF
Asparagales Amaryllidaceae Phycella aff. cyrtanthoides#DMIN
Asparagales Amaryllidaceae Zephyranthes candida#FC264
Asparagales Amaryllidaceae Zephyranthes minuta#F371
Asparagales Amaryllidaceae Amaryllis belladonna#LDME
Asparagales Amaryllidaceae Rhodophiala pratensis#JDTY
Asparagales Amaryllidaceae Zephyranthes treatiae#DPFW
Asparagales Amaryllidaceae Allium hookeri#F857
Asparagales Amaryllidaceae Allium paradoxum#NC 039661
Asparagales Amaryllidaceae Allium ursinum#MH157875
Asparagales Amaryllidaceae Allium prattii#NC 037432
Asparagales Amaryllidaceae Allium victorialis#NC 037240
Asparagales Amaryllidaceae Allium spicatum#F543
Asparagales Amaryllidaceae Allium sativum#GJPF
Asparagales Amaryllidaceae Allium commutatum#KBXS
Asparagales Amaryllidaceae Allium obliquum#NC 037199
Asparagales Amaryllidaceae Allium cepa#NC 024813

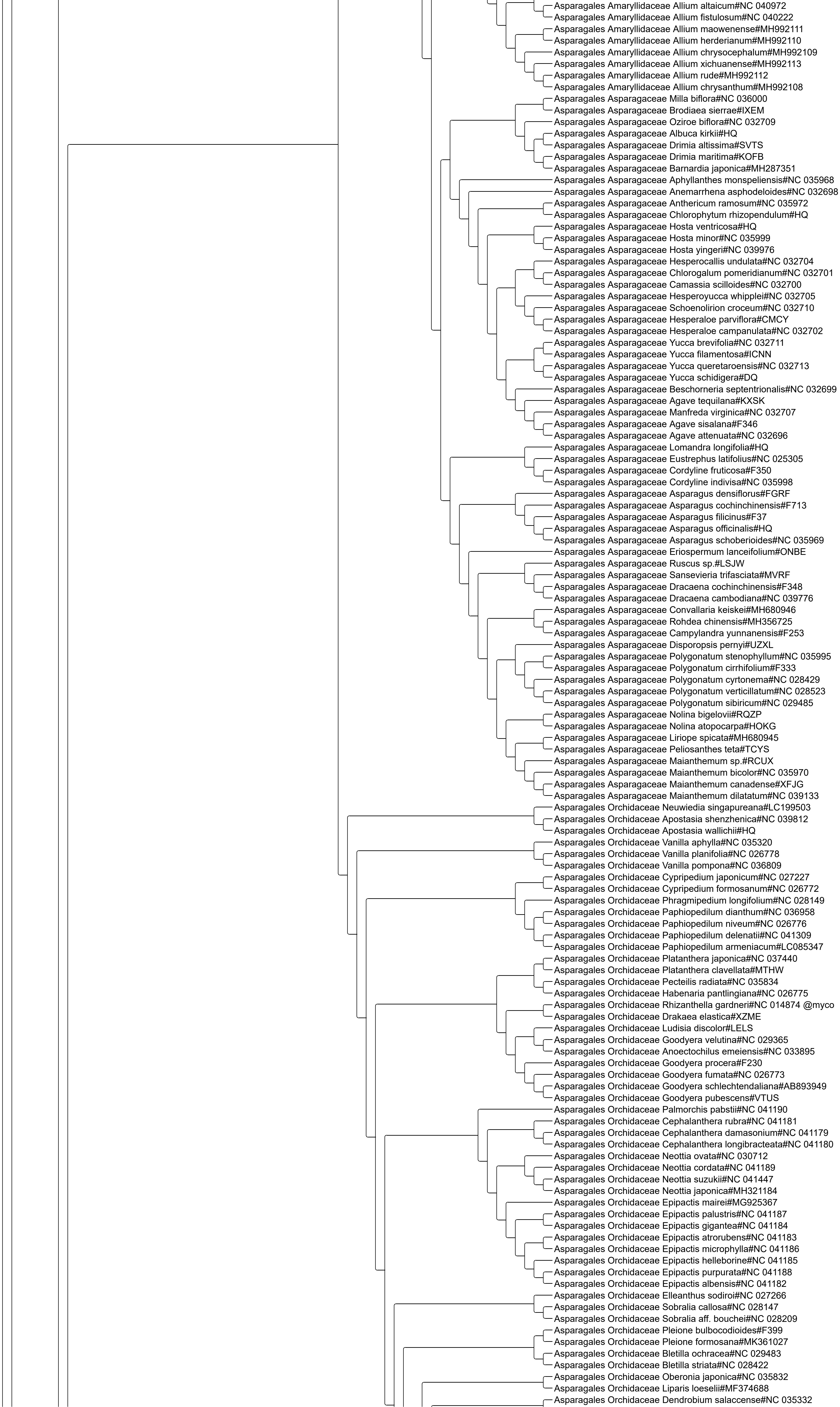
Asparagales Amaryllidaceae Allium altaicum#NC 040972
Asparagales Amaryllidaceae Allium fistulosum#NC 040222
Asparagales Amaryllidaceae Allium maowenense#MH992111
Asparagales Amaryllidaceae Allium herderianum#MH992110
Asparagales Amaryllidaceae Allium chrysocephalum#MH992109
Asparagales Amaryllidaceae Allium xichuanense#MH992113
Asparagales Amaryllidaceae Allium rude#MH992112
Asparagales Amaryllidaceae Allium chrysanthum#MH992108
Asparagales Asparagaceae Milla biflora#NC 036000
Asparagales Asparagaceae Brodiaea sierrae#IXEM
Asparagales Asparagaceae Oziroe biflora#NC 032709
Asparagales Asparagaceae Albuca kirkii#HQ
Asparagales Asparagaceae Drimia altissima#SVTS
Asparagales Asparagaceae Drimia maritima#KOFB
Asparagales Asparagaceae Barnardia japonica#MH287351
Asparagales Asparagaceae Aphyllanthes monspeliensis#NC 035968
Asparagales Asparagaceae Anemarrhena asphodeloides#NC 032698
Asparagales Asparagaceae Anthericum ramosum#NC 035972
Asparagales Asparagaceae Chlorophytum rhizopendulum#HQ
Asparagales Asparagaceae Hosta ventricosa#HQ
Asparagales Asparagaceae Hosta minor#NC 035999
Asparagales Asparagaceae Hosta yingeri#NC 039976
Asparagales Asparagaceae Hesperocallis undulata#NC 032704
Asparagales Asparagaceae Chlorogalum pomeridianum#NC 032701
Asparagales Asparagaceae Camassia scilloides#NC 032700
Asparagales Asparagaceae Hesperoyucca whipplei#NC 032705
Asparagales Asparagaceae Schoenolirion croceum#NC 032710
Asparagales Asparagaceae Hesperaloe parviflora#CMCY
Asparagales Asparagaceae Hesperaloe campanulata#NC 032702
Asparagales Asparagaceae Yucca brevifolia#NC 032711
Asparagales Asparagaceae Yucca filamentosa#ICNN
Asparagales Asparagaceae Yucca queretaroensis#NC 032713
Asparagales Asparagaceae Yucca schidigera#DQ
Asparagales Asparagaceae Beschorneria septentrionalis#NC 032699
Asparagales Asparagaceae Agave tequilana#KXSK
Asparagales Asparagaceae Manfreda virginica#NC 032707
Asparagales Asparagaceae Agave sisalana#F346
Asparagales Asparagaceae Agave attenuata#NC 032696
Asparagales Asparagaceae Lomandra longifolia#HQ
Asparagales Asparagaceae Eustrephus latifolius#NC 025305
Asparagales Asparagaceae Cordyline fruticosa#F350
Asparagales Asparagaceae Cordyline indivisa#NC 035998
Asparagales Asparagaceae Asparagus densiflorus#FGRF
Asparagales Asparagaceae Asparagus cochinchinensis#F713
Asparagales Asparagaceae Asparagus filicinus#F37
Asparagales Asparagaceae Asparagus officinalis#HQ
Asparagales Asparagaceae Asparagus schoberioides#NC 035969
Asparagales Asparagaceae Eriospermum lanceifolium#ONBE
Asparagales Asparagaceae Ruscus sp.#LSJW
Asparagales Asparagaceae Sansevieria trifasciata#MVRF
Asparagales Asparagaceae Dracaena cochinchinensis#F348
Asparagales Asparagaceae Dracaena cambodiana#NC 039776
Asparagales Asparagaceae Convallaria keiskei#MH680946
Asparagales Asparagaceae Rohdea chinensis#MH356725
Asparagales Asparagaceae Campylandra yunnanensis#F253
Asparagales Asparagaceae Disporopsis pernyi#UZXL
Asparagales Asparagaceae Polygonatum stenophyllum#NC 035995
Asparagales Asparagaceae Polygonatum cirrhifolium#F333
Asparagales Asparagaceae Polygonatum cyrtonema#NC 028429
Asparagales Asparagaceae Polygonatum verticillatum#NC 028523
Asparagales Asparagaceae Polygonatum sibiricum#NC 029485
Asparagales Asparagaceae Nolina bigelovii#RQZP
Asparagales Asparagaceae Nolina atopocarpa#HOKG
Asparagales Asparagaceae Liriope spicata#MH680945
Asparagales Asparagaceae Peliosanthes teta#TCYS
Asparagales Asparagaceae Maianthemum sp.#RCUX
Asparagales Asparagaceae Maianthemum bicolor#NC 035970
Asparagales Asparagaceae Maianthemum canadense#XFJG
Asparagales Asparagaceae Maianthemum dilatatum#NC 039133
Asparagales Orchidaceae Neuwiedia singapureana#LC199503
Asparagales Orchidaceae Apostasia shenzhenica#NC 039812
Asparagales Orchidaceae Apostasia wallichii#HQ
Asparagales Orchidaceae Vanilla aphylla#NC 035320
Asparagales Orchidaceae Vanilla planifolia#NC 026778
Asparagales Orchidaceae Vanilla pompona#NC 036809
Asparagales Orchidaceae Cypripedium japonicum#NC 027227
Asparagales Orchidaceae Cypripedium formosanum#NC 026772
Asparagales Orchidaceae Phragmipedium longifolium#NC 028149
Asparagales Orchidaceae Paphiopedilum dianthum#NC 036958
Asparagales Orchidaceae Paphiopedilum niveum#NC 026776
Asparagales Orchidaceae Paphiopedilum delenatii#NC 041309
Asparagales Orchidaceae Paphiopedilum armeniacum#LC085347
Asparagales Orchidaceae Platanthera japonica#NC 037440
Asparagales Orchidaceae Platanthera clavellata#MTHW
Asparagales Orchidaceae Pecteilis radiata#NC 035834
Asparagales Orchidaceae Habenaria pantlingiana#NC 026775
Asparagales Orchidaceae Rhizanthella gardneri#NC 014874 @myco
Asparagales Orchidaceae Drakaea elastica#XZME
Asparagales Orchidaceae Ludisia discolor#LELS
Asparagales Orchidaceae Goodyera velutina#NC 029365
Asparagales Orchidaceae Anoectochilus emeiensis#NC 033895
Asparagales Orchidaceae Goodyera procera#F230
Asparagales Orchidaceae Goodyera fumata#NC 026773
Asparagales Orchidaceae Goodyera schlechtendaliana#AB893949
Asparagales Orchidaceae Goodyera pubescens#VTUS
Asparagales Orchidaceae Palmorchis pabstii#NC 041190
Asparagales Orchidaceae Cephalanthera rubra#NC 041181
Asparagales Orchidaceae Cephalanthera damasonium#NC 041179
Asparagales Orchidaceae Cephalanthera longibracteata#NC 041180
Asparagales Orchidaceae Neottia ovata#NC 030712
Asparagales Orchidaceae Neottia cordata#NC 041189
Asparagales Orchidaceae Neottia suzukii#NC 041447
Asparagales Orchidaceae Neottia japonica#MH321184
Asparagales Orchidaceae Epipactis mairei#MG925367
Asparagales Orchidaceae Epipactis palustris#NC 041187
Asparagales Orchidaceae Epipactis gigantea#NC 041184
Asparagales Orchidaceae Epipactis atrorubens#NC 041183
Asparagales Orchidaceae Epipactis microphylla#NC 041186
Asparagales Orchidaceae Epipactis helleborine#NC 041185
Asparagales Orchidaceae Epipactis purpurata#NC 041188
Asparagales Orchidaceae Epipactis albensis#NC 041182
Asparagales Orchidaceae Elleanthus sodiroi#NC 027266
Asparagales Orchidaceae Sobralia callosa#NC 028147
Asparagales Orchidaceae Sobralia aff. bouchei#NC 028209
Asparagales Orchidaceae Pleione bulbocodioides#F399
Asparagales Orchidaceae Pleione formosana#MK361027
Asparagales Orchidaceae Bletilla ochracea#NC 029483
Asparagales Orchidaceae Bletilla striata#NC 028422
Asparagales Orchidaceae Oberonia japonica#NC 035832
Asparagales Orchidaceae Liparis loeselii#MF374688
Asparagales Orchidaceae Dendrobium salaccense#NC 035332

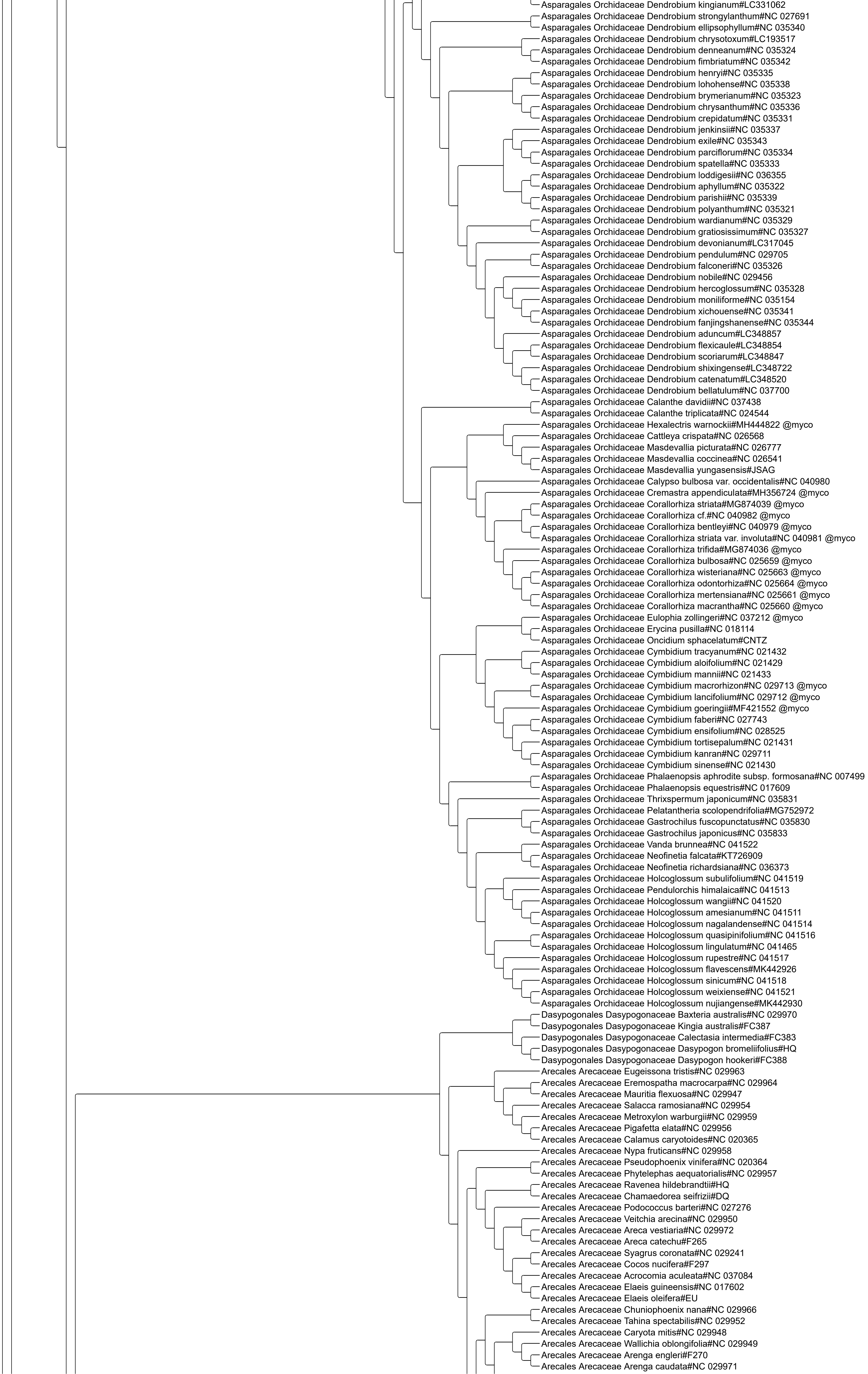
Asparagales Orchidaceae Dendrobium kingianum#LC331062
Asparagales Orchidaceae Dendrobium strongylanthum#NC 027691
Asparagales Orchidaceae Dendrobium ellipsophyllum#NC 035340
Asparagales Orchidaceae Dendrobium chrysotoxum#LC193517
Asparagales Orchidaceae Dendrobium denneanum#NC 035324
Asparagales Orchidaceae Dendrobium fimbriatum#NC 035342
Asparagales Orchidaceae Dendrobium henryi#NC 035335
Asparagales Orchidaceae Dendrobium lohohense#NC 035338
Asparagales Orchidaceae Dendrobium brymerianum#NC 035323
Asparagales Orchidaceae Dendrobium chrysanthum#NC 035336
Asparagales Orchidaceae Dendrobium crepidatum#NC 035331
Asparagales Orchidaceae Dendrobium jenkinsii#NC 035337
Asparagales Orchidaceae Dendrobium exile#NC 035343
Asparagales Orchidaceae Dendrobium parciflorum#NC 035334
Asparagales Orchidaceae Dendrobium spatella#NC 035333
Asparagales Orchidaceae Dendrobium loddigesii#NC 036355
Asparagales Orchidaceae Dendrobium aphyllum#NC 035322
Asparagales Orchidaceae Dendrobium parishii#NC 035339
Asparagales Orchidaceae Dendrobium polyanthum#NC 035321
Asparagales Orchidaceae Dendrobium wardianum#NC 035329
Asparagales Orchidaceae Dendrobium gratiosissimum#NC 035327
Asparagales Orchidaceae Dendrobium devonianum#LC317045
Asparagales Orchidaceae Dendrobium pendulum#NC 029705
Asparagales Orchidaceae Dendrobium falconeri#NC 035326
Asparagales Orchidaceae Dendrobium nobile#NC 029456
Asparagales Orchidaceae Dendrobium hercoglossum#NC 035328
Asparagales Orchidaceae Dendrobium moniliforme#NC 035154
Asparagales Orchidaceae Dendrobium xichouense#NC 035341
Asparagales Orchidaceae Dendrobium fanjingshanense#NC 035344
Asparagales Orchidaceae Dendrobium aduncum#LC348857
Asparagales Orchidaceae Dendrobium flexicaule#LC348854
Asparagales Orchidaceae Dendrobium scoriarum#LC348847
Asparagales Orchidaceae Dendrobium shixingense#LC348722
Asparagales Orchidaceae Dendrobium catenatum#LC348520
Asparagales Orchidaceae Dendrobium bellatulum#NC 037700
Asparagales Orchidaceae Calanthe davidii#NC 037438
Asparagales Orchidaceae Calanthe triplicata#NC 024544
Asparagales Orchidaceae Hexalectris warnockii#MH444822 @myco
Asparagales Orchidaceae Cattleya crispata#NC 026568
Asparagales Orchidaceae Masdevallia picturata#NC 026777
Asparagales Orchidaceae Masdevallia coccinea#NC 026541
Asparagales Orchidaceae Masdevallia yungasensis#JSAG
Asparagales Orchidaceae Calypso bulbosa var. occidentalis#NC 040980
Asparagales Orchidaceae Cremastra appendiculata#MH356724 @myco
Asparagales Orchidaceae Corallorhiza striata#MG874039 @myco
Asparagales Orchidaceae Corallorhiza cf.#NC 040982 @myco
Asparagales Orchidaceae Corallorhiza bentleyi#NC 040979 @myco
Asparagales Orchidaceae Corallorhiza striata var. involuta#NC 040981 @myco
Asparagales Orchidaceae Corallorhiza trifida#MG874036 @myco
Asparagales Orchidaceae Corallorhiza bulbosa#NC 025659 @myco
Asparagales Orchidaceae Corallorhiza wisteriana#NC 025663 @myco
Asparagales Orchidaceae Corallorhiza odontorhiza#NC 025664 @myco
Asparagales Orchidaceae Corallorhiza mertensiana#NC 025661 @myco
Asparagales Orchidaceae Corallorhiza macrantha#NC 025660 @myco
Asparagales Orchidaceae Eulophia zollingeri#NC 037212 @myco
Asparagales Orchidaceae Erycina pusilla#NC 018114
Asparagales Orchidaceae Oncidium sphacelatum#CNTZ
Asparagales Orchidaceae Cymbidium tracyanum#NC 021432
Asparagales Orchidaceae Cymbidium aloifolium#NC 021429
Asparagales Orchidaceae Cymbidium mannii#NC 021433
Asparagales Orchidaceae Cymbidium macrorhizon#NC 029713 @myco
Asparagales Orchidaceae Cymbidium lancifolium#NC 029712 @myco
Asparagales Orchidaceae Cymbidium goeringii#MF421552 @myco
Asparagales Orchidaceae Cymbidium faberi#NC 027743
Asparagales Orchidaceae Cymbidium ensifolium#NC 028525
Asparagales Orchidaceae Cymbidium tortisepalum#NC 021431
Asparagales Orchidaceae Cymbidium kanran#NC 029711
Asparagales Orchidaceae Cymbidium sinense#NC 021430
Asparagales Orchidaceae Phalaenopsis aphrodite subsp. formosana#NC 007499
Asparagales Orchidaceae Phalaenopsis equestris#NC 017609
Asparagales Orchidaceae Thrixspermum japonicum#NC 035831
Asparagales Orchidaceae Pelatantheria scolopendrifolia#MG752972
Asparagales Orchidaceae Gastrochilus fuscopunctatus#NC 035830
Asparagales Orchidaceae Gastrochilus japonicus#NC 035833
Asparagales Orchidaceae Vanda brunnea#NC 041522
Asparagales Orchidaceae Neofinetia falcata#KT726909
Asparagales Orchidaceae Neofinetia richardsiana#NC 036373
Asparagales Orchidaceae Holcoglossum subulifolium#NC 041519
Asparagales Orchidaceae Pendulorchis himalaica#NC 041513
Asparagales Orchidaceae Holcoglossum wangii#NC 041520
Asparagales Orchidaceae Holcoglossum amesianum#NC 041511
Asparagales Orchidaceae Holcoglossum nagalandense#NC 041514
Asparagales Orchidaceae Holcoglossum quasipinifolium#NC 041516
Asparagales Orchidaceae Holcoglossum lingulatum#NC 041465
Asparagales Orchidaceae Holcoglossum rupestre#NC 041517
Asparagales Orchidaceae Holcoglossum flavescens#MK442926
Asparagales Orchidaceae Holcoglossum sinicum#NC 041518
Asparagales Orchidaceae Holcoglossum weixiense#NC 041521
Asparagales Orchidaceae Holcoglossum nujiangense#MK442930
Dasypogonales Dasypogonaceae Baxteria australis#NC 029970
Dasypogonales Dasypogonaceae Kingia australis#FC387
Dasypogonales Dasypogonaceae Calectasia intermedia#FC383
Dasypogonales Dasypogonaceae Dasypogon bromeliifolius#HQ
Dasypogonales Dasypogonaceae Dasypogon hookeri#FC388
Arecales Arecaceae Eugeissona tristis#NC 029963
Arecales Arecaceae Eremospatha macrocarpa#NC 029964
Arecales Arecaceae Mauritia flexuosa#NC 029947
Arecales Arecaceae Salacca ramosiana#NC 029954
Arecales Arecaceae Metroxylon warburgii#NC 029959
Arecales Arecaceae Pigafetta elata#NC 029956
Arecales Arecaceae Calamus caryotoides#NC 020365
Arecales Arecaceae Nypa fruticans#NC 029958
Arecales Arecaceae Pseudophoenix vinifera#NC 020364
Arecales Arecaceae Phytelephas aequatorialis#NC 029957
Arecales Arecaceae Ravenea hildebrandtii#HQ
Arecales Arecaceae Chamaedorea seifrizii#DQ
Arecales Arecaceae Podococcus barteri#NC 027276
Arecales Arecaceae Veitchia arecina#NC 029950
Arecales Arecaceae Areca vestiaria#NC 029972
Arecales Arecaceae Areca catechu#F265
Arecales Arecaceae Syagrus coronata#NC 029241
Arecales Arecaceae Cocos nucifera#F297
Arecales Arecaceae Acrocomia aculeata#NC 037084
Arecales Arecaceae Elaeis guineensis#NC 017602
Arecales Arecaceae Elaeis oleifera#EU
Arecales Arecaceae Chuniophoenix nana#NC 029966
Arecales Arecaceae Tahina spectabilis#NC 029952
Arecales Arecaceae Caryota mitis#NC 029948
Arecales Arecaceae Wallichia oblongifolia#NC 029949
Arecales Arecaceae Arenga engleri#F270
Arecales Arecaceae Arenga caudata#NC 029971

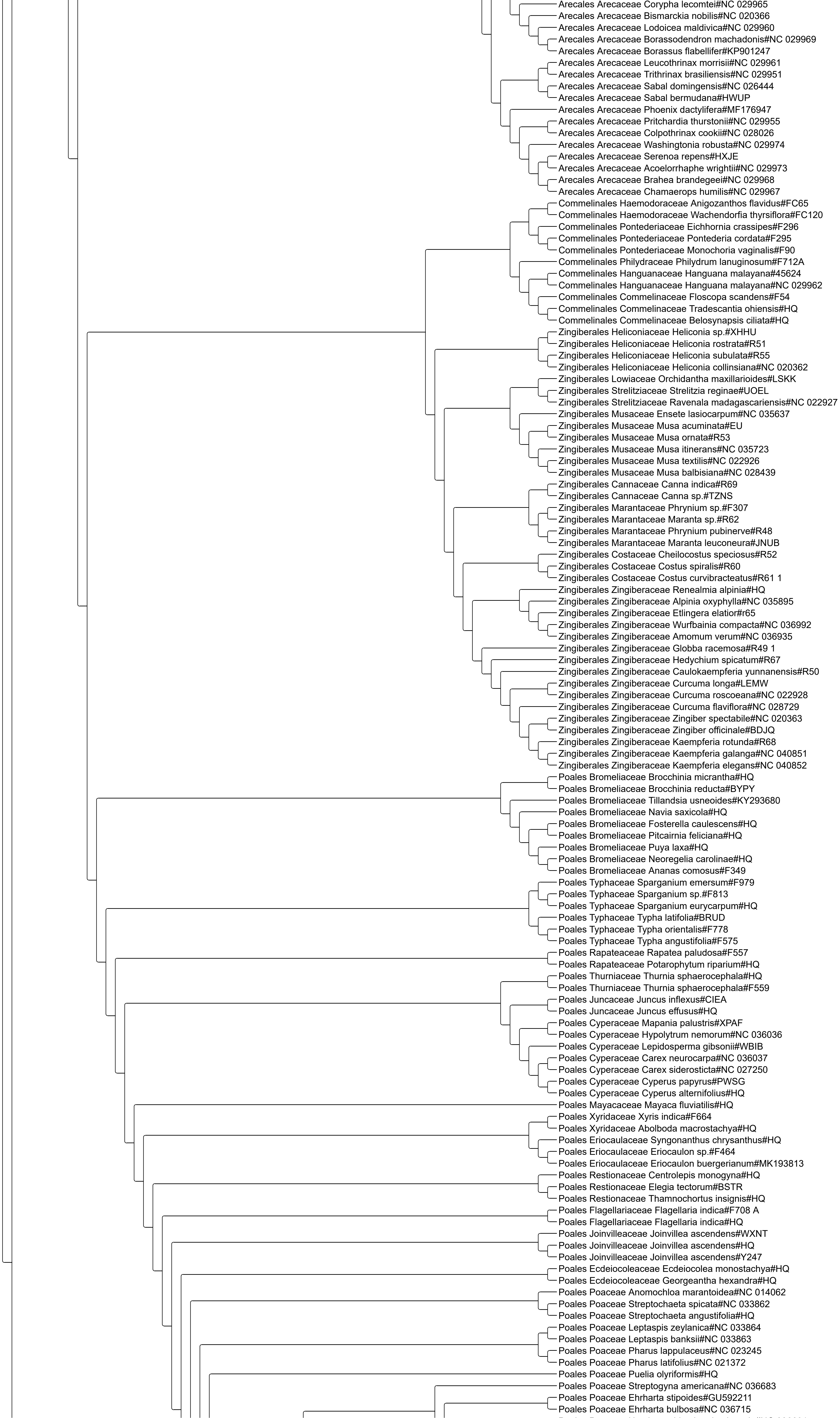
Arecales Arecaceae Corypha lecomtei#NC 029965
Arecales Arecaceae Bismarckia nobilis#NC 020366
Arecales Arecaceae Lodoicea maldivica#NC 029960
Arecales Arecaceae Borassodendron machadonis#NC 029969
Arecales Arecaceae Borassus flabellifer#KP901247
Arecales Arecaceae Leucothrinax morrisii#NC 029961
Arecales Arecaceae Trithrinax brasiliensis#NC 029951
Arecales Arecaceae Sabal domingensis#NC 026444
Arecales Arecaceae Sabal bermudana#HWUP
Arecales Arecaceae Phoenix dactylifera#MF176947
Arecales Arecaceae Pritchardia thurstonii#NC 029955
Arecales Arecaceae Colpothrinax cookii#NC 028026
Arecales Arecaceae Washingtonia robusta#NC 029974
Arecales Arecaceae Serenoa repens#HXJE
Arecales Arecaceae Acoelorrhaphe wrightii#NC 029973
Arecales Arecaceae Brahea brandegeei#NC 029968
Arecales Arecaceae Chamaerops humilis#NC 029967
Commelinales Haemodoraceae Anigozanthos flavidus#FC65
Commelinales Haemodoraceae Wachendorfia thyrsiflora#FC120
Commelinales Pontederiaceae Eichhornia crassipes#F296
Commelinales Pontederiaceae Pontederia cordata#F295
Commelinales Pontederiaceae Monochoria vaginalis#F90
Commelinales Philydraceae Philydrum lanuginosum#F712A
Commelinales Hanguanaceae Hanguana malayana#45624
Commelinales Hanguanaceae Hanguana malayana#NC 029962
Commelinales Commelinaceae Floscopa scandens#F54
Commelinales Commelinaceae Tradescantia ohiensis#HQ
Commelinales Commelinaceae Belosynapsis ciliata#HQ
Zingiberales Heliconiaceae Heliconia sp.#XHHU
Zingiberales Heliconiaceae Heliconia rostrata#R51
Zingiberales Heliconiaceae Heliconia subulata#R55
Zingiberales Heliconiaceae Heliconia collinsiana#NC 020362
Zingiberales Lowiaceae Orchidantha maxillarioides#LSKK
Zingiberales Strelitziaceae Strelitzia reginae#UOEL
Zingiberales Strelitziaceae Ravenala madagascariensis#NC 022927
Zingiberales Musaceae Ensete lasiocarpum#NC 035637
Zingiberales Musaceae Musa acuminata#EU
Zingiberales Musaceae Musa ornata#R53
Zingiberales Musaceae Musa itinerans#NC 035723
Zingiberales Musaceae Musa textilis#NC 022926
Zingiberales Musaceae Musa balbisiana#NC 028439
Zingiberales Cannaceae Canna indica#R69
Zingiberales Cannaceae Canna sp.#TZNS
Zingiberales Marantaceae Phrynium sp.#F307
Zingiberales Marantaceae Maranta sp.#R62
Zingiberales Marantaceae Phrynium pubinerve#R48
Zingiberales Marantaceae Maranta leuconeura#JNUB
Zingiberales Costaceae Cheilocostus speciosus#R52
Zingiberales Costaceae Costus spiralis#R60
Zingiberales Costaceae Costus curvibracteatus#R61 1
Zingiberales Zingiberaceae Renealmia alpinia#HQ
Zingiberales Zingiberaceae Alpinia oxyphylla#NC 035895
Zingiberales Zingiberaceae Etlingera elatior#r65
Zingiberales Zingiberaceae Wurfbainia compacta#NC 036992
Zingiberales Zingiberaceae Amomum verum#NC 036935
Zingiberales Zingiberaceae Globba racemosa#R49 1
Zingiberales Zingiberaceae Hedychium spicatum#R67
Zingiberales Zingiberaceae Caulokaempferia yunnanensis#R50
Zingiberales Zingiberaceae Curcuma longa#LEMW
Zingiberales Zingiberaceae Curcuma roscoeana#NC 022928
Zingiberales Zingiberaceae Curcuma flaviflora#NC 028729
Zingiberales Zingiberaceae Zingiber spectabile#NC 020363
Zingiberales Zingiberaceae Zingiber officinale#BDJQ
Zingiberales Zingiberaceae Kaempferia rotunda#R68
Zingiberales Zingiberaceae Kaempferia galanga#NC 040851
Zingiberales Zingiberaceae Kaempferia elegans#NC 040852
Poales Bromeliaceae Brocchinia micrantha#HQ
Poales Bromeliaceae Brocchinia reducta#BYPY
Poales Bromeliaceae Tillandsia usneoides#KY293680
Poales Bromeliaceae Navia saxicola#HQ
Poales Bromeliaceae Fosterella caulescens#HQ
Poales Bromeliaceae Pitcairnia feliciana#HQ
Poales Bromeliaceae Puya laxa#HQ
Poales Bromeliaceae Neoregelia carolinae#HQ
Poales Bromeliaceae Ananas comosus#F349
Poales Typhaceae Sparganium emersum#F979
Poales Typhaceae Sparganium sp.#F813
Poales Typhaceae Sparganium eurycarpum#HQ
Poales Typhaceae Typha latifolia#BRUD
Poales Typhaceae Typha orientalis#F778
Poales Typhaceae Typha angustifolia#F575
Poales Rapateaceae Rapatea paludosa#F557
Poales Rapateaceae Potarophytum riparium#HQ
Poales Thurniaceae Thurnia sphaerocephala#HQ
Poales Thurniaceae Thurnia sphaerocephala#F559
Poales Juncaceae Juncus inflexus#CIEA
Poales Juncaceae Juncus effusus#HQ
Poales Cyperaceae Mapania palustris#XPAF
Poales Cyperaceae Hypolytrum nemorum#NC 036036
Poales Cyperaceae Lepidosperma gibsonii#WBIB
Poales Cyperaceae Carex neurocarpa#NC 036037
Poales Cyperaceae Carex siderosticta#NC 027250
Poales Cyperaceae Cyperus papyrus#PWSG
Poales Cyperaceae Cyperus alternifolius#HQ
Poales Mayacaceae Mayaca fluviatilis#HQ
Poales Xyridaceae Xyris indica#F664
Poales Xyridaceae Abolboda macrostachya#HQ
Poales Eriocaulaceae Syngonanthus chrysanthus#HQ
Poales Eriocaulaceae Eriocaulon sp.#F464
Poales Eriocaulaceae Eriocaulon buergerianum#MK193813
Poales Restionaceae Centrolepis monogyna#HQ
Poales Restionaceae Elegia tectorum#BSTR
Poales Restionaceae Thamnochortus insignis#HQ
Poales Flagellariaceae Flagellaria indica#F708 A
Poales Flagellariaceae Flagellaria indica#HQ
Poales Joinvilleaceae Joinvillea ascendens#WXNT
Poales Joinvilleaceae Joinvillea ascendens#HQ
Poales Joinvilleaceae Joinvillea ascendens#Y247
Poales Ecdeiocoleaceae Ecdeiocolea monostachya#HQ
Poales Ecdeiocoleaceae Georgeantha hexandra#HQ
Poales Poaceae Anomochloa marantoidea#NC 014062
Poales Poaceae Streptochaeta spicata#NC 033862
Poales Poaceae Streptochaeta angustifolia#HQ
Poales Poaceae Leptaspis zeylanica#NC 033864
Poales Poaceae Leptaspis banksii#NC 033863
Poales Poaceae Pharus lappulaceus#NC 023245
Poales Poaceae Pharus latifolius#NC 021372
Poales Poaceae Puelia olyriformis#HQ
Poales Poaceae Streptogyna americana#NC 036683
Poales Poaceae Ehrharta stipoides#GU592211
Poales Poaceae Ehrharta bulbosa#NC 036715

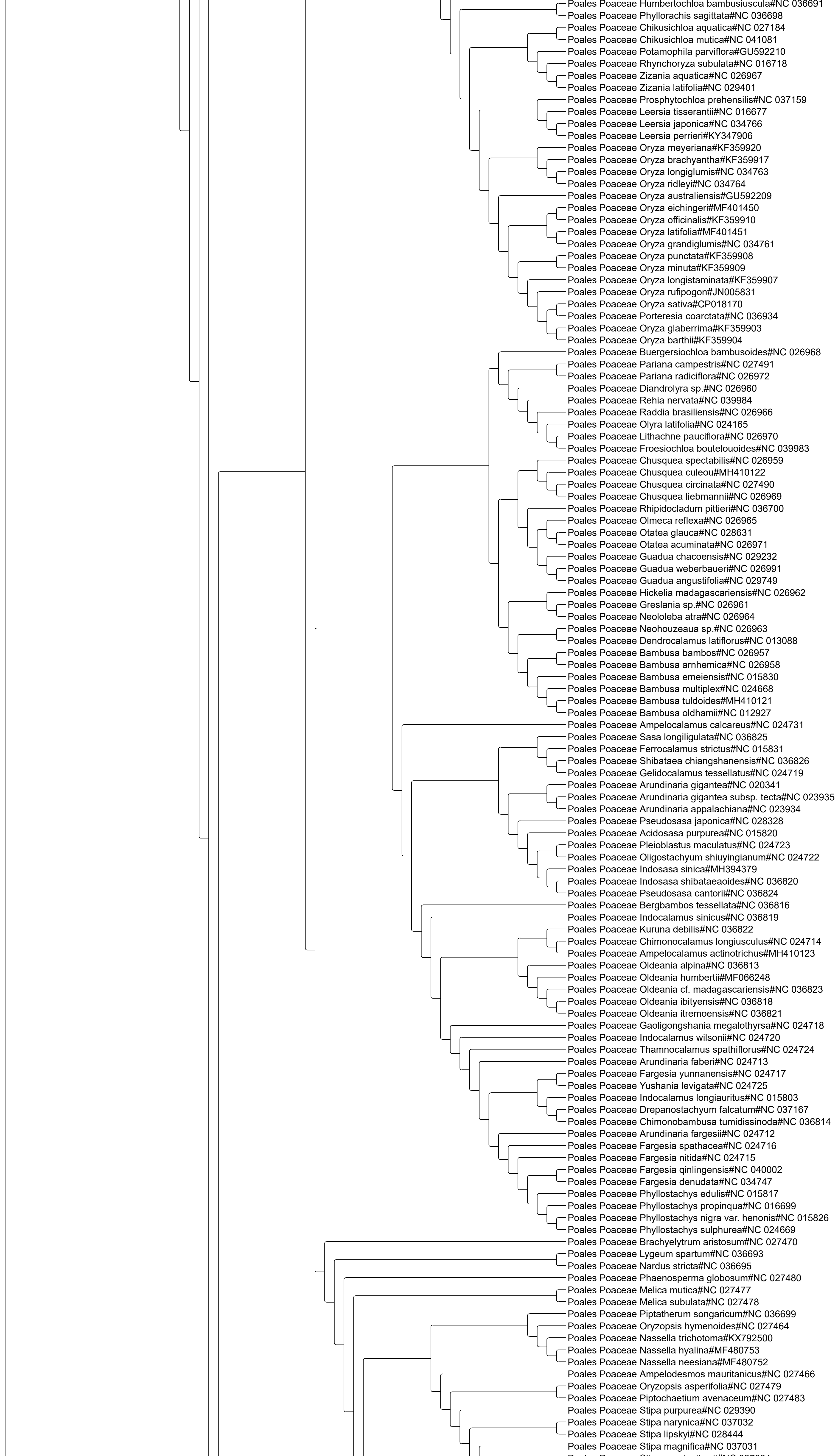
Poales Poaceae Humbertochloa bambusiuscula#NC 036691
Poales Poaceae Phyllorachis sagittata#NC 036698
Poales Poaceae Chikusichloa aquatica#NC 027184
Poales Poaceae Chikusichloa mutica#NC 041081
Poales Poaceae Potamophila parviflora#GU592210
Poales Poaceae Rhynchoryza subulata#NC 016718
Poales Poaceae Zizania aquatica#NC 026967
Poales Poaceae Zizania latifolia#NC 029401
Poales Poaceae Prosphytochloa prehensilis#NC 037159
Poales Poaceae Leersia tisserantii#NC 016677
Poales Poaceae Leersia japonica#NC 034766
Poales Poaceae Leersia perrieri#KY347906
Poales Poaceae Oryza meyeriana#KF359920
Poales Poaceae Oryza brachyantha#KF359917
Poales Poaceae Oryza longiglumis#NC 034763
Poales Poaceae Oryza ridleyi#NC 034764
Poales Poaceae Oryza australiensis#GU592209
Poales Poaceae Oryza eichingeri#MF401450
Poales Poaceae Oryza officinalis#KF359910
Poales Poaceae Oryza latifolia#MF401451
Poales Poaceae Oryza grandiglumis#NC 034761
Poales Poaceae Oryza punctata#KF359908
Poales Poaceae Oryza minuta#KF359909
Poales Poaceae Oryza longistaminata#KF359907
Poales Poaceae Oryza rufipogon#JN005831
Poales Poaceae Oryza sativa#CP018170
Poales Poaceae Porteresia coarctata#NC 036934
Poales Poaceae Oryza glaberrima#KF359903
Poales Poaceae Oryza barthii#KF359904
Poales Poaceae Buergersiochloa bambusoides#NC 026968
Poales Poaceae Pariana campestris#NC 027491
Poales Poaceae Pariana radiciflora#NC 026972
Poales Poaceae Diandrolyra sp.#NC 026960
Poales Poaceae Rehia nervata#NC 039984
Poales Poaceae Raddia brasiliensis#NC 026966
Poales Poaceae Olyra latifolia#NC 024165
Poales Poaceae Lithachne pauciflora#NC 026970
Poales Poaceae Froesiochloa boutelouoides#NC 039983
Poales Poaceae Chusquea spectabilis#NC 026959
Poales Poaceae Chusquea culeou#MH410122
Poales Poaceae Chusquea circinata#NC 027490
Poales Poaceae Chusquea liebmannii#NC 026969
Poales Poaceae Rhipidocladum pittieri#NC 036700
Poales Poaceae Olmeca reflexa#NC 026965
Poales Poaceae Otatea glauca#NC 028631
Poales Poaceae Otatea acuminata#NC 026971
Poales Poaceae Guadua chacoensis#NC 029232
Poales Poaceae Guadua weberbaueri#NC 026991
Poales Poaceae Guadua angustifolia#NC 029749
Poales Poaceae Hickelia madagascariensis#NC 026962
Poales Poaceae Greslania sp.#NC 026961
Poales Poaceae Neololeba atra#NC 026964
Poales Poaceae Neohouzeaua sp.#NC 026963
Poales Poaceae Dendrocalamus latiflorus#NC 013088
Poales Poaceae Bambusa bambos#NC 026957
Poales Poaceae Bambusa arnhemica#NC 026958
Poales Poaceae Bambusa emeiensis#NC 015830
Poales Poaceae Bambusa multiplex#NC 024668
Poales Poaceae Bambusa tuldoides#MH410121
Poales Poaceae Bambusa oldhamii#NC 012927
Poales Poaceae Ampelocalamus calcareus#NC 024731
Poales Poaceae Sasa longiligulata#NC 036825
Poales Poaceae Ferrocalamus strictus#NC 015831
Poales Poaceae Shibataea chiangshanensis#NC 036826
Poales Poaceae Gelidocalamus tessellatus#NC 024719
Poales Poaceae Arundinaria gigantea#NC 020341
Poales Poaceae Arundinaria gigantea subsp. tecta#NC 023935
Poales Poaceae Arundinaria appalachiana#NC 023934
Poales Poaceae Pseudosasa japonica#NC 028328
Poales Poaceae Acidosasa purpurea#NC 015820
Poales Poaceae Pleioblastus maculatus#NC 024723
Poales Poaceae Oligostachyum shiuyingianum#NC 024722
Poales Poaceae Indosasa sinica#MH394379
Poales Poaceae Indosasa shibataeaoides#NC 036820
Poales Poaceae Pseudosasa cantorii#NC 036824
Poales Poaceae Bergbambos tessellata#NC 036816
Poales Poaceae Indocalamus sinicus#NC 036819
Poales Poaceae Kuruna debilis#NC 036822
Poales Poaceae Chimonocalamus longiusculus#NC 024714
Poales Poaceae Ampelocalamus actinotrichus#MH410123
Poales Poaceae Oldeania alpina#NC 036813
Poales Poaceae Oldeania humbertii#MF066248
Poales Poaceae Oldeania cf. madagascariensis#NC 036823
Poales Poaceae Oldeania ibityensis#NC 036818
Poales Poaceae Oldeania itremoensis#NC 036821
Poales Poaceae Gaoligongshania megalothyrsa#NC 024718
Poales Poaceae Indocalamus wilsonii#NC 024720
Poales Poaceae Thamnocalamus spathiflorus#NC 024724
Poales Poaceae Arundinaria faberi#NC 024713
Poales Poaceae Fargesia yunnanensis#NC 024717
Poales Poaceae Yushania levigata#NC 024725
Poales Poaceae Indocalamus longiauritus#NC 015803
Poales Poaceae Drepanostachyum falcatum#NC 037167
Poales Poaceae Chimonobambusa tumidissinoda#NC 036814
Poales Poaceae Arundinaria fargesii#NC 024712
Poales Poaceae Fargesia spathacea#NC 024716
Poales Poaceae Fargesia nitida#NC 024715
Poales Poaceae Fargesia qinlingensis#NC 040002
Poales Poaceae Fargesia denudata#NC 034747
Poales Poaceae Phyllostachys edulis#NC 015817
Poales Poaceae Phyllostachys propinqua#NC 016699
Poales Poaceae Phyllostachys nigra var. henonis#NC 015826
Poales Poaceae Phyllostachys sulphurea#NC 024669
Poales Poaceae Brachyelytrum aristosum#NC 027470
Poales Poaceae Lygeum spartum#NC 036693
Poales Poaceae Nardus stricta#NC 036695
Poales Poaceae Phaenosperma globosum#NC 027480
Poales Poaceae Melica mutica#NC 027477
Poales Poaceae Melica subulata#NC 027478
Poales Poaceae Piptatherum songaricum#NC 036699
Poales Poaceae Oryzopsis hymenoides#NC 027464
Poales Poaceae Nassella trichotoma#KX792500
Poales Poaceae Nassella hyalina#MF480753
Poales Poaceae Nassella neesiana#MF480752
Poales Poaceae Ampelodesmos mauritanicus#NC 027466
Poales Poaceae Oryzopsis asperifolia#NC 027479
Poales Poaceae Piptochaetium avenaceum#NC 027483
Poales Poaceae Stipa purpurea#NC 029390
Poales Poaceae Stipa narynica#NC 037032
Poales Poaceae Stipa lipskyi#NC 028444
Poales Poaceae Stipa magnifica#NC 037031

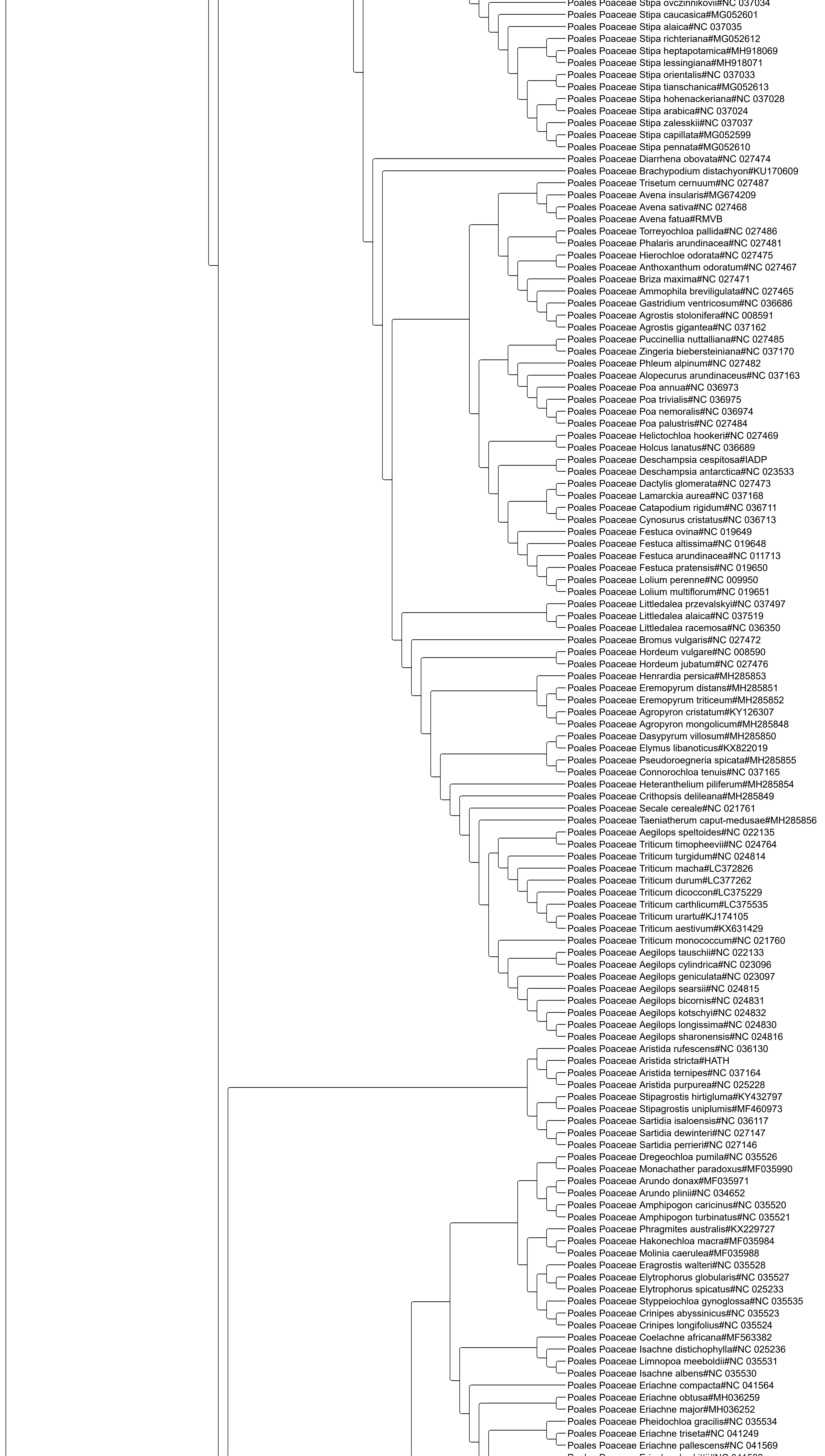
Poales Poaceae Stipa ovczinnikovii#NC 037034
Poales Poaceae Stipa caucasica#MG052601
Poales Poaceae Stipa alaica#NC 037035
Poales Poaceae Stipa richteriana#MG052612
Poales Poaceae Stipa heptapotamica#MH918069
Poales Poaceae Stipa lessingiana#MH918071
Poales Poaceae Stipa orientalis#NC 037033
Poales Poaceae Stipa tianschanica#MG052613
Poales Poaceae Stipa hohenackeriana#NC 037028
Poales Poaceae Stipa arabica#NC 037024
Poales Poaceae Stipa zalesskii#NC 037037
Poales Poaceae Stipa capillata#MG052599
Poales Poaceae Stipa pennata#MG052610
Poales Poaceae Diarrhena obovata#NC 027474
Poales Poaceae Brachypodium distachyon#KU170609
Poales Poaceae Trisetum cernuum#NC 027487
Poales Poaceae Avena insularis#MG674209
Poales Poaceae Avena sativa#NC 027468
Poales Poaceae Avena fatua#RMVB
Poales Poaceae Torreyochloa pallida#NC 027486
Poales Poaceae Phalaris arundinacea#NC 027481
Poales Poaceae Hierochloe odorata#NC 027475
Poales Poaceae Anthoxanthum odoratum#NC 027467
Poales Poaceae Briza maxima#NC 027471
Poales Poaceae Ammophila breviligulata#NC 027465
Poales Poaceae Gastridium ventricosum#NC 036686
Poales Poaceae Agrostis stolonifera#NC 008591
Poales Poaceae Agrostis gigantea#NC 037162
Poales Poaceae Puccinellia nuttalliana#NC 027485
Poales Poaceae Zingeria biebersteiniana#NC 037170
Poales Poaceae Phleum alpinum#NC 027482
Poales Poaceae Alopecurus arundinaceus#NC 037163
Poales Poaceae Poa annua#NC 036973
Poales Poaceae Poa trivialis#NC 036975
Poales Poaceae Poa nemoralis#NC 036974
Poales Poaceae Poa palustris#NC 027484
Poales Poaceae Helictochloa hookeri#NC 027469
Poales Poaceae Holcus lanatus#NC 036689
Poales Poaceae Deschampsia cespitosa#IADP
Poales Poaceae Deschampsia antarctica#NC 023533
Poales Poaceae Dactylis glomerata#NC 027473
Poales Poaceae Lamarckia aurea#NC 037168
Poales Poaceae Catapodium rigidum#NC 036711
Poales Poaceae Cynosurus cristatus#NC 036713
Poales Poaceae Festuca ovina#NC 019649
Poales Poaceae Festuca altissima#NC 019648
Poales Poaceae Festuca arundinacea#NC 011713
Poales Poaceae Festuca pratensis#NC 019650
Poales Poaceae Lolium perenne#NC 009950
Poales Poaceae Lolium multiflorum#NC 019651
Poales Poaceae Littledalea przevalskyi#NC 037497
Poales Poaceae Littledalea alaica#NC 037519
Poales Poaceae Littledalea racemosa#NC 036350
Poales Poaceae Bromus vulgaris#NC 027472
Poales Poaceae Hordeum vulgare#NC 008590
Poales Poaceae Hordeum jubatum#NC 027476
Poales Poaceae Henrardia persica#MH285853
Poales Poaceae Eremopyrum distans#MH285851
Poales Poaceae Eremopyrum triticeum#MH285852
Poales Poaceae Agropyron cristatum#KY126307
Poales Poaceae Agropyron mongolicum#MH285848
Poales Poaceae Dasypyrum villosum#MH285850
Poales Poaceae Elymus libanoticus#KX822019
Poales Poaceae Pseudoroegneria spicata#MH285855
Poales Poaceae Connorochloa tenuis#NC 037165
Poales Poaceae Heteranthelium piliferum#MH285854
Poales Poaceae Crithopsis delileana#MH285849
Poales Poaceae Secale cereale#NC 021761
Poales Poaceae Taeniatherum caput-medusae#MH285856
Poales Poaceae Aegilops speltoides#NC 022135
Poales Poaceae Triticum timopheevii#NC 024764
Poales Poaceae Triticum turgidum#NC 024814
Poales Poaceae Triticum macha#LC372826
Poales Poaceae Triticum durum#LC377262
Poales Poaceae Triticum dicoccon#LC375229
Poales Poaceae Triticum carthlicum#LC375535
Poales Poaceae Triticum urartu#KJ174105
Poales Poaceae Triticum aestivum#KX631429
Poales Poaceae Triticum monococcum#NC 021760
Poales Poaceae Aegilops tauschii#NC 022133
Poales Poaceae Aegilops cylindrica#NC 023096
Poales Poaceae Aegilops geniculata#NC 023097
Poales Poaceae Aegilops searsii#NC 024815
Poales Poaceae Aegilops bicornis#NC 024831
Poales Poaceae Aegilops kotschyi#NC 024832
Poales Poaceae Aegilops longissima#NC 024830
Poales Poaceae Aegilops sharonensis#NC 024816
Poales Poaceae Aristida rufescens#NC 036130
Poales Poaceae Aristida stricta#HATH
Poales Poaceae Aristida ternipes#NC 037164
Poales Poaceae Aristida purpurea#NC 025228
Poales Poaceae Stipagrostis hirtigluma#KY432797
Poales Poaceae Stipagrostis uniplumis#MF460973
Poales Poaceae Sartidia isaloensis#NC 036117
Poales Poaceae Sartidia dewinteri#NC 027147
Poales Poaceae Sartidia perrieri#NC 027146
Poales Poaceae Dregeochloa pumila#NC 035526
Poales Poaceae Monachather paradoxus#MF035990
Poales Poaceae Arundo donax#MF035971
Poales Poaceae Arundo plinii#NC 034652
Poales Poaceae Amphipogon caricinus#NC 035520
Poales Poaceae Amphipogon turbinatus#NC 035521
Poales Poaceae Phragmites australis#KX229727
Poales Poaceae Hakonechloa macra#MF035984
Poales Poaceae Molinia caerulea#MF035988
Poales Poaceae Eragrostis walteri#NC 035528
Poales Poaceae Elytrophorus globularis#NC 035527
Poales Poaceae Elytrophorus spicatus#NC 025233
Poales Poaceae Styppeiochloa gynoglossa#NC 035535
Poales Poaceae Crinipes abyssinicus#NC 035523
Poales Poaceae Crinipes longifolius#NC 035524
Poales Poaceae Coelachne africana#MF563382
Poales Poaceae Isachne distichophylla#NC 025236
Poales Poaceae Limnopoa meeboldii#NC 035531
Poales Poaceae Isachne albens#NC 035530
Poales Poaceae Eriachne compacta#NC 041564
Poales Poaceae Eriachne obtusa#MH036259
Poales Poaceae Eriachne major#MH036252
Poales Poaceae Pheidochloa gracilis#NC 035534
Poales Poaceae Eriachne triseta#NC 041249
Poales Poaceae Eriachne pallescens#NC 041569

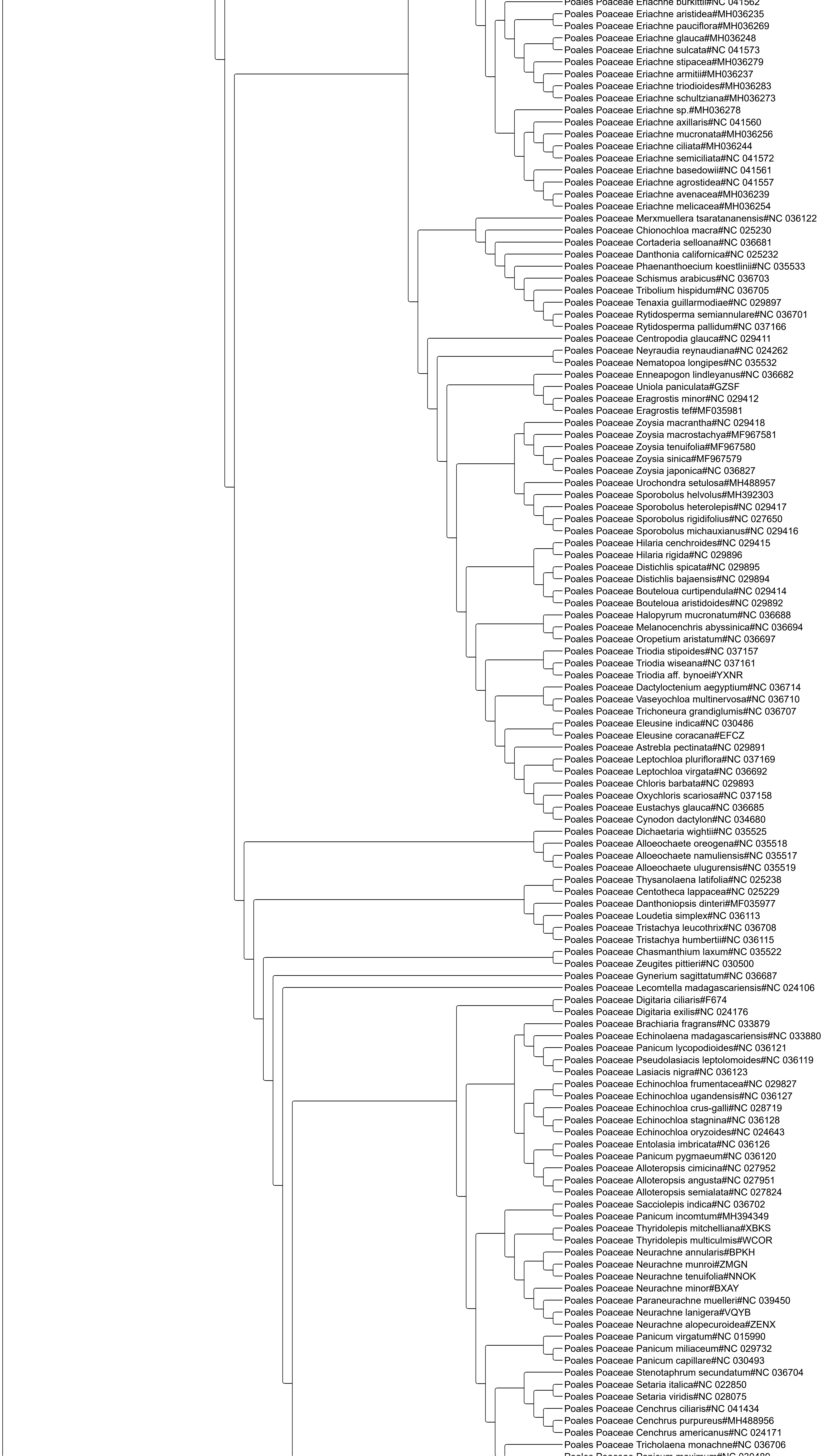

Poales Poaceae Eriachne burkittii#NC 041562
Poales Poaceae Eriachne aristidea#MH036235
Poales Poaceae Eriachne pauciflora#MH036269
Poales Poaceae Eriachne glauca#MH036248
Poales Poaceae Eriachne sulcata#NC 041573
Poales Poaceae Eriachne stipacea#MH036279
Poales Poaceae Eriachne armitii#MH036237
Poales Poaceae Eriachne triodioides#MH036283
Poales Poaceae Eriachne schultziana#MH036273
Poales Poaceae Eriachne sp.#MH036278
Poales Poaceae Eriachne axillaris#NC 041560
Poales Poaceae Eriachne mucronata#MH036256
Poales Poaceae Eriachne ciliata#MH036244
Poales Poaceae Eriachne semiciliata#NC 041572
Poales Poaceae Eriachne basedowii#NC 041561
Poales Poaceae Eriachne agrostidea#NC 041557
Poales Poaceae Eriachne avenacea#MH036239
Poales Poaceae Eriachne melicacea#MH036254
Poales Poaceae Merxmuellera tsaratananensis#NC 036122
Poales Poaceae Chionochloa macra#NC 025230
Poales Poaceae Cortaderia selloana#NC 036681
Poales Poaceae Danthonia californica#NC 025232
Poales Poaceae Phaenanthoecium koestlinii#NC 035533
Poales Poaceae Schismus arabicus#NC 036703
Poales Poaceae Tribolium hispidum#NC 036705
Poales Poaceae Tenaxia guillarmodiae#NC 029897
Poales Poaceae Rytidosperma semiannulare#NC 036701
Poales Poaceae Rytidosperma pallidum#NC 037166
Poales Poaceae Centropodia glauca#NC 029411
Poales Poaceae Neyraudia reynaudiana#NC 024262
Poales Poaceae Nematopoa longipes#NC 035532
Poales Poaceae Enneapogon lindleyanus#NC 036682
Poales Poaceae Uniola paniculata#GZSF
Poales Poaceae Eragrostis minor#NC 029412
Poales Poaceae Eragrostis tef#MF035981
Poales Poaceae Zoysia macrantha#NC 029418
Poales Poaceae Zoysia macrostachya#MF967581
Poales Poaceae Zoysia tenuifolia#MF967580
Poales Poaceae Zoysia sinica#MF967579
Poales Poaceae Zoysia japonica#NC 036827
Poales Poaceae Urochondra setulosa#MH488957
Poales Poaceae Sporobolus helvolus#MH392303
Poales Poaceae Sporobolus heterolepis#NC 029417
Poales Poaceae Sporobolus rigidifolius#NC 027650
Poales Poaceae Sporobolus michauxianus#NC 029416
Poales Poaceae Hilaria cenchroides#NC 029415
Poales Poaceae Hilaria rigida#NC 029896
Poales Poaceae Distichlis spicata#NC 029895
Poales Poaceae Distichlis bajaensis#NC 029894
Poales Poaceae Bouteloua curtipendula#NC 029414
Poales Poaceae Bouteloua aristidoides#NC 029892
Poales Poaceae Halopyrum mucronatum#NC 036688
Poales Poaceae Melanocenchris abyssinica#NC 036694
Poales Poaceae Oropetium aristatum#NC 036697
Poales Poaceae Triodia stipoides#NC 037157
Poales Poaceae Triodia wiseana#NC 037161
Poales Poaceae Triodia aff. bynoei#YXNR
Poales Poaceae Dactyloctenium aegyptium#NC 036714
Poales Poaceae Vaseyochloa multinervosa#NC 036710
Poales Poaceae Trichoneura grandiglumis#NC 036707
Poales Poaceae Eleusine indica#NC 030486
Poales Poaceae Eleusine coracana#EFCZ
Poales Poaceae Astrebla pectinata#NC 029891
Poales Poaceae Leptochloa pluriflora#NC 037169
Poales Poaceae Leptochloa virgata#NC 036692
Poales Poaceae Chloris barbata#NC 029893
Poales Poaceae Oxychloris scariosa#NC 037158
Poales Poaceae Eustachys glauca#NC 036685
Poales Poaceae Cynodon dactylon#NC 034680
Poales Poaceae Dichaetaria wightii#NC 035525
Poales Poaceae Alloeochaete oreogena#NC 035518
Poales Poaceae Alloeochaete namuliensis#NC 035517
Poales Poaceae Alloeochaete ulugurensis#NC 035519
Poales Poaceae Thysanolaena latifolia#NC 025238
Poales Poaceae Centotheca lappacea#NC 025229
Poales Poaceae Danthoniopsis dinteri#MF035977
Poales Poaceae Loudetia simplex#NC 036113
Poales Poaceae Tristachya leucothrix#NC 036708
Poales Poaceae Tristachya humbertii#NC 036115
Poales Poaceae Chasmanthium laxum#NC 035522
Poales Poaceae Zeugites pittieri#NC 030500
Poales Poaceae Gynerium sagittatum#NC 036687
Poales Poaceae Lecomtella madagascariensis#NC 024106
Poales Poaceae Digitaria ciliaris#F674
Poales Poaceae Digitaria exilis#NC 024176
Poales Poaceae Brachiaria fragrans#NC 033879
Poales Poaceae Echinolaena madagascariensis#NC 033880
Poales Poaceae Panicum lycopodioides#NC 036121
Poales Poaceae Pseudolasiacis leptolomoides#NC 036119
Poales Poaceae Lasiacis nigra#NC 036123
Poales Poaceae Echinochloa frumentacea#NC 029827
Poales Poaceae Echinochloa ugandensis#NC 036127
Poales Poaceae Echinochloa crus-galli#NC 028719
Poales Poaceae Echinochloa stagnina#NC 036128
Poales Poaceae Echinochloa oryzoides#NC 024643
Poales Poaceae Entolasia imbricata#NC 036126
Poales Poaceae Panicum pygmaeum#NC 036120
Poales Poaceae Alloteropsis cimicina#NC 027952
Poales Poaceae Alloteropsis angusta#NC 027951
Poales Poaceae Alloteropsis semialata#NC 027824
Poales Poaceae Sacciolepis indica#NC 036702
Poales Poaceae Panicum incomtum#MH394349
Poales Poaceae Thyridolepis mitchelliana#XBKS
Poales Poaceae Thyridolepis multiculmis#WCOR
Poales Poaceae Neurachne annularis#BPKH
Poales Poaceae Neurachne munroi#ZMGN
Poales Poaceae Neurachne tenuifolia#NNOK
Poales Poaceae Neurachne minor#BXAY
Poales Poaceae Paraneurachne muelleri#NC 039450
Poales Poaceae Neurachne lanigera#VQYB
Poales Poaceae Neurachne alopecuroidea#ZENX
Poales Poaceae Panicum virgatum#NC 015990
Poales Poaceae Panicum miliaceum#NC 029732
Poales Poaceae Panicum capillare#NC 030493
Poales Poaceae Stenotaphrum secundatum#NC 036704
Poales Poaceae Setaria italica#NC 022850
Poales Poaceae Setaria viridis#NC 028075
Poales Poaceae Cenchrus ciliaris#NC 041434
Poales Poaceae Cenchrus purpureus#MH488956
Poales Poaceae Cenchrus americanus#NC 024171
Poales Poaceae Tricholaena monachne#NC 036706

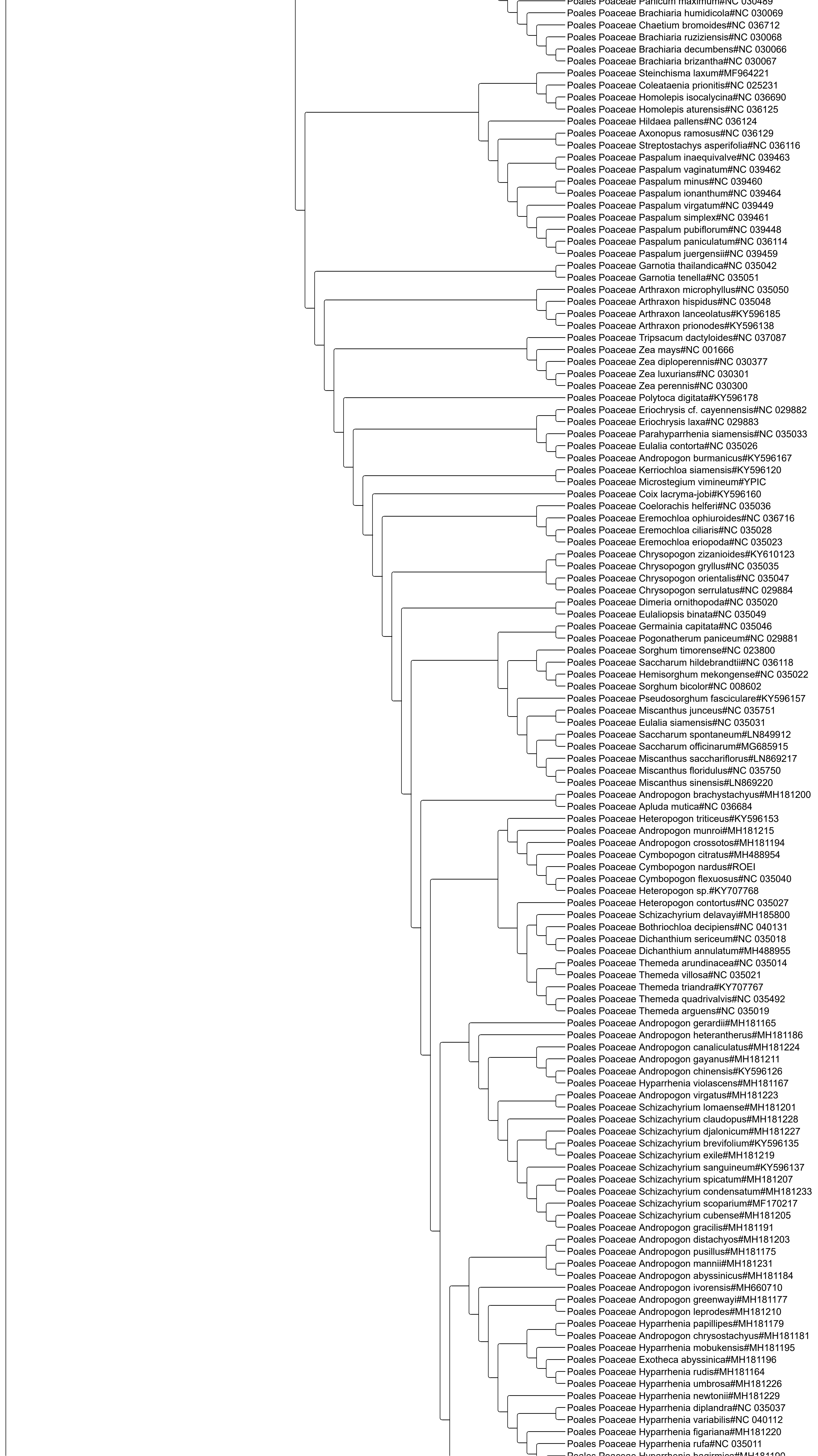
Poales Poaceae Panicum maximum#NC 030489
Poales Poaceae Brachiaria humidicola#NC 030069
Poales Poaceae Chaetium bromoides#NC 036712
Poales Poaceae Brachiaria ruziziensis#NC 030068
Poales Poaceae Brachiaria decumbens#NC 030066
Poales Poaceae Brachiaria brizantha#NC 030067
Poales Poaceae Steinchisma laxum#MF964221
Poales Poaceae Coleataenia prionitis#NC 025231
Poales Poaceae Homolepis isocalycina#NC 036690
Poales Poaceae Homolepis aturensis#NC 036125
Poales Poaceae Hildaea pallens#NC 036124
Poales Poaceae Axonopus ramosus#NC 036129
Poales Poaceae Streptostachys asperifolia#NC 036116
Poales Poaceae Paspalum inaequivalve#NC 039463
Poales Poaceae Paspalum vaginatum#NC 039462
Poales Poaceae Paspalum minus#NC 039460
Poales Poaceae Paspalum ionanthum#NC 039464
Poales Poaceae Paspalum virgatum#NC 039449
Poales Poaceae Paspalum simplex#NC 039461
Poales Poaceae Paspalum pubiflorum#NC 039448
Poales Poaceae Paspalum paniculatum#NC 036114
Poales Poaceae Paspalum juergensii#NC 039459
Poales Poaceae Garnotia thailandica#NC 035042
Poales Poaceae Garnotia tenella#NC 035051
Poales Poaceae Arthraxon microphyllus#NC 035050
Poales Poaceae Arthraxon hispidus#NC 035048
Poales Poaceae Arthraxon lanceolatus#KY596185
Poales Poaceae Arthraxon prionodes#KY596138
Poales Poaceae Tripsacum dactyloides#NC 037087
Poales Poaceae Zea mays#NC 001666
Poales Poaceae Zea diploperennis#NC 030377
Poales Poaceae Zea luxurians#NC 030301
Poales Poaceae Zea perennis#NC 030300
Poales Poaceae Polytoca digitata#KY596178
Poales Poaceae Eriochrysis cf. cayennensis#NC 029882
Poales Poaceae Eriochrysis laxa#NC 029883
Poales Poaceae Parahyparrhenia siamensis#NC 035033
Poales Poaceae Eulalia contorta#NC 035026
Poales Poaceae Andropogon burmanicus#KY596167
Poales Poaceae Kerriochloa siamensis#KY596120
Poales Poaceae Microstegium vimineum#YPIC
Poales Poaceae Coix lacryma-jobi#KY596160
Poales Poaceae Coelorachis helferi#NC 035036
Poales Poaceae Eremochloa ophiuroides#NC 036716
Poales Poaceae Eremochloa ciliaris#NC 035028
Poales Poaceae Eremochloa eriopoda#NC 035023
Poales Poaceae Chrysopogon zizanioides#KY610123
Poales Poaceae Chrysopogon gryllus#NC 035035
Poales Poaceae Chrysopogon orientalis#NC 035047
Poales Poaceae Chrysopogon serrulatus#NC 029884
Poales Poaceae Dimeria ornithopoda#NC 035020
Poales Poaceae Eulaliopsis binata#NC 035049
Poales Poaceae Germainia capitata#NC 035046
Poales Poaceae Pogonatherum paniceum#NC 029881
Poales Poaceae Sorghum timorense#NC 023800
Poales Poaceae Saccharum hildebrandtii#NC 036118
Poales Poaceae Hemisorghum mekongense#NC 035022
Poales Poaceae Sorghum bicolor#NC 008602
Poales Poaceae Pseudosorghum fasciculare#KY596157
Poales Poaceae Miscanthus junceus#NC 035751
Poales Poaceae Eulalia siamensis#NC 035031
Poales Poaceae Saccharum spontaneum#LN849912
Poales Poaceae Saccharum officinarum#MG685915
Poales Poaceae Miscanthus sacchariflorus#LN869217
Poales Poaceae Miscanthus floridulus#NC 035750
Poales Poaceae Miscanthus sinensis#LN869220
Poales Poaceae Andropogon brachystachyus#MH181200
Poales Poaceae Apluda mutica#NC 036684
Poales Poaceae Heteropogon triticeus#KY596153
Poales Poaceae Andropogon munroi#MH181215
Poales Poaceae Andropogon crossotos#MH181194
Poales Poaceae Cymbopogon citratus#MH488954
Poales Poaceae Cymbopogon nardus#ROEI
Poales Poaceae Cymbopogon flexuosus#NC 035040
Poales Poaceae Heteropogon sp.#KY707768
Poales Poaceae Heteropogon contortus#NC 035027
Poales Poaceae Schizachyrium delavayi#MH185800
Poales Poaceae Bothriochloa decipiens#NC 040131
Poales Poaceae Dichanthium sericeum#NC 035018
Poales Poaceae Dichanthium annulatum#MH488955
Poales Poaceae Themeda arundinacea#NC 035014
Poales Poaceae Themeda villosa#NC 035021
Poales Poaceae Themeda triandra#KY707767
Poales Poaceae Themeda quadrivalvis#NC 035492
Poales Poaceae Themeda arguens#NC 035019
Poales Poaceae Andropogon gerardii#MH181165
Poales Poaceae Andropogon heterantherus#MH181186
Poales Poaceae Andropogon canaliculatus#MH181224
Poales Poaceae Andropogon gayanus#MH181211
Poales Poaceae Andropogon chinensis#KY596126
Poales Poaceae Hyparrhenia violascens#MH181167
Poales Poaceae Andropogon virgatus#MH181223
Poales Poaceae Schizachyrium lomaense#MH181201
Poales Poaceae Schizachyrium claudopus#MH181228
Poales Poaceae Schizachyrium djalonicum#MH181227
Poales Poaceae Schizachyrium brevifolium#KY596135
Poales Poaceae Schizachyrium exile#MH181219
Poales Poaceae Schizachyrium sanguineum#KY596137
Poales Poaceae Schizachyrium spicatum#MH181207
Poales Poaceae Schizachyrium condensatum#MH181233
Poales Poaceae Schizachyrium scoparium#MF170217
Poales Poaceae Schizachyrium cubense#MH181205
Poales Poaceae Andropogon gracilis#MH181191
Poales Poaceae Andropogon distachyos#MH181203
Poales Poaceae Andropogon pusillus#MH181175
Poales Poaceae Andropogon mannii#MH181231
Poales Poaceae Andropogon abyssinicus#MH181184
Poales Poaceae Andropogon ivorensis#MH660710
Poales Poaceae Andropogon greenwayi#MH181177
Poales Poaceae Andropogon leprodes#MH181210
Poales Poaceae Hyparrhenia papillipes#MH181179
Poales Poaceae Andropogon chrysostachyus#MH181181
Poales Poaceae Hyparrhenia mobukensis#MH181195
Poales Poaceae Exotheca abyssinica#MH181196
Poales Poaceae Hyparrhenia rudis#MH181164
Poales Poaceae Hyparrhenia umbrosa#MH181226
Poales Poaceae Hyparrhenia newtonii#MH181229
Poales Poaceae Hyparrhenia diplandra#NC 035037
Poales Poaceae Hyparrhenia variabilis#NC 040112
Poales Poaceae Hyparrhenia figariana#MH181220
Poales Poaceae Hyparrhenia rufa#NC 035011

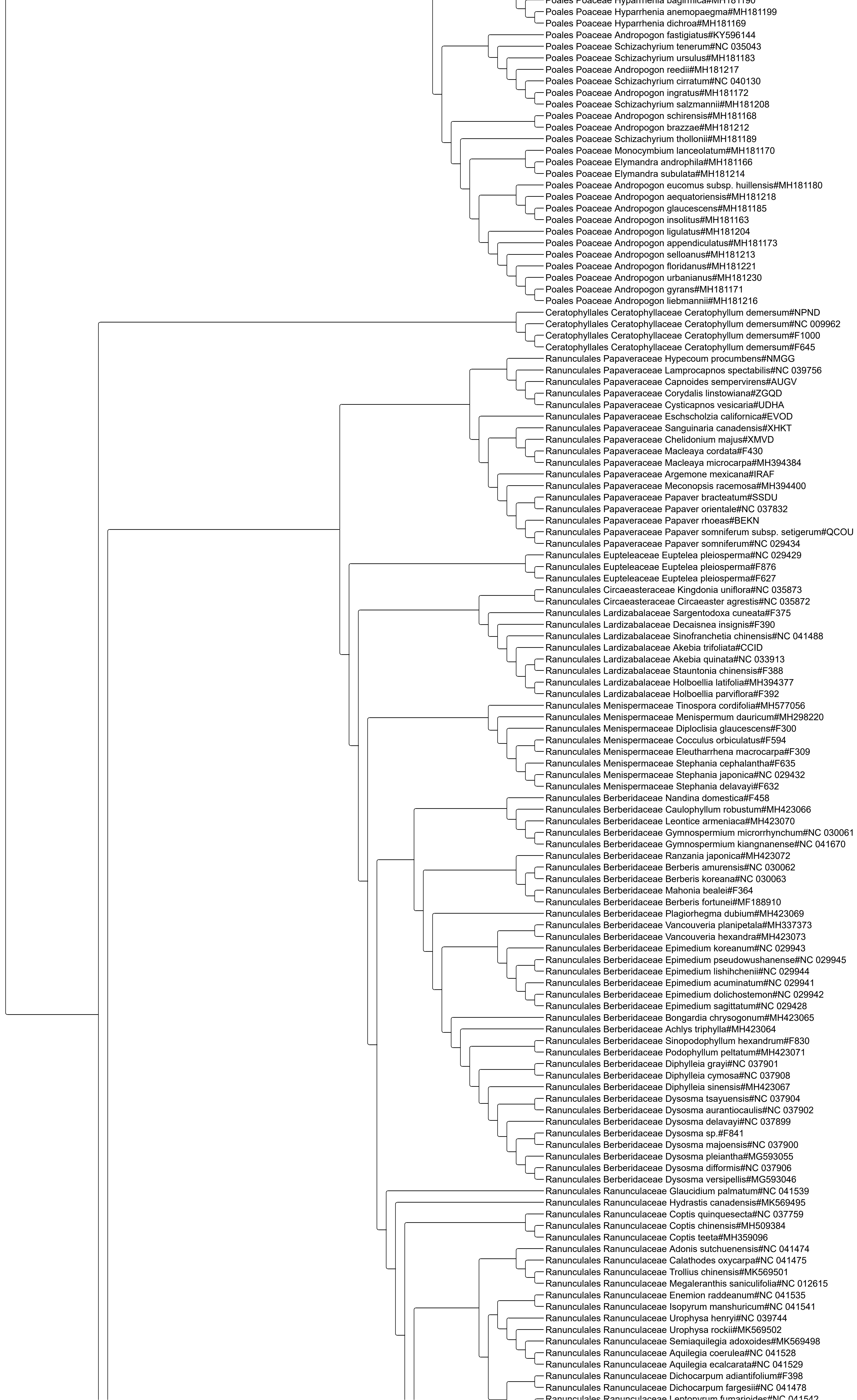

Poales Poaceae Hyparrhenia anemopaegma#MH181199
Poales Poaceae Hyparrhenia dichroa#MH181169
Poales Poaceae Andropogon fastigiatus#KY596144
Poales Poaceae Schizachyrium tenerum#NC 035043
Poales Poaceae Schizachyrium ursulus#MH181183
Poales Poaceae Andropogon reedii#MH181217
Poales Poaceae Schizachyrium cirratum#NC 040130
Poales Poaceae Andropogon ingratus#MH181172
Poales Poaceae Schizachyrium salzmannii#MH181208
Poales Poaceae Andropogon schirensis#MH181168
Poales Poaceae Andropogon brazzae#MH181212
Poales Poaceae Schizachyrium thollonii#MH181189
Poales Poaceae Monocymbium lanceolatum#MH181170
Poales Poaceae Elymandra androphila#MH181166
Poales Poaceae Elymandra subulata#MH181214
Poales Poaceae Andropogon eucomus subsp. huillensis#MH181180
Poales Poaceae Andropogon aequatoriensis#MH181218
Poales Poaceae Andropogon glaucescens#MH181185
Poales Poaceae Andropogon insolitus#MH181163
Poales Poaceae Andropogon ligulatus#MH181204
Poales Poaceae Andropogon appendiculatus#MH181173
Poales Poaceae Andropogon selloanus#MH181213
Poales Poaceae Andropogon floridanus#MH181221
Poales Poaceae Andropogon urbanianus#MH181230
Poales Poaceae Andropogon gyrans#MH181171
Poales Poaceae Andropogon liebmannii#MH181216
Ceratophyllales Ceratophyllaceae Ceratophyllum demersum#NPND
Ceratophyllales Ceratophyllaceae Ceratophyllum demersum#NC 009962
Ceratophyllales Ceratophyllaceae Ceratophyllum demersum#F1000
Ceratophyllales Ceratophyllaceae Ceratophyllum demersum#F645
Ranunculales Papaveraceae Hypecoum procumbens#NMGG
Ranunculales Papaveraceae Lamprocapnos spectabilis#NC 039756
Ranunculales Papaveraceae Capnoides sempervirens#AUGV
Ranunculales Papaveraceae Corydalis linstowiana#ZGQD
Ranunculales Papaveraceae Cysticapnos vesicaria#UDHA
Ranunculales Papaveraceae Eschscholzia californica#EVOD
Ranunculales Papaveraceae Sanguinaria canadensis#XHKT
Ranunculales Papaveraceae Chelidonium majus#XMVD
Ranunculales Papaveraceae Macleaya cordata#F430
Ranunculales Papaveraceae Macleaya microcarpa#MH394384
Ranunculales Papaveraceae Argemone mexicana#IRAF
Ranunculales Papaveraceae Meconopsis racemosa#MH394400
Ranunculales Papaveraceae Papaver bracteatum#SSDU
Ranunculales Papaveraceae Papaver orientale#NC 037832
Ranunculales Papaveraceae Papaver rhoeas#BEKN
Ranunculales Papaveraceae Papaver somniferum subsp. setigerum#QCOU
Ranunculales Papaveraceae Papaver somniferum#NC 029434
Ranunculales Eupteleaceae Euptelea pleiosperma#NC 029429
Ranunculales Eupteleaceae Euptelea pleiosperma#F876
Ranunculales Eupteleaceae Euptelea pleiosperma#F627
Ranunculales Circaeasteraceae Kingdonia uniflora#NC 035873
Ranunculales Circaeasteraceae Circaeaster agrestis#NC 035872
Ranunculales Lardizabalaceae Sargentodoxa cuneata#F375
Ranunculales Lardizabalaceae Decaisnea insignis#F390
Ranunculales Lardizabalaceae Sinofranchetia chinensis#NC 041488
Ranunculales Lardizabalaceae Akebia trifoliata#CCID
Ranunculales Lardizabalaceae Akebia quinata#NC 033913
Ranunculales Lardizabalaceae Stauntonia chinensis#F388
Ranunculales Lardizabalaceae Holboellia latifolia#MH394377
Ranunculales Lardizabalaceae Holboellia parviflora#F392
Ranunculales Menispermaceae Tinospora cordifolia#MH577056
Ranunculales Menispermaceae Menispermum dauricum#MH298220
Ranunculales Menispermaceae Diploclisia glaucescens#F300
Ranunculales Menispermaceae Cocculus orbiculatus#F594
Ranunculales Menispermaceae Eleutharrhena macrocarpa#F309
Ranunculales Menispermaceae Stephania cephalantha#F635
Ranunculales Menispermaceae Stephania japonica#NC 029432
Ranunculales Menispermaceae Stephania delavayi#F632
Ranunculales Berberidaceae Nandina domestica#F458
Ranunculales Berberidaceae Caulophyllum robustum#MH423066
Ranunculales Berberidaceae Leontice armeniaca#MH423070
Ranunculales Berberidaceae Gymnospermium microrrhynchum#NC 030061
Ranunculales Berberidaceae Gymnospermium kiangnanense#NC 041670
Ranunculales Berberidaceae Ranzania japonica#MH423072
Ranunculales Berberidaceae Berberis amurensis#NC 030062
Ranunculales Berberidaceae Berberis koreana#NC 030063
Ranunculales Berberidaceae Mahonia bealei#F364
Ranunculales Berberidaceae Berberis fortunei#MF188910
Ranunculales Berberidaceae Plagiorhegma dubium#MH423069
Ranunculales Berberidaceae Vancouveria planipetala#MH337373
Ranunculales Berberidaceae Vancouveria hexandra#MH423073
Ranunculales Berberidaceae Epimedium koreanum#NC 029943
Ranunculales Berberidaceae Epimedium pseudowushanense#NC 029945
Ranunculales Berberidaceae Epimedium lishihchenii#NC 029944
Ranunculales Berberidaceae Epimedium acuminatum#NC 029941
Ranunculales Berberidaceae Epimedium dolichostemon#NC 029942
Ranunculales Berberidaceae Epimedium sagittatum#NC 029428
Ranunculales Berberidaceae Bongardia chrysogonum#MH423065
Ranunculales Berberidaceae Achlys triphylla#MH423064
Ranunculales Berberidaceae Sinopodophyllum hexandrum#F830
Ranunculales Berberidaceae Podophyllum peltatum#MH423071
Ranunculales Berberidaceae Diphylleia grayi#NC 037901
Ranunculales Berberidaceae Diphylleia cymosa#NC 037908
Ranunculales Berberidaceae Diphylleia sinensis#MH423067
Ranunculales Berberidaceae Dysosma tsayuensis#NC 037904
Ranunculales Berberidaceae Dysosma aurantiocaulis#NC 037902
Ranunculales Berberidaceae Dysosma delavayi#NC 037899
Ranunculales Berberidaceae Dysosma sp.#F841
Ranunculales Berberidaceae Dysosma majoensis#NC 037900
Ranunculales Berberidaceae Dysosma pleiantha#MG593055
Ranunculales Berberidaceae Dysosma difformis#NC 037906
Ranunculales Berberidaceae Dysosma versipellis#MG593046
Ranunculales Ranunculaceae Glaucidium palmatum#NC 041539
Ranunculales Ranunculaceae Hydrastis canadensis#MK569495
Ranunculales Ranunculaceae Coptis quinquesecta#NC 037759
Ranunculales Ranunculaceae Coptis chinensis#MH509384
Ranunculales Ranunculaceae Coptis teeta#MH359096
Ranunculales Ranunculaceae Adonis sutchuenensis#NC 041474
Ranunculales Ranunculaceae Calathodes oxycarpa#NC 041475
Ranunculales Ranunculaceae Trollius chinensis#MK569501
Ranunculales Ranunculaceae Megaleranthis saniculifolia#NC 012615
Ranunculales Ranunculaceae Enemion raddeanum#NC 041535
Ranunculales Ranunculaceae Isopyrum manshuricum#NC 041541
Ranunculales Ranunculaceae Urophysa henryi#NC 039744
Ranunculales Ranunculaceae Urophysa rockii#MK569502
Ranunculales Ranunculaceae Semiaquilegia adoxoides#MK569498
Ranunculales Ranunculaceae Aquilegia coerulea#NC 041528
Ranunculales Ranunculaceae Aquilegia ecalcarata#NC 041529
Ranunculales Ranunculaceae Dichocarpum adiantifolium#F398
Ranunculales Ranunculaceae Dichocarpum fargesii#NC 041478

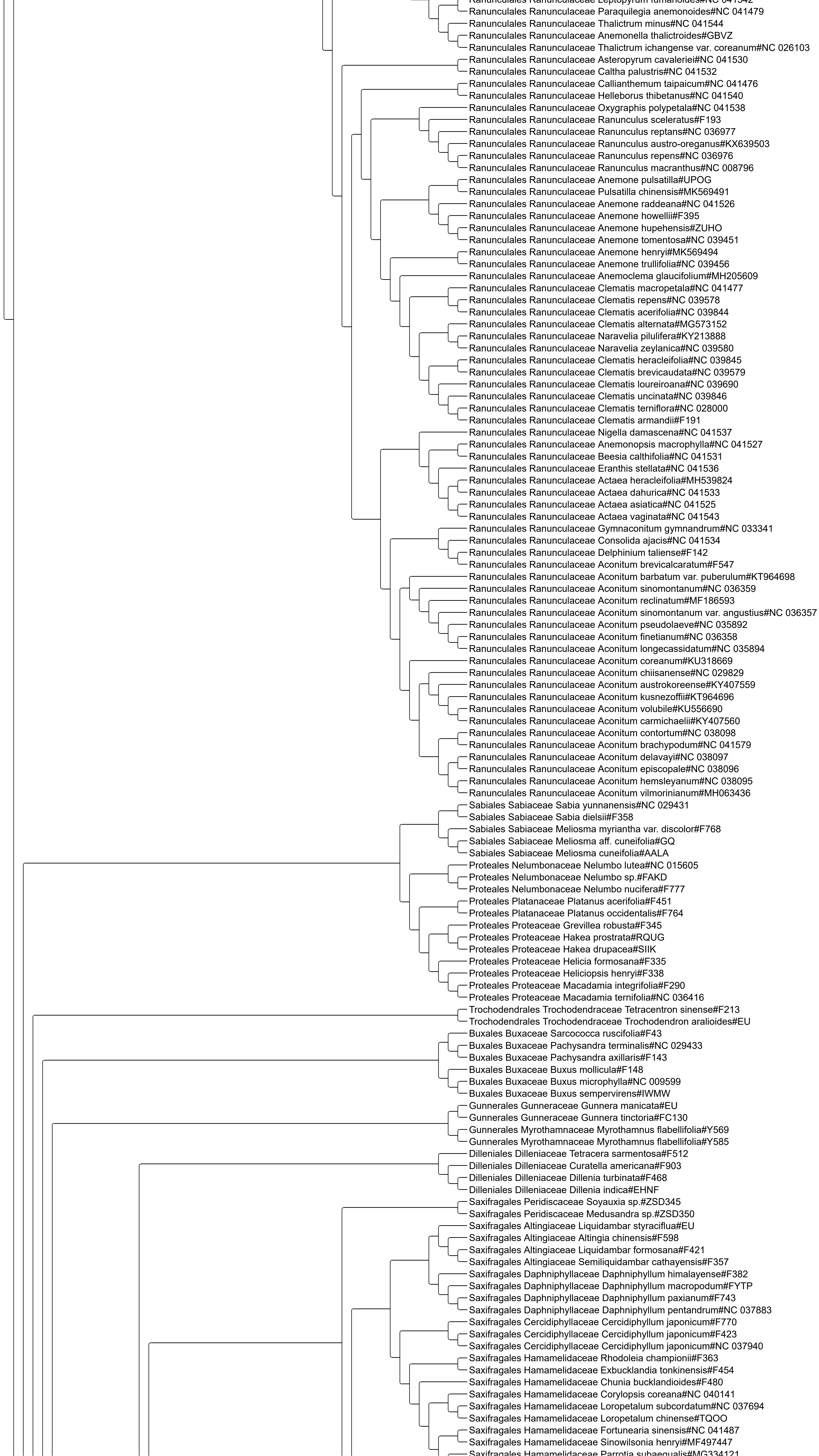
Ranunculales Ranunculaceae Leptopyrum fumarioides#NC 041542
Ranunculales Ranunculaceae Paraquilegia anemonoides#NC 041479
Ranunculales Ranunculaceae Thalictrum minus#NC 041544
Ranunculales Ranunculaceae Anemonella thalictroides#GBVZ
Ranunculales Ranunculaceae Thalictrum ichangense var. coreanum#NC 026103
Ranunculales Ranunculaceae Asteropyrum cavaleriei#NC 041530
Ranunculales Ranunculaceae Caltha palustris#NC 041532
Ranunculales Ranunculaceae Callianthemum taipaicum#NC 041476
Ranunculales Ranunculaceae Helleborus thibetanus#NC 041540
Ranunculales Ranunculaceae Oxygraphis polypetala#NC 041538
Ranunculales Ranunculaceae Ranunculus sceleratus#F193
Ranunculales Ranunculaceae Ranunculus reptans#NC 036977
Ranunculales Ranunculaceae Ranunculus austro-oreganus#KX639503
Ranunculales Ranunculaceae Ranunculus repens#NC 036976
Ranunculales Ranunculaceae Ranunculus macranthus#NC 008796
Ranunculales Ranunculaceae Anemone pulsatilla#UPOG
Ranunculales Ranunculaceae Pulsatilla chinensis#MK569491
Ranunculales Ranunculaceae Anemone raddeana#NC 041526
Ranunculales Ranunculaceae Anemone howellii#F395
Ranunculales Ranunculaceae Anemone hupehensis#ZUHO
Ranunculales Ranunculaceae Anemone tomentosa#NC 039451
Ranunculales Ranunculaceae Anemone henryi#MK569494
Ranunculales Ranunculaceae Anemone trullifolia#NC 039456
Ranunculales Ranunculaceae Anemoclema glaucifolium#MH205609
Ranunculales Ranunculaceae Clematis macropetala#NC 041477
Ranunculales Ranunculaceae Clematis repens#NC 039578
Ranunculales Ranunculaceae Clematis acerifolia#NC 039844
Ranunculales Ranunculaceae Clematis alternata#MG573152
Ranunculales Ranunculaceae Naravelia pilulifera#KY213888
Ranunculales Ranunculaceae Naravelia zeylanica#NC 039580
Ranunculales Ranunculaceae Clematis heracleifolia#NC 039845
Ranunculales Ranunculaceae Clematis brevicaudata#NC 039579
Ranunculales Ranunculaceae Clematis loureiroana#NC 039690
Ranunculales Ranunculaceae Clematis uncinata#NC 039846
Ranunculales Ranunculaceae Clematis terniflora#NC 028000
Ranunculales Ranunculaceae Clematis armandii#F191
Ranunculales Ranunculaceae Nigella damascena#NC 041537
Ranunculales Ranunculaceae Anemonopsis macrophylla#NC 041527
Ranunculales Ranunculaceae Beesia calthifolia#NC 041531
Ranunculales Ranunculaceae Eranthis stellata#NC 041536
Ranunculales Ranunculaceae Actaea heracleifolia#MH539824
Ranunculales Ranunculaceae Actaea dahurica#NC 041533
Ranunculales Ranunculaceae Actaea asiatica#NC 041525
Ranunculales Ranunculaceae Actaea vaginata#NC 041543
Ranunculales Ranunculaceae Gymnaconitum gymnandrum#NC 033341
Ranunculales Ranunculaceae Consolida ajacis#NC 041534
Ranunculales Ranunculaceae Delphinium taliense#F142
Ranunculales Ranunculaceae Aconitum brevicalcaratum#F547
Ranunculales Ranunculaceae Aconitum barbatum var. puberulum#KT964698
Ranunculales Ranunculaceae Aconitum sinomontanum#NC 036359
Ranunculales Ranunculaceae Aconitum reclinatum#MF186593
Ranunculales Ranunculaceae Aconitum sinomontanum var. angustius#NC 036357
Ranunculales Ranunculaceae Aconitum pseudolaeve#NC 035892
Ranunculales Ranunculaceae Aconitum finetianum#NC 036358
Ranunculales Ranunculaceae Aconitum longecassidatum#NC 035894
Ranunculales Ranunculaceae Aconitum coreanum#KU318669
Ranunculales Ranunculaceae Aconitum chiisanense#NC 029829
Ranunculales Ranunculaceae Aconitum austrokoreense#KY407559
Ranunculales Ranunculaceae Aconitum kusnezoffii#KT964696
Ranunculales Ranunculaceae Aconitum volubile#KU556690
Ranunculales Ranunculaceae Aconitum carmichaelii#KY407560
Ranunculales Ranunculaceae Aconitum contortum#NC 038098
Ranunculales Ranunculaceae Aconitum brachypodum#NC 041579
Ranunculales Ranunculaceae Aconitum delavayi#NC 038097
Ranunculales Ranunculaceae Aconitum episcopale#NC 038096
Ranunculales Ranunculaceae Aconitum hemsleyanum#NC 038095
Ranunculales Ranunculaceae Aconitum vilmorinianum#MH063436
Sabiales Sabiaceae Sabia yunnanensis#NC 029431
Sabiales Sabiaceae Sabia dielsii#F358
Sabiales Sabiaceae Meliosma myriantha var. discolor#F768
Sabiales Sabiaceae Meliosma aff. cuneifolia#GQ
Sabiales Sabiaceae Meliosma cuneifolia#AALA
Proteales Nelumbonaceae Nelumbo lutea#NC 015605
Proteales Nelumbonaceae Nelumbo sp.#FAKD
Proteales Nelumbonaceae Nelumbo nucifera#F777
Proteales Platanaceae Platanus acerifolia#F451
Proteales Platanaceae Platanus occidentalis#F764
Proteales Proteaceae Grevillea robusta#F345
Proteales Proteaceae Hakea prostrata#RQUG
Proteales Proteaceae Hakea drupacea#SIIK
Proteales Proteaceae Helicia formosana#F335
Proteales Proteaceae Heliciopsis henryi#F338
Proteales Proteaceae Macadamia integrifolia#F290
Proteales Proteaceae Macadamia ternifolia#NC 036416
Trochodendrales Trochodendraceae Tetracentron sinense#F213
Trochodendrales Trochodendraceae Trochodendron aralioides#EU
Buxales Buxaceae Sarcococca ruscifolia#F43
Buxales Buxaceae Pachysandra terminalis#NC 029433
Buxales Buxaceae Pachysandra axillaris#F143
Buxales Buxaceae Buxus mollicula#F148
Buxales Buxaceae Buxus microphylla#NC 009599
Buxales Buxaceae Buxus sempervirens#IWMW
Gunnerales Gunneraceae Gunnera manicata#EU
Gunnerales Gunneraceae Gunnera tinctoria#FC130
Gunnerales Myrothamnaceae Myrothamnus flabellifolia#Y569
Gunnerales Myrothamnaceae Myrothamnus flabellifolia#Y585
Dilleniales Dilleniaceae Tetracera sarmentosa#F512
Dilleniales Dilleniaceae Curatella americana#F903
Dilleniales Dilleniaceae Dillenia turbinata#F468
Dilleniales Dilleniaceae Dillenia indica#EHNF
Saxifragales Peridiscaceae Soyauxia sp.#ZSD345
Saxifragales Peridiscaceae Medusandra sp.#ZSD350
Saxifragales Altingiaceae Liquidambar styraciflua#EU
Saxifragales Altingiaceae Altingia chinensis#F598
Saxifragales Altingiaceae Liquidambar formosana#F421
Saxifragales Altingiaceae Semiliquidambar cathayensis#F357
Saxifragales Daphniphyllaceae Daphniphyllum himalayense#F382
Saxifragales Daphniphyllaceae Daphniphyllum macropodum#FYTP
Saxifragales Daphniphyllaceae Daphniphyllum paxianum#F743
Saxifragales Daphniphyllaceae Daphniphyllum pentandrum#NC 037883
Saxifragales Cercidiphyllaceae Cercidiphyllum japonicum#F770
Saxifragales Cercidiphyllaceae Cercidiphyllum japonicum#F423
Saxifragales Cercidiphyllaceae Cercidiphyllum japonicum#NC 037940
Saxifragales Hamamelidaceae Rhodoleia championii#F363
Saxifragales Hamamelidaceae Exbucklandia tonkinensis#F454
Saxifragales Hamamelidaceae Chunia bucklandioides#F480
Saxifragales Hamamelidaceae Corylopsis coreana#NC 040141
Saxifragales Hamamelidaceae Loropetalum subcordatum#NC 037694
Saxifragales Hamamelidaceae Loropetalum chinense#TQOO
Saxifragales Hamamelidaceae Fortunearia sinensis#NC 041487
Saxifragales Hamamelidaceae Sinowilsonia henryi#MF497447
Saxifragales Hamamelidaceae Parrotia subaequalis#MG334121

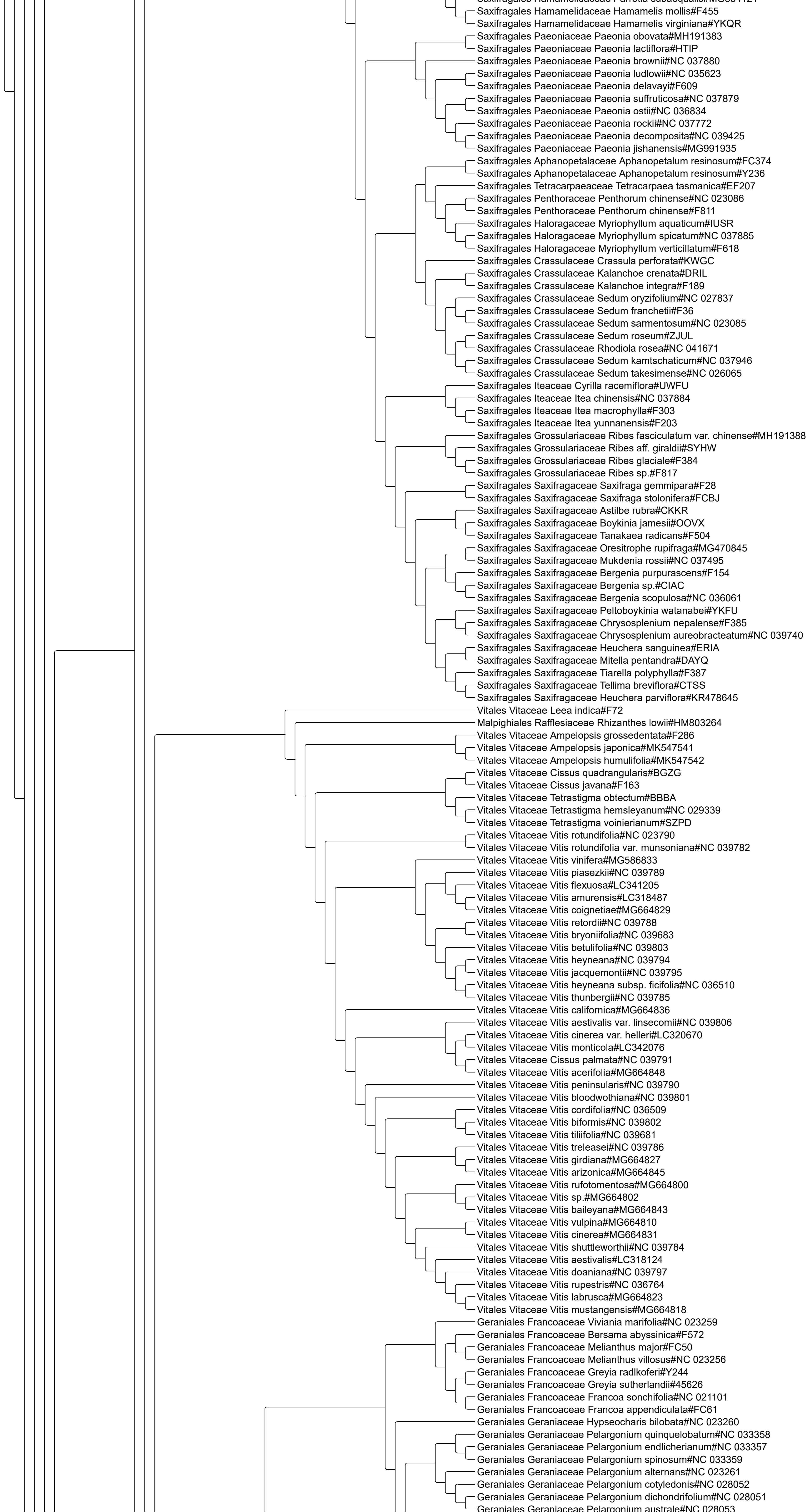
Saxifragales Hamamelidaceae Hamamelis mollis#F455
Saxifragales Hamamelidaceae Hamamelis virginiana#YKQR
Saxifragales Paeoniaceae Paeonia obovata#MH191383
Saxifragales Paeoniaceae Paeonia lactiflora#HTIP
Saxifragales Paeoniaceae Paeonia brownii#NC 037880
Saxifragales Paeoniaceae Paeonia ludlowii#NC 035623
Saxifragales Paeoniaceae Paeonia delavayi#F609
Saxifragales Paeoniaceae Paeonia suffruticosa#NC 037879
Saxifragales Paeoniaceae Paeonia ostii#NC 036834
Saxifragales Paeoniaceae Paeonia rockii#NC 037772
Saxifragales Paeoniaceae Paeonia decomposita#NC 039425
Saxifragales Paeoniaceae Paeonia jishanensis#MG991935
Saxifragales Aphanopetalaceae Aphanopetalum resinosum#FC374
Saxifragales Aphanopetalaceae Aphanopetalum resinosum#Y236
Saxifragales Tetracarpaeaceae Tetracarpaea tasmanica#EF207
Saxifragales Penthoraceae Penthorum chinense#NC 023086
Saxifragales Penthoraceae Penthorum chinense#F811
Saxifragales Haloragaceae Myriophyllum aquaticum#IUSR
Saxifragales Haloragaceae Myriophyllum spicatum#NC 037885
Saxifragales Haloragaceae Myriophyllum verticillatum#F618
Saxifragales Crassulaceae Crassula perforata#KWGC
Saxifragales Crassulaceae Kalanchoe crenata#DRIL
Saxifragales Crassulaceae Kalanchoe integra#F189
Saxifragales Crassulaceae Sedum oryzifolium#NC 027837
Saxifragales Crassulaceae Sedum franchetii#F36
Saxifragales Crassulaceae Sedum sarmentosum#NC 023085
Saxifragales Crassulaceae Sedum roseum#ZJUL
Saxifragales Crassulaceae Rhodiola rosea#NC 041671
Saxifragales Crassulaceae Sedum kamtschaticum#NC 037946
Saxifragales Crassulaceae Sedum takesimense#NC 026065
Saxifragales Iteaceae Cyrilla racemiflora#UWFU
Saxifragales Iteaceae Itea chinensis#NC 037884
Saxifragales Iteaceae Itea macrophylla#F303
Saxifragales Iteaceae Itea yunnanensis#F203
Saxifragales Grossulariaceae Ribes fasciculatum var. chinense#MH191388
Saxifragales Grossulariaceae Ribes aff. giraldii#SYHW
Saxifragales Grossulariaceae Ribes glaciale#F384
Saxifragales Grossulariaceae Ribes sp.#F817
Saxifragales Saxifragaceae Saxifraga gemmipara#F28
Saxifragales Saxifragaceae Saxifraga stolonifera#FCBJ
Saxifragales Saxifragaceae Astilbe rubra#CKKR
Saxifragales Saxifragaceae Boykinia jamesii#OOVX
Saxifragales Saxifragaceae Tanakaea radicans#F504
Saxifragales Saxifragaceae Oresitrophe rupifraga#MG470845
Saxifragales Saxifragaceae Mukdenia rossii#NC 037495
Saxifragales Saxifragaceae Bergenia purpurascens#F154
Saxifragales Saxifragaceae Bergenia sp.#CIAC
Saxifragales Saxifragaceae Bergenia scopulosa#NC 036061
Saxifragales Saxifragaceae Peltoboykinia watanabei#YKFU
Saxifragales Saxifragaceae Chrysosplenium nepalense#F385
Saxifragales Saxifragaceae Chrysosplenium aureobracteatum#NC 039740
Saxifragales Saxifragaceae Heuchera sanguinea#ERIA
Saxifragales Saxifragaceae Mitella pentandra#DAYQ
Saxifragales Saxifragaceae Tiarella polyphylla#F387
Saxifragales Saxifragaceae Tellima breviflora#CTSS
Saxifragales Saxifragaceae Heuchera parviflora#KR478645
Vitales Vitaceae Leea indica#F72
Malpighiales Rafflesiaceae Rhizanthes lowii#HM803264
Vitales Vitaceae Ampelopsis grossedentata#F286
Vitales Vitaceae Ampelopsis japonica#MK547541
Vitales Vitaceae Ampelopsis humulifolia#MK547542
Vitales Vitaceae Cissus quadrangularis#BGZG
Vitales Vitaceae Cissus javana#F163
Vitales Vitaceae Tetrastigma obtectum#BBBA
Vitales Vitaceae Tetrastigma hemsleyanum#NC 029339
Vitales Vitaceae Tetrastigma voinierianum#SZPD
Vitales Vitaceae Vitis rotundifolia#NC 023790
Vitales Vitaceae Vitis rotundifolia var. munsoniana#NC 039782
Vitales Vitaceae Vitis vinifera#MG586833
Vitales Vitaceae Vitis piasezkii#NC 039789
Vitales Vitaceae Vitis flexuosa#LC341205
Vitales Vitaceae Vitis amurensis#LC318487
Vitales Vitaceae Vitis coignetiae#MG664829
Vitales Vitaceae Vitis retordii#NC 039788
Vitales Vitaceae Vitis bryoniifolia#NC 039683
Vitales Vitaceae Vitis betulifolia#NC 039803
Vitales Vitaceae Vitis heyneana#NC 039794
Vitales Vitaceae Vitis jacquemontii#NC 039795
Vitales Vitaceae Vitis heyneana subsp. ficifolia#NC 036510
Vitales Vitaceae Vitis thunbergii#NC 039785
Vitales Vitaceae Vitis californica#MG664836
Vitales Vitaceae Vitis aestivalis var. linsecomii#NC 039806
Vitales Vitaceae Vitis cinerea var. helleri#LC320670
Vitales Vitaceae Vitis monticola#LC342076
Vitales Vitaceae Cissus palmata#NC 039791
Vitales Vitaceae Vitis acerifolia#MG664848
Vitales Vitaceae Vitis peninsularis#NC 039790
Vitales Vitaceae Vitis bloodwothiana#NC 039801
Vitales Vitaceae Vitis cordifolia#NC 036509
Vitales Vitaceae Vitis biformis#NC 039802
Vitales Vitaceae Vitis tiliifolia#NC 039681
Vitales Vitaceae Vitis treleasei#NC 039786
Vitales Vitaceae Vitis girdiana#MG664827
Vitales Vitaceae Vitis arizonica#MG664845
Vitales Vitaceae Vitis rufotomentosa#MG664800
Vitales Vitaceae Vitis sp.#MG664802
Vitales Vitaceae Vitis baileyana#MG664843
Vitales Vitaceae Vitis vulpina#MG664810
Vitales Vitaceae Vitis cinerea#MG664831
Vitales Vitaceae Vitis shuttleworthii#NC 039784
Vitales Vitaceae Vitis aestivalis#LC318124
Vitales Vitaceae Vitis doaniana#NC 039797
Vitales Vitaceae Vitis rupestris#NC 036764
Vitales Vitaceae Vitis labrusca#MG664823
Vitales Vitaceae Vitis mustangensis#MG664818
Geraniales Francoaceae Viviania marifolia#NC 023259
Geraniales Francoaceae Bersama abyssinica#F572
Geraniales Francoaceae Melianthus major#FC50
Geraniales Francoaceae Melianthus villosus#NC 023256
Geraniales Francoaceae Greyia radlkoferi#Y244
Geraniales Francoaceae Greyia sutherlandii#45626
Geraniales Francoaceae Francoa sonchifolia#NC 021101
Geraniales Francoaceae Francoa appendiculata#FC61
Geraniales Geraniaceae Hypseocharis bilobata#NC 023260
Geraniales Geraniaceae Pelargonium quinquelobatum#NC 033358
Geraniales Geraniaceae Pelargonium endlicherianum#NC 033357
Geraniales Geraniaceae Pelargonium spinosum#NC 033359
Geraniales Geraniaceae Pelargonium alternans#NC 023261
Geraniales Geraniaceae Pelargonium cotyledonis#NC 028052
Geraniales Geraniaceae Pelargonium dichondrifolium#NC 028051

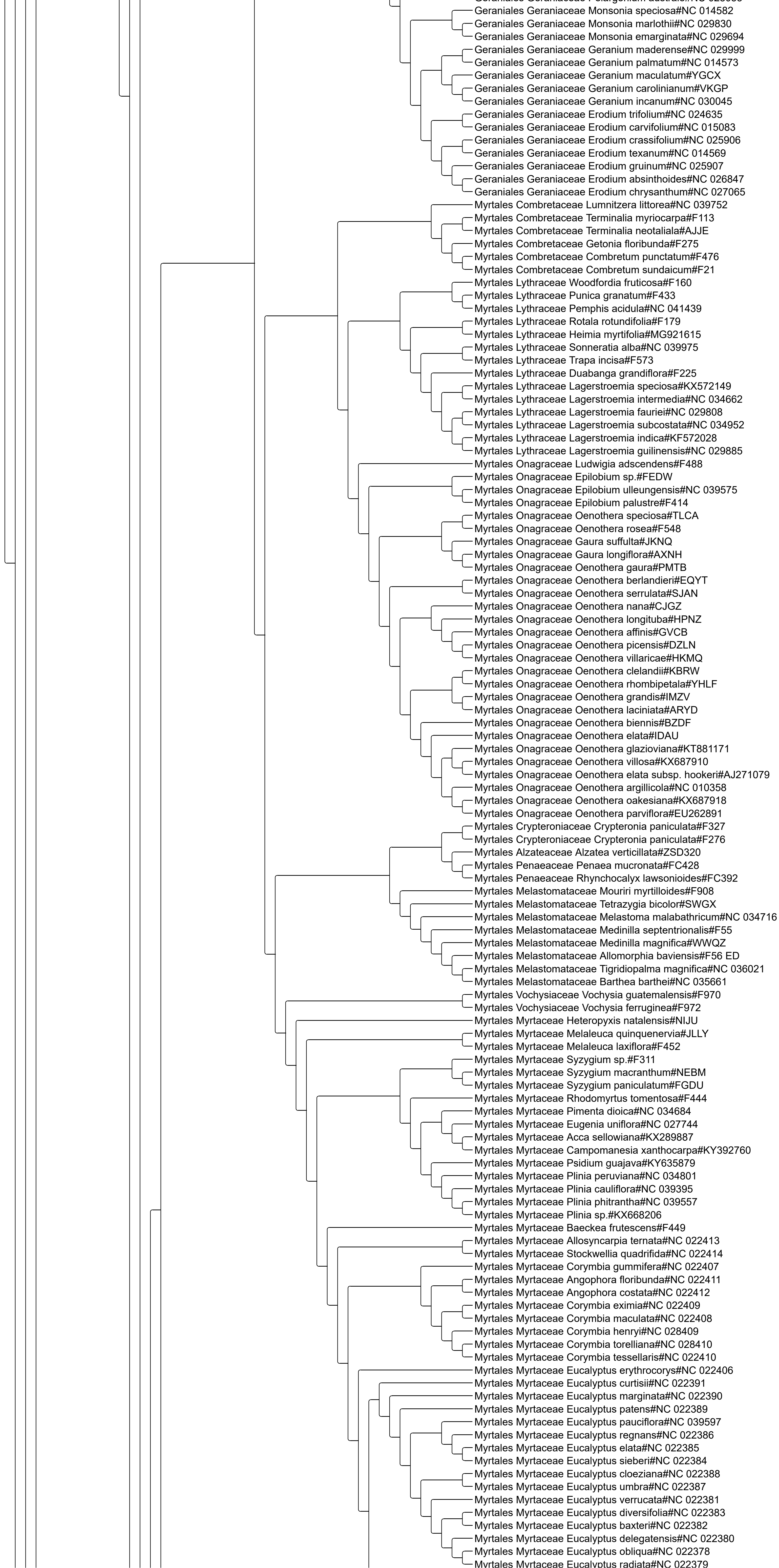
Geraniales Geraniaceae Monsonia speciosa#NC 014582
Geraniales Geraniaceae Monsonia marlothii#NC 029830
Geraniales Geraniaceae Monsonia emarginata#NC 029694
Geraniales Geraniaceae Geranium maderense#NC 029999
Geraniales Geraniaceae Geranium palmatum#NC 014573
Geraniales Geraniaceae Geranium maculatum#YGCX
Geraniales Geraniaceae Geranium carolinianum#VKGP
Geraniales Geraniaceae Geranium incanum#NC 030045
Geraniales Geraniaceae Erodium trifolium#NC 024635
Geraniales Geraniaceae Erodium carvifolium#NC 015083
Geraniales Geraniaceae Erodium crassifolium#NC 025906
Geraniales Geraniaceae Erodium texanum#NC 014569
Geraniales Geraniaceae Erodium gruinum#NC 025907
Geraniales Geraniaceae Erodium absinthoides#NC 026847
Geraniales Geraniaceae Erodium chrysanthum#NC 027065
Myrtales Combretaceae Lumnitzera littorea#NC 039752
Myrtales Combretaceae Terminalia myriocarpa#F113
Myrtales Combretaceae Terminalia neotaliala#AJJE
Myrtales Combretaceae Getonia floribunda#F275
Myrtales Combretaceae Combretum punctatum#F476
Myrtales Combretaceae Combretum sundaicum#F21
Myrtales Lythraceae Woodfordia fruticosa#F160
Myrtales Lythraceae Punica granatum#F433
Myrtales Lythraceae Pemphis acidula#NC 041439
Myrtales Lythraceae Rotala rotundifolia#F179
Myrtales Lythraceae Heimia myrtifolia#MG921615
Myrtales Lythraceae Sonneratia alba#NC 039975
Myrtales Lythraceae Trapa incisa#F573
Myrtales Lythraceae Duabanga grandiflora#F225
Myrtales Lythraceae Lagerstroemia speciosa#KX572149
Myrtales Lythraceae Lagerstroemia intermedia#NC 034662
Myrtales Lythraceae Lagerstroemia fauriei#NC 029808
Myrtales Lythraceae Lagerstroemia subcostata#NC 034952
Myrtales Lythraceae Lagerstroemia indica#KF572028
Myrtales Lythraceae Lagerstroemia guilinensis#NC 029885
Myrtales Onagraceae Ludwigia adscendens#F488
Myrtales Onagraceae Epilobium sp.#FEDW
Myrtales Onagraceae Epilobium ulleungensis#NC 039575
Myrtales Onagraceae Epilobium palustre#F414
Myrtales Onagraceae Oenothera speciosa#TLCA
Myrtales Onagraceae Oenothera rosea#F548
Myrtales Onagraceae Gaura suffulta#JKNQ
Myrtales Onagraceae Gaura longiflora#AXNH
Myrtales Onagraceae Oenothera gaura#PMTB
Myrtales Onagraceae Oenothera berlandieri#EQYT
Myrtales Onagraceae Oenothera serrulata#SJAN
Myrtales Onagraceae Oenothera nana#CJGZ
Myrtales Onagraceae Oenothera longituba#HPNZ
Myrtales Onagraceae Oenothera affinis#GVCB
Myrtales Onagraceae Oenothera picensis#DZLN
Myrtales Onagraceae Oenothera villaricae#HKMQ
Myrtales Onagraceae Oenothera clelandii#KBRW
Myrtales Onagraceae Oenothera rhombipetala#YHLF
Myrtales Onagraceae Oenothera grandis#IMZV
Myrtales Onagraceae Oenothera laciniata#ARYD
Myrtales Onagraceae Oenothera biennis#BZDF
Myrtales Onagraceae Oenothera elata#IDAU
Myrtales Onagraceae Oenothera glazioviana#KT881171
Myrtales Onagraceae Oenothera villosa#KX687910
Myrtales Onagraceae Oenothera elata subsp. hookeri#AJ271079
Myrtales Onagraceae Oenothera argillicola#NC 010358
Myrtales Onagraceae Oenothera oakesiana#KX687918
Myrtales Onagraceae Oenothera parviflora#EU262891
Myrtales Crypteroniaceae Crypteronia paniculata#F327
Myrtales Crypteroniaceae Crypteronia paniculata#F276
Myrtales Alzateaceae Alzatea verticillata#ZSD320
Myrtales Penaeaceae Penaea mucronata#FC428
Myrtales Penaeaceae Rhynchocalyx lawsonioides#FC392
Myrtales Melastomataceae Mouriri myrtilloides#F908
Myrtales Melastomataceae Tetrazygia bicolor#SWGX
Myrtales Melastomataceae Melastoma malabathricum#NC 034716
Myrtales Melastomataceae Medinilla septentrionalis#F55
Myrtales Melastomataceae Medinilla magnifica#WWQZ
Myrtales Melastomataceae Allomorphia baviensis#F56 ED
Myrtales Melastomataceae Tigridiopalma magnifica#NC 036021
Myrtales Melastomataceae Barthea barthei#NC 035661
Myrtales Vochysiaceae Vochysia guatemalensis#F970
Myrtales Vochysiaceae Vochysia ferruginea#F972
Myrtales Myrtaceae Heteropyxis natalensis#NIJU
Myrtales Myrtaceae Melaleuca quinquenervia#JLLY
Myrtales Myrtaceae Melaleuca laxiflora#F452
Myrtales Myrtaceae Syzygium sp.#F311
Myrtales Myrtaceae Syzygium macranthum#NEBM
Myrtales Myrtaceae Syzygium paniculatum#FGDU
Myrtales Myrtaceae Rhodomyrtus tomentosa#F444
Myrtales Myrtaceae Pimenta dioica#NC 034684
Myrtales Myrtaceae Eugenia uniflora#NC 027744
Myrtales Myrtaceae Acca sellowiana#KX289887
Myrtales Myrtaceae Campomanesia xanthocarpa#KY392760
Myrtales Myrtaceae Psidium guajava#KY635879
Myrtales Myrtaceae Plinia peruviana#NC 034801
Myrtales Myrtaceae Plinia cauliflora#NC 039395
Myrtales Myrtaceae Plinia phitrantha#NC 039557
Myrtales Myrtaceae Plinia sp.#KX668206
Myrtales Myrtaceae Baeckea frutescens#F449
Myrtales Myrtaceae Allosyncarpia ternata#NC 022413
Myrtales Myrtaceae Stockwellia quadrifida#NC 022414
Myrtales Myrtaceae Corymbia gummifera#NC 022407
Myrtales Myrtaceae Angophora floribunda#NC 022411
Myrtales Myrtaceae Angophora costata#NC 022412
Myrtales Myrtaceae Corymbia eximia#NC 022409
Myrtales Myrtaceae Corymbia maculata#NC 022408
Myrtales Myrtaceae Corymbia henryi#NC 028409
Myrtales Myrtaceae Corymbia torelliana#NC 028410
Myrtales Myrtaceae Corymbia tessellaris#NC 022410
Myrtales Myrtaceae Eucalyptus erythrocorys#NC 022406
Myrtales Myrtaceae Eucalyptus curtisii#NC 022391
Myrtales Myrtaceae Eucalyptus marginata#NC 022390
Myrtales Myrtaceae Eucalyptus patens#NC 022389
Myrtales Myrtaceae Eucalyptus pauciflora#NC 039597
Myrtales Myrtaceae Eucalyptus regnans#NC 022386
Myrtales Myrtaceae Eucalyptus elata#NC 022385
Myrtales Myrtaceae Eucalyptus sieberi#NC 022384
Myrtales Myrtaceae Eucalyptus cloeziana#NC 022388
Myrtales Myrtaceae Eucalyptus umbra#NC 022387
Myrtales Myrtaceae Eucalyptus verrucata#NC 022381
Myrtales Myrtaceae Eucalyptus diversifolia#NC 022383
Myrtales Myrtaceae Eucalyptus baxteri#NC 022382
Myrtales Myrtaceae Eucalyptus delegatensis#NC 022380
Myrtales Myrtaceae Eucalyptus obliqua#NC 022378
Myrtales Myrtaceae Eucalyptus radiata#NC 022379

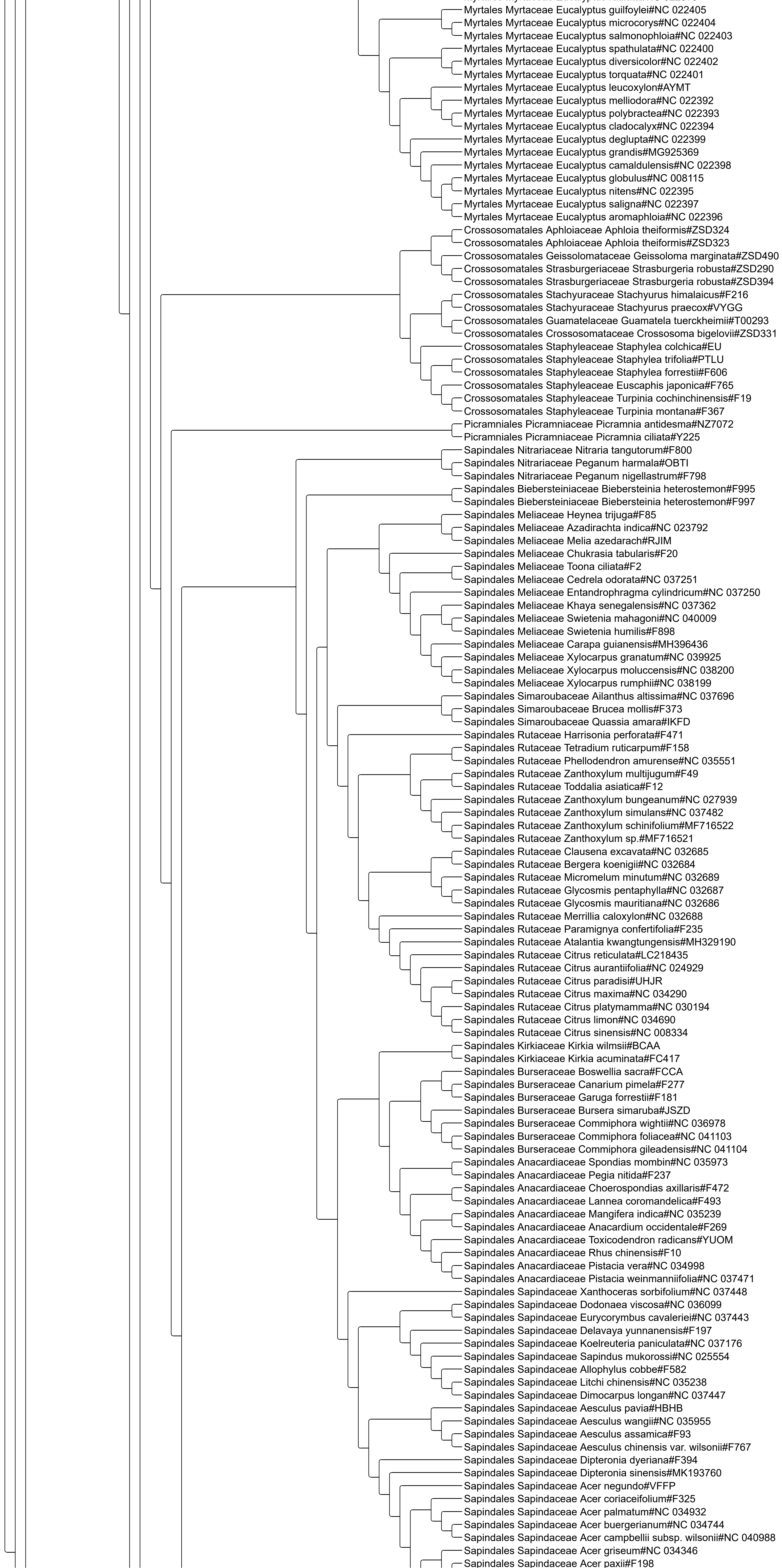

Myrtales Myrtaceae Eucalyptus guilfoylei#NC 022405
Myrtales Myrtaceae Eucalyptus microcorys#NC 022404
Myrtales Myrtaceae Eucalyptus salmonophloia#NC 022403
Myrtales Myrtaceae Eucalyptus spathulata#NC 022400
Myrtales Myrtaceae Eucalyptus diversicolor#NC 022402
Myrtales Myrtaceae Eucalyptus torquata#NC 022401
Myrtales Myrtaceae Eucalyptus leucoxylon#AYMT
Myrtales Myrtaceae Eucalyptus melliodora#NC 022392
Myrtales Myrtaceae Eucalyptus polybractea#NC 022393
Myrtales Myrtaceae Eucalyptus cladocalyx#NC 022394
Myrtales Myrtaceae Eucalyptus deglupta#NC 022399
Myrtales Myrtaceae Eucalyptus grandis#MG925369
Myrtales Myrtaceae Eucalyptus camaldulensis#NC 022398
Myrtales Myrtaceae Eucalyptus globulus#NC 008115
Myrtales Myrtaceae Eucalyptus nitens#NC 022395
Myrtales Myrtaceae Eucalyptus saligna#NC 022397
Myrtales Myrtaceae Eucalyptus aromaphloia#NC 022396
Crossosomatales Aphloiaceae Aphloia theiformis#ZSD324
Crossosomatales Aphloiaceae Aphloia theiformis#ZSD323
Crossosomatales Geissolomataceae Geissoloma marginata#ZSD490
Crossosomatales Strasburgeriaceae Strasburgeria robusta#ZSD290
Crossosomatales Strasburgeriaceae Strasburgeria robusta#ZSD394
Crossosomatales Stachyuraceae Stachyurus himalaicus#F216
Crossosomatales Stachyuraceae Stachyurus praecox#VYGG
Crossosomatales Guamatelaceae Guamatela tuerckheimii#T00293
Crossosomatales Crossosomataceae Crossosoma bigelovii#ZSD331
Crossosomatales Staphyleaceae Staphylea colchica#EU
Crossosomatales Staphyleaceae Staphylea trifolia#PTLU
Crossosomatales Staphyleaceae Staphylea forrestii#F606
Crossosomatales Staphyleaceae Euscaphis japonica#F765
Crossosomatales Staphyleaceae Turpinia cochinchinensis#F19
Crossosomatales Staphyleaceae Turpinia montana#F367
Picramniales Picramniaceae Picramnia antidesma#NZ7072
Picramniales Picramniaceae Picramnia ciliata#Y225
Sapindales Nitrariaceae Nitraria tangutorum#F800
Sapindales Nitrariaceae Peganum harmala#OBTI
Sapindales Nitrariaceae Peganum nigellastrum#F798
Sapindales Biebersteiniaceae Biebersteinia heterostemon#F995
Sapindales Biebersteiniaceae Biebersteinia heterostemon#F997
Sapindales Meliaceae Heynea trijuga#F85
Sapindales Meliaceae Azadirachta indica#NC 023792
Sapindales Meliaceae Melia azedarach#RJIM
Sapindales Meliaceae Chukrasia tabularis#F20
Sapindales Meliaceae Toona ciliata#F2
Sapindales Meliaceae Cedrela odorata#NC 037251
Sapindales Meliaceae Entandrophragma cylindricum#NC 037250
Sapindales Meliaceae Khaya senegalensis#NC 037362
Sapindales Meliaceae Swietenia mahagoni#NC 040009
Sapindales Meliaceae Swietenia humilis#F898
Sapindales Meliaceae Carapa guianensis#MH396436
Sapindales Meliaceae Xylocarpus granatum#NC 039925
Sapindales Meliaceae Xylocarpus moluccensis#NC 038200
Sapindales Meliaceae Xylocarpus rumphii#NC 038199
Sapindales Simaroubaceae Ailanthus altissima#NC 037696
Sapindales Simaroubaceae Brucea mollis#F373
Sapindales Simaroubaceae Quassia amara#IKFD
Sapindales Rutaceae Harrisonia perforata#F471
Sapindales Rutaceae Tetradium ruticarpum#F158
Sapindales Rutaceae Phellodendron amurense#NC 035551
Sapindales Rutaceae Zanthoxylum multijugum#F49
Sapindales Rutaceae Toddalia asiatica#F12
Sapindales Rutaceae Zanthoxylum bungeanum#NC 027939
Sapindales Rutaceae Zanthoxylum simulans#NC 037482
Sapindales Rutaceae Zanthoxylum schinifolium#MF716522
Sapindales Rutaceae Zanthoxylum sp.#MF716521
Sapindales Rutaceae Clausena excavata#NC 032685
Sapindales Rutaceae Bergera koenigii#NC 032684
Sapindales Rutaceae Micromelum minutum#NC 032689
Sapindales Rutaceae Glycosmis pentaphylla#NC 032687
Sapindales Rutaceae Glycosmis mauritiana#NC 032686
Sapindales Rutaceae Merrillia caloxylon#NC 032688
Sapindales Rutaceae Paramignya confertifolia#F235
Sapindales Rutaceae Atalantia kwangtungensis#MH329190
Sapindales Rutaceae Citrus reticulata#LC218435
Sapindales Rutaceae Citrus aurantiifolia#NC 024929
Sapindales Rutaceae Citrus paradisi#UHJR
Sapindales Rutaceae Citrus maxima#NC 034290
Sapindales Rutaceae Citrus platymamma#NC 030194
Sapindales Rutaceae Citrus limon#NC 034690
Sapindales Rutaceae Citrus sinensis#NC 008334
Sapindales Kirkiaceae Kirkia wilmsii#BCAA
Sapindales Kirkiaceae Kirkia acuminata#FC417
Sapindales Burseraceae Boswellia sacra#FCCA
Sapindales Burseraceae Canarium pimela#F277
Sapindales Burseraceae Garuga forrestii#F181
Sapindales Burseraceae Bursera simaruba#JSZD
Sapindales Burseraceae Commiphora wightii#NC 036978
Sapindales Burseraceae Commiphora foliacea#NC 041103
Sapindales Burseraceae Commiphora gileadensis#NC 041104
Sapindales Anacardiaceae Spondias mombin#NC 035973
Sapindales Anacardiaceae Pegia nitida#F237
Sapindales Anacardiaceae Choerospondias axillaris#F472
Sapindales Anacardiaceae Lannea coromandelica#F493
Sapindales Anacardiaceae Mangifera indica#NC 035239
Sapindales Anacardiaceae Anacardium occidentale#F269
Sapindales Anacardiaceae Toxicodendron radicans#YUOM
Sapindales Anacardiaceae Rhus chinensis#F10
Sapindales Anacardiaceae Pistacia vera#NC 034998
Sapindales Anacardiaceae Pistacia weinmanniifolia#NC 037471
Sapindales Sapindaceae Xanthoceras sorbifolium#NC 037448
Sapindales Sapindaceae Dodonaea viscosa#NC 036099
Sapindales Sapindaceae Eurycorymbus cavaleriei#NC 037443
Sapindales Sapindaceae Delavaya yunnanensis#F197
Sapindales Sapindaceae Koelreuteria paniculata#NC 037176
Sapindales Sapindaceae Sapindus mukorossi#NC 025554
Sapindales Sapindaceae Allophylus cobbe#F582
Sapindales Sapindaceae Litchi chinensis#NC 035238
Sapindales Sapindaceae Dimocarpus longan#NC 037447
Sapindales Sapindaceae Aesculus pavia#HBHB
Sapindales Sapindaceae Aesculus wangii#NC 035955
Sapindales Sapindaceae Aesculus assamica#F93
Sapindales Sapindaceae Aesculus chinensis var. wilsonii#F767
Sapindales Sapindaceae Dipteronia dyeriana#F394
Sapindales Sapindaceae Dipteronia sinensis#MK193760
Sapindales Sapindaceae Acer negundo#VFFP
Sapindales Sapindaceae Acer coriaceifolium#F325
Sapindales Sapindaceae Acer palmatum#NC 034932
Sapindales Sapindaceae Acer buergerianum#NC 034744
Sapindales Sapindaceae Acer campbellii subsp. wilsonii#NC 040988
Sapindales Sapindaceae Acer griseum#NC 034346
Sapindales Sapindaceae Acer paxii#F198

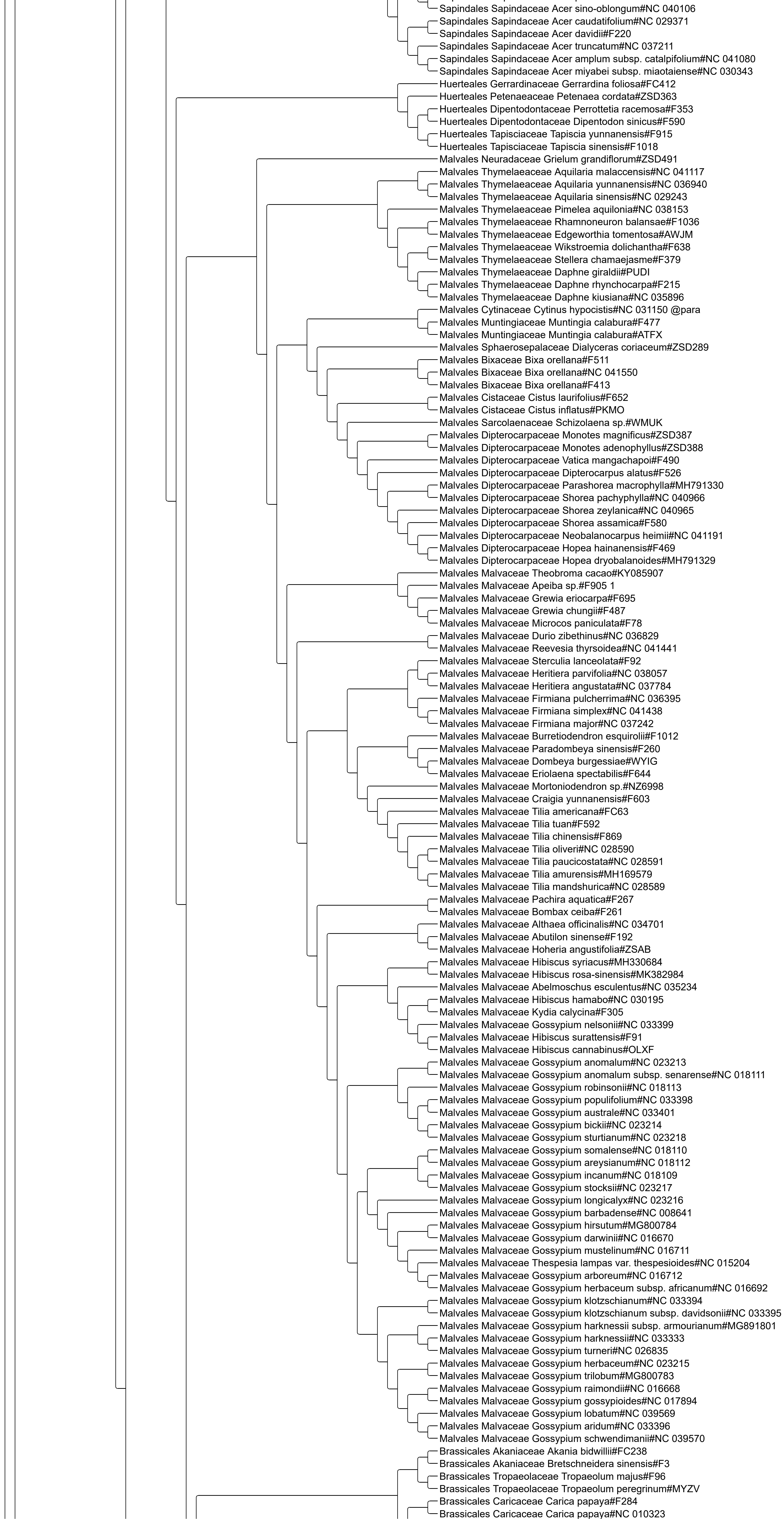
Sapindales Sapindaceae Acer sino-oblongum#NC 040106
Sapindales Sapindaceae Acer caudatifolium#NC 029371
Sapindales Sapindaceae Acer davidii#F220
Sapindales Sapindaceae Acer truncatum#NC 037211
Sapindales Sapindaceae Acer amplum subsp. catalpifolium#NC 041080
Sapindales Sapindaceae Acer miyabei subsp. miaotaiense#NC 030343
Huerteales Gerrardinaceae Gerrardina foliosa#FC412
Huerteales Petenaeaceae Petenaea cordata#ZSD363
Huerteales Dipentodontaceae Perrottetia racemosa#F353
Huerteales Dipentodontaceae Dipentodon sinicus#F590
Huerteales Tapisciaceae Tapiscia yunnanensis#F915
Huerteales Tapisciaceae Tapiscia sinensis#F1018
Malvales Neuradaceae Grielum grandiflorum#ZSD491
Malvales Thymelaeaceae Aquilaria malaccensis#NC 041117
Malvales Thymelaeaceae Aquilaria yunnanensis#NC 036940
Malvales Thymelaeaceae Aquilaria sinensis#NC 029243
Malvales Thymelaeaceae Pimelea aquilonia#NC 038153
Malvales Thymelaeaceae Rhamnoneuron balansae#F1036
Malvales Thymelaeaceae Edgeworthia tomentosa#AWJM
Malvales Thymelaeaceae Wikstroemia dolichantha#F638
Malvales Thymelaeaceae Stellera chamaejasme#F379
Malvales Thymelaeaceae Daphne giraldii#PUDI
Malvales Thymelaeaceae Daphne rhynchocarpa#F215
Malvales Thymelaeaceae Daphne kiusiana#NC 035896
Malvales Cytinaceae Cytinus hypocistis#NC 031150 @para
Malvales Muntingiaceae Muntingia calabura#F477
Malvales Muntingiaceae Muntingia calabura#ATFX
Malvales Sphaerosepalaceae Dialyceras coriaceum#ZSD289
Malvales Bixaceae Bixa orellana#F511
Malvales Bixaceae Bixa orellana#NC 041550
Malvales Bixaceae Bixa orellana#F413
Malvales Cistaceae Cistus laurifolius#F652
Malvales Cistaceae Cistus inflatus#PKMO
Malvales Sarcolaenaceae Schizolaena sp.#WMUK
Malvales Dipterocarpaceae Monotes magnificus#ZSD387
Malvales Dipterocarpaceae Monotes adenophyllus#ZSD388
Malvales Dipterocarpaceae Vatica mangachapoi#F490
Malvales Dipterocarpaceae Dipterocarpus alatus#F526
Malvales Dipterocarpaceae Parashorea macrophylla#MH791330
Malvales Dipterocarpaceae Shorea pachyphylla#NC 040966
Malvales Dipterocarpaceae Shorea zeylanica#NC 040965
Malvales Dipterocarpaceae Shorea assamica#F580
Malvales Dipterocarpaceae Neobalanocarpus heimii#NC 041191
Malvales Dipterocarpaceae Hopea hainanensis#F469
Malvales Dipterocarpaceae Hopea dryobalanoides#MH791329
Malvales Malvaceae Theobroma cacao#KY085907
Malvales Malvaceae Apeiba sp.#F905 1
Malvales Malvaceae Grewia eriocarpa#F695
Malvales Malvaceae Grewia chungii#F487
Malvales Malvaceae Microcos paniculata#F78
Malvales Malvaceae Durio zibethinus#NC 036829
Malvales Malvaceae Reevesia thyrsoidea#NC 041441
Malvales Malvaceae Sterculia lanceolata#F92
Malvales Malvaceae Heritiera parvifolia#NC 038057
Malvales Malvaceae Heritiera angustata#NC 037784
Malvales Malvaceae Firmiana pulcherrima#NC 036395
Malvales Malvaceae Firmiana simplex#NC 041438
Malvales Malvaceae Firmiana major#NC 037242
Malvales Malvaceae Burretiodendron esquirolii#F1012
Malvales Malvaceae Paradombeya sinensis#F260
Malvales Malvaceae Dombeya burgessiae#WYIG
Malvales Malvaceae Eriolaena spectabilis#F644
Malvales Malvaceae Mortoniodendron sp.#NZ6998
Malvales Malvaceae Craigia yunnanensis#F603
Malvales Malvaceae Tilia americana#FC63
Malvales Malvaceae Tilia tuan#F592
Malvales Malvaceae Tilia chinensis#F869
Malvales Malvaceae Tilia oliveri#NC 028590
Malvales Malvaceae Tilia paucicostata#NC 028591
Malvales Malvaceae Tilia amurensis#MH169579
Malvales Malvaceae Tilia mandshurica#NC 028589
Malvales Malvaceae Pachira aquatica#F267
Malvales Malvaceae Bombax ceiba#F261
Malvales Malvaceae Althaea officinalis#NC 034701
Malvales Malvaceae Abutilon sinense#F192
Malvales Malvaceae Hoheria angustifolia#ZSAB
Malvales Malvaceae Hibiscus syriacus#MH330684
Malvales Malvaceae Hibiscus rosa-sinensis#MK382984
Malvales Malvaceae Abelmoschus esculentus#NC 035234
Malvales Malvaceae Hibiscus hamabo#NC 030195
Malvales Malvaceae Kydia calycina#F305
Malvales Malvaceae Gossypium nelsonii#NC 033399
Malvales Malvaceae Hibiscus surattensis#F91
Malvales Malvaceae Hibiscus cannabinus#OLXF
Malvales Malvaceae Gossypium anomalum#NC 023213
Malvales Malvaceae Gossypium anomalum subsp. senarense#NC 018111
Malvales Malvaceae Gossypium robinsonii#NC 018113
Malvales Malvaceae Gossypium populifolium#NC 033398
Malvales Malvaceae Gossypium australe#NC 033401
Malvales Malvaceae Gossypium bickii#NC 023214
Malvales Malvaceae Gossypium sturtianum#NC 023218
Malvales Malvaceae Gossypium somalense#NC 018110
Malvales Malvaceae Gossypium areysianum#NC 018112
Malvales Malvaceae Gossypium incanum#NC 018109
Malvales Malvaceae Gossypium stocksii#NC 023217
Malvales Malvaceae Gossypium longicalyx#NC 023216
Malvales Malvaceae Gossypium barbadense#NC 008641
Malvales Malvaceae Gossypium hirsutum#MG800784
Malvales Malvaceae Gossypium darwinii#NC 016670
Malvales Malvaceae Gossypium mustelinum#NC 016711
Malvales Malvaceae Thespesia lampas var. thespesioides#NC 015204
Malvales Malvaceae Gossypium arboreum#NC 016712
Malvales Malvaceae Gossypium herbaceum subsp. africanum#NC 016692
Malvales Malvaceae Gossypium klotzschianum#NC 033394
Malvales Malvaceae Gossypium klotzschianum subsp. davidsonii#NC 033395
Malvales Malvaceae Gossypium harknessii subsp. armourianum#MG891801
Malvales Malvaceae Gossypium harknessii#NC 033333
Malvales Malvaceae Gossypium turneri#NC 026835
Malvales Malvaceae Gossypium herbaceum#NC 023215
Malvales Malvaceae Gossypium trilobum#MG800783
Malvales Malvaceae Gossypium raimondii#NC 016668
Malvales Malvaceae Gossypium gossypioides#NC 017894
Malvales Malvaceae Gossypium lobatum#NC 039569
Malvales Malvaceae Gossypium aridum#NC 033396
Malvales Malvaceae Gossypium schwendimanii#NC 039570
Brassicales Akaniaceae Akania bidwillii#FC238
Brassicales Akaniaceae Bretschneidera sinensis#F3
Brassicales Tropaeolaceae Tropaeolum majus#F96
Brassicales Tropaeolaceae Tropaeolum peregrinum#MYZV
Brassicales Caricaceae Carica papaya#F284
Brassicales Caricaceae Carica papaya#NC 010323

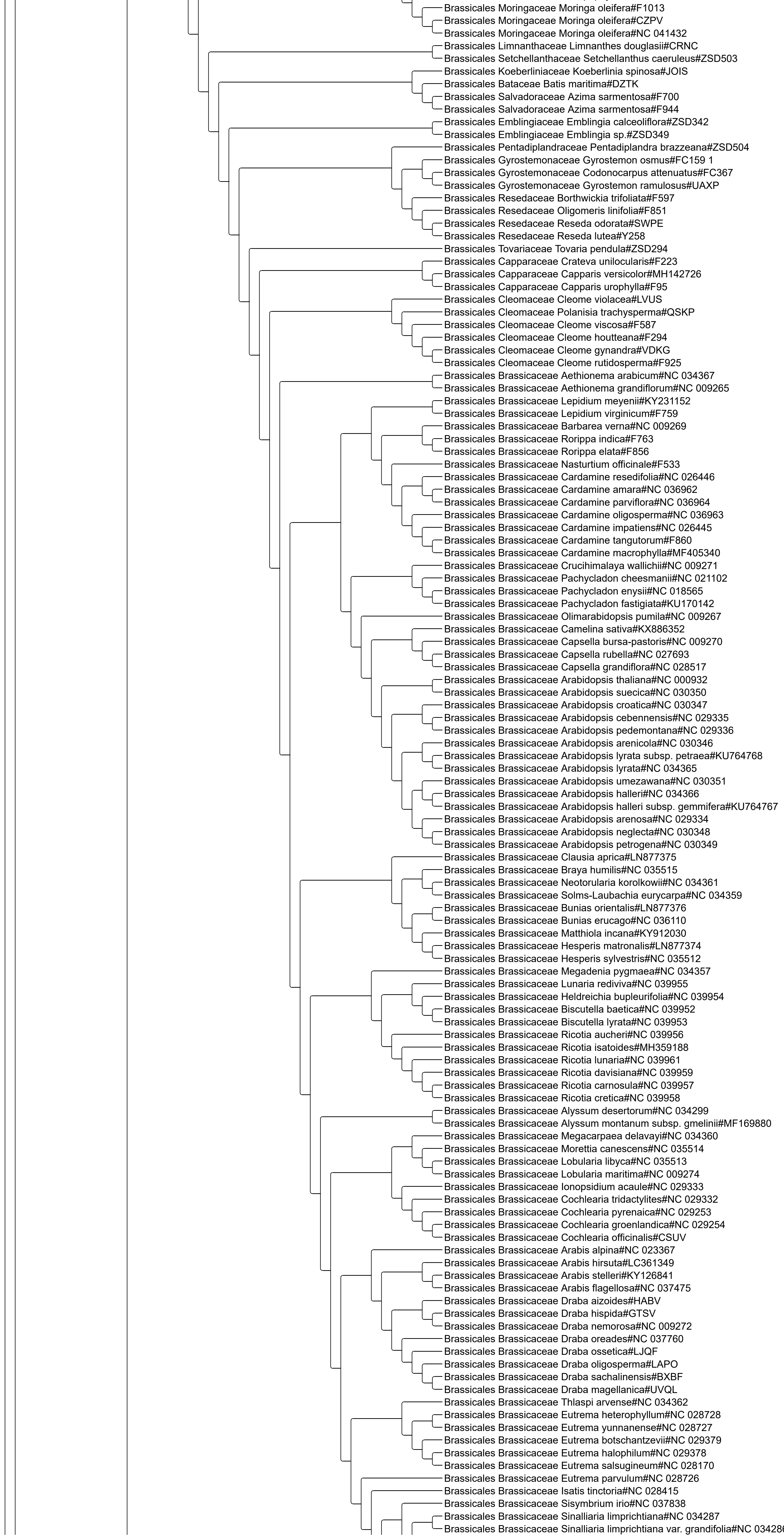

Brassicales Moringaceae Moringa oleifera#F1013
Brassicales Moringaceae Moringa oleifera#CZPV
Brassicales Moringaceae Moringa oleifera#NC 041432
Brassicales Limnanthaceae Limnanthes douglasii#CRNC
Brassicales Setchellanthaceae Setchellanthus caeruleus#ZSD503
Brassicales Koeberliniaceae Koeberlinia spinosa#JOIS
Brassicales Bataceae Batis maritima#DZTK
Brassicales Salvadoraceae Azima sarmentosa#F700
Brassicales Salvadoraceae Azima sarmentosa#F944
Brassicales Emblingiaceae Emblingia calceoliflora#ZSD342
Brassicales Emblingiaceae Emblingia sp.#ZSD349
Brassicales Pentadiplandraceae Pentadiplandra brazzeana#ZSD504
Brassicales Gyrostemonaceae Gyrostemon osmus#FC159 1
Brassicales Gyrostemonaceae Codonocarpus attenuatus#FC367
Brassicales Gyrostemonaceae Gyrostemon ramulosus#UAXP
Brassicales Resedaceae Borthwickia trifoliata#F597
Brassicales Resedaceae Oligomeris linifolia#F851
Brassicales Resedaceae Reseda odorata#SWPE
Brassicales Resedaceae Reseda lutea#Y258
Brassicales Tovariaceae Tovaria pendula#ZSD294
Brassicales Capparaceae Crateva unilocularis#F223
Brassicales Capparaceae Capparis versicolor#MH142726
Brassicales Capparaceae Capparis urophylla#F95
Brassicales Cleomaceae Cleome violacea#LVUS
Brassicales Cleomaceae Polanisia trachysperma#QSKP
Brassicales Cleomaceae Cleome viscosa#F587
Brassicales Cleomaceae Cleome houtteana#F294
Brassicales Cleomaceae Cleome gynandra#VDKG
Brassicales Cleomaceae Cleome rutidosperma#F925
Brassicales Brassicaceae Aethionema arabicum#NC 034367
Brassicales Brassicaceae Aethionema grandiflorum#NC 009265
Brassicales Brassicaceae Lepidium meyenii#KY231152
Brassicales Brassicaceae Lepidium virginicum#F759
Brassicales Brassicaceae Barbarea verna#NC 009269
Brassicales Brassicaceae Rorippa indica#F763
Brassicales Brassicaceae Rorippa elata#F856
Brassicales Brassicaceae Nasturtium officinale#F533
Brassicales Brassicaceae Cardamine resedifolia#NC 026446
Brassicales Brassicaceae Cardamine amara#NC 036962
Brassicales Brassicaceae Cardamine parviflora#NC 036964
Brassicales Brassicaceae Cardamine oligosperma#NC 036963
Brassicales Brassicaceae Cardamine impatiens#NC 026445
Brassicales Brassicaceae Cardamine tangutorum#F860
Brassicales Brassicaceae Cardamine macrophylla#MF405340
Brassicales Brassicaceae Crucihimalaya wallichii#NC 009271
Brassicales Brassicaceae Pachycladon cheesmanii#NC 021102
Brassicales Brassicaceae Pachycladon enysii#NC 018565
Brassicales Brassicaceae Pachycladon fastigiata#KU170142
Brassicales Brassicaceae Olimarabidopsis pumila#NC 009267
Brassicales Brassicaceae Camelina sativa#KX886352
Brassicales Brassicaceae Capsella bursa-pastoris#NC 009270
Brassicales Brassicaceae Capsella rubella#NC 027693
Brassicales Brassicaceae Capsella grandiflora#NC 028517
Brassicales Brassicaceae Arabidopsis thaliana#NC 000932
Brassicales Brassicaceae Arabidopsis suecica#NC 030350
Brassicales Brassicaceae Arabidopsis croatica#NC 030347
Brassicales Brassicaceae Arabidopsis cebennensis#NC 029335
Brassicales Brassicaceae Arabidopsis pedemontana#NC 029336
Brassicales Brassicaceae Arabidopsis arenicola#NC 030346
Brassicales Brassicaceae Arabidopsis lyrata subsp. petraea#KU764768
Brassicales Brassicaceae Arabidopsis lyrata#NC 034365
Brassicales Brassicaceae Arabidopsis umezawana#NC 030351
Brassicales Brassicaceae Arabidopsis halleri#NC 034366
Brassicales Brassicaceae Arabidopsis halleri subsp. gemmifera#KU764767
Brassicales Brassicaceae Arabidopsis arenosa#NC 029334
Brassicales Brassicaceae Arabidopsis neglecta#NC 030348
Brassicales Brassicaceae Arabidopsis petrogena#NC 030349
Brassicales Brassicaceae Clausia aprica#LN877375
Brassicales Brassicaceae Braya humilis#NC 035515
Brassicales Brassicaceae Neotorularia korolkowii#NC 034361
Brassicales Brassicaceae Solms-Laubachia eurycarpa#NC 034359
Brassicales Brassicaceae Bunias orientalis#LN877376
Brassicales Brassicaceae Bunias erucago#NC 036110
Brassicales Brassicaceae Matthiola incana#KY912030
Brassicales Brassicaceae Hesperis matronalis#LN877374
Brassicales Brassicaceae Hesperis sylvestris#NC 035512
Brassicales Brassicaceae Megadenia pygmaea#NC 034357
Brassicales Brassicaceae Lunaria rediviva#NC 039955
Brassicales Brassicaceae Heldreichia bupleurifolia#NC 039954
Brassicales Brassicaceae Biscutella baetica#NC 039952
Brassicales Brassicaceae Biscutella lyrata#NC 039953
Brassicales Brassicaceae Ricotia aucheri#NC 039956
Brassicales Brassicaceae Ricotia isatoides#MH359188
Brassicales Brassicaceae Ricotia lunaria#NC 039961
Brassicales Brassicaceae Ricotia davisiana#NC 039959
Brassicales Brassicaceae Ricotia carnosula#NC 039957
Brassicales Brassicaceae Ricotia cretica#NC 039958
Brassicales Brassicaceae Alyssum desertorum#NC 034299
Brassicales Brassicaceae Alyssum montanum subsp. gmelinii#MF169880
Brassicales Brassicaceae Megacarpaea delavayi#NC 034360
Brassicales Brassicaceae Morettia canescens#NC 035514
Brassicales Brassicaceae Lobularia libyca#NC 035513
Brassicales Brassicaceae Lobularia maritima#NC 009274
Brassicales Brassicaceae Ionopsidium acaule#NC 029333
Brassicales Brassicaceae Cochlearia tridactylites#NC 029332
Brassicales Brassicaceae Cochlearia pyrenaica#NC 029253
Brassicales Brassicaceae Cochlearia groenlandica#NC 029254
Brassicales Brassicaceae Cochlearia officinalis#CSUV
Brassicales Brassicaceae Arabis alpina#NC 023367
Brassicales Brassicaceae Arabis hirsuta#LC361349
Brassicales Brassicaceae Arabis stelleri#KY126841
Brassicales Brassicaceae Arabis flagellosa#NC 037475
Brassicales Brassicaceae Draba aizoides#HABV
Brassicales Brassicaceae Draba hispida#GTSV
Brassicales Brassicaceae Draba nemorosa#NC 009272
Brassicales Brassicaceae Draba oreades#NC 037760
Brassicales Brassicaceae Draba ossetica#LJQF
Brassicales Brassicaceae Draba oligosperma#LAPO
Brassicales Brassicaceae Draba sachalinensis#BXBF
Brassicales Brassicaceae Draba magellanica#UVQL
Brassicales Brassicaceae Thlaspi arvense#NC 034362
Brassicales Brassicaceae Eutrema heterophyllum#NC 028728
Brassicales Brassicaceae Eutrema yunnanense#NC 028727
Brassicales Brassicaceae Eutrema botschantzevii#NC 029379
Brassicales Brassicaceae Eutrema halophilum#NC 029378
Brassicales Brassicaceae Eutrema salsugineum#NC 028170
Brassicales Brassicaceae Eutrema parvulum#NC 028726
Brassicales Brassicaceae Isatis tinctoria#NC 028415
Brassicales Brassicaceae Sisymbrium irio#NC 037838
Brassicales Brassicaceae Sinalliaria limprichtiana#NC 034287
Brassicales Brassicaceae Sinalliaria limprichtiana var. grandifolia#NC 034286

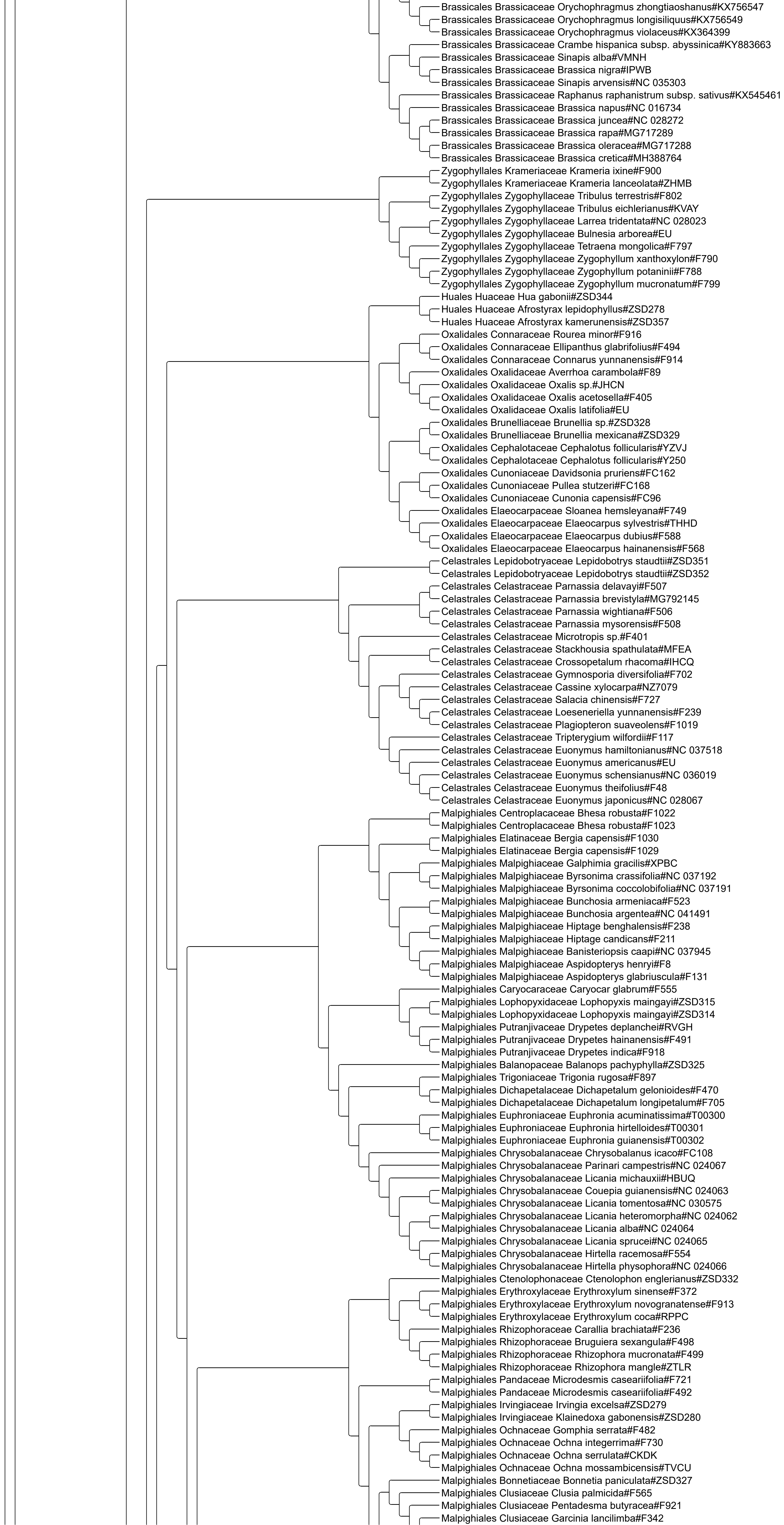
Brassicales Brassicaceae Orychophragmus zhongtiaoshanus#KX756547
Brassicales Brassicaceae Orychophragmus longisiliquus#KX756549
Brassicales Brassicaceae Orychophragmus violaceus#KX364399
Brassicales Brassicaceae Crambe hispanica subsp. abyssinica#KY883663
Brassicales Brassicaceae Sinapis alba#VMNH
Brassicales Brassicaceae Brassica nigra#IPWB
Brassicales Brassicaceae Sinapis arvensis#NC 035303
Brassicales Brassicaceae Raphanus raphanistrum subsp. sativus#KX545461
Brassicales Brassicaceae Brassica napus#NC 016734
Brassicales Brassicaceae Brassica juncea#NC 028272
Brassicales Brassicaceae Brassica rapa#MG717289
Brassicales Brassicaceae Brassica oleracea#MG717288
Brassicales Brassicaceae Brassica cretica#MH388764
Zygophyllales Krameriaceae Krameria ixine#F900
Zygophyllales Krameriaceae Krameria lanceolata#ZHMB
Zygophyllales Zygophyllaceae Tribulus terrestris#F802
Zygophyllales Zygophyllaceae Tribulus eichlerianus#KVAY
Zygophyllales Zygophyllaceae Larrea tridentata#NC 028023
Zygophyllales Zygophyllaceae Bulnesia arborea#EU
Zygophyllales Zygophyllaceae Tetraena mongolica#F797
Zygophyllales Zygophyllaceae Zygophyllum xanthoxylon#F790
Zygophyllales Zygophyllaceae Zygophyllum potaninii#F788
Zygophyllales Zygophyllaceae Zygophyllum mucronatum#F799
Huales Huaceae Hua gabonii#ZSD344
Huales Huaceae Afrostyrax lepidophyllus#ZSD278
Huales Huaceae Afrostyrax kamerunensis#ZSD357
Oxalidales Connaraceae Rourea minor#F916
Oxalidales Connaraceae Ellipanthus glabrifolius#F494
Oxalidales Connaraceae Connarus yunnanensis#F914
Oxalidales Oxalidaceae Averrhoa carambola#F89
Oxalidales Oxalidaceae Oxalis sp.#JHCN
Oxalidales Oxalidaceae Oxalis acetosella#F405
Oxalidales Oxalidaceae Oxalis latifolia#EU
Oxalidales Brunelliaceae Brunellia sp.#ZSD328
Oxalidales Brunelliaceae Brunellia mexicana#ZSD329
Oxalidales Cephalotaceae Cephalotus follicularis#YZVJ
Oxalidales Cephalotaceae Cephalotus follicularis#Y250
Oxalidales Cunoniaceae Davidsonia pruriens#FC162
Oxalidales Cunoniaceae Pullea stutzeri#FC168
Oxalidales Cunoniaceae Cunonia capensis#FC96
Oxalidales Elaeocarpaceae Sloanea hemsleyana#F749
Oxalidales Elaeocarpaceae Elaeocarpus sylvestris#THHD
Oxalidales Elaeocarpaceae Elaeocarpus dubius#F588
Oxalidales Elaeocarpaceae Elaeocarpus hainanensis#F568
Celastrales Lepidobotryaceae Lepidobotrys staudtii#ZSD351
Celastrales Lepidobotryaceae Lepidobotrys staudtii#ZSD352
Celastrales Celastraceae Parnassia delavayi#F507
Celastrales Celastraceae Parnassia brevistyla#MG792145
Celastrales Celastraceae Parnassia wightiana#F506
Celastrales Celastraceae Parnassia mysorensis#F508
Celastrales Celastraceae Microtropis sp.#F401
Celastrales Celastraceae Stackhousia spathulata#MFEA
Celastrales Celastraceae Crossopetalum rhacoma#IHCQ
Celastrales Celastraceae Gymnosporia diversifolia#F702
Celastrales Celastraceae Cassine xylocarpa#NZ7079
Celastrales Celastraceae Salacia chinensis#F727
Celastrales Celastraceae Loeseneriella yunnanensis#F239
Celastrales Celastraceae Plagiopteron suaveolens#F1019
Celastrales Celastraceae Tripterygium wilfordii#F117
Celastrales Celastraceae Euonymus hamiltonianus#NC 037518
Celastrales Celastraceae Euonymus americanus#EU
Celastrales Celastraceae Euonymus schensianus#NC 036019
Celastrales Celastraceae Euonymus theifolius#F48
Celastrales Celastraceae Euonymus japonicus#NC 028067
Malpighiales Centroplacaceae Bhesa robusta#F1022
Malpighiales Centroplacaceae Bhesa robusta#F1023
Malpighiales Elatinaceae Bergia capensis#F1030
Malpighiales Elatinaceae Bergia capensis#F1029
Malpighiales Malpighiaceae Galphimia gracilis#XPBC
Malpighiales Malpighiaceae Byrsonima crassifolia#NC 037192
Malpighiales Malpighiaceae Byrsonima coccolobifolia#NC 037191
Malpighiales Malpighiaceae Bunchosia armeniaca#F523
Malpighiales Malpighiaceae Bunchosia argentea#NC 041491
Malpighiales Malpighiaceae Hiptage benghalensis#F238
Malpighiales Malpighiaceae Hiptage candicans#F211
Malpighiales Malpighiaceae Banisteriopsis caapi#NC 037945
Malpighiales Malpighiaceae Aspidopterys henryi#F8
Malpighiales Malpighiaceae Aspidopterys glabriuscula#F131
Malpighiales Caryocaraceae Caryocar glabrum#F555
Malpighiales Lophopyxidaceae Lophopyxis maingayi#ZSD315
Malpighiales Lophopyxidaceae Lophopyxis maingayi#ZSD314
Malpighiales Putranjivaceae Drypetes deplanchei#RVGH
Malpighiales Putranjivaceae Drypetes hainanensis#F491
Malpighiales Putranjivaceae Drypetes indica#F918
Malpighiales Balanopaceae Balanops pachyphylla#ZSD325
Malpighiales Trigoniaceae Trigonia rugosa#F897
Malpighiales Dichapetalaceae Dichapetalum gelonioides#F470
Malpighiales Dichapetalaceae Dichapetalum longipetalum#F705
Malpighiales Euphroniaceae Euphronia acuminatissima#T00300
Malpighiales Euphroniaceae Euphronia hirtelloides#T00301
Malpighiales Euphroniaceae Euphronia guianensis#T00302
Malpighiales Chrysobalanaceae Chrysobalanus icaco#FC108
Malpighiales Chrysobalanaceae Parinari campestris#NC 024067
Malpighiales Chrysobalanaceae Licania michauxii#HBUQ
Malpighiales Chrysobalanaceae Couepia guianensis#NC 024063
Malpighiales Chrysobalanaceae Licania tomentosa#NC 030575
Malpighiales Chrysobalanaceae Licania heteromorpha#NC 024062
Malpighiales Chrysobalanaceae Licania alba#NC 024064
Malpighiales Chrysobalanaceae Licania sprucei#NC 024065
Malpighiales Chrysobalanaceae Hirtella racemosa#F554
Malpighiales Chrysobalanaceae Hirtella physophora#NC 024066
Malpighiales Ctenolophonaceae Ctenolophon englerianus#ZSD332
Malpighiales Erythroxylaceae Erythroxylum sinense#F372
Malpighiales Erythroxylaceae Erythroxylum novogranatense#F913
Malpighiales Erythroxylaceae Erythroxylum coca#RPPC
Malpighiales Rhizophoraceae Carallia brachiata#F236
Malpighiales Rhizophoraceae Bruguiera sexangula#F498
Malpighiales Rhizophoraceae Rhizophora mucronata#F499
Malpighiales Rhizophoraceae Rhizophora mangle#ZTLR
Malpighiales Pandaceae Microdesmis caseariifolia#F721
Malpighiales Pandaceae Microdesmis caseariifolia#F492
Malpighiales Irvingiaceae Irvingia excelsa#ZSD279
Malpighiales Irvingiaceae Klainedoxa gabonensis#ZSD280
Malpighiales Ochnaceae Gomphia serrata#F482
Malpighiales Ochnaceae Ochna integerrima#F730
Malpighiales Ochnaceae Ochna serrulata#CKDK
Malpighiales Ochnaceae Ochna mossambicensis#TVCU
Malpighiales Bonnetiaceae Bonnetia paniculata#ZSD327
Malpighiales Clusiaceae Clusia palmicida#F565
Malpighiales Clusiaceae Pentadesma butyracea#F921
Malpighiales Clusiaceae Garcinia lancilimba#F342

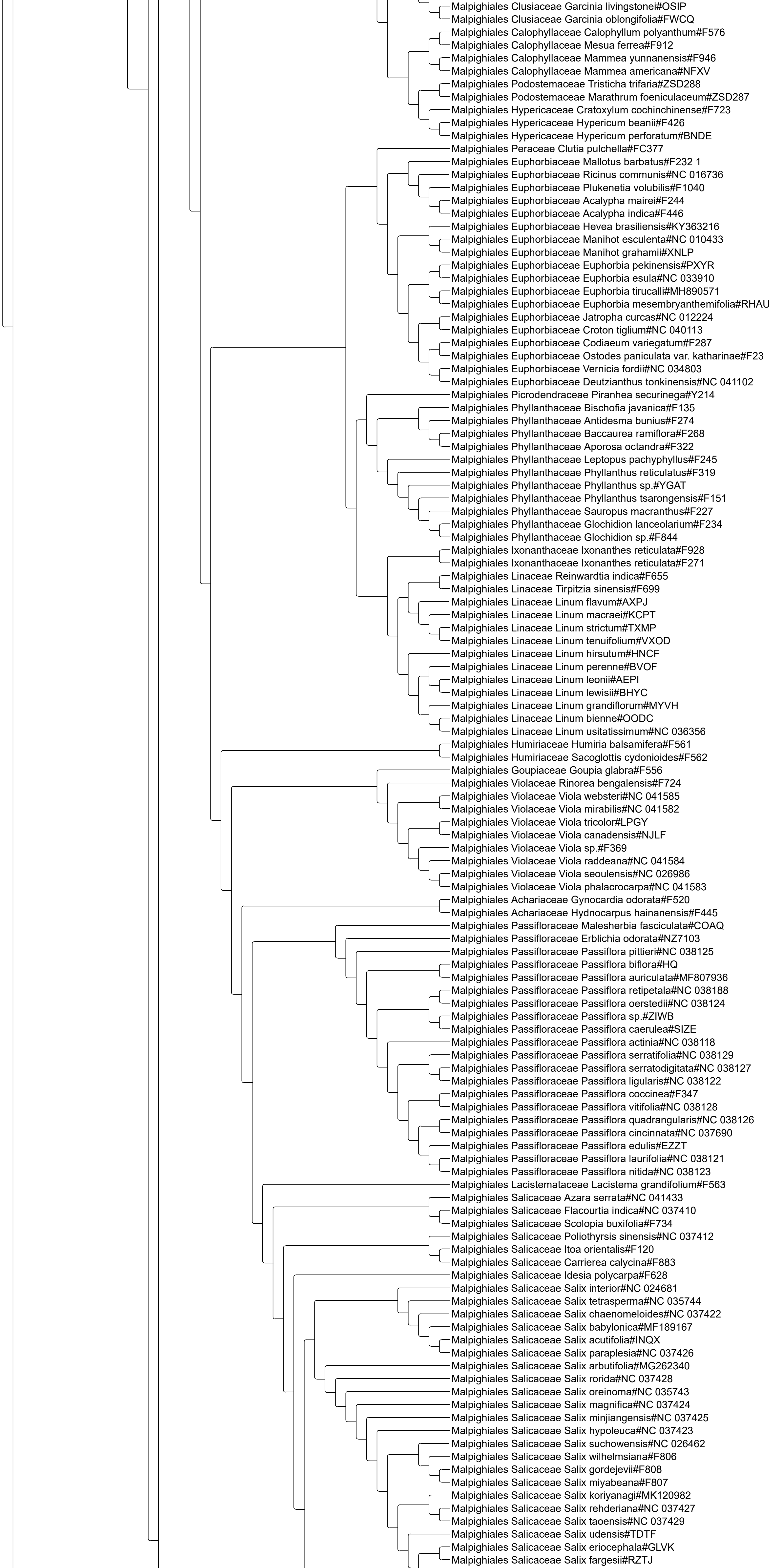

Malpighiales Clusiaceae Garcinia livingstonei#OSIP
Malpighiales Clusiaceae Garcinia oblongifolia#FWCQ
Malpighiales Calophyllaceae Calophyllum polyanthum#F576
Malpighiales Calophyllaceae Mesua ferrea#F912
Malpighiales Calophyllaceae Mammea yunnanensis#F946
Malpighiales Calophyllaceae Mammea americana#NFXV
Malpighiales Podostemaceae Tristicha trifaria#ZSD288
Malpighiales Podostemaceae Marathrum foeniculaceum#ZSD287
Malpighiales Hypericaceae Cratoxylum cochinchinense#F723
Malpighiales Hypericaceae Hypericum beanii#F426
Malpighiales Hypericaceae Hypericum perforatum#BNDE
Malpighiales Peraceae Clutia pulchella#FC377
Malpighiales Euphorbiaceae Mallotus barbatus#F232 1
Malpighiales Euphorbiaceae Ricinus communis#NC 016736
Malpighiales Euphorbiaceae Plukenetia volubilis#F1040
Malpighiales Euphorbiaceae Acalypha mairei#F244
Malpighiales Euphorbiaceae Acalypha indica#F446
Malpighiales Euphorbiaceae Hevea brasiliensis#KY363216
Malpighiales Euphorbiaceae Manihot esculenta#NC 010433
Malpighiales Euphorbiaceae Manihot grahamii#XNLP
Malpighiales Euphorbiaceae Euphorbia pekinensis#PXYR
Malpighiales Euphorbiaceae Euphorbia esula#NC 033910
Malpighiales Euphorbiaceae Euphorbia tirucalli#MH890571
Malpighiales Euphorbiaceae Euphorbia mesembryanthemifolia#RHAU
Malpighiales Euphorbiaceae Jatropha curcas#NC 012224
Malpighiales Euphorbiaceae Croton tiglium#NC 040113
Malpighiales Euphorbiaceae Codiaeum variegatum#F287
Malpighiales Euphorbiaceae Ostodes paniculata var. katharinae#F23
Malpighiales Euphorbiaceae Vernicia fordii#NC 034803
Malpighiales Euphorbiaceae Deutzianthus tonkinensis#NC 041102
Malpighiales Picrodendraceae Piranhea securinega#Y214
Malpighiales Phyllanthaceae Bischofia javanica#F135
Malpighiales Phyllanthaceae Antidesma bunius#F274
Malpighiales Phyllanthaceae Baccaurea ramiflora#F268
Malpighiales Phyllanthaceae Aporosa octandra#F322
Malpighiales Phyllanthaceae Leptopus pachyphyllus#F245
Malpighiales Phyllanthaceae Phyllanthus reticulatus#F319
Malpighiales Phyllanthaceae Phyllanthus sp.#YGAT
Malpighiales Phyllanthaceae Phyllanthus tsarongensis#F151
Malpighiales Phyllanthaceae Sauropus macranthus#F227
Malpighiales Phyllanthaceae Glochidion lanceolarium#F234
Malpighiales Phyllanthaceae Glochidion sp.#F844
Malpighiales Ixonanthaceae Ixonanthes reticulata#F928
Malpighiales Ixonanthaceae Ixonanthes reticulata#F271
Malpighiales Linaceae Reinwardtia indica#F655
Malpighiales Linaceae Tirpitzia sinensis#F699
Malpighiales Linaceae Linum flavum#AXPJ
Malpighiales Linaceae Linum macraei#KCPT
Malpighiales Linaceae Linum strictum#TXMP
Malpighiales Linaceae Linum tenuifolium#VXOD
Malpighiales Linaceae Linum hirsutum#HNCF
Malpighiales Linaceae Linum perenne#BVOF
Malpighiales Linaceae Linum leonii#AEPI
Malpighiales Linaceae Linum lewisii#BHYC
Malpighiales Linaceae Linum grandiflorum#MYVH
Malpighiales Linaceae Linum bienne#OODC
Malpighiales Linaceae Linum usitatissimum#NC 036356
Malpighiales Humiriaceae Humiria balsamifera#F561
Malpighiales Humiriaceae Sacoglottis cydonioides#F562
Malpighiales Goupiaceae Goupia glabra#F556
Malpighiales Violaceae Rinorea bengalensis#F724
Malpighiales Violaceae Viola websteri#NC 041585
Malpighiales Violaceae Viola mirabilis#NC 041582
Malpighiales Violaceae Viola tricolor#LPGY
Malpighiales Violaceae Viola canadensis#NJLF
Malpighiales Violaceae Viola sp.#F369
Malpighiales Violaceae Viola raddeana#NC 041584
Malpighiales Violaceae Viola seoulensis#NC 026986
Malpighiales Violaceae Viola phalacrocarpa#NC 041583
Malpighiales Achariaceae Gynocardia odorata#F520
Malpighiales Achariaceae Hydnocarpus hainanensis#F445
Malpighiales Passifloraceae Malesherbia fasciculata#COAQ
Malpighiales Passifloraceae Erblichia odorata#NZ7103
Malpighiales Passifloraceae Passiflora pittieri#NC 038125
Malpighiales Passifloraceae Passiflora biflora#HQ
Malpighiales Passifloraceae Passiflora auriculata#MF807936
Malpighiales Passifloraceae Passiflora retipetala#NC 038188
Malpighiales Passifloraceae Passiflora oerstedii#NC 038124
Malpighiales Passifloraceae Passiflora sp.#ZIWB
Malpighiales Passifloraceae Passiflora caerulea#SIZE
Malpighiales Passifloraceae Passiflora actinia#NC 038118
Malpighiales Passifloraceae Passiflora serratifolia#NC 038129
Malpighiales Passifloraceae Passiflora serratodigitata#NC 038127
Malpighiales Passifloraceae Passiflora ligularis#NC 038122
Malpighiales Passifloraceae Passiflora coccinea#F347
Malpighiales Passifloraceae Passiflora vitifolia#NC 038128
Malpighiales Passifloraceae Passiflora quadrangularis#NC 038126
Malpighiales Passifloraceae Passiflora cincinnata#NC 037690
Malpighiales Passifloraceae Passiflora edulis#EZZT
Malpighiales Passifloraceae Passiflora laurifolia#NC 038121
Malpighiales Passifloraceae Passiflora nitida#NC 038123
Malpighiales Lacistemataceae Lacistema grandifolium#F563
Malpighiales Salicaceae Azara serrata#NC 041433
Malpighiales Salicaceae Flacourtia indica#NC 037410
Malpighiales Salicaceae Scolopia buxifolia#F734
Malpighiales Salicaceae Poliothyrsis sinensis#NC 037412
Malpighiales Salicaceae Itoa orientalis#F120
Malpighiales Salicaceae Carrierea calycina#F883
Malpighiales Salicaceae Idesia polycarpa#F628
Malpighiales Salicaceae Salix interior#NC 024681
Malpighiales Salicaceae Salix tetrasperma#NC 035744
Malpighiales Salicaceae Salix chaenomeloides#NC 037422
Malpighiales Salicaceae Salix babylonica#MF189167
Malpighiales Salicaceae Salix acutifolia#INQX
Malpighiales Salicaceae Salix paraplesia#NC 037426
Malpighiales Salicaceae Salix arbutifolia#MG262340
Malpighiales Salicaceae Salix rorida#NC 037428
Malpighiales Salicaceae Salix oreinoma#NC 035743
Malpighiales Salicaceae Salix magnifica#NC 037424
Malpighiales Salicaceae Salix minjiangensis#NC 037425
Malpighiales Salicaceae Salix hypoleuca#NC 037423
Malpighiales Salicaceae Salix suchowensis#NC 026462
Malpighiales Salicaceae Salix wilhelmsiana#F806
Malpighiales Salicaceae Salix gordejevii#F808
Malpighiales Salicaceae Salix miyabeana#F807
Malpighiales Salicaceae Salix koriyanagi#MK120982
Malpighiales Salicaceae Salix rehderiana#NC 037427
Malpighiales Salicaceae Salix taoensis#NC 037429
Malpighiales Salicaceae Salix udensis#TDTF
Malpighiales Salicaceae Salix eriocephala#GLVK
Malpighiales Salicaceae Salix fargesii#RZTJ

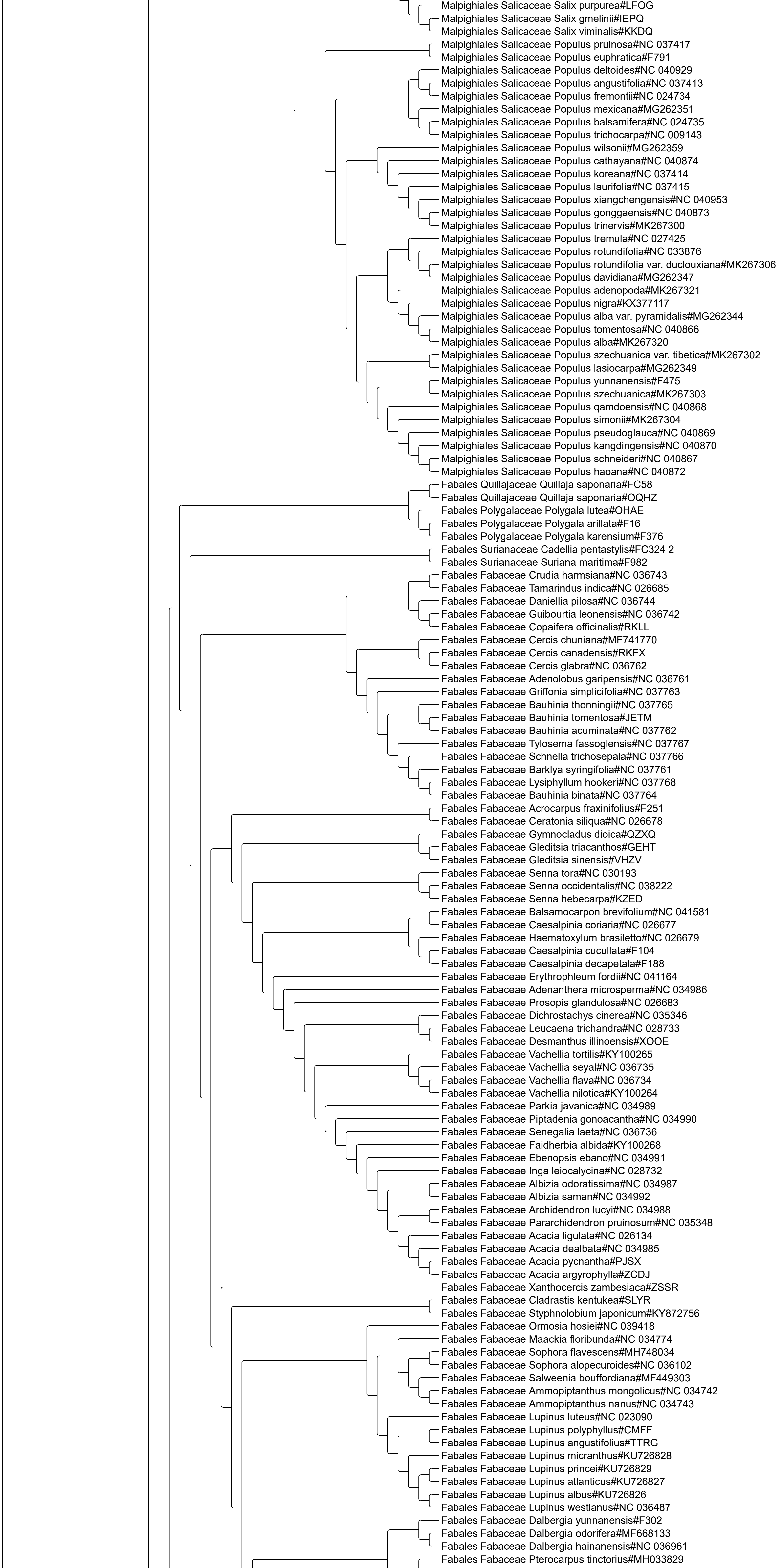

Malpighiales Salicaceae Salix purpurea#LFOG
Malpighiales Salicaceae Salix gmelinii#IEPQ
Malpighiales Salicaceae Salix viminalis#KKDQ
Malpighiales Salicaceae Populus pruinosa#NC 037417
Malpighiales Salicaceae Populus euphratica#F791
Malpighiales Salicaceae Populus deltoides#NC 040929
Malpighiales Salicaceae Populus angustifolia#NC 037413
Malpighiales Salicaceae Populus fremontii#NC 024734
Malpighiales Salicaceae Populus mexicana#MG262351
Malpighiales Salicaceae Populus balsamifera#NC 024735
Malpighiales Salicaceae Populus trichocarpa#NC 009143
Malpighiales Salicaceae Populus wilsonii#MG262359
Malpighiales Salicaceae Populus cathayana#NC 040874
Malpighiales Salicaceae Populus koreana#NC 037414
Malpighiales Salicaceae Populus laurifolia#NC 037415
Malpighiales Salicaceae Populus xiangchengensis#NC 040953
Malpighiales Salicaceae Populus gonggaensis#NC 040873
Malpighiales Salicaceae Populus trinervis#MK267300
Malpighiales Salicaceae Populus tremula#NC 027425
Malpighiales Salicaceae Populus rotundifolia#NC 033876
Malpighiales Salicaceae Populus rotundifolia var. duclouxiana#MK267306
Malpighiales Salicaceae Populus davidiana#MG262347
Malpighiales Salicaceae Populus adenopoda#MK267321
Malpighiales Salicaceae Populus nigra#KX377117
Malpighiales Salicaceae Populus alba var. pyramidalis#MG262344
Malpighiales Salicaceae Populus tomentosa#NC 040866
Malpighiales Salicaceae Populus alba#MK267320
Malpighiales Salicaceae Populus szechuanica var. tibetica#MK267302
Malpighiales Salicaceae Populus lasiocarpa#MG262349
Malpighiales Salicaceae Populus yunnanensis#F475
Malpighiales Salicaceae Populus szechuanica#MK267303
Malpighiales Salicaceae Populus qamdoensis#NC 040868
Malpighiales Salicaceae Populus simonii#MK267304
Malpighiales Salicaceae Populus pseudoglauca#NC 040869
Malpighiales Salicaceae Populus kangdingensis#NC 040870
Malpighiales Salicaceae Populus schneideri#NC 040867
Malpighiales Salicaceae Populus haoana#NC 040872
Fabales Quillajaceae Quillaja saponaria#FC58
Fabales Quillajaceae Quillaja saponaria#OQHZ
Fabales Polygalaceae Polygala lutea#OHAE
Fabales Polygalaceae Polygala arillata#F16
Fabales Polygalaceae Polygala karensium#F376
Fabales Surianaceae Cadellia pentastylis#FC324 2
Fabales Surianaceae Suriana maritima#F982
Fabales Fabaceae Crudia harmsiana#NC 036743
Fabales Fabaceae Tamarindus indica#NC 026685
Fabales Fabaceae Daniellia pilosa#NC 036744
Fabales Fabaceae Guibourtia leonensis#NC 036742
Fabales Fabaceae Copaifera officinalis#RKLL
Fabales Fabaceae Cercis chuniana#MF741770
Fabales Fabaceae Cercis canadensis#RKFX
Fabales Fabaceae Cercis glabra#NC 036762
Fabales Fabaceae Adenolobus garipensis#NC 036761
Fabales Fabaceae Griffonia simplicifolia#NC 037763
Fabales Fabaceae Bauhinia thonningii#NC 037765
Fabales Fabaceae Bauhinia tomentosa#JETM
Fabales Fabaceae Bauhinia acuminata#NC 037762
Fabales Fabaceae Tylosema fassoglensis#NC 037767
Fabales Fabaceae Schnella trichosepala#NC 037766
Fabales Fabaceae Barklya syringifolia#NC 037761
Fabales Fabaceae Lysiphyllum hookeri#NC 037768
Fabales Fabaceae Bauhinia binata#NC 037764
Fabales Fabaceae Acrocarpus fraxinifolius#F251
Fabales Fabaceae Ceratonia siliqua#NC 026678
Fabales Fabaceae Gymnocladus dioica#QZXQ
Fabales Fabaceae Gleditsia triacanthos#GEHT
Fabales Fabaceae Gleditsia sinensis#VHZV
Fabales Fabaceae Senna tora#NC 030193
Fabales Fabaceae Senna occidentalis#NC 038222
Fabales Fabaceae Senna hebecarpa#KZED
Fabales Fabaceae Balsamocarpon brevifolium#NC 041581
Fabales Fabaceae Caesalpinia coriaria#NC 026677
Fabales Fabaceae Haematoxylum brasiletto#NC 026679
Fabales Fabaceae Caesalpinia cucullata#F104
Fabales Fabaceae Caesalpinia decapetala#F188
Fabales Fabaceae Erythrophleum fordii#NC 041164
Fabales Fabaceae Adenanthera microsperma#NC 034986
Fabales Fabaceae Prosopis glandulosa#NC 026683
Fabales Fabaceae Dichrostachys cinerea#NC 035346
Fabales Fabaceae Leucaena trichandra#NC 028733
Fabales Fabaceae Desmanthus illinoensis#XOOE
Fabales Fabaceae Vachellia tortilis#KY100265
Fabales Fabaceae Vachellia seyal#NC 036735
Fabales Fabaceae Vachellia flava#NC 036734
Fabales Fabaceae Vachellia nilotica#KY100264
Fabales Fabaceae Parkia javanica#NC 034989
Fabales Fabaceae Piptadenia gonoacantha#NC 034990
Fabales Fabaceae Senegalia laeta#NC 036736
Fabales Fabaceae Faidherbia albida#KY100268
Fabales Fabaceae Ebenopsis ebano#NC 034991
Fabales Fabaceae Inga leiocalycina#NC 028732
Fabales Fabaceae Albizia odoratissima#NC 034987
Fabales Fabaceae Albizia saman#NC 034992
Fabales Fabaceae Archidendron lucyi#NC 034988
Fabales Fabaceae Pararchidendron pruinosum#NC 035348
Fabales Fabaceae Acacia ligulata#NC 026134
Fabales Fabaceae Acacia dealbata#NC 034985
Fabales Fabaceae Acacia pycnantha#PJSX
Fabales Fabaceae Acacia argyrophylla#ZCDJ
Fabales Fabaceae Xanthocercis zambesiaca#ZSSR
Fabales Fabaceae Cladrastis kentukea#SLYR
Fabales Fabaceae Styphnolobium japonicum#KY872756
Fabales Fabaceae Ormosia hosiei#NC 039418
Fabales Fabaceae Maackia floribunda#NC 034774
Fabales Fabaceae Sophora flavescens#MH748034
Fabales Fabaceae Sophora alopecuroides#NC 036102
Fabales Fabaceae Salweenia bouffordiana#MF449303
Fabales Fabaceae Ammopiptanthus mongolicus#NC 034742
Fabales Fabaceae Ammopiptanthus nanus#NC 034743
Fabales Fabaceae Lupinus luteus#NC 023090
Fabales Fabaceae Lupinus polyphyllus#CMFF
Fabales Fabaceae Lupinus angustifolius#TTRG
Fabales Fabaceae Lupinus micranthus#KU726828
Fabales Fabaceae Lupinus princei#KU726829
Fabales Fabaceae Lupinus atlanticus#KU726827
Fabales Fabaceae Lupinus albus#KU726826
Fabales Fabaceae Lupinus westianus#NC 036487
Fabales Fabaceae Dalbergia yunnanensis#F302
Fabales Fabaceae Dalbergia odorifera#MF668133
Fabales Fabaceae Dalbergia hainanensis#NC 036961
Fabales Fabaceae Pterocarpus tinctorius#MH033829

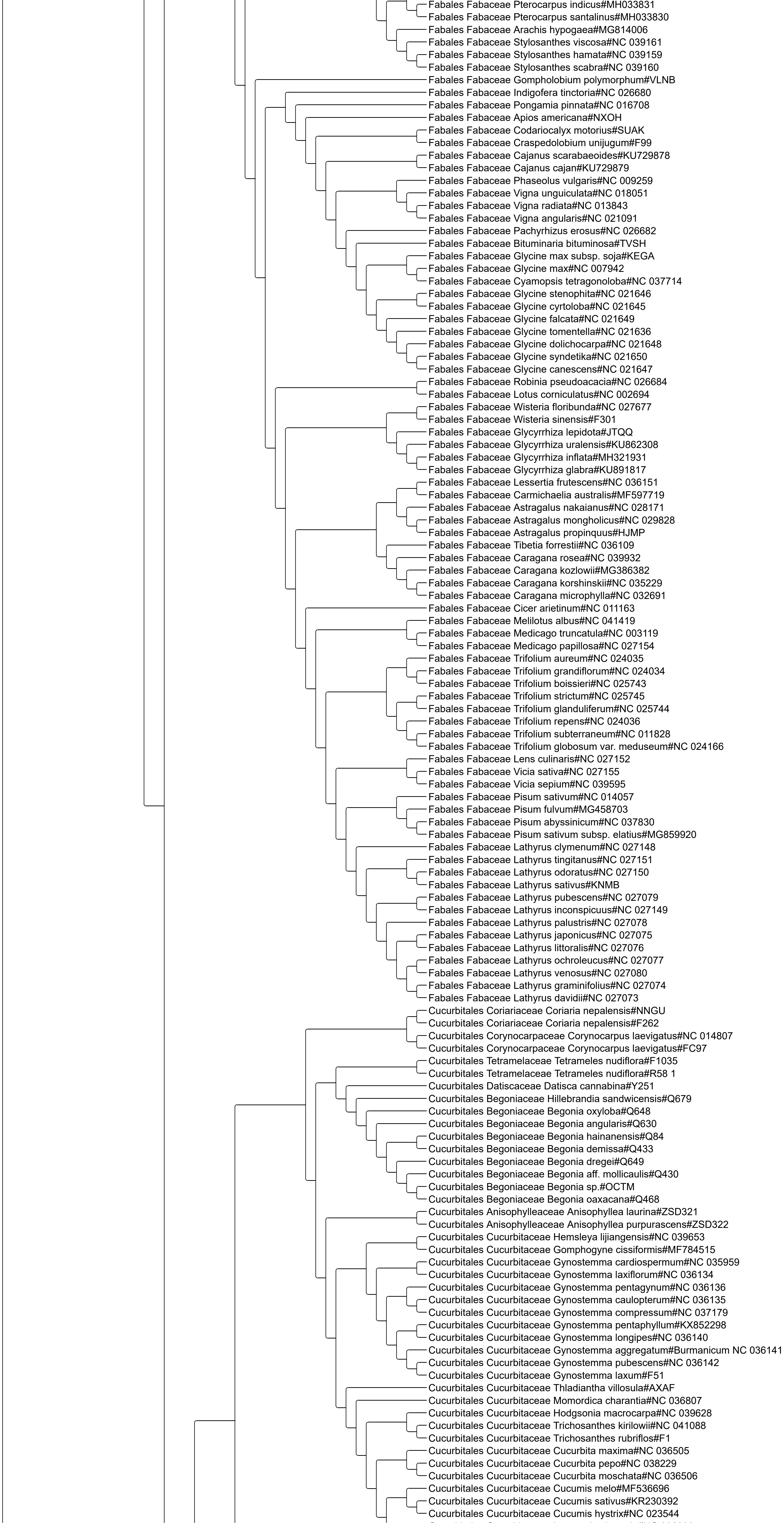
Fabales Fabaceae Pterocarpus indicus#MH033831
Fabales Fabaceae Pterocarpus santalinus#MH033830
Fabales Fabaceae Arachis hypogaea#MG814006
Fabales Fabaceae Stylosanthes viscosa#NC 039161
Fabales Fabaceae Stylosanthes hamata#NC 039159
Fabales Fabaceae Stylosanthes scabra#NC 039160
Fabales Fabaceae Gompholobium polymorphum#VLNB
Fabales Fabaceae Indigofera tinctoria#NC 026680
Fabales Fabaceae Pongamia pinnata#NC 016708
Fabales Fabaceae Apios americana#NXOH
Fabales Fabaceae Codariocalyx motorius#SUAK
Fabales Fabaceae Craspedolobium unijugum#F99
Fabales Fabaceae Cajanus scarabaeoides#KU729878
Fabales Fabaceae Cajanus cajan#KU729879
Fabales Fabaceae Phaseolus vulgaris#NC 009259
Fabales Fabaceae Vigna unguiculata#NC 018051
Fabales Fabaceae Vigna radiata#NC 013843
Fabales Fabaceae Vigna angularis#NC 021091
Fabales Fabaceae Pachyrhizus erosus#NC 026682
Fabales Fabaceae Bituminaria bituminosa#TVSH
Fabales Fabaceae Glycine max subsp. soja#KEGA
Fabales Fabaceae Glycine max#NC 007942
Fabales Fabaceae Cyamopsis tetragonoloba#NC 037714
Fabales Fabaceae Glycine stenophita#NC 021646
Fabales Fabaceae Glycine cyrtoloba#NC 021645
Fabales Fabaceae Glycine falcata#NC 021649
Fabales Fabaceae Glycine tomentella#NC 021636
Fabales Fabaceae Glycine dolichocarpa#NC 021648
Fabales Fabaceae Glycine syndetika#NC 021650
Fabales Fabaceae Glycine canescens#NC 021647
Fabales Fabaceae Robinia pseudoacacia#NC 026684
Fabales Fabaceae Lotus corniculatus#NC 002694
Fabales Fabaceae Wisteria floribunda#NC 027677
Fabales Fabaceae Wisteria sinensis#F301
Fabales Fabaceae Glycyrrhiza lepidota#JTQQ
Fabales Fabaceae Glycyrrhiza uralensis#KU862308
Fabales Fabaceae Glycyrrhiza inflata#MH321931
Fabales Fabaceae Glycyrrhiza glabra#KU891817
Fabales Fabaceae Lessertia frutescens#NC 036151
Fabales Fabaceae Carmichaelia australis#MF597719
Fabales Fabaceae Astragalus nakaianus#NC 028171
Fabales Fabaceae Astragalus mongholicus#NC 029828
Fabales Fabaceae Astragalus propinquus#HJMP
Fabales Fabaceae Tibetia forrestii#NC 036109
Fabales Fabaceae Caragana rosea#NC 039932
Fabales Fabaceae Caragana kozlowii#MG386382
Fabales Fabaceae Caragana korshinskii#NC 035229
Fabales Fabaceae Caragana microphylla#NC 032691
Fabales Fabaceae Cicer arietinum#NC 011163
Fabales Fabaceae Melilotus albus#NC 041419
Fabales Fabaceae Medicago truncatula#NC 003119
Fabales Fabaceae Medicago papillosa#NC 027154
Fabales Fabaceae Trifolium aureum#NC 024035
Fabales Fabaceae Trifolium grandiflorum#NC 024034
Fabales Fabaceae Trifolium boissieri#NC 025743
Fabales Fabaceae Trifolium strictum#NC 025745
Fabales Fabaceae Trifolium glanduliferum#NC 025744
Fabales Fabaceae Trifolium repens#NC 024036
Fabales Fabaceae Trifolium subterraneum#NC 011828
Fabales Fabaceae Trifolium globosum var. meduseum#NC 024166
Fabales Fabaceae Lens culinaris#NC 027152
Fabales Fabaceae Vicia sativa#NC 027155
Fabales Fabaceae Vicia sepium#NC 039595
Fabales Fabaceae Pisum sativum#NC 014057
Fabales Fabaceae Pisum fulvum#MG458703
Fabales Fabaceae Pisum abyssinicum#NC 037830
Fabales Fabaceae Pisum sativum subsp. elatius#MG859920
Fabales Fabaceae Lathyrus clymenum#NC 027148
Fabales Fabaceae Lathyrus tingitanus#NC 027151
Fabales Fabaceae Lathyrus odoratus#NC 027150
Fabales Fabaceae Lathyrus sativus#KNMB
Fabales Fabaceae Lathyrus pubescens#NC 027079
Fabales Fabaceae Lathyrus inconspicuus#NC 027149
Fabales Fabaceae Lathyrus palustris#NC 027078
Fabales Fabaceae Lathyrus japonicus#NC 027075
Fabales Fabaceae Lathyrus littoralis#NC 027076
Fabales Fabaceae Lathyrus ochroleucus#NC 027077
Fabales Fabaceae Lathyrus venosus#NC 027080
Fabales Fabaceae Lathyrus graminifolius#NC 027074
Fabales Fabaceae Lathyrus davidii#NC 027073
Cucurbitales Coriariaceae Coriaria nepalensis#NNGU
Cucurbitales Coriariaceae Coriaria nepalensis#F262
Cucurbitales Corynocarpaceae Corynocarpus laevigatus#NC 014807
Cucurbitales Corynocarpaceae Corynocarpus laevigatus#FC97
Cucurbitales Tetramelaceae Tetrameles nudiflora#F1035
Cucurbitales Tetramelaceae Tetrameles nudiflora#R58 1
Cucurbitales Datiscaceae Datisca cannabina#Y251
Cucurbitales Begoniaceae Hillebrandia sandwicensis#Q679
Cucurbitales Begoniaceae Begonia oxyloba#Q648
Cucurbitales Begoniaceae Begonia angularis#Q630
Cucurbitales Begoniaceae Begonia hainanensis#Q84
Cucurbitales Begoniaceae Begonia demissa#Q433
Cucurbitales Begoniaceae Begonia dregei#Q649
Cucurbitales Begoniaceae Begonia aff. mollicaulis#Q430
Cucurbitales Begoniaceae Begonia sp.#OCTM
Cucurbitales Begoniaceae Begonia oaxacana#Q468
Cucurbitales Anisophylleaceae Anisophyllea laurina#ZSD321
Cucurbitales Anisophylleaceae Anisophyllea purpurascens#ZSD322
Cucurbitales Cucurbitaceae Hemsleya lijiangensis#NC 039653
Cucurbitales Cucurbitaceae Gomphogyne cissiformis#MF784515
Cucurbitales Cucurbitaceae Gynostemma cardiospermum#NC 035959
Cucurbitales Cucurbitaceae Gynostemma laxiflorum#NC 036134
Cucurbitales Cucurbitaceae Gynostemma pentagynum#NC 036136
Cucurbitales Cucurbitaceae Gynostemma caulopterum#NC 036135
Cucurbitales Cucurbitaceae Gynostemma compressum#NC 037179
Cucurbitales Cucurbitaceae Gynostemma pentaphyllum#KX852298
Cucurbitales Cucurbitaceae Gynostemma longipes#NC 036140
Cucurbitales Cucurbitaceae Gynostemma aggregatum#Burmanicum NC 036141
Cucurbitales Cucurbitaceae Gynostemma pubescens#NC 036142
Cucurbitales Cucurbitaceae Gynostemma laxum#F51
Cucurbitales Cucurbitaceae Thladiantha villosula#AXAF
Cucurbitales Cucurbitaceae Momordica charantia#NC 036807
Cucurbitales Cucurbitaceae Hodgsonia macrocarpa#NC 039628
Cucurbitales Cucurbitaceae Trichosanthes kirilowii#NC 041088
Cucurbitales Cucurbitaceae Trichosanthes rubriflos#F1
Cucurbitales Cucurbitaceae Cucurbita maxima#NC 036505
Cucurbitales Cucurbitaceae Cucurbita pepo#NC 038229
Cucurbitales Cucurbitaceae Cucurbita moschata#NC 036506
Cucurbitales Cucurbitaceae Cucumis melo#MF536696
Cucurbitales Cucurbitaceae Cucumis sativus#KR230392
Cucurbitales Cucurbitaceae Cucumis hystrix#NC 023544

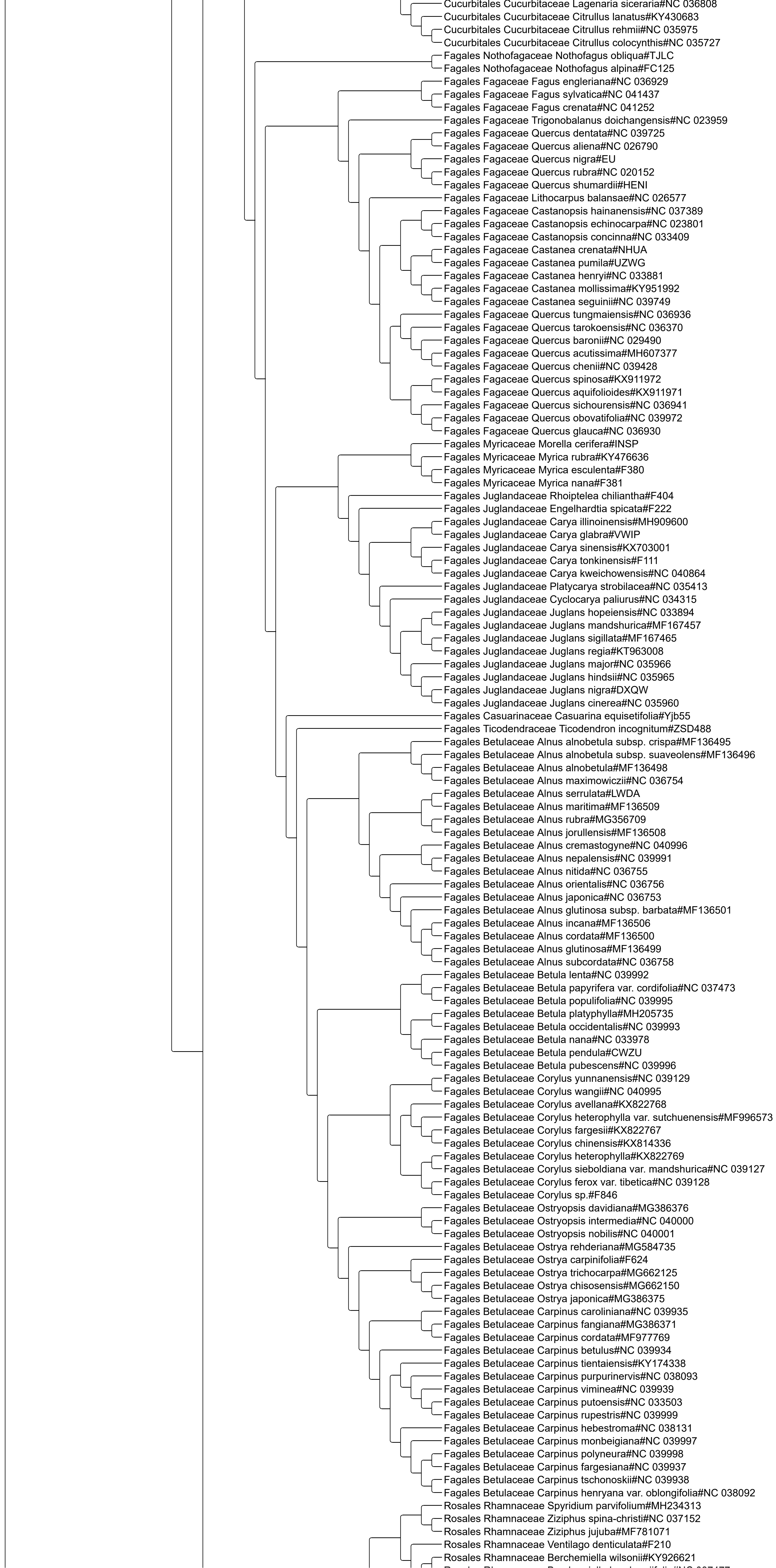
Cucurbitales Cucurbitaceae Lagenaria siceraria#NC 036808
Cucurbitales Cucurbitaceae Citrullus lanatus#KY430683
Cucurbitales Cucurbitaceae Citrullus rehmii#NC 035975
Cucurbitales Cucurbitaceae Citrullus colocynthis#NC 035727
Fagales Nothofagaceae Nothofagus obliqua#TJLC
Fagales Nothofagaceae Nothofagus alpina#FC125
Fagales Fagaceae Fagus engleriana#NC 036929
Fagales Fagaceae Fagus sylvatica#NC 041437
Fagales Fagaceae Fagus crenata#NC 041252
Fagales Fagaceae Trigonobalanus doichangensis#NC 023959
Fagales Fagaceae Quercus dentata#NC 039725
Fagales Fagaceae Quercus aliena#NC 026790
Fagales Fagaceae Quercus nigra#EU
Fagales Fagaceae Quercus rubra#NC 020152
Fagales Fagaceae Quercus shumardii#HENI
Fagales Fagaceae Lithocarpus balansae#NC 026577
Fagales Fagaceae Castanopsis hainanensis#NC 037389
Fagales Fagaceae Castanopsis echinocarpa#NC 023801
Fagales Fagaceae Castanopsis concinna#NC 033409
Fagales Fagaceae Castanea crenata#NHUA
Fagales Fagaceae Castanea pumila#UZWG
Fagales Fagaceae Castanea henryi#NC 033881
Fagales Fagaceae Castanea mollissima#KY951992
Fagales Fagaceae Castanea seguinii#NC 039749
Fagales Fagaceae Quercus tungmaiensis#NC 036936
Fagales Fagaceae Quercus tarokoensis#NC 036370
Fagales Fagaceae Quercus baronii#NC 029490
Fagales Fagaceae Quercus acutissima#MH607377
Fagales Fagaceae Quercus chenii#NC 039428
Fagales Fagaceae Quercus spinosa#KX911972
Fagales Fagaceae Quercus aquifolioides#KX911971
Fagales Fagaceae Quercus sichourensis#NC 036941
Fagales Fagaceae Quercus obovatifolia#NC 039972
Fagales Fagaceae Quercus glauca#NC 036930
Fagales Myricaceae Morella cerifera#INSP
Fagales Myricaceae Myrica rubra#KY476636
Fagales Myricaceae Myrica esculenta#F380
Fagales Myricaceae Myrica nana#F381
Fagales Juglandaceae Rhoiptelea chiliantha#F404
Fagales Juglandaceae Engelhardtia spicata#F222
Fagales Juglandaceae Carya illinoinensis#MH909600
Fagales Juglandaceae Carya glabra#VWIP
Fagales Juglandaceae Carya sinensis#KX703001
Fagales Juglandaceae Carya tonkinensis#F111
Fagales Juglandaceae Carya kweichowensis#NC 040864
Fagales Juglandaceae Platycarya strobilacea#NC 035413
Fagales Juglandaceae Cyclocarya paliurus#NC 034315
Fagales Juglandaceae Juglans hopeiensis#NC 033894
Fagales Juglandaceae Juglans mandshurica#MF167457
Fagales Juglandaceae Juglans sigillata#MF167465
Fagales Juglandaceae Juglans regia#KT963008
Fagales Juglandaceae Juglans major#NC 035966
Fagales Juglandaceae Juglans hindsii#NC 035965
Fagales Juglandaceae Juglans nigra#DXQW
Fagales Juglandaceae Juglans cinerea#NC 035960
Fagales Casuarinaceae Casuarina equisetifolia#Yjb55
Fagales Ticodendraceae Ticodendron incognitum#ZSD488
Fagales Betulaceae Alnus alnobetula subsp. crispa#MF136495
Fagales Betulaceae Alnus alnobetula subsp. suaveolens#MF136496
Fagales Betulaceae Alnus alnobetula#MF136498
Fagales Betulaceae Alnus maximowiczii#NC 036754
Fagales Betulaceae Alnus serrulata#LWDA
Fagales Betulaceae Alnus maritima#MF136509
Fagales Betulaceae Alnus rubra#MG356709
Fagales Betulaceae Alnus jorullensis#MF136508
Fagales Betulaceae Alnus cremastogyne#NC 040996
Fagales Betulaceae Alnus nepalensis#NC 039991
Fagales Betulaceae Alnus nitida#NC 036755
Fagales Betulaceae Alnus orientalis#NC 036756
Fagales Betulaceae Alnus japonica#NC 036753
Fagales Betulaceae Alnus glutinosa subsp. barbata#MF136501
Fagales Betulaceae Alnus incana#MF136506
Fagales Betulaceae Alnus cordata#MF136500
Fagales Betulaceae Alnus glutinosa#MF136499
Fagales Betulaceae Alnus subcordata#NC 036758
Fagales Betulaceae Betula lenta#NC 039992
Fagales Betulaceae Betula papyrifera var. cordifolia#NC 037473
Fagales Betulaceae Betula populifolia#NC 039995
Fagales Betulaceae Betula platyphylla#MH205735
Fagales Betulaceae Betula occidentalis#NC 039993
Fagales Betulaceae Betula nana#NC 033978
Fagales Betulaceae Betula pendula#CWZU
Fagales Betulaceae Betula pubescens#NC 039996
Fagales Betulaceae Corylus yunnanensis#NC 039129
Fagales Betulaceae Corylus wangii#NC 040995
Fagales Betulaceae Corylus avellana#KX822768
Fagales Betulaceae Corylus heterophylla var. sutchuenensis#MF996573
Fagales Betulaceae Corylus fargesii#KX822767
Fagales Betulaceae Corylus chinensis#KX814336
Fagales Betulaceae Corylus heterophylla#KX822769
Fagales Betulaceae Corylus sieboldiana var. mandshurica#NC 039127
Fagales Betulaceae Corylus ferox var. tibetica#NC 039128
Fagales Betulaceae Corylus sp.#F846
Fagales Betulaceae Ostryopsis davidiana#MG386376
Fagales Betulaceae Ostryopsis intermedia#NC 040000
Fagales Betulaceae Ostryopsis nobilis#NC 040001
Fagales Betulaceae Ostrya rehderiana#MG584735
Fagales Betulaceae Ostrya carpinifolia#F624
Fagales Betulaceae Ostrya trichocarpa#MG662125
Fagales Betulaceae Ostrya chisosensis#MG662150
Fagales Betulaceae Ostrya japonica#MG386375
Fagales Betulaceae Carpinus caroliniana#NC 039935
Fagales Betulaceae Carpinus fangiana#MG386371
Fagales Betulaceae Carpinus cordata#MF977769
Fagales Betulaceae Carpinus betulus#NC 039934
Fagales Betulaceae Carpinus tientaiensis#KY174338
Fagales Betulaceae Carpinus purpurinervis#NC 038093
Fagales Betulaceae Carpinus viminea#NC 039939
Fagales Betulaceae Carpinus putoensis#NC 033503
Fagales Betulaceae Carpinus rupestris#NC 039999
Fagales Betulaceae Carpinus hebestroma#NC 038131
Fagales Betulaceae Carpinus monbeigiana#NC 039997
Fagales Betulaceae Carpinus polyneura#NC 039998
Fagales Betulaceae Carpinus fargesiana#NC 039937
Fagales Betulaceae Carpinus tschonoskii#NC 039938
Fagales Betulaceae Carpinus henryana var. oblongifolia#NC 038092
Rosales Rhamnaceae Spyridium parvifolium#MH234313
Rosales Rhamnaceae Ziziphus spina-christi#NC 037152
Rosales Rhamnaceae Ziziphus jujuba#MF781071
Rosales Rhamnaceae Ventilago denticulata#F210
Rosales Rhamnaceae Berchemiella wilsonii#KY926621

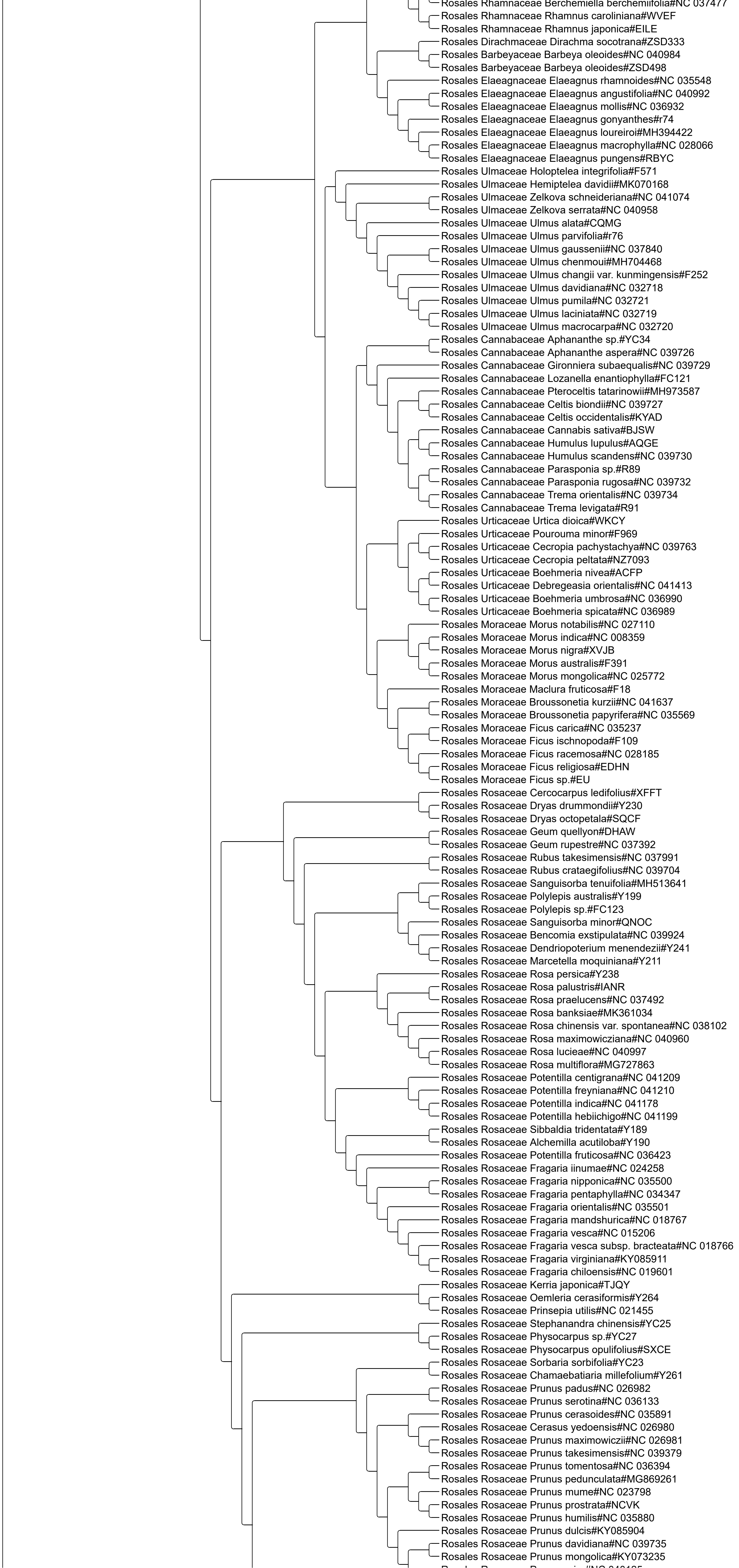

Rosales Rhamnaceae Berchemiella berchemiifolia#NC 037477
Rosales Rhamnaceae Rhamnus caroliniana#WVEF
Rosales Rhamnaceae Rhamnus japonica#EILE
Rosales Dirachmaceae Dirachma socotrana#ZSD333
Rosales Barbeyaceae Barbeya oleoides#NC 040984
Rosales Barbeyaceae Barbeya oleoides#ZSD498
Rosales Elaeagnaceae Elaeagnus rhamnoides#NC 035548
Rosales Elaeagnaceae Elaeagnus angustifolia#NC 040992
Rosales Elaeagnaceae Elaeagnus mollis#NC 036932
Rosales Elaeagnaceae Elaeagnus gonyanthes#r74
Rosales Elaeagnaceae Elaeagnus loureiroi#MH394422
Rosales Elaeagnaceae Elaeagnus macrophylla#NC 028066
Rosales Elaeagnaceae Elaeagnus pungens#RBYC
Rosales Ulmaceae Holoptelea integrifolia#F571
Rosales Ulmaceae Hemiptelea davidii#MK070168
Rosales Ulmaceae Zelkova schneideriana#NC 041074
Rosales Ulmaceae Zelkova serrata#NC 040958
Rosales Ulmaceae Ulmus alata#CQMG
Rosales Ulmaceae Ulmus parvifolia#r76
Rosales Ulmaceae Ulmus gaussenii#NC 037840
Rosales Ulmaceae Ulmus chenmoui#MH704468
Rosales Ulmaceae Ulmus changii var. kunmingensis#F252
Rosales Ulmaceae Ulmus davidiana#NC 032718
Rosales Ulmaceae Ulmus pumila#NC 032721
Rosales Ulmaceae Ulmus laciniata#NC 032719
Rosales Ulmaceae Ulmus macrocarpa#NC 032720
Rosales Cannabaceae Aphananthe sp.#YC34
Rosales Cannabaceae Aphananthe aspera#NC 039726
Rosales Cannabaceae Gironniera subaequalis#NC 039729
Rosales Cannabaceae Lozanella enantiophylla#FC121
Rosales Cannabaceae Pteroceltis tatarinowii#MH973587
Rosales Cannabaceae Celtis biondii#NC 039727
Rosales Cannabaceae Celtis occidentalis#KYAD
Rosales Cannabaceae Cannabis sativa#BJSW
Rosales Cannabaceae Humulus lupulus#AQGE
Rosales Cannabaceae Humulus scandens#NC 039730
Rosales Cannabaceae Parasponia sp.#R89
Rosales Cannabaceae Parasponia rugosa#NC 039732
Rosales Cannabaceae Trema orientalis#NC 039734
Rosales Cannabaceae Trema levigata#R91
Rosales Urticaceae Urtica dioica#WKCY
Rosales Urticaceae Pourouma minor#F969
Rosales Urticaceae Cecropia pachystachya#NC 039763
Rosales Urticaceae Cecropia peltata#NZ7093
Rosales Urticaceae Boehmeria nivea#ACFP
Rosales Urticaceae Debregeasia orientalis#NC 041413
Rosales Urticaceae Boehmeria umbrosa#NC 036990
Rosales Urticaceae Boehmeria spicata#NC 036989
Rosales Moraceae Morus notabilis#NC 027110
Rosales Moraceae Morus indica#NC 008359
Rosales Moraceae Morus nigra#XVJB
Rosales Moraceae Morus australis#F391
Rosales Moraceae Morus mongolica#NC 025772
Rosales Moraceae Maclura fruticosa#F18
Rosales Moraceae Broussonetia kurzii#NC 041637
Rosales Moraceae Broussonetia papyrifera#NC 035569
Rosales Moraceae Ficus carica#NC 035237
Rosales Moraceae Ficus ischnopoda#F109
Rosales Moraceae Ficus racemosa#NC 028185
Rosales Moraceae Ficus religiosa#EDHN
Rosales Moraceae Ficus sp.#EU
Rosales Rosaceae Cercocarpus ledifolius#XFFT
Rosales Rosaceae Dryas drummondii#Y230
Rosales Rosaceae Dryas octopetala#SQCF
Rosales Rosaceae Geum quellyon#DHAW
Rosales Rosaceae Geum rupestre#NC 037392
Rosales Rosaceae Rubus takesimensis#NC 037991
Rosales Rosaceae Rubus crataegifolius#NC 039704
Rosales Rosaceae Sanguisorba tenuifolia#MH513641
Rosales Rosaceae Polylepis australis#Y199
Rosales Rosaceae Polylepis sp.#FC123
Rosales Rosaceae Sanguisorba minor#QNOC
Rosales Rosaceae Bencomia exstipulata#NC 039924
Rosales Rosaceae Dendriopoterium menendezii#Y241
Rosales Rosaceae Marcetella moquiniana#Y211
Rosales Rosaceae Rosa persica#Y238
Rosales Rosaceae Rosa palustris#IANR
Rosales Rosaceae Rosa praelucens#NC 037492
Rosales Rosaceae Rosa banksiae#MK361034
Rosales Rosaceae Rosa chinensis var. spontanea#NC 038102
Rosales Rosaceae Rosa maximowicziana#NC 040960
Rosales Rosaceae Rosa lucieae#NC 040997
Rosales Rosaceae Rosa multiflora#MG727863
Rosales Rosaceae Potentilla centigrana#NC 041209
Rosales Rosaceae Potentilla freyniana#NC 041210
Rosales Rosaceae Potentilla indica#NC 041178
Rosales Rosaceae Potentilla hebiichigo#NC 041199
Rosales Rosaceae Sibbaldia tridentata#Y189
Rosales Rosaceae Alchemilla acutiloba#Y190
Rosales Rosaceae Potentilla fruticosa#NC 036423
Rosales Rosaceae Fragaria iinumae#NC 024258
Rosales Rosaceae Fragaria nipponica#NC 035500
Rosales Rosaceae Fragaria pentaphylla#NC 034347
Rosales Rosaceae Fragaria orientalis#NC 035501
Rosales Rosaceae Fragaria mandshurica#NC 018767
Rosales Rosaceae Fragaria vesca#NC 015206
Rosales Rosaceae Fragaria vesca subsp. bracteata#NC 018766
Rosales Rosaceae Fragaria virginiana#KY085911
Rosales Rosaceae Fragaria chiloensis#NC 019601
Rosales Rosaceae Kerria japonica#TJQY
Rosales Rosaceae Oemleria cerasiformis#Y264
Rosales Rosaceae Prinsepia utilis#NC 021455
Rosales Rosaceae Stephanandra chinensis#YC25
Rosales Rosaceae Physocarpus sp.#YC27
Rosales Rosaceae Physocarpus opulifolius#SXCE
Rosales Rosaceae Sorbaria sorbifolia#YC23
Rosales Rosaceae Chamaebatiaria millefolium#Y261
Rosales Rosaceae Prunus padus#NC 026982
Rosales Rosaceae Prunus serotina#NC 036133
Rosales Rosaceae Prunus cerasoides#NC 035891
Rosales Rosaceae Cerasus yedoensis#NC 026980
Rosales Rosaceae Prunus maximowiczii#NC 026981
Rosales Rosaceae Prunus takesimensis#NC 039379
Rosales Rosaceae Prunus tomentosa#NC 036394
Rosales Rosaceae Prunus pedunculata#MG869261
Rosales Rosaceae Prunus mume#NC 023798
Rosales Rosaceae Prunus prostrata#NCVK
Rosales Rosaceae Prunus humilis#NC 035880
Rosales Rosaceae Prunus dulcis#KY085904
Rosales Rosaceae Prunus davidiana#NC 039735
Rosales Rosaceae Prunus mongolica#KY073235

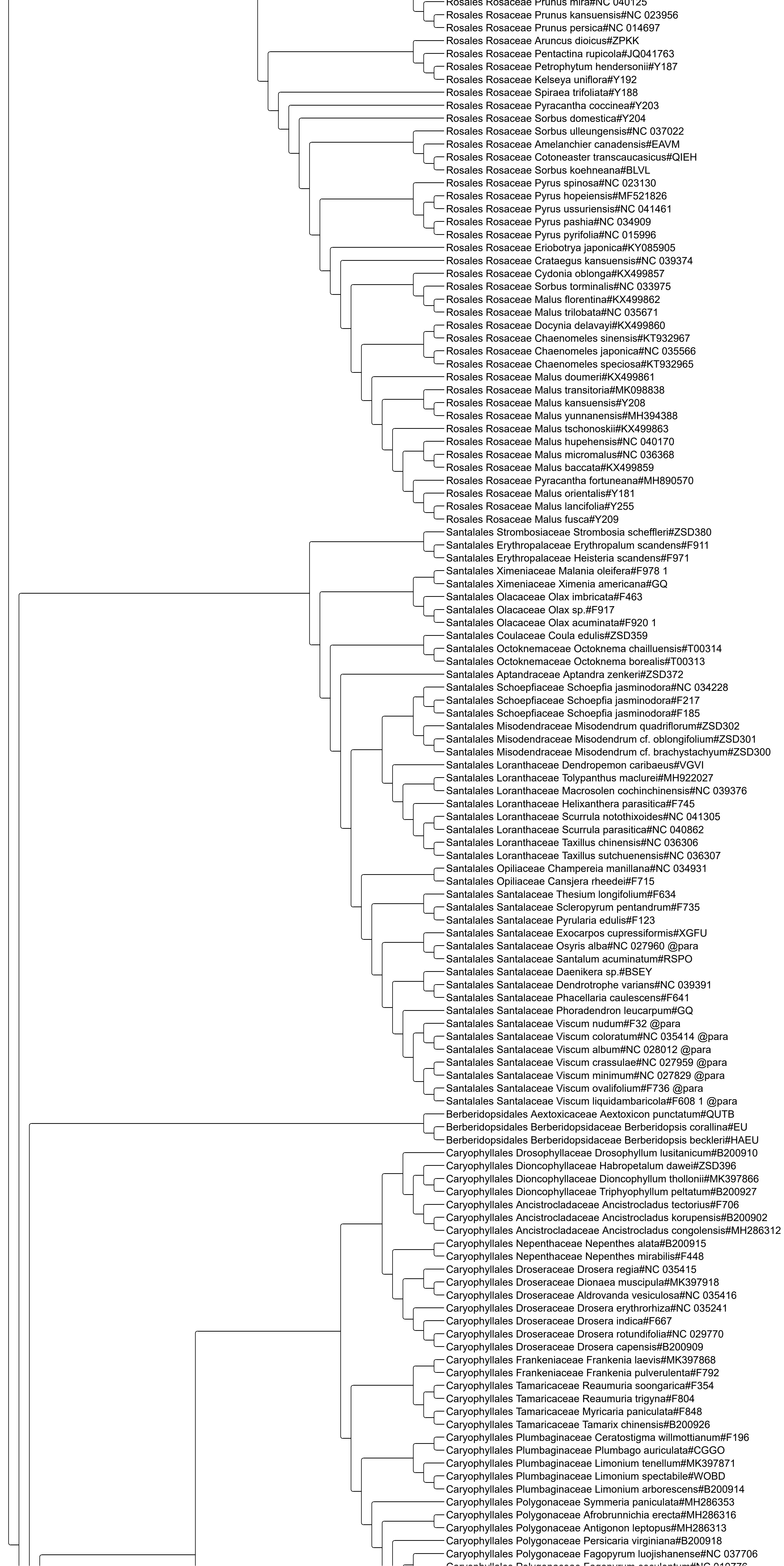

Rosales Rosaceae Prunus mira#NC 040125
Rosales Rosaceae Prunus kansuensis#NC 023956
Rosales Rosaceae Prunus persica#NC 014697
Rosales Rosaceae Aruncus dioicus#ZPKK
Rosales Rosaceae Pentactina rupicola#JQ041763
Rosales Rosaceae Petrophytum hendersonii#Y187
Rosales Rosaceae Kelseya uniflora#Y192
Rosales Rosaceae Spiraea trifoliata#Y188
Rosales Rosaceae Pyracantha coccinea#Y203
Rosales Rosaceae Sorbus domestica#Y204
Rosales Rosaceae Sorbus ulleungensis#NC 037022
Rosales Rosaceae Amelanchier canadensis#EAVM
Rosales Rosaceae Cotoneaster transcaucasicus#QIEH
Rosales Rosaceae Sorbus koehneana#BLVL
Rosales Rosaceae Pyrus spinosa#NC 023130
Rosales Rosaceae Pyrus hopeiensis#MF521826
Rosales Rosaceae Pyrus ussuriensis#NC 041461
Rosales Rosaceae Pyrus pashia#NC 034909
Rosales Rosaceae Pyrus pyrifolia#NC 015996
Rosales Rosaceae Eriobotrya japonica#KY085905
Rosales Rosaceae Crataegus kansuensis#NC 039374
Rosales Rosaceae Cydonia oblonga#KX499857
Rosales Rosaceae Sorbus torminalis#NC 033975
Rosales Rosaceae Malus florentina#KX499862
Rosales Rosaceae Malus trilobata#NC 035671
Rosales Rosaceae Docynia delavayi#KX499860
Rosales Rosaceae Chaenomeles sinensis#KT932967
Rosales Rosaceae Chaenomeles japonica#NC 035566
Rosales Rosaceae Chaenomeles speciosa#KT932965
Rosales Rosaceae Malus doumeri#KX499861
Rosales Rosaceae Malus transitoria#MK098838
Rosales Rosaceae Malus kansuensis#Y208
Rosales Rosaceae Malus yunnanensis#MH394388
Rosales Rosaceae Malus tschonoskii#KX499863
Rosales Rosaceae Malus hupehensis#NC 040170
Rosales Rosaceae Malus micromalus#NC 036368
Rosales Rosaceae Malus baccata#KX499859
Rosales Rosaceae Pyracantha fortuneana#MH890570
Rosales Rosaceae Malus orientalis#Y181
Rosales Rosaceae Malus lancifolia#Y255
Rosales Rosaceae Malus fusca#Y209
Santalales Strombosiaceae Strombosia scheffleri#ZSD380
Santalales Erythropalaceae Erythropalum scandens#F911
Santalales Erythropalaceae Heisteria scandens#F971
Santalales Ximeniaceae Malania oleifera#F978 1
Santalales Ximeniaceae Ximenia americana#GQ
Santalales Olacaceae Olax imbricata#F463
Santalales Olacaceae Olax sp.#F917
Santalales Olacaceae Olax acuminata#F920 1
Santalales Coulaceae Coula edulis#ZSD359
Santalales Octoknemaceae Octoknema chailluensis#T00314
Santalales Octoknemaceae Octoknema borealis#T00313
Santalales Aptandraceae Aptandra zenkeri#ZSD372
Santalales Schoepfiaceae Schoepfia jasminodora#NC 034228
Santalales Schoepfiaceae Schoepfia jasminodora#F217
Santalales Schoepfiaceae Schoepfia jasminodora#F185
Santalales Misodendraceae Misodendrum quadriflorum#ZSD302
Santalales Misodendraceae Misodendrum cf. oblongifolium#ZSD301
Santalales Misodendraceae Misodendrum cf. brachystachyum#ZSD300
Santalales Loranthaceae Dendropemon caribaeus#VGVI
Santalales Loranthaceae Tolypanthus maclurei#MH922027
Santalales Loranthaceae Macrosolen cochinchinensis#NC 039376
Santalales Loranthaceae Helixanthera parasitica#F745
Santalales Loranthaceae Scurrula notothixoides#NC 041305
Santalales Loranthaceae Scurrula parasitica#NC 040862
Santalales Loranthaceae Taxillus chinensis#NC 036306
Santalales Loranthaceae Taxillus sutchuenensis#NC 036307
Santalales Opiliaceae Champereia manillana#NC 034931
Santalales Opiliaceae Cansjera rheedei#F715
Santalales Santalaceae Thesium longifolium#F634
Santalales Santalaceae Scleropyrum pentandrum#F735
Santalales Santalaceae Pyrularia edulis#F123
Santalales Santalaceae Exocarpos cupressiformis#XGFU
Santalales Santalaceae Osyris alba#NC 027960 @para
Santalales Santalaceae Santalum acuminatum#RSPO
Santalales Santalaceae Daenikera sp.#BSEY
Santalales Santalaceae Dendrotrophe varians#NC 039391
Santalales Santalaceae Phacellaria caulescens#F641
Santalales Santalaceae Phoradendron leucarpum#GQ
Santalales Santalaceae Viscum nudum#F32 @para
Santalales Santalaceae Viscum coloratum#NC 035414 @para
Santalales Santalaceae Viscum album#NC 028012 @para
Santalales Santalaceae Viscum crassulae#NC 027959 @para
Santalales Santalaceae Viscum minimum#NC 027829 @para
Santalales Santalaceae Viscum ovalifolium#F736 @para
Santalales Santalaceae Viscum liquidambaricola#F608 1 @para
Berberidopsidales Aextoxicaceae Aextoxicon punctatum#QUTB
Berberidopsidales Berberidopsidaceae Berberidopsis corallina#EU
Berberidopsidales Berberidopsidaceae Berberidopsis beckleri#HAEU
Caryophyllales Drosophyllaceae Drosophyllum lusitanicum#B200910
Caryophyllales Dioncophyllaceae Habropetalum dawei#ZSD396
Caryophyllales Dioncophyllaceae Dioncophyllum thollonii#MK397866
Caryophyllales Dioncophyllaceae Triphyophyllum peltatum#B200927
Caryophyllales Ancistrocladaceae Ancistrocladus tectorius#F706
Caryophyllales Ancistrocladaceae Ancistrocladus korupensis#B200902
Caryophyllales Ancistrocladaceae Ancistrocladus congolensis#MH286312
Caryophyllales Nepenthaceae Nepenthes alata#B200915
Caryophyllales Nepenthaceae Nepenthes mirabilis#F448
Caryophyllales Droseraceae Drosera regia#NC 035415
Caryophyllales Droseraceae Dionaea muscipula#MK397918
Caryophyllales Droseraceae Aldrovanda vesiculosa#NC 035416
Caryophyllales Droseraceae Drosera erythrorhiza#NC 035241
Caryophyllales Droseraceae Drosera indica#F667
Caryophyllales Droseraceae Drosera rotundifolia#NC 029770
Caryophyllales Droseraceae Drosera capensis#B200909
Caryophyllales Frankeniaceae Frankenia laevis#MK397868
Caryophyllales Frankeniaceae Frankenia pulverulenta#F792
Caryophyllales Tamaricaceae Reaumuria soongarica#F354
Caryophyllales Tamaricaceae Reaumuria trigyna#F804
Caryophyllales Tamaricaceae Myricaria paniculata#F848
Caryophyllales Tamaricaceae Tamarix chinensis#B200926
Caryophyllales Plumbaginaceae Ceratostigma willmottianum#F196
Caryophyllales Plumbaginaceae Plumbago auriculata#CGGO
Caryophyllales Plumbaginaceae Limonium tenellum#MK397871
Caryophyllales Plumbaginaceae Limonium spectabile#WOBD
Caryophyllales Plumbaginaceae Limonium arborescens#B200914
Caryophyllales Polygonaceae Symmeria paniculata#MH286353
Caryophyllales Polygonaceae Afrobrunnichia erecta#MH286316
Caryophyllales Polygonaceae Antigonon leptopus#MH286313
Caryophyllales Polygonaceae Persicaria virginiana#B200918
Caryophyllales Polygonaceae Fagopyrum luojishanense#NC 037706

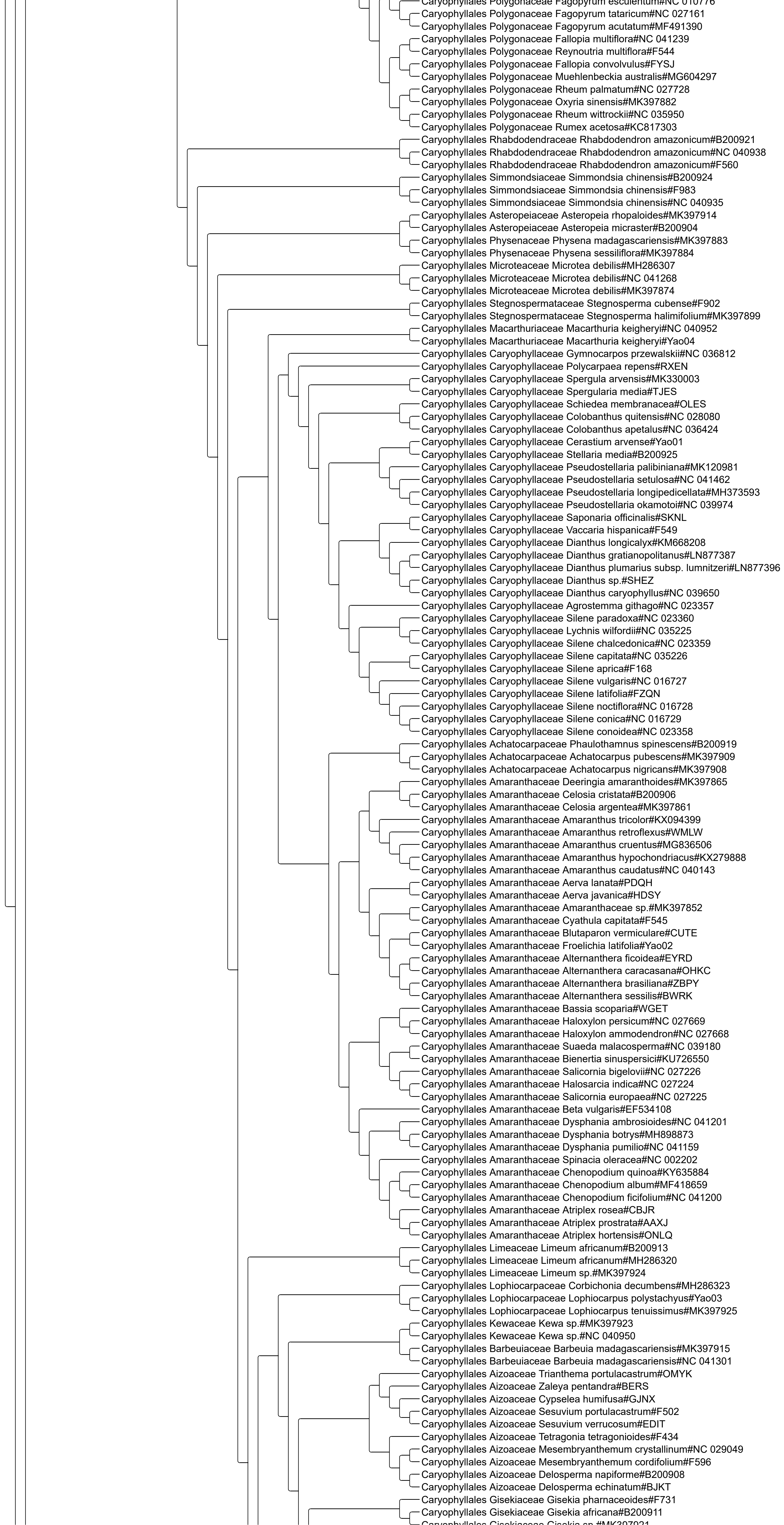
Caryophyllales Polygonaceae Fagopyrum esculentum#NC 010776
Caryophyllales Polygonaceae Fagopyrum tataricum#NC 027161
Caryophyllales Polygonaceae Fagopyrum acutatum#MF491390
Caryophyllales Polygonaceae Fallopia multiflora#NC 041239
Caryophyllales Polygonaceae Reynoutria multiflora#F544
Caryophyllales Polygonaceae Fallopia convolvulus#FYSJ
Caryophyllales Polygonaceae Muehlenbeckia australis#MG604297
Caryophyllales Polygonaceae Rheum palmatum#NC 027728
Caryophyllales Polygonaceae Oxyria sinensis#MK397882
Caryophyllales Polygonaceae Rheum wittrockii#NC 035950
Caryophyllales Polygonaceae Rumex acetosa#KC817303
Caryophyllales Rhabdodendraceae Rhabdodendron amazonicum#B200921
Caryophyllales Rhabdodendraceae Rhabdodendron amazonicum#NC 040938
Caryophyllales Rhabdodendraceae Rhabdodendron amazonicum#F560
Caryophyllales Simmondsiaceae Simmondsia chinensis#B200924
Caryophyllales Simmondsiaceae Simmondsia chinensis#F983
Caryophyllales Simmondsiaceae Simmondsia chinensis#NC 040935
Caryophyllales Asteropeiaceae Asteropeia rhopaloides#MK397914
Caryophyllales Asteropeiaceae Asteropeia micraster#B200904
Caryophyllales Physenaceae Physena madagascariensis#MK397883
Caryophyllales Physenaceae Physena sessiliflora#MK397884
Caryophyllales Microteaceae Microtea debilis#MH286307
Caryophyllales Microteaceae Microtea debilis#NC 041268
Caryophyllales Microteaceae Microtea debilis#MK397874
Caryophyllales Stegnospermataceae Stegnosperma cubense#F902
Caryophyllales Stegnospermataceae Stegnosperma halimifolium#MK397899
Caryophyllales Macarthuriaceae Macarthuria keigheryi#NC 040952
Caryophyllales Macarthuriaceae Macarthuria keigheryi#Yao04
Caryophyllales Caryophyllaceae Gymnocarpos przewalskii#NC 036812
Caryophyllales Caryophyllaceae Polycarpaea repens#RXEN
Caryophyllales Caryophyllaceae Spergula arvensis#MK330003
Caryophyllales Caryophyllaceae Spergularia media#TJES
Caryophyllales Caryophyllaceae Schiedea membranacea#OLES
Caryophyllales Caryophyllaceae Colobanthus quitensis#NC 028080
Caryophyllales Caryophyllaceae Colobanthus apetalus#NC 036424
Caryophyllales Caryophyllaceae Cerastium arvense#Yao01
Caryophyllales Caryophyllaceae Stellaria media#B200925
Caryophyllales Caryophyllaceae Pseudostellaria palibiniana#MK120981
Caryophyllales Caryophyllaceae Pseudostellaria setulosa#NC 041462
Caryophyllales Caryophyllaceae Pseudostellaria longipedicellata#MH373593
Caryophyllales Caryophyllaceae Pseudostellaria okamotoi#NC 039974
Caryophyllales Caryophyllaceae Saponaria officinalis#SKNL
Caryophyllales Caryophyllaceae Vaccaria hispanica#F549
Caryophyllales Caryophyllaceae Dianthus longicalyx#KM668208
Caryophyllales Caryophyllaceae Dianthus gratianopolitanus#LN877387
Caryophyllales Caryophyllaceae Dianthus plumarius subsp. lumnitzeri#LN877396
Caryophyllales Caryophyllaceae Dianthus sp.#SHEZ
Caryophyllales Caryophyllaceae Dianthus caryophyllus#NC 039650
Caryophyllales Caryophyllaceae Agrostemma githago#NC 023357
Caryophyllales Caryophyllaceae Silene paradoxa#NC 023360
Caryophyllales Caryophyllaceae Lychnis wilfordii#NC 035225
Caryophyllales Caryophyllaceae Silene chalcedonica#NC 023359
Caryophyllales Caryophyllaceae Silene capitata#NC 035226
Caryophyllales Caryophyllaceae Silene aprica#F168
Caryophyllales Caryophyllaceae Silene vulgaris#NC 016727
Caryophyllales Caryophyllaceae Silene latifolia#FZQN
Caryophyllales Caryophyllaceae Silene noctiflora#NC 016728
Caryophyllales Caryophyllaceae Silene conica#NC 016729
Caryophyllales Caryophyllaceae Silene conoidea#NC 023358
Caryophyllales Achatocarpaceae Phaulothamnus spinescens#B200919
Caryophyllales Achatocarpaceae Achatocarpus pubescens#MK397909
Caryophyllales Achatocarpaceae Achatocarpus nigricans#MK397908
Caryophyllales Amaranthaceae Deeringia amaranthoides#MK397865
Caryophyllales Amaranthaceae Celosia cristata#B200906
Caryophyllales Amaranthaceae Celosia argentea#MK397861
Caryophyllales Amaranthaceae Amaranthus tricolor#KX094399
Caryophyllales Amaranthaceae Amaranthus retroflexus#WMLW
Caryophyllales Amaranthaceae Amaranthus cruentus#MG836506
Caryophyllales Amaranthaceae Amaranthus hypochondriacus#KX279888
Caryophyllales Amaranthaceae Amaranthus caudatus#NC 040143
Caryophyllales Amaranthaceae Aerva lanata#PDQH
Caryophyllales Amaranthaceae Aerva javanica#HDSY
Caryophyllales Amaranthaceae Amaranthaceae sp.#MK397852
Caryophyllales Amaranthaceae Cyathula capitata#F545
Caryophyllales Amaranthaceae Blutaparon vermiculare#CUTE
Caryophyllales Amaranthaceae Froelichia latifolia#Yao02
Caryophyllales Amaranthaceae Alternanthera ficoidea#EYRD
Caryophyllales Amaranthaceae Alternanthera caracasana#OHKC
Caryophyllales Amaranthaceae Alternanthera brasiliana#ZBPY
Caryophyllales Amaranthaceae Alternanthera sessilis#BWRK
Caryophyllales Amaranthaceae Bassia scoparia#WGET
Caryophyllales Amaranthaceae Haloxylon persicum#NC 027669
Caryophyllales Amaranthaceae Haloxylon ammodendron#NC 027668
Caryophyllales Amaranthaceae Suaeda malacosperma#NC 039180
Caryophyllales Amaranthaceae Bienertia sinuspersici#KU726550
Caryophyllales Amaranthaceae Salicornia bigelovii#NC 027226
Caryophyllales Amaranthaceae Halosarcia indica#NC 027224
Caryophyllales Amaranthaceae Salicornia europaea#NC 027225
Caryophyllales Amaranthaceae Beta vulgaris#EF534108
Caryophyllales Amaranthaceae Dysphania ambrosioides#NC 041201
Caryophyllales Amaranthaceae Dysphania botrys#MH898873
Caryophyllales Amaranthaceae Dysphania pumilio#NC 041159
Caryophyllales Amaranthaceae Spinacia oleracea#NC 002202
Caryophyllales Amaranthaceae Chenopodium quinoa#KY635884
Caryophyllales Amaranthaceae Chenopodium album#MF418659
Caryophyllales Amaranthaceae Chenopodium ficifolium#NC 041200
Caryophyllales Amaranthaceae Atriplex rosea#CBJR
Caryophyllales Amaranthaceae Atriplex prostrata#AAXJ
Caryophyllales Amaranthaceae Atriplex hortensis#ONLQ
Caryophyllales Limeaceae Limeum africanum#B200913
Caryophyllales Limeaceae Limeum africanum#MH286320
Caryophyllales Limeaceae Limeum sp.#MK397924
Caryophyllales Lophiocarpaceae Corbichonia decumbens#MH286323
Caryophyllales Lophiocarpaceae Lophiocarpus polystachyus#Yao03
Caryophyllales Lophiocarpaceae Lophiocarpus tenuissimus#MK397925
Caryophyllales Kewaceae Kewa sp.#MK397923
Caryophyllales Kewaceae Kewa sp.#NC 040950
Caryophyllales Barbeuiaceae Barbeuia madagascariensis#MK397915
Caryophyllales Barbeuiaceae Barbeuia madagascariensis#NC 041301
Caryophyllales Aizoaceae Trianthema portulacastrum#OMYK
Caryophyllales Aizoaceae Zaleya pentandra#BERS
Caryophyllales Aizoaceae Cypselea humifusa#GJNX
Caryophyllales Aizoaceae Sesuvium portulacastrum#F502
Caryophyllales Aizoaceae Sesuvium verrucosum#EDIT
Caryophyllales Aizoaceae Tetragonia tetragonioides#F434
Caryophyllales Aizoaceae Mesembryanthemum crystallinum#NC 029049
Caryophyllales Aizoaceae Mesembryanthemum cordifolium#F596
Caryophyllales Aizoaceae Delosperma napiforme#B200908
Caryophyllales Aizoaceae Delosperma echinatum#BJKT
Caryophyllales Gisekiaceae Gisekia pharnaceoides#F731
Caryophyllales Gisekiaceae Gisekia africana#B200911

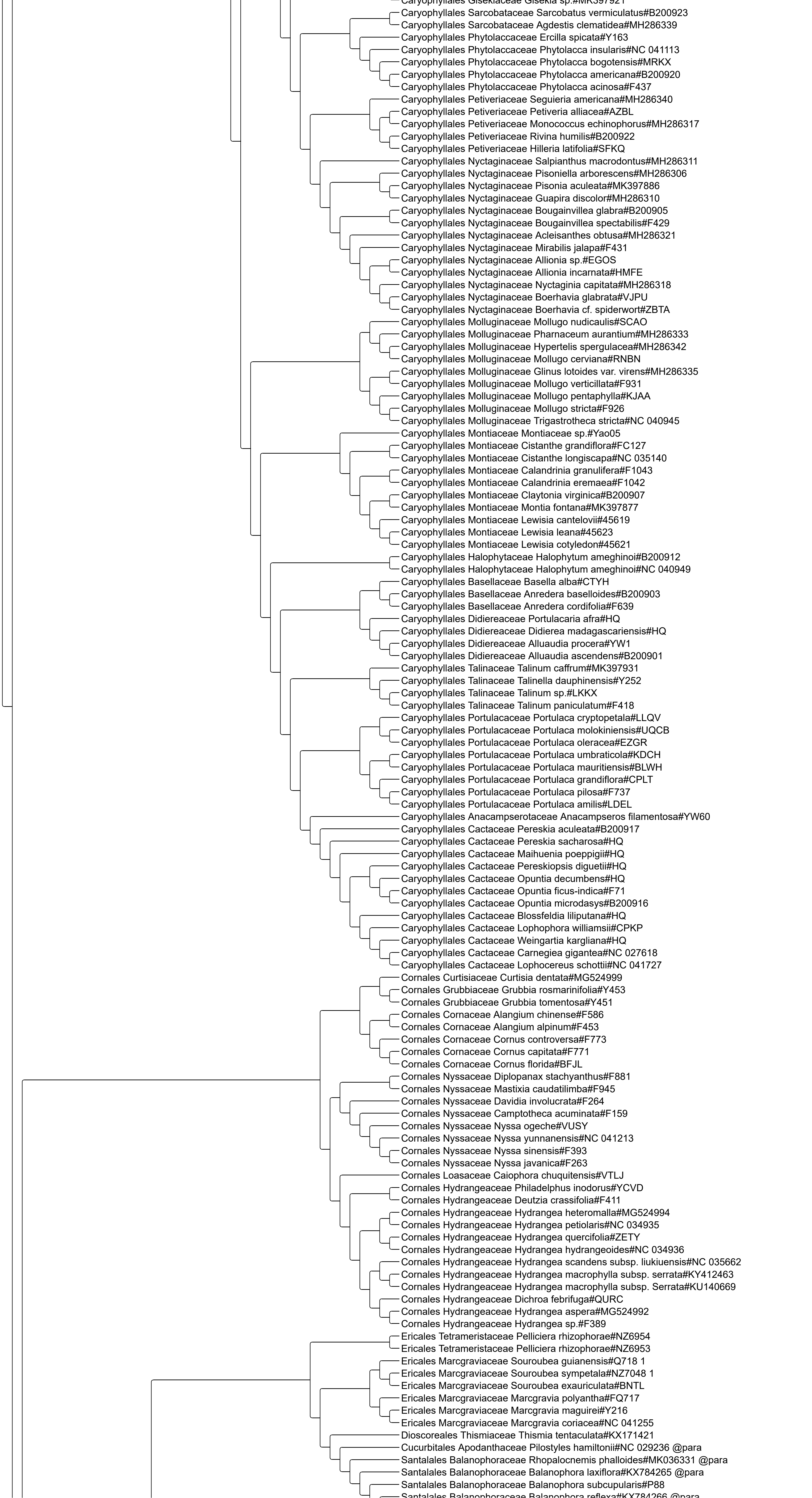
Caryophyllales Sarcobataceae Sarcobatus vermiculatus#B200923
Caryophyllales Sarcobataceae Agdestis clematidea#MH286339
Caryophyllales Phytolaccaceae Ercilla spicata#Y163
Caryophyllales Phytolaccaceae Phytolacca insularis#NC 041113
Caryophyllales Phytolaccaceae Phytolacca bogotensis#MRKX
Caryophyllales Phytolaccaceae Phytolacca americana#B200920
Caryophyllales Phytolaccaceae Phytolacca acinosa#F437
Caryophyllales Petiveriaceae Seguieria americana#MH286340
Caryophyllales Petiveriaceae Petiveria alliacea#AZBL
Caryophyllales Petiveriaceae Monococcus echinophorus#MH286317
Caryophyllales Petiveriaceae Rivina humilis#B200922
Caryophyllales Petiveriaceae Hilleria latifolia#SFKQ
Caryophyllales Nyctaginaceae Salpianthus macrodontus#MH286311
Caryophyllales Nyctaginaceae Pisoniella arborescens#MH286306
Caryophyllales Nyctaginaceae Pisonia aculeata#MK397886
Caryophyllales Nyctaginaceae Guapira discolor#MH286310
Caryophyllales Nyctaginaceae Bougainvillea glabra#B200905
Caryophyllales Nyctaginaceae Bougainvillea spectabilis#F429
Caryophyllales Nyctaginaceae Acleisanthes obtusa#MH286321
Caryophyllales Nyctaginaceae Mirabilis jalapa#F431
Caryophyllales Nyctaginaceae Allionia sp.#EGOS
Caryophyllales Nyctaginaceae Allionia incarnata#HMFE
Caryophyllales Nyctaginaceae Nyctaginia capitata#MH286318
Caryophyllales Nyctaginaceae Boerhavia glabrata#VJPU
Caryophyllales Nyctaginaceae Boerhavia cf. spiderwort#ZBTA
Caryophyllales Molluginaceae Mollugo nudicaulis#SCAO
Caryophyllales Molluginaceae Pharnaceum aurantium#MH286333
Caryophyllales Molluginaceae Hypertelis spergulacea#MH286342
Caryophyllales Molluginaceae Mollugo cerviana#RNBN
Caryophyllales Molluginaceae Glinus lotoides var. virens#MH286335
Caryophyllales Molluginaceae Mollugo verticillata#F931
Caryophyllales Molluginaceae Mollugo pentaphylla#KJAA
Caryophyllales Molluginaceae Mollugo stricta#F926
Caryophyllales Molluginaceae Trigastrotheca stricta#NC 040945
Caryophyllales Montiaceae Montiaceae sp.#Yao05
Caryophyllales Montiaceae Cistanthe grandiflora#FC127
Caryophyllales Montiaceae Cistanthe longiscapa#NC 035140
Caryophyllales Montiaceae Calandrinia granulifera#F1043
Caryophyllales Montiaceae Calandrinia eremaea#F1042
Caryophyllales Montiaceae Claytonia virginica#B200907
Caryophyllales Montiaceae Montia fontana#MK397877
Caryophyllales Montiaceae Lewisia cantelovii#45619
Caryophyllales Montiaceae Lewisia leana#45623
Caryophyllales Montiaceae Lewisia cotyledon#45621
Caryophyllales Halophytaceae Halophytum ameghinoi#B200912
Caryophyllales Halophytaceae Halophytum ameghinoi#NC 040949
Caryophyllales Basellaceae Basella alba#CTYH
Caryophyllales Basellaceae Anredera baselloides#B200903
Caryophyllales Basellaceae Anredera cordifolia#F639
Caryophyllales Didiereaceae Portulacaria afra#HQ
Caryophyllales Didiereaceae Didierea madagascariensis#HQ
Caryophyllales Didiereaceae Alluaudia procera#YW1
Caryophyllales Didiereaceae Alluaudia ascendens#B200901
Caryophyllales Talinaceae Talinum caffrum#MK397931
Caryophyllales Talinaceae Talinella dauphinensis#Y252
Caryophyllales Talinaceae Talinum sp.#LKKX
Caryophyllales Talinaceae Talinum paniculatum#F418
Caryophyllales Portulacaceae Portulaca cryptopetala#LLQV
Caryophyllales Portulacaceae Portulaca molokiniensis#UQCB
Caryophyllales Portulacaceae Portulaca oleracea#EZGR
Caryophyllales Portulacaceae Portulaca umbraticola#KDCH
Caryophyllales Portulacaceae Portulaca mauritiensis#BLWH
Caryophyllales Portulacaceae Portulaca grandiflora#CPLT
Caryophyllales Portulacaceae Portulaca pilosa#F737
Caryophyllales Portulacaceae Portulaca amilis#LDEL
Caryophyllales Anacampserotaceae Anacampseros filamentosa#YW60
Caryophyllales Cactaceae Pereskia aculeata#B200917
Caryophyllales Cactaceae Pereskia sacharosa#HQ
Caryophyllales Cactaceae Maihuenia poeppigii#HQ
Caryophyllales Cactaceae Pereskiopsis diguetii#HQ
Caryophyllales Cactaceae Opuntia decumbens#HQ
Caryophyllales Cactaceae Opuntia ficus-indica#F71
Caryophyllales Cactaceae Opuntia microdasys#B200916
Caryophyllales Cactaceae Blossfeldia liliputana#HQ
Caryophyllales Cactaceae Lophophora williamsii#CPKP
Caryophyllales Cactaceae Weingartia kargliana#HQ
Caryophyllales Cactaceae Carnegiea gigantea#NC 027618
Caryophyllales Cactaceae Lophocereus schottii#NC 041727
Cornales Curtisiaceae Curtisia dentata#MG524999
Cornales Grubbiaceae Grubbia rosmarinifolia#Y453
Cornales Grubbiaceae Grubbia tomentosa#Y451
Cornales Cornaceae Alangium chinense#F586
Cornales Cornaceae Alangium alpinum#F453
Cornales Cornaceae Cornus controversa#F773
Cornales Cornaceae Cornus capitata#F771
Cornales Cornaceae Cornus florida#BFJL
Cornales Nyssaceae Diplopanax stachyanthus#F881
Cornales Nyssaceae Mastixia caudatilimba#F945
Cornales Nyssaceae Davidia involucrata#F264
Cornales Nyssaceae Camptotheca acuminata#F159
Cornales Nyssaceae Nyssa ogeche#VUSY
Cornales Nyssaceae Nyssa yunnanensis#NC 041213
Cornales Nyssaceae Nyssa sinensis#F393
Cornales Nyssaceae Nyssa javanica#F263
Cornales Loasaceae Caiophora chuquitensis#VTLJ
Cornales Hydrangeaceae Philadelphus inodorus#YCVD
Cornales Hydrangeaceae Deutzia crassifolia#F411
Cornales Hydrangeaceae Hydrangea heteromalla#MG524994
Cornales Hydrangeaceae Hydrangea petiolaris#NC 034935
Cornales Hydrangeaceae Hydrangea quercifolia#ZETY
Cornales Hydrangeaceae Hydrangea hydrangeoides#NC 034936
Cornales Hydrangeaceae Hydrangea scandens subsp. liukiuensis#NC 035662
Cornales Hydrangeaceae Hydrangea macrophylla subsp. serrata#KY412463
Cornales Hydrangeaceae Hydrangea macrophylla subsp. Serrata#KU140669
Cornales Hydrangeaceae Dichroa febrifuga#QURC
Cornales Hydrangeaceae Hydrangea aspera#MG524992
Cornales Hydrangeaceae Hydrangea sp.#F389
Ericales Tetrameristaceae Pelliciera rhizophorae#NZ6954
Ericales Tetrameristaceae Pelliciera rhizophorae#NZ6953
Ericales Marcgraviaceae Souroubea guianensis#Q718 1
Ericales Marcgraviaceae Souroubea sympetala#NZ7048 1
Ericales Marcgraviaceae Souroubea exauriculata#BNTL
Ericales Marcgraviaceae Marcgravia polyantha#FQ717
Ericales Marcgraviaceae Marcgravia maguirei#Y216
Ericales Marcgraviaceae Marcgravia coriacea#NC 041255
Dioscoreales Thismiaceae Thismia tentaculata#KX171421
Cucurbitales Apodanthaceae Pilostyles hamiltonii#NC 029236 @para
Santalales Balanophoraceae Rhopalocnemis phalloides#MK036331 @para
Santalales Balanophoraceae Balanophora laxiflora#KX784265 @para
Santalales Balanophoraceae Balanophora subcupularis#P88

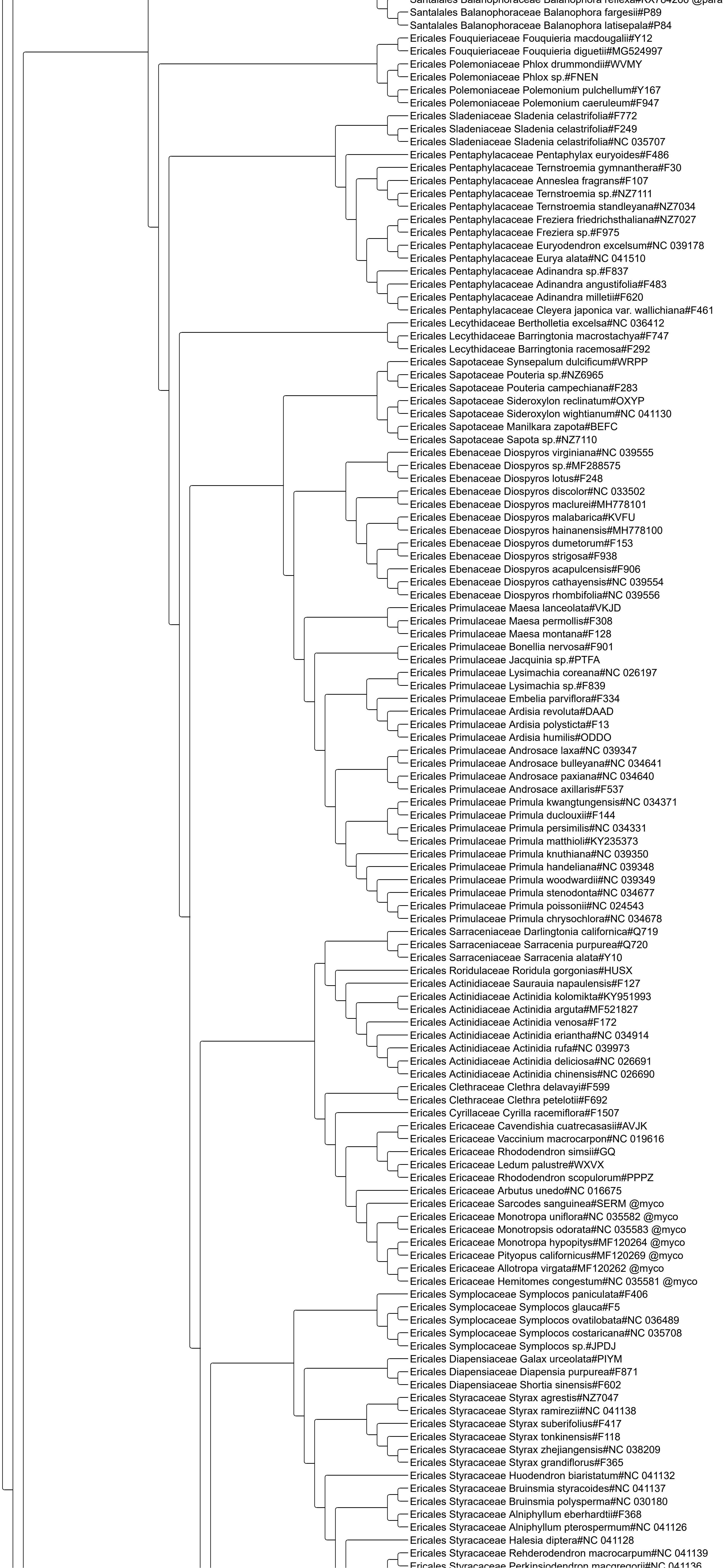
Santalales Balanophoraceae Balanophora fargesii#P89
Santalales Balanophoraceae Balanophora latisepala#P84
Ericales Fouquieriaceae Fouquieria macdougalii#Y12
Ericales Fouquieriaceae Fouquieria diguetii#MG524997
Ericales Polemoniaceae Phlox drummondii#WVMY
Ericales Polemoniaceae Phlox sp.#FNEN
Ericales Polemoniaceae Polemonium pulchellum#Y167
Ericales Polemoniaceae Polemonium caeruleum#F947
Ericales Sladeniaceae Sladenia celastrifolia#F772
Ericales Sladeniaceae Sladenia celastrifolia#F249
Ericales Sladeniaceae Sladenia celastrifolia#NC 035707
Ericales Pentaphylacaceae Pentaphylax euryoides#F486
Ericales Pentaphylacaceae Ternstroemia gymnanthera#F30
Ericales Pentaphylacaceae Anneslea fragrans#F107
Ericales Pentaphylacaceae Ternstroemia sp.#NZ7111
Ericales Pentaphylacaceae Ternstroemia standleyana#NZ7034
Ericales Pentaphylacaceae Freziera friedrichsthaliana#NZ7027
Ericales Pentaphylacaceae Freziera sp.#F975
Ericales Pentaphylacaceae Euryodendron excelsum#NC 039178
Ericales Pentaphylacaceae Eurya alata#NC 041510
Ericales Pentaphylacaceae Adinandra sp.#F837
Ericales Pentaphylacaceae Adinandra angustifolia#F483
Ericales Pentaphylacaceae Adinandra millettii#F620
Ericales Pentaphylacaceae Cleyera japonica var. wallichiana#F461
Ericales Lecythidaceae Bertholletia excelsa#NC 036412
Ericales Lecythidaceae Barringtonia macrostachya#F747
Ericales Lecythidaceae Barringtonia racemosa#F292
Ericales Sapotaceae Synsepalum dulcificum#WRPP
Ericales Sapotaceae Pouteria sp.#NZ6965
Ericales Sapotaceae Pouteria campechiana#F283
Ericales Sapotaceae Sideroxylon reclinatum#OXYP
Ericales Sapotaceae Sideroxylon wightianum#NC 041130
Ericales Sapotaceae Manilkara zapota#BEFC
Ericales Sapotaceae Sapota sp.#NZ7110
Ericales Ebenaceae Diospyros virginiana#NC 039555
Ericales Ebenaceae Diospyros sp.#MF288575
Ericales Ebenaceae Diospyros lotus#F248
Ericales Ebenaceae Diospyros discolor#NC 033502
Ericales Ebenaceae Diospyros maclurei#MH778101
Ericales Ebenaceae Diospyros malabarica#KVFU
Ericales Ebenaceae Diospyros hainanensis#MH778100
Ericales Ebenaceae Diospyros dumetorum#F153
Ericales Ebenaceae Diospyros strigosa#F938
Ericales Ebenaceae Diospyros acapulcensis#F906
Ericales Ebenaceae Diospyros cathayensis#NC 039554
Ericales Ebenaceae Diospyros rhombifolia#NC 039556
Ericales Primulaceae Maesa lanceolata#VKJD
Ericales Primulaceae Maesa permollis#F308
Ericales Primulaceae Maesa montana#F128
Ericales Primulaceae Bonellia nervosa#F901
Ericales Primulaceae Jacquinia sp.#PTFA
Ericales Primulaceae Lysimachia coreana#NC 026197
Ericales Primulaceae Lysimachia sp.#F839
Ericales Primulaceae Embelia parviflora#F334
Ericales Primulaceae Ardisia revoluta#DAAD
Ericales Primulaceae Ardisia polysticta#F13
Ericales Primulaceae Ardisia humilis#ODDO
Ericales Primulaceae Androsace laxa#NC 039347
Ericales Primulaceae Androsace bulleyana#NC 034641
Ericales Primulaceae Androsace paxiana#NC 034640
Ericales Primulaceae Androsace axillaris#F537
Ericales Primulaceae Primula kwangtungensis#NC 034371
Ericales Primulaceae Primula duclouxii#F144
Ericales Primulaceae Primula persimilis#NC 034331
Ericales Primulaceae Primula matthioli#KY235373
Ericales Primulaceae Primula knuthiana#NC 039350
Ericales Primulaceae Primula handeliana#NC 039348
Ericales Primulaceae Primula woodwardii#NC 039349
Ericales Primulaceae Primula stenodonta#NC 034677
Ericales Primulaceae Primula poissonii#NC 024543
Ericales Primulaceae Primula chrysochlora#NC 034678
Ericales Sarraceniaceae Darlingtonia californica#Q719
Ericales Sarraceniaceae Sarracenia purpurea#Q720
Ericales Sarraceniaceae Sarracenia alata#Y10
Ericales Roridulaceae Roridula gorgonias#HUSX
Ericales Actinidiaceae Saurauia napaulensis#F127
Ericales Actinidiaceae Actinidia kolomikta#KY951993
Ericales Actinidiaceae Actinidia arguta#MF521827
Ericales Actinidiaceae Actinidia venosa#F172
Ericales Actinidiaceae Actinidia eriantha#NC 034914
Ericales Actinidiaceae Actinidia rufa#NC 039973
Ericales Actinidiaceae Actinidia deliciosa#NC 026691
Ericales Actinidiaceae Actinidia chinensis#NC 026690
Ericales Clethraceae Clethra delavayi#F599
Ericales Clethraceae Clethra petelotii#F692
Ericales Cyrillaceae Cyrilla racemiflora#F1507
Ericales Ericaceae Cavendishia cuatrecasasii#AVJK
Ericales Ericaceae Vaccinium macrocarpon#NC 019616
Ericales Ericaceae Rhododendron simsii#GQ
Ericales Ericaceae Ledum palustre#WXVX
Ericales Ericaceae Rhododendron scopulorum#PPPZ
Ericales Ericaceae Arbutus unedo#NC 016675
Ericales Ericaceae Sarcodes sanguinea#SERM @myco
Ericales Ericaceae Monotropa uniflora#NC 035582 @myco
Ericales Ericaceae Monotropsis odorata#NC 035583 @myco
Ericales Ericaceae Monotropa hypopitys#MF120264 @myco
Ericales Ericaceae Pityopus californicus#MF120269 @myco
Ericales Ericaceae Allotropa virgata#MF120262 @myco
Ericales Ericaceae Hemitomes congestum#NC 035581 @myco
Ericales Symplocaceae Symplocos paniculata#F406
Ericales Symplocaceae Symplocos glauca#F5
Ericales Symplocaceae Symplocos ovatilobata#NC 036489
Ericales Symplocaceae Symplocos costaricana#NC 035708
Ericales Symplocaceae Symplocos sp.#JPDJ
Ericales Diapensiaceae Galax urceolata#PIYM
Ericales Diapensiaceae Diapensia purpurea#F871
Ericales Diapensiaceae Shortia sinensis#F602
Ericales Styracaceae Styrax agrestis#NZ7047
Ericales Styracaceae Styrax ramirezii#NC 041138
Ericales Styracaceae Styrax suberifolius#F417
Ericales Styracaceae Styrax tonkinensis#F118
Ericales Styracaceae Styrax zhejiangensis#NC 038209
Ericales Styracaceae Styrax grandiflorus#F365
Ericales Styracaceae Huodendron biaristatum#NC 041132
Ericales Styracaceae Bruinsmia styracoides#NC 041137
Ericales Styracaceae Bruinsmia polysperma#NC 030180
Ericales Styracaceae Alniphyllum eberhardtii#F368
Ericales Styracaceae Alniphyllum pterospermum#NC 041126
Ericales Styracaceae Halesia diptera#NC 041128
Ericales Styracaceae Rehderodendron macrocarpum#NC 041139

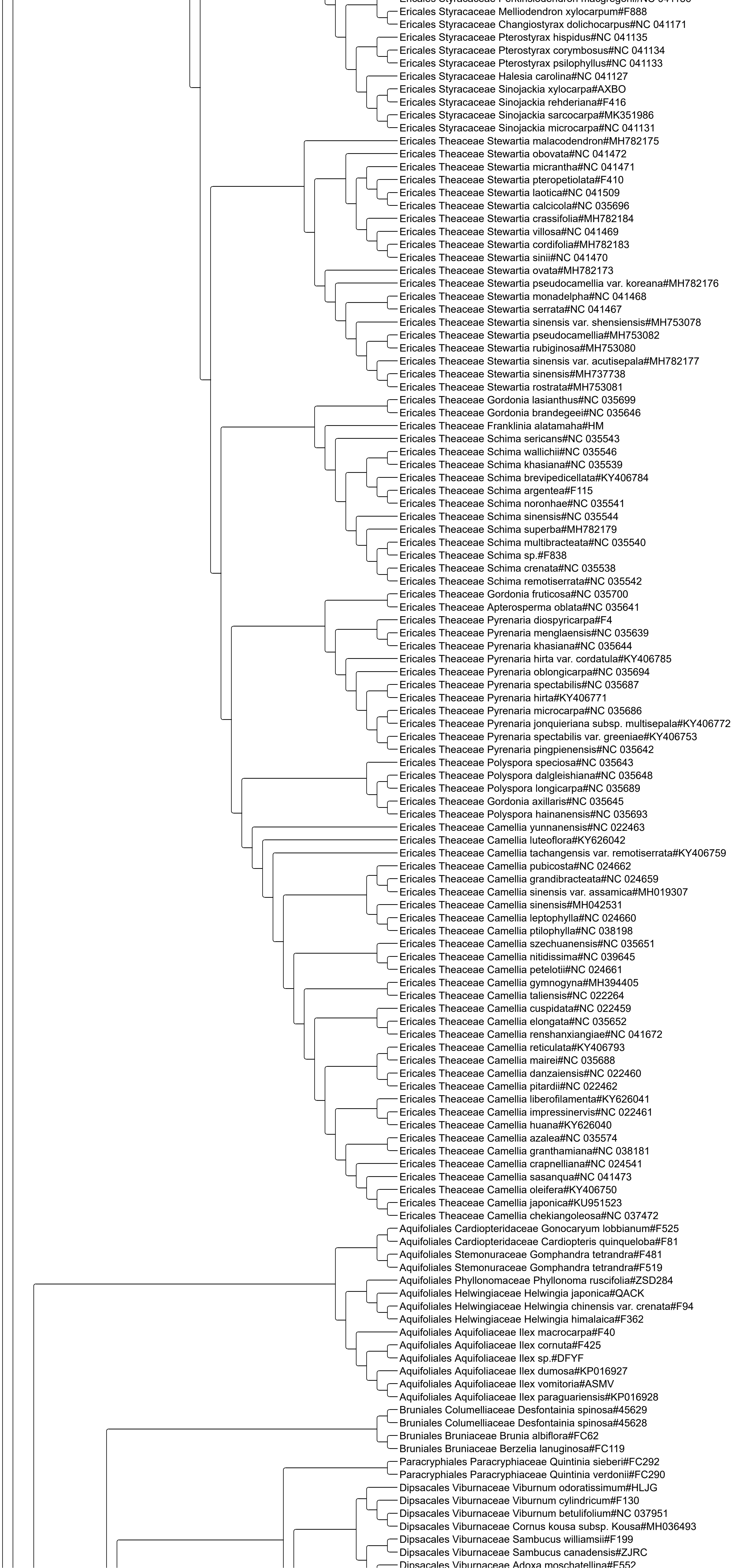
Ericales Styracaceae Melliodendron xylocarpum#F888
Ericales Styracaceae Changiostyrax dolichocarpus#NC 041171
Ericales Styracaceae Pterostyrax hispidus#NC 041135
Ericales Styracaceae Pterostyrax corymbosus#NC 041134
Ericales Styracaceae Pterostyrax psilophyllus#NC 041133
Ericales Styracaceae Halesia carolina#NC 041127
Ericales Styracaceae Sinojackia xylocarpa#AXBO
Ericales Styracaceae Sinojackia rehderiana#F416
Ericales Styracaceae Sinojackia sarcocarpa#MK351986
Ericales Styracaceae Sinojackia microcarpa#NC 041131
Ericales Theaceae Stewartia malacodendron#MH782175
Ericales Theaceae Stewartia obovata#NC 041472
Ericales Theaceae Stewartia micrantha#NC 041471
Ericales Theaceae Stewartia pteropetiolata#F410
Ericales Theaceae Stewartia laotica#NC 041509
Ericales Theaceae Stewartia calcicola#NC 035696
Ericales Theaceae Stewartia crassifolia#MH782184
Ericales Theaceae Stewartia villosa#NC 041469
Ericales Theaceae Stewartia cordifolia#MH782183
Ericales Theaceae Stewartia sinii#NC 041470
Ericales Theaceae Stewartia ovata#MH782173
Ericales Theaceae Stewartia pseudocamellia var. koreana#MH782176
Ericales Theaceae Stewartia monadelpha#NC 041468
Ericales Theaceae Stewartia serrata#NC 041467
Ericales Theaceae Stewartia sinensis var. shensiensis#MH753078
Ericales Theaceae Stewartia pseudocamellia#MH753082
Ericales Theaceae Stewartia rubiginosa#MH753080
Ericales Theaceae Stewartia sinensis var. acutisepala#MH782177
Ericales Theaceae Stewartia sinensis#MH737738
Ericales Theaceae Stewartia rostrata#MH753081
Ericales Theaceae Gordonia lasianthus#NC 035699
Ericales Theaceae Gordonia brandegeei#NC 035646
Ericales Theaceae Franklinia alatamaha#HM
Ericales Theaceae Schima sericans#NC 035543
Ericales Theaceae Schima wallichii#NC 035546
Ericales Theaceae Schima khasiana#NC 035539
Ericales Theaceae Schima brevipedicellata#KY406784
Ericales Theaceae Schima argentea#F115
Ericales Theaceae Schima noronhae#NC 035541
Ericales Theaceae Schima sinensis#NC 035544
Ericales Theaceae Schima superba#MH782179
Ericales Theaceae Schima multibracteata#NC 035540
Ericales Theaceae Schima sp.#F838
Ericales Theaceae Schima crenata#NC 035538
Ericales Theaceae Schima remotiserrata#NC 035542
Ericales Theaceae Gordonia fruticosa#NC 035700
Ericales Theaceae Apterosperma oblata#NC 035641
Ericales Theaceae Pyrenaria diospyricarpa#F4
Ericales Theaceae Pyrenaria menglaensis#NC 035639
Ericales Theaceae Pyrenaria khasiana#NC 035644
Ericales Theaceae Pyrenaria hirta var. cordatula#KY406785
Ericales Theaceae Pyrenaria oblongicarpa#NC 035694
Ericales Theaceae Pyrenaria spectabilis#NC 035687
Ericales Theaceae Pyrenaria hirta#KY406771
Ericales Theaceae Pyrenaria microcarpa#NC 035686
Ericales Theaceae Pyrenaria jonquieriana subsp. multisepala#KY406772
Ericales Theaceae Pyrenaria spectabilis var. greeniae#KY406753
Ericales Theaceae Pyrenaria pingpienensis#NC 035642
Ericales Theaceae Polyspora speciosa#NC 035643
Ericales Theaceae Polyspora dalgleishiana#NC 035648
Ericales Theaceae Polyspora longicarpa#NC 035689
Ericales Theaceae Gordonia axillaris#NC 035645
Ericales Theaceae Polyspora hainanensis#NC 035693
Ericales Theaceae Camellia yunnanensis#NC 022463
Ericales Theaceae Camellia luteoflora#KY626042
Ericales Theaceae Camellia tachangensis var. remotiserrata#KY406759
Ericales Theaceae Camellia pubicosta#NC 024662
Ericales Theaceae Camellia grandibracteata#NC 024659
Ericales Theaceae Camellia sinensis var. assamica#MH019307
Ericales Theaceae Camellia sinensis#MH042531
Ericales Theaceae Camellia leptophylla#NC 024660
Ericales Theaceae Camellia ptilophylla#NC 038198
Ericales Theaceae Camellia szechuanensis#NC 035651
Ericales Theaceae Camellia nitidissima#NC 039645
Ericales Theaceae Camellia petelotii#NC 024661
Ericales Theaceae Camellia gymnogyna#MH394405
Ericales Theaceae Camellia taliensis#NC 022264
Ericales Theaceae Camellia cuspidata#NC 022459
Ericales Theaceae Camellia elongata#NC 035652
Ericales Theaceae Camellia renshanxiangiae#NC 041672
Ericales Theaceae Camellia reticulata#KY406793
Ericales Theaceae Camellia mairei#NC 035688
Ericales Theaceae Camellia danzaiensis#NC 022460
Ericales Theaceae Camellia pitardii#NC 022462
Ericales Theaceae Camellia liberofilamenta#KY626041
Ericales Theaceae Camellia impressinervis#NC 022461
Ericales Theaceae Camellia huana#KY626040
Ericales Theaceae Camellia azalea#NC 035574
Ericales Theaceae Camellia granthamiana#NC 038181
Ericales Theaceae Camellia crapnelliana#NC 024541
Ericales Theaceae Camellia sasanqua#NC 041473
Ericales Theaceae Camellia oleifera#KY406750
Ericales Theaceae Camellia japonica#KU951523
Ericales Theaceae Camellia chekiangoleosa#NC 037472
Aquifoliales Cardiopteridaceae Gonocaryum lobbianum#F525
Aquifoliales Cardiopteridaceae Cardiopteris quinqueloba#F81
Aquifoliales Stemonuraceae Gomphandra tetrandra#F481
Aquifoliales Stemonuraceae Gomphandra tetrandra#F519
Aquifoliales Phyllonomaceae Phyllonoma ruscifolia#ZSD284
Aquifoliales Helwingiaceae Helwingia japonica#QACK
Aquifoliales Helwingiaceae Helwingia chinensis var. crenata#F94
Aquifoliales Helwingiaceae Helwingia himalaica#F362
Aquifoliales Aquifoliaceae Ilex macrocarpa#F40
Aquifoliales Aquifoliaceae Ilex cornuta#F425
Aquifoliales Aquifoliaceae Ilex sp.#DFYF
Aquifoliales Aquifoliaceae Ilex dumosa#KP016927
Aquifoliales Aquifoliaceae Ilex vomitoria#ASMV
Aquifoliales Aquifoliaceae Ilex paraguariensis#KP016928
Bruniales Columelliaceae Desfontainia spinosa#45629
Bruniales Columelliaceae Desfontainia spinosa#45628
Bruniales Bruniaceae Brunia albiflora#FC62
Bruniales Bruniaceae Berzelia lanuginosa#FC119
Paracryphiales Paracryphiaceae Quintinia sieberi#FC292
Paracryphiales Paracryphiaceae Quintinia verdonii#FC290
Dipsacales Viburnaceae Viburnum odoratissimum#HLJG
Dipsacales Viburnaceae Viburnum cylindricum#F130
Dipsacales Viburnaceae Viburnum betulifolium#NC 037951
Dipsacales Viburnaceae Cornus kousa subsp. Kousa#MH036493
Dipsacales Viburnaceae Sambucus williamsii#F199
Dipsacales Viburnaceae Sambucus canadensis#ZJRC
Dipsacales Viburnaceae Adoxa moschatellina#F552

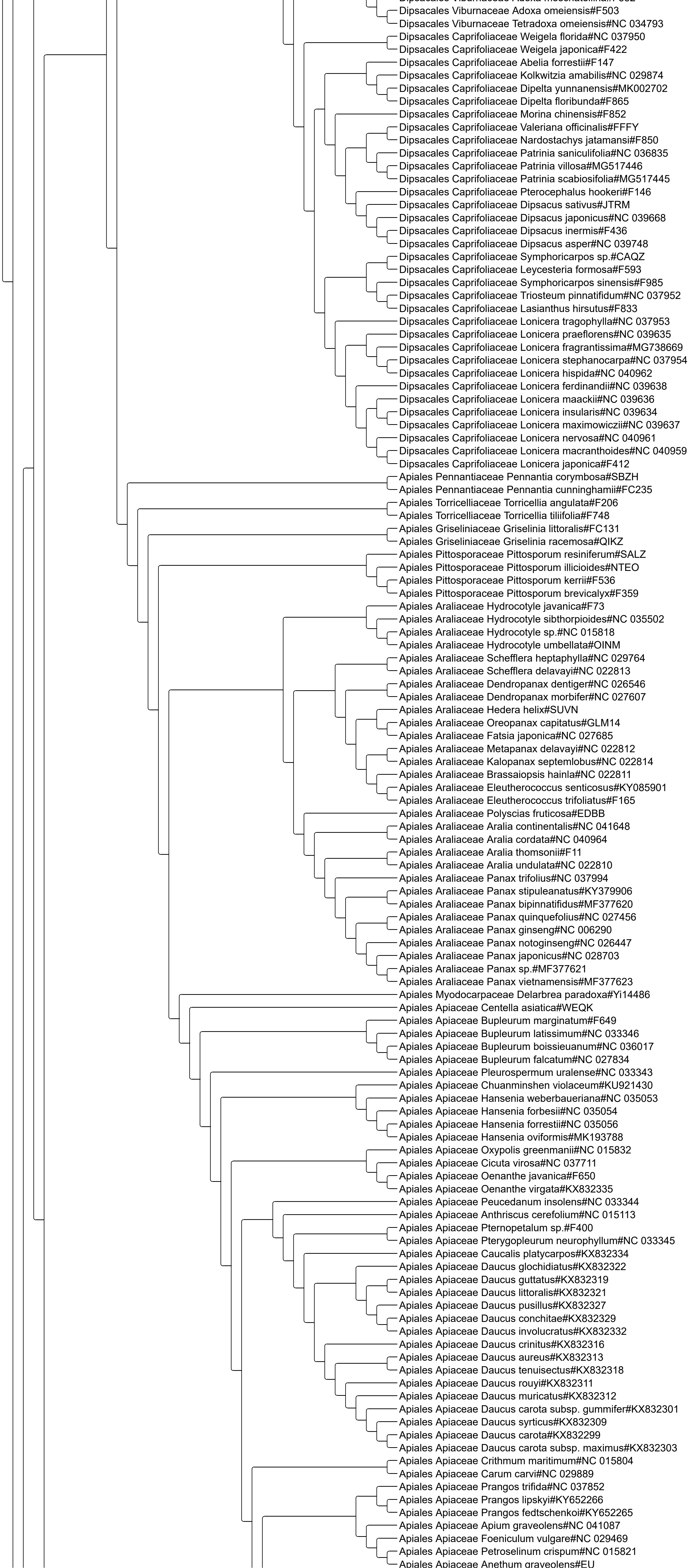

Dipsacales Viburnaceae Adoxa omeiensis#F503
Dipsacales Viburnaceae Tetradoxa omeiensis#NC 034793
Dipsacales Caprifoliaceae Weigela florida#NC 037950
Dipsacales Caprifoliaceae Weigela japonica#F422
Dipsacales Caprifoliaceae Abelia forrestii#F147
Dipsacales Caprifoliaceae Kolkwitzia amabilis#NC 029874
Dipsacales Caprifoliaceae Dipelta yunnanensis#MK002702
Dipsacales Caprifoliaceae Dipelta floribunda#F865
Dipsacales Caprifoliaceae Morina chinensis#F852
Dipsacales Caprifoliaceae Valeriana officinalis#FFFY
Dipsacales Caprifoliaceae Nardostachys jatamansi#F850
Dipsacales Caprifoliaceae Patrinia saniculifolia#NC 036835
Dipsacales Caprifoliaceae Patrinia villosa#MG517446
Dipsacales Caprifoliaceae Patrinia scabiosifolia#MG517445
Dipsacales Caprifoliaceae Pterocephalus hookeri#F146
Dipsacales Caprifoliaceae Dipsacus sativus#JTRM
Dipsacales Caprifoliaceae Dipsacus japonicus#NC 039668
Dipsacales Caprifoliaceae Dipsacus inermis#F436
Dipsacales Caprifoliaceae Dipsacus asper#NC 039748
Dipsacales Caprifoliaceae Symphoricarpos sp.#CAQZ
Dipsacales Caprifoliaceae Leycesteria formosa#F593
Dipsacales Caprifoliaceae Symphoricarpos sinensis#F985
Dipsacales Caprifoliaceae Triosteum pinnatifidum#NC 037952
Dipsacales Caprifoliaceae Lasianthus hirsutus#F833
Dipsacales Caprifoliaceae Lonicera tragophylla#NC 037953
Dipsacales Caprifoliaceae Lonicera praeflorens#NC 039635
Dipsacales Caprifoliaceae Lonicera fragrantissima#MG738669
Dipsacales Caprifoliaceae Lonicera stephanocarpa#NC 037954
Dipsacales Caprifoliaceae Lonicera hispida#NC 040962
Dipsacales Caprifoliaceae Lonicera ferdinandii#NC 039638
Dipsacales Caprifoliaceae Lonicera maackii#NC 039636
Dipsacales Caprifoliaceae Lonicera insularis#NC 039634
Dipsacales Caprifoliaceae Lonicera maximowiczii#NC 039637
Dipsacales Caprifoliaceae Lonicera nervosa#NC 040961
Dipsacales Caprifoliaceae Lonicera macranthoides#NC 040959
Dipsacales Caprifoliaceae Lonicera japonica#F412
Apiales Pennantiaceae Pennantia corymbosa#SBZH
Apiales Pennantiaceae Pennantia cunninghamii#FC235
Apiales Torricelliaceae Torricellia angulata#F206
Apiales Torricelliaceae Torricellia tiliifolia#F748
Apiales Griseliniaceae Griselinia littoralis#FC131
Apiales Griseliniaceae Griselinia racemosa#QIKZ
Apiales Pittosporaceae Pittosporum resiniferum#SALZ
Apiales Pittosporaceae Pittosporum illicioides#NTEO
Apiales Pittosporaceae Pittosporum kerrii#F536
Apiales Pittosporaceae Pittosporum brevicalyx#F359
Apiales Araliaceae Hydrocotyle javanica#F73
Apiales Araliaceae Hydrocotyle sibthorpioides#NC 035502
Apiales Araliaceae Hydrocotyle sp.#NC 015818
Apiales Araliaceae Hydrocotyle umbellata#OINM
Apiales Araliaceae Schefflera heptaphylla#NC 029764
Apiales Araliaceae Schefflera delavayi#NC 022813
Apiales Araliaceae Dendropanax dentiger#NC 026546
Apiales Araliaceae Dendropanax morbifer#NC 027607
Apiales Araliaceae Hedera helix#SUVN
Apiales Araliaceae Oreopanax capitatus#GLM14
Apiales Araliaceae Fatsia japonica#NC 027685
Apiales Araliaceae Metapanax delavayi#NC 022812
Apiales Araliaceae Kalopanax septemlobus#NC 022814
Apiales Araliaceae Brassaiopsis hainla#NC 022811
Apiales Araliaceae Eleutherococcus senticosus#KY085901
Apiales Araliaceae Eleutherococcus trifoliatus#F165
Apiales Araliaceae Polyscias fruticosa#EDBB
Apiales Araliaceae Aralia continentalis#NC 041648
Apiales Araliaceae Aralia cordata#NC 040964
Apiales Araliaceae Aralia thomsonii#F11
Apiales Araliaceae Aralia undulata#NC 022810
Apiales Araliaceae Panax trifolius#NC 037994
Apiales Araliaceae Panax stipuleanatus#KY379906
Apiales Araliaceae Panax bipinnatifidus#MF377620
Apiales Araliaceae Panax quinquefolius#NC 027456
Apiales Araliaceae Panax ginseng#NC 006290
Apiales Araliaceae Panax notoginseng#NC 026447
Apiales Araliaceae Panax japonicus#NC 028703
Apiales Araliaceae Panax sp.#MF377621
Apiales Araliaceae Panax vietnamensis#MF377623
Apiales Myodocarpaceae Delarbrea paradoxa#Yi14486
Apiales Apiaceae Centella asiatica#WEQK
Apiales Apiaceae Bupleurum marginatum#F649
Apiales Apiaceae Bupleurum latissimum#NC 033346
Apiales Apiaceae Bupleurum boissieuanum#NC 036017
Apiales Apiaceae Bupleurum falcatum#NC 027834
Apiales Apiaceae Pleurospermum uralense#NC 033343
Apiales Apiaceae Chuanminshen violaceum#KU921430
Apiales Apiaceae Hansenia weberbaueriana#NC 035053
Apiales Apiaceae Hansenia forbesii#NC 035054
Apiales Apiaceae Hansenia forrestii#NC 035056
Apiales Apiaceae Hansenia oviformis#MK193788
Apiales Apiaceae Oxypolis greenmanii#NC 015832
Apiales Apiaceae Cicuta virosa#NC 037711
Apiales Apiaceae Oenanthe javanica#F650
Apiales Apiaceae Oenanthe virgata#KX832335
Apiales Apiaceae Peucedanum insolens#NC 033344
Apiales Apiaceae Anthriscus cerefolium#NC 015113
Apiales Apiaceae Pternopetalum sp.#F400
Apiales Apiaceae Pterygopleurum neurophyllum#NC 033345
Apiales Apiaceae Caucalis platycarpos#KX832334
Apiales Apiaceae Daucus glochidiatus#KX832322
Apiales Apiaceae Daucus guttatus#KX832319
Apiales Apiaceae Daucus littoralis#KX832321
Apiales Apiaceae Daucus pusillus#KX832327
Apiales Apiaceae Daucus conchitae#KX832329
Apiales Apiaceae Daucus involucratus#KX832332
Apiales Apiaceae Daucus crinitus#KX832316
Apiales Apiaceae Daucus aureus#KX832313
Apiales Apiaceae Daucus tenuisectus#KX832318
Apiales Apiaceae Daucus rouyi#KX832311
Apiales Apiaceae Daucus muricatus#KX832312
Apiales Apiaceae Daucus carota subsp. gummifer#KX832301
Apiales Apiaceae Daucus syrticus#KX832309
Apiales Apiaceae Daucus carota#KX832299
Apiales Apiaceae Daucus carota subsp. maximus#KX832303
Apiales Apiaceae Crithmum maritimum#NC 015804
Apiales Apiaceae Carum carvi#NC 029889
Apiales Apiaceae Prangos trifida#NC 037852
Apiales Apiaceae Prangos lipskyi#KY652266
Apiales Apiaceae Prangos fedtschenkoi#KY652265
Apiales Apiaceae Apium graveolens#NC 041087
Apiales Apiaceae Foeniculum vulgare#NC 029469
Apiales Apiaceae Petroselinum crispum#NC 015821
Apiales Apiaceae Anethum graveolens#EU

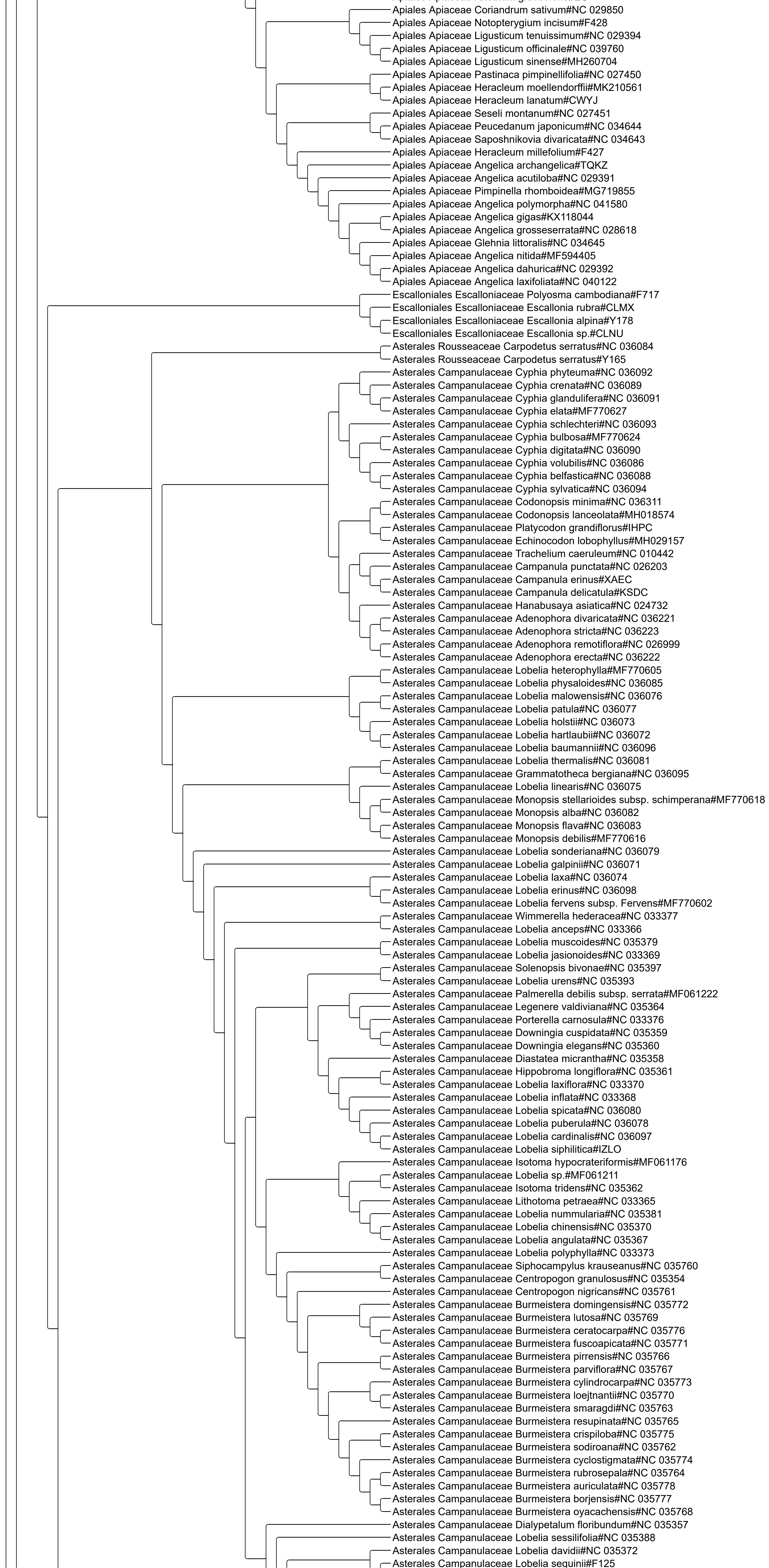

Apiales Apiaceae Coriandrum sativum#NC 029850
Apiales Apiaceae Notopterygium incisum#F428
Apiales Apiaceae Ligusticum tenuissimum#NC 029394
Apiales Apiaceae Ligusticum officinale#NC 039760
Apiales Apiaceae Ligusticum sinense#MH260704
Apiales Apiaceae Pastinaca pimpinellifolia#NC 027450
Apiales Apiaceae Heracleum moellendorffii#MK210561
Apiales Apiaceae Heracleum lanatum#CWYJ
Apiales Apiaceae Seseli montanum#NC 027451
Apiales Apiaceae Peucedanum japonicum#NC 034644
Apiales Apiaceae Saposhnikovia divaricata#NC 034643
Apiales Apiaceae Heracleum millefolium#F427
Apiales Apiaceae Angelica archangelica#TQKZ
Apiales Apiaceae Angelica acutiloba#NC 029391
Apiales Apiaceae Pimpinella rhomboidea#MG719855
Apiales Apiaceae Angelica polymorpha#NC 041580
Apiales Apiaceae Angelica gigas#KX118044
Apiales Apiaceae Angelica grosseserrata#NC 028618
Apiales Apiaceae Glehnia littoralis#NC 034645
Apiales Apiaceae Angelica nitida#MF594405
Apiales Apiaceae Angelica dahurica#NC 029392
Apiales Apiaceae Angelica laxifoliata#NC 040122
Escalloniales Escalloniaceae Polyosma cambodiana#F717
Escalloniales Escalloniaceae Escallonia rubra#CLMX
Escalloniales Escalloniaceae Escallonia alpina#Y178
Escalloniales Escalloniaceae Escallonia sp.#CLNU
Asterales Rousseaceae Carpodetus serratus#NC 036084
Asterales Rousseaceae Carpodetus serratus#Y165
Asterales Campanulaceae Cyphia phyteuma#NC 036092
Asterales Campanulaceae Cyphia crenata#NC 036089
Asterales Campanulaceae Cyphia glandulifera#NC 036091
Asterales Campanulaceae Cyphia elata#MF770627
Asterales Campanulaceae Cyphia schlechteri#NC 036093
Asterales Campanulaceae Cyphia bulbosa#MF770624
Asterales Campanulaceae Cyphia digitata#NC 036090
Asterales Campanulaceae Cyphia volubilis#NC 036086
Asterales Campanulaceae Cyphia belfastica#NC 036088
Asterales Campanulaceae Cyphia sylvatica#NC 036094
Asterales Campanulaceae Codonopsis minima#NC 036311
Asterales Campanulaceae Codonopsis lanceolata#MH018574
Asterales Campanulaceae Platycodon grandiflorus#IHPC
Asterales Campanulaceae Echinocodon lobophyllus#MH029157
Asterales Campanulaceae Trachelium caeruleum#NC 010442
Asterales Campanulaceae Campanula punctata#NC 026203
Asterales Campanulaceae Campanula erinus#XAEC
Asterales Campanulaceae Campanula delicatula#KSDC
Asterales Campanulaceae Hanabusaya asiatica#NC 024732
Asterales Campanulaceae Adenophora divaricata#NC 036221
Asterales Campanulaceae Adenophora stricta#NC 036223
Asterales Campanulaceae Adenophora remotiflora#NC 026999
Asterales Campanulaceae Adenophora erecta#NC 036222
Asterales Campanulaceae Lobelia heterophylla#MF770605
Asterales Campanulaceae Lobelia physaloides#NC 036085
Asterales Campanulaceae Lobelia malowensis#NC 036076
Asterales Campanulaceae Lobelia patula#NC 036077
Asterales Campanulaceae Lobelia holstii#NC 036073
Asterales Campanulaceae Lobelia hartlaubii#NC 036072
Asterales Campanulaceae Lobelia baumannii#NC 036096
Asterales Campanulaceae Lobelia thermalis#NC 036081
Asterales Campanulaceae Grammatotheca bergiana#NC 036095
Asterales Campanulaceae Lobelia linearis#NC 036075
Asterales Campanulaceae Monopsis stellarioides subsp. schimperana#MF770618
Asterales Campanulaceae Monopsis alba#NC 036082
Asterales Campanulaceae Monopsis flava#NC 036083
Asterales Campanulaceae Monopsis debilis#MF770616
Asterales Campanulaceae Lobelia sonderiana#NC 036079
Asterales Campanulaceae Lobelia galpinii#NC 036071
Asterales Campanulaceae Lobelia laxa#NC 036074
Asterales Campanulaceae Lobelia erinus#NC 036098
Asterales Campanulaceae Lobelia fervens subsp. Fervens#MF770602
Asterales Campanulaceae Wimmerella hederacea#NC 033377
Asterales Campanulaceae Lobelia anceps#NC 033366
Asterales Campanulaceae Lobelia muscoides#NC 035379
Asterales Campanulaceae Lobelia jasionoides#NC 033369
Asterales Campanulaceae Solenopsis bivonae#NC 035397
Asterales Campanulaceae Lobelia urens#NC 035393
Asterales Campanulaceae Palmerella debilis subsp. serrata#MF061222
Asterales Campanulaceae Legenere valdiviana#NC 035364
Asterales Campanulaceae Porterella carnosula#NC 033376
Asterales Campanulaceae Downingia cuspidata#NC 035359
Asterales Campanulaceae Downingia elegans#NC 035360
Asterales Campanulaceae Diastatea micrantha#NC 035358
Asterales Campanulaceae Hippobroma longiflora#NC 035361
Asterales Campanulaceae Lobelia laxiflora#NC 033370
Asterales Campanulaceae Lobelia inflata#NC 033368
Asterales Campanulaceae Lobelia spicata#NC 036080
Asterales Campanulaceae Lobelia puberula#NC 036078
Asterales Campanulaceae Lobelia cardinalis#NC 036097
Asterales Campanulaceae Lobelia siphilitica#IZLO
Asterales Campanulaceae Isotoma hypocrateriformis#MF061176
Asterales Campanulaceae Lobelia sp.#MF061211
Asterales Campanulaceae Isotoma tridens#NC 035362
Asterales Campanulaceae Lithotoma petraea#NC 033365
Asterales Campanulaceae Lobelia nummularia#NC 035381
Asterales Campanulaceae Lobelia chinensis#NC 035370
Asterales Campanulaceae Lobelia angulata#NC 035367
Asterales Campanulaceae Lobelia polyphylla#NC 033373
Asterales Campanulaceae Siphocampylus krauseanus#NC 035760
Asterales Campanulaceae Centropogon granulosus#NC 035354
Asterales Campanulaceae Centropogon nigricans#NC 035761
Asterales Campanulaceae Burmeistera domingensis#NC 035772
Asterales Campanulaceae Burmeistera lutosa#NC 035769
Asterales Campanulaceae Burmeistera ceratocarpa#NC 035776
Asterales Campanulaceae Burmeistera fuscoapicata#NC 035771
Asterales Campanulaceae Burmeistera pirrensis#NC 035766
Asterales Campanulaceae Burmeistera parviflora#NC 035767
Asterales Campanulaceae Burmeistera cylindrocarpa#NC 035773
Asterales Campanulaceae Burmeistera loejtnantii#NC 035770
Asterales Campanulaceae Burmeistera smaragdi#NC 035763
Asterales Campanulaceae Burmeistera resupinata#NC 035765
Asterales Campanulaceae Burmeistera crispiloba#NC 035775
Asterales Campanulaceae Burmeistera sodiroana#NC 035762
Asterales Campanulaceae Burmeistera cyclostigmata#NC 035774
Asterales Campanulaceae Burmeistera rubrosepala#NC 035764
Asterales Campanulaceae Burmeistera auriculata#NC 035778
Asterales Campanulaceae Burmeistera borjensis#NC 035777
Asterales Campanulaceae Burmeistera oyacachensis#NC 035768
Asterales Campanulaceae Dialypetalum floribundum#NC 035357
Asterales Campanulaceae Lobelia sessilifolia#NC 035388
Asterales Campanulaceae Lobelia davidii#NC 035372
Asterales Campanulaceae Lobelia seguinii#F125

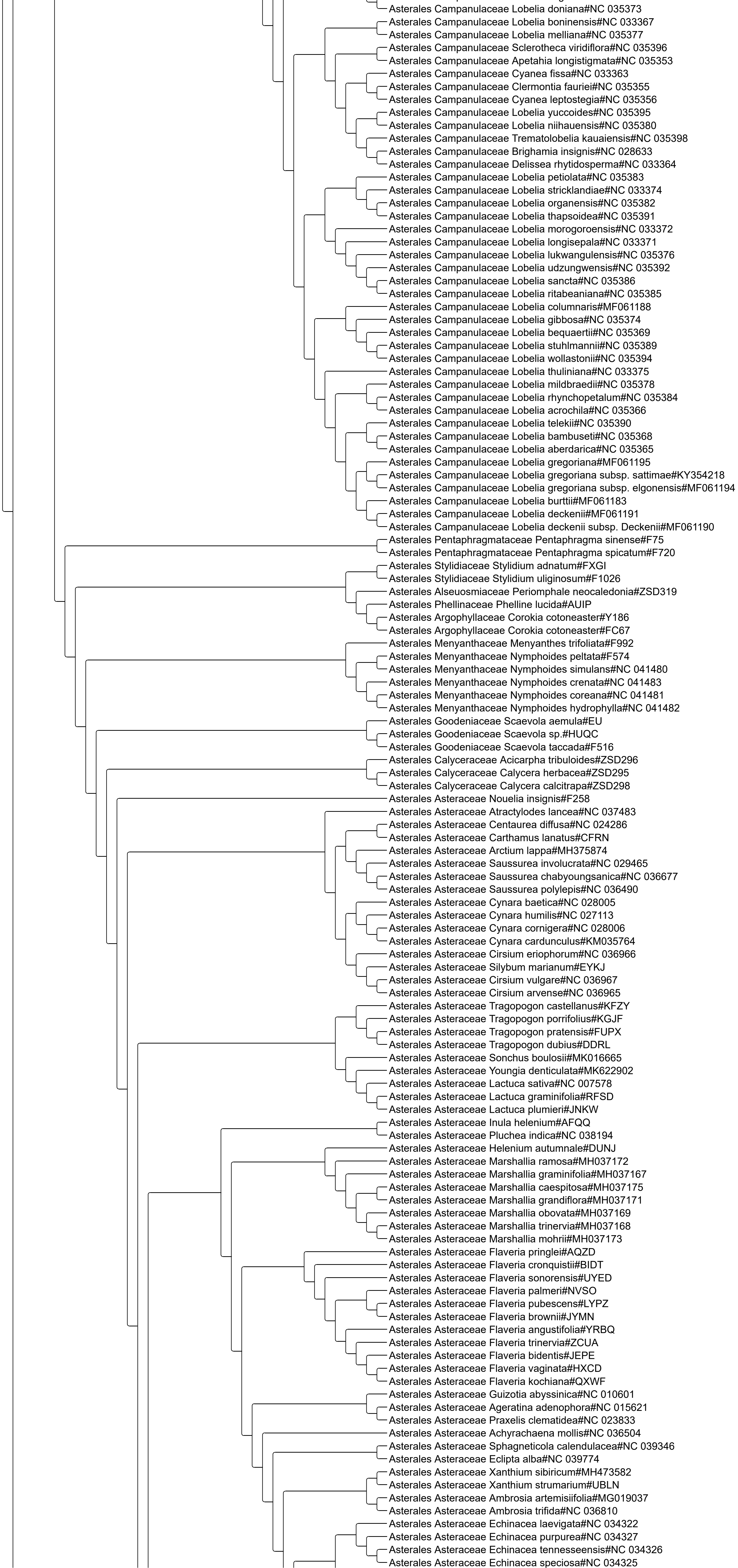

Asterales Campanulaceae Lobelia doniana#NC 035373
Asterales Campanulaceae Lobelia boninensis#NC 033367
Asterales Campanulaceae Lobelia melliana#NC 035377
Asterales Campanulaceae Sclerotheca viridiflora#NC 035396
Asterales Campanulaceae Apetahia longistigmata#NC 035353
Asterales Campanulaceae Cyanea fissa#NC 033363
Asterales Campanulaceae Clermontia fauriei#NC 035355
Asterales Campanulaceae Cyanea leptostegia#NC 035356
Asterales Campanulaceae Lobelia yuccoides#NC 035395
Asterales Campanulaceae Lobelia niihauensis#NC 035380
Asterales Campanulaceae Trematolobelia kauaiensis#NC 035398
Asterales Campanulaceae Brighamia insignis#NC 028633
Asterales Campanulaceae Delissea rhytidosperma#NC 033364
Asterales Campanulaceae Lobelia petiolata#NC 035383
Asterales Campanulaceae Lobelia stricklandiae#NC 033374
Asterales Campanulaceae Lobelia organensis#NC 035382
Asterales Campanulaceae Lobelia thapsoidea#NC 035391
Asterales Campanulaceae Lobelia morogoroensis#NC 033372
Asterales Campanulaceae Lobelia longisepala#NC 033371
Asterales Campanulaceae Lobelia lukwangulensis#NC 035376
Asterales Campanulaceae Lobelia udzungwensis#NC 035392
Asterales Campanulaceae Lobelia sancta#NC 035386
Asterales Campanulaceae Lobelia ritabeaniana#NC 035385
Asterales Campanulaceae Lobelia columnaris#MF061188
Asterales Campanulaceae Lobelia gibbosa#NC 035374
Asterales Campanulaceae Lobelia bequaertii#NC 035369
Asterales Campanulaceae Lobelia stuhlmannii#NC 035389
Asterales Campanulaceae Lobelia wollastonii#NC 035394
Asterales Campanulaceae Lobelia thuliniana#NC 033375
Asterales Campanulaceae Lobelia mildbraedii#NC 035378
Asterales Campanulaceae Lobelia rhynchopetalum#NC 035384
Asterales Campanulaceae Lobelia acrochila#NC 035366
Asterales Campanulaceae Lobelia telekii#NC 035390
Asterales Campanulaceae Lobelia bambuseti#NC 035368
Asterales Campanulaceae Lobelia aberdarica#NC 035365
Asterales Campanulaceae Lobelia gregoriana#MF061195
Asterales Campanulaceae Lobelia gregoriana subsp. sattimae#KY354218
Asterales Campanulaceae Lobelia gregoriana subsp. elgonensis#MF061194
Asterales Campanulaceae Lobelia burttii#MF061183
Asterales Campanulaceae Lobelia deckenii#MF061191
Asterales Campanulaceae Lobelia deckenii subsp. Deckenii#MF061190
Asterales Pentaphragmataceae Pentaphragma sinense#F75
Asterales Pentaphragmataceae Pentaphragma spicatum#F720
Asterales Stylidiaceae Stylidium adnatum#FXGI
Asterales Stylidiaceae Stylidium uliginosum#F1026
Asterales Alseuosmiaceae Periomphale neocaledonia#ZSD319
Asterales Phellinaceae Phelline lucida#AUIP
Asterales Argophyllaceae Corokia cotoneaster#Y186
Asterales Argophyllaceae Corokia cotoneaster#FC67
Asterales Menyanthaceae Menyanthes trifoliata#F992
Asterales Menyanthaceae Nymphoides peltata#F574
Asterales Menyanthaceae Nymphoides simulans#NC 041480
Asterales Menyanthaceae Nymphoides crenata#NC 041483
Asterales Menyanthaceae Nymphoides coreana#NC 041481
Asterales Menyanthaceae Nymphoides hydrophylla#NC 041482
Asterales Goodeniaceae Scaevola aemula#EU
Asterales Goodeniaceae Scaevola sp.#HUQC
Asterales Goodeniaceae Scaevola taccada#F516
Asterales Calyceraceae Acicarpha tribuloides#ZSD296
Asterales Calyceraceae Calycera herbacea#ZSD295
Asterales Calyceraceae Calycera calcitrapa#ZSD298
Asterales Asteraceae Nouelia insignis#F258
Asterales Asteraceae Atractylodes lancea#NC 037483
Asterales Asteraceae Centaurea diffusa#NC 024286
Asterales Asteraceae Carthamus lanatus#CFRN
Asterales Asteraceae Arctium lappa#MH375874
Asterales Asteraceae Saussurea involucrata#NC 029465
Asterales Asteraceae Saussurea chabyoungsanica#NC 036677
Asterales Asteraceae Saussurea polylepis#NC 036490
Asterales Asteraceae Cynara baetica#NC 028005
Asterales Asteraceae Cynara humilis#NC 027113
Asterales Asteraceae Cynara cornigera#NC 028006
Asterales Asteraceae Cynara cardunculus#KM035764
Asterales Asteraceae Cirsium eriophorum#NC 036966
Asterales Asteraceae Silybum marianum#EYKJ
Asterales Asteraceae Cirsium vulgare#NC 036967
Asterales Asteraceae Cirsium arvense#NC 036965
Asterales Asteraceae Tragopogon castellanus#KFZY
Asterales Asteraceae Tragopogon porrifolius#KGJF
Asterales Asteraceae Tragopogon pratensis#FUPX
Asterales Asteraceae Tragopogon dubius#DDRL
Asterales Asteraceae Sonchus boulosii#MK016665
Asterales Asteraceae Youngia denticulata#MK622902
Asterales Asteraceae Lactuca sativa#NC 007578
Asterales Asteraceae Lactuca graminifolia#RFSD
Asterales Asteraceae Lactuca plumieri#JNKW
Asterales Asteraceae Inula helenium#AFQQ
Asterales Asteraceae Pluchea indica#NC 038194
Asterales Asteraceae Helenium autumnale#DUNJ
Asterales Asteraceae Marshallia ramosa#MH037172
Asterales Asteraceae Marshallia graminifolia#MH037167
Asterales Asteraceae Marshallia caespitosa#MH037175
Asterales Asteraceae Marshallia grandiflora#MH037171
Asterales Asteraceae Marshallia obovata#MH037169
Asterales Asteraceae Marshallia trinervia#MH037168
Asterales Asteraceae Marshallia mohrii#MH037173
Asterales Asteraceae Flaveria pringlei#AQZD
Asterales Asteraceae Flaveria cronquistii#BIDT
Asterales Asteraceae Flaveria sonorensis#UYED
Asterales Asteraceae Flaveria palmeri#NVSO
Asterales Asteraceae Flaveria pubescens#LYPZ
Asterales Asteraceae Flaveria brownii#JYMN
Asterales Asteraceae Flaveria angustifolia#YRBQ
Asterales Asteraceae Flaveria trinervia#ZCUA
Asterales Asteraceae Flaveria bidentis#JEPE
Asterales Asteraceae Flaveria vaginata#HXCD
Asterales Asteraceae Flaveria kochiana#QXWF
Asterales Asteraceae Guizotia abyssinica#NC 010601
Asterales Asteraceae Ageratina adenophora#NC 015621
Asterales Asteraceae Praxelis clematidea#NC 023833
Asterales Asteraceae Achyrachaena mollis#NC 036504
Asterales Asteraceae Sphagneticola calendulacea#NC 039346
Asterales Asteraceae Eclipta alba#NC 039774
Asterales Asteraceae Xanthium sibiricum#MH473582
Asterales Asteraceae Xanthium strumarium#UBLN
Asterales Asteraceae Ambrosia artemisiifolia#MG019037
Asterales Asteraceae Ambrosia trifida#NC 036810
Asterales Asteraceae Echinacea laevigata#NC 034322
Asterales Asteraceae Echinacea purpurea#NC 034327
Asterales Asteraceae Echinacea tennesseensis#NC 034326
Asterales Asteraceae Echinacea speciosa#NC 034325

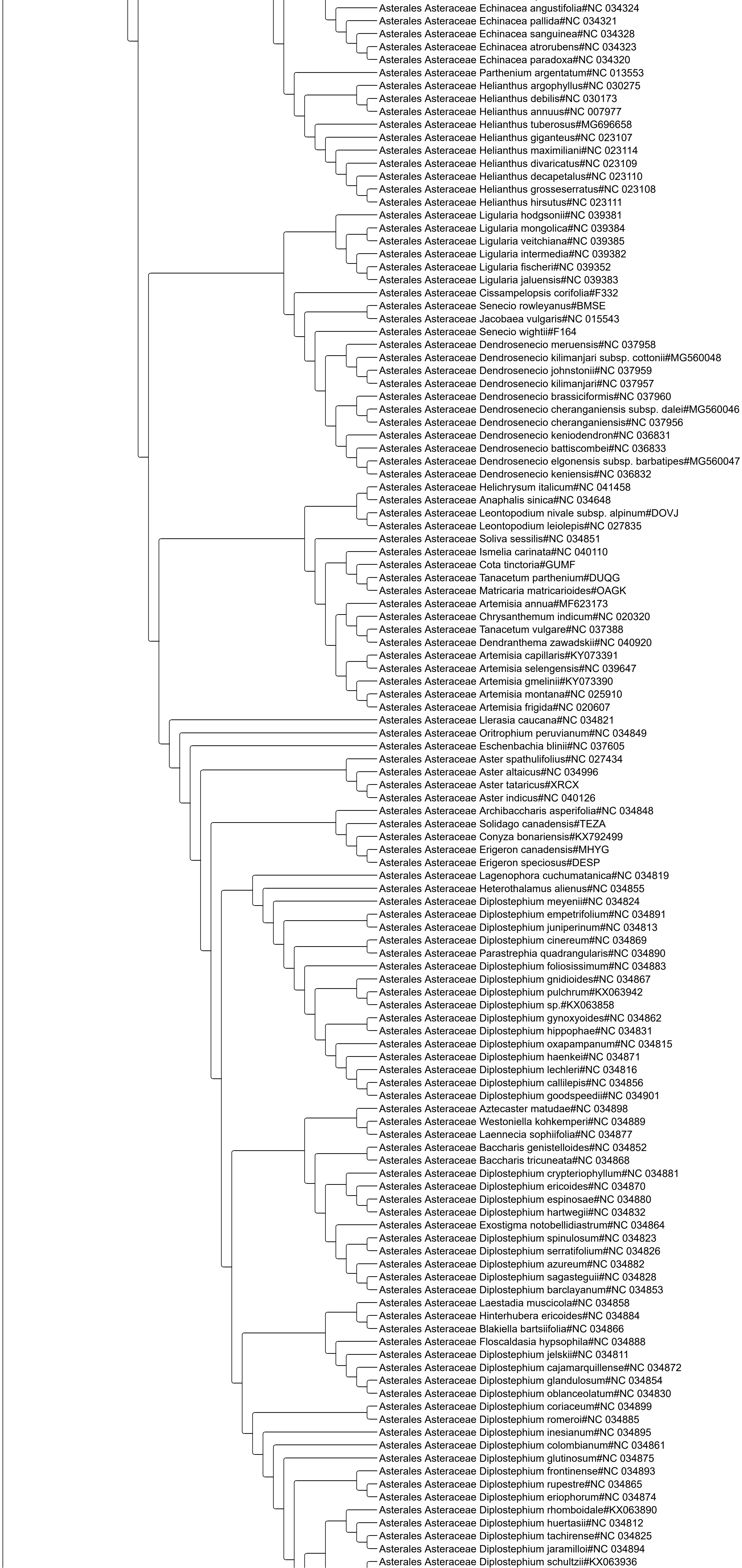
Asterales Asteraceae Echinacea angustifolia#NC 034324
Asterales Asteraceae Echinacea pallida#NC 034321
Asterales Asteraceae Echinacea sanguinea#NC 034328
Asterales Asteraceae Echinacea atrorubens#NC 034323
Asterales Asteraceae Echinacea paradoxa#NC 034320
Asterales Asteraceae Parthenium argentatum#NC 013553
Asterales Asteraceae Helianthus argophyllus#NC 030275
Asterales Asteraceae Helianthus debilis#NC 030173
Asterales Asteraceae Helianthus annuus#NC 007977
Asterales Asteraceae Helianthus tuberosus#MG696658
Asterales Asteraceae Helianthus giganteus#NC 023107
Asterales Asteraceae Helianthus maximiliani#NC 023114
Asterales Asteraceae Helianthus divaricatus#NC 023109
Asterales Asteraceae Helianthus decapetalus#NC 023110
Asterales Asteraceae Helianthus grosseserratus#NC 023108
Asterales Asteraceae Helianthus hirsutus#NC 023111
Asterales Asteraceae Ligularia hodgsonii#NC 039381
Asterales Asteraceae Ligularia mongolica#NC 039384
Asterales Asteraceae Ligularia veitchiana#NC 039385
Asterales Asteraceae Ligularia intermedia#NC 039382
Asterales Asteraceae Ligularia fischeri#NC 039352
Asterales Asteraceae Ligularia jaluensis#NC 039383
Asterales Asteraceae Cissampelopsis corifolia#F332
Asterales Asteraceae Senecio rowleyanus#BMSE
Asterales Asteraceae Jacobaea vulgaris#NC 015543
Asterales Asteraceae Senecio wightii#F164
Asterales Asteraceae Dendrosenecio meruensis#NC 037958
Asterales Asteraceae Dendrosenecio kilimanjari subsp. cottonii#MG560048
Asterales Asteraceae Dendrosenecio johnstonii#NC 037959
Asterales Asteraceae Dendrosenecio kilimanjari#NC 037957
Asterales Asteraceae Dendrosenecio brassiciformis#NC 037960
Asterales Asteraceae Dendrosenecio cheranganiensis subsp. dalei#MG560046
Asterales Asteraceae Dendrosenecio cheranganiensis#NC 037956
Asterales Asteraceae Dendrosenecio keniodendron#NC 036831
Asterales Asteraceae Dendrosenecio battiscombei#NC 036833
Asterales Asteraceae Dendrosenecio elgonensis subsp. barbatipes#MG560047
Asterales Asteraceae Dendrosenecio keniensis#NC 036832
Asterales Asteraceae Helichrysum italicum#NC 041458
Asterales Asteraceae Anaphalis sinica#NC 034648
Asterales Asteraceae Leontopodium nivale subsp. alpinum#DOVJ
Asterales Asteraceae Leontopodium leiolepis#NC 027835
Asterales Asteraceae Soliva sessilis#NC 034851
Asterales Asteraceae Ismelia carinata#NC 040110
Asterales Asteraceae Cota tinctoria#GUMF
Asterales Asteraceae Tanacetum parthenium#DUQG
Asterales Asteraceae Matricaria matricarioides#OAGK
Asterales Asteraceae Artemisia annua#MF623173
Asterales Asteraceae Chrysanthemum indicum#NC 020320
Asterales Asteraceae Tanacetum vulgare#NC 037388
Asterales Asteraceae Dendranthema zawadskii#NC 040920
Asterales Asteraceae Artemisia capillaris#KY073391
Asterales Asteraceae Artemisia selengensis#NC 039647
Asterales Asteraceae Artemisia gmelinii#KY073390
Asterales Asteraceae Artemisia montana#NC 025910
Asterales Asteraceae Artemisia frigida#NC 020607
Asterales Asteraceae Llerasia caucana#NC 034821
Asterales Asteraceae Oritrophium peruvianum#NC 034849
Asterales Asteraceae Eschenbachia blinii#NC 037605
Asterales Asteraceae Aster spathulifolius#NC 027434
Asterales Asteraceae Aster altaicus#NC 034996
Asterales Asteraceae Aster tataricus#XRCX
Asterales Asteraceae Aster indicus#NC 040126
Asterales Asteraceae Archibaccharis asperifolia#NC 034848
Asterales Asteraceae Solidago canadensis#TEZA
Asterales Asteraceae Conyza bonariensis#KX792499
Asterales Asteraceae Erigeron canadensis#MHYG
Asterales Asteraceae Erigeron speciosus#DESP
Asterales Asteraceae Lagenophora cuchumatanica#NC 034819
Asterales Asteraceae Heterothalamus alienus#NC 034855
Asterales Asteraceae Diplostephium meyenii#NC 034824
Asterales Asteraceae Diplostephium empetrifolium#NC 034891
Asterales Asteraceae Diplostephium juniperinum#NC 034813
Asterales Asteraceae Diplostephium cinereum#NC 034869
Asterales Asteraceae Parastrephia quadrangularis#NC 034890
Asterales Asteraceae Diplostephium foliosissimum#NC 034883
Asterales Asteraceae Diplostephium gnidioides#NC 034867
Asterales Asteraceae Diplostephium pulchrum#KX063942
Asterales Asteraceae Diplostephium sp.#KX063858
Asterales Asteraceae Diplostephium gynoxyoides#NC 034862
Asterales Asteraceae Diplostephium hippophae#NC 034831
Asterales Asteraceae Diplostephium oxapampanum#NC 034815
Asterales Asteraceae Diplostephium haenkei#NC 034871
Asterales Asteraceae Diplostephium lechleri#NC 034816
Asterales Asteraceae Diplostephium callilepis#NC 034856
Asterales Asteraceae Diplostephium goodspeedii#NC 034901
Asterales Asteraceae Aztecaster matudae#NC 034898
Asterales Asteraceae Westoniella kohkemperi#NC 034889
Asterales Asteraceae Laennecia sophiifolia#NC 034877
Asterales Asteraceae Baccharis genistelloides#NC 034852
Asterales Asteraceae Baccharis tricuneata#NC 034868
Asterales Asteraceae Diplostephium crypteriophyllum#NC 034881
Asterales Asteraceae Diplostephium ericoides#NC 034870
Asterales Asteraceae Diplostephium espinosae#NC 034880
Asterales Asteraceae Diplostephium hartwegii#NC 034832
Asterales Asteraceae Exostigma notobellidiastrum#NC 034864
Asterales Asteraceae Diplostephium spinulosum#NC 034823
Asterales Asteraceae Diplostephium serratifolium#NC 034826
Asterales Asteraceae Diplostephium azureum#NC 034882
Asterales Asteraceae Diplostephium sagasteguii#NC 034828
Asterales Asteraceae Diplostephium barclayanum#NC 034853
Asterales Asteraceae Laestadia muscicola#NC 034858
Asterales Asteraceae Hinterhubera ericoides#NC 034884
Asterales Asteraceae Blakiella bartsiifolia#NC 034866
Asterales Asteraceae Floscaldasia hypsophila#NC 034888
Asterales Asteraceae Diplostephium jelskii#NC 034811
Asterales Asteraceae Diplostephium cajamarquillense#NC 034872
Asterales Asteraceae Diplostephium glandulosum#NC 034854
Asterales Asteraceae Diplostephium oblanceolatum#NC 034830
Asterales Asteraceae Diplostephium coriaceum#NC 034899
Asterales Asteraceae Diplostephium romeroi#NC 034885
Asterales Asteraceae Diplostephium inesianum#NC 034895
Asterales Asteraceae Diplostephium colombianum#NC 034861
Asterales Asteraceae Diplostephium glutinosum#NC 034875
Asterales Asteraceae Diplostephium frontinense#NC 034893
Asterales Asteraceae Diplostephium rupestre#NC 034865
Asterales Asteraceae Diplostephium eriophorum#NC 034874
Asterales Asteraceae Diplostephium rhomboidale#KX063890
Asterales Asteraceae Diplostephium huertasii#NC 034812
Asterales Asteraceae Diplostephium tachirense#NC 034825
Asterales Asteraceae Diplostephium jaramilloi#NC 034894
Asterales Asteraceae Diplostephium schultzii#KX063936

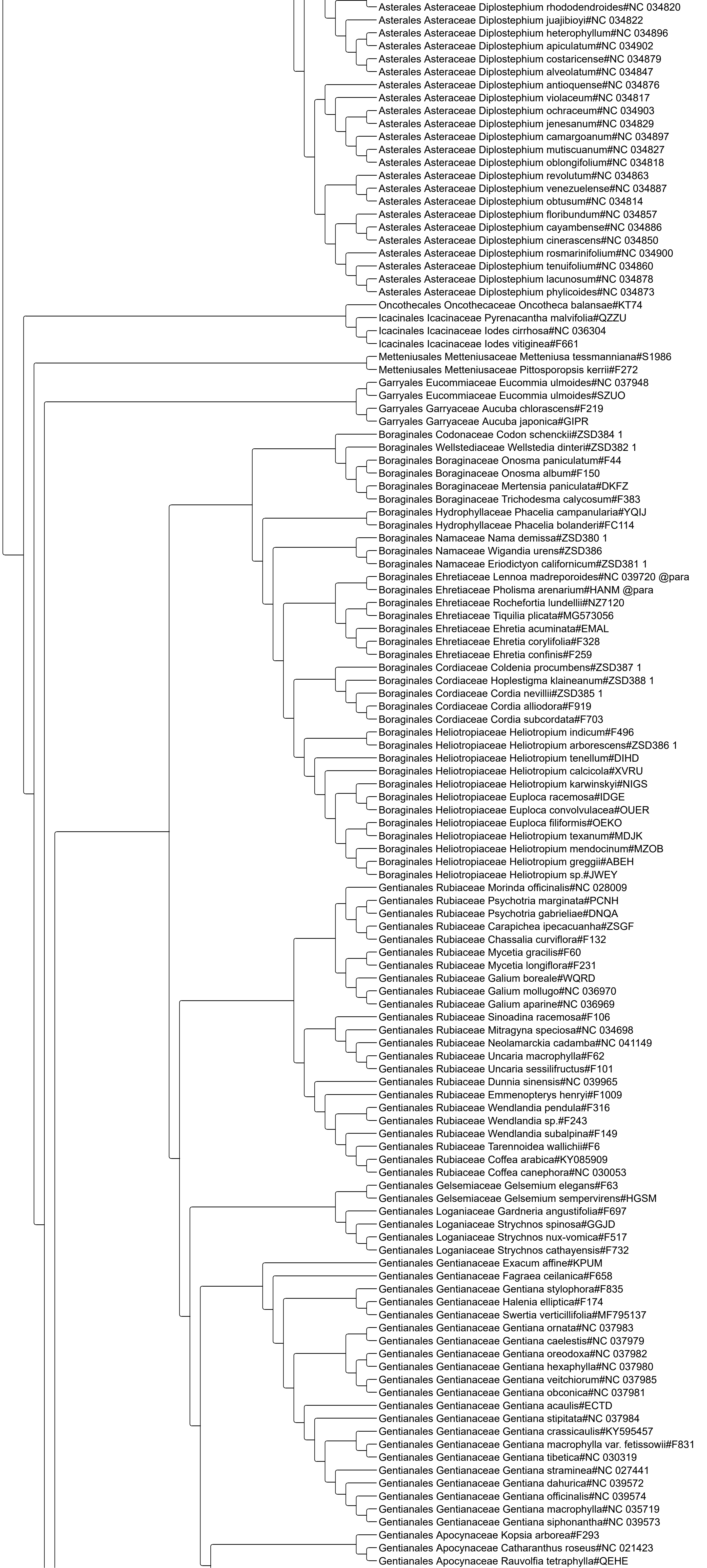
Asterales Asteraceae Diplostephium rhododendroides#NC 034820
Asterales Asteraceae Diplostephium juajibioyi#NC 034822
Asterales Asteraceae Diplostephium heterophyllum#NC 034896
Asterales Asteraceae Diplostephium apiculatum#NC 034902
Asterales Asteraceae Diplostephium costaricense#NC 034879
Asterales Asteraceae Diplostephium alveolatum#NC 034847
Asterales Asteraceae Diplostephium antioquense#NC 034876
Asterales Asteraceae Diplostephium violaceum#NC 034817
Asterales Asteraceae Diplostephium ochraceum#NC 034903
Asterales Asteraceae Diplostephium jenesanum#NC 034829
Asterales Asteraceae Diplostephium camargoanum#NC 034897
Asterales Asteraceae Diplostephium mutiscuanum#NC 034827
Asterales Asteraceae Diplostephium oblongifolium#NC 034818
Asterales Asteraceae Diplostephium revolutum#NC 034863
Asterales Asteraceae Diplostephium venezuelense#NC 034887
Asterales Asteraceae Diplostephium obtusum#NC 034814
Asterales Asteraceae Diplostephium floribundum#NC 034857
Asterales Asteraceae Diplostephium cayambense#NC 034886
Asterales Asteraceae Diplostephium cinerascens#NC 034850
Asterales Asteraceae Diplostephium rosmarinifolium#NC 034900
Asterales Asteraceae Diplostephium tenuifolium#NC 034860
Asterales Asteraceae Diplostephium lacunosum#NC 034878
Asterales Asteraceae Diplostephium phylicoides#NC 034873
Oncothecales Oncothecaceae Oncotheca balansae#KT74
Icacinales Icacinaceae Pyrenacantha malvifolia#QZZU
Icacinales Icacinaceae Iodes cirrhosa#NC 036304
Icacinales Icacinaceae Iodes vitiginea#F661
Metteniusales Metteniusaceae Metteniusa tessmanniana#S1986
Metteniusales Metteniusaceae Pittosporopsis kerrii#F272
Garryales Eucommiaceae Eucommia ulmoides#NC 037948
Garryales Eucommiaceae Eucommia ulmoides#SZUO
Garryales Garryaceae Aucuba chlorascens#F219
Garryales Garryaceae Aucuba japonica#GIPR
Boraginales Codonaceae Codon schenckii#ZSD384 1
Boraginales Wellstediaceae Wellstedia dinteri#ZSD382 1
Boraginales Boraginaceae Onosma paniculatum#F44
Boraginales Boraginaceae Onosma album#F150
Boraginales Boraginaceae Mertensia paniculata#DKFZ
Boraginales Boraginaceae Trichodesma calycosum#F383
Boraginales Hydrophyllaceae Phacelia campanularia#YQIJ
Boraginales Hydrophyllaceae Phacelia bolanderi#FC114
Boraginales Namaceae Nama demissa#ZSD380 1
Boraginales Namaceae Wigandia urens#ZSD386
Boraginales Namaceae Eriodictyon californicum#ZSD381 1
Boraginales Ehretiaceae Lennoa madreporoides#NC 039720 @para
Boraginales Ehretiaceae Pholisma arenarium#HANM @para
Boraginales Ehretiaceae Rochefortia lundellii#NZ7120
Boraginales Ehretiaceae Tiquilia plicata#MG573056
Boraginales Ehretiaceae Ehretia acuminata#EMAL
Boraginales Ehretiaceae Ehretia corylifolia#F328
Boraginales Ehretiaceae Ehretia confinis#F259
Boraginales Cordiaceae Coldenia procumbens#ZSD387 1
Boraginales Cordiaceae Hoplestigma klaineanum#ZSD388 1
Boraginales Cordiaceae Cordia nevillii#ZSD385 1
Boraginales Cordiaceae Cordia alliodora#F919
Boraginales Cordiaceae Cordia subcordata#F703
Boraginales Heliotropiaceae Heliotropium indicum#F496
Boraginales Heliotropiaceae Heliotropium arborescens#ZSD386 1
Boraginales Heliotropiaceae Heliotropium tenellum#DIHD
Boraginales Heliotropiaceae Heliotropium calcicola#XVRU
Boraginales Heliotropiaceae Heliotropium karwinskyi#NIGS
Boraginales Heliotropiaceae Euploca racemosa#IDGE
Boraginales Heliotropiaceae Euploca convolvulacea#OUER
Boraginales Heliotropiaceae Euploca filiformis#OEKO
Boraginales Heliotropiaceae Heliotropium texanum#MDJK
Boraginales Heliotropiaceae Heliotropium mendocinum#MZOB
Boraginales Heliotropiaceae Heliotropium greggii#ABEH
Boraginales Heliotropiaceae Heliotropium sp.#JWEY
Gentianales Rubiaceae Morinda officinalis#NC 028009
Gentianales Rubiaceae Psychotria marginata#PCNH
Gentianales Rubiaceae Psychotria gabrieliae#DNQA
Gentianales Rubiaceae Carapichea ipecacuanha#ZSGF
Gentianales Rubiaceae Chassalia curviflora#F132
Gentianales Rubiaceae Mycetia gracilis#F60
Gentianales Rubiaceae Mycetia longiflora#F231
Gentianales Rubiaceae Galium boreale#WQRD
Gentianales Rubiaceae Galium mollugo#NC 036970
Gentianales Rubiaceae Galium aparine#NC 036969
Gentianales Rubiaceae Sinoadina racemosa#F106
Gentianales Rubiaceae Mitragyna speciosa#NC 034698
Gentianales Rubiaceae Neolamarckia cadamba#NC 041149
Gentianales Rubiaceae Uncaria macrophylla#F62
Gentianales Rubiaceae Uncaria sessilifructus#F101
Gentianales Rubiaceae Dunnia sinensis#NC 039965
Gentianales Rubiaceae Emmenopterys henryi#F1009
Gentianales Rubiaceae Wendlandia pendula#F316
Gentianales Rubiaceae Wendlandia sp.#F243
Gentianales Rubiaceae Wendlandia subalpina#F149
Gentianales Rubiaceae Tarennoidea wallichii#F6
Gentianales Rubiaceae Coffea arabica#KY085909
Gentianales Rubiaceae Coffea canephora#NC 030053
Gentianales Gelsemiaceae Gelsemium elegans#F63
Gentianales Gelsemiaceae Gelsemium sempervirens#HGSM
Gentianales Loganiaceae Gardneria angustifolia#F697
Gentianales Loganiaceae Strychnos spinosa#GGJD
Gentianales Loganiaceae Strychnos nux-vomica#F517
Gentianales Loganiaceae Strychnos cathayensis#F732
Gentianales Gentianaceae Exacum affine#KPUM
Gentianales Gentianaceae Fagraea ceilanica#F658
Gentianales Gentianaceae Gentiana stylophora#F835
Gentianales Gentianaceae Halenia elliptica#F174
Gentianales Gentianaceae Swertia verticillifolia#MF795137
Gentianales Gentianaceae Gentiana ornata#NC 037983
Gentianales Gentianaceae Gentiana caelestis#NC 037979
Gentianales Gentianaceae Gentiana oreodoxa#NC 037982
Gentianales Gentianaceae Gentiana hexaphylla#NC 037980
Gentianales Gentianaceae Gentiana veitchiorum#NC 037985
Gentianales Gentianaceae Gentiana obconica#NC 037981
Gentianales Gentianaceae Gentiana acaulis#ECTD
Gentianales Gentianaceae Gentiana stipitata#NC 037984
Gentianales Gentianaceae Gentiana crassicaulis#KY595457
Gentianales Gentianaceae Gentiana macrophylla var. fetissowii#F831
Gentianales Gentianaceae Gentiana tibetica#NC 030319
Gentianales Gentianaceae Gentiana straminea#NC 027441
Gentianales Gentianaceae Gentiana dahurica#NC 039572
Gentianales Gentianaceae Gentiana officinalis#NC 039574
Gentianales Gentianaceae Gentiana macrophylla#NC 035719
Gentianales Gentianaceae Gentiana siphonantha#NC 039573
Gentianales Apocynaceae Kopsia arborea#F293
Gentianales Apocynaceae Catharanthus roseus#NC 021423
Gentianales Apocynaceae Rauvolfia tetraphylla#QEHE

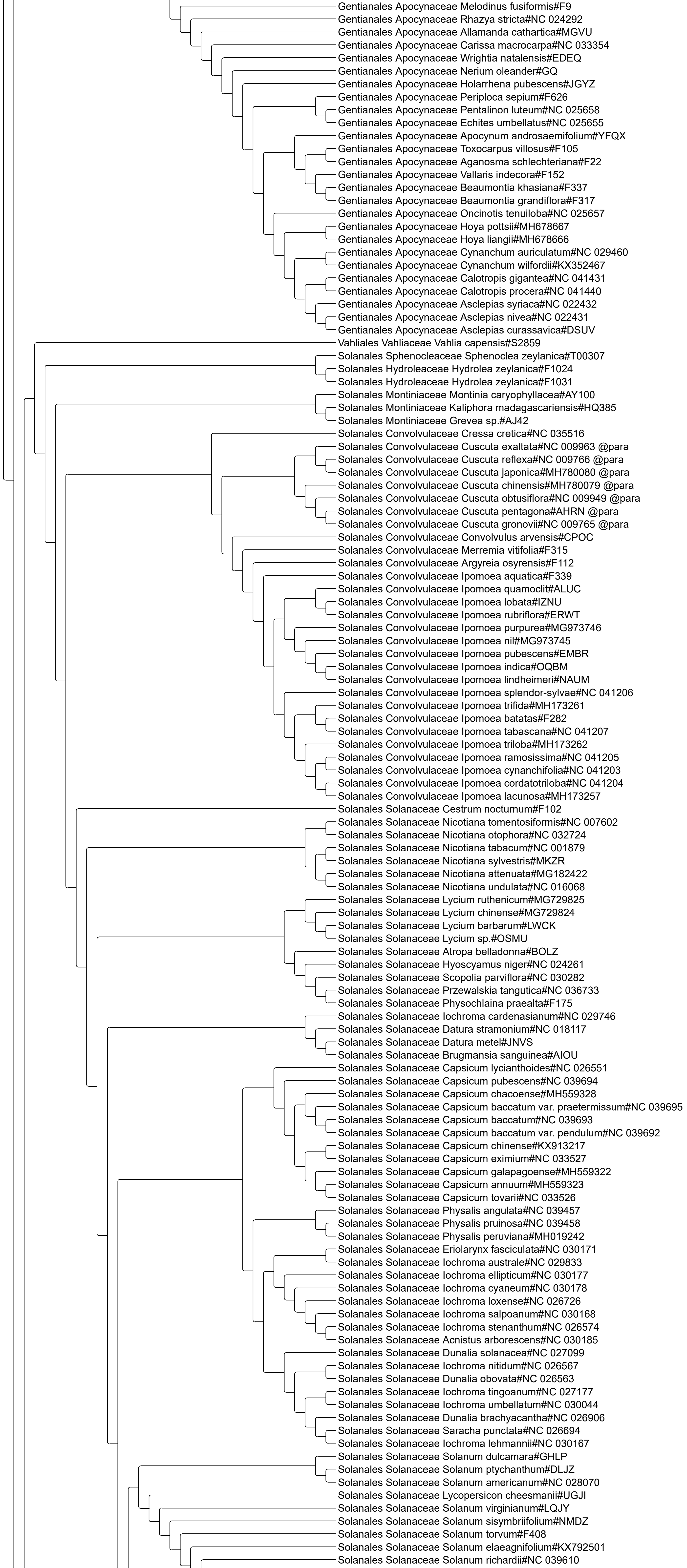

Gentianales Apocynaceae Melodinus fusiformis#F9
Gentianales Apocynaceae Rhazya stricta#NC 024292
Gentianales Apocynaceae Allamanda cathartica#MGVU
Gentianales Apocynaceae Carissa macrocarpa#NC 033354
Gentianales Apocynaceae Wrightia natalensis#EDEQ
Gentianales Apocynaceae Nerium oleander#GQ
Gentianales Apocynaceae Holarrhena pubescens#JGYZ
Gentianales Apocynaceae Periploca sepium#F626
Gentianales Apocynaceae Pentalinon luteum#NC 025658
Gentianales Apocynaceae Echites umbellatus#NC 025655
Gentianales Apocynaceae Apocynum androsaemifolium#YFQX
Gentianales Apocynaceae Toxocarpus villosus#F105
Gentianales Apocynaceae Aganosma schlechteriana#F22
Gentianales Apocynaceae Vallaris indecora#F152
Gentianales Apocynaceae Beaumontia khasiana#F337
Gentianales Apocynaceae Beaumontia grandiflora#F317
Gentianales Apocynaceae Oncinotis tenuiloba#NC 025657
Gentianales Apocynaceae Hoya pottsii#MH678667
Gentianales Apocynaceae Hoya liangii#MH678666
Gentianales Apocynaceae Cynanchum auriculatum#NC 029460
Gentianales Apocynaceae Cynanchum wilfordii#KX352467
Gentianales Apocynaceae Calotropis gigantea#NC 041431
Gentianales Apocynaceae Calotropis procera#NC 041440
Gentianales Apocynaceae Asclepias syriaca#NC 022432
Gentianales Apocynaceae Asclepias nivea#NC 022431
Gentianales Apocynaceae Asclepias curassavica#DSUV
Vahliales Vahliaceae Vahlia capensis#S2859
Solanales Sphenocleaceae Sphenoclea zeylanica#T00307
Solanales Hydroleaceae Hydrolea zeylanica#F1024
Solanales Hydroleaceae Hydrolea zeylanica#F1031
Solanales Montiniaceae Montinia caryophyllacea#AY100
Solanales Montiniaceae Kaliphora madagascariensis#HQ385
Solanales Montiniaceae Grevea sp.#AJ42
Solanales Convolvulaceae Cressa cretica#NC 035516
Solanales Convolvulaceae Cuscuta exaltata#NC 009963 @para
Solanales Convolvulaceae Cuscuta reflexa#NC 009766 @para
Solanales Convolvulaceae Cuscuta japonica#MH780080 @para
Solanales Convolvulaceae Cuscuta chinensis#MH780079 @para
Solanales Convolvulaceae Cuscuta obtusiflora#NC 009949 @para
Solanales Convolvulaceae Cuscuta pentagona#AHRN @para
Solanales Convolvulaceae Cuscuta gronovii#NC 009765 @para
Solanales Convolvulaceae Convolvulus arvensis#CPOC
Solanales Convolvulaceae Merremia vitifolia#F315
Solanales Convolvulaceae Argyreia osyrensis#F112
Solanales Convolvulaceae Ipomoea aquatica#F339
Solanales Convolvulaceae Ipomoea quamoclit#ALUC
Solanales Convolvulaceae Ipomoea lobata#IZNU
Solanales Convolvulaceae Ipomoea rubriflora#ERWT
Solanales Convolvulaceae Ipomoea purpurea#MG973746
Solanales Convolvulaceae Ipomoea nil#MG973745
Solanales Convolvulaceae Ipomoea pubescens#EMBR
Solanales Convolvulaceae Ipomoea indica#OQBM
Solanales Convolvulaceae Ipomoea lindheimeri#NAUM
Solanales Convolvulaceae Ipomoea splendor-sylvae#NC 041206
Solanales Convolvulaceae Ipomoea trifida#MH173261
Solanales Convolvulaceae Ipomoea batatas#F282
Solanales Convolvulaceae Ipomoea tabascana#NC 041207
Solanales Convolvulaceae Ipomoea triloba#MH173262
Solanales Convolvulaceae Ipomoea ramosissima#NC 041205
Solanales Convolvulaceae Ipomoea cynanchifolia#NC 041203
Solanales Convolvulaceae Ipomoea cordatotriloba#NC 041204
Solanales Convolvulaceae Ipomoea lacunosa#MH173257
Solanales Solanaceae Cestrum nocturnum#F102
Solanales Solanaceae Nicotiana tomentosiformis#NC 007602
Solanales Solanaceae Nicotiana otophora#NC 032724
Solanales Solanaceae Nicotiana tabacum#NC 001879
Solanales Solanaceae Nicotiana sylvestris#MKZR
Solanales Solanaceae Nicotiana attenuata#MG182422
Solanales Solanaceae Nicotiana undulata#NC 016068
Solanales Solanaceae Lycium ruthenicum#MG729825
Solanales Solanaceae Lycium chinense#MG729824
Solanales Solanaceae Lycium barbarum#LWCK
Solanales Solanaceae Lycium sp.#OSMU
Solanales Solanaceae Atropa belladonna#BOLZ
Solanales Solanaceae Hyoscyamus niger#NC 024261
Solanales Solanaceae Scopolia parviflora#NC 030282
Solanales Solanaceae Przewalskia tangutica#NC 036733
Solanales Solanaceae Physochlaina praealta#F175
Solanales Solanaceae Iochroma cardenasianum#NC 029746
Solanales Solanaceae Datura stramonium#NC 018117
Solanales Solanaceae Datura metel#JNVS
Solanales Solanaceae Brugmansia sanguinea#AIOU
Solanales Solanaceae Capsicum lycianthoides#NC 026551
Solanales Solanaceae Capsicum pubescens#NC 039694
Solanales Solanaceae Capsicum chacoense#MH559328
Solanales Solanaceae Capsicum baccatum var. praetermissum#NC 039695
Solanales Solanaceae Capsicum baccatum#NC 039693
Solanales Solanaceae Capsicum baccatum var. pendulum#NC 039692
Solanales Solanaceae Capsicum chinense#KX913217
Solanales Solanaceae Capsicum eximium#NC 033527
Solanales Solanaceae Capsicum galapagoense#MH559322
Solanales Solanaceae Capsicum annuum#MH559323
Solanales Solanaceae Capsicum tovarii#NC 033526
Solanales Solanaceae Physalis angulata#NC 039457
Solanales Solanaceae Physalis pruinosa#NC 039458
Solanales Solanaceae Physalis peruviana#MH019242
Solanales Solanaceae Eriolarynx fasciculata#NC 030171
Solanales Solanaceae Iochroma australe#NC 029833
Solanales Solanaceae Iochroma ellipticum#NC 030177
Solanales Solanaceae Iochroma cyaneum#NC 030178
Solanales Solanaceae Iochroma loxense#NC 026726
Solanales Solanaceae Iochroma salpoanum#NC 030168
Solanales Solanaceae Iochroma stenanthum#NC 026574
Solanales Solanaceae Acnistus arborescens#NC 030185
Solanales Solanaceae Dunalia solanacea#NC 027099
Solanales Solanaceae Iochroma nitidum#NC 026567
Solanales Solanaceae Dunalia obovata#NC 026563
Solanales Solanaceae Iochroma tingoanum#NC 027177
Solanales Solanaceae Iochroma umbellatum#NC 030044
Solanales Solanaceae Dunalia brachyacantha#NC 026906
Solanales Solanaceae Saracha punctata#NC 026694
Solanales Solanaceae Iochroma lehmannii#NC 030167
Solanales Solanaceae Solanum dulcamara#GHLP
Solanales Solanaceae Solanum ptychanthum#DLJZ
Solanales Solanaceae Solanum americanum#NC 028070
Solanales Solanaceae Lycopersicon cheesmanii#UGJI
Solanales Solanaceae Solanum virginianum#LQJY
Solanales Solanaceae Solanum sisymbriifolium#NMDZ
Solanales Solanaceae Solanum torvum#F408
Solanales Solanaceae Solanum elaeagnifolium#KX792501
Solanales Solanaceae Solanum richardii#NC 039610

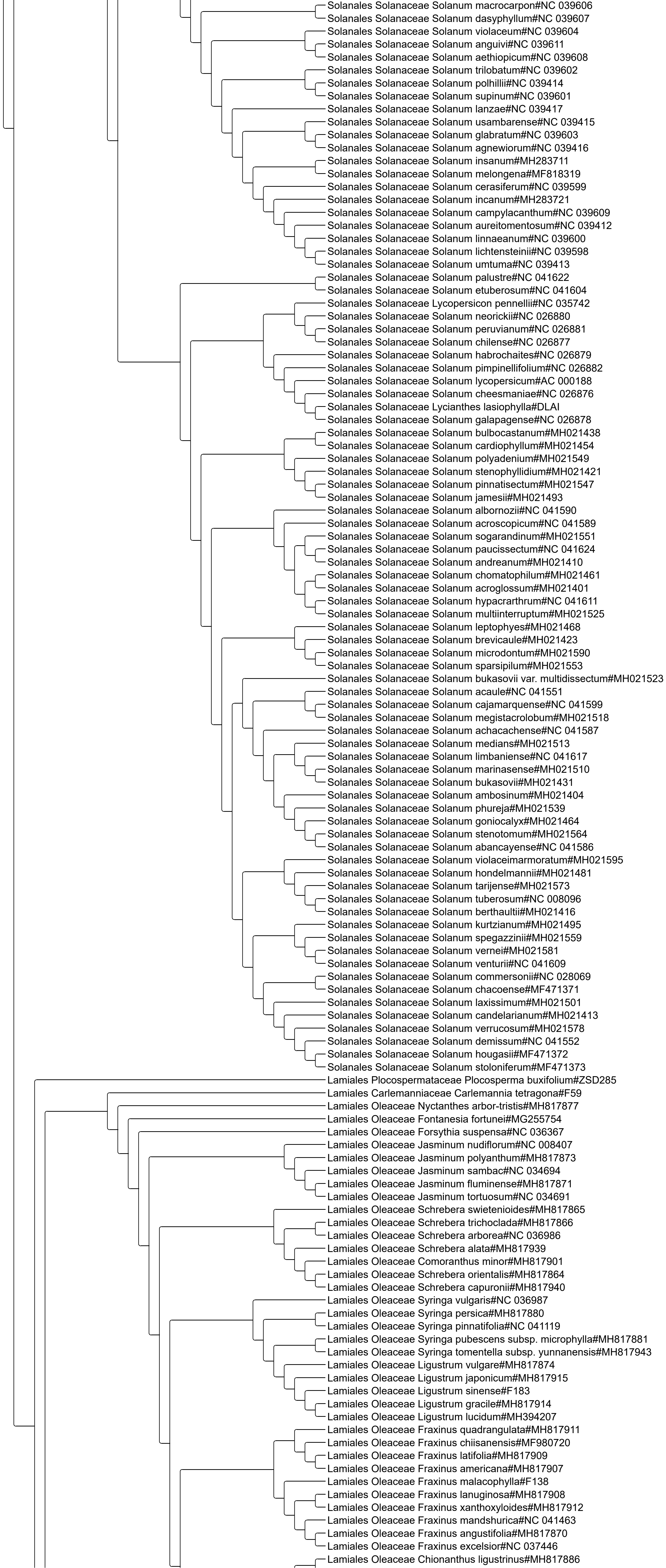

Solanales Solanaceae Solanum macrocarpon#NC 039606
Solanales Solanaceae Solanum dasyphyllum#NC 039607
Solanales Solanaceae Solanum violaceum#NC 039604
Solanales Solanaceae Solanum anguivi#NC 039611
Solanales Solanaceae Solanum aethiopicum#NC 039608
Solanales Solanaceae Solanum trilobatum#NC 039602
Solanales Solanaceae Solanum polhillii#NC 039414
Solanales Solanaceae Solanum supinum#NC 039601
Solanales Solanaceae Solanum lanzae#NC 039417
Solanales Solanaceae Solanum usambarense#NC 039415
Solanales Solanaceae Solanum glabratum#NC 039603
Solanales Solanaceae Solanum agnewiorum#NC 039416
Solanales Solanaceae Solanum insanum#MH283711
Solanales Solanaceae Solanum melongena#MF818319
Solanales Solanaceae Solanum cerasiferum#NC 039599
Solanales Solanaceae Solanum incanum#MH283721
Solanales Solanaceae Solanum campylacanthum#NC 039609
Solanales Solanaceae Solanum aureitomentosum#NC 039412
Solanales Solanaceae Solanum linnaeanum#NC 039600
Solanales Solanaceae Solanum lichtensteinii#NC 039598
Solanales Solanaceae Solanum umtuma#NC 039413
Solanales Solanaceae Solanum palustre#NC 041622
Solanales Solanaceae Solanum etuberosum#NC 041604
Solanales Solanaceae Lycopersicon pennellii#NC 035742
Solanales Solanaceae Solanum neorickii#NC 026880
Solanales Solanaceae Solanum peruvianum#NC 026881
Solanales Solanaceae Solanum chilense#NC 026877
Solanales Solanaceae Solanum habrochaites#NC 026879
Solanales Solanaceae Solanum pimpinellifolium#NC 026882
Solanales Solanaceae Solanum lycopersicum#AC 000188
Solanales Solanaceae Solanum cheesmaniae#NC 026876
Solanales Solanaceae Lycianthes lasiophylla#DLAI
Solanales Solanaceae Solanum galapagense#NC 026878
Solanales Solanaceae Solanum bulbocastanum#MH021438
Solanales Solanaceae Solanum cardiophyllum#MH021454
Solanales Solanaceae Solanum polyadenium#MH021549
Solanales Solanaceae Solanum stenophyllidium#MH021421
Solanales Solanaceae Solanum pinnatisectum#MH021547
Solanales Solanaceae Solanum jamesii#MH021493
Solanales Solanaceae Solanum albornozii#NC 041590
Solanales Solanaceae Solanum acroscopicum#NC 041589
Solanales Solanaceae Solanum sogarandinum#MH021551
Solanales Solanaceae Solanum paucissectum#NC 041624
Solanales Solanaceae Solanum andreanum#MH021410
Solanales Solanaceae Solanum chomatophilum#MH021461
Solanales Solanaceae Solanum acroglossum#MH021401
Solanales Solanaceae Solanum hypacrarthrum#NC 041611
Solanales Solanaceae Solanum multiinterruptum#MH021525
Solanales Solanaceae Solanum leptophyes#MH021468
Solanales Solanaceae Solanum brevicaule#MH021423
Solanales Solanaceae Solanum microdontum#MH021590
Solanales Solanaceae Solanum sparsipilum#MH021553
Solanales Solanaceae Solanum bukasovii var. multidissectum#MH021523
Solanales Solanaceae Solanum acaule#NC 041551
Solanales Solanaceae Solanum cajamarquense#NC 041599
Solanales Solanaceae Solanum megistacrolobum#MH021518
Solanales Solanaceae Solanum achacachense#NC 041587
Solanales Solanaceae Solanum medians#MH021513
Solanales Solanaceae Solanum limbaniense#NC 041617
Solanales Solanaceae Solanum marinasense#MH021510
Solanales Solanaceae Solanum bukasovii#MH021431
Solanales Solanaceae Solanum ambosinum#MH021404
Solanales Solanaceae Solanum phureja#MH021539
Solanales Solanaceae Solanum goniocalyx#MH021464
Solanales Solanaceae Solanum stenotomum#MH021564
Solanales Solanaceae Solanum abancayense#NC 041586
Solanales Solanaceae Solanum violaceimarmoratum#MH021595
Solanales Solanaceae Solanum hondelmannii#MH021481
Solanales Solanaceae Solanum tarijense#MH021573
Solanales Solanaceae Solanum tuberosum#NC 008096
Solanales Solanaceae Solanum berthaultii#MH021416
Solanales Solanaceae Solanum kurtzianum#MH021495
Solanales Solanaceae Solanum spegazzinii#MH021559
Solanales Solanaceae Solanum vernei#MH021581
Solanales Solanaceae Solanum venturii#NC 041609
Solanales Solanaceae Solanum commersonii#NC 028069
Solanales Solanaceae Solanum chacoense#MF471371
Solanales Solanaceae Solanum laxissimum#MH021501
Solanales Solanaceae Solanum candelarianum#MH021413
Solanales Solanaceae Solanum verrucosum#MH021578
Solanales Solanaceae Solanum demissum#NC 041552
Solanales Solanaceae Solanum hougasii#MF471372
Solanales Solanaceae Solanum stoloniferum#MF471373
Lamiales Plocospermataceae Plocosperma buxifolium#ZSD285
Lamiales Carlemanniaceae Carlemannia tetragona#F59
Lamiales Oleaceae Nyctanthes arbor-tristis#MH817877
Lamiales Oleaceae Fontanesia fortunei#MG255754
Lamiales Oleaceae Forsythia suspensa#NC 036367
Lamiales Oleaceae Jasminum nudiflorum#NC 008407
Lamiales Oleaceae Jasminum polyanthum#MH817873
Lamiales Oleaceae Jasminum sambac#NC 034694
Lamiales Oleaceae Jasminum fluminense#MH817871
Lamiales Oleaceae Jasminum tortuosum#NC 034691
Lamiales Oleaceae Schrebera swietenioides#MH817865
Lamiales Oleaceae Schrebera trichoclada#MH817866
Lamiales Oleaceae Schrebera arborea#NC 036986
Lamiales Oleaceae Schrebera alata#MH817939
Lamiales Oleaceae Comoranthus minor#MH817901
Lamiales Oleaceae Schrebera orientalis#MH817864
Lamiales Oleaceae Schrebera capuronii#MH817940
Lamiales Oleaceae Syringa vulgaris#NC 036987
Lamiales Oleaceae Syringa persica#MH817880
Lamiales Oleaceae Syringa pinnatifolia#NC 041119
Lamiales Oleaceae Syringa pubescens subsp. microphylla#MH817881
Lamiales Oleaceae Syringa tomentella subsp. yunnanensis#MH817943
Lamiales Oleaceae Ligustrum vulgare#MH817874
Lamiales Oleaceae Ligustrum japonicum#MH817915
Lamiales Oleaceae Ligustrum sinense#F183
Lamiales Oleaceae Ligustrum gracile#MH817914
Lamiales Oleaceae Ligustrum lucidum#MH394207
Lamiales Oleaceae Fraxinus quadrangulata#MH817911
Lamiales Oleaceae Fraxinus chiisanensis#MF980720
Lamiales Oleaceae Fraxinus latifolia#MH817909
Lamiales Oleaceae Fraxinus americana#MH817907
Lamiales Oleaceae Fraxinus malacophylla#F138
Lamiales Oleaceae Fraxinus lanuginosa#MH817908
Lamiales Oleaceae Fraxinus xanthoxyloides#MH817912
Lamiales Oleaceae Fraxinus mandshurica#NC 041463
Lamiales Oleaceae Fraxinus angustifolia#MH817870
Lamiales Oleaceae Fraxinus excelsior#NC 037446
Lamiales Oleaceae Chionanthus ligustrinus#MH817886

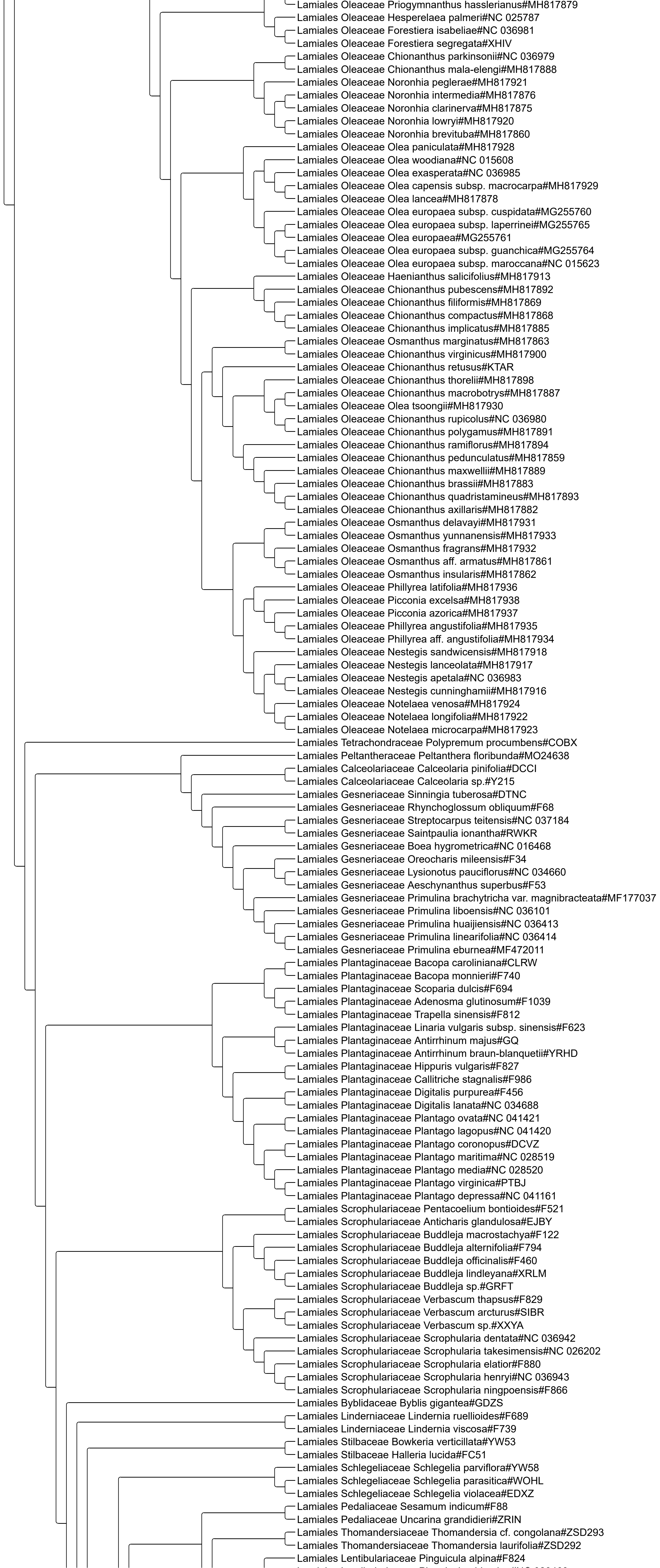
Lamiales Oleaceae Priogymnanthus hasslerianus#MH817879
Lamiales Oleaceae Hesperelaea palmeri#NC 025787
Lamiales Oleaceae Forestiera isabeliae#NC 036981
Lamiales Oleaceae Forestiera segregata#XHIV
Lamiales Oleaceae Chionanthus parkinsonii#NC 036979
Lamiales Oleaceae Chionanthus mala-elengi#MH817888
Lamiales Oleaceae Noronhia peglerae#MH817921
Lamiales Oleaceae Noronhia intermedia#MH817876
Lamiales Oleaceae Noronhia clarinerva#MH817875
Lamiales Oleaceae Noronhia lowryi#MH817920
Lamiales Oleaceae Noronhia brevituba#MH817860
Lamiales Oleaceae Olea paniculata#MH817928
Lamiales Oleaceae Olea woodiana#NC 015608
Lamiales Oleaceae Olea exasperata#NC 036985
Lamiales Oleaceae Olea capensis subsp. macrocarpa#MH817929
Lamiales Oleaceae Olea lancea#MH817878
Lamiales Oleaceae Olea europaea subsp. cuspidata#MG255760
Lamiales Oleaceae Olea europaea subsp. laperrinei#MG255765
Lamiales Oleaceae Olea europaea#MG255761
Lamiales Oleaceae Olea europaea subsp. guanchica#MG255764
Lamiales Oleaceae Olea europaea subsp. maroccana#NC 015623
Lamiales Oleaceae Haenianthus salicifolius#MH817913
Lamiales Oleaceae Chionanthus pubescens#MH817892
Lamiales Oleaceae Chionanthus filiformis#MH817869
Lamiales Oleaceae Chionanthus compactus#MH817868
Lamiales Oleaceae Chionanthus implicatus#MH817885
Lamiales Oleaceae Osmanthus marginatus#MH817863
Lamiales Oleaceae Chionanthus virginicus#MH817900
Lamiales Oleaceae Chionanthus retusus#KTAR
Lamiales Oleaceae Chionanthus thorelii#MH817898
Lamiales Oleaceae Chionanthus macrobotrys#MH817887
Lamiales Oleaceae Olea tsoongii#MH817930
Lamiales Oleaceae Chionanthus rupicolus#NC 036980
Lamiales Oleaceae Chionanthus polygamus#MH817891
Lamiales Oleaceae Chionanthus ramiflorus#MH817894
Lamiales Oleaceae Chionanthus pedunculatus#MH817859
Lamiales Oleaceae Chionanthus maxwellii#MH817889
Lamiales Oleaceae Chionanthus brassii#MH817883
Lamiales Oleaceae Chionanthus quadristamineus#MH817893
Lamiales Oleaceae Chionanthus axillaris#MH817882
Lamiales Oleaceae Osmanthus delavayi#MH817931
Lamiales Oleaceae Osmanthus yunnanensis#MH817933
Lamiales Oleaceae Osmanthus fragrans#MH817932
Lamiales Oleaceae Osmanthus aff. armatus#MH817861
Lamiales Oleaceae Osmanthus insularis#MH817862
Lamiales Oleaceae Phillyrea latifolia#MH817936
Lamiales Oleaceae Picconia excelsa#MH817938
Lamiales Oleaceae Picconia azorica#MH817937
Lamiales Oleaceae Phillyrea angustifolia#MH817935
Lamiales Oleaceae Phillyrea aff. angustifolia#MH817934
Lamiales Oleaceae Nestegis sandwicensis#MH817918
Lamiales Oleaceae Nestegis lanceolata#MH817917
Lamiales Oleaceae Nestegis apetala#NC 036983
Lamiales Oleaceae Nestegis cunninghamii#MH817916
Lamiales Oleaceae Notelaea venosa#MH817924
Lamiales Oleaceae Notelaea longifolia#MH817922
Lamiales Oleaceae Notelaea microcarpa#MH817923
Lamiales Tetrachondraceae Polypremum procumbens#COBX
Lamiales Peltantheraceae Peltanthera floribunda#MO24638
Lamiales Calceolariaceae Calceolaria pinifolia#DCCI
Lamiales Calceolariaceae Calceolaria sp.#Y215
Lamiales Gesneriaceae Sinningia tuberosa#DTNC
Lamiales Gesneriaceae Rhynchoglossum obliquum#F68
Lamiales Gesneriaceae Streptocarpus teitensis#NC 037184
Lamiales Gesneriaceae Saintpaulia ionantha#RWKR
Lamiales Gesneriaceae Boea hygrometrica#NC 016468
Lamiales Gesneriaceae Oreocharis mileensis#F34
Lamiales Gesneriaceae Lysionotus pauciflorus#NC 034660
Lamiales Gesneriaceae Aeschynanthus superbus#F53
Lamiales Gesneriaceae Primulina brachytricha var. magnibracteata#MF177037
Lamiales Gesneriaceae Primulina liboensis#NC 036101
Lamiales Gesneriaceae Primulina huaijiensis#NC 036413
Lamiales Gesneriaceae Primulina linearifolia#NC 036414
Lamiales Gesneriaceae Primulina eburnea#MF472011
Lamiales Plantaginaceae Bacopa caroliniana#CLRW
Lamiales Plantaginaceae Bacopa monnieri#F740
Lamiales Plantaginaceae Scoparia dulcis#F694
Lamiales Plantaginaceae Adenosma glutinosum#F1039
Lamiales Plantaginaceae Trapella sinensis#F812
Lamiales Plantaginaceae Linaria vulgaris subsp. sinensis#F623
Lamiales Plantaginaceae Antirrhinum majus#GQ
Lamiales Plantaginaceae Antirrhinum braun-blanquetii#YRHD
Lamiales Plantaginaceae Hippuris vulgaris#F827
Lamiales Plantaginaceae Callitriche stagnalis#F986
Lamiales Plantaginaceae Digitalis purpurea#F456
Lamiales Plantaginaceae Digitalis lanata#NC 034688
Lamiales Plantaginaceae Plantago ovata#NC 041421
Lamiales Plantaginaceae Plantago lagopus#NC 041420
Lamiales Plantaginaceae Plantago coronopus#DCVZ
Lamiales Plantaginaceae Plantago maritima#NC 028519
Lamiales Plantaginaceae Plantago media#NC 028520
Lamiales Plantaginaceae Plantago virginica#PTBJ
Lamiales Plantaginaceae Plantago depressa#NC 041161
Lamiales Scrophulariaceae Pentacoelium bontioides#F521
Lamiales Scrophulariaceae Anticharis glandulosa#EJBY
Lamiales Scrophulariaceae Buddleja macrostachya#F122
Lamiales Scrophulariaceae Buddleja alternifolia#F794
Lamiales Scrophulariaceae Buddleja officinalis#F460
Lamiales Scrophulariaceae Buddleja lindleyana#XRLM
Lamiales Scrophulariaceae Buddleja sp.#GRFT
Lamiales Scrophulariaceae Verbascum thapsus#F829
Lamiales Scrophulariaceae Verbascum arcturus#SIBR
Lamiales Scrophulariaceae Verbascum sp.#XXYA
Lamiales Scrophulariaceae Scrophularia dentata#NC 036942
Lamiales Scrophulariaceae Scrophularia takesimensis#NC 026202
Lamiales Scrophulariaceae Scrophularia elatior#F880
Lamiales Scrophulariaceae Scrophularia henryi#NC 036943
Lamiales Scrophulariaceae Scrophularia ningpoensis#F866
Lamiales Byblidaceae Byblis gigantea#GDZS
Lamiales Linderniaceae Lindernia ruellioides#F689
Lamiales Linderniaceae Lindernia viscosa#F739
Lamiales Stilbaceae Bowkeria verticillata#YW53
Lamiales Stilbaceae Halleria lucida#FC51
Lamiales Schlegeliaceae Schlegelia parviflora#YW58
Lamiales Schlegeliaceae Schlegelia parasitica#WOHL
Lamiales Schlegeliaceae Schlegelia violacea#EDXZ
Lamiales Pedaliaceae Sesamum indicum#F88
Lamiales Pedaliaceae Uncarina grandidieri#ZRIN
Lamiales Thomandersiaceae Thomandersia cf. congolana#ZSD293
Lamiales Thomandersiaceae Thomandersia laurifolia#ZSD292
Lamiales Lentibulariaceae Pinguicula alpina#F824

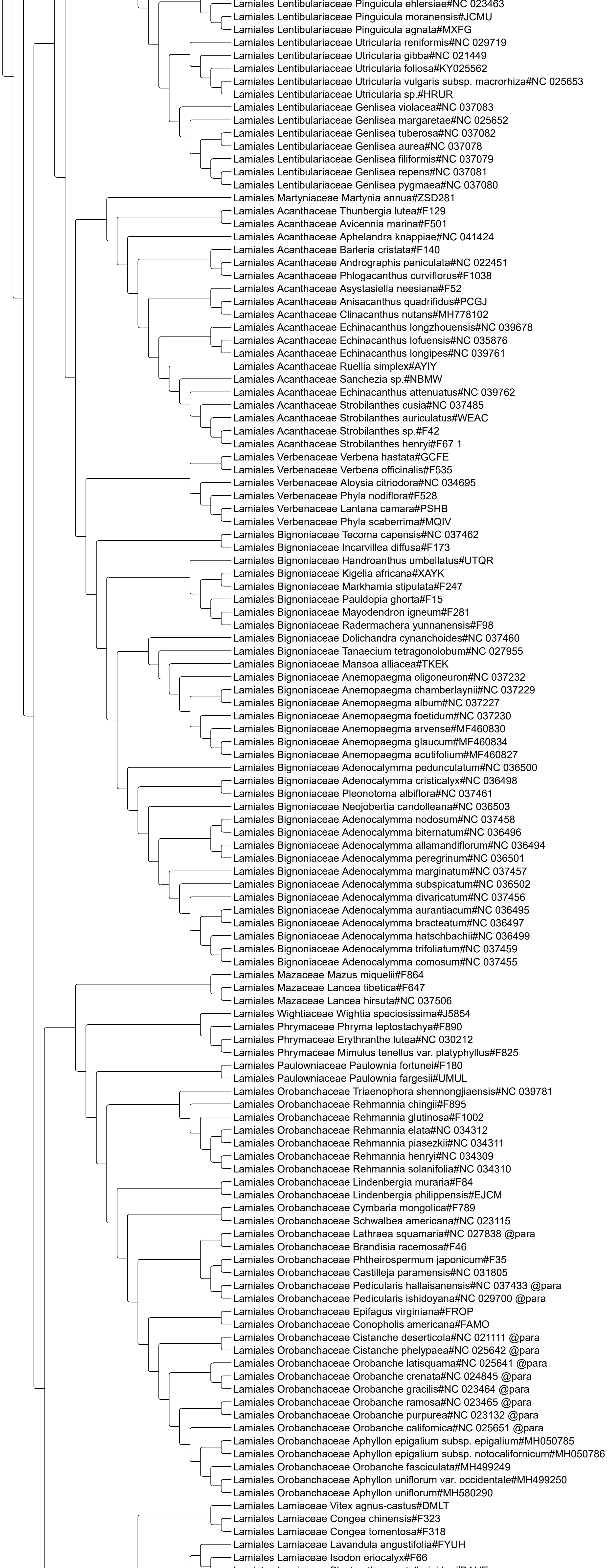

Lamiales Lentibulariaceae Pinguicula ehlersiae#NC 023463
Lamiales Lentibulariaceae Pinguicula moranensis#JCMU
Lamiales Lentibulariaceae Pinguicula agnata#MXFG
Lamiales Lentibulariaceae Utricularia reniformis#NC 029719
Lamiales Lentibulariaceae Utricularia gibba#NC 021449
Lamiales Lentibulariaceae Utricularia foliosa#KY025562
Lamiales Lentibulariaceae Utricularia vulgaris subsp. macrorhiza#NC 025653
Lamiales Lentibulariaceae Utricularia sp.#HRUR
Lamiales Lentibulariaceae Genlisea violacea#NC 037083
Lamiales Lentibulariaceae Genlisea margaretae#NC 025652
Lamiales Lentibulariaceae Genlisea tuberosa#NC 037082
Lamiales Lentibulariaceae Genlisea aurea#NC 037078
Lamiales Lentibulariaceae Genlisea filiformis#NC 037079
Lamiales Lentibulariaceae Genlisea repens#NC 037081
Lamiales Lentibulariaceae Genlisea pygmaea#NC 037080
Lamiales Martyniaceae Martynia annua#ZSD281
Lamiales Acanthaceae Thunbergia lutea#F129
Lamiales Acanthaceae Avicennia marina#F501
Lamiales Acanthaceae Aphelandra knappiae#NC 041424
Lamiales Acanthaceae Barleria cristata#F140
Lamiales Acanthaceae Andrographis paniculata#NC 022451
Lamiales Acanthaceae Phlogacanthus curviflorus#F1038
Lamiales Acanthaceae Asystasiella neesiana#F52
Lamiales Acanthaceae Anisacanthus quadrifidus#PCGJ
Lamiales Acanthaceae Clinacanthus nutans#MH778102
Lamiales Acanthaceae Echinacanthus longzhouensis#NC 039678
Lamiales Acanthaceae Echinacanthus lofuensis#NC 035876
Lamiales Acanthaceae Echinacanthus longipes#NC 039761
Lamiales Acanthaceae Ruellia simplex#AYIY
Lamiales Acanthaceae Sanchezia sp.#NBMW
Lamiales Acanthaceae Echinacanthus attenuatus#NC 039762
Lamiales Acanthaceae Strobilanthes cusia#NC 037485
Lamiales Acanthaceae Strobilanthes auriculatus#WEAC
Lamiales Acanthaceae Strobilanthes sp.#F42
Lamiales Acanthaceae Strobilanthes henryi#F67 1
Lamiales Verbenaceae Verbena hastata#GCFE
Lamiales Verbenaceae Verbena officinalis#F535
Lamiales Verbenaceae Aloysia citriodora#NC 034695
Lamiales Verbenaceae Phyla nodiflora#F528
Lamiales Verbenaceae Lantana camara#PSHB
Lamiales Verbenaceae Phyla scaberrima#MQIV
Lamiales Bignoniaceae Tecoma capensis#NC 037462
Lamiales Bignoniaceae Incarvillea diffusa#F173
Lamiales Bignoniaceae Handroanthus umbellatus#UTQR
Lamiales Bignoniaceae Kigelia africana#XAYK
Lamiales Bignoniaceae Markhamia stipulata#F247
Lamiales Bignoniaceae Pauldopia ghorta#F15
Lamiales Bignoniaceae Mayodendron igneum#F281
Lamiales Bignoniaceae Radermachera yunnanensis#F98
Lamiales Bignoniaceae Dolichandra cynanchoides#NC 037460
Lamiales Bignoniaceae Tanaecium tetragonolobum#NC 027955
Lamiales Bignoniaceae Mansoa alliacea#TKEK
Lamiales Bignoniaceae Anemopaegma oligoneuron#NC 037232
Lamiales Bignoniaceae Anemopaegma chamberlaynii#NC 037229
Lamiales Bignoniaceae Anemopaegma album#NC 037227
Lamiales Bignoniaceae Anemopaegma foetidum#NC 037230
Lamiales Bignoniaceae Anemopaegma arvense#MF460830
Lamiales Bignoniaceae Anemopaegma glaucum#MF460834
Lamiales Bignoniaceae Anemopaegma acutifolium#MF460827
Lamiales Bignoniaceae Adenocalymma pedunculatum#NC 036500
Lamiales Bignoniaceae Adenocalymma cristicalyx#NC 036498
Lamiales Bignoniaceae Pleonotoma albiflora#NC 037461
Lamiales Bignoniaceae Neojobertia candolleana#NC 036503
Lamiales Bignoniaceae Adenocalymma nodosum#NC 037458
Lamiales Bignoniaceae Adenocalymma biternatum#NC 036496
Lamiales Bignoniaceae Adenocalymma allamandiflorum#NC 036494
Lamiales Bignoniaceae Adenocalymma peregrinum#NC 036501
Lamiales Bignoniaceae Adenocalymma marginatum#NC 037457
Lamiales Bignoniaceae Adenocalymma subspicatum#NC 036502
Lamiales Bignoniaceae Adenocalymma divaricatum#NC 037456
Lamiales Bignoniaceae Adenocalymma aurantiacum#NC 036495
Lamiales Bignoniaceae Adenocalymma bracteatum#NC 036497
Lamiales Bignoniaceae Adenocalymma hatschbachii#NC 036499
Lamiales Bignoniaceae Adenocalymma trifoliatum#NC 037459
Lamiales Bignoniaceae Adenocalymma comosum#NC 037455
Lamiales Mazaceae Mazus miquelii#F864
Lamiales Mazaceae Lancea tibetica#F647
Lamiales Mazaceae Lancea hirsuta#NC 037506
Lamiales Wightiaceae Wightia speciosissima#J5854
Lamiales Phrymaceae Phryma leptostachya#F890
Lamiales Phrymaceae Erythranthe lutea#NC 030212
Lamiales Phrymaceae Mimulus tenellus var. platyphyllus#F825
Lamiales Paulowniaceae Paulownia fortunei#F180
Lamiales Paulowniaceae Paulownia fargesii#UMUL
Lamiales Orobanchaceae Triaenophora shennongjiaensis#NC 039781
Lamiales Orobanchaceae Rehmannia chingii#F895
Lamiales Orobanchaceae Rehmannia glutinosa#F1002
Lamiales Orobanchaceae Rehmannia elata#NC 034312
Lamiales Orobanchaceae Rehmannia piasezkii#NC 034311
Lamiales Orobanchaceae Rehmannia henryi#NC 034309
Lamiales Orobanchaceae Rehmannia solanifolia#NC 034310
Lamiales Orobanchaceae Lindenbergia muraria#F84
Lamiales Orobanchaceae Lindenbergia philippensis#EJCM
Lamiales Orobanchaceae Cymbaria mongolica#F789
Lamiales Orobanchaceae Schwalbea americana#NC 023115
Lamiales Orobanchaceae Lathraea squamaria#NC 027838 @para
Lamiales Orobanchaceae Brandisia racemosa#F46
Lamiales Orobanchaceae Phtheirospermum japonicum#F35
Lamiales Orobanchaceae Castilleja paramensis#NC 031805
Lamiales Orobanchaceae Pedicularis hallaisanensis#NC 037433 @para
Lamiales Orobanchaceae Pedicularis ishidoyana#NC 029700 @para
Lamiales Orobanchaceae Epifagus virginiana#FROP
Lamiales Orobanchaceae Conopholis americana#FAMO
Lamiales Orobanchaceae Cistanche deserticola#NC 021111 @para
Lamiales Orobanchaceae Cistanche phelypaea#NC 025642 @para
Lamiales Orobanchaceae Orobanche latisquama#NC 025641 @para
Lamiales Orobanchaceae Orobanche crenata#NC 024845 @para
Lamiales Orobanchaceae Orobanche gracilis#NC 023464 @para
Lamiales Orobanchaceae Orobanche ramosa#NC 023465 @para
Lamiales Orobanchaceae Orobanche purpurea#NC 023132 @para
Lamiales Orobanchaceae Orobanche californica#NC 025651 @para
Lamiales Orobanchaceae Aphyllon epigalium subsp. epigalium#MH050785
Lamiales Orobanchaceae Aphyllon epigalium subsp. notocalifornicum#MH050786
Lamiales Orobanchaceae Orobanche fasciculata#MH499249
Lamiales Orobanchaceae Aphyllon uniflorum var. occidentale#MH499250
Lamiales Orobanchaceae Aphyllon uniflorum#MH580290
Lamiales Lamiaceae Vitex agnus-castus#DMLT
Lamiales Lamiaceae Congea chinensis#F323
Lamiales Lamiaceae Congea tomentosa#F318
Lamiales Lamiaceae Lavandula angustifolia#FYUH
Lamiales Lamiaceae Isodon eriocalyx#F66

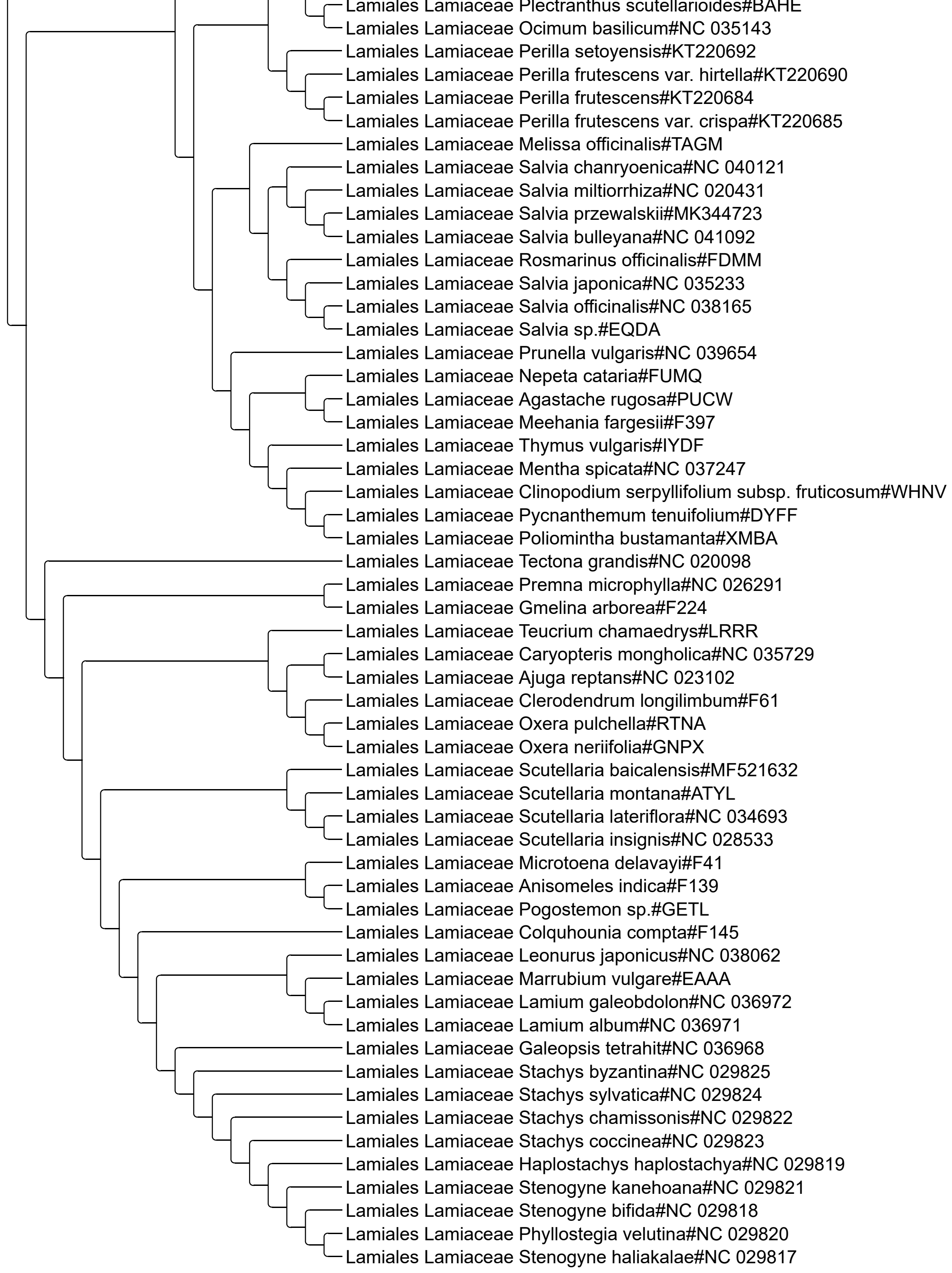

Lamiales Lamiaceae Plectranthus scutellarioides#BAHE
Lamiales Lamiaceae Ocimum basilicum#NC 035143
Lamiales Lamiaceae Perilla setoyensis#KT220692
Lamiales Lamiaceae Perilla frutescens var. hirtella#KT220690
Lamiales Lamiaceae Perilla frutescens#KT220684
Lamiales Lamiaceae Perilla frutescens var. crispa#KT220685
Lamiales Lamiaceae Melissa officinalis#TAGM
Lamiales Lamiaceae Salvia chanryoenica#NC 040121
Lamiales Lamiaceae Salvia miltiorrhiza#NC 020431
Lamiales Lamiaceae Salvia przewalskii#MK344723
Lamiales Lamiaceae Salvia bulleyana#NC 041092
Lamiales Lamiaceae Rosmarinus officinalis#FDMM
Lamiales Lamiaceae Salvia japonica#NC 035233
Lamiales Lamiaceae Salvia officinalis#NC 038165
Lamiales Lamiaceae Salvia sp.#EQDA
Lamiales Lamiaceae Prunella vulgaris#NC 039654
Lamiales Lamiaceae Nepeta cataria#FUMQ
Lamiales Lamiaceae Agastache rugosa#PUCW
Lamiales Lamiaceae Meehania fargesii#F397
Lamiales Lamiaceae Thymus vulgaris#IYDF
Lamiales Lamiaceae Mentha spicata#NC 037247
Lamiales Lamiaceae Clinopodium serpyllifolium subsp. fruticosum#WHNV
Lamiales Lamiaceae Pycnanthemum tenuifolium#DYFF
Lamiales Lamiaceae Poliomintha bustamanta#XMBA
Lamiales Lamiaceae Tectona grandis#NC 020098
Lamiales Lamiaceae Premna microphylla#NC 026291
Lamiales Lamiaceae Gmelina arborea#F224
Lamiales Lamiaceae Teucrium chamaedrys#LRRR
Lamiales Lamiaceae Caryopteris mongholica#NC 035729
Lamiales Lamiaceae Ajuga reptans#NC 023102
Lamiales Lamiaceae Clerodendrum longilimbum#F61
Lamiales Lamiaceae Oxera pulchella#RTNA
Lamiales Lamiaceae Oxera neriifolia#GNPX
Lamiales Lamiaceae Scutellaria baicalensis#MF521632
Lamiales Lamiaceae Scutellaria montana#ATYL
Lamiales Lamiaceae Scutellaria lateriflora#NC 034693
Lamiales Lamiaceae Scutellaria insignis#NC 028533
Lamiales Lamiaceae Microtoena delavayi#F41
Lamiales Lamiaceae Anisomeles indica#F139
Lamiales Lamiaceae Pogostemon sp.#GETL
Lamiales Lamiaceae Colquhounia compta#F145
Lamiales Lamiaceae Leonurus japonicus#NC 038062
Lamiales Lamiaceae Marrubium vulgare#EAAA
Lamiales Lamiaceae Lamium galeobdolon#NC 036972
Lamiales Lamiaceae Lamium album#NC 036971
Lamiales Lamiaceae Galeopsis tetrahit#NC 036968
Lamiales Lamiaceae Stachys byzantina#NC 029825
Lamiales Lamiaceae Stachys sylvatica#NC 029824
Lamiales Lamiaceae Stachys chamissonis#NC 029822
Lamiales Lamiaceae Stachys coccinea#NC 029823
Lamiales Lamiaceae Haplostachys haplostachya#NC 029819
Lamiales Lamiaceae Stenogyne kanehoana#NC 029821
Lamiales Lamiaceae Stenogyne bifida#NC 029818
Lamiales Lamiaceae Phyllostegia velutina#NC 029820
Lamiales Lamiaceae Stenogyne haliakalae#NC 029817

[Figure S8f]

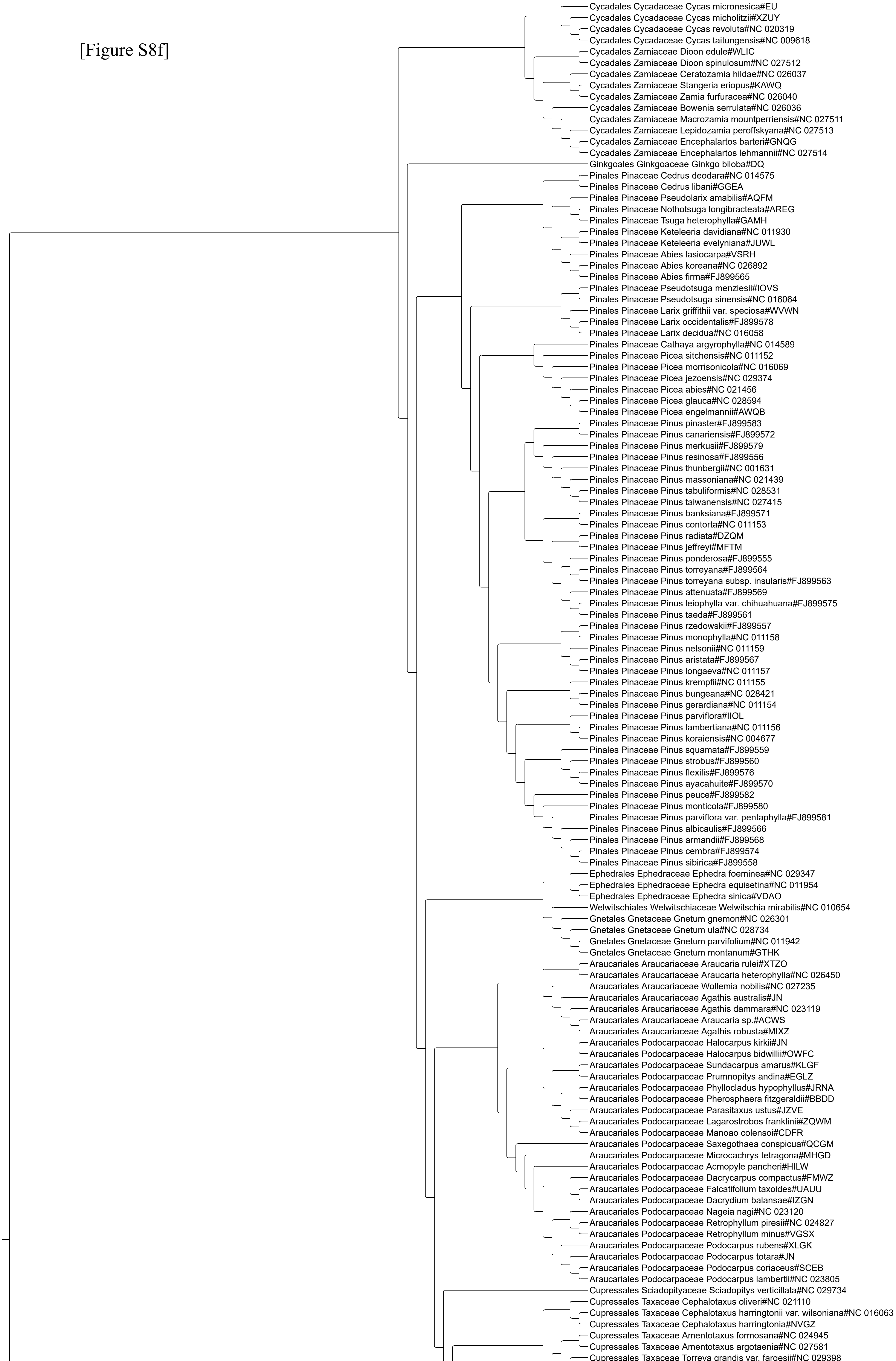
Cycadales Cycadaceae Cycas micronesica#EU
Cycadales Cycadaceae Cycas micholitzii#XZUY
Cycadales Cycadaceae Cycas revoluta#NC 020319
Cycadales Cycadaceae Cycas taitungensis#NC 009618
Cycadales Zamiaceae Dioon edule#WLIC
Cycadales Zamiaceae Dioon spinulosum#NC 027512
Cycadales Zamiaceae Ceratozamia hildae#NC 026037
Cycadales Zamiaceae Stangeria eriopus#KAWQ
Cycadales Zamiaceae Zamia furfuracea#NC 026040
Cycadales Zamiaceae Bowenia serrulata#NC 026036
Cycadales Zamiaceae Macrozamia mountperriensis#NC 027511
Cycadales Zamiaceae Lepidozamia peroffskyana#NC 027513
Cycadales Zamiaceae Encephalartos barteri#GNQG
Cycadales Zamiaceae Encephalartos lehmannii#NC 027514
Ginkgoales Ginkgoaceae Ginkgo biloba#DQ
Pinales Pinaceae Cedrus deodara#NC 014575
Pinales Pinaceae Cedrus libani#GGEA
Pinales Pinaceae Pseudolarix amabilis#AQFM
Pinales Pinaceae Nothotsuga longibracteata#AREG
Pinales Pinaceae Tsuga heterophylla#GAMH
Pinales Pinaceae Keteleeria davidiana#NC 011930
Pinales Pinaceae Keteleeria evelyniana#JUWL
Pinales Pinaceae Abies lasiocarpa#VSRH
Pinales Pinaceae Abies koreana#NC 026892
Pinales Pinaceae Abies firma#FJ899565
Pinales Pinaceae Pseudotsuga menziesii#IOVS
Pinales Pinaceae Pseudotsuga sinensis#NC 016064
Pinales Pinaceae Larix griffithii var. speciosa#WVWN
Pinales Pinaceae Larix occidentalis#FJ899578
Pinales Pinaceae Larix decidua#NC 016058
Pinales Pinaceae Cathaya argyrophylla#NC 014589
Pinales Pinaceae Picea sitchensis#NC 011152
Pinales Pinaceae Picea morrisonicola#NC 016069
Pinales Pinaceae Picea jezoensis#NC 029374
Pinales Pinaceae Picea abies#NC 021456
Pinales Pinaceae Picea glauca#NC 028594
Pinales Pinaceae Picea engelmannii#AWQB
Pinales Pinaceae Pinus pinaster#FJ899583
Pinales Pinaceae Pinus canariensis#FJ899572
Pinales Pinaceae Pinus merkusii#FJ899579
Pinales Pinaceae Pinus resinosa#FJ899556
Pinales Pinaceae Pinus thunbergii#NC 001631
Pinales Pinaceae Pinus massoniana#NC 021439
Pinales Pinaceae Pinus tabuliformis#NC 028531
Pinales Pinaceae Pinus taiwanensis#NC 027415
Pinales Pinaceae Pinus banksiana#FJ899571
Pinales Pinaceae Pinus contorta#NC 011153
Pinales Pinaceae Pinus radiata#DZQM
Pinales Pinaceae Pinus jeffreyi#MFTM
Pinales Pinaceae Pinus ponderosa#FJ899555
Pinales Pinaceae Pinus torreyana#FJ899564
Pinales Pinaceae Pinus torreyana subsp. insularis#FJ899563
Pinales Pinaceae Pinus attenuata#FJ899569
Pinales Pinaceae Pinus leiophylla var. chihuahuana#FJ899575
Pinales Pinaceae Pinus taeda#FJ899561
Pinales Pinaceae Pinus rzedowskii#FJ899557
Pinales Pinaceae Pinus monophylla#NC 011158
Pinales Pinaceae Pinus nelsonii#NC 011159
Pinales Pinaceae Pinus aristata#FJ899567
Pinales Pinaceae Pinus longaeva#NC 011157
Pinales Pinaceae Pinus krempfii#NC 011155
Pinales Pinaceae Pinus bungeana#NC 028421
Pinales Pinaceae Pinus gerardiana#NC 011154
Pinales Pinaceae Pinus parviflora#IIOL
Pinales Pinaceae Pinus lambertiana#NC 011156
Pinales Pinaceae Pinus koraiensis#NC 004677
Pinales Pinaceae Pinus squamata#FJ899559
Pinales Pinaceae Pinus strobus#FJ899560
Pinales Pinaceae Pinus flexilis#FJ899576
Pinales Pinaceae Pinus ayacahuite#FJ899570
Pinales Pinaceae Pinus peuce#FJ899582
Pinales Pinaceae Pinus monticola#FJ899580
Pinales Pinaceae Pinus parviflora var. pentaphylla#FJ899581
Pinales Pinaceae Pinus albicaulis#FJ899566
Pinales Pinaceae Pinus armandii#FJ899568
Pinales Pinaceae Pinus cembra#FJ899574
Pinales Pinaceae Pinus sibirica#FJ899558
Ephedrales Ephedraceae Ephedra foeminea#NC 029347
Ephedrales Ephedraceae Ephedra equisetina#NC 011954
Ephedrales Ephedraceae Ephedra sinica#VDAO
Welwitschiales Welwitschiaceae Welwitschia mirabilis#NC 010654
Gnetales Gnetaceae Gnetum gnemon#NC 026301
Gnetales Gnetaceae Gnetum ula#NC 028734
Gnetales Gnetaceae Gnetum parvifolium#NC 011942
Gnetales Gnetaceae Gnetum montanum#GTHK
Araucariales Araucariaceae Araucaria rulei#XTZO
Araucariales Araucariaceae Araucaria heterophylla#NC 026450
Araucariales Araucariaceae Wollemia nobilis#NC 027235
Araucariales Araucariaceae Agathis australis#JN
Araucariales Araucariaceae Agathis dammara#NC 023119
Araucariales Araucariaceae Araucaria sp.#ACWS
Araucariales Araucariaceae Agathis robusta#MIXZ
Araucariales Podocarpaceae Halocarpus kirkii#JN
Araucariales Podocarpaceae Halocarpus bidwillii#OWFC
Araucariales Podocarpaceae Sundacarpus amarus#KLGF
Araucariales Podocarpaceae Prumnopitys andina#EGLZ
Araucariales Podocarpaceae Phyllocladus hypophyllus#JRNA
Araucariales Podocarpaceae Pherosphaera fitzgeraldii#BBDD
Araucariales Podocarpaceae Parasitaxus ustus#JZVE
Araucariales Podocarpaceae Lagarostrobos franklinii#ZQWM
Araucariales Podocarpaceae Manoao colensoi#CDFR
Araucariales Podocarpaceae Saxegothaea conspicua#QCGM
Araucariales Podocarpaceae Microcachrys tetragona#MHGD
Araucariales Podocarpaceae Acmopyle pancheri#HILW
Araucariales Podocarpaceae Dacrycarpus compactus#FMWZ
Araucariales Podocarpaceae Falcatifolium taxoides#UAUU
Araucariales Podocarpaceae Dacrydium balansae#IZGN
Araucariales Podocarpaceae Nageia nagi#NC 023120
Araucariales Podocarpaceae Retrophyllum piresii#NC 024827
Araucariales Podocarpaceae Retrophyllum minus#VGSX
Araucariales Podocarpaceae Podocarpus rubens#XLGK
Araucariales Podocarpaceae Podocarpus totara#JN
Araucariales Podocarpaceae Podocarpus coriaceus#SCEB
Araucariales Podocarpaceae Podocarpus lambertii#NC 023805
Cupressales Sciadopityaceae Sciadopitys verticillata#NC 029734
Cupressales Taxaceae Cephalotaxus oliveri#NC 021110
Cupressales Taxaceae Cephalotaxus harringtonii var. wilsoniana#NC 016063
Cupressales Taxaceae Cephalotaxus harringtonia#NVGZ
Cupressales Taxaceae Amentotaxus formosana#NC 024945
Cupressales Taxaceae Amentotaxus argotaenia#NC 027581
Cupressales Taxaceae Torreya grandis var. fargesii#NC 029398

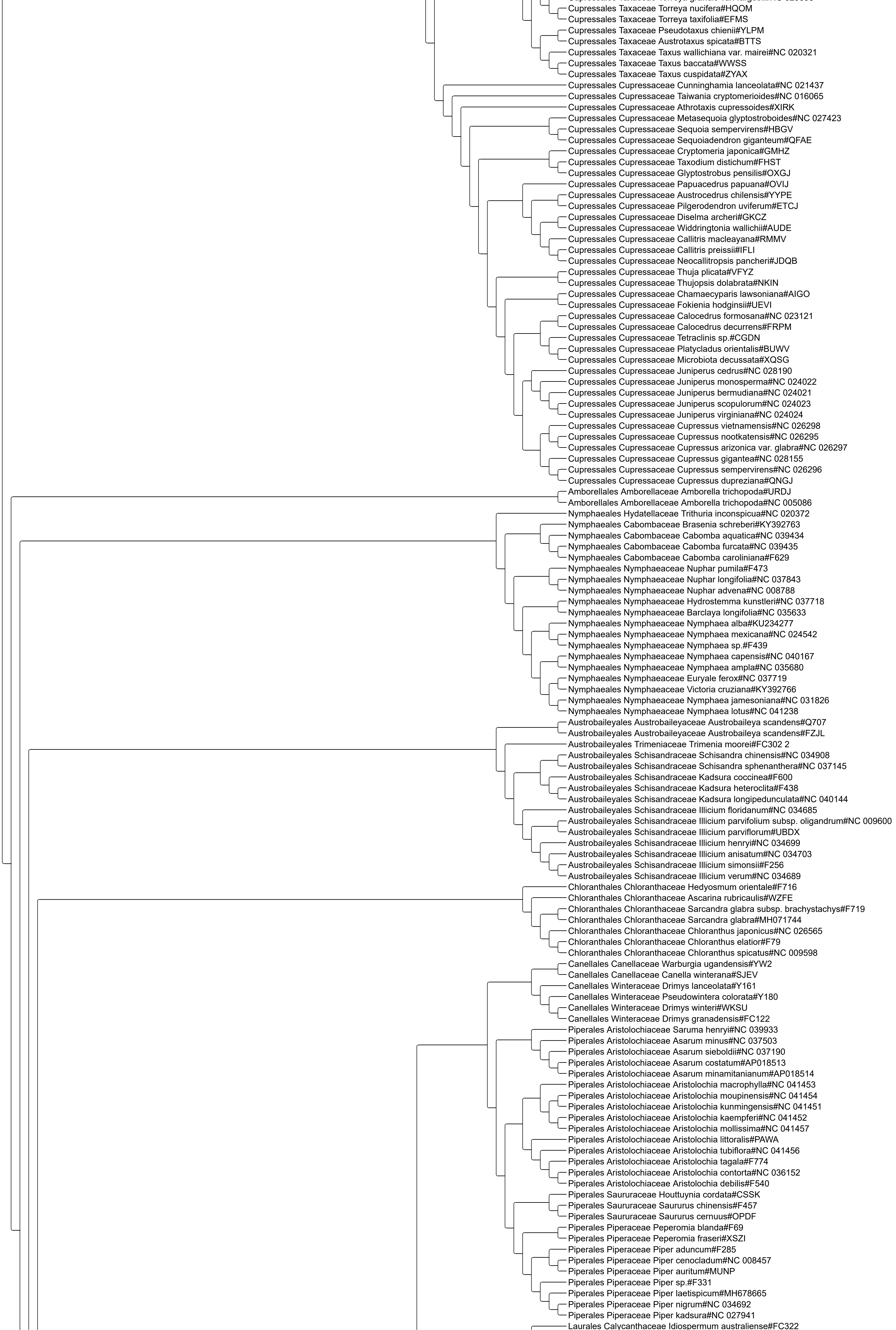
Cupressales Taxaceae Torreya nucifera#HQOM
Cupressales Taxaceae Torreya taxifolia#EFMS
Cupressales Taxaceae Pseudotaxus chienii#YLPM
Cupressales Taxaceae Austrotaxus spicata#BTTS
Cupressales Taxaceae Taxus wallichiana var. mairei#NC 020321
Cupressales Taxaceae Taxus baccata#WWSS
Cupressales Taxaceae Taxus cuspidata#ZYAX
Cupressales Cupressaceae Cunninghamia lanceolata#NC 021437
Cupressales Cupressaceae Taiwania cryptomerioides#NC 016065
Cupressales Cupressaceae Athrotaxis cupressoides#XIRK
Cupressales Cupressaceae Metasequoia glyptostroboides#NC 027423
Cupressales Cupressaceae Sequoia sempervirens#HBGV
Cupressales Cupressaceae Sequoiadendron giganteum#QFAE
Cupressales Cupressaceae Cryptomeria japonica#GMHZ
Cupressales Cupressaceae Taxodium distichum#FHST
Cupressales Cupressaceae Glyptostrobus pensilis#OXGJ
Cupressales Cupressaceae Papuacedrus papuana#OVIJ
Cupressales Cupressaceae Austrocedrus chilensis#YYPE
Cupressales Cupressaceae Pilgerodendron uviferum#ETCJ
Cupressales Cupressaceae Diselma archeri#GKCZ
Cupressales Cupressaceae Widdringtonia wallichii#AUDE
Cupressales Cupressaceae Callitris macleayana#RMMV
Cupressales Cupressaceae Callitris preissii#IFLI
Cupressales Cupressaceae Neocallitropsis pancheri#JDQB
Cupressales Cupressaceae Thuja plicata#VFYZ
Cupressales Cupressaceae Thujopsis dolabrata#NKIN
Cupressales Cupressaceae Chamaecyparis lawsoniana#AIGO
Cupressales Cupressaceae Fokienia hodginsii#UEVI
Cupressales Cupressaceae Calocedrus formosana#NC 023121
Cupressales Cupressaceae Calocedrus decurrens#FRPM
Cupressales Cupressaceae Tetraclinis sp.#CGDN
Cupressales Cupressaceae Platycladus orientalis#BUWV
Cupressales Cupressaceae Microbiota decussata#XQSG
Cupressales Cupressaceae Juniperus cedrus#NC 028190
Cupressales Cupressaceae Juniperus monosperma#NC 024022
Cupressales Cupressaceae Juniperus bermudiana#NC 024021
Cupressales Cupressaceae Juniperus scopulorum#NC 024023
Cupressales Cupressaceae Juniperus virginiana#NC 024024
Cupressales Cupressaceae Cupressus vietnamensis#NC 026298
Cupressales Cupressaceae Cupressus nootkatensis#NC 026295
Cupressales Cupressaceae Cupressus arizonica var. glabra#NC 026297
Cupressales Cupressaceae Cupressus gigantea#NC 028155
Cupressales Cupressaceae Cupressus sempervirens#NC 026296
Cupressales Cupressaceae Cupressus dupreziana#QNGJ
Amborellales Amborellaceae Amborella trichopoda#URDJ
Amborellales Amborellaceae Amborella trichopoda#NC 005086
Nymphaeales Hydatellaceae Trithuria inconspicua#NC 020372
Nymphaeales Cabombaceae Brasenia schreberi#KY392763
Nymphaeales Cabombaceae Cabomba aquatica#NC 039434
Nymphaeales Cabombaceae Cabomba furcata#NC 039435
Nymphaeales Cabombaceae Cabomba caroliniana#F629
Nymphaeales Nymphaeaceae Nuphar pumila#F473
Nymphaeales Nymphaeaceae Nuphar longifolia#NC 037843
Nymphaeales Nymphaeaceae Nuphar advena#NC 008788
Nymphaeales Nymphaeaceae Hydrostemma kunstleri#NC 037718
Nymphaeales Nymphaeaceae Barclaya longifolia#NC 035633
Nymphaeales Nymphaeaceae Nymphaea alba#KU234277
Nymphaeales Nymphaeaceae Nymphaea mexicana#NC 024542
Nymphaeales Nymphaeaceae Nymphaea sp.#F439
Nymphaeales Nymphaeaceae Nymphaea capensis#NC 040167
Nymphaeales Nymphaeaceae Nymphaea ampla#NC 035680
Nymphaeales Nymphaeaceae Euryale ferox#NC 037719
Nymphaeales Nymphaeaceae Victoria cruziana#KY392766
Nymphaeales Nymphaeaceae Nymphaea jamesoniana#NC 031826
Nymphaeales Nymphaeaceae Nymphaea lotus#NC 041238
Austrobaileyales Austrobaileyaceae Austrobaileya scandens#Q707
Austrobaileyales Austrobaileyaceae Austrobaileya scandens#FZJL
Austrobaileyales Trimeniaceae Trimenia moorei#FC302 2
Austrobaileyales Schisandraceae Schisandra chinensis#NC 034908
Austrobaileyales Schisandraceae Schisandra sphenanthera#NC 037145
Austrobaileyales Schisandraceae Kadsura coccinea#F600
Austrobaileyales Schisandraceae Kadsura heteroclita#F438
Austrobaileyales Schisandraceae Kadsura longipedunculata#NC 040144
Austrobaileyales Schisandraceae Illicium floridanum#NC 034685
Austrobaileyales Schisandraceae Illicium parvifolium subsp. oligandrum#NC 009600
Austrobaileyales Schisandraceae Illicium parviflorum#UBDX
Austrobaileyales Schisandraceae Illicium henryi#NC 034699
Austrobaileyales Schisandraceae Illicium anisatum#NC 034703
Austrobaileyales Schisandraceae Illicium simonsii#F256
Austrobaileyales Schisandraceae Illicium verum#NC 034689
Chloranthales Chloranthaceae Hedyosmum orientale#F716
Chloranthales Chloranthaceae Ascarina rubricaulis#WZFE
Chloranthales Chloranthaceae Sarcandra glabra subsp. brachystachys#F719
Chloranthales Chloranthaceae Sarcandra glabra#MH071744
Chloranthales Chloranthaceae Chloranthus japonicus#NC 026565
Chloranthales Chloranthaceae Chloranthus elatior#F79
Chloranthales Chloranthaceae Chloranthus spicatus#NC 009598
Canellales Canellaceae Warburgia ugandensis#YW2
Canellales Canellaceae Canella winterana#SJEV
Canellales Winteraceae Drimys lanceolata#Y161
Canellales Winteraceae Pseudowintera colorata#Y180
Canellales Winteraceae Drimys winteri#WKSU
Canellales Winteraceae Drimys granadensis#FC122
Piperales Aristolochiaceae Saruma henryi#NC 039933
Piperales Aristolochiaceae Asarum minus#NC 037503
Piperales Aristolochiaceae Asarum sieboldii#NC 037190
Piperales Aristolochiaceae Asarum costatum#AP018513
Piperales Aristolochiaceae Asarum minamitanianum#AP018514
Piperales Aristolochiaceae Aristolochia macrophylla#NC 041453
Piperales Aristolochiaceae Aristolochia moupinensis#NC 041454
Piperales Aristolochiaceae Aristolochia kunmingensis#NC 041451
Piperales Aristolochiaceae Aristolochia kaempferi#NC 041452
Piperales Aristolochiaceae Aristolochia mollissima#NC 041457
Piperales Aristolochiaceae Aristolochia littoralis#PAWA
Piperales Aristolochiaceae Aristolochia tubiflora#NC 041456
Piperales Aristolochiaceae Aristolochia tagala#F774
Piperales Aristolochiaceae Aristolochia contorta#NC 036152
Piperales Aristolochiaceae Aristolochia debilis#F540
Piperales Saururaceae Houttuynia cordata#CSSK
Piperales Saururaceae Saururus chinensis#F457
Piperales Saururaceae Saururus cernuus#OPDF
Piperales Piperaceae Peperomia blanda#F69
Piperales Piperaceae Peperomia fraseri#XSZI
Piperales Piperaceae Piper aduncum#F285
Piperales Piperaceae Piper cenocladum#NC 008457
Piperales Piperaceae Piper auritum#MUNP
Piperales Piperaceae Piper sp.#F331
Piperales Piperaceae Piper laetispicum#MH678665
Piperales Piperaceae Piper nigrum#NC 034692
Piperales Piperaceae Piper kadsura#NC 027941
Laurales Calycanthaceae Idiospermum australiense#FC322

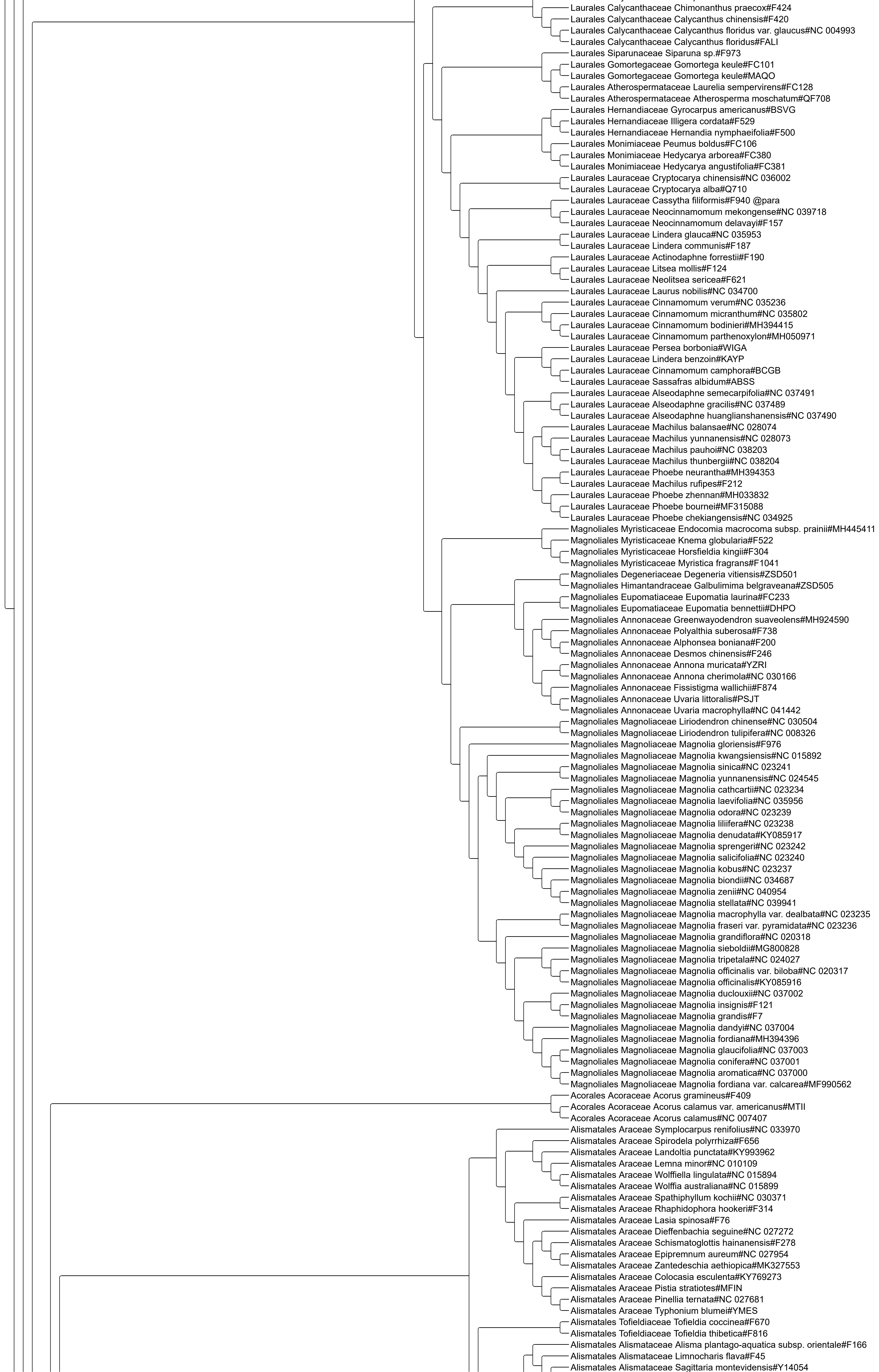

Laurales Calycanthaceae Chimonanthus praecox#F424
Laurales Calycanthaceae Calycanthus chinensis#F420
Laurales Calycanthaceae Calycanthus floridus var. glaucus#NC 004993
Laurales Calycanthaceae Calycanthus floridus#FALI
Laurales Siparunaceae Siparuna sp.#F973
Laurales Gomortegaceae Gomortega keule#FC101
Laurales Gomortegaceae Gomortega keule#MAQO
Laurales Atherospermataceae Laurelia sempervirens#FC128
Laurales Atherospermataceae Atherosperma moschatum#QF708
Laurales Hernandiaceae Gyrocarpus americanus#BSVG
Laurales Hernandiaceae Illigera cordata#F529
Laurales Hernandiaceae Hernandia nymphaeifolia#F500
Laurales Monimiaceae Peumus boldus#FC106
Laurales Monimiaceae Hedycarya arborea#FC380
Laurales Monimiaceae Hedycarya angustifolia#FC381
Laurales Lauraceae Cryptocarya chinensis#NC 036002
Laurales Lauraceae Cryptocarya alba#Q710
Laurales Lauraceae Cassytha filiformis#F940 @para
Laurales Lauraceae Neocinnamomum mekongense#NC 039718
Laurales Lauraceae Neocinnamomum delavayi#F157
Laurales Lauraceae Lindera glauca#NC 035953
Laurales Lauraceae Lindera communis#F187
Laurales Lauraceae Actinodaphne forrestii#F190
Laurales Lauraceae Litsea mollis#F124
Laurales Lauraceae Neolitsea sericea#F621
Laurales Lauraceae Laurus nobilis#NC 034700
Laurales Lauraceae Cinnamomum verum#NC 035236
Laurales Lauraceae Cinnamomum micranthum#NC 035802
Laurales Lauraceae Cinnamomum bodinieri#MH394415
Laurales Lauraceae Cinnamomum parthenoxylon#MH050971
Laurales Lauraceae Persea borbonia#WIGA
Laurales Lauraceae Lindera benzoin#KAYP
Laurales Lauraceae Cinnamomum camphora#BCGB
Laurales Lauraceae Sassafras albidum#ABSS
Laurales Lauraceae Alseodaphne semecarpifolia#NC 037491
Laurales Lauraceae Alseodaphne gracilis#NC 037489
Laurales Lauraceae Alseodaphne huanglianshanensis#NC 037490
Laurales Lauraceae Machilus balansae#NC 028074
Laurales Lauraceae Machilus yunnanensis#NC 028073
Laurales Lauraceae Machilus pauhoi#NC 038203
Laurales Lauraceae Machilus thunbergii#NC 038204
Laurales Lauraceae Phoebe neurantha#MH394353
Laurales Lauraceae Machilus rufipes#F212
Laurales Lauraceae Phoebe zhennan#MH033832
Laurales Lauraceae Phoebe bournei#MF315088
Laurales Lauraceae Phoebe chekiangensis#NC 034925
Magnoliales Myristicaceae Endocomia macrocoma subsp. prainii#MH445411
Magnoliales Myristicaceae Knema globularia#F522
Magnoliales Myristicaceae Horsfieldia kingii#F304
Magnoliales Myristicaceae Myristica fragrans#F1041
Magnoliales Degeneriaceae Degeneria vitiensis#ZSD501
Magnoliales Himantandraceae Galbulimima belgraveana#ZSD505
Magnoliales Eupomatiaceae Eupomatia laurina#FC233
Magnoliales Eupomatiaceae Eupomatia bennettii#DHPO
Magnoliales Annonaceae Greenwayodendron suaveolens#MH924590
Magnoliales Annonaceae Polyalthia suberosa#F738
Magnoliales Annonaceae Alphonsea boniana#F200
Magnoliales Annonaceae Desmos chinensis#F246
Magnoliales Annonaceae Annona muricata#YZRI
Magnoliales Annonaceae Annona cherimola#NC 030166
Magnoliales Annonaceae Fissistigma wallichii#F874
Magnoliales Annonaceae Uvaria littoralis#PSJT
Magnoliales Annonaceae Uvaria macrophylla#NC 041442
Magnoliales Magnoliaceae Liriodendron chinense#NC 030504
Magnoliales Magnoliaceae Liriodendron tulipifera#NC 008326
Magnoliales Magnoliaceae Magnolia gloriensis#F976
Magnoliales Magnoliaceae Magnolia kwangsiensis#NC 015892
Magnoliales Magnoliaceae Magnolia sinica#NC 023241
Magnoliales Magnoliaceae Magnolia yunnanensis#NC 024545
Magnoliales Magnoliaceae Magnolia cathcartii#NC 023234
Magnoliales Magnoliaceae Magnolia laevifolia#NC 035956
Magnoliales Magnoliaceae Magnolia odora#NC 023239
Magnoliales Magnoliaceae Magnolia liliifera#NC 023238
Magnoliales Magnoliaceae Magnolia denudata#KY085917
Magnoliales Magnoliaceae Magnolia sprengeri#NC 023242
Magnoliales Magnoliaceae Magnolia salicifolia#NC 023240
Magnoliales Magnoliaceae Magnolia kobus#NC 023237
Magnoliales Magnoliaceae Magnolia biondii#NC 034687
Magnoliales Magnoliaceae Magnolia zenii#NC 040954
Magnoliales Magnoliaceae Magnolia stellata#NC 039941
Magnoliales Magnoliaceae Magnolia macrophylla var. dealbata#NC 023235
Magnoliales Magnoliaceae Magnolia fraseri var. pyramidata#NC 023236
Magnoliales Magnoliaceae Magnolia grandiflora#NC 020318
Magnoliales Magnoliaceae Magnolia sieboldii#MG800828
Magnoliales Magnoliaceae Magnolia tripetala#NC 024027
Magnoliales Magnoliaceae Magnolia officinalis var. biloba#NC 020317
Magnoliales Magnoliaceae Magnolia officinalis#KY085916
Magnoliales Magnoliaceae Magnolia duclouxii#NC 037002
Magnoliales Magnoliaceae Magnolia insignis#F121
Magnoliales Magnoliaceae Magnolia grandis#F7
Magnoliales Magnoliaceae Magnolia dandyi#NC 037004
Magnoliales Magnoliaceae Magnolia fordiana#MH394396
Magnoliales Magnoliaceae Magnolia glaucifolia#NC 037003
Magnoliales Magnoliaceae Magnolia conifera#NC 037001
Magnoliales Magnoliaceae Magnolia aromatica#NC 037000
Magnoliales Magnoliaceae Magnolia fordiana var. calcarea#MF990562
Acorales Acoraceae Acorus gramineus#F409
Acorales Acoraceae Acorus calamus var. americanus#MTII
Acorales Acoraceae Acorus calamus#NC 007407
Alismatales Araceae Symplocarpus renifolius#NC 033970
Alismatales Araceae Spirodela polyrrhiza#F656
Alismatales Araceae Landoltia punctata#KY993962
Alismatales Araceae Lemna minor#NC 010109
Alismatales Araceae Wolffiella lingulata#NC 015894
Alismatales Araceae Wolffia australiana#NC 015899
Alismatales Araceae Spathiphyllum kochii#NC 030371
Alismatales Araceae Rhaphidophora hookeri#F314
Alismatales Araceae Lasia spinosa#F76
Alismatales Araceae Dieffenbachia seguine#NC 027272
Alismatales Araceae Schismatoglottis hainanensis#F278
Alismatales Araceae Epipremnum aureum#NC 027954
Alismatales Araceae Zantedeschia aethiopica#MK327553
Alismatales Araceae Colocasia esculenta#KY769273
Alismatales Araceae Pistia stratiotes#MFIN
Alismatales Araceae Pinellia ternata#NC 027681
Alismatales Araceae Typhonium blumei#YMES
Alismatales Tofieldiaceae Tofieldia coccinea#F670
Alismatales Tofieldiaceae Tofieldia thibetica#F816
Alismatales Alismataceae Alisma plantago-aquatica subsp. orientale#F166
Alismatales Alismataceae Limnocharis flava#F45
Alismatales Alismataceae Sagittaria montevidensis#Y14054

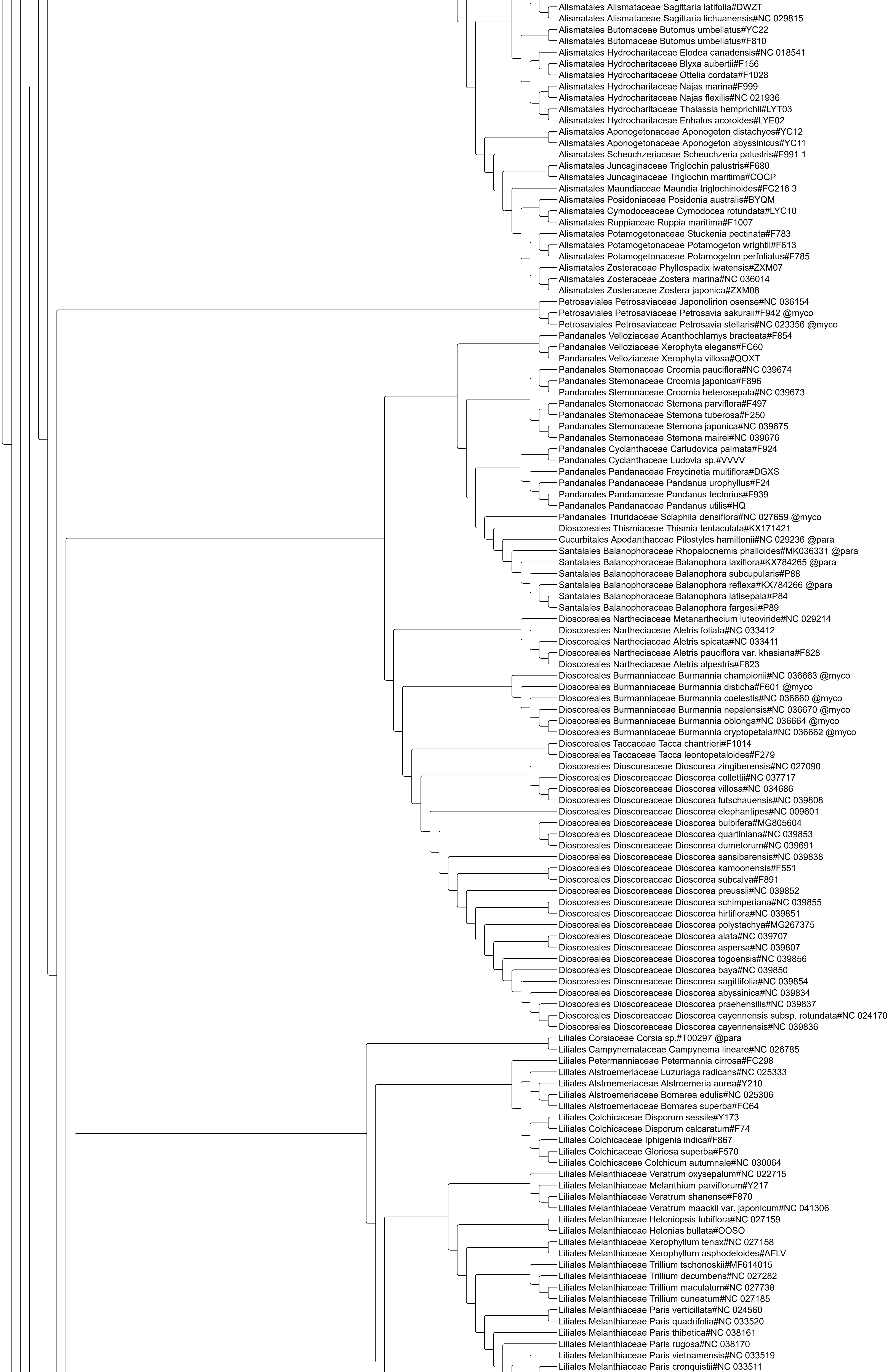
Alismatales Alismataceae Sagittaria latifolia#DWZT
Alismatales Alismataceae Sagittaria lichuanensis#NC 029815
Alismatales Butomaceae Butomus umbellatus#YC22
Alismatales Butomaceae Butomus umbellatus#F810
Alismatales Hydrocharitaceae Elodea canadensis#NC 018541
Alismatales Hydrocharitaceae Blyxa aubertii#F156
Alismatales Hydrocharitaceae Ottelia cordata#F1028
Alismatales Hydrocharitaceae Najas marina#F999
Alismatales Hydrocharitaceae Najas flexilis#NC 021936
Alismatales Hydrocharitaceae Thalassia hemprichii#LYT03
Alismatales Hydrocharitaceae Enhalus acoroides#LYE02
Alismatales Aponogetonaceae Aponogeton distachyos#YC12
Alismatales Aponogetonaceae Aponogeton abyssinicus#YC11
Alismatales Scheuchzeriaceae Scheuchzeria palustris#F991 1
Alismatales Juncaginaceae Triglochin palustris#F680
Alismatales Juncaginaceae Triglochin maritima#COCP
Alismatales Maundiaceae Maundia triglochinoides#FC216 3
Alismatales Posidoniaceae Posidonia australis#BYQM
Alismatales Cymodoceaceae Cymodocea rotundata#LYC10
Alismatales Ruppiaceae Ruppia maritima#F1007
Alismatales Potamogetonaceae Stuckenia pectinata#F783
Alismatales Potamogetonaceae Potamogeton wrightii#F613
Alismatales Potamogetonaceae Potamogeton perfoliatus#F785
Alismatales Zosteraceae Phyllospadix iwatensis#ZXM07
Alismatales Zosteraceae Zostera marina#NC 036014
Alismatales Zosteraceae Zostera japonica#ZXM08
Petrosaviales Petrosaviaceae Japonolirion osense#NC 036154
Petrosaviales Petrosaviaceae Petrosavia sakuraii#F942 @myco
Petrosaviales Petrosaviaceae Petrosavia stellaris#NC 023356 @myco
Pandanales Velloziaceae Acanthochlamys bracteata#F854
Pandanales Velloziaceae Xerophyta elegans#FC60
Pandanales Velloziaceae Xerophyta villosa#QOXT
Pandanales Stemonaceae Croomia pauciflora#NC 039674
Pandanales Stemonaceae Croomia japonica#F896
Pandanales Stemonaceae Croomia heterosepala#NC 039673
Pandanales Stemonaceae Stemona parviflora#F497
Pandanales Stemonaceae Stemona tuberosa#F250
Pandanales Stemonaceae Stemona japonica#NC 039675
Pandanales Stemonaceae Stemona mairei#NC 039676
Pandanales Cyclanthaceae Carludovica palmata#F924
Pandanales Cyclanthaceae Ludovia sp.#VVVV
Pandanales Pandanaceae Freycinetia multiflora#DGXS
Pandanales Pandanaceae Pandanus urophyllus#F24
Pandanales Pandanaceae Pandanus tectorius#F939
Pandanales Pandanaceae Pandanus utilis#HQ
Pandanales Triuridaceae Sciaphila densiflora#NC 027659 @myco
Dioscoreales Thismiaceae Thismia tentaculata#KX171421
Cucurbitales Apodanthaceae Pilostyles hamiltonii#NC 029236 @para
Santalales Balanophoraceae Rhopalocnemis phalloides#MK036331 @para
Santalales Balanophoraceae Balanophora laxiflora#KX784265 @para
Santalales Balanophoraceae Balanophora subcupularis#P88
Santalales Balanophoraceae Balanophora reflexa#KX784266 @para
Santalales Balanophoraceae Balanophora latisepala#P84
Santalales Balanophoraceae Balanophora fargesii#P89
Dioscoreales Nartheciaceae Metanarthecium luteoviride#NC 029214
Dioscoreales Nartheciaceae Aletris foliata#NC 033412
Dioscoreales Nartheciaceae Aletris spicata#NC 033411
Dioscoreales Nartheciaceae Aletris pauciflora var. khasiana#F828
Dioscoreales Nartheciaceae Aletris alpestris#F823
Dioscoreales Burmanniaceae Burmannia championii#NC 036663 @myco
Dioscoreales Burmanniaceae Burmannia disticha#F601 @myco
Dioscoreales Burmanniaceae Burmannia coelestis#NC 036660 @myco
Dioscoreales Burmanniaceae Burmannia nepalensis#NC 036670 @myco
Dioscoreales Burmanniaceae Burmannia oblonga#NC 036664 @myco
Dioscoreales Burmanniaceae Burmannia cryptopetala#NC 036662 @myco
Dioscoreales Taccaceae Tacca chantrieri#F1014
Dioscoreales Taccaceae Tacca leontopetaloides#F279
Dioscoreales Dioscoreaceae Dioscorea zingiberensis#NC 027090
Dioscoreales Dioscoreaceae Dioscorea collettii#NC 037717
Dioscoreales Dioscoreaceae Dioscorea villosa#NC 034686
Dioscoreales Dioscoreaceae Dioscorea futschauensis#NC 039808
Dioscoreales Dioscoreaceae Dioscorea elephantipes#NC 009601
Dioscoreales Dioscoreaceae Dioscorea bulbifera#MG805604
Dioscoreales Dioscoreaceae Dioscorea quartiniana#NC 039853
Dioscoreales Dioscoreaceae Dioscorea dumetorum#NC 039691
Dioscoreales Dioscoreaceae Dioscorea sansibarensis#NC 039838
Dioscoreales Dioscoreaceae Dioscorea kamoonensis#F551
Dioscoreales Dioscoreaceae Dioscorea subcalva#F891
Dioscoreales Dioscoreaceae Dioscorea preussii#NC 039852
Dioscoreales Dioscoreaceae Dioscorea schimperiana#NC 039855
Dioscoreales Dioscoreaceae Dioscorea hirtiflora#NC 039851
Dioscoreales Dioscoreaceae Dioscorea polystachya#MG267375
Dioscoreales Dioscoreaceae Dioscorea alata#NC 039707
Dioscoreales Dioscoreaceae Dioscorea aspersa#NC 039807
Dioscoreales Dioscoreaceae Dioscorea togoensis#NC 039856
Dioscoreales Dioscoreaceae Dioscorea baya#NC 039850
Dioscoreales Dioscoreaceae Dioscorea sagittifolia#NC 039854
Dioscoreales Dioscoreaceae Dioscorea abyssinica#NC 039834
Dioscoreales Dioscoreaceae Dioscorea praehensilis#NC 039837
Dioscoreales Dioscoreaceae Dioscorea cayennensis subsp. rotundata#NC 024170
Dioscoreales Dioscoreaceae Dioscorea cayennensis#NC 039836
Liliales Corsiaceae Corsia sp.#T00297 @para
Liliales Campynemataceae Campynema lineare#NC 026785
Liliales Petermanniaceae Petermannia cirrosa#FC298
Liliales Alstroemeriaceae Luzuriaga radicans#NC 025333
Liliales Alstroemeriaceae Alstroemeria aurea#Y210
Liliales Alstroemeriaceae Bomarea edulis#NC 025306
Liliales Alstroemeriaceae Bomarea superba#FC64
Liliales Colchicaceae Disporum sessile#Y173
Liliales Colchicaceae Disporum calcaratum#F74
Liliales Colchicaceae Iphigenia indica#F867
Liliales Colchicaceae Gloriosa superba#F570
Liliales Colchicaceae Colchicum autumnale#NC 030064
Liliales Melanthiaceae Veratrum oxysepalum#NC 022715
Liliales Melanthiaceae Melanthium parviflorum#Y217
Liliales Melanthiaceae Veratrum shanense#F870
Liliales Melanthiaceae Veratrum maackii var. japonicum#NC 041306
Liliales Melanthiaceae Heloniopsis tubiflora#NC 027159
Liliales Melanthiaceae Helonias bullata#OOSO
Liliales Melanthiaceae Xerophyllum tenax#NC 027158
Liliales Melanthiaceae Xerophyllum asphodeloides#AFLV
Liliales Melanthiaceae Trillium tschonoskii#MF614015
Liliales Melanthiaceae Trillium decumbens#NC 027282
Liliales Melanthiaceae Trillium maculatum#NC 027738
Liliales Melanthiaceae Trillium cuneatum#NC 027185
Liliales Melanthiaceae Paris verticillata#NC 024560
Liliales Melanthiaceae Paris quadrifolia#NC 033520
Liliales Melanthiaceae Paris thibetica#NC 038161
Liliales Melanthiaceae Paris rugosa#NC 038170
Liliales Melanthiaceae Paris vietnamensis#NC 033519
Liliales Melanthiaceae Paris cronquistii#NC 033511

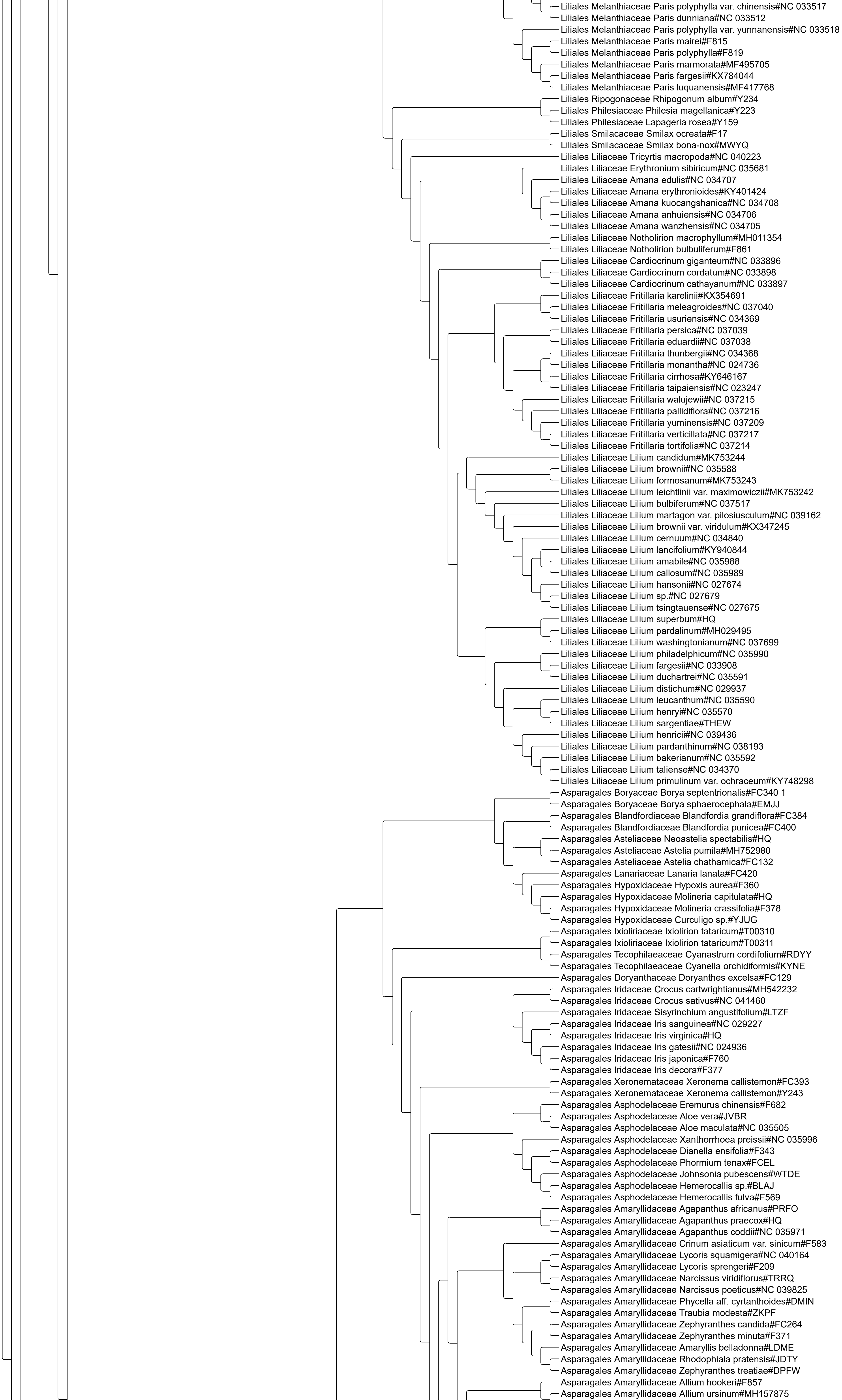

Liliales Melanthiaceae Paris polyphylla var. chinensis#NC 033517
Liliales Melanthiaceae Paris dunniana#NC 033512
Liliales Melanthiaceae Paris polyphylla var. yunnanensis#NC 033518
Liliales Melanthiaceae Paris mairei#F815
Liliales Melanthiaceae Paris polyphylla#F819
Liliales Melanthiaceae Paris marmorata#MF495705
Liliales Melanthiaceae Paris fargesii#KX784044
Liliales Melanthiaceae Paris luquanensis#MF417768
Liliales Ripogonaceae Rhipogonum album#Y234
Liliales Philesiaceae Philesia magellanica#Y223
Liliales Philesiaceae Lapageria rosea#Y159
Liliales Smilacaceae Smilax ocreata#F17
Liliales Smilacaceae Smilax bona-nox#MWYQ
Liliales Liliaceae Tricyrtis macropoda#NC 040223
Liliales Liliaceae Erythronium sibiricum#NC 035681
Liliales Liliaceae Amana edulis#NC 034707
Liliales Liliaceae Amana erythronioides#KY401424
Liliales Liliaceae Amana kuocangshanica#NC 034708
Liliales Liliaceae Amana anhuiensis#NC 034706
Liliales Liliaceae Amana wanzhensis#NC 034705
Liliales Liliaceae Notholirion macrophyllum#MH011354
Liliales Liliaceae Notholirion bulbuliferum#F861
Liliales Liliaceae Cardiocrinum giganteum#NC 033896
Liliales Liliaceae Cardiocrinum cordatum#NC 033898
Liliales Liliaceae Cardiocrinum cathayanum#NC 033897
Liliales Liliaceae Fritillaria karelinii#KX354691
Liliales Liliaceae Fritillaria meleagroides#NC 037040
Liliales Liliaceae Fritillaria usuriensis#NC 034369
Liliales Liliaceae Fritillaria persica#NC 037039
Liliales Liliaceae Fritillaria eduardii#NC 037038
Liliales Liliaceae Fritillaria thunbergii#NC 034368
Liliales Liliaceae Fritillaria monantha#NC 024736
Liliales Liliaceae Fritillaria cirrhosa#KY646167
Liliales Liliaceae Fritillaria taipaiensis#NC 023247
Liliales Liliaceae Fritillaria walujewii#NC 037215
Liliales Liliaceae Fritillaria pallidiflora#NC 037216
Liliales Liliaceae Fritillaria yuminensis#NC 037209
Liliales Liliaceae Fritillaria verticillata#NC 037217
Liliales Liliaceae Fritillaria tortifolia#NC 037214
Liliales Liliaceae Lilium candidum#MK753244
Liliales Liliaceae Lilium brownii#NC 035588
Liliales Liliaceae Lilium formosanum#MK753243
Liliales Liliaceae Lilium leichtlinii var. maximowiczii#MK753242
Liliales Liliaceae Lilium bulbiferum#NC 037517
Liliales Liliaceae Lilium martagon var. pilosiusculum#NC 039162
Liliales Liliaceae Lilium brownii var. viridulum#KX347245
Liliales Liliaceae Lilium cernuum#NC 034840
Liliales Liliaceae Lilium lancifolium#KY940844
Liliales Liliaceae Lilium amabile#NC 035988
Liliales Liliaceae Lilium callosum#NC 035989
Liliales Liliaceae Lilium hansonii#NC 027674
Liliales Liliaceae Lilium sp.#NC 027679
Liliales Liliaceae Lilium tsingtauense#NC 027675
Liliales Liliaceae Lilium superbum#HQ
Liliales Liliaceae Lilium pardalinum#MH029495
Liliales Liliaceae Lilium washingtonianum#NC 037699
Liliales Liliaceae Lilium philadelphicum#NC 035990
Liliales Liliaceae Lilium fargesii#NC 033908
Liliales Liliaceae Lilium duchartrei#NC 035591
Liliales Liliaceae Lilium distichum#NC 029937
Liliales Liliaceae Lilium leucanthum#NC 035590
Liliales Liliaceae Lilium henryi#NC 035570
Liliales Liliaceae Lilium sargentiae#THEW
Liliales Liliaceae Lilium henricii#NC 039436
Liliales Liliaceae Lilium pardanthinum#NC 038193
Liliales Liliaceae Lilium bakerianum#NC 035592
Liliales Liliaceae Lilium taliense#NC 034370
Liliales Liliaceae Lilium primulinum var. ochraceum#KY748298
Asparagales Boryaceae Borya septentrionalis#FC340 1
Asparagales Boryaceae Borya sphaerocephala#EMJJ
Asparagales Blandfordiaceae Blandfordia grandiflora#FC384
Asparagales Blandfordiaceae Blandfordia punicea#FC400
Asparagales Asteliaceae Neoastelia spectabilis#HQ
Asparagales Asteliaceae Astelia pumila#MH752980
Asparagales Asteliaceae Astelia chathamica#FC132
Asparagales Lanariaceae Lanaria lanata#FC420
Asparagales Hypoxidaceae Hypoxis aurea#F360
Asparagales Hypoxidaceae Molineria capitulata#HQ
Asparagales Hypoxidaceae Molineria crassifolia#F378
Asparagales Hypoxidaceae Curculigo sp.#YJUG
Asparagales Ixioliriaceae Ixiolirion tataricum#T00310
Asparagales Ixioliriaceae Ixiolirion tataricum#T00311
Asparagales Tecophilaeaceae Cyanastrum cordifolium#RDYY
Asparagales Tecophilaeaceae Cyanella orchidiformis#KYNE
Asparagales Doryanthaceae Doryanthes excelsa#FC129
Asparagales Iridaceae Crocus cartwrightianus#MH542232
Asparagales Iridaceae Crocus sativus#NC 041460
Asparagales Iridaceae Sisyrinchium angustifolium#LTZF
Asparagales Iridaceae Iris sanguinea#NC 029227
Asparagales Iridaceae Iris virginica#HQ
Asparagales Iridaceae Iris gatesii#NC 024936
Asparagales Iridaceae Iris japonica#F760
Asparagales Iridaceae Iris decora#F377
Asparagales Xeronemataceae Xeronema callistemon#FC393
Asparagales Xeronemataceae Xeronema callistemon#Y243
Asparagales Asphodelaceae Eremurus chinensis#F682
Asparagales Asphodelaceae Aloe vera#JVBR
Asparagales Asphodelaceae Aloe maculata#NC 035505
Asparagales Asphodelaceae Xanthorrhoea preissii#NC 035996
Asparagales Asphodelaceae Dianella ensifolia#F343
Asparagales Asphodelaceae Phormium tenax#FCEL
Asparagales Asphodelaceae Johnsonia pubescens#WTDE
Asparagales Asphodelaceae Hemerocallis sp.#BLAJ
Asparagales Asphodelaceae Hemerocallis fulva#F569
Asparagales Amaryllidaceae Agapanthus africanus#PRFO
Asparagales Amaryllidaceae Agapanthus praecox#HQ
Asparagales Amaryllidaceae Agapanthus coddii#NC 035971
Asparagales Amaryllidaceae Crinum asiaticum var. sinicum#F583
Asparagales Amaryllidaceae Lycoris squamigera#NC 040164
Asparagales Amaryllidaceae Lycoris sprengeri#F209
Asparagales Amaryllidaceae Narcissus viridiflorus#TRRQ
Asparagales Amaryllidaceae Narcissus poeticus#NC 039825
Asparagales Amaryllidaceae Phycella aff. cyrtanthoides#DMIN
Asparagales Amaryllidaceae Traubia modesta#ZKPF
Asparagales Amaryllidaceae Zephyranthes candida#FC264
Asparagales Amaryllidaceae Zephyranthes minuta#F371
Asparagales Amaryllidaceae Amaryllis belladonna#LDME
Asparagales Amaryllidaceae Rhodophiala pratensis#JDTY
Asparagales Amaryllidaceae Zephyranthes treatiae#DPFW
Asparagales Amaryllidaceae Allium hookeri#F857
Asparagales Amaryllidaceae Allium ursinum#MH157875

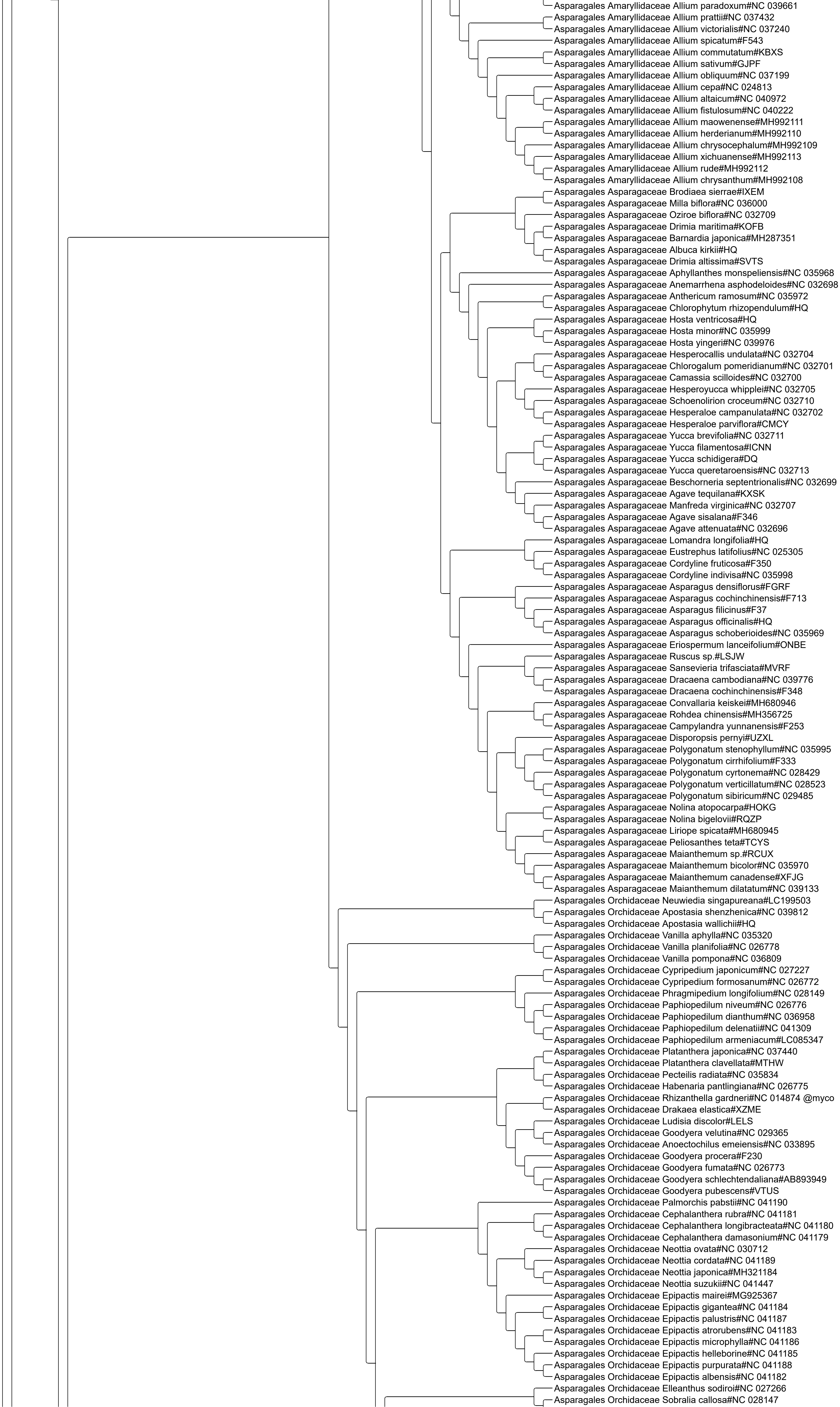

Asparagales Amaryllidaceae Allium paradoxum#NC 039661
Asparagales Amaryllidaceae Allium prattii#NC 037432
Asparagales Amaryllidaceae Allium victorialis#NC 037240
Asparagales Amaryllidaceae Allium spicatum#F543
Asparagales Amaryllidaceae Allium commutatum#KBXS
Asparagales Amaryllidaceae Allium sativum#GJPF
Asparagales Amaryllidaceae Allium obliquum#NC 037199
Asparagales Amaryllidaceae Allium cepa#NC 024813
Asparagales Amaryllidaceae Allium altaicum#NC 040972
Asparagales Amaryllidaceae Allium fistulosum#NC 040222
Asparagales Amaryllidaceae Allium maowenense#MH992111
Asparagales Amaryllidaceae Allium herderianum#MH992110
Asparagales Amaryllidaceae Allium chrysocephalum#MH992109
Asparagales Amaryllidaceae Allium xichuanense#MH992113
Asparagales Amaryllidaceae Allium rude#MH992112
Asparagales Amaryllidaceae Allium chrysanthum#MH992108
Asparagales Asparagaceae Brodiaea sierrae#IXEM
Asparagales Asparagaceae Milla biflora#NC 036000
Asparagales Asparagaceae Oziroe biflora#NC 032709
Asparagales Asparagaceae Drimia maritima#KOFB
Asparagales Asparagaceae Barnardia japonica#MH287351
Asparagales Asparagaceae Albuca kirkii#HQ
Asparagales Asparagaceae Drimia altissima#SVTS
Asparagales Asparagaceae Aphyllanthes monspeliensis#NC 035968
Asparagales Asparagaceae Anemarrhena asphodeloides#NC 032698
Asparagales Asparagaceae Anthericum ramosum#NC 035972
Asparagales Asparagaceae Chlorophytum rhizopendulum#HQ
Asparagales Asparagaceae Hosta ventricosa#HQ
Asparagales Asparagaceae Hosta minor#NC 035999
Asparagales Asparagaceae Hosta yingeri#NC 039976
Asparagales Asparagaceae Hesperocallis undulata#NC 032704
Asparagales Asparagaceae Chlorogalum pomeridianum#NC 032701
Asparagales Asparagaceae Camassia scilloides#NC 032700
Asparagales Asparagaceae Hesperoyucca whipplei#NC 032705
Asparagales Asparagaceae Schoenolirion croceum#NC 032710
Asparagales Asparagaceae Hesperaloe campanulata#NC 032702
Asparagales Asparagaceae Hesperaloe parviflora#CMCY
Asparagales Asparagaceae Yucca brevifolia#NC 032711
Asparagales Asparagaceae Yucca filamentosa#ICNN
Asparagales Asparagaceae Yucca schidigera#DQ
Asparagales Asparagaceae Yucca queretaroensis#NC 032713
Asparagales Asparagaceae Beschorneria septentrionalis#NC 032699
Asparagales Asparagaceae Agave tequilana#KXSK
Asparagales Asparagaceae Manfreda virginica#NC 032707
Asparagales Asparagaceae Agave sisalana#F346
Asparagales Asparagaceae Agave attenuata#NC 032696
Asparagales Asparagaceae Lomandra longifolia#HQ
Asparagales Asparagaceae Eustrephus latifolius#NC 025305
Asparagales Asparagaceae Cordyline fruticosa#F350
Asparagales Asparagaceae Cordyline indivisa#NC 035998
Asparagales Asparagaceae Asparagus densiflorus#FGRF
Asparagales Asparagaceae Asparagus cochinchinensis#F713
Asparagales Asparagaceae Asparagus filicinus#F37
Asparagales Asparagaceae Asparagus officinalis#HQ
Asparagales Asparagaceae Asparagus schoberioides#NC 035969
Asparagales Asparagaceae Eriospermum lanceifolium#ONBE
Asparagales Asparagaceae Ruscus sp.#LSJW
Asparagales Asparagaceae Sansevieria trifasciata#MVRF
Asparagales Asparagaceae Dracaena cambodiana#NC 039776
Asparagales Asparagaceae Dracaena cochinchinensis#F348
Asparagales Asparagaceae Convallaria keiskei#MH680946
Asparagales Asparagaceae Rohdea chinensis#MH356725
Asparagales Asparagaceae Campylandra yunnanensis#F253
Asparagales Asparagaceae Disporopsis pernyi#UZXL
Asparagales Asparagaceae Polygonatum stenophyllum#NC 035995
Asparagales Asparagaceae Polygonatum cirrhifolium#F333
Asparagales Asparagaceae Polygonatum cyrtonema#NC 028429
Asparagales Asparagaceae Polygonatum verticillatum#NC 028523
Asparagales Asparagaceae Polygonatum sibiricum#NC 029485
Asparagales Asparagaceae Nolina atopocarpa#HOKG
Asparagales Asparagaceae Nolina bigelovii#RQZP
Asparagales Asparagaceae Liriope spicata#MH680945
Asparagales Asparagaceae Peliosanthes teta#TCYS
Asparagales Asparagaceae Maianthemum sp.#RCUX
Asparagales Asparagaceae Maianthemum bicolor#NC 035970
Asparagales Asparagaceae Maianthemum canadense#XFJG
Asparagales Asparagaceae Maianthemum dilatatum#NC 039133
Asparagales Orchidaceae Neuwiedia singapureana#LC199503
Asparagales Orchidaceae Apostasia shenzhenica#NC 039812
Asparagales Orchidaceae Apostasia wallichii#HQ
Asparagales Orchidaceae Vanilla aphylla#NC 035320
Asparagales Orchidaceae Vanilla planifolia#NC 026778
Asparagales Orchidaceae Vanilla pompona#NC 036809
Asparagales Orchidaceae Cypripedium japonicum#NC 027227
Asparagales Orchidaceae Cypripedium formosanum#NC 026772
Asparagales Orchidaceae Phragmipedium longifolium#NC 028149
Asparagales Orchidaceae Paphiopedilum niveum#NC 026776
Asparagales Orchidaceae Paphiopedilum dianthum#NC 036958
Asparagales Orchidaceae Paphiopedilum delenatii#NC 041309
Asparagales Orchidaceae Paphiopedilum armeniacum#LC085347
Asparagales Orchidaceae Platanthera japonica#NC 037440
Asparagales Orchidaceae Platanthera clavellata#MTHW
Asparagales Orchidaceae Pecteilis radiata#NC 035834
Asparagales Orchidaceae Habenaria pantlingiana#NC 026775
Asparagales Orchidaceae Rhizanthella gardneri#NC 014874 @myco
Asparagales Orchidaceae Drakaea elastica#XZME
Asparagales Orchidaceae Ludisia discolor#LELS
Asparagales Orchidaceae Goodyera velutina#NC 029365
Asparagales Orchidaceae Anoectochilus emeiensis#NC 033895
Asparagales Orchidaceae Goodyera procera#F230
Asparagales Orchidaceae Goodyera fumata#NC 026773
Asparagales Orchidaceae Goodyera schlechtendaliana#AB893949
Asparagales Orchidaceae Goodyera pubescens#VTUS
Asparagales Orchidaceae Palmorchis pabstii#NC 041190
Asparagales Orchidaceae Cephalanthera rubra#NC 041181
Asparagales Orchidaceae Cephalanthera longibracteata#NC 041180
Asparagales Orchidaceae Cephalanthera damasonium#NC 041179
Asparagales Orchidaceae Neottia ovata#NC 030712
Asparagales Orchidaceae Neottia cordata#NC 041189
Asparagales Orchidaceae Neottia japonica#MH321184
Asparagales Orchidaceae Neottia suzukii#NC 041447
Asparagales Orchidaceae Epipactis mairei#MG925367
Asparagales Orchidaceae Epipactis gigantea#NC 041184
Asparagales Orchidaceae Epipactis palustris#NC 041187
Asparagales Orchidaceae Epipactis atrorubens#NC 041183
Asparagales Orchidaceae Epipactis microphylla#NC 041186
Asparagales Orchidaceae Epipactis helleborine#NC 041185
Asparagales Orchidaceae Epipactis purpurata#NC 041188
Asparagales Orchidaceae Epipactis albensis#NC 041182
Asparagales Orchidaceae Elleanthus sodiroi#NC 027266
Asparagales Orchidaceae Sobralia callosa#NC 028147

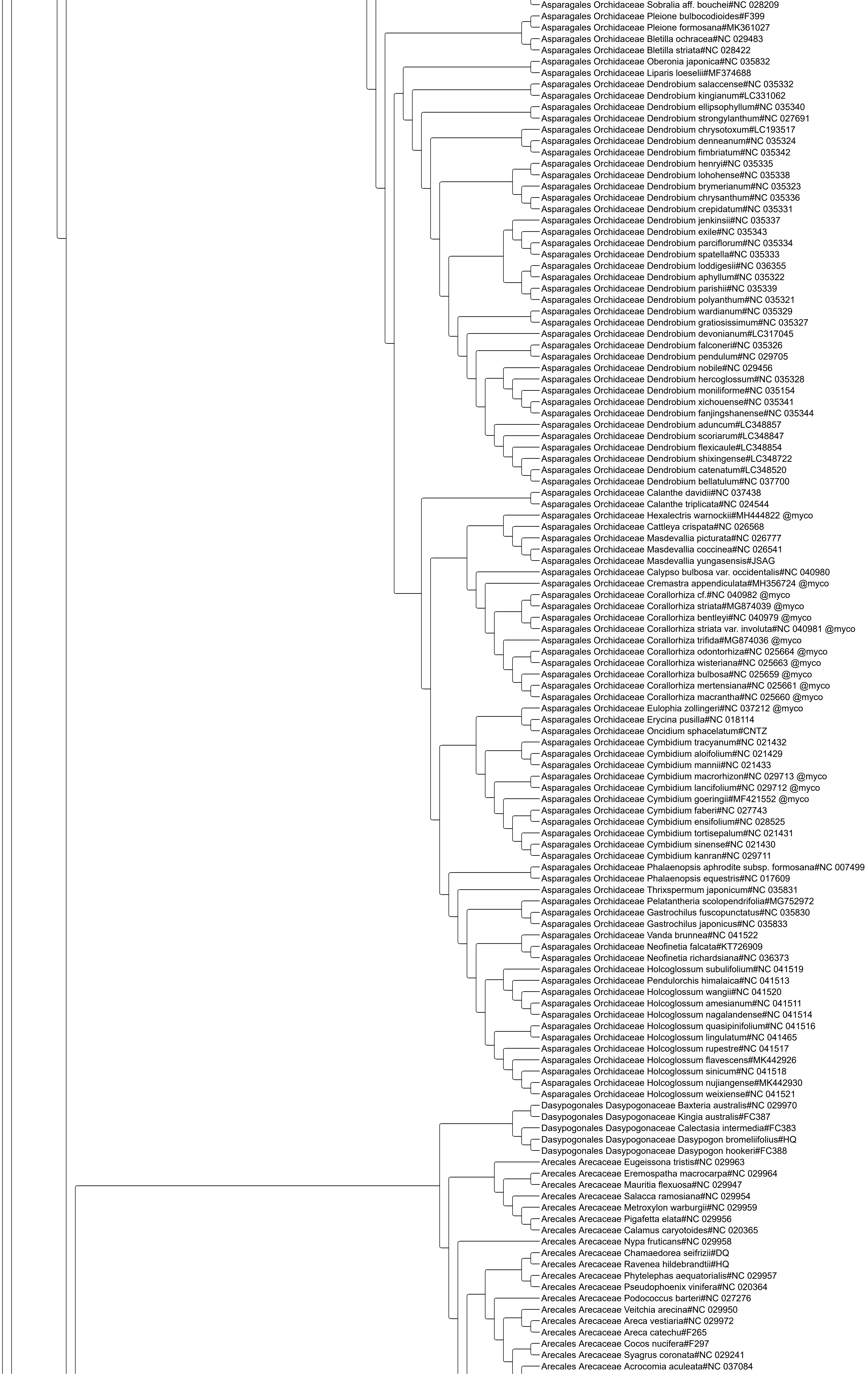
Asparagales Orchidaceae Sobralia aff. bouchei#NC 028209
Asparagales Orchidaceae Pleione bulbocodioides#F399
Asparagales Orchidaceae Pleione formosana#MK361027
Asparagales Orchidaceae Bletilla ochracea#NC 029483
Asparagales Orchidaceae Bletilla striata#NC 028422
Asparagales Orchidaceae Oberonia japonica#NC 035832
Asparagales Orchidaceae Liparis loeselii#MF374688
Asparagales Orchidaceae Dendrobium salaccense#NC 035332
Asparagales Orchidaceae Dendrobium kingianum#LC331062
Asparagales Orchidaceae Dendrobium ellipsophyllum#NC 035340
Asparagales Orchidaceae Dendrobium strongylanthum#NC 027691
Asparagales Orchidaceae Dendrobium chrysotoxum#LC193517
Asparagales Orchidaceae Dendrobium denneanum#NC 035324
Asparagales Orchidaceae Dendrobium fimbriatum#NC 035342
Asparagales Orchidaceae Dendrobium henryi#NC 035335
Asparagales Orchidaceae Dendrobium lohohense#NC 035338
Asparagales Orchidaceae Dendrobium brymerianum#NC 035323
Asparagales Orchidaceae Dendrobium chrysanthum#NC 035336
Asparagales Orchidaceae Dendrobium crepidatum#NC 035331
Asparagales Orchidaceae Dendrobium jenkinsii#NC 035337
Asparagales Orchidaceae Dendrobium exile#NC 035343
Asparagales Orchidaceae Dendrobium parciflorum#NC 035334
Asparagales Orchidaceae Dendrobium spatella#NC 035333
Asparagales Orchidaceae Dendrobium loddigesii#NC 036355
Asparagales Orchidaceae Dendrobium aphyllum#NC 035322
Asparagales Orchidaceae Dendrobium parishii#NC 035339
Asparagales Orchidaceae Dendrobium polyanthum#NC 035321
Asparagales Orchidaceae Dendrobium wardianum#NC 035329
Asparagales Orchidaceae Dendrobium gratiosissimum#NC 035327
Asparagales Orchidaceae Dendrobium devonianum#LC317045
Asparagales Orchidaceae Dendrobium falconeri#NC 035326
Asparagales Orchidaceae Dendrobium pendulum#NC 029705
Asparagales Orchidaceae Dendrobium nobile#NC 029456
Asparagales Orchidaceae Dendrobium hercoglossum#NC 035328
Asparagales Orchidaceae Dendrobium moniliforme#NC 035154
Asparagales Orchidaceae Dendrobium xichouense#NC 035341
Asparagales Orchidaceae Dendrobium fanjingshanense#NC 035344
Asparagales Orchidaceae Dendrobium aduncum#LC348857
Asparagales Orchidaceae Dendrobium scoriarum#LC348847
Asparagales Orchidaceae Dendrobium flexicaule#LC348854
Asparagales Orchidaceae Dendrobium shixingense#LC348722
Asparagales Orchidaceae Dendrobium catenatum#LC348520
Asparagales Orchidaceae Dendrobium bellatulum#NC 037700
Asparagales Orchidaceae Calanthe davidii#NC 037438
Asparagales Orchidaceae Calanthe triplicata#NC 024544
Asparagales Orchidaceae Hexalectris warnockii#MH444822 @myco
Asparagales Orchidaceae Cattleya crispata#NC 026568
Asparagales Orchidaceae Masdevallia picturata#NC 026777
Asparagales Orchidaceae Masdevallia coccinea#NC 026541
Asparagales Orchidaceae Masdevallia yungasensis#JSAG
Asparagales Orchidaceae Calypso bulbosa var. occidentalis#NC 040980
Asparagales Orchidaceae Cremastra appendiculata#MH356724 @myco
Asparagales Orchidaceae Corallorhiza cf.#NC 040982 @myco
Asparagales Orchidaceae Corallorhiza striata#MG874039 @myco
Asparagales Orchidaceae Corallorhiza bentleyi#NC 040979 @myco
Asparagales Orchidaceae Corallorhiza striata var. involuta#NC 040981 @myco
Asparagales Orchidaceae Corallorhiza trifida#MG874036 @myco
Asparagales Orchidaceae Corallorhiza odontorhiza#NC 025664 @myco
Asparagales Orchidaceae Corallorhiza wisteriana#NC 025663 @myco
Asparagales Orchidaceae Corallorhiza bulbosa#NC 025659 @myco
Asparagales Orchidaceae Corallorhiza mertensiana#NC 025661 @myco
Asparagales Orchidaceae Corallorhiza macrantha#NC 025660 @myco
Asparagales Orchidaceae Eulophia zollingeri#NC 037212 @myco
Asparagales Orchidaceae Erycina pusilla#NC 018114
Asparagales Orchidaceae Oncidium sphacelatum#CNTZ
Asparagales Orchidaceae Cymbidium tracyanum#NC 021432
Asparagales Orchidaceae Cymbidium aloifolium#NC 021429
Asparagales Orchidaceae Cymbidium mannii#NC 021433
Asparagales Orchidaceae Cymbidium macrorhizon#NC 029713 @myco
Asparagales Orchidaceae Cymbidium lancifolium#NC 029712 @myco
Asparagales Orchidaceae Cymbidium goeringii#MF421552 @myco
Asparagales Orchidaceae Cymbidium faberi#NC 027743
Asparagales Orchidaceae Cymbidium ensifolium#NC 028525
Asparagales Orchidaceae Cymbidium tortisepalum#NC 021431
Asparagales Orchidaceae Cymbidium sinense#NC 021430
Asparagales Orchidaceae Cymbidium kanran#NC 029711
Asparagales Orchidaceae Phalaenopsis aphrodite subsp. formosana#NC 007499
Asparagales Orchidaceae Phalaenopsis equestris#NC 017609
Asparagales Orchidaceae Thrixspermum japonicum#NC 035831
Asparagales Orchidaceae Pelatantheria scolopendrifolia#MG752972
Asparagales Orchidaceae Gastrochilus fuscopunctatus#NC 035830
Asparagales Orchidaceae Gastrochilus japonicus#NC 035833
Asparagales Orchidaceae Vanda brunnea#NC 041522
Asparagales Orchidaceae Neofinetia falcata#KT726909
Asparagales Orchidaceae Neofinetia richardsiana#NC 036373
Asparagales Orchidaceae Holcoglossum subulifolium#NC 041519
Asparagales Orchidaceae Pendulorchis himalaica#NC 041513
Asparagales Orchidaceae Holcoglossum wangii#NC 041520
Asparagales Orchidaceae Holcoglossum amesianum#NC 041511
Asparagales Orchidaceae Holcoglossum nagalandense#NC 041514
Asparagales Orchidaceae Holcoglossum quasipinifolium#NC 041516
Asparagales Orchidaceae Holcoglossum lingulatum#NC 041465
Asparagales Orchidaceae Holcoglossum rupestre#NC 041517
Asparagales Orchidaceae Holcoglossum flavescens#MK442926
Asparagales Orchidaceae Holcoglossum sinicum#NC 041518
Asparagales Orchidaceae Holcoglossum nujiangense#MK442930
Asparagales Orchidaceae Holcoglossum weixiense#NC 041521
Dasypogonales Dasypogonaceae Baxteria australis#NC 029970
Dasypogonales Dasypogonaceae Kingia australis#FC387
Dasypogonales Dasypogonaceae Calectasia intermedia#FC383
Dasypogonales Dasypogonaceae Dasypogon bromeliifolius#HQ
Dasypogonales Dasypogonaceae Dasypogon hookeri#FC388
Arecales Arecaceae Eugeissona tristis#NC 029963
Arecales Arecaceae Eremospatha macrocarpa#NC 029964
Arecales Arecaceae Mauritia flexuosa#NC 029947
Arecales Arecaceae Salacca ramosiana#NC 029954
Arecales Arecaceae Metroxylon warburgii#NC 029959
Arecales Arecaceae Pigafetta elata#NC 029956
Arecales Arecaceae Calamus caryotoides#NC 020365
Arecales Arecaceae Nypa fruticans#NC 029958
Arecales Arecaceae Chamaedorea seifrizii#DQ
Arecales Arecaceae Ravenea hildebrandtii#HQ
Arecales Arecaceae Phytelephas aequatorialis#NC 029957
Arecales Arecaceae Pseudophoenix vinifera#NC 020364
Arecales Arecaceae Podococcus barteri#NC 027276
Arecales Arecaceae Veitchia arecina#NC 029950
Arecales Arecaceae Areca vestiaria#NC 029972
Arecales Arecaceae Areca catechu#F265
Arecales Arecaceae Cocos nucifera#F297
Arecales Arecaceae Syagrus coronata#NC 029241
Arecales Arecaceae Acrocomia aculeata#NC 037084

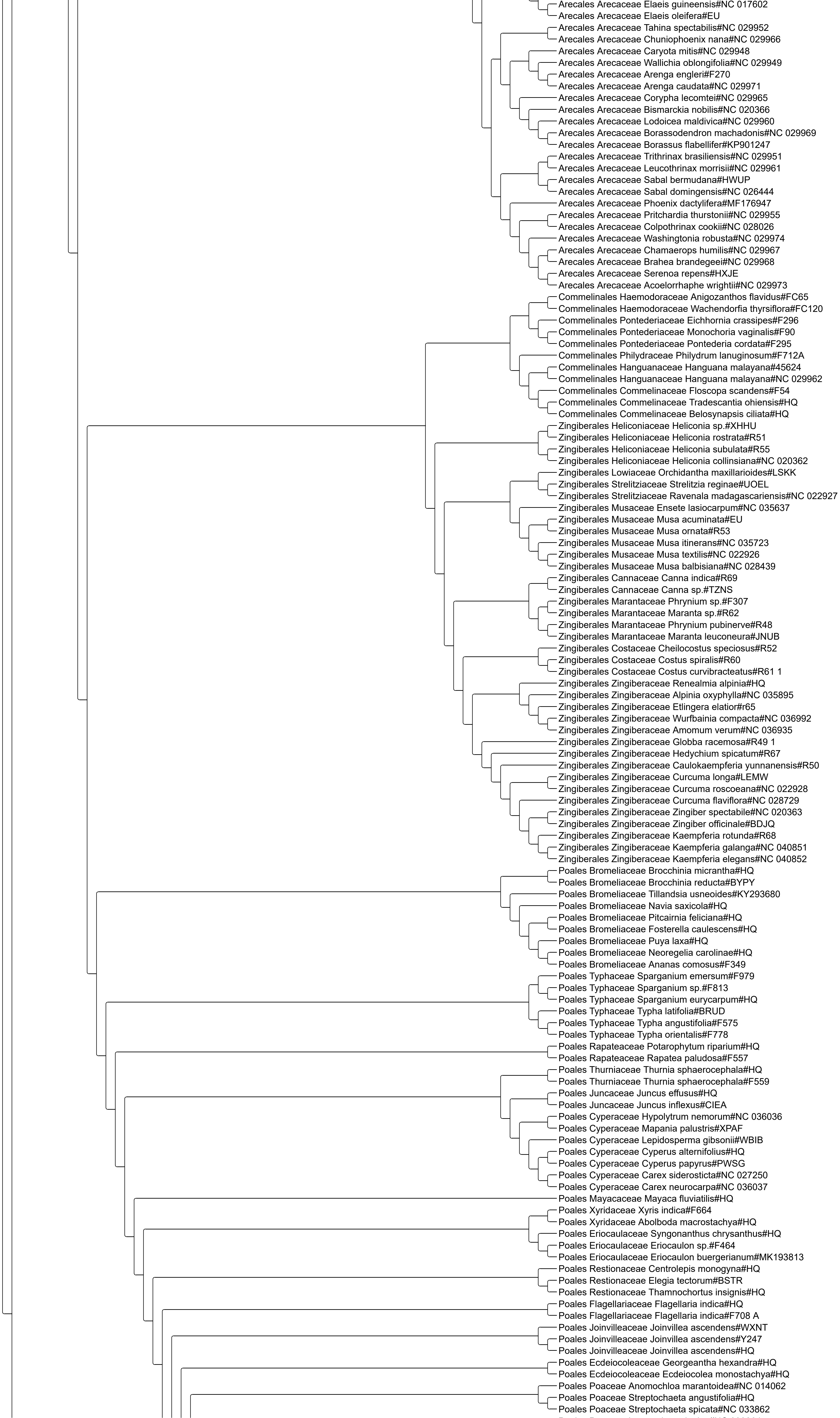

Arecales Arecaceae Elaeis guineensis#NC 017602
Arecales Arecaceae Elaeis oleifera#EU
Arecales Arecaceae Tahina spectabilis#NC 029952
Arecales Arecaceae Chuniophoenix nana#NC 029966
Arecales Arecaceae Caryota mitis#NC 029948
Arecales Arecaceae Wallichia oblongifolia#NC 029949
Arecales Arecaceae Arenga engleri#F270
Arecales Arecaceae Arenga caudata#NC 029971
Arecales Arecaceae Corypha lecomtei#NC 029965
Arecales Arecaceae Bismarckia nobilis#NC 020366
Arecales Arecaceae Lodoicea maldivica#NC 029960
Arecales Arecaceae Borassodendron machadonis#NC 029969
Arecales Arecaceae Borassus flabellifer#KP901247
Arecales Arecaceae Trithrinax brasiliensis#NC 029951
Arecales Arecaceae Leucothrinax morrisii#NC 029961
Arecales Arecaceae Sabal bermudana#HWUP
Arecales Arecaceae Sabal domingensis#NC 026444
Arecales Arecaceae Phoenix dactylifera#MF176947
Arecales Arecaceae Pritchardia thurstonii#NC 029955
Arecales Arecaceae Colpothrinax cookii#NC 028026
Arecales Arecaceae Washingtonia robusta#NC 029974
Arecales Arecaceae Chamaerops humilis#NC 029967
Arecales Arecaceae Brahea brandegeei#NC 029968
Arecales Arecaceae Serenoa repens#HXJE
Arecales Arecaceae Acoelorrhaphe wrightii#NC 029973
Commelinales Haemodoraceae Anigozanthos flavidus#FC65
Commelinales Haemodoraceae Wachendorfia thyrsiflora#FC120
Commelinales Pontederiaceae Eichhornia crassipes#F296
Commelinales Pontederiaceae Monochoria vaginalis#F90
Commelinales Pontederiaceae Pontederia cordata#F295
Commelinales Philydraceae Philydrum lanuginosum#F712A
Commelinales Hanguanaceae Hanguana malayana#45624
Commelinales Hanguanaceae Hanguana malayana#NC 029962
Commelinales Commelinaceae Floscopa scandens#F54
Commelinales Commelinaceae Tradescantia ohiensis#HQ
Commelinales Commelinaceae Belosynapsis ciliata#HQ
Zingiberales Heliconiaceae Heliconia sp.#XHHU
Zingiberales Heliconiaceae Heliconia rostrata#R51
Zingiberales Heliconiaceae Heliconia subulata#R55
Zingiberales Heliconiaceae Heliconia collinsiana#NC 020362
Zingiberales Lowiaceae Orchidantha maxillarioides#LSKK
Zingiberales Strelitziaceae Strelitzia reginae#UOEL
Zingiberales Strelitziaceae Ravenala madagascariensis#NC 022927
Zingiberales Musaceae Ensete lasiocarpum#NC 035637
Zingiberales Musaceae Musa acuminata#EU
Zingiberales Musaceae Musa ornata#R53
Zingiberales Musaceae Musa itinerans#NC 035723
Zingiberales Musaceae Musa textilis#NC 022926
Zingiberales Musaceae Musa balbisiana#NC 028439
Zingiberales Cannaceae Canna indica#R69
Zingiberales Cannaceae Canna sp.#TZNS
Zingiberales Marantaceae Phrynium sp.#F307
Zingiberales Marantaceae Maranta sp.#R62
Zingiberales Marantaceae Phrynium pubinerve#R48
Zingiberales Marantaceae Maranta leuconeura#JNUB
Zingiberales Costaceae Cheilocostus speciosus#R52
Zingiberales Costaceae Costus spiralis#R60
Zingiberales Costaceae Costus curvibracteatus#R61 1
Zingiberales Zingiberaceae Renealmia alpinia#HQ
Zingiberales Zingiberaceae Alpinia oxyphylla#NC 035895
Zingiberales Zingiberaceae Etlingera elatior#r65
Zingiberales Zingiberaceae Wurfbainia compacta#NC 036992
Zingiberales Zingiberaceae Amomum verum#NC 036935
Zingiberales Zingiberaceae Globba racemosa#R49 1
Zingiberales Zingiberaceae Hedychium spicatum#R67
Zingiberales Zingiberaceae Caulokaempferia yunnanensis#R50
Zingiberales Zingiberaceae Curcuma longa#LEMW
Zingiberales Zingiberaceae Curcuma roscoeana#NC 022928
Zingiberales Zingiberaceae Curcuma flaviflora#NC 028729
Zingiberales Zingiberaceae Zingiber spectabile#NC 020363
Zingiberales Zingiberaceae Zingiber officinale#BDJQ
Zingiberales Zingiberaceae Kaempferia rotunda#R68
Zingiberales Zingiberaceae Kaempferia galanga#NC 040851
Zingiberales Zingiberaceae Kaempferia elegans#NC 040852
Poales Bromeliaceae Brocchinia micrantha#HQ
Poales Bromeliaceae Brocchinia reducta#BYPY
Poales Bromeliaceae Tillandsia usneoides#KY293680
Poales Bromeliaceae Navia saxicola#HQ
Poales Bromeliaceae Pitcairnia feliciana#HQ
Poales Bromeliaceae Fosterella caulescens#HQ
Poales Bromeliaceae Puya laxa#HQ
Poales Bromeliaceae Neoregelia carolinae#HQ
Poales Bromeliaceae Ananas comosus#F349
Poales Typhaceae Sparganium emersum#F979
Poales Typhaceae Sparganium sp.#F813
Poales Typhaceae Sparganium eurycarpum#HQ
Poales Typhaceae Typha latifolia#BRUD
Poales Typhaceae Typha angustifolia#F575
Poales Typhaceae Typha orientalis#F778
Poales Rapateaceae Potarophytum riparium#HQ
Poales Rapateaceae Rapatea paludosa#F557
Poales Thurniaceae Thurnia sphaerocephala#HQ
Poales Thurniaceae Thurnia sphaerocephala#F559
Poales Juncaceae Juncus effusus#HQ
Poales Juncaceae Juncus inflexus#CIEA
Poales Cyperaceae Hypolytrum nemorum#NC 036036
Poales Cyperaceae Mapania palustris#XPAF
Poales Cyperaceae Lepidosperma gibsonii#WBIB
Poales Cyperaceae Cyperus alternifolius#HQ
Poales Cyperaceae Cyperus papyrus#PWSG
Poales Cyperaceae Carex siderosticta#NC 027250
Poales Cyperaceae Carex neurocarpa#NC 036037
Poales Mayacaceae Mayaca fluviatilis#HQ
Poales Xyridaceae Xyris indica#F664
Poales Xyridaceae Abolboda macrostachya#HQ
Poales Eriocaulaceae Syngonanthus chrysanthus#HQ
Poales Eriocaulaceae Eriocaulon sp.#F464
Poales Eriocaulaceae Eriocaulon buergerianum#MK193813
Poales Restionaceae Centrolepis monogyna#HQ
Poales Restionaceae Elegia tectorum#BSTR
Poales Restionaceae Thamnochortus insignis#HQ
Poales Flagellariaceae Flagellaria indica#HQ
Poales Flagellariaceae Flagellaria indica#F708 A
Poales Joinvilleaceae Joinvillea ascendens#WXNT
Poales Joinvilleaceae Joinvillea ascendens#Y247
Poales Joinvilleaceae Joinvillea ascendens#HQ
Poales Ecdeiocoleaceae Georgeantha hexandra#HQ
Poales Ecdeiocoleaceae Ecdeiocolea monostachya#HQ
Poales Poaceae Anomochloa marantoidea#NC 014062
Poales Poaceae Streptochaeta angustifolia#HQ
Poales Poaceae Streptochaeta spicata#NC 033862

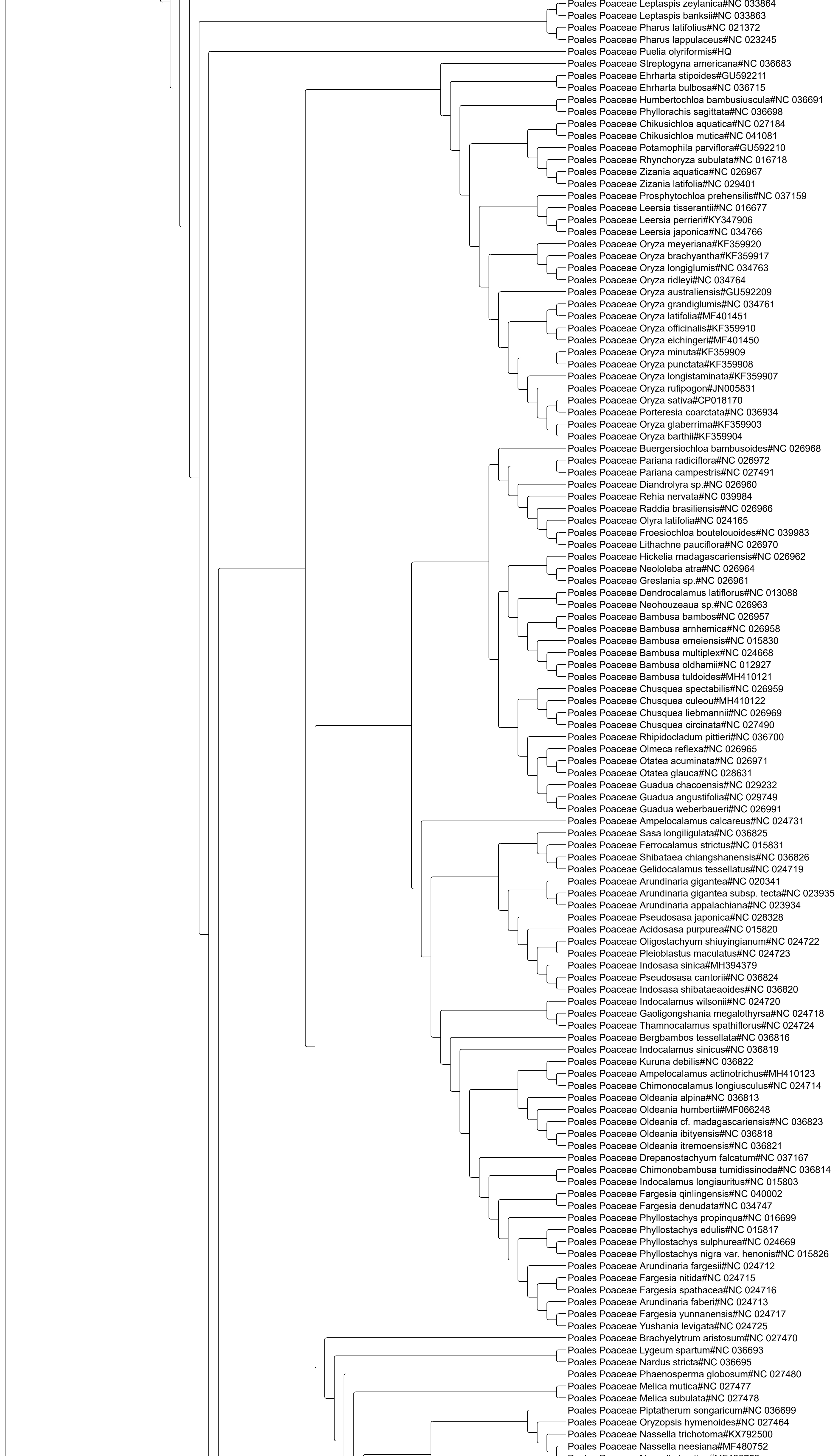

Poales Poaceae Leptaspis zeylanica#NC 033864
Poales Poaceae Leptaspis banksii#NC 033863
Poales Poaceae Pharus latifolius#NC 021372
Poales Poaceae Pharus lappulaceus#NC 023245
Poales Poaceae Puelia olyriformis#HQ
Poales Poaceae Streptogyna americana#NC 036683
Poales Poaceae Ehrharta stipoides#GU592211
Poales Poaceae Ehrharta bulbosa#NC 036715
Poales Poaceae Humbertochloa bambusiuscula#NC 036691
Poales Poaceae Phyllorachis sagittata#NC 036698
Poales Poaceae Chikusichloa aquatica#NC 027184
Poales Poaceae Chikusichloa mutica#NC 041081
Poales Poaceae Potamophila parviflora#GU592210
Poales Poaceae Rhynchoryza subulata#NC 016718
Poales Poaceae Zizania aquatica#NC 026967
Poales Poaceae Zizania latifolia#NC 029401
Poales Poaceae Prosphytochloa prehensilis#NC 037159
Poales Poaceae Leersia tisserantii#NC 016677
Poales Poaceae Leersia perrieri#KY347906
Poales Poaceae Leersia japonica#NC 034766
Poales Poaceae Oryza meyeriana#KF359920
Poales Poaceae Oryza brachyantha#KF359917
Poales Poaceae Oryza longiglumis#NC 034763
Poales Poaceae Oryza ridleyi#NC 034764
Poales Poaceae Oryza australiensis#GU592209
Poales Poaceae Oryza grandiglumis#NC 034761
Poales Poaceae Oryza latifolia#MF401451
Poales Poaceae Oryza officinalis#KF359910
Poales Poaceae Oryza eichingeri#MF401450
Poales Poaceae Oryza minuta#KF359909
Poales Poaceae Oryza punctata#KF359908
Poales Poaceae Oryza longistaminata#KF359907
Poales Poaceae Oryza rufipogon#JN005831
Poales Poaceae Oryza sativa#CP018170
Poales Poaceae Porteresia coarctata#NC 036934
Poales Poaceae Oryza glaberrima#KF359903
Poales Poaceae Oryza barthii#KF359904
Poales Poaceae Buergersiochloa bambusoides#NC 026968
Poales Poaceae Pariana radiciflora#NC 026972
Poales Poaceae Pariana campestris#NC 027491
Poales Poaceae Diandrolyra sp.#NC 026960
Poales Poaceae Rehia nervata#NC 039984
Poales Poaceae Raddia brasiliensis#NC 026966
Poales Poaceae Olyra latifolia#NC 024165
Poales Poaceae Froesiochloa boutelouoides#NC 039983
Poales Poaceae Lithachne pauciflora#NC 026970
Poales Poaceae Hickelia madagascariensis#NC 026962
Poales Poaceae Neololeba atra#NC 026964
Poales Poaceae Greslania sp.#NC 026961
Poales Poaceae Dendrocalamus latiflorus#NC 013088
Poales Poaceae Neohouzeaua sp.#NC 026963
Poales Poaceae Bambusa bambos#NC 026957
Poales Poaceae Bambusa arnhemica#NC 026958
Poales Poaceae Bambusa emeiensis#NC 015830
Poales Poaceae Bambusa multiplex#NC 024668
Poales Poaceae Bambusa oldhamii#NC 012927
Poales Poaceae Bambusa tuldoides#MH410121
Poales Poaceae Chusquea spectabilis#NC 026959
Poales Poaceae Chusquea culeou#MH410122
Poales Poaceae Chusquea liebmannii#NC 026969
Poales Poaceae Chusquea circinata#NC 027490
Poales Poaceae Rhipidocladum pittieri#NC 036700
Poales Poaceae Olmeca reflexa#NC 026965
Poales Poaceae Otatea acuminata#NC 026971
Poales Poaceae Otatea glauca#NC 028631
Poales Poaceae Guadua chacoensis#NC 029232
Poales Poaceae Guadua angustifolia#NC 029749
Poales Poaceae Guadua weberbaueri#NC 026991
Poales Poaceae Ampelocalamus calcareus#NC 024731
Poales Poaceae Sasa longiligulata#NC 036825
Poales Poaceae Ferrocalamus strictus#NC 015831
Poales Poaceae Shibataea chiangshanensis#NC 036826
Poales Poaceae Gelidocalamus tessellatus#NC 024719
Poales Poaceae Arundinaria gigantea#NC 020341
Poales Poaceae Arundinaria gigantea subsp. tecta#NC 023935
Poales Poaceae Arundinaria appalachiana#NC 023934
Poales Poaceae Pseudosasa japonica#NC 028328
Poales Poaceae Acidosasa purpurea#NC 015820
Poales Poaceae Oligostachyum shiuyingianum#NC 024722
Poales Poaceae Pleioblastus maculatus#NC 024723
Poales Poaceae Indosasa sinica#MH394379
Poales Poaceae Pseudosasa cantorii#NC 036824
Poales Poaceae Indosasa shibataeaoides#NC 036820
Poales Poaceae Indocalamus wilsonii#NC 024720
Poales Poaceae Gaoligongshania megalothyrsa#NC 024718
Poales Poaceae Thamnocalamus spathiflorus#NC 024724
Poales Poaceae Bergbambos tessellata#NC 036816
Poales Poaceae Indocalamus sinicus#NC 036819
Poales Poaceae Kuruna debilis#NC 036822
Poales Poaceae Ampelocalamus actinotrichus#MH410123
Poales Poaceae Chimonocalamus longiusculus#NC 024714
Poales Poaceae Oldeania alpina#NC 036813
Poales Poaceae Oldeania humbertii#MF066248
Poales Poaceae Oldeania cf. madagascariensis#NC 036823
Poales Poaceae Oldeania ibityensis#NC 036818
Poales Poaceae Oldeania itremoensis#NC 036821
Poales Poaceae Drepanostachyum falcatum#NC 037167
Poales Poaceae Chimonobambusa tumidissinoda#NC 036814
Poales Poaceae Indocalamus longiauritus#NC 015803
Poales Poaceae Fargesia qinlingensis#NC 040002
Poales Poaceae Fargesia denudata#NC 034747
Poales Poaceae Phyllostachys propinqua#NC 016699
Poales Poaceae Phyllostachys edulis#NC 015817
Poales Poaceae Phyllostachys sulphurea#NC 024669
Poales Poaceae Phyllostachys nigra var. henonis#NC 015826
Poales Poaceae Arundinaria fargesii#NC 024712
Poales Poaceae Fargesia nitida#NC 024715
Poales Poaceae Fargesia spathacea#NC 024716
Poales Poaceae Arundinaria faberi#NC 024713
Poales Poaceae Fargesia yunnanensis#NC 024717
Poales Poaceae Yushania levigata#NC 024725
Poales Poaceae Brachyelytrum aristosum#NC 027470
Poales Poaceae Lygeum spartum#NC 036693
Poales Poaceae Nardus stricta#NC 036695
Poales Poaceae Phaenosperma globosum#NC 027480
Poales Poaceae Melica mutica#NC 027477
Poales Poaceae Melica subulata#NC 027478
Poales Poaceae Piptatherum songaricum#NC 036699
Poales Poaceae Oryzopsis hymenoides#NC 027464
Poales Poaceae Nassella trichotoma#KX792500
Poales Poaceae Nassella neesiana#MF480752

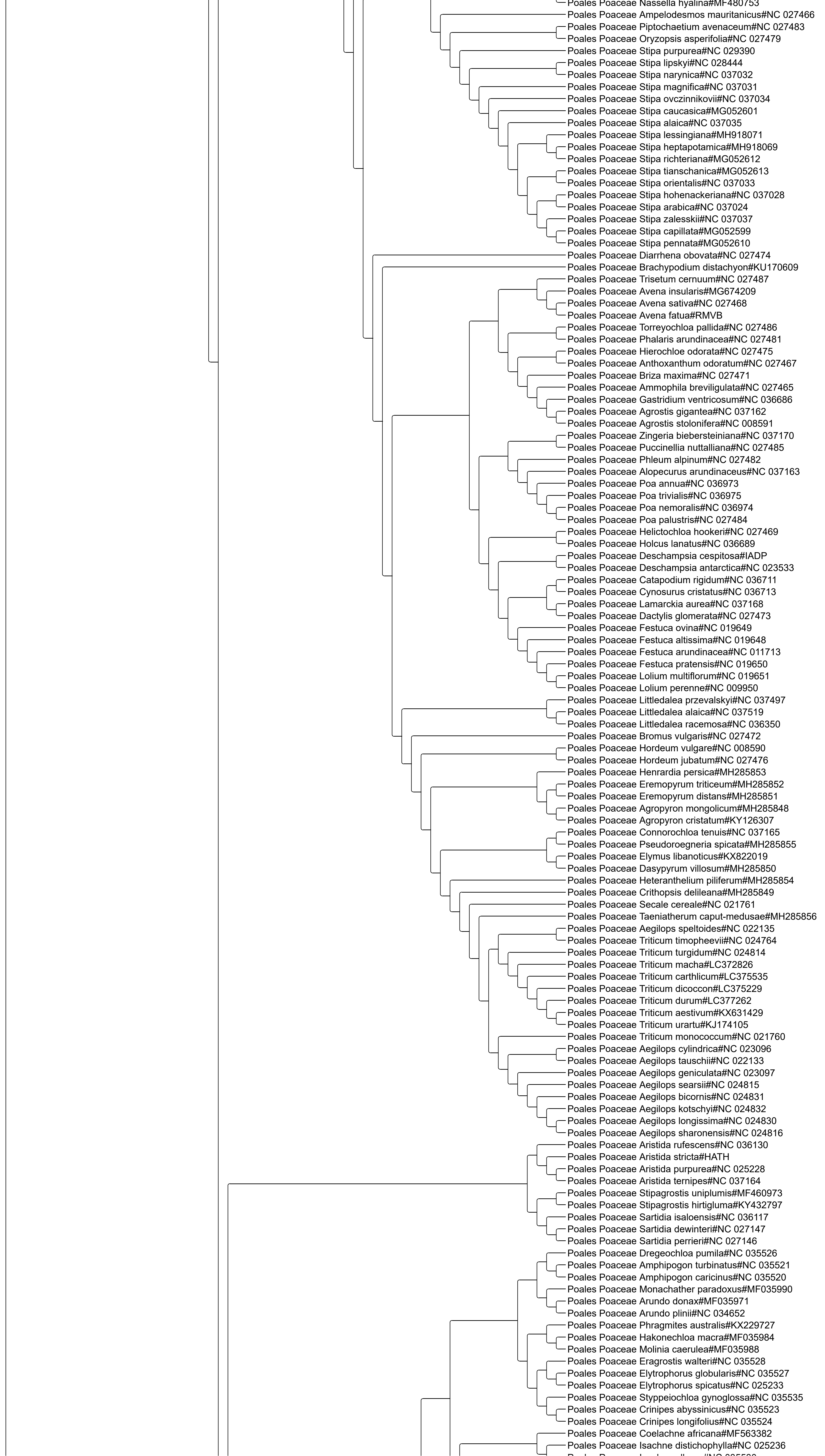

Poales Poaceae Ampelodesmos mauritanicus#NC 027466
Poales Poaceae Piptochaetium avenaceum#NC 027483
Poales Poaceae Oryzopsis asperifolia#NC 027479
Poales Poaceae Stipa purpurea#NC 029390
Poales Poaceae Stipa lipskyi#NC 028444
Poales Poaceae Stipa narynica#NC 037032
Poales Poaceae Stipa magnifica#NC 037031
Poales Poaceae Stipa ovczinnikovii#NC 037034
Poales Poaceae Stipa caucasica#MG052601
Poales Poaceae Stipa alaica#NC 037035
Poales Poaceae Stipa lessingiana#MH918071
Poales Poaceae Stipa heptapotamica#MH918069
Poales Poaceae Stipa richteriana#MG052612
Poales Poaceae Stipa tianschanica#MG052613
Poales Poaceae Stipa orientalis#NC 037033
Poales Poaceae Stipa hohenackeriana#NC 037028
Poales Poaceae Stipa arabica#NC 037024
Poales Poaceae Stipa zalesskii#NC 037037
Poales Poaceae Stipa capillata#MG052599
Poales Poaceae Stipa pennata#MG052610
Poales Poaceae Diarrhena obovata#NC 027474
Poales Poaceae Brachypodium distachyon#KU170609
Poales Poaceae Trisetum cernuum#NC 027487
Poales Poaceae Avena insularis#MG674209
Poales Poaceae Avena sativa#NC 027468
Poales Poaceae Avena fatua#RMVB
Poales Poaceae Torreyochloa pallida#NC 027486
Poales Poaceae Phalaris arundinacea#NC 027481
Poales Poaceae Hierochloe odorata#NC 027475
Poales Poaceae Anthoxanthum odoratum#NC 027467
Poales Poaceae Briza maxima#NC 027471
Poales Poaceae Ammophila breviligulata#NC 027465
Poales Poaceae Gastridium ventricosum#NC 036686
Poales Poaceae Agrostis gigantea#NC 037162
Poales Poaceae Agrostis stolonifera#NC 008591
Poales Poaceae Zingeria biebersteiniana#NC 037170
Poales Poaceae Puccinellia nuttalliana#NC 027485
Poales Poaceae Phleum alpinum#NC 027482
Poales Poaceae Alopecurus arundinaceus#NC 037163
Poales Poaceae Poa annua#NC 036973
Poales Poaceae Poa trivialis#NC 036975
Poales Poaceae Poa nemoralis#NC 036974
Poales Poaceae Poa palustris#NC 027484
Poales Poaceae Helictochloa hookeri#NC 027469
Poales Poaceae Holcus lanatus#NC 036689
Poales Poaceae Deschampsia cespitosa#IADP
Poales Poaceae Deschampsia antarctica#NC 023533
Poales Poaceae Catapodium rigidum#NC 036711
Poales Poaceae Cynosurus cristatus#NC 036713
Poales Poaceae Lamarckia aurea#NC 037168
Poales Poaceae Dactylis glomerata#NC 027473
Poales Poaceae Festuca ovina#NC 019649
Poales Poaceae Festuca altissima#NC 019648
Poales Poaceae Festuca arundinacea#NC 011713
Poales Poaceae Festuca pratensis#NC 019650
Poales Poaceae Lolium multiflorum#NC 019651
Poales Poaceae Lolium perenne#NC 009950
Poales Poaceae Littledalea przevalskyi#NC 037497
Poales Poaceae Littledalea alaica#NC 037519
Poales Poaceae Littledalea racemosa#NC 036350
Poales Poaceae Bromus vulgaris#NC 027472
Poales Poaceae Hordeum vulgare#NC 008590
Poales Poaceae Hordeum jubatum#NC 027476
Poales Poaceae Henrardia persica#MH285853
Poales Poaceae Eremopyrum triticeum#MH285852
Poales Poaceae Eremopyrum distans#MH285851
Poales Poaceae Agropyron mongolicum#MH285848
Poales Poaceae Agropyron cristatum#KY126307
Poales Poaceae Connorochloa tenuis#NC 037165
Poales Poaceae Pseudoroegneria spicata#MH285855
Poales Poaceae Elymus libanoticus#KX822019
Poales Poaceae Dasypyrum villosum#MH285850
Poales Poaceae Heteranthelium piliferum#MH285854
Poales Poaceae Crithopsis delileana#MH285849
Poales Poaceae Secale cereale#NC 021761
Poales Poaceae Taeniatherum caput-medusae#MH285856
Poales Poaceae Aegilops speltoides#NC 022135
Poales Poaceae Triticum timopheevii#NC 024764
Poales Poaceae Triticum turgidum#NC 024814
Poales Poaceae Triticum macha#LC372826
Poales Poaceae Triticum carthlicum#LC375535
Poales Poaceae Triticum dicoccon#LC375229
Poales Poaceae Triticum durum#LC377262
Poales Poaceae Triticum aestivum#KX631429
Poales Poaceae Triticum urartu#KJ174105
Poales Poaceae Triticum monococcum#NC 021760
Poales Poaceae Aegilops cylindrica#NC 023096
Poales Poaceae Aegilops tauschii#NC 022133
Poales Poaceae Aegilops geniculata#NC 023097
Poales Poaceae Aegilops searsii#NC 024815
Poales Poaceae Aegilops bicornis#NC 024831
Poales Poaceae Aegilops kotschyi#NC 024832
Poales Poaceae Aegilops longissima#NC 024830
Poales Poaceae Aegilops sharonensis#NC 024816
Poales Poaceae Aristida rufescens#NC 036130
Poales Poaceae Aristida stricta#HATH
Poales Poaceae Aristida purpurea#NC 025228
Poales Poaceae Aristida ternipes#NC 037164
Poales Poaceae Stipagrostis uniplumis#MF460973
Poales Poaceae Stipagrostis hirtigluma#KY432797
Poales Poaceae Sartidia isaloensis#NC 036117
Poales Poaceae Sartidia dewinteri#NC 027147
Poales Poaceae Sartidia perrieri#NC 027146
Poales Poaceae Dregeochloa pumila#NC 035526
Poales Poaceae Amphipogon turbinatus#NC 035521
Poales Poaceae Amphipogon caricinus#NC 035520
Poales Poaceae Monachather paradoxus#MF035990
Poales Poaceae Arundo donax#MF035971
Poales Poaceae Arundo plinii#NC 034652
Poales Poaceae Phragmites australis#KX229727
Poales Poaceae Hakonechloa macra#MF035984
Poales Poaceae Molinia caerulea#MF035988
Poales Poaceae Eragrostis walteri#NC 035528
Poales Poaceae Elytrophorus globularis#NC 035527
Poales Poaceae Elytrophorus spicatus#NC 025233
Poales Poaceae Styppeiochloa gynoglossa#NC 035535
Poales Poaceae Crinipes abyssinicus#NC 035523
Poales Poaceae Crinipes longifolius#NC 035524
Poales Poaceae Coelachne africana#MF563382
Poales Poaceae Isachne distichophylla#NC 025236

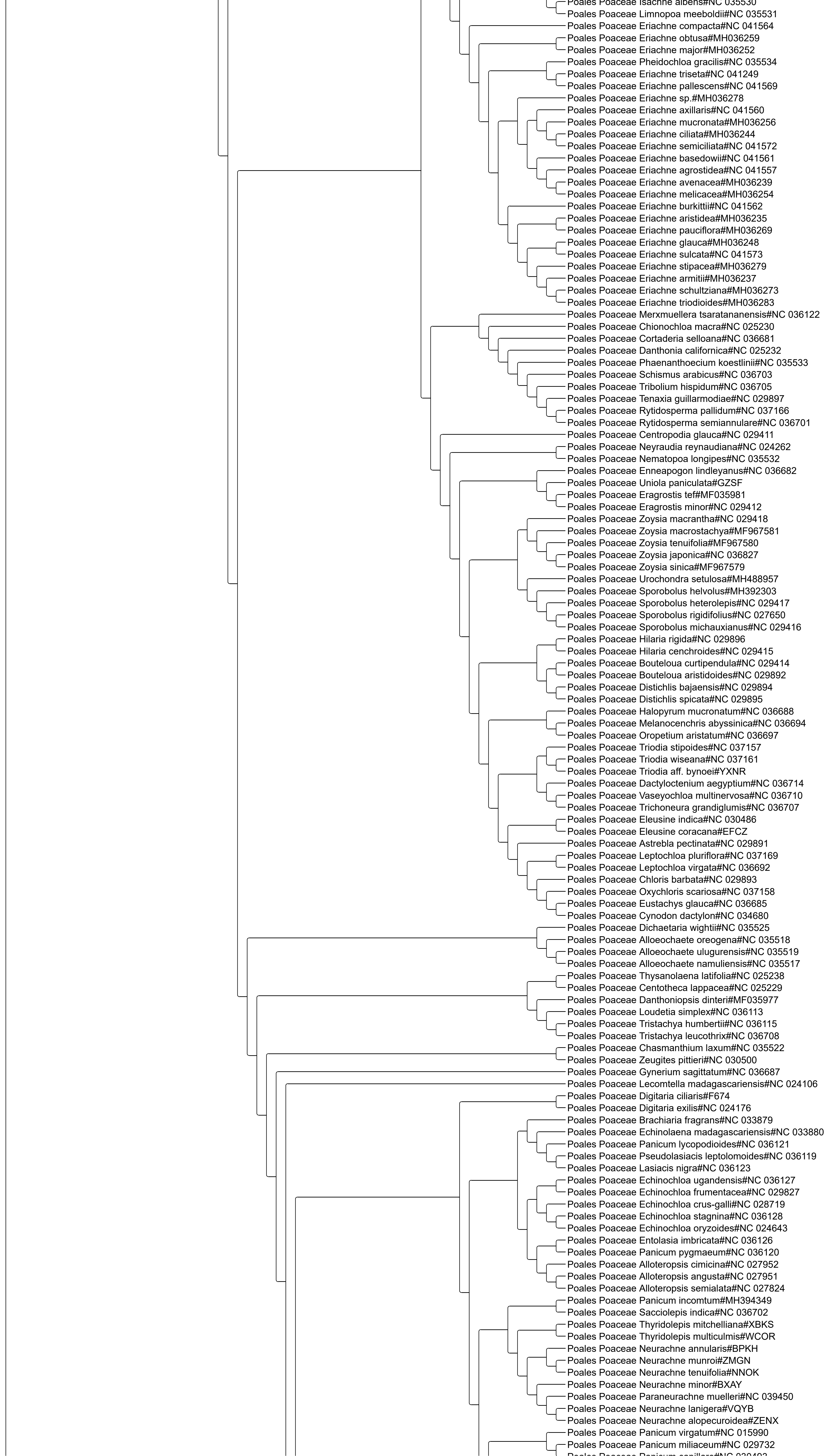
Poales Poaceae Isachne albens#NC 035530
Poales Poaceae Limnopoa meeboldii#NC 035531
Poales Poaceae Eriachne compacta#NC 041564
Poales Poaceae Eriachne obtusa#MH036259
Poales Poaceae Eriachne major#MH036252
Poales Poaceae Pheidochloa gracilis#NC 035534
Poales Poaceae Eriachne triseta#NC 041249
Poales Poaceae Eriachne pallescens#NC 041569
Poales Poaceae Eriachne sp.#MH036278
Poales Poaceae Eriachne axillaris#NC 041560
Poales Poaceae Eriachne mucronata#MH036256
Poales Poaceae Eriachne ciliata#MH036244
Poales Poaceae Eriachne semiciliata#NC 041572
Poales Poaceae Eriachne basedowii#NC 041561
Poales Poaceae Eriachne agrostidea#NC 041557
Poales Poaceae Eriachne avenacea#MH036239
Poales Poaceae Eriachne melicacea#MH036254
Poales Poaceae Eriachne burkittii#NC 041562
Poales Poaceae Eriachne aristidea#MH036235
Poales Poaceae Eriachne pauciflora#MH036269
Poales Poaceae Eriachne glauca#MH036248
Poales Poaceae Eriachne sulcata#NC 041573
Poales Poaceae Eriachne stipacea#MH036279
Poales Poaceae Eriachne armitii#MH036237
Poales Poaceae Eriachne schultziana#MH036273
Poales Poaceae Eriachne triodioides#MH036283
Poales Poaceae Merxmuellera tsaratananensis#NC 036122
Poales Poaceae Chionochloa macra#NC 025230
Poales Poaceae Cortaderia selloana#NC 036681
Poales Poaceae Danthonia californica#NC 025232
Poales Poaceae Phaenanthoecium koestlinii#NC 035533
Poales Poaceae Schismus arabicus#NC 036703
Poales Poaceae Tribolium hispidum#NC 036705
Poales Poaceae Tenaxia guillarmodiae#NC 029897
Poales Poaceae Rytidosperma pallidum#NC 037166
Poales Poaceae Rytidosperma semiannulare#NC 036701
Poales Poaceae Centropodia glauca#NC 029411
Poales Poaceae Neyraudia reynaudiana#NC 024262
Poales Poaceae Nematopoa longipes#NC 035532
Poales Poaceae Enneapogon lindleyanus#NC 036682
Poales Poaceae Uniola paniculata#GZSF
Poales Poaceae Eragrostis tef#MF035981
Poales Poaceae Eragrostis minor#NC 029412
Poales Poaceae Zoysia macrantha#NC 029418
Poales Poaceae Zoysia macrostachya#MF967581
Poales Poaceae Zoysia tenuifolia#MF967580
Poales Poaceae Zoysia japonica#NC 036827
Poales Poaceae Zoysia sinica#MF967579
Poales Poaceae Urochondra setulosa#MH488957
Poales Poaceae Sporobolus helvolus#MH392303
Poales Poaceae Sporobolus heterolepis#NC 029417
Poales Poaceae Sporobolus rigidifolius#NC 027650
Poales Poaceae Sporobolus michauxianus#NC 029416
Poales Poaceae Hilaria rigida#NC 029896
Poales Poaceae Hilaria cenchroides#NC 029415
Poales Poaceae Bouteloua curtipendula#NC 029414
Poales Poaceae Bouteloua aristidoides#NC 029892
Poales Poaceae Distichlis bajaensis#NC 029894
Poales Poaceae Distichlis spicata#NC 029895
Poales Poaceae Halopyrum mucronatum#NC 036688
Poales Poaceae Melanocenchris abyssinica#NC 036694
Poales Poaceae Oropetium aristatum#NC 036697
Poales Poaceae Triodia stipoides#NC 037157
Poales Poaceae Triodia wiseana#NC 037161
Poales Poaceae Triodia aff. bynoei#YXNR
Poales Poaceae Dactyloctenium aegyptium#NC 036714
Poales Poaceae Vaseyochloa multinervosa#NC 036710
Poales Poaceae Trichoneura grandiglumis#NC 036707
Poales Poaceae Eleusine indica#NC 030486
Poales Poaceae Eleusine coracana#EFCZ
Poales Poaceae Astrebla pectinata#NC 029891
Poales Poaceae Leptochloa pluriflora#NC 037169
Poales Poaceae Leptochloa virgata#NC 036692
Poales Poaceae Chloris barbata#NC 029893
Poales Poaceae Oxychloris scariosa#NC 037158
Poales Poaceae Eustachys glauca#NC 036685
Poales Poaceae Cynodon dactylon#NC 034680
Poales Poaceae Dichaetaria wightii#NC 035525
Poales Poaceae Alloeochaete oreogena#NC 035518
Poales Poaceae Alloeochaete ulugurensis#NC 035519
Poales Poaceae Alloeochaete namuliensis#NC 035517
Poales Poaceae Thysanolaena latifolia#NC 025238
Poales Poaceae Centotheca lappacea#NC 025229
Poales Poaceae Danthoniopsis dinteri#MF035977
Poales Poaceae Loudetia simplex#NC 036113
Poales Poaceae Tristachya humbertii#NC 036115
Poales Poaceae Tristachya leucothrix#NC 036708
Poales Poaceae Chasmanthium laxum#NC 035522
Poales Poaceae Zeugites pittieri#NC 030500
Poales Poaceae Gynerium sagittatum#NC 036687
Poales Poaceae Lecomtella madagascariensis#NC 024106
Poales Poaceae Digitaria ciliaris#F674
Poales Poaceae Digitaria exilis#NC 024176
Poales Poaceae Brachiaria fragrans#NC 033879
Poales Poaceae Echinolaena madagascariensis#NC 033880
Poales Poaceae Panicum lycopodioides#NC 036121
Poales Poaceae Pseudolasiacis leptolomoides#NC 036119
Poales Poaceae Lasiacis nigra#NC 036123
Poales Poaceae Echinochloa ugandensis#NC 036127
Poales Poaceae Echinochloa frumentacea#NC 029827
Poales Poaceae Echinochloa crus-galli#NC 028719
Poales Poaceae Echinochloa stagnina#NC 036128
Poales Poaceae Echinochloa oryzoides#NC 024643
Poales Poaceae Entolasia imbricata#NC 036126
Poales Poaceae Panicum pygmaeum#NC 036120
Poales Poaceae Alloteropsis cimicina#NC 027952
Poales Poaceae Alloteropsis angusta#NC 027951
Poales Poaceae Alloteropsis semialata#NC 027824
Poales Poaceae Panicum incomtum#MH394349
Poales Poaceae Sacciolepis indica#NC 036702
Poales Poaceae Thyridolepis mitchelliana#XBKS
Poales Poaceae Thyridolepis multiculmis#WCOR
Poales Poaceae Neurachne annularis#BPKH
Poales Poaceae Neurachne munroi#ZMGN
Poales Poaceae Neurachne tenuifolia#NNOK
Poales Poaceae Neurachne minor#BXAY
Poales Poaceae Paraneurachne muelleri#NC 039450
Poales Poaceae Neurachne lanigera#VQYB
Poales Poaceae Neurachne alopecuroidea#ZENX
Poales Poaceae Panicum virgatum#NC 015990
Poales Poaceae Panicum miliaceum#NC 029732

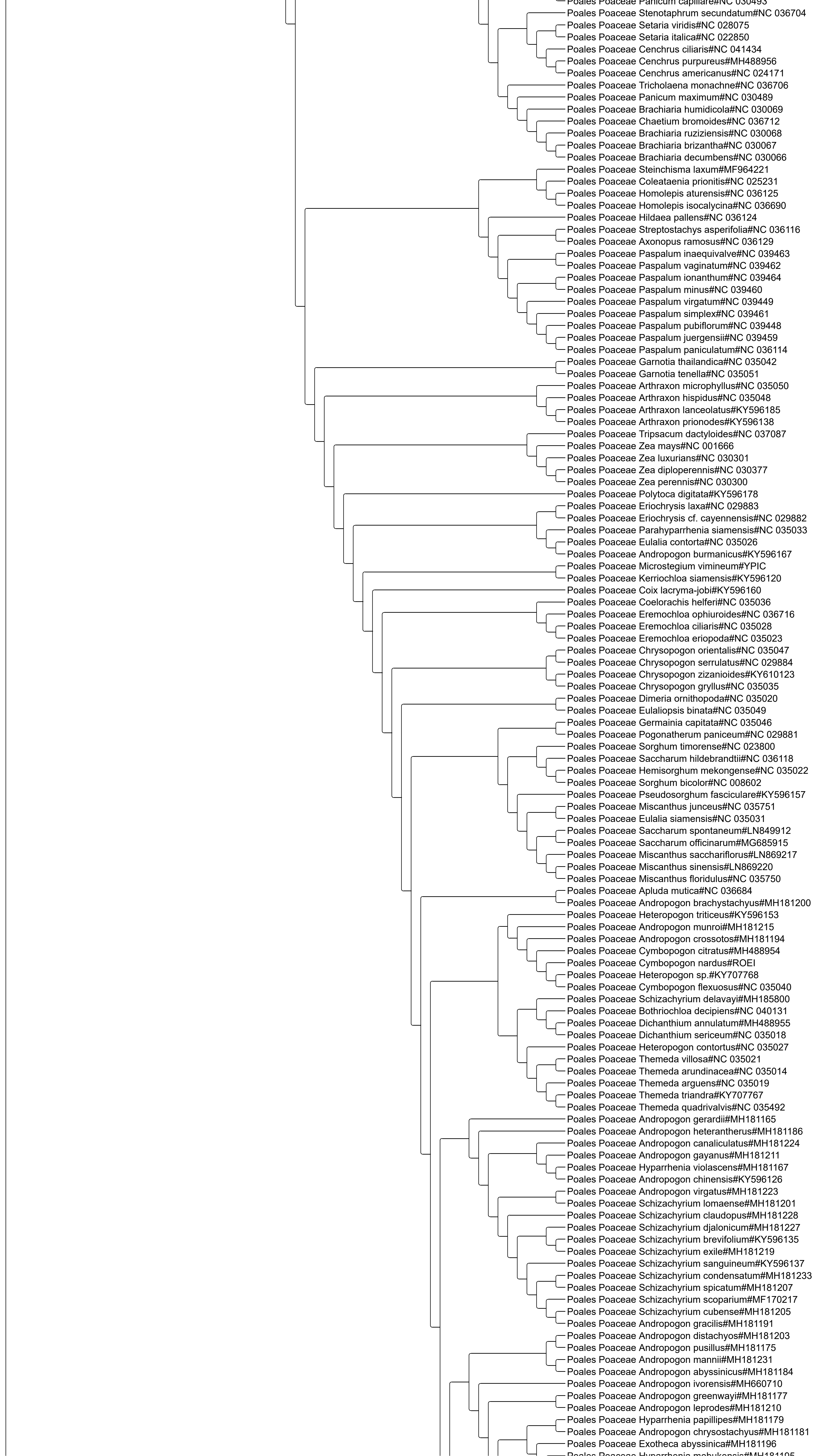

Poales Poaceae Stenotaphrum secundatum#NC 036704
Poales Poaceae Setaria viridis#NC 028075
Poales Poaceae Setaria italica#NC 022850
Poales Poaceae Cenchrus ciliaris#NC 041434
Poales Poaceae Cenchrus purpureus#MH488956
Poales Poaceae Cenchrus americanus#NC 024171
Poales Poaceae Tricholaena monachne#NC 036706
Poales Poaceae Panicum maximum#NC 030489
Poales Poaceae Brachiaria humidicola#NC 030069
Poales Poaceae Chaetium bromoides#NC 036712
Poales Poaceae Brachiaria ruziziensis#NC 030068
Poales Poaceae Brachiaria brizantha#NC 030067
Poales Poaceae Brachiaria decumbens#NC 030066
Poales Poaceae Steinchisma laxum#MF964221
Poales Poaceae Coleataenia prionitis#NC 025231
Poales Poaceae Homolepis aturensis#NC 036125
Poales Poaceae Homolepis isocalycina#NC 036690
Poales Poaceae Hildaea pallens#NC 036124
Poales Poaceae Streptostachys asperifolia#NC 036116
Poales Poaceae Axonopus ramosus#NC 036129
Poales Poaceae Paspalum inaequivalve#NC 039463
Poales Poaceae Paspalum vaginatum#NC 039462
Poales Poaceae Paspalum ionanthum#NC 039464
Poales Poaceae Paspalum minus#NC 039460
Poales Poaceae Paspalum virgatum#NC 039449
Poales Poaceae Paspalum simplex#NC 039461
Poales Poaceae Paspalum pubiflorum#NC 039448
Poales Poaceae Paspalum juergensii#NC 039459
Poales Poaceae Paspalum paniculatum#NC 036114
Poales Poaceae Garnotia thailandica#NC 035042
Poales Poaceae Garnotia tenella#NC 035051
Poales Poaceae Arthraxon microphyllus#NC 035050
Poales Poaceae Arthraxon hispidus#NC 035048
Poales Poaceae Arthraxon lanceolatus#KY596185
Poales Poaceae Arthraxon prionodes#KY596138
Poales Poaceae Tripsacum dactyloides#NC 037087
Poales Poaceae Zea mays#NC 001666
Poales Poaceae Zea luxurians#NC 030301
Poales Poaceae Zea diploperennis#NC 030377
Poales Poaceae Zea perennis#NC 030300
Poales Poaceae Polytoca digitata#KY596178
Poales Poaceae Eriochrysis laxa#NC 029883
Poales Poaceae Eriochrysis cf. cayennensis#NC 029882
Poales Poaceae Parahyparrhenia siamensis#NC 035033
Poales Poaceae Eulalia contorta#NC 035026
Poales Poaceae Andropogon burmanicus#KY596167
Poales Poaceae Microstegium vimineum#YPIC
Poales Poaceae Kerriochloa siamensis#KY596120
Poales Poaceae Coix lacryma-jobi#KY596160
Poales Poaceae Coelorachis helferi#NC 035036
Poales Poaceae Eremochloa ophiuroides#NC 036716
Poales Poaceae Eremochloa ciliaris#NC 035028
Poales Poaceae Eremochloa eriopoda#NC 035023
Poales Poaceae Chrysopogon orientalis#NC 035047
Poales Poaceae Chrysopogon serrulatus#NC 029884
Poales Poaceae Chrysopogon zizanioides#KY610123
Poales Poaceae Chrysopogon gryllus#NC 035035
Poales Poaceae Dimeria ornithopoda#NC 035020
Poales Poaceae Eulaliopsis binata#NC 035049
Poales Poaceae Germainia capitata#NC 035046
Poales Poaceae Pogonatherum paniceum#NC 029881
Poales Poaceae Sorghum timorense#NC 023800
Poales Poaceae Saccharum hildebrandtii#NC 036118
Poales Poaceae Hemisorghum mekongense#NC 035022
Poales Poaceae Sorghum bicolor#NC 008602
Poales Poaceae Pseudosorghum fasciculare#KY596157
Poales Poaceae Miscanthus junceus#NC 035751
Poales Poaceae Eulalia siamensis#NC 035031
Poales Poaceae Saccharum spontaneum#LN849912
Poales Poaceae Saccharum officinarum#MG685915
Poales Poaceae Miscanthus sacchariflorus#LN869217
Poales Poaceae Miscanthus sinensis#LN869220
Poales Poaceae Miscanthus floridulus#NC 035750
Poales Poaceae Apluda mutica#NC 036684
Poales Poaceae Andropogon brachystachyus#MH181200
Poales Poaceae Heteropogon triticeus#KY596153
Poales Poaceae Andropogon munroi#MH181215
Poales Poaceae Andropogon crossotos#MH181194
Poales Poaceae Cymbopogon citratus#MH488954
Poales Poaceae Cymbopogon nardus#ROEI
Poales Poaceae Heteropogon sp.#KY707768
Poales Poaceae Cymbopogon flexuosus#NC 035040
Poales Poaceae Schizachyrium delavayi#MH185800
Poales Poaceae Bothriochloa decipiens#NC 040131
Poales Poaceae Dichanthium annulatum#MH488955
Poales Poaceae Dichanthium sericeum#NC 035018
Poales Poaceae Heteropogon contortus#NC 035027
Poales Poaceae Themeda villosa#NC 035021
Poales Poaceae Themeda arundinacea#NC 035014
Poales Poaceae Themeda arguens#NC 035019
Poales Poaceae Themeda triandra#KY707767
Poales Poaceae Themeda quadrivalvis#NC 035492
Poales Poaceae Andropogon gerardii#MH181165
Poales Poaceae Andropogon heterantherus#MH181186
Poales Poaceae Andropogon canaliculatus#MH181224
Poales Poaceae Andropogon gayanus#MH181211
Poales Poaceae Hyparrhenia violascens#MH181167
Poales Poaceae Andropogon chinensis#KY596126
Poales Poaceae Andropogon virgatus#MH181223
Poales Poaceae Schizachyrium lomaense#MH181201
Poales Poaceae Schizachyrium claudopus#MH181228
Poales Poaceae Schizachyrium djalonicum#MH181227
Poales Poaceae Schizachyrium brevifolium#KY596135
Poales Poaceae Schizachyrium exile#MH181219
Poales Poaceae Schizachyrium sanguineum#KY596137
Poales Poaceae Schizachyrium condensatum#MH181233
Poales Poaceae Schizachyrium spicatum#MH181207
Poales Poaceae Schizachyrium scoparium#MF170217
Poales Poaceae Schizachyrium cubense#MH181205
Poales Poaceae Andropogon gracilis#MH181191
Poales Poaceae Andropogon distachyos#MH181203
Poales Poaceae Andropogon pusillus#MH181175
Poales Poaceae Andropogon mannii#MH181231
Poales Poaceae Andropogon abyssinicus#MH181184
Poales Poaceae Andropogon ivorensis#MH660710
Poales Poaceae Andropogon greenwayi#MH181177
Poales Poaceae Andropogon leprodes#MH181210
Poales Poaceae Hyparrhenia papillipes#MH181179
Poales Poaceae Andropogon chrysostachyus#MH181181
Poales Poaceae Exotheca abyssinica#MH181196

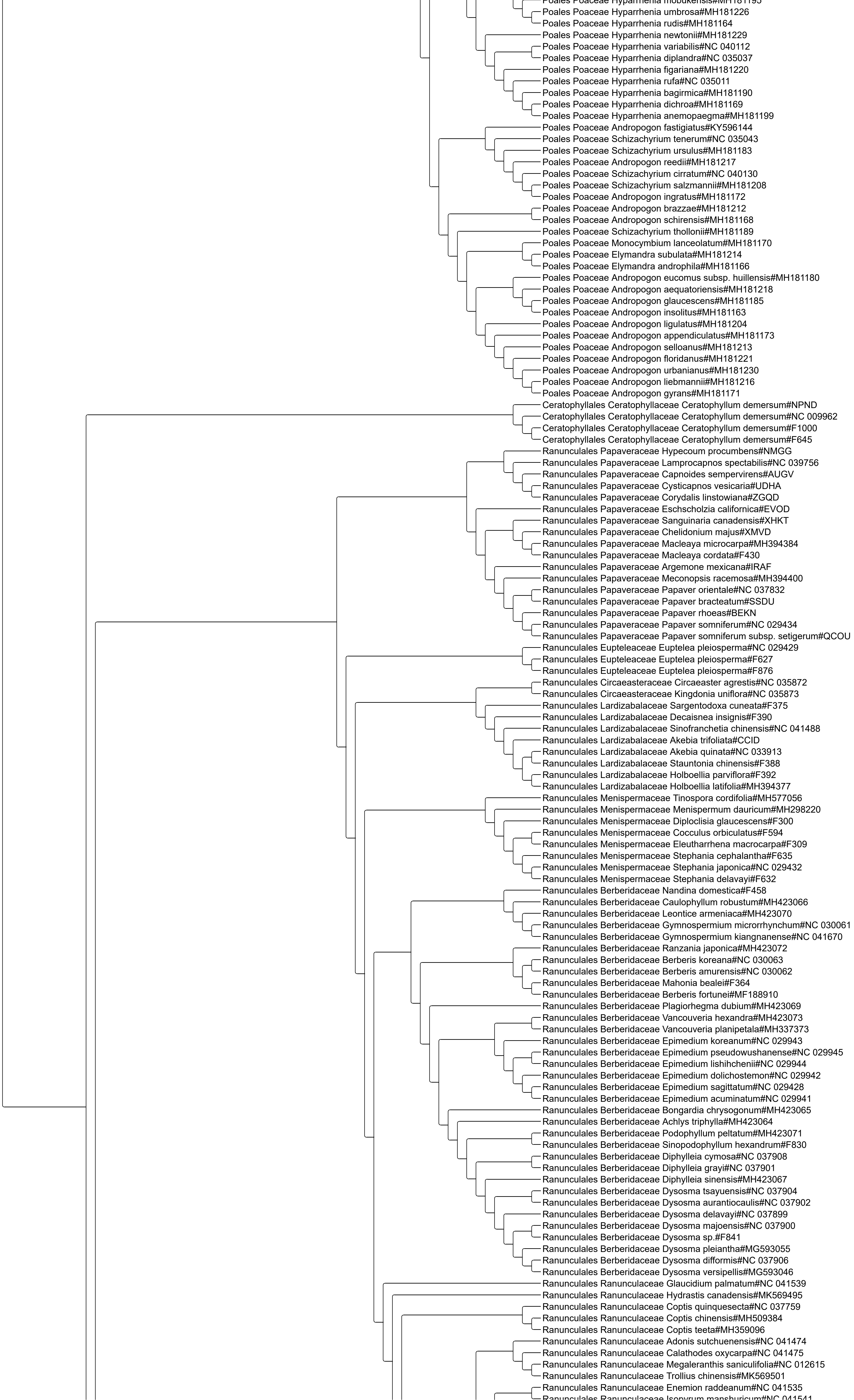

Poales Poaceae Hyparrhenia umbrosa#MH181226
Poales Poaceae Hyparrhenia rudis#MH181164
Poales Poaceae Hyparrhenia newtonii#MH181229
Poales Poaceae Hyparrhenia variabilis#NC 040112
Poales Poaceae Hyparrhenia diplandra#NC 035037
Poales Poaceae Hyparrhenia figariana#MH181220
Poales Poaceae Hyparrhenia rufa#NC 035011
Poales Poaceae Hyparrhenia bagirmica#MH181190
Poales Poaceae Hyparrhenia dichroa#MH181169
Poales Poaceae Hyparrhenia anemopaegma#MH181199
Poales Poaceae Andropogon fastigiatus#KY596144
Poales Poaceae Schizachyrium tenerum#NC 035043
Poales Poaceae Schizachyrium ursulus#MH181183
Poales Poaceae Andropogon reedii#MH181217
Poales Poaceae Schizachyrium cirratum#NC 040130
Poales Poaceae Schizachyrium salzmannii#MH181208
Poales Poaceae Andropogon ingratus#MH181172
Poales Poaceae Andropogon brazzae#MH181212
Poales Poaceae Andropogon schirensis#MH181168
Poales Poaceae Schizachyrium thollonii#MH181189
Poales Poaceae Monocymbium lanceolatum#MH181170
Poales Poaceae Elymandra subulata#MH181214
Poales Poaceae Elymandra androphila#MH181166
Poales Poaceae Andropogon eucomus subsp. huillensis#MH181180
Poales Poaceae Andropogon aequatoriensis#MH181218
Poales Poaceae Andropogon glaucescens#MH181185
Poales Poaceae Andropogon insolitus#MH181163
Poales Poaceae Andropogon ligulatus#MH181204
Poales Poaceae Andropogon appendiculatus#MH181173
Poales Poaceae Andropogon selloanus#MH181213
Poales Poaceae Andropogon floridanus#MH181221
Poales Poaceae Andropogon urbanianus#MH181230
Poales Poaceae Andropogon liebmannii#MH181216
Poales Poaceae Andropogon gyrans#MH181171
Ceratophyllales Ceratophyllaceae Ceratophyllum demersum#NPND
Ceratophyllales Ceratophyllaceae Ceratophyllum demersum#NC 009962
Ceratophyllales Ceratophyllaceae Ceratophyllum demersum#F1000
Ceratophyllales Ceratophyllaceae Ceratophyllum demersum#F645
Ranunculales Papaveraceae Hypecoum procumbens#NMGG
Ranunculales Papaveraceae Lamprocapnos spectabilis#NC 039756
Ranunculales Papaveraceae Capnoides sempervirens#AUGV
Ranunculales Papaveraceae Cysticapnos vesicaria#UDHA
Ranunculales Papaveraceae Corydalis linstowiana#ZGQD
Ranunculales Papaveraceae Eschscholzia californica#EVOD
Ranunculales Papaveraceae Sanguinaria canadensis#XHKT
Ranunculales Papaveraceae Chelidonium majus#XMVD
Ranunculales Papaveraceae Macleaya microcarpa#MH394384
Ranunculales Papaveraceae Macleaya cordata#F430
Ranunculales Papaveraceae Argemone mexicana#IRAF
Ranunculales Papaveraceae Meconopsis racemosa#MH394400
Ranunculales Papaveraceae Papaver orientale#NC 037832
Ranunculales Papaveraceae Papaver bracteatum#SSDU
Ranunculales Papaveraceae Papaver rhoeas#BEKN
Ranunculales Papaveraceae Papaver somniferum#NC 029434
Ranunculales Papaveraceae Papaver somniferum subsp. setigerum#QCOU
Ranunculales Eupteleaceae Euptelea pleiosperma#NC 029429
Ranunculales Eupteleaceae Euptelea pleiosperma#F627
Ranunculales Eupteleaceae Euptelea pleiosperma#F876
Ranunculales Circaeasteraceae Circaeaster agrestis#NC 035872
Ranunculales Circaeasteraceae Kingdonia uniflora#NC 035873
Ranunculales Lardizabalaceae Sargentodoxa cuneata#F375
Ranunculales Lardizabalaceae Decaisnea insignis#F390
Ranunculales Lardizabalaceae Sinofranchetia chinensis#NC 041488
Ranunculales Lardizabalaceae Akebia trifoliata#CCID
Ranunculales Lardizabalaceae Akebia quinata#NC 033913
Ranunculales Lardizabalaceae Stauntonia chinensis#F388
Ranunculales Lardizabalaceae Holboellia parviflora#F392
Ranunculales Lardizabalaceae Holboellia latifolia#MH394377
Ranunculales Menispermaceae Tinospora cordifolia#MH577056
Ranunculales Menispermaceae Menispermum dauricum#MH298220
Ranunculales Menispermaceae Diploclisia glaucescens#F300
Ranunculales Menispermaceae Cocculus orbiculatus#F594
Ranunculales Menispermaceae Eleutharrhena macrocarpa#F309
Ranunculales Menispermaceae Stephania cephalantha#F635
Ranunculales Menispermaceae Stephania japonica#NC 029432
Ranunculales Menispermaceae Stephania delavayi#F632
Ranunculales Berberidaceae Nandina domestica#F458
Ranunculales Berberidaceae Caulophyllum robustum#MH423066
Ranunculales Berberidaceae Leontice armeniaca#MH423070
Ranunculales Berberidaceae Gymnospermium microrrhynchum#NC 030061
Ranunculales Berberidaceae Gymnospermium kiangnanense#NC 041670
Ranunculales Berberidaceae Ranzania japonica#MH423072
Ranunculales Berberidaceae Berberis koreana#NC 030063
Ranunculales Berberidaceae Berberis amurensis#NC 030062
Ranunculales Berberidaceae Mahonia bealei#F364
Ranunculales Berberidaceae Berberis fortunei#MF188910
Ranunculales Berberidaceae Plagiorhegma dubium#MH423069
Ranunculales Berberidaceae Vancouveria hexandra#MH423073
Ranunculales Berberidaceae Vancouveria planipetala#MH337373
Ranunculales Berberidaceae Epimedium koreanum#NC 029943
Ranunculales Berberidaceae Epimedium pseudowushanense#NC 029945
Ranunculales Berberidaceae Epimedium lishihchenii#NC 029944
Ranunculales Berberidaceae Epimedium dolichostemon#NC 029942
Ranunculales Berberidaceae Epimedium sagittatum#NC 029428
Ranunculales Berberidaceae Epimedium acuminatum#NC 029941
Ranunculales Berberidaceae Bongardia chrysogonum#MH423065
Ranunculales Berberidaceae Achlys triphylla#MH423064
Ranunculales Berberidaceae Podophyllum peltatum#MH423071
Ranunculales Berberidaceae Sinopodophyllum hexandrum#F830
Ranunculales Berberidaceae Diphylleia cymosa#NC 037908
Ranunculales Berberidaceae Diphylleia grayi#NC 037901
Ranunculales Berberidaceae Diphylleia sinensis#MH423067
Ranunculales Berberidaceae Dysosma tsayuensis#NC 037904
Ranunculales Berberidaceae Dysosma aurantiocaulis#NC 037902
Ranunculales Berberidaceae Dysosma delavayi#NC 037899
Ranunculales Berberidaceae Dysosma majoensis#NC 037900
Ranunculales Berberidaceae Dysosma sp.#F841
Ranunculales Berberidaceae Dysosma pleiantha#MG593055
Ranunculales Berberidaceae Dysosma difformis#NC 037906
Ranunculales Berberidaceae Dysosma versipellis#MG593046
Ranunculales Ranunculaceae Glaucidium palmatum#NC 041539
Ranunculales Ranunculaceae Hydrastis canadensis#MK569495
Ranunculales Ranunculaceae Coptis quinquesecta#NC 037759
Ranunculales Ranunculaceae Coptis chinensis#MH509384
Ranunculales Ranunculaceae Coptis teeta#MH359096
Ranunculales Ranunculaceae Adonis sutchuenensis#NC 041474
Ranunculales Ranunculaceae Calathodes oxycarpa#NC 041475
Ranunculales Ranunculaceae Megaleranthis saniculifolia#NC 012615
Ranunculales Ranunculaceae Trollius chinensis#MK569501
Ranunculales Ranunculaceae Enemion raddeanum#NC 041535

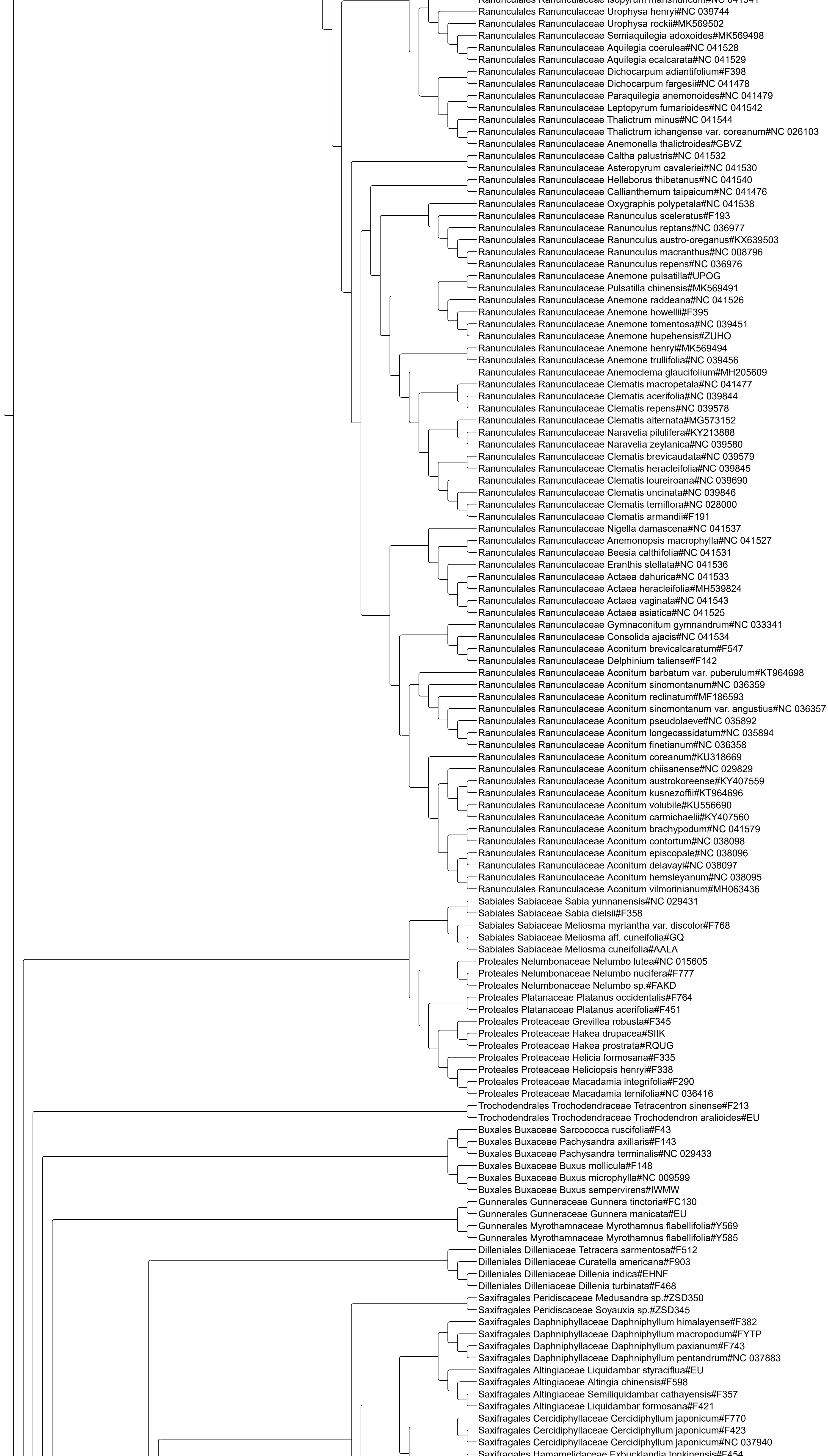

Ranunculales Ranunculaceae Urophysa henryi#NC 039744
Ranunculales Ranunculaceae Urophysa rockii#MK569502
Ranunculales Ranunculaceae Semiaquilegia adoxoides#MK569498
Ranunculales Ranunculaceae Aquilegia coerulea#NC 041528
Ranunculales Ranunculaceae Aquilegia ecalcarata#NC 041529
Ranunculales Ranunculaceae Dichocarpum adiantifolium#F398
Ranunculales Ranunculaceae Dichocarpum fargesii#NC 041478
Ranunculales Ranunculaceae Paraquilegia anemonoides#NC 041479
Ranunculales Ranunculaceae Leptopyrum fumarioides#NC 041542
Ranunculales Ranunculaceae Thalictrum minus#NC 041544
Ranunculales Ranunculaceae Thalictrum ichangense var. coreanum#NC 026103
Ranunculales Ranunculaceae Anemonella thalictroides#GBVZ
Ranunculales Ranunculaceae Caltha palustris#NC 041532
Ranunculales Ranunculaceae Asteropyrum cavaleriei#NC 041530
Ranunculales Ranunculaceae Helleborus thibetanus#NC 041540
Ranunculales Ranunculaceae Callianthemum taipaicum#NC 041476
Ranunculales Ranunculaceae Oxygraphis polypetala#NC 041538
Ranunculales Ranunculaceae Ranunculus sceleratus#F193
Ranunculales Ranunculaceae Ranunculus reptans#NC 036977
Ranunculales Ranunculaceae Ranunculus austro-oreganus#KX639503
Ranunculales Ranunculaceae Ranunculus macranthus#NC 008796
Ranunculales Ranunculaceae Ranunculus repens#NC 036976
Ranunculales Ranunculaceae Anemone pulsatilla#UPOG
Ranunculales Ranunculaceae Pulsatilla chinensis#MK569491
Ranunculales Ranunculaceae Anemone raddeana#NC 041526
Ranunculales Ranunculaceae Anemone howellii#F395
Ranunculales Ranunculaceae Anemone tomentosa#NC 039451
Ranunculales Ranunculaceae Anemone hupehensis#ZUHO
Ranunculales Ranunculaceae Anemone henryi#MK569494
Ranunculales Ranunculaceae Anemone trullifolia#NC 039456
Ranunculales Ranunculaceae Anemoclema glaucifolium#MH205609
Ranunculales Ranunculaceae Clematis macropetala#NC 041477
Ranunculales Ranunculaceae Clematis acerifolia#NC 039844
Ranunculales Ranunculaceae Clematis repens#NC 039578
Ranunculales Ranunculaceae Clematis alternata#MG573152
Ranunculales Ranunculaceae Naravelia pilulifera#KY213888
Ranunculales Ranunculaceae Naravelia zeylanica#NC 039580
Ranunculales Ranunculaceae Clematis brevicaudata#NC 039579
Ranunculales Ranunculaceae Clematis heracleifolia#NC 039845
Ranunculales Ranunculaceae Clematis loureiroana#NC 039690
Ranunculales Ranunculaceae Clematis uncinata#NC 039846
Ranunculales Ranunculaceae Clematis terniflora#NC 028000
Ranunculales Ranunculaceae Clematis armandii#F191
Ranunculales Ranunculaceae Nigella damascena#NC 041537
Ranunculales Ranunculaceae Anemonopsis macrophylla#NC 041527
Ranunculales Ranunculaceae Beesia calthifolia#NC 041531
Ranunculales Ranunculaceae Eranthis stellata#NC 041536
Ranunculales Ranunculaceae Actaea dahurica#NC 041533
Ranunculales Ranunculaceae Actaea heracleifolia#MH539824
Ranunculales Ranunculaceae Actaea vaginata#NC 041543
Ranunculales Ranunculaceae Actaea asiatica#NC 041525
Ranunculales Ranunculaceae Gymnaconitum gymnandrum#NC 033341
Ranunculales Ranunculaceae Consolida ajacis#NC 041534
Ranunculales Ranunculaceae Aconitum brevicalcaratum#F547
Ranunculales Ranunculaceae Delphinium taliense#F142
Ranunculales Ranunculaceae Aconitum barbatum var. puberulum#KT964698
Ranunculales Ranunculaceae Aconitum sinomontanum#NC 036359
Ranunculales Ranunculaceae Aconitum reclinatum#MF186593
Ranunculales Ranunculaceae Aconitum sinomontanum var. angustius#NC 036357
Ranunculales Ranunculaceae Aconitum pseudolaeve#NC 035892
Ranunculales Ranunculaceae Aconitum longecassidatum#NC 035894
Ranunculales Ranunculaceae Aconitum finetianum#NC 036358
Ranunculales Ranunculaceae Aconitum coreanum#KU318669
Ranunculales Ranunculaceae Aconitum chiisanense#NC 029829
Ranunculales Ranunculaceae Aconitum austrokoreense#KY407559
Ranunculales Ranunculaceae Aconitum kusnezoffii#KT964696
Ranunculales Ranunculaceae Aconitum volubile#KU556690
Ranunculales Ranunculaceae Aconitum carmichaelii#KY407560
Ranunculales Ranunculaceae Aconitum brachypodum#NC 041579
Ranunculales Ranunculaceae Aconitum contortum#NC 038098
Ranunculales Ranunculaceae Aconitum episcopale#NC 038096
Ranunculales Ranunculaceae Aconitum delavayi#NC 038097
Ranunculales Ranunculaceae Aconitum hemsleyanum#NC 038095
Ranunculales Ranunculaceae Aconitum vilmorinianum#MH063436
Sabiales Sabiaceae Sabia yunnanensis#NC 029431
Sabiales Sabiaceae Sabia dielsii#F358
Sabiales Sabiaceae Meliosma myriantha var. discolor#F768
Sabiales Sabiaceae Meliosma aff. cuneifolia#GQ
Sabiales Sabiaceae Meliosma cuneifolia#AALA
Proteales Nelumbonaceae Nelumbo lutea#NC 015605
Proteales Nelumbonaceae Nelumbo nucifera#F777
Proteales Nelumbonaceae Nelumbo sp.#FAKD
Proteales Platanaceae Platanus occidentalis#F764
Proteales Platanaceae Platanus acerifolia#F451
Proteales Proteaceae Grevillea robusta#F345
Proteales Proteaceae Hakea drupacea#SIIK
Proteales Proteaceae Hakea prostrata#RQUG
Proteales Proteaceae Helicia formosana#F335
Proteales Proteaceae Heliciopsis henryi#F338
Proteales Proteaceae Macadamia integrifolia#F290
Proteales Proteaceae Macadamia ternifolia#NC 036416
Trochodendrales Trochodendraceae Tetracentron sinense#F213
Trochodendrales Trochodendraceae Trochodendron aralioides#EU
Buxales Buxaceae Sarcococca ruscifolia#F43
Buxales Buxaceae Pachysandra axillaris#F143
Buxales Buxaceae Pachysandra terminalis#NC 029433
Buxales Buxaceae Buxus mollicula#F148
Buxales Buxaceae Buxus microphylla#NC 009599
Buxales Buxaceae Buxus sempervirens#IWMW
Gunnerales Gunneraceae Gunnera tinctoria#FC130
Gunnerales Gunneraceae Gunnera manicata#EU
Gunnerales Myrothamnaceae Myrothamnus flabellifolia#Y569
Gunnerales Myrothamnaceae Myrothamnus flabellifolia#Y585
Dilleniales Dilleniaceae Tetracera sarmentosa#F512
Dilleniales Dilleniaceae Curatella americana#F903
Dilleniales Dilleniaceae Dillenia indica#EHNF
Dilleniales Dilleniaceae Dillenia turbinata#F468
Saxifragales Peridiscaceae Medusandra sp.#ZSD350
Saxifragales Peridiscaceae Soyauxia sp.#ZSD345
Saxifragales Daphniphyllaceae Daphniphyllum himalayense#F382
Saxifragales Daphniphyllaceae Daphniphyllum macropodum#FYTP
Saxifragales Daphniphyllaceae Daphniphyllum paxianum#F743
Saxifragales Daphniphyllaceae Daphniphyllum pentandrum#NC 037883
Saxifragales Altingiaceae Liquidambar styraciflua#EU
Saxifragales Altingiaceae Altingia chinensis#F598
Saxifragales Altingiaceae Semiliquidambar cathayensis#F357
Saxifragales Altingiaceae Liquidambar formosana#F421
Saxifragales Cercidiphyllaceae Cercidiphyllum japonicum#F770
Saxifragales Cercidiphyllaceae Cercidiphyllum japonicum#F423
Saxifragales Cercidiphyllaceae Cercidiphyllum japonicum#NC 037940

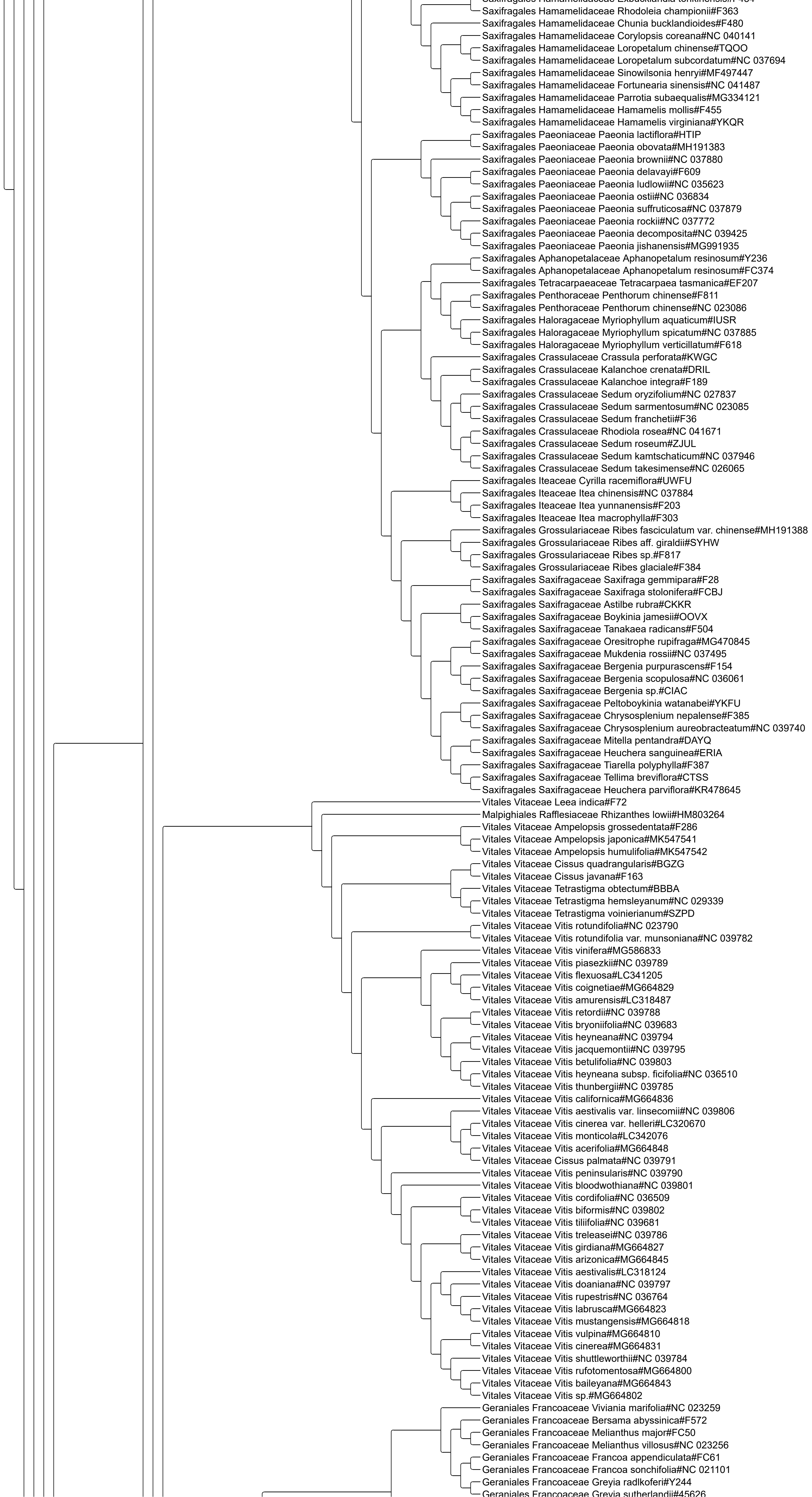

Saxifragales Hamamelidaceae Rhodoleia championii#F363
Saxifragales Hamamelidaceae Chunia bucklandioides#F480
Saxifragales Hamamelidaceae Corylopsis coreana#NC 040141
Saxifragales Hamamelidaceae Loropetalum chinense#TQOO
Saxifragales Hamamelidaceae Loropetalum subcordatum#NC 037694
Saxifragales Hamamelidaceae Sinowilsonia henryi#MF497447
Saxifragales Hamamelidaceae Fortunearia sinensis#NC 041487
Saxifragales Hamamelidaceae Parrotia subaequalis#MG334121
Saxifragales Hamamelidaceae Hamamelis mollis#F455
Saxifragales Hamamelidaceae Hamamelis virginiana#YKQR
Saxifragales Paeoniaceae Paeonia lactiflora#HTIP
Saxifragales Paeoniaceae Paeonia obovata#MH191383
Saxifragales Paeoniaceae Paeonia brownii#NC 037880
Saxifragales Paeoniaceae Paeonia delavayi#F609
Saxifragales Paeoniaceae Paeonia ludlowii#NC 035623
Saxifragales Paeoniaceae Paeonia ostii#NC 036834
Saxifragales Paeoniaceae Paeonia suffruticosa#NC 037879
Saxifragales Paeoniaceae Paeonia rockii#NC 037772
Saxifragales Paeoniaceae Paeonia decomposita#NC 039425
Saxifragales Paeoniaceae Paeonia jishanensis#MG991935
Saxifragales Aphanopetalaceae Aphanopetalum resinosum#Y236
Saxifragales Aphanopetalaceae Aphanopetalum resinosum#FC374
Saxifragales Tetracarpaeaceae Tetracarpaea tasmanica#EF207
Saxifragales Penthoraceae Penthorum chinense#F811
Saxifragales Penthoraceae Penthorum chinense#NC 023086
Saxifragales Haloragaceae Myriophyllum aquaticum#IUSR
Saxifragales Haloragaceae Myriophyllum spicatum#NC 037885
Saxifragales Haloragaceae Myriophyllum verticillatum#F618
Saxifragales Crassulaceae Crassula perforata#KWGC
Saxifragales Crassulaceae Kalanchoe crenata#DRIL
Saxifragales Crassulaceae Kalanchoe integra#F189
Saxifragales Crassulaceae Sedum oryzifolium#NC 027837
Saxifragales Crassulaceae Sedum sarmentosum#NC 023085
Saxifragales Crassulaceae Sedum franchetii#F36
Saxifragales Crassulaceae Rhodiola rosea#NC 041671
Saxifragales Crassulaceae Sedum roseum#ZJUL
Saxifragales Crassulaceae Sedum kamtschaticum#NC 037946
Saxifragales Crassulaceae Sedum takesimense#NC 026065
Saxifragales Iteaceae Cyrilla racemiflora#UWFU
Saxifragales Iteaceae Itea chinensis#NC 037884
Saxifragales Iteaceae Itea yunnanensis#F203
Saxifragales Iteaceae Itea macrophylla#F303
Saxifragales Grossulariaceae Ribes fasciculatum var. chinense#MH191388
Saxifragales Grossulariaceae Ribes aff. giraldii#SYHW
Saxifragales Grossulariaceae Ribes sp.#F817
Saxifragales Grossulariaceae Ribes glaciale#F384
Saxifragales Saxifragaceae Saxifraga gemmipara#F28
Saxifragales Saxifragaceae Saxifraga stolonifera#FCBJ
Saxifragales Saxifragaceae Astilbe rubra#CKKR
Saxifragales Saxifragaceae Boykinia jamesii#OOVX
Saxifragales Saxifragaceae Tanakaea radicans#F504
Saxifragales Saxifragaceae Oresitrophe rupifraga#MG470845
Saxifragales Saxifragaceae Mukdenia rossii#NC 037495
Saxifragales Saxifragaceae Bergenia purpurascens#F154
Saxifragales Saxifragaceae Bergenia scopulosa#NC 036061
Saxifragales Saxifragaceae Bergenia sp.#CIAC
Saxifragales Saxifragaceae Peltoboykinia watanabei#YKFU
Saxifragales Saxifragaceae Chrysosplenium nepalense#F385
Saxifragales Saxifragaceae Chrysosplenium aureobracteatum#NC 039740
Saxifragales Saxifragaceae Mitella pentandra#DAYQ
Saxifragales Saxifragaceae Heuchera sanguinea#ERIA
Saxifragales Saxifragaceae Tiarella polyphylla#F387
Saxifragales Saxifragaceae Tellima breviflora#CTSS
Saxifragales Saxifragaceae Heuchera parviflora#KR478645
Vitales Vitaceae Leea indica#F72
Malpighiales Rafflesiaceae Rhizanthes lowii#HM803264
Vitales Vitaceae Ampelopsis grossedentata#F286
Vitales Vitaceae Ampelopsis japonica#MK547541
Vitales Vitaceae Ampelopsis humulifolia#MK547542
Vitales Vitaceae Cissus quadrangularis#BGZG
Vitales Vitaceae Cissus javana#F163
Vitales Vitaceae Tetrastigma obtectum#BBBA
Vitales Vitaceae Tetrastigma hemsleyanum#NC 029339
Vitales Vitaceae Tetrastigma voinierianum#SZPD
Vitales Vitaceae Vitis rotundifolia#NC 023790
Vitales Vitaceae Vitis rotundifolia var. munsoniana#NC 039782
Vitales Vitaceae Vitis vinifera#MG586833
Vitales Vitaceae Vitis piasezkii#NC 039789
Vitales Vitaceae Vitis flexuosa#LC341205
Vitales Vitaceae Vitis coignetiae#MG664829
Vitales Vitaceae Vitis amurensis#LC318487
Vitales Vitaceae Vitis retordii#NC 039788
Vitales Vitaceae Vitis bryoniifolia#NC 039683
Vitales Vitaceae Vitis heyneana#NC 039794
Vitales Vitaceae Vitis jacquemontii#NC 039795
Vitales Vitaceae Vitis betulifolia#NC 039803
Vitales Vitaceae Vitis heyneana subsp. ficifolia#NC 036510
Vitales Vitaceae Vitis thunbergii#NC 039785
Vitales Vitaceae Vitis californica#MG664836
Vitales Vitaceae Vitis aestivalis var. linsecomii#NC 039806
Vitales Vitaceae Vitis cinerea var. helleri#LC320670
Vitales Vitaceae Vitis monticola#LC342076
Vitales Vitaceae Vitis acerifolia#MG664848
Vitales Vitaceae Cissus palmata#NC 039791
Vitales Vitaceae Vitis peninsularis#NC 039790
Vitales Vitaceae Vitis bloodwothiana#NC 039801
Vitales Vitaceae Vitis cordifolia#NC 036509
Vitales Vitaceae Vitis biformis#NC 039802
Vitales Vitaceae Vitis tiliifolia#NC 039681
Vitales Vitaceae Vitis treleasei#NC 039786
Vitales Vitaceae Vitis girdiana#MG664827
Vitales Vitaceae Vitis arizonica#MG664845
Vitales Vitaceae Vitis aestivalis#LC318124
Vitales Vitaceae Vitis doaniana#NC 039797
Vitales Vitaceae Vitis rupestris#NC 036764
Vitales Vitaceae Vitis labrusca#MG664823
Vitales Vitaceae Vitis mustangensis#MG664818
Vitales Vitaceae Vitis vulpina#MG664810
Vitales Vitaceae Vitis cinerea#MG664831
Vitales Vitaceae Vitis shuttleworthii#NC 039784
Vitales Vitaceae Vitis rufotomentosa#MG664800
Vitales Vitaceae Vitis baileyana#MG664843
Vitales Vitaceae Vitis sp.#MG664802
Geraniales Francoaceae Viviania marifolia#NC 023259
Geraniales Francoaceae Bersama abyssinica#F572
Geraniales Francoaceae Melianthus major#FC50
Geraniales Francoaceae Melianthus villosus#NC 023256
Geraniales Francoaceae Francoa appendiculata#FC61
Geraniales Francoaceae Francoa sonchifolia#NC 021101
Geraniales Francoaceae Greyia radlkoferi#Y244
Geraniales Francoaceae Greyia sutherlandii#45626

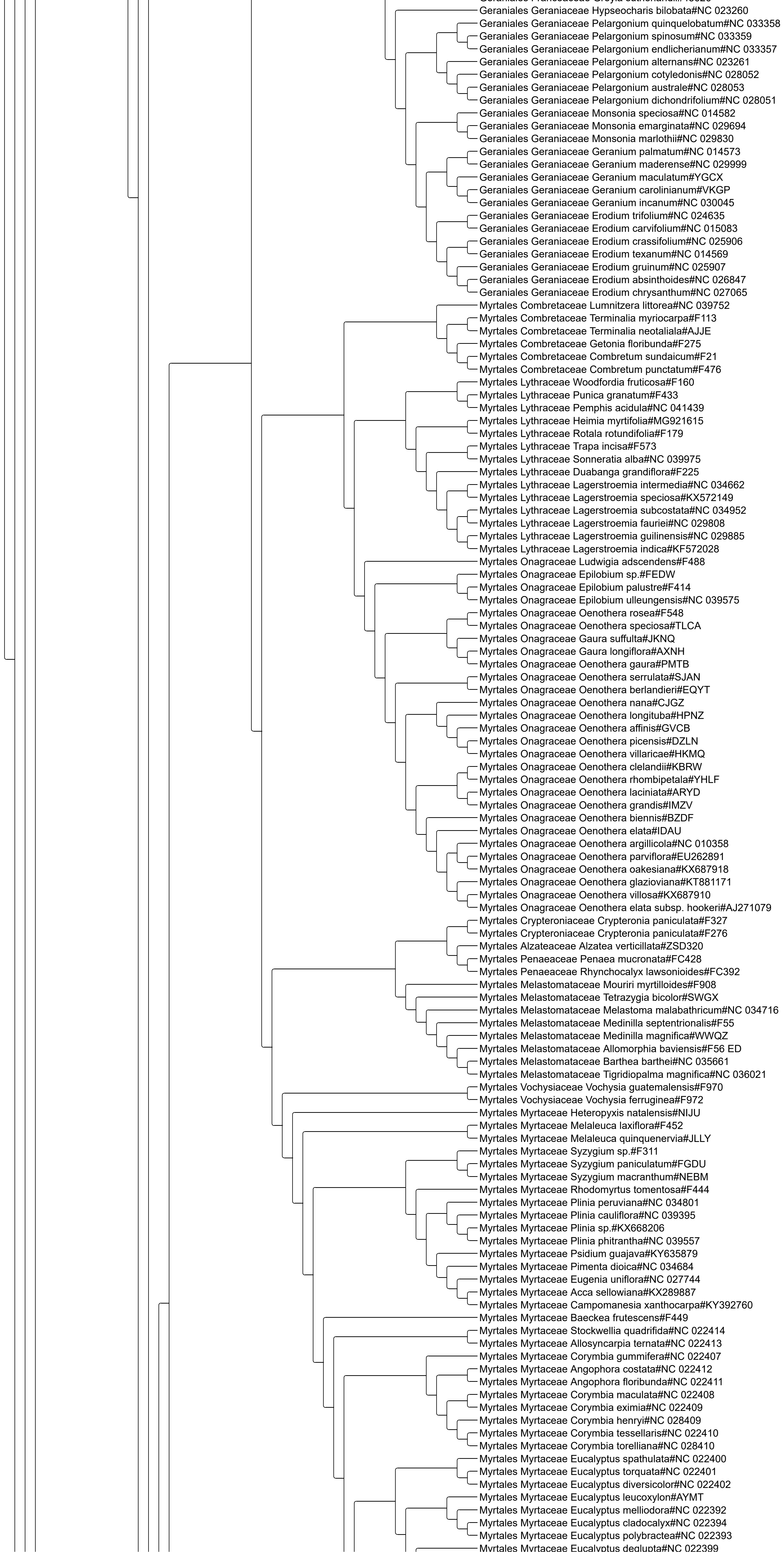
Geraniales Geraniaceae Hypseocharis bilobata#NC 023260
Geraniales Geraniaceae Pelargonium quinquelobatum#NC 033358
Geraniales Geraniaceae Pelargonium spinosum#NC 033359
Geraniales Geraniaceae Pelargonium endlicherianum#NC 033357
Geraniales Geraniaceae Pelargonium alternans#NC 023261
Geraniales Geraniaceae Pelargonium cotyledonis#NC 028052
Geraniales Geraniaceae Pelargonium australe#NC 028053
Geraniales Geraniaceae Pelargonium dichondrifolium#NC 028051
Geraniales Geraniaceae Monsonia speciosa#NC 014582
Geraniales Geraniaceae Monsonia emarginata#NC 029694
Geraniales Geraniaceae Monsonia marlothii#NC 029830
Geraniales Geraniaceae Geranium palmatum#NC 014573
Geraniales Geraniaceae Geranium maderense#NC 029999
Geraniales Geraniaceae Geranium maculatum#YGCX
Geraniales Geraniaceae Geranium carolinianum#VKGP
Geraniales Geraniaceae Geranium incanum#NC 030045
Geraniales Geraniaceae Erodium trifolium#NC 024635
Geraniales Geraniaceae Erodium carvifolium#NC 015083
Geraniales Geraniaceae Erodium crassifolium#NC 025906
Geraniales Geraniaceae Erodium texanum#NC 014569
Geraniales Geraniaceae Erodium gruinum#NC 025907
Geraniales Geraniaceae Erodium absinthoides#NC 026847
Geraniales Geraniaceae Erodium chrysanthum#NC 027065
Myrtales Combretaceae Lumnitzera littorea#NC 039752
Myrtales Combretaceae Terminalia myriocarpa#F113
Myrtales Combretaceae Terminalia neotaliala#AJJE
Myrtales Combretaceae Getonia floribunda#F275
Myrtales Combretaceae Combretum sundaicum#F21
Myrtales Combretaceae Combretum punctatum#F476
Myrtales Lythraceae Woodfordia fruticosa#F160
Myrtales Lythraceae Punica granatum#F433
Myrtales Lythraceae Pemphis acidula#NC 041439
Myrtales Lythraceae Heimia myrtifolia#MG921615
Myrtales Lythraceae Rotala rotundifolia#F179
Myrtales Lythraceae Trapa incisa#F573
Myrtales Lythraceae Sonneratia alba#NC 039975
Myrtales Lythraceae Duabanga grandiflora#F225
Myrtales Lythraceae Lagerstroemia intermedia#NC 034662
Myrtales Lythraceae Lagerstroemia speciosa#KX572149
Myrtales Lythraceae Lagerstroemia subcostata#NC 034952
Myrtales Lythraceae Lagerstroemia fauriei#NC 029808
Myrtales Lythraceae Lagerstroemia guilinensis#NC 029885
Myrtales Lythraceae Lagerstroemia indica#KF572028
Myrtales Onagraceae Ludwigia adscendens#F488
Myrtales Onagraceae Epilobium sp.#FEDW
Myrtales Onagraceae Epilobium palustre#F414
Myrtales Onagraceae Epilobium ulleungensis#NC 039575
Myrtales Onagraceae Oenothera rosea#F548
Myrtales Onagraceae Oenothera speciosa#TLCA
Myrtales Onagraceae Gaura suffulta#JKNQ
Myrtales Onagraceae Gaura longiflora#AXNH
Myrtales Onagraceae Oenothera gaura#PMTB
Myrtales Onagraceae Oenothera serrulata#SJAN
Myrtales Onagraceae Oenothera berlandieri#EQYT
Myrtales Onagraceae Oenothera nana#CJGZ
Myrtales Onagraceae Oenothera longituba#HPNZ
Myrtales Onagraceae Oenothera affinis#GVCB
Myrtales Onagraceae Oenothera picensis#DZLN
Myrtales Onagraceae Oenothera villaricae#HKMQ
Myrtales Onagraceae Oenothera clelandii#KBRW
Myrtales Onagraceae Oenothera rhombipetala#YHLF
Myrtales Onagraceae Oenothera laciniata#ARYD
Myrtales Onagraceae Oenothera grandis#IMZV
Myrtales Onagraceae Oenothera biennis#BZDF
Myrtales Onagraceae Oenothera elata#IDAU
Myrtales Onagraceae Oenothera argillicola#NC 010358
Myrtales Onagraceae Oenothera parviflora#EU262891
Myrtales Onagraceae Oenothera oakesiana#KX687918
Myrtales Onagraceae Oenothera glazioviana#KT881171
Myrtales Onagraceae Oenothera villosa#KX687910
Myrtales Onagraceae Oenothera elata subsp. hookeri#AJ271079
Myrtales Crypteroniaceae Crypteronia paniculata#F327
Myrtales Crypteroniaceae Crypteronia paniculata#F276
Myrtales Alzateaceae Alzatea verticillata#ZSD320
Myrtales Penaeaceae Penaea mucronata#FC428
Myrtales Penaeaceae Rhynchocalyx lawsonioides#FC392
Myrtales Melastomataceae Mouriri myrtilloides#F908
Myrtales Melastomataceae Tetrazygia bicolor#SWGX
Myrtales Melastomataceae Melastoma malabathricum#NC 034716
Myrtales Melastomataceae Medinilla septentrionalis#F55
Myrtales Melastomataceae Medinilla magnifica#WWQZ
Myrtales Melastomataceae Allomorphia baviensis#F56 ED
Myrtales Melastomataceae Barthea barthei#NC 035661
Myrtales Melastomataceae Tigridiopalma magnifica#NC 036021
Myrtales Vochysiaceae Vochysia guatemalensis#F970
Myrtales Vochysiaceae Vochysia ferruginea#F972
Myrtales Myrtaceae Heteropyxis natalensis#NIJU
Myrtales Myrtaceae Melaleuca laxiflora#F452
Myrtales Myrtaceae Melaleuca quinquenervia#JLLY
Myrtales Myrtaceae Syzygium sp.#F311
Myrtales Myrtaceae Syzygium paniculatum#FGDU
Myrtales Myrtaceae Syzygium macranthum#NEBM
Myrtales Myrtaceae Rhodomyrtus tomentosa#F444
Myrtales Myrtaceae Plinia peruviana#NC 034801
Myrtales Myrtaceae Plinia cauliflora#NC 039395
Myrtales Myrtaceae Plinia sp.#KX668206
Myrtales Myrtaceae Plinia phitrantha#NC 039557
Myrtales Myrtaceae Psidium guajava#KY635879
Myrtales Myrtaceae Pimenta dioica#NC 034684
Myrtales Myrtaceae Eugenia uniflora#NC 027744
Myrtales Myrtaceae Acca sellowiana#KX289887
Myrtales Myrtaceae Campomanesia xanthocarpa#KY392760
Myrtales Myrtaceae Baeckea frutescens#F449
Myrtales Myrtaceae Stockwellia quadrifida#NC 022414
Myrtales Myrtaceae Allosyncarpia ternata#NC 022413
Myrtales Myrtaceae Corymbia gummifera#NC 022407
Myrtales Myrtaceae Angophora costata#NC 022412
Myrtales Myrtaceae Angophora floribunda#NC 022411
Myrtales Myrtaceae Corymbia maculata#NC 022408
Myrtales Myrtaceae Corymbia eximia#NC 022409
Myrtales Myrtaceae Corymbia henryi#NC 028409
Myrtales Myrtaceae Corymbia tessellaris#NC 022410
Myrtales Myrtaceae Corymbia torelliana#NC 028410
Myrtales Myrtaceae Eucalyptus spathulata#NC 022400
Myrtales Myrtaceae Eucalyptus torquata#NC 022401
Myrtales Myrtaceae Eucalyptus diversicolor#NC 022402
Myrtales Myrtaceae Eucalyptus leucoxylon#AYMT
Myrtales Myrtaceae Eucalyptus melliodora#NC 022392
Myrtales Myrtaceae Eucalyptus cladocalyx#NC 022394
Myrtales Myrtaceae Eucalyptus polybractea#NC 022393
Myrtales Myrtaceae Eucalyptus deglupta#NC 022399

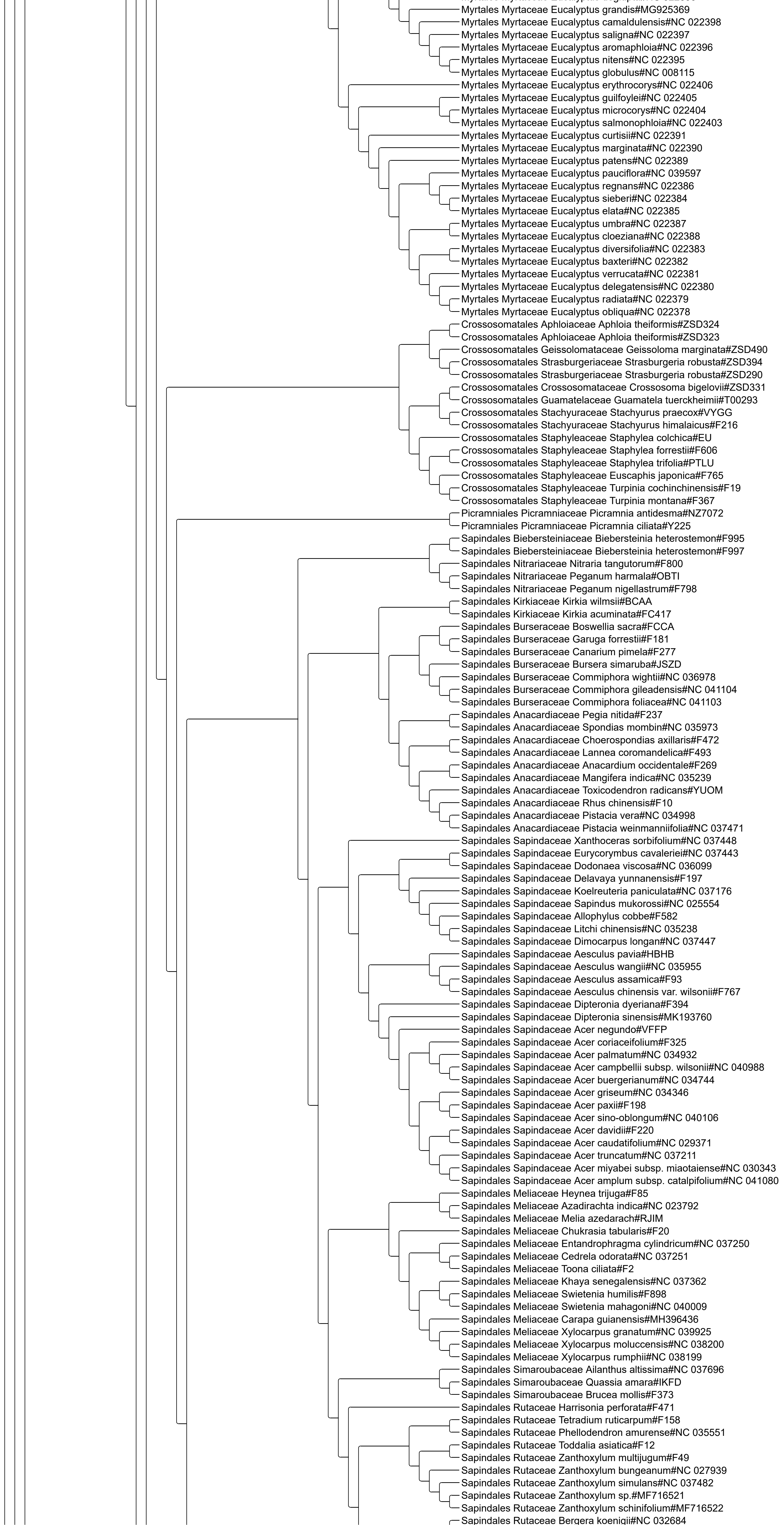
Myrtales Myrtaceae Eucalyptus grandis#MG925369
Myrtales Myrtaceae Eucalyptus camaldulensis#NC 022398
Myrtales Myrtaceae Eucalyptus saligna#NC 022397
Myrtales Myrtaceae Eucalyptus aromaphloia#NC 022396
Myrtales Myrtaceae Eucalyptus nitens#NC 022395
Myrtales Myrtaceae Eucalyptus globulus#NC 008115
Myrtales Myrtaceae Eucalyptus erythrocorys#NC 022406
Myrtales Myrtaceae Eucalyptus guilfoylei#NC 022405
Myrtales Myrtaceae Eucalyptus microcorys#NC 022404
Myrtales Myrtaceae Eucalyptus salmonophloia#NC 022403
Myrtales Myrtaceae Eucalyptus curtisii#NC 022391
Myrtales Myrtaceae Eucalyptus marginata#NC 022390
Myrtales Myrtaceae Eucalyptus patens#NC 022389
Myrtales Myrtaceae Eucalyptus pauciflora#NC 039597
Myrtales Myrtaceae Eucalyptus regnans#NC 022386
Myrtales Myrtaceae Eucalyptus sieberi#NC 022384
Myrtales Myrtaceae Eucalyptus elata#NC 022385
Myrtales Myrtaceae Eucalyptus umbra#NC 022387
Myrtales Myrtaceae Eucalyptus cloeziana#NC 022388
Myrtales Myrtaceae Eucalyptus diversifolia#NC 022383
Myrtales Myrtaceae Eucalyptus baxteri#NC 022382
Myrtales Myrtaceae Eucalyptus verrucata#NC 022381
Myrtales Myrtaceae Eucalyptus delegatensis#NC 022380
Myrtales Myrtaceae Eucalyptus radiata#NC 022379
Myrtales Myrtaceae Eucalyptus obliqua#NC 022378
Crossosomatales Aphloiaceae Aphloia theiformis#ZSD324
Crossosomatales Aphloiaceae Aphloia theiformis#ZSD323
Crossosomatales Geissolomataceae Geissoloma marginata#ZSD490
Crossosomatales Strasburgeriaceae Strasburgeria robusta#ZSD394
Crossosomatales Strasburgeriaceae Strasburgeria robusta#ZSD290
Crossosomatales Crossosomataceae Crossosoma bigelovii#ZSD331
Crossosomatales Guamatelaceae Guamatela tuerckheimii#T00293
Crossosomatales Stachyuraceae Stachyurus praecox#VYGG
Crossosomatales Stachyuraceae Stachyurus himalaicus#F216
Crossosomatales Staphyleaceae Staphylea colchica#EU
Crossosomatales Staphyleaceae Staphylea forrestii#F606
Crossosomatales Staphyleaceae Staphylea trifolia#PTLU
Crossosomatales Staphyleaceae Euscaphis japonica#F765
Crossosomatales Staphyleaceae Turpinia cochinchinensis#F19
Crossosomatales Staphyleaceae Turpinia montana#F367
Picramniales Picramniaceae Picramnia antidesma#NZ7072
Picramniales Picramniaceae Picramnia ciliata#Y225
Sapindales Biebersteiniaceae Biebersteinia heterostemon#F995
Sapindales Biebersteiniaceae Biebersteinia heterostemon#F997
Sapindales Nitrariaceae Nitraria tangutorum#F800
Sapindales Nitrariaceae Peganum harmala#OBTI
Sapindales Nitrariaceae Peganum nigellastrum#F798
Sapindales Kirkiaceae Kirkia wilmsii#BCAA
Sapindales Kirkiaceae Kirkia acuminata#FC417
Sapindales Burseraceae Boswellia sacra#FCCA
Sapindales Burseraceae Garuga forrestii#F181
Sapindales Burseraceae Canarium pimela#F277
Sapindales Burseraceae Bursera simaruba#JSZD
Sapindales Burseraceae Commiphora wightii#NC 036978
Sapindales Burseraceae Commiphora gileadensis#NC 041104
Sapindales Burseraceae Commiphora foliacea#NC 041103
Sapindales Anacardiaceae Pegia nitida#F237
Sapindales Anacardiaceae Spondias mombin#NC 035973
Sapindales Anacardiaceae Choerospondias axillaris#F472
Sapindales Anacardiaceae Lannea coromandelica#F493
Sapindales Anacardiaceae Anacardium occidentale#F269
Sapindales Anacardiaceae Mangifera indica#NC 035239
Sapindales Anacardiaceae Toxicodendron radicans#YUOM
Sapindales Anacardiaceae Rhus chinensis#F10
Sapindales Anacardiaceae Pistacia vera#NC 034998
Sapindales Anacardiaceae Pistacia weinmanniifolia#NC 037471
Sapindales Sapindaceae Xanthoceras sorbifolium#NC 037448
Sapindales Sapindaceae Eurycorymbus cavaleriei#NC 037443
Sapindales Sapindaceae Dodonaea viscosa#NC 036099
Sapindales Sapindaceae Delavaya yunnanensis#F197
Sapindales Sapindaceae Koelreuteria paniculata#NC 037176
Sapindales Sapindaceae Sapindus mukorossi#NC 025554
Sapindales Sapindaceae Allophylus cobbe#F582
Sapindales Sapindaceae Litchi chinensis#NC 035238
Sapindales Sapindaceae Dimocarpus longan#NC 037447
Sapindales Sapindaceae Aesculus pavia#HBHB
Sapindales Sapindaceae Aesculus wangii#NC 035955
Sapindales Sapindaceae Aesculus assamica#F93
Sapindales Sapindaceae Aesculus chinensis var. wilsonii#F767
Sapindales Sapindaceae Dipteronia dyeriana#F394
Sapindales Sapindaceae Dipteronia sinensis#MK193760
Sapindales Sapindaceae Acer negundo#VFFP
Sapindales Sapindaceae Acer coriaceifolium#F325
Sapindales Sapindaceae Acer palmatum#NC 034932
Sapindales Sapindaceae Acer campbellii subsp. wilsonii#NC 040988
Sapindales Sapindaceae Acer buergerianum#NC 034744
Sapindales Sapindaceae Acer griseum#NC 034346
Sapindales Sapindaceae Acer paxii#F198
Sapindales Sapindaceae Acer sino-oblongum#NC 040106
Sapindales Sapindaceae Acer davidii#F220
Sapindales Sapindaceae Acer caudatifolium#NC 029371
Sapindales Sapindaceae Acer truncatum#NC 037211
Sapindales Sapindaceae Acer miyabei subsp. miaotaiense#NC 030343
Sapindales Sapindaceae Acer amplum subsp. catalpifolium#NC 041080
Sapindales Meliaceae Heynea trijuga#F85
Sapindales Meliaceae Azadirachta indica#NC 023792
Sapindales Meliaceae Melia azedarach#RJIM
Sapindales Meliaceae Chukrasia tabularis#F20
Sapindales Meliaceae Entandrophragma cylindricum#NC 037250
Sapindales Meliaceae Cedrela odorata#NC 037251
Sapindales Meliaceae Toona ciliata#F2
Sapindales Meliaceae Khaya senegalensis#NC 037362
Sapindales Meliaceae Swietenia humilis#F898
Sapindales Meliaceae Swietenia mahagoni#NC 040009
Sapindales Meliaceae Carapa guianensis#MH396436
Sapindales Meliaceae Xylocarpus granatum#NC 039925
Sapindales Meliaceae Xylocarpus moluccensis#NC 038200
Sapindales Meliaceae Xylocarpus rumphii#NC 038199
Sapindales Simaroubaceae Ailanthus altissima#NC 037696
Sapindales Simaroubaceae Quassia amara#IKFD
Sapindales Simaroubaceae Brucea mollis#F373
Sapindales Rutaceae Harrisonia perforata#F471
Sapindales Rutaceae Tetradium ruticarpum#F158
Sapindales Rutaceae Phellodendron amurense#NC 035551
Sapindales Rutaceae Toddalia asiatica#F12
Sapindales Rutaceae Zanthoxylum multijugum#F49
Sapindales Rutaceae Zanthoxylum bungeanum#NC 027939
Sapindales Rutaceae Zanthoxylum simulans#NC 037482
Sapindales Rutaceae Zanthoxylum sp.#MF716521
Sapindales Rutaceae Zanthoxylum schinifolium#MF716522
Sapindales Rutaceae Bergera koenigii#NC 032684

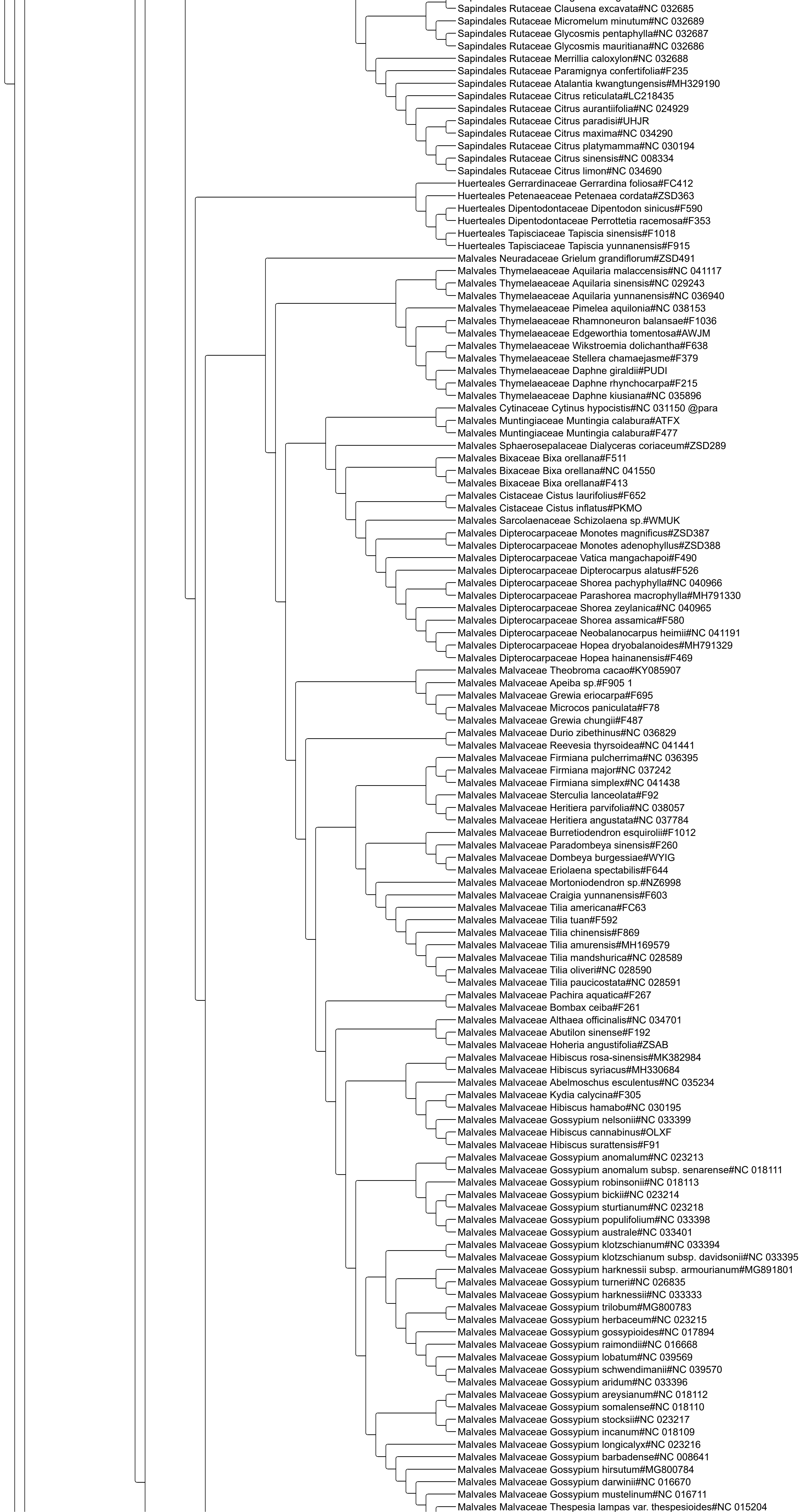

Sapindales Rutaceae Clausena excavata#NC 032685
Sapindales Rutaceae Micromelum minutum#NC 032689
Sapindales Rutaceae Glycosmis pentaphylla#NC 032687
Sapindales Rutaceae Glycosmis mauritiana#NC 032686
Sapindales Rutaceae Merrillia caloxylon#NC 032688
Sapindales Rutaceae Paramignya confertifolia#F235
Sapindales Rutaceae Atalantia kwangtungensis#MH329190
Sapindales Rutaceae Citrus reticulata#LC218435
Sapindales Rutaceae Citrus aurantiifolia#NC 024929
Sapindales Rutaceae Citrus paradisi#UHJR
Sapindales Rutaceae Citrus maxima#NC 034290
Sapindales Rutaceae Citrus platymamma#NC 030194
Sapindales Rutaceae Citrus sinensis#NC 008334
Sapindales Rutaceae Citrus limon#NC 034690
Huerteales Gerrardinaceae Gerrardina foliosa#FC412
Huerteales Petenaeaceae Petenaea cordata#ZSD363
Huerteales Dipentodontaceae Dipentodon sinicus#F590
Huerteales Dipentodontaceae Perrottetia racemosa#F353
Huerteales Tapisciaceae Tapiscia sinensis#F1018
Huerteales Tapisciaceae Tapiscia yunnanensis#F915
Malvales Neuradaceae Grielum grandiflorum#ZSD491
Malvales Thymelaeaceae Aquilaria malaccensis#NC 041117
Malvales Thymelaeaceae Aquilaria sinensis#NC 029243
Malvales Thymelaeaceae Aquilaria yunnanensis#NC 036940
Malvales Thymelaeaceae Pimelea aquilonia#NC 038153
Malvales Thymelaeaceae Rhamnoneuron balansae#F1036
Malvales Thymelaeaceae Edgeworthia tomentosa#AWJM
Malvales Thymelaeaceae Wikstroemia dolichantha#F638
Malvales Thymelaeaceae Stellera chamaejasme#F379
Malvales Thymelaeaceae Daphne giraldii#PUDI
Malvales Thymelaeaceae Daphne rhynchocarpa#F215
Malvales Thymelaeaceae Daphne kiusiana#NC 035896
Malvales Cytinaceae Cytinus hypocistis#NC 031150 @para
Malvales Muntingiaceae Muntingia calabura#ATFX
Malvales Muntingiaceae Muntingia calabura#F477
Malvales Sphaerosepalaceae Dialyceras coriaceum#ZSD289
Malvales Bixaceae Bixa orellana#F511
Malvales Bixaceae Bixa orellana#NC 041550
Malvales Bixaceae Bixa orellana#F413
Malvales Cistaceae Cistus laurifolius#F652
Malvales Cistaceae Cistus inflatus#PKMO
Malvales Sarcolaenaceae Schizolaena sp.#WMUK
Malvales Dipterocarpaceae Monotes magnificus#ZSD387
Malvales Dipterocarpaceae Monotes adenophyllus#ZSD388
Malvales Dipterocarpaceae Vatica mangachapoi#F490
Malvales Dipterocarpaceae Dipterocarpus alatus#F526
Malvales Dipterocarpaceae Shorea pachyphylla#NC 040966
Malvales Dipterocarpaceae Parashorea macrophylla#MH791330
Malvales Dipterocarpaceae Shorea zeylanica#NC 040965
Malvales Dipterocarpaceae Shorea assamica#F580
Malvales Dipterocarpaceae Neobalanocarpus heimii#NC 041191
Malvales Dipterocarpaceae Hopea dryobalanoides#MH791329
Malvales Dipterocarpaceae Hopea hainanensis#F469
Malvales Malvaceae Theobroma cacao#KY085907
Malvales Malvaceae Apeiba sp.#F905 1
Malvales Malvaceae Grewia eriocarpa#F695
Malvales Malvaceae Microcos paniculata#F78
Malvales Malvaceae Grewia chungii#F487
Malvales Malvaceae Durio zibethinus#NC 036829
Malvales Malvaceae Reevesia thyrsoidea#NC 041441
Malvales Malvaceae Firmiana pulcherrima#NC 036395
Malvales Malvaceae Firmiana major#NC 037242
Malvales Malvaceae Firmiana simplex#NC 041438
Malvales Malvaceae Sterculia lanceolata#F92
Malvales Malvaceae Heritiera parvifolia#NC 038057
Malvales Malvaceae Heritiera angustata#NC 037784
Malvales Malvaceae Burretiodendron esquirolii#F1012
Malvales Malvaceae Paradombeya sinensis#F260
Malvales Malvaceae Dombeya burgessiae#WYIG
Malvales Malvaceae Eriolaena spectabilis#F644
Malvales Malvaceae Mortoniodendron sp.#NZ6998
Malvales Malvaceae Craigia yunnanensis#F603
Malvales Malvaceae Tilia americana#FC63
Malvales Malvaceae Tilia tuan#F592
Malvales Malvaceae Tilia chinensis#F869
Malvales Malvaceae Tilia amurensis#MH169579
Malvales Malvaceae Tilia mandshurica#NC 028589
Malvales Malvaceae Tilia oliveri#NC 028590
Malvales Malvaceae Tilia paucicostata#NC 028591
Malvales Malvaceae Pachira aquatica#F267
Malvales Malvaceae Bombax ceiba#F261
Malvales Malvaceae Althaea officinalis#NC 034701
Malvales Malvaceae Abutilon sinense#F192
Malvales Malvaceae Hoheria angustifolia#ZSAB
Malvales Malvaceae Hibiscus rosa-sinensis#MK382984
Malvales Malvaceae Hibiscus syriacus#MH330684
Malvales Malvaceae Abelmoschus esculentus#NC 035234
Malvales Malvaceae Kydia calycina#F305
Malvales Malvaceae Hibiscus hamabo#NC 030195
Malvales Malvaceae Gossypium nelsonii#NC 033399
Malvales Malvaceae Hibiscus cannabinus#OLXF
Malvales Malvaceae Hibiscus surattensis#F91
Malvales Malvaceae Gossypium anomalum#NC 023213
Malvales Malvaceae Gossypium anomalum subsp. senarense#NC 018111
Malvales Malvaceae Gossypium robinsonii#NC 018113
Malvales Malvaceae Gossypium bickii#NC 023214
Malvales Malvaceae Gossypium sturtianum#NC 023218
Malvales Malvaceae Gossypium populifolium#NC 033398
Malvales Malvaceae Gossypium australe#NC 033401
Malvales Malvaceae Gossypium klotzschianum#NC 033394
Malvales Malvaceae Gossypium klotzschianum subsp. davidsonii#NC 033395
Malvales Malvaceae Gossypium harknessii subsp. armourianum#MG891801
Malvales Malvaceae Gossypium turneri#NC 026835
Malvales Malvaceae Gossypium harknessii#NC 033333
Malvales Malvaceae Gossypium trilobum#MG800783
Malvales Malvaceae Gossypium herbaceum#NC 023215
Malvales Malvaceae Gossypium gossypioides#NC 017894
Malvales Malvaceae Gossypium raimondii#NC 016668
Malvales Malvaceae Gossypium lobatum#NC 039569
Malvales Malvaceae Gossypium schwendimanii#NC 039570
Malvales Malvaceae Gossypium aridum#NC 033396
Malvales Malvaceae Gossypium areysianum#NC 018112
Malvales Malvaceae Gossypium somalense#NC 018110
Malvales Malvaceae Gossypium stocksii#NC 023217
Malvales Malvaceae Gossypium incanum#NC 018109
Malvales Malvaceae Gossypium longicalyx#NC 023216
Malvales Malvaceae Gossypium barbadense#NC 008641
Malvales Malvaceae Gossypium hirsutum#MG800784
Malvales Malvaceae Gossypium darwinii#NC 016670
Malvales Malvaceae Gossypium mustelinum#NC 016711
Malvales Malvaceae Thespesia lampas var. thespesioides#NC 015204

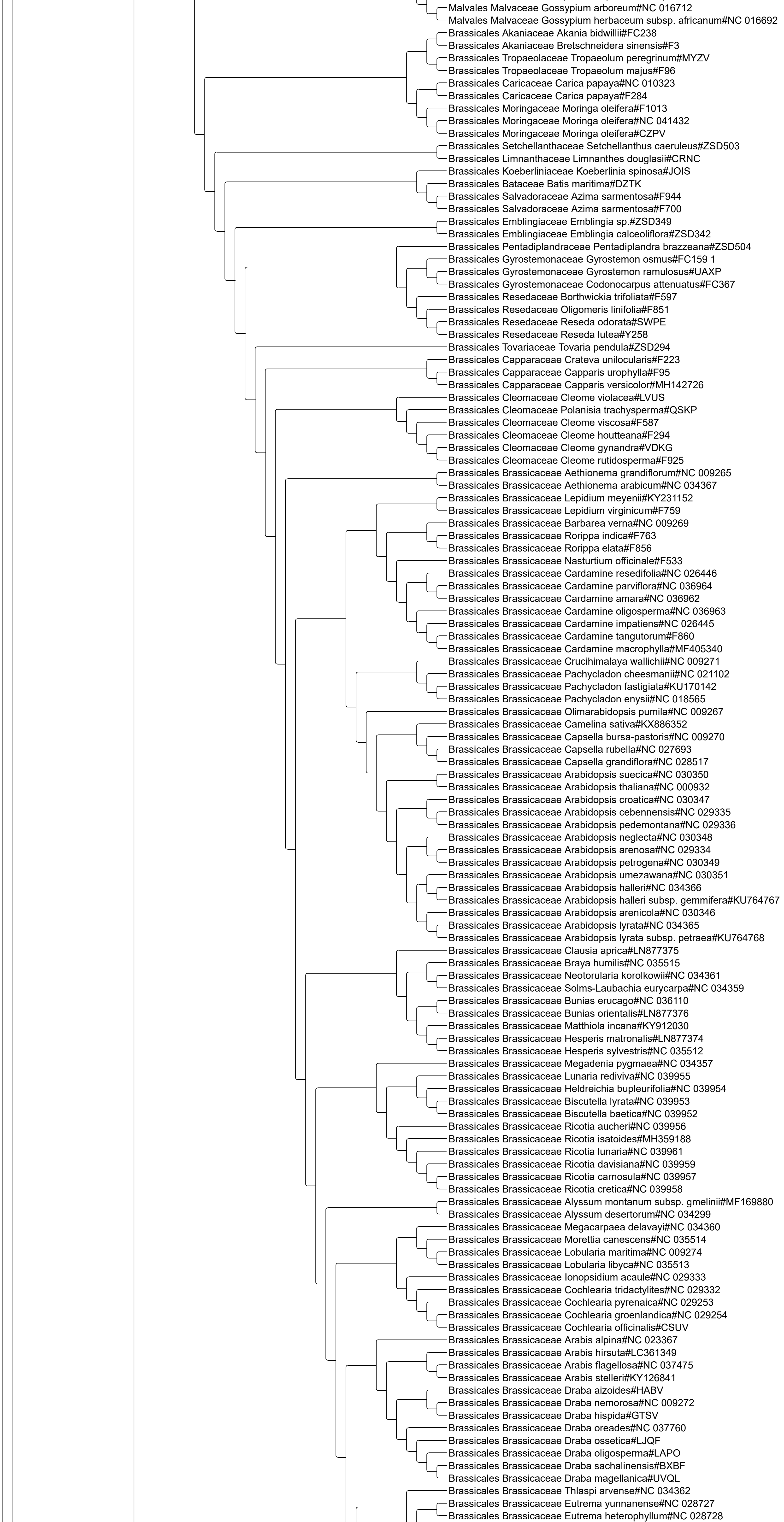
Malvales Malvaceae Gossypium arboreum#NC 016712
Malvales Malvaceae Gossypium herbaceum subsp. africanum#NC 016692
Brassicales Akaniaceae Akania bidwillii#FC238
Brassicales Akaniaceae Bretschneidera sinensis#F3
Brassicales Tropaeolaceae Tropaeolum peregrinum#MYZV
Brassicales Tropaeolaceae Tropaeolum majus#F96
Brassicales Caricaceae Carica papaya#NC 010323
Brassicales Caricaceae Carica papaya#F284
Brassicales Moringaceae Moringa oleifera#F1013
Brassicales Moringaceae Moringa oleifera#NC 041432
Brassicales Moringaceae Moringa oleifera#CZPV
Brassicales Setchellanthaceae Setchellanthus caeruleus#ZSD503
Brassicales Limnanthaceae Limnanthes douglasii#CRNC
Brassicales Koeberliniaceae Koeberlinia spinosa#JOIS
Brassicales Bataceae Batis maritima#DZTK
Brassicales Salvadoraceae Azima sarmentosa#F944
Brassicales Salvadoraceae Azima sarmentosa#F700
Brassicales Emblingiaceae Emblingia sp.#ZSD349
Brassicales Emblingiaceae Emblingia calceoliflora#ZSD342
Brassicales Pentadiplandraceae Pentadiplandra brazzeana#ZSD504
Brassicales Gyrostemonaceae Gyrostemon osmus#FC159 1
Brassicales Gyrostemonaceae Gyrostemon ramulosus#UAXP
Brassicales Gyrostemonaceae Codonocarpus attenuatus#FC367
Brassicales Resedaceae Borthwickia trifoliata#F597
Brassicales Resedaceae Oligomeris linifolia#F851
Brassicales Resedaceae Reseda odorata#SWPE
Brassicales Resedaceae Reseda lutea#Y258
Brassicales Tovariaceae Tovaria pendula#ZSD294
Brassicales Capparaceae Crateva unilocularis#F223
Brassicales Capparaceae Capparis urophylla#F95
Brassicales Capparaceae Capparis versicolor#MH142726
Brassicales Cleomaceae Cleome violacea#LVUS
Brassicales Cleomaceae Polanisia trachysperma#QSKP
Brassicales Cleomaceae Cleome viscosa#F587
Brassicales Cleomaceae Cleome houtteana#F294
Brassicales Cleomaceae Cleome gynandra#VDKG
Brassicales Cleomaceae Cleome rutidosperma#F925
Brassicales Brassicaceae Aethionema grandiflorum#NC 009265
Brassicales Brassicaceae Aethionema arabicum#NC 034367
Brassicales Brassicaceae Lepidium meyenii#KY231152
Brassicales Brassicaceae Lepidium virginicum#F759
Brassicales Brassicaceae Barbarea verna#NC 009269
Brassicales Brassicaceae Rorippa indica#F763
Brassicales Brassicaceae Rorippa elata#F856
Brassicales Brassicaceae Nasturtium officinale#F533
Brassicales Brassicaceae Cardamine resedifolia#NC 026446
Brassicales Brassicaceae Cardamine parviflora#NC 036964
Brassicales Brassicaceae Cardamine amara#NC 036962
Brassicales Brassicaceae Cardamine oligosperma#NC 036963
Brassicales Brassicaceae Cardamine impatiens#NC 026445
Brassicales Brassicaceae Cardamine tangutorum#F860
Brassicales Brassicaceae Cardamine macrophylla#MF405340
Brassicales Brassicaceae Crucihimalaya wallichii#NC 009271
Brassicales Brassicaceae Pachycladon cheesmanii#NC 021102
Brassicales Brassicaceae Pachycladon fastigiata#KU170142
Brassicales Brassicaceae Pachycladon enysii#NC 018565
Brassicales Brassicaceae Olimarabidopsis pumila#NC 009267
Brassicales Brassicaceae Camelina sativa#KX886352
Brassicales Brassicaceae Capsella bursa-pastoris#NC 009270
Brassicales Brassicaceae Capsella rubella#NC 027693
Brassicales Brassicaceae Capsella grandiflora#NC 028517
Brassicales Brassicaceae Arabidopsis suecica#NC 030350
Brassicales Brassicaceae Arabidopsis thaliana#NC 000932
Brassicales Brassicaceae Arabidopsis croatica#NC 030347
Brassicales Brassicaceae Arabidopsis cebennensis#NC 029335
Brassicales Brassicaceae Arabidopsis pedemontana#NC 029336
Brassicales Brassicaceae Arabidopsis neglecta#NC 030348
Brassicales Brassicaceae Arabidopsis arenosa#NC 029334
Brassicales Brassicaceae Arabidopsis petrogena#NC 030349
Brassicales Brassicaceae Arabidopsis umezawana#NC 030351
Brassicales Brassicaceae Arabidopsis halleri#NC 034366
Brassicales Brassicaceae Arabidopsis halleri subsp. gemmifera#KU764767
Brassicales Brassicaceae Arabidopsis arenicola#NC 030346
Brassicales Brassicaceae Arabidopsis lyrata#NC 034365
Brassicales Brassicaceae Arabidopsis lyrata subsp. petraea#KU764768
Brassicales Brassicaceae Clausia aprica#LN877375
Brassicales Brassicaceae Braya humilis#NC 035515
Brassicales Brassicaceae Neotorularia korolkowii#NC 034361
Brassicales Brassicaceae Solms-Laubachia eurycarpa#NC 034359
Brassicales Brassicaceae Bunias erucago#NC 036110
Brassicales Brassicaceae Bunias orientalis#LN877376
Brassicales Brassicaceae Matthiola incana#KY912030
Brassicales Brassicaceae Hesperis matronalis#LN877374
Brassicales Brassicaceae Hesperis sylvestris#NC 035512
Brassicales Brassicaceae Megadenia pygmaea#NC 034357
Brassicales Brassicaceae Lunaria rediviva#NC 039955
Brassicales Brassicaceae Heldreichia bupleurifolia#NC 039954
Brassicales Brassicaceae Biscutella lyrata#NC 039953
Brassicales Brassicaceae Biscutella baetica#NC 039952
Brassicales Brassicaceae Ricotia aucheri#NC 039956
Brassicales Brassicaceae Ricotia isatoides#MH359188
Brassicales Brassicaceae Ricotia lunaria#NC 039961
Brassicales Brassicaceae Ricotia davisiana#NC 039959
Brassicales Brassicaceae Ricotia carnosula#NC 039957
Brassicales Brassicaceae Ricotia cretica#NC 039958
Brassicales Brassicaceae Alyssum montanum subsp. gmelinii#MF169880
Brassicales Brassicaceae Alyssum desertorum#NC 034299
Brassicales Brassicaceae Megacarpaea delavayi#NC 034360
Brassicales Brassicaceae Morettia canescens#NC 035514
Brassicales Brassicaceae Lobularia maritima#NC 009274
Brassicales Brassicaceae Lobularia libyca#NC 035513
Brassicales Brassicaceae Ionopsidium acaule#NC 029333
Brassicales Brassicaceae Cochlearia tridactylites#NC 029332
Brassicales Brassicaceae Cochlearia pyrenaica#NC 029253
Brassicales Brassicaceae Cochlearia groenlandica#NC 029254
Brassicales Brassicaceae Cochlearia officinalis#CSUV
Brassicales Brassicaceae Arabis alpina#NC 023367
Brassicales Brassicaceae Arabis hirsuta#LC361349
Brassicales Brassicaceae Arabis flagellosa#NC 037475
Brassicales Brassicaceae Arabis stelleri#KY126841
Brassicales Brassicaceae Draba aizoides#HABV
Brassicales Brassicaceae Draba nemorosa#NC 009272
Brassicales Brassicaceae Draba hispida#GTSV
Brassicales Brassicaceae Draba oreades#NC 037760
Brassicales Brassicaceae Draba ossetica#LJQF
Brassicales Brassicaceae Draba oligosperma#LAPO
Brassicales Brassicaceae Draba sachalinensis#BXBF
Brassicales Brassicaceae Draba magellanica#UVQL
Brassicales Brassicaceae Thlaspi arvense#NC 034362
Brassicales Brassicaceae Eutrema yunnanense#NC 028727
Brassicales Brassicaceae Eutrema heterophyllum#NC 028728

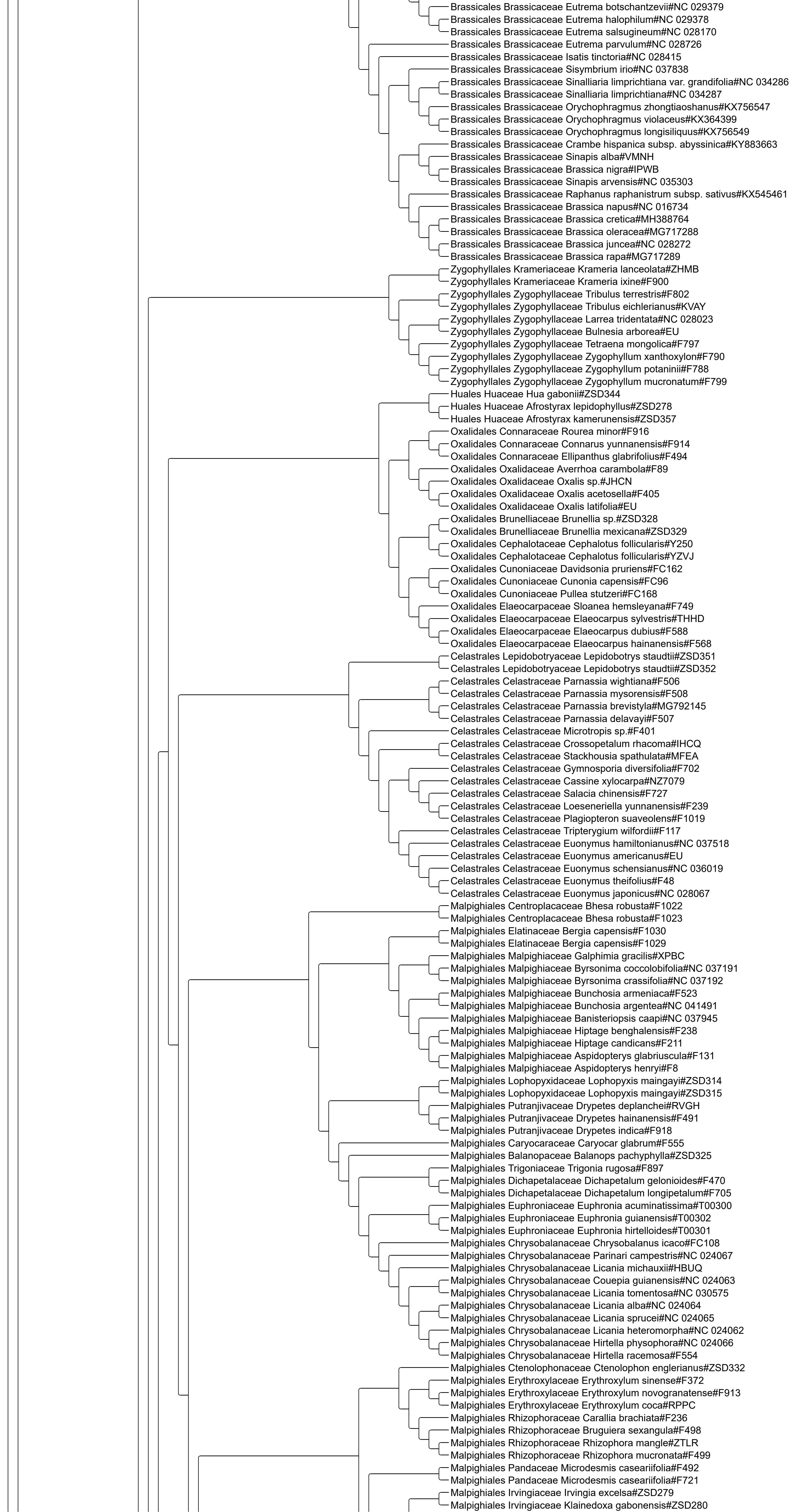
Brassicales Brassicaceae Eutrema botschantzevii#NC 029379
Brassicales Brassicaceae Eutrema halophilum#NC 029378
Brassicales Brassicaceae Eutrema salsugineum#NC 028170
Brassicales Brassicaceae Eutrema parvulum#NC 028726
Brassicales Brassicaceae Isatis tinctoria#NC 028415
Brassicales Brassicaceae Sisymbrium irio#NC 037838
Brassicales Brassicaceae Sinalliaria limprichtiana var. grandifolia#NC 034286
Brassicales Brassicaceae Sinalliaria limprichtiana#NC 034287
Brassicales Brassicaceae Orychophragmus zhongtiaoshanus#KX756547
Brassicales Brassicaceae Orychophragmus violaceus#KX364399
Brassicales Brassicaceae Orychophragmus longisiliquus#KX756549
Brassicales Brassicaceae Crambe hispanica subsp. abyssinica#KY883663
Brassicales Brassicaceae Sinapis alba#VMNH
Brassicales Brassicaceae Brassica nigra#IPWB
Brassicales Brassicaceae Sinapis arvensis#NC 035303
Brassicales Brassicaceae Raphanus raphanistrum subsp. sativus#KX545461
Brassicales Brassicaceae Brassica napus#NC 016734
Brassicales Brassicaceae Brassica cretica#MH388764
Brassicales Brassicaceae Brassica oleracea#MG717288
Brassicales Brassicaceae Brassica juncea#NC 028272
Brassicales Brassicaceae Brassica rapa#MG717289
Zygophyllales Krameriaceae Krameria lanceolata#ZHMB
Zygophyllales Krameriaceae Krameria ixine#F900
Zygophyllales Zygophyllaceae Tribulus terrestris#F802
Zygophyllales Zygophyllaceae Tribulus eichlerianus#KVAY
Zygophyllales Zygophyllaceae Larrea tridentata#NC 028023
Zygophyllales Zygophyllaceae Bulnesia arborea#EU
Zygophyllales Zygophyllaceae Tetraena mongolica#F797
Zygophyllales Zygophyllaceae Zygophyllum xanthoxylon#F790
Zygophyllales Zygophyllaceae Zygophyllum potaninii#F788
Zygophyllales Zygophyllaceae Zygophyllum mucronatum#F799
Huales Huaceae Hua gabonii#ZSD344
Huales Huaceae Afrostyrax lepidophyllus#ZSD278
Huales Huaceae Afrostyrax kamerunensis#ZSD357
Oxalidales Connaraceae Rourea minor#F916
Oxalidales Connaraceae Connarus yunnanensis#F914
Oxalidales Connaraceae Ellipanthus glabrifolius#F494
Oxalidales Oxalidaceae Averrhoa carambola#F89
Oxalidales Oxalidaceae Oxalis sp.#JHCN
Oxalidales Oxalidaceae Oxalis acetosella#F405
Oxalidales Oxalidaceae Oxalis latifolia#EU
Oxalidales Brunelliaceae Brunellia sp.#ZSD328
Oxalidales Brunelliaceae Brunellia mexicana#ZSD329
Oxalidales Cephalotaceae Cephalotus follicularis#Y250
Oxalidales Cephalotaceae Cephalotus follicularis#YZVJ
Oxalidales Cunoniaceae Davidsonia pruriens#FC162
Oxalidales Cunoniaceae Cunonia capensis#FC96
Oxalidales Cunoniaceae Pullea stutzeri#FC168
Oxalidales Elaeocarpaceae Sloanea hemsleyana#F749
Oxalidales Elaeocarpaceae Elaeocarpus sylvestris#THHD
Oxalidales Elaeocarpaceae Elaeocarpus dubius#F588
Oxalidales Elaeocarpaceae Elaeocarpus hainanensis#F568
Celastrales Lepidobotryaceae Lepidobotrys staudtii#ZSD351
Celastrales Lepidobotryaceae Lepidobotrys staudtii#ZSD352
Celastrales Celastraceae Parnassia wightiana#F506
Celastrales Celastraceae Parnassia mysorensis#F508
Celastrales Celastraceae Parnassia brevistyla#MG792145
Celastrales Celastraceae Parnassia delavayi#F507
Celastrales Celastraceae Microtropis sp.#F401
Celastrales Celastraceae Crossopetalum rhacoma#IHCQ
Celastrales Celastraceae Stackhousia spathulata#MFEA
Celastrales Celastraceae Gymnosporia diversifolia#F702
Celastrales Celastraceae Cassine xylocarpa#NZ7079
Celastrales Celastraceae Salacia chinensis#F727
Celastrales Celastraceae Loeseneriella yunnanensis#F239
Celastrales Celastraceae Plagiopteron suaveolens#F1019
Celastrales Celastraceae Tripterygium wilfordii#F117
Celastrales Celastraceae Euonymus hamiltonianus#NC 037518
Celastrales Celastraceae Euonymus americanus#EU
Celastrales Celastraceae Euonymus schensianus#NC 036019
Celastrales Celastraceae Euonymus theifolius#F48
Celastrales Celastraceae Euonymus japonicus#NC 028067
Malpighiales Centroplacaceae Bhesa robusta#F1022
Malpighiales Centroplacaceae Bhesa robusta#F1023
Malpighiales Elatinaceae Bergia capensis#F1030
Malpighiales Elatinaceae Bergia capensis#F1029
Malpighiales Malpighiaceae Galphimia gracilis#XPBC
Malpighiales Malpighiaceae Byrsonima coccolobifolia#NC 037191
Malpighiales Malpighiaceae Byrsonima crassifolia#NC 037192
Malpighiales Malpighiaceae Bunchosia armeniaca#F523
Malpighiales Malpighiaceae Bunchosia argentea#NC 041491
Malpighiales Malpighiaceae Banisteriopsis caapi#NC 037945
Malpighiales Malpighiaceae Hiptage benghalensis#F238
Malpighiales Malpighiaceae Hiptage candicans#F211
Malpighiales Malpighiaceae Aspidopterys glabriuscula#F131
Malpighiales Malpighiaceae Aspidopterys henryi#F8
Malpighiales Lophopyxidaceae Lophopyxis maingayi#ZSD314
Malpighiales Lophopyxidaceae Lophopyxis maingayi#ZSD315
Malpighiales Putranjivaceae Drypetes deplanchei#RVGH
Malpighiales Putranjivaceae Drypetes hainanensis#F491
Malpighiales Putranjivaceae Drypetes indica#F918
Malpighiales Caryocaraceae Caryocar glabrum#F555
Malpighiales Balanopaceae Balanops pachyphylla#ZSD325
Malpighiales Trigoniaceae Trigonia rugosa#F897
Malpighiales Dichapetalaceae Dichapetalum gelonioides#F470
Malpighiales Dichapetalaceae Dichapetalum longipetalum#F705
Malpighiales Euphroniaceae Euphronia acuminatissima#T00300
Malpighiales Euphroniaceae Euphronia guianensis#T00302
Malpighiales Euphroniaceae Euphronia hirtelloides#T00301
Malpighiales Chrysobalanaceae Chrysobalanus icaco#FC108
Malpighiales Chrysobalanaceae Parinari campestris#NC 024067
Malpighiales Chrysobalanaceae Licania michauxii#HBUQ
Malpighiales Chrysobalanaceae Couepia guianensis#NC 024063
Malpighiales Chrysobalanaceae Licania tomentosa#NC 030575
Malpighiales Chrysobalanaceae Licania alba#NC 024064
Malpighiales Chrysobalanaceae Licania sprucei#NC 024065
Malpighiales Chrysobalanaceae Licania heteromorpha#NC 024062
Malpighiales Chrysobalanaceae Hirtella physophora#NC 024066
Malpighiales Chrysobalanaceae Hirtella racemosa#F554
Malpighiales Ctenolophonaceae Ctenolophon englerianus#ZSD332
Malpighiales Erythroxylaceae Erythroxylum sinense#F372
Malpighiales Erythroxylaceae Erythroxylum novogranatense#F913
Malpighiales Erythroxylaceae Erythroxylum coca#RPPC
Malpighiales Rhizophoraceae Carallia brachiata#F236
Malpighiales Rhizophoraceae Bruguiera sexangula#F498
Malpighiales Rhizophoraceae Rhizophora mangle#ZTLR
Malpighiales Rhizophoraceae Rhizophora mucronata#F499
Malpighiales Pandaceae Microdesmis caseariifolia#F492
Malpighiales Pandaceae Microdesmis caseariifolia#F721
Malpighiales Irvingiaceae Irvingia excelsa#ZSD279
Malpighiales Irvingiaceae Klainedoxa gabonensis#ZSD280

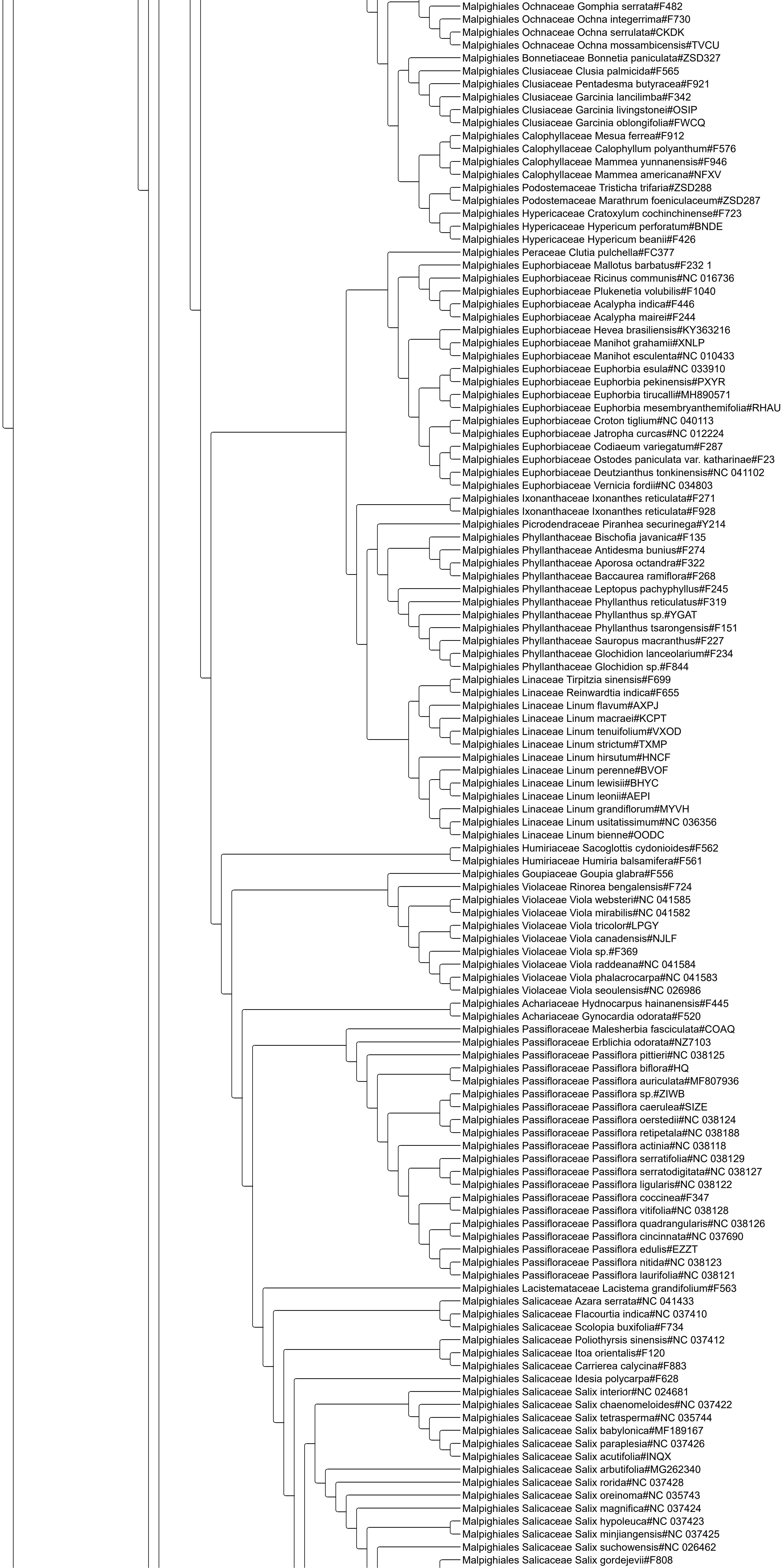
Malpighiales Ochnaceae Gomphia serrata#F482
Malpighiales Ochnaceae Ochna integerrima#F730
Malpighiales Ochnaceae Ochna serrulata#CKDK
Malpighiales Ochnaceae Ochna mossambicensis#TVCU
Malpighiales Bonnetiaceae Bonnetia paniculata#ZSD327
Malpighiales Clusiaceae Clusia palmicida#F565
Malpighiales Clusiaceae Pentadesma butyracea#F921
Malpighiales Clusiaceae Garcinia lancilimba#F342
Malpighiales Clusiaceae Garcinia livingstonei#OSIP
Malpighiales Clusiaceae Garcinia oblongifolia#FWCQ
Malpighiales Calophyllaceae Mesua ferrea#F912
Malpighiales Calophyllaceae Calophyllum polyanthum#F576
Malpighiales Calophyllaceae Mammea yunnanensis#F946
Malpighiales Calophyllaceae Mammea americana#NFXV
Malpighiales Podostemaceae Tristicha trifaria#ZSD288
Malpighiales Podostemaceae Marathrum foeniculaceum#ZSD287
Malpighiales Hypericaceae Cratoxylum cochinchinense#F723
Malpighiales Hypericaceae Hypericum perforatum#BNDE
Malpighiales Hypericaceae Hypericum beanii#F426
Malpighiales Peraceae Clutia pulchella#FC377
Malpighiales Euphorbiaceae Mallotus barbatus#F232 1
Malpighiales Euphorbiaceae Ricinus communis#NC 016736
Malpighiales Euphorbiaceae Plukenetia volubilis#F1040
Malpighiales Euphorbiaceae Acalypha indica#F446
Malpighiales Euphorbiaceae Acalypha mairei#F244
Malpighiales Euphorbiaceae Hevea brasiliensis#KY363216
Malpighiales Euphorbiaceae Manihot grahamii#XNLP
Malpighiales Euphorbiaceae Manihot esculenta#NC 010433
Malpighiales Euphorbiaceae Euphorbia esula#NC 033910
Malpighiales Euphorbiaceae Euphorbia pekinensis#PXYR
Malpighiales Euphorbiaceae Euphorbia tirucalli#MH890571
Malpighiales Euphorbiaceae Euphorbia mesembryanthemifolia#RHAU
Malpighiales Euphorbiaceae Croton tiglium#NC 040113
Malpighiales Euphorbiaceae Jatropha curcas#NC 012224
Malpighiales Euphorbiaceae Codiaeum variegatum#F287
Malpighiales Euphorbiaceae Ostodes paniculata var. katharinae#F23
Malpighiales Euphorbiaceae Deutzianthus tonkinensis#NC 041102
Malpighiales Euphorbiaceae Vernicia fordii#NC 034803
Malpighiales Ixonanthaceae Ixonanthes reticulata#F271
Malpighiales Ixonanthaceae Ixonanthes reticulata#F928
Malpighiales Picrodendraceae Piranhea securinega#Y214
Malpighiales Phyllanthaceae Bischofia javanica#F135
Malpighiales Phyllanthaceae Antidesma bunius#F274
Malpighiales Phyllanthaceae Aporosa octandra#F322
Malpighiales Phyllanthaceae Baccaurea ramiflora#F268
Malpighiales Phyllanthaceae Leptopus pachyphyllus#F245
Malpighiales Phyllanthaceae Phyllanthus reticulatus#F319
Malpighiales Phyllanthaceae Phyllanthus sp.#YGAT
Malpighiales Phyllanthaceae Phyllanthus tsarongensis#F151
Malpighiales Phyllanthaceae Sauropus macranthus#F227
Malpighiales Phyllanthaceae Glochidion lanceolarium#F234
Malpighiales Phyllanthaceae Glochidion sp.#F844
Malpighiales Linaceae Tirpitzia sinensis#F699
Malpighiales Linaceae Reinwardtia indica#F655
Malpighiales Linaceae Linum flavum#AXPJ
Malpighiales Linaceae Linum macraei#KCPT
Malpighiales Linaceae Linum tenuifolium#VXOD
Malpighiales Linaceae Linum strictum#TXMP
Malpighiales Linaceae Linum hirsutum#HNCF
Malpighiales Linaceae Linum perenne#BVOF
Malpighiales Linaceae Linum lewisii#BHYC
Malpighiales Linaceae Linum leonii#AEPI
Malpighiales Linaceae Linum grandiflorum#MYVH
Malpighiales Linaceae Linum usitatissimum#NC 036356
Malpighiales Linaceae Linum bienne#OODC
Malpighiales Humiriaceae Sacoglottis cydonioides#F562
Malpighiales Humiriaceae Humiria balsamifera#F561
Malpighiales Goupiaceae Goupia glabra#F556
Malpighiales Violaceae Rinorea bengalensis#F724
Malpighiales Violaceae Viola websteri#NC 041585
Malpighiales Violaceae Viola mirabilis#NC 041582
Malpighiales Violaceae Viola tricolor#LPGY
Malpighiales Violaceae Viola canadensis#NJLF
Malpighiales Violaceae Viola sp.#F369
Malpighiales Violaceae Viola raddeana#NC 041584
Malpighiales Violaceae Viola phalacrocarpa#NC 041583
Malpighiales Violaceae Viola seoulensis#NC 026986
Malpighiales Achariaceae Hydnocarpus hainanensis#F445
Malpighiales Achariaceae Gynocardia odorata#F520
Malpighiales Passifloraceae Malesherbia fasciculata#COAQ
Malpighiales Passifloraceae Erblichia odorata#NZ7103
Malpighiales Passifloraceae Passiflora pittieri#NC 038125
Malpighiales Passifloraceae Passiflora biflora#HQ
Malpighiales Passifloraceae Passiflora auriculata#MF807936
Malpighiales Passifloraceae Passiflora sp.#ZIWB
Malpighiales Passifloraceae Passiflora caerulea#SIZE
Malpighiales Passifloraceae Passiflora oerstedii#NC 038124
Malpighiales Passifloraceae Passiflora retipetala#NC 038188
Malpighiales Passifloraceae Passiflora actinia#NC 038118
Malpighiales Passifloraceae Passiflora serratifolia#NC 038129
Malpighiales Passifloraceae Passiflora serratodigitata#NC 038127
Malpighiales Passifloraceae Passiflora ligularis#NC 038122
Malpighiales Passifloraceae Passiflora coccinea#F347
Malpighiales Passifloraceae Passiflora vitifolia#NC 038128
Malpighiales Passifloraceae Passiflora quadrangularis#NC 038126
Malpighiales Passifloraceae Passiflora cincinnata#NC 037690
Malpighiales Passifloraceae Passiflora edulis#EZZT
Malpighiales Passifloraceae Passiflora nitida#NC 038123
Malpighiales Passifloraceae Passiflora laurifolia#NC 038121
Malpighiales Lacistemataceae Lacistema grandifolium#F563
Malpighiales Salicaceae Azara serrata#NC 041433
Malpighiales Salicaceae Flacourtia indica#NC 037410
Malpighiales Salicaceae Scolopia buxifolia#F734
Malpighiales Salicaceae Poliothyrsis sinensis#NC 037412
Malpighiales Salicaceae Itoa orientalis#F120
Malpighiales Salicaceae Carrierea calycina#F883
Malpighiales Salicaceae Idesia polycarpa#F628
Malpighiales Salicaceae Salix interior#NC 024681
Malpighiales Salicaceae Salix chaenomeloides#NC 037422
Malpighiales Salicaceae Salix tetrasperma#NC 035744
Malpighiales Salicaceae Salix babylonica#MF189167
Malpighiales Salicaceae Salix paraplesia#NC 037426
Malpighiales Salicaceae Salix acutifolia#INQX
Malpighiales Salicaceae Salix arbutifolia#MG262340
Malpighiales Salicaceae Salix rorida#NC 037428
Malpighiales Salicaceae Salix oreinoma#NC 035743
Malpighiales Salicaceae Salix magnifica#NC 037424
Malpighiales Salicaceae Salix hypoleuca#NC 037423
Malpighiales Salicaceae Salix minjiangensis#NC 037425
Malpighiales Salicaceae Salix suchowensis#NC 026462
Malpighiales Salicaceae Salix gordejevii#F808

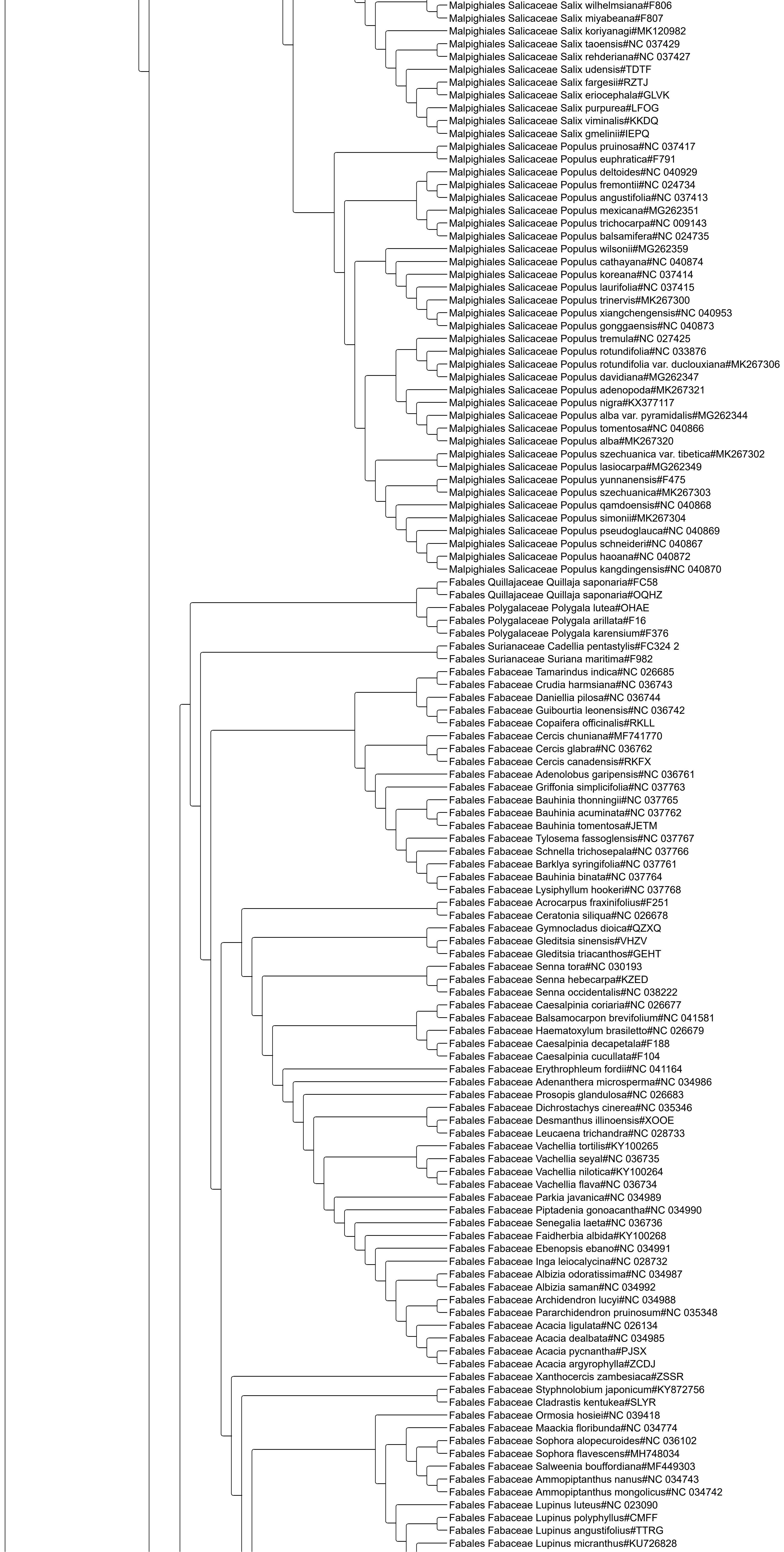
Malpighiales Salicaceae Salix wilhelmsiana#F806
Malpighiales Salicaceae Salix miyabeana#F807
Malpighiales Salicaceae Salix koriyanagi#MK120982
Malpighiales Salicaceae Salix taoensis#NC 037429
Malpighiales Salicaceae Salix rehderiana#NC 037427
Malpighiales Salicaceae Salix udensis#TDTF
Malpighiales Salicaceae Salix fargesii#RZTJ
Malpighiales Salicaceae Salix eriocephala#GLVK
Malpighiales Salicaceae Salix purpurea#LFOG
Malpighiales Salicaceae Salix viminalis#KKDQ
Malpighiales Salicaceae Salix gmelinii#IEPQ
Malpighiales Salicaceae Populus pruinosa#NC 037417
Malpighiales Salicaceae Populus euphratica#F791
Malpighiales Salicaceae Populus deltoides#NC 040929
Malpighiales Salicaceae Populus fremontii#NC 024734
Malpighiales Salicaceae Populus angustifolia#NC 037413
Malpighiales Salicaceae Populus mexicana#MG262351
Malpighiales Salicaceae Populus trichocarpa#NC 009143
Malpighiales Salicaceae Populus balsamifera#NC 024735
Malpighiales Salicaceae Populus wilsonii#MG262359
Malpighiales Salicaceae Populus cathayana#NC 040874
Malpighiales Salicaceae Populus koreana#NC 037414
Malpighiales Salicaceae Populus laurifolia#NC 037415
Malpighiales Salicaceae Populus trinervis#MK267300
Malpighiales Salicaceae Populus xiangchengensis#NC 040953
Malpighiales Salicaceae Populus gonggaensis#NC 040873
Malpighiales Salicaceae Populus tremula#NC 027425
Malpighiales Salicaceae Populus rotundifolia#NC 033876
Malpighiales Salicaceae Populus rotundifolia var. duclouxiana#MK267306
Malpighiales Salicaceae Populus davidiana#MG262347
Malpighiales Salicaceae Populus adenopoda#MK267321
Malpighiales Salicaceae Populus nigra#KX377117
Malpighiales Salicaceae Populus alba var. pyramidalis#MG262344
Malpighiales Salicaceae Populus tomentosa#NC 040866
Malpighiales Salicaceae Populus alba#MK267320
Malpighiales Salicaceae Populus szechuanica var. tibetica#MK267302
Malpighiales Salicaceae Populus lasiocarpa#MG262349
Malpighiales Salicaceae Populus yunnanensis#F475
Malpighiales Salicaceae Populus szechuanica#MK267303
Malpighiales Salicaceae Populus qamdoensis#NC 040868
Malpighiales Salicaceae Populus simonii#MK267304
Malpighiales Salicaceae Populus pseudoglauca#NC 040869
Malpighiales Salicaceae Populus schneideri#NC 040867
Malpighiales Salicaceae Populus haoana#NC 040872
Malpighiales Salicaceae Populus kangdingensis#NC 040870
Fabales Quillajaceae Quillaja saponaria#FC58
Fabales Quillajaceae Quillaja saponaria#OQHZ
Fabales Polygalaceae Polygala lutea#OHAE
Fabales Polygalaceae Polygala arillata#F16
Fabales Polygalaceae Polygala karensium#F376
Fabales Surianaceae Cadellia pentastylis#FC324 2
Fabales Surianaceae Suriana maritima#F982
Fabales Fabaceae Tamarindus indica#NC 026685
Fabales Fabaceae Crudia harmsiana#NC 036743
Fabales Fabaceae Daniellia pilosa#NC 036744
Fabales Fabaceae Guibourtia leonensis#NC 036742
Fabales Fabaceae Copaifera officinalis#RKLL
Fabales Fabaceae Cercis chuniana#MF741770
Fabales Fabaceae Cercis glabra#NC 036762
Fabales Fabaceae Cercis canadensis#RKFX
Fabales Fabaceae Adenolobus garipensis#NC 036761
Fabales Fabaceae Griffonia simplicifolia#NC 037763
Fabales Fabaceae Bauhinia thonningii#NC 037765
Fabales Fabaceae Bauhinia acuminata#NC 037762
Fabales Fabaceae Bauhinia tomentosa#JETM
Fabales Fabaceae Tylosema fassoglensis#NC 037767
Fabales Fabaceae Schnella trichosepala#NC 037766
Fabales Fabaceae Barklya syringifolia#NC 037761
Fabales Fabaceae Bauhinia binata#NC 037764
Fabales Fabaceae Lysiphyllum hookeri#NC 037768
Fabales Fabaceae Acrocarpus fraxinifolius#F251
Fabales Fabaceae Ceratonia siliqua#NC 026678
Fabales Fabaceae Gymnocladus dioica#QZXQ
Fabales Fabaceae Gleditsia sinensis#VHZV
Fabales Fabaceae Gleditsia triacanthos#GEHT
Fabales Fabaceae Senna tora#NC 030193
Fabales Fabaceae Senna hebecarpa#KZED
Fabales Fabaceae Senna occidentalis#NC 038222
Fabales Fabaceae Caesalpinia coriaria#NC 026677
Fabales Fabaceae Balsamocarpon brevifolium#NC 041581
Fabales Fabaceae Haematoxylum brasiletto#NC 026679
Fabales Fabaceae Caesalpinia decapetala#F188
Fabales Fabaceae Caesalpinia cucullata#F104
Fabales Fabaceae Erythrophleum fordii#NC 041164
Fabales Fabaceae Adenanthera microsperma#NC 034986
Fabales Fabaceae Prosopis glandulosa#NC 026683
Fabales Fabaceae Dichrostachys cinerea#NC 035346
Fabales Fabaceae Desmanthus illinoensis#XOOE
Fabales Fabaceae Leucaena trichandra#NC 028733
Fabales Fabaceae Vachellia tortilis#KY100265
Fabales Fabaceae Vachellia seyal#NC 036735
Fabales Fabaceae Vachellia nilotica#KY100264
Fabales Fabaceae Vachellia flava#NC 036734
Fabales Fabaceae Parkia javanica#NC 034989
Fabales Fabaceae Piptadenia gonoacantha#NC 034990
Fabales Fabaceae Senegalia laeta#NC 036736
Fabales Fabaceae Faidherbia albida#KY100268
Fabales Fabaceae Ebenopsis ebano#NC 034991
Fabales Fabaceae Inga leiocalycina#NC 028732
Fabales Fabaceae Albizia odoratissima#NC 034987
Fabales Fabaceae Albizia saman#NC 034992
Fabales Fabaceae Archidendron lucyi#NC 034988
Fabales Fabaceae Pararchidendron pruinosum#NC 035348
Fabales Fabaceae Acacia ligulata#NC 026134
Fabales Fabaceae Acacia dealbata#NC 034985
Fabales Fabaceae Acacia pycnantha#PJSX
Fabales Fabaceae Acacia argyrophylla#ZCDJ
Fabales Fabaceae Xanthocercis zambesiaca#ZSSR
Fabales Fabaceae Styphnolobium japonicum#KY872756
Fabales Fabaceae Cladrastis kentukea#SLYR
Fabales Fabaceae Ormosia hosiei#NC 039418
Fabales Fabaceae Maackia floribunda#NC 034774
Fabales Fabaceae Sophora alopecuroides#NC 036102
Fabales Fabaceae Sophora flavescens#MH748034
Fabales Fabaceae Salweenia bouffordiana#MF449303
Fabales Fabaceae Ammopiptanthus nanus#NC 034743
Fabales Fabaceae Ammopiptanthus mongolicus#NC 034742
Fabales Fabaceae Lupinus luteus#NC 023090
Fabales Fabaceae Lupinus polyphyllus#CMFF
Fabales Fabaceae Lupinus angustifolius#TTRG
Fabales Fabaceae Lupinus micranthus#KU726828

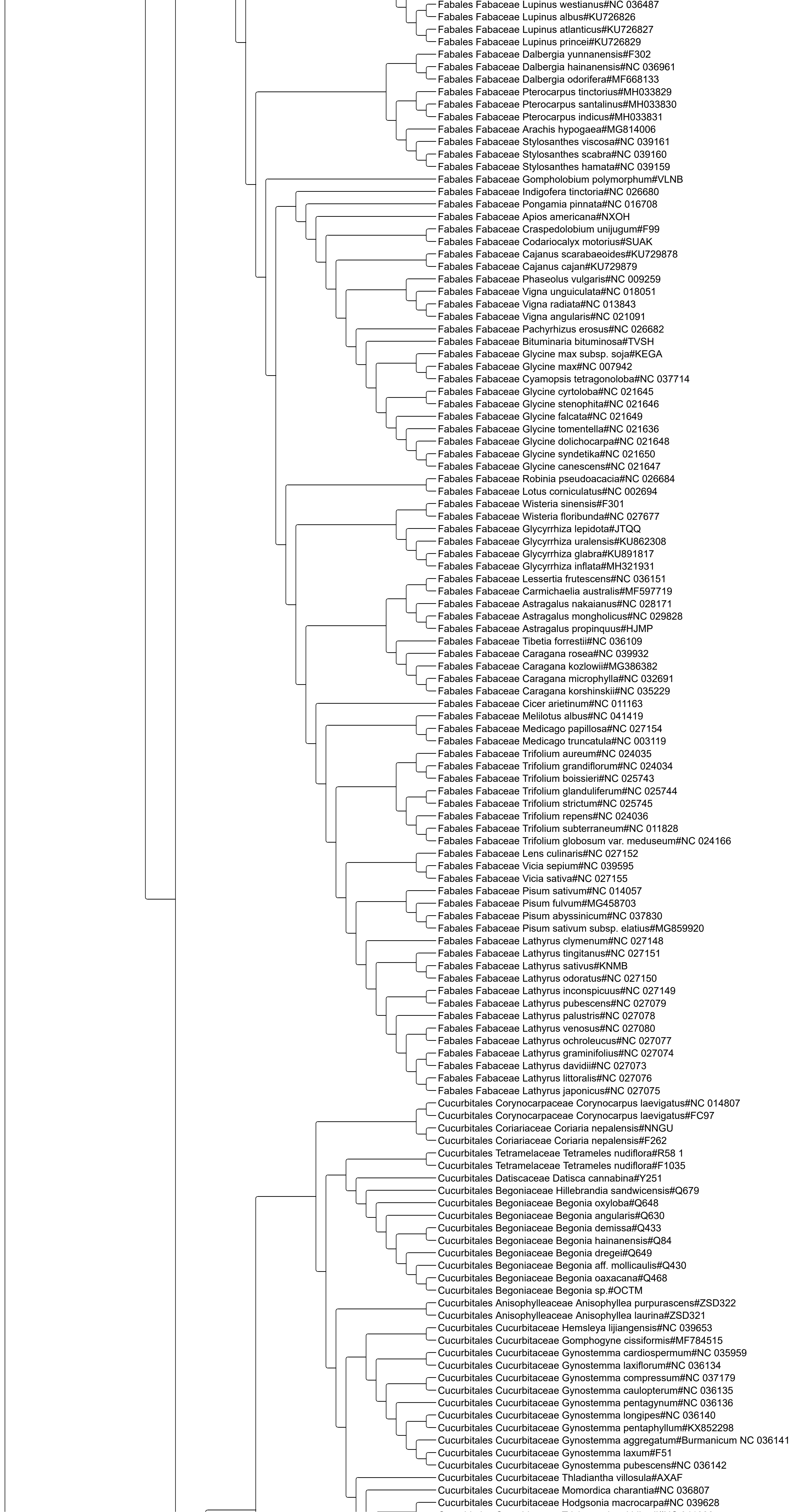

Fabales Fabaceae Lupinus westianus#NC 036487
Fabales Fabaceae Lupinus albus#KU726826
Fabales Fabaceae Lupinus atlanticus#KU726827
Fabales Fabaceae Lupinus princei#KU726829
Fabales Fabaceae Dalbergia yunnanensis#F302
Fabales Fabaceae Dalbergia hainanensis#NC 036961
Fabales Fabaceae Dalbergia odorifera#MF668133
Fabales Fabaceae Pterocarpus tinctorius#MH033829
Fabales Fabaceae Pterocarpus santalinus#MH033830
Fabales Fabaceae Pterocarpus indicus#MH033831
Fabales Fabaceae Arachis hypogaea#MG814006
Fabales Fabaceae Stylosanthes viscosa#NC 039161
Fabales Fabaceae Stylosanthes scabra#NC 039160
Fabales Fabaceae Stylosanthes hamata#NC 039159
Fabales Fabaceae Gompholobium polymorphum#VLNB
Fabales Fabaceae Indigofera tinctoria#NC 026680
Fabales Fabaceae Pongamia pinnata#NC 016708
Fabales Fabaceae Apios americana#NXOH
Fabales Fabaceae Craspedolobium unijugum#F99
Fabales Fabaceae Codariocalyx motorius#SUAK
Fabales Fabaceae Cajanus scarabaeoides#KU729878
Fabales Fabaceae Cajanus cajan#KU729879
Fabales Fabaceae Phaseolus vulgaris#NC 009259
Fabales Fabaceae Vigna unguiculata#NC 018051
Fabales Fabaceae Vigna radiata#NC 013843
Fabales Fabaceae Vigna angularis#NC 021091
Fabales Fabaceae Pachyrhizus erosus#NC 026682
Fabales Fabaceae Bituminaria bituminosa#TVSH
Fabales Fabaceae Glycine max subsp. soja#KEGA
Fabales Fabaceae Glycine max#NC 007942
Fabales Fabaceae Cyamopsis tetragonoloba#NC 037714
Fabales Fabaceae Glycine cyrtoloba#NC 021645
Fabales Fabaceae Glycine stenophita#NC 021646
Fabales Fabaceae Glycine falcata#NC 021649
Fabales Fabaceae Glycine tomentella#NC 021636
Fabales Fabaceae Glycine dolichocarpa#NC 021648
Fabales Fabaceae Glycine syndetika#NC 021650
Fabales Fabaceae Glycine canescens#NC 021647
Fabales Fabaceae Robinia pseudoacacia#NC 026684
Fabales Fabaceae Lotus corniculatus#NC 002694
Fabales Fabaceae Wisteria sinensis#F301
Fabales Fabaceae Wisteria floribunda#NC 027677
Fabales Fabaceae Glycyrrhiza lepidota#JTQQ
Fabales Fabaceae Glycyrrhiza uralensis#KU862308
Fabales Fabaceae Glycyrrhiza glabra#KU891817
Fabales Fabaceae Glycyrrhiza inflata#MH321931
Fabales Fabaceae Lessertia frutescens#NC 036151
Fabales Fabaceae Carmichaelia australis#MF597719
Fabales Fabaceae Astragalus nakaianus#NC 028171
Fabales Fabaceae Astragalus mongholicus#NC 029828
Fabales Fabaceae Astragalus propinquus#HJMP
Fabales Fabaceae Tibetia forrestii#NC 036109
Fabales Fabaceae Caragana rosea#NC 039932
Fabales Fabaceae Caragana kozlowii#MG386382
Fabales Fabaceae Caragana microphylla#NC 032691
Fabales Fabaceae Caragana korshinskii#NC 035229
Fabales Fabaceae Cicer arietinum#NC 011163
Fabales Fabaceae Melilotus albus#NC 041419
Fabales Fabaceae Medicago papillosa#NC 027154
Fabales Fabaceae Medicago truncatula#NC 003119
Fabales Fabaceae Trifolium aureum#NC 024035
Fabales Fabaceae Trifolium grandiflorum#NC 024034
Fabales Fabaceae Trifolium boissieri#NC 025743
Fabales Fabaceae Trifolium glanduliferum#NC 025744
Fabales Fabaceae Trifolium strictum#NC 025745
Fabales Fabaceae Trifolium repens#NC 024036
Fabales Fabaceae Trifolium subterraneum#NC 011828
Fabales Fabaceae Trifolium globosum var. meduseum#NC 024166
Fabales Fabaceae Lens culinaris#NC 027152
Fabales Fabaceae Vicia sepium#NC 039595
Fabales Fabaceae Vicia sativa#NC 027155
Fabales Fabaceae Pisum sativum#NC 014057
Fabales Fabaceae Pisum fulvum#MG458703
Fabales Fabaceae Pisum abyssinicum#NC 037830
Fabales Fabaceae Pisum sativum subsp. elatius#MG859920
Fabales Fabaceae Lathyrus clymenum#NC 027148
Fabales Fabaceae Lathyrus tingitanus#NC 027151
Fabales Fabaceae Lathyrus sativus#KNMB
Fabales Fabaceae Lathyrus odoratus#NC 027150
Fabales Fabaceae Lathyrus inconspicuus#NC 027149
Fabales Fabaceae Lathyrus pubescens#NC 027079
Fabales Fabaceae Lathyrus palustris#NC 027078
Fabales Fabaceae Lathyrus venosus#NC 027080
Fabales Fabaceae Lathyrus ochroleucus#NC 027077
Fabales Fabaceae Lathyrus graminifolius#NC 027074
Fabales Fabaceae Lathyrus davidii#NC 027073
Fabales Fabaceae Lathyrus littoralis#NC 027076
Fabales Fabaceae Lathyrus japonicus#NC 027075
Cucurbitales Corynocarpaceae Corynocarpus laevigatus#NC 014807
Cucurbitales Corynocarpaceae Corynocarpus laevigatus#FC97
Cucurbitales Coriariaceae Coriaria nepalensis#NNGU
Cucurbitales Coriariaceae Coriaria nepalensis#F262
Cucurbitales Tetramelaceae Tetrameles nudiflora#R58 1
Cucurbitales Tetramelaceae Tetrameles nudiflora#F1035
Cucurbitales Datiscaceae Datisca cannabina#Y251
Cucurbitales Begoniaceae Hillebrandia sandwicensis#Q679
Cucurbitales Begoniaceae Begonia oxyloba#Q648
Cucurbitales Begoniaceae Begonia angularis#Q630
Cucurbitales Begoniaceae Begonia demissa#Q433
Cucurbitales Begoniaceae Begonia hainanensis#Q84
Cucurbitales Begoniaceae Begonia dregei#Q649
Cucurbitales Begoniaceae Begonia aff. mollicaulis#Q430
Cucurbitales Begoniaceae Begonia oaxacana#Q468
Cucurbitales Begoniaceae Begonia sp.#OCTM
Cucurbitales Anisophylleaceae Anisophyllea purpurascens#ZSD322
Cucurbitales Anisophylleaceae Anisophyllea laurina#ZSD321
Cucurbitales Cucurbitaceae Hemsleya lijiangensis#NC 039653
Cucurbitales Cucurbitaceae Gomphogyne cissiformis#MF784515
Cucurbitales Cucurbitaceae Gynostemma cardiospermum#NC 035959
Cucurbitales Cucurbitaceae Gynostemma laxiflorum#NC 036134
Cucurbitales Cucurbitaceae Gynostemma compressum#NC 037179
Cucurbitales Cucurbitaceae Gynostemma caulopterum#NC 036135
Cucurbitales Cucurbitaceae Gynostemma pentagynum#NC 036136
Cucurbitales Cucurbitaceae Gynostemma longipes#NC 036140
Cucurbitales Cucurbitaceae Gynostemma pentaphyllum#KX852298
Cucurbitales Cucurbitaceae Gynostemma aggregatum#Burmanicum NC 036141
Cucurbitales Cucurbitaceae Gynostemma laxum#F51
Cucurbitales Cucurbitaceae Gynostemma pubescens#NC 036142
Cucurbitales Cucurbitaceae Thladiantha villosula#AXAF
Cucurbitales Cucurbitaceae Momordica charantia#NC 036807
Cucurbitales Cucurbitaceae Hodgsonia macrocarpa#NC 039628

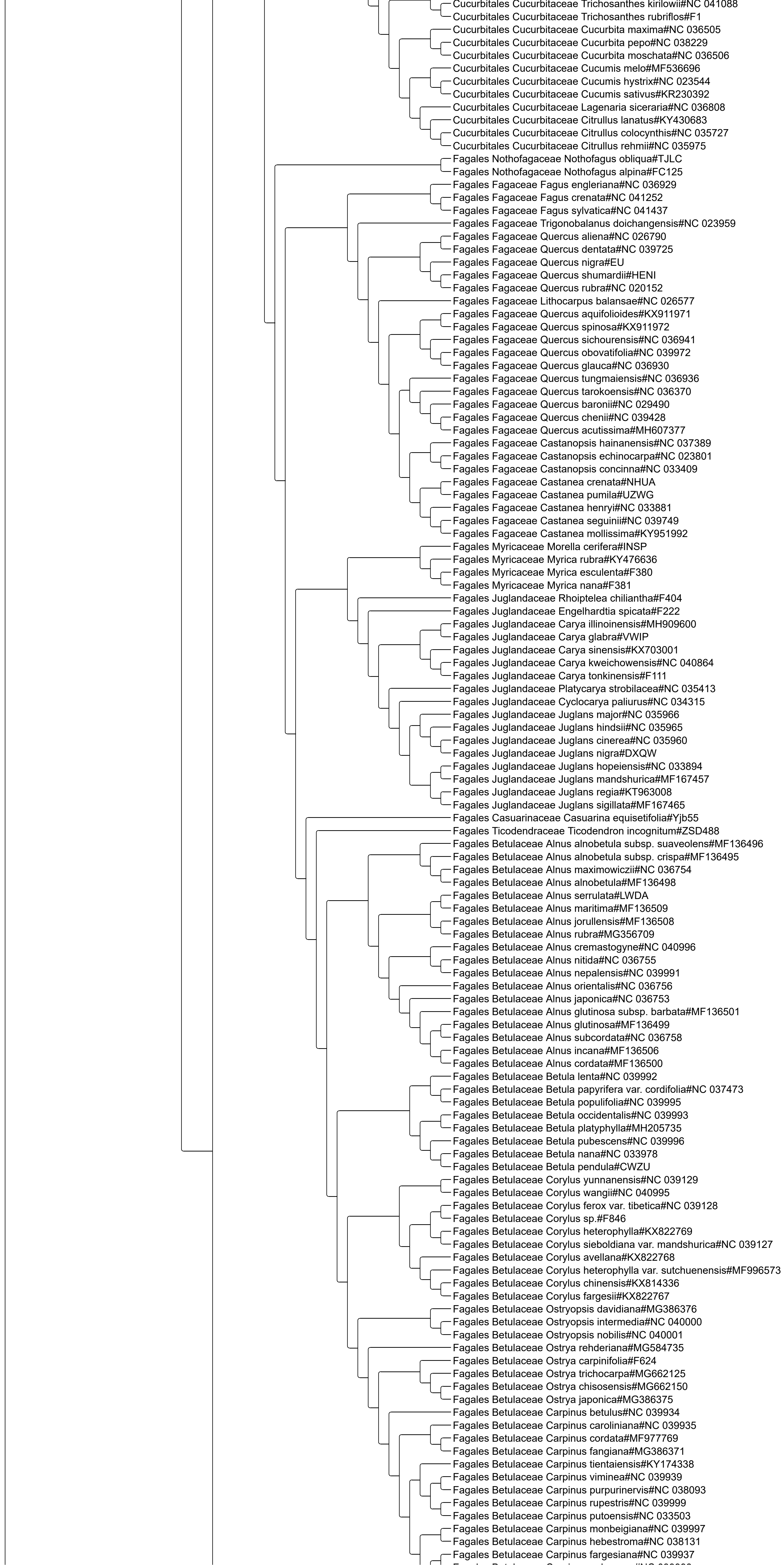
Cucurbitales Cucurbitaceae Trichosanthes kirilowii#NC 041088
Cucurbitales Cucurbitaceae Trichosanthes rubriflos#F1
Cucurbitales Cucurbitaceae Cucurbita maxima#NC 036505
Cucurbitales Cucurbitaceae Cucurbita pepo#NC 038229
Cucurbitales Cucurbitaceae Cucurbita moschata#NC 036506
Cucurbitales Cucurbitaceae Cucumis melo#MF536696
Cucurbitales Cucurbitaceae Cucumis hystrix#NC 023544
Cucurbitales Cucurbitaceae Cucumis sativus#KR230392
Cucurbitales Cucurbitaceae Lagenaria siceraria#NC 036808
Cucurbitales Cucurbitaceae Citrullus lanatus#KY430683
Cucurbitales Cucurbitaceae Citrullus colocynthis#NC 035727
Cucurbitales Cucurbitaceae Citrullus rehmii#NC 035975
Fagales Nothofagaceae Nothofagus obliqua#TJLC
Fagales Nothofagaceae Nothofagus alpina#FC125
Fagales Fagaceae Fagus engleriana#NC 036929
Fagales Fagaceae Fagus crenata#NC 041252
Fagales Fagaceae Fagus sylvatica#NC 041437
Fagales Fagaceae Trigonobalanus doichangensis#NC 023959
Fagales Fagaceae Quercus aliena#NC 026790
Fagales Fagaceae Quercus dentata#NC 039725
Fagales Fagaceae Quercus nigra#EU
Fagales Fagaceae Quercus shumardii#HENI
Fagales Fagaceae Quercus rubra#NC 020152
Fagales Fagaceae Lithocarpus balansae#NC 026577
Fagales Fagaceae Quercus aquifolioides#KX911971
Fagales Fagaceae Quercus spinosa#KX911972
Fagales Fagaceae Quercus sichourensis#NC 036941
Fagales Fagaceae Quercus obovatifolia#NC 039972
Fagales Fagaceae Quercus glauca#NC 036930
Fagales Fagaceae Quercus tungmaiensis#NC 036936
Fagales Fagaceae Quercus tarokoensis#NC 036370
Fagales Fagaceae Quercus baronii#NC 029490
Fagales Fagaceae Quercus chenii#NC 039428
Fagales Fagaceae Quercus acutissima#MH607377
Fagales Fagaceae Castanopsis hainanensis#NC 037389
Fagales Fagaceae Castanopsis echinocarpa#NC 023801
Fagales Fagaceae Castanopsis concinna#NC 033409
Fagales Fagaceae Castanea crenata#NHUA
Fagales Fagaceae Castanea pumila#UZWG
Fagales Fagaceae Castanea henryi#NC 033881
Fagales Fagaceae Castanea seguinii#NC 039749
Fagales Fagaceae Castanea mollissima#KY951992
Fagales Myricaceae Morella cerifera#INSP
Fagales Myricaceae Myrica rubra#KY476636
Fagales Myricaceae Myrica esculenta#F380
Fagales Myricaceae Myrica nana#F381
Fagales Juglandaceae Rhoiptelea chiliantha#F404
Fagales Juglandaceae Engelhardtia spicata#F222
Fagales Juglandaceae Carya illinoinensis#MH909600
Fagales Juglandaceae Carya glabra#VWIP
Fagales Juglandaceae Carya sinensis#KX703001
Fagales Juglandaceae Carya kweichowensis#NC 040864
Fagales Juglandaceae Carya tonkinensis#F111
Fagales Juglandaceae Platycarya strobilacea#NC 035413
Fagales Juglandaceae Cyclocarya paliurus#NC 034315
Fagales Juglandaceae Juglans major#NC 035966
Fagales Juglandaceae Juglans hindsii#NC 035965
Fagales Juglandaceae Juglans cinerea#NC 035960
Fagales Juglandaceae Juglans nigra#DXQW
Fagales Juglandaceae Juglans hopeiensis#NC 033894
Fagales Juglandaceae Juglans mandshurica#MF167457
Fagales Juglandaceae Juglans regia#KT963008
Fagales Juglandaceae Juglans sigillata#MF167465
Fagales Casuarinaceae Casuarina equisetifolia#Yjb55
Fagales Ticodendraceae Ticodendron incognitum#ZSD488
Fagales Betulaceae Alnus alnobetula subsp. suaveolens#MF136496
Fagales Betulaceae Alnus alnobetula subsp. crispa#MF136495
Fagales Betulaceae Alnus maximowiczii#NC 036754
Fagales Betulaceae Alnus alnobetula#MF136498
Fagales Betulaceae Alnus serrulata#LWDA
Fagales Betulaceae Alnus maritima#MF136509
Fagales Betulaceae Alnus jorullensis#MF136508
Fagales Betulaceae Alnus rubra#MG356709
Fagales Betulaceae Alnus cremastogyne#NC 040996
Fagales Betulaceae Alnus nitida#NC 036755
Fagales Betulaceae Alnus nepalensis#NC 039991
Fagales Betulaceae Alnus orientalis#NC 036756
Fagales Betulaceae Alnus japonica#NC 036753
Fagales Betulaceae Alnus glutinosa subsp. barbata#MF136501
Fagales Betulaceae Alnus glutinosa#MF136499
Fagales Betulaceae Alnus subcordata#NC 036758
Fagales Betulaceae Alnus incana#MF136506
Fagales Betulaceae Alnus cordata#MF136500
Fagales Betulaceae Betula lenta#NC 039992
Fagales Betulaceae Betula papyrifera var. cordifolia#NC 037473
Fagales Betulaceae Betula populifolia#NC 039995
Fagales Betulaceae Betula occidentalis#NC 039993
Fagales Betulaceae Betula platyphylla#MH205735
Fagales Betulaceae Betula pubescens#NC 039996
Fagales Betulaceae Betula nana#NC 033978
Fagales Betulaceae Betula pendula#CWZU
Fagales Betulaceae Corylus yunnanensis#NC 039129
Fagales Betulaceae Corylus wangii#NC 040995
Fagales Betulaceae Corylus ferox var. tibetica#NC 039128
Fagales Betulaceae Corylus sp.#F846
Fagales Betulaceae Corylus heterophylla#KX822769
Fagales Betulaceae Corylus sieboldiana var. mandshurica#NC 039127
Fagales Betulaceae Corylus avellana#KX822768
Fagales Betulaceae Corylus heterophylla var. sutchuenensis#MF996573
Fagales Betulaceae Corylus chinensis#KX814336
Fagales Betulaceae Corylus fargesii#KX822767
Fagales Betulaceae Ostryopsis davidiana#MG386376
Fagales Betulaceae Ostryopsis intermedia#NC 040000
Fagales Betulaceae Ostryopsis nobilis#NC 040001
Fagales Betulaceae Ostrya rehderiana#MG584735
Fagales Betulaceae Ostrya carpinifolia#F624
Fagales Betulaceae Ostrya trichocarpa#MG662125
Fagales Betulaceae Ostrya chisosensis#MG662150
Fagales Betulaceae Ostrya japonica#MG386375
Fagales Betulaceae Carpinus betulus#NC 039934
Fagales Betulaceae Carpinus caroliniana#NC 039935
Fagales Betulaceae Carpinus cordata#MF977769
Fagales Betulaceae Carpinus fangiana#MG386371
Fagales Betulaceae Carpinus tientaiensis#KY174338
Fagales Betulaceae Carpinus viminea#NC 039939
Fagales Betulaceae Carpinus purpurinervis#NC 038093
Fagales Betulaceae Carpinus rupestris#NC 039999
Fagales Betulaceae Carpinus putoensis#NC 033503
Fagales Betulaceae Carpinus monbeigiana#NC 039997
Fagales Betulaceae Carpinus hebestroma#NC 038131
Fagales Betulaceae Carpinus fargesiana#NC 039937

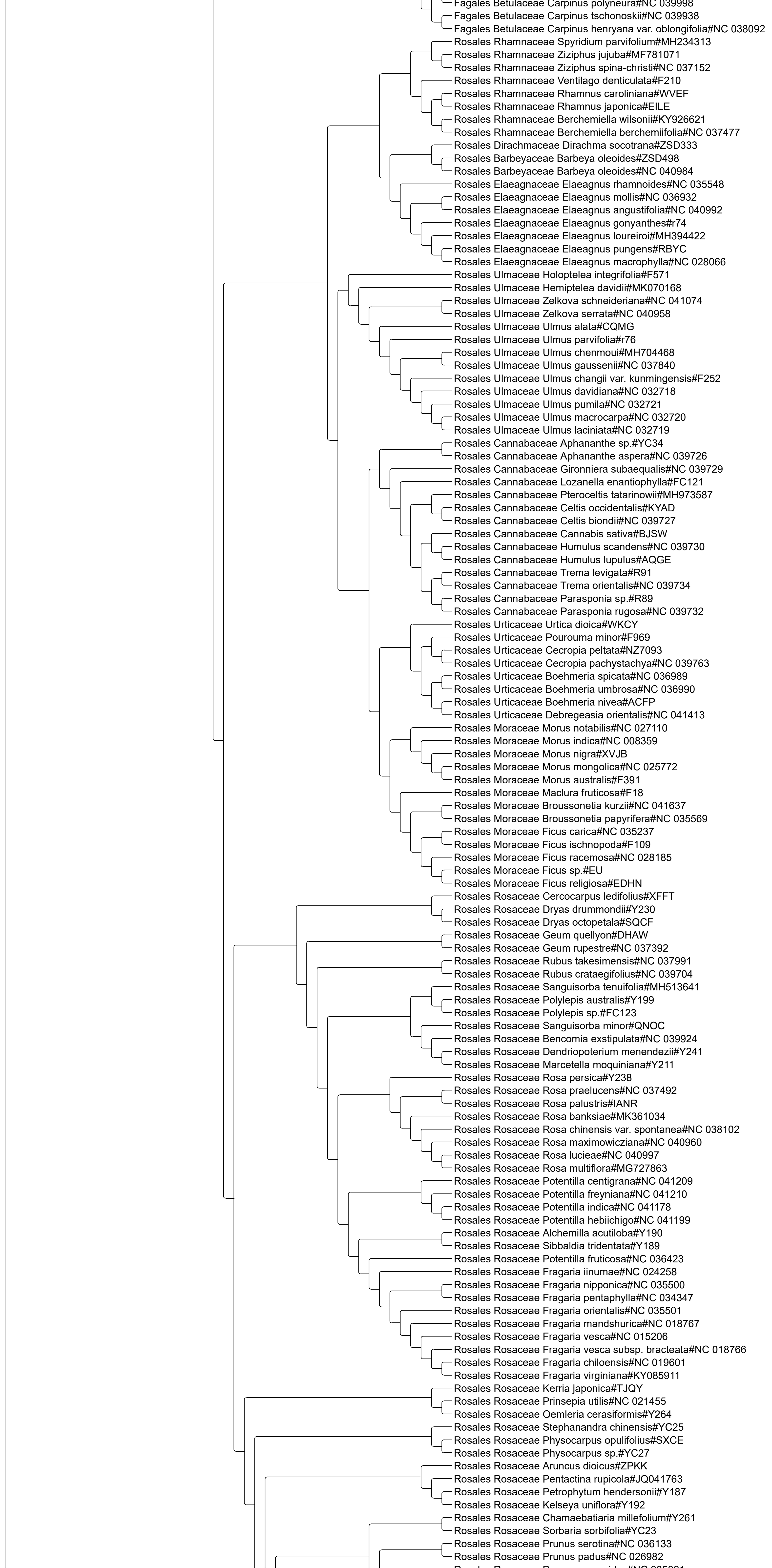
Fagales Betulaceae Carpinus polyneura#NC 039998
Fagales Betulaceae Carpinus tschonoskii#NC 039938
Fagales Betulaceae Carpinus henryana var. oblongifolia#NC 038092
Rosales Rhamnaceae Spyridium parvifolium#MH234313
Rosales Rhamnaceae Ziziphus jujuba#MF781071
Rosales Rhamnaceae Ziziphus spina-christi#NC 037152
Rosales Rhamnaceae Ventilago denticulata#F210
Rosales Rhamnaceae Rhamnus caroliniana#WVEF
Rosales Rhamnaceae Rhamnus japonica#EILE
Rosales Rhamnaceae Berchemiella wilsonii#KY926621
Rosales Rhamnaceae Berchemiella berchemiifolia#NC 037477
Rosales Dirachmaceae Dirachma socotrana#ZSD333
Rosales Barbeyaceae Barbeya oleoides#ZSD498
Rosales Barbeyaceae Barbeya oleoides#NC 040984
Rosales Elaeagnaceae Elaeagnus rhamnoides#NC 035548
Rosales Elaeagnaceae Elaeagnus mollis#NC 036932
Rosales Elaeagnaceae Elaeagnus angustifolia#NC 040992
Rosales Elaeagnaceae Elaeagnus gonyanthes#r74
Rosales Elaeagnaceae Elaeagnus loureiroi#MH394422
Rosales Elaeagnaceae Elaeagnus pungens#RBYC
Rosales Elaeagnaceae Elaeagnus macrophylla#NC 028066
Rosales Ulmaceae Holoptelea integrifolia#F571
Rosales Ulmaceae Hemiptelea davidii#MK070168
Rosales Ulmaceae Zelkova schneideriana#NC 041074
Rosales Ulmaceae Zelkova serrata#NC 040958
Rosales Ulmaceae Ulmus alata#CQMG
Rosales Ulmaceae Ulmus parvifolia#r76
Rosales Ulmaceae Ulmus chenmoui#MH704468
Rosales Ulmaceae Ulmus gaussenii#NC 037840
Rosales Ulmaceae Ulmus changii var. kunmingensis#F252
Rosales Ulmaceae Ulmus davidiana#NC 032718
Rosales Ulmaceae Ulmus pumila#NC 032721
Rosales Ulmaceae Ulmus macrocarpa#NC 032720
Rosales Ulmaceae Ulmus laciniata#NC 032719
Rosales Cannabaceae Aphananthe sp.#YC34
Rosales Cannabaceae Aphananthe aspera#NC 039726
Rosales Cannabaceae Gironniera subaequalis#NC 039729
Rosales Cannabaceae Lozanella enantiophylla#FC121
Rosales Cannabaceae Pteroceltis tatarinowii#MH973587
Rosales Cannabaceae Celtis occidentalis#KYAD
Rosales Cannabaceae Celtis biondii#NC 039727
Rosales Cannabaceae Cannabis sativa#BJSW
Rosales Cannabaceae Humulus scandens#NC 039730
Rosales Cannabaceae Humulus lupulus#AQGE
Rosales Cannabaceae Trema levigata#R91
Rosales Cannabaceae Trema orientalis#NC 039734
Rosales Cannabaceae Parasponia sp.#R89
Rosales Cannabaceae Parasponia rugosa#NC 039732
Rosales Urticaceae Urtica dioica#WKCY
Rosales Urticaceae Pourouma minor#F969
Rosales Urticaceae Cecropia peltata#NZ7093
Rosales Urticaceae Cecropia pachystachya#NC 039763
Rosales Urticaceae Boehmeria spicata#NC 036989
Rosales Urticaceae Boehmeria umbrosa#NC 036990
Rosales Urticaceae Boehmeria nivea#ACFP
Rosales Urticaceae Debregeasia orientalis#NC 041413
Rosales Moraceae Morus notabilis#NC 027110
Rosales Moraceae Morus indica#NC 008359
Rosales Moraceae Morus nigra#XVJB
Rosales Moraceae Morus mongolica#NC 025772
Rosales Moraceae Morus australis#F391
Rosales Moraceae Maclura fruticosa#F18
Rosales Moraceae Broussonetia kurzii#NC 041637
Rosales Moraceae Broussonetia papyrifera#NC 035569
Rosales Moraceae Ficus carica#NC 035237
Rosales Moraceae Ficus ischnopoda#F109
Rosales Moraceae Ficus racemosa#NC 028185
Rosales Moraceae Ficus sp.#EU
Rosales Moraceae Ficus religiosa#EDHN
Rosales Rosaceae Cercocarpus ledifolius#XFFT
Rosales Rosaceae Dryas drummondii#Y230
Rosales Rosaceae Dryas octopetala#SQCF
Rosales Rosaceae Geum quellyon#DHAW
Rosales Rosaceae Geum rupestre#NC 037392
Rosales Rosaceae Rubus takesimensis#NC 037991
Rosales Rosaceae Rubus crataegifolius#NC 039704
Rosales Rosaceae Sanguisorba tenuifolia#MH513641
Rosales Rosaceae Polylepis australis#Y199
Rosales Rosaceae Polylepis sp.#FC123
Rosales Rosaceae Sanguisorba minor#QNOC
Rosales Rosaceae Bencomia exstipulata#NC 039924
Rosales Rosaceae Dendriopoterium menendezii#Y241
Rosales Rosaceae Marcetella moquiniana#Y211
Rosales Rosaceae Rosa persica#Y238
Rosales Rosaceae Rosa praelucens#NC 037492
Rosales Rosaceae Rosa palustris#IANR
Rosales Rosaceae Rosa banksiae#MK361034
Rosales Rosaceae Rosa chinensis var. spontanea#NC 038102
Rosales Rosaceae Rosa maximowicziana#NC 040960
Rosales Rosaceae Rosa lucieae#NC 040997
Rosales Rosaceae Rosa multiflora#MG727863
Rosales Rosaceae Potentilla centigrana#NC 041209
Rosales Rosaceae Potentilla freyniana#NC 041210
Rosales Rosaceae Potentilla indica#NC 041178
Rosales Rosaceae Potentilla hebiichigo#NC 041199
Rosales Rosaceae Alchemilla acutiloba#Y190
Rosales Rosaceae Sibbaldia tridentata#Y189
Rosales Rosaceae Potentilla fruticosa#NC 036423
Rosales Rosaceae Fragaria iinumae#NC 024258
Rosales Rosaceae Fragaria nipponica#NC 035500
Rosales Rosaceae Fragaria pentaphylla#NC 034347
Rosales Rosaceae Fragaria orientalis#NC 035501
Rosales Rosaceae Fragaria mandshurica#NC 018767
Rosales Rosaceae Fragaria vesca#NC 015206
Rosales Rosaceae Fragaria vesca subsp. bracteata#NC 018766
Rosales Rosaceae Fragaria chiloensis#NC 019601
Rosales Rosaceae Fragaria virginiana#KY085911
Rosales Rosaceae Kerria japonica#TJQY
Rosales Rosaceae Prinsepia utilis#NC 021455
Rosales Rosaceae Oemleria cerasiformis#Y264
Rosales Rosaceae Stephanandra chinensis#YC25
Rosales Rosaceae Physocarpus opulifolius#SXCE
Rosales Rosaceae Physocarpus sp.#YC27
Rosales Rosaceae Aruncus dioicus#ZPKK
Rosales Rosaceae Pentactina rupicola#JQ041763
Rosales Rosaceae Petrophytum hendersonii#Y187
Rosales Rosaceae Kelseya uniflora#Y192
Rosales Rosaceae Chamaebatiaria millefolium#Y261
Rosales Rosaceae Sorbaria sorbifolia#YC23
Rosales Rosaceae Prunus serotina#NC 036133
Rosales Rosaceae Prunus padus#NC 026982

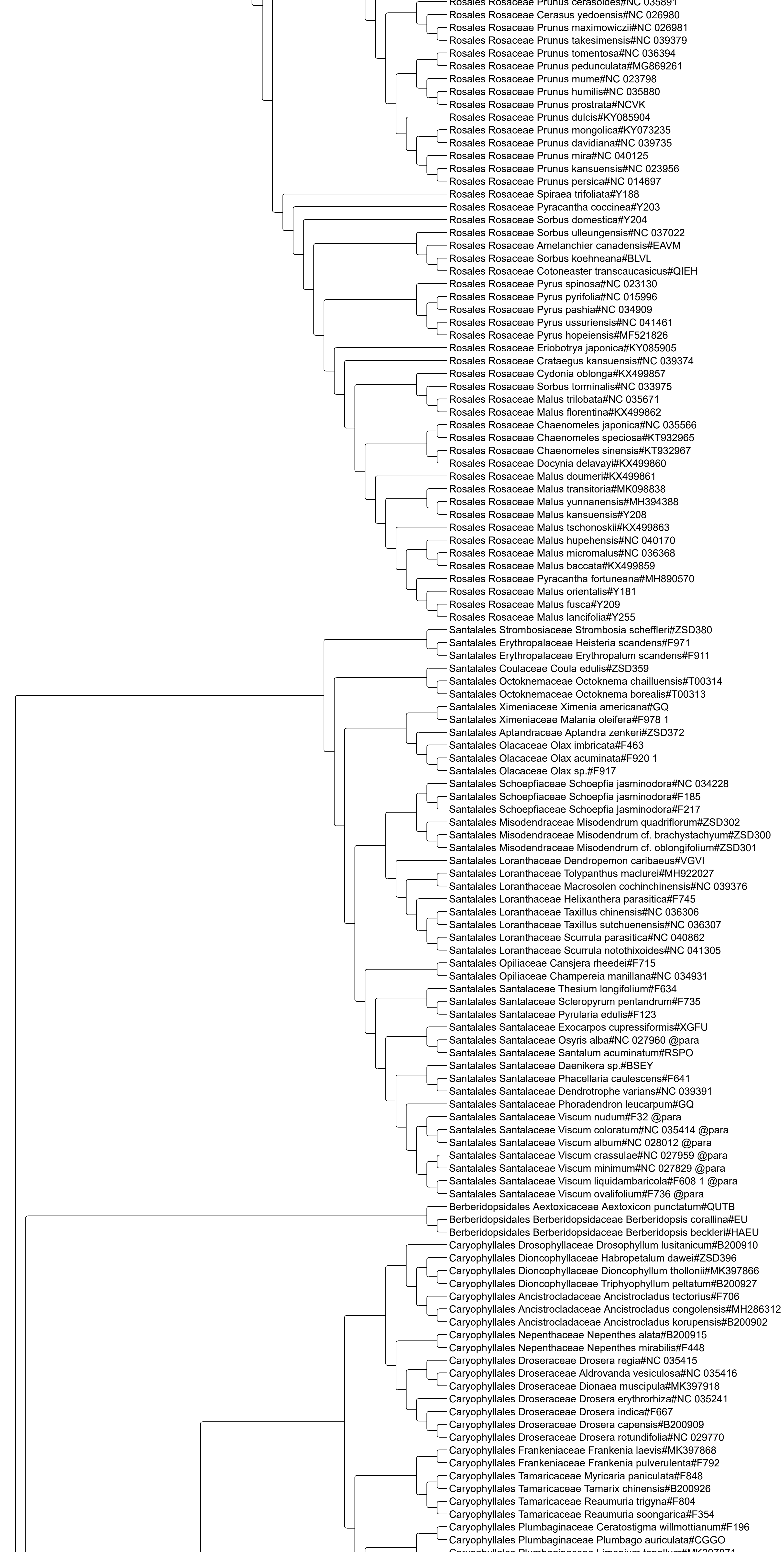
Rosales Rosaceae Prunus cerasoides#NC 035891
Rosales Rosaceae Cerasus yedoensis#NC 026980
Rosales Rosaceae Prunus maximowiczii#NC 026981
Rosales Rosaceae Prunus takesimensis#NC 039379
Rosales Rosaceae Prunus tomentosa#NC 036394
Rosales Rosaceae Prunus pedunculata#MG869261
Rosales Rosaceae Prunus mume#NC 023798
Rosales Rosaceae Prunus humilis#NC 035880
Rosales Rosaceae Prunus prostrata#NCVK
Rosales Rosaceae Prunus dulcis#KY085904
Rosales Rosaceae Prunus mongolica#KY073235
Rosales Rosaceae Prunus davidiana#NC 039735
Rosales Rosaceae Prunus mira#NC 040125
Rosales Rosaceae Prunus kansuensis#NC 023956
Rosales Rosaceae Prunus persica#NC 014697
Rosales Rosaceae Spiraea trifoliata#Y188
Rosales Rosaceae Pyracantha coccinea#Y203
Rosales Rosaceae Sorbus domestica#Y204
Rosales Rosaceae Sorbus ulleungensis#NC 037022
Rosales Rosaceae Amelanchier canadensis#EAVM
Rosales Rosaceae Sorbus koehneana#BLVL
Rosales Rosaceae Cotoneaster transcaucasicus#QIEH
Rosales Rosaceae Pyrus spinosa#NC 023130
Rosales Rosaceae Pyrus pyrifolia#NC 015996
Rosales Rosaceae Pyrus pashia#NC 034909
Rosales Rosaceae Pyrus ussuriensis#NC 041461
Rosales Rosaceae Pyrus hopeiensis#MF521826
Rosales Rosaceae Eriobotrya japonica#KY085905
Rosales Rosaceae Crataegus kansuensis#NC 039374
Rosales Rosaceae Cydonia oblonga#KX499857
Rosales Rosaceae Sorbus torminalis#NC 033975
Rosales Rosaceae Malus trilobata#NC 035671
Rosales Rosaceae Malus florentina#KX499862
Rosales Rosaceae Chaenomeles japonica#NC 035566
Rosales Rosaceae Chaenomeles speciosa#KT932965
Rosales Rosaceae Chaenomeles sinensis#KT932967
Rosales Rosaceae Docynia delavayi#KX499860
Rosales Rosaceae Malus doumeri#KX499861
Rosales Rosaceae Malus transitoria#MK098838
Rosales Rosaceae Malus yunnanensis#MH394388
Rosales Rosaceae Malus kansuensis#Y208
Rosales Rosaceae Malus tschonoskii#KX499863
Rosales Rosaceae Malus hupehensis#NC 040170
Rosales Rosaceae Malus micromalus#NC 036368
Rosales Rosaceae Malus baccata#KX499859
Rosales Rosaceae Pyracantha fortuneana#MH890570
Rosales Rosaceae Malus orientalis#Y181
Rosales Rosaceae Malus fusca#Y209
Rosales Rosaceae Malus lancifolia#Y255
Santalales Strombosiaceae Strombosia scheffleri#ZSD380
Santalales Erythropalaceae Heisteria scandens#F971
Santalales Erythropalaceae Erythropalum scandens#F911
Santalales Coulaceae Coula edulis#ZSD359
Santalales Octoknemaceae Octoknema chailluensis#T00314
Santalales Octoknemaceae Octoknema borealis#T00313
Santalales Ximeniaceae Ximenia americana#GQ
Santalales Ximeniaceae Malania oleifera#F978 1
Santalales Aptandraceae Aptandra zenkeri#ZSD372
Santalales Olacaceae Olax imbricata#F463
Santalales Olacaceae Olax acuminata#F920 1
Santalales Olacaceae Olax sp.#F917
Santalales Schoepfiaceae Schoepfia jasminodora#NC 034228
Santalales Schoepfiaceae Schoepfia jasminodora#F185
Santalales Schoepfiaceae Schoepfia jasminodora#F217
Santalales Misodendraceae Misodendrum quadriflorum#ZSD302
Santalales Misodendraceae Misodendrum cf. brachystachyum#ZSD300
Santalales Misodendraceae Misodendrum cf. oblongifolium#ZSD301
Santalales Loranthaceae Dendropemon caribaeus#VGVI
Santalales Loranthaceae Tolypanthus maclurei#MH922027
Santalales Loranthaceae Macrosolen cochinchinensis#NC 039376
Santalales Loranthaceae Helixanthera parasitica#F745
Santalales Loranthaceae Taxillus chinensis#NC 036306
Santalales Loranthaceae Taxillus sutchuenensis#NC 036307
Santalales Loranthaceae Scurrula parasitica#NC 040862
Santalales Loranthaceae Scurrula notothixoides#NC 041305
Santalales Opiliaceae Cansjera rheedei#F715
Santalales Opiliaceae Champereia manillana#NC 034931
Santalales Santalaceae Thesium longifolium#F634
Santalales Santalaceae Scleropyrum pentandrum#F735
Santalales Santalaceae Pyrularia edulis#F123
Santalales Santalaceae Exocarpos cupressiformis#XGFU
Santalales Santalaceae Osyris alba#NC 027960 @para
Santalales Santalaceae Santalum acuminatum#RSPO
Santalales Santalaceae Daenikera sp.#BSEY
Santalales Santalaceae Phacellaria caulescens#F641
Santalales Santalaceae Dendrotrophe varians#NC 039391
Santalales Santalaceae Phoradendron leucarpum#GQ
Santalales Santalaceae Viscum nudum#F32 @para
Santalales Santalaceae Viscum coloratum#NC 035414 @para
Santalales Santalaceae Viscum album#NC 028012 @para
Santalales Santalaceae Viscum crassulae#NC 027959 @para
Santalales Santalaceae Viscum minimum#NC 027829 @para
Santalales Santalaceae Viscum liquidambaricola#F608 1 @para
Santalales Santalaceae Viscum ovalifolium#F736 @para
Berberidopsidales Aextoxicaceae Aextoxicon punctatum#QUTB
Berberidopsidales Berberidopsidaceae Berberidopsis corallina#EU
Berberidopsidales Berberidopsidaceae Berberidopsis beckleri#HAEU
Caryophyllales Drosophyllaceae Drosophyllum lusitanicum#B200910
Caryophyllales Dioncophyllaceae Habropetalum dawei#ZSD396
Caryophyllales Dioncophyllaceae Dioncophyllum thollonii#MK397866
Caryophyllales Dioncophyllaceae Triphyophyllum peltatum#B200927
Caryophyllales Ancistrocladaceae Ancistrocladus tectorius#F706
Caryophyllales Ancistrocladaceae Ancistrocladus congolensis#MH286312
Caryophyllales Ancistrocladaceae Ancistrocladus korupensis#B200902
Caryophyllales Nepenthaceae Nepenthes alata#B200915
Caryophyllales Nepenthaceae Nepenthes mirabilis#F448
Caryophyllales Droseraceae Drosera regia#NC 035415
Caryophyllales Droseraceae Aldrovanda vesiculosa#NC 035416
Caryophyllales Droseraceae Dionaea muscipula#MK397918
Caryophyllales Droseraceae Drosera erythrorhiza#NC 035241
Caryophyllales Droseraceae Drosera indica#F667
Caryophyllales Droseraceae Drosera capensis#B200909
Caryophyllales Droseraceae Drosera rotundifolia#NC 029770
Caryophyllales Frankeniaceae Frankenia laevis#MK397868
Caryophyllales Frankeniaceae Frankenia pulverulenta#F792
Caryophyllales Tamaricaceae Myricaria paniculata#F848
Caryophyllales Tamaricaceae Tamarix chinensis#B200926
Caryophyllales Tamaricaceae Reaumuria trigyna#F804
Caryophyllales Tamaricaceae Reaumuria soongarica#F354
Caryophyllales Plumbaginaceae Ceratostigma willmottianum#F196
Caryophyllales Plumbaginaceae Plumbago auriculata#CGGO

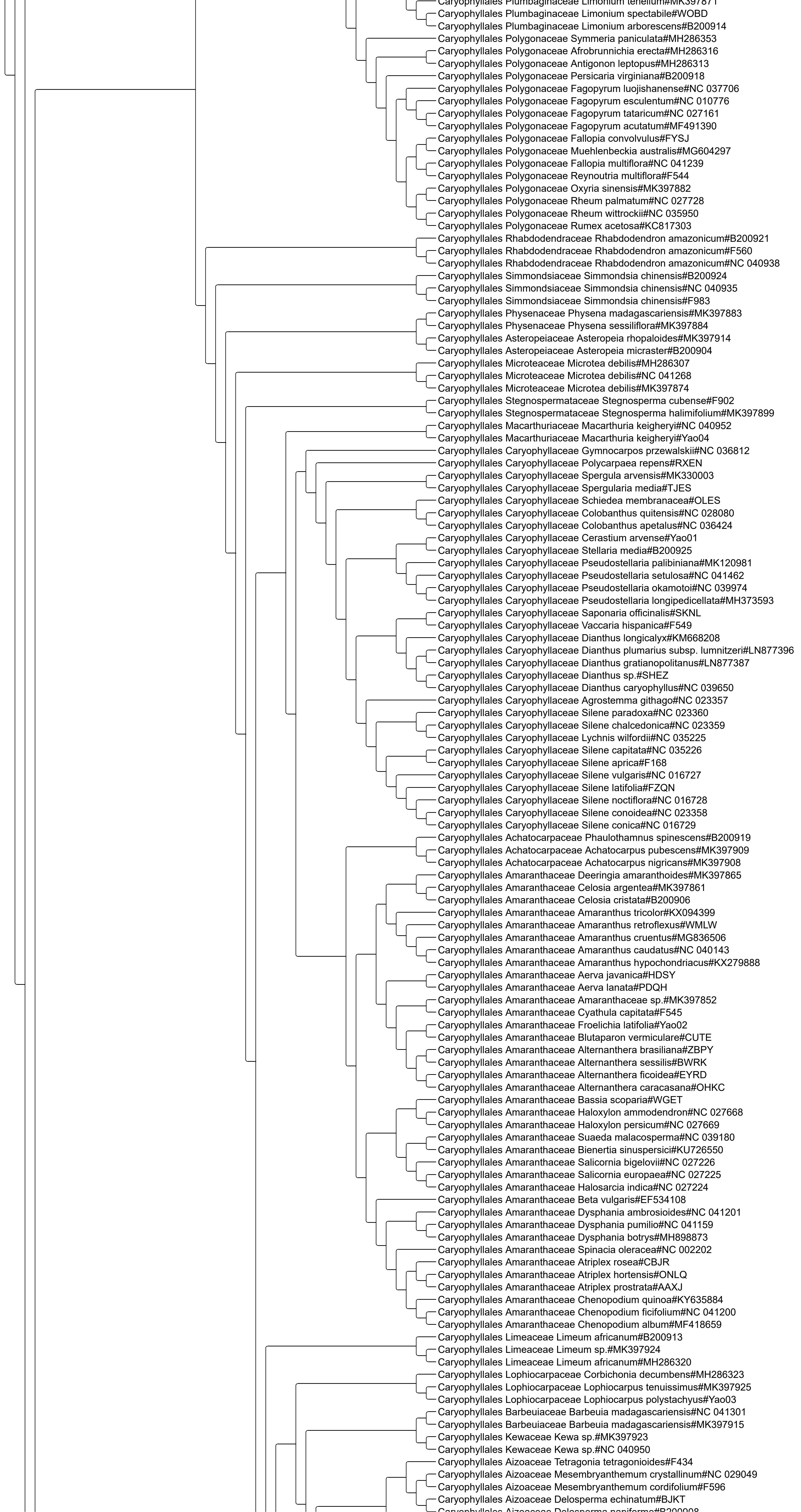
Caryophyllales Plumbaginaceae Limonium tenellum#MK397871
Caryophyllales Plumbaginaceae Limonium spectabile#WOBD
Caryophyllales Plumbaginaceae Limonium arborescens#B200914
Caryophyllales Polygonaceae Symmeria paniculata#MH286353
Caryophyllales Polygonaceae Afrobrunnichia erecta#MH286316
Caryophyllales Polygonaceae Antigonon leptopus#MH286313
Caryophyllales Polygonaceae Persicaria virginiana#B200918
Caryophyllales Polygonaceae Fagopyrum luojishanense#NC 037706
Caryophyllales Polygonaceae Fagopyrum esculentum#NC 010776
Caryophyllales Polygonaceae Fagopyrum tataricum#NC 027161
Caryophyllales Polygonaceae Fagopyrum acutatum#MF491390
Caryophyllales Polygonaceae Fallopia convolvulus#FYSJ
Caryophyllales Polygonaceae Muehlenbeckia australis#MG604297
Caryophyllales Polygonaceae Fallopia multiflora#NC 041239
Caryophyllales Polygonaceae Reynoutria multiflora#F544
Caryophyllales Polygonaceae Oxyria sinensis#MK397882
Caryophyllales Polygonaceae Rheum palmatum#NC 027728
Caryophyllales Polygonaceae Rheum wittrockii#NC 035950
Caryophyllales Polygonaceae Rumex acetosa#KC817303
Caryophyllales Rhabdodendraceae Rhabdodendron amazonicum#B200921
Caryophyllales Rhabdodendraceae Rhabdodendron amazonicum#F560
Caryophyllales Rhabdodendraceae Rhabdodendron amazonicum#NC 040938
Caryophyllales Simmondsiaceae Simmondsia chinensis#B200924
Caryophyllales Simmondsiaceae Simmondsia chinensis#NC 040935
Caryophyllales Simmondsiaceae Simmondsia chinensis#F983
Caryophyllales Physenaceae Physena madagascariensis#MK397883
Caryophyllales Physenaceae Physena sessiliflora#MK397884
Caryophyllales Asteropeiaceae Asteropeia rhopaloides#MK397914
Caryophyllales Asteropeiaceae Asteropeia micraster#B200904
Caryophyllales Microteaceae Microtea debilis#MH286307
Caryophyllales Microteaceae Microtea debilis#NC 041268
Caryophyllales Microteaceae Microtea debilis#MK397874
Caryophyllales Stegnospermataceae Stegnosperma cubense#F902
Caryophyllales Stegnospermataceae Stegnosperma halimifolium#MK397899
Caryophyllales Macarthuriaceae Macarthuria keigheryi#NC 040952
Caryophyllales Macarthuriaceae Macarthuria keigheryi#Yao04
Caryophyllales Caryophyllaceae Gymnocarpos przewalskii#NC 036812
Caryophyllales Caryophyllaceae Polycarpaea repens#RXEN
Caryophyllales Caryophyllaceae Spergula arvensis#MK330003
Caryophyllales Caryophyllaceae Spergularia media#TJES
Caryophyllales Caryophyllaceae Schiedea membranacea#OLES
Caryophyllales Caryophyllaceae Colobanthus quitensis#NC 028080
Caryophyllales Caryophyllaceae Colobanthus apetalus#NC 036424
Caryophyllales Caryophyllaceae Cerastium arvense#Yao01
Caryophyllales Caryophyllaceae Stellaria media#B200925
Caryophyllales Caryophyllaceae Pseudostellaria palibiniana#MK120981
Caryophyllales Caryophyllaceae Pseudostellaria setulosa#NC 041462
Caryophyllales Caryophyllaceae Pseudostellaria okamotoi#NC 039974
Caryophyllales Caryophyllaceae Pseudostellaria longipedicellata#MH373593
Caryophyllales Caryophyllaceae Saponaria officinalis#SKNL
Caryophyllales Caryophyllaceae Vaccaria hispanica#F549
Caryophyllales Caryophyllaceae Dianthus longicalyx#KM668208
Caryophyllales Caryophyllaceae Dianthus plumarius subsp. lumnitzeri#LN877396
Caryophyllales Caryophyllaceae Dianthus gratianopolitanus#LN877387
Caryophyllales Caryophyllaceae Dianthus sp.#SHEZ
Caryophyllales Caryophyllaceae Dianthus caryophyllus#NC 039650
Caryophyllales Caryophyllaceae Agrostemma githago#NC 023357
Caryophyllales Caryophyllaceae Silene paradoxa#NC 023360
Caryophyllales Caryophyllaceae Silene chalcedonica#NC 023359
Caryophyllales Caryophyllaceae Lychnis wilfordii#NC 035225
Caryophyllales Caryophyllaceae Silene capitata#NC 035226
Caryophyllales Caryophyllaceae Silene aprica#F168
Caryophyllales Caryophyllaceae Silene vulgaris#NC 016727
Caryophyllales Caryophyllaceae Silene latifolia#FZQN
Caryophyllales Caryophyllaceae Silene noctiflora#NC 016728
Caryophyllales Caryophyllaceae Silene conoidea#NC 023358
Caryophyllales Caryophyllaceae Silene conica#NC 016729
Caryophyllales Achatocarpaceae Phaulothamnus spinescens#B200919
Caryophyllales Achatocarpaceae Achatocarpus pubescens#MK397909
Caryophyllales Achatocarpaceae Achatocarpus nigricans#MK397908
Caryophyllales Amaranthaceae Deeringia amaranthoides#MK397865
Caryophyllales Amaranthaceae Celosia argentea#MK397861
Caryophyllales Amaranthaceae Celosia cristata#B200906
Caryophyllales Amaranthaceae Amaranthus tricolor#KX094399
Caryophyllales Amaranthaceae Amaranthus retroflexus#WMLW
Caryophyllales Amaranthaceae Amaranthus cruentus#MG836506
Caryophyllales Amaranthaceae Amaranthus caudatus#NC 040143
Caryophyllales Amaranthaceae Amaranthus hypochondriacus#KX279888
Caryophyllales Amaranthaceae Aerva javanica#HDSY
Caryophyllales Amaranthaceae Aerva lanata#PDQH
Caryophyllales Amaranthaceae Amaranthaceae sp.#MK397852
Caryophyllales Amaranthaceae Cyathula capitata#F545
Caryophyllales Amaranthaceae Froelichia latifolia#Yao02
Caryophyllales Amaranthaceae Blutaparon vermiculare#CUTE
Caryophyllales Amaranthaceae Alternanthera brasiliana#ZBPY
Caryophyllales Amaranthaceae Alternanthera sessilis#BWRK
Caryophyllales Amaranthaceae Alternanthera ficoidea#EYRD
Caryophyllales Amaranthaceae Alternanthera caracasana#OHKC
Caryophyllales Amaranthaceae Bassia scoparia#WGET
Caryophyllales Amaranthaceae Haloxylon ammodendron#NC 027668
Caryophyllales Amaranthaceae Haloxylon persicum#NC 027669
Caryophyllales Amaranthaceae Suaeda malacosperma#NC 039180
Caryophyllales Amaranthaceae Bienertia sinuspersici#KU726550
Caryophyllales Amaranthaceae Salicornia bigelovii#NC 027226
Caryophyllales Amaranthaceae Salicornia europaea#NC 027225
Caryophyllales Amaranthaceae Halosarcia indica#NC 027224
Caryophyllales Amaranthaceae Beta vulgaris#EF534108
Caryophyllales Amaranthaceae Dysphania ambrosioides#NC 041201
Caryophyllales Amaranthaceae Dysphania pumilio#NC 041159
Caryophyllales Amaranthaceae Dysphania botrys#MH898873
Caryophyllales Amaranthaceae Spinacia oleracea#NC 002202
Caryophyllales Amaranthaceae Atriplex rosea#CBJR
Caryophyllales Amaranthaceae Atriplex hortensis#ONLQ
Caryophyllales Amaranthaceae Atriplex prostrata#AAXJ
Caryophyllales Amaranthaceae Chenopodium quinoa#KY635884
Caryophyllales Amaranthaceae Chenopodium ficifolium#NC 041200
Caryophyllales Amaranthaceae Chenopodium album#MF418659
Caryophyllales Limeaceae Limeum africanum#B200913
Caryophyllales Limeaceae Limeum sp.#MK397924
Caryophyllales Limeaceae Limeum africanum#MH286320
Caryophyllales Lophiocarpaceae Corbichonia decumbens#MH286323
Caryophyllales Lophiocarpaceae Lophiocarpus tenuissimus#MK397925
Caryophyllales Lophiocarpaceae Lophiocarpus polystachyus#Yao03
Caryophyllales Barbeuiaceae Barbeuia madagascariensis#NC 041301
Caryophyllales Barbeuiaceae Barbeuia madagascariensis#MK397915
Caryophyllales Kewaceae Kewa sp.#MK397923
Caryophyllales Kewaceae Kewa sp.#NC 040950
Caryophyllales Aizoaceae Tetragonia tetragonioides#F434
Caryophyllales Aizoaceae Mesembryanthemum crystallinum#NC 029049
Caryophyllales Aizoaceae Mesembryanthemum cordifolium#F596
Caryophyllales Aizoaceae Delosperma echinatum#BJKT

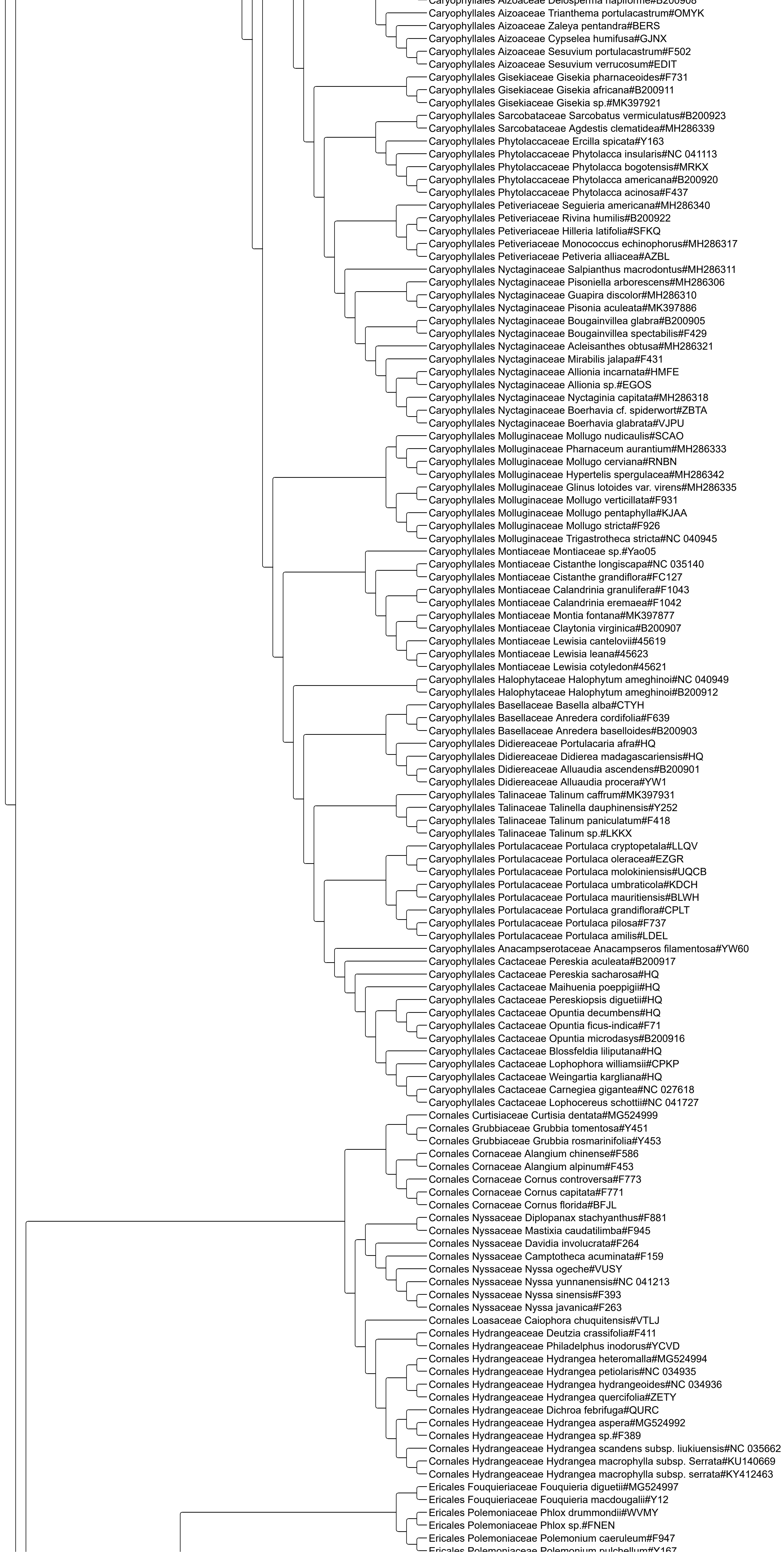

Caryophyllales Aizoaceae Trianthema portulacastrum#OMYK
Caryophyllales Aizoaceae Zaleya pentandra#BERS
Caryophyllales Aizoaceae Cypselea humifusa#GJNX
Caryophyllales Aizoaceae Sesuvium portulacastrum#F502
Caryophyllales Aizoaceae Sesuvium verrucosum#EDIT
Caryophyllales Gisekiaceae Gisekia pharnaceoides#F731
Caryophyllales Gisekiaceae Gisekia africana#B200911
Caryophyllales Gisekiaceae Gisekia sp.#MK397921
Caryophyllales Sarcobataceae Sarcobatus vermiculatus#B200923
Caryophyllales Sarcobataceae Agdestis clematidea#MH286339
Caryophyllales Phytolaccaceae Ercilla spicata#Y163
Caryophyllales Phytolaccaceae Phytolacca insularis#NC 041113
Caryophyllales Phytolaccaceae Phytolacca bogotensis#MRKX
Caryophyllales Phytolaccaceae Phytolacca americana#B200920
Caryophyllales Phytolaccaceae Phytolacca acinosa#F437
Caryophyllales Petiveriaceae Seguieria americana#MH286340
Caryophyllales Petiveriaceae Rivina humilis#B200922
Caryophyllales Petiveriaceae Hilleria latifolia#SFKQ
Caryophyllales Petiveriaceae Monococcus echinophorus#MH286317
Caryophyllales Petiveriaceae Petiveria alliacea#AZBL
Caryophyllales Nyctaginaceae Salpianthus macrodontus#MH286311
Caryophyllales Nyctaginaceae Pisoniella arborescens#MH286306
Caryophyllales Nyctaginaceae Guapira discolor#MH286310
Caryophyllales Nyctaginaceae Pisonia aculeata#MK397886
Caryophyllales Nyctaginaceae Bougainvillea glabra#B200905
Caryophyllales Nyctaginaceae Bougainvillea spectabilis#F429
Caryophyllales Nyctaginaceae Acleisanthes obtusa#MH286321
Caryophyllales Nyctaginaceae Mirabilis jalapa#F431
Caryophyllales Nyctaginaceae Allionia incarnata#HMFE
Caryophyllales Nyctaginaceae Allionia sp.#EGOS
Caryophyllales Nyctaginaceae Nyctaginia capitata#MH286318
Caryophyllales Nyctaginaceae Boerhavia cf. spiderwort#ZBTA
Caryophyllales Nyctaginaceae Boerhavia glabrata#VJPU
Caryophyllales Molluginaceae Mollugo nudicaulis#SCAO
Caryophyllales Molluginaceae Pharnaceum aurantium#MH286333
Caryophyllales Molluginaceae Mollugo cerviana#RNBN
Caryophyllales Molluginaceae Hypertelis spergulacea#MH286342
Caryophyllales Molluginaceae Glinus lotoides var. virens#MH286335
Caryophyllales Molluginaceae Mollugo verticillata#F931
Caryophyllales Molluginaceae Mollugo pentaphylla#KJAA
Caryophyllales Molluginaceae Mollugo stricta#F926
Caryophyllales Molluginaceae Trigastrotheca stricta#NC 040945
Caryophyllales Montiaceae Montiaceae sp.#Yao05
Caryophyllales Montiaceae Cistanthe longiscapa#NC 035140
Caryophyllales Montiaceae Cistanthe grandiflora#FC127
Caryophyllales Montiaceae Calandrinia granulifera#F1043
Caryophyllales Montiaceae Calandrinia eremaea#F1042
Caryophyllales Montiaceae Montia fontana#MK397877
Caryophyllales Montiaceae Claytonia virginica#B200907
Caryophyllales Montiaceae Lewisia cantelovii#45619
Caryophyllales Montiaceae Lewisia leana#45623
Caryophyllales Montiaceae Lewisia cotyledon#45621
Caryophyllales Halophytaceae Halophytum ameghinoi#NC 040949
Caryophyllales Halophytaceae Halophytum ameghinoi#B200912
Caryophyllales Basellaceae Basella alba#CTYH
Caryophyllales Basellaceae Anredera cordifolia#F639
Caryophyllales Basellaceae Anredera baselloides#B200903
Caryophyllales Didiereaceae Portulacaria afra#HQ
Caryophyllales Didiereaceae Didierea madagascariensis#HQ
Caryophyllales Didiereaceae Alluaudia ascendens#B200901
Caryophyllales Didiereaceae Alluaudia procera#YW1
Caryophyllales Talinaceae Talinum caffrum#MK397931
Caryophyllales Talinaceae Talinella dauphinensis#Y252
Caryophyllales Talinaceae Talinum paniculatum#F418
Caryophyllales Talinaceae Talinum sp.#LKKX
Caryophyllales Portulacaceae Portulaca cryptopetala#LLQV
Caryophyllales Portulacaceae Portulaca oleracea#EZGR
Caryophyllales Portulacaceae Portulaca molokiniensis#UQCB
Caryophyllales Portulacaceae Portulaca umbraticola#KDCH
Caryophyllales Portulacaceae Portulaca mauritiensis#BLWH
Caryophyllales Portulacaceae Portulaca grandiflora#CPLT
Caryophyllales Portulacaceae Portulaca pilosa#F737
Caryophyllales Portulacaceae Portulaca amilis#LDEL
Caryophyllales Anacampserotaceae Anacampseros filamentosa#YW60
Caryophyllales Cactaceae Pereskia aculeata#B200917
Caryophyllales Cactaceae Pereskia sacharosa#HQ
Caryophyllales Cactaceae Maihuenia poeppigii#HQ
Caryophyllales Cactaceae Pereskiopsis diguetii#HQ
Caryophyllales Cactaceae Opuntia decumbens#HQ
Caryophyllales Cactaceae Opuntia ficus-indica#F71
Caryophyllales Cactaceae Opuntia microdasys#B200916
Caryophyllales Cactaceae Blossfeldia liliputana#HQ
Caryophyllales Cactaceae Lophophora williamsii#CPKP
Caryophyllales Cactaceae Weingartia kargliana#HQ
Caryophyllales Cactaceae Carnegiea gigantea#NC 027618
Caryophyllales Cactaceae Lophocereus schottii#NC 041727
Cornales Curtisiaceae Curtisia dentata#MG524999
Cornales Grubbiaceae Grubbia tomentosa#Y451
Cornales Grubbiaceae Grubbia rosmarinifolia#Y453
Cornales Cornaceae Alangium chinense#F586
Cornales Cornaceae Alangium alpinum#F453
Cornales Cornaceae Cornus controversa#F773
Cornales Cornaceae Cornus capitata#F771
Cornales Cornaceae Cornus florida#BFJL
Cornales Nyssaceae Diplopanax stachyanthus#F881
Cornales Nyssaceae Mastixia caudatilimba#F945
Cornales Nyssaceae Davidia involucrata#F264
Cornales Nyssaceae Camptotheca acuminata#F159
Cornales Nyssaceae Nyssa ogeche#VUSY
Cornales Nyssaceae Nyssa yunnanensis#NC 041213
Cornales Nyssaceae Nyssa sinensis#F393
Cornales Nyssaceae Nyssa javanica#F263
Cornales Loasaceae Caiophora chuquitensis#VTLJ
Cornales Hydrangeaceae Deutzia crassifolia#F411
Cornales Hydrangeaceae Philadelphus inodorus#YCVD
Cornales Hydrangeaceae Hydrangea heteromalla#MG524994
Cornales Hydrangeaceae Hydrangea petiolaris#NC 034935
Cornales Hydrangeaceae Hydrangea hydrangeoides#NC 034936
Cornales Hydrangeaceae Hydrangea quercifolia#ZETY
Cornales Hydrangeaceae Dichroa febrifuga#QURC
Cornales Hydrangeaceae Hydrangea aspera#MG524992
Cornales Hydrangeaceae Hydrangea sp.#F389
Cornales Hydrangeaceae Hydrangea scandens subsp. liukiuensis#NC 035662
Cornales Hydrangeaceae Hydrangea macrophylla subsp. Serrata#KU140669
Cornales Hydrangeaceae Hydrangea macrophylla subsp. serrata#KY412463
Ericales Fouquieriaceae Fouquieria diguetii#MG524997
Ericales Fouquieriaceae Fouquieria macdougalii#Y12
Ericales Polemoniaceae Phlox drummondii#WVMY
Ericales Polemoniaceae Phlox sp.#FNEN
Ericales Polemoniaceae Polemonium caeruleum#F947

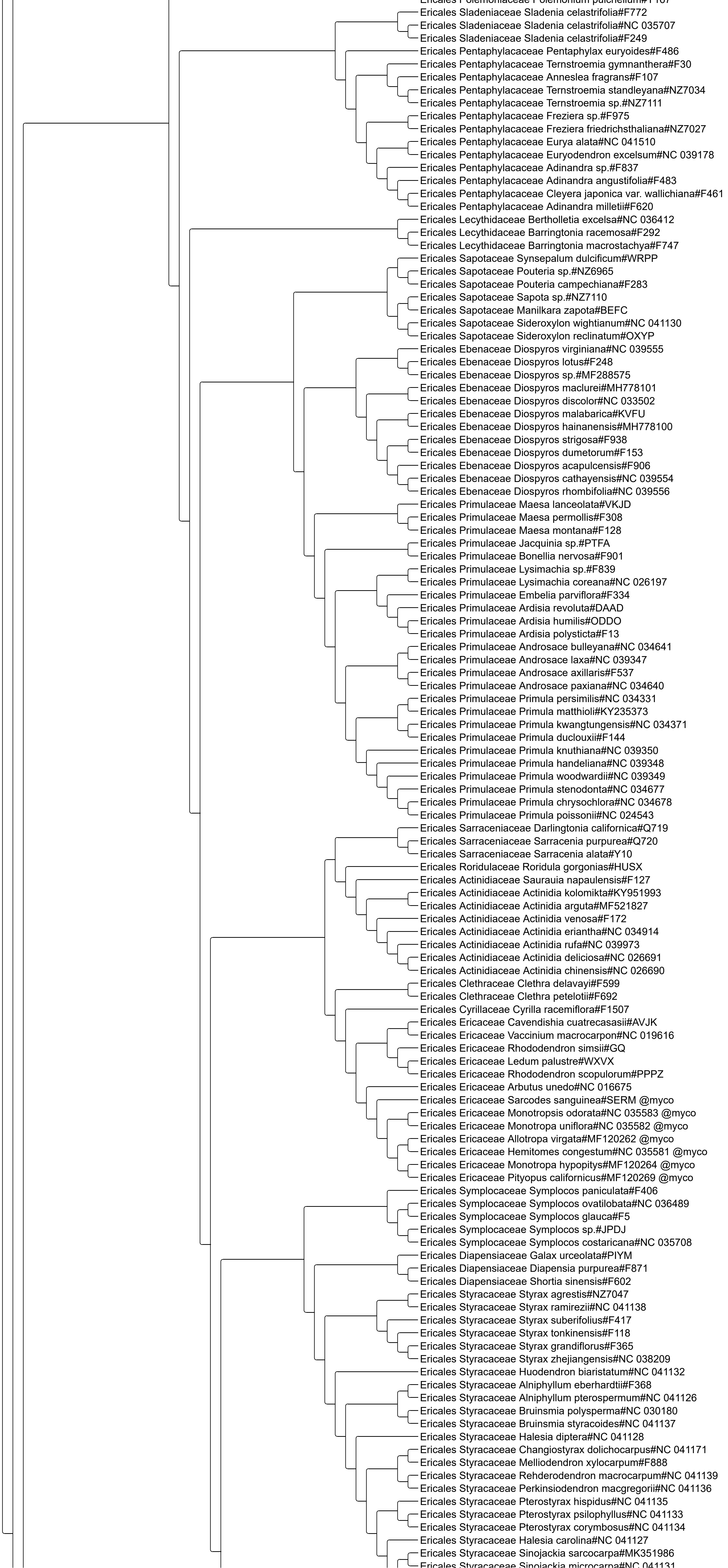
Ericales Sladeniaceae Sladenia celastrifolia#F772
Ericales Sladeniaceae Sladenia celastrifolia#NC 035707
Ericales Sladeniaceae Sladenia celastrifolia#F249
Ericales Pentaphylacaceae Pentaphylax euryoides#F486
Ericales Pentaphylacaceae Ternstroemia gymnanthera#F30
Ericales Pentaphylacaceae Anneslea fragrans#F107
Ericales Pentaphylacaceae Ternstroemia standleyana#NZ7034
Ericales Pentaphylacaceae Ternstroemia sp.#NZ7111
Ericales Pentaphylacaceae Freziera sp.#F975
Ericales Pentaphylacaceae Freziera friedrichsthaliana#NZ7027
Ericales Pentaphylacaceae Eurya alata#NC 041510
Ericales Pentaphylacaceae Euryodendron excelsum#NC 039178
Ericales Pentaphylacaceae Adinandra sp.#F837
Ericales Pentaphylacaceae Adinandra angustifolia#F483
Ericales Pentaphylacaceae Cleyera japonica var. wallichiana#F461
Ericales Pentaphylacaceae Adinandra millettii#F620
Ericales Lecythidaceae Bertholletia excelsa#NC 036412
Ericales Lecythidaceae Barringtonia racemosa#F292
Ericales Lecythidaceae Barringtonia macrostachya#F747
Ericales Sapotaceae Synsepalum dulcificum#WRPP
Ericales Sapotaceae Pouteria sp.#NZ6965
Ericales Sapotaceae Pouteria campechiana#F283
Ericales Sapotaceae Sapota sp.#NZ7110
Ericales Sapotaceae Manilkara zapota#BEFC
Ericales Sapotaceae Sideroxylon wightianum#NC 041130
Ericales Sapotaceae Sideroxylon reclinatum#OXYP
Ericales Ebenaceae Diospyros virginiana#NC 039555
Ericales Ebenaceae Diospyros lotus#F248
Ericales Ebenaceae Diospyros sp.#MF288575
Ericales Ebenaceae Diospyros maclurei#MH778101
Ericales Ebenaceae Diospyros discolor#NC 033502
Ericales Ebenaceae Diospyros malabarica#KVFU
Ericales Ebenaceae Diospyros hainanensis#MH778100
Ericales Ebenaceae Diospyros strigosa#F938
Ericales Ebenaceae Diospyros dumetorum#F153
Ericales Ebenaceae Diospyros acapulcensis#F906
Ericales Ebenaceae Diospyros cathayensis#NC 039554
Ericales Ebenaceae Diospyros rhombifolia#NC 039556
Ericales Primulaceae Maesa lanceolata#VKJD
Ericales Primulaceae Maesa permollis#F308
Ericales Primulaceae Maesa montana#F128
Ericales Primulaceae Jacquinia sp.#PTFA
Ericales Primulaceae Bonellia nervosa#F901
Ericales Primulaceae Lysimachia sp.#F839
Ericales Primulaceae Lysimachia coreana#NC 026197
Ericales Primulaceae Embelia parviflora#F334
Ericales Primulaceae Ardisia revoluta#DAAD
Ericales Primulaceae Ardisia humilis#ODDO
Ericales Primulaceae Ardisia polysticta#F13
Ericales Primulaceae Androsace bulleyana#NC 034641
Ericales Primulaceae Androsace laxa#NC 039347
Ericales Primulaceae Androsace axillaris#F537
Ericales Primulaceae Androsace paxiana#NC 034640
Ericales Primulaceae Primula persimilis#NC 034331
Ericales Primulaceae Primula matthioli#KY235373
Ericales Primulaceae Primula kwangtungensis#NC 034371
Ericales Primulaceae Primula duclouxii#F144
Ericales Primulaceae Primula knuthiana#NC 039350
Ericales Primulaceae Primula handeliana#NC 039348
Ericales Primulaceae Primula woodwardii#NC 039349
Ericales Primulaceae Primula stenodonta#NC 034677
Ericales Primulaceae Primula chrysochlora#NC 034678
Ericales Primulaceae Primula poissonii#NC 024543
Ericales Sarraceniaceae Darlingtonia californica#Q719
Ericales Sarraceniaceae Sarracenia purpurea#Q720
Ericales Sarraceniaceae Sarracenia alata#Y10
Ericales Roridulaceae Roridula gorgonias#HUSX
Ericales Actinidiaceae Saurauia napaulensis#F127
Ericales Actinidiaceae Actinidia kolomikta#KY951993
Ericales Actinidiaceae Actinidia arguta#MF521827
Ericales Actinidiaceae Actinidia venosa#F172
Ericales Actinidiaceae Actinidia eriantha#NC 034914
Ericales Actinidiaceae Actinidia rufa#NC 039973
Ericales Actinidiaceae Actinidia deliciosa#NC 026691
Ericales Actinidiaceae Actinidia chinensis#NC 026690
Ericales Clethraceae Clethra delavayi#F599
Ericales Clethraceae Clethra petelotii#F692
Ericales Cyrillaceae Cyrilla racemiflora#F1507
Ericales Ericaceae Cavendishia cuatrecasasii#AVJK
Ericales Ericaceae Vaccinium macrocarpon#NC 019616
Ericales Ericaceae Rhododendron simsii#GQ
Ericales Ericaceae Ledum palustre#WXVX
Ericales Ericaceae Rhododendron scopulorum#PPPZ
Ericales Ericaceae Arbutus unedo#NC 016675
Ericales Ericaceae Sarcodes sanguinea#SERM @myco
Ericales Ericaceae Monotropsis odorata#NC 035583 @myco
Ericales Ericaceae Monotropa uniflora#NC 035582 @myco
Ericales Ericaceae Allotropa virgata#MF120262 @myco
Ericales Ericaceae Hemitomes congestum#NC 035581 @myco
Ericales Ericaceae Monotropa hypopitys#MF120264 @myco
Ericales Ericaceae Pityopus californicus#MF120269 @myco
Ericales Symplocaceae Symplocos paniculata#F406
Ericales Symplocaceae Symplocos ovatilobata#NC 036489
Ericales Symplocaceae Symplocos glauca#F5
Ericales Symplocaceae Symplocos sp.#JPDJ
Ericales Symplocaceae Symplocos costaricana#NC 035708
Ericales Diapensiaceae Galax urceolata#PIYM
Ericales Diapensiaceae Diapensia purpurea#F871
Ericales Diapensiaceae Shortia sinensis#F602
Ericales Styracaceae Styrax agrestis#NZ7047
Ericales Styracaceae Styrax ramirezii#NC 041138
Ericales Styracaceae Styrax suberifolius#F417
Ericales Styracaceae Styrax tonkinensis#F118
Ericales Styracaceae Styrax grandiflorus#F365
Ericales Styracaceae Styrax zhejiangensis#NC 038209
Ericales Styracaceae Huodendron biaristatum#NC 041132
Ericales Styracaceae Alniphyllum eberhardtii#F368
Ericales Styracaceae Alniphyllum pterospermum#NC 041126
Ericales Styracaceae Bruinsmia polysperma#NC 030180
Ericales Styracaceae Bruinsmia styracoides#NC 041137
Ericales Styracaceae Halesia diptera#NC 041128
Ericales Styracaceae Changiostyrax dolichocarpus#NC 041171
Ericales Styracaceae Melliodendron xylocarpum#F888
Ericales Styracaceae Rehderodendron macrocarpum#NC 041139
Ericales Styracaceae Perkinsiodendron macgregorii#NC 041136
Ericales Styracaceae Pterostyrax hispidus#NC 041135
Ericales Styracaceae Pterostyrax psilophyllus#NC 041133
Ericales Styracaceae Pterostyrax corymbosus#NC 041134
Ericales Styracaceae Halesia carolina#NC 041127
Ericales Styracaceae Sinojackia sarcocarpa#MK351986

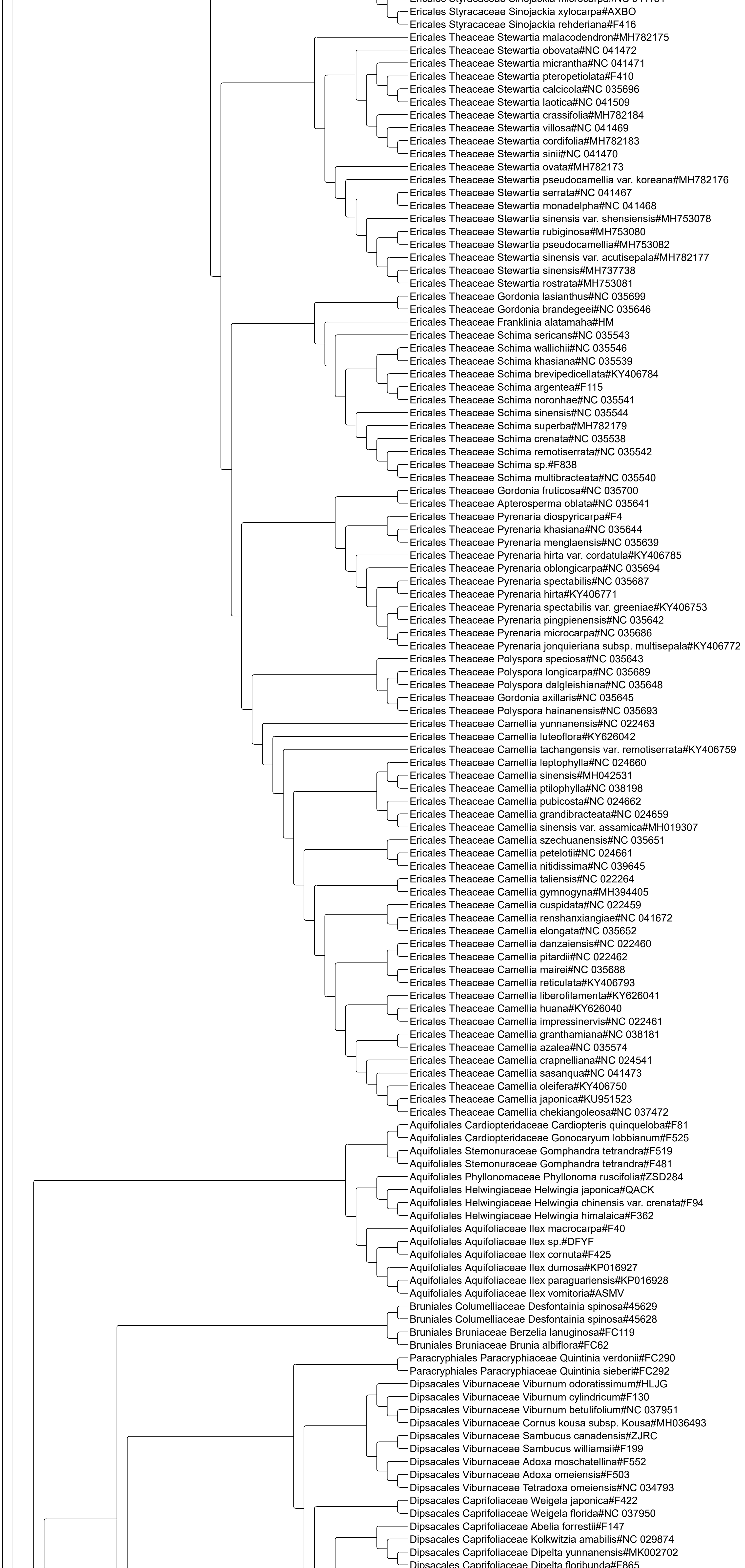

Ericales Styracaceae Sinojackia xylocarpa#AXBO
Ericales Styracaceae Sinojackia rehderiana#F416
Ericales Theaceae Stewartia malacodendron#MH782175
Ericales Theaceae Stewartia obovata#NC 041472
Ericales Theaceae Stewartia micrantha#NC 041471
Ericales Theaceae Stewartia pteropetiolata#F410
Ericales Theaceae Stewartia calcicola#NC 035696
Ericales Theaceae Stewartia laotica#NC 041509
Ericales Theaceae Stewartia crassifolia#MH782184
Ericales Theaceae Stewartia villosa#NC 041469
Ericales Theaceae Stewartia cordifolia#MH782183
Ericales Theaceae Stewartia sinii#NC 041470
Ericales Theaceae Stewartia ovata#MH782173
Ericales Theaceae Stewartia pseudocamellia var. koreana#MH782176
Ericales Theaceae Stewartia serrata#NC 041467
Ericales Theaceae Stewartia monadelpha#NC 041468
Ericales Theaceae Stewartia sinensis var. shensiensis#MH753078
Ericales Theaceae Stewartia rubiginosa#MH753080
Ericales Theaceae Stewartia pseudocamellia#MH753082
Ericales Theaceae Stewartia sinensis var. acutisepala#MH782177
Ericales Theaceae Stewartia sinensis#MH737738
Ericales Theaceae Stewartia rostrata#MH753081
Ericales Theaceae Gordonia lasianthus#NC 035699
Ericales Theaceae Gordonia brandegeei#NC 035646
Ericales Theaceae Franklinia alatamaha#HM
Ericales Theaceae Schima sericans#NC 035543
Ericales Theaceae Schima wallichii#NC 035546
Ericales Theaceae Schima khasiana#NC 035539
Ericales Theaceae Schima brevipedicellata#KY406784
Ericales Theaceae Schima argentea#F115
Ericales Theaceae Schima noronhae#NC 035541
Ericales Theaceae Schima sinensis#NC 035544
Ericales Theaceae Schima superba#MH782179
Ericales Theaceae Schima crenata#NC 035538
Ericales Theaceae Schima remotiserrata#NC 035542
Ericales Theaceae Schima sp.#F838
Ericales Theaceae Schima multibracteata#NC 035540
Ericales Theaceae Gordonia fruticosa#NC 035700
Ericales Theaceae Apterosperma oblata#NC 035641
Ericales Theaceae Pyrenaria diospyricarpa#F4
Ericales Theaceae Pyrenaria khasiana#NC 035644
Ericales Theaceae Pyrenaria menglaensis#NC 035639
Ericales Theaceae Pyrenaria hirta var. cordatula#KY406785
Ericales Theaceae Pyrenaria oblongicarpa#NC 035694
Ericales Theaceae Pyrenaria spectabilis#NC 035687
Ericales Theaceae Pyrenaria hirta#KY406771
Ericales Theaceae Pyrenaria spectabilis var. greeniae#KY406753
Ericales Theaceae Pyrenaria pingpienensis#NC 035642
Ericales Theaceae Pyrenaria microcarpa#NC 035686
Ericales Theaceae Pyrenaria jonquieriana subsp. multisepala#KY406772
Ericales Theaceae Polyspora speciosa#NC 035643
Ericales Theaceae Polyspora longicarpa#NC 035689
Ericales Theaceae Polyspora dalgleishiana#NC 035648
Ericales Theaceae Gordonia axillaris#NC 035645
Ericales Theaceae Polyspora hainanensis#NC 035693
Ericales Theaceae Camellia yunnanensis#NC 022463
Ericales Theaceae Camellia luteoflora#KY626042
Ericales Theaceae Camellia tachangensis var. remotiserrata#KY406759
Ericales Theaceae Camellia leptophylla#NC 024660
Ericales Theaceae Camellia sinensis#MH042531
Ericales Theaceae Camellia ptilophylla#NC 038198
Ericales Theaceae Camellia pubicosta#NC 024662
Ericales Theaceae Camellia grandibracteata#NC 024659
Ericales Theaceae Camellia sinensis var. assamica#MH019307
Ericales Theaceae Camellia szechuanensis#NC 035651
Ericales Theaceae Camellia petelotii#NC 024661
Ericales Theaceae Camellia nitidissima#NC 039645
Ericales Theaceae Camellia taliensis#NC 022264
Ericales Theaceae Camellia gymnogyna#MH394405
Ericales Theaceae Camellia cuspidata#NC 022459
Ericales Theaceae Camellia renshanxiangiae#NC 041672
Ericales Theaceae Camellia elongata#NC 035652
Ericales Theaceae Camellia danzaiensis#NC 022460
Ericales Theaceae Camellia pitardii#NC 022462
Ericales Theaceae Camellia mairei#NC 035688
Ericales Theaceae Camellia reticulata#KY406793
Ericales Theaceae Camellia liberofilamenta#KY626041
Ericales Theaceae Camellia huana#KY626040
Ericales Theaceae Camellia impressinervis#NC 022461
Ericales Theaceae Camellia granthamiana#NC 038181
Ericales Theaceae Camellia azalea#NC 035574
Ericales Theaceae Camellia crapnelliana#NC 024541
Ericales Theaceae Camellia sasanqua#NC 041473
Ericales Theaceae Camellia oleifera#KY406750
Ericales Theaceae Camellia japonica#KU951523
Ericales Theaceae Camellia chekiangoleosa#NC 037472
Aquifoliales Cardiopteridaceae Cardiopteris quinqueloba#F81
Aquifoliales Cardiopteridaceae Gonocaryum lobbianum#F525
Aquifoliales Stemonuraceae Gomphandra tetrandra#F519
Aquifoliales Stemonuraceae Gomphandra tetrandra#F481
Aquifoliales Phyllonomaceae Phyllonoma ruscifolia#ZSD284
Aquifoliales Helwingiaceae Helwingia japonica#QACK
Aquifoliales Helwingiaceae Helwingia chinensis var. crenata#F94
Aquifoliales Helwingiaceae Helwingia himalaica#F362
Aquifoliales Aquifoliaceae Ilex macrocarpa#F40
Aquifoliales Aquifoliaceae Ilex sp.#DFYF
Aquifoliales Aquifoliaceae Ilex cornuta#F425
Aquifoliales Aquifoliaceae Ilex dumosa#KP016927
Aquifoliales Aquifoliaceae Ilex paraguariensis#KP016928
Aquifoliales Aquifoliaceae Ilex vomitoria#ASMV
Bruniales Columelliaceae Desfontainia spinosa#45629
Bruniales Columelliaceae Desfontainia spinosa#45628
Bruniales Bruniaceae Berzelia lanuginosa#FC119
Bruniales Bruniaceae Brunia albiflora#FC62
Paracryphiales Paracryphiaceae Quintinia verdonii#FC290
Paracryphiales Paracryphiaceae Quintinia sieberi#FC292
Dipsacales Viburnaceae Viburnum odoratissimum#HLJG
Dipsacales Viburnaceae Viburnum cylindricum#F130
Dipsacales Viburnaceae Viburnum betulifolium#NC 037951
Dipsacales Viburnaceae Cornus kousa subsp. Kousa#MH036493
Dipsacales Viburnaceae Sambucus canadensis#ZJRC
Dipsacales Viburnaceae Sambucus williamsii#F199
Dipsacales Viburnaceae Adoxa moschatellina#F552
Dipsacales Viburnaceae Adoxa omeiensis#F503
Dipsacales Viburnaceae Tetradoxa omeiensis#NC 034793
Dipsacales Caprifoliaceae Weigela japonica#F422
Dipsacales Caprifoliaceae Weigela florida#NC 037950
Dipsacales Caprifoliaceae Abelia forrestii#F147
Dipsacales Caprifoliaceae Kolkwitzia amabilis#NC 029874
Dipsacales Caprifoliaceae Dipelta yunnanensis#MK002702
Dipsacales Caprifoliaceae Dipelta floribunda#F865

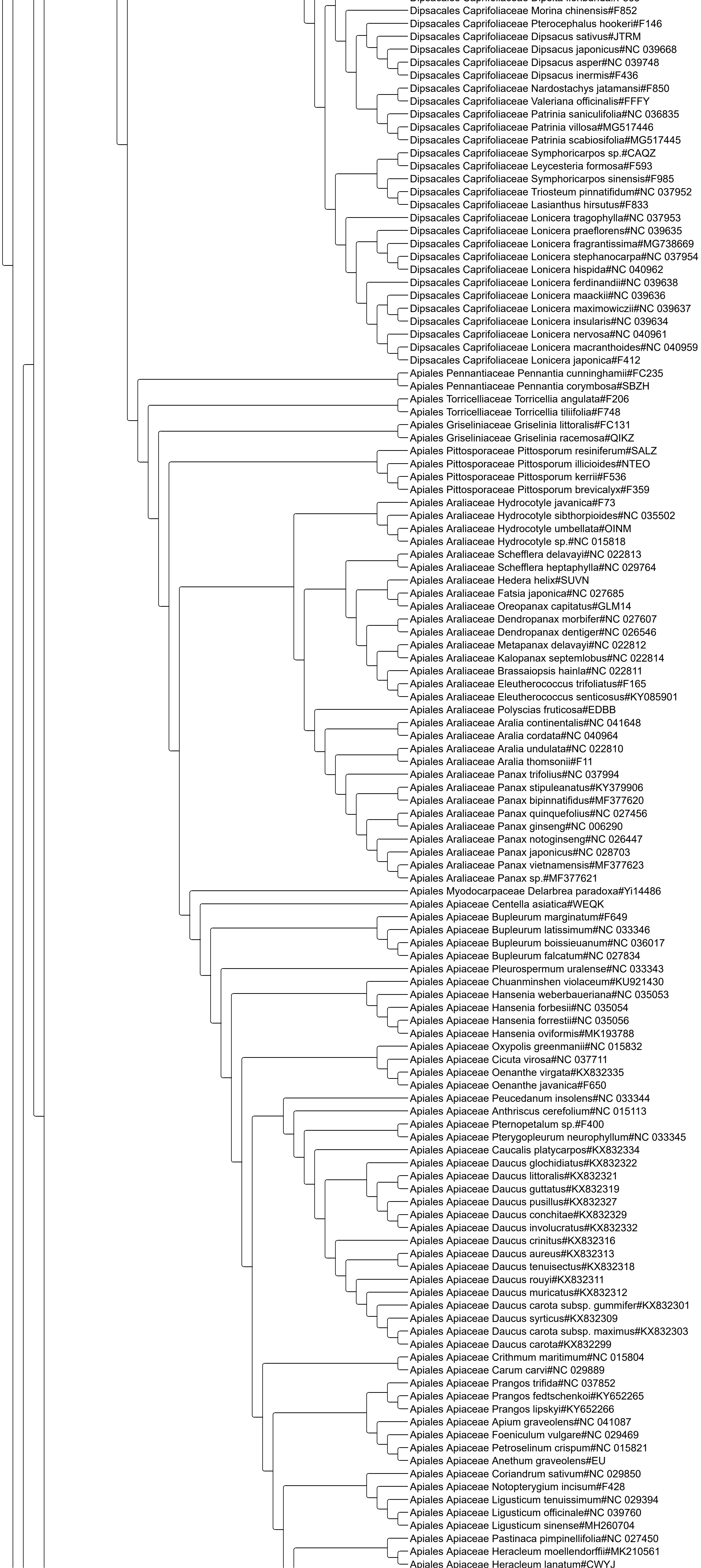
Dipsacales Caprifoliaceae Morina chinensis#F852
Dipsacales Caprifoliaceae Pterocephalus hookeri#F146
Dipsacales Caprifoliaceae Dipsacus sativus#JTRM
Dipsacales Caprifoliaceae Dipsacus japonicus#NC 039668
Dipsacales Caprifoliaceae Dipsacus asper#NC 039748
Dipsacales Caprifoliaceae Dipsacus inermis#F436
Dipsacales Caprifoliaceae Nardostachys jatamansi#F850
Dipsacales Caprifoliaceae Valeriana officinalis#FFFY
Dipsacales Caprifoliaceae Patrinia saniculifolia#NC 036835
Dipsacales Caprifoliaceae Patrinia villosa#MG517446
Dipsacales Caprifoliaceae Patrinia scabiosifolia#MG517445
Dipsacales Caprifoliaceae Symphoricarpos sp.#CAQZ
Dipsacales Caprifoliaceae Leycesteria formosa#F593
Dipsacales Caprifoliaceae Symphoricarpos sinensis#F985
Dipsacales Caprifoliaceae Triosteum pinnatifidum#NC 037952
Dipsacales Caprifoliaceae Lasianthus hirsutus#F833
Dipsacales Caprifoliaceae Lonicera tragophylla#NC 037953
Dipsacales Caprifoliaceae Lonicera praeflorens#NC 039635
Dipsacales Caprifoliaceae Lonicera fragrantissima#MG738669
Dipsacales Caprifoliaceae Lonicera stephanocarpa#NC 037954
Dipsacales Caprifoliaceae Lonicera hispida#NC 040962
Dipsacales Caprifoliaceae Lonicera ferdinandii#NC 039638
Dipsacales Caprifoliaceae Lonicera maackii#NC 039636
Dipsacales Caprifoliaceae Lonicera maximowiczii#NC 039637
Dipsacales Caprifoliaceae Lonicera insularis#NC 039634
Dipsacales Caprifoliaceae Lonicera nervosa#NC 040961
Dipsacales Caprifoliaceae Lonicera macranthoides#NC 040959
Dipsacales Caprifoliaceae Lonicera japonica#F412
Apiales Pennantiaceae Pennantia cunninghamii#FC235
Apiales Pennantiaceae Pennantia corymbosa#SBZH
Apiales Torricelliaceae Torricellia angulata#F206
Apiales Torricelliaceae Torricellia tiliifolia#F748
Apiales Griseliniaceae Griselinia littoralis#FC131
Apiales Griseliniaceae Griselinia racemosa#QIKZ
Apiales Pittosporaceae Pittosporum resiniferum#SALZ
Apiales Pittosporaceae Pittosporum illicioides#NTEO
Apiales Pittosporaceae Pittosporum kerrii#F536
Apiales Pittosporaceae Pittosporum brevicalyx#F359
Apiales Araliaceae Hydrocotyle javanica#F73
Apiales Araliaceae Hydrocotyle sibthorpioides#NC 035502
Apiales Araliaceae Hydrocotyle umbellata#OINM
Apiales Araliaceae Hydrocotyle sp.#NC 015818
Apiales Araliaceae Schefflera delavayi#NC 022813
Apiales Araliaceae Schefflera heptaphylla#NC 029764
Apiales Araliaceae Hedera helix#SUVN
Apiales Araliaceae Fatsia japonica#NC 027685
Apiales Araliaceae Oreopanax capitatus#GLM14
Apiales Araliaceae Dendropanax morbifer#NC 027607
Apiales Araliaceae Dendropanax dentiger#NC 026546
Apiales Araliaceae Metapanax delavayi#NC 022812
Apiales Araliaceae Kalopanax septemlobus#NC 022814
Apiales Araliaceae Brassaiopsis hainla#NC 022811
Apiales Araliaceae Eleutherococcus trifoliatus#F165
Apiales Araliaceae Eleutherococcus senticosus#KY085901
Apiales Araliaceae Polyscias fruticosa#EDBB
Apiales Araliaceae Aralia continentalis#NC 041648
Apiales Araliaceae Aralia cordata#NC 040964
Apiales Araliaceae Aralia undulata#NC 022810
Apiales Araliaceae Aralia thomsonii#F11
Apiales Araliaceae Panax trifolius#NC 037994
Apiales Araliaceae Panax stipuleanatus#KY379906
Apiales Araliaceae Panax bipinnatifidus#MF377620
Apiales Araliaceae Panax quinquefolius#NC 027456
Apiales Araliaceae Panax ginseng#NC 006290
Apiales Araliaceae Panax notoginseng#NC 026447
Apiales Araliaceae Panax japonicus#NC 028703
Apiales Araliaceae Panax vietnamensis#MF377623
Apiales Araliaceae Panax sp.#MF377621
Apiales Myodocarpaceae Delarbrea paradoxa#Yi14486
Apiales Apiaceae Centella asiatica#WEQK
Apiales Apiaceae Bupleurum marginatum#F649
Apiales Apiaceae Bupleurum latissimum#NC 033346
Apiales Apiaceae Bupleurum boissieuanum#NC 036017
Apiales Apiaceae Bupleurum falcatum#NC 027834
Apiales Apiaceae Pleurospermum uralense#NC 033343
Apiales Apiaceae Chuanminshen violaceum#KU921430
Apiales Apiaceae Hansenia weberbaueriana#NC 035053
Apiales Apiaceae Hansenia forbesii#NC 035054
Apiales Apiaceae Hansenia forrestii#NC 035056
Apiales Apiaceae Hansenia oviformis#MK193788
Apiales Apiaceae Oxypolis greenmanii#NC 015832
Apiales Apiaceae Cicuta virosa#NC 037711
Apiales Apiaceae Oenanthe virgata#KX832335
Apiales Apiaceae Oenanthe javanica#F650
Apiales Apiaceae Peucedanum insolens#NC 033344
Apiales Apiaceae Anthriscus cerefolium#NC 015113
Apiales Apiaceae Pternopetalum sp.#F400
Apiales Apiaceae Pterygopleurum neurophyllum#NC 033345
Apiales Apiaceae Caucalis platycarpos#KX832334
Apiales Apiaceae Daucus glochidiatus#KX832322
Apiales Apiaceae Daucus littoralis#KX832321
Apiales Apiaceae Daucus guttatus#KX832319
Apiales Apiaceae Daucus pusillus#KX832327
Apiales Apiaceae Daucus conchitae#KX832329
Apiales Apiaceae Daucus involucratus#KX832332
Apiales Apiaceae Daucus crinitus#KX832316
Apiales Apiaceae Daucus aureus#KX832313
Apiales Apiaceae Daucus tenuisectus#KX832318
Apiales Apiaceae Daucus rouyi#KX832311
Apiales Apiaceae Daucus muricatus#KX832312
Apiales Apiaceae Daucus carota subsp. gummifer#KX832301
Apiales Apiaceae Daucus syrticus#KX832309
Apiales Apiaceae Daucus carota subsp. maximus#KX832303
Apiales Apiaceae Daucus carota#KX832299
Apiales Apiaceae Crithmum maritimum#NC 015804
Apiales Apiaceae Carum carvi#NC 029889
Apiales Apiaceae Prangos trifida#NC 037852
Apiales Apiaceae Prangos fedtschenkoi#KY652265
Apiales Apiaceae Prangos lipskyi#KY652266
Apiales Apiaceae Apium graveolens#NC 041087
Apiales Apiaceae Foeniculum vulgare#NC 029469
Apiales Apiaceae Petroselinum crispum#NC 015821
Apiales Apiaceae Anethum graveolens#EU
Apiales Apiaceae Coriandrum sativum#NC 029850
Apiales Apiaceae Notopterygium incisum#F428
Apiales Apiaceae Ligusticum tenuissimum#NC 029394
Apiales Apiaceae Ligusticum officinale#NC 039760
Apiales Apiaceae Ligusticum sinense#MH260704
Apiales Apiaceae Pastinaca pimpinellifolia#NC 027450
Apiales Apiaceae Heracleum moellendorffii#MK210561
Apiales Apiaceae Heracleum lanatum#CWYJ

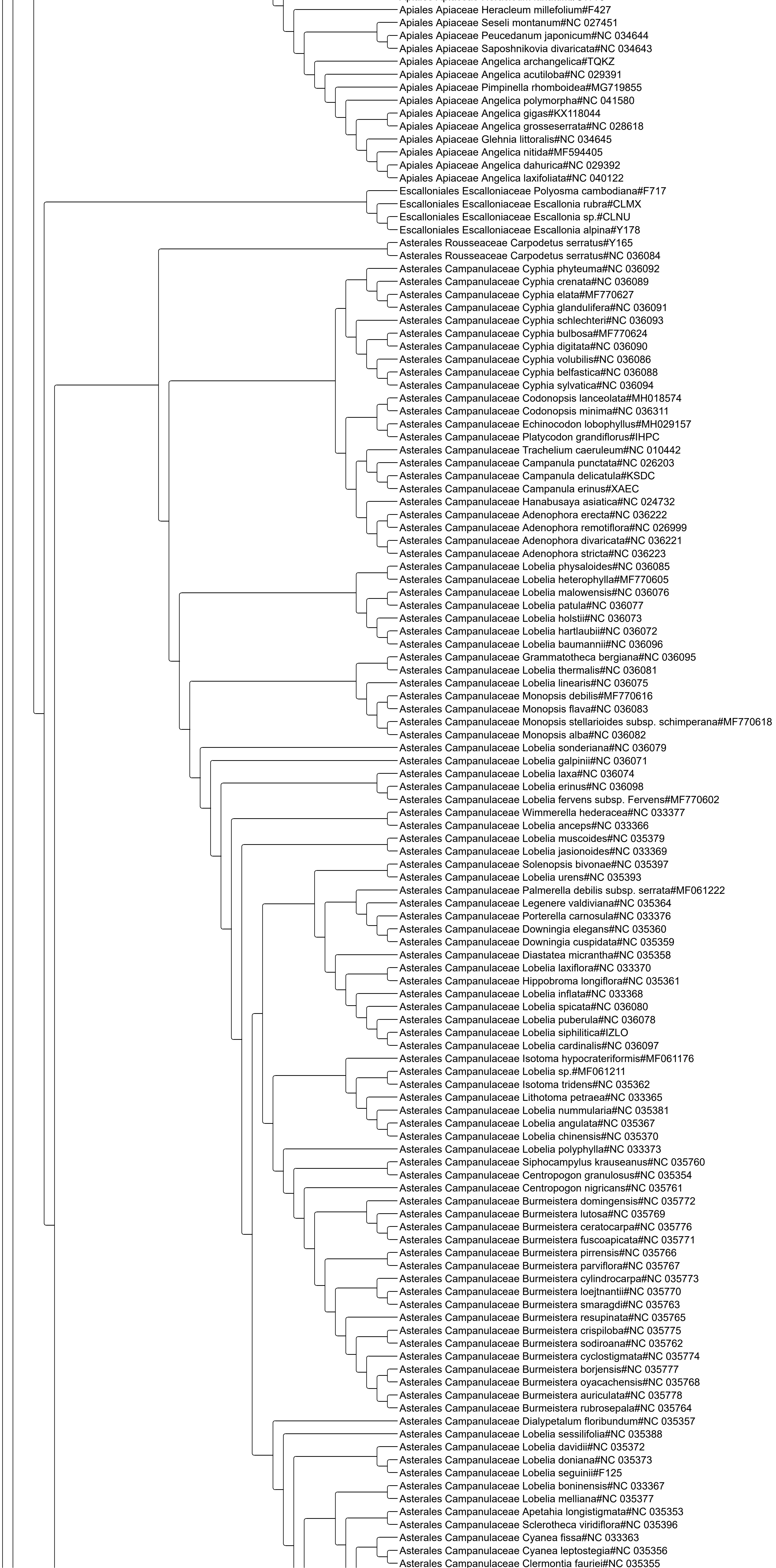

Apiales Apiaceae Heracleum millefolium#F427
Apiales Apiaceae Seseli montanum#NC 027451
Apiales Apiaceae Peucedanum japonicum#NC 034644
Apiales Apiaceae Saposhnikovia divaricata#NC 034643
Apiales Apiaceae Angelica archangelica#TQKZ
Apiales Apiaceae Angelica acutiloba#NC 029391
Apiales Apiaceae Pimpinella rhomboidea#MG719855
Apiales Apiaceae Angelica polymorpha#NC 041580
Apiales Apiaceae Angelica gigas#KX118044
Apiales Apiaceae Angelica grosseserrata#NC 028618
Apiales Apiaceae Glehnia littoralis#NC 034645
Apiales Apiaceae Angelica nitida#MF594405
Apiales Apiaceae Angelica dahurica#NC 029392
Apiales Apiaceae Angelica laxifoliata#NC 040122
Escalloniales Escalloniaceae Polyosma cambodiana#F717
Escalloniales Escalloniaceae Escallonia rubra#CLMX
Escalloniales Escalloniaceae Escallonia sp.#CLNU
Escalloniales Escalloniaceae Escallonia alpina#Y178
Asterales Rousseaceae Carpodetus serratus#Y165
Asterales Rousseaceae Carpodetus serratus#NC 036084
Asterales Campanulaceae Cyphia phyteuma#NC 036092
Asterales Campanulaceae Cyphia crenata#NC 036089
Asterales Campanulaceae Cyphia elata#MF770627
Asterales Campanulaceae Cyphia glandulifera#NC 036091
Asterales Campanulaceae Cyphia schlechteri#NC 036093
Asterales Campanulaceae Cyphia bulbosa#MF770624
Asterales Campanulaceae Cyphia digitata#NC 036090
Asterales Campanulaceae Cyphia volubilis#NC 036086
Asterales Campanulaceae Cyphia belfastica#NC 036088
Asterales Campanulaceae Cyphia sylvatica#NC 036094
Asterales Campanulaceae Codonopsis lanceolata#MH018574
Asterales Campanulaceae Codonopsis minima#NC 036311
Asterales Campanulaceae Echinocodon lobophyllus#MH029157
Asterales Campanulaceae Platycodon grandiflorus#IHPC
Asterales Campanulaceae Trachelium caeruleum#NC 010442
Asterales Campanulaceae Campanula punctata#NC 026203
Asterales Campanulaceae Campanula delicatula#KSDC
Asterales Campanulaceae Campanula erinus#XAEC
Asterales Campanulaceae Hanabusaya asiatica#NC 024732
Asterales Campanulaceae Adenophora erecta#NC 036222
Asterales Campanulaceae Adenophora remotiflora#NC 026999
Asterales Campanulaceae Adenophora divaricata#NC 036221
Asterales Campanulaceae Adenophora stricta#NC 036223
Asterales Campanulaceae Lobelia physaloides#NC 036085
Asterales Campanulaceae Lobelia heterophylla#MF770605
Asterales Campanulaceae Lobelia malowensis#NC 036076
Asterales Campanulaceae Lobelia patula#NC 036077
Asterales Campanulaceae Lobelia holstii#NC 036073
Asterales Campanulaceae Lobelia hartlaubii#NC 036072
Asterales Campanulaceae Lobelia baumannii#NC 036096
Asterales Campanulaceae Grammatotheca bergiana#NC 036095
Asterales Campanulaceae Lobelia thermalis#NC 036081
Asterales Campanulaceae Lobelia linearis#NC 036075
Asterales Campanulaceae Monopsis debilis#MF770616
Asterales Campanulaceae Monopsis flava#NC 036083
Asterales Campanulaceae Monopsis stellarioides subsp. schimperana#MF770618
Asterales Campanulaceae Monopsis alba#NC 036082
Asterales Campanulaceae Lobelia sonderiana#NC 036079
Asterales Campanulaceae Lobelia galpinii#NC 036071
Asterales Campanulaceae Lobelia laxa#NC 036074
Asterales Campanulaceae Lobelia erinus#NC 036098
Asterales Campanulaceae Lobelia fervens subsp. Fervens#MF770602
Asterales Campanulaceae Wimmerella hederacea#NC 033377
Asterales Campanulaceae Lobelia anceps#NC 033366
Asterales Campanulaceae Lobelia muscoides#NC 035379
Asterales Campanulaceae Lobelia jasionoides#NC 033369
Asterales Campanulaceae Solenopsis bivonae#NC 035397
Asterales Campanulaceae Lobelia urens#NC 035393
Asterales Campanulaceae Palmerella debilis subsp. serrata#MF061222
Asterales Campanulaceae Legenere valdiviana#NC 035364
Asterales Campanulaceae Porterella carnosula#NC 033376
Asterales Campanulaceae Downingia elegans#NC 035360
Asterales Campanulaceae Downingia cuspidata#NC 035359
Asterales Campanulaceae Diastatea micrantha#NC 035358
Asterales Campanulaceae Lobelia laxiflora#NC 033370
Asterales Campanulaceae Hippobroma longiflora#NC 035361
Asterales Campanulaceae Lobelia inflata#NC 033368
Asterales Campanulaceae Lobelia spicata#NC 036080
Asterales Campanulaceae Lobelia puberula#NC 036078
Asterales Campanulaceae Lobelia siphilitica#IZLO
Asterales Campanulaceae Lobelia cardinalis#NC 036097
Asterales Campanulaceae Isotoma hypocrateriformis#MF061176
Asterales Campanulaceae Lobelia sp.#MF061211
Asterales Campanulaceae Isotoma tridens#NC 035362
Asterales Campanulaceae Lithotoma petraea#NC 033365
Asterales Campanulaceae Lobelia nummularia#NC 035381
Asterales Campanulaceae Lobelia angulata#NC 035367
Asterales Campanulaceae Lobelia chinensis#NC 035370
Asterales Campanulaceae Lobelia polyphylla#NC 033373
Asterales Campanulaceae Siphocampylus krauseanus#NC 035760
Asterales Campanulaceae Centropogon granulosus#NC 035354
Asterales Campanulaceae Centropogon nigricans#NC 035761
Asterales Campanulaceae Burmeistera domingensis#NC 035772
Asterales Campanulaceae Burmeistera lutosa#NC 035769
Asterales Campanulaceae Burmeistera ceratocarpa#NC 035776
Asterales Campanulaceae Burmeistera fuscoapicata#NC 035771
Asterales Campanulaceae Burmeistera pirrensis#NC 035766
Asterales Campanulaceae Burmeistera parviflora#NC 035767
Asterales Campanulaceae Burmeistera cylindrocarpa#NC 035773
Asterales Campanulaceae Burmeistera loejtnantii#NC 035770
Asterales Campanulaceae Burmeistera smaragdi#NC 035763
Asterales Campanulaceae Burmeistera resupinata#NC 035765
Asterales Campanulaceae Burmeistera crispiloba#NC 035775
Asterales Campanulaceae Burmeistera sodiroana#NC 035762
Asterales Campanulaceae Burmeistera cyclostigmata#NC 035774
Asterales Campanulaceae Burmeistera borjensis#NC 035777
Asterales Campanulaceae Burmeistera oyacachensis#NC 035768
Asterales Campanulaceae Burmeistera auriculata#NC 035778
Asterales Campanulaceae Burmeistera rubrosepala#NC 035764
Asterales Campanulaceae Dialypetalum floribundum#NC 035357
Asterales Campanulaceae Lobelia sessilifolia#NC 035388
Asterales Campanulaceae Lobelia davidii#NC 035372
Asterales Campanulaceae Lobelia doniana#NC 035373
Asterales Campanulaceae Lobelia seguinii#F125
Asterales Campanulaceae Lobelia boninensis#NC 033367
Asterales Campanulaceae Lobelia melliana#NC 035377
Asterales Campanulaceae Apetahia longistigmata#NC 035353
Asterales Campanulaceae Sclerotheca viridiflora#NC 035396
Asterales Campanulaceae Cyanea fissa#NC 033363
Asterales Campanulaceae Cyanea leptostegia#NC 035356
Asterales Campanulaceae Clermontia fauriei#NC 035355

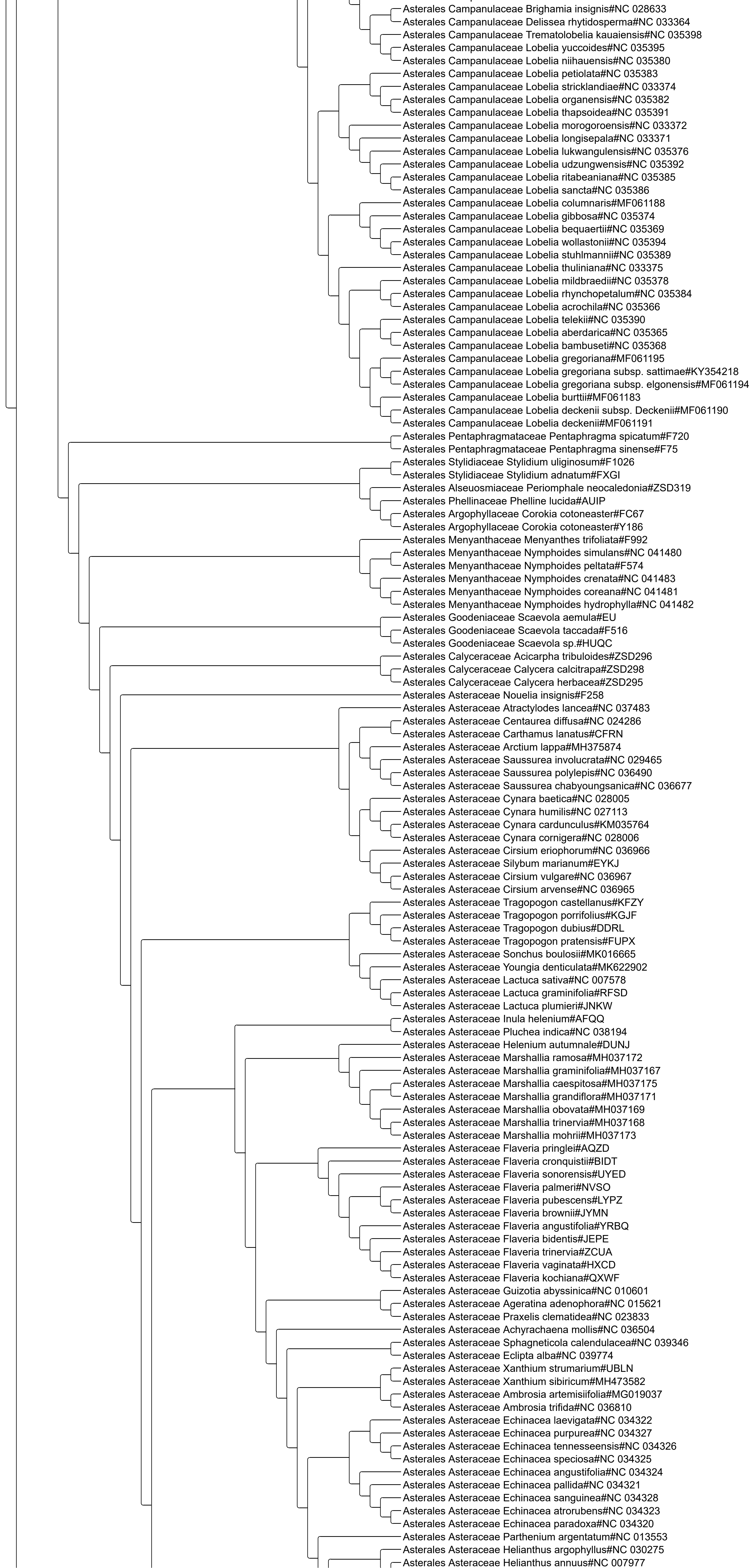

Asterales Campanulaceae Brighamia insignis#NC 028633
Asterales Campanulaceae Delissea rhytidosperma#NC 033364
Asterales Campanulaceae Trematolobelia kauaiensis#NC 035398
Asterales Campanulaceae Lobelia yuccoides#NC 035395
Asterales Campanulaceae Lobelia niihauensis#NC 035380
Asterales Campanulaceae Lobelia petiolata#NC 035383
Asterales Campanulaceae Lobelia stricklandiae#NC 033374
Asterales Campanulaceae Lobelia organensis#NC 035382
Asterales Campanulaceae Lobelia thapsoidea#NC 035391
Asterales Campanulaceae Lobelia morogoroensis#NC 033372
Asterales Campanulaceae Lobelia longisepala#NC 033371
Asterales Campanulaceae Lobelia lukwangulensis#NC 035376
Asterales Campanulaceae Lobelia udzungwensis#NC 035392
Asterales Campanulaceae Lobelia ritabeaniana#NC 035385
Asterales Campanulaceae Lobelia sancta#NC 035386
Asterales Campanulaceae Lobelia columnaris#MF061188
Asterales Campanulaceae Lobelia gibbosa#NC 035374
Asterales Campanulaceae Lobelia bequaertii#NC 035369
Asterales Campanulaceae Lobelia wollastonii#NC 035394
Asterales Campanulaceae Lobelia stuhlmannii#NC 035389
Asterales Campanulaceae Lobelia thuliniana#NC 033375
Asterales Campanulaceae Lobelia mildbraedii#NC 035378
Asterales Campanulaceae Lobelia rhynchopetalum#NC 035384
Asterales Campanulaceae Lobelia acrochila#NC 035366
Asterales Campanulaceae Lobelia telekii#NC 035390
Asterales Campanulaceae Lobelia aberdarica#NC 035365
Asterales Campanulaceae Lobelia bambuseti#NC 035368
Asterales Campanulaceae Lobelia gregoriana#MF061195
Asterales Campanulaceae Lobelia gregoriana subsp. sattimae#KY354218
Asterales Campanulaceae Lobelia gregoriana subsp. elgonensis#MF061194
Asterales Campanulaceae Lobelia burttii#MF061183
Asterales Campanulaceae Lobelia deckenii subsp. Deckenii#MF061190
Asterales Campanulaceae Lobelia deckenii#MF061191
Asterales Pentaphragmataceae Pentaphragma spicatum#F720
Asterales Pentaphragmataceae Pentaphragma sinense#F75
Asterales Stylidiaceae Stylidium uliginosum#F1026
Asterales Stylidiaceae Stylidium adnatum#FXGI
Asterales Alseuosmiaceae Periomphale neocaledonia#ZSD319
Asterales Phellinaceae Phelline lucida#AUIP
Asterales Argophyllaceae Corokia cotoneaster#FC67
Asterales Argophyllaceae Corokia cotoneaster#Y186
Asterales Menyanthaceae Menyanthes trifoliata#F992
Asterales Menyanthaceae Nymphoides simulans#NC 041480
Asterales Menyanthaceae Nymphoides peltata#F574
Asterales Menyanthaceae Nymphoides crenata#NC 041483
Asterales Menyanthaceae Nymphoides coreana#NC 041481
Asterales Menyanthaceae Nymphoides hydrophylla#NC 041482
Asterales Goodeniaceae Scaevola aemula#EU
Asterales Goodeniaceae Scaevola taccada#F516
Asterales Goodeniaceae Scaevola sp.#HUQC
Asterales Calyceraceae Acicarpha tribuloides#ZSD296
Asterales Calyceraceae Calycera calcitrapa#ZSD298
Asterales Calyceraceae Calycera herbacea#ZSD295
Asterales Asteraceae Nouelia insignis#F258
Asterales Asteraceae Atractylodes lancea#NC 037483
Asterales Asteraceae Centaurea diffusa#NC 024286
Asterales Asteraceae Carthamus lanatus#CFRN
Asterales Asteraceae Arctium lappa#MH375874
Asterales Asteraceae Saussurea involucrata#NC 029465
Asterales Asteraceae Saussurea polylepis#NC 036490
Asterales Asteraceae Saussurea chabyoungsanica#NC 036677
Asterales Asteraceae Cynara baetica#NC 028005
Asterales Asteraceae Cynara humilis#NC 027113
Asterales Asteraceae Cynara cardunculus#KM035764
Asterales Asteraceae Cynara cornigera#NC 028006
Asterales Asteraceae Cirsium eriophorum#NC 036966
Asterales Asteraceae Silybum marianum#EYKJ
Asterales Asteraceae Cirsium vulgare#NC 036967
Asterales Asteraceae Cirsium arvense#NC 036965
Asterales Asteraceae Tragopogon castellanus#KFZY
Asterales Asteraceae Tragopogon porrifolius#KGJF
Asterales Asteraceae Tragopogon dubius#DDRL
Asterales Asteraceae Tragopogon pratensis#FUPX
Asterales Asteraceae Sonchus boulosii#MK016665
Asterales Asteraceae Youngia denticulata#MK622902
Asterales Asteraceae Lactuca sativa#NC 007578
Asterales Asteraceae Lactuca graminifolia#RFSD
Asterales Asteraceae Lactuca plumieri#JNKW
Asterales Asteraceae Inula helenium#AFQQ
Asterales Asteraceae Pluchea indica#NC 038194
Asterales Asteraceae Helenium autumnale#DUNJ
Asterales Asteraceae Marshallia ramosa#MH037172
Asterales Asteraceae Marshallia graminifolia#MH037167
Asterales Asteraceae Marshallia caespitosa#MH037175
Asterales Asteraceae Marshallia grandiflora#MH037171
Asterales Asteraceae Marshallia obovata#MH037169
Asterales Asteraceae Marshallia trinervia#MH037168
Asterales Asteraceae Marshallia mohrii#MH037173
Asterales Asteraceae Flaveria pringlei#AQZD
Asterales Asteraceae Flaveria cronquistii#BIDT
Asterales Asteraceae Flaveria sonorensis#UYED
Asterales Asteraceae Flaveria palmeri#NVSO
Asterales Asteraceae Flaveria pubescens#LYPZ
Asterales Asteraceae Flaveria brownii#JYMN
Asterales Asteraceae Flaveria angustifolia#YRBQ
Asterales Asteraceae Flaveria bidentis#JEPE
Asterales Asteraceae Flaveria trinervia#ZCUA
Asterales Asteraceae Flaveria vaginata#HXCD
Asterales Asteraceae Flaveria kochiana#QXWF
Asterales Asteraceae Guizotia abyssinica#NC 010601
Asterales Asteraceae Ageratina adenophora#NC 015621
Asterales Asteraceae Praxelis clematidea#NC 023833
Asterales Asteraceae Achyrachaena mollis#NC 036504
Asterales Asteraceae Sphagneticola calendulacea#NC 039346
Asterales Asteraceae Eclipta alba#NC 039774
Asterales Asteraceae Xanthium strumarium#UBLN
Asterales Asteraceae Xanthium sibiricum#MH473582
Asterales Asteraceae Ambrosia artemisiifolia#MG019037
Asterales Asteraceae Ambrosia trifida#NC 036810
Asterales Asteraceae Echinacea laevigata#NC 034322
Asterales Asteraceae Echinacea purpurea#NC 034327
Asterales Asteraceae Echinacea tennesseensis#NC 034326
Asterales Asteraceae Echinacea speciosa#NC 034325
Asterales Asteraceae Echinacea angustifolia#NC 034324
Asterales Asteraceae Echinacea pallida#NC 034321
Asterales Asteraceae Echinacea sanguinea#NC 034328
Asterales Asteraceae Echinacea atrorubens#NC 034323
Asterales Asteraceae Echinacea paradoxa#NC 034320
Asterales Asteraceae Parthenium argentatum#NC 013553
Asterales Asteraceae Helianthus argophyllus#NC 030275
Asterales Asteraceae Helianthus annuus#NC 007977

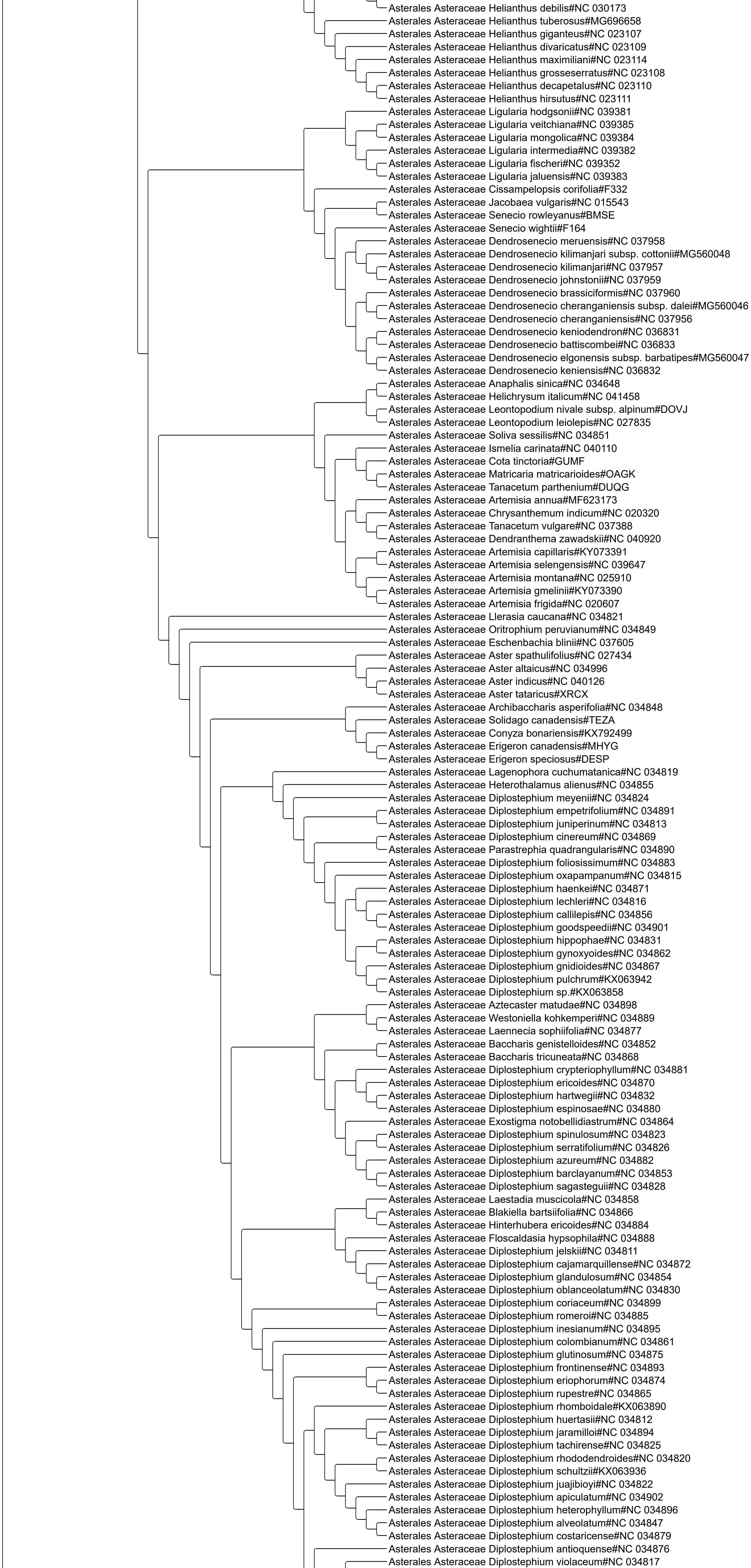
Asterales Asteraceae Helianthus debilis#NC 030173
Asterales Asteraceae Helianthus tuberosus#MG696658
Asterales Asteraceae Helianthus giganteus#NC 023107
Asterales Asteraceae Helianthus divaricatus#NC 023109
Asterales Asteraceae Helianthus maximiliani#NC 023114
Asterales Asteraceae Helianthus grosseserratus#NC 023108
Asterales Asteraceae Helianthus decapetalus#NC 023110
Asterales Asteraceae Helianthus hirsutus#NC 023111
Asterales Asteraceae Ligularia hodgsonii#NC 039381
Asterales Asteraceae Ligularia veitchiana#NC 039385
Asterales Asteraceae Ligularia mongolica#NC 039384
Asterales Asteraceae Ligularia intermedia#NC 039382
Asterales Asteraceae Ligularia fischeri#NC 039352
Asterales Asteraceae Ligularia jaluensis#NC 039383
Asterales Asteraceae Cissampelopsis corifolia#F332
Asterales Asteraceae Jacobaea vulgaris#NC 015543
Asterales Asteraceae Senecio rowleyanus#BMSE
Asterales Asteraceae Senecio wightii#F164
Asterales Asteraceae Dendrosenecio meruensis#NC 037958
Asterales Asteraceae Dendrosenecio kilimanjari subsp. cottonii#MG560048
Asterales Asteraceae Dendrosenecio kilimanjari#NC 037957
Asterales Asteraceae Dendrosenecio johnstonii#NC 037959
Asterales Asteraceae Dendrosenecio brassiciformis#NC 037960
Asterales Asteraceae Dendrosenecio cheranganiensis subsp. dalei#MG560046
Asterales Asteraceae Dendrosenecio cheranganiensis#NC 037956
Asterales Asteraceae Dendrosenecio keniodendron#NC 036831
Asterales Asteraceae Dendrosenecio battiscombei#NC 036833
Asterales Asteraceae Dendrosenecio elgonensis subsp. barbatipes#MG560047
Asterales Asteraceae Dendrosenecio keniensis#NC 036832
Asterales Asteraceae Anaphalis sinica#NC 034648
Asterales Asteraceae Helichrysum italicum#NC 041458
Asterales Asteraceae Leontopodium nivale subsp. alpinum#DOVJ
Asterales Asteraceae Leontopodium leiolepis#NC 027835
Asterales Asteraceae Soliva sessilis#NC 034851
Asterales Asteraceae Ismelia carinata#NC 040110
Asterales Asteraceae Cota tinctoria#GUMF
Asterales Asteraceae Matricaria matricarioides#OAGK
Asterales Asteraceae Tanacetum parthenium#DUQG
Asterales Asteraceae Artemisia annua#MF623173
Asterales Asteraceae Chrysanthemum indicum#NC 020320
Asterales Asteraceae Tanacetum vulgare#NC 037388
Asterales Asteraceae Dendranthema zawadskii#NC 040920
Asterales Asteraceae Artemisia capillaris#KY073391
Asterales Asteraceae Artemisia selengensis#NC 039647
Asterales Asteraceae Artemisia montana#NC 025910
Asterales Asteraceae Artemisia gmelinii#KY073390
Asterales Asteraceae Artemisia frigida#NC 020607
Asterales Asteraceae Llerasia caucana#NC 034821
Asterales Asteraceae Oritrophium peruvianum#NC 034849
Asterales Asteraceae Eschenbachia blinii#NC 037605
Asterales Asteraceae Aster spathulifolius#NC 027434
Asterales Asteraceae Aster altaicus#NC 034996
Asterales Asteraceae Aster indicus#NC 040126
Asterales Asteraceae Aster tataricus#XRCX
Asterales Asteraceae Archibaccharis asperifolia#NC 034848
Asterales Asteraceae Solidago canadensis#TEZA
Asterales Asteraceae Conyza bonariensis#KX792499
Asterales Asteraceae Erigeron canadensis#MHYG
Asterales Asteraceae Erigeron speciosus#DESP
Asterales Asteraceae Lagenophora cuchumatanica#NC 034819
Asterales Asteraceae Heterothalamus alienus#NC 034855
Asterales Asteraceae Diplostephium meyenii#NC 034824
Asterales Asteraceae Diplostephium empetrifolium#NC 034891
Asterales Asteraceae Diplostephium juniperinum#NC 034813
Asterales Asteraceae Diplostephium cinereum#NC 034869
Asterales Asteraceae Parastrephia quadrangularis#NC 034890
Asterales Asteraceae Diplostephium foliosissimum#NC 034883
Asterales Asteraceae Diplostephium oxapampanum#NC 034815
Asterales Asteraceae Diplostephium haenkei#NC 034871
Asterales Asteraceae Diplostephium lechleri#NC 034816
Asterales Asteraceae Diplostephium callilepis#NC 034856
Asterales Asteraceae Diplostephium goodspeedii#NC 034901
Asterales Asteraceae Diplostephium hippophae#NC 034831
Asterales Asteraceae Diplostephium gynoxyoides#NC 034862
Asterales Asteraceae Diplostephium gnidioides#NC 034867
Asterales Asteraceae Diplostephium pulchrum#KX063942
Asterales Asteraceae Diplostephium sp.#KX063858
Asterales Asteraceae Aztecaster matudae#NC 034898
Asterales Asteraceae Westoniella kohkemperi#NC 034889
Asterales Asteraceae Laennecia sophiifolia#NC 034877
Asterales Asteraceae Baccharis genistelloides#NC 034852
Asterales Asteraceae Baccharis tricuneata#NC 034868
Asterales Asteraceae Diplostephium crypteriophyllum#NC 034881
Asterales Asteraceae Diplostephium ericoides#NC 034870
Asterales Asteraceae Diplostephium hartwegii#NC 034832
Asterales Asteraceae Diplostephium espinosae#NC 034880
Asterales Asteraceae Exostigma notobellidiastrum#NC 034864
Asterales Asteraceae Diplostephium spinulosum#NC 034823
Asterales Asteraceae Diplostephium serratifolium#NC 034826
Asterales Asteraceae Diplostephium azureum#NC 034882
Asterales Asteraceae Diplostephium barclayanum#NC 034853
Asterales Asteraceae Diplostephium sagasteguii#NC 034828
Asterales Asteraceae Laestadia muscicola#NC 034858
Asterales Asteraceae Blakiella bartsiifolia#NC 034866
Asterales Asteraceae Hinterhubera ericoides#NC 034884
Asterales Asteraceae Floscaldasia hypsophila#NC 034888
Asterales Asteraceae Diplostephium jelskii#NC 034811
Asterales Asteraceae Diplostephium cajamarquillense#NC 034872
Asterales Asteraceae Diplostephium glandulosum#NC 034854
Asterales Asteraceae Diplostephium oblanceolatum#NC 034830
Asterales Asteraceae Diplostephium coriaceum#NC 034899
Asterales Asteraceae Diplostephium romeroi#NC 034885
Asterales Asteraceae Diplostephium inesianum#NC 034895
Asterales Asteraceae Diplostephium colombianum#NC 034861
Asterales Asteraceae Diplostephium glutinosum#NC 034875
Asterales Asteraceae Diplostephium frontinense#NC 034893
Asterales Asteraceae Diplostephium eriophorum#NC 034874
Asterales Asteraceae Diplostephium rupestre#NC 034865
Asterales Asteraceae Diplostephium rhomboidale#KX063890
Asterales Asteraceae Diplostephium huertasii#NC 034812
Asterales Asteraceae Diplostephium jaramilloi#NC 034894
Asterales Asteraceae Diplostephium tachirense#NC 034825
Asterales Asteraceae Diplostephium rhododendroides#NC 034820
Asterales Asteraceae Diplostephium schultzii#KX063936
Asterales Asteraceae Diplostephium juajibioyi#NC 034822
Asterales Asteraceae Diplostephium apiculatum#NC 034902
Asterales Asteraceae Diplostephium heterophyllum#NC 034896
Asterales Asteraceae Diplostephium alveolatum#NC 034847
Asterales Asteraceae Diplostephium costaricense#NC 034879
Asterales Asteraceae Diplostephium antioquense#NC 034876
Asterales Asteraceae Diplostephium violaceum#NC 034817

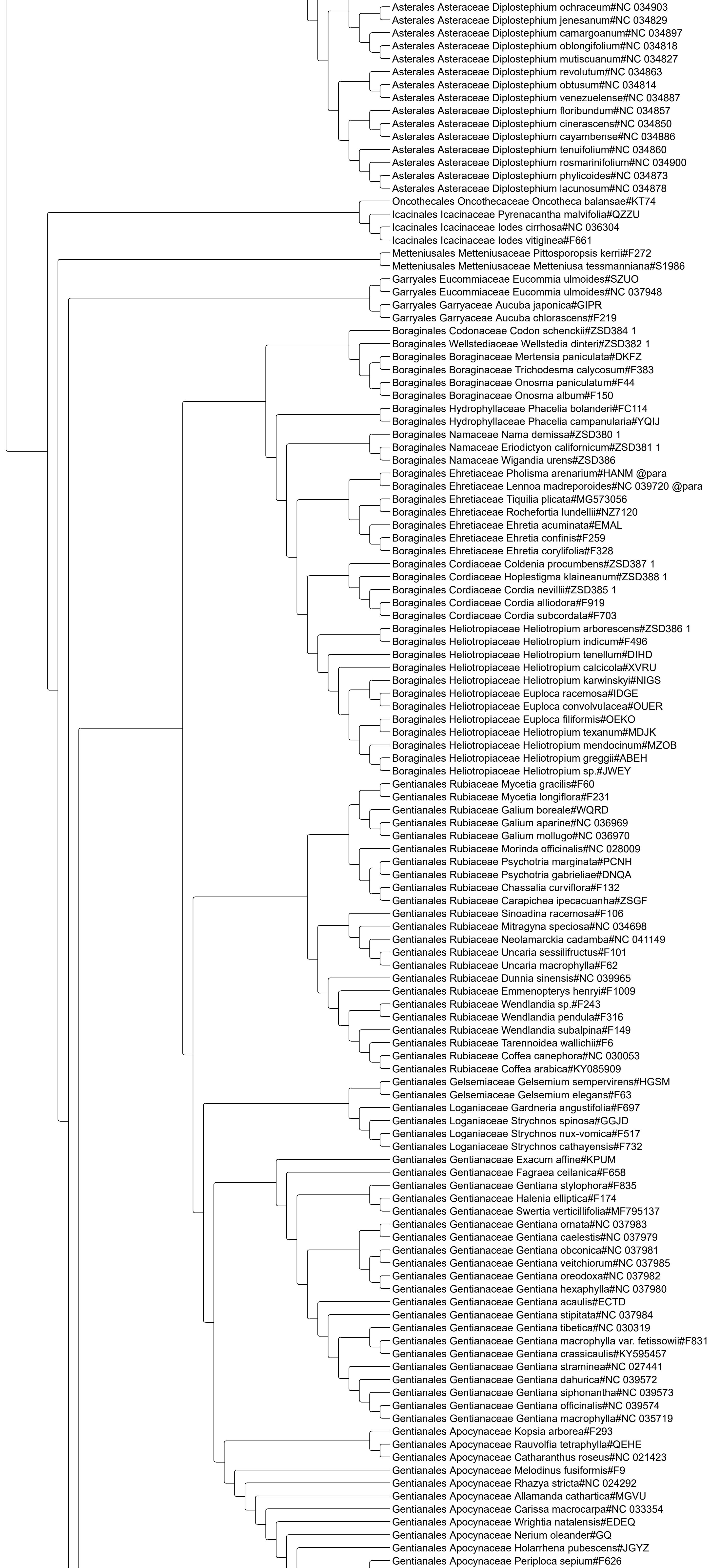

Asterales Asteraceae Diplostephium ochraceum#NC 034903
Asterales Asteraceae Diplostephium jenesanum#NC 034829
Asterales Asteraceae Diplostephium camargoanum#NC 034897
Asterales Asteraceae Diplostephium oblongifolium#NC 034818
Asterales Asteraceae Diplostephium mutiscuanum#NC 034827
Asterales Asteraceae Diplostephium revolutum#NC 034863
Asterales Asteraceae Diplostephium obtusum#NC 034814
Asterales Asteraceae Diplostephium venezuelense#NC 034887
Asterales Asteraceae Diplostephium floribundum#NC 034857
Asterales Asteraceae Diplostephium cinerascens#NC 034850
Asterales Asteraceae Diplostephium cayambense#NC 034886
Asterales Asteraceae Diplostephium tenuifolium#NC 034860
Asterales Asteraceae Diplostephium rosmarinifolium#NC 034900
Asterales Asteraceae Diplostephium phylicoides#NC 034873
Asterales Asteraceae Diplostephium lacunosum#NC 034878
Oncothecales Oncothecaceae Oncotheca balansae#KT74
Icacinales Icacinaceae Pyrenacantha malvifolia#QZZU
Icacinales Icacinaceae Iodes cirrhosa#NC 036304
Icacinales Icacinaceae Iodes vitiginea#F661
Metteniusales Metteniusaceae Pittosporopsis kerrii#F272
Metteniusales Metteniusaceae Metteniusa tessmanniana#S1986
Garryales Eucommiaceae Eucommia ulmoides#SZUO
Garryales Eucommiaceae Eucommia ulmoides#NC 037948
Garryales Garryaceae Aucuba japonica#GIPR
Garryales Garryaceae Aucuba chlorascens#F219
Boraginales Codonaceae Codon schenckii#ZSD384 1
Boraginales Wellstediaceae Wellstedia dinteri#ZSD382 1
Boraginales Boraginaceae Mertensia paniculata#DKFZ
Boraginales Boraginaceae Trichodesma calycosum#F383
Boraginales Boraginaceae Onosma paniculatum#F44
Boraginales Boraginaceae Onosma album#F150
Boraginales Hydrophyllaceae Phacelia bolanderi#FC114
Boraginales Hydrophyllaceae Phacelia campanularia#YQIJ
Boraginales Namaceae Nama demissa#ZSD380 1
Boraginales Namaceae Eriodictyon californicum#ZSD381 1
Boraginales Namaceae Wigandia urens#ZSD386
Boraginales Ehretiaceae Pholisma arenarium#HANM @para
Boraginales Ehretiaceae Lennoa madreporoides#NC 039720 @para
Boraginales Ehretiaceae Tiquilia plicata#MG573056
Boraginales Ehretiaceae Rochefortia lundellii#NZ7120
Boraginales Ehretiaceae Ehretia acuminata#EMAL
Boraginales Ehretiaceae Ehretia confinis#F259
Boraginales Ehretiaceae Ehretia corylifolia#F328
Boraginales Cordiaceae Coldenia procumbens#ZSD387 1
Boraginales Cordiaceae Hoplestigma klaineanum#ZSD388 1
Boraginales Cordiaceae Cordia nevillii#ZSD385 1
Boraginales Cordiaceae Cordia alliodora#F919
Boraginales Cordiaceae Cordia subcordata#F703
Boraginales Heliotropiaceae Heliotropium arborescens#ZSD386 1
Boraginales Heliotropiaceae Heliotropium indicum#F496
Boraginales Heliotropiaceae Heliotropium tenellum#DIHD
Boraginales Heliotropiaceae Heliotropium calcicola#XVRU
Boraginales Heliotropiaceae Heliotropium karwinskyi#NIGS
Boraginales Heliotropiaceae Euploca racemosa#IDGE
Boraginales Heliotropiaceae Euploca convolvulacea#OUER
Boraginales Heliotropiaceae Euploca filiformis#OEKO
Boraginales Heliotropiaceae Heliotropium texanum#MDJK
Boraginales Heliotropiaceae Heliotropium mendocinum#MZOB
Boraginales Heliotropiaceae Heliotropium greggii#ABEH
Boraginales Heliotropiaceae Heliotropium sp.#JWEY
Gentianales Rubiaceae Mycetia gracilis#F60
Gentianales Rubiaceae Mycetia longiflora#F231
Gentianales Rubiaceae Galium boreale#WQRD
Gentianales Rubiaceae Galium aparine#NC 036969
Gentianales Rubiaceae Galium mollugo#NC 036970
Gentianales Rubiaceae Morinda officinalis#NC 028009
Gentianales Rubiaceae Psychotria marginata#PCNH
Gentianales Rubiaceae Psychotria gabrieliae#DNQA
Gentianales Rubiaceae Chassalia curviflora#F132
Gentianales Rubiaceae Carapichea ipecacuanha#ZSGF
Gentianales Rubiaceae Sinoadina racemosa#F106
Gentianales Rubiaceae Mitragyna speciosa#NC 034698
Gentianales Rubiaceae Neolamarckia cadamba#NC 041149
Gentianales Rubiaceae Uncaria sessilifructus#F101
Gentianales Rubiaceae Uncaria macrophylla#F62
Gentianales Rubiaceae Dunnia sinensis#NC 039965
Gentianales Rubiaceae Emmenopterys henryi#F1009
Gentianales Rubiaceae Wendlandia sp.#F243
Gentianales Rubiaceae Wendlandia pendula#F316
Gentianales Rubiaceae Wendlandia subalpina#F149
Gentianales Rubiaceae Tarennoidea wallichii#F6
Gentianales Rubiaceae Coffea canephora#NC 030053
Gentianales Rubiaceae Coffea arabica#KY085909
Gentianales Gelsemiaceae Gelsemium sempervirens#HGSM
Gentianales Gelsemiaceae Gelsemium elegans#F63
Gentianales Loganiaceae Gardneria angustifolia#F697
Gentianales Loganiaceae Strychnos spinosa#GGJD
Gentianales Loganiaceae Strychnos nux-vomica#F517
Gentianales Loganiaceae Strychnos cathayensis#F732
Gentianales Gentianaceae Exacum affine#KPUM
Gentianales Gentianaceae Fagraea ceilanica#F658
Gentianales Gentianaceae Gentiana stylophora#F835
Gentianales Gentianaceae Halenia elliptica#F174
Gentianales Gentianaceae Swertia verticillifolia#MF795137
Gentianales Gentianaceae Gentiana ornata#NC 037983
Gentianales Gentianaceae Gentiana caelestis#NC 037979
Gentianales Gentianaceae Gentiana obconica#NC 037981
Gentianales Gentianaceae Gentiana veitchiorum#NC 037985
Gentianales Gentianaceae Gentiana oreodoxa#NC 037982
Gentianales Gentianaceae Gentiana hexaphylla#NC 037980
Gentianales Gentianaceae Gentiana acaulis#ECTD
Gentianales Gentianaceae Gentiana stipitata#NC 037984
Gentianales Gentianaceae Gentiana tibetica#NC 030319
Gentianales Gentianaceae Gentiana macrophylla var. fetissowii#F831
Gentianales Gentianaceae Gentiana crassicaulis#KY595457
Gentianales Gentianaceae Gentiana straminea#NC 027441
Gentianales Gentianaceae Gentiana dahurica#NC 039572
Gentianales Gentianaceae Gentiana siphonantha#NC 039573
Gentianales Gentianaceae Gentiana officinalis#NC 039574
Gentianales Gentianaceae Gentiana macrophylla#NC 035719
Gentianales Apocynaceae Kopsia arborea#F293
Gentianales Apocynaceae Rauvolfia tetraphylla#QEHE
Gentianales Apocynaceae Catharanthus roseus#NC 021423
Gentianales Apocynaceae Melodinus fusiformis#F9
Gentianales Apocynaceae Rhazya stricta#NC 024292
Gentianales Apocynaceae Allamanda cathartica#MGVU
Gentianales Apocynaceae Carissa macrocarpa#NC 033354
Gentianales Apocynaceae Wrightia natalensis#EDEQ
Gentianales Apocynaceae Nerium oleander#GQ
Gentianales Apocynaceae Holarrhena pubescens#JGYZ
Gentianales Apocynaceae Periploca sepium#F626

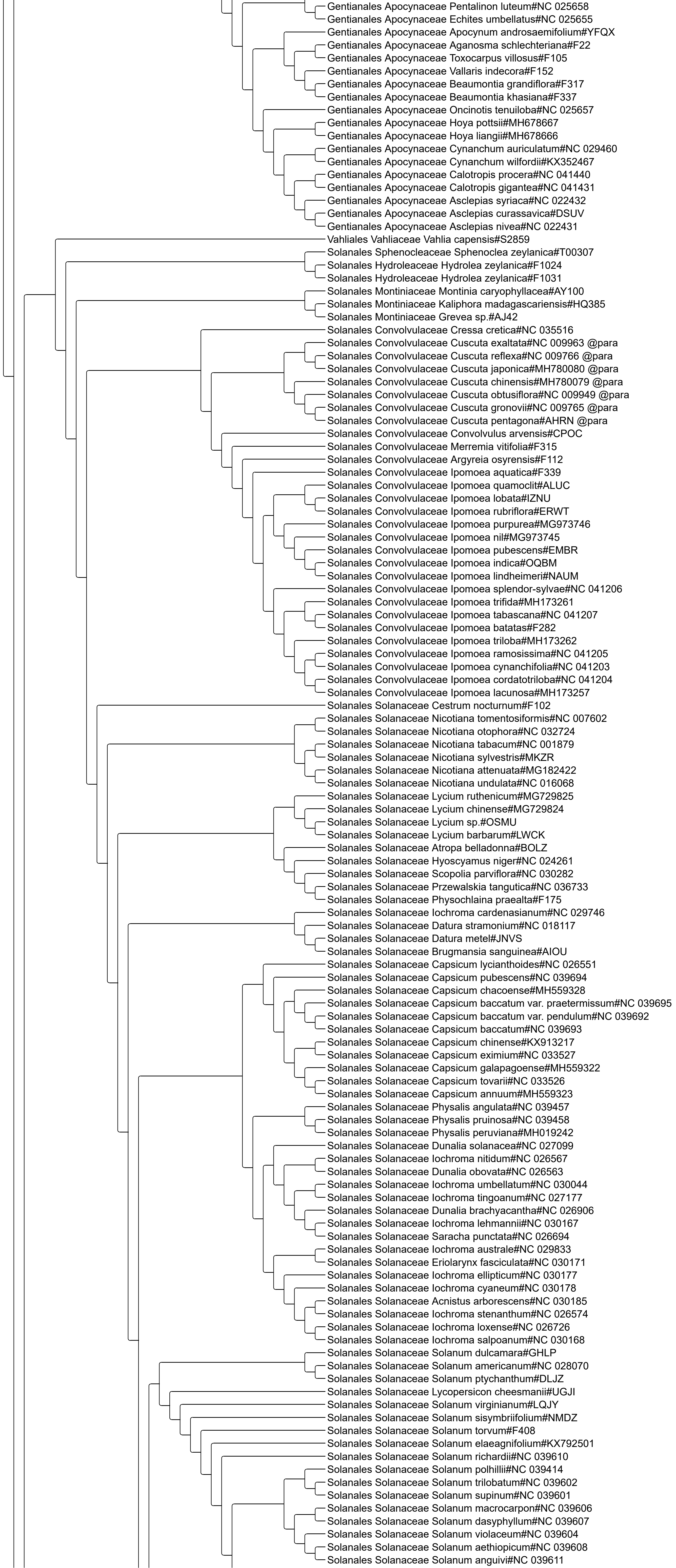
Gentianales Apocynaceae Pentalinon luteum#NC 025658
Gentianales Apocynaceae Echites umbellatus#NC 025655
Gentianales Apocynaceae Apocynum androsaemifolium#YFQX
Gentianales Apocynaceae Aganosma schlechteriana#F22
Gentianales Apocynaceae Toxocarpus villosus#F105
Gentianales Apocynaceae Vallaris indecora#F152
Gentianales Apocynaceae Beaumontia grandiflora#F317
Gentianales Apocynaceae Beaumontia khasiana#F337
Gentianales Apocynaceae Oncinotis tenuiloba#NC 025657
Gentianales Apocynaceae Hoya pottsii#MH678667
Gentianales Apocynaceae Hoya liangii#MH678666
Gentianales Apocynaceae Cynanchum auriculatum#NC 029460
Gentianales Apocynaceae Cynanchum wilfordii#KX352467
Gentianales Apocynaceae Calotropis procera#NC 041440
Gentianales Apocynaceae Calotropis gigantea#NC 041431
Gentianales Apocynaceae Asclepias syriaca#NC 022432
Gentianales Apocynaceae Asclepias curassavica#DSUV
Gentianales Apocynaceae Asclepias nivea#NC 022431
Vahliales Vahliaceae Vahlia capensis#S2859
Solanales Sphenocleaceae Sphenoclea zeylanica#T00307
Solanales Hydroleaceae Hydrolea zeylanica#F1024
Solanales Hydroleaceae Hydrolea zeylanica#F1031
Solanales Montiniaceae Montinia caryophyllacea#AY100
Solanales Montiniaceae Kaliphora madagascariensis#HQ385
Solanales Montiniaceae Grevea sp.#AJ42
Solanales Convolvulaceae Cressa cretica#NC 035516
Solanales Convolvulaceae Cuscuta exaltata#NC 009963 @para
Solanales Convolvulaceae Cuscuta reflexa#NC 009766 @para
Solanales Convolvulaceae Cuscuta japonica#MH780080 @para
Solanales Convolvulaceae Cuscuta chinensis#MH780079 @para
Solanales Convolvulaceae Cuscuta obtusiflora#NC 009949 @para
Solanales Convolvulaceae Cuscuta gronovii#NC 009765 @para
Solanales Convolvulaceae Cuscuta pentagona#AHRN @para
Solanales Convolvulaceae Convolvulus arvensis#CPOC
Solanales Convolvulaceae Merremia vitifolia#F315
Solanales Convolvulaceae Argyreia osyrensis#F112
Solanales Convolvulaceae Ipomoea aquatica#F339
Solanales Convolvulaceae Ipomoea quamoclit#ALUC
Solanales Convolvulaceae Ipomoea lobata#IZNU
Solanales Convolvulaceae Ipomoea rubriflora#ERWT
Solanales Convolvulaceae Ipomoea purpurea#MG973746
Solanales Convolvulaceae Ipomoea nil#MG973745
Solanales Convolvulaceae Ipomoea pubescens#EMBR
Solanales Convolvulaceae Ipomoea indica#OQBM
Solanales Convolvulaceae Ipomoea lindheimeri#NAUM
Solanales Convolvulaceae Ipomoea splendor-sylvae#NC 041206
Solanales Convolvulaceae Ipomoea trifida#MH173261
Solanales Convolvulaceae Ipomoea tabascana#NC 041207
Solanales Convolvulaceae Ipomoea batatas#F282
Solanales Convolvulaceae Ipomoea triloba#MH173262
Solanales Convolvulaceae Ipomoea ramosissima#NC 041205
Solanales Convolvulaceae Ipomoea cynanchifolia#NC 041203
Solanales Convolvulaceae Ipomoea cordatotriloba#NC 041204
Solanales Convolvulaceae Ipomoea lacunosa#MH173257
Solanales Solanaceae Cestrum nocturnum#F102
Solanales Solanaceae Nicotiana tomentosiformis#NC 007602
Solanales Solanaceae Nicotiana otophora#NC 032724
Solanales Solanaceae Nicotiana tabacum#NC 001879
Solanales Solanaceae Nicotiana sylvestris#MKZR
Solanales Solanaceae Nicotiana attenuata#MG182422
Solanales Solanaceae Nicotiana undulata#NC 016068
Solanales Solanaceae Lycium ruthenicum#MG729825
Solanales Solanaceae Lycium chinense#MG729824
Solanales Solanaceae Lycium sp.#OSMU
Solanales Solanaceae Lycium barbarum#LWCK
Solanales Solanaceae Atropa belladonna#BOLZ
Solanales Solanaceae Hyoscyamus niger#NC 024261
Solanales Solanaceae Scopolia parviflora#NC 030282
Solanales Solanaceae Przewalskia tangutica#NC 036733
Solanales Solanaceae Physochlaina praealta#F175
Solanales Solanaceae Iochroma cardenasianum#NC 029746
Solanales Solanaceae Datura stramonium#NC 018117
Solanales Solanaceae Datura metel#JNVS
Solanales Solanaceae Brugmansia sanguinea#AIOU
Solanales Solanaceae Capsicum lycianthoides#NC 026551
Solanales Solanaceae Capsicum pubescens#NC 039694
Solanales Solanaceae Capsicum chacoense#MH559328
Solanales Solanaceae Capsicum baccatum var. praetermissum#NC 039695
Solanales Solanaceae Capsicum baccatum var. pendulum#NC 039692
Solanales Solanaceae Capsicum baccatum#NC 039693
Solanales Solanaceae Capsicum chinense#KX913217
Solanales Solanaceae Capsicum eximium#NC 033527
Solanales Solanaceae Capsicum galapagoense#MH559322
Solanales Solanaceae Capsicum tovarii#NC 033526
Solanales Solanaceae Capsicum annuum#MH559323
Solanales Solanaceae Physalis angulata#NC 039457
Solanales Solanaceae Physalis pruinosa#NC 039458
Solanales Solanaceae Physalis peruviana#MH019242
Solanales Solanaceae Dunalia solanacea#NC 027099
Solanales Solanaceae Iochroma nitidum#NC 026567
Solanales Solanaceae Dunalia obovata#NC 026563
Solanales Solanaceae Iochroma umbellatum#NC 030044
Solanales Solanaceae Iochroma tingoanum#NC 027177
Solanales Solanaceae Dunalia brachyacantha#NC 026906
Solanales Solanaceae Iochroma lehmannii#NC 030167
Solanales Solanaceae Saracha punctata#NC 026694
Solanales Solanaceae Iochroma australe#NC 029833
Solanales Solanaceae Eriolarynx fasciculata#NC 030171
Solanales Solanaceae Iochroma ellipticum#NC 030177
Solanales Solanaceae Iochroma cyaneum#NC 030178
Solanales Solanaceae Acnistus arborescens#NC 030185
Solanales Solanaceae Iochroma stenanthum#NC 026574
Solanales Solanaceae Iochroma loxense#NC 026726
Solanales Solanaceae Iochroma salpoanum#NC 030168
Solanales Solanaceae Solanum dulcamara#GHLP
Solanales Solanaceae Solanum americanum#NC 028070
Solanales Solanaceae Solanum ptychanthum#DLJZ
Solanales Solanaceae Lycopersicon cheesmanii#UGJI
Solanales Solanaceae Solanum virginianum#LQJY
Solanales Solanaceae Solanum sisymbriifolium#NMDZ
Solanales Solanaceae Solanum torvum#F408
Solanales Solanaceae Solanum elaeagnifolium#KX792501
Solanales Solanaceae Solanum richardii#NC 039610
Solanales Solanaceae Solanum polhillii#NC 039414
Solanales Solanaceae Solanum trilobatum#NC 039602
Solanales Solanaceae Solanum supinum#NC 039601
Solanales Solanaceae Solanum macrocarpon#NC 039606
Solanales Solanaceae Solanum dasyphyllum#NC 039607
Solanales Solanaceae Solanum violaceum#NC 039604
Solanales Solanaceae Solanum aethiopicum#NC 039608
Solanales Solanaceae Solanum anguivi#NC 039611

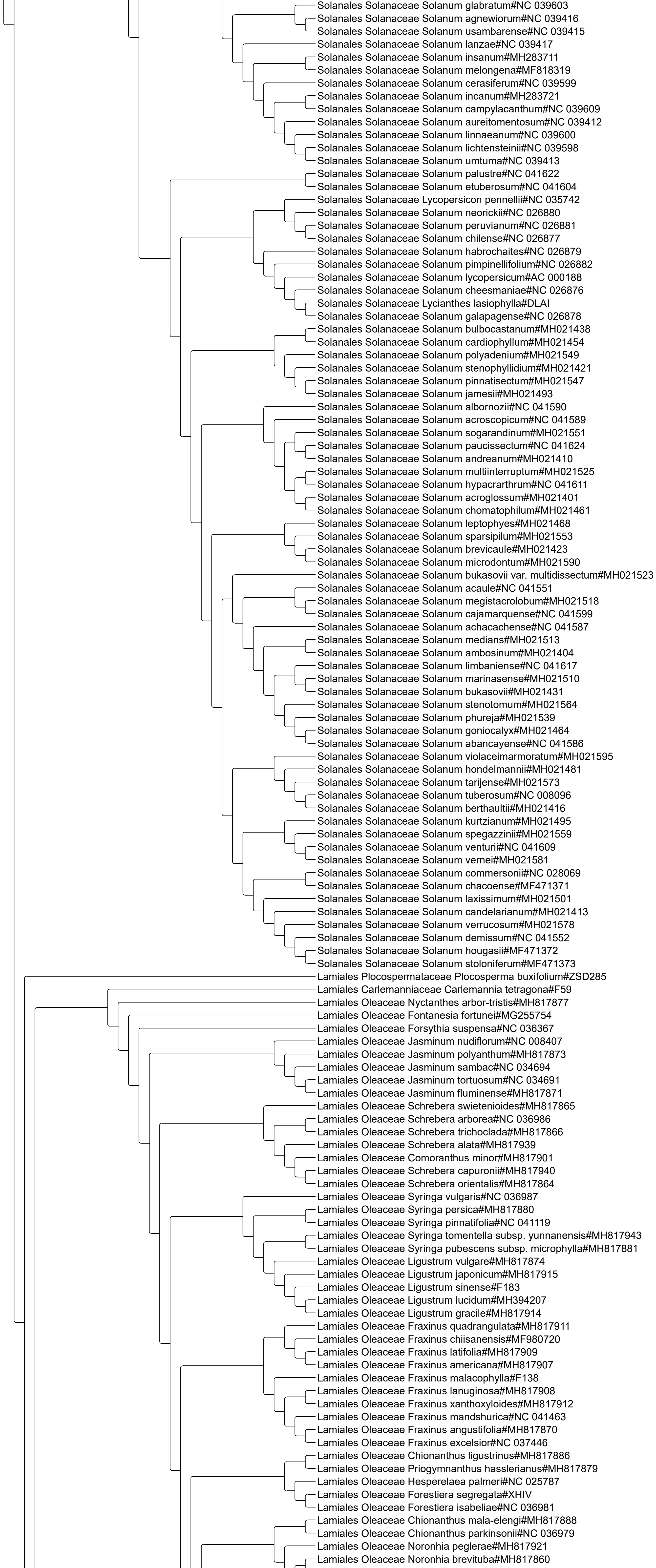
Solanales Solanaceae Solanum glabratum#NC 039603
Solanales Solanaceae Solanum agnewiorum#NC 039416
Solanales Solanaceae Solanum usambarense#NC 039415
Solanales Solanaceae Solanum lanzae#NC 039417
Solanales Solanaceae Solanum insanum#MH283711
Solanales Solanaceae Solanum melongena#MF818319
Solanales Solanaceae Solanum cerasiferum#NC 039599
Solanales Solanaceae Solanum incanum#MH283721
Solanales Solanaceae Solanum campylacanthum#NC 039609
Solanales Solanaceae Solanum aureitomentosum#NC 039412
Solanales Solanaceae Solanum linnaeanum#NC 039600
Solanales Solanaceae Solanum lichtensteinii#NC 039598
Solanales Solanaceae Solanum umtuma#NC 039413
Solanales Solanaceae Solanum palustre#NC 041622
Solanales Solanaceae Solanum etuberosum#NC 041604
Solanales Solanaceae Lycopersicon pennellii#NC 035742
Solanales Solanaceae Solanum neorickii#NC 026880
Solanales Solanaceae Solanum peruvianum#NC 026881
Solanales Solanaceae Solanum chilense#NC 026877
Solanales Solanaceae Solanum habrochaites#NC 026879
Solanales Solanaceae Solanum pimpinellifolium#NC 026882
Solanales Solanaceae Solanum lycopersicum#AC 000188
Solanales Solanaceae Solanum cheesmaniae#NC 026876
Solanales Solanaceae Lycianthes lasiophylla#DLAI
Solanales Solanaceae Solanum galapagense#NC 026878
Solanales Solanaceae Solanum bulbocastanum#MH021438
Solanales Solanaceae Solanum cardiophyllum#MH021454
Solanales Solanaceae Solanum polyadenium#MH021549
Solanales Solanaceae Solanum stenophyllidium#MH021421
Solanales Solanaceae Solanum pinnatisectum#MH021547
Solanales Solanaceae Solanum jamesii#MH021493
Solanales Solanaceae Solanum albornozii#NC 041590
Solanales Solanaceae Solanum acroscopicum#NC 041589
Solanales Solanaceae Solanum sogarandinum#MH021551
Solanales Solanaceae Solanum paucissectum#NC 041624
Solanales Solanaceae Solanum andreanum#MH021410
Solanales Solanaceae Solanum multiinterruptum#MH021525
Solanales Solanaceae Solanum hypacrarthrum#NC 041611
Solanales Solanaceae Solanum acroglossum#MH021401
Solanales Solanaceae Solanum chomatophilum#MH021461
Solanales Solanaceae Solanum leptophyes#MH021468
Solanales Solanaceae Solanum sparsipilum#MH021553
Solanales Solanaceae Solanum brevicaule#MH021423
Solanales Solanaceae Solanum microdontum#MH021590
Solanales Solanaceae Solanum bukasovii var. multidissectum#MH021523
Solanales Solanaceae Solanum acaule#NC 041551
Solanales Solanaceae Solanum megistacrolobum#MH021518
Solanales Solanaceae Solanum cajamarquense#NC 041599
Solanales Solanaceae Solanum achacachense#NC 041587
Solanales Solanaceae Solanum medians#MH021513
Solanales Solanaceae Solanum ambosinum#MH021404
Solanales Solanaceae Solanum limbaniense#NC 041617
Solanales Solanaceae Solanum marinasense#MH021510
Solanales Solanaceae Solanum bukasovii#MH021431
Solanales Solanaceae Solanum stenotomum#MH021564
Solanales Solanaceae Solanum phureja#MH021539
Solanales Solanaceae Solanum goniocalyx#MH021464
Solanales Solanaceae Solanum abancayense#NC 041586
Solanales Solanaceae Solanum violaceimarmoratum#MH021595
Solanales Solanaceae Solanum hondelmannii#MH021481
Solanales Solanaceae Solanum tarijense#MH021573
Solanales Solanaceae Solanum tuberosum#NC 008096
Solanales Solanaceae Solanum berthaultii#MH021416
Solanales Solanaceae Solanum kurtzianum#MH021495
Solanales Solanaceae Solanum spegazzinii#MH021559
Solanales Solanaceae Solanum venturii#NC 041609
Solanales Solanaceae Solanum vernei#MH021581
Solanales Solanaceae Solanum commersonii#NC 028069
Solanales Solanaceae Solanum chacoense#MF471371
Solanales Solanaceae Solanum laxissimum#MH021501
Solanales Solanaceae Solanum candelarianum#MH021413
Solanales Solanaceae Solanum verrucosum#MH021578
Solanales Solanaceae Solanum demissum#NC 041552
Solanales Solanaceae Solanum hougasii#MF471372
Solanales Solanaceae Solanum stoloniferum#MF471373
Lamiales Plocospermataceae Plocosperma buxifolium#ZSD285
Lamiales Carlemanniaceae Carlemannia tetragona#F59
Lamiales Oleaceae Nyctanthes arbor-tristis#MH817877
Lamiales Oleaceae Fontanesia fortunei#MG255754
Lamiales Oleaceae Forsythia suspensa#NC 036367
Lamiales Oleaceae Jasminum nudiflorum#NC 008407
Lamiales Oleaceae Jasminum polyanthum#MH817873
Lamiales Oleaceae Jasminum sambac#NC 034694
Lamiales Oleaceae Jasminum tortuosum#NC 034691
Lamiales Oleaceae Jasminum fluminense#MH817871
Lamiales Oleaceae Schrebera swietenioides#MH817865
Lamiales Oleaceae Schrebera arborea#NC 036986
Lamiales Oleaceae Schrebera trichoclada#MH817866
Lamiales Oleaceae Schrebera alata#MH817939
Lamiales Oleaceae Comoranthus minor#MH817901
Lamiales Oleaceae Schrebera capuronii#MH817940
Lamiales Oleaceae Schrebera orientalis#MH817864
Lamiales Oleaceae Syringa vulgaris#NC 036987
Lamiales Oleaceae Syringa persica#MH817880
Lamiales Oleaceae Syringa pinnatifolia#NC 041119
Lamiales Oleaceae Syringa tomentella subsp. yunnanensis#MH817943
Lamiales Oleaceae Syringa pubescens subsp. microphylla#MH817881
Lamiales Oleaceae Ligustrum vulgare#MH817874
Lamiales Oleaceae Ligustrum japonicum#MH817915
Lamiales Oleaceae Ligustrum sinense#F183
Lamiales Oleaceae Ligustrum lucidum#MH394207
Lamiales Oleaceae Ligustrum gracile#MH817914
Lamiales Oleaceae Fraxinus quadrangulata#MH817911
Lamiales Oleaceae Fraxinus chiisanensis#MF980720
Lamiales Oleaceae Fraxinus latifolia#MH817909
Lamiales Oleaceae Fraxinus americana#MH817907
Lamiales Oleaceae Fraxinus malacophylla#F138
Lamiales Oleaceae Fraxinus lanuginosa#MH817908
Lamiales Oleaceae Fraxinus xanthoxyloides#MH817912
Lamiales Oleaceae Fraxinus mandshurica#NC 041463
Lamiales Oleaceae Fraxinus angustifolia#MH817870
Lamiales Oleaceae Fraxinus excelsior#NC 037446
Lamiales Oleaceae Chionanthus ligustrinus#MH817886
Lamiales Oleaceae Priogymnanthus hasslerianus#MH817879
Lamiales Oleaceae Hesperelaea palmeri#NC 025787
Lamiales Oleaceae Forestiera segregata#XHIV
Lamiales Oleaceae Forestiera isabeliae#NC 036981
Lamiales Oleaceae Chionanthus mala-elengi#MH817888
Lamiales Oleaceae Chionanthus parkinsonii#NC 036979
Lamiales Oleaceae Noronhia peglerae#MH817921
Lamiales Oleaceae Noronhia brevituba#MH817860

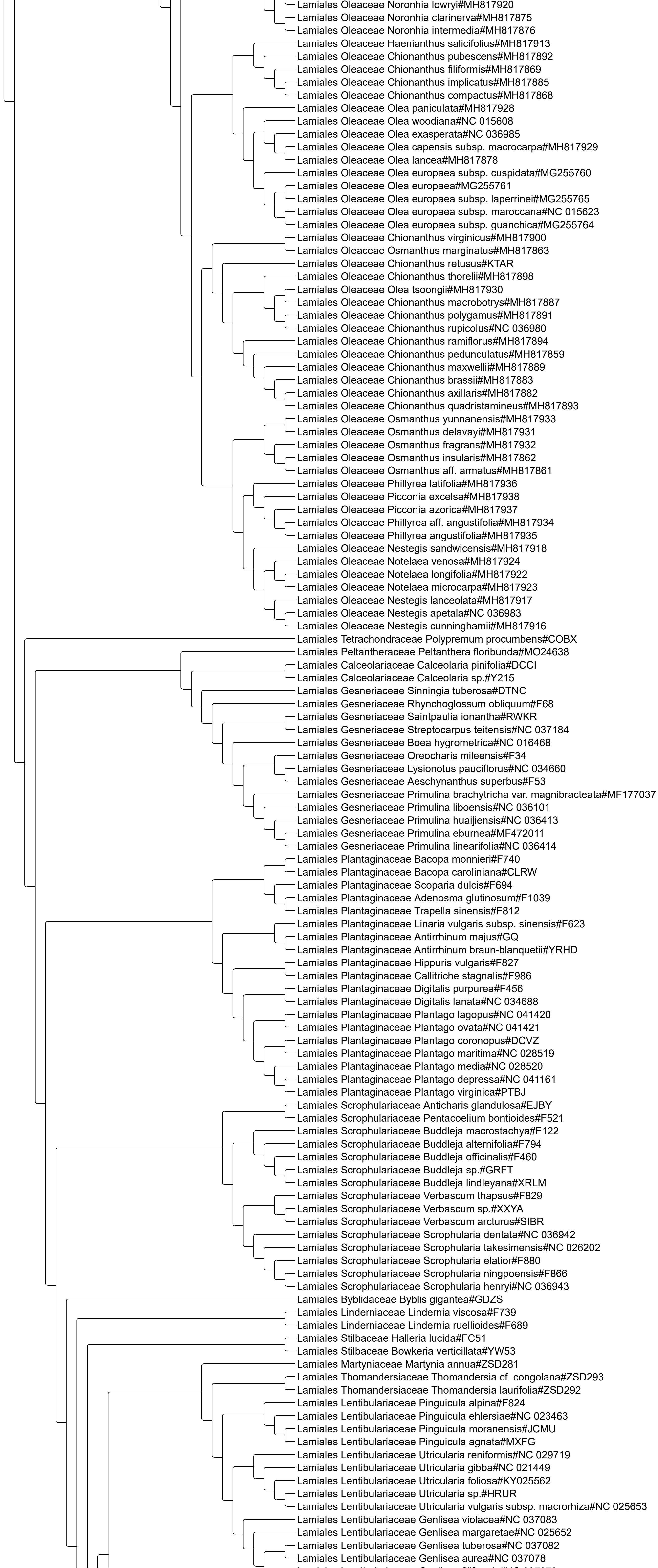
Lamiales Oleaceae Noronhia lowryi#MH817920
Lamiales Oleaceae Noronhia clarinerva#MH817875
Lamiales Oleaceae Noronhia intermedia#MH817876
Lamiales Oleaceae Haenianthus salicifolius#MH817913
Lamiales Oleaceae Chionanthus pubescens#MH817892
Lamiales Oleaceae Chionanthus filiformis#MH817869
Lamiales Oleaceae Chionanthus implicatus#MH817885
Lamiales Oleaceae Chionanthus compactus#MH817868
Lamiales Oleaceae Olea paniculata#MH817928
Lamiales Oleaceae Olea woodiana#NC 015608
Lamiales Oleaceae Olea exasperata#NC 036985
Lamiales Oleaceae Olea capensis subsp. macrocarpa#MH817929
Lamiales Oleaceae Olea lancea#MH817878
Lamiales Oleaceae Olea europaea subsp. cuspidata#MG255760
Lamiales Oleaceae Olea europaea#MG255761
Lamiales Oleaceae Olea europaea subsp. laperrinei#MG255765
Lamiales Oleaceae Olea europaea subsp. maroccana#NC 015623
Lamiales Oleaceae Olea europaea subsp. guanchica#MG255764
Lamiales Oleaceae Chionanthus virginicus#MH817900
Lamiales Oleaceae Osmanthus marginatus#MH817863
Lamiales Oleaceae Chionanthus retusus#KTAR
Lamiales Oleaceae Chionanthus thorelii#MH817898
Lamiales Oleaceae Olea tsoongii#MH817930
Lamiales Oleaceae Chionanthus macrobotrys#MH817887
Lamiales Oleaceae Chionanthus polygamus#MH817891
Lamiales Oleaceae Chionanthus rupicolus#NC 036980
Lamiales Oleaceae Chionanthus ramiflorus#MH817894
Lamiales Oleaceae Chionanthus pedunculatus#MH817859
Lamiales Oleaceae Chionanthus maxwellii#MH817889
Lamiales Oleaceae Chionanthus brassii#MH817883
Lamiales Oleaceae Chionanthus axillaris#MH817882
Lamiales Oleaceae Chionanthus quadristamineus#MH817893
Lamiales Oleaceae Osmanthus yunnanensis#MH817933
Lamiales Oleaceae Osmanthus delavayi#MH817931
Lamiales Oleaceae Osmanthus fragrans#MH817932
Lamiales Oleaceae Osmanthus insularis#MH817862
Lamiales Oleaceae Osmanthus aff. armatus#MH817861
Lamiales Oleaceae Phillyrea latifolia#MH817936
Lamiales Oleaceae Picconia excelsa#MH817938
Lamiales Oleaceae Picconia azorica#MH817937
Lamiales Oleaceae Phillyrea aff. angustifolia#MH817934
Lamiales Oleaceae Phillyrea angustifolia#MH817935
Lamiales Oleaceae Nestegis sandwicensis#MH817918
Lamiales Oleaceae Notelaea venosa#MH817924
Lamiales Oleaceae Notelaea longifolia#MH817922
Lamiales Oleaceae Notelaea microcarpa#MH817923
Lamiales Oleaceae Nestegis lanceolata#MH817917
Lamiales Oleaceae Nestegis apetala#NC 036983
Lamiales Oleaceae Nestegis cunninghamii#MH817916
Lamiales Tetrachondraceae Polypremum procumbens#COBX
Lamiales Peltantheraceae Peltanthera floribunda#MO24638
Lamiales Calceolariaceae Calceolaria pinifolia#DCCI
Lamiales Calceolariaceae Calceolaria sp.#Y215
Lamiales Gesneriaceae Sinningia tuberosa#DTNC
Lamiales Gesneriaceae Rhynchoglossum obliquum#F68
Lamiales Gesneriaceae Saintpaulia ionantha#RWKR
Lamiales Gesneriaceae Streptocarpus teitensis#NC 037184
Lamiales Gesneriaceae Boea hygrometrica#NC 016468
Lamiales Gesneriaceae Oreocharis mileensis#F34
Lamiales Gesneriaceae Lysionotus pauciflorus#NC 034660
Lamiales Gesneriaceae Aeschynanthus superbus#F53
Lamiales Gesneriaceae Primulina brachytricha var. magnibracteata#MF177037
Lamiales Gesneriaceae Primulina liboensis#NC 036101
Lamiales Gesneriaceae Primulina huaijiensis#NC 036413
Lamiales Gesneriaceae Primulina eburnea#MF472011
Lamiales Gesneriaceae Primulina linearifolia#NC 036414
Lamiales Plantaginaceae Bacopa monnieri#F740
Lamiales Plantaginaceae Bacopa caroliniana#CLRW
Lamiales Plantaginaceae Scoparia dulcis#F694
Lamiales Plantaginaceae Adenosma glutinosum#F1039
Lamiales Plantaginaceae Trapella sinensis#F812
Lamiales Plantaginaceae Linaria vulgaris subsp. sinensis#F623
Lamiales Plantaginaceae Antirrhinum majus#GQ
Lamiales Plantaginaceae Antirrhinum braun-blanquetii#YRHD
Lamiales Plantaginaceae Hippuris vulgaris#F827
Lamiales Plantaginaceae Callitriche stagnalis#F986
Lamiales Plantaginaceae Digitalis purpurea#F456
Lamiales Plantaginaceae Digitalis lanata#NC 034688
Lamiales Plantaginaceae Plantago lagopus#NC 041420
Lamiales Plantaginaceae Plantago ovata#NC 041421
Lamiales Plantaginaceae Plantago coronopus#DCVZ
Lamiales Plantaginaceae Plantago maritima#NC 028519
Lamiales Plantaginaceae Plantago media#NC 028520
Lamiales Plantaginaceae Plantago depressa#NC 041161
Lamiales Plantaginaceae Plantago virginica#PTBJ
Lamiales Scrophulariaceae Anticharis glandulosa#EJBY
Lamiales Scrophulariaceae Pentacoelium bontioides#F521
Lamiales Scrophulariaceae Buddleja macrostachya#F122
Lamiales Scrophulariaceae Buddleja alternifolia#F794
Lamiales Scrophulariaceae Buddleja officinalis#F460
Lamiales Scrophulariaceae Buddleja sp.#GRFT
Lamiales Scrophulariaceae Buddleja lindleyana#XRLM
Lamiales Scrophulariaceae Verbascum thapsus#F829
Lamiales Scrophulariaceae Verbascum sp.#XXYA
Lamiales Scrophulariaceae Verbascum arcturus#SIBR
Lamiales Scrophulariaceae Scrophularia dentata#NC 036942
Lamiales Scrophulariaceae Scrophularia takesimensis#NC 026202
Lamiales Scrophulariaceae Scrophularia elatior#F880
Lamiales Scrophulariaceae Scrophularia ningpoensis#F866
Lamiales Scrophulariaceae Scrophularia henryi#NC 036943
Lamiales Byblidaceae Byblis gigantea#GDZS
Lamiales Linderniaceae Lindernia viscosa#F739
Lamiales Linderniaceae Lindernia ruellioides#F689
Lamiales Stilbaceae Halleria lucida#FC51
Lamiales Stilbaceae Bowkeria verticillata#YW53
Lamiales Martyniaceae Martynia annua#ZSD281
Lamiales Thomandersiaceae Thomandersia cf. congolana#ZSD293
Lamiales Thomandersiaceae Thomandersia laurifolia#ZSD292
Lamiales Lentibulariaceae Pinguicula alpina#F824
Lamiales Lentibulariaceae Pinguicula ehlersiae#NC 023463
Lamiales Lentibulariaceae Pinguicula moranensis#JCMU
Lamiales Lentibulariaceae Pinguicula agnata#MXFG
Lamiales Lentibulariaceae Utricularia reniformis#NC 029719
Lamiales Lentibulariaceae Utricularia gibba#NC 021449
Lamiales Lentibulariaceae Utricularia foliosa#KY025562
Lamiales Lentibulariaceae Utricularia sp.#HRUR
Lamiales Lentibulariaceae Utricularia vulgaris subsp. macrorhiza#NC 025653
Lamiales Lentibulariaceae Genlisea violacea#NC 037083
Lamiales Lentibulariaceae Genlisea margaretae#NC 025652
Lamiales Lentibulariaceae Genlisea tuberosa#NC 037082
Lamiales Lentibulariaceae Genlisea aurea#NC 037078

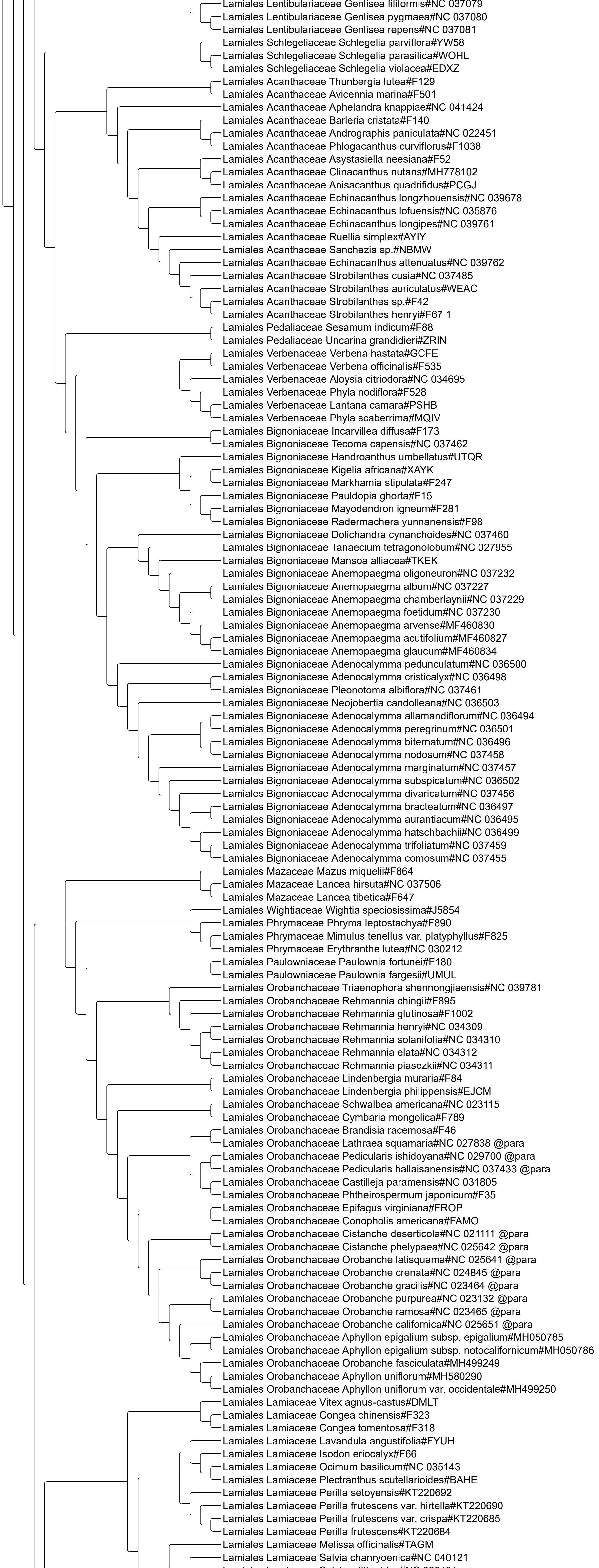

Lamiales Lentibulariaceae Genlisea filiformis#NC 037079
Lamiales Lentibulariaceae Genlisea pygmaea#NC 037080
Lamiales Lentibulariaceae Genlisea repens#NC 037081
Lamiales Schlegeliaceae Schlegelia parviflora#YW58
Lamiales Schlegeliaceae Schlegelia parasitica#WOHL
Lamiales Schlegeliaceae Schlegelia violacea#EDXZ
Lamiales Acanthaceae Thunbergia lutea#F129
Lamiales Acanthaceae Avicennia marina#F501
Lamiales Acanthaceae Aphelandra knappiae#NC 041424
Lamiales Acanthaceae Barleria cristata#F140
Lamiales Acanthaceae Andrographis paniculata#NC 022451
Lamiales Acanthaceae Phlogacanthus curviflorus#F1038
Lamiales Acanthaceae Asystasiella neesiana#F52
Lamiales Acanthaceae Clinacanthus nutans#MH778102
Lamiales Acanthaceae Anisacanthus quadrifidus#PCGJ
Lamiales Acanthaceae Echinacanthus longzhouensis#NC 039678
Lamiales Acanthaceae Echinacanthus lofuensis#NC 035876
Lamiales Acanthaceae Echinacanthus longipes#NC 039761
Lamiales Acanthaceae Ruellia simplex#AYIY
Lamiales Acanthaceae Sanchezia sp.#NBMW
Lamiales Acanthaceae Echinacanthus attenuatus#NC 039762
Lamiales Acanthaceae Strobilanthes cusia#NC 037485
Lamiales Acanthaceae Strobilanthes auriculatus#WEAC
Lamiales Acanthaceae Strobilanthes sp.#F42
Lamiales Acanthaceae Strobilanthes henryi#F67 1
Lamiales Pedaliaceae Sesamum indicum#F88
Lamiales Pedaliaceae Uncarina grandidieri#ZRIN
Lamiales Verbenaceae Verbena hastata#GCFE
Lamiales Verbenaceae Verbena officinalis#F535
Lamiales Verbenaceae Aloysia citriodora#NC 034695
Lamiales Verbenaceae Phyla nodiflora#F528
Lamiales Verbenaceae Lantana camara#PSHB
Lamiales Verbenaceae Phyla scaberrima#MQIV
Lamiales Bignoniaceae Incarvillea diffusa#F173
Lamiales Bignoniaceae Tecoma capensis#NC 037462
Lamiales Bignoniaceae Handroanthus umbellatus#UTQR
Lamiales Bignoniaceae Kigelia africana#XAYK
Lamiales Bignoniaceae Markhamia stipulata#F247
Lamiales Bignoniaceae Pauldopia ghorta#F15
Lamiales Bignoniaceae Mayodendron igneum#F281
Lamiales Bignoniaceae Radermachera yunnanensis#F98
Lamiales Bignoniaceae Dolichandra cynanchoides#NC 037460
Lamiales Bignoniaceae Tanaecium tetragonolobum#NC 027955
Lamiales Bignoniaceae Mansoa alliacea#TKEK
Lamiales Bignoniaceae Anemopaegma oligoneuron#NC 037232
Lamiales Bignoniaceae Anemopaegma album#NC 037227
Lamiales Bignoniaceae Anemopaegma chamberlaynii#NC 037229
Lamiales Bignoniaceae Anemopaegma foetidum#NC 037230
Lamiales Bignoniaceae Anemopaegma arvense#MF460830
Lamiales Bignoniaceae Anemopaegma acutifolium#MF460827
Lamiales Bignoniaceae Anemopaegma glaucum#MF460834
Lamiales Bignoniaceae Adenocalymma pedunculatum#NC 036500
Lamiales Bignoniaceae Adenocalymma cristicalyx#NC 036498
Lamiales Bignoniaceae Pleonotoma albiflora#NC 037461
Lamiales Bignoniaceae Neojobertia candolleana#NC 036503
Lamiales Bignoniaceae Adenocalymma allamandiflorum#NC 036494
Lamiales Bignoniaceae Adenocalymma peregrinum#NC 036501
Lamiales Bignoniaceae Adenocalymma biternatum#NC 036496
Lamiales Bignoniaceae Adenocalymma nodosum#NC 037458
Lamiales Bignoniaceae Adenocalymma marginatum#NC 037457
Lamiales Bignoniaceae Adenocalymma subspicatum#NC 036502
Lamiales Bignoniaceae Adenocalymma divaricatum#NC 037456
Lamiales Bignoniaceae Adenocalymma bracteatum#NC 036497
Lamiales Bignoniaceae Adenocalymma aurantiacum#NC 036495
Lamiales Bignoniaceae Adenocalymma hatschbachii#NC 036499
Lamiales Bignoniaceae Adenocalymma trifoliatum#NC 037459
Lamiales Bignoniaceae Adenocalymma comosum#NC 037455
Lamiales Mazaceae Mazus miquelii#F864
Lamiales Mazaceae Lancea hirsuta#NC 037506
Lamiales Mazaceae Lancea tibetica#F647
Lamiales Wightiaceae Wightia speciosissima#J5854
Lamiales Phrymaceae Phryma leptostachya#F890
Lamiales Phrymaceae Mimulus tenellus var. platyphyllus#F825
Lamiales Phrymaceae Erythranthe lutea#NC 030212
Lamiales Paulowniaceae Paulownia fortunei#F180
Lamiales Paulowniaceae Paulownia fargesii#UMUL
Lamiales Orobanchaceae Triaenophora shennongjiaensis#NC 039781
Lamiales Orobanchaceae Rehmannia chingii#F895
Lamiales Orobanchaceae Rehmannia glutinosa#F1002
Lamiales Orobanchaceae Rehmannia henryi#NC 034309
Lamiales Orobanchaceae Rehmannia solanifolia#NC 034310
Lamiales Orobanchaceae Rehmannia elata#NC 034312
Lamiales Orobanchaceae Rehmannia piasezkii#NC 034311
Lamiales Orobanchaceae Lindenbergia muraria#F84
Lamiales Orobanchaceae Lindenbergia philippensis#EJCM
Lamiales Orobanchaceae Schwalbea americana#NC 023115
Lamiales Orobanchaceae Cymbaria mongolica#F789
Lamiales Orobanchaceae Brandisia racemosa#F46
Lamiales Orobanchaceae Lathraea squamaria#NC 027838 @para
Lamiales Orobanchaceae Pedicularis ishidoyana#NC 029700 @para
Lamiales Orobanchaceae Pedicularis hallaisanensis#NC 037433 @para
Lamiales Orobanchaceae Castilleja paramensis#NC 031805
Lamiales Orobanchaceae Phtheirospermum japonicum#F35
Lamiales Orobanchaceae Epifagus virginiana#FROP
Lamiales Orobanchaceae Conopholis americana#FAMO
Lamiales Orobanchaceae Cistanche deserticola#NC 021111 @para
Lamiales Orobanchaceae Cistanche phelypaea#NC 025642 @para
Lamiales Orobanchaceae Orobanche latisquama#NC 025641 @para
Lamiales Orobanchaceae Orobanche crenata#NC 024845 @para
Lamiales Orobanchaceae Orobanche gracilis#NC 023464 @para
Lamiales Orobanchaceae Orobanche purpurea#NC 023132 @para
Lamiales Orobanchaceae Orobanche ramosa#NC 023465 @para
Lamiales Orobanchaceae Orobanche californica#NC 025651 @para
Lamiales Orobanchaceae Aphyllon epigalium subsp. epigalium#MH050785
Lamiales Orobanchaceae Aphyllon epigalium subsp. notocalifornicum#MH050786
Lamiales Orobanchaceae Orobanche fasciculata#MH499249
Lamiales Orobanchaceae Aphyllon uniflorum#MH580290
Lamiales Orobanchaceae Aphyllon uniflorum var. occidentale#MH499250
Lamiales Lamiaceae Vitex agnus-castus#DMLT
Lamiales Lamiaceae Congea chinensis#F323
Lamiales Lamiaceae Congea tomentosa#F318
Lamiales Lamiaceae Lavandula angustifolia#FYUH
Lamiales Lamiaceae Isodon eriocalyx#F66
Lamiales Lamiaceae Ocimum basilicum#NC 035143
Lamiales Lamiaceae Plectranthus scutellarioides#BAHE
Lamiales Lamiaceae Perilla setoyensis#KT220692
Lamiales Lamiaceae Perilla frutescens var. hirtella#KT220690
Lamiales Lamiaceae Perilla frutescens var. crispa#KT220685
Lamiales Lamiaceae Perilla frutescens#KT220684
Lamiales Lamiaceae Melissa officinalis#TAGM
Lamiales Lamiaceae Salvia chanryoenica#NC 040121

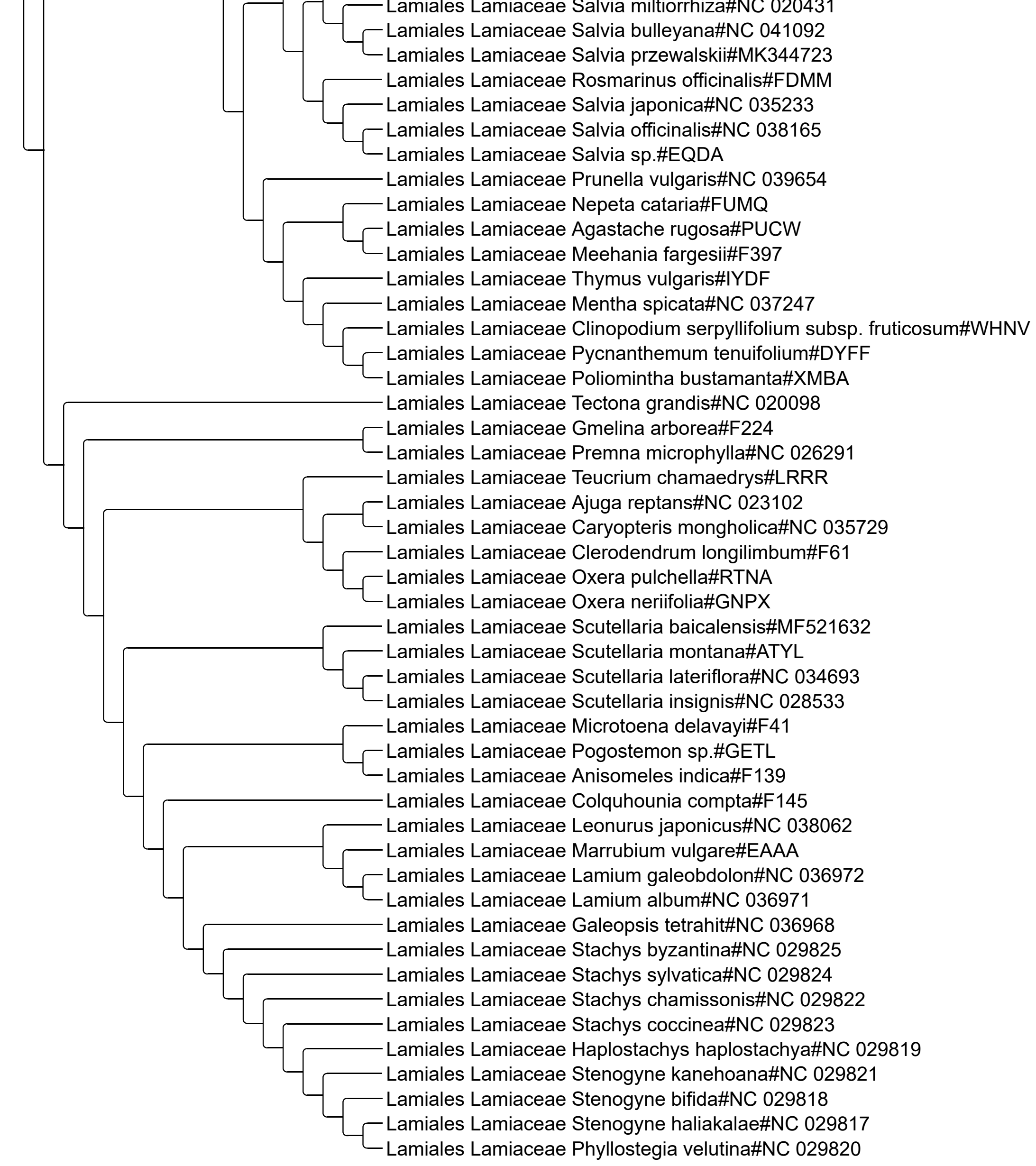
Lamiales Lamiaceae Salvia miltiorrhiza#NC 020431
Lamiales Lamiaceae Salvia bulleyana#NC 041092
Lamiales Lamiaceae Salvia przewalskii#MK344723
Lamiales Lamiaceae Rosmarinus officinalis#FDMM
Lamiales Lamiaceae Salvia japonica#NC 035233
Lamiales Lamiaceae Salvia officinalis#NC 038165
Lamiales Lamiaceae Salvia sp.#EQDA
Lamiales Lamiaceae Prunella vulgaris#NC 039654
Lamiales Lamiaceae Nepeta cataria#FUMQ
Lamiales Lamiaceae Agastache rugosa#PUCW
Lamiales Lamiaceae Meehania fargesii#F397
Lamiales Lamiaceae Thymus vulgaris#IYDF
Lamiales Lamiaceae Mentha spicata#NC 037247
Lamiales Lamiaceae Clinopodium serpyllifolium subsp. fruticosum#WHNV
Lamiales Lamiaceae Pycnanthemum tenuifolium#DYFF
Lamiales Lamiaceae Poliomintha bustamanta#XMBA
Lamiales Lamiaceae Tectona grandis#NC 020098
Lamiales Lamiaceae Gmelina arborea#F224
Lamiales Lamiaceae Premna microphylla#NC 026291
Lamiales Lamiaceae Teucrium chamaedrys#LRRR
Lamiales Lamiaceae Ajuga reptans#NC 023102
Lamiales Lamiaceae Caryopteris mongholica#NC 035729
Lamiales Lamiaceae Clerodendrum longilimbum#F61
Lamiales Lamiaceae Oxera pulchella#RTNA
Lamiales Lamiaceae Oxera neriifolia#GNPX
Lamiales Lamiaceae Scutellaria baicalensis#MF521632
Lamiales Lamiaceae Scutellaria montana#ATYL
Lamiales Lamiaceae Scutellaria lateriflora#NC 034693
Lamiales Lamiaceae Scutellaria insignis#NC 028533
Lamiales Lamiaceae Microtoena delavayi#F41
Lamiales Lamiaceae Pogostemon sp.#GETL
Lamiales Lamiaceae Anisomeles indica#F139
Lamiales Lamiaceae Colquhounia compta#F145
Lamiales Lamiaceae Leonurus japonicus#NC 038062
Lamiales Lamiaceae Marrubium vulgare#EAAA
Lamiales Lamiaceae Lamium galeobdolon#NC 036972
Lamiales Lamiaceae Lamium album#NC 036971
Lamiales Lamiaceae Galeopsis tetrahit#NC 036968
Lamiales Lamiaceae Stachys byzantina#NC 029825
Lamiales Lamiaceae Stachys sylvatica#NC 029824
Lamiales Lamiaceae Stachys chamissonis#NC 029822
Lamiales Lamiaceae Stachys coccinea#NC 029823
Lamiales Lamiaceae Haplostachys haplostachya#NC 029819
Lamiales Lamiaceae Stenogyne kanehoana#NC 029821
Lamiales Lamiaceae Stenogyne bifida#NC 029818
Lamiales Lamiaceae Stenogyne haliakalae#NC 029817
Lamiales Lamiaceae Phyllostegia velutina#NC 029820

[Figure S8g]

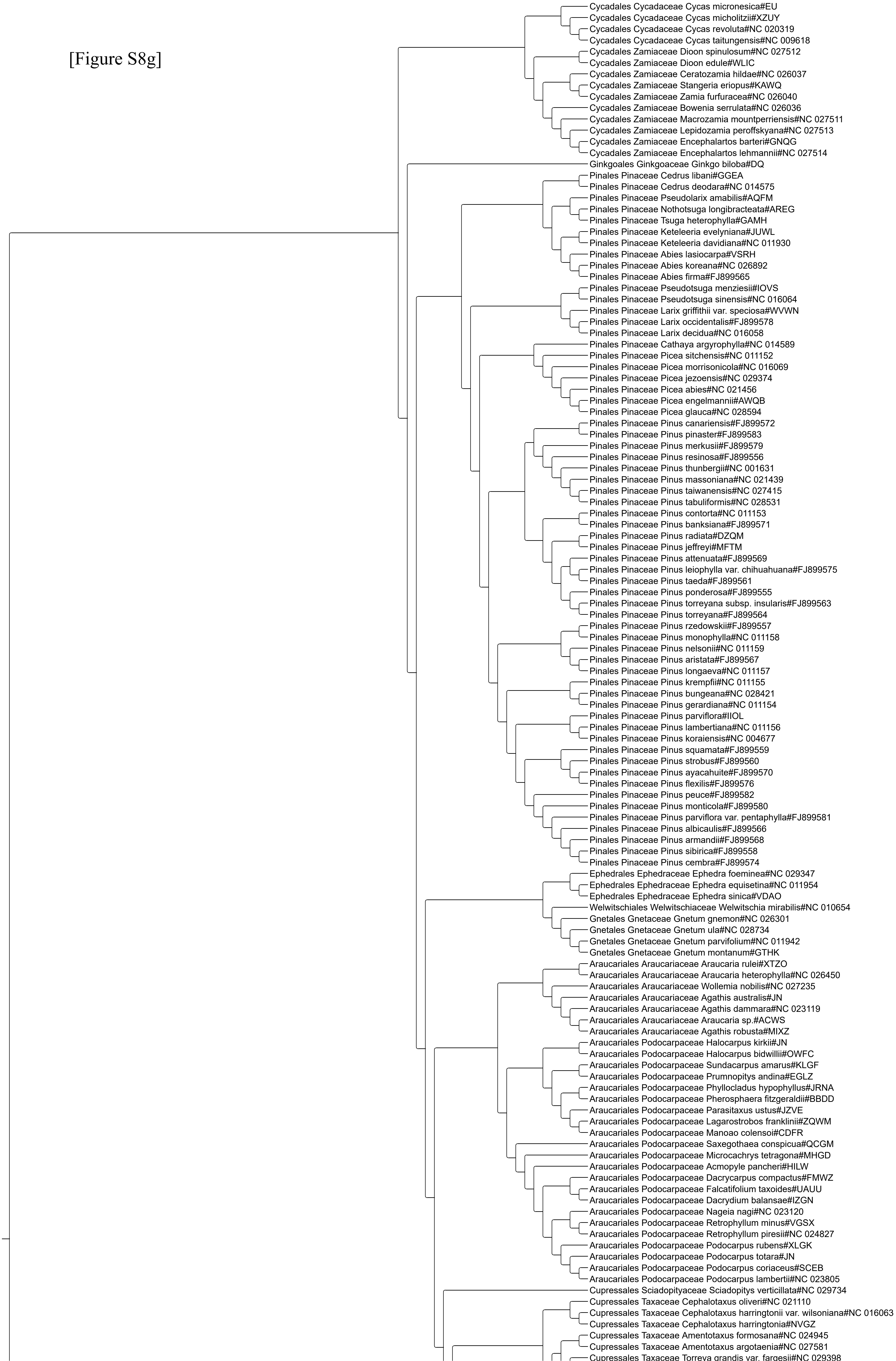

Cycadales Cycadaceae Cycas micronesica#EU
Cycadales Cycadaceae Cycas micholitzii#XZUY
Cycadales Cycadaceae Cycas revoluta#NC 020319
Cycadales Cycadaceae Cycas taitungensis#NC 009618
Cycadales Zamiaceae Dioon spinulosum#NC 027512
Cycadales Zamiaceae Dioon edule#WLIC
Cycadales Zamiaceae Ceratozamia hildae#NC 026037
Cycadales Zamiaceae Stangeria eriopus#KAWQ
Cycadales Zamiaceae Zamia furfuracea#NC 026040
Cycadales Zamiaceae Bowenia serrulata#NC 026036
Cycadales Zamiaceae Macrozamia mountperriensis#NC 027511
Cycadales Zamiaceae Lepidozamia peroffskyana#NC 027513
Cycadales Zamiaceae Encephalartos barteri#GNQG
Cycadales Zamiaceae Encephalartos lehmannii#NC 027514
Ginkgoales Ginkgoaceae Ginkgo biloba#DQ
Pinales Pinaceae Cedrus libani#GGEA
Pinales Pinaceae Cedrus deodara#NC 014575
Pinales Pinaceae Pseudolarix amabilis#AQFM
Pinales Pinaceae Nothotsuga longibracteata#AREG
Pinales Pinaceae Tsuga heterophylla#GAMH
Pinales Pinaceae Keteleeria evelyniana#JUWL
Pinales Pinaceae Keteleeria davidiana#NC 011930
Pinales Pinaceae Abies lasiocarpa#VSRH
Pinales Pinaceae Abies koreana#NC 026892
Pinales Pinaceae Abies firma#FJ899565
Pinales Pinaceae Pseudotsuga menziesii#IOVS
Pinales Pinaceae Pseudotsuga sinensis#NC 016064
Pinales Pinaceae Larix griffithii var. speciosa#WVWN
Pinales Pinaceae Larix occidentalis#FJ899578
Pinales Pinaceae Larix decidua#NC 016058
Pinales Pinaceae Cathaya argyrophylla#NC 014589
Pinales Pinaceae Picea sitchensis#NC 011152
Pinales Pinaceae Picea morrisonicola#NC 016069
Pinales Pinaceae Picea jezoensis#NC 029374
Pinales Pinaceae Picea abies#NC 021456
Pinales Pinaceae Picea engelmannii#AWQB
Pinales Pinaceae Picea glauca#NC 028594
Pinales Pinaceae Pinus canariensis#FJ899572
Pinales Pinaceae Pinus pinaster#FJ899583
Pinales Pinaceae Pinus merkusii#FJ899579
Pinales Pinaceae Pinus resinosa#FJ899556
Pinales Pinaceae Pinus thunbergii#NC 001631
Pinales Pinaceae Pinus massoniana#NC 021439
Pinales Pinaceae Pinus taiwanensis#NC 027415
Pinales Pinaceae Pinus tabuliformis#NC 028531
Pinales Pinaceae Pinus contorta#NC 011153
Pinales Pinaceae Pinus banksiana#FJ899571
Pinales Pinaceae Pinus radiata#DZQM
Pinales Pinaceae Pinus jeffreyi#MFTM
Pinales Pinaceae Pinus attenuata#FJ899569
Pinales Pinaceae Pinus leiophylla var. chihuahuana#FJ899575
Pinales Pinaceae Pinus taeda#FJ899561
Pinales Pinaceae Pinus ponderosa#FJ899555
Pinales Pinaceae Pinus torreyana subsp. insularis#FJ899563
Pinales Pinaceae Pinus torreyana#FJ899564
Pinales Pinaceae Pinus rzedowskii#FJ899557
Pinales Pinaceae Pinus monophylla#NC 011158
Pinales Pinaceae Pinus nelsonii#NC 011159
Pinales Pinaceae Pinus aristata#FJ899567
Pinales Pinaceae Pinus longaeva#NC 011157
Pinales Pinaceae Pinus krempfii#NC 011155
Pinales Pinaceae Pinus bungeana#NC 028421
Pinales Pinaceae Pinus gerardiana#NC 011154
Pinales Pinaceae Pinus parviflora#IIOL
Pinales Pinaceae Pinus lambertiana#NC 011156
Pinales Pinaceae Pinus koraiensis#NC 004677
Pinales Pinaceae Pinus squamata#FJ899559
Pinales Pinaceae Pinus strobus#FJ899560
Pinales Pinaceae Pinus ayacahuite#FJ899570
Pinales Pinaceae Pinus flexilis#FJ899576
Pinales Pinaceae Pinus peuce#FJ899582
Pinales Pinaceae Pinus monticola#FJ899580
Pinales Pinaceae Pinus parviflora var. pentaphylla#FJ899581
Pinales Pinaceae Pinus albicaulis#FJ899566
Pinales Pinaceae Pinus armandii#FJ899568
Pinales Pinaceae Pinus sibirica#FJ899558
Pinales Pinaceae Pinus cembra#FJ899574
Ephedrales Ephedraceae Ephedra foeminea#NC 029347
Ephedrales Ephedraceae Ephedra equisetina#NC 011954
Ephedrales Ephedraceae Ephedra sinica#VDAO
Welwitschiales Welwitschiaceae Welwitschia mirabilis#NC 010654
Gnetales Gnetaceae Gnetum gnemon#NC 026301
Gnetales Gnetaceae Gnetum ula#NC 028734
Gnetales Gnetaceae Gnetum parvifolium#NC 011942
Gnetales Gnetaceae Gnetum montanum#GTHK
Araucariales Araucariaceae Araucaria rulei#XTZO
Araucariales Araucariaceae Araucaria heterophylla#NC 026450
Araucariales Araucariaceae Wollemia nobilis#NC 027235
Araucariales Araucariaceae Agathis australis#JN
Araucariales Araucariaceae Agathis dammara#NC 023119
Araucariales Araucariaceae Araucaria sp.#ACWS
Araucariales Araucariaceae Agathis robusta#MIXZ
Araucariales Podocarpaceae Halocarpus kirkii#JN
Araucariales Podocarpaceae Halocarpus bidwillii#OWFC
Araucariales Podocarpaceae Sundacarpus amarus#KLGF
Araucariales Podocarpaceae Prumnopitys andina#EGLZ
Araucariales Podocarpaceae Phyllocladus hypophyllus#JRNA
Araucariales Podocarpaceae Pherosphaera fitzgeraldii#BBDD
Araucariales Podocarpaceae Parasitaxus ustus#JZVE
Araucariales Podocarpaceae Lagarostrobos franklinii#ZQWM
Araucariales Podocarpaceae Manoao colensoi#CDFR
Araucariales Podocarpaceae Saxegothaea conspicua#QCGM
Araucariales Podocarpaceae Microcachrys tetragona#MHGD
Araucariales Podocarpaceae Acmopyle pancheri#HILW
Araucariales Podocarpaceae Dacrycarpus compactus#FMWZ
Araucariales Podocarpaceae Falcatifolium taxoides#UAUU
Araucariales Podocarpaceae Dacrydium balansae#IZGN
Araucariales Podocarpaceae Nageia nagi#NC 023120
Araucariales Podocarpaceae Retrophyllum minus#VGSX
Araucariales Podocarpaceae Retrophyllum piresii#NC 024827
Araucariales Podocarpaceae Podocarpus rubens#XLGK
Araucariales Podocarpaceae Podocarpus totara#JN
Araucariales Podocarpaceae Podocarpus coriaceus#SCEB
Araucariales Podocarpaceae Podocarpus lambertii#NC 023805
Cupressales Sciadopityaceae Sciadopitys verticillata#NC 029734
Cupressales Taxaceae Cephalotaxus oliveri#NC 021110
Cupressales Taxaceae Cephalotaxus harringtonii var. wilsoniana#NC 016063
Cupressales Taxaceae Cephalotaxus harringtonia#NVGZ
Cupressales Taxaceae Amentotaxus formosana#NC 024945
Cupressales Taxaceae Amentotaxus argotaenia#NC 027581
Cupressales Taxaceae Torreya grandis var. fargesii#NC 029398

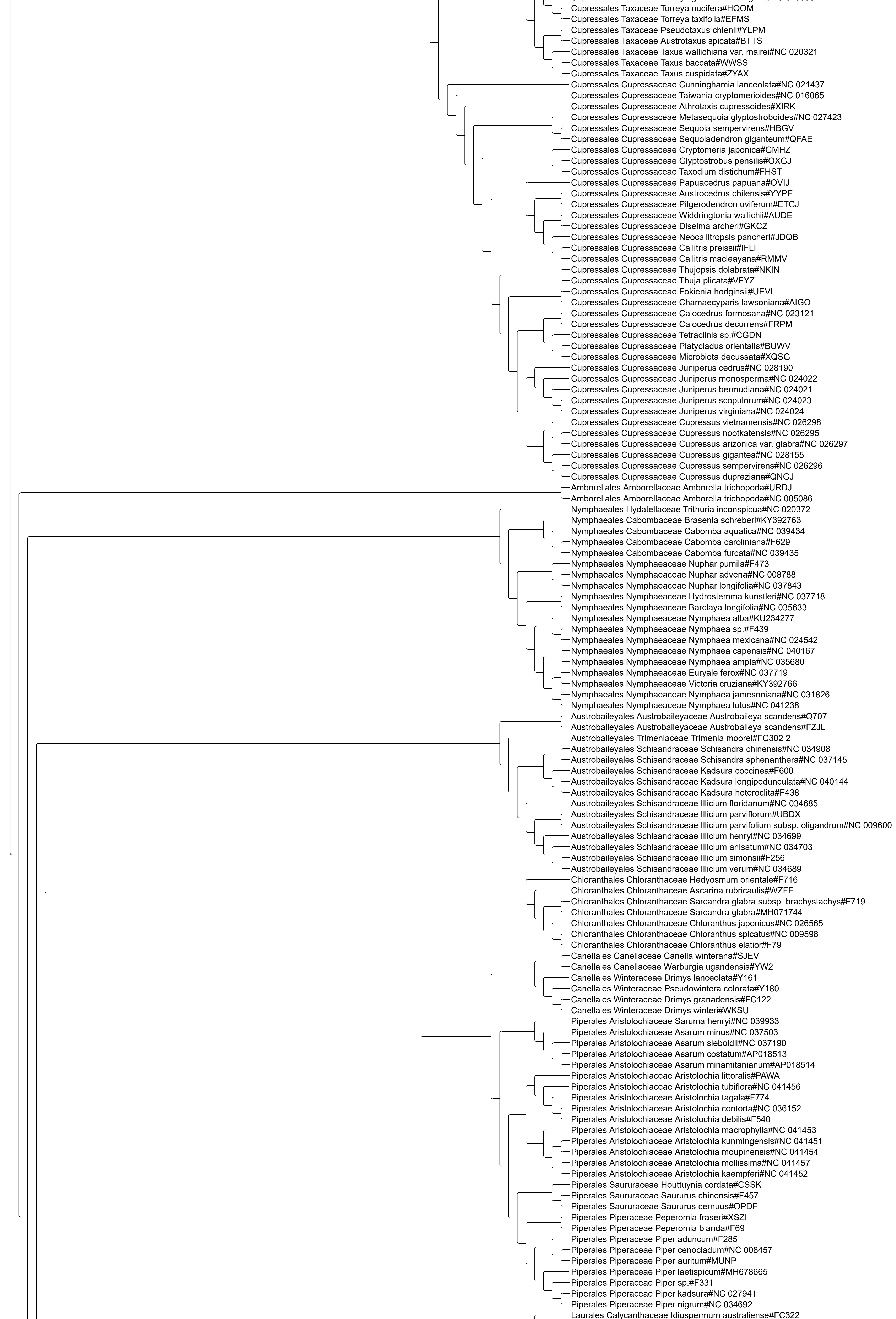
Cupressales Taxaceae Torreya nucifera#HQOM
Cupressales Taxaceae Torreya taxifolia#EFMS
Cupressales Taxaceae Pseudotaxus chienii#YLPM
Cupressales Taxaceae Austrotaxus spicata#BTTS
Cupressales Taxaceae Taxus wallichiana var. mairei#NC 020321
Cupressales Taxaceae Taxus baccata#WWSS
Cupressales Taxaceae Taxus cuspidata#ZYAX
Cupressales Cupressaceae Cunninghamia lanceolata#NC 021437
Cupressales Cupressaceae Taiwania cryptomerioides#NC 016065
Cupressales Cupressaceae Athrotaxis cupressoides#XIRK
Cupressales Cupressaceae Metasequoia glyptostroboides#NC 027423
Cupressales Cupressaceae Sequoia sempervirens#HBGV
Cupressales Cupressaceae Sequoiadendron giganteum#QFAE
Cupressales Cupressaceae Cryptomeria japonica#GMHZ
Cupressales Cupressaceae Glyptostrobus pensilis#OXGJ
Cupressales Cupressaceae Taxodium distichum#FHST
Cupressales Cupressaceae Papuacedrus papuana#OVIJ
Cupressales Cupressaceae Austrocedrus chilensis#YYPE
Cupressales Cupressaceae Pilgerodendron uviferum#ETCJ
Cupressales Cupressaceae Widdringtonia wallichii#AUDE
Cupressales Cupressaceae Diselma archeri#GKCZ
Cupressales Cupressaceae Neocallitropsis pancheri#JDQB
Cupressales Cupressaceae Callitris preissii#IFLI
Cupressales Cupressaceae Callitris macleayana#RMMV
Cupressales Cupressaceae Thujopsis dolabrata#NKIN
Cupressales Cupressaceae Thuja plicata#VFYZ
Cupressales Cupressaceae Fokienia hodginsii#UEVI
Cupressales Cupressaceae Chamaecyparis lawsoniana#AIGO
Cupressales Cupressaceae Calocedrus formosana#NC 023121
Cupressales Cupressaceae Calocedrus decurrens#FRPM
Cupressales Cupressaceae Tetraclinis sp.#CGDN
Cupressales Cupressaceae Platycladus orientalis#BUWV
Cupressales Cupressaceae Microbiota decussata#XQSG
Cupressales Cupressaceae Juniperus cedrus#NC 028190
Cupressales Cupressaceae Juniperus monosperma#NC 024022
Cupressales Cupressaceae Juniperus bermudiana#NC 024021
Cupressales Cupressaceae Juniperus scopulorum#NC 024023
Cupressales Cupressaceae Juniperus virginiana#NC 024024
Cupressales Cupressaceae Cupressus vietnamensis#NC 026298
Cupressales Cupressaceae Cupressus nootkatensis#NC 026295
Cupressales Cupressaceae Cupressus arizonica var. glabra#NC 026297
Cupressales Cupressaceae Cupressus gigantea#NC 028155
Cupressales Cupressaceae Cupressus sempervirens#NC 026296
Cupressales Cupressaceae Cupressus dupreziana#QNGJ
Amborellales Amborellaceae Amborella trichopoda#URDJ
Amborellales Amborellaceae Amborella trichopoda#NC 005086
Nymphaeales Hydatellaceae Trithuria inconspicua#NC 020372
Nymphaeales Cabombaceae Brasenia schreberi#KY392763
Nymphaeales Cabombaceae Cabomba aquatica#NC 039434
Nymphaeales Cabombaceae Cabomba caroliniana#F629
Nymphaeales Cabombaceae Cabomba furcata#NC 039435
Nymphaeales Nymphaeaceae Nuphar pumila#F473
Nymphaeales Nymphaeaceae Nuphar advena#NC 008788
Nymphaeales Nymphaeaceae Nuphar longifolia#NC 037843
Nymphaeales Nymphaeaceae Hydrostemma kunstleri#NC 037718
Nymphaeales Nymphaeaceae Barclaya longifolia#NC 035633
Nymphaeales Nymphaeaceae Nymphaea alba#KU234277
Nymphaeales Nymphaeaceae Nymphaea sp.#F439
Nymphaeales Nymphaeaceae Nymphaea mexicana#NC 024542
Nymphaeales Nymphaeaceae Nymphaea capensis#NC 040167
Nymphaeales Nymphaeaceae Nymphaea ampla#NC 035680
Nymphaeales Nymphaeaceae Euryale ferox#NC 037719
Nymphaeales Nymphaeaceae Victoria cruziana#KY392766
Nymphaeales Nymphaeaceae Nymphaea jamesoniana#NC 031826
Nymphaeales Nymphaeaceae Nymphaea lotus#NC 041238
Austrobaileyales Austrobaileyaceae Austrobaileya scandens#Q707
Austrobaileyales Austrobaileyaceae Austrobaileya scandens#FZJL
Austrobaileyales Trimeniaceae Trimenia moorei#FC302 2
Austrobaileyales Schisandraceae Schisandra chinensis#NC 034908
Austrobaileyales Schisandraceae Schisandra sphenanthera#NC 037145
Austrobaileyales Schisandraceae Kadsura coccinea#F600
Austrobaileyales Schisandraceae Kadsura longipedunculata#NC 040144
Austrobaileyales Schisandraceae Kadsura heteroclita#F438
Austrobaileyales Schisandraceae Illicium floridanum#NC 034685
Austrobaileyales Schisandraceae Illicium parviflorum#UBDX
Austrobaileyales Schisandraceae Illicium parvifolium subsp. oligandrum#NC 009600
Austrobaileyales Schisandraceae Illicium henryi#NC 034699
Austrobaileyales Schisandraceae Illicium anisatum#NC 034703
Austrobaileyales Schisandraceae Illicium simonsii#F256
Austrobaileyales Schisandraceae Illicium verum#NC 034689
Chloranthales Chloranthaceae Hedyosmum orientale#F716
Chloranthales Chloranthaceae Ascarina rubricaulis#WZFE
Chloranthales Chloranthaceae Sarcandra glabra subsp. brachystachys#F719
Chloranthales Chloranthaceae Sarcandra glabra#MH071744
Chloranthales Chloranthaceae Chloranthus japonicus#NC 026565
Chloranthales Chloranthaceae Chloranthus spicatus#NC 009598
Chloranthales Chloranthaceae Chloranthus elatior#F79
Canellales Canellaceae Canella winterana#SJEV
Canellales Canellaceae Warburgia ugandensis#YW2
Canellales Winteraceae Drimys lanceolata#Y161
Canellales Winteraceae Pseudowintera colorata#Y180
Canellales Winteraceae Drimys granadensis#FC122
Canellales Winteraceae Drimys winteri#WKSU
Piperales Aristolochiaceae Saruma henryi#NC 039933
Piperales Aristolochiaceae Asarum minus#NC 037503
Piperales Aristolochiaceae Asarum sieboldii#NC 037190
Piperales Aristolochiaceae Asarum costatum#AP018513
Piperales Aristolochiaceae Asarum minamitanianum#AP018514
Piperales Aristolochiaceae Aristolochia littoralis#PAWA
Piperales Aristolochiaceae Aristolochia tubiflora#NC 041456
Piperales Aristolochiaceae Aristolochia tagala#F774
Piperales Aristolochiaceae Aristolochia contorta#NC 036152
Piperales Aristolochiaceae Aristolochia debilis#F540
Piperales Aristolochiaceae Aristolochia macrophylla#NC 041453
Piperales Aristolochiaceae Aristolochia kunmingensis#NC 041451
Piperales Aristolochiaceae Aristolochia moupinensis#NC 041454
Piperales Aristolochiaceae Aristolochia mollissima#NC 041457
Piperales Aristolochiaceae Aristolochia kaempferi#NC 041452
Piperales Saururaceae Houttuynia cordata#CSSK
Piperales Saururaceae Saururus chinensis#F457
Piperales Saururaceae Saururus cernuus#OPDF
Piperales Piperaceae Peperomia fraseri#XSZI
Piperales Piperaceae Peperomia blanda#F69
Piperales Piperaceae Piper aduncum#F285
Piperales Piperaceae Piper cenocladum#NC 008457
Piperales Piperaceae Piper auritum#MUNP
Piperales Piperaceae Piper laetispicum#MH678665
Piperales Piperaceae Piper sp.#F331
Piperales Piperaceae Piper kadsura#NC 027941
Piperales Piperaceae Piper nigrum#NC 034692
Laurales Calycanthaceae Idiospermum australiense#FC322

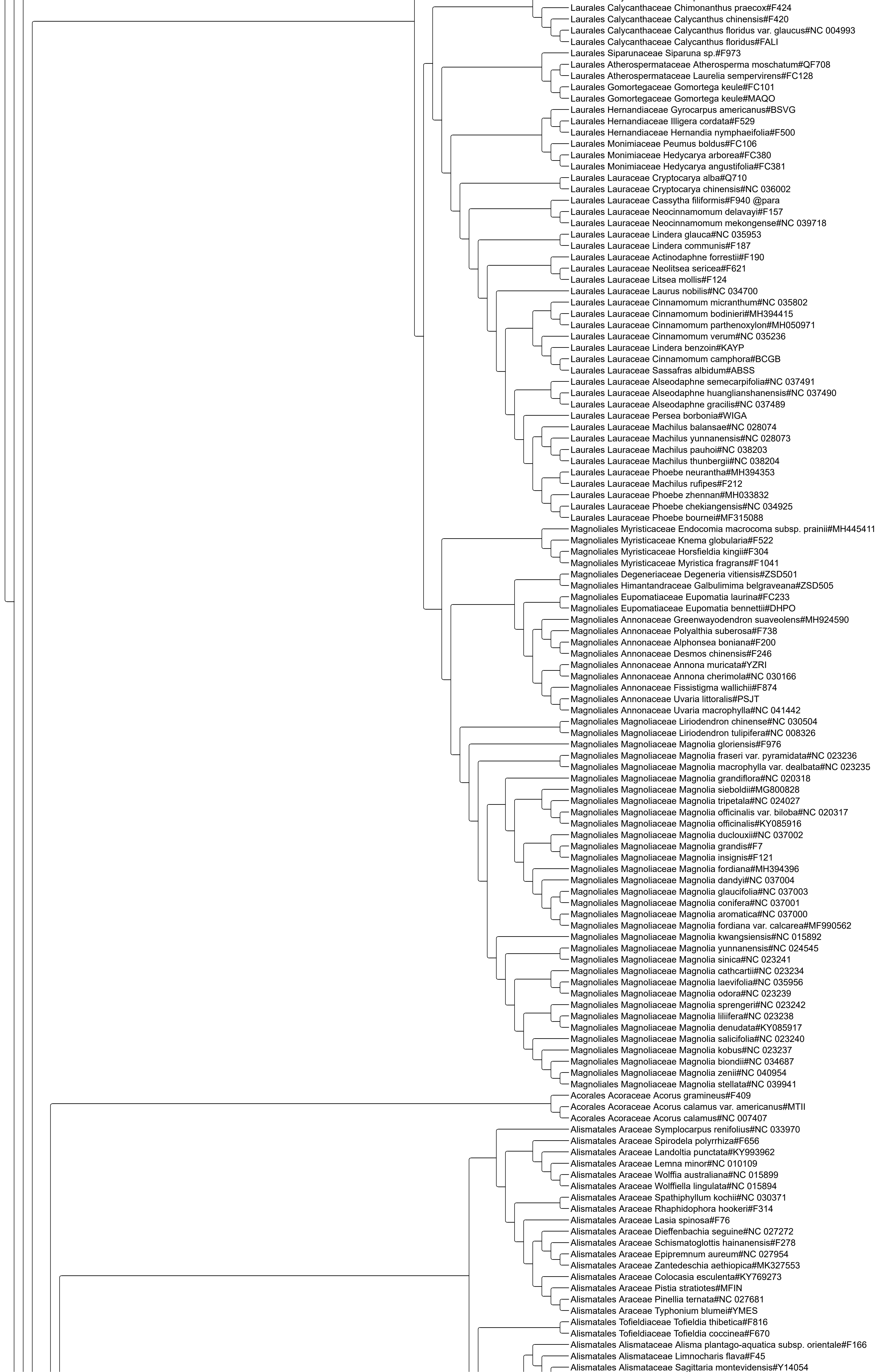
Laurales Calycanthaceae Chimonanthus praecox#F424
Laurales Calycanthaceae Calycanthus chinensis#F420
Laurales Calycanthaceae Calycanthus floridus var. glaucus#NC 004993
Laurales Calycanthaceae Calycanthus floridus#FALI
Laurales Siparunaceae Siparuna sp.#F973
Laurales Atherospermataceae Atherosperma moschatum#QF708
Laurales Atherospermataceae Laurelia sempervirens#FC128
Laurales Gomortegaceae Gomortega keule#FC101
Laurales Gomortegaceae Gomortega keule#MAQO
Laurales Hernandiaceae Gyrocarpus americanus#BSVG
Laurales Hernandiaceae Illigera cordata#F529
Laurales Hernandiaceae Hernandia nymphaeifolia#F500
Laurales Monimiaceae Peumus boldus#FC106
Laurales Monimiaceae Hedycarya arborea#FC380
Laurales Monimiaceae Hedycarya angustifolia#FC381
Laurales Lauraceae Cryptocarya alba#Q710
Laurales Lauraceae Cryptocarya chinensis#NC 036002
Laurales Lauraceae Cassytha filiformis#F940 @para
Laurales Lauraceae Neocinnamomum delavayi#F157
Laurales Lauraceae Neocinnamomum mekongense#NC 039718
Laurales Lauraceae Lindera glauca#NC 035953
Laurales Lauraceae Lindera communis#F187
Laurales Lauraceae Actinodaphne forrestii#F190
Laurales Lauraceae Neolitsea sericea#F621
Laurales Lauraceae Litsea mollis#F124
Laurales Lauraceae Laurus nobilis#NC 034700
Laurales Lauraceae Cinnamomum micranthum#NC 035802
Laurales Lauraceae Cinnamomum bodinieri#MH394415
Laurales Lauraceae Cinnamomum parthenoxylon#MH050971
Laurales Lauraceae Cinnamomum verum#NC 035236
Laurales Lauraceae Lindera benzoin#KAYP
Laurales Lauraceae Cinnamomum camphora#BCGB
Laurales Lauraceae Sassafras albidum#ABSS
Laurales Lauraceae Alseodaphne semecarpifolia#NC 037491
Laurales Lauraceae Alseodaphne huanglianshanensis#NC 037490
Laurales Lauraceae Alseodaphne gracilis#NC 037489
Laurales Lauraceae Persea borbonia#WIGA
Laurales Lauraceae Machilus balansae#NC 028074
Laurales Lauraceae Machilus yunnanensis#NC 028073
Laurales Lauraceae Machilus pauhoi#NC 038203
Laurales Lauraceae Machilus thunbergii#NC 038204
Laurales Lauraceae Phoebe neurantha#MH394353
Laurales Lauraceae Machilus rufipes#F212
Laurales Lauraceae Phoebe zhennan#MH033832
Laurales Lauraceae Phoebe chekiangensis#NC 034925
Laurales Lauraceae Phoebe bournei#MF315088
Magnoliales Myristicaceae Endocomia macrocoma subsp. prainii#MH445411
Magnoliales Myristicaceae Knema globularia#F522
Magnoliales Myristicaceae Horsfieldia kingii#F304
Magnoliales Myristicaceae Myristica fragrans#F1041
Magnoliales Degeneriaceae Degeneria vitiensis#ZSD501
Magnoliales Himantandraceae Galbulimima belgraveana#ZSD505
Magnoliales Eupomatiaceae Eupomatia laurina#FC233
Magnoliales Eupomatiaceae Eupomatia bennettii#DHPO
Magnoliales Annonaceae Greenwayodendron suaveolens#MH924590
Magnoliales Annonaceae Polyalthia suberosa#F738
Magnoliales Annonaceae Alphonsea boniana#F200
Magnoliales Annonaceae Desmos chinensis#F246
Magnoliales Annonaceae Annona muricata#YZRI
Magnoliales Annonaceae Annona cherimola#NC 030166
Magnoliales Annonaceae Fissistigma wallichii#F874
Magnoliales Annonaceae Uvaria littoralis#PSJT
Magnoliales Annonaceae Uvaria macrophylla#NC 041442
Magnoliales Magnoliaceae Liriodendron chinense#NC 030504
Magnoliales Magnoliaceae Liriodendron tulipifera#NC 008326
Magnoliales Magnoliaceae Magnolia gloriensis#F976
Magnoliales Magnoliaceae Magnolia fraseri var. pyramidata#NC 023236
Magnoliales Magnoliaceae Magnolia macrophylla var. dealbata#NC 023235
Magnoliales Magnoliaceae Magnolia grandiflora#NC 020318
Magnoliales Magnoliaceae Magnolia sieboldii#MG800828
Magnoliales Magnoliaceae Magnolia tripetala#NC 024027
Magnoliales Magnoliaceae Magnolia officinalis var. biloba#NC 020317
Magnoliales Magnoliaceae Magnolia officinalis#KY085916
Magnoliales Magnoliaceae Magnolia duclouxii#NC 037002
Magnoliales Magnoliaceae Magnolia grandis#F7
Magnoliales Magnoliaceae Magnolia insignis#F121
Magnoliales Magnoliaceae Magnolia fordiana#MH394396
Magnoliales Magnoliaceae Magnolia dandyi#NC 037004
Magnoliales Magnoliaceae Magnolia glaucifolia#NC 037003
Magnoliales Magnoliaceae Magnolia conifera#NC 037001
Magnoliales Magnoliaceae Magnolia aromatica#NC 037000
Magnoliales Magnoliaceae Magnolia fordiana var. calcarea#MF990562
Magnoliales Magnoliaceae Magnolia kwangsiensis#NC 015892
Magnoliales Magnoliaceae Magnolia yunnanensis#NC 024545
Magnoliales Magnoliaceae Magnolia sinica#NC 023241
Magnoliales Magnoliaceae Magnolia cathcartii#NC 023234
Magnoliales Magnoliaceae Magnolia laevifolia#NC 035956
Magnoliales Magnoliaceae Magnolia odora#NC 023239
Magnoliales Magnoliaceae Magnolia sprengeri#NC 023242
Magnoliales Magnoliaceae Magnolia liliifera#NC 023238
Magnoliales Magnoliaceae Magnolia denudata#KY085917
Magnoliales Magnoliaceae Magnolia salicifolia#NC 023240
Magnoliales Magnoliaceae Magnolia kobus#NC 023237
Magnoliales Magnoliaceae Magnolia biondii#NC 034687
Magnoliales Magnoliaceae Magnolia zenii#NC 040954
Magnoliales Magnoliaceae Magnolia stellata#NC 039941
Acorales Acoraceae Acorus gramineus#F409
Acorales Acoraceae Acorus calamus var. americanus#MTII
Acorales Acoraceae Acorus calamus#NC 007407
Alismatales Araceae Symplocarpus renifolius#NC 033970
Alismatales Araceae Spirodela polyrrhiza#F656
Alismatales Araceae Landoltia punctata#KY993962
Alismatales Araceae Lemna minor#NC 010109
Alismatales Araceae Wolffia australiana#NC 015899
Alismatales Araceae Wolffiella lingulata#NC 015894
Alismatales Araceae Spathiphyllum kochii#NC 030371
Alismatales Araceae Rhaphidophora hookeri#F314
Alismatales Araceae Lasia spinosa#F76
Alismatales Araceae Dieffenbachia seguine#NC 027272
Alismatales Araceae Schismatoglottis hainanensis#F278
Alismatales Araceae Epipremnum aureum#NC 027954
Alismatales Araceae Zantedeschia aethiopica#MK327553
Alismatales Araceae Colocasia esculenta#KY769273
Alismatales Araceae Pistia stratiotes#MFIN
Alismatales Araceae Pinellia ternata#NC 027681
Alismatales Araceae Typhonium blumei#YMES
Alismatales Tofieldiaceae Tofieldia thibetica#F816
Alismatales Tofieldiaceae Tofieldia coccinea#F670
Alismatales Alismataceae Alisma plantago-aquatica subsp. orientale#F166
Alismatales Alismataceae Limnocharis flava#F45
Alismatales Alismataceae Sagittaria montevidensis#Y14054

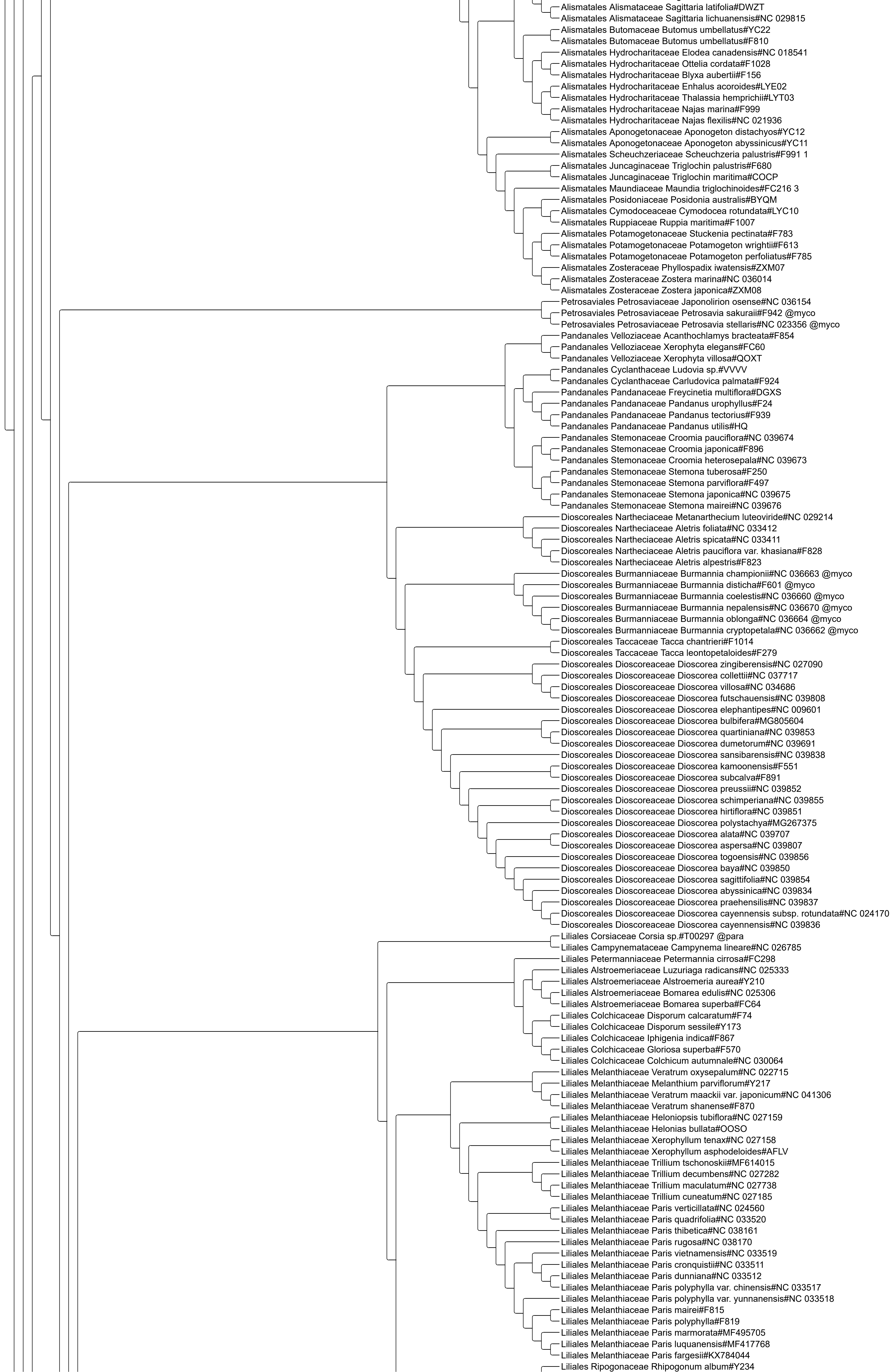

Alismatales Alismataceae Sagittaria latifolia#DWZT
Alismatales Alismataceae Sagittaria lichuanensis#NC 029815
Alismatales Butomaceae Butomus umbellatus#YC22
Alismatales Butomaceae Butomus umbellatus#F810
Alismatales Hydrocharitaceae Elodea canadensis#NC 018541
Alismatales Hydrocharitaceae Ottelia cordata#F1028
Alismatales Hydrocharitaceae Blyxa aubertii#F156
Alismatales Hydrocharitaceae Enhalus acoroides#LYE02
Alismatales Hydrocharitaceae Thalassia hemprichii#LYT03
Alismatales Hydrocharitaceae Najas marina#F999
Alismatales Hydrocharitaceae Najas flexilis#NC 021936
Alismatales Aponogetonaceae Aponogeton distachyos#YC12
Alismatales Aponogetonaceae Aponogeton abyssinicus#YC11
Alismatales Scheuchzeriaceae Scheuchzeria palustris#F991 1
Alismatales Juncaginaceae Triglochin palustris#F680
Alismatales Juncaginaceae Triglochin maritima#COCP
Alismatales Maundiaceae Maundia triglochinoides#FC216 3
Alismatales Posidoniaceae Posidonia australis#BYQM
Alismatales Cymodoceaceae Cymodocea rotundata#LYC10
Alismatales Ruppiaceae Ruppia maritima#F1007
Alismatales Potamogetonaceae Stuckenia pectinata#F783
Alismatales Potamogetonaceae Potamogeton wrightii#F613
Alismatales Potamogetonaceae Potamogeton perfoliatus#F785
Alismatales Zosteraceae Phyllospadix iwatensis#ZXM07
Alismatales Zosteraceae Zostera marina#NC 036014
Alismatales Zosteraceae Zostera japonica#ZXM08
Petrosaviales Petrosaviaceae Japonolirion osense#NC 036154
Petrosaviales Petrosaviaceae Petrosavia sakuraii#F942 @myco
Petrosaviales Petrosaviaceae Petrosavia stellaris#NC 023356 @myco
Pandanales Velloziaceae Acanthochlamys bracteata#F854
Pandanales Velloziaceae Xerophyta elegans#FC60
Pandanales Velloziaceae Xerophyta villosa#QOXT
Pandanales Cyclanthaceae Ludovia sp.#VVVV
Pandanales Cyclanthaceae Carludovica palmata#F924
Pandanales Pandanaceae Freycinetia multiflora#DGXS
Pandanales Pandanaceae Pandanus urophyllus#F24
Pandanales Pandanaceae Pandanus tectorius#F939
Pandanales Pandanaceae Pandanus utilis#HQ
Pandanales Stemonaceae Croomia pauciflora#NC 039674
Pandanales Stemonaceae Croomia japonica#F896
Pandanales Stemonaceae Croomia heterosepala#NC 039673
Pandanales Stemonaceae Stemona tuberosa#F250
Pandanales Stemonaceae Stemona parviflora#F497
Pandanales Stemonaceae Stemona japonica#NC 039675
Pandanales Stemonaceae Stemona mairei#NC 039676
Dioscoreales Nartheciaceae Metanarthecium luteoviride#NC 029214
Dioscoreales Nartheciaceae Aletris foliata#NC 033412
Dioscoreales Nartheciaceae Aletris spicata#NC 033411
Dioscoreales Nartheciaceae Aletris pauciflora var. khasiana#F828
Dioscoreales Nartheciaceae Aletris alpestris#F823
Dioscoreales Burmanniaceae Burmannia championii#NC 036663 @myco
Dioscoreales Burmanniaceae Burmannia disticha#F601 @myco
Dioscoreales Burmanniaceae Burmannia coelestis#NC 036660 @myco
Dioscoreales Burmanniaceae Burmannia nepalensis#NC 036670 @myco
Dioscoreales Burmanniaceae Burmannia oblonga#NC 036664 @myco
Dioscoreales Burmanniaceae Burmannia cryptopetala#NC 036662 @myco
Dioscoreales Taccaceae Tacca chantrieri#F1014
Dioscoreales Taccaceae Tacca leontopetaloides#F279
Dioscoreales Dioscoreaceae Dioscorea zingiberensis#NC 027090
Dioscoreales Dioscoreaceae Dioscorea collettii#NC 037717
Dioscoreales Dioscoreaceae Dioscorea villosa#NC 034686
Dioscoreales Dioscoreaceae Dioscorea futschauensis#NC 039808
Dioscoreales Dioscoreaceae Dioscorea elephantipes#NC 009601
Dioscoreales Dioscoreaceae Dioscorea bulbifera#MG805604
Dioscoreales Dioscoreaceae Dioscorea quartiniana#NC 039853
Dioscoreales Dioscoreaceae Dioscorea dumetorum#NC 039691
Dioscoreales Dioscoreaceae Dioscorea sansibarensis#NC 039838
Dioscoreales Dioscoreaceae Dioscorea kamoonensis#F551
Dioscoreales Dioscoreaceae Dioscorea subcalva#F891
Dioscoreales Dioscoreaceae Dioscorea preussii#NC 039852
Dioscoreales Dioscoreaceae Dioscorea schimperiana#NC 039855
Dioscoreales Dioscoreaceae Dioscorea hirtiflora#NC 039851
Dioscoreales Dioscoreaceae Dioscorea polystachya#MG267375
Dioscoreales Dioscoreaceae Dioscorea alata#NC 039707
Dioscoreales Dioscoreaceae Dioscorea aspersa#NC 039807
Dioscoreales Dioscoreaceae Dioscorea togoensis#NC 039856
Dioscoreales Dioscoreaceae Dioscorea baya#NC 039850
Dioscoreales Dioscoreaceae Dioscorea sagittifolia#NC 039854
Dioscoreales Dioscoreaceae Dioscorea abyssinica#NC 039834
Dioscoreales Dioscoreaceae Dioscorea praehensilis#NC 039837
Dioscoreales Dioscoreaceae Dioscorea cayennensis subsp. rotundata#NC 024170
Dioscoreales Dioscoreaceae Dioscorea cayennensis#NC 039836
Liliales Corsiaceae Corsia sp.#T00297 @para
Liliales Campynemataceae Campynema lineare#NC 026785
Liliales Petermanniaceae Petermannia cirrosa#FC298
Liliales Alstroemeriaceae Luzuriaga radicans#NC 025333
Liliales Alstroemeriaceae Alstroemeria aurea#Y210
Liliales Alstroemeriaceae Bomarea edulis#NC 025306
Liliales Alstroemeriaceae Bomarea superba#FC64
Liliales Colchicaceae Disporum calcaratum#F74
Liliales Colchicaceae Disporum sessile#Y173
Liliales Colchicaceae Iphigenia indica#F867
Liliales Colchicaceae Gloriosa superba#F570
Liliales Colchicaceae Colchicum autumnale#NC 030064
Liliales Melanthiaceae Veratrum oxysepalum#NC 022715
Liliales Melanthiaceae Melanthium parviflorum#Y217
Liliales Melanthiaceae Veratrum maackii var. japonicum#NC 041306
Liliales Melanthiaceae Veratrum shanense#F870
Liliales Melanthiaceae Heloniopsis tubiflora#NC 027159
Liliales Melanthiaceae Helonias bullata#OOSO
Liliales Melanthiaceae Xerophyllum tenax#NC 027158
Liliales Melanthiaceae Xerophyllum asphodeloides#AFLV
Liliales Melanthiaceae Trillium tschonoskii#MF614015
Liliales Melanthiaceae Trillium decumbens#NC 027282
Liliales Melanthiaceae Trillium maculatum#NC 027738
Liliales Melanthiaceae Trillium cuneatum#NC 027185
Liliales Melanthiaceae Paris verticillata#NC 024560
Liliales Melanthiaceae Paris quadrifolia#NC 033520
Liliales Melanthiaceae Paris thibetica#NC 038161
Liliales Melanthiaceae Paris rugosa#NC 038170
Liliales Melanthiaceae Paris vietnamensis#NC 033519
Liliales Melanthiaceae Paris cronquistii#NC 033511
Liliales Melanthiaceae Paris dunniana#NC 033512
Liliales Melanthiaceae Paris polyphylla var. chinensis#NC 033517
Liliales Melanthiaceae Paris polyphylla var. yunnanensis#NC 033518
Liliales Melanthiaceae Paris mairei#F815
Liliales Melanthiaceae Paris polyphylla#F819
Liliales Melanthiaceae Paris marmorata#MF495705
Liliales Melanthiaceae Paris luquanensis#MF417768
Liliales Melanthiaceae Paris fargesii#KX784044
Liliales Ripogonaceae Rhipogonum album#Y234

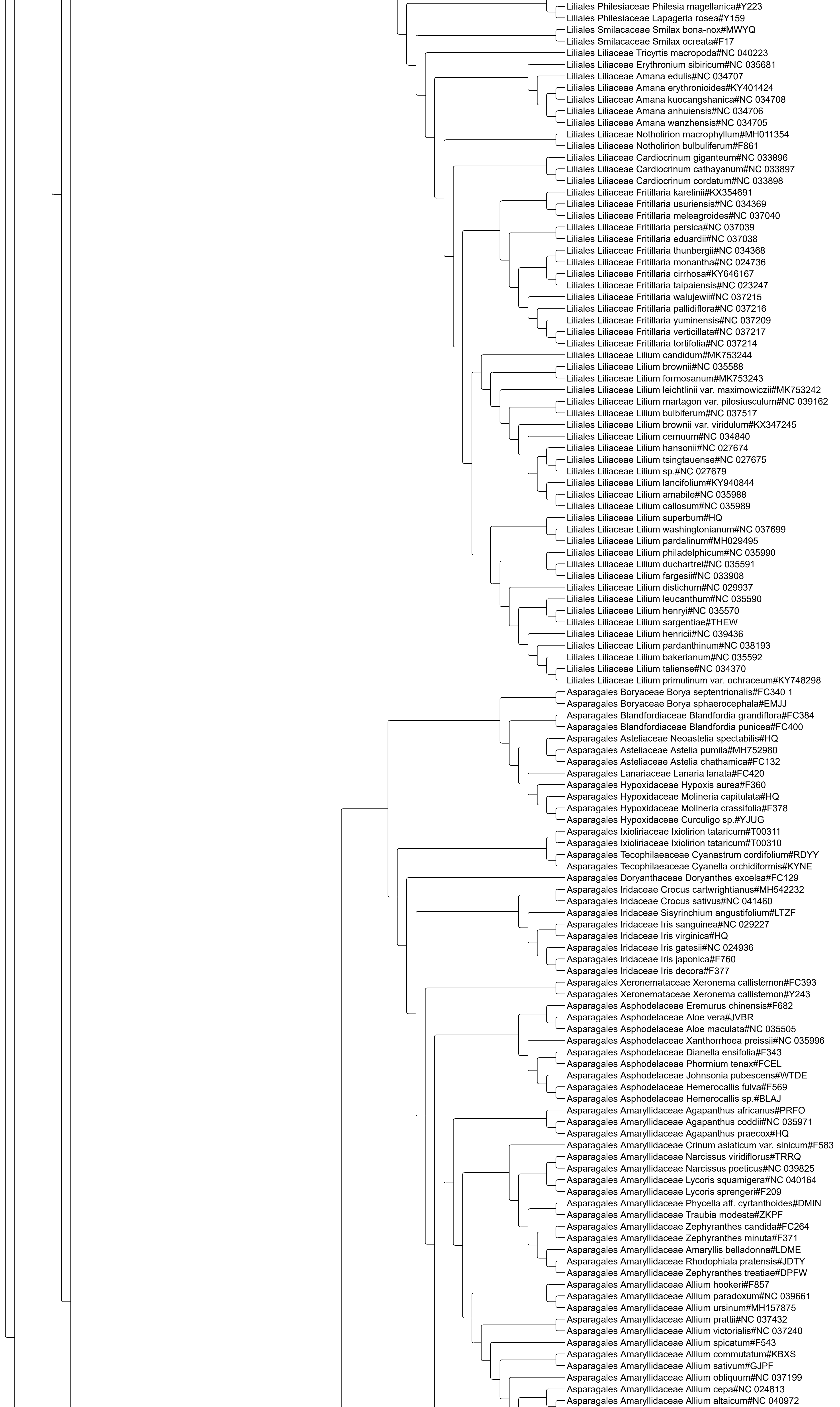
Liliales Philesiaceae Philesia magellanica#Y223
Liliales Philesiaceae Lapageria rosea#Y159
Liliales Smilacaceae Smilax bona-nox#MWYQ
Liliales Smilacaceae Smilax ocreata#F17
Liliales Liliaceae Tricyrtis macropoda#NC 040223
Liliales Liliaceae Erythronium sibiricum#NC 035681
Liliales Liliaceae Amana edulis#NC 034707
Liliales Liliaceae Amana erythronioides#KY401424
Liliales Liliaceae Amana kuocangshanica#NC 034708
Liliales Liliaceae Amana anhuiensis#NC 034706
Liliales Liliaceae Amana wanzhensis#NC 034705
Liliales Liliaceae Notholirion macrophyllum#MH011354
Liliales Liliaceae Notholirion bulbuliferum#F861
Liliales Liliaceae Cardiocrinum giganteum#NC 033896
Liliales Liliaceae Cardiocrinum cathayanum#NC 033897
Liliales Liliaceae Cardiocrinum cordatum#NC 033898
Liliales Liliaceae Fritillaria karelinii#KX354691
Liliales Liliaceae Fritillaria usuriensis#NC 034369
Liliales Liliaceae Fritillaria meleagroides#NC 037040
Liliales Liliaceae Fritillaria persica#NC 037039
Liliales Liliaceae Fritillaria eduardii#NC 037038
Liliales Liliaceae Fritillaria thunbergii#NC 034368
Liliales Liliaceae Fritillaria monantha#NC 024736
Liliales Liliaceae Fritillaria cirrhosa#KY646167
Liliales Liliaceae Fritillaria taipaiensis#NC 023247
Liliales Liliaceae Fritillaria walujewii#NC 037215
Liliales Liliaceae Fritillaria pallidiflora#NC 037216
Liliales Liliaceae Fritillaria yuminensis#NC 037209
Liliales Liliaceae Fritillaria verticillata#NC 037217
Liliales Liliaceae Fritillaria tortifolia#NC 037214
Liliales Liliaceae Lilium candidum#MK753244
Liliales Liliaceae Lilium brownii#NC 035588
Liliales Liliaceae Lilium formosanum#MK753243
Liliales Liliaceae Lilium leichtlinii var. maximowiczii#MK753242
Liliales Liliaceae Lilium martagon var. pilosiusculum#NC 039162
Liliales Liliaceae Lilium bulbiferum#NC 037517
Liliales Liliaceae Lilium brownii var. viridulum#KX347245
Liliales Liliaceae Lilium cernuum#NC 034840
Liliales Liliaceae Lilium hansonii#NC 027674
Liliales Liliaceae Lilium tsingtauense#NC 027675
Liliales Liliaceae Lilium sp.#NC 027679
Liliales Liliaceae Lilium lancifolium#KY940844
Liliales Liliaceae Lilium amabile#NC 035988
Liliales Liliaceae Lilium callosum#NC 035989
Liliales Liliaceae Lilium superbum#HQ
Liliales Liliaceae Lilium washingtonianum#NC 037699
Liliales Liliaceae Lilium pardalinum#MH029495
Liliales Liliaceae Lilium philadelphicum#NC 035990
Liliales Liliaceae Lilium duchartrei#NC 035591
Liliales Liliaceae Lilium fargesii#NC 033908
Liliales Liliaceae Lilium distichum#NC 029937
Liliales Liliaceae Lilium leucanthum#NC 035590
Liliales Liliaceae Lilium henryi#NC 035570
Liliales Liliaceae Lilium sargentiae#THEW
Liliales Liliaceae Lilium henricii#NC 039436
Liliales Liliaceae Lilium pardanthinum#NC 038193
Liliales Liliaceae Lilium bakerianum#NC 035592
Liliales Liliaceae Lilium taliense#NC 034370
Liliales Liliaceae Lilium primulinum var. ochraceum#KY748298
Asparagales Boryaceae Borya septentrionalis#FC340 1
Asparagales Boryaceae Borya sphaerocephala#EMJJ
Asparagales Blandfordiaceae Blandfordia grandiflora#FC384
Asparagales Blandfordiaceae Blandfordia punicea#FC400
Asparagales Asteliaceae Neoastelia spectabilis#HQ
Asparagales Asteliaceae Astelia pumila#MH752980
Asparagales Asteliaceae Astelia chathamica#FC132
Asparagales Lanariaceae Lanaria lanata#FC420
Asparagales Hypoxidaceae Hypoxis aurea#F360
Asparagales Hypoxidaceae Molineria capitulata#HQ
Asparagales Hypoxidaceae Molineria crassifolia#F378
Asparagales Hypoxidaceae Curculigo sp.#YJUG
Asparagales Ixioliriaceae Ixiolirion tataricum#T00311
Asparagales Ixioliriaceae Ixiolirion tataricum#T00310
Asparagales Tecophilaeaceae Cyanastrum cordifolium#RDYY
Asparagales Tecophilaeaceae Cyanella orchidiformis#KYNE
Asparagales Doryanthaceae Doryanthes excelsa#FC129
Asparagales Iridaceae Crocus cartwrightianus#MH542232
Asparagales Iridaceae Crocus sativus#NC 041460
Asparagales Iridaceae Sisyrinchium angustifolium#LTZF
Asparagales Iridaceae Iris sanguinea#NC 029227
Asparagales Iridaceae Iris virginica#HQ
Asparagales Iridaceae Iris gatesii#NC 024936
Asparagales Iridaceae Iris japonica#F760
Asparagales Iridaceae Iris decora#F377
Asparagales Xeronemataceae Xeronema callistemon#FC393
Asparagales Xeronemataceae Xeronema callistemon#Y243
Asparagales Asphodelaceae Eremurus chinensis#F682
Asparagales Asphodelaceae Aloe vera#JVBR
Asparagales Asphodelaceae Aloe maculata#NC 035505
Asparagales Asphodelaceae Xanthorrhoea preissii#NC 035996
Asparagales Asphodelaceae Dianella ensifolia#F343
Asparagales Asphodelaceae Phormium tenax#FCEL
Asparagales Asphodelaceae Johnsonia pubescens#WTDE
Asparagales Asphodelaceae Hemerocallis fulva#F569
Asparagales Asphodelaceae Hemerocallis sp.#BLAJ
Asparagales Amaryllidaceae Agapanthus africanus#PRFO
Asparagales Amaryllidaceae Agapanthus coddii#NC 035971
Asparagales Amaryllidaceae Agapanthus praecox#HQ
Asparagales Amaryllidaceae Crinum asiaticum var. sinicum#F583
Asparagales Amaryllidaceae Narcissus viridiflorus#TRRQ
Asparagales Amaryllidaceae Narcissus poeticus#NC 039825
Asparagales Amaryllidaceae Lycoris squamigera#NC 040164
Asparagales Amaryllidaceae Lycoris sprengeri#F209
Asparagales Amaryllidaceae Phycella aff. cyrtanthoides#DMIN
Asparagales Amaryllidaceae Traubia modesta#ZKPF
Asparagales Amaryllidaceae Zephyranthes candida#FC264
Asparagales Amaryllidaceae Zephyranthes minuta#F371
Asparagales Amaryllidaceae Amaryllis belladonna#LDME
Asparagales Amaryllidaceae Rhodophiala pratensis#JDTY
Asparagales Amaryllidaceae Zephyranthes treatiae#DPFW
Asparagales Amaryllidaceae Allium hookeri#F857
Asparagales Amaryllidaceae Allium paradoxum#NC 039661
Asparagales Amaryllidaceae Allium ursinum#MH157875
Asparagales Amaryllidaceae Allium prattii#NC 037432
Asparagales Amaryllidaceae Allium victorialis#NC 037240
Asparagales Amaryllidaceae Allium spicatum#F543
Asparagales Amaryllidaceae Allium commutatum#KBXS
Asparagales Amaryllidaceae Allium sativum#GJPF
Asparagales Amaryllidaceae Allium obliquum#NC 037199
Asparagales Amaryllidaceae Allium cepa#NC 024813
Asparagales Amaryllidaceae Allium altaicum#NC 040972

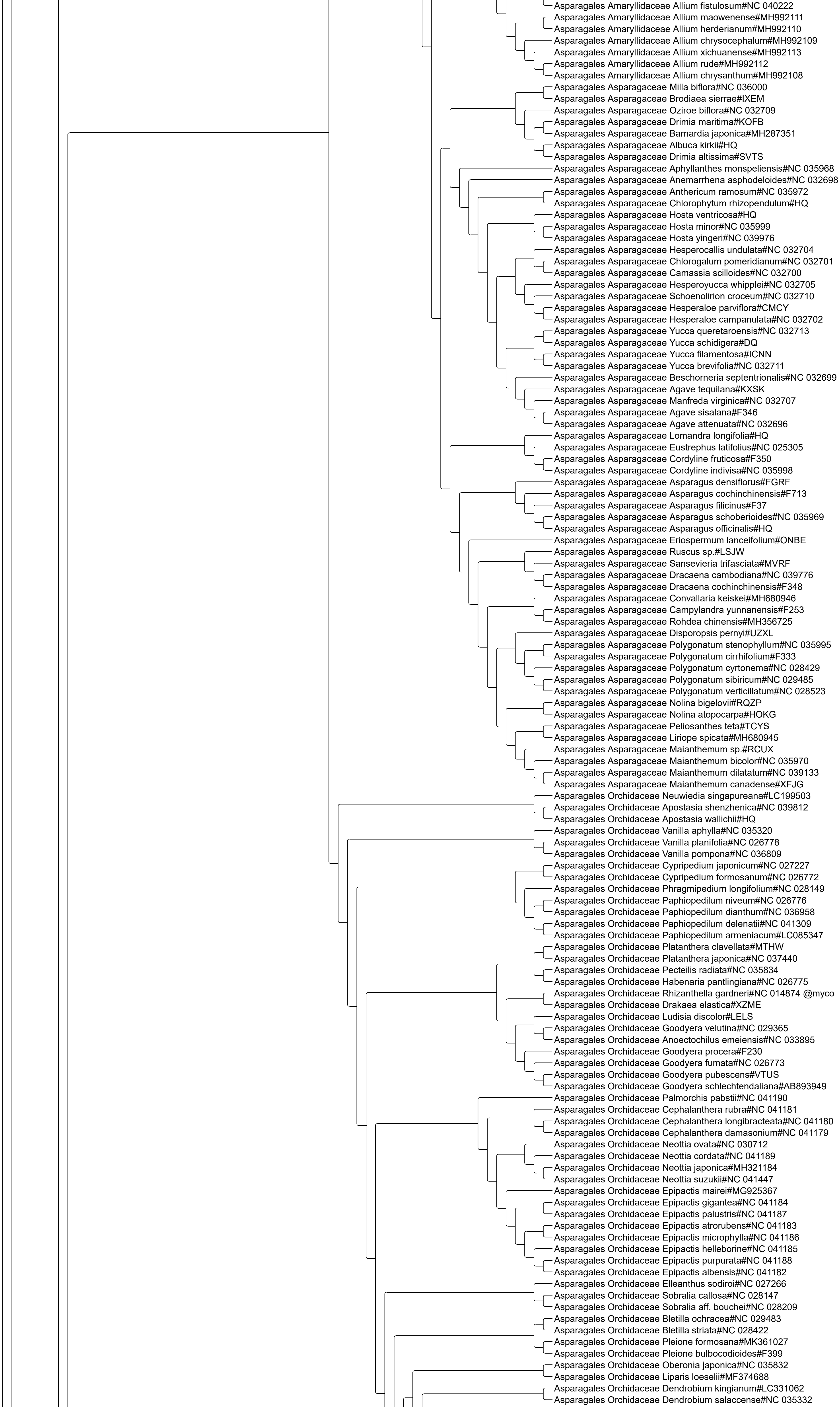

Asparagales Amaryllidaceae Allium fistulosum#NC 040222
Asparagales Amaryllidaceae Allium maowenense#MH992111
Asparagales Amaryllidaceae Allium herderianum#MH992110
Asparagales Amaryllidaceae Allium chrysocephalum#MH992109
Asparagales Amaryllidaceae Allium xichuanense#MH992113
Asparagales Amaryllidaceae Allium rude#MH992112
Asparagales Amaryllidaceae Allium chrysanthum#MH992108
Asparagales Asparagaceae Milla biflora#NC 036000
Asparagales Asparagaceae Brodiaea sierrae#IXEM
Asparagales Asparagaceae Oziroe biflora#NC 032709
Asparagales Asparagaceae Drimia maritima#KOFB
Asparagales Asparagaceae Barnardia japonica#MH287351
Asparagales Asparagaceae Albuca kirkii#HQ
Asparagales Asparagaceae Drimia altissima#SVTS
Asparagales Asparagaceae Aphyllanthes monspeliensis#NC 035968
Asparagales Asparagaceae Anemarrhena asphodeloides#NC 032698
Asparagales Asparagaceae Anthericum ramosum#NC 035972
Asparagales Asparagaceae Chlorophytum rhizopendulum#HQ
Asparagales Asparagaceae Hosta ventricosa#HQ
Asparagales Asparagaceae Hosta minor#NC 035999
Asparagales Asparagaceae Hosta yingeri#NC 039976
Asparagales Asparagaceae Hesperocallis undulata#NC 032704
Asparagales Asparagaceae Chlorogalum pomeridianum#NC 032701
Asparagales Asparagaceae Camassia scilloides#NC 032700
Asparagales Asparagaceae Hesperoyucca whipplei#NC 032705
Asparagales Asparagaceae Schoenolirion croceum#NC 032710
Asparagales Asparagaceae Hesperaloe parviflora#CMCY
Asparagales Asparagaceae Hesperaloe campanulata#NC 032702
Asparagales Asparagaceae Yucca queretaroensis#NC 032713
Asparagales Asparagaceae Yucca schidigera#DQ
Asparagales Asparagaceae Yucca filamentosa#ICNN
Asparagales Asparagaceae Yucca brevifolia#NC 032711
Asparagales Asparagaceae Beschorneria septentrionalis#NC 032699
Asparagales Asparagaceae Agave tequilana#KXSK
Asparagales Asparagaceae Manfreda virginica#NC 032707
Asparagales Asparagaceae Agave sisalana#F346
Asparagales Asparagaceae Agave attenuata#NC 032696
Asparagales Asparagaceae Lomandra longifolia#HQ
Asparagales Asparagaceae Eustrephus latifolius#NC 025305
Asparagales Asparagaceae Cordyline fruticosa#F350
Asparagales Asparagaceae Cordyline indivisa#NC 035998
Asparagales Asparagaceae Asparagus densiflorus#FGRF
Asparagales Asparagaceae Asparagus cochinchinensis#F713
Asparagales Asparagaceae Asparagus filicinus#F37
Asparagales Asparagaceae Asparagus schoberioides#NC 035969
Asparagales Asparagaceae Asparagus officinalis#HQ
Asparagales Asparagaceae Eriospermum lanceifolium#ONBE
Asparagales Asparagaceae Ruscus sp.#LSJW
Asparagales Asparagaceae Sansevieria trifasciata#MVRF
Asparagales Asparagaceae Dracaena cambodiana#NC 039776
Asparagales Asparagaceae Dracaena cochinchinensis#F348
Asparagales Asparagaceae Convallaria keiskei#MH680946
Asparagales Asparagaceae Campylandra yunnanensis#F253
Asparagales Asparagaceae Rohdea chinensis#MH356725
Asparagales Asparagaceae Disporopsis pernyi#UZXL
Asparagales Asparagaceae Polygonatum stenophyllum#NC 035995
Asparagales Asparagaceae Polygonatum cirrhifolium#F333
Asparagales Asparagaceae Polygonatum cyrtonema#NC 028429
Asparagales Asparagaceae Polygonatum sibiricum#NC 029485
Asparagales Asparagaceae Polygonatum verticillatum#NC 028523
Asparagales Asparagaceae Nolina bigelovii#RQZP
Asparagales Asparagaceae Nolina atopocarpa#HOKG
Asparagales Asparagaceae Peliosanthes teta#TCYS
Asparagales Asparagaceae Liriope spicata#MH680945
Asparagales Asparagaceae Maianthemum sp.#RCUX
Asparagales Asparagaceae Maianthemum bicolor#NC 035970
Asparagales Asparagaceae Maianthemum dilatatum#NC 039133
Asparagales Asparagaceae Maianthemum canadense#XFJG
Asparagales Orchidaceae Neuwiedia singapureana#LC199503
Asparagales Orchidaceae Apostasia shenzhenica#NC 039812
Asparagales Orchidaceae Apostasia wallichii#HQ
Asparagales Orchidaceae Vanilla aphylla#NC 035320
Asparagales Orchidaceae Vanilla planifolia#NC 026778
Asparagales Orchidaceae Vanilla pompona#NC 036809
Asparagales Orchidaceae Cypripedium japonicum#NC 027227
Asparagales Orchidaceae Cypripedium formosanum#NC 026772
Asparagales Orchidaceae Phragmipedium longifolium#NC 028149
Asparagales Orchidaceae Paphiopedilum niveum#NC 026776
Asparagales Orchidaceae Paphiopedilum dianthum#NC 036958
Asparagales Orchidaceae Paphiopedilum delenatii#NC 041309
Asparagales Orchidaceae Paphiopedilum armeniacum#LC085347
Asparagales Orchidaceae Platanthera clavellata#MTHW
Asparagales Orchidaceae Platanthera japonica#NC 037440
Asparagales Orchidaceae Pecteilis radiata#NC 035834
Asparagales Orchidaceae Habenaria pantlingiana#NC 026775
Asparagales Orchidaceae Rhizanthella gardneri#NC 014874 @myco
Asparagales Orchidaceae Drakaea elastica#XZME
Asparagales Orchidaceae Ludisia discolor#LELS
Asparagales Orchidaceae Goodyera velutina#NC 029365
Asparagales Orchidaceae Anoectochilus emeiensis#NC 033895
Asparagales Orchidaceae Goodyera procera#F230
Asparagales Orchidaceae Goodyera fumata#NC 026773
Asparagales Orchidaceae Goodyera pubescens#VTUS
Asparagales Orchidaceae Goodyera schlechtendaliana#AB893949
Asparagales Orchidaceae Palmorchis pabstii#NC 041190
Asparagales Orchidaceae Cephalanthera rubra#NC 041181
Asparagales Orchidaceae Cephalanthera longibracteata#NC 041180
Asparagales Orchidaceae Cephalanthera damasonium#NC 041179
Asparagales Orchidaceae Neottia ovata#NC 030712
Asparagales Orchidaceae Neottia cordata#NC 041189
Asparagales Orchidaceae Neottia japonica#MH321184
Asparagales Orchidaceae Neottia suzukii#NC 041447
Asparagales Orchidaceae Epipactis mairei#MG925367
Asparagales Orchidaceae Epipactis gigantea#NC 041184
Asparagales Orchidaceae Epipactis palustris#NC 041187
Asparagales Orchidaceae Epipactis atrorubens#NC 041183
Asparagales Orchidaceae Epipactis microphylla#NC 041186
Asparagales Orchidaceae Epipactis helleborine#NC 041185
Asparagales Orchidaceae Epipactis purpurata#NC 041188
Asparagales Orchidaceae Epipactis albensis#NC 041182
Asparagales Orchidaceae Elleanthus sodiroi#NC 027266
Asparagales Orchidaceae Sobralia callosa#NC 028147
Asparagales Orchidaceae Sobralia aff. bouchei#NC 028209
Asparagales Orchidaceae Bletilla ochracea#NC 029483
Asparagales Orchidaceae Bletilla striata#NC 028422
Asparagales Orchidaceae Pleione formosana#MK361027
Asparagales Orchidaceae Pleione bulbocodioides#F399
Asparagales Orchidaceae Oberonia japonica#NC 035832
Asparagales Orchidaceae Liparis loeselii#MF374688
Asparagales Orchidaceae Dendrobium kingianum#LC331062
Asparagales Orchidaceae Dendrobium salaccense#NC 035332

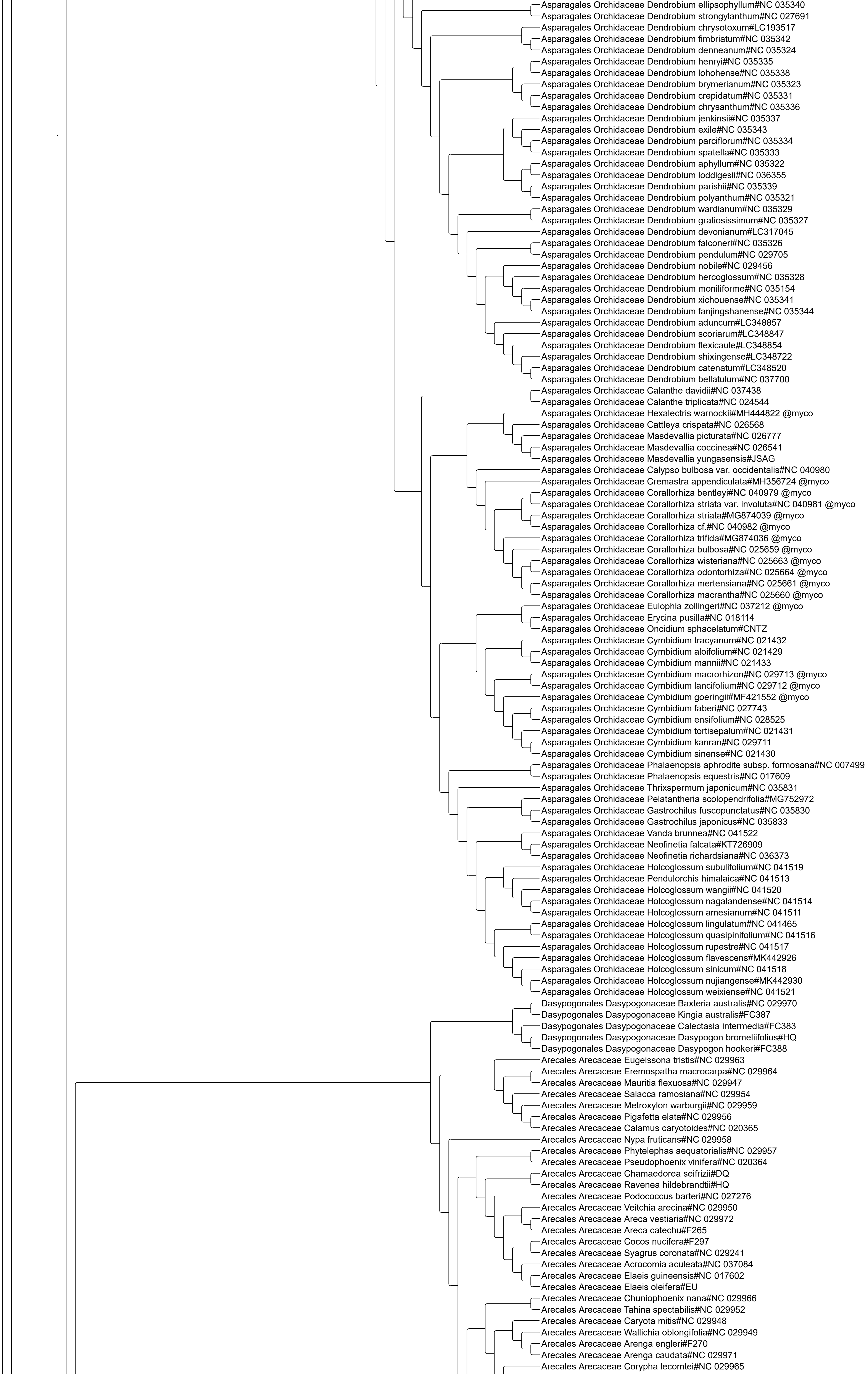

Asparagales Orchidaceae Dendrobium ellipsophyllum#NC 035340
Asparagales Orchidaceae Dendrobium strongylanthum#NC 027691
Asparagales Orchidaceae Dendrobium chrysotoxum#LC193517
Asparagales Orchidaceae Dendrobium fimbriatum#NC 035342
Asparagales Orchidaceae Dendrobium denneanum#NC 035324
Asparagales Orchidaceae Dendrobium henryi#NC 035335
Asparagales Orchidaceae Dendrobium lohohense#NC 035338
Asparagales Orchidaceae Dendrobium brymerianum#NC 035323
Asparagales Orchidaceae Dendrobium crepidatum#NC 035331
Asparagales Orchidaceae Dendrobium chrysanthum#NC 035336
Asparagales Orchidaceae Dendrobium jenkinsii#NC 035337
Asparagales Orchidaceae Dendrobium exile#NC 035343
Asparagales Orchidaceae Dendrobium parciflorum#NC 035334
Asparagales Orchidaceae Dendrobium spatella#NC 035333
Asparagales Orchidaceae Dendrobium aphyllum#NC 035322
Asparagales Orchidaceae Dendrobium loddigesii#NC 036355
Asparagales Orchidaceae Dendrobium parishii#NC 035339
Asparagales Orchidaceae Dendrobium polyanthum#NC 035321
Asparagales Orchidaceae Dendrobium wardianum#NC 035329
Asparagales Orchidaceae Dendrobium gratiosissimum#NC 035327
Asparagales Orchidaceae Dendrobium devonianum#LC317045
Asparagales Orchidaceae Dendrobium falconeri#NC 035326
Asparagales Orchidaceae Dendrobium pendulum#NC 029705
Asparagales Orchidaceae Dendrobium nobile#NC 029456
Asparagales Orchidaceae Dendrobium hercoglossum#NC 035328
Asparagales Orchidaceae Dendrobium moniliforme#NC 035154
Asparagales Orchidaceae Dendrobium xichouense#NC 035341
Asparagales Orchidaceae Dendrobium fanjingshanense#NC 035344
Asparagales Orchidaceae Dendrobium aduncum#LC348857
Asparagales Orchidaceae Dendrobium scoriarum#LC348847
Asparagales Orchidaceae Dendrobium flexicaule#LC348854
Asparagales Orchidaceae Dendrobium shixingense#LC348722
Asparagales Orchidaceae Dendrobium catenatum#LC348520
Asparagales Orchidaceae Dendrobium bellatulum#NC 037700
Asparagales Orchidaceae Calanthe davidii#NC 037438
Asparagales Orchidaceae Calanthe triplicata#NC 024544
Asparagales Orchidaceae Hexalectris warnockii#MH444822 @myco
Asparagales Orchidaceae Cattleya crispata#NC 026568
Asparagales Orchidaceae Masdevallia picturata#NC 026777
Asparagales Orchidaceae Masdevallia coccinea#NC 026541
Asparagales Orchidaceae Masdevallia yungasensis#JSAG
Asparagales Orchidaceae Calypso bulbosa var. occidentalis#NC 040980
Asparagales Orchidaceae Cremastra appendiculata#MH356724 @myco
Asparagales Orchidaceae Corallorhiza bentleyi#NC 040979 @myco
Asparagales Orchidaceae Corallorhiza striata var. involuta#NC 040981 @myco
Asparagales Orchidaceae Corallorhiza striata#MG874039 @myco
Asparagales Orchidaceae Corallorhiza cf.#NC 040982 @myco
Asparagales Orchidaceae Corallorhiza trifida#MG874036 @myco
Asparagales Orchidaceae Corallorhiza bulbosa#NC 025659 @myco
Asparagales Orchidaceae Corallorhiza wisteriana#NC 025663 @myco
Asparagales Orchidaceae Corallorhiza odontorhiza#NC 025664 @myco
Asparagales Orchidaceae Corallorhiza mertensiana#NC 025661 @myco
Asparagales Orchidaceae Corallorhiza macrantha#NC 025660 @myco
Asparagales Orchidaceae Eulophia zollingeri#NC 037212 @myco
Asparagales Orchidaceae Erycina pusilla#NC 018114
Asparagales Orchidaceae Oncidium sphacelatum#CNTZ
Asparagales Orchidaceae Cymbidium tracyanum#NC 021432
Asparagales Orchidaceae Cymbidium aloifolium#NC 021429
Asparagales Orchidaceae Cymbidium mannii#NC 021433
Asparagales Orchidaceae Cymbidium macrorhizon#NC 029713 @myco
Asparagales Orchidaceae Cymbidium lancifolium#NC 029712 @myco
Asparagales Orchidaceae Cymbidium goeringii#MF421552 @myco
Asparagales Orchidaceae Cymbidium faberi#NC 027743
Asparagales Orchidaceae Cymbidium ensifolium#NC 028525
Asparagales Orchidaceae Cymbidium tortisepalum#NC 021431
Asparagales Orchidaceae Cymbidium kanran#NC 029711
Asparagales Orchidaceae Cymbidium sinense#NC 021430
Asparagales Orchidaceae Phalaenopsis aphrodite subsp. formosana#NC 007499
Asparagales Orchidaceae Phalaenopsis equestris#NC 017609
Asparagales Orchidaceae Thrixspermum japonicum#NC 035831
Asparagales Orchidaceae Pelatantheria scolopendrifolia#MG752972
Asparagales Orchidaceae Gastrochilus fuscopunctatus#NC 035830
Asparagales Orchidaceae Gastrochilus japonicus#NC 035833
Asparagales Orchidaceae Vanda brunnea#NC 041522
Asparagales Orchidaceae Neofinetia falcata#KT726909
Asparagales Orchidaceae Neofinetia richardsiana#NC 036373
Asparagales Orchidaceae Holcoglossum subulifolium#NC 041519
Asparagales Orchidaceae Pendulorchis himalaica#NC 041513
Asparagales Orchidaceae Holcoglossum wangii#NC 041520
Asparagales Orchidaceae Holcoglossum nagalandense#NC 041514
Asparagales Orchidaceae Holcoglossum amesianum#NC 041511
Asparagales Orchidaceae Holcoglossum lingulatum#NC 041465
Asparagales Orchidaceae Holcoglossum quasipinifolium#NC 041516
Asparagales Orchidaceae Holcoglossum rupestre#NC 041517
Asparagales Orchidaceae Holcoglossum flavescens#MK442926
Asparagales Orchidaceae Holcoglossum sinicum#NC 041518
Asparagales Orchidaceae Holcoglossum nujiangense#MK442930
Asparagales Orchidaceae Holcoglossum weixiense#NC 041521
Dasypogonales Dasypogonaceae Baxteria australis#NC 029970
Dasypogonales Dasypogonaceae Kingia australis#FC387
Dasypogonales Dasypogonaceae Calectasia intermedia#FC383
Dasypogonales Dasypogonaceae Dasypogon bromeliifolius#HQ
Dasypogonales Dasypogonaceae Dasypogon hookeri#FC388
Arecales Arecaceae Eugeissona tristis#NC 029963
Arecales Arecaceae Eremospatha macrocarpa#NC 029964
Arecales Arecaceae Mauritia flexuosa#NC 029947
Arecales Arecaceae Salacca ramosiana#NC 029954
Arecales Arecaceae Metroxylon warburgii#NC 029959
Arecales Arecaceae Pigafetta elata#NC 029956
Arecales Arecaceae Calamus caryotoides#NC 020365
Arecales Arecaceae Nypa fruticans#NC 029958
Arecales Arecaceae Phytelephas aequatorialis#NC 029957
Arecales Arecaceae Pseudophoenix vinifera#NC 020364
Arecales Arecaceae Chamaedorea seifrizii#DQ
Arecales Arecaceae Ravenea hildebrandtii#HQ
Arecales Arecaceae Podococcus barteri#NC 027276
Arecales Arecaceae Veitchia arecina#NC 029950
Arecales Arecaceae Areca vestiaria#NC 029972
Arecales Arecaceae Areca catechu#F265
Arecales Arecaceae Cocos nucifera#F297
Arecales Arecaceae Syagrus coronata#NC 029241
Arecales Arecaceae Acrocomia aculeata#NC 037084
Arecales Arecaceae Elaeis guineensis#NC 017602
Arecales Arecaceae Elaeis oleifera#EU
Arecales Arecaceae Chuniophoenix nana#NC 029966
Arecales Arecaceae Tahina spectabilis#NC 029952
Arecales Arecaceae Caryota mitis#NC 029948
Arecales Arecaceae Wallichia oblongifolia#NC 029949
Arecales Arecaceae Arenga engleri#F270
Arecales Arecaceae Arenga caudata#NC 029971
Arecales Arecaceae Corypha lecomtei#NC 029965

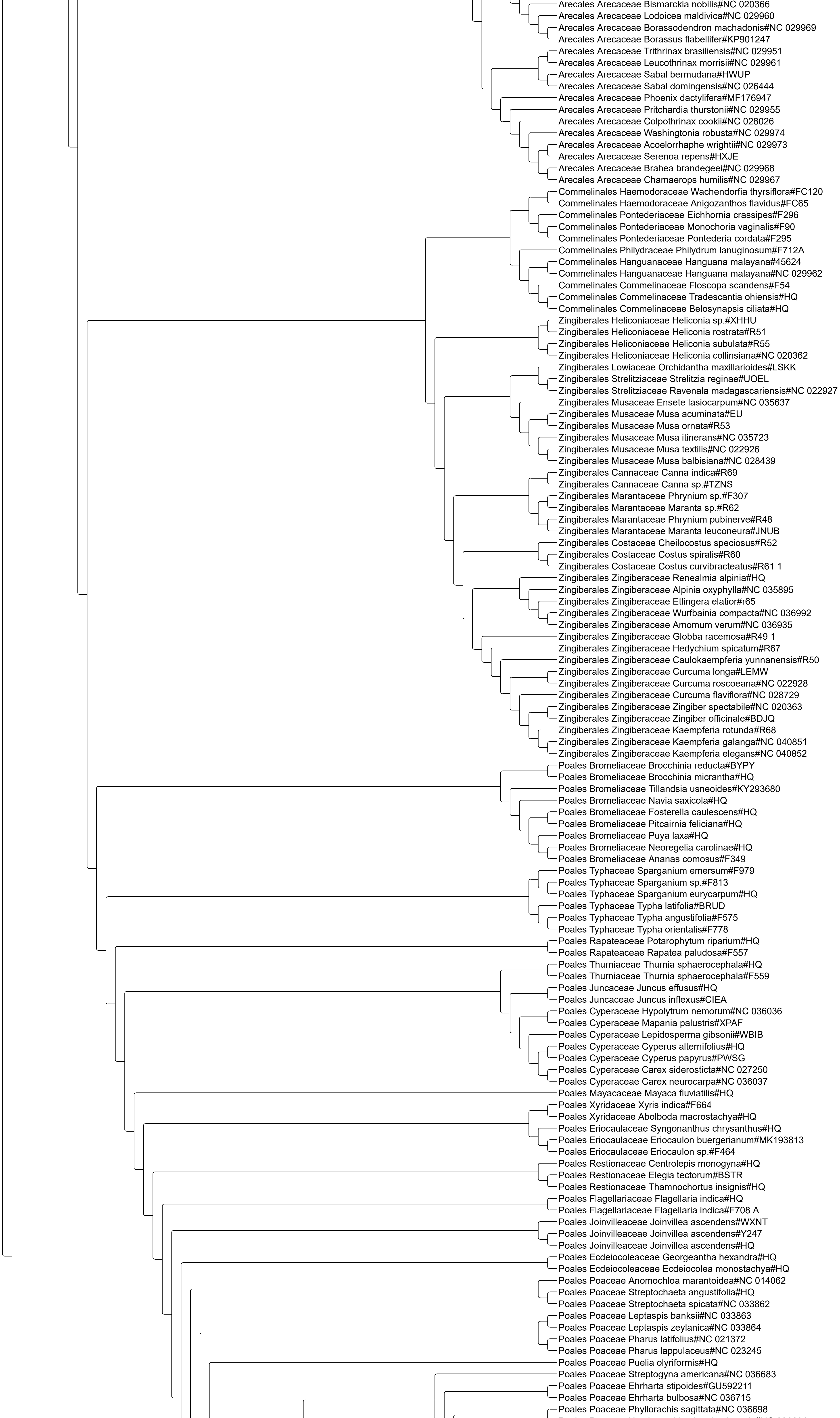
Arecales Arecaceae Bismarckia nobilis#NC 020366
Arecales Arecaceae Lodoicea maldivica#NC 029960
Arecales Arecaceae Borassodendron machadonis#NC 029969
Arecales Arecaceae Borassus flabellifer#KP901247
Arecales Arecaceae Trithrinax brasiliensis#NC 029951
Arecales Arecaceae Leucothrinax morrisii#NC 029961
Arecales Arecaceae Sabal bermudana#HWUP
Arecales Arecaceae Sabal domingensis#NC 026444
Arecales Arecaceae Phoenix dactylifera#MF176947
Arecales Arecaceae Pritchardia thurstonii#NC 029955
Arecales Arecaceae Colpothrinax cookii#NC 028026
Arecales Arecaceae Washingtonia robusta#NC 029974
Arecales Arecaceae Acoelorrhaphe wrightii#NC 029973
Arecales Arecaceae Serenoa repens#HXJE
Arecales Arecaceae Brahea brandegeei#NC 029968
Arecales Arecaceae Chamaerops humilis#NC 029967
Commelinales Haemodoraceae Wachendorfia thyrsiflora#FC120
Commelinales Haemodoraceae Anigozanthos flavidus#FC65
Commelinales Pontederiaceae Eichhornia crassipes#F296
Commelinales Pontederiaceae Monochoria vaginalis#F90
Commelinales Pontederiaceae Pontederia cordata#F295
Commelinales Philydraceae Philydrum lanuginosum#F712A
Commelinales Hanguanaceae Hanguana malayana#45624
Commelinales Hanguanaceae Hanguana malayana#NC 029962
Commelinales Commelinaceae Floscopa scandens#F54
Commelinales Commelinaceae Tradescantia ohiensis#HQ
Commelinales Commelinaceae Belosynapsis ciliata#HQ
Zingiberales Heliconiaceae Heliconia sp.#XHHU
Zingiberales Heliconiaceae Heliconia rostrata#R51
Zingiberales Heliconiaceae Heliconia subulata#R55
Zingiberales Heliconiaceae Heliconia collinsiana#NC 020362
Zingiberales Lowiaceae Orchidantha maxillarioides#LSKK
Zingiberales Strelitziaceae Strelitzia reginae#UOEL
Zingiberales Strelitziaceae Ravenala madagascariensis#NC 022927
Zingiberales Musaceae Ensete lasiocarpum#NC 035637
Zingiberales Musaceae Musa acuminata#EU
Zingiberales Musaceae Musa ornata#R53
Zingiberales Musaceae Musa itinerans#NC 035723
Zingiberales Musaceae Musa textilis#NC 022926
Zingiberales Musaceae Musa balbisiana#NC 028439
Zingiberales Cannaceae Canna indica#R69
Zingiberales Cannaceae Canna sp.#TZNS
Zingiberales Marantaceae Phrynium sp.#F307
Zingiberales Marantaceae Maranta sp.#R62
Zingiberales Marantaceae Phrynium pubinerve#R48
Zingiberales Marantaceae Maranta leuconeura#JNUB
Zingiberales Costaceae Cheilocostus speciosus#R52
Zingiberales Costaceae Costus spiralis#R60
Zingiberales Costaceae Costus curvibracteatus#R61 1
Zingiberales Zingiberaceae Renealmia alpinia#HQ
Zingiberales Zingiberaceae Alpinia oxyphylla#NC 035895
Zingiberales Zingiberaceae Etlingera elatior#r65
Zingiberales Zingiberaceae Wurfbainia compacta#NC 036992
Zingiberales Zingiberaceae Amomum verum#NC 036935
Zingiberales Zingiberaceae Globba racemosa#R49 1
Zingiberales Zingiberaceae Hedychium spicatum#R67
Zingiberales Zingiberaceae Caulokaempferia yunnanensis#R50
Zingiberales Zingiberaceae Curcuma longa#LEMW
Zingiberales Zingiberaceae Curcuma roscoeana#NC 022928
Zingiberales Zingiberaceae Curcuma flaviflora#NC 028729
Zingiberales Zingiberaceae Zingiber spectabile#NC 020363
Zingiberales Zingiberaceae Zingiber officinale#BDJQ
Zingiberales Zingiberaceae Kaempferia rotunda#R68
Zingiberales Zingiberaceae Kaempferia galanga#NC 040851
Zingiberales Zingiberaceae Kaempferia elegans#NC 040852
Poales Bromeliaceae Brocchinia reducta#BYPY
Poales Bromeliaceae Brocchinia micrantha#HQ
Poales Bromeliaceae Tillandsia usneoides#KY293680
Poales Bromeliaceae Navia saxicola#HQ
Poales Bromeliaceae Fosterella caulescens#HQ
Poales Bromeliaceae Pitcairnia feliciana#HQ
Poales Bromeliaceae Puya laxa#HQ
Poales Bromeliaceae Neoregelia carolinae#HQ
Poales Bromeliaceae Ananas comosus#F349
Poales Typhaceae Sparganium emersum#F979
Poales Typhaceae Sparganium sp.#F813
Poales Typhaceae Sparganium eurycarpum#HQ
Poales Typhaceae Typha latifolia#BRUD
Poales Typhaceae Typha angustifolia#F575
Poales Typhaceae Typha orientalis#F778
Poales Rapateaceae Potarophytum riparium#HQ
Poales Rapateaceae Rapatea paludosa#F557
Poales Thurniaceae Thurnia sphaerocephala#HQ
Poales Thurniaceae Thurnia sphaerocephala#F559
Poales Juncaceae Juncus effusus#HQ
Poales Juncaceae Juncus inflexus#CIEA
Poales Cyperaceae Hypolytrum nemorum#NC 036036
Poales Cyperaceae Mapania palustris#XPAF
Poales Cyperaceae Lepidosperma gibsonii#WBIB
Poales Cyperaceae Cyperus alternifolius#HQ
Poales Cyperaceae Cyperus papyrus#PWSG
Poales Cyperaceae Carex siderosticta#NC 027250
Poales Cyperaceae Carex neurocarpa#NC 036037
Poales Mayacaceae Mayaca fluviatilis#HQ
Poales Xyridaceae Xyris indica#F664
Poales Xyridaceae Abolboda macrostachya#HQ
Poales Eriocaulaceae Syngonanthus chrysanthus#HQ
Poales Eriocaulaceae Eriocaulon buergerianum#MK193813
Poales Eriocaulaceae Eriocaulon sp.#F464
Poales Restionaceae Centrolepis monogyna#HQ
Poales Restionaceae Elegia tectorum#BSTR
Poales Restionaceae Thamnochortus insignis#HQ
Poales Flagellariaceae Flagellaria indica#HQ
Poales Flagellariaceae Flagellaria indica#F708 A
Poales Joinvilleaceae Joinvillea ascendens#WXNT
Poales Joinvilleaceae Joinvillea ascendens#Y247
Poales Joinvilleaceae Joinvillea ascendens#HQ
Poales Ecdeiocoleaceae Georgeantha hexandra#HQ
Poales Ecdeiocoleaceae Ecdeiocolea monostachya#HQ
Poales Poaceae Anomochloa marantoidea#NC 014062
Poales Poaceae Streptochaeta angustifolia#HQ
Poales Poaceae Streptochaeta spicata#NC 033862
Poales Poaceae Leptaspis banksii#NC 033863
Poales Poaceae Leptaspis zeylanica#NC 033864
Poales Poaceae Pharus latifolius#NC 021372
Poales Poaceae Pharus lappulaceus#NC 023245
Poales Poaceae Puelia olyriformis#HQ
Poales Poaceae Streptogyna americana#NC 036683
Poales Poaceae Ehrharta stipoides#GU592211
Poales Poaceae Ehrharta bulbosa#NC 036715
Poales Poaceae Phyllorachis sagittata#NC 036698

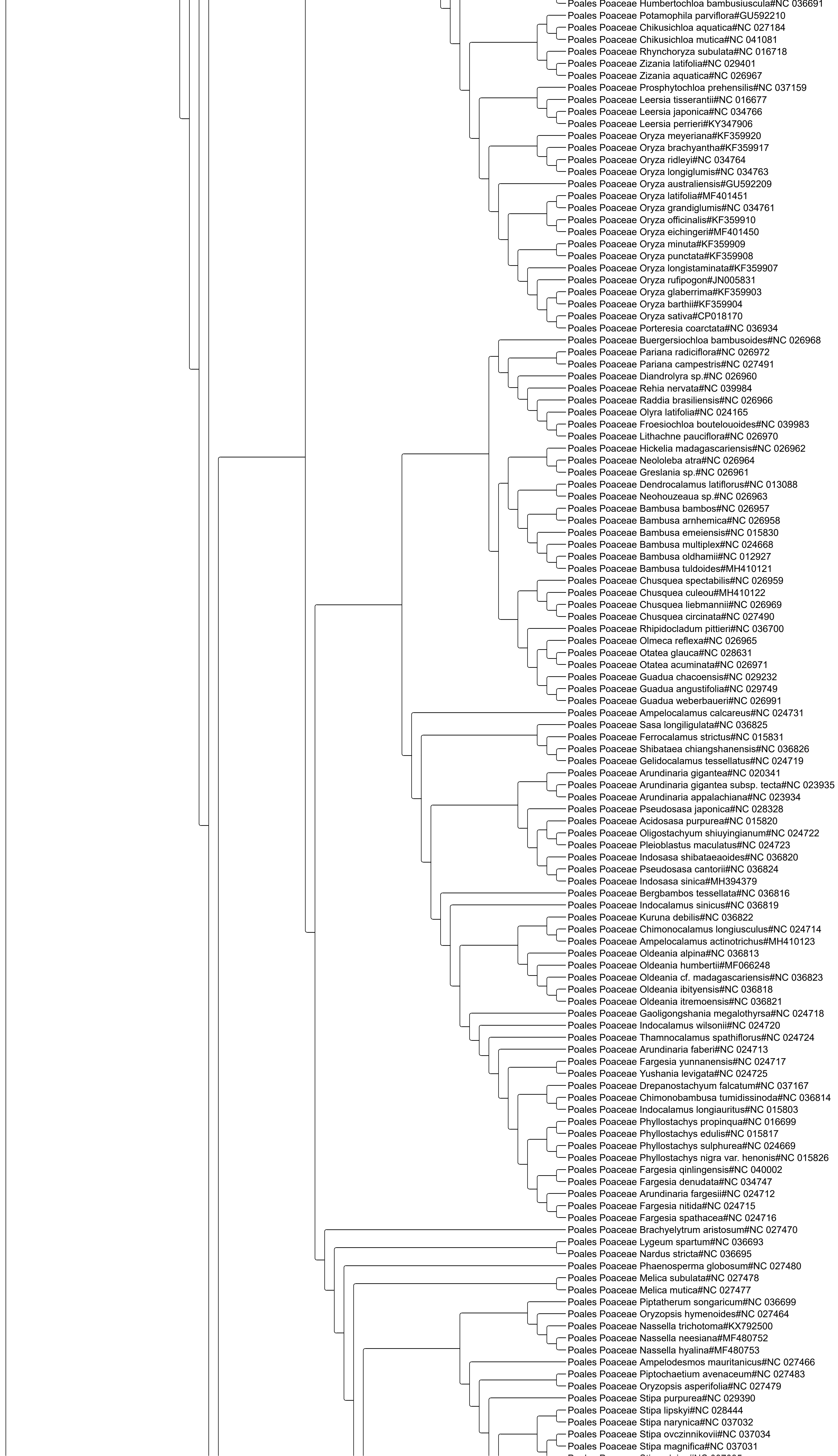
Poales Poaceae Humbertochloa bambusiuscula#NC 036691
Poales Poaceae Potamophila parviflora#GU592210
Poales Poaceae Chikusichloa aquatica#NC 027184
Poales Poaceae Chikusichloa mutica#NC 041081
Poales Poaceae Rhynchoryza subulata#NC 016718
Poales Poaceae Zizania latifolia#NC 029401
Poales Poaceae Zizania aquatica#NC 026967
Poales Poaceae Prosphytochloa prehensilis#NC 037159
Poales Poaceae Leersia tisserantii#NC 016677
Poales Poaceae Leersia japonica#NC 034766
Poales Poaceae Leersia perrieri#KY347906
Poales Poaceae Oryza meyeriana#KF359920
Poales Poaceae Oryza brachyantha#KF359917
Poales Poaceae Oryza ridleyi#NC 034764
Poales Poaceae Oryza longiglumis#NC 034763
Poales Poaceae Oryza australiensis#GU592209
Poales Poaceae Oryza latifolia#MF401451
Poales Poaceae Oryza grandiglumis#NC 034761
Poales Poaceae Oryza officinalis#KF359910
Poales Poaceae Oryza eichingeri#MF401450
Poales Poaceae Oryza minuta#KF359909
Poales Poaceae Oryza punctata#KF359908
Poales Poaceae Oryza longistaminata#KF359907
Poales Poaceae Oryza rufipogon#JN005831
Poales Poaceae Oryza glaberrima#KF359903
Poales Poaceae Oryza barthii#KF359904
Poales Poaceae Oryza sativa#CP018170
Poales Poaceae Porteresia coarctata#NC 036934
Poales Poaceae Buergersiochloa bambusoides#NC 026968
Poales Poaceae Pariana radiciflora#NC 026972
Poales Poaceae Pariana campestris#NC 027491
Poales Poaceae Diandrolyra sp.#NC 026960
Poales Poaceae Rehia nervata#NC 039984
Poales Poaceae Raddia brasiliensis#NC 026966
Poales Poaceae Olyra latifolia#NC 024165
Poales Poaceae Froesiochloa boutelouoides#NC 039983
Poales Poaceae Lithachne pauciflora#NC 026970
Poales Poaceae Hickelia madagascariensis#NC 026962
Poales Poaceae Neololeba atra#NC 026964
Poales Poaceae Greslania sp.#NC 026961
Poales Poaceae Dendrocalamus latiflorus#NC 013088
Poales Poaceae Neohouzeaua sp.#NC 026963
Poales Poaceae Bambusa bambos#NC 026957
Poales Poaceae Bambusa arnhemica#NC 026958
Poales Poaceae Bambusa emeiensis#NC 015830
Poales Poaceae Bambusa multiplex#NC 024668
Poales Poaceae Bambusa oldhamii#NC 012927
Poales Poaceae Bambusa tuldoides#MH410121
Poales Poaceae Chusquea spectabilis#NC 026959
Poales Poaceae Chusquea culeou#MH410122
Poales Poaceae Chusquea liebmannii#NC 026969
Poales Poaceae Chusquea circinata#NC 027490
Poales Poaceae Rhipidocladum pittieri#NC 036700
Poales Poaceae Olmeca reflexa#NC 026965
Poales Poaceae Otatea glauca#NC 028631
Poales Poaceae Otatea acuminata#NC 026971
Poales Poaceae Guadua chacoensis#NC 029232
Poales Poaceae Guadua angustifolia#NC 029749
Poales Poaceae Guadua weberbaueri#NC 026991
Poales Poaceae Ampelocalamus calcareus#NC 024731
Poales Poaceae Sasa longiligulata#NC 036825
Poales Poaceae Ferrocalamus strictus#NC 015831
Poales Poaceae Shibataea chiangshanensis#NC 036826
Poales Poaceae Gelidocalamus tessellatus#NC 024719
Poales Poaceae Arundinaria gigantea#NC 020341
Poales Poaceae Arundinaria gigantea subsp. tecta#NC 023935
Poales Poaceae Arundinaria appalachiana#NC 023934
Poales Poaceae Pseudosasa japonica#NC 028328
Poales Poaceae Acidosasa purpurea#NC 015820
Poales Poaceae Oligostachyum shiuyingianum#NC 024722
Poales Poaceae Pleioblastus maculatus#NC 024723
Poales Poaceae Indosasa shibataeaoides#NC 036820
Poales Poaceae Pseudosasa cantorii#NC 036824
Poales Poaceae Indosasa sinica#MH394379
Poales Poaceae Bergbambos tessellata#NC 036816
Poales Poaceae Indocalamus sinicus#NC 036819
Poales Poaceae Kuruna debilis#NC 036822
Poales Poaceae Chimonocalamus longiusculus#NC 024714
Poales Poaceae Ampelocalamus actinotrichus#MH410123
Poales Poaceae Oldeania alpina#NC 036813
Poales Poaceae Oldeania humbertii#MF066248
Poales Poaceae Oldeania cf. madagascariensis#NC 036823
Poales Poaceae Oldeania ibityensis#NC 036818
Poales Poaceae Oldeania itremoensis#NC 036821
Poales Poaceae Gaoligongshania megalothyrsa#NC 024718
Poales Poaceae Indocalamus wilsonii#NC 024720
Poales Poaceae Thamnocalamus spathiflorus#NC 024724
Poales Poaceae Arundinaria faberi#NC 024713
Poales Poaceae Fargesia yunnanensis#NC 024717
Poales Poaceae Yushania levigata#NC 024725
Poales Poaceae Drepanostachyum falcatum#NC 037167
Poales Poaceae Chimonobambusa tumidissinoda#NC 036814
Poales Poaceae Indocalamus longiauritus#NC 015803
Poales Poaceae Phyllostachys propinqua#NC 016699
Poales Poaceae Phyllostachys edulis#NC 015817
Poales Poaceae Phyllostachys sulphurea#NC 024669
Poales Poaceae Phyllostachys nigra var. henonis#NC 015826
Poales Poaceae Fargesia qinlingensis#NC 040002
Poales Poaceae Fargesia denudata#NC 034747
Poales Poaceae Arundinaria fargesii#NC 024712
Poales Poaceae Fargesia nitida#NC 024715
Poales Poaceae Fargesia spathacea#NC 024716
Poales Poaceae Brachyelytrum aristosum#NC 027470
Poales Poaceae Lygeum spartum#NC 036693
Poales Poaceae Nardus stricta#NC 036695
Poales Poaceae Phaenosperma globosum#NC 027480
Poales Poaceae Melica subulata#NC 027478
Poales Poaceae Melica mutica#NC 027477
Poales Poaceae Piptatherum songaricum#NC 036699
Poales Poaceae Oryzopsis hymenoides#NC 027464
Poales Poaceae Nassella trichotoma#KX792500
Poales Poaceae Nassella neesiana#MF480752
Poales Poaceae Nassella hyalina#MF480753
Poales Poaceae Ampelodesmos mauritanicus#NC 027466
Poales Poaceae Piptochaetium avenaceum#NC 027483
Poales Poaceae Oryzopsis asperifolia#NC 027479
Poales Poaceae Stipa purpurea#NC 029390
Poales Poaceae Stipa lipskyi#NC 028444
Poales Poaceae Stipa narynica#NC 037032
Poales Poaceae Stipa ovczinnikovii#NC 037034
Poales Poaceae Stipa magnifica#NC 037031

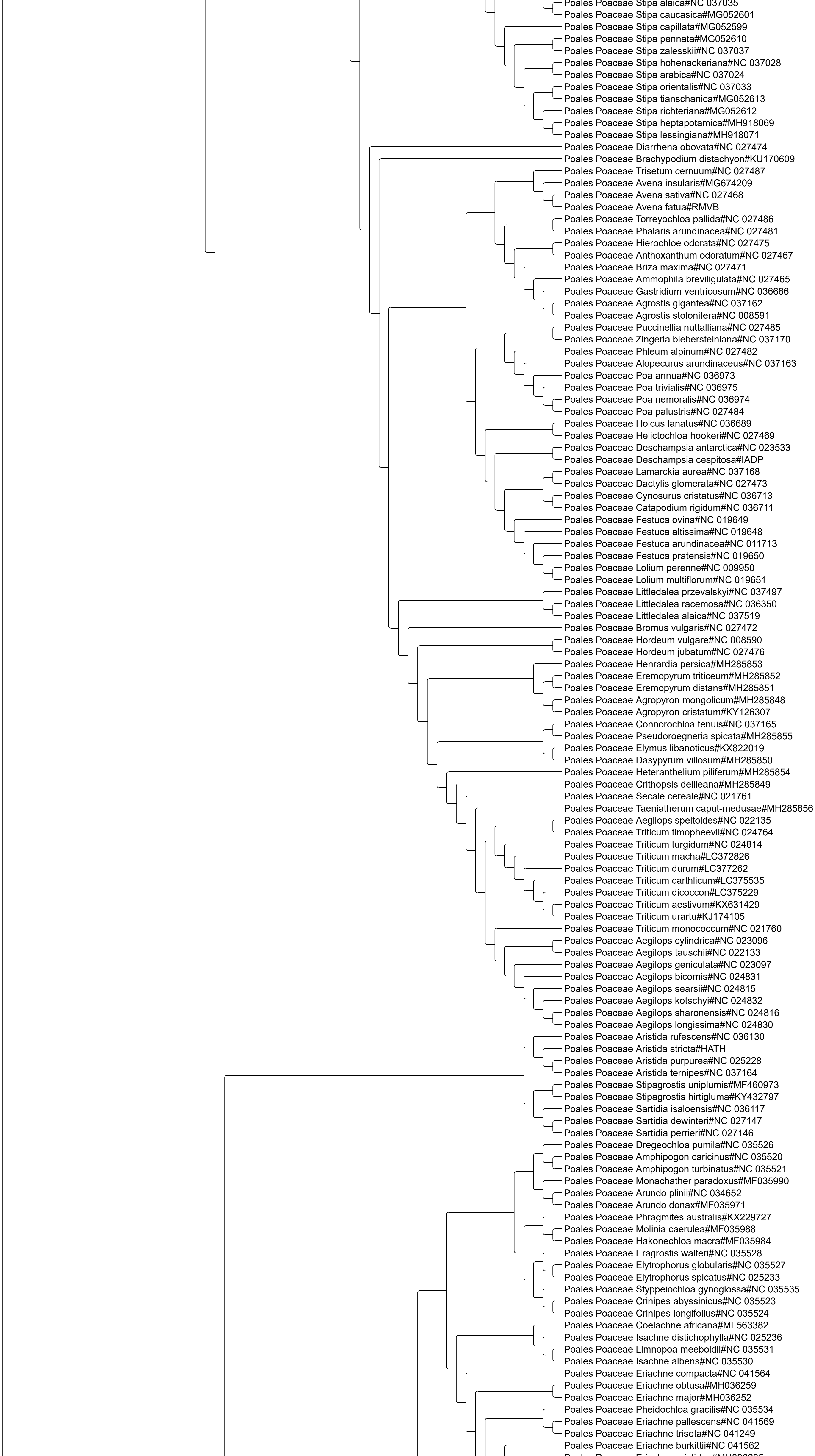
Poales Poaceae Stipa alaica#NC 037035
Poales Poaceae Stipa caucasica#MG052601
Poales Poaceae Stipa capillata#MG052599
Poales Poaceae Stipa pennata#MG052610
Poales Poaceae Stipa zalesskii#NC 037037
Poales Poaceae Stipa hohenackeriana#NC 037028
Poales Poaceae Stipa arabica#NC 037024
Poales Poaceae Stipa orientalis#NC 037033
Poales Poaceae Stipa tianschanica#MG052613
Poales Poaceae Stipa richteriana#MG052612
Poales Poaceae Stipa heptapotamica#MH918069
Poales Poaceae Stipa lessingiana#MH918071
Poales Poaceae Diarrhena obovata#NC 027474
Poales Poaceae Brachypodium distachyon#KU170609
Poales Poaceae Trisetum cernuum#NC 027487
Poales Poaceae Avena insularis#MG674209
Poales Poaceae Avena sativa#NC 027468
Poales Poaceae Avena fatua#RMVB
Poales Poaceae Torreyochloa pallida#NC 027486
Poales Poaceae Phalaris arundinacea#NC 027481
Poales Poaceae Hierochloe odorata#NC 027475
Poales Poaceae Anthoxanthum odoratum#NC 027467
Poales Poaceae Briza maxima#NC 027471
Poales Poaceae Ammophila breviligulata#NC 027465
Poales Poaceae Gastridium ventricosum#NC 036686
Poales Poaceae Agrostis gigantea#NC 037162
Poales Poaceae Agrostis stolonifera#NC 008591
Poales Poaceae Puccinellia nuttalliana#NC 027485
Poales Poaceae Zingeria biebersteiniana#NC 037170
Poales Poaceae Phleum alpinum#NC 027482
Poales Poaceae Alopecurus arundinaceus#NC 037163
Poales Poaceae Poa annua#NC 036973
Poales Poaceae Poa trivialis#NC 036975
Poales Poaceae Poa nemoralis#NC 036974
Poales Poaceae Poa palustris#NC 027484
Poales Poaceae Holcus lanatus#NC 036689
Poales Poaceae Helictochloa hookeri#NC 027469
Poales Poaceae Deschampsia antarctica#NC 023533
Poales Poaceae Deschampsia cespitosa#IADP
Poales Poaceae Lamarckia aurea#NC 037168
Poales Poaceae Dactylis glomerata#NC 027473
Poales Poaceae Cynosurus cristatus#NC 036713
Poales Poaceae Catapodium rigidum#NC 036711
Poales Poaceae Festuca ovina#NC 019649
Poales Poaceae Festuca altissima#NC 019648
Poales Poaceae Festuca arundinacea#NC 011713
Poales Poaceae Festuca pratensis#NC 019650
Poales Poaceae Lolium perenne#NC 009950
Poales Poaceae Lolium multiflorum#NC 019651
Poales Poaceae Littledalea przevalskyi#NC 037497
Poales Poaceae Littledalea racemosa#NC 036350
Poales Poaceae Littledalea alaica#NC 037519
Poales Poaceae Bromus vulgaris#NC 027472
Poales Poaceae Hordeum vulgare#NC 008590
Poales Poaceae Hordeum jubatum#NC 027476
Poales Poaceae Henrardia persica#MH285853
Poales Poaceae Eremopyrum triticeum#MH285852
Poales Poaceae Eremopyrum distans#MH285851
Poales Poaceae Agropyron mongolicum#MH285848
Poales Poaceae Agropyron cristatum#KY126307
Poales Poaceae Connorochloa tenuis#NC 037165
Poales Poaceae Pseudoroegneria spicata#MH285855
Poales Poaceae Elymus libanoticus#KX822019
Poales Poaceae Dasypyrum villosum#MH285850
Poales Poaceae Heteranthelium piliferum#MH285854
Poales Poaceae Crithopsis delileana#MH285849
Poales Poaceae Secale cereale#NC 021761
Poales Poaceae Taeniatherum caput-medusae#MH285856
Poales Poaceae Aegilops speltoides#NC 022135
Poales Poaceae Triticum timopheevii#NC 024764
Poales Poaceae Triticum turgidum#NC 024814
Poales Poaceae Triticum macha#LC372826
Poales Poaceae Triticum durum#LC377262
Poales Poaceae Triticum carthlicum#LC375535
Poales Poaceae Triticum dicoccon#LC375229
Poales Poaceae Triticum aestivum#KX631429
Poales Poaceae Triticum urartu#KJ174105
Poales Poaceae Triticum monococcum#NC 021760
Poales Poaceae Aegilops cylindrica#NC 023096
Poales Poaceae Aegilops tauschii#NC 022133
Poales Poaceae Aegilops geniculata#NC 023097
Poales Poaceae Aegilops bicornis#NC 024831
Poales Poaceae Aegilops searsii#NC 024815
Poales Poaceae Aegilops kotschyi#NC 024832
Poales Poaceae Aegilops sharonensis#NC 024816
Poales Poaceae Aegilops longissima#NC 024830
Poales Poaceae Aristida rufescens#NC 036130
Poales Poaceae Aristida stricta#HATH
Poales Poaceae Aristida purpurea#NC 025228
Poales Poaceae Aristida ternipes#NC 037164
Poales Poaceae Stipagrostis uniplumis#MF460973
Poales Poaceae Stipagrostis hirtigluma#KY432797
Poales Poaceae Sartidia isaloensis#NC 036117
Poales Poaceae Sartidia dewinteri#NC 027147
Poales Poaceae Sartidia perrieri#NC 027146
Poales Poaceae Dregeochloa pumila#NC 035526
Poales Poaceae Amphipogon caricinus#NC 035520
Poales Poaceae Amphipogon turbinatus#NC 035521
Poales Poaceae Monachather paradoxus#MF035990
Poales Poaceae Arundo plinii#NC 034652
Poales Poaceae Arundo donax#MF035971
Poales Poaceae Phragmites australis#KX229727
Poales Poaceae Molinia caerulea#MF035988
Poales Poaceae Hakonechloa macra#MF035984
Poales Poaceae Eragrostis walteri#NC 035528
Poales Poaceae Elytrophorus globularis#NC 035527
Poales Poaceae Elytrophorus spicatus#NC 025233
Poales Poaceae Styppeiochloa gynoglossa#NC 035535
Poales Poaceae Crinipes abyssinicus#NC 035523
Poales Poaceae Crinipes longifolius#NC 035524
Poales Poaceae Coelachne africana#MF563382
Poales Poaceae Isachne distichophylla#NC 025236
Poales Poaceae Limnopoa meeboldii#NC 035531
Poales Poaceae Isachne albens#NC 035530
Poales Poaceae Eriachne compacta#NC 041564
Poales Poaceae Eriachne obtusa#MH036259
Poales Poaceae Eriachne major#MH036252
Poales Poaceae Pheidochloa gracilis#NC 035534
Poales Poaceae Eriachne pallescens#NC 041569
Poales Poaceae Eriachne triseta#NC 041249
Poales Poaceae Eriachne burkittii#NC 041562

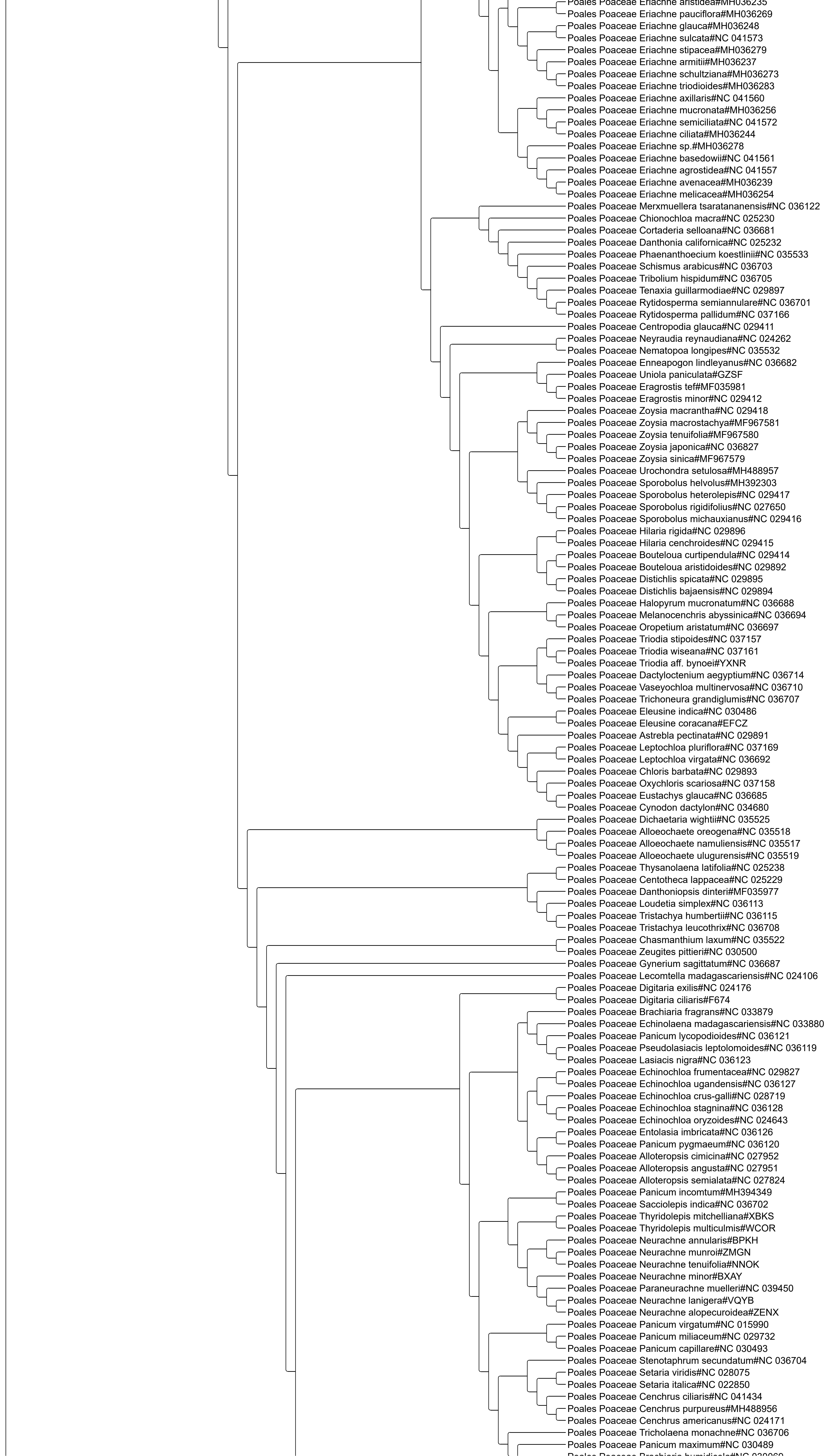
Poales Poaceae Eriachne aristidea#MH036235
Poales Poaceae Eriachne pauciflora#MH036269
Poales Poaceae Eriachne glauca#MH036248
Poales Poaceae Eriachne sulcata#NC 041573
Poales Poaceae Eriachne stipacea#MH036279
Poales Poaceae Eriachne armitii#MH036237
Poales Poaceae Eriachne schultziana#MH036273
Poales Poaceae Eriachne triodioides#MH036283
Poales Poaceae Eriachne axillaris#NC 041560
Poales Poaceae Eriachne mucronata#MH036256
Poales Poaceae Eriachne semiciliata#NC 041572
Poales Poaceae Eriachne ciliata#MH036244
Poales Poaceae Eriachne sp.#MH036278
Poales Poaceae Eriachne basedowii#NC 041561
Poales Poaceae Eriachne agrostidea#NC 041557
Poales Poaceae Eriachne avenacea#MH036239
Poales Poaceae Eriachne melicacea#MH036254
Poales Poaceae Merxmuellera tsaratananensis#NC 036122
Poales Poaceae Chionochloa macra#NC 025230
Poales Poaceae Cortaderia selloana#NC 036681
Poales Poaceae Danthonia californica#NC 025232
Poales Poaceae Phaenanthoecium koestlinii#NC 035533
Poales Poaceae Schismus arabicus#NC 036703
Poales Poaceae Tribolium hispidum#NC 036705
Poales Poaceae Tenaxia guillarmodiae#NC 029897
Poales Poaceae Rytidosperma semiannulare#NC 036701
Poales Poaceae Rytidosperma pallidum#NC 037166
Poales Poaceae Centropodia glauca#NC 029411
Poales Poaceae Neyraudia reynaudiana#NC 024262
Poales Poaceae Nematopoa longipes#NC 035532
Poales Poaceae Enneapogon lindleyanus#NC 036682
Poales Poaceae Uniola paniculata#GZSF
Poales Poaceae Eragrostis tef#MF035981
Poales Poaceae Eragrostis minor#NC 029412
Poales Poaceae Zoysia macrantha#NC 029418
Poales Poaceae Zoysia macrostachya#MF967581
Poales Poaceae Zoysia tenuifolia#MF967580
Poales Poaceae Zoysia japonica#NC 036827
Poales Poaceae Zoysia sinica#MF967579
Poales Poaceae Urochondra setulosa#MH488957
Poales Poaceae Sporobolus helvolus#MH392303
Poales Poaceae Sporobolus heterolepis#NC 029417
Poales Poaceae Sporobolus rigidifolius#NC 027650
Poales Poaceae Sporobolus michauxianus#NC 029416
Poales Poaceae Hilaria rigida#NC 029896
Poales Poaceae Hilaria cenchroides#NC 029415
Poales Poaceae Bouteloua curtipendula#NC 029414
Poales Poaceae Bouteloua aristidoides#NC 029892
Poales Poaceae Distichlis spicata#NC 029895
Poales Poaceae Distichlis bajaensis#NC 029894
Poales Poaceae Halopyrum mucronatum#NC 036688
Poales Poaceae Melanocenchris abyssinica#NC 036694
Poales Poaceae Oropetium aristatum#NC 036697
Poales Poaceae Triodia stipoides#NC 037157
Poales Poaceae Triodia wiseana#NC 037161
Poales Poaceae Triodia aff. bynoei#YXNR
Poales Poaceae Dactyloctenium aegyptium#NC 036714
Poales Poaceae Vaseyochloa multinervosa#NC 036710
Poales Poaceae Trichoneura grandiglumis#NC 036707
Poales Poaceae Eleusine indica#NC 030486
Poales Poaceae Eleusine coracana#EFCZ
Poales Poaceae Astrebla pectinata#NC 029891
Poales Poaceae Leptochloa pluriflora#NC 037169
Poales Poaceae Leptochloa virgata#NC 036692
Poales Poaceae Chloris barbata#NC 029893
Poales Poaceae Oxychloris scariosa#NC 037158
Poales Poaceae Eustachys glauca#NC 036685
Poales Poaceae Cynodon dactylon#NC 034680
Poales Poaceae Dichaetaria wightii#NC 035525
Poales Poaceae Alloeochaete oreogena#NC 035518
Poales Poaceae Alloeochaete namuliensis#NC 035517
Poales Poaceae Alloeochaete ulugurensis#NC 035519
Poales Poaceae Thysanolaena latifolia#NC 025238
Poales Poaceae Centotheca lappacea#NC 025229
Poales Poaceae Danthoniopsis dinteri#MF035977
Poales Poaceae Loudetia simplex#NC 036113
Poales Poaceae Tristachya humbertii#NC 036115
Poales Poaceae Tristachya leucothrix#NC 036708
Poales Poaceae Chasmanthium laxum#NC 035522
Poales Poaceae Zeugites pittieri#NC 030500
Poales Poaceae Gynerium sagittatum#NC 036687
Poales Poaceae Lecomtella madagascariensis#NC 024106
Poales Poaceae Digitaria exilis#NC 024176
Poales Poaceae Digitaria ciliaris#F674
Poales Poaceae Brachiaria fragrans#NC 033879
Poales Poaceae Echinolaena madagascariensis#NC 033880
Poales Poaceae Panicum lycopodioides#NC 036121
Poales Poaceae Pseudolasiacis leptolomoides#NC 036119
Poales Poaceae Lasiacis nigra#NC 036123
Poales Poaceae Echinochloa frumentacea#NC 029827
Poales Poaceae Echinochloa ugandensis#NC 036127
Poales Poaceae Echinochloa crus-galli#NC 028719
Poales Poaceae Echinochloa stagnina#NC 036128
Poales Poaceae Echinochloa oryzoides#NC 024643
Poales Poaceae Entolasia imbricata#NC 036126
Poales Poaceae Panicum pygmaeum#NC 036120
Poales Poaceae Alloteropsis cimicina#NC 027952
Poales Poaceae Alloteropsis angusta#NC 027951
Poales Poaceae Alloteropsis semialata#NC 027824
Poales Poaceae Panicum incomtum#MH394349
Poales Poaceae Sacciolepis indica#NC 036702
Poales Poaceae Thyridolepis mitchelliana#XBKS
Poales Poaceae Thyridolepis multiculmis#WCOR
Poales Poaceae Neurachne annularis#BPKH
Poales Poaceae Neurachne munroi#ZMGN
Poales Poaceae Neurachne tenuifolia#NNOK
Poales Poaceae Neurachne minor#BXAY
Poales Poaceae Paraneurachne muelleri#NC 039450
Poales Poaceae Neurachne lanigera#VQYB
Poales Poaceae Neurachne alopecuroidea#ZENX
Poales Poaceae Panicum virgatum#NC 015990
Poales Poaceae Panicum miliaceum#NC 029732
Poales Poaceae Panicum capillare#NC 030493
Poales Poaceae Stenotaphrum secundatum#NC 036704
Poales Poaceae Setaria viridis#NC 028075
Poales Poaceae Setaria italica#NC 022850
Poales Poaceae Cenchrus ciliaris#NC 041434
Poales Poaceae Cenchrus purpureus#MH488956
Poales Poaceae Cenchrus americanus#NC 024171
Poales Poaceae Tricholaena monachne#NC 036706
Poales Poaceae Panicum maximum#NC 030489

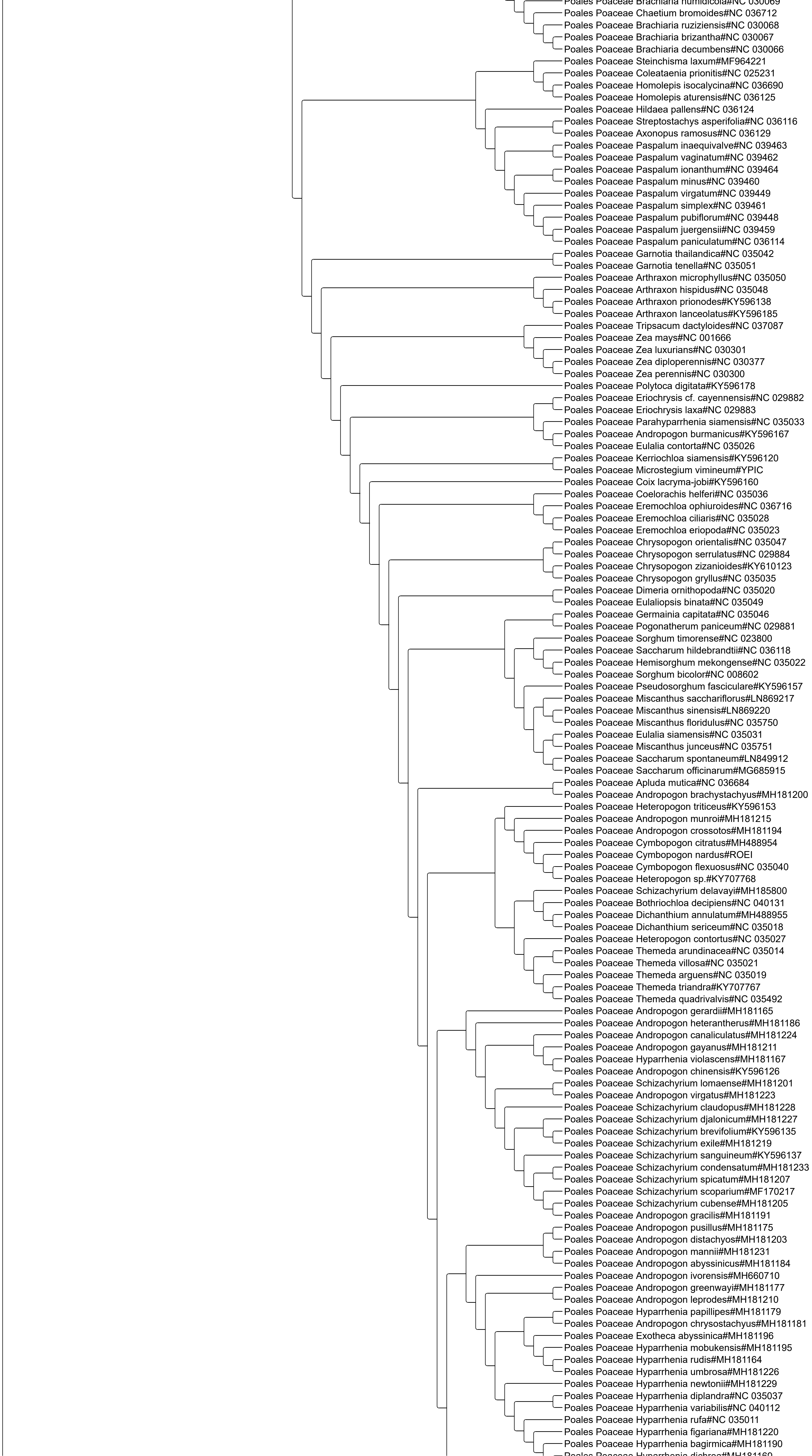
Poales Poaceae Brachiaria humidicola#NC 030069
Poales Poaceae Chaetium bromoides#NC 036712
Poales Poaceae Brachiaria ruziziensis#NC 030068
Poales Poaceae Brachiaria brizantha#NC 030067
Poales Poaceae Brachiaria decumbens#NC 030066
Poales Poaceae Steinchisma laxum#MF964221
Poales Poaceae Coleataenia prionitis#NC 025231
Poales Poaceae Homolepis isocalycina#NC 036690
Poales Poaceae Homolepis aturensis#NC 036125
Poales Poaceae Hildaea pallens#NC 036124
Poales Poaceae Streptostachys asperifolia#NC 036116
Poales Poaceae Axonopus ramosus#NC 036129
Poales Poaceae Paspalum inaequivalve#NC 039463
Poales Poaceae Paspalum vaginatum#NC 039462
Poales Poaceae Paspalum ionanthum#NC 039464
Poales Poaceae Paspalum minus#NC 039460
Poales Poaceae Paspalum virgatum#NC 039449
Poales Poaceae Paspalum simplex#NC 039461
Poales Poaceae Paspalum pubiflorum#NC 039448
Poales Poaceae Paspalum juergensii#NC 039459
Poales Poaceae Paspalum paniculatum#NC 036114
Poales Poaceae Garnotia thailandica#NC 035042
Poales Poaceae Garnotia tenella#NC 035051
Poales Poaceae Arthraxon microphyllus#NC 035050
Poales Poaceae Arthraxon hispidus#NC 035048
Poales Poaceae Arthraxon prionodes#KY596138
Poales Poaceae Arthraxon lanceolatus#KY596185
Poales Poaceae Tripsacum dactyloides#NC 037087
Poales Poaceae Zea mays#NC 001666
Poales Poaceae Zea luxurians#NC 030301
Poales Poaceae Zea diploperennis#NC 030377
Poales Poaceae Zea perennis#NC 030300
Poales Poaceae Polytoca digitata#KY596178
Poales Poaceae Eriochrysis cf. cayennensis#NC 029882
Poales Poaceae Eriochrysis laxa#NC 029883
Poales Poaceae Parahyparrhenia siamensis#NC 035033
Poales Poaceae Andropogon burmanicus#KY596167
Poales Poaceae Eulalia contorta#NC 035026
Poales Poaceae Kerriochloa siamensis#KY596120
Poales Poaceae Microstegium vimineum#YPIC
Poales Poaceae Coix lacryma-jobi#KY596160
Poales Poaceae Coelorachis helferi#NC 035036
Poales Poaceae Eremochloa ophiuroides#NC 036716
Poales Poaceae Eremochloa ciliaris#NC 035028
Poales Poaceae Eremochloa eriopoda#NC 035023
Poales Poaceae Chrysopogon orientalis#NC 035047
Poales Poaceae Chrysopogon serrulatus#NC 029884
Poales Poaceae Chrysopogon zizanioides#KY610123
Poales Poaceae Chrysopogon gryllus#NC 035035
Poales Poaceae Dimeria ornithopoda#NC 035020
Poales Poaceae Eulaliopsis binata#NC 035049
Poales Poaceae Germainia capitata#NC 035046
Poales Poaceae Pogonatherum paniceum#NC 029881
Poales Poaceae Sorghum timorense#NC 023800
Poales Poaceae Saccharum hildebrandtii#NC 036118
Poales Poaceae Hemisorghum mekongense#NC 035022
Poales Poaceae Sorghum bicolor#NC 008602
Poales Poaceae Pseudosorghum fasciculare#KY596157
Poales Poaceae Miscanthus sacchariflorus#LN869217
Poales Poaceae Miscanthus sinensis#LN869220
Poales Poaceae Miscanthus floridulus#NC 035750
Poales Poaceae Eulalia siamensis#NC 035031
Poales Poaceae Miscanthus junceus#NC 035751
Poales Poaceae Saccharum spontaneum#LN849912
Poales Poaceae Saccharum officinarum#MG685915
Poales Poaceae Apluda mutica#NC 036684
Poales Poaceae Andropogon brachystachyus#MH181200
Poales Poaceae Heteropogon triticeus#KY596153
Poales Poaceae Andropogon munroi#MH181215
Poales Poaceae Andropogon crossotos#MH181194
Poales Poaceae Cymbopogon citratus#MH488954
Poales Poaceae Cymbopogon nardus#ROEI
Poales Poaceae Cymbopogon flexuosus#NC 035040
Poales Poaceae Heteropogon sp.#KY707768
Poales Poaceae Schizachyrium delavayi#MH185800
Poales Poaceae Bothriochloa decipiens#NC 040131
Poales Poaceae Dichanthium annulatum#MH488955
Poales Poaceae Dichanthium sericeum#NC 035018
Poales Poaceae Heteropogon contortus#NC 035027
Poales Poaceae Themeda arundinacea#NC 035014
Poales Poaceae Themeda villosa#NC 035021
Poales Poaceae Themeda arguens#NC 035019
Poales Poaceae Themeda triandra#KY707767
Poales Poaceae Themeda quadrivalvis#NC 035492
Poales Poaceae Andropogon gerardii#MH181165
Poales Poaceae Andropogon heterantherus#MH181186
Poales Poaceae Andropogon canaliculatus#MH181224
Poales Poaceae Andropogon gayanus#MH181211
Poales Poaceae Hyparrhenia violascens#MH181167
Poales Poaceae Andropogon chinensis#KY596126
Poales Poaceae Schizachyrium lomaense#MH181201
Poales Poaceae Andropogon virgatus#MH181223
Poales Poaceae Schizachyrium claudopus#MH181228
Poales Poaceae Schizachyrium djalonicum#MH181227
Poales Poaceae Schizachyrium brevifolium#KY596135
Poales Poaceae Schizachyrium exile#MH181219
Poales Poaceae Schizachyrium sanguineum#KY596137
Poales Poaceae Schizachyrium condensatum#MH181233
Poales Poaceae Schizachyrium spicatum#MH181207
Poales Poaceae Schizachyrium scoparium#MF170217
Poales Poaceae Schizachyrium cubense#MH181205
Poales Poaceae Andropogon gracilis#MH181191
Poales Poaceae Andropogon pusillus#MH181175
Poales Poaceae Andropogon distachyos#MH181203
Poales Poaceae Andropogon mannii#MH181231
Poales Poaceae Andropogon abyssinicus#MH181184
Poales Poaceae Andropogon ivorensis#MH660710
Poales Poaceae Andropogon greenwayi#MH181177
Poales Poaceae Andropogon leprodes#MH181210
Poales Poaceae Hyparrhenia papillipes#MH181179
Poales Poaceae Andropogon chrysostachyus#MH181181
Poales Poaceae Exotheca abyssinica#MH181196
Poales Poaceae Hyparrhenia mobukensis#MH181195
Poales Poaceae Hyparrhenia rudis#MH181164
Poales Poaceae Hyparrhenia umbrosa#MH181226
Poales Poaceae Hyparrhenia newtonii#MH181229
Poales Poaceae Hyparrhenia diplandra#NC 035037
Poales Poaceae Hyparrhenia variabilis#NC 040112
Poales Poaceae Hyparrhenia rufa#NC 035011
Poales Poaceae Hyparrhenia figariana#MH181220
Poales Poaceae Hyparrhenia bagirmica#MH181190

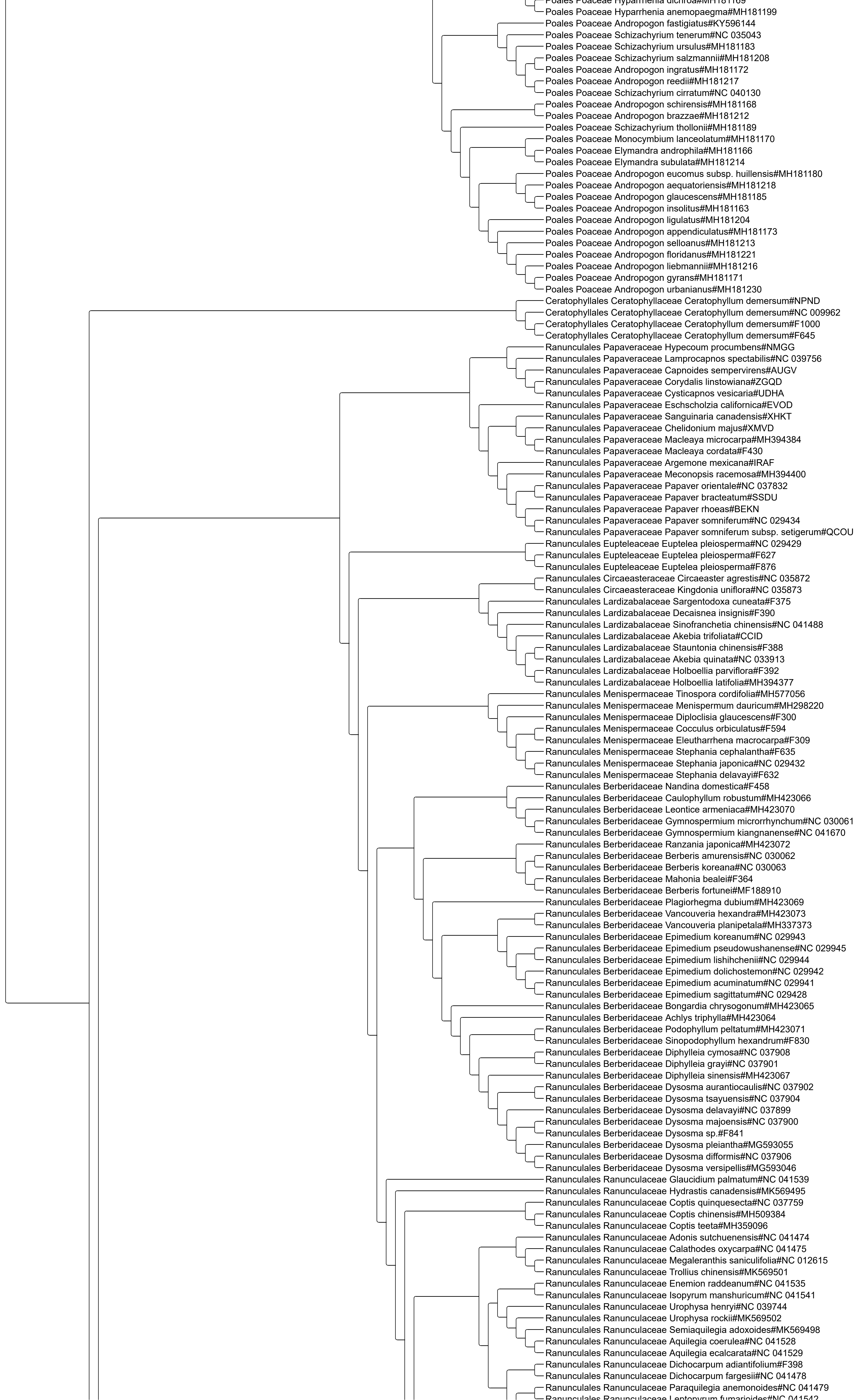

Poales Poaceae Hyparrhenia anemopaegma#MH181199
Poales Poaceae Andropogon fastigiatus#KY596144
Poales Poaceae Schizachyrium tenerum#NC 035043
Poales Poaceae Schizachyrium ursulus#MH181183
Poales Poaceae Schizachyrium salzmannii#MH181208
Poales Poaceae Andropogon ingratus#MH181172
Poales Poaceae Andropogon reedii#MH181217
Poales Poaceae Schizachyrium cirratum#NC 040130
Poales Poaceae Andropogon schirensis#MH181168
Poales Poaceae Andropogon brazzae#MH181212
Poales Poaceae Schizachyrium thollonii#MH181189
Poales Poaceae Monocymbium lanceolatum#MH181170
Poales Poaceae Elymandra androphila#MH181166
Poales Poaceae Elymandra subulata#MH181214
Poales Poaceae Andropogon eucomus subsp. huillensis#MH181180
Poales Poaceae Andropogon aequatoriensis#MH181218
Poales Poaceae Andropogon glaucescens#MH181185
Poales Poaceae Andropogon insolitus#MH181163
Poales Poaceae Andropogon ligulatus#MH181204
Poales Poaceae Andropogon appendiculatus#MH181173
Poales Poaceae Andropogon selloanus#MH181213
Poales Poaceae Andropogon floridanus#MH181221
Poales Poaceae Andropogon liebmannii#MH181216
Poales Poaceae Andropogon gyrans#MH181171
Poales Poaceae Andropogon urbanianus#MH181230
Ceratophyllales Ceratophyllaceae Ceratophyllum demersum#NPND
Ceratophyllales Ceratophyllaceae Ceratophyllum demersum#NC 009962
Ceratophyllales Ceratophyllaceae Ceratophyllum demersum#F1000
Ceratophyllales Ceratophyllaceae Ceratophyllum demersum#F645
Ranunculales Papaveraceae Hypecoum procumbens#NMGG
Ranunculales Papaveraceae Lamprocapnos spectabilis#NC 039756
Ranunculales Papaveraceae Capnoides sempervirens#AUGV
Ranunculales Papaveraceae Corydalis linstowiana#ZGQD
Ranunculales Papaveraceae Cysticapnos vesicaria#UDHA
Ranunculales Papaveraceae Eschscholzia californica#EVOD
Ranunculales Papaveraceae Sanguinaria canadensis#XHKT
Ranunculales Papaveraceae Chelidonium majus#XMVD
Ranunculales Papaveraceae Macleaya microcarpa#MH394384
Ranunculales Papaveraceae Macleaya cordata#F430
Ranunculales Papaveraceae Argemone mexicana#IRAF
Ranunculales Papaveraceae Meconopsis racemosa#MH394400
Ranunculales Papaveraceae Papaver orientale#NC 037832
Ranunculales Papaveraceae Papaver bracteatum#SSDU
Ranunculales Papaveraceae Papaver rhoeas#BEKN
Ranunculales Papaveraceae Papaver somniferum#NC 029434
Ranunculales Papaveraceae Papaver somniferum subsp. setigerum#QCOU
Ranunculales Eupteleaceae Euptelea pleiosperma#NC 029429
Ranunculales Eupteleaceae Euptelea pleiosperma#F627
Ranunculales Eupteleaceae Euptelea pleiosperma#F876
Ranunculales Circaeasteraceae Circaeaster agrestis#NC 035872
Ranunculales Circaeasteraceae Kingdonia uniflora#NC 035873
Ranunculales Lardizabalaceae Sargentodoxa cuneata#F375
Ranunculales Lardizabalaceae Decaisnea insignis#F390
Ranunculales Lardizabalaceae Sinofranchetia chinensis#NC 041488
Ranunculales Lardizabalaceae Akebia trifoliata#CCID
Ranunculales Lardizabalaceae Stauntonia chinensis#F388
Ranunculales Lardizabalaceae Akebia quinata#NC 033913
Ranunculales Lardizabalaceae Holboellia parviflora#F392
Ranunculales Lardizabalaceae Holboellia latifolia#MH394377
Ranunculales Menispermaceae Tinospora cordifolia#MH577056
Ranunculales Menispermaceae Menispermum dauricum#MH298220
Ranunculales Menispermaceae Diploclisia glaucescens#F300
Ranunculales Menispermaceae Cocculus orbiculatus#F594
Ranunculales Menispermaceae Eleutharrhena macrocarpa#F309
Ranunculales Menispermaceae Stephania cephalantha#F635
Ranunculales Menispermaceae Stephania japonica#NC 029432
Ranunculales Menispermaceae Stephania delavayi#F632
Ranunculales Berberidaceae Nandina domestica#F458
Ranunculales Berberidaceae Caulophyllum robustum#MH423066
Ranunculales Berberidaceae Leontice armeniaca#MH423070
Ranunculales Berberidaceae Gymnospermium microrrhynchum#NC 030061
Ranunculales Berberidaceae Gymnospermium kiangnanense#NC 041670
Ranunculales Berberidaceae Ranzania japonica#MH423072
Ranunculales Berberidaceae Berberis amurensis#NC 030062
Ranunculales Berberidaceae Berberis koreana#NC 030063
Ranunculales Berberidaceae Mahonia bealei#F364
Ranunculales Berberidaceae Berberis fortunei#MF188910
Ranunculales Berberidaceae Plagiorhegma dubium#MH423069
Ranunculales Berberidaceae Vancouveria hexandra#MH423073
Ranunculales Berberidaceae Vancouveria planipetala#MH337373
Ranunculales Berberidaceae Epimedium koreanum#NC 029943
Ranunculales Berberidaceae Epimedium pseudowushanense#NC 029945
Ranunculales Berberidaceae Epimedium lishihchenii#NC 029944
Ranunculales Berberidaceae Epimedium dolichostemon#NC 029942
Ranunculales Berberidaceae Epimedium acuminatum#NC 029941
Ranunculales Berberidaceae Epimedium sagittatum#NC 029428
Ranunculales Berberidaceae Bongardia chrysogonum#MH423065
Ranunculales Berberidaceae Achlys triphylla#MH423064
Ranunculales Berberidaceae Podophyllum peltatum#MH423071
Ranunculales Berberidaceae Sinopodophyllum hexandrum#F830
Ranunculales Berberidaceae Diphylleia cymosa#NC 037908
Ranunculales Berberidaceae Diphylleia grayi#NC 037901
Ranunculales Berberidaceae Diphylleia sinensis#MH423067
Ranunculales Berberidaceae Dysosma aurantiocaulis#NC 037902
Ranunculales Berberidaceae Dysosma tsayuensis#NC 037904
Ranunculales Berberidaceae Dysosma delavayi#NC 037899
Ranunculales Berberidaceae Dysosma majoensis#NC 037900
Ranunculales Berberidaceae Dysosma sp.#F841
Ranunculales Berberidaceae Dysosma pleiantha#MG593055
Ranunculales Berberidaceae Dysosma difformis#NC 037906
Ranunculales Berberidaceae Dysosma versipellis#MG593046
Ranunculales Ranunculaceae Glaucidium palmatum#NC 041539
Ranunculales Ranunculaceae Hydrastis canadensis#MK569495
Ranunculales Ranunculaceae Coptis quinquesecta#NC 037759
Ranunculales Ranunculaceae Coptis chinensis#MH509384
Ranunculales Ranunculaceae Coptis teeta#MH359096
Ranunculales Ranunculaceae Adonis sutchuenensis#NC 041474
Ranunculales Ranunculaceae Calathodes oxycarpa#NC 041475
Ranunculales Ranunculaceae Megaleranthis saniculifolia#NC 012615
Ranunculales Ranunculaceae Trollius chinensis#MK569501
Ranunculales Ranunculaceae Enemion raddeanum#NC 041535
Ranunculales Ranunculaceae Isopyrum manshuricum#NC 041541
Ranunculales Ranunculaceae Urophysa henryi#NC 039744
Ranunculales Ranunculaceae Urophysa rockii#MK569502
Ranunculales Ranunculaceae Semiaquilegia adoxoides#MK569498
Ranunculales Ranunculaceae Aquilegia coerulea#NC 041528
Ranunculales Ranunculaceae Aquilegia ecalcarata#NC 041529
Ranunculales Ranunculaceae Dichocarpum adiantifolium#F398
Ranunculales Ranunculaceae Dichocarpum fargesii#NC 041478
Ranunculales Ranunculaceae Paraquilegia anemonoides#NC 041479

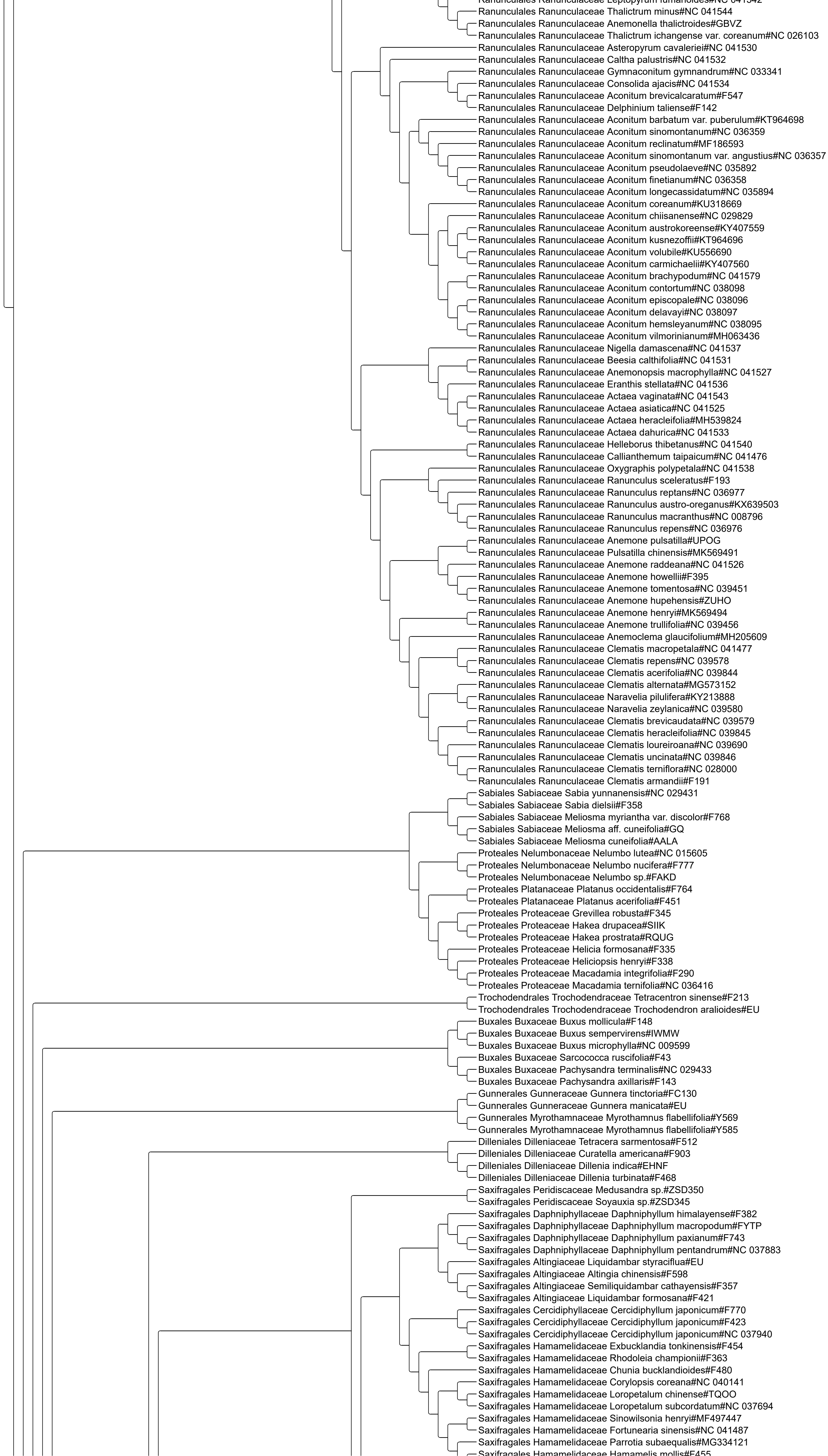
Ranunculales Ranunculaceae Leptopyrum fumarioides#NC 041542
Ranunculales Ranunculaceae Thalictrum minus#NC 041544
Ranunculales Ranunculaceae Anemonella thalictroides#GBVZ
Ranunculales Ranunculaceae Thalictrum ichangense var. coreanum#NC 026103
Ranunculales Ranunculaceae Asteropyrum cavaleriei#NC 041530
Ranunculales Ranunculaceae Caltha palustris#NC 041532
Ranunculales Ranunculaceae Gymnaconitum gymnandrum#NC 033341
Ranunculales Ranunculaceae Consolida ajacis#NC 041534
Ranunculales Ranunculaceae Aconitum brevicalcaratum#F547
Ranunculales Ranunculaceae Delphinium taliense#F142
Ranunculales Ranunculaceae Aconitum barbatum var. puberulum#KT964698
Ranunculales Ranunculaceae Aconitum sinomontanum#NC 036359
Ranunculales Ranunculaceae Aconitum reclinatum#MF186593
Ranunculales Ranunculaceae Aconitum sinomontanum var. angustius#NC 036357
Ranunculales Ranunculaceae Aconitum pseudolaeve#NC 035892
Ranunculales Ranunculaceae Aconitum finetianum#NC 036358
Ranunculales Ranunculaceae Aconitum longecassidatum#NC 035894
Ranunculales Ranunculaceae Aconitum coreanum#KU318669
Ranunculales Ranunculaceae Aconitum chiisanense#NC 029829
Ranunculales Ranunculaceae Aconitum austrokoreense#KY407559
Ranunculales Ranunculaceae Aconitum kusnezoffii#KT964696
Ranunculales Ranunculaceae Aconitum volubile#KU556690
Ranunculales Ranunculaceae Aconitum carmichaelii#KY407560
Ranunculales Ranunculaceae Aconitum brachypodum#NC 041579
Ranunculales Ranunculaceae Aconitum contortum#NC 038098
Ranunculales Ranunculaceae Aconitum episcopale#NC 038096
Ranunculales Ranunculaceae Aconitum delavayi#NC 038097
Ranunculales Ranunculaceae Aconitum hemsleyanum#NC 038095
Ranunculales Ranunculaceae Aconitum vilmorinianum#MH063436
Ranunculales Ranunculaceae Nigella damascena#NC 041537
Ranunculales Ranunculaceae Beesia calthifolia#NC 041531
Ranunculales Ranunculaceae Anemonopsis macrophylla#NC 041527
Ranunculales Ranunculaceae Eranthis stellata#NC 041536
Ranunculales Ranunculaceae Actaea vaginata#NC 041543
Ranunculales Ranunculaceae Actaea asiatica#NC 041525
Ranunculales Ranunculaceae Actaea heracleifolia#MH539824
Ranunculales Ranunculaceae Actaea dahurica#NC 041533
Ranunculales Ranunculaceae Helleborus thibetanus#NC 041540
Ranunculales Ranunculaceae Callianthemum taipaicum#NC 041476
Ranunculales Ranunculaceae Oxygraphis polypetala#NC 041538
Ranunculales Ranunculaceae Ranunculus sceleratus#F193
Ranunculales Ranunculaceae Ranunculus reptans#NC 036977
Ranunculales Ranunculaceae Ranunculus austro-oreganus#KX639503
Ranunculales Ranunculaceae Ranunculus macranthus#NC 008796
Ranunculales Ranunculaceae Ranunculus repens#NC 036976
Ranunculales Ranunculaceae Anemone pulsatilla#UPOG
Ranunculales Ranunculaceae Pulsatilla chinensis#MK569491
Ranunculales Ranunculaceae Anemone raddeana#NC 041526
Ranunculales Ranunculaceae Anemone howellii#F395
Ranunculales Ranunculaceae Anemone tomentosa#NC 039451
Ranunculales Ranunculaceae Anemone hupehensis#ZUHO
Ranunculales Ranunculaceae Anemone henryi#MK569494
Ranunculales Ranunculaceae Anemone trullifolia#NC 039456
Ranunculales Ranunculaceae Anemoclema glaucifolium#MH205609
Ranunculales Ranunculaceae Clematis macropetala#NC 041477
Ranunculales Ranunculaceae Clematis repens#NC 039578
Ranunculales Ranunculaceae Clematis acerifolia#NC 039844
Ranunculales Ranunculaceae Clematis alternata#MG573152
Ranunculales Ranunculaceae Naravelia pilulifera#KY213888
Ranunculales Ranunculaceae Naravelia zeylanica#NC 039580
Ranunculales Ranunculaceae Clematis brevicaudata#NC 039579
Ranunculales Ranunculaceae Clematis heracleifolia#NC 039845
Ranunculales Ranunculaceae Clematis loureiroana#NC 039690
Ranunculales Ranunculaceae Clematis uncinata#NC 039846
Ranunculales Ranunculaceae Clematis terniflora#NC 028000
Ranunculales Ranunculaceae Clematis armandii#F191
Sabiales Sabiaceae Sabia yunnanensis#NC 029431
Sabiales Sabiaceae Sabia dielsii#F358
Sabiales Sabiaceae Meliosma myriantha var. discolor#F768
Sabiales Sabiaceae Meliosma aff. cuneifolia#GQ
Sabiales Sabiaceae Meliosma cuneifolia#AALA
Proteales Nelumbonaceae Nelumbo lutea#NC 015605
Proteales Nelumbonaceae Nelumbo nucifera#F777
Proteales Nelumbonaceae Nelumbo sp.#FAKD
Proteales Platanaceae Platanus occidentalis#F764
Proteales Platanaceae Platanus acerifolia#F451
Proteales Proteaceae Grevillea robusta#F345
Proteales Proteaceae Hakea drupacea#SIIK
Proteales Proteaceae Hakea prostrata#RQUG
Proteales Proteaceae Helicia formosana#F335
Proteales Proteaceae Heliciopsis henryi#F338
Proteales Proteaceae Macadamia integrifolia#F290
Proteales Proteaceae Macadamia ternifolia#NC 036416
Trochodendrales Trochodendraceae Tetracentron sinense#F213
Trochodendrales Trochodendraceae Trochodendron aralioides#EU
Buxales Buxaceae Buxus mollicula#F148
Buxales Buxaceae Buxus sempervirens#IWMW
Buxales Buxaceae Buxus microphylla#NC 009599
Buxales Buxaceae Sarcococca ruscifolia#F43
Buxales Buxaceae Pachysandra terminalis#NC 029433
Buxales Buxaceae Pachysandra axillaris#F143
Gunnerales Gunneraceae Gunnera tinctoria#FC130
Gunnerales Gunneraceae Gunnera manicata#EU
Gunnerales Myrothamnaceae Myrothamnus flabellifolia#Y569
Gunnerales Myrothamnaceae Myrothamnus flabellifolia#Y585
Dilleniales Dilleniaceae Tetracera sarmentosa#F512
Dilleniales Dilleniaceae Curatella americana#F903
Dilleniales Dilleniaceae Dillenia indica#EHNF
Dilleniales Dilleniaceae Dillenia turbinata#F468
Saxifragales Peridiscaceae Medusandra sp.#ZSD350
Saxifragales Peridiscaceae Soyauxia sp.#ZSD345
Saxifragales Daphniphyllaceae Daphniphyllum himalayense#F382
Saxifragales Daphniphyllaceae Daphniphyllum macropodum#FYTP
Saxifragales Daphniphyllaceae Daphniphyllum paxianum#F743
Saxifragales Daphniphyllaceae Daphniphyllum pentandrum#NC 037883
Saxifragales Altingiaceae Liquidambar styraciflua#EU
Saxifragales Altingiaceae Altingia chinensis#F598
Saxifragales Altingiaceae Semiliquidambar cathayensis#F357
Saxifragales Altingiaceae Liquidambar formosana#F421
Saxifragales Cercidiphyllaceae Cercidiphyllum japonicum#F770
Saxifragales Cercidiphyllaceae Cercidiphyllum japonicum#F423
Saxifragales Cercidiphyllaceae Cercidiphyllum japonicum#NC 037940
Saxifragales Hamamelidaceae Exbucklandia tonkinensis#F454
Saxifragales Hamamelidaceae Rhodoleia championii#F363
Saxifragales Hamamelidaceae Chunia bucklandioides#F480
Saxifragales Hamamelidaceae Corylopsis coreana#NC 040141
Saxifragales Hamamelidaceae Loropetalum chinense#TQOO
Saxifragales Hamamelidaceae Loropetalum subcordatum#NC 037694
Saxifragales Hamamelidaceae Sinowilsonia henryi#MF497447
Saxifragales Hamamelidaceae Fortunearia sinensis#NC 041487
Saxifragales Hamamelidaceae Parrotia subaequalis#MG334121
Saxifragales Hamamelidaceae Hamamelis mollis#F455

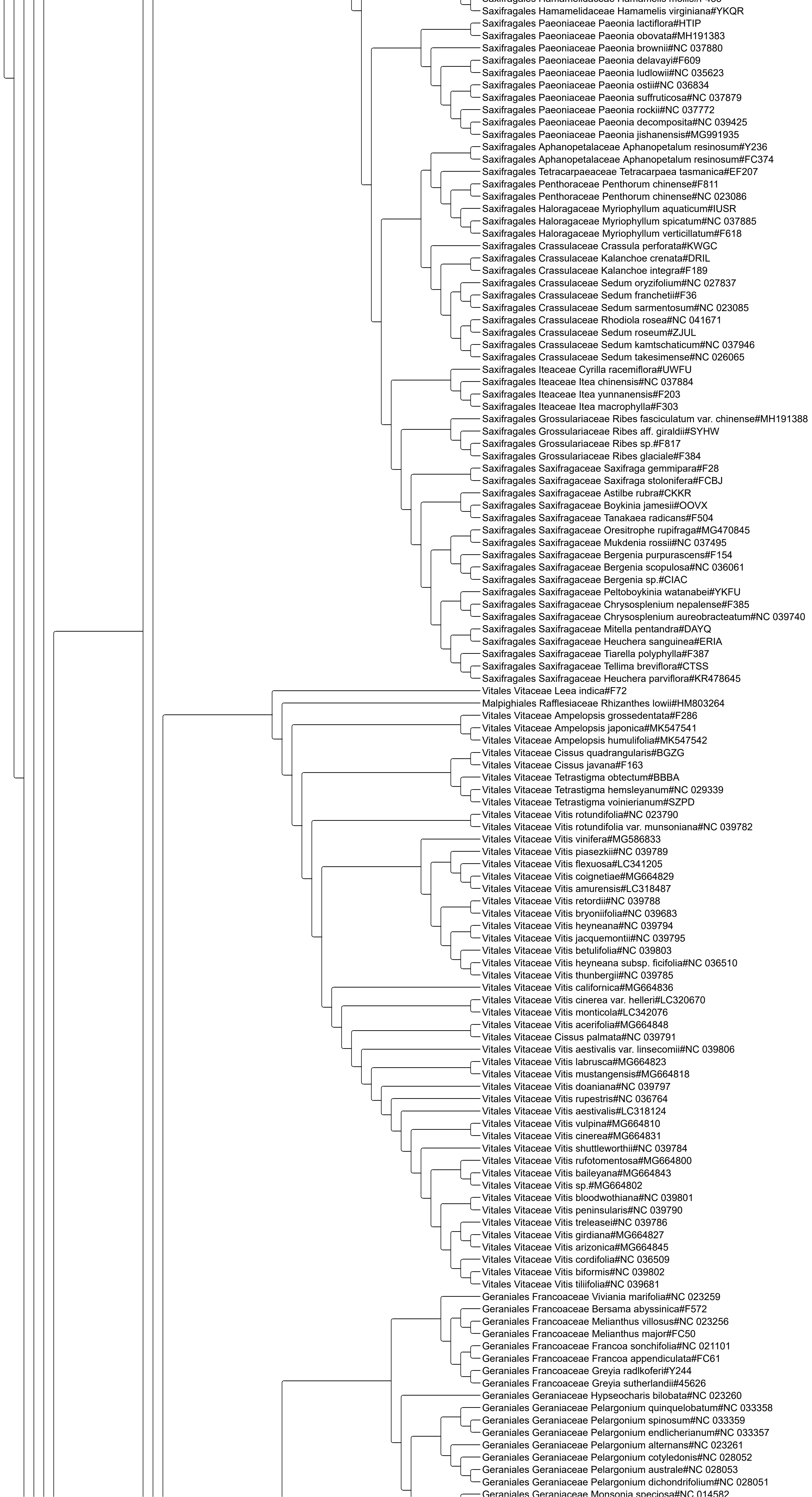
Saxifragales Hamamelidaceae Hamamelis virginiana#YKQR
Saxifragales Paeoniaceae Paeonia lactiflora#HTIP
Saxifragales Paeoniaceae Paeonia obovata#MH191383
Saxifragales Paeoniaceae Paeonia brownii#NC 037880
Saxifragales Paeoniaceae Paeonia delavayi#F609
Saxifragales Paeoniaceae Paeonia ludlowii#NC 035623
Saxifragales Paeoniaceae Paeonia ostii#NC 036834
Saxifragales Paeoniaceae Paeonia suffruticosa#NC 037879
Saxifragales Paeoniaceae Paeonia rockii#NC 037772
Saxifragales Paeoniaceae Paeonia decomposita#NC 039425
Saxifragales Paeoniaceae Paeonia jishanensis#MG991935
Saxifragales Aphanopetalaceae Aphanopetalum resinosum#Y236
Saxifragales Aphanopetalaceae Aphanopetalum resinosum#FC374
Saxifragales Tetracarpaeaceae Tetracarpaea tasmanica#EF207
Saxifragales Penthoraceae Penthorum chinense#F811
Saxifragales Penthoraceae Penthorum chinense#NC 023086
Saxifragales Haloragaceae Myriophyllum aquaticum#IUSR
Saxifragales Haloragaceae Myriophyllum spicatum#NC 037885
Saxifragales Haloragaceae Myriophyllum verticillatum#F618
Saxifragales Crassulaceae Crassula perforata#KWGC
Saxifragales Crassulaceae Kalanchoe crenata#DRIL
Saxifragales Crassulaceae Kalanchoe integra#F189
Saxifragales Crassulaceae Sedum oryzifolium#NC 027837
Saxifragales Crassulaceae Sedum franchetii#F36
Saxifragales Crassulaceae Sedum sarmentosum#NC 023085
Saxifragales Crassulaceae Rhodiola rosea#NC 041671
Saxifragales Crassulaceae Sedum roseum#ZJUL
Saxifragales Crassulaceae Sedum kamtschaticum#NC 037946
Saxifragales Crassulaceae Sedum takesimense#NC 026065
Saxifragales Iteaceae Cyrilla racemiflora#UWFU
Saxifragales Iteaceae Itea chinensis#NC 037884
Saxifragales Iteaceae Itea yunnanensis#F203
Saxifragales Iteaceae Itea macrophylla#F303
Saxifragales Grossulariaceae Ribes fasciculatum var. chinense#MH191388
Saxifragales Grossulariaceae Ribes aff. giraldii#SYHW
Saxifragales Grossulariaceae Ribes sp.#F817
Saxifragales Grossulariaceae Ribes glaciale#F384
Saxifragales Saxifragaceae Saxifraga gemmipara#F28
Saxifragales Saxifragaceae Saxifraga stolonifera#FCBJ
Saxifragales Saxifragaceae Astilbe rubra#CKKR
Saxifragales Saxifragaceae Boykinia jamesii#OOVX
Saxifragales Saxifragaceae Tanakaea radicans#F504
Saxifragales Saxifragaceae Oresitrophe rupifraga#MG470845
Saxifragales Saxifragaceae Mukdenia rossii#NC 037495
Saxifragales Saxifragaceae Bergenia purpurascens#F154
Saxifragales Saxifragaceae Bergenia scopulosa#NC 036061
Saxifragales Saxifragaceae Bergenia sp.#CIAC
Saxifragales Saxifragaceae Peltoboykinia watanabei#YKFU
Saxifragales Saxifragaceae Chrysosplenium nepalense#F385
Saxifragales Saxifragaceae Chrysosplenium aureobracteatum#NC 039740
Saxifragales Saxifragaceae Mitella pentandra#DAYQ
Saxifragales Saxifragaceae Heuchera sanguinea#ERIA
Saxifragales Saxifragaceae Tiarella polyphylla#F387
Saxifragales Saxifragaceae Tellima breviflora#CTSS
Saxifragales Saxifragaceae Heuchera parviflora#KR478645
Vitales Vitaceae Leea indica#F72
Malpighiales Rafflesiaceae Rhizanthes lowii#HM803264
Vitales Vitaceae Ampelopsis grossedentata#F286
Vitales Vitaceae Ampelopsis japonica#MK547541
Vitales Vitaceae Ampelopsis humulifolia#MK547542
Vitales Vitaceae Cissus quadrangularis#BGZG
Vitales Vitaceae Cissus javana#F163
Vitales Vitaceae Tetrastigma obtectum#BBBA
Vitales Vitaceae Tetrastigma hemsleyanum#NC 029339
Vitales Vitaceae Tetrastigma voinierianum#SZPD
Vitales Vitaceae Vitis rotundifolia#NC 023790
Vitales Vitaceae Vitis rotundifolia var. munsoniana#NC 039782
Vitales Vitaceae Vitis vinifera#MG586833
Vitales Vitaceae Vitis piasezkii#NC 039789
Vitales Vitaceae Vitis flexuosa#LC341205
Vitales Vitaceae Vitis coignetiae#MG664829
Vitales Vitaceae Vitis amurensis#LC318487
Vitales Vitaceae Vitis retordii#NC 039788
Vitales Vitaceae Vitis bryoniifolia#NC 039683
Vitales Vitaceae Vitis heyneana#NC 039794
Vitales Vitaceae Vitis jacquemontii#NC 039795
Vitales Vitaceae Vitis betulifolia#NC 039803
Vitales Vitaceae Vitis heyneana subsp. ficifolia#NC 036510
Vitales Vitaceae Vitis thunbergii#NC 039785
Vitales Vitaceae Vitis californica#MG664836
Vitales Vitaceae Vitis cinerea var. helleri#LC320670
Vitales Vitaceae Vitis monticola#LC342076
Vitales Vitaceae Vitis acerifolia#MG664848
Vitales Vitaceae Cissus palmata#NC 039791
Vitales Vitaceae Vitis aestivalis var. linsecomii#NC 039806
Vitales Vitaceae Vitis labrusca#MG664823
Vitales Vitaceae Vitis mustangensis#MG664818
Vitales Vitaceae Vitis doaniana#NC 039797
Vitales Vitaceae Vitis rupestris#NC 036764
Vitales Vitaceae Vitis aestivalis#LC318124
Vitales Vitaceae Vitis vulpina#MG664810
Vitales Vitaceae Vitis cinerea#MG664831
Vitales Vitaceae Vitis shuttleworthii#NC 039784
Vitales Vitaceae Vitis rufotomentosa#MG664800
Vitales Vitaceae Vitis baileyana#MG664843
Vitales Vitaceae Vitis sp.#MG664802
Vitales Vitaceae Vitis bloodwothiana#NC 039801
Vitales Vitaceae Vitis peninsularis#NC 039790
Vitales Vitaceae Vitis treleasei#NC 039786
Vitales Vitaceae Vitis girdiana#MG664827
Vitales Vitaceae Vitis arizonica#MG664845
Vitales Vitaceae Vitis cordifolia#NC 036509
Vitales Vitaceae Vitis biformis#NC 039802
Vitales Vitaceae Vitis tiliifolia#NC 039681
Geraniales Francoaceae Viviania marifolia#NC 023259
Geraniales Francoaceae Bersama abyssinica#F572
Geraniales Francoaceae Melianthus villosus#NC 023256
Geraniales Francoaceae Melianthus major#FC50
Geraniales Francoaceae Francoa sonchifolia#NC 021101
Geraniales Francoaceae Francoa appendiculata#FC61
Geraniales Francoaceae Greyia radlkoferi#Y244
Geraniales Francoaceae Greyia sutherlandii#45626
Geraniales Geraniaceae Hypseocharis bilobata#NC 023260
Geraniales Geraniaceae Pelargonium quinquelobatum#NC 033358
Geraniales Geraniaceae Pelargonium spinosum#NC 033359
Geraniales Geraniaceae Pelargonium endlicherianum#NC 033357
Geraniales Geraniaceae Pelargonium alternans#NC 023261
Geraniales Geraniaceae Pelargonium cotyledonis#NC 028052
Geraniales Geraniaceae Pelargonium australe#NC 028053
Geraniales Geraniaceae Pelargonium dichondrifolium#NC 028051
Geraniales Geraniaceae Monsonia speciosa#NC 014582

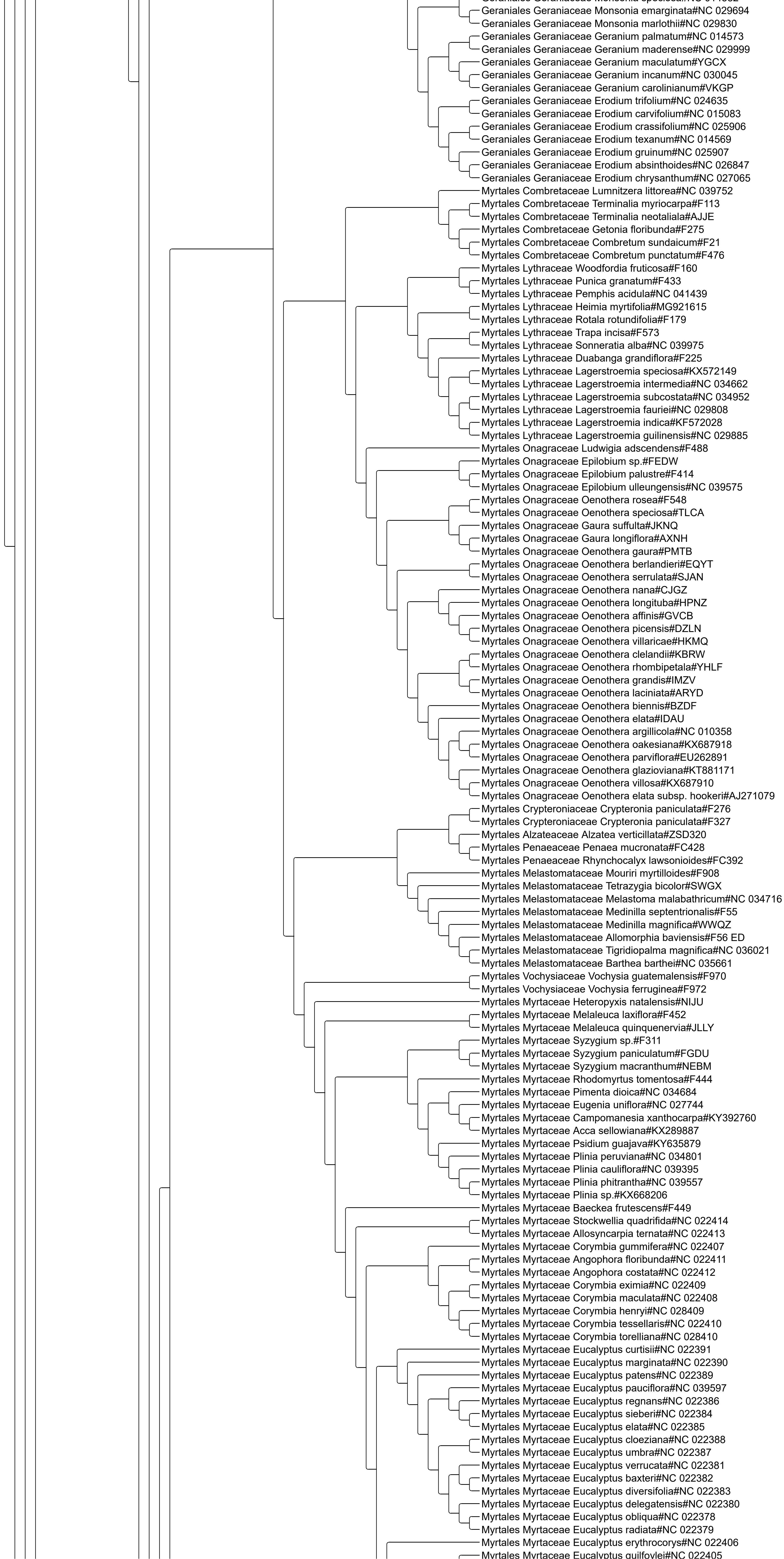
Geraniales Geraniaceae Monsonia emarginata#NC 029694
Geraniales Geraniaceae Monsonia marlothii#NC 029830
Geraniales Geraniaceae Geranium palmatum#NC 014573
Geraniales Geraniaceae Geranium maderense#NC 029999
Geraniales Geraniaceae Geranium maculatum#YGCX
Geraniales Geraniaceae Geranium incanum#NC 030045
Geraniales Geraniaceae Geranium carolinianum#VKGP
Geraniales Geraniaceae Erodium trifolium#NC 024635
Geraniales Geraniaceae Erodium carvifolium#NC 015083
Geraniales Geraniaceae Erodium crassifolium#NC 025906
Geraniales Geraniaceae Erodium texanum#NC 014569
Geraniales Geraniaceae Erodium gruinum#NC 025907
Geraniales Geraniaceae Erodium absinthoides#NC 026847
Geraniales Geraniaceae Erodium chrysanthum#NC 027065
Myrtales Combretaceae Lumnitzera littorea#NC 039752
Myrtales Combretaceae Terminalia myriocarpa#F113
Myrtales Combretaceae Terminalia neotaliala#AJJE
Myrtales Combretaceae Getonia floribunda#F275
Myrtales Combretaceae Combretum sundaicum#F21
Myrtales Combretaceae Combretum punctatum#F476
Myrtales Lythraceae Woodfordia fruticosa#F160
Myrtales Lythraceae Punica granatum#F433
Myrtales Lythraceae Pemphis acidula#NC 041439
Myrtales Lythraceae Heimia myrtifolia#MG921615
Myrtales Lythraceae Rotala rotundifolia#F179
Myrtales Lythraceae Trapa incisa#F573
Myrtales Lythraceae Sonneratia alba#NC 039975
Myrtales Lythraceae Duabanga grandiflora#F225
Myrtales Lythraceae Lagerstroemia speciosa#KX572149
Myrtales Lythraceae Lagerstroemia intermedia#NC 034662
Myrtales Lythraceae Lagerstroemia subcostata#NC 034952
Myrtales Lythraceae Lagerstroemia fauriei#NC 029808
Myrtales Lythraceae Lagerstroemia indica#KF572028
Myrtales Lythraceae Lagerstroemia guilinensis#NC 029885
Myrtales Onagraceae Ludwigia adscendens#F488
Myrtales Onagraceae Epilobium sp.#FEDW
Myrtales Onagraceae Epilobium palustre#F414
Myrtales Onagraceae Epilobium ulleungensis#NC 039575
Myrtales Onagraceae Oenothera rosea#F548
Myrtales Onagraceae Oenothera speciosa#TLCA
Myrtales Onagraceae Gaura suffulta#JKNQ
Myrtales Onagraceae Gaura longiflora#AXNH
Myrtales Onagraceae Oenothera gaura#PMTB
Myrtales Onagraceae Oenothera berlandieri#EQYT
Myrtales Onagraceae Oenothera serrulata#SJAN
Myrtales Onagraceae Oenothera nana#CJGZ
Myrtales Onagraceae Oenothera longituba#HPNZ
Myrtales Onagraceae Oenothera affinis#GVCB
Myrtales Onagraceae Oenothera picensis#DZLN
Myrtales Onagraceae Oenothera villaricae#HKMQ
Myrtales Onagraceae Oenothera clelandii#KBRW
Myrtales Onagraceae Oenothera rhombipetala#YHLF
Myrtales Onagraceae Oenothera grandis#IMZV
Myrtales Onagraceae Oenothera laciniata#ARYD
Myrtales Onagraceae Oenothera biennis#BZDF
Myrtales Onagraceae Oenothera elata#IDAU
Myrtales Onagraceae Oenothera argillicola#NC 010358
Myrtales Onagraceae Oenothera oakesiana#KX687918
Myrtales Onagraceae Oenothera parviflora#EU262891
Myrtales Onagraceae Oenothera glazioviana#KT881171
Myrtales Onagraceae Oenothera villosa#KX687910
Myrtales Onagraceae Oenothera elata subsp. hookeri#AJ271079
Myrtales Crypteroniaceae Crypteronia paniculata#F276
Myrtales Crypteroniaceae Crypteronia paniculata#F327
Myrtales Alzateaceae Alzatea verticillata#ZSD320
Myrtales Penaeaceae Penaea mucronata#FC428
Myrtales Penaeaceae Rhynchocalyx lawsonioides#FC392
Myrtales Melastomataceae Mouriri myrtilloides#F908
Myrtales Melastomataceae Tetrazygia bicolor#SWGX
Myrtales Melastomataceae Melastoma malabathricum#NC 034716
Myrtales Melastomataceae Medinilla septentrionalis#F55
Myrtales Melastomataceae Medinilla magnifica#WWQZ
Myrtales Melastomataceae Allomorphia baviensis#F56 ED
Myrtales Melastomataceae Tigridiopalma magnifica#NC 036021
Myrtales Melastomataceae Barthea barthei#NC 035661
Myrtales Vochysiaceae Vochysia guatemalensis#F970
Myrtales Vochysiaceae Vochysia ferruginea#F972
Myrtales Myrtaceae Heteropyxis natalensis#NIJU
Myrtales Myrtaceae Melaleuca laxiflora#F452
Myrtales Myrtaceae Melaleuca quinquenervia#JLLY
Myrtales Myrtaceae Syzygium sp.#F311
Myrtales Myrtaceae Syzygium paniculatum#FGDU
Myrtales Myrtaceae Syzygium macranthum#NEBM
Myrtales Myrtaceae Rhodomyrtus tomentosa#F444
Myrtales Myrtaceae Pimenta dioica#NC 034684
Myrtales Myrtaceae Eugenia uniflora#NC 027744
Myrtales Myrtaceae Campomanesia xanthocarpa#KY392760
Myrtales Myrtaceae Acca sellowiana#KX289887
Myrtales Myrtaceae Psidium guajava#KY635879
Myrtales Myrtaceae Plinia peruviana#NC 034801
Myrtales Myrtaceae Plinia cauliflora#NC 039395
Myrtales Myrtaceae Plinia phitrantha#NC 039557
Myrtales Myrtaceae Plinia sp.#KX668206
Myrtales Myrtaceae Baeckea frutescens#F449
Myrtales Myrtaceae Stockwellia quadrifida#NC 022414
Myrtales Myrtaceae Allosyncarpia ternata#NC 022413
Myrtales Myrtaceae Corymbia gummifera#NC 022407
Myrtales Myrtaceae Angophora floribunda#NC 022411
Myrtales Myrtaceae Angophora costata#NC 022412
Myrtales Myrtaceae Corymbia eximia#NC 022409
Myrtales Myrtaceae Corymbia maculata#NC 022408
Myrtales Myrtaceae Corymbia henryi#NC 028409
Myrtales Myrtaceae Corymbia tessellaris#NC 022410
Myrtales Myrtaceae Corymbia torelliana#NC 028410
Myrtales Myrtaceae Eucalyptus curtisii#NC 022391
Myrtales Myrtaceae Eucalyptus marginata#NC 022390
Myrtales Myrtaceae Eucalyptus patens#NC 022389
Myrtales Myrtaceae Eucalyptus pauciflora#NC 039597
Myrtales Myrtaceae Eucalyptus regnans#NC 022386
Myrtales Myrtaceae Eucalyptus sieberi#NC 022384
Myrtales Myrtaceae Eucalyptus elata#NC 022385
Myrtales Myrtaceae Eucalyptus cloeziana#NC 022388
Myrtales Myrtaceae Eucalyptus umbra#NC 022387
Myrtales Myrtaceae Eucalyptus verrucata#NC 022381
Myrtales Myrtaceae Eucalyptus baxteri#NC 022382
Myrtales Myrtaceae Eucalyptus diversifolia#NC 022383
Myrtales Myrtaceae Eucalyptus delegatensis#NC 022380
Myrtales Myrtaceae Eucalyptus obliqua#NC 022378
Myrtales Myrtaceae Eucalyptus radiata#NC 022379
Myrtales Myrtaceae Eucalyptus erythrocorys#NC 022406
Myrtales Myrtaceae Eucalyptus guilfoylei#NC 022405

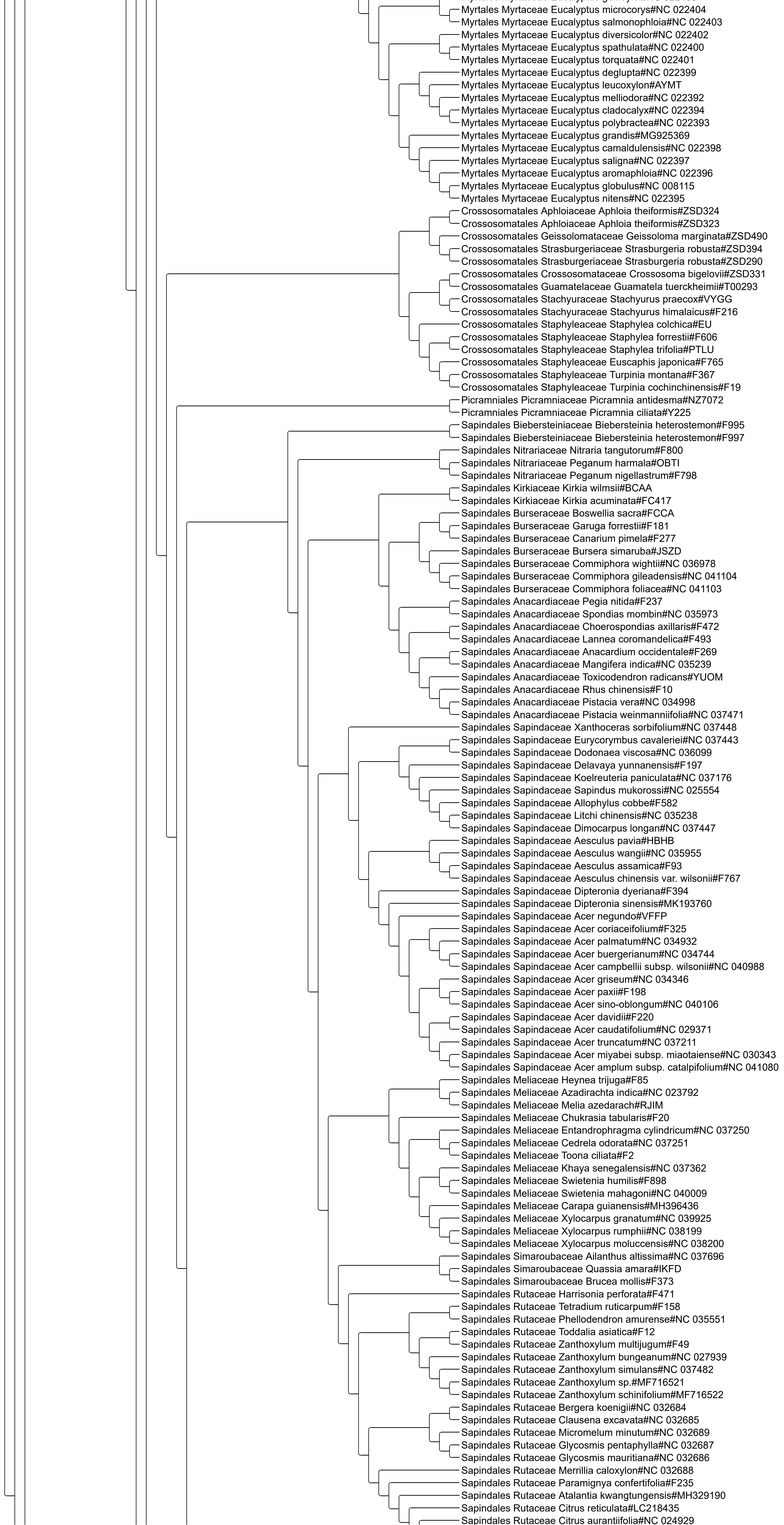
Myrtales Myrtaceae Eucalyptus microcorys#NC 022404
Myrtales Myrtaceae Eucalyptus salmonophloia#NC 022403
Myrtales Myrtaceae Eucalyptus diversicolor#NC 022402
Myrtales Myrtaceae Eucalyptus spathulata#NC 022400
Myrtales Myrtaceae Eucalyptus torquata#NC 022401
Myrtales Myrtaceae Eucalyptus deglupta#NC 022399
Myrtales Myrtaceae Eucalyptus leucoxylon#AYMT
Myrtales Myrtaceae Eucalyptus melliodora#NC 022392
Myrtales Myrtaceae Eucalyptus cladocalyx#NC 022394
Myrtales Myrtaceae Eucalyptus polybractea#NC 022393
Myrtales Myrtaceae Eucalyptus grandis#MG925369
Myrtales Myrtaceae Eucalyptus camaldulensis#NC 022398
Myrtales Myrtaceae Eucalyptus saligna#NC 022397
Myrtales Myrtaceae Eucalyptus aromaphloia#NC 022396
Myrtales Myrtaceae Eucalyptus globulus#NC 008115
Myrtales Myrtaceae Eucalyptus nitens#NC 022395
Crossosomatales Aphloiaceae Aphloia theiformis#ZSD324
Crossosomatales Aphloiaceae Aphloia theiformis#ZSD323
Crossosomatales Geissolomataceae Geissoloma marginata#ZSD490
Crossosomatales Strasburgeriaceae Strasburgeria robusta#ZSD394
Crossosomatales Strasburgeriaceae Strasburgeria robusta#ZSD290
Crossosomatales Crossosomataceae Crossosoma bigelovii#ZSD331
Crossosomatales Guamatelaceae Guamatela tuerckheimii#T00293
Crossosomatales Stachyuraceae Stachyurus praecox#VYGG
Crossosomatales Stachyuraceae Stachyurus himalaicus#F216
Crossosomatales Staphyleaceae Staphylea colchica#EU
Crossosomatales Staphyleaceae Staphylea forrestii#F606
Crossosomatales Staphyleaceae Staphylea trifolia#PTLU
Crossosomatales Staphyleaceae Euscaphis japonica#F765
Crossosomatales Staphyleaceae Turpinia montana#F367
Crossosomatales Staphyleaceae Turpinia cochinchinensis#F19
Picramniales Picramniaceae Picramnia antidesma#NZ7072
Picramniales Picramniaceae Picramnia ciliata#Y225
Sapindales Biebersteiniaceae Biebersteinia heterostemon#F995
Sapindales Biebersteiniaceae Biebersteinia heterostemon#F997
Sapindales Nitrariaceae Nitraria tangutorum#F800
Sapindales Nitrariaceae Peganum harmala#OBTI
Sapindales Nitrariaceae Peganum nigellastrum#F798
Sapindales Kirkiaceae Kirkia wilmsii#BCAA
Sapindales Kirkiaceae Kirkia acuminata#FC417
Sapindales Burseraceae Boswellia sacra#FCCA
Sapindales Burseraceae Garuga forrestii#F181
Sapindales Burseraceae Canarium pimela#F277
Sapindales Burseraceae Bursera simaruba#JSZD
Sapindales Burseraceae Commiphora wightii#NC 036978
Sapindales Burseraceae Commiphora gileadensis#NC 041104
Sapindales Burseraceae Commiphora foliacea#NC 041103
Sapindales Anacardiaceae Pegia nitida#F237
Sapindales Anacardiaceae Spondias mombin#NC 035973
Sapindales Anacardiaceae Choerospondias axillaris#F472
Sapindales Anacardiaceae Lannea coromandelica#F493
Sapindales Anacardiaceae Anacardium occidentale#F269
Sapindales Anacardiaceae Mangifera indica#NC 035239
Sapindales Anacardiaceae Toxicodendron radicans#YUOM
Sapindales Anacardiaceae Rhus chinensis#F10
Sapindales Anacardiaceae Pistacia vera#NC 034998
Sapindales Anacardiaceae Pistacia weinmanniifolia#NC 037471
Sapindales Sapindaceae Xanthoceras sorbifolium#NC 037448
Sapindales Sapindaceae Eurycorymbus cavaleriei#NC 037443
Sapindales Sapindaceae Dodonaea viscosa#NC 036099
Sapindales Sapindaceae Delavaya yunnanensis#F197
Sapindales Sapindaceae Koelreuteria paniculata#NC 037176
Sapindales Sapindaceae Sapindus mukorossi#NC 025554
Sapindales Sapindaceae Allophylus cobbe#F582
Sapindales Sapindaceae Litchi chinensis#NC 035238
Sapindales Sapindaceae Dimocarpus longan#NC 037447
Sapindales Sapindaceae Aesculus pavia#HBHB
Sapindales Sapindaceae Aesculus wangii#NC 035955
Sapindales Sapindaceae Aesculus assamica#F93
Sapindales Sapindaceae Aesculus chinensis var. wilsonii#F767
Sapindales Sapindaceae Dipteronia dyeriana#F394
Sapindales Sapindaceae Dipteronia sinensis#MK193760
Sapindales Sapindaceae Acer negundo#VFFP
Sapindales Sapindaceae Acer coriaceifolium#F325
Sapindales Sapindaceae Acer palmatum#NC 034932
Sapindales Sapindaceae Acer buergerianum#NC 034744
Sapindales Sapindaceae Acer campbellii subsp. wilsonii#NC 040988
Sapindales Sapindaceae Acer griseum#NC 034346
Sapindales Sapindaceae Acer paxii#F198
Sapindales Sapindaceae Acer sino-oblongum#NC 040106
Sapindales Sapindaceae Acer davidii#F220
Sapindales Sapindaceae Acer caudatifolium#NC 029371
Sapindales Sapindaceae Acer truncatum#NC 037211
Sapindales Sapindaceae Acer miyabei subsp. miaotaiense#NC 030343
Sapindales Sapindaceae Acer amplum subsp. catalpifolium#NC 041080
Sapindales Meliaceae Heynea trijuga#F85
Sapindales Meliaceae Azadirachta indica#NC 023792
Sapindales Meliaceae Melia azedarach#RJIM
Sapindales Meliaceae Chukrasia tabularis#F20
Sapindales Meliaceae Entandrophragma cylindricum#NC 037250
Sapindales Meliaceae Cedrela odorata#NC 037251
Sapindales Meliaceae Toona ciliata#F2
Sapindales Meliaceae Khaya senegalensis#NC 037362
Sapindales Meliaceae Swietenia humilis#F898
Sapindales Meliaceae Swietenia mahagoni#NC 040009
Sapindales Meliaceae Carapa guianensis#MH396436
Sapindales Meliaceae Xylocarpus granatum#NC 039925
Sapindales Meliaceae Xylocarpus rumphii#NC 038199
Sapindales Meliaceae Xylocarpus moluccensis#NC 038200
Sapindales Simaroubaceae Ailanthus altissima#NC 037696
Sapindales Simaroubaceae Quassia amara#IKFD
Sapindales Simaroubaceae Brucea mollis#F373
Sapindales Rutaceae Harrisonia perforata#F471
Sapindales Rutaceae Tetradium ruticarpum#F158
Sapindales Rutaceae Phellodendron amurense#NC 035551
Sapindales Rutaceae Toddalia asiatica#F12
Sapindales Rutaceae Zanthoxylum multijugum#F49
Sapindales Rutaceae Zanthoxylum bungeanum#NC 027939
Sapindales Rutaceae Zanthoxylum simulans#NC 037482
Sapindales Rutaceae Zanthoxylum sp.#MF716521
Sapindales Rutaceae Zanthoxylum schinifolium#MF716522
Sapindales Rutaceae Bergera koenigii#NC 032684
Sapindales Rutaceae Clausena excavata#NC 032685
Sapindales Rutaceae Micromelum minutum#NC 032689
Sapindales Rutaceae Glycosmis pentaphylla#NC 032687
Sapindales Rutaceae Glycosmis mauritiana#NC 032686
Sapindales Rutaceae Merrillia caloxylon#NC 032688
Sapindales Rutaceae Paramignya confertifolia#F235
Sapindales Rutaceae Atalantia kwangtungensis#MH329190
Sapindales Rutaceae Citrus reticulata#LC218435
Sapindales Rutaceae Citrus aurantiifolia#NC 024929

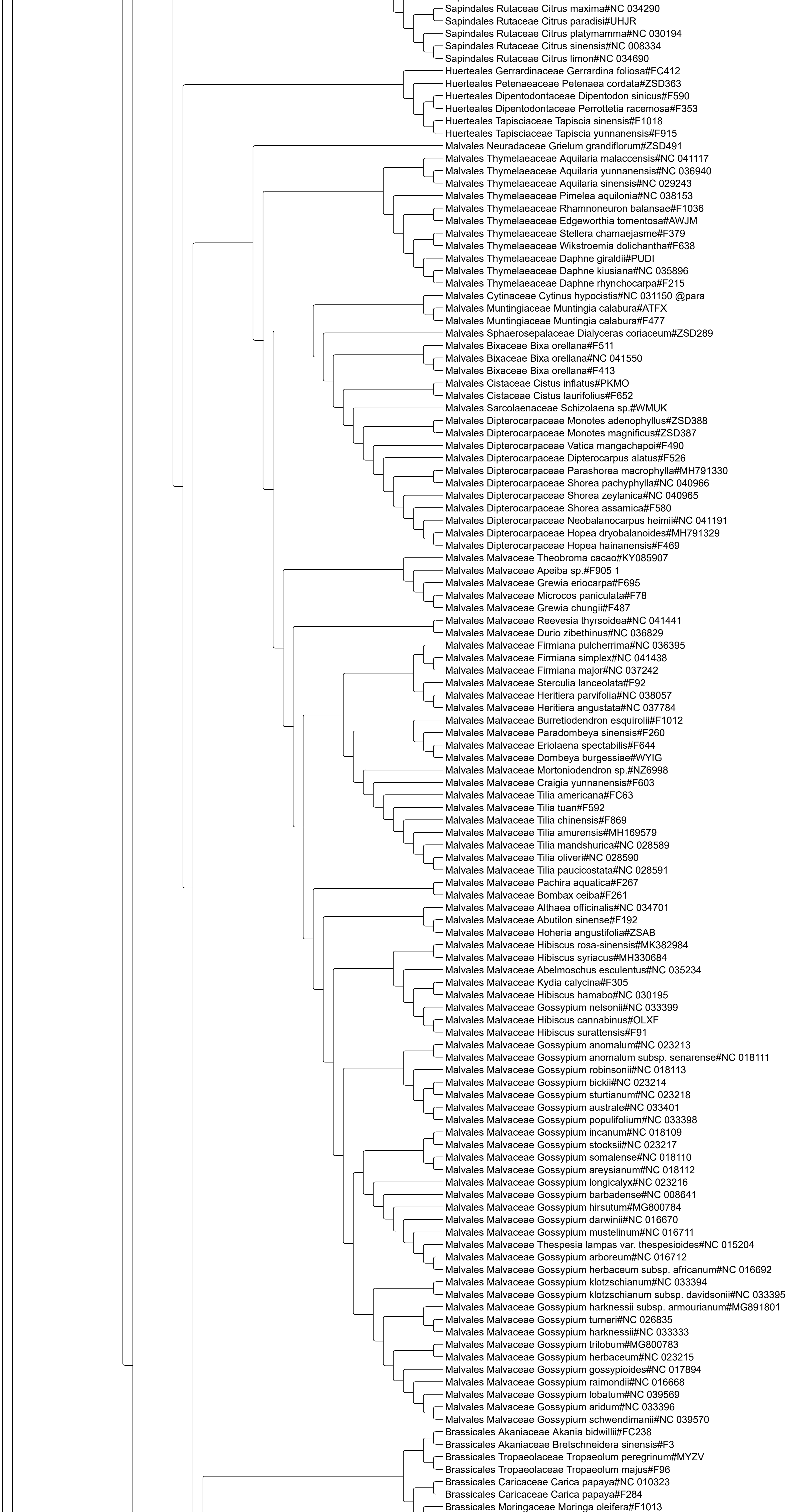

Sapindales Rutaceae Citrus maxima#NC 034290
Sapindales Rutaceae Citrus paradisi#UHJR
Sapindales Rutaceae Citrus platymamma#NC 030194
Sapindales Rutaceae Citrus sinensis#NC 008334
Sapindales Rutaceae Citrus limon#NC 034690
Huerteales Gerrardinaceae Gerrardina foliosa#FC412
Huerteales Petenaeaceae Petenaea cordata#ZSD363
Huerteales Dipentodontaceae Dipentodon sinicus#F590
Huerteales Dipentodontaceae Perrottetia racemosa#F353
Huerteales Tapisciaceae Tapiscia sinensis#F1018
Huerteales Tapisciaceae Tapiscia yunnanensis#F915
Malvales Neuradaceae Grielum grandiflorum#ZSD491
Malvales Thymelaeaceae Aquilaria malaccensis#NC 041117
Malvales Thymelaeaceae Aquilaria yunnanensis#NC 036940
Malvales Thymelaeaceae Aquilaria sinensis#NC 029243
Malvales Thymelaeaceae Pimelea aquilonia#NC 038153
Malvales Thymelaeaceae Rhamnoneuron balansae#F1036
Malvales Thymelaeaceae Edgeworthia tomentosa#AWJM
Malvales Thymelaeaceae Stellera chamaejasme#F379
Malvales Thymelaeaceae Wikstroemia dolichantha#F638
Malvales Thymelaeaceae Daphne giraldii#PUDI
Malvales Thymelaeaceae Daphne kiusiana#NC 035896
Malvales Thymelaeaceae Daphne rhynchocarpa#F215
Malvales Cytinaceae Cytinus hypocistis#NC 031150 @para
Malvales Muntingiaceae Muntingia calabura#ATFX
Malvales Muntingiaceae Muntingia calabura#F477
Malvales Sphaerosepalaceae Dialyceras coriaceum#ZSD289
Malvales Bixaceae Bixa orellana#F511
Malvales Bixaceae Bixa orellana#NC 041550
Malvales Bixaceae Bixa orellana#F413
Malvales Cistaceae Cistus inflatus#PKMO
Malvales Cistaceae Cistus laurifolius#F652
Malvales Sarcolaenaceae Schizolaena sp.#WMUK
Malvales Dipterocarpaceae Monotes adenophyllus#ZSD388
Malvales Dipterocarpaceae Monotes magnificus#ZSD387
Malvales Dipterocarpaceae Vatica mangachapoi#F490
Malvales Dipterocarpaceae Dipterocarpus alatus#F526
Malvales Dipterocarpaceae Parashorea macrophylla#MH791330
Malvales Dipterocarpaceae Shorea pachyphylla#NC 040966
Malvales Dipterocarpaceae Shorea zeylanica#NC 040965
Malvales Dipterocarpaceae Shorea assamica#F580
Malvales Dipterocarpaceae Neobalanocarpus heimii#NC 041191
Malvales Dipterocarpaceae Hopea dryobalanoides#MH791329
Malvales Dipterocarpaceae Hopea hainanensis#F469
Malvales Malvaceae Theobroma cacao#KY085907
Malvales Malvaceae Apeiba sp.#F905 1
Malvales Malvaceae Grewia eriocarpa#F695
Malvales Malvaceae Microcos paniculata#F78
Malvales Malvaceae Grewia chungii#F487
Malvales Malvaceae Reevesia thyrsoidea#NC 041441
Malvales Malvaceae Durio zibethinus#NC 036829
Malvales Malvaceae Firmiana pulcherrima#NC 036395
Malvales Malvaceae Firmiana simplex#NC 041438
Malvales Malvaceae Firmiana major#NC 037242
Malvales Malvaceae Sterculia lanceolata#F92
Malvales Malvaceae Heritiera parvifolia#NC 038057
Malvales Malvaceae Heritiera angustata#NC 037784
Malvales Malvaceae Burretiodendron esquirolii#F1012
Malvales Malvaceae Paradombeya sinensis#F260
Malvales Malvaceae Eriolaena spectabilis#F644
Malvales Malvaceae Dombeya burgessiae#WYIG
Malvales Malvaceae Mortoniodendron sp.#NZ6998
Malvales Malvaceae Craigia yunnanensis#F603
Malvales Malvaceae Tilia americana#FC63
Malvales Malvaceae Tilia tuan#F592
Malvales Malvaceae Tilia chinensis#F869
Malvales Malvaceae Tilia amurensis#MH169579
Malvales Malvaceae Tilia mandshurica#NC 028589
Malvales Malvaceae Tilia oliveri#NC 028590
Malvales Malvaceae Tilia paucicostata#NC 028591
Malvales Malvaceae Pachira aquatica#F267
Malvales Malvaceae Bombax ceiba#F261
Malvales Malvaceae Althaea officinalis#NC 034701
Malvales Malvaceae Abutilon sinense#F192
Malvales Malvaceae Hoheria angustifolia#ZSAB
Malvales Malvaceae Hibiscus rosa-sinensis#MK382984
Malvales Malvaceae Hibiscus syriacus#MH330684
Malvales Malvaceae Abelmoschus esculentus#NC 035234
Malvales Malvaceae Kydia calycina#F305
Malvales Malvaceae Hibiscus hamabo#NC 030195
Malvales Malvaceae Gossypium nelsonii#NC 033399
Malvales Malvaceae Hibiscus cannabinus#OLXF
Malvales Malvaceae Hibiscus surattensis#F91
Malvales Malvaceae Gossypium anomalum#NC 023213
Malvales Malvaceae Gossypium anomalum subsp. senarense#NC 018111
Malvales Malvaceae Gossypium robinsonii#NC 018113
Malvales Malvaceae Gossypium bickii#NC 023214
Malvales Malvaceae Gossypium sturtianum#NC 023218
Malvales Malvaceae Gossypium australe#NC 033401
Malvales Malvaceae Gossypium populifolium#NC 033398
Malvales Malvaceae Gossypium incanum#NC 018109
Malvales Malvaceae Gossypium stocksii#NC 023217
Malvales Malvaceae Gossypium somalense#NC 018110
Malvales Malvaceae Gossypium areysianum#NC 018112
Malvales Malvaceae Gossypium longicalyx#NC 023216
Malvales Malvaceae Gossypium barbadense#NC 008641
Malvales Malvaceae Gossypium hirsutum#MG800784
Malvales Malvaceae Gossypium darwinii#NC 016670
Malvales Malvaceae Gossypium mustelinum#NC 016711
Malvales Malvaceae Thespesia lampas var. thespesioides#NC 015204
Malvales Malvaceae Gossypium arboreum#NC 016712
Malvales Malvaceae Gossypium herbaceum subsp. africanum#NC 016692
Malvales Malvaceae Gossypium klotzschianum#NC 033394
Malvales Malvaceae Gossypium klotzschianum subsp. davidsonii#NC 033395
Malvales Malvaceae Gossypium harknessii subsp. armourianum#MG891801
Malvales Malvaceae Gossypium turneri#NC 026835
Malvales Malvaceae Gossypium harknessii#NC 033333
Malvales Malvaceae Gossypium trilobum#MG800783
Malvales Malvaceae Gossypium herbaceum#NC 023215
Malvales Malvaceae Gossypium gossypioides#NC 017894
Malvales Malvaceae Gossypium raimondii#NC 016668
Malvales Malvaceae Gossypium lobatum#NC 039569
Malvales Malvaceae Gossypium aridum#NC 033396
Malvales Malvaceae Gossypium schwendimanii#NC 039570
Brassicales Akaniaceae Akania bidwillii#FC238
Brassicales Akaniaceae Bretschneidera sinensis#F3
Brassicales Tropaeolaceae Tropaeolum peregrinum#MYZV
Brassicales Tropaeolaceae Tropaeolum majus#F96
Brassicales Caricaceae Carica papaya#NC 010323
Brassicales Caricaceae Carica papaya#F284
Brassicales Moringaceae Moringa oleifera#F1013

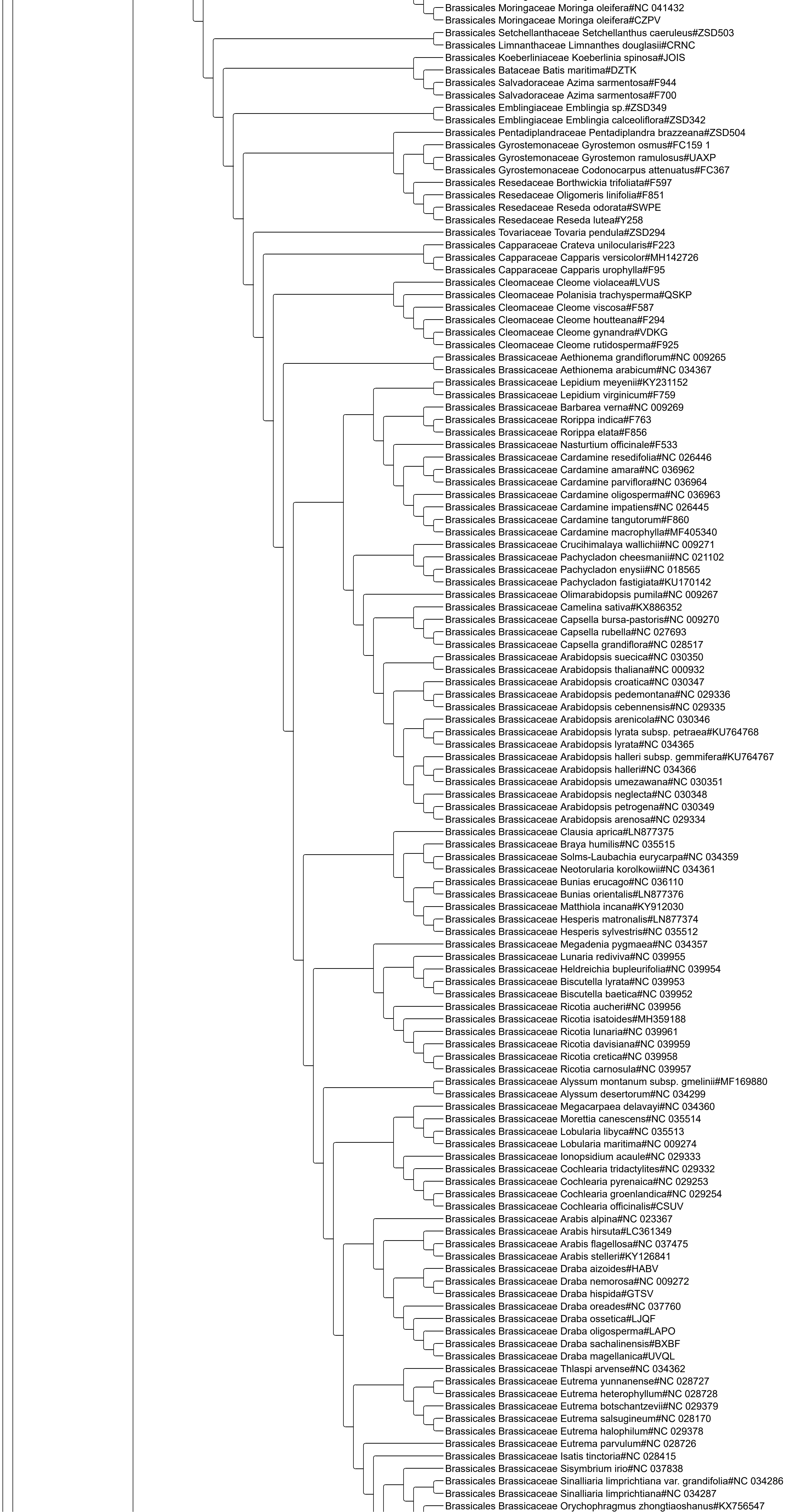

Brassicales Moringaceae Moringa oleifera#NC 041432
Brassicales Moringaceae Moringa oleifera#CZPV
Brassicales Setchellanthaceae Setchellanthus caeruleus#ZSD503
Brassicales Limnanthaceae Limnanthes douglasii#CRNC
Brassicales Koeberliniaceae Koeberlinia spinosa#JOIS
Brassicales Bataceae Batis maritima#DZTK
Brassicales Salvadoraceae Azima sarmentosa#F944
Brassicales Salvadoraceae Azima sarmentosa#F700
Brassicales Emblingiaceae Emblingia sp.#ZSD349
Brassicales Emblingiaceae Emblingia calceoliflora#ZSD342
Brassicales Pentadiplandraceae Pentadiplandra brazzeana#ZSD504
Brassicales Gyrostemonaceae Gyrostemon osmus#FC159 1
Brassicales Gyrostemonaceae Gyrostemon ramulosus#UAXP
Brassicales Gyrostemonaceae Codonocarpus attenuatus#FC367
Brassicales Resedaceae Borthwickia trifoliata#F597
Brassicales Resedaceae Oligomeris linifolia#F851
Brassicales Resedaceae Reseda odorata#SWPE
Brassicales Resedaceae Reseda lutea#Y258
Brassicales Tovariaceae Tovaria pendula#ZSD294
Brassicales Capparaceae Crateva unilocularis#F223
Brassicales Capparaceae Capparis versicolor#MH142726
Brassicales Capparaceae Capparis urophylla#F95
Brassicales Cleomaceae Cleome violacea#LVUS
Brassicales Cleomaceae Polanisia trachysperma#QSKP
Brassicales Cleomaceae Cleome viscosa#F587
Brassicales Cleomaceae Cleome houtteana#F294
Brassicales Cleomaceae Cleome gynandra#VDKG
Brassicales Cleomaceae Cleome rutidosperma#F925
Brassicales Brassicaceae Aethionema grandiflorum#NC 009265
Brassicales Brassicaceae Aethionema arabicum#NC 034367
Brassicales Brassicaceae Lepidium meyenii#KY231152
Brassicales Brassicaceae Lepidium virginicum#F759
Brassicales Brassicaceae Barbarea verna#NC 009269
Brassicales Brassicaceae Rorippa indica#F763
Brassicales Brassicaceae Rorippa elata#F856
Brassicales Brassicaceae Nasturtium officinale#F533
Brassicales Brassicaceae Cardamine resedifolia#NC 026446
Brassicales Brassicaceae Cardamine amara#NC 036962
Brassicales Brassicaceae Cardamine parviflora#NC 036964
Brassicales Brassicaceae Cardamine oligosperma#NC 036963
Brassicales Brassicaceae Cardamine impatiens#NC 026445
Brassicales Brassicaceae Cardamine tangutorum#F860
Brassicales Brassicaceae Cardamine macrophylla#MF405340
Brassicales Brassicaceae Crucihimalaya wallichii#NC 009271
Brassicales Brassicaceae Pachycladon cheesmanii#NC 021102
Brassicales Brassicaceae Pachycladon enysii#NC 018565
Brassicales Brassicaceae Pachycladon fastigiata#KU170142
Brassicales Brassicaceae Olimarabidopsis pumila#NC 009267
Brassicales Brassicaceae Camelina sativa#KX886352
Brassicales Brassicaceae Capsella bursa-pastoris#NC 009270
Brassicales Brassicaceae Capsella rubella#NC 027693
Brassicales Brassicaceae Capsella grandiflora#NC 028517
Brassicales Brassicaceae Arabidopsis suecica#NC 030350
Brassicales Brassicaceae Arabidopsis thaliana#NC 000932
Brassicales Brassicaceae Arabidopsis croatica#NC 030347
Brassicales Brassicaceae Arabidopsis pedemontana#NC 029336
Brassicales Brassicaceae Arabidopsis cebennensis#NC 029335
Brassicales Brassicaceae Arabidopsis arenicola#NC 030346
Brassicales Brassicaceae Arabidopsis lyrata subsp. petraea#KU764768
Brassicales Brassicaceae Arabidopsis lyrata#NC 034365
Brassicales Brassicaceae Arabidopsis halleri subsp. gemmifera#KU764767
Brassicales Brassicaceae Arabidopsis halleri#NC 034366
Brassicales Brassicaceae Arabidopsis umezawana#NC 030351
Brassicales Brassicaceae Arabidopsis neglecta#NC 030348
Brassicales Brassicaceae Arabidopsis petrogena#NC 030349
Brassicales Brassicaceae Arabidopsis arenosa#NC 029334
Brassicales Brassicaceae Clausia aprica#LN877375
Brassicales Brassicaceae Braya humilis#NC 035515
Brassicales Brassicaceae Solms-Laubachia eurycarpa#NC 034359
Brassicales Brassicaceae Neotorularia korolkowii#NC 034361
Brassicales Brassicaceae Bunias erucago#NC 036110
Brassicales Brassicaceae Bunias orientalis#LN877376
Brassicales Brassicaceae Matthiola incana#KY912030
Brassicales Brassicaceae Hesperis matronalis#LN877374
Brassicales Brassicaceae Hesperis sylvestris#NC 035512
Brassicales Brassicaceae Megadenia pygmaea#NC 034357
Brassicales Brassicaceae Lunaria rediviva#NC 039955
Brassicales Brassicaceae Heldreichia bupleurifolia#NC 039954
Brassicales Brassicaceae Biscutella lyrata#NC 039953
Brassicales Brassicaceae Biscutella baetica#NC 039952
Brassicales Brassicaceae Ricotia aucheri#NC 039956
Brassicales Brassicaceae Ricotia isatoides#MH359188
Brassicales Brassicaceae Ricotia lunaria#NC 039961
Brassicales Brassicaceae Ricotia davisiana#NC 039959
Brassicales Brassicaceae Ricotia cretica#NC 039958
Brassicales Brassicaceae Ricotia carnosula#NC 039957
Brassicales Brassicaceae Alyssum montanum subsp. gmelinii#MF169880
Brassicales Brassicaceae Alyssum desertorum#NC 034299
Brassicales Brassicaceae Megacarpaea delavayi#NC 034360
Brassicales Brassicaceae Morettia canescens#NC 035514
Brassicales Brassicaceae Lobularia libyca#NC 035513
Brassicales Brassicaceae Lobularia maritima#NC 009274
Brassicales Brassicaceae Ionopsidium acaule#NC 029333
Brassicales Brassicaceae Cochlearia tridactylites#NC 029332
Brassicales Brassicaceae Cochlearia pyrenaica#NC 029253
Brassicales Brassicaceae Cochlearia groenlandica#NC 029254
Brassicales Brassicaceae Cochlearia officinalis#CSUV
Brassicales Brassicaceae Arabis alpina#NC 023367
Brassicales Brassicaceae Arabis hirsuta#LC361349
Brassicales Brassicaceae Arabis flagellosa#NC 037475
Brassicales Brassicaceae Arabis stelleri#KY126841
Brassicales Brassicaceae Draba aizoides#HABV
Brassicales Brassicaceae Draba nemorosa#NC 009272
Brassicales Brassicaceae Draba hispida#GTSV
Brassicales Brassicaceae Draba oreades#NC 037760
Brassicales Brassicaceae Draba ossetica#LJQF
Brassicales Brassicaceae Draba oligosperma#LAPO
Brassicales Brassicaceae Draba sachalinensis#BXBF
Brassicales Brassicaceae Draba magellanica#UVQL
Brassicales Brassicaceae Thlaspi arvense#NC 034362
Brassicales Brassicaceae Eutrema yunnanense#NC 028727
Brassicales Brassicaceae Eutrema heterophyllum#NC 028728
Brassicales Brassicaceae Eutrema botschantzevii#NC 029379
Brassicales Brassicaceae Eutrema salsugineum#NC 028170
Brassicales Brassicaceae Eutrema halophilum#NC 029378
Brassicales Brassicaceae Eutrema parvulum#NC 028726
Brassicales Brassicaceae Isatis tinctoria#NC 028415
Brassicales Brassicaceae Sisymbrium irio#NC 037838
Brassicales Brassicaceae Sinalliaria limprichtiana var. grandifolia#NC 034286
Brassicales Brassicaceae Sinalliaria limprichtiana#NC 034287
Brassicales Brassicaceae Orychophragmus zhongtiaoshanus#KX756547

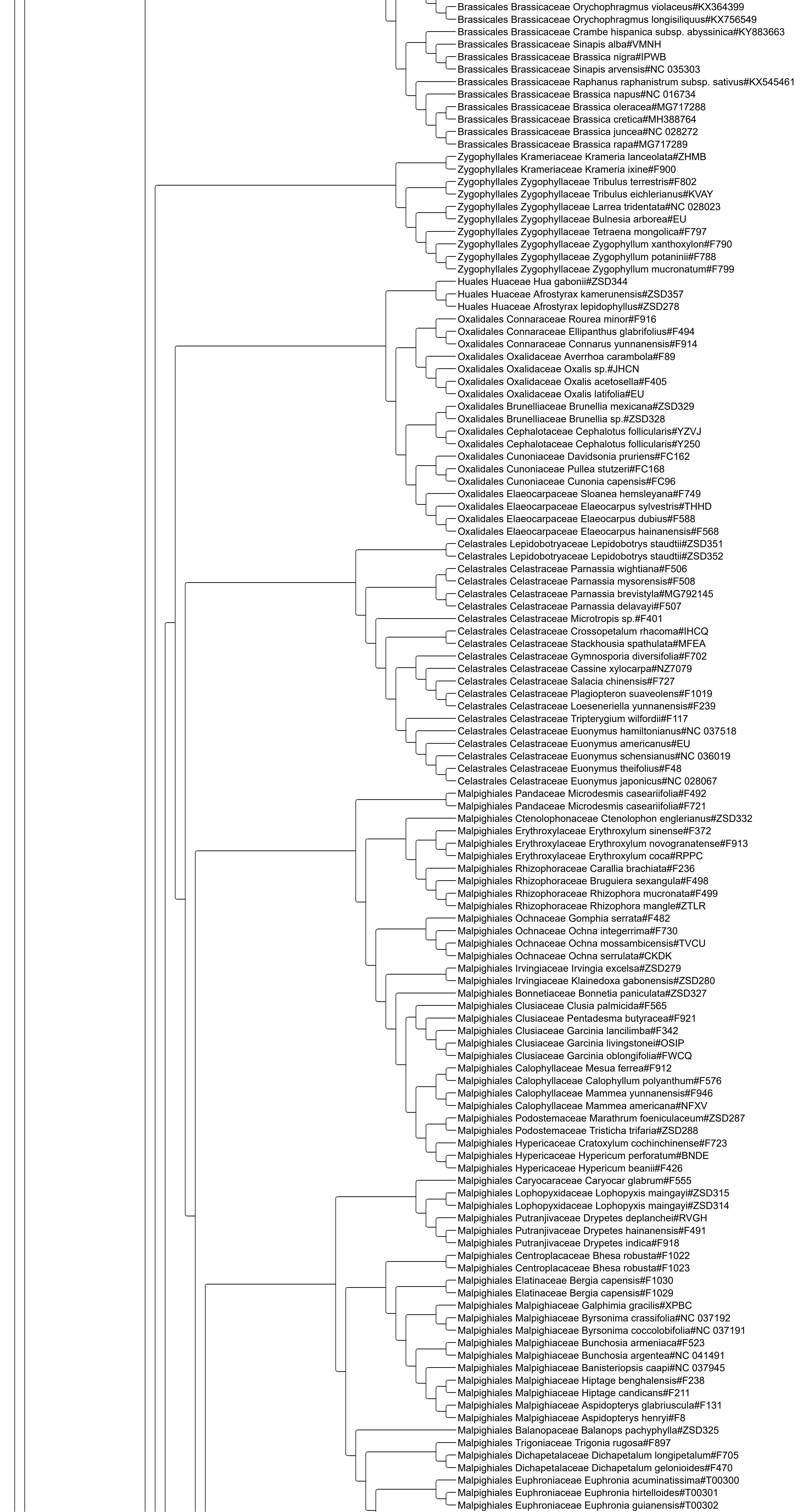
Brassicales Brassicaceae Orychophragmus violaceus#KX364399
Brassicales Brassicaceae Orychophragmus longisiliquus#KX756549
Brassicales Brassicaceae Crambe hispanica subsp. abyssinica#KY883663
Brassicales Brassicaceae Sinapis alba#VMNH
Brassicales Brassicaceae Brassica nigra#IPWB
Brassicales Brassicaceae Sinapis arvensis#NC 035303
Brassicales Brassicaceae Raphanus raphanistrum subsp. sativus#KX545461
Brassicales Brassicaceae Brassica napus#NC 016734
Brassicales Brassicaceae Brassica oleracea#MG717288
Brassicales Brassicaceae Brassica cretica#MH388764
Brassicales Brassicaceae Brassica juncea#NC 028272
Brassicales Brassicaceae Brassica rapa#MG717289
Zygophyllales Krameriaceae Krameria lanceolata#ZHMB
Zygophyllales Krameriaceae Krameria ixine#F900
Zygophyllales Zygophyllaceae Tribulus terrestris#F802
Zygophyllales Zygophyllaceae Tribulus eichlerianus#KVAY
Zygophyllales Zygophyllaceae Larrea tridentata#NC 028023
Zygophyllales Zygophyllaceae Bulnesia arborea#EU
Zygophyllales Zygophyllaceae Tetraena mongolica#F797
Zygophyllales Zygophyllaceae Zygophyllum xanthoxylon#F790
Zygophyllales Zygophyllaceae Zygophyllum potaninii#F788
Zygophyllales Zygophyllaceae Zygophyllum mucronatum#F799
Huales Huaceae Hua gabonii#ZSD344
Huales Huaceae Afrostyrax kamerunensis#ZSD357
Huales Huaceae Afrostyrax lepidophyllus#ZSD278
Oxalidales Connaraceae Rourea minor#F916
Oxalidales Connaraceae Ellipanthus glabrifolius#F494
Oxalidales Connaraceae Connarus yunnanensis#F914
Oxalidales Oxalidaceae Averrhoa carambola#F89
Oxalidales Oxalidaceae Oxalis sp.#JHCN
Oxalidales Oxalidaceae Oxalis acetosella#F405
Oxalidales Oxalidaceae Oxalis latifolia#EU
Oxalidales Brunelliaceae Brunellia mexicana#ZSD329
Oxalidales Brunelliaceae Brunellia sp.#ZSD328
Oxalidales Cephalotaceae Cephalotus follicularis#YZVJ
Oxalidales Cephalotaceae Cephalotus follicularis#Y250
Oxalidales Cunoniaceae Davidsonia pruriens#FC162
Oxalidales Cunoniaceae Pullea stutzeri#FC168
Oxalidales Cunoniaceae Cunonia capensis#FC96
Oxalidales Elaeocarpaceae Sloanea hemsleyana#F749
Oxalidales Elaeocarpaceae Elaeocarpus sylvestris#THHD
Oxalidales Elaeocarpaceae Elaeocarpus dubius#F588
Oxalidales Elaeocarpaceae Elaeocarpus hainanensis#F568
Celastrales Lepidobotryaceae Lepidobotrys staudtii#ZSD351
Celastrales Lepidobotryaceae Lepidobotrys staudtii#ZSD352
Celastrales Celastraceae Parnassia wightiana#F506
Celastrales Celastraceae Parnassia mysorensis#F508
Celastrales Celastraceae Parnassia brevistyla#MG792145
Celastrales Celastraceae Parnassia delavayi#F507
Celastrales Celastraceae Microtropis sp.#F401
Celastrales Celastraceae Crossopetalum rhacoma#IHCQ
Celastrales Celastraceae Stackhousia spathulata#MFEA
Celastrales Celastraceae Gymnosporia diversifolia#F702
Celastrales Celastraceae Cassine xylocarpa#NZ7079
Celastrales Celastraceae Salacia chinensis#F727
Celastrales Celastraceae Plagiopteron suaveolens#F1019
Celastrales Celastraceae Loeseneriella yunnanensis#F239
Celastrales Celastraceae Tripterygium wilfordii#F117
Celastrales Celastraceae Euonymus hamiltonianus#NC 037518
Celastrales Celastraceae Euonymus americanus#EU
Celastrales Celastraceae Euonymus schensianus#NC 036019
Celastrales Celastraceae Euonymus theifolius#F48
Celastrales Celastraceae Euonymus japonicus#NC 028067
Malpighiales Pandaceae Microdesmis caseariifolia#F492
Malpighiales Pandaceae Microdesmis caseariifolia#F721
Malpighiales Ctenolophonaceae Ctenolophon englerianus#ZSD332
Malpighiales Erythroxylaceae Erythroxylum sinense#F372
Malpighiales Erythroxylaceae Erythroxylum novogranatense#F913
Malpighiales Erythroxylaceae Erythroxylum coca#RPPC
Malpighiales Rhizophoraceae Carallia brachiata#F236
Malpighiales Rhizophoraceae Bruguiera sexangula#F498
Malpighiales Rhizophoraceae Rhizophora mucronata#F499
Malpighiales Rhizophoraceae Rhizophora mangle#ZTLR
Malpighiales Ochnaceae Gomphia serrata#F482
Malpighiales Ochnaceae Ochna integerrima#F730
Malpighiales Ochnaceae Ochna mossambicensis#TVCU
Malpighiales Ochnaceae Ochna serrulata#CKDK
Malpighiales Irvingiaceae Irvingia excelsa#ZSD279
Malpighiales Irvingiaceae Klainedoxa gabonensis#ZSD280
Malpighiales Bonnetiaceae Bonnetia paniculata#ZSD327
Malpighiales Clusiaceae Clusia palmicida#F565
Malpighiales Clusiaceae Pentadesma butyracea#F921
Malpighiales Clusiaceae Garcinia lancilimba#F342
Malpighiales Clusiaceae Garcinia livingstonei#OSIP
Malpighiales Clusiaceae Garcinia oblongifolia#FWCQ
Malpighiales Calophyllaceae Mesua ferrea#F912
Malpighiales Calophyllaceae Calophyllum polyanthum#F576
Malpighiales Calophyllaceae Mammea yunnanensis#F946
Malpighiales Calophyllaceae Mammea americana#NFXV
Malpighiales Podostemaceae Marathrum foeniculaceum#ZSD287
Malpighiales Podostemaceae Tristicha trifaria#ZSD288
Malpighiales Hypericaceae Cratoxylum cochinchinense#F723
Malpighiales Hypericaceae Hypericum perforatum#BNDE
Malpighiales Hypericaceae Hypericum beanii#F426
Malpighiales Caryocaraceae Caryocar glabrum#F555
Malpighiales Lophopyxidaceae Lophopyxis maingayi#ZSD315
Malpighiales Lophopyxidaceae Lophopyxis maingayi#ZSD314
Malpighiales Putranjivaceae Drypetes deplanchei#RVGH
Malpighiales Putranjivaceae Drypetes hainanensis#F491
Malpighiales Putranjivaceae Drypetes indica#F918
Malpighiales Centroplacaceae Bhesa robusta#F1022
Malpighiales Centroplacaceae Bhesa robusta#F1023
Malpighiales Elatinaceae Bergia capensis#F1030
Malpighiales Elatinaceae Bergia capensis#F1029
Malpighiales Malpighiaceae Galphimia gracilis#XPBC
Malpighiales Malpighiaceae Byrsonima crassifolia#NC 037192
Malpighiales Malpighiaceae Byrsonima coccolobifolia#NC 037191
Malpighiales Malpighiaceae Bunchosia armeniaca#F523
Malpighiales Malpighiaceae Bunchosia argentea#NC 041491
Malpighiales Malpighiaceae Banisteriopsis caapi#NC 037945
Malpighiales Malpighiaceae Hiptage benghalensis#F238
Malpighiales Malpighiaceae Hiptage candicans#F211
Malpighiales Malpighiaceae Aspidopterys glabriuscula#F131
Malpighiales Malpighiaceae Aspidopterys henryi#F8
Malpighiales Balanopaceae Balanops pachyphylla#ZSD325
Malpighiales Trigoniaceae Trigonia rugosa#F897
Malpighiales Dichapetalaceae Dichapetalum longipetalum#F705
Malpighiales Dichapetalaceae Dichapetalum gelonioides#F470
Malpighiales Euphroniaceae Euphronia acuminatissima#T00300
Malpighiales Euphroniaceae Euphronia hirtelloides#T00301
Malpighiales Euphroniaceae Euphronia guianensis#T00302

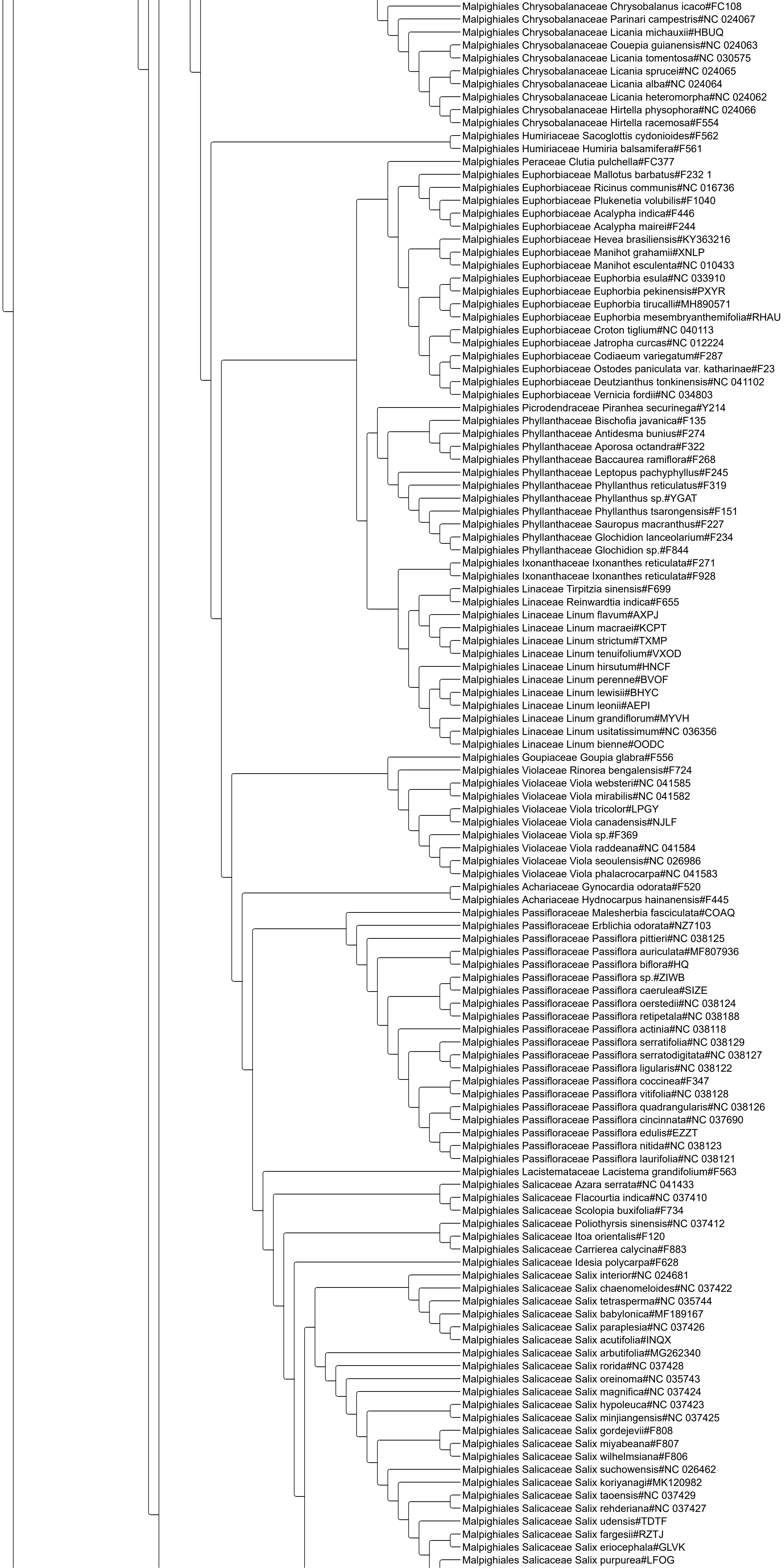
Malpighiales Chrysobalanaceae Chrysobalanus icaco#FC108
Malpighiales Chrysobalanaceae Parinari campestris#NC 024067
Malpighiales Chrysobalanaceae Licania michauxii#HBUQ
Malpighiales Chrysobalanaceae Couepia guianensis#NC 024063
Malpighiales Chrysobalanaceae Licania tomentosa#NC 030575
Malpighiales Chrysobalanaceae Licania sprucei#NC 024065
Malpighiales Chrysobalanaceae Licania alba#NC 024064
Malpighiales Chrysobalanaceae Licania heteromorpha#NC 024062
Malpighiales Chrysobalanaceae Hirtella physophora#NC 024066
Malpighiales Chrysobalanaceae Hirtella racemosa#F554
Malpighiales Humiriaceae Sacoglottis cydonioides#F562
Malpighiales Humiriaceae Humiria balsamifera#F561
Malpighiales Peraceae Clutia pulchella#FC377
Malpighiales Euphorbiaceae Mallotus barbatus#F232 1
Malpighiales Euphorbiaceae Ricinus communis#NC 016736
Malpighiales Euphorbiaceae Plukenetia volubilis#F1040
Malpighiales Euphorbiaceae Acalypha indica#F446
Malpighiales Euphorbiaceae Acalypha mairei#F244
Malpighiales Euphorbiaceae Hevea brasiliensis#KY363216
Malpighiales Euphorbiaceae Manihot grahamii#XNLP
Malpighiales Euphorbiaceae Manihot esculenta#NC 010433
Malpighiales Euphorbiaceae Euphorbia esula#NC 033910
Malpighiales Euphorbiaceae Euphorbia pekinensis#PXYR
Malpighiales Euphorbiaceae Euphorbia tirucalli#MH890571
Malpighiales Euphorbiaceae Euphorbia mesembryanthemifolia#RHAU
Malpighiales Euphorbiaceae Croton tiglium#NC 040113
Malpighiales Euphorbiaceae Jatropha curcas#NC 012224
Malpighiales Euphorbiaceae Codiaeum variegatum#F287
Malpighiales Euphorbiaceae Ostodes paniculata var. katharinae#F23
Malpighiales Euphorbiaceae Deutzianthus tonkinensis#NC 041102
Malpighiales Euphorbiaceae Vernicia fordii#NC 034803
Malpighiales Picrodendraceae Piranhea securinega#Y214
Malpighiales Phyllanthaceae Bischofia javanica#F135
Malpighiales Phyllanthaceae Antidesma bunius#F274
Malpighiales Phyllanthaceae Aporosa octandra#F322
Malpighiales Phyllanthaceae Baccaurea ramiflora#F268
Malpighiales Phyllanthaceae Leptopus pachyphyllus#F245
Malpighiales Phyllanthaceae Phyllanthus reticulatus#F319
Malpighiales Phyllanthaceae Phyllanthus sp.#YGAT
Malpighiales Phyllanthaceae Phyllanthus tsarongensis#F151
Malpighiales Phyllanthaceae Sauropus macranthus#F227
Malpighiales Phyllanthaceae Glochidion lanceolarium#F234
Malpighiales Phyllanthaceae Glochidion sp.#F844
Malpighiales Ixonanthaceae Ixonanthes reticulata#F271
Malpighiales Ixonanthaceae Ixonanthes reticulata#F928
Malpighiales Linaceae Tirpitzia sinensis#F699
Malpighiales Linaceae Reinwardtia indica#F655
Malpighiales Linaceae Linum flavum#AXPJ
Malpighiales Linaceae Linum macraei#KCPT
Malpighiales Linaceae Linum strictum#TXMP
Malpighiales Linaceae Linum tenuifolium#VXOD
Malpighiales Linaceae Linum hirsutum#HNCF
Malpighiales Linaceae Linum perenne#BVOF
Malpighiales Linaceae Linum lewisii#BHYC
Malpighiales Linaceae Linum leonii#AEPI
Malpighiales Linaceae Linum grandiflorum#MYVH
Malpighiales Linaceae Linum usitatissimum#NC 036356
Malpighiales Linaceae Linum bienne#OODC
Malpighiales Goupiaceae Goupia glabra#F556
Malpighiales Violaceae Rinorea bengalensis#F724
Malpighiales Violaceae Viola websteri#NC 041585
Malpighiales Violaceae Viola mirabilis#NC 041582
Malpighiales Violaceae Viola tricolor#LPGY
Malpighiales Violaceae Viola canadensis#NJLF
Malpighiales Violaceae Viola sp.#F369
Malpighiales Violaceae Viola raddeana#NC 041584
Malpighiales Violaceae Viola seoulensis#NC 026986
Malpighiales Violaceae Viola phalacrocarpa#NC 041583
Malpighiales Achariaceae Gynocardia odorata#F520
Malpighiales Achariaceae Hydnocarpus hainanensis#F445
Malpighiales Passifloraceae Malesherbia fasciculata#COAQ
Malpighiales Passifloraceae Erblichia odorata#NZ7103
Malpighiales Passifloraceae Passiflora pittieri#NC 038125
Malpighiales Passifloraceae Passiflora auriculata#MF807936
Malpighiales Passifloraceae Passiflora biflora#HQ
Malpighiales Passifloraceae Passiflora sp.#ZIWB
Malpighiales Passifloraceae Passiflora caerulea#SIZE
Malpighiales Passifloraceae Passiflora oerstedii#NC 038124
Malpighiales Passifloraceae Passiflora retipetala#NC 038188
Malpighiales Passifloraceae Passiflora actinia#NC 038118
Malpighiales Passifloraceae Passiflora serratifolia#NC 038129
Malpighiales Passifloraceae Passiflora serratodigitata#NC 038127
Malpighiales Passifloraceae Passiflora ligularis#NC 038122
Malpighiales Passifloraceae Passiflora coccinea#F347
Malpighiales Passifloraceae Passiflora vitifolia#NC 038128
Malpighiales Passifloraceae Passiflora quadrangularis#NC 038126
Malpighiales Passifloraceae Passiflora cincinnata#NC 037690
Malpighiales Passifloraceae Passiflora edulis#EZZT
Malpighiales Passifloraceae Passiflora nitida#NC 038123
Malpighiales Passifloraceae Passiflora laurifolia#NC 038121
Malpighiales Lacistemataceae Lacistema grandifolium#F563
Malpighiales Salicaceae Azara serrata#NC 041433
Malpighiales Salicaceae Flacourtia indica#NC 037410
Malpighiales Salicaceae Scolopia buxifolia#F734
Malpighiales Salicaceae Poliothyrsis sinensis#NC 037412
Malpighiales Salicaceae Itoa orientalis#F120
Malpighiales Salicaceae Carrierea calycina#F883
Malpighiales Salicaceae Idesia polycarpa#F628
Malpighiales Salicaceae Salix interior#NC 024681
Malpighiales Salicaceae Salix chaenomeloides#NC 037422
Malpighiales Salicaceae Salix tetrasperma#NC 035744
Malpighiales Salicaceae Salix babylonica#MF189167
Malpighiales Salicaceae Salix paraplesia#NC 037426
Malpighiales Salicaceae Salix acutifolia#INQX
Malpighiales Salicaceae Salix arbutifolia#MG262340
Malpighiales Salicaceae Salix rorida#NC 037428
Malpighiales Salicaceae Salix oreinoma#NC 035743
Malpighiales Salicaceae Salix magnifica#NC 037424
Malpighiales Salicaceae Salix hypoleuca#NC 037423
Malpighiales Salicaceae Salix minjiangensis#NC 037425
Malpighiales Salicaceae Salix gordejevii#F808
Malpighiales Salicaceae Salix miyabeana#F807
Malpighiales Salicaceae Salix wilhelmsiana#F806
Malpighiales Salicaceae Salix suchowensis#NC 026462
Malpighiales Salicaceae Salix koriyanagi#MK120982
Malpighiales Salicaceae Salix taoensis#NC 037429
Malpighiales Salicaceae Salix rehderiana#NC 037427
Malpighiales Salicaceae Salix udensis#TDTF
Malpighiales Salicaceae Salix fargesii#RZTJ
Malpighiales Salicaceae Salix eriocephala#GLVK
Malpighiales Salicaceae Salix purpurea#LFOG

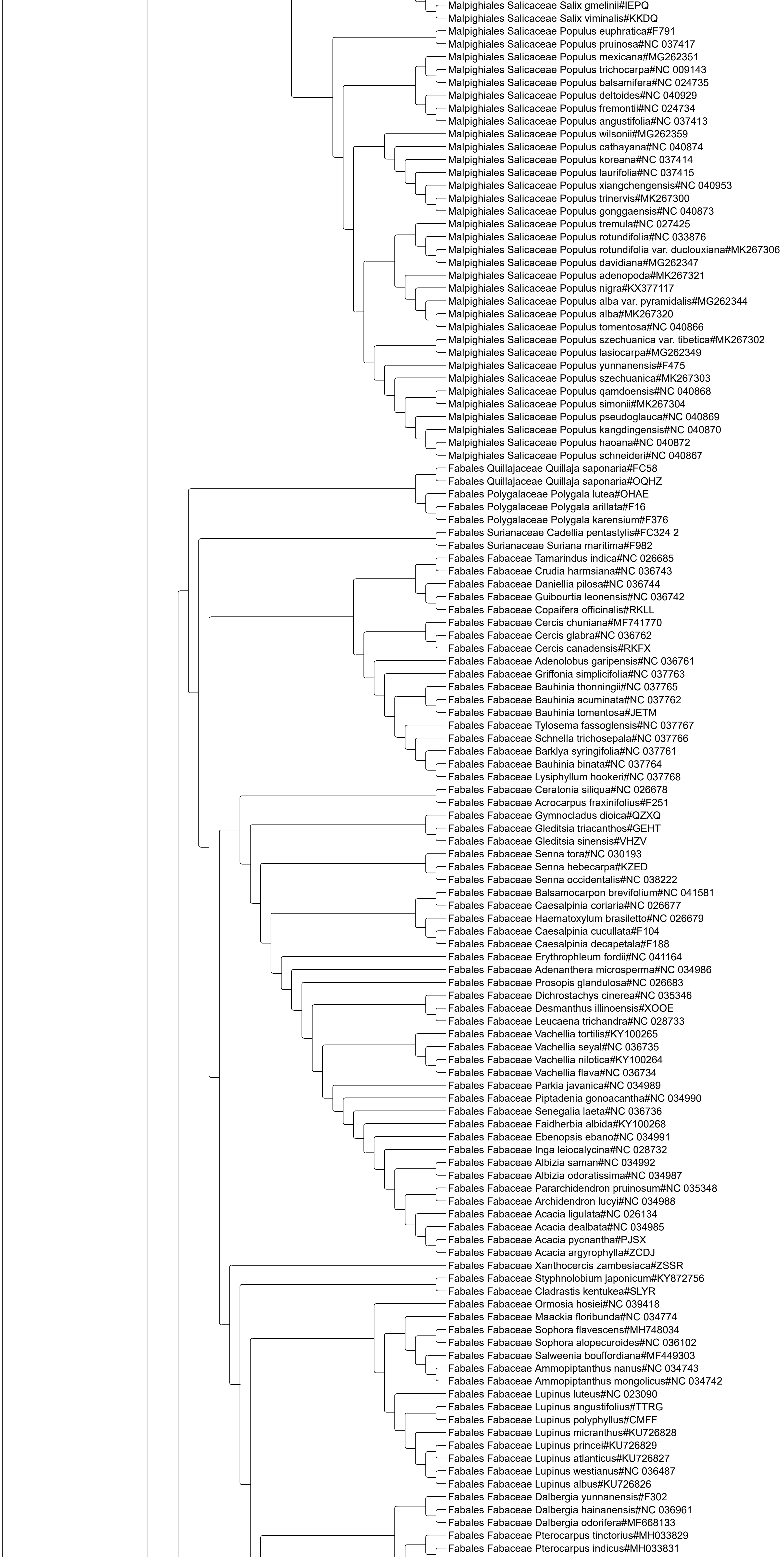
Malpighiales Salicaceae Salix gmelinii#IEPQ
Malpighiales Salicaceae Salix viminalis#KKDQ
Malpighiales Salicaceae Populus euphratica#F791
Malpighiales Salicaceae Populus pruinosa#NC 037417
Malpighiales Salicaceae Populus mexicana#MG262351
Malpighiales Salicaceae Populus trichocarpa#NC 009143
Malpighiales Salicaceae Populus balsamifera#NC 024735
Malpighiales Salicaceae Populus deltoides#NC 040929
Malpighiales Salicaceae Populus fremontii#NC 024734
Malpighiales Salicaceae Populus angustifolia#NC 037413
Malpighiales Salicaceae Populus wilsonii#MG262359
Malpighiales Salicaceae Populus cathayana#NC 040874
Malpighiales Salicaceae Populus koreana#NC 037414
Malpighiales Salicaceae Populus laurifolia#NC 037415
Malpighiales Salicaceae Populus xiangchengensis#NC 040953
Malpighiales Salicaceae Populus trinervis#MK267300
Malpighiales Salicaceae Populus gonggaensis#NC 040873
Malpighiales Salicaceae Populus tremula#NC 027425
Malpighiales Salicaceae Populus rotundifolia#NC 033876
Malpighiales Salicaceae Populus rotundifolia var. duclouxiana#MK267306
Malpighiales Salicaceae Populus davidiana#MG262347
Malpighiales Salicaceae Populus adenopoda#MK267321
Malpighiales Salicaceae Populus nigra#KX377117
Malpighiales Salicaceae Populus alba var. pyramidalis#MG262344
Malpighiales Salicaceae Populus alba#MK267320
Malpighiales Salicaceae Populus tomentosa#NC 040866
Malpighiales Salicaceae Populus szechuanica var. tibetica#MK267302
Malpighiales Salicaceae Populus lasiocarpa#MG262349
Malpighiales Salicaceae Populus yunnanensis#F475
Malpighiales Salicaceae Populus szechuanica#MK267303
Malpighiales Salicaceae Populus qamdoensis#NC 040868
Malpighiales Salicaceae Populus simonii#MK267304
Malpighiales Salicaceae Populus pseudoglauca#NC 040869
Malpighiales Salicaceae Populus kangdingensis#NC 040870
Malpighiales Salicaceae Populus haoana#NC 040872
Malpighiales Salicaceae Populus schneideri#NC 040867
Fabales Quillajaceae Quillaja saponaria#FC58
Fabales Quillajaceae Quillaja saponaria#OQHZ
Fabales Polygalaceae Polygala lutea#OHAE
Fabales Polygalaceae Polygala arillata#F16
Fabales Polygalaceae Polygala karensium#F376
Fabales Surianaceae Cadellia pentastylis#FC324 2
Fabales Surianaceae Suriana maritima#F982
Fabales Fabaceae Tamarindus indica#NC 026685
Fabales Fabaceae Crudia harmsiana#NC 036743
Fabales Fabaceae Daniellia pilosa#NC 036744
Fabales Fabaceae Guibourtia leonensis#NC 036742
Fabales Fabaceae Copaifera officinalis#RKLL
Fabales Fabaceae Cercis chuniana#MF741770
Fabales Fabaceae Cercis glabra#NC 036762
Fabales Fabaceae Cercis canadensis#RKFX
Fabales Fabaceae Adenolobus garipensis#NC 036761
Fabales Fabaceae Griffonia simplicifolia#NC 037763
Fabales Fabaceae Bauhinia thonningii#NC 037765
Fabales Fabaceae Bauhinia acuminata#NC 037762
Fabales Fabaceae Bauhinia tomentosa#JETM
Fabales Fabaceae Tylosema fassoglensis#NC 037767
Fabales Fabaceae Schnella trichosepala#NC 037766
Fabales Fabaceae Barklya syringifolia#NC 037761
Fabales Fabaceae Bauhinia binata#NC 037764
Fabales Fabaceae Lysiphyllum hookeri#NC 037768
Fabales Fabaceae Ceratonia siliqua#NC 026678
Fabales Fabaceae Acrocarpus fraxinifolius#F251
Fabales Fabaceae Gymnocladus dioica#QZXQ
Fabales Fabaceae Gleditsia triacanthos#GEHT
Fabales Fabaceae Gleditsia sinensis#VHZV
Fabales Fabaceae Senna tora#NC 030193
Fabales Fabaceae Senna hebecarpa#KZED
Fabales Fabaceae Senna occidentalis#NC 038222
Fabales Fabaceae Balsamocarpon brevifolium#NC 041581
Fabales Fabaceae Caesalpinia coriaria#NC 026677
Fabales Fabaceae Haematoxylum brasiletto#NC 026679
Fabales Fabaceae Caesalpinia cucullata#F104
Fabales Fabaceae Caesalpinia decapetala#F188
Fabales Fabaceae Erythrophleum fordii#NC 041164
Fabales Fabaceae Adenanthera microsperma#NC 034986
Fabales Fabaceae Prosopis glandulosa#NC 026683
Fabales Fabaceae Dichrostachys cinerea#NC 035346
Fabales Fabaceae Desmanthus illinoensis#XOOE
Fabales Fabaceae Leucaena trichandra#NC 028733
Fabales Fabaceae Vachellia tortilis#KY100265
Fabales Fabaceae Vachellia seyal#NC 036735
Fabales Fabaceae Vachellia nilotica#KY100264
Fabales Fabaceae Vachellia flava#NC 036734
Fabales Fabaceae Parkia javanica#NC 034989
Fabales Fabaceae Piptadenia gonoacantha#NC 034990
Fabales Fabaceae Senegalia laeta#NC 036736
Fabales Fabaceae Faidherbia albida#KY100268
Fabales Fabaceae Ebenopsis ebano#NC 034991
Fabales Fabaceae Inga leiocalycina#NC 028732
Fabales Fabaceae Albizia saman#NC 034992
Fabales Fabaceae Albizia odoratissima#NC 034987
Fabales Fabaceae Pararchidendron pruinosum#NC 035348
Fabales Fabaceae Archidendron lucyi#NC 034988
Fabales Fabaceae Acacia ligulata#NC 026134
Fabales Fabaceae Acacia dealbata#NC 034985
Fabales Fabaceae Acacia pycnantha#PJSX
Fabales Fabaceae Acacia argyrophylla#ZCDJ
Fabales Fabaceae Xanthocercis zambesiaca#ZSSR
Fabales Fabaceae Styphnolobium japonicum#KY872756
Fabales Fabaceae Cladrastis kentukea#SLYR
Fabales Fabaceae Ormosia hosiei#NC 039418
Fabales Fabaceae Maackia floribunda#NC 034774
Fabales Fabaceae Sophora flavescens#MH748034
Fabales Fabaceae Sophora alopecuroides#NC 036102
Fabales Fabaceae Salweenia bouffordiana#MF449303
Fabales Fabaceae Ammopiptanthus nanus#NC 034743
Fabales Fabaceae Ammopiptanthus mongolicus#NC 034742
Fabales Fabaceae Lupinus luteus#NC 023090
Fabales Fabaceae Lupinus angustifolius#TTRG
Fabales Fabaceae Lupinus polyphyllus#CMFF
Fabales Fabaceae Lupinus micranthus#KU726828
Fabales Fabaceae Lupinus princei#KU726829
Fabales Fabaceae Lupinus atlanticus#KU726827
Fabales Fabaceae Lupinus westianus#NC 036487
Fabales Fabaceae Lupinus albus#KU726826
Fabales Fabaceae Dalbergia yunnanensis#F302
Fabales Fabaceae Dalbergia hainanensis#NC 036961
Fabales Fabaceae Dalbergia odorifera#MF668133
Fabales Fabaceae Pterocarpus tinctorius#MH033829
Fabales Fabaceae Pterocarpus indicus#MH033831

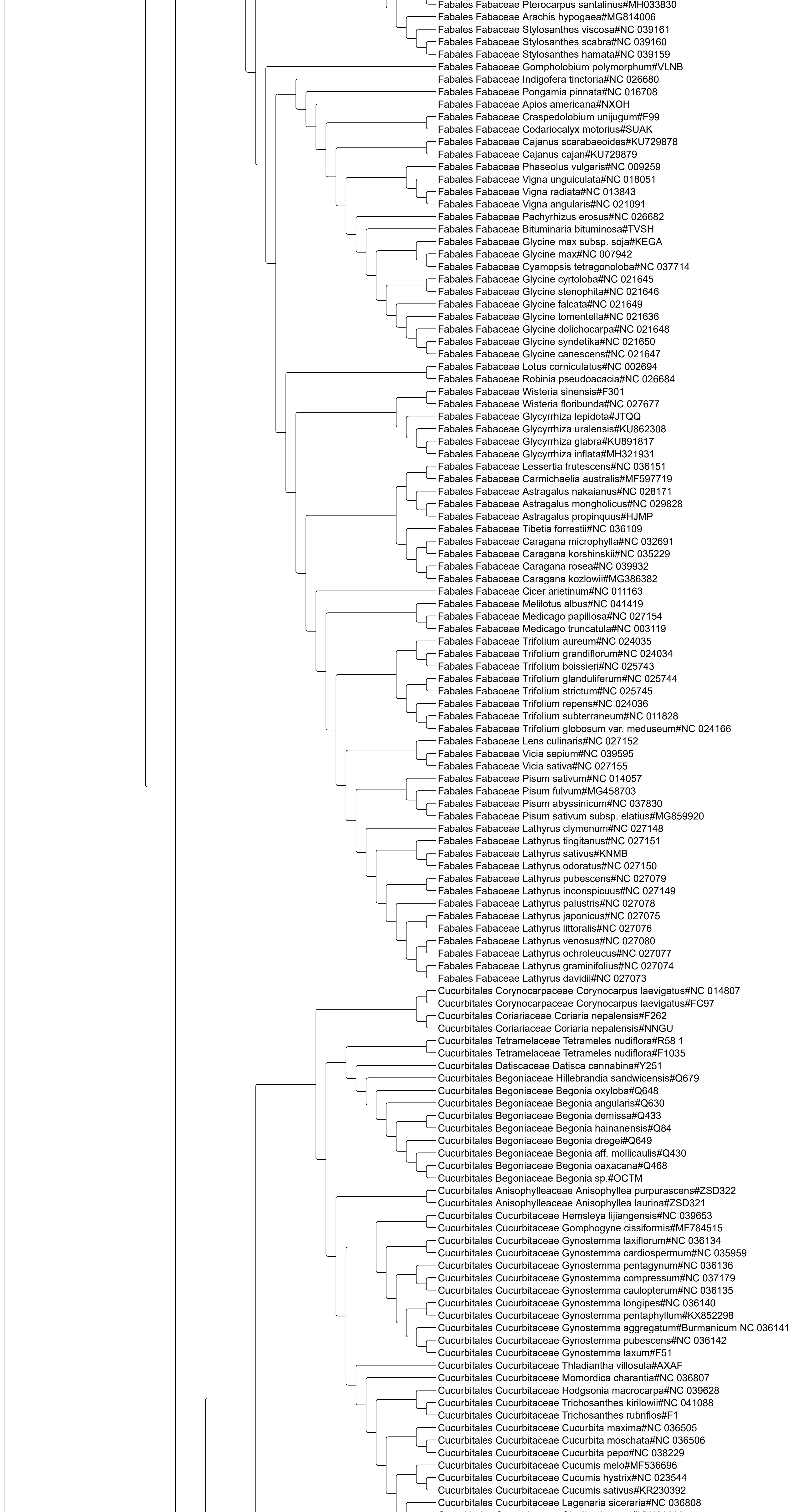

Fabales Fabaceae Pterocarpus santalinus#MH033830
Fabales Fabaceae Arachis hypogaea#MG814006
Fabales Fabaceae Stylosanthes viscosa#NC 039161
Fabales Fabaceae Stylosanthes scabra#NC 039160
Fabales Fabaceae Stylosanthes hamata#NC 039159
Fabales Fabaceae Gompholobium polymorphum#VLNB
Fabales Fabaceae Indigofera tinctoria#NC 026680
Fabales Fabaceae Pongamia pinnata#NC 016708
Fabales Fabaceae Apios americana#NXOH
Fabales Fabaceae Craspedolobium unijugum#F99
Fabales Fabaceae Codariocalyx motorius#SUAK
Fabales Fabaceae Cajanus scarabaeoides#KU729878
Fabales Fabaceae Cajanus cajan#KU729879
Fabales Fabaceae Phaseolus vulgaris#NC 009259
Fabales Fabaceae Vigna unguiculata#NC 018051
Fabales Fabaceae Vigna radiata#NC 013843
Fabales Fabaceae Vigna angularis#NC 021091
Fabales Fabaceae Pachyrhizus erosus#NC 026682
Fabales Fabaceae Bituminaria bituminosa#TVSH
Fabales Fabaceae Glycine max subsp. soja#KEGA
Fabales Fabaceae Glycine max#NC 007942
Fabales Fabaceae Cyamopsis tetragonoloba#NC 037714
Fabales Fabaceae Glycine cyrtoloba#NC 021645
Fabales Fabaceae Glycine stenophita#NC 021646
Fabales Fabaceae Glycine falcata#NC 021649
Fabales Fabaceae Glycine tomentella#NC 021636
Fabales Fabaceae Glycine dolichocarpa#NC 021648
Fabales Fabaceae Glycine syndetika#NC 021650
Fabales Fabaceae Glycine canescens#NC 021647
Fabales Fabaceae Lotus corniculatus#NC 002694
Fabales Fabaceae Robinia pseudoacacia#NC 026684
Fabales Fabaceae Wisteria sinensis#F301
Fabales Fabaceae Wisteria floribunda#NC 027677
Fabales Fabaceae Glycyrrhiza lepidota#JTQQ
Fabales Fabaceae Glycyrrhiza uralensis#KU862308
Fabales Fabaceae Glycyrrhiza glabra#KU891817
Fabales Fabaceae Glycyrrhiza inflata#MH321931
Fabales Fabaceae Lessertia frutescens#NC 036151
Fabales Fabaceae Carmichaelia australis#MF597719
Fabales Fabaceae Astragalus nakaianus#NC 028171
Fabales Fabaceae Astragalus mongholicus#NC 029828
Fabales Fabaceae Astragalus propinquus#HJMP
Fabales Fabaceae Tibetia forrestii#NC 036109
Fabales Fabaceae Caragana microphylla#NC 032691
Fabales Fabaceae Caragana korshinskii#NC 035229
Fabales Fabaceae Caragana rosea#NC 039932
Fabales Fabaceae Caragana kozlowii#MG386382
Fabales Fabaceae Cicer arietinum#NC 011163
Fabales Fabaceae Melilotus albus#NC 041419
Fabales Fabaceae Medicago papillosa#NC 027154
Fabales Fabaceae Medicago truncatula#NC 003119
Fabales Fabaceae Trifolium aureum#NC 024035
Fabales Fabaceae Trifolium grandiflorum#NC 024034
Fabales Fabaceae Trifolium boissieri#NC 025743
Fabales Fabaceae Trifolium glanduliferum#NC 025744
Fabales Fabaceae Trifolium strictum#NC 025745
Fabales Fabaceae Trifolium repens#NC 024036
Fabales Fabaceae Trifolium subterraneum#NC 011828
Fabales Fabaceae Trifolium globosum var. meduseum#NC 024166
Fabales Fabaceae Lens culinaris#NC 027152
Fabales Fabaceae Vicia sepium#NC 039595
Fabales Fabaceae Vicia sativa#NC 027155
Fabales Fabaceae Pisum sativum#NC 014057
Fabales Fabaceae Pisum fulvum#MG458703
Fabales Fabaceae Pisum abyssinicum#NC 037830
Fabales Fabaceae Pisum sativum subsp. elatius#MG859920
Fabales Fabaceae Lathyrus clymenum#NC 027148
Fabales Fabaceae Lathyrus tingitanus#NC 027151
Fabales Fabaceae Lathyrus sativus#KNMB
Fabales Fabaceae Lathyrus odoratus#NC 027150
Fabales Fabaceae Lathyrus pubescens#NC 027079
Fabales Fabaceae Lathyrus inconspicuus#NC 027149
Fabales Fabaceae Lathyrus palustris#NC 027078
Fabales Fabaceae Lathyrus japonicus#NC 027075
Fabales Fabaceae Lathyrus littoralis#NC 027076
Fabales Fabaceae Lathyrus venosus#NC 027080
Fabales Fabaceae Lathyrus ochroleucus#NC 027077
Fabales Fabaceae Lathyrus graminifolius#NC 027074
Fabales Fabaceae Lathyrus davidii#NC 027073
Cucurbitales Corynocarpaceae Corynocarpus laevigatus#NC 014807
Cucurbitales Corynocarpaceae Corynocarpus laevigatus#FC97
Cucurbitales Coriariaceae Coriaria nepalensis#F262
Cucurbitales Coriariaceae Coriaria nepalensis#NNGU
Cucurbitales Tetramelaceae Tetrameles nudiflora#R58 1
Cucurbitales Tetramelaceae Tetrameles nudiflora#F1035
Cucurbitales Datiscaceae Datisca cannabina#Y251
Cucurbitales Begoniaceae Hillebrandia sandwicensis#Q679
Cucurbitales Begoniaceae Begonia oxyloba#Q648
Cucurbitales Begoniaceae Begonia angularis#Q630
Cucurbitales Begoniaceae Begonia demissa#Q433
Cucurbitales Begoniaceae Begonia hainanensis#Q84
Cucurbitales Begoniaceae Begonia dregei#Q649
Cucurbitales Begoniaceae Begonia aff. mollicaulis#Q430
Cucurbitales Begoniaceae Begonia oaxacana#Q468
Cucurbitales Begoniaceae Begonia sp.#OCTM
Cucurbitales Anisophylleaceae Anisophyllea purpurascens#ZSD322
Cucurbitales Anisophylleaceae Anisophyllea laurina#ZSD321
Cucurbitales Cucurbitaceae Hemsleya lijiangensis#NC 039653
Cucurbitales Cucurbitaceae Gomphogyne cissiformis#MF784515
Cucurbitales Cucurbitaceae Gynostemma laxiflorum#NC 036134
Cucurbitales Cucurbitaceae Gynostemma cardiospermum#NC 035959
Cucurbitales Cucurbitaceae Gynostemma pentagynum#NC 036136
Cucurbitales Cucurbitaceae Gynostemma compressum#NC 037179
Cucurbitales Cucurbitaceae Gynostemma caulopterum#NC 036135
Cucurbitales Cucurbitaceae Gynostemma longipes#NC 036140
Cucurbitales Cucurbitaceae Gynostemma pentaphyllum#KX852298
Cucurbitales Cucurbitaceae Gynostemma aggregatum#Burmanicum NC 036141
Cucurbitales Cucurbitaceae Gynostemma pubescens#NC 036142
Cucurbitales Cucurbitaceae Gynostemma laxum#F51
Cucurbitales Cucurbitaceae Thladiantha villosula#AXAF
Cucurbitales Cucurbitaceae Momordica charantia#NC 036807
Cucurbitales Cucurbitaceae Hodgsonia macrocarpa#NC 039628
Cucurbitales Cucurbitaceae Trichosanthes kirilowii#NC 041088
Cucurbitales Cucurbitaceae Trichosanthes rubriflos#F1
Cucurbitales Cucurbitaceae Cucurbita maxima#NC 036505
Cucurbitales Cucurbitaceae Cucurbita moschata#NC 036506
Cucurbitales Cucurbitaceae Cucurbita pepo#NC 038229
Cucurbitales Cucurbitaceae Cucumis melo#MF536696
Cucurbitales Cucurbitaceae Cucumis hystrix#NC 023544
Cucurbitales Cucurbitaceae Cucumis sativus#KR230392
Cucurbitales Cucurbitaceae Lagenaria siceraria#NC 036808

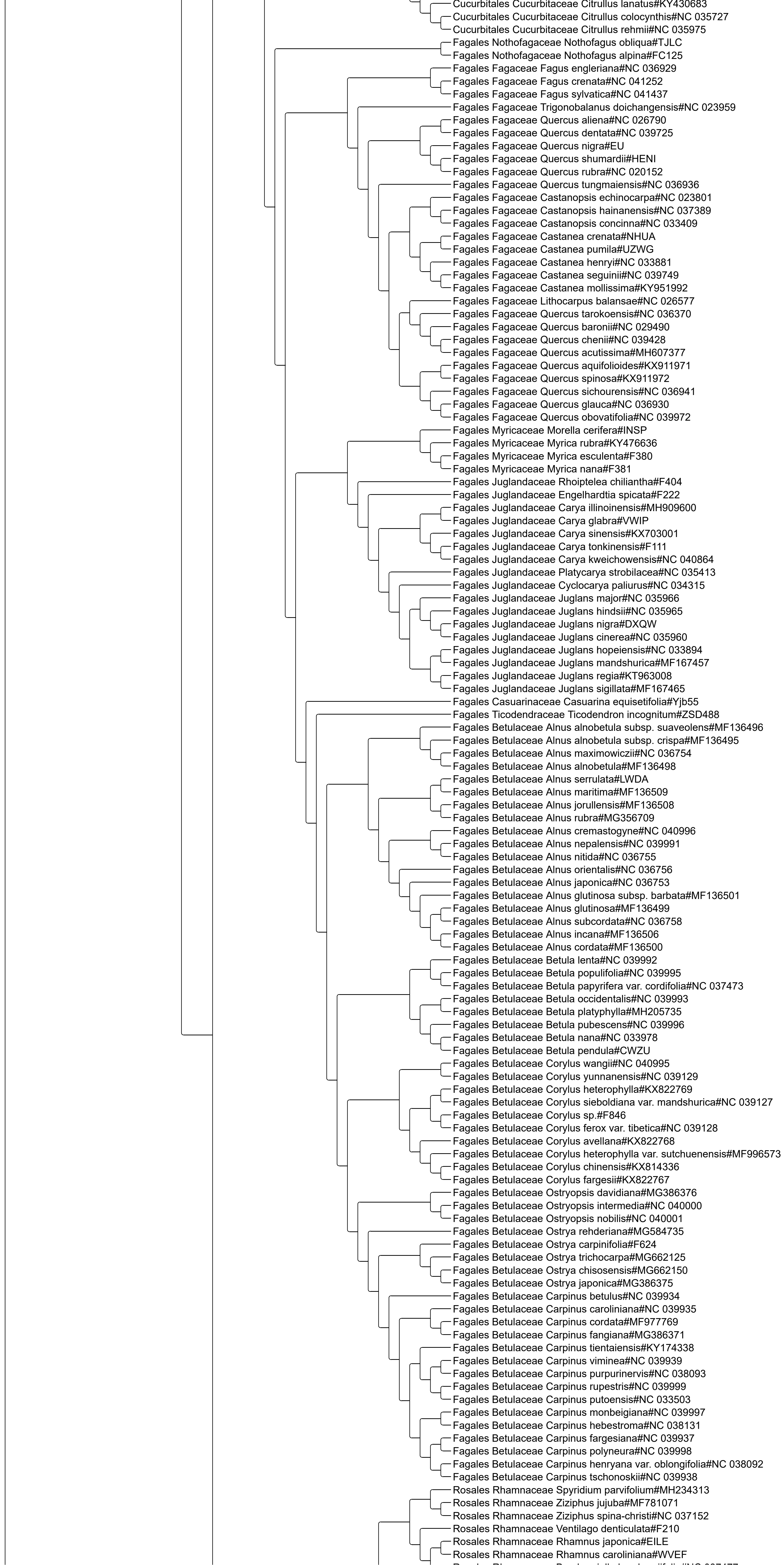
Cucurbitales Cucurbitaceae Citrullus lanatus#KY430683
Cucurbitales Cucurbitaceae Citrullus colocynthis#NC 035727
Cucurbitales Cucurbitaceae Citrullus rehmii#NC 035975
Fagales Nothofagaceae Nothofagus obliqua#TJLC
Fagales Nothofagaceae Nothofagus alpina#FC125
Fagales Fagaceae Fagus engleriana#NC 036929
Fagales Fagaceae Fagus crenata#NC 041252
Fagales Fagaceae Fagus sylvatica#NC 041437
Fagales Fagaceae Trigonobalanus doichangensis#NC 023959
Fagales Fagaceae Quercus aliena#NC 026790
Fagales Fagaceae Quercus dentata#NC 039725
Fagales Fagaceae Quercus nigra#EU
Fagales Fagaceae Quercus shumardii#HENI
Fagales Fagaceae Quercus rubra#NC 020152
Fagales Fagaceae Quercus tungmaiensis#NC 036936
Fagales Fagaceae Castanopsis echinocarpa#NC 023801
Fagales Fagaceae Castanopsis hainanensis#NC 037389
Fagales Fagaceae Castanopsis concinna#NC 033409
Fagales Fagaceae Castanea crenata#NHUA
Fagales Fagaceae Castanea pumila#UZWG
Fagales Fagaceae Castanea henryi#NC 033881
Fagales Fagaceae Castanea seguinii#NC 039749
Fagales Fagaceae Castanea mollissima#KY951992
Fagales Fagaceae Lithocarpus balansae#NC 026577
Fagales Fagaceae Quercus tarokoensis#NC 036370
Fagales Fagaceae Quercus baronii#NC 029490
Fagales Fagaceae Quercus chenii#NC 039428
Fagales Fagaceae Quercus acutissima#MH607377
Fagales Fagaceae Quercus aquifolioides#KX911971
Fagales Fagaceae Quercus spinosa#KX911972
Fagales Fagaceae Quercus sichourensis#NC 036941
Fagales Fagaceae Quercus glauca#NC 036930
Fagales Fagaceae Quercus obovatifolia#NC 039972
Fagales Myricaceae Morella cerifera#INSP
Fagales Myricaceae Myrica rubra#KY476636
Fagales Myricaceae Myrica esculenta#F380
Fagales Myricaceae Myrica nana#F381
Fagales Juglandaceae Rhoiptelea chiliantha#F404
Fagales Juglandaceae Engelhardtia spicata#F222
Fagales Juglandaceae Carya illinoinensis#MH909600
Fagales Juglandaceae Carya glabra#VWIP
Fagales Juglandaceae Carya sinensis#KX703001
Fagales Juglandaceae Carya tonkinensis#F111
Fagales Juglandaceae Carya kweichowensis#NC 040864
Fagales Juglandaceae Platycarya strobilacea#NC 035413
Fagales Juglandaceae Cyclocarya paliurus#NC 034315
Fagales Juglandaceae Juglans major#NC 035966
Fagales Juglandaceae Juglans hindsii#NC 035965
Fagales Juglandaceae Juglans nigra#DXQW
Fagales Juglandaceae Juglans cinerea#NC 035960
Fagales Juglandaceae Juglans hopeiensis#NC 033894
Fagales Juglandaceae Juglans mandshurica#MF167457
Fagales Juglandaceae Juglans regia#KT963008
Fagales Juglandaceae Juglans sigillata#MF167465
Fagales Casuarinaceae Casuarina equisetifolia#Yjb55
Fagales Ticodendraceae Ticodendron incognitum#ZSD488
Fagales Betulaceae Alnus alnobetula subsp. suaveolens#MF136496
Fagales Betulaceae Alnus alnobetula subsp. crispa#MF136495
Fagales Betulaceae Alnus maximowiczii#NC 036754
Fagales Betulaceae Alnus alnobetula#MF136498
Fagales Betulaceae Alnus serrulata#LWDA
Fagales Betulaceae Alnus maritima#MF136509
Fagales Betulaceae Alnus jorullensis#MF136508
Fagales Betulaceae Alnus rubra#MG356709
Fagales Betulaceae Alnus cremastogyne#NC 040996
Fagales Betulaceae Alnus nepalensis#NC 039991
Fagales Betulaceae Alnus nitida#NC 036755
Fagales Betulaceae Alnus orientalis#NC 036756
Fagales Betulaceae Alnus japonica#NC 036753
Fagales Betulaceae Alnus glutinosa subsp. barbata#MF136501
Fagales Betulaceae Alnus glutinosa#MF136499
Fagales Betulaceae Alnus subcordata#NC 036758
Fagales Betulaceae Alnus incana#MF136506
Fagales Betulaceae Alnus cordata#MF136500
Fagales Betulaceae Betula lenta#NC 039992
Fagales Betulaceae Betula populifolia#NC 039995
Fagales Betulaceae Betula papyrifera var. cordifolia#NC 037473
Fagales Betulaceae Betula occidentalis#NC 039993
Fagales Betulaceae Betula platyphylla#MH205735
Fagales Betulaceae Betula pubescens#NC 039996
Fagales Betulaceae Betula nana#NC 033978
Fagales Betulaceae Betula pendula#CWZU
Fagales Betulaceae Corylus wangii#NC 040995
Fagales Betulaceae Corylus yunnanensis#NC 039129
Fagales Betulaceae Corylus heterophylla#KX822769
Fagales Betulaceae Corylus sieboldiana var. mandshurica#NC 039127
Fagales Betulaceae Corylus sp.#F846
Fagales Betulaceae Corylus ferox var. tibetica#NC 039128
Fagales Betulaceae Corylus avellana#KX822768
Fagales Betulaceae Corylus heterophylla var. sutchuenensis#MF996573
Fagales Betulaceae Corylus chinensis#KX814336
Fagales Betulaceae Corylus fargesii#KX822767
Fagales Betulaceae Ostryopsis davidiana#MG386376
Fagales Betulaceae Ostryopsis intermedia#NC 040000
Fagales Betulaceae Ostryopsis nobilis#NC 040001
Fagales Betulaceae Ostrya rehderiana#MG584735
Fagales Betulaceae Ostrya carpinifolia#F624
Fagales Betulaceae Ostrya trichocarpa#MG662125
Fagales Betulaceae Ostrya chisosensis#MG662150
Fagales Betulaceae Ostrya japonica#MG386375
Fagales Betulaceae Carpinus betulus#NC 039934
Fagales Betulaceae Carpinus caroliniana#NC 039935
Fagales Betulaceae Carpinus cordata#MF977769
Fagales Betulaceae Carpinus fangiana#MG386371
Fagales Betulaceae Carpinus tientaiensis#KY174338
Fagales Betulaceae Carpinus viminea#NC 039939
Fagales Betulaceae Carpinus purpurinervis#NC 038093
Fagales Betulaceae Carpinus rupestris#NC 039999
Fagales Betulaceae Carpinus putoensis#NC 033503
Fagales Betulaceae Carpinus monbeigiana#NC 039997
Fagales Betulaceae Carpinus hebestroma#NC 038131
Fagales Betulaceae Carpinus fargesiana#NC 039937
Fagales Betulaceae Carpinus polyneura#NC 039998
Fagales Betulaceae Carpinus henryana var. oblongifolia#NC 038092
Fagales Betulaceae Carpinus tschonoskii#NC 039938
Rosales Rhamnaceae Spyridium parvifolium#MH234313
Rosales Rhamnaceae Ziziphus jujuba#MF781071
Rosales Rhamnaceae Ziziphus spina-christi#NC 037152
Rosales Rhamnaceae Ventilago denticulata#F210
Rosales Rhamnaceae Rhamnus japonica#EILE
Rosales Rhamnaceae Rhamnus caroliniana#WVEF

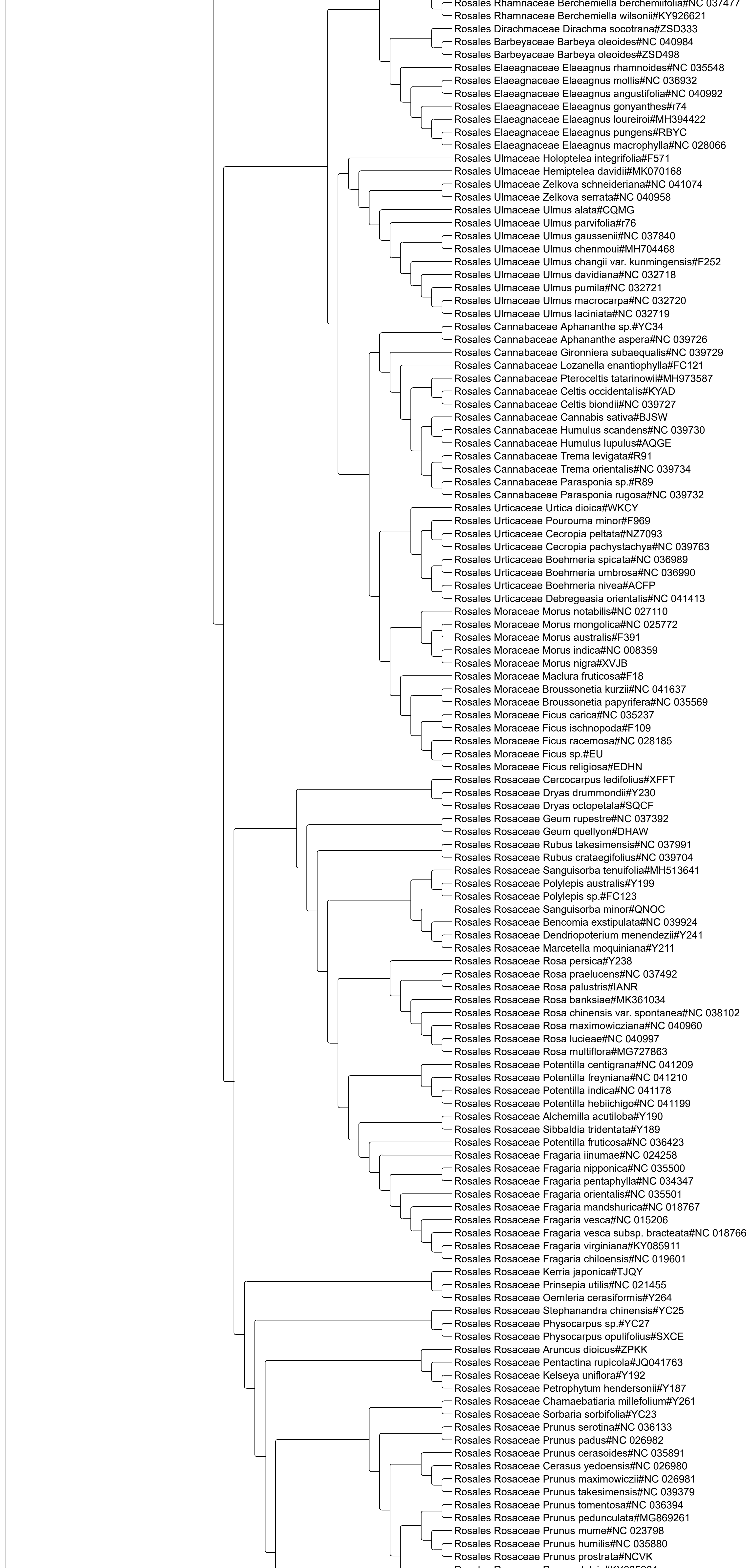
Rosales Rhamnaceae Berchemiella berchemiifolia#NC 037477
Rosales Rhamnaceae Berchemiella wilsonii#KY926621
Rosales Dirachmaceae Dirachma socotrana#ZSD333
Rosales Barbeyaceae Barbeya oleoides#NC 040984
Rosales Barbeyaceae Barbeya oleoides#ZSD498
Rosales Elaeagnaceae Elaeagnus rhamnoides#NC 035548
Rosales Elaeagnaceae Elaeagnus mollis#NC 036932
Rosales Elaeagnaceae Elaeagnus angustifolia#NC 040992
Rosales Elaeagnaceae Elaeagnus gonyanthes#r74
Rosales Elaeagnaceae Elaeagnus loureiroi#MH394422
Rosales Elaeagnaceae Elaeagnus pungens#RBYC
Rosales Elaeagnaceae Elaeagnus macrophylla#NC 028066
Rosales Ulmaceae Holoptelea integrifolia#F571
Rosales Ulmaceae Hemiptelea davidii#MK070168
Rosales Ulmaceae Zelkova schneideriana#NC 041074
Rosales Ulmaceae Zelkova serrata#NC 040958
Rosales Ulmaceae Ulmus alata#CQMG
Rosales Ulmaceae Ulmus parvifolia#r76
Rosales Ulmaceae Ulmus gaussenii#NC 037840
Rosales Ulmaceae Ulmus chenmoui#MH704468
Rosales Ulmaceae Ulmus changii var. kunmingensis#F252
Rosales Ulmaceae Ulmus davidiana#NC 032718
Rosales Ulmaceae Ulmus pumila#NC 032721
Rosales Ulmaceae Ulmus macrocarpa#NC 032720
Rosales Ulmaceae Ulmus laciniata#NC 032719
Rosales Cannabaceae Aphananthe sp.#YC34
Rosales Cannabaceae Aphananthe aspera#NC 039726
Rosales Cannabaceae Gironniera subaequalis#NC 039729
Rosales Cannabaceae Lozanella enantiophylla#FC121
Rosales Cannabaceae Pteroceltis tatarinowii#MH973587
Rosales Cannabaceae Celtis occidentalis#KYAD
Rosales Cannabaceae Celtis biondii#NC 039727
Rosales Cannabaceae Cannabis sativa#BJSW
Rosales Cannabaceae Humulus scandens#NC 039730
Rosales Cannabaceae Humulus lupulus#AQGE
Rosales Cannabaceae Trema levigata#R91
Rosales Cannabaceae Trema orientalis#NC 039734
Rosales Cannabaceae Parasponia sp.#R89
Rosales Cannabaceae Parasponia rugosa#NC 039732
Rosales Urticaceae Urtica dioica#WKCY
Rosales Urticaceae Pourouma minor#F969
Rosales Urticaceae Cecropia peltata#NZ7093
Rosales Urticaceae Cecropia pachystachya#NC 039763
Rosales Urticaceae Boehmeria spicata#NC 036989
Rosales Urticaceae Boehmeria umbrosa#NC 036990
Rosales Urticaceae Boehmeria nivea#ACFP
Rosales Urticaceae Debregeasia orientalis#NC 041413
Rosales Moraceae Morus notabilis#NC 027110
Rosales Moraceae Morus mongolica#NC 025772
Rosales Moraceae Morus australis#F391
Rosales Moraceae Morus indica#NC 008359
Rosales Moraceae Morus nigra#XVJB
Rosales Moraceae Maclura fruticosa#F18
Rosales Moraceae Broussonetia kurzii#NC 041637
Rosales Moraceae Broussonetia papyrifera#NC 035569
Rosales Moraceae Ficus carica#NC 035237
Rosales Moraceae Ficus ischnopoda#F109
Rosales Moraceae Ficus racemosa#NC 028185
Rosales Moraceae Ficus sp.#EU
Rosales Moraceae Ficus religiosa#EDHN
Rosales Rosaceae Cercocarpus ledifolius#XFFT
Rosales Rosaceae Dryas drummondii#Y230
Rosales Rosaceae Dryas octopetala#SQCF
Rosales Rosaceae Geum rupestre#NC 037392
Rosales Rosaceae Geum quellyon#DHAW
Rosales Rosaceae Rubus takesimensis#NC 037991
Rosales Rosaceae Rubus crataegifolius#NC 039704
Rosales Rosaceae Sanguisorba tenuifolia#MH513641
Rosales Rosaceae Polylepis australis#Y199
Rosales Rosaceae Polylepis sp.#FC123
Rosales Rosaceae Sanguisorba minor#QNOC
Rosales Rosaceae Bencomia exstipulata#NC 039924
Rosales Rosaceae Dendriopoterium menendezii#Y241
Rosales Rosaceae Marcetella moquiniana#Y211
Rosales Rosaceae Rosa persica#Y238
Rosales Rosaceae Rosa praelucens#NC 037492
Rosales Rosaceae Rosa palustris#IANR
Rosales Rosaceae Rosa banksiae#MK361034
Rosales Rosaceae Rosa chinensis var. spontanea#NC 038102
Rosales Rosaceae Rosa maximowicziana#NC 040960
Rosales Rosaceae Rosa lucieae#NC 040997
Rosales Rosaceae Rosa multiflora#MG727863
Rosales Rosaceae Potentilla centigrana#NC 041209
Rosales Rosaceae Potentilla freyniana#NC 041210
Rosales Rosaceae Potentilla indica#NC 041178
Rosales Rosaceae Potentilla hebiichigo#NC 041199
Rosales Rosaceae Alchemilla acutiloba#Y190
Rosales Rosaceae Sibbaldia tridentata#Y189
Rosales Rosaceae Potentilla fruticosa#NC 036423
Rosales Rosaceae Fragaria iinumae#NC 024258
Rosales Rosaceae Fragaria nipponica#NC 035500
Rosales Rosaceae Fragaria pentaphylla#NC 034347
Rosales Rosaceae Fragaria orientalis#NC 035501
Rosales Rosaceae Fragaria mandshurica#NC 018767
Rosales Rosaceae Fragaria vesca#NC 015206
Rosales Rosaceae Fragaria vesca subsp. bracteata#NC 018766
Rosales Rosaceae Fragaria virginiana#KY085911
Rosales Rosaceae Fragaria chiloensis#NC 019601
Rosales Rosaceae Kerria japonica#TJQY
Rosales Rosaceae Prinsepia utilis#NC 021455
Rosales Rosaceae Oemleria cerasiformis#Y264
Rosales Rosaceae Stephanandra chinensis#YC25
Rosales Rosaceae Physocarpus sp.#YC27
Rosales Rosaceae Physocarpus opulifolius#SXCE
Rosales Rosaceae Aruncus dioicus#ZPKK
Rosales Rosaceae Pentactina rupicola#JQ041763
Rosales Rosaceae Kelseya uniflora#Y192
Rosales Rosaceae Petrophytum hendersonii#Y187
Rosales Rosaceae Chamaebatiaria millefolium#Y261
Rosales Rosaceae Sorbaria sorbifolia#YC23
Rosales Rosaceae Prunus serotina#NC 036133
Rosales Rosaceae Prunus padus#NC 026982
Rosales Rosaceae Prunus cerasoides#NC 035891
Rosales Rosaceae Cerasus yedoensis#NC 026980
Rosales Rosaceae Prunus maximowiczii#NC 026981
Rosales Rosaceae Prunus takesimensis#NC 039379
Rosales Rosaceae Prunus tomentosa#NC 036394
Rosales Rosaceae Prunus pedunculata#MG869261
Rosales Rosaceae Prunus mume#NC 023798
Rosales Rosaceae Prunus humilis#NC 035880
Rosales Rosaceae Prunus prostrata#NCVK

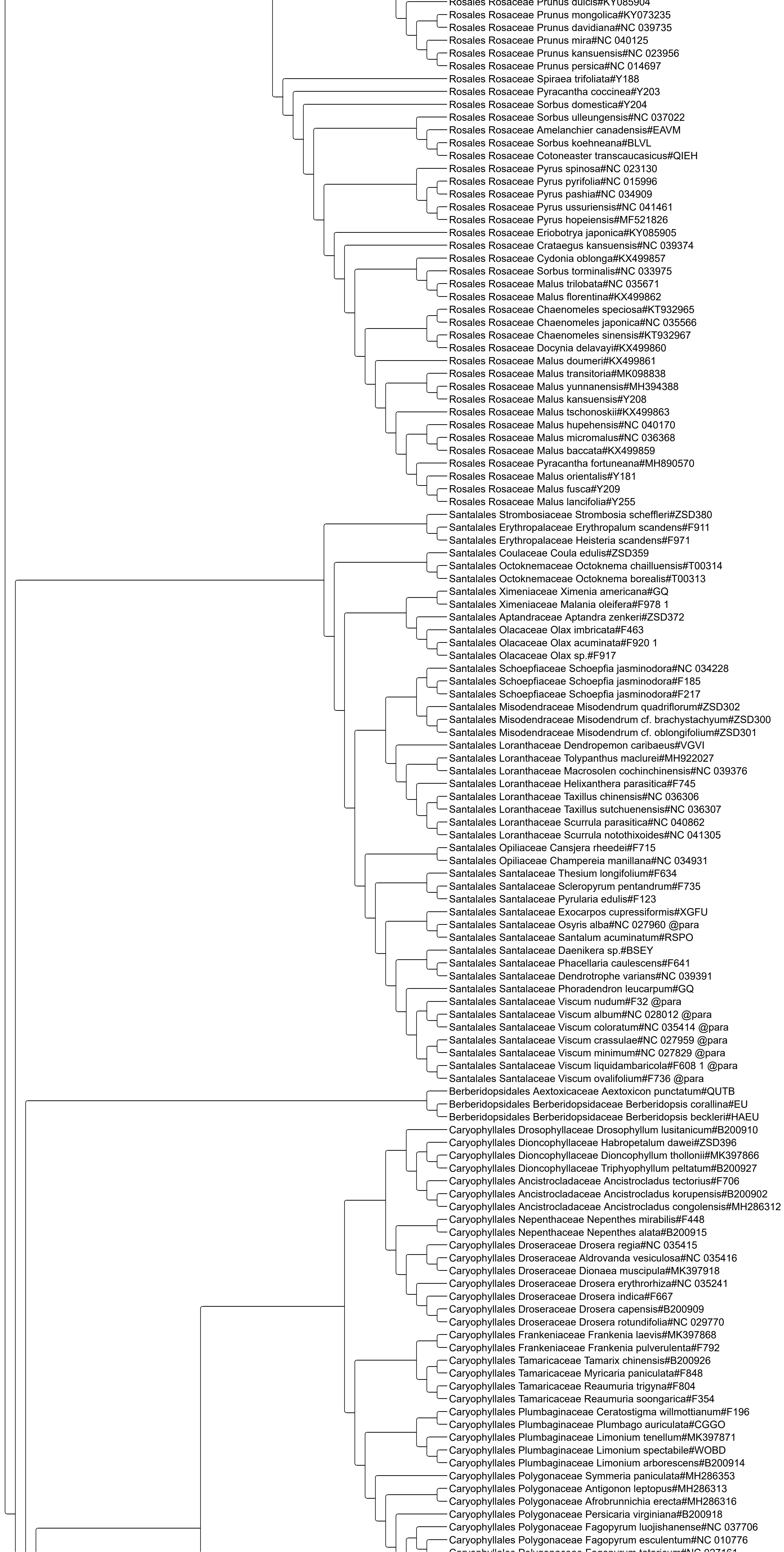

Rosales Rosaceae Prunus dulcis#KY085904
Rosales Rosaceae Prunus mongolica#KY073235
Rosales Rosaceae Prunus davidiana#NC 039735
Rosales Rosaceae Prunus mira#NC 040125
Rosales Rosaceae Prunus kansuensis#NC 023956
Rosales Rosaceae Prunus persica#NC 014697
Rosales Rosaceae Spiraea trifoliata#Y188
Rosales Rosaceae Pyracantha coccinea#Y203
Rosales Rosaceae Sorbus domestica#Y204
Rosales Rosaceae Sorbus ulleungensis#NC 037022
Rosales Rosaceae Amelanchier canadensis#EAVM
Rosales Rosaceae Sorbus koehneana#BLVL
Rosales Rosaceae Cotoneaster transcaucasicus#QIEH
Rosales Rosaceae Pyrus spinosa#NC 023130
Rosales Rosaceae Pyrus pyrifolia#NC 015996
Rosales Rosaceae Pyrus pashia#NC 034909
Rosales Rosaceae Pyrus ussuriensis#NC 041461
Rosales Rosaceae Pyrus hopeiensis#MF521826
Rosales Rosaceae Eriobotrya japonica#KY085905
Rosales Rosaceae Crataegus kansuensis#NC 039374
Rosales Rosaceae Cydonia oblonga#KX499857
Rosales Rosaceae Sorbus torminalis#NC 033975
Rosales Rosaceae Malus trilobata#NC 035671
Rosales Rosaceae Malus florentina#KX499862
Rosales Rosaceae Chaenomeles speciosa#KT932965
Rosales Rosaceae Chaenomeles japonica#NC 035566
Rosales Rosaceae Chaenomeles sinensis#KT932967
Rosales Rosaceae Docynia delavayi#KX499860
Rosales Rosaceae Malus doumeri#KX499861
Rosales Rosaceae Malus transitoria#MK098838
Rosales Rosaceae Malus yunnanensis#MH394388
Rosales Rosaceae Malus kansuensis#Y208
Rosales Rosaceae Malus tschonoskii#KX499863
Rosales Rosaceae Malus hupehensis#NC 040170
Rosales Rosaceae Malus micromalus#NC 036368
Rosales Rosaceae Malus baccata#KX499859
Rosales Rosaceae Pyracantha fortuneana#MH890570
Rosales Rosaceae Malus orientalis#Y181
Rosales Rosaceae Malus fusca#Y209
Rosales Rosaceae Malus lancifolia#Y255
Santalales Strombosiaceae Strombosia scheffleri#ZSD380
Santalales Erythropalaceae Erythropalum scandens#F911
Santalales Erythropalaceae Heisteria scandens#F971
Santalales Coulaceae Coula edulis#ZSD359
Santalales Octoknemaceae Octoknema chailluensis#T00314
Santalales Octoknemaceae Octoknema borealis#T00313
Santalales Ximeniaceae Ximenia americana#GQ
Santalales Ximeniaceae Malania oleifera#F978 1
Santalales Aptandraceae Aptandra zenkeri#ZSD372
Santalales Olacaceae Olax imbricata#F463
Santalales Olacaceae Olax acuminata#F920 1
Santalales Olacaceae Olax sp.#F917
Santalales Schoepfiaceae Schoepfia jasminodora#NC 034228
Santalales Schoepfiaceae Schoepfia jasminodora#F185
Santalales Schoepfiaceae Schoepfia jasminodora#F217
Santalales Misodendraceae Misodendrum quadriflorum#ZSD302
Santalales Misodendraceae Misodendrum cf. brachystachyum#ZSD300
Santalales Misodendraceae Misodendrum cf. oblongifolium#ZSD301
Santalales Loranthaceae Dendropemon caribaeus#VGVI
Santalales Loranthaceae Tolypanthus maclurei#MH922027
Santalales Loranthaceae Macrosolen cochinchinensis#NC 039376
Santalales Loranthaceae Helixanthera parasitica#F745
Santalales Loranthaceae Taxillus chinensis#NC 036306
Santalales Loranthaceae Taxillus sutchuenensis#NC 036307
Santalales Loranthaceae Scurrula parasitica#NC 040862
Santalales Loranthaceae Scurrula notothixoides#NC 041305
Santalales Opiliaceae Cansjera rheedei#F715
Santalales Opiliaceae Champereia manillana#NC 034931
Santalales Santalaceae Thesium longifolium#F634
Santalales Santalaceae Scleropyrum pentandrum#F735
Santalales Santalaceae Pyrularia edulis#F123
Santalales Santalaceae Exocarpos cupressiformis#XGFU
Santalales Santalaceae Osyris alba#NC 027960 @para
Santalales Santalaceae Santalum acuminatum#RSPO
Santalales Santalaceae Daenikera sp.#BSEY
Santalales Santalaceae Phacellaria caulescens#F641
Santalales Santalaceae Dendrotrophe varians#NC 039391
Santalales Santalaceae Phoradendron leucarpum#GQ
Santalales Santalaceae Viscum nudum#F32 @para
Santalales Santalaceae Viscum album#NC 028012 @para
Santalales Santalaceae Viscum coloratum#NC 035414 @para
Santalales Santalaceae Viscum crassulae#NC 027959 @para
Santalales Santalaceae Viscum minimum#NC 027829 @para
Santalales Santalaceae Viscum liquidambaricola#F608 1 @para
Santalales Santalaceae Viscum ovalifolium#F736 @para
Berberidopsidales Aextoxicaceae Aextoxicon punctatum#QUTB
Berberidopsidales Berberidopsidaceae Berberidopsis corallina#EU
Berberidopsidales Berberidopsidaceae Berberidopsis beckleri#HAEU
Caryophyllales Drosophyllaceae Drosophyllum lusitanicum#B200910
Caryophyllales Dioncophyllaceae Habropetalum dawei#ZSD396
Caryophyllales Dioncophyllaceae Dioncophyllum thollonii#MK397866
Caryophyllales Dioncophyllaceae Triphyophyllum peltatum#B200927
Caryophyllales Ancistrocladaceae Ancistrocladus tectorius#F706
Caryophyllales Ancistrocladaceae Ancistrocladus korupensis#B200902
Caryophyllales Ancistrocladaceae Ancistrocladus congolensis#MH286312
Caryophyllales Nepenthaceae Nepenthes mirabilis#F448
Caryophyllales Nepenthaceae Nepenthes alata#B200915
Caryophyllales Droseraceae Drosera regia#NC 035415
Caryophyllales Droseraceae Aldrovanda vesiculosa#NC 035416
Caryophyllales Droseraceae Dionaea muscipula#MK397918
Caryophyllales Droseraceae Drosera erythrorhiza#NC 035241
Caryophyllales Droseraceae Drosera indica#F667
Caryophyllales Droseraceae Drosera capensis#B200909
Caryophyllales Droseraceae Drosera rotundifolia#NC 029770
Caryophyllales Frankeniaceae Frankenia laevis#MK397868
Caryophyllales Frankeniaceae Frankenia pulverulenta#F792
Caryophyllales Tamaricaceae Tamarix chinensis#B200926
Caryophyllales Tamaricaceae Myricaria paniculata#F848
Caryophyllales Tamaricaceae Reaumuria trigyna#F804
Caryophyllales Tamaricaceae Reaumuria soongarica#F354
Caryophyllales Plumbaginaceae Ceratostigma willmottianum#F196
Caryophyllales Plumbaginaceae Plumbago auriculata#CGGO
Caryophyllales Plumbaginaceae Limonium tenellum#MK397871
Caryophyllales Plumbaginaceae Limonium spectabile#WOBD
Caryophyllales Plumbaginaceae Limonium arborescens#B200914
Caryophyllales Polygonaceae Symmeria paniculata#MH286353
Caryophyllales Polygonaceae Antigonon leptopus#MH286313
Caryophyllales Polygonaceae Afrobrunnichia erecta#MH286316
Caryophyllales Polygonaceae Persicaria virginiana#B200918
Caryophyllales Polygonaceae Fagopyrum luojishanense#NC 037706
Caryophyllales Polygonaceae Fagopyrum esculentum#NC 010776

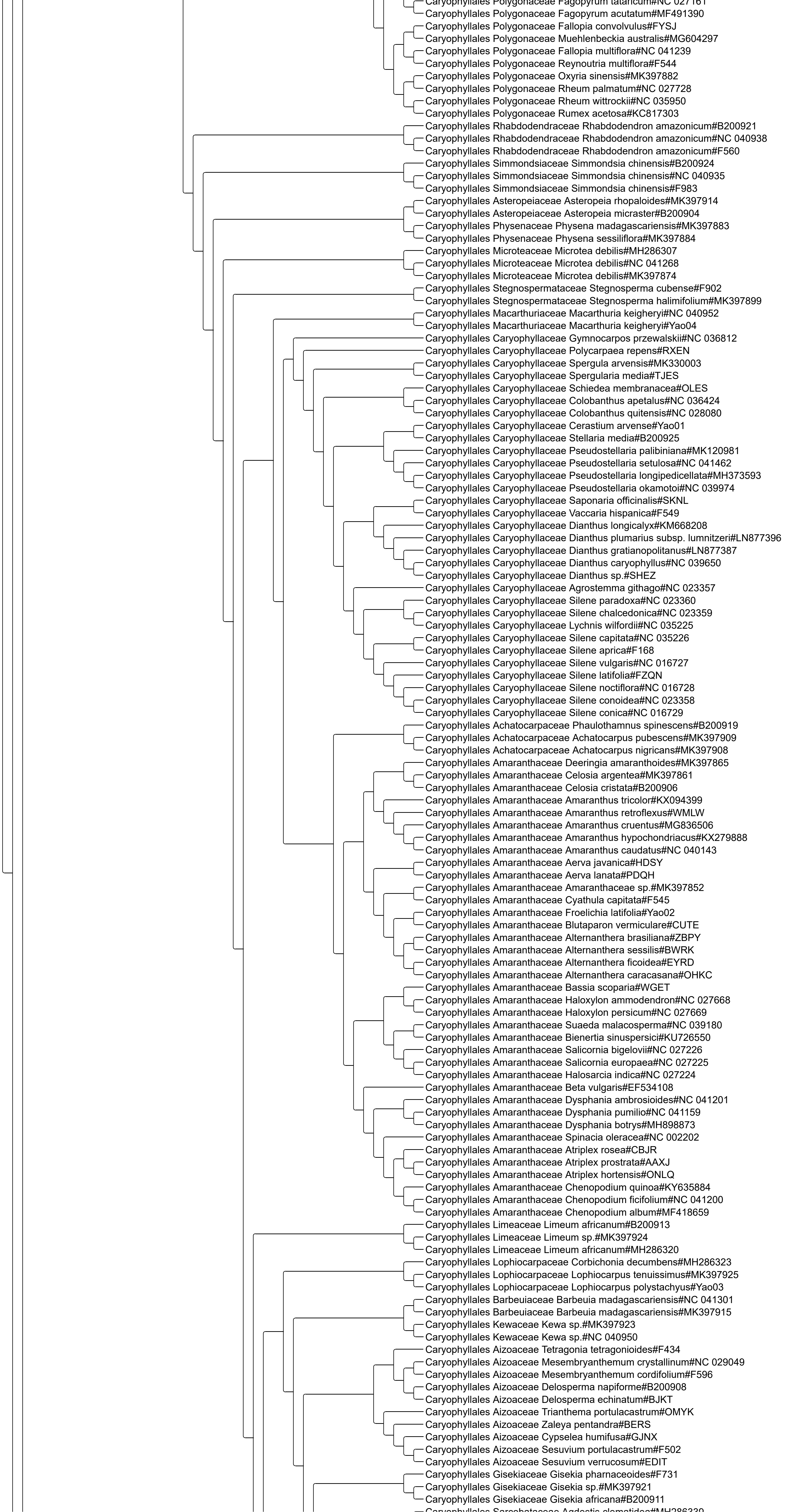
Caryophyllales Polygonaceae Fagopyrum acutatum#MF491390
Caryophyllales Polygonaceae Fallopia convolvulus#FYSJ
Caryophyllales Polygonaceae Muehlenbeckia australis#MG604297
Caryophyllales Polygonaceae Fallopia multiflora#NC 041239
Caryophyllales Polygonaceae Reynoutria multiflora#F544
Caryophyllales Polygonaceae Oxyria sinensis#MK397882
Caryophyllales Polygonaceae Rheum palmatum#NC 027728
Caryophyllales Polygonaceae Rheum wittrockii#NC 035950
Caryophyllales Polygonaceae Rumex acetosa#KC817303
Caryophyllales Rhabdodendraceae Rhabdodendron amazonicum#B200921
Caryophyllales Rhabdodendraceae Rhabdodendron amazonicum#NC 040938
Caryophyllales Rhabdodendraceae Rhabdodendron amazonicum#F560
Caryophyllales Simmondsiaceae Simmondsia chinensis#B200924
Caryophyllales Simmondsiaceae Simmondsia chinensis#NC 040935
Caryophyllales Simmondsiaceae Simmondsia chinensis#F983
Caryophyllales Asteropeiaceae Asteropeia rhopaloides#MK397914
Caryophyllales Asteropeiaceae Asteropeia micraster#B200904
Caryophyllales Physenaceae Physena madagascariensis#MK397883
Caryophyllales Physenaceae Physena sessiliflora#MK397884
Caryophyllales Microteaceae Microtea debilis#MH286307
Caryophyllales Microteaceae Microtea debilis#NC 041268
Caryophyllales Microteaceae Microtea debilis#MK397874
Caryophyllales Stegnospermataceae Stegnosperma cubense#F902
Caryophyllales Stegnospermataceae Stegnosperma halimifolium#MK397899
Caryophyllales Macarthuriaceae Macarthuria keigheryi#NC 040952
Caryophyllales Macarthuriaceae Macarthuria keigheryi#Yao04
Caryophyllales Caryophyllaceae Gymnocarpos przewalskii#NC 036812
Caryophyllales Caryophyllaceae Polycarpaea repens#RXEN
Caryophyllales Caryophyllaceae Spergula arvensis#MK330003
Caryophyllales Caryophyllaceae Spergularia media#TJES
Caryophyllales Caryophyllaceae Schiedea membranacea#OLES
Caryophyllales Caryophyllaceae Colobanthus apetalus#NC 036424
Caryophyllales Caryophyllaceae Colobanthus quitensis#NC 028080
Caryophyllales Caryophyllaceae Cerastium arvense#Yao01
Caryophyllales Caryophyllaceae Stellaria media#B200925
Caryophyllales Caryophyllaceae Pseudostellaria palibiniana#MK120981
Caryophyllales Caryophyllaceae Pseudostellaria setulosa#NC 041462
Caryophyllales Caryophyllaceae Pseudostellaria longipedicellata#MH373593
Caryophyllales Caryophyllaceae Pseudostellaria okamotoi#NC 039974
Caryophyllales Caryophyllaceae Saponaria officinalis#SKNL
Caryophyllales Caryophyllaceae Vaccaria hispanica#F549
Caryophyllales Caryophyllaceae Dianthus longicalyx#KM668208
Caryophyllales Caryophyllaceae Dianthus plumarius subsp. lumnitzeri#LN877396
Caryophyllales Caryophyllaceae Dianthus gratianopolitanus#LN877387
Caryophyllales Caryophyllaceae Dianthus caryophyllus#NC 039650
Caryophyllales Caryophyllaceae Dianthus sp.#SHEZ
Caryophyllales Caryophyllaceae Agrostemma githago#NC 023357
Caryophyllales Caryophyllaceae Silene paradoxa#NC 023360
Caryophyllales Caryophyllaceae Silene chalcedonica#NC 023359
Caryophyllales Caryophyllaceae Lychnis wilfordii#NC 035225
Caryophyllales Caryophyllaceae Silene capitata#NC 035226
Caryophyllales Caryophyllaceae Silene aprica#F168
Caryophyllales Caryophyllaceae Silene vulgaris#NC 016727
Caryophyllales Caryophyllaceae Silene latifolia#FZQN
Caryophyllales Caryophyllaceae Silene noctiflora#NC 016728
Caryophyllales Caryophyllaceae Silene conoidea#NC 023358
Caryophyllales Caryophyllaceae Silene conica#NC 016729
Caryophyllales Achatocarpaceae Phaulothamnus spinescens#B200919
Caryophyllales Achatocarpaceae Achatocarpus pubescens#MK397909
Caryophyllales Achatocarpaceae Achatocarpus nigricans#MK397908
Caryophyllales Amaranthaceae Deeringia amaranthoides#MK397865
Caryophyllales Amaranthaceae Celosia argentea#MK397861
Caryophyllales Amaranthaceae Celosia cristata#B200906
Caryophyllales Amaranthaceae Amaranthus tricolor#KX094399
Caryophyllales Amaranthaceae Amaranthus retroflexus#WMLW
Caryophyllales Amaranthaceae Amaranthus cruentus#MG836506
Caryophyllales Amaranthaceae Amaranthus hypochondriacus#KX279888
Caryophyllales Amaranthaceae Amaranthus caudatus#NC 040143
Caryophyllales Amaranthaceae Aerva javanica#HDSY
Caryophyllales Amaranthaceae Aerva lanata#PDQH
Caryophyllales Amaranthaceae Amaranthaceae sp.#MK397852
Caryophyllales Amaranthaceae Cyathula capitata#F545
Caryophyllales Amaranthaceae Froelichia latifolia#Yao02
Caryophyllales Amaranthaceae Blutaparon vermiculare#CUTE
Caryophyllales Amaranthaceae Alternanthera brasiliana#ZBPY
Caryophyllales Amaranthaceae Alternanthera sessilis#BWRK
Caryophyllales Amaranthaceae Alternanthera ficoidea#EYRD
Caryophyllales Amaranthaceae Alternanthera caracasana#OHKC
Caryophyllales Amaranthaceae Bassia scoparia#WGET
Caryophyllales Amaranthaceae Haloxylon ammodendron#NC 027668
Caryophyllales Amaranthaceae Haloxylon persicum#NC 027669
Caryophyllales Amaranthaceae Suaeda malacosperma#NC 039180
Caryophyllales Amaranthaceae Bienertia sinuspersici#KU726550
Caryophyllales Amaranthaceae Salicornia bigelovii#NC 027226
Caryophyllales Amaranthaceae Salicornia europaea#NC 027225
Caryophyllales Amaranthaceae Halosarcia indica#NC 027224
Caryophyllales Amaranthaceae Beta vulgaris#EF534108
Caryophyllales Amaranthaceae Dysphania ambrosioides#NC 041201
Caryophyllales Amaranthaceae Dysphania pumilio#NC 041159
Caryophyllales Amaranthaceae Dysphania botrys#MH898873
Caryophyllales Amaranthaceae Spinacia oleracea#NC 002202
Caryophyllales Amaranthaceae Atriplex rosea#CBJR
Caryophyllales Amaranthaceae Atriplex prostrata#AAXJ
Caryophyllales Amaranthaceae Atriplex hortensis#ONLQ
Caryophyllales Amaranthaceae Chenopodium quinoa#KY635884
Caryophyllales Amaranthaceae Chenopodium ficifolium#NC 041200
Caryophyllales Amaranthaceae Chenopodium album#MF418659
Caryophyllales Limeaceae Limeum africanum#B200913
Caryophyllales Limeaceae Limeum sp.#MK397924
Caryophyllales Limeaceae Limeum africanum#MH286320
Caryophyllales Lophiocarpaceae Corbichonia decumbens#MH286323
Caryophyllales Lophiocarpaceae Lophiocarpus tenuissimus#MK397925
Caryophyllales Lophiocarpaceae Lophiocarpus polystachyus#Yao03
Caryophyllales Barbeuiaceae Barbeuia madagascariensis#NC 041301
Caryophyllales Barbeuiaceae Barbeuia madagascariensis#MK397915
Caryophyllales Kewaceae Kewa sp.#MK397923
Caryophyllales Kewaceae Kewa sp.#NC 040950
Caryophyllales Aizoaceae Tetragonia tetragonioides#F434
Caryophyllales Aizoaceae Mesembryanthemum crystallinum#NC 029049
Caryophyllales Aizoaceae Mesembryanthemum cordifolium#F596
Caryophyllales Aizoaceae Delosperma napiforme#B200908
Caryophyllales Aizoaceae Delosperma echinatum#BJKT
Caryophyllales Aizoaceae Trianthema portulacastrum#OMYK
Caryophyllales Aizoaceae Zaleya pentandra#BERS
Caryophyllales Aizoaceae Cypselea humifusa#GJNX
Caryophyllales Aizoaceae Sesuvium portulacastrum#F502
Caryophyllales Aizoaceae Sesuvium verrucosum#EDIT
Caryophyllales Gisekiaceae Gisekia pharnaceoides#F731
Caryophyllales Gisekiaceae Gisekia sp.#MK397921
Caryophyllales Gisekiaceae Gisekia africana#B200911

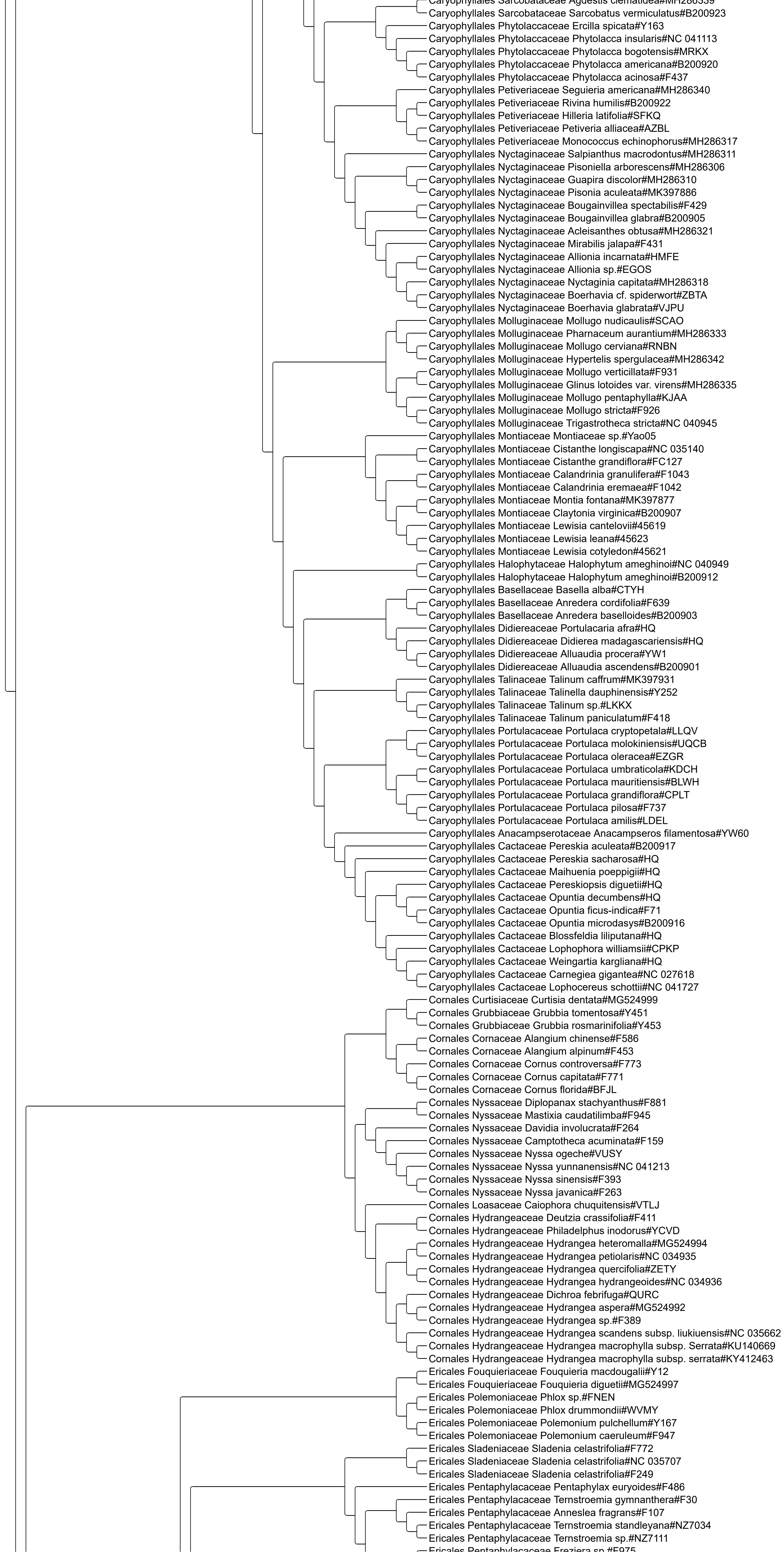
Caryophyllales Sarcobataceae Agdestis clematidea#MH286339
Caryophyllales Sarcobataceae Sarcobatus vermiculatus#B200923
Caryophyllales Phytolaccaceae Ercilla spicata#Y163
Caryophyllales Phytolaccaceae Phytolacca insularis#NC 041113
Caryophyllales Phytolaccaceae Phytolacca bogotensis#MRKX
Caryophyllales Phytolaccaceae Phytolacca americana#B200920
Caryophyllales Phytolaccaceae Phytolacca acinosa#F437
Caryophyllales Petiveriaceae Seguieria americana#MH286340
Caryophyllales Petiveriaceae Rivina humilis#B200922
Caryophyllales Petiveriaceae Hilleria latifolia#SFKQ
Caryophyllales Petiveriaceae Petiveria alliacea#AZBL
Caryophyllales Petiveriaceae Monococcus echinophorus#MH286317
Caryophyllales Nyctaginaceae Salpianthus macrodontus#MH286311
Caryophyllales Nyctaginaceae Pisoniella arborescens#MH286306
Caryophyllales Nyctaginaceae Guapira discolor#MH286310
Caryophyllales Nyctaginaceae Pisonia aculeata#MK397886
Caryophyllales Nyctaginaceae Bougainvillea spectabilis#F429
Caryophyllales Nyctaginaceae Bougainvillea glabra#B200905
Caryophyllales Nyctaginaceae Acleisanthes obtusa#MH286321
Caryophyllales Nyctaginaceae Mirabilis jalapa#F431
Caryophyllales Nyctaginaceae Allionia incarnata#HMFE
Caryophyllales Nyctaginaceae Allionia sp.#EGOS
Caryophyllales Nyctaginaceae Nyctaginia capitata#MH286318
Caryophyllales Nyctaginaceae Boerhavia cf. spiderwort#ZBTA
Caryophyllales Nyctaginaceae Boerhavia glabrata#VJPU
Caryophyllales Molluginaceae Mollugo nudicaulis#SCAO
Caryophyllales Molluginaceae Pharnaceum aurantium#MH286333
Caryophyllales Molluginaceae Mollugo cerviana#RNBN
Caryophyllales Molluginaceae Hypertelis spergulacea#MH286342
Caryophyllales Molluginaceae Mollugo verticillata#F931
Caryophyllales Molluginaceae Glinus lotoides var. virens#MH286335
Caryophyllales Molluginaceae Mollugo pentaphylla#KJAA
Caryophyllales Molluginaceae Mollugo stricta#F926
Caryophyllales Molluginaceae Trigastrotheca stricta#NC 040945
Caryophyllales Montiaceae Montiaceae sp.#Yao05
Caryophyllales Montiaceae Cistanthe longiscapa#NC 035140
Caryophyllales Montiaceae Cistanthe grandiflora#FC127
Caryophyllales Montiaceae Calandrinia granulifera#F1043
Caryophyllales Montiaceae Calandrinia eremaea#F1042
Caryophyllales Montiaceae Montia fontana#MK397877
Caryophyllales Montiaceae Claytonia virginica#B200907
Caryophyllales Montiaceae Lewisia cantelovii#45619
Caryophyllales Montiaceae Lewisia leana#45623
Caryophyllales Montiaceae Lewisia cotyledon#45621
Caryophyllales Halophytaceae Halophytum ameghinoi#NC 040949
Caryophyllales Halophytaceae Halophytum ameghinoi#B200912
Caryophyllales Basellaceae Basella alba#CTYH
Caryophyllales Basellaceae Anredera cordifolia#F639
Caryophyllales Basellaceae Anredera baselloides#B200903
Caryophyllales Didiereaceae Portulacaria afra#HQ
Caryophyllales Didiereaceae Didierea madagascariensis#HQ
Caryophyllales Didiereaceae Alluaudia procera#YW1
Caryophyllales Didiereaceae Alluaudia ascendens#B200901
Caryophyllales Talinaceae Talinum caffrum#MK397931
Caryophyllales Talinaceae Talinella dauphinensis#Y252
Caryophyllales Talinaceae Talinum sp.#LKKX
Caryophyllales Talinaceae Talinum paniculatum#F418
Caryophyllales Portulacaceae Portulaca cryptopetala#LLQV
Caryophyllales Portulacaceae Portulaca molokiniensis#UQCB
Caryophyllales Portulacaceae Portulaca oleracea#EZGR
Caryophyllales Portulacaceae Portulaca umbraticola#KDCH
Caryophyllales Portulacaceae Portulaca mauritiensis#BLWH
Caryophyllales Portulacaceae Portulaca grandiflora#CPLT
Caryophyllales Portulacaceae Portulaca pilosa#F737
Caryophyllales Portulacaceae Portulaca amilis#LDEL
Caryophyllales Anacampserotaceae Anacampseros filamentosa#YW60
Caryophyllales Cactaceae Pereskia aculeata#B200917
Caryophyllales Cactaceae Pereskia sacharosa#HQ
Caryophyllales Cactaceae Maihuenia poeppigii#HQ
Caryophyllales Cactaceae Pereskiopsis diguetii#HQ
Caryophyllales Cactaceae Opuntia decumbens#HQ
Caryophyllales Cactaceae Opuntia ficus-indica#F71
Caryophyllales Cactaceae Opuntia microdasys#B200916
Caryophyllales Cactaceae Blossfeldia liliputana#HQ
Caryophyllales Cactaceae Lophophora williamsii#CPKP
Caryophyllales Cactaceae Weingartia kargliana#HQ
Caryophyllales Cactaceae Carnegiea gigantea#NC 027618
Caryophyllales Cactaceae Lophocereus schottii#NC 041727
Cornales Curtisiaceae Curtisia dentata#MG524999
Cornales Grubbiaceae Grubbia tomentosa#Y451
Cornales Grubbiaceae Grubbia rosmarinifolia#Y453
Cornales Cornaceae Alangium chinense#F586
Cornales Cornaceae Alangium alpinum#F453
Cornales Cornaceae Cornus controversa#F773
Cornales Cornaceae Cornus capitata#F771
Cornales Cornaceae Cornus florida#BFJL
Cornales Nyssaceae Diplopanax stachyanthus#F881
Cornales Nyssaceae Mastixia caudatilimba#F945
Cornales Nyssaceae Davidia involucrata#F264
Cornales Nyssaceae Camptotheca acuminata#F159
Cornales Nyssaceae Nyssa ogeche#VUSY
Cornales Nyssaceae Nyssa yunnanensis#NC 041213
Cornales Nyssaceae Nyssa sinensis#F393
Cornales Nyssaceae Nyssa javanica#F263
Cornales Loasaceae Caiophora chuquitensis#VTLJ
Cornales Hydrangeaceae Deutzia crassifolia#F411
Cornales Hydrangeaceae Philadelphus inodorus#YCVD
Cornales Hydrangeaceae Hydrangea heteromalla#MG524994
Cornales Hydrangeaceae Hydrangea petiolaris#NC 034935
Cornales Hydrangeaceae Hydrangea quercifolia#ZETY
Cornales Hydrangeaceae Hydrangea hydrangeoides#NC 034936
Cornales Hydrangeaceae Dichroa febrifuga#QURC
Cornales Hydrangeaceae Hydrangea aspera#MG524992
Cornales Hydrangeaceae Hydrangea sp.#F389
Cornales Hydrangeaceae Hydrangea scandens subsp. liukiuensis#NC 035662
Cornales Hydrangeaceae Hydrangea macrophylla subsp. Serrata#KU140669
Cornales Hydrangeaceae Hydrangea macrophylla subsp. serrata#KY412463
Ericales Fouquieriaceae Fouquieria macdougalii#Y12
Ericales Fouquieriaceae Fouquieria diguetii#MG524997
Ericales Polemoniaceae Phlox sp.#FNEN
Ericales Polemoniaceae Phlox drummondii#WVMY
Ericales Polemoniaceae Polemonium pulchellum#Y167
Ericales Polemoniaceae Polemonium caeruleum#F947
Ericales Sladeniaceae Sladenia celastrifolia#F772
Ericales Sladeniaceae Sladenia celastrifolia#NC 035707
Ericales Sladeniaceae Sladenia celastrifolia#F249
Ericales Pentaphylacaceae Pentaphylax euryoides#F486
Ericales Pentaphylacaceae Ternstroemia gymnanthera#F30
Ericales Pentaphylacaceae Anneslea fragrans#F107
Ericales Pentaphylacaceae Ternstroemia standleyana#NZ7034
Ericales Pentaphylacaceae Ternstroemia sp.#NZ7111

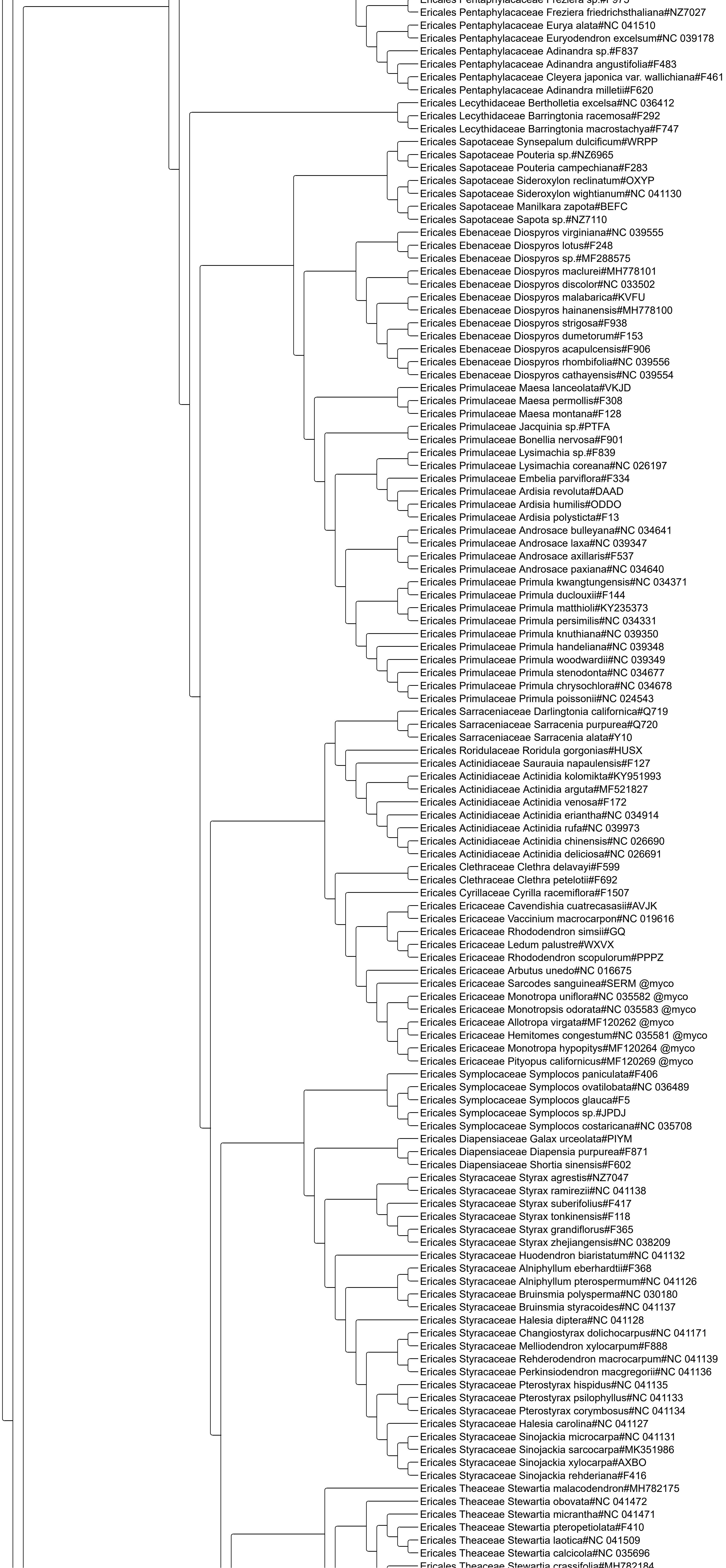
Ericales Pentaphylacaceae Freziera friedrichsthaliana#NZ7027
Ericales Pentaphylacaceae Eurya alata#NC 041510
Ericales Pentaphylacaceae Euryodendron excelsum#NC 039178
Ericales Pentaphylacaceae Adinandra sp.#F837
Ericales Pentaphylacaceae Adinandra angustifolia#F483
Ericales Pentaphylacaceae Cleyera japonica var. wallichiana#F461
Ericales Pentaphylacaceae Adinandra milletii#F620
Ericales Lecythidaceae Bertholletia excelsa#NC 036412
Ericales Lecythidaceae Barringtonia racemosa#F292
Ericales Lecythidaceae Barringtonia macrostachya#F747
Ericales Sapotaceae Synsepalum dulcificum#WRPP
Ericales Sapotaceae Pouteria sp.#NZ6965
Ericales Sapotaceae Pouteria campechiana#F283
Ericales Sapotaceae Sideroxylon reclinatum#OXYP
Ericales Sapotaceae Sideroxylon wightianum#NC 041130
Ericales Sapotaceae Manilkara zapota#BEFC
Ericales Sapotaceae Sapota sp.#NZ7110
Ericales Ebenaceae Diospyros virginiana#NC 039555
Ericales Ebenaceae Diospyros lotus#F248
Ericales Ebenaceae Diospyros sp.#MF288575
Ericales Ebenaceae Diospyros maclurei#MH778101
Ericales Ebenaceae Diospyros discolor#NC 033502
Ericales Ebenaceae Diospyros malabarica#KVFU
Ericales Ebenaceae Diospyros hainanensis#MH778100
Ericales Ebenaceae Diospyros strigosa#F938
Ericales Ebenaceae Diospyros dumetorum#F153
Ericales Ebenaceae Diospyros acapulcensis#F906
Ericales Ebenaceae Diospyros rhombifolia#NC 039556
Ericales Ebenaceae Diospyros cathayensis#NC 039554
Ericales Primulaceae Maesa lanceolata#VKJD
Ericales Primulaceae Maesa permollis#F308
Ericales Primulaceae Maesa montana#F128
Ericales Primulaceae Jacquinia sp.#PTFA
Ericales Primulaceae Bonellia nervosa#F901
Ericales Primulaceae Lysimachia sp.#F839
Ericales Primulaceae Lysimachia coreana#NC 026197
Ericales Primulaceae Embelia parviflora#F334
Ericales Primulaceae Ardisia revoluta#DAAD
Ericales Primulaceae Ardisia humilis#ODDO
Ericales Primulaceae Ardisia polysticta#F13
Ericales Primulaceae Androsace bulleyana#NC 034641
Ericales Primulaceae Androsace laxa#NC 039347
Ericales Primulaceae Androsace axillaris#F537
Ericales Primulaceae Androsace paxiana#NC 034640
Ericales Primulaceae Primula kwangtungensis#NC 034371
Ericales Primulaceae Primula duclouxii#F144
Ericales Primulaceae Primula matthioli#KY235373
Ericales Primulaceae Primula persimilis#NC 034331
Ericales Primulaceae Primula knuthiana#NC 039350
Ericales Primulaceae Primula handeliana#NC 039348
Ericales Primulaceae Primula woodwardii#NC 039349
Ericales Primulaceae Primula stenodonta#NC 034677
Ericales Primulaceae Primula chrysochlora#NC 034678
Ericales Primulaceae Primula poissonii#NC 024543
Ericales Sarraceniaceae Darlingtonia californica#Q719
Ericales Sarraceniaceae Sarracenia purpurea#Q720
Ericales Sarraceniaceae Sarracenia alata#Y10
Ericales Roridulaceae Roridula gorgonias#HUSX
Ericales Actinidiaceae Saurauia napaulensis#F127
Ericales Actinidiaceae Actinidia kolomikta#KY951993
Ericales Actinidiaceae Actinidia arguta#MF521827
Ericales Actinidiaceae Actinidia venosa#F172
Ericales Actinidiaceae Actinidia eriantha#NC 034914
Ericales Actinidiaceae Actinidia rufa#NC 039973
Ericales Actinidiaceae Actinidia chinensis#NC 026690
Ericales Actinidiaceae Actinidia deliciosa#NC 026691
Ericales Clethraceae Clethra delavayi#F599
Ericales Clethraceae Clethra petelotii#F692
Ericales Cyrillaceae Cyrilla racemiflora#F1507
Ericales Ericaceae Cavendishia cuatrecasasii#AVJK
Ericales Ericaceae Vaccinium macrocarpon#NC 019616
Ericales Ericaceae Rhododendron simsii#GQ
Ericales Ericaceae Ledum palustre#WXVX
Ericales Ericaceae Rhododendron scopulorum#PPPZ
Ericales Ericaceae Arbutus unedo#NC 016675
Ericales Ericaceae Sarcodes sanguinea#SERM @myco
Ericales Ericaceae Monotropa uniflora#NC 035582 @myco
Ericales Ericaceae Monotropsis odorata#NC 035583 @myco
Ericales Ericaceae Allotropa virgata#MF120262 @myco
Ericales Ericaceae Hemitomes congestum#NC 035581 @myco
Ericales Ericaceae Monotropa hypopitys#MF120264 @myco
Ericales Ericaceae Pityopus californicus#MF120269 @myco
Ericales Symplocaceae Symplocos paniculata#F406
Ericales Symplocaceae Symplocos ovatilobata#NC 036489
Ericales Symplocaceae Symplocos glauca#F5
Ericales Symplocaceae Symplocos sp.#JPDJ
Ericales Symplocaceae Symplocos costaricana#NC 035708
Ericales Diapensiaceae Galax urceolata#PIYM
Ericales Diapensiaceae Diapensia purpurea#F871
Ericales Diapensiaceae Shortia sinensis#F602
Ericales Styracaceae Styrax agrestis#NZ7047
Ericales Styracaceae Styrax ramirezii#NC 041138
Ericales Styracaceae Styrax suberifolius#F417
Ericales Styracaceae Styrax tonkinensis#F118
Ericales Styracaceae Styrax grandiflorus#F365
Ericales Styracaceae Styrax zhejiangensis#NC 038209
Ericales Styracaceae Huodendron biaristatum#NC 041132
Ericales Styracaceae Alniphyllum eberhardtii#F368
Ericales Styracaceae Alniphyllum pterospermum#NC 041126
Ericales Styracaceae Bruinsmia polysperma#NC 030180
Ericales Styracaceae Bruinsmia styracoides#NC 041137
Ericales Styracaceae Halesia diptera#NC 041128
Ericales Styracaceae Changiostyrax dolichocarpus#NC 041171
Ericales Styracaceae Melliodendron xylocarpum#F888
Ericales Styracaceae Rehderodendron macrocarpum#NC 041139
Ericales Styracaceae Perkinsiodendron macgregorii#NC 041136
Ericales Styracaceae Pterostyrax hispidus#NC 041135
Ericales Styracaceae Pterostyrax psilophyllus#NC 041133
Ericales Styracaceae Pterostyrax corymbosus#NC 041134
Ericales Styracaceae Halesia carolina#NC 041127
Ericales Styracaceae Sinojackia microcarpa#NC 041131
Ericales Styracaceae Sinojackia sarcocarpa#MK351986
Ericales Styracaceae Sinojackia xylocarpa#AXBO
Ericales Styracaceae Sinojackia rehderiana#F416
Ericales Theaceae Stewartia malacodendron#MH782175
Ericales Theaceae Stewartia obovata#NC 041472
Ericales Theaceae Stewartia micrantha#NC 041471
Ericales Theaceae Stewartia pteropetiolata#F410
Ericales Theaceae Stewartia laotica#NC 041509
Ericales Theaceae Stewartia calcicola#NC 035696
Ericales Theaceae Stewartia crassifolia#MH782184

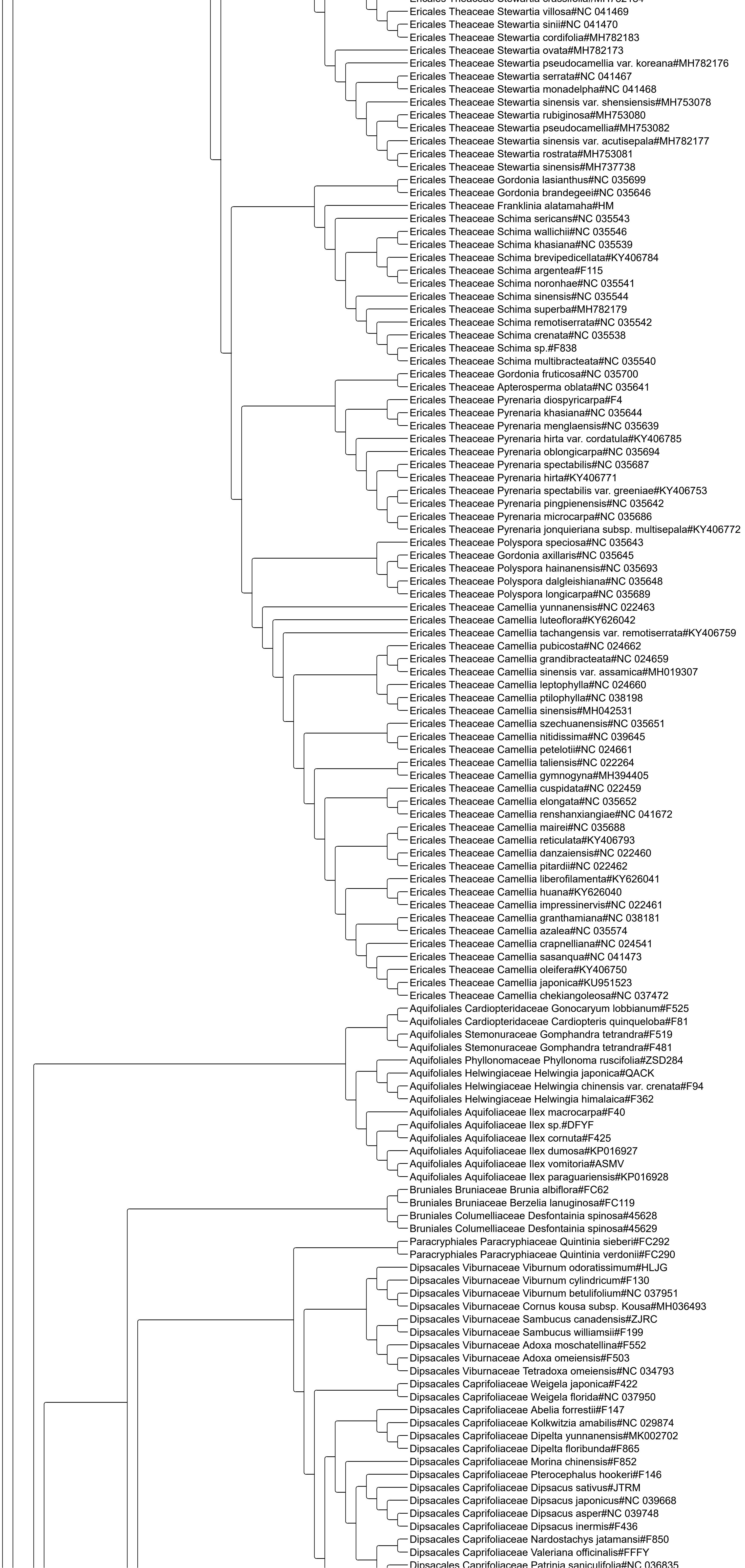
Ericales Theaceae Stewartia villosa#NC 041469
Ericales Theaceae Stewartia sinii#NC 041470
Ericales Theaceae Stewartia cordifolia#MH782183
Ericales Theaceae Stewartia ovata#MH782173
Ericales Theaceae Stewartia pseudocamellia var. koreana#MH782176
Ericales Theaceae Stewartia serrata#NC 041467
Ericales Theaceae Stewartia monadelpha#NC 041468
Ericales Theaceae Stewartia sinensis var. shensiensis#MH753078
Ericales Theaceae Stewartia rubiginosa#MH753080
Ericales Theaceae Stewartia pseudocamellia#MH753082
Ericales Theaceae Stewartia sinensis var. acutisepala#MH782177
Ericales Theaceae Stewartia rostrata#MH753081
Ericales Theaceae Stewartia sinensis#MH737738
Ericales Theaceae Gordonia lasianthus#NC 035699
Ericales Theaceae Gordonia brandegeei#NC 035646
Ericales Theaceae Franklinia alatamaha#HM
Ericales Theaceae Schima sericans#NC 035543
Ericales Theaceae Schima wallichii#NC 035546
Ericales Theaceae Schima khasiana#NC 035539
Ericales Theaceae Schima brevipedicellata#KY406784
Ericales Theaceae Schima argentea#F115
Ericales Theaceae Schima noronhae#NC 035541
Ericales Theaceae Schima sinensis#NC 035544
Ericales Theaceae Schima superba#MH782179
Ericales Theaceae Schima remotiserrata#NC 035542
Ericales Theaceae Schima crenata#NC 035538
Ericales Theaceae Schima sp.#F838
Ericales Theaceae Schima multibracteata#NC 035540
Ericales Theaceae Gordonia fruticosa#NC 035700
Ericales Theaceae Apterosperma oblata#NC 035641
Ericales Theaceae Pyrenaria diospyricarpa#F4
Ericales Theaceae Pyrenaria khasiana#NC 035644
Ericales Theaceae Pyrenaria menglaensis#NC 035639
Ericales Theaceae Pyrenaria hirta var. cordatula#KY406785
Ericales Theaceae Pyrenaria oblongicarpa#NC 035694
Ericales Theaceae Pyrenaria spectabilis#NC 035687
Ericales Theaceae Pyrenaria hirta#KY406771
Ericales Theaceae Pyrenaria spectabilis var. greeniae#KY406753
Ericales Theaceae Pyrenaria pingpienensis#NC 035642
Ericales Theaceae Pyrenaria microcarpa#NC 035686
Ericales Theaceae Pyrenaria jonquieriana subsp. multisepala#KY406772
Ericales Theaceae Polyspora speciosa#NC 035643
Ericales Theaceae Gordonia axillaris#NC 035645
Ericales Theaceae Polyspora hainanensis#NC 035693
Ericales Theaceae Polyspora dalgleishiana#NC 035648
Ericales Theaceae Polyspora longicarpa#NC 035689
Ericales Theaceae Camellia yunnanensis#NC 022463
Ericales Theaceae Camellia luteoflora#KY626042
Ericales Theaceae Camellia tachangensis var. remotiserrata#KY406759
Ericales Theaceae Camellia pubicosta#NC 024662
Ericales Theaceae Camellia grandibracteata#NC 024659
Ericales Theaceae Camellia sinensis var. assamica#MH019307
Ericales Theaceae Camellia leptophylla#NC 024660
Ericales Theaceae Camellia ptilophylla#NC 038198
Ericales Theaceae Camellia sinensis#MH042531
Ericales Theaceae Camellia szechuanensis#NC 035651
Ericales Theaceae Camellia nitidissima#NC 039645
Ericales Theaceae Camellia petelotii#NC 024661
Ericales Theaceae Camellia taliensis#NC 022264
Ericales Theaceae Camellia gymnogyna#MH394405
Ericales Theaceae Camellia cuspidata#NC 022459
Ericales Theaceae Camellia elongata#NC 035652
Ericales Theaceae Camellia renshanxiangiae#NC 041672
Ericales Theaceae Camellia mairei#NC 035688
Ericales Theaceae Camellia reticulata#KY406793
Ericales Theaceae Camellia danzaiensis#NC 022460
Ericales Theaceae Camellia pitardii#NC 022462
Ericales Theaceae Camellia liberofilamenta#KY626041
Ericales Theaceae Camellia huana#KY626040
Ericales Theaceae Camellia impressinervis#NC 022461
Ericales Theaceae Camellia granthamiana#NC 038181
Ericales Theaceae Camellia azalea#NC 035574
Ericales Theaceae Camellia crapnelliana#NC 024541
Ericales Theaceae Camellia sasanqua#NC 041473
Ericales Theaceae Camellia oleifera#KY406750
Ericales Theaceae Camellia japonica#KU951523
Ericales Theaceae Camellia chekiangoleosa#NC 037472
Aquifoliales Cardiopteridaceae Gonocaryum lobbianum#F525
Aquifoliales Cardiopteridaceae Cardiopteris quinqueloba#F81
Aquifoliales Stemonuraceae Gomphandra tetrandra#F519
Aquifoliales Stemonuraceae Gomphandra tetrandra#F481
Aquifoliales Phyllonomaceae Phyllonoma ruscifolia#ZSD284
Aquifoliales Helwingiaceae Helwingia japonica#QACK
Aquifoliales Helwingiaceae Helwingia chinensis var. crenata#F94
Aquifoliales Helwingiaceae Helwingia himalaica#F362
Aquifoliales Aquifoliaceae Ilex macrocarpa#F40
Aquifoliales Aquifoliaceae Ilex sp.#DFYF
Aquifoliales Aquifoliaceae Ilex cornuta#F425
Aquifoliales Aquifoliaceae Ilex dumosa#KP016927
Aquifoliales Aquifoliaceae Ilex vomitoria#ASMV
Aquifoliales Aquifoliaceae Ilex paraguariensis#KP016928
Bruniales Bruniaceae Brunia albiflora#FC62
Bruniales Bruniaceae Berzelia lanuginosa#FC119
Bruniales Columelliaceae Desfontainia spinosa#45628
Bruniales Columelliaceae Desfontainia spinosa#45629
Paracryphiales Paracryphiaceae Quintinia sieberi#FC292
Paracryphiales Paracryphiaceae Quintinia verdonii#FC290
Dipsacales Viburnaceae Viburnum odoratissimum#HLJG
Dipsacales Viburnaceae Viburnum cylindricum#F130
Dipsacales Viburnaceae Viburnum betulifolium#NC 037951
Dipsacales Viburnaceae Cornus kousa subsp. Kousa#MH036493
Dipsacales Viburnaceae Sambucus canadensis#ZJRC
Dipsacales Viburnaceae Sambucus williamsii#F199
Dipsacales Viburnaceae Adoxa moschatellina#F552
Dipsacales Viburnaceae Adoxa omeiensis#F503
Dipsacales Viburnaceae Tetradoxa omeiensis#NC 034793
Dipsacales Caprifoliaceae Weigela japonica#F422
Dipsacales Caprifoliaceae Weigela florida#NC 037950
Dipsacales Caprifoliaceae Abelia forrestii#F147
Dipsacales Caprifoliaceae Kolkwitzia amabilis#NC 029874
Dipsacales Caprifoliaceae Dipelta yunnanensis#MK002702
Dipsacales Caprifoliaceae Dipelta floribunda#F865
Dipsacales Caprifoliaceae Morina chinensis#F852
Dipsacales Caprifoliaceae Pterocephalus hookeri#F146
Dipsacales Caprifoliaceae Dipsacus sativus#JTRM
Dipsacales Caprifoliaceae Dipsacus japonicus#NC 039668
Dipsacales Caprifoliaceae Dipsacus asper#NC 039748
Dipsacales Caprifoliaceae Dipsacus inermis#F436
Dipsacales Caprifoliaceae Nardostachys jatamansi#F850
Dipsacales Caprifoliaceae Valeriana officinalis#FFFY
Dipsacales Caprifoliaceae Patrinia saniculifolia#NC 036835

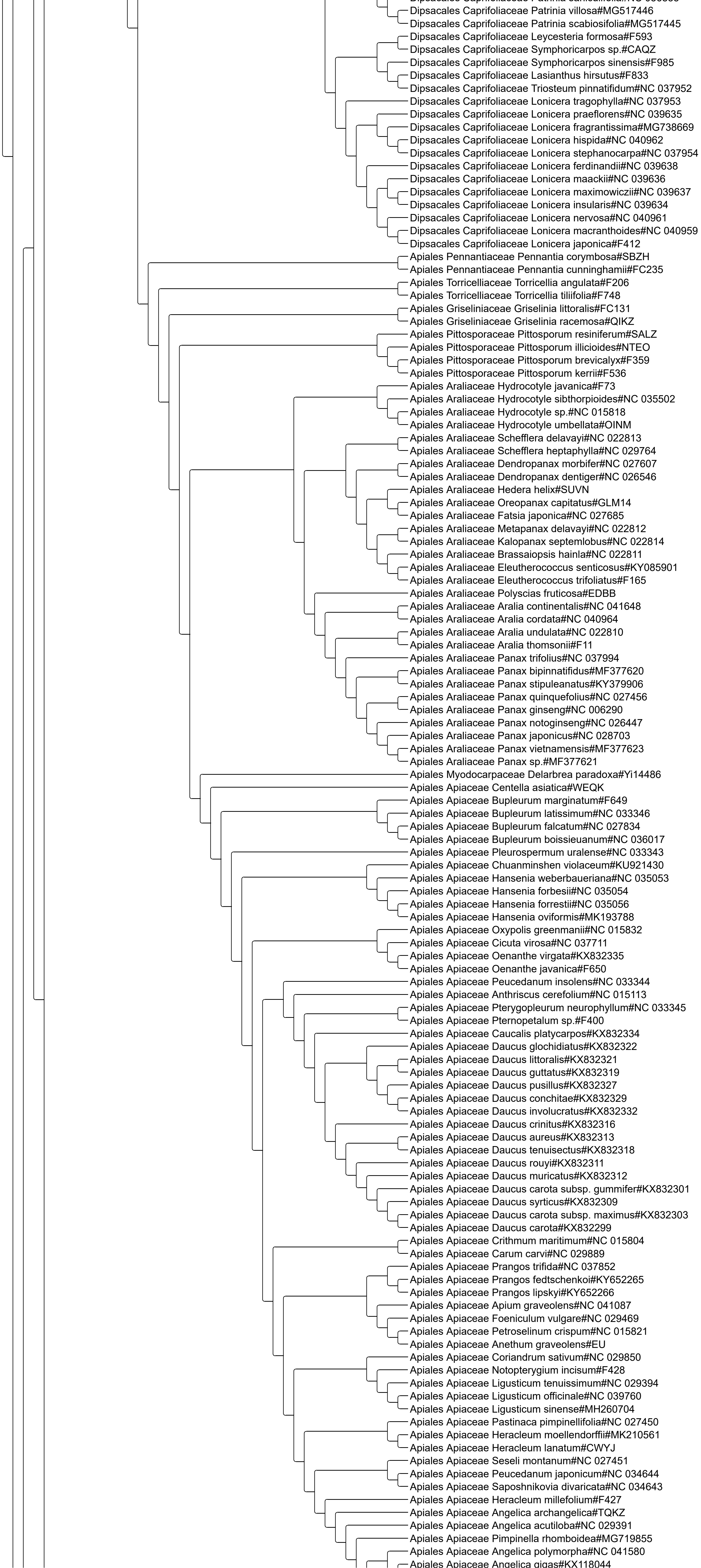

Dipsacales Caprifoliaceae Patrinia villosa#MG517446
Dipsacales Caprifoliaceae Patrinia scabiosifolia#MG517445
Dipsacales Caprifoliaceae Leycesteria formosa#F593
Dipsacales Caprifoliaceae Symphoricarpos sp.#CAQZ
Dipsacales Caprifoliaceae Symphoricarpos sinensis#F985
Dipsacales Caprifoliaceae Lasianthus hirsutus#F833
Dipsacales Caprifoliaceae Triosteum pinnatifidum#NC 037952
Dipsacales Caprifoliaceae Lonicera tragophylla#NC 037953
Dipsacales Caprifoliaceae Lonicera praeflorens#NC 039635
Dipsacales Caprifoliaceae Lonicera fragrantissima#MG738669
Dipsacales Caprifoliaceae Lonicera hispida#NC 040962
Dipsacales Caprifoliaceae Lonicera stephanocarpa#NC 037954
Dipsacales Caprifoliaceae Lonicera ferdinandii#NC 039638
Dipsacales Caprifoliaceae Lonicera maackii#NC 039636
Dipsacales Caprifoliaceae Lonicera maximowiczii#NC 039637
Dipsacales Caprifoliaceae Lonicera insularis#NC 039634
Dipsacales Caprifoliaceae Lonicera nervosa#NC 040961
Dipsacales Caprifoliaceae Lonicera macranthoides#NC 040959
Dipsacales Caprifoliaceae Lonicera japonica#F412
Apiales Pennantiaceae Pennantia corymbosa#SBZH
Apiales Pennantiaceae Pennantia cunninghamii#FC235
Apiales Torricelliaceae Torricellia angulata#F206
Apiales Torricelliaceae Torricellia tiliifolia#F748
Apiales Griseliniaceae Griselinia littoralis#FC131
Apiales Griseliniaceae Griselinia racemosa#QIKZ
Apiales Pittosporaceae Pittosporum resiniferum#SALZ
Apiales Pittosporaceae Pittosporum illicioides#NTEO
Apiales Pittosporaceae Pittosporum brevicalyx#F359
Apiales Pittosporaceae Pittosporum kerrii#F536
Apiales Araliaceae Hydrocotyle javanica#F73
Apiales Araliaceae Hydrocotyle sibthorpioides#NC 035502
Apiales Araliaceae Hydrocotyle sp.#NC 015818
Apiales Araliaceae Hydrocotyle umbellata#OINM
Apiales Araliaceae Schefflera delavayi#NC 022813
Apiales Araliaceae Schefflera heptaphylla#NC 029764
Apiales Araliaceae Dendropanax morbifer#NC 027607
Apiales Araliaceae Dendropanax dentiger#NC 026546
Apiales Araliaceae Hedera helix#SUVN
Apiales Araliaceae Oreopanax capitatus#GLM14
Apiales Araliaceae Fatsia japonica#NC 027685
Apiales Araliaceae Metapanax delavayi#NC 022812
Apiales Araliaceae Kalopanax septemlobus#NC 022814
Apiales Araliaceae Brassaiopsis hainla#NC 022811
Apiales Araliaceae Eleutherococcus senticosus#KY085901
Apiales Araliaceae Eleutherococcus trifoliatus#F165
Apiales Araliaceae Polyscias fruticosa#EDBB
Apiales Araliaceae Aralia continentalis#NC 041648
Apiales Araliaceae Aralia cordata#NC 040964
Apiales Araliaceae Aralia undulata#NC 022810
Apiales Araliaceae Aralia thomsonii#F11
Apiales Araliaceae Panax trifolius#NC 037994
Apiales Araliaceae Panax bipinnatifidus#MF377620
Apiales Araliaceae Panax stipuleanatus#KY379906
Apiales Araliaceae Panax quinquefolius#NC 027456
Apiales Araliaceae Panax ginseng#NC 006290
Apiales Araliaceae Panax notoginseng#NC 026447
Apiales Araliaceae Panax japonicus#NC 028703
Apiales Araliaceae Panax vietnamensis#MF377623
Apiales Araliaceae Panax sp.#MF377621
Apiales Myodocarpaceae Delarbrea paradoxa#Yi14486
Apiales Apiaceae Centella asiatica#WEQK
Apiales Apiaceae Bupleurum marginatum#F649
Apiales Apiaceae Bupleurum latissimum#NC 033346
Apiales Apiaceae Bupleurum falcatum#NC 027834
Apiales Apiaceae Bupleurum boissieuanum#NC 036017
Apiales Apiaceae Pleurospermum uralense#NC 033343
Apiales Apiaceae Chuanminshen violaceum#KU921430
Apiales Apiaceae Hansenia weberbaueriana#NC 035053
Apiales Apiaceae Hansenia forbesii#NC 035054
Apiales Apiaceae Hansenia forrestii#NC 035056
Apiales Apiaceae Hansenia oviformis#MK193788
Apiales Apiaceae Oxypolis greenmanii#NC 015832
Apiales Apiaceae Cicuta virosa#NC 037711
Apiales Apiaceae Oenanthe virgata#KX832335
Apiales Apiaceae Oenanthe javanica#F650
Apiales Apiaceae Peucedanum insolens#NC 033344
Apiales Apiaceae Anthriscus cerefolium#NC 015113
Apiales Apiaceae Pterygopleurum neurophyllum#NC 033345
Apiales Apiaceae Pternopetalum sp.#F400
Apiales Apiaceae Caucalis platycarpos#KX832334
Apiales Apiaceae Daucus glochidiatus#KX832322
Apiales Apiaceae Daucus littoralis#KX832321
Apiales Apiaceae Daucus guttatus#KX832319
Apiales Apiaceae Daucus pusillus#KX832327
Apiales Apiaceae Daucus conchitae#KX832329
Apiales Apiaceae Daucus involucratus#KX832332
Apiales Apiaceae Daucus crinitus#KX832316
Apiales Apiaceae Daucus aureus#KX832313
Apiales Apiaceae Daucus tenuisectus#KX832318
Apiales Apiaceae Daucus rouyi#KX832311
Apiales Apiaceae Daucus muricatus#KX832312
Apiales Apiaceae Daucus carota subsp. gummifer#KX832301
Apiales Apiaceae Daucus syrticus#KX832309
Apiales Apiaceae Daucus carota subsp. maximus#KX832303
Apiales Apiaceae Daucus carota#KX832299
Apiales Apiaceae Crithmum maritimum#NC 015804
Apiales Apiaceae Carum carvi#NC 029889
Apiales Apiaceae Prangos trifida#NC 037852
Apiales Apiaceae Prangos fedtschenkoi#KY652265
Apiales Apiaceae Prangos lipskyi#KY652266
Apiales Apiaceae Apium graveolens#NC 041087
Apiales Apiaceae Foeniculum vulgare#NC 029469
Apiales Apiaceae Petroselinum crispum#NC 015821
Apiales Apiaceae Anethum graveolens#EU
Apiales Apiaceae Coriandrum sativum#NC 029850
Apiales Apiaceae Notopterygium incisum#F428
Apiales Apiaceae Ligusticum tenuissimum#NC 029394
Apiales Apiaceae Ligusticum officinale#NC 039760
Apiales Apiaceae Ligusticum sinense#MH260704
Apiales Apiaceae Pastinaca pimpinellifolia#NC 027450
Apiales Apiaceae Heracleum moellendorffii#MK210561
Apiales Apiaceae Heracleum lanatum#CWYJ
Apiales Apiaceae Seseli montanum#NC 027451
Apiales Apiaceae Peucedanum japonicum#NC 034644
Apiales Apiaceae Saposhnikovia divaricata#NC 034643
Apiales Apiaceae Heracleum millefolium#F427
Apiales Apiaceae Angelica archangelica#TQKZ
Apiales Apiaceae Angelica acutiloba#NC 029391
Apiales Apiaceae Pimpinella rhomboidea#MG719855
Apiales Apiaceae Angelica polymorpha#NC 041580
Apiales Apiaceae Angelica gigas#KX118044

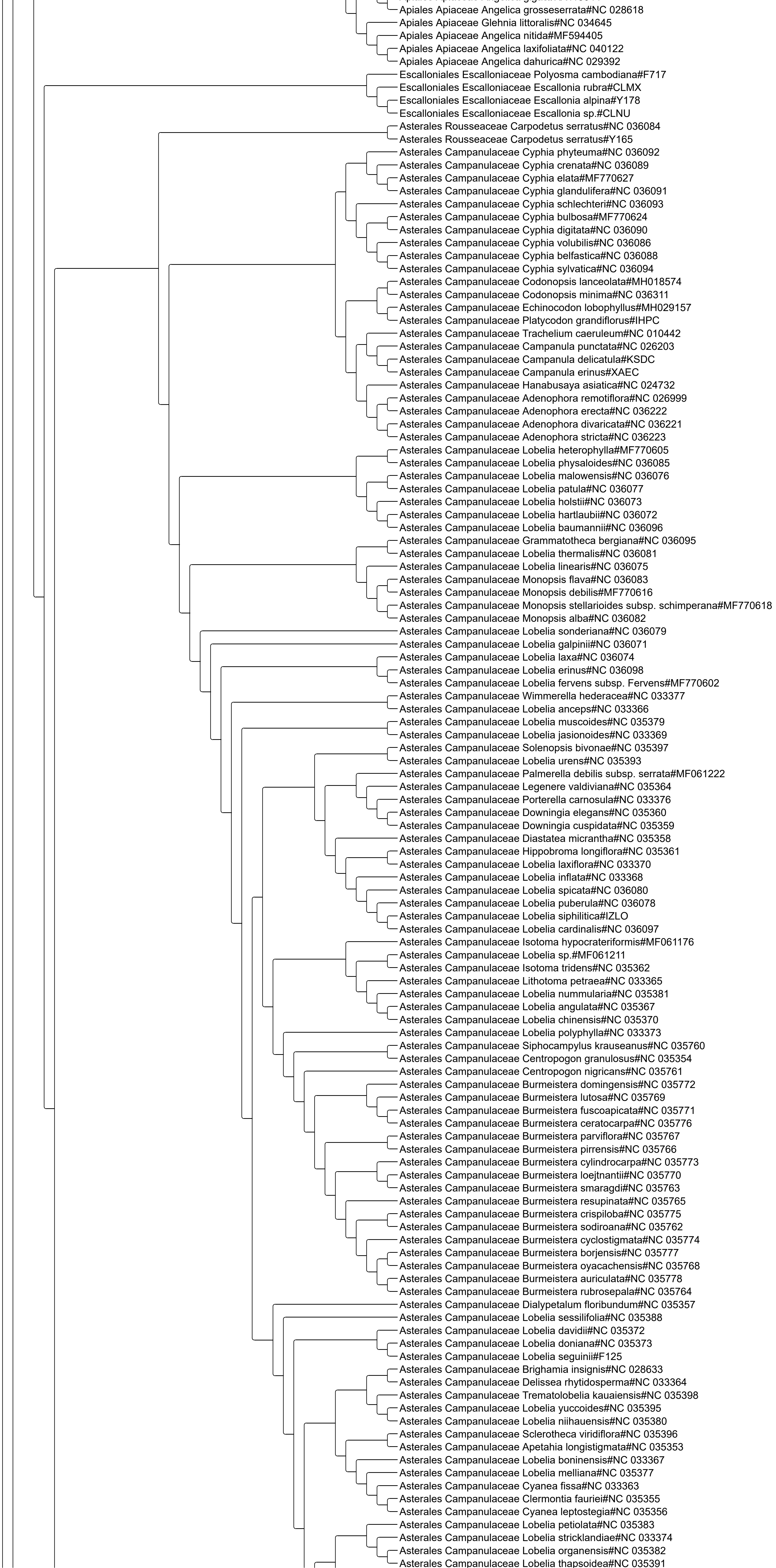

Apiales Apiaceae Angelica grosseserrata#NC 028618
Apiales Apiaceae Glehnia littoralis#NC 034645
Apiales Apiaceae Angelica nitida#MF594405
Apiales Apiaceae Angelica laxifoliata#NC 040122
Apiales Apiaceae Angelica dahurica#NC 029392
Escalloniales Escalloniaceae Polyosma cambodiana#F717
Escalloniales Escalloniaceae Escallonia rubra#CLMX
Escalloniales Escalloniaceae Escallonia alpina#Y178
Escalloniales Escalloniaceae Escallonia sp.#CLNU
Asterales Rousseaceae Carpodetus serratus#NC 036084
Asterales Rousseaceae Carpodetus serratus#Y165
Asterales Campanulaceae Cyphia phyteuma#NC 036092
Asterales Campanulaceae Cyphia crenata#NC 036089
Asterales Campanulaceae Cyphia elata#MF770627
Asterales Campanulaceae Cyphia glandulifera#NC 036091
Asterales Campanulaceae Cyphia schlechteri#NC 036093
Asterales Campanulaceae Cyphia bulbosa#MF770624
Asterales Campanulaceae Cyphia digitata#NC 036090
Asterales Campanulaceae Cyphia volubilis#NC 036086
Asterales Campanulaceae Cyphia belfastica#NC 036088
Asterales Campanulaceae Cyphia sylvatica#NC 036094
Asterales Campanulaceae Codonopsis lanceolata#MH018574
Asterales Campanulaceae Codonopsis minima#NC 036311
Asterales Campanulaceae Echinocodon lobophyllus#MH029157
Asterales Campanulaceae Platycodon grandiflorus#IHPC
Asterales Campanulaceae Trachelium caeruleum#NC 010442
Asterales Campanulaceae Campanula punctata#NC 026203
Asterales Campanulaceae Campanula delicatula#KSDC
Asterales Campanulaceae Campanula erinus#XAEC
Asterales Campanulaceae Hanabusaya asiatica#NC 024732
Asterales Campanulaceae Adenophora remotiflora#NC 026999
Asterales Campanulaceae Adenophora erecta#NC 036222
Asterales Campanulaceae Adenophora divaricata#NC 036221
Asterales Campanulaceae Adenophora stricta#NC 036223
Asterales Campanulaceae Lobelia heterophylla#MF770605
Asterales Campanulaceae Lobelia physaloides#NC 036085
Asterales Campanulaceae Lobelia malowensis#NC 036076
Asterales Campanulaceae Lobelia patula#NC 036077
Asterales Campanulaceae Lobelia holstii#NC 036073
Asterales Campanulaceae Lobelia hartlaubii#NC 036072
Asterales Campanulaceae Lobelia baumannii#NC 036096
Asterales Campanulaceae Grammatotheca bergiana#NC 036095
Asterales Campanulaceae Lobelia thermalis#NC 036081
Asterales Campanulaceae Lobelia linearis#NC 036075
Asterales Campanulaceae Monopsis flava#NC 036083
Asterales Campanulaceae Monopsis debilis#MF770616
Asterales Campanulaceae Monopsis stellarioides subsp. schimperana#MF770618
Asterales Campanulaceae Monopsis alba#NC 036082
Asterales Campanulaceae Lobelia sonderiana#NC 036079
Asterales Campanulaceae Lobelia galpinii#NC 036071
Asterales Campanulaceae Lobelia laxa#NC 036074
Asterales Campanulaceae Lobelia erinus#NC 036098
Asterales Campanulaceae Lobelia fervens subsp. Fervens#MF770602
Asterales Campanulaceae Wimmerella hederacea#NC 033377
Asterales Campanulaceae Lobelia anceps#NC 033366
Asterales Campanulaceae Lobelia muscoides#NC 035379
Asterales Campanulaceae Lobelia jasionoides#NC 033369
Asterales Campanulaceae Solenopsis bivonae#NC 035397
Asterales Campanulaceae Lobelia urens#NC 035393
Asterales Campanulaceae Palmerella debilis subsp. serrata#MF061222
Asterales Campanulaceae Legenere valdiviana#NC 035364
Asterales Campanulaceae Porterella carnosula#NC 033376
Asterales Campanulaceae Downingia elegans#NC 035360
Asterales Campanulaceae Downingia cuspidata#NC 035359
Asterales Campanulaceae Diastatea micrantha#NC 035358
Asterales Campanulaceae Hippobroma longiflora#NC 035361
Asterales Campanulaceae Lobelia laxiflora#NC 033370
Asterales Campanulaceae Lobelia inflata#NC 033368
Asterales Campanulaceae Lobelia spicata#NC 036080
Asterales Campanulaceae Lobelia puberula#NC 036078
Asterales Campanulaceae Lobelia siphilitica#IZLO
Asterales Campanulaceae Lobelia cardinalis#NC 036097
Asterales Campanulaceae Isotoma hypocrateriformis#MF061176
Asterales Campanulaceae Lobelia sp.#MF061211
Asterales Campanulaceae Isotoma tridens#NC 035362
Asterales Campanulaceae Lithotoma petraea#NC 033365
Asterales Campanulaceae Lobelia nummularia#NC 035381
Asterales Campanulaceae Lobelia angulata#NC 035367
Asterales Campanulaceae Lobelia chinensis#NC 035370
Asterales Campanulaceae Lobelia polyphylla#NC 033373
Asterales Campanulaceae Siphocampylus krauseanus#NC 035760
Asterales Campanulaceae Centropogon granulosus#NC 035354
Asterales Campanulaceae Centropogon nigricans#NC 035761
Asterales Campanulaceae Burmeistera domingensis#NC 035772
Asterales Campanulaceae Burmeistera lutosa#NC 035769
Asterales Campanulaceae Burmeistera fuscoapicata#NC 035771
Asterales Campanulaceae Burmeistera ceratocarpa#NC 035776
Asterales Campanulaceae Burmeistera parviflora#NC 035767
Asterales Campanulaceae Burmeistera pirrensis#NC 035766
Asterales Campanulaceae Burmeistera cylindrocarpa#NC 035773
Asterales Campanulaceae Burmeistera loejtnantii#NC 035770
Asterales Campanulaceae Burmeistera smaragdi#NC 035763
Asterales Campanulaceae Burmeistera resupinata#NC 035765
Asterales Campanulaceae Burmeistera crispiloba#NC 035775
Asterales Campanulaceae Burmeistera sodiroana#NC 035762
Asterales Campanulaceae Burmeistera cyclostigmata#NC 035774
Asterales Campanulaceae Burmeistera borjensis#NC 035777
Asterales Campanulaceae Burmeistera oyacachensis#NC 035768
Asterales Campanulaceae Burmeistera auriculata#NC 035778
Asterales Campanulaceae Burmeistera rubrosepala#NC 035764
Asterales Campanulaceae Dialypetalum floribundum#NC 035357
Asterales Campanulaceae Lobelia sessilifolia#NC 035388
Asterales Campanulaceae Lobelia davidii#NC 035372
Asterales Campanulaceae Lobelia doniana#NC 035373
Asterales Campanulaceae Lobelia seguinii#F125
Asterales Campanulaceae Brighamia insignis#NC 028633
Asterales Campanulaceae Delissea rhytidosperma#NC 033364
Asterales Campanulaceae Trematolobelia kauaiensis#NC 035398
Asterales Campanulaceae Lobelia yuccoides#NC 035395
Asterales Campanulaceae Lobelia niihauensis#NC 035380
Asterales Campanulaceae Sclerotheca viridiflora#NC 035396
Asterales Campanulaceae Apetahia longistigmata#NC 035353
Asterales Campanulaceae Lobelia boninensis#NC 033367
Asterales Campanulaceae Lobelia melliana#NC 035377
Asterales Campanulaceae Cyanea fissa#NC 033363
Asterales Campanulaceae Clermontia fauriei#NC 035355
Asterales Campanulaceae Cyanea leptostegia#NC 035356
Asterales Campanulaceae Lobelia petiolata#NC 035383
Asterales Campanulaceae Lobelia stricklandiae#NC 033374
Asterales Campanulaceae Lobelia organensis#NC 035382
Asterales Campanulaceae Lobelia thapsoidea#NC 035391

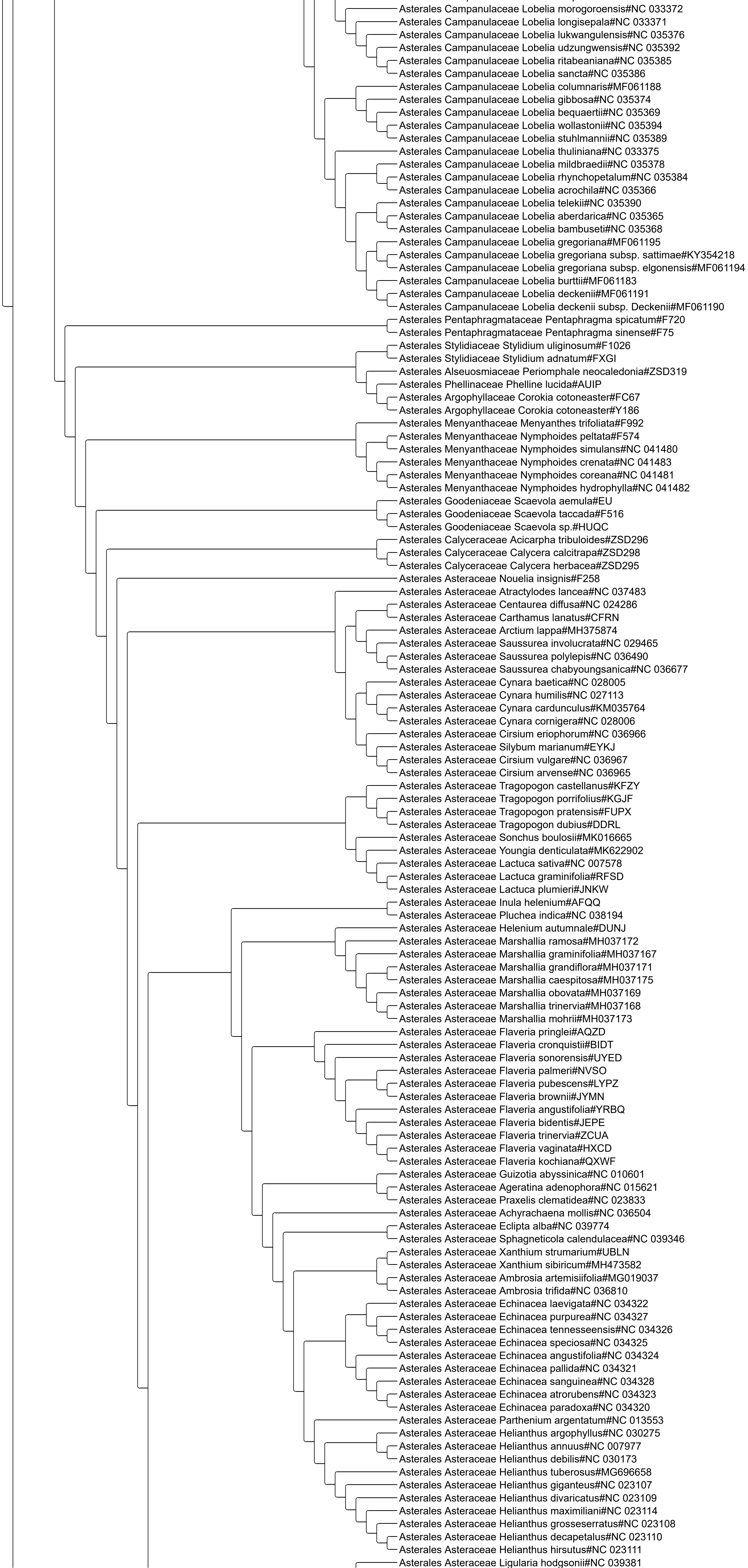

Asterales Campanulaceae Lobelia morogoroensis#NC 033372
Asterales Campanulaceae Lobelia longisepala#NC 033371
Asterales Campanulaceae Lobelia lukwangulensis#NC 035376
Asterales Campanulaceae Lobelia udzungwensis#NC 035392
Asterales Campanulaceae Lobelia ritabeaniana#NC 035385
Asterales Campanulaceae Lobelia sancta#NC 035386
Asterales Campanulaceae Lobelia columnaris#MF061188
Asterales Campanulaceae Lobelia gibbosa#NC 035374
Asterales Campanulaceae Lobelia bequaertii#NC 035369
Asterales Campanulaceae Lobelia wollastonii#NC 035394
Asterales Campanulaceae Lobelia stuhlmannii#NC 035389
Asterales Campanulaceae Lobelia thuliniana#NC 033375
Asterales Campanulaceae Lobelia mildbraedii#NC 035378
Asterales Campanulaceae Lobelia rhynchopetalum#NC 035384
Asterales Campanulaceae Lobelia acrochila#NC 035366
Asterales Campanulaceae Lobelia telekii#NC 035390
Asterales Campanulaceae Lobelia aberdarica#NC 035365
Asterales Campanulaceae Lobelia bambuseti#NC 035368
Asterales Campanulaceae Lobelia gregoriana#MF061195
Asterales Campanulaceae Lobelia gregoriana subsp. sattimae#KY354218
Asterales Campanulaceae Lobelia gregoriana subsp. elgonensis#MF061194
Asterales Campanulaceae Lobelia burttii#MF061183
Asterales Campanulaceae Lobelia deckenii#MF061191
Asterales Campanulaceae Lobelia deckenii subsp. Deckenii#MF061190
Asterales Pentaphragmataceae Pentaphragma spicatum#F720
Asterales Pentaphragmataceae Pentaphragma sinense#F75
Asterales Stylidiaceae Stylidium uliginosum#F1026
Asterales Stylidiaceae Stylidium adnatum#FXGI
Asterales Alseuosmiaceae Periomphale neocaledonia#ZSD319
Asterales Phellinaceae Phelline lucida#AUIP
Asterales Argophyllaceae Corokia cotoneaster#FC67
Asterales Argophyllaceae Corokia cotoneaster#Y186
Asterales Menyanthaceae Menyanthes trifoliata#F992
Asterales Menyanthaceae Nymphoides peltata#F574
Asterales Menyanthaceae Nymphoides simulans#NC 041480
Asterales Menyanthaceae Nymphoides crenata#NC 041483
Asterales Menyanthaceae Nymphoides coreana#NC 041481
Asterales Menyanthaceae Nymphoides hydrophylla#NC 041482
Asterales Goodeniaceae Scaevola aemula#EU
Asterales Goodeniaceae Scaevola taccada#F516
Asterales Goodeniaceae Scaevola sp.#HUQC
Asterales Calyceraceae Acicarpha tribuloides#ZSD296
Asterales Calyceraceae Calycera calcitrapa#ZSD298
Asterales Calyceraceae Calycera herbacea#ZSD295
Asterales Asteraceae Nouelia insignis#F258
Asterales Asteraceae Atractylodes lancea#NC 037483
Asterales Asteraceae Centaurea diffusa#NC 024286
Asterales Asteraceae Carthamus lanatus#CFRN
Asterales Asteraceae Arctium lappa#MH375874
Asterales Asteraceae Saussurea involucrata#NC 029465
Asterales Asteraceae Saussurea polylepis#NC 036490
Asterales Asteraceae Saussurea chabyoungsanica#NC 036677
Asterales Asteraceae Cynara baetica#NC 028005
Asterales Asteraceae Cynara humilis#NC 027113
Asterales Asteraceae Cynara cardunculus#KM035764
Asterales Asteraceae Cynara cornigera#NC 028006
Asterales Asteraceae Cirsium eriophorum#NC 036966
Asterales Asteraceae Silybum marianum#EYKJ
Asterales Asteraceae Cirsium vulgare#NC 036967
Asterales Asteraceae Cirsium arvense#NC 036965
Asterales Asteraceae Tragopogon castellanus#KFZY
Asterales Asteraceae Tragopogon porrifolius#KGJF
Asterales Asteraceae Tragopogon pratensis#FUPX
Asterales Asteraceae Tragopogon dubius#DDRL
Asterales Asteraceae Sonchus boulosii#MK016665
Asterales Asteraceae Youngia denticulata#MK622902
Asterales Asteraceae Lactuca sativa#NC 007578
Asterales Asteraceae Lactuca graminifolia#RFSD
Asterales Asteraceae Lactuca plumieri#JNKW
Asterales Asteraceae Inula helenium#AFQQ
Asterales Asteraceae Pluchea indica#NC 038194
Asterales Asteraceae Helenium autumnale#DUNJ
Asterales Asteraceae Marshallia ramosa#MH037172
Asterales Asteraceae Marshallia graminifolia#MH037167
Asterales Asteraceae Marshallia grandiflora#MH037171
Asterales Asteraceae Marshallia caespitosa#MH037175
Asterales Asteraceae Marshallia obovata#MH037169
Asterales Asteraceae Marshallia trinervia#MH037168
Asterales Asteraceae Marshallia mohrii#MH037173
Asterales Asteraceae Flaveria pringlei#AQZD
Asterales Asteraceae Flaveria cronquistii#BIDT
Asterales Asteraceae Flaveria sonorensis#UYED
Asterales Asteraceae Flaveria palmeri#NVSO
Asterales Asteraceae Flaveria pubescens#LYPZ
Asterales Asteraceae Flaveria brownii#JYMN
Asterales Asteraceae Flaveria angustifolia#YRBQ
Asterales Asteraceae Flaveria bidentis#JEPE
Asterales Asteraceae Flaveria trinervia#ZCUA
Asterales Asteraceae Flaveria vaginata#HXCD
Asterales Asteraceae Flaveria kochiana#QXWF
Asterales Asteraceae Guizotia abyssinica#NC 010601
Asterales Asteraceae Ageratina adenophora#NC 015621
Asterales Asteraceae Praxelis clematidea#NC 023833
Asterales Asteraceae Achyrachaena mollis#NC 036504
Asterales Asteraceae Eclipta alba#NC 039774
Asterales Asteraceae Sphagneticola calendulacea#NC 039346
Asterales Asteraceae Xanthium strumarium#UBLN
Asterales Asteraceae Xanthium sibiricum#MH473582
Asterales Asteraceae Ambrosia artemisiifolia#MG019037
Asterales Asteraceae Ambrosia trifida#NC 036810
Asterales Asteraceae Echinacea laevigata#NC 034322
Asterales Asteraceae Echinacea purpurea#NC 034327
Asterales Asteraceae Echinacea tennesseensis#NC 034326
Asterales Asteraceae Echinacea speciosa#NC 034325
Asterales Asteraceae Echinacea angustifolia#NC 034324
Asterales Asteraceae Echinacea pallida#NC 034321
Asterales Asteraceae Echinacea sanguinea#NC 034328
Asterales Asteraceae Echinacea atrorubens#NC 034323
Asterales Asteraceae Echinacea paradoxa#NC 034320
Asterales Asteraceae Parthenium argentatum#NC 013553
Asterales Asteraceae Helianthus argophyllus#NC 030275
Asterales Asteraceae Helianthus annuus#NC 007977
Asterales Asteraceae Helianthus debilis#NC 030173
Asterales Asteraceae Helianthus tuberosus#MG696658
Asterales Asteraceae Helianthus giganteus#NC 023107
Asterales Asteraceae Helianthus divaricatus#NC 023109
Asterales Asteraceae Helianthus maximiliani#NC 023114
Asterales Asteraceae Helianthus grosseserratus#NC 023108
Asterales Asteraceae Helianthus decapetalus#NC 023110
Asterales Asteraceae Helianthus hirsutus#NC 023111
Asterales Asteraceae Ligularia hodgsonii#NC 039381

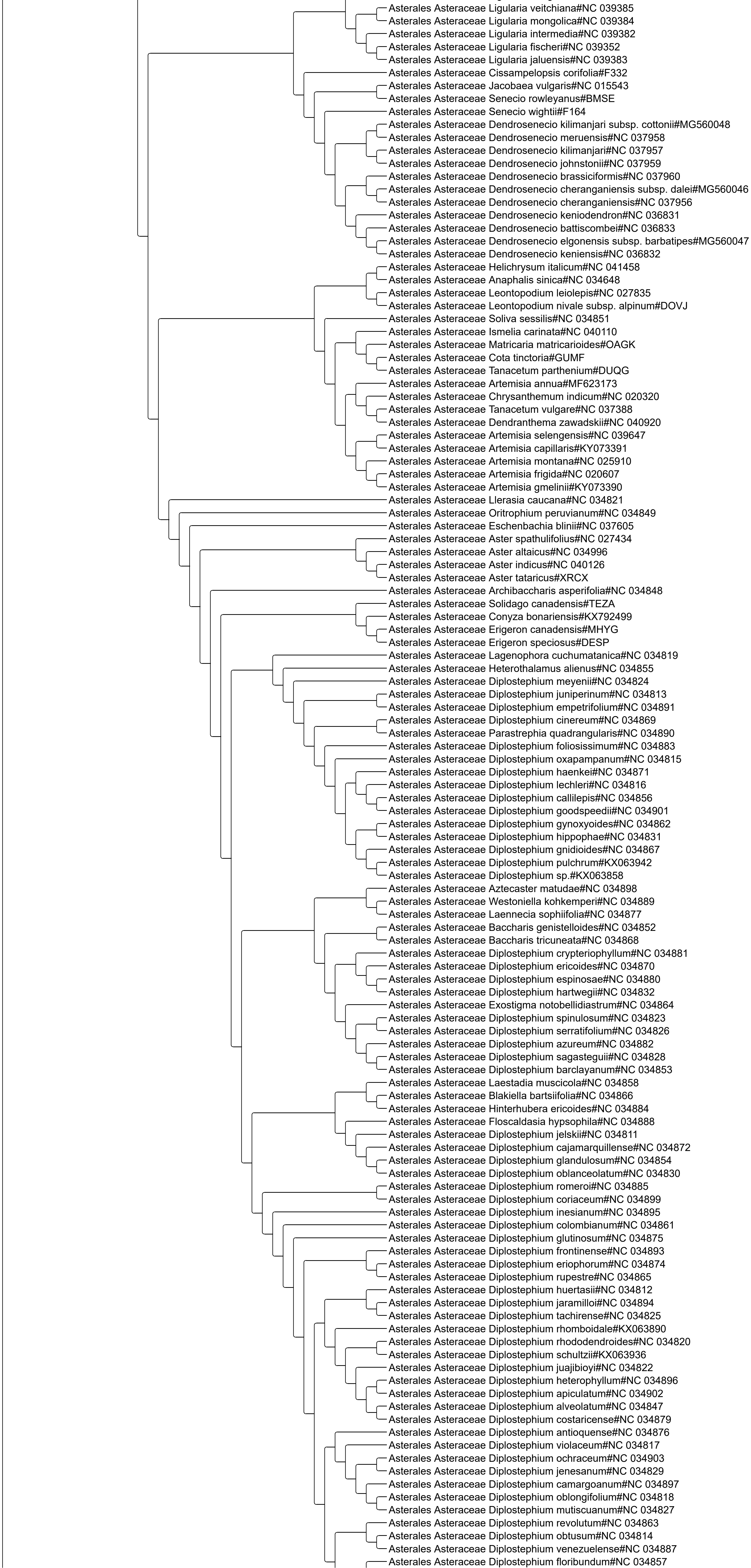
Asterales Asteraceae Ligularia veitchiana#NC 039385
Asterales Asteraceae Ligularia mongolica#NC 039384
Asterales Asteraceae Ligularia intermedia#NC 039382
Asterales Asteraceae Ligularia fischeri#NC 039352
Asterales Asteraceae Ligularia jaluensis#NC 039383
Asterales Asteraceae Cissampelopsis corifolia#F332
Asterales Asteraceae Jacobaea vulgaris#NC 015543
Asterales Asteraceae Senecio rowleyanus#BMSE
Asterales Asteraceae Senecio wightii#F164
Asterales Asteraceae Dendrosenecio kilimanjari subsp. cottonii#MG560048
Asterales Asteraceae Dendrosenecio meruensis#NC 037958
Asterales Asteraceae Dendrosenecio kilimanjari#NC 037957
Asterales Asteraceae Dendrosenecio johnstonii#NC 037959
Asterales Asteraceae Dendrosenecio brassiciformis#NC 037960
Asterales Asteraceae Dendrosenecio cheranganiensis subsp. dalei#MG560046
Asterales Asteraceae Dendrosenecio cheranganiensis#NC 037956
Asterales Asteraceae Dendrosenecio keniodendron#NC 036831
Asterales Asteraceae Dendrosenecio battiscombei#NC 036833
Asterales Asteraceae Dendrosenecio elgonensis subsp. barbatipes#MG560047
Asterales Asteraceae Dendrosenecio keniensis#NC 036832
Asterales Asteraceae Helichrysum italicum#NC 041458
Asterales Asteraceae Anaphalis sinica#NC 034648
Asterales Asteraceae Leontopodium leiolepis#NC 027835
Asterales Asteraceae Leontopodium nivale subsp. alpinum#DOVJ
Asterales Asteraceae Soliva sessilis#NC 034851
Asterales Asteraceae Ismelia carinata#NC 040110
Asterales Asteraceae Matricaria matricarioides#OAGK
Asterales Asteraceae Cota tinctoria#GUMF
Asterales Asteraceae Tanacetum parthenium#DUQG
Asterales Asteraceae Artemisia annua#MF623173
Asterales Asteraceae Chrysanthemum indicum#NC 020320
Asterales Asteraceae Tanacetum vulgare#NC 037388
Asterales Asteraceae Dendranthema zawadskii#NC 040920
Asterales Asteraceae Artemisia selengensis#NC 039647
Asterales Asteraceae Artemisia capillaris#KY073391
Asterales Asteraceae Artemisia montana#NC 025910
Asterales Asteraceae Artemisia frigida#NC 020607
Asterales Asteraceae Artemisia gmelinii#KY073390
Asterales Asteraceae Llerasia caucana#NC 034821
Asterales Asteraceae Oritrophium peruvianum#NC 034849
Asterales Asteraceae Eschenbachia blinii#NC 037605
Asterales Asteraceae Aster spathulifolius#NC 027434
Asterales Asteraceae Aster altaicus#NC 034996
Asterales Asteraceae Aster indicus#NC 040126
Asterales Asteraceae Aster tataricus#XRCX
Asterales Asteraceae Archibaccharis asperifolia#NC 034848
Asterales Asteraceae Solidago canadensis#TEZA
Asterales Asteraceae Conyza bonariensis#KX792499
Asterales Asteraceae Erigeron canadensis#MHYG
Asterales Asteraceae Erigeron speciosus#DESP
Asterales Asteraceae Lagenophora cuchumatanica#NC 034819
Asterales Asteraceae Heterothalamus alienus#NC 034855
Asterales Asteraceae Diplostephium meyenii#NC 034824
Asterales Asteraceae Diplostephium juniperinum#NC 034813
Asterales Asteraceae Diplostephium empetrifolium#NC 034891
Asterales Asteraceae Diplostephium cinereum#NC 034869
Asterales Asteraceae Parastrephia quadrangularis#NC 034890
Asterales Asteraceae Diplostephium foliosissimum#NC 034883
Asterales Asteraceae Diplostephium oxapampanum#NC 034815
Asterales Asteraceae Diplostephium haenkei#NC 034871
Asterales Asteraceae Diplostephium lechleri#NC 034816
Asterales Asteraceae Diplostephium callilepis#NC 034856
Asterales Asteraceae Diplostephium goodspeedii#NC 034901
Asterales Asteraceae Diplostephium gynoxyoides#NC 034862
Asterales Asteraceae Diplostephium hippophae#NC 034831
Asterales Asteraceae Diplostephium gnidioides#NC 034867
Asterales Asteraceae Diplostephium pulchrum#KX063942
Asterales Asteraceae Diplostephium sp.#KX063858
Asterales Asteraceae Aztecaster matudae#NC 034898
Asterales Asteraceae Westoniella kohkemperi#NC 034889
Asterales Asteraceae Laennecia sophiifolia#NC 034877
Asterales Asteraceae Baccharis genistelloides#NC 034852
Asterales Asteraceae Baccharis tricuneata#NC 034868
Asterales Asteraceae Diplostephium crypteriophyllum#NC 034881
Asterales Asteraceae Diplostephium ericoides#NC 034870
Asterales Asteraceae Diplostephium espinosae#NC 034880
Asterales Asteraceae Diplostephium hartwegii#NC 034832
Asterales Asteraceae Exostigma notobellidiastrum#NC 034864
Asterales Asteraceae Diplostephium spinulosum#NC 034823
Asterales Asteraceae Diplostephium serratifolium#NC 034826
Asterales Asteraceae Diplostephium azureum#NC 034882
Asterales Asteraceae Diplostephium sagasteguii#NC 034828
Asterales Asteraceae Diplostephium barclayanum#NC 034853
Asterales Asteraceae Laestadia muscicola#NC 034858
Asterales Asteraceae Blakiella bartsiifolia#NC 034866
Asterales Asteraceae Hinterhubera ericoides#NC 034884
Asterales Asteraceae Floscaldasia hypsophila#NC 034888
Asterales Asteraceae Diplostephium jelskii#NC 034811
Asterales Asteraceae Diplostephium cajamarquillense#NC 034872
Asterales Asteraceae Diplostephium glandulosum#NC 034854
Asterales Asteraceae Diplostephium oblanceolatum#NC 034830
Asterales Asteraceae Diplostephium romeroi#NC 034885
Asterales Asteraceae Diplostephium coriaceum#NC 034899
Asterales Asteraceae Diplostephium inesianum#NC 034895
Asterales Asteraceae Diplostephium colombianum#NC 034861
Asterales Asteraceae Diplostephium glutinosum#NC 034875
Asterales Asteraceae Diplostephium frontinense#NC 034893
Asterales Asteraceae Diplostephium eriophorum#NC 034874
Asterales Asteraceae Diplostephium rupestre#NC 034865
Asterales Asteraceae Diplostephium huertasii#NC 034812
Asterales Asteraceae Diplostephium jaramilloi#NC 034894
Asterales Asteraceae Diplostephium tachirense#NC 034825
Asterales Asteraceae Diplostephium rhomboidale#KX063890
Asterales Asteraceae Diplostephium rhododendroides#NC 034820
Asterales Asteraceae Diplostephium schultzii#KX063936
Asterales Asteraceae Diplostephium juajibioyi#NC 034822
Asterales Asteraceae Diplostephium heterophyllum#NC 034896
Asterales Asteraceae Diplostephium apiculatum#NC 034902
Asterales Asteraceae Diplostephium alveolatum#NC 034847
Asterales Asteraceae Diplostephium costaricense#NC 034879
Asterales Asteraceae Diplostephium antioquense#NC 034876
Asterales Asteraceae Diplostephium violaceum#NC 034817
Asterales Asteraceae Diplostephium ochraceum#NC 034903
Asterales Asteraceae Diplostephium jenesanum#NC 034829
Asterales Asteraceae Diplostephium camargoanum#NC 034897
Asterales Asteraceae Diplostephium oblongifolium#NC 034818
Asterales Asteraceae Diplostephium mutiscuanum#NC 034827
Asterales Asteraceae Diplostephium revolutum#NC 034863
Asterales Asteraceae Diplostephium obtusum#NC 034814
Asterales Asteraceae Diplostephium venezuelense#NC 034887
Asterales Asteraceae Diplostephium floribundum#NC 034857

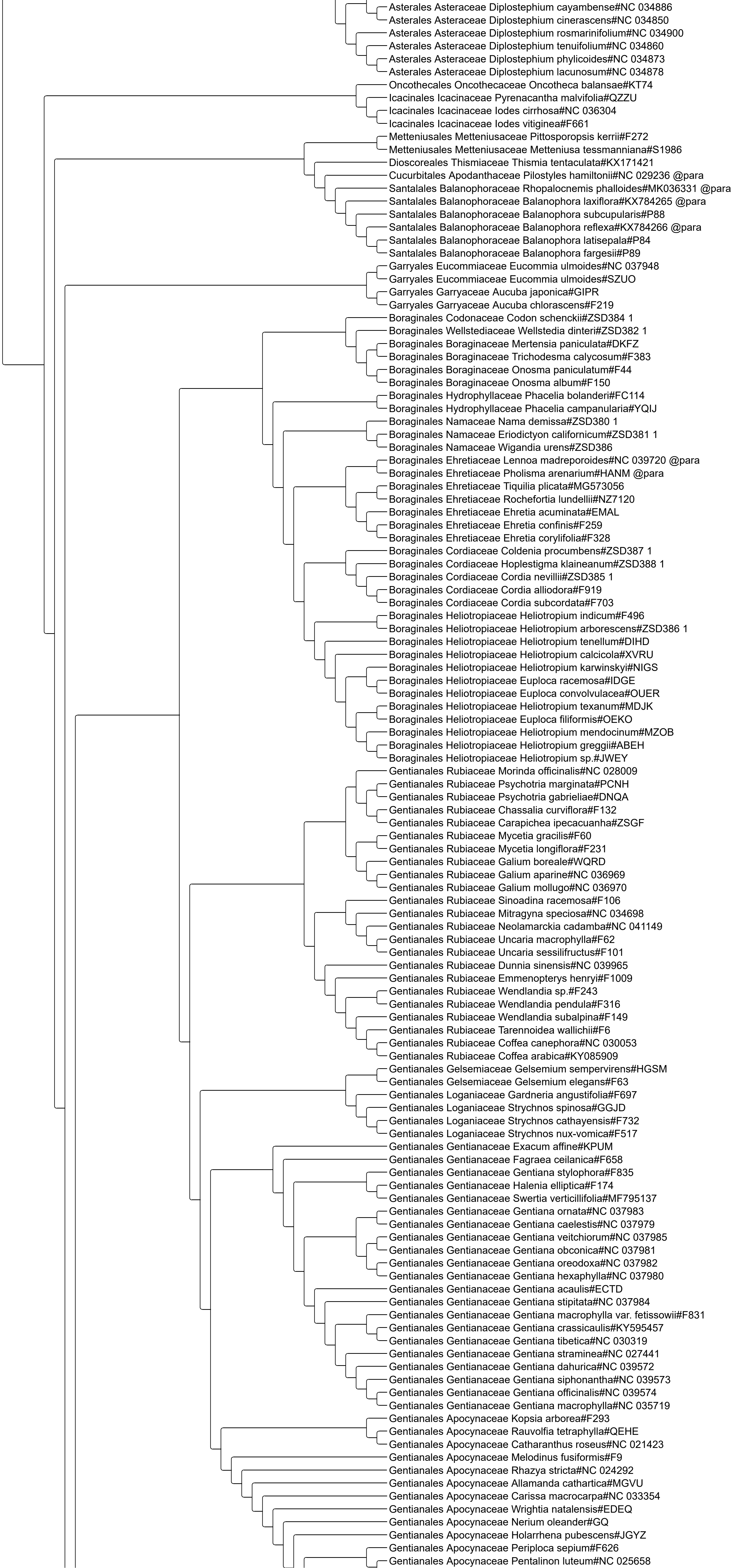

Asterales Asteraceae Diplostephium cayambense#NC 034886
Asterales Asteraceae Diplostephium cinerascens#NC 034850
Asterales Asteraceae Diplostephium rosmarinifolium#NC 034900
Asterales Asteraceae Diplostephium tenuifolium#NC 034860
Asterales Asteraceae Diplostephium phylicoides#NC 034873
Asterales Asteraceae Diplostephium lacunosum#NC 034878
Oncothecales Oncothecaceae Oncotheca balansae#KT74
Icacinales Icacinaceae Pyrenacantha malvifolia#QZZU
Icacinales Icacinaceae Iodes cirrhosa#NC 036304
Icacinales Icacinaceae Iodes vitiginea#F661
Metteniusales Metteniusaceae Pittosporopsis kerrii#F272
Metteniusales Metteniusaceae Metteniusa tessmanniana#S1986
Dioscoreales Thismiaceae Thismia tentaculata#KX171421
Cucurbitales Apodanthaceae Pilostyles hamiltonii#NC 029236 @para
Santalales Balanophoraceae Rhopalocnemis phalloides#MK036331 @para
Santalales Balanophoraceae Balanophora laxiflora#KX784265 @para
Santalales Balanophoraceae Balanophora subcupularis#P88
Santalales Balanophoraceae Balanophora reflexa#KX784266 @para
Santalales Balanophoraceae Balanophora latisepala#P84
Santalales Balanophoraceae Balanophora fargesii#P89
Garryales Eucommiaceae Eucommia ulmoides#NC 037948
Garryales Eucommiaceae Eucommia ulmoides#SZUO
Garryales Garryaceae Aucuba japonica#GIPR
Garryales Garryaceae Aucuba chlorascens#F219
Boraginales Codonaceae Codon schenckii#ZSD384 1
Boraginales Wellstediaceae Wellstedia dinteri#ZSD382 1
Boraginales Boraginaceae Mertensia paniculata#DKFZ
Boraginales Boraginaceae Trichodesma calycosum#F383
Boraginales Boraginaceae Onosma paniculatum#F44
Boraginales Boraginaceae Onosma album#F150
Boraginales Hydrophyllaceae Phacelia bolanderi#FC114
Boraginales Hydrophyllaceae Phacelia campanularia#YQIJ
Boraginales Namaceae Nama demissa#ZSD380 1
Boraginales Namaceae Eriodictyon californicum#ZSD381 1
Boraginales Namaceae Wigandia urens#ZSD386
Boraginales Ehretiaceae Lennoa madreporoides#NC 039720 @para
Boraginales Ehretiaceae Pholisma arenarium#HANM @para
Boraginales Ehretiaceae Tiquilia plicata#MG573056
Boraginales Ehretiaceae Rochefortia lundellii#NZ7120
Boraginales Ehretiaceae Ehretia acuminata#EMAL
Boraginales Ehretiaceae Ehretia confinis#F259
Boraginales Ehretiaceae Ehretia corylifolia#F328
Boraginales Cordiaceae Coldenia procumbens#ZSD387 1
Boraginales Cordiaceae Hoplestigma klaineanum#ZSD388 1
Boraginales Cordiaceae Cordia nevillii#ZSD385 1
Boraginales Cordiaceae Cordia alliodora#F919
Boraginales Cordiaceae Cordia subcordata#F703
Boraginales Heliotropiaceae Heliotropium indicum#F496
Boraginales Heliotropiaceae Heliotropium arborescens#ZSD386 1
Boraginales Heliotropiaceae Heliotropium tenellum#DIHD
Boraginales Heliotropiaceae Heliotropium calcicola#XVRU
Boraginales Heliotropiaceae Heliotropium karwinskyi#NIGS
Boraginales Heliotropiaceae Euploca racemosa#IDGE
Boraginales Heliotropiaceae Euploca convolvulacea#OUER
Boraginales Heliotropiaceae Heliotropium texanum#MDJK
Boraginales Heliotropiaceae Euploca filiformis#OEKO
Boraginales Heliotropiaceae Heliotropium mendocinum#MZOB
Boraginales Heliotropiaceae Heliotropium greggii#ABEH
Boraginales Heliotropiaceae Heliotropium sp.#JWEY
Gentianales Rubiaceae Morinda officinalis#NC 028009
Gentianales Rubiaceae Psychotria marginata#PCNH
Gentianales Rubiaceae Psychotria gabrieliae#DNQA
Gentianales Rubiaceae Chassalia curviflora#F132
Gentianales Rubiaceae Carapichea ipecacuanha#ZSGF
Gentianales Rubiaceae Mycetia gracilis#F60
Gentianales Rubiaceae Mycetia longiflora#F231
Gentianales Rubiaceae Galium boreale#WQRD
Gentianales Rubiaceae Galium aparine#NC 036969
Gentianales Rubiaceae Galium mollugo#NC 036970
Gentianales Rubiaceae Sinoadina racemosa#F106
Gentianales Rubiaceae Mitragyna speciosa#NC 034698
Gentianales Rubiaceae Neolamarckia cadamba#NC 041149
Gentianales Rubiaceae Uncaria macrophylla#F62
Gentianales Rubiaceae Uncaria sessilifructus#F101
Gentianales Rubiaceae Dunnia sinensis#NC 039965
Gentianales Rubiaceae Emmenopterys henryi#F1009
Gentianales Rubiaceae Wendlandia sp.#F243
Gentianales Rubiaceae Wendlandia pendula#F316
Gentianales Rubiaceae Wendlandia subalpina#F149
Gentianales Rubiaceae Tarennoidea wallichii#F6
Gentianales Rubiaceae Coffea canephora#NC 030053
Gentianales Rubiaceae Coffea arabica#KY085909
Gentianales Gelsemiaceae Gelsemium sempervirens#HGSM
Gentianales Gelsemiaceae Gelsemium elegans#F63
Gentianales Loganiaceae Gardneria angustifolia#F697
Gentianales Loganiaceae Strychnos spinosa#GGJD
Gentianales Loganiaceae Strychnos cathayensis#F732
Gentianales Loganiaceae Strychnos nux-vomica#F517
Gentianales Gentianaceae Exacum affine#KPUM
Gentianales Gentianaceae Fagraea ceilanica#F658
Gentianales Gentianaceae Gentiana stylophora#F835
Gentianales Gentianaceae Halenia elliptica#F174
Gentianales Gentianaceae Swertia verticillifolia#MF795137
Gentianales Gentianaceae Gentiana ornata#NC 037983
Gentianales Gentianaceae Gentiana caelestis#NC 037979
Gentianales Gentianaceae Gentiana veitchiorum#NC 037985
Gentianales Gentianaceae Gentiana obconica#NC 037981
Gentianales Gentianaceae Gentiana oreodoxa#NC 037982
Gentianales Gentianaceae Gentiana hexaphylla#NC 037980
Gentianales Gentianaceae Gentiana acaulis#ECTD
Gentianales Gentianaceae Gentiana stipitata#NC 037984
Gentianales Gentianaceae Gentiana macrophylla var. fetissowii#F831
Gentianales Gentianaceae Gentiana crassicaulis#KY595457
Gentianales Gentianaceae Gentiana tibetica#NC 030319
Gentianales Gentianaceae Gentiana straminea#NC 027441
Gentianales Gentianaceae Gentiana dahurica#NC 039572
Gentianales Gentianaceae Gentiana siphonantha#NC 039573
Gentianales Gentianaceae Gentiana officinalis#NC 039574
Gentianales Gentianaceae Gentiana macrophylla#NC 035719
Gentianales Apocynaceae Kopsia arborea#F293
Gentianales Apocynaceae Rauvolfia tetraphylla#QEHE
Gentianales Apocynaceae Catharanthus roseus#NC 021423
Gentianales Apocynaceae Melodinus fusiformis#F9
Gentianales Apocynaceae Rhazya stricta#NC 024292
Gentianales Apocynaceae Allamanda cathartica#MGVU
Gentianales Apocynaceae Carissa macrocarpa#NC 033354
Gentianales Apocynaceae Wrightia natalensis#EDEQ
Gentianales Apocynaceae Nerium oleander#GQ
Gentianales Apocynaceae Holarrhena pubescens#JGYZ
Gentianales Apocynaceae Periploca sepium#F626
Gentianales Apocynaceae Pentalinon luteum#NC 025658

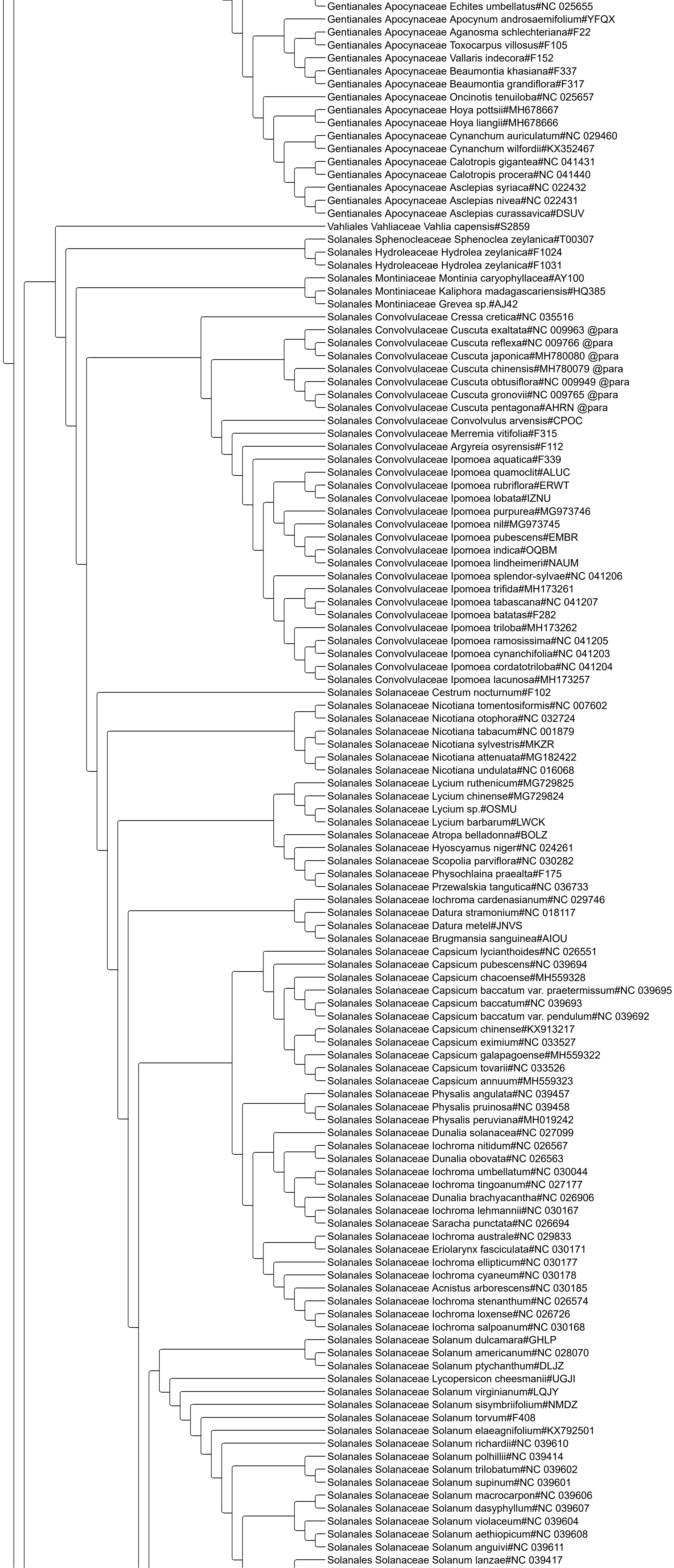
Gentianales Apocynaceae Echites umbellatus#NC 025655
Gentianales Apocynaceae Apocynum androsaemifolium#YFQX
Gentianales Apocynaceae Aganosma schlechteriana#F22
Gentianales Apocynaceae Toxocarpus villosus#F105
Gentianales Apocynaceae Vallaris indecora#F152
Gentianales Apocynaceae Beaumontia khasiana#F337
Gentianales Apocynaceae Beaumontia grandiflora#F317
Gentianales Apocynaceae Oncinotis tenuiloba#NC 025657
Gentianales Apocynaceae Hoya pottsii#MH678667
Gentianales Apocynaceae Hoya liangii#MH678666
Gentianales Apocynaceae Cynanchum auriculatum#NC 029460
Gentianales Apocynaceae Cynanchum wilfordii#KX352467
Gentianales Apocynaceae Calotropis gigantea#NC 041431
Gentianales Apocynaceae Calotropis procera#NC 041440
Gentianales Apocynaceae Asclepias syriaca#NC 022432
Gentianales Apocynaceae Asclepias nivea#NC 022431
Gentianales Apocynaceae Asclepias curassavica#DSUV
Vahliales Vahliaceae Vahlia capensis#S2859
Solanales Sphenocleaceae Sphenoclea zeylanica#T00307
Solanales Hydroleaceae Hydrolea zeylanica#F1024
Solanales Hydroleaceae Hydrolea zeylanica#F1031
Solanales Montiniaceae Montinia caryophyllacea#AY100
Solanales Montiniaceae Kaliphora madagascariensis#HQ385
Solanales Montiniaceae Grevea sp.#AJ42
Solanales Convolvulaceae Cressa cretica#NC 035516
Solanales Convolvulaceae Cuscuta exaltata#NC 009963 @para
Solanales Convolvulaceae Cuscuta reflexa#NC 009766 @para
Solanales Convolvulaceae Cuscuta japonica#MH780080 @para
Solanales Convolvulaceae Cuscuta chinensis#MH780079 @para
Solanales Convolvulaceae Cuscuta obtusiflora#NC 009949 @para
Solanales Convolvulaceae Cuscuta gronovii#NC 009765 @para
Solanales Convolvulaceae Cuscuta pentagona#AHRN @para
Solanales Convolvulaceae Convolvulus arvensis#CPOC
Solanales Convolvulaceae Merremia vitifolia#F315
Solanales Convolvulaceae Argyreia osyrensis#F112
Solanales Convolvulaceae Ipomoea aquatica#F339
Solanales Convolvulaceae Ipomoea quamoclit#ALUC
Solanales Convolvulaceae Ipomoea rubriflora#ERWT
Solanales Convolvulaceae Ipomoea lobata#IZNU
Solanales Convolvulaceae Ipomoea purpurea#MG973746
Solanales Convolvulaceae Ipomoea nil#MG973745
Solanales Convolvulaceae Ipomoea pubescens#EMBR
Solanales Convolvulaceae Ipomoea indica#OQBM
Solanales Convolvulaceae Ipomoea lindheimeri#NAUM
Solanales Convolvulaceae Ipomoea splendor-sylvae#NC 041206
Solanales Convolvulaceae Ipomoea trifida#MH173261
Solanales Convolvulaceae Ipomoea tabascana#NC 041207
Solanales Convolvulaceae Ipomoea batatas#F282
Solanales Convolvulaceae Ipomoea triloba#MH173262
Solanales Convolvulaceae Ipomoea ramosissima#NC 041205
Solanales Convolvulaceae Ipomoea cynanchifolia#NC 041203
Solanales Convolvulaceae Ipomoea cordatotriloba#NC 041204
Solanales Convolvulaceae Ipomoea lacunosa#MH173257
Solanales Solanaceae Cestrum nocturnum#F102
Solanales Solanaceae Nicotiana tomentosiformis#NC 007602
Solanales Solanaceae Nicotiana otophora#NC 032724
Solanales Solanaceae Nicotiana tabacum#NC 001879
Solanales Solanaceae Nicotiana sylvestris#MKZR
Solanales Solanaceae Nicotiana attenuata#MG182422
Solanales Solanaceae Nicotiana undulata#NC 016068
Solanales Solanaceae Lycium ruthenicum#MG729825
Solanales Solanaceae Lycium chinense#MG729824
Solanales Solanaceae Lycium sp.#OSMU
Solanales Solanaceae Lycium barbarum#LWCK
Solanales Solanaceae Atropa belladonna#BOLZ
Solanales Solanaceae Hyoscyamus niger#NC 024261
Solanales Solanaceae Scopolia parviflora#NC 030282
Solanales Solanaceae Physochlaina praealta#F175
Solanales Solanaceae Przewalskia tangutica#NC 036733
Solanales Solanaceae Iochroma cardenasianum#NC 029746
Solanales Solanaceae Datura stramonium#NC 018117
Solanales Solanaceae Datura metel#JNVS
Solanales Solanaceae Brugmansia sanguinea#AIOU
Solanales Solanaceae Capsicum lycianthoides#NC 026551
Solanales Solanaceae Capsicum pubescens#NC 039694
Solanales Solanaceae Capsicum chacoense#MH559328
Solanales Solanaceae Capsicum baccatum var. praetermissum#NC 039695
Solanales Solanaceae Capsicum baccatum#NC 039693
Solanales Solanaceae Capsicum baccatum var. pendulum#NC 039692
Solanales Solanaceae Capsicum chinense#KX913217
Solanales Solanaceae Capsicum eximium#NC 033527
Solanales Solanaceae Capsicum galapagoense#MH559322
Solanales Solanaceae Capsicum tovarii#NC 033526
Solanales Solanaceae Capsicum annuum#MH559323
Solanales Solanaceae Physalis angulata#NC 039457
Solanales Solanaceae Physalis pruinosa#NC 039458
Solanales Solanaceae Physalis peruviana#MH019242
Solanales Solanaceae Dunalia solanacea#NC 027099
Solanales Solanaceae Iochroma nitidum#NC 026567
Solanales Solanaceae Dunalia obovata#NC 026563
Solanales Solanaceae Iochroma umbellatum#NC 030044
Solanales Solanaceae Iochroma tingoanum#NC 027177
Solanales Solanaceae Dunalia brachyacantha#NC 026906
Solanales Solanaceae Iochroma lehmannii#NC 030167
Solanales Solanaceae Saracha punctata#NC 026694
Solanales Solanaceae Iochroma australe#NC 029833
Solanales Solanaceae Eriolarynx fasciculata#NC 030171
Solanales Solanaceae Iochroma ellipticum#NC 030177
Solanales Solanaceae Iochroma cyaneum#NC 030178
Solanales Solanaceae Acnistus arborescens#NC 030185
Solanales Solanaceae Iochroma stenanthum#NC 026574
Solanales Solanaceae Iochroma loxense#NC 026726
Solanales Solanaceae Iochroma salpoanum#NC 030168
Solanales Solanaceae Solanum dulcamara#GHLP
Solanales Solanaceae Solanum americanum#NC 028070
Solanales Solanaceae Solanum ptychanthum#DLJZ
Solanales Solanaceae Lycopersicon cheesmanii#UGJI
Solanales Solanaceae Solanum virginianum#LQJY
Solanales Solanaceae Solanum sisymbriifolium#NMDZ
Solanales Solanaceae Solanum torvum#F408
Solanales Solanaceae Solanum elaeagnifolium#KX792501
Solanales Solanaceae Solanum richardii#NC 039610
Solanales Solanaceae Solanum polhillii#NC 039414
Solanales Solanaceae Solanum trilobatum#NC 039602
Solanales Solanaceae Solanum supinum#NC 039601
Solanales Solanaceae Solanum macrocarpon#NC 039606
Solanales Solanaceae Solanum dasyphyllum#NC 039607
Solanales Solanaceae Solanum violaceum#NC 039604
Solanales Solanaceae Solanum aethiopicum#NC 039608
Solanales Solanaceae Solanum anguivi#NC 039611
Solanales Solanaceae Solanum lanzae#NC 039417

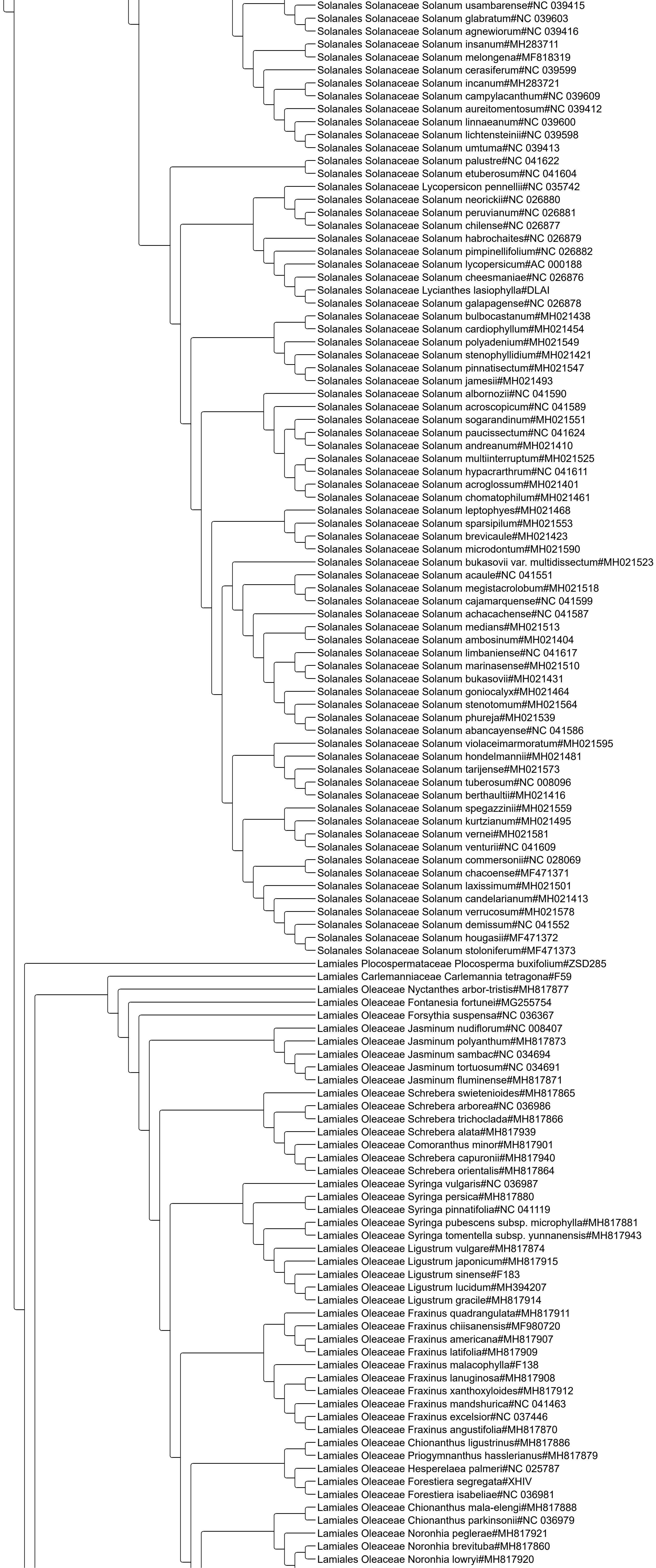
Solanales Solanaceae Solanum usambarense#NC 039415
Solanales Solanaceae Solanum glabratum#NC 039603
Solanales Solanaceae Solanum agnewiorum#NC 039416
Solanales Solanaceae Solanum insanum#MH283711
Solanales Solanaceae Solanum melongena#MF818319
Solanales Solanaceae Solanum cerasiferum#NC 039599
Solanales Solanaceae Solanum incanum#MH283721
Solanales Solanaceae Solanum campylacanthum#NC 039609
Solanales Solanaceae Solanum aureitomentosum#NC 039412
Solanales Solanaceae Solanum linnaeanum#NC 039600
Solanales Solanaceae Solanum lichtensteinii#NC 039598
Solanales Solanaceae Solanum umtuma#NC 039413
Solanales Solanaceae Solanum palustre#NC 041622
Solanales Solanaceae Solanum etuberosum#NC 041604
Solanales Solanaceae Lycopersicon pennellii#NC 035742
Solanales Solanaceae Solanum neorickii#NC 026880
Solanales Solanaceae Solanum peruvianum#NC 026881
Solanales Solanaceae Solanum chilense#NC 026877
Solanales Solanaceae Solanum habrochaites#NC 026879
Solanales Solanaceae Solanum pimpinellifolium#NC 026882
Solanales Solanaceae Solanum lycopersicum#AC 000188
Solanales Solanaceae Solanum cheesmaniae#NC 026876
Solanales Solanaceae Lycianthes lasiophylla#DLAI
Solanales Solanaceae Solanum galapagense#NC 026878
Solanales Solanaceae Solanum bulbocastanum#MH021438
Solanales Solanaceae Solanum cardiophyllum#MH021454
Solanales Solanaceae Solanum polyadenium#MH021549
Solanales Solanaceae Solanum stenophyllidium#MH021421
Solanales Solanaceae Solanum pinnatisectum#MH021547
Solanales Solanaceae Solanum jamesii#MH021493
Solanales Solanaceae Solanum albornozii#NC 041590
Solanales Solanaceae Solanum acroscopicum#NC 041589
Solanales Solanaceae Solanum sogarandinum#MH021551
Solanales Solanaceae Solanum paucissectum#NC 041624
Solanales Solanaceae Solanum andreanum#MH021410
Solanales Solanaceae Solanum multiinterruptum#MH021525
Solanales Solanaceae Solanum hypacrarthrum#NC 041611
Solanales Solanaceae Solanum acroglossum#MH021401
Solanales Solanaceae Solanum chomatophilum#MH021461
Solanales Solanaceae Solanum leptophyes#MH021468
Solanales Solanaceae Solanum sparsipilum#MH021553
Solanales Solanaceae Solanum brevicaule#MH021423
Solanales Solanaceae Solanum microdontum#MH021590
Solanales Solanaceae Solanum bukasovii var. multidissectum#MH021523
Solanales Solanaceae Solanum acaule#NC 041551
Solanales Solanaceae Solanum megistacrolobum#MH021518
Solanales Solanaceae Solanum cajamarquense#NC 041599
Solanales Solanaceae Solanum achacachense#NC 041587
Solanales Solanaceae Solanum medians#MH021513
Solanales Solanaceae Solanum ambosinum#MH021404
Solanales Solanaceae Solanum limbaniense#NC 041617
Solanales Solanaceae Solanum marinasense#MH021510
Solanales Solanaceae Solanum bukasovii#MH021431
Solanales Solanaceae Solanum goniocalyx#MH021464
Solanales Solanaceae Solanum stenotomum#MH021564
Solanales Solanaceae Solanum phureja#MH021539
Solanales Solanaceae Solanum abancayense#NC 041586
Solanales Solanaceae Solanum violaceimarmoratum#MH021595
Solanales Solanaceae Solanum hondelmannii#MH021481
Solanales Solanaceae Solanum tarijense#MH021573
Solanales Solanaceae Solanum tuberosum#NC 008096
Solanales Solanaceae Solanum berthaultii#MH021416
Solanales Solanaceae Solanum spegazzinii#MH021559
Solanales Solanaceae Solanum kurtzianum#MH021495
Solanales Solanaceae Solanum vernei#MH021581
Solanales Solanaceae Solanum venturii#NC 041609
Solanales Solanaceae Solanum commersonii#NC 028069
Solanales Solanaceae Solanum chacoense#MF471371
Solanales Solanaceae Solanum laxissimum#MH021501
Solanales Solanaceae Solanum candelarianum#MH021413
Solanales Solanaceae Solanum verrucosum#MH021578
Solanales Solanaceae Solanum demissum#NC 041552
Solanales Solanaceae Solanum hougasii#MF471372
Solanales Solanaceae Solanum stoloniferum#MF471373
Lamiales Plocospermataceae Plocosperma buxifolium#ZSD285
Lamiales Carlemanniaceae Carlemannia tetragona#F59
Lamiales Oleaceae Nyctanthes arbor-tristis#MH817877
Lamiales Oleaceae Fontanesia fortunei#MG255754
Lamiales Oleaceae Forsythia suspensa#NC 036367
Lamiales Oleaceae Jasminum nudiflorum#NC 008407
Lamiales Oleaceae Jasminum polyanthum#MH817873
Lamiales Oleaceae Jasminum sambac#NC 034694
Lamiales Oleaceae Jasminum tortuosum#NC 034691
Lamiales Oleaceae Jasminum fluminense#MH817871
Lamiales Oleaceae Schrebera swietenioides#MH817865
Lamiales Oleaceae Schrebera arborea#NC 036986
Lamiales Oleaceae Schrebera trichoclada#MH817866
Lamiales Oleaceae Schrebera alata#MH817939
Lamiales Oleaceae Comoranthus minor#MH817901
Lamiales Oleaceae Schrebera capuronii#MH817940
Lamiales Oleaceae Schrebera orientalis#MH817864
Lamiales Oleaceae Syringa vulgaris#NC 036987
Lamiales Oleaceae Syringa persica#MH817880
Lamiales Oleaceae Syringa pinnatifolia#NC 041119
Lamiales Oleaceae Syringa pubescens subsp. microphylla#MH817881
Lamiales Oleaceae Syringa tomentella subsp. yunnanensis#MH817943
Lamiales Oleaceae Ligustrum vulgare#MH817874
Lamiales Oleaceae Ligustrum japonicum#MH817915
Lamiales Oleaceae Ligustrum sinense#F183
Lamiales Oleaceae Ligustrum lucidum#MH394207
Lamiales Oleaceae Ligustrum gracile#MH817914
Lamiales Oleaceae Fraxinus quadrangulata#MH817911
Lamiales Oleaceae Fraxinus chiisanensis#MF980720
Lamiales Oleaceae Fraxinus americana#MH817907
Lamiales Oleaceae Fraxinus latifolia#MH817909
Lamiales Oleaceae Fraxinus malacophylla#F138
Lamiales Oleaceae Fraxinus lanuginosa#MH817908
Lamiales Oleaceae Fraxinus xanthoxyloides#MH817912
Lamiales Oleaceae Fraxinus mandshurica#NC 041463
Lamiales Oleaceae Fraxinus excelsior#NC 037446
Lamiales Oleaceae Fraxinus angustifolia#MH817870
Lamiales Oleaceae Chionanthus ligustrinus#MH817886
Lamiales Oleaceae Priogymnanthus hasslerianus#MH817879
Lamiales Oleaceae Hesperelaea palmeri#NC 025787
Lamiales Oleaceae Forestiera segregata#XHIV
Lamiales Oleaceae Forestiera isabeliae#NC 036981
Lamiales Oleaceae Chionanthus mala-elengi#MH817888
Lamiales Oleaceae Chionanthus parkinsonii#NC 036979
Lamiales Oleaceae Noronhia peglerae#MH817921
Lamiales Oleaceae Noronhia brevituba#MH817860
Lamiales Oleaceae Noronhia lowryi#MH817920

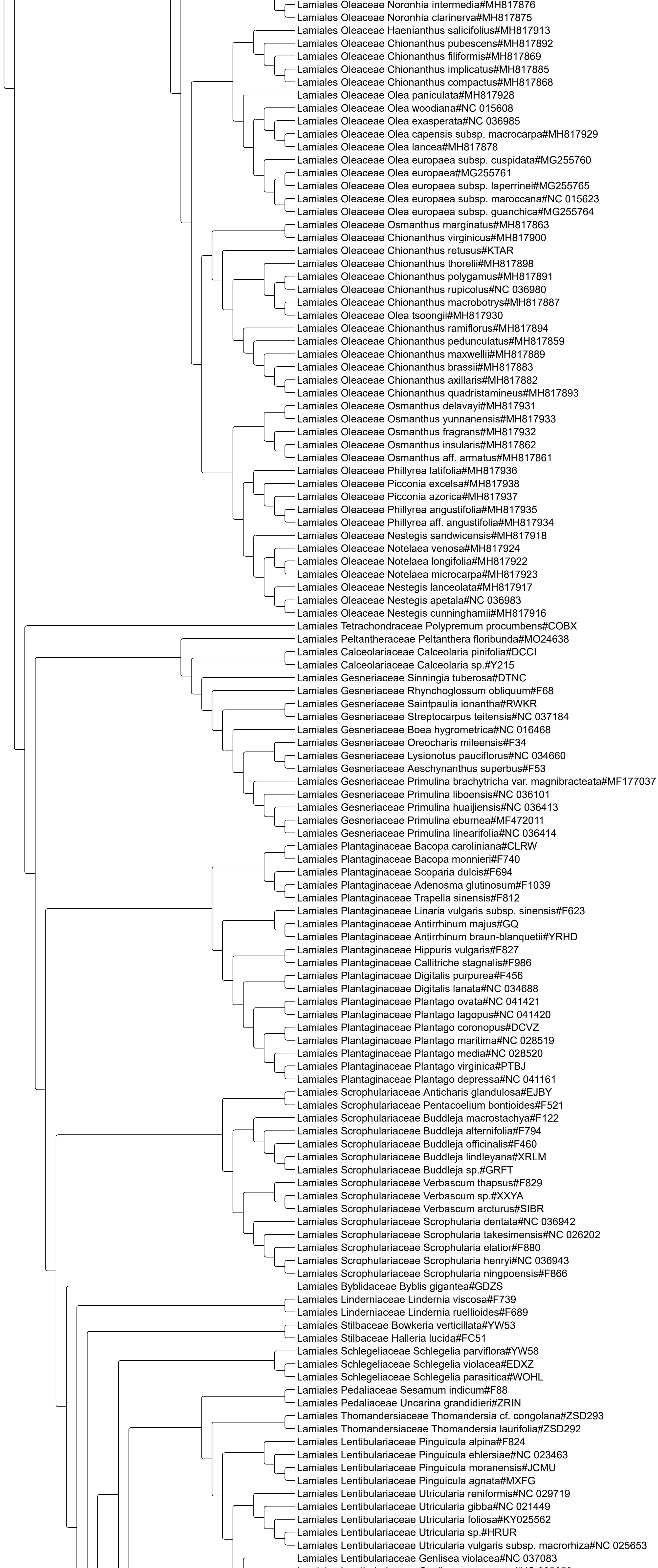
Lamiales Oleaceae Noronhia intermedia#MH817876
Lamiales Oleaceae Noronhia clarinerva#MH817875
Lamiales Oleaceae Haenianthus salicifolius#MH817913
Lamiales Oleaceae Chionanthus pubescens#MH817892
Lamiales Oleaceae Chionanthus filiformis#MH817869
Lamiales Oleaceae Chionanthus implicatus#MH817885
Lamiales Oleaceae Chionanthus compactus#MH817868
Lamiales Oleaceae Olea paniculata#MH817928
Lamiales Oleaceae Olea woodiana#NC 015608
Lamiales Oleaceae Olea exasperata#NC 036985
Lamiales Oleaceae Olea capensis subsp. macrocarpa#MH817929
Lamiales Oleaceae Olea lancea#MH817878
Lamiales Oleaceae Olea europaea subsp. cuspidata#MG255760
Lamiales Oleaceae Olea europaea#MG255761
Lamiales Oleaceae Olea europaea subsp. laperrinei#MG255765
Lamiales Oleaceae Olea europaea subsp. maroccana#NC 015623
Lamiales Oleaceae Olea europaea subsp. guanchica#MG255764
Lamiales Oleaceae Osmanthus marginatus#MH817863
Lamiales Oleaceae Chionanthus virginicus#MH817900
Lamiales Oleaceae Chionanthus retusus#KTAR
Lamiales Oleaceae Chionanthus thorelii#MH817898
Lamiales Oleaceae Chionanthus polygamus#MH817891
Lamiales Oleaceae Chionanthus rupicolus#NC 036980
Lamiales Oleaceae Chionanthus macrobotrys#MH817887
Lamiales Oleaceae Olea tsoongii#MH817930
Lamiales Oleaceae Chionanthus ramiflorus#MH817894
Lamiales Oleaceae Chionanthus pedunculatus#MH817859
Lamiales Oleaceae Chionanthus maxwellii#MH817889
Lamiales Oleaceae Chionanthus brassii#MH817883
Lamiales Oleaceae Chionanthus axillaris#MH817882
Lamiales Oleaceae Chionanthus quadristamineus#MH817893
Lamiales Oleaceae Osmanthus delavayi#MH817931
Lamiales Oleaceae Osmanthus yunnanensis#MH817933
Lamiales Oleaceae Osmanthus fragrans#MH817932
Lamiales Oleaceae Osmanthus insularis#MH817862
Lamiales Oleaceae Osmanthus aff. armatus#MH817861
Lamiales Oleaceae Phillyrea latifolia#MH817936
Lamiales Oleaceae Picconia excelsa#MH817938
Lamiales Oleaceae Picconia azorica#MH817937
Lamiales Oleaceae Phillyrea angustifolia#MH817935
Lamiales Oleaceae Phillyrea aff. angustifolia#MH817934
Lamiales Oleaceae Nestegis sandwicensis#MH817918
Lamiales Oleaceae Notelaea venosa#MH817924
Lamiales Oleaceae Notelaea longifolia#MH817922
Lamiales Oleaceae Notelaea microcarpa#MH817923
Lamiales Oleaceae Nestegis lanceolata#MH817917
Lamiales Oleaceae Nestegis apetala#NC 036983
Lamiales Oleaceae Nestegis cunninghamii#MH817916
Lamiales Tetrachondraceae Polypremum procumbens#COBX
Lamiales Peltantheraceae Peltanthera floribunda#MO24638
Lamiales Calceolariaceae Calceolaria pinifolia#DCCI
Lamiales Calceolariaceae Calceolaria sp.#Y215
Lamiales Gesneriaceae Sinningia tuberosa#DTNC
Lamiales Gesneriaceae Rhynchoglossum obliquum#F68
Lamiales Gesneriaceae Saintpaulia ionantha#RWKR
Lamiales Gesneriaceae Streptocarpus teitensis#NC 037184
Lamiales Gesneriaceae Boea hygrometrica#NC 016468
Lamiales Gesneriaceae Oreocharis mileensis#F34
Lamiales Gesneriaceae Lysionotus pauciflorus#NC 034660
Lamiales Gesneriaceae Aeschynanthus superbus#F53
Lamiales Gesneriaceae Primulina brachytricha var. magnibracteata#MF177037
Lamiales Gesneriaceae Primulina liboensis#NC 036101
Lamiales Gesneriaceae Primulina huaijiensis#NC 036413
Lamiales Gesneriaceae Primulina eburnea#MF472011
Lamiales Gesneriaceae Primulina linearifolia#NC 036414
Lamiales Plantaginaceae Bacopa caroliniana#CLRW
Lamiales Plantaginaceae Bacopa monnieri#F740
Lamiales Plantaginaceae Scoparia dulcis#F694
Lamiales Plantaginaceae Adenosma glutinosum#F1039
Lamiales Plantaginaceae Trapella sinensis#F812
Lamiales Plantaginaceae Linaria vulgaris subsp. sinensis#F623
Lamiales Plantaginaceae Antirrhinum majus#GQ
Lamiales Plantaginaceae Antirrhinum braun-blanquetii#YRHD
Lamiales Plantaginaceae Hippuris vulgaris#F827
Lamiales Plantaginaceae Callitriche stagnalis#F986
Lamiales Plantaginaceae Digitalis purpurea#F456
Lamiales Plantaginaceae Digitalis lanata#NC 034688
Lamiales Plantaginaceae Plantago ovata#NC 041421
Lamiales Plantaginaceae Plantago lagopus#NC 041420
Lamiales Plantaginaceae Plantago coronopus#DCVZ
Lamiales Plantaginaceae Plantago maritima#NC 028519
Lamiales Plantaginaceae Plantago media#NC 028520
Lamiales Plantaginaceae Plantago virginica#PTBJ
Lamiales Plantaginaceae Plantago depressa#NC 041161
Lamiales Scrophulariaceae Anticharis glandulosa#EJBY
Lamiales Scrophulariaceae Pentacoelium bontioides#F521
Lamiales Scrophulariaceae Buddleja macrostachya#F122
Lamiales Scrophulariaceae Buddleja alternifolia#F794
Lamiales Scrophulariaceae Buddleja officinalis#F460
Lamiales Scrophulariaceae Buddleja lindleyana#XRLM
Lamiales Scrophulariaceae Buddleja sp.#GRFT
Lamiales Scrophulariaceae Verbascum thapsus#F829
Lamiales Scrophulariaceae Verbascum sp.#XXYA
Lamiales Scrophulariaceae Verbascum arcturus#SIBR
Lamiales Scrophulariaceae Scrophularia dentata#NC 036942
Lamiales Scrophulariaceae Scrophularia takesimensis#NC 026202
Lamiales Scrophulariaceae Scrophularia elatior#F880
Lamiales Scrophulariaceae Scrophularia henryi#NC 036943
Lamiales Scrophulariaceae Scrophularia ningpoensis#F866
Lamiales Byblidaceae Byblis gigantea#GDZS
Lamiales Linderniaceae Lindernia viscosa#F739
Lamiales Linderniaceae Lindernia ruellioides#F689
Lamiales Stilbaceae Bowkeria verticillata#YW53
Lamiales Stilbaceae Halleria lucida#FC51
Lamiales Schlegeliaceae Schlegelia parviflora#YW58
Lamiales Schlegeliaceae Schlegelia violacea#EDXZ
Lamiales Schlegeliaceae Schlegelia parasitica#WOHL
Lamiales Pedaliaceae Sesamum indicum#F88
Lamiales Pedaliaceae Uncarina grandidieri#ZRIN
Lamiales Thomandersiaceae Thomandersia cf. congolana#ZSD293
Lamiales Thomandersiaceae Thomandersia laurifolia#ZSD292
Lamiales Lentibulariaceae Pinguicula alpina#F824
Lamiales Lentibulariaceae Pinguicula ehlersiae#NC 023463
Lamiales Lentibulariaceae Pinguicula moranensis#JCMU
Lamiales Lentibulariaceae Pinguicula agnata#MXFG
Lamiales Lentibulariaceae Utricularia reniformis#NC 029719
Lamiales Lentibulariaceae Utricularia gibba#NC 021449
Lamiales Lentibulariaceae Utricularia foliosa#KY025562
Lamiales Lentibulariaceae Utricularia sp.#HRUR
Lamiales Lentibulariaceae Utricularia vulgaris subsp. macrorhiza#NC 025653
Lamiales Lentibulariaceae Genlisea violacea#NC 037083

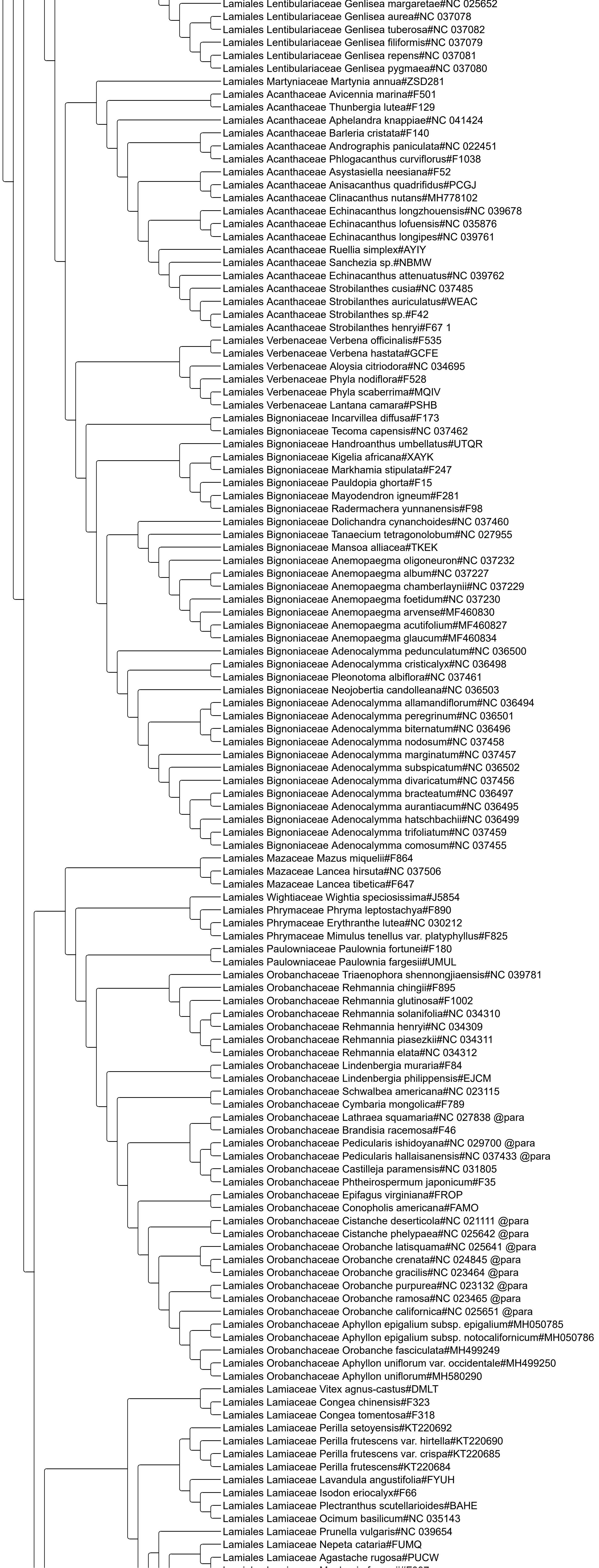

Lamiales Lentibulariaceae Genlisea margaretae#NC 025652
Lamiales Lentibulariaceae Genlisea aurea#NC 037078
Lamiales Lentibulariaceae Genlisea tuberosa#NC 037082
Lamiales Lentibulariaceae Genlisea filiformis#NC 037079
Lamiales Lentibulariaceae Genlisea repens#NC 037081
Lamiales Lentibulariaceae Genlisea pygmaea#NC 037080
Lamiales Martyniaceae Martynia annua#ZSD281
Lamiales Acanthaceae Avicennia marina#F501
Lamiales Acanthaceae Thunbergia lutea#F129
Lamiales Acanthaceae Aphelandra knappiae#NC 041424
Lamiales Acanthaceae Barleria cristata#F140
Lamiales Acanthaceae Andrographis paniculata#NC 022451
Lamiales Acanthaceae Phlogacanthus curviflorus#F1038
Lamiales Acanthaceae Asystasiella neesiana#F52
Lamiales Acanthaceae Anisacanthus quadrifidus#PCGJ
Lamiales Acanthaceae Clinacanthus nutans#MH778102
Lamiales Acanthaceae Echinacanthus longzhouensis#NC 039678
Lamiales Acanthaceae Echinacanthus lofuensis#NC 035876
Lamiales Acanthaceae Echinacanthus longipes#NC 039761
Lamiales Acanthaceae Ruellia simplex#AYIY
Lamiales Acanthaceae Sanchezia sp.#NBMW
Lamiales Acanthaceae Echinacanthus attenuatus#NC 039762
Lamiales Acanthaceae Strobilanthes cusia#NC 037485
Lamiales Acanthaceae Strobilanthes auriculatus#WEAC
Lamiales Acanthaceae Strobilanthes sp.#F42
Lamiales Acanthaceae Strobilanthes henryi#F67 1
Lamiales Verbenaceae Verbena officinalis#F535
Lamiales Verbenaceae Verbena hastata#GCFE
Lamiales Verbenaceae Aloysia citriodora#NC 034695
Lamiales Verbenaceae Phyla nodiflora#F528
Lamiales Verbenaceae Phyla scaberrima#MQIV
Lamiales Verbenaceae Lantana camara#PSHB
Lamiales Bignoniaceae Incarvillea diffusa#F173
Lamiales Bignoniaceae Tecoma capensis#NC 037462
Lamiales Bignoniaceae Handroanthus umbellatus#UTQR
Lamiales Bignoniaceae Kigelia africana#XAYK
Lamiales Bignoniaceae Markhamia stipulata#F247
Lamiales Bignoniaceae Pauldopia ghorta#F15
Lamiales Bignoniaceae Mayodendron igneum#F281
Lamiales Bignoniaceae Radermachera yunnanensis#F98
Lamiales Bignoniaceae Dolichandra cynanchoides#NC 037460
Lamiales Bignoniaceae Tanaecium tetragonolobum#NC 027955
Lamiales Bignoniaceae Mansoa alliacea#TKEK
Lamiales Bignoniaceae Anemopaegma oligoneuron#NC 037232
Lamiales Bignoniaceae Anemopaegma album#NC 037227
Lamiales Bignoniaceae Anemopaegma chamberlaynii#NC 037229
Lamiales Bignoniaceae Anemopaegma foetidum#NC 037230
Lamiales Bignoniaceae Anemopaegma arvense#MF460830
Lamiales Bignoniaceae Anemopaegma acutifolium#MF460827
Lamiales Bignoniaceae Anemopaegma glaucum#MF460834
Lamiales Bignoniaceae Adenocalymma pedunculatum#NC 036500
Lamiales Bignoniaceae Adenocalymma cristicalyx#NC 036498
Lamiales Bignoniaceae Pleonotoma albiflora#NC 037461
Lamiales Bignoniaceae Neojobertia candolleana#NC 036503
Lamiales Bignoniaceae Adenocalymma allamandiflorum#NC 036494
Lamiales Bignoniaceae Adenocalymma peregrinum#NC 036501
Lamiales Bignoniaceae Adenocalymma biternatum#NC 036496
Lamiales Bignoniaceae Adenocalymma nodosum#NC 037458
Lamiales Bignoniaceae Adenocalymma marginatum#NC 037457
Lamiales Bignoniaceae Adenocalymma subspicatum#NC 036502
Lamiales Bignoniaceae Adenocalymma divaricatum#NC 037456
Lamiales Bignoniaceae Adenocalymma bracteatum#NC 036497
Lamiales Bignoniaceae Adenocalymma aurantiacum#NC 036495
Lamiales Bignoniaceae Adenocalymma hatschbachii#NC 036499
Lamiales Bignoniaceae Adenocalymma trifoliatum#NC 037459
Lamiales Bignoniaceae Adenocalymma comosum#NC 037455
Lamiales Mazaceae Mazus miquelii#F864
Lamiales Mazaceae Lancea hirsuta#NC 037506
Lamiales Mazaceae Lancea tibetica#F647
Lamiales Wightiaceae Wightia speciosissima#J5854
Lamiales Phrymaceae Phryma leptostachya#F890
Lamiales Phrymaceae Erythranthe lutea#NC 030212
Lamiales Phrymaceae Mimulus tenellus var. platyphyllus#F825
Lamiales Paulowniaceae Paulownia fortunei#F180
Lamiales Paulowniaceae Paulownia fargesii#UMUL
Lamiales Orobanchaceae Triaenophora shennongjiaensis#NC 039781
Lamiales Orobanchaceae Rehmannia chingii#F895
Lamiales Orobanchaceae Rehmannia glutinosa#F1002
Lamiales Orobanchaceae Rehmannia solanifolia#NC 034310
Lamiales Orobanchaceae Rehmannia henryi#NC 034309
Lamiales Orobanchaceae Rehmannia piasezkii#NC 034311
Lamiales Orobanchaceae Rehmannia elata#NC 034312
Lamiales Orobanchaceae Lindenbergia muraria#F84
Lamiales Orobanchaceae Lindenbergia philippensis#EJCM
Lamiales Orobanchaceae Schwalbea americana#NC 023115
Lamiales Orobanchaceae Cymbaria mongolica#F789
Lamiales Orobanchaceae Lathraea squamaria#NC 027838 @para
Lamiales Orobanchaceae Brandisia racemosa#F46
Lamiales Orobanchaceae Pedicularis ishidoyana#NC 029700 @para
Lamiales Orobanchaceae Pedicularis hallaisanensis#NC 037433 @para
Lamiales Orobanchaceae Castilleja paramensis#NC 031805
Lamiales Orobanchaceae Phtheirospermum japonicum#F35
Lamiales Orobanchaceae Epifagus virginiana#FROP
Lamiales Orobanchaceae Conopholis americana#FAMO
Lamiales Orobanchaceae Cistanche deserticola#NC 021111 @para
Lamiales Orobanchaceae Cistanche phelypaea#NC 025642 @para
Lamiales Orobanchaceae Orobanche latisquama#NC 025641 @para
Lamiales Orobanchaceae Orobanche crenata#NC 024845 @para
Lamiales Orobanchaceae Orobanche gracilis#NC 023464 @para
Lamiales Orobanchaceae Orobanche purpurea#NC 023132 @para
Lamiales Orobanchaceae Orobanche ramosa#NC 023465 @para
Lamiales Orobanchaceae Orobanche californica#NC 025651 @para
Lamiales Orobanchaceae Aphyllon epigalium subsp. epigalium#MH050785
Lamiales Orobanchaceae Aphyllon epigalium subsp. notocalifornicum#MH050786
Lamiales Orobanchaceae Orobanche fasciculata#MH499249
Lamiales Orobanchaceae Aphyllon uniflorum var. occidentale#MH499250
Lamiales Orobanchaceae Aphyllon uniflorum#MH580290
Lamiales Lamiaceae Vitex agnus-castus#DMLT
Lamiales Lamiaceae Congea chinensis#F323
Lamiales Lamiaceae Congea tomentosa#F318
Lamiales Lamiaceae Perilla setoyensis#KT220692
Lamiales Lamiaceae Perilla frutescens var. hirtella#KT220690
Lamiales Lamiaceae Perilla frutescens var. crispa#KT220685
Lamiales Lamiaceae Perilla frutescens#KT220684
Lamiales Lamiaceae Lavandula angustifolia#FYUH
Lamiales Lamiaceae Isodon eriocalyx#F66
Lamiales Lamiaceae Plectranthus scutellarioides#BAHE
Lamiales Lamiaceae Ocimum basilicum#NC 035143
Lamiales Lamiaceae Prunella vulgaris#NC 039654
Lamiales Lamiaceae Nepeta cataria#FUMQ
Lamiales Lamiaceae Agastache rugosa#PUCW

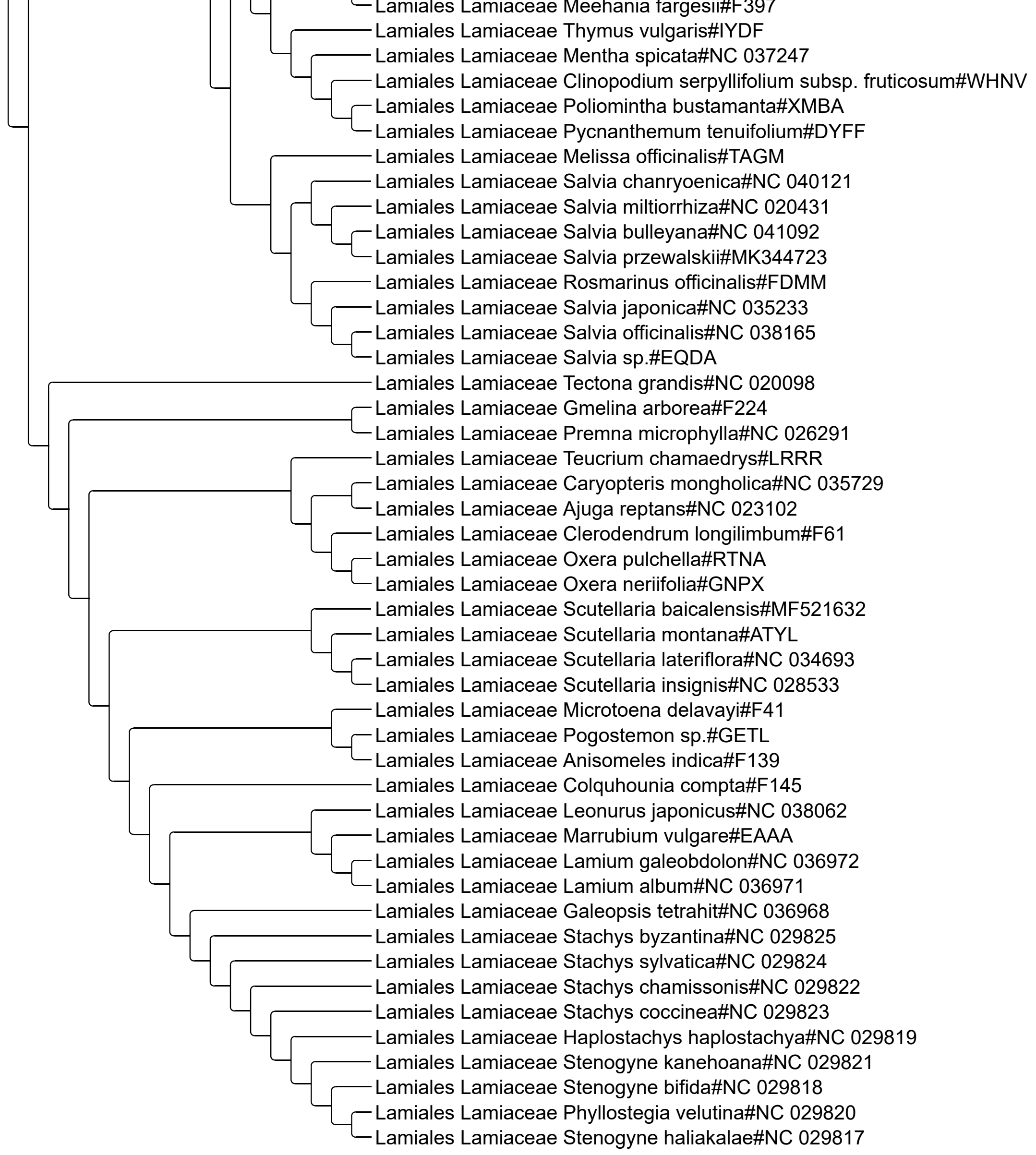

Lamiales Lamiaceae Meehania fargesii#F397
Lamiales Lamiaceae Thymus vulgaris#IYDF
Lamiales Lamiaceae Mentha spicata#NC 037247
Lamiales Lamiaceae Clinopodium serpyllifolium subsp. fruticosum#WHNV
Lamiales Lamiaceae Poliomintha bustamanta#XMBA
Lamiales Lamiaceae Pycnanthemum tenuifolium#DYFF
Lamiales Lamiaceae Melissa officinalis#TAGM
Lamiales Lamiaceae Salvia chanryoenica#NC 040121
Lamiales Lamiaceae Salvia miltiorrhiza#NC 020431
Lamiales Lamiaceae Salvia bulleyana#NC 041092
Lamiales Lamiaceae Salvia przewalskii#MK344723
Lamiales Lamiaceae Rosmarinus officinalis#FDMM
Lamiales Lamiaceae Salvia japonica#NC 035233
Lamiales Lamiaceae Salvia officinalis#NC 038165
Lamiales Lamiaceae Salvia sp.#EQDA
Lamiales Lamiaceae Tectona grandis#NC 020098
Lamiales Lamiaceae Gmelina arborea#F224
Lamiales Lamiaceae Premna microphylla#NC 026291
Lamiales Lamiaceae Teucrium chamaedrys#LRRR
Lamiales Lamiaceae Caryopteris mongholica#NC 035729
Lamiales Lamiaceae Ajuga reptans#NC 023102
Lamiales Lamiaceae Clerodendrum longilimbum#F61
Lamiales Lamiaceae Oxera pulchella#RTNA
Lamiales Lamiaceae Oxera neriifolia#GNPX
Lamiales Lamiaceae Scutellaria baicalensis#MF521632
Lamiales Lamiaceae Scutellaria montana#ATYL
Lamiales Lamiaceae Scutellaria lateriflora#NC 034693
Lamiales Lamiaceae Scutellaria insignis#NC 028533
Lamiales Lamiaceae Microtoena delavayi#F41
Lamiales Lamiaceae Pogostemon sp.#GETL
Lamiales Lamiaceae Anisomeles indica#F139
Lamiales Lamiaceae Colquhounia compta#F145
Lamiales Lamiaceae Leonurus japonicus#NC 038062
Lamiales Lamiaceae Marrubium vulgare#EAAA
Lamiales Lamiaceae Lamium galeobdolon#NC 036972
Lamiales Lamiaceae Lamium album#NC 036971
Lamiales Lamiaceae Galeopsis tetrahit#NC 036968
Lamiales Lamiaceae Stachys byzantina#NC 029825
Lamiales Lamiaceae Stachys sylvatica#NC 029824
Lamiales Lamiaceae Stachys chamissonis#NC 029822
Lamiales Lamiaceae Stachys coccinea#NC 029823
Lamiales Lamiaceae Haplostachys haplostachya#NC 029819
Lamiales Lamiaceae Stenogyne kanehoana#NC 029821
Lamiales Lamiaceae Stenogyne bifida#NC 029818
Lamiales Lamiaceae Phyllostegia velutina#NC 029820
Lamiales Lamiaceae Stenogyne haliakalae#NC 029817

[Figure S8h]

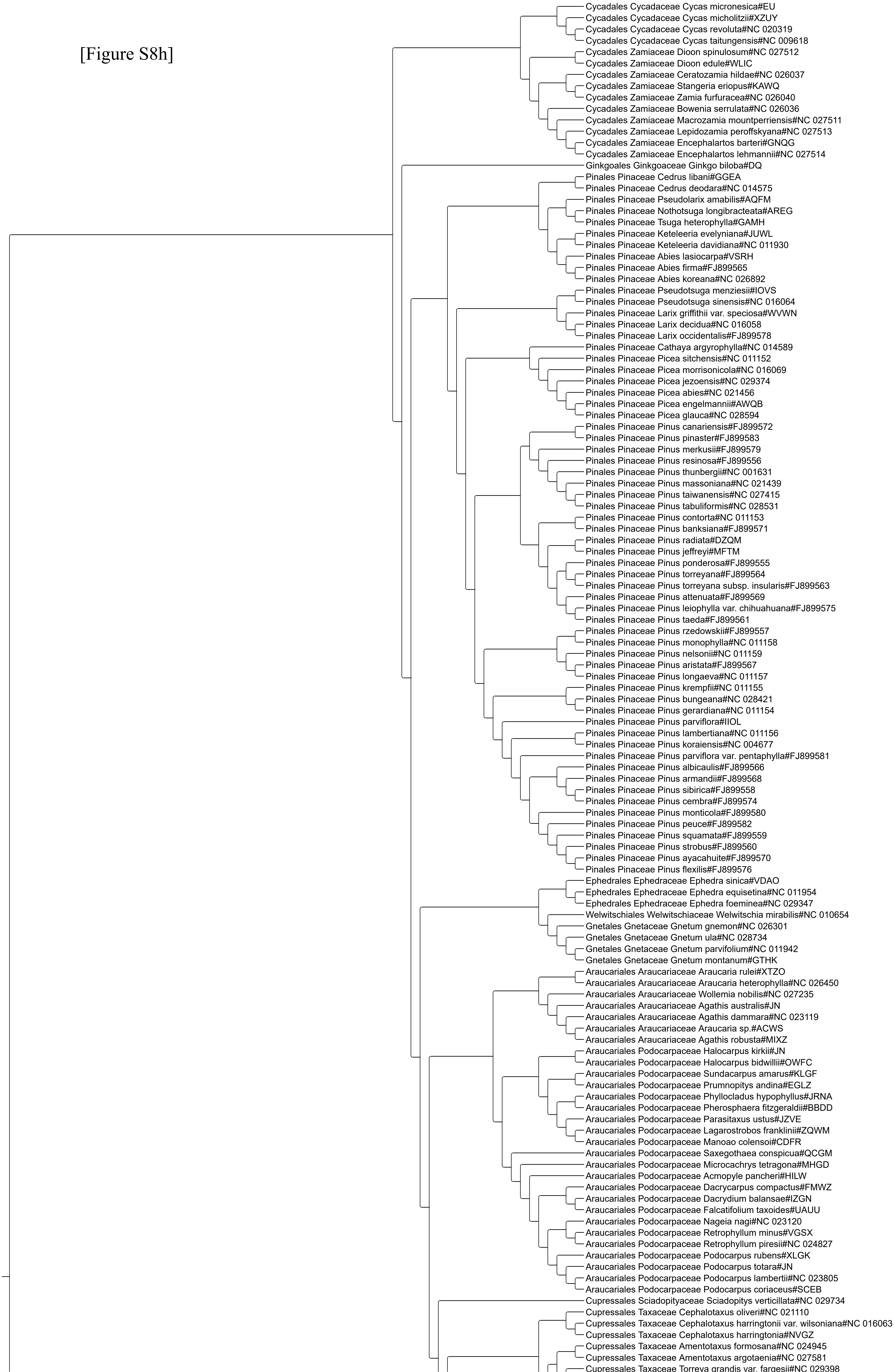
Cycadales Cycadaceae Cycas micronesica#EU
Cycadales Cycadaceae Cycas micholitzii#XZUY
Cycadales Cycadaceae Cycas revoluta#NC 020319
Cycadales Cycadaceae Cycas taitungensis#NC 009618
Cycadales Zamiaceae Dioon spinulosum#NC 027512
Cycadales Zamiaceae Dioon edule#WLIC
Cycadales Zamiaceae Ceratozamia hildae#NC 026037
Cycadales Zamiaceae Stangeria eriopus#KAWQ
Cycadales Zamiaceae Zamia furfuracea#NC 026040
Cycadales Zamiaceae Bowenia serrulata#NC 026036
Cycadales Zamiaceae Macrozamia mountperriensis#NC 027511
Cycadales Zamiaceae Lepidozamia peroffskyana#NC 027513
Cycadales Zamiaceae Encephalartos barteri#GNQG
Cycadales Zamiaceae Encephalartos lehmannii#NC 027514
Ginkgoales Ginkgoaceae Ginkgo biloba#DQ
Pinales Pinaceae Cedrus libani#GGEA
Pinales Pinaceae Cedrus deodara#NC 014575
Pinales Pinaceae Pseudolarix amabilis#AQFM
Pinales Pinaceae Nothotsuga longibracteata#AREG
Pinales Pinaceae Tsuga heterophylla#GAMH
Pinales Pinaceae Keteleeria evelyniana#JUWL
Pinales Pinaceae Keteleeria davidiana#NC 011930
Pinales Pinaceae Abies lasiocarpa#VSRH
Pinales Pinaceae Abies firma#FJ899565
Pinales Pinaceae Abies koreana#NC 026892
Pinales Pinaceae Pseudotsuga menziesii#IOVS
Pinales Pinaceae Pseudotsuga sinensis#NC 016064
Pinales Pinaceae Larix griffithii var. speciosa#WVWN
Pinales Pinaceae Larix decidua#NC 016058
Pinales Pinaceae Larix occidentalis#FJ899578
Pinales Pinaceae Cathaya argyrophylla#NC 014589
Pinales Pinaceae Picea sitchensis#NC 011152
Pinales Pinaceae Picea morrisonicola#NC 016069
Pinales Pinaceae Picea jezoensis#NC 029374
Pinales Pinaceae Picea abies#NC 021456
Pinales Pinaceae Picea engelmannii#AWQB
Pinales Pinaceae Picea glauca#NC 028594
Pinales Pinaceae Pinus canariensis#FJ899572
Pinales Pinaceae Pinus pinaster#FJ899583
Pinales Pinaceae Pinus merkusii#FJ899579
Pinales Pinaceae Pinus resinosa#FJ899556
Pinales Pinaceae Pinus thunbergii#NC 001631
Pinales Pinaceae Pinus massoniana#NC 021439
Pinales Pinaceae Pinus taiwanensis#NC 027415
Pinales Pinaceae Pinus tabuliformis#NC 028531
Pinales Pinaceae Pinus contorta#NC 011153
Pinales Pinaceae Pinus banksiana#FJ899571
Pinales Pinaceae Pinus radiata#DZQM
Pinales Pinaceae Pinus jeffreyi#MFTM
Pinales Pinaceae Pinus ponderosa#FJ899555
Pinales Pinaceae Pinus torreyana#FJ899564
Pinales Pinaceae Pinus torreyana subsp. insularis#FJ899563
Pinales Pinaceae Pinus attenuata#FJ899569
Pinales Pinaceae Pinus leiophylla var. chihuahuana#FJ899575
Pinales Pinaceae Pinus taeda#FJ899561
Pinales Pinaceae Pinus rzedowskii#FJ899557
Pinales Pinaceae Pinus monophylla#NC 011158
Pinales Pinaceae Pinus nelsonii#NC 011159
Pinales Pinaceae Pinus aristata#FJ899567
Pinales Pinaceae Pinus longaeva#NC 011157
Pinales Pinaceae Pinus krempfii#NC 011155
Pinales Pinaceae Pinus bungeana#NC 028421
Pinales Pinaceae Pinus gerardiana#NC 011154
Pinales Pinaceae Pinus parviflora#IIOL
Pinales Pinaceae Pinus lambertiana#NC 011156
Pinales Pinaceae Pinus koraiensis#NC 004677
Pinales Pinaceae Pinus parviflora var. pentaphylla#FJ899581
Pinales Pinaceae Pinus albicaulis#FJ899566
Pinales Pinaceae Pinus armandii#FJ899568
Pinales Pinaceae Pinus sibirica#FJ899558
Pinales Pinaceae Pinus cembra#FJ899574
Pinales Pinaceae Pinus monticola#FJ899580
Pinales Pinaceae Pinus peuce#FJ899582
Pinales Pinaceae Pinus squamata#FJ899559
Pinales Pinaceae Pinus strobus#FJ899560
Pinales Pinaceae Pinus ayacahuite#FJ899570
Pinales Pinaceae Pinus flexilis#FJ899576
Ephedrales Ephedraceae Ephedra sinica#VDAO
Ephedrales Ephedraceae Ephedra equisetina#NC 011954
Ephedrales Ephedraceae Ephedra foeminea#NC 029347
Welwitschiales Welwitschiaceae Welwitschia mirabilis#NC 010654
Gnetales Gnetaceae Gnetum gnemon#NC 026301
Gnetales Gnetaceae Gnetum ula#NC 028734
Gnetales Gnetaceae Gnetum parvifolium#NC 011942
Gnetales Gnetaceae Gnetum montanum#GTHK
Araucariales Araucariaceae Araucaria rulei#XTZO
Araucariales Araucariaceae Araucaria heterophylla#NC 026450
Araucariales Araucariaceae Wollemia nobilis#NC 027235
Araucariales Araucariaceae Agathis australis#JN
Araucariales Araucariaceae Agathis dammara#NC 023119
Araucariales Araucariaceae Araucaria sp.#ACWS
Araucariales Araucariaceae Agathis robusta#MIXZ
Araucariales Podocarpaceae Halocarpus kirkii#JN
Araucariales Podocarpaceae Halocarpus bidwillii#OWFC
Araucariales Podocarpaceae Sundacarpus amarus#KLGF
Araucariales Podocarpaceae Prumnopitys andina#EGLZ
Araucariales Podocarpaceae Phyllocladus hypophyllus#JRNA
Araucariales Podocarpaceae Pherosphaera fitzgeraldii#BBDD
Araucariales Podocarpaceae Parasitaxus ustus#JZVE
Araucariales Podocarpaceae Lagarostrobos franklinii#ZQWM
Araucariales Podocarpaceae Manoao colensoi#CDFR
Araucariales Podocarpaceae Saxegothaea conspicua#QCGM
Araucariales Podocarpaceae Microcachrys tetragona#MHGD
Araucariales Podocarpaceae Acmopyle pancheri#HILW
Araucariales Podocarpaceae Dacrycarpus compactus#FMWZ
Araucariales Podocarpaceae Dacrydium balansae#IZGN
Araucariales Podocarpaceae Falcatifolium taxoides#UAUU
Araucariales Podocarpaceae Nageia nagi#NC 023120
Araucariales Podocarpaceae Retrophyllum minus#VGSX
Araucariales Podocarpaceae Retrophyllum piresii#NC 024827
Araucariales Podocarpaceae Podocarpus rubens#XLGK
Araucariales Podocarpaceae Podocarpus totara#JN
Araucariales Podocarpaceae Podocarpus lambertii#NC 023805
Araucariales Podocarpaceae Podocarpus coriaceus#SCEB
Cupressales Sciadopityaceae Sciadopitys verticillata#NC 029734
Cupressales Taxaceae Cephalotaxus oliveri#NC 021110
Cupressales Taxaceae Cephalotaxus harringtonii var. wilsoniana#NC 016063
Cupressales Taxaceae Cephalotaxus harringtonia#NVGZ
Cupressales Taxaceae Amentotaxus formosana#NC 024945
Cupressales Taxaceae Amentotaxus argotaenia#NC 027581
Cupressales Taxaceae Torreya grandis var. fargesii#NC 029398

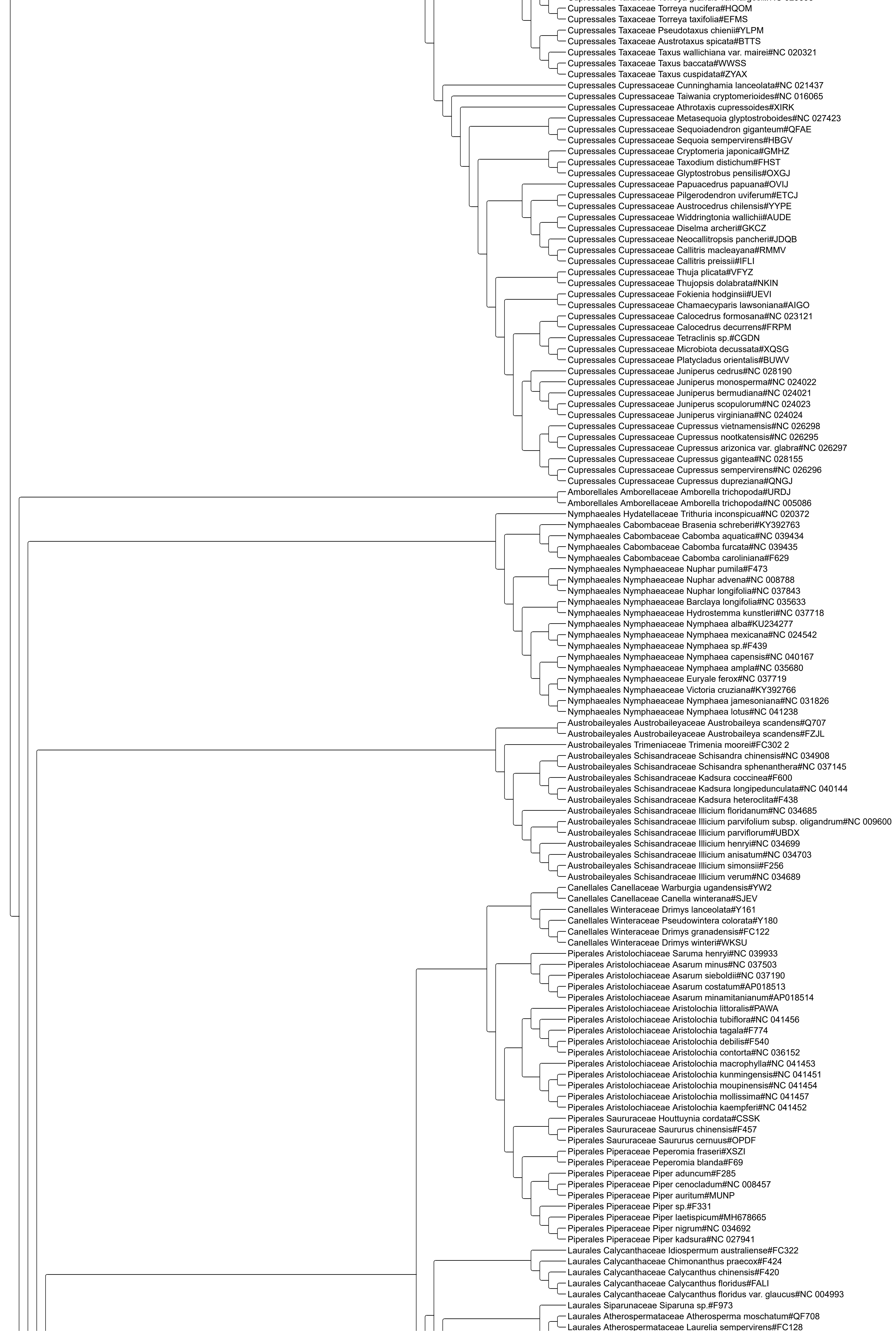

Cupressales Taxaceae Torreya nucifera#HQOM
Cupressales Taxaceae Torreya taxifolia#EFMS
Cupressales Taxaceae Pseudotaxus chienii#YLPM
Cupressales Taxaceae Austrotaxus spicata#BTTS
Cupressales Taxaceae Taxus wallichiana var. mairei#NC 020321
Cupressales Taxaceae Taxus baccata#WWSS
Cupressales Taxaceae Taxus cuspidata#ZYAX
Cupressales Cupressaceae Cunninghamia lanceolata#NC 021437
Cupressales Cupressaceae Taiwania cryptomerioides#NC 016065
Cupressales Cupressaceae Athrotaxis cupressoides#XIRK
Cupressales Cupressaceae Metasequoia glyptostroboides#NC 027423
Cupressales Cupressaceae Sequoiadendron giganteum#QFAE
Cupressales Cupressaceae Sequoia sempervirens#HBGV
Cupressales Cupressaceae Cryptomeria japonica#GMHZ
Cupressales Cupressaceae Taxodium distichum#FHST
Cupressales Cupressaceae Glyptostrobus pensilis#OXGJ
Cupressales Cupressaceae Papuacedrus papuana#OVIJ
Cupressales Cupressaceae Pilgerodendron uviferum#ETCJ
Cupressales Cupressaceae Austrocedrus chilensis#YYPE
Cupressales Cupressaceae Widdringtonia wallichii#AUDE
Cupressales Cupressaceae Diselma archeri#GKCZ
Cupressales Cupressaceae Neocallitropsis pancheri#JDQB
Cupressales Cupressaceae Callitris macleayana#RMMV
Cupressales Cupressaceae Callitris preissii#IFLI
Cupressales Cupressaceae Thuja plicata#VFYZ
Cupressales Cupressaceae Thujopsis dolabrata#NKIN
Cupressales Cupressaceae Fokienia hodginsii#UEVI
Cupressales Cupressaceae Chamaecyparis lawsoniana#AIGO
Cupressales Cupressaceae Calocedrus formosana#NC 023121
Cupressales Cupressaceae Calocedrus decurrens#FRPM
Cupressales Cupressaceae Tetraclinis sp.#CGDN
Cupressales Cupressaceae Microbiota decussata#XQSG
Cupressales Cupressaceae Platycladus orientalis#BUWV
Cupressales Cupressaceae Juniperus cedrus#NC 028190
Cupressales Cupressaceae Juniperus monosperma#NC 024022
Cupressales Cupressaceae Juniperus bermudiana#NC 024021
Cupressales Cupressaceae Juniperus scopulorum#NC 024023
Cupressales Cupressaceae Juniperus virginiana#NC 024024
Cupressales Cupressaceae Cupressus vietnamensis#NC 026298
Cupressales Cupressaceae Cupressus nootkatensis#NC 026295
Cupressales Cupressaceae Cupressus arizonica var. glabra#NC 026297
Cupressales Cupressaceae Cupressus gigantea#NC 028155
Cupressales Cupressaceae Cupressus sempervirens#NC 026296
Cupressales Cupressaceae Cupressus dupreziana#QNGJ
Amborellales Amborellaceae Amborella trichopoda#URDJ
Amborellales Amborellaceae Amborella trichopoda#NC 005086
Nymphaeales Hydatellaceae Trithuria inconspicua#NC 020372
Nymphaeales Cabombaceae Brasenia schreberi#KY392763
Nymphaeales Cabombaceae Cabomba aquatica#NC 039434
Nymphaeales Cabombaceae Cabomba furcata#NC 039435
Nymphaeales Cabombaceae Cabomba caroliniana#F629
Nymphaeales Nymphaeaceae Nuphar pumila#F473
Nymphaeales Nymphaeaceae Nuphar advena#NC 008788
Nymphaeales Nymphaeaceae Nuphar longifolia#NC 037843
Nymphaeales Nymphaeaceae Barclaya longifolia#NC 035633
Nymphaeales Nymphaeaceae Hydrostemma kunstleri#NC 037718
Nymphaeales Nymphaeaceae Nymphaea alba#KU234277
Nymphaeales Nymphaeaceae Nymphaea mexicana#NC 024542
Nymphaeales Nymphaeaceae Nymphaea sp.#F439
Nymphaeales Nymphaeaceae Nymphaea capensis#NC 040167
Nymphaeales Nymphaeaceae Nymphaea ampla#NC 035680
Nymphaeales Nymphaeaceae Euryale ferox#NC 037719
Nymphaeales Nymphaeaceae Victoria cruziana#KY392766
Nymphaeales Nymphaeaceae Nymphaea jamesoniana#NC 031826
Nymphaeales Nymphaeaceae Nymphaea lotus#NC 041238
Austrobaileyales Austrobaileyaceae Austrobaileya scandens#Q707
Austrobaileyales Austrobaileyaceae Austrobaileya scandens#FZJL
Austrobaileyales Trimeniaceae Trimenia moorei#FC302 2
Austrobaileyales Schisandraceae Schisandra chinensis#NC 034908
Austrobaileyales Schisandraceae Schisandra sphenanthera#NC 037145
Austrobaileyales Schisandraceae Kadsura coccinea#F600
Austrobaileyales Schisandraceae Kadsura longipedunculata#NC 040144
Austrobaileyales Schisandraceae Kadsura heteroclita#F438
Austrobaileyales Schisandraceae Illicium floridanum#NC 034685
Austrobaileyales Schisandraceae Illicium parvifolium subsp. oligandrum#NC 009600
Austrobaileyales Schisandraceae Illicium parviflorum#UBDX
Austrobaileyales Schisandraceae Illicium henryi#NC 034699
Austrobaileyales Schisandraceae Illicium anisatum#NC 034703
Austrobaileyales Schisandraceae Illicium simonsii#F256
Austrobaileyales Schisandraceae Illicium verum#NC 034689
Canellales Canellaceae Warburgia ugandensis#YW2
Canellales Canellaceae Canella winterana#SJEV
Canellales Winteraceae Drimys lanceolata#Y161
Canellales Winteraceae Pseudowintera colorata#Y180
Canellales Winteraceae Drimys granadensis#FC122
Canellales Winteraceae Drimys winteri#WKSU
Piperales Aristolochiaceae Saruma henryi#NC 039933
Piperales Aristolochiaceae Asarum minus#NC 037503
Piperales Aristolochiaceae Asarum sieboldii#NC 037190
Piperales Aristolochiaceae Asarum costatum#AP018513
Piperales Aristolochiaceae Asarum minamitanianum#AP018514
Piperales Aristolochiaceae Aristolochia littoralis#PAWA
Piperales Aristolochiaceae Aristolochia tubiflora#NC 041456
Piperales Aristolochiaceae Aristolochia tagala#F774
Piperales Aristolochiaceae Aristolochia debilis#F540
Piperales Aristolochiaceae Aristolochia contorta#NC 036152
Piperales Aristolochiaceae Aristolochia macrophylla#NC 041453
Piperales Aristolochiaceae Aristolochia kunmingensis#NC 041451
Piperales Aristolochiaceae Aristolochia moupinensis#NC 041454
Piperales Aristolochiaceae Aristolochia mollissima#NC 041457
Piperales Aristolochiaceae Aristolochia kaempferi#NC 041452
Piperales Saururaceae Houttuynia cordata#CSSK
Piperales Saururaceae Saururus chinensis#F457
Piperales Saururaceae Saururus cernuus#OPDF
Piperales Piperaceae Peperomia fraseri#XSZI
Piperales Piperaceae Peperomia blanda#F69
Piperales Piperaceae Piper aduncum#F285
Piperales Piperaceae Piper cenocladum#NC 008457
Piperales Piperaceae Piper auritum#MUNP
Piperales Piperaceae Piper sp.#F331
Piperales Piperaceae Piper laetispicum#MH678665
Piperales Piperaceae Piper nigrum#NC 034692
Piperales Piperaceae Piper kadsura#NC 027941
Laurales Calycanthaceae Idiospermum australiense#FC322
Laurales Calycanthaceae Chimonanthus praecox#F424
Laurales Calycanthaceae Calycanthus chinensis#F420
Laurales Calycanthaceae Calycanthus floridus#FALI
Laurales Calycanthaceae Calycanthus floridus var. glaucus#NC 004993
Laurales Siparunaceae Siparuna sp.#F973
Laurales Atherospermataceae Atherosperma moschatum#QF708
Laurales Atherospermataceae Laurelia sempervirens#FC128

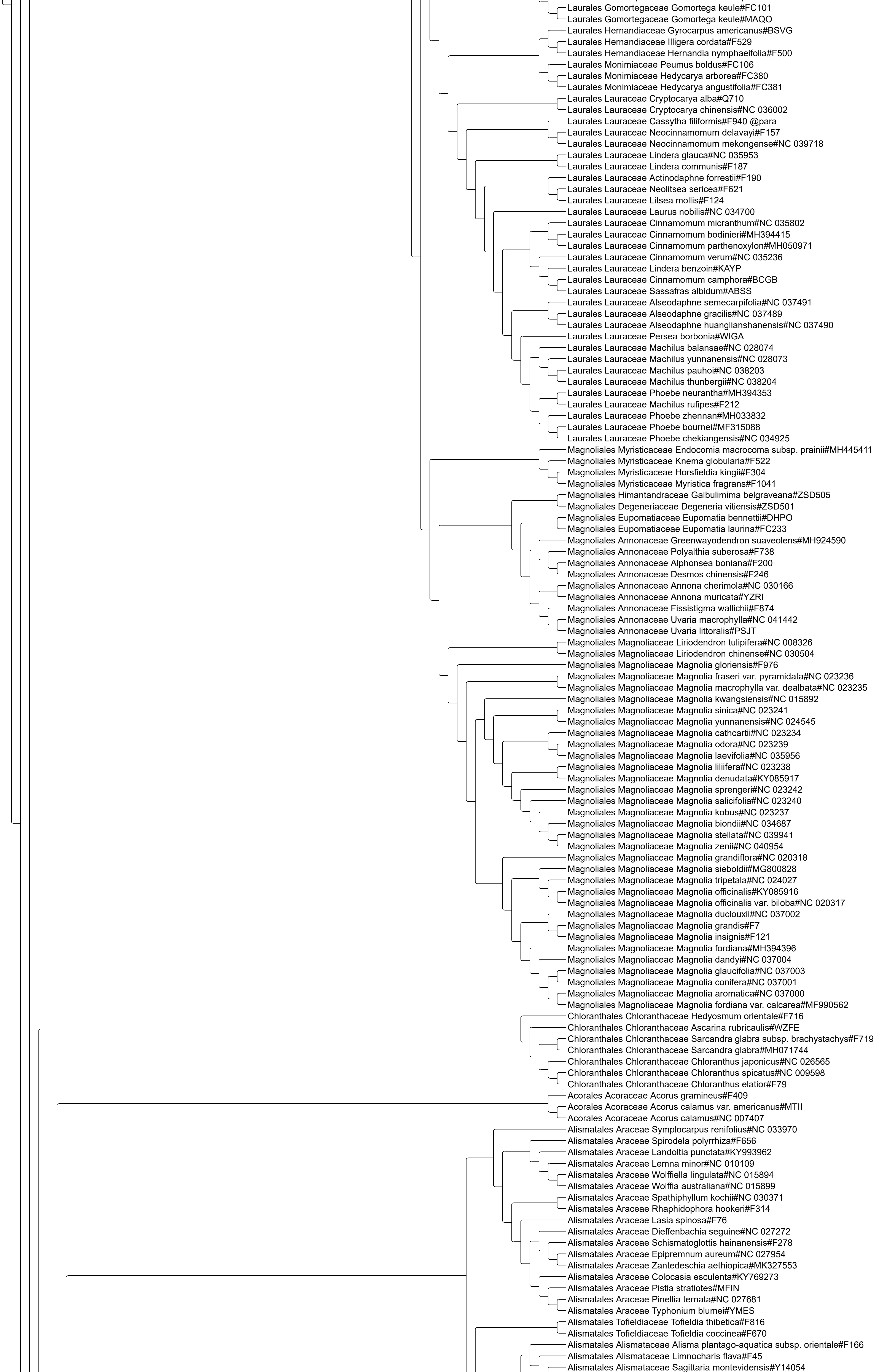

Laurales Gomortegaceae Gomortega keule#FC101
Laurales Gomortegaceae Gomortega keule#MAQO
Laurales Hernandiaceae Gyrocarpus americanus#BSVG
Laurales Hernandiaceae Illigera cordata#F529
Laurales Hernandiaceae Hernandia nymphaeifolia#F500
Laurales Monimiaceae Peumus boldus#FC106
Laurales Monimiaceae Hedycarya arborea#FC380
Laurales Monimiaceae Hedycarya angustifolia#FC381
Laurales Lauraceae Cryptocarya alba#Q710
Laurales Lauraceae Cryptocarya chinensis#NC 036002
Laurales Lauraceae Cassytha filiformis#F940 @para
Laurales Lauraceae Neocinnamomum delavayi#F157
Laurales Lauraceae Neocinnamomum mekongense#NC 039718
Laurales Lauraceae Lindera glauca#NC 035953
Laurales Lauraceae Lindera communis#F187
Laurales Lauraceae Actinodaphne forrestii#F190
Laurales Lauraceae Neolitsea sericea#F621
Laurales Lauraceae Litsea mollis#F124
Laurales Lauraceae Laurus nobilis#NC 034700
Laurales Lauraceae Cinnamomum micranthum#NC 035802
Laurales Lauraceae Cinnamomum bodinieri#MH394415
Laurales Lauraceae Cinnamomum parthenoxylon#MH050971
Laurales Lauraceae Cinnamomum verum#NC 035236
Laurales Lauraceae Lindera benzoin#KAYP
Laurales Lauraceae Cinnamomum camphora#BCGB
Laurales Lauraceae Sassafras albidum#ABSS
Laurales Lauraceae Alseodaphne semecarpifolia#NC 037491
Laurales Lauraceae Alseodaphne gracilis#NC 037489
Laurales Lauraceae Alseodaphne huanglianshanensis#NC 037490
Laurales Lauraceae Persea borbonia#WIGA
Laurales Lauraceae Machilus balansae#NC 028074
Laurales Lauraceae Machilus yunnanensis#NC 028073
Laurales Lauraceae Machilus pauhoi#NC 038203
Laurales Lauraceae Machilus thunbergii#NC 038204
Laurales Lauraceae Phoebe neurantha#MH394353
Laurales Lauraceae Machilus rufipes#F212
Laurales Lauraceae Phoebe zhennan#MH033832
Laurales Lauraceae Phoebe bournei#MF315088
Laurales Lauraceae Phoebe chekiangensis#NC 034925
Magnoliales Myristicaceae Endocomia macrocoma subsp. prainii#MH445411
Magnoliales Myristicaceae Knema globularia#F522
Magnoliales Myristicaceae Horsfieldia kingii#F304
Magnoliales Myristicaceae Myristica fragrans#F1041
Magnoliales Himantandraceae Galbulimima belgraveana#ZSD505
Magnoliales Degeneriaceae Degeneria vitiensis#ZSD501
Magnoliales Eupomatiaceae Eupomatia bennettii#DHPO
Magnoliales Eupomatiaceae Eupomatia laurina#FC233
Magnoliales Annonaceae Greenwayodendron suaveolens#MH924590
Magnoliales Annonaceae Polyalthia suberosa#F738
Magnoliales Annonaceae Alphonsea boniana#F200
Magnoliales Annonaceae Desmos chinensis#F246
Magnoliales Annonaceae Annona cherimola#NC 030166
Magnoliales Annonaceae Annona muricata#YZRI
Magnoliales Annonaceae Fissistigma wallichii#F874
Magnoliales Annonaceae Uvaria macrophylla#NC 041442
Magnoliales Annonaceae Uvaria littoralis#PSJT
Magnoliales Magnoliaceae Liriodendron tulipifera#NC 008326
Magnoliales Magnoliaceae Liriodendron chinense#NC 030504
Magnoliales Magnoliaceae Magnolia gloriensis#F976
Magnoliales Magnoliaceae Magnolia fraseri var. pyramidata#NC 023236
Magnoliales Magnoliaceae Magnolia macrophylla var. dealbata#NC 023235
Magnoliales Magnoliaceae Magnolia kwangsiensis#NC 015892
Magnoliales Magnoliaceae Magnolia sinica#NC 023241
Magnoliales Magnoliaceae Magnolia yunnanensis#NC 024545
Magnoliales Magnoliaceae Magnolia cathcartii#NC 023234
Magnoliales Magnoliaceae Magnolia odora#NC 023239
Magnoliales Magnoliaceae Magnolia laevifolia#NC 035956
Magnoliales Magnoliaceae Magnolia liliifera#NC 023238
Magnoliales Magnoliaceae Magnolia denudata#KY085917
Magnoliales Magnoliaceae Magnolia sprengeri#NC 023242
Magnoliales Magnoliaceae Magnolia salicifolia#NC 023240
Magnoliales Magnoliaceae Magnolia kobus#NC 023237
Magnoliales Magnoliaceae Magnolia biondii#NC 034687
Magnoliales Magnoliaceae Magnolia stellata#NC 039941
Magnoliales Magnoliaceae Magnolia zenii#NC 040954
Magnoliales Magnoliaceae Magnolia grandiflora#NC 020318
Magnoliales Magnoliaceae Magnolia sieboldii#MG800828
Magnoliales Magnoliaceae Magnolia tripetala#NC 024027
Magnoliales Magnoliaceae Magnolia officinalis#KY085916
Magnoliales Magnoliaceae Magnolia officinalis var. biloba#NC 020317
Magnoliales Magnoliaceae Magnolia duclouxii#NC 037002
Magnoliales Magnoliaceae Magnolia grandis#F7
Magnoliales Magnoliaceae Magnolia insignis#F121
Magnoliales Magnoliaceae Magnolia fordiana#MH394396
Magnoliales Magnoliaceae Magnolia dandyi#NC 037004
Magnoliales Magnoliaceae Magnolia glaucifolia#NC 037003
Magnoliales Magnoliaceae Magnolia conifera#NC 037001
Magnoliales Magnoliaceae Magnolia aromatica#NC 037000
Magnoliales Magnoliaceae Magnolia fordiana var. calcarea#MF990562
Chloranthales Chloranthaceae Hedyosmum orientale#F716
Chloranthales Chloranthaceae Ascarina rubricaulis#WZFE
Chloranthales Chloranthaceae Sarcandra glabra subsp. brachystachys#F719
Chloranthales Chloranthaceae Sarcandra glabra#MH071744
Chloranthales Chloranthaceae Chloranthus japonicus#NC 026565
Chloranthales Chloranthaceae Chloranthus spicatus#NC 009598
Chloranthales Chloranthaceae Chloranthus elatior#F79
Acorales Acoraceae Acorus gramineus#F409
Acorales Acoraceae Acorus calamus var. americanus#MTII
Acorales Acoraceae Acorus calamus#NC 007407
Alismatales Araceae Symplocarpus renifolius#NC 033970
Alismatales Araceae Spirodela polyrrhiza#F656
Alismatales Araceae Landoltia punctata#KY993962
Alismatales Araceae Lemna minor#NC 010109
Alismatales Araceae Wolffiella lingulata#NC 015894
Alismatales Araceae Wolffia australiana#NC 015899
Alismatales Araceae Spathiphyllum kochii#NC 030371
Alismatales Araceae Rhaphidophora hookeri#F314
Alismatales Araceae Lasia spinosa#F76
Alismatales Araceae Dieffenbachia seguine#NC 027272
Alismatales Araceae Schismatoglottis hainanensis#F278
Alismatales Araceae Epipremnum aureum#NC 027954
Alismatales Araceae Zantedeschia aethiopica#MK327553
Alismatales Araceae Colocasia esculenta#KY769273
Alismatales Araceae Pistia stratiotes#MFIN
Alismatales Araceae Pinellia ternata#NC 027681
Alismatales Araceae Typhonium blumei#YMES
Alismatales Tofieldiaceae Tofieldia thibetica#F816
Alismatales Tofieldiaceae Tofieldia coccinea#F670
Alismatales Alismataceae Alisma plantago-aquatica subsp. orientale#F166
Alismatales Alismataceae Limnocharis flava#F45
Alismatales Alismataceae Sagittaria montevidensis#Y14054

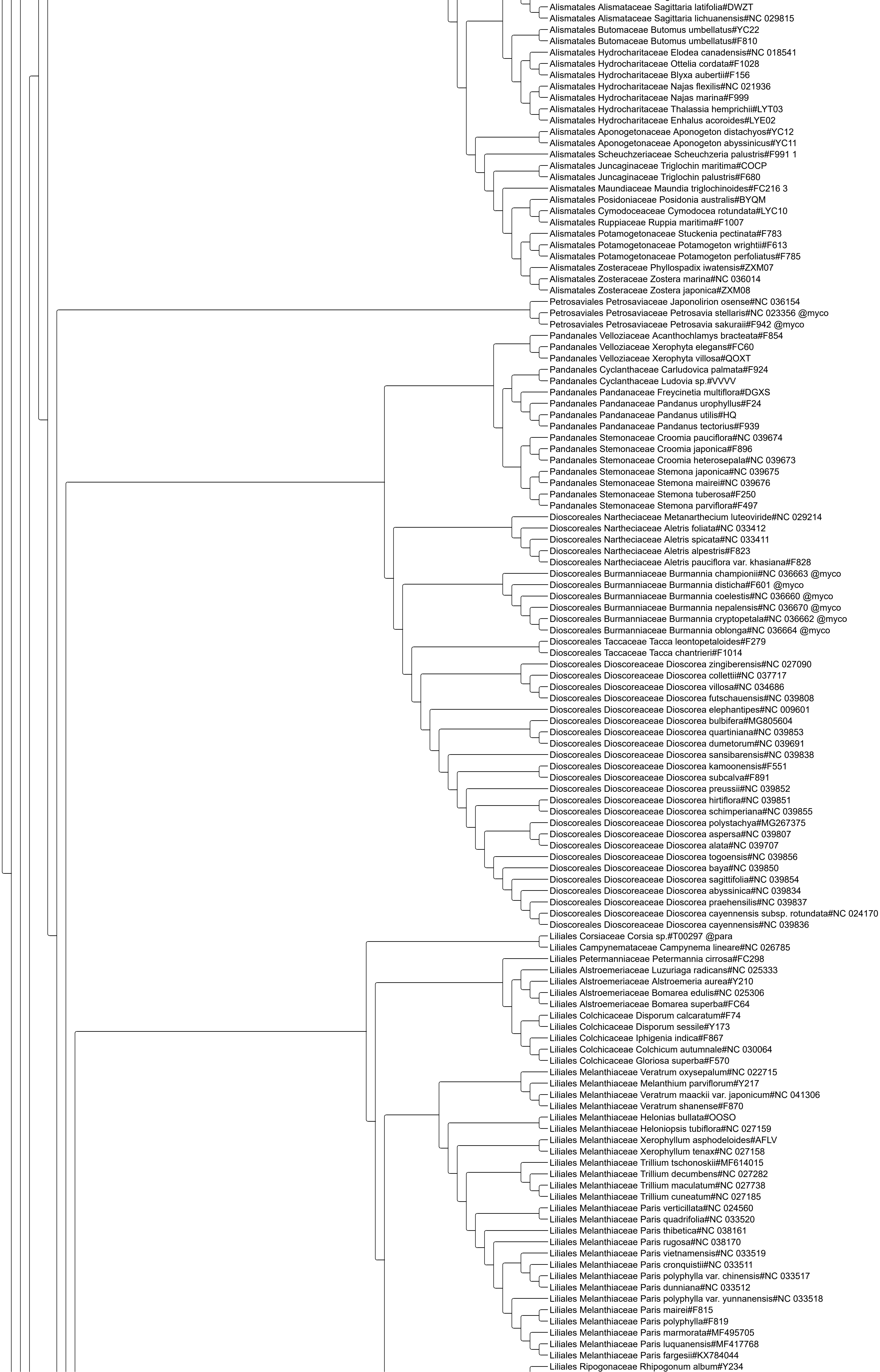
Alismatales Alismataceae Sagittaria latifolia#DWZT
Alismatales Alismataceae Sagittaria lichuanensis#NC 029815
Alismatales Butomaceae Butomus umbellatus#YC22
Alismatales Butomaceae Butomus umbellatus#F810
Alismatales Hydrocharitaceae Elodea canadensis#NC 018541
Alismatales Hydrocharitaceae Ottelia cordata#F1028
Alismatales Hydrocharitaceae Blyxa aubertii#F156
Alismatales Hydrocharitaceae Najas flexilis#NC 021936
Alismatales Hydrocharitaceae Najas marina#F999
Alismatales Hydrocharitaceae Thalassia hemprichii#LYT03
Alismatales Hydrocharitaceae Enhalus acoroides#LYE02
Alismatales Aponogetonaceae Aponogeton distachyos#YC12
Alismatales Aponogetonaceae Aponogeton abyssinicus#YC11
Alismatales Scheuchzeriaceae Scheuchzeria palustris#F991 1
Alismatales Juncaginaceae Triglochin maritima#COCP
Alismatales Juncaginaceae Triglochin palustris#F680
Alismatales Maundiaceae Maundia triglochinoides#FC216 3
Alismatales Posidoniaceae Posidonia australis#BYQM
Alismatales Cymodoceaceae Cymodocea rotundata#LYC10
Alismatales Ruppiaceae Ruppia maritima#F1007
Alismatales Potamogetonaceae Stuckenia pectinata#F783
Alismatales Potamogetonaceae Potamogeton wrightii#F613
Alismatales Potamogetonaceae Potamogeton perfoliatus#F785
Alismatales Zosteraceae Phyllospadix iwatensis#ZXM07
Alismatales Zosteraceae Zostera marina#NC 036014
Alismatales Zosteraceae Zostera japonica#ZXM08
Petrosaviales Petrosaviaceae Japonolirion osense#NC 036154
Petrosaviales Petrosaviaceae Petrosavia stellaris#NC 023356 @myco
Petrosaviales Petrosaviaceae Petrosavia sakuraii#F942 @myco
Pandanales Velloziaceae Acanthochlamys bracteata#F854
Pandanales Velloziaceae Xerophyta elegans#FC60
Pandanales Velloziaceae Xerophyta villosa#QOXT
Pandanales Cyclanthaceae Carludovica palmata#F924
Pandanales Cyclanthaceae Ludovia sp.#VVVV
Pandanales Pandanaceae Freycinetia multiflora#DGXS
Pandanales Pandanaceae Pandanus urophyllus#F24
Pandanales Pandanaceae Pandanus utilis#HQ
Pandanales Pandanaceae Pandanus tectorius#F939
Pandanales Stemonaceae Croomia pauciflora#NC 039674
Pandanales Stemonaceae Croomia japonica#F896
Pandanales Stemonaceae Croomia heterosepala#NC 039673
Pandanales Stemonaceae Stemona japonica#NC 039675
Pandanales Stemonaceae Stemona mairei#NC 039676
Pandanales Stemonaceae Stemona tuberosa#F250
Pandanales Stemonaceae Stemona parviflora#F497
Dioscoreales Nartheciaceae Metanarthecium luteoviride#NC 029214
Dioscoreales Nartheciaceae Aletris foliata#NC 033412
Dioscoreales Nartheciaceae Aletris spicata#NC 033411
Dioscoreales Nartheciaceae Aletris alpestris#F823
Dioscoreales Nartheciaceae Aletris pauciflora var. khasiana#F828
Dioscoreales Burmanniaceae Burmannia championii#NC 036663 @myco
Dioscoreales Burmanniaceae Burmannia disticha#F601 @myco
Dioscoreales Burmanniaceae Burmannia coelestis#NC 036660 @myco
Dioscoreales Burmanniaceae Burmannia nepalensis#NC 036670 @myco
Dioscoreales Burmanniaceae Burmannia cryptopetala#NC 036662 @myco
Dioscoreales Burmanniaceae Burmannia oblonga#NC 036664 @myco
Dioscoreales Taccaceae Tacca leontopetaloides#F279
Dioscoreales Taccaceae Tacca chantrieri#F1014
Dioscoreales Dioscoreaceae Dioscorea zingiberensis#NC 027090
Dioscoreales Dioscoreaceae Dioscorea collettii#NC 037717
Dioscoreales Dioscoreaceae Dioscorea villosa#NC 034686
Dioscoreales Dioscoreaceae Dioscorea futschauensis#NC 039808
Dioscoreales Dioscoreaceae Dioscorea elephantipes#NC 009601
Dioscoreales Dioscoreaceae Dioscorea bulbifera#MG805604
Dioscoreales Dioscoreaceae Dioscorea quartiniana#NC 039853
Dioscoreales Dioscoreaceae Dioscorea dumetorum#NC 039691
Dioscoreales Dioscoreaceae Dioscorea sansibarensis#NC 039838
Dioscoreales Dioscoreaceae Dioscorea kamoonensis#F551
Dioscoreales Dioscoreaceae Dioscorea subcalva#F891
Dioscoreales Dioscoreaceae Dioscorea preussii#NC 039852
Dioscoreales Dioscoreaceae Dioscorea hirtiflora#NC 039851
Dioscoreales Dioscoreaceae Dioscorea schimperiana#NC 039855
Dioscoreales Dioscoreaceae Dioscorea polystachya#MG267375
Dioscoreales Dioscoreaceae Dioscorea aspersa#NC 039807
Dioscoreales Dioscoreaceae Dioscorea alata#NC 039707
Dioscoreales Dioscoreaceae Dioscorea togoensis#NC 039856
Dioscoreales Dioscoreaceae Dioscorea baya#NC 039850
Dioscoreales Dioscoreaceae Dioscorea sagittifolia#NC 039854
Dioscoreales Dioscoreaceae Dioscorea abyssinica#NC 039834
Dioscoreales Dioscoreaceae Dioscorea praehensilis#NC 039837
Dioscoreales Dioscoreaceae Dioscorea cayennensis subsp. rotundata#NC 024170
Dioscoreales Dioscoreaceae Dioscorea cayennensis#NC 039836
Liliales Corsiaceae Corsia sp.#T00297 @para
Liliales Campynemataceae Campynema lineare#NC 026785
Liliales Petermanniaceae Petermannia cirrosa#FC298
Liliales Alstroemeriaceae Luzuriaga radicans#NC 025333
Liliales Alstroemeriaceae Alstroemeria aurea#Y210
Liliales Alstroemeriaceae Bomarea edulis#NC 025306
Liliales Alstroemeriaceae Bomarea superba#FC64
Liliales Colchicaceae Disporum calcaratum#F74
Liliales Colchicaceae Disporum sessile#Y173
Liliales Colchicaceae Iphigenia indica#F867
Liliales Colchicaceae Colchicum autumnale#NC 030064
Liliales Colchicaceae Gloriosa superba#F570
Liliales Melanthiaceae Veratrum oxysepalum#NC 022715
Liliales Melanthiaceae Melanthium parviflorum#Y217
Liliales Melanthiaceae Veratrum maackii var. japonicum#NC 041306
Liliales Melanthiaceae Veratrum shanense#F870
Liliales Melanthiaceae Helonias bullata#OOSO
Liliales Melanthiaceae Heloniopsis tubiflora#NC 027159
Liliales Melanthiaceae Xerophyllum asphodeloides#AFLV
Liliales Melanthiaceae Xerophyllum tenax#NC 027158
Liliales Melanthiaceae Trillium tschonoskii#MF614015
Liliales Melanthiaceae Trillium decumbens#NC 027282
Liliales Melanthiaceae Trillium maculatum#NC 027738
Liliales Melanthiaceae Trillium cuneatum#NC 027185
Liliales Melanthiaceae Paris verticillata#NC 024560
Liliales Melanthiaceae Paris quadrifolia#NC 033520
Liliales Melanthiaceae Paris thibetica#NC 038161
Liliales Melanthiaceae Paris rugosa#NC 038170
Liliales Melanthiaceae Paris vietnamensis#NC 033519
Liliales Melanthiaceae Paris cronquistii#NC 033511
Liliales Melanthiaceae Paris polyphylla var. chinensis#NC 033517
Liliales Melanthiaceae Paris dunniana#NC 033512
Liliales Melanthiaceae Paris polyphylla var. yunnanensis#NC 033518
Liliales Melanthiaceae Paris mairei#F815
Liliales Melanthiaceae Paris polyphylla#F819
Liliales Melanthiaceae Paris marmorata#MF495705
Liliales Melanthiaceae Paris luquanensis#MF417768
Liliales Melanthiaceae Paris fargesii#KX784044
Liliales Ripogonaceae Rhipogonum album#Y234

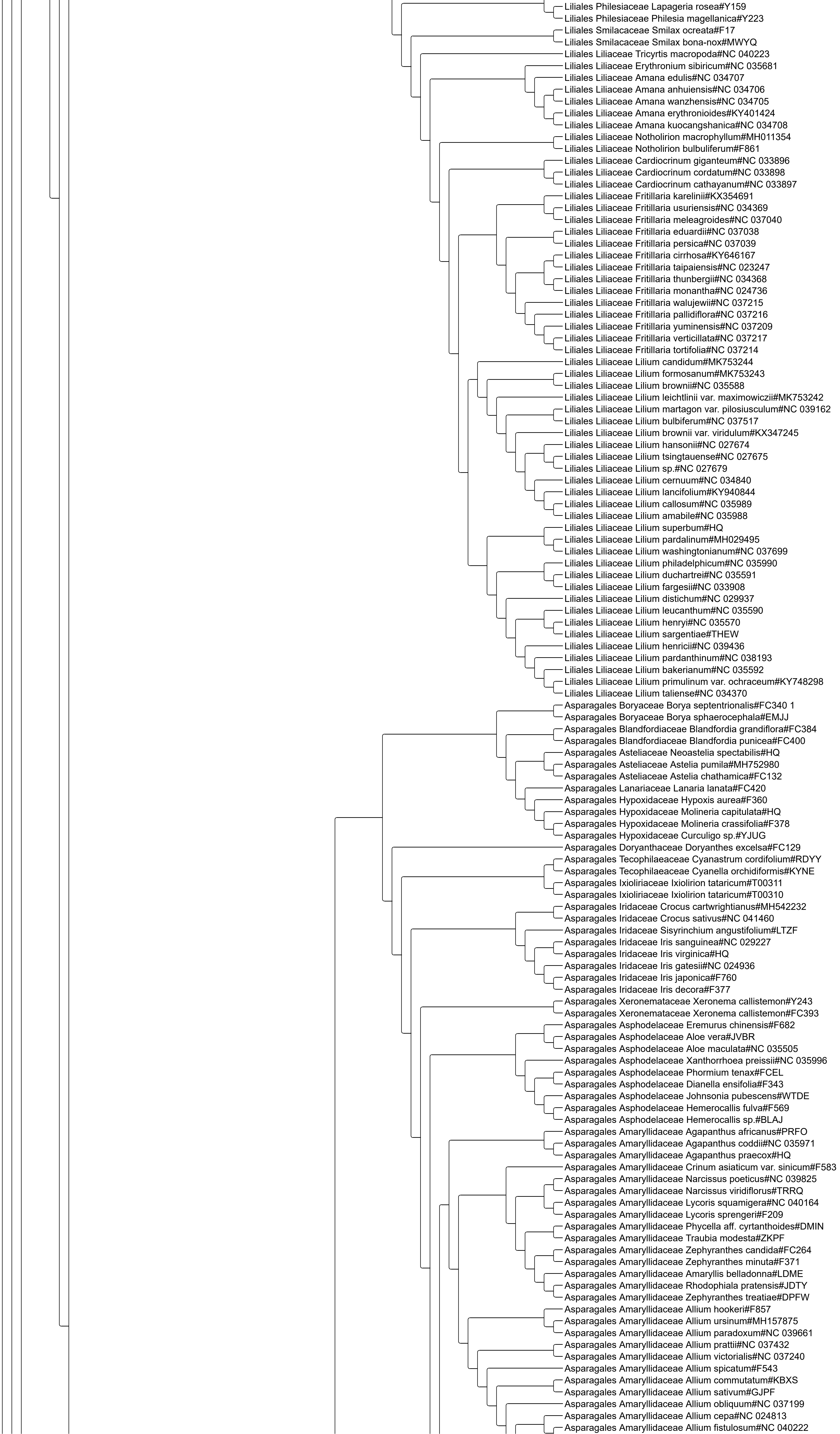
Liliales Philesiaceae Lapageria rosea#Y159
Liliales Philesiaceae Philesia magellanica#Y223
Liliales Smilacaceae Smilax ocreata#F17
Liliales Smilacaceae Smilax bona-nox#MWYQ
Liliales Liliaceae Tricyrtis macropoda#NC 040223
Liliales Liliaceae Erythronium sibiricum#NC 035681
Liliales Liliaceae Amana edulis#NC 034707
Liliales Liliaceae Amana anhuiensis#NC 034706
Liliales Liliaceae Amana wanzhensis#NC 034705
Liliales Liliaceae Amana erythronioides#KY401424
Liliales Liliaceae Amana kuocangshanica#NC 034708
Liliales Liliaceae Notholirion macrophyllum#MH011354
Liliales Liliaceae Notholirion bulbuliferum#F861
Liliales Liliaceae Cardiocrinum giganteum#NC 033896
Liliales Liliaceae Cardiocrinum cordatum#NC 033898
Liliales Liliaceae Cardiocrinum cathayanum#NC 033897
Liliales Liliaceae Fritillaria karelinii#KX354691
Liliales Liliaceae Fritillaria usuriensis#NC 034369
Liliales Liliaceae Fritillaria meleagroides#NC 037040
Liliales Liliaceae Fritillaria eduardii#NC 037038
Liliales Liliaceae Fritillaria persica#NC 037039
Liliales Liliaceae Fritillaria cirrhosa#KY646167
Liliales Liliaceae Fritillaria taipaiensis#NC 023247
Liliales Liliaceae Fritillaria thunbergii#NC 034368
Liliales Liliaceae Fritillaria monantha#NC 024736
Liliales Liliaceae Fritillaria walujewii#NC 037215
Liliales Liliaceae Fritillaria pallidiflora#NC 037216
Liliales Liliaceae Fritillaria yuminensis#NC 037209
Liliales Liliaceae Fritillaria verticillata#NC 037217
Liliales Liliaceae Fritillaria tortifolia#NC 037214
Liliales Liliaceae Lilium candidum#MK753244
Liliales Liliaceae Lilium formosanum#MK753243
Liliales Liliaceae Lilium brownii#NC 035588
Liliales Liliaceae Lilium leichtlinii var. maximowiczii#MK753242
Liliales Liliaceae Lilium martagon var. pilosiusculum#NC 039162
Liliales Liliaceae Lilium bulbiferum#NC 037517
Liliales Liliaceae Lilium brownii var. viridulum#KX347245
Liliales Liliaceae Lilium hansonii#NC 027674
Liliales Liliaceae Lilium tsingtauense#NC 027675
Liliales Liliaceae Lilium sp.#NC 027679
Liliales Liliaceae Lilium cernuum#NC 034840
Liliales Liliaceae Lilium lancifolium#KY940844
Liliales Liliaceae Lilium callosum#NC 035989
Liliales Liliaceae Lilium amabile#NC 035988
Liliales Liliaceae Lilium superbum#HQ
Liliales Liliaceae Lilium pardalinum#MH029495
Liliales Liliaceae Lilium washingtonianum#NC 037699
Liliales Liliaceae Lilium philadelphicum#NC 035990
Liliales Liliaceae Lilium duchartrei#NC 035591
Liliales Liliaceae Lilium fargesii#NC 033908
Liliales Liliaceae Lilium distichum#NC 029937
Liliales Liliaceae Lilium leucanthum#NC 035590
Liliales Liliaceae Lilium henryi#NC 035570
Liliales Liliaceae Lilium sargentiae#THEW
Liliales Liliaceae Lilium henricii#NC 039436
Liliales Liliaceae Lilium pardanthinum#NC 038193
Liliales Liliaceae Lilium bakerianum#NC 035592
Liliales Liliaceae Lilium primulinum var. ochraceum#KY748298
Liliales Liliaceae Lilium taliense#NC 034370
Asparagales Boryaceae Borya septentrionalis#FC340 1
Asparagales Boryaceae Borya sphaerocephala#EMJJ
Asparagales Blandfordiaceae Blandfordia grandiflora#FC384
Asparagales Blandfordiaceae Blandfordia punicea#FC400
Asparagales Asteliaceae Neoastelia spectabilis#HQ
Asparagales Asteliaceae Astelia pumila#MH752980
Asparagales Asteliaceae Astelia chathamica#FC132
Asparagales Lanariaceae Lanaria lanata#FC420
Asparagales Hypoxidaceae Hypoxis aurea#F360
Asparagales Hypoxidaceae Molineria capitulata#HQ
Asparagales Hypoxidaceae Molineria crassifolia#F378
Asparagales Hypoxidaceae Curculigo sp.#YJUG
Asparagales Doryanthaceae Doryanthes excelsa#FC129
Asparagales Tecophilaeaceae Cyanastrum cordifolium#RDYY
Asparagales Tecophilaeaceae Cyanella orchidiformis#KYNE
Asparagales Ixioliriaceae Ixiolirion tataricum#T00311
Asparagales Ixioliriaceae Ixiolirion tataricum#T00310
Asparagales Iridaceae Crocus cartwrightianus#MH542232
Asparagales Iridaceae Crocus sativus#NC 041460
Asparagales Iridaceae Sisyrinchium angustifolium#LTZF
Asparagales Iridaceae Iris sanguinea#NC 029227
Asparagales Iridaceae Iris virginica#HQ
Asparagales Iridaceae Iris gatesii#NC 024936
Asparagales Iridaceae Iris japonica#F760
Asparagales Iridaceae Iris decora#F377
Asparagales Xeronemataceae Xeronema callistemon#Y243
Asparagales Xeronemataceae Xeronema callistemon#FC393
Asparagales Asphodelaceae Eremurus chinensis#F682
Asparagales Asphodelaceae Aloe vera#JVBR
Asparagales Asphodelaceae Aloe maculata#NC 035505
Asparagales Asphodelaceae Xanthorrhoea preissii#NC 035996
Asparagales Asphodelaceae Phormium tenax#FCEL
Asparagales Asphodelaceae Dianella ensifolia#F343
Asparagales Asphodelaceae Johnsonia pubescens#WTDE
Asparagales Asphodelaceae Hemerocallis fulva#F569
Asparagales Asphodelaceae Hemerocallis sp.#BLAJ
Asparagales Amaryllidaceae Agapanthus africanus#PRFO
Asparagales Amaryllidaceae Agapanthus coddii#NC 035971
Asparagales Amaryllidaceae Agapanthus praecox#HQ
Asparagales Amaryllidaceae Crinum asiaticum var. sinicum#F583
Asparagales Amaryllidaceae Narcissus poeticus#NC 039825
Asparagales Amaryllidaceae Narcissus viridiflorus#TRRQ
Asparagales Amaryllidaceae Lycoris squamigera#NC 040164
Asparagales Amaryllidaceae Lycoris sprengeri#F209
Asparagales Amaryllidaceae Phycella aff. cyrtanthoides#DMIN
Asparagales Amaryllidaceae Traubia modesta#ZKPF
Asparagales Amaryllidaceae Zephyranthes candida#FC264
Asparagales Amaryllidaceae Zephyranthes minuta#F371
Asparagales Amaryllidaceae Amaryllis belladonna#LDME
Asparagales Amaryllidaceae Rhodophiala pratensis#JDTY
Asparagales Amaryllidaceae Zephyranthes treatiae#DPFW
Asparagales Amaryllidaceae Allium hookeri#F857
Asparagales Amaryllidaceae Allium ursinum#MH157875
Asparagales Amaryllidaceae Allium paradoxum#NC 039661
Asparagales Amaryllidaceae Allium prattii#NC 037432
Asparagales Amaryllidaceae Allium victorialis#NC 037240
Asparagales Amaryllidaceae Allium spicatum#F543
Asparagales Amaryllidaceae Allium commutatum#KBXS
Asparagales Amaryllidaceae Allium sativum#GJPF
Asparagales Amaryllidaceae Allium obliquum#NC 037199
Asparagales Amaryllidaceae Allium cepa#NC 024813
Asparagales Amaryllidaceae Allium fistulosum#NC 040222

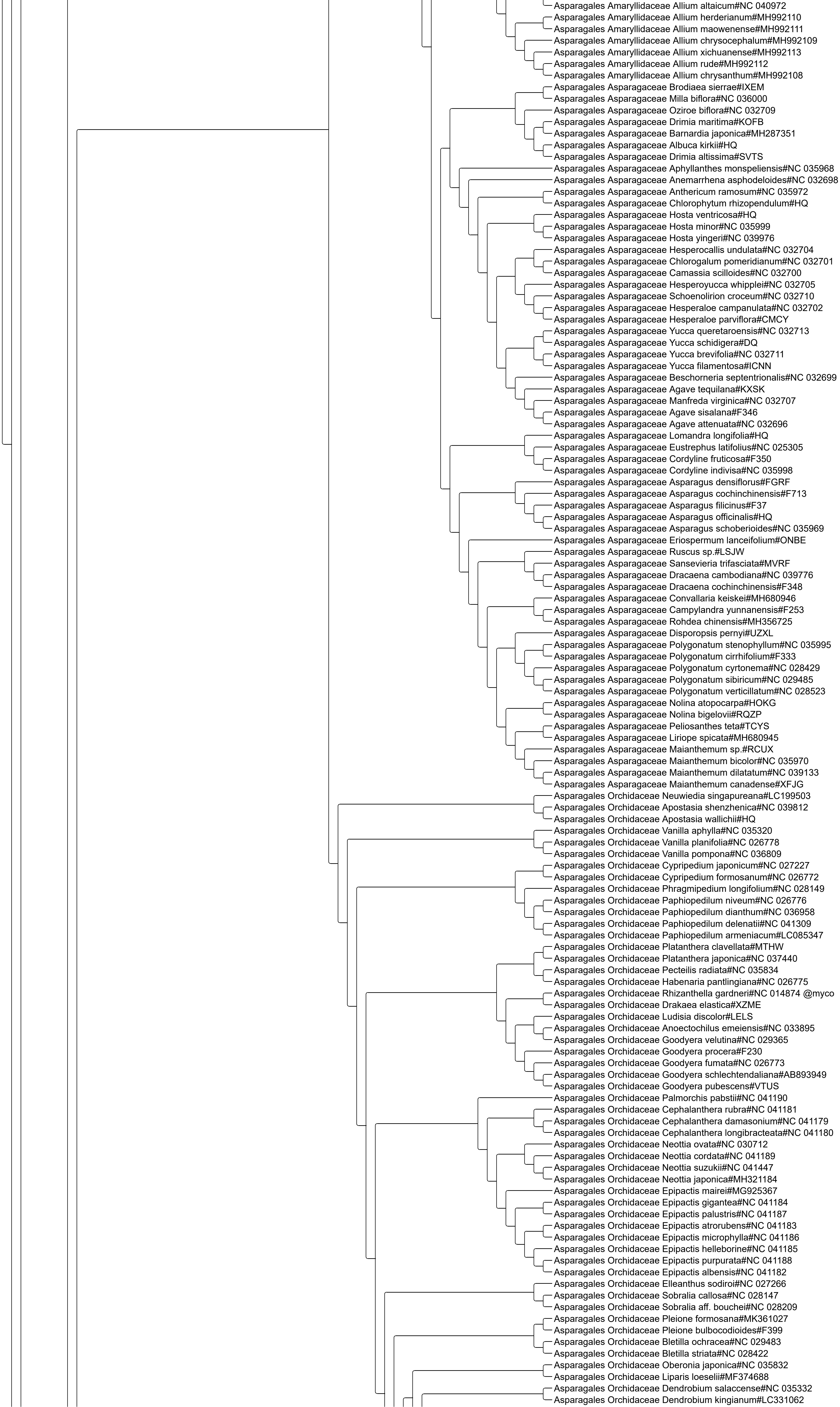

Asparagales Amaryllidaceae Allium altaicum#NC 040972
Asparagales Amaryllidaceae Allium herderianum#MH992110
Asparagales Amaryllidaceae Allium maowenense#MH992111
Asparagales Amaryllidaceae Allium chrysocephalum#MH992109
Asparagales Amaryllidaceae Allium xichuanense#MH992113
Asparagales Amaryllidaceae Allium rude#MH992112
Asparagales Amaryllidaceae Allium chrysanthum#MH992108
Asparagales Asparagaceae Brodiaea sierrae#IXEM
Asparagales Asparagaceae Milla biflora#NC 036000
Asparagales Asparagaceae Oziroe biflora#NC 032709
Asparagales Asparagaceae Drimia maritima#KOFB
Asparagales Asparagaceae Barnardia japonica#MH287351
Asparagales Asparagaceae Albuca kirkii#HQ
Asparagales Asparagaceae Drimia altissima#SVTS
Asparagales Asparagaceae Aphyllanthes monspeliensis#NC 035968
Asparagales Asparagaceae Anemarrhena asphodeloides#NC 032698
Asparagales Asparagaceae Anthericum ramosum#NC 035972
Asparagales Asparagaceae Chlorophytum rhizopendulum#HQ
Asparagales Asparagaceae Hosta ventricosa#HQ
Asparagales Asparagaceae Hosta minor#NC 035999
Asparagales Asparagaceae Hosta yingeri#NC 039976
Asparagales Asparagaceae Hesperocallis undulata#NC 032704
Asparagales Asparagaceae Chlorogalum pomeridianum#NC 032701
Asparagales Asparagaceae Camassia scilloides#NC 032700
Asparagales Asparagaceae Hesperoyucca whipplei#NC 032705
Asparagales Asparagaceae Schoenolirion croceum#NC 032710
Asparagales Asparagaceae Hesperaloe campanulata#NC 032702
Asparagales Asparagaceae Hesperaloe parviflora#CMCY
Asparagales Asparagaceae Yucca queretaroensis#NC 032713
Asparagales Asparagaceae Yucca schidigera#DQ
Asparagales Asparagaceae Yucca brevifolia#NC 032711
Asparagales Asparagaceae Yucca filamentosa#ICNN
Asparagales Asparagaceae Beschorneria septentrionalis#NC 032699
Asparagales Asparagaceae Agave tequilana#KXSK
Asparagales Asparagaceae Manfreda virginica#NC 032707
Asparagales Asparagaceae Agave sisalana#F346
Asparagales Asparagaceae Agave attenuata#NC 032696
Asparagales Asparagaceae Lomandra longifolia#HQ
Asparagales Asparagaceae Eustrephus latifolius#NC 025305
Asparagales Asparagaceae Cordyline fruticosa#F350
Asparagales Asparagaceae Cordyline indivisa#NC 035998
Asparagales Asparagaceae Asparagus densiflorus#FGRF
Asparagales Asparagaceae Asparagus cochinchinensis#F713
Asparagales Asparagaceae Asparagus filicinus#F37
Asparagales Asparagaceae Asparagus officinalis#HQ
Asparagales Asparagaceae Asparagus schoberioides#NC 035969
Asparagales Asparagaceae Eriospermum lanceifolium#ONBE
Asparagales Asparagaceae Ruscus sp.#LSJW
Asparagales Asparagaceae Sansevieria trifasciata#MVRF
Asparagales Asparagaceae Dracaena cambodiana#NC 039776
Asparagales Asparagaceae Dracaena cochinchinensis#F348
Asparagales Asparagaceae Convallaria keiskei#MH680946
Asparagales Asparagaceae Campylandra yunnanensis#F253
Asparagales Asparagaceae Rohdea chinensis#MH356725
Asparagales Asparagaceae Disporopsis pernyi#UZXL
Asparagales Asparagaceae Polygonatum stenophyllum#NC 035995
Asparagales Asparagaceae Polygonatum cirrhifolium#F333
Asparagales Asparagaceae Polygonatum cyrtonema#NC 028429
Asparagales Asparagaceae Polygonatum sibiricum#NC 029485
Asparagales Asparagaceae Polygonatum verticillatum#NC 028523
Asparagales Asparagaceae Nolina atopocarpa#HOKG
Asparagales Asparagaceae Nolina bigelovii#RQZP
Asparagales Asparagaceae Peliosanthes teta#TCYS
Asparagales Asparagaceae Liriope spicata#MH680945
Asparagales Asparagaceae Maianthemum sp.#RCUX
Asparagales Asparagaceae Maianthemum bicolor#NC 035970
Asparagales Asparagaceae Maianthemum dilatatum#NC 039133
Asparagales Asparagaceae Maianthemum canadense#XFJG
Asparagales Orchidaceae Neuwiedia singapureana#LC199503
Asparagales Orchidaceae Apostasia shenzhenica#NC 039812
Asparagales Orchidaceae Apostasia wallichii#HQ
Asparagales Orchidaceae Vanilla aphylla#NC 035320
Asparagales Orchidaceae Vanilla planifolia#NC 026778
Asparagales Orchidaceae Vanilla pompona#NC 036809
Asparagales Orchidaceae Cypripedium japonicum#NC 027227
Asparagales Orchidaceae Cypripedium formosanum#NC 026772
Asparagales Orchidaceae Phragmipedium longifolium#NC 028149
Asparagales Orchidaceae Paphiopedilum niveum#NC 026776
Asparagales Orchidaceae Paphiopedilum dianthum#NC 036958
Asparagales Orchidaceae Paphiopedilum delenatii#NC 041309
Asparagales Orchidaceae Paphiopedilum armeniacum#LC085347
Asparagales Orchidaceae Platanthera clavellata#MTHW
Asparagales Orchidaceae Platanthera japonica#NC 037440
Asparagales Orchidaceae Pecteilis radiata#NC 035834
Asparagales Orchidaceae Habenaria pantlingiana#NC 026775
Asparagales Orchidaceae Rhizanthella gardneri#NC 014874 @myco
Asparagales Orchidaceae Drakaea elastica#XZME
Asparagales Orchidaceae Ludisia discolor#LELS
Asparagales Orchidaceae Anoectochilus emeiensis#NC 033895
Asparagales Orchidaceae Goodyera velutina#NC 029365
Asparagales Orchidaceae Goodyera procera#F230
Asparagales Orchidaceae Goodyera fumata#NC 026773
Asparagales Orchidaceae Goodyera schlechtendaliana#AB893949
Asparagales Orchidaceae Goodyera pubescens#VTUS
Asparagales Orchidaceae Palmorchis pabstii#NC 041190
Asparagales Orchidaceae Cephalanthera rubra#NC 041181
Asparagales Orchidaceae Cephalanthera damasonium#NC 041179
Asparagales Orchidaceae Cephalanthera longibracteata#NC 041180
Asparagales Orchidaceae Neottia ovata#NC 030712
Asparagales Orchidaceae Neottia cordata#NC 041189
Asparagales Orchidaceae Neottia suzukii#NC 041447
Asparagales Orchidaceae Neottia japonica#MH321184
Asparagales Orchidaceae Epipactis mairei#MG925367
Asparagales Orchidaceae Epipactis gigantea#NC 041184
Asparagales Orchidaceae Epipactis palustris#NC 041187
Asparagales Orchidaceae Epipactis atrorubens#NC 041183
Asparagales Orchidaceae Epipactis microphylla#NC 041186
Asparagales Orchidaceae Epipactis helleborine#NC 041185
Asparagales Orchidaceae Epipactis purpurata#NC 041188
Asparagales Orchidaceae Epipactis albensis#NC 041182
Asparagales Orchidaceae Elleanthus sodiroi#NC 027266
Asparagales Orchidaceae Sobralia callosa#NC 028147
Asparagales Orchidaceae Sobralia aff. bouchei#NC 028209
Asparagales Orchidaceae Pleione formosana#MK361027
Asparagales Orchidaceae Pleione bulbocodioides#F399
Asparagales Orchidaceae Bletilla ochracea#NC 029483
Asparagales Orchidaceae Bletilla striata#NC 028422
Asparagales Orchidaceae Oberonia japonica#NC 035832
Asparagales Orchidaceae Liparis loeselii#MF374688
Asparagales Orchidaceae Dendrobium salaccense#NC 035332
Asparagales Orchidaceae Dendrobium kingianum#LC331062

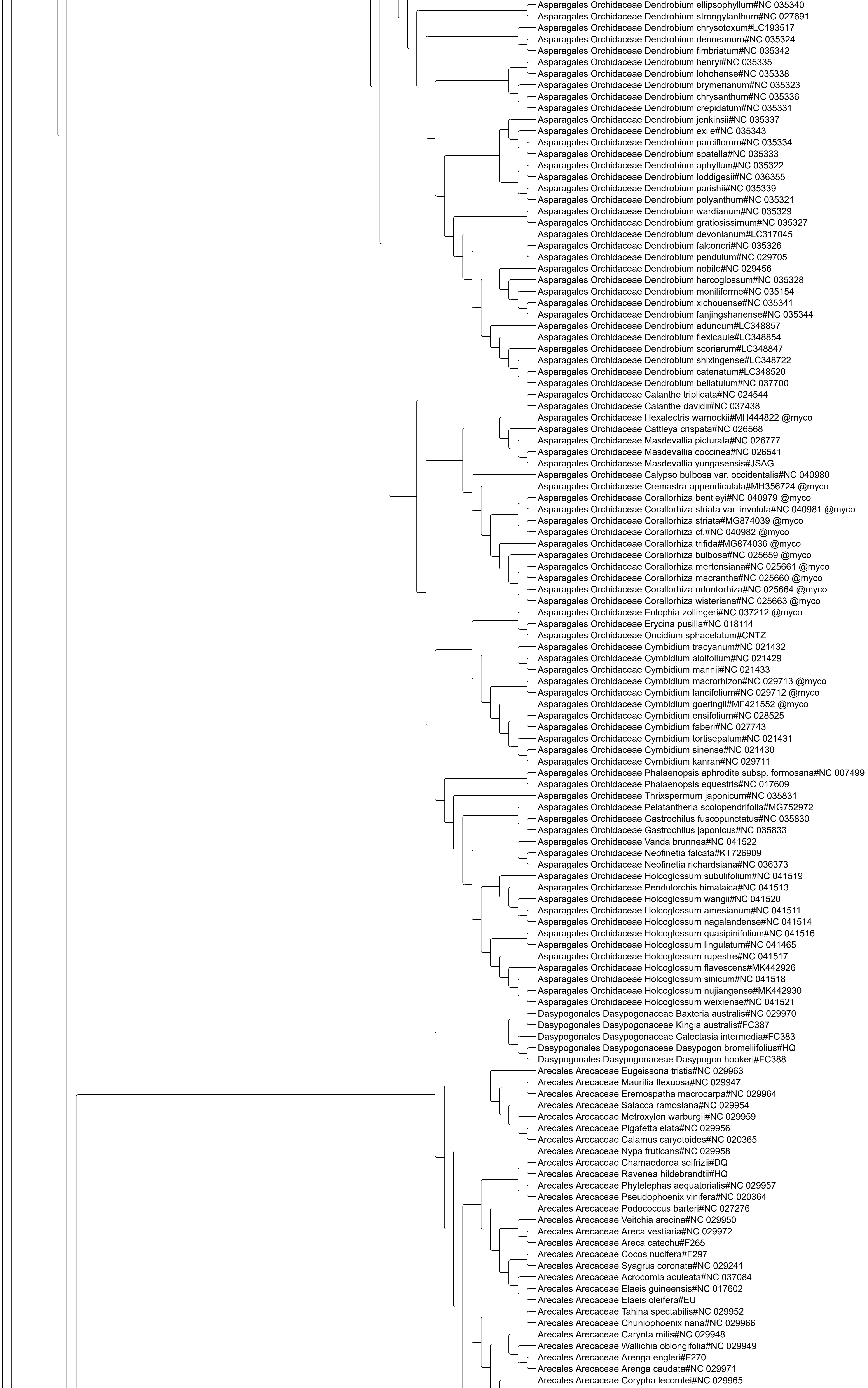

Asparagales Orchidaceae Dendrobium ellipsophyllum#NC 035340
Asparagales Orchidaceae Dendrobium strongylanthum#NC 027691
Asparagales Orchidaceae Dendrobium chrysotoxum#LC193517
Asparagales Orchidaceae Dendrobium denneanum#NC 035324
Asparagales Orchidaceae Dendrobium fimbriatum#NC 035342
Asparagales Orchidaceae Dendrobium henryi#NC 035335
Asparagales Orchidaceae Dendrobium lohohense#NC 035338
Asparagales Orchidaceae Dendrobium brymerianum#NC 035323
Asparagales Orchidaceae Dendrobium chrysanthum#NC 035336
Asparagales Orchidaceae Dendrobium crepidatum#NC 035331
Asparagales Orchidaceae Dendrobium jenkinsii#NC 035337
Asparagales Orchidaceae Dendrobium exile#NC 035343
Asparagales Orchidaceae Dendrobium parciflorum#NC 035334
Asparagales Orchidaceae Dendrobium spatella#NC 035333
Asparagales Orchidaceae Dendrobium aphyllum#NC 035322
Asparagales Orchidaceae Dendrobium loddigesii#NC 036355
Asparagales Orchidaceae Dendrobium parishii#NC 035339
Asparagales Orchidaceae Dendrobium polyanthum#NC 035321
Asparagales Orchidaceae Dendrobium wardianum#NC 035329
Asparagales Orchidaceae Dendrobium gratiosissimum#NC 035327
Asparagales Orchidaceae Dendrobium devonianum#LC317045
Asparagales Orchidaceae Dendrobium falconeri#NC 035326
Asparagales Orchidaceae Dendrobium pendulum#NC 029705
Asparagales Orchidaceae Dendrobium nobile#NC 029456
Asparagales Orchidaceae Dendrobium hercoglossum#NC 035328
Asparagales Orchidaceae Dendrobium moniliforme#NC 035154
Asparagales Orchidaceae Dendrobium xichouense#NC 035341
Asparagales Orchidaceae Dendrobium fanjingshanense#NC 035344
Asparagales Orchidaceae Dendrobium aduncum#LC348857
Asparagales Orchidaceae Dendrobium flexicaule#LC348854
Asparagales Orchidaceae Dendrobium scoriarum#LC348847
Asparagales Orchidaceae Dendrobium shixingense#LC348722
Asparagales Orchidaceae Dendrobium catenatum#LC348520
Asparagales Orchidaceae Dendrobium bellatulum#NC 037700
Asparagales Orchidaceae Calanthe triplicata#NC 024544
Asparagales Orchidaceae Calanthe davidii#NC 037438
Asparagales Orchidaceae Hexalectris warnockii#MH444822 @myco
Asparagales Orchidaceae Cattleya crispata#NC 026568
Asparagales Orchidaceae Masdevallia picturata#NC 026777
Asparagales Orchidaceae Masdevallia coccinea#NC 026541
Asparagales Orchidaceae Masdevallia yungasensis#JSAG
Asparagales Orchidaceae Calypso bulbosa var. occidentalis#NC 040980
Asparagales Orchidaceae Cremastra appendiculata#MH356724 @myco
Asparagales Orchidaceae Corallorhiza bentleyi#NC 040979 @myco
Asparagales Orchidaceae Corallorhiza striata var. involuta#NC 040981 @myco
Asparagales Orchidaceae Corallorhiza striata#MG874039 @myco
Asparagales Orchidaceae Corallorhiza cf.#NC 040982 @myco
Asparagales Orchidaceae Corallorhiza trifida#MG874036 @myco
Asparagales Orchidaceae Corallorhiza bulbosa#NC 025659 @myco
Asparagales Orchidaceae Corallorhiza mertensiana#NC 025661 @myco
Asparagales Orchidaceae Corallorhiza macrantha#NC 025660 @myco
Asparagales Orchidaceae Corallorhiza odontorhiza#NC 025664 @myco
Asparagales Orchidaceae Corallorhiza wisteriana#NC 025663 @myco
Asparagales Orchidaceae Eulophia zollingeri#NC 037212 @myco
Asparagales Orchidaceae Erycina pusilla#NC 018114
Asparagales Orchidaceae Oncidium sphacelatum#CNTZ
Asparagales Orchidaceae Cymbidium tracyanum#NC 021432
Asparagales Orchidaceae Cymbidium aloifolium#NC 021429
Asparagales Orchidaceae Cymbidium mannii#NC 021433
Asparagales Orchidaceae Cymbidium macrorhizon#NC 029713 @myco
Asparagales Orchidaceae Cymbidium lancifolium#NC 029712 @myco
Asparagales Orchidaceae Cymbidium goeringii#MF421552 @myco
Asparagales Orchidaceae Cymbidium ensifolium#NC 028525
Asparagales Orchidaceae Cymbidium faberi#NC 027743
Asparagales Orchidaceae Cymbidium tortisepalum#NC 021431
Asparagales Orchidaceae Cymbidium sinense#NC 021430
Asparagales Orchidaceae Cymbidium kanran#NC 029711
Asparagales Orchidaceae Phalaenopsis aphrodite subsp. formosana#NC 007499
Asparagales Orchidaceae Phalaenopsis equestris#NC 017609
Asparagales Orchidaceae Thrixspermum japonicum#NC 035831
Asparagales Orchidaceae Pelatantheria scolopendrifolia#MG752972
Asparagales Orchidaceae Gastrochilus fuscopunctatus#NC 035830
Asparagales Orchidaceae Gastrochilus japonicus#NC 035833
Asparagales Orchidaceae Vanda brunnea#NC 041522
Asparagales Orchidaceae Neofinetia falcata#KT726909
Asparagales Orchidaceae Neofinetia richardsiana#NC 036373
Asparagales Orchidaceae Holcoglossum subulifolium#NC 041519
Asparagales Orchidaceae Pendulorchis himalaica#NC 041513
Asparagales Orchidaceae Holcoglossum wangii#NC 041520
Asparagales Orchidaceae Holcoglossum amesianum#NC 041511
Asparagales Orchidaceae Holcoglossum nagalandense#NC 041514
Asparagales Orchidaceae Holcoglossum quasipinifolium#NC 041516
Asparagales Orchidaceae Holcoglossum lingulatum#NC 041465
Asparagales Orchidaceae Holcoglossum rupestre#NC 041517
Asparagales Orchidaceae Holcoglossum flavescens#MK442926
Asparagales Orchidaceae Holcoglossum sinicum#NC 041518
Asparagales Orchidaceae Holcoglossum nujiangense#MK442930
Asparagales Orchidaceae Holcoglossum weixiense#NC 041521
Dasypogonales Dasypogonaceae Baxteria australis#NC 029970
Dasypogonales Dasypogonaceae Kingia australis#FC387
Dasypogonales Dasypogonaceae Calectasia intermedia#FC383
Dasypogonales Dasypogonaceae Dasypogon bromeliifolius#HQ
Dasypogonales Dasypogonaceae Dasypogon hookeri#FC388
Arecales Arecaceae Eugeissona tristis#NC 029963
Arecales Arecaceae Mauritia flexuosa#NC 029947
Arecales Arecaceae Eremospatha macrocarpa#NC 029964
Arecales Arecaceae Salacca ramosiana#NC 029954
Arecales Arecaceae Metroxylon warburgii#NC 029959
Arecales Arecaceae Pigafetta elata#NC 029956
Arecales Arecaceae Calamus caryotoides#NC 020365
Arecales Arecaceae Nypa fruticans#NC 029958
Arecales Arecaceae Chamaedorea seifrizii#DQ
Arecales Arecaceae Ravenea hildebrandtii#HQ
Arecales Arecaceae Phytelephas aequatorialis#NC 029957
Arecales Arecaceae Pseudophoenix vinifera#NC 020364
Arecales Arecaceae Podococcus barteri#NC 027276
Arecales Arecaceae Veitchia arecina#NC 029950
Arecales Arecaceae Areca vestiaria#NC 029972
Arecales Arecaceae Areca catechu#F265
Arecales Arecaceae Cocos nucifera#F297
Arecales Arecaceae Syagrus coronata#NC 029241
Arecales Arecaceae Acrocomia aculeata#NC 037084
Arecales Arecaceae Elaeis guineensis#NC 017602
Arecales Arecaceae Elaeis oleifera#EU
Arecales Arecaceae Tahina spectabilis#NC 029952
Arecales Arecaceae Chuniophoenix nana#NC 029966
Arecales Arecaceae Caryota mitis#NC 029948
Arecales Arecaceae Wallichia oblongifolia#NC 029949
Arecales Arecaceae Arenga engleri#F270
Arecales Arecaceae Arenga caudata#NC 029971
Arecales Arecaceae Corypha lecomtei#NC 029965

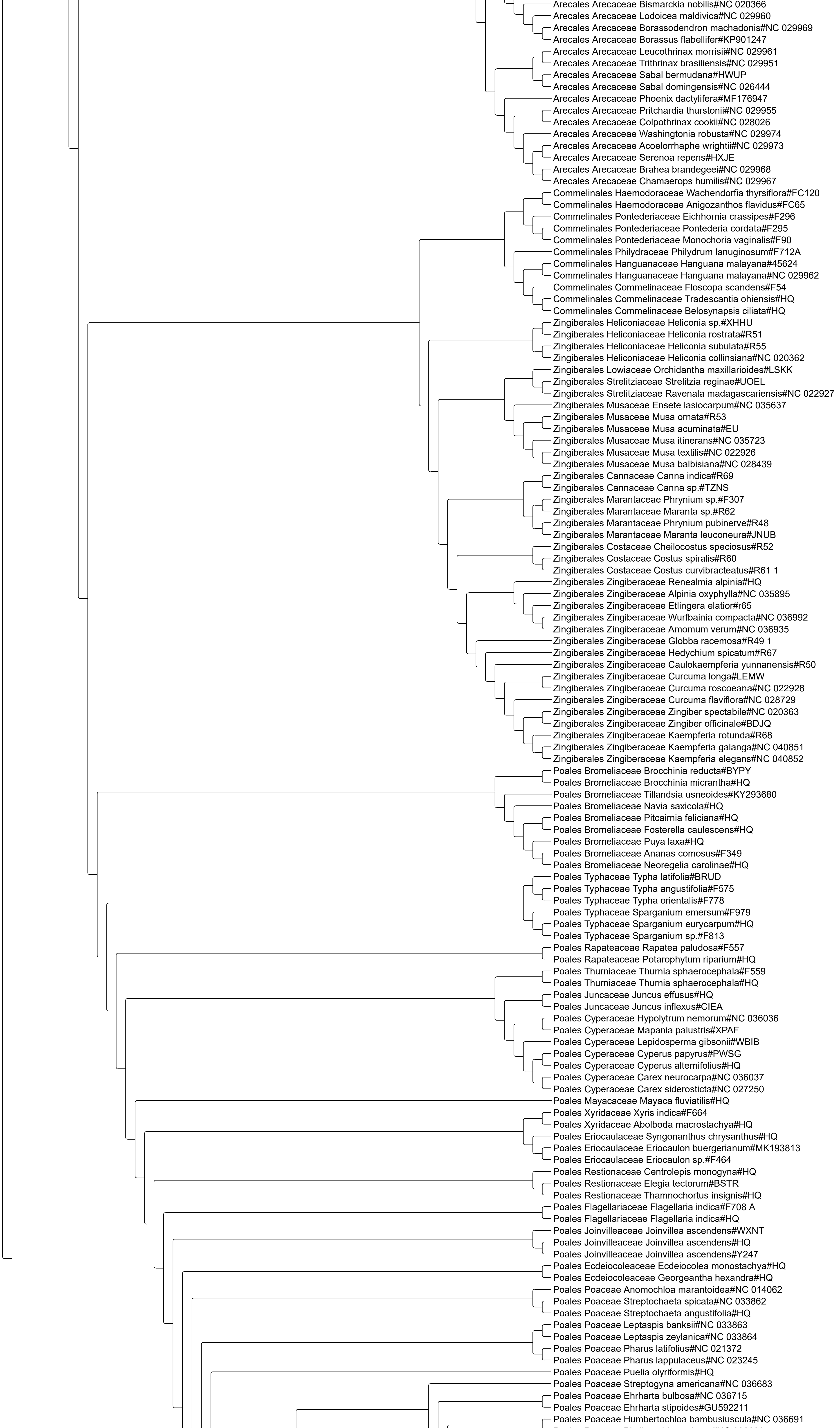
Arecales Arecaceae Bismarckia nobilis#NC 020366
Arecales Arecaceae Lodoicea maldivica#NC 029960
Arecales Arecaceae Borassodendron machadonis#NC 029969
Arecales Arecaceae Borassus flabellifer#KP901247
Arecales Arecaceae Leucothrinax morrisii#NC 029961
Arecales Arecaceae Trithrinax brasiliensis#NC 029951
Arecales Arecaceae Sabal bermudana#HWUP
Arecales Arecaceae Sabal domingensis#NC 026444
Arecales Arecaceae Phoenix dactylifera#MF176947
Arecales Arecaceae Pritchardia thurstonii#NC 029955
Arecales Arecaceae Colpothrinax cookii#NC 028026
Arecales Arecaceae Washingtonia robusta#NC 029974
Arecales Arecaceae Acoelorrhaphe wrightii#NC 029973
Arecales Arecaceae Serenoa repens#HXJE
Arecales Arecaceae Brahea brandegeei#NC 029968
Arecales Arecaceae Chamaerops humilis#NC 029967
Commelinales Haemodoraceae Wachendorfia thyrsiflora#FC120
Commelinales Haemodoraceae Anigozanthos flavidus#FC65
Commelinales Pontederiaceae Eichhornia crassipes#F296
Commelinales Pontederiaceae Pontederia cordata#F295
Commelinales Pontederiaceae Monochoria vaginalis#F90
Commelinales Philydraceae Philydrum lanuginosum#F712A
Commelinales Hanguanaceae Hanguana malayana#45624
Commelinales Hanguanaceae Hanguana malayana#NC 029962
Commelinales Commelinaceae Floscopa scandens#F54
Commelinales Commelinaceae Tradescantia ohiensis#HQ
Commelinales Commelinaceae Belosynapsis ciliata#HQ
Zingiberales Heliconiaceae Heliconia sp.#XHHU
Zingiberales Heliconiaceae Heliconia rostrata#R51
Zingiberales Heliconiaceae Heliconia subulata#R55
Zingiberales Heliconiaceae Heliconia collinsiana#NC 020362
Zingiberales Lowiaceae Orchidantha maxillarioides#LSKK
Zingiberales Strelitziaceae Strelitzia reginae#UOEL
Zingiberales Strelitziaceae Ravenala madagascariensis#NC 022927
Zingiberales Musaceae Ensete lasiocarpum#NC 035637
Zingiberales Musaceae Musa ornata#R53
Zingiberales Musaceae Musa acuminata#EU
Zingiberales Musaceae Musa itinerans#NC 035723
Zingiberales Musaceae Musa textilis#NC 022926
Zingiberales Musaceae Musa balbisiana#NC 028439
Zingiberales Cannaceae Canna indica#R69
Zingiberales Cannaceae Canna sp.#TZNS
Zingiberales Marantaceae Phrynium sp.#F307
Zingiberales Marantaceae Maranta sp.#R62
Zingiberales Marantaceae Phrynium pubinerve#R48
Zingiberales Marantaceae Maranta leuconeura#JNUB
Zingiberales Costaceae Cheilocostus speciosus#R52
Zingiberales Costaceae Costus spiralis#R60
Zingiberales Costaceae Costus curvibracteatus#R61 1
Zingiberales Zingiberaceae Renealmia alpinia#HQ
Zingiberales Zingiberaceae Alpinia oxyphylla#NC 035895
Zingiberales Zingiberaceae Etlingera elatior#r65
Zingiberales Zingiberaceae Wurfbainia compacta#NC 036992
Zingiberales Zingiberaceae Amomum verum#NC 036935
Zingiberales Zingiberaceae Globba racemosa#R49 1
Zingiberales Zingiberaceae Hedychium spicatum#R67
Zingiberales Zingiberaceae Caulokaempferia yunnanensis#R50
Zingiberales Zingiberaceae Curcuma longa#LEMW
Zingiberales Zingiberaceae Curcuma roscoeana#NC 022928
Zingiberales Zingiberaceae Curcuma flaviflora#NC 028729
Zingiberales Zingiberaceae Zingiber spectabile#NC 020363
Zingiberales Zingiberaceae Zingiber officinale#BDJQ
Zingiberales Zingiberaceae Kaempferia rotunda#R68
Zingiberales Zingiberaceae Kaempferia galanga#NC 040851
Zingiberales Zingiberaceae Kaempferia elegans#NC 040852
Poales Bromeliaceae Brocchinia reducta#BYPY
Poales Bromeliaceae Brocchinia micrantha#HQ
Poales Bromeliaceae Tillandsia usneoides#KY293680
Poales Bromeliaceae Navia saxicola#HQ
Poales Bromeliaceae Pitcairnia feliciana#HQ
Poales Bromeliaceae Fosterella caulescens#HQ
Poales Bromeliaceae Puya laxa#HQ
Poales Bromeliaceae Ananas comosus#F349
Poales Bromeliaceae Neoregelia carolinae#HQ
Poales Typhaceae Typha latifolia#BRUD
Poales Typhaceae Typha angustifolia#F575
Poales Typhaceae Typha orientalis#F778
Poales Typhaceae Sparganium emersum#F979
Poales Typhaceae Sparganium eurycarpum#HQ
Poales Typhaceae Sparganium sp.#F813
Poales Rapateaceae Rapatea paludosa#F557
Poales Rapateaceae Potarophytum riparium#HQ
Poales Thurniaceae Thurnia sphaerocephala#F559
Poales Thurniaceae Thurnia sphaerocephala#HQ
Poales Juncaceae Juncus effusus#HQ
Poales Juncaceae Juncus inflexus#CIEA
Poales Cyperaceae Hypolytrum nemorum#NC 036036
Poales Cyperaceae Mapania palustris#XPAF
Poales Cyperaceae Lepidosperma gibsonii#WBIB
Poales Cyperaceae Cyperus papyrus#PWSG
Poales Cyperaceae Cyperus alternifolius#HQ
Poales Cyperaceae Carex neurocarpa#NC 036037
Poales Cyperaceae Carex siderosticta#NC 027250
Poales Mayacaceae Mayaca fluviatilis#HQ
Poales Xyridaceae Xyris indica#F664
Poales Xyridaceae Abolboda macrostachya#HQ
Poales Eriocaulaceae Syngonanthus chrysanthus#HQ
Poales Eriocaulaceae Eriocaulon buergerianum#MK193813
Poales Eriocaulaceae Eriocaulon sp.#F464
Poales Restionaceae Centrolepis monogyna#HQ
Poales Restionaceae Elegia tectorum#BSTR
Poales Restionaceae Thamnochortus insignis#HQ
Poales Flagellariaceae Flagellaria indica#F708 A
Poales Flagellariaceae Flagellaria indica#HQ
Poales Joinvilleaceae Joinvillea ascendens#WXNT
Poales Joinvilleaceae Joinvillea ascendens#HQ
Poales Joinvilleaceae Joinvillea ascendens#Y247
Poales Ecdeiocoleaceae Ecdeiocolea monostachya#HQ
Poales Ecdeiocoleaceae Georgeantha hexandra#HQ
Poales Poaceae Anomochloa marantoidea#NC 014062
Poales Poaceae Streptochaeta spicata#NC 033862
Poales Poaceae Streptochaeta angustifolia#HQ
Poales Poaceae Leptaspis banksii#NC 033863
Poales Poaceae Leptaspis zeylanica#NC 033864
Poales Poaceae Pharus latifolius#NC 021372
Poales Poaceae Pharus lappulaceus#NC 023245
Poales Poaceae Puelia olyriformis#HQ
Poales Poaceae Streptogyna americana#NC 036683
Poales Poaceae Ehrharta bulbosa#NC 036715
Poales Poaceae Ehrharta stipoides#GU592211
Poales Poaceae Humbertochloa bambusiuscula#NC 036691

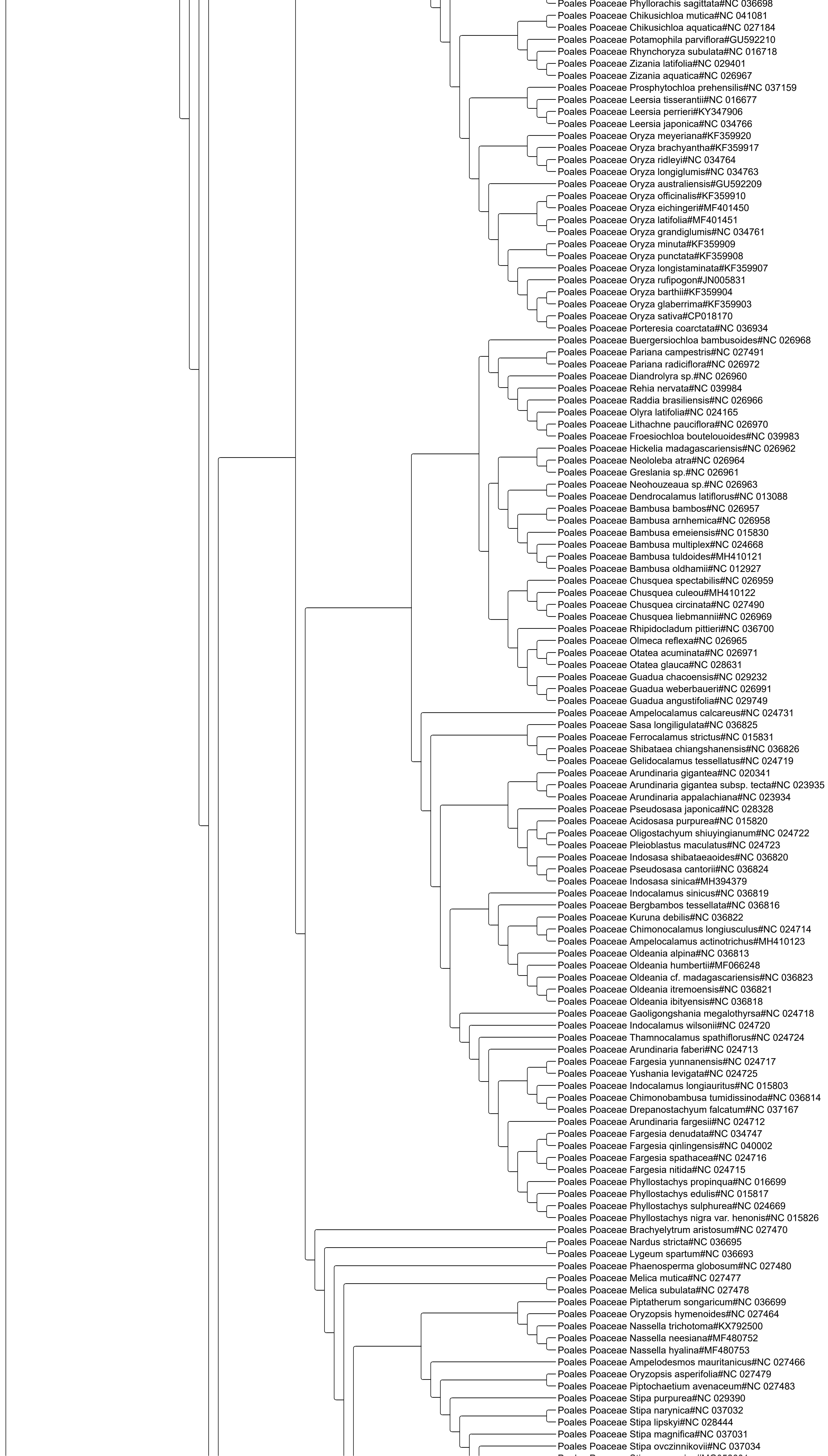
Poales Poaceae Phyllorachis sagittata#NC 036698
Poales Poaceae Chikusichloa mutica#NC 041081
Poales Poaceae Chikusichloa aquatica#NC 027184
Poales Poaceae Potamophila parviflora#GU592210
Poales Poaceae Rhynchoryza subulata#NC 016718
Poales Poaceae Zizania latifolia#NC 029401
Poales Poaceae Zizania aquatica#NC 026967
Poales Poaceae Prosphytochloa prehensilis#NC 037159
Poales Poaceae Leersia tisserantii#NC 016677
Poales Poaceae Leersia perrieri#KY347906
Poales Poaceae Leersia japonica#NC 034766
Poales Poaceae Oryza meyeriana#KF359920
Poales Poaceae Oryza brachyantha#KF359917
Poales Poaceae Oryza ridleyi#NC 034764
Poales Poaceae Oryza longiglumis#NC 034763
Poales Poaceae Oryza australiensis#GU592209
Poales Poaceae Oryza officinalis#KF359910
Poales Poaceae Oryza eichingeri#MF401450
Poales Poaceae Oryza latifolia#MF401451
Poales Poaceae Oryza grandiglumis#NC 034761
Poales Poaceae Oryza minuta#KF359909
Poales Poaceae Oryza punctata#KF359908
Poales Poaceae Oryza longistaminata#KF359907
Poales Poaceae Oryza rufipogon#JN005831
Poales Poaceae Oryza barthii#KF359904
Poales Poaceae Oryza glaberrima#KF359903
Poales Poaceae Oryza sativa#CP018170
Poales Poaceae Porteresia coarctata#NC 036934
Poales Poaceae Buergersiochloa bambusoides#NC 026968
Poales Poaceae Pariana campestris#NC 027491
Poales Poaceae Pariana radiciflora#NC 026972
Poales Poaceae Diandrolyra sp.#NC 026960
Poales Poaceae Rehia nervata#NC 039984
Poales Poaceae Raddia brasiliensis#NC 026966
Poales Poaceae Olyra latifolia#NC 024165
Poales Poaceae Lithachne pauciflora#NC 026970
Poales Poaceae Froesiochloa boutelouoides#NC 039983
Poales Poaceae Hickelia madagascariensis#NC 026962
Poales Poaceae Neololeba atra#NC 026964
Poales Poaceae Greslania sp.#NC 026961
Poales Poaceae Neohouzeaua sp.#NC 026963
Poales Poaceae Dendrocalamus latiflorus#NC 013088
Poales Poaceae Bambusa bambos#NC 026957
Poales Poaceae Bambusa arnhemica#NC 026958
Poales Poaceae Bambusa emeiensis#NC 015830
Poales Poaceae Bambusa multiplex#NC 024668
Poales Poaceae Bambusa tuldoides#MH410121
Poales Poaceae Bambusa oldhamii#NC 012927
Poales Poaceae Chusquea spectabilis#NC 026959
Poales Poaceae Chusquea culeou#MH410122
Poales Poaceae Chusquea circinata#NC 027490
Poales Poaceae Chusquea liebmannii#NC 026969
Poales Poaceae Rhipidocladum pittieri#NC 036700
Poales Poaceae Olmeca reflexa#NC 026965
Poales Poaceae Otatea acuminata#NC 026971
Poales Poaceae Otatea glauca#NC 028631
Poales Poaceae Guadua chacoensis#NC 029232
Poales Poaceae Guadua weberbaueri#NC 026991
Poales Poaceae Guadua angustifolia#NC 029749
Poales Poaceae Ampelocalamus calcareus#NC 024731
Poales Poaceae Sasa longiligulata#NC 036825
Poales Poaceae Ferrocalamus strictus#NC 015831
Poales Poaceae Shibataea chiangshanensis#NC 036826
Poales Poaceae Gelidocalamus tessellatus#NC 024719
Poales Poaceae Arundinaria gigantea#NC 020341
Poales Poaceae Arundinaria gigantea subsp. tecta#NC 023935
Poales Poaceae Arundinaria appalachiana#NC 023934
Poales Poaceae Pseudosasa japonica#NC 028328
Poales Poaceae Acidosasa purpurea#NC 015820
Poales Poaceae Oligostachyum shiuyingianum#NC 024722
Poales Poaceae Pleioblastus maculatus#NC 024723
Poales Poaceae Indosasa shibataeaoides#NC 036820
Poales Poaceae Pseudosasa cantorii#NC 036824
Poales Poaceae Indosasa sinica#MH394379
Poales Poaceae Indocalamus sinicus#NC 036819
Poales Poaceae Bergbambos tessellata#NC 036816
Poales Poaceae Kuruna debilis#NC 036822
Poales Poaceae Chimonocalamus longiusculus#NC 024714
Poales Poaceae Ampelocalamus actinotrichus#MH410123
Poales Poaceae Oldeania alpina#NC 036813
Poales Poaceae Oldeania humbertii#MF066248
Poales Poaceae Oldeania cf. madagascariensis#NC 036823
Poales Poaceae Oldeania itremoensis#NC 036821
Poales Poaceae Oldeania ibityensis#NC 036818
Poales Poaceae Gaoligongshania megalothyrsa#NC 024718
Poales Poaceae Indocalamus wilsonii#NC 024720
Poales Poaceae Thamnocalamus spathiflorus#NC 024724
Poales Poaceae Arundinaria faberi#NC 024713
Poales Poaceae Fargesia yunnanensis#NC 024717
Poales Poaceae Yushania levigata#NC 024725
Poales Poaceae Indocalamus longiauritus#NC 015803
Poales Poaceae Chimonobambusa tumidissinoda#NC 036814
Poales Poaceae Drepanostachyum falcatum#NC 037167
Poales Poaceae Arundinaria fargesii#NC 024712
Poales Poaceae Fargesia denudata#NC 034747
Poales Poaceae Fargesia qinlingensis#NC 040002
Poales Poaceae Fargesia spathacea#NC 024716
Poales Poaceae Fargesia nitida#NC 024715
Poales Poaceae Phyllostachys propinqua#NC 016699
Poales Poaceae Phyllostachys edulis#NC 015817
Poales Poaceae Phyllostachys sulphurea#NC 024669
Poales Poaceae Phyllostachys nigra var. henonis#NC 015826
Poales Poaceae Brachyelytrum aristosum#NC 027470
Poales Poaceae Nardus stricta#NC 036695
Poales Poaceae Lygeum spartum#NC 036693
Poales Poaceae Phaenosperma globosum#NC 027480
Poales Poaceae Melica mutica#NC 027477
Poales Poaceae Melica subulata#NC 027478
Poales Poaceae Piptatherum songaricum#NC 036699
Poales Poaceae Oryzopsis hymenoides#NC 027464
Poales Poaceae Nassella trichotoma#KX792500
Poales Poaceae Nassella neesiana#MF480752
Poales Poaceae Nassella hyalina#MF480753
Poales Poaceae Ampelodesmos mauritanicus#NC 027466
Poales Poaceae Oryzopsis asperifolia#NC 027479
Poales Poaceae Piptochaetium avenaceum#NC 027483
Poales Poaceae Stipa purpurea#NC 029390
Poales Poaceae Stipa narynica#NC 037032
Poales Poaceae Stipa lipskyi#NC 028444
Poales Poaceae Stipa magnifica#NC 037031
Poales Poaceae Stipa ovczinnikovii#NC 037034

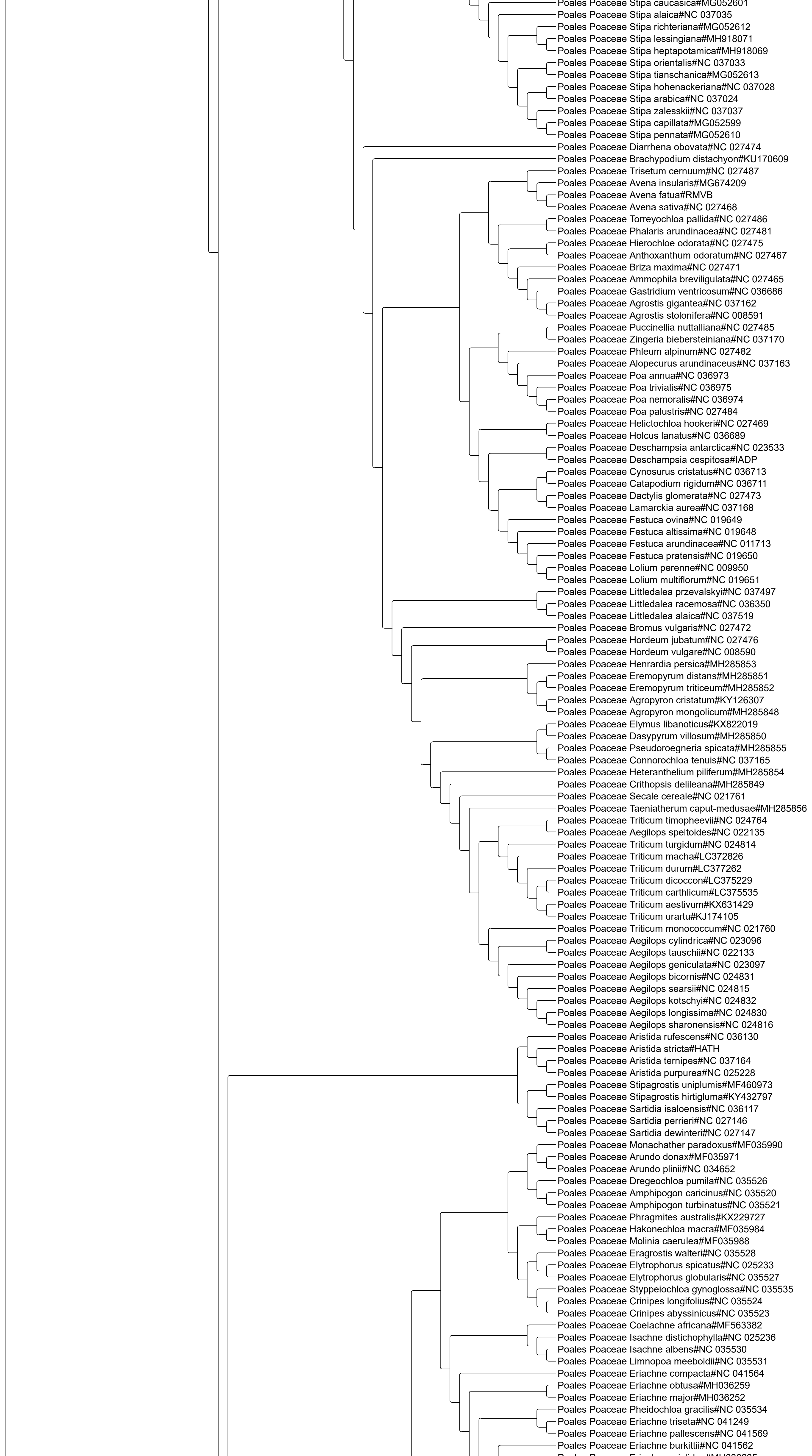

Poales Poaceae Stipa caucasica#MG052601
Poales Poaceae Stipa alaica#NC 037035
Poales Poaceae Stipa richteriana#MG052612
Poales Poaceae Stipa lessingiana#MH918071
Poales Poaceae Stipa heptapotamica#MH918069
Poales Poaceae Stipa orientalis#NC 037033
Poales Poaceae Stipa tianschanica#MG052613
Poales Poaceae Stipa hohenackeriana#NC 037028
Poales Poaceae Stipa arabica#NC 037024
Poales Poaceae Stipa zalesskii#NC 037037
Poales Poaceae Stipa capillata#MG052599
Poales Poaceae Stipa pennata#MG052610
Poales Poaceae Diarrhena obovata#NC 027474
Poales Poaceae Brachypodium distachyon#KU170609
Poales Poaceae Trisetum cernuum#NC 027487
Poales Poaceae Avena insularis#MG674209
Poales Poaceae Avena fatua#RMVB
Poales Poaceae Avena sativa#NC 027468
Poales Poaceae Torreyochloa pallida#NC 027486
Poales Poaceae Phalaris arundinacea#NC 027481
Poales Poaceae Hierochloe odorata#NC 027475
Poales Poaceae Anthoxanthum odoratum#NC 027467
Poales Poaceae Briza maxima#NC 027471
Poales Poaceae Ammophila breviligulata#NC 027465
Poales Poaceae Gastridium ventricosum#NC 036686
Poales Poaceae Agrostis gigantea#NC 037162
Poales Poaceae Agrostis stolonifera#NC 008591
Poales Poaceae Puccinellia nuttalliana#NC 027485
Poales Poaceae Zingeria biebersteiniana#NC 037170
Poales Poaceae Phleum alpinum#NC 027482
Poales Poaceae Alopecurus arundinaceus#NC 037163
Poales Poaceae Poa annua#NC 036973
Poales Poaceae Poa trivialis#NC 036975
Poales Poaceae Poa nemoralis#NC 036974
Poales Poaceae Poa palustris#NC 027484
Poales Poaceae Helictochloa hookeri#NC 027469
Poales Poaceae Holcus lanatus#NC 036689
Poales Poaceae Deschampsia antarctica#NC 023533
Poales Poaceae Deschampsia cespitosa#IADP
Poales Poaceae Cynosurus cristatus#NC 036713
Poales Poaceae Catapodium rigidum#NC 036711
Poales Poaceae Dactylis glomerata#NC 027473
Poales Poaceae Lamarckia aurea#NC 037168
Poales Poaceae Festuca ovina#NC 019649
Poales Poaceae Festuca altissima#NC 019648
Poales Poaceae Festuca arundinacea#NC 011713
Poales Poaceae Festuca pratensis#NC 019650
Poales Poaceae Lolium perenne#NC 009950
Poales Poaceae Lolium multiflorum#NC 019651
Poales Poaceae Littledalea przevalskyi#NC 037497
Poales Poaceae Littledalea racemosa#NC 036350
Poales Poaceae Littledalea alaica#NC 037519
Poales Poaceae Bromus vulgaris#NC 027472
Poales Poaceae Hordeum jubatum#NC 027476
Poales Poaceae Hordeum vulgare#NC 008590
Poales Poaceae Henrardia persica#MH285853
Poales Poaceae Eremopyrum distans#MH285851
Poales Poaceae Eremopyrum triticeum#MH285852
Poales Poaceae Agropyron cristatum#KY126307
Poales Poaceae Agropyron mongolicum#MH285848
Poales Poaceae Elymus libanoticus#KX822019
Poales Poaceae Dasypyrum villosum#MH285850
Poales Poaceae Pseudoroegneria spicata#MH285855
Poales Poaceae Connorochloa tenuis#NC 037165
Poales Poaceae Heteranthelium piliferum#MH285854
Poales Poaceae Crithopsis delileana#MH285849
Poales Poaceae Secale cereale#NC 021761
Poales Poaceae Taeniatherum caput-medusae#MH285856
Poales Poaceae Triticum timopheevii#NC 024764
Poales Poaceae Aegilops speltoides#NC 022135
Poales Poaceae Triticum turgidum#NC 024814
Poales Poaceae Triticum macha#LC372826
Poales Poaceae Triticum durum#LC377262
Poales Poaceae Triticum dicoccon#LC375229
Poales Poaceae Triticum carthlicum#LC375535
Poales Poaceae Triticum aestivum#KX631429
Poales Poaceae Triticum urartu#KJ174105
Poales Poaceae Triticum monococcum#NC 021760
Poales Poaceae Aegilops cylindrica#NC 023096
Poales Poaceae Aegilops tauschii#NC 022133
Poales Poaceae Aegilops geniculata#NC 023097
Poales Poaceae Aegilops bicornis#NC 024831
Poales Poaceae Aegilops searsii#NC 024815
Poales Poaceae Aegilops kotschyi#NC 024832
Poales Poaceae Aegilops longissima#NC 024830
Poales Poaceae Aegilops sharonensis#NC 024816
Poales Poaceae Aristida rufescens#NC 036130
Poales Poaceae Aristida stricta#HATH
Poales Poaceae Aristida ternipes#NC 037164
Poales Poaceae Aristida purpurea#NC 025228
Poales Poaceae Stipagrostis uniplumis#MF460973
Poales Poaceae Stipagrostis hirtigluma#KY432797
Poales Poaceae Sartidia isaloensis#NC 036117
Poales Poaceae Sartidia perrieri#NC 027146
Poales Poaceae Sartidia dewinteri#NC 027147
Poales Poaceae Monachather paradoxus#MF035990
Poales Poaceae Arundo donax#MF035971
Poales Poaceae Arundo plinii#NC 034652
Poales Poaceae Dregeochloa pumila#NC 035526
Poales Poaceae Amphipogon caricinus#NC 035520
Poales Poaceae Amphipogon turbinatus#NC 035521
Poales Poaceae Phragmites australis#KX229727
Poales Poaceae Hakonechloa macra#MF035984
Poales Poaceae Molinia caerulea#MF035988
Poales Poaceae Eragrostis walteri#NC 035528
Poales Poaceae Elytrophorus spicatus#NC 025233
Poales Poaceae Elytrophorus globularis#NC 035527
Poales Poaceae Styppeiochloa gynoglossa#NC 035535
Poales Poaceae Crinipes longifolius#NC 035524
Poales Poaceae Crinipes abyssinicus#NC 035523
Poales Poaceae Coelachne africana#MF563382
Poales Poaceae Isachne distichophylla#NC 025236
Poales Poaceae Isachne albens#NC 035530
Poales Poaceae Limnopoa meeboldii#NC 035531
Poales Poaceae Eriachne compacta#NC 041564
Poales Poaceae Eriachne obtusa#MH036259
Poales Poaceae Eriachne major#MH036252
Poales Poaceae Pheidochloa gracilis#NC 035534
Poales Poaceae Eriachne triseta#NC 041249
Poales Poaceae Eriachne pallescens#NC 041569
Poales Poaceae Eriachne burkittii#NC 041562

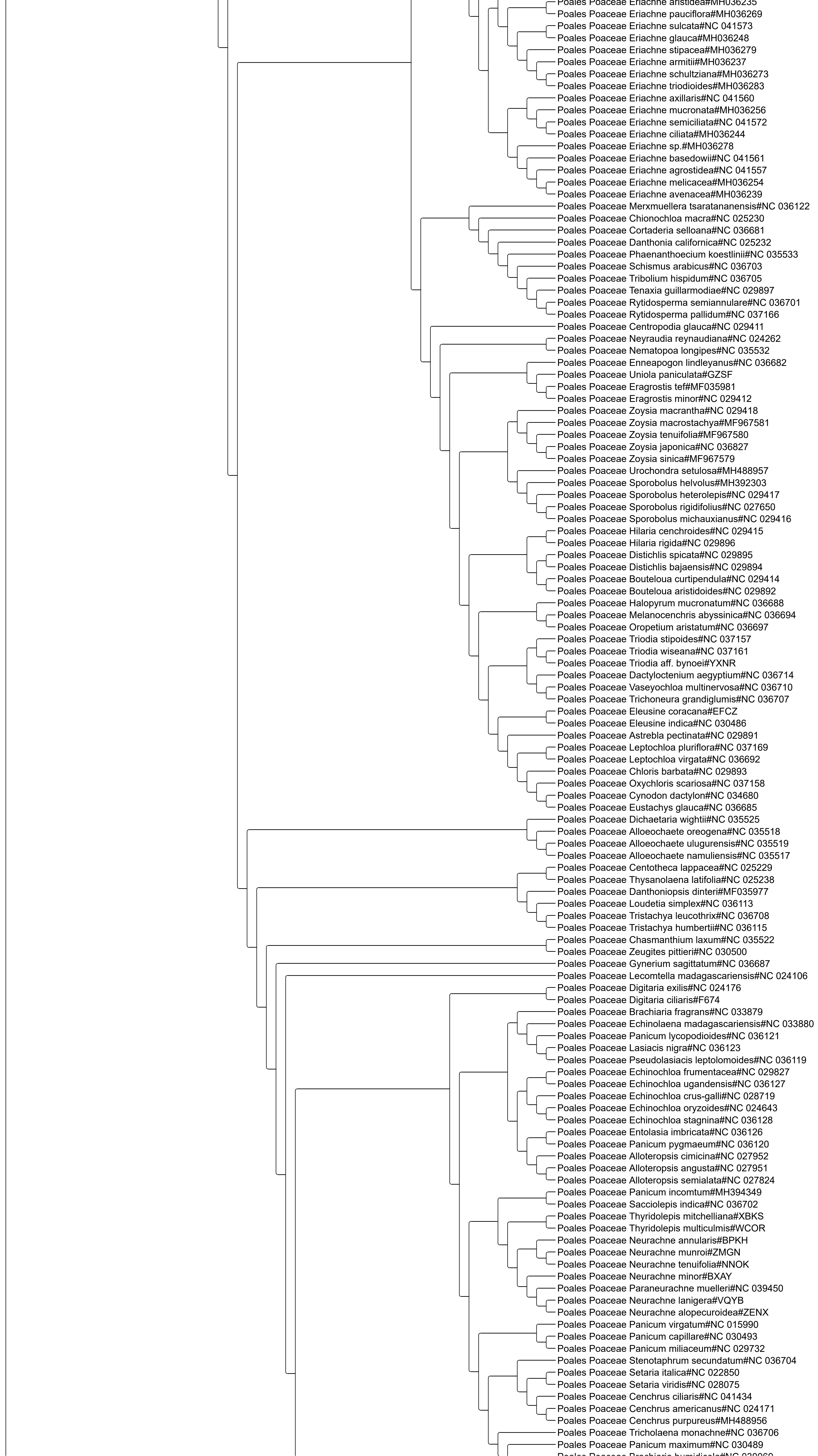

Poales Poaceae Eriachne aristidea#MH036235
Poales Poaceae Eriachne pauciflora#MH036269
Poales Poaceae Eriachne sulcata#NC 041573
Poales Poaceae Eriachne glauca#MH036248
Poales Poaceae Eriachne stipacea#MH036279
Poales Poaceae Eriachne armitii#MH036237
Poales Poaceae Eriachne schultziana#MH036273
Poales Poaceae Eriachne triodioides#MH036283
Poales Poaceae Eriachne axillaris#NC 041560
Poales Poaceae Eriachne mucronata#MH036256
Poales Poaceae Eriachne semiciliata#NC 041572
Poales Poaceae Eriachne ciliata#MH036244
Poales Poaceae Eriachne sp.#MH036278
Poales Poaceae Eriachne basedowii#NC 041561
Poales Poaceae Eriachne agrostidea#NC 041557
Poales Poaceae Eriachne melicacea#MH036254
Poales Poaceae Eriachne avenacea#MH036239
Poales Poaceae Merxmuellera tsaratananensis#NC 036122
Poales Poaceae Chionochloa macra#NC 025230
Poales Poaceae Cortaderia selloana#NC 036681
Poales Poaceae Danthonia californica#NC 025232
Poales Poaceae Phaenanthoecium koestlinii#NC 035533
Poales Poaceae Schismus arabicus#NC 036703
Poales Poaceae Tribolium hispidum#NC 036705
Poales Poaceae Tenaxia guillarmodiae#NC 029897
Poales Poaceae Rytidosperma semiannulare#NC 036701
Poales Poaceae Rytidosperma pallidum#NC 037166
Poales Poaceae Centropodia glauca#NC 029411
Poales Poaceae Neyraudia reynaudiana#NC 024262
Poales Poaceae Nematopoa longipes#NC 035532
Poales Poaceae Enneapogon lindleyanus#NC 036682
Poales Poaceae Uniola paniculata#GZSF
Poales Poaceae Eragrostis tef#MF035981
Poales Poaceae Eragrostis minor#NC 029412
Poales Poaceae Zoysia macrantha#NC 029418
Poales Poaceae Zoysia macrostachya#MF967581
Poales Poaceae Zoysia tenuifolia#MF967580
Poales Poaceae Zoysia japonica#NC 036827
Poales Poaceae Zoysia sinica#MF967579
Poales Poaceae Urochondra setulosa#MH488957
Poales Poaceae Sporobolus helvolus#MH392303
Poales Poaceae Sporobolus heterolepis#NC 029417
Poales Poaceae Sporobolus rigidifolius#NC 027650
Poales Poaceae Sporobolus michauxianus#NC 029416
Poales Poaceae Hilaria cenchroides#NC 029415
Poales Poaceae Hilaria rigida#NC 029896
Poales Poaceae Distichlis spicata#NC 029895
Poales Poaceae Distichlis bajaensis#NC 029894
Poales Poaceae Bouteloua curtipendula#NC 029414
Poales Poaceae Bouteloua aristidoides#NC 029892
Poales Poaceae Halopyrum mucronatum#NC 036688
Poales Poaceae Melanocenchris abyssinica#NC 036694
Poales Poaceae Oropetium aristatum#NC 036697
Poales Poaceae Triodia stipoides#NC 037157
Poales Poaceae Triodia wiseana#NC 037161
Poales Poaceae Triodia aff. bynoei#YXNR
Poales Poaceae Dactyloctenium aegyptium#NC 036714
Poales Poaceae Vaseyochloa multinervosa#NC 036710
Poales Poaceae Trichoneura grandiglumis#NC 036707
Poales Poaceae Eleusine coracana#EFCZ
Poales Poaceae Eleusine indica#NC 030486
Poales Poaceae Astrebla pectinata#NC 029891
Poales Poaceae Leptochloa pluriflora#NC 037169
Poales Poaceae Leptochloa virgata#NC 036692
Poales Poaceae Chloris barbata#NC 029893
Poales Poaceae Oxychloris scariosa#NC 037158
Poales Poaceae Cynodon dactylon#NC 034680
Poales Poaceae Eustachys glauca#NC 036685
Poales Poaceae Dichaetaria wightii#NC 035525
Poales Poaceae Alloeochaete oreogena#NC 035518
Poales Poaceae Alloeochaete ulugurensis#NC 035519
Poales Poaceae Alloeochaete namuliensis#NC 035517
Poales Poaceae Centotheca lappacea#NC 025229
Poales Poaceae Thysanolaena latifolia#NC 025238
Poales Poaceae Danthoniopsis dinteri#MF035977
Poales Poaceae Loudetia simplex#NC 036113
Poales Poaceae Tristachya leucothrix#NC 036708
Poales Poaceae Tristachya humbertii#NC 036115
Poales Poaceae Chasmanthium laxum#NC 035522
Poales Poaceae Zeugites pittieri#NC 030500
Poales Poaceae Gynerium sagittatum#NC 036687
Poales Poaceae Lecomtella madagascariensis#NC 024106
Poales Poaceae Digitaria exilis#NC 024176
Poales Poaceae Digitaria ciliaris#F674
Poales Poaceae Brachiaria fragrans#NC 033879
Poales Poaceae Echinolaena madagascariensis#NC 033880
Poales Poaceae Panicum lycopodioides#NC 036121
Poales Poaceae Lasiacis nigra#NC 036123
Poales Poaceae Pseudolasiacis leptolomoides#NC 036119
Poales Poaceae Echinochloa frumentacea#NC 029827
Poales Poaceae Echinochloa ugandensis#NC 036127
Poales Poaceae Echinochloa crus-galli#NC 028719
Poales Poaceae Echinochloa oryzoides#NC 024643
Poales Poaceae Echinochloa stagnina#NC 036128
Poales Poaceae Entolasia imbricata#NC 036126
Poales Poaceae Panicum pygmaeum#NC 036120
Poales Poaceae Alloteropsis cimicina#NC 027952
Poales Poaceae Alloteropsis angusta#NC 027951
Poales Poaceae Alloteropsis semialata#NC 027824
Poales Poaceae Panicum incomtum#MH394349
Poales Poaceae Sacciolepis indica#NC 036702
Poales Poaceae Thyridolepis mitchelliana#XBKS
Poales Poaceae Thyridolepis multiculmis#WCOR
Poales Poaceae Neurachne annularis#BPKH
Poales Poaceae Neurachne munroi#ZMGN
Poales Poaceae Neurachne tenuifolia#NNOK
Poales Poaceae Neurachne minor#BXAY
Poales Poaceae Paraneurachne muelleri#NC 039450
Poales Poaceae Neurachne lanigera#VQYB
Poales Poaceae Neurachne alopecuroidea#ZENX
Poales Poaceae Panicum virgatum#NC 015990
Poales Poaceae Panicum capillare#NC 030493
Poales Poaceae Panicum miliaceum#NC 029732
Poales Poaceae Stenotaphrum secundatum#NC 036704
Poales Poaceae Setaria italica#NC 022850
Poales Poaceae Setaria viridis#NC 028075
Poales Poaceae Cenchrus ciliaris#NC 041434
Poales Poaceae Cenchrus americanus#NC 024171
Poales Poaceae Cenchrus purpureus#MH488956
Poales Poaceae Tricholaena monachne#NC 036706
Poales Poaceae Panicum maximum#NC 030489

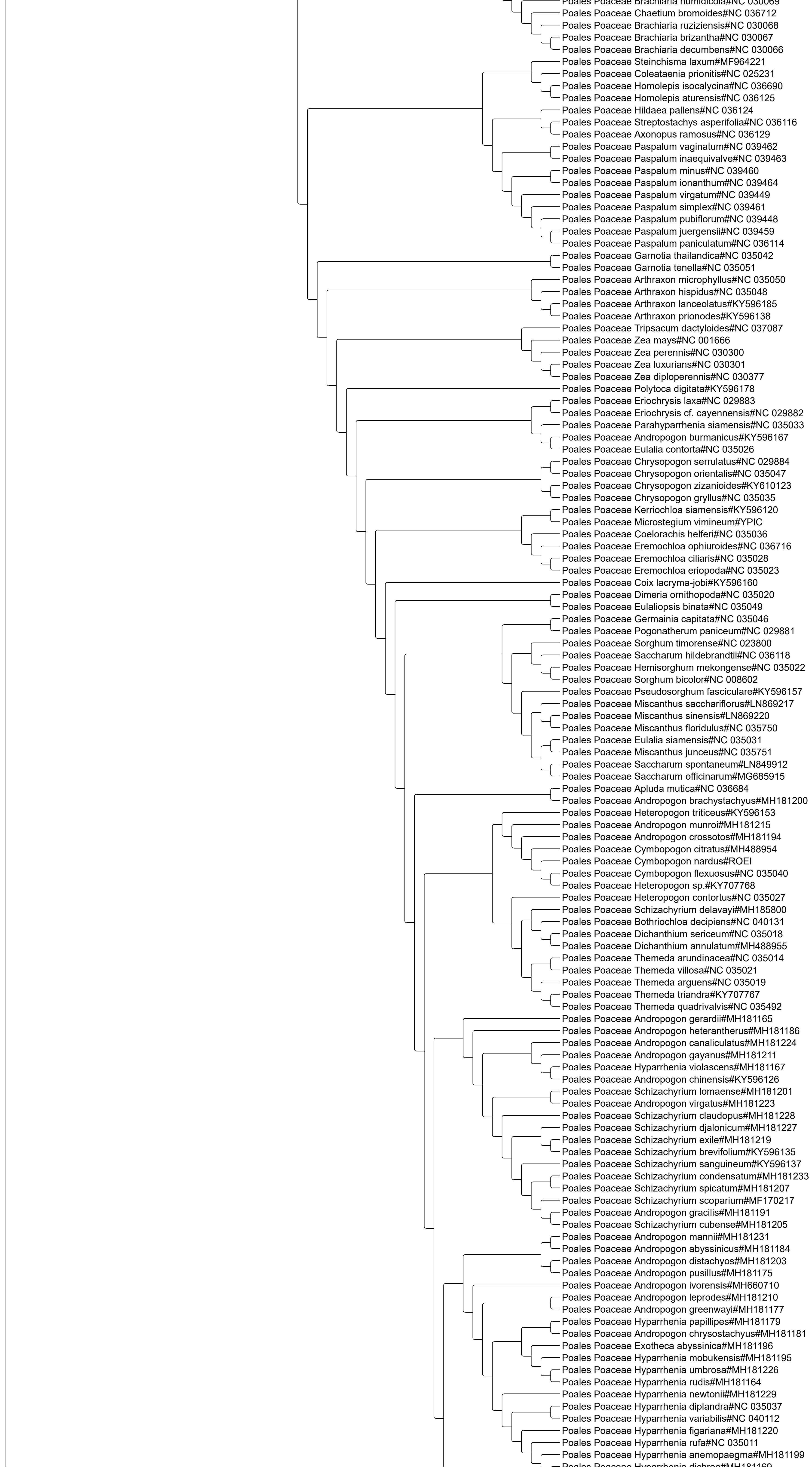
Poales Poaceae Brachiaria humidicola#NC 030069
Poales Poaceae Chaetium bromoides#NC 036712
Poales Poaceae Brachiaria ruziziensis#NC 030068
Poales Poaceae Brachiaria brizantha#NC 030067
Poales Poaceae Brachiaria decumbens#NC 030066
Poales Poaceae Steinchisma laxum#MF964221
Poales Poaceae Coleataenia prionitis#NC 025231
Poales Poaceae Homolepis isocalycina#NC 036690
Poales Poaceae Homolepis aturensis#NC 036125
Poales Poaceae Hildaea pallens#NC 036124
Poales Poaceae Streptostachys asperifolia#NC 036116
Poales Poaceae Axonopus ramosus#NC 036129
Poales Poaceae Paspalum vaginatum#NC 039462
Poales Poaceae Paspalum inaequivalve#NC 039463
Poales Poaceae Paspalum minus#NC 039460
Poales Poaceae Paspalum ionanthum#NC 039464
Poales Poaceae Paspalum virgatum#NC 039449
Poales Poaceae Paspalum simplex#NC 039461
Poales Poaceae Paspalum pubiflorum#NC 039448
Poales Poaceae Paspalum juergensii#NC 039459
Poales Poaceae Paspalum paniculatum#NC 036114
Poales Poaceae Garnotia thailandica#NC 035042
Poales Poaceae Garnotia tenella#NC 035051
Poales Poaceae Arthraxon microphyllus#NC 035050
Poales Poaceae Arthraxon hispidus#NC 035048
Poales Poaceae Arthraxon lanceolatus#KY596185
Poales Poaceae Arthraxon prionodes#KY596138
Poales Poaceae Tripsacum dactyloides#NC 037087
Poales Poaceae Zea mays#NC 001666
Poales Poaceae Zea perennis#NC 030300
Poales Poaceae Zea luxurians#NC 030301
Poales Poaceae Zea diploperennis#NC 030377
Poales Poaceae Polytoca digitata#KY596178
Poales Poaceae Eriochrysis laxa#NC 029883
Poales Poaceae Eriochrysis cf. cayennensis#NC 029882
Poales Poaceae Parahyparrhenia siamensis#NC 035033
Poales Poaceae Andropogon burmanicus#KY596167
Poales Poaceae Eulalia contorta#NC 035026
Poales Poaceae Chrysopogon serrulatus#NC 029884
Poales Poaceae Chrysopogon orientalis#NC 035047
Poales Poaceae Chrysopogon zizanioides#KY610123
Poales Poaceae Chrysopogon gryllus#NC 035035
Poales Poaceae Kerriochloa siamensis#KY596120
Poales Poaceae Microstegium vimineum#YPIC
Poales Poaceae Coelorachis helferi#NC 035036
Poales Poaceae Eremochloa ophiuroides#NC 036716
Poales Poaceae Eremochloa ciliaris#NC 035028
Poales Poaceae Eremochloa eriopoda#NC 035023
Poales Poaceae Coix lacryma-jobi#KY596160
Poales Poaceae Dimeria ornithopoda#NC 035020
Poales Poaceae Eulaliopsis binata#NC 035049
Poales Poaceae Germainia capitata#NC 035046
Poales Poaceae Pogonatherum paniceum#NC 029881
Poales Poaceae Sorghum timorense#NC 023800
Poales Poaceae Saccharum hildebrandtii#NC 036118
Poales Poaceae Hemisorghum mekongense#NC 035022
Poales Poaceae Sorghum bicolor#NC 008602
Poales Poaceae Pseudosorghum fasciculare#KY596157
Poales Poaceae Miscanthus sacchariflorus#LN869217
Poales Poaceae Miscanthus sinensis#LN869220
Poales Poaceae Miscanthus floridulus#NC 035750
Poales Poaceae Eulalia siamensis#NC 035031
Poales Poaceae Miscanthus junceus#NC 035751
Poales Poaceae Saccharum spontaneum#LN849912
Poales Poaceae Saccharum officinarum#MG685915
Poales Poaceae Apluda mutica#NC 036684
Poales Poaceae Andropogon brachystachyus#MH181200
Poales Poaceae Heteropogon triticeus#KY596153
Poales Poaceae Andropogon munroi#MH181215
Poales Poaceae Andropogon crossotos#MH181194
Poales Poaceae Cymbopogon citratus#MH488954
Poales Poaceae Cymbopogon nardus#ROEI
Poales Poaceae Cymbopogon flexuosus#NC 035040
Poales Poaceae Heteropogon sp.#KY707768
Poales Poaceae Heteropogon contortus#NC 035027
Poales Poaceae Schizachyrium delavayi#MH185800
Poales Poaceae Bothriochloa decipiens#NC 040131
Poales Poaceae Dichanthium sericeum#NC 035018
Poales Poaceae Dichanthium annulatum#MH488955
Poales Poaceae Themeda arundinacea#NC 035014
Poales Poaceae Themeda villosa#NC 035021
Poales Poaceae Themeda arguens#NC 035019
Poales Poaceae Themeda triandra#KY707767
Poales Poaceae Themeda quadrivalvis#NC 035492
Poales Poaceae Andropogon gerardii#MH181165
Poales Poaceae Andropogon heterantherus#MH181186
Poales Poaceae Andropogon canaliculatus#MH181224
Poales Poaceae Andropogon gayanus#MH181211
Poales Poaceae Hyparrhenia violascens#MH181167
Poales Poaceae Andropogon chinensis#KY596126
Poales Poaceae Schizachyrium lomaense#MH181201
Poales Poaceae Andropogon virgatus#MH181223
Poales Poaceae Schizachyrium claudopus#MH181228
Poales Poaceae Schizachyrium djalonicum#MH181227
Poales Poaceae Schizachyrium exile#MH181219
Poales Poaceae Schizachyrium brevifolium#KY596135
Poales Poaceae Schizachyrium sanguineum#KY596137
Poales Poaceae Schizachyrium condensatum#MH181233
Poales Poaceae Schizachyrium spicatum#MH181207
Poales Poaceae Schizachyrium scoparium#MF170217
Poales Poaceae Andropogon gracilis#MH181191
Poales Poaceae Schizachyrium cubense#MH181205
Poales Poaceae Andropogon mannii#MH181231
Poales Poaceae Andropogon abyssinicus#MH181184
Poales Poaceae Andropogon distachyos#MH181203
Poales Poaceae Andropogon pusillus#MH181175
Poales Poaceae Andropogon ivorensis#MH660710
Poales Poaceae Andropogon leprodes#MH181210
Poales Poaceae Andropogon greenwayi#MH181177
Poales Poaceae Hyparrhenia papillipes#MH181179
Poales Poaceae Andropogon chrysostachyus#MH181181
Poales Poaceae Exotheca abyssinica#MH181196
Poales Poaceae Hyparrhenia mobukensis#MH181195
Poales Poaceae Hyparrhenia umbrosa#MH181226
Poales Poaceae Hyparrhenia rudis#MH181164
Poales Poaceae Hyparrhenia newtonii#MH181229
Poales Poaceae Hyparrhenia diplandra#NC 035037
Poales Poaceae Hyparrhenia variabilis#NC 040112
Poales Poaceae Hyparrhenia figariana#MH181220
Poales Poaceae Hyparrhenia rufa#NC 035011
Poales Poaceae Hyparrhenia anemopaegma#MH181199

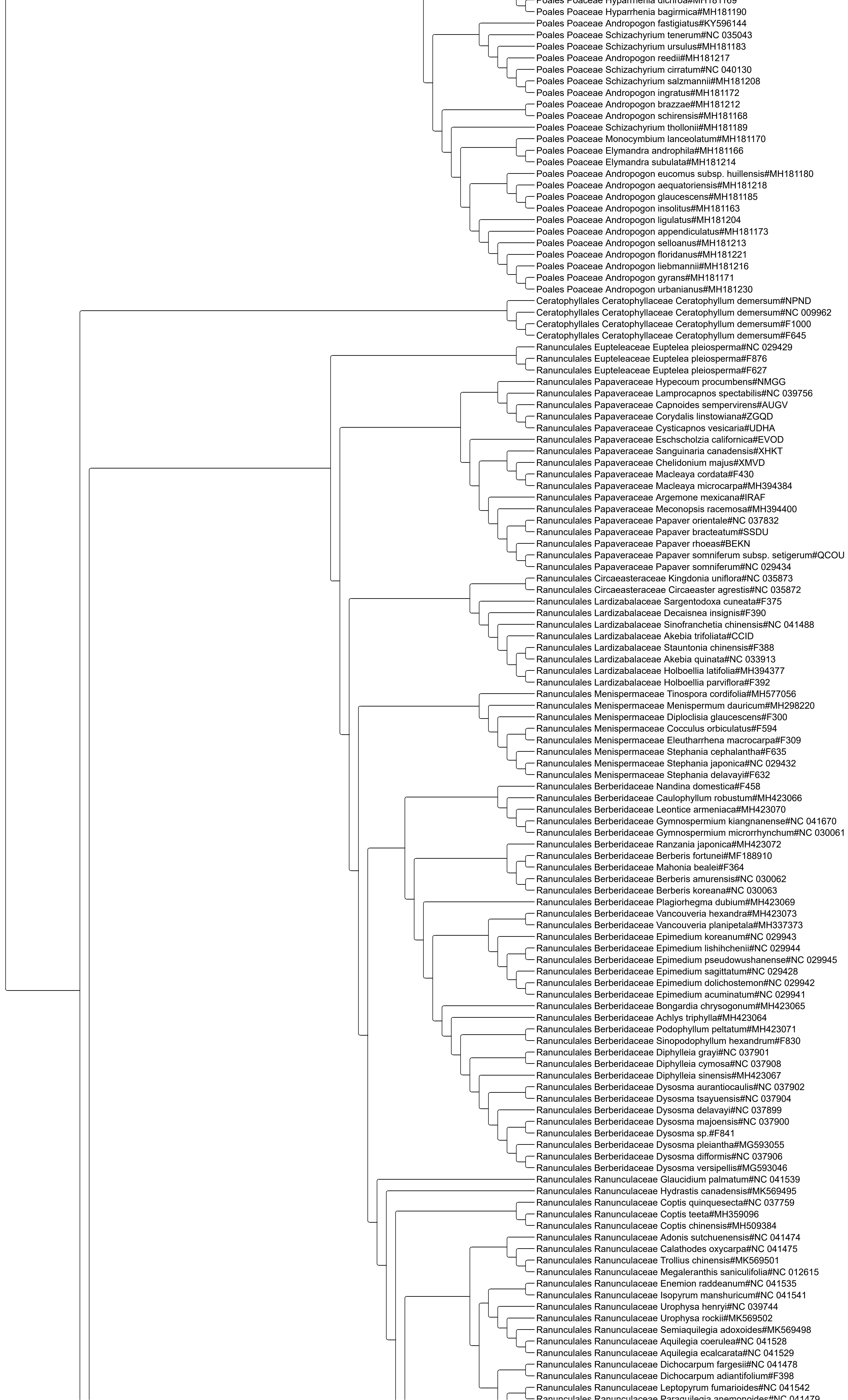
Poales Poaceae Hyparrhenia dichroa#MH181169
Poales Poaceae Hyparrhenia bagirmica#MH181190
Poales Poaceae Andropogon fastigiatus#KY596144
Poales Poaceae Schizachyrium tenerum#NC 035043
Poales Poaceae Schizachyrium ursulus#MH181183
Poales Poaceae Andropogon reedii#MH181217
Poales Poaceae Schizachyrium cirratum#NC 040130
Poales Poaceae Schizachyrium salzmannii#MH181208
Poales Poaceae Andropogon ingratus#MH181172
Poales Poaceae Andropogon brazzae#MH181212
Poales Poaceae Andropogon schirensis#MH181168
Poales Poaceae Schizachyrium thollonii#MH181189
Poales Poaceae Monocymbium lanceolatum#MH181170
Poales Poaceae Elymandra androphila#MH181166
Poales Poaceae Elymandra subulata#MH181214
Poales Poaceae Andropogon eucomus subsp. huillensis#MH181180
Poales Poaceae Andropogon aequatoriensis#MH181218
Poales Poaceae Andropogon glaucescens#MH181185
Poales Poaceae Andropogon insolitus#MH181163
Poales Poaceae Andropogon ligulatus#MH181204
Poales Poaceae Andropogon appendiculatus#MH181173
Poales Poaceae Andropogon selloanus#MH181213
Poales Poaceae Andropogon floridanus#MH181221
Poales Poaceae Andropogon liebmannii#MH181216
Poales Poaceae Andropogon gyrans#MH181171
Poales Poaceae Andropogon urbanianus#MH181230
Ceratophyllales Ceratophyllaceae Ceratophyllum demersum#NPND
Ceratophyllales Ceratophyllaceae Ceratophyllum demersum#NC 009962
Ceratophyllales Ceratophyllaceae Ceratophyllum demersum#F1000
Ceratophyllales Ceratophyllaceae Ceratophyllum demersum#F645
Ranunculales Eupteleaceae Euptelea pleiosperma#NC 029429
Ranunculales Eupteleaceae Euptelea pleiosperma#F876
Ranunculales Eupteleaceae Euptelea pleiosperma#F627
Ranunculales Papaveraceae Hypecoum procumbens#NMGG
Ranunculales Papaveraceae Lamprocapnos spectabilis#NC 039756
Ranunculales Papaveraceae Capnoides sempervirens#AUGV
Ranunculales Papaveraceae Corydalis linstowiana#ZGQD
Ranunculales Papaveraceae Cysticapnos vesicaria#UDHA
Ranunculales Papaveraceae Eschscholzia californica#EVOD
Ranunculales Papaveraceae Sanguinaria canadensis#XHKT
Ranunculales Papaveraceae Chelidonium majus#XMVD
Ranunculales Papaveraceae Macleaya cordata#F430
Ranunculales Papaveraceae Macleaya microcarpa#MH394384
Ranunculales Papaveraceae Argemone mexicana#IRAF
Ranunculales Papaveraceae Meconopsis racemosa#MH394400
Ranunculales Papaveraceae Papaver orientale#NC 037832
Ranunculales Papaveraceae Papaver bracteatum#SSDU
Ranunculales Papaveraceae Papaver rhoeas#BEKN
Ranunculales Papaveraceae Papaver somniferum subsp. setigerum#QCOU
Ranunculales Papaveraceae Papaver somniferum#NC 029434
Ranunculales Circaeasteraceae Kingdonia uniflora#NC 035873
Ranunculales Circaeasteraceae Circaeaster agrestis#NC 035872
Ranunculales Lardizabalaceae Sargentodoxa cuneata#F375
Ranunculales Lardizabalaceae Decaisnea insignis#F390
Ranunculales Lardizabalaceae Sinofranchetia chinensis#NC 041488
Ranunculales Lardizabalaceae Akebia trifoliata#CCID
Ranunculales Lardizabalaceae Stauntonia chinensis#F388
Ranunculales Lardizabalaceae Akebia quinata#NC 033913
Ranunculales Lardizabalaceae Holboellia latifolia#MH394377
Ranunculales Lardizabalaceae Holboellia parviflora#F392
Ranunculales Menispermaceae Tinospora cordifolia#MH577056
Ranunculales Menispermaceae Menispermum dauricum#MH298220
Ranunculales Menispermaceae Diploclisia glaucescens#F300
Ranunculales Menispermaceae Cocculus orbiculatus#F594
Ranunculales Menispermaceae Eleutharrhena macrocarpa#F309
Ranunculales Menispermaceae Stephania cephalantha#F635
Ranunculales Menispermaceae Stephania japonica#NC 029432
Ranunculales Menispermaceae Stephania delavayi#F632
Ranunculales Berberidaceae Nandina domestica#F458
Ranunculales Berberidaceae Caulophyllum robustum#MH423066
Ranunculales Berberidaceae Leontice armeniaca#MH423070
Ranunculales Berberidaceae Gymnospermium kiangnanense#NC 041670
Ranunculales Berberidaceae Gymnospermium microrrhynchum#NC 030061
Ranunculales Berberidaceae Ranzania japonica#MH423072
Ranunculales Berberidaceae Berberis fortunei#MF188910
Ranunculales Berberidaceae Mahonia bealei#F364
Ranunculales Berberidaceae Berberis amurensis#NC 030062
Ranunculales Berberidaceae Berberis koreana#NC 030063
Ranunculales Berberidaceae Plagiorhegma dubium#MH423069
Ranunculales Berberidaceae Vancouveria hexandra#MH423073
Ranunculales Berberidaceae Vancouveria planipetala#MH337373
Ranunculales Berberidaceae Epimedium koreanum#NC 029943
Ranunculales Berberidaceae Epimedium lishihchenii#NC 029944
Ranunculales Berberidaceae Epimedium pseudowushanense#NC 029945
Ranunculales Berberidaceae Epimedium sagittatum#NC 029428
Ranunculales Berberidaceae Epimedium dolichostemon#NC 029942
Ranunculales Berberidaceae Epimedium acuminatum#NC 029941
Ranunculales Berberidaceae Bongardia chrysogonum#MH423065
Ranunculales Berberidaceae Achlys triphylla#MH423064
Ranunculales Berberidaceae Podophyllum peltatum#MH423071
Ranunculales Berberidaceae Sinopodophyllum hexandrum#F830
Ranunculales Berberidaceae Diphylleia grayi#NC 037901
Ranunculales Berberidaceae Diphylleia cymosa#NC 037908
Ranunculales Berberidaceae Diphylleia sinensis#MH423067
Ranunculales Berberidaceae Dysosma aurantiocaulis#NC 037902
Ranunculales Berberidaceae Dysosma tsayuensis#NC 037904
Ranunculales Berberidaceae Dysosma delavayi#NC 037899
Ranunculales Berberidaceae Dysosma majoensis#NC 037900
Ranunculales Berberidaceae Dysosma sp.#F841
Ranunculales Berberidaceae Dysosma pleiantha#MG593055
Ranunculales Berberidaceae Dysosma difformis#NC 037906
Ranunculales Berberidaceae Dysosma versipellis#MG593046
Ranunculales Ranunculaceae Glaucidium palmatum#NC 041539
Ranunculales Ranunculaceae Hydrastis canadensis#MK569495
Ranunculales Ranunculaceae Coptis quinquesecta#NC 037759
Ranunculales Ranunculaceae Coptis teeta#MH359096
Ranunculales Ranunculaceae Coptis chinensis#MH509384
Ranunculales Ranunculaceae Adonis sutchuenensis#NC 041474
Ranunculales Ranunculaceae Calathodes oxycarpa#NC 041475
Ranunculales Ranunculaceae Trollius chinensis#MK569501
Ranunculales Ranunculaceae Megaleranthis saniculifolia#NC 012615
Ranunculales Ranunculaceae Enemion raddeanum#NC 041535
Ranunculales Ranunculaceae Isopyrum manshuricum#NC 041541
Ranunculales Ranunculaceae Urophysa henryi#NC 039744
Ranunculales Ranunculaceae Urophysa rockii#MK569502
Ranunculales Ranunculaceae Semiaquilegia adoxoides#MK569498
Ranunculales Ranunculaceae Aquilegia coerulea#NC 041528
Ranunculales Ranunculaceae Aquilegia ecalcarata#NC 041529
Ranunculales Ranunculaceae Dichocarpum fargesii#NC 041478
Ranunculales Ranunculaceae Dichocarpum adiantifolium#F398
Ranunculales Ranunculaceae Leptopyrum fumarioides#NC 041542
Ranunculales Ranunculaceae Paraquilegia anemonoides#NC 041479

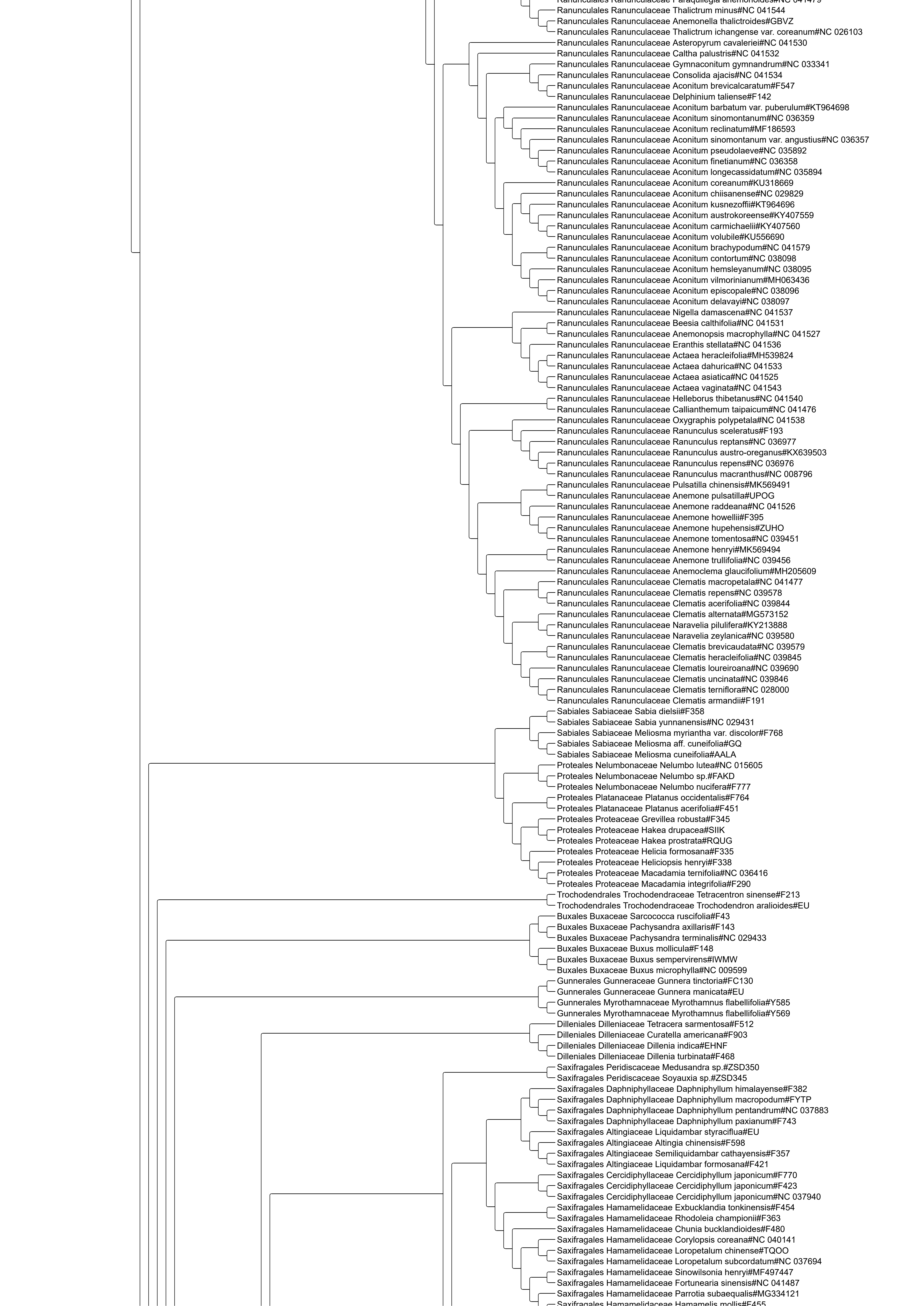
Ranunculales Ranunculaceae Paraquilegia anemonoides#NC 041479
Ranunculales Ranunculaceae Thalictrum minus#NC 041544
Ranunculales Ranunculaceae Anemonella thalictroides#GBVZ
Ranunculales Ranunculaceae Thalictrum ichangense var. coreanum#NC 026103
Ranunculales Ranunculaceae Asteropyrum cavaleriei#NC 041530
Ranunculales Ranunculaceae Caltha palustris#NC 041532
Ranunculales Ranunculaceae Gymnaconitum gymnandrum#NC 033341
Ranunculales Ranunculaceae Consolida ajacis#NC 041534
Ranunculales Ranunculaceae Aconitum brevicalcaratum#F547
Ranunculales Ranunculaceae Delphinium taliense#F142
Ranunculales Ranunculaceae Aconitum barbatum var. puberulum#KT964698
Ranunculales Ranunculaceae Aconitum sinomontanum#NC 036359
Ranunculales Ranunculaceae Aconitum reclinatum#MF186593
Ranunculales Ranunculaceae Aconitum sinomontanum var. angustius#NC 036357
Ranunculales Ranunculaceae Aconitum pseudolaeve#NC 035892
Ranunculales Ranunculaceae Aconitum finetianum#NC 036358
Ranunculales Ranunculaceae Aconitum longecassidatum#NC 035894
Ranunculales Ranunculaceae Aconitum coreanum#KU318669
Ranunculales Ranunculaceae Aconitum chiisanense#NC 029829
Ranunculales Ranunculaceae Aconitum kusnezoffii#KT964696
Ranunculales Ranunculaceae Aconitum austrokoreense#KY407559
Ranunculales Ranunculaceae Aconitum carmichaelii#KY407560
Ranunculales Ranunculaceae Aconitum volubile#KU556690
Ranunculales Ranunculaceae Aconitum brachypodum#NC 041579
Ranunculales Ranunculaceae Aconitum contortum#NC 038098
Ranunculales Ranunculaceae Aconitum hemsleyanum#NC 038095
Ranunculales Ranunculaceae Aconitum vilmorinianum#MH063436
Ranunculales Ranunculaceae Aconitum episcopale#NC 038096
Ranunculales Ranunculaceae Aconitum delavayi#NC 038097
Ranunculales Ranunculaceae Nigella damascena#NC 041537
Ranunculales Ranunculaceae Beesia calthifolia#NC 041531
Ranunculales Ranunculaceae Anemonopsis macrophylla#NC 041527
Ranunculales Ranunculaceae Eranthis stellata#NC 041536
Ranunculales Ranunculaceae Actaea heracleifolia#MH539824
Ranunculales Ranunculaceae Actaea dahurica#NC 041533
Ranunculales Ranunculaceae Actaea asiatica#NC 041525
Ranunculales Ranunculaceae Actaea vaginata#NC 041543
Ranunculales Ranunculaceae Helleborus thibetanus#NC 041540
Ranunculales Ranunculaceae Callianthemum taipaicum#NC 041476
Ranunculales Ranunculaceae Oxygraphis polypetala#NC 041538
Ranunculales Ranunculaceae Ranunculus sceleratus#F193
Ranunculales Ranunculaceae Ranunculus reptans#NC 036977
Ranunculales Ranunculaceae Ranunculus austro-oreganus#KX639503
Ranunculales Ranunculaceae Ranunculus repens#NC 036976
Ranunculales Ranunculaceae Ranunculus macranthus#NC 008796
Ranunculales Ranunculaceae Pulsatilla chinensis#MK569491
Ranunculales Ranunculaceae Anemone pulsatilla#UPOG
Ranunculales Ranunculaceae Anemone raddeana#NC 041526
Ranunculales Ranunculaceae Anemone howellii#F395
Ranunculales Ranunculaceae Anemone hupehensis#ZUHO
Ranunculales Ranunculaceae Anemone tomentosa#NC 039451
Ranunculales Ranunculaceae Anemone henryi#MK569494
Ranunculales Ranunculaceae Anemone trullifolia#NC 039456
Ranunculales Ranunculaceae Anemoclema glaucifolium#MH205609
Ranunculales Ranunculaceae Clematis macropetala#NC 041477
Ranunculales Ranunculaceae Clematis repens#NC 039578
Ranunculales Ranunculaceae Clematis acerifolia#NC 039844
Ranunculales Ranunculaceae Clematis alternata#MG573152
Ranunculales Ranunculaceae Naravelia pilulifera#KY213888
Ranunculales Ranunculaceae Naravelia zeylanica#NC 039580
Ranunculales Ranunculaceae Clematis brevicaudata#NC 039579
Ranunculales Ranunculaceae Clematis heracleifolia#NC 039845
Ranunculales Ranunculaceae Clematis loureiroana#NC 039690
Ranunculales Ranunculaceae Clematis uncinata#NC 039846
Ranunculales Ranunculaceae Clematis terniflora#NC 028000
Ranunculales Ranunculaceae Clematis armandii#F191
Sabiales Sabiaceae Sabia dielsii#F358
Sabiales Sabiaceae Sabia yunnanensis#NC 029431
Sabiales Sabiaceae Meliosma myriantha var. discolor#F768
Sabiales Sabiaceae Meliosma aff. cuneifolia#GQ
Sabiales Sabiaceae Meliosma cuneifolia#AALA
Proteales Nelumbonaceae Nelumbo lutea#NC 015605
Proteales Nelumbonaceae Nelumbo sp.#FAKD
Proteales Nelumbonaceae Nelumbo nucifera#F777
Proteales Platanaceae Platanus occidentalis#F764
Proteales Platanaceae Platanus acerifolia#F451
Proteales Proteaceae Grevillea robusta#F345
Proteales Proteaceae Hakea drupacea#SIIK
Proteales Proteaceae Hakea prostrata#RQUG
Proteales Proteaceae Helicia formosana#F335
Proteales Proteaceae Heliciopsis henryi#F338
Proteales Proteaceae Macadamia ternifolia#NC 036416
Proteales Proteaceae Macadamia integrifolia#F290
Trochodendrales Trochodendraceae Tetracentron sinense#F213
Trochodendrales Trochodendraceae Trochodendron aralioides#EU
Buxales Buxaceae Sarcococca ruscifolia#F43
Buxales Buxaceae Pachysandra axillaris#F143
Buxales Buxaceae Pachysandra terminalis#NC 029433
Buxales Buxaceae Buxus mollicula#F148
Buxales Buxaceae Buxus sempervirens#IWMW
Buxales Buxaceae Buxus microphylla#NC 009599
Gunnerales Gunneraceae Gunnera tinctoria#FC130
Gunnerales Gunneraceae Gunnera manicata#EU
Gunnerales Myrothamnaceae Myrothamnus flabellifolia#Y585
Gunnerales Myrothamnaceae Myrothamnus flabellifolia#Y569
Dilleniales Dilleniaceae Tetracera sarmentosa#F512
Dilleniales Dilleniaceae Curatella americana#F903
Dilleniales Dilleniaceae Dillenia indica#EHNF
Dilleniales Dilleniaceae Dillenia turbinata#F468
Saxifragales Peridiscaceae Medusandra sp.#ZSD350
Saxifragales Peridiscaceae Soyauxia sp.#ZSD345
Saxifragales Daphniphyllaceae Daphniphyllum himalayense#F382
Saxifragales Daphniphyllaceae Daphniphyllum macropodum#FYTP
Saxifragales Daphniphyllaceae Daphniphyllum pentandrum#NC 037883
Saxifragales Daphniphyllaceae Daphniphyllum paxianum#F743
Saxifragales Altingiaceae Liquidambar styraciflua#EU
Saxifragales Altingiaceae Altingia chinensis#F598
Saxifragales Altingiaceae Semiliquidambar cathayensis#F357
Saxifragales Altingiaceae Liquidambar formosana#F421
Saxifragales Cercidiphyllaceae Cercidiphyllum japonicum#F770
Saxifragales Cercidiphyllaceae Cercidiphyllum japonicum#F423
Saxifragales Cercidiphyllaceae Cercidiphyllum japonicum#NC 037940
Saxifragales Hamamelidaceae Exbucklandia tonkinensis#F454
Saxifragales Hamamelidaceae Rhodoleia championii#F363
Saxifragales Hamamelidaceae Chunia bucklandioides#F480
Saxifragales Hamamelidaceae Corylopsis coreana#NC 040141
Saxifragales Hamamelidaceae Loropetalum chinense#TQOO
Saxifragales Hamamelidaceae Loropetalum subcordatum#NC 037694
Saxifragales Hamamelidaceae Sinowilsonia henryi#MF497447
Saxifragales Hamamelidaceae Fortunearia sinensis#NC 041487
Saxifragales Hamamelidaceae Parrotia subaequalis#MG334121
Saxifragales Hamamelidaceae Hamamelis mollis#F455

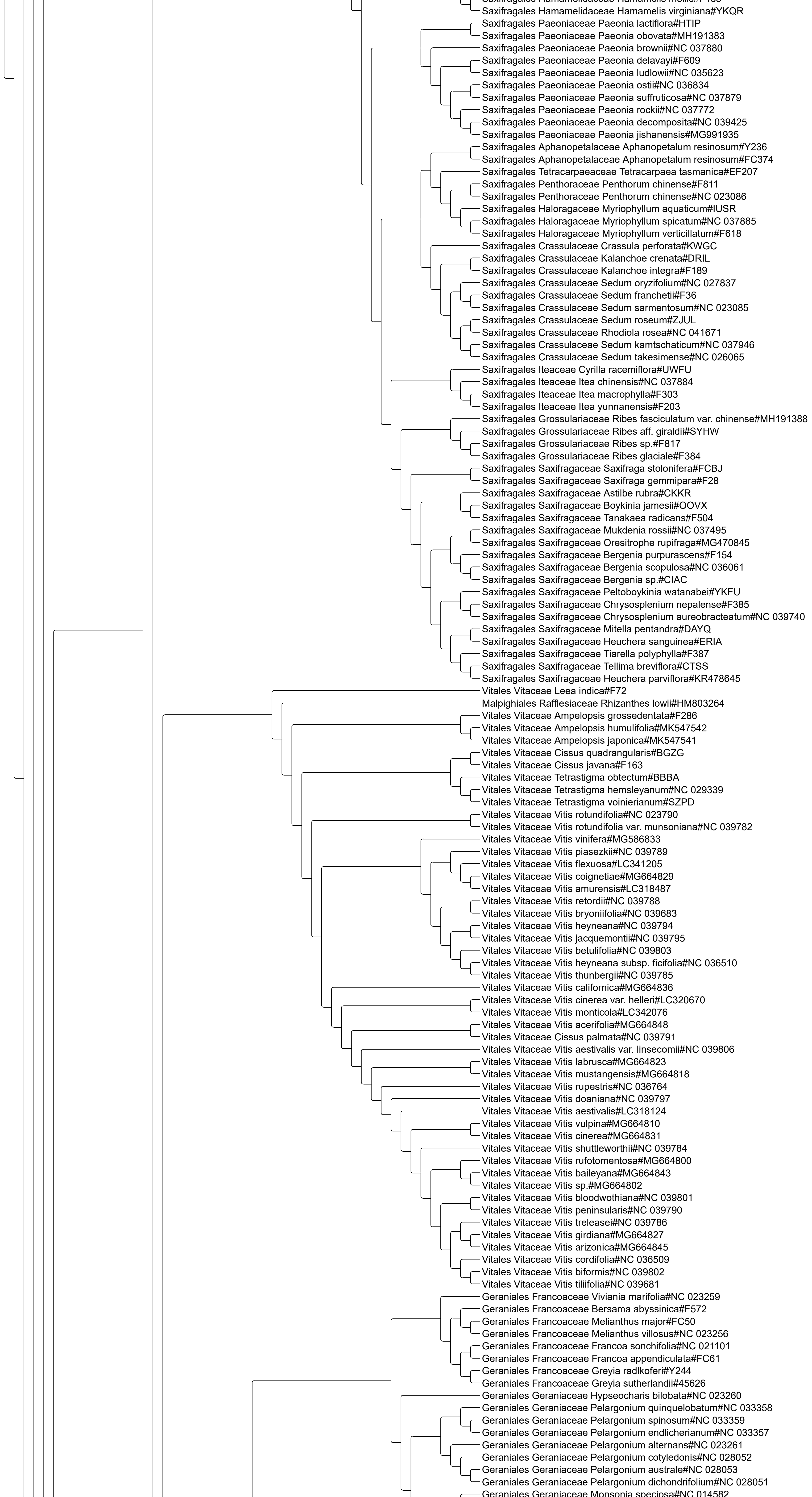
Saxifragales Hamamelidaceae Hamamelis virginiana#YKQR
Saxifragales Paeoniaceae Paeonia lactiflora#HTIP
Saxifragales Paeoniaceae Paeonia obovata#MH191383
Saxifragales Paeoniaceae Paeonia brownii#NC 037880
Saxifragales Paeoniaceae Paeonia delavayi#F609
Saxifragales Paeoniaceae Paeonia ludlowii#NC 035623
Saxifragales Paeoniaceae Paeonia ostii#NC 036834
Saxifragales Paeoniaceae Paeonia suffruticosa#NC 037879
Saxifragales Paeoniaceae Paeonia rockii#NC 037772
Saxifragales Paeoniaceae Paeonia decomposita#NC 039425
Saxifragales Paeoniaceae Paeonia jishanensis#MG991935
Saxifragales Aphanopetalaceae Aphanopetalum resinosum#Y236
Saxifragales Aphanopetalaceae Aphanopetalum resinosum#FC374
Saxifragales Tetracarpaeaceae Tetracarpaea tasmanica#EF207
Saxifragales Penthoraceae Penthorum chinense#F811
Saxifragales Penthoraceae Penthorum chinense#NC 023086
Saxifragales Haloragaceae Myriophyllum aquaticum#IUSR
Saxifragales Haloragaceae Myriophyllum spicatum#NC 037885
Saxifragales Haloragaceae Myriophyllum verticillatum#F618
Saxifragales Crassulaceae Crassula perforata#KWGC
Saxifragales Crassulaceae Kalanchoe crenata#DRIL
Saxifragales Crassulaceae Kalanchoe integra#F189
Saxifragales Crassulaceae Sedum oryzifolium#NC 027837
Saxifragales Crassulaceae Sedum franchetii#F36
Saxifragales Crassulaceae Sedum sarmentosum#NC 023085
Saxifragales Crassulaceae Sedum roseum#ZJUL
Saxifragales Crassulaceae Rhodiola rosea#NC 041671
Saxifragales Crassulaceae Sedum kamtschaticum#NC 037946
Saxifragales Crassulaceae Sedum takesimense#NC 026065
Saxifragales Iteaceae Cyrilla racemiflora#UWFU
Saxifragales Iteaceae Itea chinensis#NC 037884
Saxifragales Iteaceae Itea macrophylla#F303
Saxifragales Iteaceae Itea yunnanensis#F203
Saxifragales Grossulariaceae Ribes fasciculatum var. chinense#MH191388
Saxifragales Grossulariaceae Ribes aff. giraldii#SYHW
Saxifragales Grossulariaceae Ribes sp.#F817
Saxifragales Grossulariaceae Ribes glaciale#F384
Saxifragales Saxifragaceae Saxifraga stolonifera#FCBJ
Saxifragales Saxifragaceae Saxifraga gemmipara#F28
Saxifragales Saxifragaceae Astilbe rubra#CKKR
Saxifragales Saxifragaceae Boykinia jamesii#OOVX
Saxifragales Saxifragaceae Tanakaea radicans#F504
Saxifragales Saxifragaceae Mukdenia rossii#NC 037495
Saxifragales Saxifragaceae Oresitrophe rupifraga#MG470845
Saxifragales Saxifragaceae Bergenia purpurascens#F154
Saxifragales Saxifragaceae Bergenia scopulosa#NC 036061
Saxifragales Saxifragaceae Bergenia sp.#CIAC
Saxifragales Saxifragaceae Peltoboykinia watanabei#YKFU
Saxifragales Saxifragaceae Chrysosplenium nepalense#F385
Saxifragales Saxifragaceae Chrysosplenium aureobracteatum#NC 039740
Saxifragales Saxifragaceae Mitella pentandra#DAYQ
Saxifragales Saxifragaceae Heuchera sanguinea#ERIA
Saxifragales Saxifragaceae Tiarella polyphylla#F387
Saxifragales Saxifragaceae Tellima breviflora#CTSS
Saxifragales Saxifragaceae Heuchera parviflora#KR478645
Vitales Vitaceae Leea indica#F72
Malpighiales Rafflesiaceae Rhizanthes lowii#HM803264
Vitales Vitaceae Ampelopsis grossedentata#F286
Vitales Vitaceae Ampelopsis humulifolia#MK547542
Vitales Vitaceae Ampelopsis japonica#MK547541
Vitales Vitaceae Cissus quadrangularis#BGZG
Vitales Vitaceae Cissus javana#F163
Vitales Vitaceae Tetrastigma obtectum#BBBA
Vitales Vitaceae Tetrastigma hemsleyanum#NC 029339
Vitales Vitaceae Tetrastigma voinierianum#SZPD
Vitales Vitaceae Vitis rotundifolia#NC 023790
Vitales Vitaceae Vitis rotundifolia var. munsoniana#NC 039782
Vitales Vitaceae Vitis vinifera#MG586833
Vitales Vitaceae Vitis piasezkii#NC 039789
Vitales Vitaceae Vitis flexuosa#LC341205
Vitales Vitaceae Vitis coignetiae#MG664829
Vitales Vitaceae Vitis amurensis#LC318487
Vitales Vitaceae Vitis retordii#NC 039788
Vitales Vitaceae Vitis bryoniifolia#NC 039683
Vitales Vitaceae Vitis heyneana#NC 039794
Vitales Vitaceae Vitis jacquemontii#NC 039795
Vitales Vitaceae Vitis betulifolia#NC 039803
Vitales Vitaceae Vitis heyneana subsp. ficifolia#NC 036510
Vitales Vitaceae Vitis thunbergii#NC 039785
Vitales Vitaceae Vitis californica#MG664836
Vitales Vitaceae Vitis cinerea var. helleri#LC320670
Vitales Vitaceae Vitis monticola#LC342076
Vitales Vitaceae Vitis acerifolia#MG664848
Vitales Vitaceae Cissus palmata#NC 039791
Vitales Vitaceae Vitis aestivalis var. linsecomii#NC 039806
Vitales Vitaceae Vitis labrusca#MG664823
Vitales Vitaceae Vitis mustangensis#MG664818
Vitales Vitaceae Vitis rupestris#NC 036764
Vitales Vitaceae Vitis doaniana#NC 039797
Vitales Vitaceae Vitis aestivalis#LC318124
Vitales Vitaceae Vitis vulpina#MG664810
Vitales Vitaceae Vitis cinerea#MG664831
Vitales Vitaceae Vitis shuttleworthii#NC 039784
Vitales Vitaceae Vitis rufotomentosa#MG664800
Vitales Vitaceae Vitis baileyana#MG664843
Vitales Vitaceae Vitis sp.#MG664802
Vitales Vitaceae Vitis bloodwothiana#NC 039801
Vitales Vitaceae Vitis peninsularis#NC 039790
Vitales Vitaceae Vitis treleasei#NC 039786
Vitales Vitaceae Vitis girdiana#MG664827
Vitales Vitaceae Vitis arizonica#MG664845
Vitales Vitaceae Vitis cordifolia#NC 036509
Vitales Vitaceae Vitis biformis#NC 039802
Vitales Vitaceae Vitis tiliifolia#NC 039681
Geraniales Francoaceae Viviania marifolia#NC 023259
Geraniales Francoaceae Bersama abyssinica#F572
Geraniales Francoaceae Melianthus major#FC50
Geraniales Francoaceae Melianthus villosus#NC 023256
Geraniales Francoaceae Francoa sonchifolia#NC 021101
Geraniales Francoaceae Francoa appendiculata#FC61
Geraniales Francoaceae Greyia radlkoferi#Y244
Geraniales Francoaceae Greyia sutherlandii#45626
Geraniales Geraniaceae Hypseocharis bilobata#NC 023260
Geraniales Geraniaceae Pelargonium quinquelobatum#NC 033358
Geraniales Geraniaceae Pelargonium spinosum#NC 033359
Geraniales Geraniaceae Pelargonium endlicherianum#NC 033357
Geraniales Geraniaceae Pelargonium alternans#NC 023261
Geraniales Geraniaceae Pelargonium cotyledonis#NC 028052
Geraniales Geraniaceae Pelargonium australe#NC 028053
Geraniales Geraniaceae Pelargonium dichondrifolium#NC 028051
Geraniales Geraniaceae Monsonia speciosa#NC 014582

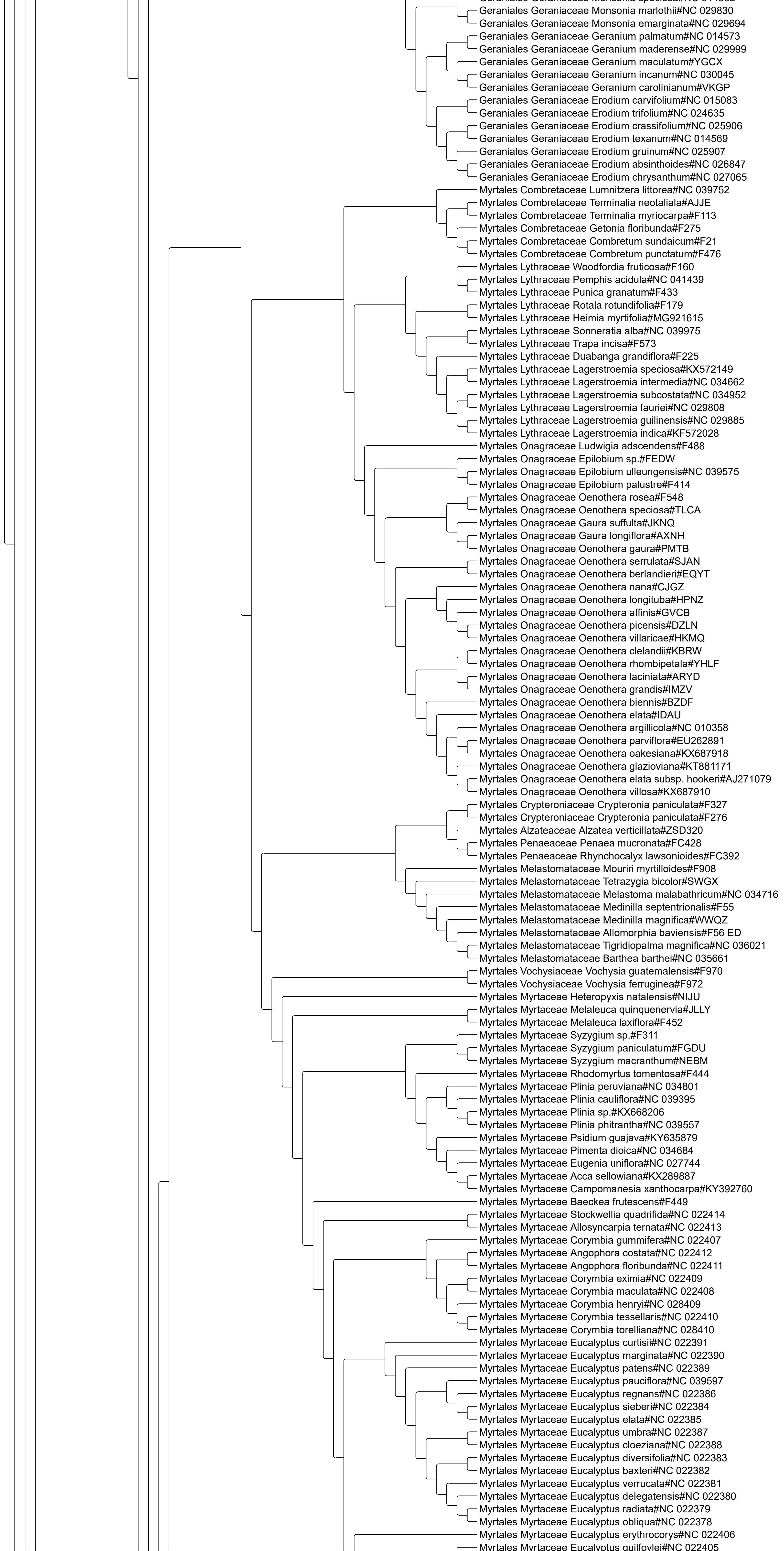

Geraniales Geraniaceae Monsonia marlothii#NC 029830
Geraniales Geraniaceae Monsonia emarginata#NC 029694
Geraniales Geraniaceae Geranium palmatum#NC 014573
Geraniales Geraniaceae Geranium maderense#NC 029999
Geraniales Geraniaceae Geranium maculatum#YGCX
Geraniales Geraniaceae Geranium incanum#NC 030045
Geraniales Geraniaceae Geranium carolinianum#VKGP
Geraniales Geraniaceae Erodium carvifolium#NC 015083
Geraniales Geraniaceae Erodium trifolium#NC 024635
Geraniales Geraniaceae Erodium crassifolium#NC 025906
Geraniales Geraniaceae Erodium texanum#NC 014569
Geraniales Geraniaceae Erodium gruinum#NC 025907
Geraniales Geraniaceae Erodium absinthoides#NC 026847
Geraniales Geraniaceae Erodium chrysanthum#NC 027065
Myrtales Combretaceae Lumnitzera littorea#NC 039752
Myrtales Combretaceae Terminalia neotaliala#AJJE
Myrtales Combretaceae Terminalia myriocarpa#F113
Myrtales Combretaceae Getonia floribunda#F275
Myrtales Combretaceae Combretum sundaicum#F21
Myrtales Combretaceae Combretum punctatum#F476
Myrtales Lythraceae Woodfordia fruticosa#F160
Myrtales Lythraceae Pemphis acidula#NC 041439
Myrtales Lythraceae Punica granatum#F433
Myrtales Lythraceae Rotala rotundifolia#F179
Myrtales Lythraceae Heimia myrtifolia#MG921615
Myrtales Lythraceae Sonneratia alba#NC 039975
Myrtales Lythraceae Trapa incisa#F573
Myrtales Lythraceae Duabanga grandiflora#F225
Myrtales Lythraceae Lagerstroemia speciosa#KX572149
Myrtales Lythraceae Lagerstroemia intermedia#NC 034662
Myrtales Lythraceae Lagerstroemia subcostata#NC 034952
Myrtales Lythraceae Lagerstroemia fauriei#NC 029808
Myrtales Lythraceae Lagerstroemia guilinensis#NC 029885
Myrtales Lythraceae Lagerstroemia indica#KF572028
Myrtales Onagraceae Ludwigia adscendens#F488
Myrtales Onagraceae Epilobium sp.#FEDW
Myrtales Onagraceae Epilobium ulleungensis#NC 039575
Myrtales Onagraceae Epilobium palustre#F414
Myrtales Onagraceae Oenothera rosea#F548
Myrtales Onagraceae Oenothera speciosa#TLCA
Myrtales Onagraceae Gaura suffulta#JKNQ
Myrtales Onagraceae Gaura longiflora#AXNH
Myrtales Onagraceae Oenothera gaura#PMTB
Myrtales Onagraceae Oenothera serrulata#SJAN
Myrtales Onagraceae Oenothera berlandieri#EQYT
Myrtales Onagraceae Oenothera nana#CJGZ
Myrtales Onagraceae Oenothera longituba#HPNZ
Myrtales Onagraceae Oenothera affinis#GVCB
Myrtales Onagraceae Oenothera picensis#DZLN
Myrtales Onagraceae Oenothera villaricae#HKMQ
Myrtales Onagraceae Oenothera clelandii#KBRW
Myrtales Onagraceae Oenothera rhombipetala#YHLF
Myrtales Onagraceae Oenothera laciniata#ARYD
Myrtales Onagraceae Oenothera grandis#IMZV
Myrtales Onagraceae Oenothera biennis#BZDF
Myrtales Onagraceae Oenothera elata#IDAU
Myrtales Onagraceae Oenothera argillicola#NC 010358
Myrtales Onagraceae Oenothera parviflora#EU262891
Myrtales Onagraceae Oenothera oakesiana#KX687918
Myrtales Onagraceae Oenothera glazioviana#KT881171
Myrtales Onagraceae Oenothera elata subsp. hookeri#AJ271079
Myrtales Onagraceae Oenothera villosa#KX687910
Myrtales Crypteroniaceae Crypteronia paniculata#F327
Myrtales Crypteroniaceae Crypteronia paniculata#F276
Myrtales Alzateaceae Alzatea verticillata#ZSD320
Myrtales Penaeaceae Penaea mucronata#FC428
Myrtales Penaeaceae Rhynchocalyx lawsonioides#FC392
Myrtales Melastomataceae Mouriri myrtilloides#F908
Myrtales Melastomataceae Tetrazygia bicolor#SWGX
Myrtales Melastomataceae Melastoma malabathricum#NC 034716
Myrtales Melastomataceae Medinilla septentrionalis#F55
Myrtales Melastomataceae Medinilla magnifica#WWQZ
Myrtales Melastomataceae Allomorphia baviensis#F56 ED
Myrtales Melastomataceae Tigridiopalma magnifica#NC 036021
Myrtales Melastomataceae Barthea barthei#NC 035661
Myrtales Vochysiaceae Vochysia guatemalensis#F970
Myrtales Vochysiaceae Vochysia ferruginea#F972
Myrtales Myrtaceae Heteropyxis natalensis#NIJU
Myrtales Myrtaceae Melaleuca quinquenervia#JLLY
Myrtales Myrtaceae Melaleuca laxiflora#F452
Myrtales Myrtaceae Syzygium sp.#F311
Myrtales Myrtaceae Syzygium paniculatum#FGDU
Myrtales Myrtaceae Syzygium macranthum#NEBM
Myrtales Myrtaceae Rhodomyrtus tomentosa#F444
Myrtales Myrtaceae Plinia peruviana#NC 034801
Myrtales Myrtaceae Plinia cauliflora#NC 039395
Myrtales Myrtaceae Plinia sp.#KX668206
Myrtales Myrtaceae Plinia phitrantha#NC 039557
Myrtales Myrtaceae Psidium guajava#KY635879
Myrtales Myrtaceae Pimenta dioica#NC 034684
Myrtales Myrtaceae Eugenia uniflora#NC 027744
Myrtales Myrtaceae Acca sellowiana#KX289887
Myrtales Myrtaceae Campomanesia xanthocarpa#KY392760
Myrtales Myrtaceae Baeckea frutescens#F449
Myrtales Myrtaceae Stockwellia quadrifida#NC 022414
Myrtales Myrtaceae Allosyncarpia ternata#NC 022413
Myrtales Myrtaceae Corymbia gummifera#NC 022407
Myrtales Myrtaceae Angophora costata#NC 022412
Myrtales Myrtaceae Angophora floribunda#NC 022411
Myrtales Myrtaceae Corymbia eximia#NC 022409
Myrtales Myrtaceae Corymbia maculata#NC 022408
Myrtales Myrtaceae Corymbia henryi#NC 028409
Myrtales Myrtaceae Corymbia tessellaris#NC 022410
Myrtales Myrtaceae Corymbia torelliana#NC 028410
Myrtales Myrtaceae Eucalyptus curtisii#NC 022391
Myrtales Myrtaceae Eucalyptus marginata#NC 022390
Myrtales Myrtaceae Eucalyptus patens#NC 022389
Myrtales Myrtaceae Eucalyptus pauciflora#NC 039597
Myrtales Myrtaceae Eucalyptus regnans#NC 022386
Myrtales Myrtaceae Eucalyptus sieberi#NC 022384
Myrtales Myrtaceae Eucalyptus elata#NC 022385
Myrtales Myrtaceae Eucalyptus umbra#NC 022387
Myrtales Myrtaceae Eucalyptus cloeziana#NC 022388
Myrtales Myrtaceae Eucalyptus diversifolia#NC 022383
Myrtales Myrtaceae Eucalyptus baxteri#NC 022382
Myrtales Myrtaceae Eucalyptus verrucata#NC 022381
Myrtales Myrtaceae Eucalyptus delegatensis#NC 022380
Myrtales Myrtaceae Eucalyptus radiata#NC 022379
Myrtales Myrtaceae Eucalyptus obliqua#NC 022378
Myrtales Myrtaceae Eucalyptus erythrocorys#NC 022406
Myrtales Myrtaceae Eucalyptus guilfoylei#NC 022405

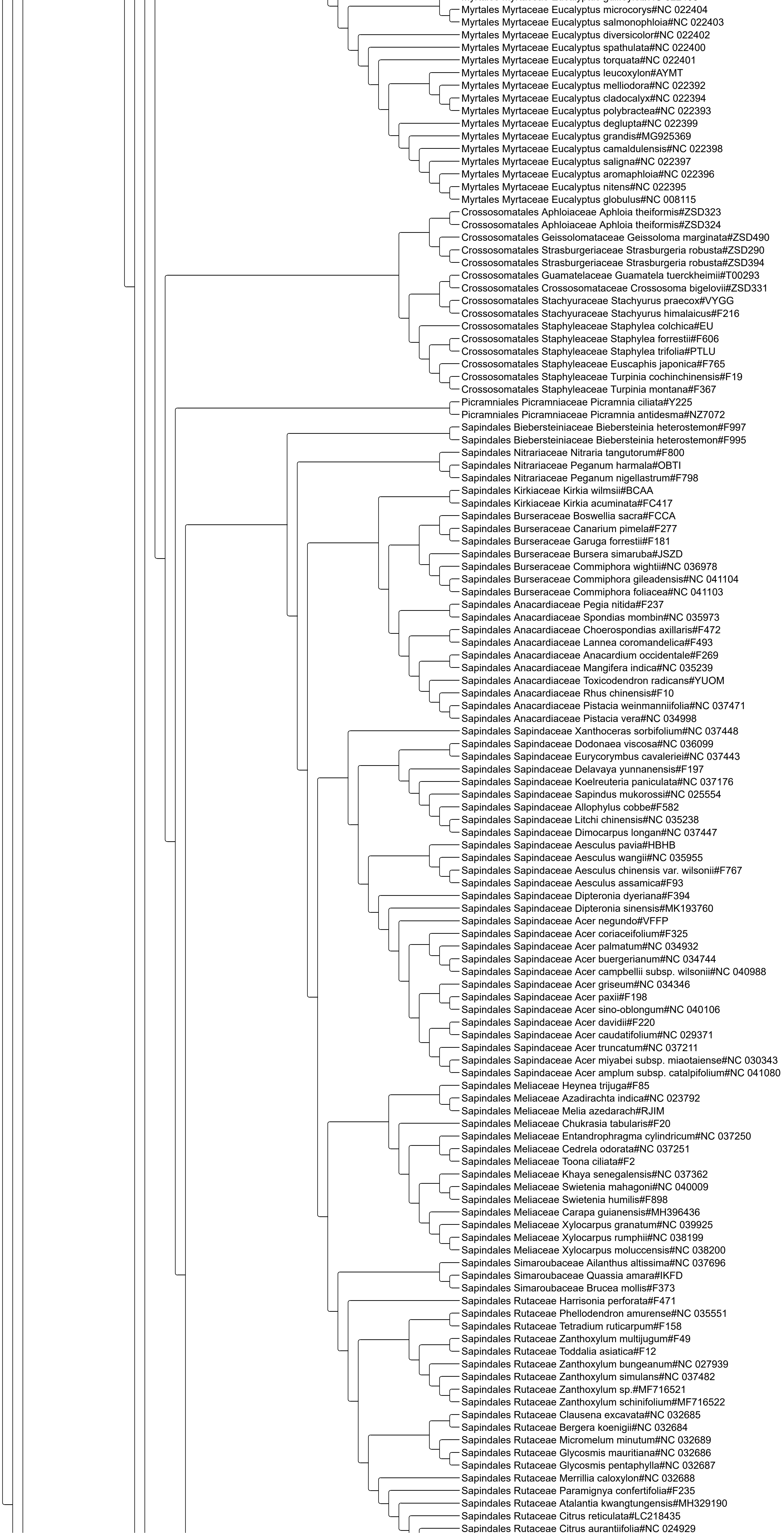

Myrtales Myrtaceae Eucalyptus microcorys#NC 022404
Myrtales Myrtaceae Eucalyptus salmonophloia#NC 022403
Myrtales Myrtaceae Eucalyptus diversicolor#NC 022402
Myrtales Myrtaceae Eucalyptus spathulata#NC 022400
Myrtales Myrtaceae Eucalyptus torquata#NC 022401
Myrtales Myrtaceae Eucalyptus leucoxylon#AYMT
Myrtales Myrtaceae Eucalyptus melliodora#NC 022392
Myrtales Myrtaceae Eucalyptus cladocalyx#NC 022394
Myrtales Myrtaceae Eucalyptus polybractea#NC 022393
Myrtales Myrtaceae Eucalyptus deglupta#NC 022399
Myrtales Myrtaceae Eucalyptus grandis#MG925369
Myrtales Myrtaceae Eucalyptus camaldulensis#NC 022398
Myrtales Myrtaceae Eucalyptus saligna#NC 022397
Myrtales Myrtaceae Eucalyptus aromaphloia#NC 022396
Myrtales Myrtaceae Eucalyptus nitens#NC 022395
Myrtales Myrtaceae Eucalyptus globulus#NC 008115
Crossosomatales Aphloiaceae Aphloia theiformis#ZSD323
Crossosomatales Aphloiaceae Aphloia theiformis#ZSD324
Crossosomatales Geissolomataceae Geissoloma marginata#ZSD490
Crossosomatales Strasburgeriaceae Strasburgeria robusta#ZSD290
Crossosomatales Strasburgeriaceae Strasburgeria robusta#ZSD394
Crossosomatales Guamatelaceae Guamatela tuerckheimii#T00293
Crossosomatales Crossosomataceae Crossosoma bigelovii#ZSD331
Crossosomatales Stachyuraceae Stachyurus praecox#VYGG
Crossosomatales Stachyuraceae Stachyurus himalaicus#F216
Crossosomatales Staphyleaceae Staphylea colchica#EU
Crossosomatales Staphyleaceae Staphylea forrestii#F606
Crossosomatales Staphyleaceae Staphylea trifolia#PTLU
Crossosomatales Staphyleaceae Euscaphis japonica#F765
Crossosomatales Staphyleaceae Turpinia cochinchinensis#F19
Crossosomatales Staphyleaceae Turpinia montana#F367
Picramniales Picramniaceae Picramnia ciliata#Y225
Picramniales Picramniaceae Picramnia antidesma#NZ7072
Sapindales Biebersteiniaceae Biebersteinia heterostemon#F997
Sapindales Biebersteiniaceae Biebersteinia heterostemon#F995
Sapindales Nitrariaceae Nitraria tangutorum#F800
Sapindales Nitrariaceae Peganum harmala#OBTI
Sapindales Nitrariaceae Peganum nigellastrum#F798
Sapindales Kirkiaceae Kirkia wilmsii#BCAA
Sapindales Kirkiaceae Kirkia acuminata#FC417
Sapindales Burseraceae Boswellia sacra#FCCA
Sapindales Burseraceae Canarium pimela#F277
Sapindales Burseraceae Garuga forrestii#F181
Sapindales Burseraceae Bursera simaruba#JSZD
Sapindales Burseraceae Commiphora wightii#NC 036978
Sapindales Burseraceae Commiphora gileadensis#NC 041104
Sapindales Burseraceae Commiphora foliacea#NC 041103
Sapindales Anacardiaceae Pegia nitida#F237
Sapindales Anacardiaceae Spondias mombin#NC 035973
Sapindales Anacardiaceae Choerospondias axillaris#F472
Sapindales Anacardiaceae Lannea coromandelica#F493
Sapindales Anacardiaceae Anacardium occidentale#F269
Sapindales Anacardiaceae Mangifera indica#NC 035239
Sapindales Anacardiaceae Toxicodendron radicans#YUOM
Sapindales Anacardiaceae Rhus chinensis#F10
Sapindales Anacardiaceae Pistacia weinmanniifolia#NC 037471
Sapindales Anacardiaceae Pistacia vera#NC 034998
Sapindales Sapindaceae Xanthoceras sorbifolium#NC 037448
Sapindales Sapindaceae Dodonaea viscosa#NC 036099
Sapindales Sapindaceae Eurycorymbus cavaleriei#NC 037443
Sapindales Sapindaceae Delavaya yunnanensis#F197
Sapindales Sapindaceae Koelreuteria paniculata#NC 037176
Sapindales Sapindaceae Sapindus mukorossi#NC 025554
Sapindales Sapindaceae Allophylus cobbe#F582
Sapindales Sapindaceae Litchi chinensis#NC 035238
Sapindales Sapindaceae Dimocarpus longan#NC 037447
Sapindales Sapindaceae Aesculus pavia#HBHB
Sapindales Sapindaceae Aesculus wangii#NC 035955
Sapindales Sapindaceae Aesculus chinensis var. wilsonii#F767
Sapindales Sapindaceae Aesculus assamica#F93
Sapindales Sapindaceae Dipteronia dyeriana#F394
Sapindales Sapindaceae Dipteronia sinensis#MK193760
Sapindales Sapindaceae Acer negundo#VFFP
Sapindales Sapindaceae Acer coriaceifolium#F325
Sapindales Sapindaceae Acer palmatum#NC 034932
Sapindales Sapindaceae Acer buergerianum#NC 034744
Sapindales Sapindaceae Acer campbellii subsp. wilsonii#NC 040988
Sapindales Sapindaceae Acer griseum#NC 034346
Sapindales Sapindaceae Acer paxii#F198
Sapindales Sapindaceae Acer sino-oblongum#NC 040106
Sapindales Sapindaceae Acer davidii#F220
Sapindales Sapindaceae Acer caudatifolium#NC 029371
Sapindales Sapindaceae Acer truncatum#NC 037211
Sapindales Sapindaceae Acer miyabei subsp. miaotaiense#NC 030343
Sapindales Sapindaceae Acer amplum subsp. catalpifolium#NC 041080
Sapindales Meliaceae Heynea trijuga#F85
Sapindales Meliaceae Azadirachta indica#NC 023792
Sapindales Meliaceae Melia azedarach#RJIM
Sapindales Meliaceae Chukrasia tabularis#F20
Sapindales Meliaceae Entandrophragma cylindricum#NC 037250
Sapindales Meliaceae Cedrela odorata#NC 037251
Sapindales Meliaceae Toona ciliata#F2
Sapindales Meliaceae Khaya senegalensis#NC 037362
Sapindales Meliaceae Swietenia mahagoni#NC 040009
Sapindales Meliaceae Swietenia humilis#F898
Sapindales Meliaceae Carapa guianensis#MH396436
Sapindales Meliaceae Xylocarpus granatum#NC 039925
Sapindales Meliaceae Xylocarpus rumphii#NC 038199
Sapindales Meliaceae Xylocarpus moluccensis#NC 038200
Sapindales Simaroubaceae Ailanthus altissima#NC 037696
Sapindales Simaroubaceae Quassia amara#IKFD
Sapindales Simaroubaceae Brucea mollis#F373
Sapindales Rutaceae Harrisonia perforata#F471
Sapindales Rutaceae Phellodendron amurense#NC 035551
Sapindales Rutaceae Tetradium ruticarpum#F158
Sapindales Rutaceae Zanthoxylum multijugum#F49
Sapindales Rutaceae Toddalia asiatica#F12
Sapindales Rutaceae Zanthoxylum bungeanum#NC 027939
Sapindales Rutaceae Zanthoxylum simulans#NC 037482
Sapindales Rutaceae Zanthoxylum sp.#MF716521
Sapindales Rutaceae Zanthoxylum schinifolium#MF716522
Sapindales Rutaceae Clausena excavata#NC 032685
Sapindales Rutaceae Bergera koenigii#NC 032684
Sapindales Rutaceae Micromelum minutum#NC 032689
Sapindales Rutaceae Glycosmis mauritiana#NC 032686
Sapindales Rutaceae Glycosmis pentaphylla#NC 032687
Sapindales Rutaceae Merrillia caloxylon#NC 032688
Sapindales Rutaceae Paramignya confertifolia#F235
Sapindales Rutaceae Atalantia kwangtungensis#MH329190
Sapindales Rutaceae Citrus reticulata#LC218435
Sapindales Rutaceae Citrus aurantiifolia#NC 024929

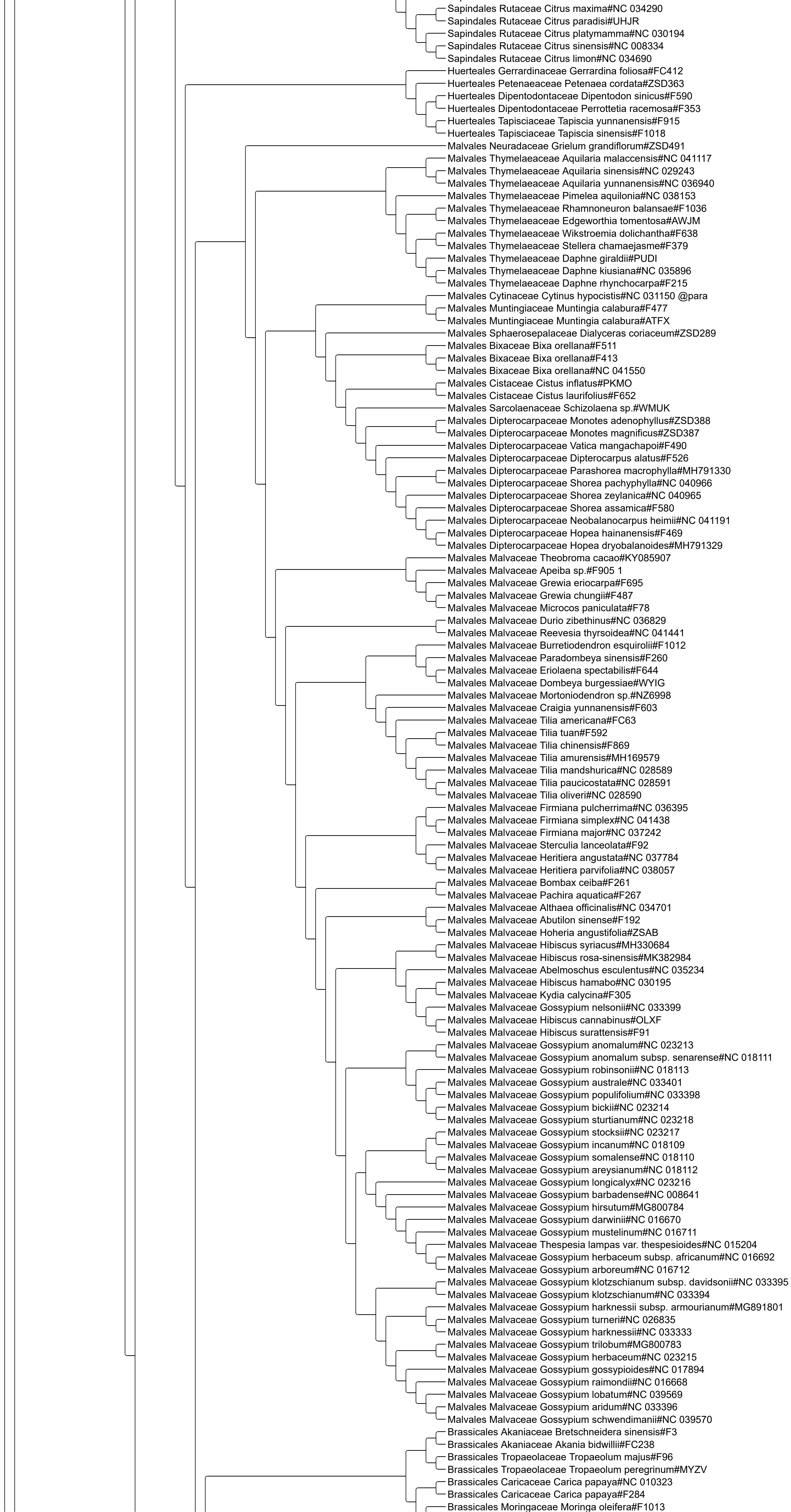
Sapindales Rutaceae Citrus maxima#NC 034290
Sapindales Rutaceae Citrus paradisi#UHJR
Sapindales Rutaceae Citrus platymamma#NC 030194
Sapindales Rutaceae Citrus sinensis#NC 008334
Sapindales Rutaceae Citrus limon#NC 034690
Huerteales Gerrardinaceae Gerrardina foliosa#FC412
Huerteales Petenaeaceae Petenaea cordata#ZSD363
Huerteales Dipentodontaceae Dipentodon sinicus#F590
Huerteales Dipentodontaceae Perrottetia racemosa#F353
Huerteales Tapisciaceae Tapiscia yunnanensis#F915
Huerteales Tapisciaceae Tapiscia sinensis#F1018
Malvales Neuradaceae Grielum grandiflorum#ZSD491
Malvales Thymelaeaceae Aquilaria malaccensis#NC 041117
Malvales Thymelaeaceae Aquilaria sinensis#NC 029243
Malvales Thymelaeaceae Aquilaria yunnanensis#NC 036940
Malvales Thymelaeaceae Pimelea aquilonia#NC 038153
Malvales Thymelaeaceae Rhamnoneuron balansae#F1036
Malvales Thymelaeaceae Edgeworthia tomentosa#AWJM
Malvales Thymelaeaceae Wikstroemia dolichantha#F638
Malvales Thymelaeaceae Stellera chamaejasme#F379
Malvales Thymelaeaceae Daphne giraldii#PUDI
Malvales Thymelaeaceae Daphne kiusiana#NC 035896
Malvales Thymelaeaceae Daphne rhynchocarpa#F215
Malvales Cytinaceae Cytinus hypocistis#NC 031150 @para
Malvales Muntingiaceae Muntingia calabura#F477
Malvales Muntingiaceae Muntingia calabura#ATFX
Malvales Sphaerosepalaceae Dialyceras coriaceum#ZSD289
Malvales Bixaceae Bixa orellana#F511
Malvales Bixaceae Bixa orellana#F413
Malvales Bixaceae Bixa orellana#NC 041550
Malvales Cistaceae Cistus inflatus#PKMO
Malvales Cistaceae Cistus laurifolius#F652
Malvales Sarcolaenaceae Schizolaena sp.#WMUK
Malvales Dipterocarpaceae Monotes adenophyllus#ZSD388
Malvales Dipterocarpaceae Monotes magnificus#ZSD387
Malvales Dipterocarpaceae Vatica mangachapoi#F490
Malvales Dipterocarpaceae Dipterocarpus alatus#F526
Malvales Dipterocarpaceae Parashorea macrophylla#MH791330
Malvales Dipterocarpaceae Shorea pachyphylla#NC 040966
Malvales Dipterocarpaceae Shorea zeylanica#NC 040965
Malvales Dipterocarpaceae Shorea assamica#F580
Malvales Dipterocarpaceae Neobalanocarpus heimii#NC 041191
Malvales Dipterocarpaceae Hopea hainanensis#F469
Malvales Dipterocarpaceae Hopea dryobalanoides#MH791329
Malvales Malvaceae Theobroma cacao#KY085907
Malvales Malvaceae Apeiba sp.#F905 1
Malvales Malvaceae Grewia eriocarpa#F695
Malvales Malvaceae Grewia chungii#F487
Malvales Malvaceae Microcos paniculata#F78
Malvales Malvaceae Durio zibethinus#NC 036829
Malvales Malvaceae Reevesia thyrsoidea#NC 041441
Malvales Malvaceae Burretiodendron esquirolii#F1012
Malvales Malvaceae Paradombeya sinensis#F260
Malvales Malvaceae Eriolaena spectabilis#F644
Malvales Malvaceae Dombeya burgessiae#WYIG
Malvales Malvaceae Mortoniodendron sp.#NZ6998
Malvales Malvaceae Craigia yunnanensis#F603
Malvales Malvaceae Tilia americana#FC63
Malvales Malvaceae Tilia tuan#F592
Malvales Malvaceae Tilia chinensis#F869
Malvales Malvaceae Tilia amurensis#MH169579
Malvales Malvaceae Tilia mandshurica#NC 028589
Malvales Malvaceae Tilia paucicostata#NC 028591
Malvales Malvaceae Tilia oliveri#NC 028590
Malvales Malvaceae Firmiana pulcherrima#NC 036395
Malvales Malvaceae Firmiana simplex#NC 041438
Malvales Malvaceae Firmiana major#NC 037242
Malvales Malvaceae Sterculia lanceolata#F92
Malvales Malvaceae Heritiera angustata#NC 037784
Malvales Malvaceae Heritiera parvifolia#NC 038057
Malvales Malvaceae Bombax ceiba#F261
Malvales Malvaceae Pachira aquatica#F267
Malvales Malvaceae Althaea officinalis#NC 034701
Malvales Malvaceae Abutilon sinense#F192
Malvales Malvaceae Hoheria angustifolia#ZSAB
Malvales Malvaceae Hibiscus syriacus#MH330684
Malvales Malvaceae Hibiscus rosa-sinensis#MK382984
Malvales Malvaceae Abelmoschus esculentus#NC 035234
Malvales Malvaceae Hibiscus hamabo#NC 030195
Malvales Malvaceae Kydia calycina#F305
Malvales Malvaceae Gossypium nelsonii#NC 033399
Malvales Malvaceae Hibiscus cannabinus#OLXF
Malvales Malvaceae Hibiscus surattensis#F91
Malvales Malvaceae Gossypium anomalum#NC 023213
Malvales Malvaceae Gossypium anomalum subsp. senarense#NC 018111
Malvales Malvaceae Gossypium robinsonii#NC 018113
Malvales Malvaceae Gossypium australe#NC 033401
Malvales Malvaceae Gossypium populifolium#NC 033398
Malvales Malvaceae Gossypium bickii#NC 023214
Malvales Malvaceae Gossypium sturtianum#NC 023218
Malvales Malvaceae Gossypium stocksii#NC 023217
Malvales Malvaceae Gossypium incanum#NC 018109
Malvales Malvaceae Gossypium somalense#NC 018110
Malvales Malvaceae Gossypium areysianum#NC 018112
Malvales Malvaceae Gossypium longicalyx#NC 023216
Malvales Malvaceae Gossypium barbadense#NC 008641
Malvales Malvaceae Gossypium hirsutum#MG800784
Malvales Malvaceae Gossypium darwinii#NC 016670
Malvales Malvaceae Gossypium mustelinum#NC 016711
Malvales Malvaceae Thespesia lampas var. thespesioides#NC 015204
Malvales Malvaceae Gossypium herbaceum subsp. africanum#NC 016692
Malvales Malvaceae Gossypium arboreum#NC 016712
Malvales Malvaceae Gossypium klotzschianum subsp. davidsonii#NC 033395
Malvales Malvaceae Gossypium klotzschianum#NC 033394
Malvales Malvaceae Gossypium harknessii subsp. armourianum#MG891801
Malvales Malvaceae Gossypium turneri#NC 026835
Malvales Malvaceae Gossypium harknessii#NC 033333
Malvales Malvaceae Gossypium trilobum#MG800783
Malvales Malvaceae Gossypium herbaceum#NC 023215
Malvales Malvaceae Gossypium gossypioides#NC 017894
Malvales Malvaceae Gossypium raimondii#NC 016668
Malvales Malvaceae Gossypium lobatum#NC 039569
Malvales Malvaceae Gossypium aridum#NC 033396
Malvales Malvaceae Gossypium schwendimanii#NC 039570
Brassicales Akaniaceae Bretschneidera sinensis#F3
Brassicales Akaniaceae Akania bidwillii#FC238
Brassicales Tropaeolaceae Tropaeolum majus#F96
Brassicales Tropaeolaceae Tropaeolum peregrinum#MYZV
Brassicales Caricaceae Carica papaya#NC 010323
Brassicales Caricaceae Carica papaya#F284
Brassicales Moringaceae Moringa oleifera#F1013

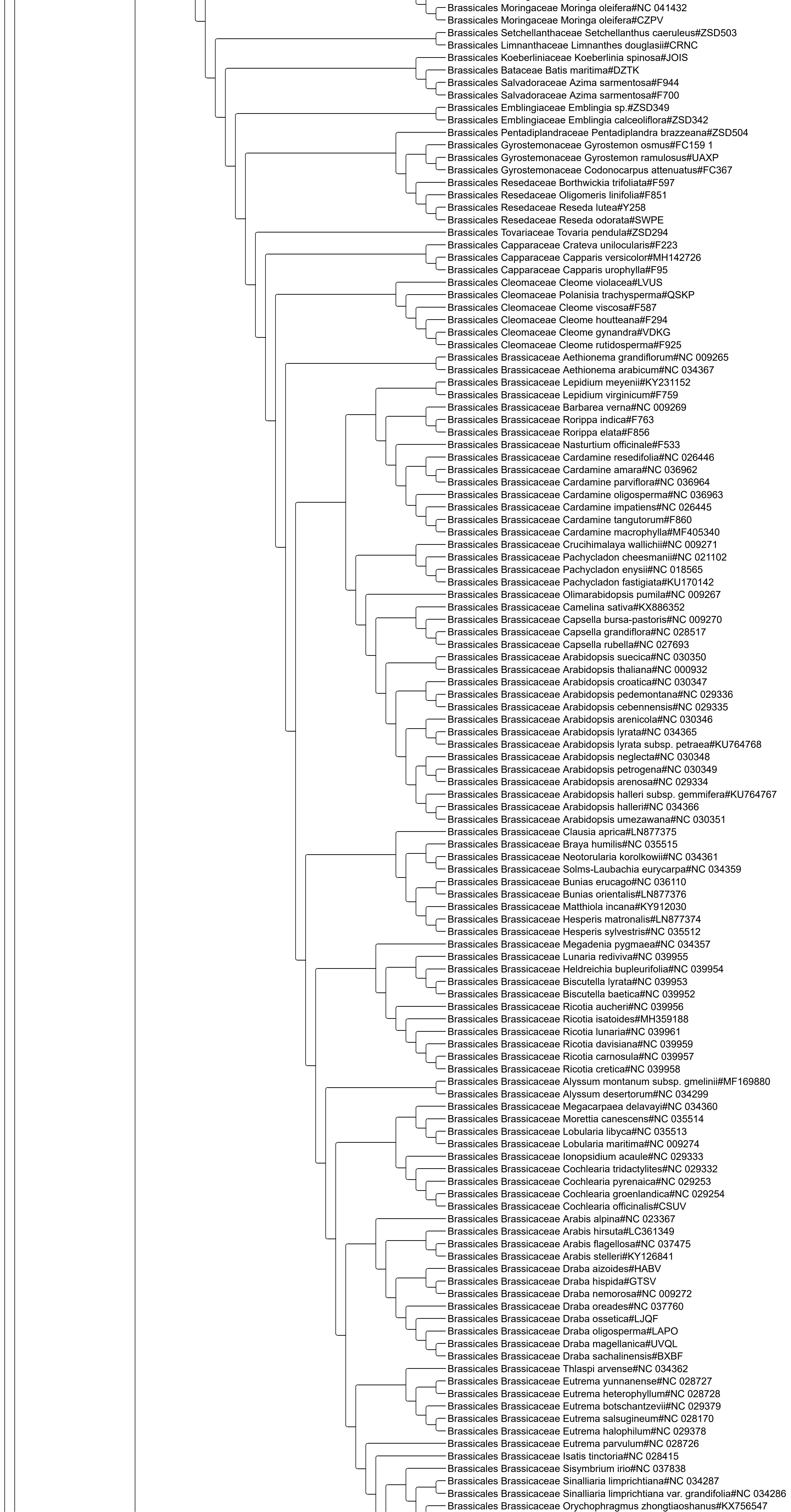
Brassicales Moringaceae Moringa oleifera#NC 041432
Brassicales Moringaceae Moringa oleifera#CZPV
Brassicales Setchellanthaceae Setchellanthus caeruleus#ZSD503
Brassicales Limnanthaceae Limnanthes douglasii#CRNC
Brassicales Koeberliniaceae Koeberlinia spinosa#JOIS
Brassicales Bataceae Batis maritima#DZTK
Brassicales Salvadoraceae Azima sarmentosa#F944
Brassicales Salvadoraceae Azima sarmentosa#F700
Brassicales Emblingiaceae Emblingia sp.#ZSD349
Brassicales Emblingiaceae Emblingia calceoliflora#ZSD342
Brassicales Pentadiplandraceae Pentadiplandra brazzeana#ZSD504
Brassicales Gyrostemonaceae Gyrostemon osmus#FC159 1
Brassicales Gyrostemonaceae Gyrostemon ramulosus#UAXP
Brassicales Gyrostemonaceae Codonocarpus attenuatus#FC367
Brassicales Resedaceae Borthwickia trifoliata#F597
Brassicales Resedaceae Oligomeris linifolia#F851
Brassicales Resedaceae Reseda lutea#Y258
Brassicales Resedaceae Reseda odorata#SWPE
Brassicales Tovariaceae Tovaria pendula#ZSD294
Brassicales Capparaceae Crateva unilocularis#F223
Brassicales Capparaceae Capparis versicolor#MH142726
Brassicales Capparaceae Capparis urophylla#F95
Brassicales Cleomaceae Cleome violacea#LVUS
Brassicales Cleomaceae Polanisia trachysperma#QSKP
Brassicales Cleomaceae Cleome viscosa#F587
Brassicales Cleomaceae Cleome houtteana#F294
Brassicales Cleomaceae Cleome gynandra#VDKG
Brassicales Cleomaceae Cleome rutidosperma#F925
Brassicales Brassicaceae Aethionema grandiflorum#NC 009265
Brassicales Brassicaceae Aethionema arabicum#NC 034367
Brassicales Brassicaceae Lepidium meyenii#KY231152
Brassicales Brassicaceae Lepidium virginicum#F759
Brassicales Brassicaceae Barbarea verna#NC 009269
Brassicales Brassicaceae Rorippa indica#F763
Brassicales Brassicaceae Rorippa elata#F856
Brassicales Brassicaceae Nasturtium officinale#F533
Brassicales Brassicaceae Cardamine resedifolia#NC 026446
Brassicales Brassicaceae Cardamine amara#NC 036962
Brassicales Brassicaceae Cardamine parviflora#NC 036964
Brassicales Brassicaceae Cardamine oligosperma#NC 036963
Brassicales Brassicaceae Cardamine impatiens#NC 026445
Brassicales Brassicaceae Cardamine tangutorum#F860
Brassicales Brassicaceae Cardamine macrophylla#MF405340
Brassicales Brassicaceae Crucihimalaya wallichii#NC 009271
Brassicales Brassicaceae Pachycladon cheesmanii#NC 021102
Brassicales Brassicaceae Pachycladon enysii#NC 018565
Brassicales Brassicaceae Pachycladon fastigiata#KU170142
Brassicales Brassicaceae Olimarabidopsis pumila#NC 009267
Brassicales Brassicaceae Camelina sativa#KX886352
Brassicales Brassicaceae Capsella bursa-pastoris#NC 009270
Brassicales Brassicaceae Capsella grandiflora#NC 028517
Brassicales Brassicaceae Capsella rubella#NC 027693
Brassicales Brassicaceae Arabidopsis suecica#NC 030350
Brassicales Brassicaceae Arabidopsis thaliana#NC 000932
Brassicales Brassicaceae Arabidopsis croatica#NC 030347
Brassicales Brassicaceae Arabidopsis pedemontana#NC 029336
Brassicales Brassicaceae Arabidopsis cebennensis#NC 029335
Brassicales Brassicaceae Arabidopsis arenicola#NC 030346
Brassicales Brassicaceae Arabidopsis lyrata#NC 034365
Brassicales Brassicaceae Arabidopsis lyrata subsp. petraea#KU764768
Brassicales Brassicaceae Arabidopsis neglecta#NC 030348
Brassicales Brassicaceae Arabidopsis petrogena#NC 030349
Brassicales Brassicaceae Arabidopsis arenosa#NC 029334
Brassicales Brassicaceae Arabidopsis halleri subsp. gemmifera#KU764767
Brassicales Brassicaceae Arabidopsis halleri#NC 034366
Brassicales Brassicaceae Arabidopsis umezawana#NC 030351
Brassicales Brassicaceae Clausia aprica#LN877375
Brassicales Brassicaceae Braya humilis#NC 035515
Brassicales Brassicaceae Neotorularia korolkowii#NC 034361
Brassicales Brassicaceae Solms-Laubachia eurycarpa#NC 034359
Brassicales Brassicaceae Bunias erucago#NC 036110
Brassicales Brassicaceae Bunias orientalis#LN877376
Brassicales Brassicaceae Matthiola incana#KY912030
Brassicales Brassicaceae Hesperis matronalis#LN877374
Brassicales Brassicaceae Hesperis sylvestris#NC 035512
Brassicales Brassicaceae Megadenia pygmaea#NC 034357
Brassicales Brassicaceae Lunaria rediviva#NC 039955
Brassicales Brassicaceae Heldreichia bupleurifolia#NC 039954
Brassicales Brassicaceae Biscutella lyrata#NC 039953
Brassicales Brassicaceae Biscutella baetica#NC 039952
Brassicales Brassicaceae Ricotia aucheri#NC 039956
Brassicales Brassicaceae Ricotia isatoides#MH359188
Brassicales Brassicaceae Ricotia lunaria#NC 039961
Brassicales Brassicaceae Ricotia davisiana#NC 039959
Brassicales Brassicaceae Ricotia carnosula#NC 039957
Brassicales Brassicaceae Ricotia cretica#NC 039958
Brassicales Brassicaceae Alyssum montanum subsp. gmelinii#MF169880
Brassicales Brassicaceae Alyssum desertorum#NC 034299
Brassicales Brassicaceae Megacarpaea delavayi#NC 034360
Brassicales Brassicaceae Morettia canescens#NC 035514
Brassicales Brassicaceae Lobularia libyca#NC 035513
Brassicales Brassicaceae Lobularia maritima#NC 009274
Brassicales Brassicaceae Ionopsidium acaule#NC 029333
Brassicales Brassicaceae Cochlearia tridactylites#NC 029332
Brassicales Brassicaceae Cochlearia pyrenaica#NC 029253
Brassicales Brassicaceae Cochlearia groenlandica#NC 029254
Brassicales Brassicaceae Cochlearia officinalis#CSUV
Brassicales Brassicaceae Arabis alpina#NC 023367
Brassicales Brassicaceae Arabis hirsuta#LC361349
Brassicales Brassicaceae Arabis flagellosa#NC 037475
Brassicales Brassicaceae Arabis stelleri#KY126841
Brassicales Brassicaceae Draba aizoides#HABV
Brassicales Brassicaceae Draba hispida#GTSV
Brassicales Brassicaceae Draba nemorosa#NC 009272
Brassicales Brassicaceae Draba oreades#NC 037760
Brassicales Brassicaceae Draba ossetica#LJQF
Brassicales Brassicaceae Draba oligosperma#LAPO
Brassicales Brassicaceae Draba magellanica#UVQL
Brassicales Brassicaceae Draba sachalinensis#BXBF
Brassicales Brassicaceae Thlaspi arvense#NC 034362
Brassicales Brassicaceae Eutrema yunnanense#NC 028727
Brassicales Brassicaceae Eutrema heterophyllum#NC 028728
Brassicales Brassicaceae Eutrema botschantzevii#NC 029379
Brassicales Brassicaceae Eutrema salsugineum#NC 028170
Brassicales Brassicaceae Eutrema halophilum#NC 029378
Brassicales Brassicaceae Eutrema parvulum#NC 028726
Brassicales Brassicaceae Isatis tinctoria#NC 028415
Brassicales Brassicaceae Sisymbrium irio#NC 037838
Brassicales Brassicaceae Sinalliaria limprichtiana#NC 034287
Brassicales Brassicaceae Sinalliaria limprichtiana var. grandifolia#NC 034286
Brassicales Brassicaceae Orychophragmus zhongtiaoshanus#KX756547

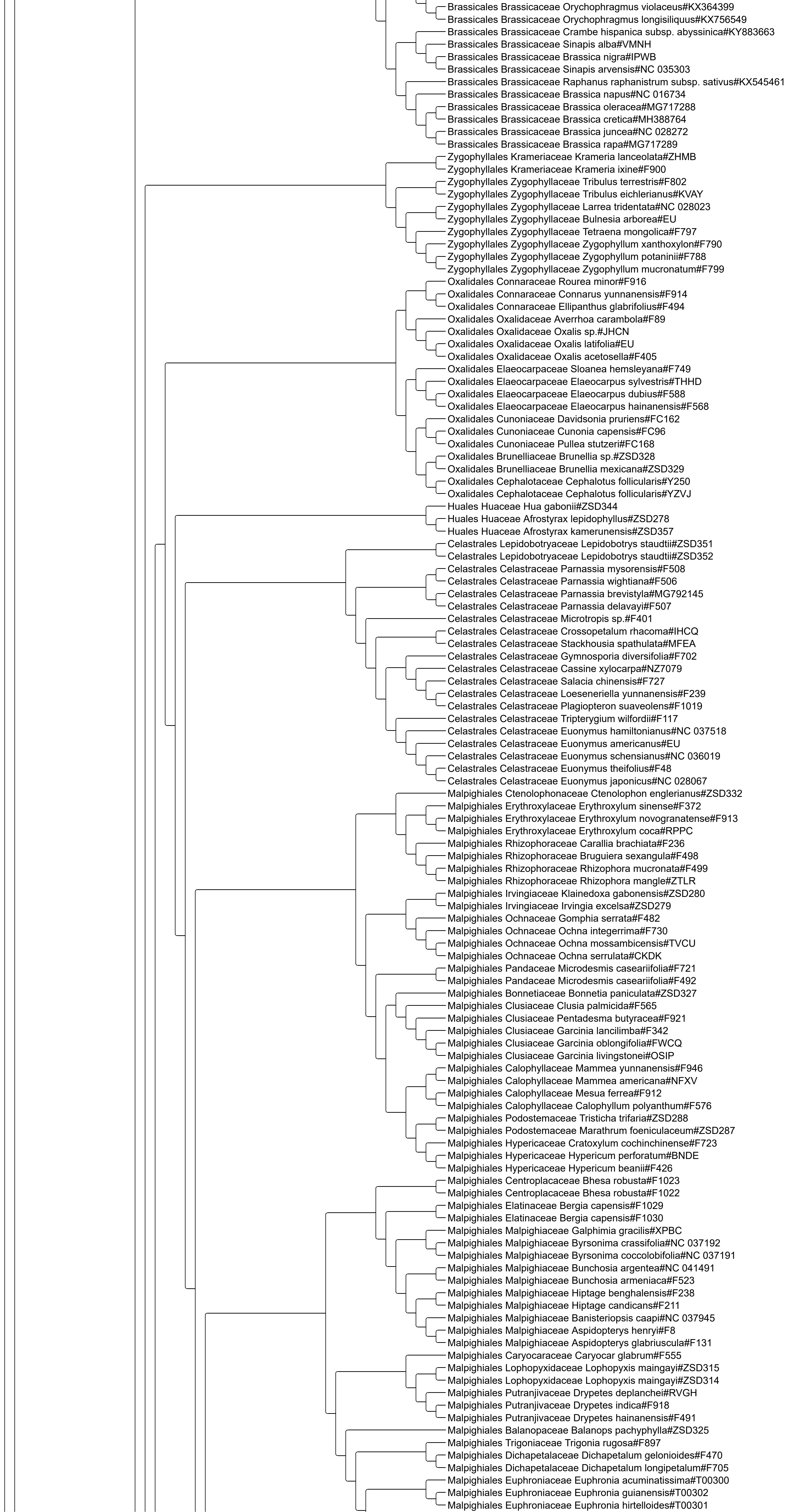
Brassicales Brassicaceae Orychophragmus violaceus#KX364399
Brassicales Brassicaceae Orychophragmus longisiliquus#KX756549
Brassicales Brassicaceae Crambe hispanica subsp. abyssinica#KY883663
Brassicales Brassicaceae Sinapis alba#VMNH
Brassicales Brassicaceae Brassica nigra#IPWB
Brassicales Brassicaceae Sinapis arvensis#NC 035303
Brassicales Brassicaceae Raphanus raphanistrum subsp. sativus#KX545461
Brassicales Brassicaceae Brassica napus#NC 016734
Brassicales Brassicaceae Brassica oleracea#MG717288
Brassicales Brassicaceae Brassica cretica#MH388764
Brassicales Brassicaceae Brassica juncea#NC 028272
Brassicales Brassicaceae Brassica rapa#MG717289
Zygophyllales Krameriaceae Krameria lanceolata#ZHMB
Zygophyllales Krameriaceae Krameria ixine#F900
Zygophyllales Zygophyllaceae Tribulus terrestris#F802
Zygophyllales Zygophyllaceae Tribulus eichlerianus#KVAY
Zygophyllales Zygophyllaceae Larrea tridentata#NC 028023
Zygophyllales Zygophyllaceae Bulnesia arborea#EU
Zygophyllales Zygophyllaceae Tetraena mongolica#F797
Zygophyllales Zygophyllaceae Zygophyllum xanthoxylon#F790
Zygophyllales Zygophyllaceae Zygophyllum potaninii#F788
Zygophyllales Zygophyllaceae Zygophyllum mucronatum#F799
Oxalidales Connaraceae Rourea minor#F916
Oxalidales Connaraceae Connarus yunnanensis#F914
Oxalidales Connaraceae Ellipanthus glabrifolius#F494
Oxalidales Oxalidaceae Averrhoa carambola#F89
Oxalidales Oxalidaceae Oxalis sp.#JHCN
Oxalidales Oxalidaceae Oxalis latifolia#EU
Oxalidales Oxalidaceae Oxalis acetosella#F405
Oxalidales Elaeocarpaceae Sloanea hemsleyana#F749
Oxalidales Elaeocarpaceae Elaeocarpus sylvestris#THHD
Oxalidales Elaeocarpaceae Elaeocarpus dubius#F588
Oxalidales Elaeocarpaceae Elaeocarpus hainanensis#F568
Oxalidales Cunoniaceae Davidsonia pruriens#FC162
Oxalidales Cunoniaceae Cunonia capensis#FC96
Oxalidales Cunoniaceae Pullea stutzeri#FC168
Oxalidales Brunelliaceae Brunellia sp.#ZSD328
Oxalidales Brunelliaceae Brunellia mexicana#ZSD329
Oxalidales Cephalotaceae Cephalotus follicularis#Y250
Oxalidales Cephalotaceae Cephalotus follicularis#YZVJ
Huales Huaceae Hua gabonii#ZSD344
Huales Huaceae Afrostyrax lepidophyllus#ZSD278
Huales Huaceae Afrostyrax kamerunensis#ZSD357
Celastrales Lepidobotryaceae Lepidobotrys staudtii#ZSD351
Celastrales Lepidobotryaceae Lepidobotrys staudtii#ZSD352
Celastrales Celastraceae Parnassia mysorensis#F508
Celastrales Celastraceae Parnassia wightiana#F506
Celastrales Celastraceae Parnassia brevistyla#MG792145
Celastrales Celastraceae Parnassia delavayi#F507
Celastrales Celastraceae Microtropis sp.#F401
Celastrales Celastraceae Crossopetalum rhacoma#IHCQ
Celastrales Celastraceae Stackhousia spathulata#MFEA
Celastrales Celastraceae Gymnosporia diversifolia#F702
Celastrales Celastraceae Cassine xylocarpa#NZ7079
Celastrales Celastraceae Salacia chinensis#F727
Celastrales Celastraceae Loeseneriella yunnanensis#F239
Celastrales Celastraceae Plagiopteron suaveolens#F1019
Celastrales Celastraceae Tripterygium wilfordii#F117
Celastrales Celastraceae Euonymus hamiltonianus#NC 037518
Celastrales Celastraceae Euonymus americanus#EU
Celastrales Celastraceae Euonymus schensianus#NC 036019
Celastrales Celastraceae Euonymus theifolius#F48
Celastrales Celastraceae Euonymus japonicus#NC 028067
Malpighiales Ctenolophonaceae Ctenolophon englerianus#ZSD332
Malpighiales Erythroxylaceae Erythroxylum sinense#F372
Malpighiales Erythroxylaceae Erythroxylum novogranatense#F913
Malpighiales Erythroxylaceae Erythroxylum coca#RPPC
Malpighiales Rhizophoraceae Carallia brachiata#F236
Malpighiales Rhizophoraceae Bruguiera sexangula#F498
Malpighiales Rhizophoraceae Rhizophora mucronata#F499
Malpighiales Rhizophoraceae Rhizophora mangle#ZTLR
Malpighiales Irvingiaceae Klainedoxa gabonensis#ZSD280
Malpighiales Irvingiaceae Irvingia excelsa#ZSD279
Malpighiales Ochnaceae Gomphia serrata#F482
Malpighiales Ochnaceae Ochna integerrima#F730
Malpighiales Ochnaceae Ochna mossambicensis#TVCU
Malpighiales Ochnaceae Ochna serrulata#CKDK
Malpighiales Pandaceae Microdesmis caseariifolia#F721
Malpighiales Pandaceae Microdesmis caseariifolia#F492
Malpighiales Bonnetiaceae Bonnetia paniculata#ZSD327
Malpighiales Clusiaceae Clusia palmicida#F565
Malpighiales Clusiaceae Pentadesma butyracea#F921
Malpighiales Clusiaceae Garcinia lancilimba#F342
Malpighiales Clusiaceae Garcinia oblongifolia#FWCQ
Malpighiales Clusiaceae Garcinia livingstonei#OSIP
Malpighiales Calophyllaceae Mammea yunnanensis#F946
Malpighiales Calophyllaceae Mammea americana#NFXV
Malpighiales Calophyllaceae Mesua ferrea#F912
Malpighiales Calophyllaceae Calophyllum polyanthum#F576
Malpighiales Podostemaceae Tristicha trifaria#ZSD288
Malpighiales Podostemaceae Marathrum foeniculaceum#ZSD287
Malpighiales Hypericaceae Cratoxylum cochinchinense#F723
Malpighiales Hypericaceae Hypericum perforatum#BNDE
Malpighiales Hypericaceae Hypericum beanii#F426
Malpighiales Centroplacaceae Bhesa robusta#F1023
Malpighiales Centroplacaceae Bhesa robusta#F1022
Malpighiales Elatinaceae Bergia capensis#F1029
Malpighiales Elatinaceae Bergia capensis#F1030
Malpighiales Malpighiaceae Galphimia gracilis#XPBC
Malpighiales Malpighiaceae Byrsonima crassifolia#NC 037192
Malpighiales Malpighiaceae Byrsonima coccolobifolia#NC 037191
Malpighiales Malpighiaceae Bunchosia argentea#NC 041491
Malpighiales Malpighiaceae Bunchosia armeniaca#F523
Malpighiales Malpighiaceae Hiptage benghalensis#F238
Malpighiales Malpighiaceae Hiptage candicans#F211
Malpighiales Malpighiaceae Banisteriopsis caapi#NC 037945
Malpighiales Malpighiaceae Aspidopterys henryi#F8
Malpighiales Malpighiaceae Aspidopterys glabriuscula#F131
Malpighiales Caryocaraceae Caryocar glabrum#F555
Malpighiales Lophopyxidaceae Lophopyxis maingayi#ZSD315
Malpighiales Lophopyxidaceae Lophopyxis maingayi#ZSD314
Malpighiales Putranjivaceae Drypetes deplanchei#RVGH
Malpighiales Putranjivaceae Drypetes indica#F918
Malpighiales Putranjivaceae Drypetes hainanensis#F491
Malpighiales Balanopaceae Balanops pachyphylla#ZSD325
Malpighiales Trigoniaceae Trigonia rugosa#F897
Malpighiales Dichapetalaceae Dichapetalum gelonioides#F470
Malpighiales Dichapetalaceae Dichapetalum longipetalum#F705
Malpighiales Euphroniaceae Euphronia acuminatissima#T00300
Malpighiales Euphroniaceae Euphronia guianensis#T00302
Malpighiales Euphroniaceae Euphronia hirtelloides#T00301

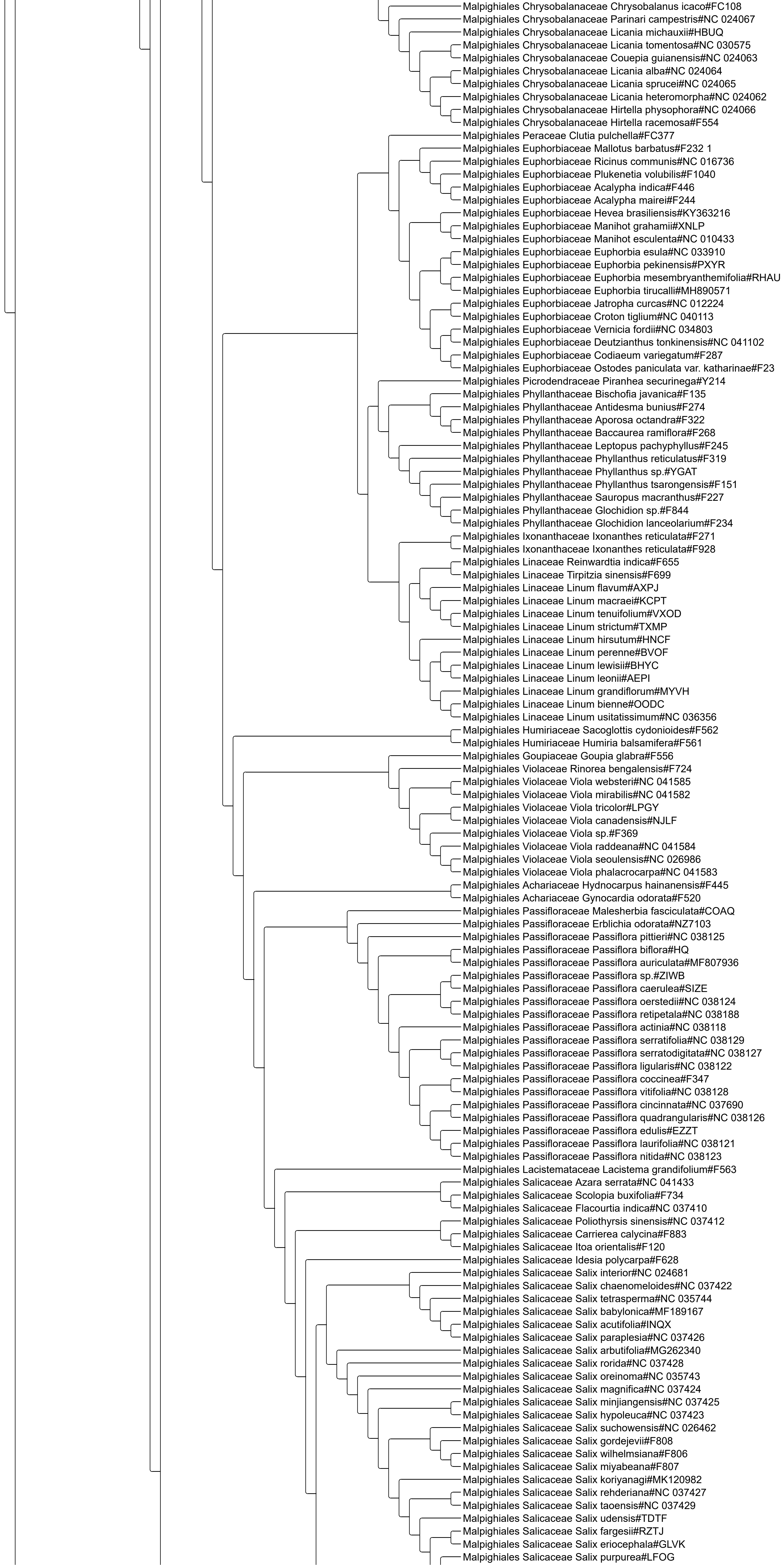
Malpighiales Chrysobalanaceae Chrysobalanus icaco#FC108
Malpighiales Chrysobalanaceae Parinari campestris#NC 024067
Malpighiales Chrysobalanaceae Licania michauxii#HBUQ
Malpighiales Chrysobalanaceae Licania tomentosa#NC 030575
Malpighiales Chrysobalanaceae Couepia guianensis#NC 024063
Malpighiales Chrysobalanaceae Licania alba#NC 024064
Malpighiales Chrysobalanaceae Licania sprucei#NC 024065
Malpighiales Chrysobalanaceae Licania heteromorpha#NC 024062
Malpighiales Chrysobalanaceae Hirtella physophora#NC 024066
Malpighiales Chrysobalanaceae Hirtella racemosa#F554
Malpighiales Peraceae Clutia pulchella#FC377
Malpighiales Euphorbiaceae Mallotus barbatus#F232 1
Malpighiales Euphorbiaceae Ricinus communis#NC 016736
Malpighiales Euphorbiaceae Plukenetia volubilis#F1040
Malpighiales Euphorbiaceae Acalypha indica#F446
Malpighiales Euphorbiaceae Acalypha mairei#F244
Malpighiales Euphorbiaceae Hevea brasiliensis#KY363216
Malpighiales Euphorbiaceae Manihot grahamii#XNLP
Malpighiales Euphorbiaceae Manihot esculenta#NC 010433
Malpighiales Euphorbiaceae Euphorbia esula#NC 033910
Malpighiales Euphorbiaceae Euphorbia pekinensis#PXYR
Malpighiales Euphorbiaceae Euphorbia mesembryanthemifolia#RHAU
Malpighiales Euphorbiaceae Euphorbia tirucalli#MH890571
Malpighiales Euphorbiaceae Jatropha curcas#NC 012224
Malpighiales Euphorbiaceae Croton tiglium#NC 040113
Malpighiales Euphorbiaceae Vernicia fordii#NC 034803
Malpighiales Euphorbiaceae Deutzianthus tonkinensis#NC 041102
Malpighiales Euphorbiaceae Codiaeum variegatum#F287
Malpighiales Euphorbiaceae Ostodes paniculata var. katharinae#F23
Malpighiales Picrodendraceae Piranhea securinega#Y214
Malpighiales Phyllanthaceae Bischofia javanica#F135
Malpighiales Phyllanthaceae Antidesma bunius#F274
Malpighiales Phyllanthaceae Aporosa octandra#F322
Malpighiales Phyllanthaceae Baccaurea ramiflora#F268
Malpighiales Phyllanthaceae Leptopus pachyphyllus#F245
Malpighiales Phyllanthaceae Phyllanthus reticulatus#F319
Malpighiales Phyllanthaceae Phyllanthus sp.#YGAT
Malpighiales Phyllanthaceae Phyllanthus tsarongensis#F151
Malpighiales Phyllanthaceae Sauropus macranthus#F227
Malpighiales Phyllanthaceae Glochidion sp.#F844
Malpighiales Phyllanthaceae Glochidion lanceolarium#F234
Malpighiales Ixonanthaceae Ixonanthes reticulata#F271
Malpighiales Ixonanthaceae Ixonanthes reticulata#F928
Malpighiales Linaceae Reinwardtia indica#F655
Malpighiales Linaceae Tirpitzia sinensis#F699
Malpighiales Linaceae Linum flavum#AXPJ
Malpighiales Linaceae Linum macraei#KCPT
Malpighiales Linaceae Linum tenuifolium#VXOD
Malpighiales Linaceae Linum strictum#TXMP
Malpighiales Linaceae Linum hirsutum#HNCF
Malpighiales Linaceae Linum perenne#BVOF
Malpighiales Linaceae Linum lewisii#BHYC
Malpighiales Linaceae Linum leonii#AEPI
Malpighiales Linaceae Linum grandiflorum#MYVH
Malpighiales Linaceae Linum bienne#OODC
Malpighiales Linaceae Linum usitatissimum#NC 036356
Malpighiales Humiriaceae Sacoglottis cydonioides#F562
Malpighiales Humiriaceae Humiria balsamifera#F561
Malpighiales Goupiaceae Goupia glabra#F556
Malpighiales Violaceae Rinorea bengalensis#F724
Malpighiales Violaceae Viola websteri#NC 041585
Malpighiales Violaceae Viola mirabilis#NC 041582
Malpighiales Violaceae Viola tricolor#LPGY
Malpighiales Violaceae Viola canadensis#NJLF
Malpighiales Violaceae Viola sp.#F369
Malpighiales Violaceae Viola raddeana#NC 041584
Malpighiales Violaceae Viola seoulensis#NC 026986
Malpighiales Violaceae Viola phalacrocarpa#NC 041583
Malpighiales Achariaceae Hydnocarpus hainanensis#F445
Malpighiales Achariaceae Gynocardia odorata#F520
Malpighiales Passifloraceae Malesherbia fasciculata#COAQ
Malpighiales Passifloraceae Erblichia odorata#NZ7103
Malpighiales Passifloraceae Passiflora pittieri#NC 038125
Malpighiales Passifloraceae Passiflora biflora#HQ
Malpighiales Passifloraceae Passiflora auriculata#MF807936
Malpighiales Passifloraceae Passiflora sp.#ZIWB
Malpighiales Passifloraceae Passiflora caerulea#SIZE
Malpighiales Passifloraceae Passiflora oerstedii#NC 038124
Malpighiales Passifloraceae Passiflora retipetala#NC 038188
Malpighiales Passifloraceae Passiflora actinia#NC 038118
Malpighiales Passifloraceae Passiflora serratifolia#NC 038129
Malpighiales Passifloraceae Passiflora serratodigitata#NC 038127
Malpighiales Passifloraceae Passiflora ligularis#NC 038122
Malpighiales Passifloraceae Passiflora coccinea#F347
Malpighiales Passifloraceae Passiflora vitifolia#NC 038128
Malpighiales Passifloraceae Passiflora cincinnata#NC 037690
Malpighiales Passifloraceae Passiflora quadrangularis#NC 038126
Malpighiales Passifloraceae Passiflora edulis#EZZT
Malpighiales Passifloraceae Passiflora laurifolia#NC 038121
Malpighiales Passifloraceae Passiflora nitida#NC 038123
Malpighiales Lacistemataceae Lacistema grandifolium#F563
Malpighiales Salicaceae Azara serrata#NC 041433
Malpighiales Salicaceae Scolopia buxifolia#F734
Malpighiales Salicaceae Flacourtia indica#NC 037410
Malpighiales Salicaceae Poliothyrsis sinensis#NC 037412
Malpighiales Salicaceae Carrierea calycina#F883
Malpighiales Salicaceae Itoa orientalis#F120
Malpighiales Salicaceae Idesia polycarpa#F628
Malpighiales Salicaceae Salix interior#NC 024681
Malpighiales Salicaceae Salix chaenomeloides#NC 037422
Malpighiales Salicaceae Salix tetrasperma#NC 035744
Malpighiales Salicaceae Salix babylonica#MF189167
Malpighiales Salicaceae Salix acutifolia#INQX
Malpighiales Salicaceae Salix paraplesia#NC 037426
Malpighiales Salicaceae Salix arbutifolia#MG262340
Malpighiales Salicaceae Salix rorida#NC 037428
Malpighiales Salicaceae Salix oreinoma#NC 035743
Malpighiales Salicaceae Salix magnifica#NC 037424
Malpighiales Salicaceae Salix minjiangensis#NC 037425
Malpighiales Salicaceae Salix hypoleuca#NC 037423
Malpighiales Salicaceae Salix suchowensis#NC 026462
Malpighiales Salicaceae Salix gordejevii#F808
Malpighiales Salicaceae Salix wilhelmsiana#F806
Malpighiales Salicaceae Salix miyabeana#F807
Malpighiales Salicaceae Salix koriyanagi#MK120982
Malpighiales Salicaceae Salix rehderiana#NC 037427
Malpighiales Salicaceae Salix taoensis#NC 037429
Malpighiales Salicaceae Salix udensis#TDTF
Malpighiales Salicaceae Salix fargesii#RZTJ
Malpighiales Salicaceae Salix eriocephala#GLVK
Malpighiales Salicaceae Salix purpurea#LFOG

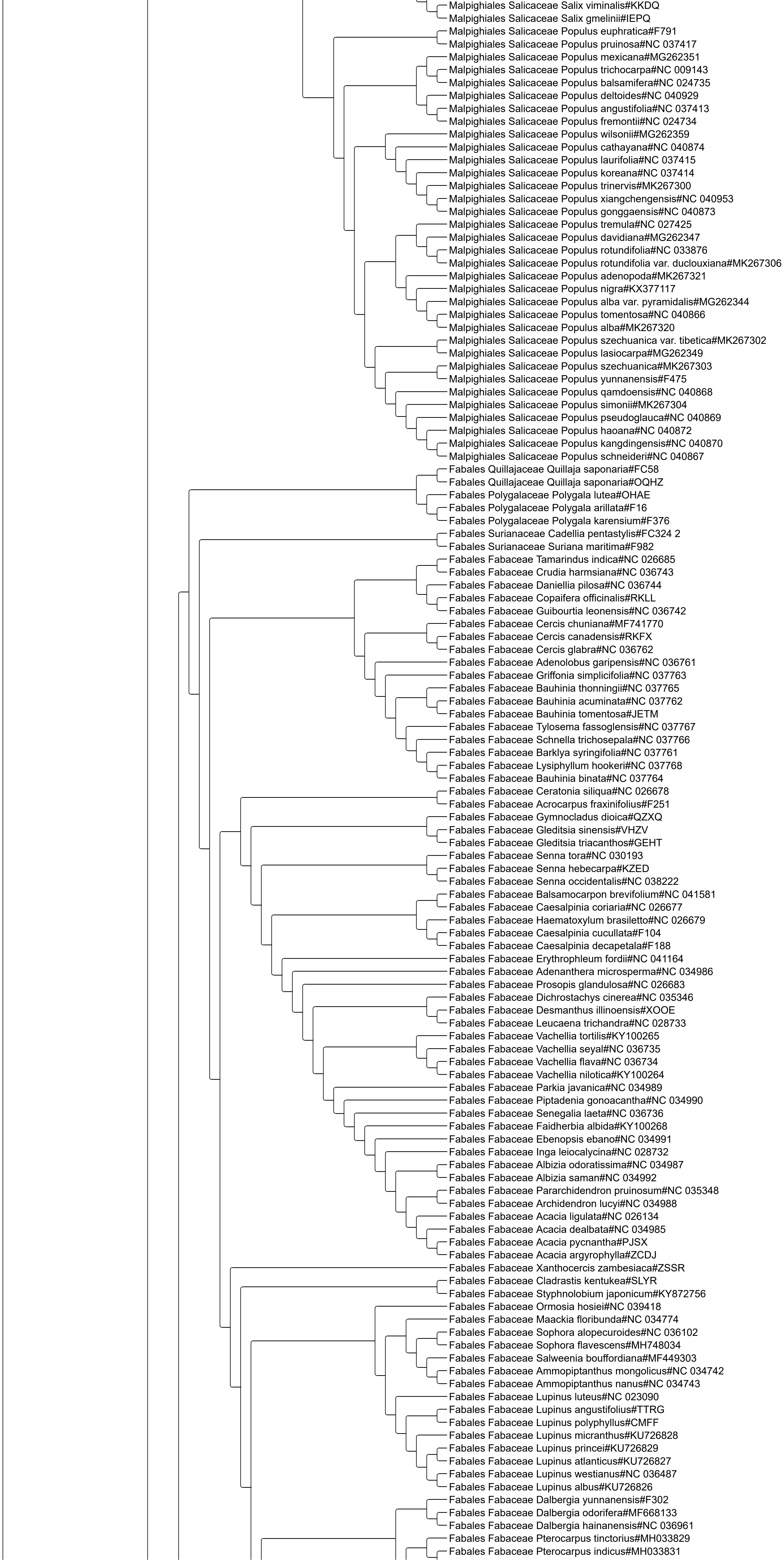
Malpighiales Salicaceae Salix viminalis#KKDQ
Malpighiales Salicaceae Salix gmelinii#IEPQ
Malpighiales Salicaceae Populus euphratica#F791
Malpighiales Salicaceae Populus pruinosa#NC 037417
Malpighiales Salicaceae Populus mexicana#MG262351
Malpighiales Salicaceae Populus trichocarpa#NC 009143
Malpighiales Salicaceae Populus balsamifera#NC 024735
Malpighiales Salicaceae Populus deltoides#NC 040929
Malpighiales Salicaceae Populus angustifolia#NC 037413
Malpighiales Salicaceae Populus fremontii#NC 024734
Malpighiales Salicaceae Populus wilsonii#MG262359
Malpighiales Salicaceae Populus cathayana#NC 040874
Malpighiales Salicaceae Populus laurifolia#NC 037415
Malpighiales Salicaceae Populus koreana#NC 037414
Malpighiales Salicaceae Populus trinervis#MK267300
Malpighiales Salicaceae Populus xiangchengensis#NC 040953
Malpighiales Salicaceae Populus gonggaensis#NC 040873
Malpighiales Salicaceae Populus tremula#NC 027425
Malpighiales Salicaceae Populus davidiana#MG262347
Malpighiales Salicaceae Populus rotundifolia#NC 033876
Malpighiales Salicaceae Populus rotundifolia var. duclouxiana#MK267306
Malpighiales Salicaceae Populus adenopoda#MK267321
Malpighiales Salicaceae Populus nigra#KX377117
Malpighiales Salicaceae Populus alba var. pyramidalis#MG262344
Malpighiales Salicaceae Populus tomentosa#NC 040866
Malpighiales Salicaceae Populus alba#MK267320
Malpighiales Salicaceae Populus szechuanica var. tibetica#MK267302
Malpighiales Salicaceae Populus lasiocarpa#MG262349
Malpighiales Salicaceae Populus szechuanica#MK267303
Malpighiales Salicaceae Populus yunnanensis#F475
Malpighiales Salicaceae Populus qamdoensis#NC 040868
Malpighiales Salicaceae Populus simonii#MK267304
Malpighiales Salicaceae Populus pseudoglauca#NC 040869
Malpighiales Salicaceae Populus haoana#NC 040872
Malpighiales Salicaceae Populus kangdingensis#NC 040870
Malpighiales Salicaceae Populus schneideri#NC 040867
Fabales Quillajaceae Quillaja saponaria#FC58
Fabales Quillajaceae Quillaja saponaria#OQHZ
Fabales Polygalaceae Polygala lutea#OHAE
Fabales Polygalaceae Polygala arillata#F16
Fabales Polygalaceae Polygala karensium#F376
Fabales Surianaceae Cadellia pentastylis#FC324 2
Fabales Surianaceae Suriana maritima#F982
Fabales Fabaceae Tamarindus indica#NC 026685
Fabales Fabaceae Crudia harmsiana#NC 036743
Fabales Fabaceae Daniellia pilosa#NC 036744
Fabales Fabaceae Copaifera officinalis#RKLL
Fabales Fabaceae Guibourtia leonensis#NC 036742
Fabales Fabaceae Cercis chuniana#MF741770
Fabales Fabaceae Cercis canadensis#RKFX
Fabales Fabaceae Cercis glabra#NC 036762
Fabales Fabaceae Adenolobus garipensis#NC 036761
Fabales Fabaceae Griffonia simplicifolia#NC 037763
Fabales Fabaceae Bauhinia thonningii#NC 037765
Fabales Fabaceae Bauhinia acuminata#NC 037762
Fabales Fabaceae Bauhinia tomentosa#JETM
Fabales Fabaceae Tylosema fassoglensis#NC 037767
Fabales Fabaceae Schnella trichosepala#NC 037766
Fabales Fabaceae Barklya syringifolia#NC 037761
Fabales Fabaceae Lysiphyllum hookeri#NC 037768
Fabales Fabaceae Bauhinia binata#NC 037764
Fabales Fabaceae Ceratonia siliqua#NC 026678
Fabales Fabaceae Acrocarpus fraxinifolius#F251
Fabales Fabaceae Gymnocladus dioica#QZXQ
Fabales Fabaceae Gleditsia sinensis#VHZV
Fabales Fabaceae Gleditsia triacanthos#GEHT
Fabales Fabaceae Senna tora#NC 030193
Fabales Fabaceae Senna hebecarpa#KZED
Fabales Fabaceae Senna occidentalis#NC 038222
Fabales Fabaceae Balsamocarpon brevifolium#NC 041581
Fabales Fabaceae Caesalpinia coriaria#NC 026677
Fabales Fabaceae Haematoxylum brasiletto#NC 026679
Fabales Fabaceae Caesalpinia cucullata#F104
Fabales Fabaceae Caesalpinia decapetala#F188
Fabales Fabaceae Erythrophleum fordii#NC 041164
Fabales Fabaceae Adenanthera microsperma#NC 034986
Fabales Fabaceae Prosopis glandulosa#NC 026683
Fabales Fabaceae Dichrostachys cinerea#NC 035346
Fabales Fabaceae Desmanthus illinoensis#XOOE
Fabales Fabaceae Leucaena trichandra#NC 028733
Fabales Fabaceae Vachellia tortilis#KY100265
Fabales Fabaceae Vachellia seyal#NC 036735
Fabales Fabaceae Vachellia flava#NC 036734
Fabales Fabaceae Vachellia nilotica#KY100264
Fabales Fabaceae Parkia javanica#NC 034989
Fabales Fabaceae Piptadenia gonoacantha#NC 034990
Fabales Fabaceae Senegalia laeta#NC 036736
Fabales Fabaceae Faidherbia albida#KY100268
Fabales Fabaceae Ebenopsis ebano#NC 034991
Fabales Fabaceae Inga leiocalycina#NC 028732
Fabales Fabaceae Albizia odoratissima#NC 034987
Fabales Fabaceae Albizia saman#NC 034992
Fabales Fabaceae Pararchidendron pruinosum#NC 035348
Fabales Fabaceae Archidendron lucyi#NC 034988
Fabales Fabaceae Acacia ligulata#NC 026134
Fabales Fabaceae Acacia dealbata#NC 034985
Fabales Fabaceae Acacia pycnantha#PJSX
Fabales Fabaceae Acacia argyrophylla#ZCDJ
Fabales Fabaceae Xanthocercis zambesiaca#ZSSR
Fabales Fabaceae Cladrastis kentukea#SLYR
Fabales Fabaceae Styphnolobium japonicum#KY872756
Fabales Fabaceae Ormosia hosiei#NC 039418
Fabales Fabaceae Maackia floribunda#NC 034774
Fabales Fabaceae Sophora alopecuroides#NC 036102
Fabales Fabaceae Sophora flavescens#MH748034
Fabales Fabaceae Salweenia bouffordiana#MF449303
Fabales Fabaceae Ammopiptanthus mongolicus#NC 034742
Fabales Fabaceae Ammopiptanthus nanus#NC 034743
Fabales Fabaceae Lupinus luteus#NC 023090
Fabales Fabaceae Lupinus angustifolius#TTRG
Fabales Fabaceae Lupinus polyphyllus#CMFF
Fabales Fabaceae Lupinus micranthus#KU726828
Fabales Fabaceae Lupinus princei#KU726829
Fabales Fabaceae Lupinus atlanticus#KU726827
Fabales Fabaceae Lupinus westianus#NC 036487
Fabales Fabaceae Lupinus albus#KU726826
Fabales Fabaceae Dalbergia yunnanensis#F302
Fabales Fabaceae Dalbergia odorifera#MF668133
Fabales Fabaceae Dalbergia hainanensis#NC 036961
Fabales Fabaceae Pterocarpus tinctorius#MH033829
Fabales Fabaceae Pterocarpus indicus#MH033831

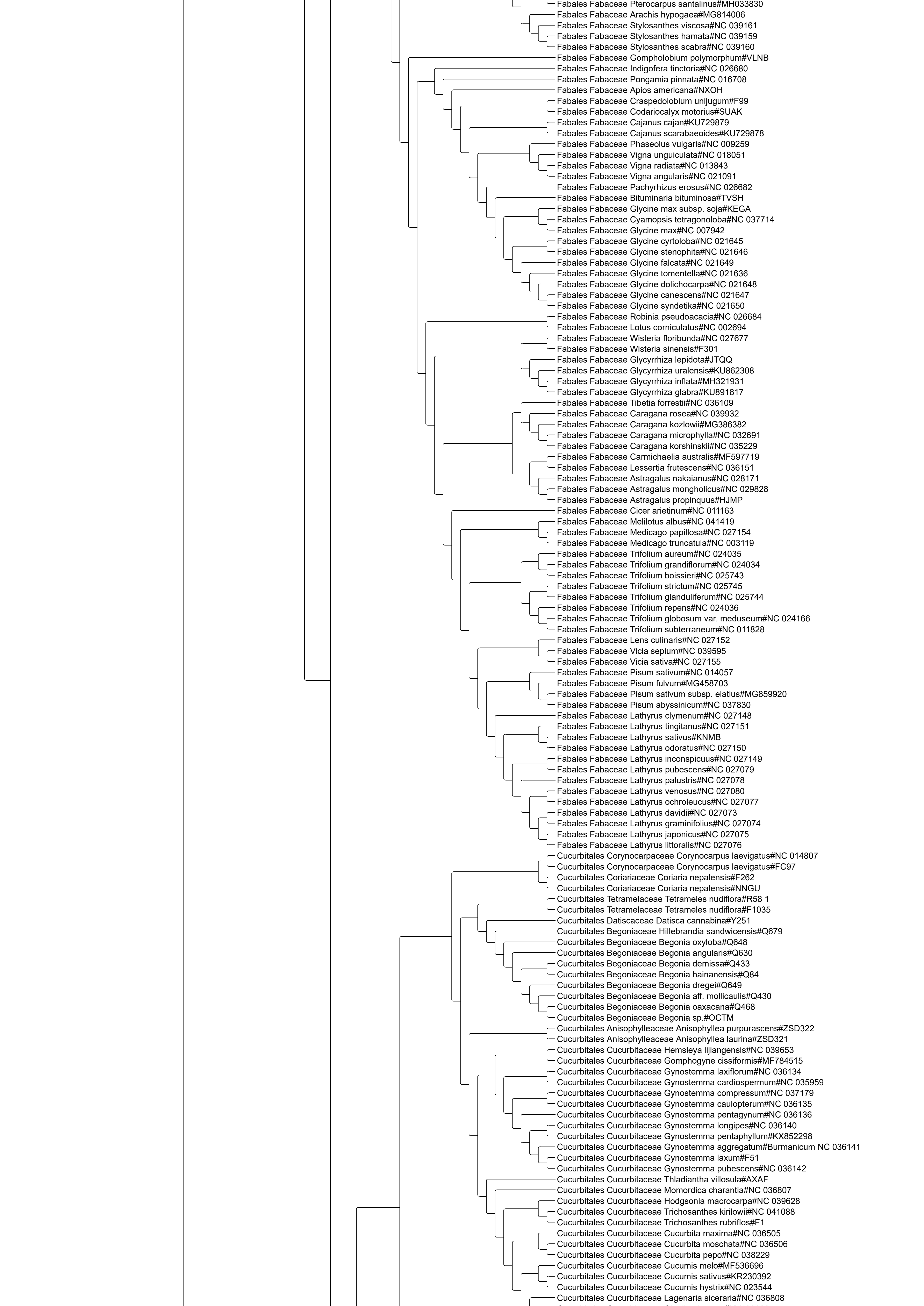

Fabales Fabaceae Pterocarpus santalinus#MH033830
Fabales Fabaceae Arachis hypogaea#MG814006
Fabales Fabaceae Stylosanthes viscosa#NC 039161
Fabales Fabaceae Stylosanthes hamata#NC 039159
Fabales Fabaceae Stylosanthes scabra#NC 039160
Fabales Fabaceae Gompholobium polymorphum#VLNB
Fabales Fabaceae Indigofera tinctoria#NC 026680
Fabales Fabaceae Pongamia pinnata#NC 016708
Fabales Fabaceae Apios americana#NXOH
Fabales Fabaceae Craspedolobium unijugum#F99
Fabales Fabaceae Codariocalyx motorius#SUAK
Fabales Fabaceae Cajanus cajan#KU729879
Fabales Fabaceae Cajanus scarabaeoides#KU729878
Fabales Fabaceae Phaseolus vulgaris#NC 009259
Fabales Fabaceae Vigna unguiculata#NC 018051
Fabales Fabaceae Vigna radiata#NC 013843
Fabales Fabaceae Vigna angularis#NC 021091
Fabales Fabaceae Pachyrhizus erosus#NC 026682
Fabales Fabaceae Bituminaria bituminosa#TVSH
Fabales Fabaceae Glycine max subsp. soja#KEGA
Fabales Fabaceae Cyamopsis tetragonoloba#NC 037714
Fabales Fabaceae Glycine max#NC 007942
Fabales Fabaceae Glycine cyrtoloba#NC 021645
Fabales Fabaceae Glycine stenophita#NC 021646
Fabales Fabaceae Glycine falcata#NC 021649
Fabales Fabaceae Glycine tomentella#NC 021636
Fabales Fabaceae Glycine dolichocarpa#NC 021648
Fabales Fabaceae Glycine canescens#NC 021647
Fabales Fabaceae Glycine syndetika#NC 021650
Fabales Fabaceae Robinia pseudoacacia#NC 026684
Fabales Fabaceae Lotus corniculatus#NC 002694
Fabales Fabaceae Wisteria floribunda#NC 027677
Fabales Fabaceae Wisteria sinensis#F301
Fabales Fabaceae Glycyrrhiza lepidota#JTQQ
Fabales Fabaceae Glycyrrhiza uralensis#KU862308
Fabales Fabaceae Glycyrrhiza inflata#MH321931
Fabales Fabaceae Glycyrrhiza glabra#KU891817
Fabales Fabaceae Tibetia forrestii#NC 036109
Fabales Fabaceae Caragana rosea#NC 039932
Fabales Fabaceae Caragana kozlowii#MG386382
Fabales Fabaceae Caragana microphylla#NC 032691
Fabales Fabaceae Caragana korshinskii#NC 035229
Fabales Fabaceae Carmichaelia australis#MF597719
Fabales Fabaceae Lessertia frutescens#NC 036151
Fabales Fabaceae Astragalus nakaianus#NC 028171
Fabales Fabaceae Astragalus mongholicus#NC 029828
Fabales Fabaceae Astragalus propinquus#HJMP
Fabales Fabaceae Cicer arietinum#NC 011163
Fabales Fabaceae Melilotus albus#NC 041419
Fabales Fabaceae Medicago papillosa#NC 027154
Fabales Fabaceae Medicago truncatula#NC 003119
Fabales Fabaceae Trifolium aureum#NC 024035
Fabales Fabaceae Trifolium grandiflorum#NC 024034
Fabales Fabaceae Trifolium boissieri#NC 025743
Fabales Fabaceae Trifolium strictum#NC 025745
Fabales Fabaceae Trifolium glanduliferum#NC 025744
Fabales Fabaceae Trifolium repens#NC 024036
Fabales Fabaceae Trifolium globosum var. meduseum#NC 024166
Fabales Fabaceae Trifolium subterraneum#NC 011828
Fabales Fabaceae Lens culinaris#NC 027152
Fabales Fabaceae Vicia sepium#NC 039595
Fabales Fabaceae Vicia sativa#NC 027155
Fabales Fabaceae Pisum sativum#NC 014057
Fabales Fabaceae Pisum fulvum#MG458703
Fabales Fabaceae Pisum sativum subsp. elatius#MG859920
Fabales Fabaceae Pisum abyssinicum#NC 037830
Fabales Fabaceae Lathyrus clymenum#NC 027148
Fabales Fabaceae Lathyrus tingitanus#NC 027151
Fabales Fabaceae Lathyrus sativus#KNMB
Fabales Fabaceae Lathyrus odoratus#NC 027150
Fabales Fabaceae Lathyrus inconspicuus#NC 027149
Fabales Fabaceae Lathyrus pubescens#NC 027079
Fabales Fabaceae Lathyrus palustris#NC 027078
Fabales Fabaceae Lathyrus venosus#NC 027080
Fabales Fabaceae Lathyrus ochroleucus#NC 027077
Fabales Fabaceae Lathyrus davidii#NC 027073
Fabales Fabaceae Lathyrus graminifolius#NC 027074
Fabales Fabaceae Lathyrus japonicus#NC 027075
Fabales Fabaceae Lathyrus littoralis#NC 027076
Cucurbitales Corynocarpaceae Corynocarpus laevigatus#NC 014807
Cucurbitales Corynocarpaceae Corynocarpus laevigatus#FC97
Cucurbitales Coriariaceae Coriaria nepalensis#F262
Cucurbitales Coriariaceae Coriaria nepalensis#NNGU
Cucurbitales Tetramelaceae Tetrameles nudiflora#R58 1
Cucurbitales Tetramelaceae Tetrameles nudiflora#F1035
Cucurbitales Datiscaceae Datisca cannabina#Y251
Cucurbitales Begoniaceae Hillebrandia sandwicensis#Q679
Cucurbitales Begoniaceae Begonia oxyloba#Q648
Cucurbitales Begoniaceae Begonia angularis#Q630
Cucurbitales Begoniaceae Begonia demissa#Q433
Cucurbitales Begoniaceae Begonia hainanensis#Q84
Cucurbitales Begoniaceae Begonia dregei#Q649
Cucurbitales Begoniaceae Begonia aff. mollicaulis#Q430
Cucurbitales Begoniaceae Begonia oaxacana#Q468
Cucurbitales Begoniaceae Begonia sp.#OCTM
Cucurbitales Anisophylleaceae Anisophyllea purpurascens#ZSD322
Cucurbitales Anisophylleaceae Anisophyllea laurina#ZSD321
Cucurbitales Cucurbitaceae Hemsleya lijiangensis#NC 039653
Cucurbitales Cucurbitaceae Gomphogyne cissiformis#MF784515
Cucurbitales Cucurbitaceae Gynostemma laxiflorum#NC 036134
Cucurbitales Cucurbitaceae Gynostemma cardiospermum#NC 035959
Cucurbitales Cucurbitaceae Gynostemma compressum#NC 037179
Cucurbitales Cucurbitaceae Gynostemma caulopterum#NC 036135
Cucurbitales Cucurbitaceae Gynostemma pentagynum#NC 036136
Cucurbitales Cucurbitaceae Gynostemma longipes#NC 036140
Cucurbitales Cucurbitaceae Gynostemma pentaphyllum#KX852298
Cucurbitales Cucurbitaceae Gynostemma aggregatum#Burmanicum NC 036141
Cucurbitales Cucurbitaceae Gynostemma laxum#F51
Cucurbitales Cucurbitaceae Gynostemma pubescens#NC 036142
Cucurbitales Cucurbitaceae Thladiantha villosula#AXAF
Cucurbitales Cucurbitaceae Momordica charantia#NC 036807
Cucurbitales Cucurbitaceae Hodgsonia macrocarpa#NC 039628
Cucurbitales Cucurbitaceae Trichosanthes kirilowii#NC 041088
Cucurbitales Cucurbitaceae Trichosanthes rubriflos#F1
Cucurbitales Cucurbitaceae Cucurbita maxima#NC 036505
Cucurbitales Cucurbitaceae Cucurbita moschata#NC 036506
Cucurbitales Cucurbitaceae Cucurbita pepo#NC 038229
Cucurbitales Cucurbitaceae Cucumis melo#MF536696
Cucurbitales Cucurbitaceae Cucumis sativus#KR230392
Cucurbitales Cucurbitaceae Cucumis hystrix#NC 023544
Cucurbitales Cucurbitaceae Lagenaria siceraria#NC 036808

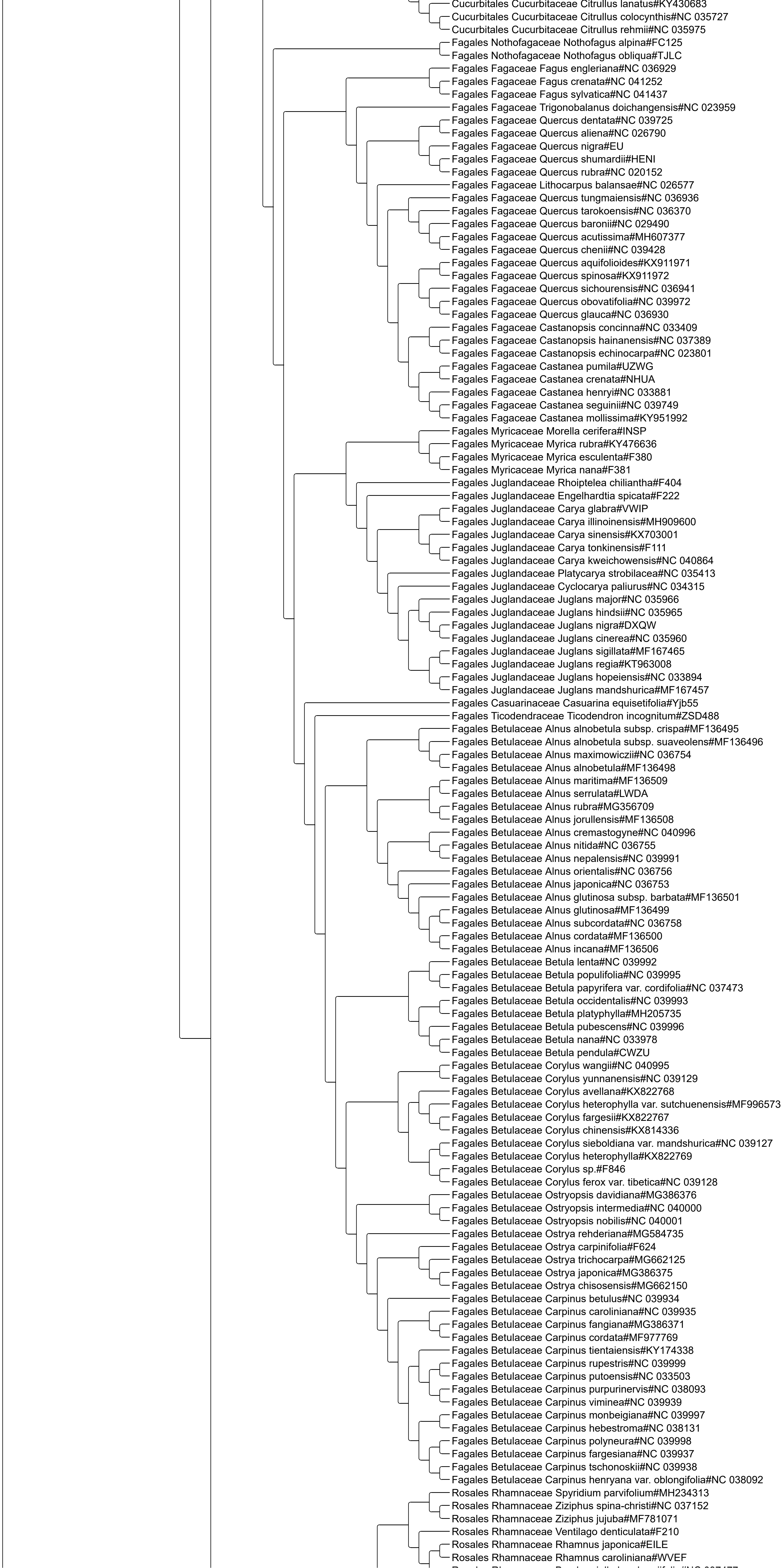
Cucurbitales Cucurbitaceae Citrullus lanatus#KY430683
Cucurbitales Cucurbitaceae Citrullus colocynthis#NC 035727
Cucurbitales Cucurbitaceae Citrullus rehmii#NC 035975
Fagales Nothofagaceae Nothofagus alpina#FC125
Fagales Nothofagaceae Nothofagus obliqua#TJLC
Fagales Fagaceae Fagus engleriana#NC 036929
Fagales Fagaceae Fagus crenata#NC 041252
Fagales Fagaceae Fagus sylvatica#NC 041437
Fagales Fagaceae Trigonobalanus doichangensis#NC 023959
Fagales Fagaceae Quercus dentata#NC 039725
Fagales Fagaceae Quercus aliena#NC 026790
Fagales Fagaceae Quercus nigra#EU
Fagales Fagaceae Quercus shumardii#HENI
Fagales Fagaceae Quercus rubra#NC 020152
Fagales Fagaceae Lithocarpus balansae#NC 026577
Fagales Fagaceae Quercus tungmaiensis#NC 036936
Fagales Fagaceae Quercus tarokoensis#NC 036370
Fagales Fagaceae Quercus baronii#NC 029490
Fagales Fagaceae Quercus acutissima#MH607377
Fagales Fagaceae Quercus chenii#NC 039428
Fagales Fagaceae Quercus aquifolioides#KX911971
Fagales Fagaceae Quercus spinosa#KX911972
Fagales Fagaceae Quercus sichourensis#NC 036941
Fagales Fagaceae Quercus obovatifolia#NC 039972
Fagales Fagaceae Quercus glauca#NC 036930
Fagales Fagaceae Castanopsis concinna#NC 033409
Fagales Fagaceae Castanopsis hainanensis#NC 037389
Fagales Fagaceae Castanopsis echinocarpa#NC 023801
Fagales Fagaceae Castanea pumila#UZWG
Fagales Fagaceae Castanea crenata#NHUA
Fagales Fagaceae Castanea henryi#NC 033881
Fagales Fagaceae Castanea seguinii#NC 039749
Fagales Fagaceae Castanea mollissima#KY951992
Fagales Myricaceae Morella cerifera#INSP
Fagales Myricaceae Myrica rubra#KY476636
Fagales Myricaceae Myrica esculenta#F380
Fagales Myricaceae Myrica nana#F381
Fagales Juglandaceae Rhoiptelea chiliantha#F404
Fagales Juglandaceae Engelhardtia spicata#F222
Fagales Juglandaceae Carya glabra#VWIP
Fagales Juglandaceae Carya illinoinensis#MH909600
Fagales Juglandaceae Carya sinensis#KX703001
Fagales Juglandaceae Carya tonkinensis#F111
Fagales Juglandaceae Carya kweichowensis#NC 040864
Fagales Juglandaceae Platycarya strobilacea#NC 035413
Fagales Juglandaceae Cyclocarya paliurus#NC 034315
Fagales Juglandaceae Juglans major#NC 035966
Fagales Juglandaceae Juglans hindsii#NC 035965
Fagales Juglandaceae Juglans nigra#DXQW
Fagales Juglandaceae Juglans cinerea#NC 035960
Fagales Juglandaceae Juglans sigillata#MF167465
Fagales Juglandaceae Juglans regia#KT963008
Fagales Juglandaceae Juglans hopeiensis#NC 033894
Fagales Juglandaceae Juglans mandshurica#MF167457
Fagales Casuarinaceae Casuarina equisetifolia#Yjb55
Fagales Ticodendraceae Ticodendron incognitum#ZSD488
Fagales Betulaceae Alnus alnobetula subsp. crispa#MF136495
Fagales Betulaceae Alnus alnobetula subsp. suaveolens#MF136496
Fagales Betulaceae Alnus maximowiczii#NC 036754
Fagales Betulaceae Alnus alnobetula#MF136498
Fagales Betulaceae Alnus maritima#MF136509
Fagales Betulaceae Alnus serrulata#LWDA
Fagales Betulaceae Alnus rubra#MG356709
Fagales Betulaceae Alnus jorullensis#MF136508
Fagales Betulaceae Alnus cremastogyne#NC 040996
Fagales Betulaceae Alnus nitida#NC 036755
Fagales Betulaceae Alnus nepalensis#NC 039991
Fagales Betulaceae Alnus orientalis#NC 036756
Fagales Betulaceae Alnus japonica#NC 036753
Fagales Betulaceae Alnus glutinosa subsp. barbata#MF136501
Fagales Betulaceae Alnus glutinosa#MF136499
Fagales Betulaceae Alnus subcordata#NC 036758
Fagales Betulaceae Alnus cordata#MF136500
Fagales Betulaceae Alnus incana#MF136506
Fagales Betulaceae Betula lenta#NC 039992
Fagales Betulaceae Betula populifolia#NC 039995
Fagales Betulaceae Betula papyrifera var. cordifolia#NC 037473
Fagales Betulaceae Betula occidentalis#NC 039993
Fagales Betulaceae Betula platyphylla#MH205735
Fagales Betulaceae Betula pubescens#NC 039996
Fagales Betulaceae Betula nana#NC 033978
Fagales Betulaceae Betula pendula#CWZU
Fagales Betulaceae Corylus wangii#NC 040995
Fagales Betulaceae Corylus yunnanensis#NC 039129
Fagales Betulaceae Corylus avellana#KX822768
Fagales Betulaceae Corylus heterophylla var. sutchuenensis#MF996573
Fagales Betulaceae Corylus fargesii#KX822767
Fagales Betulaceae Corylus chinensis#KX814336
Fagales Betulaceae Corylus sieboldiana var. mandshurica#NC 039127
Fagales Betulaceae Corylus heterophylla#KX822769
Fagales Betulaceae Corylus sp.#F846
Fagales Betulaceae Corylus ferox var. tibetica#NC 039128
Fagales Betulaceae Ostryopsis davidiana#MG386376
Fagales Betulaceae Ostryopsis intermedia#NC 040000
Fagales Betulaceae Ostryopsis nobilis#NC 040001
Fagales Betulaceae Ostrya rehderiana#MG584735
Fagales Betulaceae Ostrya carpinifolia#F624
Fagales Betulaceae Ostrya trichocarpa#MG662125
Fagales Betulaceae Ostrya japonica#MG386375
Fagales Betulaceae Ostrya chisosensis#MG662150
Fagales Betulaceae Carpinus betulus#NC 039934
Fagales Betulaceae Carpinus caroliniana#NC 039935
Fagales Betulaceae Carpinus fangiana#MG386371
Fagales Betulaceae Carpinus cordata#MF977769
Fagales Betulaceae Carpinus tientaiensis#KY174338
Fagales Betulaceae Carpinus rupestris#NC 039999
Fagales Betulaceae Carpinus putoensis#NC 033503
Fagales Betulaceae Carpinus purpurinervis#NC 038093
Fagales Betulaceae Carpinus viminea#NC 039939
Fagales Betulaceae Carpinus monbeigiana#NC 039997
Fagales Betulaceae Carpinus hebestroma#NC 038131
Fagales Betulaceae Carpinus polyneura#NC 039998
Fagales Betulaceae Carpinus fargesiana#NC 039937
Fagales Betulaceae Carpinus tschonoskii#NC 039938
Fagales Betulaceae Carpinus henryana var. oblongifolia#NC 038092
Rosales Rhamnaceae Spyridium parvifolium#MH234313
Rosales Rhamnaceae Ziziphus spina-christi#NC 037152
Rosales Rhamnaceae Ziziphus jujuba#MF781071
Rosales Rhamnaceae Ventilago denticulata#F210
Rosales Rhamnaceae Rhamnus japonica#EILE
Rosales Rhamnaceae Rhamnus caroliniana#WVEF

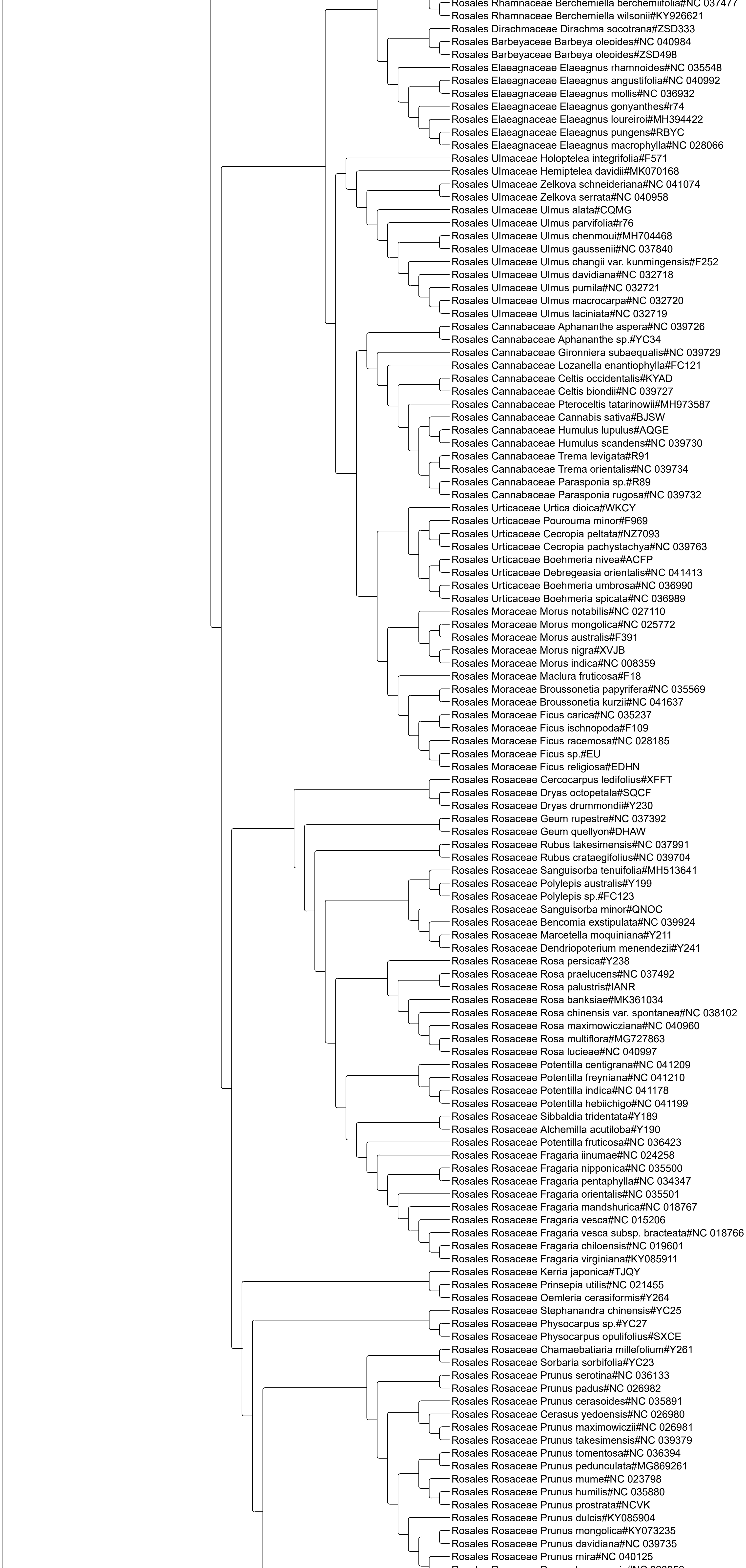
Rosales Rhamnaceae Berchemiella berchemiifolia#NC 037477
Rosales Rhamnaceae Berchemiella wilsonii#KY926621
Rosales Dirachmaceae Dirachma socotrana#ZSD333
Rosales Barbeyaceae Barbeya oleoides#NC 040984
Rosales Barbeyaceae Barbeya oleoides#ZSD498
Rosales Elaeagnaceae Elaeagnus rhamnoides#NC 035548
Rosales Elaeagnaceae Elaeagnus angustifolia#NC 040992
Rosales Elaeagnaceae Elaeagnus mollis#NC 036932
Rosales Elaeagnaceae Elaeagnus gonyanthes#r74
Rosales Elaeagnaceae Elaeagnus loureiroi#MH394422
Rosales Elaeagnaceae Elaeagnus pungens#RBYC
Rosales Elaeagnaceae Elaeagnus macrophylla#NC 028066
Rosales Ulmaceae Holoptelea integrifolia#F571
Rosales Ulmaceae Hemiptelea davidii#MK070168
Rosales Ulmaceae Zelkova schneideriana#NC 041074
Rosales Ulmaceae Zelkova serrata#NC 040958
Rosales Ulmaceae Ulmus alata#CQMG
Rosales Ulmaceae Ulmus parvifolia#r76
Rosales Ulmaceae Ulmus chenmoui#MH704468
Rosales Ulmaceae Ulmus gaussenii#NC 037840
Rosales Ulmaceae Ulmus changii var. kunmingensis#F252
Rosales Ulmaceae Ulmus davidiana#NC 032718
Rosales Ulmaceae Ulmus pumila#NC 032721
Rosales Ulmaceae Ulmus macrocarpa#NC 032720
Rosales Ulmaceae Ulmus laciniata#NC 032719
Rosales Cannabaceae Aphananthe aspera#NC 039726
Rosales Cannabaceae Aphananthe sp.#YC34
Rosales Cannabaceae Gironniera subaequalis#NC 039729
Rosales Cannabaceae Lozanella enantiophylla#FC121
Rosales Cannabaceae Celtis occidentalis#KYAD
Rosales Cannabaceae Celtis biondii#NC 039727
Rosales Cannabaceae Pteroceltis tatarinowii#MH973587
Rosales Cannabaceae Cannabis sativa#BJSW
Rosales Cannabaceae Humulus lupulus#AQGE
Rosales Cannabaceae Humulus scandens#NC 039730
Rosales Cannabaceae Trema levigata#R91
Rosales Cannabaceae Trema orientalis#NC 039734
Rosales Cannabaceae Parasponia sp.#R89
Rosales Cannabaceae Parasponia rugosa#NC 039732
Rosales Urticaceae Urtica dioica#WKCY
Rosales Urticaceae Pourouma minor#F969
Rosales Urticaceae Cecropia peltata#NZ7093
Rosales Urticaceae Cecropia pachystachya#NC 039763
Rosales Urticaceae Boehmeria nivea#ACFP
Rosales Urticaceae Debregeasia orientalis#NC 041413
Rosales Urticaceae Boehmeria umbrosa#NC 036990
Rosales Urticaceae Boehmeria spicata#NC 036989
Rosales Moraceae Morus notabilis#NC 027110
Rosales Moraceae Morus mongolica#NC 025772
Rosales Moraceae Morus australis#F391
Rosales Moraceae Morus nigra#XVJB
Rosales Moraceae Morus indica#NC 008359
Rosales Moraceae Maclura fruticosa#F18
Rosales Moraceae Broussonetia papyrifera#NC 035569
Rosales Moraceae Broussonetia kurzii#NC 041637
Rosales Moraceae Ficus carica#NC 035237
Rosales Moraceae Ficus ischnopoda#F109
Rosales Moraceae Ficus racemosa#NC 028185
Rosales Moraceae Ficus sp.#EU
Rosales Moraceae Ficus religiosa#EDHN
Rosales Rosaceae Cercocarpus ledifolius#XFFT
Rosales Rosaceae Dryas octopetala#SQCF
Rosales Rosaceae Dryas drummondii#Y230
Rosales Rosaceae Geum rupestre#NC 037392
Rosales Rosaceae Geum quellyon#DHAW
Rosales Rosaceae Rubus takesimensis#NC 037991
Rosales Rosaceae Rubus crataegifolius#NC 039704
Rosales Rosaceae Sanguisorba tenuifolia#MH513641
Rosales Rosaceae Polylepis australis#Y199
Rosales Rosaceae Polylepis sp.#FC123
Rosales Rosaceae Sanguisorba minor#QNOC
Rosales Rosaceae Bencomia exstipulata#NC 039924
Rosales Rosaceae Marcetella moquiniana#Y211
Rosales Rosaceae Dendriopoterium menendezii#Y241
Rosales Rosaceae Rosa persica#Y238
Rosales Rosaceae Rosa praelucens#NC 037492
Rosales Rosaceae Rosa palustris#IANR
Rosales Rosaceae Rosa banksiae#MK361034
Rosales Rosaceae Rosa chinensis var. spontanea#NC 038102
Rosales Rosaceae Rosa maximowicziana#NC 040960
Rosales Rosaceae Rosa multiflora#MG727863
Rosales Rosaceae Rosa lucieae#NC 040997
Rosales Rosaceae Potentilla centigrana#NC 041209
Rosales Rosaceae Potentilla freyniana#NC 041210
Rosales Rosaceae Potentilla indica#NC 041178
Rosales Rosaceae Potentilla hebiichigo#NC 041199
Rosales Rosaceae Sibbaldia tridentata#Y189
Rosales Rosaceae Alchemilla acutiloba#Y190
Rosales Rosaceae Potentilla fruticosa#NC 036423
Rosales Rosaceae Fragaria iinumae#NC 024258
Rosales Rosaceae Fragaria nipponica#NC 035500
Rosales Rosaceae Fragaria pentaphylla#NC 034347
Rosales Rosaceae Fragaria orientalis#NC 035501
Rosales Rosaceae Fragaria mandshurica#NC 018767
Rosales Rosaceae Fragaria vesca#NC 015206
Rosales Rosaceae Fragaria vesca subsp. bracteata#NC 018766
Rosales Rosaceae Fragaria chiloensis#NC 019601
Rosales Rosaceae Fragaria virginiana#KY085911
Rosales Rosaceae Kerria japonica#TJQY
Rosales Rosaceae Prinsepia utilis#NC 021455
Rosales Rosaceae Oemleria cerasiformis#Y264
Rosales Rosaceae Stephanandra chinensis#YC25
Rosales Rosaceae Physocarpus sp.#YC27
Rosales Rosaceae Physocarpus opulifolius#SXCE
Rosales Rosaceae Chamaebatiaria millefolium#Y261
Rosales Rosaceae Sorbaria sorbifolia#YC23
Rosales Rosaceae Prunus serotina#NC 036133
Rosales Rosaceae Prunus padus#NC 026982
Rosales Rosaceae Prunus cerasoides#NC 035891
Rosales Rosaceae Cerasus yedoensis#NC 026980
Rosales Rosaceae Prunus maximowiczii#NC 026981
Rosales Rosaceae Prunus takesimensis#NC 039379
Rosales Rosaceae Prunus tomentosa#NC 036394
Rosales Rosaceae Prunus pedunculata#MG869261
Rosales Rosaceae Prunus mume#NC 023798
Rosales Rosaceae Prunus humilis#NC 035880
Rosales Rosaceae Prunus prostrata#NCVK
Rosales Rosaceae Prunus dulcis#KY085904
Rosales Rosaceae Prunus mongolica#KY073235
Rosales Rosaceae Prunus davidiana#NC 039735
Rosales Rosaceae Prunus mira#NC 040125

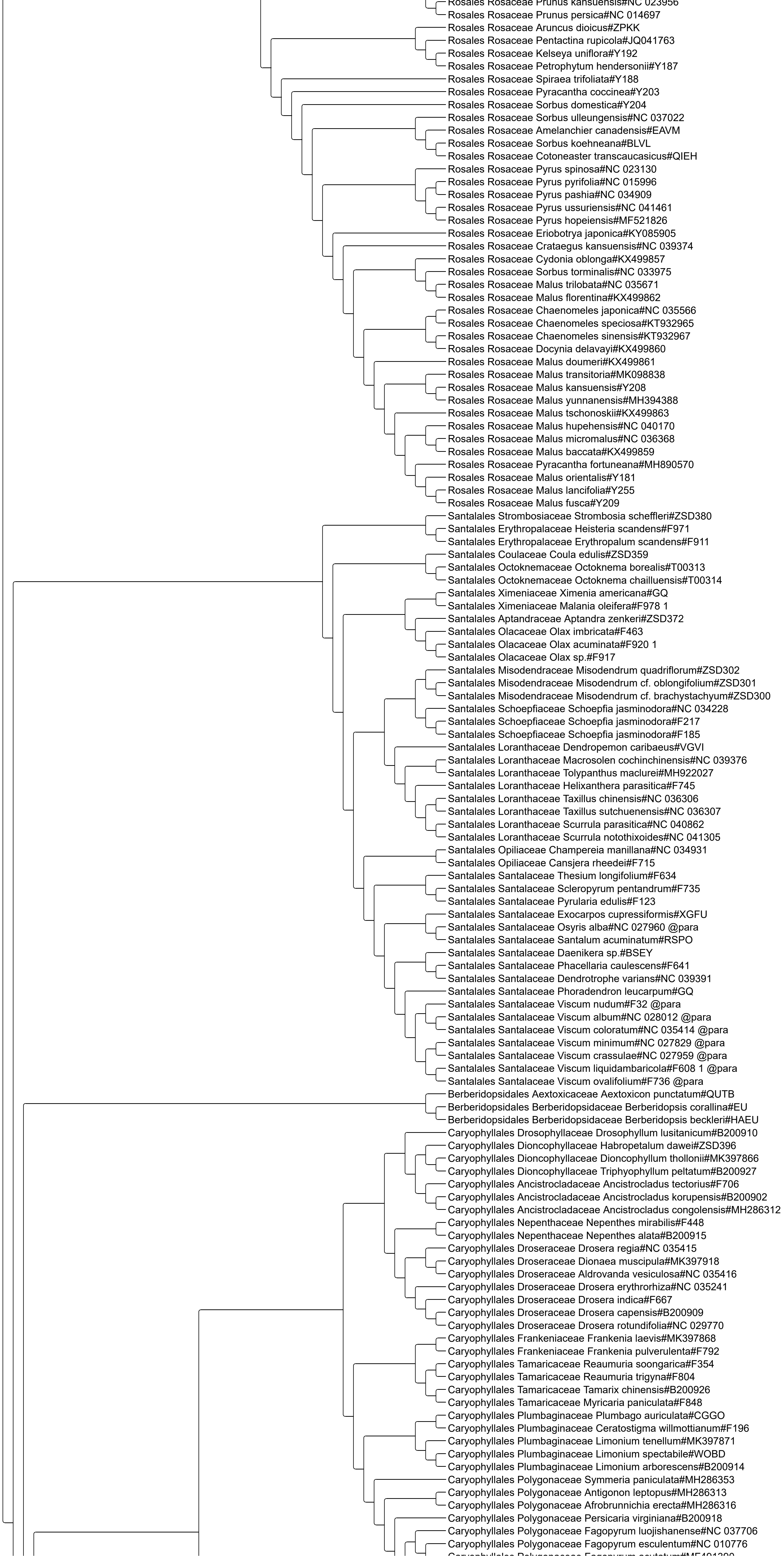

Rosales Rosaceae Prunus kansuensis#NC 023956
Rosales Rosaceae Prunus persica#NC 014697
Rosales Rosaceae Aruncus dioicus#ZPKK
Rosales Rosaceae Pentactina rupicola#JQ041763
Rosales Rosaceae Kelseya uniflora#Y192
Rosales Rosaceae Petrophytum hendersonii#Y187
Rosales Rosaceae Spiraea trifoliata#Y188
Rosales Rosaceae Pyracantha coccinea#Y203
Rosales Rosaceae Sorbus domestica#Y204
Rosales Rosaceae Sorbus ulleungensis#NC 037022
Rosales Rosaceae Amelanchier canadensis#EAVM
Rosales Rosaceae Sorbus koehneana#BLVL
Rosales Rosaceae Cotoneaster transcaucasicus#QIEH
Rosales Rosaceae Pyrus spinosa#NC 023130
Rosales Rosaceae Pyrus pyrifolia#NC 015996
Rosales Rosaceae Pyrus pashia#NC 034909
Rosales Rosaceae Pyrus ussuriensis#NC 041461
Rosales Rosaceae Pyrus hopeiensis#MF521826
Rosales Rosaceae Eriobotrya japonica#KY085905
Rosales Rosaceae Crataegus kansuensis#NC 039374
Rosales Rosaceae Cydonia oblonga#KX499857
Rosales Rosaceae Sorbus torminalis#NC 033975
Rosales Rosaceae Malus trilobata#NC 035671
Rosales Rosaceae Malus florentina#KX499862
Rosales Rosaceae Chaenomeles japonica#NC 035566
Rosales Rosaceae Chaenomeles speciosa#KT932965
Rosales Rosaceae Chaenomeles sinensis#KT932967
Rosales Rosaceae Docynia delavayi#KX499860
Rosales Rosaceae Malus doumeri#KX499861
Rosales Rosaceae Malus transitoria#MK098838
Rosales Rosaceae Malus kansuensis#Y208
Rosales Rosaceae Malus yunnanensis#MH394388
Rosales Rosaceae Malus tschonoskii#KX499863
Rosales Rosaceae Malus hupehensis#NC 040170
Rosales Rosaceae Malus micromalus#NC 036368
Rosales Rosaceae Malus baccata#KX499859
Rosales Rosaceae Pyracantha fortuneana#MH890570
Rosales Rosaceae Malus orientalis#Y181
Rosales Rosaceae Malus lancifolia#Y255
Rosales Rosaceae Malus fusca#Y209
Santalales Strombosiaceae Strombosia scheffleri#ZSD380
Santalales Erythropalaceae Heisteria scandens#F971
Santalales Erythropalaceae Erythropalum scandens#F911
Santalales Coulaceae Coula edulis#ZSD359
Santalales Octoknemaceae Octoknema borealis#T00313
Santalales Octoknemaceae Octoknema chailluensis#T00314
Santalales Ximeniaceae Ximenia americana#GQ
Santalales Ximeniaceae Malania oleifera#F978 1
Santalales Aptandraceae Aptandra zenkeri#ZSD372
Santalales Olacaceae Olax imbricata#F463
Santalales Olacaceae Olax acuminata#F920 1
Santalales Olacaceae Olax sp.#F917
Santalales Misodendraceae Misodendrum quadriflorum#ZSD302
Santalales Misodendraceae Misodendrum cf. oblongifolium#ZSD301
Santalales Misodendraceae Misodendrum cf. brachystachyum#ZSD300
Santalales Schoepfiaceae Schoepfia jasminodora#NC 034228
Santalales Schoepfiaceae Schoepfia jasminodora#F217
Santalales Schoepfiaceae Schoepfia jasminodora#F185
Santalales Loranthaceae Dendropemon caribaeus#VGVI
Santalales Loranthaceae Macrosolen cochinchinensis#NC 039376
Santalales Loranthaceae Tolypanthus maclurei#MH922027
Santalales Loranthaceae Helixanthera parasitica#F745
Santalales Loranthaceae Taxillus chinensis#NC 036306
Santalales Loranthaceae Taxillus sutchuenensis#NC 036307
Santalales Loranthaceae Scurrula parasitica#NC 040862
Santalales Loranthaceae Scurrula notothixoides#NC 041305
Santalales Opiliaceae Champereia manillana#NC 034931
Santalales Opiliaceae Cansjera rheedei#F715
Santalales Santalaceae Thesium longifolium#F634
Santalales Santalaceae Scleropyrum pentandrum#F735
Santalales Santalaceae Pyrularia edulis#F123
Santalales Santalaceae Exocarpos cupressiformis#XGFU
Santalales Santalaceae Osyris alba#NC 027960 @para
Santalales Santalaceae Santalum acuminatum#RSPO
Santalales Santalaceae Daenikera sp.#BSEY
Santalales Santalaceae Phacellaria caulescens#F641
Santalales Santalaceae Dendrotrophe varians#NC 039391
Santalales Santalaceae Phoradendron leucarpum#GQ
Santalales Santalaceae Viscum nudum#F32 @para
Santalales Santalaceae Viscum album#NC 028012 @para
Santalales Santalaceae Viscum coloratum#NC 035414 @para
Santalales Santalaceae Viscum minimum#NC 027829 @para
Santalales Santalaceae Viscum crassulae#NC 027959 @para
Santalales Santalaceae Viscum liquidambaricola#F608 1 @para
Santalales Santalaceae Viscum ovalifolium#F736 @para
Berberidopsidales Aextoxicaceae Aextoxicon punctatum#QUTB
Berberidopsidales Berberidopsidaceae Berberidopsis corallina#EU
Berberidopsidales Berberidopsidaceae Berberidopsis beckleri#HAEU
Caryophyllales Drosophyllaceae Drosophyllum lusitanicum#B200910
Caryophyllales Dioncophyllaceae Habropetalum dawei#ZSD396
Caryophyllales Dioncophyllaceae Dioncophyllum thollonii#MK397866
Caryophyllales Dioncophyllaceae Triphyophyllum peltatum#B200927
Caryophyllales Ancistrocladaceae Ancistrocladus tectorius#F706
Caryophyllales Ancistrocladaceae Ancistrocladus korupensis#B200902
Caryophyllales Ancistrocladaceae Ancistrocladus congolensis#MH286312
Caryophyllales Nepenthaceae Nepenthes mirabilis#F448
Caryophyllales Nepenthaceae Nepenthes alata#B200915
Caryophyllales Droseraceae Drosera regia#NC 035415
Caryophyllales Droseraceae Dionaea muscipula#MK397918
Caryophyllales Droseraceae Aldrovanda vesiculosa#NC 035416
Caryophyllales Droseraceae Drosera erythrorhiza#NC 035241
Caryophyllales Droseraceae Drosera indica#F667
Caryophyllales Droseraceae Drosera capensis#B200909
Caryophyllales Droseraceae Drosera rotundifolia#NC 029770
Caryophyllales Frankeniaceae Frankenia laevis#MK397868
Caryophyllales Frankeniaceae Frankenia pulverulenta#F792
Caryophyllales Tamaricaceae Reaumuria soongarica#F354
Caryophyllales Tamaricaceae Reaumuria trigyna#F804
Caryophyllales Tamaricaceae Tamarix chinensis#B200926
Caryophyllales Tamaricaceae Myricaria paniculata#F848
Caryophyllales Plumbaginaceae Plumbago auriculata#CGGO
Caryophyllales Plumbaginaceae Ceratostigma willmottianum#F196
Caryophyllales Plumbaginaceae Limonium tenellum#MK397871
Caryophyllales Plumbaginaceae Limonium spectabile#WOBD
Caryophyllales Plumbaginaceae Limonium arborescens#B200914
Caryophyllales Polygonaceae Symmeria paniculata#MH286353
Caryophyllales Polygonaceae Antigonon leptopus#MH286313
Caryophyllales Polygonaceae Afrobrunnichia erecta#MH286316
Caryophyllales Polygonaceae Persicaria virginiana#B200918
Caryophyllales Polygonaceae Fagopyrum luojishanense#NC 037706
Caryophyllales Polygonaceae Fagopyrum esculentum#NC 010776

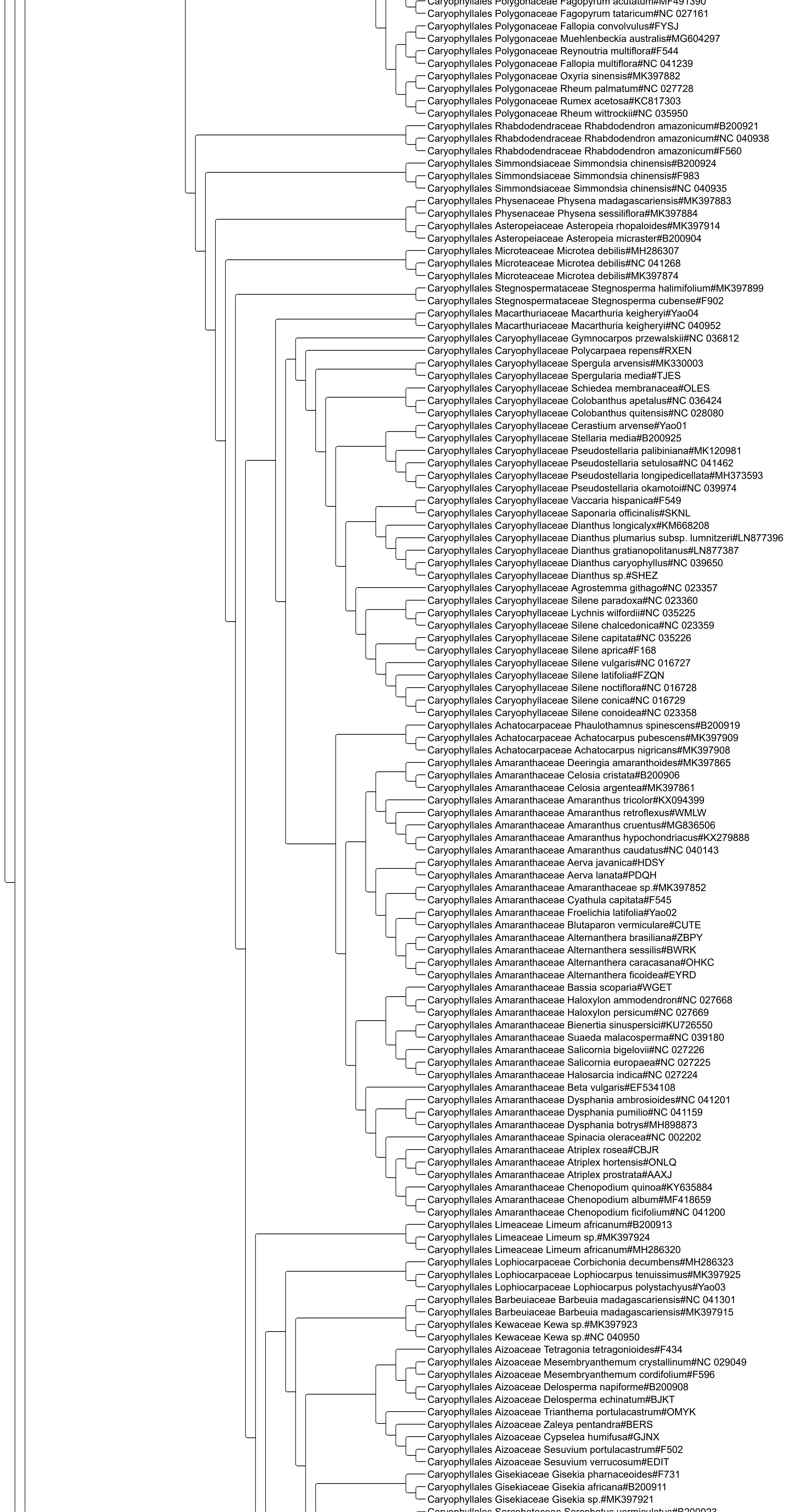

Caryophyllales Polygonaceae Fagopyrum acutatum#MF491390
Caryophyllales Polygonaceae Fagopyrum tataricum#NC 027161
Caryophyllales Polygonaceae Fallopia convolvulus#FYSJ
Caryophyllales Polygonaceae Muehlenbeckia australis#MG604297
Caryophyllales Polygonaceae Reynoutria multiflora#F544
Caryophyllales Polygonaceae Fallopia multiflora#NC 041239
Caryophyllales Polygonaceae Oxyria sinensis#MK397882
Caryophyllales Polygonaceae Rheum palmatum#NC 027728
Caryophyllales Polygonaceae Rumex acetosa#KC817303
Caryophyllales Polygonaceae Rheum wittrockii#NC 035950
Caryophyllales Rhabdodendraceae Rhabdodendron amazonicum#B200921
Caryophyllales Rhabdodendraceae Rhabdodendron amazonicum#NC 040938
Caryophyllales Rhabdodendraceae Rhabdodendron amazonicum#F560
Caryophyllales Simmondsiaceae Simmondsia chinensis#B200924
Caryophyllales Simmondsiaceae Simmondsia chinensis#F983
Caryophyllales Simmondsiaceae Simmondsia chinensis#NC 040935
Caryophyllales Physenaceae Physena madagascariensis#MK397883
Caryophyllales Physenaceae Physena sessiliflora#MK397884
Caryophyllales Asteropeiaceae Asteropeia rhopaloides#MK397914
Caryophyllales Asteropeiaceae Asteropeia micraster#B200904
Caryophyllales Microteaceae Microtea debilis#MH286307
Caryophyllales Microteaceae Microtea debilis#NC 041268
Caryophyllales Microteaceae Microtea debilis#MK397874
Caryophyllales Stegnospermataceae Stegnosperma halimifolium#MK397899
Caryophyllales Stegnospermataceae Stegnosperma cubense#F902
Caryophyllales Macarthuriaceae Macarthuria keigheryi#Yao04
Caryophyllales Macarthuriaceae Macarthuria keigheryi#NC 040952
Caryophyllales Caryophyllaceae Gymnocarpos przewalskii#NC 036812
Caryophyllales Caryophyllaceae Polycarpaea repens#RXEN
Caryophyllales Caryophyllaceae Spergula arvensis#MK330003
Caryophyllales Caryophyllaceae Spergularia media#TJES
Caryophyllales Caryophyllaceae Schiedea membranacea#OLES
Caryophyllales Caryophyllaceae Colobanthus apetalus#NC 036424
Caryophyllales Caryophyllaceae Colobanthus quitensis#NC 028080
Caryophyllales Caryophyllaceae Cerastium arvense#Yao01
Caryophyllales Caryophyllaceae Stellaria media#B200925
Caryophyllales Caryophyllaceae Pseudostellaria palibiniana#MK120981
Caryophyllales Caryophyllaceae Pseudostellaria setulosa#NC 041462
Caryophyllales Caryophyllaceae Pseudostellaria longipedicellata#MH373593
Caryophyllales Caryophyllaceae Pseudostellaria okamotoi#NC 039974
Caryophyllales Caryophyllaceae Vaccaria hispanica#F549
Caryophyllales Caryophyllaceae Saponaria officinalis#SKNL
Caryophyllales Caryophyllaceae Dianthus longicalyx#KM668208
Caryophyllales Caryophyllaceae Dianthus plumarius subsp. lumnitzeri#LN877396
Caryophyllales Caryophyllaceae Dianthus gratianopolitanus#LN877387
Caryophyllales Caryophyllaceae Dianthus caryophyllus#NC 039650
Caryophyllales Caryophyllaceae Dianthus sp.#SHEZ
Caryophyllales Caryophyllaceae Agrostemma githago#NC 023357
Caryophyllales Caryophyllaceae Silene paradoxa#NC 023360
Caryophyllales Caryophyllaceae Lychnis wilfordii#NC 035225
Caryophyllales Caryophyllaceae Silene chalcedonica#NC 023359
Caryophyllales Caryophyllaceae Silene capitata#NC 035226
Caryophyllales Caryophyllaceae Silene aprica#F168
Caryophyllales Caryophyllaceae Silene vulgaris#NC 016727
Caryophyllales Caryophyllaceae Silene latifolia#FZQN
Caryophyllales Caryophyllaceae Silene noctiflora#NC 016728
Caryophyllales Caryophyllaceae Silene conica#NC 016729
Caryophyllales Caryophyllaceae Silene conoidea#NC 023358
Caryophyllales Achatocarpaceae Phaulothamnus spinescens#B200919
Caryophyllales Achatocarpaceae Achatocarpus pubescens#MK397909
Caryophyllales Achatocarpaceae Achatocarpus nigricans#MK397908
Caryophyllales Amaranthaceae Deeringia amaranthoides#MK397865
Caryophyllales Amaranthaceae Celosia cristata#B200906
Caryophyllales Amaranthaceae Celosia argentea#MK397861
Caryophyllales Amaranthaceae Amaranthus tricolor#KX094399
Caryophyllales Amaranthaceae Amaranthus retroflexus#WMLW
Caryophyllales Amaranthaceae Amaranthus cruentus#MG836506
Caryophyllales Amaranthaceae Amaranthus hypochondriacus#KX279888
Caryophyllales Amaranthaceae Amaranthus caudatus#NC 040143
Caryophyllales Amaranthaceae Aerva javanica#HDSY
Caryophyllales Amaranthaceae Aerva lanata#PDQH
Caryophyllales Amaranthaceae Amaranthaceae sp.#MK397852
Caryophyllales Amaranthaceae Cyathula capitata#F545
Caryophyllales Amaranthaceae Froelichia latifolia#Yao02
Caryophyllales Amaranthaceae Blutaparon vermiculare#CUTE
Caryophyllales Amaranthaceae Alternanthera brasiliana#ZBPY
Caryophyllales Amaranthaceae Alternanthera sessilis#BWRK
Caryophyllales Amaranthaceae Alternanthera caracasana#OHKC
Caryophyllales Amaranthaceae Alternanthera ficoidea#EYRD
Caryophyllales Amaranthaceae Bassia scoparia#WGET
Caryophyllales Amaranthaceae Haloxylon ammodendron#NC 027668
Caryophyllales Amaranthaceae Haloxylon persicum#NC 027669
Caryophyllales Amaranthaceae Bienertia sinuspersici#KU726550
Caryophyllales Amaranthaceae Suaeda malacosperma#NC 039180
Caryophyllales Amaranthaceae Salicornia bigelovii#NC 027226
Caryophyllales Amaranthaceae Salicornia europaea#NC 027225
Caryophyllales Amaranthaceae Halosarcia indica#NC 027224
Caryophyllales Amaranthaceae Beta vulgaris#EF534108
Caryophyllales Amaranthaceae Dysphania ambrosioides#NC 041201
Caryophyllales Amaranthaceae Dysphania pumilio#NC 041159
Caryophyllales Amaranthaceae Dysphania botrys#MH898873
Caryophyllales Amaranthaceae Spinacia oleracea#NC 002202
Caryophyllales Amaranthaceae Atriplex rosea#CBJR
Caryophyllales Amaranthaceae Atriplex hortensis#ONLQ
Caryophyllales Amaranthaceae Atriplex prostrata#AAXJ
Caryophyllales Amaranthaceae Chenopodium quinoa#KY635884
Caryophyllales Amaranthaceae Chenopodium album#MF418659
Caryophyllales Amaranthaceae Chenopodium ficifolium#NC 041200
Caryophyllales Limeaceae Limeum africanum#B200913
Caryophyllales Limeaceae Limeum sp.#MK397924
Caryophyllales Limeaceae Limeum africanum#MH286320
Caryophyllales Lophiocarpaceae Corbichonia decumbens#MH286323
Caryophyllales Lophiocarpaceae Lophiocarpus tenuissimus#MK397925
Caryophyllales Lophiocarpaceae Lophiocarpus polystachyus#Yao03
Caryophyllales Barbeuiaceae Barbeuia madagascariensis#NC 041301
Caryophyllales Barbeuiaceae Barbeuia madagascariensis#MK397915
Caryophyllales Kewaceae Kewa sp.#MK397923
Caryophyllales Kewaceae Kewa sp.#NC 040950
Caryophyllales Aizoaceae Tetragonia tetragonioides#F434
Caryophyllales Aizoaceae Mesembryanthemum crystallinum#NC 029049
Caryophyllales Aizoaceae Mesembryanthemum cordifolium#F596
Caryophyllales Aizoaceae Delosperma napiforme#B200908
Caryophyllales Aizoaceae Delosperma echinatum#BJKT
Caryophyllales Aizoaceae Trianthema portulacastrum#OMYK
Caryophyllales Aizoaceae Zaleya pentandra#BERS
Caryophyllales Aizoaceae Cypselea humifusa#GJNX
Caryophyllales Aizoaceae Sesuvium portulacastrum#F502
Caryophyllales Aizoaceae Sesuvium verrucosum#EDIT
Caryophyllales Gisekiaceae Gisekia pharnaceoides#F731
Caryophyllales Gisekiaceae Gisekia africana#B200911
Caryophyllales Gisekiaceae Gisekia sp.#MK397921

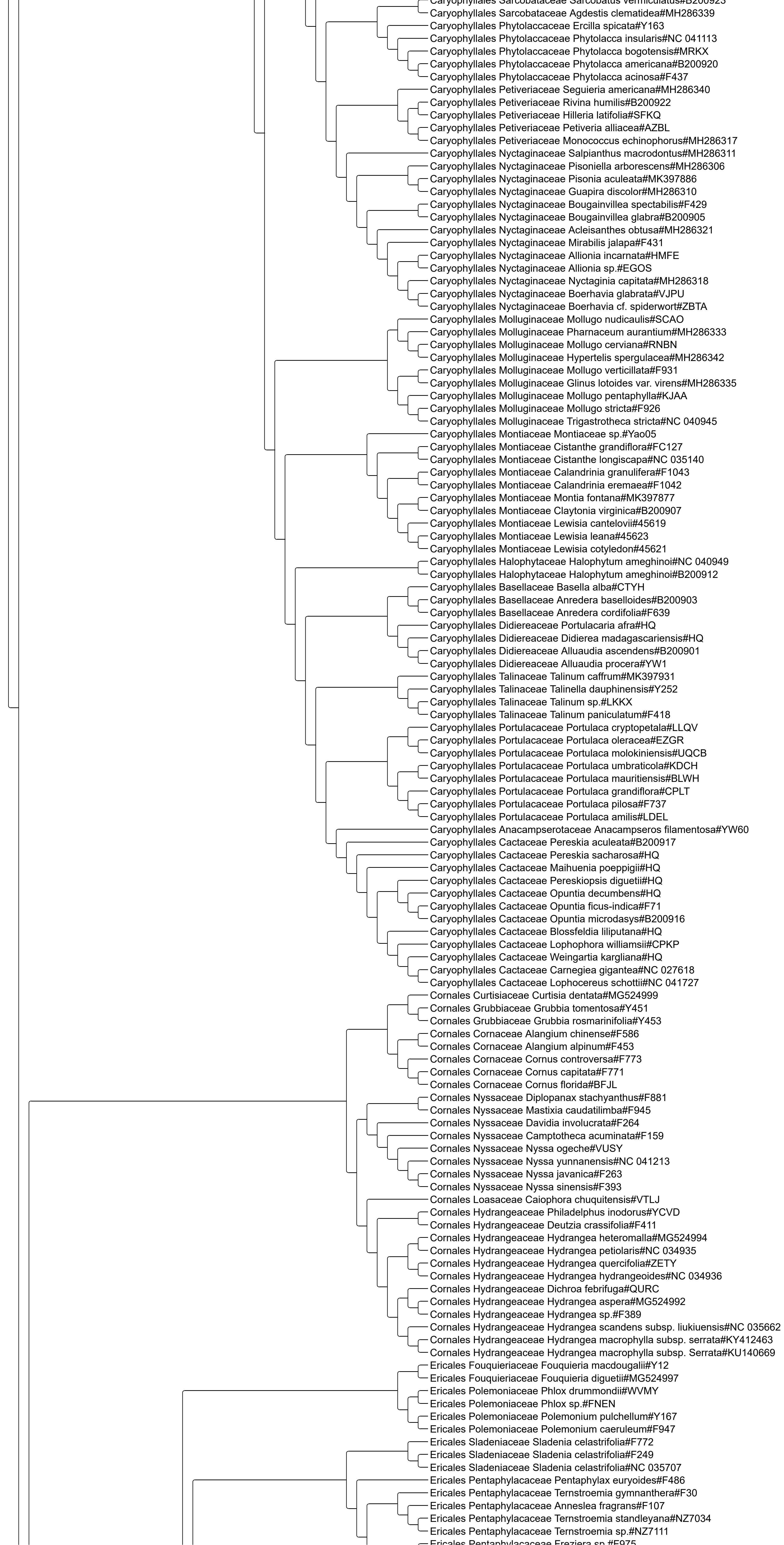
Caryophyllales Sarcobataceae Agdestis clematidea#MH286339
Caryophyllales Phytolaccaceae Ercilla spicata#Y163
Caryophyllales Phytolaccaceae Phytolacca insularis#NC 041113
Caryophyllales Phytolaccaceae Phytolacca bogotensis#MRKX
Caryophyllales Phytolaccaceae Phytolacca americana#B200920
Caryophyllales Phytolaccaceae Phytolacca acinosa#F437
Caryophyllales Petiveriaceae Seguieria americana#MH286340
Caryophyllales Petiveriaceae Rivina humilis#B200922
Caryophyllales Petiveriaceae Hilleria latifolia#SFKQ
Caryophyllales Petiveriaceae Petiveria alliacea#AZBL
Caryophyllales Petiveriaceae Monococcus echinophorus#MH286317
Caryophyllales Nyctaginaceae Salpianthus macrodontus#MH286311
Caryophyllales Nyctaginaceae Pisoniella arborescens#MH286306
Caryophyllales Nyctaginaceae Pisonia aculeata#MK397886
Caryophyllales Nyctaginaceae Guapira discolor#MH286310
Caryophyllales Nyctaginaceae Bougainvillea spectabilis#F429
Caryophyllales Nyctaginaceae Bougainvillea glabra#B200905
Caryophyllales Nyctaginaceae Acleisanthes obtusa#MH286321
Caryophyllales Nyctaginaceae Mirabilis jalapa#F431
Caryophyllales Nyctaginaceae Allionia incarnata#HMFE
Caryophyllales Nyctaginaceae Allionia sp.#EGOS
Caryophyllales Nyctaginaceae Nyctaginia capitata#MH286318
Caryophyllales Nyctaginaceae Boerhavia glabrata#VJPU
Caryophyllales Nyctaginaceae Boerhavia cf. spiderwort#ZBTA
Caryophyllales Molluginaceae Mollugo nudicaulis#SCAO
Caryophyllales Molluginaceae Pharnaceum aurantium#MH286333
Caryophyllales Molluginaceae Mollugo cerviana#RNBN
Caryophyllales Molluginaceae Hypertelis spergulacea#MH286342
Caryophyllales Molluginaceae Mollugo verticillata#F931
Caryophyllales Molluginaceae Glinus lotoides var. virens#MH286335
Caryophyllales Molluginaceae Mollugo pentaphylla#KJAA
Caryophyllales Molluginaceae Mollugo stricta#F926
Caryophyllales Molluginaceae Trigastrotheca stricta#NC 040945
Caryophyllales Montiaceae Montiaceae sp.#Yao05
Caryophyllales Montiaceae Cistanthe grandiflora#FC127
Caryophyllales Montiaceae Cistanthe longiscapa#NC 035140
Caryophyllales Montiaceae Calandrinia granulifera#F1043
Caryophyllales Montiaceae Calandrinia eremaea#F1042
Caryophyllales Montiaceae Montia fontana#MK397877
Caryophyllales Montiaceae Claytonia virginica#B200907
Caryophyllales Montiaceae Lewisia cantelovii#45619
Caryophyllales Montiaceae Lewisia leana#45623
Caryophyllales Montiaceae Lewisia cotyledon#45621
Caryophyllales Halophytaceae Halophytum ameghinoi#NC 040949
Caryophyllales Halophytaceae Halophytum ameghinoi#B200912
Caryophyllales Basellaceae Basella alba#CTYH
Caryophyllales Basellaceae Anredera baselloides#B200903
Caryophyllales Basellaceae Anredera cordifolia#F639
Caryophyllales Didiereaceae Portulacaria afra#HQ
Caryophyllales Didiereaceae Didierea madagascariensis#HQ
Caryophyllales Didiereaceae Alluaudia ascendens#B200901
Caryophyllales Didiereaceae Alluaudia procera#YW1
Caryophyllales Talinaceae Talinum caffrum#MK397931
Caryophyllales Talinaceae Talinella dauphinensis#Y252
Caryophyllales Talinaceae Talinum sp.#LKKX
Caryophyllales Talinaceae Talinum paniculatum#F418
Caryophyllales Portulacaceae Portulaca cryptopetala#LLQV
Caryophyllales Portulacaceae Portulaca oleracea#EZGR
Caryophyllales Portulacaceae Portulaca molokiniensis#UQCB
Caryophyllales Portulacaceae Portulaca umbraticola#KDCH
Caryophyllales Portulacaceae Portulaca mauritiensis#BLWH
Caryophyllales Portulacaceae Portulaca grandiflora#CPLT
Caryophyllales Portulacaceae Portulaca pilosa#F737
Caryophyllales Portulacaceae Portulaca amilis#LDEL
Caryophyllales Anacampserotaceae Anacampseros filamentosa#YW60
Caryophyllales Cactaceae Pereskia aculeata#B200917
Caryophyllales Cactaceae Pereskia sacharosa#HQ
Caryophyllales Cactaceae Maihuenia poeppigii#HQ
Caryophyllales Cactaceae Pereskiopsis diguetii#HQ
Caryophyllales Cactaceae Opuntia decumbens#HQ
Caryophyllales Cactaceae Opuntia ficus-indica#F71
Caryophyllales Cactaceae Opuntia microdasys#B200916
Caryophyllales Cactaceae Blossfeldia liliputana#HQ
Caryophyllales Cactaceae Lophophora williamsii#CPKP
Caryophyllales Cactaceae Weingartia kargliana#HQ
Caryophyllales Cactaceae Carnegiea gigantea#NC 027618
Caryophyllales Cactaceae Lophocereus schottii#NC 041727
Cornales Curtisiaceae Curtisia dentata#MG524999
Cornales Grubbiaceae Grubbia tomentosa#Y451
Cornales Grubbiaceae Grubbia rosmarinifolia#Y453
Cornales Cornaceae Alangium chinense#F586
Cornales Cornaceae Alangium alpinum#F453
Cornales Cornaceae Cornus controversa#F773
Cornales Cornaceae Cornus capitata#F771
Cornales Cornaceae Cornus florida#BFJL
Cornales Nyssaceae Diplopanax stachyanthus#F881
Cornales Nyssaceae Mastixia caudatilimba#F945
Cornales Nyssaceae Davidia involucrata#F264
Cornales Nyssaceae Camptotheca acuminata#F159
Cornales Nyssaceae Nyssa ogeche#VUSY
Cornales Nyssaceae Nyssa yunnanensis#NC 041213
Cornales Nyssaceae Nyssa javanica#F263
Cornales Nyssaceae Nyssa sinensis#F393
Cornales Loasaceae Caiophora chuquitensis#VTLJ
Cornales Hydrangeaceae Philadelphus inodorus#YCVD
Cornales Hydrangeaceae Deutzia crassifolia#F411
Cornales Hydrangeaceae Hydrangea heteromalla#MG524994
Cornales Hydrangeaceae Hydrangea petiolaris#NC 034935
Cornales Hydrangeaceae Hydrangea quercifolia#ZETY
Cornales Hydrangeaceae Hydrangea hydrangeoides#NC 034936
Cornales Hydrangeaceae Dichroa febrifuga#QURC
Cornales Hydrangeaceae Hydrangea aspera#MG524992
Cornales Hydrangeaceae Hydrangea sp.#F389
Cornales Hydrangeaceae Hydrangea scandens subsp. liukiuensis#NC 035662
Cornales Hydrangeaceae Hydrangea macrophylla subsp. serrata#KY412463
Cornales Hydrangeaceae Hydrangea macrophylla subsp. Serrata#KU140669
Ericales Fouquieriaceae Fouquieria macdougalii#Y12
Ericales Fouquieriaceae Fouquieria diguetii#MG524997
Ericales Polemoniaceae Phlox drummondii#WVMY
Ericales Polemoniaceae Phlox sp.#FNEN
Ericales Polemoniaceae Polemonium pulchellum#Y167
Ericales Polemoniaceae Polemonium caeruleum#F947
Ericales Sladeniaceae Sladenia celastrifolia#F772
Ericales Sladeniaceae Sladenia celastrifolia#F249
Ericales Sladeniaceae Sladenia celastrifolia#NC 035707
Ericales Pentaphylacaceae Pentaphylax euryoides#F486
Ericales Pentaphylacaceae Ternstroemia gymnanthera#F30
Ericales Pentaphylacaceae Anneslea fragrans#F107
Ericales Pentaphylacaceae Ternstroemia standleyana#NZ7034
Ericales Pentaphylacaceae Ternstroemia sp.#NZ7111

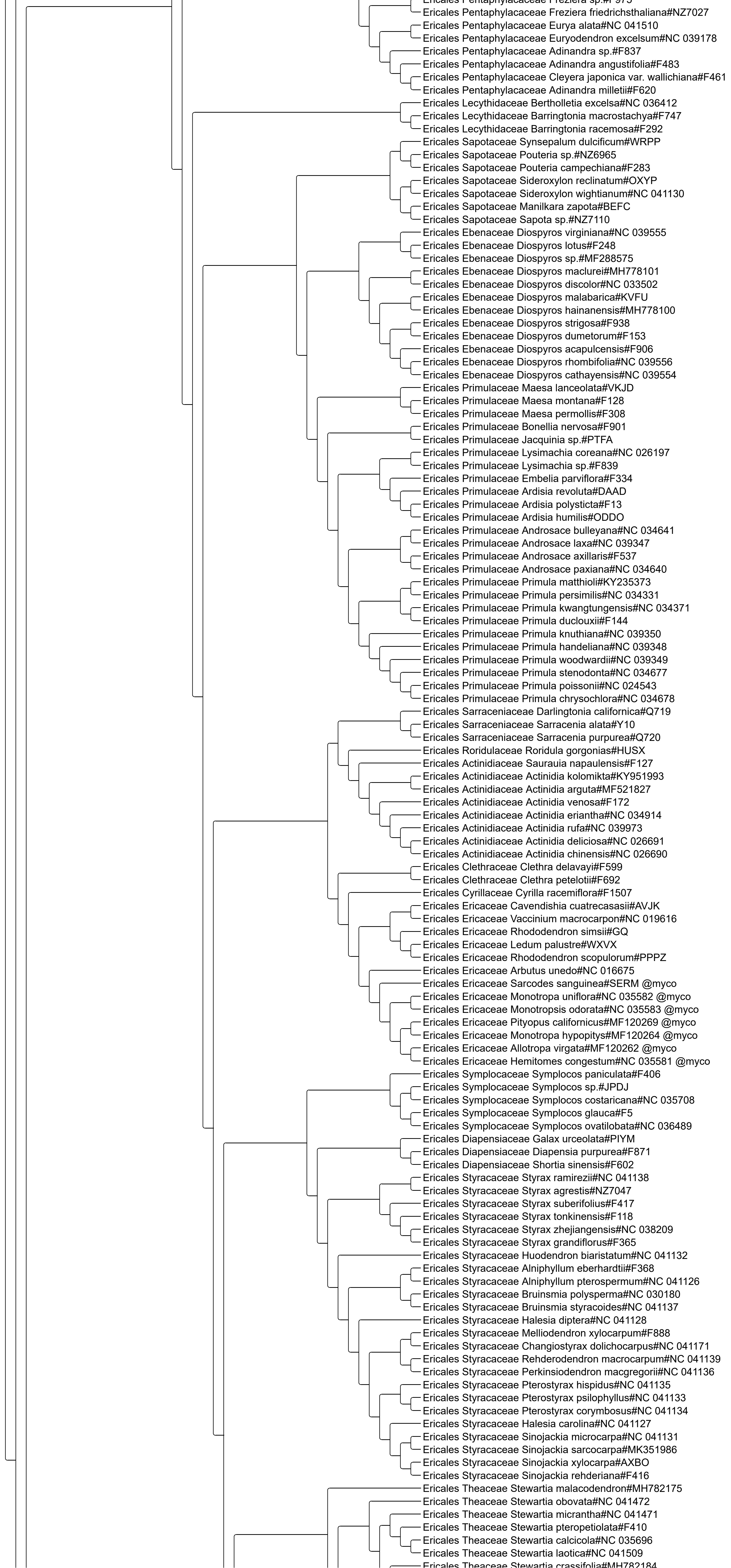

Ericales Pentaphylacaceae Freziera friedrichsthaliana#NZ7027
Ericales Pentaphylacaceae Eurya alata#NC 041510
Ericales Pentaphylacaceae Euryodendron excelsum#NC 039178
Ericales Pentaphylacaceae Adinandra sp.#F837
Ericales Pentaphylacaceae Adinandra angustifolia#F483
Ericales Pentaphylacaceae Cleyera japonica var. wallichiana#F461
Ericales Pentaphylacaceae Adinandra milletii#F620
Ericales Lecythidaceae Bertholletia excelsa#NC 036412
Ericales Lecythidaceae Barringtonia macrostachya#F747
Ericales Lecythidaceae Barringtonia racemosa#F292
Ericales Sapotaceae Synsepalum dulcificum#WRPP
Ericales Sapotaceae Pouteria sp.#NZ6965
Ericales Sapotaceae Pouteria campechiana#F283
Ericales Sapotaceae Sideroxylon reclinatum#OXYP
Ericales Sapotaceae Sideroxylon wightianum#NC 041130
Ericales Sapotaceae Manilkara zapota#BEFC
Ericales Sapotaceae Sapota sp.#NZ7110
Ericales Ebenaceae Diospyros virginiana#NC 039555
Ericales Ebenaceae Diospyros lotus#F248
Ericales Ebenaceae Diospyros sp.#MF288575
Ericales Ebenaceae Diospyros maclurei#MH778101
Ericales Ebenaceae Diospyros discolor#NC 033502
Ericales Ebenaceae Diospyros malabarica#KVFU
Ericales Ebenaceae Diospyros hainanensis#MH778100
Ericales Ebenaceae Diospyros strigosa#F938
Ericales Ebenaceae Diospyros dumetorum#F153
Ericales Ebenaceae Diospyros acapulcensis#F906
Ericales Ebenaceae Diospyros rhombifolia#NC 039556
Ericales Ebenaceae Diospyros cathayensis#NC 039554
Ericales Primulaceae Maesa lanceolata#VKJD
Ericales Primulaceae Maesa montana#F128
Ericales Primulaceae Maesa permollis#F308
Ericales Primulaceae Bonellia nervosa#F901
Ericales Primulaceae Jacquinia sp.#PTFA
Ericales Primulaceae Lysimachia coreana#NC 026197
Ericales Primulaceae Lysimachia sp.#F839
Ericales Primulaceae Embelia parviflora#F334
Ericales Primulaceae Ardisia revoluta#DAAD
Ericales Primulaceae Ardisia polysticta#F13
Ericales Primulaceae Ardisia humilis#ODDO
Ericales Primulaceae Androsace bulleyana#NC 034641
Ericales Primulaceae Androsace laxa#NC 039347
Ericales Primulaceae Androsace axillaris#F537
Ericales Primulaceae Androsace paxiana#NC 034640
Ericales Primulaceae Primula matthioli#KY235373
Ericales Primulaceae Primula persimilis#NC 034331
Ericales Primulaceae Primula kwangtungensis#NC 034371
Ericales Primulaceae Primula duclouxii#F144
Ericales Primulaceae Primula knuthiana#NC 039350
Ericales Primulaceae Primula handeliana#NC 039348
Ericales Primulaceae Primula woodwardii#NC 039349
Ericales Primulaceae Primula stenodonta#NC 034677
Ericales Primulaceae Primula poissonii#NC 024543
Ericales Primulaceae Primula chrysochlora#NC 034678
Ericales Sarraceniaceae Darlingtonia californica#Q719
Ericales Sarraceniaceae Sarracenia alata#Y10
Ericales Sarraceniaceae Sarracenia purpurea#Q720
Ericales Roridulaceae Roridula gorgonias#HUSX
Ericales Actinidiaceae Saurauia napaulensis#F127
Ericales Actinidiaceae Actinidia kolomikta#KY951993
Ericales Actinidiaceae Actinidia arguta#MF521827
Ericales Actinidiaceae Actinidia venosa#F172
Ericales Actinidiaceae Actinidia eriantha#NC 034914
Ericales Actinidiaceae Actinidia rufa#NC 039973
Ericales Actinidiaceae Actinidia deliciosa#NC 026691
Ericales Actinidiaceae Actinidia chinensis#NC 026690
Ericales Clethraceae Clethra delavayi#F599
Ericales Clethraceae Clethra petelotii#F692
Ericales Cyrillaceae Cyrilla racemiflora#F1507
Ericales Ericaceae Cavendishia cuatrecasasii#AVJK
Ericales Ericaceae Vaccinium macrocarpon#NC 019616
Ericales Ericaceae Rhododendron simsii#GQ
Ericales Ericaceae Ledum palustre#WXVX
Ericales Ericaceae Rhododendron scopulorum#PPPZ
Ericales Ericaceae Arbutus unedo#NC 016675
Ericales Ericaceae Sarcodes sanguinea#SERM @myco
Ericales Ericaceae Monotropa uniflora#NC 035582 @myco
Ericales Ericaceae Monotropsis odorata#NC 035583 @myco
Ericales Ericaceae Pityopus californicus#MF120269 @myco
Ericales Ericaceae Monotropa hypopitys#MF120264 @myco
Ericales Ericaceae Allotropa virgata#MF120262 @myco
Ericales Ericaceae Hemitomes congestum#NC 035581 @myco
Ericales Symplocaceae Symplocos paniculata#F406
Ericales Symplocaceae Symplocos sp.#JPDJ
Ericales Symplocaceae Symplocos costaricana#NC 035708
Ericales Symplocaceae Symplocos glauca#F5
Ericales Symplocaceae Symplocos ovatilobata#NC 036489
Ericales Diapensiaceae Galax urceolata#PIYM
Ericales Diapensiaceae Diapensia purpurea#F871
Ericales Diapensiaceae Shortia sinensis#F602
Ericales Styracaceae Styrax ramirezii#NC 041138
Ericales Styracaceae Styrax agrestis#NZ7047
Ericales Styracaceae Styrax suberifolius#F417
Ericales Styracaceae Styrax tonkinensis#F118
Ericales Styracaceae Styrax zhejiangensis#NC 038209
Ericales Styracaceae Styrax grandiflorus#F365
Ericales Styracaceae Huodendron biaristatum#NC 041132
Ericales Styracaceae Alniphyllum eberhardtii#F368
Ericales Styracaceae Alniphyllum pterospermum#NC 041126
Ericales Styracaceae Bruinsmia polysperma#NC 030180
Ericales Styracaceae Bruinsmia styracoides#NC 041137
Ericales Styracaceae Halesia diptera#NC 041128
Ericales Styracaceae Melliodendron xylocarpum#F888
Ericales Styracaceae Changiostyrax dolichocarpus#NC 041171
Ericales Styracaceae Rehderodendron macrocarpum#NC 041139
Ericales Styracaceae Perkinsiodendron macgregorii#NC 041136
Ericales Styracaceae Pterostyrax hispidus#NC 041135
Ericales Styracaceae Pterostyrax psilophyllus#NC 041133
Ericales Styracaceae Pterostyrax corymbosus#NC 041134
Ericales Styracaceae Halesia carolina#NC 041127
Ericales Styracaceae Sinojackia microcarpa#NC 041131
Ericales Styracaceae Sinojackia sarcocarpa#MK351986
Ericales Styracaceae Sinojackia xylocarpa#AXBO
Ericales Styracaceae Sinojackia rehderiana#F416
Ericales Theaceae Stewartia malacodendron#MH782175
Ericales Theaceae Stewartia obovata#NC 041472
Ericales Theaceae Stewartia micrantha#NC 041471
Ericales Theaceae Stewartia pteropetiolata#F410
Ericales Theaceae Stewartia calcicola#NC 035696
Ericales Theaceae Stewartia laotica#NC 041509
Ericales Theaceae Stewartia crassifolia#MH782184

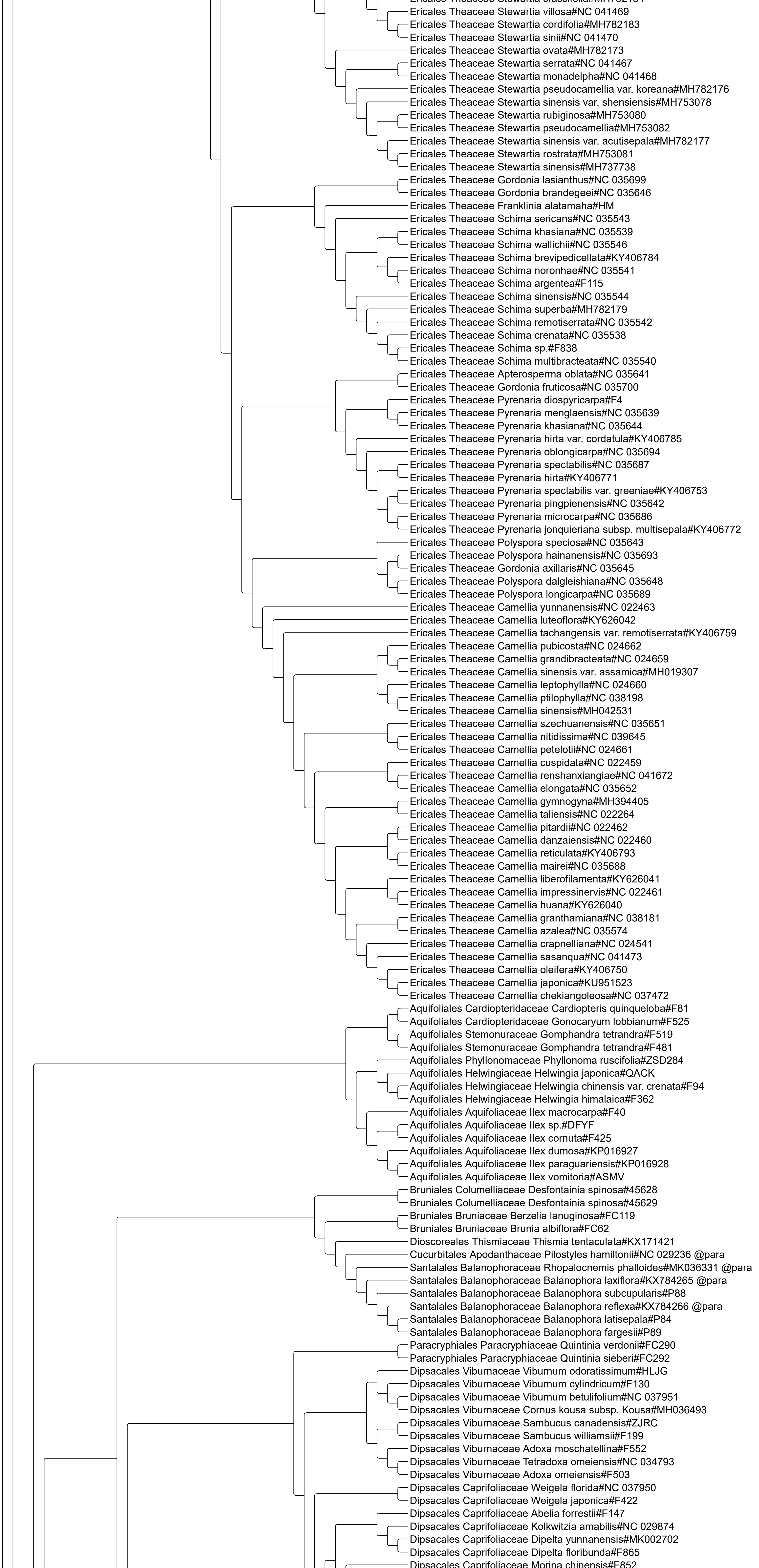

Ericales Theaceae Stewartia villosa#NC 041469
Ericales Theaceae Stewartia cordifolia#MH782183
Ericales Theaceae Stewartia sinii#NC 041470
Ericales Theaceae Stewartia ovata#MH782173
Ericales Theaceae Stewartia serrata#NC 041467
Ericales Theaceae Stewartia monadelpha#NC 041468
Ericales Theaceae Stewartia pseudocamellia var. koreana#MH782176
Ericales Theaceae Stewartia sinensis var. shensiensis#MH753078
Ericales Theaceae Stewartia rubiginosa#MH753080
Ericales Theaceae Stewartia pseudocamellia#MH753082
Ericales Theaceae Stewartia sinensis var. acutisepala#MH782177
Ericales Theaceae Stewartia rostrata#MH753081
Ericales Theaceae Stewartia sinensis#MH737738
Ericales Theaceae Gordonia lasianthus#NC 035699
Ericales Theaceae Gordonia brandegeei#NC 035646
Ericales Theaceae Franklinia alatamaha#HM
Ericales Theaceae Schima sericans#NC 035543
Ericales Theaceae Schima khasiana#NC 035539
Ericales Theaceae Schima wallichii#NC 035546
Ericales Theaceae Schima brevipedicellata#KY406784
Ericales Theaceae Schima noronhae#NC 035541
Ericales Theaceae Schima argentea#F115
Ericales Theaceae Schima sinensis#NC 035544
Ericales Theaceae Schima superba#MH782179
Ericales Theaceae Schima remotiserrata#NC 035542
Ericales Theaceae Schima crenata#NC 035538
Ericales Theaceae Schima sp.#F838
Ericales Theaceae Schima multibracteata#NC 035540
Ericales Theaceae Apterosperma oblata#NC 035641
Ericales Theaceae Gordonia fruticosa#NC 035700
Ericales Theaceae Pyrenaria diospyricarpa#F4
Ericales Theaceae Pyrenaria menglaensis#NC 035639
Ericales Theaceae Pyrenaria khasiana#NC 035644
Ericales Theaceae Pyrenaria hirta var. cordatula#KY406785
Ericales Theaceae Pyrenaria oblongicarpa#NC 035694
Ericales Theaceae Pyrenaria spectabilis#NC 035687
Ericales Theaceae Pyrenaria hirta#KY406771
Ericales Theaceae Pyrenaria spectabilis var. greeniae#KY406753
Ericales Theaceae Pyrenaria pingpienensis#NC 035642
Ericales Theaceae Pyrenaria microcarpa#NC 035686
Ericales Theaceae Pyrenaria jonquieriana subsp. multisepala#KY406772
Ericales Theaceae Polyspora speciosa#NC 035643
Ericales Theaceae Polyspora hainanensis#NC 035693
Ericales Theaceae Gordonia axillaris#NC 035645
Ericales Theaceae Polyspora dalgleishiana#NC 035648
Ericales Theaceae Polyspora longicarpa#NC 035689
Ericales Theaceae Camellia yunnanensis#NC 022463
Ericales Theaceae Camellia luteoflora#KY626042
Ericales Theaceae Camellia tachangensis var. remotiserrata#KY406759
Ericales Theaceae Camellia pubicosta#NC 024662
Ericales Theaceae Camellia grandibracteata#NC 024659
Ericales Theaceae Camellia sinensis var. assamica#MH019307
Ericales Theaceae Camellia leptophylla#NC 024660
Ericales Theaceae Camellia ptilophylla#NC 038198
Ericales Theaceae Camellia sinensis#MH042531
Ericales Theaceae Camellia szechuanensis#NC 035651
Ericales Theaceae Camellia nitidissima#NC 039645
Ericales Theaceae Camellia petelotii#NC 024661
Ericales Theaceae Camellia cuspidata#NC 022459
Ericales Theaceae Camellia renshanxiangiae#NC 041672
Ericales Theaceae Camellia elongata#NC 035652
Ericales Theaceae Camellia gymnogyna#MH394405
Ericales Theaceae Camellia taliensis#NC 022264
Ericales Theaceae Camellia pitardii#NC 022462
Ericales Theaceae Camellia danzaiensis#NC 022460
Ericales Theaceae Camellia reticulata#KY406793
Ericales Theaceae Camellia mairei#NC 035688
Ericales Theaceae Camellia liberofilamenta#KY626041
Ericales Theaceae Camellia impressinervis#NC 022461
Ericales Theaceae Camellia huana#KY626040
Ericales Theaceae Camellia granthamiana#NC 038181
Ericales Theaceae Camellia azalea#NC 035574
Ericales Theaceae Camellia crapnelliana#NC 024541
Ericales Theaceae Camellia sasanqua#NC 041473
Ericales Theaceae Camellia oleifera#KY406750
Ericales Theaceae Camellia japonica#KU951523
Ericales Theaceae Camellia chekiangoleosa#NC 037472
Aquifoliales Cardiopteridaceae Cardiopteris quinqueloba#F81
Aquifoliales Cardiopteridaceae Gonocaryum lobbianum#F525
Aquifoliales Stemonuraceae Gomphandra tetrandra#F519
Aquifoliales Stemonuraceae Gomphandra tetrandra#F481
Aquifoliales Phyllonomaceae Phyllonoma ruscifolia#ZSD284
Aquifoliales Helwingiaceae Helwingia japonica#QACK
Aquifoliales Helwingiaceae Helwingia chinensis var. crenata#F94
Aquifoliales Helwingiaceae Helwingia himalaica#F362
Aquifoliales Aquifoliaceae Ilex macrocarpa#F40
Aquifoliales Aquifoliaceae Ilex sp.#DFYF
Aquifoliales Aquifoliaceae Ilex cornuta#F425
Aquifoliales Aquifoliaceae Ilex dumosa#KP016927
Aquifoliales Aquifoliaceae Ilex paraguariensis#KP016928
Aquifoliales Aquifoliaceae Ilex vomitoria#ASMV
Bruniales Columelliaceae Desfontainia spinosa#45628
Bruniales Columelliaceae Desfontainia spinosa#45629
Bruniales Bruniaceae Berzelia lanuginosa#FC119
Bruniales Bruniaceae Brunia albiflora#FC62
Dioscoreales Thismiaceae Thismia tentaculata#KX171421
Cucurbitales Apodanthaceae Pilostyles hamiltonii#NC 029236 @para
Santalales Balanophoraceae Rhopalocnemis phalloides#MK036331 @para
Santalales Balanophoraceae Balanophora laxiflora#KX784265 @para
Santalales Balanophoraceae Balanophora subcupularis#P88
Santalales Balanophoraceae Balanophora reflexa#KX784266 @para
Santalales Balanophoraceae Balanophora latisepala#P84
Santalales Balanophoraceae Balanophora fargesii#P89
Paracryphiales Paracryphiaceae Quintinia verdonii#FC290
Paracryphiales Paracryphiaceae Quintinia sieberi#FC292
Dipsacales Viburnaceae Viburnum odoratissimum#HLJG
Dipsacales Viburnaceae Viburnum cylindricum#F130
Dipsacales Viburnaceae Viburnum betulifolium#NC 037951
Dipsacales Viburnaceae Cornus kousa subsp. Kousa#MH036493
Dipsacales Viburnaceae Sambucus canadensis#ZJRC
Dipsacales Viburnaceae Sambucus williamsii#F199
Dipsacales Viburnaceae Adoxa moschatellina#F552
Dipsacales Viburnaceae Tetradoxa omeiensis#NC 034793
Dipsacales Viburnaceae Adoxa omeiensis#F503
Dipsacales Caprifoliaceae Weigela florida#NC 037950
Dipsacales Caprifoliaceae Weigela japonica#F422
Dipsacales Caprifoliaceae Abelia forrestii#F147
Dipsacales Caprifoliaceae Kolkwitzia amabilis#NC 029874
Dipsacales Caprifoliaceae Dipelta yunnanensis#MK002702
Dipsacales Caprifoliaceae Dipelta floribunda#F865

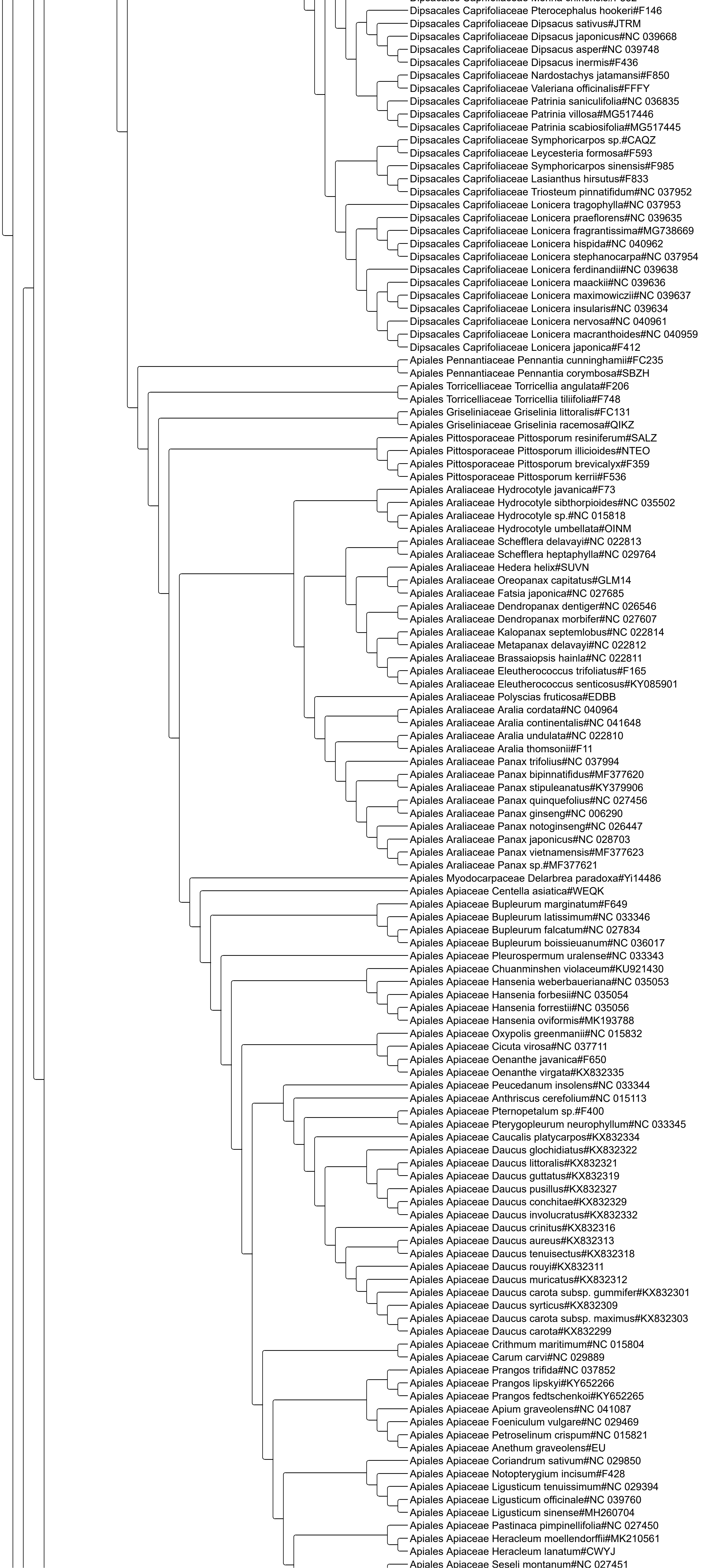
Dipsacales Caprifoliaceae Pterocephalus hookeri#F146
Dipsacales Caprifoliaceae Dipsacus sativus#JTRM
Dipsacales Caprifoliaceae Dipsacus japonicus#NC 039668
Dipsacales Caprifoliaceae Dipsacus asper#NC 039748
Dipsacales Caprifoliaceae Dipsacus inermis#F436
Dipsacales Caprifoliaceae Nardostachys jatamansi#F850
Dipsacales Caprifoliaceae Valeriana officinalis#FFFY
Dipsacales Caprifoliaceae Patrinia saniculifolia#NC 036835
Dipsacales Caprifoliaceae Patrinia villosa#MG517446
Dipsacales Caprifoliaceae Patrinia scabiosifolia#MG517445
Dipsacales Caprifoliaceae Symphoricarpos sp.#CAQZ
Dipsacales Caprifoliaceae Leycesteria formosa#F593
Dipsacales Caprifoliaceae Symphoricarpos sinensis#F985
Dipsacales Caprifoliaceae Lasianthus hirsutus#F833
Dipsacales Caprifoliaceae Triosteum pinnatifidum#NC 037952
Dipsacales Caprifoliaceae Lonicera tragophylla#NC 037953
Dipsacales Caprifoliaceae Lonicera praeflorens#NC 039635
Dipsacales Caprifoliaceae Lonicera fragrantissima#MG738669
Dipsacales Caprifoliaceae Lonicera hispida#NC 040962
Dipsacales Caprifoliaceae Lonicera stephanocarpa#NC 037954
Dipsacales Caprifoliaceae Lonicera ferdinandii#NC 039638
Dipsacales Caprifoliaceae Lonicera maackii#NC 039636
Dipsacales Caprifoliaceae Lonicera maximowiczii#NC 039637
Dipsacales Caprifoliaceae Lonicera insularis#NC 039634
Dipsacales Caprifoliaceae Lonicera nervosa#NC 040961
Dipsacales Caprifoliaceae Lonicera macranthoides#NC 040959
Dipsacales Caprifoliaceae Lonicera japonica#F412
Apiales Pennantiaceae Pennantia cunninghamii#FC235
Apiales Pennantiaceae Pennantia corymbosa#SBZH
Apiales Torricelliaceae Torricellia angulata#F206
Apiales Torricelliaceae Torricellia tiliifolia#F748
Apiales Griseliniaceae Griselinia littoralis#FC131
Apiales Griseliniaceae Griselinia racemosa#QIKZ
Apiales Pittosporaceae Pittosporum resiniferum#SALZ
Apiales Pittosporaceae Pittosporum illicioides#NTEO
Apiales Pittosporaceae Pittosporum brevicalyx#F359
Apiales Pittosporaceae Pittosporum kerrii#F536
Apiales Araliaceae Hydrocotyle javanica#F73
Apiales Araliaceae Hydrocotyle sibthorpioides#NC 035502
Apiales Araliaceae Hydrocotyle sp.#NC 015818
Apiales Araliaceae Hydrocotyle umbellata#OINM
Apiales Araliaceae Schefflera delavayi#NC 022813
Apiales Araliaceae Schefflera heptaphylla#NC 029764
Apiales Araliaceae Hedera helix#SUVN
Apiales Araliaceae Oreopanax capitatus#GLM14
Apiales Araliaceae Fatsia japonica#NC 027685
Apiales Araliaceae Dendropanax dentiger#NC 026546
Apiales Araliaceae Dendropanax morbifer#NC 027607
Apiales Araliaceae Kalopanax septemlobus#NC 022814
Apiales Araliaceae Metapanax delavayi#NC 022812
Apiales Araliaceae Brassaiopsis hainla#NC 022811
Apiales Araliaceae Eleutherococcus trifoliatus#F165
Apiales Araliaceae Eleutherococcus senticosus#KY085901
Apiales Araliaceae Polyscias fruticosa#EDBB
Apiales Araliaceae Aralia cordata#NC 040964
Apiales Araliaceae Aralia continentalis#NC 041648
Apiales Araliaceae Aralia undulata#NC 022810
Apiales Araliaceae Aralia thomsonii#F11
Apiales Araliaceae Panax trifolius#NC 037994
Apiales Araliaceae Panax bipinnatifidus#MF377620
Apiales Araliaceae Panax stipuleanatus#KY379906
Apiales Araliaceae Panax quinquefolius#NC 027456
Apiales Araliaceae Panax ginseng#NC 006290
Apiales Araliaceae Panax notoginseng#NC 026447
Apiales Araliaceae Panax japonicus#NC 028703
Apiales Araliaceae Panax vietnamensis#MF377623
Apiales Araliaceae Panax sp.#MF377621
Apiales Myodocarpaceae Delarbrea paradoxa#Yi14486
Apiales Apiaceae Centella asiatica#WEQK
Apiales Apiaceae Bupleurum marginatum#F649
Apiales Apiaceae Bupleurum latissimum#NC 033346
Apiales Apiaceae Bupleurum falcatum#NC 027834
Apiales Apiaceae Bupleurum boissieuanum#NC 036017
Apiales Apiaceae Pleurospermum uralense#NC 033343
Apiales Apiaceae Chuanminshen violaceum#KU921430
Apiales Apiaceae Hansenia weberbaueriana#NC 035053
Apiales Apiaceae Hansenia forbesii#NC 035054
Apiales Apiaceae Hansenia forrestii#NC 035056
Apiales Apiaceae Hansenia oviformis#MK193788
Apiales Apiaceae Oxypolis greenmanii#NC 015832
Apiales Apiaceae Cicuta virosa#NC 037711
Apiales Apiaceae Oenanthe javanica#F650
Apiales Apiaceae Oenanthe virgata#KX832335
Apiales Apiaceae Peucedanum insolens#NC 033344
Apiales Apiaceae Anthriscus cerefolium#NC 015113
Apiales Apiaceae Pternopetalum sp.#F400
Apiales Apiaceae Pterygopleurum neurophyllum#NC 033345
Apiales Apiaceae Caucalis platycarpos#KX832334
Apiales Apiaceae Daucus glochidiatus#KX832322
Apiales Apiaceae Daucus littoralis#KX832321
Apiales Apiaceae Daucus guttatus#KX832319
Apiales Apiaceae Daucus pusillus#KX832327
Apiales Apiaceae Daucus conchitae#KX832329
Apiales Apiaceae Daucus involucratus#KX832332
Apiales Apiaceae Daucus crinitus#KX832316
Apiales Apiaceae Daucus aureus#KX832313
Apiales Apiaceae Daucus tenuisectus#KX832318
Apiales Apiaceae Daucus rouyi#KX832311
Apiales Apiaceae Daucus muricatus#KX832312
Apiales Apiaceae Daucus carota subsp. gummifer#KX832301
Apiales Apiaceae Daucus syrticus#KX832309
Apiales Apiaceae Daucus carota subsp. maximus#KX832303
Apiales Apiaceae Daucus carota#KX832299
Apiales Apiaceae Crithmum maritimum#NC 015804
Apiales Apiaceae Carum carvi#NC 029889
Apiales Apiaceae Prangos trifida#NC 037852
Apiales Apiaceae Prangos lipskyi#KY652266
Apiales Apiaceae Prangos fedtschenkoi#KY652265
Apiales Apiaceae Apium graveolens#NC 041087
Apiales Apiaceae Foeniculum vulgare#NC 029469
Apiales Apiaceae Petroselinum crispum#NC 015821
Apiales Apiaceae Anethum graveolens#EU
Apiales Apiaceae Coriandrum sativum#NC 029850
Apiales Apiaceae Notopterygium incisum#F428
Apiales Apiaceae Ligusticum tenuissimum#NC 029394
Apiales Apiaceae Ligusticum officinale#NC 039760
Apiales Apiaceae Ligusticum sinense#MH260704
Apiales Apiaceae Pastinaca pimpinellifolia#NC 027450
Apiales Apiaceae Heracleum moellendorffii#MK210561
Apiales Apiaceae Heracleum lanatum#CWYJ
Apiales Apiaceae Seseli montanum#NC 027451

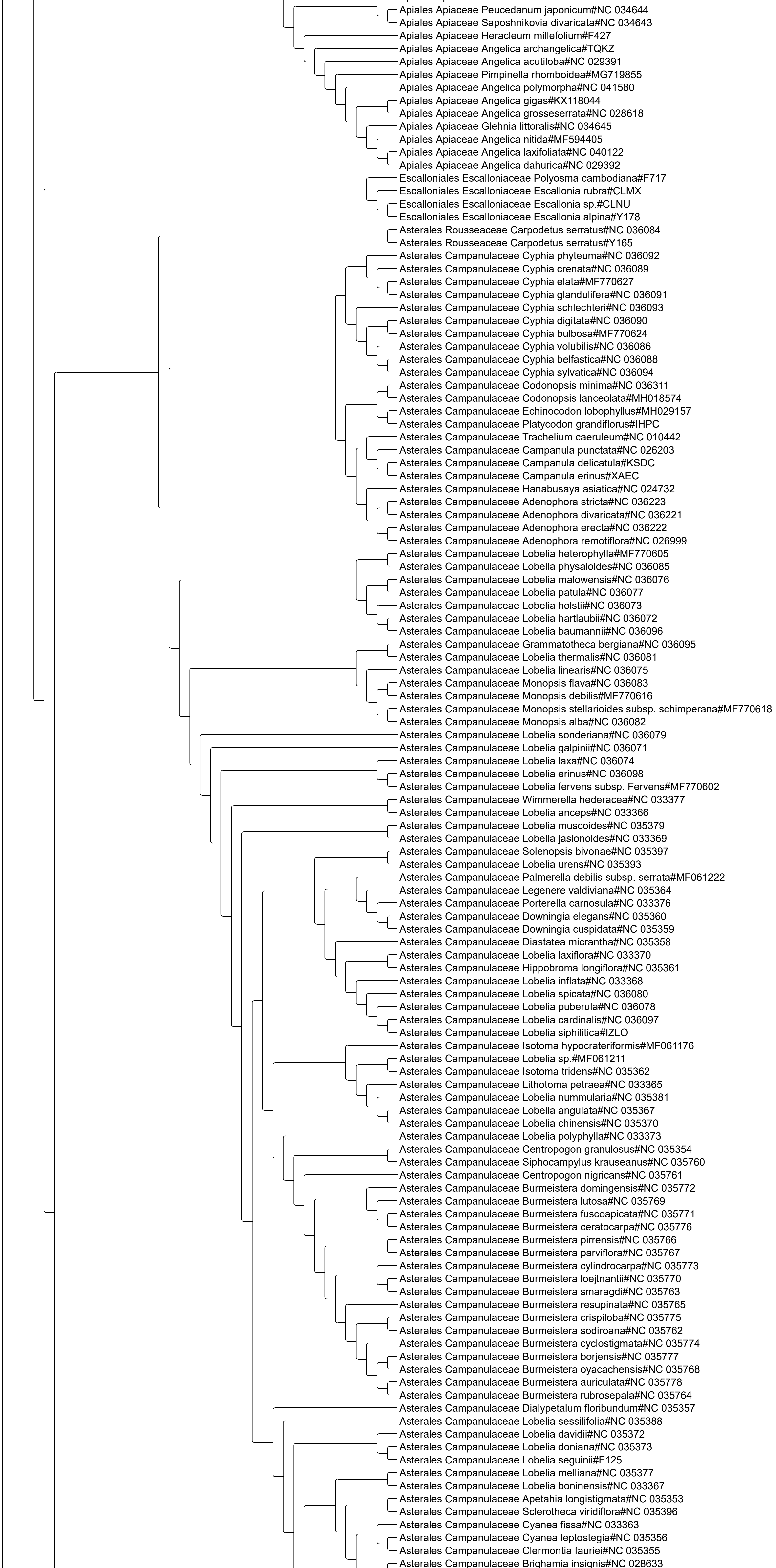
Apiales Apiaceae Peucedanum japonicum#NC 034644
Apiales Apiaceae Saposhnikovia divaricata#NC 034643
Apiales Apiaceae Heracleum millefolium#F427
Apiales Apiaceae Angelica archangelica#TQKZ
Apiales Apiaceae Angelica acutiloba#NC 029391
Apiales Apiaceae Pimpinella rhomboidea#MG719855
Apiales Apiaceae Angelica polymorpha#NC 041580
Apiales Apiaceae Angelica gigas#KX118044
Apiales Apiaceae Angelica grosseserrata#NC 028618
Apiales Apiaceae Glehnia littoralis#NC 034645
Apiales Apiaceae Angelica nitida#MF594405
Apiales Apiaceae Angelica laxifoliata#NC 040122
Apiales Apiaceae Angelica dahurica#NC 029392
Escalloniales Escalloniaceae Polyosma cambodiana#F717
Escalloniales Escalloniaceae Escallonia rubra#CLMX
Escalloniales Escalloniaceae Escallonia sp.#CLNU
Escalloniales Escalloniaceae Escallonia alpina#Y178
Asterales Rousseaceae Carpodetus serratus#NC 036084
Asterales Rousseaceae Carpodetus serratus#Y165
Asterales Campanulaceae Cyphia phyteuma#NC 036092
Asterales Campanulaceae Cyphia crenata#NC 036089
Asterales Campanulaceae Cyphia elata#MF770627
Asterales Campanulaceae Cyphia glandulifera#NC 036091
Asterales Campanulaceae Cyphia schlechteri#NC 036093
Asterales Campanulaceae Cyphia digitata#NC 036090
Asterales Campanulaceae Cyphia bulbosa#MF770624
Asterales Campanulaceae Cyphia volubilis#NC 036086
Asterales Campanulaceae Cyphia belfastica#NC 036088
Asterales Campanulaceae Cyphia sylvatica#NC 036094
Asterales Campanulaceae Codonopsis minima#NC 036311
Asterales Campanulaceae Codonopsis lanceolata#MH018574
Asterales Campanulaceae Echinocodon lobophyllus#MH029157
Asterales Campanulaceae Platycodon grandiflorus#IHPC
Asterales Campanulaceae Trachelium caeruleum#NC 010442
Asterales Campanulaceae Campanula punctata#NC 026203
Asterales Campanulaceae Campanula delicatula#KSDC
Asterales Campanulaceae Campanula erinus#XAEC
Asterales Campanulaceae Hanabusaya asiatica#NC 024732
Asterales Campanulaceae Adenophora stricta#NC 036223
Asterales Campanulaceae Adenophora divaricata#NC 036221
Asterales Campanulaceae Adenophora erecta#NC 036222
Asterales Campanulaceae Adenophora remotiflora#NC 026999
Asterales Campanulaceae Lobelia heterophylla#MF770605
Asterales Campanulaceae Lobelia physaloides#NC 036085
Asterales Campanulaceae Lobelia malowensis#NC 036076
Asterales Campanulaceae Lobelia patula#NC 036077
Asterales Campanulaceae Lobelia holstii#NC 036073
Asterales Campanulaceae Lobelia hartlaubii#NC 036072
Asterales Campanulaceae Lobelia baumannii#NC 036096
Asterales Campanulaceae Grammatotheca bergiana#NC 036095
Asterales Campanulaceae Lobelia thermalis#NC 036081
Asterales Campanulaceae Lobelia linearis#NC 036075
Asterales Campanulaceae Monopsis flava#NC 036083
Asterales Campanulaceae Monopsis debilis#MF770616
Asterales Campanulaceae Monopsis stellarioides subsp. schimperana#MF770618
Asterales Campanulaceae Monopsis alba#NC 036082
Asterales Campanulaceae Lobelia sonderiana#NC 036079
Asterales Campanulaceae Lobelia galpinii#NC 036071
Asterales Campanulaceae Lobelia laxa#NC 036074
Asterales Campanulaceae Lobelia erinus#NC 036098
Asterales Campanulaceae Lobelia fervens subsp. Fervens#MF770602
Asterales Campanulaceae Wimmerella hederacea#NC 033377
Asterales Campanulaceae Lobelia anceps#NC 033366
Asterales Campanulaceae Lobelia muscoides#NC 035379
Asterales Campanulaceae Lobelia jasionoides#NC 033369
Asterales Campanulaceae Solenopsis bivonae#NC 035397
Asterales Campanulaceae Lobelia urens#NC 035393
Asterales Campanulaceae Palmerella debilis subsp. serrata#MF061222
Asterales Campanulaceae Legenere valdiviana#NC 035364
Asterales Campanulaceae Porterella carnosula#NC 033376
Asterales Campanulaceae Downingia elegans#NC 035360
Asterales Campanulaceae Downingia cuspidata#NC 035359
Asterales Campanulaceae Diastatea micrantha#NC 035358
Asterales Campanulaceae Lobelia laxiflora#NC 033370
Asterales Campanulaceae Hippobroma longiflora#NC 035361
Asterales Campanulaceae Lobelia inflata#NC 033368
Asterales Campanulaceae Lobelia spicata#NC 036080
Asterales Campanulaceae Lobelia puberula#NC 036078
Asterales Campanulaceae Lobelia cardinalis#NC 036097
Asterales Campanulaceae Lobelia siphilitica#IZLO
Asterales Campanulaceae Isotoma hypocrateriformis#MF061176
Asterales Campanulaceae Lobelia sp.#MF061211
Asterales Campanulaceae Isotoma tridens#NC 035362
Asterales Campanulaceae Lithotoma petraea#NC 033365
Asterales Campanulaceae Lobelia nummularia#NC 035381
Asterales Campanulaceae Lobelia angulata#NC 035367
Asterales Campanulaceae Lobelia chinensis#NC 035370
Asterales Campanulaceae Lobelia polyphylla#NC 033373
Asterales Campanulaceae Centropogon granulosus#NC 035354
Asterales Campanulaceae Siphocampylus krauseanus#NC 035760
Asterales Campanulaceae Centropogon nigricans#NC 035761
Asterales Campanulaceae Burmeistera domingensis#NC 035772
Asterales Campanulaceae Burmeistera lutosa#NC 035769
Asterales Campanulaceae Burmeistera fuscoapicata#NC 035771
Asterales Campanulaceae Burmeistera ceratocarpa#NC 035776
Asterales Campanulaceae Burmeistera pirrensis#NC 035766
Asterales Campanulaceae Burmeistera parviflora#NC 035767
Asterales Campanulaceae Burmeistera cylindrocarpa#NC 035773
Asterales Campanulaceae Burmeistera loejtnantii#NC 035770
Asterales Campanulaceae Burmeistera smaragdi#NC 035763
Asterales Campanulaceae Burmeistera resupinata#NC 035765
Asterales Campanulaceae Burmeistera crispiloba#NC 035775
Asterales Campanulaceae Burmeistera sodiroana#NC 035762
Asterales Campanulaceae Burmeistera cyclostigmata#NC 035774
Asterales Campanulaceae Burmeistera borjensis#NC 035777
Asterales Campanulaceae Burmeistera oyacachensis#NC 035768
Asterales Campanulaceae Burmeistera auriculata#NC 035778
Asterales Campanulaceae Burmeistera rubrosepala#NC 035764
Asterales Campanulaceae Dialypetalum floribundum#NC 035357
Asterales Campanulaceae Lobelia sessilifolia#NC 035388
Asterales Campanulaceae Lobelia davidii#NC 035372
Asterales Campanulaceae Lobelia doniana#NC 035373
Asterales Campanulaceae Lobelia seguinii#F125
Asterales Campanulaceae Lobelia melliana#NC 035377
Asterales Campanulaceae Lobelia boninensis#NC 033367
Asterales Campanulaceae Apetahia longistigmata#NC 035353
Asterales Campanulaceae Sclerotheca viridiflora#NC 035396
Asterales Campanulaceae Cyanea fissa#NC 033363
Asterales Campanulaceae Cyanea leptostegia#NC 035356
Asterales Campanulaceae Clermontia fauriei#NC 035355
Asterales Campanulaceae Brighamia insignis#NC 028633

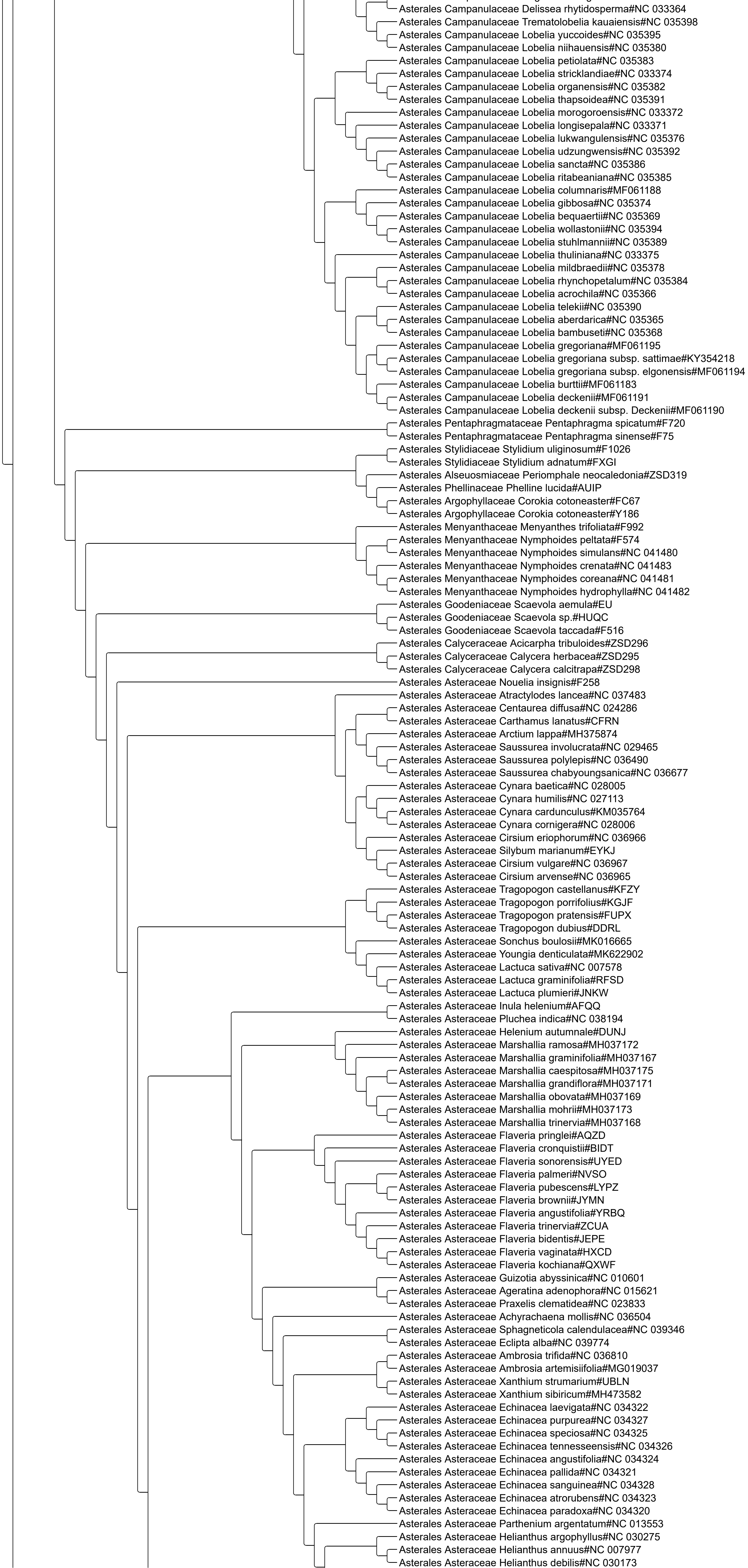
Asterales Campanulaceae Delissea rhytidosperma#NC 033364
Asterales Campanulaceae Trematolobelia kauaiensis#NC 035398
Asterales Campanulaceae Lobelia yuccoides#NC 035395
Asterales Campanulaceae Lobelia niihauensis#NC 035380
Asterales Campanulaceae Lobelia petiolata#NC 035383
Asterales Campanulaceae Lobelia stricklandiae#NC 033374
Asterales Campanulaceae Lobelia organensis#NC 035382
Asterales Campanulaceae Lobelia thapsoidea#NC 035391
Asterales Campanulaceae Lobelia morogoroensis#NC 033372
Asterales Campanulaceae Lobelia longisepala#NC 033371
Asterales Campanulaceae Lobelia lukwangulensis#NC 035376
Asterales Campanulaceae Lobelia udzungwensis#NC 035392
Asterales Campanulaceae Lobelia sancta#NC 035386
Asterales Campanulaceae Lobelia ritabeaniana#NC 035385
Asterales Campanulaceae Lobelia columnaris#MF061188
Asterales Campanulaceae Lobelia gibbosa#NC 035374
Asterales Campanulaceae Lobelia bequaertii#NC 035369
Asterales Campanulaceae Lobelia wollastonii#NC 035394
Asterales Campanulaceae Lobelia stuhlmannii#NC 035389
Asterales Campanulaceae Lobelia thuliniana#NC 033375
Asterales Campanulaceae Lobelia mildbraedii#NC 035378
Asterales Campanulaceae Lobelia rhynchopetalum#NC 035384
Asterales Campanulaceae Lobelia acrochila#NC 035366
Asterales Campanulaceae Lobelia telekii#NC 035390
Asterales Campanulaceae Lobelia aberdarica#NC 035365
Asterales Campanulaceae Lobelia bambuseti#NC 035368
Asterales Campanulaceae Lobelia gregoriana#MF061195
Asterales Campanulaceae Lobelia gregoriana subsp. sattimae#KY354218
Asterales Campanulaceae Lobelia gregoriana subsp. elgonensis#MF061194
Asterales Campanulaceae Lobelia burttii#MF061183
Asterales Campanulaceae Lobelia deckenii#MF061191
Asterales Campanulaceae Lobelia deckenii subsp. Deckenii#MF061190
Asterales Pentaphragmataceae Pentaphragma spicatum#F720
Asterales Pentaphragmataceae Pentaphragma sinense#F75
Asterales Stylidiaceae Stylidium uliginosum#F1026
Asterales Stylidiaceae Stylidium adnatum#FXGI
Asterales Alseuosmiaceae Periomphale neocaledonia#ZSD319
Asterales Phellinaceae Phelline lucida#AUIP
Asterales Argophyllaceae Corokia cotoneaster#FC67
Asterales Argophyllaceae Corokia cotoneaster#Y186
Asterales Menyanthaceae Menyanthes trifoliata#F992
Asterales Menyanthaceae Nymphoides peltata#F574
Asterales Menyanthaceae Nymphoides simulans#NC 041480
Asterales Menyanthaceae Nymphoides crenata#NC 041483
Asterales Menyanthaceae Nymphoides coreana#NC 041481
Asterales Menyanthaceae Nymphoides hydrophylla#NC 041482
Asterales Goodeniaceae Scaevola aemula#EU
Asterales Goodeniaceae Scaevola sp.#HUQC
Asterales Goodeniaceae Scaevola taccada#F516
Asterales Calyceraceae Acicarpha tribuloides#ZSD296
Asterales Calyceraceae Calycera herbacea#ZSD295
Asterales Calyceraceae Calycera calcitrapa#ZSD298
Asterales Asteraceae Nouelia insignis#F258
Asterales Asteraceae Atractylodes lancea#NC 037483
Asterales Asteraceae Centaurea diffusa#NC 024286
Asterales Asteraceae Carthamus lanatus#CFRN
Asterales Asteraceae Arctium lappa#MH375874
Asterales Asteraceae Saussurea involucrata#NC 029465
Asterales Asteraceae Saussurea polylepis#NC 036490
Asterales Asteraceae Saussurea chabyoungsanica#NC 036677
Asterales Asteraceae Cynara baetica#NC 028005
Asterales Asteraceae Cynara humilis#NC 027113
Asterales Asteraceae Cynara cardunculus#KM035764
Asterales Asteraceae Cynara cornigera#NC 028006
Asterales Asteraceae Cirsium eriophorum#NC 036966
Asterales Asteraceae Silybum marianum#EYKJ
Asterales Asteraceae Cirsium vulgare#NC 036967
Asterales Asteraceae Cirsium arvense#NC 036965
Asterales Asteraceae Tragopogon castellanus#KFZY
Asterales Asteraceae Tragopogon porrifolius#KGJF
Asterales Asteraceae Tragopogon pratensis#FUPX
Asterales Asteraceae Tragopogon dubius#DDRL
Asterales Asteraceae Sonchus boulosii#MK016665
Asterales Asteraceae Youngia denticulata#MK622902
Asterales Asteraceae Lactuca sativa#NC 007578
Asterales Asteraceae Lactuca graminifolia#RFSD
Asterales Asteraceae Lactuca plumieri#JNKW
Asterales Asteraceae Inula helenium#AFQQ
Asterales Asteraceae Pluchea indica#NC 038194
Asterales Asteraceae Helenium autumnale#DUNJ
Asterales Asteraceae Marshallia ramosa#MH037172
Asterales Asteraceae Marshallia graminifolia#MH037167
Asterales Asteraceae Marshallia caespitosa#MH037175
Asterales Asteraceae Marshallia grandiflora#MH037171
Asterales Asteraceae Marshallia obovata#MH037169
Asterales Asteraceae Marshallia mohrii#MH037173
Asterales Asteraceae Marshallia trinervia#MH037168
Asterales Asteraceae Flaveria pringlei#AQZD
Asterales Asteraceae Flaveria cronquistii#BIDT
Asterales Asteraceae Flaveria sonorensis#UYED
Asterales Asteraceae Flaveria palmeri#NVSO
Asterales Asteraceae Flaveria pubescens#LYPZ
Asterales Asteraceae Flaveria brownii#JYMN
Asterales Asteraceae Flaveria angustifolia#YRBQ
Asterales Asteraceae Flaveria trinervia#ZCUA
Asterales Asteraceae Flaveria bidentis#JEPE
Asterales Asteraceae Flaveria vaginata#HXCD
Asterales Asteraceae Flaveria kochiana#QXWF
Asterales Asteraceae Guizotia abyssinica#NC 010601
Asterales Asteraceae Ageratina adenophora#NC 015621
Asterales Asteraceae Praxelis clematidea#NC 023833
Asterales Asteraceae Achyrachaena mollis#NC 036504
Asterales Asteraceae Sphagneticola calendulacea#NC 039346
Asterales Asteraceae Eclipta alba#NC 039774
Asterales Asteraceae Ambrosia trifida#NC 036810
Asterales Asteraceae Ambrosia artemisiifolia#MG019037
Asterales Asteraceae Xanthium strumarium#UBLN
Asterales Asteraceae Xanthium sibiricum#MH473582
Asterales Asteraceae Echinacea laevigata#NC 034322
Asterales Asteraceae Echinacea purpurea#NC 034327
Asterales Asteraceae Echinacea speciosa#NC 034325
Asterales Asteraceae Echinacea tennesseensis#NC 034326
Asterales Asteraceae Echinacea angustifolia#NC 034324
Asterales Asteraceae Echinacea pallida#NC 034321
Asterales Asteraceae Echinacea sanguinea#NC 034328
Asterales Asteraceae Echinacea atrorubens#NC 034323
Asterales Asteraceae Echinacea paradoxa#NC 034320
Asterales Asteraceae Parthenium argentatum#NC 013553
Asterales Asteraceae Helianthus argophyllus#NC 030275
Asterales Asteraceae Helianthus annuus#NC 007977
Asterales Asteraceae Helianthus debilis#NC 030173

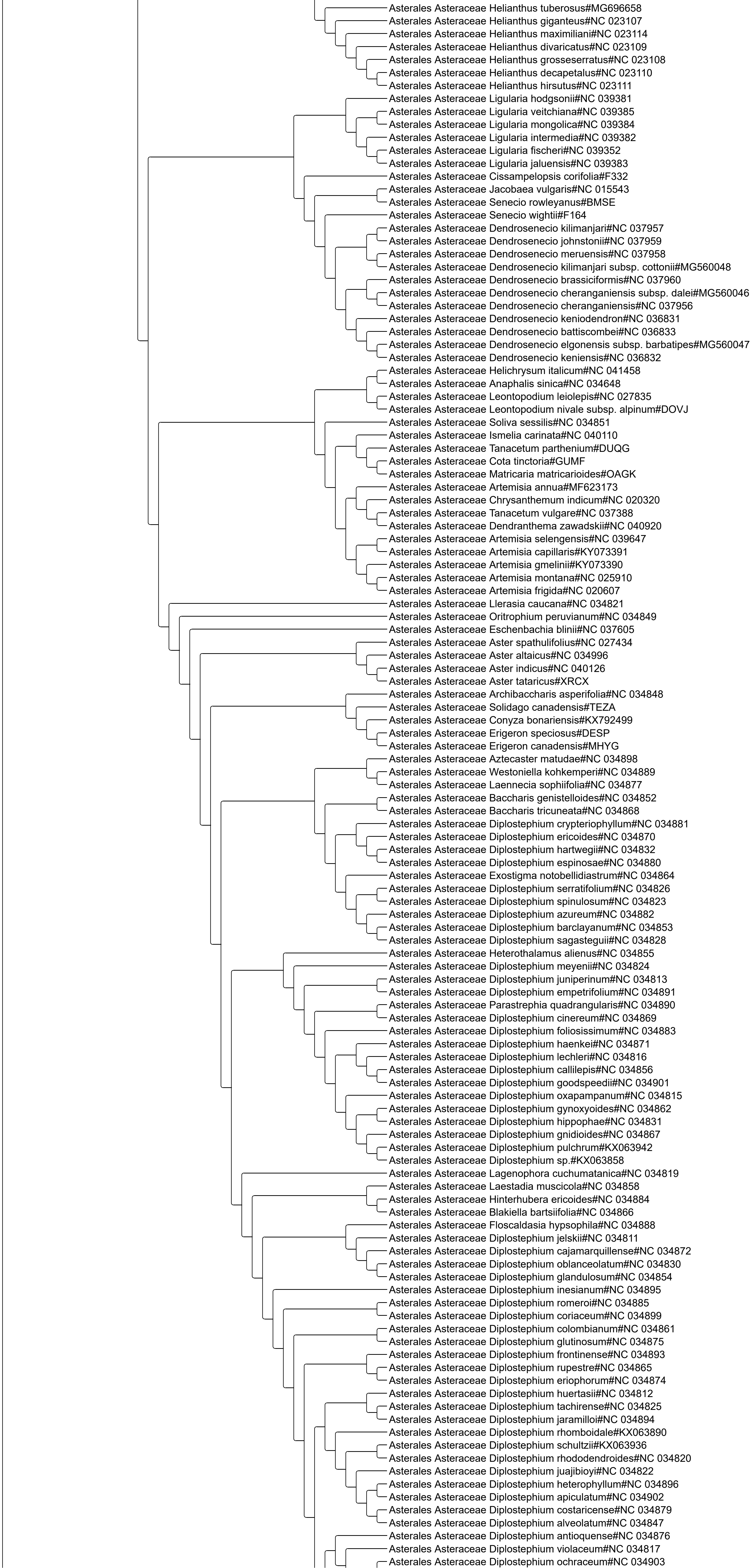
Asterales Asteraceae Helianthus tuberosus#MG696658
Asterales Asteraceae Helianthus giganteus#NC 023107
Asterales Asteraceae Helianthus maximiliani#NC 023114
Asterales Asteraceae Helianthus divaricatus#NC 023109
Asterales Asteraceae Helianthus grosseserratus#NC 023108
Asterales Asteraceae Helianthus decapetalus#NC 023110
Asterales Asteraceae Helianthus hirsutus#NC 023111
Asterales Asteraceae Ligularia hodgsonii#NC 039381
Asterales Asteraceae Ligularia veitchiana#NC 039385
Asterales Asteraceae Ligularia mongolica#NC 039384
Asterales Asteraceae Ligularia intermedia#NC 039382
Asterales Asteraceae Ligularia fischeri#NC 039352
Asterales Asteraceae Ligularia jaluensis#NC 039383
Asterales Asteraceae Cissampelopsis corifolia#F332
Asterales Asteraceae Jacobaea vulgaris#NC 015543
Asterales Asteraceae Senecio rowleyanus#BMSE
Asterales Asteraceae Senecio wightii#F164
Asterales Asteraceae Dendrosenecio kilimanjari#NC 037957
Asterales Asteraceae Dendrosenecio johnstonii#NC 037959
Asterales Asteraceae Dendrosenecio meruensis#NC 037958
Asterales Asteraceae Dendrosenecio kilimanjari subsp. cottonii#MG560048
Asterales Asteraceae Dendrosenecio brassiciformis#NC 037960
Asterales Asteraceae Dendrosenecio cheranganiensis subsp. dalei#MG560046
Asterales Asteraceae Dendrosenecio cheranganiensis#NC 037956
Asterales Asteraceae Dendrosenecio keniodendron#NC 036831
Asterales Asteraceae Dendrosenecio battiscombei#NC 036833
Asterales Asteraceae Dendrosenecio elgonensis subsp. barbatipes#MG560047
Asterales Asteraceae Dendrosenecio keniensis#NC 036832
Asterales Asteraceae Helichrysum italicum#NC 041458
Asterales Asteraceae Anaphalis sinica#NC 034648
Asterales Asteraceae Leontopodium leiolepis#NC 027835
Asterales Asteraceae Leontopodium nivale subsp. alpinum#DOVJ
Asterales Asteraceae Soliva sessilis#NC 034851
Asterales Asteraceae Ismelia carinata#NC 040110
Asterales Asteraceae Tanacetum parthenium#DUQG
Asterales Asteraceae Cota tinctoria#GUMF
Asterales Asteraceae Matricaria matricarioides#OAGK
Asterales Asteraceae Artemisia annua#MF623173
Asterales Asteraceae Chrysanthemum indicum#NC 020320
Asterales Asteraceae Tanacetum vulgare#NC 037388
Asterales Asteraceae Dendranthema zawadskii#NC 040920
Asterales Asteraceae Artemisia selengensis#NC 039647
Asterales Asteraceae Artemisia capillaris#KY073391
Asterales Asteraceae Artemisia gmelinii#KY073390
Asterales Asteraceae Artemisia montana#NC 025910
Asterales Asteraceae Artemisia frigida#NC 020607
Asterales Asteraceae Llerasia caucana#NC 034821
Asterales Asteraceae Oritrophium peruvianum#NC 034849
Asterales Asteraceae Eschenbachia blinii#NC 037605
Asterales Asteraceae Aster spathulifolius#NC 027434
Asterales Asteraceae Aster altaicus#NC 034996
Asterales Asteraceae Aster indicus#NC 040126
Asterales Asteraceae Aster tataricus#XRCX
Asterales Asteraceae Archibaccharis asperifolia#NC 034848
Asterales Asteraceae Solidago canadensis#TEZA
Asterales Asteraceae Conyza bonariensis#KX792499
Asterales Asteraceae Erigeron speciosus#DESP
Asterales Asteraceae Erigeron canadensis#MHYG
Asterales Asteraceae Aztecaster matudae#NC 034898
Asterales Asteraceae Westoniella kohkemperi#NC 034889
Asterales Asteraceae Laennecia sophiifolia#NC 034877
Asterales Asteraceae Baccharis genistelloides#NC 034852
Asterales Asteraceae Baccharis tricuneata#NC 034868
Asterales Asteraceae Diplostephium crypteriophyllum#NC 034881
Asterales Asteraceae Diplostephium ericoides#NC 034870
Asterales Asteraceae Diplostephium hartwegii#NC 034832
Asterales Asteraceae Diplostephium espinosae#NC 034880
Asterales Asteraceae Exostigma notobellidiastrum#NC 034864
Asterales Asteraceae Diplostephium serratifolium#NC 034826
Asterales Asteraceae Diplostephium spinulosum#NC 034823
Asterales Asteraceae Diplostephium azureum#NC 034882
Asterales Asteraceae Diplostephium barclayanum#NC 034853
Asterales Asteraceae Diplostephium sagasteguii#NC 034828
Asterales Asteraceae Heterothalamus alienus#NC 034855
Asterales Asteraceae Diplostephium meyenii#NC 034824
Asterales Asteraceae Diplostephium juniperinum#NC 034813
Asterales Asteraceae Diplostephium empetrifolium#NC 034891
Asterales Asteraceae Parastrephia quadrangularis#NC 034890
Asterales Asteraceae Diplostephium cinereum#NC 034869
Asterales Asteraceae Diplostephium foliosissimum#NC 034883
Asterales Asteraceae Diplostephium haenkei#NC 034871
Asterales Asteraceae Diplostephium lechleri#NC 034816
Asterales Asteraceae Diplostephium callilepis#NC 034856
Asterales Asteraceae Diplostephium goodspeedii#NC 034901
Asterales Asteraceae Diplostephium oxapampanum#NC 034815
Asterales Asteraceae Diplostephium gynoxyoides#NC 034862
Asterales Asteraceae Diplostephium hippophae#NC 034831
Asterales Asteraceae Diplostephium gnidioides#NC 034867
Asterales Asteraceae Diplostephium pulchrum#KX063942
Asterales Asteraceae Diplostephium sp.#KX063858
Asterales Asteraceae Lagenophora cuchumatanica#NC 034819
Asterales Asteraceae Laestadia muscicola#NC 034858
Asterales Asteraceae Hinterhubera ericoides#NC 034884
Asterales Asteraceae Blakiella bartsiifolia#NC 034866
Asterales Asteraceae Floscaldasia hypsophila#NC 034888
Asterales Asteraceae Diplostephium jelskii#NC 034811
Asterales Asteraceae Diplostephium cajamarquillense#NC 034872
Asterales Asteraceae Diplostephium oblanceolatum#NC 034830
Asterales Asteraceae Diplostephium glandulosum#NC 034854
Asterales Asteraceae Diplostephium inesianum#NC 034895
Asterales Asteraceae Diplostephium romeroi#NC 034885
Asterales Asteraceae Diplostephium coriaceum#NC 034899
Asterales Asteraceae Diplostephium colombianum#NC 034861
Asterales Asteraceae Diplostephium glutinosum#NC 034875
Asterales Asteraceae Diplostephium frontinense#NC 034893
Asterales Asteraceae Diplostephium rupestre#NC 034865
Asterales Asteraceae Diplostephium eriophorum#NC 034874
Asterales Asteraceae Diplostephium huertasii#NC 034812
Asterales Asteraceae Diplostephium tachirense#NC 034825
Asterales Asteraceae Diplostephium jaramilloi#NC 034894
Asterales Asteraceae Diplostephium rhomboidale#KX063890
Asterales Asteraceae Diplostephium schultzii#KX063936
Asterales Asteraceae Diplostephium rhododendroides#NC 034820
Asterales Asteraceae Diplostephium juajibioyi#NC 034822
Asterales Asteraceae Diplostephium heterophyllum#NC 034896
Asterales Asteraceae Diplostephium apiculatum#NC 034902
Asterales Asteraceae Diplostephium costaricense#NC 034879
Asterales Asteraceae Diplostephium alveolatum#NC 034847
Asterales Asteraceae Diplostephium antioquense#NC 034876
Asterales Asteraceae Diplostephium violaceum#NC 034817
Asterales Asteraceae Diplostephium ochraceum#NC 034903

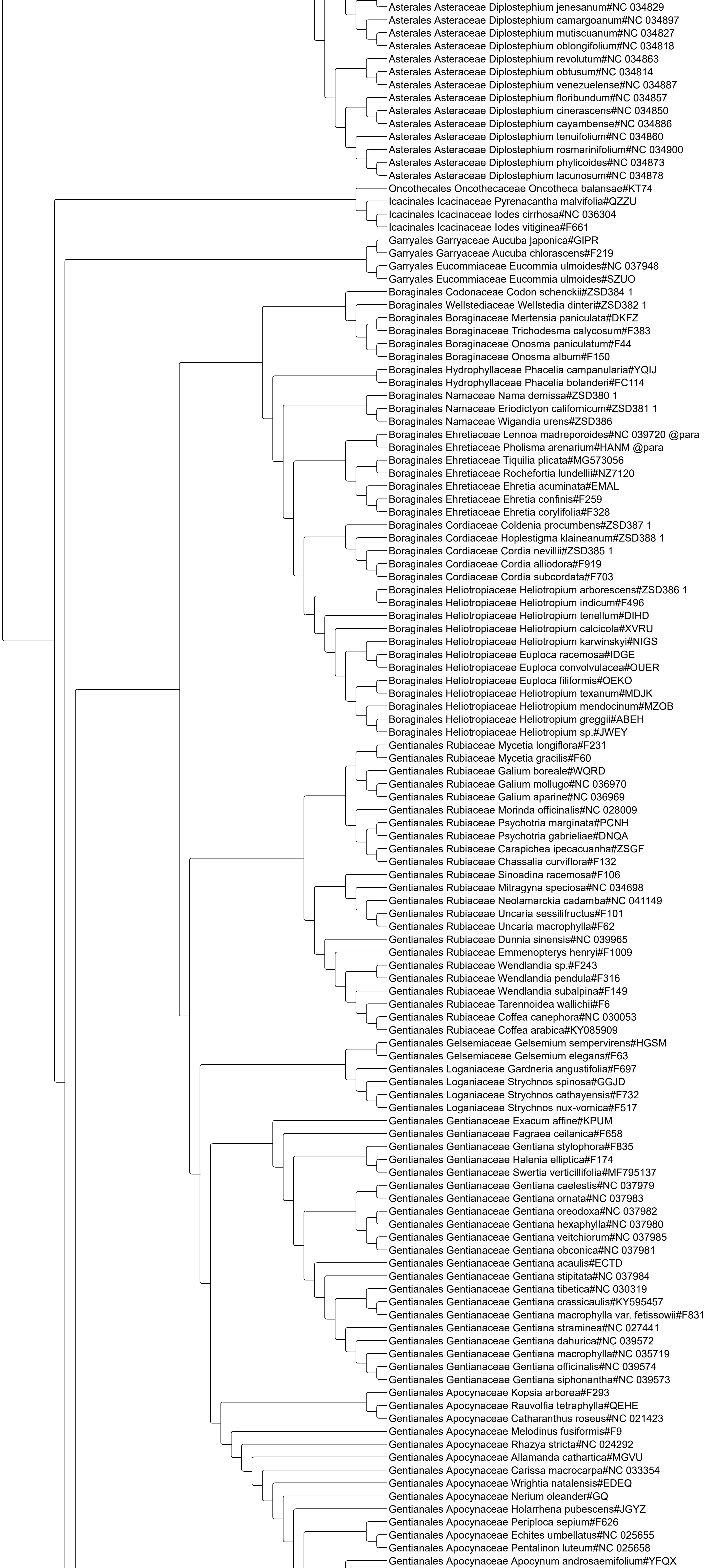
Asterales Asteraceae Diplostephium jenesanum#NC 034829
Asterales Asteraceae Diplostephium camargoanum#NC 034897
Asterales Asteraceae Diplostephium mutiscuanum#NC 034827
Asterales Asteraceae Diplostephium oblongifolium#NC 034818
Asterales Asteraceae Diplostephium revolutum#NC 034863
Asterales Asteraceae Diplostephium obtusum#NC 034814
Asterales Asteraceae Diplostephium venezuelense#NC 034887
Asterales Asteraceae Diplostephium floribundum#NC 034857
Asterales Asteraceae Diplostephium cinerascens#NC 034850
Asterales Asteraceae Diplostephium cayambense#NC 034886
Asterales Asteraceae Diplostephium tenuifolium#NC 034860
Asterales Asteraceae Diplostephium rosmarinifolium#NC 034900
Asterales Asteraceae Diplostephium phylicoides#NC 034873
Asterales Asteraceae Diplostephium lacunosum#NC 034878
Oncothecales Oncothecaceae Oncotheca balansae#KT74
Icacinales Icacinaceae Pyrenacantha malvifolia#QZZU
Icacinales Icacinaceae Iodes cirrhosa#NC 036304
Icacinales Icacinaceae Iodes vitiginea#F661
Garryales Garryaceae Aucuba japonica#GIPR
Garryales Garryaceae Aucuba chlorascens#F219
Garryales Eucommiaceae Eucommia ulmoides#NC 037948
Garryales Eucommiaceae Eucommia ulmoides#SZUO
Boraginales Codonaceae Codon schenckii#ZSD384 1
Boraginales Wellstediaceae Wellstedia dinteri#ZSD382 1
Boraginales Boraginaceae Mertensia paniculata#DKFZ
Boraginales Boraginaceae Trichodesma calycosum#F383
Boraginales Boraginaceae Onosma paniculatum#F44
Boraginales Boraginaceae Onosma album#F150
Boraginales Hydrophyllaceae Phacelia campanularia#YQIJ
Boraginales Hydrophyllaceae Phacelia bolanderi#FC114
Boraginales Namaceae Nama demissa#ZSD380 1
Boraginales Namaceae Eriodictyon californicum#ZSD381 1
Boraginales Namaceae Wigandia urens#ZSD386
Boraginales Ehretiaceae Lennoa madreporoides#NC 039720 @para
Boraginales Ehretiaceae Pholisma arenarium#HANM @para
Boraginales Ehretiaceae Tiquilia plicata#MG573056
Boraginales Ehretiaceae Rochefortia lundellii#NZ7120
Boraginales Ehretiaceae Ehretia acuminata#EMAL
Boraginales Ehretiaceae Ehretia confinis#F259
Boraginales Ehretiaceae Ehretia corylifolia#F328
Boraginales Cordiaceae Coldenia procumbens#ZSD387 1
Boraginales Cordiaceae Hoplestigma klaineanum#ZSD388 1
Boraginales Cordiaceae Cordia nevillii#ZSD385 1
Boraginales Cordiaceae Cordia alliodora#F919
Boraginales Cordiaceae Cordia subcordata#F703
Boraginales Heliotropiaceae Heliotropium arborescens#ZSD386 1
Boraginales Heliotropiaceae Heliotropium indicum#F496
Boraginales Heliotropiaceae Heliotropium tenellum#DIHD
Boraginales Heliotropiaceae Heliotropium calcicola#XVRU
Boraginales Heliotropiaceae Heliotropium karwinskyi#NIGS
Boraginales Heliotropiaceae Euploca racemosa#IDGE
Boraginales Heliotropiaceae Euploca convolvulacea#OUER
Boraginales Heliotropiaceae Euploca filiformis#OEKO
Boraginales Heliotropiaceae Heliotropium texanum#MDJK
Boraginales Heliotropiaceae Heliotropium mendocinum#MZOB
Boraginales Heliotropiaceae Heliotropium greggii#ABEH
Boraginales Heliotropiaceae Heliotropium sp.#JWEY
Gentianales Rubiaceae Mycetia longiflora#F231
Gentianales Rubiaceae Mycetia gracilis#F60
Gentianales Rubiaceae Galium boreale#WQRD
Gentianales Rubiaceae Galium mollugo#NC 036970
Gentianales Rubiaceae Galium aparine#NC 036969
Gentianales Rubiaceae Morinda officinalis#NC 028009
Gentianales Rubiaceae Psychotria marginata#PCNH
Gentianales Rubiaceae Psychotria gabrieliae#DNQA
Gentianales Rubiaceae Carapichea ipecacuanha#ZSGF
Gentianales Rubiaceae Chassalia curviflora#F132
Gentianales Rubiaceae Sinoadina racemosa#F106
Gentianales Rubiaceae Mitragyna speciosa#NC 034698
Gentianales Rubiaceae Neolamarckia cadamba#NC 041149
Gentianales Rubiaceae Uncaria sessilifructus#F101
Gentianales Rubiaceae Uncaria macrophylla#F62
Gentianales Rubiaceae Dunnia sinensis#NC 039965
Gentianales Rubiaceae Emmenopterys henryi#F1009
Gentianales Rubiaceae Wendlandia sp.#F243
Gentianales Rubiaceae Wendlandia pendula#F316
Gentianales Rubiaceae Wendlandia subalpina#F149
Gentianales Rubiaceae Tarennoidea wallichii#F6
Gentianales Rubiaceae Coffea canephora#NC 030053
Gentianales Rubiaceae Coffea arabica#KY085909
Gentianales Gelsemiaceae Gelsemium sempervirens#HGSM
Gentianales Gelsemiaceae Gelsemium elegans#F63
Gentianales Loganiaceae Gardneria angustifolia#F697
Gentianales Loganiaceae Strychnos spinosa#GGJD
Gentianales Loganiaceae Strychnos cathayensis#F732
Gentianales Loganiaceae Strychnos nux-vomica#F517
Gentianales Gentianaceae Exacum affine#KPUM
Gentianales Gentianaceae Fagraea ceilanica#F658
Gentianales Gentianaceae Gentiana stylophora#F835
Gentianales Gentianaceae Halenia elliptica#F174
Gentianales Gentianaceae Swertia verticillifolia#MF795137
Gentianales Gentianaceae Gentiana caelestis#NC 037979
Gentianales Gentianaceae Gentiana ornata#NC 037983
Gentianales Gentianaceae Gentiana oreodoxa#NC 037982
Gentianales Gentianaceae Gentiana hexaphylla#NC 037980
Gentianales Gentianaceae Gentiana veitchiorum#NC 037985
Gentianales Gentianaceae Gentiana obconica#NC 037981
Gentianales Gentianaceae Gentiana acaulis#ECTD
Gentianales Gentianaceae Gentiana stipitata#NC 037984
Gentianales Gentianaceae Gentiana tibetica#NC 030319
Gentianales Gentianaceae Gentiana crassicaulis#KY595457
Gentianales Gentianaceae Gentiana macrophylla var. fetissowii#F831
Gentianales Gentianaceae Gentiana straminea#NC 027441
Gentianales Gentianaceae Gentiana dahurica#NC 039572
Gentianales Gentianaceae Gentiana macrophylla#NC 035719
Gentianales Gentianaceae Gentiana officinalis#NC 039574
Gentianales Gentianaceae Gentiana siphonantha#NC 039573
Gentianales Apocynaceae Kopsia arborea#F293
Gentianales Apocynaceae Rauvolfia tetraphylla#QEHE
Gentianales Apocynaceae Catharanthus roseus#NC 021423
Gentianales Apocynaceae Melodinus fusiformis#F9
Gentianales Apocynaceae Rhazya stricta#NC 024292
Gentianales Apocynaceae Allamanda cathartica#MGVU
Gentianales Apocynaceae Carissa macrocarpa#NC 033354
Gentianales Apocynaceae Wrightia natalensis#EDEQ
Gentianales Apocynaceae Nerium oleander#GQ
Gentianales Apocynaceae Holarrhena pubescens#JGYZ
Gentianales Apocynaceae Periploca sepium#F626
Gentianales Apocynaceae Echites umbellatus#NC 025655
Gentianales Apocynaceae Pentalinon luteum#NC 025658
Gentianales Apocynaceae Apocynum androsaemifolium#YFQX

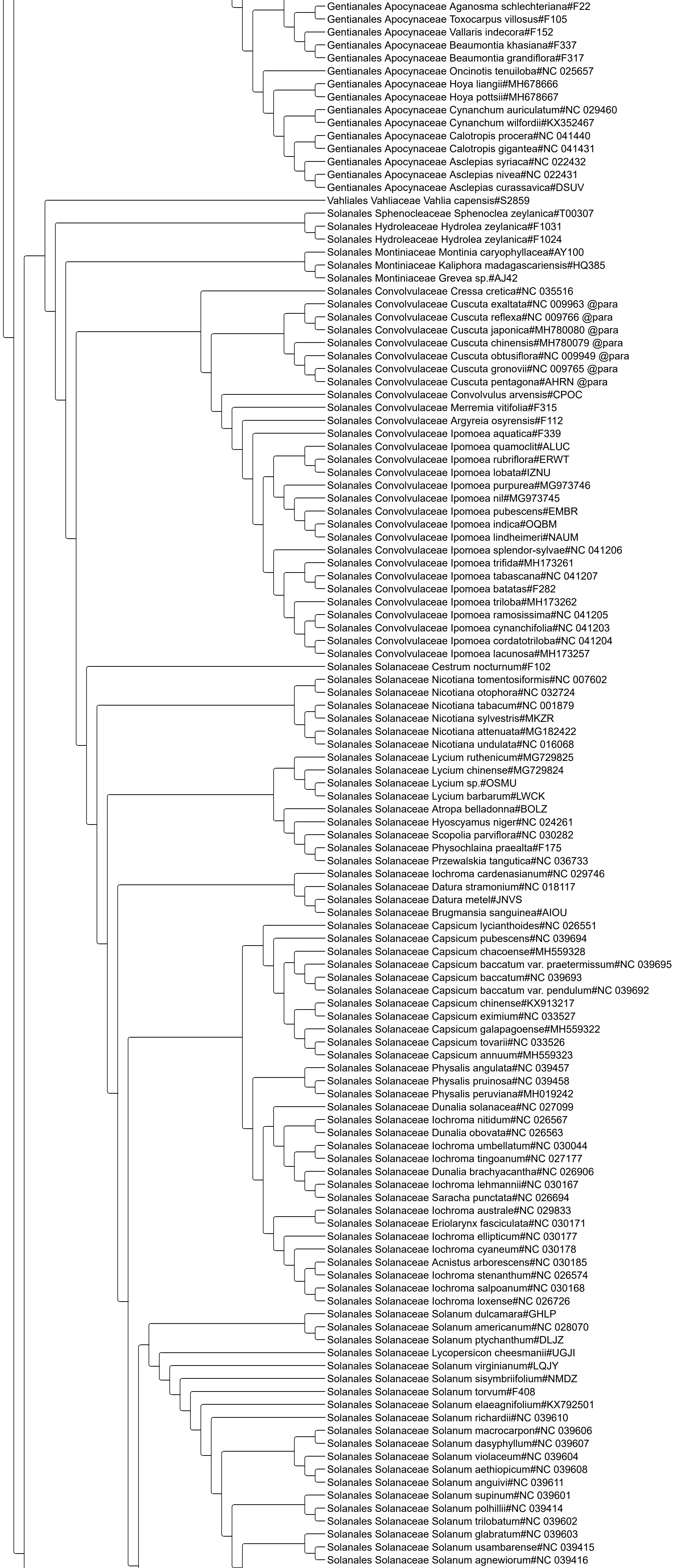
Gentianales Apocynaceae Aganosma schlechteriana#F22
Gentianales Apocynaceae Toxocarpus villosus#F105
Gentianales Apocynaceae Vallaris indecora#F152
Gentianales Apocynaceae Beaumontia khasiana#F337
Gentianales Apocynaceae Beaumontia grandiflora#F317
Gentianales Apocynaceae Oncinotis tenuiloba#NC 025657
Gentianales Apocynaceae Hoya liangii#MH678666
Gentianales Apocynaceae Hoya pottsii#MH678667
Gentianales Apocynaceae Cynanchum auriculatum#NC 029460
Gentianales Apocynaceae Cynanchum wilfordii#KX352467
Gentianales Apocynaceae Calotropis procera#NC 041440
Gentianales Apocynaceae Calotropis gigantea#NC 041431
Gentianales Apocynaceae Asclepias syriaca#NC 022432
Gentianales Apocynaceae Asclepias nivea#NC 022431
Gentianales Apocynaceae Asclepias curassavica#DSUV
Vahliales Vahliaceae Vahlia capensis#S2859
Solanales Sphenocleaceae Sphenoclea zeylanica#T00307
Solanales Hydroleaceae Hydrolea zeylanica#F1031
Solanales Hydroleaceae Hydrolea zeylanica#F1024
Solanales Montiniaceae Montinia caryophyllacea#AY100
Solanales Montiniaceae Kaliphora madagascariensis#HQ385
Solanales Montiniaceae Grevea sp.#AJ42
Solanales Convolvulaceae Cressa cretica#NC 035516
Solanales Convolvulaceae Cuscuta exaltata#NC 009963 @para
Solanales Convolvulaceae Cuscuta reflexa#NC 009766 @para
Solanales Convolvulaceae Cuscuta japonica#MH780080 @para
Solanales Convolvulaceae Cuscuta chinensis#MH780079 @para
Solanales Convolvulaceae Cuscuta obtusiflora#NC 009949 @para
Solanales Convolvulaceae Cuscuta gronovii#NC 009765 @para
Solanales Convolvulaceae Cuscuta pentagona#AHRN @para
Solanales Convolvulaceae Convolvulus arvensis#CPOC
Solanales Convolvulaceae Merremia vitifolia#F315
Solanales Convolvulaceae Argyreia osyrensis#F112
Solanales Convolvulaceae Ipomoea aquatica#F339
Solanales Convolvulaceae Ipomoea quamoclit#ALUC
Solanales Convolvulaceae Ipomoea rubriflora#ERWT
Solanales Convolvulaceae Ipomoea lobata#IZNU
Solanales Convolvulaceae Ipomoea purpurea#MG973746
Solanales Convolvulaceae Ipomoea nil#MG973745
Solanales Convolvulaceae Ipomoea pubescens#EMBR
Solanales Convolvulaceae Ipomoea indica#OQBM
Solanales Convolvulaceae Ipomoea lindheimeri#NAUM
Solanales Convolvulaceae Ipomoea splendor-sylvae#NC 041206
Solanales Convolvulaceae Ipomoea trifida#MH173261
Solanales Convolvulaceae Ipomoea tabascana#NC 041207
Solanales Convolvulaceae Ipomoea batatas#F282
Solanales Convolvulaceae Ipomoea triloba#MH173262
Solanales Convolvulaceae Ipomoea ramosissima#NC 041205
Solanales Convolvulaceae Ipomoea cynanchifolia#NC 041203
Solanales Convolvulaceae Ipomoea cordatotriloba#NC 041204
Solanales Convolvulaceae Ipomoea lacunosa#MH173257
Solanales Solanaceae Cestrum nocturnum#F102
Solanales Solanaceae Nicotiana tomentosiformis#NC 007602
Solanales Solanaceae Nicotiana otophora#NC 032724
Solanales Solanaceae Nicotiana tabacum#NC 001879
Solanales Solanaceae Nicotiana sylvestris#MKZR
Solanales Solanaceae Nicotiana attenuata#MG182422
Solanales Solanaceae Nicotiana undulata#NC 016068
Solanales Solanaceae Lycium ruthenicum#MG729825
Solanales Solanaceae Lycium chinense#MG729824
Solanales Solanaceae Lycium sp.#OSMU
Solanales Solanaceae Lycium barbarum#LWCK
Solanales Solanaceae Atropa belladonna#BOLZ
Solanales Solanaceae Hyoscyamus niger#NC 024261
Solanales Solanaceae Scopolia parviflora#NC 030282
Solanales Solanaceae Physochlaina praealta#F175
Solanales Solanaceae Przewalskia tangutica#NC 036733
Solanales Solanaceae Iochroma cardenasianum#NC 029746
Solanales Solanaceae Datura stramonium#NC 018117
Solanales Solanaceae Datura metel#JNVS
Solanales Solanaceae Brugmansia sanguinea#AIOU
Solanales Solanaceae Capsicum lycianthoides#NC 026551
Solanales Solanaceae Capsicum pubescens#NC 039694
Solanales Solanaceae Capsicum chacoense#MH559328
Solanales Solanaceae Capsicum baccatum var. praetermissum#NC 039695
Solanales Solanaceae Capsicum baccatum#NC 039693
Solanales Solanaceae Capsicum baccatum var. pendulum#NC 039692
Solanales Solanaceae Capsicum chinense#KX913217
Solanales Solanaceae Capsicum eximium#NC 033527
Solanales Solanaceae Capsicum galapagoense#MH559322
Solanales Solanaceae Capsicum tovarii#NC 033526
Solanales Solanaceae Capsicum annuum#MH559323
Solanales Solanaceae Physalis angulata#NC 039457
Solanales Solanaceae Physalis pruinosa#NC 039458
Solanales Solanaceae Physalis peruviana#MH019242
Solanales Solanaceae Dunalia solanacea#NC 027099
Solanales Solanaceae Iochroma nitidum#NC 026567
Solanales Solanaceae Dunalia obovata#NC 026563
Solanales Solanaceae Iochroma umbellatum#NC 030044
Solanales Solanaceae Iochroma tingoanum#NC 027177
Solanales Solanaceae Dunalia brachyacantha#NC 026906
Solanales Solanaceae Iochroma lehmannii#NC 030167
Solanales Solanaceae Saracha punctata#NC 026694
Solanales Solanaceae Iochroma australe#NC 029833
Solanales Solanaceae Eriolarynx fasciculata#NC 030171
Solanales Solanaceae Iochroma ellipticum#NC 030177
Solanales Solanaceae Iochroma cyaneum#NC 030178
Solanales Solanaceae Acnistus arborescens#NC 030185
Solanales Solanaceae Iochroma stenanthum#NC 026574
Solanales Solanaceae Iochroma salpoanum#NC 030168
Solanales Solanaceae Iochroma loxense#NC 026726
Solanales Solanaceae Solanum dulcamara#GHLP
Solanales Solanaceae Solanum americanum#NC 028070
Solanales Solanaceae Solanum ptychanthum#DLJZ
Solanales Solanaceae Lycopersicon cheesmanii#UGJI
Solanales Solanaceae Solanum virginianum#LQJY
Solanales Solanaceae Solanum sisymbriifolium#NMDZ
Solanales Solanaceae Solanum torvum#F408
Solanales Solanaceae Solanum elaeagnifolium#KX792501
Solanales Solanaceae Solanum richardii#NC 039610
Solanales Solanaceae Solanum macrocarpon#NC 039606
Solanales Solanaceae Solanum dasyphyllum#NC 039607
Solanales Solanaceae Solanum violaceum#NC 039604
Solanales Solanaceae Solanum aethiopicum#NC 039608
Solanales Solanaceae Solanum anguivi#NC 039611
Solanales Solanaceae Solanum supinum#NC 039601
Solanales Solanaceae Solanum polhillii#NC 039414
Solanales Solanaceae Solanum trilobatum#NC 039602
Solanales Solanaceae Solanum glabratum#NC 039603
Solanales Solanaceae Solanum usambarense#NC 039415
Solanales Solanaceae Solanum agnewiorum#NC 039416

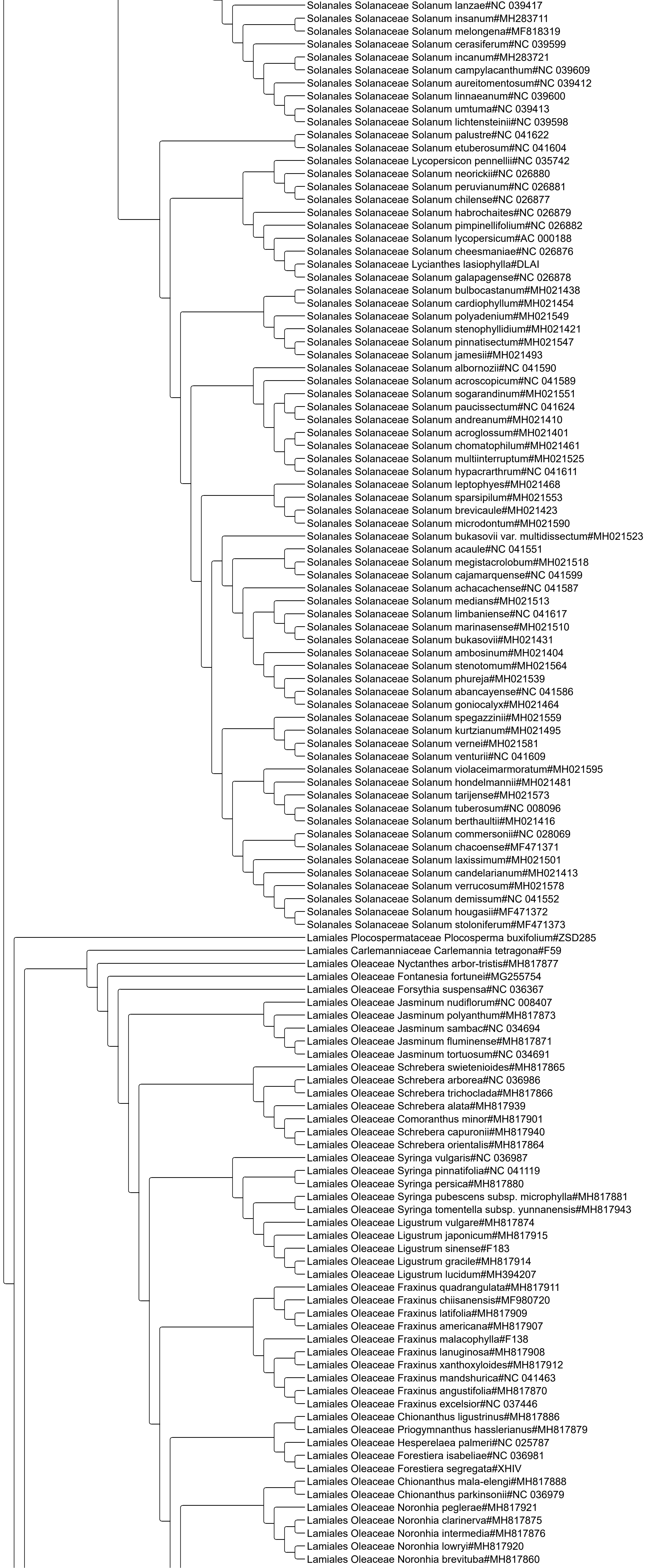
Solanales Solanaceae Solanum lanzae#NC 039417
Solanales Solanaceae Solanum insanum#MH283711
Solanales Solanaceae Solanum melongena#MF818319
Solanales Solanaceae Solanum cerasiferum#NC 039599
Solanales Solanaceae Solanum incanum#MH283721
Solanales Solanaceae Solanum campylacanthum#NC 039609
Solanales Solanaceae Solanum aureitomentosum#NC 039412
Solanales Solanaceae Solanum linnaeanum#NC 039600
Solanales Solanaceae Solanum umtuma#NC 039413
Solanales Solanaceae Solanum lichtensteinii#NC 039598
Solanales Solanaceae Solanum palustre#NC 041622
Solanales Solanaceae Solanum etuberosum#NC 041604
Solanales Solanaceae Lycopersicon pennellii#NC 035742
Solanales Solanaceae Solanum neorickii#NC 026880
Solanales Solanaceae Solanum peruvianum#NC 026881
Solanales Solanaceae Solanum chilense#NC 026877
Solanales Solanaceae Solanum habrochaites#NC 026879
Solanales Solanaceae Solanum pimpinellifolium#NC 026882
Solanales Solanaceae Solanum lycopersicum#AC 000188
Solanales Solanaceae Solanum cheesmaniae#NC 026876
Solanales Solanaceae Lycianthes lasiophylla#DLAI
Solanales Solanaceae Solanum galapagense#NC 026878
Solanales Solanaceae Solanum bulbocastanum#MH021438
Solanales Solanaceae Solanum cardiophyllum#MH021454
Solanales Solanaceae Solanum polyadenium#MH021549
Solanales Solanaceae Solanum stenophyllidium#MH021421
Solanales Solanaceae Solanum pinnatisectum#MH021547
Solanales Solanaceae Solanum jamesii#MH021493
Solanales Solanaceae Solanum albornozii#NC 041590
Solanales Solanaceae Solanum acroscopicum#NC 041589
Solanales Solanaceae Solanum sogarandinum#MH021551
Solanales Solanaceae Solanum paucissectum#NC 041624
Solanales Solanaceae Solanum andreanum#MH021410
Solanales Solanaceae Solanum acroglossum#MH021401
Solanales Solanaceae Solanum chomatophilum#MH021461
Solanales Solanaceae Solanum multiinterruptum#MH021525
Solanales Solanaceae Solanum hypacrarthrum#NC 041611
Solanales Solanaceae Solanum leptophyes#MH021468
Solanales Solanaceae Solanum sparsipilum#MH021553
Solanales Solanaceae Solanum brevicaule#MH021423
Solanales Solanaceae Solanum microdontum#MH021590
Solanales Solanaceae Solanum bukasovii var. multidissectum#MH021523
Solanales Solanaceae Solanum acaule#NC 041551
Solanales Solanaceae Solanum megistacrolobum#MH021518
Solanales Solanaceae Solanum cajamarquense#NC 041599
Solanales Solanaceae Solanum achacachense#NC 041587
Solanales Solanaceae Solanum medians#MH021513
Solanales Solanaceae Solanum limbaniense#NC 041617
Solanales Solanaceae Solanum marinasense#MH021510
Solanales Solanaceae Solanum bukasovii#MH021431
Solanales Solanaceae Solanum ambosinum#MH021404
Solanales Solanaceae Solanum stenotomum#MH021564
Solanales Solanaceae Solanum phureja#MH021539
Solanales Solanaceae Solanum abancayense#NC 041586
Solanales Solanaceae Solanum goniocalyx#MH021464
Solanales Solanaceae Solanum spegazzinii#MH021559
Solanales Solanaceae Solanum kurtzianum#MH021495
Solanales Solanaceae Solanum vernei#MH021581
Solanales Solanaceae Solanum venturii#NC 041609
Solanales Solanaceae Solanum violaceimarmoratum#MH021595
Solanales Solanaceae Solanum hondelmannii#MH021481
Solanales Solanaceae Solanum tarijense#MH021573
Solanales Solanaceae Solanum tuberosum#NC 008096
Solanales Solanaceae Solanum berthaultii#MH021416
Solanales Solanaceae Solanum commersonii#NC 028069
Solanales Solanaceae Solanum chacoense#MF471371
Solanales Solanaceae Solanum laxissimum#MH021501
Solanales Solanaceae Solanum candelarianum#MH021413
Solanales Solanaceae Solanum verrucosum#MH021578
Solanales Solanaceae Solanum demissum#NC 041552
Solanales Solanaceae Solanum hougasii#MF471372
Solanales Solanaceae Solanum stoloniferum#MF471373
Lamiales Plocospermataceae Plocosperma buxifolium#ZSD285
Lamiales Carlemanniaceae Carlemannia tetragona#F59
Lamiales Oleaceae Nyctanthes arbor-tristis#MH817877
Lamiales Oleaceae Fontanesia fortunei#MG255754
Lamiales Oleaceae Forsythia suspensa#NC 036367
Lamiales Oleaceae Jasminum nudiflorum#NC 008407
Lamiales Oleaceae Jasminum polyanthum#MH817873
Lamiales Oleaceae Jasminum sambac#NC 034694
Lamiales Oleaceae Jasminum fluminense#MH817871
Lamiales Oleaceae Jasminum tortuosum#NC 034691
Lamiales Oleaceae Schrebera swietenioides#MH817865
Lamiales Oleaceae Schrebera arborea#NC 036986
Lamiales Oleaceae Schrebera trichoclada#MH817866
Lamiales Oleaceae Schrebera alata#MH817939
Lamiales Oleaceae Comoranthus minor#MH817901
Lamiales Oleaceae Schrebera capuronii#MH817940
Lamiales Oleaceae Schrebera orientalis#MH817864
Lamiales Oleaceae Syringa vulgaris#NC 036987
Lamiales Oleaceae Syringa pinnatifolia#NC 041119
Lamiales Oleaceae Syringa persica#MH817880
Lamiales Oleaceae Syringa pubescens subsp. microphylla#MH817881
Lamiales Oleaceae Syringa tomentella subsp. yunnanensis#MH817943
Lamiales Oleaceae Ligustrum vulgare#MH817874
Lamiales Oleaceae Ligustrum japonicum#MH817915
Lamiales Oleaceae Ligustrum sinense#F183
Lamiales Oleaceae Ligustrum gracile#MH817914
Lamiales Oleaceae Ligustrum lucidum#MH394207
Lamiales Oleaceae Fraxinus quadrangulata#MH817911
Lamiales Oleaceae Fraxinus chiisanensis#MF980720
Lamiales Oleaceae Fraxinus latifolia#MH817909
Lamiales Oleaceae Fraxinus americana#MH817907
Lamiales Oleaceae Fraxinus malacophylla#F138
Lamiales Oleaceae Fraxinus lanuginosa#MH817908
Lamiales Oleaceae Fraxinus xanthoxyloides#MH817912
Lamiales Oleaceae Fraxinus mandshurica#NC 041463
Lamiales Oleaceae Fraxinus angustifolia#MH817870
Lamiales Oleaceae Fraxinus excelsior#NC 037446
Lamiales Oleaceae Chionanthus ligustrinus#MH817886
Lamiales Oleaceae Priogymnanthus hasslerianus#MH817879
Lamiales Oleaceae Hesperelaea palmeri#NC 025787
Lamiales Oleaceae Forestiera isabeliae#NC 036981
Lamiales Oleaceae Forestiera segregata#XHIV
Lamiales Oleaceae Chionanthus mala-elengi#MH817888
Lamiales Oleaceae Chionanthus parkinsonii#NC 036979
Lamiales Oleaceae Noronhia peglerae#MH817921
Lamiales Oleaceae Noronhia clarinerva#MH817875
Lamiales Oleaceae Noronhia intermedia#MH817876
Lamiales Oleaceae Noronhia lowryi#MH817920
Lamiales Oleaceae Noronhia brevituba#MH817860

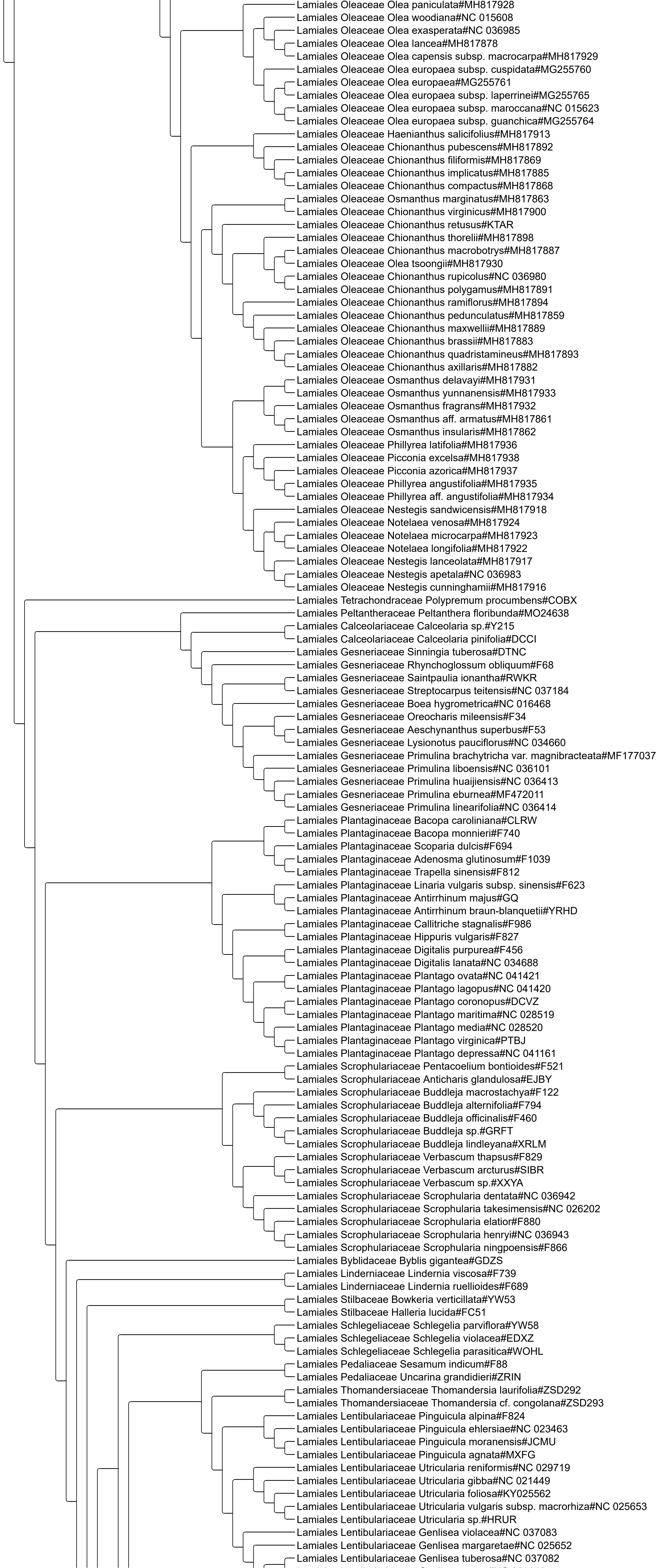

Lamiales Oleaceae Olea paniculata#MH817928
Lamiales Oleaceae Olea woodiana#NC 015608
Lamiales Oleaceae Olea exasperata#NC 036985
Lamiales Oleaceae Olea lancea#MH817878
Lamiales Oleaceae Olea capensis subsp. macrocarpa#MH817929
Lamiales Oleaceae Olea europaea subsp. cuspidata#MG255760
Lamiales Oleaceae Olea europaea#MG255761
Lamiales Oleaceae Olea europaea subsp. laperrinei#MG255765
Lamiales Oleaceae Olea europaea subsp. maroccana#NC 015623
Lamiales Oleaceae Olea europaea subsp. guanchica#MG255764
Lamiales Oleaceae Haenianthus salicifolius#MH817913
Lamiales Oleaceae Chionanthus pubescens#MH817892
Lamiales Oleaceae Chionanthus filiformis#MH817869
Lamiales Oleaceae Chionanthus implicatus#MH817885
Lamiales Oleaceae Chionanthus compactus#MH817868
Lamiales Oleaceae Osmanthus marginatus#MH817863
Lamiales Oleaceae Chionanthus virginicus#MH817900
Lamiales Oleaceae Chionanthus retusus#KTAR
Lamiales Oleaceae Chionanthus thorelii#MH817898
Lamiales Oleaceae Chionanthus macrobotrys#MH817887
Lamiales Oleaceae Olea tsoongii#MH817930
Lamiales Oleaceae Chionanthus rupicolus#NC 036980
Lamiales Oleaceae Chionanthus polygamus#MH817891
Lamiales Oleaceae Chionanthus ramiflorus#MH817894
Lamiales Oleaceae Chionanthus pedunculatus#MH817859
Lamiales Oleaceae Chionanthus maxwellii#MH817889
Lamiales Oleaceae Chionanthus brassii#MH817883
Lamiales Oleaceae Chionanthus quadristamineus#MH817893
Lamiales Oleaceae Chionanthus axillaris#MH817882
Lamiales Oleaceae Osmanthus delavayi#MH817931
Lamiales Oleaceae Osmanthus yunnanensis#MH817933
Lamiales Oleaceae Osmanthus fragrans#MH817932
Lamiales Oleaceae Osmanthus aff. armatus#MH817861
Lamiales Oleaceae Osmanthus insularis#MH817862
Lamiales Oleaceae Phillyrea latifolia#MH817936
Lamiales Oleaceae Picconia excelsa#MH817938
Lamiales Oleaceae Picconia azorica#MH817937
Lamiales Oleaceae Phillyrea angustifolia#MH817935
Lamiales Oleaceae Phillyrea aff. angustifolia#MH817934
Lamiales Oleaceae Nestegis sandwicensis#MH817918
Lamiales Oleaceae Notelaea venosa#MH817924
Lamiales Oleaceae Notelaea microcarpa#MH817923
Lamiales Oleaceae Notelaea longifolia#MH817922
Lamiales Oleaceae Nestegis lanceolata#MH817917
Lamiales Oleaceae Nestegis apetala#NC 036983
Lamiales Oleaceae Nestegis cunninghamii#MH817916
Lamiales Tetrachondraceae Polypremum procumbens#COBX
Lamiales Peltantheraceae Peltanthera floribunda#MO24638
Lamiales Calceolariaceae Calceolaria sp.#Y215
Lamiales Calceolariaceae Calceolaria pinifolia#DCCI
Lamiales Gesneriaceae Sinningia tuberosa#DTNC
Lamiales Gesneriaceae Rhynchoglossum obliquum#F68
Lamiales Gesneriaceae Saintpaulia ionantha#RWKR
Lamiales Gesneriaceae Streptocarpus teitensis#NC 037184
Lamiales Gesneriaceae Boea hygrometrica#NC 016468
Lamiales Gesneriaceae Oreocharis mileensis#F34
Lamiales Gesneriaceae Aeschynanthus superbus#F53
Lamiales Gesneriaceae Lysionotus pauciflorus#NC 034660
Lamiales Gesneriaceae Primulina brachytricha var. magnibracteata#MF177037
Lamiales Gesneriaceae Primulina liboensis#NC 036101
Lamiales Gesneriaceae Primulina huaijiensis#NC 036413
Lamiales Gesneriaceae Primulina eburnea#MF472011
Lamiales Gesneriaceae Primulina linearifolia#NC 036414
Lamiales Plantaginaceae Bacopa caroliniana#CLRW
Lamiales Plantaginaceae Bacopa monnieri#F740
Lamiales Plantaginaceae Scoparia dulcis#F694
Lamiales Plantaginaceae Adenosma glutinosum#F1039
Lamiales Plantaginaceae Trapella sinensis#F812
Lamiales Plantaginaceae Linaria vulgaris subsp. sinensis#F623
Lamiales Plantaginaceae Antirrhinum majus#GQ
Lamiales Plantaginaceae Antirrhinum braun-blanquetii#YRHD
Lamiales Plantaginaceae Callitriche stagnalis#F986
Lamiales Plantaginaceae Hippuris vulgaris#F827
Lamiales Plantaginaceae Digitalis purpurea#F456
Lamiales Plantaginaceae Digitalis lanata#NC 034688
Lamiales Plantaginaceae Plantago ovata#NC 041421
Lamiales Plantaginaceae Plantago lagopus#NC 041420
Lamiales Plantaginaceae Plantago coronopus#DCVZ
Lamiales Plantaginaceae Plantago maritima#NC 028519
Lamiales Plantaginaceae Plantago media#NC 028520
Lamiales Plantaginaceae Plantago virginica#PTBJ
Lamiales Plantaginaceae Plantago depressa#NC 041161
Lamiales Scrophulariaceae Pentacoelium bontioides#F521
Lamiales Scrophulariaceae Anticharis glandulosa#EJBY
Lamiales Scrophulariaceae Buddleja macrostachya#F122
Lamiales Scrophulariaceae Buddleja alternifolia#F794
Lamiales Scrophulariaceae Buddleja officinalis#F460
Lamiales Scrophulariaceae Buddleja sp.#GRFT
Lamiales Scrophulariaceae Buddleja lindleyana#XRLM
Lamiales Scrophulariaceae Verbascum thapsus#F829
Lamiales Scrophulariaceae Verbascum arcturus#SIBR
Lamiales Scrophulariaceae Verbascum sp.#XXYA
Lamiales Scrophulariaceae Scrophularia dentata#NC 036942
Lamiales Scrophulariaceae Scrophularia takesimensis#NC 026202
Lamiales Scrophulariaceae Scrophularia elatior#F880
Lamiales Scrophulariaceae Scrophularia henryi#NC 036943
Lamiales Scrophulariaceae Scrophularia ningpoensis#F866
Lamiales Byblidaceae Byblis gigantea#GDZS
Lamiales Linderniaceae Lindernia viscosa#F739
Lamiales Linderniaceae Lindernia ruellioides#F689
Lamiales Stilbaceae Bowkeria verticillata#YW53
Lamiales Stilbaceae Halleria lucida#FC51
Lamiales Schlegeliaceae Schlegelia parviflora#YW58
Lamiales Schlegeliaceae Schlegelia violacea#EDXZ
Lamiales Schlegeliaceae Schlegelia parasitica#WOHL
Lamiales Pedaliaceae Sesamum indicum#F88
Lamiales Pedaliaceae Uncarina grandidieri#ZRIN
Lamiales Thomandersiaceae Thomandersia laurifolia#ZSD292
Lamiales Thomandersiaceae Thomandersia cf. congolana#ZSD293
Lamiales Lentibulariaceae Pinguicula alpina#F824
Lamiales Lentibulariaceae Pinguicula ehlersiae#NC 023463
Lamiales Lentibulariaceae Pinguicula moranensis#JCMU
Lamiales Lentibulariaceae Pinguicula agnata#MXFG
Lamiales Lentibulariaceae Utricularia reniformis#NC 029719
Lamiales Lentibulariaceae Utricularia gibba#NC 021449
Lamiales Lentibulariaceae Utricularia foliosa#KY025562
Lamiales Lentibulariaceae Utricularia vulgaris subsp. macrorhiza#NC 025653
Lamiales Lentibulariaceae Utricularia sp.#HRUR
Lamiales Lentibulariaceae Genlisea violacea#NC 037083
Lamiales Lentibulariaceae Genlisea margaretae#NC 025652
Lamiales Lentibulariaceae Genlisea tuberosa#NC 037082

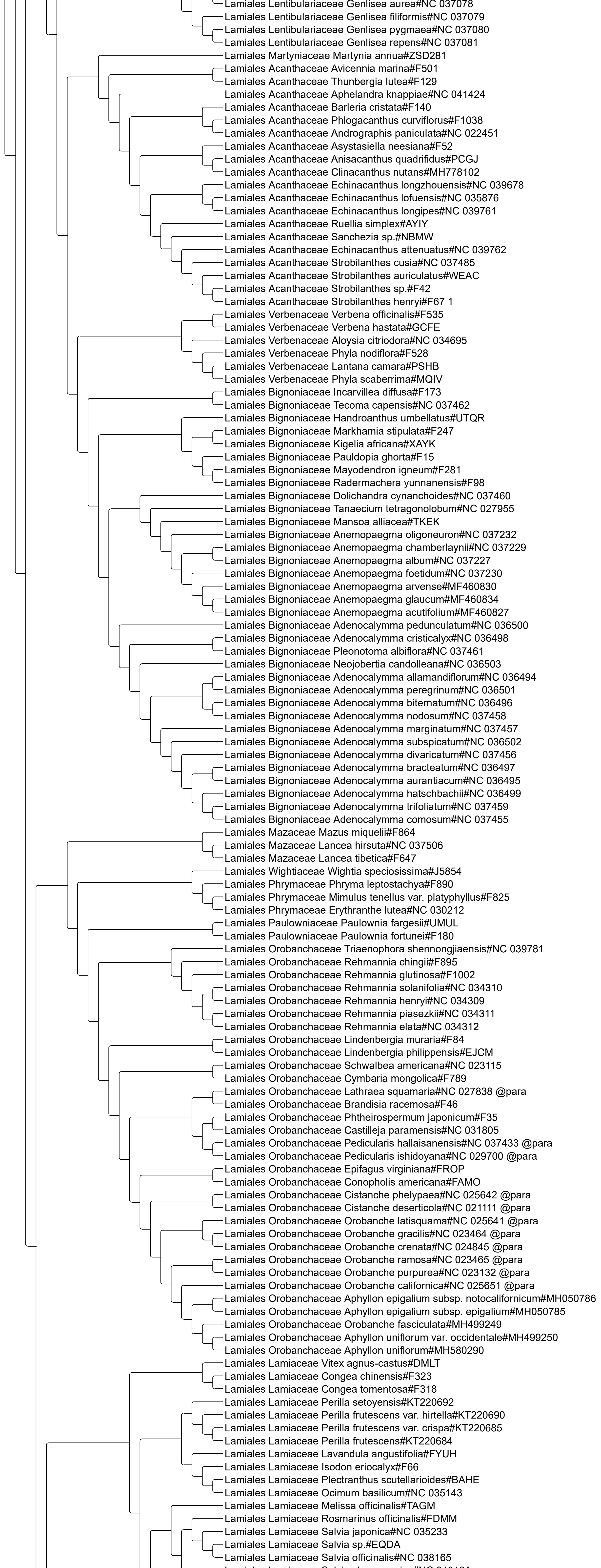

Lamiales Lentibulariaceae Genlisea aurea#NC 037078
Lamiales Lentibulariaceae Genlisea filiformis#NC 037079
Lamiales Lentibulariaceae Genlisea pygmaea#NC 037080
Lamiales Lentibulariaceae Genlisea repens#NC 037081
Lamiales Martyniaceae Martynia annua#ZSD281
Lamiales Acanthaceae Avicennia marina#F501
Lamiales Acanthaceae Thunbergia lutea#F129
Lamiales Acanthaceae Aphelandra knappiae#NC 041424
Lamiales Acanthaceae Barleria cristata#F140
Lamiales Acanthaceae Phlogacanthus curviflorus#F1038
Lamiales Acanthaceae Andrographis paniculata#NC 022451
Lamiales Acanthaceae Asystasiella neesiana#F52
Lamiales Acanthaceae Anisacanthus quadrifidus#PCGJ
Lamiales Acanthaceae Clinacanthus nutans#MH778102
Lamiales Acanthaceae Echinacanthus longzhouensis#NC 039678
Lamiales Acanthaceae Echinacanthus lofuensis#NC 035876
Lamiales Acanthaceae Echinacanthus longipes#NC 039761
Lamiales Acanthaceae Ruellia simplex#AYIY
Lamiales Acanthaceae Sanchezia sp.#NBMW
Lamiales Acanthaceae Echinacanthus attenuatus#NC 039762
Lamiales Acanthaceae Strobilanthes cusia#NC 037485
Lamiales Acanthaceae Strobilanthes auriculatus#WEAC
Lamiales Acanthaceae Strobilanthes sp.#F42
Lamiales Acanthaceae Strobilanthes henryi#F67 1
Lamiales Verbenaceae Verbena officinalis#F535
Lamiales Verbenaceae Verbena hastata#GCFE
Lamiales Verbenaceae Aloysia citriodora#NC 034695
Lamiales Verbenaceae Phyla nodiflora#F528
Lamiales Verbenaceae Lantana camara#PSHB
Lamiales Verbenaceae Phyla scaberrima#MQIV
Lamiales Bignoniaceae Incarvillea diffusa#F173
Lamiales Bignoniaceae Tecoma capensis#NC 037462
Lamiales Bignoniaceae Handroanthus umbellatus#UTQR
Lamiales Bignoniaceae Markhamia stipulata#F247
Lamiales Bignoniaceae Kigelia africana#XAYK
Lamiales Bignoniaceae Pauldopia ghorta#F15
Lamiales Bignoniaceae Mayodendron igneum#F281
Lamiales Bignoniaceae Radermachera yunnanensis#F98
Lamiales Bignoniaceae Dolichandra cynanchoides#NC 037460
Lamiales Bignoniaceae Tanaecium tetragonolobum#NC 027955
Lamiales Bignoniaceae Mansoa alliacea#TKEK
Lamiales Bignoniaceae Anemopaegma oligoneuron#NC 037232
Lamiales Bignoniaceae Anemopaegma chamberlaynii#NC 037229
Lamiales Bignoniaceae Anemopaegma album#NC 037227
Lamiales Bignoniaceae Anemopaegma foetidum#NC 037230
Lamiales Bignoniaceae Anemopaegma arvense#MF460830
Lamiales Bignoniaceae Anemopaegma glaucum#MF460834
Lamiales Bignoniaceae Anemopaegma acutifolium#MF460827
Lamiales Bignoniaceae Adenocalymma pedunculatum#NC 036500
Lamiales Bignoniaceae Adenocalymma cristicalyx#NC 036498
Lamiales Bignoniaceae Pleonotoma albiflora#NC 037461
Lamiales Bignoniaceae Neojobertia candolleana#NC 036503
Lamiales Bignoniaceae Adenocalymma allamandiflorum#NC 036494
Lamiales Bignoniaceae Adenocalymma peregrinum#NC 036501
Lamiales Bignoniaceae Adenocalymma biternatum#NC 036496
Lamiales Bignoniaceae Adenocalymma nodosum#NC 037458
Lamiales Bignoniaceae Adenocalymma marginatum#NC 037457
Lamiales Bignoniaceae Adenocalymma subspicatum#NC 036502
Lamiales Bignoniaceae Adenocalymma divaricatum#NC 037456
Lamiales Bignoniaceae Adenocalymma bracteatum#NC 036497
Lamiales Bignoniaceae Adenocalymma aurantiacum#NC 036495
Lamiales Bignoniaceae Adenocalymma hatschbachii#NC 036499
Lamiales Bignoniaceae Adenocalymma trifoliatum#NC 037459
Lamiales Bignoniaceae Adenocalymma comosum#NC 037455
Lamiales Mazaceae Mazus miquelii#F864
Lamiales Mazaceae Lancea hirsuta#NC 037506
Lamiales Mazaceae Lancea tibetica#F647
Lamiales Wightiaceae Wightia speciosissima#J5854
Lamiales Phrymaceae Phryma leptostachya#F890
Lamiales Phrymaceae Mimulus tenellus var. platyphyllus#F825
Lamiales Phrymaceae Erythranthe lutea#NC 030212
Lamiales Paulowniaceae Paulownia fargesii#UMUL
Lamiales Paulowniaceae Paulownia fortunei#F180
Lamiales Orobanchaceae Triaenophora shennongjiaensis#NC 039781
Lamiales Orobanchaceae Rehmannia chingii#F895
Lamiales Orobanchaceae Rehmannia glutinosa#F1002
Lamiales Orobanchaceae Rehmannia solanifolia#NC 034310
Lamiales Orobanchaceae Rehmannia henryi#NC 034309
Lamiales Orobanchaceae Rehmannia piasezkii#NC 034311
Lamiales Orobanchaceae Rehmannia elata#NC 034312
Lamiales Orobanchaceae Lindenbergia muraria#F84
Lamiales Orobanchaceae Lindenbergia philippensis#EJCM
Lamiales Orobanchaceae Schwalbea americana#NC 023115
Lamiales Orobanchaceae Cymbaria mongolica#F789
Lamiales Orobanchaceae Lathraea squamaria#NC 027838 @para
Lamiales Orobanchaceae Brandisia racemosa#F46
Lamiales Orobanchaceae Phtheirospermum japonicum#F35
Lamiales Orobanchaceae Castilleja paramensis#NC 031805
Lamiales Orobanchaceae Pedicularis hallaisanensis#NC 037433 @para
Lamiales Orobanchaceae Pedicularis ishidoyana#NC 029700 @para
Lamiales Orobanchaceae Epifagus virginiana#FROP
Lamiales Orobanchaceae Conopholis americana#FAMO
Lamiales Orobanchaceae Cistanche phelypaea#NC 025642 @para
Lamiales Orobanchaceae Cistanche deserticola#NC 021111 @para
Lamiales Orobanchaceae Orobanche latisquama#NC 025641 @para
Lamiales Orobanchaceae Orobanche gracilis#NC 023464 @para
Lamiales Orobanchaceae Orobanche crenata#NC 024845 @para
Lamiales Orobanchaceae Orobanche ramosa#NC 023465 @para
Lamiales Orobanchaceae Orobanche purpurea#NC 023132 @para
Lamiales Orobanchaceae Orobanche californica#NC 025651 @para
Lamiales Orobanchaceae Aphyllon epigalium subsp. notocalifornicum#MH050786
Lamiales Orobanchaceae Aphyllon epigalium subsp. epigalium#MH050785
Lamiales Orobanchaceae Orobanche fasciculata#MH499249
Lamiales Orobanchaceae Aphyllon uniflorum var. occidentale#MH499250
Lamiales Orobanchaceae Aphyllon uniflorum#MH580290
Lamiales Lamiaceae Vitex agnus-castus#DMLT
Lamiales Lamiaceae Congea chinensis#F323
Lamiales Lamiaceae Congea tomentosa#F318
Lamiales Lamiaceae Perilla setoyensis#KT220692
Lamiales Lamiaceae Perilla frutescens var. hirtella#KT220690
Lamiales Lamiaceae Perilla frutescens var. crispa#KT220685
Lamiales Lamiaceae Perilla frutescens#KT220684
Lamiales Lamiaceae Lavandula angustifolia#FYUH
Lamiales Lamiaceae Isodon eriocalyx#F66
Lamiales Lamiaceae Plectranthus scutellarioides#BAHE
Lamiales Lamiaceae Ocimum basilicum#NC 035143
Lamiales Lamiaceae Melissa officinalis#TAGM
Lamiales Lamiaceae Rosmarinus officinalis#FDMM
Lamiales Lamiaceae Salvia japonica#NC 035233
Lamiales Lamiaceae Salvia sp.#EQDA
Lamiales Lamiaceae Salvia officinalis#NC 038165

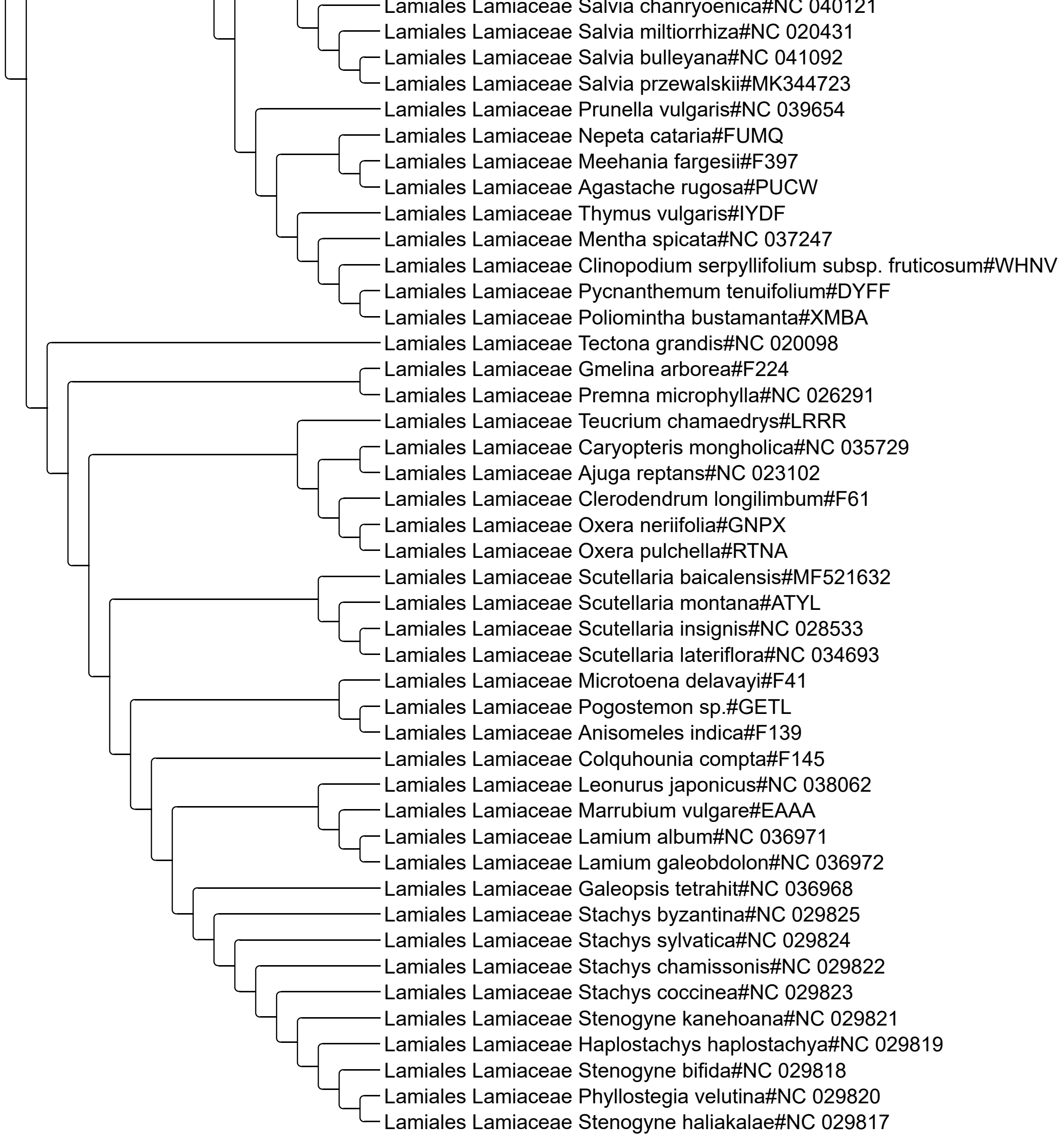
Lamiales Lamiaceae Salvia chanryoenica#NC 040121
Lamiales Lamiaceae Salvia miltiorrhiza#NC 020431
Lamiales Lamiaceae Salvia bulleyana#NC 041092
Lamiales Lamiaceae Salvia przewalskii#MK344723
Lamiales Lamiaceae Prunella vulgaris#NC 039654
Lamiales Lamiaceae Nepeta cataria#FUMQ
Lamiales Lamiaceae Meehania fargesii#F397
Lamiales Lamiaceae Agastache rugosa#PUCW
Lamiales Lamiaceae Thymus vulgaris#IYDF
Lamiales Lamiaceae Mentha spicata#NC 037247
Lamiales Lamiaceae Clinopodium serpyllifolium subsp. fruticosum#WHNV
Lamiales Lamiaceae Pycnanthemum tenuifolium#DYFF
Lamiales Lamiaceae Poliomintha bustamanta#XMBA
Lamiales Lamiaceae Tectona grandis#NC 020098
Lamiales Lamiaceae Gmelina arborea#F224
Lamiales Lamiaceae Premna microphylla#NC 026291
Lamiales Lamiaceae Teucrium chamaedrys#LRRR
Lamiales Lamiaceae Caryopteris mongholica#NC 035729
Lamiales Lamiaceae Ajuga reptans#NC 023102
Lamiales Lamiaceae Clerodendrum longilimbum#F61
Lamiales Lamiaceae Oxera neriifolia#GNPX
Lamiales Lamiaceae Oxera pulchella#RTNA
Lamiales Lamiaceae Scutellaria baicalensis#MF521632
Lamiales Lamiaceae Scutellaria montana#ATYL
Lamiales Lamiaceae Scutellaria insignis#NC 028533
Lamiales Lamiaceae Scutellaria lateriflora#NC 034693
Lamiales Lamiaceae Microtoena delavayi#F41
Lamiales Lamiaceae Pogostemon sp.#GETL
Lamiales Lamiaceae Anisomeles indica#F139
Lamiales Lamiaceae Colquhounia compta#F145
Lamiales Lamiaceae Leonurus japonicus#NC 038062
Lamiales Lamiaceae Marrubium vulgare#EAAA
Lamiales Lamiaceae Lamium album#NC 036971
Lamiales Lamiaceae Lamium galeobdolon#NC 036972
Lamiales Lamiaceae Galeopsis tetrahit#NC 036968
Lamiales Lamiaceae Stachys byzantina#NC 029825
Lamiales Lamiaceae Stachys sylvatica#NC 029824
Lamiales Lamiaceae Stachys chamissonis#NC 029822
Lamiales Lamiaceae Stachys coccinea#NC 029823
Lamiales Lamiaceae Stenogyne kanehoana#NC 029821
Lamiales Lamiaceae Haplostachys haplostachya#NC 029819
Lamiales Lamiaceae Stenogyne bifida#NC 029818
Lamiales Lamiaceae Phyllostegia velutina#NC 029820
Lamiales Lamiaceae Stenogyne haliakalae#NC 029817

[Figure S8i]

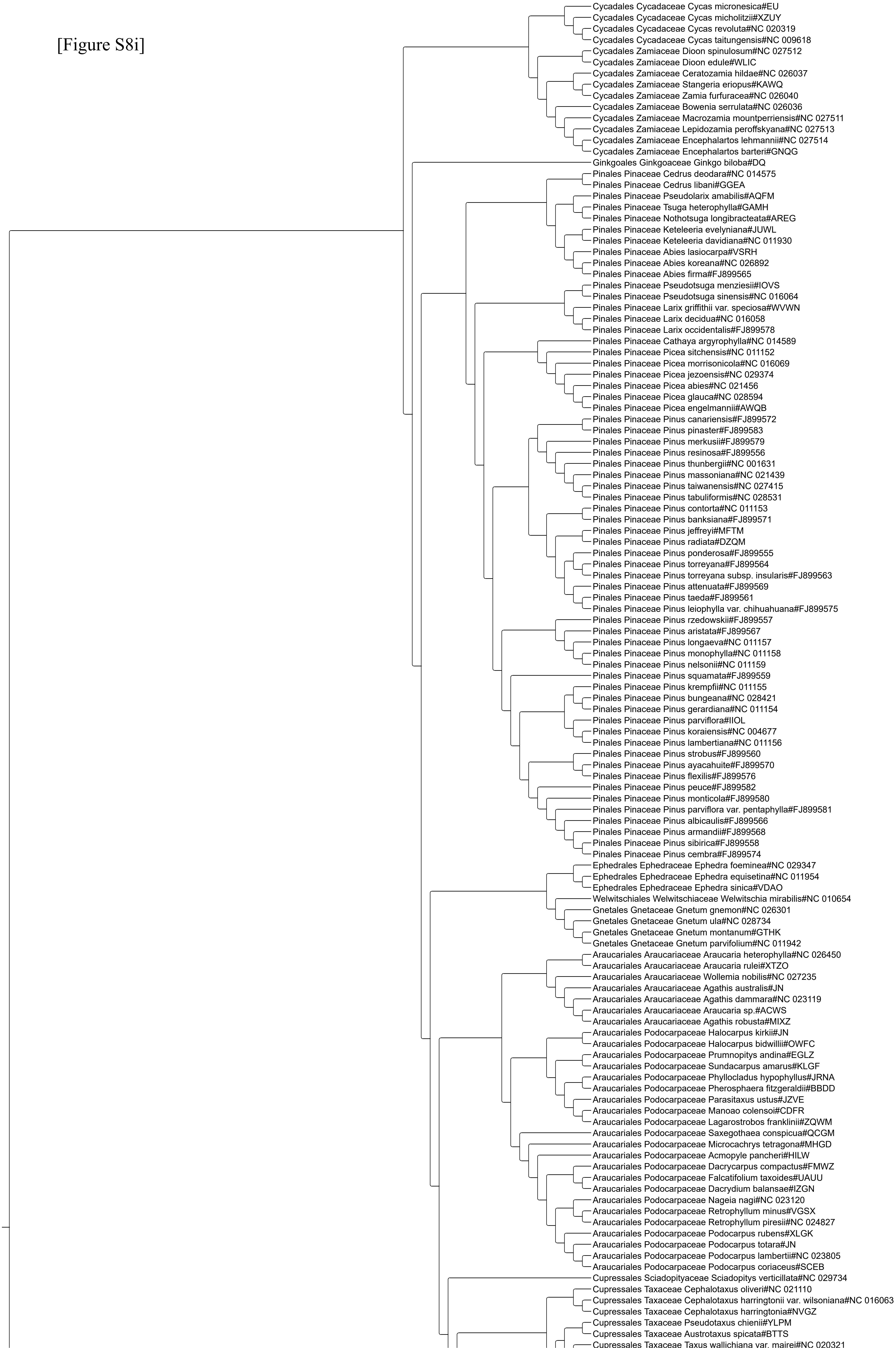
Cycadales Cycadaceae Cycas micronesica#EU
Cycadales Cycadaceae Cycas micholitzii#XZUY
Cycadales Cycadaceae Cycas revoluta#NC 020319
Cycadales Cycadaceae Cycas taitungensis#NC 009618
Cycadales Zamiaceae Dioon spinulosum#NC 027512
Cycadales Zamiaceae Dioon edule#WLIC
Cycadales Zamiaceae Ceratozamia hildae#NC 026037
Cycadales Zamiaceae Stangeria eriopus#KAWQ
Cycadales Zamiaceae Zamia furfuracea#NC 026040
Cycadales Zamiaceae Bowenia serrulata#NC 026036
Cycadales Zamiaceae Macrozamia mountperriensis#NC 027511
Cycadales Zamiaceae Lepidozamia peroffskyana#NC 027513
Cycadales Zamiaceae Encephalartos lehmannii#NC 027514
Cycadales Zamiaceae Encephalartos barteri#GNQG
Ginkgoales Ginkgoaceae Ginkgo biloba#DQ
Pinales Pinaceae Cedrus deodara#NC 014575
Pinales Pinaceae Cedrus libani#GGEA
Pinales Pinaceae Pseudolarix amabilis#AQFM
Pinales Pinaceae Tsuga heterophylla#GAMH
Pinales Pinaceae Nothotsuga longibracteata#AREG
Pinales Pinaceae Keteleeria evelyniana#JUWL
Pinales Pinaceae Keteleeria davidiana#NC 011930
Pinales Pinaceae Abies lasiocarpa#VSRH
Pinales Pinaceae Abies koreana#NC 026892
Pinales Pinaceae Abies firma#FJ899565
Pinales Pinaceae Pseudotsuga menziesii#IOVS
Pinales Pinaceae Pseudotsuga sinensis#NC 016064
Pinales Pinaceae Larix griffithii var. speciosa#WVWN
Pinales Pinaceae Larix decidua#NC 016058
Pinales Pinaceae Larix occidentalis#FJ899578
Pinales Pinaceae Cathaya argyrophylla#NC 014589
Pinales Pinaceae Picea sitchensis#NC 011152
Pinales Pinaceae Picea morrisonicola#NC 016069
Pinales Pinaceae Picea jezoensis#NC 029374
Pinales Pinaceae Picea abies#NC 021456
Pinales Pinaceae Picea glauca#NC 028594
Pinales Pinaceae Picea engelmannii#AWQB
Pinales Pinaceae Pinus canariensis#FJ899572
Pinales Pinaceae Pinus pinaster#FJ899583
Pinales Pinaceae Pinus merkusii#FJ899579
Pinales Pinaceae Pinus resinosa#FJ899556
Pinales Pinaceae Pinus thunbergii#NC 001631
Pinales Pinaceae Pinus massoniana#NC 021439
Pinales Pinaceae Pinus taiwanensis#NC 027415
Pinales Pinaceae Pinus tabuliformis#NC 028531
Pinales Pinaceae Pinus contorta#NC 011153
Pinales Pinaceae Pinus banksiana#FJ899571
Pinales Pinaceae Pinus jeffreyi#MFTM
Pinales Pinaceae Pinus radiata#DZQM
Pinales Pinaceae Pinus ponderosa#FJ899555
Pinales Pinaceae Pinus torreyana#FJ899564
Pinales Pinaceae Pinus torreyana subsp. insularis#FJ899563
Pinales Pinaceae Pinus attenuata#FJ899569
Pinales Pinaceae Pinus taeda#FJ899561
Pinales Pinaceae Pinus leiophylla var. chihuahuana#FJ899575
Pinales Pinaceae Pinus rzedowskii#FJ899557
Pinales Pinaceae Pinus aristata#FJ899567
Pinales Pinaceae Pinus longaeva#NC 011157
Pinales Pinaceae Pinus monophylla#NC 011158
Pinales Pinaceae Pinus nelsonii#NC 011159
Pinales Pinaceae Pinus squamata#FJ899559
Pinales Pinaceae Pinus krempfii#NC 011155
Pinales Pinaceae Pinus bungeana#NC 028421
Pinales Pinaceae Pinus gerardiana#NC 011154
Pinales Pinaceae Pinus parviflora#IIOL
Pinales Pinaceae Pinus koraiensis#NC 004677
Pinales Pinaceae Pinus lambertiana#NC 011156
Pinales Pinaceae Pinus strobus#FJ899560
Pinales Pinaceae Pinus ayacahuite#FJ899570
Pinales Pinaceae Pinus flexilis#FJ899576
Pinales Pinaceae Pinus peuce#FJ899582
Pinales Pinaceae Pinus monticola#FJ899580
Pinales Pinaceae Pinus parviflora var. pentaphylla#FJ899581
Pinales Pinaceae Pinus albicaulis#FJ899566
Pinales Pinaceae Pinus armandii#FJ899568
Pinales Pinaceae Pinus sibirica#FJ899558
Pinales Pinaceae Pinus cembra#FJ899574
Ephedrales Ephedraceae Ephedra foeminea#NC 029347
Ephedrales Ephedraceae Ephedra equisetina#NC 011954
Ephedrales Ephedraceae Ephedra sinica#VDAO
Welwitschiales Welwitschiaceae Welwitschia mirabilis#NC 010654
Gnetales Gnetaceae Gnetum gnemon#NC 026301
Gnetales Gnetaceae Gnetum ula#NC 028734
Gnetales Gnetaceae Gnetum montanum#GTHK
Gnetales Gnetaceae Gnetum parvifolium#NC 011942
Araucariales Araucariaceae Araucaria heterophylla#NC 026450
Araucariales Araucariaceae Araucaria rulei#XTZO
Araucariales Araucariaceae Wollemia nobilis#NC 027235
Araucariales Araucariaceae Agathis australis#JN
Araucariales Araucariaceae Agathis dammara#NC 023119
Araucariales Araucariaceae Araucaria sp.#ACWS
Araucariales Araucariaceae Agathis robusta#MIXZ
Araucariales Podocarpaceae Halocarpus kirkii#JN
Araucariales Podocarpaceae Halocarpus bidwillii#OWFC
Araucariales Podocarpaceae Prumnopitys andina#EGLZ
Araucariales Podocarpaceae Sundacarpus amarus#KLGF
Araucariales Podocarpaceae Phyllocladus hypophyllus#JRNA
Araucariales Podocarpaceae Pherosphaera fitzgeraldii#BBDD
Araucariales Podocarpaceae Parasitaxus ustus#JZVE
Araucariales Podocarpaceae Manoao colensoi#CDFR
Araucariales Podocarpaceae Lagarostrobos franklinii#ZQWM
Araucariales Podocarpaceae Saxegothaea conspicua#QCGM
Araucariales Podocarpaceae Microcachrys tetragona#MHGD
Araucariales Podocarpaceae Acmopyle pancheri#HILW
Araucariales Podocarpaceae Dacrycarpus compactus#FMWZ
Araucariales Podocarpaceae Falcatifolium taxoides#UAUU
Araucariales Podocarpaceae Dacrydium balansae#IZGN
Araucariales Podocarpaceae Nageia nagi#NC 023120
Araucariales Podocarpaceae Retrophyllum minus#VGSX
Araucariales Podocarpaceae Retrophyllum piresii#NC 024827
Araucariales Podocarpaceae Podocarpus rubens#XLGK
Araucariales Podocarpaceae Podocarpus totara#JN
Araucariales Podocarpaceae Podocarpus lambertii#NC 023805
Araucariales Podocarpaceae Podocarpus coriaceus#SCEB
Cupressales Sciadopityaceae Sciadopitys verticillata#NC 029734
Cupressales Taxaceae Cephalotaxus oliveri#NC 021110
Cupressales Taxaceae Cephalotaxus harringtonii var. wilsoniana#NC 016063
Cupressales Taxaceae Cephalotaxus harringtonia#NVGZ
Cupressales Taxaceae Pseudotaxus chienii#YLPM
Cupressales Taxaceae Austrotaxus spicata#BTTS
Cupressales Taxaceae Taxus wallichiana var. mairei#NC 020321

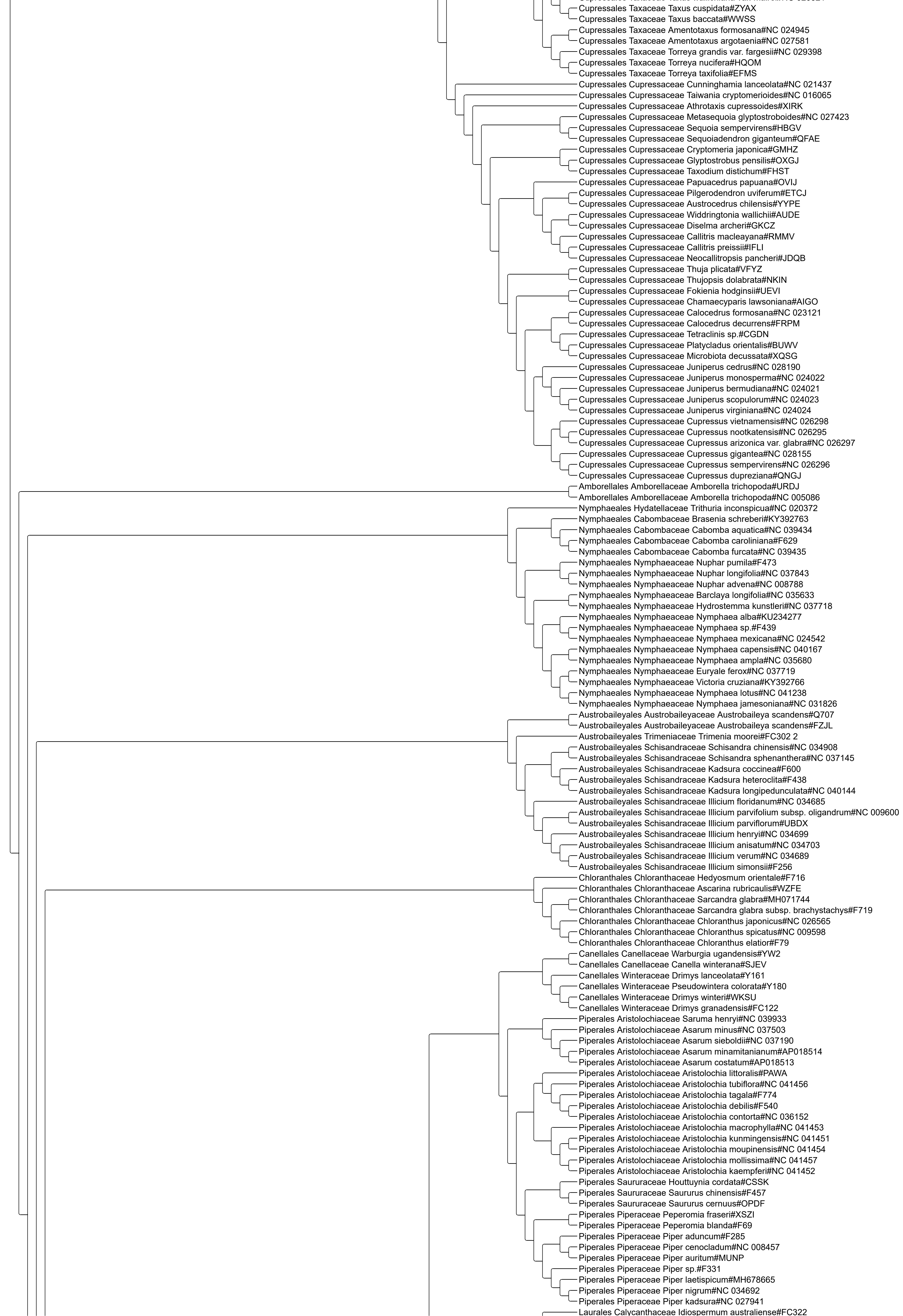

Cupressales Taxaceae Taxus cuspidata#ZYAX
Cupressales Taxaceae Taxus baccata#WWSS
Cupressales Taxaceae Amentotaxus formosana#NC 024945
Cupressales Taxaceae Amentotaxus argotaenia#NC 027581
Cupressales Taxaceae Torreya grandis var. fargesii#NC 029398
Cupressales Taxaceae Torreya nucifera#HQOM
Cupressales Taxaceae Torreya taxifolia#EFMS
Cupressales Cupressaceae Cunninghamia lanceolata#NC 021437
Cupressales Cupressaceae Taiwania cryptomerioides#NC 016065
Cupressales Cupressaceae Athrotaxis cupressoides#XIRK
Cupressales Cupressaceae Metasequoia glyptostroboides#NC 027423
Cupressales Cupressaceae Sequoia sempervirens#HBGV
Cupressales Cupressaceae Sequoiadendron giganteum#QFAE
Cupressales Cupressaceae Cryptomeria japonica#GMHZ
Cupressales Cupressaceae Glyptostrobus pensilis#OXGJ
Cupressales Cupressaceae Taxodium distichum#FHST
Cupressales Cupressaceae Papuacedrus papuana#OVIJ
Cupressales Cupressaceae Pilgerodendron uviferum#ETCJ
Cupressales Cupressaceae Austrocedrus chilensis#YYPE
Cupressales Cupressaceae Widdringtonia wallichii#AUDE
Cupressales Cupressaceae Diselma archeri#GKCZ
Cupressales Cupressaceae Callitris macleayana#RMMV
Cupressales Cupressaceae Callitris preissii#IFLI
Cupressales Cupressaceae Neocallitropsis pancheri#JDQB
Cupressales Cupressaceae Thuja plicata#VFYZ
Cupressales Cupressaceae Thujopsis dolabrata#NKIN
Cupressales Cupressaceae Fokienia hodginsii#UEVI
Cupressales Cupressaceae Chamaecyparis lawsoniana#AIGO
Cupressales Cupressaceae Calocedrus formosana#NC 023121
Cupressales Cupressaceae Calocedrus decurrens#FRPM
Cupressales Cupressaceae Tetraclinis sp.#CGDN
Cupressales Cupressaceae Platycladus orientalis#BUWV
Cupressales Cupressaceae Microbiota decussata#XQSG
Cupressales Cupressaceae Juniperus cedrus#NC 028190
Cupressales Cupressaceae Juniperus monosperma#NC 024022
Cupressales Cupressaceae Juniperus bermudiana#NC 024021
Cupressales Cupressaceae Juniperus scopulorum#NC 024023
Cupressales Cupressaceae Juniperus virginiana#NC 024024
Cupressales Cupressaceae Cupressus vietnamensis#NC 026298
Cupressales Cupressaceae Cupressus nootkatensis#NC 026295
Cupressales Cupressaceae Cupressus arizonica var. glabra#NC 026297
Cupressales Cupressaceae Cupressus gigantea#NC 028155
Cupressales Cupressaceae Cupressus sempervirens#NC 026296
Cupressales Cupressaceae Cupressus dupreziana#QNGJ
Amborellales Amborellaceae Amborella trichopoda#URDJ
Amborellales Amborellaceae Amborella trichopoda#NC 005086
Nymphaeales Hydatellaceae Trithuria inconspicua#NC 020372
Nymphaeales Cabombaceae Brasenia schreberi#KY392763
Nymphaeales Cabombaceae Cabomba aquatica#NC 039434
Nymphaeales Cabombaceae Cabomba caroliniana#F629
Nymphaeales Cabombaceae Cabomba furcata#NC 039435
Nymphaeales Nymphaeaceae Nuphar pumila#F473
Nymphaeales Nymphaeaceae Nuphar longifolia#NC 037843
Nymphaeales Nymphaeaceae Nuphar advena#NC 008788
Nymphaeales Nymphaeaceae Barclaya longifolia#NC 035633
Nymphaeales Nymphaeaceae Hydrostemma kunstleri#NC 037718
Nymphaeales Nymphaeaceae Nymphaea alba#KU234277
Nymphaeales Nymphaeaceae Nymphaea sp.#F439
Nymphaeales Nymphaeaceae Nymphaea mexicana#NC 024542
Nymphaeales Nymphaeaceae Nymphaea capensis#NC 040167
Nymphaeales Nymphaeaceae Nymphaea ampla#NC 035680
Nymphaeales Nymphaeaceae Euryale ferox#NC 037719
Nymphaeales Nymphaeaceae Victoria cruziana#KY392766
Nymphaeales Nymphaeaceae Nymphaea lotus#NC 041238
Nymphaeales Nymphaeaceae Nymphaea jamesoniana#NC 031826
Austrobaileyales Austrobaileyaceae Austrobaileya scandens#Q707
Austrobaileyales Austrobaileyaceae Austrobaileya scandens#FZJL
Austrobaileyales Trimeniaceae Trimenia moorei#FC302 2
Austrobaileyales Schisandraceae Schisandra chinensis#NC 034908
Austrobaileyales Schisandraceae Schisandra sphenanthera#NC 037145
Austrobaileyales Schisandraceae Kadsura coccinea#F600
Austrobaileyales Schisandraceae Kadsura heteroclita#F438
Austrobaileyales Schisandraceae Kadsura longipedunculata#NC 040144
Austrobaileyales Schisandraceae Illicium floridanum#NC 034685
Austrobaileyales Schisandraceae Illicium parvifolium subsp. oligandrum#NC 009600
Austrobaileyales Schisandraceae Illicium parviflorum#UBDX
Austrobaileyales Schisandraceae Illicium henryi#NC 034699
Austrobaileyales Schisandraceae Illicium anisatum#NC 034703
Austrobaileyales Schisandraceae Illicium verum#NC 034689
Austrobaileyales Schisandraceae Illicium simonsii#F256
Chloranthales Chloranthaceae Hedyosmum orientale#F716
Chloranthales Chloranthaceae Ascarina rubricaulis#WZFE
Chloranthales Chloranthaceae Sarcandra glabra#MH071744
Chloranthales Chloranthaceae Sarcandra glabra subsp. brachystachys#F719
Chloranthales Chloranthaceae Chloranthus japonicus#NC 026565
Chloranthales Chloranthaceae Chloranthus spicatus#NC 009598
Chloranthales Chloranthaceae Chloranthus elatior#F79
Canellales Canellaceae Warburgia ugandensis#YW2
Canellales Canellaceae Canella winterana#SJEV
Canellales Winteraceae Drimys lanceolata#Y161
Canellales Winteraceae Pseudowintera colorata#Y180
Canellales Winteraceae Drimys winteri#WKSU
Canellales Winteraceae Drimys granadensis#FC122
Piperales Aristolochiaceae Saruma henryi#NC 039933
Piperales Aristolochiaceae Asarum minus#NC 037503
Piperales Aristolochiaceae Asarum sieboldii#NC 037190
Piperales Aristolochiaceae Asarum minamitanianum#AP018514
Piperales Aristolochiaceae Asarum costatum#AP018513
Piperales Aristolochiaceae Aristolochia littoralis#PAWA
Piperales Aristolochiaceae Aristolochia tubiflora#NC 041456
Piperales Aristolochiaceae Aristolochia tagala#F774
Piperales Aristolochiaceae Aristolochia debilis#F540
Piperales Aristolochiaceae Aristolochia contorta#NC 036152
Piperales Aristolochiaceae Aristolochia macrophylla#NC 041453
Piperales Aristolochiaceae Aristolochia kunmingensis#NC 041451
Piperales Aristolochiaceae Aristolochia moupinensis#NC 041454
Piperales Aristolochiaceae Aristolochia mollissima#NC 041457
Piperales Aristolochiaceae Aristolochia kaempferi#NC 041452
Piperales Saururaceae Houttuynia cordata#CSSK
Piperales Saururaceae Saururus chinensis#F457
Piperales Saururaceae Saururus cernuus#OPDF
Piperales Piperaceae Peperomia fraseri#XSZI
Piperales Piperaceae Peperomia blanda#F69
Piperales Piperaceae Piper aduncum#F285
Piperales Piperaceae Piper cenocladum#NC 008457
Piperales Piperaceae Piper auritum#MUNP
Piperales Piperaceae Piper sp.#F331
Piperales Piperaceae Piper laetispicum#MH678665
Piperales Piperaceae Piper nigrum#NC 034692
Piperales Piperaceae Piper kadsura#NC 027941
Laurales Calycanthaceae Idiospermum australiense#FC322

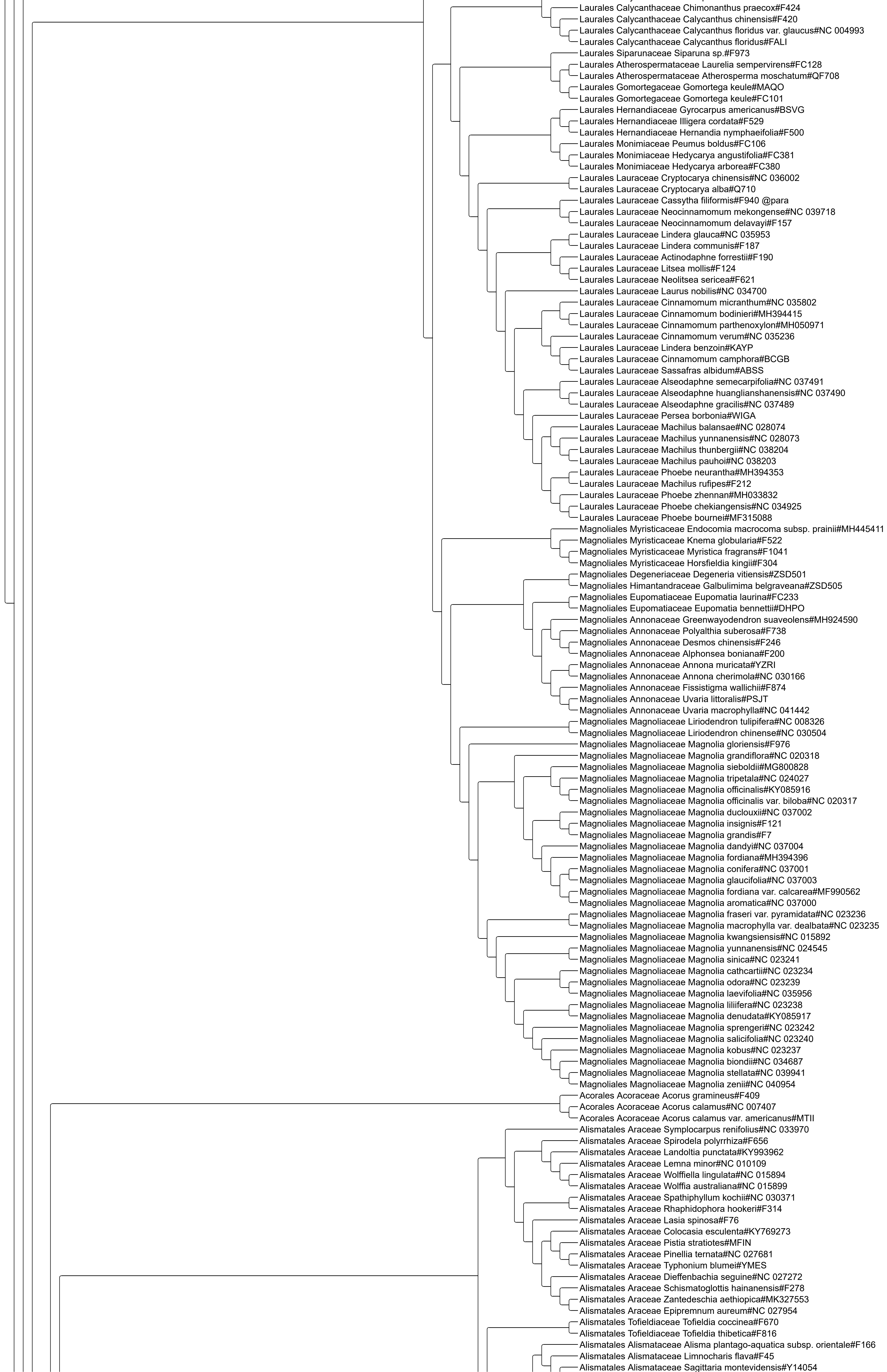

Laurales Calycanthaceae Chimonanthus praecox#F424
Laurales Calycanthaceae Calycanthus chinensis#F420
Laurales Calycanthaceae Calycanthus floridus var. glaucus#NC 004993
Laurales Calycanthaceae Calycanthus floridus#FALI
Laurales Siparunaceae Siparuna sp.#F973
Laurales Atherospermataceae Laurelia sempervirens#FC128
Laurales Atherospermataceae Atherosperma moschatum#QF708
Laurales Gomortegaceae Gomortega keule#MAQO
Laurales Gomortegaceae Gomortega keule#FC101
Laurales Hernandiaceae Gyrocarpus americanus#BSVG
Laurales Hernandiaceae Illigera cordata#F529
Laurales Hernandiaceae Hernandia nymphaeifolia#F500
Laurales Monimiaceae Peumus boldus#FC106
Laurales Monimiaceae Hedycarya angustifolia#FC381
Laurales Monimiaceae Hedycarya arborea#FC380
Laurales Lauraceae Cryptocarya chinensis#NC 036002
Laurales Lauraceae Cryptocarya alba#Q710
Laurales Lauraceae Cassytha filiformis#F940 @para
Laurales Lauraceae Neocinnamomum mekongense#NC 039718
Laurales Lauraceae Neocinnamomum delavayi#F157
Laurales Lauraceae Lindera glauca#NC 035953
Laurales Lauraceae Lindera communis#F187
Laurales Lauraceae Actinodaphne forrestii#F190
Laurales Lauraceae Litsea mollis#F124
Laurales Lauraceae Neolitsea sericea#F621
Laurales Lauraceae Laurus nobilis#NC 034700
Laurales Lauraceae Cinnamomum micranthum#NC 035802
Laurales Lauraceae Cinnamomum bodinieri#MH394415
Laurales Lauraceae Cinnamomum parthenoxylon#MH050971
Laurales Lauraceae Cinnamomum verum#NC 035236
Laurales Lauraceae Lindera benzoin#KAYP
Laurales Lauraceae Cinnamomum camphora#BCGB
Laurales Lauraceae Sassafras albidum#ABSS
Laurales Lauraceae Alseodaphne semecarpifolia#NC 037491
Laurales Lauraceae Alseodaphne huanglianshanensis#NC 037490
Laurales Lauraceae Alseodaphne gracilis#NC 037489
Laurales Lauraceae Persea borbonia#WIGA
Laurales Lauraceae Machilus balansae#NC 028074
Laurales Lauraceae Machilus yunnanensis#NC 028073
Laurales Lauraceae Machilus thunbergii#NC 038204
Laurales Lauraceae Machilus pauhoi#NC 038203
Laurales Lauraceae Phoebe neurantha#MH394353
Laurales Lauraceae Machilus rufipes#F212
Laurales Lauraceae Phoebe zhennan#MH033832
Laurales Lauraceae Phoebe chekiangensis#NC 034925
Laurales Lauraceae Phoebe bournei#MF315088
Magnoliales Myristicaceae Endocomia macrocoma subsp. prainii#MH445411
Magnoliales Myristicaceae Knema globularia#F522
Magnoliales Myristicaceae Myristica fragrans#F1041
Magnoliales Myristicaceae Horsfieldia kingii#F304
Magnoliales Degeneriaceae Degeneria vitiensis#ZSD501
Magnoliales Himantandraceae Galbulimima belgraveana#ZSD505
Magnoliales Eupomatiaceae Eupomatia laurina#FC233
Magnoliales Eupomatiaceae Eupomatia bennettii#DHPO
Magnoliales Annonaceae Greenwayodendron suaveolens#MH924590
Magnoliales Annonaceae Polyalthia suberosa#F738
Magnoliales Annonaceae Desmos chinensis#F246
Magnoliales Annonaceae Alphonsea boniana#F200
Magnoliales Annonaceae Annona muricata#YZRI
Magnoliales Annonaceae Annona cherimola#NC 030166
Magnoliales Annonaceae Fissistigma wallichii#F874
Magnoliales Annonaceae Uvaria littoralis#PSJT
Magnoliales Annonaceae Uvaria macrophylla#NC 041442
Magnoliales Magnoliaceae Liriodendron tulipifera#NC 008326
Magnoliales Magnoliaceae Liriodendron chinense#NC 030504
Magnoliales Magnoliaceae Magnolia gloriensis#F976
Magnoliales Magnoliaceae Magnolia grandiflora#NC 020318
Magnoliales Magnoliaceae Magnolia sieboldii#MG800828
Magnoliales Magnoliaceae Magnolia tripetala#NC 024027
Magnoliales Magnoliaceae Magnolia officinalis#KY085916
Magnoliales Magnoliaceae Magnolia officinalis var. biloba#NC 020317
Magnoliales Magnoliaceae Magnolia duclouxii#NC 037002
Magnoliales Magnoliaceae Magnolia insignis#F121
Magnoliales Magnoliaceae Magnolia grandis#F7
Magnoliales Magnoliaceae Magnolia dandyi#NC 037004
Magnoliales Magnoliaceae Magnolia fordiana#MH394396
Magnoliales Magnoliaceae Magnolia conifera#NC 037001
Magnoliales Magnoliaceae Magnolia glaucifolia#NC 037003
Magnoliales Magnoliaceae Magnolia fordiana var. calcarea#MF990562
Magnoliales Magnoliaceae Magnolia aromatica#NC 037000
Magnoliales Magnoliaceae Magnolia fraseri var. pyramidata#NC 023236
Magnoliales Magnoliaceae Magnolia macrophylla var. dealbata#NC 023235
Magnoliales Magnoliaceae Magnolia kwangsiensis#NC 015892
Magnoliales Magnoliaceae Magnolia yunnanensis#NC 024545
Magnoliales Magnoliaceae Magnolia sinica#NC 023241
Magnoliales Magnoliaceae Magnolia cathcartii#NC 023234
Magnoliales Magnoliaceae Magnolia odora#NC 023239
Magnoliales Magnoliaceae Magnolia laevifolia#NC 035956
Magnoliales Magnoliaceae Magnolia liliifera#NC 023238
Magnoliales Magnoliaceae Magnolia denudata#KY085917
Magnoliales Magnoliaceae Magnolia sprengeri#NC 023242
Magnoliales Magnoliaceae Magnolia salicifolia#NC 023240
Magnoliales Magnoliaceae Magnolia kobus#NC 023237
Magnoliales Magnoliaceae Magnolia biondii#NC 034687
Magnoliales Magnoliaceae Magnolia stellata#NC 039941
Magnoliales Magnoliaceae Magnolia zenii#NC 040954
Acorales Acoraceae Acorus gramineus#F409
Acorales Acoraceae Acorus calamus#NC 007407
Acorales Acoraceae Acorus calamus var. americanus#MTII
Alismatales Araceae Symplocarpus renifolius#NC 033970
Alismatales Araceae Spirodela polyrrhiza#F656
Alismatales Araceae Landoltia punctata#KY993962
Alismatales Araceae Lemna minor#NC 010109
Alismatales Araceae Wolffiella lingulata#NC 015894
Alismatales Araceae Wolffia australiana#NC 015899
Alismatales Araceae Spathiphyllum kochii#NC 030371
Alismatales Araceae Rhaphidophora hookeri#F314
Alismatales Araceae Lasia spinosa#F76
Alismatales Araceae Colocasia esculenta#KY769273
Alismatales Araceae Pistia stratiotes#MFIN
Alismatales Araceae Pinellia ternata#NC 027681
Alismatales Araceae Typhonium blumei#YMES
Alismatales Araceae Dieffenbachia seguine#NC 027272
Alismatales Araceae Schismatoglottis hainanensis#F278
Alismatales Araceae Zantedeschia aethiopica#MK327553
Alismatales Araceae Epipremnum aureum#NC 027954
Alismatales Tofieldiaceae Tofieldia coccinea#F670
Alismatales Tofieldiaceae Tofieldia thibetica#F816
Alismatales Alismataceae Alisma plantago-aquatica subsp. orientale#F166
Alismatales Alismataceae Limnocharis flava#F45
Alismatales Alismataceae Sagittaria montevidensis#Y14054

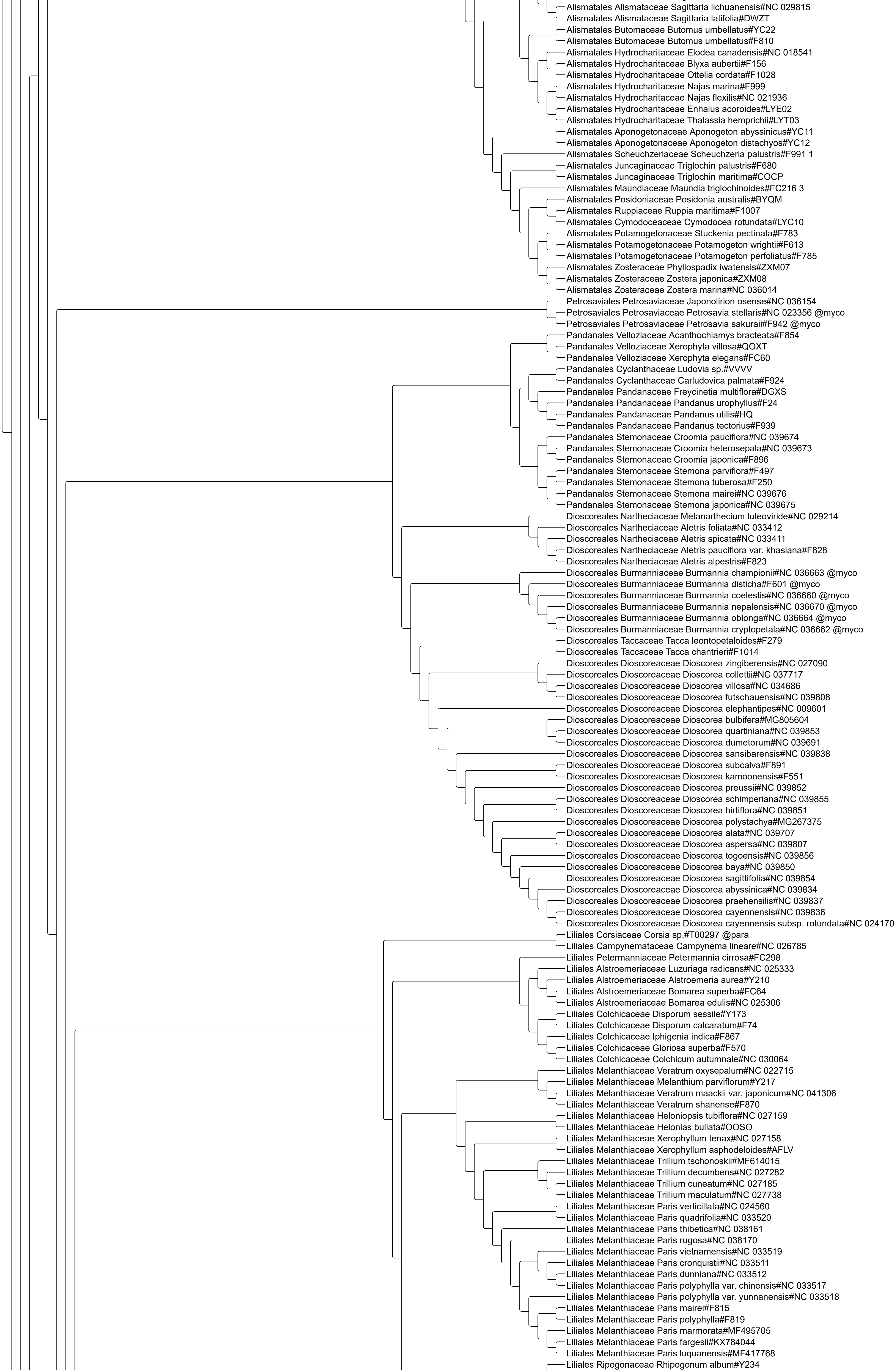

Alismatales Alismataceae Sagittaria lichuanensis#NC 029815
Alismatales Alismataceae Sagittaria latifolia#DWZT
Alismatales Butomaceae Butomus umbellatus#YC22
Alismatales Butomaceae Butomus umbellatus#F810
Alismatales Hydrocharitaceae Elodea canadensis#NC 018541
Alismatales Hydrocharitaceae Blyxa aubertii#F156
Alismatales Hydrocharitaceae Ottelia cordata#F1028
Alismatales Hydrocharitaceae Najas marina#F999
Alismatales Hydrocharitaceae Najas flexilis#NC 021936
Alismatales Hydrocharitaceae Enhalus acoroides#LYE02
Alismatales Hydrocharitaceae Thalassia hemprichii#LYT03
Alismatales Aponogetonaceae Aponogeton abyssinicus#YC11
Alismatales Aponogetonaceae Aponogeton distachyos#YC12
Alismatales Scheuchzeriaceae Scheuchzeria palustris#F991 1
Alismatales Juncaginaceae Triglochin palustris#F680
Alismatales Juncaginaceae Triglochin maritima#COCP
Alismatales Maundiaceae Maundia triglochinoides#FC216 3
Alismatales Posidoniaceae Posidonia australis#BYQM
Alismatales Ruppiaceae Ruppia maritima#F1007
Alismatales Cymodoceaceae Cymodocea rotundata#LYC10
Alismatales Potamogetonaceae Stuckenia pectinata#F783
Alismatales Potamogetonaceae Potamogeton wrightii#F613
Alismatales Potamogetonaceae Potamogeton perfoliatus#F785
Alismatales Zosteraceae Phyllospadix iwatensis#ZXM07
Alismatales Zosteraceae Zostera japonica#ZXM08
Alismatales Zosteraceae Zostera marina#NC 036014
Petrosaviales Petrosaviaceae Japonolirion osense#NC 036154
Petrosaviales Petrosaviaceae Petrosavia stellaris#NC 023356 @myco
Petrosaviales Petrosaviaceae Petrosavia sakuraii#F942 @myco
Pandanales Velloziaceae Acanthochlamys bracteata#F854
Pandanales Velloziaceae Xerophyta villosa#QOXT
Pandanales Velloziaceae Xerophyta elegans#FC60
Pandanales Cyclanthaceae Ludovia sp.#VVVV
Pandanales Cyclanthaceae Carludovica palmata#F924
Pandanales Pandanaceae Freycinetia multiflora#DGXS
Pandanales Pandanaceae Pandanus urophyllus#F24
Pandanales Pandanaceae Pandanus utilis#HQ
Pandanales Pandanaceae Pandanus tectorius#F939
Pandanales Stemonaceae Croomia pauciflora#NC 039674
Pandanales Stemonaceae Croomia heterosepala#NC 039673
Pandanales Stemonaceae Croomia japonica#F896
Pandanales Stemonaceae Stemona parviflora#F497
Pandanales Stemonaceae Stemona tuberosa#F250
Pandanales Stemonaceae Stemona mairei#NC 039676
Pandanales Stemonaceae Stemona japonica#NC 039675
Dioscoreales Nartheciaceae Metanarthecium luteoviride#NC 029214
Dioscoreales Nartheciaceae Aletris foliata#NC 033412
Dioscoreales Nartheciaceae Aletris spicata#NC 033411
Dioscoreales Nartheciaceae Aletris pauciflora var. khasiana#F828
Dioscoreales Nartheciaceae Aletris alpestris#F823
Dioscoreales Burmanniaceae Burmannia championii#NC 036663 @myco
Dioscoreales Burmanniaceae Burmannia disticha#F601 @myco
Dioscoreales Burmanniaceae Burmannia coelestis#NC 036660 @myco
Dioscoreales Burmanniaceae Burmannia nepalensis#NC 036670 @myco
Dioscoreales Burmanniaceae Burmannia oblonga#NC 036664 @myco
Dioscoreales Burmanniaceae Burmannia cryptopetala#NC 036662 @myco
Dioscoreales Taccaceae Tacca leontopetaloides#F279
Dioscoreales Taccaceae Tacca chantrieri#F1014
Dioscoreales Dioscoreaceae Dioscorea zingiberensis#NC 027090
Dioscoreales Dioscoreaceae Dioscorea collettii#NC 037717
Dioscoreales Dioscoreaceae Dioscorea villosa#NC 034686
Dioscoreales Dioscoreaceae Dioscorea futschauensis#NC 039808
Dioscoreales Dioscoreaceae Dioscorea elephantipes#NC 009601
Dioscoreales Dioscoreaceae Dioscorea bulbifera#MG805604
Dioscoreales Dioscoreaceae Dioscorea quartiniana#NC 039853
Dioscoreales Dioscoreaceae Dioscorea dumetorum#NC 039691
Dioscoreales Dioscoreaceae Dioscorea sansibarensis#NC 039838
Dioscoreales Dioscoreaceae Dioscorea subcalva#F891
Dioscoreales Dioscoreaceae Dioscorea kamoonensis#F551
Dioscoreales Dioscoreaceae Dioscorea preussii#NC 039852
Dioscoreales Dioscoreaceae Dioscorea schimperiana#NC 039855
Dioscoreales Dioscoreaceae Dioscorea hirtiflora#NC 039851
Dioscoreales Dioscoreaceae Dioscorea polystachya#MG267375
Dioscoreales Dioscoreaceae Dioscorea alata#NC 039707
Dioscoreales Dioscoreaceae Dioscorea aspersa#NC 039807
Dioscoreales Dioscoreaceae Dioscorea togoensis#NC 039856
Dioscoreales Dioscoreaceae Dioscorea baya#NC 039850
Dioscoreales Dioscoreaceae Dioscorea sagittifolia#NC 039854
Dioscoreales Dioscoreaceae Dioscorea abyssinica#NC 039834
Dioscoreales Dioscoreaceae Dioscorea praehensilis#NC 039837
Dioscoreales Dioscoreaceae Dioscorea cayennensis#NC 039836
Dioscoreales Dioscoreaceae Dioscorea cayennensis subsp. rotundata#NC 024170
Liliales Corsiaceae Corsia sp.#T00297 @para
Liliales Campynemataceae Campynema lineare#NC 026785
Liliales Petermanniaceae Petermannia cirrosa#FC298
Liliales Alstroemeriaceae Luzuriaga radicans#NC 025333
Liliales Alstroemeriaceae Alstroemeria aurea#Y210
Liliales Alstroemeriaceae Bomarea superba#FC64
Liliales Alstroemeriaceae Bomarea edulis#NC 025306
Liliales Colchicaceae Disporum sessile#Y173
Liliales Colchicaceae Disporum calcaratum#F74
Liliales Colchicaceae Iphigenia indica#F867
Liliales Colchicaceae Gloriosa superba#F570
Liliales Colchicaceae Colchicum autumnale#NC 030064
Liliales Melanthiaceae Veratrum oxysepalum#NC 022715
Liliales Melanthiaceae Melanthium parviflorum#Y217
Liliales Melanthiaceae Veratrum maackii var. japonicum#NC 041306
Liliales Melanthiaceae Veratrum shanense#F870
Liliales Melanthiaceae Heloniopsis tubiflora#NC 027159
Liliales Melanthiaceae Helonias bullata#OOSO
Liliales Melanthiaceae Xerophyllum tenax#NC 027158
Liliales Melanthiaceae Xerophyllum asphodeloides#AFLV
Liliales Melanthiaceae Trillium tschonoskii#MF614015
Liliales Melanthiaceae Trillium decumbens#NC 027282
Liliales Melanthiaceae Trillium cuneatum#NC 027185
Liliales Melanthiaceae Trillium maculatum#NC 027738
Liliales Melanthiaceae Paris verticillata#NC 024560
Liliales Melanthiaceae Paris quadrifolia#NC 033520
Liliales Melanthiaceae Paris thibetica#NC 038161
Liliales Melanthiaceae Paris rugosa#NC 038170
Liliales Melanthiaceae Paris vietnamensis#NC 033519
Liliales Melanthiaceae Paris cronquistii#NC 033511
Liliales Melanthiaceae Paris dunniana#NC 033512
Liliales Melanthiaceae Paris polyphylla var. chinensis#NC 033517
Liliales Melanthiaceae Paris polyphylla var. yunnanensis#NC 033518
Liliales Melanthiaceae Paris mairei#F815
Liliales Melanthiaceae Paris polyphylla#F819
Liliales Melanthiaceae Paris marmorata#MF495705
Liliales Melanthiaceae Paris fargesii#KX784044
Liliales Melanthiaceae Paris luquanensis#MF417768
Liliales Ripogonaceae Rhipogonum album#Y234

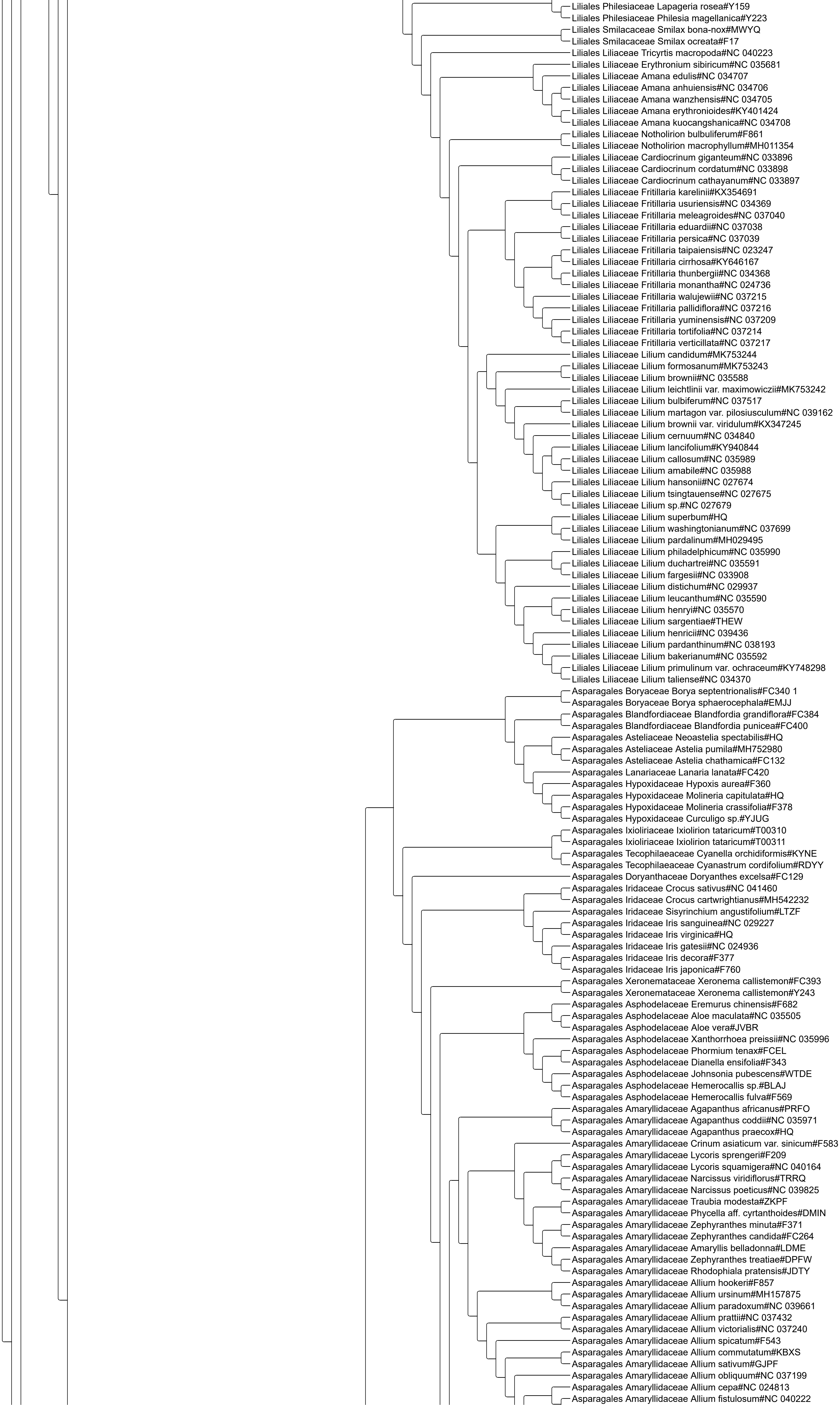

Liliales Philesiaceae Lapageria rosea#Y159
Liliales Philesiaceae Philesia magellanica#Y223
Liliales Smilacaceae Smilax bona-nox#MWYQ
Liliales Smilacaceae Smilax ocreata#F17
Liliales Liliaceae Tricyrtis macropoda#NC 040223
Liliales Liliaceae Erythronium sibiricum#NC 035681
Liliales Liliaceae Amana edulis#NC 034707
Liliales Liliaceae Amana anhuiensis#NC 034706
Liliales Liliaceae Amana wanzhensis#NC 034705
Liliales Liliaceae Amana erythronioides#KY401424
Liliales Liliaceae Amana kuocangshanica#NC 034708
Liliales Liliaceae Notholirion bulbuliferum#F861
Liliales Liliaceae Notholirion macrophyllum#MH011354
Liliales Liliaceae Cardiocrinum giganteum#NC 033896
Liliales Liliaceae Cardiocrinum cordatum#NC 033898
Liliales Liliaceae Cardiocrinum cathayanum#NC 033897
Liliales Liliaceae Fritillaria karelinii#KX354691
Liliales Liliaceae Fritillaria usuriensis#NC 034369
Liliales Liliaceae Fritillaria meleagroides#NC 037040
Liliales Liliaceae Fritillaria eduardii#NC 037038
Liliales Liliaceae Fritillaria persica#NC 037039
Liliales Liliaceae Fritillaria taipaiensis#NC 023247
Liliales Liliaceae Fritillaria cirrhosa#KY646167
Liliales Liliaceae Fritillaria thunbergii#NC 034368
Liliales Liliaceae Fritillaria monantha#NC 024736
Liliales Liliaceae Fritillaria walujewii#NC 037215
Liliales Liliaceae Fritillaria pallidiflora#NC 037216
Liliales Liliaceae Fritillaria yuminensis#NC 037209
Liliales Liliaceae Fritillaria tortifolia#NC 037214
Liliales Liliaceae Fritillaria verticillata#NC 037217
Liliales Liliaceae Lilium candidum#MK753244
Liliales Liliaceae Lilium formosanum#MK753243
Liliales Liliaceae Lilium brownii#NC 035588
Liliales Liliaceae Lilium leichtlinii var. maximowiczii#MK753242
Liliales Liliaceae Lilium bulbiferum#NC 037517
Liliales Liliaceae Lilium martagon var. pilosiusculum#NC 039162
Liliales Liliaceae Lilium brownii var. viridulum#KX347245
Liliales Liliaceae Lilium cernuum#NC 034840
Liliales Liliaceae Lilium lancifolium#KY940844
Liliales Liliaceae Lilium callosum#NC 035989
Liliales Liliaceae Lilium amabile#NC 035988
Liliales Liliaceae Lilium hansonii#NC 027674
Liliales Liliaceae Lilium tsingtauense#NC 027675
Liliales Liliaceae Lilium sp.#NC 027679
Liliales Liliaceae Lilium superbum#HQ
Liliales Liliaceae Lilium washingtonianum#NC 037699
Liliales Liliaceae Lilium pardalinum#MH029495
Liliales Liliaceae Lilium philadelphicum#NC 035990
Liliales Liliaceae Lilium duchartrei#NC 035591
Liliales Liliaceae Lilium fargesii#NC 033908
Liliales Liliaceae Lilium distichum#NC 029937
Liliales Liliaceae Lilium leucanthum#NC 035590
Liliales Liliaceae Lilium henryi#NC 035570
Liliales Liliaceae Lilium sargentiae#THEW
Liliales Liliaceae Lilium henricii#NC 039436
Liliales Liliaceae Lilium pardanthinum#NC 038193
Liliales Liliaceae Lilium bakerianum#NC 035592
Liliales Liliaceae Lilium primulinum var. ochraceum#KY748298
Liliales Liliaceae Lilium taliense#NC 034370
Asparagales Boryaceae Borya septentrionalis#FC340 1
Asparagales Boryaceae Borya sphaerocephala#EMJJ
Asparagales Blandfordiaceae Blandfordia grandiflora#FC384
Asparagales Blandfordiaceae Blandfordia punicea#FC400
Asparagales Asteliaceae Neoastelia spectabilis#HQ
Asparagales Asteliaceae Astelia pumila#MH752980
Asparagales Asteliaceae Astelia chathamica#FC132
Asparagales Lanariaceae Lanaria lanata#FC420
Asparagales Hypoxidaceae Hypoxis aurea#F360
Asparagales Hypoxidaceae Molineria capitulata#HQ
Asparagales Hypoxidaceae Molineria crassifolia#F378
Asparagales Hypoxidaceae Curculigo sp.#YJUG
Asparagales Ixioliriaceae Ixiolirion tataricum#T00310
Asparagales Ixioliriaceae Ixiolirion tataricum#T00311
Asparagales Tecophilaeaceae Cyanella orchidiformis#KYNE
Asparagales Tecophilaeaceae Cyanastrum cordifolium#RDYY
Asparagales Doryanthaceae Doryanthes excelsa#FC129
Asparagales Iridaceae Crocus sativus#NC 041460
Asparagales Iridaceae Crocus cartwrightianus#MH542232
Asparagales Iridaceae Sisyrinchium angustifolium#LTZF
Asparagales Iridaceae Iris sanguinea#NC 029227
Asparagales Iridaceae Iris virginica#HQ
Asparagales Iridaceae Iris gatesii#NC 024936
Asparagales Iridaceae Iris decora#F377
Asparagales Iridaceae Iris japonica#F760
Asparagales Xeronemataceae Xeronema callistemon#FC393
Asparagales Xeronemataceae Xeronema callistemon#Y243
Asparagales Asphodelaceae Eremurus chinensis#F682
Asparagales Asphodelaceae Aloe maculata#NC 035505
Asparagales Asphodelaceae Aloe vera#JVBR
Asparagales Asphodelaceae Xanthorrhoea preissii#NC 035996
Asparagales Asphodelaceae Phormium tenax#FCEL
Asparagales Asphodelaceae Dianella ensifolia#F343
Asparagales Asphodelaceae Johnsonia pubescens#WTDE
Asparagales Asphodelaceae Hemerocallis sp.#BLAJ
Asparagales Asphodelaceae Hemerocallis fulva#F569
Asparagales Amaryllidaceae Agapanthus africanus#PRFO
Asparagales Amaryllidaceae Agapanthus coddii#NC 035971
Asparagales Amaryllidaceae Agapanthus praecox#HQ
Asparagales Amaryllidaceae Crinum asiaticum var. sinicum#F583
Asparagales Amaryllidaceae Lycoris sprengeri#F209
Asparagales Amaryllidaceae Lycoris squamigera#NC 040164
Asparagales Amaryllidaceae Narcissus viridiflorus#TRRQ
Asparagales Amaryllidaceae Narcissus poeticus#NC 039825
Asparagales Amaryllidaceae Traubia modesta#ZKPF
Asparagales Amaryllidaceae Phycella aff. cyrtanthoides#DMIN
Asparagales Amaryllidaceae Zephyranthes minuta#F371
Asparagales Amaryllidaceae Zephyranthes candida#FC264
Asparagales Amaryllidaceae Amaryllis belladonna#LDME
Asparagales Amaryllidaceae Zephyranthes treatiae#DPFW
Asparagales Amaryllidaceae Rhodophiala pratensis#JDTY
Asparagales Amaryllidaceae Allium hookeri#F857
Asparagales Amaryllidaceae Allium ursinum#MH157875
Asparagales Amaryllidaceae Allium paradoxum#NC 039661
Asparagales Amaryllidaceae Allium prattii#NC 037432
Asparagales Amaryllidaceae Allium victorialis#NC 037240
Asparagales Amaryllidaceae Allium spicatum#F543
Asparagales Amaryllidaceae Allium commutatum#KBXS
Asparagales Amaryllidaceae Allium sativum#GJPF
Asparagales Amaryllidaceae Allium obliquum#NC 037199
Asparagales Amaryllidaceae Allium cepa#NC 024813
Asparagales Amaryllidaceae Allium fistulosum#NC 040222

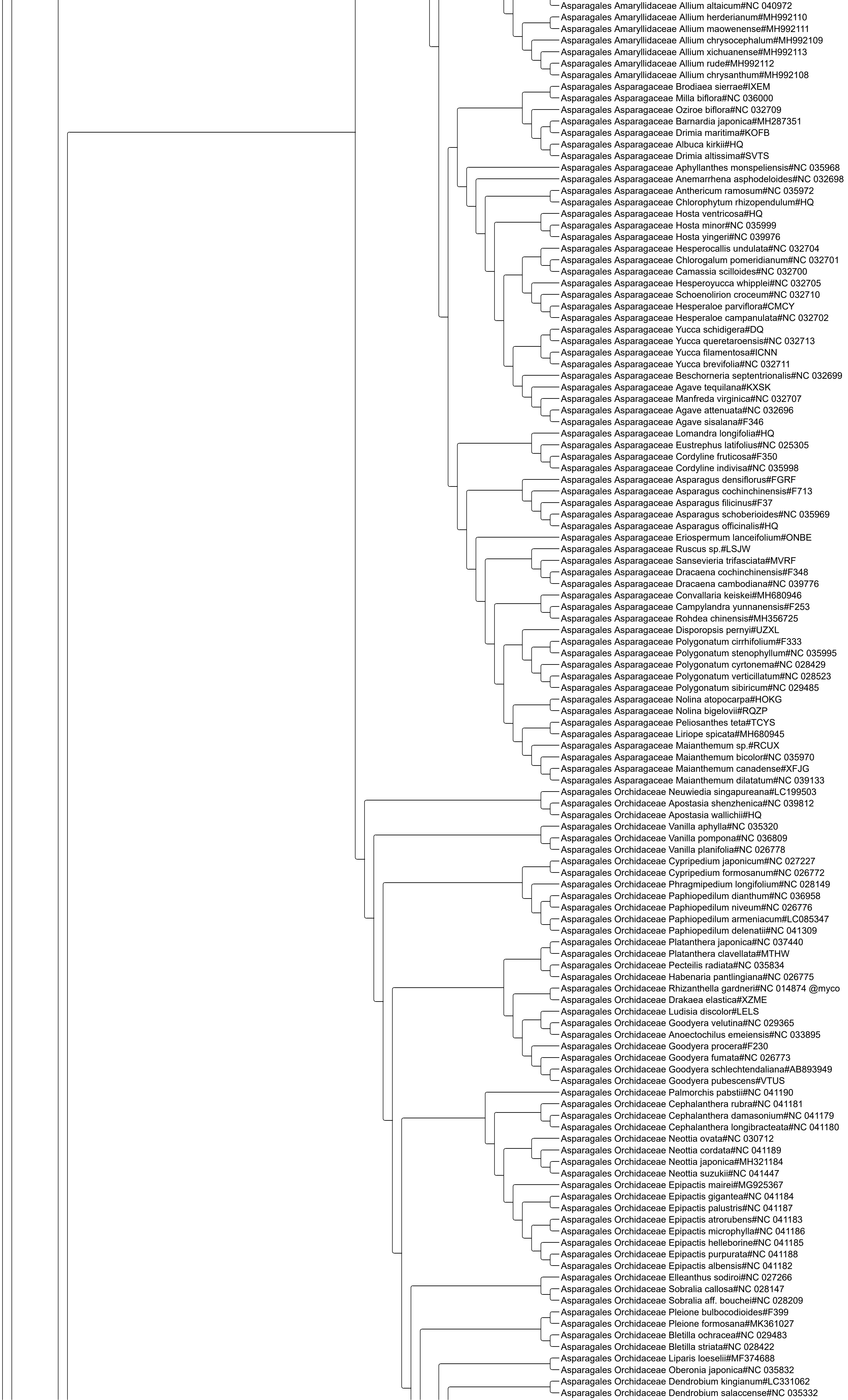

Asparagales Amaryllidaceae Allium altaicum#NC 040972
Asparagales Amaryllidaceae Allium herderianum#MH992110
Asparagales Amaryllidaceae Allium maowenense#MH992111
Asparagales Amaryllidaceae Allium chrysocephalum#MH992109
Asparagales Amaryllidaceae Allium xichuanense#MH992113
Asparagales Amaryllidaceae Allium rude#MH992112
Asparagales Amaryllidaceae Allium chrysanthum#MH992108
Asparagales Asparagaceae Brodiaea sierrae#IXEM
Asparagales Asparagaceae Milla biflora#NC 036000
Asparagales Asparagaceae Oziroe biflora#NC 032709
Asparagales Asparagaceae Barnardia japonica#MH287351
Asparagales Asparagaceae Drimia maritima#KOFB
Asparagales Asparagaceae Albuca kirkii#HQ
Asparagales Asparagaceae Drimia altissima#SVTS
Asparagales Asparagaceae Aphyllanthes monspeliensis#NC 035968
Asparagales Asparagaceae Anemarrhena asphodeloides#NC 032698
Asparagales Asparagaceae Anthericum ramosum#NC 035972
Asparagales Asparagaceae Chlorophytum rhizopendulum#HQ
Asparagales Asparagaceae Hosta ventricosa#HQ
Asparagales Asparagaceae Hosta minor#NC 035999
Asparagales Asparagaceae Hosta yingeri#NC 039976
Asparagales Asparagaceae Hesperocallis undulata#NC 032704
Asparagales Asparagaceae Chlorogalum pomeridianum#NC 032701
Asparagales Asparagaceae Camassia scilloides#NC 032700
Asparagales Asparagaceae Hesperoyucca whipplei#NC 032705
Asparagales Asparagaceae Schoenolirion croceum#NC 032710
Asparagales Asparagaceae Hesperaloe parviflora#CMCY
Asparagales Asparagaceae Hesperaloe campanulata#NC 032702
Asparagales Asparagaceae Yucca schidigera#DQ
Asparagales Asparagaceae Yucca queretaroensis#NC 032713
Asparagales Asparagaceae Yucca filamentosa#ICNN
Asparagales Asparagaceae Yucca brevifolia#NC 032711
Asparagales Asparagaceae Beschorneria septentrionalis#NC 032699
Asparagales Asparagaceae Agave tequilana#KXSK
Asparagales Asparagaceae Manfreda virginica#NC 032707
Asparagales Asparagaceae Agave attenuata#NC 032696
Asparagales Asparagaceae Agave sisalana#F346
Asparagales Asparagaceae Lomandra longifolia#HQ
Asparagales Asparagaceae Eustrephus latifolius#NC 025305
Asparagales Asparagaceae Cordyline fruticosa#F350
Asparagales Asparagaceae Cordyline indivisa#NC 035998
Asparagales Asparagaceae Asparagus densiflorus#FGRF
Asparagales Asparagaceae Asparagus cochinchinensis#F713
Asparagales Asparagaceae Asparagus filicinus#F37
Asparagales Asparagaceae Asparagus schoberioides#NC 035969
Asparagales Asparagaceae Asparagus officinalis#HQ
Asparagales Asparagaceae Eriospermum lanceifolium#ONBE
Asparagales Asparagaceae Ruscus sp.#LSJW
Asparagales Asparagaceae Sansevieria trifasciata#MVRF
Asparagales Asparagaceae Dracaena cochinchinensis#F348
Asparagales Asparagaceae Dracaena cambodiana#NC 039776
Asparagales Asparagaceae Convallaria keiskei#MH680946
Asparagales Asparagaceae Campylandra yunnanensis#F253
Asparagales Asparagaceae Rohdea chinensis#MH356725
Asparagales Asparagaceae Disporopsis pernyi#UZXL
Asparagales Asparagaceae Polygonatum cirrhifolium#F333
Asparagales Asparagaceae Polygonatum stenophyllum#NC 035995
Asparagales Asparagaceae Polygonatum cyrtonema#NC 028429
Asparagales Asparagaceae Polygonatum verticillatum#NC 028523
Asparagales Asparagaceae Polygonatum sibiricum#NC 029485
Asparagales Asparagaceae Nolina atopocarpa#HOKG
Asparagales Asparagaceae Nolina bigelovii#RQZP
Asparagales Asparagaceae Peliosanthes teta#TCYS
Asparagales Asparagaceae Liriope spicata#MH680945
Asparagales Asparagaceae Maianthemum sp.#RCUX
Asparagales Asparagaceae Maianthemum bicolor#NC 035970
Asparagales Asparagaceae Maianthemum canadense#XFJG
Asparagales Asparagaceae Maianthemum dilatatum#NC 039133
Asparagales Orchidaceae Neuwiedia singapureana#LC199503
Asparagales Orchidaceae Apostasia shenzhenica#NC 039812
Asparagales Orchidaceae Apostasia wallichii#HQ
Asparagales Orchidaceae Vanilla aphylla#NC 035320
Asparagales Orchidaceae Vanilla pompona#NC 036809
Asparagales Orchidaceae Vanilla planifolia#NC 026778
Asparagales Orchidaceae Cypripedium japonicum#NC 027227
Asparagales Orchidaceae Cypripedium formosanum#NC 026772
Asparagales Orchidaceae Phragmipedium longifolium#NC 028149
Asparagales Orchidaceae Paphiopedilum dianthum#NC 036958
Asparagales Orchidaceae Paphiopedilum niveum#NC 026776
Asparagales Orchidaceae Paphiopedilum armeniacum#LC085347
Asparagales Orchidaceae Paphiopedilum delenatii#NC 041309
Asparagales Orchidaceae Platanthera japonica#NC 037440
Asparagales Orchidaceae Platanthera clavellata#MTHW
Asparagales Orchidaceae Pecteilis radiata#NC 035834
Asparagales Orchidaceae Habenaria pantlingiana#NC 026775
Asparagales Orchidaceae Rhizanthella gardneri#NC 014874 @myco
Asparagales Orchidaceae Drakaea elastica#XZME
Asparagales Orchidaceae Ludisia discolor#LELS
Asparagales Orchidaceae Goodyera velutina#NC 029365
Asparagales Orchidaceae Anoectochilus emeiensis#NC 033895
Asparagales Orchidaceae Goodyera procera#F230
Asparagales Orchidaceae Goodyera fumata#NC 026773
Asparagales Orchidaceae Goodyera schlechtendaliana#AB893949
Asparagales Orchidaceae Goodyera pubescens#VTUS
Asparagales Orchidaceae Palmorchis pabstii#NC 041190
Asparagales Orchidaceae Cephalanthera rubra#NC 041181
Asparagales Orchidaceae Cephalanthera damasonium#NC 041179
Asparagales Orchidaceae Cephalanthera longibracteata#NC 041180
Asparagales Orchidaceae Neottia ovata#NC 030712
Asparagales Orchidaceae Neottia cordata#NC 041189
Asparagales Orchidaceae Neottia japonica#MH321184
Asparagales Orchidaceae Neottia suzukii#NC 041447
Asparagales Orchidaceae Epipactis mairei#MG925367
Asparagales Orchidaceae Epipactis gigantea#NC 041184
Asparagales Orchidaceae Epipactis palustris#NC 041187
Asparagales Orchidaceae Epipactis atrorubens#NC 041183
Asparagales Orchidaceae Epipactis microphylla#NC 041186
Asparagales Orchidaceae Epipactis helleborine#NC 041185
Asparagales Orchidaceae Epipactis purpurata#NC 041188
Asparagales Orchidaceae Epipactis albensis#NC 041182
Asparagales Orchidaceae Elleanthus sodiroi#NC 027266
Asparagales Orchidaceae Sobralia callosa#NC 028147
Asparagales Orchidaceae Sobralia aff. bouchei#NC 028209
Asparagales Orchidaceae Pleione bulbocodioides#F399
Asparagales Orchidaceae Pleione formosana#MK361027
Asparagales Orchidaceae Bletilla ochracea#NC 029483
Asparagales Orchidaceae Bletilla striata#NC 028422
Asparagales Orchidaceae Liparis loeselii#MF374688
Asparagales Orchidaceae Oberonia japonica#NC 035832
Asparagales Orchidaceae Dendrobium kingianum#LC331062
Asparagales Orchidaceae Dendrobium salaccense#NC 035332

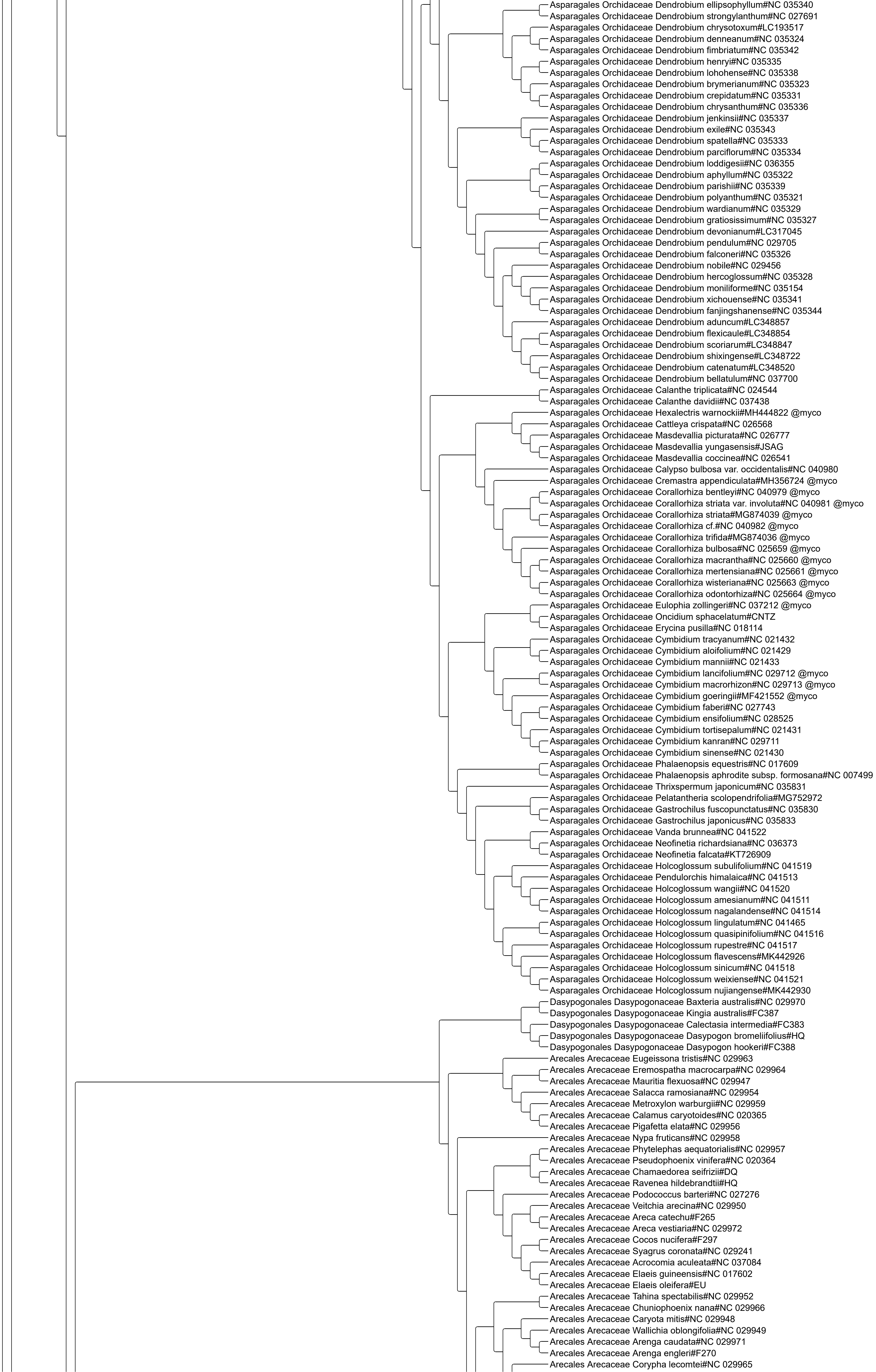

Asparagales Orchidaceae Dendrobium ellipsophyllum#NC 035340
Asparagales Orchidaceae Dendrobium strongylanthum#NC 027691
Asparagales Orchidaceae Dendrobium chrysotoxum#LC193517
Asparagales Orchidaceae Dendrobium denneanum#NC 035324
Asparagales Orchidaceae Dendrobium fimbriatum#NC 035342
Asparagales Orchidaceae Dendrobium henryi#NC 035335
Asparagales Orchidaceae Dendrobium lohohense#NC 035338
Asparagales Orchidaceae Dendrobium brymerianum#NC 035323
Asparagales Orchidaceae Dendrobium crepidatum#NC 035331
Asparagales Orchidaceae Dendrobium chrysanthum#NC 035336
Asparagales Orchidaceae Dendrobium jenkinsii#NC 035337
Asparagales Orchidaceae Dendrobium exile#NC 035343
Asparagales Orchidaceae Dendrobium spatella#NC 035333
Asparagales Orchidaceae Dendrobium parciflorum#NC 035334
Asparagales Orchidaceae Dendrobium loddigesii#NC 036355
Asparagales Orchidaceae Dendrobium aphyllum#NC 035322
Asparagales Orchidaceae Dendrobium parishii#NC 035339
Asparagales Orchidaceae Dendrobium polyanthum#NC 035321
Asparagales Orchidaceae Dendrobium wardianum#NC 035329
Asparagales Orchidaceae Dendrobium gratiosissimum#NC 035327
Asparagales Orchidaceae Dendrobium devonianum#LC317045
Asparagales Orchidaceae Dendrobium pendulum#NC 029705
Asparagales Orchidaceae Dendrobium falconeri#NC 035326
Asparagales Orchidaceae Dendrobium nobile#NC 029456
Asparagales Orchidaceae Dendrobium hercoglossum#NC 035328
Asparagales Orchidaceae Dendrobium moniliforme#NC 035154
Asparagales Orchidaceae Dendrobium xichouense#NC 035341
Asparagales Orchidaceae Dendrobium fanjingshanense#NC 035344
Asparagales Orchidaceae Dendrobium aduncum#LC348857
Asparagales Orchidaceae Dendrobium flexicaule#LC348854
Asparagales Orchidaceae Dendrobium scoriarum#LC348847
Asparagales Orchidaceae Dendrobium shixingense#LC348722
Asparagales Orchidaceae Dendrobium catenatum#LC348520
Asparagales Orchidaceae Dendrobium bellatulum#NC 037700
Asparagales Orchidaceae Calanthe triplicata#NC 024544
Asparagales Orchidaceae Calanthe davidii#NC 037438
Asparagales Orchidaceae Hexalectris warnockii#MH444822 @myco
Asparagales Orchidaceae Cattleya crispata#NC 026568
Asparagales Orchidaceae Masdevallia picturata#NC 026777
Asparagales Orchidaceae Masdevallia yungasensis#JSAG
Asparagales Orchidaceae Masdevallia coccinea#NC 026541
Asparagales Orchidaceae Calypso bulbosa var. occidentalis#NC 040980
Asparagales Orchidaceae Cremastra appendiculata#MH356724 @myco
Asparagales Orchidaceae Corallorhiza bentleyi#NC 040979 @myco
Asparagales Orchidaceae Corallorhiza striata var. involuta#NC 040981 @myco
Asparagales Orchidaceae Corallorhiza striata#MG874039 @myco
Asparagales Orchidaceae Corallorhiza cf.#NC 040982 @myco
Asparagales Orchidaceae Corallorhiza trifida#MG874036 @myco
Asparagales Orchidaceae Corallorhiza bulbosa#NC 025659 @myco
Asparagales Orchidaceae Corallorhiza macrantha#NC 025660 @myco
Asparagales Orchidaceae Corallorhiza mertensiana#NC 025661 @myco
Asparagales Orchidaceae Corallorhiza wisteriana#NC 025663 @myco
Asparagales Orchidaceae Corallorhiza odontorhiza#NC 025664 @myco
Asparagales Orchidaceae Eulophia zollingeri#NC 037212 @myco
Asparagales Orchidaceae Oncidium sphacelatum#CNTZ
Asparagales Orchidaceae Erycina pusilla#NC 018114
Asparagales Orchidaceae Cymbidium tracyanum#NC 021432
Asparagales Orchidaceae Cymbidium aloifolium#NC 021429
Asparagales Orchidaceae Cymbidium mannii#NC 021433
Asparagales Orchidaceae Cymbidium lancifolium#NC 029712 @myco
Asparagales Orchidaceae Cymbidium macrorhizon#NC 029713 @myco
Asparagales Orchidaceae Cymbidium goeringii#MF421552 @myco
Asparagales Orchidaceae Cymbidium faberi#NC 027743
Asparagales Orchidaceae Cymbidium ensifolium#NC 028525
Asparagales Orchidaceae Cymbidium tortisepalum#NC 021431
Asparagales Orchidaceae Cymbidium kanran#NC 029711
Asparagales Orchidaceae Cymbidium sinense#NC 021430
Asparagales Orchidaceae Phalaenopsis equestris#NC 017609
Asparagales Orchidaceae Phalaenopsis aphrodite subsp. formosana#NC 007499
Asparagales Orchidaceae Thrixspermum japonicum#NC 035831
Asparagales Orchidaceae Pelatantheria scolopendrifolia#MG752972
Asparagales Orchidaceae Gastrochilus fuscopunctatus#NC 035830
Asparagales Orchidaceae Gastrochilus japonicus#NC 035833
Asparagales Orchidaceae Vanda brunnea#NC 041522
Asparagales Orchidaceae Neofinetia richardsiana#NC 036373
Asparagales Orchidaceae Neofinetia falcata#KT726909
Asparagales Orchidaceae Holcoglossum subulifolium#NC 041519
Asparagales Orchidaceae Pendulorchis himalaica#NC 041513
Asparagales Orchidaceae Holcoglossum wangii#NC 041520
Asparagales Orchidaceae Holcoglossum amesianum#NC 041511
Asparagales Orchidaceae Holcoglossum nagalandense#NC 041514
Asparagales Orchidaceae Holcoglossum lingulatum#NC 041465
Asparagales Orchidaceae Holcoglossum quasipinifolium#NC 041516
Asparagales Orchidaceae Holcoglossum rupestre#NC 041517
Asparagales Orchidaceae Holcoglossum flavescens#MK442926
Asparagales Orchidaceae Holcoglossum sinicum#NC 041518
Asparagales Orchidaceae Holcoglossum weixiense#NC 041521
Asparagales Orchidaceae Holcoglossum nujiangense#MK442930
Dasypogonales Dasypogonaceae Baxteria australis#NC 029970
Dasypogonales Dasypogonaceae Kingia australis#FC387
Dasypogonales Dasypogonaceae Calectasia intermedia#FC383
Dasypogonales Dasypogonaceae Dasypogon bromeliifolius#HQ
Dasypogonales Dasypogonaceae Dasypogon hookeri#FC388
Arecales Arecaceae Eugeissona tristis#NC 029963
Arecales Arecaceae Eremospatha macrocarpa#NC 029964
Arecales Arecaceae Mauritia flexuosa#NC 029947
Arecales Arecaceae Salacca ramosiana#NC 029954
Arecales Arecaceae Metroxylon warburgii#NC 029959
Arecales Arecaceae Calamus caryotoides#NC 020365
Arecales Arecaceae Pigafetta elata#NC 029956
Arecales Arecaceae Nypa fruticans#NC 029958
Arecales Arecaceae Phytelephas aequatorialis#NC 029957
Arecales Arecaceae Pseudophoenix vinifera#NC 020364
Arecales Arecaceae Chamaedorea seifrizii#DQ
Arecales Arecaceae Ravenea hildebrandtii#HQ
Arecales Arecaceae Podococcus barteri#NC 027276
Arecales Arecaceae Veitchia arecina#NC 029950
Arecales Arecaceae Areca catechu#F265
Arecales Arecaceae Areca vestiaria#NC 029972
Arecales Arecaceae Cocos nucifera#F297
Arecales Arecaceae Syagrus coronata#NC 029241
Arecales Arecaceae Acrocomia aculeata#NC 037084
Arecales Arecaceae Elaeis guineensis#NC 017602
Arecales Arecaceae Elaeis oleifera#EU
Arecales Arecaceae Tahina spectabilis#NC 029952
Arecales Arecaceae Chuniophoenix nana#NC 029966
Arecales Arecaceae Caryota mitis#NC 029948
Arecales Arecaceae Wallichia oblongifolia#NC 029949
Arecales Arecaceae Arenga caudata#NC 029971
Arecales Arecaceae Arenga engleri#F270
Arecales Arecaceae Corypha lecomtei#NC 029965

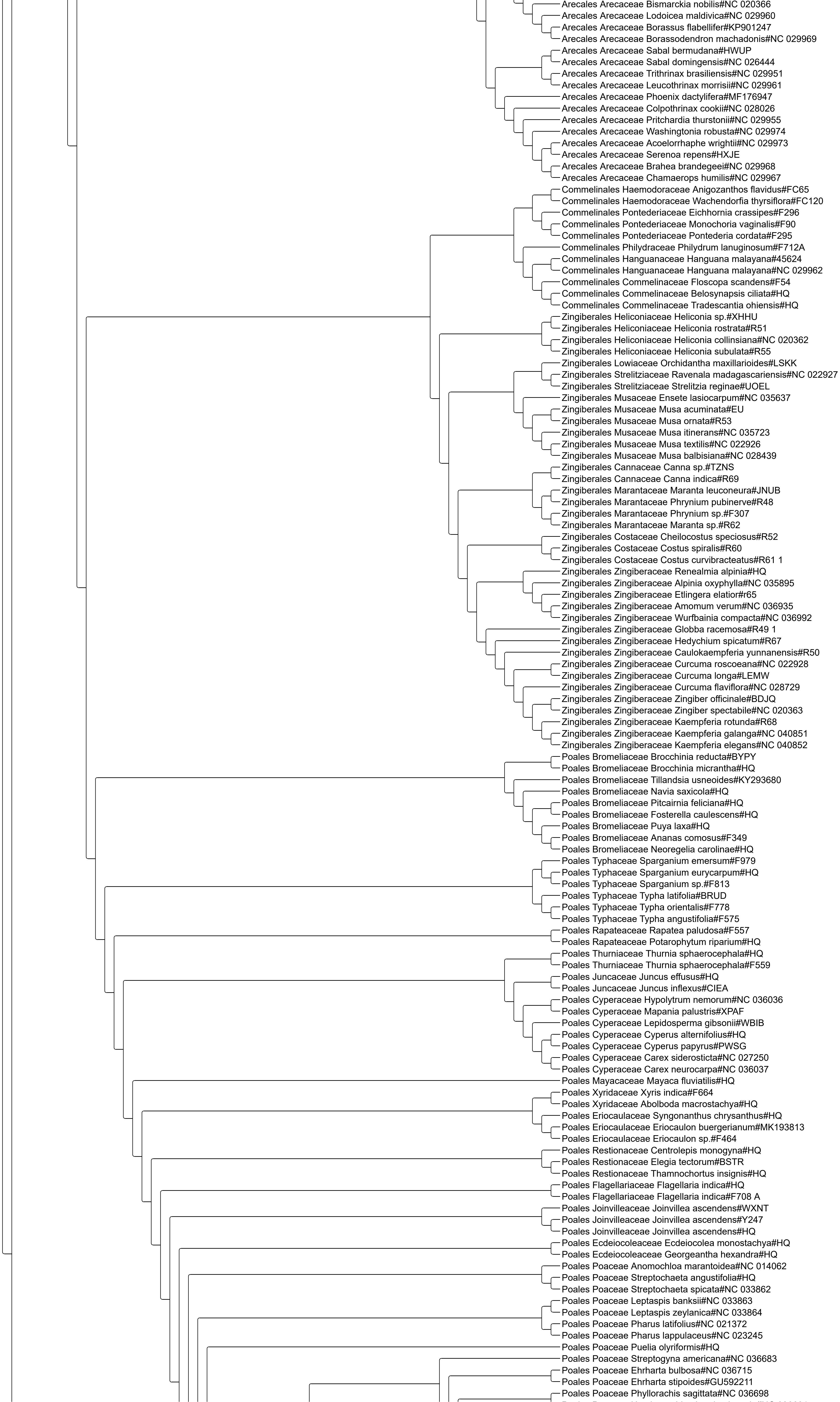
Arecales Arecaceae Bismarckia nobilis#NC 020366
Arecales Arecaceae Lodoicea maldivica#NC 029960
Arecales Arecaceae Borassus flabellifer#KP901247
Arecales Arecaceae Borassodendron machadonis#NC 029969
Arecales Arecaceae Sabal bermudana#HWUP
Arecales Arecaceae Sabal domingensis#NC 026444
Arecales Arecaceae Trithrinax brasiliensis#NC 029951
Arecales Arecaceae Leucothrinax morrisii#NC 029961
Arecales Arecaceae Phoenix dactylifera#MF176947
Arecales Arecaceae Colpothrinax cookii#NC 028026
Arecales Arecaceae Pritchardia thurstonii#NC 029955
Arecales Arecaceae Washingtonia robusta#NC 029974
Arecales Arecaceae Acoelorrhaphe wrightii#NC 029973
Arecales Arecaceae Serenoa repens#HXJE
Arecales Arecaceae Brahea brandegeei#NC 029968
Arecales Arecaceae Chamaerops humilis#NC 029967
Commelinales Haemodoraceae Anigozanthos flavidus#FC65
Commelinales Haemodoraceae Wachendorfia thyrsiflora#FC120
Commelinales Pontederiaceae Eichhornia crassipes#F296
Commelinales Pontederiaceae Monochoria vaginalis#F90
Commelinales Pontederiaceae Pontederia cordata#F295
Commelinales Philydraceae Philydrum lanuginosum#F712A
Commelinales Hanguanaceae Hanguana malayana#45624
Commelinales Hanguanaceae Hanguana malayana#NC 029962
Commelinales Commelinaceae Floscopa scandens#F54
Commelinales Commelinaceae Belosynapsis ciliata#HQ
Commelinales Commelinaceae Tradescantia ohiensis#HQ
Zingiberales Heliconiaceae Heliconia sp.#XHHU
Zingiberales Heliconiaceae Heliconia rostrata#R51
Zingiberales Heliconiaceae Heliconia collinsiana#NC 020362
Zingiberales Heliconiaceae Heliconia subulata#R55
Zingiberales Lowiaceae Orchidantha maxillarioides#LSKK
Zingiberales Strelitziaceae Ravenala madagascariensis#NC 022927
Zingiberales Strelitziaceae Strelitzia reginae#UOEL
Zingiberales Musaceae Ensete lasiocarpum#NC 035637
Zingiberales Musaceae Musa acuminata#EU
Zingiberales Musaceae Musa ornata#R53
Zingiberales Musaceae Musa itinerans#NC 035723
Zingiberales Musaceae Musa textilis#NC 022926
Zingiberales Musaceae Musa balbisiana#NC 028439
Zingiberales Cannaceae Canna sp.#TZNS
Zingiberales Cannaceae Canna indica#R69
Zingiberales Marantaceae Maranta leuconeura#JNUB
Zingiberales Marantaceae Phrynium pubinerve#R48
Zingiberales Marantaceae Phrynium sp.#F307
Zingiberales Marantaceae Maranta sp.#R62
Zingiberales Costaceae Cheilocostus speciosus#R52
Zingiberales Costaceae Costus spiralis#R60
Zingiberales Costaceae Costus curvibracteatus#R61 1
Zingiberales Zingiberaceae Renealmia alpinia#HQ
Zingiberales Zingiberaceae Alpinia oxyphylla#NC 035895
Zingiberales Zingiberaceae Etlingera elatior#r65
Zingiberales Zingiberaceae Amomum verum#NC 036935
Zingiberales Zingiberaceae Wurfbainia compacta#NC 036992
Zingiberales Zingiberaceae Globba racemosa#R49 1
Zingiberales Zingiberaceae Hedychium spicatum#R67
Zingiberales Zingiberaceae Caulokaempferia yunnanensis#R50
Zingiberales Zingiberaceae Curcuma roscoeana#NC 022928
Zingiberales Zingiberaceae Curcuma longa#LEMW
Zingiberales Zingiberaceae Curcuma flaviflora#NC 028729
Zingiberales Zingiberaceae Zingiber officinale#BDJQ
Zingiberales Zingiberaceae Zingiber spectabile#NC 020363
Zingiberales Zingiberaceae Kaempferia rotunda#R68
Zingiberales Zingiberaceae Kaempferia galanga#NC 040851
Zingiberales Zingiberaceae Kaempferia elegans#NC 040852
Poales Bromeliaceae Brocchinia reducta#BYPY
Poales Bromeliaceae Brocchinia micrantha#HQ
Poales Bromeliaceae Tillandsia usneoides#KY293680
Poales Bromeliaceae Navia saxicola#HQ
Poales Bromeliaceae Pitcairnia feliciana#HQ
Poales Bromeliaceae Fosterella caulescens#HQ
Poales Bromeliaceae Puya laxa#HQ
Poales Bromeliaceae Ananas comosus#F349
Poales Bromeliaceae Neoregelia carolinae#HQ
Poales Typhaceae Sparganium emersum#F979
Poales Typhaceae Sparganium eurycarpum#HQ
Poales Typhaceae Sparganium sp.#F813
Poales Typhaceae Typha latifolia#BRUD
Poales Typhaceae Typha orientalis#F778
Poales Typhaceae Typha angustifolia#F575
Poales Rapateaceae Rapatea paludosa#F557
Poales Rapateaceae Potarophytum riparium#HQ
Poales Thurniaceae Thurnia sphaerocephala#HQ
Poales Thurniaceae Thurnia sphaerocephala#F559
Poales Juncaceae Juncus effusus#HQ
Poales Juncaceae Juncus inflexus#CIEA
Poales Cyperaceae Hypolytrum nemorum#NC 036036
Poales Cyperaceae Mapania palustris#XPAF
Poales Cyperaceae Lepidosperma gibsonii#WBIB
Poales Cyperaceae Cyperus alternifolius#HQ
Poales Cyperaceae Cyperus papyrus#PWSG
Poales Cyperaceae Carex siderosticta#NC 027250
Poales Cyperaceae Carex neurocarpa#NC 036037
Poales Mayacaceae Mayaca fluviatilis#HQ
Poales Xyridaceae Xyris indica#F664
Poales Xyridaceae Abolboda macrostachya#HQ
Poales Eriocaulaceae Syngonanthus chrysanthus#HQ
Poales Eriocaulaceae Eriocaulon buergerianum#MK193813
Poales Eriocaulaceae Eriocaulon sp.#F464
Poales Restionaceae Centrolepis monogyna#HQ
Poales Restionaceae Elegia tectorum#BSTR
Poales Restionaceae Thamnochortus insignis#HQ
Poales Flagellariaceae Flagellaria indica#HQ
Poales Flagellariaceae Flagellaria indica#F708 A
Poales Joinvilleaceae Joinvillea ascendens#WXNT
Poales Joinvilleaceae Joinvillea ascendens#Y247
Poales Joinvilleaceae Joinvillea ascendens#HQ
Poales Ecdeiocoleaceae Ecdeiocolea monostachya#HQ
Poales Ecdeiocoleaceae Georgeantha hexandra#HQ
Poales Poaceae Anomochloa marantoidea#NC 014062
Poales Poaceae Streptochaeta angustifolia#HQ
Poales Poaceae Streptochaeta spicata#NC 033862
Poales Poaceae Leptaspis banksii#NC 033863
Poales Poaceae Leptaspis zeylanica#NC 033864
Poales Poaceae Pharus latifolius#NC 021372
Poales Poaceae Pharus lappulaceus#NC 023245
Poales Poaceae Puelia olyriformis#HQ
Poales Poaceae Streptogyna americana#NC 036683
Poales Poaceae Ehrharta bulbosa#NC 036715
Poales Poaceae Ehrharta stipoides#GU592211
Poales Poaceae Phyllorachis sagittata#NC 036698

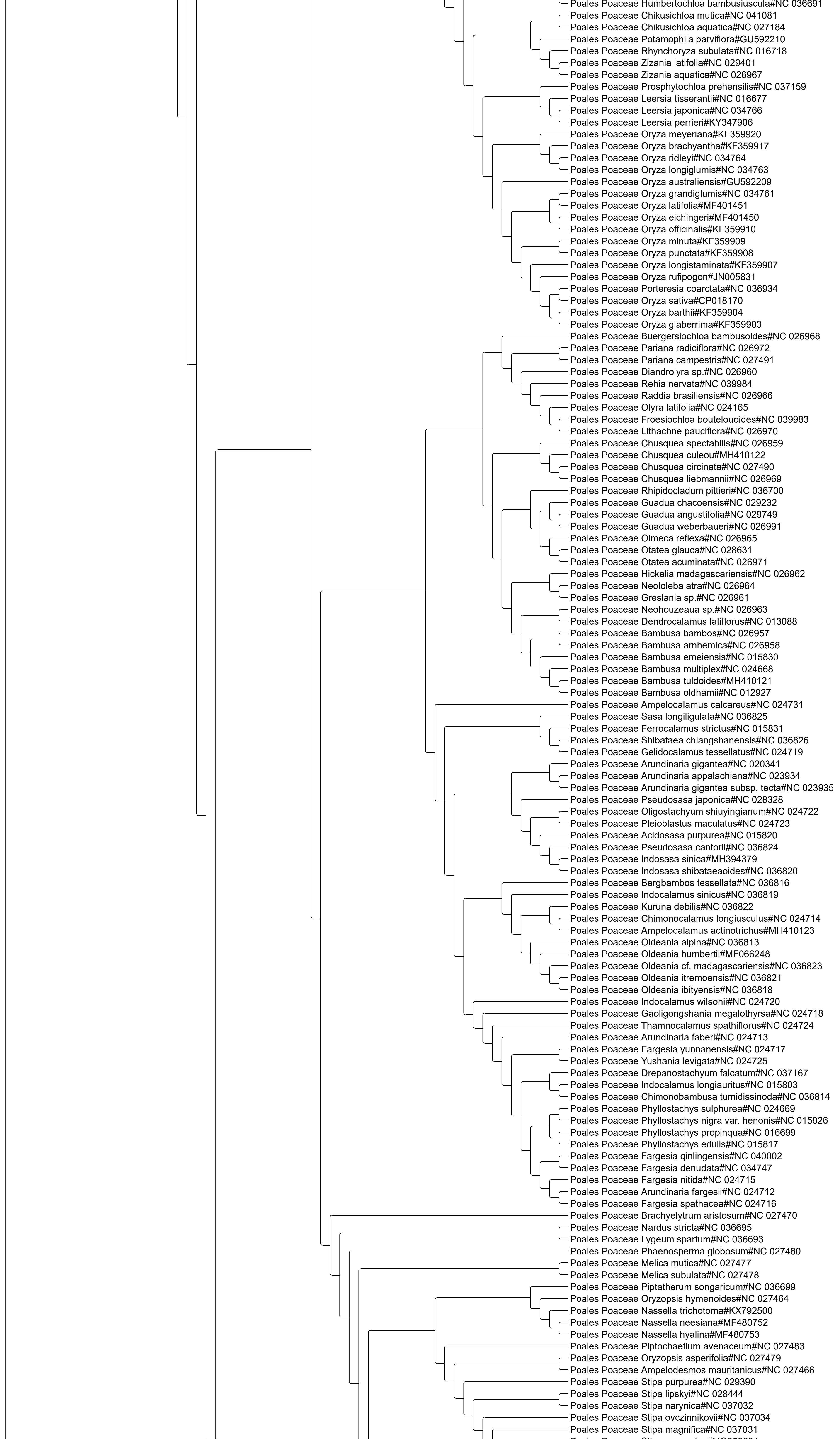

Poales Poaceae Humbertochloa bambusiuscula#NC 036691
Poales Poaceae Chikusichloa mutica#NC 041081
Poales Poaceae Chikusichloa aquatica#NC 027184
Poales Poaceae Potamophila parviflora#GU592210
Poales Poaceae Rhynchoryza subulata#NC 016718
Poales Poaceae Zizania latifolia#NC 029401
Poales Poaceae Zizania aquatica#NC 026967
Poales Poaceae Prosphytochloa prehensilis#NC 037159
Poales Poaceae Leersia tisserantii#NC 016677
Poales Poaceae Leersia japonica#NC 034766
Poales Poaceae Leersia perrieri#KY347906
Poales Poaceae Oryza meyeriana#KF359920
Poales Poaceae Oryza brachyantha#KF359917
Poales Poaceae Oryza ridleyi#NC 034764
Poales Poaceae Oryza longiglumis#NC 034763
Poales Poaceae Oryza australiensis#GU592209
Poales Poaceae Oryza grandiglumis#NC 034761
Poales Poaceae Oryza latifolia#MF401451
Poales Poaceae Oryza eichingeri#MF401450
Poales Poaceae Oryza officinalis#KF359910
Poales Poaceae Oryza minuta#KF359909
Poales Poaceae Oryza punctata#KF359908
Poales Poaceae Oryza longistaminata#KF359907
Poales Poaceae Oryza rufipogon#JN005831
Poales Poaceae Porteresia coarctata#NC 036934
Poales Poaceae Oryza sativa#CP018170
Poales Poaceae Oryza barthii#KF359904
Poales Poaceae Oryza glaberrima#KF359903
Poales Poaceae Buergersiochloa bambusoides#NC 026968
Poales Poaceae Pariana radiciflora#NC 026972
Poales Poaceae Pariana campestris#NC 027491
Poales Poaceae Diandrolyra sp.#NC 026960
Poales Poaceae Rehia nervata#NC 039984
Poales Poaceae Raddia brasiliensis#NC 026966
Poales Poaceae Olyra latifolia#NC 024165
Poales Poaceae Froesiochloa boutelouoides#NC 039983
Poales Poaceae Lithachne pauciflora#NC 026970
Poales Poaceae Chusquea spectabilis#NC 026959
Poales Poaceae Chusquea culeou#MH410122
Poales Poaceae Chusquea circinata#NC 027490
Poales Poaceae Chusquea liebmannii#NC 026969
Poales Poaceae Rhipidocladum pittieri#NC 036700
Poales Poaceae Guadua chacoensis#NC 029232
Poales Poaceae Guadua angustifolia#NC 029749
Poales Poaceae Guadua weberbaueri#NC 026991
Poales Poaceae Olmeca reflexa#NC 026965
Poales Poaceae Otatea glauca#NC 028631
Poales Poaceae Otatea acuminata#NC 026971
Poales Poaceae Hickelia madagascariensis#NC 026962
Poales Poaceae Neololeba atra#NC 026964
Poales Poaceae Greslania sp.#NC 026961
Poales Poaceae Neohouzeaua sp.#NC 026963
Poales Poaceae Dendrocalamus latiflorus#NC 013088
Poales Poaceae Bambusa bambos#NC 026957
Poales Poaceae Bambusa arnhemica#NC 026958
Poales Poaceae Bambusa emeiensis#NC 015830
Poales Poaceae Bambusa multiplex#NC 024668
Poales Poaceae Bambusa tuldoides#MH410121
Poales Poaceae Bambusa oldhamii#NC 012927
Poales Poaceae Ampelocalamus calcareus#NC 024731
Poales Poaceae Sasa longiligulata#NC 036825
Poales Poaceae Ferrocalamus strictus#NC 015831
Poales Poaceae Shibataea chiangshanensis#NC 036826
Poales Poaceae Gelidocalamus tessellatus#NC 024719
Poales Poaceae Arundinaria gigantea#NC 020341
Poales Poaceae Arundinaria appalachiana#NC 023934
Poales Poaceae Arundinaria gigantea subsp. tecta#NC 023935
Poales Poaceae Pseudosasa japonica#NC 028328
Poales Poaceae Oligostachyum shiuyingianum#NC 024722
Poales Poaceae Pleioblastus maculatus#NC 024723
Poales Poaceae Acidosasa purpurea#NC 015820
Poales Poaceae Pseudosasa cantorii#NC 036824
Poales Poaceae Indosasa sinica#MH394379
Poales Poaceae Indosasa shibataeaoides#NC 036820
Poales Poaceae Bergbambos tessellata#NC 036816
Poales Poaceae Indocalamus sinicus#NC 036819
Poales Poaceae Kuruna debilis#NC 036822
Poales Poaceae Chimonocalamus longiusculus#NC 024714
Poales Poaceae Ampelocalamus actinotrichus#MH410123
Poales Poaceae Oldeania alpina#NC 036813
Poales Poaceae Oldeania humbertii#MF066248
Poales Poaceae Oldeania cf. madagascariensis#NC 036823
Poales Poaceae Oldeania itremoensis#NC 036821
Poales Poaceae Oldeania ibityensis#NC 036818
Poales Poaceae Indocalamus wilsonii#NC 024720
Poales Poaceae Gaoligongshania megalothyrsa#NC 024718
Poales Poaceae Thamnocalamus spathiflorus#NC 024724
Poales Poaceae Arundinaria faberi#NC 024713
Poales Poaceae Fargesia yunnanensis#NC 024717
Poales Poaceae Yushania levigata#NC 024725
Poales Poaceae Drepanostachyum falcatum#NC 037167
Poales Poaceae Indocalamus longiauritus#NC 015803
Poales Poaceae Chimonobambusa tumidissinoda#NC 036814
Poales Poaceae Phyllostachys sulphurea#NC 024669
Poales Poaceae Phyllostachys nigra var. henonis#NC 015826
Poales Poaceae Phyllostachys propinqua#NC 016699
Poales Poaceae Phyllostachys edulis#NC 015817
Poales Poaceae Fargesia qinlingensis#NC 040002
Poales Poaceae Fargesia denudata#NC 034747
Poales Poaceae Fargesia nitida#NC 024715
Poales Poaceae Arundinaria fargesii#NC 024712
Poales Poaceae Fargesia spathacea#NC 024716
Poales Poaceae Brachyelytrum aristosum#NC 027470
Poales Poaceae Nardus stricta#NC 036695
Poales Poaceae Lygeum spartum#NC 036693
Poales Poaceae Phaenosperma globosum#NC 027480
Poales Poaceae Melica mutica#NC 027477
Poales Poaceae Melica subulata#NC 027478
Poales Poaceae Piptatherum songaricum#NC 036699
Poales Poaceae Oryzopsis hymenoides#NC 027464
Poales Poaceae Nassella trichotoma#KX792500
Poales Poaceae Nassella neesiana#MF480752
Poales Poaceae Nassella hyalina#MF480753
Poales Poaceae Piptochaetium avenaceum#NC 027483
Poales Poaceae Oryzopsis asperifolia#NC 027479
Poales Poaceae Ampelodesmos mauritanicus#NC 027466
Poales Poaceae Stipa purpurea#NC 029390
Poales Poaceae Stipa lipskyi#NC 028444
Poales Poaceae Stipa narynica#NC 037032
Poales Poaceae Stipa ovczinnikovii#NC 037034
Poales Poaceae Stipa magnifica#NC 037031

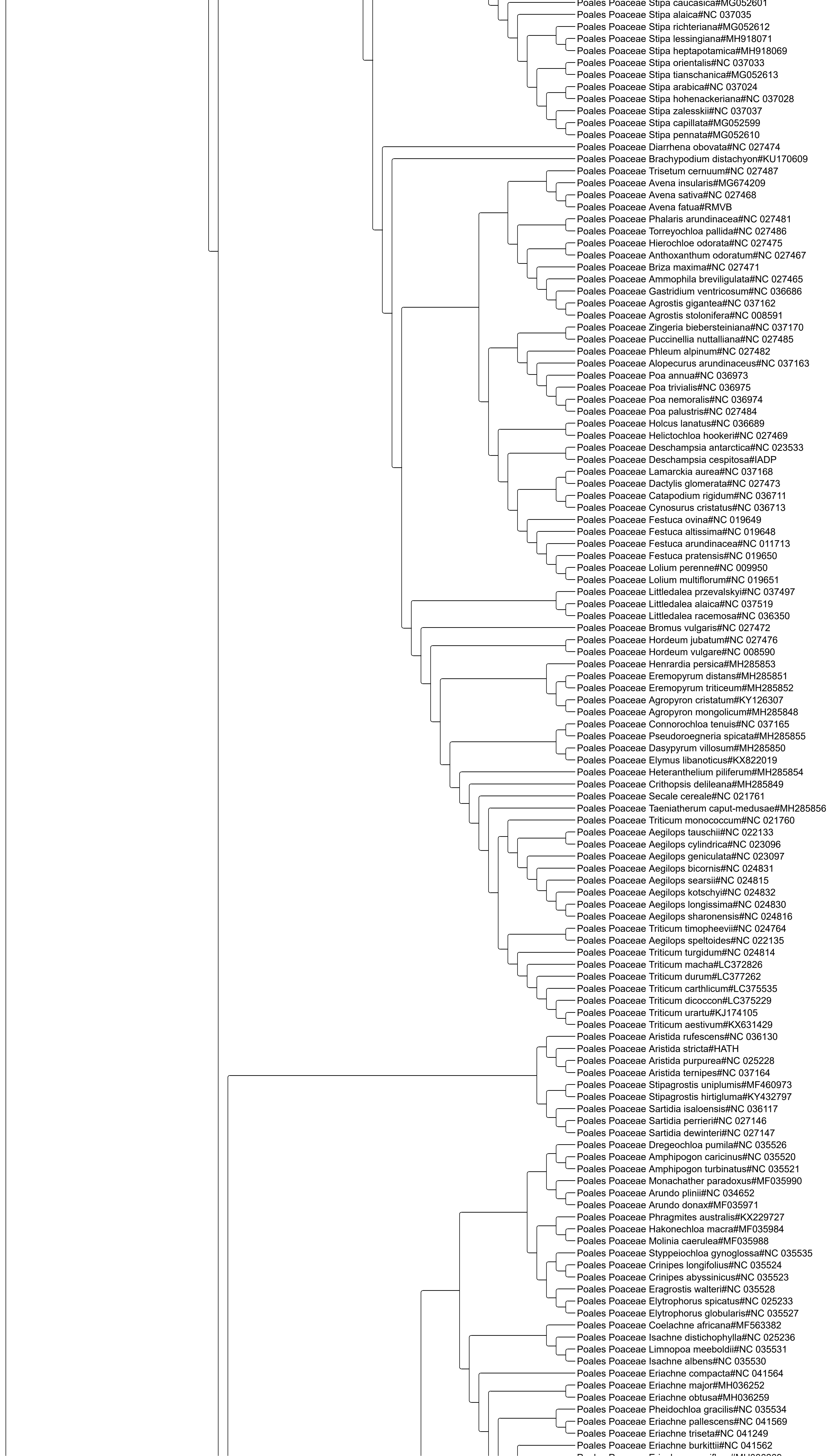
Poales Poaceae Stipa caucasica#MG052601
Poales Poaceae Stipa alaica#NC 037035
Poales Poaceae Stipa richteriana#MG052612
Poales Poaceae Stipa lessingiana#MH918071
Poales Poaceae Stipa heptapotamica#MH918069
Poales Poaceae Stipa orientalis#NC 037033
Poales Poaceae Stipa tianschanica#MG052613
Poales Poaceae Stipa arabica#NC 037024
Poales Poaceae Stipa hohenackeriana#NC 037028
Poales Poaceae Stipa zalesskii#NC 037037
Poales Poaceae Stipa capillata#MG052599
Poales Poaceae Stipa pennata#MG052610
Poales Poaceae Diarrhena obovata#NC 027474
Poales Poaceae Brachypodium distachyon#KU170609
Poales Poaceae Trisetum cernuum#NC 027487
Poales Poaceae Avena insularis#MG674209
Poales Poaceae Avena sativa#NC 027468
Poales Poaceae Avena fatua#RMVB
Poales Poaceae Phalaris arundinacea#NC 027481
Poales Poaceae Torreyochloa pallida#NC 027486
Poales Poaceae Hierochloe odorata#NC 027475
Poales Poaceae Anthoxanthum odoratum#NC 027467
Poales Poaceae Briza maxima#NC 027471
Poales Poaceae Ammophila breviligulata#NC 027465
Poales Poaceae Gastridium ventricosum#NC 036686
Poales Poaceae Agrostis gigantea#NC 037162
Poales Poaceae Agrostis stolonifera#NC 008591
Poales Poaceae Zingeria biebersteiniana#NC 037170
Poales Poaceae Puccinellia nuttalliana#NC 027485
Poales Poaceae Phleum alpinum#NC 027482
Poales Poaceae Alopecurus arundinaceus#NC 037163
Poales Poaceae Poa annua#NC 036973
Poales Poaceae Poa trivialis#NC 036975
Poales Poaceae Poa nemoralis#NC 036974
Poales Poaceae Poa palustris#NC 027484
Poales Poaceae Holcus lanatus#NC 036689
Poales Poaceae Helictochloa hookeri#NC 027469
Poales Poaceae Deschampsia antarctica#NC 023533
Poales Poaceae Deschampsia cespitosa#IADP
Poales Poaceae Lamarckia aurea#NC 037168
Poales Poaceae Dactylis glomerata#NC 027473
Poales Poaceae Catapodium rigidum#NC 036711
Poales Poaceae Cynosurus cristatus#NC 036713
Poales Poaceae Festuca ovina#NC 019649
Poales Poaceae Festuca altissima#NC 019648
Poales Poaceae Festuca arundinacea#NC 011713
Poales Poaceae Festuca pratensis#NC 019650
Poales Poaceae Lolium perenne#NC 009950
Poales Poaceae Lolium multiflorum#NC 019651
Poales Poaceae Littledalea przevalskyi#NC 037497
Poales Poaceae Littledalea alaica#NC 037519
Poales Poaceae Littledalea racemosa#NC 036350
Poales Poaceae Bromus vulgaris#NC 027472
Poales Poaceae Hordeum jubatum#NC 027476
Poales Poaceae Hordeum vulgare#NC 008590
Poales Poaceae Henrardia persica#MH285853
Poales Poaceae Eremopyrum distans#MH285851
Poales Poaceae Eremopyrum triticeum#MH285852
Poales Poaceae Agropyron cristatum#KY126307
Poales Poaceae Agropyron mongolicum#MH285848
Poales Poaceae Connorochloa tenuis#NC 037165
Poales Poaceae Pseudoroegneria spicata#MH285855
Poales Poaceae Dasypyrum villosum#MH285850
Poales Poaceae Elymus libanoticus#KX822019
Poales Poaceae Heteranthelium piliferum#MH285854
Poales Poaceae Crithopsis delileana#MH285849
Poales Poaceae Secale cereale#NC 021761
Poales Poaceae Taeniatherum caput-medusae#MH285856
Poales Poaceae Triticum monococcum#NC 021760
Poales Poaceae Aegilops tauschii#NC 022133
Poales Poaceae Aegilops cylindrica#NC 023096
Poales Poaceae Aegilops geniculata#NC 023097
Poales Poaceae Aegilops bicornis#NC 024831
Poales Poaceae Aegilops searsii#NC 024815
Poales Poaceae Aegilops kotschyi#NC 024832
Poales Poaceae Aegilops longissima#NC 024830
Poales Poaceae Aegilops sharonensis#NC 024816
Poales Poaceae Triticum timopheevii#NC 024764
Poales Poaceae Aegilops speltoides#NC 022135
Poales Poaceae Triticum turgidum#NC 024814
Poales Poaceae Triticum macha#LC372826
Poales Poaceae Triticum durum#LC377262
Poales Poaceae Triticum carthlicum#LC375535
Poales Poaceae Triticum dicoccon#LC375229
Poales Poaceae Triticum urartu#KJ174105
Poales Poaceae Triticum aestivum#KX631429
Poales Poaceae Aristida rufescens#NC 036130
Poales Poaceae Aristida stricta#HATH
Poales Poaceae Aristida purpurea#NC 025228
Poales Poaceae Aristida ternipes#NC 037164
Poales Poaceae Stipagrostis uniplumis#MF460973
Poales Poaceae Stipagrostis hirtigluma#KY432797
Poales Poaceae Sartidia isaloensis#NC 036117
Poales Poaceae Sartidia perrieri#NC 027146
Poales Poaceae Sartidia dewinteri#NC 027147
Poales Poaceae Dregeochloa pumila#NC 035526
Poales Poaceae Amphipogon caricinus#NC 035520
Poales Poaceae Amphipogon turbinatus#NC 035521
Poales Poaceae Monachather paradoxus#MF035990
Poales Poaceae Arundo plinii#NC 034652
Poales Poaceae Arundo donax#MF035971
Poales Poaceae Phragmites australis#KX229727
Poales Poaceae Hakonechloa macra#MF035984
Poales Poaceae Molinia caerulea#MF035988
Poales Poaceae Styppeiochloa gynoglossa#NC 035535
Poales Poaceae Crinipes longifolius#NC 035524
Poales Poaceae Crinipes abyssinicus#NC 035523
Poales Poaceae Eragrostis walteri#NC 035528
Poales Poaceae Elytrophorus spicatus#NC 025233
Poales Poaceae Elytrophorus globularis#NC 035527
Poales Poaceae Coelachne africana#MF563382
Poales Poaceae Isachne distichophylla#NC 025236
Poales Poaceae Limnopoa meeboldii#NC 035531
Poales Poaceae Isachne albens#NC 035530
Poales Poaceae Eriachne compacta#NC 041564
Poales Poaceae Eriachne major#MH036252
Poales Poaceae Eriachne obtusa#MH036259
Poales Poaceae Pheidochloa gracilis#NC 035534
Poales Poaceae Eriachne pallescens#NC 041569
Poales Poaceae Eriachne triseta#NC 041249
Poales Poaceae Eriachne burkittii#NC 041562

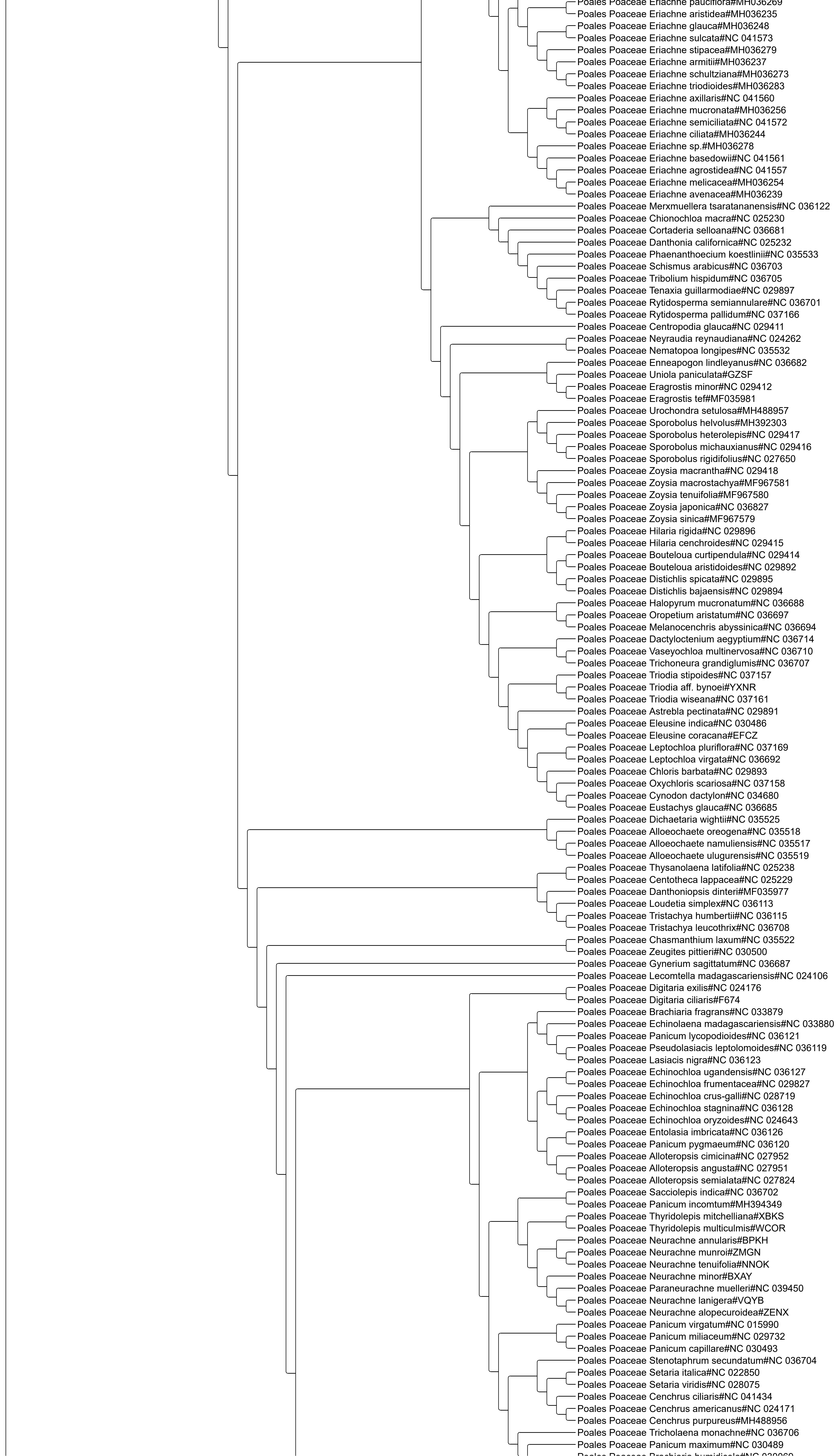

Poales Poaceae Eriachne pauciflora#MH036269
Poales Poaceae Eriachne aristidea#MH036235
Poales Poaceae Eriachne glauca#MH036248
Poales Poaceae Eriachne sulcata#NC 041573
Poales Poaceae Eriachne stipacea#MH036279
Poales Poaceae Eriachne armitii#MH036237
Poales Poaceae Eriachne schultziana#MH036273
Poales Poaceae Eriachne triodioides#MH036283
Poales Poaceae Eriachne axillaris#NC 041560
Poales Poaceae Eriachne mucronata#MH036256
Poales Poaceae Eriachne semiciliata#NC 041572
Poales Poaceae Eriachne ciliata#MH036244
Poales Poaceae Eriachne sp.#MH036278
Poales Poaceae Eriachne basedowii#NC 041561
Poales Poaceae Eriachne agrostidea#NC 041557
Poales Poaceae Eriachne melicacea#MH036254
Poales Poaceae Eriachne avenacea#MH036239
Poales Poaceae Merxmuellera tsaratananensis#NC 036122
Poales Poaceae Chionochloa macra#NC 025230
Poales Poaceae Cortaderia selloana#NC 036681
Poales Poaceae Danthonia californica#NC 025232
Poales Poaceae Phaenanthoecium koestlinii#NC 035533
Poales Poaceae Schismus arabicus#NC 036703
Poales Poaceae Tribolium hispidum#NC 036705
Poales Poaceae Tenaxia guillarmodiae#NC 029897
Poales Poaceae Rytidosperma semiannulare#NC 036701
Poales Poaceae Rytidosperma pallidum#NC 037166
Poales Poaceae Centropodia glauca#NC 029411
Poales Poaceae Neyraudia reynaudiana#NC 024262
Poales Poaceae Nematopoa longipes#NC 035532
Poales Poaceae Enneapogon lindleyanus#NC 036682
Poales Poaceae Uniola paniculata#GZSF
Poales Poaceae Eragrostis minor#NC 029412
Poales Poaceae Eragrostis tef#MF035981
Poales Poaceae Urochondra setulosa#MH488957
Poales Poaceae Sporobolus helvolus#MH392303
Poales Poaceae Sporobolus heterolepis#NC 029417
Poales Poaceae Sporobolus michauxianus#NC 029416
Poales Poaceae Sporobolus rigidifolius#NC 027650
Poales Poaceae Zoysia macrantha#NC 029418
Poales Poaceae Zoysia macrostachya#MF967581
Poales Poaceae Zoysia tenuifolia#MF967580
Poales Poaceae Zoysia japonica#NC 036827
Poales Poaceae Zoysia sinica#MF967579
Poales Poaceae Hilaria rigida#NC 029896
Poales Poaceae Hilaria cenchroides#NC 029415
Poales Poaceae Bouteloua curtipendula#NC 029414
Poales Poaceae Bouteloua aristidoides#NC 029892
Poales Poaceae Distichlis spicata#NC 029895
Poales Poaceae Distichlis bajaensis#NC 029894
Poales Poaceae Halopyrum mucronatum#NC 036688
Poales Poaceae Oropetium aristatum#NC 036697
Poales Poaceae Melanocenchris abyssinica#NC 036694
Poales Poaceae Dactyloctenium aegyptium#NC 036714
Poales Poaceae Vaseyochloa multinervosa#NC 036710
Poales Poaceae Trichoneura grandiglumis#NC 036707
Poales Poaceae Triodia stipoides#NC 037157
Poales Poaceae Triodia aff. bynoei#YXNR
Poales Poaceae Triodia wiseana#NC 037161
Poales Poaceae Astrebla pectinata#NC 029891
Poales Poaceae Eleusine indica#NC 030486
Poales Poaceae Eleusine coracana#EFCZ
Poales Poaceae Leptochloa pluriflora#NC 037169
Poales Poaceae Leptochloa virgata#NC 036692
Poales Poaceae Chloris barbata#NC 029893
Poales Poaceae Oxychloris scariosa#NC 037158
Poales Poaceae Cynodon dactylon#NC 034680
Poales Poaceae Eustachys glauca#NC 036685
Poales Poaceae Dichaetaria wightii#NC 035525
Poales Poaceae Alloeochaete oreogena#NC 035518
Poales Poaceae Alloeochaete namuliensis#NC 035517
Poales Poaceae Alloeochaete ulugurensis#NC 035519
Poales Poaceae Thysanolaena latifolia#NC 025238
Poales Poaceae Centotheca lappacea#NC 025229
Poales Poaceae Danthoniopsis dinteri#MF035977
Poales Poaceae Loudetia simplex#NC 036113
Poales Poaceae Tristachya humbertii#NC 036115
Poales Poaceae Tristachya leucothrix#NC 036708
Poales Poaceae Chasmanthium laxum#NC 035522
Poales Poaceae Zeugites pittieri#NC 030500
Poales Poaceae Gynerium sagittatum#NC 036687
Poales Poaceae Lecomtella madagascariensis#NC 024106
Poales Poaceae Digitaria exilis#NC 024176
Poales Poaceae Digitaria ciliaris#F674
Poales Poaceae Brachiaria fragrans#NC 033879
Poales Poaceae Echinolaena madagascariensis#NC 033880
Poales Poaceae Panicum lycopodioides#NC 036121
Poales Poaceae Pseudolasiacis leptolomoides#NC 036119
Poales Poaceae Lasiacis nigra#NC 036123
Poales Poaceae Echinochloa ugandensis#NC 036127
Poales Poaceae Echinochloa frumentacea#NC 029827
Poales Poaceae Echinochloa crus-galli#NC 028719
Poales Poaceae Echinochloa stagnina#NC 036128
Poales Poaceae Echinochloa oryzoides#NC 024643
Poales Poaceae Entolasia imbricata#NC 036126
Poales Poaceae Panicum pygmaeum#NC 036120
Poales Poaceae Alloteropsis cimicina#NC 027952
Poales Poaceae Alloteropsis angusta#NC 027951
Poales Poaceae Alloteropsis semialata#NC 027824
Poales Poaceae Sacciolepis indica#NC 036702
Poales Poaceae Panicum incomtum#MH394349
Poales Poaceae Thyridolepis mitchelliana#XBKS
Poales Poaceae Thyridolepis multiculmis#WCOR
Poales Poaceae Neurachne annularis#BPKH
Poales Poaceae Neurachne munroi#ZMGN
Poales Poaceae Neurachne tenuifolia#NNOK
Poales Poaceae Neurachne minor#BXAY
Poales Poaceae Paraneurachne muelleri#NC 039450
Poales Poaceae Neurachne lanigera#VQYB
Poales Poaceae Neurachne alopecuroidea#ZENX
Poales Poaceae Panicum virgatum#NC 015990
Poales Poaceae Panicum miliaceum#NC 029732
Poales Poaceae Panicum capillare#NC 030493
Poales Poaceae Stenotaphrum secundatum#NC 036704
Poales Poaceae Setaria italica#NC 022850
Poales Poaceae Setaria viridis#NC 028075
Poales Poaceae Cenchrus ciliaris#NC 041434
Poales Poaceae Cenchrus americanus#NC 024171
Poales Poaceae Cenchrus purpureus#MH488956
Poales Poaceae Tricholaena monachne#NC 036706
Poales Poaceae Panicum maximum#NC 030489

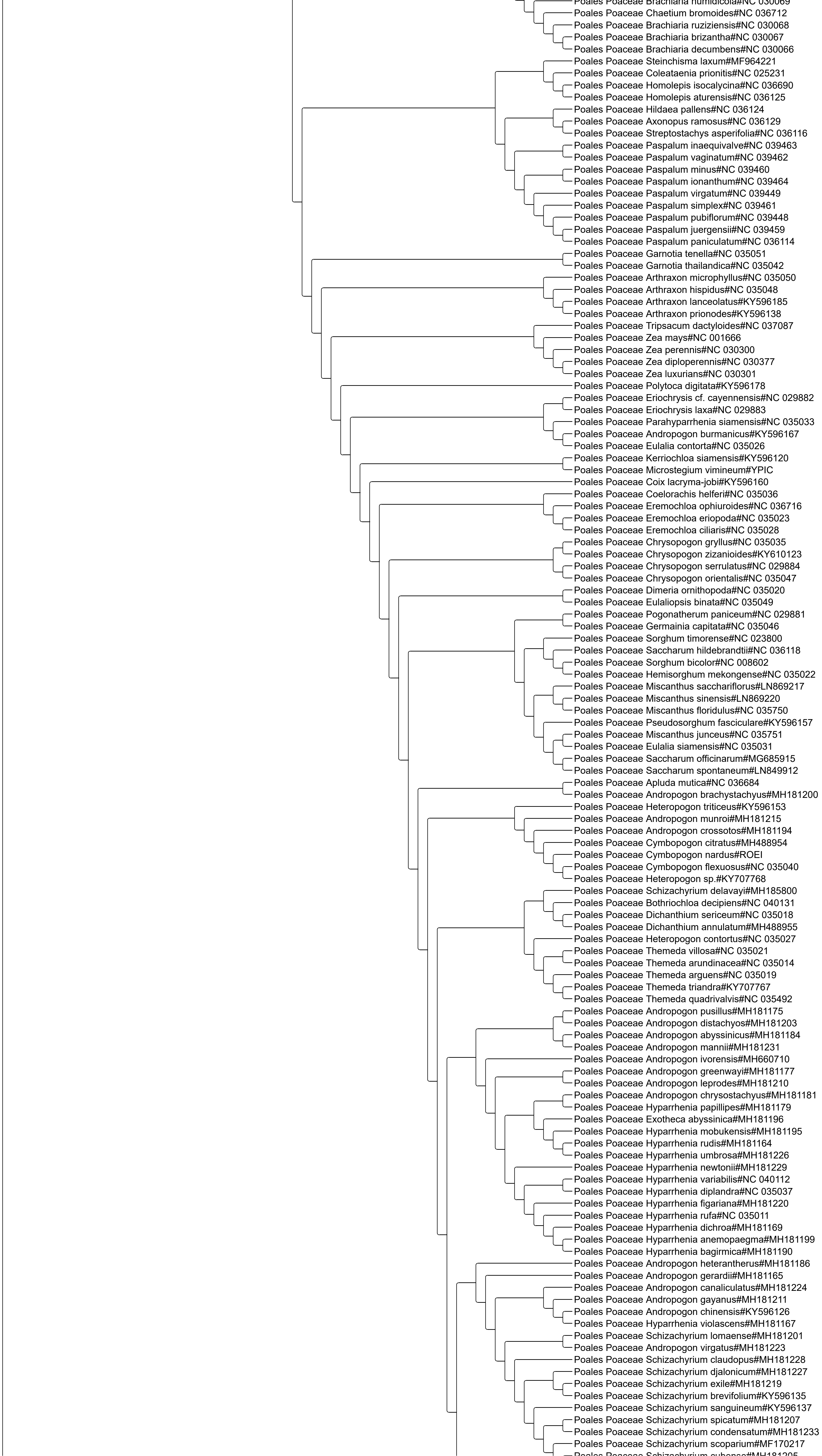

Poales Poaceae Brachiaria humidicola#NC 030069
Poales Poaceae Chaetium bromoides#NC 036712
Poales Poaceae Brachiaria ruziziensis#NC 030068
Poales Poaceae Brachiaria brizantha#NC 030067
Poales Poaceae Brachiaria decumbens#NC 030066
Poales Poaceae Steinchisma laxum#MF964221
Poales Poaceae Coleataenia prionitis#NC 025231
Poales Poaceae Homolepis isocalycina#NC 036690
Poales Poaceae Homolepis aturensis#NC 036125
Poales Poaceae Hildaea pallens#NC 036124
Poales Poaceae Axonopus ramosus#NC 036129
Poales Poaceae Streptostachys asperifolia#NC 036116
Poales Poaceae Paspalum inaequivalve#NC 039463
Poales Poaceae Paspalum vaginatum#NC 039462
Poales Poaceae Paspalum minus#NC 039460
Poales Poaceae Paspalum ionanthum#NC 039464
Poales Poaceae Paspalum virgatum#NC 039449
Poales Poaceae Paspalum simplex#NC 039461
Poales Poaceae Paspalum pubiflorum#NC 039448
Poales Poaceae Paspalum juergensii#NC 039459
Poales Poaceae Paspalum paniculatum#NC 036114
Poales Poaceae Garnotia tenella#NC 035051
Poales Poaceae Garnotia thailandica#NC 035042
Poales Poaceae Arthraxon microphyllus#NC 035050
Poales Poaceae Arthraxon hispidus#NC 035048
Poales Poaceae Arthraxon lanceolatus#KY596185
Poales Poaceae Arthraxon prionodes#KY596138
Poales Poaceae Tripsacum dactyloides#NC 037087
Poales Poaceae Zea mays#NC 001666
Poales Poaceae Zea perennis#NC 030300
Poales Poaceae Zea diploperennis#NC 030377
Poales Poaceae Zea luxurians#NC 030301
Poales Poaceae Polytoca digitata#KY596178
Poales Poaceae Eriochrysis cf. cayennensis#NC 029882
Poales Poaceae Eriochrysis laxa#NC 029883
Poales Poaceae Parahyparrhenia siamensis#NC 035033
Poales Poaceae Andropogon burmanicus#KY596167
Poales Poaceae Eulalia contorta#NC 035026
Poales Poaceae Kerriochloa siamensis#KY596120
Poales Poaceae Microstegium vimineum#YPIC
Poales Poaceae Coix lacryma-jobi#KY596160
Poales Poaceae Coelorachis helferi#NC 035036
Poales Poaceae Eremochloa ophiuroides#NC 036716
Poales Poaceae Eremochloa eriopoda#NC 035023
Poales Poaceae Eremochloa ciliaris#NC 035028
Poales Poaceae Chrysopogon gryllus#NC 035035
Poales Poaceae Chrysopogon zizanioides#KY610123
Poales Poaceae Chrysopogon serrulatus#NC 029884
Poales Poaceae Chrysopogon orientalis#NC 035047
Poales Poaceae Dimeria ornithopoda#NC 035020
Poales Poaceae Eulaliopsis binata#NC 035049
Poales Poaceae Pogonatherum paniceum#NC 029881
Poales Poaceae Germainia capitata#NC 035046
Poales Poaceae Sorghum timorense#NC 023800
Poales Poaceae Saccharum hildebrandtii#NC 036118
Poales Poaceae Sorghum bicolor#NC 008602
Poales Poaceae Hemisorghum mekongense#NC 035022
Poales Poaceae Miscanthus sacchariflorus#LN869217
Poales Poaceae Miscanthus sinensis#LN869220
Poales Poaceae Miscanthus floridulus#NC 035750
Poales Poaceae Pseudosorghum fasciculare#KY596157
Poales Poaceae Miscanthus junceus#NC 035751
Poales Poaceae Eulalia siamensis#NC 035031
Poales Poaceae Saccharum officinarum#MG685915
Poales Poaceae Saccharum spontaneum#LN849912
Poales Poaceae Apluda mutica#NC 036684
Poales Poaceae Andropogon brachystachyus#MH181200
Poales Poaceae Heteropogon triticeus#KY596153
Poales Poaceae Andropogon munroi#MH181215
Poales Poaceae Andropogon crossotos#MH181194
Poales Poaceae Cymbopogon citratus#MH488954
Poales Poaceae Cymbopogon nardus#ROEI
Poales Poaceae Cymbopogon flexuosus#NC 035040
Poales Poaceae Heteropogon sp.#KY707768
Poales Poaceae Schizachyrium delavayi#MH185800
Poales Poaceae Bothriochloa decipiens#NC 040131
Poales Poaceae Dichanthium sericeum#NC 035018
Poales Poaceae Dichanthium annulatum#MH488955
Poales Poaceae Heteropogon contortus#NC 035027
Poales Poaceae Themeda villosa#NC 035021
Poales Poaceae Themeda arundinacea#NC 035014
Poales Poaceae Themeda arguens#NC 035019
Poales Poaceae Themeda triandra#KY707767
Poales Poaceae Themeda quadrivalvis#NC 035492
Poales Poaceae Andropogon pusillus#MH181175
Poales Poaceae Andropogon distachyos#MH181203
Poales Poaceae Andropogon abyssinicus#MH181184
Poales Poaceae Andropogon mannii#MH181231
Poales Poaceae Andropogon ivorensis#MH660710
Poales Poaceae Andropogon greenwayi#MH181177
Poales Poaceae Andropogon leprodes#MH181210
Poales Poaceae Andropogon chrysostachyus#MH181181
Poales Poaceae Hyparrhenia papillipes#MH181179
Poales Poaceae Exotheca abyssinica#MH181196
Poales Poaceae Hyparrhenia mobukensis#MH181195
Poales Poaceae Hyparrhenia rudis#MH181164
Poales Poaceae Hyparrhenia umbrosa#MH181226
Poales Poaceae Hyparrhenia newtonii#MH181229
Poales Poaceae Hyparrhenia variabilis#NC 040112
Poales Poaceae Hyparrhenia diplandra#NC 035037
Poales Poaceae Hyparrhenia figariana#MH181220
Poales Poaceae Hyparrhenia rufa#NC 035011
Poales Poaceae Hyparrhenia dichroa#MH181169
Poales Poaceae Hyparrhenia anemopaegma#MH181199
Poales Poaceae Hyparrhenia bagirmica#MH181190
Poales Poaceae Andropogon heterantherus#MH181186
Poales Poaceae Andropogon gerardii#MH181165
Poales Poaceae Andropogon canaliculatus#MH181224
Poales Poaceae Andropogon gayanus#MH181211
Poales Poaceae Andropogon chinensis#KY596126
Poales Poaceae Hyparrhenia violascens#MH181167
Poales Poaceae Schizachyrium lomaense#MH181201
Poales Poaceae Andropogon virgatus#MH181223
Poales Poaceae Schizachyrium claudopus#MH181228
Poales Poaceae Schizachyrium djalonicum#MH181227
Poales Poaceae Schizachyrium exile#MH181219
Poales Poaceae Schizachyrium brevifolium#KY596135
Poales Poaceae Schizachyrium sanguineum#KY596137
Poales Poaceae Schizachyrium spicatum#MH181207
Poales Poaceae Schizachyrium condensatum#MH181233
Poales Poaceae Schizachyrium scoparium#MF170217

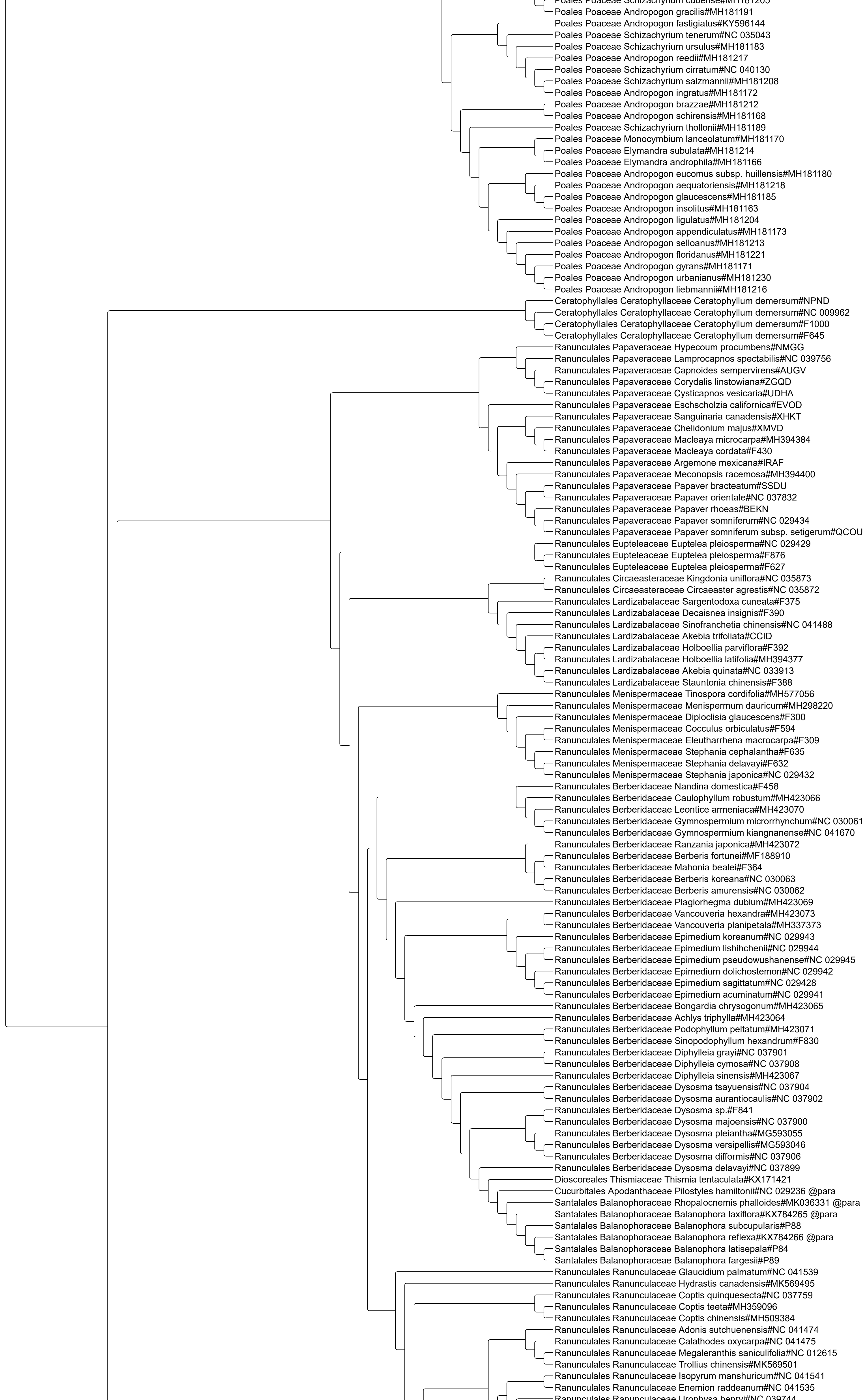
Poales Poaceae Andropogon gracilis#MH181191
Poales Poaceae Andropogon fastigiatus#KY596144
Poales Poaceae Schizachyrium tenerum#NC 035043
Poales Poaceae Schizachyrium ursulus#MH181183
Poales Poaceae Andropogon reedii#MH181217
Poales Poaceae Schizachyrium cirratum#NC 040130
Poales Poaceae Schizachyrium salzmannii#MH181208
Poales Poaceae Andropogon ingratus#MH181172
Poales Poaceae Andropogon brazzae#MH181212
Poales Poaceae Andropogon schirensis#MH181168
Poales Poaceae Schizachyrium thollonii#MH181189
Poales Poaceae Monocymbium lanceolatum#MH181170
Poales Poaceae Elymandra subulata#MH181214
Poales Poaceae Elymandra androphila#MH181166
Poales Poaceae Andropogon eucomus subsp. huillensis#MH181180
Poales Poaceae Andropogon aequatoriensis#MH181218
Poales Poaceae Andropogon glaucescens#MH181185
Poales Poaceae Andropogon insolitus#MH181163
Poales Poaceae Andropogon ligulatus#MH181204
Poales Poaceae Andropogon appendiculatus#MH181173
Poales Poaceae Andropogon selloanus#MH181213
Poales Poaceae Andropogon floridanus#MH181221
Poales Poaceae Andropogon gyrans#MH181171
Poales Poaceae Andropogon urbanianus#MH181230
Poales Poaceae Andropogon liebmannii#MH181216
Ceratophyllales Ceratophyllaceae Ceratophyllum demersum#NPND
Ceratophyllales Ceratophyllaceae Ceratophyllum demersum#NC 009962
Ceratophyllales Ceratophyllaceae Ceratophyllum demersum#F1000
Ceratophyllales Ceratophyllaceae Ceratophyllum demersum#F645
Ranunculales Papaveraceae Hypecoum procumbens#NMGG
Ranunculales Papaveraceae Lamprocapnos spectabilis#NC 039756
Ranunculales Papaveraceae Capnoides sempervirens#AUGV
Ranunculales Papaveraceae Corydalis linstowiana#ZGQD
Ranunculales Papaveraceae Cysticapnos vesicaria#UDHA
Ranunculales Papaveraceae Eschscholzia californica#EVOD
Ranunculales Papaveraceae Sanguinaria canadensis#XHKT
Ranunculales Papaveraceae Chelidonium majus#XMVD
Ranunculales Papaveraceae Macleaya microcarpa#MH394384
Ranunculales Papaveraceae Macleaya cordata#F430
Ranunculales Papaveraceae Argemone mexicana#IRAF
Ranunculales Papaveraceae Meconopsis racemosa#MH394400
Ranunculales Papaveraceae Papaver bracteatum#SSDU
Ranunculales Papaveraceae Papaver orientale#NC 037832
Ranunculales Papaveraceae Papaver rhoeas#BEKN
Ranunculales Papaveraceae Papaver somniferum#NC 029434
Ranunculales Papaveraceae Papaver somniferum subsp. setigerum#QCOU
Ranunculales Eupteleaceae Euptelea pleiosperma#NC 029429
Ranunculales Eupteleaceae Euptelea pleiosperma#F876
Ranunculales Eupteleaceae Euptelea pleiosperma#F627
Ranunculales Circaeasteraceae Kingdonia uniflora#NC 035873
Ranunculales Circaeasteraceae Circaeaster agrestis#NC 035872
Ranunculales Lardizabalaceae Sargentodoxa cuneata#F375
Ranunculales Lardizabalaceae Decaisnea insignis#F390
Ranunculales Lardizabalaceae Sinofranchetia chinensis#NC 041488
Ranunculales Lardizabalaceae Akebia trifoliata#CCID
Ranunculales Lardizabalaceae Holboellia parviflora#F392
Ranunculales Lardizabalaceae Holboellia latifolia#MH394377
Ranunculales Lardizabalaceae Akebia quinata#NC 033913
Ranunculales Lardizabalaceae Stauntonia chinensis#F388
Ranunculales Menispermaceae Tinospora cordifolia#MH577056
Ranunculales Menispermaceae Menispermum dauricum#MH298220
Ranunculales Menispermaceae Diploclisia glaucescens#F300
Ranunculales Menispermaceae Cocculus orbiculatus#F594
Ranunculales Menispermaceae Eleutharrhena macrocarpa#F309
Ranunculales Menispermaceae Stephania cephalantha#F635
Ranunculales Menispermaceae Stephania delavayi#F632
Ranunculales Menispermaceae Stephania japonica#NC 029432
Ranunculales Berberidaceae Nandina domestica#F458
Ranunculales Berberidaceae Caulophyllum robustum#MH423066
Ranunculales Berberidaceae Leontice armeniaca#MH423070
Ranunculales Berberidaceae Gymnospermium microrrhynchum#NC 030061
Ranunculales Berberidaceae Gymnospermium kiangnanense#NC 041670
Ranunculales Berberidaceae Ranzania japonica#MH423072
Ranunculales Berberidaceae Berberis fortunei#MF188910
Ranunculales Berberidaceae Mahonia bealei#F364
Ranunculales Berberidaceae Berberis koreana#NC 030063
Ranunculales Berberidaceae Berberis amurensis#NC 030062
Ranunculales Berberidaceae Plagiorhegma dubium#MH423069
Ranunculales Berberidaceae Vancouveria hexandra#MH423073
Ranunculales Berberidaceae Vancouveria planipetala#MH337373
Ranunculales Berberidaceae Epimedium koreanum#NC 029943
Ranunculales Berberidaceae Epimedium lishihchenii#NC 029944
Ranunculales Berberidaceae Epimedium pseudowushanense#NC 029945
Ranunculales Berberidaceae Epimedium dolichostemon#NC 029942
Ranunculales Berberidaceae Epimedium sagittatum#NC 029428
Ranunculales Berberidaceae Epimedium acuminatum#NC 029941
Ranunculales Berberidaceae Bongardia chrysogonum#MH423065
Ranunculales Berberidaceae Achlys triphylla#MH423064
Ranunculales Berberidaceae Podophyllum peltatum#MH423071
Ranunculales Berberidaceae Sinopodophyllum hexandrum#F830
Ranunculales Berberidaceae Diphylleia grayi#NC 037901
Ranunculales Berberidaceae Diphylleia cymosa#NC 037908
Ranunculales Berberidaceae Diphylleia sinensis#MH423067
Ranunculales Berberidaceae Dysosma tsayuensis#NC 037904
Ranunculales Berberidaceae Dysosma aurantiocaulis#NC 037902
Ranunculales Berberidaceae Dysosma sp.#F841
Ranunculales Berberidaceae Dysosma majoensis#NC 037900
Ranunculales Berberidaceae Dysosma pleiantha#MG593055
Ranunculales Berberidaceae Dysosma versipellis#MG593046
Ranunculales Berberidaceae Dysosma difformis#NC 037906
Ranunculales Berberidaceae Dysosma delavayi#NC 037899
Dioscoreales Thismiaceae Thismia tentaculata#KX171421
Cucurbitales Apodanthaceae Pilostyles hamiltonii#NC 029236 @para
Santalales Balanophoraceae Rhopalocnemis phalloides#MK036331 @para
Santalales Balanophoraceae Balanophora laxiflora#KX784265 @para
Santalales Balanophoraceae Balanophora subcupularis#P88
Santalales Balanophoraceae Balanophora reflexa#KX784266 @para
Santalales Balanophoraceae Balanophora latisepala#P84
Santalales Balanophoraceae Balanophora fargesii#P89
Ranunculales Ranunculaceae Glaucidium palmatum#NC 041539
Ranunculales Ranunculaceae Hydrastis canadensis#MK569495
Ranunculales Ranunculaceae Coptis quinquesecta#NC 037759
Ranunculales Ranunculaceae Coptis teeta#MH359096
Ranunculales Ranunculaceae Coptis chinensis#MH509384
Ranunculales Ranunculaceae Adonis sutchuenensis#NC 041474
Ranunculales Ranunculaceae Calathodes oxycarpa#NC 041475
Ranunculales Ranunculaceae Megaleranthis saniculifolia#NC 012615
Ranunculales Ranunculaceae Trollius chinensis#MK569501
Ranunculales Ranunculaceae Isopyrum manshuricum#NC 041541
Ranunculales Ranunculaceae Enemion raddeanum#NC 041535

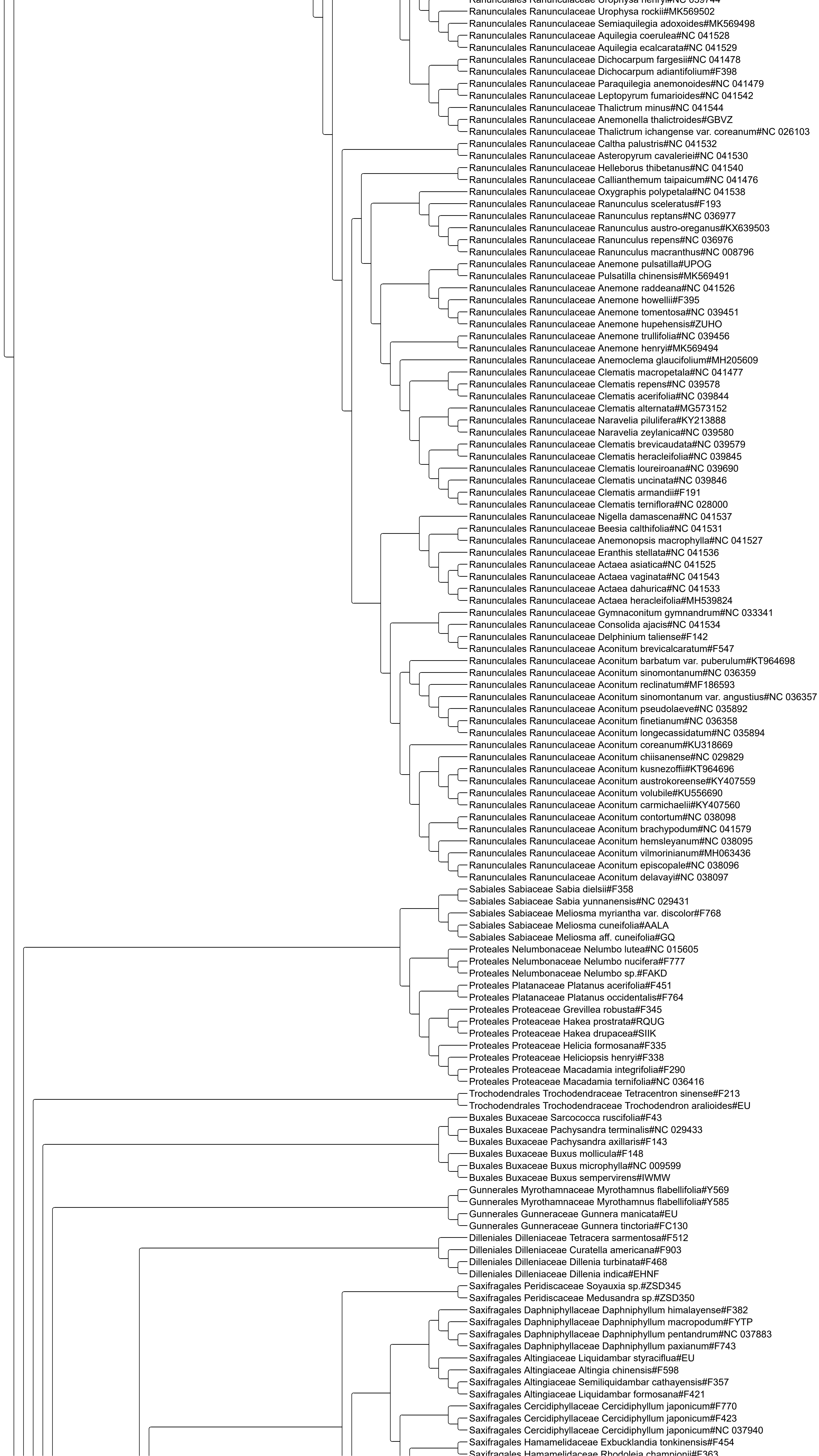
Ranunculales Ranunculaceae Urophysa rockii#MK569502
Ranunculales Ranunculaceae Semiaquilegia adoxoides#MK569498
Ranunculales Ranunculaceae Aquilegia coerulea#NC 041528
Ranunculales Ranunculaceae Aquilegia ecalcarata#NC 041529
Ranunculales Ranunculaceae Dichocarpum fargesii#NC 041478
Ranunculales Ranunculaceae Dichocarpum adiantifolium#F398
Ranunculales Ranunculaceae Paraquilegia anemonoides#NC 041479
Ranunculales Ranunculaceae Leptopyrum fumarioides#NC 041542
Ranunculales Ranunculaceae Thalictrum minus#NC 041544
Ranunculales Ranunculaceae Anemonella thalictroides#GBVZ
Ranunculales Ranunculaceae Thalictrum ichangense var. coreanum#NC 026103
Ranunculales Ranunculaceae Caltha palustris#NC 041532
Ranunculales Ranunculaceae Asteropyrum cavaleriei#NC 041530
Ranunculales Ranunculaceae Helleborus thibetanus#NC 041540
Ranunculales Ranunculaceae Callianthemum taipaicum#NC 041476
Ranunculales Ranunculaceae Oxygraphis polypetala#NC 041538
Ranunculales Ranunculaceae Ranunculus sceleratus#F193
Ranunculales Ranunculaceae Ranunculus reptans#NC 036977
Ranunculales Ranunculaceae Ranunculus austro-oreganus#KX639503
Ranunculales Ranunculaceae Ranunculus repens#NC 036976
Ranunculales Ranunculaceae Ranunculus macranthus#NC 008796
Ranunculales Ranunculaceae Anemone pulsatilla#UPOG
Ranunculales Ranunculaceae Pulsatilla chinensis#MK569491
Ranunculales Ranunculaceae Anemone raddeana#NC 041526
Ranunculales Ranunculaceae Anemone howellii#F395
Ranunculales Ranunculaceae Anemone tomentosa#NC 039451
Ranunculales Ranunculaceae Anemone hupehensis#ZUHO
Ranunculales Ranunculaceae Anemone trullifolia#NC 039456
Ranunculales Ranunculaceae Anemone henryi#MK569494
Ranunculales Ranunculaceae Anemoclema glaucifolium#MH205609
Ranunculales Ranunculaceae Clematis macropetala#NC 041477
Ranunculales Ranunculaceae Clematis repens#NC 039578
Ranunculales Ranunculaceae Clematis acerifolia#NC 039844
Ranunculales Ranunculaceae Clematis alternata#MG573152
Ranunculales Ranunculaceae Naravelia pilulifera#KY213888
Ranunculales Ranunculaceae Naravelia zeylanica#NC 039580
Ranunculales Ranunculaceae Clematis brevicaudata#NC 039579
Ranunculales Ranunculaceae Clematis heracleifolia#NC 039845
Ranunculales Ranunculaceae Clematis loureiroana#NC 039690
Ranunculales Ranunculaceae Clematis uncinata#NC 039846
Ranunculales Ranunculaceae Clematis armandii#F191
Ranunculales Ranunculaceae Clematis terniflora#NC 028000
Ranunculales Ranunculaceae Nigella damascena#NC 041537
Ranunculales Ranunculaceae Beesia calthifolia#NC 041531
Ranunculales Ranunculaceae Anemonopsis macrophylla#NC 041527
Ranunculales Ranunculaceae Eranthis stellata#NC 041536
Ranunculales Ranunculaceae Actaea asiatica#NC 041525
Ranunculales Ranunculaceae Actaea vaginata#NC 041543
Ranunculales Ranunculaceae Actaea dahurica#NC 041533
Ranunculales Ranunculaceae Actaea heracleifolia#MH539824
Ranunculales Ranunculaceae Gymnaconitum gymnandrum#NC 033341
Ranunculales Ranunculaceae Consolida ajacis#NC 041534
Ranunculales Ranunculaceae Delphinium taliense#F142
Ranunculales Ranunculaceae Aconitum brevicalcaratum#F547
Ranunculales Ranunculaceae Aconitum barbatum var. puberulum#KT964698
Ranunculales Ranunculaceae Aconitum sinomontanum#NC 036359
Ranunculales Ranunculaceae Aconitum reclinatum#MF186593
Ranunculales Ranunculaceae Aconitum sinomontanum var. angustius#NC 036357
Ranunculales Ranunculaceae Aconitum pseudolaeve#NC 035892
Ranunculales Ranunculaceae Aconitum finetianum#NC 036358
Ranunculales Ranunculaceae Aconitum longecassidatum#NC 035894
Ranunculales Ranunculaceae Aconitum coreanum#KU318669
Ranunculales Ranunculaceae Aconitum chiisanense#NC 029829
Ranunculales Ranunculaceae Aconitum kusnezoffii#KT964696
Ranunculales Ranunculaceae Aconitum austrokoreense#KY407559
Ranunculales Ranunculaceae Aconitum volubile#KU556690
Ranunculales Ranunculaceae Aconitum carmichaelii#KY407560
Ranunculales Ranunculaceae Aconitum contortum#NC 038098
Ranunculales Ranunculaceae Aconitum brachypodum#NC 041579
Ranunculales Ranunculaceae Aconitum hemsleyanum#NC 038095
Ranunculales Ranunculaceae Aconitum vilmorinianum#MH063436
Ranunculales Ranunculaceae Aconitum episcopale#NC 038096
Ranunculales Ranunculaceae Aconitum delavayi#NC 038097
Sabiales Sabiaceae Sabia dielsii#F358
Sabiales Sabiaceae Sabia yunnanensis#NC 029431
Sabiales Sabiaceae Meliosma myriantha var. discolor#F768
Sabiales Sabiaceae Meliosma cuneifolia#AALA
Sabiales Sabiaceae Meliosma aff. cuneifolia#GQ
Proteales Nelumbonaceae Nelumbo lutea#NC 015605
Proteales Nelumbonaceae Nelumbo nucifera#F777
Proteales Nelumbonaceae Nelumbo sp.#FAKD
Proteales Platanaceae Platanus acerifolia#F451
Proteales Platanaceae Platanus occidentalis#F764
Proteales Proteaceae Grevillea robusta#F345
Proteales Proteaceae Hakea prostrata#RQUG
Proteales Proteaceae Hakea drupacea#SIIK
Proteales Proteaceae Helicia formosana#F335
Proteales Proteaceae Heliciopsis henryi#F338
Proteales Proteaceae Macadamia integrifolia#F290
Proteales Proteaceae Macadamia ternifolia#NC 036416
Trochodendrales Trochodendraceae Tetracentron sinense#F213
Trochodendrales Trochodendraceae Trochodendron aralioides#EU
Buxales Buxaceae Sarcococca ruscifolia#F43
Buxales Buxaceae Pachysandra terminalis#NC 029433
Buxales Buxaceae Pachysandra axillaris#F143
Buxales Buxaceae Buxus mollicula#F148
Buxales Buxaceae Buxus microphylla#NC 009599
Buxales Buxaceae Buxus sempervirens#IWMW
Gunnerales Myrothamnaceae Myrothamnus flabellifolia#Y569
Gunnerales Myrothamnaceae Myrothamnus flabellifolia#Y585
Gunnerales Gunneraceae Gunnera manicata#EU
Gunnerales Gunneraceae Gunnera tinctoria#FC130
Dilleniales Dilleniaceae Tetracera sarmentosa#F512
Dilleniales Dilleniaceae Curatella americana#F903
Dilleniales Dilleniaceae Dillenia turbinata#F468
Dilleniales Dilleniaceae Dillenia indica#EHNF
Saxifragales Peridiscaceae Soyauxia sp.#ZSD345
Saxifragales Peridiscaceae Medusandra sp.#ZSD350
Saxifragales Daphniphyllaceae Daphniphyllum himalayense#F382
Saxifragales Daphniphyllaceae Daphniphyllum macropodum#FYTP
Saxifragales Daphniphyllaceae Daphniphyllum pentandrum#NC 037883
Saxifragales Daphniphyllaceae Daphniphyllum paxianum#F743
Saxifragales Altingiaceae Liquidambar styraciflua#EU
Saxifragales Altingiaceae Altingia chinensis#F598
Saxifragales Altingiaceae Semiliquidambar cathayensis#F357
Saxifragales Altingiaceae Liquidambar formosana#F421
Saxifragales Cercidiphyllaceae Cercidiphyllum japonicum#F770
Saxifragales Cercidiphyllaceae Cercidiphyllum japonicum#F423
Saxifragales Cercidiphyllaceae Cercidiphyllum japonicum#NC 037940
Saxifragales Hamamelidaceae Exbucklandia tonkinensis#F454

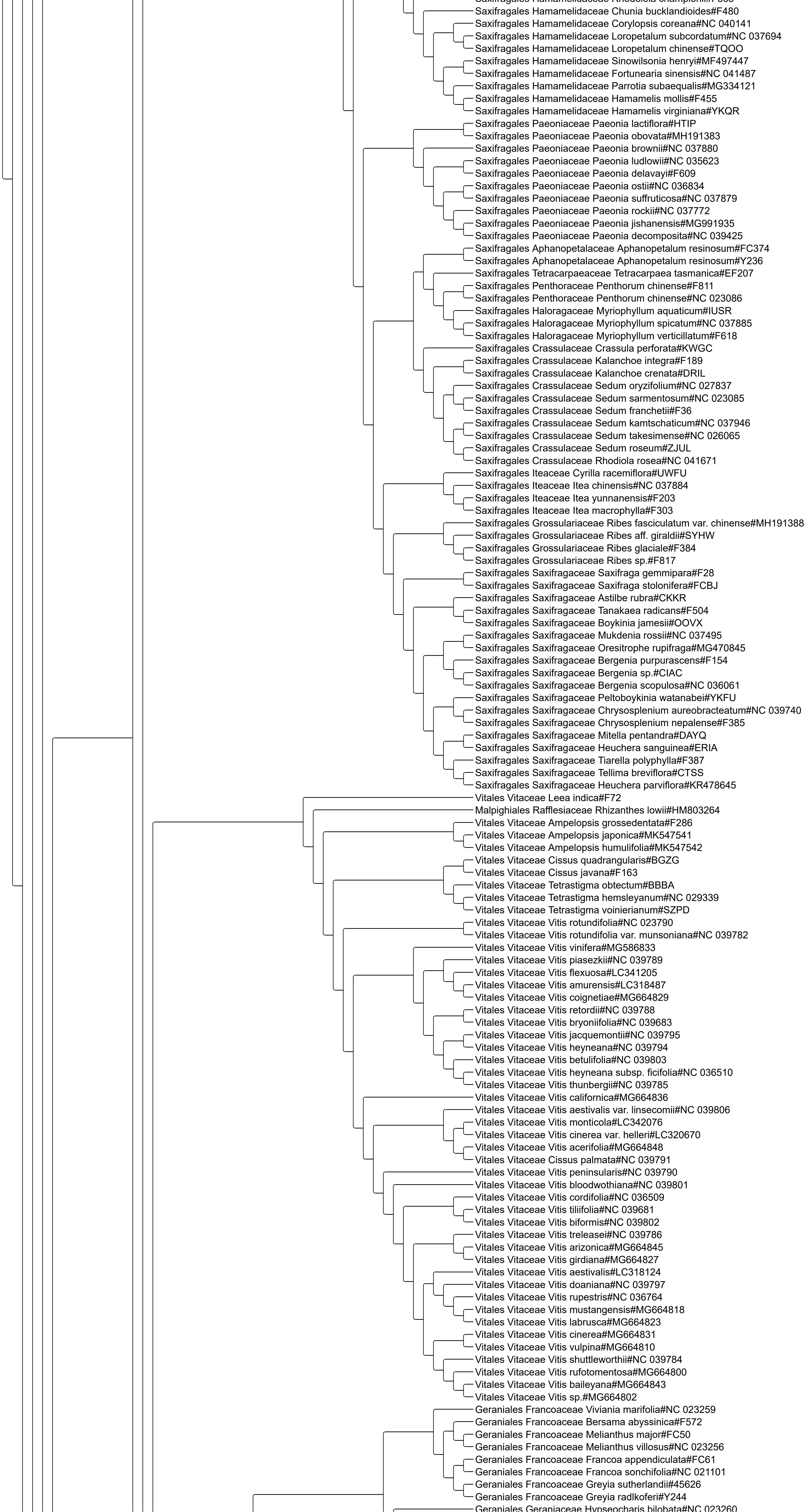
Saxifragales Hamamelidaceae Chunia bucklandioides#F480
Saxifragales Hamamelidaceae Corylopsis coreana#NC 040141
Saxifragales Hamamelidaceae Loropetalum subcordatum#NC 037694
Saxifragales Hamamelidaceae Loropetalum chinense#TQOO
Saxifragales Hamamelidaceae Sinowilsonia henryi#MF497447
Saxifragales Hamamelidaceae Fortunearia sinensis#NC 041487
Saxifragales Hamamelidaceae Parrotia subaequalis#MG334121
Saxifragales Hamamelidaceae Hamamelis mollis#F455
Saxifragales Hamamelidaceae Hamamelis virginiana#YKQR
Saxifragales Paeoniaceae Paeonia lactiflora#HTIP
Saxifragales Paeoniaceae Paeonia obovata#MH191383
Saxifragales Paeoniaceae Paeonia brownii#NC 037880
Saxifragales Paeoniaceae Paeonia ludlowii#NC 035623
Saxifragales Paeoniaceae Paeonia delavayi#F609
Saxifragales Paeoniaceae Paeonia ostii#NC 036834
Saxifragales Paeoniaceae Paeonia suffruticosa#NC 037879
Saxifragales Paeoniaceae Paeonia rockii#NC 037772
Saxifragales Paeoniaceae Paeonia jishanensis#MG991935
Saxifragales Paeoniaceae Paeonia decomposita#NC 039425
Saxifragales Aphanopetalaceae Aphanopetalum resinosum#FC374
Saxifragales Aphanopetalaceae Aphanopetalum resinosum#Y236
Saxifragales Tetracarpaeaceae Tetracarpaea tasmanica#EF207
Saxifragales Penthoraceae Penthorum chinense#F811
Saxifragales Penthoraceae Penthorum chinense#NC 023086
Saxifragales Haloragaceae Myriophyllum aquaticum#IUSR
Saxifragales Haloragaceae Myriophyllum spicatum#NC 037885
Saxifragales Haloragaceae Myriophyllum verticillatum#F618
Saxifragales Crassulaceae Crassula perforata#KWGC
Saxifragales Crassulaceae Kalanchoe integra#F189
Saxifragales Crassulaceae Kalanchoe crenata#DRIL
Saxifragales Crassulaceae Sedum oryzifolium#NC 027837
Saxifragales Crassulaceae Sedum sarmentosum#NC 023085
Saxifragales Crassulaceae Sedum franchetii#F36
Saxifragales Crassulaceae Sedum kamtschaticum#NC 037946
Saxifragales Crassulaceae Sedum takesimense#NC 026065
Saxifragales Crassulaceae Sedum roseum#ZJUL
Saxifragales Crassulaceae Rhodiola rosea#NC 041671
Saxifragales Iteaceae Cyrilla racemiflora#UWFU
Saxifragales Iteaceae Itea chinensis#NC 037884
Saxifragales Iteaceae Itea yunnanensis#F203
Saxifragales Iteaceae Itea macrophylla#F303
Saxifragales Grossulariaceae Ribes fasciculatum var. chinense#MH191388
Saxifragales Grossulariaceae Ribes aff. giraldii#SYHW
Saxifragales Grossulariaceae Ribes glaciale#F384
Saxifragales Grossulariaceae Ribes sp.#F817
Saxifragales Saxifragaceae Saxifraga gemmipara#F28
Saxifragales Saxifragaceae Saxifraga stolonifera#FCBJ
Saxifragales Saxifragaceae Astilbe rubra#CKKR
Saxifragales Saxifragaceae Tanakaea radicans#F504
Saxifragales Saxifragaceae Boykinia jamesii#OOVX
Saxifragales Saxifragaceae Mukdenia rossii#NC 037495
Saxifragales Saxifragaceae Oresitrophe rupifraga#MG470845
Saxifragales Saxifragaceae Bergenia purpurascens#F154
Saxifragales Saxifragaceae Bergenia sp.#CIAC
Saxifragales Saxifragaceae Bergenia scopulosa#NC 036061
Saxifragales Saxifragaceae Peltoboykinia watanabei#YKFU
Saxifragales Saxifragaceae Chrysosplenium aureobracteatum#NC 039740
Saxifragales Saxifragaceae Chrysosplenium nepalense#F385
Saxifragales Saxifragaceae Mitella pentandra#DAYQ
Saxifragales Saxifragaceae Heuchera sanguinea#ERIA
Saxifragales Saxifragaceae Tiarella polyphylla#F387
Saxifragales Saxifragaceae Tellima breviflora#CTSS
Saxifragales Saxifragaceae Heuchera parviflora#KR478645
Vitales Vitaceae Leea indica#F72
Malpighiales Rafflesiaceae Rhizanthes lowii#HM803264
Vitales Vitaceae Ampelopsis grossedentata#F286
Vitales Vitaceae Ampelopsis japonica#MK547541
Vitales Vitaceae Ampelopsis humulifolia#MK547542
Vitales Vitaceae Cissus quadrangularis#BGZG
Vitales Vitaceae Cissus javana#F163
Vitales Vitaceae Tetrastigma obtectum#BBBA
Vitales Vitaceae Tetrastigma hemsleyanum#NC 029339
[truncated: 155,528 more chars]
