# Supplementary material for: Plastid phylogenomic insights into relationships of all flowering plant families
Source: BMC Biol. 2021 Oct 29;19:232. doi: 10.1186/s12915-021-01166-2 (PMC8555322; doi:10.1186/s12915-021-01166-2)
Supplement: Supplementary file 10 — Additional file 10: Figure S9. Topologies of a pruned Maximum Likelihood phylogeny of 431 representative species (“ML431_pruned”) and ASTRAL analysis (“astral431_BS10”) of a 43-species subdataset of angiosperms using 80 plastid genes. The ML bootstrap percentages and ASTRAL local posterior probabilities are shown, respectively. [file 12915_2021_1166_MOESM10_ESM.pdf]

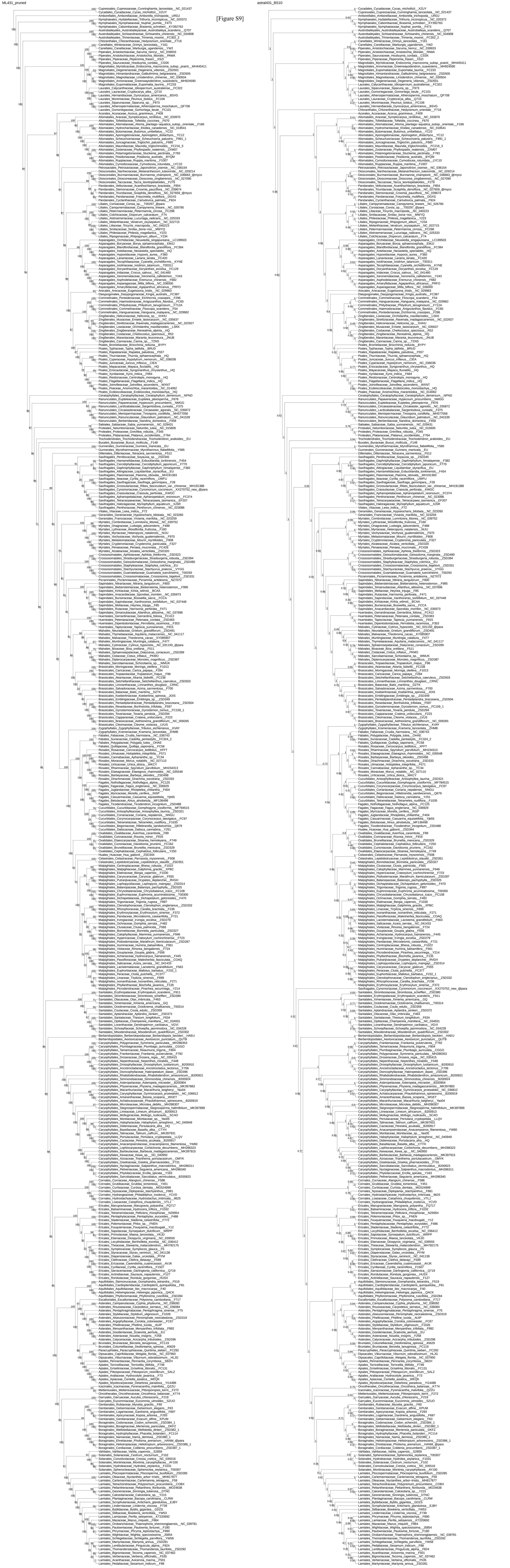

[Figure S9]
